# Dynamic expression of small non-coding RNAs, including novel microRNAs and piRNAs/21U-RNAs, during *Caenorhabditis elegans *development

**DOI:** 10.1186/gb-2009-10-5-r54

**Published:** 2009-05-21

**Authors:** Masaomi Kato, Alexandre de Lencastre, Zachary Pincus, Frank J Slack

**Affiliations:** 1 Department of Molecular, Cellular and Developmental Biology, Yale University, New Haven, CT 06520, USA

## Abstract

**Background:**

Small non-coding RNAs, including microRNAs (miRNAs), serve an important role in controlling gene expression during development and disease. However, little detailed information exists concerning the relative expression patterns of small RNAs during development of animals such as *Caenorhabditis elegans*.

**Results:**

We performed a deep analysis of small RNA expression in *C. elegans *using recent advances in sequencing technology, and found that a significant number of known miRNAs showed major changes in expression during development and between males and hermaphrodites. Additionally, we identified 66 novel miRNA candidates, about 35% of which showed transcripts from their 'star sequence', suggesting that they are *bona fide *miRNAs. Also, hundreds of novel Piwi-interacting RNAs (piRNAs)/21U-RNAs with dynamic expression during development, together with many longer transcripts encompassing 21U-RNA sequences, were detected in our libraries.

**Conclusions:**

Our analysis reveals extensive regulation of non-coding small RNAs during development of hermaphrodites and between different genders of *C. elegans*, and suggests that these RNAs, including novel miRNA candidates, are involved in developmental processes. These findings should lead to a better understanding of the biological roles of small RNAs in *C. elegans *development.

## Abstract 2

A deep-sequencing approach to profiling gender-specific developmental regulation of small non-coding RNA expression in C. elegans reveals dynamic temporal expression and novel miRNAs and 21U RNAs.

## Background

Proper control of gene expression is required for normal development, health maintenance, and successful reproduction. Until recently it had been believed that gene regulatory networks consisted solely of protein-coding genes, and, in particular, those encoding transcription factors. However, the complete sequencing of many organisms has revealed that only a small fraction of most genomes encodes proteins (reviewed in [1,2]). On the other hand, recent in-depth genome-wide efforts, including full-length cDNA cloning and tiling microarray analysis, have shown that a large fraction of the remaining non-coding regions are much more extensively transcribed into stable RNAs than previously appreciated (reviewed in [1-3]). Notably, significant portions of these transcripts are small, non-coding RNAs, including microRNAs (miRNAs) and Piwi-interacting RNAs (piRNAs).

miRNAs, first discovered in *C. elegans *[4-6], negatively regulate gene expression by binding to complementary sequences in the 3' untranslated region of their target mRNAs in an Argonaute-protein-dependent manner (reviewed in [7]). Mature miRNA products, approximately 22 nucleotides in length, are processed from hairpin-loops of larger primary transcripts. The importance of these RNAs is evidenced by their evolutionary conservation across species and by the many biological events in which they are involved, including cell proliferation, apoptosis and metabolism (reviewed in [8,9]).

piRNAs, another recently discovered class of small non-coding RNAs that are 24 to 30 nucleotides in length, were found in *Drosophila*, zebrafish and mammals and so named because they interact with Piwi proteins [10-16]. These proteins, in the Argonaute family, are required for germline development [17,18] and are important for transposon silencing in the germline of several different organisms [11,14,19-21]; this suggests that at least one role of piRNAs is to protect the germline genome against transposons. Indeed, many piRNA sequences map to transposon-like repetitive sequences [22]. Recently, a related class of 21-nucleotide RNAs starting with a uracil (21U-RNA) was identified in *C. elegans *[23]; these RNAs were subsequently confirmed to be piRNAs [24-26]. Specifically, *C. elegans *piwi-related gene (*prg*) mutants display a dramatic reduction of 21U-RNA expression and a significant up-regulation of the mRNA of Tc3 family transposons with concomitant transposition [24-26].

Previous work has demonstrated that expression of some of these small RNA genes is tightly regulated during development. For example, the expression in *C. elegans *of the two founding miRNAs, *lin-4 *and *let-7*, are specifically up-regulated at the second larval (L2) and the fourth larval (L4) stages, respectively, and are necessary for the normal transition from the first to the second larval stage and from the fourth larval stage to the adult, respectively. Additionally, a Piwi-related protein and numerous piRNAs/21U-RNAs were shown to be most abundant in the young adult stage [24-26]. This implies that Piwi protein and piRNAs/21U-RNAs function in the control of gene expression, in addition to suppressing transposon activity, in germline development. These observations suggest that expression of other miRNAs and piRNAs/21U-RNAs is temporally regulated during development. However, few studies have measured temporal patterns in expression of all these small RNAs in parallel.

Here we use recent advances in high-throughput sequencing technology to quantify the expression of non-coding small RNAs, including miRNAs and piRNAs/21U-RNAs, and demonstrate dynamic and sex-specific expression pattern changes during development of *C. elegans*. Additionally, we identify many novel miRNA candidates and hundreds of novel piRNAs/21U-RNAs, as well as longer 21U-RNA transcripts encompassing mature 21U-RNAs. These results should lead to a better understanding of the expression and function of small RNAs in *C. elegans *development.

## Results and discussion

**Figure 1:**
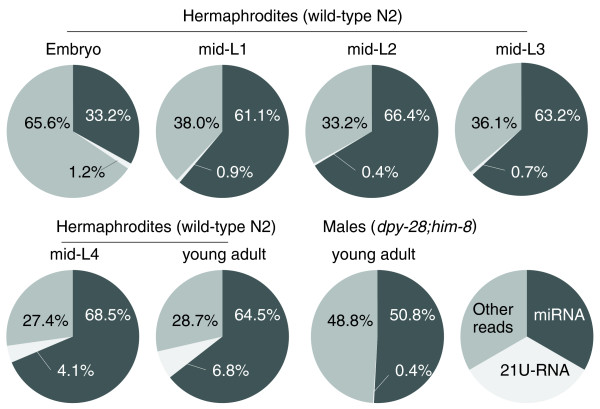
Proportions of miRNA and 21U-RNA reads at each developmental stage of hermaphrodites and in males. Details are shown in Additional data file 1.

To examine the changes in expression levels of non-coding RNA populations in development and in the different sexes of *C. elegans*, and to identify additional non-coding small RNAs, we generated cDNA libraries of small RNAs purified from six developmental stages of hermaphrodites (embryo, mid-L1, -L2, -L3, -L4 and young adult) and young adult males (generated from a *dpy-28(y1);him-8(e1489*) strain). Sequencing these samples using Solexa technology [27] produced 73,678,102 total sequence reads of which 42,005,206 matched to the *C. elegans *genome (Additional data file 1). Approximately 60% of the aligned reads in each sample consisted of known miRNAs and 21U-RNAs, while in the remaining set, categorized as 'Other reads' in Figure 1, we detected many hits to rRNAs (ribosomal RNAs), tRNAs (transfer RNAs), and snoRNAs (small nucleolar RNAs) (Additional data file 1; for these non-coding RNAs in *C. elegans*, see [28]). As purification was specific for 18- to 30-nucleotide RNAs during cDNA library preparation, we speculate that most of these are degradation products. In addition to these known functional non-coding RNA species, we identified many novel miRNA candidates and novel piRNAs/21U-RNAs in the 'Other reads' fraction (described below).

### Deep sequencing detects the majority of known miRNAs

From our libraries, we detected the expression of 133 of the 154 previously annotated *C. elegans *miRNAs (miRbase release 11.0; Additional data file 2). While we did not detect 21 of the previously reported miRNAs (we suspect that most of these undetected miRNAs may not actually encode miRNAs at all [23,29] or may be annotated incorrectly; detailed results are shown in Additional data file 3), we did obtain 125 clones of a very rare miRNA, *lsy-6*, expressed in only one pair of neurons in the *C. elegans *head [30]. These findings demonstrate the significant sequencing depth of our survey. Conversely, the maximum number of clones we obtained for a single miRNA was 12,295,951 (miR-58; Additional data file 2), which highlights the high dynamic range of miRNA expression that can be surveyed using deep-sequencing technology such as that from Solexa.

**Figure 2:**
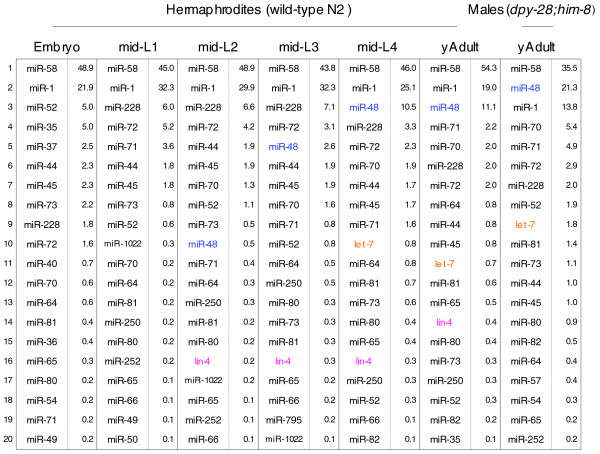
The top 20 highest expressed miRNAs in each sample. The numbers shown on the right side of the miRNAs represent the percentage of reads of each miRNA compared to all miRNA reads in that sample. The founding miRNA genes, *lin-4 *and *let-7*, and miR-48, another *let-7 *family member, are highlighted in color and in bold and are expressed at the times expected from the literature.

Two miRNAs, miR-58 and miR-1, which showed the highest expression in our total libraries, were abundantly expressed in animals of all developmental stages we examined, from embryo to young adult of hermaphrodites, and in young adult males (Figure 2). Although the function of *mir-58 *in *C. elegans *remains unknown, we speculate that it has a general housekeeping role. Similarly, *C. elegans *miR-1 has a broad and generalized role, as it is involved in the function of neuromuscular junctions [31], and a *mir-1 *homologue in *Drosophila *has an important role in muscle development [32].

### Temporal regulation of miRNA expression during development

**Figure 3:**
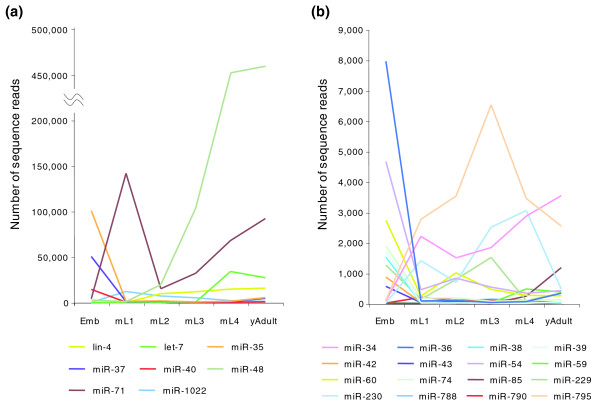
miRNAs showing major changes in expression between any two stages during development. The number of reads of each miRNA was plotted after normalization (see Materials and methods). miRNAs expressed in **(a) **high abundance (more than 10,000 reads at any stage) and **(b) **lower abundance are shown separately. For clarity, miRNAs with fewer than 200 reads are not shown. Emb, embryo; mL, mid-larval stage; yAdult, young adult.

The number of sequence reads for a particular miRNA is known to be proportional to the molecular abundance of that species [33]. Thus, the number of sequence reads of each unique miRNA in each sample is a reasonable measure of stage-specific expression during development (Figure 3). We controlled for library differences by normalizing these values to the total number of reads that matched to the *C. elegans *genome in each sample (Additional data file 4). The raw data for the number of reads is available in Additional data file 2. Finally, we confirmed by RT-PCR the relative stage-specific expression levels of the ten known miRNAs with highly dynamic expression patterns (Additional data file 5).

About 16% of known miRNAs showed major changes in expression at some point during development (for example, between embryo and the mid-L1 stage; Figure 3a, b). We define here 'a major change' as more than a tenfold difference in the number of reads. For example, the *let-7 *miRNA exhibited a major increase in expression around the mid-L4 stage, as did one of the *let-7 *family members, miR-48, from the mid-L3 stage (Figures 2 and 3a). Additionally, another well-characterized miRNA, *lin-4*, showed a large increase in expression from the mid-L2 stage (Figures 2 and 3a). These observations correspond to previously published results [34,35] and support the validity and reliability for our small RNA libraries and our analysis.

It is interesting that we were able to clone multiple members of the *let-7 *and *lin-4 *families from stages where they were not previously known to be expressed (Additional data file 4). For example, we detected small numbers of clones to both *let-7 *and *lin-4 *in embryonic stages, many hours earlier than they had been observed previously. It is unclear if these miRNAs function during these earlier stages, since no embryonic phenotypes are known for *let-7 *or *lin-4 *null mutants [6,36]. Conceivably, this could also represent maternal inheritance or a small bleed-through from the adults to the embryos during preparation.

Of the 24 miRNAs with major changes in expression, some had particularly dynamic expression patterns. For example, miR-71 is dramatically up-regulated from the embryo to the mid-L1 stage and then quickly down-regulated at the mid-L2 stage, and again gradually but significantly up-regulated after the mid-L4 stage (Figure 3a; Additional data file 5). Given its temporal regulation, this miRNA might be involved in control of developmental timing, like *lin-4 *and *let-7*. Another interesting case is the expression of miR-77, miR-85, miR-240 and miR-246, which is very low or completely absent in earlier developmental stages but increases after the mid-L4 and young adult stages (Figure 3b; Additional data files 4 and 5), implying a potential role in adult functions like reproduction, metabolism or aging. A recent report by Martinez *et al*. [37] also mentioned that some of these miRNAs, including miR-85 and miR-240, are temporally regulated during development, mirroring our results. We highlight additional developmentally regulated miRNAs in Additional data file 4.

### Male-specific miRNA expression

**Figure 4:**
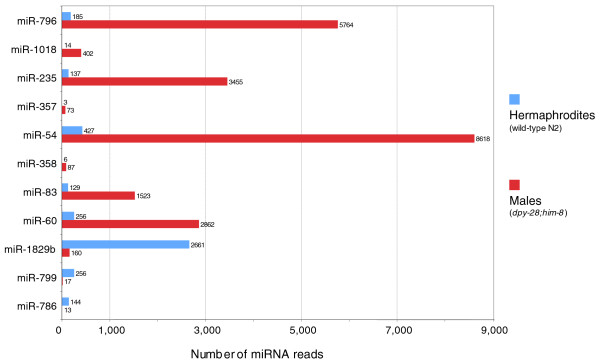
Differential expression of miRNAs in hermaphrodites and males at the young adult stage. For clarity, miRNAs with fewer than 50 reads in both hermaphrodites and males are not shown.

The different sexes of animals result from different developmental pathways, which specify and maintain cell differentiation of the animal as male rather than female or hermaphrodite. Males in *C. elegans *have several distinct features and tissues, including mating organs in the tail and a male-specific germline, generating only sperm. In addition, males exhibit a smaller overall body size and different behavior compared to hermaphrodites. To assess those miRNAs preferentially expressed in males or in hermaphrodites, we generated and sequenced a cDNA library from small RNAs of young adult males (*him-8 *(*e1489*) mutants crossed with *dpy-28 *(*y1*); see Materials and methods). We found that about 12% of known miRNAs exhibited major differences in expression in hermaphrodites and in males (Figure 4; Additional data file 4). The correlation between miRNA expression levels in males and hermaphrodites is shown in Additional data file 6. Interestingly, most of the differentially expressed miRNAs are more abundant in males than hermaphrodites, which may reflect their expression in male-specific organs, for example, the rays used in copulation.

### Identification and characterization of novel miRNA candidates

**Figure 5:**
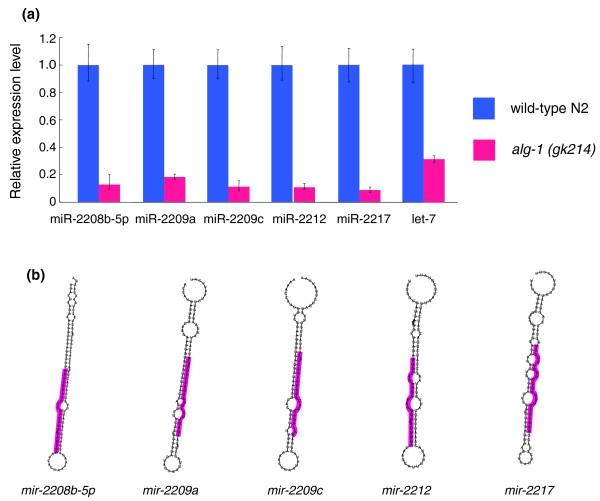
Validation of the expression of novel miRNAs. **(a) **Validation of the expression of novel miRNAs by RT-PCR. Error bars represent standard deviation. **(b) **Computationally predicted secondary structure of the primary miRNA transcripts.

In order to identify novel miRNAs, we first filtered out sequence reads corresponding to all annotated RNA molecules, including miRNAs, mRNAs and other small non-coding RNAs. We then used the miRDeep program [38] to predict which of the remaining sequence reads might be miRNAs. This analysis revealed 66 novel miRNA candidates (Additional data file 7). In addition, we found the 'star sequence' for 24 of these candidates in our sequence reads (highlighted in red in Additional data file 7). Mature miRNAs are processed from the stem of a hairpin precursor, and the star sequence corresponds to the section of this hairpin that remains hybridized to the mature form (with approximately 2-nucleotide 3' overhangs) throughout much of miRNA biogenesis [33]. The presence of these star sequence reads thus strongly suggests that at least these 24 novel candidates are *bona fide *miRNAs. We further examined the expression of five of these candidates using RT-PCR in both wild-type N2 and *alg-1(gk214) *mutant backgrounds. It is known that the two Argonaute family members *alg-1 *and *alg-2 *are essential for miRNA processing, but have no role in the RNA interference (RNAi)-mediated silencing pathway including siRNA (small interfering RNA) production [39,40]. Indeed, mature *let-7 *miRNA transcripts were less abundant in the *alg-1 *mutant background, as were those of all five novel miRNA candidates tested (Figure 5a). This was also confirmed in the *alg-1 *RNAi background (data not shown). These observations indicate that these five candidates are indeed true miRNAs. Computationally predicted secondary structures of the primary miRNA transcripts (pri-miRNAs) of these novel miRNAs are shown in Figure 5b.

**Figure 6:**
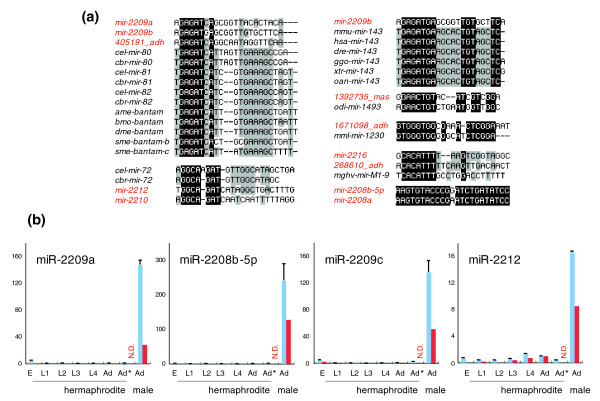
Characterization of novel miRNAs. **(a) **Sequence alignment of the novel miRNA candidates. Highly conserved 'seed' regions are highlighted in black and gray. Novel miRNAs are colored in red. **(b) **The expression of some novel miRNAs during development. Blue-colored and red-colored bars represent the results of quantitative RT-PCR and Solexa sequencing, respectively. The vertical axis indicates the relative expression level. The data were standardized to the expression in young adult hermaphrodites as 1. 'Ad' (young adult hermaphrodites) marked with an asterisk were cultured at 23°C, under the same condition as males, in order to rule out the possibility that male-enriched expression of these novel miRNAs is due to a higher culture temperature. Since Solexa sequencing was not performed for young adult hermaphrodites cultured at 23°C, this was shown as N.D. Error bars represent standard error. E, embryo; L, larval stage.

**Figure 7:**
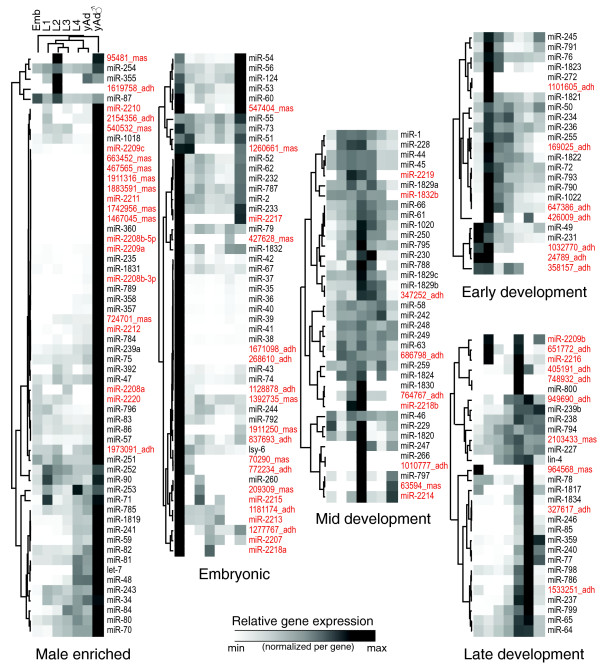
Expression clustering of known and novel miRNAs; the latter class is labeled in red. Expression levels were normalized per gene (retaining the relative shape but not the absolute magnitude of the temporal expression profiles), and the genes and time-points were clustered with complete linkage using the centered correlation coefficient. Five high-level clusters emerged and are shown here (The base of the tree, showing the relationships between these clusters, is not particularly informative and is not shown.). Emb, embryo; L, larval stage; yAd, young adult.

Furthermore, of the 66 novel miRNA candidates, 20 may fall into known miRNA families since they had the same core target-binding ('seed') sequence found in other miRNAs in other species (Figure 6a; Additional data file 7). One of the novel miRNAs verified by RT-PCR, miR-2209a, has the seed sequence common to the bantam miRNA family, which is known to function in apoptosis [41]. Further, we found that this novel miRNA is clustered on chromosome IV together with another four novel miRNA members, including miR-2208b-5p, miR-2208b-3p and miR-2209c (Additional data file 7). Also, these clustered novel miRNAs had similar expression patterns, falling into the male-enriched group (see below; Figures 6b and 7; Additional data file 8). Moreover, another validated novel miRNA, miR-2212, was genomically clustered on chromosome X with a known miRNA, miR-1819, and both showed male-enriched expression (Figures 6b and 7; Additional data files 4 and 8).

### miRNA expression cluster analysis

To visualize broad trends in the temporal expression of both previously identified and our newly identified miRNAs, we performed a simple hierarchical clustering. (Figure 7). We found that the 199 miRNAs detectable in our analysis assort into roughly five groups: those expressed primarily at the embryonic stage, those enriched in males, and those primarily expressed in early, middle, and late larval development.

Interestingly, we found that genomically clustered miRNAs are not necessarily co-expressed at the same levels. Some sets of miRNA map to specific chromosomal clusters, as in the case of miR-35 to miR-41, which have redundant functions in embryonic development [42] and are abundantly expressed in the embryonic stage (Figures 3b and 7). Genomically clustered miRNAs are thought to be transcribed as a single transcript and then individual pre-miRNA are subsequently processed out. We found that although these miRNAs have generally similar expression patterns during development (Figure 7), the absolute expression levels are strikingly different (Additional data file 4). Perhaps, then, clustered miRNAs may be differentially controlled at the transcriptional level and/or during subsequent processing.

Our analysis of the changes of miRNA expression during development may provide helpful information in identifying the target genes for these miRNAs. Coupling this data set with several of the studies describing mRNA expression profiles during development and aging of *C. elegans *[43,44] could provide correlations pointing to potential miRNA-target pairs, since changes in expression of miRNAs may cause reciprocal expression patterns of their target genes during development of *C. elegans*. (Although miRNAs that form imperfect duplexes with their targets inhibit protein production in animals, miRNA binding can also result in degradation of the target mRNA in *C. elegans *[45]; indeed, microarray analysis has proven to be an effective way to find genes modulated by miRNAs [46].)

### Expression of piRNAs/21U-RNAs during development and in the germline

Another class of *C. elegans *non-coding small RNAs, 21U-RNAs, have important functions in transposon silencing in the germline and maturation of gametes [24-26]. More than 15,000 unique 21U-RNA sequences have been reported in *C. elegans*, the vast majority of which map to either intergenic or intronic regions on chromosome IV [23,25]. As expected from their function in germline development, our results confirmed recent studies that show prominent accumulation of 21U-RNAs in the young adult stage (Figure 1; Additional data files 1 and 9) [24-26].

**Figure 8:**
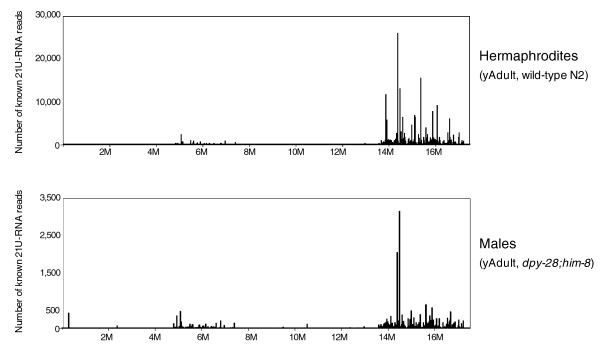
Expression of piRNAs/21U-RNAs in hermaphrodite and male germlines. The vertical and horizontal axes represent the number of reads of 21U-RNAs and their position on chromosome IV, respectively. Note the significantly higher expression of 21U-RNAs in wild-type N2 hermaphrodites compared to males at the young adult (yAdult) stage. The number of 21U-RNA reads was plotted after normalizing to the total number of reads that matched to the *C. elegans *genome in each sample.

To test if there are functional differences with regard to 21U-RNAs in the sperm, we further examined the expression of 21U-RNA in wild-type hermaphrodites together with males (*dpy-28*(*y1*)*;him-8*(*e1489*)) at the young adult stage. Although the overall mapping pattern of 21U-RNAs on chromosome IV seemed unchanged in each strain, their abundance was significantly decreased in males (*dpy-28;him-8*) compared to wild-type hermaphrodites (Figure 8 - note that the scale in wild-type (top) is tenfold greater than that in male (bottom); Additional data file 9). This reveals that sperm and/or their progenitors produce a number of the piRNAs/21U-RNAs, but the level may be lower than that in the oocyte germline in *C. elegans*.

Approximately 44% of known 21U-RNAs on chromosome IV are genomically clustered within 10 bp with other 21U-RNAs (see below), implying that expression of 21U-RNAs in each cluster is controlled in a similar manner, and one would expect that these clustered 21U-RNAs might show similar changes in expression in both male and hermaphrodite germlines compared to 21U-RNAs mapping outside the clusters. Interestingly, though, we did not detect common patterns in expression of 21U-RNAs in the clusters; that is, 21U-RNA abundance was routinely different for 21U-RNAs in the same cluster, although 21U-RNAs in a genomic cluster appears to be transcribed from the same strand (data not shown).

### Identification and characterization of additional piRNA/21U-RNA sequences

**Figure 9:**
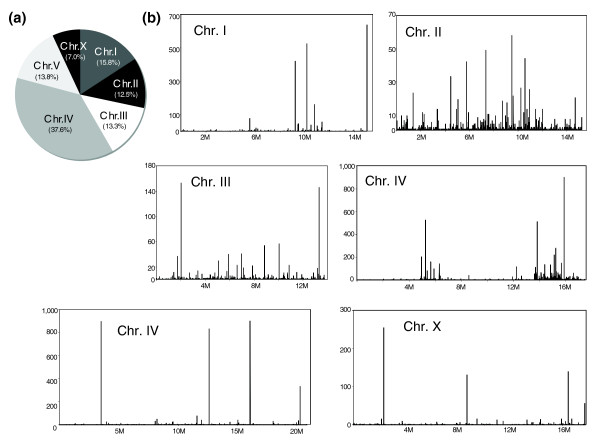
Characterization of 21nt-U-RNA reads. **(a) **Proportion of 21nt-U-RNA reads in each chromosome (some map to multiple loci; details are shown in Additional data file 10). **(b) **The expression pattern of 21nt-U-RNA reads on each chromosome. Axes are as in Figure 8.

In the course of our analysis, we identified approximately 10,000 21-nucleotide sequence reads starting with a uracil that have not been previously annotated (Additional data file 10). These reads are referred to here as 21nt-U-RNA for descriptive purposes to differentiate them from previously identified 21U-RNAs. Of these 21nt-U-RNA sequence reads, about 40% mapped to chromosome IV while the remaining approximately 6,100 reads mapped to other chromosomes, ranging from 7% of reads in chromosome X to nearly 16% in chromosome I (Figure 9; Additional data file 10). While many of the 21nt-U-RNA reads on chromosome IV mapped to the two distinct regions observed for known piRNAs/21U-RNAs, similar clustering was not apparent on other chromosomes (Figure 9). To determine whether these sequence reads represent new members of the piRNA/21U-RNA family, we searched for characteristic features of previously described 21U-RNAs. Although 21U-RNAs generally share little sequence identity other than the uracil at their 5' termini and specific localization on chromosome IV, it has been shown that the sequences upstream of 21U-RNAs contain an 8-nucleotide core consensus motif, CTGTTTCA, centered within a larger motif [23]. About 14% (562), of our 21nt-U-RNAs on chromosome IV had a complete consensus motif in their upstream larger motif (the 43-nucleotide regions, -20 to -63 bp upstream from 5' termini of each 21nt-U-RNA, were analyzed.), whereas only a few 21nt-U-RNAs on other chromosomes had this 8-nucleotide motif (Additional data file 10). This result is consistent with the chromosome IV-biased localization of known piRNAs/21U-RNAs. We therefore believe that the 21nt-U-RNAs reads that map to chromosome IV and contain the core motif are indeed new piRNA/21U-RNAs (Additional data file 11; note that 10 of the 562 novel 21U-RNAs (21nt-U-RNAs) map to multiple loci on chromosome IV).

While we have not shown that these RNAs associate with Piwi proteins like PRG-1, we suspect that these are very likely to be novel piRNAs/21U-RNAs for several reasons: first, these RNAs are abundantly expressed in the L4 and young adult stages (Additional data file 12; consistent with known 21U-RNAs); second, they are transcribed from the same two distinct regions of chromosome IV as known 21U-RNAs (Additional data file 12); third, they contain the core motif associated with *bone fide *21U-RNAs; and fourth, most of them partially overlap with known or other novel 21U-RNAs (see below). Also, approximately 8% of these novel 21U-RNAs were detectable in other libraries obtained by 454 sequencing from different biological sources (ADL and FS, unpublished result).

### Identification of larger reads corresponding to piRNAs/21U-RNAs

**Figure 10:**
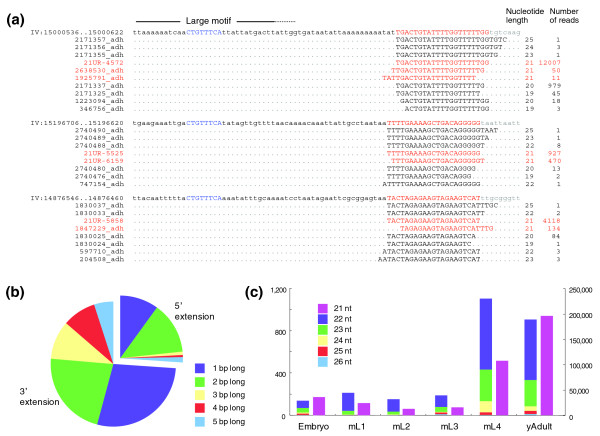
Characterization of the longer transcripts of 21U-RNAs. **(a) **A view of the longer and overlapping 21U-RNA reads. The number of reads shown in this figure was based on the computational output of the SOAP program [52] followed by removal of redundant sequences, and samples of all six developmental stages (embryo to young adult of hermaphrodites) were used as the input. The core consensus motif 'CTGTTTCA' and the mature 21U-RNA sequences are capitalized and highlighted in blue and red, respectively. **(b) **The proportion of longer 21U-RNAs of different length. The number of reads of each transcript was reflected in the result; for example, the length of an extension in a longer 21U-RNA with 3 bp extension to its 3' side and with 4 reads was calculated as 12 (3 × 4). **(c) **The abundance of longer 21U-RNAs during development. The left and right vertical axes represent the number of longer 21U-RNA reads (22 to 26 nucleotides) and that of mature 21U-RNAs with longer transcripts detected, respectively.

Of the 562 novel piRNAs/21U-RNAs we identified, 438 partially overlap other 21U-RNAs; either of their termini is located within 10 bp of another 21U-RNA terminus (although not separated by 10 nucleotides as in the case of *Drosophila *piRNAs; Figure 10a; Additional data file 11). Note also that approximately 43% of the 21U-RNAs on chromosome IV recently reported in Batista *et al*. [25] partially overlap (Figure 10a; Additional data file 9 - reads that overlap other 21U-RNAs are marked with a dagger). Interestingly, we noticed longer sequence reads in our libraries that encompassed mature 21U-RNAs (Figure 10a; a list of all longer transcripts detected is available in Additional data file 13). In total, 910 21U-RNAs were found in such longer reads, which corresponds to about 6% of the previously annotated and novel 21U-RNAs. These 21U-RNAs are marked with an asterisk in Additional data files 9 and 11. Similar longer reads were also detected in other small RNA libraries from 454 sequencing (ADL and FS, unpublished result), suggesting that they are biological products but not artifacts of Solexa sequencing. One possible explanation for the presence of longer 21U-RNA transcripts could be that they are by-products due to errors in 21U-RNA biogenesis - for example, read-through transcription and/or aberrant processing. For example, in the case of miRNAs, we also detected various larger sequence variants in our libraries (Additional data file 3). Alternatively, they may represent intermediates in 21U-RNA biogenesis. For example, original 21U-RNA transcripts may be longer in length and are processed to 21 nucleotides by an unknown mechanism. Indeed, in all cases we examined, the most abundant sequences were 21 nucleotides in length (Figure 10a; Additional data file 13), and a significant portion of these longer transcripts had an extension to their 3' side rather than the 5' side (Figure 10b). Additionally, the production of these longer 21U-RNA reads also appeared to be temporally regulated during development; they were abundant at the later stages of development, as in the case of 21-nucleotide mature 21U-RNAs (Figure 10c). Although the mechanism controlling 21U-RNA expression is still not clear, these observations lead us to speculate that precursor 21U-RNA transcripts are longer in length.

## Conclusions

Our analysis reveals extensive regulation of small, non-coding RNAs during development of *C. elegans *hermaphrodites and in males, and suggests that these RNAs are involved in developmental processes. Our results also illustrate the extreme diversity of miRNA and piRNA expression in *C. elegans*. In addition, our deep sequencing approach revealed the presence of tens more miRNAs and hundreds more piRNAs than were previously known. Since the information content of the genome is more complex than previously imagined - for example, most of both strands of the genome appear to be transcribed in human [47], and approximately 80% of transcripts map to unannotated regions [48] - it seems likely that additional non-coding RNA genes remain to be discovered and characterized in other animals as well. For instance, in our study, numerous sequence variants of miRNAs were found corresponding to their hairpin sequences, which include many 'star sequences' (Additional data file 3). Identification of further transcripts and their biological roles will lead to a better understanding of animal biology and will shed light on control of gene expression during development and disease.

## Materials and methods

### *C. elegans *strains and small RNA purification

Wild-type N2 strains were cultured under standard conditions [49] at 20°C and used to prepare RNAs from each developmental stage (time after stage L1: mid-L1 (4 h), mid-L2 (14 h), mid-L3 (25 h), mid-L4 (36 h); and young adult (48 h). RNAs enriched for small RNA species (less than 200 nucleotides) were prepared using the *mirVana *miRNA Isolation kit (Ambion/Applied Biosystems, Austin, TX, USA) with the small RNA enrichment procedure. For library preparation from young adult males, *dpy-28 (y1);him-8 (e1489) *double mutants cultured at 23°C were used to obtain male populations after backcrossing six times to wild-type N2, and RNAs were purified at 40 h after stage L1. *him-8 (e1489) *mutants produce XO males and XXX hermaphrodites at 37% and 6% frequency, respectively, in addition to XX hermaphrodites [50]. However, XX and XXX hermaphrodites can not survive at 23°C in the *dpy-28 *(*y1*) background [51], and the resulting surviving population of *dpy-28;him-8 *double mutants is almost all XO males at this temperature. For validating novel miRNA expression, total RNAs were isolated from N2 wild-type worms, *alg-1 *(*gk214*) mutants and N2 wild-type worms on both L4440 (empty vector) and *alg-1 *RNAi at the young adult stage.

### cDNA library preparation and sequencing

cDNA libraries for small RNAs were made from 10 μg of RNA from an enriched small RNA fraction using the DGE-Small RNA Sample Prep Kit (Illumina, San Diego, CA, USA) according to the manufacturer's instructions. The same amount of cDNA was sequenced on a Genetic Analyzer from Illumina. The data from the miRNA reads we mentioned above were uploaded to the Genome Expression Omnibus database together with the raw Solexa sequence results [GEO:GSE13339]. The 66 novel miRNA candidates and the 552 unique piRNAs/21U-RNAs have GenBank accession numbers (shown in Additional data files 7 and 11).

### Quantitative RT-PCR

The expression of some of the known miRNAs were confirmed by quantitative RT-PCR using a TaqMan Small RNA Assay (Applied Biosystems, Foster City, CA, USA) with the RNAs at concentrations of 0.4 ng/μl (enriched small RNAs) and 2 ng/μl (total RNAs), according to the manufacture's instruction. For validating the expression of novel miRNA candidates, 10 ng/μl of total RNAs was used, and the results were normalized to the expression level of U18. The results were further confirmed using independently prepared RNA samples.

### Computational data analysis

The number of sequence reads for miRNAs and 21U-RNAs was assessed from the raw sequence data from Solexa sequencing using perfect sequence matching to known miRNAs (miRBase release 11.0) and 21U-RNAs [25] (Additional data files 2, 4 and 9). For examining the proportion of each non-coding RNA species, including rRNAs, tRNAs, snRNAs, and snoRNAs, sequence reads that matched to the *C. elegans *genome (WS190) were extracted by the SOAP program (a maximum of 2 bp mismatches were allowed in the alignment) [52], and the number of sequence reads perfectly corresponding to each RNA species was determined using BLASTN against a database of non-coding RNAs from WormBase [53]. To compare the differential expression of small RNAs across development, the number of reads in each sample was normalized to the total number of reads that matched to the *C. elegans *genome in each sample. The Cluster 3.0 program was used to cluster the miRNAs (after normalizing each gene's expression vector to have a 2-norm of 1). The Java TreeView program [54] was then used to visualize these clusters. The miRDeep program [38] was used for finding novel miRNA candidates, and the RNA fold program was used for predicting secondary structure of primary miRNA transcripts of novel miRNAs.

## Abbreviations

L: larval stage; miRNA: microRNA; piRNA: Piwi-interacting RNA; RNAi: RNA interference.

## Authors' contributions

MK carried out sample preparation, computational analysis and experimental validation. ADL supported the computational analysis. ZP carried out the expression clustering analysis. FJS and MK conceived of the study, and participated in its design and coordination and wrote the manuscript. All authors read and approved the final manuscript.

## Additional data files

The following additional data are available with the online version of this paper: the total number of sequence reads and number of reads of each non-coding RNA species in each sample (Additional data file 1); raw data showing the number of miRNA reads in each developmental stage of hermaphrodites and in young adult males (Additional data file 2); sequence variants expressed from miRNA hairpins (Additional data file 3); normalized data of the number of miRNA reads by the total number of reads that matched to the *C. elegans *genome (Additional data file 4); confirmation of miRNA expression changes during development of hermaphrodites and in young adult males using quantitative RT-PCR (Additional data file 5); the correlation between miRNA expression levels in males and hermaphrodites (Additional data file 6); a list of novel miRNA candidates (Additional data file 7); the number of reads of novel miRNA candidates in each sample (Additional data file 8); the number of known 21U-RNA reads in each sample (Additional data file 9); sequence of 21nt-U-RNA reads and their chromosomal position (Additional data file 10); sequence of novel 21U-RNAs (Additional data file 11); changes in expression of novel 21U-RNAs during development and their position on chromosome IV (Additional data file 12); a list of all 21U-RNA longer transcripts detected in our library (Additional data file 13).

## Supplementary Material

Additional data file 1 The number of each RNA species that satisfies the following conditions was counted after searching by the BLASTN program in the pool of sequence reads that aligned to the *C. elegans *genome; the length of the query is equal to that of the match and the percentage of identical bases in the match is 100%. Click here for file

Additional data file 2 Raw data showing the number of miRNA reads in each developmental stage of hermaphrodites and in young adult males. Click here for file

Additional data file 3 The SOAP-processed aligned reads were searched against miRNA hairpin sequences. Each number represents the total number of reads detected in all developmental stages of hermaphrodites and young adult males. Annotated mature miRNAs are marked with a hatch mark and highlighted in red, and annotated novel miRNAs we report here are colored in green. Click here for file

Additional data file 4 The names of miRNAs with more than a fivefold difference in the number of reads at some point during development and/or between genders are labeled in red and their numbers of reads are compared. The miRNAs with lower numbers of reads (less than ten in the sum of reads in any two stages compared) were not highlighted since their significant changes are not clear due to extremely low reads. Click here for file

Additional data file 5 Vertical axis indicates the relative expression level. The data from both RT-PCR and Solexa sequencing were standardized to the expression level in the embryonic sample as 1. The results were further confirmed using independently prepared RNAs. Click here for file

Additional data file 6 **(a) **The correlation diagram of all known miRNAs between males and hermaphrodites. **(b) **The correlation diagram of miRNAs with relatively low abundance (less than 20 × 10^4 ^reads in males, yellow-colored area in (a)). **(c) **Correlation diagram of miRNAs with lower abundance ((less than 10 × 10^3 ^reads in males, red-colored area in (a)). Click here for file

Additional data file 7 The numbers of reads were obtained from all developmental stages of hermaphrodites and young adult males. The *bona fide *novel RNAs with transcripts from their 'star sequence' are highlighted in red. 'Genomically clustered' is defined here as localization within 1.0 kb on the same chromosome. Click here for file

Additional data file 8 Data were normalized by the total number of reads that matched to the *C. elegans *genome. The miRNAs and their number of reads were highlighted in red as mentioned in the legend for Additional data file 4. Click here for file

Additional data file 9 21U-RNAs in which we found larger transcripts and overlapping ones within 10 bp of other 21U-RNAs, including novel ones we found, are marked with an asterisk and a dagger, respectively. Click here for file

Additional data file 10 The presence of the core consensus motif CTGTTTCA was examined in their possible larger motif regions (-20 to -63 bp upstream of the 5' terminus of each 21nt-U-RNA). This list also contains the novel 21U-RNAs shown in Additional data file 11. Click here for file

Additional data file 11 A larger motif of novel 21U-RNAs represents the region -20 to -63 bp upstream of the 5' terminus of each novel 21U-RNA. 21U-RNAs in which we found larger transcripts and overlapping ones within 10 bp of other 21U-RNAs are marked with an asterisk and a dagger, respectively. Click here for file

Additional data file 12 **(a) **Number of novel 21U-RNA reads was plotted after normalizing. **(b) **Total number of reads shown in (a) that mapped on chromosome IV. Click here for file

Additional data file 13 The number of reads was obtained from computational output of the SOAP program followed by removal of redundant sequences, and samples of all six developmental stages (embryo to young adult of hermaphrodites) were used as the input. The sequences of longer transcripts, their nucleotide lengths and the number of reads are highlighted in red. Click here for file
