# Supplementary material for: Dynamic expression of small non-coding RNAs, including novel microRNAs and piRNAs/21U-RNAs, during Caenorhabditis elegans development
Source: Genome Biol. 2009 May 21;10(5):R54. doi: 10.1186/gb-2009-10-5-r54 (PMC2718520; doi:10.1186/gb-2009-10-5-r54)
Supplement: Additional data file 1 — The number of each RNA species that satisfies the following conditions was counted after searching by the BLASTN program in the pool of sequence reads that aligned to the C. elegans genome; the length of the query is equal to that of the match and the percentage of identical bases in the match is 100%. [file gb-2009-10-5-r54-S1.pdf]

Samples		Number of reads (raw sequence)	Number of reads that matched to the *C. elegans* genome	Known miRNAs	Known 21U-RNAs	tRNAs	rRNAs	snoRNAs	Other reads
wild-type N2 hermaphrodites	Embryo	14251981	5742750	1905997	70888	10308	1220025	5532	2530000
			%	33.19	1.23	0.18	21.24	0.10	44.00
	mid-L1	10013600	5617234	3432825	48300	4274	293218	2462	1836155
			%	61.11	0.86	0.08	5.22	0.04	32.69
	mid-L2	10031813	6047597	4016452	25978	2702	300577	1652	1700236
			%	66.41	0.43	0.04	4.97	0.03	28.11
	mid-L3	9191014	4948026	3125869	32624	2135	213152	1723	1572523
			%	63.17	0.66	0.04	4.31	0.03	31.78
	mid-L4	8917357	6072252	4162442	249374	1890	83359	611	1574576
			%	68.55	4.11	0.03	1.37	0.01	25.93
	young adult	9287316	5975243	3854485	407042	4925	102615	702	1605474
			%	64.51	6.81	0.08	1.72	0.01	26.87
males *dpy-28;him-8*	young adult	11985021	7602104	3863173	28273	47937	1134185	5532	2523004
			%	50.82	0.37	0.63	14.92	0.07	33.19
Total number of sequence reads		73678102	42005206						
