# Supplementary material for: Dynamic expression of small non-coding RNAs, including novel microRNAs and piRNAs/21U-RNAs, during Caenorhabditis elegans development
Source: Genome Biol. 2009 May 21;10(5):R54. doi: 10.1186/gb-2009-10-5-r54 (PMC2718520; doi:10.1186/gb-2009-10-5-r54)
Supplement: Additional data file 2 — Raw data showing the number of miRNA reads in each developmental stage of hermaphrodites and in young adult males. [file gb-2009-10-5-r54-S2.pdf]

	Hermaphrodites (wild-type N2)						Males (*dpy-28;him-8*)	
	Embryo	mid-L1	mid-L2	mid-L3	mid-L4	young adult	young adult	Total number of each miRNA read
let-7	51	72	75	432	36000	28286	77319	142235
lin-4	349	40	10207	10169	15604	16246	6322	58937
miR-1	440434	1243660	1304285	1102762	1146031	819409	590166	6646747
miR-2	2087	1020	1014	414	559	726	2028	7848
miR-34	87	2161	1577	1584	3036	3691	6311	18447
miR-35	100771	1317	1800	416	1325	5577	2365	113571
miR-36	7957	86	101	29	71	353	150	8747
miR-37	50376	993	1399	501	1225	4747	1763	61004
miR-38	1522	20	32	14	25	36	35	1684
miR-39	1731	20	19	7	10	63	29	1879
miR-40	14389	286	302	77	209	697	418	16378
miR-41	40	0	0	0	0	0	1	41
miR-42	871	35	134	14	65	4	60	1183
miR-43	566	33	50	17	7	16	96	785
miR-44	47098	70131	81389	63658	79461	33848	44434	420019
miR-45	47098	70131	81389	63658	79461	33848	44434	420019
miR-46	108	35	122	87	94	76	110	632
miR-47	293	156	547	264	395	469	2102	4226
miR-48	2632	959	20494	89204	478888	478833	910447	1981457
miR-49	3914	3382	1868	694	561	588	2184	13191
miR-50	1514	3061	2353	2316	1793	1459	3443	15939
miR-51	1280	1530	574	381	862	446	2039	7112
miR-52	101004	24171	46249	27142	11524	10962	80101	301153
miR-53	2267	417	1180	785	335	305	3730	9019
miR-54	4663	445	877	462	359	444	11408	18658
miR-55	1820	1877	1368	999	1954	1099	3649	12766
miR-56	986	549	544	263	528	495	2812	6177
miR-57	1938	492	822	568	728	1282	16122	21952
miR-58	981820	1733530	2128856	1493158	2104698	2337654	1516235	12295951
miR-59	12	9	7	11	512	400	2619	3570
miR-60	2731	243	1065	404	287	266	3788	8784
miR-61	634	929	1369	1605	1839	1308	1288	8972
miR-62	67	17	28	15	9	6	56	198
miR-63	539	2469	1946	2179	1866	2208	1288	12495
miR-64	12425	8298	14389	16980	35305	34427	19152	140976
miR-65	5595	3566	6476	8213	18523	20476	9732	72581
miR-66	3226	3515	5140	6259	6591	5541	5959	36231
miR-67	279	35	67	37	25	22	56	521
miR-70	13000	8357	55438	54046	87699	86012	232628	537180
miR-71	4243	138542	15834	27691	72029	95699	207289	561327
miR-72	31238	199926	183445	105199	103701	84250	125474	833233
miR-73	44145	29486	22642	10434	25240	12794	45444	190185
miR-74	1887	173	192	65	114	129	401	2961
miR-75	316	289	513	384	475	805	3741	6523
miR-76	56	119	102	43	55	47	51	473
miR-77	8	5	37	155	566	1608	1343	3722
miR-78	6	6	1	2	1	16	2	34
miR-79	1946	313	851	582	359	892	875	5818
miR-227	161	144	246	242	377	285	403	1858
miR-80	4720	6656	10633	10500	18844	15076	39677	106106
miR-81	8065	7711	10729	10290	30288	25121	61631	153835
miR-82	3221	2419	2630	1804	5067	8258	21319	44718
miR-83	337	213	280	116	126	134	2016	3222
miR-84	70	243	637	2735	2916	2057	6869	15527
miR-85	7	2	0	0	255	1227	277	1768

miR-86	398	204	286	206	206	224	2137	3661
miR-87	798	452	868	267	356	377	764	3882
miR-90	525	1726	1151	725	779	497	2538	7941
miR-124	433	209	148	92	184	228	768	2062
miR-228	35195	232732	289701	243644	149607	84966	84954	1120799
miR-229	1263	142	834	1305	96	360	986	4986
miR-230	21	1376	737	2161	3230	500	600	8625
miR-231	1000	664	403	256	172	109	111	2715
miR-232	219	42	77	42	15	20	176	591
miR-233	575	196	245	102	152	134	668	2072
miR-234	8	38	33	31	18	7	22	157
miR-235	58	92	38	14	23	143	4574	4942
miR-236	1089	2134	1498	1336	1197	1336	1129	9719
miR-237	12	23	36	284	1148	1383	357	3243
miR-238	178	1174	1354	1398	3079	2564	3169	12916
miR-239a	16	29	44	31	69	86	513	788
miR-239b	13	38	38	121	298	293	425	1226
miR-240	3	0	2	0	54	113	82	254
miR-241	8	3	84	306	1212	1171	5331	8115
miR-242	23	39	41	41	54	67	27	292
miR-243	25	186	174	106	230	209	604	1534
miR-244	345	44	121	91	36	28	94	759
miR-245	33	76	64	27	10	20	77	307
miR-246	0	0	0	0	103	366	48	517
miR-247	0	0	0	8	6	1	6	21
miR-248	142	1257	1333	1269	1091	929	1623	7644
miR-249	1	5	5	5	5	6	7	34
miR-250	2643	6853	11994	15599	12655	12159	4661	66564
miR-251	10	6	11	5	6	6	27	71
miR-252	1542	6180	5462	4031	3296	3262	9016	32789
miR-253	54	165	85	45	305	164	319	1137
miR-254	45	57	83	32	47	52	119	435
miR-255	190	1234	1109	946	604	369	73	4525
miR-256	0	0	0	0	0	0	0	0
miR-257	0	0	0	0	0	0	0	0
miR-258	0	0	0	0	0	0	0	0
miR-259	23	20	36	32	22	39	20	192
miR-260	3	0	1	0	0	0	0	4
miR-261	0	0	0	0	0	0	0	0
miR-262	0	0	0	0	0	0	0	0
miR-264	0	0	0	0	0	0	0	0
miR-265	0	0	0	0	0	0	0	0
miR-266	0	0	0	1	0	0	0	1
miR-267	0	0	0	0	0	0	0	0
miR-268	0	0	0	0	0	0	0	0
miR-269	0	0	0	0	0	0	0	0
miR-270	0	0	0	0	0	0	0	0
miR-271	0	0	0	0	0	0	0	0
miR-272	0	1	0	0	0	0	0	1
miR-273	0	0	0	0	0	0	0	0
miR-353	0	0	0	0	0	0	0	0
miR-354	0	0	0	0	0	0	0	0
miR-355	0	1	3	0	0	2	2	8
miR-356	0	0	0	0	0	0	0	0
miR-357	0	0	0	0	4	3	97	104
miR-358	0	0	0	0	5	6	115	126
miR-359	2	1	0	2	6	27	27	65
miR-360	0	0	0	0	0	0	2	2
lsy-6	60	24	15	7	9	5	5	125
miR-392	0	0	2	1	0	1	10	14
miR-784	0	0	3	0	4	6	63	76
miR-785	16	54	43	34	72	98	317	634
miR-786	1	1	0	2	81	150	17	252
miR-787	45	12	24	10	16	10	42	159

miR-788	30	12	88	126	113	2	42	413
miR-789	0	0	0	0	0	1	37	38
miR-790	17	200	127	96	68	49	59	616
miR-791	9	51	25	2	3	6	26	122
miR-792	21	4	5	2	0	0	3	35
miR-793	16	140	116	49	59	44	68	492
miR-794	5	10	18	18	16	21	29	117
miR-795	114	2707	3717	5629	3651	2653	3591	22062
miR-796	1413	1640	679	319	254	193	7630	12128
miR-797	8	0	6	40	7	1	33	95
miR-798	7	5	3	7	28	46	4	100
miR-799	4	4	69	101	173	266	22	639
miR-800	0	0	0	0	1	0	0	1
miR-1018	24	58	81	43	18	15	532	771
miR-1019	0	0	0	0	0	0	0	0
miR-1020	2	9	14	26	27	21	11	110
miR-1021	0	0	0	0	0	0	0	0
miR-1022	248	11710	7365	4386	2136	1367	1098	28310
miR-1817	1	0	0	0	1	2	1	5
miR-1818	0	0	0	0	0	0	0	0
miR-1819	116	100	97	352	904	847	4374	6790
miR-1820	102	48	141	182	16	10	94	593
miR-1821	11	52	26	16	18	8	21	152
miR-1822	3	19	13	11	12	8	2	68
miR-1823	3	12	8	0	2	1	0	26
miR-1824	0	1	1	1	0	1	0	4
miR-1828	0	0	0	0	0	0	0	0
miR-1829a	4	4	7	4	7	4	2	32
miR-1829b	1041	1115	1221	3015	3133	2769	212	12506
miR-1829c	306	273	325	555	457	343	163	2422
miR-1830	0	0	1	2	0	0	0	3
miR-1831	1	0	1	0	0	1	33	36
miR-1832	11	2	5	0	2	6	8	34
miR-1833	0	0	0	0	0	0	0	0
miR-1834	1	1	0	2	9	22	2	37
Total number of miRNA reads in each sample	2009133	3849961	4356669	3409665	4570554	4301926	4268510	26766418
