# Supplementary material for: Dynamic expression of small non-coding RNAs, including novel microRNAs and piRNAs/21U-RNAs, during Caenorhabditis elegans development
Source: Genome Biol. 2009 May 21;10(5):R54. doi: 10.1186/gb-2009-10-5-r54 (PMC2718520; doi:10.1186/gb-2009-10-5-r54)
Supplement: Additional data file 3 — The SOAP-processed aligned reads were searched against miRNA hairpin sequences. Each number represents the total number of reads detected in all developmental stages of hermaphrodites and young adult males. Annotated mature miRNAs are marked with a hatch mark and highlighted in red, and annotated novel miRNAs we report here are colored in green. [file gb-2009-10-5-r54-S3.pdf]

```
          let-7
          UACACUGUGGAUCCGGUGAGGUAGUAGGUUGUAUAGUUUGGAAUAUUACCACCGGUGAACUAUGCAAUUUUCUACCUUACCGGAGACAGAACUCUUCGA
          ....((((...(((((((((((((.((((((((((((((((..........))...)))))))))))))).))))))))))))).))))..........
       26 ................UGAGGUAGUAGGUUGUA..................................................................
       62 ................UGAGGUAGUAGGUUGUAU.................................................................
      123 ................UGAGGUAGUAGGUUGUAUA................................................................
      422 ................UGAGGUAGUAGGUUGUAUAG...............................................................
     3953 ................UGAGGUAGUAGGUUGUAUAGU..............................................................
        2 .................GAGGUAGUAGGUUGUAUAGU..............................................................
        2 ..................AGGUAGUAGGUUGUAUAGU..............................................................
#  122991 ................UGAGGUAGUAGGUUGUAUAGUU.............................................................
      122 .................GAGGUAGUAGGUUGUAUAGUU.............................................................
       10 ..................AGGUAGUAGGUUGUAUAGUU.............................................................
        3 ...................GGUAGUAGGUUGUAUAGUU.............................................................
        3 ....................GUAGUAGGUUGUAUAGUU.............................................................
    11395 ................UGAGGUAGUAGGUUGUAUAGUUU............................................................
       16 .................GAGGUAGUAGGUUGUAUAGUUU............................................................
        3 ..................AGGUAGUAGGUUGUAUAGUUU............................................................
        2 ...................GGUAGUAGGUUGUAUAGUUU............................................................
        7 ................UGAGGUAGUAGGUUGUAUAGUUUG...........................................................
        1 ................UGAGGUAGUAGGUUGUAUAGUUUGG..........................................................
        1 .........................................................AACUAUGCAAUUUUCUACCUUA....................
        1 ...........................................................CUAUGCAAUUUUCUACCUUA....................
        2 ...........................................................CUAUGCAAUUUUCUACCUUAC...................
       14 ...........................................................CUAUGCAAUUUUCUACCUUACC..................
        2 ............................................................UAUGCAAUUUUCUACCUUACC..................
        1 .............................................................AUGCAAUUUUCUACCUUACC..................

          lin-4
          AUGCUUCCGGCCUGUUCCCUGAGACCUCAAGUGUGAGUGUACUAUUGAUGCUUCACACCUGGGCUCUCCGGGUACCAGGACGGUUUGAGCAGAU
          .((((((((.((((..(((.((((.((((.(((((((.((((....).)))))))))).)))).)))).)))...)))).)))...)))))...
        7 .............GUUCCCUGAGACCUCAAGUGUG...........................................................
        5 .............GUUCCCUGAGACCUCAAGUGUGA..........................................................
        2 ..............UUCCCUGAGACCUCAAGUGUGA..........................................................
       22 ...............UCCCUGAGACCUCAAGU..............................................................
       97 ...............UCCCUGAGACCUCAAGUG.............................................................
       10 ...............UCCCUGAGACCUCAAGUGU............................................................
      308 ...............UCCCUGAGACCUCAAGUGUG...........................................................
#   54557 ...............UCCCUGAGACCUCAAGUGUGA..........................................................
        3 ...............UCCCUGAGACCUCAAGUGUGAG.........................................................
        1 ...............UCCCUGAGACCUCAAGUGUGAGU........................................................
       51 ................CCCUGAGACCUCAAGUGUGA..........................................................
        1 .................CCUGAGACCUCAAGUGU............................................................
        9 .................CCUGAGACCUCAAGUGUG...........................................................
      892 .................CCUGAGACCUCAAGUGUGA..........................................................
        1 .................CCUGAGACCUCAAGUGUGAG.........................................................
        1 ..................CUGAGACCUCAAGUGUG...........................................................
```

```
    140 ..................CUGAGACCUCAAGUGUGA..........................................................
    321 ...................UGAGACCUCAAGUGUGA..........................................................
      1 ....................GAGACCUCAAGUGUGA..........................................................
      1 ....................................GUGUACUAUUGAUGCUU.........................................
    155 ......................................................ACACCUGGGCUCUCCGGGUA....................
     31 ......................................................ACACCUGGGCUCUCCGGGUAC...................
      4 ......................................................ACACCUGGGCUCUCCGGGUACC..................
      1 ......................................................ACACCUGGGCUCUCCGGGUACCA.................

        miR-1
        AAAGUGACCGUACCGAGCUGCAUACUUCCUUACAUGCCCAUACUAUAUCAUAAAUGGAUAUGGAAUGUAAAGAAGUAUGUAGAACGGGGUGGUAGU
        ......((((..(((..(((((((((((.((((((..(((((((((.......)))).))))).)))))).)))))))))))..)))..))))...
      1 ....................CAUACUUCCUUACAUGC...........................................................
      1 ..................UGCAUACUUCCUUACAUGCCC.........................................................
      1 ....................CAUACUUCCUUACAUGCCC.........................................................
      1 ....................CAUACUUCCUUACAUGCCCA........................................................
     22 ....................CAUACUUCCUUACAUGCCCAU.......................................................
    565 ....................CAUACUUCCUUACAUGCCCAUA......................................................
      4 .....................AUACUUCCUUACAUGCCCAUA......................................................
      1 ............................................................UGGAAUGUAAAGAAGUA...................
     13 ..............................................................GAAUGUAAAGAAGUA...................
     28 ..............................................................GAAUGUAAAGAAGUAU..................
      2 ...............................................................AAUGUAAAGAAGUAU..................
      1 ..........................................................UAUGGAAUGUAAAGAAGU....................
      1 ..........................................................UAUGGAAUGUAAAGAAGUA...................
      6 ...........................................................AUGGAAUGUAAAGAAGUA...................
   3655 ............................................................UGGAAUGUAAAGAAGUA...................
    661 ............................................................UGGAAUGUAAAGAAGUAU..................
     65 ..........................................................UAUGGAAUGUAAAGAAGUAUG.................
     12 ...........................................................AUGGAAUGUAAAGAAGUAUG.................
  42824 ............................................................UGGAAUGUAAAGAAGUAUG.................
     31 .............................................................GGAAUGUAAAGAAGUAUG.................
      3 ..............................................................GAAUGUAAAGAAGUAUG.................
      2 .........................................................AUAUGGAAUGUAAAGAAGUAUGU................
      4 ..........................................................UAUGGAAUGUAAAGAAGUAUGU................
    840 ...........................................................AUGGAAUGUAAAGAAGUAUGU................
 382735 ............................................................UGGAAUGUAAAGAAGUAUGU................
    320 .............................................................GGAAUGUAAAGAAGUAUGU................
     62 ..............................................................GAAUGUAAAGAAGUAUGU................
      1 ...............................................................AAUGUAAAGAAGUAUGU................
      1 .......................................................GGAUAUGGAAUGUAAAGAAGUAUGUA...............
      5 ........................................................GAUAUGGAAUGUAAAGAAGUAUGUA...............
      8 .........................................................AUAUGGAAUGUAAAGAAGUAUGUA...............
     47 ..........................................................UAUGGAAUGUAAAGAAGUAUGUA...............
   1417 ...........................................................AUGGAAUGUAAAGAAGUAUGUA...............
# 5945138 ............................................................UGGAAUGUAAAGAAGUAUGUA...............
   4068 .............................................................GGAAUGUAAAGAAGUAUGUA...............
```

```
 1014 ..............................................................GAAUGUAAAGAAGUAUGUA...............
   18 ...............................................................AAUGUAAAGAAGUAUGUA...............
    8 ................................................................AUGUAAAGAAGUAUGUA...............
    1 ...........................................................AUGGAAUGUAAAGAAGUAUGUAG..............
12373 ............................................................UGGAAUGUAAAGAAGUAUGUAG..............
    8 .............................................................GGAAUGUAAAGAAGUAUGUAG..............
    4 ..............................................................GAAUGUAAAGAAGUAUGUAG..............
  339 ............................................................UGGAAUGUAAAGAAGUAUGUAGA.............
   99 ............................................................UGGAAUGUAAAGAAGUAUGUAGAA............
    1 ..............................................................GAAUGUAAAGAAGUAUGUAGAA............
    5 ............................................................UGGAAUGUAAAGAAGUAUGUAGAAC...........
    5 ..............................................................GAAUGUAAAGAAGUAUGUAGAAC...........
    2 ...............................................................AAUGUAAAGAAGUAUGUAGAAC...........

      miR-2
      UAAACAGUAUACAGAAAGCCAUCAAAGCGGUGGUUGAUGUGUUGCAAAUUAUGACUUUCAUAUCACAGCCAGCUUUGAUGUGCUGCCUGUUGCACUGU
      ...(((((..((((..(((((((((((((((...(((((((..(((.....)).)...)))))))..))).))))))))).)))..))))...)))))
    2 ...................CAUCAAAGCGGUGGUUGAU............................................................
    1 ...................CAUCAAAGCGGUGGUUGAUG...........................................................
    7 ...................CAUCAAAGCGGUGGUUGAUGU..........................................................
   17 ...................CAUCAAAGCGGUGGUUGAUGUG.........................................................
    1 ......................CAAAGCGGUGGUUGAUGUG.........................................................
    2 ...................CAUCAAAGCGGUGGUUGAUGUGU........................................................
    2 .................................UUGAUGUGUUGCAAAUUAUGACUUUC.......................................
    1 .......................................UGUUGCAAAUUAUGACUUUC.......................................
    7 .........................................UUGCAAAUUAUGACUUUCA......................................
    2 ............................................................UAUCACAGCCAGCUUUGA....................
    3 ............................................................UAUCACAGCCAGCUUUGAU...................
   24 ............................................................UAUCACAGCCAGCUUUGAUG..................
   63 ............................................................UAUCACAGCCAGCUUUGAUGU.................
    1 ...........................................................AUAUCACAGCCAGCUUUGAUGUG................
  505 ............................................................UAUCACAGCCAGCUUUGAUGUG................
    8 .............................................................AUCACAGCCAGCUUUGAUGUG................
    3 ................................................................ACAGCCAGCUUUGAUGUG................
    5 ...........................................................AUAUCACAGCCAGCUUUGAUGUGC...............
#  7046 ............................................................UAUCACAGCCAGCUUUGAUGUGC...............
   38 .............................................................AUCACAGCCAGCUUUGAUGUGC...............
    1 ..............................................................UCACAGCCAGCUUUGAUGUGC...............
  123 ................................................................ACAGCCAGCUUUGAUGUGC...............
  168 ............................................................UAUCACAGCCAGCUUUGAUGUGCU..............
   10 .............................................................AUCACAGCCAGCUUUGAUGUGCU..............
    2 ..............................................................UCACAGCCAGCUUUGAUGUGCU..............
    1 ................................................................ACAGCCAGCUUUGAUGUGCU..............
    1 .................................................................CAGCCAGCUUUGAUGUGCUGCC...........

      miR-34
      CGGACAAUGCUCGAGAGGCAGUGUGGUUAGCUGGUUGCAUAUUUCCUUGACAACGGCUACCUUCACUGCCACCCCGAACAUGUCGUCCAUCUUUGAA
```

```
          .((((.(((.(((.(.(((((((.(((.(((((.(((((........)).)))))))))))..))))))).)..))).)))...)))).........
       11 ...............AGGCAGUGUGGUUAGCU.................................................................
       16 ...............AGGCAGUGUGGUUAGCUG................................................................
      246 ...............AGGCAGUGUGGUUAGCUGG...............................................................
     1829 ...............AGGCAGUGUGGUUAGCUGGU..............................................................
    11533 ...............AGGCAGUGUGGUUAGCUGGUU.............................................................
#   16973 ...............AGGCAGUGUGGUUAGCUGGUUG............................................................
        1 ................GGCAGUGUGGUUAGCUGGUUG............................................................
        3 ...............AGGCAGUGUGGUUAGCUGGUUGC...........................................................
        2 ...............AGGCAGUGUGGUUAGCUGGUUGCA..........................................................
        4 ....................................................ACGGCUACCUUCACUGCCAC.........................
        1 ...................................................AACGGCUACCUUCACUGCCACC........................
      277 ....................................................ACGGCUACCUUCACUGCCACC........................
        2 .....................................................CGGCUACCUUCACUGCCACC........................
      505 ....................................................ACGGCUACCUUCACUGCCACCC.......................
        2 .....................................................CGGCUACCUUCACUGCCACCC.......................

          miR-35
          UCUCGGAUCAGAUCGAGCCAUUGCUGGUUUCUUCCACAGUGGUACUUUCCAUUAGAACUAUCACCGGGUGGAAACUAGCAGUGGCUCGAUCUUUUCC
          ....(((..(((((((((((((((((((((((.((.(.((((((.(((......))).)))))).))).)))))))))))))))))))))))..)))
       12 .....................UGCUGGUUUCUUCCACAGUGGUA.....................................................
        1 ...........................................................AUCACCGGGUGGAAACUAGCA.................
        2 ...........................................................AUCACCGGGUGGAAACUAGCAG................
       33 ...........................................................AUCACCGGGUGGAAACUAGCAGU...............
       10 ............................................................UCACCGGGUGGAAACUA....................
       72 ............................................................UCACCGGGUGGAAACUAG...................
       48 ............................................................UCACCGGGUGGAAACUAGC..................
     1121 ............................................................UCACCGGGUGGAAACUAGCA.................
     6569 ............................................................UCACCGGGUGGAAACUAGCAG................
#   97572 ............................................................UCACCGGGUGGAAACUAGCAGU...............
       43 ............................................................UCACCGGGUGGAAACUAGCAGUG..............
        1 ............................................................UCACCGGGUGGAAACUAGCAGUGG.............
        1 ............................................................UCACCGGGUGGAAACUAGCAGUGGC............
        1 ............................................................UCACCGGGUGGAAACUAGCAGUGGCU...........
        1 .............................................................CACCGGGUGGAAACUAGCA.................
       13 .............................................................CACCGGGUGGAAACUAGCAG................
       84 .............................................................CACCGGGUGGAAACUAGCAGU...............
        1 ..............................................................ACCGGGUGGAAACUAG...................
        2 ..............................................................ACCGGGUGGAAACUAGC..................
        9 ..............................................................ACCGGGUGGAAACUAGCA.................
       69 ..............................................................ACCGGGUGGAAACUAGCAG................
      917 ..............................................................ACCGGGUGGAAACUAGCAGU...............
        1 ..............................................................ACCGGGUGGAAACUAGCAGUG..............
        2 ...............................................................CCGGGUGGAAACUAGCAG................
        9 ...............................................................CCGGGUGGAAACUAGCAGU...............
        1 ...............................................................CCGGGUGGAAACUAGCAGUGGCU...........
        2 ................................................................CGGGUGGAAACUAGCAG................
```

```
      7 ................................................................CGGGUGGAAACUAGCAGU...............
     11 .................................................................GGGUGGAAACUAGCAGU...............

        miR-36
        CACCGCUGUCGGGGAACCGCGCCAAUUUUCGCUUCAGUGCUAGACCAUCCAAAGUGUCUAUCACCGGGUGAAAAUUCGCAUGGGUCCCCGACGCGGA
        ..((((.((((((((.(((.((.(((((((((((..(((.((((((.......).))))).))).))))))))))).)).))).)))))))))))).
      2 ...................CGCCAAUUUUCGCUUCAGUGCU........................................................
     31 ...................CGCCAAUUUUCGCUUCAGUGCUA.......................................................
      1 ..........................................GACCAUCCAAAGUGUCUA.....................................
      1 ...........................................ACCAUCCAAAGUGUCUAUCACC................................
     11 ...........................................................AUCACCGGGUGAAAAUUCGCAUG...............
      1 ............................................................UCACCGGGUGAAAAUUC....................
      1 ............................................................UCACCGGGUGAAAAUUCGC..................
     23 ............................................................UCACCGGGUGAAAAUUCGCA.................
    169 ............................................................UCACCGGGUGAAAAUUCGCAU................
#  8486 ............................................................UCACCGGGUGAAAAUUCGCAUG...............
      6 .............................................................CACCGGGUGAAAAUUCGCAUG...............
      1 ..............................................................ACCGGGUGAAAAUUCGCA.................
      1 ..............................................................ACCGGGUGAAAAUUCGCAU................
     96 ..............................................................ACCGGGUGAAAAUUCGCAUG...............
      1 ...............................................................CCGGGUGAAAAUUCGCAUG...............
      1 ................................................................CGGGUGAAAAUUCGCAUG...............
      2 .................................................................GGGUGAAAAUUCGCAUG...............

        miR-37
        UUCUAGAAACCCUUGGACCAGUGUGGGUGUCCGUUGCGGUGCUACAUUCUCUAAUCUGUAUCACCGGGUGAACACUUGCAGUGGUCCUCGUGGUUUCU
        ....((((((((..((((((.((..(((((....(.(((((.((((..........)))).))))).)...)))))..)).))))))..).)))))))
      1 ...................AGUGUGGGUGUCCGUUGCGGUGCUA......................................................
      3 ....................GUGUGGGUGUCCGUUGCGGUGCU.......................................................
      1 .....................UGUGGGUGUCCGUUGCGG...........................................................
      1 .....................UGUGGGUGUCCGUUGCGGUG.........................................................
      1 .....................UGUGGGUGUCCGUUGCGGUGC........................................................
     14 .....................UGUGGGUGUCCGUUGCGGUGCU.......................................................
     77 .....................UGUGGGUGUCCGUUGCGGUGCUA......................................................
      1 ......................................GUGCUACAUUCUCUAAUCUGU.......................................
      1 ..........................................................UAUCACCGGGUGAACACUUGCA..................
      4 ..........................................................UAUCACCGGGUGAACACUUGCAG.................
      1 ..........................................................UAUCACCGGGUGAACACUUGCAGU................
      3 ...........................................................AUCACCGGGUGAACACUUGCA..................
     14 ...........................................................AUCACCGGGUGAACACUUGCAG.................
    151 ...........................................................AUCACCGGGUGAACACUUGCAGU................
     15 ............................................................UCACCGGGUGAACACUUG....................
     21 ............................................................UCACCGGGUGAACACUUGC...................
   1179 ............................................................UCACCGGGUGAACACUUGCA..................
   2778 ............................................................UCACCGGGUGAACACUUGCAG.................
# 53218 ............................................................UCACCGGGUGAACACUUGCAGU................
     18 ............................................................UCACCGGGUGAACACUUGCAGUG...............
```

```
   1 ............................................................UCACCGGGUGAACACUUGCAGUGG..............
   1 ............................................................UCACCGGGUGAACACUUGCAGUGGU.............
   1 .............................................................CACCGGGUGAACACUUGCA..................
  24 .............................................................CACCGGGUGAACACUUGCAG.................
 264 .............................................................CACCGGGUGAACACUUGCAGU................
  15 .............................................................CACCGGGUGAACACUUGCAGUG...............
  11 ..............................................................ACCGGGUGAACACUUGCA..................
  44 ..............................................................ACCGGGUGAACACUUGCAG.................
 634 ..............................................................ACCGGGUGAACACUUGCAGU................
   1 ...............................................................CCGGGUGAACACUUGCAG.................
   2 ...............................................................CCGGGUGAACACUUGCAGU................
   1 ................................................................CGGGUGAACACUUGCAG.................
   4 ................................................................CGGGUGAACACUUGCAGU................
   5 .................................................................GGGUGAACACUUGCAGU................

     miR-38
     UCUGUGAGCCAGGUCCUGUUCCGGUUUUUUCCGUGGUGAUAACGCAUCCAAAAGUCUCUAUCACCGGGAGAAAAACUGGAGUAGGACCUGUGACUCAU
     ...((((((((((((((((((((((((((..(.((((((((..((........))...)))))))).)..))))))))))))))))))))).).)))))
   8 .........................................ACGCAUCCAAAAGUCUCUA......................................
   1 ...........................................................AUCACCGGGAGAAAAAC......................
   1 ...........................................................AUCACCGGGAGAAAAACUGGAG.................
  10 ...........................................................AUCACCGGGAGAAAAACUGGAGU................
   5 ............................................................UCACCGGGAGAAAAACU.....................
   4 ............................................................UCACCGGGAGAAAAACUGGA..................
 140 ............................................................UCACCGGGAGAAAAACUGGAG.................
#   1250 ............................................................UCACCGGGAGAAAAACUGGAGU................
  17 ............................................................UCACCGGGAGAAAAACUGGAGUA...............
   2 .............................................................CACCGGGAGAAAAACUGGAGU................
   3 ..............................................................ACCGGGAGAAAAACUGGAG.................
   9 ..............................................................ACCGGGAGAAAAACUGGAGU................
   1 ................................................................CGGGAGAAAAACUGGAGU................

     miR-39
     UAUACCGAGAGCCCAGCUGAUUUCGUCUUGGUAAUAAGCUCGUCAUUGAGAUUAUCACCGGGUGUAAAUCAGCUUGGCUCUGGUGUC
     .(((((.((((((.(((((((((((.((((((.((((.((((....)))).)))).)))))))).))))))))).))))))))))).
   1 .............CAGCUGAUUUCGUCUUGGUAAU....................................................
   6 .............CAGCUGAUUUCGUCUUGGUAAUA...................................................
   1 ..............AGCUGAUUUCGUCUUGGU.......................................................
   8 ..............AGCUGAUUUCGUCUUGGUA......................................................
  17 ..............AGCUGAUUUCGUCUUGGUAA.....................................................
  49 ..............AGCUGAUUUCGUCUUGGUAAU....................................................
 633 ..............AGCUGAUUUCGUCUUGGUAAUA...................................................
   5 ..............AGCUGAUUUCGUCUUGGUAAUAA..................................................
   1 ................CUGAUUUCGUCUUGGUAAUA...................................................
   1 ..................GAUUUCGUCUUGGUAAUA...................................................
   1 ....................................AGCUCGUCAUUGAGAUUA.................................
   3 .....................................................AUCACCGGGUGUAAAUCAGCUUG...........
```

```
         1 ......................................................UCACCGGGUGUAAAUCA................
         3 ......................................................UCACCGGGUGUAAAUCAG...............
        11 ......................................................UCACCGGGUGUAAAUCAGC..............
        20 ......................................................UCACCGGGUGUAAAUCAGCU.............
        77 ......................................................UCACCGGGUGUAAAUCAGCUU............
#     1751 ......................................................UCACCGGGUGUAAAUCAGCUUG...........
         3 ......................................................UCACCGGGUGUAAAUCAGCUUGG..........
         1 ......................................................UCACCGGGUGUAAAUCAGCUUGGC.........
         2 .......................................................CACCGGGUGUAAAUCAGCUUG...........
         1 ........................................................ACCGGGUGUAAAUCAGC..............
        39 ........................................................ACCGGGUGUAAAUCAGCUUG...........
         2 ...........................................................GGGUGUAAAUCAGCUUG...........

           miR-40
           UCCUGUCCGCACCUCAGUGGAUGUAUGCCAUGAUGAUAAGAUAUCAGAAAUCCUAUCACCGGGUGUACAUCAGCUAAGGUGCGGGUACAGGU
           .(((((((((((((.(((.((((((((((.((.(((((.(((.......))).))))).)))))))))))).))).))))))))..))))).
         2 ...............AGUGGAUGUAUGCCAUGAUGAU.......................................................
        27 ...............AGUGGAUGUAUGCCAUGAUGAUA......................................................
         1 ................GUGGAUGUAUGCCAUGAUGAUAAGA...................................................
         7 ....................................................CUAUCACCGGGUGUACAUCAGC..................
         1 ....................................................CUAUCACCGGGUGUACAUCAGCU.................
         1 .....................................................UAUCACCGGGUGUACAUCAGCUAA...............
         6 ......................................................AUCACCGGGUGUACAUCAGCUA................
        35 ......................................................AUCACCGGGUGUACAUCAGCUAA...............
         3 .......................................................UCACCGGGUGUACAUCA....................
        11 .......................................................UCACCGGGUGUACAUCAG...................
        34 .......................................................UCACCGGGUGUACAUCAGC..................
       124 .......................................................UCACCGGGUGUACAUCAGCU.................
       748 .......................................................UCACCGGGUGUACAUCAGCUA................
#    15418 .......................................................UCACCGGGUGUACAUCAGCUAA...............
        10 .......................................................UCACCGGGUGUACAUCAGCUAAG..............
         1 .......................................................UCACCGGGUGUACAUCAGCUAAGG.............
         7 ........................................................CACCGGGUGUACAUCAGCUA................
        74 ........................................................CACCGGGUGUACAUCAGCUAA...............
         2 ........................................................CACCGGGUGUACAUCAGCUAAG..............
         1 .........................................................ACCGGGUGUACAUCAGC..................
         1 .........................................................ACCGGGUGUACAUCAGCU.................
         4 .........................................................ACCGGGUGUACAUCAGCUA................
       201 .........................................................ACCGGGUGUACAUCAGCUAA...............
         1 ..........................................................CCGGGUGUACAUCAGCUA................
         2 ..........................................................CCGGGUGUACAUCAGCUAA...............
         3 ............................................................GGGUGUACAUCAGCUAA...............

           miR-41
           GGGUCCCAGAGACCUUGGUGGUUUUUCUCUGCAGUGAUAGAUACUUCUAACAACUCGCUAUCACCGGGUGAAAAAUCACCUAGGUCUGGAGCCUCCU
           ((.(((...((((((.(((((((((((((((..(((((((.................))))))))))).))))))))))).))))))))).))....
         1 ..............UUGGUGGUUUUUCUCUGCAGUGA............................................................
```

```
        1 ...............UGGUGGUUUUUCUCUGCAGUGA............................................................
        2 ................GGUGGUUUUUCUCUGCAGUGAU...........................................................
       30 ................GGUGGUUUUUCUCUGCAGUGAUA..........................................................
        1 ................GGUGGUUUUUCUCUGCAGUGAUAG.........................................................
        1 ..................UGGUUUUUCUCUGCAGUGAUA..........................................................
        1 ..................UGGUUUUUCUCUGCAGUGAUAGA........................................................
        8 .......................................GAUACUUCUAACAACUCGCUA.....................................
        1 ..........................................................UAUCACCGGGUGAAAAAUCACCU................
        8 ...........................................................AUCACCGGGUGAAAAAUCACCU................
       11 ...........................................................AUCACCGGGUGAAAAAUCACCUA...............
        2 ............................................................UCACCGGGUGAAAAAUCACCU................
#      22 ............................................................UCACCGGGUGAAAAAUCACCUA...............
        2 .............................................................CACCGGGUGAAAAAUCACCUA...............
        5 ..............................................................ACCGGGUGAAAAAUCACCUA...............
        1 ...............................................................CCGGGUGAAAAAUCACCUA...............
        1 ................................................................CGGGUGAAAAAUCACCUA...............

          miR-42
          UUCGCGGACCUUUGUGGGUGUUUGCUUUUUCGGUGAAGUUGUCUUCCGUAGCUUCUUCUUCACCGGGUUAACAUCUACAGAGGUCCAAAAAGGGG
          .....(((((((((((((((((.(((....((((((((..((........)).....))))))))))).)))))))))))))))))).........
        2 ............UGUGGGUGUUUGCUUUUUCGGU.............................................................
        6 ............UGUGGGUGUUUGCUUUUUCGGUGAAG.........................................................
        1 .............GUGGGUGUUUGCUUUUUCG...............................................................
        1 .............GUGGGUGUUUGCUUUUUCGG..............................................................
        3 .............GUGGGUGUUUGCUUUUUCGGU.............................................................
        3 .............GUGGGUGUUUGCUUUUUCGGUG............................................................
       16 .............GUGGGUGUUUGCUUUUUCGGUGAAG.........................................................
        4 ...........................................................UCACCGGGUUAACAUCUACA................
       43 ...........................................................UCACCGGGUUAACAUCUACAG...............
#    1002 ...........................................................UCACCGGGUUAACAUCUACAGA..............
        2 ...........................................................UCACCGGGUUAACAUCUACAGAG.............
        3 ............................................................CACCGGGUUAACAUCUACAGA..............
        1 .............................................................ACCGGGUUAACAUCUACAG...............
       20 .............................................................ACCGGGUUAACAUCUACAGA..............

          miR-43
          UAUUGGCACUAGUCGCCCGUGACAUCAAGAAACUAGUGAUUAUGCCAAACCACAGGGACAUAUCACAGUUUACUUGCUGUCGCGGGCGGUGCUGAGUU
          ..(..((((....(((((((((((.(((((((((.(((((.(((((.........)).))))))))))))).)))).)))))))))))))))..)...
        1 ....................GACAUCAAGAAACUAGUGAU..........................................................
        1 ....................GACAUCAAGAAACUAGUGAUU.........................................................
        2 ....................GACAUCAAGAAACUAGUGAUUA........................................................
        6 ....................GACAUCAAGAAACUAGUGAUUAU.......................................................
       34 ....................GACAUCAAGAAACUAGUGAUUAUG......................................................
        2 .....................ACAUCAAGAAACUAGUGAUUAUG......................................................
        1 ...........................................................AUAUCACAGUUUACUUGCUGUCGC...............
        6 ............................................................UAUCACAGUUUACUUGCUG...................
        2 ............................................................UAUCACAGUUUACUUGCUGUC.................
```

```
      23 ............................................................UAUCACAGUUUACUUGCUGUCG................
#    558 ............................................................UAUCACAGUUUACUUGCUGUCGC...............
       3 ............................................................UAUCACAGUUUACUUGCUGUCGCG..............
       4 ................................................................ACAGUUUACUUGCUGUCGC...............
       1 ..................................................................AGUUUACUUGCUGUCGC...............

         miR-44
         GAGAAAAUGGCCAAUCUGGAUGUGCUCGUUGGUCAUAGACGUCAACACGAACUGUUCAUAUGACUAGAGACACAUUCAGCUUGGCCUGCUUCUCUA
         (((((...((((((.((((((((((((..(((((((((((.((.....))...)))..))))))))))).))))))))).))))))...))))).. 
       2 ............AAUCUGGAUGUGCUCGUUGGUCAUA...........................................................
       1 .............AUCUGGAUGUGCUCGUUGGUCAUA...........................................................
       4 ..............UCUGGAUGUGCUCGUUGGUCAU............................................................
      15 ..............UCUGGAUGUGCUCGUUGGUCAUA...........................................................
       2 ...............CUGGAUGUGCUCGUUGG................................................................
       5 ...............CUGGAUGUGCUCGUUGGU...............................................................
       2 ...............CUGGAUGUGCUCGUUGGUC..............................................................
      13 ...............CUGGAUGUGCUCGUUGGUCA.............................................................
     155 ...............CUGGAUGUGCUCGUUGGUCAU............................................................
    2864 ...............CUGGAUGUGCUCGUUGGUCAUA...........................................................
       1 ................UGGAUGUGCUCGUUGGUCA.............................................................
       1 ................UGGAUGUGCUCGUUGGUCAU............................................................
       7 ................UGGAUGUGCUCGUUGGUCAUA...........................................................
       1 .................GGAUGUGCUCGUUGGUCAUA...........................................................
       1 ..................GAUGUGCUCGUUGGUCAUA...........................................................
       1 ....................UGUGCUCGUUGGUCAUA...........................................................
       1 .....................................GACGUCAACACGAACUGUUCA......................................
       1 .....................................GACGUCAACACGAACUGUUCAU.....................................
       7 .....................................GACGUCAACACGAACUGUUCAUA....................................
       1 .........................................................AUAUGACUAGAGACACAUUCAGC................
      23 .........................................................AUAUGACUAGAGACACAUUCAGCU...............
       2 .........................................................AUAUGACUAGAGACACAUUCAGCUU..............
       5 ..........................................................UAUGACUAGAGACACAUUCAGCU...............
       1 ...........................................................AUGACUAGAGACACAUUC...................
       1 ...........................................................AUGACUAGAGACACAUUCAG.................
       7 ...........................................................AUGACUAGAGACACAUUCAGC................
     370 ...........................................................AUGACUAGAGACACAUUCAGCU...............
      27 ...........................................................AUGACUAGAGACACAUUCAGCUU..............
      20 ............................................................UGACUAGAGACACAUUC...................
      81 ............................................................UGACUAGAGACACAUUCA..................
     456 ............................................................UGACUAGAGACACAUUCAG.................
    2996 ............................................................UGACUAGAGACACAUUCAGC................
# 183079 ............................................................UGACUAGAGACACAUUCAGCU...............
   15357 ............................................................UGACUAGAGACACAUUCAGCUU..............
       8 ............................................................UGACUAGAGACACAUUCAGCUUG.............
       1 .............................................................GACUAGAGACACAUUCAG.................
      17 .............................................................GACUAGAGACACAUUCAGC................
     722 .............................................................GACUAGAGACACAUUCAGCU...............
```

```
      53 .............................................................GACUAGAGACACAUUCAGCUU..............
       6 ..............................................................ACUAGAGACACAUUCAGC................
     165 ..............................................................ACUAGAGACACAUUCAGCU...............
      23 ..............................................................ACUAGAGACACAUUCAGCUU..............
       1 ...............................................................CUAGAGACACAUUCAGCU...............
       1 ................................................................UAGAGACACAUUCAGCU...............
       1 ................................................................UAGAGACACAUUCAGCUUGG............
       1 .................................................................AGAGACACAUUCAGCUU..............
      25 ............................................................UGACUAGAGACACAUUC...................
      90 ............................................................UGACUAGAGACACAUUCA..................
     454 ............................................................UGACUAGAGACACAUUCAG.................
       6 ...........................................................AUGACUAGAGACACAUUCAGC................
    3091 ............................................................UGACUAGAGACACAUUCAGC................
      14 .............................................................GACUAGAGACACAUUCAGC................
       3 ..............................................................ACUAGAGACACAUUCAGC................
       4 ..........................................................UAUGACUAGAGACACAUUCAGCU...............
     409 ...........................................................AUGACUAGAGACACAUUCAGCU...............
#  184208 ............................................................UGACUAGAGACACAUUCAGCU...............
     581 .............................................................GACUAGAGACACAUUCAGCU...............
     131 ..............................................................ACUAGAGACACAUUCAGCU...............
       1 ...............................................................CUAGAGACACAUUCAGCU...............
       3 ................................................................UAGAGACACAUUCAGCU...............
      35 ...........................................................AUGACUAGAGACACAUUCAGCUU..............
   15266 ............................................................UGACUAGAGACACAUUCAGCUU..............
      48 .............................................................GACUAGAGACACAUUCAGCUU..............
      11 ..............................................................ACUAGAGACACAUUCAGCUU..............
       1 ................................................................UAGAGACACAUUCAGCUU..............
       8 ............................................................UGACUAGAGACACAUUCAGCUUG.............

         miR-45
         CACCAUGUGCCACGCUGGAUGUGCUCGUUAGUCAUAAUAUCCUCCACAAAGCAAGGACUAUGACUAGAGACACAUUCAGCUUGGCGCCGAAUGCAU
         ......((((((.(((((((((((((..((((((((...((((..........)))).))))))))))).)))))))))).)))))).........
       2 ..............CUGGAUGUGCUCGUUAG.................................................................
       2 ..............CUGGAUGUGCUCGUUAGUC...............................................................
       1 ..........CACGCUGGAUGUGCUCGUUAGUCA..............................................................
       2 ..............CUGGAUGUGCUCGUUAGUCA..............................................................
      18 ..............CUGGAUGUGCUCGUUAGUCAU.............................................................
     117 ..............CUGGAUGUGCUCGUUAGUCAUA............................................................
       2 ..............CUGGAUGUGCUCGUUAGUCAUAA...........................................................
       3 ....................................AUAUCCUCCACAAAGCAAGGACU.....................................
       1 ......................................AUCCUCCACAAAGCAAGGACU.....................................
       5 ..........................................................UAUGACUAGAGACACAUUCAGCU...............
      28 ....................................AUAUCCUCCACAAAGCAAGGACUA....................................
       1 .........................................CUCCACAAAGCAAGGACUA....................................
       1 ...........................................................AUGACUAGAGACACAUUC...................
       1 ...........................................................AUGACUAGAGACACAUUCAG.................
       7 ...........................................................AUGACUAGAGACACAUUCAGC................
```

```
     370 ...........................................................AUGACUAGAGACACAUUCAGCU...............
      27 ...........................................................AUGACUAGAGACACAUUCAGCUU..............
       1 ....................................AUAUCCUCCACAAAGCAAGGACUAU...................................
      20 ............................................................UGACUAGAGACACAUUC...................
      81 ............................................................UGACUAGAGACACAUUCA..................
     456 ............................................................UGACUAGAGACACAUUCAG.................
    2996 ............................................................UGACUAGAGACACAUUCAGC................
#  183079 ............................................................UGACUAGAGACACAUUCAGCU...............
   15357 ............................................................UGACUAGAGACACAUUCAGCUU..............
       8 ............................................................UGACUAGAGACACAUUCAGCUUG.............
       1 .............................................................GACUAGAGACACAUUCAG.................
      17 .............................................................GACUAGAGACACAUUCAGC................
     722 .............................................................GACUAGAGACACAUUCAGCU...............
      53 .............................................................GACUAGAGACACAUUCAGCUU..............
       6 ..............................................................ACUAGAGACACAUUCAGC................
     165 ..............................................................ACUAGAGACACAUUCAGCU...............
      23 ..............................................................ACUAGAGACACAUUCAGCUU..............
       1 ...............................................................CUAGAGACACAUUCAGCU...............
       1 ................................................................UAGAGACACAUUCAGCU...............
       1 ................................................................UAGAGACACAUUCAGCUUGG............
       1 .................................................................AGAGACACAUUCAGCUU..............
       1 .......................................................GACUAUGACUAGAGACACAUU....................
       9 ........................................................ACUAUGACUAGAGACACAUUC...................
      25 ............................................................UGACUAGAGACACAUUC...................
      14 ........................................................ACUAUGACUAGAGACACAUUCA..................
      90 ............................................................UGACUAGAGACACAUUCA..................
      33 ........................................................ACUAUGACUAGAGACACAUUCAG.................
     454 ............................................................UGACUAGAGACACAUUCAG.................
     181 ........................................................ACUAUGACUAGAGACACAUUCAGC................
       6 ...........................................................AUGACUAGAGACACAUUCAGC................
    3091 ............................................................UGACUAGAGACACAUUCAGC................
      14 .............................................................GACUAGAGACACAUUCAGC................
       3 ..............................................................ACUAGAGACACAUUCAGC................
      51 ........................................................ACUAUGACUAGAGACACAUUCAGCU...............
       1 .........................................................CUAUGACUAGAGACACAUUCAGCU...............
       4 ..........................................................UAUGACUAGAGACACAUUCAGCU...............
     409 ...........................................................AUGACUAGAGACACAUUCAGCU...............
#  184208 ............................................................UGACUAGAGACACAUUCAGCU...............
     581 .............................................................GACUAGAGACACAUUCAGCU...............
     131 ..............................................................ACUAGAGACACAUUCAGCU...............
       1 ...............................................................CUAGAGACACAUUCAGCU...............
       3 ................................................................UAGAGACACAUUCAGCU...............
       1 ........................................................ACUAUGACUAGAGACACAUUCAGCUU..............
      35 ...........................................................AUGACUAGAGACACAUUCAGCUU..............
   15266 ............................................................UGACUAGAGACACAUUCAGCUU..............
      48 .............................................................GACUAGAGACACAUUCAGCUU..............
      11 ..............................................................ACUAGAGACACAUUCAGCUU..............
```

```
   1 ................................................................UAGAGACACAUUCAGCUU..............
   8 ............................................................UGACUAGAGACACAUUCAGCUUG.............
   2 ............................................................UGACUAGAGACACAUUCAGCUUGGCG..........
   1 ..............................................................ACUAGAGACACAUUCAGCUUGGCG..........

     miR-46
     CUGAGGUGAAGCUGAAGAGAGCCGUCUAUUGACAGUUCAAGACCACGAGUCGUUGUGUGCUGUCAUGGAGUCGCUCUCUUCAGAUGAUCCGGUCAAU
     ....((.....(((((((((((..(((((.((((((.((((((.....))).)))...)))))))))))...))))))))))).....)).......
   1 ..............AAGAGAGCCGUCUAUUG..................................................................
   1 ..............AAGAGAGCCGUCUAUUGAC................................................................
   3 ..............AAGAGAGCCGUCUAUUGACA...............................................................
  23 ..............AAGAGAGCCGUCUAUUGACAG..............................................................
1293 ..............AAGAGAGCCGUCUAUUGACAGU.............................................................
  19 ..............AAGAGAGCCGUCUAUUGACAGUU............................................................
   1 ...............AGAGAGCCGUCUAUUGACAGU.............................................................
   1 ....................................UCAAGACCACGAGUCGUUGUGUG......................................
   2 ....................................UCAAGACCACGAGUCGUUGUGUGC.....................................
   1 ...........................................................CUGUCAUGGAGUCGCUCUCUUC................
   3 ...........................................................CUGUCAUGGAGUCGCUCUCUUCA...............
   4 ............................................................UGUCAUGGAGUCGCUCUCU..................
  11 ............................................................UGUCAUGGAGUCGCUCUCUUC................
# 600 ............................................................UGUCAUGGAGUCGCUCUCUUCA...............
   2 ...............................................................CAUGGAGUCGCUCUCUUCA...............
   7 ................................................................AUGGAGUCGCUCUCUUCA...............

     miR-47
     GAGAGCCGACUGAAACUGAAGAGAGCAGUCUAUUGACAGUCGGUUACUCGAAAUCUUUACUGUCAUGGAGGCGCUCUCUUCAGAUGAUGUCUGGCCC
     ....((((((.....(((((((((((.((((..(((((((.((((......))))...)))))))...))))))))))))))).....))).))).. 
   1 ..................AAGAGAGCAGUCUAUUGAC............................................................
   7 ..................AAGAGAGCAGUCUAUUGACA...........................................................
  54 ..................AAGAGAGCAGUCUAUUGACAG..........................................................
1699 ..................AAGAGAGCAGUCUAUUGACAGU.........................................................
   5 ...................AGAGAGCAGUCUAUUGACAGU.........................................................
   1 ......................GAGCAGUCUAUUGACAGU.........................................................
   1 ........................................CGGUUACUCGAAAUCUUUA......................................
   1 ........................................CGGUUACUCGAAAUCUUUAC.....................................
   5 ...........................................................CUGUCAUGGAGGCGCUCUCUU.................
   2 ...........................................................CUGUCAUGGAGGCGCUCUCUUC................
   6 ...........................................................CUGUCAUGGAGGCGCUCUCUUCA...............
   1 ............................................................UGUCAUGGAGGCGCUCU....................
   6 ............................................................UGUCAUGGAGGCGCUCUC...................
  51 ............................................................UGUCAUGGAGGCGCUCUCU..................
 161 ............................................................UGUCAUGGAGGCGCUCUCUU.................
 392 ............................................................UGUCAUGGAGGCGCUCUCUUC................
# 4063 ............................................................UGUCAUGGAGGCGCUCUCUUCA...............
   2 ............................................................UGUCAUGGAGGCGCUCUCUUCAG..............
   1 .............................................................GUCAUGGAGGCGCUCUCU..................
```

```
      2 .............................................................GUCAUGGAGGCGCUCUCUU.................
      2 .............................................................GUCAUGGAGGCGCUCUCUUC................
      9 .............................................................GUCAUGGAGGCGCUCUCUUCA...............
      1 ...............................................................CAUGGAGGCGCUCUCUUCA...............
      1 ................................................................AUGGAGGCGCUCUCUUC................
     13 ................................................................AUGGAGGCGCUCUCUUCA...............
      1 .................................................................UGGAGGCGCUCUCUUC................

        miR-48
        AAACUCCCGGGAACUUGAGGUAGGCUCAGUAGAUGCGAAUUGAACGGUAUCUCACAUCCACCAGCCUAGCUCGCAUUCCCAGAGUUUACGGUAUUGUU
        ((((((..(((((..((((.((((((..((.((((.((..((.....))..)).)))).)).)))))).))))..))))).)))))).........
      1 ..............UUGAGGUAGGCUCAGUA...................................................................
     53 ...............UGAGGUAGGCUCAGUAG..................................................................
      2 ..............UUGAGGUAGGCUCAGUAGA.................................................................
    775 ...............UGAGGUAGGCUCAGUAGA.................................................................
      1 ..............UUGAGGUAGGCUCAGUAGAU................................................................
    341 ...............UGAGGUAGGCUCAGUAGAU................................................................
      1 ................GAGGUAGGCUCAGUAGAU................................................................
      4 ..............UUGAGGUAGGCUCAGUAGAUG...............................................................
   1193 ...............UGAGGUAGGCUCAGUAGAUG...............................................................
      1 ................GAGGUAGGCUCAGUAGAUG...............................................................
      1 .................AGGUAGGCUCAGUAGAUG...............................................................
      1 ..................GGUAGGCUCAGUAGAUG...............................................................
     29 ..............UUGAGGUAGGCUCAGUAGAUGC..............................................................
   3614 ...............UGAGGUAGGCUCAGUAGAUGC..............................................................
      4 ................GAGGUAGGCUCAGUAGAUGC..............................................................
      1 .................AGGUAGGCUCAGUAGAUGC..............................................................
      1 .............CUUGAGGUAGGCUCAGUAGAUGCG.............................................................
    184 ..............UUGAGGUAGGCUCAGUAGAUGCG.............................................................
  27433 ...............UGAGGUAGGCUCAGUAGAUGCG.............................................................
     49 ................GAGGUAGGCUCAGUAGAUGCG.............................................................
      7 .................AGGUAGGCUCAGUAGAUGCG.............................................................
      1 ...................GUAGGCUCAGUAGAUGCG.............................................................
      1 ....................UAGGCUCAGUAGAUGCG.............................................................
     24 .............CUUGAGGUAGGCUCAGUAGAUGCGA............................................................
   2797 ..............UUGAGGUAGGCUCAGUAGAUGCGA............................................................
# 1908831 ...............UGAGGUAGGCUCAGUAGAUGCGA............................................................
   2584 ................GAGGUAGGCUCAGUAGAUGCGA............................................................
    280 .................AGGUAGGCUCAGUAGAUGCGA............................................................
     27 ..................GGUAGGCUCAGUAGAUGCGA............................................................
     65 ...................GUAGGCUCAGUAGAUGCGA............................................................
     58 ....................UAGGCUCAGUAGAUGCGA............................................................
    116 .....................AGGCUCAGUAGAUGCGA............................................................
      3 ..............UUGAGGUAGGCUCAGUAGAUGCGAA...........................................................
   4538 ...............UGAGGUAGGCUCAGUAGAUGCGAA...........................................................
      6 ................GAGGUAGGCUCAGUAGAUGCGAA...........................................................
      2 .................AGGUAGGCUCAGUAGAUGCGAA...........................................................
```

```
       1 ..................GGUAGGCUCAGUAGAUGCGAA...........................................................
      66 ...............UGAGGUAGGCUCAGUAGAUGCGAAU..........................................................
      13 ...............UGAGGUAGGCUCAGUAGAUGCGAAUU.........................................................
       1 ........................CUCAGUAGAUGCGAAUUGAACGGUA.................................................
       1 ....................................GAAUUGAACGGUAUCUCAC...........................................
       3 .....................................................ACAUCCACCAGCCUAGC............................
       2 .....................................................ACAUCCACCAGCCUAGCUC..........................
      14 .....................................................ACAUCCACCAGCCUAGCUCGC........................
       1 .......................................................AUCCACCAGCCUAGCUCGC........................
     372 .....................................................ACAUCCACCAGCCUAGCUCGCA.......................
       1 ......................................................CAUCCACCAGCCUAGCUCGCA.......................
      47 .....................................................ACAUCCACCAGCCUAGCUCGCAU......................
      22 .......................................................AUCCACCAGCCUAGCUCGCAU......................
       3 .....................................................ACAUCCACCAGCCUAGCUCGCAUU.....................
       2 .......................................................AUCCACCAGCCUAGCUCGCAUU.....................

         miR-49
         UUUUGAAAAAGACCACCGUCCGCAGUUUGUUGUGAUGUGCUCCAAGCAAUCAUGAGUCUGAAGCACCACGAGAAGCUGCAGAUGGAGGUUCUGAUUU
         ..........((((.(((((.(((((((.(((((..(((((.((..(........)..)).)))))))))).))))))).))))).)))).......
       7 ....................CGCAGUUUGUUGUGAUGUG..........................................................
       4 ....................CGCAGUUUGUUGUGAUGUGCU........................................................
       5 ....................CGCAGUUUGUUGUGAUGUGCUC.......................................................
      12 ....................CGCAGUUUGUUGUGAUGUGCUCC......................................................
       9 ....................CGCAGUUUGUUGUGAUGUGCUCCA.....................................................
       1 .....................GCAGUUUGUUGUGAUGUGCUCC......................................................
       1 ............................................................AAGCACCACGAGAAGCU....................
       3 ............................................................AAGCACCACGAGAAGCUG...................
      22 ............................................................AAGCACCACGAGAAGCUGC..................
     269 ............................................................AAGCACCACGAGAAGCUGCA.................
     892 ............................................................AAGCACCACGAGAAGCUGCAG................
#  12333 ............................................................AAGCACCACGAGAAGCUGCAGA...............
      25 ............................................................AAGCACCACGAGAAGCUGCAGAU..............
      12 .............................................................AGCACCACGAGAAGCUGCAG................
     136 .............................................................AGCACCACGAGAAGCUGCAGA...............
       1 .............................................................AGCACCACGAGAAGCUGCAGAU..............
       8 ..............................................................GCACCACGAGAAGCUGCAGA...............
       4 ...............................................................CACCACGAGAAGCUGCAGA...............
       1 ................................................................ACCACGAGAAGCUGCAG................
       9 ................................................................ACCACGAGAAGCUGCAGA...............
       3 .................................................................CCACGAGAAGCUGCAGA...............

         miR-50
         CUGCCCGCCGGCCGCUGAUAUGUCUGGUAUUCUUGGGUUUGAACUUCCAGCGUUGAACCCGCAUAUUAGACGUAUCGACGGCCGGCGGGGCAGGUAAUG
         ((((((((((((((.(((((((((((((((...(((((((.(((.......))))))))))).))))))))))))))).)))))))).))))))......
       2 ..............CUGAUAUGUCUGGUAUUCUUG................................................................
       1 ..............CUGAUAUGUCUGGUAUUCUUGGG..............................................................
       3 ..............CUGAUAUGUCUGGUAUUCUUGGGUU............................................................
```

```
     2 ..............CUGAUAUGUCUGGUAUUCUUGGGUUU...........................................................
     4 ...............UGAUAUGUCUGGUAUUC...................................................................
     8 ...............UGAUAUGUCUGGUAUUCU..................................................................
    56 ...............UGAUAUGUCUGGUAUUCUU.................................................................
   978 ...............UGAUAUGUCUGGUAUUCUUG................................................................
  2401 ...............UGAUAUGUCUGGUAUUCUUGG...............................................................
#  2493 ...............UGAUAUGUCUGGUAUUCUUGGG..............................................................
  2209 ...............UGAUAUGUCUGGUAUUCUUGGGU.............................................................
  9362 ...............UGAUAUGUCUGGUAUUCUUGGGUU............................................................
  1174 ...............UGAUAUGUCUGGUAUUCUUGGGUUU...........................................................
     1 ...............UGAUAUGUCUGGUAUUCUUGGGUUUG..........................................................
     1 ................GAUAUGUCUGGUAUUCUU.................................................................
     1 ................GAUAUGUCUGGUAUUCUUG................................................................
     1 ................GAUAUGUCUGGUAUUCUUGG...............................................................
     1 ................GAUAUGUCUGGUAUUCUUGGG..............................................................
     8 ................GAUAUGUCUGGUAUUCUUGGGUU............................................................
     4 ................GAUAUGUCUGGUAUUCUUGGGUUU...........................................................
     1 .................AUAUGUCUGGUAUUCUUGG...............................................................
     1 .................AUAUGUCUGGUAUUCUUGGG..............................................................
     5 .................AUAUGUCUGGUAUUCUUGGGUU............................................................
     1 .................AUAUGUCUGGUAUUCUUGGGUUUG..........................................................
     2 ...................AUGUCUGGUAUUCUUGGGUU............................................................
     1 .........................................................CCCGCAUAUUAGACGUAU........................
    11 .........................................................CCCGCAUAUUAGACGUAUCGAC....................
     1 ...........................................................CGCAUAUUAGACGUAUCGAC....................

       miR-51
       GUCCGAAAAGUCCGUCUACCCGUAGCUCCUAUCCAUGUUACUGGUCAAAAAGUGAACAUGGAAGCAGGUACAGGUGCACGGCGAGUAGGGUCAUGAAGCU
       ((((.....(.((((.((((.(((.((.((.(((((((((((........)))).))))))))).)).))).)))).))))).....))).)........
     6 ................UACCCGUAGCUCCUAUC...................................................................
    21 ................UACCCGUAGCUCCUAUCC..................................................................
     8 ................UACCCGUAGCUCCUAUCCA.................................................................
     1 ..............UCUACCCGUAGCUCCUAUCCAU................................................................
    12 ................UACCCGUAGCUCCUAUCCAU................................................................
     1 .................ACCCGUAGCUCCUAUCCAU................................................................
    29 ................UACCCGUAGCUCCUAUCCAUG...............................................................
   407 ................UACCCGUAGCUCCUAUCCAUGU..............................................................
     1 .................ACCCGUAGCUCCUAUCCAUGU..............................................................
     2 ...............CUACCCGUAGCUCCUAUCCAUGUU.............................................................
#  5804 ................UACCCGUAGCUCCUAUCCAUGUU.............................................................
    11 .................ACCCGUAGCUCCUAUCCAUGUU.............................................................
     6 ..................CCCGUAGCUCCUAUCCAUGUU.............................................................
     2 ....................CGUAGCUCCUAUCCAUGUU.............................................................
     2 .....................GUAGCUCCUAUCCAUGUU.............................................................
    92 ................UACCCGUAGCUCCUAUCCAUGUUA............................................................
     1 ................UACCCGUAGCUCCUAUCCAUGUUACU..........................................................
     1 ..................................................AGUGAACAUGGAAGCAGGUACA............................
```

```
       1 ........................................................CAUGGAAGCAGGUACAGG..........................
       5 ........................................................CAUGGAAGCAGGUACAGGU.........................
      14 ........................................................CAUGGAAGCAGGUACAGGUG........................
      36 ........................................................CAUGGAAGCAGGUACAGGUGC.......................
       3 .........................................................AUGGAAGCAGGUACAGGUGC.......................
       3 ......................................................AACAUGGAAGCAGGUACAGGUGCA......................
       1 .......................................................ACAUGGAAGCAGGUACAGGUGCA......................
     550 ........................................................CAUGGAAGCAGGUACAGGUGCA......................
       4 .........................................................AUGGAAGCAGGUACAGGUGCA......................

         miR-52
         UCCAACUCUAACAGUCCACCCGUACAUAUGUUUCCGUGCUUGACAGCGAAGCUCAAUCACGUUACAAUGAAAGGGUAGCCGGUUAUUGAAGUUGGGUCUU
         ((((((((((((.(.(.((((...(((.(((...((((.(((((......).)))).))))..))))))...)))).).).))))..).))))))).... 
       1 ..............UCCACCCGUACAUAUGUUUCCGUGCU............................................................
       6 ...............CCACCCGUACAUAUGUUUCCGUGCU............................................................
      95 ................CACCCGUACAUAUGUUU...................................................................
     105 ................CACCCGUACAUAUGUUUC..................................................................
     205 ................CACCCGUACAUAUGUUUCC.................................................................
     676 ................CACCCGUACAUAUGUUUCCG................................................................
    1325 ................CACCCGUACAUAUGUUUCCGU...............................................................
    2511 ................CACCCGUACAUAUGUUUCCGUG..............................................................
    9385 ................CACCCGUACAUAUGUUUCCGUGC.............................................................
#  278560 ................CACCCGUACAUAUGUUUCCGUGCU............................................................
    5824 ................CACCCGUACAUAUGUUUCCGUGCUU...........................................................
      23 ................CACCCGUACAUAUGUUUCCGUGCUUG..........................................................
       1 .................ACCCGUACAUAUGUUUC..................................................................
       1 .................ACCCGUACAUAUGUUUCC.................................................................
       7 .................ACCCGUACAUAUGUUUCCG................................................................
      11 .................ACCCGUACAUAUGUUUCCGU...............................................................
      25 .................ACCCGUACAUAUGUUUCCGUG..............................................................
      84 .................ACCCGUACAUAUGUUUCCGUGC.............................................................
    2347 .................ACCCGUACAUAUGUUUCCGUGCU............................................................
      62 .................ACCCGUACAUAUGUUUCCGUGCUU...........................................................
       3 ..................CCCGUACAUAUGUUUCCGUGC.............................................................
      70 ..................CCCGUACAUAUGUUUCCGUGCU............................................................
       1 ...................CCGUACAUAUGUUUCCGUG..............................................................
       2 ...................CCGUACAUAUGUUUCCGUGC.............................................................
      51 ...................CCGUACAUAUGUUUCCGUGCU............................................................
       1 ....................CGUACAUAUGUUUCCGU...............................................................
       4 ....................CGUACAUAUGUUUCCGUG..............................................................
       7 ....................CGUACAUAUGUUUCCGUGC.............................................................
     309 ....................CGUACAUAUGUUUCCGUGCU............................................................
       7 ....................CGUACAUAUGUUUCCGUGCUU...........................................................
       1 .....................GUACAUAUGUUUCCGU...............................................................
       2 .....................GUACAUAUGUUUCCGUGC.............................................................
      57 .....................GUACAUAUGUUUCCGUGCU............................................................
       1 ......................UACAUAUGUUUCCGUGC.............................................................
```

```
      8 ......................UACAUAUGUUUCCGUGCU............................................................
      6 .......................ACAUAUGUUUCCGUGCU............................................................
     40 ........................................UGACAGCGAAGCUCAAU...........................................
      1 ........................................UGACAGCGAAGCUCAAUCACGUUACA..................................
      1 ......................................................AAUCACGUUACAAUGAAAGGGUAGC.....................
      3 .........................................................CACGUUACAAUGAAAGGGUA.......................
     17 .........................................................CACGUUACAAUGAAAGGGUAG......................
    181 .........................................................CACGUUACAAUGAAAGGGUAGC.....................
      1 .........................................................CACGUUACAAUGAAAGGGUAGCC....................
      2 ..........................................................ACGUUACAAUGAAAGGGUAGC.....................
      4 ...........................................................CGUUACAAUGAAAGGGUAGC.....................
      1 ............................................................GUUACAAUGAAAGGGUAGC.....................

        miR-53
        CCGAUUCUGACAGUCCACCCGUACAUUUGUUUCCGUGCUUGACUUCAAAGCUCAAUCACGGCACAAUAUAUGGGUCGCCAGUCAUUGUAGUCGGAAUUU
        (((((((((((.(.(.(((((((...((((..(((((.((((((....)).)))).))))).))))..))))))).).).))))..).)))))).....
      6 ...............CACCCGUACAUUUGUUU...................................................................
      3 ...............CACCCGUACAUUUGUUUC..................................................................
      5 ...............CACCCGUACAUUUGUUUCC.................................................................
     17 ...............CACCCGUACAUUUGUUUCCG................................................................
     53 ...............CACCCGUACAUUUGUUUCCGU...............................................................
     83 ...............CACCCGUACAUUUGUUUCCGUG..............................................................
      1 ................ACCCGUACAUUUGUUUCCGUG..............................................................
    229 ...............CACCCGUACAUUUGUUUCCGUGC.............................................................
      3 ................ACCCGUACAUUUGUUUCCGUGC.............................................................
#  8369 ...............CACCCGUACAUUUGUUUCCGUGCU............................................................
     61 ................ACCCGUACAUUUGUUUCCGUGCU............................................................
      1 .................CCCGUACAUUUGUUUCCGUGCU............................................................
      1 ..................CCGUACAUUUGUUUCCGUGCU............................................................
      4 ...................CGUACAUUUGUUUCCGUGCU............................................................
    181 ...............CACCCGUACAUUUGUUUCCGUGCUU...........................................................
      5 ................ACCCGUACAUUUGUUUCCGUGCUU...........................................................
      1 ........................................................CACGGCACAAUAUAUGGG.........................
      3 ........................................................CACGGCACAAUAUAUGGGU........................
      1 ........................................................CACGGCACAAUAUAUGGGUC.......................
      3 ........................................................CACGGCACAAUAUAUGGGUCG......................
     10 ........................................................CACGGCACAAUAUAUGGGUCGC.....................
      1 ...............................................................................AGUCAUUGUAGUCGGAAU..

        miR-54
        GUGAGUCGCGCUCUGACUAGGAUAUGAGACGACGAGAACAUUGCUUUUUUAAAAGACUUGUACCCGUAAUCUUCAUAAUCCGAGUCAGGGCUAGCUGAC
        ((.(((...(((((((((.((((((((((..(((.(.(((...((((....))))...))).).)))..)).)))).)))).)))))))))..))).))
      1 ...........UCUGACUAGGAUAUGAGACGAC..................................................................
      2 ...........UCUGACUAGGAUAUGAGACGACG.................................................................
     58 ..................AGGAUAUGAGACGACGA................................................................
     35 ..................AGGAUAUGAGACGACGAG...............................................................
     94 ..................AGGAUAUGAGACGACGAGA..............................................................
```

```
       309 ..................AGGAUAUGAGACGACGAGAA.............................................................
         7 .................UAGGAUAUGAGACGACGAGAAC............................................................
       305 ..................AGGAUAUGAGACGACGAGAAC............................................................
         1 ....................GAUAUGAGACGACGAGAAC............................................................
         1 .....................AUAUGAGACGACGAGAAC............................................................
        28 .................UAGGAUAUGAGACGACGAGAACA...........................................................
     12043 ..................AGGAUAUGAGACGACGAGAACA...........................................................
         3 ...................GGAUAUGAGACGACGAGAACA...........................................................
         3 .....................AUAUGAGACGACGAGAACA...........................................................
         5 .......................AUGAGACGACGAGAACA...........................................................
       300 ..................AGGAUAUGAGACGACGAGAACAU..........................................................
         1 ....................GAUAUGAGACGACGAGAACAU..........................................................
         1 .................UAGGAUAUGAGACGACGAGAACAUU.........................................................
        52 ..................AGGAUAUGAGACGACGAGAACAUU.........................................................
         1 ........................UGAGACGACGAGAACAUUG........................................................
         3 ............................................................UACCCGUAAUCUUCAUA......................
        14 ............................................................UACCCGUAAUCUUCAUAA.....................
        13 ............................................................UACCCGUAAUCUUCAUAAU....................
        17 ............................................................UACCCGUAAUCUUCAUAAUC...................
        85 ............................................................UACCCGUAAUCUUCAUAAUCC..................
         1 .............................................................ACCCGUAAUCUUCAUAAUCC..................
       165 ............................................................UACCCGUAAUCUUCAUAAUCCG.................
         1 .............................................................ACCCGUAAUCUUCAUAAUCCG.................
         3 ...........................................................GUACCCGUAAUCUUCAUAAUCCGA................
      1167 ............................................................UACCCGUAAUCUUCAUAAUCCGA................
         5 .............................................................ACCCGUAAUCUUCAUAAUCCGA................
#    17515 ............................................................UACCCGUAAUCUUCAUAAUCCGAG...............
        33 .............................................................ACCCGUAAUCUUCAUAAUCCGAG...............
         8 ..............................................................CCCGUAAUCUUCAUAAUCCGAG...............
       137 ............................................................UACCCGUAAUCUUCAUAAUCCGAGU..............

           miR-55
           AGAUUUGGAACUUAAUGGGACUCGGCAGAAACCUAUCGGUUAUACUUUUUGGAUAUGCUAUACCCGUAUAAGUUUCUGCUGAGCCCCUUAUUCCUGUU
           ......((((..(((.(((.((((((((((((.((((((.(((((...........).)))).))).))).))))))))))))))).)))))))....
         4 ......................CGGCAGAAACCUAUCGGU..........................................................
         1 ....................ACUCGGCAGAAACCUAUCGGUU........................................................
         8 ......................CGGCAGAAACCUAUCGGUU.........................................................
        24 ......................CGGCAGAAACCUAUCGGUUA........................................................
        67 ......................CGGCAGAAACCUAUCGGUUAU.......................................................
       189 ......................CGGCAGAAACCUAUCGGUUAUA......................................................
         3 .......................GGCAGAAACCUAUCGGUUAUA......................................................
         1 ......................CGGCAGAAACCUAUCGGUUAUACU....................................................
         1 ...........................................ACUUUUUGGAUAUGCUAU.....................................
         4 ............................................................UACCCGUAUAAGUUUCU.....................
         1 ............................................................UACCCGUAUAAGUUUCUG....................
        22 ............................................................UACCCGUAUAAGUUUCUGC...................
         1 ...........................................................AUACCCGUAUAAGUUUCUGCU..................
```

```
       15 ............................................................UACCCGUAUAAGUUUCUGCU..................
       23 ............................................................UACCCGUAUAAGUUUCUGCUG.................
        4 ...........................................................AUACCCGUAUAAGUUUCUGCUGA................
      186 ............................................................UACCCGUAUAAGUUUCUGCUGA................
        2 .............................................................ACCCGUAUAAGUUUCUGCUGA................
        1 ...............................................................CCGUAUAAGUUUCUGCUGA................
      124 ...........................................................AUACCCGUAUAAGUUUCUGCUGAG...............
#   11626 ............................................................UACCCGUAUAAGUUUCUGCUGAG...............
       62 .............................................................ACCCGUAUAAGUUUCUGCUGAG...............
       12 ..............................................................CCCGUAUAAGUUUCUGCUGAG...............
        5 ...............................................................CCGUAUAAGUUUCUGCUGAG...............
        3 ................................................................CGUAUAAGUUUCUGCUGAG...............
        2 .................................................................GUAUAAGUUUCUGCUGAG...............
        2 ...........................................................AUACCCGUAUAAGUUUCUGCUGAGC..............
        7 ............................................................UACCCGUAUAAGUUUCUGCUGAGC..............
        2 .............................................................ACCCGUAUAAGUUUCUGCUGAGC..............

          miR-56
          GAGCCAGUGUCUGUUCUUGGCGGAUCCAUUUUGGGUUGUACCUCAUCCUAAAUUUGACGGUACCCGUAAUGUUUCCGCUGAGAACCGACUGUGCACC
          ..((((..(((.(((((..(((((..((((.(((((...((((((.........))).)))))))).))))..)))))..))))).))))).))...
        2 .................UGGCGGAUCCAUUUUGGGUUGUA.........................................................
        2 ..................GGCGGAUCCAUUUUGGGUUGUA.........................................................
        5 .................UGGCGGAUCCAUUUUGGGUUGUACC.......................................................
        1 ..........................................UCAUCCUAAAUUUGACGG.....................................
        2 ............................................................UACCCGUAAUGUUUCCG....................
       14 ............................................................UACCCGUAAUGUUUCCGC...................
       13 ............................................................UACCCGUAAUGUUUCCGCU..................
       14 ............................................................UACCCGUAAUGUUUCCGCUG.................
        4 ...........................................................GUACCCGUAAUGUUUCCGCUGA................
      160 ............................................................UACCCGUAAUGUUUCCGCUGA................
       13 ...........................................................GUACCCGUAAUGUUUCCGCUGAG...............
#    5750 ............................................................UACCCGUAAUGUUUCCGCUGAG...............
       14 .............................................................ACCCGUAAUGUUUCCGCUGAG...............
        3 ..............................................................CCCGUAAUGUUUCCGCUGAG...............
       51 ............................................................UACCCGUAAUGUUUCCGCUGAGA..............
       48 ............................................................UACCCGUAAUGUUUCCGCUGAGAA.............
        1 ..........................................................................CCGCUGAGAACCGACUG......

          miR-57
          AUCGACAUGCUCGUCUACCCUGUAGAUCGAGCUGUGUGUUUGAAACAAUCAUACACGAGCUAGACUACAAGGUGCACGAACAAACCGAAGAUUUAUGAA
          .(((...((.((((.((((.(((((.((.((((.(((((.(((....))).))))))))).))))))).)))).)))).))...)))............
        1 ..............CUACCCUGUAGAUCGAGC...................................................................
       74 ...............UACCCUGUAGAUCGAGC...................................................................
       60 ...............UACCCUGUAGAUCGAGCU..................................................................
      169 ...............UACCCUGUAGAUCGAGCUG.................................................................
        1 ................ACCCUGUAGAUCGAGCUG.................................................................
      189 ...............UACCCUGUAGAUCGAGCUGU................................................................
```

```
    1 .................CCCUGUAGAUCGAGCUGU................................................................
  255 ...............UACCCUGUAGAUCGAGCUGUG...............................................................
    1 ...................CUGUAGAUCGAGCUGUG...............................................................
    1 ..............CUACCCUGUAGAUCGAGCUGUGU..............................................................
  524 ...............UACCCUGUAGAUCGAGCUGUGU..............................................................
    3 ................ACCCUGUAGAUCGAGCUGUGU..............................................................
    1 ....................UGUAGAUCGAGCUGUGU..............................................................
    2 ..............CUACCCUGUAGAUCGAGCUGUGUG.............................................................
  861 ...............UACCCUGUAGAUCGAGCUGUGUG.............................................................
    2 ................ACCCUGUAGAUCGAGCUGUGUG.............................................................
    1 ..................CCUGUAGAUCGAGCUGUGUG.............................................................
   47 ..............CUACCCUGUAGAUCGAGCUGUGUGU............................................................
#   20538 ...............UACCCUGUAGAUCGAGCUGUGUGU............................................................
  108 ................ACCCUGUAGAUCGAGCUGUGUGU............................................................
   13 .................CCCUGUAGAUCGAGCUGUGUGU............................................................
   22 ..................CCUGUAGAUCGAGCUGUGUGU............................................................
   21 ...................CUGUAGAUCGAGCUGUGUGU............................................................
    8 ....................UGUAGAUCGAGCUGUGUGU............................................................
    4 .....................GUAGAUCGAGCUGUGUGU............................................................
    1 ......................UAGAUCGAGCUGUGUGU............................................................
    1 ..............CUACCCUGUAGAUCGAGCUGUGUGUU...........................................................
  154 ...............UACCCUGUAGAUCGAGCUGUGUGUU...........................................................
   16 ................ACCCUGUAGAUCGAGCUGUGUGUU...........................................................
   29 ...............UACCCUGUAGAUCGAGCUGUGUGUUU..........................................................
    2 ......................................................ACGAGCUAGACUACAAGG...........................
    2 ......................................................ACGAGCUAGACUACAAGGUG.........................
   12 ......................................................ACGAGCUAGACUACAAGGUGC........................
    2 .....................................................CACGAGCUAGACUACAAGGUGCA.......................
  325 ......................................................ACGAGCUAGACUACAAGGUGCA.......................
    1 .......................................................CGAGCUAGACUACAAGGUGCA.......................
    1 ......................................................ACGAGCUAGACUACAAGGUGCAC......................

      miR-58
      GCUCGUCAUAUCCAUUGCCCUACUCUUCGCAUCUCAUCACUUCGUCCAAUACCAUAGGGAUGAGAUCGUUCAGUACGGCAAUGGACUGAGCUAGAGU
      (((((.....(((((((((.((((...((.((((((((.((..((.....))...)).))))))))))...)))).))))))))).)))))......
    1 ..............UUGCCCUACUCUUCGCAUCUCA.............................................................
   11 ..............UUGCCCUACUCUUCGCAUCUCAUC...........................................................
    1 ...............UGCCCUACUCUUCGCAUC................................................................
    4 ...............UGCCCUACUCUUCGCAUCUCA.............................................................
   18 ...............UGCCCUACUCUUCGCAUCUCAU............................................................
  218 ...............UGCCCUACUCUUCGCAUCUCAUC...........................................................
    1 ................GCCCUACUCUUCGCAUC................................................................
    1 ................GCCCUACUCUUCGCAUCUC..............................................................
   10 ................GCCCUACUCUUCGCAUCUCA.............................................................
   39 ................GCCCUACUCUUCGCAUCUCAU............................................................
  113 ................GCCCUACUCUUCGCAUCUCAUC...........................................................
    2 ................GCCCUACUCUUCGCAUCUCAUCA..........................................................
```

```
        1 .................CCCUACUCUUCGCAUCUCAUC...........................................................
        1 ..................CCUACUCUUCGCAUCUCAUC...........................................................
       22 ..................CCUACUCUUCGCAUCUCAUCACU........................................................
        1 ..................CCUACUCUUCGCAUCUCAUCACUU.......................................................
        1 ...................CUACUCUUCGCAUCUCAUCACU........................................................
        1 .....................ACUCUUCGCAUCUCAUC...........................................................
        1 ......................................ACUUCGUCCAAUACCAUA.........................................
        1 ......................................ACUUCGUCCAAUACCAUAGG.......................................
        1 ......................................ACUUCGUCCAAUACCAUAGGG......................................
        2 ......................................ACUUCGUCCAAUACCAUAGGGA.....................................
        1 .....................................................AUAGGGAUGAGAUCGUUCAGUACGG...................
        1 ........................................................GGGAUGAGAUCGUUCAGUACG....................
        2 ........................................................GGGAUGAGAUCGUUCAGUACGG...................
        2 ........................................................GGGAUGAGAUCGUUCAGUACGGCAA................
        1 ........................................................GGGAUGAGAUCGUUCAGUACGGCAAU...............
        2 .........................................................GGAUGAGAUCGUUCAGUACG....................
       12 .........................................................GGAUGAGAUCGUUCAGUACGG...................
        5 .........................................................GGAUGAGAUCGUUCAGUACGGC..................
      209 .........................................................GGAUGAGAUCGUUCAGUACGGCA.................
       10 .........................................................GGAUGAGAUCGUUCAGUACGGCAA................
        3 .........................................................GGAUGAGAUCGUUCAGUACGGCAAU...............
       15 ..........................................................GAUGAGAUCGUUCAGUACGGCAA................
        6 ..........................................................GAUGAGAUCGUUCAGUACGGCAAU...............
        1 ...........................................................AUGAGAUCGUUCAGUACG....................
        7 ...........................................................AUGAGAUCGUUCAGUACGG...................
        4 ...........................................................AUGAGAUCGUUCAGUACGGC..................
      427 ...........................................................AUGAGAUCGUUCAGUACGGCA.................
     2927 ...........................................................AUGAGAUCGUUCAGUACGGCAA................
     1141 ...........................................................AUGAGAUCGUUCAGUACGGCAAU...............
        3 ...........................................................AUGAGAUCGUUCAGUACGGCAAUG..............
      307 ............................................................UGAGAUCGUUCAGUACG....................
     1615 ............................................................UGAGAUCGUUCAGUACGG...................
     3885 ............................................................UGAGAUCGUUCAGUACGGC..................
    44002 ............................................................UGAGAUCGUUCAGUACGGCA.................
  6964792 ............................................................UGAGAUCGUUCAGUACGGCAA................
# 4126329 ............................................................UGAGAUCGUUCAGUACGGCAAU...............
     4277 ............................................................UGAGAUCGUUCAGUACGGCAAUG..............
       92 ............................................................UGAGAUCGUUCAGUACGGCAAUGG.............
       10 ............................................................UGAGAUCGUUCAGUACGGCAAUGGA............
        2 ............................................................UGAGAUCGUUCAGUACGGCAAUGGAC...........
        4 .............................................................GAGAUCGUUCAGUACG....................
        4 .............................................................GAGAUCGUUCAGUACGG...................
       10 .............................................................GAGAUCGUUCAGUACGGC..................
      129 .............................................................GAGAUCGUUCAGUACGGCA.................
    21162 .............................................................GAGAUCGUUCAGUACGGCAA................
    12089 .............................................................GAGAUCGUUCAGUACGGCAAU...............
        8 .............................................................GAGAUCGUUCAGUACGGCAAUG..............
```

```
   1 ..............................................................AGAUCGUUCAGUACG....................
   1 ..............................................................AGAUCGUUCAGUACGG...................
   2 ..............................................................AGAUCGUUCAGUACGGC..................
  24 ..............................................................AGAUCGUUCAGUACGGCA.................
2488 ..............................................................AGAUCGUUCAGUACGGCAA................
1335 ..............................................................AGAUCGUUCAGUACGGCAAU...............
   2 ..............................................................AGAUCGUUCAGUACGGCAAUG..............
   8 ...............................................................GAUCGUUCAGUACGGCA.................
  58 ...............................................................GAUCGUUCAGUACGGCAA................
  37 ...............................................................GAUCGUUCAGUACGGCAAU...............
   1 ................................................................AUCGUUCAGUACGGC..................
   7 ................................................................AUCGUUCAGUACGGCA.................
  76 ................................................................AUCGUUCAGUACGGCAA................
  39 ................................................................AUCGUUCAGUACGGCAAU...............
   4 .................................................................UCGUUCAGUACGGCA.................
 250 .................................................................UCGUUCAGUACGGCAA................
 194 .................................................................UCGUUCAGUACGGCAAU...............
   1 .......................................................................AGUACGGCAAUGGACUGAGCUAGA..

     miR-59
     AGUUGAUCUAGAUAUGACAUCGUCCUGAAAACGAAACGGAACAAAAGUUCAAGAUAUUGAUUUCGAAUCGUUUAUCAGGAUGAUGUGAUUAAAAUCAACU
     (((((((......((.((((((((((((((((((..(((((.......((((....)))))))))..)))))).))))))))))))).))....)))))))
   4 ...................UCGUCCUGAAAACGAAACGGA............................................................
   1 ...................UCGUCCUGAAAACGAAACGGAA...........................................................
   1 ..............................................................UCGAAUCGUUUAUCAGG.....................
   5 ..............................................................UCGAAUCGUUUAUCAGGAU...................
   3 ..............................................................UCGAAUCGUUUAUCAGGAUG..................
  27 ..............................................................UCGAAUCGUUUAUCAGGAUGA.................
  93 ..............................................................UCGAAUCGUUUAUCAGGAUGAU................
#   3331 ..............................................................UCGAAUCGUUUAUCAGGAUGAUG...............
   3 ...............................................................CGAAUCGUUUAUCAGGAUGAUG...............
  10 ................................................................GAAUCGUUUAUCAGGAUGAUG...............
 158 ..............................................................UCGAAUCGUUUAUCAGGAUGAUGU..............
   1 ...............................................................CGAAUCGUUUAUCAGGAUGAUGU..............

     miR-60
     CUCGAAAACCGCUUGUUCUUGAACUGGAAGAGUGCCAUAAAAUCAUGACAAAGUACGUGAUAUUAUGCACAUUUUCUAGUUCAAGACUUGAGAAAUCG
     ................(((((((((((((((((((.((((.((((((........)))))).)))))))).)))))))))))))))...((....)).
   3 .....................AACUGGAAGAGUGCCAU............................................................
   1 ................UCUUGAACUGGAAGAGUGCCAUA...........................................................
   2 .....................AACUGGAAGAGUGCCAUA...........................................................
   3 .....................AACUGGAAGAGUGCCAUAA..........................................................
   1 .....................AACUGGAAGAGUGCCAUAAA.........................................................
  19 .....................AACUGGAAGAGUGCCAUAAAA........................................................
  26 .....................AACUGGAAGAGUGCCAUAAAAU.......................................................
 437 .....................AACUGGAAGAGUGCCAUAAAAUC......................................................
   3 ......................ACUGGAAGAGUGCCAUAAAAUC......................................................
```

```
     29 .....................AACUGGAAGAGUGCCAUAAAAUCA.....................................................
      2 .....................AACUGGAAGAGUGCCAUAAAAUCAU....................................................
      1 .....................AACUGGAAGAGUGCCAUAAAAUCAUG...................................................
      1 ...........................................................AUAUUAUGCACAUUUUC......................
      5 ............................................................UAUUAUGCACAUUUUCU.....................
      9 ............................................................UAUUAUGCACAUUUUCUA....................
      5 ............................................................UAUUAUGCACAUUUUCUAG...................
      2 ...........................................................AUAUUAUGCACAUUUUCUAGU..................
     87 ............................................................UAUUAUGCACAUUUUCUAGU..................
      1 .............................................................AUUAUGCACAUUUUCUAGU..................
     25 ............................................................UAUUAUGCACAUUUUCUAGUU.................
     25 ...........................................................AUAUUAUGCACAUUUUCUAGUUC................
    104 ............................................................UAUUAUGCACAUUUUCUAGUUC................
      2 .............................................................AUUAUGCACAUUUUCUAGUUC................
      1 ..............................................................UUAUGCACAUUUUCUAGUUC................
    126 ...........................................................AUAUUAUGCACAUUUUCUAGUUCA...............
#  8353 ............................................................UAUUAUGCACAUUUUCUAGUUCA...............
     12 .............................................................AUUAUGCACAUUUUCUAGUUCA...............
      4 ..............................................................UUAUGCACAUUUUCUAGUUCA...............
      1 ...............................................................UAUGCACAUUUUCUAGUUCA...............
      6 ................................................................AUGCACAUUUUCUAGUUCA...............
      3 ..................................................................GCACAUUUUCUAGUUCA...............
     23 ............................................................UAUUAUGCACAUUUUCUAGUUCAA..............
      1 ...............................................................UAUGCACAUUUUCUAGUUCAA..............
```

miR-61

```
        UUCCAUUAUCGCUGAACCUCGAGAUGGGUUACGGGGCUUAGUCCUUCCUCCGUAUGGCAAUGACUAGAACCGUUACUCAUCUCGAGGUUUCGGUGAU
        .......((((((((((((((((((((((.((((..((.((((.((((.......)).)).))))))..)))).))))))))))))).)))))))))
      1 .....................AGAUGGGUUACGGGGCUUAGUC......................................................
      2 ........................UGGGUUACGGGGCUUAGUCC.....................................................
      2 .......................AUGGGUUACGGGGCUUAGUCCU....................................................
      8 ........................UGGGUUACGGGGCUUAGUCCU....................................................
      1 .....................AGAUGGGUUACGGGGCUUAGUCCUU...................................................
      1 .......................AUGGGUUACGGGGCUUAGUCCUU...................................................
     26 ........................UGGGUUACGGGGCUUAGUCCUU...................................................
      1 .........................GGGUUACGGGGCUUAGUCCUU...................................................
      1 ............................................................UGACUAGAACCGUUACU....................
     12 ............................................................UGACUAGAACCGUUACUC...................
      1 ...........................................................AUGACUAGAACCGUUACUCA..................
    146 ............................................................UGACUAGAACCGUUACUCA..................
      1 ..........................................................AAUGACUAGAACCGUUACUCAU.................
      2 ...........................................................AUGACUAGAACCGUUACUCAU.................
    582 ............................................................UGACUAGAACCGUUACUCAU.................
      1 .............................................................GACUAGAACCGUUACUCAU.................
      3 ..........................................................AAUGACUAGAACCGUUACUCAUC................
     20 ...........................................................AUGACUAGAACCGUUACUCAUC................
#  7212 ............................................................UGACUAGAACCGUUACUCAUC................
```

```
  10 .............................................................GACUAGAACCGUUACUCAUC................
   7 ..............................................................ACUAGAACCGUUACUCAUC................
   2 ..........................................................AAUGACUAGAACCGUUACUCAUCU...............
 878 ............................................................UGACUAGAACCGUUACUCAUCU...............
   2 .............................................................GACUAGAACCGUUACUCAUCU...............
   2 ............................................................UGACUAGAACCGUUACUCAUCUC..............
   2 ............................................................UGACUAGAACCGUUACUCAUCUCG.............
  11 ...............................................................CUAGAACCGUUACUCAUCUCG.............
   1 ...................................................................AACCGUUACUCAUCUCG.............

     miR-62
     GUGAGUUAGAUCUCAUAUCCUUCCGCAAAAUGGAAAUGAUAUGUAAUCUAGCUUACAG
     (((((((((((..((((((.(((((.....)))))..))))))..)))))))))))..
   3 GUGAGUUAGAUCUCAUAUCCUU....................................
   1 ..................................AAUGAUAUGUAAUCUAGCUUACAG
   2 ...................................AUGAUAUGUAAUCUAGCUUACAG
   1 ....................................UGAUAUGUAAUCUAGCUU....
   1 ....................................UGAUAUGUAAUCUAGCUUA...
  10 ....................................UGAUAUGUAAUCUAGCUUACA.
#    144 ....................................UGAUAUGUAAUCUAGCUUACAG

     miR-63
     UCAACAAGCAGACACAAUUUCUAACUCGUCGGUAGUCAUCGUUCUAGCUGAAAAGGACACUAUGACACUGAAGCGAGUUGGAAAUAGUGGUUCUACUUGAGCAA
     ....((((.((((((.(((((((((((((((((.(((((.(((((........)))))...)))))))))..))))))))))))).)))..))).)))).....
   3 ...............AAUUUCUAACUCGUCGGUAGUCA..................................................................
  20 ...................UCUAACUCGUCGGUAGUCA..................................................................
   4 ...................UCUAACUCGUCGGUAGUCAU.................................................................
  17 ...................UCUAACUCGUCGGUAGUCAUC................................................................
 105 ...................UCUAACUCGUCGGUAGUCAUCG...............................................................
1101 ...................UCUAACUCGUCGGUAGUCAUCGU..............................................................
   3 .....................UAACUCGUCGGUAGUCAUCGU..............................................................
   2 .........................UCGUCGGUAGUCAUCGU..............................................................
  27 ...................UCUAACUCGUCGGUAGUCAUCGUU.............................................................
   1 ....................CUAACUCGUCGGUAGUCAUCGUU.............................................................
   2 .....................UAACUCGUCGGUAGUCAUCGUU.............................................................
   1 .....................UAACUCGUCGGUAGUCAUCGUUC............................................................
   3 ......................AACUCGUCGGUAGUCAUCGUUCU...........................................................
   2 .......................ACUCGUCGGUAGUCAUCGUUCUA..........................................................
  73 ............................................................UAUGACACUGAAGCGAG...........................
  59 ............................................................UAUGACACUGAAGCGAGU..........................
   1 ...........................................................CUAUGACACUGAAGCGAGUU.........................
 160 ............................................................UAUGACACUGAAGCGAGUU.........................
 166 ............................................................UAUGACACUGAAGCGAGUUG........................
 149 ............................................................UAUGACACUGAAGCGAGUUGG.......................
   1 .............................................................AUGACACUGAAGCGAGUUGG.......................
   3 ...........................................................CUAUGACACUGAAGCGAGUUGGA......................
1564 ............................................................UAUGACACUGAAGCGAGUUGGA......................
```

```
       8 .............................................................AUGACACUGAAGCGAGUUGGA......................
       3 ...........................................................CUAUGACACUGAAGCGAGUUGGAA.....................
    3790 ............................................................UAUGACACUGAAGCGAGUUGGAA.....................
      16 .............................................................AUGACACUGAAGCGAGUUGGAA.....................
       2 ..............................................................UGACACUGAAGCGAGUUGGAA.....................
      10 ...........................................................CUAUGACACUGAAGCGAGUUGGAAA....................
#  11852 ............................................................UAUGACACUGAAGCGAGUUGGAAA....................
      43 .............................................................AUGACACUGAAGCGAGUUGGAAA....................
       2 ..............................................................UGACACUGAAGCGAGUUGGAAA....................
     140 ............................................................UAUGACACUGAAGCGAGUUGGAAAU...................
      31 .............................................................AUGACACUGAAGCGAGUUGGAAAU...................
       1 ............................................................UAUGACACUGAAGCGAGUUGGAAAUA..................
       1 ..............................................................UGACACUGAAGCGAGUUGGAAAUA..................

         miR-64
         CUCCCCGCUGACCUCGCCGAAUAUGACACUGAAGCGUUACCGAACCGUUUUCCCACACCUGGAUUCGGUGCAACGAUCAGUGGCAUGCUCGGCUAGCGCCAGUUAAGUAU
         ......((((.(((.(((((.((((.((((((..(((((((((((((............))).)))))))..))).)))))).)))).))))).)).).)))).......
       1 ................CCGAAUAUGACACUGAAGCGUU........................................................................
      10 ................CCGAAUAUGACACUGAAGCGUUA.......................................................................
      11 .................CGAAUAUGACACUGAAGCGUUAC......................................................................
       1 .................CGAAUAUGACACUGAAGCGUUACCGA...................................................................
       1 ..................GAAUAUGACACUGAAGC...........................................................................
       9 ..................GAAUAUGACACUGAAGCGUUACC.....................................................................
       3 ...................AAUAUGACACUGAAGCGUUACCG....................................................................
      10 ...................AAUAUGACACUGAAGCGUUACCGA...................................................................
     126 ...................AAUAUGACACUGAAGCGUUACCGAA..................................................................
       1 ....................AUAUGACACUGAAGCGUUA.......................................................................
       2 ....................AUAUGACACUGAAGCGUUAC......................................................................
       1 ....................AUAUGACACUGAAGCGUUACC.....................................................................
       7 ....................AUAUGACACUGAAGCGUUACCG....................................................................
     115 ....................AUAUGACACUGAAGCGUUACCGA...................................................................
      81 ....................AUAUGACACUGAAGCGUUACCGAA..................................................................
       1 ....................AUAUGACACUGAAGCGUUACCGAAC.................................................................
       2 .....................UAUGACACUGAAGCGUU........................................................................
      75 .....................UAUGACACUGAAGCGUUA.......................................................................
      44 .....................UAUGACACUGAAGCGUUAC......................................................................
      70 .....................UAUGACACUGAAGCGUUACC.....................................................................
     210 .....................UAUGACACUGAAGCGUUACCG....................................................................
    6478 .....................UAUGACACUGAAGCGUUACCGA...................................................................
# 115831 .....................UAUGACACUGAAGCGUUACCGAA..................................................................
     213 .....................UAUGACACUGAAGCGUUACCGAAC.................................................................
     215 .....................UAUGACACUGAAGCGUUACCGAACC................................................................
     522 .....................UAUGACACUGAAGCGUUACCGAACCG...............................................................
       1 ......................AUGACACUGAAGCGUUAC......................................................................
       1 ......................AUGACACUGAAGCGUUACC.....................................................................
       1 ......................AUGACACUGAAGCGUUACCG....................................................................
      27 ......................AUGACACUGAAGCGUUACCGA...................................................................
```

```
  387 ......................AUGACACUGAAGCGUUACCGAA..................................................................
    2 ......................AUGACACUGAAGCGUUACCGAAC.................................................................
    1 ......................AUGACACUGAAGCGUUACCGAACC................................................................
    3 ......................AUGACACUGAAGCGUUACCGAACCG...............................................................
    1 ......................AUGACACUGAAGCGUUACCGAACCGU..............................................................
    5 .......................UGACACUGAAGCGUUACCGA...................................................................
   27 .......................UGACACUGAAGCGUUACCGAA..................................................................
    5 ........................GACACUGAAGCGUUACCGAA..................................................................
   15 .........................ACACUGAAGCGUUACCGAA..................................................................
    1 ..........................CACUGAAGCGUUACCGA...................................................................
    1 ...........................ACUGAAGCGUUACCGAA..................................................................
    1 ............................................CCGUUUUCCCACACCUGGAU..............................................
    4 .................................................................CGGUGCAACGAUCAGUGGCAUG.......................
    4 .................................................................CGGUGCAACGAUCAGUGGCAUGC......................
    8 .................................................................CGGUGCAACGAUCAGUGGCAUGCU.....................
    2 ...................................................................GUGCAACGAUCAGUGGCAUGCU.....................

      miR-65
      AUGGAGCCUUCGCCGAUUAUGACACUGAAGCGUAACCGAACACCAUAUUUUGAGAUUCUGCUACGCGCAGUGCCAUGCUCGGCGCGUUGGCUCCAUUAAA
      ((((((((..((((((.((((.(((((..(((((.(.(((...((.....))...))).).))))).))))).)))).))))))....))))))))....
    1 ..........CGCCGAUUAUGACACUGAAGCGU...................................................................
    1 ................UUAUGACACUGAAGCGUAACC...............................................................
    1 ................UUAUGACACUGAAGCGUAACCGA.............................................................
    6 ................UUAUGACACUGAAGCGUAACCGAA............................................................
    4 .................UAUGACACUGAAGCGUA..................................................................
  137 .................UAUGACACUGAAGCGUAA.................................................................
  248 .................UAUGACACUGAAGCGUAAC................................................................
  279 .................UAUGACACUGAAGCGUAACC...............................................................
  278 .................UAUGACACUGAAGCGUAACCG..............................................................
 5595 .................UAUGACACUGAAGCGUAACCGA.............................................................
#   64231 .................UAUGACACUGAAGCGUAACCGAA............................................................
   27 .................UAUGACACUGAAGCGUAACCGAAC...........................................................
   18 .................UAUGACACUGAAGCGUAACCGAACA..........................................................
    2 .................UAUGACACUGAAGCGUAACCGAACAC.........................................................
    2 ..................AUGACACUGAAGCGUAAC................................................................
    3 ..................AUGACACUGAAGCGUAACC...............................................................
    1 ..................AUGACACUGAAGCGUAACCG..............................................................
   12 ..................AUGACACUGAAGCGUAACCGA.............................................................
  189 ..................AUGACACUGAAGCGUAACCGAA............................................................
    1 ..................AUGACACUGAAGCGUAACCGAAC...........................................................
    1 ...................UGACACUGAAGCGUAACCGA.............................................................
   18 ...................UGACACUGAAGCGUAACCGAA............................................................
    1 ....................GACACUGAAGCGUAACCGAA............................................................
    1 .....................ACACUGAAGCGUAACCG..............................................................
    1 .....................ACACUGAAGCGUAACCGA.............................................................
    8 .....................ACACUGAAGCGUAACCGAA............................................................
    1 ......................CACUGAAGCGUAACCGAA............................................................
```

```
    3 .........................................................CUGCUACGCGCAGUGCCAUGC......................
  196 .........................................................CUGCUACGCGCAGUGCCAUGCU.....................
    3 ..........................................................UGCUACGCGCAGUGCCAUGCU.....................
    1 ...........................................................GCUACGCGCAGUGCCAUGCU.....................

      miR-66
      CCACAAAAAUGCCAUACAUGACACUGAUUAGGGAUGUGAUGAAUGUUAAGAUCCCGAUCAAAUUCCUAACGGUGUCAAACAUGGCGUAUGUGGUUGUAG
      (((((...(((((((...((((((((.((((((((.((((................)))).))))))))))))))))...))))))).))))).....
    1 ..............UACAUGACACUGAUUAGGGAUGUGA............................................................
    1 ...............ACAUGACACUGAUUAGGGAUGUG.............................................................
   12 ...............ACAUGACACUGAUUAGGGAUGUGA............................................................
    1 ...............ACAUGACACUGAUUAGGGAUGUGAU...........................................................
    7 ................CAUGACACUGAUUAGGG..................................................................
   16 ................CAUGACACUGAUUAGGGA.................................................................
   28 ................CAUGACACUGAUUAGGGAU................................................................
   77 ................CAUGACACUGAUUAGGGAUG...............................................................
   65 ................CAUGACACUGAUUAGGGAUGU..............................................................
  332 ................CAUGACACUGAUUAGGGAUGUG.............................................................
#   31407 ................CAUGACACUGAUUAGGGAUGUGA............................................................
 3364 ................CAUGACACUGAUUAGGGAUGUGAU...........................................................
   22 ................CAUGACACUGAUUAGGGAUGUGAUG..........................................................
    1 .................AUGACACUGAUUAGGGAUGU..............................................................
    3 .................AUGACACUGAUUAGGGAUGUG.............................................................
  239 .................AUGACACUGAUUAGGGAUGUGA............................................................
   62 .................AUGACACUGAUUAGGGAUGUGAU...........................................................
    1 .................AUGACACUGAUUAGGGAUGUGAUG..........................................................
    1 ..................UGACACUGAUUAGGGAUGUGA............................................................
    1 ..................UGACACUGAUUAGGGAUGUGAU...........................................................
    6 ...................GACACUGAUUAGGGAUGUGA............................................................
    6 ....................ACACUGAUUAGGGAUGUGA............................................................
    2 ......................ACUGAUUAGGGAUGUGA............................................................
    1 .........................................................UCAAAUUCCUAACGGUGUCAA.....................
    2 .........................................................UCAAAUUCCUAACGGUGUCAAA....................
    1 ..........................................................CAAAUUCCUAACGGUGUCAAA....................
    1 ..........................................................CAAAUUCCUAACGGUGUCAAACA..................
    3 ...........................................................AAAUUCCUAACGGUGUCAAA....................
    6 ...........................................................AAAUUCCUAACGGUGUCAAAC...................
   52 ...........................................................AAAUUCCUAACGGUGUCAAACA..................
    6 ...........................................................AAAUUCCUAACGGUGUCAAACAU.................
    2 ..............................................................UUCCUAACGGUGUCAAAC...................

      miR-67
      GAUCAAAGAUUCGUCGAUCCGCUCAUUCUGCCGGUUGUUAUGCUAUUAUCAGAUUAAGCAUCACAACCUCCUAGAAAGAGUAGAUCGAUUUUAAAACUU
      ............(((((((..(((.(((((..((((((.(((((............))))).))))))...))))).)))..)))))))..........
    1 ...................CGCUCAUUCUGCCGGUUGUUAU..........................................................
   25 ...................CGCUCAUUCUGCCGGUUGUUAUG.........................................................
    5 ..........................................CUAUUAUCAGAUUAAGCA.......................................
```

```
         9 ...........................................................AUCACAACCUCCUAGAAAGAGUAG................
         4 ............................................................UCACAACCUCCUAGAAAGAGUAG................
        57 ...........................................................AUCACAACCUCCUAGAAAGAGUAGA...............
#      420 ............................................................UCACAACCUCCUAGAAAGAGUAGA...............
         2 .............................................................CACAACCUCCUAGAAAGAGUAGA...............
        16 ..............................................................ACAACCUCCUAGAAAGAGUAGA...............
         1 ............................................................UCACAACCUCCUAGAAAGAGUAGAU..............

           miR-70
           UCAAAAUAAAACGAUGAAAACUAUCGAAAUACUAUCGACGAAUAACACUUAUGAAGAAAUGUAAUACGUCGUUGGUGUUUCCAUAGUUUGAAUUGUUUAU
           ........(((((((..(((((((.(((((((((.(((((.((.(((.((......)).))).)).))))).))))))))).)))))))..))))))).. 
         4 ........................CGAAAUACUAUCGACGAAUAA.......................................................
         3 ........................CGAAAUACUAUCGACGAAUAAC......................................................
        75 ........................CGAAAUACUAUCGACGAAUAACA.....................................................
         2 ..............................................ACUUAUGAAGAAAUGUAAUACGUCG.............................
        36 .............................................................UAAUACGUCGUUGGUGU......................
       473 .............................................................UAAUACGUCGUUGGUGUU.....................
         1 ..............................................................AAUACGUCGUUGGUGUU.....................
       339 .............................................................UAAUACGUCGUUGGUGUUU....................
       871 .............................................................UAAUACGUCGUUGGUGUUUC...................
         5 ..............................................................AAUACGUCGUUGGUGUUUC...................
         2 ...........................................................UGUAAUACGUCGUUGGUGUUUCC..................
      1424 .............................................................UAAUACGUCGUUGGUGUUUCC..................
         4 ..............................................................AAUACGUCGUUGGUGUUUCC..................
         1 ...............................................................AUACGUCGUUGGUGUUUCC..................
     19307 .............................................................UAAUACGUCGUUGGUGUUUCCA.................
        56 ..............................................................AAUACGUCGUUGGUGUUUCCA.................
         5 ...............................................................AUACGUCGUUGGUGUUUCCA.................
         1 ..................................................................CGUCGUUGGUGUUUCCA.................
         2 ............................................................GUAAUACGUCGUUGGUGUUUCCAU................
#   499225 .............................................................UAAUACGUCGUUGGUGUUUCCAU................
      1236 ..............................................................AAUACGUCGUUGGUGUUUCCAU................
       224 ...............................................................AUACGUCGUUGGUGUUUCCAU................
        32 ................................................................UACGUCGUUGGUGUUUCCAU................
         8 .................................................................ACGUCGUUGGUGUUUCCAU................
         8 ..................................................................CGUCGUUGGUGUUUCCAU................
      1115 .............................................................UAAUACGUCGUUGGUGUUUCCAUA...............
         4 ..............................................................AAUACGUCGUUGGUGUUUCCAUA...............
         3 ...............................................................AUACGUCGUUGGUGUUUCCAUA...............
         2 .............................................................UAAUACGUCGUUGGUGUUUCCAUAG..............
         2 ................................................................UACGUCGUUGGUGUUUCCAUAG..............
         1 .............................................................UAAUACGUCGUUGGUGUUUCCAUAGU.............
         1 ...............................................................AUACGUCGUUGGUGUUUCCAUAGU.............
        25 ................................................................UACGUCGUUGGUGUUUCCAUAGU.............

           miR-71
           GUCUGCUCUGAACGAUGAAAGACAUGGGUAGUGAGACGUCGGAGCCUCGUCGUAUCACUAUUCUGUUUUUCGCCGUCGGGAUCGUGACCUGGAA
```

```
        (((((.(((..(((.(((((((((.(((((((((.(((.(((....))).))).)))))))))))))))))).))).)))..))).)).)))......
    70  ...............UGAAAGACAUGGGUAGU..............................................................
     1  .................AAAGACAUGGGUAGUG.............................................................
     3  .............GAUGAAAGACAUGGGUAGU..............................................................
    80  ...............UGAAAGACAUGGGUAGU..............................................................
     6  ..............AUGAAAGACAUGGGUAGUG.............................................................
  1135  ...............UGAAAGACAUGGGUAGUG.............................................................
     1  ................GAAAGACAUGGGUAGUG.............................................................
     3  .............GAUGAAAGACAUGGGUAGUGA............................................................
    10  ..............AUGAAAGACAUGGGUAGUGA............................................................
# 39852 ...............UGAAAGACAUGGGUAGUGA............................................................
    26  ................GAAAGACAUGGGUAGUGA............................................................
     3  .................AAAGACAUGGGUAGUGA............................................................
     6  ..............AUGAAAGACAUGGGUAGUGAG...........................................................
  5186  ...............UGAAAGACAUGGGUAGUGAG...........................................................
     3  ................GAAAGACAUGGGUAGUGAG...........................................................
     1  ..................AAGACAUGGGUAGUGAG...........................................................
     3  ..............AUGAAAGACAUGGGUAGUGAGA..........................................................
 15197  ...............UGAAAGACAUGGGUAGUGAGA..........................................................
     4  ................GAAAGACAUGGGUAGUGAGA..........................................................
     3  .................AAAGACAUGGGUAGUGAGA..........................................................
 14439  ...............UGAAAGACAUGGGUAGUGAGAC.........................................................
     5  ................GAAAGACAUGGGUAGUGAGAC.........................................................
     2  .................AAAGACAUGGGUAGUGAGAC.........................................................
     2  ..................AAGACAUGGGUAGUGAGAC.........................................................
     1  ............CGAUGAAAGACAUGGGUAGUGAGACG........................................................
     1  .............GAUGAAAGACAUGGGUAGUGAGACG........................................................
    71  ..............AUGAAAGACAUGGGUAGUGAGACG........................................................
422370  ...............UGAAAGACAUGGGUAGUGAGACG........................................................
   211  ................GAAAGACAUGGGUAGUGAGACG........................................................
    62  .................AAAGACAUGGGUAGUGAGACG........................................................
     9  ..................AAGACAUGGGUAGUGAGACG........................................................
    10  ...................AGACAUGGGUAGUGAGACG........................................................
    15  ....................GACAUGGGUAGUGAGACG........................................................
     3  .....................ACAUGGGUAGUGAGACG........................................................
     1  .............GAUGAAAGACAUGGGUAGUGAGACGU.......................................................
     2  ..............AUGAAAGACAUGGGUAGUGAGACGU.......................................................
 11131  ...............UGAAAGACAUGGGUAGUGAGACGU.......................................................
    26  ................GAAAGACAUGGGUAGUGAGACGU.......................................................
     2  .................AAAGACAUGGGUAGUGAGACGU.......................................................
    13  ...............UGAAAGACAUGGGUAGUGAGACGUC......................................................
   130  ...............UGAAAGACAUGGGUAGUGAGACGUCG.....................................................
     1  ................GAAAGACAUGGGUAGUGAGACGUCGG....................................................
     7  ....................................................UAUCACUAUUCUGUUUUUCGC.....................
    46  ....................................................UAUCACUAUUCUGUUUUUCGCC....................
     2  ......................................................UCACUAUUCUGUUUUUCGCC....................
     2  ........................................................ACUAUUCUGUUUUUCGCC....................
```

```
         miR-72
         AGGUCCCGUCAGAGCUAGGCAAGAUGUUGGCAUAGCUGAAUGAUCGCUAUAACAACUAUCAGCUUCGCCACAUUCUGCCACGCACUGAUGUGAGGACCU
         ((((((((((((.((..((((..((((.(((..((((((...................))))))..)))))))..))))..)).))))))...))))))
       2 ..............CUAGGCAAGAUGUUGGCAUAGCUG.............................................................
       1 ..............CUAGGCAAGAUGUUGGCAUAGCUGA............................................................
       2 ...............UAGGCAAGAUGUUGGCAUAGC...............................................................
       1 ...............UAGGCAAGAUGUUGGCAUAGCU..............................................................
       1 ...............UAGGCAAGAUGUUGGCAUAGCUG.............................................................
      25 ...............UAGGCAAGAUGUUGGCAUAGCUGA............................................................
       4 ...............UAGGCAAGAUGUUGGCAUAGCUGAA...........................................................
      47 ................AGGCAAGAUGUUGGCAU..................................................................
     333 ................AGGCAAGAUGUUGGCAUA.................................................................
   21879 ................AGGCAAGAUGUUGGCAUAG................................................................
   23667 ................AGGCAAGAUGUUGGCAUAGC...............................................................
   53476 ................AGGCAAGAUGUUGGCAUAGCU..............................................................
   39056 ................AGGCAAGAUGUUGGCAUAGCUG.............................................................
# 675217 ................AGGCAAGAUGUUGGCAUAGCUGA............................................................
   45833 ................AGGCAAGAUGUUGGCAUAGCUGAA...........................................................
     275 ................AGGCAAGAUGUUGGCAUAGCUGAAU..........................................................
      18 ................AGGCAAGAUGUUGGCAUAGCUGAAUG.........................................................
       2 .................GGCAAGAUGUUGGCAUA.................................................................
      38 .................GGCAAGAUGUUGGCAUAG................................................................
      40 .................GGCAAGAUGUUGGCAUAGC...............................................................
     100 .................GGCAAGAUGUUGGCAUAGCU..............................................................
      87 .................GGCAAGAUGUUGGCAUAGCUG.............................................................
    1074 .................GGCAAGAUGUUGGCAUAGCUGA............................................................
     182 .................GGCAAGAUGUUGGCAUAGCUGAA...........................................................
       7 ..................GCAAGAUGUUGGCAU..................................................................
       7 ..................GCAAGAUGUUGGCAUAG................................................................
      11 ..................GCAAGAUGUUGGCAUAGC...............................................................
      22 ..................GCAAGAUGUUGGCAUAGCU..............................................................
      22 ..................GCAAGAUGUUGGCAUAGCUG.............................................................
     265 ..................GCAAGAUGUUGGCAUAGCUGA............................................................
      19 ..................GCAAGAUGUUGGCAUAGCUGAA...........................................................
       1 ...................CAAGAUGUUGGCAUAGC...............................................................
       4 ...................CAAGAUGUUGGCAUAGCU..............................................................
       3 ...................CAAGAUGUUGGCAUAGCUG.............................................................
      32 ...................CAAGAUGUUGGCAUAGCUGA............................................................
       1 ...................CAAGAUGUUGGCAUAGCUGAA...........................................................
       2 ...................CAAGAUGUUGGCAUAGCUGAAU..........................................................
       1 ....................AAGAUGUUGGCAUAGC...............................................................
       6 ....................AAGAUGUUGGCAUAGCU..............................................................
       5 ....................AAGAUGUUGGCAUAGCUG.............................................................
     107 ....................AAGAUGUUGGCAUAGCUGA............................................................
       8 ....................AAGAUGUUGGCAUAGCUGAA...........................................................
       5 .....................AGAUGUUGGCAUAGCU..............................................................
```

```
      5 .....................AGAUGUUGGCAUAGCUG.............................................................
     79 .....................AGAUGUUGGCAUAGCUGA............................................................
      1 .....................AGAUGUUGGCAUAGCUGAA...........................................................
      9 ......................GAUGUUGGCAUAGCUGA............................................................
      1 .......................................AUGAUCGCUAUAACAACUAU........................................
      4 .......................................AUGAUCGCUAUAACAACUAUC.......................................
      1 ..........................................................UCAGCUUCGCCACAUUCUGCCAC..................
      1 ............................................................AGCUUCGCCACAUUCUGCCA...................
      3 ............................................................AGCUUCGCCACAUUCUGCCAC..................
     29 ............................................................AGCUUCGCCACAUUCUGCCACG.................
      1 ............................................................AGCUUCGCCACAUUCUGCCACGC................

        miR-73
        CAGUGAGAGUCCCACACACGACUGGACUUCCAUAUCGAGCCACAGCUAUCAACGAAUUUGCUGGCAAGAUGUAGGCAGUUCAGUUGUGCGUUUAUGGAG
        .........(((.((.((((((((((((.(((((((..((((..((.............))))))..))))).)).)))))))))))).))....))).
      1 ......................UGGACUUCCAUAUCGAGCCA.........................................................
      5 ......................UGGACUUCCAUAUCGAGCCACA.......................................................
      5 ......................UGGACUUCCAUAUCGAGCCACAG......................................................
     21 ......................UGGACUUCCAUAUCGAGCCACAGC.....................................................
      2 ......................UGGACUUCCAUAUCGAGCCACAGCU....................................................
      1 ...........................................................GCUGGCAAGAUGUAGGCAGUUCAGU...............
      3 ............................................................CUGGCAAGAUGUAGGCAGUUC..................
      1 ............................................................CUGGCAAGAUGUAGGCAGUUCA.................
      6 ............................................................CUGGCAAGAUGUAGGCAGUUCAG................
    158 ............................................................CUGGCAAGAUGUAGGCAGUUCAGU...............
      4 ............................................................CUGGCAAGAUGUAGGCAGUUCAGUU..............
     19 .............................................................UGGCAAGAUGUAGGCAG.....................
    661 .............................................................UGGCAAGAUGUAGGCAGU....................
    108 .............................................................UGGCAAGAUGUAGGCAGUU...................
    219 .............................................................UGGCAAGAUGUAGGCAGUUC..................
   3015 .............................................................UGGCAAGAUGUAGGCAGUUCA.................
   7558 .............................................................UGGCAAGAUGUAGGCAGUUCAG................
# 170397 .............................................................UGGCAAGAUGUAGGCAGUUCAGU...............
   4178 .............................................................UGGCAAGAUGUAGGCAGUUCAGUU..............
      2 .............................................................UGGCAAGAUGUAGGCAGUUCAGUUG.............
      2 .............................................................UGGCAAGAUGUAGGCAGUUCAGUUGU............
      3 ..............................................................GGCAAGAUGUAGGCAGU....................
      3 ..............................................................GGCAAGAUGUAGGCAGUUC..................
      2 ..............................................................GGCAAGAUGUAGGCAGUUCA.................
      9 ..............................................................GGCAAGAUGUAGGCAGUUCAG................
    323 ..............................................................GGCAAGAUGUAGGCAGUUCAGU...............
     19 ..............................................................GGCAAGAUGUAGGCAGUUCAGUU..............
      2 ...............................................................GCAAGAUGUAGGCAGUUCA.................
      5 ...............................................................GCAAGAUGUAGGCAGUUCAG................
    108 ...............................................................GCAAGAUGUAGGCAGUUCAGU...............
     23 ...............................................................GCAAGAUGUAGGCAGUUCAGUU..............
      2 ...............................................................GCAAGAUGUAGGCAGUUCAGUUG.............
```

```
   1 ...............................................................GCAAGAUGUAGGCAGUUCAGUUGUG...........
   1 ................................................................CAAGAUGUAGGCAGUUCAG................
  16 ................................................................CAAGAUGUAGGCAGUUCAGU...............
   1 ................................................................CAAGAUGUAGGCAGUUCAGUUG.............
   8 ................................................................CAAGAUGUAGGCAGUUCAGUUGU............
   3 ................................................................CAAGAUGUAGGCAGUUCAGUUGUG...........
   3 ................................................................CAAGAUGUAGGCAGUUCAGUUGUGC..........
   3 .................................................................AAGAUGUAGGCAGUUCA.................
   3 .................................................................AAGAUGUAGGCAGUUCAG................
  25 .................................................................AAGAUGUAGGCAGUUCAGU...............
   1 ..................................................................AGAUGUAGGCAGUUCAG................
   3 ..................................................................AGAUGUAGGCAGUUCAGU...............

     miR-74
     AAAUGGUUCAAAAAACGUUCGGGCUUCCAUCUCUUUCCCAGCCUACAUCUCAACCUGGGCUGGCAAGAAAUGGCAGUCUACACGUUUUUCAACCAAA
     ...(((((..((((((((..(((((.((((.((((..((((((((..........)))))))).)))).)))).)))))..)))))))).)))))..
   1 .................UUCGGGCUUCCAUCUCUUUCCCAGC.......................................................
   1 ..................UCGGGCUUCCAUCUCUUUCCCAGC.......................................................
   8 ...................CGGGCUUCCAUCUCUUUCCCAGC.......................................................
   1 ...................CGGGCUUCCAUCUCUUUCCCAGCC......................................................
   1 ..........................................CUACAUCUCAACCUGGGC.....................................
   1 ...........................................................CUGGCAAGAAAUGGCAGUCUAC................
  16 ...........................................................CUGGCAAGAAAUGGCAGUCUACA...............
   2 ............................................................UGGCAAGAAAUGGCAGU....................
   1 ............................................................UGGCAAGAAAUGGCAGUC...................
   2 ............................................................UGGCAAGAAAUGGCAGUCU..................
   3 ............................................................UGGCAAGAAAUGGCAGUCUA.................
  52 ............................................................UGGCAAGAAAUGGCAGUCUAC................
#    2741 ............................................................UGGCAAGAAAUGGCAGUCUACA...............
   2 ..............................................................GCAAGAAAUGGCAGUCUACA...............
   1 ...............................................................CAAGAAAUGGCAGUCUACA...............
   1 ................................................................AAGAAAUGGCAGUCUACA...............

     miR-75
     UUCUUGUUGCUUUGAAGAAUUGCAGUCGGUUGCAAGCUUAAAUACAAAUCCGAAUUGUUAUUAAAGCUACCAACCGGCUUCAAGUCUGAAAGAGCAG
     .......((((((..(((.(((.(((((((((..(((((.(((((((.......))).)))).)))))..))))))))).))).)))...)))))).
   1 .....................GCAGUCGGUUGCAAGCUUAAAUA.....................................................
  13 ......................CAGUCGGUUGCAAGCUUAA........................................................
  47 ......................CAGUCGGUUGCAAGCUUAAA.......................................................
 114 ......................CAGUCGGUUGCAAGCUUAAAU......................................................
 126 ......................CAGUCGGUUGCAAGCUUAAAUA.....................................................
   1 .......................AGUCGGUUGCAAGCUUAA........................................................
   2 .......................AGUCGGUUGCAAGCUUAAA.......................................................
   1 .......................AGUCGGUUGCAAGCUUAAAU......................................................
   1 .......................AGUCGGUUGCAAGCUUAAAUA.....................................................
   1 ...........................GGUUGCAAGCUUAAAUA.....................................................
   1 ..........................................................UAUUAAAGCUACCAACCGGCUUCA...............
```

```
        1 ...........................................................AUUAAAGCUACCAACCGGCUUC................
       12 ...........................................................AUUAAAGCUACCAACCGGCUUCA...............
        1 ............................................................UUAAAGCUACCAACCGG....................
        1 ............................................................UUAAAGCUACCAACCGGC...................
       54 ............................................................UUAAAGCUACCAACCGGCU..................
       10 ............................................................UUAAAGCUACCAACCGGCUU.................
      102 ............................................................UUAAAGCUACCAACCGGCUUC................
#    6277 ............................................................UUAAAGCUACCAACCGGCUUCA...............
        2 ............................................................UUAAAGCUACCAACCGGCUUCAA..............
        1 .............................................................UAAAGCUACCAACCGGCUUC................
       21 .............................................................UAAAGCUACCAACCGGCUUCA...............
        4 ..............................................................AAAGCUACCAACCGGCUUCA...............

          miR-76
          AUUUCAGCUCCUGUCUGGGCUUCACAAUAGUCGAAUACCUUAAAUUUCAAAAUUUGGAUAUUCGUUGUUGAUGAAGCCUUGAUGGGGGUGAGAAAGA
          .((((.(((((((((.((((((((((((((.((((((((..(((((....))))))).)))))))))))).)))))))).)))))))))..))))..
        1 ...........................................................AUUCGUUGUUGAUGAAGCCUUGA...............
        1 ............................................................UUCGUUGUUGAUGAAGCC...................
        3 ............................................................UUCGUUGUUGAUGAAGCCU..................
        1 ............................................................UUCGUUGUUGAUGAAGCCUU.................
        4 ............................................................UUCGUUGUUGAUGAAGCCUUG................
#     392 ............................................................UUCGUUGUUGAUGAAGCCUUGA...............
        3 ............................................................UUCGUUGUUGAUGAAGCCUUGAU..............
        1 ............................................................UUCGUUGUUGAUGAAGCCUUGAUGG............
        1 .............................................................UCGUUGUUGAUGAAGCCUUGA...............
        6 ...............................................................GUUGUUGAUGAAGCCUUGA...............

          miR-77
          GCAUCUGCCAAACCGCCCGUUUGGAUGGUUGUGCUCUGAGGAAAUACGCACAGAAUGUCAUUUCAUCAGGCCAUAGCUGUCCAAAUUGGUAUAGAGUUUG
          ........(((((((((.(((((((((((((((..((((.(((((..(((.....))).))))).))))..))))))))))))))).)))...).)))))
        1 .......................GAUGGUUGUGCUCUGAG............................................................
        8 .......................GAUGGUUGUGCUCUGAGGA..........................................................
       20 .......................GAUGGUUGUGCUCUGAGGAA.........................................................
      237 .......................GAUGGUUGUGCUCUGAGGAAA........................................................
      979 .......................GAUGGUUGUGCUCUGAGGAAAU.......................................................
        9 .......................GAUGGUUGUGCUCUGAGGAAAUA......................................................
        1 ........................AUGGUUGUGCUCUGAGGAAA........................................................
        2 .............................................................UUCAUCAGGCCAUAGCU......................
        7 .............................................................UUCAUCAGGCCAUAGCUG.....................
       11 .............................................................UUCAUCAGGCCAUAGCUGU....................
       15 .............................................................UUCAUCAGGCCAUAGCUGUC...................
      107 .............................................................UUCAUCAGGCCAUAGCUGUCC..................
#    3508 .............................................................UUCAUCAGGCCAUAGCUGUCCA.................
       15 .............................................................UUCAUCAGGCCAUAGCUGUCCAA................
        6 .............................................................UUCAUCAGGCCAUAGCUGUCCAAA...............
        2 ..............................................................UCAUCAGGCCAUAGCUGUCCA.................
        7 ...............................................................CAUCAGGCCAUAGCUGUCCA.................
```

```
        9 ................................................................AUCAGGCCAUAGCUGUCCA.................
        2 .................................................................UCAGGCCAUAGCUGUCCA.................
        2 ..................................................................CAGGCCAUAGCUGUCCA.................

          miR-78
          AAUAAAAUAUAUUGUUUCAUAGUGUCCGUAAAAUAACUAGAUUUAUUUUGUAAAAACUAUUGGAGGCCUGGUUGUUUGUGCUGGAAUGUUUCGAGA
          ..............((((..((..((((((((((((((((.(((.....((....)).....)))..)))))))))).))).)).)..))..))))
        1 .........................................................UAUUGGAGGCCUGGUUGUU....................
        3 .........................................................UAUUGGAGGCCUGGUUGUUUG..................
        1 ...........................................................UUGGAGGCCUGGUUGUUUGUG................
        3 ............................................................UGGAGGCCUGGUUGUUUGUG................
#      31 ............................................................UGGAGGCCUGGUUGUUUGUGC...............

          miR-79
          UAGUAGACAUUCUCCGAUCUUUGGUGAUUCAGCUUCAAUGAUUGGCUACAGGUUUCUUUCAUAAAGCUAGGUUACCAAAGCUCGGCGUCUUGAUCUAC
          ..(((((((..(.((((.((((((((((..(((((..((((..(((.....)))....)))).)))))..)))))))))).)))).)...)).)))))
        1 .................UCUUUGGUGAUUCAGCUUCAAU...........................................................
        1 .................UCUUUGGUGAUUCAGCUUCAAUGA.........................................................
        1 ..................CUUUGGUGAUUCAGCUUCAA............................................................
        5 ..................CUUUGGUGAUUCAGCUUCAAU...........................................................
        5 ..................CUUUGGUGAUUCAGCUUCAAUG..........................................................
       68 ..................CUUUGGUGAUUCAGCUUCAAUGA.........................................................
        1 ...................UUUGGUGAUUCAGCUUCAAUGA.........................................................
        1 ....................UUGGUGAUUCAGCUUCAAUGA.........................................................
        6 .........................................UUGGCUACAGGUUUCUUUC......................................
        2 ............................................................AUAAAGCUAGGUUACCA.....................
        1 ............................................................AUAAAGCUAGGUUACCAA....................
        1 ............................................................AUAAAGCUAGGUUACCAAA...................
       41 ............................................................AUAAAGCUAGGUUACCAAAG..................
      103 ............................................................AUAAAGCUAGGUUACCAAAGC.................
#    5184 ............................................................AUAAAGCUAGGUUACCAAAGCU................
       13 ............................................................AUAAAGCUAGGUUACCAAAGCUC...............
        6 .............................................................UAAAGCUAGGUUACCAAAGCU................
        3 ..............................................................AAAGCUAGGUUACCAAAGCU................
        3 ...............................................................AAGCUAGGUUACCAAAGCU................
        1 ................................................................AGCUAGGUUACCAAAGCU................

          miR-227
          AUGGACACUCGUUCGCUCAGCUUUCGACAUGAUUCUGAACAAUCCGCAAGCCCAUGUUGUUGAGAUCAUUAGUUGAAAGCCGAAUGAUCAGAGAUAUC
          .......(((..(((.((.((((((((((((((.((.((((((.(....).....)))))).)))))))..))))))))).)).)))...))).....
        6 ..................AGCUUUCGACAUGAUUC...............................................................
       12 ..................AGCUUUCGACAUGAUUCU..............................................................
        2 ..................AGCUUUCGACAUGAUUCUG.............................................................
       16 ..................AGCUUUCGACAUGAUUCUGA............................................................
       44 ..................AGCUUUCGACAUGAUUCUGAA...........................................................
#     970 ..................AGCUUUCGACAUGAUUCUGAAC..........................................................
        3 ...................GCUUUCGACAUGAUUCUGAAC..........................................................
```

```
    2 ....................CUUUCGACAUGAUUCUGAAC..........................................................
    1 ......................UUCGACAUGAUUCUGAAC..........................................................
   62 ..................AGCUUUCGACAUGAUUCUGAACA.........................................................
  690 ..................AGCUUUCGACAUGAUUCUGAACAA........................................................
    1 .....................UUUCGACAUGAUUCUGAACAA........................................................
    1 ..................AGCUUUCGACAUGAUUCUGAACAAU.......................................................
    1 ........................................AAUCCGCAAGCCCAUGUUG.......................................
   19 ........................................AAUCCGCAAGCCCAUGUUGU......................................
    2 ........................................AAUCCGCAAGCCCAUGUUGUU.....................................
    5 ............................................................UGAGAUCAUUAGUUGAA.....................
   12 ............................................................UGAGAUCAUUAGUUGAAA....................
  382 ............................................................UGAGAUCAUUAGUUGAAAG...................
    1 ...........................................................UUGAGAUCAUUAGUUGAAAGC..................
  191 ............................................................UGAGAUCAUUAGUUGAAAGC..................
    1 ...............................................................GAUCAUUAGUUGAAAGC..................
  789 ............................................................UGAGAUCAUUAGUUGAAAGCC.................
    1 .............................................................GAGAUCAUUAGUUGAAAGCC.................
   24 ...........................................................UUGAGAUCAUUAGUUGAAAGCCG................
 6142 ............................................................UGAGAUCAUUAGUUGAAAGCCG................
    4 .............................................................GAGAUCAUUAGUUGAAAGCCG................
    1 ...............................................................GAUCAUUAGUUGAAAGCCG................
    1 ..........................................................GUUGAGAUCAUUAGUUGAAAGCCGA...............
  105 ...........................................................UUGAGAUCAUUAGUUGAAAGCCGA...............
96258 ............................................................UGAGAUCAUUAGUUGAAAGCCGA...............
   46 .............................................................GAGAUCAUUAGUUGAAAGCCGA...............
   14 ..............................................................AGAUCAUUAGUUGAAAGCCGA...............
    2 ...............................................................GAUCAUUAGUUGAAAGCCGA...............
    3 ................................................................AUCAUUAGUUGAAAGCCGA...............
    3 ..................................................................CAUUAGUUGAAAGCCGA...............
    1 ...........................................................UUGAGAUCAUUAGUUGAAAGCCGAA..............
 2918 ............................................................UGAGAUCAUUAGUUGAAAGCCGAA..............
    2 .............................................................GAGAUCAUUAGUUGAAAGCCGAA..............
    1 ..................................................................CAUUAGUUGAAAGCCGAA..............
    9 ...................................................................AUUAGUUGAAAGCCGAA..............
   23 ............................................................UGAGAUCAUUAGUUGAAAGCCGAAU.............

      miR-80
      AUGGACACUCGUUCGCUCAGCUUUCGACAUGAUUCUGAACAAUCCGCAAGCCCAUGUUGUUGAGAUCAUUAGUUGAAAGCCGAAUGAUCAGAGAUAUC
      .......(((..(((.((.((((((((((((((.((.((((((.(....).....)))))).)))))))..))))))))).)).)))...))).....
    6 ..................AGCUUUCGACAUGAUUC...............................................................
   12 ..................AGCUUUCGACAUGAUUCU..............................................................
    2 ..................AGCUUUCGACAUGAUUCUG.............................................................
   16 ..................AGCUUUCGACAUGAUUCUGA............................................................
   44 ..................AGCUUUCGACAUGAUUCUGAA...........................................................
  970 ..................AGCUUUCGACAUGAUUCUGAAC..........................................................
    3 ...................GCUUUCGACAUGAUUCUGAAC..........................................................
    2 ....................CUUUCGACAUGAUUCUGAAC..........................................................
```

```
       1 ......................UUCGACAUGAUUCUGAAC..........................................................
      62 ..................AGCUUUCGACAUGAUUCUGAACA.........................................................
     690 ..................AGCUUUCGACAUGAUUCUGAACAA........................................................
       1 .....................UUUCGACAUGAUUCUGAACAA........................................................
       1 ..................AGCUUUCGACAUGAUUCUGAACAAU.......................................................
       1 ........................................AAUCCGCAAGCCCAUGUUG.......................................
      19 ........................................AAUCCGCAAGCCCAUGUUGU......................................
       2 ........................................AAUCCGCAAGCCCAUGUUGUU.....................................
       5 ............................................................UGAGAUCAUUAGUUGAA.....................
      12 ............................................................UGAGAUCAUUAGUUGAAA....................
     382 ............................................................UGAGAUCAUUAGUUGAAAG...................
       1 ...........................................................UUGAGAUCAUUAGUUGAAAGC..................
     191 ............................................................UGAGAUCAUUAGUUGAAAGC..................
       1 ...............................................................GAUCAUUAGUUGAAAGC..................
     789 ............................................................UGAGAUCAUUAGUUGAAAGCC.................
       1 .............................................................GAGAUCAUUAGUUGAAAGCC.................
      24 ...........................................................UUGAGAUCAUUAGUUGAAAGCCG................
    6142 ............................................................UGAGAUCAUUAGUUGAAAGCCG................
       4 .............................................................GAGAUCAUUAGUUGAAAGCCG................
       1 ...............................................................GAUCAUUAGUUGAAAGCCG................
       1 ..........................................................GUUGAGAUCAUUAGUUGAAAGCCGA...............
     105 ...........................................................UUGAGAUCAUUAGUUGAAAGCCGA...............
#  96258 ............................................................UGAGAUCAUUAGUUGAAAGCCGA...............
      46 .............................................................GAGAUCAUUAGUUGAAAGCCGA...............
      14 ..............................................................AGAUCAUUAGUUGAAAGCCGA...............
       2 ...............................................................GAUCAUUAGUUGAAAGCCGA...............
       3 ................................................................AUCAUUAGUUGAAAGCCGA...............
       3 ..................................................................CAUUAGUUGAAAGCCGA...............
       1 ...........................................................UUGAGAUCAUUAGUUGAAAGCCGAA..............
    2918 ............................................................UGAGAUCAUUAGUUGAAAGCCGAA..............
       2 .............................................................GAGAUCAUUAGUUGAAAGCCGAA..............
       1 ..................................................................CAUUAGUUGAAAGCCGAA..............
       9 ...................................................................AUUAGUUGAAAGCCGAA..............
      23 ............................................................UGAGAUCAUUAGUUGAAAGCCGAAU.............

         miR-81
         AUCAGUGCCAUCGUGCCCAACAGUCGGUUUUCACCGUGAUCUGAGAGCAAUCCAAAAAUGCUUUUCUGAGAUCAUCGUGAAAGCUAGUUGUUGGUCUACGGGCUUUUG
         ......(((..((((.(((((((..(((((((((.(((((((.((((((.........)))...))).))))))).)))))))))..)))))))..))))))).....
       1 ..............GCCCAACAGUCGGUUUUC............................................................................
       4 ........................CGGUUUUCACCGUGAUCUGAG...............................................................
      93 ........................CGGUUUUCACCGUGAUCUGAGA..............................................................
      14 .................................................................CUGAGAUCAUCGUGAAAGCUAGU....................
     127 ..................................................................UGAGAUCAUCGUGAAAG.........................
     453 ..................................................................UGAGAUCAUCGUGAAAGC........................
     173 ..................................................................UGAGAUCAUCGUGAAAGCU.......................
     791 ..................................................................UGAGAUCAUCGUGAAAGCUA......................
    4589 ..................................................................UGAGAUCAUCGUGAAAGCUAG.....................
```

```
#  137038 ..................................................................UGAGAUCAUCGUGAAAGCUAGU....................
     2136 ..................................................................UGAGAUCAUCGUGAAAGCUAGUU...................
       10 ..................................................................UGAGAUCAUCGUGAAAGCUAGUUG..................
        1 ..................................................................UGAGAUCAUCGUGAAAGCUAGUUGU.................
        2 ...................................................................GAGAUCAUCGUGAAAGC........................
        1 ...................................................................GAGAUCAUCGUGAAAGCU.......................
        1 ...................................................................GAGAUCAUCGUGAAAGCUA......................
       10 ...................................................................GAGAUCAUCGUGAAAGCUAG.....................
      199 ...................................................................GAGAUCAUCGUGAAAGCUAGU....................
       11 ...................................................................GAGAUCAUCGUGAAAGCUAGUU...................
        2 ....................................................................AGAUCAUCGUGAAAGCUAG.....................
       26 ....................................................................AGAUCAUCGUGAAAGCUAGU....................
        2 ....................................................................AGAUCAUCGUGAAAGCUAGUU...................
        3 ....................................................................AGAUCAUCGUGAAAGCUAGUUG..................
        3 .....................................................................GAUCAUCGUGAAAGCUAGU....................
        7 ......................................................................AUCAUCGUGAAAGCUAGU....................
        8 .......................................................................UCAUCGUGAAAGCUAGU....................
      120 ..................................................................UGAGAUCAUCGUGAAAG.........................
      466 ..................................................................UGAGAUCAUCGUGAAAGC........................
        1 ...................................................................GAGAUCAUCGUGAAAGC........................

          miR-82
          GAAAUAGGUUCUUUUAGCAACCGGUUUUCUCUGUGAUCUACAGAAUGACAGCUAAUCGUCUGAGAUCAUCGUGAAAGCCAGUUGUUUUUAUGAACUC
          ......(((((....((((((.(((((((.(.(((((((.((((.(((.......))))))))))))))).).))))))).)))))).....))))).
        1 ..................AACCGGUUUUCUCUGUGAUCU..........................................................
        1 ...................ACCGGUUUUCUCUGUGAUCUA.........................................................
        1 .....................CGGUUUUCUCUGUGAUCUAC........................................................
        1 ...................ACCGGUUUUCUCUGUGAUCUACA.......................................................
        9 .....................CGGUUUUCUCUGUGAUCUACA.......................................................
        4 .....................CGGUUUUCUCUGUGAUCUACAG......................................................
       12 .....................CGGUUUUCUCUGUGAUCUACAGA.....................................................
      127 ............................................................UGAGAUCAUCGUGAAAG....................
      453 ............................................................UGAGAUCAUCGUGAAAGC...................
        2 .............................................................GAGAUCAUCGUGAAAGC...................
      120 ............................................................UGAGAUCAUCGUGAAAG....................
      466 ............................................................UGAGAUCAUCGUGAAAGC...................
        1 .............................................................GAGAUCAUCGUGAAAGC...................
        1 ...........................................................CUGAGAUCAUCGUGAAAGCC..................
      292 ............................................................UGAGAUCAUCGUGAAAGCC..................
        1 ...........................................................CUGAGAUCAUCGUGAAAGCCA.................
     1737 ............................................................UGAGAUCAUCGUGAAAGCCA.................
        1 .............................................................GAGAUCAUCGUGAAAGCCA.................
     2461 ............................................................UGAGAUCAUCGUGAAAGCCAG................
        2 .............................................................GAGAUCAUCGUGAAAGCCAG................
        1 ..............................................................AGAUCAUCGUGAAAGCCAG................
        4 ...........................................................CUGAGAUCAUCGUGAAAGCCAGU...............
#   38053 ............................................................UGAGAUCAUCGUGAAAGCCAGU...............
```

```
  32 .............................................................GAGAUCAUCGUGAAAGCCAGU...............
  27 ..............................................................AGAUCAUCGUGAAAGCCAGU...............
   1 ................................................................AUCAUCGUGAAAGCCAGU...............
 431 ............................................................UGAGAUCAUCGUGAAAGCCAGUU..............
   8 .............................................................GAGAUCAUCGUGAAAGCCAGUU..............
   1 ...............................................................GAUCAUCGUGAAAGCCAGUU..............
   3 ............................................................UGAGAUCAUCGUGAAAGCCAGUUG.............

     miR-83
     AGCACCACUCGGAACCACUGAAUUUAUGUGUGUACUUGACGGCCAACAAGAGCAUCGAUCUAGCACCAUAUAAAUUCAGUAAUUUCGCGUCGAGAGCU
     (((.((((.(((((..((((((((((((((.((.((.((((((........))..)).)).)).))))))))))))))))..))))).)).).).)))
   1 ...............CACUGAAUUUAUGUGUGUACUUGA...........................................................
   2 ................ACUGAAUUUAUGUGUGUACUUG............................................................
 288 ................ACUGAAUUUAUGUGUGUACUUGA...........................................................
   1 ..................UGAAUUUAUGUGUGUACUUGA...........................................................
   1 .....................AUUUAUGUGUGUACUUGA...........................................................
   4 .......................................CGGCCAACAAGAGCAUCGAUC......................................
   1 .......................................CGGCCAACAAGAGCAUCGAUCU.....................................
   1 ............................................................UAGCACCAUAUAAAUUC.....................
   1 ............................................................UAGCACCAUAUAAAUUCA....................
  10 ............................................................UAGCACCAUAUAAAUUCAG...................
   8 ............................................................UAGCACCAUAUAAAUUCAGU..................
  76 ............................................................UAGCACCAUAUAAAUUCAGUA.................
#   3142 ............................................................UAGCACCAUAUAAAUUCAGUAA................
  14 ............................................................UAGCACCAUAUAAAUUCAGUAAU...............
   2 .............................................................AGCACCAUAUAAAUUCAGUA.................
   8 .............................................................AGCACCAUAUAAAUUCAGUAA................
   1 .............................................................AGCACCAUAUAAAUUCAGUAAU...............
   6 ..............................................................GCACCAUAUAAAUUCAGUAA................
   1 ................................................................ACCAUAUAAAUUCAGUAA................

     miR-84
     GUGGCAUCUGAGGUAGUAUGUAAUAUUGUAGACUGUCUAUAAUGUCCACAAUGUUUCAACUAACUCGGCUGUUCU
     ..((((.(((((.((((.((.((((((((.(((..........))).)))))))).)))))).))))).))))..
  61 ........UGAGGUAGUAUGUAAUA..................................................
   4 ........UGAGGUAGUAUGUAAUAU.................................................
  54 ........UGAGGUAGUAUGUAAUAUU................................................
  93 ........UGAGGUAGUAUGUAAUAUUG...............................................
 168 ........UGAGGUAGUAUGUAAUAUUGU..............................................
   1 .........GAGGUAGUAUGUAAUAUUGUA.............................................
   1 .............UAGUAUGUAAUAUUGUA.............................................
   1 .......CUGAGGUAGUAUGUAAUAUUGUA.............................................
#   1874 ........UGAGGUAGUAUGUAAUAUUGUA.............................................
   7 .........GAGGUAGUAUGUAAUAUUGUAG............................................
   1 ..........AGGUAGUAUGUAAUAUUGUAG............................................
   1 ...........GGUAGUAUGUAAUAUUGUAG............................................
   1 .......CUGAGGUAGUAUGUAAUAUUGUAG............................................
```

```
3857 ........UGAGGUAGUAUGUAAUAUUGUAG............................................
   5 .........GAGGUAGUAUGUAAUAUUGUAGA...........................................
   2 ..........AGGUAGUAUGUAAUAUUGUAGA...........................................
   1 ............GUAGUAUGUAAUAUUGUAGA...........................................
   1 .............UAGUAUGUAAUAUUGUAGA...........................................
   1 ..............AGUAUGUAAUAUUGUAGA...........................................
   1 ...............GUAUGUAAUAUUGUAGA...........................................
   5 .......CUGAGGUAGUAUGUAAUAUUGUAGA...........................................
8427 ........UGAGGUAGUAUGUAAUAUUGUAGA...........................................
 277 ........UGAGGUAGUAUGUAAUAUUGUAGAC..........................................
   6 ........UGAGGUAGUAUGUAAUAUUGUAGACU.........................................
   2 ..............................................CACAAUGUUUCAACUAACUCG........
   2 .............................................CCACAAUGUUUCAACUAACUCGG.......
  55 ..............................................CACAAUGUUUCAACUAACUCGG.......
   1 ...............................................ACAAUGUUUCAACUAACUCGG.......
   2 .............................................CCACAAUGUUUCAACUAACUCGGC......
 483 ..............................................CACAAUGUUUCAACUAACUCGGC......
  10 ...............................................ACAAUGUUUCAACUAACUCGGC......
   9 ................................................CAAUGUUUCAACUAACUCGGC......
   2 .................................................AAUGUUUCAACUAACUCGGC......
   2 .............................................CCACAAUGUUUCAACUAACUCGGCU.....
  24 ..............................................CACAAUGUUUCAACUAACUCGGCU.....
   1 ...............................................ACAAUGUUUCAACUAACUCGGCU.....
   2 ................................................CAAUGUUUCAACUAACUCGGCU.....
   2 .................................................AAUGUUUCAACUAACUCGGCU.....

     miR-85
     UAUAGAAUUUUGGCGUCGGAGCCCGAUUUUUCAAUAGUUUGAAACCAGUGUACACAUAAAUGGUUACAAAGUAUUUGAAAAGUCGUGCUCUGAAAAUGAAUUCUUA
     ...(((((((.....(((((((.(((((((((((((.((((.((((((((....)))...))))).)))).)).))))))))))).)))))))....)))))))..
   1 ...................AGCCCGAUUUUUCAAUAGUUUG.................................................................
   1 ....................GCCCGAUUUUUCAAUAGUUUGA................................................................
   1 .............................................CAGUGUACACAUAAAUGGU..........................................
   3 ...............................................................UUACAAAGUAUUUGAAAAGUCGU....................
  13 ...............................................................UUACAAAGUAUUUGAAAAGUCGUG...................
  48 ...............................................................UUACAAAGUAUUUGAAAAGUCGUGC..................
   2 ................................................................UACAAAGUAUUUGAAAA.........................
   7 ................................................................UACAAAGUAUUUGAAAAG........................
   3 ................................................................UACAAAGUAUUUGAAAAGUCG.....................
   2 ................................................................UACAAAGUAUUUGAAAAGUCGU....................
  75 ................................................................UACAAAGUAUUUGAAAAGUCGUG...................
# 1602 ................................................................UACAAAGUAUUUGAAAAGUCGUGC..................
  14 ................................................................UACAAAGUAUUUGAAAAGUCGUGCU.................
   3 .................................................................ACAAAGUAUUUGAAAAGUCGUGC..................

     miR-86
     CGUGUCCACGCCGUCUAAGUGAAUGCUUUGCCACAGUCUUCGAUGUUCUGAAAUGAAGCCUGGGCUCAGAUUCGCUUAGGCCGGAGUUUGACACGGCA
     (((((.((.(((((((((((((((.((..(((.(((.(((((.(.......).))))).))))))..)))))))))))))).))).))..)))))...
```

```
        1 ...............UAAGUGAAUGCUUUGCC..................................................................
        1 ...............UAAGUGAAUGCUUUGCCA.................................................................
        1 ...............UAAGUGAAUGCUUUGCCACA...............................................................
       41 ...............UAAGUGAAUGCUUUGCCACAG..............................................................
        1 ................AAGUGAAUGCUUUGCCACAG..............................................................
        2 ..............CUAAGUGAAUGCUUUGCCACAGU.............................................................
      131 ...............UAAGUGAAUGCUUUGCCACAGU.............................................................
        5 ..............CUAAGUGAAUGCUUUGCCACAGUC............................................................
#    2439 ...............UAAGUGAAUGCUUUGCCACAGUC............................................................
       12 ................AAGUGAAUGCUUUGCCACAGUC............................................................
        1 .................AGUGAAUGCUUUGCCACAGUC............................................................
      940 ...............UAAGUGAAUGCUUUGCCACAGUCU...........................................................
        2 ................AAGUGAAUGCUUUGCCACAGUCU...........................................................
        1 .................AGUGAAUGCUUUGCCACAGUCU...........................................................
        1 ....................GAAUGCUUUGCCACAGUCU...........................................................
       39 ...............UAAGUGAAUGCUUUGCCACAGUCUU..........................................................
        1 ................AAGUGAAUGCUUUGCCACAGUCUU..........................................................
        1 ......................AUGCUUUGCCACAGUCUUCGAUGUUC..................................................
        1 .................................................GAAAUGAAGCCUGGGCUCAGAUU..........................
        1 .......................................................AAGCCUGGGCUCAGAUUCGCUU.....................
        1 ........................................................AGCCUGGGCUCAGAUUCGCUUA....................
        1 .........................................................GCCUGGGCUCAGAUUCGCUUA....................
        9 ..........................................................CCUGGGCUCAGAUUCGCUUA....................
        3 ...........................................................CUGGGCUCAGAUUCGCUUA....................
        1 .........................................................GCCUGGGCUCAGAUUCGCUUAG...................
        3 ..........................................................CCUGGGCUCAGAUUCGCUUAG...................
        2 ...........................................................CUGGGCUCAGAUUCGCUUAG...................
        1 .........................................................GCCUGGGCUCAGAUUCGCUUAGG..................
        3 ..........................................................CCUGGGCUCAGAUUCGCUUAGG..................
        8 ...........................................................CUGGGCUCAGAUUCGCUUAGG..................
        3 ..........................................................CCUGGGCUCAGAUUCGCUUAGGC.................
      167 ...........................................................CUGGGCUCAGAUUCGCUUAGGC.................
        1 ............................................................UGGGCUCAGAUUCGCUUAGGC.................

          miR-87
          GGUUGUGCCAUCCGGCCGCCUGAUACUUUCGUCUCAACCUCGCUGUCAGAUUGGUCGUAGGUGAGCAAAGUUUCAGGUGUGCCGGAACACACCC
          (((.(((...(((((((((((((.(((((.(.((((.((((((((......))).)).))))))))))))).))))))).)))))).)))))).
       15 ................CGCCUGAUACUUUCGUCUCAACCU......................................................
        1 ........................................CGCUGUCAGAUUGGUCGUA...................................
       41 ........................................CGCUGUCAGAUUGGUCGUAG..................................
        1 ..........................................CUGUCAGAUUGGUCGUAG..................................
        3 ............................................................GUGAGCAAAGUUUCAGG.................
       13 ............................................................GUGAGCAAAGUUUCAGGU................
       53 ............................................................GUGAGCAAAGUUUCAGGUG...............
       68 ............................................................GUGAGCAAAGUUUCAGGUGU..............
     1157 ............................................................GUGAGCAAAGUUUCAGGUGUG.............
#    3357 ............................................................GUGAGCAAAGUUUCAGGUGUGC............
```

```
     1 ..............................................................GAGCAAAGUUUCAGGUGUGC............
     1 ................................................................GCAAAGUUUCAGGUGUGC............
     3 ............................................................GUGAGCAAAGUUUCAGGUGUGCC...........

       miR-90
       GGGCGCCAUUUCGAGCGGCUUUCAACGACGAUAUCAACCGACAACUCACACUUUUGCGUGUUGAUAUGUUGUUUGAAUGCCCCUUGAAUUGGAUGCCA
       .(((((((.((((((.(((.(((((((((.((((((((((.(((.........))))).)))))))))))).))))).))).)))))).))).)))).
     1 ...............CGGCUUUCAACGACGAU..................................................................
     2 ...............CGGCUUUCAACGACGAUA.................................................................
     1 ...............CGGCUUUCAACGACGAUAUC...............................................................
     1 ............GAGCGGCUUUCAACGACGAUAUCA..............................................................
     1 .............AGCGGCUUUCAACGACGAUAUCA..............................................................
     3 ...............CGGCUUUCAACGACGAUAUCA..............................................................
    27 ...............CGGCUUUCAACGACGAUAUCAA.............................................................
     1 ................GGCUUUCAACGACGAUAUCAA.............................................................
   152 ...............CGGCUUUCAACGACGAUAUCAAC............................................................
     6 ................GGCUUUCAACGACGAUAUCAAC............................................................
     5 .................GCUUUCAACGACGAUAUCAAC............................................................
     6 ..................CUUUCAACGACGAUAUCAAC............................................................
    39 ...................UUUCAACGACGAUAUCAAC............................................................
    10 ....................UUCAACGACGAUAUCAAC............................................................
     1 ...............CGGCUUUCAACGACGAUAUCAACC...........................................................
    13 .............................................................UGAUAUGUUGUUUGAAU....................
     4 .............................................................UGAUAUGUUGUUUGAAUG...................
    65 .............................................................UGAUAUGUUGUUUGAAUGC..................
     2 ...............................................................AUAUGUUGUUUGAAUGC..................
   129 .............................................................UGAUAUGUUGUUUGAAUGCC.................
     2 ...............................................................AUAUGUUGUUUGAAUGCC.................
   268 .............................................................UGAUAUGUUGUUUGAAUGCCC................
     1 ..............................................................GAUAUGUUGUUUGAAUGCCC................
     5 ...............................................................AUAUGUUGUUUGAAUGCCC................
     1 ............................................................UUGAUAUGUUGUUUGAAUGCCCC...............
  1329 .............................................................UGAUAUGUUGUUUGAAUGCCCC...............
     2 ..............................................................GAUAUGUUGUUUGAAUGCCCC...............
    19 ...............................................................AUAUGUUGUUUGAAUGCCCC...............
     1 .................................................................AUGUUGUUUGAAUGCCCC...............
#   6215 .............................................................UGAUAUGUUGUUUGAAUGCCCCU..............
    16 ..............................................................GAUAUGUUGUUUGAAUGCCCCU..............
  1006 ...............................................................AUAUGUUGUUUGAAUGCCCCU..............
     4 .................................................................AUGUUGUUUGAAUGCCCCU..............
     1 ..................................................................UGUUGUUUGAAUGCCCCU..............
   140 .............................................................UGAUAUGUUGUUUGAAUGCCCCUU.............
    33 ...............................................................AUAUGUUGUUUGAAUGCCCCUU.............
     2 ...............................................................AUAUGUUGUUUGAAUGCCCCUUG............

       miR-124
       GUCCCACUUGUCAUCUGGCAUGCACCCUAGUGACUUUAGUGGACAUCUAAGUCUUCCAACUAAGGCACGCGGUGAAUGCCACGUGGCCAUGAUGGG
```

```
          ..(((((..(((((.((((((.((((...(((.(((((((((((......))))....))))))))))..)))).)))))).)))))...).))))
        1 .................GCAUGCACCCUAGUGACUUUA..........................................................
        9 .................GCAUGCACCCUAGUGACUUUAG.........................................................
      106 .................GCAUGCACCCUAGUGACUUUAGU........................................................
        2 .................GCAUGCACCCUAGUGACUUUAGUG.......................................................
        1 .................GCAUGCACCCUAGUGACUUUAGUGG......................................................
        1 .......................................UGGACAUCUAAGUCUUCCAAC....................................
        2 ...........................................................CUAAGGCACGCGGUGAAUGCCA...............
        2 ............................................................UAAGGCACGCGGUGAAU...................
        4 ............................................................UAAGGCACGCGGUGAAUG..................
        2 ............................................................UAAGGCACGCGGUGAAUGC.................
        7 ............................................................UAAGGCACGCGGUGAAUGCC................
#    1844 ............................................................UAAGGCACGCGGUGAAUGCCA...............
        2 ............................................................UAAGGCACGCGGUGAAUGCCAC..............
       36 .............................................................AAGGCACGCGGUGAAUGCCA...............
        4 ..............................................................AGGCACGCGGUGAAUGCCA...............
        1 ..................................................................ACGCGGUGAAUGCCA...............

          miR-228
          CCUUAUCCCGUUCGCAAUGGCACUGCAUGAAUUCACGGCUAUGCAUAACGACAGACCGCGGAUCAUACGGUACCAUAGCGGACGGUGAUGAGGUUAAU
          (((((((((((((((.((((.((((.((((.(((.(((((...(.....)..)).))).))))))).)))).)))).)))))))).))))))).....
        1 ..............CAAUGGCACUGCAUGAAUUCA...............................................................
        2 ..............CAAUGGCACUGCAUGAAUUCACG.............................................................
      342 ..............CAAUGGCACUGCAUGAAUUCACGG............................................................
      137 ...............AAUGGCACUGCAUGAAU..................................................................
       28 ...............AAUGGCACUGCAUGAAUU.................................................................
      258 ...............AAUGGCACUGCAUGAAUUC................................................................
     1243 ...............AAUGGCACUGCAUGAAUUCA...............................................................
     2249 ...............AAUGGCACUGCAUGAAUUCAC..............................................................
    12792 ...............AAUGGCACUGCAUGAAUUCACG.............................................................
# 1004705 ...............AAUGGCACUGCAUGAAUUCACGG............................................................
     1447 ...............AAUGGCACUGCAUGAAUUCACGGC...........................................................
      271 ...............AAUGGCACUGCAUGAAUUCACGGCU..........................................................
       17 ...............AAUGGCACUGCAUGAAUUCACGGCUA.........................................................
        2 ................AUGGCACUGCAUGAAUUCA...............................................................
        5 ................AUGGCACUGCAUGAAUUCAC..............................................................
       37 ................AUGGCACUGCAUGAAUUCACG.............................................................
     2198 ................AUGGCACUGCAUGAAUUCACGG............................................................
        6 ................AUGGCACUGCAUGAAUUCACGGC...........................................................
        4 .................UGGCACUGCAUGAAUUCACG.............................................................
      358 .................UGGCACUGCAUGAAUUCACGG............................................................
        8 .................UGGCACUGCAUGAAUUCACGGCU..........................................................
        1 .................UGGCACUGCAUGAAUUCACGGCUA.........................................................
       32 ..................GGCACUGCAUGAAUUCACGG............................................................
        1 ...................GCACUGCAUGAAUUCA...............................................................
       28 ...................GCACUGCAUGAAUUCACGG............................................................
       41 ....................CACUGCAUGAAUUCACGG............................................................
```

```
  10 .....................ACUGCAUGAAUUCACGG............................................................
   2 ......................................CUAUGCAUAACGACAGACC.........................................
   1 ........................................AUGCAUAACGACAGACC.........................................
   3 .........................................................GCGGAUCAUACGGUACC........................
   7 .........................................................GCGGAUCAUACGGUACCA.......................
   6 .........................................................GCGGAUCAUACGGUACCAU......................
  39 .........................................................GCGGAUCAUACGGUACCAUA.....................
 499 .........................................................GCGGAUCAUACGGUACCAUAG....................
 514 .........................................................GCGGAUCAUACGGUACCAUAGC...................
   1 ..........................................................CGGAUCAUACGGUACCAUAG....................
   1 ...........................................................GGAUCAUACGGUACCAUAGC...................
   1 .............................................................................GCGGACGGUGAUGAGGUU...

     miR-229
     CGCCGGCAAUGACACUGGUUAUCUUUUCCAUCGUGGAAUGCCCCCCAUUGAUUUUUUCCCCUUUUCGGGGGGAAAAAAUUGGAAACGAGAAAGGUAUCGGGUGUCAUAGCCGGCG
     (((((((.((((((((.(.((((((((...((((((........)).(..((((((((((((.....))))))))))))..)..)))))))))))).).)))))))).)))))))
   2 .........UGACACUGGUUAUCUUUUCCAUCG..................................................................................
   1 ..........GACACUGGUUAUCUUUUCCAUCG..................................................................................
   1 ...........ACACUGGUUAUCUUUUCCAUCG..................................................................................
   1 ............CACUGGUUAUCUUUUCCAUCG..................................................................................
   1 ..............CUGGUUAUCUUUUCCAUCGU.................................................................................
   2 ......CAAUGACACUGGUUAUCUUUUCCAU....................................................................................
   2 ......CAAUGACACUGGUUAUCUUUUCCAUC...................................................................................
   9 ......................................................................................GAGAAAGGUAUCGGGUG............
  76 ......................................................................................GAGAAAGGUAUCGGGUGU...........
 157 ......................................................................................GAGAAAGGUAUCGGGUGUC..........
 184 ......................................................................................GAGAAAGGUAUCGGGUGUCA.........
  66 ......................................................................................GAGAAAGGUAUCGGGUGUCAU........
 103 ......................................................................................GAGAAAGGUAUCGGGUGUCAUA.......
 153 ......................................................................................GAGAAAGGUAUCGGGUGUCAUAG......
  52 ......................................................................................GAGAAAGGUAUCGGGUGUCAUAGC.....
3592 .......................................................................................AGAAAGGUAUCGGGUGU...........
2789 .......................................................................................AGAAAGGUAUCGGGUGUC..........
2128 .......................................................................................AGAAAGGUAUCGGGUGUCA.........
 477 .......................................................................................AGAAAGGUAUCGGGUGUCAU........
 662 .......................................................................................AGAAAGGUAUCGGGUGUCAUA.......
1067 .......................................................................................AGAAAGGUAUCGGGUGUCAUAG......
 535 .......................................................................................AGAAAGGUAUCGGGUGUCAUAGC.....
   6 ........................................................................................GAAAGGUAUCGGGUGU...........
  10 ........................................................................................GAAAGGUAUCGGGUGUC..........
  10 ........................................................................................GAAAGGUAUCGGGUGUCA.........
   2 ........................................................................................GAAAGGUAUCGGGUGUCAU........
   1 ........................................................................................GAAAGGUAUCGGGUGUCAUA.......
   1 ........................................................................................GAAAGGUAUCGGGUGUCAUAG......
   1 ........................................................................................GAAAGGUAUCGGGUGUCAUAGC.....
   2 .......AAUGACACUGGUUAUCU...........................................................................................
   3 .......AAUGACACUGGUUAUCUU..........................................................................................
```

```
      1 .......AAUGACACUGGUUAUCUUU.........................................................................................
      2 .......AAUGACACUGGUUAUCUUUU........................................................................................
     21 .......AAUGACACUGGUUAUCUUUUC.......................................................................................
     25 .......AAUGACACUGGUUAUCUUUUCC......................................................................................
    146 .......AAUGACACUGGUUAUCUUUUCCA.....................................................................................
     94 .......AAUGACACUGGUUAUCUUUUCCAU....................................................................................
    325 .......AAUGACACUGGUUAUCUUUUCCAUC...................................................................................
#  3883 .......AAUGACACUGGUUAUCUUUUCCAUCG..................................................................................
      6 .........................................................................................AAAGGUAUCGGGUGU...........
      2 .........................................................................................AAAGGUAUCGGGUGUC..........
      1 .........................................................................................AAAGGUAUCGGGUGUCAUA.......
      1 .........................................................................................AAAGGUAUCGGGUGUCAUAG......
      9 .........................................................................................AAAGGUAUCGGGUGUCAUAGC.....
      1 ..........................................................................................AAGGUAUCGGGUGUCAUAGC.....
      1 ..........................................................................................AAGGUAUCGGGUGUCAUAGCC....
      1 ........AUGACACUGGUUAUCUUUUC.......................................................................................
      1 ........AUGACACUGGUUAUCUUUUCCAU....................................................................................
      1 ........AUGACACUGGUUAUCUUUUCCAUC...................................................................................
     11 ........AUGACACUGGUUAUCUUUUCCAUCG..................................................................................

        miR-230
        UUGAUGUAAUGCCGUCACUUGGUCGGCGAUUUAAUAUUAUCAGAACAUAGGAAAUUGUUAGUAUUAGUUGUGCGACCAGGAGACGGUAUUCGCAUAUU
        ...(((((((((((((.((((((((((((((.(((((((.(((...........))).))))))))))))).)))))))).))))))))).))))...
      1 ...............CACUUGGUCGGCGAUUUAAUAUU............................................................
      2 ...............CACUUGGUCGGCGAUUUAAUAUUA...........................................................
      1 ................ACUUGGUCGGCGAUUUA.................................................................
      4 ................ACUUGGUCGGCGAUUUAA................................................................
     22 ................ACUUGGUCGGCGAUUUAAU...............................................................
      3 ................ACUUGGUCGGCGAUUUAAUA..............................................................
     26 ................ACUUGGUCGGCGAUUUAAUAU.............................................................
    301 ................ACUUGGUCGGCGAUUUAAUAUU............................................................
   1841 ................ACUUGGUCGGCGAUUUAAUAUUA...........................................................
    134 ................ACUUGGUCGGCGAUUUAAUAUUAU..........................................................
      2 ................ACUUGGUCGGCGAUUUAAUAUUAUCA........................................................
      1 .................CUUGGUCGGCGAUUUAAU...............................................................
      1 .................CUUGGUCGGCGAUUUAAUAUU............................................................
      3 .................CUUGGUCGGCGAUUUAAUAUUA...........................................................
      1 .................CUUGGUCGGCGAUUUAAUAUUAU..........................................................
      1 .......................................UCAGAACAUAGGAAAUUGUUA......................................
      1 ...........................................................AGUAUUAGUUGUGCGACCAGGAG................
      1 ...........................................................AGUAUUAGUUGUGCGACCAGGAGA...............
      2 ............................................................GUAUUAGUUGUGCGACCA....................
      4 ............................................................GUAUUAGUUGUGCGACCAG...................
      4 ............................................................GUAUUAGUUGUGCGACCAGG..................
    110 ............................................................GUAUUAGUUGUGCGACCAGGA.................
    409 ............................................................GUAUUAGUUGUGCGACCAGGAG................
#  8341 ............................................................GUAUUAGUUGUGCGACCAGGAGA...............
```

```
   4 ............................................................GUAUUAGUUGUGCGACCAGGAGAC..............
   1 .............................................................UAUUAGUUGUGCGACCAGGAG................
 108 .............................................................UAUUAGUUGUGCGACCAGGAGA...............
   3 .............................................................UAUUAGUUGUGCGACCAGGAGAC..............
   2 ..............................................................AUUAGUUGUGCGACCAGGAGA...............

     miR-231
     UAGCACCACAGGUUGUUCUGACUGUUUCAAAAGCUUGUAGUAUCUUAAUAAAUAAACAUAUAAGCUCGUGAUCAACAGGCAGAACAACUCGGUUUUGUG
     ..(((..((.((((((((((.((((((((..((((((((.(((........)))....))))))))..)))..))))).))))))))))..))..))).
   1 .................CUGACUGUUUCAAAAGCUUG..............................................................
  33 .................CUGACUGUUUCAAAAGCUUGU.............................................................
   1 ................UCUGACUGUUUCAAAAGCUUGUA............................................................
  71 .................CUGACUGUUUCAAAAGCUUGUA............................................................
   2 ..................UGACUGUUUCAAAAGCUUGUA............................................................
   2 ............................................................UAAGCUCGUGAUCAACAGG....................
  33 ............................................................UAAGCUCGUGAUCAACAGGCA..................
  25 ............................................................UAAGCUCGUGAUCAACAGGCAG.................
   2 ...........................................................AUAAGCUCGUGAUCAACAGGCAGA................
 220 ............................................................UAAGCUCGUGAUCAACAGGCAGA................
   1 .............................................................AAGCUCGUGAUCAACAGGCAGA................
   1 ...........................................................AUAAGCUCGUGAUCAACAGGCAGAA...............
#    2583 ............................................................UAAGCUCGUGAUCAACAGGCAGAA...............
  13 .............................................................AAGCUCGUGAUCAACAGGCAGAA...............
   1 ..............................................................AGCUCGUGAUCAACAGGCAGAA...............
   1 ................................................................CUCGUGAUCAACAGGCAGAA...............
   1 ............................................................................AGGCAGAACAACUCGGUU.....

     miR-232
     AAGUUCAAUUUUUGGAUCCCUGCAGUUUCGAUGAUUUUAUCCUUAAUUCUGAAGAUGUGAUAAAUGCAUCUUAACUGCGGUGAUCUAGAUCAUGAACA
     ..(((((...((((((((.((((((((..((((..(((((((.....((....)).).))))))..))))..)))))))).))))))))...))))).
   2 ..................CCUGCAGUUUCGAUGAU...............................................................
   2 ..................CCUGCAGUUUCGAUGAUU..............................................................
   5 ..................CCUGCAGUUUCGAUGAUUU.............................................................
   1 ...................CUGCAGUUUCGAUGAUUU.............................................................
   2 .................CCCUGCAGUUUCGAUGAUUUU............................................................
  16 ..................CCUGCAGUUUCGAUGAUUUU............................................................
   1 ...................CUGCAGUUUCGAUGAUUUU............................................................
   1 ...............AUCCCUGCAGUUUCGAUGAUUUUA...........................................................
   4 .................CCCUGCAGUUUCGAUGAUUUUA...........................................................
 165 ..................CCUGCAGUUUCGAUGAUUUUA...........................................................
   3 ...................CUGCAGUUUCGAUGAUUUUA...........................................................
  13 .................CCCUGCAGUUUCGAUGAUUUUAU..........................................................
 353 ..................CCUGCAGUUUCGAUGAUUUUAU..........................................................
   4 ...................CUGCAGUUUCGAUGAUUUUAU..........................................................
   1 ....................UGCAGUUUCGAUGAUUUUAU..........................................................
   1 .................CCCUGCAGUUUCGAUGAUUUUAUC.........................................................
 559 ..................CCUGCAGUUUCGAUGAUUUUAUC.........................................................
```

```
     6 ...................CUGCAGUUUCGAUGAUUUUAUC.........................................................
     2 .....................GCAGUUUCGAUGAUUUUAUC.........................................................
     3 .........................................CUUAAUUCUGAAGAUGUGA......................................
     4 ............................................................UAAAUGCAUCUUAACUGCGG..................
    21 ............................................................UAAAUGCAUCUUAACUGCGGU.................
     1 ...........................................................AUAAAUGCAUCUUAACUGCGGUG................
    34 ............................................................UAAAUGCAUCUUAACUGCGGUG................
     1 ...........................................................AUAAAUGCAUCUUAACUGCGGUGA...............
#  561 ............................................................UAAAUGCAUCUUAACUGCGGUGA...............
    11 .............................................................AAAUGCAUCUUAACUGCGGUGA...............
     1 ..............................................................AAUGCAUCUUAACUGCGGUGA...............
     4 ............................................................UAAAUGCAUCUUAACUGCGGUGAU..............
     1 .............................................................AAAUGCAUCUUAACUGCGGUGAU..............

       miR-233
       AUAUAGCAUCUUUCUGUCUCGCCCAUCCCGUUGCUCCAAUAUUCUAACAACAAGUGAUUAUUGAGCAAUGCGCAUGUGCGGGAUAGACUGAUGGCUGC
       ...(((((((..((((((((((.(((.(((((((((.((((.((...........)).))))))))))))).).))).))))))))))..))).)))).
     1 AUAUAGCAUCUUUCUGUC................................................................................
     1 ..................UCGCCCAUCCCGUUGCUCCAAU..........................................................
     1 ..................UCGCCCAUCCCGUUGCUCCAAUA.........................................................
     3 .........................................UUCUAACAACAAGUGAUUA......................................
     1 ............................................................UUGAGCAAUGCGCAUGUG....................
    16 ............................................................UUGAGCAAUGCGCAUGUGC...................
     2 ...........................................................AUUGAGCAAUGCGCAUGUGCG..................
   108 ............................................................UUGAGCAAUGCGCAUGUGCG..................
     1 ..............................................................GAGCAAUGCGCAUGUGCG..................
     8 ...........................................................AUUGAGCAAUGCGCAUGUGCGG.................
#  636 ............................................................UUGAGCAAUGCGCAUGUGCGG.................
     4 ..............................................................GAGCAAUGCGCAUGUGCGG.................
     6 ...........................................................AUUGAGCAAUGCGCAUGUGCGGG................
   175 ............................................................UUGAGCAAUGCGCAUGUGCGGG................
     2 ..............................................................GAGCAAUGCGCAUGUGCGGG................
     2 ...........................................................AUUGAGCAAUGCGCAUGUGCGGGA...............
  1040 ............................................................UUGAGCAAUGCGCAUGUGCGGGA...............
     2 .............................................................UGAGCAAUGCGCAUGUGCGGGA...............
     4 ..............................................................GAGCAAUGCGCAUGUGCGGGA...............
    24 ............................................................UUGAGCAAUGCGCAUGUGCGGGAU..............

       miR-234
       CAAAAAAUGAUCAAACGGUAUUCCAGAGUUGAUAAUAAAAAUGCGUCAGUCCGCAAUCUAUUAUUGCUCGAGAAUACCCUUUGACUACUAUGUGUA
       ..........(((((.(((((((..((((.(((((((....((((......))))...)))))))))))..))))))).)))))............
     3 ...............CGGUAUUCCAGAGUUGAUAAUA...........................................................
     2 ............................................................UUAUUGCUCGAGAAUAC...................
     1 ............................................................UUAUUGCUCGAGAAUAC...................
     1 ............................................................UUAUUGCUCGAGAAUACCC.................
     4 ............................................................UUAUUGCUCGAGAAUACCCU................
#  134 ............................................................UUAUUGCUCGAGAAUACCCUU...............
```

```
   1 ..............................................................AUUGCUCGAGAAUACCCUU...............
   4 ............................................................UUAUUGCUCGAGAAUACCCUUU..............

     miR-235
     UCCGAAGAUAUCAGGAUCAGGCCUUGGCUGAUUGCAAAAUUGUUCACCGUGAAAAUUAAAUAUUGCACUCUCCCCGGCCUGAUCUGAGAGUAAGGCG
     .((.......((((.((((((((..((..((.(((((.(((.(((.....))).....))).))))).))..)).))))))))))))......))..
   1 ..................AGGCCUUGGCUGAUUGCAAA...........................................................
   2 ..................AGGCCUUGGCUGAUUGCAAAA..........................................................
   4 ..................AGGCCUUGGCUGAUUGCAAAAU.........................................................
  51 ..................AGGCCUUGGCUGAUUGCAAAAUU........................................................
   1 .........................................GUUCACCGUGAAAAUUAAA.....................................
   6 ............................................................UAUUGCACUCUCCCCGGC...................
  14 ............................................................UAUUGCACUCUCCCCGGCC..................
  11 ............................................................UAUUGCACUCUCCCCGGCCU.................
  33 ............................................................UAUUGCACUCUCCCCGGCCUG................
   7 ..........................................................AAUAUUGCACUCUCCCCGGCCUGA...............
  11 ...........................................................AUAUUGCACUCUCCCCGGCCUGA...............
# 4762 ............................................................UAUUGCACUCUCCCCGGCCUGA...............
  12 .............................................................AUUGCACUCUCCCCGGCCUGA...............
  11 ............................................................UAUUGCACUCUCCCCGGCCUGAU..............
   4 .............................................................AUUGCACUCUCCCCGGCCUGAU..............

     miR-236
     UCGGUGACCGAUGUCCAGCGUCUUACCUGUUCAAUAUUUAGACUGACUAUCAAAGAGAUCUAAUACUGUCAGGUAAUGACGCUGGAUUGUCAUGUCAU
     ...(((((.((((((((((((((((((((..((.((((.(((((...........)).))))))).)).))))))).))))))))).))))..)))))
   1 ...........UGUCCAGCGUCUUACCUG.....................................................................
  10 ..................CGUCUUACCUGUUCAAUAUUUAGA........................................................
   2 ............................................................UAAUACUGUCAGGUAAU.....................
  16 ............................................................UAAUACUGUCAGGUAAUGA...................
  42 ............................................................UAAUACUGUCAGGUAAUGAC..................
 139 ............................................................UAAUACUGUCAGGUAAUGACG.................
   1 ...........................................................CUAAUACUGUCAGGUAAUGACGC................
 447 ............................................................UAAUACUGUCAGGUAAUGACGC................
   1 .............................................................AAUACUGUCAGGUAAUGACGC................
   1 ..............................................................AUACUGUCAGGUAAUGACGC................
   2 ...........................................................CUAAUACUGUCAGGUAAUGACGCU...............
# 7806 ............................................................UAAUACUGUCAGGUAAUGACGCU...............
  31 .............................................................AAUACUGUCAGGUAAUGACGCU...............
   5 ..............................................................AUACUGUCAGGUAAUGACGCU...............
   1 ...............................................................UACUGUCAGGUAAUGACGCU...............
   1 ................................................................CUGUCAGGUAAUGACGCU...............
   9 ............................................................UAAUACUGUCAGGUAAUGACGCUG..............

     miR-237
     UUCUACAUUGCGUGGUCCCUGAGAAUUCUCGAACAGCUUCAAAGUGUUCAAGCUGUCGAGUUUUGUCAAGGACCAAACAAUAGAAGAUCACUUGGGAAC
     (((((..(((..((((((.(((.((..(((((.((((((.((....)).)))))))))))..)).))).))))))..))))))))..............
   4 ...............UCCCUGAGAAUUCUCGAAC.................................................................
```

```
        1 ...............UCCCUGAGAAUUCUCGAACA................................................................
       23 ...............UCCCUGAGAAUUCUCGAACAG...............................................................
      259 ...............UCCCUGAGAAUUCUCGAACAGC..............................................................
#    2558 ...............UCCCUGAGAAUUCUCGAACAGCU.............................................................
      317 ...............UCCCUGAGAAUUCUCGAACAGCUU............................................................
        1 ................CCCUGAGAAUUCUCGAACAGC..............................................................
        1 ................CCCUGAGAAUUCUCGAACAGCU.............................................................
        1 ................CCCUGAGAAUUCUCGAACAGCUU............................................................
        4 .................CCUGAGAAUUCUCGAACAGC..............................................................
       28 .................CCUGAGAAUUCUCGAACAGCU.............................................................
        1 .................CCUGAGAAUUCUCGAACAGCUU............................................................
        1 ..................CUGAGAAUUCUCGAACAGC..............................................................
        4 ..................CUGAGAAUUCUCGAACAGCU.............................................................
        1 ...................UGAGAAUUCUCGAACAGC..............................................................
       11 ...................UGAGAAUUCUCGAACAGCU.............................................................
        1 ...................UGAGAAUUCUCGAACAGCUU............................................................
        2 ...................................................GCUGUCGAGUUUUGUCAAGGAC..........................
        1 ...................................................GCUGUCGAGUUUUGUCAAGGACC.........................
        4 ....................................................CUGUCGAGUUUUGUCAAGGAC..........................
       19 ....................................................CUGUCGAGUUUUGUCAAGGACC.........................
        1 .....................................................UGUCGAGUUUUGUCAAGGACC.........................

          miR-238
          UCUCCAUUGACUGUUUGGAUGUUCUCGGACGUUCAAAGCUACAUCCAACAAAUUGGUAGCUUUGUACUCCGAUGCCAUUCAGAUAGUUAUGAGCCAUG
          .(((...((((((((((((((...(((((.((.((((((((...((((....))))))))))))).)))))))...))))))))))))))).))).....
        1 ...............UGGAUGUUCUCGGACGU..................................................................
        5 ...............UGGAUGUUCUCGGACGUU.................................................................
        4 ...............UGGAUGUUCUCGGACGUUC................................................................
        6 ...............UGGAUGUUCUCGGACGUUCA...............................................................
       10 ...............UGGAUGUUCUCGGACGUUCAA..............................................................
        1 .................GAUGUUCUCGGACGUUCAA..............................................................
       26 ...............UGGAUGUUCUCGGACGUUCAAA.............................................................
       51 ...............UGGAUGUUCUCGGACGUUCAAAG............................................................
       31 ...............UGGAUGUUCUCGGACGUUCAAAGC...........................................................
        2 ...............UGGAUGUUCUCGGACGUUCAAAGCU..........................................................
        1 ..................AUGUUCUCGGACGUUCAAAGCUA.........................................................
        1 .......................................UACAUCCAACAAAUUGGUAGCU.....................................
       34 ............................................................UUUGUACUCCGAUGCCAU....................
        1 ...........................................................CUUUGUACUCCGAUGCCAUU...................
        1 ............................................................UUUGUACUCCGAUGCCAUU...................
        1 ............................................................UUUGUACUCCGAUGCCAUUC..................
        3 ...........................................................CUUUGUACUCCGAUGCCAUUCA.................
        7 ............................................................UUUGUACUCCGAUGCCAUUCA.................
        3 ...........................................................CUUUGUACUCCGAUGCCAUUCAG................
      138 ............................................................UUUGUACUCCGAUGCCAUUCAG................
        4 .............................................................UUGUACUCCGAUGCCAUUCAG................
       40 ...........................................................CUUUGUACUCCGAUGCCAUUCAGA...............
```

```
#  11435 ............................................................UUUGUACUCCGAUGCCAUUCAGA...............
     300 .............................................................UUGUACUCCGAUGCCAUUCAGA...............
      29 ..............................................................UGUACUCCGAUGCCAUUCAGA...............
       3 ...............................................................GUACUCCGAUGCCAUUCAGA...............
      47 ............................................................UUUGUACUCCGAUGCCAUUCAGAU..............
       9 .............................................................UUGUACUCCGAUGCCAUUCAGAU..............
       3 ..............................................................UGUACUCCGAUGCCAUUCAGAU..............
       2 ............................................................UUUGUACUCCGAUGCCAUUCAGAUA.............

         miR-239a
         UACACGUUUGCAAUUUUUGUACUACACAUAGGUACUGGACAAUUUUCAAAAUAUAUCCAGUGUCUAGUCUAGUGCAAACAUUGCUCGGUGUUGUUUGA
         .((((....(((((.(((((((((.((.(((..((((((................))))))..))))).))))))))).)))))...)))).......
       1 ...............UUUGUACUACACAUAGGU.................................................................
       6 ...............UUUGUACUACACAUAGGUAC...............................................................
       7 ...............UUUGUACUACACAUAGGUACU..............................................................
     460 ...............UUUGUACUACACAUAGGUACUG.............................................................
#    703 ...............UUUGUACUACACAUAGGUACUGG............................................................
      42 ...............UUUGUACUACACAUAGGUACUGGA...........................................................
       2 ................UUGUACUACACAUAGGUACUG.............................................................
       3 ................UUGUACUACACAUAGGUACUGG............................................................
       1 ................UUGUACUACACAUAGGUACUGGA...........................................................
       1 .................UGUACUACACAUAGGUACUG.............................................................
       1 ..................GUACUACACAUAGGUACUG.............................................................
       1 ....................ACUACACAUAGGUACUGGA...........................................................
       1 .......................................................UCCAGUGUCUAGUCUAGUGCAA.....................
       1 .........................................................CAGUGUCUAGUCUAGUGCA......................
       1 .........................................................CAGUGUCUAGUCUAGUGCAA.....................
       1 .........................................................CAGUGUCUAGUCUAGUGCAAA....................
       5 .........................................................CAGUGUCUAGUCUAGUGCAAAC...................
       3 .........................................................CAGUGUCUAGUCUAGUGCAAACA..................
      48 ..........................................................AGUGUCUAGUCUAGUGCAAACA..................
       1 ..........................................................AGUGUCUAGUCUAGUGCAAACAU.................

         miR-239b
         GCGACAGAUGCAAUUUUUGUACUACACAAAAGUACUGGUCAUUUAAGUUGAGGCUCAGCACUUUUGUGGUGUGCAAAAAUGGCAAGUUGCUUUUAUCU
         (((((...(((.((((((((((..(((((((((.((((((.((......))))).))).)))))))))..)))))))))).))).)))))........
       1 ...............UUUGUACUACACAAAAGUA................................................................
      11 ...............UUUGUACUACACAAAAGUAC...............................................................
       5 ...............UUUGUACUACACAAAAGUACU..............................................................
#   1157 ...............UUUGUACUACACAAAAGUACUG.............................................................
       1 ................UUGUACUACACAAAAGUACUG.............................................................
      26 .....................................GUCAUUUAAGUUGAGGCUCA.........................................
       1 .......................................................CAGCACUUUUGUGGUGUGCAAA.....................
       1 .........................................................GCACUUUUGUGGUGUGCAAAA....................
       3 .........................................................GCACUUUUGUGGUGUGCAAAAA...................

         miR-240
```

```
          UUGUUAGAAACUUUUUCAAAUCGAGGAUUUUGAGACUAGAAUGCUUGAAUUGUACUAGCAUACUGGCCCCCAAAUCUUCGCUUAGAAAUACAGUUCA
          ......(((....((((.((.(((((((((.(.(.((((.(((((...........))))).)))).).).))))))))).)).)))).....))).
        2 .....................CGAGGAUUUUGAGACUAGA.........................................................
        8 .....................CGAGGAUUUUGAGACUAGAAU.......................................................
       16 .....................CGAGGAUUUUGAGACUAGAAUG......................................................
      112 .....................CGAGGAUUUUGAGACUAGAAUGC.....................................................
       15 .....................CGAGGAUUUUGAGACUAGAAUGCU....................................................
        3 ..........................................................CAUACUGGCCCCCAAAUCUUCGCU...............
        2 ...........................................................AUACUGGCCCCCAAAUCUUCGC................
      223 ...........................................................AUACUGGCCCCCAAAUCUUCGCU...............
        3 ...........................................................AUACUGGCCCCCAAAUCUUCGCUU..............
#       9 ............................................................UACUGGCCCCCAAAUCUUCGCU...............

          miR-241
          CGGGGGUGUCAAAGUUGAGGUAGGUGCGAGAAAUGACGGCAUCCAUAUAGUAAUCGUUCAUUGUCUCUCAGCUGCUUCAUCUGUGACAUGGCUACG
          .((..((((((.((.((((((((.((.((((((((((((...(......)...))).))))).)))).)).)))))))).)).))))))..))...
        5 ...............UGAGGUAGGUGCGAGAA................................................................
       17 ...............UGAGGUAGGUGCGAGAAA...............................................................
       26 ...............UGAGGUAGGUGCGAGAAAU..............................................................
      112 ...............UGAGGUAGGUGCGAGAAAUG.............................................................
        1 ................GAGGUAGGUGCGAGAAAUG.............................................................
#    7764 ...............UGAGGUAGGUGCGAGAAAUGA............................................................
       10 ................GAGGUAGGUGCGAGAAAUGA............................................................
        1 .................AGGUAGGUGCGAGAAAUGA............................................................
        2 ...............UGAGGUAGGUGCGAGAAAUGAC...........................................................
        6 ...............UGAGGUAGGUGCGAGAAAUGACG..........................................................
        5 ....................................CGGCAUCCAUAUAGUAAUCGUUC.....................................
        1 ...........................................................AUUGUCUCUCAGCUGCUUC..................
        2 ...........................................................AUUGUCUCUCAGCUGCUUCA.................
        8 ...........................................................AUUGUCUCUCAGCUGCUUCAU................
        1 ..........................................................CAUUGUCUCUCAGCUGCUUCAUC...............
      107 ...........................................................AUUGUCUCUCAGCUGCUUCAUC...............
        1 ............................................................UUGUCUCUCAGCUGCUUCAUC...............
        2 .............................................................UGUCUCUCAGCUGCUUCAUC...............
        1 ..............................................................GUCUCUCAGCUGCUUCAUC...............
        6 ...........................................................AUUGUCUCUCAGCUGCUUCAUCU..............

          miR-242
          UGGCUCGCGAGAGUAUUGCGUAGGCCUUUGCUUCGAGAGAGAAGCUUCAGUUCGCAGCAAUAUCCUCCGCAAAACUUUCCCCGGCUAUGCAAAAAA
          (((((.(.((((((.(((((.(((...(((((.((((.(((....)))..)))).)))))...))).))))).)))))).).)))))).........
        3 ..............AUUGCGUAGGCCUUUGCUUCGA............................................................
        1 ...............UUGCGUAGGCCUUUGCU................................................................
#     251 ...............UUGCGUAGGCCUUUGCUUCGA............................................................
       16 ...............UUGCGUAGGCCUUUGCUUCGAGA..........................................................
        1 .................GCGUAGGCCUUUGCUUCGA............................................................
        1 ....................................GAGAGAAGCUUCAGUUC...........................................
        1 ...................................................UCGCAGCAAUAUCCUCCGCAAAA......................
```

```
         miR-243
         UGUUCACGCAUAAGCCUGAUAUCUCGGUGCGAUCGUACCGUAUCGCUCACACUUAGAUUACGGUACGAUCGCGGCGGGAUAUCAGGUACGUGAUUGGA
         ...(((((.....(((((((((((((.(((((((((((((((((...........)).))))))))))))))).))))))))))))).))))).....
      1  ...................UAUCUCGGUGCGAUCGU..............................................................
      1  ...................UAUCUCGGUGCGAUCGUAC............................................................
      1  ...................UAUCUCGGUGCGAUCGUACC...........................................................
      7  ...................UAUCUCGGUGCGAUCGUACCGUA........................................................
      2  ...........................................................ACGGUACGAUCGCGGCGGGAUAUC...............
      1  ............................................................CGGUACGAUCGCGGCGGG....................
      6  ............................................................CGGUACGAUCGCGGCGGGA...................
     12  ............................................................CGGUACGAUCGCGGCGGGAU..................
    110  ............................................................CGGUACGAUCGCGGCGGGAUA.................
    213  ............................................................CGGUACGAUCGCGGCGGGAUAU................
#  1280  ............................................................CGGUACGAUCGCGGCGGGAUAUC...............
     24  ............................................................CGGUACGAUCGCGGCGGGAUAUCA..............
      4  .............................................................GGUACGAUCGCGGCGGGAUAU................
     31  .............................................................GGUACGAUCGCGGCGGGAUAUC...............
      2  .............................................................GGUACGAUCGCGGCGGGAUAUCA..............
      1  ..............................................................GUACGAUCGCGGCGGGAUA.................
      7  ..............................................................GUACGAUCGCGGCGGGAUAU................
     60  ..............................................................GUACGAUCGCGGCGGGAUAUC...............

         miR-244
         CUCCAUAUCUCAAUCUCUUUGGUUGUACAAAGUGGUAUGGCUCAUCGAAUAAGCACAUACUGCUUUUCAGCUAAAGGAAUUGAGAUUUUGUAGGCUUUU
         ((.((.((((((((..(((((((((...((((..((((((((.........))).)))))..)))).)))))))))..))))))))..)).))......
      2  ...............UCUUUGGUUGUACAAAG...................................................................
      1  ...............UCUUUGGUUGUACAAAGUG.................................................................
     10  ...............UCUUUGGUUGUACAAAGUGG................................................................
      5  ...............UCUUUGGUUGUACAAAGUGGU...............................................................
      8  ...............UCUUUGGUUGUACAAAGUGGUA..............................................................
     95  ...............UCUUUGGUUGUACAAAGUGGUAU.............................................................
      1  .............UCUCUUUGGUUGUACAAAGUGGUAUG............................................................
      1  ..............CUCUUUGGUUGUACAAAGUGGUAUG............................................................
#   679  ...............UCUUUGGUUGUACAAAGUGGUAUG............................................................
      4  ................CUUUGGUUGUACAAAGUGGUAUG............................................................
     29  .................UUUGGUUGUACAAAGUGGUAUG............................................................
      1  ..................UUGGUUGUACAAAGUGGUAUG............................................................
      1  ...............UCUUUGGUUGUACAAAGUGGUAUGG...........................................................
      1  ........................................................AUACUGCUUUUCAGCUAAAGGA.....................
      8  .........................................................UACUGCUUUUCAGCUAAAGGAA....................

         miR-245
         CUGAAUUCAAUGUUGGAGAGCUAUUUGCAAGGUACCUAAUUGUUUGAUUAUUGAUUCUCAAUUGGUCCCCUCCAAGUAGCUCUAUUGCAUUGUUUGC
         .......((((((...(((((((((((...((.(((.(((((...((........)).)))))))).))...)))))))))))...)))))).....
      3  ...................GCUAUUUGCAAGGUACCUAAUUG.......................................................
      1  ............................................................AUUGGUCCCCUCCAAGUA...................
```

```
         4 ............................................................AUUGGUCCCCUCCAAGUAGC.................
        17 ............................................................AUUGGUCCCCUCCAAGUAGCU................
#      266 ............................................................AUUGGUCCCCUCCAAGUAGCUC...............
        17 ............................................................AUUGGUCCCCUCCAAGUAGCUCU..............
         2 .............................................................UUGGUCCCCUCCAAGUAGCUCU..............
         1 ..............................................................UGGUCCCCUCCAAGUAGCUC...............

           miR-246
           AUCUGAAUUAUAAAACAUCGCCUAACCGUUGUCAUGUAAUAUUUCCCAGAGAAAAUGAUAUUACAUGUUUCGGGUAGGAGCUGUUCAAACUUUGGAC
           .((..((......((((.(.((((.(((....((((((((((...............))))))))))...))).)))).).)))).....))..)).
         2 ............................................................UUACAUGUUUCGGGUAG....................
         1 ............................................................UUACAUGUUUCGGGUAGG...................
        79 ............................................................UUACAUGUUUCGGGUAGGA..................
       240 ............................................................UUACAUGUUUCGGGUAGGAG.................
#      155 ............................................................UUACAUGUUUCGGGUAGGAGC................
       302 ............................................................UUACAUGUUUCGGGUAGGAGCU...............
        13 .............................................................UACAUGUUUCGGGUAGGAG.................
         6 .............................................................UACAUGUUUCGGGUAGGAGC................
        28 .............................................................UACAUGUUUCGGGUAGGAGCU...............

           miR-247
           AUUACCAGCUAUUUUCCAAGUAGAGAAAAGUUUCUAAUUACCCAUCAUGCACAAAUGUGGUGACUAGAGCCUAUUCUCUUCUUGGAAAAGUGGCGACU
           .......(((((((((((((.((((((.((.(((((.(((((...(((......))).))))).))))).)).)))))).))))).))))))))....
         1 ............................................................UGACUAGAGCCUAUUCUCUUC.................
#       20 ............................................................UGACUAGAGCCUAUUCUCUUCU................

           miR-248
           UUUCCCGGCUGCAACUACGGUAAGCGGUAUCCAGCCGAUGUUUUCAAUACUGCAUUUGAAUACACGUGCACGGAUAACGCUCAUUGUUUUUCGCAUGC
           .......(((((((..(((((.((((.(((((.((((.(((.(((((........))))).))))).))..))))).)))).)))))..)).))).))
         1 ...........................................................AUACACGUGCACGGAUAAC....................
       115 ...........................................................AUACACGUGCACGGAUAACGC..................
         3 ............................................................UACACGUGCACGGAUAACGC..................
        41 ...........................................................AUACACGUGCACGGAUAACGCU.................
        11 ............................................................UACACGUGCACGGAUAACGCU.................
         1 ..........................................................AAUACACGUGCACGGAUAACGCUC................
       366 ...........................................................AUACACGUGCACGGAUAACGCUC................
        11 ............................................................UACACGUGCACGGAUAACGCUC................
         1 ..........................................................AAUACACGUGCACGGAUAACGCUCA...............
#     7377 ...........................................................AUACACGUGCACGGAUAACGCUCA...............
       133 ............................................................UACACGUGCACGGAUAACGCUCA...............
         1 .............................................................ACACGUGCACGGAUAACGCUCA...............
         2 ...............................................................ACGUGCACGGAUAACGCUCA...............
       120 ...........................................................AUACACGUGCACGGAUAACGCUCAU..............
         2 ............................................................UACACGUGCACGGAUAACGCUCAU..............
         4 ...........................................................AUACACGUGCACGGAUAACGCUCAUU.............

           miR-249
```

```
         AUACUCUUGAACGACUAGCAACGCACAAACGUCUUCUGUGCGACAACAUCUGAAUGUUUGUCACAGGACUUUUGAGCGUUGCCAGUCGAAAGAGGAA
         ...(((((...(((((.(((((((.((((.(((..((((((((..((((....))))))).)))))))).)))).))))))).))))).)))))...
       1 ................AGCAACGCACAAACGUCUUCUGUG.........................................................
       1 ..................CAACGCACAAACGUCUUCUGUGCGAC.....................................................
       1 ...................AACGCACAAACGUCUUCUGUGCGAC.....................................................
       2 ............................................................UCACAGGACUUUUGAGCGUUG................
      22 ............................................................UCACAGGACUUUUGAGCGUUGC...............
       2 ..............................................................ACAGGACUUUUGAGCGUUGC...............
#     29 ............................................................UCACAGGACUUUUGAGCGUUGCC..............
       1 ..............................................................ACAGGACUUUUGAGCGUUGCC..............

         miR-250
         AGGGUCUUCGGACCACGCCUUCAGUUGCCUCGUGAUCCGCCAAACACAAUAAAUGGACGAAUCACAGUCAACUGUUGGCAUGGUGCUCGUACCCAUUUU
         .((((....(.((((.(((..((((((.((.(((((.(((((...........))).)).))))))).))))))..))).)))).)....)))).....
       1 ...........ACCACGCCUUCAGUUGCCUCG...................................................................
       1 .................CCUUCAGUUGCCUCGUGA................................................................
       2 .................CCUUCAGUUGCCUCGUGAUCC.............................................................
      21 .................CCUUCAGUUGCCUCGUGAUCCG............................................................
       1 ..................CUUCAGUUGCCUCGUGAUCCG............................................................
       1 ................................UGAUCCGCCAAACACAAUAAAU.............................................
      15 .......................................CCAAACACAAUAAAUGGACG........................................
       1 ........................................CAAACACAAUAAAUGGACG........................................
      31 ...........................................................AAUCACAGUCAACUGUUG......................
      74 ...........................................................AAUCACAGUCAACUGUUGG.....................
       1 .........................................................CGAAUCACAGUCAACUGUUGGC....................
     592 ...........................................................AAUCACAGUCAACUGUUGGC....................
       2 ............................................................AUCACAGUCAACUGUUGGC....................
       2 ..........................................................GAAUCACAGUCAACUGUUGGCA...................
#  49337 ...........................................................AAUCACAGUCAACUGUUGGCA...................
      16 ............................................................AUCACAGUCAACUGUUGGCA...................
       1 ..............................................................CACAGUCAACUGUUGGCA...................
     159 ...............................................................ACAGUCAACUGUUGGCA...................
   11829 ...........................................................AAUCACAGUCAACUGUUGGCAU..................
       6 ............................................................AUCACAGUCAACUGUUGGCAU..................
       1 ..............................................................CACAGUCAACUGUUGGCAU..................
      34 ...............................................................ACAGUCAACUGUUGGCAU..................
       2 ................................................................CAGUCAACUGUUGGCAU..................
      61 ...........................................................AAUCACAGUCAACUGUUGGCAUG.................
       1 .............................................................UCACAGUCAACUGUUGGCAUGGU...............

         miR-251
         ACUUUCAAUGACCCCUUGUUAAGUAGUGGUGCCGCUCUUAUUAGGUUGAAAAGUAAUAAGAGUAGUUCACUACUUAUCAAGGUGAAAUUUGAAAUUU
         ..((((((....((((((.((((((((((.((..(((((((((..((....)))))))))))..))))))))))))).))))).)....))))))...
       1 ..................UUAAGUAGUGGUGCCGCUCU...........................................................
       3 ..................UUAAGUAGUGGUGCCGCUCUU..........................................................
     138 ..................UUAAGUAGUGGUGCCGCUCUUA.........................................................
      21 ..................UUAAGUAGUGGUGCCGCUCUUAU........................................................
```

```
#      45 ..................UUAAGUAGUGGUGCCGCUCUUAUU.......................................................
        2 ....................AAGUAGUGGUGCCGCUCUUAU........................................................

          miR-252
          UCCAGGGGUCUCUUUCAGUUAUAAGUAGUAGUGCCGCAGGUAACCGCCAGUCUAAAAGGCUUACCUACUGCCUUCUGCUUAAAAUUGAAAUAUUGAUG
          .......(((..((((((((.(((((((.((.((.(.((((((..(((.........))))))))).).)))).))))))).))))))))....))).
        1 ....................AUAAGUAGUAGUGCCGC.............................................................
       11 ....................AUAAGUAGUAGUGCCGCA............................................................
        8 ....................AUAAGUAGUAGUGCCGCAG...........................................................
       96 ....................AUAAGUAGUAGUGCCGCAGG..........................................................
        1 .....................UAAGUAGUAGUGCCGCAGG..........................................................
        1 ...................UAUAAGUAGUAGUGCCGCAGGU.........................................................
       61 ....................AUAAGUAGUAGUGCCGCAGGU.........................................................
        1 ...................UAUAAGUAGUAGUGCCGCAGGUA........................................................
     1692 ....................AUAAGUAGUAGUGCCGCAGGUA........................................................
        1 ...................UAUAAGUAGUAGUGCCGCAGGUAA.......................................................
#   31796 ....................AUAAGUAGUAGUGCCGCAGGUAA.......................................................
        4 .....................UAAGUAGUAGUGCCGCAGGUAA.......................................................
        1 ......................AAGUAGUAGUGCCGCAGGUAA.......................................................
        8 .......................AGUAGUAGUGCCGCAGGUAA.......................................................
        1 ..........................AGUAGUGCCGCAGGUAA.......................................................
       16 ....................AUAAGUAGUAGUGCCGCAGGUAAC......................................................
        1 ......................AAGUAGUAGUGCCGCAGGUAAC......................................................
        3 ....................AUAAGUAGUAGUGCCGCAGGUAACC.....................................................
        1 ....................AUAAGUAGUAGUGCCGCAGGUAACCG....................................................
        2 ...........................................................CUUACCUACUGCCUUCUGCUUAA................
        2 ..............................................................ACCUACUGCCUUCUGCUUAAA...............
        5 ..............................................................ACCUACUGCCUUCUGCUUAAAA..............

          miR-253
          GAAUUGUUCCGAUCGCCGCUCUUUUCACACACCUCACUAACACUGACCACCGAUGCAUAGAUGUUAGUAGGCGUUGUGGGAAGGGCGGCGACAGUGAACGUCGUUUC
          ((...(((((..(((((((((((..(((((.(((.((((((((((..((....))..))).)))))))))).).))))..))))))))))).).)))).)).....
        7 ....................CUUUUCACACACCUCACUAACA.................................................................
        1 ..........................................CUGACCACCGAUGCAUAGAUG............................................
        6 ...............................................................UUAGUAGGCGUUGUGGG...........................
       17 ...............................................................UUAGUAGGCGUUGUGGGA..........................
      188 ...............................................................UUAGUAGGCGUUGUGGGAA.........................
        1 ..............................................................GUUAGUAGGCGUUGUGGGAAG........................
      246 ...............................................................UUAGUAGGCGUUGUGGGAAG........................
#     431 ...............................................................UUAGUAGGCGUUGUGGGAAGG.......................
        2 ................................................................UAGUAGGCGUUGUGGGAAGG.......................
      548 ...............................................................UUAGUAGGCGUUGUGGGAAGGG......................

          miR-254
          ACUAUGCAUAUUGCCGCCUACAGAAGCAUAGAUUUCCACAAACCAUUCGAGGUGUUUGUGCAAAUCUUUCGCGACUGUAGGCGGCGAACAUGCAGUUUUU
          ....(((((.(((((((((((((..((..((((((.((((((((.......).)))))))).))))))...))..)))))))))))))..)))))......
       14 ..................UACAGAAGCAUAGAUUUCCACA............................................................
```

```
       1 .......................................................UUGUGCAAAUCUUUCGCGACUGU......................
       3 ..........................................................UGCAAAUCUUUCGCGACUGUA.....................
       1 .........................................................GUGCAAAUCUUUCGCGACUGUAG....................
      45 ..........................................................UGCAAAUCUUUCGCGACUGUAG....................
       2 ........................................................UGUGCAAAUCUUUCGCGACUGUAGG...................
#    410 ..........................................................UGCAAAUCUUUCGCGACUGUAGG...................
      10 ...........................................................GCAAAUCUUUCGCGACUGUAGG...................

         miR-255
         CAGUGGUUCGACUAAAUUUUGGAGGUAAGAAAUCUUUGUAGUUCUCCGUAUUGUGACGUGAAAACUGAAGAGAUUUUUUACAGACUUCACAAAUUUGAAAUAUAUUCCUG
         (((..((.....(((((((((((((((((((((((((.(((((.(((((......))).)).))))).)))))))))))))...))))).)))))))......))..)))
       8 ........................GUAAGAAAUCUUUGUAGUUCUC................................................................
       1 .........................UAAGAAAUCUUUGUAGUUCUC................................................................
       1 ............................................................AAAACUGAAGAGAUUUUUUACA............................
       5 .............................................................AAACUGAAGAGAUUUUUU...............................
       6 .............................................................AAACUGAAGAGAUUUUUUA..............................
      15 .............................................................AAACUGAAGAGAUUUUUUAC.............................
     771 .............................................................AAACUGAAGAGAUUUUUUACA............................
#   3804 .............................................................AAACUGAAGAGAUUUUUUACAG...........................
      94 .............................................................AAACUGAAGAGAUUUUUUACAGA..........................
       5 ..............................................................AACUGAAGAGAUUUUUUACAG...........................
       1 ...............................................................ACUGAAGAGAUUUUUUACAG...........................

         miR-256
         GCAAGGAGUGCAGAGGCUUACUAUGAGACGGUCAAGAGGCGCCUCCGUCACGUGGAUACGUCGGGUGGAAUGCAUAGAAGACUGUACUCAAAAAAUUUUAC
         .....((((((((...(((.(((((......(((...((((..((((.....))))..))))...)))....)))))))).))))))))............
         Note: Annotated mature miR-256 UGGAAUGCAUAGAAGACUGUA was not detectable.

         miR-257
         AUAAUAUUUCCCGCUGAGUAUCAGGAGUACCCAGUGAUCGCCUUCACAUAUUGGGAAGAAGUAUGUGAUUGCGUCCUGCAGUUCUUCCAUGAUGUACUCAGG
         .............((((((((((((((.((.(((.((.(((..(((((((((.......)))))))))..))))))))..))..)))).)))..))))))).
       2 ...............................................AUAUUGGGAAGAAGUAUGUGAU.................................
       1 .....................................................................UGCGUCCUGCAGUUCUUCCAUGAUGU.......
         Note: Annotated mature miR-257 GAGUAUCAGGAGUACCCAGUGA was not detectable.

         miR-258
         GCAAUGGUUUUGAGAGGAAUCCUUUUACAUAUUUGUUGAAGUUUUCGCUCGAAUUUGUGGUCGAAUACUGUAGAAGGAAGCCUGCACGAAGUUUUG
         .......(((((.((((..((((((((((((((((.....(..(((....)))..).....)))))).))))))))))..))).).))))).....
       1 .....................................GAAGUUUUCGCUCGAAUUUGU......................................
       1 ..................................................GAAUUUGUGGUCGAAUACUGUA........................
       1 .....................................................UUUGUGGUCGAAUACUGUA........................
       2 ......................................................UUGUGGUCGAAUACUGU.........................
       2 ......................................................UUGUGGUCGAAUACUGUA........................
       1 ......................................................UUGUGGUCGAAUACUGUAG.......................
       1 ........................................................GUGGUCGAAUACUGUAGAAGGA..................
       1 ..............................................................GAAUACUGUAGAAGGAAGCCUGC...........
       1 .........................................................................AAGGAAGCCUGCACGAAGU....
```

Note: Annotated mature miR-258 GGUUUUGAGAGGAAUCCUUUU was not detectable.

```
         miR-259
         UCAGUCAUUGAAACGAGUAAAUCUCAUCCUAAUCUGGUAGCAUCUUUCGAACCUUGCCACCGAUUUGGCAUGGGAUUGACUUGUUAAAUAGUGACUUUUUU
         ..((((((((.(((((((.((((((((((.((((.(((.(((............))).))))))).)).)))))))).)))))))...)))))))).....
       5 ..................AAAUCUCAUCCUAAUCUGGUAG.............................................................
       5 ..................AAAUCUCAUCCUAAUCUGGUAGC............................................................
       2 .................UAAAUCUCAUCCUAAUCUGGUAGCA...........................................................
#    177 ..................AAAUCUCAUCCUAAUCUGGUAGCA...........................................................
       2 ......................CUCAUCCUAAUCUGGUAGCA...........................................................
       4 ..................AAAUCUCAUCCUAAUCUGGUAGCAU..........................................................
      18 ........................................................CCACCGAUUUGGCAUGGGAUUGA......................
       5 ........................................................CCACCGAUUUGGCAUGGGAUUGAC.....................
       3 .........................................................CACCGAUUUGGCAUGGGAUUGAC.....................
       5 ........................................................CCACCGAUUUGGCAUGGGAUUGACU....................

         miR-260
         CGUACAACAAAAUUAUUGCGAGAGAAUUUUUUAACAUGGCGUUUGCUUUACCAGGUCGAUAGACUAUCGUGAUGUCGAACUCUUGUAGGACAAUCUGGUAUUU
         .((((..........(((((((((.....((..((((.(((............((((....))))..))).))))..)))))))))))).........))))..
       4 .................GCGAGAGAAUUUUUUAACAUGGC...............................................................
      11 .................GCGAGAGAAUUUUUUAACAUGGCG..............................................................
      39 .................GCGAGAGAAUUUUUUAACAUGGCGU.............................................................
     131 .................GCGAGAGAAUUUUUUAACAUGGCGUU............................................................
       2 ...................GAGAGAAUUUUUUAACAUGGCG..............................................................
       2 ...................GAGAGAAUUUUUUAACAUGGCGU.............................................................
       1 ...................GAGAGAAUUUUUUAACAUGGCGUU............................................................
       1 ...................GAGAGAAUUUUUUAACAUGGCGUUU...........................................................
       2 ...................GAGAGAAUUUUUUAACAUGGCGUUUG..........................................................
       1 ......................AGAAUUUUUUAACAUGGCGUUUG..........................................................
       1 ...........................UUUUUAACAUGGCGUUUG..........................................................
       1 ........................................GUUUGCUUUACCAGGUCGAUAGAC.......................................
      11 ........................................GUUUGCUUUACCAGGUCGAUAGACUA.....................................
       1 ............................................GCUUUACCAGGUCGAUAGACUAUCGU.................................
       1 ....................................................................GUGAUGUCGAACUCUUGUAGG..............
       2 ....................................................................GUGAUGUCGAACUCUUGUAGGA.............
       1 ............................................................................GAACUCUUGUAGGACAAUCUGGUAUU.
       1 ......ACAAAAUUAUUGCGAGA................................................................................
         Note: Reads that partially matched to annotated mature miR-260 GUGAUGUCGAACUCUUGUAG were detectable.

         miR-261
         UGAUGGCUUUUCUGGAUUUGUUCCCUUCACGUGUAGAAAGAAACGAGCAAAAAAAAUCUUAGUGUUAGCUUUUUAGUUUUCACGGUGAAUAUGGAUAUU
         .....(((((((((.((.((.......)).)).)))))......))))........((...(((..((((....))))..)))...))...........
```

Note: Annotated mature miR-261 UAGCUUUUUAGUUUUCACG was not detectable.

```
         miR-262
         GUGCAAAAUUGUAUUAAAAUAAACUUUAUUGGAUUACAAAAAAAAUGUAUCAAUUUUUCUUUUUCAUUUUUACGUUUCUCGAUGUUUUCUGAUACUGUUGCUAGCAGU
         (((((....))))).......(((..(((..((..(((....((((((.......................)))))).....)))..))..)))..))).........
```

```
4 ..........GUAUUAAAAUAAACUUUAUUGGAUUA.................................................................................
  Note: Anontated mature miR-262 GUUUCUCGAUGUUUUCUGAU was not detectable.

  miR-264
  UGGCGGCGGUCGACAUGUACAUAUCACCAGUCUGGCUUUUCCCGUUUCCGCGAGCAAGCGGCGGGUGGUUGUUGUUAUGGGAUACCGGAGCCA
  .(((..((((...((((.(((.((((((.(((..(((((((.((....)).))).))))))).))))))...)))))))....))))..))).
  Note: Annotated mature miR-264 GGCGGGUGGUUGUUGUUAUG was not detectable.

  miR-265
  GGUUUGACUUACCCCCUCCUUCCGCUGGCCGCCGUUUUUAUUAUGAACUUGAUGUGGUUUGAGGGAGGAAGGGUGGUAUUUGAACC
  ((((..(..(((((((((((((.(..(((((.((((.......)))).....)))))..).)))))).)))).)))).)..))))
  Note: Annotated mature miR-265 UGAGGGAGGAAGGGUGGUAU was not detectable.

  miR-266
  UGUCUAAACUUGGGCAAAAGUUAGGCAAGACUUUGGCAAAGCUUGAAUCCAAGUUUUGCCAAAGUUUUGCCCAACUUUUGCCCAAAUUCAGCCA
  .(.((.((.((((((((((((.((((((((((((((((((((((....)))))))))))))))))))))).))))))))))))).)).)).).
1 .....................AGGCAAGACUUUGGCAAAGCU...................................................
  Note: Reads that partially matched to annotated mature miR-266 AGGCAAGACUUUGGCAAAGC were detectable.

  miR-267
  UAAAAGUUUCAAAACCCCGUGAAGUGUCUGCUGCAAUCUAAAUUUUUAGAUAUAGAUGCAGUCGACUCUUCAUUGGGUUUUCAGGAACCCGUA
  .....((((((((((.((((((.(((.((((((.((((.(((...))).))))))))))).))).)))))).)))))))..))))).....
1 ..................................................UAGAUGCAGUCGACUCUUCAUUG..................
  Note: Annotated mature miR-267 CCCGUGAAGUGUCUGCUGCA was not detectable.

  miR-268
  GGAAGUGGCUUUGAAGCGGCAAGAAUUAGAAGCAGUUUGGUGUCAGACACACUACUCACUCACUGCUUCUUGUUUUUUCUGCUUUCUUUGCUUUU
  .((((..(....((((((.((((((.(((((((((..((((..((.....))...)))).))))))))).)))))).)))))))..)..)))).
  Note: Annotated mature miR-268 GGCAAGAAUUAGAAGCAGUUUGGU was not detectable.

  miR-269
  GGGCAAAAGUUGGGCAAGACUCUGGCAAAACUUGGAUUUAUGCUGCGCCAAAGUUUAACCCAAGUUUGACCCAACUCUUGUCAAAUUUUUGUCC
  ((((((((((.(((((((...(((..((((((((.((...(((.......)))..)).))))))))...)))..))))))).))))))))))
  Note: Annotated mature miR-269 GGCAAGACUCUGGCAAAACU was not detectable.

  miR-270
  GUCGCUAGCCUCUUUACCUGCCUACACACCUUUCUGCCUACGUGGCACAUUGGUAGGCAAGAGGGCAUGAUGUAGCAGUGGAGAUGGACUGCCAC
  ...((.((((((((((((.(((.((((((((((..(((((((...........)))))))..)))))..)).))))))))))))))).)).)))))...
  Note: Annotated mature miR-270 GGCAUGAUGUAGCAGUGGAG was not detectable.

  miR-271
  AAUAGAAAACGGGGCCCGCCGGCUCGCCGGGUGGGAAAGCAUUCGACAGCAAGACGUGUUUUUCAAUCUGCGACUCGGCGAUUCCCAUCAUCACU
  ..........((((..(((((..((((.((((.(((((((((((........)).)))))))))).))))))))..)))))..)))).........
  Note: Annotated mature miR-271 UCGCCGGGUGGGAAAGCAUU was not detectable.

  miR-272
  CGCAGGCACGUGCAGGUAUGUAGGCAGGCGUAGGCCCGUAGGCAAGUGUAGGUCUGCAGGCAUGAAUGUAGGCAUGGGUGUUUGGAAGACCUGCG
```

```
 (((..((.(.((((....))))))))..)))...(((....)))....(((((((.(((((((..(((....)))..)))))))..))))))).
 Note: Annotated mature miR-272 UGUAGGCAUGGGUGUUUG was not detectable.

 miR-273
 ACCUCGUUUUGGGAGCAGCCGGCAUUUUACGGGCUACACUUUUUUUAAAAUUGAUGUGUGCCCGUACUGUGUCGGCUGCUUUGAAAAUUUCGGU
 (((..(((((.((((((((((((((..(((((((.((((................)))))))))))..)))))))))))))).)))))...)))
 Note: Annotated mature miR-273 UGCCCGUACUGUGUCGGCUG was not detectable.

 miR-353
 UGUUGAAGUAUUGCAAAAGCAAAGGACGGGCACAAUUGCCAUGUGUUGGUAUUAUUGCUUCAAGUUAUUUGAAGCUGUAAUAUCAAUAAGCAUGUCUCGUGUGAAGUCCG
 ..........((((....)))).((((...((((...(.((((((((((((((((.((((((((...)))))))).)))))))))))..))))).)...))))..)))).
1 .............CAAAAGCAAAGGACGGGCACA............................................................................
1 ..............AAAAGCAAAGGACGGGCACAAU..........................................................................
1 ..................GCAAAGGACGGGCACAAUU.........................................................................
1 ...................CAAAGGACGGGCACAAUUG........................................................................
1 ....................AAAGGACGGGCACAAUU.........................................................................
1 ....................AAAGGACGGGCACAAUUGCCA.....................................................................
1 .....................AAGGACGGGCACAAUUGC.......................................................................
1 .....................AAGGACGGGCACAAUUGCC......................................................................
1 .....................AAGGACGGGCACAAUUGCCAU....................................................................
1 ......................AGGACGGGCACAAUUGC.......................................................................
2 ........................................AUGUGUUGGUAUUAUUGCUU..................................................
1 .......................................................................AAGCUGUAAUAUCAAUAAG....................
 Note: Annotated mature miR-353 CAAUUGCCAUGUGUUGGUAUU was not detectable.

 miR-354
 CAGAGCCGACUAAGCACCUUGGUGCGGCUGCAGACGGGUAUCCGGCUCGACGUUCAUACAUCACGACUUCUUUCCUUUUACCUUGUUUGUUGCUGCUCCUAUUGGUUUUG
 (((((((((...........((.(((((.(((((((((((...((...((.(((..........))).))...))...)))).))))))).))))).))..)))))))))
1 ..............CACCUUGGUGCGGCUGCAGACGG.........................................................................
1 ...............ACCUUGGUGCGGCUGCAGACGGGU.......................................................................
1 .....................GUGCGGCUGCAGACGGGU.......................................................................
1 ....................GGUGCGGCUGCAGACGGGUAUCC...................................................................
1 ......................UGCGGCUGCAGACGGGUAUCC...................................................................
1 ....................................GGUAUCCGGCUCGACGUUCAU.....................................................
1 .......................................AUCCGGCUCGACGUUCAU.....................................................
1 ...............................................CGACGUUCAUACAUCACGAC...........................................
1 .........................................................ACAUCACGACUUCUUUCCUUUUACC............................
1 ...........................................................................UUUUACCUUGUUUGUUGCUGCU.............
 Note: Annotated mature miR-354 ACCUUGUUUGUUGCUGCUCCU was not detectable.

 miR-355
 AAUAAUGUCAAGAAUUGAAUGGUUUGUUUUAGCCUGAGCUAUGAGUCAUCGGAAUAUGCAUAGCUUCUUGCUAAAACAUGCCAAUCAAUCUUAAUUGACGAAGUUUUUGA
 .....((((((..(((((.((((.(((((((((..((((((((..((....)).....))))))))...))))))))).)))).))))).....))))))..........
1 ....................GGUUUGUUUUAGCCUGAGCUAU....................................................................
1 ......................UUUGUUUUAGCCUGAGCUAU....................................................................
# 8 ......................UUUGUUUUAGCCUGAGCUAUG...................................................................
1 ...........................................AGUCAUCGGAAUAUGCA..................................................
```

```
      1 .............................................................AGCUUCUUGCUAAAACAUGCCAA..........................

        miR-356
        UAAAGUUUCCUUGAGAACAAUGUGGUUGAGCAACGCGAACAAAUCAUCAAUGUAAAUUUCAGCGGAUUCGUUACGACGUGUCUCACCACAUCGUUAGUAGGAUUUCAGAA
        .......((((....(((.(((((((.(((..((((((((.((((.....((.......))...)))).))).)).)))..))))))))))).)))...))))........
      2 ....................UGUGGUUGAGCAACGCGAACAA....................................................................
      8 ....................UGUGGUUGAGCAACGCGAACAAA...................................................................
      1 .....................GUGGUUGAGCAACGCGAACAA....................................................................
      1 .....................GUGGUUGAGCAACGCGAACAAA...................................................................
      1 ..........................................................UCAGCGGAUUCGUUACGACG................................
      4 .................................................................AUUCGUUACGACGUGUCUCACCA......................
        Note: Annotated mature miR-356 UUGAGCAACGCGAACAAAUCA was not detectable.

        miR-357
        ACGCUGUACUACUCACCAGCGGAUCCCUACAACGCUGCGCAUAUGCAUGAACACAAUGAAAAUGUAAAUGCCAGUCGUUGCAGGAGUUCGCAUACAGUAGUAAAAUGCGC
        .(((..(((((((.....(((((..(((.((((((((.((((.(((((.............))))).))))))).))))).)))..)))))....)))))))....))).
      1 ........................CCCUACAACGCUGCGCAUAUG.................................................................
      5 ........................CCCUACAACGCUGCGCAUAUGC................................................................
      1 ................................................................UAAAUGCCAGUCGUUGCAGG..........................
      2 .................................................................AAAUGCCAGUCGUUGCAGG..........................
      2 ................................................................UAAAUGCCAGUCGUUGCAGGA.........................
     12 .................................................................AAAUGCCAGUCGUUGCAGGA.........................
     16 ................................................................UAAAUGCCAGUCGUUGCAGGAG........................
     43 .................................................................AAAUGCCAGUCGUUGCAGGAG........................
#    86 ................................................................UAAAUGCCAGUCGUUGCAGGAGU.......................
    624 .................................................................AAAUGCCAGUCGUUGCAGGAGU.......................
      1 ................................................................UAAAUGCCAGUCGUUGCAGGAGUU......................
     38 .................................................................AAAUGCCAGUCGUUGCAGGAGUU......................

        miR-358
        AAACUCGCGACGGCUGGCGACCUGGCCAGGCAUUCCAACUGUCAAACUUACAAAGCUUUCUCGACAAUUGGUAUCCCUGUCAAGGUCUCAAUCCGUUCAGCGAAUUCGUU
        ....(((((((((...(.((((((((.(((.((.((((.((((...................)))).)))).)).)))))).))))).)...)))))..)))).......
      1 ...................ACCUGGCCAGGCAUUCCAA........................................................................
      3 ...................ACCUGGCCAGGCAUUCCAAC.......................................................................
     17 ...................ACCUGGCCAGGCAUUCCAACU......................................................................
     27 ...................ACCUGGCCAGGCAUUCCAACUG.....................................................................
    136 ...................ACCUGGCCAGGCAUUCCAACUGU....................................................................
      8 ...................ACCUGGCCAGGCAUUCCAACUGUC...................................................................
      1 ...................ACCUGGCCAGGCAUUCCAACUGUCAA.................................................................
      1 ..........................................CAAACUUACAAAGCUUUCUCGAC.............................................
      1 .........................................UCAAACUUACAAAGCUUUCUCGACA............................................
      1 .................................................................AAUUGGUAUCCCUGUCAAGG.........................
      1 ..................................................................AUUGGUAUCCCUGUCAAGG.........................
#     1 ..................................................................AUUGGUAUCCCUGUCAAGGUC.......................
     36 .................................................................AAUUGGUAUCCCUGUCAAGGUCU......................
     75 ..................................................................AUUGGUAUCCCUGUCAAGGUCU......................
```

```
         miR-359
         AAUGCUCCUUGAAAUUUCAAUCGUUAGAGUAACACACAGUUACACGACCUCAUCAAUCGUGUCACUGGUCUUUCUCUGACGAAUUGAAGUUCUGGAGACAAUUUUGGUUG
         ..((((((..(((.((((((((((((((.((.((.((((.((((((.........)))))).)))))).)).)))))))).)))))))))).)))).))...........
       1 .............................................................UCACUGGUCUUUCUCUGACGA............................
#     38 .............................................................UCACUGGUCUUUCUCUGACGAA...........................
      25 .............................................................UCACUGGUCUUUCUCUGACGAAU..........................
       1 ..............................................................CACUGGUCUUUCUCUGACGAAU..........................
       1 ...............................................................ACUGGUCUUUCUCUGACGAAU..........................

         miR-360
         CAUUCUGUUAGGAAGCAUCAAUGUGUUGUGACCGUUGUUACGGUCAAUUUGCAAAUUGAAAAAAUGACCGUAAUCCCGUUCACAAUACAUUGUUCGUUUUCUCCAAAGGU
         ...(((...(((((((..((((((((((((.((..((((((((((.(((............))).))))))))))..)).))))))))))))...)))))))....))).
       1 .........................UUGUGACCGUUGUUACGGUCAA...............................................................
       4 .........................UUGUGACCGUUGUUACGGUCAAU..............................................................
       1 ..............................................AUUUGCAAAUUGAAAAAAUG............................................
       1 ...............................................................AUGACCGUAAUCCCGUUCACAAU........................
       1 ................................................................UGACCGUAAUCCCGUUCACAAU........................
       1 ..................................................................ACCGUAAUCCCGUUCACAAUA.......................
      11 .........................UUGUGACCGUUGUUACGGUCA................................................................

         lsy-6
         CCAUCAAAUGCGUCUAGUAUCAAAAUCAUGUAAAAACUGUAAAACAGAUUUUGUAUGAGACGCAUUUCGAUGA
         .(((((((((((((.(((.(((((((.(((..............)))))))))))).)))))))))).)))).
       1 ................................................UUUUGUAUGAGACGCAUU.......
#     33 ................................................UUUUGUAUGAGACGCAUUUCG....
      73 ................................................UUUUGUAUGAGACGCAUUUCGA...
       2 .................................................UUUGUAUGAGACGCAUUUCGA...
       1 .................................................UUUGUAUGAGACGCAUUUCGAU..
       1 .....AAAUGCGUCUAGUAUCAAAAUC..............................................

         miR-392
         UUUUUGAAAAUCUCGCAGACGUGUUCAGUCAGCAUUCGUGGUUGAGGAUAUCGAACACAAAAAAAGAUAUCAUCGAUCACGUGUGAUGACAGAUUUUCUGCGACUAACAG
         ............((((((.....((.((((.(((.(((((((((.((((((..............)))))).))))))))).))).)))).))...))))))........
      25 ..............................AGCAUUCGUGGUUGAGGAUAU...........................................................
      12 ..............................AGCAUUCGUGGUUGAGGAUAUC..........................................................
       1 ...................................................................UAUCAUCGAUCACGUGUGAUG......................
#     14 ...................................................................UAUCAUCGAUCACGUGUGAUGA.....................
       4 ....................................................................AUCAUCGAUCACGUGUGAUGA.....................

         miR-784
         UUCGACGUCCACGUGGCACAAUCUGCGUACGUAGAAAGAUCAAAAAGUCACUUCUAUGUACAAAUGUUGCGCUGCCUGGCACAGUGAA
         .(((..(((((.(..((.((((.(..((((((((((.(((......)))..))))))))))..).)))).))..).))).))..))).
       2 .............UGGCACAAUCUGCGUACGUAG......................................................
#     61 .............UGGCACAAUCUGCGUACGUAGA.....................................................
       1 ...............GCACAAUCUGCGUACGUAGA.....................................................
      11 .............UGGCACAAUCUGCGUACGUAGAA....................................................
       1 .............UGGCACAAUCUGCGUACGUAGAAA...................................................
```

```
      1 ......................................................UAUGUACAAAUGUUGCGCUGCCU...........

        miR-785
        GCUCUUUUUCUCACCCAUCAGCACAGAAUUUUUCGCUAACAGAAACCUCAAAACAAUGUAAGUGAAUUGUUUUGUGUAGAUGGUGGAAAUGAGC
        ((((..((((.((((.(((.(((((((((..((((((.(((...............))).))))))..))))))))).))))))))))).))))
      1 ..........................................................UAAGUGAAUUGUUUUGU...................
      3 ..........................................................UAAGUGAAUUGUUUUGUG..................
     11 ..........................................................UAAGUGAAUUGUUUUGUGU.................
     32 ..........................................................UAAGUGAAUUGUUUUGUGUA................
     10 ..........................................................UAAGUGAAUUGUUUUGUGUAG...............
#   581 ..........................................................UAAGUGAAUUGUUUUGUGUAGA..............
      1 ...........................................................AAGUGAAUUGUUUUGUGUAGA..............

        miR-786
        GGGAUAGUUCCCGAUCGAAUAUCAGUUGGGGUAUUUACAAAAAGACAAAAUUGUAAUGCCCUGAAUGAUGUUCAAUCGGUGAUGAAUCUC
        (((((.((..(((((.((((((((.(..(((((((.((((..........)))))))))))..).)))))))).)))))..))..)))))
      2 .............AUCGAAUAUCAGUUGGGGUAUUU......................................................
      6 ...............CGAAUAUCAGUUGGGGUAUUUA.....................................................
      2 ...............CGAAUAUCAGUUGGGGUAUUUAC....................................................
      4 ...............CGAAUAUCAGUUGGGGUAUUUACA...................................................
      1 ................GAAUAUCAGUUGGGGUAUUUA.....................................................
      1 ....................................................GUAAUGCCCUGAAUGAUGUUCA................
      3 .....................................................UAAUGCCCUGAAUGAUGU...................
      6 .....................................................UAAUGCCCUGAAUGAUGUUCAA...............
#   233 .....................................................UAAUGCCCUGAAUGAUGUUCAAU..............
      5 ......................................................AAUGCCCUGAAUGAUGU...................
      2 ......................................................AAUGCCCUGAAUGAUGUUCAA...............
    210 ......................................................AAUGCCCUGAAUGAUGUUCAAU..............
      1 .......................................................AUGCCCUGAAUGAUGUUCAAU..............

        miR-787
        GAUGUAUCAGUGGACGAAAGAUACAUACGAUCUUACAUCUUCAAAAUGGUAUGUAAGCUCGUUUUAGUAUCUUUCGUCUCUGAUCACAUU
        ((((((((((.(((((((((((((..((((.(((((((((.......)).))))))).))))....)))))))))))))))))).)))))
      7 ................AAAGAUACAUACGAUCUUACA.....................................................
      1 ................AAAGAUACAUACGAUCUUACAU....................................................
      1 ....................................................GUAAGCUCGUUUUAGUAUCUU.................
      1 ....................................................GUAAGCUCGUUUUAGUAUCUUUCG..............
      7 .....................................................UAAGCUCGUUUUAGUAUCUU.................
      1 .....................................................UAAGCUCGUUUUAGUAUCUUU................
      1 .....................................................UAAGCUCGUUUUAGUAUCUUUC...............
#   131 .....................................................UAAGCUCGUUUUAGUAUCUUUCG..............
      9 .....................................................UAAGCUCGUUUUAGUAUCUUUCGU.............
      2 ......................................................AAGCUCGUUUUAGUAUCUUUCG..............

        miR-788
        GCUCACUUUUCCGCUUCUAACUUCCAUUUGCAGAGUUCAAGUAAUCUGGAAAUGGAUUAGAAUCGUGGAAAAGUUAGU
        (((.((((((((((((((((..(((((((.((((..........)))).))))))))))))..))))))))))).)))
```

```
       1 .........UCCGCUUCUAACUUCCAUUUGC...............................................
      10 .........UCCGCUUCUAACUUCCAUUUGCA..............................................
       4 ..........CCGCUUCUAACUUCCAUUUGCA..............................................
#    347 .........UCCGCUUCUAACUUCCAUUUGCAG.............................................
      88 ..........CCGCUUCUAACUUCCAUUUGCAG.............................................
      19 ...........CGCUUCUAACUUCCAUUUGCAG.............................................
       5 ............GCUUCUAACUUCCAUUUGCAG.............................................
       3 ........UUCCGCUUCUAACUUCCAUUUGCAG.............................................
      31 .........UCCGCUUCUAACUUCCAUUUGCAGA............................................
      40 ..........CCGCUUCUAACUUCCAUUUGCAGA............................................
       2 ...........CGCUUCUAACUUCCAUUUGCAGA............................................
       1 ............GCUUCUAACUUCCAUUUGCAGA............................................
       1 ....................CUUCCAUUUGCAGAGUUCAAGUAAUC................................
       1 ...............................................GGAAAUGGAUUAGAAUCG.............
       4 ...............................................GGAAAUGGAUUAGAAUCGU............
       1 ..............................................UGGAAAUGGAUUAGAAUCGUG...........
       9 ...............................................GGAAAUGGAUUAGAAUCGUG...........
       1 .................................................AAAUGGAUUAGAAUCGUG...........
       2 ..............................................UGGAAAUGGAUUAGAAUCGUGG..........
       7 ...............................................GGAAAUGGAUUAGAAUCGUGG..........
       1 ................................................GAAAUGGAUUAGAAUCGUGG..........
       1 .............................................CUGGAAAUGGAUUAGAAUCGUGGA.........
      58 ..............................................UGGAAAUGGAUUAGAAUCGUGGA.........
     344 ...............................................GGAAAUGGAUUAGAAUCGUGGA.........
       7 ................................................GAAAUGGAUUAGAAUCGUGGA.........
       1 .................................................AAAUGGAUUAGAAUCGUGGA.........
       1 .............................................CUGGAAAUGGAUUAGAAUCGUGGAA........
      87 ..............................................UGGAAAUGGAUUAGAAUCGUGGAA........
     894 ...............................................GGAAAUGGAUUAGAAUCGUGGAA........
      41 ................................................GAAAUGGAUUAGAAUCGUGGAA........
       1 .................................................AAAUGGAUUAGAAUCGUGGAA........
       1 ..................................................AAUGGAUUAGAAUCGUGGAA........
      38 ..............................................UGGAAAUGGAUUAGAAUCGUGGAAA.......
     796 ...............................................GGAAAUGGAUUAGAAUCGUGGAAA.......
      70 ................................................GAAAUGGAUUAGAAUCGUGGAAA.......
      13 .................................................AAAUGGAUUAGAAUCGUGGAAA.......
       2 ..............................................UGGAAAUGGAUUAGAAUCGUGGAAAA......
      30 ...............................................GGAAAUGGAUUAGAAUCGUGGAAAA......
      10 ................................................GAAAUGGAUUAGAAUCGUGGAAAA......
      15 .................................................AAAUGGAUUAGAAUCGUGGAAAA......
       2 ....................................................UGGAUUAGAAUCGUGGAAAAGUUA..

         miR-789-1
         UGUCCUGAAGGCAGGCAAUUGAUGACCCAGACAAGGACUAAUCAAGAUUGUCGAUCUAGUCCCUGCCUGGGUCACCAAUUGUCGGCCCCCGCGGGCA
         .((((((..(((.((((((((.((((((((.((.(((((((((.........))).)))))).)).)))))))).)))))))).)))..)).)))).
       3 ...........CAGGCAAUUGAUGACCCAGACA................................................................
       4 ................AAUUGAUGACCCAGACAAGGA............................................................
```

```
   9 ................AAUUGAUGACCCAGACAAGGACU..........................................................
   7 ..................UUGAUGACCCAGACAAGGACU..........................................................
   1 ..................UUGAUGACCCAGACAAGGACUA.........................................................
   2 ...................UGAUGACCCAGACAAGGACUA.........................................................
   1 ..................UUGAUGACCCAGACAAGGACUAA........................................................
   1 ...................UGAUGACCCAGACAAGGACUAA........................................................
   1 .......................................AAUCAAGAUUGUCGAUCUAG......................................
   2 ..........................................................GUCCCUGCCUGGGUCACCA....................
   3 ...........................................................UCCCUGCCUGGGUCACCA....................
  31 ............................................................CCCUGCCUGGGUCACCA....................
   2 ...........................................................UCCCUGCCUGGGUCACCAAU..................
   2 ...........................................................UCCCUGCCUGGGUCACCAAUUG................
#        32 ...........................................................UCCCUGCCUGGGUCACCAAUUGU...............
   1 .............................................................CCUGCCUGGGUCACCAAUUGU...............
   7 ..............................................................CUGCCUGGGUCACCAAUUGUCGG............
   4 ..............................................................CUGCCUGGGUCACCAAUUGUCGGC...........
   2 .................................................................CCUGGGUCACCAAUUGUCGGC...........
  15 .................................................................CCUGGGUCACCAAUUGUCGGCC..........

     miR-789-2
     UGUCCUGAAGGCGGACAAUUGAUGACUCAGGCAGGGACUAAUCAUGAUUGUCGAUUUAGUCCCUGCCUGGGUCACCAAUUGUCGGCCCCCGCGGACA
     .((((((..(((.((((((((.(((((((((((((((((((...((.....))..))))))))))))))))))).)))))))).)))..)).)))).
   4 ................AAUUGAUGACUCAGGCAG...............................................................
   1 ............GGACAAUUGAUGACUCAGGCAGG..............................................................
   3 ................AAUUGAUGACUCAGGCAGG..............................................................
   9 ................AAUUGAUGACUCAGGCAGGG.............................................................
  31 ................AAUUGAUGACUCAGGCAGGGA............................................................
  56 ................AAUUGAUGACUCAGGCAGGGAC...........................................................
   1 ..................UUGAUGACUCAGGCAGGGAC...........................................................
   1 ....................GAUGACUCAGGCAGGGAC...........................................................
   1 ...............CAAUUGAUGACUCAGGCAGGGACU..........................................................
2730 ................AAUUGAUGACUCAGGCAGGGACU..........................................................
   6 .................AUUGAUGACUCAGGCAGGGACU..........................................................
  24 ..................UUGAUGACUCAGGCAGGGACU..........................................................
   1 ....................GAUGACUCAGGCAGGGACU..........................................................
   1 .....................AUGACUCAGGCAGGGACU..........................................................
  42 ................AAUUGAUGACUCAGGCAGGGACUA.........................................................
   2 ..................UUGAUGACUCAGGCAGGGACUA.........................................................
  25 ................AAUUGAUGACUCAGGCAGGGACUAA........................................................
   3 ..................UUGAUGACUCAGGCAGGGACUAA........................................................
   4 ...................UGAUGACUCAGGCAGGGACUAA........................................................
  27 .......................................AAUCAUGAUUGUCGAUUUAG......................................
   2 ..........................................................GUCCCUGCCUGGGUCACCA....................
   3 ...........................................................UCCCUGCCUGGGUCACCA....................
  31 ............................................................CCCUGCCUGGGUCACCA....................
   2 ...........................................................UCCCUGCCUGGGUCACCAAU..................
   2 ...........................................................UCCCUGCCUGGGUCACCAAUUG................
```

```
#      32 ...........................................................UCCCUGCCUGGGUCACCAAUUGU...............
        1 .............................................................CCUGCCUGGGUCACCAAUUGU...............
        7 ..............................................................CUGCCUGGGUCACCAAUUGUCGG............
        4 ..............................................................CUGCCUGGGUCACCAAUUGUCGGC...........
        2 .................................................................CCUGGGUCACCAAUUGUCGGC...........
       15 .................................................................CCUGGGUCACCAAUUGUCGGCC..........

          miR-790
          GCCGGCUUGGCACUCGCGAACACCGCGAUUUCACUUAUAACUCGCGGCGUUAGCUCUGUGUCAAACCGGC
          (((((.(((((((..((.(((.((((((.............)))))).))).))...))))))).)))))
        1 ............................................CGGCGUUAGCUCUGUGUCAAAC....
        5 ............................................CGGCGUUAGCUCUGUGUCAAACC...
        1 .....CUUGGCACUCGCGAACAC...............................................
        2 .....CUUGGCACUCGCGAACACC..............................................
        3 .....CUUGGCACUCGCGAACACCG.............................................
       49 .....CUUGGCACUCGCGAACACCGC............................................
#     404 .....CUUGGCACUCGCGAACACCGCG...........................................
       14 .....CUUGGCACUCGCGAACACCGCGA..........................................
        1 ......UUGGCACUCGCGAACACCGCG...........................................

          miR-791
          GAACCUUCGAUUACCUUAUCCGUUGUAGCCAAAGUUCAACGAAGCGGCAACUUUGGCACUCCGCAGAUAAGGCAAUCGAUUGUUU
          ((((..((((((.(((((((((..((.(((((((((...((...))..)))))))))))..))..))))))).))))))..))))
        1 .........AUUACCUUAUCCGUUGUAGCCAAA....................................................
        1 ...........UACCUUAUCCGUUGUAGCCAAAGU..................................................
        3 ............ACCUUAUCCGUUGUAGCCAAAG...................................................
       25 ............ACCUUAUCCGUUGUAGCCAAAGU..................................................
        4 ............ACCUUAUCCGUUGUAGCCAAAGUU.................................................
        1 ...............UUAUCCGUUGUAGCCAAAGU..................................................
        1 .................................GUUCAACGAAGCGGCAACUU................................
        1 .................................................ACUUUGGCACUCCGCAGAUAAGGCAA..........
        2 ...................................................UUUGGCACUCCGCAGAUAAGGC............
#      14 ...................................................UUUGGCACUCCGCAGAUAAGGCA...........
       99 ...................................................UUUGGCACUCCGCAGAUAAGGCAA..........
        1 ...................................................UUUGGCACUCCGCAGAUAAGGCAAU.........
        5 ....................................................UUGGCACUCCGCAGAUAAGGCAA..........
        5 .....................................................UGGCACUCCGCAGAUAAGGCAA..........

          miR-792
          AAGUUGGUCAACGUUUGAGAGUUCAAAAGAUUUAGCAAUUUUAUACGAGUGAAAUUGAAAUCUCUUCAACUUUCAGACGUUUUCUGAUUU
          ((((..(..((((((((((((((.((.(((((...(((((((((....))))))))).))))).)).))))))))))))))..)..))))
        1 ...............UGAGAGUUCAAAAGAUUUAGCAAU...................................................
        1 ...............UGAGAGUUCAAAAGAUUUAGCAAUU..................................................
#      33 ......................................................UUGAAAUCUCUUCAACUUUCAGA.............
        1 .......................................................UGAAAUCUCUUCAACUUUCAGA.............

          miR-793
```

```
         UGGUUGGUAAAAGUCUGUUUAACUAUUGUACAUCACAAUUUUCACUGCGAAAAAUUGAGGUAUCUUAGUUAGACAGACAUAUACUACAACCA
         .((((((((...(((((((((((((..((((.(((...(((((.....)))))..))).))))..)))))))))))))...)))..))))).
      2  ......................................................UUGAGGUAUCUUAGUUAGACAGA...............
      1  .......................................................UGAGGUAUCUUAGUUAGAC..................
      6  .......................................................UGAGGUAUCUUAGUUAGACAG................
#   474  .......................................................UGAGGUAUCUUAGUUAGACAGA...............
      3  ........................................................GAGGUAUCUUAGUUAGACAGA...............

         miR-794
         CUGAGCGUGUCUCUUGAGGUAAUCAUCGUUGUCACUUCGCAAAGUUGGAAUUGAAAACGUUGUCUAUCUCGAAAGGCUUAUCAG
         ((((....((((.((((((((..((.((((.(((.(((.((....))))).))).)))).))..)))))))).))))...))))
#   111  ..............UGAGGUAAUCAUCGUUGUCACU................................................
      3  ..............UGAGGUAAUCAUCGUUGUCACUU...............................................
      2  ...............GAGGUAAUCAUCGUUGUCACU................................................
      1  ................AGGUAAUCAUCGUUGUCACU................................................
      1  ...................................................UGAAAACGUUGUCUAUC................
      4  ...................................................UGAAAACGUUGUCUAUCUCGAA...........
      1  ......................................................AAACGUUGUCUAUCUCGAA...........

         miR-795
         GAAACGUUACCUGCUGAGGUAGAUUGAUCAGCGAGCUUGAUACCCCGUUCAAAAUCGUGAUCAGUAUACUUCGUCAGGCAGCUUGUUUU
         ((((((((.((((.(((((((.((((((((.(((..((((........))))..))))))))))).))))))).)))).)))..)))))
      2  .............CUGAGGUAGAUUGAUCAGCGAGCU....................................................
     21  .............CUGAGGUAGAUUGAUCAGCGAGCUU...................................................
      3  ..............UGAGGUAGAUUGAUCAGC.........................................................
      7  ..............UGAGGUAGAUUGAUCAGCG........................................................
     32  ..............UGAGGUAGAUUGAUCAGCGA.......................................................
     44  ..............UGAGGUAGAUUGAUCAGCGAG......................................................
    149  ..............UGAGGUAGAUUGAUCAGCGAGC.....................................................
    991  ..............UGAGGUAGAUUGAUCAGCGAGCU....................................................
#  18942 ..............UGAGGUAGAUUGAUCAGCGAGCUU...................................................
     16  ..............UGAGGUAGAUUGAUCAGCGAGCUUG..................................................
      2  ..............UGAGGUAGAUUGAUCAGCGAGCUUGA.................................................
      1  ...............GAGGUAGAUUGAUCAGCGA.......................................................
      5  ...............GAGGUAGAUUGAUCAGCGAGCU....................................................
     65  ...............GAGGUAGAUUGAUCAGCGAGCUU...................................................
      7  ................AGGUAGAUUGAUCAGCGAGCUU...................................................
      1  ...................UAGAUUGAUCAGCGAGCUU...................................................
      1  ...................................................AAAUCGUGAUCAGUAUACUUCGUC..............
      5  ....................................................AAUCGUGAUCAGUAUACUUCG................
     19  ....................................................AAUCGUGAUCAGUAUACUUCGU...............
    299  ....................................................AAUCGUGAUCAGUAUACUUCGUC..............

         miR-796
         ACUCUAAUGACAAGCUGGAAUGUAGUUGAGGUUAGUAAAAGCACUAUACUUGUUACAAACCUUGCCACGUUCCGACUUGUCAAAAGAGU
         (((((..(((((((.((((((((.(..((((((.(((((((.......))).)))).))))))..)))))))))).)))))))..)))))
      4  ...............UGGAAUGUAGUUGAGGU.........................................................
```

```
       4 ...............UGGAAUGUAGUUGAGGUU........................................................
       5 ...............UGGAAUGUAGUUGAGGUUA.......................................................
      54 ...............UGGAAUGUAGUUGAGGUUAG......................................................
       1 ..............CUGGAAUGUAGUUGAGGUUAGU.....................................................
     215 ...............UGGAAUGUAGUUGAGGUUAGU.....................................................
    1164 ...............UGGAAUGUAGUUGAGGUUAGUA....................................................
       1 ................GGAAUGUAGUUGAGGUUAGUA....................................................
       4 ..............CUGGAAUGUAGUUGAGGUUAGUAA...................................................
#  11895 ...............UGGAAUGUAGUUGAGGUUAGUAA...................................................
       7 ................GGAAUGUAGUUGAGGUUAGUAA...................................................
       1 .................GAAUGUAGUUGAGGUUAGUAA...................................................
      33 ...............UGGAAUGUAGUUGAGGUUAGUAAA..................................................
      23 ...............UGGAAUGUAGUUGAGGUUAGUAAAA.................................................
       4 ......................................................ACAAACCUUGCCACGUUCCGAC.............

         miR-797
         CGUCCAAUGGACGAUAUCACAGCAAUCACAAUGAGAAGAAUACAUCUAGAUACUCUUUUCAUUGGUUUCUGUGAAAUCGUAUUUGAAUG
         (((.(((...(((((.((((((.((...(((((((((((.((........)).)))))))))))..))))))))).)))))..))).)))
       1 ..............UAUCACAGCAAUCACAAUGAGA.....................................................
       8 ..............UAUCACAGCAAUCACAAUGAGAA....................................................
      21 ..............UAUCACAGCAAUCACAAUGAGAAG...................................................
#     91 ..............UAUCACAGCAAUCACAAUGAGAAGA..................................................
       1 ..............UAUCACAGCAAUCACAAUGAGAAGAA.................................................
       3 ...............AUCACAGCAAUCACAAUGAGAAGA..................................................
       1 ................UCACAGCAAUCACAAUGAGAAGA..................................................
       3 ..................ACAGCAAUCACAAUGAGAAGA..................................................
       3 .......................................................UUUUCAUUGGUUUCUGUGAAAU............
       1 .......................................................UUUUCAUUGGUUUCUGUGAAAUC...........

         miR-798
         UACUGUUUCAUGUAGGUGUUAAAGUUUGUACAAGAAGUUAGUUUUCUGUUAAGCCUUACAUAUUGACUGAGCAGUA
         (((((((.((.((..(((((((.(((((.(((.((((.....)))))))))))).))).))))..)))))))))))
       1 .................................................UAAGCCUUACAUAUUGA..........
       2 .................................................UAAGCCUUACAUAUUGACUG.......
#     96 .................................................UAAGCCUUACAUAUUGACUGA......

         miR-799
         GUUUUUCAAUUUUUUUCACCGGUUUUAAAAAGUGGUUUCACAUUCAAUAAAGACAUGUGAACCCUGAUAAAGCUAGUGGAAAUAUCGGAAAAC
         (((((((.((..(((((((..((((((...((.((((.(((((((......)).)))))))))))..))))))..))))))).)).)))))))
       8 .........................................................UGAACCCUGAUAAAGCUAGUG...............
#    515 .........................................................UGAACCCUGAUAAAGCUAGUGG..............
       9 ..........................................................GAACCCUGAUAAAGCUAGUGG..............
       7 ..............................................................CCUGAUAAAGCUAGUGG..............
      21 .........................................................UGAACCCUGAUAAAGCUAGUGGA.............
       1 ..............................................................CCUGAUAAAGCUAGUGGA.............
      53 .........................................................UGAACCCUGAUAAAGCUAGUGGAA............
       2 ..........................................................GAACCCUGAUAAAGCUAGUGGAA............
```

```
       miR-800
       ACGGCGGCUGACAAUUUCCGAGUUAGGCCACUCUUAUAAUCAGUGGCCAAACUCGGAAAUUGUCUGCCGCCGU
       ((((((((.(((((((((((((((.(((((((.........))))))).))))))))))))))).))))))))
     1 ..........................................GUGGCCAAACUCGGAAAUUGU..........
     4 .............................................GCCAAACUCGGAAAUUGUCUGC......
       Note: Reads that partially matched to annotated mature miR-800 CAAACUCGGAAAUUGUCUGCCG were detectable.

       miR-1018
       GUAAGUUCAUGAUUUCUCCCAUAUAUUUUUCAUGAGAGAGAUCAUUGGACUUACAG
       ((((((((((((((((((.(((.........))).))))))))).)))))))))..
     1 GUAAGUUCAUGAUUUCUCCCA...................................
     2 .................................GAGAGAGAUCAUUGGACUUACA.
     3 ..................................AGAGAGAUCAUUGGACUUA...
     5 ..................................AGAGAGAUCAUUGGACUUAC..
   150 ..................................AGAGAGAUCAUUGGACUUACA.
#  443 ..................................AGAGAGAUCAUUGGACUUACAG
     2 ...................................GAGAGAUCAUUGGACUUACAG
     1 ....................................AGAGAUCAUUGGACUUACAG

       miR-1019
       GUGAGCAUUGUUCGAGUUUCAUUUUUAAUAAAAUUUAUUUAAAAACUGUAAUUCCACAUUGCUUUCCAG
       ..(((((.(((..(((((.(((((((((..........))))))).)).))))).))).))))).....
     1 GUGAGCAUUGUUCGAGUUUCAU...............................................
     1 GUGAGCAUUGUUCGAGUUUCAUU..............................................
     9 GUGAGCAUUGUUCGAGUUUCAUUU.............................................
    15 GUGAGCAUUGUUCGAGUUUCAUUUU............................................
     1 GUGAGCAUUGUUCGAGUUUCAUUUUU...........................................
       Note: Annotated mature miR-1019 CUGUAAUUCCACAUUGCUUUCCAG was not detectable.

       miR-1020
       GUAAGUGUUACAGAAUAAUCUUAGACAAAACAACUAAAAUUAAUGAAAAAUUAUUCUGUGACACUUUCAG
       (.(((((((((((((((((..............................))))))))))))))))).)..
     4 GUAAGUGUUACAGAAUAAUCU.................................................
    12 GUAAGUGUUACAGAAUAAUCUU................................................
     1 ................................................AAUUAUUCUGUGACACU.....
     1 ...............................................AAAUUAUUCUGUGACACUUUCA.
     7 ................................................AAUUAUUCUGUGACACUUUCA.
     6 .................................................AUUAUUCUGUGACACUUUCA.
    15 ................................................AAUUAUUCUGUGACACUUUCAG
#   59 .................................................AUUAUUCUGUGACACUUUCAG

       miR-1021
       UACUGUUUUGAAACAAGUGAGAUCAUGUGAAAUCCUCGGAGCUCGGAGCUUUGAAUUUUCCAAAUAUUCAUAUUUAAAUUAUUUGUUUCAAACAGUA
       (((((((.((((((((((((....(((((((....(((((((.....)))))))............))))))).....)))))))))))))))))))
       Note: Annotated mature miR-1021 AAGUGAGAUCAUGUGAAAUCCUCGG was not detectable.
```

```
        miR-1022
        GGUCCAAAAUCGGCAAGAUCAUUGUUAGGACGCCAUCUUGAAGCAAUAUAAAGAUGAUAGUCCAAUGAUGAUCCAGCUGUUCAAGGCU
        ((((......((((..((((((..((.((((..((((((...........))))))...))))))..))))))..)))).....))))
      1 ..............AAGAUCAUUGUUAGGACG........................................................
      2 ..............AAGAUCAUUGUUAGGACGC.......................................................
     28 ..............AAGAUCAUUGUUAGGACGCC......................................................
    627 ..............AAGAUCAUUGUUAGGACGCCA.....................................................
      7 .............CAAGAUCAUUGUUAGGACGCCAU....................................................
   2039 ..............AAGAUCAUUGUUAGGACGCCAU....................................................
      3 .............CAAGAUCAUUGUUAGGACGCCAUC...................................................
#  16698 ..............AAGAUCAUUGUUAGGACGCCAUC...................................................
      4 ...............AGAUCAUUGUUAGGACGCCAUC...................................................
     11 ....................AUUGUUAGGACGCCAUC...................................................
      3 .............CAAGAUCAUUGUUAGGACGCCAUCU..................................................
   9796 ..............AAGAUCAUUGUUAGGACGCCAUCU..................................................
      5 ...............AGAUCAUUGUUAGGACGCCAUCU..................................................
      1 ................GAUCAUUGUUAGGACGCCAUCU..................................................
      1 .................AUCAUUGUUAGGACGCCAUCU..................................................
      3 ....................AUUGUUAGGACGCCAUCU..................................................
    145 ..............AAGAUCAUUGUUAGGACGCCAUCUU.................................................
      2 ..............AAGAUCAUUGUUAGGACGCCAUCUUG................................................
      1 .......................................................GAUAGUCCAAUGAUGAUCCAGC...........

        miR-1817
        UGUACAUUUCAAUUUUCGAGUAGCCAAUGUCUUCUCUAUCAUGCAUUUUACAAAUAAUGAGUACAUGAUAGUGAAAUAUUUGCUUCCUGAAUUUCAGAGAUGUUU
        ...(((((((....((((.(.(((.(((((.(((.((((((((.((((..........)))).)))))))).)))))))).))).).)))).....))))))).. 
      1 ....................UAGCCAAUGUCUUCUCUA...................................................................
#      3 ....................UAGCCAAUGUCUUCUCUAUCAUG..............................................................

        miR-1818
        CUGGAAAGAGUGGCCUUUUGUGGUCUUCAUGCCAUGAUUUUAUCACUCAAACUGAUAAAAUCAUAGUUUGGAAACCUCGACAGGCUUUUCUUUUCUU
        ..((((((((.(((((.(((.(((.((((.((.((((((((((((.......)))))))))))).)).)))).))).))).))))).))))))))..
        Note: Annotated mature miR-1818 UGUGGUCUUCAUGCCAUGAUUUU was not detectable.

        miR-1819
        AAUCAGUGAUCAAUCAUGCUCAAAACAUUCGACAUAACUUAAUUUCUUUGUGGAAUGAUUGAGCUUGAUGGAUCGAUGAAA
        ..(((.(((((.((((.((((((..(((((.(((..............))).))))).)))))).)))).))))).)))..
      4 ...........AAUCAUGCUCAAAACAUUCGACA...............................................
      7 ..................................................UGGAAUGAUUGAGCUUGA.............
      1 ..................................................UGGAAUGAUUGAGCUUGAU............
      4 ..................................................UGGAAUGAUUGAGCUUGAUG...........
    136 ..................................................UGGAAUGAUUGAGCUUGAUGG..........
#   3824 ..................................................UGGAAUGAUUGAGCUUGAUGGA.........
   2719 ..................................................UGGAAUGAUUGAGCUUGAUGGAU........
      1 ..................................................UGGAAUGAUUGAGCUUGAUGGAUC.......
      4 ...................................................GGAAUGAUUGAGCUUGAUGGA.........
      2 ....................................................GAAUGAUUGAGCUUGAUGGAU........
```

```
      1 .......................................................UGAUUGAGCUUGAUGG..........
      3 .......................................................UGAUUGAGCUUGAUGGA.........

        miR-1820
        UUCAAAAAUUGCAUUUUCCAUCUUUUGAUUGUUUUUCGAUGAUGUUCGUUAAAUCGGUAUAAGCGAACCAUUGUAAACAAUCAAAGAAUGGAGAAUCAAUUUAUGAU
        .(((.((((((.(((((((((..(((((((((((..(((((..(((((((...........)))))))))))).)))))))))))..))))))))))))))).))).
      1 .....................CUUUUGAUUGUUUUUCGAUGAUGU..............................................................
      1 ......................UUUUGAUUGUUUUUCGAUGA.................................................................
      1 ......................UUUUGAUUGUUUUUCGAUGAUG...............................................................
     20 ......................UUUUGAUUGUUUUUCGAUGAUGU..............................................................
     37 ......................UUUUGAUUGUUUUUCGAUGAUGUU.............................................................
#   169 ......................UUUUGAUUGUUUUUCGAUGAUGUUC............................................................
    367 ......................UUUUGAUUGUUUUUCGAUGAUGUUCG...........................................................
      4 .......................UUUGAUUGUUUUUCGAUGAUGUUCG...........................................................
      1 ........................UUGAUUGUUUUUCGAUGAUGUUC............................................................
      2 ........................UUGAUUGUUUUUCGAUGAUGUUCG...........................................................
      1 .................................................................AACCAUUGUAAACAAUCAAAGA....................
      1 ..................................................................ACCAUUGUAAACAAUCAAAGA....................
      5 ..................................................................ACCAUUGUAAACAAUCAAAGAA...................
      2 ..................................................................ACCAUUGUAAACAAUCAAAGAAU..................

        miR-1821
        AUAUGUAGUUGGUACAAGUUUGCCCAACUUGCAGACUUUUCAAUUUUCGGCAAGUGGAAUUGAGGUCUUAUAGUUAGGUAGACAUUUACUACAACUAAUAUAUA
        ((((((((((((((...(((((((.((((...(((((..((((((((........)))))))))))))...)))).)))))))...)))..))))).)))))).
      1 ....................UGCCCAACUUGCAGACUUUUCAAUU...........................................................
      1 ............................................................UGAGGUCUUAUAGUUAGGU.........................
      2 ............................................................UGAGGUCUUAUAGUUAGGUA........................
#     3 ............................................................UGAGGUCUUAUAGUUAGGUAG.......................
      3 ...........................................................UUGAGGUCUUAUAGUUAGGUAGA......................
    127 ............................................................UGAGGUCUUAUAGUUAGGUAGA......................

        miR-1822
        AUUCUUGAAAACUCCAAUAGUUUCUCUGGGAAAGCUAUCGGCCAAAUUUAACUGUCCGAGCUGCCCUCAGAAAAACUCUUGGCUCAUCGAGAAA
        .(((((((.....((((.(((((.((((((..(((..((((.((........)).)))))))..)).)))).))))).))))....))))))).
      3 ..................AGUUUCUCUGGGAAAGCUAUCGG.....................................................
      2 ..................AGUUUCUCUGGGAAAGCUAUCGGC....................................................
      1 ..................AGUUUCUCUGGGAAAGCUAUCGGCC...................................................
      1 ....................UUCUCUGGGAAAGCUAUCGGC.....................................................
      2 ....................UUCUCUGGGAAAGCUAUCGGCC....................................................
      2 .......................................................CCGAGCUGCCCUCAGAAAAACUCU...............
      1 ........................................................CGAGCUGCCCUCAGAAAAA...................
      4 ........................................................CGAGCUGCCCUCAGAAAAAC..................
     24 ........................................................CGAGCUGCCCUCAGAAAAACUCU...............
      1 .........................................................GAGCUGCCCUCAGAAAAACU.................
      1 .........................................................GAGCUGCCCUCAGAAAAACUC................
#    32 .........................................................GAGCUGCCCUCAGAAAAACUCU...............
      1 .........................................................GAGCUGCCCUCAGAAAAACUCUU..............
```

```
          miR-1823
          CUUUGACCUGAACGUCACCCCUAACCCUAUGCAGUAUUUGGAGCUCAUAAAAUACUGGAAGUGUUUAGGAGUAAUGCUCAGUUGUCAGAA
          .((((((((((.(((.((.(((((.(((.(.((((((((.(....)...)))))))).))).).))))).)).))).))))..)))))).
        1 ...................................................AUACUGGAAGUGUUUAGGAGU..................
        1 ...................................................AUACUGGAAGUGUUUAGGAGUA.................
       23 ...................................................AUACUGGAAGUGUUUAGGAGUAA................
#       2 ....................................................UACUGGAAGUGUUUAGGAGUAA................

          miR-1824
          GUUCACCAGAGUUGGCAGUGUUUCUCCCCCAACUUUUGUUACAGAAUUGCAAAAAUGUUGGCCGUGGUGAACACUUCCGCCUCUGGUUUGAAG
          .((((((((((.(((.(((((((..((((((((((((((.........))))))..)))))..).)).))))))).))).)))))))..))).
#       4 ............UGGCAGUGUUUCUCCCCCAACUU..........................................................

        1 ....ACUUUUAUCGGUUCCGGUC........................................
          miR-1828
          GAUCACUUUUAUCGGUUCCGGUCCCUCUGCAAAAAAGUGGACUGGAAGCAUUUAAGUGAUAGU
          .(((((((..((.(.(((((((((..((.......)).))))))))).)))..)))))))...
          Note: Annotated mature miR-1828 ACUGGAAGCAUUUAAGUGAUAGU was not detectable.

          miR-1829a
          AAGGGGACUUCUAAUUGUUUGUAAAAAAUCGAGUAUUACAACCAUUGGAAUUUCUCUAUU
          .((((((.(((((((.(((.((((...........))))))).))))))).))))))...
        1 AAGGGGACUUCUAAUUGU..........................................
        1 AAGGGGACUUCUAAUUGUU.........................................
        5 AAGGGGACUUCUAAUUGUUUG.......................................
       39 AAGGGGACUUCUAAUUGUUUGU......................................
      248 AAGGGGACUUCUAAUUGUUUGUA.....................................
        1 AAGGGGACUUCUAAUUGUUUGUAA....................................
        1 AAGGGGACUUCUAAUUGUUUGUAAA...................................
        1 ......................................CAACCAUUGGAAUUUCUCUA..
       11 ......................................CAACCAUUGGAAUUUCUCUAU.
#      21 ......................................CAACCAUUGGAAUUUCUCUAUU

          miR-1829b
          AAGCGAUCUUCUAGAUGGUUGUAAAACAUCGAGUAUUACAACCACUGGAAUUUCUCUAUU
          .((.((..((((((.(((((((((.((.....)).)))))))))))))))..)).))...
       33 AAGCGAUCUUCUAGAUGG..........................................
        7 AAGCGAUCUUCUAGAUGGU.........................................
       18 AAGCGAUCUUCUAGAUGGUU........................................
      105 AAGCGAUCUUCUAGAUGGUUG.......................................
      272 AAGCGAUCUUCUAGAUGGUUGU......................................
#   11929 AAGCGAUCUUCUAGAUGGUUGUA.....................................
       50 AAGCGAUCUUCUAGAUGGUUGUAA....................................
       11 AAGCGAUCUUCUAGAUGGUUGUAAA...................................
       27 .AGCGAUCUUCUAGAUGGUUGUA.....................................
        1 ......................................CAACCACUGGAAUUUCUC....
```

```
          4 ......................................CAACCACUGGAAUUUCUCUAU.
          2 ......................................CAACCACUGGAAUUUCUCUAUU
          2 ..GCGAUCUUCUAGAUGGUUGUA.....................................
          1 .......................................AACCACUGGAAUUUCUCUAU.
          5 ......................................CAACCACUGGAAUUUCUCUAUU

            miR-1829c
            AAGCGAAAUUCAAGAUGGUUGUAAAACAUCGAGUACUACAACCACUGGAAUUUCUCUAUUGC
            .((.(((((((.((.((((((((..((.....))..)))))))))).))))))).)).....
         11 AAGCGAAAUUCAAGAUGG............................................
          4 AAGCGAAAUUCAAGAUGGU...........................................
          2 AAGCGAAAUUCAAGAUGGUU..........................................
         23 AAGCGAAAUUCAAGAUGGUUG.........................................
        127 AAGCGAAAUUCAAGAUGGUUGU........................................
#      2143 AAGCGAAAUUCAAGAUGGUUGUA.......................................
          4 .AGCGAAAUUCAAGAUGGUUGUA.......................................
          1 ..GCGAAAUUCAAGAUGGUUGUA.......................................
         22 AAGCGAAAUUCAAGAUGGUUGUAA......................................
          6 AAGCGAAAUUCAAGAUGGUUGUAAA.....................................
          1 ......................................CAACCACUGGAAUUUCUC......
          4 ......................................CAACCACUGGAAUUUCUCUAU...
          2 ......................................CAACCACUGGAAUUUCUCUAUU..
          1 .......................................AACCACUGGAAUUUCUCUAU...
          5 ......................................CAACCACUGGAAUUUCUCUAUU..

            miR-1830
            CGAGGUUUCACGUUUUCUAGGCCACGCCGCAAAUAGCCAGCUUGUGGCCUAGGAAAUGAGAAAACUCGGC
            ((((.((((.(((((((((((((((((.((.....))..))..))))))))))))))).)))).))))..
          2 CGAGGUUUCACGUUUUCUAGGCC...............................................
          1 ................................................CUAGGAAAUGAGAAAAC.....
          6 ...............................................CCUAGGAAAUGAGAAAAC.....
          4 ...............................................CCUAGGAAAUGAGAAAACUCG..
          1 ................................................CUAGGAAAUGAGAAAACUCG..
          1 ..............................................GCCUAGGAAAUGAGAAAACUCGG.
         21 ...............................................CCUAGGAAAUGAGAAAACUCGG.
          4 ................................................CUAGGAAAUGAGAAAACUCGG.
        809 ...............................................CCUAGGAAAUGAGAAAACUCGGC
        136 ................................................CUAGGAAAUGAGAAAACUCGGC
          1 .................................................UAGGAAAUGAGAAAACUCGGC
            Note: Reads that partially matched to annotated mature miR-1830 CGAGGUUUCACGUUUUCUAGGC were detectable.

            miR-1831
            ACCUGGCUGGGGGUAUCUCGUGAUCAUGAAGACGGGAUCCCCAUGGUGA
            .....((((((((.(((((((..........))))))))))).))))..
          1 .CCUGGCUGGGGGUAUCUC..............................
          1 .CCUGGCUGGGGGUAUCUCGU............................
#         2 ACCUGGCUGGGGGUAUCUCGUG...........................
```

```
 1 .CCUGGCUGGGGGUAUCUCGUG...........................
 1 ..CUGGCUGGGGGUAUCUCGUG...........................
 4 ACCUGGCUGGGGGUAUCUCGUGA..........................
 5 .CCUGGCUGGGGGUAUCUCGUGA..........................
12 ACCUGGCUGGGGGUAUCUCGUGAU.........................
 5 .CCUGGCUGGGGGUAUCUCGUGAU.........................
 1 ...UGGCUGGGGGUAUCUCGUGAU.........................
 2 ACCUGGCUGGGGGUAUCUCGUGAUC........................
 5 .CCUGGCUGGGGGUAUCUCGUGAUC........................
 1 ACCUGGCUGGGGGUAUCUCGUGAUCA.......................
 1 ......CUGGGGGUAUCUCGUGAUCA.......................
 1 ......CUGGGGGUAUCUCGUGAUCAUGA....................
 1 .......UGGGGGUAUCUCGUGAUCAUGAA...................
 1 ......CUGGGGGUAUCUCGUGAUCAUGAAG..................
 2 .........GGGGUAUCUCGUGAUCAUGAAGA.................
 2 ........GGGGGUAUCUCGUGAUCAUGAAGA.................
 3 .........GGGGUAUCUCGUGAUCAUGAAGAC................
 2 ............GUAUCUCGUGAUCAUGAAGAC................
 1 .........GGGGUAUCUCGUGAUCAUGAAGACG...............
 1 ..........GGGUAUCUCGUGAUCAUGAAGACG...............
 1 ...........GGUAUCUCGUGAUCAUGAAGACG...............
 2 ............GUAUCUCGUGAUCAUGAAGACG...............
 1 ...............UCUCGUGAUCAUGAAGACG...............
 2 ................CUCGUGAUCAUGAAGACG...............
 3 .................UCGUGAUCAUGAAGACG...............
 1 ..............AUCUCGUGAUCAUGAAGACGG..............
 3 ................CUCGUGAUCAUGAAGACGG..............
 2 ..................CGUGAUCAUGAAGACGG..............
 2 ................CUCGUGAUCAUGAAGACGGG.............
 2 ..............AUCUCGUGAUCAUGAAGACGGGA............
 1 ................CUCGUGAUCAUGAAGACGGGAU...........
 1 ..................CGUGAUCAUGAAGACGGGAUCC.........
 1 .......................UCAUGAAGACGGGAUCC.........
 1 ..................CGUGAUCAUGAAGACGGGAUCCC........
 1 .........................AUGAAGACGGGAUCCCC.......
 1 ..................CGUGAUCAUGAAGACGGGAUCCCCAU.....
 1 ........................CAUGAAGACGGGAUCCCCAU.....
 1 ........................CAUGAAGACGGGAUCCCCAUGGUG.
 1 .........................AUGAAGACGGGAUCCCCAUGGUG.

   miR-1832
   CAGCGAUUCGAACUCCGCCCACUGCACCUGAUUGGUUGACAAGUGGGCGGAGCGAAUCGAUGAU
   ((.(((((((..(((((((((((.((((.....)).))...)))))))))))))))))).))..
 2 .AGCGAUUCGAACUCCGCCCAC..........................................
 2 .AGCGAUUCGAACUCCGCCCACU.........................................
#  4 ...........................................UGGGCGGAGCGAAUCGAUGAU
 1 ...........................................UGGGCGGAGCGAAUCGA....
```

```
       miR-1833
       ACGCUUACUUCGCAAGCCUCGCGCGUUUUCUUUUCAAGAGAAAAAGCGUGCGAGGCUUGCGAAAUAAGUGUGC
       ((((((.(((((((((((((((((((((((((.....))))))..))))))))))))))))))).))))))..
     2 ..................................................CGAGGCUUGCGAAAUAAGUGUG.
       Note: Annotated mature miR-1833 CGAGGCUUGCGAAAUAAGUGUGC was not detectable.

       miR-1834
       UGACUCGGUGUGUGAUCUCUUACCGUUUUAUGGAUAAGAGAUCAACCAUUGAGAUCCAA
       ...(((((((..((((((((((((((...)))).))))))))))..)))))))......
     2 .GACUCGGUGUGUGAUCUCUUA.....................................
     1 ..............AUCUCUUACCGUUUUAUG...........................
#   29 ....................................AGAGAUCAACCAUUGAGAUCCAA

       miR-1832b
       ATTTTGACTAACTTCGACCAATCAGCGAATCGCTCGGCCCACTTTTGTGAACCAATCAGCGTCAAAAGTGGGCAGAGCGATTCGCTGATTGGTCGTTATCATTTTGAAAGG
       .(((..(.......(((((((((((((((((((((.((((((((((((((.....)))....))))))))))).)))))))))))))))))))))........)..)))..
     1 ......................CAGCGAATCGCTCGGCCCACTTT..................................................................
     1 ..........................GAATCGCTCGGCCCACTTTTG................................................................
     1 ................................................................AAAGTGGGCAGAGCGATTCGC..........................
     2 ..................................................................AGTGGGCAGAGCGATTCGCTGA.......................
#    4 ..................................................................AGTGGGCAGAGCGATTCGCTGAT......................
     1 ...................................................................GTGGGCAGAGCGATTCGCTGAT......................

       miR-2207
       ATATTCTTTGAACCACATGTTTTGTGTGAATTGAGACTGTGTATAAGAATGTAAGGAGGAGAGGGGCCGTTCGTATCGTTCGTCTTATGCACAGGCTCAATGCACACAAA
       ....................((((((((.((((((.((((((((((((.((.((.((...(((......)))...)).)))))))))))))))).)))))).))))))))
     1 .......................GTGTGAATTGAGACTGTGTATA.................................................................
     2 ........................TGTGAATTGAGACTGTGTATA.................................................................
     1 ........................TGTGAATTGAGACTGTGTATAA................................................................
#    4 ........................TGTGAATTGAGACTGTGTATAAG...............................................................
     1 ......................................................................................ATGCACAGGCTCAATGCACACAA.

       miR-2208a
       TATTCGGGGACCCGCCAGTGGTAAGTGTACCCGAATCTGATATCCTATCATCTGAAAAAGGATGCAGTTTCTGGTATACTTCACCATTGGCTGGGTCTCTTAGCAC
       .....((((((((((((((((((((((((((.(((.(((.((((((.((....))...))))))))).))).))))))))).))))))))).))))))))......
#    8 ......................AAGTGTACCCGAATCTGATATCC.............................................................
     4 .............................................................ATGCAGTTTCTGGTATACTTCA.......................

       miR-2208b-5p
       CTACTGAGGAACCCGCCAGTGGTAAGTGTACCCGGATCTGATATCCTATCACCAAAAAGAGGATGCAGATTTTGGTACACTTCACCACTGGCTGGGTCCCTGAATAGGA
       (((.(.(((.((((((((((((((((((((((..((((((.((((((............))))))))))))..))))))))).))))))))).)))).))).).)))..
     3 ......................TAAGTGTACCCGGATCTGATATC................................................................
     3 ......................TAAGTGTACCCGGATCTGATATCC...............................................................
     1 .......................AAGTGTACCCGGATCTGA....................................................................
     1 .......................AAGTGTACCCGGATCTGATA..................................................................
     6 .......................AAGTGTACCCGGATCTGATAT.................................................................
```

```
     46 .......................AAGTGTACCCGGATCTGATATC................................................................
#   495 .......................AAGTGTACCCGGATCTGATATCC...............................................................
     10 .......................AAGTGTACCCGGATCTGATATCCT..............................................................
      1 ..............................................................ATGCAGATTTTGGTACAC.............................
      3 ..............................................................ATGCAGATTTTGGTACACT............................
      1 ..............................................................ATGCAGATTTTGGTACACTT...........................
      4 ..............................................................ATGCAGATTTTGGTACACTTC..........................
    305 ..............................................................ATGCAGATTTTGGTACACTTCA.........................
    174 ..............................................................ATGCAGATTTTGGTACACTTCAC........................
      1 ..............................................................ATGCAGATTTTGGTACACTTCACCA......................
      7 ...............................................................TGCAGATTTTGGTACACTTCA.........................
      3 ...............................................................TGCAGATTTTGGTACACTTCAC........................
      2 ..................................................................AGATTTTGGTACACTTCA.........................

        miR-2208b-3p
        TACTGAGGAACCCGCCAGTGGTAAGTGTACCCGGATCTGATATCCTATCACCAAAAAGAGGATGCAGATTTTGGTACACTTCACCACTGGCTGGGTCCCTGAATAG
        ...(.(((.((((((((((((((((((((((..((((((.((((((............))))))))))))..)))))))))).))))))))).)))).))).)....
      3 .....................TAAGTGTACCCGGATCTGATATC..............................................................
      3 .....................TAAGTGTACCCGGATCTGATATCC.............................................................
      1 ......................AAGTGTACCCGGATCTGA..................................................................
      1 ......................AAGTGTACCCGGATCTGATA................................................................
      6 ......................AAGTGTACCCGGATCTGATAT...............................................................
     46 ......................AAGTGTACCCGGATCTGATATC..............................................................
    495 ......................AAGTGTACCCGGATCTGATATCC.............................................................
     10 ......................AAGTGTACCCGGATCTGATATCCT............................................................
      1 .............................................................ATGCAGATTTTGGTACAC...........................
      3 .............................................................ATGCAGATTTTGGTACACT..........................
      1 .............................................................ATGCAGATTTTGGTACACTT.........................
      4 .............................................................ATGCAGATTTTGGTACACTTC........................
#   305 .............................................................ATGCAGATTTTGGTACACTTCA.......................
    174 .............................................................ATGCAGATTTTGGTACACTTCAC......................
      1 .............................................................ATGCAGATTTTGGTACACTTCACCA....................
      7 ..............................................................TGCAGATTTTGGTACACTTCA.......................
      3 ..............................................................TGCAGATTTTGGTACACTTCAC......................
      2 .................................................................AGATTTTGGTACACTTCA.......................

        miR-2209a
        ACCATGACTCTCAGGAATGGTGAGTGTAACCACTCTTCTCCTTCCGAGTATTGATTATCGAGAAGAGATCAGCGGTTACACTACATTATTAGTAAGAGTCCTTATTCAT
        .....((((((....((((((((((((((((.((..((((.((((((..........)))).)))))))..)).))))))))).)))))))....)))))).........
      4 ......................AGTGTAACCACTCTTCTCCTT..................................................................
      6 ......................AGTGTAACCACTCTTCTCCTTC.................................................................
      1 ...............................................................AGAGATCAGCGGTTACA.............................
      3 ...............................................................AGAGATCAGCGGTTACACT...........................
     23 ...............................................................AGAGATCAGCGGTTACACTA..........................
    398 ...............................................................AGAGATCAGCGGTTACACTAC.........................
#  2776 ...............................................................AGAGATCAGCGGTTACACTACA........................
     47 ...............................................................AGAGATCAGCGGTTACACTACAT.......................
```

```
     1 ...............................................................AGAGATCAGCGGTTACACTACATT......................
     2 ................................................................GAGATCAGCGGTTACACTACA........................
     1 ................................................................GAGATCAGCGGTTACACTACAT.......................
     1 .................................................................AGATCAGCGGTTACACTACA........................
     2 ...................................................................ATCAGCGGTTACACTACA........................
     1 ....................................................................TCAGCGGTTACACTACA........................

       miR-2209b
       ACAGCCCTGGCTCCCGGGAATGGTGAGTGTAACAACTCTTCTCCTTCCGAAAACCAATAATCGAGAAGAGATGAGCGGTTGTGCTTCACCATTGGTAGGGAGTCTCCACC
       ........(((((((...(((((((((..((((..(((.((((.((((((..........))).))))))).)))..))))..).))))))))....)))))))......
     1 .........................AGTGTAACAACTCTTCTCCTTC...............................................................
     1 ..................................................................AGAGATGAGCGGTTGTGCTTC.......................
#    3 ..................................................................AGAGATGAGCGGTTGTGCTTCA......................

       miR-2209c
       AAAACCATGCCTCCCAATGGTGAGTGTAACCGCACGTCTTGTTTCAAAACTCAATATATGGAAAAAGACCACCGGTTACACTACATTATTGGTTGGAGTCTGATTAA
       .........((..(((((((((((((((((((...(((((.(((((............))))).)))))...)))))))))).)))))))))..))...........
     4 ......................AGTGTAACCGCACGTCTTGTTT...............................................................
     1 ......................AGTGTAACCGCACGTCTTGTTTC..............................................................
     1 ..............................................................AAAAGACCACCGGTTACAC..........................
     2 ..............................................................AAAAGACCACCGGTTACACTA........................
    49 ..............................................................AAAAGACCACCGGTTACACTAC.......................
#  145 ..............................................................AAAAGACCACCGGTTACACTACA......................
     1 ...............................................................AAAGACCACCGGTTACA...........................
     2 ...............................................................AAAGACCACCGGTTACACTAC.......................
     9 ...............................................................AAAGACCACCGGTTACACTACA......................

       miR-2210
       TTGTTTAGTCCATTCAGTTTGTAGGCAGATCAATCAATTTTTAGGTTTATATAAATAATCCTAAAGTCGATTGCTCTACCCACAAAAAGTAATGTTGACTTGAAGTT
       ...((.(((((((((..(((((.((.(((.(((((...(((((((.((((....)))).)))))))..))))).))).)).)))))..).)))).)))).))....
#   18 ......................AGGCAGATCAATCAATTTTTAGG..............................................................
     1 .............................................................TAAAGTCGATTGCTCTACCCA.........................
    16 .............................................................TAAAGTCGATTGCTCTACCCAC........................
     2 .............................................................TAAAGTCGATTGCTCTACCCACA.......................
     1 .............................................................TAAAGTCGATTGCTCTACCCACAA......................

       miR-2211
       GGGCTAAATGTATGTCTCGGTCTCTCCGCCTCCATCTATTCTCCATCTGACGACGTCCCGTCCCGTCAGGTAGAATTTAGAGGAGAAAAGAGTCATAGTTGCGCGGGGGG
       ((((.........))))....((((((((((((.((((...((.(((((((((((...))))..))))))).))...))))))))........((....)).))))))))
     1 .............................CTCCATCTATTCTCCATCT..............................................................
     4 .................................................................TCAGGTAGAATTTAGAGGAGAA.......................
#    5 .................................................................TCAGGTAGAATTTAGAGGAGAAA......................
     1 ..................................................................CAGGTAGAATTTAGAGGAGAAA......................
     1 ...................................................................AGGTAGAATTTAGAGGAGAAA......................

       miR-2212
       TATTTTGATGAAATATGCTCAGATGGCAGATCATAGGCTGACTTTGCAAGCATTTTATACAAAGTGGCATTTGATAAGCCATCTGACTATCATCTAATTCGTATT
```

```
         ......(((((....((.(((((((((..((((...(((.((((((.............)))))))))...))))..))))))))).)))))))...........
   2     ......................ATGGCAGATCATAGGCTGACTTTG...........................................................
   2     .......................TGGCAGATCATAGGCTGACT..............................................................
   3     .......................TGGCAGATCATAGGCTGACTT.............................................................
  38     .......................TGGCAGATCATAGGCTGACTTT............................................................
# 148    .......................TGGCAGATCATAGGCTGACTTTG...........................................................
  27     .......................TGGCAGATCATAGGCTGACTTTGC..........................................................
   1     .......................TGGCAGATCATAGGCTGACTTTGCA.........................................................
   2     .......................TGGCAGATCATAGGCTGACTTTGCAA........................................................
   4     ............................................................AAAGTGGCATTTGATAAGCCATC......................
   2     .............................................................AAGTGGCATTTGATAAGCCATC......................

         miR-2213
         GCCACAGAGATCAAACACGTTAATTGGCGGACTCTTCACAGTTTGACCTCGGAGATCAAGCTGTAAGAGGACTGCCTAAATATACGTGGTCAATTCCTATTC
         .....((..((....(((((.....(((((.(((((.(((((((((.(.....).))))))))))))))..))))).......)))))....))..))....
   2     ......................ATTGGCGGACTCTTCACAGTTTGA........................................................
   1     .........................................................AAGCTGTAAGAGGACTGCCTA........................
#  7     .........................................................AAGCTGTAAGAGGACTGCCTAA.......................
   1     .........................................................AAGCTGTAAGAGGACTGCCTAAA......................
   1     ..........................................................AGCTGTAAGAGGACTGCCTAA.......................

         miR-2214
         CAACCAAGAGAAGACAACTTTCAATTCGGTCCGGAGTCAATGGGTTATCTTTCAAAACCCCCCATTGACAACAACTTGACCGGCGTTTTTTGCAATTTTGTATATTATTAGG
         ....(((((((.((......))...((((((.((.(((((((((................))))))))).....)).))))))..)))))))....................
  15     ......................AATTCGGTCCGGAGTCA.........................................................................
   9     ......................AATTCGGTCCGGAGTCAA........................................................................
  14     ......................AATTCGGTCCGGAGTCAAT.......................................................................
   5     ......................AATTCGGTCCGGAGTCAATG......................................................................
   9     ......................AATTCGGTCCGGAGTCAATGG.....................................................................
   3     ......................AATTCGGTCCGGAGTCAATGGG....................................................................
   5     ......................AATTCGGTCCGGAGTCAATGGGT...................................................................
  10     ......................AATTCGGTCCGGAGTCAATGGGTT..................................................................
   3     ......................AATTCGGTCCGGAGTCAATGGGTTA.................................................................
  14     ......................AATTCGGTCCGGAGTCAATGGGTTAT................................................................
   2     .......................ATTCGGTCCGGAGTCAAT.......................................................................
   2     .......................ATTCGGTCCGGAGTCAATG......................................................................
   1     .......................ATTCGGTCCGGAGTCAATGGGT...................................................................
   1     .......................ATTCGGTCCGGAGTCAATGGGTTATC...............................................................
   1     ........................TTCGGTCCGGAGTCAATGGGT...................................................................
   1     ........................TTCGGTCCGGAGTCAATGGGTTATC...............................................................
   1     ...............................CGGAGTCAATGGGTTAT................................................................
   2     ......................................AATGGGTTATCTTTCAA.........................................................
   2     ......................................AATGGGTTATCTTTCAAA........................................................
   1     .....................................................AAAACCCCCCATTGACAACAACT....................................
   1     .............................................................CCATTGACAACAACTTGACCGGCG...........................
   6     .............................................................CCATTGACAACAACTTGACCGGCGT..........................
   5     .............................................................CCATTGACAACAACTTGACCGGCGTT.........................
```

```
        1 ..............................................................CATTGACAACAACTTGACCG..............................
        2 ..............................................................CATTGACAACAACTTGACCGGCG...........................
       14 ..............................................................CATTGACAACAACTTGACCGGCGT..........................
#      23 ..............................................................CATTGACAACAACTTGACCGGCGTT.........................
        1 ..............................................................CATTGACAACAACTTGACCGGCGTTT........................
        3 ...............................................................ATTGACAACAACTTGACCGGCG...........................
       15 ...............................................................ATTGACAACAACTTGACCGGCGT..........................
        6 ...............................................................ATTGACAACAACTTGACCGGCGTT.........................
        1 ................................................................TTGACAACAACTTGACCGGCG...........................
        8 ................................................................TTGACAACAACTTGACCGGCGT..........................
        4 ................................................................TTGACAACAACTTGACCGGCGTT.........................
        1 .................................................................TGACAACAACTTGACCGGCG...........................
        1 .................................................................TGACAACAACTTGACCGGCGT..........................
        3 .................................................................TGACAACAACTTGACCGGCGTT.........................
        4 ..................................................................GACAACAACTTGACCGGCGT..........................
        1 ..................................................................GACAACAACTTGACCGGCGTTTT.......................
        1 ...................................................................ACAACAACTTGACCGGC............................
        6 ...................................................................ACAACAACTTGACCGGCGT..........................
        5 ...................................................................ACAACAACTTGACCGGCGTT.........................
        1 ...................................................................ACAACAACTTGACCGGCGTTTT.......................
        4 ....................................................................CAACAACTTGACCGGCGT..........................
        4 ....................................................................CAACAACTTGACCGGCGTT.........................
        1 ....................................................................CAACAACTTGACCGGCGTTT........................
       15 .....................................................................AACAACTTGACCGGCGT..........................
       16 .....................................................................AACAACTTGACCGGCGTT.........................
        1 .....................................................................AACAACTTGACCGGCGTTT........................
        1 .....................................................................AACAACTTGACCGGCGTTTT.......................
        1 .....................................................................AACAACTTGACCGGCGTTTTT......................
       21 ......................................................................ACAACTTGACCGGCGTT.........................
        1 ......................................................................ACAACTTGACCGGCGTTTT.......................
        2 .......................................................................CAACTTGACCGGCGTTT........................
        2 ........................................................................AACTTGACCGGCGTTTTT......................

          miR-2215
          TATTGATTGAAATTTCACGCTAAACAGCACGTGTTACGATGCTCCGTTAGTTCGGAGTTTTTACGGAGAATCGTAGCGCGTGTTGTTTAGCGCGAATTTTTTAATTTTTTT
          ....((((((((.(((.(((((((((((((((((((((((.((((((...............)))))).))))))))))))))))))))))).)))..)))))))).....
        1 ......................AACAGCACGTGTTACGATGCTCC..................................................................
#       3 ..................................................................AGAATCGTAGCGCGTGTTGTTT.......................
        1 ...................................................................GAATCGTAGCGCGTGTTGTTT.......................
        2 ..................................................................AGAATCGTAGCGCGTGTTGTTTA......................
        3 ...................................................................GAATCGTAGCGCGTGTTGTTTA......................

          miR-2216
          TTGCATATTTTGGTTGATTTAGGCACATTTTAAGTCGGTAGGCGTCCAATTTTGATGCCTATCTACTTAAAATGTGCCTAAATCAATGAATTAATTAGAGATAAATGACC
          .........((.(((((((((((((((((((((((.((((((((((.......)))))))))).))))))))))))))))))))))).))....................
        2 ......................GCACATTTTAAGTCGGTAGG....................................................................
#       5 ......................GCACATTTTAAGTCGGTAGGC...................................................................
```

```
   3 ......................GCACATTTTAAGTCGGTAGGCG..................................................................
   2 .......................CACATTTTAAGTCGGTAGGCG..................................................................
   1 ..........................................................CTATCTACTTAAAATGTGCCTA..............................

     miR-2217
     TATTTTTCCGACATGTATGACCAGAGTGGGCAGTCGGTGTCGATCTACATCATTCGCAGGTCGACCCTTGTGCCTGTTTCGGTGGTACCAGGAAGCTTTTCCTTC
     ....(((((.....((((.(((..((..((((...((.((((((((.(.......).))))))))))...))))..))..))).))))..)))))..........
   1 ......................AGAGTGGGCAGTCGGTGTCGA..............................................................
#  1 ............................................................TCGACCCTTGTGCCTGTTTCGGT......................

     miR-2218a
     AGCTTAGGTGAGGCTCACACTACAAACTACAAGTTTTAAGCCTCACCAAAAGTTGGGTGAGGCCAGAATAGTGTAGTTTGTAGTGTGAGAGCCTCAACCAAAAA
     ......(((((((((((((((((((((((((...(((..((((((((........))))))))..)))...)))))))))))))))..))))))).))).....
#  3 ......................CAAACTACAAGTTTTAAGCCTCA...........................................................
   2 ...........................................................AGGCCAGAATAGTGTAGTTTGTA......................

     miR-2218b
     CAAAATCCGCTGGTTGAGGCTCAGACTACAAACTACATCATTTTCCATCTCTTTGGTGAGGCTAGAAAATTTGTAGTTTGTAGTGAGAGCCTCAACCAACCGCTTTTAAA
     ........((((((((((((((..(((((((((((((..((((((..((((......))))...)))))).)))))))))))))..))))))))))))...)).......
#  2 ......................AGACTACAAACTACATCATTTTC.................................................................
   1 ..................................................................AAATTTGTAGTTTGTAGTGAGA......................

     miR-2219
     TAGTTCAACTCGGCATATCGCCACAGCTTTCTCTCGCACATCGTCAGCTAGATTTGAACTCTGTATGAAAGCTGACGAAGTGCGAGGGAAAGCTGAAGAGGTGCGACGAGGTGCGACGAA
     ..(((((.((((((((.((....((((((((((((((((.((((((((((((.......))).......))))))))).))))))))))))))))..)).))))..)))).)).)))...
   1 ......................ACAGCTTTCTCTCGCACATCGTC...........................................................................
   1 ......................ACAGCTTTCTCTCGCACATCGTCA..........................................................................
   2 ...........................TTTCTCTCGCACATCGTCAGCT.......................................................................
   2 ...........................................................................CGAAGTGCGAGGGAAAGCTGA........................
# 10 ...........................................................................CGAAGTGCGAGGGAAAGCTGAAG......................
   1 ...........................................................................CGAAGTGCGAGGGAAAGCTGAAGA.....................

     miR-2220
     CAACTTGGCATTTACAGAGAAAGTAAGACCATAAACTATTTATCAATTCATCATTTGATCAATTGTTTGTGGACTTACAGGCTGCAAAATTTGAAGTACACT
     ..((((.(.((((.(((.....(((((.((((((((.(((.(((((........))))).))).)))))))).)))))...)))..)))).).)))).....
   1 ......................GTAAGACCATAAACTATTTATC..........................................................
  10 ..........................................................TCAATTGTTTGTGGACTTACA.......................
# 51 ..........................................................TCAATTGTTTGTGGACTTACAG......................
```
