# Supplementary material for: Dynamic expression of small non-coding RNAs, including novel microRNAs and piRNAs/21U-RNAs, during Caenorhabditis elegans development
Source: Genome Biol. 2009 May 21;10(5):R54. doi: 10.1186/gb-2009-10-5-r54 (PMC2718520; doi:10.1186/gb-2009-10-5-r54)
Supplement: Additional data file 4 — The names of miRNAs with more than a fivefold difference in the number of reads at some point during development and/or between genders are labeled in red and their numbers of reads are compared. The miRNAs with lower numbers of reads (less than ten in the sum of reads in any two stages compared) were not highlighted since their significant changes are not clear due to extremely low reads. [file gb-2009-10-5-r54-S4.pdf]

	Hermaphrodites (wild-type N2)						Males (*dpy-28;him-8*)
	Embryo	mid-L1	mid-L2	mid-L3	mid-L4	young adult	young adult
Total number of reads that matched to the *C. elegans* genome	5742750	5617234	6047597	4948026	6072252	5975243	7602104
let-7	51	74	71	501	34047	27185	58408
lin-4	349	41	9692	11802	14757	15614	4776
miR-1	440434	1271449	1238539	1279881	1083843	787526	445821
miR-2	2087	1043	963	480	529	698	1532
miR-34	87	2209	1498	1838	2871	3547	4767
miR-35	100771	1346	1709	483	1253	5360	1787
miR-36	7957	88	96	34	67	339	113
miR-37	50376	1015	1328	581	1159	4562	1332
miR-38	1522	20	30	16	24	35	26
miR-39	1731	20	18	8	9	61	22
miR-40	14389	292	287	89	198	670	316
miR-41	40	0	0	0	0	0	1
miR-42	871	36	127	16	61	4	45
miR-43	566	34	47	20	7	15	73
miR-44	47098	71698	77286	73882	75149	32531	33566
miR-45	47098	71698	77286	73882	75149	32531	33566
miR-46	108	36	116	101	89	73	83
miR-47	293	159	519	306	374	451	1588
miR-48	2632	980	19461	103531	452902	460202	687766
miR-49	3914	3458	1774	805	531	565	1650
miR-50	1514	3129	2234	2688	1696	1402	2601
miR-51	1280	1564	545	442	815	429	1540
miR-52	101004	24711	43918	31501	10899	10535	60510
miR-53	2267	426	1121	911	317	293	2818
miR-54	4663	455	833	536	340	427	8618
miR-55	1820	1919	1299	1159	1848	1056	2757
miR-56	986	561	517	305	499	476	2124
miR-57	1938	503	781	659	688	1232	12179
miR-58	981820	1772265	2021545	1732981	1990490	2246697	1145388
miR-59	12	9	7	13	484	384	1978
miR-60	2731	248	1011	469	271	256	2862
miR-61	634	950	1300	1863	1739	1257	973
miR-62	67	17	27	17	9	6	42
miR-63	539	2524	1848	2529	1765	2122	973
miR-64	12425	8483	13664	19707	33389	33087	14468
miR-65	5595	3646	6150	9532	17518	19679	7352
miR-66	3226	3594	4881	7264	6233	5325	4502
miR-67	279	36	64	43	24	21	42
miR-70	13000	8544	52643	62727	82940	82665	175731
miR-71	4243	141638	15036	32139	68120	91975	156589
miR-72	31238	204393	174198	122095	98074	80972	94785
miR-73	44145	30145	21501	12110	23870	12296	34329
miR-74	1887	177	182	75	108	124	303
miR-75	316	295	487	446	449	774	2826
miR-76	56	122	97	50	52	45	39
miR-77	8	5	35	180	535	1545	1015
miR-78	6	6	1	2	1	15	2
miR-79	1946	320	808	675	340	857	661
miR-227	161	147	234	281	357	274	304
miR-80	4720	6805	10097	12186	17821	14489	29973
miR-81	8065	7883	10188	11943	28644	24144	46557
miR-82	3221	2473	2497	2094	4792	7937	16105
miR-83	337	218	266	135	119	129	1523
miR-84	70	248	605	3174	2758	1977	5189
miR-85	7	2	0	0	241	1179	209

miR-86	398	209	272	239	195	215	1614
miR-87	798	462	824	310	337	362	577
miR-90	525	1765	1093	841	737	478	1917
miR-124	433	214	141	107	174	219	580
miR-228	35195	237932	275098	282777	141489	81660	64176
miR-229	1263	145	792	1515	91	346	745
miR-230	21	1407	700	2508	3055	481	453
miR-231	1000	679	383	297	163	105	84
miR-232	219	43	73	49	14	19	133
miR-233	575	200	233	118	144	129	505
miR-234	8	39	31	36	17	7	17
miR-235	58	94	36	16	22	137	3455
miR-236	1089	2182	1422	1551	1132	1284	853
miR-237	12	24	34	330	1086	1329	270
miR-238	178	1200	1286	1623	2912	2464	2394
miR-239a	16	30	42	36	65	83	388
miR-239b	13	39	36	140	282	282	321
miR-240	3	0	2	0	51	109	62
miR-241	8	3	80	355	1146	1125	4027
miR-242	23	40	39	48	51	64	20
miR-243	25	190	165	123	218	201	456
miR-244	345	45	115	106	34	27	71
miR-245	33	78	61	31	9	19	58
miR-246	0	0	0	0	97	352	36
miR-247	0	0	0	9	6	1	5
miR-248	142	1285	1266	1473	1032	893	1226
miR-249	1	5	5	6	5	6	5
miR-250	2643	7006	11389	18104	11968	11686	3521
miR-251	10	6	10	6	6	6	20
miR-252	1542	6318	5187	4678	3117	3135	6811
miR-253	54	169	81	52	288	158	241
miR-254	45	58	79	37	44	50	90
miR-255	190	1262	1053	1098	571	355	55
miR-256	0	0	0	0	0	0	0
miR-257	0	0	0	0	0	0	0
miR-258	0	0	0	0	0	0	0
miR-259	23	20	34	37	21	37	15
miR-260	3	0	1	0	0	0	0
miR-261	0	0	0	0	0	0	0
miR-262	0	0	0	0	0	0	0
miR-264	0	0	0	0	0	0	0
miR-265	0	0	0	0	0	0	0
miR-266	0	0	0	1	0	0	0
miR-267	0	0	0	0	0	0	0
miR-268	0	0	0	0	0	0	0
miR-269	0	0	0	0	0	0	0
miR-270	0	0	0	0	0	0	0
miR-271	0	0	0	0	0	0	0
miR-272	0	1	0	0	0	0	0
miR-273	0	0	0	0	0	0	0
miR-353	0	0	0	0	0	0	0
miR-354	0	0	0	0	0	0	0
miR-355	0	1	3	0	0	2	2
miR-356	0	0	0	0	0	0	0
miR-357	0	0	0	0	4	3	73
miR-358	0	0	0	0	5	6	87
miR-359	2	1	0	2	6	26	20
miR-360	0	0	0	0	0	0	2
lsy-6	60	25	14	8	9	5	4
miR-392	0	0	2	1	0	1	8
miR-784	0	0	3	0	4	6	48
miR-785	16	55	41	39	68	94	239
miR-786	1	1	0	2	77	144	13
miR-787	45	12	23	12	15	10	32

miR-788	30	12	84	146	107	2	32
miR-789	0	0	0	0	0	1	28
miR-790	17	204	121	111	64	47	45
miR-791	9	52	24	2	3	6	20
miR-792	21	4	5	2	0	0	2
miR-793	16	143	110	57	56	42	51
miR-794	5	10	17	21	15	20	22
miR-795	114	2767	3530	6533	3453	2550	2713
miR-796	1413	1677	645	370	240	185	5764
miR-797	8	0	6	46	7	1	25
miR-798	7	5	3	8	26	44	3
miR-799	4	4	66	117	164	256	17
miR-800	0	0	0	0	1	0	0
miR-1018	24	59	77	50	17	14	402
miR-1019	0	0	0	0	0	0	0
miR-1020	2	9	13	30	26	20	8
miR-1021	0	0	0	0	0	0	0
miR-1022	248	11972	6994	5090	2020	1314	829
miR-1817	1	0	0	0	1	2	1
miR-1818	0	0	0	0	0	0	0
miR-1819	116	102	92	409	855	814	3304
miR-1820	102	49	134	211	15	10	71
miR-1821	11	53	25	19	17	8	16
miR-1822	3	19	12	13	11	8	2
miR-1823	3	12	8	0	2	1	0
miR-1824	0	1	1	1	0	1	0
miR-1828	0	0	0	0	0	0	0
miR-1829a	4	4	7	5	7	4	2
miR-1829b	1041	1140	1159	3499	2963	2661	160
miR-1829c	306	279	309	644	432	330	123
miR-1830	0	0	1	2	0	0	0
miR-1831	1	0	1	0	0	1	25
miR-1832	11	2	5	0	2	6	6
miR-1833	0	0	0	0	0	0	0
miR-1834	1	1	0	2	9	21	2
