# Supplementary figures and images for: Dynamic expression of small non-coding RNAs, including novel microRNAs and piRNAs/21U-RNAs, during Caenorhabditis elegans development
Source: Genome Biol. 2009 May 21;10(5):R54. doi: 10.1186/gb-2009-10-5-r54 (PMC2718520; doi:10.1186/gb-2009-10-5-r54)

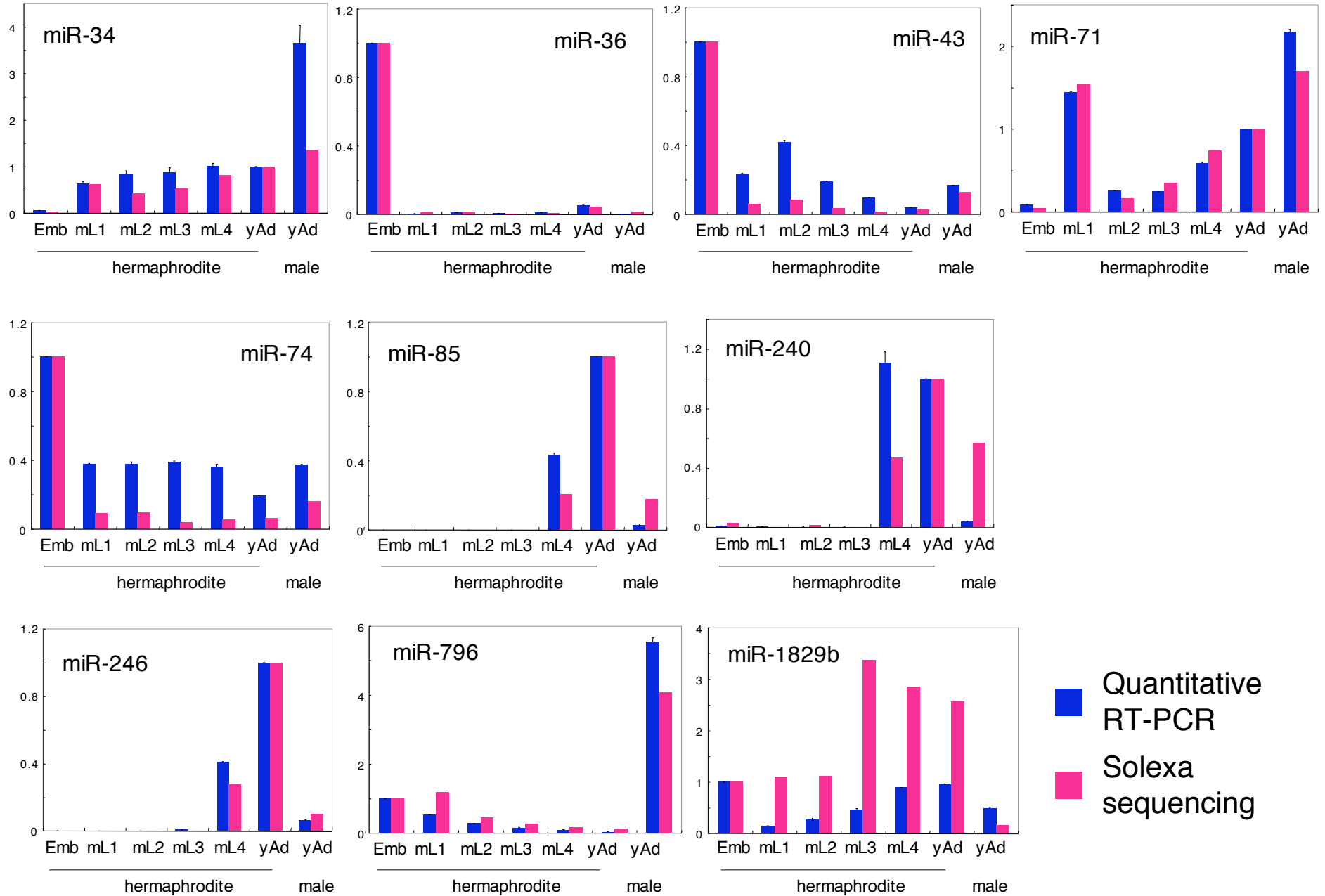

Supplement: Additional data file 5 — Vertical axis indicates the relative expression level. The data from both RT-PCR and Solexa sequencing were standardized to the expression level in the embryonic sample as 1. The results were further confirmed using independently prepared RNAs. [file gb-2009-10-5-r54-S5.pdf]

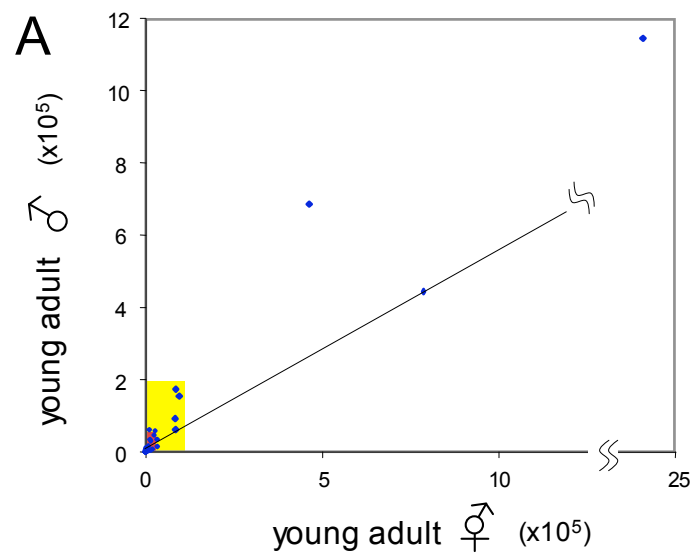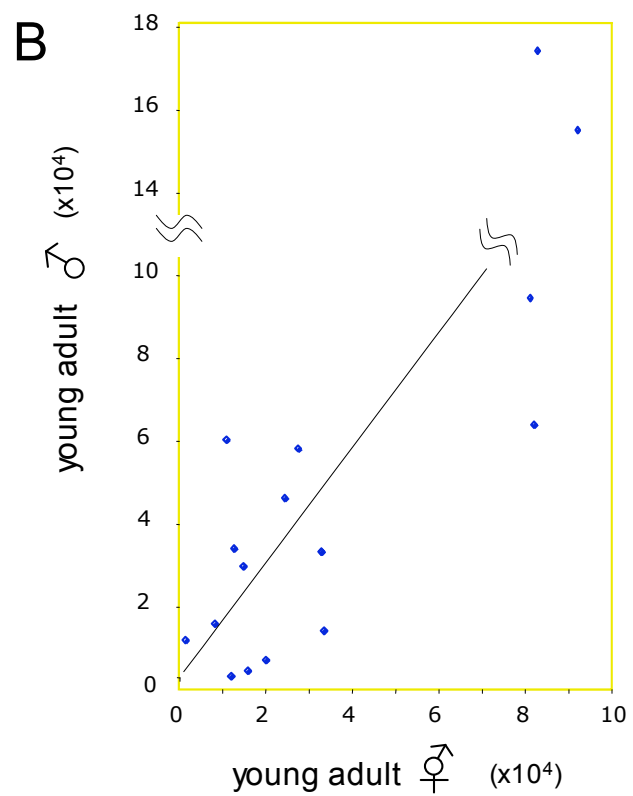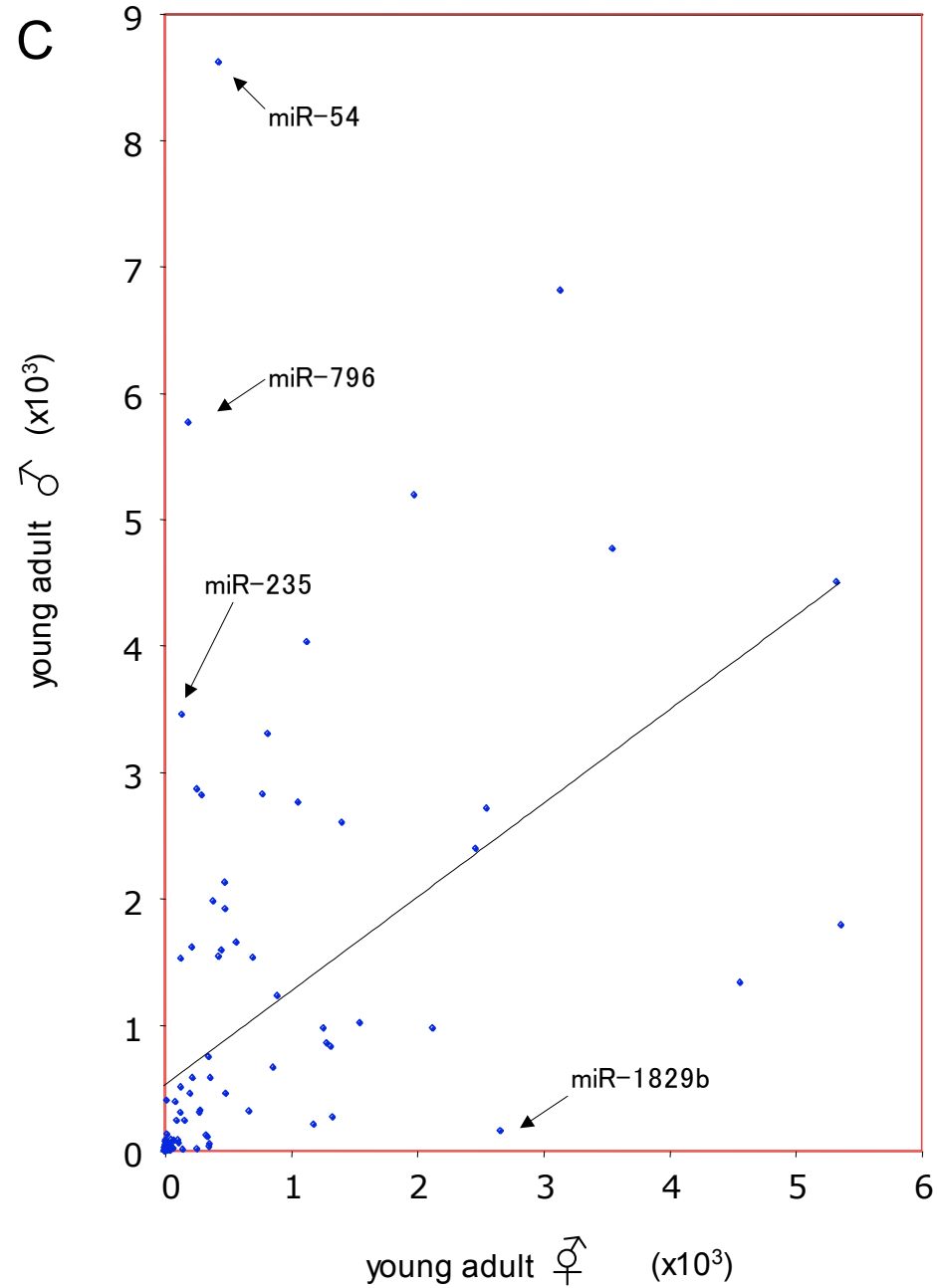

Supplement: Additional data file 6 — (a) The correlation diagram of all known miRNAs between males and hermaphrodites. (b) The correlation diagram of miRNAs with relatively low abundance (less than 20 × 104 reads in males, yellow-colored area in (a)). (c) Correlation diagram of miRNAs with lower abundance ((less than 10 × 103 reads in males, red-colored area in (a)). [file gb-2009-10-5-r54-S6.pdf]

A

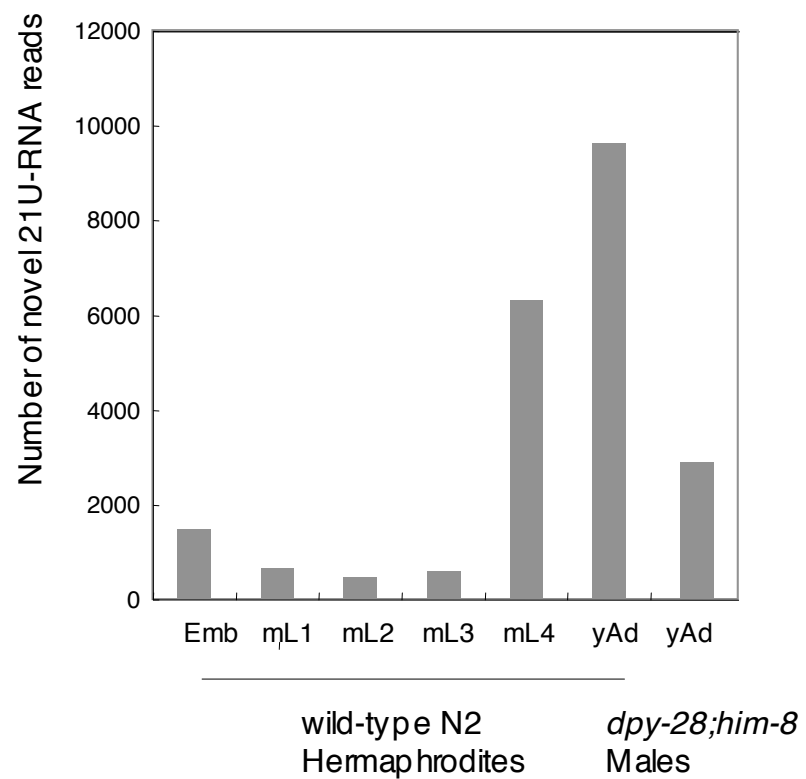

B

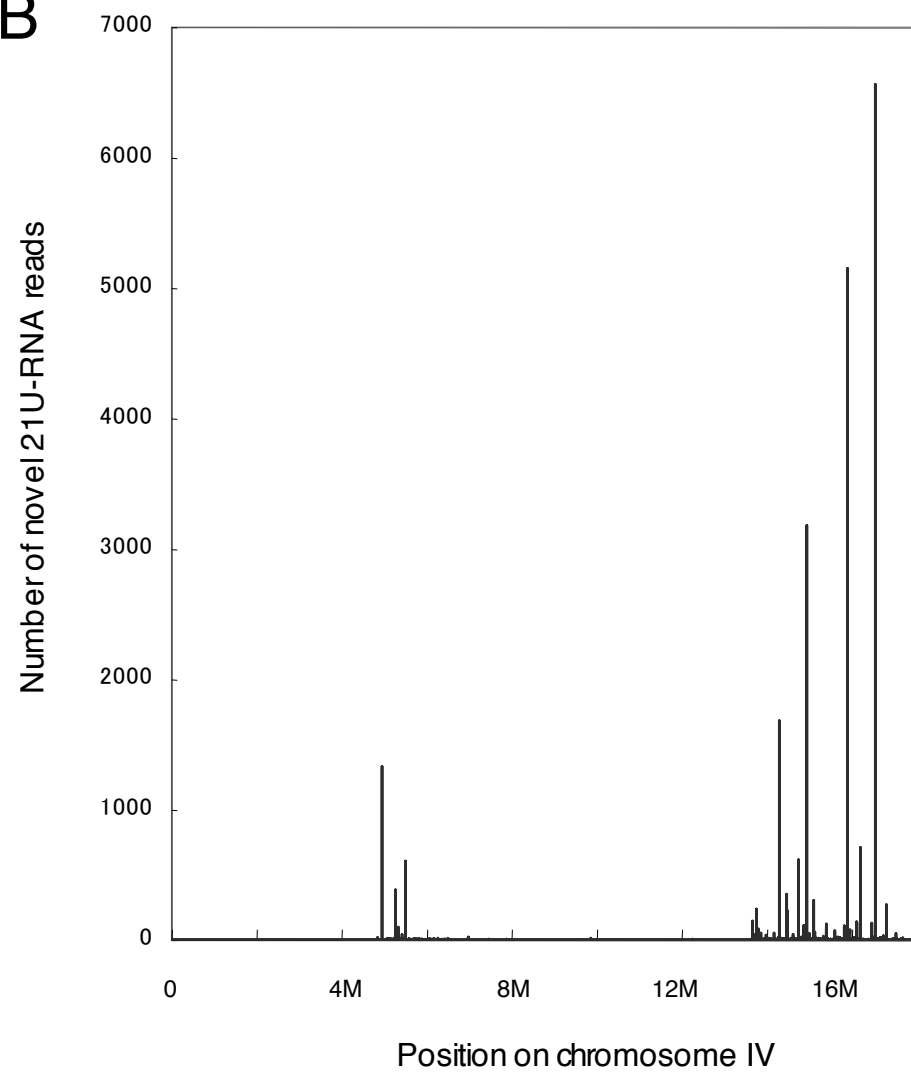

Supplement: Additional data file 12 — (a) Number of novel 21U-RNA reads was plotted after normalizing. (b) Total number of reads shown in (a) that mapped on chromosome IV. [file gb-2009-10-5-r54-S12.pdf]
