# Supplementary material for: Dynamic expression of small non-coding RNAs, including novel microRNAs and piRNAs/21U-RNAs, during Caenorhabditis elegans development
Source: Genome Biol. 2009 May 21;10(5):R54. doi: 10.1186/gb-2009-10-5-r54 (PMC2718520; doi:10.1186/gb-2009-10-5-r54)
Supplement: Additional data file 7 — The numbers of reads were obtained from all developmental stages of hermaphrodites and young adult males. The bona fide novel RNAs with transcripts from their 'star sequence' are highlighted in red. 'Genomically clustered' is defined here as localization within 1.0 kb on the same chromosome. [file gb-2009-10-5-r54-S7.pdf]

	Read#	
Novel miRNA candidate		540532_mas
mature_seq	8	AGTGTGTGGGGTGAGGCGAGACG
mature_arm		first
star_seq	0	TCTCCTCTCTCCCCACACTCTCT
loop_seq		CAGCGCACTGTCTGCG
pre_seq		AGTGTGTGGGGTGAGGCGAGACGCAGCGCACTGTCTGCGTCTCCTCTCTCCCCACACTCTCT
pre_struct		((.((((((((.((((.((((((((((.....).))))))))).)))).)))))))).))))
Genomic position/strand		III:9577..9599 +
Genomic Cluster?		No
Possible miRNA family?		Yes (mmu-mir-466d-5p,mmu-mir-466k)
Accession number		FJ589793
Notes		
Novel miRNA candidate		964568_mas
mature_seq	2	CGGGAGTGGCACGGGTTGGGGT
mature_arm		first
star_seq	0	CGCTGGTTCGTGCCCCGAC
loop_seq		TCTAAAGGC
pre_seq		CGGGAGTGGCACGGGTTGGGGTTCTAAAGGCCGCTGGTTCGTGCCCCGAC
pre_struct		((((....(((((..(..(((((.....)))).)..)..))))))))).)
Genomic position/strand		III:1122438..1122459 +
Genomic Cluster?		No
Possible miRNA family?		No
Accession number		FJ589794
Notes		
Novel miRNA candidate		95481_mas
mature_seq	2	AACGACGGATAGAAGAAGAAGA
mature_arm		second
star_seq	0	TTCTTCTTCTACTCCGAGGATTC
loop_seq		TTCCTAGGCGCGGTGGTCCTGGAA
pre_seq		TTCTTCTTCTACTCCGAGGATTCTTCCTAGGCGCGGTGGTCCTGGAAAACGACGGATAGAAGAAGAAGA
pre_struct		(((((((((((.((((.......((((.(((.((....))))))))).....)))))))))))))))))
Genomic position/strand		III:10515939..10515960 +
Genomic Cluster?		No
Possible miRNA family?		No
Accession number		FJ589795
Notes		

	Read#	
Novel miRNA candidate		1883591_mas
mature_seq	1	TCGTAAATCGGCACAAATCGTCT
mature_arm		second
star_seq	0	ACGATTTGTGTCGATTTACAGAG
loop_seq		TTTTCCTCGTTGTCATAATT
pre_seq		ACGATTTGTGTCGATTTACAGAGTTTTCCTCGTTGTCATAATTTCGTAAATCGGCACAAATCGTCT
pre_struct		(((((((((((((((((((..((((..............))))..)))))))))))))))))))))
Genomic position/strand		III:301118..301140 +
Genomic Cluster?		No
Possible miRNA family?		No
Accession number		FJ589797
Notes		
Novel miRNA candidate		1260661_mas
mature_seq	3	GATGATGAGGTCGTCGTT
mature_arm		first
star_seq	0	CGTAAGAAGTTCGTTATCCA
loop_seq		ATGCGCGGACGCCATAAGGGAAACACTGGACGTGTCCTCAGATGCTAC
pre_seq		GATGATGAGGTCGTCGTTATGCGCGGACGCCATAAGGGAAACACTGGACGTGTCCTCAGATGCTACCGTAAGAAGTTCGTTATCCA
pre_struct		((((((((.((..((.((.((((((((((....(((....).))....))))))...).))))).))...))..))))))))))..
Genomic position/strand		II:8603050..8603067 +
Genomic Cluster?		No
Possible miRNA family?		Yes (hsa-mir-1272,ptr-mir-1272)
Accession number		FJ589798
Notes		
Novel miRNA candidate		70290_mas
mature_seq	11	AAATTTTGAGATTTTCCGCACA
mature_arm		second
star_seq	0	TGCGGCAAATTTGCCGAATTTGC
loop_seq		CGTTTGTCGAGCTCGGC
pre_seq		TGCGGCAAATTTGCCGAATTTGCCGTTTGTCGAGCTCGGCAAATTTTGAGATTTTCCGCACA
pre_struct		(((((.((((((.(.((((((((((...(.....).)))))))))).))))))).)))))))
Genomic position/strand		II:1061711..1061732 +
Genomic Cluster?		No
Possible miRNA family?		No
Accession number		FJ589799
Notes		

	Read#	
Novel miRNA candidate		mir-2207
mature_seq	6	TGTGAATTGAGACTGTGTATAAG
mature_arm		first
star_seq	1	ATGCACAGGCTCAATGCACACAA
loop_seq		GAATGTAAGGAGGAGAGGGGCCGTTCGTATCGTTCGTCTT
pre_seq		TGTGAATTGAGACTGTGTATAAGAATGTAAGGAGGAGAGGGGCCGTTCGTATCGTTCGTCTTATGCACAGGCTCAATGCACACA
pre_struct		((((.((((((.((((((((((((.((.((.((...(((......)))...)).))))))))))))))))).)))))).))))))
Genomic position/strand		II:11600214..11600235 +
Genomic Cluster?		No
Possible miRNA family?		No
Accession number		FJ589801
Notes		

Novel miRNA candidate		209309_mas
mature_seq	3	AATTACATTATTGATCCAGAGA
mature_arm		second
star_seq	0	TCTGGATCGATAATGTAAACCT
loop_seq		ATTGAATCTATAG
pre_seq		TCTGGATCGATAATGTAAACCTATTGAATCTATAGAATTACATTATTGATCCAGAGA
pre_struct		((((((((((((((((((..((((.......))))..))))))))))))))))))))
Genomic position/strand		IV:6294598..6294619 +
Genomic Cluster?		No
Possible miRNA family?		No
Accession number		FJ589803
Notes		

Novel miRNA candidate		mir-2208a
mature_seq	7	AAGTGTACCCGAATCTGATATCC
mature_arm		first
star_seq	4	ATGCAGTTTCTGGTATACTTCA
loop_seq		TATCATCTGAAAAAGG
pre_seq		AAGTGTACCCGAATCTGATATCCTATCATCTGAAAAAGGATGCAGTTTCTGGTATACTTCA
pre_struct		(((((((((.(((.(((.((((((.((....))...))))))))).))).)))))))))).)
Genomic position/strand		IV:1021772..1021794 +
Genomic Cluster?		Yes (mir-2209b)
Possible miRNA family?		No
Accession number		FJ589804
Notes		

	Read#	
Novel miRNA candidate		mir-2208b-5p
mature_seq	393	AAGTGTACCCGGATCTGATATCC
mature_arm		first
star_seq	512	ATGCAGATTTTGGTACACTTCA
loop_seq		TATCACCAAAAAGAGG
pre_seq		AAGTGTACCCGGATCTGATATCCTATCACCAAAAAGAGGATGCAGATTTTGGTACACTTCA
pre_struct		(((((((((..((((((.((((((............))))))))))))..))))))))).)
Genomic position/strand		IV:1026580..1026602 +
Genomic Cluster?		Yes (1742956_mas,mir-2209a,mir-2209c)
Possible miRNA family?		No
Accession number		FJ589805
Notes		The expression was confirmed by RT-PCR in alg-1 mutant background.

Novel miRNA candidate		1742956_mas
mature_seq	9	TATCAAGGACGCGATCCACTAAA
mature_arm		second
star_seq	0	GAGTGTATTGGTCTTTTTGATATC
loop_seq		GACGGAGTACTGA
pre_seq		GAGTGTATTGGTCTTTTTGATATCGACGGAGTACTGATATCAAGGACGCGATCCACTAAA
pre_struct		.((((.((((((((..(((((((((.........)))))))))))))).)))).))))..)
Genomic position/strand		IV:1026714..1026736 +
Genomic Cluster?		Yes (mir-2208b-5p,mir-2208b-3p,mir-2209a,mir-2209c)
Possible miRNA family?		No
Accession number		FJ589806
Notes		

Novel miRNA candidate		mir-2209a
mature_seq	2291	AGAGATCAGCGGTTACACTACA
mature_arm		second
star_seq	10	GAGTGTAACCACTCTTCTCCTTC
loop_seq		CGAGTATTGATTATCGAGA
pre_seq		GAGTGTAACCACTCTTCTCCTTCCGAGTATTGATTATCGAGAAGAGATCAGCGGTTACACTACA
pre_struct		((((((((((.((..((((.((((((..........))).)))))))..)).))))))))).))
Genomic position/strand		IV:1027143..1027164 +
Genomic Cluster?		Yes (mir-2208b-5p,mir-2208b-3p,1742956_mas,mir-2209c)
Possible miRNA family?		Yes (dme-bantam,cbr-mir-80,cbr-mir-81,cbr-mir-82,ame-bantam)
Accession number		FJ589807
Notes		The expression was confirmed by RT-PCR in alg-1 mutant background.

	Read#	
Novel miRNA candidate		mir-2209c
mature_seq	113	AAAAGACCACCGGTTACACTACA
mature_arm		second
star_seq	4	GAGTGTAACCGCACGTCTTGTTT
loop_seq		CAAAACTCAATATATGGA
pre_seq		GAGTGTAACCGCACGTCTTGTTTCAAAACTCAATATATGGAAAAAGACCACCGGTTACACTACA
pre_struct		(((((((((((...(((((.(((((............))))).)))))...))))))))))).))
Genomic position/strand		IV:1027259..1027281 +
Genomic Cluster?		Yes (mir-2208b-5p,mir-2208b-3p,1742956_mas,mir-2209a)
Possible miRNA family?		No
Accession number		FJ589808
Notes		The expression was confirmed by RT-PCR in alg-1 mutant background.

Novel miRNA candidate		2103433_mas
mature_seq	672	TGTAAATGGTTGGAATCTGGTAT
mature_arm		first
star_seq	0	ACCAATTTCTTACCATTAGCATC
loop_seq		AATGGCTTCGAGACTTTTTAT
pre_seq		TGTAAATGGTTGGAATCTGGTATAATGGCTTCGAGACTTTTTATACCAATTTCTTACCATTAGCATC
pre_struct		(((.((((((.((((..((((((((.(((.....).))..))))))))..)))).)))))).))).)
Genomic position/strand		IV:9757504..9757526 +
Genomic Cluster?		No
Possible miRNA family?		No
Accession number		FJ589809
Notes		

Novel miRNA candidate		mir-2210
mature_seq	15	AGGCAGATCAATCAATTTTTAGG
mature_arm		first
star_seq	19	TAAAGTCGATTGCTCTACCCAC
loop_seq		TTTATATAAATAATCC
pre_seq		AGGCAGATCAATCAATTTTTAGGTTTATATAAATAATCCTAAAGTCGATTGCTCTACCCAC
pre_struct		.((.(((.(((((...(((((((.((((....)))).)))))))..))))).))).)).))
Genomic position/strand		IV:10914588..10914610 +
Genomic Cluster?		No
Possible miRNA family?		Yes (cbr-mir-72)
Accession number		FJ589810
Notes		

	Read#	
Novel miRNA candidate		1911250_mas
mature_seq	4	TCTGCATTTTTTCGTGGGATCGG
mature_arm		second
star_seq	0	GATTTTACGACAAAATGCAGAAA
loop_seq		ATGAGCCACAATCCAACTTT
pre_seq		GATTTTACGACAAAATGCAGAAAATGAGCCACAATCCAACTTTTCTGCATTTTTTCGTGGGATCGG
pre_struct		((((((((((.((((((((((((((.((.......))....))))))))))))).)))))))))).)
Genomic position/strand		IV:1183883..1183905 +
Genomic Cluster?		No
Possible miRNA family?		Yes (dre-mir-460-5p,gga-mir-460,oan-mir-460)
Accession number		FJ589811
Notes		
Novel miRNA candidate		663452_mas
mature_seq	1	ATTATTGATCGAAATTTGGACC
mature_arm		first
star_seq	0	TCAATCGAAGATTTTGTCAATGGAAA
loop_seq		TTCTCGTGTGAATGGAGG
pre_seq		ATTATTGATCGAAATTTGGACCTTCTCGTGTGAATGGAGGTCAATCGAAGATTTTGTCAATGGAAA
pre_struct		.(((((((.((((((((.(((((((..........)))))))......)))))))))))))))..)
Genomic position/strand		IV:1430223..1430244 +
Genomic Cluster?		No
Possible miRNA family?		Yes (rno-mir-325-3p,mmu-mir-325)
Accession number		FJ589812
Notes		
Novel miRNA candidate		63594_mas
mature_seq	2	AAATGAAATCGTGGGCGGGTCT
mature_arm		second
star_seq	0	CCCCGCCCAAGATTTCATTTGC
loop_seq		ACTGAAATGTGTCGGCCGCAGTGC
pre_seq		CCCCGCCCAAGATTTCATTTGCACTGAAATGTGTCGGCCGCAGTGCAAATGAAATCGTGGGCGGGTCT
pre_struct		.(((((((.((((((((((((((((....((....))..))))))))))))))))).))))))))..)
Genomic position/strand		IV:409829..409850 +
Genomic Cluster?		No
Possible miRNA family?		No
Accession number		FJ589813
Notes		

	Read#	
Novel miRNA candidate		mir-2211
mature_seq	4	TCAGGTAGAATTTAGAGGAGAAA
mature_arm		second
star_seq	1	CCTCCATCTATTCTCCATCTGACG
loop_seq		ACGTCCCGTCCCG
pre_seq		CCTCCATCTATTCTCCATCTGACGACGTCCCGTCCCGTCAGGTAGAATTTAGAGGAGAAA
pre_struct		(((((.((((...((.(((((((((((...))))..))))))).))...)))))))))...
Genomic position/strand		I:9447391..9447413 +
Genomic Cluster?		No
Possible miRNA family?		No
Accession number		FJ589815
Notes		
Novel miRNA candidate		1392735_mas
mature_seq	19	GGAACTGTACATCGTCGGA
mature_arm		second
star_seq	1	CGATGATGTCCAGAGCCAA
loop_seq		TTGAA
pre_seq		CGATGATGTCCAGAGCCAATTGAAGGAACTGTACATCGTCGGA
pre_struct		(((((((((.(((..((.......))..))).)))))))))..
Genomic position/strand		I:10331001..10331019 +
Genomic Cluster?		No
Possible miRNA family?		Yes (odi-mir-1493)
Accession number		FJ589816
Notes		
Novel miRNA candidate		427628_mas
mature_seq	4	AGCATGAGACTTGTGGAAATGT
mature_arm		first
star_seq	0	ATTTCTACAAGTCTCATGTTGA
loop_seq		TCGAAAAAAGCCTAAATTACGTCTTTTCGAAC
pre_seq		AGCATGAGACTTGTGGAAATGTTCGAAAAAAGCCTAAATTACGTCTTTTCGAACATTTCTACAAGTCTCATGTTGA
pre_struct		(((((((((((((((((((((((((((((................)))))))))))))))))))))))))))))))
Genomic position/strand		I:13283058..13283079 +
Genomic Cluster?		No
Possible miRNA family?		No
Accession number		FJ589817
Notes		

	Read#	
Novel miRNA candidate		1911316_mas
mature_seq	1	TCTGCCAGACTACAAACCACACA
mature_arm		second
star_seq	8	TGTAGTTTGTAGTCTAGCAGACC
loop_seq		AATACCAAAAGCTTTGGG
pre_seq		TGTAGTTTGTAGTCTAGCAGACCAATACCAAAAGCTTTGGGTCTGCCAGACTACAAACCACACA
pre_struct		(((.(((((((((((.(((((((.....(....).....))))))).))))))))))).))).)
Genomic position/strand		V:18036755..18036777 +
Genomic Cluster?		No
Possible miRNA family?		No
Accession number		FJ589818
Notes		The star sequence mapped multiple loci.
Novel miRNA candidate		1467045_mas
mature_seq	1	GGGGACAGAGAGCTCAGTCGGTAG
mature_arm		first
star_seq	0	TCAAGCCCGGCCTCACCCCCTA
loop_seq		TGGTGGCCGCTAGCAGTCTGGAGGTCACGAGT
pre_seq		GGGGACAGAGAGCTCAGTCGGTAGTGGTGGCCGCTAGCAGTCTGGAGGTCACGAGTTCAAGCCCGGCCTCACCCCCTA
pre_struct		((((...(((.(((..((..(.(.(.((((((.((((....)))).)))))).).).)..))..))))))..))))).
Genomic position/strand		X:11741394..11741417 +
Genomic Cluster?		No
Possible miRNA family?		Yes (gga-mir-1566,gga-mir-1608)
Accession number		FJ589820
Notes		
Novel miRNA candidate		467565_mas
mature_seq	1	AGGACTTTGACGGTATGTCTGAG
mature_arm		first
star_seq	1	CAGACATACCGTCAAAGTTCTAT
loop_seq		CCTTGTAAAAGCT
pre_seq		AGGACTTTGACGGTATGTCTGAGCCTTGTAAAAGCTCAGACATACCGTCAAAGTTCTAT
pre_struct		((((((((((((((((((((((((.((....))))))))))))))))))))))))))
Genomic position/strand		X:13828520..13828542 +
Genomic Cluster?		No
Possible miRNA family?		No
Accession number		FJ589821
Notes		

	Read#	
Novel miRNA candidate		724701_mas
mature_seq	15	CAAGTGATACCAGACCGCTAGT
mature_arm		second
star_seq	0	TGGCGGTTTGCATTCACTTACA
loop_seq		TTTATAAGACAAAAATG
pre_seq		TGGCGGTTTGCATTCACTTACATTTATAAGACAAAAATGCAAGTGATACCAGACCGCTAGT
pre_struct		((((((((((...((((((.(((((.........))))).))))))...))))))))))))
Genomic position/strand		X:1390914..1390935 +
Genomic Cluster?		No
Possible miRNA family?		No
Accession number		FJ589822
Notes		

Novel miRNA candidate		mir-2212
mature_seq	164	TGGCAGATCATAGGCTGACTTTG
mature_arm		first
star_seq	6	AAGTGGCATTTGATAAGCCATC
loop_seq		CAAGCATTTTATACA
pre_seq		TGGCAGATCATAGGCTGACTTTGCAAGCATTTTATACAAAGTGGCATTTGATAAGCCATC
pre_struct		((((..((((...(((.((((((.............)))))))))...))))..))))))
Genomic position/strand		X:16207747..16207769 +
Genomic Cluster?		Yes (mir-1819)
Possible miRNA family?		Yes (cbr-mir-72)
Accession number		FJ589823
Notes		The expression was confirmed by RT-PCR in alg-1 mutant background.

Novel miRNA candidate		547404_mas
mature_seq	78	AGTTGGTGATCGTACATTT
mature_arm		second
star_seq	0	ATGTGCTGAACTCCAAAGAG
loop_seq		TCAAAACTG
pre_seq		ATGTGCTGAACTCCAAAGAGTCAAAACTGAGTTGGTGATCGTACATTT
pre_struct		((((((.((.(.((((..(((....)))...)))).).))))))))).
Genomic position/strand		X:1891606..1891624 +
Genomic Cluster?		No
Possible miRNA family?		Yes (sly-mir1918)
Accession number		FJ589824
Notes		

	Read#	
Novel miRNA candidate		24789_adh
mature_seq	2	AAAAGACGTAGATCGTGGCGAGA
mature_arm		second
star_seq	0	TCGTCACGAATTGCGTGATTTTGG
loop_seq		ACGCTTCTAGGATCC
pre_seq		TCGTCACGAATTGCGTGATTTTGGACGCTTCTAGGATCCAAAAGACGTAGATCGTGGCGAGA
pre_struct		(((((((((.((((((..(((((((..((....)).))))))).)))))).)))))))))))
Genomic position/strand		III:11115521..11115543 +
Genomic Cluster?		No
Possible miRNA family?		Yes (rno-mir-743a)
Accession number		FJ589826
Notes		
Novel miRNA candidate		1010777_adh
mature_seq	1	CGTGAGCTCTACGTGACGGCTGA
mature_arm		first
star_seq	0	GGACGAATGTAGAGCTATGGT
loop_seq		AGCCAAATT
pre_seq		CGTGAGCTCTACGTGACGGCTGAAGCCAAATTGGACGAATGTAGAGCTATGGT
pre_struct		(((.((((((((((..((.(..(.......)..).)).))))))))))))))..
Genomic position/strand		III:2166784..2166806 +
Genomic Cluster?		No
Possible miRNA family?		No
Accession number		FJ589827
Notes		
Novel miRNA candidate		1032770_adh
mature_seq	9	CTAGAAACTCTCAATTCCGGCAGA
mature_arm		second
star_seq	0	TGCCGGAAATGCTCAGTTTCGGCA
loop_seq		ATTTGCCAATTG
pre_seq		TGCCGGAAATGCTCAGTTTCGGCAATTTGCCAATTTGCTAGAAACTCTCAATTCCGGCAGA
pre_struct		((((((((.((...(((((((((((.........))))).))))))..)).))))))))))
Genomic position/strand		III:12091768..12091745 -
Genomic Cluster?		No
Possible miRNA family?		No
Accession number		FJ589828
Notes		

	Read#	
Novel miRNA candidate		1973091_adh
mature_seq	62	TCATGTTGGTTTTTTGTTGGT
mature_arm		second
star_seq	0	CTTGTGAAATTAACGTGAGT
loop_seq		TTTCTGGTCATCTGACTAA
pre_seq		CTTGTGAAATTAACGTGAGTTTTCTGGTCATCTGACTAATCATGTTGGTTTTTTGTTGGT
pre_struct		(....(((((((((((((......(((((....))))).))))))))))))).....)))
Genomic position/strand		III:6971955..6971935 -
Genomic Cluster?		No
Possible miRNA family?		Yes (ppt-mir1066)
Accession number		FJ589829
Notes		

Novel miRNA candidate		mir-2213
mature_seq	9	AAGCTGTAAGAGGACTGCCTAA
mature_arm		second
star_seq	2	TGGCGGACTCTTCACAGTTTGA
loop_seq		CCTCGGAGATC
pre_seq		TGGCGGACTCTTCACAGTTTGACCTCGGAGATCAAGCTGTAAGAGGACTGCCTAA
pre_struct		.(((((.(((((.(((((((((.(.....).))))))))))))))..)))))....
Genomic position/strand		III:6968839..6968860 +
Genomic Cluster?		No
Possible family?		No
Accession number		FJ589830
Notes		

Novel miRNA candidate		mir-2214
mature_seq	56	CATTGACAACAACTTGACCGGCGTT
mature_arm		second
star_seq	43	ATTCGGTCCGGAGTCAATGGG
loop_seq		TTATCTTTCAAAACCCCC
pre_seq		ATTCGGTCCGGAGTCAATGGGTTATCTTTCAAAACCCCCCATTGACAACAACTTGACCGGCGTT
pre_struct		..((((((.((.(((((((((................))))))))).....)).))))))..))
Genomic position/strand		III:7278681..7278705 +
Genomic Cluster?		No
Possible miRNA family?		No
Accession number		FJ589831
Notes		

	Read#	
Novel miRNA candidate		764767_adh
mature_seq	43	CAACTTTTAGACCAATAGGCAT
mature_arm		first
star_seq	0	GCCTATCGGCCTAAAAGTTGTC
loop_seq		CCAAAAATGATCAAAATTGGAT
pre_seq		CAACTTTTAGACCAATAGGCATCCAAAAATGATCAAAATTGGATGCCTATCGGCCTAAAAGTTGTC
pre_struct		((((((((((.((.((((((((((((..............)))))))))))).)).))))))))))
Genomic position/strand		II:12238539..12238560 +
Genomic Cluster?		No
Possible miRNA family?		No
Accession number		FJ589832
Notes		
Novel miRNA candidate		mir-2215
mature_seq	6	AGAATCGTAGCGCGTGTTGTTT
mature_arm		second
star_seq	1	ACAGCACGTGTTACGATGCTCC
loop_seq		GTTAGTTCGGAGTTTTTACGG
pre_seq		ACAGCACGTGTTACGATGCTCCGTTAGTTCGGAGTTTTTACGGAGAATCGTAGCGCGTGTTGTTT
pre_struct		(((((((((((((((((.((((((.................)))))).)))))))))))))))))
Genomic position/strand		II:13333692..13333671 -
Genomic Cluster?		No
Possible miRNA family?		No
Accession number		FJ589833
Notes		
Novel miRNA candidate		347252_adh
mature_seq	184	ACTGTGACGGCTTATATCTCG
mature_arm		first
star_seq	0	AGATATAAGCTGTCAAAGTGA
loop_seq		GTTGTATCAGGCCAAAGATATCCG
pre_seq		ACTGTGACGGCTTATATCTCGGTTGTATCAGGCCAAAGATATCCGAGATATAAGCTGTCAAAGTGA
pre_struct		(((.((((((((((((((((((..(((((..........))))))))))))))))))))))))).)))))
Genomic position/strand		II:11616589..11616569 -
Genomic Cluster?		No
Possible miRNA family?		No
Accession number		FJ589834
Notes		

	Read#	
Novel miRNA candidate		mir-2216
mature_seq	17	GCACATTTTAAGTCGGTAGGC
mature_arm		first
star_seq	1	CTATCTACTTAAAATGTGCCT
loop_seq		GTCCAATTTTGATGC
pre_seq		GCACATTTTAAGTCGGTAGGCGTCCAATTTTGATGCCTATCTACTTAAAATGTGCCT
pre_struct		(((((((((((((.((((((((((.......)))))))))).)))))))))))))))
Genomic position/strand		II:10867094..10867074 -
Genomic Cluster?		No
Possible miRNA family?		Yes (mghv-mir-M1-9)
Accession number		FJ589835
Notes		
Novel miRNA candidate		1619758_adh
mature_seq	1	GTCAGTGTGATGATTGTC
mature_arm		second
star_seq	2	CAATTATGGCACTGAAGG
loop_seq		CAGCTGCTGCGAGGCTT
pre_seq		CAATTATGGCACTGAAGGCAGCTGCTGCGAGGCTTGTCAGTGTGATGATTGTC
pre_struct		(((((((.((((((((((.((....))...)))).))))))).)))))))).
Genomic position/strand		II:8281031..8281014 -
Genomic Cluster?		No
Possible miRNA family?		No
Accession number		FJ589836
Notes		
Novel miRNA candidate		748932_adh
mature_seq	1	ATTTTTGTAGATCACACCGTGA
mature_arm		second
star_seq	0	ACGGGTTGATGTACAAGAAATGC
loop_seq		GGGATTTCTGCGTCTCGCTCACCGC
pre_seq		ACGGGTTGATGTACAAGAAATGCGGGATTTCTGCGTCTCGCTCACCGCATTTTTGTAGATCACACCGTGA
pre_struct		((((..((((.((((((((.(((((((.....(((...))))).))))))))))))).))))..))))))
Genomic position/strand		II:92365..92344 -
Genomic Cluster?		No
Possible miRNA family?		No
Accession number		FJ589837
Notes		

	Read#	
Novel miRNA candidate		686798_adh
mature_seq	48	ATGGCCTAGTGGATAAGAGGGA
mature_arm		second
star_seq	0	TCTCTTATCCACTAGGCCACGA
loop_seq		GTGCCAGCGCCCTCAAGGAGTCGGGTCCGC
pre_seq		TCTCTTATCCACTAGGCCACGAGTGCCAGCGCCCTCAAGGAGTCGGGTCCGCATGGCCTAGTGGATAAGAGGGA
pre_struct		((((((((((((((((((((.(.(..((...((((....)).))..))..).).))))))))))))))))))))
Genomic position/strand		IV:2079060..2079081 +
Genomic Cluster?		No
Possible miRNA family?		No
Accession number		FJ589838
Notes		

Novel miRNA candidate		mir-1832b
mature_seq	6	AGTGGGCAGAGCGATTCGCTGAT
mature_arm		second
star_seq	1	CAGCGAATCGCTCGGCCCACTTT
loop_seq		TGTGAACCAATCAGCGTCAAA
pre_seq		CAGCGAATCGCTCGGCCCACTTTTGTGAACCAATCAGCGTCAAAAGTGGGCAGAGCGATTCGCTGAT
pre_struct		(((((((((((((.((((((((((((((.....)))....))))))))))).)))))))))))))))
Genomic position/strand		IV:17092840..17092818 -
Genomic Cluster?		No
Possible miRNA family?		No
Accession number		FJ589839
Notes		

Novel miRNA candidate		169025_adh
mature_seq	17	AAGGAGAAAAGTAGAAGACGATT
mature_arm		first
star_seq	0	ACGGCTTCTTCTTTTCTTCTTCT
loop_seq		CTGTTATTTTTTGGA
pre_seq		AAGGAGAAAAGTAGAAGACGATTCTGTTATTTTTTGGAACGGCTTCTTCTTTTCTTCTTCT
pre_struct		((((((((((((.(((((.((.(((((........))))))).))))).))))))))))))
Genomic position/strand		IV:11456094..11456072 -
Genomic Cluster?		No
Possible miRNA family?		No
Accession number		FJ589840
Notes		

	Read#	
Novel miRNA candidate		405191_adh
mature_seq	344	AGAGATCAGGCAATAGGTTCAA
mature_arm		second
star_seq	0	GCAACCTGTTGGTTGATCTCCTA
loop_seq		CCAAATAATGGATA
pre_seq		GCAACCTGTTGGTTGATCTCCTACCAAATAATGGATAAGAGATCAGGCAATAGGTTCAA
pre_struct		(.(((((((((.((((((((.(((((.....))).)).)))))))).))))))))))))
Genomic position/strand		IV:6660649..6660628 -
Genomic Cluster?		Yes (mir-1834)
Possible miRNA family?		Yes (dme-bantam,cbr-mir-80,cbr-mir-81,cbr-mir-82,ame-bantam)
Accession number		FJ589841
Notes		

Novel miRNA candidate		327617_adh
mature_seq	10	ACGTTTAACTCGCAGCGGGCG
mature_arm		first
star_seq	0	CCCACAGCGAGTTTAACATAG
loop_seq		GAGACAGCTCGCCACG
pre_seq		ACGTTTAACTCGCAGCGGGCGGAGACAGCTCGCCACGCCCACAGCGAGTTTAACATAG
pre_struct		(.(((.(((((((.(.((((((((....)))....))))).).))))))).))).)))
Genomic position/strand		V:6350509..6350489 -
Genomic Cluster?		No
Possible miRNA family?		No
Accession number		FJ589842
Notes		

Novel miRNA candidate		837693_adh
mature_seq	99	CATCTCATCCTGATCGCCA
mature_arm		first
star_seq	0	GCCGAACAAGGAGGCGAAGAC
loop_seq		ATGTTATATATTCGAG
pre_seq		CATCTCATCCTGATCGCCAATGTTATATATTCGAGGCCGAACAAGGAGGCGAAGAC
pre_struct		(.((.(.((((..((((((((((...)))))...)).)))...)))).).)).)))
Genomic position/strand		IV:5793766..5793748 -
Genomic Cluster?		No
Possible miRNA family?		No
Accession number		FJ589843
Notes		

	Read#	
Novel miRNA candidate		mir-2217
mature_seq	2	TCGACCCTTGTGCCTGTTTCGGT
mature_arm		second
star_seq	1	CAGAGTGGGCAGTCGGTGTCGATC
loop_seq		TACATCATTCGCAGG
pre_seq		CAGAGTGGGCAGTCGGTGTCGATCTACATCATTCGCAGGTCGACCCTTGTGCCTGTTTCGGT
pre_struct		(..((..((((...((.((((((((.(.......).))))))))))...))))..))..)))
Genomic position/strand		IV:5280086..5280064 -
Genomic Cluster?		No
Possible miRNA family?		No
Accession number		FJ589844
Notes		The expression was confirmed by RT-PCR in alg-1 mutant background.

Novel miRNA candidate		1533251_adh
mature_seq	25	GGGGTTGTAGATATAGAAGAC
mature_arm		second
star_seq	0	TTTCTCTGTTTCAGCCAGTT
loop_seq		ATGTTAAAGAGTTGGGATTTTGCTTTGAATAGT
pre_seq		TTTCTCTGTTTCAGCCAGTTATGTTAAAGAGTTGGGATTTTGCTTTGAATAGTGGGGTTGTAGATATAGAAGAC
pre_struct		(((((.((((((((((.....((((..(((((.........))))).))))....))))).))))).)))))))
Genomic position/strand		IV:5101214..5101194 -
Genomic Cluster?		No
Possible miRNA family?		Yes (ppt-mir1074)
Accession number		FJ589845
Notes		

Novel miRNA candidate		651772_adh
mature_seq	18	ATCTAGAAACTCAATATATGGAA
mature_arm		first
star_seq	0	CTATATAGTGCGCTTCTAGATAA
loop_seq		AACTGAAAAACACCACTGTTTT
pre_seq		ATCTAGAAACTCAATATATGGAAAACTGAAAAACACCACTGTTTTCTATATAGTGCGCTTCTAGATAA
pre_struct		((((((((.(.((.(((((((((((((...............))))))))))))).)).).)))))))).)
Genomic position/strand		IV:3231200..3231178 -
Genomic Cluster?		No
Possible miRNA family?		No
Accession number		FJ589846
Notes		

	Read#	
Novel miRNA candidate		mir-2209b
mature_seq	3	AGAGATGAGCGGTTGTGCTTCA
mature_arm		second
star_seq	1	AGTGTAACAACTCTTCTCCTTC
loop_seq		CGAAAACCAATAATCGAGA
pre_seq		AGTGTAACAACTCTTCTCCTTCCGAAAACCAATAATCGAGAAGAGATGAGCGGTTGTGCTTCA
pre_struct		((..((((..(((.((((.((((((...........))).))))))).)))..))))..).)))
Genomic position/strand		IV:1021324..1021303 -
Genomic Cluster?		Yes (mir-2208a)
Possible miRNA family?		Yes (mmu-mir-143,hsa-mir-143,rno-mir-143,dre-mir-143,ptr-mir-143,ppa-mir-143,xtr-mir-143)
Accession number		FJ589847
Notes		

Novel miRNA candidate		mir-2208b-3p
mature_seq	386	ATGCAGATTTTGGTACACTTCA
mature_arm		second
star_seq	518	AAGTGTACCCGGATCTGATATCC
loop_seq		TATCACCAAAAAGAGG
pre_seq		AAGTGTACCCGGATCTGATATCCTATCACCAAAAAGAGGATGCAGATTTTGGTACACTTCA
pre_struct		((((((((..((((((.((((((.............))))))))))))..))))))))).)
Genomic position/strand		IV:1026619..1026640 +
Genomic Cluster?		Yes (1742956_mas,mir-2209a,mir-2209c)
Possible miRNA family?		No
Accession number		FJ589848
Notes		

Novel miRNA candidate		1671098_adh
mature_seq	1	GTGGGTGGCGAAACTCGGAAAT
mature_arm		second
star_seq	1	TTCCGAGTTTCGCCACTCACTA
loop_seq		TAAAATTGCTGATTAAGTATA
pre_seq		TTCCGAGTTTCGCCACTCACTATAAAATTGCTGATTAAGTATAGTGGGTGGCGAAACTCGGAAAT
pre_struct		((((((((((((((((((((((...(((.....))).))))))))))))))))))))))
Genomic position/strand		I:11453615..11453636 +
Genomic Cluster?		No
Possible miRNA family?		Yes (mml-mir-1230)
Accession number		FJ589849
Notes		The star sequence mapped multiple loci.

	Read#	
Novel miRNA candidate		358157_adh
mature_seq	15	ACTTTTGTAGATCAAACCGACAT
mature_arm		second
star_seq	0	GCCGGTTTGATCTACAAAAAAATGC
loop_seq		GGGAGCATAGAAAAATCCCGC
pre_seq		GCCGGTTTGATCTACAAAAAAATGCGGGAGCATAGAAAAATCCCGCACTTTTGTAGATCAAACCGACAT
pre_struct		(.(((((((((((((((((..(((((((............))))))).))))))))))))))))).)))
Genomic position/strand		I:11885650..11885672 +
Genomic Cluster?		No
Possible miRNA family?		No
Accession number		FJ589850
Notes		

Novel miRNA candidate		mir-2218a
mature_seq	3	CAAACTACAAGTTTTAAGCCTCA
mature_arm		first
star_seq	2	AGGCCAGAATAGTGTAGTTTGTA
loop_seq		CCAAAAGTTGGGTG
pre_seq		CAAACTACAAGTTTTAAGCCTCACCAAAAGTTGGGTGAGGCCAGAATAGTGTAGTTTGTA
pre_struct		(((((((((...(((..((((((((........))))))))..)))...)))))))))))
Genomic position/strand		I:13812583..13812605 +
Genomic Cluster?		No
Possible miRNA family?		No
Accession number		FJ589851
Notes		

Novel miRNA candidate		mir-2218b
mature_seq	2	AGACTACAAACTACATCATTTTC
mature_arm		first
star_seq	1	AAATTTGTAGTTTGTAGTGAGA
loop_seq		CATCTCTTTGGTGAGGCTAGA
pre_seq		AGACTACAAACTACATCATTTTCCATCTCTTTGGTGAGGCTAGAAAATTTGTAGTTTGTAGTGAGA
pre_struct		..(((((((((((((..((((((..((((......))))...)))))).)))))))))))))..))
Genomic position/strand		I:13834928..13834906 -
Genomic Cluster?		No
Possible miRNA family?		No
Accession number		FJ589852
Notes		

	Read#	
Novel miRNA candidate		268610_adh
mature_seq	4	ACACATTTTTCAAGTTGACAACT
mature_arm		second
star_seq	0	TGGTCAACTAGAAAAGTGTTTAA
loop_seq		AAATTAATTTT
pre_seq		TGGTCAACTAGAAAAGTGTTTAAAAATTAATTTTACACATTTTTCAAGTTGACAACT
pre_struct		(.(((((((.(((((((((.(((((.....))))).))))))))).))))))).)))
Genomic position/strand		I:11878022..11878000 -
Genomic Cluster?		No
Possible miRNA family?		Yes (mghv-mir-M1-9)
Accession number		FJ589853
Notes		

Novel miRNA candidate		949690_adh
mature_seq	15	CGCGGGACTAGTCAAGTGTCGGCT
mature_arm		first
star_seq	0	CCGACACTTTCCGGTTTTGCGAA
loop_seq		GCAACCAGAGCAG
pre_seq		CGCGGGACTAGTCAAGTGTCGGCTGCAACCAGAGCAGCCGACACTTTCCGGTTTTGCGAA
pre_struct		((((((((..(..(((((((((((((.......))))))))))))).)..))))))))))
Genomic position/strand		I:2538612..2538635 +
Genomic Cluster?		No
Possible miRNA family?		No
Accession number		FJ589854
Notes		

Novel miRNA candidate		647386_adh
mature_seq	14	ATCGGGTAAGGATTTTTGTTGGG
mature_arm		first
star_seq	0	CAACAAAAATCCTTGCCCGATGT
loop_seq		TTTCTAATGATTTCATAACGTTTTCCCGTTATGAAATCATTAAAAACC
pre_seq		ATCGGGTAAGGATTTTTGTTGGGTTTCTAATGATTTCATAACGTTTTCCCGTTATGAAATCATTAAAAACCCAACAAAAATCCTTGCCC[illegible]
pre_struct		((((((((((((((((((((((((((.((((((((((((((((......)))))))))))))))).)))))))))))))))))))))))
Genomic position/strand		I:6339802..6339780 -
Genomic Cluster?		No
Possible miRNA family?		No
Accession number		FJ589855
Notes		

	Read#	
Novel miRNA candidate		1128878_adh
mature_seq	23	GAAAAATTGCGCGCATGGGTTG
mature_arm		second
star_seq	0	ACCCTTTCGCCAATTTTTTTCGT
loop_seq		GAAAAAACGTGTAATTTTAC
pre_seq		ACCCTTTCGCCAATTTTTTTCGTGAAAAAACGTGTAATTTTACGAAAAATTGCGCGCATGGGTTG
pre_struct		((((...((((((((((..((((((((..........))))))))))))))).)))...))))))
Genomic position/strand		I:2888492..2888471 -
Genomic Cluster?		No
Possible miRNA family?		No
Accession number		FJ589856
Notes		

Novel miRNA candidate		1181174_adh
mature_seq	8	GAAGGGCAAAGAGTGCGATATC
mature_arm		second
star_seq	1	TATCGCACTCTTTGCCCTTCCG
loop_seq		GCATGACTCATGGCG
pre_seq		TATCGCACTCTTTGCCCTTCCGGCATGACTCATGGCGGAAGGGCAAAGAGTGCGATATC
pre_struct		((((((((((((((((((((((.((((...)))).))))))))))))))))))))))))
Genomic position/strand		I:3632164..3632185 +
Genomic Cluster?		No
Possible miRNA family?		No
Accession number		FJ589857
Notes		The star sequence mapped multiple loci.

Novel miRNA candidate		772234_adh
mature_seq	4	CAAGGCACAGTTCTGTGGGCGG
mature_arm		second
star_seq	0	GCCCACAGAACTGTACCTTGCA
loop_seq		AAGCGGTTTCAAAAATTTT
pre_seq		GCCCACAGAACTGTACCTTGCAAAGCGGTTTCAAAAATTTTCAAGGCACAGTTCTGTGGGCGG
pre_struct		((((((((((((((.(((((.(((.............))).))))).))))))))))))))))
Genomic position/strand		V:13647931..13647910 -
Genomic Cluster?		No
Possible miRNA family?		Yes (mmu-mir-124,dme-mir-124,hsa-mir-124,cbr-mir-124,rno-mir-124,aga-mir-124,hsa-mir-506)
Accession number		FJ589858
Notes		

	Read#	
Novel miRNA candidate		2154356_adh
mature_seq	16	TGACAGGAGGTAGTAGGCCAAG
mature_arm		second
star_seq	0	TGGCGTACTTCTAACTGTCCATC
loop_seq		TAAAACACAATTATGA
pre_seq		TGGCGTACTTCTAACTGTCCATCTAAAACACAATTATGATGACAGGAGGTAGTAGGCCAAG
pre_struct		((((.((((.((..(((((.(((..............))))))))..)).)))).))))))
Genomic position/strand		V:7661081..7661060 -
Genomic Cluster?		No
Possible miRNA family?		Yes (smo-mir1092)
Accession number		FJ589859
Notes		

Novel miRNA candidate		mir-2219
mature_seq	12	CGAAGTGCGAGGGAAAGCTGAAG
mature_arm		second
star_seq	2	ACAGCTTTCTCTCGCACATCGTC
loop_seq		AGCTAGATTTGAACTCTGTATGAAAGCTGA
pre_seq		ACAGCTTTCTCTCGCACATCGTCAGCTAGATTTGAACTCTGTATGAAAGCTGACGAAGTGCGAGGGAAAGCTGAAG
pre_struct		.((((((((((((((((.((((((((((((.......))).......))))))))).))))))))))))))))..)
Genomic position/strand		V:2924601..2924579 -
Genomic Cluster?		No
Possible miRNA family?		No
Accession number		FJ589860
Notes		

Novel miRNA candidate		1277767_adh
mature_seq	14	GAGTGGAGTTGTAGCACCGGCA
mature_arm		second
star_seq	0	CAGCTTTTGATGGCTCCTTCGT
loop_seq		CTTGAC
pre_seq		CAGCTTTTGATGGCTCCTTCGTCTTGACGAGTGGAGTTGTAGCACCGGCA
pre_struct		(.(((....(..((((((((((....)))))).)))))..)))....)))
Genomic position/strand		V:8484706..8484727 +
Genomic Cluster?		No
Possible miRNA family?		No
Accession number		FJ589861
Notes		

	Read#	
Novel miRNA candidate		mir-2220
mature_seq	63	TCAATTGTTTGTGGACTTACAG
mature_arm		second
star_seq	1	GTAAGACCATAAACTATTTATC
loop_seq		AATTCATCATTTGA
pre_seq		GTAAGACCATAAACTATTTATCAATTCATCATTTGATCAATTGTTTGTGGACTTACAG
pre_struct		(((((.((((((((.(((.(((((........))))).))).)))))))).)))))..
Genomic position/strand		X:1873858..1873879 +
Genomic Cluster?		No
Possible miRNA family?		No
Accession number		FJ589862
Notes		
Novel miRNA candidate		426009_adh
mature_seq	6	AGATGACTTACTCTATATTCTTT
mature_arm		first
star_seq	0	AGAATATAGAGTAAGTCATCTCA
loop_seq		ATAAATATTATGTGTTATAA
pre_seq		AGATGACTTACTCTATATTCTTTATAAATATTATGTGTTATAAAGAATATAGAGTAAGTCATCTCA
pre_struct		(((((((((((((((((((((((((((..........)))))))))))))))))))))))))))))
Genomic position/strand		X:15699909..15699931 +
Genomic Cluster?		No
Possible miRNA family?		No
Accession number		FJ589863
Notes		
Novel miRNA candidate		1101605_adh
mature_seq	1	CTTCATGGAGCACCAACGAGCTGA
mature_arm		first
star_seq	1	GGATTGGACTGTTCGGATGATGAG
loop_seq		GAAGAGAAATCTTTTC
pre_seq		CTTCATGGAGCACCAACGAGCTGAGAAGAGAAATCTTTTCGGATTGGACTGTTCGGATGATGAG
pre_struct		(.((((.(((((((((....(((((((((....))))))))).))))..))))).).))).)))
Genomic position/strand		X:5065946..5065969 +
Genomic Cluster?		No
Possible miRNA family?		No
Accession number		FJ589864
Notes		
