# Supplementary material for: Dynamic expression of small non-coding RNAs, including novel microRNAs and piRNAs/21U-RNAs, during Caenorhabditis elegans development
Source: Genome Biol. 2009 May 21;10(5):R54. doi: 10.1186/gb-2009-10-5-r54 (PMC2718520; doi:10.1186/gb-2009-10-5-r54)
Supplement: Additional data file 8 — Data were normalized by the total number of reads that matched to the C. elegans genome. The miRNAs and their number of reads were highlighted in red as mentioned in the legend for Additional data file 4. [file gb-2009-10-5-r54-S8.pdf]

		Hermaphrodites (wild-type N2)						Males *(dpy-28;him-8)*
		Embryo	mid-L1	mid-L2	mid-L3	mid-L4	young adult	young adult
Total number of reads that matched to the *C. elegans* genome	Accession number	5742750	5617234	6047597	4948026	6072252	5975243	7602104
540532_mas	FJ589793	0	1	1	1	0	0	5
964568_mas	FJ589794	1	0	0	0	0	1	0
95481_mas	FJ589795	0	0	1	0	0	0	1
1883591_mas	FJ589797	0	0	0	0	0	0	1
1260661_mas	FJ589798	1	1	0	0	0	0	1
70290_mas	FJ589799	9	2	0	0	0	0	0
mir-2207	FJ589801	3	0	0	1	1	0	1
209309_mas	FJ589803	2	0	1	0	0	0	0
mir-2208a	FJ589804	1	0	0	1	0	0	5
mir-2208b-5p	FJ589805	0	1	0	1	3	3	385
1742956_mas	FJ589806	0	0	0	0	0	0	9
mir-2209a	FJ589807	23	55	17	6	48	73	2069
mir-2209c	FJ589808	5	2	0	0	1	2	103
2103433_mas	FJ589809	41	71	82	130	145	99	104
mir-2210	FJ589810	0	2	0	1	0	1	11
1911250_mas	FJ589811	3	0	0	0	0	0	1
663452_mas	FJ589812	0	0	0	0	0	0	1
63594_mas	FJ589813	0	0	0	1	0	0	1
mir-2211	FJ589815	0	0	0	0	0	0	4
1392735_mas	FJ589816	8	1	3	1	2	2	2
427628_mas	FJ589817	3	0	0	0	0	1	0
1911316_mas	FJ589818	0	0	0	0	0	0	1
1467045_mas	FJ589820	0	0	0	0	0	0	1
467565_mas	FJ589821	0	0	0	0	0	0	1
724701_mas	FJ589822	0	0	1	1	1	1	11
mir-2212	FJ589823	1	3	0	6	11	15	128
547404_mas	FJ589824	33	0	0	7	0	2	36
24789_adh	FJ589826	1	1	0	0	0	0	0
1010777_adh	FJ589827	0	0	0	1	0	0	0
1032770_adh	FJ589828	3	4	1	1	0	0	0
1973091_adh	FJ589829	6	6	9	1	7	8	25
mir-2213	FJ589830	4	1	1	1	2	0	0
mir-2214	FJ589831	4	0	9	19	5	4	15
764767_adh	FJ589832	0	4	10	19	5	5	0
mir-2215	FJ589833	3	0	2	0	1	0	0
347252_adh	FJ589834	11	23	12	49	47	37	5
mir-2216	FJ589835	0	6	0	1	7	1	2
1619758_adh	FJ589836	0	0	1	0	0	0	0
748932_adh	FJ589837	0	0	0	0	1	0	0
686798_adh	FJ589838	0	10	9	8	8	8	5
mir-1832b	FJ589839	1	1	1	1	1	1	0
169025_adh	FJ589840	1	6	4	3	2	1	0
405191_adh	FJ589841	0	6	6	29	183	106	14
327617_adh	FJ589842	0	0	0	0	2	8	0
837693_adh	FJ589843	48	5	15	8	1	6	16
mir-2217	FJ589844	1	0	0	0	0	0	1
1533251_adh	FJ589845	2	0	0	2	8	11	2
651772_adh	FJ589846	0	8	2	0	7	0	1

mir-2209b	FJ589847	0	1	0	0	1	0	1
mir-2208b-3p	FJ589848	0	1	1	1	4	19	360
1671098_adh	FJ589849	1	0	0	0	0	0	0
358157_adh	FJ589850	4	4	1	3	3	0	0
mir-2218a	FJ589851	2	0	0	1	0	0	0
mir-2218b	FJ589852	0	0	1	1	0	0	0
268610_adh	FJ589853	4	0	0	0	0	0	0
949690_adh	FJ589854	0	1	0	3	4	5	2
647386_adh	FJ589855	0	5	4	2	1	1	1
1128878_adh	FJ589856	20	2	1	0	0	0	0
1181174_adh	FJ589857	4	1	1	1	1	0	0
772234_adh	FJ589858	3	1	0	0	0	0	0
2154356_adh	FJ589859	0	3	0	0	1	1	11
mir-2219	FJ589860	0	2	4	3	3	0	0
1277767_adh	FJ589861	6	0	1	2	1	1	3
mir-2220	FJ589862	8	5	8	6	0	0	36
426009_adh	FJ589863	0	3	0	1	0	2	0
1101605_adh	FJ589864	0	1	0	0	0	0	0
miRNA reads in each library		271	250	210	324	518	425	3382
