# Supplementary material for: Dynamic expression of small non-coding RNAs, including novel microRNAs and piRNAs/21U-RNAs, during Caenorhabditis elegans development
Source: Genome Biol. 2009 May 21;10(5):R54. doi: 10.1186/gb-2009-10-5-r54 (PMC2718520; doi:10.1186/gb-2009-10-5-r54)
Supplement: Additional data file 9 — 21U-RNAs in which we found larger transcripts and overlapping ones within 10 bp of other 21U-RNAs, including novel ones we found, are marked with an asterisk and a dagger, respectively. [file gb-2009-10-5-r54-S9.pdf]

	Name	Sequence	wild-type N2 hermaphrodites						*dpy-28;him-8* males	Total number of each 21U-RNA read
			Embryo	mL1	mL2	mL3	mL4	yAdult	yAdult	
†	21UR-1	TGGTACGTACGTTAACCGTGC	0	0	0	0	0	0	0	0
	21UR-2	TGGGAAATTCGAATAATATAT	0	0	0	1	2	1	1	5
	21UR-3	TGCCTCAATAGATTGCTCACT	3	0	0	1	5	9	8	26
†	21UR-4	TAGAAGCTTTTTTTGGGTTTA	0	0	0	0	0	0	0	0
*	21UR-5	TAGATGTTCTGGAATTTCTGC	0	0	0	0	4	10	0	14
	21UR-6	TATGTTAAGAAAACCCTCGAC	1	0	0	0	7	6	3	17
	21UR-7	TAACTTATGTTTCATCGTTTT	0	0	1	2	18	12	7	40
	21UR-8	TCATGGACGTATGTGAATGTG	0	0	0	3	8	8	3	22
	21UR-9	TAATTTTTTAGTTTGGTGAGT	10	0	0	0	0	8	1	19
	21UR-10	TGCTCCTTTCAACTCTATTAG	0	0	0	0	0	0	0	0
†	21UR-11	TGTTAACTATGGGTTGTGCAC	0	0	0	0	2	5	2	9
†	21UR-12	TGGTTTTGTCCTCTTTGTACC	0	0	0	0	1	1	1	3
	21UR-13	TACTATTCCATTTAATCATTG	0	0	0	0	1	0	0	1
	21UR-14	TCGGAAACCAGATATATTTTG	1	1	0	0	1	0	0	3
* †	21UR-15	TCTGCGATTGTTAGTTTTGCA	1	0	0	0	12	12	2	27
* †	21UR-16	TAGTGTAGAAAAGTGGTTGCT	346	276	130	88	250	1129	33	2252
†	21UR-17	TGTTCGTGTAATGAAGTTTGC	0	0	0	1	10	10	1	22
	21UR-18	TGAAAGTTTCTCTGTGGTCTC	0	0	0	0	2	6	0	8
†	21UR-19	TAATTCTTCTTTCTTGGTAAT	12	2	0	1	2	5	0	22
†	21UR-20	TTTCGAAATATTAGGTCTCCA	0	0	0	0	8	2	4	14
†	21UR-21	TGTTGCATAGATTTATTTTCT	0	0	0	0	0	0	0	0
	21UR-22	TGTTATCATTGTATTTACCGA	0	0	0	0	0	0	1	1
	21UR-23	TATAATTAGGAGTATATCAAT	0	0	0	1	7	6	3	17
†	21UR-24	TAGTCAGCTAAAATGGTTCAG	16	3	1	4	29	37	7	97
†	21UR-25	TGAACTCTTGGTCTACGTTTT	0	2	2	0	7	3	1	15
	21UR-26	TAGAAATCCACTATGCTTTGG	0	0	0	0	0	1	0	1
	21UR-27	TGTGACTTGGAAACGAAAATT	0	1	0	0	2	1	0	4
†	21UR-28	TAATTCGACGATTGTTGTTCT	1	0	0	1	5	9	2	18
	21UR-29	TCGTAATTAATTCAAAATAAT	0	0	0	0	0	0	0	0
*	21UR-30	TGAATAATGTTTTCGTCGTAA	73	7	24	22	94	123	32	375
	21UR-31	TGAATTTAGTGACATTGATAC	2	5	5	5	3	25	1	46
	21UR-32	TAGAACTTCATCTTTAGAACA	2	3	1	5	6	9	1	27
	21UR-33	TATCGAATCAATTCAAATTTC	0	0	0	0	0	0	0	0
†	21UR-34	TGAAGGATGCGATACTCTCTC	4	7	11	3	25	75	5	130
	21UR-35	TAAAGTAGTTTATCTCAGGGA	2	7	2	0	5	18	1	35
	21UR-36	TGCGTCATCGTCGCGTCGTGA	3	0	0	0	30	51	141	225
	21UR-37	TAAAAAAGGATGAAATTTATC	0	0	0	0	0	0	0	0
*	21UR-38	TCATAATATAGTTGGGACCTA	17	6	5	1	14	21	8	72
	21UR-39	TTCGAACCTCTTGGTTTTGGA	0	0	0	0	2	2	1	5
†	21UR-40	TATTATTGGCAACTTACGTTA	0	0	0	1	1	0	0	2
	21UR-41	TATTCAATTCAGTTGGTTTAA	0	1	1	1	10	10	2	25
	21UR-42	TGAAACCTCAGAAATAGTTTC	0	0	0	0	1	0	0	1
†	21UR-43	TATTCCTTTAGGAGCGTAACA	1	0	0	0	1	7	1	10
	21UR-44	TACATTGAACTTTTTCGAAAA	1	1	1	6	43	31	4	87
	21UR-45	TTACATCAAAATTAAGTTGGG	20	3	3	2	5	10	8	51
†	21UR-46	TGTTTAGAAACCAGTAATAGA	3	1	0	0	0	2	0	6
	21UR-47	TGAATCTCTGTTTGGACACTC	13	1	0	3	7	42	4	70
	21UR-48	TGGGTTGTTATAACAATTTTA	1	1	0	0	3	2	0	7
†	21UR-49	TCGGTGTTCTCTAAGGCTTTA	0	1	1	0	1	11	0	14
†	21UR-50	TATTGTGTTTGCCGCGAGAGA	2	0	0	0	0	1	1	4
†	21UR-51	TAATTAGAAGGCCCGGTGGAA	18	16	7	9	56	164	52	322
†	21UR-52	TGGGAATGGAAAAATACGATC	1	1	0	1	43	35	2	83
	21UR-53	TCAATGACTTTCAAATGTTGT	0	0	0	0	1	2	1	4
	21UR-54	TACCCCTGTTGTTTGAACAAG	4	9	0	0	7	41	2	63
†	21UR-55	TGCCATTTTCCATCAATGATC	0	0	0	0	0	0	0	0
	21UR-56	TCCGTCGCAGGATGTATCTTT	0	0	0	0	1	0	0	1
	21UR-57	TATGTAGTCCGAGATCAATGA	1	0	0	1	12	13	3	30
†	21UR-58	TGGTCGGAGAACTTAATTTTG	1	0	0	0	0	0	0	1

*	†	21UR-59	TTGGATGAGTACATATGTGAT	18	26	11	13	95	164	9	336
*	†	21UR-60	TTAGTCTGATAGTAACAAAAC	13	10	8	9	162	143	19	364
		21UR-61	TACGCTGATGAAATTATTCTA	32	17	16	13	35	186	2	301
		21UR-62	TAAGACGTAAACGTATATATT	3	3	3	0	13	5	0	27
		21UR-63	TGCATTTTTAACAGCCTGAAA	2	0	0	1	2	3	0	8
		21UR-64	TGATGTTCCTTGAGACTTTAA	0	0	0	0	3	1	0	4
		21UR-65	TCAGACTTCGCTTCTGCTTGA	0	1	0	0	6	4	1	12
*	†	21UR-66	TATTGTAGATTTCTCCGGCGG	70	30	7	36	534	804	342	1823
		21UR-67	TCTTCTTCGAAACGTTTGCAA	1	0	0	0	1	0	0	2
*	†	21UR-68	TGTGATCTCTACCGGTATAAC	1	4	0	12	177	190	12	396
		21UR-69	TACTTGAATAGTGTTTATAAA	0	0	0	0	1	0	0	1
		21UR-70	TGATCCCACTTTTTTCACCTT	0	0	0	0	0	0	0	0
	†	21UR-71	TTGGGATTTCGCGAAAGCATT	0	0	0	0	7	3	1	11
	†	21UR-72	TGATTGCACCAGATGTAATGA	19	2	3	0	3	14	6	47
	†	21UR-73	TTCGTCGTGTGTAGTTTCGCC	5	3	1	1	13	21	2	46
*	†	21UR-74	TTCTCAGAATTCCAGATGTGT	2	6	3	3	27	46	5	92
		21UR-75	TTATTCGCTCTTACGGCACTC	0	0	0	2	50	34	8	94
	†	21UR-76	TCTGTCTGAGAAACCCATTGT	0	0	0	2	3	6	5	16
	†	21UR-77	TGACATCCTAGCAAAATGTTC	0	0	0	0	0	0	3	3
*	†	21UR-78	TAAAGATGGAGTACTGTACTC	5	3	3	19	318	264	13	625
*		21UR-79	TGCATTGTGTGAGCCGGAATC	2	3	0	7	39	59	9	119
	†	21UR-80	TTGACAAAAGTATAATGTAAA	0	0	0	0	0	0	0	0
	†	21UR-81	TAGTGCTAATTATTCCAACCC	1	0	0	0	0	1	0	2
		21UR-82	TAACTTTCCGTCTACGTTAAC	6	2	1	0	3	29	4	45
	†	21UR-83	TTAATTGAGCTGTGATGTGAT	7	25	9	11	72	128	14	266
		21UR-84	TGAAAAATTGCACCCGACAAC	0	0	0	0	0	0	0	0
		21UR-85	TCAACGGGTTTTCGATAGGTT	17	5	0	1	3	27	2	55
		21UR-86	TGCTCATACCATGTTACTTTT	0	0	0	0	0	0	0	0
		21UR-87	TGGTCCCATCGTTCTATTTAT	2	0	0	0	1	0	5	8
	†	21UR-88	TTGGGCATCTGTATTCAGAAC	1	5	0	2	3	14	0	25
	†	21UR-89	TAGTATATTGACCTAGTAAAT	0	0	0	0	1	1	0	2
		21UR-90	TGGTATGTGTGATTCAAACAG	3	0	0	0	0	11	0	14
	†	21UR-91	TAGTTGATCAAAAAATTTTCA	0	0	0	0	0	1	0	1
	†	21UR-92	TTCAGACTCAGACTACAGTTC	0	1	0	1	30	20	1	53
*	†	21UR-93	TTTATTCGGATTAGCGTTAGG	11	8	4	4	18	70	12	127
	†	21UR-94	TTGGGGTAAACTTGAGGGAAA	6	3	0	2	12	32	7	62
	†	21UR-95	TACCAATGTAAGGTTCACTCC	0	0	0	0	0	0	0	0
		21UR-96	TGTGACAAAATAGGCCAACTA	5	0	1	2	4	5	5	22
*		21UR-97	TCAGAATGACTAGAAAGTTGG	0	0	0	0	11	6	7	24
		21UR-98	TAGTTCAGGTCCTAGTGAAAG	0	0	0	0	0	7	0	7
		21UR-99	TAATTTGACCATCATCTTTCC	0	1	0	0	9	4	6	20
		21UR-100	TCTTTTCTTCTTATATGCCTG	0	0	0	0	0	0	0	0
	†	21UR-101	TAGTAGCCGCATCATTCAAAA	1	0	0	1	6	6	3	17
	†	21UR-102	TGTTCGACGTCTCTTGCTTTC	0	0	0	0	13	5	0	18
*		21UR-103	TGCACCTTGAGGGGAACTAGA	2	2	0	0	1	2	3	10
		21UR-104	TTAGTCATTAATTACGGTTCC	21	2	7	5	13	37	4	89
	†	21UR-105	TGAATGATATGCCAGATCACT	2	0	0	0	1	1	2	6
		21UR-106	TAAGTGTATCTTTCACATTTT	0	0	0	0	0	0	0	0
		21UR-107	TTTGTTTTCCATTGTGAGAAA	0	0	1	0	0	1	0	2
*		21UR-108	TAAGTTCGAATTCGAGAAAGG	3	0	5	4	106	98	1	217
		21UR-109	TCTTAATTCATTTTTATGTCA	0	0	0	0	0	0	0	0
	†	21UR-110	TTGAAGACTTATTGAAAATGT	0	1	0	2	37	14	0	54
		21UR-111	TACAAAATTATTTGGCATATT	0	0	0	1	1	2	1	5
		21UR-112	TCGCAACTGATTTTGATTTGA	0	0	0	0	5	5	1	11
		21UR-113	TCACACTTTTTCAATAGTAAC	4	0	0	0	0	0	0	4
	†	21UR-114	TGAGTAATTAAAAGACAGAAG	0	1	0	2	13	10	1	27
		21UR-115	TTTTGTTGTAACTCTTTTGAT	0	0	0	0	0	0	0	0
*	†	21UR-116	TGGGGTTGTAGATATAGAAGA	173	182	66	136	1948	2405	608	5518
		21UR-117	TGCATCCTTTGAGAAGAAAAA	0	0	0	0	0	0	0	0
	†	21UR-118	TGACACCGGCAAAAATCAGAA	11	2	1	2	14	32	27	89
	†	21UR-119	TGGGTTTAGGCGATAATGACT	12	19	9	6	28	80	2	156
	†	21UR-120	TTAAAAATTTGACCGGCAGAG	18	11	5	6	40	102	25	207
	†	21UR-121	TTCAACGGCTGCCCCATAAGA	7	2	1	0	2	12	2	26
		21UR-122	TAACCGGCTTTGAAATGACAA	0	0	1	2	21	21	0	45

		21UR-123	TTCATCTGACACTTAGATGCA	0	0	0	1	21	12	3	37
*	†	21UR-124	TACTTCTGGATAAAAGTTACA	1	6	2	4	24	12	1	50
		21UR-125	TAAAATGCATGCTTCCTGTGA	0	0	0	2	10	18	9	39
		21UR-126	TAATTGTGAACTCCTTTTCAT	1	0	0	0	2	6	0	9
		21UR-127	TGGGGTTTCGATCCTTTGTGA	0	0	0	0	0	0	0	0
		21UR-128	TGCTGTCTTCCTTCTCAAATT	0	0	0	0	0	0	0	0
	†	21UR-129	TAAAACGTACGGAAAAAATTA	7	1	0	3	4	4	14	33
*		21UR-130	TAAAAATAGCGTGTATGTACA	11	3	2	1	5	17	1	40
		21UR-131	TTGGTTTGCAACACTTGTTAC	0	0	0	0	1	1	0	2
		21UR-132	TCCATAAAATTCTAAATATTT	0	0	0	0	1	6	0	7
		21UR-133	TCGTTCGATAATGGTTTTATC	0	0	0	0	0	0	0	0
	†	21UR-134	TAATGACTTTGGATTTTCTCG	0	0	0	0	1	4	2	7
		21UR-135	TATACCACATGGGTAGATGTT	0	0	0	0	0	0	0	0
	†	21UR-136	TCTCAGATGGTATTTGAATCC	0	4	1	9	171	110	15	310
		21UR-137	TCCAGTCTCTAAGAACAGTTT	0	0	0	0	1	0	1	2
	†	21UR-138	TCTGCAATCGATTTCTGCAAT	0	1	0	0	62	46	3	112
		21UR-139	TCTAACTTGCAATGCCTCGAG	0	0	0	0	0	0	1	1
*	†	21UR-140	TGGTGTTGCCAGAAGATATAT	11	1	1	0	0	12	1	26
		21UR-141	TATATACGGTTATTCCCAATC	0	0	0	0	1	7	1	9
	†	21UR-142	TGTTATCATAGTAGATTTCGT	0	0	0	1	19	16	0	36
		21UR-143	TGCGTCCTCACTATCAGCTTG	0	1	0	0	1	1	0	3
		21UR-144	TCTGCAAATCACACTATCTGA	0	0	0	0	0	0	0	0
	†	21UR-145	TGGTTATAGAGTATGTATGCG	0	0	0	0	8	3	0	11
		21UR-146	TCGTGAGTAACAGTAGTTTTT	0	0	0	0	2	0	0	2
		21UR-147	TCTGATATCTCTAATAGGTAA	0	0	0	0	1	0	0	1
*	†	21UR-148	TCCGAAAAATCGATTATGGAC	1	1	0	3	38	51	16	110
		21UR-149	TGGTGTTGAAAAAAATAGTTT	0	0	0	0	0	0	0	0
		21UR-150	TATAGTACCTTGCTGATGTGA	0	0	0	0	0	3	0	3
	†	21UR-151	TACGAGGGACGAAATTTGCTT	31	12	7	5	12	89	61	217
		21UR-152	TGTTGAGGAATGCAATTGAAA	0	1	0	0	1	9	4	15
		21UR-153	TATGTCAGGTTCCTTTGTGCA	1	0	0	1	17	13	0	32
*	†	21UR-154	TACCATGGACATTACCACAAT	0	0	0	0	11	4	3	18
	†	21UR-155	TTGAAGAAACATAAGACAAAA	1	1	0	2	18	24	7	53
		21UR-156	TTAAGTCTTTCCAATCTCTGA	10	5	3	6	10	15	2	51
	†	21UR-157	TACTATACTTCTCCATTCTGA	0	1	1	1	8	6	4	21
		21UR-158	TTCACTGACCTCCAAAAAGTG	0	0	0	0	0	0	0	0
		21UR-159	TCTATTTTACATTATATGTGA	1	0	0	0	0	0	0	1
	†	21UR-160	TAGTGAACTGTCTGTCTTCAT	1	2	1	0	2	5	1	12
*	†	21UR-161	TTCTCATCCGGTCCAAGAGGT	5	0	0	0	3	6	6	20
		21UR-162	TGTCATGGGATTCTCAATTAA	0	0	0	0	0	0	0	0
*	†	21UR-163	TTTCGCGTCGTCATCACAAAC	96	32	28	22	121	191	115	605
		21UR-164	TCCATCTCCTGAAAGTACGTT	0	1	0	1	25	13	3	43
	†	21UR-165	TTAAACCATATTACAATAAAG	0	0	1	0	4	2	0	7
*		21UR-166	TACATCAATGACAGAAAGATC	1	0	1	3	34	27	2	68
		21UR-167	TGTGCTTTCAGGATCAGTATG	1	0	0	0	1	8	2	12
		21UR-168	TGAAGCGTTGATTAAAATTAT	0	0	0	0	1	2	0	3
		21UR-169	TGAGCCCGCTTATTGAAAAGA	0	0	0	0	0	0	1	1
		21UR-170	TGGGCCCGGCACAAAACTAAA	0	0	0	0	2	0	5	7
	†	21UR-171	TTATGACGGTTTCATTTGGAT	0	0	0	0	1	1	0	2
	†	21UR-172	TCCTGTTTTGAATCGGCTGCA	29	18	15	8	43	247	17	377
	†	21UR-173	TAACTAAGTAAAGGGCATTCA	10	1	2	3	34	36	14	100
	†	21UR-174	TAATAGGCGTCTTTCAATTCA	1	1	1	1	15	17	4	40
	†	21UR-175	TGAGTGAGGAATATTCGTAGA	0	0	0	1	34	35	5	75
		21UR-176	TGTGAGTTGTACCTGCACATC	0	0	0	0	3	2	0	5
		21UR-177	TGGAATTGACAATTTTTCTCA	0	0	0	0	0	0	0	0
	†	21UR-178	TGCATGTCTTTCTCCGTTTGG	2	0	0	0	1	6	5	14
	†	21UR-179	TAGTATTTCTTCCTCTGATTC	0	0	0	0	0	1	0	1
		21UR-180	TTTGCAAAGAAATTAGACACC	3	0	0	0	0	8	0	11
		21UR-181	TGTTCACAATAATTTCAGTCA	0	0	0	0	0	0	0	0
		21UR-182	TCCGAGATGTAATACAAGAAG	6	2	1	0	39	46	6	100
*		21UR-183	TTAAACAGCAATATGAACGAT	76	137	55	74	192	490	7	1031
		21UR-184	TAGTCATGCTTAACTTCAAAA	0	0	0	0	3	2	0	5
		21UR-185	TAACTTTATGATGAGTTCTAA	1	0	0	0	1	2	0	4
		21UR-186	TGACAATTCTATCTAATGAGA	0	0	0	0	0	0	0	0

†	21UR-187	TCGATACAGAAATGTCAAACA	1	1	0	0	1	1	0	4
	21UR-188	TTACAGCCAAAAAATGCATTC	0	0	0	1	11	11	2	25
†	21UR-189	TCAATATTTGCATAGGGTATC	12	2	2	1	2	15	2	36
†	21UR-190	TGCACTATTCTTTGAACTGTT	1	0	0	0	0	1	0	2
†	21UR-191	TCAAAATTCCCACCCGATCTG	0	0	0	0	0	1	0	1
†	21UR-192	TACAAAGTTGCTTCAATGTGA	10	0	2	0	0	14	0	26
	21UR-193	TAGGAAATTCCGCCCAACATA	0	0	0	0	0	0	0	0
†	21UR-194	TTAAACAAGGGACTGTTAGGG	159	35	39	28	356	593	97	1307
†	21UR-195	TATATGTGTTGCATAGGATGC	0	0	0	0	0	1	0	1
†	21UR-196	TACCAACATCTGCTTTGGAAA	0	0	0	1	7	9	3	20
	21UR-197	TCATGTTCTGTTATAAAAACC	1	1	0	0	6	4	1	13
	21UR-198	TTATTCCGAATTGTTGCATCG	0	1	0	0	1	4	0	6
	21UR-199	TCGAACTTGATTTCCAGTGAG	0	0	0	0	0	0	0	0
	21UR-200	TGCCAAGTAAATTGAAATTCA	0	0	0	0	2	0	0	2
†	21UR-201	TGCACAATTGATAAGGATAAA	0	0	0	0	2	2	3	7
	21UR-202	TACCCAAATATTCTAAAAAAT	0	0	0	0	0	0	1	1
†	21UR-203	TTCAACCGTTGCCGTTATATC	0	0	0	0	1	6	2	9
	21UR-204	TAAACTATAATTATTTGTTAC	0	0	0	0	0	2	1	3
†	21UR-205	TTCGGAAGCTAAACTCTGCTT	0	0	0	0	3	2	0	5
	21UR-206	TGCTATCCAATTATTCTTGTC	0	0	0	0	1	0	1	2
	21UR-207	TACATTTGCTATTCAATCCAC	0	0	0	1	11	8	5	25
	21UR-208	TATGTGTTCCTTAAGCAGATG	16	2	1	0	1	11	0	31
	21UR-209	TGAAAATCAAGTTCTCTAAGG	0	1	0	4	24	15	0	44
	21UR-210	TAAGTCACCTAAAACTACTTT	0	0	0	0	2	0	0	2
†	21UR-211	TCGGATTTGCTCACACCGGAT	7	0	2	1	12	19	49	90
	21UR-212	TGCATAATGGTTAATGTAGTT	1	2	0	0	6	5	0	14
	21UR-213	TAGAACGCGGTATAGCAAGAC	8	1	2	4	55	66	9	145
	21UR-214	TTGACTTGCTAAAAATGGAAA	2	7	1	3	5	6	2	26
	21UR-215	TAAAGTATTTGGGAACTTGGC	1	0	1	1	1	4	0	8
†	21UR-216	TTCAGCATTGATCATGTCAGA	9	1	0	3	7	17	6	43
†	21UR-217	TATATCCTATCAACTGAATTT	0	0	0	0	0	1	0	1
	21UR-218	TTCAACAACGTTTCTTCATGA	0	0	0	0	0	0	0	0
†	21UR-219	TTCGGGAGTAAAATGTTGTAT	0	4	0	2	55	34	2	97
	21UR-220	TTACTATGTATTTCCTTTAAA	0	0	0	0	4	2	0	6
	21UR-221	TCTATAGGTGCATCTAATAAC	0	0	0	0	2	2	1	5
*	21UR-222	TCATCTTATCGTGTACAATCC	5	0	2	1	6	20	2	36
	21UR-223	TCAATTGGCATTATTTCATGC	0	0	0	0	0	0	0	0
	21UR-224	TGTATAACTCTTGAATCCCAT	0	0	0	0	3	2	0	5
*	21UR-225	TGTATGATCAGTATGGAAAAT	128	74	68	75	513	633	186	1677
*	21UR-226	TATGTCCATTAGAAGAATTCT	2	0	1	0	6	10	0	19
†	21UR-227	TGCGATGTGGGTTCAATCACA	2	0	0	0	0	0	0	2
*	21UR-228	TGAAACTGTAAAAATTATGGC	40	36	26	36	215	362	15	730
	21UR-229	TCCGTGACCAACTTCCACTGT	2	1	0	0	0	0	0	3
†	21UR-230	TTCCGTAATGTTGTATTTTCC	0	1	0	1	2	1	1	6
	21UR-231	TATTGCGAATTTTTAATATTT	0	1	0	1	4	1	1	8
†	21UR-232	TGAAGATGCCTTTTATGTTCC	12	7	4	2	5	30	3	63
*	21UR-233	TAAACTTTTGATCCGGTTGCC	0	0	0	0	8	24	22	54
	21UR-234	TAAACTAGCACTGCTCGTAAA	5	0	1	0	1	8	0	15
†	21UR-235	TATTGTGGTTATAAAGGTTTT	7	2	6	1	3	5	1	25
†	21UR-236	TGATTCATCATTGTTTTCGGA	52	5	7	1	4	27	7	103
†	21UR-237	TTGGTTGATGTTTCGTCTTTA	3	0	0	0	9	8	3	23
	21UR-238	TATAACTATGGGATCGATGTT	2	0	0	1	20	19	36	78
†	21UR-239	TTTATGGAACAGTTTCCAATT	0	0	1	0	0	0	0	1
	21UR-240	TTTGATTGTTCTATGGAAAGT	24	12	5	8	36	84	12	181
	21UR-241	TTCGAGTGCAGTTATAAATGA	11	1	1	0	4	8	0	25
†	21UR-242	TGCTGCCTTGAAACACTTGAG	1	0	0	0	0	2	0	3
†	21UR-243	TATTTGTGTGCCGTCGTTGGT	291	43	33	19	222	520	296	1424
	21UR-244	TTCCTTCACTATGCACATAAT	0	0	0	0	0	0	4	4
†	21UR-245	TCACGAACTGAAACTACATTT	1	1	1	0	4	7	1	15
	21UR-246	TGGACTATCTGGACATTTGGG	1	1	0	0	8	15	2	27
†	21UR-247	TTCGACCTGCAATGACAAATC	7	5	0	3	16	46	3	80
	21UR-248	TCGTGAGACTCCTGTCACAGC	0	0	0	0	0	3	0	3
	21UR-249	TCGTCATGGCTTTAACCGTAA	0	0	0	0	0	0	0	0
	21UR-250	TCACACTTTTGGTAGTAGAAT	0	0	0	0	0	0	0	0

	21UR-251	TTCGTCGGAAATACTTGAATC	2	1	3	2	12	21	0	41
	21UR-252	TAATTTCGCTATTTCAGAGCG	0	0	0	0	0	0	0	0
†	21UR-253	TAAAATATGTGTCGATAAAGC	1	0	0	0	10	19	12	42
	21UR-254	TGATCTGTTCGTTTCTAAGGT	2	0	1	1	2	6	0	12
	21UR-255	TGAAGAAGTCATAATTATATA	0	0	0	0	1	2	0	3
	21UR-256	TGAATAGCGCTATTGTAATTG	0	1	0	0	1	0	0	2
	21UR-257	TGTTCTGATTAAAAGCCATTC	0	0	0	0	0	1	0	1
	21UR-258	TCCTGACTGCTTCAACTAATC	2	7	3	3	10	28	3	56
†	21UR-259	TAATCCGTTTCTGTATAACAT	2	1	1	0	3	2	0	9
	21UR-260	TCTGCAAGCAGTGGCTTAGCA	3	2	0	0	2	7	1	15
	21UR-261	TTATTGATGCCTCTCAAGCCC	0	0	0	0	0	0	0	0
†	21UR-262	TAGTCCTATCAACTGAGATAC	2	0	0	1	3	0	0	6
	21UR-263	TGCTCTGCCTGTTATTGATAT	0	0	0	0	0	1	0	1
	21UR-264	TTGTATTACCAATCCTAGAAA	0	0	0	0	0	0	1	1
	21UR-265	TCATGTACGAAACTAGCGGTT	4	8	9	2	10	33	1	67
* †	21UR-266	TCACACACAACAAAGGCTAAA	1	0	1	3	16	16	12	49
	21UR-267	TATTCTATACAATGGGTATAC	1	0	0	0	0	2	0	3
	21UR-268	TGCGTTCTCGAAAATTACAAA	0	0	0	0	0	1	2	3
* †	21UR-269	TGTTTGCGTTGTTGTACATAT	0	2	1	6	60	43	22	134
	21UR-270	TGTCGTATTACGATAATTTTC	0	0	0	0	0	0	0	0
†	21UR-271	TCTTGTCAATTTGTGTTAAAG	0	0	0	1	0	0	0	1
	21UR-272	TCTTCATGATGTGTTAATGGA	1	0	0	0	0	2	0	3
†	21UR-273	TCACATCTCTCTCAACCTCAA	0	0	0	0	0	1	0	1
	21UR-274	TCGGTGCGGTCACTACTCCAA	0	0	0	0	0	0	0	0
†	21UR-275	TGATATATAATTAAAGAACAT	0	1	0	1	2	1	0	5
†	21UR-276	TTCAACGTACATAACTTCCTT	0	0	0	2	12	6	0	20
†	21UR-277	TGTATCATCTACTATGTCTAC	0	0	0	0	0	0	0	0
	21UR-278	TGTCCTGCTCTTTTTATTTCC	0	0	0	0	0	0	0	0
*	21UR-279	TACGACCGAACGAATACTGAA	2	2	4	6	26	61	17	118
	21UR-280	TGGATTATGTGACTCTGTCAG	0	0	0	0	1	3	1	5
	21UR-281	TGTTTGATTTGACATTCAATA	0	0	0	0	0	0	0	0
†	21UR-282	TGTCATCTTGATTTCTAAAAT	0	0	0	0	0	0	0	0
	21UR-283	TAAACTTGGAAACCAAAAAAG	0	0	0	0	0	1	0	1
†	21UR-284	TGGTTGTGTTTACAAATGTGC	0	0	1	1	0	0	0	2
†	21UR-285	TGGATATGCCTTGCTGAATTG	2	2	0	0	9	25	0	38
†	21UR-286	TATGTGTTCAGCTCTCAGAAT	0	0	0	0	7	8	1	16
†	21UR-287	TGCACTTGTCATCAACAAACA	1	0	0	0	0	1	0	2
†	21UR-288	TGAAATAATCCATTGCTCTTT	1	0	0	0	0	0	2	3
	21UR-289	TAGTAACCAATATATAGGCGG	0	0	0	0	0	0	0	0
†	21UR-290	TTGAATTCGAACTCCGCAAGT	0	0	0	0	4	1	1	6
†	21UR-291	TCACTTGCAGAGAAATCAAAA	4	1	0	5	13	17	9	49
	21UR-292	TCGTCATAATTTTTTTAAACT	2	0	0	2	0	3	0	7
	21UR-293	TCATTCTTTCAAGTTTGTCAC	0	0	0	0	0	1	0	1
*	21UR-294	TACGACTGGCGTACGTATTCT	154	207	97	62	288	1536	41	2385
†	21UR-295	TATTGTATCGAATGAAATTTG	3	0	0	1	2	4	0	10
*	21UR-296	TCTACTTGTTGAATAATGGTA	127	198	137	143	335	1168	27	2135
	21UR-297	TGTTACCGTCTTGTTTTCGGT	0	0	0	0	0	0	0	0
	21UR-298	TGTGAGAAGTTTACATATTGA	0	0	0	0	2	0	0	2
	21UR-299	TTCGAGAAAAGTTTGTATTTC	0	0	1	0	4	1	0	6
†	21UR-300	TGCAAGTGTAAAATTCACTGC	0	0	0	0	2	0	0	2
	21UR-301	TAATATGACTCCGCCGTTGAC	0	0	0	0	1	7	3	11
	21UR-302	TGGAATGGAGAAATTTAATCC	0	0	0	0	2	2	0	4
†	21UR-303	TGAAACGAACAACTACTAATA	0	0	0	0	0	0	0	0
	21UR-304	TCGGGTTTCCGAAATGGTAGA	0	1	0	0	0	1	0	2
	21UR-305	TACCAATCTACCAACAATTCA	0	0	0	0	0	0	0	0
	21UR-306	TAAAAATAGTCACAAAACGGT	0	0	1	0	13	24	5	43
*	21UR-307	TACTTGACGGGACCAATTAAA	0	0	0	1	16	15	43	75
	21UR-308	TTGATAACTATTTAACATTAC	0	0	0	0	0	0	0	0
†	21UR-309	TGTTGTTGTCGAAAGTGTACT	1	1	1	0	1	2	3	9
†	21UR-310	TAATGCTAGTGGATGAGAAGC	0	2	3	2	61	69	14	151
†	21UR-311	TTGTTTCCTGCGAATATCGAC	0	0	0	0	2	0	1	3
* †	21UR-312	TGTTCCAAGCATGTGTGATGG	1	0	0	0	4	13	2	20
*	21UR-313	TTGGTTTAGAATTGGATGCTT	29	24	20	15	95	154	18	355
†	21UR-314	TGAGACTGAGAACTTACTTCT	0	0	0	0	0	1	0	1

		21UR-315	TGAACAACTTGAAAGTCATTC	0	0	0	0	3	0	1	4
	†	21UR-316	TGATTCCTTCATATCAGTGAT	2	0	0	0	1	2	0	5
		21UR-317	TAGGCCATCAAAATTTATTCA	3	3	2	0	5	11	2	26
		21UR-318	TGCCTTTCTCGTCTCTCTTAC	0	1	0	0	0	1	0	2
		21UR-319	TAAAATAAACATAATCTTGAG	2	0	0	1	1	0	1	5
	†	21UR-320	TTCTTCTATTTCTCCATGGGA	1	1	0	0	0	0	2	4
		21UR-321	TATCAGGCTATACCTTAACAA	0	0	1	1	10	13	1	26
		21UR-322	TATAAATTTCTGCAATTAAAA	0	0	0	0	0	5	0	5
*		21UR-323	TACTGCTTTTTCACTTCACGA	5	0	0	0	0	9	2	16
		21UR-324	TCGGCTTGAATTTGAGTAAGC	0	0	0	0	1	6	0	7
		21UR-325	TAATTGGTGCACACCATCTAT	0	0	0	0	1	0	0	1
*	†	21UR-326	TCTGCAGTTTTTGAACGCGTC	21	12	6	8	36	153	11	247
		21UR-327	TAATCTTGAGTTATACAAACA	0	0	0	0	1	1	0	2
		21UR-328	TGAACTTTTCAGTTTTTCAAA	0	0	0	0	0	0	0	0
		21UR-329	TTCGCGAACTGGTTCCAGTCA	0	0	0	0	2	4	1	7
		21UR-330	TTGCAAAGTGGGAATTGAAAG	16	20	10	2	21	89	8	166
	†	21UR-331	TTGTGTGTTAGTCATGTGTTC	1	0	0	0	1	1	0	3
	†	21UR-332	TGCTCAATTGAAAAAAACAGG	6	0	1	2	2	2	1	14
		21UR-333	TCCAGTTTTGAAAGTTTCTCT	0	0	0	0	0	0	0	0
		21UR-334	TACACAGTAAATTATTTTGAA	1	0	0	7	37	121	17	183
		21UR-335	TAAATGGCTTTTTTCCAAACT	3	0	0	1	0	1	1	6
		21UR-336	TAAACGGTTCAAGTCTGTTTC	1	0	0	1	2	3	0	7
*	†	21UR-337	TAGTGGAAACCTCAACGCTGA	0	1	0	0	16	14	0	31
	†	21UR-338	TGCCACTGTTTAATATTCATT	1	0	0	0	3	1	0	5
		21UR-339	TTTTGACGTAAAATCCACAAA	0	0	0	1	1	3	1	6
		21UR-340	TCTATTGATTTAGGAGACAAG	0	0	0	0	0	0	0	0
		21UR-341	TAAAATATGCGTTACAATTTA	1	0	1	0	0	0	0	2
	†	21UR-342	TTGTGCATTGTTAAAAAGATT	11	5	3	2	7	12	2	42
		21UR-343	TAAGAAGTGATACTTTTTCTT	4	0	0	1	4	13	0	22
		21UR-344	TAAGTGTCCTTTTTTTGTTGA	1	0	1	0	1	3	5	11
	†	21UR-345	TGCAGCCCAAATCATCACACA	0	0	0	0	0	0	2	2
		21UR-346	TGTAAGATGACATTTCGAGAA	0	0	0	1	6	14	1	22
		21UR-347	TCGAGATTCTTTGCATCTTTA	0	0	0	1	2	1	0	4
*		21UR-348	TCGGACAAGAAGAATAGATCG	3	1	0	3	29	35	7	78
		21UR-349	TGTCTTCTCTTTTTTGTGTAC	0	0	0	1	0	1	0	2
	†	21UR-350	TGATGAGTATGTACTCCATTT	0	1	0	0	2	1	0	4
		21UR-351	TGCCAATCCTGCTTTTTTTAA	0	0	0	0	0	0	0	0
		21UR-352	TAACCCATAATTCTGCACAAG	0	0	1	0	1	2	0	4
	†	21UR-353	TGCATTGGGATTTGCTTTCTT	2	0	1	0	2	3	0	8
		21UR-354	TATAGATAACATTTCTTTCCA	1	0	0	0	7	2	0	10
		21UR-355	TCAACAGTAAAAATTCAGAGA	0	0	0	0	9	10	2	21
	†	21UR-356	TATAAAATTTGTGTATTTAGA	0	0	0	0	0	0	0	0
		21UR-357	TGGGTTACAATTTTTCAATTA	0	0	0	0	0	1	0	1
		21UR-358	TAGTAAACTCAATCAATGCGA	0	0	0	0	11	8	1	20
	†	21UR-359	TTGGCAATTTTTTTCCATTGG	0	0	0	0	1	0	0	1
		21UR-360	TGGTAAGTCCATATACTTATC	0	0	0	0	1	0	0	1
	†	21UR-361	TGTCATAGCGATACGTTCTTA	49	7	6	3	17	49	6	137
	†	21UR-362	TGCCTCGGATGTACCATGGTT	0	4	0	3	82	74	28	191
		21UR-363	TGAGTACAGCTCACATTCAAA	3	0	0	0	0	0	2	5
		21UR-364	TATTCCGCTAGGAATCAGTGG	0	0	0	0	0	0	0	0
	†	21UR-365	TGTTAACTCGATCCATGCTTT	0	1	2	1	2	3	0	9
		21UR-366	TTAGAAAATGAAAAGGTCACA	1	0	0	1	0	2	1	5
		21UR-367	TAGGCTCGTTCTTAAATGTGG	0	0	0	0	1	1	0	2
		21UR-368	TGAATCTGGGTTCCTCGGGTT	0	0	0	0	0	1	0	1
		21UR-369	TAAGGCACTGTTCTGTTCATG	0	0	0	0	0	0	0	0
		21UR-370	TTGATCTTACTATGTACGTAT	0	0	0	0	0	0	0	0
		21UR-371	TGACACCCTTGACGGCAAAAG	0	0	0	1	3	5	5	14
	†	21UR-372	TTCCACTGATGCTTATGTTAA	1	1	0	1	4	7	0	14
		21UR-373	TACGGAAAAAAACAATTCCTA	1	0	1	2	8	6	0	18
		21UR-374	TGTTATATTAGCTTTTCTTGA	3	0	0	0	0	0	0	3
		21UR-375	TGGTGCAATTTGAAAAATTGG	0	0	0	0	0	0	1	1
		21UR-376	TGGGGGTATCCAATATTTTCA	0	0	0	0	0	0	0	0
		21UR-377	TGTTAAATTCCTTTAGGTCAA	0	0	0	0	0	0	0	0
	†	21UR-378	TAAGTGTGTTTTCTGTTTTTC	0	0	0	0	2	3	0	5

	†	21UR-379	TGTAACAGTGGCATGAGATGT	53	34	11	3	17	147	7	272
		21UR-380	TTCTCCTATTTCTATGCATTT	0	0	0	0	0	0	0	0
	†	21UR-381	TATTAAGTTTGGTGACCGTTT	0	0	0	0	7	0	2	9
	†	21UR-382	TACTCTCAAATTATCATTTAC	0	0	0	0	0	0	0	0
	†	21UR-383	TTGTTTCAGGATTGTAGGTTT	5	0	0	1	12	9	11	38
		21UR-384	TAAATGAGAATGATAAGGTGA	29	39	12	6	24	86	0	196
	†	21UR-385	TGATAATTTGATTCAAGGTGG	0	1	0	1	3	3	1	9
	†	21UR-386	TGTACGCACATTGGGTAAACT	0	0	0	0	0	0	0	0
		21UR-387	TCATTTTATCGGTTGGTTGCA	2	2	4	9	70	65	22	174
*		21UR-388	TCGGGAGGAAATATTCGGGAC	16	5	5	1	3	40	6	76
	†	21UR-389	TGAACACAATTTTTGCAATTT	0	0	0	0	0	0	0	0
		21UR-390	TAGGAACAGTGATGTTTGTAC	0	0	0	0	1	2	0	3
		21UR-391	TTGTTAGGAGTCCGCAAACGT	4	0	0	0	1	9	0	14
		21UR-392	TACTTCAGTAGTAACCCCCAG	0	0	0	0	1	5	0	6
	†	21UR-393	TATGATGTTTGTTTTGTATGG	0	0	0	1	9	6	3	19
		21UR-394	TATGTTTTTGCATACGTGTAC	1	0	0	0	1	3	0	5
		21UR-395	TCCCTTCACTATTACCACAAC	0	0	0	0	2	0	2	4
	†	21UR-396	TAGTAAATTCTGTTGTTTTAC	0	0	0	0	0	0	0	0
	†	21UR-397	TCCACAGTAGCGTACAAATAC	19	3	4	3	12	117	4	162
*	†	21UR-398	TTCCACTGATATCCATGGACA	1	2	1	17	154	110	23	308
*	†	21UR-399	TTTACAGAAGGACAAACAGAG	2	5	1	7	142	153	29	339
	†	21UR-400	TCGCAGTAGGATATTAATAAA	36	96	36	41	160	303	48	720
		21UR-401	TCCGCTCTAAATATCCCCCAA	0	0	0	0	1	1	0	2
	†	21UR-402	TAGAAACTCTATCTCAGATAA	0	0	0	2	12	10	1	25
		21UR-403	TACACAGGTTTTCCGCCAAAA	0	0	0	0	2	11	3	16
		21UR-404	TTGCTTTAGAACGCTCACAGA	2	8	6	13	245	296	20	590
*		21UR-405	TACGAAGGATACATAGATCGT	6	1	1	6	84	135	69	302
		21UR-406	TGCGAGCTCACTTCGTTTTGA	0	0	0	0	0	0	0	0
		21UR-407	TAGTTTTTCGACATCTCACCA	2	0	0	0	0	4	0	6
		21UR-408	TGGAGATTACTTTATCCGAGA	0	0	0	0	0	0	0	0
		21UR-409	TCGCACATTGGAAAGATTTGT	1	0	0	0	0	0	0	1
	†	21UR-410	TGGTACTCCATCAATGGTTTC	0	0	0	0	6	1	1	8
		21UR-411	TGCATTCTGCTATTAGATGTT	1	0	0	0	0	1	0	2
	†	21UR-412	TAGTTTGCATTGTTGTATTGA	0	0	0	0	3	2	3	8
		21UR-413	TCCGTCGTTTTCATATAAATA	0	0	1	0	0	0	2	3
		21UR-414	TAGTATTACTCACTATTATGT	0	0	0	1	0	0	0	1
		21UR-415	TTTCCACGATTTTTGTCACTC	0	0	0	0	0	0	0	0
		21UR-416	TAACATTCATTTATTCATAAA	5	0	1	2	0	1	0	9
	†	21UR-417	TGGGTCGTCGTTTGAGACTGT	1	0	0	0	46	45	88	180
	†	21UR-418	TATTTCGCGCCATCTAGGAAA	10	2	3	1	2	4	7	29
	†	21UR-419	TGTTTTGGAATTTCTTTCGGC	1	4	2	4	79	105	11	206
	†	21UR-420	TCCGCTTTATTTTTGCATTCT	0	0	1	0	1	1	0	3
*		21UR-421	TGGGAAAAAACGATTCTGCAT	74	107	45	43	157	711	14	1151
	†	21UR-422	TGATTGGTTTCCTGATATTCA	0	0	0	0	1	1	0	2
		21UR-423	TCGTCTCTAGAAAGTAAAGTA	1	0	0	0	1	4	1	7
		21UR-424	TCCATGATAATAAACTCTTCC	1	0	0	2	4	0	0	7
	†	21UR-425	TCACTAAGGCGATTAGGTGAA	1	0	0	0	0	1	0	2
		21UR-426	TACTCATCTTAAATTCGAATA	5	0	1	2	29	20	2	59
		21UR-427	TAAGTAGTGTTTATAAAATAA	0	0	1	1	2	7	0	11
		21UR-428	TGCATCTCTGCCTGTTTTTTC	0	0	0	0	1	0	0	1
		21UR-429	TCAACGGATCAATTTAGTTAA	0	1	0	0	13	12	4	30
	†	21UR-430	TCAATCACCACCCATCATCAT	0	0	0	0	0	0	1	1
		21UR-431	TAAGATAAACGTAAATACAGC	0	0	0	1	2	1	0	4
		21UR-432	TCCCAGCCTATACTATTGTGC	0	0	0	0	0	0	1	1
		21UR-433	TCATAAGGAAGCCTGATTGAA	3	2	1	0	9	24	0	39
		21UR-434	TAGATCTGTTTAGATTTCTCA	6	5	3	6	7	14	0	41
	†	21UR-435	TAATTAGTGCGTAGCAATGAA	0	0	0	0	0	0	0	0
	†	21UR-436	TTCTGAGTTGCAACTAGTTGA	26	10	6	6	33	57	3	141
	†	21UR-437	TGACAAACAACGAAAAAAAAG	3	0	0	0	2	3	0	8
	†	21UR-438	TCATCATTCATTGCGTGGGTC	1	0	0	0	1	3	1	6
		21UR-439	TAATTGAGCTACATACCAGGG	1	1	0	0	1	5	4	12
		21UR-440	TAACTTAGACAAACTTCAGGA	1	0	0	1	17	20	2	41
	†	21UR-441	TGATACAGAAGAAACAGAAGA	1	0	1	1	26	28	0	57
		21UR-442	TCAACATCAAGTATTTTGAGA	0	0	0	0	4	1	0	5

	†	21UR-443	TGCTAGAGAAAATTTAGTTTC	0	0	0	0	0	0	0	0
		21UR-444	TGGATTCTTTATACCAGACTG	1	1	0	0	0	1	0	3
		21UR-445	TAGTTTCTTTTTTATGTAGTC	1	1	3	0	1	5	0	11
	†	21UR-446	TTACAGAATAATTGCTTCGAT	10	3	3	1	10	14	0	41
*		21UR-447	TAAGAGAGAAATAAGAGACTT	0	0	0	1	6	6	0	13
	†	21UR-448	TGTGCTTTACTTTAGTTGGGC	0	0	0	0	0	0	0	0
	†	21UR-449	TGAAGCTTTTGCAAAAACACA	2	0	0	0	1	1	0	4
		21UR-450	TACATCGCAGTGAAAGTACCC	0	0	0	0	0	9	3	12
		21UR-451	TAATTGAATTTAAATATAATT	0	0	0	0	0	0	0	0
		21UR-452	TGAACAACTTTTACAACTTTG	0	0	0	0	3	0	0	3
*		21UR-453	TATGACAACAACGATGACAAC	81	105	87	304	3540	3720	633	8470
		21UR-454	TAACTGTTGTTTTGCTAGACA	0	0	0	0	3	1	0	4
*	†	21UR-455	TACAATAACGTGTTTTTATCT	6	0	2	1	4	13	0	26
		21UR-456	TGTGCAAGGAATCTTATATAG	0	0	0	0	0	0	0	0
	†	21UR-457	TTATTTCACTTCATTGATGCA	13	0	1	1	1	3	3	22
		21UR-458	TATAATAAAACACTGTCAGGG	0	0	0	0	0	3	1	4
*	†	21UR-459	TCTGTCACATGCGAGACCCGG	14	1	2	3	0	10	541	571
		21UR-460	TAAATACGACTACAAATTCAA	0	0	0	0	0	0	1	1
	†	21UR-461	TGGGCTCCAAATATTTTCAGA	0	0	0	1	3	1	0	5
	†	21UR-462	TGACATTCGACATCTTTAAAA	2	0	0	0	0	1	3	6
		21UR-463	TACGAGATTAAAGACAATTTT	0	0	1	1	15	15	4	36
	†	21UR-464	TGCGTATGCAGTGCACCAAGA	0	0	0	0	1	0	0	1
	†	21UR-465	TGCTGAAGATACTTGGTTCTT	1	1	0	1	35	41	1	80
		21UR-466	TCATGAGTAGTCTTTTTTTCA	1	2	1	1	7	7	0	19
	†	21UR-467	TGTGAACAGATAGTCAACATT	13	7	5	4	13	37	10	89
		21UR-468	TGAGTGCAGTAAAATCTTTCC	0	1	0	1	1	0	1	4
*		21UR-469	TATATATTCCCGCGTCAAGAA	5	0	0	0	0	1	1	7
		21UR-470	TCACGTGTATCTTTATATAGC	1	0	0	0	0	0	1	2
		21UR-471	TCTGAATGAGCTTCAACTAAG	0	0	0	0	8	2	3	13
	†	21UR-472	TCATTGGTAAAAGGTACTTCA	8	11	6	7	33	29	2	96
	†	21UR-473	TCGAAAGTATTTCACAAACGA	0	0	0	0	0	0	0	0
		21UR-474	TGTGTTTATTTTCGAACAGTA	0	0	0	0	0	0	1	1
		21UR-475	TAAGCTTGCTTCATTCGAAAA	0	0	0	0	0	0	0	0
		21UR-476	TGACAATAGGTGCAAGGTATC	0	0	0	0	0	0	0	0
		21UR-477	TTAACCCTTCATACATTGAAA	53	1	2	1	0	29	5	91
	†	21UR-478	TTCACAGATCATTCAGCGTGA	1	0	0	0	0	1	1	3
		21UR-479	TCTGCAGCTATAATGCCACGA	0	0	0	0	0	0	0	0
*	†	21UR-480	TGTTGCAACTGAAGAATCAAA	0	3	2	13	165	166	11	360
		21UR-481	TTGATACGGAAGTGTTTCTGT	0	0	0	0	1	0	0	1
*	†	21UR-482	TCAGTAGACATCTCTAACAGC	0	0	1	2	22	32	1	58
	†	21UR-483	TGGTATTCCTCGTGACTGAGA	0	0	0	0	3	4	2	9
		21UR-484	TTTATGCCATGCAGCAGTTGA	4	0	0	0	3	15	5	27
		21UR-485	TTTCTGAAAATGTTCGTATTT	0	2	0	0	0	2	0	4
		21UR-486	TAATGTGCATCCTCATGGAAA	1	0	0	0	2	3	2	8
		21UR-487	TCGTTCTTGATTCATACAATC	0	0	0	0	0	0	0	0
*	†	21UR-488	TGCGAATGGATTTTAGCTTGA	1	1	1	1	93	85	5	187
		21UR-489	TAACAATAACTATGGAAAAAA	0	0	1	1	1	7	1	11
		21UR-490	TCCATAGCAAGGTTCTAACAC	2	0	1	0	17	11	4	35
		21UR-491	TCAAGTTGCTGAATTTGACCT	0	0	0	0	0	0	0	0
	†	21UR-492	TAAATGTTGGACAACGTGTGA	28	15	5	8	17	65	17	155
		21UR-493	TCTCATTTTACCAGTTATTTA	0	0	0	0	0	0	0	0
		21UR-494	TACGTCAGGAAGATAAGAAAT	2	0	0	3	8	23	1	37
		21UR-495	TGTTCCGATGTAACTATTTAG	0	0	0	0	0	0	0	0
		21UR-496	TGCGAGTATTCGTTGCTGTAG	0	0	0	0	6	1	5	12
		21UR-497	TCTGTACAACCATTTTATCTC	0	0	0	0	0	0	0	0
		21UR-498	TAAATTCTTCCTAAATATTCC	0	0	0	0	0	1	0	1
		21UR-499	TATACAAATAACTCAATAAAA	0	5	3	2	8	9	0	27
	†	21UR-500	TTCTGAATCGTATGTTATTCC	0	0	0	0	0	4	2	6
*	†	21UR-501	TACATACTCATTGAATGGTAG	0	0	0	0	9	13	3	25
		21UR-502	TAACTTTTCTTTTTTATGGCA	3	0	0	3	11	16	2	35
*	†	21UR-503	TAAACAGGACTTAATCTACAT	2	6	4	3	43	41	5	104
		21UR-504	TGAACGTTTCATTGGAATACT	0	0	0	0	1	0	0	1
		21UR-505	TAGAGATAATATTCAACCTGT	0	0	1	0	0	0	0	1
	†	21UR-506	TTACGCTGGATCTATCAAAAT	0	0	0	0	3	0	1	4

		21UR-507	TGAACATAAGATTTCGAGAGT	10	13	9	2	25	72	8	139
	†	21UR-508	TAATTCGTCCTTCATGGCTGG	0	0	0	1	1	1	0	3
		21UR-509	TTCGTCAGAACAATTATTATT	0	0	0	1	0	1	0	2
		21UR-510	TCCCACTAATAATAAAAACTC	0	0	0	0	0	0	0	0
		21UR-511	TAACCAATCTTTCCATTTCTA	0	0	0	0	0	0	0	0
	†	21UR-512	TTGAGTTGATATTCGATTGCG	0	0	1	0	0	2	1	4
		21UR-513	TTCTGCATTTCCGAGAAAATC	0	0	0	0	0	0	0	0
		21UR-514	TGGATCAGTCATTTCATTATT	0	0	0	1	0	0	0	1
	†	21UR-515	TGATTGATATCTATTGATGAT	0	0	0	0	0	0	0	0
*	†	21UR-516	TCAATCTGAGGCTGCAAATGA	8	0	0	3	5	21	0	37
		21UR-517	TGTAGTTATCAATGTTTTTCA	0	0	0	0	0	0	0	0
	†	21UR-518	TATACCTACCATGTTCTGAGG	25	0	1	1	2	14	7	50
	†	21UR-519	TCCAACCAAAACAATTTGTAT	0	0	0	0	0	0	0	0
		21UR-520	TTAATTTATTTATTTAATGGG	0	0	0	0	2	0	0	2
		21UR-521	TAGGAAAATACTTGATGCGCG	6	6	0	0	1	6	1	20
		21UR-522	TCCACGCGGTAATTTCATTTT	0	0	0	0	0	0	0	0
	†	21UR-523	TAATTCCATTTGTGATCTTTT	0	0	0	0	1	0	0	1
		21UR-524	TGAATTTAATTATTAATCAGA	1	0	0	0	1	1	0	3
		21UR-525	TCTTGCGTAAATGACTTTGAA	3	2	0	0	18	26	3	52
		21UR-526	TCGATCTTCAAAACTCCTTCA	0	0	0	0	0	0	0	0
		21UR-527	TGGGGTCATCTTACTTTGAAG	1	1	1	1	2	9	3	18
		21UR-528	TGAATCTTCATTTGCGGTTGA	9	0	2	0	1	11	1	24
		21UR-529	TCATATTTCGGTGGGTTATAT	0	0	0	0	0	1	6	7
	†	21UR-530	TCGGTTATGGGACTCCTTTTA	0	0	1	0	0	0	0	1
		21UR-531	TTGAATTGTCACGATACTGAA	12	4	3	2	10	26	1	58
		21UR-532	TAAACCGACCCTGTTGTCATG	0	0	0	0	0	0	0	0
	†	21UR-533	TAATAACGAAATTCTGCTGTT	2	1	0	0	4	3	0	10
*		21UR-534	TAGAAAGCATATTCATAGTTA	1	0	0	4	25	18	3	51
	†	21UR-535	TGGCACTACCACTCACCACTT	0	0	0	0	1	0	0	1
		21UR-536	TCTTCCTCTACGTTTGTAAAG	0	0	0	0	0	0	0	0
		21UR-537	TGTGGCATTGGAATAATTTTG	1	0	0	0	23	10	3	37
*		21UR-538	TAGGAATGAGTTCAGTAAAGG	18	34	16	12	53	177	0	310
		21UR-539	TCCGAAATAATGGATATAAAG	0	0	0	0	0	0	0	0
		21UR-540	TGTAGATAATATTTTAGTTGT	0	0	0	0	0	0	0	0
		21UR-541	TATTATAACTTGAGGTATTCA	0	0	0	0	0	0	0	0
		21UR-542	TATTATGATTAGTTTCTCATC	0	0	0	0	0	0	0	0
	†	21UR-543	TGATCCAAAATATTCCAAAAA	0	0	0	0	0	0	0	0
		21UR-544	TGGGAAAGTGTCTTCAGTTAT	1	0	0	0	0	0	0	1
	†	21UR-545	TTGTACTACGATGCAGTAAAT	1	0	1	0	3	6	0	11
		21UR-546	TAAAGTTTCCTTGATTTGAAT	0	0	0	0	0	0	0	0
	†	21UR-547	TATCGATGTTAGAAATTGTGC	0	0	0	1	4	4	0	9
*	†	21UR-548	TGAAGCTCGGCATCAAGTAGA	56	48	19	23	324	574	77	1121
		21UR-549	TCAATATGACAAGAACACTTA	0	0	0	0	0	0	0	0
		21UR-550	TAAGCACGAGCTATAAAAAAA	0	0	0	0	2	3	5	10
	†	21UR-551	TTGGACAGATATTGGAACAAA	5	1	0	0	15	22	1	44
*		21UR-552	TCAAAGCTCTGAAGATTTGCC	37	65	27	30	175	510	4	848
	†	21UR-553	TGAAAGAAGGTCAAGTGGGAA	2	4	2	3	101	112	65	289
	†	21UR-554	TTGGACTTGTGCTGTTTTCGC	12	5	1	3	10	44	2	77
		21UR-555	TCATTGAGTGCCTTGAAAGTG	0	0	0	0	0	0	0	0
		21UR-556	TATATAAGGAAGTACCTGCTT	6	5	4	6	7	47	0	75
		21UR-557	TTAACCATTTTCAATTGACTG	2	0	0	0	8	5	1	16
	†	21UR-558	TTCCTTCTATCAAACCACCAA	3	0	2	0	0	4	1	10
		21UR-559	TACGGAATCCTCTTAATTCAT	0	1	0	0	1	2	0	4
	†	21UR-560	TGGTATCCATGTGTTAAATCA	0	0	0	0	0	0	0	0
		21UR-561	TGCATCGTTTTCATGCTACTT	0	0	0	0	0	0	0	0
	†	21UR-562	TCAGTAACGGCATTTAGTTTG	92	22	21	13	37	169	5	359
		21UR-563	TGCATGAGTGGTAACTACGTT	0	0	0	0	5	7	2	14
		21UR-564	TTTGCTCATTTTCCTAACCAA	0	0	0	0	0	1	0	1
		21UR-565	TGTGAAGCGAACAACAAATTG	0	0	1	1	6	5	1	14
		21UR-566	TTTCGTGGCGTTTCTCTCTTC	0	0	0	0	0	0	0	0
		21UR-567	TTGCAAAATACCTCAAGTGTG	0	0	0	0	0	3	1	4
		21UR-568	TTCATAGTGTACAAATATATC	1	0	0	1	3	6	2	13
	†	21UR-569	TATTTTTCAGAGAGTCAGGAT	124	15	11	10	11	85	60	316
	†	21UR-570	TACGACTCAACGACTAACTTC	0	0	0	0	1	1	0	2

	†	21UR-571	TATTTTCGTTTTGCACCAAGA	1	0	0	0	2	3	1	7
		21UR-572	TTTGCTTGTTTCTTTGTCTCT	3	4	0	2	1	0	0	10
	†	21UR-573	TATTGCTACGCGAATGTTATT	1	0	0	0	1	0	1	3
	†	21UR-574	TTTTCAGCCAGTTGTTGTACA	12	1	0	2	2	5	0	22
	†	21UR-575	TGTGCCGATTGAATATTTATT	0	0	0	0	0	0	0	0
		21UR-576	TAAAACATTTGAATTTCCAAA	2	1	0	0	0	1	0	4
	†	21UR-577	TCGAAGAAAATTAAGTTTTCA	0	0	0	0	0	0	0	0
		21UR-578	TTCGGTCAATTCAAGTATTCA	0	1	0	1	12	8	0	22
		21UR-579	TGTATTTTTCCAGTTCTTGGA	0	0	0	0	0	0	0	0
	†	21UR-580	TTCAAATGGACAACTAGCAAA	0	1	0	3	37	33	9	83
	†	21UR-581	TGTCATAATGTTGGGAGAAGT	10	4	4	7	41	59	18	143
		21UR-582	TCACTTTACATTACAACCGAA	1	0	0	0	1	8	1	11
		21UR-583	TGAAACTGGTGCTTCTTATTC	0	0	0	0	0	0	0	0
		21UR-584	TCAATCAGTCAACATCAAACT	0	0	0	0	0	0	0	0
	†	21UR-585	TATTGATAGTACAGTGCGTGC	2	0	0	0	1	3	0	6
		21UR-586	TGAACAGAAAAAGTCGTGTGT	20	5	4	5	10	54	0	98
		21UR-587	TTCCAATATCTTCTAATAATC	5	0	1	2	0	0	0	8
	†	21UR-588	TGATCTATGCTTTTTTGTTCA	0	0	0	0	0	0	0	0
	†	21UR-589	TGATCTCCAATTAAGGATAAA	0	0	0	0	0	0	0	0
	†	21UR-590	TTCCCATCTCAAATTAGCCAA	1	0	0	0	0	2	2	5
	†	21UR-591	TGAATGTGCAAAGTAAGTACC	0	1	0	0	1	0	0	2
	†	21UR-592	TCGAATCGAAAGGAGATTCAA	2	0	0	0	11	8	1	22
		21UR-593	TCCCAGAAAGTGCACATATGC	5	0	1	0	1	4	1	12
		21UR-594	TAAATCATCTGTTCTGGAATT	1	1	0	0	12	8	0	22
	†	21UR-595	TTAGTCGATTAATTCTACAAG	0	0	0	0	5	4	0	9
	†	21UR-596	TAAATCACACAGAGTAATGAA	7	1	2	0	1	7	3	21
	†	21UR-597	TAAGAATGTAAAACCATGTCA	2	4	1	6	8	41	4	66
		21UR-598	TCCAACAAGAACAATGTGTTC	0	0	0	0	1	1	0	2
		21UR-599	TGTGTAAATAATTAAAGTGCT	0	0	0	0	0	0	0	0
	†	21UR-600	TCCATCGTGATAATCAAATTA	0	0	0	0	0	1	0	1
		21UR-601	TCCAAATCTCCACCAGCCCTA	1	0	0	0	0	0	0	1
		21UR-602	TTGGCGATAGGAATTGGTAGT	2	1	4	0	3	20	7	37
*	†	21UR-603	TGTTTCGAACTTCAATGCAGG	35	44	35	33	150	442	4	743
	†	21UR-604	TTCAGTCATGTTAATCAAGTC	0	1	0	0	1	2	1	5
		21UR-605	TAAACGTCACATTTGTTTCTA	1	0	0	1	2	2	0	6
		21UR-606	TTAAAATGACTTCTTTTATCC	0	0	0	0	2	1	0	3
*	†	21UR-607	TGAAAGAACGGAAGGATTTGT	37	37	15	12	52	270	19	442
	†	21UR-608	TGTCCAGTTGCGAAAAAGATC	1	1	0	2	4	6	1	15
*	†	21UR-609	TGAATCAGAACAATGGTACCC	681	1572	548	623	1734	6674	67	11899
	†	21UR-610	TAGTCCAACGAGATGTCAAGG	1	0	1	0	0	0	2	4
*		21UR-611	TGTCGATAACCATACGCATGT	6	2	0	1	9	21	2	41
	†	21UR-612	TGTCGAACTGTTTTTGAAAAT	0	0	0	0	0	0	0	0
		21UR-613	TTTGTTCATATAACACAGAAA	0	0	0	0	0	0	0	0
		21UR-614	TGGTGATTACCAACTCAATTT	0	0	0	0	1	0	0	1
		21UR-615	TCAATCACCTTCTCTGTTATT	1	0	0	0	0	1	0	2
	†	21UR-616	TAATTGTAGTGATTGATCAAT	12	8	3	4	9	19	2	57
	†	21UR-617	TTCTAGCCCGGCTTTGCTTAA	0	0	0	0	1	2	0	3
	†	21UR-618	TATTGATTCTGTAAAATTTAT	0	1	0	1	0	0	0	2
		21UR-619	TGTCTCTTTCGCTCCCTTCAG	1	0	0	0	0	0	0	1
		21UR-620	TATAAGAGATAAAACCATAGC	10	1	0	1	2	7	2	23
	†	21UR-621	TTCACGACTTGAAGGACTCGA	3	0	0	1	4	7	0	15
		21UR-622	TGAATGCCGTGTAATGGTGAA	0	0	0	0	0	0	0	0
		21UR-623	TAGCTTTATTAAACGAGTAGT	6	0	0	0	0	3	1	10
	†	21UR-624	TGGTCGATGACAATAGAAATA	1	0	0	1	15	6	3	26
	†	21UR-625	TAAATTCTGTTGTTTTACAAA	0	0	0	0	0	0	0	0
	†	21UR-626	TATCGTTGGATTAAAAAGTTT	10	0	0	3	9	21	6	49
		21UR-627	TGTCAAGAGTTGTTCACAAAT	0	0	0	0	1	1	0	2
		21UR-628	TATGATCTTGGTTTGAATTTG	4	0	0	0	0	0	0	4
		21UR-629	TGAATTTTTGAATGACTTTTT	1	0	0	1	0	2	0	4
		21UR-630	TAACGCTACGCCCTGTTTTCC	0	0	0	0	0	0	0	0
		21UR-631	TTGGACTTCAATATTCGTTCT	1	0	2	1	15	5	1	25
*		21UR-632	TAGAACATGCAGCTATGATGA	3	5	3	9	211	179	12	422
		21UR-633	TACTGACTTTCTCTTTGCATT	0	1	0	2	17	13	0	33
	†	21UR-634	TTTATTGCACTATTCTTTGAA	0	0	0	1	0	1	0	2

		21UR-635	TGTAGCAATCTAGTGAAGCAA	3	7	3	2	14	32	1	62
	†	21UR-636	TCCTGCGAATTTCCGGTTGTC	0	0	0	0	3	1	1	5
	†	21UR-637	TACGTGTTGAGAACTGTTGTG	0	0	0	0	4	2	1	7
	†	21UR-638	TCTTTTCGTTCCTCTATCTAA	1	0	0	1	0	1	0	3
		21UR-639	TTGTAAACTAAATTGGTTACA	3	3	0	1	6	14	0	27
		21UR-640	TACTTAATTCAATCTTACCTA	1	0	1	1	4	6	1	14
	†	21UR-641	TCCTTGGGAAATTTATCGTCT	63	22	8	9	93	185	108	488
	†	21UR-642	TACACATAACAGTAATAATAG	0	0	0	0	1	1	0	2
		21UR-643	TGTGAGTTTTGTGACAGTTTT	0	0	0	0	0	0	0	0
		21UR-644	TGGTCCATTCCAATGCCTCAG	0	0	0	0	0	0	0	0
		21UR-645	TGCTGCTCGATCTGCCTCATG	0	0	0	0	3	0	0	3
		21UR-646	TCTATAAATAACAAATTTTGA	0	0	0	0	2	0	0	2
		21UR-647	TTCAAAGAATTTTCCAAATTT	0	0	0	0	1	0	0	1
	†	21UR-648	TATTAGATTGCCCGGTACATG	30	10	6	4	12	67	33	162
	†	21UR-649	TAGTTTAAAAGTATAAACCAC	0	0	0	0	1	1	0	2
	†	21UR-650	TCAATTTTGGGAATGTTCGGT	29	39	29	67	868	933	158	2123
		21UR-651	TGCAAGGCCACCCTGTTACCC	0	0	0	0	0	0	0	0
		21UR-652	TATATTTGTAATATGATTGGA	0	0	0	0	8	2	9	19
		21UR-653	TAGTATCATCCTAAAACAATA	2	1	0	0	0	0	0	3
	†	21UR-654	TATTTCCAATGTACCATAAAA	2	0	0	0	2	0	2	6
	†	21UR-655	TTGATGCACGATATTTTTTGC	2	0	0	0	0	3	0	5
	†	21UR-656	TGGGATGTTAGTTTGTTGAAC	0	0	1	0	0	0	0	1
	†	21UR-657	TCCGTTGTTCATTATAGCTAA	0	0	0	0	0	4	0	4
		21UR-658	TAATCCTTGAGCTCTTCCAAG	1	0	0	0	0	1	0	2
		21UR-659	TAGGATAATCAACTAAAATGC	0	0	0	0	2	2	1	5
		21UR-660	TGATTTCAAAATTTGCAGCGA	2	0	0	0	0	1	0	3
	†	21UR-661	TTAATGTGCTTTACTTTAGTT	0	0	0	0	0	1	0	1
*	†	21UR-662	TACATGGTCATTTTATTCTGG	0	0	0	0	8	4	1	13
	†	21UR-663	TACGATGGCTACAATAATAGC	0	0	0	0	1	1	0	2
		21UR-664	TGTGTTTTTTGTTTGATGGCC	0	0	0	1	8	13	2	24
		21UR-665	TAGTTCGTTATTTCTCAACTT	0	0	0	0	0	0	0	0
	†	21UR-666	TGCTTTGGCTTTTCTTTTAAT	2	0	0	1	0	3	1	7
		21UR-667	TATCAGGTTTAATATCCCGAT	0	0	0	0	0	1	0	1
	†	21UR-668	TGAATTAGTTGTTATTGTTGA	12	7	4	1	4	28	2	58
		21UR-669	TTGTCGATCTTTTGAATTCTT	0	0	0	0	1	0	0	1
	†	21UR-670	TATTGTTACTTAACCTTTGAA	0	0	0	0	0	0	0	0
		21UR-671	TCCATGTTTCCTGATTTTTAT	0	0	0	0	0	0	0	0
	†	21UR-672	TATTGAAAGCAGAGGTGGGCG	6	1	1	2	1	20	0	31
		21UR-673	TAGTGTACAAGGATATTTTAA	11	1	0	1	1	22	3	39
	†	21UR-674	TGGAAAGTTTTAATTAATTTG	0	1	0	0	0	2	0	3
		21UR-675	TAGTTTGTTGATATGTTTTTT	1	0	0	0	1	1	0	3
	†	21UR-676	TAATCTGAGTCAACCATGCTG	0	0	0	0	0	0	2	2
	†	21UR-677	TGTTTTCGAATTGATCCTGCG	0	0	0	0	3	1	1	5
		21UR-678	TGTGAAAAGCCCACTATTTTC	0	0	0	0	0	0	0	0
	†	21UR-679	TGATTCATCGCCATTAAAAAT	0	0	0	0	0	0	1	1
		21UR-680	TGACATGAATCACCTTTACCA	2	0	0	1	0	1	0	4
*	†	21UR-681	TACTACGACAGGAGATGAATA	3	10	1	27	339	332	63	775
		21UR-682	TGAAATGTTTGACCACACATT	0	0	0	0	0	0	0	0
		21UR-683	TATCTTCTATGAATTACTGAA	0	0	0	0	3	2	0	5
		21UR-684	TCTAGCATTTGTATCAGTCAT	0	0	0	0	3	5	2	10
	†	21UR-685	TCATTTATCAGCAATCAGAGT	14	1	0	1	2	4	1	23
	†	21UR-686	TATGAGGAACATTATCATAAC	0	0	0	2	13	13	0	28
	†	21UR-687	TGGTCTCTATGCTTTTACAAA	0	0	0	0	0	3	0	3
		21UR-688	TTAGTCAGATTAGCTTTTTCG	0	3	2	3	40	24	0	72
	†	21UR-689	TCTGAGTCAACCATGCTGGAA	0	1	0	0	1	5	0	7
*		21UR-690	TGACAATGGTTTATGAACGGT	1124	1365	574	727	3956	9559	349	17654
*	†	21UR-691	TAAATGAAGTAGAAAAAATAC	11	19	4	13	112	104	2	265
*		21UR-692	TATGGAGTAGTATAACCAAAA	44	72	40	42	198	381	15	792
		21UR-693	TGACCACTGTTTCCTATGCTC	1	1	0	0	0	1	0	3
	†	21UR-694	TATTCTCCATCTGACGTTATG	3	3	2	4	3	14	2	31
		21UR-695	TGCAAAATCTATATTGTCATC	0	0	0	0	0	2	1	3
		21UR-696	TCACTCATTTGTATGTTAAGA	0	0	0	0	0	0	0	0
	†	21UR-697	TTTCAGAAAAAAATTGGTATC	0	1	0	0	6	8	0	15
	†	21UR-698	TTACACCAGTTCCGCAAACAC	8	0	0	0	0	8	3	19

		21UR-699	TAGTCCACCACACTTCCTCGT	0	0	0	0	0	0	0	0
*		21UR-700	TACTGTATTTGTAAGCCATGA	0	2	0	1	17	21	3	44
	†	21UR-701	TTACTTACATTAAGTCGGTGT	0	0	0	0	0	0	0	0
	†	21UR-702	TAATAAAAACTTCGACAGGAA	1	1	0	0	1	8	4	15
*	†	21UR-703	TCTGACGAGTAGAGTTTAGAA	80	56	41	162	3529	4102	843	8813
	†	21UR-704	TCTGTGCATTCTTTTTTTGGC	0	1	0	0	3	6	0	10
		21UR-705	TGCACTGAGAAGGTCTGAAAG	2	2	2	4	19	57	3	89
		21UR-706	TCTTGCCTTCACACCATTTTC	0	0	0	0	0	0	0	0
	†	21UR-707	TGTGTTTGCCGCGAGAGAGAA	2	0	0	0	1	5	4	12
	†	21UR-708	TGGGCTGATTTTTCGGAATAA	2	0	1	0	38	59	19	119
		21UR-709	TGGTTCGATTGTCTATTGATC	5	3	0	1	5	3	2	19
		21UR-710	TAGATCTGATCTAATTGTTCC	1	1	0	1	1	3	0	7
*		21UR-711	TCCAGTAGCCTTGATTATCGG	15	3	9	5	163	307	119	621
*	†	21UR-712	TGGTTTGTTACGTCGATTCAC	6	5	1	1	63	44	18	138
	†	21UR-713	TATTGTAGCCTCTATCATCAC	0	0	0	0	3	2	0	5
		21UR-714	TCATGTTTTCCGCACTTGTGA	1	0	0	0	0	1	0	2
*	†	21UR-715	TATGATCAGGCATCCTAAACT	308	211	122	112	327	1064	98	2242
		21UR-716	TGACTACGTTCAAAAAAAATC	4	0	0	0	3	1	3	11
		21UR-717	TAGAAGTTCCGTATAGCTTCC	9	5	7	3	17	37	3	81
		21UR-718	TGGAGACTTTATACACATGAT	1	25	4	20	284	215	86	635
		21UR-719	TTCCATTTTTGCTTGCTTCAA	0	0	0	0	0	2	1	3
	†	21UR-720	TAAAAAATTTTTTGAGAATGG	1	0	1	1	0	3	0	6
		21UR-721	TATAAGAGACATTTCGAATCA	18	4	4	3	10	24	2	65
		21UR-722	TCTAGGAAACGTGATATCTTC	0	3	1	3	4	12	0	23
		21UR-723	TCAGAAACATGTTTAGCAAAA	0	0	0	0	0	0	0	0
	†	21UR-724	TTAAGCAATTGCAGGTAATAG	3	3	0	1	2	11	3	23
		21UR-725	TTCCTCTTCTCTAAAAATCAT	0	0	0	0	0	1	0	1
	†	21UR-726	TAGTTAGAAAATTTGTGACGT	33	51	30	19	70	256	2	461
		21UR-727	TCCAAATATTTTCAATAGAAG	0	0	0	1	0	3	0	4
		21UR-728	TCATACGTGCGCCTTGAATTT	0	0	0	0	0	0	0	0
	†	21UR-729	TACTTTTCGTATTTATCTTTA	6	0	1	1	2	4	1	15
		21UR-730	TGAGTGAACGAACATCTGAAG	0	0	0	1	14	25	2	42
	†	21UR-731	TGTCTGATTGCCTAGATTTAC	0	0	0	0	0	0	0	0
		21UR-732	TTAAACATTCAATACTCAAAT	2	0	2	1	1	3	0	9
		21UR-733	TATCGAAGTTGTTGTTATTGA	1	0	0	0	0	3	1	5
	†	21UR-734	TCATTTCATAGCACATTTGTG	0	0	0	0	0	1	0	1
*		21UR-735	TGCGTGGTGGAAAGGTTCTGT	3	3	2	3	83	80	7	181
	†	21UR-736	TAGTAATTGGACTTGGTGATA	27	18	8	22	288	265	160	788
	†	21UR-737	TAACAACTGCATATTGATGAT	5	1	1	1	62	46	8	124
		21UR-738	TGGTTTTTTTCTTTCCAGTGA	0	0	0	0	0	0	0	0
	†	21UR-739	TTTGTGTGCGAATGTCATAAC	3	3	1	1	5	16	3	32
	†	21UR-740	TGGTTATCATAAATTGGTGAA	2	0	0	1	3	4	0	10
		21UR-741	TGCAATTTGTTTCTACCGTGA	1	0	0	1	0	2	2	6
		21UR-742	TGTAATGTAGGTGTTCAGATA	2	1	1	12	104	86	79	285
		21UR-743	TCGATAGACTTTGTTCAAATT	0	0	0	0	0	0	0	0
		21UR-744	TAGATTGTGCTTTTTTATTCA	0	3	2	2	19	13	0	39
		21UR-745	TCGATCAATCATAACATTTTC	0	0	0	0	1	0	0	1
		21UR-746	TTAGACTCTTTTATGTGCTAT	0	0	0	0	0	0	0	0
		21UR-747	TAGAAGTCGTATACGCAGCCC	80	26	19	16	60	206	12	419
		21UR-748	TGGGTCTGGGATACTCTTTAT	0	0	0	0	3	0	0	3
	†	21UR-749	TGATAAAGGCAGAATTTTATC	9	8	3	4	11	24	1	60
	†	21UR-750	TTGTTACGAAAATGGAACGGA	0	0	0	0	0	0	0	0
	†	21UR-751	TTGGTGTAGTTTGAAAGTAGG	3	2	0	1	3	9	2	20
		21UR-752	TGGTGAAAATTTTCACTATAT	0	0	0	0	0	0	0	0
		21UR-753	TAAACAAGATTAGCTATGATT	0	0	0	0	6	6	2	14
	†	21UR-754	TCAGTCATGTTAATCAAGTCA	0	0	0	0	1	1	0	2
		21UR-755	TATTTTCCATGTTTTGGCAAA	1	0	0	0	0	2	0	3
		21UR-756	TAGACGCCATCCTCCAAATAC	0	0	1	0	0	3	12	16
	†	21UR-757	TATTGTGAACAGATAGTCAAC	16	5	2	1	1	38	8	71
		21UR-758	TGTATTGCTGCTGTTCGATTT	0	0	0	0	0	0	0	0
		21UR-759	TTACACTAACAGCAGGCCAAA	2	0	0	1	2	6	10	21
	†	21UR-760	TTCGGCCCATCAATTTTAGCA	1	0	0	1	5	4	1	12
		21UR-761	TGCATCGCGAATTTAGTGAGG	2	0	0	0	1	3	0	6
	†	21UR-762	TGCAGCTCGTGCGATTGAAGC	0	0	1	0	7	4	1	13

		21UR-763	TACTTTTTCCGATATGTTTCC	0	0	0	0	1	0	0	1
		21UR-764	TAAAACTATTGAGCTATCAAA	0	0	0	0	0	0	0	0
		21UR-765	TACTCTACTGAAACGTGAAAA	3	1	0	1	23	44	9	81
		21UR-766	TTCTCGAAGAAAATTTAAAAG	9	1	3	1	0	4	0	18
		21UR-767	TTCCTTTCATTTTTCCAGCTA	0	0	0	0	0	1	0	1
	†	21UR-768	TAAGAGGAAGCCAGCCATTAA	48	7	3	2	11	51	23	145
	†	21UR-769	TTAAATCACTGTGGTATCTAT	0	0	0	1	2	0	0	3
		21UR-770	TAAACGATTTGGCTTTTGTAC	1	0	1	3	52	45	11	113
		21UR-771	TGCGAGTGATTTGACAAGAAA	4	2	2	1	2	7	1	19
	†	21UR-772	TTCTTTGATAATTTTTATAAA	0	0	0	0	1	0	0	1
		21UR-773	TATTTAATCAGAAAAGACTGA	3	1	2	2	4	11	0	23
	†	21UR-774	TGATTTTAATTCTATCAGAAA	0	0	0	0	0	0	0	0
	†	21UR-775	TTCCAGAAATATTTGTACTCC	0	0	0	0	1	1	0	2
	†	21UR-776	TGGTAAGAATGTATAAGAATG	2	3	0	3	16	8	0	32
		21UR-777	TAGAATCGGCTTTGTCAGATT	4	1	1	0	6	24	1	37
	†	21UR-778	TGACGATTCAGAAAAGTCGAC	1	0	0	0	4	5	3	13
		21UR-779	TGCAACGAAGCCTTGACTTCA	0	0	0	0	0	0	0	0
	†	21UR-780	TTAGTTCAGCTAATGCTATCC	0	0	0	0	0	0	0	0
		21UR-781	TTTCTTGAATCTCCTCTACTC	1	0	0	1	0	0	1	3
	†	21UR-782	TACACATTTATTTAGAACAAG	2	0	0	2	35	24	9	72
		21UR-783	TAGGACACATCCCAGCCTGTC	0	0	0	0	5	7	13	25
		21UR-784	TAAACAATGGTAAACTTTTTC	0	0	0	0	7	6	0	13
		21UR-785	TATAATTTCTCTAAAGACATC	1	4	3	5	8	10	0	31
		21UR-786	TGTGTTGGTAGAGTTTTTACT	0	0	0	0	0	1	0	1
		21UR-787	TCCTTGCGGCATGAGCCATCA	0	0	0	0	15	4	4	23
		21UR-788	TACCAGGCTTTTAAAGACATC	2	0	0	1	45	52	9	109
	†	21UR-789	TAAAGTACTATTGCCGTTGTA	1	0	0	0	0	1	5	7
	†	21UR-790	TAGTCTATTTCTGGTTAATGC	0	0	0	0	0	1	0	1
*	†	21UR-791	TAGTAGATCTTGACAGAAGAA	48	26	13	24	255	405	47	818
		21UR-792	TTATGATCTGACAACTCGTTT	0	0	0	0	0	0	0	0
	†	21UR-793	TCATCAGGGCGAAAGTTCAAG	0	0	0	0	4	3	1	8
		21UR-794	TGAATCTGGACCCCAAATTTC	0	0	0	0	0	0	0	0
		21UR-795	TCCAGACTAAACATATTTCCT	0	0	0	1	13	6	2	22
*		21UR-796	TACAATGAGAAGCAATGTGAA	14	11	2	3	4	29	5	68
		21UR-797	TGTACCGATTGTTTGCAGGGT	0	0	0	1	0	1	2	4
*		21UR-798	TGGATTTAGCAAGAAACCGAG	56	141	67	127	1379	1643	201	3614
	†	21UR-799	TGGTGAAATCCACAACTACAA	0	0	0	0	0	0	0	0
	†	21UR-800	TAATAGTGCAATTGGTCCTCA	0	0	0	0	0	0	0	0
*		21UR-801	TGAGACGAGAAGAAAAGATTT	2	0	0	1	19	14	27	63
		21UR-802	TAATTTCTGAATTAATTGCTC	0	0	0	1	2	0	1	4
*		21UR-803	TCGGTGGTTCGAGCCCGCCCG	36	1	0	20	3	8	177	245
		21UR-804	TGTAATTAGGTCTAGCTACAT	0	0	0	0	2	1	0	3
		21UR-805	TATAATAATCCTATTGTGTGA	0	0	0	1	3	5	0	9
		21UR-806	TTTCGTATACTGTTGACTCGG	1	0	0	1	0	2	0	4
	†	21UR-807	TTTTAAGGTATTGATCATGTG	9	2	1	1	3	6	3	25
	†	21UR-808	TCGTGTTTAGAAAAAATTTCA	0	0	0	0	2	2	1	5
		21UR-809	TCTGGATTTGTTTTTCATTTA	0	0	0	0	0	0	0	0
		21UR-810	TGTGATTCATCCAGTTCTTCA	0	0	0	0	1	0	2	3
		21UR-811	TCTATGCTTCCTTTTAGCAAT	0	0	0	0	1	0	0	1
*		21UR-812	TTACAACTGTGTATGCTATGA	24	26	13	9	43	110	4	229
	†	21UR-813	TTTCGTCATTTGTATAAGACA	0	0	0	0	0	0	0	0
		21UR-814	TGGAAGTAAATTTTTCGTCAC	0	0	0	0	0	3	0	3
		21UR-815	TTCCGTTTTGTTTTTACTCGC	0	0	0	0	0	1	0	1
		21UR-816	TTATTGTTAGATTGATTTTAT	0	0	0	0	3	2	1	6
		21UR-817	TGTGTTGTGGACCTGAGCTTA	0	0	0	0	0	0	0	0
		21UR-818	TCAAAGACACGCCCGAATTAA	1	0	0	0	0	0	1	2
	†	21UR-819	TAGTTTCTTTTAGATTTTGTG	1	0	1	0	1	1	1	5
		21UR-820	TGGATTATCTACCATATGTAC	0	0	0	0	0	0	2	2
		21UR-821	TGCGAGCAAATTTAATATTCA	1	0	1	0	0	1	0	3
	†	21UR-822	TTAAGTCATAGTTATAACTGA	0	0	0	0	0	0	0	0
		21UR-823	TGCTAACTCCTGTAGATTCTA	1	0	0	0	0	2	0	3
		21UR-824	TTAAACCATGAAATCACTTGT	0	0	0	0	3	2	1	6
	†	21UR-825	TTCACGAAAAACAGGTGGTTT	18	3	0	0	1	28	0	50
	†	21UR-826	TTAGTATTCTGTGCCTTGGCA	0	0	0	0	20	13	8	41

		21UR-827	TAATTATCATTTTTCTTCGTA	3	0	0	1	0	0	1	5
	†	21UR-828	TGATCTTTACGGGCTTACGAA	0	0	0	0	6	4	4	14
		21UR-829	TGTGAACTTAGCTTTGCTCAT	1	0	0	1	4	5	0	11
		21UR-830	TAATTTGTACGACAGTAAAAA	2	1	0	0	3	3	0	9
		21UR-831	TGAAACGTTACAACGTATAAA	0	0	0	0	0	0	0	0
	†	21UR-832	TGTCGTTGTTTTTGAGCGTTT	0	0	0	0	0	0	0	0
		21UR-833	TGGAAGATCCTGGATTTTAAC	1	2	0	0	16	22	4	45
	†	21UR-834	TGATCGGATCGGGTCATACCG	0	0	0	0	0	0	0	0
		21UR-835	TAACGCCAATGACCGTTCCCC	0	0	0	0	0	2	1	3
	†	21UR-836	TGGTCCTTGATTAATTTTCAT	0	0	0	0	0	1	0	1
		21UR-837	TATACCGAACTGGTCCAAGTT	4	1	0	1	4	14	3	27
		21UR-838	TCACTAGACAGTATTTTATGG	0	1	1	0	13	5	4	24
		21UR-839	TCGACATTTTTTTCTATGAAT	0	0	0	0	0	0	0	0
		21UR-840	TACGCCCCCAATTTGTATTGC	0	0	0	0	0	1	2	3
	†	21UR-841	TATAGGCGGCCTCTTAATTAA	41	26	6	5	31	94	7	210
		21UR-842	TGTGAAGATTTTGAATACTTT	0	0	0	0	2	5	1	8
		21UR-843	TAACTGCACTAAAATTGCAAC	0	0	0	0	2	4	1	7
	†	21UR-844	TGCTCGTGTAGATCGAACTAG	9	1	1	1	40	52	25	129
*		21UR-845	TCAGGAAAGCAAGAACTCGAA	13	32	14	18	248	308	20	653
		21UR-846	TCCTGTTCAATCCTACTTTGG	0	0	0	0	0	0	0	0
	†	21UR-847	TATAGTTACCATCAGAGGTGG	2	1	1	0	1	1	1	7
	†	21UR-848	TGAACCAATTGCTAAGTCATC	0	0	0	0	0	0	0	0
		21UR-849	TAGGAAGGTTTTCAAGAGAAC	12	21	10	8	36	109	6	202
	†	21UR-850	TGTTCTCGGTTTTTGGTAGTG	1	2	2	0	4	3	1	13
	†	21UR-851	TCCGTATGGTACAATAGGAAG	0	0	0	0	6	3	0	9
		21UR-852	TGGTTTGTGTTTTGGCTACTA	5	5	1	1	2	14	1	29
	†	21UR-853	TGTGCTACTCTTTGGTCATAG	0	0	0	0	0	0	0	0
		21UR-854	TACTTCGAAAAAAATAAATTCA	19	1	1	2	7	25	6	61
		21UR-855	TCTGGACATTTCTCCATTTCT	0	0	0	0	1	1	0	2
		21UR-856	TGGTGTGCGTTTTTTTTTTGA	0	0	0	1	2	4	1	8
		21UR-857	TGCACGAAGTGTTTTTTCAAG	1	0	1	0	1	2	0	5
		21UR-858	TCATCAGTTACTTTGTTTTTG	0	1	0	0	0	0	0	1
	†	21UR-859	TGCGTCGTTGAATCTCCTTTG	0	0	0	0	0	0	0	0
		21UR-860	TGCATAGCCTGAATCTGCCTG	0	0	0	0	1	6	0	7
	†	21UR-861	TTTGGTGATAGTCTGCGTAAT	0	0	0	0	3	1	1	5
	†	21UR-862	TACTGTGTGTCCGTTAGGAAA	0	0	0	0	23	8	3	34
		21UR-863	TAATAGCCAAATGAAAAATGA	1	0	0	0	0	0	0	1
		21UR-864	TCTTGTGTTGGACGGAAATCA	136	63	35	26	179	462	31	932
*	†	21UR-865	TGATTTCGGTTTGTAACTTTG	5	9	2	1	15	20	0	52
		21UR-866	TAAACATAGATTTCTGTGATG	2	1	0	2	4	9	0	18
	†	21UR-867	TCATAATGAGAGCTGATCGAT	30	19	18	33	357	366	201	1024
		21UR-868	TTCCATTAGCATTTATATGCA	0	0	0	0	1	1	0	2
		21UR-869	TACAACTTTCATTTTATTTTA	9	0	0	1	0	3	0	13
		21UR-870	TAGCTTGCATTCTTCGGCAAC	0	0	0	0	1	2	1	4
	†	21UR-871	TCCTAGCAGAATTTTCACCAA	0	1	0	2	1	1	0	5
		21UR-872	TGAAATAAAACCACGACTCAC	0	0	0	0	0	0	2	2
	†	21UR-873	TATGGCTGATTTTGTAGTAAC	1	1	0	1	34	24	3	64
		21UR-874	TAACTGGTCGAAGCTTGTCGA	0	0	0	0	0	1	1	2
		21UR-875	TACGAAAATGAAATTTCAAGA	0	0	0	0	0	1	0	1
	†	21UR-876	TTCCTGCCATACCTTTACAAC	6	0	1	1	0	8	5	21
	†	21UR-877	TAAAAATATCTAGGAATGCGA	6	3	2	3	9	29	14	66
		21UR-878	TGGGTTTGCAATTTGAGTTGT	0	0	0	0	0	1	0	1
		21UR-879	TATAGGACCTTTTTTCAGACA	0	0	0	0	0	0	0	0
	†	21UR-880	TATGATGCCTAATGGAAATCA	1	0	0	1	19	32	2	55
	†	21UR-881	TGTACATGATCAATTGTGCCA	0	0	0	0	0	1	0	1
*	†	21UR-882	TGCGGGAGACAGACTTTGCAA	28	50	19	9	64	329	18	517
		21UR-883	TGTGTTCACAAACAACCTATT	0	0	0	0	0	0	0	0
		21UR-884	TGACTCGGTGAAAACATAACG	0	0	0	0	2	2	0	4
		21UR-885	TACAATGCGGAAAAAAAATACT	0	0	1	2	21	29	4	57
	†	21UR-886	TAGTGAGCTACATGATTCTGT	0	0	0	0	0	0	0	0
		21UR-887	TGCACAGATAGTATTCATTGG	5	7	7	17	115	87	13	251
*	†	21UR-888	TTGTCGGATAACCACATTGGA	132	11	14	15	83	128	133	516
		21UR-889	TGTGTTTCGTATAGTAACTAC	0	1	0	0	5	2	0	8
	†	21UR-890	TTGATCCCGAATTGGAAAAGA	2	0	0	0	2	4	1	9

		21UR-891	TCGCATACTGCATGTATTTTC	0	0	0	0	9	7	0	16
	†	21UR-892	TGGGCCAACGTTTTCAATGAA	0	0	0	0	1	3	8	12
		21UR-893	TGAAATCTTCAAGTTGATGGG	0	0	0	2	15	35	1	53
		21UR-894	TGTCTATGAAAACTACATTAC	0	0	0	0	0	0	0	0
	†	21UR-895	TACCTGATGTACTGACTCGAA	4	5	2	4	14	65	4	98
		21UR-896	TCAGTCGTTTTCAAAATATTG	0	0	0	0	0	0	0	0
		21UR-897	TGCTTCTGACTTTTATTTTCA	0	0	0	0	2	0	0	2
		21UR-898	TCTAGCCGATTCTTCTGCATT	14	15	9	11	75	122	8	254
		21UR-899	TGCAATGCTTAATGAATATCA	0	0	0	0	3	2	0	5
	†	21UR-900	TCGATAGATGATAATGATAAG	0	1	0	0	0	2	0	3
	†	21UR-901	TTCCTTACATGCACTTGAATA	0	1	0	4	35	31	4	75
		21UR-902	TCAAAAGCAATTTTCTCCTAA	0	0	0	0	0	0	1	1
	†	21UR-903	TTGGTTTGGTGTGATTGATTT	0	0	0	0	2	1	0	3
	†	21UR-904	TGAAGGTTCAAAATCCACTAA	0	0	0	0	0	0	0	0
	†	21UR-905	TGATCATTCATGTAACGGTTC	0	0	0	1	2	2	0	5
		21UR-906	TGTTATTATACCATTGTTTCA	0	0	0	0	1	0	0	1
		21UR-907	TGTAATTCGAGAAGTGCGTTT	0	0	0	0	0	0	0	0
		21UR-908	TTGAATCGTAGTTTTAGTGGC	190	136	78	43	161	766	48	1422
		21UR-909	TTTTTTGGTGCTTTGCAAAAA	0	0	0	0	2	1	0	3
	†	21UR-910	TAATTTCAACAGGTGATCTTC	1	1	1	0	2	1	0	6
*		21UR-911	TAAGATTTTGGTCCCAATGAA	3	4	1	2	7	24	4	45
	†	21UR-912	TGGCAAATCGATAAATATTGC	0	0	0	0	0	2	1	3
	†	21UR-913	TTTAGCGGATTTTTTACAGCC	28	15	6	6	27	94	2	178
*		21UR-914	TAGATATTCTATTACTTGCGG	1	0	0	1	1	2	2	7
		21UR-915	TATATAAATCGAATCCCTTAA	12	2	1	0	3	6	6	30
*	†	21UR-916	TCAATTTAGTCTAAGCGGCAA	7	0	0	3	30	40	14	94
	†	21UR-917	TGTTTTGTTCAGAAATGCGTG	48	9	5	4	11	37	4	118
		21UR-918	TCAGAAAACTTTTTAGTCATG	0	0	0	0	1	1	0	2
		21UR-919	TATGAAGTTTTCAACCTACAT	1	0	0	1	0	1	0	3
*	†	21UR-920	TAATCAAGTAGTATGAAATTA	131	246	112	167	445	931	11	2043
	†	21UR-921	TAGTTCAATATTATTTCACCA	0	0	0	0	0	0	0	0
	†	21UR-922	TAATTAAAAATTGTTACTGTT	0	0	0	0	1	0	0	1
		21UR-923	TCTGCAAATTGTAATGAAATG	0	0	0	0	0	0	0	0
		21UR-924	TCGCAATAGTGATTAATAAAT	0	0	0	1	0	3	0	4
	†	21UR-925	TAATAGATGTTGCTTTGTACA	0	0	0	0	0	0	0	0
	†	21UR-926	TGGTGGTTTGATAGAAGGAAC	5	4	5	4	8	20	1	47
		21UR-927	TCCTATGTAAATCCCACATAA	0	0	0	0	0	1	0	1
*	†	21UR-928	TTCAGGAACGTTACTCATTAG	56	216	108	231	537	1237	52	2437
*		21UR-929	TGCATTTGCTGTCACCTGAAA	1	2	0	2	35	37	2	79
	†	21UR-930	TGTTGTGTACTCATGGGAATG	0	0	0	0	0	0	0	0
	†	21UR-931	TGATTTACTTTCGCGGTTTTC	0	0	0	0	0	6	1	7
		21UR-932	TGCCGCTGTCTTTAAAATCAT	0	0	0	0	0	0	0	0
	†	21UR-933	TCTCTTGCGTTTACCTGTAGA	2	1	2	1	17	29	4	56
	†	21UR-934	TCAGGTCTATCAGGTCTAAAA	1	0	0	0	0	0	0	1
		21UR-935	TCTAGTTGTGAATCCTTATAA	0	0	0	0	0	0	1	1
		21UR-936	TGGTATCTTCACGGGTATTCT	0	0	0	0	2	0	0	2
		21UR-937	TCGTACTCTGTGCTCAAATTT	0	1	0	0	0	0	0	1
	†	21UR-938	TATTCAATTGGTTGCAGTCGT	1	0	0	1	3	1	4	10
	†	21UR-939	TATTCATCAATTCCAGATCAT	1	0	0	0	0	0	0	1
		21UR-940	TGCGCCACTTGTCGCTTTTAA	0	0	0	0	0	0	0	0
		21UR-941	TCAATTGTATTCAATTACGTA	0	0	0	0	1	0	0	1
	†	21UR-942	TGTAATTATACATACAACAGT	1	0	0	0	0	0	0	1
		21UR-943	TGCGATGTCTTAATCCATTCG	2	1	0	0	0	2	3	8
*	†	21UR-944	TATGTCACGAGATTGGTTTAC	0	4	2	12	144	125	19	306
		21UR-945	TGTCATACTATGTTCCGGAAA	11	1	0	0	2	8	3	25
	†	21UR-946	TAATGTCAATGTCAGAAAAAT	0	2	2	7	11	20	0	42
	†	21UR-947	TGTTGTTGTGTTGTTCTTAAC	0	0	0	0	0	0	0	0
		21UR-948	TATCGATATGAATGCATTTAT	5	0	0	2	8	16	0	31
		21UR-949	TCGGGAAAGTTTCCATTTATG	0	0	0	0	0	0	0	0
*		21UR-950	TAATTGTGACTAAATTTTTGA	0	0	0	0	5	10	0	15
	†	21UR-951	TCGATGTCCAATACTCTCAAA	0	1	0	1	4	8	0	14
		21UR-952	TGAACATTTCCTTTTCTTTGA	0	0	0	0	0	0	0	0
		21UR-953	TCAAATGGAATCTTTCATATC	0	0	0	0	3	1	0	4
		21UR-954	TGTAGACGTATTTTTTTAGTG	0	0	0	0	0	0	0	0

		21UR-955	TAAATGCCACAAACTATTCAC	0	0	0	0	0	1	0	1
		21UR-956	TGTCCAAAAATTCAGAATCCA	0	0	0	0	1	1	0	2
		21UR-957	TAACATCTTGAAGCAGGTCCT	3	1	0	0	2	40	0	46
		21UR-958	TTCAAATTTCATGTAGTTTGA	1	0	1	1	4	10	0	17
		21UR-959	TTCAGAATCTGCTGACTTGTT	0	0	0	0	8	7	0	15
		21UR-960	TACGACCAATTACTGACTCCA	0	0	0	1	18	16	6	41
	†	21UR-961	TGCTATTGGCAAAACATCGAT	1	0	0	0	2	1	0	4
*		21UR-962	TAGGCTTCGCAGTAGATTAAG	29	17	13	5	14	119	11	208
		21UR-963	TGAACTGTACTAACACATATA	0	0	0	0	4	1	1	6
		21UR-964	TCGGCGAGTGGCTGAATTTGA	0	0	0	0	1	1	0	2
		21UR-965	TATTATTTTAGTCTTGTTTGT	1	0	1	0	15	7	13	37
		21UR-966	TGATAGCTGGAAAAAGTATTT	1	0	0	0	0	0	0	1
		21UR-967	TCTAAGCATGGCTTTTGTAAA	0	0	0	0	0	1	0	1
		21UR-968	TAACTTTTTACCATAGATGTT	0	0	0	0	1	3	0	4
		21UR-969	TTCTGTATTTCCAATCCCAGC	0	1	0	0	1	1	0	3
*	†	21UR-970	TATGAAGATGATGAGCAATCT	11	12	5	5	13	43	2	91
		21UR-971	TCAGTTTTGGTTTCTAAACTT	0	0	0	0	0	1	0	1
	†	21UR-972	TACTACCAAAAATCGCTTTCA	0	0	0	0	0	0	0	0
		21UR-973	TGCCAAGAAAACATATTTTCG	0	0	0	1	0	0	0	1
		21UR-974	TAATTTAACTGATTTTTCCGA	0	0	0	0	0	1	1	2
*		21UR-975	TAAAACCGTAGAATAGAATCA	9	4	1	3	6	13	1	37
		21UR-976	TAGTGTGTTTGTCGAATTAAA	8	9	4	13	224	137	62	457
		21UR-977	TTCGTGACACATCGCTAGTCC	1	1	0	0	1	4	4	11
		21UR-978	TGGTGTCAAAATCGGACGACC	10	2	3	1	3	18	34	71
	†	21UR-979	TGATCATCAATGTTTTCGACG	1	0	0	0	4	4	1	10
		21UR-980	TATAAGTATTATTGCTTCTTA	0	0	0	0	2	0	1	3
*	†	21UR-981	TCACTCCATACTACTAGAACA	0	4	2	7	113	78	4	208
		21UR-982	TAGAGAGAAACTACTCAAGAT	0	0	0	0	0	0	0	0
		21UR-983	TGCCAAAACACCACTGAATCC	0	0	0	0	0	0	0	0
		21UR-984	TAGGTTTCAGGTCGATTGTAA	0	0	0	1	10	10	17	38
		21UR-985	TAAGGTGAATTTTATTTTTCC	0	0	0	0	1	0	1	2
	†	21UR-986	TCTCGTCAAGCGATGAATCAA	3	1	3	4	5	15	0	31
	†	21UR-987	TCTGCTCTGTACTTGAAGCTA	0	0	0	0	6	2	7	15
		21UR-988	TAGTTACATCCTTTTTGTGGC	0	0	0	0	3	1	3	7
		21UR-989	TTCGTGCAGTTTCATGATTCC	0	0	0	0	2	2	0	4
		21UR-990	TTTGTTTCGTATCCGCAAAAT	1	0	0	0	1	0	0	2
	†	21UR-991	TGGATAGTCTCACGGTATACC	0	0	0	0	4	3	0	7
	†	21UR-992	TGCATTACCGACTCAACATAA	3	1	0	0	3	4	1	12
		21UR-993	TGTATTCCTTATACTTAGCGA	1	0	0	0	0	0	0	1
		21UR-994	TCCGTTTGAATTATGGCCAGT	0	1	1	1	9	11	2	25
		21UR-995	TAGTTGTGATTTACGTACTAG	0	0	0	1	3	3	2	9
		21UR-996	TGTAAAAATTACCTAACTGCG	0	0	0	0	0	0	0	0
	†	21UR-997	TTCGGTCCACGAGATGATTCT	5	0	2	1	4	8	2	22
*	†	21UR-998	TTTACACACTTTAGTCGGGAT	12	2	3	11	152	196	76	452
*	†	21UR-999	TAGTTGAACAGTAATCTCAAA	2	2	2	3	4	17	0	30
		21UR-1000	TCCTCTAGACGCTCTCGTTTC	3	1	1	2	41	50	5	103
		21UR-1001	TGTCGATCCTGCTTTTATGAG	0	0	0	0	0	0	0	0
		21UR-1002	TTCCCAATTTCTGCATGACTG	0	0	0	0	14	21	15	50
	†	21UR-1003	TTTCAAAACTTACCAAATTGT	1	0	0	0	0	1	0	2
		21UR-1004	TGCTTTCTTCTTATCAGAGTA	0	0	0	0	0	0	0	0
*		21UR-1005	TGAATGAGCAAATTAGAATTC	14	17	6	13	30	75	1	156
*		21UR-1006	TGAAGCAGTTCAAATCTGATG	1	0	0	2	0	6	0	9
	†	21UR-1007	TGATCTTTTCTCAAACTAGTG	0	0	0	0	0	0	0	0
	†	21UR-1008	TGATGTTGTTGTGTTGTTCTT	1	0	0	0	1	2	1	5
		21UR-1009	TGGTGTTAAAATCGGACGACC	7	5	5	1	6	24	11	59
	†	21UR-1010	TATTCGCGGAAAATATTTTGA	19	4	3	5	13	34	1	79
*		21UR-1011	TCGGTGAATTAATTCGGTGTC	12	8	5	11	371	305	41	753
	†	21UR-1012	TTGATAATTTGATTCAAGGTG	0	1	0	0	0	0	0	1
		21UR-1013	TGAATTCTGATTTATTCGACA	0	0	0	0	16	5	2	23
		21UR-1014	TGAATCCAATTTAGATGTAGC	0	0	0	0	6	5	0	11
		21UR-1015	TGGATAAAGAATCTTTAAACA	0	0	0	0	1	3	0	4
*	†	21UR-1016	TGACTCGTATTGGACTAAAAA	48	26	24	41	298	448	148	1033
*	†	21UR-1017	TGAAATCTTCATGGTCGACGA	51	5	5	13	69	137	126	406
	†	21UR-1018	TGCCTTGTCTGTCCTAAAATC	2	1	0	0	1	3	0	7

	†	21UR-1019	TGATATGCCTATTTCGCCATT	0	0	0	0	1	0	0	1
	†	21UR-1020	TCTAATTGCACATTACTTACG	0	0	0	0	0	0	0	0
*	†	21UR-1021	TTCTTTTGGGGTTTGATCGAA	17	3	1	1	13	38	12	85
*		21UR-1022	TATGGATTCCAGAAAATTGTC	10	7	6	2	6	24	6	61
		21UR-1023	TGAATTTGACATTGCATTCTC	5	0	5	3	13	14	3	43
		21UR-1024	TCTCTAAGAAAAAATTATTGA	0	0	0	0	0	0	0	0
	†	21UR-1025	TTCGTATCGATTATTTGTGAC	155	62	38	68	395	590	85	1393
	†	21UR-1026	TGGGAGAATTATTTTACGTTC	0	0	0	0	0	0	0	0
	†	21UR-1027	TATTGAGTTATACGTCGGCAG	0	1	0	0	23	42	81	147
		21UR-1028	TGATTCCATTCCTTGCCCACA	0	0	0	0	0	0	2	2
	†	21UR-1029	TCCGTCTTTCATCAGGTTGTG	0	0	0	0	0	0	0	0
	†	21UR-1030	TTCGTTTGATGAAGGCGACAT	22	13	11	7	17	152	1	223
	†	21UR-1031	TCTCACAGGTCTTAACTATGC	0	0	0	0	1	0	0	1
*	†	21UR-1032	TGTGAACTGTTGATTTCGTGC	1	0	0	0	13	19	4	37
		21UR-1033	TAATCCAATGGATGGGTTTAT	0	0	0	0	1	0	0	1
		21UR-1034	TTAACCATAAGTTCCGTATCA	0	0	0	0	2	5	3	10
		21UR-1035	TGGAGAGATATTTACAATATT	0	0	0	0	0	0	0	0
*		21UR-1036	TCATCTGTAGGACAACATTTA	1	0	0	1	8	3	0	13
		21UR-1037	TCCGCAATAATTATGGTTACT	0	0	0	0	0	0	0	0
	†	21UR-1038	TTTCACAGGAGATTTTGCTCA	0	0	0	0	1	3	0	4
	†	21UR-1039	TGATCAAATCAAGTCCAATGC	0	0	0	0	0	0	0	0
		21UR-1040	TCCAATTATAATTTTTGGTTC	0	0	0	0	0	0	0	0
		21UR-1041	TACATAGCCGTTTTGGTCAGT	1	0	0	1	8	14	1	25
		21UR-1042	TTGCAAATCTTGATGAGTGAA	2	0	0	0	3	5	2	12
		21UR-1043	TGTATCTATATTAAACGAAAT	0	0	0	0	0	0	0	0
		21UR-1044	TGATTGTGCATTTAAGACAAA	0	0	1	3	36	17	3	60
		21UR-1045	TCACATAGCAGAATCAACCTC	0	0	0	0	0	0	0	0
		21UR-1046	TGCATAGTCTATCGAAAATAA	1	0	0	0	16	13	4	34
		21UR-1047	TGTGCAATGCAATGAGTTTTG	0	0	0	0	0	0	0	0
		21UR-1048	TCTTCACTTTTTCTAAAGGTT	0	0	0	0	0	0	0	0
	†	21UR-1049	TATATTTCAGTAGTTGTCATG	7	5	1	2	3	6	3	27
		21UR-1050	TCAGCGTGTTGTTCTATTCGA	2	1	0	0	2	6	0	11
*	†	21UR-1051	TTTATTGTGAAGACTGTGACG	2	0	0	3	30	37	8	80
		21UR-1052	TGAATTTAACTTCGATCAGGG	1	0	1	0	6	9	16	33
		21UR-1053	TGCATCGTTGTCATTGAAACC	0	0	0	0	3	4	0	7
		21UR-1054	TGAAAAATTTTCAATTTGTCG	1	0	0	0	3	11	7	22
		21UR-1055	TGTTAAGAGAATTGCTTTCCC	2	1	0	0	4	2	0	9
		21UR-1056	TTCGCCTGCATTTATATTCAT	9	1	1	2	3	9	1	26
		21UR-1057	TGCTTGCTGTGTTTTAAAGGA	0	1	0	0	12	21	2	36
		21UR-1058	TGGTTTTCAGTTTCAAATCAT	1	0	0	0	0	0	0	1
		21UR-1059	TGCAACGTATTGACCTGAATA	0	1	2	4	44	50	1	102
		21UR-1060	TAGTTTCGTGACATTACCGTC	0	0	0	1	4	3	2	10
	†	21UR-1061	TGTCAGTGGCTTTGATTCCGT	0	0	0	0	1	2	0	3
	†	21UR-1062	TCATATTCCTAGTTGGTATAA	1	1	1	0	0	6	0	9
*		21UR-1063	TGAGCGCATTTGTATACACTG	2	2	0	1	17	15	8	45
		21UR-1064	TAAACACCTCGTTATTTGTTC	0	0	0	0	0	0	0	0
		21UR-1065	TGACAGTATGAGGAATTGAGT	23	39	21	21	54	232	9	399
		21UR-1066	TCTATTTTCACACCGTTATTA	0	0	0	0	0	0	0	0
		21UR-1067	TAATTCAAATTCATATCGAAA	1	0	0	0	1	1	0	3
		21UR-1068	TCGAAAAGGAGTTTTGCCGGT	1	0	0	1	25	21	4	52
		21UR-1069	TAATTCGTTGTTTTGATGAGC	0	0	0	0	1	6	0	7
		21UR-1070	TAATCTGTCATCTGTTTCAAA	0	0	1	1	9	2	3	16
		21UR-1071	TGTCAATTGCGGGATAACTTC	0	0	0	0	7	3	1	11
		21UR-1072	TTATTGTCAAAGTGTTTTAGC	2	0	1	1	2	1	2	9
		21UR-1073	TTGAGTTATTGCAGGTGTGTG	0	0	0	0	0	2	0	2
	†	21UR-1074	TATAGTGCTGTTTCAACCAAT	1	1	0	0	5	4	0	11
		21UR-1075	TACGTCCTTAATTAGCATTAA	8	2	1	5	9	14	2	41
		21UR-1076	TACAGAGTCCAAAATTTCTTC	5	1	0	0	0	6	2	14
		21UR-1077	TAGAACTGCTTTTATTTATTG	0	0	0	1	0	0	0	1
		21UR-1078	TAGGTGTAGTTTTCTGATTGG	0	0	0	0	0	3	0	3
*		21UR-1079	TATACAGCTTAACTACCGATC	40	9	1	7	10	32	9	108
	†	21UR-1080	TAAGAGTACTATAATTAGATG	0	0	0	0	0	0	0	0
		21UR-1081	TCTTCCTTTGTACTTATCAAG	0	0	0	0	0	0	0	0
		21UR-1082	TCAAGATTTCACTTAGTGGTA	0	0	0	0	0	0	0	0

		21UR-1083	TCTGTGCGGAACTCAGAAACA	4	0	1	1	13	11	2	32
		21UR-1084	TGGTACGCGCCAATTACATAC	0	0	0	0	0	0	0	0
	†	21UR-1085	TTAGCGAGTAAGTGTGCGTTA	18	7	0	0	6	22	0	53
	†	21UR-1086	TACAAAATCCTACTTTCAATG	3	0	0	0	0	0	2	5
*		21UR-1087	TACAACGTCGGTCAAACACAC	68	11	16	11	54	85	26	271
*	†	21UR-1088	TTAATCTGAATGTCGTGATGA	20	9	6	15	53	92	27	222
		21UR-1089	TCAATAAACACTGAGATATTC	0	1	0	3	29	17	1	51
		21UR-1090	TCGGGGTTTTGCTGATTCTTC	0	0	0	0	0	1	0	1
*	†	21UR-1091	TGTATGAAAACGTGCCGTCTT	8	2	1	0	7	27	2	47
		21UR-1092	TACAATCAACAATTGTTGAGA	4	2	3	6	47	37	4	103
		21UR-1093	TAAACTGGTGTTTGAGAGAGA	0	0	0	0	2	4	4	10
		21UR-1094	TGAAAGTTTTCATTGTACTTC	0	0	0	0	2	0	4	6
	†	21UR-1095	TGCTTTACGTCCTTCAGGAAC	1	0	0	1	17	6	0	25
	†	21UR-1096	TATGGAGTTTTCAATATAATA	0	0	0	0	0	0	0	0
		21UR-1097	TATCGCGCTCCCCGCTTCGAA	0	0	0	0	1	1	0	2
	†	21UR-1098	TGTCCTTTTGATATGATGACT	0	0	0	0	1	1	0	2
		21UR-1099	TGATGCAAACACCTCTTATTC	0	0	0	0	0	0	0	0
	†	21UR-1100	TATAAATTGTGAAAAATTGTG	0	1	0	0	7	2	1	11
		21UR-1101	TTACAACTGTTATTTGTTGTT	0	0	0	0	1	0	0	1
	†	21UR-1102	TAAGAAAATAATTCGTCCAAA	4	0	0	0	4	5	11	24
		21UR-1103	TGCCACTTTGTATATTGGAAT	0	0	0	0	0	0	0	0
		21UR-1104	TGCGTGTTCAGTTGCCACTAA	3	0	0	0	0	1	0	4
	†	21UR-1105	TGCATTTTTCCATTGCATGAA	2	0	0	0	0	1	0	3
		21UR-1106	TCACCTTCTTTTTAATTTCTC	0	0	0	0	0	0	0	0
		21UR-1107	TCACGCAATAAATTTTATCGG	2	0	0	0	1	0	0	3
	†	21UR-1108	TGTTCCTCCTGGTCTATTCAC	0	0	0	0	0	0	0	0
		21UR-1109	TCACAGATTCTCCAGAAGTCT	2	0	0	0	1	2	0	5
	†	21UR-1110	TTTGTGTGTCCATTTTACAGT	0	0	0	0	0	0	0	0
		21UR-1111	TAAGCCTTACATATTGACTGA	7	5	3	7	28	46	4	100
	†	21UR-1112	TTCGAACACAATTTGCCAAAA	1	0	0	1	2	0	0	4
	†	21UR-1113	TTAGCAATCGTTCTACAAATT	17	1	4	0	12	15	4	53
		21UR-1114	TGAAAATTTTGCTGAACACCT	1	0	0	0	1	3	0	5
		21UR-1115	TTCCAGCAGTTACTAGAAATG	5	1	0	0	3	5	0	14
	†	21UR-1116	TATTGTGAGAAATAAAGAAAT	1	4	2	1	6	13	0	27
		21UR-1117	TCATGAGACGCTAAAAACATG	1	2	0	0	83	65	5	156
		21UR-1118	TGTCACTAATATTTCGGATTC	0	1	0	0	0	0	0	1
		21UR-1119	TCCAAAAACAATATTCGAATA	0	0	0	0	0	0	0	0
	†	21UR-1120	TGTTTCAACCGCTTGTTAGGG	0	0	1	0	0	4	3	8
		21UR-1121	TAACCTACCTGAATCTGCAAA	1	0	2	0	3	14	2	22
*		21UR-1122	TATCGTTGGGTAGACCATGGT	233	97	81	62	327	759	86	1645
		21UR-1123	TGATGTTTTAAAAATCATCAA	0	0	0	0	0	0	0	0
*		21UR-1124	TAACTTGTTGAATTTCCGAAT	0	1	1	2	2	10	0	16
*	†	21UR-1125	TGGGAATGTGAAATTTTTCAG	0	0	0	1	43	37	14	95
		21UR-1126	TAATTCCGTACAGCTTTTAAT	8	4	1	1	5	18	1	38
	†	21UR-1127	TAATCGATATGCTATTTTTCC	2	0	0	1	4	3	4	14
		21UR-1128	TGGGCGGACATAACTTTTTTG	1	0	0	0	0	1	1	3
	†	21UR-1129	TCTGTTGTCGATGGGTCAATA	13	3	0	2	8	29	51	106
*	†	21UR-1130	TGTAATTGGAAGAATCGTTGC	2	2	1	4	77	81	45	212
		21UR-1131	TCTGATGCAATTTTATGCAAT	0	0	0	0	0	0	0	0
	†	21UR-1132	TACTTTGACAATTAGTGTTTT	1	0	3	0	0	2	0	6
		21UR-1133	TCTGTGGTTTTCTATGCACGG	0	3	0	0	31	32	3	69
*		21UR-1134	TATGTTTGGAAAAACCGGTTG	10	3	4	3	86	97	53	256
		21UR-1135	TGCTGTGTCCCTTTCTTTTCT	1	0	0	1	0	0	0	2
		21UR-1136	TCAAGAATAAATAGTGAATAG	0	0	0	0	2	0	0	2
	†	21UR-1137	TTTAGACGAGGATGGTGAATA	45	25	12	7	30	166	12	297
		21UR-1138	TGCTCTTCTTTTTTCAATGAA	0	0	0	0	0	0	0	0
	†	21UR-1139	TACTTATTGTTCTTGTAAGTG	0	0	0	0	0	1	0	1
		21UR-1140	TGCCCAATGATGCAACTGAAG	0	0	0	0	0	5	0	5
		21UR-1141	TAACCTGTTCAATAAATTGTA	0	0	0	0	0	0	0	0
	†	21UR-1142	TGACTCAACTAACCTGAATTT	0	0	0	0	0	2	0	2
	†	21UR-1143	TATTAGAAGTTCATCGGGTGA	12	3	2	6	8	32	7	70
	†	21UR-1144	TGATCCACCTGTTCTCTCAAA	0	0	0	0	1	0	0	1
		21UR-1145	TTTAACATTTTTGGTGAGCAC	0	0	0	0	0	1	0	1
*	†	21UR-1146	TGGTTGGATTGTCGCAGTCGG	43	26	17	11	462	728	244	1531

*	†	21UR-1147	TAATAGGATTTTTCGGGGGAA	5	0	3	1	32	52	126	219
	†	21UR-1148	TGACTTAGTATTTTGTTGAGA	5	4	0	4	2	5	1	21
	†	21UR-1149	TACTCCGTGATTGCCACCAGA	2	0	0	0	0	1	0	3
		21UR-1150	TACATTTTGATTTATGAACTG	2	2	0	3	16	20	2	45
	†	21UR-1151	TATCCCATGTGAATCGTGTTA	0	0	0	0	0	0	1	1
		21UR-1152	TTTGATTATGTTAGGAAAATT	1	0	4	2	17	17	17	58
		21UR-1153	TATTTGCATTAATTAAAATAG	1	0	0	0	0	0	0	1
	†	21UR-1154	TGATTGTGTTGTTCATTGTGT	1	1	0	0	1	4	0	7
	†	21UR-1155	TGACTAATTTGGTACTTCAAC	0	2	2	0	15	10	3	32
		21UR-1156	TATGTAAATGGTTGGCAAATA	10	5	1	1	12	40	16	85
		21UR-1157	TAAGATGCCCGTTATAATACA	2	0	0	0	0	0	1	3
	†	21UR-1158	TTGGAACTAGAGGAATCTTAA	1	1	0	1	17	21	5	46
	†	21UR-1159	TTGCCACAATTCTGAGAATCG	1	0	0	0	0	1	0	2
		21UR-1160	TAAAAATGACAATTAAAAAAA	0	0	0	0	0	1	1	2
	†	21UR-1161	TATTCGGTAAACATGCTGTAG	1	0	0	0	0	3	0	4
	†	21UR-1162	TACCAGAAATCTACAAACTTT	2	2	3	3	12	24	0	46
		21UR-1163	TCGCAAAATTGACTAAGTTGC	0	0	0	0	2	0	0	2
		21UR-1164	TTCCAAAAATTGTTTCAAAAA	0	0	0	0	2	1	2	5
		21UR-1165	TAAGTGGTTAACTTGTTCTTC	0	0	0	0	2	1	1	4
*		21UR-1166	TGTAATAAGTGGAGGTAGCTA	311	223	101	67	487	1435	211	2835
*		21UR-1167	TCTGTACGAAGAATGGTTGTT	10	21	16	32	832	739	87	1737
		21UR-1168	TGAGTAATCTTCATTGGAGAA	0	0	0	1	2	5	3	11
	†	21UR-1169	TGTGTAGGGCTATAATAAAAC	10	3	3	1	16	18	7	58
	†	21UR-1170	TCAGTCGTCTAGGTTTCTACA	2	1	0	2	114	73	15	207
		21UR-1171	TATCTATACAAAACTGCCGAC	0	0	0	0	2	4	3	9
	†	21UR-1172	TCAGTAAGGATTGCGCAAATC	0	0	0	0	0	2	0	2
		21UR-1173	TTTTGATTGAAATACAAAACA	1	0	0	1	11	13	2	28
		21UR-1174	TGGTACAACTTTTCTGTTTTT	0	0	0	0	0	0	0	0
	†	21UR-1175	TGTTCCTCTGTTTTCCATCTG	0	0	0	0	2	4	1	7
	†	21UR-1176	TATTATCCATATGATGTTAAA	0	0	0	0	0	0	0	0
		21UR-1177	TGGGTAAGTTAGCAGCATATC	0	0	0	0	6	5	0	11
	†	21UR-1178	TGTACTGATTTAAATGAAGTT	0	0	0	0	0	0	0	0
		21UR-1179	TGCATTGTTTTCAATTAGGGC	2	0	0	0	0	1	0	3
	†	21UR-1180	TGCTTGGACAATTTAGCTGTT	0	0	0	0	2	1	0	3
		21UR-1181	TAAATATGATTTATGTTAGGA	23	1	7	3	6	12	6	58
		21UR-1182	TCAGTCTATTTTAATCATCCT	0	0	0	0	0	0	0	0
		21UR-1183	TACAAACAACAGTTTTTGTGA	0	1	0	0	0	5	0	6
		21UR-1184	TCAGCAAAACCATATAGCAGC	5	2	1	0	0	12	1	21
		21UR-1185	TGCATCCAATTTTTTGACATT	0	0	0	0	2	2	0	4
	†	21UR-1186	TGGAGTTTGGTTTAAAATGCA	1	0	0	0	0	0	0	1
		21UR-1187	TCTCACTACCTGTTTTAATTT	0	0	0	0	0	0	0	0
		21UR-1188	TATGCATGTCTTAAGTTTTTC	11	0	4	1	6	9	0	31
	†	21UR-1189	TACCATGCCAGCACGACTATA	9	2	3	0	1	6	4	25
*	†	21UR-1190	TTAGGACATAATATGATCTTA	2	4	0	4	51	29	3	93
		21UR-1191	TCCTGTTACTAATTTCAATTG	0	0	0	0	1	1	0	2
		21UR-1192	TGTTCTGCCTATATTTTCCAA	0	0	0	0	0	0	0	0
*		21UR-1193	TCATAACAAACTGGGCATAAA	26	15	6	10	39	95	9	200
*		21UR-1194	TCGAATCAACTTGATCAAATC	0	0	0	0	0	1	0	1
		21UR-1195	TCAATATGTTGTTGTCTGTGA	0	0	0	0	7	15	6	28
		21UR-1196	TGCTATCCGCTTTAACAATGA	0	0	0	1	4	2	3	10
		21UR-1197	TAAAAATGTGTAAGTGGCGTA	2	1	1	2	1	12	0	19
*		21UR-1198	TGATTCCGCAATAAGGCCTTG	0	1	0	0	0	0	0	1
		21UR-1199	TCAGTGACTCGTATTAGCGTC	0	0	0	0	5	1	0	6
*		21UR-1200	TGAACGGCTTTTTTCTGATGG	443	517	194	142	429	3119	71	4915
		21UR-1201	TCAAGTGAATTCGATTCGCAG	13	13	8	6	15	104	1	160
		21UR-1202	TCACTGCACCCACTTGTTCAG	0	2	0	1	0	3	2	8
		21UR-1203	TAACAATGCAGTATTATTATA	17	2	2	1	4	12	3	41
		21UR-1204	TTGCGATGCTTCTCCATGTTG	1	0	0	0	0	1	1	3
*		21UR-1205	TAAGACGGCCAAATTATTTTC	44	9	4	4	12	27	13	113
		21UR-1206	TTTAGTCAGTGGTGAGTCACG	5	1	0	1	1	5	2	15
		21UR-1207	TCCTTCTACGTGCATACTAGC	2	0	2	0	3	9	4	20
	†	21UR-1208	TAATGTTTTATCATCCCCACA	2	0	1	0	0	2	1	6
	†	21UR-1209	TAATATATCCTATCAACTGAA	0	0	0	0	0	0	0	0
	†	21UR-1210	TTGTGCTAGAAAAATGTTTCT	0	0	0	0	4	0	0	4

		21UR-1211	TCTTAAGGTTTTTGTTACGAG	5	0	0	1	0	0	0	6
	†	21UR-1212	TCCATAATTATGTAGTTTTAT	0	0	0	0	0	0	0	0
*		21UR-1213	TAAATGATAAGTTAGACCACA	55	20	14	17	29	97	12	244
		21UR-1214	TCCGTCTTCTTTCAAAGCTGA	0	0	0	0	0	0	0	0
*		21UR-1215	TTCTGTCTCCCAAGCCAAGTA	6	2	0	0	1	7	2	18
		21UR-1216	TTGACGGATTTTTTTGTATTC	7	6	2	7	10	29	3	64
*	†	21UR-1217	TATCATCAGGTTGAAGATTTG	68	36	25	30	99	211	3	472
		21UR-1218	TAGGTTATCAGTTGGGTTTGC	9	8	7	3	20	67	13	127
	†	21UR-1219	TCAATCATTTCGTCTTTAGGC	1	0	0	0	4	4	1	10
		21UR-1220	TGCAACAATTGTGCGTTTAAA	0	0	0	0	0	0	0	0
		21UR-1221	TCCGGCAATCATATTCTCTGT	0	0	0	0	3	2	0	5
	†	21UR-1222	TTGCAGTGAAGCTCTGGATTG	19	4	3	0	11	36	2	75
		21UR-1223	TAATTCATTTTTTTCATAAAA	0	0	0	0	0	1	0	1
	†	21UR-1224	TGTTTGGTTTCTATCAATCTG	0	0	0	0	0	0	0	0
		21UR-1225	TGTATATTTCTCAGCATTTTC	0	0	0	0	0	1	0	1
		21UR-1226	TCGTAGAACAACAAATAAATC	1	1	0	2	2	4	0	10
		21UR-1227	TCATCCAGTTAATTTGTATTA	0	0	0	0	0	0	3	3
	†	21UR-1228	TATTACGCATTGTGATTTTCG	1	0	0	1	4	1	1	8
		21UR-1229	TCGAGTATTGAACATCATTGT	1	0	1	0	0	3	0	5
		21UR-1230	TCGATGAGCCGCCTATGCAAG	0	0	0	0	0	0	0	0
		21UR-1231	TCTGTGATTAAATTGTGGTTA	0	0	0	0	1	0	0	1
	†	21UR-1232	TTTCGAATCGATCTACGAACC	1	1	0	2	9	6	0	19
*	†	21UR-1233	TATCGCTAAGCTTTTTCTGAA	1	3	2	2	4	28	0	40
	†	21UR-1234	TGATCTCATCTCTCTCTTTTC	0	0	0	0	0	0	0	0
		21UR-1235	TACGATCCATACGATGTTCAT	0	1	0	0	5	5	0	11
		21UR-1236	TACCTACATCACATTTGTAAG	0	1	0	0	4	5	3	13
	†	21UR-1237	TACGTGATCTAGTTCCGAATA	1	2	2	2	8	24	1	40
		21UR-1238	TAACGTAACTACTCAGCAAGT	6	6	3	4	9	27	3	58
	†	21UR-1239	TCAAACATAGTTCTCCCATGA	0	0	0	0	0	0	0	0
		21UR-1240	TCCTTTCTTATGACGGTTTGA	3	2	1	2	25	35	6	74
		21UR-1241	TGAATGATGTATGCATAAAAA	2	1	0	1	7	5	1	17
	†	21UR-1242	TATGAAAGGATACAGCTGCGC	2	6	7	1	29	68	9	122
		21UR-1243	TAAAACCCAAATGTTGATATA	0	0	0	0	0	0	0	0
	†	21UR-1244	TCACTCGTCATTAGTATATGC	0	0	0	0	3	2	0	5
	†	21UR-1245	TTGGAGACATCTGGTAGGTGA	1	0	2	2	2	5	1	13
		21UR-1246	TTATTTGACCAGAATACTTCC	3	0	1	0	2	4	1	11
		21UR-1247	TGGACTGCCAAGATGTTTTTT	1	0	0	1	7	10	8	27
		21UR-1248	TGCAGGTATTATTGCAAGTGG	2	0	0	0	2	3	1	8
	†	21UR-1249	TTCGCGGCTTGTCATACATTC	3	3	4	1	5	38	5	59
		21UR-1250	TACATGTGGTTTTTGCTGAAC	0	0	0	0	0	2	0	2
*		21UR-1251	TGAATACAATCAATGTTGACG	17	12	7	10	28	94	1	169
		21UR-1252	TATCGTTTTTATGAGAAGCGA	2	2	0	0	0	9	0	13
		21UR-1253	TTTGAGGAGTGACACAAATAA	0	0	0	0	1	5	3	9
		21UR-1254	TTTTCGCGTGTTTAGGTTAAA	1	0	0	0	5	7	3	16
*		21UR-1255	TCAAAATAAACACAATTCTGG	0	0	0	0	1	1	0	2
	†	21UR-1256	TTGGTATCTGATCGGTTGAAA	11	4	5	8	156	185	149	518
*	†	21UR-1257	TACTAATGTGTTGTAGGGTAA	0	1	1	2	64	52	20	140
*		21UR-1258	TAGACTTGAGTTAGAACGGTT	894	648	397	1401	18614	27106	2710	51770
		21UR-1259	TACCATTTCATTCCGACAAAA	3	0	1	0	0	1	0	5
		21UR-1260	TCGTGTTGTTATCTTGTTACT	0	0	0	0	1	0	0	1
	†	21UR-1261	TTCCATGTAGACTGTATTTTT	0	0	1	1	38	33	2	75
	†	21UR-1262	TCAGATCAGAAAAAAAATGTC	0	0	0	0	4	4	2	10
	†	21UR-1263	TGCTGCTGACTTTTTAGTTGC	0	0	0	0	0	2	0	2
		21UR-1264	TTCGTCTTTGCACAAAATCAA	1	0	0	0	0	3	0	4
	†	21UR-1265	TGATTGTGCATTGTTAAAAAG	1	0	0	0	0	2	0	3
		21UR-1266	TATGCGTATCCATGTGTATTG	0	0	0	0	0	0	0	0
		21UR-1267	TAGGAACGAAATGAACAAAAT	10	4	1	2	14	36	0	67
	†	21UR-1268	TGAATATTGTACGTTATTTTA	0	0	0	0	1	2	0	3
	†	21UR-1269	TGATACAGTGATTTGGATTTT	27	17	12	3	18	53	10	140
		21UR-1270	TCTGATGAATGTAATTTTTCA	0	0	0	0	0	0	0	0
	†	21UR-1271	TATTGGCAACTTACGTTAACT	1	0	1	5	45	61	11	124
		21UR-1272	TAGAATTAGGACTCCCGAATT	0	0	0	0	1	1	0	2
		21UR-1273	TACGTACTTATTTTGCCCAGA	14	3	4	3	23	24	12	83
		21UR-1274	TGTAATCCATCGTTAATGTTT	1	0	0	0	1	2	1	5

	†	21UR-1275	TCATGTTGTATTTATCTTGAT	0	0	0	0	0	0	0	0
		21UR-1276	TGGATCTTTTGAAATCGTACA	0	0	0	0	0	4	1	5
	†	21UR-1277	TTCGACAGGTTTCTGACAAAA	0	0	0	2	9	7	0	18
	†	21UR-1278	TATTGACGTTTTCAGAATTGA	2	1	0	2	11	9	2	27
	†	21UR-1279	TCAGTACCAAACAAAATCTCA	1	0	0	0	0	0	0	1
		21UR-1280	TTTGAAGTCCTAGCAATTTTA	1	1	0	1	0	3	1	7
	†	21UR-1281	TGTTGATAGCACGAAAAAAGC	2	0	0	1	5	9	11	28
		21UR-1282	TCAACATTTCTGTTCAATTAA	0	0	0	0	0	0	0	0
		21UR-1283	TGGTCCGTTCTTCATCTGACA	0	0	0	0	2	4	0	6
		21UR-1284	TAGTGGGCCCATAACTCATAG	0	0	0	0	0	0	0	0
*		21UR-1285	TAAATCGGTCATATAAGATAA	87	128	61	82	258	479	13	1108
	†	21UR-1286	TTTGAGGTGTTATCAGAAAGT	0	0	0	0	0	0	0	0
		21UR-1287	TGATTTTAACTTTATTATCTT	0	0	0	0	0	0	0	0
		21UR-1288	TATTCTTCTTGTATTCATAAC	2	0	0	1	0	0	0	3
	†	21UR-1289	TCTACTGTTACAGTGAGATAA	0	0	0	0	0	0	0	0
*		21UR-1290	TTAGTTGTCCATACGTTTCTC	14	8	5	5	10	33	3	78
	†	21UR-1291	TGGTTCCATCGATCTCTTATC	3	0	0	1	12	8	2	26
	†	21UR-1292	TCTTTCGGTATTATTTTTAGC	0	0	0	5	40	27	7	79
	†	21UR-1293	TTCCTCAATTGCAATGTCTTT	2	0	0	0	1	1	0	4
	†	21UR-1294	TCTGACTAAGTTCTGTGAGTC	2	4	1	3	4	10	4	28
	†	21UR-1295	TGATTGAAACGTAACGTTCAG	0	0	0	0	0	0	0	0
		21UR-1296	TAACAGCCATATTTGCGCGCC	0	0	0	0	1	5	1	7
		21UR-1297	TTTCATAAAACCCATTTTGAA	0	0	0	0	3	3	1	7
		21UR-1298	TGATCTTGTACAAAGTGTTTC	0	0	0	0	0	0	0	0
	†	21UR-1299	TTAGTTGATTTCCTAGTTGAT	1	1	0	0	1	4	0	7
*		21UR-1300	TATCCAGAAAAAGTTCAGTTT	11	0	2	2	2	6	6	29
		21UR-1301	TGCATTGTTGCCCCACCGGAA	0	0	0	0	0	3	4	7
		21UR-1302	TAAATGCCAACCATCATAATA	1	0	0	0	0	1	1	3
	†	21UR-1303	TGAGCTTCTACGAAAACAGTT	0	1	0	0	0	1	0	2
	†	21UR-1304	TGCGAACTATGTTTCCTTCCA	1	0	0	1	0	0	0	2
		21UR-1305	TGCTATTCATGGAGTCTTTTA	0	0	0	0	0	0	0	0
	†	21UR-1306	TCAAGAGGACTTCTCAGGACC	2	4	1	2	18	36	0	63
	†	21UR-1307	TGATAAGCTCCAATCGTGTCC	0	0	0	0	0	0	0	0
		21UR-1308	TGCAAGCTTAATAAACAAGTA	0	1	0	0	10	12	5	28
		21UR-1309	TAGGGTTCATGAATGGACTGA	0	0	1	0	25	30	1	57
	†	21UR-1310	TGATGAAATCAAAGTATTGCT	0	0	0	0	2	0	0	2
		21UR-1311	TTTGTACTTTGTCTGTCCCGC	2	1	1	0	0	4	0	8
*		21UR-1312	TTGACCGAAGATTTTGAGAAC	0	2	2	6	82	107	7	206
	†	21UR-1313	TAATAAACAAATGTTCGGTTA	0	2	1	6	75	52	16	152
	†	21UR-1314	TAAGTAGTGTGATGTTATAGA	1	2	3	9	100	75	10	200
	†	21UR-1315	TGGTTAAGAACATTTGGAAAA	4	0	0	0	1	3	0	8
		21UR-1316	TCCACACCACTTACTGAAAAT	1	0	0	0	1	1	1	4
*	†	21UR-1317	TCCGTAGCAGATATTTATCGT	22	21	14	21	46	119	3	246
		21UR-1318	TGACGCCACGTTTTTTTCCAT	0	0	0	0	0	0	1	1
		21UR-1319	TGGTGAATTATTTTAGCAATG	0	0	0	0	0	0	0	0
		21UR-1320	TATCATATTAGATGTCACGTA	0	0	0	0	3	1	0	4
		21UR-1321	TACGCGTAAATTCGTCAATAA	0	0	0	0	1	1	0	2
		21UR-1322	TGTAATCCATTCATAATGCAT	0	0	0	0	3	0	0	3
	†	21UR-1323	TTCAACATGGTGCTCCTTTAA	0	0	0	0	1	2	1	4
		21UR-1324	TTTATCTTATGTCGGTGTGCT	42	6	7	5	20	65	15	160
		21UR-1325	TGCCAAGTTACTTTTTTGAAT	0	0	0	0	0	0	0	0
		21UR-1326	TACGACTGTGCTTTGACAAGG	1	0	1	3	17	27	0	49
		21UR-1327	TATCACGAGAATGCAATTGAT	5	3	1	2	2	9	1	23
		21UR-1328	TACTGGAAACAAAAACAAAAC	0	0	1	1	7	9	4	22
		21UR-1329	TGCAATTTTTTTGATATTTCC	0	0	0	1	2	0	0	3
*	†	21UR-1330	TGGGACTTCCTAAACGGTTTT	0	2	0	2	151	129	23	307
		21UR-1331	TATGACTACTTTTCTTCCGTT	1	0	0	0	3	1	1	6
	†	21UR-1332	TAGAATAGAGTTTGCAGAAGA	12	6	3	3	20	60	5	109
		21UR-1333	TATACAGGCCATTTTTAGTTG	1	0	0	2	1	3	13	20
		21UR-1334	TGCAACTGCTCTTTTTCCATC	0	0	0	0	1	0	0	1
		21UR-1335	TTACAATTTTCAACTCACACT	0	1	0	0	1	4	1	7
*		21UR-1336	TCAGAAATACTTCATGAGCAA	0	2	0	5	42	36	4	89
	†	21UR-1337	TATAATGAATATTTTGGACAA	0	1	0	4	30	25	7	67
		21UR-1338	TTTGAATCTCATTATGAATGC	0	0	0	0	1	1	0	2

*		21UR-1339	TATATGTACTACATCCACCGG	14	0	0	0	4	16	2	36
		21UR-1340	TGACTAAACGTTAACTATTAC	0	0	0	0	0	1	0	1
	†	21UR-1341	TCGATATCTTGACAAGTCACA	0	0	0	0	2	0	1	3
	†	21UR-1342	TGATATTCCTATTGACATGGA	0	0	0	0	0	1	0	1
*		21UR-1343	TGAAGGAAGAGTACGAAACTT	153	222	71	71	704	975	73	2269
	†	21UR-1344	TGATTAGGACACTTCATCTCC	3	2	0	0	1	7	0	13
	†	21UR-1345	TATTATTGTAGCCTCTATCAT	0	0	0	0	0	0	0	0
	†	21UR-1346	TAATGCTTTTGTGCCATGAAT	0	0	0	0	0	0	0	0
		21UR-1347	TCGAAATAACAATTGTACTTG	0	0	0	0	0	0	0	0
	†	21UR-1348	TAACGCTTGGTTATGGTTATA	0	0	1	1	2	3	1	8
*	†	21UR-1349	TATTCATAGTTTAAGAGCAAT	4	2	2	2	8	14	1	33
		21UR-1350	TAATAACTGCATGTTCAATTC	0	0	0	0	7	6	0	13
		21UR-1351	TGTATAATTGATAGTTAAAAA	0	0	0	0	0	0	0	0
*		21UR-1352	TATACAGAAGACGTCTTGTTA	38	4	4	7	15	137	4	209
		21UR-1353	TCAGAACATCACATGATGGTA	28	29	8	13	46	96	1	221
	†	21UR-1354	TATTGTTTCCTTTTTGCGATA	2	0	1	0	1	0	2	6
*	†	21UR-1355	TGAAACTCTACTTTCAGGAGG	8	8	5	7	29	45	3	105
		21UR-1356	TGGACTTGTGAGGAACTAAAG	1	0	1	1	11	10	1	25
		21UR-1357	TAGGGTTCACTTTTTATGTTG	0	0	0	0	1	2	0	3
	†	21UR-1358	TGCTTCGTGGTAGAGCTTTGA	1	1	0	1	18	21	6	48
		21UR-1359	TAGCAATCCTATATAAACTGA	0	0	0	0	3	2	1	6
*	†	21UR-1360	TTCTCTCAGCCTACGACCAAA	3	0	2	1	15	74	20	115
	†	21UR-1361	TAATCCTCTTGCAATTTTAAC	0	0	0	0	0	1	0	1
		21UR-1362	TGGTGCATGGCGTGGCTTTGT	0	0	0	0	2	8	0	10
		21UR-1363	TCAACAGGCAAAACAAGGGCA	1	1	1	0	2	7	3	15
		21UR-1364	TGTTTTGATTTTACAAAATAT	0	0	0	0	0	0	0	0
	†	21UR-1365	TGTGAAGTTGGAATTTAATTT	4	11	1	5	47	43	8	119
		21UR-1366	TGTATCGCTGTTTTTTTCAAA	0	1	0	0	1	1	1	4
	†	21UR-1367	TTGGAGTTTGGTTTAAAATGC	12	6	1	4	13	34	1	71
	†	21UR-1368	TGTTATTTTGTCTAAAATTTC	0	0	0	0	0	0	0	0
		21UR-1369	TAAAGCGATAATACAACATTA	3	2	1	2	9	3	4	24
		21UR-1370	TATAATCATAATTTGTGCACC	0	0	0	0	4	1	0	5
		21UR-1371	TGGAAATGCTGCCTATTGAAA	0	0	0	1	2	1	3	7
*		21UR-1372	TCGGATATTAGCAAACATTAA	8	7	5	15	168	89	26	318
	†	21UR-1373	TTGTTTGCTTAAATCTGATGA	1	0	0	0	1	3	0	5
		21UR-1374	TATTGCACAGTAGAACTTAAC	0	1	0	1	15	11	1	29
*	†	21UR-1375	TAGAATCGGATTATTTATTAT	22	8	12	6	15	47	1	111
		21UR-1376	TAAGAAAAATTAAAATTCGGA	0	0	0	0	0	0	0	0
		21UR-1377	TGCGTAAGCTAGCCGAATTGG	7	1	1	0	4	4	1	18
		21UR-1378	TAAATGTTTCCTGCGTGCCCA	0	0	0	0	1	0	0	1
		21UR-1379	TATAACACTCCCATAGTTAAA	0	0	0	0	2	0	1	3
	†	21UR-1380	TGAAGCGTATTTAAGATTGTT	1	0	1	2	21	17	3	45
		21UR-1381	TACTGCTTTTGGATGTTACAG	0	0	0	1	4	9	0	14
		21UR-1382	TCGATGGTAAAAATTAAAAAA	0	0	0	0	4	0	1	5
	†	21UR-1383	TGCTTATATCGTTCACAGAAC	1	0	0	1	21	11	8	42
	†	21UR-1384	TTCTTATGCGTTACTCGAAAG	0	0	0	2	8	6	2	18
	†	21UR-1385	TATCCATATGATGTTAAAAGG	0	0	0	0	0	2	1	3
		21UR-1386	TATATTTTCCAATTCCAGTTA	0	0	0	1	0	0	2	3
		21UR-1387	TCTAGTGTTTCTCGATTGCAA	0	0	0	0	1	0	0	1
	†	21UR-1388	TATCGTCTTCTGAGCATAAGC	0	0	0	0	1	2	1	4
		21UR-1389	TTCATTCAGCTCACCATCAGC	3	1	0	0	0	5	4	13
		21UR-1390	TCCGCTTTTTCTGATTTATAA	0	0	0	0	0	1	0	1
		21UR-1391	TAGGAATTCGTCTGAACAAAA	11	5	3	2	8	40	3	72
		21UR-1392	TATGCTGTATACTGAATACTC	0	1	0	0	1	6	1	9
		21UR-1393	TGGATTAGTGGCCGTTTCATA	0	0	0	0	1	1	1	3
	†	21UR-1394	TACTTCTTTATGTCCAAAACT	0	0	0	1	9	6	2	18
	†	21UR-1395	TTAATCTCTTTGATCACAAAG	0	0	0	0	0	2	2	4
	†	21UR-1396	TAATAAACAATATGCGGGTCA	0	0	0	0	1	2	0	3
	†	21UR-1397	TTGCCAGTGAAATTTTGATAC	0	0	0	1	0	0	0	1
		21UR-1398	TTAAACATTTTAATGGAATTC	0	0	0	0	0	4	0	4
	†	21UR-1399	TCAATATCCAAAAAAACTGGC	3	0	0	0	0	3	0	6
		21UR-1400	TGTTAACTACTTTACCTTAGG	0	0	0	0	0	0	0	0
		21UR-1401	TGCGTGAGGTTTCACCATTTC	0	0	0	0	0	0	0	0
	†	21UR-1402	TCACAAAAAACGAACATGGAA	11	40	5	19	37	117	1	230

		21UR-1403	TTCCCATATTTTTTGATTTGC	1	0	0	0	1	1	2	5
*	†	21UR-1404	TTGAACTGGTCGCTGTGATGG	157	49	55	29	99	508	39	936
	†	21UR-1405	TGCAAGAGAAGCAGTATATCA	91	8	7	9	17	54	3	189
		21UR-1406	TAAGCGAGGCGTTTCATCATG	1	0	1	0	6	12	10	30
		21UR-1407	TATATTGAGTACAAAGTTCAG	7	2	2	3	29	29	1	73
	†	21UR-1408	TTGCAAATAGAAAGGTGGCAA	2	1	0	1	16	19	6	45
	†	21UR-1409	TCCTTGATTAATTTTCATTGC	0	0	0	0	0	0	0	0
		21UR-1410	TGTGTTTGTCAATTTCATAAA	1	0	0	0	0	0	1	2
		21UR-1411	TGAGATCTTTGTATTCTTCAT	0	0	0	0	1	0	0	1
	†	21UR-1412	TGTTTTGTAATTGTTCTTGGT	3	2	0	0	2	8	0	15
		21UR-1413	TTGAATCTGATTCAGGCTGTT	0	0	0	1	12	10	6	29
		21UR-1414	TTGAACGTCGATAAAATTTAT	0	0	0	0	4	0	3	7
*	†	21UR-1415	TACATTAATATTGTTCGGAAA	139	28	43	87	330	418	142	1187
	†	21UR-1416	TAGTCGCCCTAAATGCTATCA	5	0	0	0	1	2	0	8
		21UR-1417	TGGAATTCGTGGCAGTTTAAT	9	7	4	2	12	39	9	82
		21UR-1418	TCAGACTACCAAGTAGGCAGC	0	0	0	0	0	1	0	1
	†	21UR-1419	TGAAGACAGAATGTTAGCAAA	12	42	5	12	43	128	12	254
	†	21UR-1420	TAGTAAATTGTTATTAGTGGC	1	0	0	0	0	4	0	5
		21UR-1421	TGGGAAAATTTTGAAACTGCT	1	0	0	0	0	0	0	1
	†	21UR-1422	TACTGAGCTTCTACGAAAACA	0	0	0	0	0	2	0	2
		21UR-1423	TGCACTTTTGATAGTTACTTA	0	0	0	0	0	0	0	0
	†	21UR-1424	TGCTATCTCCGGAAAATCTGC	1	3	2	1	0	4	0	11
	†	21UR-1425	TCTTCCAGTCAAAAGTGAAAA	0	0	0	0	0	0	0	0
	†	21UR-1426	TCAACCCAAAACATGACAGCC	1	0	0	0	1	3	1	6
	†	21UR-1427	TGGAGCTAGGTTCCGGAGAAA	0	1	0	0	0	0	0	1
		21UR-1428	TATTGATTCAAATAGTATAAA	0	0	0	0	0	0	0	0
	†	21UR-1429	TTATTGGACCAAAAAGCTAAA	1	0	0	0	1	3	1	6
*		21UR-1430	TTGGCACTCGCGAACACCGCG	18	201	129	97	70	49	60	624
	†	21UR-1431	TGTTCAAATCATTCAAAATAA	0	0	0	0	0	0	1	1
	†	21UR-1432	TAGTCGAGAACGGAACTTCAA	1	3	0	1	25	19	5	54
	†	21UR-1433	TGGGCATATCTCCGGTCACAA	3	0	0	0	1	10	1	15
	†	21UR-1434	TAGGCTTAGGATACCTTCAAA	0	1	0	0	0	2	0	3
		21UR-1435	TCGCATTTTTTGTTCAGTTAA	0	0	0	0	0	1	0	1
	†	21UR-1436	TCAATACTCCACTGTACACCC	0	0	0	0	1	0	0	1
	†	21UR-1437	TCCGACCGTTGTTACAGCTAA	2	1	1	1	7	20	2	34
		21UR-1438	TGCCTTTTTGGGAAAAAAAAG	0	0	0	0	0	0	0	0
		21UR-1439	TGATCTGGGTTTGATCTGAAT	0	0	0	0	5	1	0	6
	†	21UR-1440	TGATAAATTGTGCGAATTTTG	0	1	1	0	1	9	2	14
		21UR-1441	TGCATCATTTGATCTAAGTCA	1	0	1	2	6	14	1	25
*		21UR-1442	TCATGCACGGCTAAAAATTTC	70	12	7	7	15	78	8	197
	†	21UR-1443	TAATACGCCCTGTAGTCTTCA	0	0	0	1	6	2	1	10
	†	21UR-1444	TGGCGTGGTCTTTTTAATTTC	0	0	0	0	0	1	0	1
	†	21UR-1445	TATGCCATTTTCGATTTCATC	3	0	0	0	0	3	1	7
	†	21UR-1446	TTATGAACCCGCAATGCGAGC	0	0	0	0	4	4	5	13
	†	21UR-1447	TTGCTTGTTGAGACGAATTGA	61	73	33	47	282	495	96	1087
	†	21UR-1448	TGCGCATATCAATTGATCTTT	2	1	1	0	7	12	0	23
	†	21UR-1449	TGCCACAACAGCTCAGGGAAA	5	1	0	0	0	4	2	12
		21UR-1450	TAACAAAGCATTTCGTGAATC	1	1	1	4	64	48	10	129
		21UR-1451	TTACTTTGTTGCTTTGTTTTT	1	0	0	0	0	0	0	1
		21UR-1452	TACAGATCAGTTTTTATTCCA	4	0	0	0	21	11	12	48
		21UR-1453	TACAGCTTCTCTGTCATCTGC	2	2	0	1	1	14	0	20
	†	21UR-1454	TGACACATCGGTTTAATGAGA	1	0	0	2	12	12	5	32
		21UR-1455	TAACGTTCACTGATTATTGTC	0	0	0	0	0	0	0	0
	†	21UR-1456	TCAGTGCCACTATTATATATG	0	0	0	0	0	0	0	0
	†	21UR-1457	TATTATTTTGAATGGGCGCAG	0	0	0	0	2	6	12	20
		21UR-1458	TCTCAACCTTGTTCTGATTCT	0	0	0	0	2	1	0	3
	†	21UR-1459	TAATACTATGTGGTGTGTTGT	0	0	0	0	0	2	1	3
		21UR-1460	TCGAAACTTCAAGTTTGAAAA	0	0	0	0	0	1	0	1
		21UR-1461	TCAAATATCCTTGTTGACTAA	1	0	1	0	20	15	8	45
		21UR-1462	TCGACAGATGTTTATGATTTT	0	0	0	0	0	1	0	1
	†	21UR-1463	TCGTTAAAAAATCCTCCAAAG	1	0	0	0	1	1	0	3
	†	21UR-1464	TAGTGATGGACTTCTACTTGG	0	0	1	0	1	1	0	3
*		21UR-1465	TACATAGGAATCGAAATATGC	0	0	0	0	9	3	3	15
		21UR-1466	TGGGGTATGGGAATCATATGT	0	1	0	1	1	10	0	13

		21UR-1467	TTGGTTGCATTTAACTCTGAT	0	0	0	0	1	0	0	1
	†	21UR-1468	TTTGTACGCCATCGAATCCAA	0	0	0	0	0	2	0	2
		21UR-1469	TACGAGTGGAAAACACAAGCT	4	2	2	5	71	91	0	175
	†	21UR-1470	TGGGTTTGATTGATCAACAGT	0	0	0	0	1	1	0	2
	†	21UR-1471	TCCACAGCTTTACTATTTGCT	2	1	0	0	0	6	0	9
	†	21UR-1472	TAATTTGAAATCACCAACCAC	1	0	0	0	0	1	1	3
		21UR-1473	TCTAAACTGTTTCACAACCTT	0	0	0	0	0	0	0	0
	†	21UR-1474	TGCTGTCCATAGAATTATATA	1	1	0	0	2	3	0	7
*	†	21UR-1475	TGGTGGATCGTCATTTGGTGG	12	3	3	1	16	95	23	153
		21UR-1476	TGACTAGTTGCTGATCCTCGC	2	6	0	4	94	127	47	280
	†	21UR-1477	TTGCTTTACGTCCTTCAGGAA	0	0	0	0	0	1	0	1
	†	21UR-1478	TCTTCATCATCAGTCTCTAAT	0	0	0	2	1	0	2	5
*		21UR-1479	TATGTACGTAACAACACACTG	0	2	0	2	22	9	1	36
	†	21UR-1480	TGAACTCCACATATCGTTCTC	0	1	1	0	3	2	1	8
	†	21UR-1481	TGCCATTAATTCATCCGATAT	0	0	0	0	0	0	0	0
	†	21UR-1482	TTATGATGTTGTTCATTTGCA	1	0	0	0	2	3	0	6
*	†	21UR-1483	TCAATCACGGTATTTATTTTG	20	3	7	5	18	27	0	80
		21UR-1484	TAACATCTTAGCAGAGTTTTC	13	0	1	0	1	6	1	22
	†	21UR-1485	TCATTGATGTTGTTGTGTTGT	1	0	0	0	0	0	0	1
		21UR-1486	TCTATGAACGGTAGCCAAACT	3	13	8	17	34	147	21	243
		21UR-1487	TCATTTACTAAGTTGCGAATA	6	0	0	0	0	3	1	10
	†	21UR-1488	TTACACTGCTGTTGGAAAATT	22	9	11	12	34	69	28	185
*		21UR-1489	TGCAGTTTTTGATGACAGCAA	1	0	0	1	2	12	0	16
		21UR-1490	TGAGGATTTTGTTGTAGATTA	16	3	2	3	4	30	0	58
		21UR-1491	TAGGATGCAAGTTTTTTTTCT	0	0	0	0	0	1	0	1
		21UR-1492	TCTTAGCGTATTGTCAATTTC	0	0	0	0	7	1	1	9
		21UR-1493	TGGAAAATGCAATTTGGTAAT	9	5	1	1	6	14	1	37
	†	21UR-1494	TACCAAACAAAATCTCAAACT	0	1	0	0	1	0	0	2
	†	21UR-1495	TCAGGCTCAGTCTTATCATCA	0	0	0	0	1	0	0	1
	†	21UR-1496	TGGATGACTAATTTTGACTAA	4	7	1	1	27	40	1	81
	†	21UR-1497	TGAGTTGATATTCGATTGCGG	70	7	9	2	17	73	32	210
*		21UR-1498	TATAATGAGATTTGACTTTGT	0	0	2	3	9	9	3	26
		21UR-1499	TCTTTTCATTTTTTTATGGCA	0	0	0	0	0	1	0	1
		21UR-1500	TGAAAAGTGTGAACCTATTTT	0	2	0	3	5	10	1	21
		21UR-1501	TAATCTTTTCAGAAAACAATC	0	0	0	0	0	1	0	1
		21UR-1502	TTCTGTCTTTTCGGAACTTAA	8	2	2	1	11	15	5	44
		21UR-1503	TACATGTGTTCTTCTACATAA	27	15	9	18	55	76	9	209
		21UR-1504	TTATTGGAACTTTATGTCCCC	0	0	0	0	3	0	1	4
		21UR-1505	TGGATTTATTCATTTTTTTCA	0	0	2	0	0	1	0	3
		21UR-1506	TACCGACGCTCTTCTTTTGAA	0	0	0	0	0	1	3	4
		21UR-1507	TCTGATTGCACAACTTTAATT	1	0	0	0	1	8	0	10
		21UR-1508	TCCACTCCTCTCATGCACATA	0	0	0	0	0	0	2	2
		21UR-1509	TCTACGTGTTTGTTTGTTTGT	0	0	0	0	0	0	0	0
		21UR-1510	TTGAATATTCGTGGTGAAAGT	0	0	0	0	2	0	1	3
*		21UR-1511	TGAATAATAGAAAATGCTGGC	159	353	137	115	381	1075	35	2255
		21UR-1512	TGAGATATCAATCCAATTCTG	0	0	0	1	0	0	0	1
		21UR-1513	TAGGTATCAAGGTATCAATAC	1	0	0	0	0	0	0	1
	†	21UR-1514	TATCATCTTGTTCAACTTAAA	1	1	0	1	2	3	1	9
	†	21UR-1515	TATCAATTTTTCGAACACATT	0	0	0	2	1	3	0	6
		21UR-1516	TGTGGTGCTTGAACTTATCTC	0	0	0	0	2	3	0	5
*	†	21UR-1517	TCGATTCGTATAATGCAGAAA	65	118	66	30	161	469	19	928
	†	21UR-1518	TGGAAACACAACTTTCAGAGC	0	0	0	0	0	1	0	1
	†	21UR-1519	TAGTGGTTTGAGTCGATTTTA	15	2	0	0	6	11	2	36
		21UR-1520	TCATAAGATCTTTCCCCTGCT	0	0	0	0	1	1	1	3
		21UR-1521	TCTTGGTTTTTATATCACTGA	0	0	0	0	0	0	0	0
		21UR-1522	TCCGGTAATATTTCATTTCCA	2	0	1	0	3	1	0	7
	†	21UR-1523	TGTGAAGATTTCTCATAGAAA	0	0	0	0	0	1	0	1
		21UR-1524	TTTAGTGGTCACTTTGGTGTA	0	1	0	0	5	5	3	14
		21UR-1525	TACCGTTGACAACTTAATGGT	0	0	0	0	2	1	1	4
		21UR-1526	TCATGTTTCGAATTATTCACC	2	0	0	0	1	0	0	3
	†	21UR-1527	TTATCCTTCTGTATTACATCC	0	1	0	0	2	3	0	6
		21UR-1528	TGAATCGATTTGTAAAATTAA	0	0	1	0	3	2	0	6
		21UR-1529	TGATAACTCTTGAAGAGGTTT	0	0	0	0	34	23	1	58
		21UR-1530	TCGCTGTGCATTCATCATCAC	1	0	0	0	14	9	1	25

*		21UR-1531	TTTGATGTTGCGTGAAGCATA	0	0	0	1	2	3	0	6
	†	21UR-1532	TCGATTCTCAAGCTCTTGTTC	1	0	0	0	0	0	0	1
	†	21UR-1533	TAATTCCGGAAAAAAACGAAT	1	2	0	4	16	8	2	33
		21UR-1534	TATGATTGGTGATAAATATCC	0	1	0	0	0	1	0	2
		21UR-1535	TCACAATAAATTCACTGAGGG	1	0	2	0	0	0	0	3
		21UR-1536	TACCAAAGAGTCAAAAGAATG	0	0	0	0	0	0	1	1
	†	21UR-1537	TGATTACACAGCCAATCTTGA	3	0	0	0	0	0	4	7
	†	21UR-1538	TGGTACATGATTTTCCAAATA	1	0	0	0	0	0	1	2
	†	21UR-1539	TATTCCACGCTGGGTTAAATA	0	0	0	0	0	0	0	0
		21UR-1540	TCTTATCGAACTTTATTCACC	0	0	0	0	0	2	0	2
		21UR-1541	TAAGAATTAAAAATCCAAATG	0	0	0	0	0	0	0	0
	†	21UR-1542	TGAAATCAAAGTATTGCTAGT	0	0	0	0	0	0	0	0
	†	21UR-1543	TGCTAATAAATGTAAATAGTG	0	0	0	0	0	0	0	0
		21UR-1544	TGACAATGTAGTTTTCCAATA	19	25	17	31	204	195	12	503
*		21UR-1545	TTCGTATGTTGAGTTAATGGC	9	25	8	53	537	478	69	1179
		21UR-1546	TAGCATGTAATCAATACGACA	0	0	0	0	0	0	0	0
*	†	21UR-1547	TACGGTCGATCTTGTTTTAGG	3	1	8	3	22	53	9	99
		21UR-1548	TGAGAACTATTTTTCCGTTCC	0	0	0	0	0	1	2	3
		21UR-1549	TGTATTAATGGCTATATTGAT	0	1	0	0	3	12	2	18
	†	21UR-1550	TGCCACTATTATATATGTTTA	1	0	1	0	0	1	2	5
		21UR-1551	TACCAATTCAAGGTGCGTAGC	3	0	0	0	2	5	3	13
	†	21UR-1552	TGTTGAAGGTTCAAAATCCAC	1	0	0	0	0	2	0	3
		21UR-1553	TTCCAAGACTCATTTAGAAAT	0	0	0	0	0	1	0	1
		21UR-1554	TCTATGGAGATTATAATTTGC	0	0	0	0	0	0	0	0
		21UR-1555	TTATATGATTAGTTCTTGATT	0	0	0	0	6	1	0	7
*		21UR-1556	TACAGAAGTGGACGTATTTAG	35	39	16	20	71	213	41	435
		21UR-1557	TACATATCACTTTCATAACCC	0	0	0	0	3	3	1	7
	†	21UR-1558	TGAATCCAATATTCGGTAAAG	0	0	0	0	3	0	0	3
	†	21UR-1559	TCCAGGGGAAAAGTTGCTGAA	3	4	2	0	12	26	2	49
*		21UR-1560	TACAAAACCCAAAACTTTTGA	4	1	1	1	24	25	3	59
	†	21UR-1561	TTGGATTGTTATTATGATTCC	1	1	0	0	5	9	0	16
		21UR-1562	TTGAAATCACAGTTTGATTGG	15	3	4	0	9	43	1	75
		21UR-1563	TGCGATCAGGTCACCCAGGTC	0	0	0	0	0	1	0	1
		21UR-1564	TCCTGATCCTTTAATATATGC	0	0	0	0	0	0	0	0
		21UR-1565	TATGAAAGTCTCACTTATGAA	0	0	0	0	2	3	2	7
	†	21UR-1566	TGTAAGGTTGGACATCTCTTG	1	1	0	0	3	10	1	16
	†	21UR-1567	TGGGAGATCGATGCTCAAGAG	0	0	0	0	3	12	3	18
		21UR-1568	TGTATCAATATTATTATTAAT	0	0	0	0	0	1	0	1
	†	21UR-1569	TGTTCGCGTCTGTCGATATTC	4	6	4	2	6	23	2	47
		21UR-1570	TAAAAAATGAATGTCCAAAAA	4	0	0	0	1	1	2	8
		21UR-1571	TGATCCCTTGAATATATATTT	0	0	0	0	1	0	0	1
		21UR-1572	TGTGGAGACAATTCTTTCAAA	1	0	1	0	5	2	2	11
	†	21UR-1573	TACTGTTTTATTGAGTACTTT	0	1	1	2	8	6	0	18
		21UR-1574	TGATCTGATATACCAAACTGT	0	0	0	0	1	0	0	1
	†	21UR-1575	TGAAGCACTGATCCGGTTAAA	80	50	11	16	58	195	17	427
	†	21UR-1576	TCAAAAACTCCCTAAAACAAA	0	0	0	0	0	0	0	0
		21UR-1577	TGGTGTTTTCATTCAACAATT	0	1	0	0	0	1	1	3
		21UR-1578	TCAGTTCATTAGAAAGCAGAA	2	0	0	0	25	28	2	57
		21UR-1579	TCCGCTTGCTCTCACCGTAAT	0	0	0	0	0	2	0	2
		21UR-1580	TAACTCTGTCCTTCATTTTAA	0	0	0	0	3	2	1	6
		21UR-1581	TCTTGTTGTAGTATAGATGAA	119	115	54	89	619	666	14	1676
	†	21UR-1582	TGAATTCGTGCTTTGGATAAC	1	3	2	0	0	7	0	13
		21UR-1583	TACCAATTGGTTTCCGATTGA	0	0	0	0	0	0	0	0
		21UR-1584	TAGGCTGTGGAATATCACATG	1	1	1	0	1	2	0	6
*	†	21UR-1585	TGATGGGTAGTTGATTTTGGT	12	35	13	28	662	514	89	1353
		21UR-1586	TACTAGATATTCTATTATACT	1	0	0	0	2	2	0	5
*		21UR-1587	TGCTGATTGAACATTTCAAAA	0	1	0	3	41	31	5	81
		21UR-1588	TCCGAATTCGACTTTCACCAA	3	2	1	2	7	7	0	22
		21UR-1589	TTTGAAATAGATTACTGAGCC	6	5	5	1	7	26	2	52
	†	21UR-1590	TCGTTGTTTTCTTCAGGTAGT	0	0	0	1	0	0	0	1
	†	21UR-1591	TGCGATTGATGACATTGCATG	10	7	3	4	34	102	2	162
		21UR-1592	TGGAAAGATCAAATCTGCCAA	0	0	0	0	0	0	0	0
		21UR-1593	TACAAACTAGAAATTGATTGT	1	1	0	0	1	3	0	6
*	†	21UR-1594	TAACGCTTGTGAACGCATGAT	1	7	2	2	14	29	0	55

*		21UR-1595	TAGAACTTCTCAAAAAATAAC	2	3	2	4	6	12	0	29
	†	21UR-1596	TATTAACCACGATCAGTACTG	0	0	1	0	2	2	4	9
		21UR-1597	TCGACGCTTTAATAACATATT	0	0	0	0	0	0	0	0
*		21UR-1598	TAGATAGGCAGAAATTTGATG	11	16	9	48	565	554	73	1276
		21UR-1599	TAGTCCATTAACTTACAAAAC	4	0	0	0	4	5	0	13
		21UR-1600	TAATACAATACAAGTTTTTCC	0	0	0	0	0	0	0	0
	†	21UR-1601	TGCTAAATGATTGATTGCTAG	0	0	0	0	11	7	1	19
		21UR-1602	TTGTCTTTACCTGTCAGCGCA	1	0	0	0	5	3	1	10
		21UR-1603	TCGACATTTTCAGGTTTTGTA	0	0	0	0	1	2	1	4
*	†	21UR-1604	TATTAGTAGGTCAGCTTTGAA	2	1	1	1	3	13	4	25
	†	21UR-1605	TCGATAGAGATACATAAAAAG	1	1	0	0	5	8	1	16
		21UR-1606	TATGGATGCTACATTTTCTTT	0	0	0	1	19	11	1	32
	†	21UR-1607	TCTATGCGAGATCATCTTAAA	4	0	1	0	1	5	0	11
	†	21UR-1608	TCGCTTACCCAATTCGAAAGT	1	0	0	0	1	0	3	5
		21UR-1609	TAACTGGTATTGCCGTTTGAT	1	0	0	0	0	1	0	2
	†	21UR-1610	TCCATGGTTAATAACGCAAAT	0	0	0	0	0	6	1	7
*	†	21UR-1611	TGAAAGCAGAGGTGGGCGGAT	35	4	2	1	6	48	7	103
		21UR-1612	TCATTTTTAGTCATTTCACGG	12	6	1	2	4	18	2	45
		21UR-1613	TGCGAGTGGCTAACTCAAGCA	1	3	2	3	59	52	6	126
		21UR-1614	TACCGATTGAAATCACATTTC	1	0	0	1	3	1	0	6
		21UR-1615	TAGGGACTATTAGCAATTATC	4	2	0	2	8	18	2	36
		21UR-1616	TGCGTTTATCAGTAGTGAGGT	0	0	0	0	2	5	0	7
		21UR-1617	TAGATTCTGTTATTGAAGAAA	0	0	0	1	1	0	0	2
*		21UR-1618	TTGGCCGTACGTAATATGAAT	9	12	6	4	65	128	18	242
	†	21UR-1619	TTTTCTCTCCTATCGAGTGGT	5	1	0	0	1	3	1	11
		21UR-1620	TACTACGATATTATTTTTTGT	0	1	0	1	3	1	0	6
		21UR-1621	TACGAACTCGAGCGCTTTTTG	0	0	0	0	1	5	1	7
		21UR-1622	TTCAGTTTCTATGAAATCAAT	0	0	0	0	0	0	0	0
*	†	21UR-1623	TATGGAAATGCGGTTATTTGA	84	96	45	50	248	596	86	1205
	†	21UR-1624	TCTATTCCTGACCGGCATTCT	11	8	3	6	41	81	5	155
		21UR-1625	TAGATGTTGATTTTCATTACA	0	0	1	1	0	0	0	2
		21UR-1626	TAGCAAGGCTTCTATCGTTTG	2	0	0	2	0	16	5	25
		21UR-1627	TAACGCTTTCTCAGGGAAAAC	0	0	0	0	0	3	1	4
		21UR-1628	TAAAAATTTGACCTACTGAAA	0	0	0	1	0	0	0	1
	†	21UR-1629	TCATTGGTGCATTGTTAAGAA	11	10	4	2	17	22	4	70
	†	21UR-1630	TCAACCATTTTGAAGATTCGT	2	1	2	5	21	18	22	71
		21UR-1631	TGAAATCAGTTTCAATTATTC	0	0	0	0	0	0	0	0
	†	21UR-1632	TTATAAATAATGGTGTAGATC	11	3	3	4	8	19	1	49
		21UR-1633	TATAGCAATTTTTCCTGTTCC	0	0	0	0	0	1	0	1
*	†	21UR-1634	TCAGAAAAGGTGTTGTACAAC	4	3	0	5	110	106	6	234
		21UR-1635	TCTGCAGGAGTATCGATTGAA	2	2	1	0	15	17	8	45
		21UR-1636	TTATATGTGTTTTCACAGTGT	0	1	0	0	0	3	0	4
	†	21UR-1637	TGTGTAAAGCGTGTACTTCAA	9	4	3	1	6	25	3	51
		21UR-1638	TACACTTCGACATCTTCGAGT	1	0	0	0	3	0	3	7
	†	21UR-1639	TGCTTGTATGTATACTTTGTG	0	2	0	6	67	44	7	126
	†	21UR-1640	TCTTACTGAGATATGTATTCG	0	0	0	0	1	0	0	1
	†	21UR-1641	TAGTATATTGTATATTGATGA	1	0	1	0	3	3	0	8
*		21UR-1642	TGTAGTAGCATCTTATTTCAA	13	9	5	8	21	39	3	98
	†	21UR-1643	TGAAAGTGCTGTTGGGCTGAA	0	0	0	0	16	22	14	52
		21UR-1644	TATAAATTGGAGAAAATTGTC	1	2	0	0	16	4	4	27
	†	21UR-1645	TGTTAGCATAGATTAATGATT	0	1	2	1	2	6	0	12
	†	21UR-1646	TTTCTTGACAAATACAGCAAC	0	0	0	0	0	0	2	2
		21UR-1647	TAGAAGGATTGTTCGCGTAAG	2	1	2	5	154	152	66	382
	†	21UR-1648	TTCAATCCTAATTCGATATAC	1	0	0	0	4	0	1	6
*	†	21UR-1649	TAGACGATTGGTTCAATTTGC	1	3	1	0	5	13	1	24
		21UR-1650	TCCCATCACTTCCCCCTCAAC	0	0	0	0	0	1	1	2
	†	21UR-1651	TAGTGCTTTGATCAATTCCCG	0	0	0	0	0	0	0	0
	†	21UR-1652	TGGCAATATGTACAAACAATG	51	79	12	29	195	336	28	730
		21UR-1653	TAGTCGAAAAAAATTGCATTT	4	1	0	0	0	2	0	7
	†	21UR-1654	TCAGTACAACTTAGGGTTGAA	0	0	0	0	3	1	0	4
		21UR-1655	TACTCCAAAAGAATGTACAAA	0	0	0	0	0	0	1	1
		21UR-1656	TCTGAACATTACATTATTTTC	0	1	0	0	2	1	1	5
		21UR-1657	TAAGCGCCCTTTCTTTGGTGA	0	0	0	0	2	0	1	3
	†	21UR-1658	TCCGTTGAACATTTGTCAAAG	0	0	0	0	1	0	0	1

	†	21UR-1659	TAACGGCATTTAGTTTGTGCA	0	0	0	0	0	0	0	0
	†	21UR-1660	TATCACAGACTTAGCAGTACT	1	3	0	4	100	67	4	179
		21UR-1661	TAGAGTATTATTTCAATATAA	0	0	0	0	0	1	0	1
		21UR-1662	TACATGAACTACATACTTTAC	1	1	0	2	56	18	4	82
		21UR-1663	TTCGTCATTTTATCAGAAGCA	1	1	1	1	4	7	0	15
	†	21UR-1664	TTCTACTGTTACAGTGAGATA	0	0	0	0	0	0	0	0
		21UR-1665	TTGTGAAATGCTAAGAATTCA	1	3	0	2	1	8	0	15
		21UR-1666	TACGACAATCTCGCTATTTTC	0	0	0	0	0	1	0	1
		21UR-1667	TTATGATGAGATGATTTTGGG	2	1	0	2	24	37	14	80
*		21UR-1668	TAAAGTCGTGATGACAAATGA	11	4	3	1	7	24	2	52
	†	21UR-1669	TATTTTTTCTTCAATATCATC	0	0	0	0	0	0	0	0
		21UR-1670	TAAAATACGTTGGTTGAATAA	14	1	1	2	10	19	3	50
		21UR-1671	TTGAATCCACACGTGTTTTCA	7	0	0	0	3	16	3	29
		21UR-1672	TAATTCTTCAATGATTGATTA	0	0	0	1	0	0	0	1
	†	21UR-1673	TTCACAGGAGATTTTGCTCAC	1	0	0	0	14	27	1	43
	†	21UR-1674	TAAGGAAGTTATATGTATATC	1	0	0	1	15	12	3	32
	†	21UR-1675	TAATGTTTGTTCAGCTTTTAA	4	1	0	2	3	6	0	16
	†	21UR-1676	TGTTTGCAGCATTCGGACAGA	75	61	19	18	54	369	44	640
		21UR-1677	TGAGTTCTACATTGTATGAAA	0	0	0	0	2	0	0	2
		21UR-1678	TACAAGGACTGCTAGTTTTGA	8	10	3	4	13	69	6	113
		21UR-1679	TAAACATAACTTCAGTTCTTT	0	0	0	0	0	0	1	1
	†	21UR-1680	TACTGAATGTTTCAATACCTC	2	0	0	0	0	4	0	6
		21UR-1681	TGTGCATGAATTGATCTGAAA	1	1	0	0	16	17	0	35
		21UR-1682	TCTTGAGTACTATCTATTTAA	0	0	0	0	0	0	0	0
*		21UR-1683	TCCAAGAGTCAATGATTCGTT	2	0	0	1	0	2	1	6
*	†	21UR-1684	TGTTTCAACTCTACGGTGCCT	4	23	5	13	32	151	16	244
		21UR-1685	TATTGATCTAATCGTGGCCGC	0	0	0	0	0	1	1	2
		21UR-1686	TATGATTTCGTCATTTAAGCC	8	1	1	1	3	20	1	35
	†	21UR-1687	TGGTTCAATCGTTGCTGAATG	3	2	0	0	5	2	3	15
		21UR-1688	TCAAATTTATTTTGCAGTAGG	1	0	1	0	2	1	2	7
		21UR-1689	TCCGGAATTCTTTCTGTATTT	1	0	0	0	1	0	0	2
		21UR-1690	TAGGTAGAAACATTTCTTTCG	2	1	2	1	19	27	1	53
	†	21UR-1691	TAGGAAACTCGGCTCCAGCGA	3	2	1	2	6	23	1	38
		21UR-1692	TATGTGTTTTGTCAAATTGTC	0	0	0	0	4	1	4	9
	†	21UR-1693	TCATTCGATTGTTTGCCTCTA	0	0	0	0	0	0	0	0
*	†	21UR-1694	TATTGACTATGAGATCTGAAC	0	0	0	1	11	18	0	30
		21UR-1695	TAAAATTCCTAAACTTTAAAC	0	0	0	0	0	0	0	0
	†	21UR-1696	TTAGCAACTTTTCATCATGAA	2	0	0	0	0	1	0	3
	†	21UR-1697	TTCAGTAAGATTTGTCACTCG	1	4	2	2	55	46	23	133
		21UR-1698	TGTACTCATTCCGCAATTTTC	0	0	0	0	0	0	0	0
	†	21UR-1699	TCGTCAGCTTCTATAAATAAA	0	0	0	0	1	0	0	1
		21UR-1700	TATGCAGCGAATATTTATTAC	2	0	0	0	1	1	0	4
		21UR-1701	TTAATCAATCAATTTACTATA	0	0	0	0	0	0	1	1
*	†	21UR-1702	TGGGTTGTCGATATTCGCAGG	1	0	0	2	15	12	3	33
		21UR-1703	TCTGATTTTTTTCTCGTTTCT	0	0	0	0	0	0	0	0
		21UR-1704	TAAAATACTATTTGATGTGTC	0	0	0	0	1	0	1	2
		21UR-1705	TATATGAACAATTTACGCTGC	1	1	0	2	0	3	0	7
	†	21UR-1706	TCTCATGCGATAGAATTAGAC	4	6	1	1	8	17	0	37
		21UR-1707	TCAAATCCTTTTTTTGTCACT	1	0	0	0	1	0	0	2
	†	21UR-1708	TGATGTGATTATTTCCTGGAA	0	2	0	0	1	1	0	4
	†	21UR-1709	TGGAATCCAACGTATCTAAAT	0	0	0	0	0	0	0	0
	†	21UR-1710	TCACTTTGGAATAGCCGCATG	7	6	6	17	169	156	57	418
		21UR-1711	TATTCAGTTGTTCTTTTTAAT	1	0	0	0	2	5	0	8
*	†	21UR-1712	TATCCGCGTCTGTCACATGCG	2	0	0	0	0	0	2	4
		21UR-1713	TATGATATTTCCTCGACCGTT	2	0	0	0	0	0	0	2
		21UR-1714	TGGGCTTCTTGAAAACTGTAC	0	0	0	1	13	9	1	24
		21UR-1715	TAAGACCTAAAAATTCATAAA	0	0	0	0	0	4	0	4
	†	21UR-1716	TTGCTCTGCCGCAATTAAAGC	0	0	0	0	1	0	0	1
	†	21UR-1717	TGTTCTTGGAATATTGTTAAC	0	0	0	1	7	4	2	14
	†	21UR-1718	TGGGCGATGTGTGATCAATTC	0	0	0	0	6	7	6	19
*	†	21UR-1719	TACTTTTGTCGTGAAAATATG	16	4	0	2	12	34	14	82
	†	21UR-1720	TATTGGTATCCATGTGTTAAA	0	0	0	0	1	1	1	3
		21UR-1721	TGTTTGCCTTGTAATTTTAAA	0	0	0	0	0	0	0	0
		21UR-1722	TTAGTCTCACTCTTTTTCCCA	0	0	0	0	0	0	0	0

	21UR-1723	TTCTGTAGAGAAAAACAAAAA	8	3	2	5	4	28	0	50
	21UR-1724	TGTGACTTCTTCATTTGTTGT	0	0	0	0	0	0	0	0
	21UR-1725	TACTGCGCAAAAATTGAAACA	1	0	0	0	0	0	0	1
	21UR-1726	TGAATTTGCGAAATGAGTGAA	0	0	0	0	0	3	1	4
†	21UR-1727	TAAGTCCTGTAACTGAAACCA	1	0	1	0	1	7	0	10
†	21UR-1728	TGTGTTCCGCGGTTTGAGGTT	2	0	1	0	0	1	0	4
	21UR-1729	TGTTGTTGTTACTTCGCAAAT	0	0	0	0	0	0	0	0
†	21UR-1730	TGGAATGGCGAAAATACATTA	5	4	0	3	20	21	9	62
†	21UR-1731	TTCCTGTTTTGAATCGGCTGC	0	0	1	0	0	4	0	5
†	21UR-1732	TATTCCTAGTGTCCCCACAAC	0	0	0	0	0	2	1	3
	21UR-1733	TCTAAATGAGAGTCTTCATTA	0	1	0	0	0	0	1	2
	21UR-1734	TACATGTTCTCCATTTTATCG	6	1	0	2	5	16	4	34
†	21UR-1735	TGATGTTAATTGTCCCATTAA	0	0	0	0	0	0	0	0
†	21UR-1736	TATCGAAAGATCTAAAGGTAA	13	1	0	2	15	23	10	64
	21UR-1737	TGACTTTTTTAGCCTTTTATT	0	0	0	1	0	0	0	1
	21UR-1738	TACACTTGTAAGGTAATCAAA	11	0	1	1	2	3	1	19
†	21UR-1739	TAGGATTCCAGGCGATCTGAT	0	1	0	1	22	18	3	45
	21UR-1740	TGGAAAATATTTGTGGATATA	5	3	2	1	5	11	3	30
†	21UR-1741	TGGGCTTATATAAAAATTCTA	0	0	0	0	0	0	0	0
†	21UR-1742	TGGTTTCTCCGAATGCATCTA	0	0	0	0	0	0	0	0
	21UR-1743	TAGTTAATATTTTCAGACCAC	0	0	0	0	0	0	0	0
†	21UR-1744	TTGTAGTGAGTACTATTGAAG	0	0	0	0	0	4	0	4
†	21UR-1745	TTACTCCGTGATTGCCACCAG	0	0	0	0	0	0	0	0
* †	21UR-1746	TTCCAGACACACAGAAGGAAG	16	4	6	3	21	34	6	90
†	21UR-1747	TGGTTCCTGCTATTAGAACCC	0	0	0	0	0	0	0	0
	21UR-1748	TGTCGAAAAACTTTTCGGGAA	1	0	0	0	0	0	1	2
*	21UR-1749	TACATGCCATCGACGGCCATC	12	1	2	0	11	33	8	67
	21UR-1750	TGGTTTGCATATATATCTTAC	1	0	0	1	15	11	8	36
	21UR-1751	TTCTGCTTCTGAGTTCATTAA	0	0	0	0	3	3	0	6
	21UR-1752	TGAAGTGTGTTTCGATAATAC	0	0	0	0	0	0	0	0
†	21UR-1753	TAGTCCCTTGCCTGTATTCTT	0	0	0	1	1	0	0	2
	21UR-1754	TATAGCCAGCGATGGAGGAAA	0	0	0	0	0	1	0	1
	21UR-1755	TGTTTCAGAATTACATGAATA	0	0	0	0	0	0	0	0
†	21UR-1756	TGTTACACCGGAGCACTAAGG	0	0	0	0	1	0	0	1
†	21UR-1757	TTAAGAAAGGAAACGCAACCT	1	0	0	2	12	30	2	47
	21UR-1758	TACAGCAACCTGCCAAGTCGT	11	7	2	0	2	24	14	60
†	21UR-1759	TCTGTCTTTAGGTTGTATTCC	1	0	0	0	0	1	0	2
	21UR-1760	TAAGAAAATACTCCTTCATCT	0	0	0	0	2	4	0	6
†	21UR-1761	TGATGATGTGAAACACTCAAA	2	4	1	0	10	7	2	26
†	21UR-1762	TGCAGTCGTTGAATGGTGACT	13	4	3	3	5	30	0	58
†	21UR-1763	TGATTCACTCGTTTGAAGAGA	0	0	0	0	1	1	0	2
	21UR-1764	TCCCATTAAAGCTTGTTGAGC	1	0	0	0	1	2	0	4
	21UR-1765	TTATAAATCGCATTTTATTTT	1	0	0	0	0	0	0	1
	21UR-1766	TGGTTGAGTATTCCTAACTGC	0	0	0	0	0	1	0	1
	21UR-1767	TCACTACTTCAATTGTTGTAA	0	0	0	0	1	0	0	1
	21UR-1768	TCCTGGAGATTTAATTATCAC	0	0	0	0	4	3	0	7
	21UR-1769	TACGTGATTGCCACAGGGAAC	0	0	0	0	8	8	5	21
	21UR-1770	TGTAGACTTTGCCATGGGAAA	4	2	1	1	9	17	8	42
	21UR-1771	TACAATGTTAAAAAATTGAAA	1	1	1	0	0	4	0	7
†	21UR-1772	TGAAATTGGGTTGTATCCAAA	0	0	0	0	0	1	0	1
	21UR-1773	TAACCTAAATTTGGTGTCACC	1	0	1	0	0	0	0	2
	21UR-1774	TAGCCAACACACTATGAATCA	0	0	0	0	0	0	0	0
†	21UR-1775	TCAATACATTTGTTGTGAAAA	0	0	0	0	0	0	0	0
†	21UR-1776	TGGTGATAGGTTTTTTGAGTT	1	1	0	1	1	5	0	9
	21UR-1777	TGCTTGCCTGAAACAGTTTTA	0	0	0	0	0	0	0	0
	21UR-1778	TCTTGAAATTTTACTGTTTAG	0	0	0	0	0	1	0	1
	21UR-1779	TGGATTGAAGATTTTCTATAA	1	5	2	3	2	15	0	28
	21UR-1780	TCTTTTCTAATAATGACGTCA	17	8	6	4	10	35	1	81
	21UR-1781	TAAACATTTTTGAATCCAAAA	0	0	0	0	0	0	0	0
	21UR-1782	TAGTTTATTAAAGTATTGTGC	0	0	0	0	0	1	0	1
	21UR-1783	TTTTGTTTTAATTCGCCTTTA	0	0	0	1	0	2	0	3
†	21UR-1784	TGGACGGTTTAAATTAACTTC	22	20	7	7	35	94	6	191
	21UR-1785	TCAGGAAATAAAATTATATTC	0	0	0	0	0	0	0	0
	21UR-1786	TGTGAATTACAGTAAAGCTGC	0	0	0	0	4	6	2	12

		21UR-1787	TACAATAGCACGAATACGATT	14	4	7	9	57	67	21	179
		21UR-1788	TATTAACAAACAATTTGACTT	0	0	0	0	0	0	0	0
		21UR-1789	TAACATGGTTATTTCTAAAGA	1	0	0	0	2	0	0	3
*		21UR-1790	TACGCTGGTAGAAAGAAAAAA	62	63	39	43	139	710	33	1089
	†	21UR-1791	TGATGCCCTTCGTTGTCATGG	2	0	0	0	1	1	0	4
		21UR-1792	TTCAATCCACTTTTTCCATTC	0	0	0	0	0	0	0	0
*		21UR-1793	TACAACGTAGAGTACTGAACT	1	4	2	0	19	40	3	69
*		21UR-1794	TAATGAACTGACAAAATTTGT	0	0	0	2	1	2	0	5
		21UR-1795	TTATAATTTTAGTTATGAATA	0	0	0	0	1	0	0	1
		21UR-1796	TGTCATTGGATTAGACGCTAA	11	12	2	11	199	109	15	359
	†	21UR-1797	TATTTTGTGTGTTTTTGAAGA	0	0	1	2	5	9	0	17
	†	21UR-1798	TAGGAAACCGATTCCCTAAAC	0	0	0	0	0	0	0	0
		21UR-1799	TCCATTCTTGGTGATAACTTC	0	0	0	0	0	0	0	0
*		21UR-1800	TTTTGCAGTTGCTGGAAATGG	43	11	6	7	18	229	3	317
	†	21UR-1801	TGATGATGCCTTGGTACTTCT	0	0	0	0	1	0	3	4
		21UR-1802	TCGAGAACGATTTTTGTGGTT	0	0	0	0	0	0	0	0
		21UR-1803	TCAAAAACATGATAATTGAAG	0	0	0	0	0	0	0	0
		21UR-1804	TCGAGATGTAGTTTTATATTC	0	0	0	0	11	8	1	20
		21UR-1805	TGTTTTGAAACCTTTAATCAC	0	0	0	0	0	0	0	0
	†	21UR-1806	TCTTAGTACTGTGCTTTGTGG	0	0	0	0	1	1	0	2
		21UR-1807	TACTCCTGATTTCAAAAAAGA	0	0	0	0	0	0	0	0
		21UR-1808	TGAGAATGGCCAACATTTTAT	12	1	0	0	0	6	1	20
	†	21UR-1809	TGTATCTTGGATTCTATCGGA	0	0	1	2	20	22	21	66
		21UR-1810	TCTGTAATCGATTTCAGATTG	0	0	0	0	0	0	0	0
		21UR-1811	TGTTGTAATCAAAATAATCCT	0	0	0	0	0	0	0	0
		21UR-1812	TCAAATGAAGCATTATTAAAA	2	0	0	0	0	10	1	13
		21UR-1813	TACATGAATTATCAGTATGTG	0	0	0	0	0	0	0	0
	†	21UR-1814	TAATACACTTGATCGTGTTTT	0	0	0	0	0	1	0	1
		21UR-1815	TGCTCTTCTAATGCTATCAAA	0	0	0	0	0	1	0	1
	†	21UR-1816	TGATTCGAAGGTTGTCAGATC	6	5	1	2	13	30	13	70
		21UR-1817	TTCCTTTGCACATTTGACAAA	1	0	0	0	0	0	0	1
		21UR-1818	TGACATACAGTTAGAGTATCA	0	0	0	0	9	5	2	16
		21UR-1819	TGAATCTTCAATCTTCAAAAG	0	0	0	0	0	0	0	0
		21UR-1820	TTGGTTTTCGGGGATAATTGC	0	0	0	0	3	4	4	11
*		21UR-1821	TTTATCCACACGGCTATCAGT	56	6	5	6	6	41	6	126
		21UR-1822	TGCTATAGACGTTGTACCACC	7	6	5	25	373	306	45	767
		21UR-1823	TATTCACTCGTGAGCTTCACT	0	0	0	0	0	0	0	0
*	†	21UR-1824	TTGTGAGGACAGTGGCATGGG	1	3	0	0	40	39	38	121
*	†	21UR-1825	TATTGTTTGTGGAAGCGTGAG	303	408	210	145	839	2873	297	5075
	†	21UR-1826	TGCAATAATCACGCTGCGATT	0	0	0	0	0	0	0	0
		21UR-1827	TATGTCATCAAAAAATAATTA	0	0	0	1	1	5	0	7
		21UR-1828	TGCAATTGGCGACTTTCTTGT	1	0	1	0	5	7	4	18
		21UR-1829	TCCATGTTCCTTTTTACACAT	0	0	0	0	0	2	1	3
	†	21UR-1830	TAAAAAATCCAACTGAAACCA	0	0	0	0	0	0	0	0
	†	21UR-1831	TTAAGTCCTGTAACTGAAACC	0	0	0	0	0	0	0	0
*		21UR-1832	TTTAACAAATGACGGTAAATC	70	11	7	9	153	191	50	491
*		21UR-1833	TGTGAAGCGAGATTGTTCAAC	12	14	0	41	626	745	146	1584
		21UR-1834	TGATAGCGTTGAGTTTATTAA	0	0	0	0	0	1	0	1
		21UR-1835	TAGACTTCGATTAATGTCCTC	0	0	0	0	0	0	0	0
	†	21UR-1836	TTGCGTTTCCAATTCTGAGGA	1	1	1	0	21	21	4	49
		21UR-1837	TCTATGTCTTGTTTATTGTTG	0	0	1	0	1	0	0	2
*	†	21UR-1838	TTGTTCTTCGTTCGGTCCAAA	659	261	238	141	298	1262	241	3100
		21UR-1839	TCGCACATTGATTTTCACTGG	0	0	0	0	0	0	0	0
		21UR-1840	TATCACAATTTTACGAATGAT	0	0	0	0	0	0	0	0
		21UR-1841	TATACATATTTGCTGTGTTTG	0	0	0	0	0	1	1	2
		21UR-1842	TGTTAGAATTTATCCAGAGTC	0	0	0	0	3	1	0	4
	†	21UR-1843	TGGGATGTTCAGCTCATATAC	0	0	0	0	0	1	0	1
		21UR-1844	TTCCGTTTTCTTTTTTGGAGT	2	0	0	0	0	0	0	2
	†	21UR-1845	TGTTGATGATGTGAAACACTC	0	0	0	0	1	1	0	2
	†	21UR-1846	TTCAATTGTTTAATTCAGGCT	0	0	0	0	23	8	1	32
		21UR-1847	TGGATTGGCCAATTGCGGACA	21	5	5	3	6	30	5	75
*	†	21UR-1848	TAAAGGCAGAATTTTATCAAC	146	200	94	86	150	556	42	1274
		21UR-1849	TGCCATTCAGCAAGTTTTTGT	2	0	0	0	1	2	0	5
*		21UR-1850	TGCGGTAGATAAAGAAGTACT	12	9	12	23	328	190	19	593

	†	21UR-1851	TTCGTCATTTGTATAAGACAC	1	2	0	1	4	2	0	10
		21UR-1852	TGTGTTTTCTCCTCCTAGGCA	0	0	0	0	2	5	12	19
	†	21UR-1853	TAATGAAATAAATTGGTCAAG	0	0	0	0	0	0	0	0
		21UR-1854	TATCATCGCTTTTATGACAAG	1	0	0	0	9	11	4	25
	†	21UR-1855	TTGCGAATCAGGTGTTTTCGA	0	0	0	0	0	1	0	1
		21UR-1856	TATAATATTTTCTTGTTCCTA	0	0	0	0	0	0	1	1
	†	21UR-1857	TCTGATGAAGATAACTAAAAA	0	0	0	0	1	7	0	8
		21UR-1858	TGTTGTGTTGTATCGAGTTGT	0	0	0	0	2	2	1	5
		21UR-1859	TAATTTCCCATTTTTTAGATA	1	0	0	0	1	2	0	4
		21UR-1860	TTCTATGTCGACATTGTCAGC	0	0	0	0	2	3	4	9
*	†	21UR-1861	TGCGTTGAAGACTACTCCTGA	3	6	3	9	163	222	28	434
		21UR-1862	TACACAATTAACGAGAATCAC	0	0	0	0	10	15	2	27
	†	21UR-1863	TAATACATCCTTCTAATCCAA	12	3	0	0	1	6	3	25
		21UR-1864	TATAGCAAGATGAATAATCAT	0	0	0	1	12	7	1	21
	†	21UR-1865	TAAAAATCGAAATATGGGAAA	11	4	2	0	5	28	8	58
		21UR-1866	TGTGTCAAATGTCCTATCAAA	0	0	0	0	0	0	0	0
		21UR-1867	TATGATAAATCCAAAACGTTT	0	0	0	0	0	0	2	2
		21UR-1868	TACAGGCTGATAACTCGCAGG	1	2	0	1	10	18	1	33
	†	21UR-1869	TCGAACACAATTTGCCAAAAA	0	0	0	0	0	0	0	0
		21UR-1870	TCGTGGCTATTTGCAAATACC	0	0	0	0	0	0	0	0
	†	21UR-1871	TCGAAAGAGTTGGTTCTGAAG	8	1	2	1	5	35	2	54
		21UR-1872	TTTACGATGGTAATAATTGTT	1	0	0	1	1	2	0	5
	†	21UR-1873	TGCTCGGGGTTTTTTGTATCT	0	0	0	0	0	0	0	0
*	†	21UR-1874	TCAATGTACTCGTTGGTTTCT	76	38	32	27	53	223	11	460
		21UR-1875	TAACTAGTTGGTCGCTTAAGT	0	0	1	1	3	3	4	12
*		21UR-1876	TATGGAGTCGGTATATTTTGA	74	50	29	19	55	266	37	530
	†	21UR-1877	TATTTCTAATCTTTGTGAATC	0	0	0	0	3	1	0	4
		21UR-1878	TAGAATGCTGAATTCACTATT	2	0	0	0	0	0	0	2
		21UR-1879	TCGGTGATTTGGTTATTGATT	0	0	0	0	0	0	0	0
	†	21UR-1880	TGGTTCTTCAGCCTGTGGAGT	0	0	0	0	1	0	0	1
	†	21UR-1881	TTTGATTGCTTCTTGCCTTCA	1	1	0	1	18	18	6	45
*	†	21UR-1882	TTACAGTATTGGTAAGGTGGA	19	1	5	2	4	36	4	71
		21UR-1883	TACAGTTCTGTTTAAAGCTGT	0	0	2	2	25	14	3	46
*	†	21UR-1884	TGCATTGAAACGAACAACTAC	2	3	2	1	25	19	0	52
		21UR-1885	TCATCCTTTCGTGTTCAATTG	1	0	0	0	0	0	0	1
		21UR-1886	TAGTAATTTTTCCTATGCTCC	1	0	0	0	0	2	0	3
	†	21UR-1887	TGATTTATTCTACAAGGACAC	0	0	0	0	0	0	0	0
		21UR-1888	TCAAAGCAAAACGTTTTCTGG	1	3	0	4	48	41	0	97
		21UR-1889	TAATAATAAATTTCACCCAAA	11	0	0	0	0	0	1	12
		21UR-1890	TATCATTACATGTTCAGAATC	0	0	0	0	1	1	0	2
		21UR-1891	TCAACATTTCTCTGTTTCAGC	0	0	0	0	0	0	0	0
*	†	21UR-1892	TATATAATTAAAGAACATTGA	1	2	1	0	21	16	1	42
		21UR-1893	TCACCGCTTTCAGAATATATA	14	1	1	1	5	10	0	32
	†	21UR-1894	TAATAAAGTACTATTGCCGTT	1	0	0	1	2	4	4	12
*	†	21UR-1895	TAGAAAGTTTGGACAATTTTT	0	0	0	0	9	2	2	13
	†	21UR-1896	TACTCCTAAGATTTTTGTGTT	12	3	1	2	4	6	1	29
		21UR-1897	TAATAAAACTGCGTGGTGTGT	0	0	0	0	0	0	0	0
		21UR-1898	TGAACTTCTTTCCTAGTATCC	0	0	0	0	0	0	0	0
		21UR-1899	TGACAACTTGTTACTTATAGT	0	0	0	0	0	0	0	0
		21UR-1900	TCCCTTACCATATTGACACTT	0	0	0	0	0	0	0	0
		21UR-1901	TGAAACAACAGTTAAATACTT	0	0	1	0	0	0	0	1
	†	21UR-1902	TCTTTTCGATCACGGCTGACA	1	1	0	2	7	8	4	23
		21UR-1903	TTGGATCCATTTAAAAGAATA	0	0	0	2	0	4	0	6
	†	21UR-1904	TATTCATGTTGTATTTATCTT	1	0	1	1	0	0	0	3
		21UR-1905	TAGGTAGCGGTTCCTATCTTT	0	0	0	0	0	1	0	1
		21UR-1906	TAGGTTCCGAACTTTCCGTTT	0	0	0	0	0	3	0	3
	†	21UR-1907	TTGGTGCATGTTGATTTTTTG	0	0	1	1	2	1	0	5
		21UR-1908	TACAACCCACGACAGGCTTGT	0	0	0	0	0	1	3	4
		21UR-1909	TCCGGAAATATTGCTAAAAAA	2	8	1	1	10	25	1	48
		21UR-1910	TAGTTCTCACTCACTCTTTGG	0	0	0	0	0	1	0	1
	†	21UR-1911	TTAACGTCTGAATCATGCGAA	12	9	16	10	42	111	2	202
		21UR-1912	TACCTCGCTGTTCGGAAAACA	2	2	1	6	114	79	31	235
	†	21UR-1913	TCCAGCCATATTTAAATCAAT	1	0	0	0	2	1	3	7
		21UR-1914	TAAAGCCGACAGATTTCGAAC	1	0	0	1	1	0	0	3

	†	21UR-1915	TATTGATTTGATTACAACAAT	0	0	0	0	0	1	0	1
		21UR-1916	TCAAGAATACATTTAAACTGG	0	0	0	0	1	0	0	1
	†	21UR-1917	TTCGCCGAAATATTTGAATCC	0	0	0	0	0	0	0	0
		21UR-1918	TACCGTAAGCAAATGTAAAAA	3	0	0	0	1	6	2	12
*		21UR-1919	TAGGGGCATGGTTTATTGAAC	47	25	4	4	28	207	24	339
		21UR-1920	TTCCGTTTGAATATACTCAAC	0	0	0	0	1	2	0	3
		21UR-1921	TGCATCCTTTATTTTTTATAC	0	1	0	1	4	3	4	13
	†	21UR-1922	TCCGCCTTTTGCTAATATAGC	0	0	0	0	0	0	0	0
	†	21UR-1923	TCACGTAAAGTTTTTTTTAAC	0	0	0	0	0	0	0	0
	†	21UR-1924	TACTTTGGCAAACCTAATACA	1	0	0	1	6	8	2	18
	†	21UR-1925	TTGTTTGTACAAGTTTTTGAC	0	0	0	0	0	0	0	0
		21UR-1926	TCTCATCTTTATTGGAAATTT	0	0	0	0	0	0	0	0
		21UR-1927	TAAAACATTAAAACTTTGCAA	0	0	1	0	2	3	0	6
		21UR-1928	TGATGTTCTCGCATTTATGTT	0	0	0	0	0	0	0	0
		21UR-1929	TCTGTATCACTGGGAAGTTCA	5	4	2	3	11	52	17	94
		21UR-1930	TAGGAAAACTTATAGGAAAAA	0	2	0	1	3	3	0	9
		21UR-1931	TCAAGCTACCAATCCTTGTAA	0	0	0	0	0	0	0	0
	†	21UR-1932	TTCAAGAATTCTCCGACGTAC	1	1	0	2	6	18	2	30
		21UR-1933	TCGGGAAATTGATATTTACCG	0	0	0	0	5	1	0	6
	†	21UR-1934	TACGAGGCACGAATTGTTGTT	3	3	1	0	5	12	0	24
	†	21UR-1935	TTTAATGTCTCTTTGTTTGAG	0	0	0	0	0	0	0	0
		21UR-1936	TCCGATTAGAAGGAAACCAAA	0	0	0	0	0	2	0	2
		21UR-1937	TCGAATCAGTTAAAATGTTTG	0	0	0	0	0	0	0	0
		21UR-1938	TGTAAATCCAAATTTTTATAG	0	0	0	0	0	0	0	0
	†	21UR-1939	TCAGCAACTGAAATTTTCGAA	5	2	1	6	26	43	3	86
		21UR-1940	TTTGGTAGATCAGATATAACA	6	4	1	4	8	18	4	45
*		21UR-1941	TATGAACTTCGTAAGAAAATA	0	0	0	1	5	4	0	10
		21UR-1942	TACATTGATTTGTTTTTTCTT	4	0	0	1	2	0	0	7
*		21UR-1943	TCAAGTCAGACAAATGTGCTT	39	79	56	102	1054	1236	68	2634
	†	21UR-1944	TTCGACAAGATTTTTCTGCTG	7	9	3	18	134	159	18	348
	†	21UR-1945	TACATATGAATATTAGCGTTT	9	0	0	1	7	8	1	26
		21UR-1946	TGAGGATGGTATTTAATATTA	33	36	21	25	108	185	16	424
	†	21UR-1947	TCAATTAGTGTTGAAGGTACA	4	0	0	0	3	9	0	16
		21UR-1948	TACTACTTTTATTTCAATTAT	0	0	0	0	2	1	0	3
*	†	21UR-1949	TACGAGGGTGACAAAGAATGG	97	70	36	25	127	601	121	1077
	†	21UR-1950	TCGAAGGTTTTTTTTTGGGAT	1	0	0	0	1	1	0	3
	†	21UR-1951	TTACATTCAGAATATCCATAA	52	15	7	14	26	45	2	161
	†	21UR-1952	TAACATTGGTGCAATCATTGT	15	0	2	0	3	4	3	27
		21UR-1953	TCCTTTGTATACTTCATCTTC	0	0	0	1	6	2	0	9
		21UR-1954	TCAGTCTTTGAACGCAGCAAT	0	0	0	0	0	5	0	5
		21UR-1955	TCTACGTCTTTCTTCATTTAC	0	0	0	0	0	0	0	0
*	†	21UR-1956	TTGGTTCCTGCTATTAGAACC	15	11	14	12	138	113	8	311
		21UR-1957	TATGGTGTTTCCTCTATTACA	0	0	0	1	2	5	2	10
		21UR-1958	TACCACGATTTTTATCACAAA	1	0	0	0	0	5	1	7
		21UR-1959	TATTGTATTCATGGAACTATA	0	0	0	0	12	1	2	15
*		21UR-1960	TGGAAAAATCCTGCATTGTAA	9	4	5	2	7	24	7	58
	†	21UR-1961	TGTGCATCCGTTTGGCAATAG	12	7	4	3	90	109	170	395
	†	21UR-1962	TAGTCGTATGTCGTATGGAAT	2	1	0	5	69	46	23	146
		21UR-1963	TGCGAACAAGAAAATGTATTA	1	0	0	1	12	5	3	22
	†	21UR-1964	TGATTTATGATTTTTGGGTGT	0	0	0	1	9	7	4	21
		21UR-1965	TAAATGATCACTAATTTTCCT	0	0	0	1	0	2	0	3
	†	21UR-1966	TTCCAGTGCGTTTGACATCTT	1	2	0	3	36	20	10	72
	†	21UR-1967	TATCTGTTTTATCCTGTAAAA	0	0	1	0	7	3	1	12
		21UR-1968	TCACTCATCAAGATTTTTCTC	0	0	0	1	0	1	0	2
	†	21UR-1969	TGCTTTGTTGATGTTCTGAAC	1	0	0	4	23	27	2	57
		21UR-1970	TCGCACTGCTCCTTTAATTGT	0	0	0	0	1	0	0	1
		21UR-1971	TACAGTTTCAATCGCATTTCA	1	1	1	2	10	53	1	69
	†	21UR-1972	TTCTTCCATTATATCAACGGT	46	0	3	1	3	13	1	67
		21UR-1973	TCGAATTATCACGTGTATTCA	1	0	1	0	3	1	0	6
		21UR-1974	TGCTTTTACGTTTTTTTGATA	3	0	1	0	0	0	0	4
	†	21UR-1975	TGTTGATTTTCTTTACGTAGC	0	0	0	0	1	1	0	2
	†	21UR-1976	TTCAATGTTTCAATGTTGGAA	0	0	0	1	4	4	0	9
		21UR-1977	TTGTCAACTTCAATTAAAGAC	2	0	0	0	0	0	0	2
		21UR-1978	TGCCAAAACTTCAGCTTTGGT	0	0	0	0	0	1	0	1

		21UR-1979	TACTCCTCAAATTGGCAACAC	9	0	1	0	0	5	1	16
		21UR-1980	TGTTTCACATTACTGTTATCA	0	0	0	0	0	0	0	0
		21UR-1981	TTCGTTTCTTGCCTATTGTGC	0	0	0	0	1	0	4	5
		21UR-1982	TACCTTCAGAGTGTTTTGCAA	0	0	0	0	0	0	0	0
	†	21UR-1983	TCAAGCAAGGTTACCGAAATA	64	13	7	7	16	126	7	240
		21UR-1984	TAGGTTTTAAATATGGAACTT	3	3	6	3	15	41	1	72
		21UR-1985	TAAAGTGGTTATTTTGGTGCG	1	0	0	0	2	4	0	7
		21UR-1986	TGAAGGCGAATAAATTTAAAA	6	1	1	0	1	5	3	17
	†	21UR-1987	TTCCTACGAATTATTTATTAT	11	0	1	1	2	0	0	15
	†	21UR-1988	TTTGAAATGAATAAATACAGG	0	0	0	0	0	0	0	0
		21UR-1989	TCAAATACTAAATTATCTTCA	0	0	0	0	0	0	0	0
		21UR-1990	TGCATACTTTTTTATGTTGAA	0	0	0	0	3	2	0	5
		21UR-1991	TCACATGGCCGTTATTTATCA	0	0	0	0	0	0	6	6
		21UR-1992	TGGGATCGATGTTCTGGCTGA	0	0	0	1	25	31	17	74
		21UR-1993	TCCAATCATGAAACTACACCA	0	0	0	0	0	0	0	0
		21UR-1994	TTGATCTGCCTCTATACTAAT	0	0	0	0	1	0	0	1
*		21UR-1995	TATCACTCCATTGAAATATTG	3	1	1	0	5	7	1	18
	†	21UR-1996	TGATGCTAATGACAAATATCC	0	0	0	0	0	0	0	0
		21UR-1997	TAATTAAAAGGTATCCAACGA	8	0	1	2	2	0	1	14
		21UR-1998	TTCTGCCTTCTTTTCAGATTC	0	0	0	0	1	0	0	1
		21UR-1999	TGATGTAGGGAATTAACCAAA	0	0	0	0	5	3	1	9
*		21UR-2000	TGCGTTCTGTATTGTATACCG	2	10	3	4	52	82	3	156
		21UR-2001	TCCAAACTACAAAATGTTTCA	0	0	0	0	1	0	0	1
		21UR-2002	TAGGAAAAAAACTAATTTTCC	0	1	0	0	0	2	0	3
		21UR-2003	TAAACTATGTATGTGTTTCAA	0	0	0	0	2	0	0	2
		21UR-2004	TGAGATCTTCAAATGCTTTCA	0	0	0	0	0	0	0	0
	†	21UR-2005	TAAAAAATCAAAAACTCTGGG	0	1	0	0	1	1	0	3
	†	21UR-2006	TGTTAATATCTTGTGGGCATA	1	0	1	2	41	38	10	93
		21UR-2007	TGCTTCAATTTCAATTTTTCC	0	0	0	0	0	0	0	0
*		21UR-2008	TAGGTGTTGAAGAAAATTAAC	0	2	0	0	6	6	0	14
		21UR-2009	TATGGTAATCCATCCTATTTT	0	0	0	0	0	0	0	0
	†	21UR-2010	TAACTGAGTCTGAATGTTAAT	0	0	1	0	4	4	0	9
		21UR-2011	TTGTCTTTCATTTCAGACGTG	0	0	0	0	1	2	0	3
		21UR-2012	TATGTTTTTCGATTACATTCC	0	0	0	0	0	0	1	1
		21UR-2013	TAAAGGTCAAGTTTTCACGCT	3	1	0	0	0	1	0	5
*	†	21UR-2014	TCACCAAGAATTCGGTTACTC	20	29	13	64	1014	961	34	2135
		21UR-2015	TGCTCCATTTTTTCCCCAACT	0	0	0	0	0	0	0	0
		21UR-2016	TTCAAGACTGTGCCAGTTAAA	6	3	3	2	8	52	4	78
		21UR-2017	TGAGGTTTTTCTTTGTTGTTC	0	0	0	0	1	1	1	3
		21UR-2018	TTATTTGTATTATTTTTTTAT	0	0	0	0	0	0	0	0
	†	21UR-2019	TCTCTTTAACTTTTTAGTCAA	0	0	0	0	0	0	0	0
	†	21UR-2020	TATCTTTCTAGTTCTCTCTTC	0	0	0	0	0	0	0	0
		21UR-2021	TGAAAATCGACTAAATCTGCC	0	1	0	0	12	10	1	24
		21UR-2022	TAATATTTCAGCTCACCCAGT	0	0	0	0	0	0	1	1
		21UR-2023	TGTGCAGTTTTTCGAAACTTA	0	0	0	0	0	1	0	1
	†	21UR-2024	TAAATTCGTGTCCTATTCCTA	0	0	0	0	0	0	0	0
	†	21UR-2025	TATTAAAAAATTCAATGACAA	0	1	0	0	1	4	0	6
	†	21UR-2026	TTAGTGAATTCGGATTCGTTC	2	0	1	1	1	6	1	12
	†	21UR-2027	TATTCATTGGTTCTATTTTTC	0	0	0	0	0	0	0	0
		21UR-2028	TATGTCCAACGTTCCTGTTAT	0	0	0	0	7	4	1	12
	†	21UR-2029	TCAATGGAATCCAACGTATCT	0	0	0	0	0	2	0	2
		21UR-2030	TAATTTACCCCTGGAGATGGC	5	1	2	2	2	19	0	31
		21UR-2031	TCCGTTTAATTTTGCAAACAA	0	0	0	0	2	4	0	6
	†	21UR-2032	TTAATTCATTGATGTTGGTTT	3	0	0	0	0	1	0	4
		21UR-2033	TCCAGTGTGCACAAAATATGG	0	0	0	0	3	0	0	3
	†	21UR-2034	TGGACTATTGAGAGTTGTATT	1	0	1	1	17	19	13	52
	†	21UR-2035	TAAATTTCACTCACCTCCAAA	0	0	0	0	0	0	0	0
		21UR-2036	TGAGCTATTCGATTCGATATA	0	0	0	0	0	0	0	0
		21UR-2037	TCGTGGCATAACATACTTTGA	11	6	7	11	43	87	16	181
	†	21UR-2038	TAAGGATTGCGCAAATCTACC	1	0	0	0	1	4	0	6
	†	21UR-2039	TTCCACAGCTTTACTATTTGC	3	1	0	0	0	6	0	10
	†	21UR-2040	TAGAAGAATATAATTTTTGGA	1	1	0	0	17	9	1	29
		21UR-2041	TACCTCTTCTTCTATCTTCAC	0	0	0	0	0	0	0	0
	†	21UR-2042	TGATTTTAATTTTTTCGTCGC	27	11	4	23	154	169	180	568

		21UR-2043	TGAACACTTTGGTGGTACTGA	0	0	0	0	0	1	0	1
		21UR-2044	TCACATTGCGTAAAACATGAA	0	0	0	0	4	8	0	12
	†	21UR-2045	TTCCATAACATTCTTCTTTCC	1	0	0	0	1	0	0	2
	†	21UR-2046	TTCATTAACTTCCAAGGCCTT	0	0	0	0	0	0	1	1
		21UR-2047	TGTTACAAATTTTTAGGAAAA	0	0	0	0	0	1	1	2
		21UR-2048	TATTTCTCTCAGTATACCTTC	0	0	0	0	2	0	0	2
	†	21UR-2049	TCTTACATTATTGACAGTTAG	0	0	0	0	0	1	0	1
	†	21UR-2050	TTCTCTCAATCTCGTTTGTAA	0	0	0	0	0	1	2	3
		21UR-2051	TGGTATAGATGTTTTTTTACC	2	0	0	1	9	8	0	20
		21UR-2052	TTGGGATAATTGTAGACTACT	0	0	0	0	0	0	1	1
		21UR-2053	TATAATTGGCAAGTTGTTGGA	1	0	0	0	7	8	6	22
	†	21UR-2054	TCGGAGGATTCAGCCTGCAAT	0	0	0	0	1	0	0	1
*		21UR-2055	TCTACAGAACACTATGGCGAG	0	8	2	4	136	179	64	393
		21UR-2056	TGGGTACTGTAACTTATTCGG	12	2	0	1	6	34	0	55
	†	21UR-2057	TAAAAAATCCTCCAAAGTTAA	0	0	0	0	0	0	0	0
	†	21UR-2058	TCACACGGCGACTATTTTGAC	0	6	1	7	113	123	71	321
	†	21UR-2059	TGAGACTTGAGACTACCGTTG	3	2	2	3	23	38	4	75
	†	21UR-2060	TGCGAAATTTTCAAAGTCTTG	0	0	0	0	0	0	0	0
*		21UR-2061	TCACATACAAGGCGCGGCTTA	51	5	5	2	28	72	33	196
*	†	21UR-2062	TGATCCTTTTGCAATTGACGA	24	2	2	5	9	37	1	80
*	†	21UR-2063	TAATGGTGTTGCCAGAAGATA	7	0	1	0	2	4	0	14
		21UR-2064	TCCAGAAATTCATGTATGGCA	0	0	0	0	19	13	1	33
		21UR-2065	TTCAGGGAATTGATGCTGACT	0	0	0	0	0	0	0	0
	†	21UR-2066	TCCAAAAATTGAACTTTTCCA	0	0	0	0	1	0	0	1
	†	21UR-2067	TTCCAACTAACTTTTTACGAA	0	0	0	0	0	0	0	0
	†	21UR-2068	TCAGAGTATTTTATTCAGAAA	0	0	0	0	0	0	0	0
	†	21UR-2069	TGTGTTCGTAACTGAATTGAG	0	0	0	0	0	0	0	0
*		21UR-2070	TAAATCTATTGAAGAGGACTG	5	5	1	19	232	261	89	612
	†	21UR-2071	TGAGTAATTGATAAATGAGAG	2	0	0	0	0	2	1	5
		21UR-2072	TAATGCTTTTAAGTTTGTCGA	1	0	0	1	14	22	3	41
	†	21UR-2073	TGAGTAGTTGATGTAAAAAGT	56	64	30	35	235	341	18	779
		21UR-2074	TCTTAACTTATGATGGGTATG	3	0	2	3	35	53	5	101
		21UR-2075	TCTCGTTTTCGGGTTGTGCTG	0	0	0	0	3	1	0	4
	†	21UR-2076	TTCGAACCATGCCTTTGCCAT	0	0	0	1	3	4	3	11
		21UR-2077	TTCCTCACTTCCAGCTTTTTA	0	0	0	0	0	0	0	0
	†	21UR-2078	TTTATTGATACTTTTGCTACA	0	2	0	1	4	1	0	8
		21UR-2079	TAAAAAAATGTCTAACTTCAA	1	1	2	0	3	7	0	14
	†	21UR-2080	TACATGAGTCATATCATTACT	0	0	0	0	7	1	0	8
		21UR-2081	TGACAATTGTATTTTTCCAAC	0	0	0	0	0	2	1	3
	†	21UR-2082	TAGTACCAATGTAAGGTTCAC	0	0	1	0	0	0	0	1
	†	21UR-2083	TGTTACTCGTAATTTTACACC	0	0	0	0	0	0	0	0
		21UR-2084	TTCTACGCATTTTTGAATTGC	1	0	0	0	0	0	0	1
	†	21UR-2085	TCTCAAACTTCTATATGATAA	0	0	0	0	0	0	0	0
		21UR-2086	TGAGAAATCAAAATGTGGAAA	0	1	3	3	20	20	8	55
		21UR-2087	TCGGATGGTTTACGAAGTTTC	0	0	0	0	23	12	3	38
		21UR-2088	TAGTACGTTTTTAATATTTTT	0	0	0	0	2	1	0	3
		21UR-2089	TTCGTTTTCACTATTTTTCTC	0	0	0	0	0	0	0	0
	†	21UR-2090	TTAAGACGAGTGCAGTCAATG	1	0	0	1	0	3	1	6
*	†	21UR-2091	TCAGTTGAGTTGAAAAAAGGT	11	2	2	2	1	34	1	53
	†	21UR-2092	TTGTGTCCACGAACAGAAAAC	0	0	0	0	0	0	0	0
	†	21UR-2093	TGTGTTTCTTGTAAAACAATC	0	0	0	0	1	0	0	1
*		21UR-2094	TTTCAATATCATTTTTGAGTC	1	0	0	1	0	0	0	2
		21UR-2095	TGGGTCTCATATCTTAATTCA	1	0	0	0	2	3	1	7
	†	21UR-2096	TGTGCTTCTTTCGAATGATAA	0	0	0	0	0	0	0	0
		21UR-2097	TACTCCAATTCCAGCTATTTG	0	0	0	0	0	0	3	3
		21UR-2098	TATTCTACCAGTTTCAAAGTG	0	0	0	0	0	1	6	7
		21UR-2099	TACTTCAGCAACGTTCTCGCG	1	1	0	0	0	1	1	4
	†	21UR-2100	TGTGACTCAATTGTGACTCAA	2	1	0	0	2	7	0	12
		21UR-2101	TGCCCCCAGATTATTTCGAAA	4	0	0	0	0	0	2	6
*	†	21UR-2102	TGGGCAGGTATTAATTCGAGT	10	8	3	11	125	191	32	380
	†	21UR-2103	TGTTCTGAGTTCTATATTGGG	0	0	0	0	0	0	0	0
*		21UR-2104	TAGCAAATCTGTAAATTTTGC	0	1	0	2	26	18	2	49
		21UR-2105	TAACTTTTGGAACTCAACAAT	0	0	0	0	0	0	0	0
	†	21UR-2106	TCGTATTGTTGGATTATAATT	39	9	8	14	109	105	40	324

	†	21UR-2107	TACTATGTGGTGTGTTGTAAA	0	0	0	0	0	1	0	1
		21UR-2108	TAGCCTCCAAGAACATTGACT	0	0	1	0	0	4	0	5
	†	21UR-2109	TGTTTGCTTTTGAGCCGTAGC	0	0	0	0	0	0	0	0
	†	21UR-2110	TGGTTGGACCACACCCAAATT	3	1	1	0	6	4	6	21
		21UR-2111	TAATCTATGCATCTCAACAGT	2	0	0	1	5	1	1	10
		21UR-2112	TGTATGCGGTTGATTTTGAGG	1	5	0	4	174	166	30	380
	†	21UR-2113	TCCACCTGATGCGGGAGTGAA	0	0	0	0	0	4	0	4
		21UR-2114	TATATATGCAGATGCTGTCGT	2	1	0	1	16	24	7	51
		21UR-2115	TAGAATAATGTATTGTGTCAC	0	0	0	0	0	0	0	0
	†	21UR-2116	TGAGTAAAAATAATAACCCTT	0	0	0	0	0	0	0	0
		21UR-2117	TGAATTATTGATTTGGTGATC	1	0	0	0	0	2	1	4
		21UR-2118	TGAAGCTAGTGTCTTTGCAGT	1	4	0	1	9	14	1	30
		21UR-2119	TAACTCTTGTGCCTCGTTTTT	0	0	0	0	1	1	0	2
		21UR-2120	TGCTTTGCGCAGTTACGAAGC	1	0	0	0	5	4	0	10
		21UR-2121	TGGATACGCTCTTCTTGGTTA	0	0	0	0	0	0	0	0
		21UR-2122	TGACAATAATATTCGTCTGAT	2	0	1	1	4	7	0	15
	†	21UR-2123	TTGGCTGTGAAAAATACTAAC	11	8	3	3	96	46	9	176
		21UR-2124	TGGTCGTGGATATGTCGGAAC	6	2	0	1	1	6	0	16
	†	21UR-2125	TTCTGAGGGTTTTGCAATTGC	0	0	0	0	10	6	1	17
	†	21UR-2126	TAGTCGTTGAATTGGCTGAAA	1	0	0	0	6	1	0	8
*	†	21UR-2127	TTGGTAGGCGTCATTAAAACC	6	5	0	2	100	112	24	249
		21UR-2128	TAACTTTATTCCATTCCTCAA	0	0	0	0	0	0	0	0
	†	21UR-2129	TAGAAGTTCATCGGGTGATTT	9	2	4	2	7	22	8	54
	†	21UR-2130	TTTCAAAATCTGATCAAGTGC	4	1	1	1	6	16	1	30
		21UR-2131	TAGCTTTTTCGAAGTTGTCGA	9	0	0	0	0	1	2	12
		21UR-2132	TATTGTGGTAGCTCGATTATT	1	0	0	0	2	1	1	5
		21UR-2133	TCTTAGCATGTAACCGTATCA	0	0	0	0	4	3	0	7
	†	21UR-2134	TGAATTGGCATTAGATGTTTA	0	0	1	2	46	24	4	77
		21UR-2135	TATTCGTCTAATGAAACTTTA	0	0	0	0	2	1	0	3
		21UR-2136	TGCCAAATCTATCCGACCCAA	0	0	0	0	0	0	0	0
		21UR-2137	TACTGATTCTTCATTGGACTG	0	0	0	0	0	0	0	0
		21UR-2138	TATTTGCTGATATCTTTCCTT	0	0	0	0	3	0	0	3
		21UR-2139	TACAAACTTGTAATCGCTATC	10	1	0	2	7	20	2	42
		21UR-2140	TACGAGTCACAAATATGAAAA	0	0	0	0	1	0	0	1
	†	21UR-2141	TTGGTTAAGAACATTTGGAAA	6	3	3	0	20	45	4	81
		21UR-2142	TGGTTCATTTGTAGTCTACCA	1	2	2	6	40	25	3	79
	†	21UR-2143	TGCTTACAGTTTATAAGCCAC	0	0	0	0	0	0	0	0
		21UR-2144	TATGTATTACCTAAAATATAC	2	0	0	1	2	3	0	8
	†	21UR-2145	TCATACGCTCAAATTGTATTT	21	3	2	3	17	21	5	72
	†	21UR-2146	TCCATCTTTCTGCGACCAGAA	0	0	0	0	1	1	1	3
*		21UR-2147	TATGGAATCGCACGGTGTAGA	7	3	3	2	39	71	44	169
*		21UR-2148	TCATGTTATACTATTAGCATA	0	0	0	1	8	6	1	16
		21UR-2149	TAAAAAAAGAGCATCCGTAAC	33	17	12	6	23	97	9	197
		21UR-2150	TGATCCTTTATTTGACCTTAA	0	0	0	0	0	0	0	0
		21UR-2151	TGGGCGAATGTAAAACTTTCA	0	0	0	0	0	1	0	1
		21UR-2152	TGAAATTTGCGGCCTTGTTGG	0	0	0	0	4	4	1	9
		21UR-2153	TGCACTTCAAGTTTTTTTCTG	0	0	0	0	0	0	0	0
		21UR-2154	TCCGATATTTTAATATTTTCA	0	0	0	0	0	0	0	0
	†	21UR-2155	TACACGACTCATGTACTGATT	6	10	2	6	21	54	1	100
*		21UR-2156	TAGCTTAGTTCTGTTACTCAT	0	0	0	0	11	8	1	20
*		21UR-2157	TGTGTATGTCGTCCCAAAAAA	21	4	5	2	3	19	12	66
		21UR-2158	TATCAGAAGTGATGTTTTCGA	0	0	0	0	0	0	0	0
		21UR-2159	TAAGATTGTAAAATTTAATAT	0	0	1	2	15	13	1	32
		21UR-2160	TCATCTTGTTTGCTAATTTAA	0	0	0	0	0	3	1	4
*		21UR-2161	TTTGGGTTTGTCGGCTATTGC	0	0	0	1	36	68	124	229
		21UR-2162	TTCTAACAACTAGAGAGGGAT	1	0	0	0	9	16	7	33
*	†	21UR-2163	TTGGTTGTGTTTACAAATGTG	0	0	1	0	0	0	0	1
*	†	21UR-2164	TAGGAATATAGCATTAAATAA	202	523	184	247	1981	2477	139	5753
		21UR-2165	TATCTCGAACCAAGACACCAA	0	0	1	1	6	5	5	18
	†	21UR-2166	TCAGTTTTGAAATGTCACGAT	0	0	0	1	0	1	0	2
		21UR-2167	TGTGCCCTTACCTGTGCATCT	0	0	0	0	0	0	2	2
	†	21UR-2168	TCTAGCCCGGCTTTGCTTAAT	1	2	0	1	2	3	3	12
		21UR-2169	TAAATGTTAACCAATACGTTT	0	0	0	0	5	2	0	7
		21UR-2170	TTCAACGTTCAAATTCAAAGG	1	0	0	0	0	1	0	2

	†	21UR-2171	TAGTCACGTAAATTGATAGTC	3	2	1	0	1	8	0	15
*		21UR-2172	TAACATTGTCGTAGAGGAACA	1	4	1	6	97	68	3	180
*	†	21UR-2173	TAATGTCAGTTGAAAATGTGC	10	3	2	2	3	22	0	42
		21UR-2174	TCCGACGTATGTACACTGTCC	0	0	0	1	17	21	1	40
		21UR-2175	TGTACCACATTTCTTCTAAAG	0	0	0	0	0	0	2	2
		21UR-2176	TTCGTGTATGCATATTCCGAC	0	0	1	1	17	19	0	38
		21UR-2177	TGCACCGTTTTTTCTGTTTCT	1	0	0	0	0	1	0	2
	†	21UR-2178	TGTAGAGATTCCAGTTCCCAA	0	0	0	0	2	0	0	2
*		21UR-2179	TAGTACTGGCACTCTAGAGAA	10	11	7	0	21	55	3	107
	†	21UR-2180	TACTTAGGCATTGGTGTACTT	0	0	0	0	16	4	0	20
	†	21UR-2181	TAAATGTCATATCTTGTGGTG	0	0	0	0	7	2	0	9
		21UR-2182	TCGTTCAAGCACAAGATGAAT	2	0	0	0	18	50	14	84
		21UR-2183	TGGGCAGCCCGTGCTTAAACC	0	0	0	0	0	0	2	2
		21UR-2184	TATGACAACTATTTAAAAAAA	4	0	0	0	0	1	0	5
		21UR-2185	TGTGACAAGACGTTCCCGTAC	0	0	0	0	0	0	1	1
		21UR-2186	TGATCAATTCTAAAGTTCCAA	0	0	0	0	0	0	0	0
*	†	21UR-2187	TAACTGACGATACGGCAATAT	43	21	20	50	554	545	74	1307
	†	21UR-2188	TAATAAGTGTGAGGGTATTTC	22	12	4	7	45	96	25	211
		21UR-2189	TTATCCATTTTTCTACATTCC	0	0	0	0	0	0	1	1
		21UR-2190	TCGCTTTATTTAAGAAAAGCA	0	0	0	1	11	8	3	23
		21UR-2191	TTGTGTTTTCATAGGACTGTT	15	0	1	1	11	15	4	47
	†	21UR-2192	TCTTTTGTGCACGTCAGGGGT	14	0	1	1	3	10	8	37
		21UR-2193	TTATGATTCGTTTTTTGTTCA	15	0	2	3	9	17	8	54
		21UR-2194	TGCGAGTCTGGTCTCTTTTGT	0	0	0	0	0	9	0	9
		21UR-2195	TGCTATATGGCAGCAATATAA	7	0	0	0	1	5	1	14
		21UR-2196	TACTGCTTCTTTGTATAGGAC	0	0	0	0	1	1	0	2
	†	21UR-2197	TGACATTGGCATATGATTATA	0	1	0	0	8	4	1	14
	†	21UR-2198	TTGGTGGATCGTCATTTGGTG	18	3	2	0	6	25	4	58
	†	21UR-2199	TGGGTAACAATTCTAAAGTGA	0	0	0	0	2	5	1	8
*		21UR-2200	TTTATGACATGTTGGAGTTTT	234	54	54	56	254	399	230	1281
		21UR-2201	TGGGTTTCGATTCCCTTCAAA	0	0	0	0	0	0	0	0
	†	21UR-2202	TCGGAGAATCGGTTTTTCAAA	1	2	0	0	16	26	3	48
		21UR-2203	TCTATGGTTCAGTCAATCTTA	0	0	0	0	1	1	1	3
	†	21UR-2204	TTGATTTCCTAGTTGATTTCA	1	0	1	1	1	1	2	7
		21UR-2205	TCATGTTTTGATATATTTTGA	1	0	0	0	0	1	0	2
*		21UR-2206	TATCTCACATCTATTATCATC	0	0	0	0	2	0	0	2
*	†	21UR-2207	TACAGGATCAGTGAAAGGTGA	19	12	6	6	60	108	14	225
		21UR-2208	TGGAATAGATGATTATTAAAT	0	0	0	0	9	4	0	13
	†	21UR-2209	TAGACAGATTGGCCTGTAATT	5	7	3	9	25	53	5	107
	†	21UR-2210	TAAGTTTCAGGCATTATTCGA	12	1	3	2	26	42	7	93
	†	21UR-2211	TCATGTAAGCAGTGTGAAAAA	1	0	2	1	13	8	4	29
		21UR-2212	TGAGTACCAACTTCAATTTTG	0	0	0	0	0	0	0	0
		21UR-2213	TAAATCCAAGGTATCTGGAAA	0	1	0	2	4	1	0	8
	†	21UR-2214	TTAAGTGATCGTTACACTCAA	11	7	7	3	4	33	5	70
	†	21UR-2215	TTACACAGCCAATCTTGAGAT	0	0	0	0	0	0	0	0
		21UR-2216	TAATTTGAAAATATTGCACCA	0	0	0	0	0	0	0	0
		21UR-2217	TGGGGCAACAACAATTTCGTT	0	0	0	0	9	10	6	25
		21UR-2218	TTTGTTATTCTTTTCATGAAC	0	0	0	0	0	1	1	2
		21UR-2219	TCGTCAATTCATACCTTGTTA	1	0	0	0	0	1	5	7
		21UR-2220	TACTGTTCTAATCTTTTCTTT	0	0	1	1	1	0	0	3
		21UR-2221	TGCATTAGTTACCGTGAAATG	0	0	0	0	1	3	0	4
	†	21UR-2222	TTGGGAGAAATCTACGGAAAT	0	0	0	0	6	3	5	14
		21UR-2223	TCCAAGGTTTAAAATCATGAG	3	0	0	1	1	1	2	8
		21UR-2224	TTCTTCACCGTCCGGTTACAA	5	0	0	2	20	36	12	75
		21UR-2225	TTCCATAATTGGTAGAAAACG	0	0	0	2	21	19	0	42
		21UR-2226	TACGATTCGCGTTTCTTCAGG	0	0	0	0	0	0	1	1
		21UR-2227	TTATATTTAATGAACTCATAA	1	0	0	0	0	3	0	4
		21UR-2228	TGTGAAACTACAAATAATGTA	0	0	0	0	0	0	0	0
		21UR-2229	TACTGGCATCTTTTTTTTAAT	0	1	0	0	0	0	0	1
	†	21UR-2230	TTGAACTGTGGCGTGACTTAT	1	0	1	4	27	28	6	67
	†	21UR-2231	TTCCACATTCGCATTCGAAAA	9	0	1	0	2	3	0	15
		21UR-2232	TGCAGAGTTCAAGTGAAAAAA	0	1	0	0	1	1	0	3
	†	21UR-2233	TGTGCTGTGATTTTGTGTGCT	4	8	6	4	6	35	4	67
		21UR-2234	TGGTCCCGCATAGTTGACAAG	0	0	0	0	0	2	2	4

*		21UR-2235	TAGATAGAGACTGCATGATTT	1412	1112	513	492	1341	6357	230	11457
*		21UR-2236	TCATCCAAGGACAATGCGGAT	19	1	4	1	14	38	3	80
	†	21UR-2237	TCGCAAATCTGTCCAGCTTTA	1	0	0	0	4	3	1	9
		21UR-2238	TATGGCAATTATTATGGCAAG	1	0	0	0	3	6	0	10
		21UR-2239	TAAATGCAAGTTTAACTGTTG	0	0	0	0	0	1	0	1
*	†	21UR-2240	TGCTGTCATGTTTCACTGGGT	2	0	0	0	3	7	1	13
		21UR-2241	TACGTTGTGTTTATTTTTGTG	0	0	0	0	0	0	0	0
		21UR-2242	TATTCCCTCGGAGTAAATATT	1	0	1	0	2	7	3	14
*	†	21UR-2243	TCAAAAGAAAGCTTGACGGCA	20	7	6	8	19	76	1	137
	†	21UR-2244	TTCTAATTGCACATTACTTAC	3	0	0	1	1	4	4	13
		21UR-2245	TTTGTCGACGTTATGGAAAGT	0	1	1	1	24	22	43	92
*	†	21UR-2246	TGAAACTCTTTTCTGATCGTC	0	0	0	1	8	8	3	20
		21UR-2247	TGCGTCTTCGAAATTTATGAT	0	0	0	0	0	0	0	0
		21UR-2248	TTTATAACAAGTAGTTGGTTG	0	0	0	0	7	9	3	19
	†	21UR-2249	TTAACATCCCGCCCGCTCAGA	0	0	0	0	0	0	0	0
	†	21UR-2250	TCAACGTGTATGTTCGGGTTG	2	0	0	0	8	6	0	16
		21UR-2251	TAAACAAAAGTCCAGTTTTTA	1	0	0	0	0	1	0	2
	†	21UR-2252	TGAAATGTGGATTTTTTTACA	0	0	0	0	0	1	0	1
	†	21UR-2253	TTTGAAAGTTCAGATTTTCAA	1	2	2	2	3	4	1	15
		21UR-2254	TCGGAGCATATTTTGGACAAT	3	4	2	1	70	64	7	151
		21UR-2255	TTCCAACAGGAGAGAATTAAA	2	2	2	0	7	10	2	25
		21UR-2256	TTTAGTGTACATTAGTAAACA	0	0	0	0	1	1	0	2
		21UR-2257	TAACACCTTACCATAGGATCC	0	0	1	0	0	0	3	4
		21UR-2258	TGAATGCACATAAACCTTCAA	0	0	0	0	0	1	3	4
		21UR-2259	TGATTGTGTATTTTTCGATAA	0	0	0	0	1	0	0	1
		21UR-2260	TCTGTGTTCAATTTTCACTGC	0	0	0	0	0	0	0	0
*		21UR-2261	TTAACATTTTTGGCATTTTCG	8	3	1	3	4	9	2	30
		21UR-2262	TAGTACGAGCACTGAATACAA	0	0	0	0	5	6	0	11
	†	21UR-2263	TGTTCAATGGATAGATCCCGC	4	2	0	1	5	16	9	37
	†	21UR-2264	TGTTGCTCTTACATTCGTGAA	0	0	1	0	3	0	0	4
	†	21UR-2265	TATTTCGGCAATGTTTCGAAA	0	0	0	3	7	7	0	17
		21UR-2266	TGGGTTTTTTATTGTGGAACT	2	1	0	1	8	15	4	31
	†	21UR-2267	TAGTTAACTACAACTGAATGC	0	0	0	0	0	0	0	0
*	†	21UR-2268	TACTGAAGAAGACGGACAAAG	31	29	8	46	708	1566	168	2556
		21UR-2269	TGCCAAAAAATAGAAAAGTCA	0	0	0	0	1	2	2	5
		21UR-2270	TCCAGATTGGTCACAATTGTA	1	1	0	2	20	35	4	63
		21UR-2271	TAAACACACTTCAGATGTCGT	7	0	3	3	34	33	13	93
		21UR-2272	TCCAAACGAAAAAAGCCCATG	0	0	0	0	0	0	0	0
	†	21UR-2273	TCCTATCCTGATTATTGGTCA	5	1	1	2	5	8	0	22
		21UR-2274	TCAATACTTCAAGATTGGGAA	2	1	0	2	55	59	4	123
*	†	21UR-2275	TACAGATTCTGACAAAAATAA	5	3	4	4	8	51	0	75
		21UR-2276	TCAAGCTTCCATATCTTCTAA	6	1	2	2	13	22	15	61
		21UR-2277	TAACTCATTTTTTAGCCTTTC	0	0	0	0	3	1	1	5
		21UR-2278	TGGAATGGCATGGTTTCCAGA	0	0	0	0	0	0	0	0
	†	21UR-2279	TCAATAACGCTTGTGAACGCA	0	0	0	0	0	4	0	4
		21UR-2280	TGAAAAAGGATTACATTATAA	0	0	2	2	22	13	10	49
		21UR-2281	TATTGACTTGCTAAAGTCTCT	0	0	0	0	0	0	0	0
		21UR-2282	TGTGAGTGATTAATACATCAA	1	0	0	0	0	0	0	1
	†	21UR-2283	TGGTGCAATCATTGTTTTGTT	0	0	0	0	0	0	0	0
		21UR-2284	TGAATGCTGACCCACAAGTAA	13	12	3	0	12	35	1	76
		21UR-2285	TCTGAAGGTTTTTTATGCTCC	0	0	0	0	0	0	0	0
*		21UR-2286	TCGGTCGTTGTGCATAAAGTT	9	1	4	1	25	28	12	80
*		21UR-2287	TTAAATCACCATTATCGTCTC	11	5	1	16	126	124	117	400
		21UR-2288	TTATCGAGTCAAAATAAGTCA	1	0	0	0	2	1	1	5
	†	21UR-2289	TCTTGCTAGTCCTCTGTCGAG	0	0	0	0	6	7	1	14
		21UR-2290	TAGGATCTCTGCTCTGTTGAG	0	0	0	0	0	1	0	1
		21UR-2291	TGCGTGTTCGAAAACGGAAAA	7	0	0	0	0	2	0	9
		21UR-2292	TTAACTGTCTGCTTTCAATTC	1	0	1	1	2	4	0	9
*		21UR-2293	TCCGGGAGATTTAATTATCAC	0	0	0	0	1	2	4	7
		21UR-2294	TTGTTTTACCATTATTTTAAA	0	0	0	0	0	0	0	0
		21UR-2295	TTGTTTTATAGAATTGTAAAC	1	0	1	1	11	4	1	19
	†	21UR-2296	TGGTGTTGAGGTAAAGCCAAG	0	0	0	0	1	1	3	5
	†	21UR-2297	TAGTATTAGTATCTAAGGGCT	2	0	0	0	14	17	8	41
		21UR-2298	TGTAAGCACTGTTCCCGTTTG	0	0	0	0	0	0	0	0

	†	21UR-2299	TTCCTACCAATTAAAACTTTA	0	0	0	0	2	2	3	7
		21UR-2300	TTCGAAGGATACATGACCAAA	8	8	7	7	22	60	8	120
		21UR-2301	TGTGAAAACAAAAAAACCAAA	0	0	0	0	0	0	0	0
		21UR-2302	TGAAGCGAATCTTTTTAATGG	1	0	0	0	0	3	0	4
		21UR-2303	TGTAGAATGTATAACGTTCAA	0	0	0	1	34	13	1	49
		21UR-2304	TGTTCATCGCATTGTTAAAAT	0	0	0	1	9	7	1	18
*		21UR-2305	TAGCACTCGGCATGTTTTTCT	107	262	78	75	289	819	23	1653
		21UR-2306	TACTACTATATAATTACCACA	0	0	0	0	8	3	2	13
	†	21UR-2307	TACGTTTTTTTCGGGTTAGTT	6	0	2	2	5	14	1	30
*	†	21UR-2308	TCGCAGCTGAAAGATGTTTTG	145	179	87	95	484	1151	64	2205
		21UR-2309	TGCTCTGTGGATAAATCTGTG	0	0	0	0	1	0	0	1
		21UR-2310	TTATATCACATTTGATGACCA	1	0	0	0	3	0	0	4
	†	21UR-2311	TTTCAGCGATCGAAAGACAAC	38	22	21	9	53	112	8	263
		21UR-2312	TAAAGCGCGTTTACGTTTTGA	3	0	2	0	0	3	0	8
		21UR-2313	TCTTGATGTGATATTCAAACA	0	0	0	0	1	0	0	1
		21UR-2314	TGTGTTGCGTTTTGAATCATG	0	1	1	0	0	1	0	3
		21UR-2315	TATCTTTCACTTGAAGAATGA	6	1	2	2	18	17	3	49
	†	21UR-2316	TTCGGATTTCGACAAAACGAT	1	1	1	1	3	14	2	23
		21UR-2317	TGTATATACGAAACCCGTTAT	0	0	0	0	0	0	0	0
		21UR-2318	TGTTATGGCATTACGCTTGAT	1	1	1	1	2	8	0	14
		21UR-2319	TAAGTTTTTATTTAACCAAAA	2	0	0	0	0	1	0	3
*		21UR-2320	TGCAATGTACAACAGGACAAT	1	2	1	14	160	126	20	324
		21UR-2321	TAACATGTGCCTAAAATAGAA	0	0	0	0	6	1	1	8
*	†	21UR-2322	TATAGCACTCGTTTGGGCATT	11	3	1	8	123	192	28	366
		21UR-2323	TCCTGCTAGGTCATTTTTTAA	0	0	0	0	0	1	0	1
*	†	21UR-2324	TGAATAGGCTGGCTTGATGGA	2	5	4	5	255	540	85	896
*	†	21UR-2325	TATTAGAAGGAACGGCGGACA	24	20	8	24	605	831	203	1715
	†	21UR-2326	TTATACAGTCCGTTTGTGAAA	0	0	0	0	5	3	2	10
*		21UR-2327	TAAGAACTGGCTTCTGTGTGG	1	0	0	0	11	30	4	46
	†	21UR-2328	TTCAAGCAAACGTTGCATGAT	9	2	1	0	5	18	0	35
		21UR-2329	TCCTCTGATCGTGAACGTGAA	1	1	0	0	2	10	3	17
*		21UR-2330	TCAGTGGATGTTAATTTCTGG	1	1	0	0	1	3	0	6
		21UR-2331	TACTCCAGTCATTTCGCCTTT	1	0	0	0	1	0	6	8
	†	21UR-2332	TGTTGAAAGAATTCATGACTG	0	0	1	0	0	0	0	1
		21UR-2333	TATGTATGTCTTTGATCTCAT	0	0	0	0	2	0	0	2
	†	21UR-2334	TGGGTGAATCCGACAACTTCT	0	0	0	0	0	1	0	1
		21UR-2335	TGGACTGAAAAAAGGCCCAGA	1	2	1	1	12	50	3	70
	†	21UR-2336	TGGACTGCTAAATTGTTAGAC	4	0	0	1	40	45	8	98
		21UR-2337	TCAACAAACATTTGGCAACTA	1	3	0	0	3	0	0	7
		21UR-2338	TCAAATTTTCAATATCTCTCA	1	0	0	0	0	0	0	1
	†	21UR-2339	TTTGATGTCCATTCAAAATAA	0	0	0	0	0	0	0	0
		21UR-2340	TAGAATGGAGAAATTTAAGCC	0	0	0	0	2	2	0	4
	†	21UR-2341	TGGTTCGATCGATACGCTAGG	0	0	0	0	9	18	16	43
		21UR-2342	TCTTCGGTTGTTGTGAAGAAG	0	0	0	0	0	0	0	0
*		21UR-2343	TAGAATTCGTGATTACAAAAA	0	1	0	0	12	6	8	27
		21UR-2344	TGGAAGGCCAAAAGAAAAAAA	2	0	0	0	3	10	8	23
	†	21UR-2345	TCTGCTTGATTGTCTATCCTA	0	0	0	2	1	4	1	8
		21UR-2346	TATGCGAAGTGTAAGTATTAA	0	2	1	0	34	36	5	78
		21UR-2347	TTCTATTCCAGTAACATTGCG	0	0	0	0	1	1	1	3
		21UR-2348	TGATGTCCCATGTCGCAATTC	0	0	0	0	0	0	0	0
		21UR-2349	TTAAATCCTTCACACAAACCC	0	0	0	1	3	2	2	8
	†	21UR-2350	TCTCCATCTGACGTTATGAGA	5	1	1	1	2	22	1	33
	†	21UR-2351	TCCTTGGCTCTTGATCTGAAT	1	0	0	0	6	13	0	20
	†	21UR-2352	TGATTCATGTTGACTTGTCAA	0	0	1	1	0	2	0	4
	†	21UR-2353	TGAAAACTCCATATTATATTC	0	0	1	1	5	1	2	10
		21UR-2354	TCCTGCCTTTAAACTCATCAA	0	0	0	0	0	1	0	1
	†	21UR-2355	TCGTCGTTGAAGTTGTTCAAT	1	0	0	0	2	0	1	4
		21UR-2356	TCTCAGAGTAAGTGTTTCAGT	0	0	1	0	0	2	0	3
		21UR-2357	TCCGTTCTAAATTCTCTTTTC	0	0	0	0	0	0	0	0
		21UR-2358	TGTCGAAAAAGTTAGTGGGGA	0	0	0	0	1	4	11	16
		21UR-2359	TTCATGCGCTATGCCTTCGAC	1	0	0	0	2	3	2	8
	†	21UR-2360	TAAGCACGGCCTCTGTGAAAT	98	161	65	68	459	1037	59	1947
	†	21UR-2361	TGCTGTTAGCATAGATTAATG	2	2	0	3	9	10	1	27
		21UR-2362	TACAATATACACTTTTTCGTA	0	0	0	0	1	0	4	5

	†	21UR-2363	TCAATCTTGCGTACTTTTTGA	6	1	0	0	3	8	0	18
		21UR-2364	TGAATTTCCTTGTTGTCTTTG	0	0	0	0	0	1	0	1
	†	21UR-2365	TAATGACACTATCTTTGGAAA	3	4	1	6	17	15	3	49
*		21UR-2366	TGATATTGCATATTTTGGTCG	1	2	0	3	46	55	10	117
	†	21UR-2367	TGCTATAGCGAAATCTTTCAA	6	0	1	0	2	2	1	12
	†	21UR-2368	TAGACGAATCTTCAAAATGGT	23	2	2	0	2	9	6	44
	†	21UR-2369	TTGCAGTTTCTATCTATAACA	0	0	0	1	3	1	1	6
		21UR-2370	TCTGTTGATTTATGTTATTGA	0	0	0	0	0	0	0	0
	†	21UR-2371	TGGTCGAATCGAAGTTAATTA	0	0	0	0	0	0	0	0
	†	21UR-2372	TACCATGATGTATACGGCATT	7	1	1	2	0	16	2	29
		21UR-2373	TTCTGCCAATAAATGTTATGA	0	0	0	0	0	0	0	0
		21UR-2374	TATAAGTCTCTAATTCTCTTC	0	0	0	1	0	1	0	2
		21UR-2375	TAGATGGTGCAACAGAACCCG	6	2	2	0	1	17	0	28
		21UR-2376	TAGTATTAATAATTCTGCAGG	0	0	0	0	0	2	0	2
	†	21UR-2377	TACTATCGCTTGAATTTAGAA	0	0	0	0	3	3	0	6
*		21UR-2378	TGAGGTCTGCAACTTGTTCCA	11	41	16	6	42	160	1	277
		21UR-2379	TCTTCCATTTTTTTCTCTTCA	0	1	0	0	0	0	0	1
		21UR-2380	TACACAGTAAACAGCCAAATT	3	0	0	0	2	63	3	71
		21UR-2381	TGATTGTTTGGTCAAGTTTGC	0	0	0	1	9	9	7	26
	†	21UR-2382	TCATTGTATAACAACTGCTTA	0	0	0	0	7	2	0	9
	†	21UR-2383	TGTGTAAGTTCGATGGTTTGA	8	15	4	7	21	78	4	137
		21UR-2384	TGAGTAGATTACATTTCTTAT	1	2	0	0	5	7	0	15
		21UR-2385	TAAACTGTGAATTTAGTTTCA	1	1	0	0	2	5	0	9
		21UR-2386	TTCCAAAGAAACCCATTCTTT	0	0	0	0	0	0	0	0
		21UR-2387	TAACACCGCTTCATCGCTTCA	0	0	0	0	2	0	2	4
*	†	21UR-2388	TCATTTCGTCTTTAGGCTAAA	21	12	5	14	106	137	23	318
	†	21UR-2389	TGGATCAGGGGTACCCCAATA	0	0	0	0	2	0	3	5
		21UR-2390	TATTCATGGTGCAAAACTGGT	0	0	0	0	0	2	0	2
	†	21UR-2391	TGGTTTATTCAGTCGTTGTTA	333	54	37	17	62	257	111	871
*		21UR-2392	TCCGTCGATTATTTTTGCATA	2	2	1	9	183	86	17	300
	†	21UR-2393	TGCGTATGGCGTATCAGTTCT	3	1	0	4	107	64	14	193
		21UR-2394	TCGTATAATTGCATTTATTAA	0	0	0	0	1	1	0	2
		21UR-2395	TGCTCCATCCTCTGAACCAAA	1	1	0	0	1	1	0	4
		21UR-2396	TTCGATAATAACCGAAGGTTC	0	0	0	0	0	1	0	1
		21UR-2397	TGCGGTAAACTTTCAGCAACA	0	0	0	1	6	2	0	9
	†	21UR-2398	TATCATCTAGTCATTGATTCA	0	0	0	0	0	0	0	0
		21UR-2399	TAGGTCCTCTCCTATATAAAA	1	0	0	0	0	0	0	1
	†	21UR-2400	TTGGTGCCTGTTGCTAGAATC	1	0	1	0	9	12	1	24
		21UR-2401	TCAAGTGGTGTCGTGAAATTA	2	0	2	0	1	5	2	12
		21UR-2402	TTCATAAAGTGGTTGAATGAA	2	1	0	0	2	4	0	9
	†	21UR-2403	TGAGCAAATGCGAAAAACATT	0	0	0	0	0	0	0	0
		21UR-2404	TATGACTCACGAAATTCCAAA	0	0	0	0	0	1	0	1
	†	21UR-2405	TACTAGACGAATGCTAATAGA	0	0	0	0	0	0	0	0
	†	21UR-2406	TCATAAACTACTTTTGCTGCT	0	0	0	0	1	1	0	2
*	†	21UR-2407	TTATTGGCATATTTTGACAAT	31	37	42	38	122	211	11	492
		21UR-2408	TAGCGCAATTTTTTAAGGTTT	1	0	0	0	1	1	0	3
	†	21UR-2409	TACTTGTATGATAGCTGTAAT	3	16	11	26	373	375	15	819
	†	21UR-2410	TTAACTCGTATTGGTTTTTGG	0	0	0	0	0	1	2	3
		21UR-2411	TCCTTTGCCAACTTCATTTTT	1	0	0	0	0	0	0	1
	†	21UR-2412	TGCACCTTCCGGAAATCAAGA	1	0	0	0	0	4	8	13
		21UR-2413	TCAACTAGTAAAACATTTTTA	3	3	0	0	2	5	0	13
		21UR-2414	TTCTTTTCCGTGGTACCTAAC	0	0	0	0	7	7	2	16
	†	21UR-2415	TAGGTCTATCCTTTTCAAATT	0	0	0	0	0	0	0	0
	†	21UR-2416	TCGATAAGCGTTTCAACGAAG	0	2	1	1	0	1	0	5
	†	21UR-2417	TAACGAGATAAATTAATCTGC	0	0	0	0	0	0	0	0
		21UR-2418	TAGAATTGACAAGTTGGGATT	3	3	1	0	4	12	1	24
	†	21UR-2419	TGCAGACTTAAATTGAGATTA	0	0	0	0	3	1	0	4
	†	21UR-2420	TTATTCAAACATAGTTCTCCC	0	1	0	0	2	2	1	6
		21UR-2421	TACCATTAGGCTTCATTTTGG	1	0	0	0	2	2	2	7
*	†	21UR-2422	TAAGTCTTTGATGGTCATTTC	30	18	11	15	143	175	14	406
*	†	21UR-2423	TTGCAACTTTGGTGTCATAGT	4	1	1	2	4	9	0	21
		21UR-2424	TGTGCATTCACTGTAATCAAC	0	0	0	0	4	1	1	6
	†	21UR-2425	TAAGCTACCGTTTTCGCTCAA	1	0	0	0	3	4	2	10
	†	21UR-2426	TCGTATGTCAACAATTGTAAC	0	0	0	0	4	0	0	4

		21UR-2427	TAACAACACCAATTTTATAAA	0	0	0	0	0	1	0	1
		21UR-2428	TGATATATACTGCCGTCTTCC	1	2	0	0	1	3	1	8
	†	21UR-2429	TCCATTTGTCATTCGGCGAAA	1	0	0	1	3	1	0	6
		21UR-2430	TCTTCGTGAATTCTCCACAAA	0	0	0	0	2	2	0	4
	†	21UR-2431	TTGATCATTATTTTGCGGTCC	5	2	0	0	3	6	2	18
		21UR-2432	TATAGTAATATCAAAAACTCT	0	1	0	0	2	1	0	4
*	†	21UR-2433	TATGCCCTTCACTGTGTGAAT	6	0	4	3	10	33	6	62
		21UR-2434	TACAGAGTGTAATCACGACTG	0	0	0	0	2	2	0	4
	†	21UR-2435	TAGTGAAATTTGAGTCGGTTC	52	15	16	13	174	230	74	574
*		21UR-2436	TGCATACAACGTTGCCGTATG	7	0	4	4	17	100	1	133
	†	21UR-2437	TATAATACTGCTTCCTTCTGT	1	0	0	0	0	2	2	5
		21UR-2438	TAAAAGTATAGGACTGATTTA	2	1	1	2	2	6	0	14
		21UR-2439	TGCAATCGTGAATTTCAACTT	1	2	0	1	5	9	0	18
		21UR-2440	TGAACTTTCGAACTAACCCAA	0	0	1	0	0	3	0	4
		21UR-2441	TAAGACCTATTTGTGCAAAAT	0	0	0	0	0	1	0	1
		21UR-2442	TTACGTGCTTAAAAAATTGAA	0	0	0	0	9	6	3	18
		21UR-2443	TAGATAGATCCTTCGACCTTT	9	7	1	5	10	34	5	71
*		21UR-2444	TATGTGAGAGAAAGTGACTCT	3	3	2	2	6	22	2	40
		21UR-2445	TGGTATCCTCAAAAAGCTTTC	0	0	0	0	0	3	0	3
		21UR-2446	TTGTCTGTTGTGTTCAGAAAC	0	1	0	0	1	6	0	8
		21UR-2447	TCTGTGCTTAGCCGCGTTATC	1	0	0	0	0	2	1	4
		21UR-2448	TGCGCCAAGCCTATATATTTT	0	0	0	0	1	1	4	6
*		21UR-2449	TGACGTAAAAATACTTTCTAA	0	0	0	1	18	16	3	38
	†	21UR-2450	TAGAACACTGCCGTTTTTAAA	0	0	0	0	0	1	0	1
	†	21UR-2451	TGATACGAGTTTTGTGTACAG	0	0	0	0	0	0	0	0
	†	21UR-2452	TAATACTCCCTGTTTGGTTAA	1	0	0	0	0	0	0	1
	†	21UR-2453	TAAGCCACTTTGTGCAATGGT	0	0	0	0	0	0	0	0
		21UR-2454	TCAAAGTTCCCTTTGGTTTGA	0	0	0	0	1	2	1	4
	†	21UR-2455	TCATTGTCAGTTCTATGCTCC	0	1	0	0	1	1	0	3
		21UR-2456	TGCTTTCTGTCCCTTTATATC	0	0	0	0	0	0	0	0
		21UR-2457	TGCAATTTAGTGCATCTGAGC	0	0	0	0	0	0	0	0
		21UR-2458	TCTAAATCCCACAGGTGATTC	0	0	0	0	1	2	0	3
	†	21UR-2459	TACTCCTGTGTTTTGTAAAAA	1	1	0	0	1	4	2	9
		21UR-2460	TCTCTCTTCAGATATCGTTTC	4	1	4	0	7	13	3	32
		21UR-2461	TATACCGGATATTTTCTACGA	0	2	1	2	27	15	4	51
		21UR-2462	TACGATGATGTTTTGTTCAAT	3	2	0	1	11	20	0	37
	†	21UR-2463	TAATTTCCGATATTTAGTTTC	0	0	0	0	0	2	0	2
		21UR-2464	TATCAAAATATATGCCGTTCG	0	0	0	0	0	1	4	5
		21UR-2465	TAACACTTGTGCTAAACGAAA	0	0	0	0	3	1	0	4
	†	21UR-2466	TCTCAACCAGCTTCGAAATTT	1	0	0	0	0	0	1	2
	†	21UR-2467	TAATGTAGTAATTTTTGCAAT	1	3	1	0	5	10	0	20
	†	21UR-2468	TGATTTCAATCTTATTGGTAC	0	0	0	0	0	0	0	0
		21UR-2469	TCCACCTTTCATTGTCTCTCG	0	0	0	0	0	0	0	0
		21UR-2470	TCTGACCATGCCACTGCCAGA	0	0	0	0	1	1	1	3
		21UR-2471	TTAAGCATATCCTTGATTAAT	13	3	1	0	1	10	1	29
	†	21UR-2472	TAATTCCAGATTTTCAAAGTG	1	0	0	0	0	0	0	1
	†	21UR-2473	TGATCTGTGAAACTATTCAAA	0	4	2	2	11	19	3	41
		21UR-2474	TGCCACTCGTCTACAATTTTT	4	0	0	0	1	1	2	8
	†	21UR-2475	TATGACTTGCGTTGATTTGGT	93	31	29	19	90	325	21	608
	†	21UR-2476	TAGTGCAATTGGTCCTCAAAA	1	0	0	0	1	0	0	2
	†	21UR-2477	TACAGTATTGGTAAGGTGGAA	1	0	0	0	0	2	0	3
		21UR-2478	TAAATTCTATGTTTGCATTCC	1	0	1	1	1	1	0	5
	†	21UR-2479	TAATGCACGGGCTGATATTAT	1	1	0	0	1	3	2	8
	†	21UR-2480	TGCAAAAGTAACAGGCGATGA	1	0	1	0	3	5	1	11
	†	21UR-2481	TTAAAGTTCTCTCGCATTTCT	0	0	0	0	0	0	0	0
		21UR-2482	TCCAAGTTTCTTTAACGCAAA	1	0	0	0	0	1	1	3
		21UR-2483	TTCAATAGAGTCCGAGAGATT	1	0	0	0	4	2	0	7
		21UR-2484	TGTCATAAACGTAGAATCATC	0	0	0	0	0	2	0	2
		21UR-2485	TGGTGTGATGTTGCTGCTATC	0	0	0	0	9	3	1	13
	†	21UR-2486	TAGAGCTAGGTTCCGAAAAGC	0	0	1	0	0	1	0	2
	†	21UR-2487	TGTTGTTCTTTTGCCGCTTGT	0	1	0	0	0	0	0	1
	†	21UR-2488	TCACATAGACGTATCTTCCTC	0	0	1	5	44	35	9	94
		21UR-2489	TCATTCTTTATAGTTTTTGGG	0	0	0	0	0	0	0	0
	†	21UR-2490	TGCATTCCGAATAAGCAGATC	0	0	0	0	1	5	0	6

		21UR-2491	TGGGAACCATTTAAAAAAGAA	1	0	0	0	1	0	0	2
	†	21UR-2492	TAGATCATTAGAGTACGAAAC	0	0	0	1	5	3	0	9
	†	21UR-2493	TAACCATATGATAATTAAAAA	1	0	0	0	0	1	0	2
	†	21UR-2494	TATTTTGCTCATTTGACTAAA	1	0	0	0	0	0	0	1
	†	21UR-2495	TGTTATTGTTCTTCAAGGATA	0	0	0	0	0	0	0	0
	†	21UR-2496	TAGTCGATTGAACTTTTCTGT	0	1	0	0	0	1	0	2
		21UR-2497	TCATCCAATAATTTCATTTAT	0	0	0	1	0	0	0	1
	†	21UR-2498	TTCGTTGGACAAATTATAAGG	30	4	3	2	12	27	7	85
		21UR-2499	TAAGAAAACTCGTTTGTTCCA	1	1	0	2	12	9	1	26
		21UR-2500	TCTATTTAACAAGTATCCCAT	0	0	0	0	0	0	0	0
		21UR-2501	TGTGTATCTCATGAGAAATTC	0	0	0	0	0	0	0	0
*		21UR-2502	TGAAATTGTAGTAGACTGCTG	5152	2556	1252	1171	2729	16185	498	29543
		21UR-2503	TGTAGAATCTGCTCTACCGTA	1	0	0	0	8	2	0	11
*		21UR-2504	TATCAAACAGATGGCCCAAAA	11	2	1	3	6	18	6	47
		21UR-2505	TCACTTTACGTTACAATCAAA	3	0	2	0	2	9	1	17
		21UR-2506	TAAAGTATGTGCCAAAAATCG	0	0	0	0	0	0	0	0
	†	21UR-2507	TTTGGTTTTTATTTAACATAA	0	0	0	0	0	0	0	0
	†	21UR-2508	TCGCATTTAAAAATTTAACGA	1	1	0	1	2	0	0	5
		21UR-2509	TCCTTCAGCCCCCATTGAGTC	0	0	0	0	0	0	0	0
		21UR-2510	TATCAAAAATTTCTCACATTT	3	1	1	1	3	4	0	13
		21UR-2511	TTTCTCCTGTTGAACGTTTCT	0	0	0	0	0	0	0	0
		21UR-2512	TAGGTTGGTCGAAAAGTCTTT	3	2	1	0	5	7	2	20
	†	21UR-2513	TAGTAGTTCTGAAAGATCCCA	0	1	0	0	12	15	10	38
	†	21UR-2514	TGCTGTTAGTAGATCCATTGA	1	3	7	12	314	229	72	638
		21UR-2515	TCTCATATGGTAACTTTATTT	0	0	0	0	3	0	0	3
*		21UR-2516	TAGGAAAGCCCCACTATGCTG	1	0	0	0	1	1	1	4
	†	21UR-2517	TTCATACATTGCAATTACCAG	12	0	0	3	9	8	10	42
		21UR-2518	TAGACGATTCATTCCTTAAAA	3	0	0	0	1	2	1	7
	†	21UR-2519	TAAGTTCGATGCTCGTTTTCA	5	1	1	2	8	15	1	33
		21UR-2520	TAACATACAACAAAAAAATTT	35	7	3	10	11	36	3	105
		21UR-2521	TACCGTTTCAATATCTTCTTA	0	0	0	0	0	0	0	0
		21UR-2522	TATATCCGTGTATTTCGTAGA	1	0	0	0	4	7	6	18
*	†	21UR-2523	TGGCATTGCAAAATCTAGGGT	16	3	0	2	36	42	22	121
		21UR-2524	TTACTCTTAACATGTGCAATG	0	0	0	0	4	4	1	9
		21UR-2525	TTTGGCTTTAAATTTGTTGGA	30	7	5	8	99	105	50	304
	†	21UR-2526	TTCAGCTGCAAATTTGAACTT	2	1	0	2	1	3	0	9
	†	21UR-2527	TGATGAGCAATCTATTTATGC	0	0	0	0	0	0	0	0
		21UR-2528	TACTAACATATTAAATGTCAT	9	1	2	2	13	9	1	37
		21UR-2529	TGGAAATTTCATACTGTTTCA	0	2	0	4	38	21	2	67
		21UR-2530	TCGTTCCTGTTCAAAATCCAA	0	0	0	0	0	1	0	1
		21UR-2531	TTGGTTTACAACTTTTTTATT	4	0	0	0	1	2	0	7
	†	21UR-2532	TTCGACTGTCATCGTTAACCA	38	20	26	26	130	229	13	482
*	†	21UR-2533	TAAGAACCCGTTAACAAGATC	1	0	0	0	2	4	0	7
		21UR-2534	TGCAAGATATCAATTCAAGAA	0	0	0	0	0	0	0	0
		21UR-2535	TAACGAAGTATTCCCAACAGA	2	1	1	0	2	20	0	26
	†	21UR-2536	TAAAATTGGGACATGGGATGA	0	0	0	0	5	6	10	21
	†	21UR-2537	TAAGCTATGAAAAGAACGGCT	0	0	0	2	0	3	0	5
	†	21UR-2538	TATTTAACTAGTTCGCTAATT	2	1	0	0	1	1	1	6
	†	21UR-2539	TCATACGATGGAACTGAGATA	0	1	0	6	47	48	7	109
		21UR-2540	TAGAATTTTCGATGCAGGTGT	0	0	0	1	30	25	6	62
		21UR-2541	TAAGCTCAGAATAATTGTTGA	1	0	0	0	1	2	0	4
	†	21UR-2542	TCTGATTCTTTCGTAATGATG	0	0	0	0	0	0	0	0
*		21UR-2543	TGAGACAAGAAAGTTTTGGTT	233	329	162	175	399	1527	65	2890
	†	21UR-2544	TCTTCTGTCATCATGCAACTC	0	0	0	0	1	0	0	1
		21UR-2545	TGAACTGTGAAGTGACTAGCT	21	19	12	8	41	140	12	253
		21UR-2546	TAGGTCAATGATTCATAAATT	0	0	0	0	0	0	0	0
	†	21UR-2547	TCTGGCACATGATTTAAAAAA	1	0	0	0	0	1	1	3
	†	21UR-2548	TGATCGTGGTGTTTTCTGAAA	1	3	0	1	3	9	0	17
*		21UR-2549	TAAATCCATACCAGTAGGACA	3	4	7	5	100	81	19	219
	†	21UR-2550	TTAATGTTCCAAAGTTGTTTT	0	0	0	0	0	0	0	0
	†	21UR-2551	TCAACATCCAGTTTTTGAACA	0	0	0	0	2	6	0	8
		21UR-2552	TCTTTCAGTGGGAATAATTTT	0	0	0	0	0	0	0	0
	†	21UR-2553	TAGTCATTGTAGAATCCGCCA	1	1	0	3	36	24	6	71
	†	21UR-2554	TCCGTCACCATCATTAATCAT	1	0	0	0	0	3	0	4

*	†	21UR-2555	TAGACTGATCTGACTGTAAGA	1	0	1	2	14	22	12	52
		21UR-2556	TCAATCGTATGGAAACTTCAC	0	0	0	2	45	23	6	76
	†	21UR-2557	TTCAAACAGGATGAAAATGAA	1	4	3	0	3	10	0	21
		21UR-2558	TTCCACTGTACTGAACATGGA	8	3	0	4	98	119	10	242
		21UR-2559	TATATTTTCACGGTTATTTGC	3	0	1	0	0	4	2	10
	†	21UR-2560	TCACATGCTTGGACTCGATAG	1	2	0	1	31	39	16	90
	†	21UR-2561	TGCTGAAAAATGAAACAATTA	0	0	0	0	0	1	0	1
	†	21UR-2562	TCGCCCCCTCATGGATTTTCA	2	0	0	0	0	0	0	2
	†	21UR-2563	TTGACATACAATCAAAACTCC	14	5	4	4	23	51	1	102
		21UR-2564	TGCTTGCTTCTTGCATAAAAA	0	1	0	0	0	0	0	1
		21UR-2565	TAACGTATTGATTACTAGGTT	1	0	0	2	4	5	0	12
		21UR-2566	TCCAAATTCATCAATCAACTC	0	0	0	1	0	2	0	3
	†	21UR-2567	TACTGATTCCATTTGATTGAA	2	0	0	0	1	1	0	4
	†	21UR-2568	TCATCCCTCTCGTCAACATAA	5	0	1	3	29	20	16	74
	†	21UR-2569	TCGGAATGGGTTCAAGTCAGC	30	16	11	8	32	171	28	296
		21UR-2570	TGATTTTGCATAAAATTTGAC	2	0	0	0	1	4	4	11
		21UR-2571	TCCTATCATACTTTCAAAAAC	0	0	0	0	2	4	1	7
*	†	21UR-2572	TTCAAGGAACTAACGTTGGGA	7	8	11	17	308	394	135	880
		21UR-2573	TGAGTTTTAGATAAGTTTGCC	0	1	0	3	5	3	4	16
		21UR-2574	TGAGCGAATATCATTATTGAT	0	0	0	0	0	0	0	0
		21UR-2575	TTCCACGAAGAGAAATTTATG	0	0	1	0	1	1	2	5
		21UR-2576	TCATGTCGAGAATTCAACTGT	8	2	2	1	3	16	1	33
		21UR-2577	TAAAGTCCGAAAATTTTCACA	0	0	0	0	0	0	2	2
	†	21UR-2578	TATTACATTTGTTTCACACAG	4	0	0	1	8	9	2	24
	†	21UR-2579	TTAGTTTGCGGAGGTAATTTC	0	1	0	0	16	15	15	47
		21UR-2580	TAAAATCTTTGAAATCAGTTG	0	0	0	0	4	2	1	7
	†	21UR-2581	TTTTAAATTCTTGAAACAAAT	0	0	0	0	0	1	0	1
*	†	21UR-2582	TTGGTAGGGAGGCAAACTAGT	49	18	6	8	18	100	21	220
	†	21UR-2583	TGATTACGTTGATCGACTAGT	0	0	0	0	0	3	0	3
		21UR-2584	TACAGATCTTTCACATATAGA	20	5	1	7	4	15	7	59
		21UR-2585	TGATTTTTCCACATAATTCCA	0	0	0	0	1	0	2	3
	†	21UR-2586	TTCGGTTGGTTTAGTTCAAAA	1	1	1	5	70	58	5	141
		21UR-2587	TCCTGAGTTTAAACAATTTCA	0	0	0	0	0	0	0	0
		21UR-2588	TATGACCGCAGTCTCTTTTTT	0	0	0	0	0	0	0	0
*		21UR-2589	TGGTTTTATAGTAGTAGGCTC	52	36	19	74	1023	969	134	2307
		21UR-2590	TGCTTTCACCTTCTTCGTACA	0	0	1	0	3	3	5	12
	†	21UR-2591	TGGCAAATTATCATCCTCAGT	0	0	0	0	0	0	0	0
		21UR-2592	TCCGATTGTAACGAGACTTTA	0	1	0	6	49	34	9	99
	†	21UR-2593	TTTATCTATTAAATGATTGGC	0	0	0	0	0	1	0	1
		21UR-2594	TGGGTCCTTAGCTTGATTAGG	0	0	0	0	0	0	0	0
		21UR-2595	TACAGCTCTTTCAAATTTTTC	0	0	0	0	1	1	0	2
*		21UR-2596	TAGAGAATTTGTAGATGTCTC	14	6	4	24	201	215	4	468
		21UR-2597	TTTTTGGCCTATGATGATGCA	3	2	0	0	9	21	1	36
		21UR-2598	TGCAAAATCAGTTATGTATTT	1	0	0	0	0	0	0	1
	†	21UR-2599	TATCTCCTTCGTTCAGCTAAC	0	0	0	0	5	3	5	13
		21UR-2600	TCAAAGCATGTGTGTAATATG	0	0	0	0	0	0	0	0
	†	21UR-2601	TCTGCAACTCACAAAGTGTCA	0	0	0	0	1	2	0	3
	†	21UR-2602	TTGGTTCCTCATTGAAGATTG	9	1	3	5	23	31	13	85
		21UR-2603	TCAGGCATTTCAATGAGATGA	1	0	0	0	0	1	0	2
*		21UR-2604	TTGAACACGGTTTCGGAATGT	0	0	0	0	12	17	10	39
	†	21UR-2605	TTTCATTACACTTATGGAAAC	0	0	0	0	0	0	0	0
	†	21UR-2606	TCTCTTTTTGTGGCAAAAAAA	0	0	0	0	0	0	0	0
		21UR-2607	TCTGTCTCTTATGATGATAAC	0	0	0	0	0	0	0	0
	†	21UR-2608	TAAGTTCATCATCGGAAAACG	41	32	14	15	127	175	44	448
		21UR-2609	TGCCATGATTTTACTAAAAGC	1	0	0	0	3	1	0	5
		21UR-2610	TGTATTTTTCAAAAACTGCGA	0	0	0	0	0	0	0	0
		21UR-2611	TGCATTTTGCAAGGGTTTTTG	0	0	0	0	1	1	0	2
		21UR-2612	TCCAGTTATAATCGCAAGAAA	0	0	0	0	0	2	0	2
		21UR-2613	TAATGTTGTTTCCATCATAAT	0	0	0	0	0	0	0	0
		21UR-2614	TGAACCTCTAAATTCAGTACC	0	0	0	0	0	0	0	0
		21UR-2615	TACCAACATGCATTACCAACA	0	0	0	0	0	4	0	4
		21UR-2616	TCATTCATTCAAACCGGTTCA	1	0	0	0	2	4	10	17
		21UR-2617	TGCTTTATTTATCATTGATTC	1	0	0	0	0	1	0	2
		21UR-2618	TGAAAATCAAAAATGCTGAAT	3	0	0	1	1	7	1	13

		21UR-2619	TCCAAGGAGTTTTCGTATTTC	1	0	0	0	0	1	8	10
		21UR-2620	TGTGTTGAGTCTTTTGGCGAG	0	0	0	0	2	0	0	2
*		21UR-2621	TGAAAGGCAAAATAAGGATAA	22	18	7	4	21	57	8	137
		21UR-2622	TGTCATTTTTTCCATGAGGTC	0	0	0	0	0	0	0	0
	†	21UR-2623	TCCACAATGACTGTTTCCTTT	0	1	0	2	12	3	0	18
		21UR-2624	TTGTACAATGTTACTTGTTGC	0	0	0	0	0	0	0	0
*		21UR-2625	TAAATAGGTCGAACAAAAAAT	1	0	0	1	27	22	36	87
		21UR-2626	TGGTTGCTTGTTCATCTTTTT	0	1	0	0	0	0	0	1
	†	21UR-2627	TGGTTGTACATTACCCACCAA	2	0	1	0	2	0	0	5
*		21UR-2628	TAAGTCTTTTACTCCCGATTT	2	1	0	0	1	3	0	7
	†	21UR-2629	TAGTTTGCCGTCTTTCATAAT	32	1	1	3	2	18	8	65
	†	21UR-2630	TGATCAACAGATTTACCAAAA	0	0	1	0	0	0	0	1
		21UR-2631	TCAGACCCAGTTGGTTTAGTT	0	0	0	0	0	0	0	0
		21UR-2632	TCAAACTATTTTGCACGATTT	0	0	0	0	0	0	0	0
	†	21UR-2633	TGAAACATCGGATTATCTGAC	0	0	0	0	4	4	0	8
		21UR-2634	TTCTTGCAAGTTGTCACGATT	7	1	0	0	6	17	0	31
	†	21UR-2635	TGCTACGCGAATGTTATTGCT	0	0	0	0	1	3	1	5
		21UR-2636	TCCATCCATTTTTCTACAATG	0	0	0	0	0	0	0	0
*		21UR-2637	TCGGAGAGTGCGTTTAGTTTT	12	1	2	0	3	6	1	25
		21UR-2638	TGTAATGCTGATGTTTTTCTG	0	0	0	0	1	0	0	1
	†	21UR-2639	TGGTATTCCAGTGAACGATTA	1	6	1	0	13	11	2	34
		21UR-2640	TTCACGCATTATTTCCAAAGA	0	0	0	0	0	3	2	5
		21UR-2641	TAAGATAATCCAGATAGGTTT	0	2	0	0	2	3	0	7
	†	21UR-2642	TAATAAGCCACTTTGTGCAAT	1	1	0	2	7	6	0	17
		21UR-2643	TTAATTCGTTTAATTCGTCAT	4	0	0	4	0	10	0	18
	†	21UR-2644	TTGATTACGGCTCCATTACAG	19	12	5	1	40	141	9	227
		21UR-2645	TAACTTCTTCTAAAATTAAAA	0	0	0	0	0	0	0	0
		21UR-2646	TCTGTCTCCCATCACAATATC	0	0	0	0	0	0	0	0
	†	21UR-2647	TGAGAGAACGCAATTGATACA	0	0	0	0	11	8	0	19
		21UR-2648	TGGTCAGATGTTATTTGTTTA	0	0	0	0	1	1	0	2
		21UR-2649	TGTGTAGTTCATTGCTCCCGC	0	0	0	0	0	1	0	1
	†	21UR-2650	TGATAAGAACGTCCAAACAAT	2	1	5	2	10	11	1	32
		21UR-2651	TACTCAGCACTTATGTATAAA	0	0	0	0	4	2	5	11
		21UR-2652	TGCTTTGTTTTTCTGACTCAA	0	0	0	0	1	0	0	1
	†	21UR-2653	TTCCCATTCAAATAACTTACG	0	0	0	0	0	1	0	1
	†	21UR-2654	TATTCATAATTTGTACAGAAA	0	0	1	0	1	1	0	3
		21UR-2655	TACTGCTTTTAATGCAACATG	0	0	1	1	4	2	0	8
*		21UR-2656	TAGAGGAATGATTATGTTAGC	0	0	0	1	68	72	6	147
*	†	21UR-2657	TCAAGGAACTAACGTTGGGAC	1	0	1	1	17	15	9	44
		21UR-2658	TCAAACATTTAATTTGATTCA	0	0	0	0	0	0	0	0
	†	21UR-2659	TACATACATTAAATTCAGAAC	2	0	0	1	26	7	11	47
	†	21UR-2660	TCTGATTAGACTAAAAAGAAA	1	8	4	5	21	39	4	82
		21UR-2661	TGGAGAATCCTATAAGCAGTT	0	0	0	0	0	3	1	4
		21UR-2662	TCAACTTGAATTCAGATTTAT	2	2	0	1	4	11	0	20
*	†	21UR-2663	TTCGGTTTCTCCATACAAATT	9	9	2	8	48	57	13	146
	†	21UR-2664	TTAGCAAGTATTCTGACATTC	0	0	0	0	0	0	0	0
		21UR-2665	TCAATCGTGTTCTCCATGAAT	0	0	0	0	0	0	0	0
		21UR-2666	TTTACAACTAGCACGGTTAAG	0	0	0	0	11	8	6	25
		21UR-2667	TAAACTGGGTTTTTTCTATGG	0	1	0	0	4	3	1	9
		21UR-2668	TTCAAGCTCCAAATTCATTTC	0	0	0	0	1	0	0	1
		21UR-2669	TATCCAGCAATGGGAACGGTT	13	12	5	5	9	31	3	78
		21UR-2670	TAAAAAAACAGTAGCCAAGAA	2	0	0	1	25	21	2	51
		21UR-2671	TAGAAACTGATCTCTGAAAGT	0	0	0	1	0	2	0	3
	†	21UR-2672	TGGGTATTGGAATTGAATTGG	0	0	0	0	0	0	0	0
		21UR-2673	TACTAGACTTCCTGTCGAAAC	0	0	0	0	7	7	2	16
*		21UR-2674	TACGTTGACTTTGTGCAGTGA	1	3	2	8	79	112	8	213
	†	21UR-2675	TCCGTTGTTGATGATTATAAA	1	0	1	0	1	4	1	8
		21UR-2676	TCGTGGTCATTTTTTACATAA	2	0	0	0	1	0	0	3
		21UR-2677	TGCCATCTTTACTCACTATCA	0	0	0	0	0	0	0	0
		21UR-2678	TGACTCTTTTTGTTTTTCGGT	3	5	0	1	13	34	1	57
		21UR-2679	TACATGTGGACCTCCAATTTA	0	0	0	0	2	3	0	5
		21UR-2680	TTTCAGGTTATGACAGCTCAA	0	0	0	0	0	3	1	4
		21UR-2681	TGTTCGTGGCGGCAACATAAT	4	0	0	1	11	11	13	40
	†	21UR-2682	TACTTTTCTGCTGACCATTTA	2	1	0	2	4	3	1	13

*		21UR-2683	TATGTAGTAACGGTTGCTGTT	108	79	58	82	1026	1564	97	3014
	†	21UR-2684	TAGAGGTGCACTGTGAGTTAA	0	0	1	1	1	2	1	6
		21UR-2685	TCATTTTCAGTTGTTTTCGTA	2	0	0	0	2	7	1	12
*	†	21UR-2686	TCCGGCATTGATCTGTGGAGG	90	114	46	43	242	935	39	1509
		21UR-2687	TATATTCGTAATATTCTCAAG	0	0	0	0	0	1	0	1
	†	21UR-2688	TTCAGTCGTAGACTCATCAAC	1	0	1	1	11	7	3	24
	†	21UR-2689	TTTGGACTGTTCGCAAGAGTC	8	1	2	0	0	14	3	28
		21UR-2690	TGATTGCCTTGCTTTTTTCAT	0	0	0	0	0	3	2	5
		21UR-2691	TGGTTGAGTGAAATTCCACAA	0	0	0	0	0	0	0	0
		21UR-2692	TAACATGTTATCTAACTGTAC	0	0	0	0	2	0	0	2
	†	21UR-2693	TTGTAAATTTGCCTTTATGAT	1	1	0	0	2	0	1	5
		21UR-2694	TTCACAAATACCAATAACAAC	1	0	0	0	2	0	0	3
		21UR-2695	TAAAATTGTATAACATAAAAA	0	11	2	3	9	10	0	35
		21UR-2696	TGAATCTTATTTTGGCACCTC	0	0	0	0	0	0	0	0
	†	21UR-2697	TGGGTTTAATCAATATTTCTA	0	0	0	0	0	0	0	0
	†	21UR-2698	TATACCGATTCTTAGTTCCAT	3	0	0	0	1	4	0	8
	†	21UR-2699	TTTCATTTCCGTCTTATCATC	1	0	0	0	8	2	6	17
		21UR-2700	TGCATTAACATTGTCTTCATC	0	0	0	0	0	0	1	1
	†	21UR-2701	TTTGTGAACTGCAATTGATGT	13	36	15	43	817	719	81	1724
		21UR-2702	TACATTTCCTCAATTTCATCG	1	0	0	0	0	1	2	4
	†	21UR-2703	TTTTGAAAATTGAAATGAGAA	0	0	0	0	2	2	0	4
		21UR-2704	TCGGATACTCGTAATTTTCCT	0	0	0	1	0	1	0	2
		21UR-2705	TGAGTCATTAAACAATAAGTG	0	0	0	0	0	1	0	1
*	†	21UR-2706	TTCCAAAGAACGCGGGATTTC	17	11	6	6	12	60	8	120
	†	21UR-2707	TCTTAACGTCTTGATATATGA	0	0	0	0	0	0	0	0
	†	21UR-2708	TGGGTGAAGTTTGGTCTTTGT	1	2	4	2	37	67	3	116
		21UR-2709	TAACAATCTCTTGAAATCTTT	0	0	0	0	0	0	0	0
*	†	21UR-2710	TGGATAAGTTGTATCTTCGGG	503	186	114	99	902	1501	212	3517
*		21UR-2711	TCAAACACGCTGATTTCGTGG	1	0	0	0	2	4	1	8
	†	21UR-2712	TCGATTGTTTGCCTCTAAACC	0	0	0	0	1	1	0	2
	†	21UR-2713	TGATGACTCTGTAACGTCGCA	0	1	1	1	8	7	4	22
	†	21UR-2714	TCATTGTAACAGTGGCATGAG	0	0	0	0	0	0	0	0
	†	21UR-2715	TAAAGATCAAAAGCACATATT	1	0	0	2	17	14	27	61
	†	21UR-2716	TATTCATGGACATCGAGGAAT	4	5	7	2	8	27	5	58
	†	21UR-2717	TCGAAAAATTGGACTGATTCA	0	0	0	0	2	1	0	3
	†	21UR-2718	TGTGGTTATAAAGGTTTTAGA	2	0	1	0	1	2	0	6
	†	21UR-2719	TCACTGAAGTCACTCACAATG	0	0	1	1	12	17	0	31
		21UR-2720	TGATCCTTACATCACTTTGAA	8	0	3	0	1	9	4	25
	†	21UR-2721	TTTAGATCAGTGCTGTGCGGC	475	297	184	89	981	2901	360	5287
	†	21UR-2722	TGCTAATTATTCCAACCCCGA	0	0	0	0	0	1	0	1
	†	21UR-2723	TCATTATGAAGCTTCACAAAA	0	0	0	0	3	8	1	12
		21UR-2724	TATCCTTCTGAATGACCAATG	2	0	1	0	12	19	1	35
	†	21UR-2725	TCCTGTTAGCAATGATTATTT	2	2	3	3	9	8	0	27
		21UR-2726	TGTTTGAAGCAAATTGCGCAA	1	0	0	0	2	4	0	7
		21UR-2727	TTCCGAAGTATTCAACATTAG	0	1	0	0	4	2	0	7
		21UR-2728	TGACTGAGGACTTTATTTAAA	2	5	0	2	4	18	0	31
*	†	21UR-2729	TAGACACAGTTGATACGATTT	4	2	2	1	9	88	2	108
		21UR-2730	TCTAAAAGCGAAATAGTGATC	0	0	0	0	3	1	0	4
		21UR-2731	TGGAGCGTTTGATTTGTTCAA	1	1	0	0	7	7	9	25
		21UR-2732	TATGCCATCTGTTTCCTCAAA	1	0	0	1	0	1	0	3
		21UR-2733	TTCAAAATCTTTGTTCTTCAA	0	0	0	1	0	2	1	4
		21UR-2734	TGAATAATCATGCAACTTCAA	4	1	1	1	15	12	8	42
		21UR-2735	TGCAGCCAAATATAGTTTGTG	0	0	0	0	0	1	0	1
		21UR-2736	TCCACGACGATACGATTTTCG	2	0	0	0	0	5	0	7
		21UR-2737	TACTTGGACCAAATTTTCAAC	0	0	1	0	12	5	0	18
		21UR-2738	TAGCTCATTTCGATTTATTAT	0	0	0	0	0	1	0	1
		21UR-2739	TGCACTCTGCAGCCTAATAGT	0	0	0	2	3	6	1	12
*	†	21UR-2740	TAATCTTAGGCTGTGAAAAGA	12	2	4	1	4	17	1	41
	†	21UR-2741	TATGTTCAACAAACTAACATC	0	0	0	0	4	2	0	6
*		21UR-2742	TAGGAAAGGGGACTGCATTGT	48	21	13	10	39	166	11	308
	†	21UR-2743	TGAAATGAAGGCATACATTAG	19	3	2	2	3	27	2	58
		21UR-2744	TAGTCGTATTTCTCTTCTGAC	0	0	0	1	3	6	2	12
	†	21UR-2745	TTACACAAGTTTTATCCAGTC	2	0	0	0	2	7	0	11
	†	21UR-2746	TAGTTTCGAGTTTGGTTTTTG	0	0	0	0	12	10	4	26

	†	21UR-2747	TAGATCGAAGCTCAAACTATT	0	0	0	0	4	1	0	5
	†	21UR-2748	TTCATGTTGACTTGTCAAAAG	0	2	0	0	0	3	0	5
		21UR-2749	TCGCAGTTTGGATCTTTTATC	2	2	0	6	86	201	14	311
	†	21UR-2750	TCTGATCAATGAAATATTATC	0	0	0	0	0	0	0	0
		21UR-2751	TGAATTTGGTGAATGTCGAAT	0	0	1	0	1	5	0	7
	†	21UR-2752	TTAGAGGTGCACTGTGAGTTA	3	2	4	0	9	16	2	36
		21UR-2753	TCACAAAACTCATAGCCCTAA	1	1	0	1	1	0	0	4
	†	21UR-2754	TCGTTGATTTTTGGATATAGG	2	2	1	2	59	51	65	182
		21UR-2755	TTAAAAGAAATAGATTTGATG	0	0	0	1	2	0	0	3
		21UR-2756	TACAGCTGTTTTAGTTGGTTT	1	0	0	0	0	1	0	2
		21UR-2757	TGTGCGGGCAGAGCTTTTATG	0	0	0	0	0	0	0	0
		21UR-2758	TGCAATCGGAGGATTACCCCA	1	0	0	0	31	43	22	97
		21UR-2759	TACACTTATCCCATTTCTTCC	0	1	0	0	0	1	1	3
		21UR-2760	TTCGTAGTCTACCGTTTTACC	0	0	0	0	0	3	0	3
		21UR-2761	TGGATTTTGTCTCTCGTAGGA	91	31	25	26	94	253	75	595
		21UR-2762	TAAATTTTCTGCTCATTTATA	0	0	0	0	0	0	0	0
		21UR-2763	TGAAATCGTAATTTCTTCTGG	0	0	0	0	1	0	0	1
	†	21UR-2764	TTGGAAGAGAACTCGATAAAT	1	0	0	0	0	1	0	2
		21UR-2765	TCGTGCATTGCAAATTTTTCA	0	0	0	0	10	1	1	12
		21UR-2766	TTCACGAAAAGTTTTGGAGAA	1	0	0	0	6	3	3	13
		21UR-2767	TATGTTTCCTACAGCGATGAA	3	0	0	0	2	6	1	12
		21UR-2768	TGTATGCGATTTGTACATTTC	0	1	0	0	4	1	0	6
	†	21UR-2769	TAATGCCATTTTCCATCAATG	5	1	1	0	0	5	2	14
	†	21UR-2770	TAAGTCACTGCTTCTGAATTT	0	0	0	0	0	1	0	1
	†	21UR-2771	TGAAATGGTGAAAGTATTGTC	2	0	0	0	2	1	5	10
	†	21UR-2772	TACTTCTTCGACTAAACCCAA	0	0	0	0	10	1	0	11
	†	21UR-2773	TGGGAGGAAAATTGTTCAGAT	2	0	0	0	3	1	2	8
	†	21UR-2774	TCAATGATCAAAGCTTGCAAC	4	1	4	3	20	32	14	78
		21UR-2775	TCTAATTGGTTTTGTCTTTAT	0	0	0	0	2	3	1	6
		21UR-2776	TGAATCGTTTTTTTTTTGTTC	0	0	0	0	0	0	0	0
		21UR-2777	TAAATAATCACTAGGGGAAGA	2	0	0	0	0	4	1	7
		21UR-2778	TGGTATTCCTGCATACAGATC	1	2	0	0	9	7	1	20
	†	21UR-2779	TACTTGGGGTTCAGCATTTAT	17	2	1	1	2	14	11	48
		21UR-2780	TTCACGATCGTTCAAATCTTG	4	1	0	0	4	8	4	21
		21UR-2781	TGGAATTGGAAATTGGACAGA	2	1	1	1	23	23	5	56
		21UR-2782	TATAGCCTTTTGCATTTAACT	0	0	0	0	0	0	0	0
		21UR-2783	TAAACAGTATAATTGTTAAAC	0	0	0	0	0	0	0	0
		21UR-2784	TAGAACGAATTTTTATCCACT	1	0	0	0	0	2	0	3
		21UR-2785	TAATATCGTAAAAAGTTGTGT	2	0	0	0	0	0	1	3
		21UR-2786	TACAATTTATACCATTCTGCC	1	0	0	0	0	4	1	6
	†	21UR-2787	TGTCAAATAAACAGTCGAAAA	1	0	0	0	2	3	0	6
	†	21UR-2788	TGAGTGGAAGTTTTAAATTTT	0	0	0	2	4	1	0	7
		21UR-2789	TACTTTCTCTTGAATCCTGAA	1	0	0	2	0	1	0	4
	†	21UR-2790	TCGATATAGATCTGCCGATTC	3	3	1	11	133	116	34	301
		21UR-2791	TAGTTTCTCTTTGTTGCAAAA	0	0	0	0	0	1	0	1
		21UR-2792	TCCGTGTGTTAATCCTGTGTT	0	0	0	0	0	0	0	0
		21UR-2793	TCAATCGAGTTTGAAATGCAC	0	0	2	1	15	14	2	34
	†	21UR-2794	TGCTGAATAAATTTGAAATAG	0	0	0	0	1	2	0	3
		21UR-2795	TCAAATTGTTGAATTAAACAA	0	0	0	0	1	0	0	1
	†	21UR-2796	TACACGCGATTGATTTTTATC	0	0	0	0	0	1	0	1
		21UR-2797	TGGGTTTCCATCAATATGCAT	0	0	0	0	1	3	6	10
	†	21UR-2798	TGTTAGACATATTAGGTCACC	1	0	1	1	7	11	1	22
*	†	21UR-2799	TTGGACGAAATTGCAATATGT	48	43	18	36	112	249	29	535
		21UR-2800	TCATAGTTCATTTTGAAAATA	0	0	0	0	6	3	1	10
	†	21UR-2801	TAAACCATATTACAATAAAGT	0	0	1	0	4	2	0	7
		21UR-2802	TCACATATCGAAATCTTTCAG	0	0	0	0	0	2	2	4
		21UR-2803	TCAACTATTGCCATAGCACAG	1	0	0	0	0	0	0	1
		21UR-2804	TATCCTAATGGCTACTGCAAT	0	0	0	0	9	19	2	30
		21UR-2805	TAGATACAATCATGAATTAAG	1	0	0	0	0	2	0	3
		21UR-2806	TAGACGAAATTCTCCTTGTAC	0	0	0	1	13	4	2	20
	†	21UR-2807	TACATATAGCATCGAAATGTG	0	0	0	0	7	7	1	15
		21UR-2808	TTGGTGGAGCAAATTTAATTC	1	0	0	1	1	2	0	5
		21UR-2809	TGTGAATCTGAAAATGTCCTA	3	3	0	0	0	3	0	9
	†	21UR-2810	TGCTGGAATTCCTGTTGCAAT	0	1	0	0	0	2	0	3

		21UR-2811	TTCGATGGGAACATTGTTTTA	0	0	0	0	1	2	0	3
	†	21UR-2812	TAATTGTTTTTCAGCGTGTCC	1	0	0	0	5	8	7	21
	†	21UR-2813	TCAATGTTTCAAATCCTTCAT	0	0	0	0	0	1	0	1
		21UR-2814	TTTGTCGATCCACGTGGGATT	2	0	1	0	1	5	0	9
	†	21UR-2815	TCAATGTACTATGCTACAAAA	0	0	0	0	0	0	0	0
*		21UR-2816	TAAGGTTGGCGGGAGACAGTA	198	77	56	23	157	843	176	1530
	†	21UR-2817	TCATAAATCAATCCATGTACG	0	0	0	0	0	0	0	0
*	†	21UR-2818	TCAGATTTTGGACTACGATCG	12	1	3	2	15	29	3	65
		21UR-2819	TGTAAAGATTAAAGAATGGAA	5	7	4	3	33	40	11	103
	†	21UR-2820	TTCATTGTCGATTATCATTGC	0	0	0	0	3	1	0	4
		21UR-2821	TGACAACAAAAAAAAACCAAT	0	0	0	0	1	1	0	2
	†	21UR-2822	TTGAAGACTGTTGAACATATC	5	2	1	0	4	19	0	31
	†	21UR-2823	TGCTATTTCGGTTATAACTGA	4	1	0	0	1	5	4	15
		21UR-2824	TCTCAAAAAATAGAAGCATTA	0	0	0	3	10	9	2	24
	†	21UR-2825	TAATGTTTCCTTCCATTTTGC	0	0	0	0	0	0	0	0
	†	21UR-2826	TAATTAATTCATTGATGTTGG	57	2	5	6	10	30	25	135
	†	21UR-2827	TATAATTTGAAACGCTAGGGA	19	2	2	2	2	26	16	69
		21UR-2828	TCAAAGGATAATGTTTTTTAA	1	0	0	1	7	6	0	15
*		21UR-2829	TACGACCAAAAATGAGATTGA	2	8	7	24	340	349	41	771
	†	21UR-2830	TCTCAATGTAGTAGTAAAAAG	1	1	0	4	20	18	1	45
		21UR-2831	TATGATCAAGCCTTATGATGG	1	0	0	1	18	27	5	52
		21UR-2832	TAACCAGCAAACCCTTCGAAA	0	0	0	0	0	0	0	0
		21UR-2833	TTGGGAAGTTCCTTTTGCAAC	1	0	0	0	2	1	0	4
*	†	21UR-2834	TTCCACGGCTGAACAGGACAA	133	80	58	37	245	546	66	1165
	†	21UR-2835	TGTAATGTAACTGTATGCCTA	0	0	0	0	8	4	1	13
		21UR-2836	TATCGACAAACTCTGATCGAT	0	0	1	0	2	1	0	4
	†	21UR-2837	TAATTTTGCTGAATTAAAAAA	0	0	0	1	1	1	0	3
		21UR-2838	TAGTGCAGGCATTCGAAAATC	1	0	0	0	6	13	2	22
	†	21UR-2839	TTTGATATGCCTATACTGTTC	0	0	0	0	0	1	0	1
		21UR-2840	TCGGGCACAATTTTTCTCGAT	0	0	0	0	0	0	0	0
		21UR-2841	TCTGACTTTCGCCTGCCTGGA	0	0	0	0	1	0	2	3
	†	21UR-2842	TGAAATAAATTGGTCAAGAAA	0	0	0	0	2	0	0	2
		21UR-2843	TGCGTAACCGTTTCTGTGTGA	1	0	1	0	6	17	3	28
		21UR-2844	TCTTCAGTATGGGTCAATCTC	0	0	0	0	1	0	2	3
		21UR-2845	TAGGTTGAATGGAAATATGTC	5	6	1	4	5	21	0	42
		21UR-2846	TCGAATCGCCGTCGAAAATGA	0	0	0	0	1	1	2	4
		21UR-2847	TACAGCGTAGTTACTTAGTTT	0	0	0	0	6	1	0	7
		21UR-2848	TCAGAGAGGTAATTTATTAGG	3	0	1	1	0	4	0	9
		21UR-2849	TTGAGCACGTTCTTGGTAATA	0	0	0	0	0	0	0	0
		21UR-2850	TCTTGTTGATTTGATACGTGC	0	0	0	0	9	11	2	22
		21UR-2851	TGTTAAGTTGTGATAAATAGT	0	0	0	0	0	0	0	0
	†	21UR-2852	TTTGATGTATAATGTCCAATA	0	0	0	0	0	0	0	0
	†	21UR-2853	TAATTGACGCTGCGGGTGTAC	6	4	2	1	2	15	1	31
*	†	21UR-2854	TAATCTACAAAGCAGGGAAAA	28	6	2	2	12	52	5	107
		21UR-2855	TGCAATCTCGTGACTTCCTGC	0	0	0	0	0	0	0	0
		21UR-2856	TATGACGAAGAAGTTCAAAAA	0	4	2	5	23	49	2	85
*		21UR-2857	TCAGGCTTCGCTTCTAGTTGG	0	0	0	0	4	7	9	20
		21UR-2858	TAAGCCTTTCAATGGTACAAA	0	0	0	0	0	1	0	1
	†	21UR-2859	TCAGCGTGTTTTTGCATAAAT	1	0	0	0	1	0	0	2
		21UR-2860	TTGATAGTTTCTCTATATAGC	0	0	0	0	1	0	0	1
*		21UR-2861	TACATTAGAATGAGAAATTAA	13	14	7	13	36	93	5	181
*		21UR-2862	TACAGCCCACTGCCATCTAGT	2	0	0	0	0	2	18	22
*		21UR-2863	TGCAATTCAAGAAGATAACGC	2	0	1	2	49	70	6	130
	†	21UR-2864	TACGTCGGTTGATTGAATTGG	1	0	0	0	1	0	1	3
	†	21UR-2865	TTCTTCGAAGTCTTTGTTGTG	25	8	5	8	16	29	10	101
		21UR-2866	TGTTTCGTCTGCAATTGATGG	15	3	4	1	10	26	0	59
	†	21UR-2867	TTCCTCGATCTTCTTATAAAC	8	1	1	0	6	5	7	28
	†	21UR-2868	TAATTACGGATACTGTTCAAA	15	18	19	17	45	134	22	270
		21UR-2869	TAACATATATTCAAACTGTTA	0	0	0	0	1	0	0	1
		21UR-2870	TTAACGAAATAGCTAGCTGTT	17	1	0	3	2	22	0	45
*		21UR-2871	TTCCTTACGAAATCTCCATAA	7	0	0	1	16	11	6	41
*	†	21UR-2872	TGCTGTGTAGGGCTATAATAA	142	78	43	31	271	489	34	1088
	†	21UR-2873	TCGTTCATATTAGCTATTATC	0	0	0	0	0	0	0	0
		21UR-2874	TTGAGGATCTGCCAGAGGTAA	0	1	0	0	0	2	0	3

	†	21UR-2875	TATTTCGATGGACTTTGGGTT	105	32	24	19	93	221	92	586
		21UR-2876	TAACATGCCAAAATGTCCCTG	0	0	0	0	0	0	1	1
		21UR-2877	TAAAAATCGTGTTTTAATAGT	1	0	0	1	7	5	8	22
	†	21UR-2878	TTGTGTCGGTAAAAATTAAGC	2	2	0	1	19	3	5	32
		21UR-2879	TATGTGAATAATTTCAGTACA	0	0	0	0	0	1	1	2
		21UR-2880	TCAGTCTAGAAGAATTTTGAA	0	1	0	3	32	38	2	76
		21UR-2881	TGCTATGGCTATGCCCACATT	3	0	0	0	1	0	0	4
		21UR-2882	TGAAACCAACTGTTAATTAAC	0	0	0	0	0	0	0	0
	†	21UR-2883	TACGAAAAAAACACTCTAACT	1	0	2	3	2	5	1	14
		21UR-2884	TGCATTTGCTGAATTTGACAC	3	5	1	7	33	45	2	96
	†	21UR-2885	TCAGTTGGTATCTTACTTATT	0	0	2	4	3	13	0	22
		21UR-2886	TCTATTGACAATCTCCAAAAT	0	0	0	0	0	0	0	0
	†	21UR-2887	TCTTCGAATCCATGCAAAGCT	0	2	0	0	1	2	0	5
		21UR-2888	TATAGTCGCTATACAAACAAT	6	6	3	13	146	104	22	300
		21UR-2889	TTAGTTGATGTGATTGTTCCA	6	6	2	5	20	32	6	77
	†	21UR-2890	TCCTTCAAACGTGTCCATTAC	0	0	0	0	1	0	0	1
	†	21UR-2891	TAAGACCGTGGTAAATATACG	0	0	0	1	5	4	0	10
	†	21UR-2892	TGATAAGGTACAGAATAGTTG	0	0	0	0	0	0	0	0
		21UR-2893	TTGTCTTTTGTAGATCACGTC	6	2	3	25	259	244	35	574
		21UR-2894	TCACGTTGAAACTAAAATGTC	1	0	1	1	4	5	3	15
		21UR-2895	TGCGTATTCTACCATCAGATA	0	0	0	1	2	5	6	14
		21UR-2896	TTCACGGACTACATAAGTTGC	3	0	0	0	1	4	0	8
	†	21UR-2897	TGAAATCCACAACTACAATGC	1	0	0	0	0	1	1	3
	†	21UR-2898	TCATTGTTCTTTAAAGGGCTG	2	0	3	0	0	3	0	8
		21UR-2899	TATGCAAATTATTAACCGAAA	0	0	0	0	0	1	1	2
*		21UR-2900	TGAACTACTAAAGACAGGAAA	3	1	2	20	180	130	10	346
	†	21UR-2901	TCGTTGAATTGGCTGAAATAA	2	2	0	1	21	32	0	58
	†	21UR-2902	TGCTTGAGGTGAATTGTAGTC	1	1	0	4	29	32	4	71
		21UR-2903	TCCTATAGAAATCCGAGTCTG	5	3	6	2	8	31	14	69
*		21UR-2904	TACTGTTTGCTTCTTTTTTGA	0	3	1	2	3	6	0	15
	†	21UR-2905	TTCAATCAGATTGATTTGTCC	0	0	0	0	2	0	0	2
*	†	21UR-2906	TGGGTGGTGGATGTGTACAAT	44	18	8	4	15	94	26	209
		21UR-2907	TTAGGATTTGGAAATGACCGA	2	4	2	7	58	31	1	105
	†	21UR-2908	TGATTTGGAATACGTTGATTT	21	26	6	13	32	62	0	160
*		21UR-2909	TGAAGCATGCATATCGGTCTG	37	82	40	94	1860	2375	135	4623
*	†	21UR-2910	TGCAGTTAAGGCTATTATTTC	1	2	2	3	14	42	2	66
	†	21UR-2911	TCATATACTTGGACAGGGAAC	0	1	1	3	33	63	24	125
		21UR-2912	TCAATCCGAATGTTTTTGGAA	0	0	0	0	0	0	0	0
		21UR-2913	TAACTGATGTGTTCAACTTAA	0	0	0	0	4	6	0	10
	†	21UR-2914	TTCCTAGTCTCTTAATTAGCT	0	0	0	1	0	1	0	2
		21UR-2915	TTGGCGTTTTTCAACCATTTC	0	0	0	0	0	0	0	0
*		21UR-2916	TACGAGTTGTGCACTTTGGAA	1	0	2	0	6	13	0	22
*	†	21UR-2917	TGTGACGTTGAAATGAGTGGC	96	157	76	35	272	1312	63	2011
		21UR-2918	TGCATTTTGGACATGTGTTAC	2	1	0	2	3	7	2	17
	†	21UR-2919	TGATGGCATTTTACATGGCAC	5	1	0	0	2	7	0	15
		21UR-2920	TCTTGAGTTGTCATAATCAAT	0	0	0	0	0	0	0	0
	†	21UR-2921	TCGATTTACTGGTGTCGATTT	1	0	0	0	0	1	1	3
	†	21UR-2922	TTGTATAGCCAATAAAACGCA	0	0	0	0	0	1	0	1
	†	21UR-2923	TGCTATTGTCTTTAATACAAT	1	0	0	0	2	1	0	4
		21UR-2924	TCGGACCCTCTTCACCTTGTC	0	0	0	0	0	0	0	0
	†	21UR-2925	TCAGACTCAGACTACAGTTCA	0	0	0	0	2	3	0	5
		21UR-2926	TTCGGTAAATATTGTCATTTT	4	0	1	0	3	3	1	12
		21UR-2927	TAATTAGTGCTAAATAAATAG	0	0	0	0	0	0	0	0
	†	21UR-2928	TGATTGTTTTTGGTGCTTATG	0	0	0	0	10	9	3	22
*	†	21UR-2929	TTGGTTCAAAAATGAAGCTGT	17	28	11	16	57	180	2	311
	†	21UR-2930	TTAGCATTACGTAATGCTTGA	0	0	0	0	0	0	0	0
	†	21UR-2931	TCCAACAAATGTTGTTTTTTT	0	0	0	0	1	0	0	1
		21UR-2932	TATGTGAACGTTTCATGAGGT	3	0	0	1	3	9	0	16
	†	21UR-2933	TAACAAGAATACATCGGAGGT	4	2	2	1	18	17	78	122
*	†	21UR-2934	TAATTTTGTAGGACTTCAATT	10	1	1	2	12	18	12	56
		21UR-2935	TTAGTATGGAATTAATGTATT	0	4	2	0	11	12	0	29
		21UR-2936	TATATGTTCATTTATTAAGAA	1	0	0	0	0	0	0	1
		21UR-2937	TACTCATTGGATGTGCGTTCT	6	18	10	8	42	142	3	229
	†	21UR-2938	TCAAAGCACAGTGCAAAACAT	0	0	0	0	0	0	1	1

		21UR-2939	TAAAACAACGGGCTAGTTGGC	2	1	2	0	2	10	1	18
*		21UR-2940	TAAAGATAGCTGTTTATATTT	21	15	9	15	37	50	3	150
		21UR-2941	TTATTGTCCTTATTCCACCGA	0	0	0	0	0	2	3	5
		21UR-2942	TAAATTTTCCTTCGGTACAAC	4	2	1	1	24	31	6	69
*		21UR-2943	TGCGACCGCGATACTTCTAAA	5	30	15	7	43	140	37	277
	†	21UR-2944	TGCTTTGATCAATTCCCGATA	3	0	0	0	0	1	0	4
		21UR-2945	TGATCGCAATAAATTAATAAA	0	0	0	0	0	0	0	0
	†	21UR-2946	TACTTGTCATTTTATCGAAGA	0	0	0	0	5	1	2	8
	†	21UR-2947	TGGTGAATCTTTTAAGAAGTA	0	0	0	0	0	0	0	0
		21UR-2948	TGAGTTTGCTGGATATGGTCC	2	1	0	0	6	11	3	23
*	†	21UR-2949	TATAGTTTCTCGACGGTGTGT	129	189	76	68	397	1290	65	2214
		21UR-2950	TCATGTATTTTCTAGTTTTCC	0	0	0	0	0	0	0	0
		21UR-2951	TCTATAATTCTTTTTTTGTTG	0	0	0	0	0	0	0	0
		21UR-2952	TGAATCTGACTCATTGAACAA	17	7	2	12	41	71	0	150
		21UR-2953	TGAAGAGTTATATTGACTTAT	1	0	0	0	1	1	0	3
		21UR-2954	TACATGCTGTTTTTCCGTTAA	16	16	9	11	30	64	3	149
		21UR-2955	TCATATCGCTTTTACTGTATT	0	0	0	0	4	0	0	4
	†	21UR-2956	TTCAGGAGGTTTACCTTTGCA	0	3	0	1	15	23	0	42
		21UR-2957	TGACAAATCCGTTCGGCTATG	0	0	0	1	21	29	25	76
		21UR-2958	TACTTCTTTAATCTTGCCTAA	1	0	1	2	2	12	4	22
		21UR-2959	TTTACCTTCTGCCTTTTTGAA	4	0	0	0	3	4	1	12
		21UR-2960	TAAAAGCTTATTTATTGACGC	1	1	0	0	8	11	1	22
		21UR-2961	TGAACAAAACTGGTAAATGAA	0	0	0	0	0	0	0	0
		21UR-2962	TGTGACACATGGTAATTGAAT	0	1	2	2	75	67	22	169
		21UR-2963	TGTGTCTAAAACATACCTAGG	0	0	0	0	0	0	0	0
		21UR-2964	TACAACAGAATTATGTGAGGA	1	2	0	1	11	12	1	28
		21UR-2965	TAATTCCGTAACTGTATGATG	0	0	0	0	4	0	0	4
	†	21UR-2966	TCATATTTTAGGCTCGATTCG	2	1	1	3	14	24	9	54
	†	21UR-2967	TGCTTGGATAAAAAGCGTTGC	0	0	0	0	1	3	0	4
		21UR-2968	TATAAGTATTGCTTTTGTTTT	0	0	0	0	0	0	0	0
	†	21UR-2969	TGAGAGGACCATGTCAAATGT	0	0	0	0	1	1	0	2
		21UR-2970	TAAATTCTTGAAACTGGTTTT	0	0	0	0	0	1	0	1
		21UR-2971	TGAAGTGGGTGTGTAACAGAA	3	1	0	0	2	25	2	33
		21UR-2972	TGTGTTTCGAGTACTTTTGGG	0	0	0	0	0	2	0	2
		21UR-2973	TGCTACTCGTATTTTAGTGAA	1	1	0	0	0	0	0	2
	†	21UR-2974	TTTCGTAATAGTAGAATACAG	37	59	18	23	50	165	0	352
	†	21UR-2975	TAGTCCATCGATTTTATATAG	0	0	0	0	1	0	2	3
	†	21UR-2976	TCGTTGATCTCATCTCTCTCT	1	0	0	0	0	0	0	1
*		21UR-2977	TATGATTAGGCGTTAGCAAAA	2	0	1	1	28	19	11	62
	†	21UR-2978	TTGAAGGACTCCTAGATTTCA	0	0	0	0	0	0	0	0
	†	21UR-2979	TCCAAGAATTCCAGTTTTCCA	0	0	0	0	1	0	1	2
		21UR-2980	TCCAGTTGTTATTGGCATATC	0	0	0	0	2	1	1	4
		21UR-2981	TCAATATGTCCATAAAATTCA	0	0	0	0	0	0	0	0
		21UR-2982	TGATGTTCACATTTTTGCCTT	0	0	0	0	0	0	5	5
		21UR-2983	TGTGGGCCTTATTTTCTGTGG	1	0	0	0	0	1	0	2
		21UR-2984	TGCGTCGTTGAACTTCAAACA	0	0	0	0	0	0	0	0
		21UR-2985	TACTTATATAGTAGTTGAACC	0	1	0	1	5	7	0	14
		21UR-2986	TGAAATAGCTTTATTGATTGG	1	1	3	5	24	21	4	59
		21UR-2987	TGCTAATACGATATGTTTGCC	0	0	0	0	1	0	0	1
		21UR-2988	TTTTAAGATCACACCTCCAAA	0	0	0	0	0	0	0	0
	†	21UR-2989	TGGTAGAAACACATTTAGAAT	7	10	13	22	261	177	11	501
		21UR-2990	TAGTTGAATTGGTGCAAACAT	0	1	0	0	1	2	0	4
	†	21UR-2991	TTGATGTGACGAAAACTGACG	0	0	0	0	15	10	1	26
		21UR-2992	TTTCACAGAAATACTATGATC	0	0	0	0	15	12	0	27
	†	21UR-2993	TTCGAGGGTTATTGATTTAAA	0	0	0	0	0	3	2	5
	†	21UR-2994	TTCGCTCGTGTTGGCAGATAA	9	2	0	1	7	20	1	40
		21UR-2995	TAGGATTTTCGAAAATGTTAC	0	0	0	1	4	1	3	9
		21UR-2996	TATGTACAACCATATTGTGCG	0	0	0	0	2	2	0	4
	†	21UR-2997	TTTCCGATATTTAGTTTCAGA	1	0	0	0	3	2	2	8
	†	21UR-2998	TTAAGAATTGTAAAGCCTTCT	0	0	2	3	30	21	3	59
	†	21UR-2999	TTGAAATCGTTTTCTGTGTGC	0	0	0	0	6	4	7	17
	†	21UR-3000	TCAATTGGTTGCAGTCGTAAA	0	0	0	0	3	4	0	7
		21UR-3001	TGCAAATTTTGAAAATGATGT	0	0	0	0	0	1	0	1
		21UR-3002	TCGTAATCTGTTTTAGACAAA	0	0	1	0	5	7	0	13

		21UR-3003	TAGATAGAATGGAACTTTACC	2	0	0	1	3	5	0	11
	†	21UR-3004	TAGTACCGTGATTTGATTTGG	1	0	0	0	2	3	1	7
		21UR-3005	TATCCATATAATGTTAAAAGG	0	0	0	0	1	0	0	1
		21UR-3006	TTCACCTGTGATCGCGATGTT	16	5	5	7	66	99	116	314
		21UR-3007	TTAACAATAGTTAATGGGAAG	0	0	0	0	0	0	1	1
		21UR-3008	TGAGATGGTGATTCTTAATTT	7	8	6	6	37	41	1	106
	†	21UR-3009	TATTGGTCTCTATGCTTTTAC	0	0	0	0	0	0	0	0
	†	21UR-3010	TAGGTTGTTTGATGAGGCGCC	14	6	7	2	10	16	2	57
		21UR-3011	TTGTACTAGTATTTGGGTACA	0	1	0	3	10	3	0	17
	†	21UR-3012	TGGAGGTATCCTATCGTGTGT	0	0	1	0	4	3	16	24
		21UR-3013	TAATAAGGGTGTCCCGTAAAA	1	0	0	0	1	2	1	5
		21UR-3014	TGGCTTGTATGATACACAATT	8	8	2	2	55	52	4	131
	†	21UR-3015	TGATCCCGTTGTTTATATTGT	0	0	0	0	1	2	1	4
*	†	21UR-3016	TGTTATTTGAATCAGACGACT	140	96	71	115	906	1290	291	2909
*	†	21UR-3017	TTCAGAAAGTAAGTGAAGCAA	4	4	0	2	17	23	1	51
	†	21UR-3018	TCTTCTCGTTCAATGCATTTC	0	0	1	0	0	0	0	1
		21UR-3019	TATGGATTTTGGAGACATAAT	0	5	0	4	91	70	4	174
	†	21UR-3020	TTGATTGACTTCCTTCTAATC	6	3	2	0	1	6	0	18
		21UR-3021	TGAACTAAACTTATGAAATAT	0	0	0	0	0	1	0	1
		21UR-3022	TTAATATAATTGATTGTGTTT	1	0	0	0	0	0	0	1
	†	21UR-3023	TCGGTGAATCCTCGGATTGAT	4	2	0	0	5	12	9	32
		21UR-3024	TAAACATCTCACATCTGGTCG	21	10	9	8	64	73	28	213
		21UR-3025	TCCCACAGATTTGCAATAGAA	0	0	0	0	0	0	0	0
		21UR-3026	TGTAGTTAAAATTCAGTGTAG	0	0	0	0	1	0	0	1
		21UR-3027	TATGTTGTTCTCGGAATGGTT	7	3	0	4	72	77	25	188
	†	21UR-3028	TGTAAGTGCTACAACTGATTA	0	0	0	0	0	0	0	0
	†	21UR-3029	TTGTAGTGATTGATCAATTAG	4	0	0	1	5	11	0	21
	†	21UR-3030	TTATGACATTCCTTGTAGAAA	65	74	39	48	100	250	2	578
	†	21UR-3031	TTAAAACTCCGAAACAAATGA	0	0	0	0	1	0	0	1
		21UR-3032	TCTGATCGGTTTGTTCTTTTT	16	1	1	3	13	11	6	51
		21UR-3033	TAAATGATTGAAATATTAATT	0	0	0	0	0	1	0	1
		21UR-3034	TGTTTTAATTAGTCTTTACAG	0	0	0	0	0	0	0	0
	†	21UR-3035	TAGATCATATATAGGAATGTT	0	0	1	1	2	4	1	9
	†	21UR-3036	TCGATACTGGGGCTTTTTGAA	4	3	4	0	4	11	2	28
	†	21UR-3037	TAATCCGAAGCAATTGAAAGG	0	0	0	0	0	9	6	15
		21UR-3038	TATCTCAGGATGTTGTGTATA	0	0	0	6	70	36	16	128
	†	21UR-3039	TGTTCGAGACATCGTGGAAAA	4	0	1	2	1	8	1	17
		21UR-3040	TAAGATTACATTAGCGATTCA	0	0	0	0	4	1	1	6
	†	21UR-3041	TAATCCAGTGTGAACTGATTG	0	0	0	0	1	0	0	1
		21UR-3042	TGACTGCGCTCTCAATCAGTT	0	0	1	0	9	0	0	10
		21UR-3043	TGAGTGGCTAGTTGCAAAGAC	0	0	1	0	7	21	6	35
		21UR-3044	TGTAGCCATTTTTTGGGTCCA	0	0	0	0	0	0	0	0
		21UR-3045	TTACTGTGCTTAAAATTTTAC	0	0	0	1	7	4	1	13
	†	21UR-3046	TGATACCATGGACATTACCAC	0	0	0	0	0	0	1	1
	†	21UR-3047	TAGCACTTCAAAAATTAGGAG	1	0	0	0	1	1	2	5
		21UR-3048	TACAAAACCCTACATAAAAAT	0	1	0	0	1	11	5	18
		21UR-3049	TGAGTACACTTGTCTTTCTGT	0	0	0	0	1	2	1	4
	†	21UR-3050	TTTTCTGAGCATCAATCATCA	0	0	0	2	3	3	0	8
*	†	21UR-3051	TGCGACTTACGTTCTGTCGGA	14	3	2	2	32	108	31	192
*		21UR-3052	TCAACTTCAGATACGTAGTGA	30	31	24	33	133	222	23	496
		21UR-3053	TACAATTCATTTTTTGTTGTC	0	0	0	0	0	0	0	0
*		21UR-3054	TACTACATTTTCACGAAATCA	54	4	9	5	11	51	3	137
		21UR-3055	TTTAGACTTCGATAAAATTAT	0	1	0	1	3	2	0	7
		21UR-3056	TGGATTGAATCTATAAATTAA	0	0	1	0	4	3	1	9
		21UR-3057	TAGAGGCATTCCTGATGTTTT	1	1	2	3	14	7	4	32
		21UR-3058	TCCGTTGCTTATTATGCATGT	0	0	0	0	0	0	0	0
*	†	21UR-3059	TTCAACGTGTATGTTCGGGTT	1	1	1	0	11	13	6	33
		21UR-3060	TATTCATCAATTACAATTATT	0	0	1	1	0	2	1	5
		21UR-3061	TAAATATGAATTTTTGATATG	0	1	0	0	7	3	0	11
		21UR-3062	TCTACCGCTGCTTATCACGTT	0	0	0	0	0	0	0	0
	†	21UR-3063	TAATTTGTTCAGTGTAGGATA	6	0	2	1	3	10	0	22
		21UR-3064	TCGAACGTTTTTTTTGCACAT	0	0	0	0	0	1	0	1
		21UR-3065	TGCCATTATTCTACTGTAGTT	0	0	0	1	0	1	0	2
		21UR-3066	TCCTTATTTTCTACGACATCA	1	0	0	0	0	1	0	2

		21UR-3067	TGGTGTGTTTTTTTTTAAATT	0	0	0	0	3	2	1	6
	†	21UR-3068	TGCTAGTATTCCTCTGTCTCG	0	0	0	0	0	0	0	0
	†	21UR-3069	TAATTTGATTCAAGGTGGTCG	1	1	0	1	3	5	3	14
	†	21UR-3070	TATTTTGAAGATACAAGTTCA	2	0	0	0	1	5	1	9
*		21UR-3071	TCCGCTTCCATTGCTGAATGA	2	0	0	1	2	1	0	6
		21UR-3072	TATTTTCCATTATTGTAGGTT	1	1	1	1	2	7	3	16
		21UR-3073	TGCCTCTTCAAAAAATGACAA	0	0	0	0	0	1	0	1
*	†	21UR-3074	TTCAACGGCTGTACAAAAAGA	34	10	3	8	62	103	36	256
		21UR-3075	TGAAACAAGTATAGTTTGAAT	0	0	1	0	15	13	0	29
		21UR-3076	TCCAGTTGCACAATTGTTTAG	1	0	0	0	1	3	0	5
		21UR-3077	TAGCTTATGAAAATATGGCAC	4	1	3	1	5	24	0	38
*		21UR-3078	TCTATGTTGGCAATAAGCGGA	39	35	17	14	121	280	42	548
*	†	21UR-3079	TATTCGGAGGATTCAGCCTGC	0	0	0	0	1	2	0	3
		21UR-3080	TGTTTTGTTTCATCTCTTCAG	0	0	0	0	0	0	0	0
		21UR-3081	TGAGTCGTGTACAAGATTTAT	2	6	3	4	46	37	9	107
		21UR-3082	TACAACTATGGCTAAACTAAT	7	1	0	0	1	1	0	10
	†	21UR-3083	TAGTAATGGTATTTCTAATAA	8	1	0	2	1	7	0	19
*		21UR-3084	TGCAGTCACAAGAGGATTCAC	1	1	1	1	76	101	3	184
*		21UR-3085	TACGTAGACGCCTTTAAAAGC	35	48	26	27	85	272	10	503
	†	21UR-3086	TCTTAGTTGGCTGTTCTATTT	0	0	0	0	1	0	0	1
	†	21UR-3087	TACGATGAGTGACTTCACATA	2	2	1	6	130	97	13	251
	†	21UR-3088	TAATAACCATATGATAATTAA	2	0	2	2	4	8	0	18
		21UR-3089	TCACCTTTTACCTGTTTTGAA	0	0	0	0	0	0	0	0
	†	21UR-3090	TCTAAGGTGATCATTAATTGA	0	0	0	0	0	0	0	0
		21UR-3091	TGATATCTTTATTCTTTTAGT	0	0	0	0	0	0	0	0
		21UR-3092	TGAAGAAGATGGAAAGTGAAT	61	63	23	15	46	141	7	356
		21UR-3093	TGGTGATCGTGTAAATTCTTG	0	0	0	0	0	0	1	1
		21UR-3094	TGTTCAGCTTGTTAACTGTGA	0	0	0	0	0	0	0	0
		21UR-3095	TATGCAAACGTTTTTTACTAA	5	1	1	0	6	13	1	27
	†	21UR-3096	TATGTAGGCTATAGGAATTCC	0	0	0	0	1	1	0	2
	†	21UR-3097	TACAACTATAAAGTGATAATG	118	3	4	0	7	23	13	168
		21UR-3098	TTGATTTGGACAGCTGAAAAT	1	4	1	3	50	43	0	102
	†	21UR-3099	TACGCTTGTTTTTTGAGAAGC	2	1	0	0	1	0	0	4
	†	21UR-3100	TAATGCATTTTTAATGTCGGA	60	15	7	26	242	323	335	1008
	†	21UR-3101	TACGGACTGGATAAAACTTGT	0	0	0	0	0	0	0	0
		21UR-3102	TCATCTCGAAACTAATGAAGG	0	0	0	0	8	9	0	17
	†	21UR-3103	TCAATGCTCTGGACAGAACGT	2	0	1	0	6	8	0	17
		21UR-3104	TTATTTGATGATGAAAATTGA	0	0	0	0	0	0	0	0
		21UR-3105	TTCTTCTTTCAATTGATTTAC	0	0	0	0	1	0	1	2
*		21UR-3106	TATGTAGTAATATGGGGATGG	288	294	119	75	257	1387	57	2477
		21UR-3107	TGATGAAAATGCAAAACCAAA	6	0	0	0	0	3	0	9
		21UR-3108	TGAATTTGGCTACCGGCGAAC	0	1	0	1	8	14	3	27
		21UR-3109	TTTCAATGCGCTTGTCAATGA	1	1	0	0	7	13	2	24
		21UR-3110	TGCATTATTGAATTGAAAACA	2	2	1	4	4	7	1	21
		21UR-3111	TCTCCTACTCACTCTGGTTTT	0	1	0	0	1	0	0	2
*	†	21UR-3112	TGATGTGCAGGTCGCTTATGA	8	0	0	0	4	10	12	34
		21UR-3113	TGTTCCGTCTACTATATGCAA	0	0	0	0	0	0	0	0
*	†	21UR-3114	TAGTAGTTTGTCGATATATAT	90	63	34	39	150	190	38	604
		21UR-3115	TGATGAAAAATTATAATCTTG	0	0	0	0	1	0	0	1
		21UR-3116	TGGAATCTGCTTCAATCAGGG	0	0	0	0	0	0	0	0
		21UR-3117	TAGTGGAAAATTTATAGAATT	13	12	6	6	16	28	0	81
	†	21UR-3118	TGGAGAGAATATTATAGAGAA	0	1	2	3	38	35	16	95
*		21UR-3119	TGCACAGGAGCAGCTTATTGT	5	1	0	1	3	12	2	24
		21UR-3120	TACGATACATCTTCTCTTGAC	12	1	0	1	12	27	1	54
		21UR-3121	TGGTCATATTAGGTGAGTAGA	0	0	0	2	0	2	1	5
*		21UR-3122	TCTCAGTCGTTGATACATTGA	145	54	56	54	190	510	89	1098
*		21UR-3123	TAATCTGTTAACATCCGAATA	0	0	0	0	3	0	1	4
*	†	21UR-3124	TCAGATACTGGTCCTTGGAAA	24	23	21	10	41	128	23	270
		21UR-3125	TGGGACCGTCTCTTCATTTTC	0	0	0	0	2	2	1	5
		21UR-3126	TGACACGCAAATTCGTGAAGC	0	0	0	0	0	0	0	0
		21UR-3127	TCAGCAAAATATTTGTCGCTG	0	0	0	0	0	2	0	2
	†	21UR-3128	TGATCATATTTTAGGCTCGAT	2	3	1	2	11	24	11	54
*		21UR-3129	TGTATGTAAAACTTTACGGCA	1017	777	413	444	1652	6071	129	10503
		21UR-3130	TATTTTGAAGCTATGTTTCAG	0	0	0	0	1	1	0	2

		21UR-3131	TCATTTCTTGATACATTACTT	0	1	0	0	3	0	1	5
	†	21UR-3132	TAGACACATCTCATCGTCTGA	0	0	0	1	32	19	9	61
	†	21UR-3133	TGGGAATAATAAATAGTCTAA	0	0	0	0	1	0	0	1
		21UR-3134	TCCGTCATCGCTATCTCATGC	0	0	0	0	0	0	0	0
	†	21UR-3135	TTAATGAGCGTTAAATATGTA	2	1	0	0	7	13	8	31
	†	21UR-3136	TAACTTCGATGTTCGTTTTCA	3	0	1	0	10	6	12	32
		21UR-3137	TAATGTAAGTTAAATTGGTGT	1	1	0	0	3	1	2	8
		21UR-3138	TCAAGTAGTCTTTTAAATGTA	0	10	6	13	167	74	2	272
		21UR-3139	TGGCTCATATTTGCTAATTTC	0	0	0	0	0	0	0	0
		21UR-3140	TCTGGGTGTTTCTCGGTTGGT	0	0	0	0	0	0	0	0
		21UR-3141	TCCACCGATTGAACACAGGTG	0	0	0	0	1	1	0	2
		21UR-3142	TAGGGTCGTCTCTTGAGAGCA	1	0	2	0	13	4	1	21
		21UR-3143	TAACGATCTTTTTAACACGTT	11	1	2	0	2	7	2	25
	†	21UR-3144	TGACAAATTCAATGGGTTAAC	0	0	0	0	2	0	0	2
		21UR-3145	TGGTTACTCACTATTGCTTCC	0	0	0	0	0	2	0	2
		21UR-3146	TTTCATGTGCAATGATTTTGA	0	0	0	0	0	2	1	3
		21UR-3147	TTTTCCAATGGCCTTTTGATG	0	0	0	0	0	0	0	0
	†	21UR-3148	TCAGCTTTCTGAAAAATCAAT	0	0	0	0	3	4	1	8
	†	21UR-3149	TATTTCTTTGCTGGCATGTGG	8	3	1	0	7	36	5	60
	†	21UR-3150	TATTACAAAGTTGCTTCAATG	0	0	0	0	0	0	0	0
		21UR-3151	TTATTTCGACAGTTCGCGATA	0	0	0	0	2	6	7	15
	†	21UR-3152	TCAATGATAGTTACAATTAAA	0	0	1	1	8	8	0	18
		21UR-3153	TGACTCACAAATGACTCAGAA	0	2	2	11	172	187	34	408
		21UR-3154	TAGTCAATGCCCATGCCCAGC	0	0	0	0	0	1	8	9
	†	21UR-3155	TAATAAGATCACTGTCGTTGA	0	0	0	2	7	8	29	46
		21UR-3156	TTGTTCATCAGTGGCTGTAAA	1	0	0	0	0	0	3	4
	†	21UR-3157	TTTAGGACCGTTTGTGATGAT	1	3	4	6	140	146	189	489
	†	21UR-3158	TTAGGATTTCACTAGAAGAAG	0	1	0	0	0	0	0	1
		21UR-3159	TATTGCGTTTGCCGTATTCAT	0	0	0	0	0	1	0	1
		21UR-3160	TACAAATCCATTTGATCATTG	0	0	0	0	1	3	1	5
	†	21UR-3161	TGTTGATATGAGGAAATCGGG	3	3	0	3	70	90	48	217
		21UR-3162	TAATCTATTCTAATGTTACTC	0	0	0	0	2	0	0	2
		21UR-3163	TGCCGCATGTATTTGACAAAA	0	0	0	0	2	2	0	4
		21UR-3164	TGATTCAGGTTACTTTTTTAC	0	0	0	0	3	0	0	3
		21UR-3165	TCCGTGATCTATATCTTCTTC	0	0	0	0	0	0	1	1
	†	21UR-3166	TCTATTGGAAAAGAGGGACTA	3	3	1	1	16	19	16	59
	†	21UR-3167	TCCATGGAAATATATGCTGAC	0	0	1	0	3	5	0	9
*	†	21UR-3168	TATTACGGCCGTACAGCAAAT	9	12	1	6	33	20	4	85
		21UR-3169	TTATACTATTTTTCCGTTAAA	3	1	0	0	0	0	0	4
	†	21UR-3170	TGATTGACGACGTGGCTATCA	0	2	1	3	43	34	23	106
	†	21UR-3171	TTAATGCATGGGCGGATTTCA	0	0	1	1	5	12	2	21
	†	21UR-3172	TCTCAACCAATCCTAATTTTT	0	0	0	0	0	0	0	0
*		21UR-3173	TAGGTTGTTCGTAGCAGTGCT	0	1	0	0	22	19	9	51
		21UR-3174	TGGGATGGCTCATCACTCGAA	0	0	0	0	0	2	0	2
		21UR-3175	TACTGGAATCACTCTGATATC	0	0	0	0	1	1	0	2
		21UR-3176	TATTTGATTGATGAAATGCAA	3	0	0	0	1	3	0	7
		21UR-3177	TATTCGCTTAATATAACGGGA	0	0	0	0	2	1	3	6
	†	21UR-3178	TAATTCTAATGGGTTTTTGCA	1	0	0	0	0	0	0	1
		21UR-3179	TATGTCGCATGATGGACTTGA	1	2	0	1	0	5	0	9
*		21UR-3180	TCGGAACATAAAGAATGAACA	39	9	8	10	19	83	7	175
	†	21UR-3181	TCGGCCCATCAATTTTAGCAA	1	0	0	0	1	2	1	5
		21UR-3182	TCCGACTTTCCGCAGTATTCC	0	0	0	0	0	0	1	1
*		21UR-3183	TGCGAAGACTTTACATATCGT	2	8	5	9	222	208	19	473
*		21UR-3184	TCTTTCTGTGGAAGCGTTTGA	165	112	55	60	164	968	56	1580
		21UR-3185	TGCCAATTTCTCTATTTGTGA	0	0	0	0	0	0	1	1
		21UR-3186	TCTTCGTGGTATAACGGATAC	1	0	0	0	14	5	0	20
		21UR-3187	TCCTCTCAACCGATACGTCAG	1	0	1	2	10	5	5	24
		21UR-3188	TCCTACGTTCAACAACAACAA	0	0	0	0	0	0	1	1
	†	21UR-3189	TGAGTAGCATCCGAAGAAAAT	11	38	8	42	641	566	10	1316
		21UR-3190	TAGCAATACGATTACGTTTCA	0	1	0	0	0	0	0	1
*		21UR-3191	TTCGGCTACATTTCTCTCTTC	0	1	0	7	77	56	2	143
		21UR-3192	TAATGACGTTCTGTTTTTTTC	0	1	0	0	7	4	1	13
		21UR-3193	TACTTGTGAAATATTTGCCTT	0	0	0	0	0	0	0	0
		21UR-3194	TGCGGCTGTGTTTTAAAAGAT	0	0	0	0	1	0	0	1

	†	21UR-3195	TGTATAATACCCTGAACTTCC	0	0	0	0	1	0	0	1
*	†	21UR-3196	TTACGTTGATCGACTAGTTCC	3	2	0	3	46	38	0	92
	†	21UR-3197	TTTTCTGCTGACCATTTAAAT	1	0	0	0	0	1	0	2
*		21UR-3198	TACATTATTGGCATATATAAC	1	2	1	0	0	4	0	8
	†	21UR-3199	TACAATCGTCAAAGAAGTTCG	38	31	23	35	111	270	5	513
*	†	21UR-3200	TGGTTCCTCATTGAAGATTGA	2	0	0	0	0	4	0	6
	†	21UR-3201	TCGGAGTGGCGTTTTGTTGAC	0	0	0	0	11	24	6	41
		21UR-3202	TGGTAAATATATATCTCACGG	0	1	0	0	1	4	0	6
*		21UR-3203	TGGGACACACTTTTGGTTTTA	3	1	1	2	10	27	2	46
	†	21UR-3204	TTAAAGCTATTTAAACTATGG	2	1	1	0	2	3	0	9
		21UR-3205	TCCGATTTCGATTTTCACCGA	4	0	0	0	0	0	0	4
		21UR-3206	TGCAAGAAAAGAAAGAAAATC	0	0	0	0	1	4	1	6
		21UR-3207	TCGTTGTGCAGTATGTATCAC	0	0	0	0	0	0	0	0
		21UR-3208	TTGTCCAGACGGTTGGATCTC	0	0	0	0	14	12	0	26
		21UR-3209	TCCTTCAACAATCGAAATAAA	0	0	0	0	0	1	0	1
		21UR-3210	TAGACTCAGTGAAAAAATGCG	0	0	0	0	0	2	0	2
	†	21UR-3211	TGCCAAGTAAAAAGTTTCTAA	0	0	0	0	3	2	2	7
		21UR-3212	TAAAAATTTGCATTAATGAAC	2	0	0	3	13	7	7	32
*		21UR-3213	TAAGTAACGGACTTTTCAATT	113	43	29	31	80	266	10	572
		21UR-3214	TCTTTCGCAGATTGTTTCTGA	0	0	0	1	2	2	0	5
		21UR-3215	TAATTCGTCGTCTTCTGAAGA	2	0	0	3	8	16	4	33
	†	21UR-3216	TCGTATGATGCTTTGAATACT	1	0	0	0	0	4	0	5
		21UR-3217	TCAATTCTTTTCAATTACCAA	0	0	0	0	1	0	0	1
		21UR-3218	TGCTCCACTATTTATTAGAAC	2	0	0	0	0	1	0	3
*	†	21UR-3219	TAGGAGGAAGCATTGTGCATA	164	122	36	54	292	694	23	1385
	†	21UR-3220	TTGAAAATTCAGTTGATTAAT	12	4	1	2	10	120	5	154
		21UR-3221	TACGCTTGGTAAACTTTGAGT	0	0	0	0	5	6	0	11
		21UR-3222	TGCGTTAACGGTTTGCAGACA	0	0	0	0	0	0	0	0
		21UR-3223	TAAAGAAACCTTGGACTTTTA	0	0	1	2	32	29	7	71
*		21UR-3224	TGAACAAGCGACTGCATAAAG	92	138	56	43	126	802	32	1289
		21UR-3225	TGGAACATCATTTCTCGACCA	1	0	0	0	1	4	0	6
		21UR-3226	TGAACTTCACGTTAGCAAAAG	0	1	0	0	4	5	6	16
	†	21UR-3227	TGCGCAGTGAAATAGTATGCG	0	1	0	0	1	4	0	6
		21UR-3228	TCCACTGTTGAACTACTTTCC	1	0	1	0	1	5	0	8
		21UR-3229	TGCTTTTATCACATGTGTAAG	1	0	0	0	0	1	0	2
		21UR-3230	TATCTAGTTCTTCAATTAATG	1	0	0	0	1	1	1	4
		21UR-3231	TGAAAATATTGTTCTGTTTCA	0	0	0	0	0	0	0	0
	†	21UR-3232	TTTGACAATTAGTGTTTTGAG	0	0	0	0	0	0	0	0
	†	21UR-3233	TTCAATGCTACCCATTTCTCG	1	0	0	0	0	1	0	2
		21UR-3234	TCCTCTGTTTTTCTGTATGAG	0	0	0	0	17	4	1	22
		21UR-3235	TCTTAACAAAAAAGCTCGATC	0	1	0	0	1	2	0	4
	†	21UR-3236	TACGTTCAGATAAGTAGTTCC	0	1	0	1	11	11	0	24
		21UR-3237	TGTTGAGCAAAAAGTCTTTGC	0	0	0	0	0	0	0	0
*	†	21UR-3238	TAGATTAAGTGTAGGCTATGA	3	0	0	6	69	59	17	154
		21UR-3239	TGTCAATAAAGATGTGTTCAA	1	0	1	1	0	3	0	6
		21UR-3240	TAAATTCTTTTACCCACCTCT	0	0	0	0	0	0	0	0
	†	21UR-3241	TGAAACACAAATGTTTGTTGT	0	0	0	0	0	1	0	1
*	†	21UR-3242	TAAGTTTGAAAGATTTGAAGG	12	2	0	0	3	14	0	31
		21UR-3243	TAGTGTTATTCAAATTTCAAG	0	0	0	0	0	1	0	1
		21UR-3244	TCATAGTTTCTGATATATACG	0	1	0	0	0	1	0	2
		21UR-3245	TTTTCTTGACTCGAATTCTTC	0	0	0	0	0	0	0	0
*	†	21UR-3246	TGAGTGAACTTATCAGCAGGC	9	12	7	2	141	194	6	371
		21UR-3247	TTTCACGCCAAGTATACATTT	0	0	0	0	1	0	2	3
*	†	21UR-3248	TATGGCACGTCATTTTATAAA	7	2	0	0	1	10	1	21
	†	21UR-3249	TGTTCATCTCGTATTTATTCA	0	0	0	0	0	0	0	0
	†	21UR-3250	TGGCAGGTTCTGATTTTAATC	1	0	0	1	1	1	0	4
	†	21UR-3251	TAGTTCCATTTTTCACGCACG	0	0	0	0	1	0	0	1
	†	21UR-3252	TGTGTTGTGACTACAATGTTC	0	0	0	0	0	0	0	0
		21UR-3253	TGAAATAACCTTATTATCTCC	0	0	0	0	0	0	0	0
		21UR-3254	TACCTACCTATTACCTATGCG	1	1	0	0	0	6	4	12
	†	21UR-3255	TGATAGTTTTGTTATCCGGTG	0	0	0	0	0	0	1	1
		21UR-3256	TGCAATTTGTCATGTTGATGA	0	0	0	0	0	0	0	0
	†	21UR-3257	TCCTTCCTTTTGATCCCCATA	1	0	0	0	0	0	1	2
		21UR-3258	TACCAGGGAGCAATCGTGTAT	2	0	0	0	0	7	7	16

		21UR-3259	TATCCATGATATATAATGACC	3	0	0	0	3	4	1	11
*	†	21UR-3260	TATTTCATGTCTTCGGTTTCA	128	49	47	44	187	336	60	851
	†	21UR-3261	TGATCACTAGGTTGACAAAAA	1	0	0	0	1	3	1	6
		21UR-3262	TCGAGGATTAATCGAGAATGA	4	1	0	4	43	61	16	129
		21UR-3263	TCCACGGCACGAATTTACTAA	0	3	2	6	132	108	51	302
	†	21UR-3264	TGGTTAAATTCTCCGTTAATA	1	0	0	0	0	0	0	1
	†	21UR-3265	TGTTCCAGAGTTCCAACCTCA	0	0	0	0	0	1	0	1
		21UR-3266	TACGTTAAGTGAATAATCCCG	0	0	1	0	2	2	0	5
*	†	21UR-3267	TATCGCGCCTTGGAATGGATA	0	0	0	2	28	22	11	63
		21UR-3268	TGGAGCGATTCTTTGAAACTC	1	0	0	0	0	0	0	1
*	†	21UR-3269	TAGTTTCCTCCAAACACGGCA	13	3	2	1	2	14	0	35
		21UR-3270	TCAGTTGTTTTAATTTTGGAT	0	2	1	0	3	4	1	11
		21UR-3271	TTTGGGTGGTTCAATTTATTT	3	0	0	0	0	0	1	4
	†	21UR-3272	TGTTGAAGCTCGGCATCAAGT	21	46	14	23	270	575	46	995
		21UR-3273	TGGGTCCTTTCTCAAAGCTGA	1	1	0	0	3	8	1	14
		21UR-3274	TCACAAAACACACCTTGATTC	0	0	0	0	0	1	0	1
		21UR-3275	TCGATCGAATTTTTTTTCGGT	0	0	0	0	0	0	0	0
		21UR-3276	TAGTAGTAACCTTGTCAGTAT	4	4	2	0	6	24	1	41
	†	21UR-3277	TCAAGAATTGATAGCGCTTGC	6	14	8	9	26	96	1	160
		21UR-3278	TGCATTCTTCAAATTAAAGTT	3	0	0	0	0	0	0	3
		21UR-3279	TGATGGTATGTTTTAAACCAA	0	1	0	0	0	3	0	4
		21UR-3280	TCTCATTGTGATGTAGACCAA	0	0	0	0	2	0	0	2
		21UR-3281	TAGACATAAAATGCATTTGAA	0	0	1	0	4	4	1	10
*		21UR-3282	TGCGTCTGACAAGGAAAGGAA	0	6	1	5	80	101	51	244
	†	21UR-3283	TATCGAAATTTACGTTGTCCA	12	2	1	0	3	20	4	42
	†	21UR-3284	TCTTTTTGTTGCCAAATTTCA	0	0	0	0	0	0	0	0
		21UR-3285	TGGGAAAAATCATTCCAGCGG	0	0	0	0	0	0	0	0
		21UR-3286	TGTGCAAAGCATTGTTTTGTA	0	0	0	0	3	1	2	6
		21UR-3287	TGGTCAATCTGAAATGTATTG	0	0	0	0	0	1	0	1
	†	21UR-3288	TTGTACATGATCAATTGTGCC	10	0	1	1	6	20	3	41
		21UR-3289	TTATGTCTTGTTATTCTTTAG	0	0	0	0	7	1	1	9
	†	21UR-3290	TTCCTTCCGTTATGGGTCGAA	3	0	0	0	0	1	0	4
	†	21UR-3291	TGCTTGTTTCTGCCGCTCTTT	0	0	0	0	0	0	0	0
*	†	21UR-3292	TGGGAAGTTTGGTCTTACGGC	36	19	11	10	50	202	8	336
		21UR-3293	TATTCATTTGGCAAATTCAAA	1	0	0	0	1	0	0	2
		21UR-3294	TTTCAATACTATTCAAATACT	0	0	0	0	1	0	0	1
		21UR-3295	TCACAAAATACAATGAGGCTC	0	0	0	0	5	6	0	11
		21UR-3296	TCGATATTCGAAATGACAAAC	0	1	0	0	0	9	0	10
		21UR-3297	TCGGGATCATGCTCGACCTCT	0	0	1	0	2	4	0	7
		21UR-3298	TACGGATAAACCAGCAAACTT	0	0	0	0	2	1	5	8
		21UR-3299	TAACAGTTGTTGTGGAATTGT	20	12	7	9	25	82	3	158
	†	21UR-3300	TTACTTAGGCATTGGTGTACT	0	0	0	1	15	3	0	19
		21UR-3301	TGGTGATCACTTCATGGTGAC	0	0	0	0	0	1	0	1
	†	21UR-3302	TCGGACAGGCAAGATTTTGTC	5	2	1	4	65	89	19	185
		21UR-3303	TAGCACTTAAACAAATCTCAC	0	0	0	0	1	0	0	1
		21UR-3304	TGGGCGATGGAAACTAATTCG	0	0	0	0	4	3	4	11
		21UR-3305	TCGACTCAGTGCAATAAACAA	2	0	1	2	0	14	0	19
	†	21UR-3306	TTGGCATCATCTTTTTGTTGC	0	0	0	0	0	0	0	0
	†	21UR-3307	TCGGTCTCAATTATTAAACTG	0	0	0	0	0	0	0	0
		21UR-3308	TCGTTGACTGACTATAGGGTT	2	0	1	0	5	8	2	18
	†	21UR-3309	TCCTCTCCGTTTGTTATGACT	1	0	0	0	0	0	0	1
	†	21UR-3310	TGAGCTACATGATTCTGTATA	4	6	2	2	8	29	1	52
		21UR-3311	TGACCATATAGACGAAATAGA	12	33	12	14	36	88	6	201
	†	21UR-3312	TAGAAAGTGGATGAAGTGGTT	120	212	98	48	271	639	48	1436
	†	21UR-3313	TCTACCGTTTTTGTCACAGTC	0	0	0	0	1	0	0	1
		21UR-3314	TGGTTTTGCTATATTACTACA	0	0	0	0	1	1	0	2
		21UR-3315	TCGTCTATTTTGTTTGTGAAA	0	0	0	0	0	0	1	1
		21UR-3316	TTCCTTACTTCCTCATTAAAT	7	1	1	2	0	10	5	26
	†	21UR-3317	TGAAAATGGCATCAATTGTAT	36	7	6	5	13	55	5	127
		21UR-3318	TCGTTCGGAAACTCTTTGTAT	0	0	0	0	1	0	0	1
		21UR-3319	TACAATTTTAATTTTAATGTC	6	2	2	5	4	6	2	27
	†	21UR-3320	TTCGTCTTGGAAAAAACAACA	7	5	0	2	29	15	7	65
	†	21UR-3321	TGATTTGAGTGAATGTGTTAA	3	1	2	3	5	9	1	24
	†	21UR-3322	TGAGTGACACACACGGCGTGG	5	0	0	0	8	8	11	32

		21UR-3323	TCAAATAGTATAAGCATCCAA	13	4	3	3	4	19	1	47
*		21UR-3324	TGACTGACTGACACTACTAGA	4	13	7	3	12	47	0	86
	†	21UR-3325	TAAATGCTGCATTTTAATGAC	1	0	0	0	0	1	0	2
	†	21UR-3326	TGAAATCGTTTTCTGTGTGCA	0	0	0	0	6	4	7	17
		21UR-3327	TAAGTACCGATAGCCTTCAAC	16	2	1	2	4	7	2	34
	†	21UR-3328	TCTGAGCGGGCGGGATGTTAA	5	1	0	0	3	12	11	32
		21UR-3329	TGAACTTTAAATACAATTTCC	0	0	1	1	2	3	2	9
*	†	21UR-3330	TGATTGTTGATTGTCGTTTTC	42	17	19	36	621	632	197	1564
	†	21UR-3331	TACATCTTTTCTGTTTGGAAA	2	0	0	1	0	3	0	6
	†	21UR-3332	TGTACTTCTCCTTATCAGAGG	0	0	0	0	0	0	0	0
	†	21UR-3333	TTCCTGCCTCTCATATCGATT	0	0	0	0	0	0	0	0
		21UR-3334	TCCTGTTACATGATTTGTCCC	0	0	0	0	0	0	0	0
		21UR-3335	TCATTTCCCAACTCATCGACG	0	0	0	0	0	2	0	2
*		21UR-3336	TATCTTCGTTTTGGCAATAAC	70	73	61	43	85	288	19	639
		21UR-3337	TATGGTTTTTTGAACATTGCA	1	0	0	0	11	11	1	24
	†	21UR-3338	TAATTCATTAACTTCCAAGGC	1	0	0	0	0	0	3	4
*	†	21UR-3339	TCCAGATGATGAACGGTAATT	16	64	26	114	2169	1706	89	4184
	†	21UR-3340	TGGTGCTTCAACATTTTCCCA	0	0	0	0	0	2	0	2
	†	21UR-3341	TCATTCATAGACAATCGTGAA	0	0	0	0	1	0	0	1
	†	21UR-3342	TAATTTAGTCATTGGTGTTAC	1	0	0	2	18	23	12	56
	†	21UR-3343	TCATTCATATTCCTAGTTGGT	0	0	0	0	0	1	0	1
*		21UR-3344	TATGACTAGTGCAAATTTCTG	1	0	0	3	10	6	1	21
		21UR-3345	TACACAATGAATTTTCCGATA	10	10	9	8	14	47	4	102
	†	21UR-3346	TCCGTTGCTCAAATCAGGATC	0	0	0	0	0	0	0	0
		21UR-3347	TCAGGCTTTCATTTGCTTTTC	1	0	0	0	0	0	0	1
		21UR-3348	TGCACTGCGATGTTGTAGAAA	0	1	0	0	23	33	7	64
	†	21UR-3349	TGTTCTAACTTCAATGCATGT	0	0	0	0	0	0	0	0
*		21UR-3350	TCAGCAAATGATACAAAATTG	1	0	1	0	0	0	0	2
		21UR-3351	TAATTAAGGTGGATGTGTACA	1	0	0	3	16	13	3	36
	†	21UR-3352	TTCATCGCTTGACGAGATTGA	3	0	1	0	3	15	0	22
		21UR-3353	TGAGACTATAATGTCCGCGCT	0	0	0	0	1	0	0	1
	†	21UR-3354	TCATTCATACTTTTATTTATG	0	0	0	0	0	0	0	0
	†	21UR-3355	TAAACACCAGTAACTTGTTGT	0	0	0	0	4	0	0	4
*		21UR-3356	TATGATTGACCGAATAATTGA	0	0	0	1	11	15	0	27
	†	21UR-3357	TGCAACGTAAGGTTAACTACT	2	0	0	0	1	1	1	5
		21UR-3358	TCCATGTTTTTTTTTTCGGAA	3	0	0	1	1	5	0	10
		21UR-3359	TCTTATTGTGAAATTGGTAGG	0	0	0	1	6	4	9	20
		21UR-3360	TAACTCGTTCAGTTGGCTATT	12	2	5	4	16	55	3	97
		21UR-3361	TGGTTAGCTAAATCCACAATT	0	0	0	0	0	0	0	0
		21UR-3362	TGTGTGTTTTGTGCAATTATA	1	0	0	0	0	0	0	1
*	†	21UR-3363	TCGATGGCTATGTGGACTATT	14	10	6	5	25	55	1	116
		21UR-3364	TTAAATTATGAAATGAGAAAA	0	0	0	0	1	0	0	1
	†	21UR-3365	TGGAGGTCCATTATACGCAAG	0	0	0	0	2	1	0	3
	†	21UR-3366	TCAGTCAATCTGAGGCTGCAA	0	0	0	0	0	0	0	0
	†	21UR-3367	TAATGGAGGTCCATTATACGC	0	0	0	0	0	0	0	0
	†	21UR-3368	TATTCCGTCTTTCATCAGGTT	0	0	0	0	0	0	1	1
		21UR-3369	TCGGTGTAGTTTGAGGTAGGT	0	0	0	0	0	0	0	0
		21UR-3370	TAACGGACAATTCTTGAGTTA	0	1	0	0	0	5	0	6
		21UR-3371	TGCTTCATTGCCATTCTGCAT	6	0	1	1	0	9	0	17
	†	21UR-3372	TGCATCTAAAGTTGATTGAAG	0	0	0	0	2	3	0	5
		21UR-3373	TTGCACATCTGCTTTAGTTAA	0	1	0	0	0	4	0	5
		21UR-3374	TAAGACTCTCATTCTTGTAAA	0	0	0	0	1	2	0	3
*	†	21UR-3375	TTATTTGTGTGCCGTCGTTGG	297	47	34	19	225	529	300	1451
		21UR-3376	TATGAGAGTCCAATGGATTAA	4	1	0	0	16	14	1	36
		21UR-3377	TATAGAACTCTTACCTTTGAG	0	1	0	1	3	5	3	13
	†	21UR-3378	TAAGTATTTCGGAATTGCAAC	1	0	0	1	34	37	30	103
*	†	21UR-3379	TACTAGTCCTATCAACTGAGA	3	0	1	0	6	8	3	21
		21UR-3380	TGTGAATAACTGTCCGGTTGG	2	0	1	0	2	9	3	17
*	†	21UR-3381	TGCTCTGGACAGAACGTTAGT	18	10	9	10	109	136	17	309
		21UR-3382	TAATCCAACGTTTATTTCAGA	0	0	0	0	0	1	3	4
	†	21UR-3383	TTCATTCGAAATAACAATTAG	0	0	0	0	3	2	0	5
	†	21UR-3384	TGTCATTACGTCTCATACTTT	0	0	0	1	2	1	0	4
		21UR-3385	TGTTCACAAAATTCATTGAAA	2	0	0	0	0	0	0	2
*		21UR-3386	TCAATTTTGGTAATTAATCCG	0	0	0	0	0	0	0	0

*		21UR-3387	TGAACTTAGAGCTAGACAATC	37	22	17	19	59	189	13	356
	†	21UR-3388	TTTTTGAGTCACGGTGATTAA	23	4	1	0	9	38	5	80
		21UR-3389	TAACGCCTTTTTAAAGTCAAA	0	0	0	0	0	0	0	0
		21UR-3390	TGATTTACCAACATGGAGAAA	0	0	0	0	0	0	0	0
		21UR-3391	TGAAATGTTGGTTCACAAAAA	0	0	0	0	0	7	1	8
	†	21UR-3392	TGAGCAATCTATTTATGCAGA	1	0	0	0	2	5	1	9
	†	21UR-3393	TAGCTATTCCGTTATCGATGA	2	2	0	0	0	1	0	5
	†	21UR-3394	TTACCTTGTTATGATCGAAGA	4	0	0	2	11	15	16	48
*	†	21UR-3395	TTGATGATTGAAAGTTGTGCG	14	8	0	3	5	11	5	46
		21UR-3396	TGTGGTTTTAATATTGATAAA	0	0	0	0	0	0	0	0
		21UR-3397	TAAATTTGCAATATATGTTAC	1	0	0	0	0	2	0	3
		21UR-3398	TCCTAAACTTAAATAACAAAA	0	0	0	0	0	0	0	0
*	†	21UR-3399	TGATAAGCTATGAAAAGAACG	21	31	13	12	32	105	0	214
		21UR-3400	TTCGTTGTTTTTGCGAATCAA	0	0	0	0	0	1	0	1
		21UR-3401	TGGTCGCTCAAAACTTATGCG	0	0	0	0	0	1	0	1
	†	21UR-3402	TTAGTAAGCGGTCTAAGAAAG	1	1	0	0	19	25	10	56
		21UR-3403	TGATAATTTTGGGCAATTTTT	0	0	0	0	0	0	0	0
		21UR-3404	TGAAATCAGTTCATTTTGTTT	0	0	0	0	0	0	1	1
*	†	21UR-3405	TGGATCTAAAATTTGTAGAAC	31	15	9	5	16	71	5	152
	†	21UR-3406	TAGTACGATGTCCACTTAAAA	0	0	0	0	0	1	1	2
*	†	21UR-3407	TTCATCAAGCTAAAATCAGAA	13	2	3	0	1	39	3	61
		21UR-3408	TCATCAAATTATAAATTTCGC	0	0	0	0	0	0	0	0
		21UR-3409	TGAATCATTAGGTTAGAAAAG	0	0	0	0	0	1	0	1
		21UR-3410	TGCAAACGTTTCTTTCCCATT	0	0	0	0	1	1	1	3
	†	21UR-3411	TACAAATGTGTAAACTGAAAA	11	15	11	6	38	89	6	176
		21UR-3412	TATGCTCGAGATTTTCATCAT	4	6	2	2	6	13	0	33
		21UR-3413	TCTACCATTGATAGGTCATAG	1	0	0	0	2	3	0	6
	†	21UR-3414	TATGTAGATTAAGTCCTGTTT	1	2	1	0	0	6	1	11
	†	21UR-3415	TCATTAGGTTGTAAGCAAACT	0	1	0	0	17	8	0	26
		21UR-3416	TCCACAGGTCAATTTTGCATT	0	0	0	0	2	16	6	24
	†	21UR-3417	TGTTAAATTTCCGCGACTTAA	2	2	0	1	2	4	2	13
		21UR-3418	TAGATTTTTCCAGCAGAAAAA	2	0	1	0	2	2	2	9
		21UR-3419	TGCAAATCGTCAATCGTAGAA	0	0	0	0	1	2	1	4
	†	21UR-3420	TAATCAAACCAATCTTTTTCG	1	0	0	0	0	0	0	1
	†	21UR-3421	TCATCCTGATTTTGAACTACA	2	1	0	1	3	12	0	19
	†	21UR-3422	TCGATTTCGCAAAACATCGGA	1	0	1	0	2	5	3	12
		21UR-3423	TCCTCCGAAAATAGTCTAAAA	0	0	0	0	0	0	1	1
	†	21UR-3424	TAATTCGTCGTAAAACCAAAA	6	1	1	10	91	43	48	200
		21UR-3425	TCATCGTTTTTTTTCACAGAA	4	0	1	0	1	2	1	9
	†	21UR-3426	TTTCCAAAATGCTGGTGCTTC	2	0	0	0	0	0	0	2
	†	21UR-3427	TTGTAGTCTGAAGTTTGCACA	1	0	1	0	11	6	1	20
		21UR-3428	TAAATTTGCCAGTTATAGGAA	0	0	0	0	0	0	0	0
	†	21UR-3429	TAATATTATGTGGATCTGAAT	0	0	0	0	0	0	0	0
	†	21UR-3430	TATTGTACCTTTGTAGATTGC	2	0	1	1	1	5	1	11
	†	21UR-3431	TCCCCGTTCACACGTTCTACC	1	0	0	0	0	1	0	2
		21UR-3432	TGAATTTGCTGAATTGAAAAA	1	0	0	1	21	18	1	42
		21UR-3433	TGAACGAGCCATGAAAGTGTG	0	0	0	0	2	2	0	4
	†	21UR-3434	TTATGCGACAGTACGTATGAA	2	0	3	5	52	66	10	138
		21UR-3435	TTCGTATGAGTGCTTAACTTA	1	0	0	0	0	3	0	4
		21UR-3436	TCATATCTTGATTTCACCCAA	3	0	0	1	3	7	6	20
		21UR-3437	TTGGTAGAAAAAAATATGCGC	0	2	1	1	30	11	2	47
		21UR-3438	TCACTACAGTATATGACAGAT	4	1	5	1	3	15	0	29
	†	21UR-3439	TCCCTTTCCGACGTAGAATAT	3	0	0	0	3	8	0	14
	†	21UR-3440	TCTCAGAATTCCAGATGTGTT	1	7	3	5	25	50	5	96
	†	21UR-3441	TAAGCGTTCGCTTGCACACAC	0	0	0	0	1	4	6	11
	†	21UR-3442	TACTAGAGTGTTGAGATTGTG	36	6	6	5	20	64	10	147
		21UR-3443	TGCACGCCATCTCCGTTTCGG	0	0	0	0	1	0	2	3
		21UR-3444	TTATGAGAATTTTAATTATGC	0	0	0	0	3	0	1	4
*		21UR-3445	TAGGGAATGACTTTTTTCCCT	1	0	0	0	2	6	0	9
	†	21UR-3446	TAGGATATTTGGGTAGGATAA	2	0	2	1	39	39	3	86
		21UR-3447	TACATCCGAGCCAATTTTGAA	0	1	0	0	3	4	2	10
		21UR-3448	TGTGACTCATCCTGTGGAAAC	1	1	0	4	76	94	20	196
		21UR-3449	TAAGTGAGTAAAATAAATGTA	0	0	1	1	1	4	1	8
	†	21UR-3450	TCGAGCCGTTTTTAATGTGCC	0	0	0	0	0	0	0	0

		21UR-3451	TTCTTCTATATATTTCTGGGC	0	3	0	2	25	18	9	57
		21UR-3452	TCCTTTTGGTTCAACATGAGC	1	0	0	0	0	1	0	2
		21UR-3453	TAATGAGCATAACTGTTAAAC	2	1	2	3	39	19	1	67
	†	21UR-3454	TGATTCTGGATCAATGTTTCC	2	0	0	0	1	0	0	3
		21UR-3455	TGACTAATACAGGAAGTACGG	0	0	0	0	8	13	3	24
		21UR-3456	TGCTCATTCATTTTTACATTC	0	0	0	0	0	0	0	0
		21UR-3457	TGCACATTTCAGATCTTGATG	0	0	0	0	0	1	1	2
		21UR-3458	TTTGTACCGATTATTTGTGAC	1	0	1	0	0	2	0	4
	†	21UR-3459	TTCATACAATGGGTCTTTTGG	9	1	0	0	1	5	1	17
		21UR-3460	TGCTTTCAACATTTTTCACAT	0	0	0	0	2	2	0	4
	†	21UR-3461	TGTAATCATGTTCCAATTCAG	0	0	0	0	1	1	0	2
		21UR-3462	TTGATGAATTGGCCTTGATAT	0	1	0	0	18	22	3	44
	†	21UR-3463	TATTGATTGGATTTTTGTTAA	10	7	2	4	11	19	11	64
	†	21UR-3464	TCTAAGTGTTTACAGATCAAA	0	0	0	0	0	0	0	0
		21UR-3465	TAAAATCATGAGGAAATCTAA	0	0	0	1	4	5	6	16
	†	21UR-3466	TAATTGTGCTACTCTTTGGTC	0	0	0	0	0	0	0	0
*		21UR-3467	TGGGAATGATAAATGAATGAA	1	0	0	0	4	2	2	9
*		21UR-3468	TAGGATGGTACTATTTCATTC	34	44	32	42	122	225	10	509
	†	21UR-3469	TGGATCGTGACATTTCAAAAC	2	2	0	1	2	8	1	16
*		21UR-3470	TAAGAGGAGATAACGAACTGA	97	275	96	118	471	1147	36	2240
		21UR-3471	TTGAACTGACGGGCTTTTGTT	36	23	12	13	43	195	18	340
	†	21UR-3472	TAATGCGTTGTCGCTATAGCA	0	0	0	0	0	0	0	0
	†	21UR-3473	TATTGAGTTTTGTGTATCCAA	6	1	0	1	9	8	1	26
		21UR-3474	TGCTTTTATCGAAAAGTTGTC	0	0	0	0	1	1	0	2
		21UR-3475	TGCTCGCCTTGTTTAATATAC	0	0	0	0	0	0	0	0
		21UR-3476	TTGTACATTGCTACGTGAGAG	0	0	1	0	1	0	1	3
	†	21UR-3477	TCAATTTTCCGGTGGCGATTC	0	0	0	0	2	0	2	4
		21UR-3478	TAAAGTCGATAGTGAACTGAT	0	0	0	0	0	1	0	1
	†	21UR-3479	TGATATCCGTGCTAAAAAAGT	0	0	0	0	0	0	2	2
		21UR-3480	TGCTGCGACATTTTCTTTCAC	0	0	0	0	0	2	0	2
		21UR-3481	TGCTGTCATGAAATTGAACAG	3	0	0	1	14	22	1	41
	†	21UR-3482	TTATGCGAGTTTTCTCATAGT	0	0	0	0	0	1	0	1
		21UR-3483	TGATCTTTTTGCATACATTAG	32	6	4	1	2	11	0	56
	†	21UR-3484	TATTGTAATCATGTTCCAATT	0	0	0	0	0	0	0	0
		21UR-3485	TCAGATTCTTAAGTTGCATCA	1	0	1	0	0	1	0	3
*		21UR-3486	TACCAATCTTCTTATTATTGG	0	0	0	0	2	5	1	8
	†	21UR-3487	TACGTTTTCAATGCTGAGCCC	0	0	1	0	4	8	0	13
		21UR-3488	TAAAGCGTCAATTTATTCGCG	0	0	0	0	2	1	0	3
		21UR-3489	TAAAGTGGACAATAATATCAT	1	0	0	1	31	18	2	53
		21UR-3490	TAGAAAAACATAAAATCGACA	0	0	0	0	0	0	0	0
		21UR-3491	TGTAGAACTTGGATGTGTTCC	4	1	0	2	16	16	3	42
		21UR-3492	TATAAATTGAATCAGTTTTCC	1	0	0	0	1	1	0	3
		21UR-3493	TAACCAATATATAGAAACACG	0	0	0	1	2	2	0	5
		21UR-3494	TAGAAATATGCAAGTTTCACT	0	2	0	1	1	4	0	8
		21UR-3495	TCTAGATACATGTTTACTTCA	1	0	0	2	0	2	0	5
		21UR-3496	TCGGCATATTGCCTCCTTTCC	0	0	0	0	0	0	0	0
		21UR-3497	TGGTAACGATTTTTGGAATAA	5	5	4	2	75	67	28	186
*	†	21UR-3498	TACTATTCTGGCCAACTTTGA	0	0	1	0	10	15	3	29
		21UR-3499	TTGTACTCCACTGAGGAAATT	0	0	0	0	1	0	0	1
		21UR-3500	TGGAAAGTTATGTTTACCCAA	0	0	0	0	0	0	1	1
	†	21UR-3501	TAACAATTGCAGACGGCTAAT	12	0	2	4	25	87	17	147
*	†	21UR-3502	TATTGTTATTTGATTGGCATG	219	109	69	125	548	1150	60	2280
		21UR-3503	TCTGTGAGTCTTGTTCTGTGA	0	1	1	1	4	8	3	18
		21UR-3504	TCTGAGCACCAGAATTGCAAA	0	0	0	0	6	6	1	13
		21UR-3505	TGATGTTTTAGTCACTTCTGA	2	1	0	1	10	16	0	30
		21UR-3506	TCTGACAGCCGTTCTAATGAA	0	0	0	0	0	0	0	0
		21UR-3507	TGTATAGTACCATGCTGCATA	0	0	0	0	12	12	7	31
	†	21UR-3508	TAGTGACCCATAATCGCCAAA	1	0	0	0	0	0	0	1
*	†	21UR-3509	TTAGTAAACTGGTAGACAATA	5	3	0	1	6	7	0	22
	†	21UR-3510	TATTCCTTCTATTTCACTTGA	0	0	0	0	0	0	0	0
	†	21UR-3511	TACCAGATCTTGAACGTTTCT	1	1	0	1	11	19	0	33
	†	21UR-3512	TGATCATGTAAGTTTCAAAAA	0	0	0	0	1	0	0	1
		21UR-3513	TTAGTTCTTTGCATAGTACCT	1	0	0	2	2	4	0	9
	†	21UR-3514	TTTGATGATTATGTTGACCAA	0	1	0	0	0	4	1	6

		21UR-3515	TCCGTCTGATGTTTTTTTAAT	0	0	0	1	8	4	1	14
		21UR-3516	TGCAGTGCTTTTTTCCAAACC	0	0	0	0	0	0	0	0
		21UR-3517	TTTGTGTTCTGTTTTCTGCCA	0	0	0	0	0	0	0	0
	†	21UR-3518	TCAATAACATTTAAATGAGAC	2	0	1	1	0	1	0	5
		21UR-3519	TTGGTACAATTATACCGAAAC	0	0	0	0	0	0	1	1
	†	21UR-3520	TCAACTCTGATGTTTCCTTGA	1	0	0	1	8	3	1	14
	†	21UR-3521	TGGGCTAAACGACTGAGCTAT	1	0	0	0	1	2	0	4
*	†	21UR-3522	TGTACCTTTGTAGATTGCTTT	35	30	20	15	47	200	1	348
	†	21UR-3523	TTACATATCCTTCAAACCAAT	29	3	4	3	15	19	18	91
	†	21UR-3524	TACTTTCCGCATTCAAAGTGA	0	0	1	1	2	2	2	8
		21UR-3525	TCTGCCGATTCCCAATGTGAA	0	0	0	0	0	0	0	0
		21UR-3526	TACATGGGAATTGTTTTCAGA	1	0	0	0	15	7	0	23
*		21UR-3527	TGAGACTGCCGAAACAACTCA	4	9	3	2	17	39	0	74
		21UR-3528	TAAACGATATTTTTAGAAGGT	0	0	0	0	5	2	0	7
	†	21UR-3529	TCCACTTGTCTTGCCAATTCA	0	0	0	0	0	0	0	0
*		21UR-3530	TGAGACATAGTTAGGCACAAA	12	24	12	20	174	232	84	558
		21UR-3531	TGTTGTTTCCTTTTACTAAGC	2	0	0	0	0	1	0	3
	†	21UR-3532	TCGGAAAAGTGTAGAAGTTTC	12	1	2	4	9	25	0	53
	†	21UR-3533	TAAGACGAGTGCAGTCAATGG	0	0	1	0	0	5	0	6
	†	21UR-3534	TCCTGAAGAAATTGTGGAAAT	0	4	3	1	33	35	4	80
	†	21UR-3535	TTGGAGGTATCCTATCGTGTG	0	0	1	0	3	3	14	21
	†	21UR-3536	TGGTGCAGAATAGCTGATCCC	1	2	0	1	3	7	1	15
		21UR-3537	TATGTCAAAGTTATAAAAACC	3	0	0	0	0	4	0	7
*		21UR-3538	TACTGGAATTTTATTGTAACT	1	1	1	5	13	8	4	33
	†	21UR-3539	TACTTTGATTCCGGCCTGAAA	1	1	0	1	6	11	2	22
	†	21UR-3540	TAACTTGCCTTGTTCGAATGA	27	4	1	1	0	12	9	54
	†	21UR-3541	TCCACCAAACATGATAGAACT	0	2	0	0	66	43	9	120
	†	21UR-3542	TTGGTGGTTTGATAGAAGGAA	0	1	0	0	3	1	0	5
		21UR-3543	TCACTACTCATTTTTTGTATT	0	0	0	0	0	0	0	0
	†	21UR-3544	TGGAATAACAAATACGATAAG	0	0	0	0	0	0	0	0
		21UR-3545	TCATAGAGTAAGATTTGTTCT	2	0	0	1	0	2	0	5
		21UR-3546	TTCGTTTTGGCTCTCTTTTTT	0	0	0	0	1	0	0	1
		21UR-3547	TACACTTGTGCATATGTACTG	18	2	4	2	25	43	1	95
	†	21UR-3548	TGCTGATGTAAAATGGTTGGA	0	0	0	0	13	15	6	34
		21UR-3549	TATAAGAGAGTGCTCCCTCAC	0	0	0	1	1	1	1	4
	†	21UR-3550	TCGACCTATAAACCTTTGTGT	0	2	0	0	3	4	0	9
	†	21UR-3551	TATATTTGAGTGGAATAACAA	2	0	0	0	2	8	3	15
		21UR-3552	TTGGGATTTGGATCAACATTT	0	1	2	0	4	4	0	11
		21UR-3553	TATAATTGTTACATTAGGAGA	14	3	2	2	6	18	1	46
		21UR-3554	TGTGAAAGATATTCTTGTAGA	0	1	0	3	16	17	3	40
		21UR-3555	TCTGTATCGAATGTGTGACTA	26	6	8	8	10	66	5	129
		21UR-3556	TGAGCATGTATTTAATGAACC	0	0	0	0	1	2	1	4
	†	21UR-3557	TTGTAAGACTGAAATTTGAAA	1	1	0	0	2	5	0	9
	†	21UR-3558	TGTATTATTACTTAACCACTC	0	0	0	0	0	0	0	0
*		21UR-3559	TGAATAAAGGCCGAAGGCAAA	0	0	1	1	24	27	3	56
	†	21UR-3560	TGGGTAATGAAGTTAAAAAAG	1	0	3	0	6	9	0	19
		21UR-3561	TGGAACCACATTAATAATGCA	1	0	0	0	0	2	0	3
	†	21UR-3562	TAATTTAATGGTATTGTAATA	0	0	1	1	21	19	1	43
		21UR-3563	TAGATATAACTAAATCAAGAA	0	0	0	0	0	0	0	0
		21UR-3564	TTCATCACTTCTACAAATTAA	3	0	0	1	2	2	0	8
	†	21UR-3565	TAGAACAGCGTGTTGATGCAT	15	11	0	8	66	141	5	246
		21UR-3566	TCAAGTTTCCATCTACCTGCA	0	0	0	0	0	0	0	0
	†	21UR-3567	TGTAACCGTGTATATTACATA	0	0	0	0	0	0	0	0
	†	21UR-3568	TTTCACTTTTTTGCTTGCCTA	0	0	1	0	0	0	0	1
		21UR-3569	TAGAAAGCACTAAAGTAGTTT	3	0	0	0	1	5	0	9
		21UR-3570	TGCCGATGAGTCTCTATCCAA	0	0	1	0	2	5	0	8
		21UR-3571	TTGGGTGATAATCGAAATTTT	1	0	0	0	0	1	0	2
	†	21UR-3572	TCCTGCGTTCGCAAGACTTGC	0	0	0	0	1	1	1	3
*	†	21UR-3573	TATAACGGTTAACTTAGAGCC	1	0	0	3	11	26	0	41
	†	21UR-3574	TATGTTATGTATTTGCAGTTA	0	0	0	0	0	2	0	2
		21UR-3575	TATCTGTAGACCTCCAATTTG	0	0	0	0	1	2	0	3
	†	21UR-3576	TAACTCGTTTTCGATCGGAAA	58	6	16	19	175	214	69	557
		21UR-3577	TCTTATCATTTCTTTGACAGA	0	0	1	0	1	2	0	4
		21UR-3578	TGCAAAGTTTAAATAAATTAA	0	0	0	0	0	0	2	2

		21UR-3579	TAAAAATATCCAATGTTTCGA	0	0	0	0	0	0	0	0
	†	21UR-3580	TAATTAAGTCATAGTTATAAC	0	0	0	0	0	1	0	1
*		21UR-3581	TACGATGGATATTTTGTTGGT	2	3	3	5	91	121	29	254
		21UR-3582	TCGAACATTCGCTGATGATAT	4	3	0	7	82	50	21	167
	†	21UR-3583	TACGTCTGAGGTTTTCATTTC	0	1	0	2	4	3	1	11
		21UR-3584	TATCTCGTCTATTTGCCTACT	2	0	0	1	2	0	3	8
		21UR-3585	TCTGAACAACTTTCTCATTGG	1	1	0	0	2	3	0	7
		21UR-3586	TAAGTTGCAATATGCAAAAAT	0	0	0	0	1	0	0	1
		21UR-3587	TCCCGTGACTTTATGATGTTT	0	0	0	0	3	1	0	4
*	†	21UR-3588	TGAATCATGTAGAATTGTCAA	7507	3440	1826	1752	3489	12115	199	30328
		21UR-3589	TCATCATTGCAGGAAATCGGT	35	10	6	5	11	46	5	118
	†	21UR-3590	TCAGCCTGTGCATTGAAATGG	6	1	2	0	5	31	2	47
	†	21UR-3591	TAAGAACGTCCAAACAATGAC	0	0	0	0	0	0	0	0
	†	21UR-3592	TTGCTGCATCGTTTTTATTGT	1	0	0	0	0	1	0	2
		21UR-3593	TATGCTCCAACCAAAATCGTA	1	1	0	0	5	8	7	22
	†	21UR-3594	TATTTAGTTTTCAGTATAGGC	2	0	1	1	2	2	1	9
	†	21UR-3595	TCGGTTACAAAAGTCCATTTA	0	2	0	0	3	6	0	11
	†	21UR-3596	TGGCAATTTTTTTCCATTGGT	0	0	0	0	1	0	0	1
	†	21UR-3597	TTTGATAGACTTCCTTAACGC	0	0	0	0	0	0	0	0
	†	21UR-3598	TTGGGTGAAAACTTTATGAAT	0	0	0	0	0	0	0	0
		21UR-3599	TCCAGTTTCCCTTCCTTTCGT	0	0	0	0	0	0	0	0
	†	21UR-3600	TTGAATGTCGCTATGGGTGAA	0	0	0	0	3	7	4	14
*	†	21UR-3601	TCTACGGCTTGGAGAAAAACC	10	15	5	4	20	334	9	397
		21UR-3602	TCATAAAAATGAAGCAATCAC	2	0	0	0	7	9	2	20
		21UR-3603	TACATCCATCATCTATTCGGT	0	0	0	0	0	2	0	2
*		21UR-3604	TGAAAGAATGCAGAAATTATG	187	199	79	109	519	786	28	1907
		21UR-3605	TGTGACTTCTTTTCATGTTAA	0	0	0	0	1	0	0	1
		21UR-3606	TTCTGTTCTCTAAAAATCAAC	0	1	0	0	5	3	1	10
		21UR-3607	TCAGATTGGGTTTCTAATTGG	2	0	0	0	0	2	0	4
	†	21UR-3608	TAAGTTTCCAATATGAGAATA	0	0	0	0	0	1	0	1
	†	21UR-3609	TGGAGTTTTGATTGTATGTCA	8	3	4	4	30	31	5	85
		21UR-3610	TGAAGCTTCATTATGCAGATG	0	0	0	0	2	2	1	5
		21UR-3611	TACGATATTGAAAAATGTTGA	0	0	0	1	4	0	0	5
		21UR-3612	TTATCCAGATTTCATATTTCC	0	0	0	0	4	3	1	8
*		21UR-3613	TTTGTTGACGTATACGCAATT	110	106	49	72	319	615	16	1287
		21UR-3614	TGTGCACGGACTGCGTTTTTC	2	2	0	4	43	40	4	95
	†	21UR-3615	TGCTGTAAAAACGAGTTGATT	15	13	4	10	17	65	3	127
		21UR-3616	TCATAAATAGTGGTCAGTTAA	0	0	0	0	0	0	0	0
	†	21UR-3617	TGTCAATGGTAACTTGGAAAT	0	0	0	0	0	0	0	0
		21UR-3618	TTCATAATGAAACACAATTGA	1	0	0	0	10	2	3	16
		21UR-3619	TCACAAGTGGTTTGGGATCAG	0	0	0	0	0	5	1	6
		21UR-3620	TATTGGTGTATAAGTCAAAAA	7	3	0	4	0	23	2	39
	†	21UR-3621	TAGTAATATTCGGTGGTTATT	2	0	0	0	16	13	12	43
		21UR-3622	TGGTGGGTCTGACTTGATATT	0	1	0	0	1	0	1	3
		21UR-3623	TGTCTATCCTGGAAGTTGTCA	0	1	0	0	3	1	1	6
	†	21UR-3624	TTCAGCAATATTGTGATCTAT	1	1	0	0	1	1	0	4
		21UR-3625	TAATTGTGTCATATTCAAACT	13	3	3	5	5	20	2	51
		21UR-3626	TGCCTTCAAAAATTTGTTGGC	0	0	0	0	0	0	0	0
	†	21UR-3627	TATTACGACTCAACGACTAAC	12	14	9	9	57	99	15	215
	†	21UR-3628	TCGGAAGCTAAACTCTGCTTG	0	0	0	0	0	0	0	0
		21UR-3629	TATGTCACCTGTATAGCTACC	0	0	0	0	0	1	0	1
		21UR-3630	TTTGAAATGTTGTGTAAATTG	3	0	0	0	0	0	0	3
		21UR-3631	TACTTCTACCATTTTAGTAGC	0	0	0	0	2	0	3	5
		21UR-3632	TCACTACAATCGAAACAACTA	2	0	1	0	4	1	1	9
		21UR-3633	TAGTCCCATCTTTACTTCACC	0	0	0	0	0	4	0	4
		21UR-3634	TAGCAGCCTCTTAACTACACA	0	0	0	0	5	5	1	11
		21UR-3635	TCGAGAAATAAACTTGATAAT	0	0	0	0	0	1	0	1
*		21UR-3636	TAAAACGAGAACTGGCATGAT	3	5	2	8	274	261	60	613
		21UR-3637	TGACTATTACATTCTCAAGAA	1	0	0	0	0	1	0	2
		21UR-3638	TAAATTATGTATGTTGTCAAA	0	0	0	0	1	1	0	2
*		21UR-3639	TAGGAGAAACAAGTGTGTTGT	8	1	0	3	21	40	6	79
		21UR-3640	TCATAACCAGGCAAAAACCAC	1	0	0	1	7	12	10	31
		21UR-3641	TCTCCAGACAACTGATGTCTC	0	0	0	0	0	0	0	0
*		21UR-3642	TGGAACGATGTGCAACGAAAA	154	241	85	56	220	1061	28	1845

		21UR-3643	TTAGTCCAGCGTCCCACGAAA	0	0	0	0	0	4	6	10
*		21UR-3644	TACATAAGCGGTAAGAGTCCA	5	1	1	5	80	76	8	176
	†	21UR-3645	TGGAGAGCGAAATAGTCTCTA	0	0	0	0	0	1	0	1
	†	21UR-3646	TAGATGAGTGTGTCAGTTCAT	6	3	2	0	0	11	2	24
		21UR-3647	TTAATACTTTTGGTGCTTTCG	2	0	0	1	3	0	1	7
		21UR-3648	TTACCTAAAGATATCAACTTC	1	1	0	0	1	3	0	6
*		21UR-3649	TCAGCAACCATCGTGTCCCAA	1	1	0	0	0	0	1	3
	†	21UR-3650	TGCATTGAAGGGTTCCATAGA	22	6	3	2	13	48	10	104
*		21UR-3651	TCTCGTGGATAAATAAGCATT	121	118	62	72	161	444	33	1011
	†	21UR-3652	TTGCAGCCCAAATCATCACAC	0	0	0	0	0	1	0	1
		21UR-3653	TCCATTTTGATTTTGGCATAA	0	1	1	1	10	12	0	25
		21UR-3654	TAGAGCCATCGTTCATCGAAT	1	0	0	0	1	0	1	3
		21UR-3655	TCACTTTGTTGTTGTTGTATA	0	0	0	0	1	0	0	1
	†	21UR-3656	TCGCGTTTACTTCACGAGATG	0	0	0	0	0	0	0	0
	†	21UR-3657	TAATTGCACTGTATTAATTTG	1	0	1	1	0	2	0	5
	†	21UR-3658	TATTACAACTATAAAGTGATA	0	0	0	0	0	0	0	0
	†	21UR-3659	TTGATAGTGTCACATGGTGTT	0	0	0	0	0	1	0	1
*	†	21UR-3660	TGCGGCGTAGATATGACATGA	0	2	0	0	8	19	3	32
	†	21UR-3661	TGCCCTTCGTTGTCATGGAAT	0	0	0	0	0	0	0	0
	†	21UR-3662	TTATAATTCAAGACAGCGGAA	2	1	0	2	40	19	9	73
		21UR-3663	TATGCATTCTCGCATTGCTCA	0	0	0	0	0	0	0	0
		21UR-3664	TAAAATTTTATAAAGTGTTTC	0	0	0	0	2	2	0	4
		21UR-3665	TAGAGTAGTAATATGGTAGTA	3	4	1	7	149	80	16	260
		21UR-3666	TACATATGGCTCCATACTTTA	0	0	0	0	3	5	2	10
*		21UR-3667	TTCTCCTACTTTCCAGGCAGA	1	0	0	0	17	25	7	50
		21UR-3668	TGATCTGTTCTTTTCATTTCA	0	1	0	0	0	2	0	3
		21UR-3669	TAGGCCTAAAATATCAACACA	5	0	1	3	7	11	0	27
		21UR-3670	TCAGTTTCATAGTTGATTGCA	29	19	10	8	15	226	1	308
		21UR-3671	TGAATTATGTATACCTAATTC	0	0	0	1	2	2	0	5
		21UR-3672	TAATCGGAATTAATCGGAAAA	12	5	3	5	35	35	3	98
		21UR-3673	TCGCGATGACATAAACAGTGC	0	1	0	0	7	5	8	21
		21UR-3674	TTCCTGTTTTTTTGTACACAC	0	0	0	1	6	6	0	13
	†	21UR-3675	TAAGGTATCGCAGTACAGGGG	12	1	4	1	3	13	4	38
		21UR-3676	TACAGCAAATTTTCTGAAGAG	6	5	0	0	1	21	0	33
		21UR-3677	TGAACCTATATCCGTATTTAA	0	0	0	0	0	0	0	0
	†	21UR-3678	TAATTGGTATTAATTATCAGG	0	0	0	0	5	5	1	11
		21UR-3679	TATAGCTCGTATTGATAATTA	1	0	1	1	4	1	3	11
	†	21UR-3680	TCACATCAAGAAAGTAATTTC	1	0	0	0	0	1	0	2
*		21UR-3681	TCTAATGCAGAAATTTTGTTC	24	39	8	36	385	338	9	839
		21UR-3682	TTCAACAACAACATTATTTTA	0	0	0	0	1	0	0	1
	†	21UR-3683	TACGATTTGTCCATTTTTTGG	1	0	0	1	0	1	0	3
	†	21UR-3684	TAAACAAGGCATGTGTAGGAA	37	68	39	52	465	910	76	1647
*	†	21UR-3685	TACAGTTAGAGAAGCATTAAC	0	0	0	1	6	26	2	35
	†	21UR-3686	TTCTGATTCTTTCGTAATGAT	0	0	0	0	0	0	0	0
	†	21UR-3687	TGCTGATTCTTTTGCTCTCTG	1	0	0	0	0	3	0	4
		21UR-3688	TGGAAATGTGACAATAGCTAA	0	2	1	4	47	35	19	108
		21UR-3689	TGGATCTTTCAAAAGTTTGTG	0	0	0	0	2	0	0	2
		21UR-3690	TAACTGATTCGCATCCTGCAA	2	2	3	5	55	31	4	102
		21UR-3691	TAAAAGTGTTTCAAACATAAA	0	0	0	1	2	0	0	3
*	†	21UR-3692	TAGCAACGGTATTGTAATCAA	17	17	8	20	351	327	34	774
		21UR-3693	TGGGTATTTGACAATTTTTGA	0	0	0	0	6	13	2	21
		21UR-3694	TGAACCGTTAATTTTTGGGAG	0	0	0	0	0	0	0	0
	†	21UR-3695	TTAATCGAAGTTCGTGGTATT	16	5	4	2	5	24	6	62
		21UR-3696	TCAGTATGTGGGTTGATGTTT	0	0	0	0	3	0	9	12
	†	21UR-3697	TGTTACAGCATATGAAAACAA	0	0	0	0	0	0	0	0
		21UR-3698	TCGAATGCATTCTTAGGTACA	0	0	0	0	1	2	1	4
		21UR-3699	TTTAAACATAATATATGTAGC	0	2	0	1	1	5	0	9
		21UR-3700	TAGGGAATAGCTTCAACACTA	2	1	0	0	0	6	0	9
		21UR-3701	TAATCTCTTGTTTTCCATTTC	1	0	0	0	0	2	0	3
	†	21UR-3702	TCGGATTATAAATGGATTCCA	0	0	0	0	0	0	0	0
	†	21UR-3703	TGTAAGGTTGCTCTAGAAATT	2	0	0	0	0	0	0	2
	†	21UR-3704	TGCAGAATAGCTGATCCCAAA	0	0	0	2	6	7	1	16
	†	21UR-3705	TGTCGCTGTCATGAAGCAAAA	0	0	1	0	0	1	0	2
		21UR-3706	TATGCGGTTTTTTTTGGGAGC	0	0	0	0	2	3	1	6

		21UR-3707	TCGAGAAAAATCATATCCTTT	0	0	0	0	0	3	0	3
*	†	21UR-3708	TGACAATGTAGCGTTAGCAAT	15	17	12	12	119	94	23	292
		21UR-3709	TCTATGTTCCGTGTTGATGTC	0	0	1	0	7	9	6	23
	†	21UR-3710	TGCAGGTTTGATTTTCTGTGG	1	1	0	0	8	2	0	12
		21UR-3711	TCATCTCTTCTTCTTTCCAAG	0	0	0	0	0	1	2	3
	†	21UR-3712	TACGCTAGTTCCAATATAAAC	0	0	0	0	1	0	2	3
	†	21UR-3713	TGGTGCATGTTGATTTTTTGC	0	1	0	0	0	0	0	1
		21UR-3714	TGCTTGTCATCATTACAGTGA	0	0	0	0	0	0	0	0
	†	21UR-3715	TAGATTGAGTGAATCGAAAAC	0	0	0	0	0	0	0	0
*		21UR-3716	TACTTCGTGGCTAACTATCGT	4	1	1	0	12	13	5	36
*		21UR-3717	TAGGGCTTTCTACTTTTTTGA	0	1	0	0	3	3	1	8
	†	21UR-3718	TACTGCGATGGATTTACAGAA	0	0	0	0	19	16	14	49
		21UR-3719	TCATCTTACGAAATTCTTTTA	0	1	1	0	1	0	0	3
	†	21UR-3720	TATTATTATCGTCTTCTGAGC	0	0	0	0	0	0	0	0
	†	21UR-3721	TGTATAGATCATCGTTTAACA	11	3	0	1	2	13	1	31
		21UR-3722	TGGTGTGATACTTCTTCGTTT	0	0	0	0	0	0	0	0
		21UR-3723	TCTGTGCGGTTCACATTTCGT	0	0	0	0	0	0	0	0
		21UR-3724	TGGTACAATAACTCCTAAATC	0	0	0	0	4	0	1	5
		21UR-3725	TCACATCAAGAATTGAACACC	0	0	1	1	11	4	0	17
		21UR-3726	TACTTCGTGAACAGTTGTTAA	0	1	0	1	4	7	0	13
		21UR-3727	TGTGACTTGTGTCTTGTAAAT	0	0	0	0	0	0	0	0
	†	21UR-3728	TATTTCGCATTACGCAGAAAA	0	0	0	0	3	3	0	6
		21UR-3729	TGTCAATAGAATTTAGTTTTT	0	0	0	0	0	1	0	1
*	†	21UR-3730	TTTTCGTTGTGGACAATTTGG	254	54	46	41	316	628	155	1494
	†	21UR-3731	TGATTATATGGTATTTGTTTC	0	1	1	1	9	6	3	21
	†	21UR-3732	TAGTATTTAACTAGTTCGCTA	0	0	0	0	0	0	0	0
		21UR-3733	TAAACGAATTGTGGTTAATCG	7	2	2	0	4	11	0	26
	†	21UR-3734	TCACTCTCATTGTTTAGGATC	0	0	1	0	6	0	0	7
		21UR-3735	TGTGTCATCTTTGCTATAAAC	0	0	0	0	0	0	0	0
	†	21UR-3736	TAGTTGAGTAGTTAAAATGAG	1	0	0	0	2	2	0	5
		21UR-3737	TGCTGACGTATCATAGGTAAA	1	1	1	2	14	24	5	48
		21UR-3738	TGTAAGCCTTGGTTCTGTTCC	0	0	0	0	0	2	0	2
	†	21UR-3739	TCACATTAATCAAAGCCAGTA	0	0	0	0	0	0	0	0
	†	21UR-3740	TGGTTCTTGCATGTATGAATA	3	5	1	2	7	26	1	45
		21UR-3741	TGTAGCAGATCCATGCTTTTT	0	0	0	1	2	0	1	4
*	†	21UR-3742	TAAGTTAGGCGTAATACTTGT	3	8	1	18	255	213	4	502
	†	21UR-3743	TTGGACTAGAACTTTGAATGC	0	0	0	0	2	17	0	19
		21UR-3744	TGACTTGATTTTTATCAAATT	0	0	0	0	0	0	0	0
		21UR-3745	TGGCTCTCCATTCGAGGGTAA	0	0	0	0	4	2	0	6
	†	21UR-3746	TAAAGTGCACGATTTATGAAT	1	0	0	0	0	1	0	2
		21UR-3747	TCCTGCCCCTCATTCCAATTC	0	0	0	0	0	0	0	0
		21UR-3748	TATCCTATCCGAAGTTTGTTG	0	0	0	0	0	1	2	3
	†	21UR-3749	TCCTACTATGGCGTTTATGAA	0	0	0	0	0	0	0	0
		21UR-3750	TCCATGTGCCTTACAAGTTAG	2	0	1	0	3	6	3	15
	†	21UR-3751	TCCGTAGCGGCTGTAATGCAA	0	0	0	0	3	0	0	3
		21UR-3752	TGCTTCACCAGTCACGAATCT	0	0	0	0	0	0	0	0
	†	21UR-3753	TGTTATTACGGGACGAACAAC	0	1	0	0	43	42	17	103
		21UR-3754	TATGGATATCTATGTTATGGA	0	0	0	0	1	0	1	2
	†	21UR-3755	TTGTTTGGACATTTTTAAAAC	3	2	1	4	37	15	9	71
		21UR-3756	TGGCTATTTTTTCGAACCAGT	1	2	1	2	9	13	2	30
		21UR-3757	TTTCAATATTCCAAATTGCCT	0	0	0	0	0	0	0	0
		21UR-3758	TTATTGATTCATTTTAACGCA	0	1	0	0	0	0	0	1
		21UR-3759	TCCAATGGGGTGTCCAATCGA	0	0	0	0	0	2	0	2
	†	21UR-3760	TAATAAATTTCACTCACCTCC	0	0	0	1	0	0	0	1
	†	21UR-3761	TTGAATTTAGTCTGTGGAAGG	13	6	6	12	150	161	17	365
		21UR-3762	TCAGTCTGCATTTACAAGCTG	0	0	0	0	0	1	0	1
	†	21UR-3763	TAATCGTTTCGAGGAAACAGT	0	0	1	0	2	3	0	6
		21UR-3764	TACACACTGTTCTTACTGAAA	1	0	0	1	0	2	0	4
		21UR-3765	TTAATTATTTTATTTTCCGAG	0	0	0	0	1	0	1	2
*	†	21UR-3766	TGGAAGCTTGATGGAAAATGC	12	20	3	14	273	345	47	714
	†	21UR-3767	TGATTTGTATCTAAACTTGCA	0	0	0	0	3	0	1	4
	†	21UR-3768	TGTAGTTTGAAAGTAGGTGAT	1	2	1	1	4	13	5	27
	†	21UR-3769	TTAATGGAGGTCCATTATACG	0	0	0	0	0	0	0	0
		21UR-3770	TCTTCGATGAACTGTCCTATT	0	0	0	0	1	1	2	4

		21UR-3771	TTCGTTTATTCTTTGCGGTAC	0	0	0	0	4	5	2	11
		21UR-3772	TCAAAAGAAATTAGAAAGAAA	0	0	0	0	0	1	0	1
		21UR-3773	TAGAGTTTTCACAACAAAATT	0	0	0	0	0	3	0	3
		21UR-3774	TCGCAAATAGAACAAAAATCA	0	0	0	0	2	2	0	4
*		21UR-3775	TGACAACGGCTTTGTGAGAAA	32	64	19	25	288	536	42	1006
	†	21UR-3776	TAATGTTTTGAAACACCACAT	0	0	0	0	6	2	1	9
	†	21UR-3777	TGAAACAAGTATAGTTTAAAG	0	0	0	0	2	1	2	5
	†	21UR-3778	TGGAAGATTAATTTTCTAGAC	0	0	0	0	0	0	0	0
*	†	21UR-3779	TCGAAGATGACGATGACCTTA	50	136	58	52	294	743	39	1372
	†	21UR-3780	TGGTTACAACAGAGTCACAAA	1	1	1	1	4	8	2	18
		21UR-3781	TGTGAAGTGAGTTACGATTTA	1	1	0	0	4	5	1	12
		21UR-3782	TAGTCCTCTCATATAGTTGAG	2	0	0	1	0	1	2	6
		21UR-3783	TAGTCATATTCTGCGCAATTC	0	0	0	0	1	1	2	4
	†	21UR-3784	TCGACTATTGATTTTGTTTGG	72	24	33	35	137	251	55	607
	†	21UR-3785	TGACAATTTGGATTGCTCAAT	1	0	0	0	6	7	1	15
	†	21UR-3786	TATTATGATGTTTGTTTTGTA	0	1	0	0	1	1	0	3
		21UR-3787	TCTGGGTTGGAGTCACACGTT	3	0	0	2	6	7	6	24
*	†	21UR-3788	TGCGACACCGATAAGAGAACA	1	2	1	1	4	13	3	25
		21UR-3789	TGGGATTTCCTTTCCTCCACT	0	0	0	0	0	1	0	1
	†	21UR-3790	TTCGTCTGTGCTAATTATCGT	0	1	0	0	9	5	1	16
	†	21UR-3791	TAGGAAAATCGTTCACATTCC	2	1	0	4	51	22	32	112
		21UR-3792	TGGCTGATATTCCTACAATGA	0	0	0	0	0	1	0	1
		21UR-3793	TATGGAAAGTTTAGATTCTTC	1	0	0	4	40	25	2	72
	†	21UR-3794	TCTAATGCCTTTGGGAAGTTG	1	0	0	0	0	0	0	1
	†	21UR-3795	TTGGAGTTTCAATTGGAATGA	0	0	0	0	0	0	0	0
		21UR-3796	TGCCTTTAGCATTTTTCGTAA	1	0	0	0	0	0	0	1
		21UR-3797	TGCATCATCATTTGTTCCCTT	0	0	0	0	0	0	0	0
*	†	21UR-3798	TATGATTTCATCAGATGGTTC	4	0	0	0	2	0	0	6
		21UR-3799	TTACACATTCTGTACCACTTT	0	0	0	0	0	0	0	0
	†	21UR-3800	TGATGTAGAGATTCCAGTTCC	0	6	4	3	42	47	6	108
		21UR-3801	TTTACGTAACTGAATAAACTC	9	5	2	4	7	19	1	47
*	†	21UR-3802	TGGACCAAGGATAATGTCAGC	131	127	60	29	212	922	22	1503
	†	21UR-3803	TCGGAAATCATTAAAATAAAA	0	0	0	0	0	0	0	0
	†	21UR-3804	TCAATATGATCAGGCATCCTA	0	0	0	0	1	3	0	4
*	†	21UR-3805	TTAGGATTAGTTGTACAGCCC	104	96	57	41	230	429	46	1003
	†	21UR-3806	TACTTTTGCAATTCAAAACTG	2	0	0	1	1	4	0	8
		21UR-3807	TCCTTCTTTTATTCTCACTTC	0	0	0	0	0	0	0	0
*	†	21UR-3808	TTGGTCGTCGATCAGGCAAAG	1	0	0	0	6	11	1	19
	†	21UR-3809	TGTTCTGTAATTTCTGATTGA	0	0	0	0	0	0	0	0
	†	21UR-3810	TAAAATTCTTCCATTGTTCCA	0	0	0	0	2	0	0	2
	†	21UR-3811	TATTCATACGATGGAACTGAG	0	0	0	0	0	0	0	0
		21UR-3812	TCCTTCATTCGAAATTGATCA	1	0	0	0	0	1	0	2
		21UR-3813	TGGGATACGAATACCAAATTC	0	0	0	0	0	1	0	1
		21UR-3814	TAATCCAGGTGTTTTCCATGA	0	0	0	0	0	2	0	2
	†	21UR-3815	TAGCTCGCAAAACTTTTTTGC	0	0	0	0	0	0	0	0
		21UR-3816	TTAGAAAACCTCGTTTAGTGA	1	0	0	0	0	2	0	3
		21UR-3817	TGCAATATTGAAATGTCAATC	0	0	0	0	0	0	0	0
		21UR-3818	TAAAAATGCTTTTGTTTCAAT	0	0	1	0	0	1	1	3
*	†	21UR-3819	TGGTCTGCCAAAGAACACGTT	10	18	7	13	40	93	1	182
	†	21UR-3820	TCGTTTTTCACATCAGATAAA	0	0	0	0	0	0	0	0
		21UR-3821	TAAAGGAGACATTCTGCTTAC	11	25	8	4	18	61	0	127
		21UR-3822	TAATAAATCTTTTGGACCAGT	2	1	3	7	110	107	71	301
	†	21UR-3823	TGTTCAGTTTATCGTACAAAT	2	0	0	0	0	2	0	4
	†	21UR-3824	TATAAGTTCTAGGTATTATTA	0	0	0	0	0	0	1	1
	†	21UR-3825	TGACGAGGAATTTTTACAAAT	0	0	0	0	0	2	1	3
		21UR-3826	TGGCAAAGTTGCAAAGGTAGA	1	0	0	0	27	31	6	65
	†	21UR-3827	TCGACGTCTCTTGCTTTCTCA	0	0	0	0	1	1	1	3
	†	21UR-3828	TGAAAATTTGAGTCTAACATC	0	0	0	0	6	2	0	8
		21UR-3829	TCGATGTTCTAAAATAATTGC	1	0	0	0	0	1	0	2
		21UR-3830	TTGAGCAATGATTACCTTTGT	0	0	0	0	0	1	0	1
		21UR-3831	TCACTTTCTGCAAAATGATTA	0	0	0	0	0	1	0	1
		21UR-3832	TATCCGTACAGTAACCTGATG	0	0	0	0	0	0	0	0
		21UR-3833	TAAATGAATTCCTTTAACATC	0	0	0	0	1	0	1	2
	†	21UR-3834	TTAACCACCATTTTCCAAAAT	0	0	0	0	0	0	2	2

		21UR-3835	TTCATCCTTTTTTCCAGTTAA	0	0	0	0	0	1	0	1
	†	21UR-3836	TGAGTCTTTGGTATTTTTCCT	0	0	0	0	0	0	0	0
		21UR-3837	TCATACTCGTACCAATTTGGG	13	1	1	0	7	12	1	35
	†	21UR-3838	TGCTAGATTTATTCCAGTTTT	0	0	0	0	0	0	0	0
		21UR-3839	TAATATACTTGGGTTCGGAAA	1	1	0	1	39	40	13	95
	†	21UR-3840	TGCAATTGTGCTTGATCGAAT	1	0	0	0	16	41	5	63
		21UR-3841	TATGGGTTTATTCAATTTACC	1	0	0	0	3	3	0	7
		21UR-3842	TAAGATCGCAGTTGAACATTT	23	12	6	5	15	41	1	103
	†	21UR-3843	TCGCACTGCTAGTTGAAAGCA	3	1	0	0	2	0	1	7
		21UR-3844	TCTATCGCACTCACAACTAAT	1	0	0	0	0	1	0	2
	†	21UR-3845	TATTCGTTTTTCACATCAGAT	0	0	0	0	1	1	1	3
	†	21UR-3846	TTAGTATGTTCGTGGTGAAAG	18	5	2	2	38	68	25	158
		21UR-3847	TAGACTACTATTTGACTCGAG	0	0	0	0	1	6	0	7
		21UR-3848	TGAATATTGGGCAAACACTCA	0	0	0	0	1	0	1	2
		21UR-3849	TCTGTATTTCGCTGCCAATAA	0	1	0	1	0	1	1	4
	†	21UR-3850	TAATTCCGGGACGCTGAATAT	0	1	0	0	1	2	0	4
		21UR-3851	TACTCTCCACACCACGAAGTT	0	0	0	0	0	0	1	1
	†	21UR-3852	TTAGAAAGGATTGTTTATTAA	48	19	19	19	51	141	32	329
	†	21UR-3853	TTTTGGGAAATCAAAATGTGC	0	1	0	0	0	1	0	2
	†	21UR-3854	TAGGTAGTGAATTTCACTGTA	2	0	0	3	4	3	0	12
		21UR-3855	TACATCAGGTTTCTAACCAAT	1	0	1	0	9	5	0	16
		21UR-3856	TGTAACAGGCAATTTCTTGAG	0	1	0	0	16	23	7	47
		21UR-3857	TCTGCTGTTAATTTCAATTTC	0	0	1	1	1	0	0	3
		21UR-3858	TCCTAACTTCAAATTATCTCA	0	0	0	0	0	0	0	0
	†	21UR-3859	TGATTTGATTACAACAATTTA	0	1	0	0	1	0	0	2
	†	21UR-3860	TAGTTTCATAATTTTTCTGAC	0	0	1	0	0	0	0	1
		21UR-3861	TATGACGTCTAAACGTAAAGC	1	0	0	0	1	2	1	5
		21UR-3862	TGCCATTTGGATGATATAACG	2	0	0	0	3	2	1	8
*	†	21UR-3863	TTGGACTGTTCCACGTGGAAA	107	25	18	43	502	452	142	1289
		21UR-3864	TAAGTCAGTCCATTGTATCGA	0	0	0	0	1	1	0	2
		21UR-3865	TATAGCTGTTGTTTTCTTCGT	7	7	3	4	4	11	3	39
*		21UR-3866	TATGATAATTTGGTTACTCGA	2	1	1	2	18	13	2	39
		21UR-3867	TCTGAGACATCTTCTATTTTC	0	0	0	0	0	0	0	0
		21UR-3868	TGTTGCTTTATGAGTGGAAAT	0	0	0	0	0	3	0	3
		21UR-3869	TGAAGTTACATAACCTGTTGA	1	0	1	1	39	29	13	84
		21UR-3870	TCGTTCTGTGACTCTCATTTT	0	0	0	0	0	1	0	1
	†	21UR-3871	TTGATTCATCATTGTTTTCGG	0	0	0	0	0	0	0	0
	†	21UR-3872	TGGAATTCCTGTTGCAATGCA	1	0	0	0	0	1	1	3
		21UR-3873	TGCACGCTCAATATATTCTCA	0	0	0	0	0	0	0	0
		21UR-3874	TGTTGCTACAACTGTGCTTTA	0	0	0	0	0	0	0	0
	†	21UR-3875	TGTTGCACCAGGTGGAATTGA	1	0	0	0	1	1	1	4
	†	21UR-3876	TTCCAACTGACACATTTTTAT	2	1	0	0	0	6	1	10
	†	21UR-3877	TGATCGCCTTCTACCAAAATG	0	0	0	0	0	0	0	0
		21UR-3878	TGAAATTGATATTTGAGACAT	0	0	0	0	0	0	0	0
	†	21UR-3879	TGCTACTTTCACAACAAAACC	0	0	0	1	1	1	0	3
		21UR-3880	TCTTGCAAGGTCAATAATAAT	0	0	0	0	0	1	0	1
		21UR-3881	TGCTTTGCATACATGCGAACG	1	0	0	0	8	10	1	20
		21UR-3882	TCGGATAACTGGAAACCCACA	0	0	0	0	1	3	1	5
	†	21UR-3883	TCCGAAGTCGATCAGGTTTAC	0	0	0	0	12	15	7	34
		21UR-3884	TTACTCCTAGTGCAATTTTTA	3	0	0	0	0	1	0	4
	†	21UR-3885	TAATTGGTCGGCTGCTTCAAT	0	0	0	0	5	1	11	17
	†	21UR-3886	TGAAGATGGCAAAAACTTTCA	6	7	3	1	5	22	0	44
	†	21UR-3887	TTAAATGCTGCATTTTAATGA	1	0	0	0	0	1	0	2
		21UR-3888	TGGAAAAATAAACTTAAATTC	0	0	0	0	0	0	0	0
	†	21UR-3889	TTTCACATTCCATTGAAATAG	0	0	0	0	0	0	0	0
	†	21UR-3890	TATGTACGGGCTTCTGTTTGG	0	0	0	1	5	5	3	14
*	†	21UR-3891	TGTTGCGAAATTTTCAAAGTC	0	1	0	0	1	0	0	2
		21UR-3892	TGAGTCTTCTTCCTTTAGGTT	2	0	0	0	0	0	1	3
	†	21UR-3893	TTAGTCCGTTTTTTCGTCAAC	0	0	1	1	6	12	2	22
	†	21UR-3894	TCTTTGCGACAAGAATTGTGA	0	1	0	5	48	43	8	105
	†	21UR-3895	TTCATAATGATAACGGATCTA	0	0	0	0	0	1	0	1
	†	21UR-3896	TGCAAGCGATTTTCAAACAAT	1	0	0	0	3	5	2	11
	†	21UR-3897	TTTCAATTGAATGGAACTGTC	28	16	9	8	14	65	11	151
*		21UR-3898	TGAGTAGGGCATTGGATTACT	2	2	1	5	152	129	14	305

		21UR-3899	TATTCCTAGCTTTATTACTAA	1	0	0	0	2	2	3	8
	†	21UR-3900	TTGATATGGTAAATTGTGAAC	0	1	0	1	39	38	2	81
*		21UR-3901	TGTAAGAGTTGAAAAGGAGAA	38	77	25	15	52	244	2	453
*	†	21UR-3902	TCACATTCTTGTCCTCTGTAC	1	1	0	2	5	8	0	17
	†	21UR-3903	TGTTTCCTTTTTGCGATAAAT	0	0	0	0	0	1	0	1
*	†	21UR-3904	TTGGAATCAATTGTTGTTGGA	54	6	2	8	15	66	18	169
		21UR-3905	TAATTCTTCACAGTCCTTAAA	0	0	0	0	0	2	1	3
		21UR-3906	TTCCGTTTGTTCATAAATGTA	2	0	0	1	2	5	0	10
	†	21UR-3907	TGATGAAAAGTGAATATTTAG	0	0	0	1	0	0	0	1
		21UR-3908	TGATTCCATAACATGTGTTTT	0	0	0	0	0	0	0	0
		21UR-3909	TCGCATAAGACACAATTTTCA	3	0	1	0	0	0	0	4
		21UR-3910	TAGATACTTGCATTTTCCCTC	1	1	1	0	0	5	1	9
	†	21UR-3911	TCGAAATTTCTTTTTGCTTCA	0	0	0	0	0	1	0	1
		21UR-3912	TCATTTGGGTGAAGAAATCAA	0	0	0	2	3	3	1	9
	†	21UR-3913	TCAACATAATCATCAAAATCA	0	0	0	0	0	0	0	0
		21UR-3914	TCAACAAGCCAATTTTGTTCT	0	0	0	0	0	1	0	1
	†	21UR-3915	TCGCTTTTTTAATCCAAAGTG	0	0	0	0	0	1	1	2
*		21UR-3916	TAAGGGTACCATTTTTTCTAC	0	0	0	0	5	2	3	10
	†	21UR-3917	TAATCCGCCTTTTGCTAATAT	0	0	0	0	0	1	0	1
		21UR-3918	TTTCAACTAACATCTAGTTCC	0	1	1	0	1	2	0	5
	†	21UR-3919	TGAAGCTCTGACTGTCAACGC	0	0	0	0	0	0	0	0
*	†	21UR-3920	TTCGAAGGTCAACAAAATATT	5	1	0	0	1	9	2	18
	†	21UR-3921	TTGGTGAAATCCACAACTACA	0	0	0	0	0	0	0	0
	†	21UR-3922	TCGGATCGGGTCATACCGGAT	33	2	5	1	26	30	19	116
		21UR-3923	TAAATGTTGAGTCCCTGACCA	0	0	0	0	1	1	0	2
		21UR-3924	TCTCGATCCAATTCCAATTTT	0	0	0	0	0	0	0	0
		21UR-3925	TAACTTTAACTTGCTCAGAAA	1	2	0	0	2	9	0	14
*		21UR-3926	TAGAGTGATATGTTGAGAACA	42	35	18	6	24	96	10	231
*		21UR-3927	TCGAATACTTTAAAAATTCAA	0	1	0	1	10	4	0	16
		21UR-3928	TACCGGTACAGAGAGTGTAGA	4	0	0	0	4	19	2	29
*	†	21UR-3929	TTGCAAGGATTTTGGTGATAA	42	54	22	13	62	250	9	452
	†	21UR-3930	TCAATGCTTACAGTTTATAAG	0	0	0	0	0	1	0	1
	†	21UR-3931	TGAGAACATAGCTTTTTTTCA	0	0	0	0	3	0	0	3
	†	21UR-3932	TGTTTCTTTTTGCCAATCGCT	0	0	0	0	0	0	0	0
		21UR-3933	TCGACCAAAATTTATTTCTTG	0	0	0	0	0	0	0	0
		21UR-3934	TTTTCTTTGATCACACTCCTC	1	0	0	0	0	1	1	3
		21UR-3935	TAAACAATTAATGTAGGGCAA	18	18	4	17	18	89	3	167
		21UR-3936	TACGTTTGCAAGTTTTAGCGG	40	8	2	3	22	63	7	145
*		21UR-3937	TAATGACGTTTTTTCCTCTGA	0	0	1	4	23	29	0	57
		21UR-3938	TGACAAGTGCCCTTGAATTTT	0	0	0	0	0	1	0	1
		21UR-3939	TAGATCTACCGGAGTTTTGAA	2	0	0	1	0	6	3	12
		21UR-3940	TCTCATAGGGTCTCGTTGACA	21	21	9	8	98	114	49	320
		21UR-3941	TAATTCCCAACTTTGTAATCC	0	0	0	0	1	1	0	2
		21UR-3942	TGCATAGAACGAACCTTTTTC	5	2	2	1	3	15	0	28
		21UR-3943	TCATCGGGTTTGTCTCATTGA	1	0	0	0	5	4	0	10
		21UR-3944	TGCTCGCATGATATAATTGAT	0	0	0	0	1	3	1	5
	†	21UR-3945	TTCGTGGATTTTCTATTAGGT	0	0	1	0	7	3	2	13
	†	21UR-3946	TGGTAGTTCAATATTATTTCA	0	0	0	1	1	3	0	5
	†	21UR-3947	TTGTTCCACTGATTAGTCATT	4	1	3	2	18	9	1	38
		21UR-3948	TGCAGATATATTTTTAATGCA	0	0	0	0	0	1	0	1
*		21UR-3949	TTCGTAGCCTTGTCAGTTAAT	2	2	1	0	3	4	0	12
	†	21UR-3950	TACTGTTTTCCTAATCATGGT	0	0	0	0	2	7	0	9
		21UR-3951	TGCCAGTTTCGAGCAAGCCCA	0	0	0	0	0	0	1	1
		21UR-3952	TGAGTGGTTTGCTATGAGTGA	0	1	0	1	3	1	0	6
		21UR-3953	TAAAATAGTCACCATTACAAA	1	0	0	0	0	1	1	3
		21UR-3954	TATGGAATAAAATTATGCAGC	0	0	0	1	11	8	4	24
		21UR-3955	TACGAATGTGTTTTTCTTTTT	0	1	2	0	8	6	0	17
		21UR-3956	TGCAAAACATGTCCCGGAATT	0	0	0	1	0	0	4	5
*	†	21UR-3957	TAACGACGTTGTATAGGAATA	40	49	20	16	170	196	8	499
		21UR-3958	TAGTCTCGCCATTGCTGTAGG	0	0	0	0	3	2	3	8
*		21UR-3959	TCCAACACAGAAATTGCCAGG	0	0	0	0	1	2	3	6
	†	21UR-3960	TCGATGTCGTTCTTATGTCGT	5	2	2	2	13	16	1	41
	†	21UR-3961	TGATACACGCGATTGATTTTT	2	1	0	0	1	1	0	5
		21UR-3962	TGTGCCAATGGCTAAACAAAA	0	0	0	0	0	0	1	1

		21UR-3963	TCTGAAGAAACGTTATTGAAA	1	0	1	0	24	18	1	45
*	†	21UR-3964	TAGTGAATTCGGATTCGTTCT	22	21	6	8	26	109	3	195
		21UR-3965	TATATAACAAGCAACAAATAC	55	11	4	10	19	35	6	140
		21UR-3966	TACTATTGTCAGTCTCACAGG	0	0	1	1	9	11	8	30
		21UR-3967	TGGGATTGATGCAGTTTCACT	29	35	17	15	43	159	15	313
		21UR-3968	TGGTCTGAAAGTGAGTAAACA	1	0	1	0	0	4	0	6
	†	21UR-3969	TATTACCAATTCGTCATGTTT	9	1	3	2	17	10	8	50
		21UR-3970	TCCGCGTGCAGACTTCTTATT	0	0	0	0	35	21	8	64
*		21UR-3971	TGCACTTATTGAATCGACTGA	0	1	2	4	37	52	5	101
*		21UR-3972	TGTGAAGTTGGGATAGGTATC	84	91	47	44	502	923	168	1859
	†	21UR-3973	TAAGGGACCTCTAATATTTCT	0	0	0	0	0	2	0	2
	†	21UR-3974	TAGTGTGAAAATGAAAAATAG	0	2	1	0	0	0	0	3
		21UR-3975	TCGCGTTTGTGATCTCTTCTT	0	0	0	0	0	0	0	0
		21UR-3976	TAAAAAAGTTAAATGCCTGAA	1	0	0	1	0	5	0	7
		21UR-3977	TATATTCATTCTTGGTACTTG	2	0	1	0	3	4	0	10
	†	21UR-3978	TGGATTGTAAAAACAATGATA	0	0	1	0	1	2	0	4
*	†	21UR-3979	TGTATTGCATAAATGAACGGT	3	9	1	7	173	176	10	379
	†	21UR-3980	TGTGAATATTTTTTTCACGAC	0	0	0	0	0	1	0	1
	†	21UR-3981	TGTTCTCTAGATGGGACCTGA	6	4	6	1	19	76	2	114
	†	21UR-3982	TATATGTATGACATCGAGTGT	0	0	0	0	9	4	0	13
	†	21UR-3983	TAAACGAACCTTGGTGATTGG	0	0	0	0	0	0	1	1
		21UR-3984	TGGAAATTTCTATCACAGTTT	0	0	0	0	1	1	0	2
	†	21UR-3985	TTGTTAACTGCTTTTTGTCTC	0	1	0	1	3	3	0	8
	†	21UR-3986	TTTGATACATAGCTTTTCCAG	0	0	0	0	0	0	0	0
	†	21UR-3987	TCAGACGACGATCCGGTTATT	7	6	5	10	104	133	74	339
		21UR-3988	TAAGATTGTGACAGACATGAG	3	2	1	1	2	15	1	25
	†	21UR-3989	TCGGATTTCGACAAAACGATT	1	2	0	3	9	24	2	41
	†	21UR-3990	TGGACATGACGTTTAACGGAA	1	2	2	1	36	47	2	91
		21UR-3991	TCGAATAACCATATGCTCAGT	1	0	0	0	0	0	0	1
		21UR-3992	TATAAGAAACCTGTCCACTCA	0	0	0	0	0	3	0	3
	†	21UR-3993	TTCTTTTCTGAAAGGTGTCCA	0	0	0	0	8	19	16	43
	†	21UR-3994	TAATCCGTTTACTTTTAGATT	0	1	0	0	0	2	0	3
	†	21UR-3995	TAGTATAAGTTCTAGGTATTA	0	0	0	1	1	1	4	7
	†	21UR-3996	TTGGATCAAAAAACAAAAACT	0	1	0	0	0	0	0	1
		21UR-3997	TCCAGATATTATAATCCCAAC	0	0	0	0	2	0	0	2
		21UR-3998	TCACAATATAAATACAATTGT	0	0	0	0	0	0	0	0
	†	21UR-3999	TGCAATGAATTGAATGTTCTT	2	0	0	0	1	1	0	4
		21UR-4000	TTGGACAACGTTATAGATTCA	2	0	1	7	75	41	4	130
		21UR-4001	TGTGTCGCAGCTAAATTGTTT	0	0	0	0	0	0	0	0
	†	21UR-4002	TTTTGTAGCCAAAACAAAAGT	0	0	0	0	0	1	0	1
	†	21UR-4003	TATTCGATTCGAACGATTGAA	0	0	0	0	4	4	1	9
*		21UR-4004	TGAATCGGTAATACTAGAATT	74	59	43	136	2049	1769	138	4268
		21UR-4005	TCCATGAGTCGTTTTGATAGC	0	0	0	0	6	10	2	18
		21UR-4006	TAAATTCATGATTCCGATAAA	0	0	0	0	0	0	2	2
		21UR-4007	TGCTCGACTATTCATCTTCAA	0	0	0	0	1	1	0	2
	†	21UR-4008	TGGAATTTTCTTGAGAAAGAT	0	0	0	0	0	0	0	0
*		21UR-4009	TGGAAATCATGAGAATACTTC	8	34	18	66	696	446	117	1385
	†	21UR-4010	TGGTTGATTCAAGAATGTTTG	0	1	0	0	1	0	0	2
		21UR-4011	TCGTACGCTTAAACTTGCAAA	2	3	5	2	5	15	1	33
		21UR-4012	TATAACTTCACAAGAAATTAT	2	0	0	0	0	2	0	4
	†	21UR-4013	TTGTCCTTTCATGGGAGAACT	0	0	0	0	0	2	0	2
	†	21UR-4014	TAAAAAACATCAGGCTGGTAA	15	9	6	7	12	50	4	103
		21UR-4015	TGAGCTTGATCCTCTATTTGA	0	0	0	0	0	0	0	0
		21UR-4016	TTACTAGTTAGATAGGTGTGT	1	0	1	2	19	28	7	58
	†	21UR-4017	TTTGTCCTATGGAATCGCAGT	0	0	0	0	4	3	1	8
	†	21UR-4018	TAAATTGCTCGATTTCACAAA	0	0	0	0	0	5	1	6
		21UR-4019	TATGTATAATAAGTAGTCTTA	0	0	0	0	3	0	1	4
		21UR-4020	TGAATTAAAAGTTTGTTTCAA	0	0	0	0	0	0	0	0
*		21UR-4021	TAGTGGAATTTTGCTCGACGT	11	5	7	4	36	71	4	138
		21UR-4022	TCTCCATTCATTACTTGCTAC	1	0	1	0	1	2	0	5
		21UR-4023	TGGTCTGTGGTAAGTCACTGT	0	0	1	0	6	3	1	11
		21UR-4024	TCCACTTATCTCTTTGGTGTC	0	0	0	0	0	0	0	0
	†	21UR-4025	TTGAATATGGAAGCTCATTTT	0	0	0	0	2	4	0	6
		21UR-4026	TGCGGTCAGAACTTTATGAAA	0	0	0	0	3	6	1	10

*		21UR-4027	TGAGACGCGAAACGACATAAT	13	21	10	29	256	421	88	838
		21UR-4028	TCCGTTGATTTCATGCCTTAA	0	0	0	1	18	13	5	37
	†	21UR-4029	TGCATTTCCTTCTAGGTCGTT	2	1	1	1	2	5	3	15
*		21UR-4030	TATATCAAGACCAGCCGCTGC	0	0	0	0	2	2	1	5
*	†	21UR-4031	TGGACACAACACAAGAACCAA	27	63	24	109	2537	1682	449	4891
	†	21UR-4032	TCAAGAATTCTCCGACGTACT	1	1	0	2	6	18	2	30
	†	21UR-4033	TGATCATAACGTTTTGATTTC	0	0	0	0	0	0	0	0
		21UR-4034	TGTGTTAGGTTTTCCTGCAAA	0	1	0	0	0	3	0	4
		21UR-4035	TAGTTGCAAGTTCTCGAAAAC	0	0	0	2	15	13	2	32
		21UR-4036	TTAACCATTTCCAACTATAGT	0	0	0	0	0	0	0	0
		21UR-4037	TGTGACTGCCTTTCAAATTCA	0	2	1	1	39	21	4	68
	†	21UR-4038	TAACACAGTTGTTCGAATTAA	7	0	0	1	52	531	21	612
	†	21UR-4039	TCACTCAGAATACTCCCATTA	0	1	0	1	19	11	4	36
		21UR-4040	TCTGGTTCCTCCAATGCCATA	0	0	0	0	0	0	0	0
	†	21UR-4041	TGAATGGTTGGCCCGATCGAA	2	0	0	0	1	18	6	27
		21UR-4042	TCAAAAGGAATCCTCTACAGA	1	1	0	0	3	4	0	9
		21UR-4043	TTGACAAATGTTAAATAAATG	0	0	0	0	2	2	0	4
		21UR-4044	TATATAGAACAAATTCTTGGT	1	1	2	7	69	40	8	128
		21UR-4045	TGCATATTTCATTGCTAGGCA	9	0	1	1	8	18	6	43
		21UR-4046	TCATTTAAATTTTATGATTAA	0	0	0	0	0	0	0	0
	†	21UR-4047	TCATAATGCCTTAATGTATGC	0	0	0	0	1	0	0	1
		21UR-4048	TCTCGCTTTGACTTCAATTGC	0	0	0	0	0	0	0	0
*	†	21UR-4049	TGGTCCTTGGCTATAGCCGGC	2	1	1	0	6	17	1	28
*	†	21UR-4050	TTTGAGTGTTGATTTTGTCCA	1	0	0	1	9	6	2	19
	†	21UR-4051	TGAATCTTTTAAGAAGTATTG	0	0	0	0	0	1	0	1
		21UR-4052	TAAACTTGAATGTGGAGTAGC	0	0	0	3	43	40	5	91
	†	21UR-4053	TAATTTTACTGCATTAAGAAA	0	0	0	0	2	0	0	2
		21UR-4054	TGCCATTTTGTATGCATTTGT	0	0	0	0	0	0	0	0
		21UR-4055	TAGGTTCATGATCCTCAAGCT	0	0	0	0	0	2	0	2
	†	21UR-4056	TAATGCTTTACGAAAGTCAAA	1	0	0	0	1	2	0	4
		21UR-4057	TGAACCAATTTTCTTCGTAAT	0	0	0	0	0	0	0	0
*		21UR-4058	TAGGCACTACATAACTGAACA	14	4	5	3	13	75	4	118
	†	21UR-4059	TGGGTTTCGAAGCAAGAGCCA	2	0	0	1	0	13	2	18
		21UR-4060	TGTACACACACCTGACAATCA	0	0	0	1	0	1	0	2
	†	21UR-4061	TTAATCTGGTGTGGGTAAAAT	1	0	0	1	0	6	0	8
	†	21UR-4062	TGACAGGCGTAAACGTAAGAG	2	6	4	9	120	142	36	319
*	†	21UR-4063	TTTGGAGACTTTATGCAGAAC	10	51	22	83	1455	1590	202	3413
	†	21UR-4064	TATGAAAAGAAACAAGCAGCA	7	3	2	5	83	169	19	288
		21UR-4065	TAAGTAAGAAAATTAGAACTG	4	1	1	1	27	22	2	58
	†	21UR-4066	TGGTTTGATCAAAAGTGAAAA	1	0	1	0	0	1	0	3
	†	21UR-4067	TTCCACAGTAGCGTACAAATA	17	5	3	2	17	108	6	158
*		21UR-4068	TAGAGAATCCATAGCTGTTAC	0	0	0	0	3	7	0	10
	†	21UR-4069	TTCTATGGTTTCATTCAGTGC	0	0	0	0	1	2	0	3
		21UR-4070	TACAATGCACGCTTCTGCGAA	0	0	0	0	4	6	9	19
	†	21UR-4071	TTAACGGGCTGAAATACTAAA	0	0	0	1	34	23	24	82
		21UR-4072	TGTAAATCGAACAAAGCCTAA	0	0	0	0	1	0	2	3
*	†	21UR-4073	TGGACACTAGTGAATTATTGC	6	3	2	11	221	140	66	449
		21UR-4074	TGCCCTATCGGTTTCCTTATG	0	0	0	0	0	0	0	0
	†	21UR-4075	TCATTATATATACATTGGATA	0	0	0	0	0	0	0	0
*		21UR-4076	TGTCTCGCCAACTGCAAGCCA	0	1	0	0	0	1	0	2
		21UR-4077	TAACCTTTCATTAATACTTCG	0	0	0	0	2	0	1	3
	†	21UR-4078	TCTTTGAACTACTTGCACCAA	3	1	3	1	13	15	5	41
		21UR-4079	TGACAGAGGGACCACGCCAAA	2	1	1	0	0	8	1	13
		21UR-4080	TAGATTGTTTTTATTAGTCAA	1	1	0	0	16	2	0	20
	†	21UR-4081	TTCACGGATGCAATTTGTGAT	0	0	0	0	2	0	0	2
		21UR-4082	TGTGTGATGCGAAATATTTTT	0	0	0	0	1	0	1	2
		21UR-4083	TATCAAAATCACCAAAAAAAA	0	0	0	1	0	0	0	1
		21UR-4084	TAAACGTGGTCCTCAATGATC	0	0	0	0	1	4	0	5
*	†	21UR-4085	TGCTTGATTGTTAAATTGGAT	230	70	55	43	106	363	50	917
	†	21UR-4086	TTATATTTCAGTAGTTGTCAT	6	2	2	2	3	5	0	20
		21UR-4087	TGGTGTCTTTTTTTCATAAGT	0	0	0	0	1	0	0	1
		21UR-4088	TATGTGTGCGTCGCATCGAAT	0	0	0	0	2	4	4	10
		21UR-4089	TATTCAAATTTCCGTATGTCA	0	0	1	0	2	1	1	5
		21UR-4090	TGTGTATAAGAAATCCATTTC	0	1	0	0	1	1	0	3

*	†	21UR-4091	TCAATCGAAGCAATCGAACTC	2	4	1	0	4	17	1	29
	†	21UR-4092	TGTTAGAACTATTGAGAGTTA	0	0	0	0	0	0	0	0
	†	21UR-4093	TTGAAATTGGTATCTTGTTCT	1	0	0	0	1	1	0	3
	†	21UR-4094	TGCATTGACTTCTAAAGCAAT	1	0	0	0	0	1	1	3
		21UR-4095	TTCCAGCTCCGTAGAGTGTTT	0	0	0	0	1	1	1	3
	†	21UR-4096	TATTAAGTCCTGTAACTGAAA	0	0	0	1	0	0	0	1
	†	21UR-4097	TACAGTGATTTGGATTTTTAC	1	0	1	0	2	1	0	5
		21UR-4098	TATGATCGACTATAATAGGAT	0	0	0	0	0	0	0	0
	†	21UR-4099	TACTTCTTCTTCTGGAATCGA	1	0	0	0	2	2	1	6
	†	21UR-4100	TATACACCAGAGTGAACATTA	0	0	0	0	0	0	1	1
	†	21UR-4101	TTGTTGTGTTGTGACTACAAT	0	0	0	3	17	13	3	36
		21UR-4102	TGCTCTCGACTTAGTTTTTAG	1	0	0	0	4	12	0	17
		21UR-4103	TGTTTTCAAATCAGAAGAGCT	0	1	0	2	27	25	1	56
	†	21UR-4104	TCTCGCGTTAGCACCAAAAGA	0	0	0	0	0	0	0	0
		21UR-4105	TTGAGTTTGATATTTCATTCC	2	1	0	1	0	5	0	9
		21UR-4106	TCAAGTTACGATATTTACAGT	6	2	1	0	0	5	0	14
		21UR-4107	TGAGATTTTGCTTACAATTAG	7	3	4	2	12	13	0	41
	†	21UR-4108	TTGGACTGTTCGCAAGAGTCC	2	2	0	2	2	15	1	24
	†	21UR-4109	TCTGCCCAGTGGTGTCAAGAT	0	0	0	0	0	0	0	0
*	†	21UR-4110	TGAGTGGTTGTAATTTGGTTG	65	93	35	18	84	134	9	438
		21UR-4111	TATATCATCATTGTCTAGTGG	2	0	0	1	4	2	0	9
		21UR-4112	TGAGTATGGATTTTATACCAA	0	0	0	1	0	0	0	1
	†	21UR-4113	TCAACATCCTCGATGGAGTAG	0	0	0	0	1	1	0	2
		21UR-4114	TCGTTGGTCTCCTTCATCTTC	0	0	0	0	0	0	0	0
	†	21UR-4115	TAATTTTTCGATCAATCAAAT	1	0	1	1	1	4	0	8
		21UR-4116	TATAGACACGACAAAAACATC	1	0	0	0	0	0	0	1
	†	21UR-4117	TGCTTCGTTTGGTGTCAATCG	1	0	0	0	2	2	0	5
		21UR-4118	TATATATGCGCATTCCACTAG	7	1	1	1	7	6	12	35
		21UR-4119	TTGATTTCGCATTTTAAAAAG	6	1	1	1	1	4	1	15
	†	21UR-4120	TACTCAGTCAGCGATCGAGAA	1	0	0	1	4	13	2	21
	†	21UR-4121	TGCGGCTTCTCCTTTCATGAG	0	0	0	0	3	3	0	6
	†	21UR-4122	TGTGAAGCCATCGTATTTATT	0	0	0	0	3	3	2	8
		21UR-4123	TGCAAAAATAATGGTAAAAAT	0	0	0	0	2	0	1	3
		21UR-4124	TCACATTGAAAATTAGGAACG	0	0	1	0	8	0	1	10
	†	21UR-4125	TTCACTGATTAACTGAATTAG	0	1	1	4	23	10	1	40
*	†	21UR-4126	TAATTTGGTTGGTGTATGAAG	0	0	1	1	8	6	0	16
		21UR-4127	TTTAGCGAATATTCCTAATAA	0	0	0	0	0	0	0	0
		21UR-4128	TCATCCTTCAAATTTAATATG	0	0	0	0	0	0	0	0
		21UR-4129	TTGAATGCGTTCAACTCACAA	1	1	0	0	1	3	1	7
		21UR-4130	TGAACATACCGTTAGCAAAAG	0	0	0	0	4	2	2	8
		21UR-4131	TCCCATGTTTCAATATTTTTA	0	0	0	0	0	0	0	0
		21UR-4132	TAGGCACAGCTTATATTCTTC	0	0	0	0	0	2	0	2
		21UR-4133	TGGGTGAACTGTGCTGCAAAA	0	0	0	1	7	22	4	34
		21UR-4134	TACTCTTTAATCTTATGTGCG	0	1	0	0	3	2	3	9
		21UR-4135	TGTGCTTTCAAAAAAATGCAG	0	0	0	0	0	0	0	0
	†	21UR-4136	TGTATAGACTGTGACTGTAGT	7	1	1	1	2	15	0	27
*		21UR-4137	TGCTATCTCGGCTTCAATCTA	4	9	5	6	28	56	1	109
	†	21UR-4138	TATTCGAATCGAAAGGAGATT	5	1	1	1	6	11	1	26
		21UR-4139	TCGTCGTTCCAGATTGTAATT	0	0	0	0	0	1	0	1
	†	21UR-4140	TGCTGTTTCAACCAATGAAAT	0	0	0	0	0	0	0	0
*		21UR-4141	TATACTTTGACATTGACGTTC	2	2	1	6	55	44	4	114
		21UR-4142	TAGCTATTCGTAGCAGCAAAA	0	0	0	0	0	0	0	0
	†	21UR-4143	TGATATTAAAAAATTCAATGA	0	0	0	0	0	1	0	1
		21UR-4144	TCGAAAGTCGATGGTGCACAA	0	0	0	1	24	16	6	47
		21UR-4145	TAACATTGAAAGAGCTTTTTG	0	2	1	5	15	21	3	47
*		21UR-4146	TTCTCTCGAAAGTTGGTTTTT	13	2	3	7	15	32	7	79
*		21UR-4147	TAAGCAGAATGGAAACGATTT	1	4	0	0	4	27	0	36
	†	21UR-4148	TGTAAGACTGAAATTTGAAAT	1	4	3	2	10	23	1	44
*	†	21UR-4149	TTAACTCAGTCAAAGCGATCC	0	0	2	0	2	2	1	7
	†	21UR-4150	TGATGATTTATTCTACAAGGA	0	0	0	0	0	0	0	0
		21UR-4151	TATACTGTGTAGTAACTGTGT	0	0	0	1	1	3	0	5
	†	21UR-4152	TCAGAACTATTCGACTGCTTC	0	1	0	0	8	12	0	21
		21UR-4153	TATTGTTGCTGCGTTGTGGAG	0	0	0	0	0	0	1	1
		21UR-4154	TTACCCAACACTAGAAGTTTC	0	0	0	0	0	0	0	0

		21UR-4155	TTCCTTCACATCATCATTTAA	0	0	0	0	3	1	0	4
	†	21UR-4156	TGCGTGTTTTCGTTTAAATAA	0	0	0	0	1	3	0	4
		21UR-4157	TACGCACAGGGCTTTTTTCAA	8	8	1	1	16	26	10	70
	†	21UR-4158	TAGAACAAAAATTGAAATAAA	0	0	0	0	1	1	0	2
	†	21UR-4159	TACTTCATTGAATTGGATACA	0	0	0	0	1	3	0	4
		21UR-4160	TCACATTCGAGTTTTAAAGCT	0	0	0	0	0	1	0	1
		21UR-4161	TCACGCGATAGTTCTCTATAA	0	0	0	0	0	2	0	2
*	†	21UR-4162	TTCAGCGATCGAAAGACAACA	2962	1122	962	801	4149	7978	745	18719
	†	21UR-4163	TAATACATGGTAATTTGAAAT	2	1	1	0	1	7	0	12
		21UR-4164	TGTCTTGATCTTCTCCAAAAT	2	0	0	0	1	0	1	4
		21UR-4165	TCACCTCATAATTCCATAATT	1	0	0	0	0	0	0	1
	†	21UR-4166	TGATTCATACTCCACCATCAA	1	0	0	0	0	0	0	1
		21UR-4167	TAAACTCACCGACAAAACGAG	0	0	0	0	1	0	0	1
		21UR-4168	TAAGCATTTTTCAAGGTTTTA	0	0	1	0	5	1	0	7
	†	21UR-4169	TTCCATTGATAATTGATGATT	0	0	0	0	0	0	0	0
	†	21UR-4170	TATTGCAAGACTTCGAGTGCA	0	0	0	1	7	18	1	27
	†	21UR-4171	TGAGAAATTTTGAATAGATTT	0	0	0	0	0	0	0	0
	†	21UR-4172	TGCTAGATGTGCAAATATCTG	1	0	0	0	0	0	0	1
	†	21UR-4173	TGCAATTCCGAAATACTTATT	2	0	1	1	8	4	3	19
		21UR-4174	TCAGATTCGTTTTCAATCTCA	0	0	0	1	4	3	0	8
	†	21UR-4175	TGAGAGTGATTTAGTTGGCGG	8	10	1	4	72	85	34	214
	†	21UR-4176	TACATCTGAAACAGGATGAAT	0	1	0	0	1	3	0	5
	†	21UR-4177	TCTTGGTGAAATTTTCTTTGT	0	0	0	0	0	0	0	0
	†	21UR-4178	TAATGGGTAACAATTCTAAAG	0	0	0	0	0	0	0	0
		21UR-4179	TCAAACTTAACGTGTTTCCTC	0	0	0	0	0	0	0	0
	†	21UR-4180	TGGTTTGATAGAAGGAACTTT	1	0	0	1	17	15	0	34
		21UR-4181	TATGTATAGATTGGATTTTGG	18	8	7	4	21	56	14	128
		21UR-4182	TGATGTCCTTCTATTTCCATA	0	0	0	0	0	0	0	0
		21UR-4183	TGACGTGTTTTCTCAACTACG	0	1	1	1	1	1	0	5
		21UR-4184	TCCAAGACAAATTTCAGTGGA	0	0	4	1	1	7	0	13
		21UR-4185	TTTGTCATCGTCGTCATGTTT	113	14	20	8	30	79	33	297
	†	21UR-4186	TCATAATGTCGAACCGATAAA	7	8	9	6	41	56	19	146
		21UR-4187	TCCTTTCTTCGATTTGATCTG	1	0	0	2	4	4	1	12
		21UR-4188	TAAGTGGATCTATTATCAATT	0	5	1	3	3	5	0	17
	†	21UR-4189	TGTGTGCGCTGTTTTGGTCAC	0	0	0	0	0	0	1	1
*	†	21UR-4190	TTGGGATCCAATGTGAAATGC	0	0	1	0	2	3	0	6
		21UR-4191	TTCACAGGTAAGACGATGGAC	8	4	4	0	3	35	0	54
		21UR-4192	TCTTCACCTCGATCTTCAACA	2	0	2	0	0	3	2	9
		21UR-4193	TTCAACGGCCACTTGCCTTCA	0	0	0	0	5	7	4	16
	†	21UR-4194	TAACGCAGGATGCAAATTTAA	2	1	0	1	12	20	9	45
	†	21UR-4195	TAATTATGAACCCGCAATGCG	0	0	0	0	0	0	0	0
	†	21UR-4196	TAAAGGCTTGAATCAACAACT	0	0	0	0	0	0	0	0
	†	21UR-4197	TCGCGGCTTGTCATACATTCA	1	2	1	0	5	10	1	20
		21UR-4198	TCAATACATTGATGTTGGCAC	18	6	3	3	9	60	4	103
		21UR-4199	TAACATGTTCAGTAAAAGGTG	29	10	11	3	16	48	6	123
		21UR-4200	TGCCGTAGCTAGTTGCCCTGA	0	0	0	0	1	1	0	2
		21UR-4201	TGCCGTCTTTGTTTTGGCTTT	1	0	0	0	0	0	0	1
		21UR-4202	TCATTTCATTTACTAGTGAAG	0	0	0	0	0	2	0	2
		21UR-4203	TCGTCTTGTTGATTACCTAAG	0	0	0	0	2	0	0	2
		21UR-4204	TAAGAATGTTGCAATTTTCGG	52	34	33	22	91	250	10	492
		21UR-4205	TGCCGTCAATATTGCCAATCT	0	0	0	0	0	0	0	0
	†	21UR-4206	TATTGAAATGATTATCTTTAT	1	1	0	0	1	0	0	3
	†	21UR-4207	TGCATTTAATAGACCAGCAGC	0	0	0	0	0	3	3	6
*		21UR-4208	TTGTACACAAACATGTTAGGC	4	1	0	3	30	40	2	80
	†	21UR-4209	TGTCAAGGAAAAATATCCACA	0	0	0	0	0	1	1	2
		21UR-4210	TAGTTCTTTCGTATATTTGAC	0	0	0	0	6	4	0	10
		21UR-4211	TAACAAAGTCATGTTTTTGTA	0	0	0	1	2	3	1	7
		21UR-4212	TAAAAATTGGTTCTCCATGAA	0	0	0	0	4	2	0	6
		21UR-4213	TATGACTTTGCAAGGTTTACC	0	0	0	0	0	0	0	0
		21UR-4214	TTAAATCCATTTTTTCATTGA	1	0	1	0	2	2	2	8
	†	21UR-4215	TTTGTTACCCTGTTTGTTCAA	1	0	0	0	0	0	0	1
*		21UR-4216	TAAGCGACAAACCTTTAGTGA	4	2	0	1	5	6	2	20
		21UR-4217	TGGTAACAAATTTTTCATGAT	0	1	0	0	1	1	2	5
	†	21UR-4218	TTGAACAGTAATCTCAAACAT	1	0	0	0	0	0	0	1

	†	21UR-4219	TGGACTTGTGCTGTTTTCGCG	1	0	0	0	2	3	0	6
		21UR-4220	TATATAATATATAATATATAA	0	0	0	0	1	0	0	1
		21UR-4221	TATATATATGGTATGAGACAA	16	5	2	2	10	21	1	57
*	†	21UR-4222	TGCTAAGACAAGATGGGTAAA	47	12	6	2	9	61	4	141
		21UR-4223	TTATTTTCGTAGCTTTACCGC	0	0	0	1	3	5	1	10
	†	21UR-4224	TTAGCTTTGCTCTTGCTATTC	0	0	0	0	0	0	0	0
		21UR-4225	TCCTCCTTGTGTAATCGGTTT	10	3	0	2	6	23	0	44
	†	21UR-4226	TCTTTGGATCTTATTCTTGGA	0	0	0	0	0	0	0	0
		21UR-4227	TGGTCGTTTTCCAAATTGTGT	0	0	0	0	0	0	0	0
	†	21UR-4228	TACCCATGTTTCATCACAAGA	14	1	3	2	4	18	14	56
	†	21UR-4229	TAGTTTTGAAATGTGTATAGA	2	1	0	1	1	2	1	8
*	†	21UR-4230	TTGATGCCTGCTATTAGAATT	16	22	7	6	56	75	2	184
*	†	21UR-4231	TATTCTGACAAGCTATGCCCG	2	0	1	0	21	25	13	62
		21UR-4232	TCTCTCTATGTAATTGACACC	0	0	0	0	4	4	1	9
*	†	21UR-4233	TTGGATAGACTAATTAGCAAG	6	13	7	11	164	148	8	357
		21UR-4234	TAAATTCTTTATGTACTTTGG	0	0	0	1	14	3	1	19
		21UR-4235	TTATGACTTTTGTTTAATTGT	1	0	1	0	5	2	0	9
		21UR-4236	TTAAAGTTTGAAAACATAGGT	0	0	0	0	1	2	0	3
	†	21UR-4237	TCTCGAATATACATGTACTGA	0	0	0	1	0	0	0	1
*		21UR-4238	TAGACTACAGAAATAGATTGG	18	3	3	6	7	40	5	82
	†	21UR-4239	TGACTTAGCAATTGGTTCAAT	1	0	0	0	13	10	0	24
	†	21UR-4240	TTGCAAACCACCACAACATTG	0	0	0	0	0	0	0	0
		21UR-4241	TCAGCCGAGCCGTTTTTGATG	0	0	0	0	0	0	0	0
		21UR-4242	TAGTGAAATTGGCCTATAATT	10	2	1	1	2	8	4	28
		21UR-4243	TCAGCCAAGTGCATTAACAGT	0	0	0	0	1	0	0	1
		21UR-4244	TGGTTCTATCTTACTATTAAA	1	0	0	0	0	1	1	3
		21UR-4245	TGGCGTTCTCATCCACTGAAC	0	0	0	0	0	0	0	0
	†	21UR-4246	TATCCAATGTATATATAATGA	1	0	1	1	2	5	0	10
		21UR-4247	TTGATATGCTTCTGCAATTGC	0	0	0	0	0	0	0	0
	†	21UR-4248	TCTGTAATTTCTGATTGATGT	9	3	6	6	44	56	1	125
	†	21UR-4249	TGATTGAACGAAAATGATATT	0	0	0	0	1	0	0	1
		21UR-4250	TAGACTACGAATTCCAAAAAA	0	0	0	6	35	29	9	79
		21UR-4251	TGTGTTCGTAAGTCAGAGATC	0	0	1	1	7	1	2	12
		21UR-4252	TAGATAGTCGAAAACTCAATG	1	1	0	1	2	3	0	8
		21UR-4253	TGGTATCTGTGATTTGCGAAT	7	11	1	1	18	58	3	99
	†	21UR-4254	TACTCGGATGAAATTGAAATC	1	0	0	1	3	15	0	20
		21UR-4255	TCTCTATGGATTTTTTGCTTG	0	0	0	0	0	0	0	0
		21UR-4256	TACCGGTACAGAGAATGTAGA	1	0	0	1	1	6	0	9
		21UR-4257	TCAACTGGAAATATTTATCAA	0	0	0	0	0	1	0	1
		21UR-4258	TGGATTTTAATTAGAATAATT	2	0	3	1	6	8	0	20
	†	21UR-4259	TATTTCACACGCTCCTTAACA	2	0	0	0	2	4	19	27
		21UR-4260	TACATACCTGCAAATATCCAT	0	0	0	0	0	1	0	1
	†	21UR-4261	TTAGTAATGGTATTTCTAATA	0	0	0	0	0	0	0	0
		21UR-4262	TATACTCATCGAATTTCCGTA	16	23	8	11	32	62	2	154
		21UR-4263	TCATATGTTATAAATAAAGCA	0	0	0	0	0	0	0	0
*		21UR-4264	TAACTTAAGTAGATAAATGAT	78	173	103	103	282	688	12	1439
	†	21UR-4265	TCGCATGTTGCATCCAATTGA	1	0	0	0	2	3	0	6
		21UR-4266	TATCTTCCGAGTGTTCAAAAA	0	0	0	0	0	2	0	2
		21UR-4267	TTACAAGTTTTTGCTTACCGT	1	0	0	0	0	1	1	3
	†	21UR-4268	TAGTCTATGCTTTGTACCCAA	0	0	0	0	1	0	0	1
	†	21UR-4269	TGGGATCCAATGTGAAATGCC	0	0	0	0	1	0	0	1
		21UR-4270	TAAGACAAATAAGGCAAGAAA	0	0	0	0	0	0	0	0
	†	21UR-4271	TTTCGTTTGCTTTTGCTTGAT	0	0	0	0	1	0	0	1
	†	21UR-4272	TTAATTCATTTGAGTTTCGAG	0	0	1	0	0	2	0	3
	†	21UR-4273	TAATGATAAGCTATGAAAAGA	0	0	0	0	0	1	0	1
*		21UR-4274	TATATATGATTGTGATCCCTC	1	1	2	4	59	32	16	115
		21UR-4275	TATGACTTTCACTACAGCGAT	0	0	0	0	2	1	0	3
		21UR-4276	TACCGTACTATTTTCAAAAGC	0	0	0	0	5	1	0	6
		21UR-4277	TTATTCTTGCTTTCTGTTTTG	0	0	0	2	2	4	0	8
	†	21UR-4278	TTCCGAAATAATAGATATAAG	0	0	0	0	0	2	1	3
		21UR-4279	TACGGCTACTCATTTCGGTTT	30	73	32	60	743	839	49	1826
		21UR-4280	TCATGAGCGAATTTAGGCAGC	0	3	0	0	6	9	6	24
	†	21UR-4281	TAGTACCGACATATCAACGGA	1	0	0	0	1	1	0	3
		21UR-4282	TGAAAATGAGTCTTCGTTTCC	1	0	0	0	1	1	1	4

	†	21UR-4283	TAATTCTAGGCGGATCAAATC	1	0	1	4	46	45	12	109
	†	21UR-4284	TGATACAATAACGTGTTTTTA	0	0	0	0	0	1	0	1
	†	21UR-4285	TTGTCAATGTTTACAGATAAG	0	0	0	0	0	0	0	0
	†	21UR-4286	TAGAGTGTTGAGATTGTGTTT	1	1	1	0	2	2	0	7
	†	21UR-4287	TAAAAAAATATTGTGATAAGG	4	0	2	1	0	3	1	11
	†	21UR-4288	TAAATATCGAGTTCTTGAGAG	0	0	0	1	1	3	0	5
*		21UR-4289	TCAAGAAGAATTTCAAATACA	5	19	3	11	25	46	2	111
*	†	21UR-4290	TTGCGACTTACGTTCTGTCGG	13	6	2	7	23	77	23	151
		21UR-4291	TCGAAAGGTTGTAAAATTGAA	0	1	1	3	10	4	1	20
	†	21UR-4292	TCAATACGAGGGTGACAAAGA	0	0	0	0	0	1	0	1
	†	21UR-4293	TTCCATAGTTTGGTTGACTAA	1	0	0	0	0	2	0	3
	†	21UR-4294	TACCATTAAGAATCTTTGCGA	3	1	0	3	6	31	2	46
	†	21UR-4295	TCCACCTTTGTCCATGAGTAT	0	0	0	0	0	0	1	1
	†	21UR-4296	TGAAATTGGTATCTTGTTCTA	2	1	1	1	2	5	3	15
		21UR-4297	TATACACTTGGTGATCTCGTA	1	0	0	0	1	2	0	4
		21UR-4298	TCCGTTCAGAGATTTTTGGAT	3	2	3	7	86	85	29	215
		21UR-4299	TCATACAATAATATCATTTCA	0	0	0	2	7	0	1	10
	†	21UR-4300	TGGTACAACGGTATTTCAAGT	19	5	2	0	9	36	2	73
		21UR-4301	TGACAGCCTCAATTTTTTTGC	0	0	0	0	0	1	0	1
	†	21UR-4302	TTGGTTTGTGAACATCTCATG	1	1	0	0	0	2	0	4
		21UR-4303	TAGAAGCCATTGACAAAATCG	0	0	0	0	0	0	0	0
*		21UR-4304	TGTGCAGTATGCAACTCTCAA	2	6	3	2	21	30	0	64
	†	21UR-4305	TTGTTTTGTCGCTTTAGGTTC	1	0	0	1	4	1	0	7
	†	21UR-4306	TCTCACTCAAAAATAGGCACT	0	0	0	0	1	3	0	4
	†	21UR-4307	TATACGATGTTCTCTTAAAAA	5	3	1	3	27	42	1	82
		21UR-4308	TAGAGATGTGTTTAATGCAAA	0	1	1	3	24	27	3	59
	†	21UR-4309	TGATCATTATTTTGCGGTCCA	1	0	0	0	0	0	0	1
		21UR-4310	TGTGGCGCTGTCTTGTATTCC	0	0	0	0	0	0	0	0
	†	21UR-4311	TAGTTCGTTTGTATGTGTTGT	0	0	0	0	4	0	0	4
		21UR-4312	TGTGTAGGCCAACCTCATCTC	1	0	0	0	0	3	0	4
	†	21UR-4313	TACTGGCAAATAATACTGGTG	0	1	0	2	15	14	0	32
	†	21UR-4314	TCCGGACTCCGAACGTTCAGC	1	0	1	1	15	9	2	29
		21UR-4315	TAGGATGTTGTCTCGTTTACC	0	0	0	0	0	0	0	0
*		21UR-4316	TGCAATTGATCCCACAATTGA	1	0	0	0	5	5	3	14
		21UR-4317	TGACAATGTCTTAGGCAATTA	0	0	0	0	1	0	0	1
	†	21UR-4318	TGCTTTTTCATTTATTATCTG	0	0	0	0	0	0	0	0
	†	21UR-4319	TATCTCCGGAAAATCTGCAGG	1	5	2	2	3	14	1	28
	†	21UR-4320	TAGATCCTCAAATTATGCAAA	0	0	0	0	0	0	0	0
		21UR-4321	TCAGCTATTGATTAAGATAAT	0	0	0	0	1	0	0	1
	†	21UR-4322	TCATGTCGTTAAATGTATCGA	0	0	0	0	8	2	4	14
*		21UR-4323	TCTTTTTGTACTAACGGTTGA	15	3	0	2	35	46	15	116
		21UR-4324	TCTGTCGTTTGTTGTGATCCA	0	1	0	0	11	9	1	22
		21UR-4325	TTTGCCGTACGACAGTATTAA	0	0	0	1	4	5	1	11
	†	21UR-4326	TAGTAAATGTCATATCTTGTG	0	1	0	0	2	1	0	4
	†	21UR-4327	TAAACGACTTGTATGAACAAT	0	0	0	2	6	6	0	14
	†	21UR-4328	TATTGTGGAAAAAAACATTTA	2	1	0	1	4	6	1	15
*	†	21UR-4329	TACTGATGGCATTTTACATGG	87	26	11	13	41	196	10	384
	†	21UR-4330	TGAAACCTTGAGAACTATGAG	0	0	0	0	0	9	0	9
	†	21UR-4331	TTCATGTCTTAATTGCGTGGC	0	0	0	0	0	0	1	1
		21UR-4332	TGTCATTGTGGGATATTTTTG	0	0	0	0	5	0	0	5
		21UR-4333	TGCGAATTCGACAAGTAATAA	15	24	10	20	59	110	0	238
*		21UR-4334	TGAGTTTATTTGCACTAAAGT	7	0	1	2	0	2	1	13
		21UR-4335	TCTACTTATACTACGATGTTT	0	0	0	0	0	0	0	0
		21UR-4336	TGAATAATATGTAGTATTTAA	0	0	0	0	0	0	0	0
	†	21UR-4337	TATGTAGTAACATTTATGGAC	0	0	0	0	1	0	0	1
	†	21UR-4338	TTAATGTCGTTGTTTTTGAGC	1	2	0	1	1	5	2	12
		21UR-4339	TGCCTTTAAACGCGAAATTGT	0	0	0	0	0	0	1	1
		21UR-4340	TGACTGCTAGAGATAAGACAT	2	3	0	0	5	14	0	24
		21UR-4341	TCATGGTAGGCCTTTCGATAA	0	0	0	1	1	11	1	14
	†	21UR-4342	TTTACCTAAAGAGATTAGTAA	0	0	0	0	0	0	0	0
	†	21UR-4343	TGATCCACAATGACTGTTTCC	0	0	0	0	3	6	1	10
		21UR-4344	TGAAACGTTGCATAAAAACAG	13	1	4	5	6	24	2	55
		21UR-4345	TATGATCCACTTTTTATTTTC	0	0	0	0	1	0	0	1
		21UR-4346	TTCTGTTGGTATTCCTGTCAT	2	0	0	2	6	1	1	12

	†	21UR-4347	TGATATTTCATCTAATCGTAG	4	1	0	0	0	1	0	6
		21UR-4348	TATGATGTTCTGAACATTACA	0	0	0	0	0	2	0	2
*		21UR-4349	TAGGATAATAATAGGATGATA	6	13	16	33	605	331	46	1050
		21UR-4350	TTCCGTGAACACTCCCAGGCT	0	1	0	0	2	3	1	7
*		21UR-4351	TATGCACTTGAGTAAGCTAAC	0	0	0	0	2	3	3	8
	†	21UR-4352	TATTGCATGTCTTTCTCCGTT	0	0	0	0	0	0	1	1
		21UR-4353	TATTCCGCAATTATTTATATC	0	0	0	0	0	2	3	5
	†	21UR-4354	TAATCTCGCGTTAGCACCAAA	0	0	0	0	0	0	0	0
*		21UR-4355	TAAAAATACATCGGAATCCAA	2	2	0	3	34	22	21	84
		21UR-4356	TTCGTCAATGGGTAACAACTC	2	0	0	0	1	7	0	10
	†	21UR-4357	TGGTTTCACTGCACTCGAACT	0	0	0	0	0	2	0	2
	†	21UR-4358	TCTATTTCTGGTTAATGCGCA	0	1	0	2	2	12	1	18
		21UR-4359	TTATACCGAATTCAAAGGGGG	0	0	0	0	0	6	0	6
	†	21UR-4360	TTGATTCCCTTAGCACATACC	0	0	0	0	1	0	1	2
	†	21UR-4361	TTCACTTCTTTTTTGGTCATT	4	1	0	0	1	0	1	7
		21UR-4362	TAAAATAGGACAGTTAAATTT	2	1	1	2	7	12	0	25
		21UR-4363	TATTACCAACGAAGTAGGTTC	0	0	0	1	5	3	2	11
*		21UR-4364	TCAGTCGTATGGGTTTATCCA	0	0	0	0	3	1	1	5
	†	21UR-4365	TATTGAAGTCCAATTGGGAAT	2	1	0	2	9	8	0	22
		21UR-4366	TGAATCAGAGTTTGAGTTTGT	0	0	0	0	1	1	1	3
		21UR-4367	TAAATCTTCTTTTGAACGTGC	0	1	0	0	3	2	0	6
	†	21UR-4368	TCGGTCTCTACTCACTCCCAC	0	0	0	0	0	0	0	0
	†	21UR-4369	TGGAATCAATTGTTGTTGGAA	11	7	2	7	17	37	5	86
	†	21UR-4370	TATTGACATTCGACATCTTTA	0	0	0	0	2	3	1	6
*	†	21UR-4371	TAAGATGAAGACATCGTTTGA	38	9	6	12	24	78	6	173
		21UR-4372	TACCGTCAGCTTTTTTTGGAA	0	1	1	0	2	2	1	7
	†	21UR-4373	TCATTATCACAGACTTAGCAG	0	0	0	0	1	1	0	2
*	†	21UR-4374	TAACAACAAGGAATTTACAAC	1	2	1	1	10	11	0	26
		21UR-4375	TAGGATCTATTTTAATTTTTT	0	1	0	1	2	0	1	5
		21UR-4376	TGATCATTATTTTCAGATGAC	1	1	0	0	8	5	0	15
	†	21UR-4377	TTTCGGAGATTCAAACACGAG	0	5	1	4	5	12	0	27
		21UR-4378	TAGCACACCTGTCCTTTATTT	0	0	0	0	0	0	0	0
		21UR-4379	TATGATTCTTTTCAGTTCTTT	1	0	0	0	0	1	0	2
	†	21UR-4380	TAATAGTGTCTTTTCGGTCTA	12	6	1	3	7	19	6	54
		21UR-4381	TGAATTAAGATCCAATACCAA	0	0	0	0	2	1	2	5
*	†	21UR-4382	TGGATCGCGGAAATATGCAAA	7	6	2	7	293	268	54	637
		21UR-4383	TATCACACTAATAGAGTGAAA	0	0	0	0	2	0	0	2
	†	21UR-4384	TAATGCGAACTTTTCTCTATT	0	0	1	1	9	5	0	16
		21UR-4385	TAATAGTAGTAAAAAGCCGGA	27	19	9	2	21	80	12	170
		21UR-4386	TGGACGTCAAGATTTGGTAAA	0	1	1	0	48	43	8	101
		21UR-4387	TAAATAGATATAGTAATTAGT	4	1	0	0	3	2	0	10
	†	21UR-4388	TTTTACGTTCTCTGGTTGAAA	0	0	0	0	5	6	0	11
		21UR-4389	TTACACCTTGAATTCAGCATT	6	4	3	9	216	114	19	371
		21UR-4390	TCGTTTTCCATAATTACAGTC	0	0	0	0	0	0	0	0
	†	21UR-4391	TTGATATGCCTAAGCATATGG	3	1	0	0	0	4	0	8
	†	21UR-4392	TTTTGATGATTATGTTGACCA	11	5	3	4	4	21	1	49
		21UR-4393	TGAGTTTATTGGAGATTGAAA	1	0	0	0	15	10	2	28
		21UR-4394	TAAATTTCGATAAATTGGAGT	1	0	0	0	0	0	0	1
		21UR-4395	TCGTGTCACTATTGGATCAAT	0	0	0	0	2	0	1	3
		21UR-4396	TCTAGGCTGTGCCTCACCTGC	0	0	0	0	0	0	0	0
	†	21UR-4397	TATTGAACTCTTGGTCTACGT	0	0	0	0	0	5	0	5
		21UR-4398	TCTTTTAGATGTTCCGAGAAA	4	1	0	0	0	2	2	9
		21UR-4399	TACCTTTGGAATCGATTACAA	0	0	0	0	1	16	3	20
		21UR-4400	TGCGAATTGCTTATTTCCTGA	1	1	1	2	13	13	1	32
		21UR-4401	TAGAAAGGTTTTTCGCAAGCG	2	0	0	0	1	2	0	5
		21UR-4402	TGGTATTCTGAATGTTTTTGA	0	0	0	0	0	0	0	0
		21UR-4403	TTATTCGAAATTTTAGTCAGT	6	0	0	0	1	5	0	12
		21UR-4404	TGATCCGATTGGAAAATAGGG	0	0	0	0	0	0	0	0
	†	21UR-4405	TGTTGACTGCCTCTCTTGGAA	2	6	2	0	3	25	0	38
*	†	21UR-4406	TATCATTGGTACACTAATTTC	0	0	0	0	10	4	1	15
*	†	21UR-4407	TACTGTGATGGAAAAACGGTA	1	0	0	4	59	57	6	127
		21UR-4408	TAATTGGTACAGATTCTTATT	2	3	2	0	1	4	0	12
		21UR-4409	TCTCAGCTAAATTAATTCAGT	0	0	0	0	0	0	0	0
		21UR-4410	TTGGTGTCTTTTATCCAAAAA	2	0	0	0	1	0	0	3

	†	21UR-4411	TGGTAATGATATTTGAACAAT	0	1	1	0	0	2	0	4
		21UR-4412	TGTTCTGTTATTCAGTTGTTC	0	0	0	0	0	0	0	0
		21UR-4413	TGACACAGAAGTTTAATAAAA	0	0	0	0	7	4	1	12
		21UR-4414	TTGATTTGGATTTTGACCTAT	0	0	0	1	0	2	0	3
*		21UR-4415	TCAAGAATTACGGATGCCGCC	0	3	3	6	102	140	69	323
	†	21UR-4416	TTCGAAAGTATTTCACAAACG	0	0	0	0	1	0	0	1
		21UR-4417	TATGGTACGAACCTTCTTTTG	0	0	0	0	0	3	0	3
	†	21UR-4418	TACCAGTGGTTTTTGGGATCG	72	12	1	3	25	139	11	263
	†	21UR-4419	TAGTTTCACATTAAAGGTGGT	0	0	0	0	0	2	0	2
		21UR-4420	TAACCCGACTTCTATCTTAAC	0	0	0	0	1	2	1	4
	†	21UR-4421	TGATACTTTTGCTACACTTTG	0	0	0	0	4	0	2	6
	†	21UR-4422	TAGTTCAGCTAATGCTATCCG	0	1	0	0	0	0	1	2
	†	21UR-4423	TCCGCAGAAATCTCTCTTTGT	0	0	0	1	10	4	1	16
	†	21UR-4424	TCGTATTTATTTCAGTTTATC	0	0	0	0	0	0	0	0
		21UR-4425	TTTGAGATTGTGATGTAGTAC	2	2	0	6	51	59	20	140
	†	21UR-4426	TCAGTTGGTTAATAAAGATAG	5	8	2	6	12	20	5	58
		21UR-4427	TGATTTCCTTTCATTTTGATA	0	0	0	1	1	0	0	2
		21UR-4428	TCATGAAAAAATTTGTCGTGGT	2	0	1	2	5	15	2	27
*		21UR-4429	TACGGTTAATACGGTTAGGGG	40	3	0	0	5	35	4	87
		21UR-4430	TTCCACTTGCACATCGAGTTC	0	1	0	0	0	0	0	1
	†	21UR-4431	TCGATAACTTAAGGTGGAAAT	0	0	1	4	20	29	2	56
		21UR-4432	TGCCATCCAATAATTATGTGG	0	0	0	0	0	0	1	1
		21UR-4433	TTAGATCTTGGACAAAAAAAT	146	99	42	55	119	379	97	937
		21UR-4434	TCTGAATGCTATATTTTTTGG	0	0	0	0	1	0	0	1
	†	21UR-4435	TTGTATAGATCATCGTTTAAC	38	5	14	10	14	57	11	149
		21UR-4436	TCTCAATGAAGCACAACTTTG	0	0	0	0	4	7	0	11
*		21UR-4437	TAAAGCTGGCTACCGTACTTC	33	17	13	3	25	79	7	177
	†	21UR-4438	TACATCACAAACAACTGTAGT	0	0	0	1	8	6	1	16
	†	21UR-4439	TACCAAAAATCGCTTTCAAAT	0	1	1	1	0	1	1	5
	†	21UR-4440	TATTCAATATTTGCATAGGGT	4	0	0	0	0	0	0	4
	†	21UR-4441	TGATTGAGGGACTCTCGAAAA	3	4	1	0	3	19	0	30
		21UR-4442	TGCAATTGCAAGTGACATTTC	0	0	1	0	8	4	2	15
	†	21UR-4443	TGGAGTCTCGCTAACGATTCA	0	0	0	0	0	0	0	0
		21UR-4444	TAGACATATTTGCAATGTGAC	0	0	0	0	0	2	0	2
		21UR-4445	TCCGTAATCGATGATAAAGAT	0	0	0	0	0	1	0	1
	†	21UR-4446	TTTATTGTGCCGATTGAATAT	0	0	0	0	0	0	0	0
*		21UR-4447	TAAATTCAAATTGCTTGAAGA	3	0	2	1	9	22	5	42
		21UR-4448	TATTTTCGAGTTGATGCGTGT	0	0	0	0	0	2	0	2
		21UR-4449	TTCCAACCACACATTAGCATA	4	0	1	2	20	9	32	68
	†	21UR-4450	TTTCAAACTTGTGCCTTTGAA	1	1	0	0	18	21	3	44
		21UR-4451	TGAGTCGGGTTTTTTGCTTTT	0	0	0	0	0	0	0	0
	†	21UR-4452	TATTTGTGATAACTACGTTTC	0	0	0	0	4	1	1	6
		21UR-4453	TATGGAAAAGCAACTATTGTT	0	1	0	0	12	8	2	23
		21UR-4454	TAAATTATCTTTCTGATGTTC	0	1	0	0	0	0	0	1
		21UR-4455	TATCAGTTCAAATGAAGTCAT	0	0	0	0	0	2	1	3
		21UR-4456	TGGGTACGTCTTTTAATTTAA	5	2	1	0	2	9	0	19
	†	21UR-4457	TGCAAATCAGAATATTAGGAA	1	0	0	2	17	21	5	46
		21UR-4458	TCTTGAGGCACCTTTGTTGAA	1	0	0	0	0	0	0	1
*		21UR-4459	TGGTCTAGTGGTATGTGCGGG	73	89	34	6	101	464	36	803
		21UR-4460	TGTCTAAGAGTTTTTATTGAA	0	0	0	0	0	0	0	0
	†	21UR-4461	TATCATGTCGAGTTCGTGCAT	1	3	3	1	58	51	2	119
		21UR-4462	TAAATAAATACCGTATTAGCA	0	1	0	0	1	4	3	9
		21UR-4463	TCCAATAATGCACGACAATGG	1	1	0	1	4	8	12	27
		21UR-4464	TATTTCGCCCGCTTCCCAAAC	3	0	0	0	0	0	1	4
	†	21UR-4465	TTTCGAAATGTTCTGTGGTGT	5	4	4	4	8	21	10	56
*		21UR-4466	TGAAGCGACTAAAATTACAAC	0	2	2	5	77	52	19	157
*	†	21UR-4467	TACTTGGATAGACTAATTAGC	0	5	2	5	113	64	6	195
		21UR-4468	TATAAAAACATTTGTTCACTT	0	0	0	0	1	1	0	2
	†	21UR-4469	TTTCGGGCAGATCCATTTTTC	3	0	1	0	1	7	2	14
	†	21UR-4470	TGTTGCAACTGTTTTCAAGGG	0	0	0	0	0	0	0	0
*		21UR-4471	TCACGAAATTGTATTAACTGG	1	3	0	2	49	48	8	111
	†	21UR-4472	TTAGGATGCCTGATCATATTG	0	0	0	0	0	3	0	3
		21UR-4473	TACTTCTGAACCTTTGTAACT	1	0	0	0	7	5	2	15
*		21UR-4474	TTCTACATCGTAGCGGAAATC	29	4	13	17	125	201	116	505

	21UR-4475	TACTTTTTGAGGCTTGAAAAC	6	9	2	3	17	62	2	101
†	21UR-4476	TTAATAAGCAAATTCGCAATA	22	3	3	1	15	36	2	82
†	21UR-4477	TCGATATGACGATTAAAAAAG	1	0	0	1	1	0	0	3
	21UR-4478	TGATATTTTTGTGTGCCAATT	0	0	0	0	0	0	0	0
	21UR-4479	TGCCGCTTTTGCAAGGTTGAA	0	0	1	0	0	0	1	2
	21UR-4480	TAAAAATCTTAGTAAAAGAAC	4	0	0	0	1	1	0	6
	21UR-4481	TATCAAATGCGAATGCAAGAT	0	0	0	0	10	2	1	13
†	21UR-4482	TAATGCCTTGATATTTGAGGT	0	0	0	0	0	0	0	0
†	21UR-4483	TTGAAACTCTACTTTCAGGAG	0	1	0	1	0	5	0	7
	21UR-4484	TACCTTAAAACTAATGAACTG	1	0	0	0	2	4	0	7
*	21UR-4485	TCTCATGACGGTTTCCATTTT	63	9	8	12	19	67	8	186
	21UR-4486	TCGATTTCGACATTTTCCTCT	0	0	0	0	0	0	0	0
	21UR-4487	TCATTGAAGTTTTTTAAATCA	0	0	0	0	0	0	0	0
†	21UR-4488	TCCGAAATAATAGATATAAGT	0	0	0	0	0	2	1	3
†	21UR-4489	TTTGTCGACTTCAAATGACAC	5	1	2	4	32	26	5	75
	21UR-4490	TCAATCATCTTCAATGTTTCT	0	0	0	0	0	0	1	1
	21UR-4491	TGAGAAACATTTTTAGAACAA	0	0	0	0	9	4	0	13
	21UR-4492	TCTTCCATGCCCTGTTTTATA	0	0	0	0	0	1	0	1
	21UR-4493	TCAGTCTTTTCAATCTGTTCC	0	0	0	0	1	1	0	2
†	21UR-4494	TCCAGTGTGAACTGATTGTGA	0	1	0	0	0	1	0	2
†	21UR-4495	TTCGGGCCTTGAGTTTCTTTA	0	0	0	0	0	0	0	0
	21UR-4496	TAAGGCATAGACTGTTCTGAC	3	2	2	0	5	34	0	46
*	21UR-4497	TCGACTCATTACAGCTGATTA	0	1	1	0	35	19	1	57
	21UR-4498	TGTATTTATTAAAATTGAACA	0	0	0	0	0	0	0	0
†	21UR-4499	TCGAAATATCCTTGATGTTTT	0	0	0	0	0	0	0	0
†	21UR-4500	TCCATGCATTTACATTTGAAT	2	0	0	2	7	6	4	21
†	21UR-4501	TGCACATGTACTTTCTTGTAC	0	0	0	0	0	0	0	0
†	21UR-4502	TTAGATTACGTGAATAAAGAG	6	9	3	8	23	52	0	101
	21UR-4503	TGACTTTGATCCTTTTTCAAC	0	0	0	1	0	1	0	2
	21UR-4504	TAGTGCAGCAATGTATGGAAA	0	0	0	0	0	0	0	0
	21UR-4505	TCTTGCTCTTTTATTAACTTC	0	0	0	0	0	0	0	0
	21UR-4506	TGTCACTGTGATATTGAAGTT	0	0	0	0	2	0	1	3
	21UR-4507	TGATCGGGAGGTCCTGATATC	0	0	0	0	0	0	0	0
	21UR-4508	TGTTGAGTTCTCGTTATTGAG	0	0	0	0	0	0	0	0
*	21UR-4509	TGAAGCGGAAAATATAGAAAG	79	158	61	109	1623	1597	853	4480
*	21UR-4510	TAAAGTAGACGTTGCCATGAT	260	477	228	240	693	2505	140	4543
	21UR-4511	TTGGTATCGCTCCAGATTTCC	7	2	1	0	3	6	0	19
†	21UR-4512	TTCCATTTCATAATGATAACG	0	0	0	0	0	1	1	2
†	21UR-4513	TGCATCGTTTTTATTGTAAAT	0	1	0	0	2	2	1	6
*	21UR-4514	TATGATTGCGTAATTGTTTGA	2	3	2	3	23	21	9	63
	21UR-4515	TCCACGCCTTGATAATTTTTG	0	0	0	0	0	0	0	0
	21UR-4516	TAAACGAACAATGGTTGAACC	0	0	0	1	12	13	0	26
	21UR-4517	TGAGATAAGCCAACAACAAAA	4	0	1	0	0	1	3	9
	21UR-4518	TGCAGTTTTTCTTTGGTTGCC	0	0	0	0	3	4	0	7
	21UR-4519	TCAGAGTAGATCTTCTGGTAA	8	2	1	6	120	133	64	334
†	21UR-4520	TAATGCATCGATAATTGTCAA	0	0	0	0	3	1	0	4
	21UR-4521	TGAGCATTTGCACCCCATTGT	0	0	0	0	0	0	0	0
	21UR-4522	TCCACCGTGTCCTTTATGTGA	0	0	0	0	0	0	0	0
†	21UR-4523	TAATCTTACATTATTGACAGT	3	0	1	0	0	1	2	7
	21UR-4524	TGTTCATTGTAAATATCGAAA	0	0	0	1	0	0	0	1
†	21UR-4525	TGTTGATTTTGCCTGACTACG	0	0	0	0	1	0	0	1
	21UR-4526	TAACTATAAAAAATAAAATCA	0	0	0	1	0	0	0	1
†	21UR-4527	TGATGTCCGATTTAGTTGAAG	1	2	2	5	43	40	5	98
	21UR-4528	TAAATGCAGCTTTGGATTTCC	0	0	0	0	0	3	1	4
	21UR-4529	TCATGCGATTATATCTTCAGT	0	0	0	0	2	1	0	3
	21UR-4530	TGTCAGTTTTGAATTAACAGT	0	0	0	0	0	0	0	0
	21UR-4531	TAAGTGAACGTATCCGAATCC	0	0	0	0	0	0	0	0
†	21UR-4532	TGGGACATTAAAAAGTGATTT	3	1	1	1	0	4	0	10
†	21UR-4533	TCTATCGCGGTTATACTATTT	49	49	15	24	76	208	7	428
	21UR-4534	TCGGCACATAACAAATGTTGA	1	0	0	2	18	7	3	31
†	21UR-4535	TAGTATTACTTCATGATTTTC	0	0	0	1	1	0	0	2
†	21UR-4536	TGGCTGTACTTGTGAATTCAA	1	1	0	0	0	3	1	6
†	21UR-4537	TTCCGATTCCGACTAGAAGGC	39	28	18	12	34	178	5	314
	21UR-4538	TGTATATCACCAAGTATAATA	3	0	0	0	0	1	0	4

		21UR-4539	TAACACTGAGTTTTTGAATAA	3	0	0	1	0	5	0	9
*		21UR-4540	TCAGCTTAGTAAGTATTTTCA	1	0	0	0	1	4	1	7
		21UR-4541	TACAATGATTAAATAATGTTA	0	0	0	0	5	4	0	9
		21UR-4542	TCGCACGTTCGCGTTTCGAAG	0	0	0	0	0	0	0	0
*	†	21UR-4543	TTGGAGCAACTTCGGTTTTAA	0	1	0	2	27	40	5	75
*		21UR-4544	TATGGTATTTCTCCCTCTGTG	0	1	0	1	1	1	0	4
*		21UR-4545	TAGTAGATACTATAGTAGAAG	3	7	5	18	160	166	11	370
		21UR-4546	TGGGTGACGTTTTGTTTTCTA	0	0	0	0	0	0	0	0
	†	21UR-4547	TTCTTCATTGGGGGGTCGAAA	0	0	0	0	1	0	1	2
	†	21UR-4548	TTTTAAGCATGGCTTTTGTAA	1	0	0	0	3	2	1	7
		21UR-4549	TGAAATTGCTTCACAAGATTC	0	0	0	0	0	0	0	0
		21UR-4550	TGACAGTGTTTTTCCTATTGT	0	0	0	0	0	0	0	0
	†	21UR-4551	TGCATAACGGCTTGCATTTTC	1	0	2	1	10	19	11	44
		21UR-4552	TGACCACCATTGCTTACTATT	0	0	0	0	0	0	2	2
		21UR-4553	TAACTCATTGAAACAGTTCTG	0	0	0	0	0	1	0	1
		21UR-4554	TCTGAACACTTTTCGATTTTC	0	0	0	0	3	2	0	5
	†	21UR-4555	TTGTGCTACTCTTTGGTCATA	2	1	2	1	0	8	0	14
	†	21UR-4556	TTAATTACACTGCTGTTGGAA	0	1	0	0	0	0	0	1
*		21UR-4557	TATGTGGAGTAATGGTTGATG	71	116	49	32	105	352	15	740
	†	21UR-4558	TACATGCTCTTCTCATTAAAC	1	0	0	0	5	12	6	24
	†	21UR-4559	TGGAATGTAAATCGCGCCTAA	1	0	0	0	42	42	22	107
	†	21UR-4560	TGATTGCAAGGATTTTGGTGA	0	0	0	0	0	0	0	0
		21UR-4561	TGAGTCCACGAATTCTGGAAG	0	0	0	1	2	5	1	9
	†	21UR-4562	TCACAATGGCATGGTGGTATT	0	1	2	0	15	22	4	44
		21UR-4563	TGATTTTACTTCAATATGTGG	0	0	0	0	0	0	0	0
	†	21UR-4564	TGTGATCTGCCATCGTTCCAT	0	0	0	0	0	2	1	3
	†	21UR-4565	TATTGATCGCCTTCTACCAAA	14	0	3	0	3	9	1	30
		21UR-4566	TAAACTTGGGAAAAAATGGTC	0	0	0	2	12	13	2	29
	†	21UR-4567	TAAAGACAATAGTTTGATTCA	4	5	4	7	10	30	2	62
*	†	21UR-4568	TAGTTAGGACATAATATGATC	4	0	4	2	42	23	6	81
		21UR-4569	TCGGGTTATTGATTTACCACT	0	0	0	0	4	9	1	14
*	†	21UR-4570	TAAGGGAATCAAATGTCGAAT	3	2	2	1	4	7	3	22
		21UR-4571	TGTATTTTGATATCTGGAAGA	2	0	0	3	1	15	2	23
*	†	21UR-4572	TGACTGTATTTTGGTTTTTGG	681	462	314	372	2734	4785	621	9969
*		21UR-4573	TATGATTCCATTTCGTCCTCA	25	3	3	3	17	26	16	93
		21UR-4574	TCCACATCATCATCTAATCCT	0	0	0	0	0	2	0	2
	†	21UR-4575	TCAATATCTCAGTAGGGAATC	0	0	0	0	2	0	1	3
		21UR-4576	TACTGTCATTATAAATTAAGT	0	2	1	0	8	2	1	14
		21UR-4577	TGTGCGATCACTAGAATACAT	0	0	0	0	1	0	0	1
	†	21UR-4578	TCTAGCCATGTCGAACATGTT	0	0	0	0	0	1	0	1
		21UR-4579	TAAACATCACCACTGTGAAAA	0	0	0	0	2	1	1	4
	†	21UR-4580	TTAAGAACCCGTTAACAAGAT	0	0	0	0	1	0	0	1
	†	21UR-4581	TACGCCATAAAAAGTAACATA	0	0	0	1	0	1	0	2
	†	21UR-4582	TTAGTTCCATTTTTCACGCAC	0	0	0	0	0	0	0	0
	†	21UR-4583	TGCGTCTCTTTGTTGGAGAAA	0	0	0	0	4	2	1	7
		21UR-4584	TAATCTTGTTCTGAAGCAAAA	0	2	0	2	9	24	0	37
	†	21UR-4585	TTCGCATTCTGAAAGGTTGGT	5	2	2	3	42	62	3	119
		21UR-4586	TAAGTACCTAAAGGACAAAGC	2	1	0	1	20	28	4	56
		21UR-4587	TGATTGCTCTCTAATAAGTTT	0	1	0	2	0	0	0	3
	†	21UR-4588	TACTTGCTTAAAATAAAAAAT	0	0	0	0	0	1	0	1
		21UR-4589	TTACTTCTCGTTTGGAATGGA	21	7	10	3	7	34	8	90
		21UR-4590	TACAGTAACACCGGTCGAAAA	3	1	1	0	4	27	8	44
		21UR-4591	TACAATATGGTAGCTCGATCT	0	3	1	2	70	76	57	209
		21UR-4592	TGGTCAAGGCATCATTATTCA	2	1	1	0	10	14	8	36
		21UR-4593	TGTCTAATTCTAAGTAAGTCA	0	0	0	0	0	0	0	0
		21UR-4594	TGTTGACCTTATATTTTTTTA	0	0	0	0	0	0	0	0
	†	21UR-4595	TCGGGGTTTTTTGTATCTATT	0	0	0	0	1	0	0	1
	†	21UR-4596	TGAGTCTCACAGTTTTTAGCA	2	0	0	1	0	1	0	4
		21UR-4597	TGTATTCAGTCAGTCTAAGAT	0	0	0	0	0	0	0	0
		21UR-4598	TGGACCGCTTTTCGTTCATAT	0	0	0	0	1	1	2	4
	†	21UR-4599	TTCTTCATTGGCTGTGCTTGC	0	0	0	0	0	0	0	0
		21UR-4600	TGAAGACATGCAAAACAGGGG	7	0	0	2	1	21	4	35
	†	21UR-4601	TCCGTATCTATAATGAATACA	1	2	0	0	1	7	0	11
	†	21UR-4602	TAATAGATTGAGTGAATCGAA	0	0	0	0	1	1	0	2

	21UR-4603	TGTCATGACACAAGTCGTCAG	2	0	0	0	2	1	1	6
	21UR-4604	TGCGCGCCTTTTACAAGTTCA	0	0	0	0	0	1	0	1
*	21UR-4605	TCGACATAGATGGCTTGTATG	20	9	8	12	220	362	33	664
	21UR-4606	TCCTACTATCGGTCTATTTCG	1	0	1	0	4	1	1	8
†	21UR-4607	TTCTGATTCTCCTCCCATAGT	0	0	0	0	0	1	0	1
†	21UR-4608	TAAGGGCGTGTTACCATGTCG	0	0	0	0	2	0	0	2
	21UR-4609	TGCACTTCCCATTAACTGCAA	1	0	1	0	1	0	3	6
†	21UR-4610	TGTTTGGTAAAATATATCTAA	0	0	0	0	0	0	0	0
* †	21UR-4611	TCTATGTTGAGGACGTCATTG	2	0	1	3	60	66	53	185
†	21UR-4612	TAGTGAAATGGTGAAAGTATT	0	0	0	0	1	3	0	4
†	21UR-4613	TGCCCATTGAAAATTCGACAT	0	1	0	0	21	8	1	31
†	21UR-4614	TTGGTTGTTGGCGTGTTTTTC	5	4	5	2	7	27	0	50
	21UR-4615	TTATACGGATATCTTTATAGT	3	5	2	4	42	41	4	101
†	21UR-4616	TGCCTATTCGTCAAATTTGAA	0	1	1	0	0	1	3	6
	21UR-4617	TACGTACTCCAGATTTGTTTT	0	0	0	0	2	0	0	2
*	21UR-4618	TAGAAAAAATTTGCAATTGAAG	1	0	0	2	1	11	2	17
†	21UR-4619	TATTCGATAACTTAAGGTGGA	2	0	1	3	13	31	3	53
†	21UR-4620	TTAAACGACAGTTTGATCAGC	0	0	0	0	0	0	1	1
* †	21UR-4621	TTGACTGTCGAAGACCATTTC	38	41	21	96	1751	1803	303	4053
†	21UR-4622	TCGATCTTCAAAACAGTTGCT	0	0	2	0	2	3	1	8
	21UR-4623	TATTTAACACTTGTTGAAATT	0	0	0	0	3	3	0	6
†	21UR-4624	TAATATTTTTCAAGAAGGTGA	7	0	1	0	6	3	0	17
* †	21UR-4625	TAGAGTCCAGTTGATGTCCAT	103	30	16	9	33	438	15	644
	21UR-4626	TTCAAAGTTTCTCTGTTTGCG	0	0	0	1	3	3	0	7
	21UR-4627	TCATAGAAAAGAACCTATTTT	0	1	1	1	1	8	0	12
†	21UR-4628	TTGAGGAATACAAAACACGAT	0	0	0	0	0	0	0	0
	21UR-4629	TGTAGTTTTTCGATTATATCC	0	0	0	0	0	0	0	0
†	21UR-4630	TTGAGGTGAATTGTAGTCCGT	0	0	0	0	0	0	0	0
†	21UR-4631	TGGATCCAATAGATTCGCCAA	0	0	0	0	2	1	1	4
*	21UR-4632	TCTTAGTATTGTGAGAAGGGT	6	2	1	4	76	64	11	164
	21UR-4633	TCGTACTTCTGCAATATTTTC	0	0	0	0	1	1	0	2
	21UR-4634	TGTGCCGCTATGTTCAACATC	0	0	0	0	0	0	0	0
†	21UR-4635	TATTAAATCTTCCCCACCAAT	0	0	0	0	0	0	1	1
	21UR-4636	TAGTTCTATTCCATCCATCTC	0	0	0	0	0	0	0	0
	21UR-4637	TCTATTAGTTTGTCTTTGTGA	0	0	0	0	0	0	0	0
	21UR-4638	TAGGATAGATATATGATAAGT	12	27	14	19	62	122	2	258
†	21UR-4639	TCAACAGATTTACCAAAACCA	0	0	0	0	0	0	0	0
	21UR-4640	TTATGAGTTTGTAAGTATTGA	6	3	2	3	8	6	3	31
†	21UR-4641	TGAGTAATGAAATGGGGAATT	1	0	0	0	2	0	3	6
	21UR-4642	TGACATGCTGTTTTGTTTTGT	0	0	1	0	5	3	2	11
†	21UR-4643	TTATACACTTGTTTTCAAACA	12	1	1	0	1	3	1	19
†	21UR-4644	TATTCCTACCTTTAAAGTGTT	14	0	0	0	0	2	5	21
†	21UR-4645	TCGAATGCATACAGTCCAAAA	7	4	3	0	5	22	1	42
	21UR-4646	TCAAGAGTGACGTTGTTTATG	1	0	0	0	8	16	11	36
*	21UR-4647	TATAACGAGTAGTAGATGGTA	2	2	2	4	118	141	40	309
†	21UR-4648	TACTCAATTGATGGTTTTTTG	0	0	0	2	5	5	0	12
* †	21UR-4649	TAGGAATTGTGTTCACCAACG	6	2	1	5	27	43	10	94
†	21UR-4650	TACTTGATTGTAAATCTAGAT	0	1	0	0	0	3	0	4
	21UR-4651	TATACACATTTATGGTATTCT	1	0	0	0	1	1	0	3
	21UR-4652	TGCCAGGTTCGAATTAGTTGA	1	0	0	0	4	2	0	7
* †	21UR-4653	TAACTAGTGCAGTACACGGTG	4	3	0	9	123	141	6	286
	21UR-4654	TCTGCAAATTGAATGGTGTCG	0	0	0	0	1	4	3	8
* †	21UR-4655	TGCTTCGAGCGGATAGTTAAT	35	9	12	17	212	262	169	716
	21UR-4656	TCCGTTCATTAAGCCTCGATC	0	0	0	0	0	0	0	0
†	21UR-4657	TCTGGCGTGGTCCCATAACTT	0	0	0	0	0	0	0	0
	21UR-4658	TAAATGAAAAGCTGGCTATGG	4	4	3	2	17	42	4	76
* †	21UR-4659	TTGTTGAAACGGTATGAAATT	817	574	281	258	689	2775	126	5520
	21UR-4660	TACGCATGGTTTATATGCACA	0	0	0	0	8	10	0	18
†	21UR-4661	TAATAACGACACTTTAGCAAA	1	0	0	1	3	2	2	9
	21UR-4662	TCGCACAGTTGTACTATTTTT	0	0	0	0	9	36	0	45
†	21UR-4663	TCCAAGCATGTGTGATGGCTC	3	1	1	1	20	45	1	72
†	21UR-4664	TGCCATTTTCCATAGACGCAG	0	0	1	0	0	0	1	2
	21UR-4665	TAAGGTGATGAACCCCAAAAA	0	0	0	0	0	0	0	0
†	21UR-4666	TATTCCCTCTTTATTCCAACA	1	0	0	0	0	0	0	1

*	†	21UR-4667	TCCGTGAAGAGTTTCGGCGTA	6	3	2	1	4	45	1	62
*		21UR-4668	TAAAGTAATAAGAATGAGTAG	27	26	13	18	42	85	5	216
		21UR-4669	TAAGTCCTTTGATGAATCCTT	0	0	0	0	0	0	0	0
		21UR-4670	TGCAGAGGCTGGAAAAAGTGC	1	1	0	0	1	5	1	9
	†	21UR-4671	TTGAATGTTGAAATGTATTTT	2	1	0	1	1	4	1	10
	†	21UR-4672	TGCTGATTTACTTTCGCGGTT	1	1	1	0	0	8	0	11
		21UR-4673	TCATGTCAATTTTAGGAAAAC	0	0	0	0	0	1	0	1
		21UR-4674	TGCATTCTTTTTAAAATTTCT	0	0	0	0	0	0	0	0
	†	21UR-4675	TACACACTTGACTTAGGTCTC	0	0	0	0	1	0	0	1
		21UR-4676	TGTATCGTCTGATATATGTAT	0	0	0	1	1	1	1	4
		21UR-4677	TACCGTTGCTGCCGATGGAAA	0	0	0	0	0	2	1	3
		21UR-4678	TCTCAGGTATCGTGCAGCATA	0	0	0	0	38	33	14	85
		21UR-4679	TACAGAGAGCGCGCCCAAGCC	0	0	0	0	0	0	0	0
	†	21UR-4680	TCGGTAGCTAATTTTCGTACG	1	1	0	1	8	7	0	18
		21UR-4681	TGAATTGTTTGTACAATCGTA	0	0	0	0	1	0	0	1
	†	21UR-4682	TGATCGCACTGCTAGTTGAAA	7	6	3	5	19	42	4	86
	†	21UR-4683	TTAGTGTTGAAGGTACACTTA	0	0	0	0	0	0	0	0
		21UR-4684	TAAAATAGCTGTAGAAAAGTT	0	1	0	2	2	6	0	11
		21UR-4685	TTGAGTAGCTCCAATTATACC	1	0	0	0	4	2	0	7
		21UR-4686	TCATCTGTTGGTATTCAGAAA	0	0	0	0	1	1	0	2
		21UR-4687	TAACATCATGGTATCATCGTT	0	0	0	0	13	5	22	40
		21UR-4688	TTTCTTAAGGAATAACAACAC	0	0	0	0	0	5	0	5
	†	21UR-4689	TGATCCAGATTTGATGTTCAA	2	0	0	0	5	11	1	19
	†	21UR-4690	TATTGCATTACCGACTCAACA	5	4	1	2	4	17	2	35
		21UR-4691	TCCAAGCGGCCCCTATGTTCA	0	0	0	0	3	3	8	14
	†	21UR-4692	TATTGGTAGAATGAAAATTTT	1	0	0	0	8	9	0	18
	†	21UR-4693	TGGGTAGTTGATTTTGGTTTT	2	8	3	9	383	214	35	654
	†	21UR-4694	TTAAAAGAAACTCAAACAGCG	0	0	0	0	4	3	0	7
	†	21UR-4695	TAGCAAGTATTCTGACATTCG	0	0	0	1	10	9	0	20
		21UR-4696	TAATTATGATTAAAGAAAAAG	1	0	0	1	6	7	1	16
		21UR-4697	TTAAAAAACCAGATCAAACCA	0	0	0	0	0	0	0	0
*		21UR-4698	TACGATCGTTGGGGGTGAGAA	13	4	0	0	7	32	7	63
		21UR-4699	TGATTGCTTTTTGAATTACCA	0	0	0	0	1	2	0	3
		21UR-4700	TACGATATTTTTGAAATTTCA	1	0	1	0	3	1	0	6
	†	21UR-4701	TTGCACAATTGATAAGGATAA	0	0	0	0	2	1	0	3
	†	21UR-4702	TTAAAGTTTAATATACAATTA	1	0	1	1	1	1	0	5
	†	21UR-4703	TTCGATTCGAGTGGTTGAAGA	16	14	7	13	83	244	22	399
	†	21UR-4704	TCTTGCTTTTGCAGATGTTGT	0	0	0	0	1	4	0	5
	†	21UR-4705	TACTGGCAAATTATCATCCTC	0	0	0	0	0	3	1	4
		21UR-4706	TTGGGCATCTTTTCAGAAGTA	0	0	0	0	13	14	1	28
		21UR-4707	TTCCACAATGAATTCCACAAA	0	0	0	0	1	2	1	4
		21UR-4708	TGTCAACGTATTTTTACACGA	1	0	0	0	0	1	0	2
*	†	21UR-4709	TAAGACTAGCCTATAATTTAA	4	4	1	8	89	56	20	182
	†	21UR-4710	TGAAATGATTATCTTTATTTC	2	0	0	0	0	0	0	2
*		21UR-4711	TCTCACAAATGGACTTTGGTG	6	15	12	47	883	1024	57	2044
		21UR-4712	TAAACAGCTACTTTTCACAGT	6	1	0	0	1	1	1	10
		21UR-4713	TCAATACTGCTCCATCGAGAA	0	0	0	0	1	0	2	3
	†	21UR-4714	TGGTAAAATCTCTGTCTCTCA	0	0	0	0	1	0	0	1
		21UR-4715	TATAATATCAATCAAATCAAT	2	0	1	0	1	1	2	7
*		21UR-4716	TCAACAATCACTATATCCACT	0	0	0	0	1	0	1	2
		21UR-4717	TAAGTATTTTAGATTTATTTT	0	0	0	0	0	0	0	0
*	†	21UR-4718	TACAGGAAGAAATGGCACTAC	3	3	3	3	87	114	20	233
	†	21UR-4719	TGAAACTTCTCATAAAGTCCA	0	0	0	0	2	1	1	4
		21UR-4720	TACTCGTCCCATTAAGGAGCA	0	0	0	2	2	0	0	4
	†	21UR-4721	TAATAGGAAGTACTCAAATAC	0	0	0	0	2	3	0	5
	†	21UR-4722	TCCTAATTCGATATACTTTAT	0	0	0	0	0	0	0	0
		21UR-4723	TTCAGTCTGAGATAAAAACAT	5	5	3	1	15	26	1	56
	†	21UR-4724	TTAGTTTAGTTCTGTACGCAC	0	0	1	1	18	15	5	40
		21UR-4725	TAAGTCTTGATGATGTATCCG	1	0	0	0	5	4	2	12
	†	21UR-4726	TGTAACTCTAAAGGGTCAAAA	0	0	0	0	1	1	0	2
	†	21UR-4727	TAGGCCTAGGATTTCTCTAGT	0	0	0	0	2	2	2	6
*	†	21UR-4728	TGGGCAAAAGTTAGGCAGGCA	0	0	0	0	26	50	8	84
*		21UR-4729	TATGTTGATCGTGTTTAAATA	0	0	1	2	25	32	77	137
*	†	21UR-4730	TCCTTCGGCTATATTTACAAT	19	88	32	74	894	616	33	1756

	†	21UR-4731	TTAATAAACGAACCTTGGTGA	0	0	0	0	3	2	1	6
	†	21UR-4732	TTGAAGTTGTGTGCAGTTTGC	1	0	0	0	1	0	0	2
		21UR-4733	TGCAATAAGTCTCTCTTACTG	0	0	0	0	0	1	0	1
		21UR-4734	TAAAACAGGCTCTCCAGTGTA	0	1	0	0	16	4	2	23
		21UR-4735	TCGTCCAATAGGAATCAGGTT	6	1	0	1	1	10	1	20
		21UR-4736	TAACGAGAAAGAAACGAAAAC	2	0	0	1	5	14	1	23
*		21UR-4737	TATGTATGTGATTCTAGGTGG	13	7	3	8	9	47	4	91
		21UR-4738	TCGAATTTCTAAAATTGCCAA	0	0	0	0	0	2	0	2
		21UR-4739	TTTAATCAACACATTGAATGG	7	1	3	1	0	4	0	16
		21UR-4740	TGAAAAACCACATCAAGTTTC	1	0	0	0	0	0	0	1
		21UR-4741	TGCCAAACACATCCGCTCTTA	0	0	0	0	0	0	0	0
		21UR-4742	TGAAATTTGTGCCCCAAAAAA	0	0	0	0	0	0	0	0
		21UR-4743	TGCGAACGCTACACTTTCATG	0	1	0	0	12	14	2	29
*	†	21UR-4744	TGACAATGACGTAGATTATAA	43	58	26	29	95	211	24	486
*		21UR-4745	TAAGTTCAAAAAATTGTCAAG	1	0	0	0	2	6	3	12
	†	21UR-4746	TGAGTATGTACTCCATTTTCA	0	0	0	0	0	0	0	0
	†	21UR-4747	TTGTAACTCTCTGGTTGTTGA	7	11	5	7	15	63	3	111
*		21UR-4748	TACAACGTAGAGTACTGGCTA	1583	771	583	1097	7530	13563	4174	29301
	†	21UR-4749	TTCAGTTAAAGACTTTGGAAA	0	0	0	1	14	17	1	33
	†	21UR-4750	TGACGAAACATTATGATCACT	2	2	2	0	6	5	0	17
		21UR-4751	TCATGTAACATAGGTGATTCG	0	0	0	0	2	2	0	4
	†	21UR-4752	TGCATCGTTCCACTCTTGGTA	8	2	1	2	15	34	3	65
		21UR-4753	TATCAACATGGTTTTTCCAGT	2	0	0	0	1	1	1	5
		21UR-4754	TTGAATGCCATTTTTTATAAA	0	0	0	0	0	0	0	0
*	†	21UR-4755	TAAGGTACAGAATAGTTGAAA	27	46	23	32	78	190	9	405
	†	21UR-4756	TACTTGTAATGACATTTCAAG	0	0	0	0	2	0	0	2
		21UR-4757	TCGGGTTAAATTTTTGCGATA	0	0	0	0	0	1	0	1
	†	21UR-4758	TGTGATTCAAGCTGGTATAAT	1	0	0	2	48	28	8	87
		21UR-4759	TGCATAATTTGAAATACAGTT	0	0	0	0	0	0	0	0
		21UR-4760	TGAAACAAAATTTAAAAAAAG	0	0	0	0	0	0	0	0
	†	21UR-4761	TTCCAGTCCACGTATGGTATT	0	0	0	0	0	0	1	1
		21UR-4762	TAACATTATGCTTTCTGCAAT	0	0	0	0	1	1	1	3
	†	21UR-4763	TTGTACGCCATCGAATCCAAT	2	0	0	0	0	5	2	9
		21UR-4764	TACCAGTTGTTTCTCTGGGGA	0	0	0	1	28	13	0	42
	†	21UR-4765	TCATTAAAGGCTTGAATCAAC	13	23	5	17	32	136	2	228
	†	21UR-4766	TTGAAATGAATAAATACAGGG	0	3	1	1	0	5	1	11
		21UR-4767	TGTTATTACGACGATTGGTGA	0	1	0	0	0	0	0	1
		21UR-4768	TGCAGTCATCACCCACTTTTC	0	0	0	0	1	0	1	2
		21UR-4769	TACCACTTGCTCTTTTGCTCA	24	6	1	5	9	29	8	82
		21UR-4770	TATGATTGTAATTCTCATTTC	0	0	0	0	2	0	0	2
		21UR-4771	TGGCTGCTGATAAGAGTTTCG	0	0	0	0	1	1	0	2
	†	21UR-4772	TCAATTTGTGTGCGAATGTCA	0	0	0	0	0	0	0	0
*		21UR-4773	TAGAATGCTCGTAGATCTTGG	50	22	11	32	687	887	274	1963
	†	21UR-4774	TTAGGACACTTCATCTCCCTA	0	0	0	0	1	1	0	2
	†	21UR-4775	TATCTGATCGACATTGATTGG	0	0	0	0	2	0	1	3
	†	21UR-4776	TGATAGGTTTTTTGAGTTCTA	1	0	0	1	0	1	0	3
		21UR-4777	TCCTTTTTTTTCGATACCTTC	0	0	0	0	0	0	0	0
		21UR-4778	TACCATTCTAACTCTGTGAAA	0	0	1	7	71	54	43	176
		21UR-4779	TATGGTGTTTACTTGAGTAAT	0	0	0	2	6	4	2	14
		21UR-4780	TGCACGTAATGGATGTGTCGA	4	1	2	1	2	28	3	41
	†	21UR-4781	TCAATCGTAAATTTGAAGTGT	0	0	1	0	0	0	0	1
*		21UR-4782	TCGAAAAAATTGGAATTGGACA	97	67	29	35	124	405	22	779
*	†	21UR-4783	TGTCAGTTGAAAATGTGCACT	14	2	2	0	2	59	0	79
		21UR-4784	TTTTCCTTCCGTTAGAATTTA	0	0	0	0	0	4	0	4
		21UR-4785	TGTCACGAATTCATTAAATGA	0	0	0	1	2	0	0	3
		21UR-4786	TCATCCATTTTTCGATTTTGG	0	1	0	0	0	0	0	1
		21UR-4787	TTCCACTATAATACTCCCTAC	0	0	0	0	4	2	3	9
	†	21UR-4788	TATTATTACTTCTTCGCAAAA	0	0	0	0	0	0	0	0
		21UR-4789	TGAGCAGGATCATCGCCTAAC	1	0	0	1	5	7	2	16
		21UR-4790	TAGATTTGCTAACCGTATATT	4	1	0	0	1	2	2	10
		21UR-4791	TGAATTTTGCTTAATGTGAAG	0	0	0	0	0	0	0	0
	†	21UR-4792	TTTTGGTGTCGAAAAGTTTCA	3	1	0	0	1	1	2	8
		21UR-4793	TGCATGTTTGTATTTTCTCAA	4	0	2	3	32	18	2	61
		21UR-4794	TAGGGTTTCAACCTCATTTCA	0	0	0	0	0	0	0	0

		21UR-4795	TAACTTATGTTCATTGACAGC	0	0	0	3	89	67	6	165
	†	21UR-4796	TAACTCTTCTATTCGATTGGT	1	0	0	1	5	8	7	22
*	†	21UR-4797	TTGTGACAGAACGCATTTTTT	49	173	76	88	640	969	75	2070
		21UR-4798	TCATGCTTCCAGTTTCTTGAT	0	0	0	0	0	0	1	1
	†	21UR-4799	TGCCACAATTCTGAGAATCGT	1	0	0	0	0	1	0	2
		21UR-4800	TGATTTAAGTGCCTGTCCTTT	0	0	0	0	0	0	0	0
		21UR-4801	TAGTTGTGCTTTATACACTCG	0	0	0	0	0	0	0	0
	†	21UR-4802	TAAATGCCTTTTTTCAAATGT	0	1	0	0	0	2	1	4
	†	21UR-4803	TTAACTACAACTGAATGCCAA	1	1	0	1	34	22	5	64
		21UR-4804	TGTTTGATCAGCATTGAAAAC	4	4	1	1	12	15	5	42
	†	21UR-4805	TTTAATGGTATTGTAATAATT	0	0	0	0	5	0	2	7
	†	21UR-4806	TATACAGTCCGTTTGTGAAAA	0	0	0	0	0	1	0	1
*		21UR-4807	TGGGAGAATAGTAAGGACGTG	13	19	6	21	683	848	52	1642
		21UR-4808	TTTTCCTTCACCCATCCTGAC	0	0	0	0	1	0	1	2
		21UR-4809	TACGTAGGGTTTCGAAACTAA	2	4	2	7	163	135	15	328
		21UR-4810	TTCATGGAAAAATTCAATGGT	1	0	0	0	2	3	0	6
		21UR-4811	TGTCGAATCGCCTTCTGTAAA	0	0	0	0	3	1	3	7
		21UR-4812	TGCATGTGTCTGGAATTAAGA	0	1	0	0	1	1	0	3
		21UR-4813	TGGGCTAATTTTATCTCAACA	1	0	0	1	4	12	1	19
	†	21UR-4814	TGGCATCTCCGGAAAAAACGA	28	13	6	1	17	76	15	156
	†	21UR-4815	TAAGCAAAGAAGCAAAAGCAA	2	0	0	0	7	19	0	28
	†	21UR-4816	TAACATACGGAAGATACCAAA	4	0	1	1	16	16	2	40
		21UR-4817	TGGGTGAATTCTGTCCCGAAC	14	7	2	1	10	35	0	69
		21UR-4818	TTATTGCTTTATTGAATCCTG	0	0	0	0	0	0	0	0
		21UR-4819	TAAAAATGTAGAATGAGAATG	0	0	0	1	2	12	0	15
	†	21UR-4820	TGCTTAATGGAAGATAGGGAA	15	4	7	8	52	69	4	159
	†	21UR-4821	TATTTTCGACGAGATCAAAAG	4	1	0	0	0	1	0	6
	†	21UR-4822	TTTCAGCAATATTGTGATCTA	0	0	0	0	1	0	0	1
		21UR-4823	TTCGGTGAAGGAAAATTGAAA	2	5	0	0	4	14	0	25
		21UR-4824	TGATCAAAAACTGCTATCAAA	0	0	0	0	0	0	0	0
	†	21UR-4825	TATTGATGAAAAGTGAATATT	0	0	0	0	4	0	0	4
		21UR-4826	TAGACAACACTTTGAGTTTCA	0	0	0	0	6	2	0	8
		21UR-4827	TATAGCGTGTAACAAAAATGG	0	0	0	0	1	4	3	8
		21UR-4828	TGAAATGTCCAATAAAATTCC	1	0	0	0	0	0	0	1
		21UR-4829	TCCGAAGACCGTTACCAGTTT	0	0	1	1	14	10	1	27
		21UR-4830	TCTCTTTCCTATTTTGAAACC	0	0	0	0	0	0	0	0
	†	21UR-4831	TTTATGACTCGTGAGGAAAAT	8	3	2	3	33	59	30	138
		21UR-4832	TGGACTTGCCATTCAAATTGC	0	0	0	0	0	0	4	4
		21UR-4833	TGAGTTGGTAAAAACAATACA	0	0	0	0	0	0	0	0
*		21UR-4834	TGACTCATCGAATTGCATAAT	2	3	1	4	60	71	21	162
	†	21UR-4835	TATTGTTTTTAGAATTTTAGT	0	0	0	0	2	0	0	2
	†	21UR-4836	TTAATGAAATAATCCATTGCT	0	0	0	0	0	0	0	0
		21UR-4837	TCCTCTCGTTTTGCCAATATT	1	0	0	0	2	0	0	3
	†	21UR-4838	TTGGTCAAAGATATTGTAAGG	13	6	8	2	9	44	9	91
		21UR-4839	TAGATGCCTTTATTTTTCATT	0	0	0	0	1	0	0	1
		21UR-4840	TAGGAAATTCGAGTTCTATCA	0	0	0	2	16	11	0	29
		21UR-4841	TATCATTTTCATACTTTGTTT	2	1	2	4	1	5	1	16
		21UR-4842	TTTAATTATCCAATTTTTGCG	0	0	0	0	0	0	0	0
*		21UR-4843	TATGAAACTACTTGCATTACC	0	1	0	1	3	3	1	9
		21UR-4844	TCGCCGATTCTTTCACTTTGG	0	0	0	1	3	1	1	6
		21UR-4845	TGATTGTTTGGCCACAACATT	0	0	0	0	0	1	1	2
		21UR-4846	TGTCAAGGTAGAAAATTCTTG	0	0	0	0	1	1	0	2
		21UR-4847	TCACACAAAAAGATCATCCAA	0	2	0	0	3	10	7	22
		21UR-4848	TTTGCACCTAATTTGAATTCA	0	0	0	0	1	1	0	2
*	†	21UR-4849	TAGACTTAGATAAAGCTAAAC	0	0	0	1	17	10	4	32
		21UR-4850	TGCAATTGTTTTGCTTGTTAC	0	0	0	0	0	0	0	0
	†	21UR-4851	TGAAATTTTCACATGCTGTTT	0	0	0	0	3	1	1	5
*		21UR-4852	TACATCAGCCGCTCGAAGAGG	31	1	4	2	4	91	3	136
		21UR-4853	TGGTTTCTTCAATTTTCTTAA	0	0	0	0	0	0	0	0
	†	21UR-4854	TCTCATTGTATTGTAAAATGA	4	6	3	7	9	21	1	51
*		21UR-4855	TCACAGCTATTTGGCAACACC	9	3	1	2	1	15	1	32
		21UR-4856	TTGTTCGAGCAAACACTTTTC	1	0	0	0	1	1	0	3
*	†	21UR-4857	TTCGAGTAGCATTTGAGTGGC	53	40	20	12	51	348	5	529
	†	21UR-4858	TGGTTTGAGTCGATTTTATTA	0	0	0	0	0	0	0	0

	†	21UR-4859	TCAACATGTACACCTTTTGAA	0	0	0	0	4	6	1	11
	†	21UR-4860	TCGTTCAGTTTTTGTTGGAAA	21	4	2	3	21	32	7	90
	†	21UR-4861	TCACATCTTCACTTTTAGAGC	0	0	0	1	7	9	0	17
		21UR-4862	TGATTTGCACAATGTTTGCAT	0	0	0	0	0	0	0	0
	†	21UR-4863	TTTTCAGTTGATTGATGATGA	6	0	1	0	5	15	0	27
		21UR-4864	TAGCTCCTGGATATGTCTAGC	2	1	0	0	1	6	0	10
		21UR-4865	TACAGCAACTGCAGTTAATCG	8	0	3	1	8	28	0	48
	†	21UR-4866	TTTCTGTCTGTGTGTTTGAAC	6	8	3	9	50	67	6	149
		21UR-4867	TATCAATACGTTTTTCTGTAA	0	1	0	0	12	3	1	17
		21UR-4868	TGCGCTACATTTCATGTTCAA	1	0	0	0	0	2	0	3
		21UR-4869	TTGGCGATGCTCTCAGTTGAG	1	0	0	0	2	3	1	7
	†	21UR-4870	TTCAAACCATTTGGGATTAAT	0	0	0	0	0	2	0	2
		21UR-4871	TGGATTGGCTTTTAATCCTTG	0	1	0	0	4	1	0	6
	†	21UR-4872	TCTCCGAATGACAAATTTTTC	0	0	0	0	0	0	0	0
	†	21UR-4873	TAGTTTGTTTGGACATATAAA	13	3	9	5	37	68	15	150
		21UR-4874	TGCACTTGTAGTTTCGCCCTC	12	7	3	3	18	78	18	139
		21UR-4875	TGGCCCTTGAATCTATATGAA	0	0	0	0	3	1	1	5
		21UR-4876	TAGACTCGAATTGTGCGTTAA	0	0	1	0	4	9	0	14
	†	21UR-4877	TGGTGTTACGATAAAATGAAG	1	2	1	0	2	0	0	6
		21UR-4878	TCCATAAGTTTTCCCGACAAT	0	0	0	0	1	1	0	2
	†	21UR-4879	TTCTTAGTACATAGTAGGTGA	1	2	4	4	67	39	6	123
	†	21UR-4880	TACTCTGGCACATGATTTAAA	0	0	0	0	2	2	1	5
*	†	21UR-4881	TGATGGACTTCTACTTGGAAA	25	18	10	17	16	78	2	166
	†	21UR-4882	TGTTTTCAGTTTGGACTTTGG	5	5	1	2	13	23	13	62
	†	21UR-4883	TGGAGATATATAGTGGTTTGA	54	58	33	22	119	215	31	532
		21UR-4884	TCGACGCCAGTTTATGTAGAA	0	0	0	0	3	1	0	4
		21UR-4885	TAATCGTAACACTTTTCTAAT	0	0	0	0	0	0	0	0
		21UR-4886	TGGATACTCTTACAATTAACG	1	1	0	0	6	9	5	22
		21UR-4887	TCATTTCAATCTTTTCAAAAA	0	0	0	0	0	0	0	0
	†	21UR-4888	TGGTTTATGGACTTAACTTCA	1	0	0	0	6	7	0	14
		21UR-4889	TGTTTGGTCCAATTTAATTCC	0	0	0	0	0	0	0	0
	†	21UR-4890	TAAACCGTATGTAGTAAAAGA	2	6	1	5	45	45	8	112
		21UR-4891	TCATCTAAGGTTCCAATGCAA	1	0	1	0	1	0	2	5
		21UR-4892	TCAAGCTTTTTTTATAATAAC	1	0	0	1	2	1	0	5
*		21UR-4893	TCTCAATTGGATAGCATAACA	34	19	12	14	56	115	8	258
		21UR-4894	TGTCCTTTCTAAATAGCATTT	1	0	0	0	0	2	0	3
*	†	21UR-4895	TTGGATGGTTGAAGAAGGAGT	728	816	407	229	833	3447	152	6612
		21UR-4896	TATGCTGAAACAGTTTATGGT	0	0	0	0	0	1	0	1
		21UR-4897	TCGTGATGTTTAACTCATGAA	0	2	0	1	5	2	0	10
	†	21UR-4898	TTCGTTAGTGAGAAGGTAATC	1	1	1	0	8	11	4	26
	†	21UR-4899	TGGGCATCTGTATTCAGAACA	0	0	0	0	1	2	0	3
		21UR-4900	TTGAGCATCAATTATCATTCT	0	0	0	0	4	2	3	9
	†	21UR-4901	TCGTATCGTTTTACTTACAAT	13	4	1	6	10	24	2	60
*	†	21UR-4902	TTTCGTCAGCATGGCTAGTAC	0	0	0	0	2	3	2	7
		21UR-4903	TCTACGGTTCATTTTACATCA	0	0	0	0	0	0	0	0
		21UR-4904	TATCAGTAAGATGTTGACCAT	4	11	8	17	153	182	18	393
	†	21UR-4905	TTGCATCGTTGAAATACCCAA	19	4	5	2	10	27	6	73
	†	21UR-4906	TCTATCTATATTGTGAACAAA	0	0	0	0	0	0	0	0
	†	21UR-4907	TAACGGTATTGGTTGCTTTAT	0	0	0	0	14	8	3	25
*		21UR-4908	TTCGATGCACGCGCATGTACC	4	0	1	0	1	5	2	13
		21UR-4909	TTAGACAAAAAGGGTAATGAA	1	0	2	0	31	24	7	65
		21UR-4910	TGCCATTTGTTTCAGATTAAT	4	0	0	0	0	1	0	5
	†	21UR-4911	TAATACTGAACTAATTATACA	0	2	2	2	20	10	1	37
	†	21UR-4912	TTGCCTTTTTCACCACTTCGA	11	0	1	0	0	1	3	16
	†	21UR-4913	TATAAGGTGCAATAGATGATT	0	0	0	0	2	1	2	5
	†	21UR-4914	TAGTCTCCAGGCGTTTGTTTA	0	0	0	0	0	10	7	17
		21UR-4915	TCTCAAGTTGCATTTAGGATT	0	1	1	1	5	13	0	21
		21UR-4916	TTGACTGGCTGAAATAAAATC	0	0	0	0	0	0	0	0
		21UR-4917	TAATAAGCAAACCCAATATTA	1	0	0	0	0	0	1	2
		21UR-4918	TACTCGGCCCGATTTATGATT	0	0	0	0	13	8	0	21
		21UR-4919	TTCCATAATGAAAATACCCTT	1	0	0	0	0	1	0	2
		21UR-4920	TCATCGAGAAATAACATTTAC	1	1	1	0	0	2	0	5
		21UR-4921	TTAATTCATCAGACCAGAAAA	0	0	0	0	0	1	2	3
		21UR-4922	TTGTATTTCGTTATGGGGTTA	46	10	8	6	4	42	10	126

		21UR-4923	TGTAACATTTCACCGCATACA	1	0	0	0	0	0	0	1
		21UR-4924	TCAACCAGTAACTGATTGTGA	0	0	0	0	4	7	0	11
	†	21UR-4925	TGAGCATACTCGAAGTGCATC	1	0	1	1	20	22	5	50
	†	21UR-4926	TTCTCAAATTGTTGTAAGGTT	0	0	0	0	0	3	0	3
		21UR-4927	TTCTTGGGTCATTAAACCTAA	1	0	0	0	0	1	0	2
	†	21UR-4928	TCGTATTCGTTAATAGTTCCG	0	0	0	0	2	0	0	2
		21UR-4929	TGGTTTTGCAGTTATTTAATC	0	0	0	0	4	3	3	10
		21UR-4930	TGCCTCATTCACCAACGGAAG	20	4	3	1	3	10	4	45
		21UR-4931	TACTCTCATAAGATTCATTTG	10	2	2	0	2	9	1	26
		21UR-4932	TATGACTTTGATAGCTTTCAA	0	0	0	0	8	3	2	13
		21UR-4933	TCACATAGGTGTTTCTTTTTT	0	0	0	0	0	0	0	0
		21UR-4934	TGGGTTCAGTGTGTGTATCTT	3	1	0	1	3	9	1	18
		21UR-4935	TGAAACATCTTCAATCGAATC	1	0	0	0	0	1	1	3
		21UR-4936	TCTTTCTATGATGGTAATCGC	3	0	0	0	15	21	6	45
	†	21UR-4937	TGGGGAAAAATAACTGCAAGA	0	1	0	0	0	0	0	1
	†	21UR-4938	TAAACGATCCTTCAAATGTGA	0	0	0	0	3	2	1	6
		21UR-4939	TGGGACAGCTTCAAACTTAAA	1	3	3	12	225	200	88	532
	†	21UR-4940	TAGAATTGTCACAACCGTGAA	0	0	0	0	0	1	1	2
		21UR-4941	TGCACTGTAATCGTGAATTGT	0	0	1	0	15	15	3	34
		21UR-4942	TAGGGTTTTGAAATCTTGAAT	1	0	0	1	0	0	0	2
		21UR-4943	TCGTAGTTGGAATATGATTTC	20	8	3	7	25	52	4	119
	†	21UR-4944	TACTCTTGCTTTTGCAGATGT	0	0	0	0	0	2	0	2
	†	21UR-4945	TGGCTTCTAAATTTGATGTCC	0	0	0	0	0	1	0	1
		21UR-4946	TGAAGCCCTTGCGAACAGGAT	0	0	0	0	0	0	0	0
	†	21UR-4947	TCAGAAGATAAATTAAAACCA	0	1	0	2	42	42	5	92
		21UR-4948	TACCAATGGTTTAGACTTGGA	0	0	0	2	11	10	3	26
		21UR-4949	TTATAATCCAGCACATGTTAT	5	0	1	1	2	7	8	24
	†	21UR-4950	TATAATGTCGAAACAGACAGA	2	1	1	1	8	11	0	24
		21UR-4951	TATTGTAACTTTAGTAATGTC	0	0	1	0	2	4	0	7
	†	21UR-4952	TGACAAAAGTATAATGTAAAG	1	1	0	0	2	0	0	4
*	†	21UR-4953	TCGACAAAATGCAGGCAATGA	23	15	12	4	20	100	6	180
	†	21UR-4954	TTTGGGTTTCTTTAAATATCC	0	0	0	0	0	0	0	0
		21UR-4955	TAAGACTTGCCTATCCTTGCC	1	1	0	3	7	9	11	32
		21UR-4956	TGACACATTTTTTGGAATATT	0	0	0	0	0	0	0	0
	†	21UR-4957	TTAGTGGAATTTTCTTGAGAA	0	0	1	1	2	1	0	5
		21UR-4958	TGAGGTTTCAACAAAATTTGG	0	0	0	0	0	0	0	0
		21UR-4959	TTTTGCAAGCTTTATGATAGC	1	0	0	1	3	3	0	8
	†	21UR-4960	TTAATGGCACGTTTGATTTCC	17	3	4	4	5	21	8	62
	†	21UR-4961	TAGTCGTAATCGTATGTTTCA	0	0	0	0	0	0	0	0
	†	21UR-4962	TTGATATTCTAGTTGGAATTC	13	4	5	3	36	56	4	121
		21UR-4963	TGCATCAATAGATTTCTCACT	0	1	1	2	10	3	0	17
		21UR-4964	TCCCATTCGCAAATACTAAAA	0	0	0	0	0	2	7	9
		21UR-4965	TCGGTTTTTATTTATCTCTGT	0	0	0	0	0	0	0	0
	†	21UR-4966	TGATGCCTTGGTACTTCTTGT	0	0	0	0	0	0	0	0
	†	21UR-4967	TGCTGTAGTTGATATAATGAA	6	4	4	9	48	58	9	138
		21UR-4968	TAGCAAAACAAAAAATTTTTC	0	1	2	0	6	8	1	18
		21UR-4969	TGAAGCATTGATTGACTAGTA	5	7	1	7	341	240	15	616
*		21UR-4970	TACATTCGGGATTGTTGGTAA	110	24	23	19	60	201	49	486
	†	21UR-4971	TGAATACTGCCTTTGTGAATC	0	0	0	0	5	8	0	13
		21UR-4972	TAAGCGGAAAGTGTGAAAATC	45	53	16	15	42	263	14	448
		21UR-4973	TAAAGATAGCTTTTCAAAACA	1	0	0	0	4	1	0	6
	†	21UR-4974	TGGTCCGTTGAACATTTGTCA	0	0	0	0	2	8	0	10
		21UR-4975	TCAAATCCAAAGCTGGAGCAA	1	0	1	0	0	0	0	2
*		21UR-4976	TAGCGATTGCAGAGTAAGATT	27	35	12	23	175	256	15	543
		21UR-4977	TACGATTAGTCTTTTTTACAT	0	0	0	0	0	1	0	1
		21UR-4978	TGTGGATTCGATAGGTTGAAC	46	17	9	7	20	141	35	275
	†	21UR-4979	TCCGTTTACTTTTAGATTTCA	0	0	0	0	0	0	0	0
	†	21UR-4980	TCGTTTTTCGCCATGAGTCAT	0	0	0	0	0	0	0	0
		21UR-4981	TGTAACAAATATCAACCGAAA	0	0	0	0	0	0	1	1
	†	21UR-4982	TCCACATTTTCAAACTGATCA	0	0	0	0	4	1	1	6
		21UR-4983	TAATCGTTCGATTTTCAGAGT	1	0	0	0	0	0	0	1
	†	21UR-4984	TCTTGCGAGTTATTTTTCTAT	0	0	0	0	0	0	0	0
*		21UR-4985	TGGACGACCTGGAGTAATAGA	7	2	5	4	45	59	63	185
		21UR-4986	TGAATATTTACGCTGTATCCT	0	0	0	0	1	1	0	2

		21UR-4987	TCGTTCTGAAAATTCAGGTTA	0	0	0	0	1	4	0	5
		21UR-4988	TCCACGCTTCCCACTCTTTCC	1	0	0	0	0	0	0	1
*	†	21UR-4989	TAGTTGTTTAGCGAAAATAGG	3	3	0	1	0	2	0	9
	†	21UR-4990	TGCTCGGAAATCATTAAAATA	0	0	0	2	6	2	2	12
	†	21UR-4991	TGGTTCGACGCTTCTATTCTC	2	1	2	1	44	45	21	116
		21UR-4992	TCCAATATGAATAGAATTTTT	0	0	0	0	0	0	0	0
	†	21UR-4993	TCAACAAAAAAAATCAACAAA	0	0	0	0	0	0	0	0
	†	21UR-4994	TTAATGGCTCAAAGAAATTTT	0	0	0	0	2	0	0	2
	†	21UR-4995	TACGCCACACGCTCATTCCTC	0	0	0	0	0	0	0	0
	†	21UR-4996	TCGAATCGAAGTTAATTACTT	0	0	0	0	0	0	1	1
		21UR-4997	TATGAACAACAAAAAACACAA	0	0	0	0	5	2	0	7
	†	21UR-4998	TGTGAATATCACAAATGACAA	0	0	0	0	0	0	0	0
		21UR-4999	TGGGGATCAAGAGCACGATCA	0	1	0	1	3	2	1	8
	†	21UR-5000	TTGAGCGATCGTTAGGAGTAG	6	1	2	1	1	21	2	34
	†	21UR-5001	TTATGAAGCTTCTAACTGATA	0	1	0	0	5	4	0	10
		21UR-5002	TGGTAATGTGTTGTACCAAAT	0	0	0	0	0	0	0	0
		21UR-5003	TTAACTTGTAATTCTAGCTTT	0	0	0	1	4	3	0	8
	†	21UR-5004	TATCATCAACTTTGCTCTCGA	0	0	0	0	4	2	0	6
		21UR-5005	TTGGTCGAAAATGGCGAGGTC	0	0	0	0	1	0	0	1
	†	21UR-5006	TGCGAATCAGGTGTTTTCGAA	0	0	0	1	0	5	0	6
	†	21UR-5007	TTAGTTAGCTTTGCTCTTGCT	0	0	0	0	1	0	0	1
		21UR-5008	TATGATTGTCTTAATTTAGCT	0	1	0	0	16	11	1	29
	†	21UR-5009	TTATTATATTGTTCTGGGATC	0	0	0	2	16	13	19	50
		21UR-5010	TAAATAAGCCATCATAGTTAA	0	0	0	0	1	1	10	12
		21UR-5011	TAACGATTTAAGATGTTTCAG	0	0	0	1	2	2	0	5
		21UR-5012	TGCTCCGTTATTATAAAGCTC	0	0	0	0	0	1	0	1
		21UR-5013	TCGAACTTTTATGTATAAATT	0	0	0	0	1	0	1	2
		21UR-5014	TGGTGTCTTTTCATGCTCCAA	0	0	0	0	1	0	0	1
		21UR-5015	TGTAGTACACACACCATAGAA	0	0	0	0	1	0	2	3
		21UR-5016	TGCTTACATTATTCTTATGGC	0	0	0	0	0	5	0	5
	†	21UR-5017	TAGTCGCAGTAGGATATTAAT	1	2	1	0	3	8	0	15
	†	21UR-5018	TTCGGATAGTGCTAAATATTC	34	16	9	5	25	81	3	173
	†	21UR-5019	TATGTCCTGTAACTGAAACCA	0	1	0	0	0	2	0	3
		21UR-5020	TGTCTCATTGTTAACATTTTC	1	0	0	0	0	0	0	1
		21UR-5021	TTGCTTCTTTGAAAACTTCCA	0	0	0	0	4	6	0	10
	†	21UR-5022	TGGTCCTGTTCTTAACTTATC	0	0	0	0	3	0	0	3
*		21UR-5023	TAGCAACTAAGAATTCCGTTA	0	5	0	8	46	42	3	104
		21UR-5024	TCCCAAGCCAATCCACTGAGA	0	0	0	0	0	0	0	0
		21UR-5025	TGAATTGGGATGATCGTAGTT	75	28	21	26	203	278	601	1232
	†	21UR-5026	TGCGTGTATAGTACGACTTAA	5	1	1	1	8	22	1	39
		21UR-5027	TAACGCTTTTAAAAGATTTGC	0	0	0	0	0	0	0	0
*		21UR-5028	TAGGACAGTGGAACGAACGAT	0	0	0	0	0	9	1	10
		21UR-5029	TGGATTTGCAAAAAGTAGAAT	17	13	8	40	350	273	33	734
		21UR-5030	TAACAGAAGACTTGAAAAAGG	6	3	3	1	9	33	0	55
*		21UR-5031	TAACGATACAGCCTTACATGT	19	2	0	0	3	10	2	36
	†	21UR-5032	TCCAGTATTTTGTAGATGGAT	11	21	9	27	247	222	12	549
		21UR-5033	TGCGAGTTTTTAACTTTTAGA	0	0	0	0	1	1	1	3
		21UR-5034	TGAACTGGACGTTTTTCGAAT	0	8	8	13	250	254	79	612
	†	21UR-5035	TTCTGGTTCGTTTAAATTGAA	0	0	0	0	1	2	0	3
	†	21UR-5036	TTGATAAGCAAAGAAGCAAAA	0	0	0	0	0	0	0	0
	†	21UR-5037	TTAATGGAAAGTTTTAATTAA	0	0	0	0	0	0	0	0
		21UR-5038	TGTACCGTCGGTATAAATATC	1	1	1	1	29	18	4	55
		21UR-5039	TCTACAAATCATTTACTATAT	0	0	0	0	0	1	0	1
	†	21UR-5040	TACTTCTTTGTTTGTATCAAT	0	0	0	0	1	1	0	2
*	†	21UR-5041	TATAGTATGTTCCAGAGGTGG	16	4	1	2	11	23	3	60
*		21UR-5042	TCAACGTATTGTATTCAACTT	0	0	0	0	31	13	0	44
		21UR-5043	TAAATACTAATAATCCAACAA	0	0	0	0	0	0	0	0
	†	21UR-5044	TGATATTTCTACACAGATTGA	0	0	0	0	0	0	0	0
*		21UR-5045	TGCCAAACTCCATTTAACGGC	48	18	6	8	27	130	2	239
		21UR-5046	TACCAGTAAATATGCCAGGAA	0	0	0	0	2	3	3	8
	†	21UR-5047	TCAGTTCTTCTTCGTGTAAAC	0	0	0	0	1	0	0	1
	†	21UR-5048	TCGGGCCTTGAGTTTCTTTAA	0	0	0	0	1	2	0	3
		21UR-5049	TATTTTTCATCTTTCTGCTGC	1	0	0	0	0	0	0	1
	†	21UR-5050	TAATCTTTTTCTGATATTTGC	0	0	0	0	0	0	0	0

		21UR-5051	TAAAGTCAATCTTTCTTGTTT	0	0	1	0	0	0	0	1
*	†	21UR-5052	TGGTCACACACAACAAAGGCT	0	1	0	6	39	62	24	132
	†	21UR-5053	TGTTGTATATTAATGGTGTAA	67	6	11	6	7	19	6	122
	†	21UR-5054	TAATCCGGTTATCATAGCTAG	5	0	0	0	2	9	2	18
		21UR-5055	TGTCAGTAATGAATAATCACA	0	0	0	0	0	0	0	0
		21UR-5056	TACAGTTTTTTCCTATTCGGT	1	0	0	1	0	9	2	13
		21UR-5057	TATGAAGACTTCATTGACCCA	0	1	0	1	2	5	0	9
	†	21UR-5058	TCGTAATCGTATGTTTCAACT	6	1	0	2	7	8	1	25
		21UR-5059	TAGGTCCGGTTGTGGAATATC	0	0	0	0	5	5	0	10
		21UR-5060	TACTTACTCTTCAATTTTTGC	5	2	1	0	9	11	1	29
*		21UR-5061	TGTGCATTTCTCTGTTCGTCA	2	0	1	0	3	6	0	12
		21UR-5062	TAAGCGGATTGCATTATTTCG	0	0	0	0	12	9	2	23
		21UR-5063	TTCGTCATCGCTATCAATCAA	0	0	1	1	1	0	2	5
		21UR-5064	TCATGTTGCTGGTCATCTTTT	0	0	0	0	1	0	0	1
		21UR-5065	TCGAACACTGGTGGTCCAACT	0	0	0	1	4	2	3	10
	†	21UR-5066	TGTGACTATATTTACAATCAA	0	1	0	1	7	1	3	13
		21UR-5067	TACTTTCAGATCCCCTATAAA	3	0	0	0	4	5	3	15
	†	21UR-5068	TTATAGAAGTCATTTTGCTCA	0	0	1	0	1	6	0	8
*		21UR-5069	TGATTCCATTGTATTGGCATA	0	0	1	3	31	29	3	67
		21UR-5070	TGACTTACCTGTTTTTTCCTG	0	0	0	0	0	1	0	1
		21UR-5071	TCAACAAGTCAACAAATTGAA	0	0	1	0	2	0	0	3
	†	21UR-5072	TCATTCCTACTATGGCGTTTA	15	1	0	1	1	2	3	23
		21UR-5073	TTTGACTTTATTGAAGATTCT	1	0	0	3	5	16	1	26
		21UR-5074	TCTCAATTTTGAAGCATTATT	0	0	0	0	0	0	0	0
		21UR-5075	TCGTATGAACCAGTTGAATAT	0	0	0	0	17	11	2	30
	†	21UR-5076	TGTTAAGGTGATAAAACATTG	0	0	0	0	0	0	0	0
		21UR-5077	TGGGCTGTTGGACATGTAGAA	29	8	4	0	4	54	11	110
		21UR-5078	TCTTTGCACTTTTATTTTTAT	0	0	0	0	0	0	0	0
		21UR-5079	TAACCGTTGTTTTGTCATGGT	0	0	0	0	5	10	0	15
	†	21UR-5080	TAACTTCCGCATTTGTGAAAC	0	0	0	0	4	5	3	12
		21UR-5081	TCTGGTGTTGAATAGGCTTCC	2	5	0	2	50	49	14	122
	†	21UR-5082	TCCTCGACTATTTCGCCAAAA	1	0	0	0	0	0	0	1
	†	21UR-5083	TATTATGTGGATCTGAATGTC	2	1	1	0	2	8	0	14
	†	21UR-5084	TATTCGACTCATAATTTCAAA	0	0	0	1	1	4	1	7
		21UR-5085	TAGATCGTTATGGAATGTTCT	2	1	1	3	8	7	1	23
	†	21UR-5086	TACCAATTCGTCATGTTTATG	3	0	1	1	3	8	5	21
		21UR-5087	TTGGATTCTGGTCCTACATTA	0	0	0	0	1	0	0	1
	†	21UR-5088	TGATGTATTCATTTGGCGAGG	0	0	0	0	2	5	4	11
	†	21UR-5089	TTGGGCTCCAAATATTTTCAG	0	0	0	0	1	1	0	2
		21UR-5090	TTGTATTAGATCAACTTCAAG	3	2	0	0	1	2	2	10
		21UR-5091	TAGTATTGCCAAGTATTTGTT	0	0	0	0	0	2	0	2
*		21UR-5092	TCGATATAACTGAACTTCAAA	0	1	0	0	9	6	1	17
		21UR-5093	TAAACGGTTTTTTTATGTACT	0	1	1	0	14	17	0	33
		21UR-5094	TGTGAAATACTTAGGCTGTGA	2	0	0	0	15	11	7	35
	†	21UR-5095	TTTCTGGACGTTTTCAGCAAT	0	0	0	0	2	0	2	4
	†	21UR-5096	TAAAAGGGCATTTGAACTTAA	9	6	3	6	31	49	0	104
*	†	21UR-5097	TTGTCTCTGTGTGGGTATATC	19	9	4	7	36	58	11	144
	†	21UR-5098	TTACGCATACTTGTTGGTTGA	4	1	1	2	12	15	1	36
		21UR-5099	TCGTGCTTCATTTATATGCTC	0	0	0	0	0	0	0	0
		21UR-5100	TCTCCGAAAATATTCCGCATT	0	0	0	0	0	0	0	0
*	†	21UR-5101	TAGGTACTACTTTGAACTTGG	0	0	0	1	0	9	0	10
		21UR-5102	TAGTTGAACAGTTTCAATTTG	0	0	0	0	1	1	0	2
		21UR-5103	TGAATTGTTACGTATTGTCTG	0	0	0	2	17	10	10	39
		21UR-5104	TAGAAGCATATTTAGATGTTC	0	1	0	1	11	7	1	21
*	†	21UR-5105	TATTATCGTCTTCTGAGCATA	58	41	30	27	85	187	20	448
	†	21UR-5106	TTGGTTGCCAGTGAAATTTTG	0	0	0	1	3	0	0	4
	†	21UR-5107	TCCGAATTCCGAATTGAAACT	1	0	0	0	4	8	0	13
		21UR-5108	TGCTACGTTTAGCCAATTTGT	1	0	0	0	0	0	0	1
*	†	21UR-5109	TAGGATTTCACTAGAAGAAGC	221	275	100	108	294	1203	43	2244
		21UR-5110	TTCCATTACCGTTGAATGCAT	4	0	1	4	11	27	10	57
	†	21UR-5111	TTGATGTAAAGAAAGAAATAG	5	1	2	0	8	13	4	33
		21UR-5112	TCATCTATAGATACGTCAGCA	8	2	4	15	100	72	29	230
		21UR-5113	TATAACATCTGACTCAGTTCT	0	0	0	0	1	1	0	2
		21UR-5114	TCATTTGTTTTATTACAGTTG	0	0	0	0	0	0	0	0

		21UR-5115	TGGGAATTCTTTGATTATCAA	0	1	0	0	2	1	1	5
		21UR-5116	TAACCCATGTTGATTAGATGA	0	0	0	0	0	0	0	0
		21UR-5117	TATCAACGCAAGAAAAATATG	0	0	0	0	4	2	3	9
*	†	21UR-5118	TGGCACAATTGTATTCAATTT	0	0	0	0	4	9	4	17
	†	21UR-5119	TCTGTGCGTGGTTTTCAATAT	0	0	0	0	0	0	0	0
	†	21UR-5120	TTCATTGGAAGCATAACTCGA	1	3	2	3	32	40	0	81
		21UR-5121	TAAATACCTTCACAATTTCTA	0	0	0	0	0	0	0	0
*	†	21UR-5122	TAGGCCAAGTCGGTTAGCTAA	17	1	2	2	7	31	10	70
		21UR-5123	TACATTTCGGTTATGGTTTTC	1	0	1	0	2	9	0	13
	†	21UR-5124	TAACAAATCATCATGCTATAA	9	0	1	0	2	5	3	20
		21UR-5125	TAGACTCCAAAGTGTCAAAGC	0	0	0	0	0	1	0	1
		21UR-5126	TTTCAATAAACTTATGCCTGC	0	0	0	0	2	1	0	3
		21UR-5127	TATCGAATTCATCTTCCTTTC	0	0	0	0	0	0	1	1
		21UR-5128	TGCTGTGTTTTTCTCTCTCTG	0	0	0	0	0	0	0	0
	†	21UR-5129	TAGTCACGTAAAGTTTTTTTT	0	0	0	0	0	2	0	2
		21UR-5130	TGGGAACTTTGGTCTTCAAAA	1	0	1	0	8	7	1	18
		21UR-5131	TGAAAAAAATTAAATCAAGCTT	1	0	0	0	0	2	0	3
	†	21UR-5132	TAATAAACGAACCTTGGTGAT	0	0	0	0	3	2	1	6
	†	21UR-5133	TACGAAGAGATAATACTAGAA	38	16	12	19	56	111	11	263
		21UR-5134	TGCCTTACTTAGTTTCCCCTT	0	0	1	0	2	5	2	10
	†	21UR-5135	TTAGTTTCCAAACGTTTAAAA	0	0	0	0	0	1	0	1
	†	21UR-5136	TCGAAATAAGATTGCATTAGC	0	1	0	0	29	14	3	47
	†	21UR-5137	TTTTACTTTATTGTGCAATAC	0	0	0	1	2	1	0	4
		21UR-5138	TTTCAAGAGTTTACTACAAAA	1	1	0	0	0	1	0	3
		21UR-5139	TCTGTGACTGATGTTTAATAT	1	0	1	2	1	10	1	16
		21UR-5140	TAATTTTAAGAATGAATGGCA	166	261	84	168	391	1082	31	2183
		21UR-5141	TTAGTTCAACAGTTCAATCGT	1	0	0	0	2	0	0	3
		21UR-5142	TCTTTTGGATGTAGGCTTATG	0	1	0	1	5	10	3	20
*	†	21UR-5143	TAGTAGGTGTTCAAGTTAGGG	13	2	0	1	10	14	5	45
		21UR-5144	TATATTACCAATCGTCTGACT	0	0	1	2	24	36	29	92
	†	21UR-5145	TTGAGAACATCTGGTTTTAAT	0	0	0	0	4	6	0	10
		21UR-5146	TCTACAATAGGAAATCCGGCA	0	0	0	0	1	3	0	4
	†	21UR-5147	TTCAATGTACTATGCTACAAA	0	1	0	0	0	0	0	1
		21UR-5148	TCTAGAGTCAATTCCAATGAA	0	0	0	0	0	0	0	0
		21UR-5149	TGTGATGTCTATTAGACTGAC	0	0	0	0	5	4	0	9
	†	21UR-5150	TCGATTCGCATGTTGCTATAG	2	3	1	1	1	6	0	14
		21UR-5151	TCAACCGACTGGATGCCTATC	0	0	0	0	4	5	7	16
	†	21UR-5152	TTATTTAGCAATCGTTCTACA	6	0	1	1	0	1	1	10
		21UR-5153	TAAAAAAGTTCAAGTAGGGAA	0	0	1	1	14	9	5	30
		21UR-5154	TGAATTCGGCCAATTATTCGA	1	0	0	0	2	5	1	9
	†	21UR-5155	TACTCCTGTCCTCTCCAACAT	2	9	0	5	45	35	3	99
*	†	21UR-5156	TAGTTTGGTGACTAAGGAAGA	0	1	1	0	49	43	11	105
		21UR-5157	TAAGGTATTGCATTTTAAATC	2	0	1	3	4	14	0	24
		21UR-5158	TGATTGAGGAAAACACACAAT	6	2	1	0	9	15	0	33
		21UR-5159	TGCATTTGAATTATTTTTCCA	0	0	0	0	6	6	1	13
	†	21UR-5160	TGGTTTTGCCCTGATCTTGGA	8	1	3	4	44	82	22	164
		21UR-5161	TATGAAAACTATTTTACAAAT	0	0	0	0	1	0	0	1
	†	21UR-5162	TGGTGTAGATTGCCTAGCTTG	0	0	0	0	1	2	0	3
		21UR-5163	TGCATCCATCATCTACGGTTG	0	0	0	0	0	0	0	0
	†	21UR-5164	TAGTGTGCAAAAAATTGAATAC	0	2	0	1	25	21	3	52
		21UR-5165	TGCATGGTTCTGCTTTAGACG	3	11	0	6	169	203	6	398
		21UR-5166	TAACATAGCATTGATTTCGCC	78	5	6	6	13	45	2	155
		21UR-5167	TGGTTTTCCCGATGGCACATG	2	0	0	0	0	5	1	8
	†	21UR-5168	TGACAATCGTAAATTATTTTA	0	0	0	0	0	0	0	0
*		21UR-5169	TAACATATATTTCTGATTAAA	2	2	1	9	45	52	9	120
*	†	21UR-5170	TTAACTAAAGAGCAGAATATG	379	381	162	166	393	1742	100	3323
		21UR-5171	TAAGACATTAATAACTTCAGC	0	0	0	0	1	0	0	1
	†	21UR-5172	TCAAAAAATTGAAGGTGTTCAC	12	2	0	4	18	25	10	71
		21UR-5173	TATAGTGTTCACTTAAGCTTG	1	0	0	1	1	6	1	10
*	†	21UR-5174	TCAGTAGAAGAAGACTCTCAA	735	794	368	624	5099	7065	385	15070
		21UR-5175	TACAATTATTACAAGACGCGT	2	0	0	1	12	25	9	49
	†	21UR-5176	TTCAGTTGGTAAAATTGAAAA	0	0	0	1	1	2	1	5
	†	21UR-5177	TTGGATATCTCAAAGATAAAC	1	1	2	1	8	10	1	24
*		21UR-5178	TTTATGGTTAGAAAAACTATA	10	3	1	2	18	31	0	65

*		21UR-5179	TGTATCGTCGAATGCTCCATC	5	0	1	0	8	10	0	24
*	†	21UR-5180	TGATGAATAGGTGATTAGGGA	7	2	1	2	48	54	14	128
	†	21UR-5181	TGATTATTCTATTTTTCACAA	0	0	0	0	0	0	0	0
	†	21UR-5182	TTCAATGTTCTCGGTTTTTGG	2	0	0	2	1	4	0	9
	†	21UR-5183	TCTGAGTAGGTATTATCGTGA	1	0	1	0	9	14	4	29
		21UR-5184	TAGTTTCACTGGAATATTTTC	1	0	0	0	0	1	0	2
	†	21UR-5185	TAATTCTTGCAAAATCGGTGT	121	36	19	15	23	211	9	434
	†	21UR-5186	TCTGTGTGTCATATTGCCTAC	0	0	0	0	2	3	1	6
		21UR-5187	TGTAATAATGCATCTTTGCTC	0	0	0	0	2	0	0	2
*		21UR-5188	TAAGGGAGTATGCTTAGTAGT	0	0	0	1	12	17	5	35
		21UR-5189	TCACGGCATTTAACACCGAAT	0	0	0	0	0	1	0	1
		21UR-5190	TGCGAGCTTCGCCATTTTAGA	1	0	0	0	0	0	0	1
		21UR-5191	TGTAAAAAGTTTTTTGATGTA	0	0	1	0	9	5	0	15
	†	21UR-5192	TGGGCATCCAAATTTTACAAA	1	0	0	0	0	1	0	2
		21UR-5193	TAACGATTTCAATGAAAGTAC	0	1	0	0	0	1	0	2
		21UR-5194	TAATAAATTTATATGATTGTG	0	0	0	0	5	3	1	9
*	†	21UR-5195	TAATGACAATGACGTAGATTA	37	23	10	15	40	101	2	228
		21UR-5196	TGACAATAACATGTGTGTATC	0	0	0	0	0	0	0	0
*	†	21UR-5197	TTGATTTCGACAGTACTCAAA	18	23	10	27	127	182	9	396
		21UR-5198	TCATGATCAGATATGCTCATC	0	0	0	0	9	3	0	12
	†	21UR-5199	TCCGAAATAGATAACCATCAG	0	0	0	1	0	0	1	2
	†	21UR-5200	TATTCATTGTTCTTTAAAGGG	2	1	0	0	0	4	1	8
		21UR-5201	TGTGGTATTAGTGCAATGTTT	0	0	0	1	39	14	5	59
		21UR-5202	TCAGACGTGACACAAATGGTT	0	0	0	3	22	12	3	40
		21UR-5203	TGCGTAAGTTTTAAATATCAG	0	0	0	0	0	0	0	0
		21UR-5204	TAACTTCGTAAATTTCTTCGT	4	0	1	1	1	2	0	9
		21UR-5205	TTTGCCAGTAGATTTCTTGAA	1	1	0	2	43	36	7	90
		21UR-5206	TAACTCCATGTATGCAATTAG	2	0	0	0	13	7	5	27
		21UR-5207	TCAAAATTCCAGTAGATTCTT	0	2	0	0	7	4	0	13
	†	21UR-5208	TGGTGCTATTGTCTTTAATAC	1	0	0	0	0	0	0	1
		21UR-5209	TTCTTTTAGTTATGCTGCCTA	0	0	0	1	1	5	0	7
		21UR-5210	TATGCAATGACACTATGCAAA	0	0	0	3	10	13	11	37
	†	21UR-5211	TTAGGCATTCATCGGCTTTGG	3	0	0	0	12	18	12	45
	†	21UR-5212	TGATAGTGATAGAACATACAA	1	1	1	0	9	5	0	17
*		21UR-5213	TCCGTGAACTTTTTTGCTTGC	0	0	0	0	5	5	0	10
	†	21UR-5214	TCATTCCTAGCAGAATTTTCA	52	9	8	4	27	66	5	171
		21UR-5215	TGGTCTAATAAATTTTTGAAA	0	0	0	0	4	2	1	7
		21UR-5216	TAAAGTGAATTTCAATGCATT	4	1	0	0	5	5	2	17
		21UR-5217	TGTTGTCCTCCTGGCCTTTGA	0	0	0	0	0	1	2	3
		21UR-5218	TCAGATATTTGAATGCTATGA	0	0	0	1	6	4	0	11
	†	21UR-5219	TCATTAAATGCCTTTTTTCAA	0	0	0	0	0	0	0	0
*		21UR-5220	TGATCTGATTGAGAACGTGAA	14	5	4	1	12	46	0	82
		21UR-5221	TAGCATAAGGCGTTTTATTAT	0	1	0	0	2	5	2	10
	†	21UR-5222	TCAATTGAAAGTCTTCAGTCA	2	0	0	2	0	3	3	10
		21UR-5223	TACTTTCTCTTTTAACAAGCT	2	1	2	6	13	14	1	39
		21UR-5224	TGTGACTCTATAGTCGTTGTA	0	0	0	0	1	1	0	2
	†	21UR-5225	TCCAACTAACTTTTTACGAAT	0	0	0	0	2	0	0	2
*	†	21UR-5226	TCTGTGTTCGGTGGACTTTCA	237	64	45	37	250	736	257	1626
*	†	21UR-5227	TCTTTCGAGTACCATAGGACG	29	29	24	71	912	963	339	2367
	†	21UR-5228	TTCATTGGATGATGGAAGAAC	15	17	12	13	201	260	17	535
		21UR-5229	TATAATCATCTTCAATTTTCC	0	0	0	0	1	0	0	1
	†	21UR-5230	TAGGAGTTCAGTACTGTTTGA	2	3	1	0	26	26	12	70
*		21UR-5231	TATGTCGTTTTGAATCACATA	0	0	2	0	2	3	0	7
		21UR-5232	TAACACTATTGCATTATGGGG	0	0	0	0	1	0	1	2
		21UR-5233	TACAAAGGTTCCTAGAAGAAT	21	4	7	5	4	31	6	78
	†	21UR-5234	TGTTATCGAAAGATCTAAAGG	3	1	0	0	6	7	7	24
		21UR-5235	TGGACTACTGATTCTCCTCAC	1	0	0	0	7	0	1	9
*	†	21UR-5236	TGGTTTTCGGATTAAAGCAAT	17	9	5	3	22	65	5	126
	†	21UR-5237	TTGCGTGTATAGTACGACTTA	1	2	1	0	3	7	0	14
		21UR-5238	TAGTTTCCCCGTAATCGGAAT	1	0	0	0	0	2	1	4
	†	21UR-5239	TGTTTCCTACGAATTATTTAT	3	0	0	0	1	2	0	6
	†	21UR-5240	TATTCCGTGTTTGTATGTAAA	0	0	0	0	3	1	0	4
	†	21UR-5241	TCTCGTCCCTCACGGATAAGA	0	0	0	0	0	0	0	0
		21UR-5242	TCGGATCAACATCAATAGAAT	2	3	1	2	35	12	12	67

	†	21UR-5243	TGACATTCATAATACATTTCT	0	0	0	0	0	0	0	0
*	†	21UR-5244	TTTGATGTCCAAACGTTATTA	0	0	0	0	1	1	1	3
		21UR-5245	TATGTCGCAACAATGCTATCC	0	0	0	1	8	6	1	16
*	†	21UR-5246	TAGGTAACGTGTAGGCGATGC	7	3	4	1	7	44	1	67
	†	21UR-5247	TCAAACGTTTATATCTTAAAC	0	0	0	0	0	0	0	0
	†	21UR-5248	TCATACTTTAATCAAAATACC	4	2	3	4	42	30	2	87
		21UR-5249	TCAAATTATATGCTTATAAAC	1	0	0	1	0	0	0	2
		21UR-5250	TGACATCAAAAATTAGTTGTT	0	0	0	0	0	0	1	1
	†	21UR-5251	TTGCATCGTTCCACTCTTGGT	1	0	0	0	0	0	0	1
		21UR-5252	TAACACCATAATGTAATGAAA	0	0	0	0	1	0	1	2
	†	21UR-5253	TGATCAGGTCTATCAGGTCTA	0	0	0	0	0	0	0	0
		21UR-5254	TACGCTATCCAAGCTACAATC	0	0	0	0	0	0	0	0
*		21UR-5255	TCACAAGGTAGAAAGCTATCA	15	26	9	11	33	141	5	240
*		21UR-5256	TGACGCAAACTGTTTATTTCC	0	0	0	0	6	0	0	6
	†	21UR-5257	TGATTGAAGGACTCCTAGATT	0	0	0	0	0	0	0	0
		21UR-5258	TATACAACAAAAATTTGCTCA	0	0	0	2	8	12	3	25
		21UR-5259	TCCAATTGCTATATAATTTCC	0	0	0	0	0	2	1	3
	†	21UR-5260	TCGTTTCCAATAAATCGTTAA	2	2	0	2	2	6	0	14
		21UR-5261	TGTATAGATTCATTTATCAAT	0	0	0	0	0	0	0	0
	†	21UR-5262	TGTAAAAACGAGTTGATTATA	0	0	0	0	0	0	0	0
		21UR-5263	TGTGACTACCAATGTTTTATA	0	0	0	0	0	0	0	0
	†	21UR-5264	TTTAATGCAAATGGTTGTTGT	0	0	1	2	6	7	4	20
	†	21UR-5265	TTATCATCTTGTTCAACTTAA	0	0	0	0	2	4	0	6
		21UR-5266	TCCATCCATCTAAAAATAAAA	3	0	0	0	0	1	2	6
		21UR-5267	TCACTAAGTCAACCAACCCAA	0	0	0	0	2	2	4	8
		21UR-5268	TGCAACATCAGCACCTTATTA	0	0	0	0	0	1	0	1
	†	21UR-5269	TAATAGGATATCAGAATTCAT	0	0	0	1	11	6	0	18
		21UR-5270	TACAGTCGTTTTTCTTTCAAT	5	2	1	2	3	12	3	28
*		21UR-5271	TAACTTTGAACATGAAGTAAG	3	1	1	11	193	189	10	408
		21UR-5272	TACCAAGTACGCATTTTTCCA	45	6	11	7	65	114	23	271
*		21UR-5273	TTGACGCAATCCTTAAAATCA	0	0	1	1	0	6	0	8
	†	21UR-5274	TTGGAGAGCGAAATAGTCTCT	0	1	0	1	1	4	0	7
		21UR-5275	TGAGTCTCCACTACAACGAAC	0	0	0	0	3	3	2	8
		21UR-5276	TTCCATTGGTGTTTCTTTTCT	0	0	0	0	0	0	0	0
		21UR-5277	TACAAATATCCAATTGTTCCA	0	0	0	0	0	0	0	0
		21UR-5278	TCGTAACTTATTGTCGTCCTT	6	3	3	4	30	33	3	82
		21UR-5279	TACCTATCTGTACACTTTTCA	0	0	0	2	10	7	0	19
		21UR-5280	TGCAGTTGGTTCAAATGCAAT	0	0	0	0	0	1	0	1
		21UR-5281	TAACGAACGTTCTGTGAAAAT	0	0	0	0	4	2	3	9
	†	21UR-5282	TGCATACACGGTACTCAGAGG	4	2	2	3	74	73	36	194
		21UR-5283	TGGGATATTCAATTGCAATTT	0	1	0	0	0	2	0	3
		21UR-5284	TATCTTACAGCTATTCAGACA	0	1	0	0	9	4	1	15
		21UR-5285	TAGGTCCCTTCTTGGAAGTAA	1	0	0	0	0	0	0	1
	†	21UR-5286	TTCGTTTCGGTCCAACTCAAA	0	0	0	0	1	0	1	2
		21UR-5287	TGTTCATCAGCTGAACTTGAA	0	0	0	0	0	0	0	0
		21UR-5288	TACCATTCCGAATCTGTAAAA	3	1	0	0	3	3	0	10
		21UR-5289	TTGATAGATTATTTTGTCGCA	9	3	3	2	34	17	1	69
		21UR-5290	TAGATGTGCTAAATAGAATGA	2	3	1	0	51	44	6	107
		21UR-5291	TCGTAGTACGATCAATCACCA	1	1	0	0	6	6	0	14
	†	21UR-5292	TTCAGCGTGTTTTTGCATAAA	0	0	0	0	0	0	0	0
		21UR-5293	TAGTTGAAGCACAACTTTTCA	1	1	1	0	0	4	0	7
		21UR-5294	TAGGTTTTTTACTTATTTGTT	0	0	0	0	0	0	0	0
		21UR-5295	TGGAATAGCGTAAACAAAAGA	1	5	2	1	7	15	0	31
	†	21UR-5296	TGACTAACCACGATTCCTCAA	0	0	0	0	0	0	0	0
		21UR-5297	TGACATGAGTCGTTTGTTGAC	17	1	4	1	11	26	15	75
		21UR-5298	TATGATGAAACGTTTCAGTGC	3	1	0	1	0	0	0	5
		21UR-5299	TGTATACCAATTTTTGATCTA	0	0	0	0	0	1	0	1
		21UR-5300	TCTATTTCACATTTTTATGTT	0	0	0	0	1	0	0	1
	†	21UR-5301	TCTCCTTTCTTTCTAATTCCA	0	0	0	1	0	0	0	1
	†	21UR-5302	TTGAAGAAACATCTTCTGCCA	0	0	0	0	0	0	0	0
		21UR-5303	TGGAATGTATGGAACTGTCCG	0	1	1	0	2	2	0	6
	†	21UR-5304	TGATCTCTCAGGTGGAAGTTA	6	5	0	4	17	22	2	56
*	†	21UR-5305	TTGAGTAGAAATGCAAGGTTG	25	18	11	12	99	109	13	287
		21UR-5306	TCGCATCCGTGTGTCAACTTC	0	0	0	0	1	2	0	3

*	21UR-5307	TAAGTCTCTTTGTCATGCATA	14	5	4	8	14	45	3	93
	21UR-5308	TCCAAATTTGAAACTTATAGG	1	0	0	0	0	0	0	1
†	21UR-5309	TTTCAATCCTAACGAAAATGC	0	0	0	0	0	1	0	1
	21UR-5310	TAGCACATCCTTTTGTGTAGC	0	0	0	0	0	1	0	1
	21UR-5311	TCTGAGAGCGGTTCAAAAAAG	3	1	0	0	9	14	5	32
	21UR-5312	TATTGTATTTTGAGAACTTTT	0	0	1	1	2	2	0	6
	21UR-5313	TCATCCTCGAATCAAATGTTA	5	0	0	0	0	0	0	5
†	21UR-5314	TATCTCTTCGTAATAAAACAA	0	1	0	0	0	2	0	3
	21UR-5315	TTCATGACTCAATTTTTTTTC	0	0	0	0	0	0	0	0
†	21UR-5316	TGGATCGTCATTTGGTGGTAG	0	1	0	0	1	3	0	5
	21UR-5317	TCGAATCAATTTTTTTGTTCT	0	0	0	0	0	0	0	0
	21UR-5318	TATTTCTCCTTTAAAGCAGAT	0	0	0	0	0	1	0	1
	21UR-5319	TGGTGCAAAAAATGAAGAAAA	9	6	2	0	3	18	2	40
* †	21UR-5320	TCAGTTTTGAACAGCGGTAAC	37	67	48	26	161	694	3	1036
	21UR-5321	TTCCATCAACTTACTAATTCT	5	2	0	0	4	11	0	22
*	21UR-5322	TGCGGAAGAATACCGAGTAGT	1	5	1	1	63	82	19	172
	21UR-5323	TTTGCTGTTCTCTGAGTTATC	0	1	0	0	3	2	0	6
†	21UR-5324	TACAGAAGTAGAATTCGAACC	0	0	0	0	1	5	0	6
	21UR-5325	TGGGTTTATCAAAGTGCATAC	1	0	0	0	1	1	0	3
†	21UR-5326	TGTAAAACTGCAGTCAGAACG	0	0	0	0	22	17	8	47
	21UR-5327	TTCTTTGTCAACTTTCATGTA	0	0	0	0	1	1	1	3
	21UR-5328	TACAACTACTCCAAACAATAA	1	0	0	0	8	9	3	21
* †	21UR-5329	TACGGAGATTGAACAAAAAAT	32	37	16	17	110	270	9	491
	21UR-5330	TGTTTCTGTCTGCTTCTTTTC	0	0	0	0	0	0	0	0
†	21UR-5331	TGTTATGTCGATAGGCTTTTT	2	2	1	6	41	19	9	80
†	21UR-5332	TACACCGGAGCACTAAGGTAG	0	0	0	0	0	0	1	1
†	21UR-5333	TGTCAATGTTTACAGATAAGT	0	0	0	0	0	0	0	0
	21UR-5334	TCGGTGGACTTTCTTAAAATA	3	0	1	1	5	7	0	17
*	21UR-5335	TATTCGAATGTACATTTCATC	2	3	2	1	4	9	1	22
	21UR-5336	TGAGGTATCTTGATGTTTTGT	0	0	0	0	0	1	0	1
†	21UR-5337	TTTCATTAGGATGATGGTTTT	7	4	4	25	264	187	26	517
*	21UR-5338	TTTGATTGACAGAATTGGGAT	640	460	253	217	536	2692	127	4925
†	21UR-5339	TCCTACCAATTAAAACTTTAC	0	0	0	0	0	0	0	0
	21UR-5340	TCCAATTTTCAACAAGTTGCT	0	0	0	0	0	0	0	0
	21UR-5341	TGACAGTGAGTCCATTGCAAT	0	0	0	0	0	1	0	1
* †	21UR-5342	TGAATATGAGTATGACGGTAG	1413	919	386	405	3437	6636	476	13672
	21UR-5343	TGCACCAGTTAAAAGTAGATC	0	0	0	1	7	3	2	13
	21UR-5344	TGGGTTGTTTGCATTTGATTT	1	0	0	1	19	13	0	34
†	21UR-5345	TGTCCTTTCCATAGCATTACT	24	1	0	1	3	4	8	41
	21UR-5346	TGCATCGTGGAATCGATTCAA	1	0	0	0	1	3	0	5
†	21UR-5347	TCTTTCAGTTGACTGAAATGT	1	0	0	1	1	5	1	9
†	21UR-5348	TTAATTACGGATACTGTTCAA	10	5	1	8	17	66	23	130
†	21UR-5349	TGGTATCCGACAAAAAATTGT	0	0	0	0	0	3	1	4
*	21UR-5350	TTTGCAATTGGAGCAGTGGAA	78	57	22	19	145	459	62	842
	21UR-5351	TCACTATGAGTAGTTTAACCA	2	1	0	1	2	1	0	7
	21UR-5352	TGACGTTGCCTAAAATTCACA	0	0	0	0	0	0	0	0
	21UR-5353	TGAATTCGCGAATGCAAAATA	0	0	0	0	6	3	0	9
	21UR-5354	TGTCACGTTTTGTTGTATTTA	0	0	0	0	0	0	0	0
†	21UR-5355	TGGTGTACGCCTATATCCCAC	4	0	0	0	0	3	2	9
†	21UR-5356	TGTTGCCAGAAGATATATAGT	0	0	0	0	0	0	0	0
*	21UR-5357	TAGAATTGGAAGACGAGAAAA	228	281	138	96	263	1312	55	2373
†	21UR-5358	TAGTGTCACATGGTGTTTAGT	15	2	1	1	3	12	1	35
	21UR-5359	TTTGATGATGATACACCTAAA	0	0	0	1	8	5	2	16
	21UR-5360	TACTCCAGTCTTCTACTTCAA	0	0	0	0	0	0	1	1
	21UR-5361	TGCGCTTCAGTTTTATGTATT	0	0	0	0	0	1	0	1
	21UR-5362	TGGTTTAGTCGGTTTAGTTTT	11	5	3	2	4	19	0	44
	21UR-5363	TTCGTTGGTTTATTACTTTGA	9	0	2	2	27	24	1	65
	21UR-5364	TCAGCATTGTCAAAATATATT	7	3	0	1	6	7	4	28
†	21UR-5365	TGATATTGATTGTGAGTTCAA	0	0	0	0	1	2	2	5
†	21UR-5366	TACGATCTTCCTTCAATGTGT	1	1	0	0	2	8	0	12
†	21UR-5367	TATTTCCATTATTCCCCTGTT	0	0	0	0	0	0	0	0
	21UR-5368	TGTATCTCGCTTCGACCTTGA	0	0	0	0	0	1	0	1
	21UR-5369	TCGCTCTCTGCACTCAACAAA	0	0	0	0	7	5	1	13
	21UR-5370	TCGTAAATGTGCCAAGCAGTC	0	0	0	1	8	12	0	21

		21UR-5371	TACATTGAGTGAATGATTTGT	0	0	0	0	3	0	0	3
	†	21UR-5372	TTCTAATCCAAAATTCTAATC	0	0	0	0	2	1	0	3
		21UR-5373	TCGGTACTGTCAGAGACTTGG	0	0	0	0	0	0	0	0
		21UR-5374	TCGAATTTTGGTTCTCGCTGA	1	1	0	1	0	5	1	9
		21UR-5375	TTGGCCGTCCATACTCACGTA	0	0	0	0	1	2	4	7
	†	21UR-5376	TAATACTGCGATTTGAGTTGC	2	0	0	2	10	10	3	27
	†	21UR-5377	TAGATGTTGCTTTGTACATGC	0	0	0	0	0	1	0	1
	†	21UR-5378	TACGTTGTTAATCTAATCATG	3	0	0	1	10	7	1	22
		21UR-5379	TAGTAAGTGGTGAAAACTCAA	0	0	0	1	16	9	1	27
	†	21UR-5380	TAGTGCATTACCTCATATTCC	1	0	0	0	0	0	0	1
	†	21UR-5381	TAATACAGTTCTGTGAAAGTA	0	2	0	0	0	3	0	5
		21UR-5382	TGCCAGTTACACAGTTACACA	0	0	0	0	0	0	1	1
	†	21UR-5383	TTCCATATTCACTAAAAATGC	0	0	0	1	2	0	8	11
	†	21UR-5384	TTTATAACGATTTTGGACGAT	96	26	29	30	50	231	32	494
*	†	21UR-5385	TCTATGTACAACGGTTCTCTC	2	40	3	32	388	311	37	813
	†	21UR-5386	TGTGGAAAAAAACATTTAAAC	0	0	0	0	0	0	0	0
		21UR-5387	TCTGTACAACTTTCGACTCAA	8	4	3	5	89	80	41	230
	†	21UR-5388	TAAACTGATAGTTGGTAAAAA	1	0	1	1	16	13	13	45
*	†	21UR-5389	TACAGTGTTGAAAATGAAGGT	3	3	3	5	95	85	18	212
		21UR-5390	TGCGTTTTTTTATGCAGTTGG	8	0	0	0	0	3	0	11
	†	21UR-5391	TCGCGATCTTCTTTATGGTAT	5	1	2	2	31	34	3	78
		21UR-5392	TATAGAAAAAATAAAAGATTT	0	0	0	1	9	4	0	14
		21UR-5393	TAGAATATCGAAATTTTCAAA	0	0	0	0	0	0	0	0
*	†	21UR-5394	TTGTTGCACAGAATCAAAAAA	0	1	1	2	6	3	1	14
		21UR-5395	TTGAATGTTGATATTTTGAAT	2	0	0	1	2	15	0	20
	†	21UR-5396	TGATAAATTTATATGATTGTG	0	0	0	0	2	0	0	2
		21UR-5397	TGCTGTCGCTTGTAATAAACG	0	0	0	0	0	3	0	3
	†	21UR-5398	TATTGAGAGAACGCAATTGAT	0	0	0	1	5	6	1	13
		21UR-5399	TAGCGTTTTCGGTGAAGTACT	0	3	0	2	29	29	4	67
		21UR-5400	TATACTCACTTCTTTATAGGT	15	5	0	2	4	9	3	38
		21UR-5401	TCGTGTTACAATTGACAAACA	0	1	0	0	2	2	0	5
	†	21UR-5402	TCGATAGGGTGGCAAATGATC	3	0	0	1	14	16	12	46
	†	21UR-5403	TTGATCAACAGATTTACCAAA	1	0	0	0	0	0	0	1
	†	21UR-5404	TATTGGAATGTAAATCGCGCC	0	1	0	2	22	27	10	62
		21UR-5405	TCCAGTTCGCCTACATGTCCA	1	0	0	0	2	1	5	9
		21UR-5406	TGAAAATTTTCAGTTGGTAAC	0	0	0	0	0	0	0	0
		21UR-5407	TCCTATCCAAATCACAGCTCT	0	0	0	0	0	0	0	0
		21UR-5408	TCAGCAATGCATAAGTCAAAG	14	1	1	0	2	12	2	32
*	†	21UR-5409	TTAGTGCATTACCTCATATTC	15	3	3	1	6	8	2	38
*	†	21UR-5410	TCTTTTTGGAGTGTGGTCATC	374	120	119	86	178	1043	129	2049
		21UR-5411	TAGTAATGCTCACGTTGATCA	1	0	0	0	2	2	0	5
	†	21UR-5412	TGCGTGAGTAAGGTTTTTTTG	0	0	2	1	5	11	1	20
*	†	21UR-5413	TAAGACATCGGAACACAAATC	1	5	1	18	257	201	11	494
	†	21UR-5414	TGCTTATTCAAGGTTTCCAAC	0	0	0	0	1	0	0	1
		21UR-5415	TCGTAAATTTTCACATGTAAA	0	0	0	0	0	0	0	0
*	†	21UR-5416	TATTGCTAGATTTTGGGTCCC	15	3	6	6	66	104	45	245
	†	21UR-5417	TAGTGATTTGCACGGATTTTC	29	13	9	19	203	225	99	597
		21UR-5418	TCTATTAATAACTATTTGTGC	0	0	0	0	0	0	0	0
	†	21UR-5419	TGCTTTTGTGCCATGAATAAA	0	0	0	1	2	0	0	3
		21UR-5420	TGCTTTTTTAGAATTTTTCAG	0	0	0	0	2	2	0	4
	†	21UR-5421	TGTATCGAATAAGCAATCTTT	0	0	0	0	0	0	0	0
		21UR-5422	TACACCGTAATAAATATTTTC	0	0	0	0	2	0	2	4
		21UR-5423	TCTCACTGAATGTTTTGGGAA	0	0	0	0	0	4	0	4
	†	21UR-5424	TGAGGAATACAAAACACGATT	0	0	0	1	11	8	1	21
	†	21UR-5425	TGCTTTGGTGGAACTTAAAAC	0	2	0	0	1	9	1	13
*	†	21UR-5426	TTGGTTGAGATCTCACGCGGA	13	10	12	7	213	253	159	667
	†	21UR-5427	TTTGGAGACATCTGGTAGGTG	0	0	0	0	1	1	1	3
		21UR-5428	TATATCTGGTTTCTCGATTTT	2	0	0	2	3	6	0	13
		21UR-5429	TCAAGAGCACGATCATAATTT	0	0	0	0	2	2	2	6
		21UR-5430	TGAGTTTGTGGAAGTATATTC	0	4	0	5	119	55	15	198
		21UR-5431	TTCGCCAGTTTCTATAATTTA	1	0	0	1	1	0	2	5
*		21UR-5432	TTCTTCTTCCTGATCGTTTGA	1049	132	136	151	329	1136	230	3163
		21UR-5433	TACCACGAATTCCTAGAGAAC	0	0	0	0	19	7	6	32
	†	21UR-5434	TCGTCAACAAAGTTTTTCTTC	0	0	0	0	0	0	0	0

*	†	21UR-5435	TTTAGTGCATACAATGGAGAA	0	0	1	1	21	25	7	55
	†	21UR-5436	TCATCAATGTTTTCGACGCAA	11	0	1	0	3	17	7	39
*		21UR-5437	TGAATAATTCGGTGTTGTAGA	116	45	40	46	202	440	76	965
	†	21UR-5438	TCTCGCTCTTGAACAAAACGC	6	2	2	1	6	14	0	31
		21UR-5439	TTACATTTTCTGGAAAATTTG	0	0	0	0	5	7	0	12
		21UR-5440	TGGAAGCAAGTAAGTTGTCTA	0	1	0	0	0	1	0	2
		21UR-5441	TCCCGCTTTCTTTCTACACAC	0	0	0	0	2	0	0	2
	†	21UR-5442	TAAGTTTCTCTATATTTTTGC	1	0	0	1	1	0	0	3
	†	21UR-5443	TAGTGAGTTGTTAAAACAGTA	1	0	1	0	4	6	0	12
	†	21UR-5444	TGAAGTTGTGTGCAGTTTGCA	9	1	0	1	0	4	4	19
	†	21UR-5445	TGATAGTACAGTGCGTGCAAC	0	0	0	0	1	1	1	3
		21UR-5446	TATATACAAGAACCCAAACTA	2	2	1	0	26	11	2	44
	†	21UR-5447	TGATTTTCAAAAAGTTGTTTA	0	0	0	0	0	0	0	0
		21UR-5448	TGGGTTGCCTTTTAATTTACA	0	0	0	0	1	0	0	1
	†	21UR-5449	TAATAAGCTAGCAAAAATCAT	24	4	1	1	3	20	2	55
*	†	21UR-5450	TGTGATAGGGAAATAAAGACG	126	110	60	46	319	560	104	1325
		21UR-5451	TGCTTCTAGCAATTCCACAAT	1	0	0	0	0	1	0	2
	†	21UR-5452	TCTATTCCTCAGAATTTAAAA	0	0	0	0	0	0	0	0
		21UR-5453	TGGGTGAAGCTTTTGAATTAA	1	1	1	1	0	3	0	7
		21UR-5454	TATACATAGCAAGTGTTTGGC	19	1	1	0	3	11	0	35
		21UR-5455	TCCAAAAAGAAGAGAAAACTT	3	1	0	1	6	11	1	23
		21UR-5456	TCGAATGAGAATTTAAAAAAA	0	0	0	0	0	5	0	5
	†	21UR-5457	TCTACGATACGTAAAAAACTG	6	1	1	1	4	14	0	27
		21UR-5458	TGGGCGAATATTTTTTTTAAT	6	0	1	1	2	10	0	20
		21UR-5459	TCAGATTGAAATCTAAAATCT	1	0	0	0	1	1	0	3
		21UR-5460	CACACAAACAATTCAAACCAA	0	0	0	0	0	2	0	2
		21UR-5461	GGAAAATTAAAACGCAAAAAA	0	0	0	0	0	0	0	0
*	†	21UR-5462	TTATTTTTCAGAGAGTCAGGA	119	15	11	10	9	84	59	307
		21UR-5463	TGTAATAATAGTTTTAAAAAA	0	0	0	0	0	0	0	0
*		21UR-5464	TTTTGAACAATCGGAAACACA	32	57	10	23	83	214	44	463
		21UR-5465	TCAGCTGATTTTTAAAATTTG	0	0	0	1	0	1	0	2
		21UR-5466	TTCGACGTGTTTTTTAATACA	1	1	0	0	4	1	1	8
		21UR-5467	TCATTTGTTTTTTTAATATGA	0	0	0	0	0	0	0	0
	†	21UR-5468	TCAGATAATTTTTACGAATTA	0	0	0	0	0	0	0	0
		21UR-5469	TCCCATTGATTTTTTTTTGAA	0	0	0	0	0	1	0	1
		21UR-5470	CGAAAAAAAAAATTACTAATTT	1	1	0	0	0	1	0	3
		21UR-5471	TCTCAAGATTGATTTATATTT	0	0	0	0	0	0	0	0
		21UR-5472	TGAGATAAATTTTTGAAAAAA	0	0	1	0	0	0	0	1
		21UR-5473	TGAATGATGAATTTTAATCAA	0	0	0	0	1	1	0	2
		21UR-5474	TTCAGCAAATATTTTATCAAA	0	0	0	0	0	0	0	0
		21UR-5475	TCCATCAGTCAATTGAAAACA	0	0	0	0	0	0	0	0
	†	21UR-5476	TCTCCTATTTTTTTTAACACC	0	0	0	0	0	1	0	1
	†	21UR-5477	TTCTAAGGGCTTTGAAAAATC	0	0	0	0	4	5	0	9
		21UR-5478	TGGCAATTTTATTTTATAAAA	0	0	0	0	0	0	0	0
		21UR-5479	TGTTATTGAAATTAAAAAAAA	0	0	0	0	0	0	0	0
		21UR-5480	TGATAGAATCATTCTATTTCA	0	0	0	0	0	0	0	0
		21UR-5481	TCATAACACTAATTTAAGATT	0	0	0	0	0	1	1	2
		21UR-5482	TCTGATGACGCATTTATAATA	2	0	0	0	6	17	2	27
	†	21UR-5483	TGGAGGCCTGGTTGTTTGTGC	6	6	1	2	1	16	2	34
	†	21UR-5484	TCAATAAGGACATTGAATAAA	2	3	3	2	23	9	5	47
		21UR-5485	TGGGAAAAATTCTAGAAATTT	0	0	0	0	0	1	0	1
*	†	21UR-5486	TATTGGATTTAGATCTGGAGA	10	2	3	6	18	66	5	110
	†	21UR-5487	TCACATCGAAATGAAAAAAAA	0	0	0	0	0	0	0	0
*	†	21UR-5488	TTGGTGCCTATTGCTAGAATC	3	4	0	1	9	23	1	41
	†	21UR-5489	TTTTAACGTTAGTAGATGGTT	69	74	41	20	124	324	19	671
		21UR-5490	TCAGCAATTCCAAAAAATTCA	0	0	0	0	0	0	0	0
		21UR-5491	TTTTGATGATGAGGATGTATT	52	13	5	6	24	89	21	210
		21UR-5492	TTGTGTTAAAAAAAGTAGAAT	61	65	32	41	141	198	11	549
		21UR-5493	TTTGCCGACTGCGTAAAGCTT	3	0	2	1	5	14	2	27
		21UR-5494	TTCCAAAAGAATTTCATTGTT	5	4	2	0	29	17	9	66
		21UR-5495	TGCGTTGATTATTTGCAAAAC	1	1	0	1	2	5	0	10
		21UR-5496	TCATATCACATTTATGACAAA	0	0	0	0	0	1	0	1
	†	21UR-5497	TAATCCATATGATCTCAAACT	2	2	1	3	5	14	1	28
		21UR-5498	TTCGTAAAATTTTTATATTGA	0	0	1	0	0	1	0	2

		21UR-5499	TGGATACCTAAACTTAGGAAA	0	0	0	0	2	3	0	5
	†	21UR-5500	TCTGTCAAGCATTTGTTTGAA	0	0	0	0	0	0	0	0
		21UR-5501	TCATTCAATCTTTTAATGATT	0	0	0	0	0	0	0	0
	†	21UR-5502	TATCAAAAATAATGATATTGA	0	0	0	0	0	0	0	0
*	†	21UR-5503	TTTCAATTGTAGACTGAGGGC	252	122	61	97	1591	2315	466	4904
	†	21UR-5504	TCTTGAACGGCTAGGATCACT	13	37	7	5	37	218	17	334
		21UR-5505	TGATTATAGTGTACAATAATA	0	0	1	2	1	1	0	5
		21UR-5506	CAAAAAAAACAGTTTCAACAA	0	0	0	0	0	0	0	0
	†	21UR-5507	TGCTCGGCAAGTTAGTAAAAA	2	0	1	2	50	35	12	102
		21UR-5508	TTGCGAATTGATTTTTGCAAG	0	0	0	0	0	0	0	0
		21UR-5509	TGTTTTCCAAATTTTCGAAAA	0	0	0	0	0	0	0	0
		21UR-5510	GGAATATATATTTATTTGATT	0	0	0	0	0	0	0	0
	†	21UR-5511	TTAGATTGTTGAAATAGTACT	1	0	1	0	0	2	2	6
		21UR-5512	TCAAAGAAACAGTTACTCTAA	1	2	1	0	1	5	0	10
	†	21UR-5513	TATTTTAGACGAGGATGGTGA	61	21	16	7	22	148	21	296
		21UR-5514	TCAGTGATCAATTTAAACAAA	0	0	0	0	0	0	0	0
		21UR-5515	TTTCCACTCCAGAAGAAAAGA	30	2	2	1	3	38	9	85
		21UR-5516	TCCTGGGATCATATAAAAAAA	4	1	1	0	4	3	2	15
		21UR-5517	TGTGAATACAATATATAAAAA	0	0	0	0	0	0	0	0
	†	21UR-5518	TCTGTAAAAACACTAGTTTTT	0	0	0	0	0	0	0	0
	†	21UR-5519	TGATCATATATGATCAAAAAA	0	0	0	0	0	0	0	0
		21UR-5520	TCTGATGATTTTTCTATATTG	0	0	0	0	0	0	0	0
	†	21UR-5521	TGGTCCGTTGTATTATTCAGA	3	0	0	0	7	13	1	24
*	†	21UR-5522	TTGGTGCCTACTACTGGAATC	1	0	0	2	7	12	1	23
	†	21UR-5523	TTGTATTGGCGGAGATGAACT	1	0	0	0	5	8	2	16
	†	21UR-5524	TTCTATCGACGTGTAATATTT	2	2	1	0	12	15	3	35
*	†	21UR-5525	TTTTGAAAAGCTGACAGGGGG	40	41	13	18	106	355	43	616
		21UR-5526	TGACTGGATTTTTTTGAGTTT	1	0	0	0	0	1	0	2
*	†	21UR-5527	TCGAAGACTCAAAAGTGTAGA	32	27	13	13	53	208	5	351
		21UR-5528	TGTTCGAATTTTCGAATTGGT	0	0	0	0	0	0	0	0
	†	21UR-5529	TGATGATAAAAAACACCAAAA	2	0	0	0	2	3	6	13
		21UR-5530	TCGGAGATGACCTTTTAACCA	0	0	0	0	3	6	0	9
	†	21UR-5531	TTCGAAAAAAAACTAAAACAC	0	0	0	0	1	1	0	2
	†	21UR-5532	TCAGTGACAAAAAAACATCTA	0	3	1	2	17	6	2	31
		21UR-5533	TGGGAAAAATGGTTGACATTT	0	0	0	0	0	0	0	0
		21UR-5534	TCAGCAATGTTTTTTTTTCAA	0	0	0	0	1	0	0	1
	†	21UR-5535	TTTTCTTATAACGCTGTTGCT	2	0	0	0	0	3	0	5
		21UR-5536	TCGTTTGAACAAAAAAAAGGT	0	0	0	0	1	0	0	1
	†	21UR-5537	TTGGTAAGGATTATCAAATAA	0	0	0	0	1	1	0	2
	†	21UR-5538	TGACTGAATTTTTTTGATAGT	0	0	0	0	2	1	0	3
	†	21UR-5539	TTTCGTTTCGGTCCAACTCAA	1	3	0	0	3	1	1	9
		21UR-5540	TTGATGTGATTTAGTAATCCT	0	0	0	0	3	2	0	5
*		21UR-5541	TGCTCGTCTAATTTGGTAAGA	0	2	1	0	4	10	3	20
		21UR-5542	TAACTAAAAAATAAAAAAAAA	0	0	0	0	0	1	0	1
*	†	21UR-5543	TGAATTCGGCTGATTATTATC	74	88	53	45	147	455	52	914
	†	21UR-5544	TGATTCCGGGAATTCAAATTT	0	0	0	0	0	1	0	1
		21UR-5545	TATGTTTTTTATAAAAACCTT	1	0	0	0	0	1	0	2
	†	21UR-5546	TATTTCGGAAGTTATTGAATT	0	2	1	1	3	9	2	18
		21UR-5547	TGACAAATTCTATTACAAAAT	0	0	0	0	0	0	0	0
*	†	21UR-5548	TTTCGGATCGAAAGTAATACT	9	10	8	12	254	184	188	665
	†	21UR-5549	TAGACGTGTTGAAAAAATGCT	3	1	0	1	10	18	0	33
		21UR-5550	TGGTTCGGCATTTTTTTTAAT	124	52	38	33	71	248	25	591
	†	21UR-5551	TTGGCCGAATATTTTATATTT	0	0	0	0	4	1	4	9
*	†	21UR-5552	TGGAATTCACATGTGGAATTT	1	2	4	0	13	28	1	49
		21UR-5553	TGAAATGAAATATTTACCTTT	0	0	0	0	0	0	0	0
	†	21UR-5554	TTCAAAAAACATGTAGTGATT	3	3	2	1	33	25	0	67
		21UR-5555	TCAGGAAAGCATTGCAATAAA	0	0	1	0	10	10	5	26
	†	21UR-5556	TAATTTTAAATCATCGTGAAG	26	8	6	0	16	34	13	103
	†	21UR-5557	TTGGCGTGGTCTTTTTAATTT	0	0	0	0	4	7	2	13
	†	21UR-5558	TTGTACATCTCTATGAAGGGT	1	0	0	0	0	2	0	3
		21UR-5559	TAGAAAGATAAGTATTAAAGA	1	1	0	0	0	3	1	6
	†	21UR-5560	TTCTGTCGCGCTTTTGACTTT	2	0	0	0	0	1	0	3
		21UR-5561	TGTTCATATTTTGATCAAACA	0	0	0	0	0	0	0	0
		21UR-5562	TGGAAAAAATAATGTACAGTTC	0	0	0	0	0	1	0	1

	21UR-5563	TGAATTTGAAATACAAAAATT	0	0	0	0	0	0	0	0
*	21UR-5564	TCAGAGATCAAACTACATAGG	1	0	1	0	1	2	6	11
	21UR-5565	TAGACGTACTGAAGAAAAACT	0	1	0	3	50	60	5	119
	21UR-5566	TCAGTCCTTTTTTTTTGATAA	0	0	0	0	0	0	0	0
†	21UR-5567	TTCTATATCGAATGACGTATT	12	6	3	6	17	21	7	72
†	21UR-5568	TTTCGTTCTGTTTCTTGTGAA	6	2	1	5	9	37	1	61
†	21UR-5569	TTTTGTGCCATTAAGAGAAGC	11	1	1	0	2	11	6	32
	21UR-5570	TGTGCAATAGCTCAAAAAAGA	0	0	0	0	0	0	0	0
	21UR-5571	TCTCAAGAAACTCAATTAAAA	0	0	0	0	11	3	0	14
	21UR-5572	TCTCAAAGCAAGTTATTTTTT	0	0	0	0	1	0	0	1
	21UR-5573	TGGATTTGGAATTTGTAGACT	0	0	0	0	0	0	0	0
* †	21UR-5574	TTTGATGTTACTGGAATATGG	110	117	62	77	486	660	54	1566
	21UR-5575	TATCATATGAAATTAAAGTTT	1	0	0	1	1	2	0	5
†	21UR-5576	TTCACAAAAAAGAGATTTGAA	0	0	0	2	5	6	1	14
	21UR-5577	TATCCATTCCGTGATTAAAGC	0	0	0	0	0	0	0	0
†	21UR-5578	TTCGTATGATGCTTTGAATAC	1	0	0	0	0	4	0	5
†	21UR-5579	TTCTTCGTTTGGGTGAAAAAA	0	0	0	1	23	45	21	90
†	21UR-5580	TGTTTGATGTTTCGTAAAACT	0	0	0	0	0	0	0	0
	21UR-5581	TTACAAAATTGAAATAAAATT	2	1	0	1	5	6	0	15
	21UR-5582	TCGATTTTATTTATCGATTCC	0	0	0	0	0	0	0	0
	21UR-5583	TATGGAAATTTGTGTTGCATT	8	7	4	6	48	24	5	102
	21UR-5584	TGAACATAATTTTTGTTTCAC	0	0	0	0	0	0	0	0
†	21UR-5585	TATTTCAACGGCTGTACAAAA	2	1	0	1	10	19	3	36
†	21UR-5586	TAAAACTTTGATTGGAAAAAA	0	0	2	0	2	7	1	12
	21UR-5587	TCACTATTTATTTTTCAGAAA	0	0	0	0	0	0	0	0
*	21UR-5588	TTTGATTCCATTAGGTCATCA	0	0	0	0	3	3	3	9
	21UR-5589	TGAAAAAAAGATGATTAAAAA	0	0	0	0	0	0	0	0
	21UR-5590	TTTCTTCGAGTTTCTAGATTC	0	0	0	0	0	0	0	0
	21UR-5591	TCTTCCTTCGACCTCGAAAGT	0	0	0	0	0	0	0	0
†	21UR-5592	TTCATTGAATTGGATACACAT	1	1	0	1	5	7	1	16
†	21UR-5593	TAGCAAGTTGAAGTACGAGCG	13	8	7	4	62	179	11	284
	21UR-5594	TCTTGCTTGGACATGGAAATT	0	1	1	0	0	6	3	11
†	21UR-5595	TTCATTCTGAGTCGAAAAAAA	8	0	2	1	7	19	3	40
	21UR-5596	TCTGTACGGATTTTTTCGAAT	0	0	0	3	9	5	0	17
	21UR-5597	TGTGATTGTGATTTCCGAATT	0	0	0	0	0	0	0	0
	21UR-5598	TTTGATATTATTTTTCACACA	1	0	0	0	0	0	1	2
* †	21UR-5599	TTGATGTTGCACCAGGTGGAA	0	2	0	3	21	42	14	82
	21UR-5600	TGGTAAATCATATTCAAAAAA	0	0	0	0	1	3	0	4
†	21UR-5601	TAAGTTCTTCTTCGGGGTAAA	0	1	0	0	9	8	22	40
†	21UR-5602	TTTTGACACGCTATAAAAATG	1	0	0	0	1	2	3	7
	21UR-5603	TTCGATTGTTTGTATAATTCC	4	0	0	2	2	9	1	18
†	21UR-5604	TTTGGATTTGTTGTTAACATT	2	0	0	1	11	10	5	29
†	21UR-5605	TTCTCGGTATGACTTTTTTCT	2	6	2	2	12	28	0	52
	21UR-5606	TGGATTGAATGCTTCAGAATT	0	1	0	0	3	4	1	9
* †	21UR-5607	TCTCACTCAAGTTCAGACGGT	1	0	0	4	33	63	8	109
	21UR-5608	TAACTAGGGACAGGGCAGTTT	12	6	2	6	21	58	122	227
†	21UR-5609	TCAATTGAAAAAAACAGGAAT	0	0	0	0	0	0	0	0
*	21UR-5610	TTAAAGAGAAGAACGATTAAA	2	24	12	39	405	317	27	826
	21UR-5611	TGCATGAAGTTTTTGATTGCG	0	0	0	0	1	0	0	1
	21UR-5612	TCTATCGTTTTTTTAATCTAA	0	0	0	0	0	0	0	0
	21UR-5613	TGCATTGTATTGTGTAAACTG	0	0	0	1	31	19	0	51
	21UR-5614	TTTAAAGAAAAGGTGTAGAGT	12	5	1	3	7	42	2	72
†	21UR-5615	TGCTAAATTGAGAAATTTATA	1	0	0	0	0	1	0	2
	21UR-5616	TAACAAAATTAATAACAAAAT	0	0	0	0	0	1	0	1
	21UR-5617	TCTCAAGGAGAATTCAATTTC	0	1	1	1	39	34	0	76
	21UR-5618	TCAGCTATGAATTAAAAATGA	0	0	0	0	2	1	0	3
†	21UR-5619	TTCGTACCTCACACTGTCAAG	0	0	0	0	5	2	0	7
†	21UR-5620	TTGATCTCTCAGGTGGAAGTT	10	12	6	8	42	101	4	183
	21UR-5621	TGAATTGTTCCTTCGTAAAAA	0	0	0	0	1	1	1	3
	21UR-5622	TACGTGTGTCGAGGGTTGTGA	2	1	1	0	1	3	4	12
†	21UR-5623	TGGAGTTGTTTCAGTCAGGTC	0	0	0	0	0	1	0	1
	21UR-5624	TTCGAAACAACTCAATTGCTT	0	1	0	0	35	22	1	59
†	21UR-5625	TCTGTGTTTTTTTAATCAAAA	0	0	0	0	0	0	0	0
†	21UR-5626	TGTTTCAAATAAACAAAAAAG	0	0	0	0	0	0	0	0

		21UR-5627	TTTTCCCTGCACACGTGAAGA	3	0	1	1	10	19	6	40
	†	21UR-5628	TTATGATAGATCCTCAAATTA	23	16	12	20	116	115	77	379
	†	21UR-5629	TCACTTAAACATTATCGAGTT	0	0	0	0	0	0	0	0
		21UR-5630	TTCATCCCGGATAAGAACATC	1	0	0	0	1	9	0	11
		21UR-5631	TCAAATGTTAATTTTACAAGA	0	0	0	0	0	0	0	0
	†	21UR-5632	TTTTGATTTTGGGCTGATAAC	2	0	1	1	2	15	1	22
		21UR-5633	TATTTGATGAGTTGTTAAAAC	5	0	0	0	0	0	0	5
	†	21UR-5634	TTCGACTGCAGTTTACTCGCT	0	0	0	0	24	32	1	57
		21UR-5635	TGGATATTCCGTGTAGGTTTT	0	0	0	0	0	1	0	1
	†	21UR-5636	TCGGTATAAGATTTCGGTGCT	7	6	5	4	16	54	3	95
		21UR-5637	TTTGAATGATATGACAAATTT	0	0	0	0	0	0	0	0
*	†	21UR-5638	TTCTCTTCAAGTAGATATCGG	3	1	1	8	88	109	42	252
		21UR-5639	TCATCTCGAAAAAGATTGAGA	0	0	0	0	0	0	0	0
		21UR-5640	TACAGATCAGCTGATGAACAT	3	3	0	11	196	180	8	401
		21UR-5641	TCGAAATATAAAGAAAATTCA	0	0	0	0	3	1	4	8
		21UR-5642	TTCGAATAGCCCCTTTACAAA	0	0	0	0	0	0	0	0
		21UR-5643	TTTTTCCGTTACGATATCAAT	20	0	2	0	5	11	2	40
		21UR-5644	TGCTTCGATTGATTGCTCACT	0	0	0	0	0	2	0	2
	†	21UR-5645	TTCCAATAATTTTTGTTCGAA	0	0	0	0	2	0	0	2
	†	21UR-5646	TCCACTGAGAAATCCATTCAC	0	1	0	0	1	0	0	2
		21UR-5647	TCCAAAAATAATTTTCAAAAA	0	0	0	0	0	0	0	0
		21UR-5648	TATCGTTGTAAACTAAAAAGC	4	0	0	1	4	2	0	11
*	†	21UR-5649	TACGGATTCAGTCAACAGTTT	11	14	5	3	61	94	41	229
	†	21UR-5650	TGAGAAATATAGGAACTTTTT	0	0	0	0	0	1	0	1
	†	21UR-5651	TGTCACTATTCTTTCGTTGAC	1	0	0	0	0	2	2	5
		21UR-5652	TCTAATTAAGAATCAATTAGA	0	0	0	0	0	0	0	0
	†	21UR-5653	TATAAGAACTCATAAAAATAG	4	1	2	0	5	16	0	28
	†	21UR-5654	TCTTCATCCTGAACCAAAAAT	0	0	0	1	3	1	1	6
		21UR-5655	TCTTCTCGTGAATGTCGCTCT	0	0	0	1	1	2	1	5
	†	21UR-5656	TTGAAGATCACGATGTTCAAA	0	0	1	0	28	31	0	60
	†	21UR-5657	TTCTGTTCTCTCCTAGAAACT	0	0	0	0	1	1	0	2
		21UR-5658	TTCTGCTAATGCCAATTGGAA	0	0	0	0	0	0	0	0
		21UR-5659	TTCGTTGAAAGCTGAACACTT	0	0	0	0	1	0	1	2
	†	21UR-5660	TTGTTTGGTCATGATAAAAAT	4	0	3	1	4	9	2	23
	†	21UR-5661	TAATTAAATTTACTTTATGAC	0	0	0	0	1	1	0	2
		21UR-5662	TATCCTTTCTTAGAATCCAAG	3	0	0	0	2	3	0	8
		21UR-5663	TTGTACGGATTGACTTCGAAA	0	0	0	1	14	12	5	32
		21UR-5664	TATCATGACCAGCTAACCATT	0	0	0	0	0	0	0	0
*		21UR-5665	TTTGTACGGTGCATTATGTAT	13	15	8	6	39	69	3	153
		21UR-5666	TCCGAGATCAATTGACTGATT	0	0	0	0	0	0	0	0
	†	21UR-5667	TACAGTTCTGTGAAAGTAGAC	4	2	1	3	7	15	1	33
	†	21UR-5668	TCTATTGAGCGGTCTCAAGTA	1	0	0	0	11	4	5	21
		21UR-5669	TAACAACGGTTTTCAAAATTC	14	7	3	4	10	15	1	54
	†	21UR-5670	TTTTTGTGGCTTATCTAAATC	8	2	4	3	7	25	2	51
	†	21UR-5671	TTTCTTCGAGGCAAAGCAAGA	0	0	0	0	4	6	0	10
	†	21UR-5672	TATTTCTCGTACTCAAAAGTC	0	0	0	1	1	1	0	3
		21UR-5673	TCATTTGCATATTATAGCAAA	0	0	0	0	0	0	0	0
		21UR-5674	TGAAATCAGAAAATGTTGAAT	2	1	4	7	162	81	24	281
*	†	21UR-5675	TTGAGTAGTTAAAATGAGCAT	628	625	287	355	1744	2941	157	6737
		21UR-5676	TGAGAGCACATTTTAGAAAAA	0	0	0	0	0	1	0	1
		21UR-5677	TCTGGCTAATGAAAAAAATGT	0	0	0	0	1	0	0	1
		21UR-5678	TCTTTTCAATTTTTTATTTGA	0	0	0	0	0	0	0	0
		21UR-5679	TTTCTTGTCGAAAATGCTAGT	6	0	1	0	1	8	0	16
		21UR-5680	TCCCGAAAAGAAAAAATTCTA	0	0	0	0	0	0	0	0
*	†	21UR-5681	TTTGCTTGGAATCGTCACTTT	6	3	1	6	78	79	110	283
	†	21UR-5682	TTTAACTGAGATGAAAAGGTT	4	0	1	1	9	28	1	44
		21UR-5683	TGGAATAAGATAAATTATCCG	0	0	0	0	0	0	0	0
	†	21UR-5684	TACATAAGAAATAAAAAAAAA	0	0	0	0	3	2	0	5
	†	21UR-5685	TCTGCCTAAAAAAAATCATCT	0	0	0	0	0	0	0	0
		21UR-5686	TTCGAAATTCAATGTAAAAAG	0	0	0	0	0	0	0	0
		21UR-5687	TGTCAATAAAAGAAATTCAGA	0	0	0	0	0	0	0	0
		21UR-5688	TCACTAATTTTTTCGATAACT	0	0	0	0	0	1	0	1
	†	21UR-5689	TCGACGAATATTTTTACTTGA	0	0	0	0	4	1	0	5
		21UR-5690	TATGATTGATAAATATAACAC	0	0	0	0	6	4	0	10

		21UR-5691	TTTGCAACGAACTTATAGTTC	0	0	0	0	0	0	0	0
	†	21UR-5692	TTTTTTTTAGTTGGCGAAGGA	12	3	2	5	48	94	171	335
		21UR-5693	TTTTTTTAAGAAGTTGGTGTT	0	1	0	13	51	70	54	189
	†	21UR-5694	TTGATAGACTAGAACAAATCA	0	0	0	0	3	2	0	5
		21UR-5695	TTTGTTGTATTTTTTCGGAGT	21	6	2	4	9	21	10	73
	†	21UR-5696	TCCTACGATGGTGAATGTGAT	0	2	0	0	7	12	4	25
		21UR-5697	TCACTTATCTATCTCGAGATA	1	0	0	0	0	1	1	3
		21UR-5698	TCACATTTTCTATCTATATAA	1	0	0	0	0	0	0	1
	†	21UR-5699	TTTCAACGGCTGTACAAAAAG	3	1	2	1	2	13	3	25
		21UR-5700	TGAGTATATATTGATTGTCCT	32	9	8	6	9	33	18	115
		21UR-5701	TATTTGAAGTAAAATAATACA	1	1	1	0	2	4	2	11
		21UR-5702	TATTATGACGTTAAAAAAAAC	6	1	1	1	3	9	4	25
		21UR-5703	TACTCGACGAAAAAATTGGTT	3	5	0	0	5	17	1	31
	†	21UR-5704	TTTAGAGCTACTCAAAAAAAG	0	0	0	0	2	2	0	4
		21UR-5705	TGAATCAGATTTTCAAAAAAA	0	0	0	0	0	1	0	1
		21UR-5706	TCTTACTGAATTAGAAAACGG	2	5	3	0	8	32	2	52
	†	21UR-5707	TTCTGACATTTCTTCTGAAAG	0	0	0	0	0	0	0	0
	†	21UR-5708	TTTTAACAGCGGAGAGTTTGT	2	0	0	0	0	3	0	5
	†	21UR-5709	TTCTTGAGTGTTAATATCATG	4	0	0	0	2	4	0	10
		21UR-5710	TTCATAGCGTTTTTTTCACAA	2	0	0	0	0	1	0	3
	†	21UR-5711	TTCCATGGAAATATATGCTGA	1	1	0	1	7	6	2	18
		21UR-5712	TCCTTGAAAGGAAAGGTAAAA	0	0	0	0	0	0	0	0
	†	21UR-5713	TCATCATTGAATTGTCTTTCT	1	0	2	0	4	2	1	10
		21UR-5714	TTATGATTTGGAATTTTTGGC	26	13	10	22	168	213	26	478
	†	21UR-5715	TCTCATTAGGATATCATGTTC	1	0	0	1	9	4	2	17
		21UR-5716	TCTCAACATCAAAACTTAATC	0	0	0	0	0	0	0	0
		21UR-5717	TATCAATGGATGTTGCCCACT	1	0	0	0	6	5	2	14
*		21UR-5718	TTCAGAAGAAGAAATTTGGAA	1	5	2	8	168	146	19	349
	†	21UR-5719	TACAAAATCGATTGAAGTTCT	1	3	1	0	5	10	0	20
	†	21UR-5720	TGCTATATTCCTAGTGAAAAA	1	0	0	0	0	1	0	2
	†	21UR-5721	TGAGAAATCCATTCACAGTCT	0	0	0	0	0	1	0	1
		21UR-5722	TTAAAGTCACAAGAGAACAAC	7	13	3	2	4	28	0	57
	†	21UR-5723	TCTCAAGATAACTTATGAATT	0	0	0	0	2	0	0	2
	†	21UR-5724	TATTTTGATACGAGATGGAAA	3	3	1	0	8	11	0	26
	†	21UR-5725	TTACAAAGCTGATTTCGAGTT	20	2	0	1	1	23	0	47
		21UR-5726	TCACACCTTAATAAATAATAA	0	1	0	0	0	1	1	3
	†	21UR-5727	TTTTGGTACCATATAAGCAGA	8	4	2	2	1	8	3	28
*		21UR-5728	TTTGGGCTGATTTGTCGGAAA	28	7	3	1	9	68	11	127
		21UR-5729	TTTAGATCCGAACTCCCCCCT	1	0	0	0	0	0	0	1
	†	21UR-5730	TTGAGTGAACTTATCAGCAGG	31	37	20	14	262	390	25	779
		21UR-5731	GAAACAGCAATATTTGGACAA	1	5	0	0	1	2	0	9
*	†	21UR-5732	TTCTAATCGGTCTCAATCAAT	5	8	1	3	40	58	19	134
	†	21UR-5733	TTCATCTCTGGGCCAAACTTT	0	0	0	0	4	3	0	7
		21UR-5734	TGGAAGTCTGGTTAAAAAAAA	0	1	0	1	22	23	2	49
	†	21UR-5735	TAGGAAAAAAATTTAAAAAAA	0	0	0	0	0	1	1	2
		21UR-5736	TCGTTTAACAATTTTTTTAAA	0	0	0	0	0	0	0	0
	†	21UR-5737	TTATGCGTCCCTAAAAACTGA	0	0	0	0	1	3	0	4
	†	21UR-5738	TTTACGGCTAACTTTTGTCAT	25	32	10	20	104	232	8	431
		21UR-5739	TTTACATGTTGAATTGCGGGT	3	5	2	12	186	264	47	519
	†	21UR-5740	TTAACAGGATTAACAGGATTC	2	0	1	2	14	28	10	57
		21UR-5741	TCTTATTTTCATGTCAGAAAC	2	1	1	1	0	1	0	6
		21UR-5742	TCTTTTGTGATTTGAAAACCC	1	0	0	0	0	7	0	8
*	†	21UR-5743	TGCTCGAGAATACAACGAAAA	2	1	2	0	8	17	3	33
		21UR-5744	TCGATACCAAATTATCAGTTC	0	0	0	0	0	0	0	0
		21UR-5745	TTTCGGATTCGGTCTTGTTGG	0	1	0	1	7	4	2	15
		21UR-5746	TTGTCATATATTTTATGTACA	0	0	0	0	0	0	0	0
	†	21UR-5747	TCCGAAAACCAATAGAATTTA	0	0	0	0	0	0	0	0
	†	21UR-5748	TGTTGTTTTTAAGTAACAGAA	1	0	0	0	1	5	0	7
		21UR-5749	TGCGAAAATATAGTGTGGCAA	2	0	0	0	2	5	4	13
		21UR-5750	TTCGTCTTTTATTTTTTTAAA	1	1	0	0	0	1	0	3
	†	21UR-5751	TTCATACAAAATGAACAGAAG	0	0	0	0	1	4	0	5
	†	21UR-5752	TCCTGAATCAGCACTTTTCTT	0	0	0	0	4	3	2	9
	†	21UR-5753	CGAAACATTATGATCACTGAT	1	1	0	0	3	1	1	7
		21UR-5754	TGCGTTAAACGTTCTATCAAA	0	0	0	0	0	0	0	0

		21UR-5755	TATTTTCTTTATCATCCGCGC	10	0	1	0	0	8	11	30
		21UR-5756	TTGGGCCCAAAAATTGTCTTT	0	0	0	0	8	2	1	11
	†	21UR-5757	TCTCACAAAAAAGATTTTCTA	1	0	0	0	1	2	0	4
*	†	21UR-5758	TTCGTACGTTGGAAGCTAAAT	38	14	14	24	329	336	271	1026
		21UR-5759	TGACTAATAGTAAGCAAAATT	0	1	1	0	1	0	1	4
*		21UR-5760	TGACCAAAACTCTCGGCATTT	17	31	7	11	62	204	5	337
	†	21UR-5761	TCGATATGATATCGAAGAGCT	0	0	0	0	0	0	0	0
		21UR-5762	TCCATTGTTTGACTATAAAAA	0	0	1	2	6	4	3	16
	†	21UR-5763	TTGTCCGAGTAGATGTTCTAA	2	4	0	3	10	13	1	33
	†	21UR-5764	TAATTCATTTGAGTTTCGAGC	0	0	0	0	0	0	0	0
		21UR-5765	TTGTTCTGATAAAAAAGCGAA	6	1	2	4	28	33	10	84
		21UR-5766	TTGTTCCATCCGGTTTAAACA	9	0	0	1	0	3	2	15
	†	21UR-5767	TTCTACACTCTGCTCAATTCC	0	0	0	0	0	1	0	1
		21UR-5768	TTAGTTGTGATTTTTTTGTCA	0	2	0	0	2	3	1	8
	†	21UR-5769	TGGTTCTTCCAAATGAAAAAA	0	0	0	0	0	0	0	0
	†	21UR-5770	TATTCTCTCGCCGTGTGCTTC	0	0	0	1	6	3	4	14
	†	21UR-5771	TAGACTTACAAAAAACTCATA	0	1	2	2	9	6	0	20
		21UR-5772	CACAAAGTAAACTAGTAATAA	0	0	0	0	0	0	0	0
		21UR-5773	TGTTCTTTTCTTCAACCAAAA	0	0	0	0	0	0	0	0
	†	21UR-5774	GGTTCTTGCATGTATGAATAA	0	0	0	0	0	0	0	0
	†	21UR-5775	TTCTACGATCGTATTGAGGTT	59	13	13	10	29	157	32	313
		21UR-5776	TGAAATTTTATTATTTAAAAA	0	0	0	1	0	0	0	1
		21UR-5777	TTATTTCATCCATAAATCAGA	4	0	0	0	4	1	0	9
		21UR-5778	TTCGATATGAGAACAATAATT	2	3	0	2	4	13	0	24
	†	21UR-5779	TGTGATCATCTGTCAACACTC	0	0	0	0	2	0	0	2
		21UR-5780	TGTCTGTCTGAAAAAAACAAT	0	0	1	1	6	4	0	12
		21UR-5781	TGCTCGTGTCTCCTCAAAAAT	0	0	0	0	0	0	0	0
		21UR-5782	TATGATATCGCCCTGTATTGG	0	0	0	0	2	0	3	5
		21UR-5783	TCCATTTATTGATAATGGGAA	0	0	0	0	0	0	0	0
	†	21UR-5784	TTGTTTGTTCCTACAACAAAA	0	1	0	0	1	2	0	4
	†	21UR-5785	TTGAAGACCATTTTGATAGAA	1	0	0	0	32	36	1	70
	†	21UR-5786	TCTTTCTTTGGCCTTGGGAAG	0	0	0	0	0	2	0	2
	†	21UR-5787	TTGGATCCAATTGTTGAATTT	2	0	1	2	13	16	1	35
	†	21UR-5788	TCTTGCCGGTATTGATGTTGA	2	4	2	6	56	88	87	245
		21UR-5789	TACAGAAGAGATTTTATAATT	0	0	0	2	14	21	0	37
	†	21UR-5790	TAATTCAGTCAGGAGAAAAAC	0	0	0	0	25	25	5	55
	†	21UR-5791	TTCCTCTATATCTCTGACTTT	0	1	1	0	1	1	2	6
		21UR-5792	TTCCAACAGTTTAATCGTAAC	0	0	0	0	0	3	4	7
		21UR-5793	TGATTTCAAAGGTATAAGGCA	0	1	0	0	20	16	1	38
	†	21UR-5794	TTTAAAACGAAGTTTAGGAGA	3	3	3	1	9	14	5	38
	†	21UR-5795	TGAGCAATCGATTCGGGTTAT	13	8	6	11	232	289	438	997
	†	21UR-5796	TTCTTTTCCAAATCCAATAGA	0	0	0	0	0	0	0	0
		21UR-5797	TTATCGAATATTGAAAAATTC	0	0	0	0	0	0	0	0
		21UR-5798	TCGATTAATGCCTCTACAATT	0	0	0	0	0	0	0	0
	†	21UR-5799	TATTTACTACGAGGATGAAAA	3	0	2	0	5	6	6	22
*		21UR-5800	TCTCAAATTGATCTGTTGAGG	0	0	0	0	13	5	5	23
		21UR-5801	TTTTCAAGGTACGTAACTTAA	0	1	2	0	3	11	0	17
		21UR-5802	TTGAAAAAAAGGAGAAAACTC	5	2	1	2	17	10	4	41
		21UR-5803	TGTATGATCAATAAAAATAGA	0	0	0	0	0	0	0	0
	†	21UR-5804	TTGTGAATGTTTTTTACGAAA	0	0	0	0	0	1	0	1
		21UR-5805	TTTTTCACAGATTAAACCAAA	3	1	1	0	0	3	0	8
		21UR-5806	TCTAAATTTAAAAACAAAGGT	0	0	0	0	0	1	0	1
		21UR-5807	TTGGTTGTCAGAGCTATTGCT	1	0	0	0	0	2	0	3
	†	21UR-5808	TTCGCCCAAACTTCTAAAAAC	0	0	0	0	0	3	0	3
		21UR-5809	TGCTCCTTTGGCTTATACAAA	1	0	0	0	0	1	0	2
*	†	21UR-5810	TTTTGCACGGTTTCGTATTAA	51	10	4	8	38	47	13	171
		21UR-5811	TGGATAAAGATTTAGTTTTTC	0	0	0	0	1	0	0	1
		21UR-5812	TCAGAAATTTTTTGGGCTTTT	0	0	0	2	9	4	1	16
	†	21UR-5813	TTCTTCAGCATATGGATCAAA	3	0	0	0	2	3	0	8
	†	21UR-5814	TGGATAAAATAAAAAAATAAA	0	0	0	0	0	0	0	0
		21UR-5815	TGAGATCGCTCAACATGAAGA	7	14	9	23	431	309	55	848
		21UR-5816	TCTTACAAGATTATGTATGCA	0	0	0	1	4	4	1	10
		21UR-5817	TATGGTTTTTTTTTAATAAGT	2	0	0	0	0	0	0	2
	†	21UR-5818	TTCCTAGGATCGTTGCACAAA	55	30	10	43	455	598	242	1433

	†	21UR-5819	TGGTCCTGCTGTAGAATAAAA	5	3	0	4	39	59	15	125
	†	21UR-5820	TGGATGATTGAATTGCAATTT	0	0	0	0	2	2	0	4
	†	21UR-5821	TTTTCGCGAATTGGTTTCTAA	1	1	0	0	3	6	0	11
	†	21UR-5822	TTGAAGTAGGATTTTGAAATA	0	0	0	0	9	4	0	13
		21UR-5823	TTCCATGCTATCTGACTTGAG	0	1	0	0	3	2	2	8
		21UR-5824	TGTAAGAAACATTGTATAAAA	0	0	0	1	5	0	1	7
		21UR-5825	TGCATTTTTTGCTCCAAAAAA	0	0	0	0	0	0	0	0
	†	21UR-5826	TTTGTTTGGTCATTAGAATAT	3	2	0	0	2	7	0	14
	†	21UR-5827	TTTCGTGATTTTGTCGTTTAC	59	12	13	14	83	105	45	331
		21UR-5828	TCTTTTCAAAACTTAACGTTT	0	0	0	0	0	0	0	0
	†	21UR-5829	TCAGAAATTTTTTCGTATGAT	0	0	0	0	0	1	0	1
		21UR-5830	TCAACAGAATTATAAGTTTTT	0	0	0	0	0	0	0	0
	†	21UR-5831	TATTCTAGCCATGTCGAACAT	2	1	1	0	3	7	0	14
	†	21UR-5832	TACACGATTGATCTCACTTTA	6	12	3	6	22	41	4	94
		21UR-5833	TTTGGCCTAGAAATTTAAGAT	0	0	0	0	0	0	0	0
		21UR-5834	TTCTCGTGAATTCTTTTTCAA	0	0	0	0	0	2	0	2
	†	21UR-5835	TCTATAGATGACATTCAAAAA	0	0	0	0	10	5	1	16
		21UR-5836	TCAGATTGTTTCTCGGGTTAC	0	0	3	4	68	62	11	148
		21UR-5837	TTGAAAGAGGAGAGTAATGAT	3	1	0	1	19	29	7	60
		21UR-5838	TTCGATTGGTTTTTTTGTGTA	0	0	0	0	2	2	0	4
		21UR-5839	TTAGAAACTTTCAAAACTCAA	0	0	0	0	1	0	0	1
		21UR-5840	TGTGTATTTGTTTTTCAATGT	0	0	0	0	0	0	0	0
		21UR-5841	TCTTTAAACTTTAATTTTCAA	0	0	0	0	0	0	0	0
		21UR-5842	CGAGATGTCGTAAAAACTGTA	3	11	3	0	13	24	5	59
	†	21UR-5843	TTTAGGCTGAATTGTAATATT	0	0	0	0	0	2	0	2
		21UR-5844	TGATAAATCAGTTTTAAATAG	0	0	0	0	0	0	0	0
		21UR-5845	TATGAGATTTTAATAAAATCA	0	1	1	2	0	1	0	5
		21UR-5846	TAGAACTTCTCCCGTGGTTTT	0	0	0	0	0	0	0	0
	†	21UR-5847	TTTATTGGGTATTGGAATTGA	17	6	7	6	13	47	15	111
		21UR-5848	TCTTTGAAGATATTAAGATAT	0	0	0	0	1	0	0	1
		21UR-5849	TTTGAAGAACTAAAAAAAGAA	1	4	1	7	28	20	3	64
	†	21UR-5850	TGATTATTTCTCCGAAATTCT	0	0	1	0	0	2	0	3
	†	21UR-5851	TCCACGCTGGGTTAAATAAAA	0	0	0	0	1	0	1	2
	†	21UR-5852	TTTAATCGAAATGTCCACTGT	0	1	0	1	10	7	6	25
		21UR-5853	TCGGAAAAAGTATGGGCTTCT	0	0	0	0	0	0	0	0
	†	21UR-5854	TTTTTGGCCCTACGAAATTCA	0	1	0	2	4	9	0	16
	†	21UR-5855	TTTCAATCGAGATAAATAGGC	0	3	1	6	69	50	11	140
		21UR-5856	TGATCCTTTCGGTATAGATAA	13	8	8	9	51	57	11	157
		21UR-5857	TCTTTTCCGTGATTTAAAGAA	0	0	0	0	1	2	0	3
*	†	21UR-5858	TACTAGAGAAGTAGAAGTCAT	23	35	17	105	1618	1286	357	3441
		21UR-5859	TGCACTCCAGCCAGACTTGGT	0	0	0	0	1	2	2	5
		21UR-5860	TCCTTCCTAGATTTTGAAATT	0	0	0	0	6	3	0	9
		21UR-5861	TAGAAAATATTGATTGAATCG	18	2	1	3	4	11	5	44
		21UR-5862	TAATTCATCTAATCGAGATTT	1	0	0	0	0	6	0	7
		21UR-5863	AGATTATGCTAATGTGAAAGA	0	0	0	0	0	0	0	0
		21UR-5864	TTGCAAAAAAAAAAATTCCGA	1	0	0	0	1	2	0	4
		21UR-5865	TTCGCATCTTCCACCGGGTCT	0	0	0	0	0	0	0	0
		21UR-5866	TGGGTTACGAGTTTTAAAATT	0	0	0	0	0	1	0	1
		21UR-5867	TCCTGGGATTTCAATTTCAAA	0	0	0	0	1	0	0	1
		21UR-5868	TAGATTTGAATGATATGTTAG	0	0	0	0	3	5	0	8
		21UR-5869	TTCCTGTGTTATATATAAAAA	1	0	0	0	1	0	0	2
	†	21UR-5870	TTCATGTGAGTACCAGAAAAT	1	1	0	1	1	3	2	9
		21UR-5871	TGTGAAACTATAGAAACACAG	0	0	1	2	0	4	0	7
	†	21UR-5872	TAATAGCAGAGATAGAGTAAA	0	0	0	1	6	7	6	20
	†	21UR-5873	TTCTCATATTCTAATGACCAA	0	0	0	0	0	0	0	0
	†	21UR-5874	TTCGGAAAAAGTTGGGTAGTG	2	1	0	0	1	5	2	11
	†	21UR-5875	TTCACATTGTCAGAGAAAACT	2	0	1	6	57	49	2	117
	†	21UR-5876	TTCGTTTGTCGTCAGCGAAAG	1	0	0	3	13	33	13	63
	†	21UR-5877	TCGGATAGGGAAACAGCAAAA	3	2	4	8	148	135	87	387
*		21UR-5878	TAGGATATAGAATGAATCATT	0	0	0	1	20	7	1	29
	†	21UR-5879	CGATAATAAATTTGAAGTGAC	0	0	0	0	0	0	0	0
		21UR-5880	TTTTATCGAAGGAAACCCATT	5	1	1	4	55	41	98	205
	†	21UR-5881	TTCAAACCAAATTGATACTTC	0	0	0	0	0	1	0	1
	†	21UR-5882	TGATAGTGCGTACGGAAATTA	1	0	0	2	16	8	19	46

*		21UR-5883	TGACTCGATAGATTGCTCACT	1	1	0	1	11	20	5	39
		21UR-5884	TGAATTTTTTTCACAATCATT	0	0	0	0	0	0	0	0
	†	21UR-5885	TCGAAATCGAAATTGCCCATT	0	0	0	0	0	0	0	0
*		21UR-5886	TAGGGATATTGCTAGGAAGAC	9	28	9	26	544	672	76	1364
		21UR-5887	TTCCGATTCCTATTTCAAAAA	4	0	0	0	2	4	1	11
	†	21UR-5888	TAGAAGATTTTGGATGGAAAC	9	6	6	9	21	55	8	114
	†	21UR-5889	TGCAAAGGCGAAATTTTTCTA	55	4	5	3	21	31	19	138
	†	21UR-5890	TTTAAAGAGGTTTTGAACAAT	1	0	0	2	3	10	0	16
	†	21UR-5891	TTGCTGGATAAAATAAAAAAA	0	0	1	1	0	7	0	9
	†	21UR-5892	TTCCTGATTGTTTGAGTAAGG	2	0	0	3	14	14	14	47
		21UR-5893	TCCATTCAATATGGCAAAAAA	0	0	0	0	0	0	0	0
		21UR-5894	TCAACTCGTTGAATTTTAAGA	1	0	0	2	7	12	5	27
		21UR-5895	TCAAAATGAAGACACAATTTT	10	2	3	1	9	24	0	49
		21UR-5896	TATATGCGCATCAAGGGTTGC	0	0	0	0	1	1	1	3
*		21UR-5897	TAGGAAAGTATGTAGGAAAGT	7	9	7	13	328	282	9	655
		21UR-5898	TCAGTAGGTGGCATTAACAAT	1	2	1	1	2	11	0	18
	†	21UR-5899	TTCTCAATGTAGTAGTAAAAA	0	0	0	0	1	0	0	1
		21UR-5900	TTAAAAAATATTGTCTATAAA	1	0	1	0	2	1	1	6
	†	21UR-5901	TGTTACGATAAAATGAAGACA	6	6	4	5	21	37	2	81
		21UR-5902	TGCTGAAGCAAATCAAGAAAT	0	0	0	0	3	22	0	25
		21UR-5903	TGCACATCCTATTTTCAAAAA	0	0	0	0	1	1	1	3
		21UR-5904	TCCACAATCTGTAGCCTTTTT	0	2	0	0	0	0	0	2
		21UR-5905	TATGCATATTTTTAATGTTTC	0	0	0	2	3	4	0	9
	†	21UR-5906	TCTTCCTTTGTAACCGCCAAC	0	0	0	0	0	1	1	2
	†	21UR-5907	TTTGTCCTCAATCACCGTGTT	0	0	0	0	0	0	0	0
		21UR-5908	TTCTCCCATTACATTGTACTA	0	0	0	0	1	3	5	9
	†	21UR-5909	TTCCATGAGAGGAAATTAGAA	0	1	0	0	4	2	1	8
		21UR-5910	TGTAATTTTAATTTTCTTAAA	0	0	0	0	0	0	0	0
		21UR-5911	TCGAAGATCTTTAGATTGTCC	0	0	0	0	4	2	0	6
	†	21UR-5912	TTCTCTTCGACAGACCTGATA	0	0	0	0	0	0	0	0
*	†	21UR-5913	TGCATGGGCGGATTTCAGTTT	4	1	0	0	22	29	9	65
		21UR-5914	TCGAATACGAATTTCACATAA	0	0	0	0	0	0	1	1
		21UR-5915	TAGATATTTTGTAAACAAATT	0	0	0	0	0	1	0	1
		21UR-5916	GGACACTTTAAGATTTCTAAA	0	0	0	0	0	0	0	0
	†	21UR-5917	TTGTTCCATTCCTGGTTGCAA	0	0	0	0	1	0	0	1
		21UR-5918	TTGGATAATTTTACTTAAAAA	0	0	0	0	14	5	2	21
		21UR-5919	TTCAAGCAGAAAAAAATATCT	3	0	1	3	8	8	2	25
		21UR-5920	TGGAAGGTTTTTAATACACTC	1	0	0	0	0	0	0	1
*		21UR-5921	TGCACACACGAGGTACGGCAT	19	1	3	6	35	50	12	126
	†	21UR-5922	TTTTCTCTCAACAGTTGCTGA	1	0	0	2	13	18	2	36
	†	21UR-5923	TTCTTCTGTCATCATGCAACT	0	1	0	2	1	3	0	7
	†	21UR-5924	TTATGAAAGGAGACAAAAAAA	0	0	0	0	4	3	2	9
		21UR-5925	TGGTCTTTGAGAAATCCCAAA	0	0	0	0	1	1	0	2
		21UR-5926	TGCCCAGATCCATCAGAGTCC	0	0	0	0	0	2	0	2
	†	21UR-5927	TGATACTCGTCGAATCGCATA	363	145	127	82	339	852	145	2053
		21UR-5928	TTCTTGCACTACCAAGGAAGT	1	1	0	0	0	1	0	3
*		21UR-5929	TTAGAGAGAGTTTCGGAAATT	6	0	4	4	24	34	41	113
		21UR-5930	TGGTTGGTTTCTCTTCGCTTT	0	0	0	0	0	1	0	1
		21UR-5931	TCGTTCTCCTAAATGCAAAAA	0	0	0	0	1	0	0	1
		21UR-5932	TCACAGAACTTTCGTCCTCAT	0	0	0	0	11	5	1	17
	†	21UR-5933	TCAATTATTAACTGAAAATTC	0	0	0	0	0	0	0	0
*	†	21UR-5934	TACTTGGACAGATGAAGTTTT	6	9	3	6	13	49	2	88
	†	21UR-5935	TTCTACTTTACTCTGATGTAA	7	1	1	1	5	13	3	31
	†	21UR-5936	TTCATCTGTGATCCCCTCAAT	0	0	0	0	5	0	3	8
*	†	21UR-5937	TTAAACAGTGGAATTAGGAAT	7	6	4	19	392	265	9	702
		21UR-5938	TCCTAATCTAAATTCAAGCCA	0	0	0	0	0	0	0	0
		21UR-5939	TAGACTAGGCCCTTTTAGAGA	0	0	0	0	1	5	6	12
		21UR-5940	TTTGAAAGAGTTGTTTATGCA	0	2	0	1	6	6	2	17
*	†	21UR-5941	TTCGGGGCACGAACGGTTAAT	1	2	0	1	64	117	73	258
		21UR-5942	TGATCATGTATCCCGAGGTAT	0	0	0	0	2	1	4	7
		21UR-5943	TTCTTCTTTGTTCGCATCAAA	0	0	0	0	0	0	1	1
		21UR-5944	TGTTGATAAAGCATCCTTCAA	3	1	0	1	13	9	9	36
		21UR-5945	TCATAAACAACTGATCATAAA	0	0	0	0	1	1	0	2
*	†	21UR-5946	TATCACAATGCAGCTAGAATT	37	16	6	9	87	128	21	304

	21UR-5947	TAAACTTCAATCAAAAAAAAC	0	0	0	0	0	1	0	1
	21UR-5948	TTTGCGGCGCAGTGTCTTATT	0	0	0	0	3	2	7	12
	21UR-5949	TTGTTATATTATTTTGGAAAT	0	0	0	3	7	2	1	13
*	21UR-5950	TTCTCCAGTTGACTCTATTTC	2	14	5	26	345	165	11	568
	21UR-5951	TTACTTTGCTATTAGAATAAA	5	5	1	0	2	7	0	20
†	21UR-5952	TGTTTGATTTAAACGTTCGAG	0	0	0	0	0	1	0	1
*	21UR-5953	TCTACGGAGCAAGAGGGAAAA	3	1	1	3	60	80	29	177
†	21UR-5954	TCGGAAATTTGAAAAAATCAG	2	1	1	0	7	5	1	17
†	21UR-5955	TCCTTTAGGAGCGTAACAAAA	0	0	0	0	6	2	3	11
	21UR-5956	TCAGATGAATACATATGTATC	0	0	0	0	1	0	1	2
†	21UR-5957	TTTGACAGCGTTACGATAAAA	0	0	0	0	6	5	1	12
	21UR-5958	TTTCAGAAATAGTTGTATGTT	1	0	0	0	2	0	0	3
†	21UR-5959	TTGATAGGGATTTCCTCGTTC	4	0	1	0	1	9	0	15
†	21UR-5960	TTATTTCAACACTAAGAGCCA	1	0	2	1	3	3	0	10
*	21UR-5961	TACTTTGATTATTCCGGCTTC	0	0	0	3	19	20	6	48
†	21UR-5962	TTTCGTTTCTGTCCAAGGATT	2	0	0	0	5	2	0	9
†	21UR-5963	TTGGAGCATGATCAAATAGAA	0	0	0	0	2	4	1	7
	21UR-5964	TTGATCCTTCTCATTCGGAAA	0	0	0	1	8	1	5	15
†	21UR-5965	TGTTATGATTTAGTAGTAACA	0	1	0	0	0	0	0	1
	21UR-5966	TAAAATTGTCCCAAGAGAAAC	0	0	0	0	1	1	0	2
	21UR-5967	TCTTTCGGTCGATAGGTTGAC	0	0	0	1	9	17	12	39
†	21UR-5968	TCGATTCGAACGATTGAAAAA	0	0	0	0	3	7	0	10
	21UR-5969	TATGTCAACAAGATGACAGAA	0	0	0	0	0	0	0	0
	21UR-5970	TTTCCAAAATGTGCCATGGAT	1	0	1	0	0	1	1	4
	21UR-5971	TTGCATAACTAATTGCATAAC	0	0	0	0	0	0	0	0
	21UR-5972	TTGAATTCTCGAATTCAAAAA	0	0	0	0	0	0	0	0
†	21UR-5973	TTAGTTCTCTCTCCGTTGTTA	0	0	0	0	1	0	0	1
†	21UR-5974	TCTTCTGAGCGGGTTAGATCT	0	0	0	1	14	16	22	53
*	21UR-5975	TCTGAGTAGCAATTTTTGAAA	1	12	6	26	382	319	35	781
†	21UR-5976	CAAATTCAATGGGTTAACAAA	0	0	0	0	0	0	0	0
†	21UR-5977	TTAGCATCTTCCACCGGGTCT	0	0	0	0	1	2	2	5
†	21UR-5978	TATGACGGTTTCATTTGGATT	24	26	27	51	672	584	134	1518
	21UR-5979	TTTGAAAACTCCTCGACTATC	0	0	0	0	0	0	0	0
	21UR-5980	TTTGACGTTTCTGAGTAAAAG	0	0	0	0	6	2	0	8
	21UR-5981	TTCTGCGATCCAACTTATTTA	0	0	0	0	0	1	0	1
	21UR-5982	TTCTCGTGGTATATTCAAACC	2	0	0	0	3	4	0	9
	21UR-5983	TTCCTGGTCCGACTTAAACCT	0	0	0	0	0	2	1	3
	21UR-5984	TTCACACCCAATCTTTCATCA	2	0	1	0	0	0	7	10
†	21UR-5985	TTATAGTACTCTTAATGTAGT	0	0	0	0	0	0	0	0
†	21UR-5986	TGCTATGAACTTAGGTACAAC	0	0	0	0	5	2	0	7
†	21UR-5987	TGGGAATCCGATTTGATCGAA	0	0	0	0	0	2	0	2
	21UR-5988	TCTAAGAAGTTTTTTCAAATA	0	0	0	0	0	0	0	0
†	21UR-5989	TAATGCTTCTCTAACTGTAAT	0	0	0	0	3	2	0	5
†	21UR-5990	TTTCAATAAACTCTTTAGAAC	1	2	0	0	3	8	0	14
	21UR-5991	TGTGTTAATCATAATACAATC	0	1	0	0	0	0	0	1
	21UR-5992	TGGAGACGACATTGAGAGTTG	1	0	0	0	13	18	0	32
†	21UR-5993	TGATCGATTTTTTTGCTTTTT	0	0	0	0	0	0	0	0
†	21UR-5994	TCCATATGATCTCAAACTGAT	0	1	0	0	0	2	1	4
	21UR-5995	TGTTCAGATATTAACTGATAA	0	0	0	0	0	0	0	0
	21UR-5996	TGGGTCATTATACTGCTGCAA	1	0	2	0	1	5	1	10
	21UR-5997	TCACTCGCAGCTTTTAGAACA	0	1	0	1	29	19	5	55
	21UR-5998	TTTTGCTACATCCGCGTTAGT	0	0	0	0	0	0	1	1
	21UR-5999	TTCCAAATTCACGAAAGGATG	0	0	0	0	1	0	0	1
	21UR-6000	TGAGAAATAATAATAGAACTT	0	0	0	0	0	1	0	1
†	21UR-6001	TCTTTTGGGGTTTGATCGAAG	8	2	0	0	9	13	7	39
	21UR-6002	TAGGGACAATATAAATTTTTG	0	1	0	0	7	1	2	11
	21UR-6003	CGAAAATAAAATGTGCAAACT	0	0	0	0	0	0	0	0
	21UR-6004	TTGGATTGCTTCTTTCAACGT	0	0	0	0	0	2	0	2
	21UR-6005	TTCACGAATGAGAAAATTTGA	0	1	1	1	8	10	0	21
	21UR-6006	TAGAGAAACAATCGTAATTTC	0	0	0	0	0	1	0	1
	21UR-6007	TAATAAGTTTCTGGACAACTC	8	3	5	0	8	18	2	44
*	21UR-6008	TTTGTCAAGATAGAAGATAGT	4	7	3	20	249	232	36	551
	21UR-6009	TCTTGTTGGATTTCTTCAATT	0	0	0	0	0	0	0	0
	21UR-6010	TACGCATCTTGTCTATTCTGA	0	0	0	3	32	38	1	74

	†	21UR-6011	TTTTAAATGAAAAATTGGAGA	2	1	0	0	0	3	1	7
		21UR-6012	TTCATCGTGGAACCGTTCCCA	0	0	0	2	3	5	1	11
		21UR-6013	TGAGATTGGAAAAAAAAATTC	0	1	1	0	1	2	0	5
		21UR-6014	TCTCGATCAATCTCGTATACC	0	0	0	0	0	0	0	0
		21UR-6015	TCCGATTCTCCCTGTTACTTT	0	0	0	0	0	0	0	0
	†	21UR-6016	TATTTGAATCAGACGACTGTA	2	1	0	0	6	8	0	17
		21UR-6017	TTCTTCTATGATTCCACAAAC	0	0	0	0	3	2	2	7
		21UR-6018	TGCTTCTTTCGTGTGTTTAGC	0	0	1	0	0	0	0	1
	†	21UR-6019	TCGTTTGTGGCACTAGGATCT	0	0	0	0	1	0	0	1
*		21UR-6020	TGAGACAAGAATACTTATTCA	2	1	1	9	117	65	14	209
		21UR-6021	TTTGTTTTGCCAATAATAAAG	0	0	0	0	0	0	0	0
	†	21UR-6022	TTTAACAGTTAGTAAGCGGCA	1	0	0	3	55	154	5	218
		21UR-6023	TTATCACGATTCTTTCCAAAT	6	0	0	1	3	5	4	19
	†	21UR-6024	TTACAAGAACATCGAACCAAA	0	1	0	2	60	55	3	121
	†	21UR-6025	TGTTCTCTAGCTGGGACTATG	1	2	1	1	1	5	0	11
		21UR-6026	TCTTATACAATTATGAAAACA	0	0	0	0	2	0	0	2
		21UR-6027	TCAACTCCCAATTGTTGAACA	0	0	0	0	4	3	0	7
		21UR-6028	TATGAAAATTAATCCAAATTC	0	0	0	0	0	0	1	1
	†	21UR-6029	TTTTTGTGAAATTTTCGGTTC	2	2	1	1	27	22	37	92
	†	21UR-6030	TTTTTGGAATTTTTCGTGACA	13	7	5	4	17	42	6	94
	†	21UR-6031	TTTGATGTGAGTATAGAAAGT	1	1	1	1	13	15	2	34
		21UR-6032	TTGTTCGTTTTCATCTAACAA	0	0	0	0	0	0	0	0
	†	21UR-6033	TTGTCGTACACTATCGCCTCG	0	0	0	0	10	8	5	23
		21UR-6034	TGTGATGGATGAGTAATTTTG	0	2	2	1	2	4	0	11
	†	21UR-6035	TTCTTAGTCCATTCGTGTTGA	0	0	0	0	1	4	1	6
	†	21UR-6036	TTATGCACGGTCTTGTACATT	7	10	1	4	9	19	1	51
		21UR-6037	TGCTAATTGTGAACTCAATTC	0	0	0	1	6	6	0	13
	†	21UR-6038	TGCACGGGCTGATATTATTTA	0	0	0	0	0	0	0	0
		21UR-6039	TCATAAATTTCAATTTGAATG	0	0	0	1	0	0	0	1
		21UR-6040	TAGAATTGGAATAATACCCGT	0	0	0	0	0	0	0	0
*	†	21UR-6041	TTCTTTTTGGAGTGTGGTCAT	17	6	4	5	5	50	19	106
		21UR-6042	TGTAAACAAAACCAATAATAA	0	0	0	0	0	0	0	0
		21UR-6043	TGCTAATGGGCAACACAAAAA	0	0	0	0	4	2	5	11
		21UR-6044	TCTCCTTCCGTACATATCTCA	0	0	0	1	2	1	1	5
*	†	21UR-6045	TCAGATTGGAATTAAAATTGG	3	0	0	0	1	11	0	15
		21UR-6046	TTTTGGTTTTTTGTGTGTAAC	0	0	0	0	4	4	0	8
	†	21UR-6047	TTTACTACGGACACCGTTCAA	4	2	0	1	9	16	7	39
		21UR-6048	TTACTATTACTCTCGAAGAAG	1	0	0	3	21	20	0	45
		21UR-6049	TTTTCACGGCTAATCTCGTTG	1	0	1	1	2	4	0	9
		21UR-6050	TTTGTAATGAATGGAGACTCT	2	1	2	3	57	61	5	131
		21UR-6051	TTTCATCAACAATCATCAACA	1	0	0	0	2	2	1	6
		21UR-6052	TGCTTGATTTAGTTTGATTTA	0	1	1	0	22	18	2	44
		21UR-6053	TCGACCTGTTGTTCAATCGAC	0	0	0	0	3	4	1	8
	†	21UR-6054	TATGATCAAAATTCGAGTAGT	0	1	0	3	22	23	3	52
	†	21UR-6055	TAAGCACAATAAGTCATGTGA	0	0	0	0	2	1	3	6
		21UR-6056	TTTGACTTTCGTTGAAGTTCC	1	0	1	0	0	0	0	2
	†	21UR-6057	TTTCAAACAGGATGAAAATGA	6	7	7	9	5	49	3	86
	†	21UR-6058	TTAATAGAGGATTTTTGAAAA	3	6	3	8	103	72	1	196
*		21UR-6059	TGTGAGAAGGGAATTTGTCGA	1	3	1	1	15	34	17	72
		21UR-6060	TGTCAAATTAAACTCATCATT	0	0	0	0	0	0	0	0
	†	21UR-6061	TGATGAACTTATAATCGAATT	0	0	0	0	1	0	0	1
*		21UR-6062	TCTTCGAAGGCATTATTTTCT	10	5	2	3	27	35	12	94
	†	21UR-6063	TCATGCAAAAAAAATAGCTCG	1	1	0	0	0	0	0	2
		21UR-6064	TATGTCCTTAGCACGCGTGTT	11	1	0	0	1	6	2	21
		21UR-6065	TATCAGAATTGTTTTGGATTT	1	0	1	4	22	10	4	42
		21UR-6066	TTTTTGTAAAAAACTGAGATT	2	0	1	0	2	2	0	7
	†	21UR-6067	TGTTTGTTGTGTGTTGTTGTA	1	1	1	2	7	1	5	18
		21UR-6068	TGGATGAATCTTGGGGATTTA	0	0	0	0	1	0	1	2
		21UR-6069	TTTTTCATGGTGATACATAGG	0	0	0	0	1	2	2	5
	†	21UR-6070	TTGATTTTGGCAAGATAAACT	6	1	0	0	3	5	0	15
	†	21UR-6071	TTCTTTGTAGTTTTCTTAGAA	2	1	1	0	18	8	3	33
	†	21UR-6072	TTCTAGTATGAAAATAATAGG	0	1	0	0	0	1	0	2
		21UR-6073	TTCCAACTACAATTTTTCGAA	0	0	0	0	0	0	0	0
		21UR-6074	TTCATTTTGTAGAAATGATGA	0	2	0	2	73	42	1	120

		21UR-6075	TCTGATGATAGTTTGAATAGA	0	0	0	0	7	5	1	13
		21UR-6076	TATATTAAAGATTTTGATATA	0	0	0	0	0	0	0	0
*	†	21UR-6077	TAGCAGTTCGAGGAAATAAAA	0	0	0	0	4	7	0	11
	†	21UR-6078	TAATGTGATTTTGGGTTGAAA	0	1	0	0	2	5	3	11
	†	21UR-6079	TTTCACTGGCGATGAATGTAA	6	1	2	1	3	13	5	31
		21UR-6080	TTCTGAATCAAACTTCTGAAT	3	1	0	4	6	15	1	30
	†	21UR-6081	TGCGATCTAATTTTTTCAATC	0	0	0	0	1	1	0	2
	†	21UR-6082	TGATTAGATTGTTGAAATAGT	8	6	2	3	12	19	8	58
		21UR-6083	TCTCTTTCTCAGTTTTCTAAC	1	0	0	0	0	0	0	1
		21UR-6084	TCGTATCCAAATTTTATACAA	0	0	0	0	1	1	0	2
*		21UR-6085	TAAGATTGTTAGAGAAATTGG	107	31	27	39	433	484	189	1310
		21UR-6086	TTTGGAAAGTTCGAGATGAAA	2	6	4	2	12	26	4	56
	†	21UR-6087	TTTCATTTAATCTTTAGGAAC	1	0	1	1	41	20	6	70
	†	21UR-6088	TTCTGCCAAAGCCAATTGGAA	0	0	0	0	2	0	1	3
		21UR-6089	TTCGGATCGGATTTTAGTTTA	0	0	0	0	1	2	0	3
		21UR-6090	TTCCTAGATTGTTCCCACATG	1	0	0	0	2	4	1	8
	†	21UR-6091	TTCCGTAATTTCCTTGTTGTT	1	0	0	0	1	2	0	4
		21UR-6092	TGCAAAGATTTTATTAAAATC	0	0	0	0	0	0	0	0
	†	21UR-6093	TGATAATAATCAATCAATACT	0	0	0	0	0	0	0	0
		21UR-6094	TCTTATTGATCCAGAACTTTG	1	0	0	0	2	2	0	5
	†	21UR-6095	TATTTGCTTTTGTAGAACTGA	5	0	1	0	7	8	0	21
	†	21UR-6096	TTTGAAGATTTGTTTTGCGCT	0	1	0	5	19	19	12	56
		21UR-6097	TTCTCATGCCAGCAAACATTT	0	0	0	0	0	0	0	0
*		21UR-6098	TTCGATTTTGAGAAAACTGGC	8	23	13	49	863	1224	95	2275
		21UR-6099	TTCACCTTCTAGTCCAGTTTT	0	0	0	0	0	0	1	1
		21UR-6100	TGCAGAACGATTTTTAATGTT	0	0	0	0	3	1	0	4
		21UR-6101	TCGAGGTCTGGCTGAAACTAA	1	0	0	0	3	1	0	5
		21UR-6102	TCAGTTATCTTTGGCAGCAAA	0	0	0	1	7	4	2	14
		21UR-6103	CTCTGAATGGATTTGATGAAC	2	4	0	0	1	13	3	23
	†	21UR-6104	TTTTTTCTGCAGCAAAGAATC	0	0	0	1	4	5	1	11
		21UR-6105	TTTGATTCCAATTTGAAAAAA	0	0	0	0	0	0	0	0
*		21UR-6106	TTTAAATGGGCAGAAAAAAGT	8	0	0	1	39	56	39	143
		21UR-6107	TTTAAAAAAACTTGAGCATTGA	1	0	0	1	17	15	0	34
		21UR-6108	TGTTGTTGTTTAGAATCTACT	0	0	0	0	1	1	0	2
		21UR-6109	TGGACACAATTTTTGTTTTCA	0	0	0	0	0	0	0	0
	†	21UR-6110	TCTTAAAAAAGAATTTCAAGA	0	0	0	0	0	0	0	0
		21UR-6111	TCTGCTTGCAACTTAAGTAGC	0	0	0	0	5	2	1	8
	†	21UR-6112	TCGGTAATTTTATTGTCAATT	0	0	0	0	0	0	0	0
	†	21UR-6113	TATTACCAGATCTTGAACGTT	12	1	5	2	10	28	15	73
	†	21UR-6114	TACCTCTCTTTTTTCGAAACG	5	0	0	2	0	5	1	13
		21UR-6115	TTTTGCCACTCCAACATCAAG	0	0	0	0	0	0	0	0
	†	21UR-6116	TTTGATAAAAAGAAAAAAATT	1	0	0	0	0	0	0	1
	†	21UR-6117	TTAGTTTCCTCCAAACACGGC	4	1	2	1	1	8	0	17
		21UR-6118	TGCACTGGGATATTTATATGT	0	0	0	1	0	1	2	4
		21UR-6119	TGATCCAAAGTCTGGACAAAA	7	3	2	2	4	19	1	38
		21UR-6120	TGAGAATTTTTAATTTTTAAA	0	0	0	0	0	0	0	0
		21UR-6121	TCTTCATCTTTATTTTTCACC	0	0	0	0	0	0	0	0
		21UR-6122	TCCAGAAAAATTAAAATGTTT	0	0	0	0	0	0	0	0
*	†	21UR-6123	TTTCTTAGGGCTTTTGGCAAA	91	42	25	22	92	329	22	623
		21UR-6124	TTGAAAAAGTTCCAAATTGGA	1	0	0	0	0	1	0	2
	†	21UR-6125	TTCGAAATTTCTTTTTGCTTC	0	0	0	0	0	0	0	0
	†	21UR-6126	TTATGTTGACGTAGAGCGAAT	0	0	0	0	5	10	0	15
		21UR-6127	TGTCGCTCTCTTTTCAAAACT	0	0	0	0	1	0	0	1
	†	21UR-6128	TCTTCAAATTGTTTGATTTTC	0	0	0	0	2	2	0	4
	†	21UR-6129	TAATTTTGGATTGATTTGTGC	6	2	0	6	106	91	46	257
	†	21UR-6130	TTTTGTTGTCGAGTGATGATC	0	0	0	2	5	6	3	16
		21UR-6131	TTTGTCCCCTGTTTTGTATTA	0	0	0	0	2	1	1	4
		21UR-6132	TTTAAACATTCCCTGTAGAAA	6	1	1	1	1	10	0	20
	†	21UR-6133	TTGGTTTCGATGTTGAAGTAG	0	0	0	0	5	4	1	10
	†	21UR-6134	TTGGCTAGTTGGTCCCTCTTT	0	0	0	0	0	0	0	0
		21UR-6135	TTAAGAACTTTGATTCGTTCT	0	0	0	0	3	0	0	3
	†	21UR-6136	TGATTTTATTTTCAAAAATGA	0	0	0	0	0	1	0	1
		21UR-6137	TGATCATTTTCATTCAAAATT	0	0	0	0	0	0	0	0
	†	21UR-6138	TGATAATTCCATATAATATTT	1	0	1	0	4	1	2	9

		21UR-6139	TCTTCCCAGAATGTACGGAAA	4	6	2	4	16	44	2	78
		21UR-6140	TCTTATAGATTAGGCAATAAC	0	0	0	0	0	1	0	1
		21UR-6141	TCGAATTCTAATGTGGTAAGT	0	0	0	1	2	3	1	7
		21UR-6142	TCATAAAATAATAAAATTCCA	0	0	0	0	0	1	0	1
	†	21UR-6143	TCAGAATATGGGTTATATCAA	0	0	0	0	3	5	1	9
		21UR-6144	TCAGAAACATTTTCTCAAAGT	0	0	0	0	0	0	0	0
		21UR-6145	TAGAGCTGAAATTCAATGTGC	0	0	0	0	1	0	0	1
	†	21UR-6146	TAAGAAAAAGGGGAATTTTTT	0	0	0	0	1	4	3	8
		21UR-6147	TTGATATAAATAAATACAAGA	0	0	0	0	0	0	0	0
		21UR-6148	TTCTTTTTGTTCGGATTTCTG	0	1	2	2	22	25	6	58
		21UR-6149	TGTGACATACTCTGTAAAAAT	0	0	0	0	8	4	0	12
		21UR-6150	TGAACCCGAGTAAACTCAAAT	0	0	0	0	0	1	2	3
	†	21UR-6151	TCTTTGATAATTTTTATAAAA	0	0	0	0	0	0	0	0
	†	21UR-6152	TCGAAGTTCGTGGTATTCTGT	1	1	0	0	0	5	0	7
		21UR-6153	TATTTTTCGAAATCATTGAAA	2	0	0	0	2	0	0	4
	†	21UR-6154	TATTTGTATAACAATCACTAG	0	0	0	0	3	1	0	4
	†	21UR-6155	TACTGGGCTTATATAAAAATT	0	2	0	0	5	0	1	8
*	†	21UR-6156	TTTTGCAAGGATATATACGGA	1	1	1	2	61	44	27	137
*		21UR-6157	TTTTGAGTCTAATGAAACAAT	0	0	0	0	2	1	0	3
		21UR-6158	TTTTCAACTATAAAACCAAGC	0	0	0	0	2	4	1	7
*	†	21UR-6159	TTTGAAAAGCTGACAGGGGGT	59	65	28	22	149	525	49	897
	†	21UR-6160	TTGCACATAAGTTCTTGGATT	0	0	0	1	2	1	0	4
		21UR-6161	TTCGCCATATGCACCAGATCC	0	0	0	0	1	0	2	3
	†	21UR-6162	TTCGATATCTTGACAAGTCAC	0	0	0	1	3	6	0	10
		21UR-6163	TTCCCTTCACGATAACACATC	2	0	0	0	4	6	2	14
	†	21UR-6164	TTCAGATCCTTGTACTACATC	1	0	0	2	20	14	13	50
		21UR-6165	TTATGTTAAGATCCATGATTC	0	0	0	1	4	4	2	11
	†	21UR-6166	TCTTTGAGCTAACCCTAAATA	0	0	0	0	0	0	0	0
		21UR-6167	TCTACGACGAGACGCATTTTC	7	8	5	5	63	96	30	214
		21UR-6168	TCGCGTTGATTTTCGAAGCTT	0	0	0	0	10	10	2	22
		21UR-6169	TATTTCGTTTATGTTTTGAAC	2	1	1	2	0	7	0	13
	†	21UR-6170	TATCATAGTAGATTTCGTATC	0	1	1	1	46	26	6	81
	†	21UR-6171	TTTTGATCTACGGAGAAAATA	40	16	12	9	25	108	10	220
		21UR-6172	TTCATCAGAAAAAAATGAAAG	0	0	0	0	0	0	0	0
		21UR-6173	TTCAGATACCCGTCCCTGCCA	0	0	0	0	0	0	0	0
		21UR-6174	TGTTGAAAATTATAAAAATTT	0	0	0	0	0	0	0	0
	†	21UR-6175	TGTTCGAAGAAAATTAAGTTT	0	0	0	0	0	1	0	1
		21UR-6176	TGTTCCGACAGTTCTTTTTGA	1	0	0	0	10	4	2	17
		21UR-6177	TCATCTCAAAATTGAAGCAGG	1	2	0	1	9	48	2	63
		21UR-6178	TATTATGGACTTTTTAACTTG	0	0	0	0	1	5	0	6
	†	21UR-6179	TATGTATTTTTGAACGTCTTT	12	3	2	3	19	24	4	67
	†	21UR-6180	TTAAGAAACTTTTTCGAGAGT	2	0	0	0	0	0	0	2
		21UR-6181	TGTTCAAAATTAAAATTTGCT	0	0	0	0	0	0	0	0
	†	21UR-6182	TGTATGCGTAAAAAAAAATGT	1	0	1	0	1	0	0	3
		21UR-6183	TGGAAATATGAGTTGAAAACC	2	2	0	0	19	21	1	45
		21UR-6184	TCTTCTGGCAAAATCAGCAAA	0	0	0	0	0	4	1	5
	†	21UR-6185	TATGCGAGTTTTCTCATAGTA	0	0	0	0	0	0	0	0
		21UR-6186	TAAGGCAAGGAAACAAAAAAT	0	0	1	1	4	5	0	11
		21UR-6187	TTTGCAACTATAGCGAACTCC	0	2	1	7	91	61	11	173
	†	21UR-6188	TTCTTTTGTGCACGTCAGGGG	13	0	1	0	4	9	6	33
		21UR-6189	TCTGAAGTTTTCTTCCGAAGA	0	0	0	0	0	1	0	1
		21UR-6190	TCTCAAGCTTGCTCCAAAAAT	0	0	0	0	0	0	0	0
	†	21UR-6191	TCGTTGAATACTTTGATTGAT	0	0	0	0	1	2	0	3
	†	21UR-6192	TATTGAAAGAACGGAAGGATT	0	0	0	0	0	1	0	1
		21UR-6193	TATATGAGAAGAGCGGTCTAT	15	4	4	1	3	25	2	54
		21UR-6194	TAGCAATTTCGAACACGTACT	0	1	0	0	8	16	1	26
	†	21UR-6195	TTTGCACGATTTCGGTGATGA	0	0	0	0	1	3	0	4
*		21UR-6196	TTCTTGGATTAAACCGTGAAA	0	0	0	0	0	3	0	3
		21UR-6197	TGAAAACTCAGAACTGGAAAA	0	3	0	4	77	55	8	147
		21UR-6198	TCTCAAGTGTAAACTGGCATT	2	2	1	2	39	50	13	109
		21UR-6199	TCGGAAAGAAAGCTGAAAACC	0	0	0	0	4	5	0	9
	†	21UR-6200	TCATCAGAGGAAAAAACATGA	0	0	0	0	14	6	2	22
		21UR-6201	TATTTGCACCACTTCTCAAGA	1	0	0	0	0	2	4	7
*	†	21UR-6202	TAGTTTTTATCCGGACAACCT	111	44	43	34	79	312	66	689

*		21UR-6203	TACGGTCAATTATTTAAACTC	0	2	0	2	53	31	0	88
	†	21UR-6204	TGGATTGATAAGATGCAAGAG	2	3	3	3	121	136	20	288
		21UR-6205	TGAAATTTTAATTTCCACATT	0	0	0	0	0	0	0	0
		21UR-6206	TAGTATGTTTCCGATGAAAAC	0	0	0	0	0	1	0	1
*	†	21UR-6207	TAGGGTTGATGAGTTAAGTTT	3	4	4	7	152	110	17	297
	†	21UR-6208	TTGTTTTCTTGCTAGAACACA	0	0	0	1	16	12	2	31
		21UR-6209	TTGAAGCATTATTCGACAAGG	0	0	0	0	2	1	0	3
	†	21UR-6210	TTCTGCATTGAAAAACTCCCC	0	0	1	1	9	4	0	15
		21UR-6211	TTATCTCATAATTTTCTGATA	0	1	0	0	2	1	2	6
	†	21UR-6212	TTTTCATAGCGACGGTTGGAA	1	1	1	3	35	50	44	135
	†	21UR-6213	TTTATCGTACGGTGATTATGT	2	0	0	0	11	13	9	35
	†	21UR-6214	TTACAATGTAATTGCTTGAAA	3	0	0	1	4	7	0	15
		21UR-6215	TGTCAACATTTTGTTCAAAAT	0	0	0	0	0	0	0	0
		21UR-6216	TCTATGATTCCAGGTACCGAG	0	0	0	0	0	1	0	1
		21UR-6217	TACGTTGAAATCAATTATTTT	1	2	0	3	27	15	1	49
	†	21UR-6218	TTTTGCCAACTTTATACTTGC	0	0	0	0	0	0	0	0
	†	21UR-6219	TTTCTAGACGACATCAATTGT	8	2	2	7	20	52	11	102
	†	21UR-6220	TTTATTAGGAATTACTAAAGA	0	0	0	0	6	6	1	13
		21UR-6221	TTTACCAAACTTCTTCAGACG	6	1	0	0	11	22	4	44
		21UR-6222	TGGAATCAATATGATGAGATC	0	1	0	0	0	0	0	1
		21UR-6223	TCTTTTTATTTATAATTAAAA	0	0	0	0	0	0	0	0
	†	21UR-6224	TCTCTGTTCTGAATTGTAGAA	3	0	0	0	0	2	0	5
		21UR-6225	GAAAAAGAGATGCAGAGAAAA	0	0	0	0	0	0	0	0
		21UR-6226	TTTTTTTCCACTTCTACGACT	0	0	0	0	0	0	0	0
	†	21UR-6227	TTTTTTGCGATCACAGTACAC	0	2	0	0	8	7	3	20
	†	21UR-6228	TTTGTACTGTGAGACATGAGT	3	0	0	2	5	20	4	34
		21UR-6229	TTTGCCTCGTATTATACCAGT	0	0	0	0	0	1	0	1
	†	21UR-6230	TTTCACGACTTGAAGGACTCG	1	1	0	0	1	8	1	12
		21UR-6231	TGCATTTGAGAAAATTTTTGA	1	1	0	0	3	3	0	8
		21UR-6232	TGACTAAGTCATATTTTTCCA	0	0	1	0	1	1	0	3
		21UR-6233	TCCGTGATTGGTATCTTTTTC	0	0	0	0	25	17	2	44
		21UR-6234	TCATAATCAAAGAAAATTTGC	0	0	0	0	0	0	0	0
	†	21UR-6235	TATTCGTTGAATTATGCCTAG	0	0	0	0	0	0	0	0
	†	21UR-6236	TATGATTTAGTAGTAACACTA	0	0	0	0	1	0	0	1
		21UR-6237	TAAATTCTGTCTGCTGTCTTG	0	0	0	0	0	0	0	0
		21UR-6238	TTTGTATCCAGTCGAAATTTC	2	1	1	0	4	4	8	20
	†	21UR-6239	TTTATAGATGTCCTCCGAAAA	0	0	0	0	3	1	1	5
	†	21UR-6240	TTGTAAAAAAGGATTACAGGA	4	1	0	4	76	72	15	172
	†	21UR-6241	TTCGTTCGCGGTATTAAAAGT	24	6	6	3	27	79	15	160
		21UR-6242	TTCGATTGTTTCATTCAACAT	0	0	0	0	19	13	1	33
	†	21UR-6243	TTCAATAGCAACACGGGTAAT	2	1	0	1	33	37	16	90
		21UR-6244	TGTTGAATGTTCACTATGGAT	0	0	0	2	1	4	2	9
		21UR-6245	TGATTTTTCAATTGGGCGTTC	0	0	0	0	0	0	0	0
	†	21UR-6246	TATAGATGCAAGATAAACTTC	1	1	0	0	1	1	0	4
		21UR-6247	TAGTTAAATCTTACTTTAGAA	0	0	0	0	0	0	0	0
		21UR-6248	TAACGATGTTGTGTTAAAGAA	0	0	0	0	0	0	0	0
	†	21UR-6249	TTTTGTTTGTTTTCCTCTGAA	1	0	0	0	0	3	0	4
	†	21UR-6250	TTTGATATGATTACTTAATCC	0	0	0	0	0	0	0	0
	†	21UR-6251	TTTGAAGACTGTTGAACATAT	1	1	0	0	2	3	0	7
		21UR-6252	TTTCTCGACTAATCTACGACC	0	1	0	0	1	0	0	2
		21UR-6253	TTCTTCGAAGAGTGTTTCACC	0	0	0	0	5	4	1	10
		21UR-6254	TTATGGAACTGGAAGTGCTTC	0	0	0	0	2	6	0	8
	†	21UR-6255	TGAAGTTCTTCGCTGAGTTTT	0	0	0	0	1	1	0	2
		21UR-6256	TCCGCTTGTAGCTTCACCATG	0	0	0	0	1	2	0	3
		21UR-6257	TCCCAGCTCGATTTTCAAAAA	0	0	0	0	0	1	0	1
		21UR-6258	TTTAAAACAGTTGAGCATTGA	2	3	1	5	43	97	4	155
		21UR-6259	TTCTGTTTTTCTGTCACGACA	0	0	0	3	20	10	6	39
	†	21UR-6260	TTAATACCAGGAGGGAATCCA	0	0	0	2	18	13	6	39
	†	21UR-6261	TGTTTGATCATACCGCATTTT	0	0	0	0	9	5	4	18
		21UR-6262	TGGGCGTACAGTTTGAACTTC	0	0	0	0	3	5	0	8
	†	21UR-6263	TGATATTCATTCATTGGTTCT	0	0	0	1	2	1	0	4
		21UR-6264	TGAATAAACTATTTTTTCCCG	0	0	0	0	0	0	0	0
		21UR-6265	TCCCTGCAGCGATTGGAAGAT	0	0	0	0	5	5	0	10
		21UR-6266	TCCATGGATTTATAGCGAAAT	0	1	0	2	20	15	10	48

	21UR-6267	TATGTAGTCCTTTTTATACAA	0	0	0	1	10	4	0	15
†	21UR-6268	TATGACAAGGGGAGAAAAAAT	2	5	1	0	1	12	2	23
	21UR-6269	TAAATATGACCGAAAAAAAAA	0	0	0	0	0	1	0	1
	21UR-6270	TTTGTATGTGGGCCACTGGGT	0	0	0	0	0	0	1	1
†	21UR-6271	TTTGACGATAGTTTCGATTGA	0	0	0	0	0	3	0	3
	21UR-6272	TTTCCTTAGACTGCGTGTTTC	0	0	0	0	22	37	1	60
	21UR-6273	TTTCAAAAATTTCTTCCATGA	1	0	1	0	2	1	1	6
†	21UR-6274	TTGTAGATTGGAGAGTAATTT	2	5	0	4	46	38	10	105
†	21UR-6275	TTGGTGCCTGCTATTAGAATC	4	5	1	2	12	41	0	65
†	21UR-6276	TTGGTAAAATATATCTAAAAC	0	0	0	0	0	0	0	0
†	21UR-6277	TTCAGAACTTTTTCATGAGAA	0	1	1	1	3	4	0	10
	21UR-6278	TTATGTTCCATCAATTTGAGA	6	0	0	0	1	5	0	12
	21UR-6279	TGGCTTTTTTTTTGTAATGAG	0	0	0	0	0	0	0	0
	21UR-6280	TGAATAATATTTTCTGTCTAA	1	0	0	0	0	2	0	3
	21UR-6281	TCTTTCATTCATTGAAGAAAT	0	0	0	0	0	0	0	0
†	21UR-6282	TCTTCTTCATGTAATTCGGGG	1	0	0	0	1	1	0	3
	21UR-6283	TCAAAAGAAAAATGAATTGAA	0	0	0	0	1	1	0	2
	21UR-6284	TATTATTTGTGATAGTTGAGA	2	1	0	2	5	4	1	15
	21UR-6285	GAAATTATGAAATATACAATT	0	0	0	0	0	0	0	0
†	21UR-6286	TTTTCTGGCAACACACAAAAA	0	0	0	1	5	2	2	10
	21UR-6287	TTTCCGCGTGAAATAATTGTT	0	0	0	0	0	2	0	2
	21UR-6288	TTTCAAAAATGACAATAGGGT	0	0	0	0	8	6	7	21
†	21UR-6289	TTGGAGTCTCGCTAACGATTC	1	0	0	0	1	1	1	4
	21UR-6290	TTCTTTTGGATGATATTGCGT	7	1	2	11	51	43	39	154
	21UR-6291	TTCACTTCATTAGAGAGTAGG	0	0	0	0	4	1	6	11
*	21UR-6292	TCGCTACGCTTTTTCCTTTTA	0	0	0	0	2	1	0	3
†	21UR-6293	TCATGATGTAAAATATCCAAA	0	0	0	0	0	0	0	0
†	21UR-6294	TCAAGTGCATGTAAGGAAACA	2	0	0	0	0	3	0	5
†	21UR-6295	TTTTCAATGTGACTGTTCGAG	1	0	0	0	0	0	0	1
†	21UR-6296	TTTCGCAAGACAAAAAACCTC	0	0	0	0	4	4	0	8
	21UR-6297	TTTCAAAGTGATTTTTGTTCA	0	0	0	0	0	0	0	0
†	21UR-6298	TTCGAGTAACCATTGGATACG	0	0	0	0	10	7	3	20
†	21UR-6299	TTCAGATGTTAGATAGGTGAA	1	1	2	4	74	77	44	203
	21UR-6300	TGAGTGACAACTTTCCCGATT	0	0	0	0	0	0	1	1
	21UR-6301	TGAAGAATGTAAATGGAGAAA	0	7	0	0	2	8	2	19
	21UR-6302	TGAAATCTTGATCGCATGTTT	0	0	1	1	17	5	8	32
	21UR-6303	TCTCTCTAATGGAAACAAAGC	0	0	0	0	0	0	0	0
†	21UR-6304	TCGGTTATCGATGATTTATAA	0	1	1	0	0	1	0	3
†	21UR-6305	TTTAATGGTGAAAAATCAGGC	0	0	0	0	0	3	0	3
†	21UR-6306	TTGCAAATCCAAAAATCGAAA	1	0	0	0	0	1	0	2
	21UR-6307	TTCTCTGAAATTTGCATCAGT	24	4	6	2	13	44	9	102
†	21UR-6308	TGGCCTTCGAACGGAACAAGA	0	1	0	0	2	5	0	8
†	21UR-6309	TGACTCAATCATCGATAATGA	0	0	0	0	0	0	0	0
	21UR-6310	TGAAAGAAATATCGAAAATTC	0	0	0	0	0	0	0	0
	21UR-6311	TCTTAACAGCCGTTTAATTCT	0	0	0	0	0	0	0	0
	21UR-6312	TCACAAAGAAGGTTCTTTTTT	0	0	0	0	0	0	0	0
	21UR-6313	TAAAGTTCAATTTCAAGAAAA	1	0	0	0	0	3	0	4
	21UR-6314	TAAACTTAGACTCAAATATCT	0	0	0	0	0	2	0	2
	21UR-6315	TTTTATGGCCATATGAAAAAG	0	0	0	0	1	1	7	9
†	21UR-6316	TTTTACGAACAATAACAAAAG	1	1	0	0	0	0	0	2
	21UR-6317	TTCTGATTGATCATATTAAGA	0	0	0	0	7	5	1	13
†	21UR-6318	TTCAAGATCTGAGAGTTCTAA	14	6	10	4	15	25	7	81
†	21UR-6319	TGCATATGGATTAGGCGTCAT	1	2	2	1	23	29	17	75
	21UR-6320	TGCAATGGTAAATTGCAGTGG	0	0	0	0	0	0	0	0
	21UR-6321	TGCAAAGAAGATGAAAACGTT	0	0	1	0	1	3	0	5
†	21UR-6322	TCTTCTAGAGTCCAACAATTG	0	0	0	0	0	0	0	0
	21UR-6323	TCCTGTAAAAAGTAAAAACAA	0	0	0	0	1	0	0	1
†	21UR-6324	TCAGAAATTTGGTAAAATTAA	0	0	0	1	9	8	2	20
	21UR-6325	TAGACTGAAAAAATTTTTTTT	0	1	1	0	12	10	1	25
†	21UR-6326	TACTGCCAGTCCTCTCCAAAT	0	0	0	0	2	0	0	2
†	21UR-6327	TTTCTCATCCGGTCCAAGAGG	2	0	0	0	0	1	0	3
*	21UR-6328	TTTATATATCGTGTTGACTTA	6	2	2	6	107	70	68	261
	21UR-6329	TTTACACGATTTTCTGCCTGA	0	0	0	0	0	3	0	3
†	21UR-6330	TTCTTCTTGCGTGTCATGTAA	0	0	0	0	1	0	0	1

	†	21UR-6331	TTCTGATAGTGATCCGCTTTC	0	0	0	0	0	1	0	1
	†	21UR-6332	TTCTCTTTGCTTTTGAGTTGA	0	0	0	0	3	1	1	5
		21UR-6333	TTATCAGGTTCTGTGGAAAAA	0	0	0	0	5	7	1	13
		21UR-6334	TCTGAAGTTACATCAAAAACT	0	0	0	0	0	0	0	0
	†	21UR-6335	TAGCTCTTGGTTTTTAGGAAA	1	0	0	0	0	0	0	1
		21UR-6336	TACCAAAAAATTATTCAACTC	0	0	0	0	0	0	0	0
		21UR-6337	TTTACCGTTTACTGTACCCTC	0	0	0	0	3	3	0	6
		21UR-6338	TTCTCCGTTCATATAGAAAGG	1	0	0	0	1	0	0	2
	†	21UR-6339	TTCATTCCTGTTTTGCAAGAC	0	0	0	0	3	3	0	6
		21UR-6340	TTCAAACACTGTTTCAACAAA	0	0	0	0	0	0	0	0
	†	21UR-6341	TTATAATAAAATTGTCGTAAA	18	0	7	1	8	15	6	55
	†	21UR-6342	TGTATTGTATGTGTACAATTT	0	0	0	0	2	3	0	5
		21UR-6343	TGGGATTTTTCACAATCTTTC	0	0	0	0	0	0	0	0
		21UR-6344	TGAGTTGATTTTATCACGAAT	0	0	0	0	1	1	0	2
	†	21UR-6345	TGACACTATCTTTGGAAATTC	2	0	0	2	4	0	0	8
		21UR-6346	TATTTTCGGAAAATTTGATTT	1	1	0	2	16	8	0	28
		21UR-6347	TATATCAGACCGTTTCAAATA	1	0	0	1	5	12	15	34
		21UR-6348	TAGTGAATCGAAAACAAATTT	0	0	0	0	0	0	0	0
		21UR-6349	TACAATGTGATAAAAAAAAAG	0	0	0	0	4	4	0	8
	†	21UR-6350	TTTTTTGACTATACTCTGTGA	0	0	0	1	1	3	0	5
		21UR-6351	TTTGGACTTTTGAATGAAATT	0	0	0	0	0	0	0	0
		21UR-6352	TTTACAAAAAAATTAGTGTTA	0	0	0	0	3	4	0	7
		21UR-6353	TTTAAAGCCGGAATGCTGAAG	0	0	0	0	27	18	0	45
		21UR-6354	TTGTGGGGCTATTCGCGAAAT	0	0	0	0	9	9	6	24
		21UR-6355	TTGTGCCTTGATTTGATGATT	1	0	0	1	0	2	0	4
	†	21UR-6356	TTGAGCAGTGATTTAAATTGG	1	2	0	0	2	8	1	14
		21UR-6357	TTCTGATCCTTTTTTATTGTT	0	0	0	0	0	0	0	0
		21UR-6358	TTCTGACACGACATTTACGAG	1	2	2	1	33	48	2	89
*		21UR-6359	TTCGCGAGGGCGCTTTTTACC	0	0	0	0	13	15	6	34
		21UR-6360	TGTGTCAGGGGAAATAGAACA	0	0	0	0	9	4	0	13
	†	21UR-6361	TGGTTCGCTTGGAAAGTAACT	0	0	0	0	5	0	0	5
		21UR-6362	TGGGCGGTTTATTTTTTAATC	1	0	0	0	3	2	6	12
	†	21UR-6363	TGGATCTGATGATTTAATCGT	0	0	0	0	7	4	1	12
*	†	21UR-6364	TGGACAAATTATAAGGAGACA	43	113	42	33	78	242	17	568
		21UR-6365	TCCAAATTTTGTTTGGAAAAT	0	0	0	0	0	0	0	0
		21UR-6366	TAGGTTAGATAATTAGGTAGA	0	1	4	1	2	7	0	15
*	†	21UR-6367	TAAGCTCTCGGAATTTTCTTA	2	4	2	2	5	14	2	31
		21UR-6368	TAACTTAAATAGATATACAGA	1	0	0	0	1	0	1	3
		21UR-6369	TTTGTACTTATTGAAAGTTGC	0	0	0	1	2	1	0	4
		21UR-6370	TGATATATTTTTTAAGCATGT	0	0	0	0	0	1	1	2
		21UR-6371	TGAAGAATTATGTACAAAAAT	0	0	0	0	1	3	0	4
		21UR-6372	TCCTTCTTAGATTTAACATCA	0	0	0	0	1	1	3	5
		21UR-6373	TATATTGGAAAACATGAGGAT	0	1	0	0	0	4	0	5
		21UR-6374	TTTGTTGATGATTGATTTTGT	2	3	3	4	7	15	2	36
	†	21UR-6375	TTTCTGAATTGTGAAAGGAAA	1	0	0	0	3	2	0	6
	†	21UR-6376	TTTAGAAAAGGAATTGCCTAA	2	0	0	0	0	5	0	7
	†	21UR-6377	TTTAAGGCACGTAAAGAAATG	1	0	0	0	3	4	3	11
		21UR-6378	TTCTTCTACAATTCTCGAACT	0	0	0	1	0	0	0	1
	†	21UR-6379	TTCTCAAGAATGTTTGACAAT	10	7	3	6	102	90	23	241
	†	21UR-6380	TTCCTAGGTTTGTACTCGAAT	0	0	1	0	1	0	0	2
	†	21UR-6381	TTCAAGTTCGATAGCACGTTG	2	0	0	0	12	19	9	42
	†	21UR-6382	TTACTGTCAGTGGCTTTGATT	0	1	0	0	0	0	0	1
	†	21UR-6383	TGATATTTTGAACCTGAAACT	0	0	0	0	2	0	0	2
	†	21UR-6384	TCCTTTTTCAAAAGAACAAGA	0	0	0	0	0	0	0	0
		21UR-6385	TCAATTTAAACTTGAAGGGCA	0	0	0	0	2	3	2	7
*	†	21UR-6386	TAGCAGCCTCTTAACTACAAA	0	0	0	0	7	2	0	9
	†	21UR-6387	TTTTTTTCGATTGATGTGCTA	3	3	2	11	105	95	9	228
	†	21UR-6388	TTTCTCTGAGCTTTTTTCCAA	1	0	0	0	0	2	1	4
	†	21UR-6389	TTTAGGAATAATTTTAGGGAT	2	0	0	1	0	1	0	4
		21UR-6390	TTTACTGGATGTATTAATCTC	0	0	0	1	19	15	1	36
	†	21UR-6391	TTCCCGTTAACTTTCTCTGTT	0	0	0	0	0	0	0	0
		21UR-6392	TGTTCACGTCGTTCGTCTTTC	14	5	5	4	10	34	11	83
		21UR-6393	TGTGTTAGATATTTACGTTAC	1	0	0	0	1	1	1	4
*		21UR-6394	TGTATAGAAGTCCGGATGATC	1	1	0	3	36	48	53	142

	†	21UR-6395	TGGAAAAATGGGAATTACCAC	0	0	0	0	4	1	0	5
		21UR-6396	TGCGCAACTCATATTTATCAA	0	0	0	0	0	0	1	1
		21UR-6397	TGAAAGATTGAAAAAACATTC	0	0	0	0	0	0	0	0
		21UR-6398	TCTACTTGCAATTTCAAATTA	0	0	1	1	4	2	2	10
		21UR-6399	TCCACTCCCAATTTCTCGCCA	0	0	0	0	0	0	1	1
		21UR-6400	TCAGATCAAAAATTTTTAAAG	0	0	0	0	0	0	0	0
		21UR-6401	TACTGGTTGTGAAATTGAAAA	0	0	0	1	15	15	10	41
		21UR-6402	TAATCAGACTTTATTGAGACA	2	1	0	2	18	15	0	38
	†	21UR-6403	TAACCAGTGGAATGAAAAAAA	19	31	5	13	46	138	5	257
		21UR-6404	TAAATGTTTCAGATTTGTTTC	0	0	0	0	2	1	1	4
*		21UR-6405	TTTTTACGTATCTGATTTCCA	6	9	1	4	7	16	0	43
		21UR-6406	TTTTGAGGTTCTTAACATTGA	0	1	0	0	0	1	0	2
		21UR-6407	TTGGCGTTCCATTCAGACTTT	0	0	0	0	7	10	1	18
		21UR-6408	TTGATGATTCAATAAAGTTTG	0	0	0	0	1	0	0	1
*	†	21UR-6409	TTATTGCAGAATATGGACTAC	40	35	13	51	831	1003	75	2048
		21UR-6410	TTAGAGAAATATCAAACGCTA	0	1	0	0	0	2	1	4
		21UR-6411	TCTGTACGGCCAGATCAAATC	0	0	0	0	1	0	2	3
	†	21UR-6412	TCTATGCTTTGTACCCAATAA	0	0	0	0	2	1	0	3
		21UR-6413	TCCAAGAATAGAACATATATC	0	5	1	2	16	10	0	34
		21UR-6414	TCATGCAATCGTGTAAGTTGA	0	0	0	0	2	0	0	2
	†	21UR-6415	TATTTGTGTTCATGAAGGAAT	1	0	0	0	4	2	2	9
	†	21UR-6416	TATTGGACACTAGTGAATTAT	0	0	0	0	3	3	0	6
*	†	21UR-6417	TATTCATATGGTAGAAAAAAG	6	2	3	6	64	54	7	142
	†	21UR-6418	TAGGAACATTTTAGTCAAATT	0	0	0	0	1	0	0	1
		21UR-6419	TAAATCCTTCGTTTAAATTGT	4	1	0	0	3	8	0	16
	†	21UR-6420	TTTTTTCCGTCGTCTTGGACT	2	0	1	5	78	34	31	151
	†	21UR-6421	TTTTCTGTTCATGGAGACAAT	12	1	4	6	15	35	19	92
		21UR-6422	TTTGTCTGTTGTATGGTTGTA	1	0	2	5	35	28	7	78
		21UR-6423	TTTGTAGTTCAATTTCATTGT	0	0	0	0	0	1	0	1
*		21UR-6424	TTTGAATGTCGGCAACGTTTC	46	24	11	10	61	252	17	421
		21UR-6425	TTTCGCACTTGTTCTCTGTCA	0	0	0	0	0	0	0	0
		21UR-6426	TTGTCGTGGGGCTCACCAATT	0	0	0	0	7	3	4	14
	†	21UR-6427	TTCTTTCATAGTCAGAGCCTG	1	2	0	0	4	8	1	16
		21UR-6428	TTCTGATCCGAATTTCAAGTC	0	0	0	0	0	1	1	2
		21UR-6429	TTCGCATTTTCTTCAGCTCTT	0	0	0	0	1	1	0	2
	†	21UR-6430	TTCCGGCCTGAAAGGTAACTA	1	1	1	0	1	5	0	9
	†	21UR-6431	TTATTTGCCGTCAAGCTTTCT	13	0	1	1	3	6	3	27
	†	21UR-6432	TTAGTCGCAGTAGGATATTAA	1	0	1	0	3	6	0	11
	†	21UR-6433	TGTAGAATAGAATAAACAATA	0	0	0	0	4	5	0	9
	†	21UR-6434	TGGTTTGGTTTTCCATTGCTT	0	0	0	0	0	0	0	0
		21UR-6435	TGAACAAATTGTGAACTTTTT	0	0	0	0	0	0	0	0
		21UR-6436	TCTGCTGACCTTTTTATTATG	0	0	0	0	0	0	0	0
	†	21UR-6437	TCTGCGTGTCATCGTCATCAT	0	0	0	0	2	0	1	3
	†	21UR-6438	TCTCTAGTCTAGAATTTGAAA	0	0	0	1	9	4	1	15
		21UR-6439	TCTCGAATCCACACATACTTT	0	0	0	0	0	0	0	0
		21UR-6440	TCAGAATTGGAATCGACAACG	0	0	0	0	1	4	1	6
	†	21UR-6441	TATTCATGATGTAAAATATCC	0	0	0	0	0	0	0	0
		21UR-6442	TATATGAATTGTTTTAGGAGA	1	1	0	6	27	24	14	73
*		21UR-6443	TAGATCCAAATTAATCGGCAT	5	3	1	11	100	135	21	276
		21UR-6444	TACGGATACGATCTCTATTTT	0	0	0	0	4	1	0	5
*		21UR-6445	TAATTGGTCGAGATAAACTTT	0	0	0	0	8	6	2	16
	†	21UR-6446	TAATTCAAGCAATCCAAACGA	4	0	1	0	12	15	1	33
	†	21UR-6447	TAATGAAGTGATCCAGCAACT	1	0	0	0	2	4	2	9
		21UR-6448	TAATACTTCGGAAAAAGAACC	0	0	0	0	3	1	0	4
*		21UR-6449	TAAGATAATTCTGCCTACTTT	0	0	0	2	6	4	0	12
		21UR-6450	TTTTTTTTCAAGCGGAGTGTC	3	1	1	10	96	134	71	316
	†	21UR-6451	TTTTGATGATGATCCAGCTAA	0	0	0	0	0	1	2	3
*		21UR-6452	TTTGTGGCACACATGTAAATT	6	1	0	1	10	11	8	37
	†	21UR-6453	TTGAGACTTGAGACTACCGTT	0	0	0	0	0	0	0	0
		21UR-6454	TGGATGTTCTTAAAAAAGATC	0	0	0	0	0	0	0	0
		21UR-6455	TGCGTGAGCAATTTTTTTAAG	0	0	0	0	0	0	0	0
		21UR-6456	TGAAACGCATAGCGAAAATCT	1	0	0	1	1	4	0	7
	†	21UR-6457	TCTTGAGATCGCAACGATTCT	0	0	0	0	2	2	1	5
	†	21UR-6458	TCGTGTTTAGAAAAATTTCAA	0	0	0	0	0	1	0	1

		21UR-6459	TCACTTCTTTTTAGTCGACAT	0	0	0	2	7	4	1	14
		21UR-6460	TATCTAATTGCAAAAACATCA	0	1	0	0	3	1	0	5
		21UR-6461	TATATTTCTTTGTGAGCGATT	2	0	0	0	0	1	0	3
		21UR-6462	TTTAGAGCTTTCAAAACTAAA	0	2	0	0	7	2	0	11
	†	21UR-6463	TTGAAATTTATCGCCTGAAAA	0	0	0	0	1	0	1	2
		21UR-6464	TTCCATCACTCAGAAATCTGT	1	0	0	0	10	9	1	21
	†	21UR-6465	TTCATCTTTGATTCTTTGACT	0	0	0	2	8	4	0	14
	†	21UR-6466	TTCAAGCAACAATACCTCTTT	3	0	0	2	44	27	8	84
		21UR-6467	TGATTTGCAAAATCAAATTCA	0	0	0	0	0	0	1	1
	†	21UR-6468	TGAAAAGTAACGGAGTATGCA	6	2	3	2	12	25	0	50
		21UR-6469	TCTCTTACCATTTCAAAAACT	0	0	0	0	0	0	0	0
	†	21UR-6470	TCGACCTGAACTCAGAAATCT	0	0	0	0	0	0	0	0
		21UR-6471	TCCGAGTTTCTTTTCAAAAAA	0	0	0	0	0	0	0	0
		21UR-6472	TCACATCAGATCTACATATAT	0	0	0	0	8	2	4	14
	†	21UR-6473	TCAATTAGTCGATTTTGATCT	2	3	1	0	1	5	0	12
		21UR-6474	TCAAATTTTGTTTTTCGCAAA	0	0	0	0	0	0	0	0
*	†	21UR-6475	TATTTACTCACTGGAAAATTG	1	0	0	0	4	3	0	8
*		21UR-6476	TATGACGAACAATTCAACTCT	0	0	0	1	12	13	2	28
		21UR-6477	TATAGATGCAACCATAAAAAC	1	0	0	0	0	0	1	2
		21UR-6478	TAGTGAGAGTGTTTTCACTTG	0	0	0	0	0	0	0	0
		21UR-6479	TAGAGTATTTTTTAAATATCC	4	0	2	4	15	10	2	37
	†	21UR-6480	TAATTTTGCACGGTTTCTGGT	0	0	0	0	20	23	1	44
		21UR-6481	TAATATTAGTCCAGTCCAATT	0	0	0	0	3	0	11	14
		21UR-6482	TAAGCAAAATAAATCAAAGTT	0	0	0	0	0	0	0	0
	†	21UR-6483	GAGGCAAATTATATCAAAATT	0	0	0	0	0	0	0	0
		21UR-6484	TTTTGATATCTGTCTCATGTT	0	0	0	0	2	1	0	3
*	†	21UR-6485	TTTTCTGTTCGTGGACACAAT	254	76	84	124	721	964	313	2536
	†	21UR-6486	TTTTCAAGAATAAATTTCAGA	1	0	0	0	0	1	0	2
	†	21UR-6487	TTTTATGTTCGTGGGACTATA	51	13	6	7	19	92	36	224
	†	21UR-6488	TTTCCAGAAACTATTGAAGCA	0	0	1	1	22	9	2	35
	†	21UR-6489	TTGCTTTCTTGTGAATGATGG	0	3	0	3	33	70	4	113
		21UR-6490	TTGATGGTAAAAAAAAATCCG	0	0	1	0	2	1	0	4
		21UR-6491	TTCGCTTGAACCCTTCCAGAT	0	0	0	0	0	0	0	0
	†	21UR-6492	TTACTTGTTTGTTGAAAGACT	0	0	0	0	6	9	0	15
		21UR-6493	TGGCAGAAGCAGCAGTCAAAA	0	0	0	0	1	6	0	7
		21UR-6494	TGATTCTATTAAATAAAGTAT	0	0	0	0	0	0	0	0
		21UR-6495	TCCTCTGTCTGTTTTTCCTCG	0	0	0	0	0	0	0	0
		21UR-6496	TCAGTCTTGAGGACCCGAAAG	0	0	0	0	1	0	1	2
*		21UR-6497	TATGGTCCCGGAATCAATCAG	2	0	0	0	2	1	7	12
		21UR-6498	TATCCAACCAATCCAACGAGA	0	0	0	0	0	0	0	0
		21UR-6499	TAGCTCAACTATAATTCGAGA	0	0	0	0	1	8	1	10
		21UR-6500	TAGATTCAGTTTTGAATCGAC	0	0	0	1	0	0	0	1
		21UR-6501	TTTTTTTAATATCATTGTTGG	28	3	6	8	25	36	71	177
		21UR-6502	TTTTGATGGGAATGTCATGCT	0	1	0	0	3	4	0	8
		21UR-6503	TTTGTAACCAGTGTGAACAGA	0	1	0	1	23	17	9	51
		21UR-6504	TTTCATCACAGCTTCAGATGA	0	0	0	1	9	4	3	17
		21UR-6505	TTGCGTTTGTTAAAAAAACAT	0	0	1	0	4	0	1	6
	†	21UR-6506	TTCACTTAGAATTTTCTGACT	0	0	0	0	0	0	0	0
	†	21UR-6507	TTATTAGAAAACCGGCTCTCA	2	1	0	1	35	34	10	83
		21UR-6508	TTAACTTTTTGATCAACAAAG	0	0	0	0	1	0	0	1
	†	21UR-6509	TGGACAGTTTTGAAAAAATTT	0	0	0	0	0	0	0	0
	†	21UR-6510	TGATTCTCCGATATTATGCAT	0	0	0	1	1	4	4	10
	†	21UR-6511	TGATGATGATAAAAAACACCA	0	0	0	0	0	0	0	0
	†	21UR-6512	TCGTTCACGACTTTTATTTGA	0	0	0	0	0	1	0	1
		21UR-6513	TCGTTATCGGGGTTTCACAAA	0	0	0	1	3	2	3	9
	†	21UR-6514	TCGCGAATATAAAAACTTCAA	0	0	0	0	12	0	1	13
		21UR-6515	TCCAATCTTTCAGAACTGAAG	0	0	0	0	4	3	0	7
		21UR-6516	TCATTCCATACATTTTAACAA	3	0	0	0	0	1	1	5
		21UR-6517	TCATAATAATAATAATGTGAA	0	0	0	0	0	0	0	0
		21UR-6518	TACAAAAAACAATTTCTACAA	2	0	0	0	1	1	0	4
	†	21UR-6519	TAATTTCAGGATTTGAATGCT	0	1	1	0	20	13	2	37
*		21UR-6520	TAAGTCCTGGATTGAAATTTT	0	0	0	0	3	1	1	5
	†	21UR-6521	GGCTAAACCTATTTATCGGAT	2	1	0	0	2	2	2	9
	†	21UR-6522	TTTAGTTAGCTTTGCTCTTGC	0	0	0	0	0	0	0	0

	†	21UR-6523	TTTAATCCTACTTAATACTGA	2	0	0	0	2	1	5	10
		21UR-6524	TTGGCATTGTACATATGGAAT	3	4	2	1	5	21	0	36
	†	21UR-6525	TTGATTTCAAAATCGGGATTT	0	0	0	0	1	0	0	1
		21UR-6526	TTGAAAACATAGTTTCAGATA	0	0	0	0	0	0	0	0
*		21UR-6527	TTCTTCGAAGATCTTCAAGGG	1	0	0	0	7	15	5	28
	†	21UR-6528	TGGATAGTTTTTTTGGACACT	0	0	0	0	6	4	1	11
	†	21UR-6529	TGCTTTTTCTGCAAAGTTGTC	1	0	0	1	0	0	0	2
		21UR-6530	TGAATGGGCAAAAAATAGTTT	0	0	0	0	0	0	0	0
		21UR-6531	TCCAAGTCGCTTTTTGATCAG	0	0	0	0	0	0	1	1
		21UR-6532	TCAATCTGTAGTTTTTAATTC	1	1	1	0	5	6	0	14
		21UR-6533	TATTGTGCCTAGTTTGTCGTA	2	0	1	2	11	16	11	43
		21UR-6534	TAGTTAAAACTTTTCCTCTTA	0	0	0	0	0	0	0	0
*	†	21UR-6535	TACTAGTGCTTCGGAAAACAA	0	0	0	0	24	19	8	51
	†	21UR-6536	TAATGATTGAATTGTGGAAAT	0	0	0	0	0	1	1	2
		21UR-6537	TTTTATTGTTCTGGAATACTC	2	4	4	8	12	31	1	62
	†	21UR-6538	TTTTATGACTCGTGAGGAAAA	3	1	1	2	17	35	19	78
		21UR-6539	TTTTACTAACCGCTCAATATC	0	0	0	0	0	0	1	1
	†	21UR-6540	TTTGACAGTTGCTAGGTTTTT	2	1	0	0	6	14	1	24
		21UR-6541	TTTATCAAAATTGTTGTGAAT	0	0	0	0	12	4	10	26
		21UR-6542	TTTAGCAAGATCTAGAGAAAT	0	1	0	0	0	3	1	5
	†	21UR-6543	TTGACTTAAAATGAAACTACG	0	0	0	1	3	1	0	5
	†	21UR-6544	TTCTGAAGACATTTTGATTGG	0	1	1	1	31	37	2	73
	†	21UR-6545	TTCCGATCTAGATTTCATCTA	0	0	0	0	2	0	0	2
	†	21UR-6546	TTAAATGGATTGAAATTTTCT	0	1	1	0	0	3	0	5
		21UR-6547	TGTTCTGTGGTTTTTCGTTCC	0	0	0	0	0	2	0	2
		21UR-6548	TGATTCTGTACTTTTGAAGTT	0	0	0	0	0	2	0	2
	†	21UR-6549	TGATCTCTCTTTTTAAATTTT	0	0	0	0	0	0	0	0
	†	21UR-6550	TGATAGAAGTAGATTTTACAG	3	3	3	4	30	35	1	79
		21UR-6551	TCGTGATTTAAATTTTAAAAA	0	0	0	0	0	0	0	0
		21UR-6552	TCGAAACAAAAGTACTGTTTT	0	0	0	0	0	0	0	0
		21UR-6553	TCATGTTACGATTTTTCTCGG	0	0	0	0	3	5	2	10
	†	21UR-6554	TATTTCATCTGAAAGAAAACC	1	0	0	0	0	2	0	3
		21UR-6555	TATAATCGTGGTATTTGCTCG	1	3	0	1	3	1	0	9
		21UR-6556	TACATGAGGGAGATCTTTTTT	0	0	0	3	27	21	9	60
	†	21UR-6557	TAATTAAATGTCCGGGAATGA	0	0	0	0	5	10	19	34
		21UR-6558	TTTGTTGAATAATTGTGTTTT	0	0	0	0	1	1	0	2
	†	21UR-6559	TTGACAATAAAATTACCGATT	0	0	0	0	2	0	1	3
	†	21UR-6560	TTCTGCTGGCTATTTAGTAGC	0	0	0	0	0	1	0	1
		21UR-6561	TTCATGATTGAGTGACGAGAA	0	0	0	0	0	1	0	1
		21UR-6562	TTCAAATACAAATTTTGTACA	2	0	0	1	1	1	1	6
		21UR-6563	TTATCATAGTTTTTTCATAGT	7	0	0	0	0	6	0	13
		21UR-6564	TGGTACTCCCATATAATGTTT	0	0	0	0	2	0	0	2
		21UR-6565	TGGTAAACTTTCATTCAATTT	0	0	0	0	0	0	0	0
	†	21UR-6566	TCGTAGTTTGATAAAATTCAA	0	0	1	0	0	1	0	2
		21UR-6567	TCGAATGAGTACTTTATGATT	0	0	0	1	1	0	0	2
		21UR-6568	TATGGTTCCCATAGGCGATGA	0	0	0	0	2	7	6	15
		21UR-6569	TAGAGACTGTCATCAGGTTCT	0	0	0	0	22	14	1	37
	†	21UR-6570	TTGGTGAATCGGTCCCGGTTT	0	0	0	0	0	0	8	8
	†	21UR-6571	TTCTTCGGTTTTGAAATTTTC	1	0	0	0	1	2	0	4
	†	21UR-6572	TTCTTCATTGTTTTCGTTTGA	2	0	0	0	2	2	3	9
	†	21UR-6573	TTCGCTTTTTTAATCCAAAGT	2	1	0	0	1	1	0	5
		21UR-6574	TTCCTTTTTAATTATTGCACA	0	0	0	0	0	0	0	0
	†	21UR-6575	TTATTCCCAGGACCGATAATA	1	1	0	0	0	1	1	4
*	†	21UR-6576	TTAATTTAAAGCACGAGACTT	88	12	13	13	31	133	13	303
		21UR-6577	TGCGACACTTTTTTGAGTCTT	0	0	0	0	0	0	0	0
		21UR-6578	TGATTGACGACCACATAATCG	1	0	0	0	1	3	2	7
	†	21UR-6579	TGAAGAATTTATTTCGGGAAT	0	0	0	1	0	0	0	1
	†	21UR-6580	TCGGCTCAACTTTTTAACTTT	0	0	0	0	2	0	0	2
	†	21UR-6581	TCCACGGCTGAACAGGACAAT	134	80	57	37	249	554	64	1175
		21UR-6582	TAAGAATTCTTTTTTTATAAA	0	0	0	0	1	0	0	1
	†	21UR-6583	TAACTTTAATAAAAAGTAAAG	0	0	1	0	0	0	0	1
		21UR-6584	TAAAATGAATAGCAAACCATT	1	0	0	0	2	2	1	6
		21UR-6585	GGGAATTAAACGTTATATAAA	0	0	0	0	0	0	0	0
		21UR-6586	TTTTTCATGTACGTCACACAC	0	0	2	1	5	0	17	25

		21UR-6587	TTTTGCGCGAGACTTTAAAAA	1	0	0	0	8	8	4	21
	†	21UR-6588	TTTGGTTTGTCATTAATGTAG	1	1	2	0	10	9	5	28
	†	21UR-6589	TTTGATCTTTCAAACATCACA	1	0	0	0	0	1	0	2
	†	21UR-6590	TTTATCTGAAGTTGCGATAAC	0	0	0	1	5	5	1	12
		21UR-6591	TTCTTTCTGTTCCTTGAGTGT	0	0	0	0	2	0	0	2
	†	21UR-6592	TTCTTAGTTGGCTGTTCTATT	0	0	0	0	2	0	0	2
		21UR-6593	TTCGCCCGTTAACTTGAACGA	0	0	0	0	2	3	0	5
	†	21UR-6594	TGTTGACGAGGAATTTTTACA	2	0	0	0	7	8	3	20
		21UR-6595	TGTCGAAAAAAAAATATTTTT	0	0	0	0	0	0	0	0
	†	21UR-6596	TGGACAGATATTGGAACAAAA	8	5	1	2	13	13	1	43
		21UR-6597	TGCTACGTTGGAAACATTATT	4	5	2	6	40	33	2	92
		21UR-6598	TGATCATAGCTTTACAATAGA	0	0	0	0	0	0	0	0
		21UR-6599	TGAAGTAGTTAGTTCAAATAA	0	0	0	0	1	0	0	1
		21UR-6600	TCTTGACATTCCATAATCCTT	0	0	0	0	0	0	0	0
		21UR-6601	TCTCTTCACATCTCTGTCTCC	0	0	0	0	4	1	0	5
		21UR-6602	TCTAATTATTTTCTGAAAAAA	0	0	0	0	0	0	0	0
		21UR-6603	TCGAATTTTAAATGTGTTTTA	0	0	0	0	0	0	0	0
	†	21UR-6604	TATTGTGGACTTTTCTACATT	0	0	0	0	0	1	0	1
	†	21UR-6605	TATTCGAGGAAATTTTTTGAC	1	0	0	1	1	1	1	5
		21UR-6606	TATGTTTTTCTCCCAACAATA	0	0	0	0	1	0	0	1
		21UR-6607	TAATCTATTGAAAACTTTAGA	0	0	0	0	1	1	0	2
		21UR-6608	TTTTATGCAAGAACTGGTTGA	0	0	2	3	51	33	8	97
		21UR-6609	TTTCAACCTTCCAGGAAAAAA	0	0	0	0	0	2	0	2
		21UR-6610	TTGTGGACTTGTTGAACGCTC	0	0	0	0	0	0	0	0
	†	21UR-6611	TTCCGTTCTTATTTCAACAGC	0	0	0	0	0	2	1	3
*	†	21UR-6612	TTAGAATCTAGAGTTGAGCAA	1	0	0	3	25	25	3	57
	†	21UR-6613	TGGACTTTGATTGGAAATTGG	1	2	1	1	28	32	26	91
	†	21UR-6614	TGATAGGGATTTCCTCGTTCA	0	0	0	0	0	1	0	1
		21UR-6615	TATCACTTTTTCGTTTTTAGG	0	0	0	0	0	3	0	3
	†	21UR-6616	TACGTCTTGGTCCTCAAGCTA	1	0	0	0	0	3	0	4
		21UR-6617	TTTCTAGAATTGTTTTGGAGA	0	0	1	2	6	1	4	14
		21UR-6618	TTTCAATGCACTTGGCAATAA	2	0	0	0	33	26	10	71
	†	21UR-6619	TTCTTGGATATGTGTTGCTAA	0	0	1	0	0	1	0	2
		21UR-6620	TTCTGACTCCAAGCTATTTTT	0	0	0	0	1	2	1	4
	†	21UR-6621	TGCTACGCTAGTTCCAATATA	0	0	1	0	4	6	2	13
		21UR-6622	TGATGTTTTTTGCAAAAGCAA	0	0	0	0	0	0	0	0
		21UR-6623	TGATATAGTAATATTAGCCTG	0	0	0	0	0	0	0	0
	†	21UR-6624	TCTTGATTGGTTTCGGATTCT	13	10	3	3	11	58	7	105
	†	21UR-6625	TCAATATTTACGTTGATGCTT	0	0	0	0	0	2	0	2
		21UR-6626	TATAATTATCTTTCGGATGTT	33	13	16	14	35	120	30	261
	†	21UR-6627	TACATGATTTTCCAAATAAAA	0	0	0	0	3	2	3	8
	†	21UR-6628	TTTTCAATTGAATGGAACTGT	7	0	0	2	7	11	3	30
		21UR-6629	TTTGAAAGGTTGTCAATGCTC	1	1	2	1	9	13	7	34
	†	21UR-6630	TTGTCCTTTTGATATGATGAC	0	0	0	0	1	1	0	2
		21UR-6631	TTCAGTTGAACATCTGGTTTT	0	0	0	0	9	6	1	16
		21UR-6632	TTATAGAAATATACTAAAATT	1	0	1	0	0	3	0	5
		21UR-6633	TGTTTTTGATTCATAAAAAAC	0	0	0	0	1	1	0	2
		21UR-6634	TGTTTGATGTTTTTGTGAACG	0	0	0	0	1	0	0	1
		21UR-6635	TGTTTCAGATTTAAACAGATT	0	0	0	0	0	0	0	0
	†	21UR-6636	TGTCTGTTCGATTTAGCATTT	4	8	1	4	15	12	1	45
		21UR-6637	TCGTCCATCTCATTCGGGTTT	0	0	0	0	1	2	0	3
*		21UR-6638	TCAGGTAAGTAAGAAAACTAA	17	21	11	43	490	378	30	990
		21UR-6639	TAGGGAATCTGGAATGGAAAT	0	0	0	0	2	3	1	6
*		21UR-6640	TACGTTTCCTATATGGAGATC	0	1	0	0	5	6	0	12
		21UR-6641	TACACAAAGTCTTCTCAAAAA	0	0	0	0	0	0	0	0
	†	21UR-6642	TAATCCAATACGAGAAACAAA	0	0	0	0	0	1	0	1
		21UR-6643	TTTTTACTCCTCATTCAGATC	2	0	0	0	0	1	0	3
		21UR-6644	TTTTCGCAAAACAAGCAGTAA	0	0	0	0	0	0	0	0
	†	21UR-6645	TTGTTTAGAAATGCGGGCGAC	4	3	1	2	7	29	7	53
	†	21UR-6646	TTGATTTAGTCTGGTGGCTTC	3	0	0	0	2	5	0	10
		21UR-6647	TTGATACATATATCTATAATA	0	0	0	0	0	0	0	0
		21UR-6648	TTCTCGTATAACAGAAAAAAA	0	0	0	0	4	0	0	4
*		21UR-6649	TTACGCACACGGTGAAGATTT	7	2	2	0	8	10	17	46
	†	21UR-6650	TTAAAGCAGCATTTTGAAAAA	2	4	0	3	27	19	4	59

	21UR-6651	TGGGGAAAATAAAAATAATTT	0	0	0	0	0	0	1	1
†	21UR-6652	TGCCTCGTATGATACCTTTGT	0	0	0	0	12	2	1	15
	21UR-6653	TGAGCTACTGAGCGATGACAA	0	0	0	2	112	80	17	211
†	21UR-6654	TGAAGAAAAATGCACTGGAAA	0	0	0	0	1	1	1	3
	21UR-6655	TGAAACCTATGTAAACGTTTT	0	0	0	0	2	0	2	4
*	21UR-6656	TGAAAAATTCCTAAGATGGTG	22	12	5	7	14	67	2	129
	21UR-6657	TCCACCTTCATTGCGTACAGT	0	0	0	0	0	0	0	0
	21UR-6658	TCAGACATGTGTTATCGTATG	0	0	0	0	0	0	0	0
	21UR-6659	TCACATTTTCAATCAATCCAA	0	0	0	0	2	1	0	3
	21UR-6660	TCAATCATCATTAACTATATA	0	0	0	0	0	1	0	1
	21UR-6661	TATTTATTTGATATTTGATGA	0	0	0	0	0	0	0	0
	21UR-6662	TATATTCAAAGGTTTTTGGAA	0	0	0	0	4	2	0	6
	21UR-6663	TAGATGATGTTCTGAACCATT	0	0	0	0	0	0	0	0
†	21UR-6664	TTTTGAGAAACCTTTACCGAT	0	0	0	0	0	0	0	0
	21UR-6665	TTTGTACACTGCAACAAGTAA	0	2	2	3	55	40	1	103
†	21UR-6666	TTTGAATAATCTGTCGCTGCT	0	0	0	0	2	2	3	7
†	21UR-6667	TTTCCAATTAAATTGATTTGG	0	0	0	0	0	1	1	2
†	21UR-6668	TTTATTGAAATTGGGTTGTAT	1	0	0	1	1	0	0	3
†	21UR-6669	TTTAAGCATGGCTTTTGTAAA	0	0	0	0	1	3	0	4
†	21UR-6670	TTGGGTTTAGGCGATAATGAC	1	2	1	0	4	7	0	15
†	21UR-6671	TTGATCTTCTGTTCTCTCTTC	0	0	0	0	0	0	0	0
	21UR-6672	TTCCCTTTTGGCCTGTTGTAT	0	0	0	0	4	3	2	9
	21UR-6673	TTACAATCAGGTTCTATAAAA	0	0	0	0	0	1	0	1
	21UR-6674	TGTCATTACATTAGACCGTTT	1	0	0	0	15	4	2	22
	21UR-6675	TGCCTTTGTCATTCGTTGCCC	0	0	0	0	0	1	0	1
	21UR-6676	TCTTTTTTGGGTGAGCATGTT	0	0	0	1	0	1	0	2
	21UR-6677	TCTCACTCAGATATTGTTAAA	0	0	0	0	0	0	2	2
†	21UR-6678	TCAGATCAACGAAACAATAAT	0	0	0	0	3	1	0	4
	21UR-6679	TATTCATAGAATAAAGGTAAT	1	1	1	3	2	8	0	16
†	21UR-6680	TATCTAATAATCGGCCCCTTC	3	0	0	0	1	1	2	7
	21UR-6681	TAACCTTCCATTTCAATCAGA	0	0	0	0	1	2	1	4
	21UR-6682	CTGACTTAGATATTCTGCATG	0	2	0	0	0	3	0	5
	21UR-6683	TTTTTGAAGAGTGTTCACGGG	4	1	0	0	10	23	19	57
†	21UR-6684	TTTTGTGAATTTATCGGAAAG	1	0	0	2	10	12	8	33
	21UR-6685	TTTTGCACGCACGAGGTTCAA	0	0	0	0	1	4	7	12
	21UR-6686	TTTTGCAAATTCCGTGCTGAC	0	0	0	0	0	1	2	3
	21UR-6687	TTTGTAAATCGAAAAAGTCAA	0	0	0	1	0	2	2	5
†	21UR-6688	TTTGATGTTGCACCAGGTGGA	3	2	1	0	13	25	2	46
*	21UR-6689	TTGTTTCTACATCGTTGGGGC	2	1	2	4	57	84	35	185
	21UR-6690	TTGTCAATTCAAAAATTTCTT	0	0	0	0	0	0	0	0
	21UR-6691	TGGATTTCCGCATTTCCGTAA	0	0	0	0	0	0	0	0
†	21UR-6692	TGCTCGACTACTATCCTATTT	0	0	0	0	0	0	0	0
†	21UR-6693	TCTTTGTTCCTTATGTTGCAA	0	0	0	0	0	0	0	0
†	21UR-6694	TCTGATTTGCTCCGGTGTCAA	0	0	0	0	0	2	0	2
	21UR-6695	TCGTTTTGTCCAATTGTCTTT	0	0	0	0	0	1	0	1
†	21UR-6696	TCCGATTTCTTTTCATCATCT	0	0	0	0	0	0	0	0
	21UR-6697	TCAAAAAAACAGTGTAAAACA	0	0	0	0	0	0	0	0
†	21UR-6698	TATTTTGGTGATAGTCTGCGT	0	1	2	2	69	62	42	178
	21UR-6699	TATACCTCACAAGGGAAGGCT	2	1	0	2	11	19	7	42
*	21UR-6700	TACACCCAGAACTAAATTCAG	0	0	0	0	9	1	6	16
	21UR-6701	TTTGCTTTGGATGTATGATAA	4	2	0	0	4	16	0	26
†	21UR-6702	TTTCTGAATCGCATACGCATT	0	1	1	4	23	25	1	55
	21UR-6703	TTTCATTTTGTCGCAACATAC	0	0	1	2	10	2	2	17
	21UR-6704	TTTACTCTTCATTTGCGGTAA	1	2	1	1	5	5	4	19
	21UR-6705	TTGTGCACAACTTTTAAAAAT	0	0	0	0	0	1	0	1
	21UR-6706	TTCTGAGCTGATAATCATTTC	0	0	0	0	0	0	0	0
	21UR-6707	TTCCTGACTTGAGTATTTAAA	0	0	0	0	2	0	1	3
	21UR-6708	TTAATCGTTGATTGTATAAAA	1	0	0	0	20	8	1	30
†	21UR-6709	TGTAAAAAAGGATTACAGGAA	1	2	0	0	17	17	3	40
	21UR-6710	TGCGTAGTATGTTTTTAATTT	0	1	0	2	6	8	1	18
	21UR-6711	TCTCACAGTTTATTGGGTGAT	0	0	0	0	1	0	0	1
*	21UR-6712	TCCAGGAACTCAAGGGACGAT	5	8	1	3	161	178	65	421
†	21UR-6713	TATTTGCGGCAAAACTCTATG	0	0	0	0	1	1	0	2
	21UR-6714	TATATCCCTCCTCGTCGTGTT	7	2	1	2	66	69	98	245

	21UR-6715	TAATTTTTGCAATCTTTGGAC	13	7	6	46	407	362	125	966
	21UR-6716	TAATTTCCAGTACCATATGAC	1	0	0	0	1	2	1	5
	21UR-6717	TAAGTATTCACAAGGGAAGCT	1	2	0	1	1	8	1	14
*	21UR-6718	TAAGAAAACTGGACGTTCACA	1	0	0	0	7	3	1	12
†	21UR-6719	TTTTGTCTGAAAATAAGGAGT	1	2	1	0	2	13	1	20
†	21UR-6720	TTTTCAATTAGTAGGTTGTTC	0	0	0	0	1	0	0	1
†	21UR-6721	TTTGTTCGCAAATCTGTCCAG	0	0	0	1	1	4	1	7
	21UR-6722	TTTCTTTCTTGTAGTGGGTTC	0	0	0	1	14	18	7	40
	21UR-6723	TTTCGTTTTTTCTGTAGTTAG	3	0	1	0	0	1	0	5
†	21UR-6724	TTGTTTTTTTCCGAGGAACAT	0	0	0	0	0	0	4	4
†	21UR-6725	TTGATAGCTTTCATCTATTCA	0	0	0	0	1	0	0	1
	21UR-6726	TTCTCTTTCCGGTTCTAGAGC	0	0	0	0	0	1	0	1
†	21UR-6727	TTCTCTTCTAGTTAGTGTGTG	1	0	0	0	2	3	3	9
	21UR-6728	TTCCTACTGATTTGGCATTAC	2	2	1	8	44	41	10	108
†	21UR-6729	TTACTCTATAGAATTTACAAT	0	1	1	6	61	25	2	96
†	21UR-6730	TGTTGATTTGATTTTGTGTCC	0	0	0	0	0	0	0	0
	21UR-6731	TGTAGAAGTTGAAAAACTAAA	0	0	0	0	1	3	0	4
†	21UR-6732	TGATTCTTTGCATTCTTCGCA	0	0	0	0	0	0	0	0
	21UR-6733	TGAGCAATGTTTTCCTAACTA	0	0	0	0	0	0	1	1
†	21UR-6734	TGAAATTCCAAAGTCCATAAG	0	0	0	0	0	0	0	0
†	21UR-6735	TCTTGACATTGTCAGATAATC	0	0	0	0	1	1	0	2
	21UR-6736	TCTCTTATAGAGACTGGAATC	0	0	0	1	19	20	6	46
* †	21UR-6737	TCTCTCTGCATATATCTCATT	2	13	4	22	400	130	7	578
	21UR-6738	TCTCATTGCGATTTCTTTCTT	0	0	0	0	0	0	0	0
	21UR-6739	TCATTTTGTAAGATATTTCAA	0	0	0	0	0	0	0	0
	21UR-6740	TATCGAGTTTCTATACTTATT	0	0	0	0	0	2	0	2
	21UR-6741	TTTCGCATGCATATAACTAGA	1	1	2	0	10	8	4	26
†	21UR-6742	TTTAGCTACCACCGGAAATTA	0	0	0	0	2	0	1	3
	21UR-6743	TTGTTTGTGCAATTGCTAAAA	0	0	0	0	0	1	0	1
†	21UR-6744	TTGGAACAAAGCAAATTTAAG	0	2	1	7	46	44	1	101
†	21UR-6745	TTGATTTTCATTTGTCAGTTA	3	0	1	0	0	4	1	9
	21UR-6746	TTCAGCGTTAACATGCCAAAT	0	0	0	0	2	1	1	4
	21UR-6747	TGGTAAACATTTTTGAATCCA	0	0	0	0	0	0	0	0
	21UR-6748	TGCTATTGCGAATTGACCTGA	0	0	0	0	2	2	0	4
†	21UR-6749	TGCGATGGATTTACAGAAACT	0	0	0	0	7	6	0	13
	21UR-6750	TGAATACATATCTCAATAGGC	1	0	0	0	1	2	0	4
	21UR-6751	TCTTTTGTCCGGATATTAACT	0	1	0	0	3	8	3	15
†	21UR-6752	TCTCGGCGACGATTTTTAACT	0	0	0	1	18	24	2	45
	21UR-6753	TCTCAATACGAAGATTAAACT	0	0	0	1	4	0	0	5
* †	21UR-6754	TATTTTTGTCGAACTTCTTGA	4	2	2	6	12	21	3	50
	21UR-6755	TACCTTTTTTTTGCGTGACCG	0	0	0	0	6	3	1	10
	21UR-6756	TACAATGTGAAAAGTTTCATT	1	0	0	2	12	24	2	41
	21UR-6757	TAATGTAAATATGAGTGTATT	1	0	0	0	1	0	0	2
†	21UR-6758	TTTTGAATTTCTCGGTGCTGG	0	0	0	0	6	5	7	18
	21UR-6759	TTTTCAAAAAGTGTGAGATAC	1	0	1	0	1	6	0	9
†	21UR-6760	TTTCCGAAATTTGTGGTTCTT	0	1	1	0	0	0	0	2
†	21UR-6761	TTTACAAAATTTTGAAGGATC	0	0	0	0	0	2	2	4
†	21UR-6762	TTCTTCAAACGCCCATCAGAT	0	0	0	0	0	0	0	0
	21UR-6763	TTCGTATAGAATATAAATAAA	0	0	0	0	1	1	0	2
†	21UR-6764	TTCCTTTGTGATCTCCTATTA	0	2	0	1	2	3	0	8
	21UR-6765	TTCCCCAAAGACCATACACCA	1	1	0	1	19	17	13	52
* †	21UR-6766	TTCCATGCATTTACATTTGAA	2	0	0	2	2	4	2	12
†	21UR-6767	TTATCCAGAGTCGGTATAACT	5	2	1	11	205	171	72	467
* †	21UR-6768	TTAGGCATTGGTGTACTTGCG	1	0	0	0	8	18	3	30
	21UR-6769	TGTTTATTTTAGAACAAATTT	0	0	0	0	0	0	0	0
	21UR-6770	TGTTAGAGACCCCATAATTGC	0	0	0	0	1	1	1	3
	21UR-6771	TGGAAGATTGATACTTTTTTA	6	23	9	3	14	78	0	133
†	21UR-6772	TGCCACGTAGTTCTCAATTTT	0	0	0	0	0	0	0	0
	21UR-6773	TGCATTCAGAAAAGTCTTGTT	0	0	0	0	6	6	0	12
	21UR-6774	TGCATTATCTATTCTAGAAGC	0	0	1	1	1	1	0	4
†	21UR-6775	TGATCCATAAAAATAGGATAA	0	0	0	0	0	0	1	1
	21UR-6776	TGACCAGATTTTAAAGAGATT	0	0	0	0	1	1	0	2
†	21UR-6777	TCTTTCCTGTCCTCTAGTCAG	0	0	0	0	1	0	0	1
*	21UR-6778	TCAGTAACGTCTCTTTTTCCT	0	2	0	1	8	5	0	16

		21UR-6779	TCACACGGCGACTATTTTGAG	1	1	1	5	78	74	39	199
	†	21UR-6780	TATTTTTTCTGATATAACGGA	4	1	2	0	2	5	0	14
		21UR-6781	TACTTCGAACAACAAAAAAAT	0	0	0	0	1	4	1	6
		21UR-6782	TAATTTAGTTATTTTACGACT	0	0	0	0	1	0	0	1
		21UR-6783	TTTTCCTCTGCCTGTTTTTAG	0	0	0	0	0	0	0	0
	†	21UR-6784	TTTCATCGTACACAATGCCAT	0	0	0	0	6	6	2	14
	†	21UR-6785	TTGGTAGGTAGAGAACTTTTT	1	1	1	0	0	4	0	7
	†	21UR-6786	TTCTACTCCATTCTTGATTCG	0	0	0	0	0	0	0	0
	†	21UR-6787	TTCGTCGTTGAAGTTGTTCAA	1	0	0	0	2	0	1	4
		21UR-6788	TTCCTCGTTGCTATTAAAAAT	0	0	0	0	0	2	1	3
	†	21UR-6789	TTCATCTCGTTCCAAAATTCA	1	0	0	0	1	0	0	2
	†	21UR-6790	TTCAGTTTGAACCGCCGTGTA	1	1	0	0	5	3	4	14
	†	21UR-6791	TTCAGCTTCAAAGTGCCCGTC	0	0	0	0	0	0	2	2
		21UR-6792	TTATAATCTCACATGCGATGA	1	0	0	1	4	5	3	14
	†	21UR-6793	TTAATTCCTTCTGAAATAACA	0	0	0	1	3	3	0	7
		21UR-6794	TGGTATTGTTTTCATGAAAAA	1	0	1	1	7	5	1	16
	†	21UR-6795	TGCTTCTGCAGTAGCGTGTTT	0	0	0	0	9	5	2	16
		21UR-6796	TGATTGATTTGAAATCCTTCT	0	0	0	0	0	1	0	1
	†	21UR-6797	TGATGAGTAATTGATAAATGA	0	0	0	0	0	0	0	0
		21UR-6798	TGAATATGATTCTTTTTGATG	0	0	0	0	0	0	0	0
*	†	21UR-6799	TGAATATGAATGACGAACGGA	17	26	14	52	600	808	108	1625
		21UR-6800	TCTTGCGATCATTTGATTAAA	0	0	0	0	1	0	0	1
		21UR-6801	TCTGTTGTGCATATATTCACT	1	1	0	1	15	14	1	33
		21UR-6802	TCTGCTCTAATCACCAACAAG	0	0	0	1	4	2	0	7
		21UR-6803	TCGTAGTTTTTTCGGAATTCC	0	1	1	0	24	30	1	57
*		21UR-6804	TCAATGATTAACGGTACTACT	2	0	2	5	4	17	1	31
		21UR-6805	TCAATAAGTTCGATTTGTTTT	0	0	0	0	1	1	0	2
		21UR-6806	TAGCAAAAAAATCTGGTTGAA	0	0	0	2	17	11	3	33
		21UR-6807	TAGAGAAGTTTTTGAAAAAAT	1	0	0	3	2	5	0	11
		21UR-6808	TACAAATTTGGTAAACGTTTG	0	0	0	0	1	4	2	7
	†	21UR-6809	TTTGATTGGTTTTTTAAGTAA	0	0	0	0	0	0	0	0
		21UR-6810	TTTATGATTGAGACAGTCGAA	1	0	0	0	1	3	0	5
	†	21UR-6811	TTGGTCTGTGGTCTGTATCTT	0	0	0	0	0	1	1	2
		21UR-6812	TTGCTTTGAAAGGGCCATGAA	0	0	0	0	2	5	2	9
		21UR-6813	TTCACTCAAGAAATACTAGGG	2	0	0	1	2	3	0	8
		21UR-6814	TTCAAATAACAGAGACTGTGA	8	2	1	1	6	24	3	45
		21UR-6815	TTACATCTGATTTTGAATGAT	0	1	1	0	0	2	0	4
		21UR-6816	TGGTCTGTGTGTGTGTGTTTG	0	2	0	2	84	50	24	162
	†	21UR-6817	TATTCAAGTAGGTTTAATTTT	0	1	0	1	5	4	0	11
*		21UR-6818	TAGAACGACGCCAACGATATT	0	2	2	0	13	20	0	37
	†	21UR-6819	TAATCGCGTTCTAGAATTTCT	0	0	0	0	1	1	0	2
	†	21UR-6820	TTTTAAAAAATCTCCTTAGCC	2	0	0	0	0	2	0	4
	†	21UR-6821	TTCAATCGCAGTCTGTATTCC	0	0	0	1	8	11	1	21
		21UR-6822	TTAGATCCAGTTTTTAGAAAA	0	0	0	0	0	4	0	4
	†	21UR-6823	TTAAAAAGGTAATCTTTAAAA	0	0	0	0	0	1	0	1
		21UR-6824	TGGAAAAAGATAATCAAACAG	1	1	0	1	2	8	0	13
		21UR-6825	TGCAAAGCTTAATTTTTTCTA	0	0	0	0	1	0	0	1
	†	21UR-6826	TGACATCCAAGTTATCATGGC	0	0	0	0	5	1	1	7
		21UR-6827	TCTTATAGGCTTTTGCAAAAA	0	0	1	0	5	5	0	11
*		21UR-6828	TCCGCGGTCGTACGGCAGATT	3	1	0	1	114	124	123	366
		21UR-6829	TCACTCATCAGTCATTAGTAT	0	0	0	0	0	0	0	0
	†	21UR-6830	TATTGCACTATTCTTTGAACT	0	0	0	1	0	0	0	1
	†	21UR-6831	TATTAGATGAGTGTGTCAGTT	3	0	0	0	1	6	0	10
		21UR-6832	TATAAATACGTTCATTGAGAT	0	0	0	0	0	0	0	0
		21UR-6833	TAGGGAAATTAAACAAAAAAA	0	0	0	0	0	0	0	0
*	†	21UR-6834	TAGGCAAGAGCGGGTGAAGAA	2	3	0	1	50	94	24	174
		21UR-6835	TAAGTTTGTGTTAGTCAAGTC	1	1	0	0	0	0	4	6
		21UR-6836	TAAAAAAATGATGGAACTACG	0	0	0	0	6	3	6	15
		21UR-6837	TTTTTCTTCAACATTCGTACC	2	2	0	0	1	1	1	7
	†	21UR-6838	TTTTGCAGGCGGAAAATAAAT	0	0	0	2	5	5	5	17
		21UR-6839	TTTTCCCATAAATCTTGCCAG	0	0	0	0	1	1	2	4
		21UR-6840	TTTGGATATTGGTCAACTTTT	2	0	0	0	5	4	2	13
	†	21UR-6841	TTTGATTTCTATTTCAAATTA	0	1	0	0	0	0	0	1
	†	21UR-6842	TTTCACATTGTCAGAGAAAAC	1	0	0	1	4	7	0	13

†	21UR-6843	TTGTTATGTTCTCGTCACATA	0	0	0	0	0	0	0	0
*	21UR-6844	TTGCAAGTACGACCACGAAAG	0	0	0	0	10	7	3	20
†	21UR-6845	TTCGTTTGTGGCACTAGGATC	0	0	0	0	1	0	0	1
	21UR-6846	TTCCCACAGCAGTTTTGATCA	3	1	1	6	79	84	9	183
	21UR-6847	TGCGGATCTCCTGCCTTTCTT	0	0	0	0	0	0	0	0
	21UR-6848	TGAGTAATTGTAATGCATGTT	0	0	0	0	2	0	0	2
	21UR-6849	TGAGATAGAAATTTTTTTATC	0	0	0	0	0	0	0	0
	21UR-6850	TGAATCAATGCGTTTTTCTCT	0	0	0	0	0	0	0	0
	21UR-6851	TGAAATCTGCGTTTTTTATTT	0	0	0	0	1	0	0	1
	21UR-6852	TGAAACATGCACTGGAATTAT	2	0	0	1	13	19	31	66
†	21UR-6853	TCTTTTGCGTTTTGGAGCCAT	0	0	0	0	2	3	8	13
	21UR-6854	TCGTTTAAGTCTACTTTGTGA	0	0	0	0	2	1	0	3
	21UR-6855	TCGAAAAACGAATGGAAAGTT	0	0	0	0	0	0	0	0
	21UR-6856	TATCAAAAAATTTAGGCACAA	2	0	0	0	7	4	3	16
	21UR-6857	TAGTTCGTAAAAAACCCAAAT	0	0	0	0	0	0	0	0
	21UR-6858	TAAAGACTGTATATTTTTTGA	1	6	4	21	203	134	8	377
	21UR-6859	TAAAAAAGAAATTCAAAACAT	0	2	1	2	0	2	0	7
	21UR-6860	TTTTCCGGCCATATTATTAAA	4	1	0	1	1	4	0	11
	21UR-6861	TTTGTTTGGTGGCCTTATTGA	1	0	0	3	5	6	10	25
†	21UR-6862	TTCCATTAAAAAACCCAAAAA	0	0	0	0	1	0	0	1
†	21UR-6863	TTATGATTCTTTTTTTGTGAT	0	0	0	0	0	0	1	1
†	21UR-6864	TGTTGTATTGCACTAAAGGGT	0	0	0	0	0	0	1	1
†	21UR-6865	TGTTATTTGATTGGCATGTAC	5	7	2	6	30	53	0	103
	21UR-6866	TGTCTCTGTCTTATTCCTAAA	0	0	0	0	1	1	0	2
	21UR-6867	TGTCTAGGGGGAACTTAAAAC	0	0	0	0	2	3	1	6
†	21UR-6868	TGCTACACTGATGCAAACAAG	0	1	0	0	0	2	0	3
	21UR-6869	TGAAGCTCCCGAAATTTCGAA	0	0	0	0	0	0	1	1
	21UR-6870	TCCATCTTTTGGAAAAATTGC	0	0	0	0	0	0	0	0
	21UR-6871	TCAAGATTTTAACATCTCATT	0	0	0	0	0	1	0	1
	21UR-6872	TCAAAAGTGAAACATTGATTT	0	0	0	0	0	0	0	0
†	21UR-6873	TAGACGAATGCTAATAGAATT	0	0	0	0	2	3	0	5
	21UR-6874	TAACACATGTATAAAAAATGA	1	1	1	0	0	0	0	3
	21UR-6875	TAAACTGCAGTTTTATGAGTC	0	0	0	2	30	27	3	62
	21UR-6876	TTTTTCAGCTCTTTTGACTTT	0	0	0	0	2	1	3	6
	21UR-6877	TTTTGAATGGATTTGTGTCTT	0	0	0	0	0	0	0	0
†	21UR-6878	TTTCCTTGCTCTTTGAAGTTT	1	0	0	0	2	1	0	4
†	21UR-6879	TTTACTACAACCAGTTTGAAT	1	0	0	0	0	0	1	2
	21UR-6880	TTTACGAAATTACGAAGTTCT	0	0	0	0	0	0	0	0
	21UR-6881	TTGATCTGATAGAATGTCATC	1	1	1	7	109	70	3	192
	21UR-6882	TTCTTCTCTTTTTTAAATCCT	0	0	0	0	1	0	0	1
	21UR-6883	TTCTCCTTGACTTCAATTTGG	0	0	0	0	1	0	0	1
†	21UR-6884	TTCTCCGTACTTTTGGATGAA	0	0	0	0	1	0	0	1
†	21UR-6885	TTCCAATGTTCTTTGCTTGTT	0	0	0	0	1	0	0	1
	21UR-6886	TTCATTGTTCGTTCAATGGTT	0	0	0	1	0	0	0	1
	21UR-6887	TTCATTAGCTGTACTTCTATA	0	1	0	0	0	6	0	7
†	21UR-6888	TTATTAGATGAGTGTGTCAGT	0	0	1	0	0	2	0	3
†	21UR-6889	TTAGGAAGACATAAATAATTG	5	7	0	5	25	28	2	72
	21UR-6890	TGTTTACAACATATCAAATTA	0	0	0	0	0	0	0	0
†	21UR-6891	TGTTGAGATAGTAATTTTATC	0	0	0	0	0	0	0	0
†	21UR-6892	TGTTAAGATCAACTAGAAGTT	0	0	0	0	0	0	0	0
†	21UR-6893	TGTTAAAAAAAATAGGAGAAC	1	0	0	0	0	0	0	1
	21UR-6894	TGGATCTGTAGTTTGTTTGTT	0	0	0	0	4	2	0	6
	21UR-6895	TGATGATAGCATTAAAATTGG	3	0	0	0	6	1	1	11
	21UR-6896	TGATCACATCTTTTAATGTTT	0	0	0	0	0	0	0	0
	21UR-6897	TGAGACATTACAATGAATAAT	1	0	0	1	21	20	3	46
	21UR-6898	TGAAAATAGGCGGTTACATTC	4	1	0	0	2	0	1	8
†	21UR-6899	TCTTGTTCTCTCCCAAAGAAT	0	0	0	0	0	0	0	0
†	21UR-6900	TCTATCGATCAAAACATATCA	0	0	0	0	0	1	0	1
†	21UR-6901	TCGTACAAAACTCAATCAAAA	0	0	0	0	8	2	2	12
†	21UR-6902	TCGATTCTGGATCCACAGTGG	0	0	0	0	1	1	0	2
	21UR-6903	TCCTCGAGAAAATTTTGTTAT	0	0	0	0	0	0	0	0
†	21UR-6904	TCAACGGACCAATAAAAATTT	0	0	0	0	1	1	1	3
†	21UR-6905	TATAATTCAAGACAGCGGAAT	2	1	0	3	42	22	10	80
	21UR-6906	TAGGTTTTTGATTCTATAGAG	0	0	0	0	1	0	0	1

		21UR-6907	TACTTGACCAAACCAATTAAA	0	0	1	0	3	2	1	7
		21UR-6908	TACATTATTTTGTAGGGTTTC	0	0	0	0	6	2	0	8
	†	21UR-6909	TAATTTTCCACGAGCGGTACA	5	1	2	1	6	12	10	37
*	†	21UR-6910	TAATTTCGTCAGCATGGCTAG	11	6	7	11	216	156	126	533
		21UR-6911	TAAAAAAATTTTGAGTTATGC	0	0	1	0	0	0	0	1
	†	21UR-6912	TTTTCAGGAATACCGTATTTC	2	0	0	0	2	0	1	5
*		21UR-6913	TTGTATTGATGGTGATGGGCT	3	2	1	1	28	36	11	82
	†	21UR-6914	TTGTAACAGTGGCATGAGATG	8	3	2	2	5	15	1	36
		21UR-6915	TTGGTATCCTTTCTTAAGTTT	0	0	0	0	0	0	0	0
*	†	21UR-6916	TTGATCATTCATGTAACGGTT	0	6	0	2	52	44	6	110
		21UR-6917	TTCTACCATCGTGCATCTTTT	0	0	0	0	4	1	1	6
	†	21UR-6918	TTCGTACAAAACTCAATCAAA	0	1	0	2	27	13	2	45
		21UR-6919	TTCCTAGTAATATTTTGTTCG	2	1	1	0	0	5	0	9
	†	21UR-6920	TTCAACTCTGATGTTTCCTTG	0	0	0	0	3	0	1	4
	†	21UR-6921	TTATGAGTCCTATTTAATCGA	0	0	1	0	2	1	0	4
		21UR-6922	TGTTATTGTTTCCAGCTTTGA	1	1	2	0	2	3	0	9
		21UR-6923	TGTGTAGGGGTGTGTTAGATT	0	0	2	1	17	14	5	39
	†	21UR-6924	TGTGAGAAATAAAGAAATCGA	0	1	0	1	1	0	0	3
	†	21UR-6925	TGATAAAAAACATCAGGCTGG	0	0	1	0	0	3	0	4
		21UR-6926	TGAGTTTTTTGATAAAGAATA	0	0	0	0	0	0	0	0
		21UR-6927	TGAGTCATCATATCAGGTATC	0	0	0	0	0	1	0	1
		21UR-6928	TGAATATAGTCAATAAATTCG	0	0	0	0	0	0	0	0
		21UR-6929	TCTGATTTCATTCCTGGTGTT	0	0	0	0	0	0	1	1
	†	21UR-6930	TCTCAATTGGCTATATATGAA	0	0	0	0	4	2	4	10
	†	21UR-6931	TCATGTGAACTCATCAAAGTT	0	0	0	0	0	2	1	3
	†	21UR-6932	TATTACTTCTTCGCAAAAAAG	0	0	0	0	0	5	1	6
*	†	21UR-6933	TATTAAATGAAGTAGAAAAAA	2	7	5	1	32	35	2	84
	†	21UR-6934	TACTTCGTACCTCACACTGTC	0	0	0	0	2	0	0	2
		21UR-6935	TACATTAATAATTTGGACAAA	0	0	0	1	26	18	3	48
		21UR-6936	TAAGGTTACGATAATCGAATC	0	0	0	0	0	0	0	0
*	†	21UR-6937	TAAGCGTAATGTGTCGGACGC	0	0	0	1	8	16	36	61
		21UR-6938	TAAAACAGAGTTTTCAATGTT	2	0	0	0	4	3	0	9
*	†	21UR-6939	CAAAAAAGAGTAGAAGGAATC	25	69	22	17	66	221	7	427
	†	21UR-6940	TTTGAAAAACAAAACCAAAAC	1	0	0	0	0	0	0	1
	†	21UR-6941	TTTACTAAAATTCATGCCAGT	6	1	0	0	2	6	0	15
		21UR-6942	TTGTTTTTCCTTGAAGCTGAC	0	0	0	0	1	3	0	4
		21UR-6943	TTGTTGCAGAAAATTGAAAAA	0	2	2	3	74	70	12	163
		21UR-6944	TTGATTCTTCACAACTTCACG	0	0	0	0	1	0	0	1
	†	21UR-6945	TTGAAAGTAGGATTTTGTATC	1	0	0	0	1	1	1	4
		21UR-6946	TTCTAGTAAAGTTTTTATGCA	0	0	0	0	0	0	0	0
	†	21UR-6947	TTCTAACCAAAACTAATGATT	0	0	0	0	0	0	0	0
		21UR-6948	TTCCACAAAACTCTGGAAATC	0	1	0	1	2	6	1	11
		21UR-6949	TTCATGTGCAAAAAAAGAATT	0	0	0	0	0	0	0	0
		21UR-6950	TTATGTATTGGCCTTTTTGTT	0	0	2	0	3	7	1	13
	†	21UR-6951	TTAGTATCAAGAAAAATACGA	3	1	1	0	2	1	0	8
		21UR-6952	TTACTTTTCCGCTGTTTTCAA	0	0	0	0	3	1	1	5
*	†	21UR-6953	TGGATGGTTGAAGAAGGAGTA	174	195	98	49	152	533	19	1220
		21UR-6954	TGAATTCCAGTGAATTACACT	0	0	0	0	0	0	0	0
*		21UR-6955	TGAAGCGCTGAAAATCATCAA	0	0	0	1	6	6	0	13
		21UR-6956	TGAACACTGCTCTCCAAAACG	0	0	0	0	0	0	0	0
		21UR-6957	TCTTCTTCTATTTTAAGAAAA	0	0	0	0	0	0	0	0
		21UR-6958	TCTGAAAGCGGAGTGTTAAAA	0	0	0	0	4	3	1	8
		21UR-6959	TCATTTTCAAAAACCGTATCG	0	0	0	2	15	11	4	32
	†	21UR-6960	TATTTCTCCTATCAACAAGCA	5	1	0	3	24	59	14	106
		21UR-6961	TAAGGATATATATGATATATG	0	0	0	0	3	1	0	4
	†	21UR-6962	CGTAATCGCATATTTATTCCA	6	0	1	0	1	7	0	15
	†	21UR-6963	TTTTTTTCAGAGAAAAGTAAA	0	0	0	0	0	1	1	2
		21UR-6964	TTTTTAGTTGGTGGTGAAAAA	1	1	0	0	2	9	1	14
		21UR-6965	TTTGGAAATGCTAGTAAAGAA	0	1	1	3	45	32	19	101
		21UR-6966	TTTCTTCAGCTTTTTGGTTTC	0	0	0	0	0	0	0	0
		21UR-6967	TTTCCATACGCTTCAAGCAAA	1	0	0	1	5	5	3	15
		21UR-6968	TTTCACTTGCATTGTTTCTTC	0	0	0	1	3	1	1	6
	†	21UR-6969	TTGTATTTTATTCTGGTAAGA	1	0	0	0	0	2	1	4
	†	21UR-6970	TTCGCTGTATATTAGGCTCCG	1	1	4	4	28	29	3	70

		21UR-6971	TTCAAGTTCGATAGCACGTCG	0	0	0	1	3	7	5	16
	†	21UR-6972	TGCTCCTCTTTTCCCTTTGTT	0	0	0	0	0	0	0	0
		21UR-6973	TGATTTAAAAAACGAAAAAAA	0	0	0	0	1	0	2	3
		21UR-6974	TCGGAATTTTATGAACACACT	0	0	0	0	0	0	0	0
		21UR-6975	TCAGATTTCTGGATTAGTGTT	0	0	0	0	3	2	0	5
		21UR-6976	TCAAAATTGTATTTCGCAAAG	2	0	0	0	0	0	0	2
		21UR-6977	TATTGCATACTTGAGAAAAAA	0	0	0	0	8	11	5	24
	†	21UR-6978	TATCGCTTGAATTTAGAACAA	0	1	0	0	1	4	0	6
		21UR-6979	TACCTCTCTGATGTTTTCATT	0	0	0	0	4	5	0	9
		21UR-6980	TAAGTTACAATTGGTAACCGA	0	0	0	0	0	0	0	0
	†	21UR-6981	TAACGCAATTGATCGAAATTT	0	0	0	0	0	0	1	1
		21UR-6982	TAAATAAATGAAATCAAGAAA	0	0	0	0	3	0	1	4
	†	21UR-6983	TTTTGATAGACTTCCTTAACG	0	2	0	1	3	4	0	10
		21UR-6984	TTTGATTTGATAATTGATAAT	0	0	0	0	0	0	0	0
		21UR-6985	TTTCGATATCACTCTCGAATT	0	0	0	0	0	0	1	1
		21UR-6986	TTTCATTAAATTTTGTGAAGA	0	0	0	0	1	1	0	2
		21UR-6987	TTTAGGATTTTTGATTTTAGT	0	1	0	1	1	1	1	5
	†	21UR-6988	TTGCTTAAGATGCCGTTTTTT	2	2	1	0	15	20	4	44
	†	21UR-6989	TTGCAAAAGCATAGATAAAAC	0	0	0	0	0	0	0	0
		21UR-6990	TTAGCCCGGTTTTCTACTTCG	0	0	0	0	0	0	1	1
	†	21UR-6991	TTACGGATACTGTTCAAAAAT	4	7	2	1	7	23	1	45
	†	21UR-6992	TTACATTAAGTCGGTGTGAAA	5	6	2	1	7	20	3	44
	†	21UR-6993	TGTGTCCACGAACAGAAAACT	0	0	0	0	0	0	0	0
		21UR-6994	TGTATGATCGATTTTTCCTTT	2	0	0	0	0	0	0	2
		21UR-6995	TGGATCAATGTTTGAAAGCAA	0	3	1	0	3	10	0	17
		21UR-6996	TGCGATTTTTTTAAAATTAAA	0	0	0	0	0	0	0	0
		21UR-6997	TGATTAGTTGAGTGTAGGCCA	5	5	1	1	6	17	0	35
	†	21UR-6998	TGAATAAGACAAATTAGACAA	1	4	0	4	49	36	7	101
		21UR-6999	TCGAGCTTTCGTTTGAAGTTT	0	0	0	0	0	0	0	0
		21UR-7000	TCGACTTGACTTGACATAACT	0	0	0	1	0	3	0	4
		21UR-7001	TCCACTATTTTCAATTTCGAA	0	0	0	0	0	0	0	0
	†	21UR-7002	TCAGCATTTAAAACTCCTTGC	0	0	0	0	2	0	0	2
*		21UR-7003	TCAGCATAACTCATTTTTTAA	0	2	0	3	28	11	0	44
		21UR-7004	TCAAAAACGAATAGCTGAATT	0	1	0	1	1	2	0	5
		21UR-7005	TAGTGATATCTTATTACTAAA	0	0	0	0	0	0	0	0
	†	21UR-7006	TAATCTCTTTAGGTAAATTAA	0	0	0	0	0	0	0	0
		21UR-7007	TAAAGCTTTTTTAGTAAATGA	0	0	0	2	0	0	0	2
		21UR-7008	TAAACAATCAGAAAAACGGTA	1	2	1	4	47	99	19	173
	†	21UR-7009	TTTATTGCTCGAGAATACAAC	0	0	0	0	0	0	1	1
		21UR-7010	TTTAACCGATATCAGAAAAAT	0	0	0	0	1	0	0	1
		21UR-7011	TTGTCTTAAGTTTTAGCTGGT	0	0	0	0	9	9	0	18
	†	21UR-7012	TTCTATAGAAAAACGCTTAAG	2	1	0	3	34	23	2	65
		21UR-7013	TTCCGTTCCATTGCTAGTTTT	0	0	0	0	0	0	0	0
*	†	21UR-7014	TTATTCATTCGGACAAAAATT	105	36	20	40	91	210	71	573
		21UR-7015	TGTTTTCTGTTCTATTCAGTT	1	0	0	0	0	0	0	1
	†	21UR-7016	TGTTGTGCATCGATTTGTCAT	0	0	0	0	1	0	0	1
	†	21UR-7017	TGTGTTGCTAAAAACTTTACA	0	0	0	2	1	1	0	4
		21UR-7018	TGATCTGACGAGAAAGTTTTT	1	0	0	0	0	1	0	2
		21UR-7019	TGATAAATTTTCAAGACTAAC	0	0	0	0	0	0	0	0
		21UR-7020	TGAGTCCCAATTAAGTAATGT	0	0	0	0	0	0	0	0
	†	21UR-7021	TGAATGAACCTTAGAAAAAAG	0	0	0	0	5	1	0	6
		21UR-7022	TGAAATTGATATGTAAAAACT	0	0	0	0	0	0	0	0
		21UR-7023	TCCTGCTGTCATTGTGTTGTC	0	0	0	0	0	0	1	1
		21UR-7024	TATGAATAGCGGTTTTTTTCA	1	1	0	2	27	21	1	53
*		21UR-7025	TACCTGTGTGTTTGTTACAAA	0	0	0	1	7	4	2	14
		21UR-7026	TAATCTTTTCAAAAAAGTTTA	0	0	0	0	0	0	0	0
	†	21UR-7027	TAAGAAACTTTTTCGAGAGTT	2	0	0	0	0	0	0	2
		21UR-7028	TAACGCCTTAAAAATCGTGCA	0	0	0	0	0	2	0	2
*		21UR-7029	TAACAGACTTATAAAGACATT	2	6	2	3	46	41	5	105
		21UR-7030	TAAAAAGCTTTTGGATAAACT	0	0	0	1	9	11	1	22
		21UR-7031	CAAATTAAAATTTCAGTTGAA	0	0	0	0	0	0	0	0
	†	21UR-7032	TTTTCTTAGGGCTTTTGGCAA	87	50	28	25	108	334	16	648
		21UR-7033	TTTTCCCACGAACCTCCACAA	0	0	0	0	0	1	0	1
		21UR-7034	TTTGATCAATTTTCTAGTAGT	0	0	0	1	4	3	0	8

	21UR-7035	TTTAATGAAGACAAGAAAAAA	0	0	1	1	9	6	0	17
	21UR-7036	TTCTTCGTCTCTAGTTTCTTC	0	0	0	0	1	0	0	1
†	21UR-7037	TTATTATTATAGAACGGACGA	9	3	2	4	76	72	137	303
	21UR-7038	TGTTCTAGAATAAAATAAAAT	0	0	0	0	0	0	0	0
	21UR-7039	TGTGTTTTTTTTTCTGTGGAC	24	15	10	4	27	80	6	166
	21UR-7040	TGATTTTTCAGAAATTCAGAA	0	0	0	0	0	0	0	0
	21UR-7041	TGAGATGAGTGCATCAATTCA	0	0	0	0	0	0	0	0
	21UR-7042	TGAAATCGTTATCCCTATACT	0	0	0	0	0	1	0	1
	21UR-7043	TCTTGAAGATGAATTGGGCTC	2	3	1	0	3	6	2	17
	21UR-7044	TATTTTTGCAATAGTGATGAA	0	0	0	0	0	2	0	2
* †	21UR-7045	TATTTAATTTTGCGAGTAGAA	3	4	4	3	55	74	28	171
	21UR-7046	TAGTTAATAATTATTTAATGA	0	1	0	0	0	0	0	1
†	21UR-7047	TACTTTTTTTTGAAAGTAAGT	0	0	0	0	0	0	0	0
*	21UR-7048	TACGATCTTCGGCAAGCTTTC	1	0	1	0	14	9	3	28
	21UR-7049	TAAGTGGTTCACGAATTTTTT	1	0	0	1	2	11	0	15
	21UR-7050	TAAGCAATAAAATGATAAAGA	0	0	0	0	0	0	0	0
†	21UR-7051	TTTGATTGGAATCCCTGTCAC	0	0	0	0	3	5	0	8
†	21UR-7052	TTTCTTGATTTTCTATGATTA	2	0	0	0	0	0	0	2
	21UR-7053	TTTCTTCTTAGCTCTACAACA	0	0	0	0	1	0	0	1
†	21UR-7054	TTTAGGATGCCTGATCATATT	0	0	1	0	0	0	0	1
†	21UR-7055	TTTAACTCAGTCTAACTCATA	0	0	0	0	0	0	0	0
†	21UR-7056	TTGTAGAACGACAGGAAAATT	2	0	0	4	42	35	4	87
	21UR-7057	TTGAGTCGATGTTTATAGTCC	0	0	0	0	20	11	0	31
†	21UR-7058	TTCGTGTGTTTCATCTTCTCT	2	0	0	0	0	1	0	3
†	21UR-7059	TTCAAGAGTACGATCAGTTTT	4	2	1	1	1	1	0	10
	21UR-7060	TTATTGCGCTCTTAAAAAAAG	1	0	0	0	1	1	0	3
†	21UR-7061	TTATGATTGCTTAGTGGAAGA	2	6	5	3	115	137	34	302
	21UR-7062	TTATAACTCAATTCAATATCC	0	0	0	0	1	1	0	2
†	21UR-7063	TTACAATGTTCCAAAAAAAAG	5	1	0	1	6	6	3	22
	21UR-7064	TGTAATTGCTATCATGAAAAA	0	0	0	0	0	0	0	0
	21UR-7065	TGGTGCGAATATTTTGTTCCA	0	0	0	0	0	1	0	1
†	21UR-7066	TGATTTACGCGCTAATCTGAG	0	0	0	0	6	2	3	11
	21UR-7067	TGAGAGATTTTGTTTTGTTTT	0	0	0	0	0	0	0	0
	21UR-7068	TGAACGAGAATCAGTAAGGAA	0	0	1	0	1	3	0	5
	21UR-7069	TCTCGAAAACTCATCGAGCAT	1	0	1	1	7	10	1	21
†	21UR-7070	TCCGTTGTATTATTCAGAAAA	0	0	0	0	0	0	0	0
†	21UR-7071	TATTACACGATTGATCTCACT	0	0	0	1	3	3	0	7
	21UR-7072	TATCTAAGGAACTTGAAACTT	0	1	0	0	0	0	0	1
†	21UR-7073	TAGAACGTTTGGAAAATTTTT	0	0	0	0	2	1	1	4
	21UR-7074	TAATTTTTGAAGTCTCACACA	0	0	0	3	18	10	1	32
	21UR-7075	TAACGTGTCGAAAAAATATGA	0	0	0	0	3	4	0	7
	21UR-7076	TTTTCTGAACCAACTACAAAA	0	1	0	0	10	10	8	29
	21UR-7077	TTTGCATCGTTTTGTCACTCT	0	0	0	0	0	0	0	0
	21UR-7078	TTTCGATTCAATTTAGTGAAA	0	0	0	0	0	0	0	0
†	21UR-7079	TTTATTCTCTTCAGAGATGGC	0	0	0	1	3	10	2	16
	21UR-7080	TTTAAAACTGGCCTGCAATCT	0	0	0	0	2	2	0	4
†	21UR-7081	TTGTGTGTTCTTCTGGAATTT	0	0	0	0	0	0	0	0
	21UR-7082	TTGCTTTGTATTAACCGTTTA	0	0	1	0	1	0	0	2
†	21UR-7083	TTGAACGTTTACTATCGCCAT	0	0	0	0	1	2	0	3
†	21UR-7084	TTCTTGATTGTCAATTGTTTT	1	0	0	0	5	4	0	10
	21UR-7085	TTCTGTTGTGGATGCTAATTT	1	0	0	3	30	13	4	51
	21UR-7086	TTCTCGCAGGTCTTTGAAAAT	0	0	0	1	4	3	2	10
	21UR-7087	TTCCATGTTGCTTTTTGCAAA	0	0	0	0	1	0	1	2
†	21UR-7088	TTCCAGCCATATTTAAATCAA	0	0	0	0	2	1	3	6
†	21UR-7089	TTCATCCACTAATTACGAAAA	0	0	0	0	0	1	0	1
†	21UR-7090	TTCAGATAATTTTTACGAATT	0	0	1	0	0	0	0	1
†	21UR-7091	TTCAACGAGTACATTTGATAT	0	0	1	1	17	7	0	26
†	21UR-7092	TTACACTCCGTTTTGAACGTA	12	1	3	0	1	8	2	27
	21UR-7093	TGTTTAACCAATGTTTACCAT	0	0	0	0	0	0	0	0
	21UR-7094	TGTTCTCGTCGCATCAACTTT	1	0	1	0	5	5	2	14
†	21UR-7095	TGTTCGTTTTTCGCCATGAGT	0	0	0	0	0	0	0	0
	21UR-7096	TGTGTTAGTTACTCCTCTGAA	0	0	0	0	4	11	6	21
†	21UR-7097	TGTGTAGTTTGTTCATTAAAA	0	0	0	0	1	1	0	2
†	21UR-7098	TGGTTCATCAAAAAATGTAAA	0	0	0	0	0	0	0	0

		21UR-7099	TGATCTTATTTTTGATCATCA	0	0	0	0	0	0	0	0
		21UR-7100	TGAAAGAAAGATGCGATTAGT	3	0	0	2	1	3	0	9
		21UR-7101	TCCGTAATGATGCAAGACTAA	0	0	0	0	0	1	0	1
		21UR-7102	TCAGTTTCACAACTTCCACTT	0	0	0	0	0	1	0	1
	†	21UR-7103	TCAGAAAATTGCTATGAATAT	0	2	2	6	72	41	9	132
		21UR-7104	TCACATAATCATATAGCTTTT	0	0	0	0	1	1	0	2
		21UR-7105	TCAACTTTTTTTTTCAGAAAT	0	0	0	0	0	0	0	0
	†	21UR-7106	TATATCTGGTTTTTTTGGTAT	0	0	0	1	10	6	2	19
		21UR-7107	TAATGATTTTATCTGAAAACC	0	0	0	1	0	1	0	2
		21UR-7108	TAACGTTTTTTTGTTGTGGTA	0	0	0	0	4	2	0	6
		21UR-7109	TTTTTTTCTGGCATTAAATGG	4	4	1	8	81	78	11	187
	†	21UR-7110	TTTTCATGAGTTTCTGGATTG	9	3	1	4	38	68	7	130
	†	21UR-7111	TTGTTAATTTTTGGCAATGGG	0	0	0	0	0	2	0	2
	†	21UR-7112	TTGGTGAAGTTTCAAGTAGGT	1	1	1	0	4	4	1	12
	†	21UR-7113	TTGAGTAATGAAATGGGGAAT	0	0	0	0	1	0	2	3
		21UR-7114	TTCTTCTGCCTCCAACACACC	0	1	0	0	0	0	0	1
		21UR-7115	TGTTGTATTTTCGTTCAATTT	2	0	0	0	0	0	0	2
		21UR-7116	TGTTCAACGCTAAAAATTTTT	0	0	0	0	0	0	0	0
	†	21UR-7117	TGCTCTGTGTTTTTTTAATCA	0	0	0	0	0	0	0	0
		21UR-7118	TGCATTCATTGAGTTTTGAGA	0	0	0	0	0	2	0	2
	†	21UR-7119	TGATTTTTATTTTGTCTGATT	0	0	1	1	7	5	4	18
		21UR-7120	TGAAGAGCAATGCATTTTTGT	0	0	0	0	8	16	1	25
	†	21UR-7121	TGAAAACCGAGTTTCTAACAA	0	0	0	0	0	0	0	0
	†	21UR-7122	TCGTTTATCCCGTCGATAATA	2	0	0	0	1	2	3	8
	†	21UR-7123	TCATCAACTCCGGAATAAAAT	0	0	0	0	0	0	0	0
	†	21UR-7124	TATTGACAAATTCAGGTAAAT	0	0	0	0	1	1	0	2
	†	21UR-7125	TATTGAATGAACCTTAGAAAA	0	0	0	0	4	0	0	4
*		21UR-7126	TATGAAGTTGAATGGGCATTT	1	5	1	0	1	16	0	24
	†	21UR-7127	TATATCGTTAATCTGGCAATC	0	0	0	0	18	7	4	29
		21UR-7128	TAGCGATTCTTTTTTCCTTCT	0	0	0	0	0	0	0	0
		21UR-7129	TAGATTAGTAACTTAAGAATG	0	0	0	0	0	4	0	4
	†	21UR-7130	TACATGGTAATTTGAAATTCC	0	0	1	0	1	1	0	3
*	†	21UR-7131	TAATTGATTGTCTCCGGTCTT	0	1	0	0	4	3	5	13
	†	21UR-7132	TAATCCTTGCCTGTTTATTCT	0	0	0	0	0	0	0	0
		21UR-7133	TAATAACACCGATCTTTTGAT	0	0	0	0	0	0	1	1
		21UR-7134	TAACAGAACTGTTTCATAATT	0	1	0	1	31	12	0	45
		21UR-7135	TTTTTTTGCCATCACCCGACG	1	0	0	0	0	0	2	3
		21UR-7136	TTTTTAGCTCGATTTTCACTT	2	0	0	0	0	0	0	2
	†	21UR-7137	TTTTGATGTGAGTATAGAAAG	0	0	1	0	5	8	0	14
	†	21UR-7138	TTTTCCTGCCAAATTTAAGAA	0	0	0	0	0	0	0	0
		21UR-7139	TTTGCTCAACCAAATATCAAC	0	0	0	0	0	0	0	0
	†	21UR-7140	TTTGAGACTTTCCTGACTCAT	0	0	0	0	8	2	0	10
*		21UR-7141	TTTGACGTGACTTCGTTCTGA	0	1	3	2	6	19	1	32
		21UR-7142	TTTGAAAATTCTAAGAAGATT	0	0	0	0	0	1	0	1
		21UR-7143	TTTCTTATCCTTTTTGACAAC	0	0	0	0	1	1	1	3
	†	21UR-7144	TTTCTACTTTACTCTGATGTA	0	0	0	0	1	0	0	1
	†	21UR-7145	TTGTTGATCTCCTGGTAATTC	0	0	0	0	3	2	0	5
		21UR-7146	TTCTTTGAATGAAAAAAACAC	0	0	0	0	0	1	0	1
	†	21UR-7147	TTCTGTTCGGTTCATGTGAAA	0	0	0	0	3	6	3	12
		21UR-7148	TTCGCAAGTGCCCCCAAAAAT	0	0	0	0	0	0	0	0
	†	21UR-7149	TTCACTATCTTATTGACCAAC	1	0	0	0	1	0	2	4
		21UR-7150	TGTTCATAGTTTTTCAAACTT	0	0	0	0	0	0	0	0
		21UR-7151	TGTAAGCAATTCAAAAGCATG	0	0	0	0	7	5	0	12
	†	21UR-7152	TGGGTGAAAACTTTATGAATT	0	0	0	0	0	0	0	0
		21UR-7153	TGGATCATTCTGAAATGAGAA	0	0	1	0	1	2	0	4
	†	21UR-7154	TGATCCTTTTCAGTGAGAACT	0	0	0	0	1	2	0	3
		21UR-7155	TCTTGCTACCTTTTGAAGTCA	0	1	0	1	1	5	1	9
	†	21UR-7156	TCTTAGCAATATGAAAAAACA	0	0	0	0	0	0	0	0
	†	21UR-7157	TCTGCTCATATCTTGTAATTT	0	0	0	0	0	0	0	0
		21UR-7158	TCTAAAATTGAAGTTAACACA	0	0	0	0	0	0	0	0
		21UR-7159	TCCTGTCTTTTCTTGTATTTC	0	0	0	0	0	0	1	1
		21UR-7160	TCCGAATCCAATAAAGATTCC	0	0	0	0	3	0	0	3
	†	21UR-7161	TCCAGAATCATCACAAACGGT	0	0	0	0	3	1	0	4
		21UR-7162	TCCAATGTAGGTATTTGTTTT	0	0	1	0	10	5	0	16

	21UR-7163	TCAGAAACAAATGAGAAAAAA	0	0	0	0	0	1	0	1
†	21UR-7164	TCACTAAGGCCGAAATTACAA	0	0	0	0	0	0	1	1
	21UR-7165	TCACATTCCCGTTTAAAAAAT	0	0	0	0	0	0	2	2
	21UR-7166	TATGCTCGAAAAACAAAAGAG	0	0	0	1	0	1	0	2
	21UR-7167	TAGGTTTGTCCAGTTCACGAC	1	0	0	0	0	0	0	1
†	21UR-7168	TAATCATAAAAACTTGGATAA	2	0	1	2	6	10	0	21
	21UR-7169	TAACACAGTATTATAGGAAAC	0	0	0	1	1	14	1	17
	21UR-7170	TAAATTTGATCGTTCACAATT	16	6	2	15	236	148	270	693
	21UR-7171	TAAACGAGTTACAATGAAATT	1	0	0	1	13	4	1	20
	21UR-7172	TTTTGTTTGAAAAAACACTGC	0	0	0	0	0	0	0	0
	21UR-7173	TTTTCTTTTTTGAATCACCTT	0	0	0	0	0	0	0	0
	21UR-7174	TTTTCTCGTCTCAATTACTTG	0	0	0	0	2	0	0	2
†	21UR-7175	TTTTCAAAAAAAAACGTATGA	3	0	0	0	1	2	3	9
	21UR-7176	TTTGTAGTTGTATCACTATAT	2	2	0	4	4	3	0	15
	21UR-7177	TTTGCAATTTTGAATTGCGGA	0	0	0	0	2	1	0	3
†	21UR-7178	TTTGCAAATAGAAAGGTGGCA	2	0	0	1	8	8	2	21
	21UR-7179	TTTCGCACAAGTACTTCAAAA	2	0	1	0	3	8	0	14
	21UR-7180	TTTCAACTTTATTTTCAATAC	0	0	0	0	0	0	0	0
	21UR-7181	TTTACGCCTTGGGTTTGTTTT	0	0	0	0	0	0	1	1
†	21UR-7182	TTGTTTACCCATCTTACTGTA	0	0	0	1	3	6	1	11
	21UR-7183	TTGCACTAAAACATTCGTATA	1	0	0	0	4	3	2	10
	21UR-7184	TTGCAATTTTCAAATTCTTGT	2	0	1	0	0	2	0	5
†	21UR-7185	TTGACTTTTCGTTTCTAAATA	2	0	0	0	2	1	2	7
†	21UR-7186	TTCGCCCATCTCCTAGGAAGT	0	0	0	0	0	0	4	4
†	21UR-7187	TTCAACAAAAAAATTTTCTCA	0	0	0	0	0	0	0	0
	21UR-7188	TTATTCGGTTTTCCCAGCATC	1	0	0	0	4	11	8	24
	21UR-7189	TTATAGTGGGATTTTATTTCC	0	0	0	0	0	0	0	0
	21UR-7190	TTAGATTGTCTAGGAACCAAA	0	1	0	2	35	16	1	55
*	21UR-7191	TTAATTTGCGGTACACGAATT	0	1	0	0	2	1	0	4
* †	21UR-7192	TGTTTTTAGAATTTTAGTCGG	240	57	28	26	77	297	41	766
	21UR-7193	TGTATATGTAGTAACGATAGT	2	4	0	4	30	37	2	79
†	21UR-7194	TGATTGGATTTTTGTTAAAAA	0	0	0	0	0	0	0	0
	21UR-7195	TGACTATCCAACAAAATCTTC	0	0	0	0	0	0	0	0
	21UR-7196	TGACACTTGTTTAAGCCAATT	0	0	0	0	2	0	0	2
	21UR-7197	TCTACTTCGAGTCTTAACTTG	0	0	0	0	0	2	0	2
	21UR-7198	TCTAATTTTTACAGCAACACA	0	0	0	0	0	0	0	0
*	21UR-7199	TCGGCCTGTCATATCGTCATC	6	2	9	15	312	313	149	806
†	21UR-7200	TCATAAAAACTTGGATAAAGA	0	0	0	0	0	0	0	0
†	21UR-7201	TATTAAAAATCGAAATATGGG	2	0	0	1	2	1	0	6
	21UR-7202	TATCCAATTGTTTTGTATTTC	0	0	0	0	0	0	0	0
	21UR-7203	TATATACGCCGACTAGAGAAT	0	0	0	0	0	0	0	0
†	21UR-7204	TATACAAGCATTTTCCATGTA	3	0	1	0	4	2	2	12
*	21UR-7205	TAGGTTGTAAATTTTTATCGC	0	0	1	1	13	9	4	28
	21UR-7206	TAACACTCTCATGGTATGCTT	0	0	0	0	0	1	1	2
	21UR-7207	TAAAGCCTGGCTTTGAAAATC	0	0	0	0	1	1	0	2
†	21UR-7208	GGCGTAGATATGACATGATAT	0	0	0	0	1	0	0	1
†	21UR-7209	TTTTGAAGCTTACTGGAAAAA	0	1	0	0	2	3	1	7
†	21UR-7210	TTTTATTGTCGAAATTTAATT	1	1	0	0	7	6	4	19
†	21UR-7211	TTTGATCGGTTTTTCAATTCT	0	0	0	2	6	2	0	10
	21UR-7212	TTGTTTTCGTATAATACAACA	0	0	0	0	2	2	0	4
	21UR-7213	TTGTGAATTGAAGTTGCATCA	0	0	0	0	1	0	0	1
†	21UR-7214	TTGATAAAGGCAGAATTTTAT	2	4	5	3	9	26	0	49
	21UR-7215	TTCTGAAATGTCTCCCAAAAA	0	0	0	0	0	0	0	0
†	21UR-7216	TTCCAGAAGAAGAAGTACTAA	0	0	0	2	7	6	0	15
	21UR-7217	TTCACTTGTACTTTCAGATTC	0	0	0	1	2	3	0	6
	21UR-7218	TTCAAACGATTAAAGCGTTTA	0	2	0	0	7	6	1	16
	21UR-7219	TTAGAACATAAAGGGAAATGT	0	0	0	0	2	0	0	2
†	21UR-7220	TGTTTGTTTTGAACCATAACA	0	0	0	0	0	0	0	0
†	21UR-7221	TGTTGAATTATCTTCCACCAC	0	0	0	0	2	0	0	2
†	21UR-7222	TGTTGAAAAAATGTACTGTCA	0	0	0	0	0	1	0	1
†	21UR-7223	TGTTCCATTGTATCAAATTCA	0	0	0	0	0	0	0	0
	21UR-7224	TGCGAGCTGCTGTTTCAGGTT	0	0	0	0	8	4	1	13
*	21UR-7225	TGCACCACATCGTCGAAATTA	161	24	18	17	117	287	83	707
†	21UR-7226	TGATAAAAGGAATAGTCTCCA	0	0	0	0	0	0	0	0

	†	21UR-7227	TGAGTCGGTCAATTTAAACAT	0	2	0	2	38	25	11	78
		21UR-7228	TGAGCACTAAACTGTTTTTCT	0	0	0	0	1	0	0	1
		21UR-7229	TCTTTTTCGAATTACCTAGGA	0	0	0	0	0	1	0	1
		21UR-7230	TCTCATCACACTCCACACTCT	0	0	0	0	0	0	0	0
	†	21UR-7231	TCGATTGAACTTTTCTGTAAG	0	0	0	0	0	0	0	0
		21UR-7232	TCCACGTCTTACACTAATTTT	0	0	0	1	1	1	0	3
		21UR-7233	TCATGCTCTTTCAAAAATATG	1	1	1	0	0	4	0	7
		21UR-7234	TCACAACACTTGAATAACTTA	0	0	0	0	0	0	0	0
	†	21UR-7235	TATTGAGACTTGAGACTACCG	0	0	0	0	0	2	0	2
	†	21UR-7236	TATGAGAAAATCAAATTTAGG	0	0	0	0	0	0	0	0
		21UR-7237	TATGACAGCCTTACTTACATT	0	0	0	0	0	0	1	1
		21UR-7238	TATCTAAAACTCATTATTTCC	0	0	0	0	3	1	1	5
		21UR-7239	TATCAAACCCGATACAACCAA	0	0	0	0	2	2	3	7
*	†	21UR-7240	TATATGGGAAATGGGAAATAA	0	1	0	1	33	17	12	64
		21UR-7241	TAATACGTCTGGGCGTTTACT	0	0	0	1	8	6	2	17
		21UR-7242	TAAAGTTTTTCGGAGCTAATG	0	0	0	0	1	4	2	7
		21UR-7243	TAAAATTAATATACCTCTAAA	4	1	2	1	1	3	5	17
		21UR-7244	TTTTTTGTGTATCTCACCTGA	0	0	0	0	1	0	0	1
	†	21UR-7245	TTTTAGACGAGGATGGTGAAT	4	1	0	0	1	8	3	17
		21UR-7246	TTTGTTTCTTTTTCGGAATTA	2	0	0	2	3	5	0	12
		21UR-7247	TTTGATTTTTTTCCATCTTCA	1	0	0	0	1	0	0	2
	†	21UR-7248	TTTGAAAAGTCAGATTTGCAT	2	4	1	11	109	110	23	260
	†	21UR-7249	TTTAACCTAAGCAGGAGTGAA	3	0	0	0	0	2	0	5
*		21UR-7250	TTGTTCCAATCTTCATGGAAT	0	1	1	1	6	13	2	24
		21UR-7251	TTGCGCAATTGTTCTTAAAAA	0	0	0	1	0	0	0	1
	†	21UR-7252	TTCGTAGAGCCAACGTACTTT	0	0	0	0	1	0	0	1
	†	21UR-7253	TTCGACGATTGTTGTTCTCAA	0	0	0	0	0	0	0	0
		21UR-7254	TTCATACATAGTCAATAAATT	9	0	1	6	6	9	3	34
*	†	21UR-7255	TTAGAATTTTAGTTGACTATG	1	0	0	2	3	6	1	13
	†	21UR-7256	TGTTCCATCGTGATAATCAAA	0	0	0	0	0	0	0	0
		21UR-7257	TGGTACTTCGTTTCCGTATTT	0	0	0	0	0	0	0	0
	†	21UR-7258	TGGATTCTACTATATGGTACA	0	0	0	0	0	1	0	1
		21UR-7259	TGATCTAATGGCCGTTGCAAA	0	0	0	0	0	0	1	1
	†	21UR-7260	TGAGAGGGACGAGTTTATTTT	2	5	0	1	28	18	17	71
*		21UR-7261	TGACTGAACATCAAAGGAGAA	4	8	5	18	216	268	30	549
	†	21UR-7262	TGAACAAGAAAAATATGGGTT	1	0	0	1	7	3	0	12
		21UR-7263	TCTTTTCAAATTACATAAAAA	0	0	0	0	0	0	0	0
		21UR-7264	TCTGAAAATATCACTCAATAA	0	0	0	0	0	0	0	0
		21UR-7265	TATGATATCACGATGTATTTT	0	0	0	2	2	3	0	7
	†	21UR-7266	TAGTTTTAGAATCGTATAGTT	4	0	1	2	17	10	9	43
*		21UR-7267	TAGTGAGACTGTTCTGAAAAA	0	1	0	0	13	17	3	34
		21UR-7268	TAGCGATCAATCATTTGTTTT	0	0	0	0	1	0	0	1
		21UR-7269	TAAAGTTACGGTATGAAAACA	0	1	1	2	14	20	0	38
		21UR-7270	CTCACAAAGTAGATCGATTCC	1	7	2	2	10	49	2	73
		21UR-7271	TTTTTTTTCATGAGTTGACTC	0	0	0	0	3	2	0	5
		21UR-7272	TTTTCCAGCAATTCAACCATT	0	0	0	0	0	1	1	2
	†	21UR-7273	TTTGTTTGTTTTCCTCTGAAT	1	0	0	0	0	3	0	4
	†	21UR-7274	TTTGGTTGGTGTATGAAGCAA	1	3	1	1	89	71	4	170
	†	21UR-7275	TTTGGATTGTTATTATGATTC	1	0	0	0	1	1	0	3
	†	21UR-7276	TTTCTTACGACGAAACAGTAG	0	0	0	0	1	0	1	2
	†	21UR-7277	TTTCGCTGTATATTAGGCTCC	1	0	1	1	25	15	3	46
		21UR-7278	TTTACGGTAGATTGCAAAAAA	0	2	0	0	13	20	2	37
	†	21UR-7279	TTTACGAACACAATTAAATTT	0	0	0	0	0	0	0	0
	†	21UR-7280	TTTAAAGCAGCATTTTGAAAA	0	0	0	1	9	15	1	26
		21UR-7281	TTGGTAATTAAAAAAAAATAA	0	0	0	0	1	0	0	1
	†	21UR-7282	TTCTGCAGTAGCGTGTTTAGA	0	0	0	0	1	0	0	1
	†	21UR-7283	TTCTCGGGAAAAAAATATTGG	0	0	0	0	5	1	2	8
		21UR-7284	TTCGATTTTTTCTTTCAATAG	0	0	0	0	0	2	0	2
		21UR-7285	TTCAGTTCAGAAATTGAAAAC	0	0	0	0	1	2	0	3
		21UR-7286	TTATTTTGCTTTCCTTGGATC	0	0	0	0	2	3	3	8
	†	21UR-7287	TTATTCACTCTAAAGGAAACT	0	1	0	1	3	6	0	11
	†	21UR-7288	TTATGTCAGATGAAAAAAAAA	0	1	1	0	0	11	0	13
	†	21UR-7289	TTAATGTTCATTTTTAAGCGA	0	0	0	0	0	0	0	0
		21UR-7290	TGTTTGTAATTTCTTGAGAAA	0	0	0	0	0	0	0	0

		21UR-7291	TGTTGATTTCAAATTACTTAC	0	0	0	0	0	0	0	0
		21UR-7292	TGTGGCTCCTGGTTTTAAATT	0	0	0	0	0	0	0	0
		21UR-7293	TGTCGAATTATTGACAAAAAT	0	0	0	0	0	0	0	0
	†	21UR-7294	TGGCTCAGGAAATTAGTTATT	0	0	0	0	6	5	2	13
		21UR-7295	TGCTGAAGTTTTTTAACGAAA	0	0	0	0	3	2	1	6
		21UR-7296	TGCATTTTCTCTGTAAGTTGG	0	0	0	0	1	1	0	2
	†	21UR-7297	TGAGCTTTCTTTATTAATTAA	0	0	0	0	0	0	0	0
		21UR-7298	TGAATTTTTCGTATACATTTT	0	0	0	0	8	4	1	13
	†	21UR-7299	TGAAAGTAGGATTTTGTATCG	3	0	0	0	2	0	0	5
		21UR-7300	TCTTCGCATCTACTAATTATT	0	0	0	0	0	1	0	1
		21UR-7301	TCGGATCTTTGTCTACTCCTT	0	0	0	0	0	0	0	0
	†	21UR-7302	TCGAAAACGCATTAAAATTGA	0	0	0	0	0	0	0	0
		21UR-7303	TCATTTCTTATTTTAGAATTG	0	0	0	0	0	0	0	0
		21UR-7304	TCAGTTGAAACGTCATTTAAA	1	0	0	0	0	1	0	2
	†	21UR-7305	TATGACTACACTTAATGATCA	1	4	0	0	10	7	1	23
	†	21UR-7306	TATCTATACAGGATTAGCTTG	0	0	0	0	0	0	0	0
		21UR-7307	TATCGTTTTGGTTCTAAATGA	0	0	0	0	1	0	1	2
		21UR-7308	TATCCAAAAAAAATTGTTCTT	2	0	0	0	1	0	1	4
		21UR-7309	TAGTAATTCTTTTAAAGTATT	0	0	0	0	0	0	0	0
*	†	21UR-7310	TAGATCTTGACAGAAGAACAA	15	43	33	94	1180	1002	62	2429
		21UR-7311	TACAGAGCATATCAATTTATT	5	5	2	7	57	47	9	132
*		21UR-7312	TAAGGAAATTATTAGAATGAA	3	6	8	14	205	117	3	356
		21UR-7313	TTTTAGCAGGAAATTGAAAAA	0	1	0	0	0	0	0	1
		21UR-7314	TTTGATCCTGTTTTTGAGCAA	0	0	0	2	2	2	1	7
		21UR-7315	TTTGATAATTACTTCACTCAA	0	0	0	0	0	0	0	0
	†	21UR-7316	TTTCAGTGTTTTCTCGAGATT	0	0	0	0	0	0	0	0
		21UR-7317	TTTCAAATGACTTTTCAAAAA	0	0	0	0	1	0	0	1
		21UR-7318	TTTATATCGAGTTCCGACATT	5	0	2	2	2	10	3	24
		21UR-7319	TTTAAACTTTATTGAGACAGA	0	0	0	0	4	3	0	7
		21UR-7320	TTGTGCATTTTCATTTTTATA	1	0	0	1	2	3	0	7
	†	21UR-7321	TTGTAATGACATTTCAAGATT	0	0	0	0	0	0	0	0
	†	21UR-7322	TTGAGTTCGTTAAAAAAAAAG	0	0	0	0	6	8	8	22
	†	21UR-7323	TTGACAGCGTTACGATAAAAA	0	0	0	0	1	1	0	2
		21UR-7324	TTCGCTATTTCTATGCCATGT	2	0	0	2	9	14	2	29
		21UR-7325	TTCATTCCAAAGTGAAAAGGT	0	0	0	0	1	0	0	1
		21UR-7326	TTCAGAGGTAAAAAAAAAACG	0	0	0	0	2	4	1	7
	†	21UR-7327	TTACGATAAATTTAAAACTGA	0	1	1	0	1	2	0	5
		21UR-7328	TTAAATTTATTGATCTCAGAG	9	10	1	3	10	18	2	53
		21UR-7329	TGTTTTGGCAATTTTTTGTTC	1	0	1	0	1	2	1	6
		21UR-7330	TGTGTCTTTTCTTTGAAGCAT	0	0	0	0	1	1	0	2
		21UR-7331	TGGTTGATGAGGTTAGTGAGT	1	0	0	0	5	4	0	10
		21UR-7332	TGCTTTGTTCTGAGAGCTTTT	0	0	0	0	2	1	0	3
		21UR-7333	TGATTCCTTCATTTGTAATAC	0	0	0	0	2	0	0	2
		21UR-7334	TGAAATTGTATTGTATTGTAT	2	0	1	3	9	6	3	24
		21UR-7335	TCTTCCGTATTTGCTACAAAT	0	0	0	0	0	0	0	0
	†	21UR-7336	TCTCGTTAAATAGTAACTAAT	0	0	0	0	0	0	0	0
		21UR-7337	TCTCCTAGGGGCTTTAAACAC	0	0	0	0	5	5	6	16
	†	21UR-7338	TCTCATCTTAAATTTTCAAAT	0	0	0	0	0	0	0	0
		21UR-7339	TCGGTACGTATTATAGTTATT	0	0	0	0	6	0	0	6
		21UR-7340	TCCTCTCGTAACTTTGAATTT	0	0	0	0	0	0	0	0
	†	21UR-7341	TCCATGAGAGGAAATTAGAAA	0	0	0	0	1	4	1	6
		21UR-7342	TCCAACAAAAATACATCATAC	0	0	0	0	0	0	0	0
	†	21UR-7343	TCATATGGTAGAAAAAAGCAT	7	13	5	8	103	76	9	221
	†	21UR-7344	TATTGAAATTGGGTTGTATCC	1	0	0	0	0	0	0	1
	†	21UR-7345	TATTACCAAAAATGGACCAAA	0	0	0	0	0	0	0	0
		21UR-7346	TATTAAATGATAAAATAAAAT	0	0	0	0	0	0	0	0
*	†	21UR-7347	TATGGAGAATGTATGTATTGT	0	3	0	5	119	77	8	212
*		21UR-7348	TAGCAAAAGTGAGCTGAAATA	10	11	6	32	389	366	50	864
*	†	21UR-7349	TAGATAGGCAGGATTGCAAAA	0	0	0	0	17	16	7	40
		21UR-7350	TAGAAAAGGATCATGACGAAT	2	1	0	0	13	20	1	37
		21UR-7351	TACTGGTGAAGAATTTGAAAA	1	0	0	2	16	19	6	44
		21UR-7352	TACTGAAATCATGACACTATT	0	0	0	0	5	9	4	18
		21UR-7353	TAATTTTCTTTTTTTTATGCA	0	0	0	0	0	3	0	3
	†	21UR-7354	TAATGGTATTTCTAATAAATA	1	0	0	1	0	0	0	2

†	21UR-7355	TAATATCAAAAAGTTCCAGAT	0	0	0	0	0	0	0	0
	21UR-7356	TAATAAACTTCTAAATTGTAG	1	0	0	0	0	1	0	2
†	21UR-7357	TAAGATCACTGTCGTTGAAAA	0	0	0	0	2	5	6	13
	21UR-7358	CAGATGGTTATATAATTTGAT	0	0	0	0	0	0	0	0
†	21UR-7359	TTTTCTAAGACCTGTTTATTA	0	0	0	0	6	2	0	8
†	21UR-7360	TTTTACAAACTCCGCGCACAA	0	0	0	0	0	0	0	0
	21UR-7361	TTTGTTCCTTGTTTCTTTGTT	0	0	0	0	0	0	0	0
†	21UR-7362	TTTCAACAGGTGATCTTCAAA	0	0	1	0	0	1	0	2
	21UR-7363	TTGTCCTAATGTATTTGTTTC	0	0	0	0	1	4	0	5
	21UR-7364	TTGCTACTTTTCGAGCTGTTA	0	0	0	0	0	0	0	0
* †	21UR-7365	TTGATCTCCAATTAAGGATAA	2	0	0	0	1	2	0	5
†	21UR-7366	TTCTGGGAGACGGAGAATAAG	5	7	5	2	5	45	5	74
	21UR-7367	TTCCGTAACTTCCGTAAAAAT	0	0	0	0	0	1	2	3
†	21UR-7368	TTCATATCGAAGATTAAATTA	0	0	0	0	0	0	0	0
†	21UR-7369	TTATTGGTTTATTTAACAGGA	1	2	0	0	6	4	1	14
†	21UR-7370	TTAAGCACGGCCTCTGTGAAA	0	2	1	1	4	17	0	25
†	21UR-7371	TTAACCTAAGCAGGAGTGAAT	4	0	1	0	0	3	0	8
	21UR-7372	TGTTTTGACCACATTATAAAT	0	0	0	1	0	1	0	2
†	21UR-7373	TGTATCTGATTTGTGCCTACA	0	0	0	0	0	0	0	0
	21UR-7374	TGTATACACAAAAAAAAACTA	7	0	0	0	2	3	0	12
	21UR-7375	TGGTTTTAAACTTTAGGTTAA	0	0	0	0	0	0	0	0
	21UR-7376	TGCGTAATCTCTACAGCAATT	0	0	0	0	0	0	0	0
	21UR-7377	TGCCCTTCTTGAAAGCATGAA	0	0	0	0	0	1	4	5
	21UR-7378	TGCAGAAGGCAAATCCAGATT	1	0	0	0	9	15	1	26
	21UR-7379	TGAGCACGTCATATTTTTATT	10	2	3	7	44	49	36	151
	21UR-7380	TGAAGTTTTTAAGCCGCAATT	0	0	0	0	0	0	1	1
†	21UR-7381	TGAAGATACTTGGTTCTTATA	0	0	0	1	1	2	0	4
†	21UR-7382	TCTTGATTTCTTCGCGAAGAT	0	0	0	0	0	0	0	0
	21UR-7383	TCTTCGTTTTTTCAGTTTGAA	0	0	0	0	0	0	0	0
	21UR-7384	TCTTCAAAGATTGAATTTTCC	0	0	0	0	0	0	0	0
	21UR-7385	TCTCGTTTCTTTTTGCTGATT	0	0	0	1	0	0	0	1
	21UR-7386	TCGTCCGAATATCGTCTTATT	2	0	0	0	4	5	2	13
	21UR-7387	TCGATTTTCATATATCTTACA	0	0	0	0	0	0	0	0
	21UR-7388	TCGACAGAAATAACTTTGTGA	0	0	0	1	0	1	0	2
	21UR-7389	TCATTGACTAATTGTTTAAGA	0	0	0	0	0	0	0	0
	21UR-7390	TATTTTTGTCAAAAGTAACCA	0	0	0	0	0	0	0	0
†	21UR-7391	TATTTTTAATTTTGGATACAA	1	1	2	2	2	3	0	11
	21UR-7392	TATTTCGTGATTTCAAAAATA	1	0	0	0	1	1	0	3
*	21UR-7393	TATTGTGTTCGAGAGATTTTT	38	7	10	13	65	65	24	222
	21UR-7394	TATTCAATTACTGACAACGAA	0	0	0	1	0	2	0	3
	21UR-7395	TAGATAATTTTTGCAATTTTA	0	2	0	0	0	3	0	5
†	21UR-7396	TACTGAATTCACTCTCAACAA	0	0	0	0	1	2	0	3
	21UR-7397	TACCAAAAAACTAAATTTCAA	0	0	0	0	0	0	0	0
†	21UR-7398	TAATCTTGTTCTCTCCCAAAG	0	0	0	0	1	3	0	4
†	21UR-7399	TAAATTGATCCAATCCAAGTT	0	0	0	0	0	0	0	0
	21UR-7400	TAAACTCGGAAGTATTATAGA	1	1	0	0	11	6	5	24
†	21UR-7401	TTTTTTTTTCGGTGTTTCCAT	9	2	1	1	6	12	4	35
	21UR-7402	TTTTCTGTTGATTATTAGAGT	0	0	0	0	2	1	0	3
	21UR-7403	TTTTATTCAATTAGAGCAAAA	0	0	0	0	0	0	0	0
	21UR-7404	TTTCAAAGTCAAAAAAAGAGC	0	0	0	0	0	0	0	0
	21UR-7405	TTTATGGTTCTTCTTGCAATA	1	0	0	0	0	0	0	1
	21UR-7406	TTTACACAACTCGATTGTATT	1	0	0	0	1	6	0	8
†	21UR-7407	TTGTTTGGGTTCCTGATCTTC	0	0	0	0	0	0	0	0
†	21UR-7408	TTGGATCTGATGATTTAATCG	0	0	0	0	4	4	1	9
†	21UR-7409	TTGATCAAAAAATTTTCAAAA	0	0	0	0	0	0	0	0
†	21UR-7410	TTCTGAATCGTGTTTGAGAAA	0	0	0	0	0	0	0	0
	21UR-7411	TTCCTTCAGAGTTTTTTTCCA	0	0	0	0	0	0	0	0
	21UR-7412	TTCCGTTGTGAGATGTAAACA	0	0	0	0	1	0	0	1
	21UR-7413	TTCCAACGTATCCTAAACTTA	0	0	0	0	1	0	0	1
	21UR-7414	TTATTCATTGATAATATCAGT	0	0	0	1	0	0	0	1
	21UR-7415	TTAGAATGAACTTCAAAAAAT	0	0	0	0	0	0	0	0
	21UR-7416	TTAGAAACTTGGGGCAGCTTA	0	0	0	0	7	13	8	28
	21UR-7417	TGTTTACAATGTCTTGTCCAC	0	0	0	0	0	0	0	0
†	21UR-7418	TGTTGTTGTTGTTGGTGTAGT	0	0	0	0	0	1	0	1

		21UR-7419	TGTTCTGAACTACTTTTTTAA	0	0	0	0	3	2	2	7
		21UR-7420	TGTCATATTCATTAAAAAAAT	1	0	0	0	0	1	1	3
		21UR-7421	TGGACTCTTCTTAGCATTAAC	0	11	2	10	243	157	64	487
		21UR-7422	TGCTGATTCCTTTATAATCCT	0	0	0	0	0	0	0	0
		21UR-7423	TGATTAATCGTCTCTCTGTGT	0	0	0	0	1	1	0	2
	†	21UR-7424	TGATGATTGATATCTATTGAT	0	0	0	0	2	0	0	2
		21UR-7425	TGATCTTTATGAAACAAAAAA	0	0	0	0	1	0	0	1
		21UR-7426	TGATAATCTAGTTTGTTGTTT	0	0	0	0	1	5	1	7
		21UR-7427	TGAGATTGTCGATTTCATTTT	0	0	0	0	26	21	9	56
		21UR-7428	TCTTCATGTTTTTCGGTTTCT	0	0	0	1	12	10	2	25
	†	21UR-7429	TCTCATCTAGGAAGAAAAAAA	0	0	0	0	1	1	0	2
		21UR-7430	TCTACACAAATATTCCAAATT	0	0	0	0	0	1	0	1
		21UR-7431	TCGTTTGGTTTTTTAAATCTG	0	0	0	0	0	0	0	0
	†	21UR-7432	TCGTTGAACACTATATGTTTT	0	0	0	0	0	0	0	0
		21UR-7433	TCACTTACAATTTGCGAATAA	0	0	0	0	0	0	0	0
		21UR-7434	TATGAAGAGTAGCTTAGCTCT	0	0	0	0	0	1	0	1
		21UR-7435	TATCAAATTTTCAAAATCTCC	0	0	0	0	0	0	0	0
*	†	21UR-7436	TAGATTACGTGAATAAAGAGA	0	1	0	0	0	6	0	7
*		21UR-7437	TACGATCACTGGTTTATTTGT	1	2	1	0	14	11	3	32
		21UR-7438	TAAGCTTGCGCTCCACCTTTT	1	0	1	0	0	0	3	5
		21UR-7439	TAAATTTAGTGATATGTAAAA	0	0	0	0	0	0	0	0
		21UR-7440	TAAACCCGTAGCAATCTTAAT	0	0	0	0	17	11	3	31
	†	21UR-7441	TTTTCTCATCATTGGAAAGGT	0	0	0	0	1	1	0	2
	†	21UR-7442	TTTTCAGACTCCTTGCTATCA	0	0	0	0	1	0	0	1
		21UR-7443	TTTCTTTGAGATATCCAAATA	0	0	0	0	0	1	0	1
	†	21UR-7444	TTTCTGATTATCTTCGTACCT	0	0	0	0	1	0	0	1
		21UR-7445	TTTCAAATCGTAAGCTCAAGT	0	0	1	1	0	2	0	4
	†	21UR-7446	TTTCAAAGCGTTAATAAAAAA	0	0	0	0	0	0	1	1
		21UR-7447	TTGCAACCATTGAATAATAGA	0	0	0	0	0	0	1	1
	†	21UR-7448	TTGATGAATGGTTGGCCCGAT	0	0	0	0	0	1	0	1
		21UR-7449	TTGAGTAGAGTCCTGAAACAA	0	0	0	0	3	5	0	8
		21UR-7450	TTGACAGTTTTTATACGTGGG	0	1	0	1	1	4	1	8
	†	21UR-7451	TTCTTTCAAAATTCCGATCAC	0	0	0	1	0	0	0	1
	†	21UR-7452	TTCTTCACAGTCTCCATTCTT	1	0	0	0	0	0	0	1
	†	21UR-7453	TTCTTAGTTAGTAGTAATAAC	0	0	0	0	1	0	0	1
	†	21UR-7454	TTCTGTCAAGCATTTGTTTGA	0	0	0	0	0	0	0	0
	†	21UR-7455	TTCTAACAGTTTTCTTTGGAT	2	1	0	3	47	28	8	89
		21UR-7456	TTCGTTTCCAATTTTAGGAAA	0	0	0	0	2	0	0	2
		21UR-7457	TTCCTGTAGTCGAATTTATTG	2	1	1	1	18	23	2	48
	†	21UR-7458	TTCCATTGATTTTTTTGCCAG	0	0	0	0	1	0	1	2
	†	21UR-7459	TTCAATTTTTTTGGTTATCCA	1	0	0	0	0	0	0	1
		21UR-7460	TTCAATAGAATGTTTTTTGAC	0	0	0	0	5	5	0	10
	†	21UR-7461	TTATTTTTGAAATCGAACTTC	5	1	2	1	7	13	1	30
		21UR-7462	TTACTTAATGAGAAAAAGTGA	0	0	0	0	0	0	0	0
	†	21UR-7463	TTAATTTACAACCACTTGTAG	4	1	0	0	2	5	2	14
		21UR-7464	TTAATACTTTTTTCTGAACAG	0	1	0	0	14	20	1	36
		21UR-7465	TGATTTCTTCTTTTTTTAAAA	1	0	0	0	0	0	0	1
		21UR-7466	TCTGATTCGCGCCTGGCTCAA	0	0	0	0	0	1	1	2
		21UR-7467	TCTCATTAAGAAACAAAACAA	0	0	0	0	0	0	0	0
		21UR-7468	TCTAACAACTTAACGTGTTTG	0	0	0	0	0	0	0	0
		21UR-7469	TCGTAGTATAGTAGAAAACCC	2	4	0	3	89	52	3	153
		21UR-7470	TCGGTTCCATGCTGCAATTTT	0	0	0	0	1	1	0	2
		21UR-7471	TCGGGGTTTGATAAACTGCAG	0	0	0	0	2	0	0	2
		21UR-7472	TCGAAGATATCAAAAAAAATT	0	1	0	0	1	1	0	3
		21UR-7473	TCAGTCGTTCAGTCCAACCAA	0	0	0	0	2	1	4	7
		21UR-7474	TATACGCCAAATGAAAAATTC	20	2	3	1	24	23	4	77
		21UR-7475	TAGGATGATTGAAATTTTTAT	1	0	0	0	1	2	0	4
		21UR-7476	TAGATTTTTTTCAGGTTCCCT	1	0	0	0	0	0	0	1
		21UR-7477	TACGGAAGTCGGCGCCTGTAT	0	1	0	0	3	4	6	14
		21UR-7478	TACACGAAATTTTATACAATT	0	0	0	0	1	1	0	2
	†	21UR-7479	TAATCAACAAAAAAAATCAAC	1	0	0	0	0	0	0	1
		21UR-7480	TAAATAAATCAATAATAATAA	0	2	0	0	1	0	0	3
	†	21UR-7481	TAAAAGTCTGAAGGAAAAAAG	1	3	2	1	2	12	1	22
	†	21UR-7482	CGATTCGTATAATGCAGAAAT	65	119	67	28	159	468	20	926

		21UR-7483	TTTTTTTTTCGCCATGTTCTT	1	0	0	0	2	2	2	7
		21UR-7484	TTTTCACTCGAAGAAAATTGA	1	0	0	0	0	0	0	1
		21UR-7485	TTTGTAGGAAATTATAACATT	0	0	0	1	0	1	0	2
	†	21UR-7486	TTTGAAGACCATTTTGATAGA	0	0	0	1	6	2	0	9
	†	21UR-7487	TTTCGTCGTCGATCCATACCA	7	1	3	8	75	47	46	187
		21UR-7488	TTTCACATCTGTTCAGATTGG	0	0	0	0	0	0	0	0
		21UR-7489	TTGATACTTGTTTATTTGATT	1	0	1	0	1	0	0	3
	†	21UR-7490	TTCTTTTTGAGAAATTAACAG	0	0	0	0	0	0	0	0
	†	21UR-7491	TTCTCCTTGAATTAAATTTAA	0	0	0	0	0	0	0	0
	†	21UR-7492	TTCGTTTGAAAAAATCGCATT	0	0	0	0	1	0	0	1
		21UR-7493	TTCGAGTTTTTATTTTTTTCG	0	0	0	0	4	1	0	5
		21UR-7494	TTCATCCGCGATTCCGTTTTT	0	0	0	0	1	0	1	2
*	†	21UR-7495	TTCAGAACTATTCGACTGCTT	3	4	5	18	221	178	28	457
	†	21UR-7496	TTCAAAAATGAAGCTGTCAAT	4	1	0	0	2	7	1	15
	†	21UR-7497	TTATTTGAACATATACCTCAT	3	2	1	2	15	11	2	36
	†	21UR-7498	TTAGTAGAAGATTTTGAAAGC	13	3	11	7	61	97	4	196
	†	21UR-7499	TTACAAGAGAACAATTAGATT	0	1	1	6	59	29	7	103
	†	21UR-7500	TTAACGTCTGAAATATGTGGA	0	0	0	1	0	1	0	2
		21UR-7501	TTAAACTTCGTCCAACATTTT	1	0	1	2	38	14	17	73
	†	21UR-7502	TGTTGTGAAAGGAAATTCAAA	0	0	0	0	1	1	0	2
		21UR-7503	TGTCATTAACCCAAAATTAAC	0	0	0	0	0	0	1	1
	†	21UR-7504	TGGATGAATTTTTTGATTTTT	0	0	0	0	0	0	0	0
		21UR-7505	TGCGATTTTTTTAAAATTTTT	0	0	0	0	0	0	0	0
		21UR-7506	TGATTTTCTGGAATTTTAAAT	0	0	0	0	3	2	0	5
		21UR-7507	TGAATTGAATTGAAAAATGTG	0	0	0	0	0	0	0	0
		21UR-7508	TGAACCAGGTAAATTTCCAGT	0	0	0	0	0	2	2	4
		21UR-7509	TCTTAAAAATAGAAAAACGTT	0	1	2	1	1	2	0	7
	†	21UR-7510	TCTGATCCATTTTTAATCGAC	0	0	0	0	0	0	0	0
		21UR-7511	TCTCAATTTAATACTAAAAGA	0	0	0	0	0	0	0	0
	†	21UR-7512	TCGGAAAAAGTTGGGTAGTGG	5	2	1	1	6	10	4	29
		21UR-7513	TATTTAGGTAGATCTTTCAGA	1	2	1	0	17	28	5	54
	†	21UR-7514	TATAGTTGGTTTGGGTTTCAC	0	0	0	0	7	12	4	23
	†	21UR-7515	TAGGCATTCATCGGCTTTGGT	4	0	0	0	12	20	11	47
	†	21UR-7516	TAGGCACATAATGTTCACTCT	0	1	0	0	16	10	0	27
		21UR-7517	TAGAGAAGTGTTTTGAAATGC	0	0	0	0	1	2	0	3
		21UR-7518	TACTCTGTGGGATAAAAATTC	0	0	0	0	3	8	6	17
		21UR-7519	TACAAGAAATGGAAATGGAAA	0	0	1	0	0	11	0	12
		21UR-7520	TAATCTTCGAATTCCAGAAGA	0	0	0	0	0	0	0	0
	†	21UR-7521	TAATATGTATTCCTCGAGTAT	0	0	0	0	9	4	1	14
		21UR-7522	TAAATTTACAATTAAATTTTA	0	0	0	0	0	0	0	0
		21UR-7523	TAAAGATGTGCTATCATATTC	3	0	3	1	5	17	2	31
		21UR-7524	TAAAAGGGAAGGAGACTTTTT	1	2	3	2	32	30	9	79
	†	21UR-7525	TAAAAAATTTTTCGTTTCAAA	0	0	0	1	1	1	0	3
		21UR-7526	CGGGCATGTTGTAAAGGTGTT	9	22	8	3	63	105	7	217
		21UR-7527	TTTTTAAACGGTTATCTCTAA	0	2	0	8	112	65	13	200
	†	21UR-7528	TTTTGCTCATTTGACTAAACA	0	0	0	0	0	1	0	1
	†	21UR-7529	TTTTCGTTTCTGTCCAAGGAT	0	0	0	0	1	1	0	2
		21UR-7530	TTTGTCTCTCTTTTTTCCTGG	0	1	0	0	0	1	0	2
	†	21UR-7531	TTTGATTTAGTCTGGTGGCTT	3	2	0	0	4	11	2	22
		21UR-7532	TTTCGGGATCTTAAGATTTGT	0	0	0	0	8	12	10	30
	†	21UR-7533	TTTCCGTTCCATTTTCGTAAC	1	0	0	0	0	0	0	1
	†	21UR-7534	TTTCCGATTCCGACTAGAAGG	0	0	0	0	0	0	0	0
		21UR-7535	TTTAGTTGTACATCCACAATG	0	0	0	0	1	1	1	3
		21UR-7536	TTTAAGATTGCTTTGAGCGAA	0	1	0	0	1	4	1	7
		21UR-7537	TTGCCGAATCTCATAAATCAG	1	0	0	0	6	7	1	15
	†	21UR-7538	TTGCAGCATTCGGACAGAAAC	8	9	2	4	5	28	7	63
		21UR-7539	TTCTCCGAAATTCAGAAATAA	0	0	0	0	0	0	0	0
		21UR-7540	TTCTATAGTTCGCTTTTATGT	1	2	0	0	1	1	1	6
		21UR-7541	TTCTAACTCATGATTCTTTGC	0	0	0	0	1	0	0	1
		21UR-7542	TTCGTAAAATCTATACATCAC	0	0	0	0	3	0	0	3
		21UR-7543	TTCAGGGATATTATGCTTCCG	2	0	1	0	2	3	0	8
	†	21UR-7544	TTCAATACAAGCAAGGGATGA	3	0	0	0	3	16	2	24
		21UR-7545	TTCAAACTCTGCAATCGTCGC	1	0	0	3	37	29	2	72
		21UR-7546	TTATTTTGTCGCAGAGAACAA	1	0	0	0	2	1	1	5

	†	21UR-7547	TTACAAAATTTTGAAGGATCG	22	6	4	4	28	79	12	155
		21UR-7548	TGTGTTATTAAATTGAATCCC	0	0	0	0	0	0	0	0
	†	21UR-7549	TGTCATATTCGTATTTAACAG	0	0	0	0	0	0	1	1
	†	21UR-7550	TGGTTGACACATTAGGATTCT	0	1	0	0	2	0	0	3
		21UR-7551	TGGTTCCTTCCTTCCGCCTTT	0	0	0	0	0	0	2	2
		21UR-7552	TGGTATTAAAATTTTTTACTC	0	0	0	0	1	1	0	2
		21UR-7553	TGCATGAGTAGATTTTCACAG	1	2	1	2	37	23	7	73
	†	21UR-7554	TGCAGCGGTTTGATGATTTCA	1	1	0	0	12	11	4	29
		21UR-7555	TGATTCTATTTGTATTTTGAT	0	0	0	0	0	0	0	0
	†	21UR-7556	TGATCTCACAAAAAAGATTTT	0	0	0	0	0	0	0	0
		21UR-7557	TGAATGATTGGACATAATCTT	0	0	0	1	7	2	0	10
		21UR-7558	TGAATCTATGGGCAACAGATT	0	0	0	0	1	2	0	3
		21UR-7559	TGAAATTGTTGTTAAGGAACA	0	0	0	0	0	0	0	0
		21UR-7560	TCTTTCAAATTTCCCCGCTTC	0	0	0	0	0	0	0	0
	†	21UR-7561	TCTTCAGTTTTAGTTTAGCTC	0	0	0	0	0	0	0	0
		21UR-7562	TCTATGAAGAAAATCTATTGG	0	0	0	0	0	1	0	1
		21UR-7563	TCGTTTTTGGAAAAAAAAACT	0	0	0	0	0	0	1	1
		21UR-7564	TCGTACAATGCTATGCCCAAA	0	0	0	0	0	1	0	1
		21UR-7565	TCCCATTTCGAATTGTGGAAT	0	0	0	0	0	0	0	0
	†	21UR-7566	TCATTAGTCATTGTAGAATCC	0	0	0	0	0	0	0	0
		21UR-7567	TCATGTTTCTTTTAGAATTTT	0	0	0	0	2	1	0	3
		21UR-7568	TCAGAAAGTTCTAAATCGCCA	0	1	0	0	0	1	0	2
*		21UR-7569	TCACATTTTAGATAACTGGTT	5	18	10	40	477	321	44	915
	†	21UR-7570	TATTGATAGGTAAATAAAAAA	0	0	0	0	0	0	0	0
		21UR-7571	TATTCGATGAATATGAATTTA	0	0	0	0	16	3	1	20
		21UR-7572	TAGTTCCACTTCTTAATTGTT	0	0	0	0	0	0	0	0
		21UR-7573	TAGTAACGTTTTTCTATAAAA	0	0	0	0	0	1	0	1
*	†	21UR-7574	TAGGAAAACTGAAAACGCATA	5	7	3	11	326	310	27	689
		21UR-7575	TAGCTTCGTTGATCGGACAAG	1	2	1	0	10	15	4	33
		21UR-7576	TACAATTAAATTTCACAAAAT	5	0	2	0	1	2	1	11
	†	21UR-7577	CTGTAACATGCGAAAAAAAAA	0	0	0	0	0	0	0	0
		21UR-7578	CACATCAACTACGAATGCTAC	0	0	0	0	0	0	0	0
	†	21UR-7579	TTTTGTAGGACTTCAATTTCA	2	0	0	0	0	3	1	6
		21UR-7580	TTTCTGAAGACCTTTTTTCAA	3	0	1	1	23	15	0	43
	†	21UR-7581	TTTCGAAATATCCTTGATGTT	0	0	0	0	0	2	1	3
		21UR-7582	TTTCATAAAAATTGAACAGAT	0	0	0	0	2	1	0	3
		21UR-7583	TTTAATCTCTAATTGCGTTTC	0	0	0	0	2	1	1	4
*	†	21UR-7584	TTTAAGGATTGAAGGTATTTT	22	9	8	34	426	333	440	1272
		21UR-7585	TTGAATAGGTGTTATTATTTG	1	0	0	1	1	0	1	4
		21UR-7586	TTATGTAATCATTTTCGATGG	1	0	0	0	0	1	0	2
		21UR-7587	TTACTTTCCTTTTTCTGATGA	1	1	0	0	21	9	4	36
		21UR-7588	TGTTGTTTCATTTATCAAAAA	0	0	0	0	0	0	0	0
		21UR-7589	TGTGTTGTGATTGAAATCTAA	0	0	1	0	3	4	0	8
		21UR-7590	TGTATCACTTTGACAGCTTTT	0	0	0	0	0	0	0	0
	†	21UR-7591	TGTAATGAAGGCCTTCCTTGG	0	0	0	0	0	2	4	6
		21UR-7592	TGGGCGATTTCTACAGGTTGG	0	0	0	0	0	1	2	3
	†	21UR-7593	TGATCCCGAATTGGAAAAGAT	2	0	0	0	2	5	1	10
	†	21UR-7594	TGACTTTGGATTTTCTCGATT	0	0	0	0	0	1	0	1
		21UR-7595	TCTTGTTTTTTTTTATTCTTG	0	0	0	0	0	0	0	0
*		21UR-7596	TCTTGTGTTTCCGGTTTCAAA	0	0	0	0	0	2	5	7
		21UR-7597	TCTCTTTTCAAAGAATCAACA	0	0	0	0	0	0	0	0
		21UR-7598	TCTCCACCTTTTTCCATTAGT	0	0	0	0	0	0	0	0
	†	21UR-7599	TCGTTGGACAAATTATAAGGA	1	3	1	2	1	7	3	18
		21UR-7600	TCCACACTAGACTAATCATAT	0	0	0	0	1	0	3	4
	†	21UR-7601	TATTGACCCGCAATGATTTCT	1	0	0	0	1	0	3	5
	†	21UR-7602	TATTCTTGAACGGCTAGGATC	0	2	1	2	0	8	0	13
		21UR-7603	TATTAGGAGGAGTTCAATTAC	0	1	0	0	5	1	2	9
	†	21UR-7604	TATTACGGTCGATCTTGTTTT	0	0	0	0	2	2	2	6
	†	21UR-7605	TATCTTTCGGAAATACATTTT	5	0	0	2	3	5	1	16
		21UR-7606	TAGATTGATTTTTTTATCATC	2	1	1	3	8	11	1	27
		21UR-7607	TACTTTTGTATTTCTTAAAAT	0	0	0	0	1	1	0	2
	†	21UR-7608	TACTGAATGGAAGAACTGGAA	80	67	34	25	99	557	17	879
	†	21UR-7609	TACTCCTTGTGATTTGGTTTT	0	0	0	0	1	0	0	1
	†	21UR-7610	TAATGCTAAGACAAGATGGGT	0	0	0	1	1	2	0	4

	†	21UR-7611	GCATTTAAAAATTTAACGACT	0	0	0	0	0	0	0	0
	†	21UR-7612	CGAAATCATCAATGGACTATT	1	2	1	2	3	3	2	14
		21UR-7613	TTTTGTTTACGTTCTATCTGG	0	0	0	0	0	1	0	1
	†	21UR-7614	TTTTGATGTCCATTCAAAATA	0	0	0	0	0	0	0	0
		21UR-7615	TTTTGATGGGAACAATATTGA	0	2	0	5	46	31	6	90
		21UR-7616	TTTTCTTTGCGATTGAGGGGG	0	0	0	0	0	0	1	1
	†	21UR-7617	TTTTCGAATCGATCTACGAAC	0	0	0	0	2	2	1	5
	†	21UR-7618	TTTGTTATTGTCGGCAGGAGA	0	1	0	0	50	90	64	205
	†	21UR-7619	TTTGTCGCTTTAGGTTCAATT	0	1	0	0	1	1	0	3
	†	21UR-7620	TTTGGAGTCTATTTCCGTAAA	0	0	0	0	0	0	0	0
		21UR-7621	TTTCCGAAGTTGCAAAACAAC	0	0	0	0	0	0	1	1
	†	21UR-7622	TTTCACGAGGTGTTTTTCAGA	0	0	0	0	0	1	1	2
	†	21UR-7623	TTTAGTCTGGTGGCTTCCTCT	0	1	0	0	0	2	0	3
		21UR-7624	TTTAGTCAAGTAAAGTAAACT	1	0	1	0	5	3	0	10
		21UR-7625	TTTAGTAATGACAATCGCTAA	0	0	0	0	1	1	0	2
		21UR-7626	TTTACGTGCCCGTTGTCTTCA	0	0	0	0	1	1	0	2
	†	21UR-7627	TTGTGTTCCGCGGTTTGAGGT	0	0	0	0	0	0	0	0
	†	21UR-7628	TTGAGCAATCGATTCGGGTTA	0	1	0	0	8	6	1	16
	†	21UR-7629	TTGAATGTTTTTTTCCTGGTA	0	0	0	0	0	0	0	0
		21UR-7630	TTGAAGGCAGGTTTTCAGTTT	3	0	0	0	11	27	9	50
		21UR-7631	TTCTTCGTTTTCAAAAAATAC	1	0	0	0	0	0	1	2
		21UR-7632	TTCTGCTTTCCTTAGTTTGTT	0	0	0	0	0	0	0	0
	†	21UR-7633	TTCGGTCTCAATTATTAAACT	1	0	0	0	8	3	0	12
		21UR-7634	TTCCTTTGTTGGTTTTTAATT	0	0	0	0	5	2	2	9
		21UR-7635	TTCATCGACTATATTTCAAAA	0	0	0	0	7	5	1	13
	†	21UR-7636	TTCAGGATACGAATATACGGA	0	0	0	0	7	6	1	14
	†	21UR-7637	TTCACTCGTTTGAAGAGAAAA	0	0	0	0	0	1	0	1
		21UR-7638	TTCACTAGTTTAAATAGTTCA	0	0	0	0	3	0	0	3
		21UR-7639	TTCACACCATATACTCATATT	0	0	0	1	3	2	6	12
	†	21UR-7640	TTAGTTAATGGCACGTTTGAT	0	0	0	0	0	1	0	1
		21UR-7641	TTAGCCGTTTTTTTAGAGGAA	0	0	0	0	4	3	0	7
		21UR-7642	TTAGCATTTAATTTGAGGAAA	0	0	0	0	2	1	0	3
	†	21UR-7643	TTACTTTTGATCAATGTCCAA	0	0	0	0	5	4	1	10
	†	21UR-7644	TTACAATCGAGTATATAGTTT	1	0	0	0	1	1	1	4
*		21UR-7645	TTAATGAACTTTTTAATCGGC	14	21	17	67	1073	1115	59	2366
	†	21UR-7646	TGTTAATAGGAAGTACTCAAA	1	0	0	1	2	5	0	9
		21UR-7647	TGTGAATACAGCTTTTTTTGA	0	0	0	0	2	2	1	5
	†	21UR-7648	TGTGAAAATGAAAAATAGATG	0	0	0	0	0	0	0	0
		21UR-7649	TGTAAATGAAATTAGGCCTAT	0	0	0	0	0	0	1	1
		21UR-7650	TGTAAACGTCATAAATCTGTT	2	0	0	0	1	3	0	6
		21UR-7651	TGGTTTTGTTTCCCACGTTTT	0	0	0	0	0	0	0	0
	†	21UR-7652	TGGAGAAAATGGTTGGAAGTT	0	0	0	0	23	21	6	50
		21UR-7653	TGCGAATCCTTTTCCTTTCTT	0	0	0	0	0	0	0	0
*		21UR-7654	TGCAGTGGACGGATCTGTTTT	145	69	38	54	793	1777	350	3226
		21UR-7655	TGCAGGAATTTTTCCACTGCG	0	0	0	0	1	0	0	1
		21UR-7656	TGATCCGAACACAGCAATGAA	0	0	0	0	0	0	0	0
	†	21UR-7657	TGAATTTCGATTTTGTTAATT	0	0	0	0	0	0	0	0
		21UR-7658	TCTTTCCAATATTTCTTTTCC	0	0	0	0	0	0	0	0
*	†	21UR-7659	TCTGGGAGACGGAGAATAAGA	12	13	7	2	12	78	1	125
		21UR-7660	TCTGATGTGTAGCTTTTTATC	0	0	0	0	0	0	0	0
		21UR-7661	TCTATTCGACATCTAATTGTA	0	0	0	0	0	0	0	0
	†	21UR-7662	TCGTTCAGTCGAATGAATTTC	0	0	0	0	1	0	0	1
	†	21UR-7663	TCATTGTGAATATCACAAATG	0	0	0	0	0	0	0	0
		21UR-7664	TCAACCACCATTTTTTGGAAG	0	0	0	0	0	0	0	0
		21UR-7665	TATTTCTGCCATTACCATAGT	0	0	0	0	2	1	4	7
		21UR-7666	TATTCAATTGACAGGGCATCG	5	0	0	0	0	6	0	11
		21UR-7667	TATCTATTCTCAGACAAACCG	0	1	0	0	2	1	1	5
*	†	21UR-7668	TAGTGAAACTCTTTTCTGATC	0	0	0	1	2	1	0	4
		21UR-7669	TAGAATCGCCATTATTTTTAA	0	0	0	0	0	1	2	3
		21UR-7670	TACTGACTGAATTTTTTTTCT	0	0	1	1	2	1	1	6
		21UR-7671	TAACTAAGATGAAAACGATAG	2	0	0	1	8	10	0	21
*	†	21UR-7672	CACGAACTGAAACTACATTTT	1	1	1	0	4	6	1	14
		21UR-7673	TTTTTGAGTAAATATCATAGG	0	1	0	0	0	0	0	1
	†	21UR-7674	TTTTTGAAAGTATTTAGAAAC	0	0	1	0	0	0	0	1

		21UR-7675	TTTTGAAAACAAGTAAGTGTT	0	0	0	0	0	0	0	0
*		21UR-7676	TTTTCATCCGGCATAACTAAA	0	0	0	0	13	11	9	33
		21UR-7677	TTTGTTTATTTAAAAAAAAAG	0	0	0	0	0	0	0	0
*		21UR-7678	TTTGATTGTATACCAAATTTG	25	27	21	25	81	176	2	357
		21UR-7679	TTTGAATTCGGAATTCGCCAC	2	3	0	2	40	31	54	132
	†	21UR-7680	TTTGAACCGTTTCCATAGTTT	0	0	0	0	0	0	0	0
		21UR-7681	TTTCAAATAAAATTTCCCATG	1	0	0	0	0	0	1	2
	†	21UR-7682	TTTAGTCATTGGTGTTACCCT	0	0	0	0	2	0	1	3
	†	21UR-7683	TTTAATTGGCATTAAAGAACG	1	1	0	2	50	39	12	105
		21UR-7684	TTTAATGTTCATCGCTAGTGT	0	0	0	0	0	0	7	7
	†	21UR-7685	TTGCTAAATGATTGATTGCTA	0	0	0	0	1	0	0	1
		21UR-7686	TTCGACATGAAATATCAAAAC	2	0	0	0	0	1	0	3
		21UR-7687	TTCCCATTTATTTAGAATTGA	0	0	0	0	2	1	5	8
	†	21UR-7688	TTCCACTCTACCTAGAGACAT	0	0	0	0	0	0	3	3
	†	21UR-7689	TTCAATCCTAGTTCCTATTTT	0	0	1	0	0	0	0	1
	†	21UR-7690	TTATGTAGTAACATTTATGGA	1	1	1	1	38	24	2	68
	†	21UR-7691	TGTTTTGAGTCAACTCCTCAA	0	0	0	0	0	0	0	0
	†	21UR-7692	TGTTTCAGTTGCAAAATTGTA	0	0	0	0	0	1	0	1
		21UR-7693	TGTTGTACTCCAAAAGCAATA	0	0	0	0	0	0	0	0
	†	21UR-7694	TGTTCTATTCCTCAGAATTTA	0	0	0	0	0	0	0	0
		21UR-7695	TGTATTATCTTTATCAAATTA	0	0	0	0	0	0	0	0
		21UR-7696	TGTAATGTAAATACGACTATC	0	0	0	2	12	11	0	25
		21UR-7697	TGCAGAATCAAATAATCATTC	0	0	0	0	0	0	0	0
	†	21UR-7698	TGCAAGGATTTTGGTGATAAT	44	56	22	14	64	252	9	461
		21UR-7699	TGATTTTTCAGATCTAAATTG	0	0	0	0	0	0	0	0
	†	21UR-7700	TGATCTCAACCAATCCTAATT	0	0	0	0	0	0	0	0
		21UR-7701	TGATCCAATCATAACAAACTG	0	0	0	0	0	0	0	0
		21UR-7702	TGACTGTATATTGAAGCGAAA	17	19	13	12	38	145	7	251
	†	21UR-7703	TGACCCGCAATGATTTCTCCA	0	0	0	0	0	0	0	0
		21UR-7704	TCTTTTACAGAATGTTTGAAA	0	0	0	0	5	4	0	9
		21UR-7705	TCTTTCAAATTATCGTTCTTT	2	0	0	0	2	1	0	5
		21UR-7706	TCTTCTTTATTCGGTATGCGT	0	0	0	0	0	0	0	0
	†	21UR-7707	TCTGAATCGTGTTTGAGAAAG	0	0	0	0	0	1	0	1
		21UR-7708	TCTACCTTTTTTTCTTTTAAA	0	0	0	0	0	0	0	0
		21UR-7709	TCTACCGTACAAAATATAATA	0	1	0	0	4	2	1	8
		21UR-7710	TCGCATACAAACTAGGAGCTT	4	6	1	1	11	30	0	53
	†	21UR-7711	TCGATTTTTTTGCTTTTTGCT	0	0	0	0	0	0	0	0
		21UR-7712	TCCAACTCATAGCACATTTTT	0	0	0	1	0	0	0	1
	†	21UR-7713	TCCAACGAGATGTCAAGGTTT	0	0	0	0	0	0	0	0
	†	21UR-7714	TCATAGTTTAAGAGCAATAAA	1	0	0	1	2	4	2	10
	†	21UR-7715	TATTGCGTCATTTGATTTTGA	0	0	0	0	1	0	0	1
		21UR-7716	TATGTCGAATGAGTTACAAAT	0	0	0	0	5	0	0	5
		21UR-7717	TATGCAAAAGTATCGATAATT	0	0	0	0	2	3	0	5
	†	21UR-7718	TATGAACATAATGAAACAGTT	0	0	0	0	1	0	0	1
		21UR-7719	TATCACTTCTTGGCTTTACAT	0	1	0	0	1	1	0	3
		21UR-7720	TATCAAAGTATCACATCAATT	1	0	0	0	3	0	1	5
	†	21UR-7721	TATAAATAATGGTGTAGATCA	2	0	1	0	0	2	0	5
	†	21UR-7722	TAGTTTAACGTCTTCCCGGGA	0	0	0	0	0	0	2	2
	†	21UR-7723	TAGACACTTTTCTTCTTTCTT	0	0	0	0	0	0	0	0
*	†	21UR-7724	TAGAAGAATTTGTGCAAAAAT	2	6	0	4	80	59	8	159
		21UR-7725	TACTTAGTTTGCATTTTCCCT	4	0	1	2	8	6	2	23
		21UR-7726	TACAATAATAAATCAAACTCA	1	0	0	2	14	3	0	20
	†	21UR-7727	TAAGTTGCTGCTTGTTTCTTT	0	0	0	0	1	0	0	1
	†	21UR-7728	TAAAGCAGAAATATTTACCAT	5	5	1	2	1	7	0	21
	†	21UR-7729	TAAACATCTAGTCTTTGAATC	0	0	0	0	0	0	0	0
		21UR-7730	TAAAAGCACTGATAACATTGA	0	0	0	0	9	6	0	15
		21UR-7731	GGCGTTTTCAATTGTGTAAAC	0	0	0	0	0	0	0	0
		21UR-7732	TTTTTTCTATTTCTGTAACAA	0	0	0	0	0	1	0	1
	†	21UR-7733	TTTTTTATGGGTGGTCATTGG	0	1	0	0	2	2	9	14
	†	21UR-7734	TTTTTCATGCTCACCATGCTC	0	0	0	0	0	0	1	1
	†	21UR-7735	TTTTGTTATAAAAATGAGGCG	0	0	0	0	0	2	1	3
	†	21UR-7736	TTTGCTCATTTGACTAAACAT	0	0	0	1	1	2	0	4
	†	21UR-7737	TTTCCTCCAAACACGGCAATT	9	0	0	2	1	7	0	19
		21UR-7738	TTTAATCTTTTTTTTAACAAA	0	0	0	0	0	0	0	0

	21UR-7739	TTTAAGTGTTTAGGGAGAAAT	5	0	0	0	10	10	6	31
	21UR-7740	TTGTGTCGTTAAACGTAATCT	0	0	0	0	1	2	3	6
	21UR-7741	TTGTAGTTTTAAAAAATAGAA	0	0	0	0	1	0	0	1
	21UR-7742	TTGGGTTTTCATATTTGAAAT	0	0	0	0	0	1	0	1
†	21UR-7743	TTGGGCAGTTTTTTGTGACAT	1	5	2	0	5	19	1	33
	21UR-7744	TTGCAAATAAATAAAATCCTC	0	0	0	0	0	0	0	0
	21UR-7745	TTGACCATATTTAATTCCAGG	2	0	0	0	0	1	0	3
	21UR-7746	TTCTTTGCTTACAGAATACTC	0	0	0	1	9	10	3	23
	21UR-7747	TTCTGGCCGCATACGTCTACC	0	0	0	0	7	7	0	14
	21UR-7748	TTCTAATTGTTACGCATTATA	0	0	1	0	1	1	0	3
	21UR-7749	TTCGATTACAATTCTTCTAAA	0	0	1	0	3	0	1	5
	21UR-7750	TTCGATCTTCAGACGAGCAAG	0	0	0	2	9	9	11	31
†	21UR-7751	TTAGTTCAAGTAGTGCTTTCT	2	4	2	1	1	2	0	12
†	21UR-7752	TTACACTGCTGTTGTAAAACT	0	1	0	0	3	3	0	7
	21UR-7753	TGTTTGGAGTTTTCTCTGTTT	0	0	0	0	0	0	0	0
	21UR-7754	TGTTGGCTCTAATTTAAGACC	0	0	0	0	0	0	0	0
†	21UR-7755	TGTTGGAGATTCCCAATTAAA	0	0	0	0	0	0	0	0
	21UR-7756	TGTGTGATCTGTATTTGTTTC	0	0	0	0	0	0	0	0
†	21UR-7757	TGCTTGGAATCTCAAAAATTG	0	0	0	0	0	0	0	0
†	21UR-7758	TGATAGTGGCGGATAAAATGA	3	0	1	0	25	20	19	68
*	21UR-7759	TGAACAGTGAAGCAGAACTTT	58	62	27	25	94	320	4	590
	21UR-7760	TGAAATGTTGTGATGAATTTT	0	0	1	0	2	0	4	7
*	21UR-7761	TCTGGATATGTTTAAAGGTGG	1	3	4	9	227	267	35	546
	21UR-7762	TCTACAGCCAGTTTTTACAGA	0	0	0	0	0	0	2	2
	21UR-7763	TCGTTTTTGAGAATAATTCAT	0	1	0	0	6	1	0	8
†	21UR-7764	TCCGTGATTGCCACCAGACCT	0	0	0	0	0	0	0	0
	21UR-7765	TCATTTGTTACTTGATTAGTA	0	0	0	0	0	0	0	0
†	21UR-7766	TCAGTTAAAGACTTTGGAAAA	0	0	0	0	4	4	1	9
†	21UR-7767	TCAGTGTAGCATTTAAACTTC	0	0	0	0	4	5	0	9
†	21UR-7768	TCAGCATTGATCATGTCAGAG	0	0	0	0	0	0	0	0
	21UR-7769	TATTGATCTTCTAAACGTTTG	0	0	0	0	1	3	0	4
†	21UR-7770	TATTACTTCATGATTTTCATT	1	0	0	0	9	9	4	23
†	21UR-7771	TAGTTGTGCTACTTTCCCACA	1	0	0	0	1	1	0	3
*	21UR-7772	TAGAAACAGTCGGAAAAAAGT	1	3	2	2	1	12	3	24
	21UR-7773	TACCGTTTGCCCCTACGCAGG	0	0	0	0	0	0	1	1
	21UR-7774	TAATACGAAAAAGAATGTTCT	0	0	0	1	5	4	0	10
*	21UR-7775	TAAAATTTGTCCATACGAACC	0	0	0	0	3	2	0	5
	21UR-7776	TAAAACTGCGTGGTGTGTACT	0	0	0	0	0	0	0	0
	21UR-7777	TAAAAATGGAATATAGTTTAG	0	0	0	0	2	2	0	4
	21UR-7778	TTTTGCAGAATTATTATCAGA	1	3	2	6	43	26	3	84
	21UR-7779	TTTTAAAAAACCCGGTAAATT	0	0	0	0	0	1	0	1
†	21UR-7780	TTTGTTTTGTCGCTTTAGGTT	2	0	0	1	8	6	2	19
	21UR-7781	TTTGTTTGTTTATCGAAGTAA	0	0	0	0	0	0	0	0
	21UR-7782	TTTGTTCATATAACATAGAAA	0	0	0	0	0	0	0	0
*	21UR-7783	TTTGAAGGATATAGGGCATTT	2	2	1	3	73	58	100	239
†	21UR-7784	TTTGAAATCACATTGATAACA	0	0	0	0	0	0	1	1
	21UR-7785	TTTCTGTTAAATTTTATTTGA	0	1	0	0	1	0	0	2
†	21UR-7786	TTGTGACTCAATTTCAAGATT	0	0	0	0	0	1	0	1
	21UR-7787	TTGATAATTTAAAGAATTTTA	0	0	0	0	2	3	0	5
†	21UR-7788	TTGAGTCGAGGTTTCAACACT	0	0	0	0	1	1	2	4
†	21UR-7789	TTGACTTAGTATTTTGTTGAG	0	0	0	0	0	0	0	0
	21UR-7790	TTGACACTTTGGATAGAAAAT	0	0	0	0	0	1	0	1
†	21UR-7791	TTGAAGAGTAATAAAAAAATG	0	0	0	1	1	3	0	5
†	21UR-7792	TTCTTAAAAAAGAATTTCAAG	0	0	0	0	0	0	0	0
†	21UR-7793	TTCGGTTCAAAATTTAAAACC	0	0	1	0	1	1	0	3
	21UR-7794	TTCCACTGTTTTAGTTTGTCT	0	0	0	0	1	2	0	3
	21UR-7795	TTCAGTTAAGCATTGCCAATT	1	0	0	1	9	16	2	29
	21UR-7796	TTCAATCATAAGAAACCAGGA	0	0	0	0	0	0	1	1
†	21UR-7797	TTCAACCATAGGGTTATTGCG	0	0	0	0	0	0	0	0
	21UR-7798	TTACTTTATTCCCATAAAAAA	0	0	0	0	0	0	0	0
	21UR-7799	TTAATATTGTCTTGTCTGTAC	0	0	0	0	2	1	0	3
	21UR-7800	TTAAAACACAGTTTGGACTGT	0	0	0	0	8	2	2	12
	21UR-7801	TGTTCGTTTTTCCCTTTTCTT	0	0	0	0	0	0	0	0
	21UR-7802	TGGAAGCTGATAATGATGAAA	0	2	0	1	18	30	4	55

	21UR-7803	TGCAGTTCTTTTTCTGTCTTT	0	0	0	0	0	0	0	0
	21UR-7804	TGACTATTAAAAAATCGGGAC	0	0	0	0	0	1	1	2
†	21UR-7805	TGAATTCGAACTCCGCAAGTA	0	0	0	0	26	20	2	48
	21UR-7806	TGAAAACTTTTTACATAATTT	0	0	0	0	0	0	0	0
†	21UR-7807	TCTGAATTCTCTCTCCAACCT	1	0	0	0	0	0	0	1
	21UR-7808	TCTCTTTGCGCGTTATAATTC	0	0	0	0	0	0	0	0
†	21UR-7809	TCTCCAGGCGTTTGTTTAAAT	0	0	0	0	0	2	1	3
	21UR-7810	TCCAATTGAAAAACAGTGCGC	0	0	0	0	0	0	0	0
†	21UR-7811	TCATCAAGCTAAAATCAGAAA	1	0	0	0	0	1	0	2
	21UR-7812	TCAGCGAATTCAGAGAAAAAC	0	0	0	0	0	1	0	1
	21UR-7813	TCACTTAGTGACAAAATAATT	0	0	0	0	0	0	0	0
	21UR-7814	TCAAATGTAGCTTTTGAACTT	0	0	0	0	0	0	0	0
	21UR-7815	TATTTGTGCAGATGTTCATGA	9	3	1	2	5	12	1	33
†	21UR-7816	TATTTCTACGTTTTGAATAGA	0	0	0	0	0	0	0	0
	21UR-7817	TATTGAAACTTCAGCTGAAAA	0	0	0	0	0	2	1	3
	21UR-7818	TATGTTTAGAGAAGTCCACCA	0	1	0	0	8	5	0	14
	21UR-7819	TATGAATTTTGCTCTATAAGC	0	0	0	0	0	0	0	0
†	21UR-7820	TATGAAAAAAAGGAACAGTTA	1	0	1	1	3	2	1	9
	21UR-7821	TAGTGAAACAATTGTTGTAAT	0	0	0	0	0	0	0	0
	21UR-7822	TAGGGAAGTTGATCCACACCT	0	0	0	0	0	0	0	0
	21UR-7823	TAGAATCACATTTTTTGGTGT	0	0	0	0	0	0	1	1
	21UR-7824	TACGTCTCTAGCCATACATTG	0	0	1	0	9	6	2	18
	21UR-7825	TAAGTCAGGGATCTGCTTTCT	2	1	0	1	49	49	1	103
	21UR-7826	TAAAATTTCCATTTTGTGCAG	0	0	0	1	2	0	1	4
	21UR-7827	TAAAACGCTCTATTATCTTCA	0	0	0	0	0	0	0	0
	21UR-7828	TAAAAAGCGTTCTTTACAGAG	0	0	0	0	2	7	3	12
†	21UR-7829	TTTTTATGTTCGTGGGACTAT	37	8	3	8	15	81	20	172
†	21UR-7830	TTTTATTCTTGGAGAAATGAA	0	0	0	0	1	1	0	2
	21UR-7831	TTTCTGTTGATTCTGACTTTC	0	0	0	0	2	0	0	2
	21UR-7832	TTTCAATATCATTTTTAGTCA	0	0	0	0	0	0	0	0
	21UR-7833	TTTATGAGTTTTCCGAATGTT	0	0	0	0	1	0	0	1
†	21UR-7834	TTTAAAAATTGAGAGTTTAAC	0	0	0	0	1	0	0	1
	21UR-7835	TTGTTTCGTCGCAGCACGCTT	1	0	0	0	14	7	13	35
	21UR-7836	TTCCAAAAGCATTACGCAAGA	0	0	0	0	0	0	0	0
	21UR-7837	TTCATGTTTGGAATCAAAGTT	0	0	0	1	0	0	1	2
†	21UR-7838	TTCATGTCTTCGGTTTCATTT	6	1	3	3	18	20	11	62
†	21UR-7839	TTATTATCAACTTTCTCCAGA	0	0	0	0	0	2	2	4
	21UR-7840	TTATGCAAACATAAAAAGCAA	0	0	1	0	3	0	1	5
†	21UR-7841	TTAGTATTCATCAAAAAAAAA	0	0	0	0	2	1	0	3
†	21UR-7842	TTAATGACCTTTTCCAAAAAA	0	0	0	0	0	0	0	0
†	21UR-7843	TTAAACGAAAAATTTCATTGA	0	0	0	1	0	0	0	1
†	21UR-7844	TGTTTTTGGATCTTTTTGTTT	1	0	0	0	1	1	1	4
†	21UR-7845	TGTTTTTCCAAATATATCGTT	5	0	0	0	0	1	2	8
	21UR-7846	TGTTTTGTCTTTCGGAAGCTT	3	0	3	0	5	5	0	16
†	21UR-7847	TGTTTAAGACATATATGTGGA	1	3	1	2	31	35	16	89
*	21UR-7848	TGGATATTTTGATCAACGGGA	2	2	1	1	32	47	39	124
	21UR-7849	TGACATTGTTTGTTACATTTC	0	0	0	0	0	0	0	0
	21UR-7850	TCTTTCGAATGTTTTCAGTTT	0	0	0	0	0	0	0	0
	21UR-7851	TCTTAGAACTACACTATGTCT	0	0	0	0	1	1	0	2
	21UR-7852	TCTCATAACCATTTACATCCT	0	0	0	0	0	0	0	0
	21UR-7853	TCCCGTCTCCAAAGACTATTT	0	0	0	0	0	0	0	0
	21UR-7854	TCCCATCTTCATCAATAATTT	0	0	0	0	0	0	0	0
	21UR-7855	TCATTTGCTTTCATTCGGAAT	0	0	2	0	4	5	0	11
†	21UR-7856	TCATTAGTATTCAAAATATCG	0	0	0	0	4	2	1	7
	21UR-7857	TCAGCAACATTATTCAAATTT	0	0	0	0	0	0	0	0
	21UR-7858	TCAGATTCTCGAAATTAAAAT	0	0	0	0	0	0	0	0
	21UR-7859	TATCATTGGTAAAAAATGTTC	0	0	0	0	1	0	0	1
	21UR-7860	TATAAAACTGGCGTTGTGGAC	3	0	0	1	14	23	11	52
	21UR-7861	TAGTAATTTAATGATATGACA	0	0	0	0	1	0	0	1
	21UR-7862	TAGTAAAGGAATTTTTAGCAT	0	3	1	6	64	49	0	123
†	21UR-7863	TACTTTCATTCCTCTTCGTAT	0	0	0	0	0	0	2	2
	21UR-7864	TACATGCTTAACATTACGCCC	2	0	0	0	3	2	6	13
†	21UR-7865	TACAAGTTTGATTTTGTAGTT	0	0	0	0	1	2	0	3
	21UR-7866	TAATTTTCGAGGCTTCAAAAC	0	0	0	0	0	1	0	1

	21UR-7867	TAAAATACTAGCTGGAGGAAT	2	0	1	2	1	4	1	11
	21UR-7868	TTTTTTCATTCGGAGATATTG	0	1	0	0	2	2	0	5
	21UR-7869	TTTTTTAAGTTATGGGAAATA	0	0	0	0	1	4	1	6
†	21UR-7870	TTTTGCCTGTAAAAAATTTTC	0	1	0	1	0	1	0	3
†	21UR-7871	TTTTCTGAATTGGCAATGTGT	0	0	0	2	39	30	18	89
†	21UR-7872	TTTTATACACCGACTCTGAAG	0	0	0	0	1	3	5	9
	21UR-7873	TTTGTGAAAATAGAACATCTT	0	0	0	0	2	2	2	6
	21UR-7874	TTTGTCTTCTTCCCAAAAGAG	0	0	0	0	0	0	0	0
	21UR-7875	TTTGTAGCATAAGCTCTCTAA	1	0	0	0	7	5	0	13
†	21UR-7876	TTTGTACACTTAAAACGTTGA	0	0	0	0	2	0	0	2
†	21UR-7877	TTTGGTTGTCTTTTAGCTTTT	0	0	0	1	3	1	0	5
	21UR-7878	TTTGATTTTTTTTCCGGTGTT	1	0	0	1	0	0	0	2
	21UR-7879	TTTCCTCGGACTCTTGACTAC	0	0	0	0	0	0	0	0
†	21UR-7880	TTTCCAGAAGACTGAGTCAAA	0	1	0	1	30	35	5	72
	21UR-7881	TTTCATAAGCTTGACACGACC	0	3	0	0	1	4	1	9
†	21UR-7882	TTTCAGTCTCATAAAGTCATC	0	0	0	0	0	1	0	1
	21UR-7883	TTTCACAATAGATGCCCTCTC	0	0	0	0	1	0	1	2
	21UR-7884	TTTATAAAAAATAATAAAAAA	0	0	0	0	0	0	0	0
	21UR-7885	TTTAAAAGTTTGAAAAGGTGA	2	0	0	0	11	10	1	24
†	21UR-7886	TTGTTTTCATAGTTATCAGAT	0	0	0	0	0	1	0	1
	21UR-7887	TTGTTATGTTGTTGCTTTTTT	0	0	0	0	1	0	0	1
	21UR-7888	TTGGTTCTTTACTTCTGGATT	0	0	0	0	0	0	0	0
	21UR-7889	TTGGAGACGGATCCTATTGTT	4	2	0	1	36	34	18	95
†	21UR-7890	TTCTTTTCAACCATTCAAGTT	0	0	0	0	0	0	0	0
	21UR-7891	TTCTCGACCATGGCCCACTTA	0	0	0	0	0	0	0	0
†	21UR-7892	TTCCTTCTGAAATAACAAGGC	0	0	0	0	0	0	0	0
†	21UR-7893	TTCAAAGCGTTAATAAAAAAA	0	0	0	0	2	1	1	4
†	21UR-7894	TTATTTTGTGTGTTTTTGAAG	0	0	0	0	2	2	0	4
	21UR-7895	TTATTGTAAAACTAACTTATC	0	0	0	0	4	2	0	6
†	21UR-7896	TTATTATGCCGTTGTCCGCTA	4	1	0	0	0	7	0	12
	21UR-7897	TTAGATAGTTTTTGTGAATTT	1	2	1	0	1	2	0	7
†	21UR-7898	TTAATTTCACCTTTCTTCGAA	0	0	0	0	0	0	4	4
	21UR-7899	TTAAATTTTTTTCAAGTTTAA	0	0	0	0	1	0	0	1
†	21UR-7900	TGTTCGAAATCGAAATTGCCC	0	0	0	0	0	0	0	0
	21UR-7901	TGTGATGTTTTTTACTACTAC	0	0	1	0	0	0	0	1
	21UR-7902	TGTATCATTATACACTAAAAT	0	0	0	0	0	0	1	1
	21UR-7903	TGCTTTCTATTCTTGTGGGAA	0	0	0	0	0	0	0	0
	21UR-7904	TGCCATGGCAAATGCGAAATT	2	0	0	0	1	1	0	4
	21UR-7905	TGCAGTTTTTGAAAAAAATCT	0	0	0	0	1	0	0	1
†	21UR-7906	TGATTAATGATCATGATAAAA	0	0	0	0	0	0	0	0
	21UR-7907	TGATGTGTTTTTTTGGAACTT	0	0	0	0	1	1	0	2
	21UR-7908	TGATAAAAACCATTCTGCTTC	0	0	0	0	0	0	0	0
	21UR-7909	TGAGTCTGTCAACAACAATTT	0	0	0	0	3	0	0	3
†	21UR-7910	TGAGAAGGTTTTTCGGAGAAT	0	1	0	0	2	6	2	11
*	21UR-7911	TCTGTTTTGTCGGAAACTGTC	0	0	0	0	2	6	2	10
	21UR-7912	TCTGAATTCTGGATTCCTAAA	0	2	1	1	2	1	0	7
	21UR-7913	TCTCTTTCTCGAAAAACTTTC	0	0	0	0	0	0	0	0
	21UR-7914	TCTCCTCATAATTTCAACTAA	0	0	0	0	0	0	0	0
	21UR-7915	TCTCAAAAAAAAAAGAGTTAA	0	0	0	0	0	0	0	0
†	21UR-7916	TCGACTGCATTTTTTTATTGC	0	0	0	0	0	0	0	0
	21UR-7917	TCGAACTTCCGGATTTTAATG	0	0	0	0	0	0	0	0
	21UR-7918	TCCTGAGCTATATTCCTAACT	0	0	0	0	0	0	0	0
†	21UR-7919	TCATCAATTCCAGATCATGCA	3	0	0	0	0	0	1	4
†	21UR-7920	TCAGTCGAGCCGTTTTTAATG	0	0	0	0	0	0	1	1
	21UR-7921	TCACAAACTGGCTTACATGTT	1	0	0	0	0	0	0	1
	21UR-7922	TATTTTTTCGATTTCGTAAAA	0	0	0	0	0	0	0	0
†	21UR-7923	TATTGTTTTGGTATAATGATC	0	0	0	0	1	0	0	1
	21UR-7924	TATTCCAAAAATTGTAACTTT	0	0	0	0	0	0	0	0
	21UR-7925	TATTAATCCATTATTGCTCTT	0	0	0	0	0	0	0	0
	21UR-7926	TATGTTCAAAAATATTGTTTC	0	0	1	0	1	0	0	2
	21UR-7927	TATGAGAAGTTCATGCGTATG	0	0	0	0	0	1	0	1
	21UR-7928	TATCCATAACGTCGCCTTCCG	0	0	0	0	5	0	2	7
†	21UR-7929	TAGTTCTTTACCATTAGCAAA	0	0	0	0	0	0	0	0
*	21UR-7930	TAGACTCAGAAAAAACATTGA	0	0	0	0	1	4	0	5

	21UR-7931	TAGAATCGTTTTAATAACTGA	0	0	0	1	8	3	1	13
	21UR-7932	TACCAAGTTGGGATTAATTTT	0	1	0	1	10	9	0	21
*	21UR-7933	TACATAAGATGTTTGTTTTCG	2	0	0	0	5	2	4	13
†	21UR-7934	TAATCCATGTGTAGTAATTTT	0	1	0	0	0	1	0	2
†	21UR-7935	TAAATTTCCGCGACTTAAATT	0	0	0	0	1	0	0	1
	21UR-7936	TAAAAAATTGGGGTAAATTTT	2	1	1	0	5	3	2	14
	21UR-7937	GAAGAGCGATTTATTTGGTTA	0	0	0	0	0	0	0	0
†	21UR-7938	TTTTCAAAATCTGATCAAGTG	2	2	0	0	0	2	1	7
†	21UR-7939	TTTTAGAAGATGACTCACCTT	7	4	2	1	1	24	2	41
	21UR-7940	TTTGAAGCAGATTTTTCTTGG	1	0	0	0	0	1	0	2
	21UR-7941	TTTCTTTTCGAAAGGTATTTC	0	0	0	0	1	0	0	1
	21UR-7942	TTTCTATCTTCTTTCAGATGG	0	0	0	0	0	0	0	0
	21UR-7943	TTTCGTTTCGTTATTCTCTTC	0	0	0	0	0	0	0	0
	21UR-7944	TTGATCTCATAGGGAATTTTT	0	0	0	0	0	0	0	0
†	21UR-7945	TTGAATCAAATTTATCACAAT	0	0	0	0	0	0	0	0
†	21UR-7946	TTCTTAGCTTGTGTATGATTT	1	0	1	0	0	1	0	3
	21UR-7947	TTCCTTTGATATTTATTCCGG	0	0	0	0	2	1	0	3
	21UR-7948	TTCCAACACTTTTTGAATGTA	0	0	0	0	1	0	0	1
†	21UR-7949	TTCACTGAAAATGAAAACAGA	0	0	0	0	0	0	0	0
†	21UR-7950	TTATTTCAACGGCTGTACAAA	0	0	0	0	0	0	0	0
	21UR-7951	TTATTGGCAAATATTTCAACA	1	0	0	0	8	2	3	14
	21UR-7952	TTATCGTGTTATTGTGATATT	0	0	0	0	0	0	0	0
	21UR-7953	TTAGTATTGTTCGTTCCTATG	0	0	0	0	6	2	4	12
	21UR-7954	TTAAAAAAAAAAAGGATTAAA	1	0	0	0	8	4	2	15
	21UR-7955	TGTTTTATGGAGTTTTATGAA	0	0	0	0	1	0	0	1
	21UR-7956	TGTTTTAATTTTTTTTCAAAA	0	0	0	0	0	0	0	0
	21UR-7957	TGTGGTATTCTTTGTACCCAA	0	0	0	0	0	0	0	0
	21UR-7958	TGGGAGTACAATGCTGTGGGA	0	0	0	0	5	6	0	11
†	21UR-7959	TGCTAACTTATTGGAAAGAAA	0	0	0	0	0	0	0	0
†	21UR-7960	TGCATTGTCTTGCTGAAATAT	0	0	0	0	2	1	0	3
	21UR-7961	TGATTTATGAAACTATGAAAC	0	1	1	0	0	1	1	4
	21UR-7962	TGAAATGTGTCAGAACTAAAA	0	0	0	1	16	5	2	24
	21UR-7963	TCTGGCTGAATTTAAGATTCT	0	0	0	0	0	0	0	0
	21UR-7964	TCTCGTATTCACTGAACATTG	0	0	0	0	1	0	0	1
	21UR-7965	TCTCGAAATCGTATTATTTTC	0	0	0	0	0	0	0	0
	21UR-7966	TCTAAAATTTCCTGTTTCAAC	0	0	0	0	0	0	0	0
	21UR-7967	TCATTTGGAATTCTTCCGCCT	0	0	0	0	2	2	0	4
	21UR-7968	TCATATCCATTTTACTGTATT	0	0	0	0	10	3	1	14
	21UR-7969	TCAGTGTTCTTTTTTCGTTTC	0	0	0	0	2	0	0	2
	21UR-7970	TCAACTTTTGAATCCCTCCTT	0	0	0	0	0	0	0	0
†	21UR-7971	TATTATTACGACTCAACGACT	0	0	0	2	0	0	1	3
	21UR-7972	TATGTTCAGGTGAATTACATT	0	0	0	0	0	0	0	0
	21UR-7973	TATGTCAATTTAGTTGGTCTT	1	0	0	0	0	4	1	6
	21UR-7974	TATAGTTAATCGATATTCGTA	0	0	0	0	1	0	0	1
	21UR-7975	TAGTCCCATAGTAAAAAGTTT	0	0	0	0	0	0	0	0
†	21UR-7976	TAGTATGTCTGATATAAAACA	0	0	0	0	0	0	1	1
	21UR-7977	TAGACTTTGTTTCCAGTTTAA	0	0	0	0	0	0	0	0
†	21UR-7978	TACACTCCGTTTTGAACGTAG	0	0	0	0	0	0	0	0
†	21UR-7979	TAATACTTCTGGATAAAAGTT	0	0	0	0	0	1	0	1
†	21UR-7980	TAAGATTTTAGTTCCCTGGCA	0	0	0	2	9	7	3	21
*	21UR-7981	TAAATGAATAAATCATTCATT	0	0	0	0	5	0	0	5
	21UR-7982	TAAACCTAGTGAAAGGAGGGG	0	0	0	0	2	3	2	7
	21UR-7983	TAAACACTTCAAAAATTGTTT	0	0	0	0	0	0	0	0
	21UR-7984	TAAAATCTATCCGTCGAAAAA	6	2	0	3	6	20	4	41
†	21UR-7985	TTTTTCTCCGAGAATCTGCGA	0	0	0	0	0	0	0	0
†	21UR-7986	TTTTTCGTACATTTTCACTGC	3	0	0	1	2	3	0	9
†	21UR-7987	TTTTGTTCAGATACTGGTCCT	0	0	0	0	0	0	0	0
†	21UR-7988	TTTTGGTCTTTTTATCAAACG	0	0	0	0	0	0	1	1
	21UR-7989	TTTTAATGTGAAATAAATAAC	0	0	0	0	0	3	0	3
	21UR-7990	TTTTAATAAGTTGTCTAAATC	5	0	0	0	3	1	2	11
†	21UR-7991	TTTGGAAGAGAACTCGATAAA	0	0	0	0	0	1	0	1
†	21UR-7992	TTTCGTTTAATTTCCGTTGGG	1	2	0	5	69	48	51	176
†	21UR-7993	TTGTTTTGCCTTTAACCTCCT	0	1	0	0	0	0	0	1
†	21UR-7994	TTGTGCGATTGTTCGTAGAAC	0	0	0	0	2	0	0	2

	†	21UR-7995	TTGGGAAATTTTGTGACTTTT	0	0	0	0	0	0	1	1
		21UR-7996	TTGGACTAGAGTTTTACGGAA	27	22	13	9	28	128	10	237
	†	21UR-7997	TTGCAAGAGAAGCAGTATATC	1	0	0	0	0	0	0	1
		21UR-7998	TTGAATTTCATGCCCGTGTAA	0	0	0	0	0	0	0	0
		21UR-7999	TTCTGTGAAATTTCGATTATC	0	0	0	0	1	0	1	2
		21UR-8000	TTCTCTTCTCAAAAGTAGTAG	2	0	0	0	0	1	0	3
		21UR-8001	TTCGTTTTCATTTTGAACGAA	0	0	0	0	1	0	0	1
		21UR-8002	TTCGTCTCTTTCTTACGAAGA	0	0	0	0	2	0	1	3
		21UR-8003	TTCGCTTCCTTTTCATGAAAT	0	0	0	0	0	0	0	0
		21UR-8004	TTCCTTCTCTCCAGTCACTTT	0	0	0	0	0	0	0	0
*		21UR-8005	TTCATTTGACGTTTATGAAAA	0	0	0	0	1	5	1	7
		21UR-8006	TTCATAAAATATTATCCTTCT	0	0	0	0	0	2	0	2
	†	21UR-8007	TTCAGAAATTTTTTCGTATGA	0	0	0	0	0	1	0	1
	†	21UR-8008	TTATTTAGAGAGATCTCGTTG	4	2	3	5	80	60	24	178
	†	21UR-8009	TTATTCGATACACTGTTTCAA	0	0	0	0	0	1	0	1
	†	21UR-8010	TTATGTGGTTATTTGAATCAA	0	0	1	0	1	3	0	5
		21UR-8011	TTATCCACTTTTGAAGCTCTG	0	0	0	0	0	0	0	0
	†	21UR-8012	TTAGAATTTTTTAAAAATGTT	0	0	0	0	0	0	0	0
	†	21UR-8013	TTAATTTGATCATCCGTTTTC	4	0	0	0	0	0	0	4
		21UR-8014	TTAAAACAGTGAATTTCGAAG	0	0	0	1	2	5	0	8
		21UR-8015	TGGATTATCTCTTTTTGATTA	0	0	0	0	0	0	0	0
		21UR-8016	TGGAAAGAGTTTAAAATAGTT	0	0	0	0	0	0	0	0
		21UR-8017	TGCTGCTTAGATTTTTTTTTT	2	2	1	0	7	1	0	13
	†	21UR-8018	TGCGTTTTCGAGTAAATATAC	1	1	0	0	0	1	0	3
		21UR-8019	TGAATCTGATCTTCTCACTCC	0	0	0	0	0	0	0	0
		21UR-8020	TGAACAAATGAAAATTTGACC	0	0	0	0	0	0	0	0
	†	21UR-8021	TCTTTCTCCTACATTTTAATC	0	0	0	0	0	3	0	3
		21UR-8022	TCTTCAATGTATGGACCCAAC	0	0	0	0	2	0	0	2
	†	21UR-8023	TCTTATGTGAATAGCGAAATG	0	0	0	0	0	1	0	1
		21UR-8024	TCTGTTCGTCCTGAATTTTGT	0	0	0	1	0	1	1	3
		21UR-8025	TCTGTCAAAGAGTTATGAAAA	0	0	0	0	0	1	0	1
		21UR-8026	TCCTTGAAACTCAAATGTTTT	0	0	0	1	0	0	0	1
		21UR-8027	TCCTCAAGGGGAAAGCGTCTC	0	0	0	0	0	1	1	2
	†	21UR-8028	TCAGATCTTGGTTTTTTCATT	0	0	0	2	17	3	0	22
		21UR-8029	TATTTTCTTAACTCAAATCAG	3	1	0	0	1	2	1	8
		21UR-8030	TATTTGACCTAAAATCATAAA	1	0	0	0	0	0	0	1
	†	21UR-8031	TATTTCAGCGAAACATTATCT	0	0	1	0	2	0	3	6
		21UR-8032	TATTTAGTTTTTTGTGGAACA	1	0	0	0	4	3	0	8
*	†	21UR-8033	TATTGAAGAAAAATGCACTGG	0	0	0	0	3	3	0	6
		21UR-8034	TATGTATTACATATTATGATC	0	0	0	0	2	0	0	2
		21UR-8035	TATGATATTTTCTCTCCCTCT	1	0	0	0	0	0	0	1
	†	21UR-8036	TATCTTAGTGAGCTGTATTTT	0	0	0	0	5	3	0	8
		21UR-8037	TATAAAAGTTAGATTGTCCGT	8	2	2	1	3	14	9	39
		21UR-8038	TAGGGGGTGTTCACCAAAATT	0	0	0	0	1	0	0	1
		21UR-8039	TAGGCTGATCTTTAGAGCATC	0	2	0	0	23	17	4	46
		21UR-8040	TACGTCGGTGAAAAAAAAAAC	2	1	1	12	114	148	78	356
		21UR-8041	TACCAATTTTTCGAATATTTT	2	1	1	2	2	2	0	10
		21UR-8042	TAACATATCGATATGCCTACA	0	0	0	0	3	2	2	7
		21UR-8043	TAAAGTCAACAGTTCTGAAAT	0	0	0	0	1	1	0	2
		21UR-8044	CACGGATGCCAGATTTTGAAA	0	0	0	0	1	0	0	1
		21UR-8045	TTTTTGTTCCACTATTTCAAA	0	0	0	0	1	0	0	1
		21UR-8046	TTTTGGCTGAAACATGAAAAT	0	0	0	0	1	2	0	3
		21UR-8047	TTTTGCTTTGCGATTTTAAAT	0	0	0	0	0	0	0	0
		21UR-8048	TTTTGATACATTAATGAAAGT	0	0	0	0	0	0	0	0
		21UR-8049	TTTTGAAGTTCCTTATGTATC	0	0	0	0	0	3	0	3
	†	21UR-8050	TTTTCCAGTCCCATTCTTTTA	0	0	0	0	0	0	0	0
	†	21UR-8051	TTTGTTATATAAAACATGGTA	0	0	0	0	0	0	0	0
		21UR-8052	TTTGTGTAGCGCATTAAATGT	0	0	0	0	10	3	2	15
*		21UR-8053	TTTGTGGCGAAGAAGCATTTT	36	30	5	11	23	143	18	266
		21UR-8054	TTTGGTGTTTGAATTGAGTTT	0	0	0	0	2	2	0	4
		21UR-8055	TTTCTTTTTTGTGTTCATAAC	0	0	0	0	0	1	0	1
		21UR-8056	TTTCTCTTGTCGACTTTTTGA	0	0	0	0	0	1	0	1
	†	21UR-8057	TTTATTCAGTCGTTGTTAGTA	2	0	1	0	1	3	1	8
	†	21UR-8058	TTTAGTACCGCTTCCCACAAA	1	0	1	0	0	0	1	3

	21UR-8059	TTGTACCGGACCTTTTGATTC	0	0	0	0	0	0	0	0
†	21UR-8060	TTGATTGTTAAATTGGATACA	0	0	0	0	0	4	0	4
	21UR-8061	TTCTTCACTCCTTTGATTCTT	0	0	0	0	0	0	0	0
	21UR-8062	TTCTGAAATTGGAATTCGAAC	0	3	0	0	3	8	0	14
	21UR-8063	TTCGTCTTACTTACTTCAAAT	0	0	0	0	5	0	0	5
†	21UR-8064	TTCCATAGCTAAAAGCCAATT	0	0	0	0	3	1	2	6
†	21UR-8065	TTCACAAAAAACGAACATGGA	0	0	0	0	0	1	0	1
	21UR-8066	TTATAGCTTTGGATATGGTGT	0	3	0	4	101	70	21	199
	21UR-8067	TTAGTTTTTGTAATTAACGAA	0	0	0	1	0	0	0	1
	21UR-8068	TTACAGCCGTTCCAGCGAATA	0	0	0	1	1	2	6	10
	21UR-8069	TGTTGCTGGCAAGTCCGTTAA	0	0	0	0	0	0	0	0
†	21UR-8070	TGGTAGGGAGGCAAACTAGTT	47	17	6	7	17	108	23	225
	21UR-8071	TGGGAAAGTTAATCTGAAATT	0	0	0	0	0	0	0	0
†	21UR-8072	TGGACCTTCCAAATTGAATTT	0	0	0	0	1	0	0	1
	21UR-8073	TGGAATATCCTTTTTTTGGTG	0	0	0	0	0	0	0	0
†	21UR-8074	TGCTAAACCGTATGTAGTAAA	0	0	0	0	0	0	0	0
†	21UR-8075	TGATTTTGCCTGACTACGAAC	0	0	0	0	3	0	0	3
†	21UR-8076	TGATTACTTTTAGGAGATTTA	5	1	0	1	0	10	0	17
	21UR-8077	TGATATATAAACTGCAACACA	0	0	0	0	0	0	0	0
	21UR-8078	TGACTACTTTTTTGTTTTGTC	0	0	0	0	0	0	0	0
	21UR-8079	TGACGTTTGCAAATTATAAGC	0	0	0	0	0	0	0	0
	21UR-8080	TGACACACCAGTTCTAAAAAA	0	0	0	0	0	0	0	0
	21UR-8081	TGAAACCTCAATTTTATAAGA	0	0	0	0	0	0	0	0
†	21UR-8082	TCTGTGAAACTATTCAAAATT	0	0	0	0	0	0	0	0
	21UR-8083	TCTGACTGTAAATTTTTTTAG	0	0	0	0	6	3	2	11
*	21UR-8084	TCGTGTTAGTAAATAACCACT	0	0	0	0	0	1	0	1
	21UR-8085	TCGAGGGATTTAACCAGAAAC	0	0	0	0	0	0	0	0
†	21UR-8086	TCCCATTCAAATAACTTACGT	0	0	0	0	0	1	0	1
	21UR-8087	TCCATGTGAGAATTACCTATT	0	0	0	3	28	6	2	39
†	21UR-8088	TCCAATACGAGAAACAAAACC	0	0	0	0	0	0	0	0
	21UR-8089	TCATAAAAAGTTTTTGAAAGA	0	0	0	0	0	0	0	0
	21UR-8090	TCAATTGTGTGGGAATTTAGA	0	0	0	0	0	1	0	1
†	21UR-8091	TATTGGCATCTCCGGAAAAAA	0	0	0	0	0	0	0	0
	21UR-8092	TATGTGCAAAATTCGAAGATT	0	0	0	0	0	0	0	0
*	21UR-8093	TATCTTTTTAAGTCGTACATA	15	5	5	26	272	159	93	575
	21UR-8094	TATCTAGTATATTAAGAGATT	0	0	0	0	0	0	0	0
	21UR-8095	TATATCCTTTATTGCCGCATT	2	0	0	3	3	3	21	32
	21UR-8096	TAGCTCTTTTTTCTGCAAGTT	0	0	0	0	0	1	0	1
	21UR-8097	TAGAACAACGTTTTTTTACGT	0	0	0	0	0	0	0	0
	21UR-8098	TAGAAAAAGCTTTCGAACAGT	0	0	0	1	3	4	0	8
	21UR-8099	TACTTTTTGTGTATCCATTCT	0	0	0	0	0	0	0	0
†	21UR-8100	TACTGGGGCTTTTTGAAGATT	0	0	0	0	0	0	0	0
†	21UR-8101	TAATTTTATTGGATAAATGAG	0	0	0	0	0	1	0	1
	21UR-8102	TAAGCCTCCGAATTTCACATT	0	0	0	0	0	0	2	2
	21UR-8103	TAAAGGATTAATAATAAAATG	0	0	0	0	1	1	0	2
	21UR-8104	CGAATACTGTTTGAATTAGTT	0	0	0	0	0	0	0	0
	21UR-8105	CGAACTCAAATATAGTCAATA	0	0	0	0	0	0	0	0
	21UR-8106	CAAGTAAAATAGAACGGAACT	4	5	4	0	2	26	1	42
*	21UR-8107	CAAAATCTTTAGACGACAAAA	0	0	0	1	7	9	0	17
†	21UR-8108	TTTTTTCACAATCGAGTCTTT	1	0	0	0	0	0	3	4
	21UR-8109	TTTTTGATTGATTCTTACTCA	1	0	0	1	1	2	0	5
†	21UR-8110	TTTTGTACTTCTACAATCTAC	0	0	0	0	0	0	0	0
	21UR-8111	TTTTGCAGTTGCAATGTTTGT	1	0	1	0	0	2	0	4
	21UR-8112	TTTTCGACGTTTTTCGACTTT	0	0	0	0	1	2	2	5
	21UR-8113	TTTTCGACCTGAATGTTTGGT	0	0	0	0	2	0	1	3
	21UR-8114	TTTGTCTTTAATGTTAGAGTA	0	0	0	0	0	0	0	0
	21UR-8115	TTTGCTGTGTGCATCTAGCAG	0	1	2	0	6	7	0	16
* †	21UR-8116	TTTGCATACCTCTAATAACCA	0	0	0	0	4	4	0	8
†	21UR-8117	TTTGAAACAGTGACTTGCTTT	0	0	0	0	0	1	0	1
	21UR-8118	TTTCTTTTTGTTGTTGATTTC	0	0	0	0	13	4	1	18
	21UR-8119	TTTCAAATATTAGGATTGAAA	0	0	0	0	0	0	0	0
†	21UR-8120	TTTATTATGCCGTTGTCCGCT	0	0	0	0	0	1	1	2
	21UR-8121	TTTAGTTGCCATATGAATTTG	1	2	2	0	4	4	0	13
	21UR-8122	TTTAATAAGAGACATATACTG	0	0	0	0	5	1	0	6

	21UR-8123	TTGGCTGATTAAATGTTTCAA	0	0	0	0	0	1	0	1
†	21UR-8124	TTGGATGACTAATTTTGACTA	0	0	0	0	0	2	0	2
	21UR-8125	TTGCAAGTTTTGTCCGAATAG	0	0	0	0	0	0	0	0
	21UR-8126	TTGAGAAACGATCACAATTTT	0	0	0	0	6	0	1	7
†	21UR-8127	TTCTTCGTTCATGCTTTTCTT	0	0	0	0	1	1	0	2
†	21UR-8128	TTCTGGGATTTGTTTATCATT	0	0	0	0	0	0	2	2
†	21UR-8129	TTCTCATCGATTTTCAACGAT	0	0	0	0	0	0	0	0
	21UR-8130	TTCGAAAAAAATTTCGTAAGT	0	0	0	0	0	0	0	0
†	21UR-8131	TTCCGGGACGCTGAATATTCG	0	1	0	0	1	2	0	4
	21UR-8132	TTCAATACATCAAACAATGAA	0	0	0	0	0	0	0	0
	21UR-8133	TTCAACTAGATTTAAATACTT	0	0	0	0	0	0	0	0
†	21UR-8134	TTATTTCATGTCTTCGGTTTC	5	2	0	2	6	9	2	26
†	21UR-8135	TTATAATAGGGTTTAAGATTT	0	0	0	0	0	1	0	1
	21UR-8136	TTAGTTTCTAATTTTTTTAAA	0	0	0	0	0	0	0	0
	21UR-8137	TTAGCTTATCAAAAAATCTTC	0	0	0	0	0	0	0	0
	21UR-8138	TTAATCTTCTCAAAAACAAAT	0	0	0	0	2	1	1	4
†	21UR-8139	TTAAATTTGTTTTTGTCCAGC	2	0	0	0	0	1	1	4
	21UR-8140	TTAAACTTGATAAAACTTGCA	1	1	0	0	2	7	0	11
	21UR-8141	TTAAACGGGAGCACTTTTTTG	3	1	0	2	3	11	6	26
†	21UR-8142	TGTTTGCATAAAATTATCGTA	2	0	0	0	9	8	21	40
†	21UR-8143	TGTTAATAAGTGTGAGGGTAT	1	0	0	1	0	3	2	7
	21UR-8144	TGTGTTTCTATCGTTTCGTAG	0	0	0	0	3	1	3	7
	21UR-8145	TGTGAATTTTGTTTAAGATTA	0	0	0	0	0	0	0	0
	21UR-8146	TGTCTCCATGTCATTTTCCAT	0	0	0	0	0	0	0	0
	21UR-8147	TGGTTCATCGTTTAGTGTATT	0	0	0	0	0	1	0	1
	21UR-8148	TGGACATATTAACTTGAGAAA	3	2	0	0	1	7	0	13
	21UR-8149	TGCTTATGGAGATAATAAAAC	0	0	0	0	0	0	0	0
†	21UR-8150	TGCTCTTACATTCGTGAAAAC	0	0	0	0	0	0	0	0
†	21UR-8151	TGCTAACGCAATTGATCGAAA	0	0	0	0	0	0	0	0
	21UR-8152	TGATTTTTTGATATACAGAAC	0	0	0	0	1	1	1	3
	21UR-8153	TGATTCTATGCCCTGCTCACT	0	0	0	0	0	0	0	0
	21UR-8154	TGATTAATTGCTTTTTTAATA	0	0	0	0	0	0	0	0
	21UR-8155	TGACTTGATCTATCTCTTACT	0	0	0	0	1	0	0	1
†	21UR-8156	TGACTCAGAAAAAATTTCATT	0	0	1	0	1	1	0	3
	21UR-8157	TGACATCTGAACATTTCTTTT	0	0	0	0	0	1	0	1
	21UR-8158	TCTGGCCATTTGCGAAAAATT	0	0	0	0	0	0	0	0
†	21UR-8159	TCTCCAGAAACTTACTTATTG	0	0	0	5	44	61	8	118
†	21UR-8160	TCTCATAGGTCTAAAAAACTG	0	0	0	0	0	1	0	1
†	21UR-8161	TCGTAGCGTAGCATGATTTTT	0	1	1	5	93	37	3	140
†	21UR-8162	TCGGACATAATATCGTAGACA	0	0	0	0	0	2	1	3
	21UR-8163	TCCCATTTCCGTACCTCAAAT	0	0	0	0	0	0	0	0
	21UR-8164	TCATGATTCGCCTCCAAGTTT	0	0	0	0	0	0	0	0
	21UR-8165	TCAGAGGCTTTTATTATCAAC	0	0	0	0	1	1	0	2
	21UR-8166	TCAATCTTCATTTCCAGACCC	0	0	0	0	0	0	0	0
†	21UR-8167	TATTGGATGATTGAATTGCAA	0	0	1	0	2	1	0	4
	21UR-8168	TATGCATATAGTGCCCCAAAA	0	0	0	0	0	0	0	0
	21UR-8169	TATGAAACAGTTGATCATTAT	1	0	0	4	28	161	5	199
	21UR-8170	TATCTTTTTTTTCTCTGCTTA	0	0	0	0	0	0	0	0
	21UR-8171	TATCAAACTTCTCTATAAATT	0	0	0	0	3	0	0	3
	21UR-8172	TATAATACATAGCGAATTGTT	0	0	1	0	1	1	0	3
	21UR-8173	TAGTACTTTGCTTTTTCATTG	0	0	0	0	0	0	0	0
*	21UR-8174	TAGCGTGATGTCATCTATTTG	3	0	2	2	55	43	8	113
†	21UR-8175	TAGAATTGATGAATGGGCAAA	0	0	0	0	3	6	5	14
	21UR-8176	TACCAGATTCATCATCAAATT	0	0	0	0	0	0	0	0
†	21UR-8177	TAATGATAATAATCAATCAAT	0	0	0	0	0	1	0	1
	21UR-8178	TAATAATTTCACTCAAAAAGA	0	0	0	0	0	1	0	1
	21UR-8179	TAAGATAAAAATTATGACCGA	0	0	0	0	0	1	0	1
	21UR-8180	TAACGTATTCTTGAAAAGATC	0	0	0	0	0	0	0	0
	21UR-8181	TAAACGCTCAAAATCACCAAA	0	0	0	0	0	0	0	0
	21UR-8182	TAAACCATTGGTCCCCGTTTA	0	0	0	0	0	0	1	1
†	21UR-8183	GATATATTTGAGTGGAATAAC	0	0	0	0	0	1	0	1
	21UR-8184	TTTTTTTGCCCGTATGTATGA	0	0	0	0	1	3	2	6
	21UR-8185	TTTTTTATAGAGATATGTCCA	2	0	0	0	3	1	2	8
†	21UR-8186	TTTTTGAAGCTTACTGGAAAA	1	0	0	3	4	7	2	17

†	21UR-8187	TTTTCAATGAATTAGAAAGAA	0	0	0	0	0	2	0	2
	21UR-8188	TTTTAACAAATTTCTGATAGA	0	2	0	0	0	1	0	3
	21UR-8189	TTTGGCCTCAAAATAAGTTTC	0	0	0	0	0	0	1	1
	21UR-8190	TTTGACCCGTTTAATTAGGCC	0	0	0	0	0	0	0	0
* †	21UR-8191	TTTGAACACCATACAATCTTT	2	1	0	6	51	31	24	115
	21UR-8192	TTTCTGGCTCTACGGGTGTTG	0	0	0	0	1	0	0	1
†	21UR-8193	TTTCTATCGTGTTGTAATTTC	0	0	0	0	4	1	0	5
	21UR-8194	TTTCGACAGAATTTAAAAAAT	0	1	0	0	5	2	1	9
	21UR-8195	TTTATTCCCCACCTCAAAAAA	2	0	0	0	0	0	0	2
	21UR-8196	TTTATCAACCAATTGAACTCC	0	0	0	0	0	1	0	1
†	21UR-8197	TTGTGAGTTGAATTTTGGAAC	0	0	0	0	2	0	0	2
†	21UR-8198	TTGTCCAGTTGCGAAAAAGAT	0	0	0	0	0	1	0	1
†	21UR-8199	TTGAGACTGAGAACTTACTTC	0	0	0	0	0	0	0	0
	21UR-8200	TTGAAAATTCGATTTTAGAAA	0	0	0	0	0	0	1	1
†	21UR-8201	TTCTGTGCGTGGTTTTCAATA	0	0	0	0	0	0	0	0
	21UR-8202	TTCGTATCTTGCGCTTTCACT	0	0	0	0	0	0	0	0
	21UR-8203	TTCGGATTTTAGTGTTCGATT	0	0	0	0	0	0	0	0
	21UR-8204	TTATTTTTTCACTTGAATAAA	0	0	0	0	1	0	0	1
†	21UR-8205	TTATTTGATGAAAACTTTGCA	0	0	0	0	0	2	0	2
	21UR-8206	TTATTCCTTCGATAAAAAAAT	0	0	0	0	0	0	0	0
†	21UR-8207	TTATTACATATGCATAACCAA	3	3	3	0	2	3	1	15
	21UR-8208	TTATGAAATGATTTATTTATT	0	0	0	0	0	0	0	0
	21UR-8209	TTATCCTAGTCTATAAAAGCC	0	0	0	0	2	0	0	2
†	21UR-8210	TTATATTATATTTCTAGGCAA	2	0	1	0	1	9	2	15
†	21UR-8211	TTAGAAGCTTTTTTTGGGTTT	0	0	0	0	0	0	0	0
†	21UR-8212	TTACTTACAATTAGTCGATCC	0	0	0	0	0	1	0	1
	21UR-8213	TTACTAAAAGCTTCTATATGA	1	0	0	0	2	2	3	8
	21UR-8214	TTAATAATTTTGTGCTTCGAA	0	0	0	0	1	0	0	1
†	21UR-8215	TTAAGAGGAAGCCAGCCATTA	12	0	2	1	2	8	12	37
†	21UR-8216	TGTTGGCCTTCGAACGGAACA	0	0	0	0	0	0	0	0
†	21UR-8217	TGTAGTAATTTTTGCAATACT	0	0	0	0	0	0	0	0
	21UR-8218	TGGTATACGGAGCTTCAGTTG	2	1	0	0	21	16	9	49
	21UR-8219	TGGTAAAAATATTAGCTACTT	0	0	0	0	1	0	0	1
	21UR-8220	TGGCAGTATTTGAAATACTTT	0	0	0	0	0	0	0	0
†	21UR-8221	TGGATTATAAATAGATATTAC	0	1	0	0	1	0	0	2
	21UR-8222	TGCTCCAATAATTTCAACTAT	0	0	0	0	0	0	0	0
	21UR-8223	TGCGGAAATTGGTAAGATTTT	2	3	1	2	3	8	0	19
	21UR-8224	TGCGAGTTTTTTGCCCAATAA	0	0	0	0	0	0	1	1
	21UR-8225	TGAGTGTTTAAAATGAGCAAA	0	0	0	0	5	1	0	6
†	21UR-8226	TGACTAAAAAAATGTAGGAAAT	0	0	0	0	1	4	1	6
*	21UR-8227	TGAAGCTGCAGAGATGAATCT	10	14	4	16	281	489	189	1003
	21UR-8228	TGAACCCATAAGTTTATGTCT	0	0	0	0	0	0	0	0
* †	21UR-8229	TCTGAGGGTTTTGCAATTGCA	0	0	0	0	1	0	0	1
	21UR-8230	TCTCTTTACTCCAAGTTTTGT	0	0	0	0	1	1	0	2
	21UR-8231	TCGGTGGATTTTCTGAGTCTT	0	0	0	0	0	0	0	0
	21UR-8232	TCCTCACCGTTTATCATTCCA	0	0	0	0	0	0	0	0
	21UR-8233	TCCCTTCCATCTTGACCAACA	0	0	0	0	0	0	0	0
†	21UR-8234	TCCAGATTTGATGTTCAAAAT	0	0	0	0	0	0	0	0
†	21UR-8235	TCCAGAAATATTTGTACTCCA	0	0	0	0	0	0	0	0
†	21UR-8236	TCATCTCGTATTTATTCAAAA	1	0	0	0	0	0	0	1
†	21UR-8237	TCAGCGAAAAAAACAAACAAA	0	0	0	0	0	0	0	0
†	21UR-8238	TCACTACGTGGTACAATAATA	0	0	0	0	2	0	0	2
†	21UR-8239	TATTTGCTTTTTAGACTATGC	0	0	0	1	4	4	1	10
†	21UR-8240	TATTATCGTCGAAATGTTATA	6	1	0	3	10	13	9	42
†	21UR-8241	TATGATTTTCTTGTCTTTTGT	0	0	0	0	0	0	0	0
	21UR-8242	TATGATGTTTTCCTTAAATAC	0	0	0	0	3	2	4	9
	21UR-8243	TATGAATTTTGAAATTTCGAA	1	0	0	0	3	3	1	8
	21UR-8244	TATAAGTTCAACTGCACATCG	0	0	0	0	5	1	0	6
	21UR-8245	TAGTTTTCAATCAGGTTTTGA	0	0	0	0	1	1	0	2
†	21UR-8246	TAGTCATAGGGGTATATCTAA	0	0	0	0	5	5	1	11
	21UR-8247	TAGATATTATATTCCGTCATA	6	0	0	0	1	7	0	14
	21UR-8248	TAGATACATTTCAGATCATTA	0	0	0	0	2	1	0	3
	21UR-8249	TAGAAAAAAGTTGTCATCATC	0	0	0	0	1	1	2	4
	21UR-8250	TACCTCGGTCGGTCAAACATT	0	0	0	1	6	7	3	17

	21UR-8251	TAATTATCAGGTGATCAGTCA	1	0	0	0	1	2	6	10
	21UR-8252	TAATGTAATTGAAATGATTTT	0	0	0	0	12	3	0	15
	21UR-8253	TAATCTTAAATTATAGAAAAA	0	0	0	0	0	0	0	0
	21UR-8254	TAATAAGAGTTTAAACTGTTT	0	0	0	0	2	2	0	4
*	21UR-8255	TAAGCTAGAAAATGGATAAAT	7	10	11	41	276	275	12	632
†	21UR-8256	TAACCACGATCAGTACTGAAC	0	0	0	0	0	1	0	1
	21UR-8257	TAAATAAGATAAAATAAATTG	0	0	0	0	0	1	0	1
	21UR-8258	CTGATATAATGGCGCCGACGA	0	0	0	0	0	1	3	4
	21UR-8259	TTTTGGAATTATTCTAATCCC	0	0	0	0	0	0	0	0
	21UR-8260	TTTTCACATAAATTGCTTTCG	1	0	0	0	3	0	0	4
	21UR-8261	TTTTCAACGAATATAAACTTT	0	0	0	0	0	0	0	0
	21UR-8262	TTTTCAAATTAATTTGACTTA	0	0	0	0	1	0	0	1
†	21UR-8263	TTTTAGATTGATAACATTCCA	0	0	0	0	1	0	0	1
	21UR-8264	TTTTACTTTGTTTGCATCAAT	3	0	0	0	0	2	1	6
	21UR-8265	TTTTAACCACTAAACCACATT	0	0	0	0	0	0	0	0
	21UR-8266	TTTGTTTAAAACGAATACAAA	0	0	0	0	0	0	0	0
	21UR-8267	TTTGAATTTTTTTTTGAGTTA	0	0	0	0	0	0	0	0
	21UR-8268	TTTCTTTGAATTTGTATTTGC	0	0	0	0	1	1	0	2
†	21UR-8269	TTTCACCTTAACTTTAGACCT	0	0	0	1	3	1	0	5
	21UR-8270	TTGTCATTTTTTTTCATTCAG	0	0	0	1	1	0	1	3
†	21UR-8271	TTGACAATTTGGATTGCTCAA	0	0	0	0	0	1	0	1
	21UR-8272	TTGAATATTGAAAAATTGAAT	0	0	0	0	0	1	0	1
	21UR-8273	TTCTTTTTTTTGTATGCAAGT	1	1	0	0	2	1	0	5
†	21UR-8274	TTCTTGTTGTGTTTTATAGTA	0	0	0	0	0	0	0	0
	21UR-8275	TTCTTGCACTGGAAAGTTGAA	0	0	0	0	2	0	1	3
†	21UR-8276	TTCTTGAACGGCTAGGATCAC	0	2	1	0	0	2	0	5
	21UR-8277	TTCGTAATTGGCAGAGCTTTG	0	0	0	0	1	1	0	2
	21UR-8278	TTCCTTGAATGGTACAGGCAT	0	0	1	0	2	2	3	8
†	21UR-8279	TTCAGCGAAAAAAACAAACAA	0	0	0	0	0	0	0	0
†	21UR-8280	TTCAAGCAATCCAAACGATTT	0	0	0	0	2	0	0	2
†	21UR-8281	TTATGTGTTGAGAAATAACAA	1	0	1	0	3	1	0	6
†	21UR-8282	TTATGGCTGATTTTGTAGTAA	0	0	0	0	8	10	6	24
†	21UR-8283	TTATCCTAGAATGACAAATCA	0	1	0	0	0	1	0	2
†	21UR-8284	TTAGGAATAATTTTAGGGATA	1	0	0	0	0	1	0	2
	21UR-8285	TTACTTATTGTTGGAAATAGT	0	0	0	0	0	0	0	0
†	21UR-8286	TTACCAATTCGGTTCAAAGAT	0	0	0	0	1	0	0	1
	21UR-8287	TTAAACTTAGAACAAAAAATA	0	1	0	0	0	1	0	2
	21UR-8288	TGTTTCTTTGTTACTAGCAAT	0	0	0	0	0	0	0	0
	21UR-8289	TGTTTCATTCTTCTTACGAAC	0	0	0	0	0	0	0	0
	21UR-8290	TGTATTTTATTTATTCAAAAA	0	0	0	0	0	0	0	0
	21UR-8291	TGTACTGATATATAAAATTGG	0	0	0	0	0	0	0	0
	21UR-8292	TGGATATTTTTTCAAAGAATA	0	0	0	0	0	0	0	0
†	21UR-8293	TGCTTCTTGAAGTTTTAGATT	0	0	0	0	0	0	0	0
†	21UR-8294	TGCTATGATTTTCTTGTCTTT	0	0	0	0	0	0	0	0
	21UR-8295	TGCGCCATCATCACTTTCGGA	0	0	0	0	0	1	1	2
	21UR-8296	TGATCCCAGTCATTCCATTTT	0	0	0	0	0	0	0	0
	21UR-8297	TGAGGATTTTTTAAGAACTGG	0	0	0	2	13	4	0	19
	21UR-8298	TGAATCCATTATTGTTATTGA	0	0	0	0	0	0	0	0
	21UR-8299	TCTTTCCTTAGATTTAAATCG	1	0	0	0	0	0	0	1
	21UR-8300	TCTTTCAACTGGATTTAGTTT	0	0	0	0	0	0	0	0
	21UR-8301	TCTTGTATTGCTTCTTTAAAA	0	0	0	0	4	0	0	4
	21UR-8302	TCTTGATTCGTTAGTTTTTTT	3	6	1	3	6	7	1	27
	21UR-8303	TCTTCGTCGCGCTCAAACAAT	0	0	0	0	2	3	1	6
†	21UR-8304	TCTTCGGTTTTGAAATTTTCA	0	0	0	0	0	1	0	1
	21UR-8305	TCTGAAGGTTTCGAAATTACC	0	0	0	0	0	0	0	0
†	21UR-8306	TCTAATTGGCATGTTATTTTG	0	0	0	0	3	5	1	9
	21UR-8307	TCTAATTGAAACATTTATAAA	0	0	0	0	0	1	0	1
†	21UR-8308	TCGTTTCGAGGAAACAGTAAA	0	0	0	0	0	0	0	0
	21UR-8309	TCGGCTTATTTCAAAATCTAA	0	0	0	0	9	4	0	13
†	21UR-8310	TCGCGGAAAATATTTTGATAT	2	1	1	0	1	2	0	7
	21UR-8311	TCCGCAATTAGAGTTTTATAT	0	0	0	0	0	0	0	0
	21UR-8312	TCCATGTGGGTTATCAAAATT	0	0	0	0	2	0	0	2
	21UR-8313	TCCATCATCTTTCTCCCAAAT	0	0	0	0	0	0	0	0
	21UR-8314	TCAGGAACACGCATTGATAAC	0	1	0	0	0	0	1	2

	†	21UR-8315	TCAATCCGTTTTGTATTCTTA	0	0	0	0	1	0	0	1
*	†	21UR-8316	TATTTTGAATGGGCGCAGGAA	0	0	0	0	9	21	12	42
		21UR-8317	TATGTTGATTTTATAATCAGT	0	0	0	1	10	7	0	18
	†	21UR-8318	TATCGTTGCAAATACGAGATT	0	0	0	0	0	0	0	0
		21UR-8319	TATCCTATTTTTTGTATTGTT	0	0	0	0	0	0	1	1
	†	21UR-8320	TATCCAAAAAAACTGGCGAGC	0	0	0	0	0	0	0	0
		21UR-8321	TAGTTTTTGTGAAATTCTCGA	0	0	0	0	2	0	1	3
		21UR-8322	TACTTCTTTTGCACTTTGTTC	0	0	0	0	4	5	0	9
		21UR-8323	TAATTGTTTCATCAAGCTTTT	2	0	0	1	3	18	0	24
		21UR-8324	TAATTGTATAAATGAAGAAGT	0	0	0	0	2	5	0	7
	†	21UR-8325	TAATTGAATTAAAAACACAGT	0	0	1	0	2	1	1	5
		21UR-8326	TAAATTTCTTTTCTAGGTTTC	0	0	0	0	1	0	2	3
	†	21UR-8327	CGAGATAAATTAATCTGCAAA	0	0	0	0	0	0	0	0
		21UR-8328	TTTTTTTAGTCAGAAAACTGT	1	0	0	2	1	5	0	9
		21UR-8329	TTTTGTTTTTAATTTTGTCCC	0	0	1	0	3	3	0	7
	†	21UR-8330	TTTTGTTGACGTACGTGGTAA	1	0	0	4	32	68	52	157
		21UR-8331	TTTTGCAAACATGTTTATTGT	0	0	0	0	0	0	0	0
	†	21UR-8332	TTTTGAAGCGAATAATTGCAA	0	0	0	1	2	1	0	4
		21UR-8333	TTTTCTAGCCCTCCGATTTAA	0	0	0	0	0	0	0	0
		21UR-8334	TTTTCAGTTGGAATTTGACAT	0	0	1	3	15	10	1	30
	†	21UR-8335	TTTTATATTAACGAAAAAAGG	0	0	0	0	0	1	0	1
	†	21UR-8336	TTTGTACTCTTTTTTGTGTTG	0	0	0	0	0	0	0	0
		21UR-8337	TTTGGTTATGATATAACAAAT	0	0	0	0	0	0	0	0
	†	21UR-8338	TTTGGCATCATCTTTTTGTTG	0	0	0	0	0	0	0	0
		21UR-8339	TTTGGATATGAGTTTGTGAAT	0	0	0	0	0	0	0	0
	†	21UR-8340	TTTGCAAACCACCACAACATT	0	0	0	0	0	0	0	0
*		21UR-8341	TTTCATACCGTTGGAAAAATT	3	0	0	1	23	29	10	66
		21UR-8342	TTTCACAATGTTTATTGATAA	0	0	0	0	0	1	0	1
		21UR-8343	TTTCAATGTAATGGAACATTT	1	1	0	1	1	1	0	5
		21UR-8344	TTTATCATAGTTGATGAAACA	0	0	0	0	0	0	0	0
		21UR-8345	TTTATAGTTTGTCTACTTTCT	1	0	1	0	0	0	0	2
		21UR-8346	TTTATACGTTTGTTTCTTGAA	0	0	0	0	3	4	4	11
	†	21UR-8347	TTGTTCCATTAAAAATTTTCC	0	0	0	0	1	0	0	1
		21UR-8348	TTGTGGACCGATTTTTTAAAG	0	0	1	0	0	0	0	1
	†	21UR-8349	TTGGGCGATGTGTGATCAATT	0	0	0	0	1	0	0	1
		21UR-8350	TTGAGTAGTATTTTTCAAAAC	0	0	0	0	2	3	0	5
		21UR-8351	TTGAAAATGAACTTCTTTTCT	0	0	0	0	3	3	0	6
	†	21UR-8352	TTCTTCCACGAACTAAGTATA	0	0	0	0	5	2	0	7
		21UR-8353	TTCTTATGATTTTCAGTTTCA	0	0	0	0	0	0	0	0
	†	21UR-8354	TTCGGATCCAATATTTTTTTG	1	0	0	0	0	1	1	3
	†	21UR-8355	TTCCACTGAGAAATCCATTCA	0	0	0	0	0	0	0	0
	†	21UR-8356	TTCATTTCGATCATCATTTAA	0	0	0	1	15	5	5	26
		21UR-8357	TTCATGCGGATTTTTCGTTAA	2	0	2	1	2	9	0	16
		21UR-8358	TTCAGTTGTCTCTGTGGATGC	0	0	0	0	0	1	0	1
		21UR-8359	TTCAATTCTTACTACATTACT	1	0	0	0	0	1	0	2
	†	21UR-8360	TTCAATTCGCTCAGCTCTCGT	0	0	0	0	0	0	0	0
		21UR-8361	TTCAATGCTAGAAACATTACA	0	0	2	1	11	8	0	22
	†	21UR-8362	TTATCCCATGTGAATCGTGTT	0	0	0	0	1	0	1	2
	†	21UR-8363	TTATAGGATAATACCGGATTT	0	0	0	1	1	0	0	2
	†	21UR-8364	TTATAAAATTCAGATTTTGCC	0	1	0	0	0	0	1	2
		21UR-8365	TTAGGATTGATCAGGAAGTTG	1	0	1	0	0	7	0	9
	†	21UR-8366	TTAACGGTAGAGCAAACATTC	0	3	1	0	2	6	0	12
		21UR-8367	TTAAATACCGAAAAAAATCAAA	0	0	0	0	0	0	0	0
		21UR-8368	TGTTTGGACTAAATTTAAAAT	3	0	0	0	6	2	2	13
		21UR-8369	TGTTGAATCTTTATTTATTAA	0	0	0	0	0	0	0	0
		21UR-8370	TGTTCAAAATTTAAAATTGTC	0	0	0	0	0	1	0	1
	†	21UR-8371	TGTTACTGCTATGCTTTTCTT	0	0	0	0	0	1	0	1
		21UR-8372	TGTGATTATCGTATCATCTTC	0	0	0	0	0	1	0	1
		21UR-8373	TGGGAAACAAAAATTCAAACG	0	0	0	0	3	6	0	9
		21UR-8374	TGATTTGAATCTGAGTGATTA	0	0	0	0	0	0	0	0
		21UR-8375	TGATAAAATTTCCAATTAATT	0	0	0	0	0	0	0	0
		21UR-8376	TGAGGAGTGGATTCGTTCAGT	1	2	0	0	5	15	1	24
		21UR-8377	TGAACCTCGGTTATTATTTTT	0	0	0	0	0	0	0	0
		21UR-8378	TGAAATGGGATCGGATAACAA	2	2	1	1	2	3	1	12

	21UR-8379	TCTTTTCGATCACTATTCTTA	0	0	0	0	0	0	0	0
	21UR-8380	TCTGGAAATTTTAGTGAATAA	0	0	0	0	0	0	0	0
	21UR-8381	TCTGAGAGCCACTTAAAACAC	0	0	0	0	0	0	0	0
	21UR-8382	TCTCTTAAAAAAATGGTTGAT	0	0	0	0	0	0	0	0
	21UR-8383	TCTCGGGTTCCAAAACGAAAA	0	0	0	0	0	0	0	0
	21UR-8384	TCTCAGGTTTTTTCACTGTAC	0	0	0	0	0	0	0	0
	21UR-8385	TCGATTTACTCTTTTATGCAA	0	0	0	0	1	0	0	1
†	21UR-8386	TCCAGCAGAAAGCAAAAAGTT	2	0	0	0	0	0	0	2
†	21UR-8387	TCAGAAAGTAAGTGAAGCAAC	0	0	0	0	0	0	0	0
	21UR-8388	TCAACCTAATTTCTGTTTCAG	0	0	0	0	0	0	0	0
	21UR-8389	TATTCATGTTTCGAAAAACTA	0	0	0	0	2	1	2	5
†	21UR-8390	TATTATTTTTCACGCAGGTTT	0	0	0	0	0	0	2	2
	21UR-8391	TATCATATTGGGTTTAGAATT	0	0	0	4	19	12	3	38
	21UR-8392	TATATTGAATGTGTATGAAGC	0	0	0	0	9	5	1	15
	21UR-8393	TATACATAGGGTTATTATAAA	0	0	0	0	0	1	0	1
	21UR-8394	TATAACAACAAAATAATAAAA	0	0	0	0	1	0	0	1
	21UR-8395	TAGTTTGTGTGCCAAAAAAAA	0	0	0	0	0	0	0	0
	21UR-8396	TAGTTTCATCACATCTATAGA	0	0	0	0	0	0	0	0
*	21UR-8397	TAGTCAGAAAGCTATCGGTAT	2	0	0	2	27	38	1	70
†	21UR-8398	TACTTCTCACTGTGATTCATT	0	0	0	0	0	0	0	0
†	21UR-8399	TACTGGATCAGGGGTACCCCA	0	0	0	0	1	0	0	1
	21UR-8400	TACGGCAAATAAATTGTTTTT	2	0	0	2	2	6	0	12
	21UR-8401	TACCATGATAAAACAATGAAA	0	0	0	2	8	2	5	17
	21UR-8402	TACAGTGGAATTCAAAAAAAT	4	3	0	1	8	8	2	26
†	21UR-8403	TAATCTGCAGTTTTTGAACGC	0	1	1	1	0	8	0	11
	21UR-8404	TAAGTGAATAATAATGTTCTC	0	0	0	0	0	0	0	0
	21UR-8405	TAACCATTCCATAGTAGACTC	0	0	0	0	6	3	0	9
*	21UR-8406	TAACACTTAGAACAGAATTCG	1	2	1	8	90	106	4	212
	21UR-8407	TAACACGAACATTAATGTAAC	0	0	0	0	6	2	1	9
	21UR-8408	TAAATTTGAAGTAAAATTACT	0	0	0	0	1	0	0	1
	21UR-8409	TAAACTTAAAAAAATCAAAAA	0	1	0	0	0	0	0	1
	21UR-8410	TAAAAGTGGCTCCGAGCTAGG	0	0	0	0	0	0	0	0
	21UR-8411	GAGCAATTTTTCGATTCAAAA	0	0	0	0	0	0	0	0
	21UR-8412	GACTCGGTGTGTGATCTCTTA	2	0	0	0	0	1	0	3
†	21UR-8413	CGATATATTTGAGTGGAATAA	2	2	0	0	0	0	0	4
	21UR-8414	TTTTTCATGCGTTCAGAATTT	0	0	0	1	0	1	0	2
†	21UR-8415	TTTTTACGTTCTCTGGTTGAA	0	0	0	0	1	2	0	3
†	21UR-8416	TTTTATTGCCAAAATTCCTGT	0	0	0	0	1	1	0	2
†	21UR-8417	TTTTAGATTGCCGAAGAGAGT	0	0	0	0	0	0	0	0
†	21UR-8418	TTTTAGAGTAAAATCGCGTTG	0	0	0	0	8	1	11	20
†	21UR-8419	TTTTAATTTTGGATACAAGAC	0	0	0	0	2	1	0	3
†	21UR-8420	TTTTAAGAGCACAAATACAAA	0	0	0	0	1	0	0	1
	21UR-8421	TTTTAACTTTTTTCAGGATTG	2	0	0	0	1	5	3	11
†	21UR-8422	TTTGTATCTGGTGGCATTAAA	1	0	0	0	7	6	2	16
	21UR-8423	TTTGCTTGATTTGTACGTTTC	0	0	0	0	1	3	1	5
†	21UR-8424	TTTGCCTGTAAAAAATTTTCA	0	0	0	0	1	0	0	1
†	21UR-8425	TTTGAGCGATCGTTAGGAGTA	3	1	0	1	0	3	1	9
	21UR-8426	TTTGAAAATTATGGAATTTGA	0	0	0	0	4	1	2	7
	21UR-8427	TTTAACGTTAATGAAGTCTAG	0	0	1	0	1	1	0	3
†	21UR-8428	TTGTTCTTTATTACTGGGCGT	1	0	1	1	2	5	1	11
	21UR-8429	TTGTTCTCTTTGTCGAGTTTT	0	0	0	0	2	0	0	2
†	21UR-8430	TTGTCGACTTCAAATGACACA	0	0	0	0	0	0	0	0
†	21UR-8431	TTGGGCAGGTATTAATTCGAG	0	1	0	0	2	1	0	4
†	21UR-8432	TTGGAATCAATTATTTTTCAA	0	0	0	0	0	0	0	0
†	21UR-8433	TTGCTGGAATTCCTGTTGCAA	0	0	0	0	0	0	0	0
	21UR-8434	TTCTTTACAGTTCAAAGTTCG	0	0	0	0	3	4	0	7
	21UR-8435	TTCTTAGCTTTTGTTGTAAAA	0	0	1	0	0	0	0	1
†	21UR-8436	TTCTGAACAACGTTTTCTTTA	0	0	1	0	1	0	0	2
	21UR-8437	TTCGTACATGCTTTTTTCAAA	2	0	0	0	0	0	0	2
†	21UR-8438	TTCGATTTACTGGTGTCGATT	0	0	0	0	0	0	0	0
†	21UR-8439	TTCCTTTTTTGCTTTCGTGGA	0	0	0	0	2	3	2	7
	21UR-8440	TTCCTTCCTTTTCAATCGGAA	0	0	0	0	0	1	0	1
†	21UR-8441	TTCATTAGGATGATGGTTTTT	8	4	4	23	270	200	22	531
†	21UR-8442	TTCATGCAAAAAAAATAGCTC	4	1	0	0	1	1	0	7

		21UR-8443	TTCACTTTTCAAAGCCAAAAC	0	0	0	0	0	0	0	0
		21UR-8444	TTCACGTTGTTTATTATCTAA	1	0	0	1	2	2	0	6
	†	21UR-8445	TTATTTAATAATGGACGCAGT	0	0	0	1	5	5	7	18
		21UR-8446	TTATGTGGTCGGTTTACGATT	0	0	0	0	0	0	0	0
	†	21UR-8447	TTATAAAATAATATGCGACAA	1	0	0	0	1	2	0	4
	†	21UR-8448	TTAGAGTTAGTTAGAAAGCAA	0	3	0	2	79	51	6	141
		21UR-8449	TTAATTTTTGAAATCTAGTAA	2	0	1	0	1	0	0	4
		21UR-8450	TTAAGAGATTGAAAAATATAA	0	0	0	0	2	1	1	4
		21UR-8451	TTAACAATTAGGATAAAAACT	0	0	0	0	1	1	0	2
	†	21UR-8452	TGTATAGCCAATAAAACGCAT	4	0	0	0	0	3	1	8
	†	21UR-8453	TGGGTAAGGGTTCGATATTTT	0	0	0	0	0	0	0	0
	†	21UR-8454	TGCTCATGTCTCACTTTCTTA	0	0	0	0	0	0	0	0
	†	21UR-8455	TGATTCCTTTGCTGGTTGGCT	0	0	0	0	0	0	0	0
		21UR-8456	TGAATTTGATGAGGTTATATT	0	0	0	0	3	1	0	4
		21UR-8457	TCTTTCTTTGTAACCCATTAA	0	0	0	0	0	0	0	0
	†	21UR-8458	TCTGTTCTCTCCTAGAAACTG	0	0	0	0	0	0	0	0
		21UR-8459	TCTGTTCCATCAATAAATACA	0	0	0	0	1	0	0	1
		21UR-8460	TCTGAGTTTAGTTTTCCTTTA	0	0	0	0	0	0	0	0
	†	21UR-8461	TCTCTCGATCGTAAACATTAC	5	6	0	13	160	105	187	476
		21UR-8462	TCTCATTAATGGGAGATTTTT	0	0	0	0	0	0	0	0
		21UR-8463	TCTAGCAGTATAAAGCAAATT	55	9	6	5	4	7	3	89
	†	21UR-8464	TCGTTTTCAAAACTTACCAAA	0	0	0	0	0	0	0	0
	†	21UR-8465	TCGTTGAAAAAATTTGGGTAA	0	0	0	0	0	0	0	0
		21UR-8466	TCGAATAATTGTGAGTTCGAC	0	0	0	0	1	1	0	2
		21UR-8467	TCCTGGTAAATAAGAAATCTA	0	0	0	0	0	0	0	0
		21UR-8468	TCCGTAGTGTTTCAAGAAATT	0	0	0	0	0	0	0	0
		21UR-8469	TCCATTTGTTCCCTTTAATTT	0	0	0	0	0	0	0	0
		21UR-8470	TCCAGCTTTTTTCCATTTTAA	0	0	0	0	1	0	0	1
	†	21UR-8471	TCAGTCGTAGACTCATCAACT	1	0	1	1	18	10	3	34
	†	21UR-8472	TCAGCCGAATTCAACAACTTG	0	0	0	0	0	0	0	0
		21UR-8473	TCAGACTTTCTTGTTCTGATT	0	0	0	0	0	0	0	0
		21UR-8474	TCAAGGTTTTTTTATTGCATT	2	2	0	0	5	2	2	13
	†	21UR-8475	TCAAAAGATTTTAACAAAAAT	0	0	0	0	0	0	0	0
	†	21UR-8476	TATTGGGCTAAACGACTGAGC	0	0	0	0	0	0	0	0
	†	21UR-8477	TATTATACCTTTATTCCCAAA	0	0	0	0	1	1	1	3
		21UR-8478	TATGTCTGTTTGGAAAGAAGT	0	1	0	0	6	7	3	17
		21UR-8479	TATGTACTTCCAAGACCCAAA	0	0	0	0	0	0	0	0
		21UR-8480	TATGAATTTAATTGTGAGATC	0	0	0	0	0	0	0	0
		21UR-8481	TATAAGATAATAAGATTTAAT	1	0	1	2	1	5	1	11
	†	21UR-8482	TAGTGAGAAGGTTTTTCGGAG	0	1	0	0	6	8	1	16
	†	21UR-8483	TAGGGAGGCAAACTAGTTTTA	3	0	0	0	0	1	1	5
		21UR-8484	TAGAAAACGTCAGTGTTTTTT	0	0	0	0	0	0	0	0
	†	21UR-8485	TACTCTTGCAGTCAAAAAAAT	1	0	0	0	0	3	0	4
	†	21UR-8486	TACTATTTCATCTGAAAGAAA	0	0	0	0	0	1	0	1
	†	21UR-8487	TACACTTGATCGTGTTTTAGC	0	0	0	0	0	0	0	0
		21UR-8488	TAATTCTAGTTTCGCTTCGCT	0	0	0	0	0	0	2	2
	†	21UR-8489	TAATGTAATTATACATACAAC	0	0	0	0	0	0	0	0
*	†	21UR-8490	TAATACAAATAGAAATATTGA	1	0	0	3	3	5	0	12
		21UR-8491	TAAGCAAATAATAGTTTGGGG	0	0	0	0	0	1	0	1
	†	21UR-8492	TAACGATGAATAAAACTTGTA	0	0	1	0	0	0	0	1
		21UR-8493	TAAATTAAAATGTTTGATTGA	0	0	0	0	0	0	0	0
		21UR-8494	TAAAGAATTTACTTTAAACTT	0	0	0	0	0	0	0	0
		21UR-8495	TAAAACTTATATATACGATTT	1	0	0	0	0	0	0	1
	†	21UR-8496	AGTGCACGATTTATGAATTCA	0	1	0	0	0	0	0	1
	†	21UR-8497	AGCTAGTGATTGAGTAGTAAT	0	0	0	0	0	0	0	0
	†	21UR-8498	TTTTTTTTTAAAAATTGGTAA	0	0	0	0	1	0	0	1
		21UR-8499	TTTTTAGTTGATTCTTTTTTC	0	0	0	0	0	0	0	0
		21UR-8500	TTTTTAATATTTGTTAGGAAA	1	0	0	0	3	0	0	4
		21UR-8501	TTTTGTATTTGAGGTAACAAT	0	1	0	1	7	5	3	17
	†	21UR-8502	TTTTCCGAAATAATTACCCTT	0	0	0	0	0	0	0	0
		21UR-8503	TTTGCTCCAATAGCAGAAAAA	0	0	0	0	0	0	0	0
		21UR-8504	TTTGCGGTTCTTTGGAATTTT	0	0	0	1	1	3	0	5
		21UR-8505	TTTGCGGTTATCGGTTTCAAA	0	0	0	0	9	10	1	20
	†	21UR-8506	TTTGCATTGTTGTATTGAAGA	0	0	0	0	0	0	0	0

	21UR-8507	TTTGCATACGTTTTTTTCCTC	0	0	0	0	2	0	0	2
†	21UR-8508	TTTGATCTTCTGTTCTCTCTT	0	0	0	0	0	0	0	0
	21UR-8509	TTTGAATAGTATATTGCAAGT	0	0	0	0	0	0	0	0
†	21UR-8510	TTTGAAGAGTAATAAAAAAAT	0	0	0	0	0	0	0	0
	21UR-8511	TTTCGTAAAGATTTTCGTAAA	0	0	0	0	0	1	0	1
	21UR-8512	TTTCGATAAAAGTGGCAAAAA	0	0	0	0	1	1	1	3
†	21UR-8513	TTTCGAGATAAAAAAATGTTT	0	0	0	0	1	0	0	1
	21UR-8514	TTTCACATTGATAAAGATTTC	0	0	0	0	0	0	0	0
	21UR-8515	TTTCAAGATATTGGCGGAATA	1	0	0	0	0	3	1	5
	21UR-8516	TTTCAAAACGATTACCGTCTG	0	0	0	1	0	0	0	1
	21UR-8517	TTTAGATAGTATTCTACAGTC	0	0	0	0	3	2	1	6
	21UR-8518	TTTAGAATTTTCAAAGAACCA	0	0	0	0	4	3	0	7
†	21UR-8519	TTTACAGTCAACCAATAATTA	0	0	0	0	0	0	0	0
†	21UR-8520	TTGTTTGTAACAATTGAAAGA	0	0	0	0	0	0	0	0
†	21UR-8521	TTGTGAATATTTTTTTCACGA	0	0	0	0	0	0	0	0
†	21UR-8522	TTGCTCACAGTAAAAACTTCT	1	0	0	0	0	1	0	2
	21UR-8523	TTCTTACAAAATCCCAACGTT	1	0	0	0	1	0	0	2
	21UR-8524	TTCTGCAATTTTCCAATCTAT	0	0	0	0	0	0	0	0
†	21UR-8525	TTCTCACTGTGATTCATTTCC	0	0	0	0	0	1	0	1
	21UR-8526	TTCTAACCGCCTCAAGAAATT	0	0	0	0	0	0	0	0
	21UR-8527	TTCGTTGTCATGTATTTTTTT	0	0	0	0	0	1	0	1
	21UR-8528	TTCGTGCAATTCGAACAAATC	0	0	0	0	0	0	0	0
	21UR-8529	TTCATTAAAACTTTCCGGACA	2	5	1	9	95	83	37	232
	21UR-8530	TTCAGTAGACTTCTTGCCACA	2	0	0	0	0	10	0	12
†	21UR-8531	TTCAGATGTGGATCAAACGCA	0	1	1	0	2	4	0	8
†	21UR-8532	TTCAGAAACGCTATGAATATC	15	19	15	12	38	99	4	202
	21UR-8533	TTCAACCAACAATTGTTTTTT	0	0	0	0	0	0	0	0
†	21UR-8534	TTAGGACTTTTGTAGACGTTT	0	0	0	2	24	27	2	55
	21UR-8535	TTACTTTCTTTCCCTGTCTTT	0	0	0	0	0	0	0	0
†	21UR-8536	TTACGTGATCTAGTTCCGAAT	0	0	0	0	1	3	0	4
†	21UR-8537	TTACCCTTCGTTTTCTAATTG	0	0	0	0	0	0	0	0
	21UR-8538	TTAATATCGAAAACGCTGAAA	1	0	0	0	1	1	0	3
	21UR-8539	TTAAATTCTTTGAACTTACAA	0	0	0	0	0	1	0	1
	21UR-8540	TTAAAGAAATTTTAAAATAAA	0	0	0	0	0	0	0	0
†	21UR-8541	TGTTATAGATCCATCAATTTC	0	0	0	0	0	1	0	1
	21UR-8542	TGTGTAGCATGTTAGCTGGCT	3	0	1	3	22	34	4	67
	21UR-8543	TGTACTGAAAGGTATAGTTGC	0	0	0	0	0	0	0	0
	21UR-8544	TGGTTTTATTTCAAAGCGGCA	1	1	0	0	1	6	1	10
	21UR-8545	TGGTAGGAATTGAAATCATCT	0	1	1	0	0	0	0	2
†	21UR-8546	TGGATTTAGATCTGGAGAGAA	2	1	2	0	1	1	0	7
	21UR-8547	TGGAATCTCAATTTTATAAGA	0	0	0	0	0	0	0	0
†	21UR-8548	TGGAAATCACAAGGAAAGTAT	1	0	0	0	10	4	18	33
	21UR-8549	TGCTTCGATTTTTGTTTAAAA	0	0	0	0	0	0	1	1
†	21UR-8550	TGCTTAATCTCTTTGATCACA	0	0	0	0	0	0	0	0
	21UR-8551	TGCTCAGTTGTATCGCTATTA	0	0	0	0	0	2	0	2
	21UR-8552	TGCGCATTGGTCTGCGTTAAA	0	0	0	0	0	0	0	0
	21UR-8553	TGCCAATTTTATTCAATAAAA	0	0	0	0	0	0	0	0
	21UR-8554	TGCACAATTGATGAAGGTGCT	0	0	0	0	2	1	0	3
	21UR-8555	TGCAAATTTCATATGTTTAAA	0	0	0	0	0	0	0	0
†	21UR-8556	TGAATGGAAGAACTGGAAATC	29	22	4	9	26	115	3	208
	21UR-8557	TGAAAAGTTTCCGAAATTCAT	0	0	0	0	0	0	0	0
	21UR-8558	TGAAAACATTTTCCACTAAAT	0	1	0	0	0	0	0	1
	21UR-8559	TGAAAAATATTTATCTGAGAA	0	0	0	0	0	0	0	0
†	21UR-8560	TCTTGTCTTAAACGTTCTCCT	0	0	0	0	1	0	0	1
†	21UR-8561	TCTTCCCGTACGCCTCGCAGA	2	0	0	0	1	2	3	8
†	21UR-8562	TCTTCATTGTTTTCGTTTGAA	0	0	0	0	0	0	0	0
†	21UR-8563	TCTTATTTTGGGGTCAGTGTT	0	0	0	0	0	1	1	2
†	21UR-8564	TCTGAAGACATTTTGATTGGA	1	0	2	2	19	14	6	44
	21UR-8565	TCGTCAGTCACTCTCCCCACT	0	0	0	0	0	0	0	0
†	21UR-8566	TCGATTCATTTTCAACAATGG	0	0	0	0	0	0	0	0
†	21UR-8567	TCGAGAACGGAACTTCAAAAC	0	0	0	0	22	13	14	49
†	21UR-8568	TCGAATTTGAAGGTATCCTAA	0	0	0	0	0	1	0	1
	21UR-8569	TCCTTAACTTATTGAAGTTCT	0	0	0	0	0	0	0	0
†	21UR-8570	TCATTGTATTGTATGTGTACA	0	0	0	0	0	0	0	0

		21UR-8571	TCATGCAAGTCTTTTCCATTC	0	0	1	0	0	1	2	4
	†	21UR-8572	TCAGTTGTCCGAGTAGATGTT	0	0	0	0	1	0	0	1
		21UR-8573	TCAGCAGCATAAATGATTTTT	0	0	0	0	4	5	7	16
		21UR-8574	TCAATTCGAAGTTGTCACATC	1	0	0	0	0	0	0	1
		21UR-8575	TATTTTTGATTTTTTGACATT	2	2	4	8	113	48	11	188
	†	21UR-8576	TATTTTTCAAGAAGGTGAACT	2	0	0	0	0	0	0	2
		21UR-8577	TATTTACTCCTTGGGTTATCT	0	0	0	0	1	0	1	2
	†	21UR-8578	TATTCCGGACTCCGAACGTTC	0	0	0	0	0	0	0	0
	†	21UR-8579	TATTCAACTCGAAAAATATTC	0	0	0	0	0	1	0	1
*	†	21UR-8580	TATTATTCGGATTTCAGATAG	0	0	1	0	8	1	1	11
	†	21UR-8581	TATGTTATGATATGTTATGTT	0	0	0	0	0	1	2	3
		21UR-8582	TATGTCTTCCTTTTTTTCAAA	0	0	0	1	0	1	0	2
		21UR-8583	TATCTGAGTATCTCGTTCTTT	0	0	0	0	2	2	0	4
		21UR-8584	TATCAATTCAGAAAATAAAAA	0	0	0	0	0	0	0	0
		21UR-8585	TATATAATTGAAAATTCCAAA	0	0	0	2	1	4	0	7
		21UR-8586	TAGGAAATGCTTTGAAAAAAT	0	0	0	0	0	1	0	1
		21UR-8587	TAGGAAACTAAATAATTTTGA	0	0	0	1	13	9	8	31
		21UR-8588	TAGAATTATCATATTTTGAAG	0	0	0	0	0	0	0	0
	†	21UR-8589	TACTCTTGTTTTATTCAGGCA	0	0	0	0	0	0	2	2
		21UR-8590	TACGTTCTCTAACTTGAAATC	0	1	0	0	1	0	0	2
		21UR-8591	TACGGCTGAATCAAGTACAGC	0	0	0	0	1	6	1	8
	†	21UR-8592	TACGCTTTTTTAATCGAGAAA	0	0	0	0	0	1	0	1
		21UR-8593	TACATATTTTTGACAATATGA	1	0	0	0	0	0	0	1
	†	21UR-8594	TAATTGCTTCTAATGGTTTTC	0	0	0	0	0	1	0	1
		21UR-8595	TAATCCGTGAAAACTCCGTGA	0	0	0	0	1	0	0	1
	†	21UR-8596	TAAGATCAACTAGAAGTTTCC	0	1	0	2	38	21	0	62
		21UR-8597	TAACTTTACTCAACGGCGAAA	0	0	0	0	12	23	2	37
		21UR-8598	TAACAACTCCTTTTTATTTAA	0	0	0	0	0	0	0	0
		21UR-8599	TAAAAATTGGACTAATAAAAA	0	0	1	1	1	0	1	4
		21UR-8600	TTTTTTGGCTGGCCTAACCAT	0	0	0	0	0	0	0	0
	†	21UR-8601	TTTTTGTAACTCCTCCAATTT	0	1	0	0	1	0	0	2
		21UR-8602	TTTTTGCTCTACGGAGAATGA	0	0	0	0	0	0	0	0
		21UR-8603	TTTTTCTGCTCTGGTGGACTT	0	0	0	0	0	0	0	0
	†	21UR-8604	TTTTGAACTGAATGCTAAACT	0	0	0	0	1	0	0	1
	†	21UR-8605	TTTTGAAATGTGTATAGAAAG	0	0	0	0	0	0	0	0
		21UR-8606	TTTTATCAGATTTTAAGTACC	1	1	0	1	2	2	1	8
		21UR-8607	TTTGTAATGTATTGAAACAAG	0	0	1	0	0	0	0	1
		21UR-8608	TTTGGATTTTCTAATTGCGAA	0	0	0	0	0	1	0	1
		21UR-8609	TTTGATTTTTCACTGTTTCTG	0	0	0	1	8	1	0	10
	†	21UR-8610	TTTGATTCCCTTAGCACATAC	0	0	0	0	0	0	0	0
		21UR-8611	TTTGATATATATTTCTTCTGG	0	1	0	0	1	0	0	2
		21UR-8612	TTTGAAAGATGTTTCATTATC	0	0	0	0	0	0	0	0
*		21UR-8613	TTTCCTTTTTGAACAACAGCA	0	1	0	0	26	12	0	39
	†	21UR-8614	TTTCCGTTCAAATTCCAGCTT	0	0	0	0	0	0	0	0
	†	21UR-8615	TTTCCATTAAAAAACCCAAAA	0	0	0	0	0	0	0	0
	†	21UR-8616	TTTCATCCACTAATTACGAAA	0	0	0	0	0	0	0	0
		21UR-8617	TTGTTTTGCATAGTTGAAACA	0	0	0	0	1	3	0	4
	†	21UR-8618	TTGTTTTACTACTAATAAACA	0	0	0	0	3	0	1	4
	†	21UR-8619	TTGTTATTCGTCAGCTTTTTG	6	2	2	1	2	9	2	24
		21UR-8620	TTGTCAGTTTGTTTCCAGGAA	0	0	0	0	0	2	0	2
	†	21UR-8621	TTGTAACAATTGAAAGAAAAC	0	0	0	0	0	0	0	0
		21UR-8622	TTGGTTCTTCTTTTTATTCAT	0	0	0	0	0	0	0	0
	†	21UR-8623	TTGGTCTACGAGTTATGAAAG	0	0	0	0	0	1	1	2
		21UR-8624	TTGGTCCAATTTCTCAATTTT	0	0	0	0	0	0	0	0
	†	21UR-8625	TTGGATATGCCTTGCTGAATT	0	0	0	0	0	0	0	0
	†	21UR-8626	TTGGAATAACAAATACGATAA	0	1	0	0	1	0	0	2
	†	21UR-8627	TTGCTCGAGAATACAACGAAA	2	2	1	1	6	6	0	18
		21UR-8628	TTGCATTTCAATTGTTATGTT	0	0	0	0	0	0	0	0
	†	21UR-8629	TTGAGTTGAAAAAAGGTTTCA	1	0	0	0	0	1	0	2
		21UR-8630	TTCTTCCACTCATGGCATTTT	3	0	0	1	1	1	1	7
		21UR-8631	TTCTCAAAATGTTTTATAACA	0	0	0	0	0	0	0	0
		21UR-8632	TTCTAACCTTTTCTGTCATTT	0	0	0	0	0	0	1	1
		21UR-8633	TTCGTTTGTGAATTTCACACA	0	0	0	0	3	5	0	8
		21UR-8634	TTCGTTTGGAAAAACCGCAAG	0	0	0	0	0	2	0	2

	21UR-8635	TTCGGTTTGATTCAGTCTCCG	0	0	0	0	0	0	0	0
	21UR-8636	TTCATTTGTACTTGGGTTTTT	0	0	0	0	4	1	0	5
	21UR-8637	TTCATTAAGTTCATTAAATTA	0	0	0	0	1	2	0	3
	21UR-8638	TTCATGCGGTATTAACAGTTT	8	1	1	1	7	15	0	33
	21UR-8639	TTCATATGAAAATTGGTTGAC	0	0	0	0	0	0	1	1
†	21UR-8640	TTCAATGATACTTCCAGTGTT	0	0	0	0	0	0	0	0
	21UR-8641	TTCAAAATTGCAAAACAAATC	0	0	0	0	7	1	4	12
†	21UR-8642	TTATTGGGTGAAGTTTGGTCT	4	0	0	0	5	4	1	14
	21UR-8643	TTATCTCTTCAATAGGTCCCT	0	0	0	0	0	0	0	0
†	21UR-8644	TTATCAGGTAGATACTTTTAG	2	4	0	7	163	101	3	280
	21UR-8645	TTATAGCTCTCATATCTTGGC	0	0	0	0	0	2	0	2
†	21UR-8646	TTAAAAATCGACATGTATTTC	0	0	0	0	7	5	1	13
	21UR-8647	TTAAAAATCATTTTTCATGGC	0	0	0	0	1	0	2	3
	21UR-8648	TGTTTTTCATATTGGTTAATT	0	0	0	0	1	0	0	1
†	21UR-8649	TGTTTGAAGCTAGAAAGAAAA	0	0	0	0	0	0	0	0
	21UR-8650	TGTTCTTCTTGCAAACAATCG	0	0	0	1	0	1	0	2
†	21UR-8651	TGTTAGCAAGTTGAAGTACGA	0	1	1	0	2	4	0	8
†	21UR-8652	TGTTACTTAACCTTTGAAATA	0	0	0	0	0	0	0	0
	21UR-8653	TGTGTTAGTGTAGTGATCATT	0	0	0	1	3	0	0	4
	21UR-8654	TGTGATCTCGAATTCCTAACC	0	0	0	0	2	1	0	3
	21UR-8655	TGTGAAAAAAACACTTTAAGT	0	0	0	0	0	0	0	0
	21UR-8656	TGTCTTTTCTTTTTTTGAATA	0	0	0	0	0	0	0	0
†	21UR-8657	TGTCGTATTCACTTCAATGTA	0	0	0	0	1	0	0	1
	21UR-8658	TGTCAGTCTTCTTGATTAGGT	0	0	0	0	0	0	0	0
†	21UR-8659	TGGTTGGTTCCTCATTGAAGA	0	0	0	0	0	1	0	1
	21UR-8660	TGGACTGATAATCTGTTGTTT	0	0	0	0	0	0	0	0
	21UR-8661	TGCAAATTGATGAAAACAGTC	0	0	0	0	0	0	0	0
	21UR-8662	TGATGATTTCATATGTAGTAG	53	86	49	60	164	323	45	780
†	21UR-8663	TGAGTATATGTCATAAAAACG	0	0	0	0	0	1	1	2
	21UR-8664	TGAACTTTTTTAAAAGGGTCT	0	0	0	0	0	0	0	0
	21UR-8665	TGAACTGTATTGATAAAAATT	0	0	1	0	0	0	1	2
	21UR-8666	TGAAACAGTGGATTTTTGGGC	1	3	1	3	64	92	11	175
†	21UR-8667	TCTTAGTTAGTAGTAATAACA	0	0	0	0	0	0	0	0
†	21UR-8668	TCTGCTGGCTATTTAGTAGCA	0	0	0	0	0	0	0	0
†	21UR-8669	TCTCTCAATCTCGTTTGTAAC	0	0	0	0	0	0	0	0
†	21UR-8670	TCTATTGGTTTTAGAAACTGA	0	0	0	0	2	0	0	2
	21UR-8671	TCTAGAGTTAAAATGATTCAA	0	0	0	0	0	0	0	0
	21UR-8672	TCTAAGCTTTATTACAATTTT	0	0	0	0	1	0	0	1
	21UR-8673	TCGGGCTAATCCATAATCTAC	0	0	0	0	5	2	2	9
	21UR-8674	TCGGAACACTGTTCCAAAATA	0	0	0	0	0	0	0	0
†	21UR-8675	TCGACTCATAATTTCAAACTT	0	0	0	0	0	0	0	0
†	21UR-8676	TCGAACCATGCCTTTGCCATT	0	0	0	1	3	5	3	12
	21UR-8677	TCATGTGAATTGTATTTCTAA	0	0	0	0	7	8	1	16
	21UR-8678	TCAGTTCGAAGGTTTTTTTTT	1	0	0	1	1	2	0	5
	21UR-8679	TCAGGAAGAAGAGCCGGATTT	2	2	2	0	2	8	3	19
†	21UR-8680	TCACTAGGTTGACAAAAAGAG	0	0	0	0	0	0	0	0
†	21UR-8681	TCACATCAAAAATTTTGTACA	0	0	0	0	2	0	0	2
	21UR-8682	TCAATTCTTGTATTCCACCCA	0	0	0	0	0	0	0	0
	21UR-8683	TATTTTACATTTGCACAATAC	0	0	0	0	2	1	3	6
	21UR-8684	TATTTGCATCATTGTTAAACG	0	0	0	0	11	5	0	16
	21UR-8685	TATTTATTTGGGGAAGACAAA	0	0	0	0	3	3	1	7
*	21UR-8686	TATTCTTCAGGCAGTTGCAAA	0	0	0	0	1	1	0	2
†	21UR-8687	TATTCTCGTTAAATAGTAACT	0	0	0	0	1	1	0	2
	21UR-8688	TATGGATTTTCTGTTTGAATG	0	0	1	0	3	1	0	5
†	21UR-8689	TATCTCTCGAAATTTAATGTT	0	0	0	0	0	0	0	0
	21UR-8690	TATCTATTTTCAAGAGCACAA	0	0	0	0	1	2	1	4
	21UR-8691	TAGTTATCAAGTAACCAAAAG	0	0	0	0	0	1	0	1
†	21UR-8692	TAGTCTAACTTTGCAAAGATT	0	0	0	0	0	1	0	1
	21UR-8693	TAGGCCATCAAAAAGTTTATT	0	0	0	0	1	0	1	2
	21UR-8694	TACTCGGTAAAAAAAAACTGT	0	0	0	0	9	4	2	15
*	21UR-8695	TACTAGCGATACTTTTTGCAG	0	0	0	0	15	11	2	28
	21UR-8696	TACATATGTAGTGTCCACTAT	0	0	0	0	7	5	1	13
	21UR-8697	TAATGAGTGTAGGCGTCTCTT	1	7	2	0	5	23	0	38
†	21UR-8698	TAACCTATCCAAAAAAGTACG	2	0	0	0	0	0	0	2

	21UR-8699	TAAATTTTCGCTACTATTATA	4	0	0	0	9	3	0	16
	21UR-8700	AGATCAAGCGTAGAATTCAAT	0	1	0	0	0	0	0	1
†	21UR-8701	TTTTTAGATTGATAACATTCC	0	0	0	1	2	2	0	5
	21UR-8702	TTTTGAATCTGGCCTGATAGT	0	0	0	0	0	1	1	2
†	21UR-8703	TTTTCGCATCGAATGGATCTA	0	0	0	1	3	5	6	15
†	21UR-8704	TTTTCCAAATATATCGTTCAG	1	0	0	0	0	0	0	1
	21UR-8705	TTTTATTAGGAATCTTCAACA	0	0	0	0	13	5	4	22
	21UR-8706	TTTTAAGAATGGTACAATTTC	4	0	1	2	2	3	1	13
†	21UR-8707	TTTGTTTGTAACAATTGAAAG	0	0	0	0	0	0	0	0
†	21UR-8708	TTTGTTTATTGCTCTGGTTGT	0	0	1	1	1	2	3	8
	21UR-8709	TTTCGAACAAATAGAAAAGTT	0	0	0	1	2	0	0	3
	21UR-8710	TTTCATCACAGAGTCCCATTT	0	0	0	0	0	0	1	1
	21UR-8711	TTTATTGGTTTTTTGTTAGCT	0	0	0	0	1	0	0	1
†	21UR-8712	TTTATTGGGTGAAGTTTGGTC	2	0	0	0	3	2	0	7
	21UR-8713	TTTATCTTCTTTTTCGCCGAA	0	0	0	0	1	3	5	9
	21UR-8714	TTTAGGTGATGGTTCGACTGA	0	0	0	0	0	1	4	5
*	21UR-8715	TTTACGTAGAAAACAATTAAA	2	5	1	13	151	80	10	262
	21UR-8716	TTTAACTGGGAAAAATTTAAA	0	0	0	0	1	1	0	2
	21UR-8717	TTGTTTTTTTTTCCCCATAAA	0	0	0	0	0	1	1	2
†	21UR-8718	TTGTTTGATCATACCGCATTT	0	0	0	0	0	0	0	0
†	21UR-8719	TTGTTATATAAAACATGGTAA	0	0	0	0	0	0	0	0
	21UR-8720	TTGTGTAGCATTATAATTTAA	0	1	3	2	13	3	0	22
	21UR-8721	TTGTACTGAAAACCCAAAATC	0	0	0	0	0	0	0	0
	21UR-8722	TTGGTTTAGAAGGGAAATTGG	0	1	0	0	0	0	2	3
†	21UR-8723	TTGAGATCTCACGCGGAAACT	0	0	1	0	1	0	0	2
†	21UR-8724	TTGACTTTGTTAAAATTTTGA	0	1	0	1	15	7	1	25
	21UR-8725	TTCTTCTCTTTTTCTGCCAAG	1	0	0	0	0	0	0	1
	21UR-8726	TTCTTATTAGATAAAATTTGA	0	0	0	1	0	0	0	1
	21UR-8727	TTCTAGGACATTTTTGCAGAA	0	3	3	12	180	200	40	438
†	21UR-8728	TTCTAAAAATCGCTGACAATA	0	0	1	3	44	24	7	79
†	21UR-8729	TTCGGTCTCTACTCACTCCCA	0	0	0	0	0	0	0	0
	21UR-8730	TTCGCGGTTATACATGTTGCT	1	1	0	0	22	20	2	46
	21UR-8731	TTCCGATAATTTTCCATGTTG	1	0	0	0	0	1	0	2
†	21UR-8732	TTCCCTTTCCGACGTAGAATA	0	0	0	0	0	0	0	0
	21UR-8733	TTCCCAAAAATAATCGTGATT	0	0	0	0	0	1	0	1
	21UR-8734	TTCATAATGTTTGAAAATATC	0	0	0	0	0	1	0	1
†	21UR-8735	TTCAGTTGAAAATTCAGTTGA	0	0	0	0	0	1	0	1
	21UR-8736	TTATTTGAGAAGATTTCATTC	0	0	0	0	0	0	0	0
†	21UR-8737	TTATCTATATGAGTTCAGTTC	0	0	0	0	1	1	1	3
†	21UR-8738	TTATCATAAATTGGTGAAAAA	0	0	0	0	0	0	0	0
†	21UR-8739	TTAGGAGGAAGCATTGTGCAT	5	1	0	2	4	6	1	19
	21UR-8740	TTAGAAAAACTCTTTTTGAGA	0	0	0	1	0	0	0	1
†	21UR-8741	TTACCCGACATCTTTTGAGAA	0	0	0	0	0	0	5	5
	21UR-8742	TTAACTTCATAAATCGAATAA	0	0	0	0	3	0	1	4
†	21UR-8743	TGTTTTCATTTTCAAAAATTC	0	0	0	0	0	0	0	0
	21UR-8744	TGTTTCGAATGTTTGAAACAG	0	0	0	0	0	1	0	1
†	21UR-8745	TGTTGAGTAAAAATAATAACC	0	0	0	0	0	0	0	0
†	21UR-8746	TGTTCCTTCAGAAGAGATTAA	0	0	0	0	0	0	0	0
†	21UR-8747	TGTTCAAAGCTATGTTCAATA	0	0	0	0	0	0	0	0
	21UR-8748	TGTTACGGTTTCCCAGATTTC	0	0	0	0	0	2	0	2
	21UR-8749	TGTGGCATTGTTTCTACAGGC	0	0	0	0	0	0	0	0
	21UR-8750	TGTAGTATACGAGGAGTAGGA	2	2	0	1	43	41	11	100
†	21UR-8751	TGGTGAAGTTTCAAGTAGGTT	1	1	0	1	17	11	2	33
	21UR-8752	TGGGGTTTTTTCTCACTTTGA	0	0	0	0	0	0	0	0
	21UR-8753	TGGCAATACATATTATCTTTT	0	0	0	0	1	2	1	4
†	21UR-8754	TGCGTTGTCGCTATAGCAATT	0	0	0	0	0	0	0	0
	21UR-8755	TGCCTACTCAACTCCTTTTGA	1	0	0	0	0	1	0	2
	21UR-8756	TGCCCCCATTAAAACGGATTT	0	0	0	0	1	0	0	1
	21UR-8757	TGATTCCTAAAAATAAAATTA	0	0	0	0	0	0	1	1
	21UR-8758	TGAATGCACTATTTTTCATCC	0	0	0	0	0	1	0	1
	21UR-8759	TGAAATTTGAGCGAATTTCCA	0	0	0	0	0	0	0	0
	21UR-8760	TCTTTTGAGTTCTATTTCTTT	0	0	0	0	0	2	0	2
	21UR-8761	TCTTGTAACTTTAAAAATCGT	0	0	0	0	0	1	0	1
	21UR-8762	TCTTGAATCAAGGGTGTCGGT	0	0	0	3	25	44	159	231

		21UR-8763	TCTTACGTTTGCTCTGAAATT	0	0	0	0	0	1	0	1
		21UR-8764	TCTGGCTTTTTTCTGTGATTC	0	0	0	0	0	0	0	0
		21UR-8765	TCTGGCTACAATTTGAAGCTT	1	0	0	0	0	0	0	1
*	†	21UR-8766	TCTCGTAGGAAAAAGCGGTTT	2	5	0	1	92	101	9	210
		21UR-8767	TCCGACCCGAAAAAACCCCAA	0	0	0	0	0	0	0	0
	†	21UR-8768	TCCCTCTTTATTCCAACAACA	0	0	0	0	0	0	0	0
		21UR-8769	TCCAAAATTTTAAGATTTTTA	0	0	0	0	0	0	0	0
	†	21UR-8770	TCAGCTGCAAATTTGAACTTC	0	0	0	0	0	0	0	0
		21UR-8771	TCAGAGAAGCATCCTGAAAAA	0	0	0	0	0	0	0	0
	†	21UR-8772	TCACCAGATCCCCAAAATTAA	2	0	0	0	0	0	0	2
		21UR-8773	TCAACTATTCCTCTTACGAAA	0	0	0	0	0	0	0	0
	†	21UR-8774	TCAACAAAAAAATTTTCTCAG	0	0	0	0	0	0	0	0
	†	21UR-8775	TATTTTTGACTAGGGAGAATT	0	0	0	0	4	4	0	8
		21UR-8776	TATTTCTATCTCATTGTTGTT	0	0	0	0	2	0	1	3
	†	21UR-8777	TATTTATATGATTTTTGCCAT	0	0	0	0	1	1	0	2
	†	21UR-8778	TATTGTGTTCGTAACTGAATT	0	0	0	0	0	0	0	0
		21UR-8779	TATGTTGATCAAAGATGGATT	1	0	0	0	1	9	1	12
		21UR-8780	TATGTTCGATTTTTTATTTCA	0	0	0	1	4	1	0	6
		21UR-8781	TATGGTATCAACATCAGGGAT	0	0	0	0	0	1	0	1
	†	21UR-8782	TATGAGAAAACAAATAATTGA	0	0	0	0	0	0	0	0
		21UR-8783	TATCACAACTTCAATTGTAAA	0	0	0	0	0	0	0	0
	†	21UR-8784	TAGTCTTTCTTTATTTTCAGG	0	0	0	0	1	1	1	3
	†	21UR-8785	TAGATCTTGAAGAATATCCTT	0	1	0	0	0	0	0	1
		21UR-8786	TAGACTATAAATCTTAGAAAG	0	0	0	0	0	0	0	0
*		21UR-8787	TAGAACAAGTAGTCTACGGCT	2	5	2	3	30	55	8	105
		21UR-8788	TACTTTGGACAAAGAAAGTTT	0	0	0	0	3	2	0	5
		21UR-8789	TACTTGATACTTGATCCACTC	0	0	0	0	1	1	0	2
	†	21UR-8790	TACTATAGATGCAAGATAAAC	0	1	0	0	1	3	0	5
		21UR-8791	TACTAAGATCTTGTGCATGCA	0	0	0	0	0	0	0	0
		21UR-8792	TACACATCGTGAAATAAATTT	1	0	0	0	1	4	0	6
		21UR-8793	TAATCAGACTAAAAGCAAAAT	1	1	0	0	0	1	0	3
	†	21UR-8794	TAATACTCTCAAATTATCATT	0	0	0	0	0	0	0	0
	†	21UR-8795	TAACTCGATCCATGCTTTGCC	0	0	0	0	0	0	0	0
		21UR-8796	TAACTCAAACATGACTTGCTC	0	2	0	0	2	2	1	7
		21UR-8797	TAAAAATCGCCCTTCGTTTTG	0	0	0	0	0	1	0	1
	†	21UR-8798	GCTGCTAATAAATGTAAATAG	0	0	0	0	0	0	0	0
		21UR-8799	TTTTTTTTGAATTTTGAAGAA	0	0	0	0	10	3	1	14
		21UR-8800	TTTTTTTGCTAAGAGATATCG	0	0	0	0	0	1	0	1
		21UR-8801	TTTTTTACCTGTATGACTGAT	0	0	0	0	0	3	0	3
	†	21UR-8802	TTTTTATTTCCTTCATCCACT	0	0	0	0	0	0	2	2
		21UR-8803	TTTTGTTTTTCTTTTGCCTCC	0	0	1	0	3	1	1	6
		21UR-8804	TTTTGTTTGATTACTGCTTCA	0	0	0	0	2	0	0	2
		21UR-8805	TTTTGTATTTAATCAATTTGG	0	0	0	0	1	0	0	1
		21UR-8806	TTTTGCACGCACTGTTACTGA	0	0	0	0	2	3	2	7
		21UR-8807	TTTTCTGGAGATTTTATATAC	0	0	0	0	5	0	0	5
	†	21UR-8808	TTTTCGTTTGCTTTTGCTTGA	0	0	0	0	0	0	0	0
		21UR-8809	TTTTCCCGACATTTTTATGAT	0	0	0	0	0	0	0	0
		21UR-8810	TTTTATGATTTTTTTCACTGT	0	0	0	0	1	1	1	3
	†	21UR-8811	TTTGATCACTATTTTGGCATA	1	2	0	5	37	34	0	79
		21UR-8812	TTTGAAAACGACTTAAGTTAA	0	0	0	0	7	2	0	9
	†	21UR-8813	TTTCGCGAATTGGTTTCTAAA	0	0	0	0	0	0	0	0
	†	21UR-8814	TTTCACCTCAAAATCCATAAC	0	0	0	0	0	0	3	3
	†	21UR-8815	TTTCAACCGTTGCCGTTATAT	0	0	1	0	1	2	1	5
	†	21UR-8816	TTTATTGGACCAAAAAGCTAA	1	0	0	0	1	1	0	3
	†	21UR-8817	TTTATGTGTTGAGAAATAACA	0	0	0	0	0	0	0	0
		21UR-8818	TTTATATATGGTGTTGACTTA	0	0	0	0	1	1	6	8
	†	21UR-8819	TTTAGTTTTTTTTCGAATTAA	0	0	0	0	0	0	0	0
	†	21UR-8820	TTTAGAGACACATTTCTAGAA	0	0	1	0	4	4	0	9
	†	21UR-8821	TTTACATTCAGAATATCCATA	11	2	0	2	11	12	1	39
	†	21UR-8822	TTTAAAGGTTTCTGAGACTTG	0	0	0	0	1	0	1	2
*	†	21UR-8823	TTGTTGGAAAAATCGTCTAAT	75	12	20	7	13	64	16	207
		21UR-8824	TTGTCCCATCACTAATTTAGC	0	0	0	0	0	0	2	2
	†	21UR-8825	TTGTATGGCTAGAATTCAAAT	0	0	0	0	0	0	0	0
		21UR-8826	TTGTAGAAATATATTAATTGG	2	0	0	1	32	16	2	53

†	21UR-8827	TTGCATAAAATTATCGTAATG	0	0	0	0	0	0	1	1
	21UR-8828	TTGATCAACCCAACTGCTAAT	0	0	0	0	0	0	0	0
	21UR-8829	TTGATATATGATTTTTCAAAA	0	0	0	0	0	0	0	0
†	21UR-8830	TTGAGTAGTTGATGTAAAAAG	0	0	1	1	7	4	0	13
	21UR-8831	TTCTTTCGACTAGAAAATTTT	0	0	0	0	0	0	0	0
	21UR-8832	TTCTTTATTCTTGCTTGTCAG	0	0	0	0	1	0	0	1
	21UR-8833	TTCTTGAATAAAAAATCAATT	0	0	0	0	0	0	0	0
	21UR-8834	TTCTCTCTGTTTGCAATTTTT	0	0	0	0	1	0	0	1
†	21UR-8835	TTCTCCGATATTATGCATGTA	0	0	1	0	0	6	0	7
†	21UR-8836	TTCTATCTATATTGTGAACAA	0	0	0	0	0	0	0	0
	21UR-8837	TTCCTTTTTGTAAACTTCAAG	0	0	0	0	0	1	0	1
†	21UR-8838	TTCCATTTGTGATCTTTTGGT	0	0	0	0	4	5	1	10
†	21UR-8839	TTCCAGAAGACTGAGTCAAAT	2	3	1	8	103	109	11	237
	21UR-8840	TTCCACTGTATCGTTAATTCA	0	0	0	2	25	7	9	43
†	21UR-8841	TTCCACTATGGTTTTTTTCCA	0	0	0	0	0	0	0	0
	21UR-8842	TTCACCAGTTTTATAAAAACT	0	0	0	0	0	0	0	0
	21UR-8843	TTCAAATGAACGGATTTTTTT	10	7	1	3	12	31	1	65
†	21UR-8844	TTATTGTTCTTGTAAGTGTAA	0	2	2	0	0	3	0	7
†	21UR-8845	TTATTGAGACTTGAGACTACC	1	1	0	0	3	3	0	8
†	21UR-8846	TTATTACATCTGAAACAGGAT	0	0	0	0	0	0	0	0
	21UR-8847	TTATGTGTTGCATTCCGAAAC	1	4	0	0	1	5	1	12
	21UR-8848	TTATACAACATTTTCACGAAT	1	0	0	1	10	6	2	20
	21UR-8849	TTAGACTAGAAAAACAAAGAA	1	1	0	0	1	8	0	11
†	21UR-8850	TTAAGTGTTCATTTGATGCAC	0	0	0	0	0	0	0	0
	21UR-8851	TTAAGGTGAAAGATTTTGCTT	0	0	0	1	7	4	0	12
	21UR-8852	TTAAGATTGAAACTTACAGAG	0	0	0	0	0	0	0	0
†	21UR-8853	TGTTTTTCTTGCTGAAAAAGG	0	0	0	0	0	0	0	0
	21UR-8854	TGTTTTCTTTTTCATCGTATG	0	0	0	1	0	0	0	1
	21UR-8855	TGTTTCAAGAAATTCAGAAAT	0	0	0	0	0	0	0	0
	21UR-8856	TGTTCCAGATTGAATATTGTA	0	0	0	0	0	0	0	0
†	21UR-8857	TGTTCACTGACAAATTACTCC	0	0	0	0	0	0	0	0
†	21UR-8858	TGTTATGTTCTCGTCACATAT	0	0	0	0	0	0	0	0
	21UR-8859	TGTCTTTTTTTCGCACGAAAT	0	0	0	0	0	0	0	0
	21UR-8860	TGTAAAAGAAAATGTTGACAA	0	0	0	0	0	0	0	0
	21UR-8861	TGGTGGAGTCTGTTCCAATTC	0	0	0	0	1	1	0	2
†	21UR-8862	TGGTGCATTGACTTCTAAAGC	0	0	0	0	0	1	0	1
	21UR-8863	TGGGCTAAAATTTGGAAAAAT	0	0	0	0	0	0	0	0
	21UR-8864	TGGATTTGATCCGCGATTTGA	0	0	0	0	0	0	1	1
†	21UR-8865	TGCTTTTGCTTCTTTGCTTAT	1	0	0	0	0	0	0	1
†	21UR-8866	TGCTTGTAACATATAATTTCA	0	0	0	0	1	1	0	2
	21UR-8867	TGCCGTTGTTTCACCTGCTTG	0	0	0	0	0	0	0	0
†	21UR-8868	TGCCAGTCCTCTCCAAATTTT	0	0	0	0	0	0	0	0
	21UR-8869	TGCATTCGGCATTTTTATATT	0	1	2	2	28	30	8	71
†	21UR-8870	TGATTCTTAGAGCCTTCACAA	0	0	0	0	0	0	0	0
	21UR-8871	TGAACAGTTCAGAAGAATTCG	6	7	2	15	240	257	55	582
†	21UR-8872	TGAAATTTGAGTCGGTTCGTC	1	0	0	0	4	2	2	9
†	21UR-8873	TCTTTTCCAAATCCAATAGAA	0	0	0	0	0	0	0	0
†	21UR-8874	TCTTCTTTTGCGTACCTTTTT	0	0	0	0	2	2	0	4
	21UR-8875	TCTTCAAAAAGTATTTTCCAG	0	0	0	0	0	0	0	0
	21UR-8876	TCTGTAATCGGAAAAGCATTT	0	1	0	2	30	27	7	67
	21UR-8877	TCTGGTTTTCGGAGAGCTATA	0	0	0	0	0	1	1	2
	21UR-8878	TCTCTCGAAAAATGTAAAGAG	0	0	0	0	0	0	0	0
	21UR-8879	TCTCATCCTATAAAGCAATTT	0	0	0	0	0	0	0	0
	21UR-8880	TCTCAGCCTAATAGTAGTTTT	0	0	0	0	0	0	0	0
	21UR-8881	TCTCAATTACAATACCTTTAA	0	0	0	0	0	0	0	0
	21UR-8882	TCTCAAAATAGAATGATCAAA	0	0	0	1	4	1	0	6
	21UR-8883	TCTATTCTTTCTTTAAAAGTT	0	0	0	0	0	0	0	0
	21UR-8884	TCTAGAGGTATTTGAGATATG	0	0	0	0	0	0	0	0
	21UR-8885	TCTAACAAAGGTTTGTTTAAA	0	0	0	0	0	0	0	0
†	21UR-8886	TCGTTCGATTCTGGATCCACA	0	0	0	0	1	0	0	1
	21UR-8887	TCGATTTTACATAAATATCGA	0	0	0	0	0	0	0	0
	21UR-8888	TCGAGATCCGTTTTGAAATTA	0	0	0	0	0	0	0	0
	21UR-8889	TCGACGGTTTATGTTTTTTTT	0	3	0	2	18	8	0	31
	21UR-8890	TCCGAGAATTTTTAAATATTT	0	0	0	0	0	0	0	0

		21UR-8891	TCCCAAACTATTTGCAAACTT	0	0	0	0	0	0	0	0
		21UR-8892	TCCAATTTTTACTGGAAAAAA	0	0	0	0	0	0	0	0
	†	21UR-8893	TCATTGGTTCTATTTTTCCAA	0	0	0	0	0	0	0	0
		21UR-8894	TCATCATTTGACGTAACTATT	0	0	0	0	14	2	1	17
		21UR-8895	TCATATCCCAATTCGTTGTAA	0	0	0	0	4	6	2	12
		21UR-8896	TCATAGATCTGAGTTTTATGA	0	0	0	0	0	0	0	0
		21UR-8897	TCATAAAACTAGAAATTACTC	0	0	0	0	0	0	0	0
		21UR-8898	TCAATTTTTCTTCCAAGTCAA	0	0	0	0	0	0	0	0
	†	21UR-8899	TCAATCTTTTTTTCGCATTCC	0	0	0	0	2	0	0	2
		21UR-8900	TCAAATTAAGGGGAGGTTATA	1	0	0	0	0	1	1	3
	†	21UR-8901	TCAAAGGAAAAACACTCACTT	0	0	0	0	0	1	0	1
		21UR-8902	TATTCTTTTCTCTATAGTTTT	0	0	0	0	0	0	0	0
		21UR-8903	TATTCTTCTAATATAGTGAGC	0	0	0	0	0	0	0	0
	†	21UR-8904	TATTCTCAGATGGTATTTGAA	0	0	0	0	3	1	0	4
	†	21UR-8905	TATTATTGGCGTTAACATTAG	2	0	0	1	1	2	2	8
		21UR-8906	TATTATCTTTGTTTGCATTCA	0	0	0	0	0	1	0	1
	†	21UR-8907	TATTAAAAAACAAAAACAGGA	0	0	0	0	0	0	0	0
		21UR-8908	TATGTTACATAATTTTGCTAA	0	0	0	0	0	0	0	0
*	†	21UR-8909	TATGAACTTAGGTACAACCAA	0	0	0	1	1	0	1	3
		21UR-8910	TATCTACACAACCCCTTCTTT	0	0	0	0	0	0	1	1
		21UR-8911	TATCGGTATTATAAAAGGGGT	2	0	0	0	5	3	3	13
		21UR-8912	TATAATTTTCCTAACGATTAC	1	0	0	0	5	0	0	6
	†	21UR-8913	TAGTTGGTTGGAATATAGAAA	1	0	0	0	1	1	0	3
		21UR-8914	TAGATAACCGATCAAATCTTG	0	0	0	0	0	0	0	0
		21UR-8915	TAGAATAATAACAATATATAT	0	0	0	0	1	1	0	2
	†	21UR-8916	TACCAGGAGGGAATCCATTTC	0	0	0	0	0	3	0	3
		21UR-8917	TAATTTTGCTGAAAAACAAAA	0	0	0	0	0	0	0	0
		21UR-8918	TAATTGATTTTATGGTTGAAT	0	0	0	0	0	0	0	0
	†	21UR-8919	TAATTCGTCGAAAACTTTGGC	0	1	0	0	0	2	0	3
		21UR-8920	TAATCCGTTTTTTTCTTGTAA	0	0	0	0	0	0	0	0
		21UR-8921	TAAGTTGATTTATTCTAGACA	0	0	0	0	0	1	0	1
	†	21UR-8922	TAAGGACATTGAATAAAAATA	0	0	0	0	0	0	0	0
		21UR-8923	TAAATAGTTGAAAAAAATAAT	2	1	0	1	4	19	1	28
	†	21UR-8924	TAAAAACTTCGACAGGAATTC	0	0	0	0	1	1	0	2
		21UR-8925	CAAGTTCAGAAAAAACTTAGA	0	0	0	0	0	1	0	1
		21UR-8926	AGAACAAACACGGTTACGGAA	0	0	0	0	0	1	1	2
		21UR-8927	TTTTTTCTTGATCAGAGCTGC	0	0	1	0	1	0	1	3
		21UR-8928	TTTTTTCATGGAGTTTCTCAT	0	0	0	0	0	2	0	2
	†	21UR-8929	TTTTTTACGCATTCAACAAAC	0	0	0	0	8	4	3	15
		21UR-8930	TTTTTAGAACTTGGTTATGAA	1	1	0	1	1	3	0	7
		21UR-8931	TTTTGTGTAAATTTGAGAAAA	0	0	0	0	1	2	1	4
		21UR-8932	TTTTGATTTCTGGTTGAGCTA	0	0	0	0	0	0	0	0
		21UR-8933	TTTTGACAGTATACGATAAAT	0	2	0	2	19	12	2	37
	†	21UR-8934	TTTTGAACATTTAATTTAAAA	0	0	0	0	0	0	0	0
	†	21UR-8935	TTTTCTTTCTAGCTTCAAACA	0	0	0	0	1	0	0	1
		21UR-8936	TTTTCCTTTCTTCGTACCCAG	1	0	0	0	0	0	0	1
	†	21UR-8937	TTTTAGCGGATTTTTTACAGC	2	0	0	0	1	2	0	5
	†	21UR-8938	TTTTACTGGTTTTCATTTTAA	1	0	0	0	0	1	0	2
	†	21UR-8939	TTTTAATTGGTAGGAATTGGA	0	0	0	0	0	0	0	0
		21UR-8940	TTTGAAAAGTTCATTTGGAAA	0	0	0	0	0	0	0	0
	†	21UR-8941	TTTCCGAAATAATTACCCTTC	0	0	0	0	0	0	0	0
		21UR-8942	TTTCCAACTTTCTCTAAGGCA	3	0	0	0	0	2	2	7
	†	21UR-8943	TTTCAGGAACGTTACTCATTA	9	23	5	24	40	65	4	170
		21UR-8944	TTTCACCCATTTTTCAGTTTG	0	0	0	0	1	0	2	3
		21UR-8945	TTTCAAATAGTTTAAAAATTT	0	0	0	0	0	0	0	0
	†	21UR-8946	TTTATTATTATAGAACGGACG	6	0	3	4	43	55	39	150
	†	21UR-8947	TTTATGCAGTTCTCTAACCGA	0	0	0	0	1	1	0	2
*	†	21UR-8948	TTTAATTTGATCATCCGTTTT	9	2	2	1	4	5	4	27
	†	21UR-8949	TTGTTGGCCTTCGAACGGAAC	0	0	0	0	0	0	0	0
		21UR-8950	TTGTTCTATAGACATCATTTC	0	0	0	1	4	0	0	5
		21UR-8951	TTGTGTCTTTTTTTTTTGAAA	0	0	0	0	0	0	0	0
	†	21UR-8952	TTGTACCTTTGTAGATTGCTT	4	5	0	3	3	19	0	34
	†	21UR-8953	TTGTACACTTAAAACGTTGAA	0	0	0	0	1	0	0	1
		21UR-8954	TTGTAATGGTAAATATCAGTA	0	0	0	0	0	2	0	2

†	21UR-8955	TTGGTCCGTTGTATTATTCAG	0	0	0	0	0	0	0	0
†	21UR-8956	TTGGGCTAAACGACTGAGCTA	0	0	0	0	0	0	0	0
	21UR-8957	TTGGCCATATTGATTATCTTC	2	1	0	0	0	0	0	3
†	21UR-8958	TTGGCACTACCACTCACCACT	0	0	0	0	0	0	0	0
†	21UR-8959	TTGGACAGTTTTGAAAAAATT	0	0	0	0	0	0	0	0
	21UR-8960	TTGCTTGAAAAAACTTTTGTA	0	0	0	0	0	0	0	0
†	21UR-8961	TTGCTCGTGTAGATCGAACTA	0	0	0	0	1	1	0	2
†	21UR-8962	TTGCGACACCGATAAGAGAAC	0	0	0	0	0	0	0	0
	21UR-8963	TTGATTTAATATTTTGGAACA	0	0	0	0	0	0	1	1
†	21UR-8964	TTGATTCATGTTGACTTGTCA	0	0	0	0	0	0	0	0
	21UR-8965	TTGATGGTGAAACACACTTCA	1	0	0	0	3	4	2	10
†	21UR-8966	TTGAGCTCGCCGATTTCTCTC	0	0	0	0	0	0	0	0
†	21UR-8967	TTGACGCTGCGGGTGTACTTC	0	0	0	0	0	1	2	3
	21UR-8968	TTGAAGTTTTGTTAATTTCAG	0	0	0	0	0	1	0	1
	21UR-8969	TTCTTGCAGATATTTGAAAAT	2	13	2	19	361	265	6	668
	21UR-8970	TTCTTGATAATATGTCCAGCT	0	0	0	0	0	0	0	0
†	21UR-8971	TTCGTTTTCCAAACTTGCAAT	0	0	0	0	1	0	0	1
	21UR-8972	TTCGTTGAATCTTTCTCTTAT	0	0	0	0	2	0	0	2
†	21UR-8973	TTCCTTCGAAAACCCCTCACT	0	0	0	0	0	0	0	0
†	21UR-8974	TTCCGAATTCCGAATTGAAAC	0	0	0	0	0	0	0	0
	21UR-8975	TTCATTTTTAATTAGTTCAAC	0	0	0	0	0	0	0	0
	21UR-8976	TTCATGCTGAATTCTAATAAA	1	2	2	1	7	14	0	27
	21UR-8977	TTCAGTCTTGTTGTGACTTGG	0	0	0	0	1	0	1	2
	21UR-8978	TTCAACTGTTTCAGAATGTTG	0	0	1	0	4	6	0	11
†	21UR-8979	TTCAACGTATGAAAAATCTGA	1	1	0	0	2	5	0	9
†	21UR-8980	TTCAACCCAAAACATGACAGC	1	0	0	0	0	4	0	5
	21UR-8981	TTCAAAACACACAAGGCAAAA	0	0	0	0	0	0	0	0
	21UR-8982	TTCAAAAACTAACTAGTAATT	0	0	0	0	2	0	0	2
†	21UR-8983	TTATTTATTTCAACACTAAGA	0	0	0	0	1	0	0	1
	21UR-8984	TTATGTAGTAGTTTTGCGGCA	53	43	25	10	38	189	11	369
	21UR-8985	TTATATTTTCAGTCAAGTCGT	1	1	0	0	1	5	0	8
†	21UR-8986	TTAGTTCGCCCATCTCCTAGG	0	0	0	0	0	0	0	0
†	21UR-8987	TTAGTCATTGTAGAATCCGCC	0	0	0	0	1	0	0	1
	21UR-8988	TTAGGAGTGTTCGAAATGTTG	0	0	0	0	1	0	1	2
	21UR-8989	TTAGATCAAAGTATAGTCCTA	0	0	0	0	0	0	0	0
	21UR-8990	TTACACAATCAGTGTTGAAGA	0	0	0	0	0	0	0	0
	21UR-8991	TTAATTTCTTTTGGTAGGTTT	0	0	0	0	0	1	0	1
	21UR-8992	TTAATTTAATTTTTGATTCTC	0	0	0	0	3	0	0	3
†	21UR-8993	TTAATCAATCCGATGATCTTA	1	0	0	0	0	1	6	8
†	21UR-8994	TTAAATGTCCGGGAATGAGTC	0	0	0	0	0	0	0	0
	21UR-8995	TGTTTTTTTGATACATAATCG	0	0	0	0	0	0	0	0
†	21UR-8996	TGTTTCTGTCTAGCTCGACAT	0	0	0	0	0	1	0	1
†	21UR-8997	TGTTCGATACAGAAATGTCAA	0	0	0	0	0	0	0	0
	21UR-8998	TGTTCAGGCGAAATTTTTCTA	0	0	0	0	0	0	1	1
†	21UR-8999	TGTTAGAAGGTTTTTCAATTG	0	0	0	0	0	0	0	0
	21UR-9000	TGTGTTTCACTTTCCTCACAT	0	0	0	0	0	0	0	0
	21UR-9001	TGTGTTCACTTTTTGTACTAT	0	0	0	0	0	0	0	0
†	21UR-9002	TGTGCAAAAATTGAATACTCA	0	0	0	0	0	0	0	0
†	21UR-9003	TGTCAATAGGAATAGGACACG	0	0	2	0	2	1	0	5
	21UR-9004	TGGTAAGAAAGGCGTAACTCC	0	0	0	3	12	27	1	43
	21UR-9005	TGGGTCGTTTTATCTTTCTTT	0	0	1	0	0	0	0	1
	21UR-9006	TGGGAGACTCTTGGCTAACAC	0	0	0	0	0	1	0	1
	21UR-9007	TGGCAAACTGTAAAATGTTAA	0	1	0	1	5	3	0	10
	21UR-9008	TGGAGAAATTTTTTTTAATCT	0	0	0	0	0	0	0	0
†	21UR-9009	TGCTTTTCTATCCCGCATTAC	0	0	0	0	0	0	0	0
	21UR-9010	TGCCTGTTATGAGTTTTTTGA	0	0	0	0	0	0	0	0
†	21UR-9011	TGCATATCCTTTTAAAATGAA	0	0	0	0	0	0	0	0
	21UR-9012	TGCAAGTGAAAACCACACTTC	0	0	0	0	0	0	0	0
	21UR-9013	TGATTCTATGCCTAGCTCACT	0	0	0	0	0	0	1	1
	21UR-9014	TGATTACTTGAAGTAAGATTC	0	0	0	0	0	0	0	0
	21UR-9015	TGAGCATTTTAGTGAAAAGGC	0	0	0	0	0	2	1	3
	21UR-9016	TGAATCGTTAGATTTACATGA	0	0	0	0	2	0	0	2
	21UR-9017	TGAAACTCAACGATTGACTAG	0	1	0	0	3	2	0	6
	21UR-9018	TCTTTTATTTTACAGTTTTGA	0	0	0	0	0	1	0	1

†	21UR-9019	TCTTTTAGAATACTGTGGAAA	1	1	0	1	1	1	0	5
	21UR-9020	TCTTTGAATAACCGCTTACCC	1	0	0	0	0	0	0	1
	21UR-9021	TCTTGGAACAGTAGGACTCAT	0	0	0	0	3	0	1	4
†	21UR-9022	TCTTCGTTCGGTCCAAAACAA	2	0	0	0	1	1	0	4
	21UR-9023	TCTGCATAAGACCTTTTTTTG	0	0	0	0	9	2	1	12
	21UR-9024	TCTCTTTGCTTCATACCCACC	0	0	0	0	1	1	0	2
	21UR-9025	TCTAGTGTTCCTATAGCGTAA	0	0	0	0	0	0	0	0
	21UR-9026	TCGTTCCATATTTTATTTGGT	0	0	0	0	1	0	0	1
	21UR-9027	TCGCACCTCTCCTTTTCTTTG	0	0	0	0	0	0	0	0
	21UR-9028	TCGAAATAACTCGTTTAATGT	0	0	0	0	0	0	0	0
	21UR-9029	TCCGTGGTATTAAACGTATTT	0	0	0	0	0	0	0	0
†	21UR-9030	TCATTCGAAATAACAATTAGA	0	0	0	0	0	0	0	0
	21UR-9031	TCATGCAATTAAAATTGCCTC	0	0	0	0	0	0	0	0
	21UR-9032	TCATATATGAAATTCGTATTC	0	0	1	1	11	4	1	18
	21UR-9033	TCATACAAATTTGGAAACCGT	0	0	0	0	7	3	0	10
	21UR-9034	TCAGTCTATGAGTGTATGTAG	0	0	0	0	1	0	0	1
	21UR-9035	TCACTAGAGATGTTAGCTAGA	0	0	0	0	0	0	0	0
	21UR-9036	TCAATATACGGAATTACATGA	0	0	0	0	4	2	1	7
†	21UR-9037	TATTTTCGTTTCTGTCCAAGG	0	0	0	0	0	0	0	0
*	21UR-9038	TATTTCAAGCTCTTGTTTTTC	0	2	0	0	0	4	0	6
	21UR-9039	TATTTAAATCCATGAACACCT	0	0	0	0	0	1	0	1
	21UR-9040	TATTGTTTTCCAATTTTACAA	0	0	0	0	3	0	0	3
†	21UR-9041	TATTGTTCAAATCATTCAAAA	0	0	0	0	0	0	0	0
†	21UR-9042	TATTGCTCGAGAATACAACGA	0	0	0	0	1	0	0	1
†	21UR-9043	TATTGATACTCGTCGAATCGC	4	4	1	0	5	5	0	19
†	21UR-9044	TATTAAAAAATCGTGTATTCA	1	0	0	0	7	3	3	14
	21UR-9045	TATGTAAAACAAACAGCAGAA	2	0	0	0	0	3	0	5
*	21UR-9046	TATGAGTAGGTGCTGAAGACG	48	18	16	5	12	140	1	240
	21UR-9047	TATCAAAGTAAAACGTTCCTG	0	0	0	0	2	0	2	4
†	21UR-9048	TATAGAAACGTTTCCCTCCTA	0	0	0	0	0	2	0	2
	21UR-9049	TATACTCCTGCAGTTCATTAG	0	0	0	0	5	0	2	7
	21UR-9050	TATAAAATACAGATTTCAGTG	6	2	1	3	0	3	2	17
	21UR-9051	TAGTTTTGAGTTTCAAGTTAA	0	0	0	0	0	0	0	0
	21UR-9052	TAGTTTATTTAAATATTTGAA	0	0	0	0	0	0	0	0
†	21UR-9053	TAGTTCTGAACAACGTTTTCT	0	0	0	1	3	6	0	10
	21UR-9054	TAGTGGACCGACTTACTTTAA	0	2	1	2	33	22	7	67
	21UR-9055	TAGGAAAACTATTCGGTCAAG	0	0	0	0	2	3	0	5
	21UR-9056	TACGATCGAATATCACACACT	0	0	0	0	0	0	2	2
	21UR-9057	TACCTTTTGAACGTCATTCTA	0	0	0	1	13	10	1	25
	21UR-9058	TACCCTGTTCAAGTAAAATGG	1	0	0	0	0	2	1	4
	21UR-9059	TAATTGGTGTTCTGCTTCAAA	0	0	0	0	4	2	0	6
	21UR-9060	TAATGATATATTTTGTGGCGA	0	0	0	0	4	1	3	8
*	21UR-9061	TAATCTGTTCCGTAGAAGAAA	1	2	1	4	33	36	5	82
	21UR-9062	TAATCAGTGAATAGAATGTAA	0	0	0	0	9	1	0	10
	21UR-9063	TAAGTTTCTCAAATTTCTCAA	0	0	0	0	0	0	0	0
	21UR-9064	TAACAAATACATCACTGTATA	0	0	0	1	2	0	0	3
	21UR-9065	TAAATCTTTCCATAAGCGCAA	0	0	0	0	0	1	0	1
†	21UR-9066	TAAATCACTGTGGTATCTATG	0	0	0	0	0	0	0	0
	21UR-9067	TAAAGTTTGCAAATTGTGATT	0	0	0	0	0	0	0	0
	21UR-9068	CGCATTTGATTATTTGATTTT	0	0	0	0	1	1	0	2
†	21UR-9069	CGATACTGGGGCTTTTTGAAG	0	0	0	0	0	1	0	1
†	21UR-9070	CAATAAAATTTAAAAGTGCAA	0	0	0	0	0	0	0	0
*	21UR-9071	AGAAGTCGAAACATACTAGAA	2	22	9	4	2	41	1	81
	21UR-9072	TTTTTTCGTGAAAGCTTTACT	0	0	0	0	3	3	1	7
†	21UR-9073	TTTTTCTCAATCGTCAGTATT	8	0	1	2	0	3	3	17
†	21UR-9074	TTTTGTTGTGTATCGAAACGA	0	0	0	0	3	5	1	9
†	21UR-9075	TTTTGTTACAGAGTATAAACA	0	0	0	0	0	1	0	1
	21UR-9076	TTTTGCTCGTAGTTAGATTTG	1	1	0	1	1	5	1	10
	21UR-9077	TTTGTATTTAAAACTTTGAAA	1	0	0	0	2	0	0	3
	21UR-9078	TTTGGATCTCGCCTCAAGACA	0	0	0	1	2	1	1	5
	21UR-9079	TTTGATATTTGTAAAAAACTG	0	0	0	1	0	0	0	1
	21UR-9080	TTTCTTTTTCATTGACACACT	0	0	0	0	0	0	0	0
	21UR-9081	TTTCGGTGGCACAATTAATCC	1	0	0	0	2	2	8	13
†	21UR-9082	TTTCATTTTTCTGAAACTACG	0	0	0	1	5	4	0	10

		21UR-9083	TTTCATAGTTATTTCATGAAT	0	0	0	0	0	0	0	0
		21UR-9084	TTTCAACAATAGAATCCCCCA	0	0	0	0	1	0	0	1
	†	21UR-9085	TTTCAAACCATTTGGGATTAA	0	0	0	0	0	2	0	2
	†	21UR-9086	TTTATTTCAACACTAAGAGCC	0	0	0	0	0	0	0	0
		21UR-9087	TTTATGGATAATGTAACACCA	0	0	0	0	0	3	0	3
	†	21UR-9088	TTTATCCTAAACCCGTTTTTA	0	0	0	0	0	0	0	0
		21UR-9089	TTTAGGGATAAAATATTGAAG	1	1	0	0	2	4	0	8
		21UR-9090	TTTACTGACGTTTTTCCAACC	0	0	0	0	3	2	1	6
		21UR-9091	TTTACCACTGTTTGTGAAAAA	0	0	0	0	0	1	0	1
	†	21UR-9092	TTTAACGCTACCTCAAAATGA	0	0	0	0	0	1	1	2
	†	21UR-9093	TTGTATTCGACAGTGGAGAAA	0	0	0	0	10	7	1	18
		21UR-9094	TTGTAGTCGCTCAATGTGAAT	0	0	0	0	1	0	0	1
		21UR-9095	TTGGTTTTTTGTTACAGGAAT	0	0	0	0	0	0	0	0
	†	21UR-9096	TTGGCATTGCAAAATCTAGGG	0	0	0	0	0	0	1	1
		21UR-9097	TTGGACATCCCAATTTACCAA	0	0	0	0	0	0	0	0
	†	21UR-9098	TTGGACAGTCTTGTGTCAAAT	0	0	0	0	0	0	0	0
	†	21UR-9099	TTGGAATGTGAACGATTTTCC	0	0	0	0	1	1	0	2
	†	21UR-9100	TTGCTCGGCAAGTTAGTAAAA	0	0	0	0	2	0	1	3
	†	21UR-9101	TTGCCTTGTCTGTCCTAAAAT	1	0	0	0	0	0	0	1
	†	21UR-9102	TTGATGTAATTTTGAACAAAA	0	0	0	0	9	6	2	17
		21UR-9103	TTGATCGTTGCGAGTTGGCTC	1	0	0	1	1	4	3	10
	†	21UR-9104	TTGATCAGAGTATGAATATTT	0	0	0	0	9	5	0	14
		21UR-9105	TTGAGGAACTTTATAGTCTTC	0	0	0	0	0	0	0	0
	†	21UR-9106	TTGACTTCCTTTAAACTTCTT	0	0	0	0	0	1	0	1
	†	21UR-9107	TTGACCGATTTTAAATTTCAC	0	0	0	0	0	0	0	0
		21UR-9108	TTGAAAAGTTGGACTATCATA	0	0	0	0	0	2	6	8
	†	21UR-9109	TTCTTGATTTTCTATGATTAA	0	0	0	0	0	0	0	0
	†	21UR-9110	TTCTTCGTTCTTTCGTATATT	0	0	0	0	0	0	0	0
	†	21UR-9111	TTCTTCAGTTTTAGTTTAGCT	0	0	0	0	0	0	0	0
	†	21UR-9112	TTCGGTGAATCCTCGGATTGA	0	0	0	0	0	0	0	0
	†	21UR-9113	TTCGAAGGTTGTCAGATCAAA	1	0	0	0	2	3	5	11
		21UR-9114	TTCCCGAAGTTTTTGTTTTTT	0	0	0	0	0	0	0	0
		21UR-9115	TTCCCAACACCGTCCCCCTTT	0	0	0	0	0	0	0	0
		21UR-9116	TTCCAAAATAATACAAGTTTC	0	0	0	0	1	1	0	2
		21UR-9117	TTCATTGAAAAATTACAAAAA	0	0	0	0	0	0	0	0
		21UR-9118	TTCATTCTTCAAATGTCTTTA	0	0	1	0	0	1	0	2
		21UR-9119	TTCATGCCATTGAAAATTACT	0	0	0	0	0	0	0	0
		21UR-9120	TTCATCGTTTCCTTTGCTTCT	0	0	0	0	0	0	0	0
		21UR-9121	TTCATAATTTGCATATCCTCA	2	0	0	1	0	1	0	4
	†	21UR-9122	TTCAGTTGGATTTTTTAATAA	2	1	0	0	2	2	0	7
	†	21UR-9123	TTCAGTTGAGTTGAAAAAAGG	0	0	0	0	0	0	0	0
	†	21UR-9124	TTATGTTCTACAATGACATCA	0	0	0	0	8	1	0	9
		21UR-9125	TTATACGTTTATCTTTTATAC	3	0	0	0	0	1	0	4
*	†	21UR-9126	TTAGAAGGCCCGGTGGAACTG	0	0	0	0	0	2	0	2
		21UR-9127	TTACTTACTGTATGAATCATT	0	1	0	1	10	7	1	20
		21UR-9128	TTACGTTCGTTTTTTTTAACT	1	4	2	3	43	32	4	89
	†	21UR-9129	TTACCAACATCTGCTTTGGAA	0	0	0	0	0	0	0	0
		21UR-9130	TTAATAAAAAATAAAATTTAT	1	1	0	0	1	0	0	3
		21UR-9131	TGTTTTCTTGGATGAAACTGA	0	0	0	0	0	3	0	3
		21UR-9132	TGTTTGTCTCCATCGACAATT	0	0	0	0	0	0	0	0
	†	21UR-9133	TGTTTCACTTAGAATTTTCTG	0	0	0	0	1	1	0	2
		21UR-9134	TGTTGTTTTTATATCGGAATC	1	0	0	1	2	1	0	5
		21UR-9135	TGTGTGTATGTTGAAGCAACA	0	0	0	0	0	0	0	0
		21UR-9136	TGTGATTGTTAATTCCACAGA	0	1	0	0	1	5	0	7
		21UR-9137	TGTCTTTTTAAACTTTCTTTT	0	0	0	0	0	0	0	0
		21UR-9138	TGTCCCAACTGCTGCATGGAC	1	0	0	0	0	0	0	1
		21UR-9139	TGTAATTGTGAGTCACTGTAT	0	0	0	0	0	2	0	2
	†	21UR-9140	TGGTTTTTTCATCATAGGTAA	0	0	0	0	0	0	0	0
		21UR-9141	TGGTTTATTTGAACTTCAACG	0	0	0	0	0	0	0	0
	†	21UR-9142	TGGATTTGAAAAATTGAAACG	0	0	0	0	6	10	1	17
		21UR-9143	TGCTTTGAGGAAGATCGTGCC	0	0	0	0	0	2	11	13
		21UR-9144	TGCTGGTTGCATTTTTTTTTA	0	0	0	0	0	0	0	0
		21UR-9145	TGCAAGAACTGTTCGATTCGC	0	0	0	0	0	0	0	0
	†	21UR-9146	TGCAACTGTTTTCAAGGGTTC	0	0	0	0	0	0	0	0

†	21UR-9147	TGCAACTGAAGAATCAAAATA	0	4	4	9	143	80	4	244
†	21UR-9148	TGATTCGTGTGTTTCATCTTC	0	0	0	0	0	0	0	0
†	21UR-9149	TGATACTGATCCTAAGGTCAA	0	0	0	0	0	0	0	0
†	21UR-9150	TGAGTTTTGTGTATCCAACTG	0	0	0	0	1	0	0	1
	21UR-9151	TGAGCTTACGAGGGCATTTTT	2	0	0	1	7	12	10	32
†	21UR-9152	TGACCCATAATCGCCAAAAAT	0	0	0	0	0	0	0	0
†	21UR-9153	TGAACTTATAATCGAATTACA	0	0	0	0	0	0	0	0
	21UR-9154	TCTTTCGCCGCATTTATTTCT	0	0	0	0	0	0	0	0
	21UR-9155	TCTTCGTTGCAAATTGTGATT	0	0	0	0	0	0	0	0
	21UR-9156	TCTCAAATAATTTCAAGTTCC	0	0	0	0	0	0	0	0
	21UR-9157	TCTATCACGGATTTTTAAGTT	0	0	0	0	3	1	0	4
	21UR-9158	TCGTTGTAGTTTTGTTTCGAC	0	0	0	0	3	6	1	10
†	21UR-9159	TCGTTGAATTATGCCTAGTAC	0	0	0	0	0	0	0	0
	21UR-9160	TCGATCAAATCGATCTTTTTG	0	0	0	0	0	0	0	0
†	21UR-9161	TCGAAGGTCAACAAAATATTA	0	0	0	0	0	0	1	1
	21UR-9162	TCCTTATGCAAAAGTTTCCCC	0	0	0	0	0	0	0	0
	21UR-9163	TCCGTTTGCTCGAAACACAAA	0	0	0	0	0	0	0	0
	21UR-9164	TCCCTGTTCCGAATTCTTTGA	0	0	0	0	0	0	0	0
	21UR-9165	TCCATCCAAACTAGTAAATTT	0	0	0	0	0	0	0	0
	21UR-9166	TCCACAATGGTTTGAGAATAC	0	0	0	0	0	0	0	0
†	21UR-9167	TCATTCTTTTTGTTGCCAAAT	0	0	0	0	0	0	0	0
	21UR-9168	TCAACTTTAAAACTTCAAACT	0	0	0	1	4	0	0	5
	21UR-9169	TCAAATTTTTCATTACCCTGA	0	0	0	0	0	0	0	0
†	21UR-9170	TATTTTTAAATCATCCGCAAA	0	0	0	0	0	1	3	4
	21UR-9171	TATTTCCAGTAAGCTTAAACT	1	0	0	0	1	0	0	2
†	21UR-9172	TATTGTGACTATATTTACAAT	0	0	0	0	10	0	1	11
†	21UR-9173	TATTGGAGATATATAGTGGTT	1	1	0	0	0	2	0	4
	21UR-9174	TATGTCTATAGTAAATTGAGT	0	0	0	1	5	3	1	10
	21UR-9175	TATGTACACATTTTATTATTA	0	0	0	1	2	3	0	6
	21UR-9176	TATGGTATTGCCAGACAGAAC	0	0	0	0	0	0	0	0
	21UR-9177	TATGAGTGCCTTACATGGATC	0	0	0	0	0	0	0	0
	21UR-9178	TATCTTTTATTTGTGTGATTC	0	0	0	0	0	0	0	0
†	21UR-9179	TATCTTTCAACTACTATAGTG	0	0	0	0	2	3	0	5
	21UR-9180	TATCTCTGTTGAAATTAACAT	0	0	0	0	1	3	0	4
†	21UR-9181	TATACCCCTATGACTATCAAC	0	0	0	0	0	0	1	1
†	21UR-9182	TAGTTGGATAGTACAAAAAAT	8	3	0	2	13	11	1	38
†	21UR-9183	TAGTGTCTTTTCGGTCTAAAA	1	0	0	0	0	1	0	2
	21UR-9184	TAGAGTAATGTTTTTTTTGAG	0	0	0	0	0	0	0	0
	21UR-9185	TAGAAAATCAAATTAGGAAAA	0	0	0	0	0	0	1	1
	21UR-9186	TACTCCGTTGATAAAAGTAAT	0	0	0	0	0	0	0	0
†	21UR-9187	TACTCCACAAATTTCTGTTTT	0	0	0	0	6	1	0	7
†	21UR-9188	TACGTTAGCGGTAATAAATTC	1	0	0	2	11	8	7	29
	21UR-9189	TACAGTGTTGAAATTTCGTTC	0	1	0	0	0	1	0	2
*	21UR-9190	TACACTGGAGTTTTTAGATTT	0	0	0	2	12	5	4	23
	21UR-9191	TAATTTTTTCATTTGCAGATT	1	2	1	2	32	13	1	52
	21UR-9192	TAATTTATCCAAATAATTGCA	0	0	0	0	0	0	0	0
†	21UR-9193	TAATTCGTCTAAACCAAACAG	2	0	0	0	1	2	1	6
	21UR-9194	TAATTCAGTGAAACTGTTAAA	0	0	0	0	0	0	0	0
†	21UR-9195	TAATGGATCTAAAATTTGTAG	0	0	0	0	0	0	0	0
	21UR-9196	TAATGAAAAGGATTCTTTATT	0	0	0	0	1	0	0	1
	21UR-9197	TAATCGTCAGGTTGGTGCTTT	0	0	0	0	0	0	1	1
†	21UR-9198	TAATCAGAATATGGGTTATAT	0	1	0	1	11	15	1	29
	21UR-9199	TAAGTTTCATCTACTCCTCGT	0	0	0	0	0	0	0	0
†	21UR-9200	TAAGATTTGTCACTCGGCAAA	0	0	0	0	0	0	0	0
	21UR-9201	TAAGAAAACGGTTCCCCATTA	2	1	0	1	15	10	10	39
†	21UR-9202	TAAATATTCTTTCGGCTAGCT	0	0	0	0	14	12	17	43
	21UR-9203	TAAATACCTTCCAAAATCTAA	2	1	0	0	0	3	1	7
†	21UR-9204	TAAAAATTCCCAGAGTTTTTG	0	0	0	0	1	0	0	1
	21UR-9205	CTCATGATGAATCATATGTAC	0	0	0	0	0	0	0	0
†	21UR-9206	CGTTCAGTTTTTGTTGGAAAT	21	4	2	3	21	32	7	90
	21UR-9207	CGCCTAAACCCGTACATAATA	0	0	0	0	0	0	0	0
	21UR-9208	CGAGACGGGTTTTCAATACAA	0	0	0	0	0	1	0	1
	21UR-9209	CGACGAAACTTTTTAAATTCC	0	0	0	0	0	0	0	0
	21UR-9210	CAAAAAAATTTATCTGACCAAA	0	0	0	0	0	0	0	0

	21UR-9211	TTTTTTTTGGTGACATCTAAT	0	0	0	0	4	8	0	12
	21UR-9212	TTTTTTTTCGAAAGAGTATGT	1	0	0	0	0	2	0	3
	21UR-9213	TTTTTTTTCATTCCCGAAGTT	0	0	0	0	0	0	0	0
	21UR-9214	TTTTTTTCGTTTCAATGCAAT	0	0	0	0	2	0	1	3
	21UR-9215	TTTTTGTGTAAATATCCCAAT	0	0	0	0	0	1	1	2
†	21UR-9216	TTTTTGCGATCACAGTACACA	0	0	0	0	0	0	0	0
	21UR-9217	TTTTTGAAAATGTGAAAGTTA	0	0	0	0	1	0	0	1
†	21UR-9218	TTTTTCTGATATAACGGAGAA	0	0	0	0	0	2	0	2
	21UR-9219	TTTTTCTATAGGATATTCAAG	0	0	0	1	1	1	0	3
	21UR-9220	TTTTTCAGAACTGGTTGTTAA	1	0	0	1	0	5	0	7
	21UR-9221	TTTTTCAATGTAGTGGAATAA	2	2	2	0	7	12	0	25
	21UR-9222	TTTTTCAAATTGAACTTCCAG	0	0	0	0	0	0	0	0
	21UR-9223	TTTTTATGGTTATTCTTCGCT	0	0	0	0	0	0	0	0
†	21UR-9224	TTTTTAAATGAAGATTAAAAT	0	0	0	0	0	1	0	1
†	21UR-9225	TTTTGTCCTCTTTGTACCCTG	0	0	0	0	0	0	0	0
†	21UR-9226	TTTTGAAGAAAAAGTACAAAT	0	0	0	1	0	0	0	1
†	21UR-9227	TTTTCTGATTTCTCTTAACTT	0	0	0	0	1	0	0	1
	21UR-9228	TTTTCTAGCCGTTACCATTTT	0	0	0	0	1	2	1	4
	21UR-9229	TTTTATTTCATCTATGAAACA	0	0	0	0	2	2	0	4
	21UR-9230	TTTTACTAGCAAATGCTATGA	0	0	0	0	2	0	0	2
	21UR-9231	TTTTAAAAAATTTGTTGACTT	0	0	0	1	2	1	0	4
†	21UR-9232	TTTGTTCCCGTCACCGTTTCC	0	0	0	0	0	0	0	0
	21UR-9233	TTTGTCGTTTTCTCTTTTATA	0	0	0	0	2	0	0	2
†	21UR-9234	TTTGCGGTTCTTAGTAATTTC	0	0	0	1	4	0	0	5
†	21UR-9235	TTTGCACTTTTTTAAACACAA	0	0	0	0	0	0	0	0
†	21UR-9236	TTTGCAAAAGCATAGATAAAA	0	0	0	0	0	0	0	0
	21UR-9237	TTTGAGTAAATTGAATAAATT	0	0	0	0	0	0	0	0
	21UR-9238	TTTGACTGCCTCTTACAGTTT	0	0	0	1	1	1	0	3
†	21UR-9239	TTTGAAGAAACATAAGACAAA	0	2	0	1	6	6	0	15
	21UR-9240	TTTCTTATTTTCTTCGATCTT	0	0	0	0	1	0	0	1
	21UR-9241	TTTCTGAAACTGTTTCGATAT	0	0	0	0	0	0	0	0
†	21UR-9242	TTTCTCATATTCTAATGACCA	1	0	0	0	0	0	0	1
	21UR-9243	TTTCGAAGCACGACAACCCAT	0	0	0	0	9	10	4	23
	21UR-9244	TTTCCTTGTTGTCATACTTTT	2	2	2	0	4	6	0	16
†	21UR-9245	TTTCCTTCGAAAACCCCTCAC	0	0	0	0	0	0	0	0
†	21UR-9246	TTTCCCATCTCAAATTAGCCA	0	0	0	0	0	0	0	0
†	21UR-9247	TTTCCACATTCGCATTCGAAA	0	0	0	0	0	0	0	0
†	21UR-9248	TTTATTGTGGAAAAGAAAAAC	2	2	0	0	5	5	0	14
	21UR-9249	TTTATTAACTTCAAGTTCAAT	1	0	0	0	0	1	0	2
†	21UR-9250	TTTAAAACTCCGAAACAAATG	0	0	0	0	0	0	0	0
	21UR-9251	TTGTTCAAATGTTGATTTCTC	0	0	0	0	0	0	0	0
†	21UR-9252	TTGTACAAGTTTTTGACCCAA	1	0	1	0	7	1	0	10
	21UR-9253	TTGGTTGTTGTTTTTTTTCGT	0	0	0	1	2	1	0	4
†	21UR-9254	TTGGAGAGTGGATCTAATTTT	0	2	0	1	26	14	1	44
†	21UR-9255	TTGGAGAAAATGGTTGGAAGT	0	0	0	0	1	3	0	4
†	21UR-9256	TTGCCTTGCAACTTTGAAAAA	1	1	0	0	0	1	0	3
†	21UR-9257	TTGCAGCTTTCATTGTCCCTT	0	0	0	0	0	0	0	0
†	21UR-9258	TTGATTGTAAATCTAGATGGG	0	0	0	0	0	4	1	5
†	21UR-9259	TTGAGTCTCACAGTTTTTAGC	0	0	0	0	0	1	0	1
†	21UR-9260	TTGAGAGAACGCAATTGATAC	0	0	0	0	7	10	0	17
	21UR-9261	TTGACTATTCACGCAATTTGT	0	0	0	0	0	0	4	4
	21UR-9262	TTCTCTGAGAATAATTTGCTA	0	0	1	0	17	12	3	33
	21UR-9263	TTCGGGAACCGTTTCGTTTTT	0	0	0	0	0	2	2	4
	21UR-9264	TTCCTTCGGAAATTTTTTTAT	1	0	0	0	0	0	0	1
†	21UR-9265	TTCCACCTTTGTCCATGAGTA	0	0	0	0	0	0	0	0
	21UR-9266	TTCCAATTTTTTATTCTGAAG	0	0	0	0	0	1	0	1
	21UR-9267	TTCATTTTTCTTGGTAGACTT	0	0	0	1	4	0	1	6
	21UR-9268	TTCAAGTTTGATTTATTTTTA	0	0	0	0	2	0	0	2
	21UR-9269	TTCAAACTTCAATAAAAAATG	0	0	0	0	0	0	0	0
†	21UR-9270	TTATTCAAGGTTTCCAACATA	0	0	0	0	2	0	0	2
	21UR-9271	TTATTATGGGATTTGGTTTTT	0	0	0	0	8	5	5	18
†	21UR-9272	TTATGTTTTGGGTGTTAACAT	0	0	0	0	12	5	0	17
†	21UR-9273	TTATGACTTGCGTTGATTTGG	0	1	0	1	0	3	1	6
	21UR-9274	TTATATGAAGGATTATTTAAA	0	1	0	0	3	0	0	4

	†	21UR-9275	TTAGTTGTTCCACTGATTAGT	0	0	0	0	0	0	0	0
*	†	21UR-9276	TTAGTTGAAGTTATGGCTAGT	8	13	5	4	25	274	5	334
	†	21UR-9277	TTAGTGAGAAGGTTTTTCGGA	0	0	1	0	5	1	1	8
		21UR-9278	TTAGGTGAAAACACTACATAC	0	0	0	0	0	0	0	0
	†	21UR-9279	TTAGAGAGATCTCGTTGTTTC	0	0	0	0	0	0	0	0
		21UR-9280	TTACAGCATATCCGCTGTTTA	0	0	0	0	0	0	0	0
		21UR-9281	TTAATGTGAATGAAAAAAAAA	0	0	0	0	1	0	0	1
	†	21UR-9282	TTAATAAACATCTAGTCTTTG	0	0	0	0	0	0	0	0
		21UR-9283	TTAACAACGACTTTTGGAAAT	0	0	0	0	0	1	0	1
*	†	21UR-9284	TGTTGAGCAGGTTGGTGTTTT	0	0	0	0	0	0	0	0
		21UR-9285	TGTTGACTTACCGGTTCTCGC	0	0	0	0	0	1	0	1
	†	21UR-9286	TGTTCTTAGCAATATGAAAAA	0	0	0	0	0	1	0	1
		21UR-9287	TGTTCCCGTGATATTCAGGGT	0	0	0	0	0	1	0	1
	†	21UR-9288	TGTCGGATAACCACATTGGAT	130	11	14	14	83	125	131	508
		21UR-9289	TGTATTTAAGTTCCCAGAAAT	0	0	0	0	0	0	1	1
	†	21UR-9290	TGGGATGCTCTATATCAGTTC	0	0	0	0	0	0	0	0
	†	21UR-9291	TGGACCAAAAAGCTAAAAAAA	0	1	0	0	1	0	0	2
*		21UR-9292	TGGAATGGACGAAACCCACAA	0	0	0	0	9	2	1	12
	†	21UR-9293	TGCTTTCGTCGTCGATCCATA	0	1	0	2	3	4	4	14
		21UR-9294	TGCCACTGCATTGCTATATTA	0	0	0	0	0	1	1	2
	†	21UR-9295	TGCAGTAGAAATTTTGAAAAA	0	0	1	0	0	1	0	2
	†	21UR-9296	TGCAGAATATGGACTACAATT	0	0	0	0	0	2	0	2
	†	21UR-9297	TGATTCAATTTCCATTGATTG	0	0	0	0	1	0	0	1
	†	21UR-9298	TGATTATGTTGACCAAATTAA	0	0	0	0	0	0	0	0
	†	21UR-9299	TGATGTTCTTTCTCTTCAAAT	0	0	0	0	0	0	0	0
		21UR-9300	TGAGGAACAAATTAGGGCTTC	0	0	0	0	0	0	0	0
		21UR-9301	TGAAATCTTATCAGATTAATA	0	0	0	1	0	0	0	1
		21UR-9302	TGAAACGATATCCATATGGAA	0	0	0	0	0	0	0	0
		21UR-9303	TGAAAATCAAATATGTTGAAT	0	0	0	0	0	1	0	1
		21UR-9304	TGAAAATAAAAAAAATACCGG	0	0	0	0	0	0	0	0
	†	21UR-9305	TCTTAGTCCATTCGTGTTGAA	0	0	0	0	1	0	0	1
		21UR-9306	TCTTACTTTTGTATAGAAGGA	0	0	0	0	0	0	1	1
		21UR-9307	TCTGTTCAAAGAATAACAACT	0	0	0	0	0	0	0	0
		21UR-9308	TCTGTGCGCAGCATCTTCAAA	0	0	0	0	0	0	0	0
		21UR-9309	TCTCTCAGCAAAGAAAATGAG	0	0	0	0	0	0	0	0
		21UR-9310	TCTCTCACAATAATGATTTTT	0	0	0	0	0	0	0	0
		21UR-9311	TCTCTAACTTTTAAGTTTTGA	0	0	0	0	0	0	0	0
		21UR-9312	TCTCCCTTTCCTCAAACCTTC	0	0	0	0	0	0	1	1
		21UR-9313	TCTAGGATTTCTTTTTGTTCT	0	0	0	0	0	0	0	0
		21UR-9314	TCGTGTTTTTTTTCCAAATAC	0	0	0	0	0	1	0	1
		21UR-9315	TCGAAACCGTAAAATTTGGTT	0	0	0	0	0	0	0	0
		21UR-9316	TCCTGTAACGCCCCCGTGCTA	0	0	0	0	0	0	0	0
	†	21UR-9317	TCCTCGATCTTCTTATAAACT	8	1	1	0	6	6	7	29
		21UR-9318	TCCTAAATATGCAAGTTTTGC	0	0	0	0	0	0	0	0
		21UR-9319	TCATCTCGATGTGTGGGATCG	0	0	0	0	14	15	8	37
		21UR-9320	TCATAAATACGTACTGAAATA	0	0	0	0	0	0	0	0
		21UR-9321	TCAGTTTTCCTTTTTTTTACA	0	0	0	0	0	0	0	0
		21UR-9322	TCACTCACCATTTGCACACGT	0	0	0	0	0	0	0	0
	†	21UR-9323	TCAAAAAACAAGCGTAATGAT	0	0	0	0	0	0	0	0
		21UR-9324	TATTTCTGTAACCACGAAAGT	0	0	0	0	0	1	0	1
		21UR-9325	TATTTAGTTAAAAATTTCAAA	0	0	0	0	0	0	0	0
		21UR-9326	TATTGCTGAAACAGCTAGTGT	0	0	0	0	0	1	0	1
	†	21UR-9327	TATTGAATCCGTTACTAATTT	0	0	0	0	2	0	1	3
		21UR-9328	TATTCCTTTTGCTGACAAGTG	0	1	1	0	2	4	0	8
	†	21UR-9329	TATGTCGATAGGCTTTTTCAA	1	2	0	0	25	16	2	46
		21UR-9330	TATCTTGATTAATATATATAT	0	0	0	0	0	0	0	0
		21UR-9331	TATCTCCTCAATTTTTTCTAG	3	0	0	0	1	0	0	4
	†	21UR-9332	TATCAAGAAAAATACGAAAAA	0	0	0	0	0	0	0	0
		21UR-9333	TATATCGGGTAACTTTTATTA	1	0	0	1	9	7	0	18
		21UR-9334	TATAAGCTGTACAGATCCCAC	0	2	0	5	26	19	3	55
		21UR-9335	TAGTTTTGACTTACTAAAAAT	0	0	0	0	0	1	0	1
		21UR-9336	TAGTTATGCCAAAACTTCAAA	0	0	0	0	0	0	1	1
		21UR-9337	TAGTAGTCATGAAACCGTGTT	0	0	0	0	0	0	0	0
		21UR-9338	TAGGTACTGAACTTGTCTGAA	0	0	0	0	1	2	0	3

†	21UR-9339	TAGGGTAGGGTTTCGCATTAT	0	2	0	0	1	6	1	10
	21UR-9340	TAGGAATACAATTCAAACTCT	0	1	0	0	6	2	0	9
	21UR-9341	TAGGAAAAACACCCAACATTG	0	0	0	0	0	0	0	0
	21UR-9342	TAGCTTTTGTCCTCCATTTTC	0	0	0	0	0	1	0	1
	21UR-9343	TAGAGAATCCTTTTAAAAAAG	0	0	0	0	0	0	0	0
†	21UR-9344	TAGAATGAATGAGATCAAATT	0	0	0	0	0	0	0	0
	21UR-9345	TAGAAATCTGTAAAAAGCACA	0	0	0	0	16	6	1	23
†	21UR-9346	TACTCTTGAGTTTAGGCGTTT	0	0	0	0	1	1	0	2
†	21UR-9347	TACCACCTAATAGAAAATCCA	0	0	0	0	0	0	0	0
	21UR-9348	TACACATCAGGGCTAGCGTTT	0	0	0	0	0	1	0	1
	21UR-9349	TACAAGTAAATTCAAAAAAAA	0	0	0	0	9	4	0	13
†	21UR-9350	TACAAAAAAGTGGATGAAGGA	0	0	0	0	5	8	4	17
	21UR-9351	TAATTTTTTATTCGTGGTTAA	2	1	1	0	1	3	0	8
†	21UR-9352	TAATTTTGACAATCCATTTAC	0	0	0	0	3	0	0	3
†	21UR-9353	TAATTTCACCTTTCTTCGAAA	0	0	0	0	0	0	0	0
†	21UR-9354	TAATAACGAAAAATTATTCTC	0	0	0	0	0	0	0	0
	21UR-9355	TAACGTTTGTAAATAAATAAC	0	0	0	0	0	0	0	0
†	21UR-9356	TAAATTAATTTTCTACGGTTT	0	0	0	0	1	2	0	3
	21UR-9357	TAAATCCGGAGTTGAAAATTA	0	0	0	0	0	0	0	0
†	21UR-9358	TAAACTGCCTACCTCAGATAA	0	0	1	0	5	5	1	12
†	21UR-9359	TAAAAACTAATACAAAATGTA	0	1	0	0	0	1	0	2
	21UR-9360	GTTGAATCCCGGCACTGAAGT	0	0	0	0	0	0	0	0
†	21UR-9361	GAGGACCATGTCAAATGTTGA	0	0	0	0	0	0	0	0
	21UR-9362	GAAGAACTATCAAAACAATAA	0	0	0	0	0	0	0	0
†	21UR-9363	CGGAAAAGTGTAGAAGTTTCC	0	0	0	0	2	0	0	2
	21UR-9364	TTTTTTTTGTTGAAAAAAATA	0	0	0	0	0	0	0	0
†	21UR-9365	TTTTTTTTCTTGCCTTTGAAA	0	0	0	0	0	0	0	0
	21UR-9366	TTTTTTAGGAGTTTTACTACT	0	1	0	0	0	0	0	1
	21UR-9367	TTTTTTAAATAACAAGGTCTT	0	0	0	0	2	0	0	2
	21UR-9368	TTTTTGCGAAATTAGCTGCAC	0	0	0	0	0	0	0	0
†	21UR-9369	TTTTTATACACCGACTCTGAA	0	0	0	0	8	6	2	16
†	21UR-9370	TTTTTAATGCTCTCGGATTGA	0	0	1	0	0	2	0	3
	21UR-9371	TTTTGATTGCTACATCAGTTT	2	0	0	0	3	1	1	7
	21UR-9372	TTTTGAACTAATAATTTTCGA	0	0	0	0	0	0	0	0
	21UR-9373	TTTTCTGAATCCTGTGTAACA	0	0	0	0	0	0	0	0
†	21UR-9374	TTTTCCTTTTCTGTCCCTCTT	0	0	0	0	0	0	0	0
	21UR-9375	TTTTCATCGTAAAACAGCATT	20	0	0	8	133	139	25	325
	21UR-9376	TTTTCATATTATTGAACGAAA	0	0	0	0	0	0	0	0
†	21UR-9377	TTTTACTACAACCAGTTTGAA	1	0	0	0	0	0	1	2
	21UR-9378	TTTTAAAAATTCACACTCTCC	0	0	0	0	1	0	0	1
	21UR-9379	TTTGTTCGTGGCTTTCATACC	0	0	0	0	6	0	0	6
	21UR-9380	TTTGTATTTCTTATTAGGAGT	0	0	0	0	0	0	0	0
	21UR-9381	TTTGGCAAATTTACACAATTT	0	0	0	0	0	1	0	1
	21UR-9382	TTTGGCAAAACTTTAAATGTT	0	0	0	0	0	0	0	0
	21UR-9383	TTTGATTCTAGCTCTAAAAAT	0	0	0	0	0	0	0	0
	21UR-9384	TTTGATAAAAAGGACTGTAAT	0	0	0	0	0	1	0	1
†	21UR-9385	TTTGAAAATTGAAATGAGAAT	0	0	0	0	1	2	0	3
†	21UR-9386	TTTCTGATAGTGATCCGCTTT	0	0	0	0	0	1	0	1
†	21UR-9387	TTTCTACACTCTGCTCAATTC	0	0	0	0	0	0	0	0
	21UR-9388	TTTCCTTTGCTTTTTCAAACT	0	0	0	0	0	2	0	2
	21UR-9389	TTTCCATTGCGTGTTTTTAAA	0	0	0	0	0	0	0	0
†	21UR-9390	TTTCCAACTGACACATTTTTA	1	0	0	0	0	1	0	2
	21UR-9391	TTTCAGTATGTTCAAAGGAAA	0	0	0	0	0	0	0	0
†	21UR-9392	TTTCAGAACTTTTTCATGAGA	0	1	0	0	0	1	0	2
	21UR-9393	TTTCAAAAATTTTTTCTTGTC	0	0	0	0	0	0	0	0
†	21UR-9394	TTTATTTGGAGCATGATCAAA	0	0	0	0	0	2	0	2
†	21UR-9395	TTTATTCTTCCCAATGGACTC	0	0	0	0	0	0	0	0
	21UR-9396	TTTAGTCACATTCTTTTGTTT	0	0	0	0	0	0	0	0
†	21UR-9397	TTTACGAAAAGTTGTTATTTT	0	0	0	0	1	1	0	2
†	21UR-9398	TTGTTTATTGCTCTGGTTGTT	0	0	1	1	1	2	3	8
†	21UR-9399	TTGTTGCTCTTACATTCGTGA	0	0	0	0	0	0	0	0
†	21UR-9400	TTGTTCGTTTTTCGCCATGAG	0	0	0	0	0	0	0	0
	21UR-9401	TTGTTCGAAATTCAATTTCTT	0	0	0	0	1	0	0	1
	21UR-9402	TTGTTCAAAACTCGATATTGA	0	0	0	0	0	0	0	0

	21UR-9403	TTGTCAAATATTCAATGATCT	0	0	0	0	1	0	0	1
	21UR-9404	TTGGTTTTACTTTTTTTTTCC	0	0	0	1	0	1	0	2
†	21UR-9405	TTGGTAGCCATTCTTGAAAAT	0	0	0	0	0	0	0	0
†	21UR-9406	TTGGTACATGATTTTCCAAAT	0	0	0	0	0	0	0	0
	21UR-9407	TTGGAAGACGAGAAAAAAATT	2	3	2	1	3	17	0	28
	21UR-9408	TTGGAAACAAATATTTGAAAA	0	0	0	0	1	2	1	4
†	21UR-9409	TTGCGGAGCATTATACAACGT	2	1	0	0	12	8	2	25
	21UR-9410	TTGCAATATCTTCTCCAACCA	0	0	0	0	0	0	1	1
†	21UR-9411	TTGCAACGTAAGGTTAACTAC	0	0	0	0	0	0	0	0
†	21UR-9412	TTGACAGGCGTAAACGTAAGA	0	0	0	0	3	2	0	5
†	21UR-9413	TTGAATTAAAAACACAGTTGC	0	0	0	0	1	6	0	7
†	21UR-9414	TTGAAGACAGAATGTTAGCAA	1	2	0	0	2	2	0	7
†	21UR-9415	TTGAAATTCACAAAGCTGGAA	0	0	0	0	0	2	0	2
	21UR-9416	TTCTTTCTGCATTTAACCATT	0	0	0	0	2	1	0	3
	21UR-9417	TTCTGGGTTTTTTTTTCGAAA	0	0	0	0	0	0	0	0
	21UR-9418	TTCTATTATTCCATACTGTTC	0	0	0	0	5	1	0	6
	21UR-9419	TTCGTCGACCCCAAATTGACT	0	0	0	0	0	0	0	0
†	21UR-9420	TTCGGCAATGTTTCGAAAAGT	0	0	0	0	0	0	0	0
†	21UR-9421	TTCGGATCGAAAGTAATACTA	1	0	0	0	5	1	1	8
†	21UR-9422	TTCGATCGATACGCTAGGTGA	0	0	0	0	0	0	1	1
	21UR-9423	TTCGACAATTTTCAAATTTAA	0	0	0	0	2	0	0	2
†	21UR-9424	TTCCGGCATTGATCTGTGGAG	1	0	0	1	0	2	0	4
	21UR-9425	TTCATGAACTTGTTGAGACAG	4	0	0	0	3	5	0	12
†	21UR-9426	TTCACACGCTCCTTAACAAAA	0	0	0	0	0	0	0	0
†	21UR-9427	TTCAATGAATTTAATAGATTT	0	0	0	0	0	0	0	0
†	21UR-9428	TTCAACAATCTAATCATTTTA	0	0	0	0	1	0	0	1
†	21UR-9429	TTCAAAGGAAAAACACTCACT	0	0	0	0	0	0	0	0
†	21UR-9430	TTATTGATTTGATTACAACAA	0	0	0	0	0	0	0	0
†	21UR-9431	TTATTCCTCTTTTGTCGGTTT	0	1	1	3	25	16	21	67
†	21UR-9432	TTATTCAATAAGAATCCTTTT	0	0	0	0	0	0	0	0
	21UR-9433	TTATACAAACAAAAAAAGCGA	0	1	0	0	4	0	0	5
†	21UR-9434	TTAGTCAGCTAAAATGGTTCA	0	0	0	1	3	1	0	5
	21UR-9435	TTAGATTTGGATTTGAAATTT	0	0	1	0	0	1	0	2
†	21UR-9436	TTACTTTTAGGAGATTTATTT	0	0	0	0	0	3	0	3
	21UR-9437	TTACTGGTTGAGAATCAAAAA	0	0	0	0	0	1	0	1
	21UR-9438	TTACGTTTTCGAATGCATTTC	0	0	0	0	0	1	0	1
†	21UR-9439	TTAATTGACGCTGCGGGTGTA	2	0	0	0	0	1	0	3
	21UR-9440	TTAATCACTCGAGGAGAATTA	0	0	0	0	0	5	0	5
	21UR-9441	TTAAAAAAAGTTAAAGTAGTT	0	0	0	0	0	2	1	3
†	21UR-9442	TGTTTTGATTATTTTATCAAG	0	0	0	0	0	0	0	0
	21UR-9443	TGTTTATATCTTATTCTAACT	0	0	0	0	0	0	0	0
†	21UR-9444	TGTTGGTAAAGCTGTGGAAAT	0	0	0	0	0	0	0	0
†	21UR-9445	TGTTCAGCATTTAAAACTCCT	0	0	0	0	3	0	0	3
	21UR-9446	TGTTCAAATATTTTTTCGGAT	1	0	0	1	12	1	1	16
	21UR-9447	TGTGATAGAAACTGTTTAAAA	0	1	0	0	0	3	0	4
	21UR-9448	TGTCTTAGTTATTCAATTTTC	0	0	0	0	0	0	0	0
	21UR-9449	TGTAATGGGTTTCAAATCGCA	0	0	0	0	0	0	0	0
	21UR-9450	TGTAATCGAAGTACTGGAAGA	0	0	0	0	0	1	0	1
†	21UR-9451	TGGTTGGTTTCTCTTCTTTTA	0	0	0	0	0	1	0	1
	21UR-9452	TGGGGTTATTCGTGTGATTTA	0	0	0	0	0	0	0	0
	21UR-9453	TGCTTAAAAAGATTTTTCTAA	0	0	0	0	0	0	0	0
	21UR-9454	TGCCGTTTTCAATTTTAGATC	0	0	0	0	0	0	0	0
†	21UR-9455	TGATTGTACTGCTTTACAGAT	0	0	1	0	0	0	0	1
†	21UR-9456	TGATGTAAAGAAAGAAATAGT	5	1	2	0	9	13	3	33
†	21UR-9457	TGAATTTTCTGAATATTCTTC	0	0	0	0	0	0	0	0
	21UR-9458	TGAAAGATTGAAAACCCATTT	0	0	0	0	0	0	0	0
†	21UR-9459	TCTTATTGCATTCTCTTTCAT	0	0	0	1	5	1	0	7
	21UR-9460	TCTTAGATTCCTTAATTTGAT	0	0	0	0	0	0	0	0
	21UR-9461	TCTGGAGGCATTCTCACACAA	0	0	0	0	0	2	0	2
	21UR-9462	TCTCTGGTCTTGAATCAAGGG	0	0	0	0	0	0	0	0
†	21UR-9463	TCTCGAGAAAACACTGAAAAT	0	0	0	0	0	0	0	0
	21UR-9464	TCTCATTTGTTCTTCTTCTAA	0	0	0	0	0	0	0	0
	21UR-9465	TCTCAAAAAACGGGGATCTTT	0	0	0	0	0	0	0	0
	21UR-9466	TCGTTCCCCGAAAATGTTATT	0	0	0	0	0	0	0	0

	†	21UR-9467	TCGTGGTGTTTTCTGAAAAAA	0	0	0	0	0	0	0	0
		21UR-9468	TCGTCTCTTAGCCATAATTGT	0	0	0	0	0	2	0	2
		21UR-9469	TCGTCCTATGTGTCAAGCAGA	0	0	0	0	0	0	0	0
	†	21UR-9470	TCGGCAAGTTAGTAAAAAGTT	0	0	0	0	2	1	0	3
	†	21UR-9471	TCGCTCTTATTGTTTATTGTA	0	0	0	0	0	1	0	1
	†	21UR-9472	TCGCATAGTTCCGATAGTCTT	0	0	0	0	3	0	0	3
		21UR-9473	TCGAGAGATCCATTTGGCAGT	0	0	0	0	0	0	1	1
		21UR-9474	TCGAAAAATGTTTGTAATTTT	0	0	0	0	0	0	0	0
		21UR-9475	TCCTGTGAAAACAAATTTATT	0	0	0	0	0	0	0	0
		21UR-9476	TCCTGATCGCCAAATTGTTAT	0	0	0	0	0	0	0	0
		21UR-9477	TCCTCTTGCAAATATAATAGG	0	0	0	0	0	0	0	0
		21UR-9478	TCCCGTTGACTTTCAGAATTG	0	0	0	0	0	0	0	0
		21UR-9479	TCCAATACGGAAACACTGATT	0	0	0	0	0	1	0	1
		21UR-9480	TCATTTCATTGAAGCAAAAAT	0	0	0	0	0	1	0	1
	†	21UR-9481	TCATTCGGATAGGGAAACAGC	0	0	0	0	0	0	0	0
	†	21UR-9482	TCACTCTCTTCAATAAACATT	0	0	1	0	0	0	0	1
	†	21UR-9483	TCACTCTAAAGGAAACTGAAC	0	0	0	0	1	0	0	1
		21UR-9484	TCACTCCTCTTTTTCTATTTG	0	0	0	0	1	0	0	1
		21UR-9485	TCAATGCTTTCATTCCAAAAC	0	0	0	0	0	0	0	0
	†	21UR-9486	TCAAATCAGATAATAAATGAA	0	0	0	0	0	0	0	0
	†	21UR-9487	TATTTTTTCGGTTCCATTTTC	1	0	0	0	0	1	2	4
	†	21UR-9488	TATTTCGGGCAGATCCATTTT	3	0	1	0	0	2	0	6
		21UR-9489	TATTTCAATTTAGCATGAATT	0	1	0	0	1	0	0	2
		21UR-9490	TATTCTCTTTTTATTCTCAGG	3	0	0	0	0	2	2	7
		21UR-9491	TATTCTCTTTTGAAAAAATTG	1	0	0	0	1	1	0	3
	†	21UR-9492	TATTCGCATGTTGCATCCAAT	0	0	0	0	4	3	2	9
	†	21UR-9493	TATTCGAAAGAGTTGGTTCTG	0	0	0	0	1	0	0	1
	†	21UR-9494	TATTCCTCGACTATTTCGCCA	0	0	0	0	0	0	0	0
	†	21UR-9495	TATTCCAGTATCCAATCATAA	0	0	0	0	0	0	2	2
	†	21UR-9496	TATTATCATCAGGTTGAAGAT	1	0	0	0	0	1	0	2
	†	21UR-9497	TATTAGGAATTGTGTTCACCA	0	0	0	0	0	2	0	2
		21UR-9498	TATTAAGTCCTTAGCTTATCA	0	0	0	0	1	0	0	1
	†	21UR-9499	TATTAAGGGACCTCTAATATT	0	0	0	0	0	0	0	0
		21UR-9500	TATGTTATATAAACGAATATA	0	0	0	0	0	0	0	0
*	†	21UR-9501	TATGCGACAGTACGTATGAAA	2	0	0	1	16	27	1	47
		21UR-9502	TATGCATTTTTTCTGCTTTAT	0	0	0	0	1	0	1	2
		21UR-9503	TATCAGTTGTTGAAATGACAT	0	0	0	1	2	4	0	7
		21UR-9504	TATCAGTAAACGTTTGCCTAT	0	0	0	0	2	2	0	4
		21UR-9505	TATAGTTTGTATTGTTAAATA	0	0	0	1	0	1	0	2
		21UR-9506	TATAATGTTGCTTACTTTGAG	0	0	0	0	3	7	0	10
	†	21UR-9507	TAGTGGCGGATAAAATGAGAG	0	0	0	0	8	7	6	21
	†	21UR-9508	TAGTAGTGATTTGCACGGATT	0	0	0	0	5	2	1	8
		21UR-9509	TAGGAGTAGGATCTAACTTTT	0	0	0	0	0	0	0	0
*	†	21UR-9510	TAGCTGAACGAAGGAGATATC	0	4	2	2	6	21	2	37
		21UR-9511	TAGATTTAATAAAAAACCCAA	0	0	0	0	1	2	0	3
	†	21UR-9512	TACTTCATGTGAGTACCAGAA	1	0	0	0	0	0	0	1
		21UR-9513	TACGAGAAAACAAGTGAATCG	0	0	1	1	7	14	0	23
		21UR-9514	TACCTTCTCTTTAATAATTCC	0	0	0	0	0	0	2	2
	†	21UR-9515	TACACGCGCATAAGTTTCTTC	0	0	0	0	1	1	5	7
		21UR-9516	TAATTGTGTGAAACAATTTTC	1	0	0	0	0	0	0	1
	†	21UR-9517	TAATGGTAAAGAACTAGTGTT	0	0	0	0	0	0	0	0
	†	21UR-9518	TAATCTTTGAGCTAACCCTAA	0	0	0	0	0	0	0	0
	†	21UR-9519	TAATATGCTGTTTGGCTCAGG	0	0	0	0	2	2	0	4
		21UR-9520	TAATACTCTATTTTTCGTTGC	0	0	0	1	6	2	4	13
	†	21UR-9521	TAATAACTGACGATACGGCAA	4	1	0	0	16	10	2	33
	†	21UR-9522	TAAGTTCAATATGTGTTTAAT	0	0	0	0	0	1	0	1
		21UR-9523	TAAGAAACTACAGTCAAAAAT	0	0	0	0	0	3	2	5
	†	21UR-9524	TAACTTTGGGTGAAAATCAAT	0	0	0	0	0	0	0	0
	†	21UR-9525	TAACTTTGATTTTCTAAGAGC	0	0	0	0	0	0	0	0
	†	21UR-9526	TAACTTATTGGAAAGAAAACA	0	0	0	0	0	1	0	1
		21UR-9527	TAAATTTGTCTTCCATAAATG	2	0	0	1	2	2	1	8
		21UR-9528	TAAATTCTGAGTTTAAGAATC	0	0	0	0	3	0	0	3
	†	21UR-9529	TAAATCCTCTAATCCTATTAA	0	0	0	0	0	0	0	0
	†	21UR-9530	TAAACAGTTTCCCGTGATTCG	0	0	0	0	0	1	0	1

	21UR-9531	TAAAACTATTCGTTAAAACTA	0	0	0	0	3	2	3	8
	21UR-9532	TAAAAAACTTTTTGTGAAATT	0	0	0	0	0	1	0	1
†	21UR-9533	TAAAAAACAAAAACAGGATTG	3	2	1	0	1	2	0	9
†	21UR-9534	GAATCCAATATTCGGTAAAGT	0	0	0	0	3	0	0	3
†	21UR-9535	CGCTGTCATGAAGCAAAAAAA	0	0	0	0	0	0	0	0
	21UR-9536	CGAAAATTCATGAATTCACTA	0	0	0	0	0	0	0	0
	21UR-9537	CAAATCGGCAAAACAAATTGA	0	0	0	0	8	3	2	13
	21UR-9538	AGTCATAGAAATTGAACTCTC	0	0	0	0	0	0	0	0
†	21UR-9539	TTTTTTTTAAAAATTGGTAAC	0	0	0	0	0	0	0	0
	21UR-9540	TTTTTTGAAATTTCTCGATGA	2	0	0	0	0	0	2	4
†	21UR-9541	TTTTTGTGAAATCGAGCAATT	0	0	0	0	4	6	2	12
†	21UR-9542	TTTTTATGGGTGGTCATTGGT	1	1	1	0	3	5	13	24
	21UR-9543	TTTTGGTCGATTTACCATATT	0	0	0	0	0	0	0	0
†	21UR-9544	TTTTGGTAATTTGGCCATTGT	0	0	0	0	0	0	0	0
	21UR-9545	TTTTGCAGACTTTTTCAATTA	0	1	0	2	10	4	0	17
	21UR-9546	TTTTCGCATTTAGCTAGCTCG	0	0	0	0	0	0	0	0
	21UR-9547	TTTTCGATATTTTTATTTATA	0	0	0	0	0	0	0	0
	21UR-9548	TTTTCGAAATGGTCAAATTTA	0	0	0	0	0	0	0	0
†	21UR-9549	TTTTCGAAATATTAGGTCTCC	0	0	0	0	0	1	0	1
†	21UR-9550	TTTTCCACGAGCGGTACACAC	0	0	0	0	0	0	0	0
†	21UR-9551	TTTTCATTGACGGAGGACTTG	0	0	0	0	0	4	3	7
†	21UR-9552	TTTTAGAGCATTTCTTTGAGC	0	0	0	0	1	0	0	1
†	21UR-9553	TTTGGTTGAGATCTCACGCGG	1	0	0	0	0	1	0	2
	21UR-9554	TTTGCAAAAGACCAAAAAGCG	0	0	0	0	0	1	1	2
	21UR-9555	TTTGATGTACGTTCCAACCAC	0	0	0	0	0	0	1	1
†	21UR-9556	TTTGATCTGCATTCAATCTTT	0	0	0	0	1	1	1	3
†	21UR-9557	TTTGAGTCGAGGTTTCAACAC	0	0	0	0	0	0	0	0
†	21UR-9558	TTTGAACATTTAATTTAAAAT	0	0	0	0	0	0	0	0
	21UR-9559	TTTCTTTTCTTTTCTTTGAAA	0	0	0	0	0	0	1	1
	21UR-9560	TTTCTTATTCAAAAATTCAAA	1	0	0	0	0	1	2	4
†	21UR-9561	TTTCGGTTCAAAATTTAAAAC	2	1	0	0	1	2	0	6
†	21UR-9562	TTTCGGATAGTGCTAAATATT	1	0	0	0	0	7	0	8
†	21UR-9563	TTTCGGAGAAATAATCATTAT	0	0	0	0	0	2	0	2
†	21UR-9564	TTTCATAATTTTTCTGACTTT	0	0	0	0	0	0	0	0
	21UR-9565	TTTCAATTCATAAATATCTCC	0	0	0	0	0	0	0	0
†	21UR-9566	TTTCAAGCAAACGTTGCATGA	0	0	0	0	0	1	0	1
†	21UR-9567	TTTATTCCCAGGACCGATAAT	0	0	0	0	0	0	0	0
	21UR-9568	TTTATGCCATTCAACAGTTGA	0	0	0	0	1	2	0	3
†	21UR-9569	TTTATAGTCTCTTATGATATC	0	0	0	0	0	0	0	0
†	21UR-9570	TTTATAAGAAATTTTATTTGC	1	0	0	1	0	1	0	3
	21UR-9571	TTTAGTACTCCATTTGTTTTG	0	0	0	0	0	0	0	0
	21UR-9572	TTTAGGTGGTTTTCGGGTTGA	0	0	0	0	0	0	0	0
†	21UR-9573	TTGTTCCCGTCACCGTTTCCG	0	0	0	0	0	0	0	0
	21UR-9574	TTGTTCAAAAATAGTAAAATA	0	0	0	0	0	0	0	0
	21UR-9575	TTGTCCGTTAGTTTTAATTTT	0	0	0	0	0	0	0	0
†	21UR-9576	TTGGTTGGACCACACCCAAAT	2	0	0	0	0	1	0	3
†	21UR-9577	TTGGTCCGTTGAACATTTGTC	0	0	0	0	0	0	0	0
	21UR-9578	TTGGAGCCCCGTATTTGACTC	0	0	0	0	0	0	0	0
	21UR-9579	TTGGAAATGTAGGACCCTTGC	0	0	0	0	0	0	0	0
	21UR-9580	TTGCCGCGCACCCCTGACTTG	0	0	0	0	0	0	0	0
	21UR-9581	TTGATTATGTATTGATTTTAA	0	0	0	0	0	0	0	0
	21UR-9582	TTGATTACTTTGAAAATTTGA	0	0	0	0	0	1	0	1
†	21UR-9583	TTGATGCTGAATAAATTTGAA	0	0	0	0	0	2	0	2
†	21UR-9584	TTGATAAATTGTGCGAATTTT	1	0	0	0	0	0	0	1
†	21UR-9585	TTGACCGATTTCTTCAATTCT	0	0	0	0	0	0	0	0
	21UR-9586	TTGAATTGAATACGAAGTCAT	0	0	0	0	2	5	0	7
	21UR-9587	TTGAATTCAATTTTCAAAATT	0	0	0	0	0	0	0	0
	21UR-9588	TTGAATACGTTATCTGGATGT	0	0	0	0	0	0	0	0
	21UR-9589	TTGAAGGAAAAGATGATGAGG	0	0	0	1	1	1	1	4
†	21UR-9590	TTGAAACAGTGACTTGCTTTT	0	0	0	0	0	0	0	0
	21UR-9591	TTCTTTTTTCAGTGAAACGTA	0	0	0	0	0	0	0	0
†	21UR-9592	TTCTTTTTGCCAATCGCTTCT	0	0	0	0	0	0	0	0
	21UR-9593	TTCTTGCTCTTCCCGCTTCTC	0	0	0	0	0	0	1	1
	21UR-9594	TTCTGGGTAATTTTTAAGAAA	0	0	0	0	0	0	0	0

	†	21UR-9595	TTCTCTGTTCTGAATTGTAGA	0	0	0	0	0	0	0	0
*	†	21UR-9596	TTCTCTGAAGGCATATTTTTG	20	25	5	59	1215	920	74	2318
	†	21UR-9597	TTCTATTGGAAAAGAGGGACT	0	0	0	0	1	1	1	3
		21UR-9598	TTCGTTCACCGAAACACTTTT	0	0	0	0	0	0	0	0
		21UR-9599	TTCGTCCACAATCGTTTCACT	0	0	0	0	1	0	0	1
	†	21UR-9600	TTCGGTGTTCTCTAAGGCTTT	0	1	0	0	0	0	0	1
		21UR-9601	TTCGATTTTTGTAATCATAAT	0	0	0	0	0	0	0	0
	†	21UR-9602	TTCCTATCCTGATTATTGGTC	1	0	0	0	1	0	0	2
	†	21UR-9603	TTCCGTTAAACGTCATGTCCA	0	0	0	0	0	0	0	0
		21UR-9604	TTCAGTTGATTCTCCTTTTTT	0	1	0	0	4	6	0	11
	†	21UR-9605	TTCAGATTTTGGACTACGATC	0	0	0	0	0	0	0	0
		21UR-9606	TTCAATAGTTTTATAGGAGCA	0	0	0	0	0	0	0	0
		21UR-9607	TTCAAAAAAGCCAATAATTGA	0	0	0	0	0	0	0	0
		21UR-9608	TTATTGGTTTCAATCTTCAGT	0	0	0	0	0	0	1	1
	†	21UR-9609	TTATTGGATTTAGATCTGGAG	0	0	0	0	0	0	0	0
	†	21UR-9610	TTATTATGTCCTGTAACTGAA	0	0	0	0	0	0	0	0
		21UR-9611	TTATTACAATTTATCATCTGA	1	0	0	1	8	1	2	13
		21UR-9612	TTATGACATGTTCCTAGTTCA	0	0	0	0	0	1	0	1
	†	21UR-9613	TTATCATCAACTTTGCTCTCG	0	0	0	0	0	0	0	0
		21UR-9614	TTATCAACTGGTAGACTATAG	0	1	0	0	0	5	0	6
		21UR-9615	TTATAGTATGTTGATAAAATC	0	1	0	2	3	4	0	10
		21UR-9616	TTAGTTTAATTTAGTTTGCCT	0	0	0	0	0	0	0	0
	†	21UR-9617	TTACGAAGAGATAATACTAGA	0	0	0	0	0	0	0	0
	†	21UR-9618	TTACCATGCCAGCACGACTAT	0	0	0	0	0	0	0	0
		21UR-9619	TTACATTTGTTTCATCATAAA	0	0	0	0	0	4	0	4
		21UR-9620	TTACATAACTGAGGGTTTTCT	0	0	0	0	3	3	2	8
		21UR-9621	TTAACCTTTCCTATCGTATTT	0	0	1	0	0	0	1	2
	†	21UR-9622	TTAAAGGTTTCTGAGACTTGA	0	0	0	1	17	14	3	35
		21UR-9623	TGTTTTTAAAACCATTATGAA	0	0	0	0	0	0	0	0
		21UR-9624	TGTTTTAATGATTTATATGTC	0	0	0	0	0	0	0	0
	†	21UR-9625	TGTTTGCTTAAATCTGATGAA	0	0	0	0	0	0	0	0
	†	21UR-9626	TGTTTAAAATTGGTGAATTCA	0	0	0	0	0	0	0	0
	†	21UR-9627	TGTTGCTCATGTCTCACTTTC	0	0	0	0	0	0	0	0
		21UR-9628	TGTCACTCATGGCAAATTTTC	0	0	0	0	0	0	0	0
	†	21UR-9629	TGGTAGGTAGAGAACTTTTTC	0	0	0	0	0	1	0	1
		21UR-9630	TGGCCCATCTCCAAAATCGTC	0	0	0	0	1	2	1	4
		21UR-9631	TGGATTTTTCTTGTAGACTTT	0	0	0	0	0	2	0	2
		21UR-9632	TGGAATTAATACAGTATTGTT	0	0	0	0	0	0	0	0
		21UR-9633	TGCTTGATTATTAAATTGGAT	1	0	0	0	0	0	0	1
	†	21UR-9634	TGCTTCTCTAACTGTAATAAC	0	0	0	0	0	0	0	0
	†	21UR-9635	TGCTCCCATAAAATGAATTGC	0	1	0	0	0	1	1	3
		21UR-9636	TGCCAAGTATAATTTTCAGAT	0	0	0	0	0	0	0	0
	†	21UR-9637	TGCATTTTTAATGTCGGAGTA	0	0	0	0	1	3	0	4
		21UR-9638	TGCACACCTTTATCTCATTTT	0	0	0	0	0	0	0	0
		21UR-9639	TGATTTGCGCAAATACTCGCA	0	0	0	0	0	0	0	0
		21UR-9640	TGAAAATGGATTCATATCTCT	0	0	0	0	0	0	0	0
		21UR-9641	TGAAAAATAAAAAGAAAAAAG	0	0	0	0	0	0	0	0
	†	21UR-9642	TCTTTCGGTACATTCTTTATT	0	0	0	3	9	5	0	17
		21UR-9643	TCTTCTGTTTGTATAATCATT	0	0	0	0	0	0	0	0
		21UR-9644	TCTTAGACGATTTTTTGTACA	0	0	0	0	0	0	1	1
		21UR-9645	TCTGTTTCACTTCCAAAGTTA	0	0	0	0	0	0	0	0
		21UR-9646	TCTGATTTATTTAGCTCAATA	0	0	0	0	0	0	0	0
		21UR-9647	TCTGACGGACAAGATGGAAAT	14	13	3	6	11	73	1	121
		21UR-9648	TCTCTCTTTGATCCACAAAAC	0	0	0	0	0	0	0	0
		21UR-9649	TCTCAGTTGTTGTGAATCGAC	0	0	0	0	0	0	0	0
		21UR-9650	TCTATACAAAGCTAATGTAAG	0	0	0	0	1	0	0	1
	†	21UR-9651	TCTAACTTCAATGCATGTGTT	0	0	0	1	1	2	0	4
		21UR-9652	TCGTAACTCCACTCAAACATT	0	0	0	0	0	0	0	0
	†	21UR-9653	TCGGTGATCAAATCAAGTCCA	0	0	0	0	0	0	0	0
		21UR-9654	TCGAAGAGGCTTCTACAGTCA	1	0	0	0	0	2	0	3
		21UR-9655	TCGAAATTTCAAAAACATTAG	0	0	0	0	0	0	0	0
		21UR-9656	TCGAAATCCTCCCAATTATTT	0	0	0	0	0	0	0	0
		21UR-9657	TCCTCCCTTCAGTTCCAAAAT	0	0	0	0	0	0	1	1
	†	21UR-9658	TCCGGTTATCATAGCTAGAGA	0	0	0	0	0	2	0	2

	†	21UR-9659	TCCATTTAAAGTTTATGTCAC	0	0	0	0	0	0	0	0
		21UR-9660	TCCAAATGGCGTTTCGTTTTC	0	0	0	0	0	0	0	0
		21UR-9661	TCATTTTATTACAATCAAACC	0	0	0	0	1	0	0	1
	†	21UR-9662	TCATTTGTCTTTCTTTACGTT	0	0	0	0	1	0	0	1
	†	21UR-9663	TCATTAGTTTGCCGTCTTTCA	3	0	1	0	1	4	1	10
	†	21UR-9664	TCATTAACTTCGATGTTCGTT	0	0	0	0	0	0	1	1
	†	21UR-9665	TCATACTTTTATTTATGAAAA	0	0	0	0	0	0	0	0
	†	21UR-9666	TCATAATTTGTACAGAAAATA	0	0	0	0	0	1	0	1
		21UR-9667	TCAGTTTTTTAAACTCGTTTT	0	0	0	0	1	0	0	1
		21UR-9668	TCAGTTTTTAAATCCCATAAC	0	0	0	0	0	0	0	0
		21UR-9669	TCAGAAAACTGAAAGCTTCTC	0	0	0	0	1	0	0	1
		21UR-9670	TCACTTTTTTCAAGAACTTAA	0	0	0	0	0	0	0	0
	†	21UR-9671	TCACTCATTTATCAGCAATCA	0	0	0	0	0	0	0	0
		21UR-9672	TCAATCACAAATTTGTTAAAA	0	0	0	0	0	0	0	0
*	†	21UR-9673	TCAACCAGTAGACATTTATTA	4	6	5	32	508	234	95	884
		21UR-9674	TCAAAATTTTAAGACCCATAT	0	0	0	0	0	0	0	0
		21UR-9675	TATTTTATAATTGCCAACGAC	0	0	0	0	0	0	0	0
		21UR-9676	TATTTCTTCTCAAGTTTCAAA	0	0	0	0	0	1	0	1
	†	21UR-9677	TATTGTGCCGATTGAATATTT	0	0	0	0	0	0	0	0
	†	21UR-9678	TATTGTACGTTGCAAAAATTT	0	0	0	0	1	0	0	1
	†	21UR-9679	TATTGCATTGGGATTTGCTTT	0	0	0	0	0	0	0	0
	†	21UR-9680	TATTGATACTTTTGCTACACT	0	0	0	0	1	0	0	1
	†	21UR-9681	TATTCTTGCCATTAACTTTCG	0	0	0	0	1	1	0	2
	†	21UR-9682	TATTCTGCTCATATCTTGTAA	1	0	0	0	3	1	0	5
	†	21UR-9683	TATTCGAAAAATTGGACTGAT	0	0	0	0	2	1	0	3
		21UR-9684	TATTCATTTGAAACAAAAGCA	0	0	0	0	2	0	0	2
		21UR-9685	TATTATCGTTATGTGTAAATC	0	0	0	0	7	2	2	11
	†	21UR-9686	TATTAGATCTTGAAGAATATC	0	0	0	0	1	0	0	1
	†	21UR-9687	TATTACATTAATATTGTTCGG	2	0	0	0	0	2	4	8
		21UR-9688	TATTACAGTAAAAACAGTAAA	0	0	0	0	1	0	0	1
		21UR-9689	TATGTACCATTTTACTTTTCC	0	0	0	0	0	0	0	0
	†	21UR-9690	TATGATATCGAAGAGCTTGAG	0	0	0	0	0	0	0	0
		21UR-9691	TATGAAAACAGGAACTTACAG	0	0	0	0	0	2	0	2
		21UR-9692	TATCTTAGACATTTTGTTTTG	1	1	1	0	2	1	0	6
		21UR-9693	TATCGTATGTTGGTTTTTCAT	0	0	0	0	3	2	0	5
	†	21UR-9694	TATAGCGAAATCTTTCAACTG	0	0	0	0	1	0	0	1
		21UR-9695	TAGTTTAATTTTGTTTTGGTT	0	0	0	0	1	2	0	3
	†	21UR-9696	TAGTTCTTCAATTACCAATTT	0	0	0	0	0	0	0	0
	†	21UR-9697	TAGTGAAACAAGTATAGTTTA	0	0	0	0	0	0	0	0
	†	21UR-9698	TAGGCGTCTTTCAATTCAAAG	0	0	0	0	0	1	0	1
		21UR-9699	TAGCTTTGGTTTTAACTATAA	0	0	0	0	0	1	0	1
	†	21UR-9700	TAGCATTACGTAATGCTTGAA	0	0	0	0	0	0	0	0
		21UR-9701	TAGCATATATCTAGTATATCA	0	0	0	2	6	0	1	9
		21UR-9702	TAGATTTACCACGATCTCTTC	0	0	0	0	0	0	0	0
		21UR-9703	TAGATACAAGATGCAAATTTC	2	1	0	0	0	4	1	8
		21UR-9704	TAGAATATTTATTAGAAAAAA	0	0	0	0	0	0	0	0
		21UR-9705	TACTTCATTTTTAAAATTTCG	0	0	1	0	1	0	0	2
		21UR-9706	TACTGTTACGAGAAAAGTTCG	0	0	0	0	1	2	1	4
	†	21UR-9707	TACTGACAAACAACGAAAAAA	0	0	0	0	0	0	0	0
		21UR-9708	TACATCTTGCATTTTAAATAG	1	0	0	0	2	5	5	13
		21UR-9709	TAATTTTATGTACGTTGTTTA	1	0	0	0	4	3	1	9
		21UR-9710	TAATTGTTTCATGAAGTTTAA	0	0	0	0	1	0	0	1
	†	21UR-9711	TAATTCTTCACAGTCTCCATT	0	0	0	0	0	0	0	0
	†	21UR-9712	TAATTCAACATGGTGCTCCTT	0	0	0	0	0	1	0	1
	†	21UR-9713	TAATTATTGGCATATTTTGAC	2	0	0	1	2	8	0	13
		21UR-9714	TAATGCTTTATTTCAAATTCA	0	0	0	0	4	0	0	4
		21UR-9715	TAATAAGCTTGGATCTCAGAG	0	0	1	0	4	14	11	30
		21UR-9716	TAAGAATTTGCATTTTAACTG	0	0	0	0	0	0	1	1
		21UR-9717	TAACTCCTCCTTCAAGTTTTT	0	0	0	0	0	0	0	0
		21UR-9718	TAACCAACTTATGAACATACT	0	0	0	0	0	0	0	0
		21UR-9719	TAACATTCTATGCCCTGTTGT	0	0	0	0	0	0	0	0
*	†	21UR-9720	TAACATATGCAAATAAGTACG	1	0	1	0	6	6	0	14
	†	21UR-9721	TAAATGGATTCTAAAAAAATA	0	0	0	0	1	0	0	1
		21UR-9722	TAAATATTGTTTCATACGGTC	0	0	0	0	0	0	0	0

		21UR-9723	TAAACAATAATACATATGATT	0	0	0	0	0	0	1	1
		21UR-9724	TAAAACTTGATGTTTTGCGTT	0	0	0	0	0	0	0	0
		21UR-9725	TAAAACTGTCGAAAATTTGTT	0	0	0	0	0	2	0	2
		21UR-9726	TAAAACTCGATTTCAACAAAA	0	0	0	0	0	0	0	0
	†	21UR-9727	GGGCCAACGTTTTCAATGAAA	0	0	0	0	0	0	0	0
	†	21UR-9728	GGAGAATGTATGTATTGTAAT	0	0	0	0	1	0	0	1
	†	21UR-9729	CGACGTCTTTTTGTAATCCTC	0	0	0	0	0	0	0	0
		21UR-9730	CATATTTACAATACAAATCGC	0	0	0	0	0	0	0	0
		21UR-9731	AAAAATCCAGAAGTAACAAAA	0	0	0	0	0	0	1	1
		21UR-9732	TTTTTTTTTAAGTCAAAAAAG	0	0	0	0	0	1	0	1
	†	21UR-9733	TTTTTTGTGGCTTATCTAAAT	0	0	0	0	0	0	0	0
	†	21UR-9734	TTTTTTGGTTTTTTCACGACT	0	0	0	0	0	0	0	0
		21UR-9735	TTTTTTCAATTATGTAAGAAA	0	0	0	0	1	0	0	1
		21UR-9736	TTTTTGCACTCACTAAATAAT	1	0	0	1	0	2	0	4
		21UR-9737	TTTTTGATTTTGAAAATTGGC	0	0	0	0	3	2	0	5
		21UR-9738	TTTTTCGCCTTTCTTATTTTC	0	0	0	0	0	0	1	1
		21UR-9739	TTTTTCAAACTGGCCGGGTAA	1	0	0	0	1	1	2	5
	†	21UR-9740	TTTTGACTAGGGAGAATTTCT	0	0	0	0	0	0	0	0
		21UR-9741	TTTTCCAATTCACATCAGGGA	0	0	0	0	0	0	0	0
	†	21UR-9742	TTTTATTGAAATCTTTAAAAA	0	0	0	0	0	0	0	0
		21UR-9743	TTTTACGGCACTAGCCAATGG	0	0	0	0	1	1	1	3
		21UR-9744	TTTTAATGGAAATTGCAGTTC	0	1	0	0	9	6	1	17
		21UR-9745	TTTTAATATCAGTATTTTACC	0	0	0	0	0	0	0	0
		21UR-9746	TTTTAAATATGATTCAAAACA	0	0	0	0	0	0	0	0
		21UR-9747	TTTGTTTTGAGTATTACTATG	0	1	0	0	6	3	1	11
	†	21UR-9748	TTTGTGCAGTGGATACATTTT	2	1	5	9	76	93	17	203
		21UR-9749	TTTGTGATAAAATCAAAAAAC	0	0	0	0	0	0	0	0
	†	21UR-9750	TTTGTCGCATATTTTTACTAA	2	1	0	8	123	33	9	176
		21UR-9751	TTTGTCCTCAATCATCATAAT	0	0	0	0	0	0	0	0
		21UR-9752	TTTGTAAGCCATAAAATGGAG	0	0	0	0	0	0	0	0
	†	21UR-9753	TTTGGTAGCCATTCTTGAAAA	0	0	0	0	0	1	0	1
	†	21UR-9754	TTTGCTGGATAAAATAAAAAA	1	0	0	0	0	1	0	2
	†	21UR-9755	TTTGCTAGACTTAGATAAAGC	0	0	0	0	1	0	0	1
		21UR-9756	TTTGCCGATTGGAGTGTTTTT	0	0	0	0	0	0	1	1
		21UR-9757	TTTGAGGTGAATTGTAGTTGA	0	0	0	0	0	0	0	0
		21UR-9758	TTTGAAAAAATCATGACGTTT	0	0	0	0	0	0	0	0
	†	21UR-9759	TTTCTTCTGTCATCATGCAAC	0	0	0	0	0	0	0	0
	†	21UR-9760	TTTCTTCCACATTCCACACTC	0	0	0	0	0	0	0	0
		21UR-9761	TTTCTTACCATTCCTCAGTCC	0	0	0	0	0	0	0	0
		21UR-9762	TTTCAGACAGAAAATTATGGT	0	0	0	0	0	1	0	1
	†	21UR-9763	TTTCAACGTATGAAAAATCTG	0	1	0	0	1	1	0	3
		21UR-9764	TTTATCTGCTCCACATTTTCC	0	0	0	0	0	0	2	2
	†	21UR-9765	TTTAGTAGAGAAATTTGAATA	0	0	0	0	0	0	0	0
	†	21UR-9766	TTTAGCAATCGTTCTACAAAT	0	0	0	0	0	1	1	2
		21UR-9767	TTTAAGTATCCAATTAAAAGA	0	0	0	1	0	0	0	1
	†	21UR-9768	TTTAAGGTATTGATCATGTGT	9	2	1	1	3	6	3	25
	†	21UR-9769	TTTAAAAAACAATTAACTGTT	0	0	0	0	0	0	0	0
		21UR-9770	TTGTTTTTGTGGTACCCAGAT	0	0	0	0	0	0	0	0
		21UR-9771	TTGTTCTGATACCTTTGAGTA	0	0	0	0	0	0	0	0
	†	21UR-9772	TTGTTAGCAAGTTGAAGTACG	0	0	1	0	0	2	0	3
	†	21UR-9773	TTGTGTTGTTCATTGTGTGTC	0	0	0	0	0	0	0	0
		21UR-9774	TTGTGTATTTGCATAAACTGC	1	0	0	0	1	2	1	5
	†	21UR-9775	TTGTGATAACTACGTTTCAAA	0	0	0	0	0	0	0	0
		21UR-9776	TTGTACAGTTACTCCGAAATA	0	0	0	0	2	1	0	3
	†	21UR-9777	TTGGGTTCCTGATCTTCTTAC	0	0	0	0	0	0	0	0
	†	21UR-9778	TTGGCATCTCCGGAAAAAACG	1	0	0	0	0	0	1	2
*	†	21UR-9779	TTGGATAAGTTGTATCTTCGG	1	1	1	0	5	3	1	12
	†	21UR-9780	TTGGAGGCCTGGTTGTTTGTG	1	0	0	0	0	1	0	2
	†	21UR-9781	TTGCGTGAGTAAGGTTTTTTT	0	0	0	0	1	0	0	1
	†	21UR-9782	TTGCGCAGTGAAATAGTATGC	0	0	0	0	0	0	0	0
	†	21UR-9783	TTGCACTGTATTAATTTGTTA	0	0	0	0	0	0	0	0
	†	21UR-9784	TTGCACCAGATGTAATGAAGT	0	0	0	0	0	0	0	0
		21UR-9785	TTGCAATTACTTCCAGCTACA	0	0	0	0	0	0	0	0
		21UR-9786	TTGCAACGGAATTTTAAATGT	0	0	0	1	13	3	1	18

	21UR-9787	TTGAATGTTTTACTCCAGATA	0	0	0	0	0	0	0	0
†	21UR-9788	TTGAAGAAAAATGCACTGGAA	0	0	0	0	0	1	0	1
	21UR-9789	TTGAAATGGGTACCCTTAACT	3	2	0	2	0	3	1	11
	21UR-9790	TTCTTTTTTCATTACTTCTTA	0	0	0	0	0	0	0	0
†	21UR-9791	TTCTGCAACTCACAAAGTGTC	0	0	0	0	0	0	0	0
	21UR-9792	TTCTCTGATATCACGTGACTT	0	0	0	0	0	0	0	0
†	21UR-9793	TTCTCTGAGTCGGTGAAAAAA	1	0	0	0	0	2	1	4
	21UR-9794	TTCTATCAGTTTACTGACCGG	0	0	0	0	2	0	1	3
	21UR-9795	TTCTAGTACATGTTTTTTAGA	0	0	0	0	0	1	0	1
†	21UR-9796	TTCTACGATACGTAAAAAACT	0	0	0	0	0	0	0	0
†	21UR-9797	TTCGTGGCGTGTGAAAGCTAT	0	0	0	0	0	0	0	0
†	21UR-9798	TTCGTCGTAAAACCAAAAAAA	0	0	0	1	2	1	4	8
†	21UR-9799	TTCGCGATCTTCTTTATGGTA	0	0	1	0	0	0	0	1
†	21UR-9800	TTCCTTTTTCAAAAGAACAAG	0	0	0	0	0	0	0	0
†	21UR-9801	TTCCTGCGAATTTCCGGTTGT	0	0	0	0	1	0	0	1
	21UR-9802	TTCATGTTGAGTTTTTGTACA	1	0	0	0	0	1	0	2
	21UR-9803	TTCATCGCTTTTAAACACATT	0	0	0	0	1	1	0	2
	21UR-9804	TTCATACGCCAATGGTTTGTA	0	0	0	0	2	5	0	7
†	21UR-9805	TTCAGTTGTCTTTCGGTGTTA	0	1	0	1	9	11	4	26
	21UR-9806	TTCAGTTGGAAAAATTGAAAA	0	0	0	0	0	0	0	0
†	21UR-9807	TTCACTTTTTTAAAAACATTT	0	0	0	0	0	0	0	0
	21UR-9808	TTCAATTGATCCATTTTTCCT	0	0	0	0	0	0	0	0
†	21UR-9809	TTCAATACATTTGTTGTGAAA	0	0	0	0	0	0	0	0
	21UR-9810	TTATTTACTACTAAATTGGAG	0	0	0	0	0	0	0	0
†	21UR-9811	TTATTCGGATTAGCGTTAGGT	13	8	5	4	18	71	11	130
	21UR-9812	TTATTAATAAAATCGAATGTT	0	0	0	0	3	2	0	5
†	21UR-9813	TTATCGATCCAAACAAAAACC	0	0	0	0	0	0	0	0
	21UR-9814	TTATATTTATAAACGTAGAAA	0	0	0	0	4	5	3	12
	21UR-9815	TTATATACTTTAAAAAAGAAA	0	0	0	0	5	3	0	8
†	21UR-9816	TTAGATGAGTGTGTCAGTTCA	1	1	0	0	0	2	0	4
†	21UR-9817	TTAGACGATTGGTTCAATTTG	0	0	0	0	1	2	0	3
†	21UR-9818	TTACTTTAATTTGAAAGGCTC	0	0	0	0	1	1	0	2
	21UR-9819	TTACTTGAAAACTAGAGGTTT	0	0	0	0	0	0	0	0
†	21UR-9820	TTACGCTTTTTTAATCGAGAA	0	0	0	0	0	0	0	0
†	21UR-9821	TTAATTGTTCCCATTGCAGAG	0	0	0	0	2	0	1	3
†	21UR-9822	TTAATTGTATGGCTAGAATTC	1	0	0	0	0	1	0	2
	21UR-9823	TTAATCGTTGAGTTTTTAAGG	0	0	0	0	0	0	0	0
†	21UR-9824	TTAATAATAAACAATATGCGG	0	0	0	0	0	1	0	1
†	21UR-9825	TTAAGAGCAACTTGTATTTTT	0	0	1	0	1	2	0	4
	21UR-9826	TGTTTCTTCCTCTGTTTATAC	0	0	0	0	0	0	0	0
†	21UR-9827	TGTTTCGATTCGAGTGGTTGA	0	0	0	0	0	0	0	0
	21UR-9828	TGTTTAGGCATTGAATATTTT	0	0	0	0	0	0	0	0
	21UR-9829	TGTTTAATTGACTTTAAATTT	0	0	0	0	0	0	0	0
†	21UR-9830	TGTTGGAATGGCGAAAATACA	1	0	0	0	2	2	1	6
	21UR-9831	TGTTCTCTATTTATTTTCCGC	0	0	0	1	0	0	0	1
†	21UR-9832	TGTTCTCTAAGGCTTTAAGTG	0	0	0	0	0	1	0	1
	21UR-9833	TGTTAGTAAAAAAATTCGAGA	0	0	0	0	0	0	0	0
†	21UR-9834	TGTTACTTGTATGATAGCTGT	0	0	1	1	25	23	0	50
	21UR-9835	TGTTAAAAATGACGAGGAATA	0	0	0	0	0	0	0	0
	21UR-9836	TGTGTCAGTTTTATTGTGGAA	0	0	1	0	0	2	0	3
	21UR-9837	TGTGCAATGACTATTTGCAGA	0	0	0	0	0	0	0	0
	21UR-9838	TGTGATCATCAGTCAATACCC	0	0	0	0	0	0	0	0
	21UR-9839	TGTGACCTCTAACTTCAGAGC	0	0	0	0	0	0	0	0
	21UR-9840	TGTCTCCTGCTCTTTTTCCCA	0	0	0	0	0	0	0	0
†	21UR-9841	TGTCCTAAGAATGCCATTTCC	0	0	0	0	0	0	0	0
	21UR-9842	TGTATTTGAATTATTCGACGG	6	0	1	0	20	56	5	88
†	21UR-9843	TGTATTGGCGGAGATGAACTT	1	0	0	0	6	8	2	17
	21UR-9844	TGTACTTTAAGTTCTCAAGGA	0	0	0	0	0	0	0	0
	21UR-9845	TGTACTGTTTTCAAACAAAAA	0	0	0	0	3	0	0	3
	21UR-9846	TGTACCTTTGAATATATATTT	0	0	0	0	0	0	0	0
	21UR-9847	TGTAAATACATTTCAATTTCA	0	0	0	0	0	1	0	1
	21UR-9848	TGGGATGTTCATTCATGAAAT	0	0	0	0	0	0	0	0
	21UR-9849	TGGATTGTTTTTTTTTTGATT	0	0	0	0	0	0	0	0
	21UR-9850	TGGACTCACGCTGAGTTATTC	0	0	0	0	0	0	0	0

*	21UR-9851	TGCTTTGACTTAAGAAGAATT	0	0	0	0	3	0	0	3
	21UR-9852	TGCTTCAAATTCATTCAAATT	0	0	0	0	0	0	0	0
†	21UR-9853	TGCTTAGAAAGGATTGTTTAT	0	0	1	0	0	1	1	3
	21UR-9854	TGCTACGTACTTTTTCAAGAA	0	0	0	0	0	0	0	0
	21UR-9855	TGCGTAATGCATACAAACATT	0	0	0	0	1	0	0	1
	21UR-9856	TGCCTTTTTGTATGAAATTTT	0	0	0	0	1	0	0	1
	21UR-9857	TGCAGACGGTGGAAAAAATGA	4	3	1	1	7	17	2	35
	21UR-9858	TGCACATCCCATTCTAACTTT	0	0	0	0	0	0	0	0
	21UR-9859	TGCAATCAATCGATAATTGGT	0	0	0	0	0	0	0	0
	21UR-9860	TGCAACATTACTCCAAGAGCC	0	0	0	0	0	0	0	0
	21UR-9861	TGCAAATGGACATGATGTATC	0	0	0	0	0	0	0	0
†	21UR-9862	TGATTCTTTTGCTCTCTGCAC	0	0	0	0	0	0	0	0
†	21UR-9863	TGATGCTTCGAGCGGATAGTT	1	0	0	0	0	1	0	2
	21UR-9864	TGATAATATCAGGATGATCTC	0	0	0	0	0	0	0	0
	21UR-9865	TGAGGAAAACTGTTCAATTCT	0	0	0	0	0	0	0	0
	21UR-9866	TGACTTTGGTAAAGGAAAAAC	0	0	0	0	0	0	0	0
	21UR-9867	TGACAGTTACATTTGGGTTCA	0	0	0	0	0	0	0	0
†	21UR-9868	TGAATAGGTGATTAGGGAATA	0	0	0	0	0	0	1	1
	21UR-9869	TGAAGTCATTTTATCCTTCAA	0	0	0	0	0	0	0	0
	21UR-9870	TGAAGCTAATTTATATCTCAA	0	0	0	0	0	0	0	0
	21UR-9871	TGAACTGGCTATTTTAAATCC	0	0	0	0	0	0	0	0
†	21UR-9872	TGAAATTCACAAAGCTGGAAT	0	0	0	0	0	2	0	2
	21UR-9873	TCTTTTCCTTTTTTCCTGTCC	0	0	0	0	0	0	0	0
†	21UR-9874	TCTTTTAGAAGCAGAATTTTT	3	6	9	6	49	31	2	106
†	21UR-9875	TCTTTCATAGTCAGAGCCTGC	2	7	3	2	12	38	2	66
	21UR-9876	TCTTCTTCCATTTTATGAATC	0	0	0	0	0	0	0	0
†	21UR-9877	TCTTATTTCGATCAAAAAATA	0	0	0	0	1	0	0	1
†	21UR-9878	TCTGACACAGAAGTTAGAATA	0	1	0	0	4	8	0	13
†	21UR-9879	TCTCTTGTCCCGCAAAAAAGT	0	0	0	0	0	0	0	0
†	21UR-9880	TCTCTTCGACAGACCTGATAA	0	1	0	0	0	0	0	1
†	21UR-9881	TCTCTCAGGTGGAAGTTATAT	0	2	0	1	3	5	0	11
†	21UR-9882	TCTCCAAGCCTTTTCACTTTT	0	0	0	0	0	0	0	0
	21UR-9883	TCTCATTTTTTTTGACTTCAA	0	0	0	0	0	0	0	0
	21UR-9884	TCTCAAACTTGGATTTGGATC	0	0	0	0	0	0	0	0
	21UR-9885	TCTATCATCAATATTTACCCG	0	0	0	0	0	0	0	0
	21UR-9886	TCTATATACGATGTTCTTGCG	0	0	0	0	0	0	0	0
†	21UR-9887	TCTAACTCCAAATATCATTTT	0	0	0	0	0	0	0	0
	21UR-9888	TCGTTTTACATGTAAATTTAC	0	0	0	0	0	0	0	0
	21UR-9889	TCGTAACTTACTATTCATTTT	0	0	0	0	0	0	0	0
	21UR-9890	TCGGTAGTGATTATTCTAACG	0	0	0	0	0	0	0	0
†	21UR-9891	TCGCTCCTTCAAACGTGTCCA	0	0	0	0	0	0	0	0
†	21UR-9892	TCGAGGAAATTTTTTGACTTT	0	0	0	0	0	0	0	0
	21UR-9893	TCCTCGGCATTAACTGAGACG	0	0	0	2	6	3	0	11
†	21UR-9894	TCCTCCTGGTCTATTCACAAG	0	0	0	0	0	0	0	0
†	21UR-9895	TCCTCATCCATACACGAGGCA	0	0	0	0	0	0	0	0
	21UR-9896	TCCGAATTTTAGATGAAAAAC	0	0	0	0	0	0	0	0
	21UR-9897	TCCATTTTGTCCTGTAACTTG	0	0	0	0	0	0	0	0
†	21UR-9898	TCCATAGTTTGGTTGACTAAT	1	0	0	0	0	2	0	3
†	21UR-9899	TCCACTGATATCCATGGACAC	0	0	0	0	1	0	1	2
†	21UR-9900	TCATTTGCTAATCCAAAAAAA	0	0	0	0	0	0	0	0
	21UR-9901	TCATTGCTTGGTCTTTTTTTT	0	0	0	0	0	0	0	0
	21UR-9902	TCATGACAAAATGATTTTTAG	0	0	0	0	0	0	0	0
	21UR-9903	TCATATTTTGATAATTTCCAC	0	0	0	0	1	0	0	1
	21UR-9904	TCAGCGCTGTGCTACACGCAA	0	0	0	0	0	0	0	0
	21UR-9905	TCAAAAATTTCCAATTTCCAG	0	0	0	0	0	0	0	0
	21UR-9906	TATTTTTGAAAAGAAATAGAC	0	0	0	0	0	0	0	0
	21UR-9907	TATTTTGATTTAAAAATTGTT	1	0	0	0	0	0	0	1
	21UR-9908	TATTTGGGGTTTCCTAACCGA	0	0	0	0	0	0	1	1
†	21UR-9909	TATTTCATCTAATCGTAGTTT	3	0	0	0	0	1	0	4
	21UR-9910	TATTTATGTGATTCTATTAGT	0	0	0	0	2	1	1	4
†	21UR-9911	TATTGTTCTCTAGATGGGACC	0	0	0	0	0	0	0	0
†	21UR-9912	TATTGCACAATTGATAAGGAT	0	0	0	0	0	0	0	0
†	21UR-9913	TATTGAGAAATATAGGAACTT	0	0	0	0	0	0	0	0
	21UR-9914	TATTGAATTCATTTCCTCTGT	0	0	0	0	0	0	0	0

†	21UR-9915	TATTCCTCGTGACTGAGAAAA	0	0	0	0	2	1	0	3
†	21UR-9916	TATTAACTTTTTTTGATTCAC	0	0	0	0	0	0	0	0
	21UR-9917	TATGTTTTTTCATTTCCGTTG	1	0	0	0	0	0	0	1
	21UR-9918	TATGTGAAGTTTTGGTGTTAC	0	1	1	0	4	2	6	14
	21UR-9919	TATGGAATCTCAAAAACCTGC	0	0	0	0	0	0	0	0
†	21UR-9920	TATGCCCTTTTCCGTTTTTTG	0	0	0	0	2	0	1	3
	21UR-9921	TATGAACGCGTTTTTGTCATT	0	0	0	0	2	2	1	5
	21UR-9922	TATGAAACAGTATTCAATTTT	0	0	0	0	0	0	0	0
	21UR-9923	TATCCATTTTATACTCATTGG	0	0	0	1	1	2	0	4
	21UR-9924	TATCACAATTTTTGTCATCAT	0	0	0	0	0	0	0	0
	21UR-9925	TATATAAATCATCATTTCTGT	0	0	0	0	3	1	1	5
	21UR-9926	TATAGAATTCTCATCGACCCT	0	0	0	0	0	0	0	0
†	21UR-9927	TATAGAAGTCATTTTGCTCAG	0	0	0	0	0	0	0	0
	21UR-9928	TATAAAATTGGAAAGACTAGA	0	0	0	0	0	0	0	0
	21UR-9929	TAGTTTTTCTGTAACTGCTTG	0	0	0	0	1	0	0	1
	21UR-9930	TAGTTATTTAGTGTTTTGTCT	0	0	0	0	0	0	0	0
†	21UR-9931	TAGTGAATTCGTGCTTTGGAT	1	1	1	0	0	0	0	3
†	21UR-9932	TAGTAGTAGCCGCATCATTCA	0	1	1	2	20	17	2	43
†	21UR-9933	TAGTAGGTCAGCTTTGAAAAA	1	0	0	0	0	2	0	3
†	21UR-9934	TAGTACGCCACACGCTCATTC	0	0	0	0	0	0	1	1
	21UR-9935	TAGGTACCCTGATATGATCAA	0	0	0	0	0	0	0	0
*	21UR-9936	TAGGCACTTCAGAGCAAATAG	0	0	0	0	4	2	0	6
	21UR-9937	TAGAAATCGAATTTTCAAAAT	0	0	0	0	0	0	0	0
	21UR-9938	TACTATGTCAAAAATAGAAGT	0	0	0	0	1	0	0	1
*	21UR-9939	TACGGCCAAATCAGATTTCTG	1	0	0	0	2	3	1	7
	21UR-9940	TACCTTTCTCGATTTAATTTC	2	0	0	0	0	0	0	2
	21UR-9941	TACCACTACACAGGAATTAAA	0	0	0	0	0	0	0	0
	21UR-9942	TAATTTCTTCAATTTCTTCAA	0	0	0	0	0	0	0	0
†	21UR-9943	TAATTGACCGATTTTAAATTT	0	0	0	0	1	0	0	1
†	21UR-9944	TAATTCATTTCGATCATCATT	0	0	0	0	1	1	1	3
†	21UR-9945	TAATTATTTGGATTCCATTCT	0	0	0	0	3	0	0	3
†	21UR-9946	TAATGTTACTAAAAATGTGAT	0	0	0	0	0	0	1	1
	21UR-9947	TAATGTATTTTGGTATCCAAA	0	0	0	0	0	0	0	0
†	21UR-9948	TAATGCTGTCCATAGAATTAT	1	0	0	0	0	1	0	2
†	21UR-9949	TAATGCTGAAAAATGAAACAA	0	1	0	0	1	1	0	3
†	21UR-9950	TAATGCAGACTTAAATTGAGA	0	0	0	0	3	3	1	7
	21UR-9951	TAATGAATCCAGCTTTTCAAA	0	0	0	0	0	0	1	1
†	21UR-9952	TAATCTGATCCATTTTTAATC	0	0	0	0	1	0	1	2
†	21UR-9953	TAATCGTTGTGTCTACTTTTT	0	0	0	0	0	0	0	0
	21UR-9954	TAATCGTGGTTTTCAACTTTT	1	0	0	0	1	0	0	2
	21UR-9955	TAATCAATTTAAATAGCTAAA	0	0	0	0	0	0	0	0
†	21UR-9956	TAATACCTCTGGGCTCTTTAA	0	0	0	0	0	0	0	0
	21UR-9957	TAAGGTTTTCATTTTCAGATT	0	0	0	0	1	0	0	1
†	21UR-9958	TAAGATGGGTAAACAACTAAA	1	0	0	0	0	0	0	1
	21UR-9959	TAAGATACTTAAATTCCATTT	2	0	1	1	8	9	0	21
	21UR-9960	TAAGAAAGAACTAAAGTTTTT	0	0	0	0	0	0	0	0
	21UR-9961	TAACGTTTGAAATCTGTACAT	0	0	0	0	0	0	0	0
	21UR-9962	TAACAACCAGTTTTTCATTTT	0	0	0	0	0	0	1	1
	21UR-9963	TAAACGTCTCGAAAATTTGAA	0	0	0	0	0	0	0	0
	21UR-9964	TAAACGGGATAGAAGGTGGGT	0	0	0	0	0	1	0	1
	21UR-9965	TAAAATCGGAAAAAAACATTT	0	0	0	0	1	1	0	2
	21UR-9966	TAAAATCCATTATTGTTTCTT	0	0	0	0	0	0	0	0
	21UR-9967	TAAAAACTTTGGGGCAGCAAA	0	0	0	0	1	0	2	3
†	21UR-9968	TAAAAAATGGTCAGTAATAAC	0	0	0	0	0	0	0	0
	21UR-9969	GCATAAAGAAAGTTGAAAAAG	0	0	0	0	0	1	0	1
†	21UR-9970	CTCAACTAACCTGAATTTAGT	0	0	0	0	0	0	0	0
†	21UR-9971	CGGATAACCACATTGGATAGA	0	0	0	0	0	0	0	0
	21UR-9972	CAGGCAAAAAATTATAAAGAT	0	0	0	0	0	0	0	0
	21UR-9973	AGCCAAACTGGAAATATAATT	0	0	0	0	0	0	0	0
†	21UR-9974	AAGAAAATAATTCGTCCAAAA	0	0	0	0	0	0	1	1
	21UR-9975	TTTTTTTTCTTGTTGTGCCTT	0	0	0	0	1	0	0	1
	21UR-9976	TTTTTTGAGCTTTTTTGTGTT	0	0	0	0	0	0	0	0
	21UR-9977	TTTTTTGAAACTACCCAAAAA	0	0	0	0	0	0	0	0
	21UR-9978	TTTTTTCTTACCAAGAAATTA	0	0	0	0	0	0	0	0

†	21UR-9979	TTTTTTAATTGATCAACAAAA	0	0	0	0	0	0	0	0
†	21UR-9980	TTTTTGACTTGTATTTTGACA	0	0	0	0	9	3	0	12
†	21UR-9981	TTTTTCTGAGCATCAATCATC	0	0	1	0	3	3	0	7
	21UR-9982	TTTTTATATTCTATCGACAAA	0	0	0	0	0	0	0	0
	21UR-9983	TTTTTAGAAGGATAATATGAA	1	1	1	0	0	1	0	4
	21UR-9984	TTTTGGAGAAAAGTGAAGATA	1	0	1	0	6	5	1	14
†	21UR-9985	TTTTGATGTATAATGTCCAAT	0	0	0	0	0	1	0	1
†	21UR-9986	TTTTCTCCGAGAATCTGCGAG	0	0	0	0	0	0	0	0
†	21UR-9987	TTTTCCTCCCAACGGACACTT	0	0	0	0	0	0	0	0
†	21UR-9988	TTTTCCAAATTGTGCATTATT	8	0	0	1	2	7	0	18
	21UR-9989	TTTTCAGACTTCAAAAATTGG	0	0	0	0	0	0	0	0
†	21UR-9990	TTTTATTAGGAATTACTAAAG	0	0	0	0	0	0	0	0
†	21UR-9991	TTTTATTAAAACGTACGGAAA	5	0	0	1	3	1	3	13
†	21UR-9992	TTTTAGGCTGAATTGTAATAT	0	0	0	0	0	0	0	0
	21UR-9993	TTTTAGATCTATAGTCAGTGG	0	0	0	0	0	0	0	0
†	21UR-9994	TTTTAACTCAGTCTAACTCAT	0	0	0	0	0	0	0	0
†	21UR-9995	TTTGTTTTCTTGCTAGAACAC	0	0	0	1	1	1	0	3
	21UR-9996	TTTGTTTGATAATTACCAAAT	0	0	0	0	1	0	1	2
	21UR-9997	TTTGTTCTGTGAAGTTCTGTT	0	0	0	0	0	0	0	0
	21UR-9998	TTTGTTAGTTGACATATTTCT	0	1	0	0	0	0	0	1
	21UR-9999	TTTGTGTAAGAAGTATTGCTA	2	3	2	1	4	9	1	22
	21UR-10000	TTTGTCTGTTTTTTACAAAGG	0	0	0	0	0	0	0	0
†	21UR-10001	TTTGTACTTCTACAATCTACA	0	0	0	0	0	0	0	0
†	21UR-10002	TTTGGATTAAGAGCCGGATTT	0	1	0	0	1	1	2	5
†	21UR-10003	TTTGGATGAGTACATATGTGA	0	2	0	1	5	1	1	10
†	21UR-10004	TTTGCATCGTTGAAATACCCA	2	0	2	0	4	3	1	12
†	21UR-10005	TTTGATGATACTGATCCTAAG	0	0	0	0	0	0	0	0
	21UR-10006	TTTGATCTCGACGTGCATTCA	1	1	1	2	9	18	9	41
	21UR-10007	TTTGAGAGTGAAACTCTTTTT	0	0	0	0	0	0	0	0
†	21UR-10008	TTTGAATTCCAAATTCATTAC	0	0	0	0	0	0	0	0
†	21UR-10009	TTTGAATATGGAAGCTCATTT	0	0	0	0	0	2	0	2
	21UR-10010	TTTGAAAAAAAATGTACTGTA	0	0	0	0	0	0	0	0
	21UR-10011	TTTCTTGAACCTCAAAATACA	0	0	0	0	0	0	0	0
	21UR-10012	TTTCTTATAGGAACGAGTTTT	2	1	0	0	1	3	0	7
†	21UR-10013	TTTCTCATCAACTTACAGGCT	0	0	0	0	0	0	1	1
†	21UR-10014	TTTCGTTTGATGAAGGCGACA	1	3	1	1	2	16	0	24
†	21UR-10015	TTTCGCCGAAATATTTGAATC	0	0	0	0	0	0	0	0
	21UR-10016	TTTCGAAAAAAAAGGCGTTCT	0	0	0	1	0	0	1	2
†	21UR-10017	TTTCCACTGATGCTTATGTTA	0	1	0	0	0	0	0	1
†	21UR-10018	TTTCAATGAATTAGAAAGAAA	0	0	0	0	0	0	0	0
	21UR-10019	TTTCAACTACAACATTTAATA	0	0	0	0	0	0	0	0
†	21UR-10020	TTTCAACGGCTGCCCCATAAG	1	0	0	0	0	0	0	1
	21UR-10021	TTTATTCACCATGGAAAATGG	0	0	0	0	0	0	0	0
	21UR-10022	TTTATCCATTGTATTCATTGA	0	0	0	0	0	0	0	0
	21UR-10023	TTTATAGCAAAGATTAATTAA	0	0	0	1	2	0	0	3
	21UR-10024	TTTAGTGCGACAGGAACTATG	0	0	0	0	0	0	0	0
†	21UR-10025	TTTAGGAAGACATAAATAATT	2	2	0	2	15	13	4	38
†	21UR-10026	TTTAGATTGCCGAAGAGAGTC	0	0	0	0	0	0	0	0
†	21UR-10027	TTTACTGGTGTCGATTTAAAT	0	0	0	0	0	0	0	0
†	21UR-10028	TTTACAAGAGAACAATTAGAT	0	2	0	1	23	16	1	43
	21UR-10029	TTTAATGGAATTTGCACACCT	0	0	0	0	2	1	0	3
	21UR-10030	TTTAACAAATATAGATTAATA	0	0	0	0	0	0	0	0
	21UR-10031	TTTAAATGAAAAATTAAAAAA	0	0	0	0	0	0	0	0
	21UR-10032	TTGTTGTCATATTTTTCGGGC	1	0	0	0	1	2	1	5
†	21UR-10033	TTGTGTGCGAATGTCATAACT	3	3	1	1	5	17	3	33
†	21UR-10034	TTGTGTAAGTTCGATGGTTTG	1	0	0	0	1	1	0	3
	21UR-10035	TTGTGCTATTTTTTGCAAGCT	0	0	0	0	0	0	0	0
†	21UR-10036	TTGTCGATCGGATTAATTTTT	0	0	0	0	5	0	0	5
	21UR-10037	TTGTATCATGTTTTTTCTCGT	0	0	0	0	1	0	0	1
	21UR-10038	TTGTAATATGAAAAATAAAAT	0	0	0	0	0	0	0	0
†	21UR-10039	TTGGGTAATGAAGTTAAAAAA	0	0	0	0	1	0	0	1
†	21UR-10040	TTGGCTTCTAAATTTGATGTC	0	0	0	0	0	0	0	0
†	21UR-10041	TTGGCAATATGTACAAACAAT	0	0	0	0	0	1	0	1
†	21UR-10042	TTGGATCGCGGAAATATGCAA	0	0	0	0	14	7	4	25

†	21UR-10043	TTGGAGAGAATATTATAGAGA	0	0	0	0	0	0	0	0
†	21UR-10044	TTGCTACGCGAATGTTATTGC	0	0	0	0	0	1	0	1
†	21UR-10045	TTGATTCTGGATCAATGTTTC	0	0	0	1	1	0	0	2
	21UR-10046	TTGATTAAGATTGCCATTTCT	0	0	0	0	0	0	0	0
†	21UR-10047	TTGATGCCCTTCGTTGTCATG	0	0	0	0	0	0	0	0
	21UR-10048	TTGAGCATTGAACAGTCTACA	0	0	0	0	0	0	0	0
†	21UR-10049	TTGACTCGTATTGGACTAAAA	1	0	0	1	10	7	1	20
†	21UR-10050	TTGAAATATGAATCGGCAGAT	0	0	0	0	1	0	1	2
†	21UR-10051	TTGAAAGTTCAGATTTTCAAA	0	0	0	0	0	0	0	0
	21UR-10052	TTCTGTCCTTGAATAGCTCAT	0	0	0	0	0	0	0	0
	21UR-10053	TTCTCTCGCAATTTTTTTTTA	0	0	0	1	0	0	1	2
	21UR-10054	TTCTAGGGTGTGTTAAAAAAT	0	0	0	0	0	0	0	0
†	21UR-10055	TTCGTTGATCTCATCTCTCTC	0	0	0	0	0	0	0	0
	21UR-10056	TTCGTATGGTAAAAATTTTCA	0	0	0	0	0	0	1	1
†	21UR-10057	TTCGTAATAGTAGAATACAGA	2	5	3	1	4	8	0	23
	21UR-10058	TTCGATAGAAATTCGTTTTTC	0	0	0	0	0	0	0	0
†	21UR-10059	TTCCTGCGAATATCGACAACC	0	0	0	0	1	1	0	2
†	21UR-10060	TTCCTCGACTATTTCGCCAAA	0	0	0	0	0	1	0	1
	21UR-10061	TTCCAGTGTTAATGAAGCTTT	0	0	0	0	0	0	0	0
†	21UR-10062	TTCCAGATTTTCAAAGTGCTA	0	0	0	0	0	0	0	0
†	21UR-10063	TTCATACTCCACCATCAAAAG	1	0	1	0	0	1	1	4
†	21UR-10064	TTCAGAAAAAAAATTGGTATCA	0	0	0	0	2	1	0	3
†	21UR-10065	TTCACTTACACGTTTTTGTCT	0	0	0	0	1	0	1	2
	21UR-10066	TTCAAAACTTTATGAAAATGG	0	2	0	0	1	2	0	5
†	21UR-10067	TTATTGGGCTAAACGACTGAG	0	0	0	0	0	0	0	0
†	21UR-10068	TTATTGCATTACCGACTCAAC	0	0	0	0	0	1	0	1
†	21UR-10069	TTATTCTATTTTTCACAAACA	1	0	0	0	1	1	0	3
†	21UR-10070	TTATTCCTTTAGGAGCGTAAC	0	0	0	0	0	0	0	0
†	21UR-10071	TTATCTCTCGAAATTTAATGT	0	0	0	0	0	0	0	0
†	21UR-10072	TTAGTTGAACAGTAATCTCAA	0	0	0	0	0	0	0	0
	21UR-10073	TTAGTCATTTTTAAATTCAAT	0	0	0	0	2	1	0	3
†	21UR-10074	TTACGAAAAAAACACTCTAAC	0	0	0	1	0	0	0	1
†	21UR-10075	TTACAAAATCGATTGAAGTTC	0	0	0	0	0	0	0	0
	21UR-10076	TTAATAAAATGAAAACTATCA	0	0	0	0	0	0	0	0
	21UR-10077	TTAAGAAAATTGTTTTTAAGT	0	0	0	0	0	0	0	0
	21UR-10078	TTAACCGTCGTTCATTATATT	0	0	0	1	8	5	5	19
	21UR-10079	TTAAAGCAGCCTAAGTAAACG	1	0	0	0	0	2	1	4
†	21UR-10080	TTAAAAGTCTGAAGGAAAAAA	0	0	0	0	0	0	0	0
	21UR-10081	TGTTTCAGGGGTTTTATTAAA	0	0	0	0	0	0	0	0
	21UR-10082	TGTTTATTTTCACTGCCAAAT	0	0	0	0	0	0	0	0
†	21UR-10083	TGTTGTGTTTCTTGTAAAACA	0	0	0	0	0	0	0	0
†	21UR-10084	TGTTATCTTAGTGAGCTGTAT	0	0	0	0	0	0	0	0
†	21UR-10085	TGTTAATTGTCCCATTAATTG	0	0	0	0	0	0	0	0
	21UR-10086	TGTGAGTTTTATACGCTTTCT	0	0	0	0	0	1	0	1
†	21UR-10087	TGTGAATGTTTTTTACGAAAC	0	0	0	0	0	0	0	0
	21UR-10088	TGTCACTTTGGGAATTTTTCT	0	0	0	0	0	0	0	0
	21UR-10089	TGTAACACGTTTTAAATTTAT	0	0	0	0	0	0	0	0
†	21UR-10090	TGGTTCACTGAAAATGAAAAC	0	0	0	0	0	0	0	0
	21UR-10091	TGGTCCTTTTAAAACATCAAA	0	0	0	0	0	0	0	0
	21UR-10092	TGGCGATCAGCAATCACTTGA	0	0	0	0	0	1	0	1
	21UR-10093	TGGCAGAATGAATAAATGAAC	1	1	1	1	3	11	2	20
	21UR-10094	TGGATTTATTCGATTTTTTAT	0	0	0	0	0	0	0	0
†	21UR-10095	TGGACTAAGAACTAGATAACC	0	0	0	0	0	0	0	0
	21UR-10096	TGGAATTTGGCTGAATGGAAT	0	0	0	0	0	1	0	1
	21UR-10097	TGCTTTCTGATTTTTTTTTTG	0	1	0	1	1	1	0	4
†	21UR-10098	TGCTTGAATTTCTAGTGGCGC	0	0	0	0	0	0	0	0
†	21UR-10099	TGCTAGTGAACTGTCTGTCTT	0	0	0	0	0	0	0	0
†	21UR-10100	TGCGAACTTTTCTCTATTTTC	0	0	0	0	0	0	0	0
	21UR-10101	TGCATTGTTCTTTTTCACGGA	0	0	0	0	0	2	0	2
	21UR-10102	TGCATTCAATGTCTGATAAGC	0	0	0	0	0	0	0	0
	21UR-10103	TGCAGCAGTCCAAATAGGTTT	0	0	0	0	2	2	1	5
	21UR-10104	TGATTGTGCAAATTTTTTCTC	0	0	0	0	0	0	0	0
	21UR-10105	TGATTGCCTGACAGCTTATTA	0	0	0	0	0	0	0	0
†	21UR-10106	TGATTCTCTGAGTCGGTGAAA	0	0	0	0	1	1	0	2

†	21UR-10107	TGATCTTGACATTGTCAGATA	0	0	0	0	0	0	0	0
	21UR-10108	TGATAATGTTAAAAGTGCTGA	0	0	0	0	1	0	0	1
	21UR-10109	TGATAACGGCTTCCAACAATT	0	0	0	0	0	4	1	5
	21UR-10110	TGAATAATCAATACTAGCAAA	0	0	0	0	0	0	0	0
	21UR-10111	TCTTTTTTCTTTTGAAAAAAT	0	0	0	0	0	0	0	0
	21UR-10112	TCTTTATAGGTTTCATTTCTG	0	0	0	0	0	0	0	0
	21UR-10113	TCTTGATGGGCTATACGAAAC	0	0	0	0	8	14	15	37
	21UR-10114	TCTTCCTTTTTTACTCTCTCT	0	0	0	0	1	0	0	1
	21UR-10115	TCTTATTGTAGGGCTGTACAA	0	0	0	0	0	0	0	0
	21UR-10116	TCTCGGGATGTTCGATTCAAT	0	0	0	0	0	0	1	1
	21UR-10117	TCTCCCCCTATATAACCATTT	0	0	0	0	0	0	0	0
	21UR-10118	TCCTTGTCCCTTTTCATATGA	0	0	0	0	0	0	0	0
	21UR-10119	TCCTTGATTCAAGCACACAAA	0	0	0	0	0	0	0	0
	21UR-10120	TCCTAAAATTTCAAATAATAC	0	0	0	0	0	0	0	0
	21UR-10121	TCCGCTCTGATGTTCATCTGG	0	0	0	0	0	0	2	2
	21UR-10122	TCCGCCCTAATAATCAACATT	0	0	0	0	0	0	0	0
†	21UR-10123	TCCATTGATTTTTTTGCCAGC	0	0	0	0	0	0	0	0
	21UR-10124	TCCAGTTTGCAGACCATATTT	0	0	0	0	0	3	0	3
	21UR-10125	TCCAGAAGTTGAAATTCCTCA	0	0	0	0	0	0	0	0
†	21UR-10126	TCCACTGATGCTTATGTTAAT	1	1	0	1	3	8	0	14
†	21UR-10127	TCATTTTTTTTTCCACGTCAA	0	0	0	0	0	0	1	1
	21UR-10128	TCATAATGGAACACGAAGGAG	0	0	0	0	0	0	0	0
	21UR-10129	TCAGCATATATTCGAATTTGA	0	0	0	0	0	0	0	0
	21UR-10130	TCACTACTCCAACCGGAAAAT	0	0	0	0	0	0	0	0
†	21UR-10131	TCACGGATGCAATTTGTGATA	0	0	0	0	0	0	0	0
	21UR-10132	TCAAATCAATCAAGGTTTTCA	0	0	0	0	0	0	0	0
	21UR-10133	TCAAAAATTAAAAAAAAACTG	0	0	0	0	1	0	0	1
	21UR-10134	TATTTTAGTACTACCTTTTTT	0	0	0	0	2	1	1	4
	21UR-10135	TATTTATTGTGAAGATTAAGA	0	0	0	0	4	3	0	7
†	21UR-10136	TATTGTTCTCTAGCTGGGACT	0	0	0	0	0	0	0	0
†	21UR-10137	TATTGTAGAATAGAATAAACA	0	0	1	1	0	3	1	6
†	21UR-10138	TATTGGCGTTAACATTAGCTT	0	1	0	1	0	0	0	2
	21UR-10139	TATTCGAAAAAAGTTACTCAA	0	0	0	0	0	0	0	0
	21UR-10140	TATTCATTAAAATTTCACAAA	0	0	0	0	1	0	0	1
	21UR-10141	TATTATCCATTTTATAAAACG	0	0	0	0	0	1	0	1
†	21UR-10142	TATTAGTAGTTTGTCGATATA	4	0	0	1	1	4	1	11
†	21UR-10143	TATTAGGCCTAGGATTTCTCT	0	0	0	0	0	0	0	0
†	21UR-10144	TATTAGACAGATTGGCCTGTA	0	0	0	0	0	0	0	0
†	21UR-10145	TATTACAAATGTGTAAACTGA	1	1	1	2	8	22	1	36
†	21UR-10146	TATTAATGTTGTAATAGCTAC	0	0	0	0	0	0	0	0
†	21UR-10147	TATTAATCTTAGGCTGTGAAA	0	0	0	0	1	0	0	1
†	21UR-10148	TATTAAAACGTACGGAAAAAA	1	1	1	0	2	2	1	8
†	21UR-10149	TATGTTGATTATTAGAGCTTT	0	0	0	1	1	1	0	3
	21UR-10150	TATGTGGGTAAATCCAGCACT	1	0	0	0	0	0	0	1
	21UR-10151	TATGGTTGCATACTGTTGCGC	0	0	0	0	0	2	0	2
†	21UR-10152	TATGATTGCTTAGTGGAAGAT	3	6	6	6	143	180	43	387
	21UR-10153	TATCTTTAAATATCACCTTTT	0	0	0	0	1	0	0	1
	21UR-10154	TATCTTACATATAGGGCTGTA	0	1	0	0	4	5	3	13
	21UR-10155	TATCATCATATATGGCTGAAT	0	0	0	1	3	2	1	7
	21UR-10156	TATATAGTTGTAAATGTATTT	0	0	0	0	0	3	0	3
	21UR-10157	TATAGTGAAAACATTATTCAA	0	0	0	0	0	0	0	0
	21UR-10158	TATAATGGAGATAATACCCCT	0	0	0	0	0	0	0	0
*	21UR-10159	TATAAGAAGAAAAACAAAGTT	0	0	0	0	2	0	0	2
†	21UR-10160	TAGTTTATATTTTATTCCCTT	0	0	0	0	3	0	1	4
†	21UR-10161	TAGTTATTTTAGAAGCCAATT	1	0	0	0	1	0	0	2
	21UR-10162	TAGTAAAAATTGACGTTTTAC	0	0	0	0	0	0	0	0
†	21UR-10163	TAGCCATTCTTGAAAATTTTT	0	0	0	0	0	0	0	0
	21UR-10164	TAGAGAGTTTAGCAATAGATT	0	0	0	0	0	0	0	0
	21UR-10165	TAGAGACAAAAACACTTTAGG	0	0	0	0	0	0	0	0
†	21UR-10166	TACTGCGATTTGAGTTGCATC	0	0	0	0	0	0	0	0
	21UR-10167	TACTGCACATTCACGAGGATT	0	0	0	0	0	0	0	0
	21UR-10168	TACTGACAAAAAAAAAATAAA	0	0	0	1	2	2	0	5
	21UR-10169	TACTCTAATATTCTTCTGATG	0	0	0	0	0	1	0	1
	21UR-10170	TACCGGTTGAATTTTTTTTTT	0	1	0	0	1	0	1	3

	21UR-10171	TACCAGAACATCAGAATTTCC	1	1	1	1	1	8	0	13
	21UR-10172	TACATGAAATCTGGAAGTTTC	1	0	0	0	0	0	0	1
	21UR-10173	TACATATATATATATGAGTCT	0	0	0	0	0	0	0	0
	21UR-10174	TACATAATCTGCATTTTTGGT	0	0	0	0	2	0	0	2
	21UR-10175	TACAAAAAGTGTACAGATTGA	0	0	0	0	0	0	0	0
	21UR-10176	TAATTTTTTGGGCTTTTTTTG	0	0	0	0	0	0	0	0
	21UR-10177	TAATTGCATTAAAAAAAGAAA	0	1	0	0	0	0	1	2
	21UR-10178	TAATTAAAGACATTGGACGGT	2	1	0	0	0	10	13	26
†	21UR-10179	TAATGTAATGAAGGCCTTCCT	1	1	0	0	8	6	1	17
	21UR-10180	TAATCTTTTTCTGTTCAATAG	0	0	0	0	0	0	0	0
	21UR-10181	TAATCTAGACATAGTCTTTTC	0	0	0	0	1	1	0	2
	21UR-10182	TAATCGACCTAGCTCCCTCCA	0	0	0	0	0	0	0	0
	21UR-10183	TAAGTTTACAAAATTTCACAA	0	0	0	0	0	0	0	0
	21UR-10184	TAACTCGTTAGTCTTCCAAAA	0	0	0	0	0	0	0	0
	21UR-10185	TAACAGCCTTCCCTTAAAACT	0	0	0	0	0	0	0	0
	21UR-10186	TAACAAATCAAAAAAAGTTTT	0	0	0	0	0	0	0	0
†	21UR-10187	TAAATTCGGCATTTTATTGAG	0	0	0	0	5	3	4	12
	21UR-10188	TAAATCAAAAAAATTCACCGAC	0	0	0	0	0	0	0	0
	21UR-10189	TAAATAGTTGTAACTTCAGCG	0	0	0	0	1	0	0	1
	21UR-10190	TAAATACCGAACAATTTTTTT	0	0	0	0	0	1	1	2
	21UR-10191	TAAAATCATTTTTTTTATCCA	0	0	0	0	1	0	0	1
†	21UR-10192	TAAAAATTTGACCGGCAGAGT	18	10	5	6	41	99	25	204
* †	21UR-10193	GAATCAGAACAATGGTACCCT	679	1572	549	626	1728	6653	66	11873
†	21UR-10194	CTCCAACCAAAACAATTTGTA	0	0	0	0	0	0	0	0
†	21UR-10195	CGATTCGCATGTTGCTATAGA	0	0	0	0	0	0	0	0
	21UR-10196	CGAGACTTTAATTTTTGAGCT	0	0	0	0	0	0	0	0
†	21UR-10197	ATTCGAGGAAATTTTTTGACT	1	0	0	0	1	1	1	4
	21UR-10198	AAATCTCAGTTCTGATCTCGA	0	0	0	0	0	0	0	0
	21UR-10199	TTTTTTTTCCGTGAACATCTA	0	0	0	0	0	1	0	1
	21UR-10200	TTTTTTTGTTGATTTAGCAAC	0	0	0	0	2	0	0	2
	21UR-10201	TTTTTTGTGTTGACGTCTTTA	0	0	0	0	17	8	21	46
	21UR-10202	TTTTTTGAAACGGTGATTCTG	3	3	0	0	0	8	0	14
	21UR-10203	TTTTTTCAGTAAATAAAATAC	0	0	0	0	0	0	0	0
†	21UR-10204	TTTTTCCTGCCAAATTTAAGA	0	0	0	0	0	0	0	0
†	21UR-10205	TTTTGTTCGCAAATCTGTCCA	0	0	0	0	0	0	0	0
†	21UR-10206	TTTTGTGTGTCCATTTTACAG	0	0	0	0	0	0	0	0
†	21UR-10207	TTTTGGAATTTTTCGTGACAA	0	0	1	0	1	2	0	4
	21UR-10208	TTTTGATTTCCCACTCAAGTC	0	0	0	0	0	0	0	0
	21UR-10209	TTTTGAATGTCAATGTTAGAT	0	0	0	0	0	0	0	0
†	21UR-10210	TTTTGAAAAACAAAACCAAAA	0	0	0	0	0	0	0	0
†	21UR-10211	TTTTCTTGCTGAAAAAGGAAT	0	0	0	0	0	0	0	0
	21UR-10212	TTTTCTCTATTTTGAATACAA	0	0	0	0	0	0	0	0
	21UR-10213	TTTTCGTTGGTTTCCTTTACG	0	0	0	0	0	0	0	0
	21UR-10214	TTTTCGGGTTTTCCTGTATCT	0	0	0	0	0	0	0	0
	21UR-10215	TTTTCCAGAAATACATTCCTC	5	0	0	1	1	5	0	12
	21UR-10216	TTTTCAAGCACTACGTTTTTA	5	0	1	0	2	8	1	17
	21UR-10217	TTTTATTGTGATGAATGCATT	0	0	0	3	33	12	3	51
	21UR-10218	TTTTAGAGAATTTGACATTAA	1	0	2	7	62	38	6	116
	21UR-10219	TTTTAGAAACGTTATAATTTT	0	1	0	0	4	5	1	11
†	21UR-10220	TTTTAATGCAAATGGTTGTTG	0	0	0	0	0	1	0	1
	21UR-10221	TTTGTTGGGTAAATCGTTATC	3	1	0	0	0	0	0	4
†	21UR-10222	TTTGGTCTTTTTATCAAACGA	0	0	0	0	0	0	0	0
†	21UR-10223	TTTGCAGCTTTCATTGTCCCT	0	0	0	0	0	0	0	0
†	21UR-10224	TTTGATTAAAGTATGACTATT	0	0	0	0	0	0	0	0
†	21UR-10225	TTTGATCATCCGTTTTCCAGT	0	0	0	0	0	0	0	0
†	21UR-10226	TTTGATACGAGATGGAAACTA	0	0	0	1	1	2	1	5
	21UR-10227	TTTGAAGTGCGAATATGTGTA	0	0	0	0	2	0	0	2
†	21UR-10228	TTTGAAAAATGAGGCCTCAGT	0	0	0	0	0	0	0	0
†	21UR-10229	TTTCTTTTCAACCATTCAAGT	0	0	0	0	0	0	0	0
	21UR-10230	TTTCTCTTGAAACACTATTTT	0	0	0	0	0	0	0	0
†	21UR-10231	TTTCTCATTCCTTTCCCAAAA	0	0	0	0	0	0	0	0
†	21UR-10232	TTTCTATCGACGTGTAATATT	0	0	0	0	0	1	1	2
†	21UR-10233	TTTCGTATCGATTATTTGTGA	0	0	0	0	0	0	0	0
	21UR-10234	TTTCGAGTAAATGAAAAATTT	0	0	0	0	0	0	0	0

†	21UR-10235	TTTCCTTGAAAAATGAGCTTC	0	0	0	0	1	0	0	1
†	21UR-10236	TTTCATTCCTCTTCGTATCTA	0	0	0	0	0	0	0	0
	21UR-10237	TTTCATGTGACCTCAAATTCG	0	0	0	0	0	0	0	0
	21UR-10238	TTTCATCCGCTGAAAAAATCT	0	0	0	0	0	0	0	0
†	21UR-10239	TTTCAGTTGATTGATGATGAT	6	0	1	0	5	14	0	26
†	21UR-10240	TTTATTGGAAACACAACTTTC	0	0	0	0	0	0	0	0
	21UR-10241	TTTATTCATTTTGTTCATTCT	1	0	0	0	1	0	0	2
†	21UR-10242	TTTATGAATACTTGCTACACT	0	0	0	0	0	0	0	0
	21UR-10243	TTTATCGATGCTTTATAATTC	0	0	0	0	0	0	0	0
†	21UR-10244	TTTAGTTTGCGGAGGTAATTT	0	0	0	0	1	2	2	5
†	21UR-10245	TTTAAGAGCAACTTGTATTTT	0	0	0	0	0	0	0	0
	21UR-10246	TTTAAAATGAATGCCAGAGTT	0	0	0	0	0	0	0	0
†	21UR-10247	TTGTTTCGAACTTCAATGCAG	2	3	1	3	7	18	0	34
	21UR-10248	TTGTTCTGAGGTTTCATATAT	0	0	0	0	5	4	2	11
	21UR-10249	TTGTCCCAGTATACCAATATT	0	0	0	0	0	1	0	1
†	21UR-10250	TTGTCAGGAAATTTAGTGTAT	1	1	0	5	51	32	9	99
†	21UR-10251	TTGGTTCACTGAAAATGAAAA	0	0	0	0	0	1	0	1
†	21UR-10252	TTGGTATTCCAGTGAACGATT	0	0	0	0	0	0	0	0
†	21UR-10253	TTGGTACTCCATCAATGGTTT	0	0	0	0	0	0	0	0
†	21UR-10254	TTGGGACATTAAAAAGTGATT	0	0	0	0	0	0	0	0
†	21UR-10255	TTGGCCTAAATCGAATCAAAC	0	0	0	0	0	0	0	0
†	21UR-10256	TTGGAATGGCGAAAATACATT	0	0	0	0	0	0	0	0
	21UR-10257	TTGGAACAACCGTTTTATCCA	0	0	0	0	0	0	0	0
†	21UR-10258	TTGCTTCGTTTGGTGTCAATC	0	0	0	1	2	1	0	4
	21UR-10259	TTGCACATTATGAAAAGTTAG	1	0	0	0	0	0	0	1
†	21UR-10260	TTGCAATTGTGCTTGATCGAA	0	0	0	0	0	0	0	0
†	21UR-10261	TTGCAAAGGCGAAATTTTTCT	2	1	0	0	1	1	1	6
†	21UR-10262	TTGATTTAAACGTTCGAGTGG	0	0	0	0	0	0	0	0
†	21UR-10263	TTGATGTCTGATTGCCTAGAT	0	0	0	0	0	0	0	0
†	21UR-10264	TTGATGATCGTAAATTTGTTC	3	0	0	0	1	4	0	8
†	21UR-10265	TTGAGTGTTGATTTTGTCCAG	0	0	0	0	1	1	0	2
†	21UR-10266	TTGAATTGGCAAAAAAACACA	0	0	0	0	2	2	1	5
†	21UR-10267	TTGAATATTGTACGTTATTTT	0	0	0	0	0	0	0	0
†	21UR-10268	TTCTTTGCTGGCATGTGGGTT	0	0	0	0	0	0	0	0
†	21UR-10269	TTCTTCTAGAGTCCAACAATT	0	0	0	0	0	0	0	0
†	21UR-10270	TTCTTCAGCCTGTGGAGTTAA	0	0	0	0	0	0	1	1
	21UR-10271	TTCTGCATCAGTATCTATTAT	0	0	0	0	1	0	1	2
	21UR-10272	TTCTAAAAATTCTGAAAGTGC	0	0	0	0	0	0	0	0
†	21UR-10273	TTCGTTTCCAATAAATCGTTA	0	0	0	0	0	0	0	0
†	21UR-10274	TTCGTATTGTTGGATTATAAT	0	1	2	1	10	2	0	16
†	21UR-10275	TTCGTATAATGCAGAAATCAG	1	0	0	1	0	2	0	4
†	21UR-10276	TTCGGGCAGATCCATTTTTCT	1	0	2	1	4	11	4	23
†	21UR-10277	TTCGATCTTCAAAACAGTTGC	0	0	0	0	0	0	0	0
†	21UR-10278	TTCGACTGCATTTTTTTATTG	0	1	0	0	19	17	3	40
	21UR-10279	TTCGAAGCTATTTGAAATTCT	0	0	0	0	2	3	0	5
†	21UR-10280	TTCCTCATCCATACACGAGGC	1	0	0	0	3	0	0	4
†	21UR-10281	TTCCGTTGTTGATGATTATAA	0	0	0	0	0	1	0	1
	21UR-10282	TTCCGCTGAAAAAATGACAAT	0	0	0	0	0	0	0	0
†	21UR-10283	TTCCATGGTTAATAACGCAAA	0	0	0	0	0	0	0	0
†	21UR-10284	TTCCACCAAACATGATAGAAC	0	0	0	0	0	1	0	1
†	21UR-10285	TTCATCATTCATTGCGTGGGT	0	0	0	0	1	0	0	1
†	21UR-10286	TTCATCAACTCCGGAATAAAA	0	0	0	0	0	0	0	0
	21UR-10287	TTCATATTTTGTCCAAGAATA	0	0	0	0	0	0	0	0
	21UR-10288	TTCAGTAACTAGAACACAATA	0	0	0	0	0	0	0	0
	21UR-10289	TTCAGGTTCGAATAGCATTTC	0	0	0	0	3	4	4	11
†	21UR-10290	TTCACTTGACCGATTTCTTCA	0	0	0	0	0	0	0	0
†	21UR-10291	TTCACTTAAACATTATCGAGT	0	0	0	0	0	0	0	0
	21UR-10292	TTCACCACGCACCATACTTCC	1	0	0	0	1	3	22	27
	21UR-10293	TTCAATTTTTTAAGTATCCGA	0	0	0	0	0	0	0	0
†	21UR-10294	TTCAATTATTAACTGAAAATT	0	0	0	0	0	1	0	1
†	21UR-10295	TTCAATCACCACCCATCATCA	0	0	0	0	0	0	0	0
	21UR-10296	TTCAAATTCAAAAAATTGCCA	0	0	0	0	0	0	0	0
	21UR-10297	TTCAAACAACACATACGGTCA	0	0	0	0	0	0	0	0
†	21UR-10298	TTATTGATTAGGTTTCAAATT	0	0	0	0	0	0	0	0

	21UR-10299	TTATTGAGCCCAACACAACTG	1	0	0	0	0	0	0	1
	21UR-10300	TTATTGAAAACAAACTTTGAC	0	0	0	0	0	0	1	1
	21UR-10301	TTATGAGATTAAATTGGAATT	0	0	0	0	0	0	0	0
	21UR-10302	TTATCGTAGTTTATTTTCCAG	0	0	0	0	0	0	0	0
	21UR-10303	TTATAATTTTGTTACTATAAC	0	0	0	0	0	0	0	0
	21UR-10304	TTAGCGACAGTTTTTTAAAGC	0	0	0	0	0	0	0	0
	21UR-10305	TTAGATCATTTGGGATATTGA	0	0	0	0	0	0	0	0
†	21UR-10306	TTACTTCATTGAATTGGATAC	0	0	0	0	0	0	0	0
†	21UR-10307	TTACCATTGCTAACAGATTTT	0	0	0	0	1	1	0	2
†	21UR-10308	TTACCAGATCTTGAACGTTTC	12	1	4	2	10	27	14	70
	21UR-10309	TTACCAAAAATATTTAATTGA	0	0	0	0	0	0	0	0
	21UR-10310	TTACATATTGAGATAGTCAAA	0	0	0	0	0	0	0	0
	21UR-10311	TTAATGGTTTATTTTAGTGAT	0	0	0	0	0	0	0	0
	21UR-10312	TTAATATTCAATGATTTAATG	0	0	0	0	0	1	0	1
†	21UR-10313	TTAATAGACGGTTTTCGTTGT	0	0	0	0	10	6	1	17
	21UR-10314	TTAATACAATATTTTACCTGT	1	0	0	0	1	0	1	3
	21UR-10315	TTAAGTTTTATTTTAGAGTAC	0	0	0	0	0	0	0	0
†	21UR-10316	TTAAGACACTATGCATACCTC	0	0	0	0	1	0	0	1
	21UR-10317	TTAAATTTCATTGTACATTGG	0	0	0	0	1	1	0	2
	21UR-10318	TTAAATTGATTTCAGTACTTG	1	0	0	0	0	1	0	2
†	21UR-10319	TTAAATGTTGGACAACGTGTG	1	0	0	0	0	3	0	4
	21UR-10320	TTAAAAAATTTTCAGATGTTC	0	0	0	0	3	2	0	5
	21UR-10321	TGTTGTGTAGTTGCTGTTCTT	0	0	0	0	0	0	0	0
†	21UR-10322	TGTTGCTTTGTCTTCTAAAGT	0	0	0	0	0	0	0	0
	21UR-10323	TGTTGCGGTATTTTCTTAAAA	1	0	0	0	0	0	0	1
* †	21UR-10324	TGTTGAAACGGTATGAAATTG	6	5	4	2	5	29	4	55
†	21UR-10325	TGTTCTGATAGGTAACGTGTA	0	0	0	0	0	0	1	1
†	21UR-10326	TGTTAGAAACCACTTATTGTG	0	0	0	0	0	0	0	0
†	21UR-10327	TGTTAAAATTGGGACATGGGA	0	0	0	0	0	0	0	0
†	21UR-10328	TGTGTGTGACCAATGAATTTG	0	0	0	0	0	0	0	0
	21UR-10329	TGTCTTAATTAAAAAAGCCCC	0	0	0	0	0	0	0	0
	21UR-10330	TGTCCTAATATCTTTCATGAA	0	0	0	0	0	0	0	0
	21UR-10331	TGTATTTATTACAAACCCTAA	0	0	0	0	0	0	0	0
	21UR-10332	TGTATCGCAGGTCCCAAATTT	0	0	0	0	0	0	0	0
†	21UR-10333	TGTATATTAATGGTGTAAGAA	2	2	2	1	0	5	0	12
* †	21UR-10334	TGTAGAAAAGTGGTTGCTTGT	2	0	2	1	2	6	0	13
†	21UR-10335	TGTAATATTTGTAGAAAAAAG	0	1	1	1	20	21	1	45
	21UR-10336	TGGTTTCTCACTCAGAGGAAC	0	0	0	0	0	2	0	2
†	21UR-10337	TGGTGCATTGTTAAGAAAATC	1	0	0	0	0	0	0	1
	21UR-10338	TGGGAGGAAAATGATAGAAAA	0	0	0	0	0	1	0	1
†	21UR-10339	TGGCAAGACAAGTGGAACATG	3	1	0	1	2	16	0	23
	21UR-10340	TGGAATTATAAGGCTTAGACA	1	2	0	0	0	3	1	7
	21UR-10341	TGGAAATCTAAAATCCGCAGT	0	0	0	0	0	0	0	0
	21UR-10342	TGCTTTGAAGGCACGCGGTTT	0	0	0	0	1	0	1	2
	21UR-10343	TGCTGGACACAACACGCATTT	0	0	0	0	5	5	4	14
	21UR-10344	TGCTCTATTGGTTTTTTTTCT	0	0	0	0	0	0	0	0
†	21UR-10345	TGCTATATCTGGTTTTTTTGG	0	0	0	0	0	1	0	1
	21UR-10346	TGCATTTCTACCACTTGTTCT	0	0	0	0	0	1	0	1
†	21UR-10347	TGCACCGTTTTAGGGTTAACC	0	0	0	0	0	0	0	0
†	21UR-10348	TGCACATAAGTTCTTGGATTT	0	0	0	1	2	1	0	4
	21UR-10349	TGCACAGCTTCGGAACATTGA	0	0	1	0	4	2	0	7
	21UR-10350	TGCAATCAACTTTTTGAGTCA	0	0	0	0	0	0	0	0
	21UR-10351	TGATTCTATGCCTTGCTCACT	0	0	0	0	0	0	3	3
†	21UR-10352	TGATTCCACTATGGTTTTTTT	0	0	0	0	0	0	0	0
†	21UR-10353	TGATGCAGTAGAAATTTTGAA	2	0	0	0	0	1	0	3
†	21UR-10354	TGATGAACATGTTCTTGGGAT	0	0	0	0	0	0	0	0
	21UR-10355	TGATATGAAAATTCCCCAACT	0	0	0	0	0	0	0	0
†	21UR-10356	TGAATTAAATTTGAAAATGTT	0	0	0	0	0	0	0	0
*	21UR-10357	TGAATCGTTGAACCAATTTTA	0	0	1	0	1	1	0	3
	21UR-10358	TGAATCCAATCAATTTTTTGG	0	0	0	0	0	0	0	0
	21UR-10359	TGAATATCTTCTCCTCTTTTC	0	0	0	0	0	0	0	0
	21UR-10360	TGAAGAGGGCGGCTATAAATC	0	0	0	0	1	1	1	3
	21UR-10361	TGAAAACCAAGTGAAACAAAT	0	0	0	0	1	1	0	2
	21UR-10362	TGAAAAAATGTCAAACTTTTC	0	0	0	0	0	1	0	1

†	21UR-10363	TGAAAAAATGCTCTTAAAAAA	0	0	0	0	0	0	0	0
†	21UR-10364	TCTTTTTGAGAAATTAACAGA	0	0	0	0	0	0	0	0
	21UR-10365	TCTTTCTTTTTCCTAATATGA	0	0	0	0	0	0	0	0
	21UR-10366	TCTTTCTTTGCTTTTTTAAGT	0	0	0	0	0	0	0	0
	21UR-10367	TCTTCTCAATATGAAGTTTTT	0	0	0	0	0	0	0	0
	21UR-10368	TCTGGTCCATTTAAACCGTTT	0	0	0	0	0	0	0	0
†	21UR-10369	TCTGGGATTTGTTTATCATTT	0	0	0	0	0	1	2	3
†	21UR-10370	TCTGAGGCTGCAAATGAGCTT	0	0	0	0	0	0	0	0
	21UR-10371	TCTCGGCTTTTTTGAATTTTG	0	0	0	0	1	2	0	3
	21UR-10372	TCTACTGTTGTTCAAGAACAT	0	0	0	0	0	0	0	0
†	21UR-10373	TCTACAAAGCAGGGAAAAGAA	0	0	0	0	0	0	0	0
†	21UR-10374	TCGTTTCAGCGATCGAAAGAC	1	0	1	0	0	1	0	3
	21UR-10375	TCGTCATTTATTGTCCATTTT	0	0	0	0	0	0	0	0
	21UR-10376	TCGTAAAAAAGAATTTCAAGA	0	0	0	0	0	0	0	0
	21UR-10377	TCGGATAATTGCGATTAAATT	0	0	0	0	3	1	0	4
	21UR-10378	TCGATCTGATTGATATTTAAT	0	0	0	0	0	0	0	0
	21UR-10379	TCGACTATAGCAAACTTTTAA	0	0	0	0	0	1	0	1
	21UR-10380	TCCTCTCTTTTCAGATTCTTC	0	0	0	0	0	0	0	0
†	21UR-10381	TCCTAGTCTCTTAATTAGCTC	0	0	0	0	0	0	0	0
	21UR-10382	TCCGTTAAAAGCATGTGCCAC	0	0	0	0	3	0	0	3
	21UR-10383	TCATTTTAGGCTATTCTGTTT	0	1	1	1	11	7	0	21
	21UR-10384	TCATTTCTTTCAAACACATAT	0	0	0	0	0	0	0	0
†	21UR-10385	TCATTGGATCGCGGAAATATG	0	0	1	1	7	5	7	21
	21UR-10386	TCATTATTGTTTTCCACTTTT	0	0	0	0	0	0	0	0
	21UR-10387	TCATGGTTTTTTCATCATTTG	0	0	0	0	0	0	0	0
	21UR-10388	TCATATGGTAATCTGGGTTTA	0	1	0	0	0	0	0	1
	21UR-10389	TCATACCATTATATTTACCAT	0	0	0	0	2	2	1	5
	21UR-10390	TCATACAGATTTATTGGAGGA	0	0	0	0	1	1	1	3
	21UR-10391	TCATAAACTACAACTTCAGCA	0	0	0	0	1	0	0	1
	21UR-10392	TCAGCGGATATGAAACTTTTA	0	0	0	0	0	0	0	0
	21UR-10393	TCAGATTTAAGATACTCGGGT	0	1	0	0	2	7	1	11
	21UR-10394	TCAGAAAATTGAAATTCCGCT	0	0	0	0	0	0	0	0
†	21UR-10395	TCACTTTTGAAGAAAAAGTAC	0	0	0	0	0	0	0	0
	21UR-10396	TCACCTTTTTTTCAAATTTTC	0	0	0	0	0	0	0	0
	21UR-10397	TCACCGTTAAACCAACATTTA	0	0	0	0	0	0	1	1
	21UR-10398	TCACACCTCTCCTTAAAATTG	0	1	0	0	0	2	1	4
	21UR-10399	TCAAGTAAAAATAATGAAAAA	0	0	0	0	0	0	0	0
	21UR-10400	TCAACTGGATAAGTTTTGTTT	0	0	0	0	0	1	0	1
†	21UR-10401	TCAAATTGGTGCATCGAGAAG	1	0	0	0	0	0	0	1
	21UR-10402	TCAAAACTACTGAATTAGACA	0	0	0	0	3	0	0	3
†	21UR-10403	TCAAAAATAGGCACTTTCAGA	0	0	0	0	0	0	0	0
	21UR-10404	TATTTTTTAAAGGTTTTTTTC	0	0	0	0	0	0	0	0
	21UR-10405	TATTTTTGAAAATATTTTTGA	1	0	0	0	0	0	0	1
	21UR-10406	TATTTTCGCATTGTCTCTGAT	0	0	0	0	0	0	0	0
†	21UR-10407	TATTTGGAGCATGATCAAATA	0	0	0	0	0	0	0	0
	21UR-10408	TATTTCGTAGCTTAATACATA	0	0	0	2	9	4	4	19
	21UR-10409	TATTTATTATGTTGGCCAATT	2	0	0	1	24	15	27	69
	21UR-10410	TATTTAAATCGTTGTGAATGA	3	0	1	0	3	3	2	12
†	21UR-10411	TATTGTGATCTGCCATCGTTC	0	0	0	0	0	0	0	0
†	21UR-10412	TATTGGTTTTTTCATCATAGG	0	0	0	0	1	0	0	1
†	21UR-10413	TATTGGGAATAATAAATAGTC	0	0	0	0	0	0	0	0
†	21UR-10414	TATTGGAGGCCTGGTTGTTTG	2	0	0	0	0	1	0	3
	21UR-10415	TATTGCTACCATTTTTGAGCA	0	0	0	0	0	0	0	0
†	21UR-10416	TATTGCGTATGGCGTATCAGT	0	0	0	0	3	2	1	6
†	21UR-10417	TATTGCCACGTAGTTCTCAAT	0	0	0	0	0	2	0	2
†	21UR-10418	TATTGCATAACGGCTTGCATT	3	0	0	0	10	9	1	23
†	21UR-10419	TATTGAGCAATCGATTCGGGT	0	0	1	0	0	2	0	3
	21UR-10420	TATTGAAACAGTTTTTGTCAT	0	0	0	0	0	0	0	0
†	21UR-10421	TATTCTGATTTGCTCCGGTGT	0	0	0	0	0	1	1	2
†	21UR-10422	TATTCGCGAATATAAAAACTT	0	0	0	0	0	0	0	0
†	21UR-10423	TATTCCTATTGACATGGACTC	0	1	0	0	0	2	0	3
†	21UR-10424	TATTCATGTGAACTCATCAAA	0	0	0	0	2	0	0	2
†	21UR-10425	TATTATTTACTACGAGGATGA	0	0	0	0	0	0	1	1
	21UR-10426	TATTATTATGATGTAAATTTC	0	0	0	0	1	0	0	1

†	21UR-10427	TATTAGACGTGTTGAAAAAAT	2	2	2	5	9	22	0	42
	21UR-10428	TATTAGAATTGTTGAAAAAAA	1	0	2	1	0	0	0	4
†	21UR-10429	TATTACATCTGAAACAGGATG	0	0	0	0	0	1	0	1
†	21UR-10430	TATTAATATGAATTTTAAAAA	0	0	0	0	1	1	0	2
†	21UR-10431	TATTAAGTTTGAAAGATTTGA	1	1	0	0	1	3	0	6
	21UR-10432	TATGTTATATAAATAGTGTGG	0	0	0	0	0	0	0	0
	21UR-10433	TATGTTAAGAAGTTTTATATT	0	1	0	1	2	1	0	5
	21UR-10434	TATGTCTCTATCATTAAAATG	0	0	0	0	0	0	0	0
	21UR-10435	TATGGTAGAAATATTCCGACT	0	0	0	1	5	1	1	8
†	21UR-10436	TATGATTCTTTTTTTGTGATA	0	0	0	0	0	0	0	0
	21UR-10437	TATGAGAAGTTATCGTGTTTT	0	0	0	0	1	0	0	1
	21UR-10438	TATCTTATAATTACTCACAAT	0	0	0	0	0	0	0	0
	21UR-10439	TATCATCCAGTTAAATATAAA	0	0	0	0	0	0	0	0
	21UR-10440	TATATGGAGACGAGTTCAAAT	1	3	2	1	1	9	1	18
	21UR-10441	TATATATTCCAGAGTAGGGAG	0	0	0	0	3	3	8	14
	21UR-10442	TATATATCTGGAATTTTTATT	0	0	0	2	0	2	0	4
	21UR-10443	TATAGAATGAATGAAACAGAA	4	4	3	1	4	8	0	24
	21UR-10444	TATAATCTTTTACTTCCATTT	0	0	0	0	0	2	1	3
	21UR-10445	TATAAAAATAATATAAATTTC	0	1	0	0	2	1	0	4
	21UR-10446	TAGTTTTGTTTCACCCCCGCC	0	0	0	0	0	0	1	1
	21UR-10447	TAGTTTGATGAATTCATTCGA	0	0	0	0	0	0	0	0
	21UR-10448	TAGTTTATTAAAAAACTTTCG	0	0	0	0	0	0	0	0
	21UR-10449	TAGTTCCTCCGAAATAGTATG	0	0	0	0	0	0	0	0
†	21UR-10450	TAGTTCAAGTAGTGCTTTCTT	2	5	1	0	1	2	0	11
	21UR-10451	TAGTGTCTGACTTTAACTTTT	0	0	0	0	0	0	0	0
†	21UR-10452	TAGTCTCTTGTCCCGCAAAAA	0	0	0	0	0	0	0	0
†	21UR-10453	TAGGATTAGTTGTACAGCCCT	104	98	59	42	230	429	45	1007
†	21UR-10454	TAGCATCTTCCACCGGGTCTA	0	0	0	0	0	0	0	0
	21UR-10455	TAGATCAATTTTGTTATTTTC	0	0	0	0	0	0	0	0
	21UR-10456	TAGAGTATGACTTAGCGCGTT	0	0	0	0	2	3	0	5
	21UR-10457	TAGACTGAGTTTTTCAAAAGT	0	0	0	0	0	0	0	0
	21UR-10458	TACGTTTTTCACGCATTTCCC	0	0	0	0	0	0	0	0
†	21UR-10459	TACGGAGAAACAATTTAAAAA	0	0	0	0	0	0	0	0
	21UR-10460	TACAACAGTTATGAGCGACGC	0	0	0	0	0	1	1	2
	21UR-10461	TACAAACAGGAAGATACGTCA	0	0	0	0	0	0	0	0
	21UR-10462	TAATTTTTTTGTATTGGGAGT	0	0	0	0	0	2	1	3
†	21UR-10463	TAATTTTGACATTAAATGTTA	0	0	0	0	2	0	0	2
	21UR-10464	TAATTTTATTCATCATGTAAC	0	0	0	0	0	0	0	0
†	21UR-10465	TAATTTCTTTGTTGTTTAAAA	0	0	0	0	0	0	0	0
	21UR-10466	TAATTCTTTCGCGTTTCAATT	0	0	0	0	0	0	0	0
†	21UR-10467	TAATTCCAGACACACAGAAGG	1	0	0	0	1	0	0	2
†	21UR-10468	TAATGTCTACCTTCTGAACCG	0	0	0	0	0	0	0	0
†	21UR-10469	TAATGGATCTAAAGATTTTTC	0	0	0	1	5	1	3	10
†	21UR-10470	TAATGCTGATGTAAAATGGTT	1	0	0	0	2	1	0	4
†	21UR-10471	TAATGCGATGTGGGTTCAATC	0	0	0	0	0	0	0	0
†	21UR-10472	TAATAGTTTTGAAAAAAAAAA	0	0	0	0	0	0	0	0
†	21UR-10473	TAATAGAATAGAGTTTGCAGA	2	5	0	1	8	15	2	33
†	21UR-10474	TAATAAAGATGGAGTACTGTA	0	0	0	0	0	0	1	1
	21UR-10475	TAATAAACAACAACAAAAATT	0	0	0	1	1	1	0	3
	21UR-10476	TAAGTGTATTGGATTTGTTCG	1	0	1	1	6	2	2	13
*	21UR-10477	TAAGTCGTTGATGATCGTTTT	11	1	3	2	42	42	98	199
	21UR-10478	TAAGATGTATTTTAGTATTTT	1	0	0	0	0	1	1	3
	21UR-10479	TAACGTAGTTTTTGTCTTTTT	0	0	0	0	1	1	2	4
	21UR-10480	TAACATATCCATAATCCATAT	0	0	0	0	1	1	3	5
	21UR-10481	TAACAGACAAAAAACCGCCCC	0	0	0	0	0	0	0	0
	21UR-10482	TAAATTTTTGGAAACGTTTCT	0	0	0	0	0	1	4	5
	21UR-10483	TAAATTGAAAATTGAATAATT	0	0	0	0	1	1	0	2
	21UR-10484	TAAATCGTAAAGGAAAATGTT	0	0	0	0	0	0	1	1
†	21UR-10485	TAAATCAACTGTTCGAAACAT	0	0	0	0	0	0	0	0
	21UR-10486	TAAATATCTTATCATGTACTC	0	0	0	0	0	1	0	1
	21UR-10487	TAAATACTGTTTTTTCAAAAC	0	1	0	2	0	4	0	7
	21UR-10488	TAAATAAATATCATAGTAACG	0	0	1	0	2	1	0	4
†	21UR-10489	TAAAAATCTGAAGAAACGCAT	1	1	1	2	19	24	1	49
	21UR-10490	TAAAAAAGAAAATTTTTAAAT	0	0	0	0	0	0	1	1

	†	21UR-10491	GTAAGTTCGATGGTTTGAATT	0	0	0	0	0	1	0	1
		21UR-10492	GAGTTTTTCTCGTCGATCGAA	0	0	0	0	0	0	0	0
	†	21UR-10493	CTTTTAGAATACTGTGGAAAT	1	1	0	1	1	1	0	5
		21UR-10494	CTCAAAACTTTATTCTTCGAA	0	0	0	0	0	0	0	0
	†	21UR-10495	CGGCGAAATCATCAATGGACT	0	0	0	1	1	3	0	5
	†	21UR-10496	CGAACTGTTTTTGAAAATTAT	0	0	0	0	0	3	0	3
*	†	21UR-10497	CCTTCAAGAACAATGCAAAAG	0	0	0	0	1	8	0	9
		21UR-10498	CAGATTACTCTTATCTCAAGT	0	0	0	0	0	0	0	0
	†	21UR-10499	CAAAAGAATCATCTCGTGGAC	0	0	1	0	2	0	0	3
		21UR-10500	TTTTTTGGGAAAATACGAATG	0	0	0	0	4	0	0	4
		21UR-10501	TTTTTTCAAAAAGAAATCATA	0	0	0	0	0	0	0	0
		21UR-10502	TTTTTCGTTTTTTTTGCAACA	0	0	0	0	0	0	0	0
	†	21UR-10503	TTTTTCAAAAAAAAACGTATG	0	0	0	0	0	0	0	0
	†	21UR-10504	TTTTTAAAAAATCTCCTTAGC	1	0	0	0	1	2	0	4
	†	21UR-10505	TTTTGTGAAATTTTCGGTTCA	0	0	1	0	0	0	0	1
	†	21UR-10506	TTTTGTCCTCAATCACCGTGT	0	0	0	0	0	0	0	0
	†	21UR-10507	TTTTGTATTTGTGCTCTTAAA	0	1	0	0	0	0	0	1
	†	21UR-10508	TTTTGTACTGTGAGACATGAG	1	0	0	0	2	1	1	5
	†	21UR-10509	TTTTGATCAATGTCCAAAAGA	0	0	0	0	0	0	0	0
		21UR-10510	TTTTCTTTCATGCAGCGAGAT	0	1	0	0	0	3	0	4
		21UR-10511	TTTTCTGTTTGTTTTAGAGTG	0	0	0	0	0	1	0	1
		21UR-10512	TTTTCTGAGCAAGACATAATA	0	0	1	0	1	5	0	7
	†	21UR-10513	TTTTCGCGTCGTCATCACAAA	0	0	2	2	1	1	1	7
	†	21UR-10514	TTTTCGCGGTGAATCCTGTTA	0	0	0	0	0	0	0	0
	†	21UR-10515	TTTTCGATCTTTGGGTTTCAA	0	0	0	0	9	11	3	23
	†	21UR-10516	TTTTCATTACATATGTTCCAC	1	1	2	0	7	3	0	14
		21UR-10517	TTTTCAATCCAATTTATTTCC	0	0	0	0	0	0	0	0
*		21UR-10518	TTTTAGCGGTAGATTCGTTTT	4	1	2	4	74	46	35	166
	†	21UR-10519	TTTTAGAGACACATTTCTAGA	0	0	0	0	1	0	0	1
		21UR-10520	TTTTAGACTGGACACAAAAAA	0	0	0	0	0	2	3	5
		21UR-10521	TTTTACGGTTTCAGAGGGTTT	2	0	0	0	0	2	0	4
	†	21UR-10522	TTTTAATGGTGAAAAATCAGG	0	0	0	0	0	0	1	1
	†	21UR-10523	TTTTAAATCATCGTGAAGAGT	0	0	0	1	2	0	0	3
		21UR-10524	TTTGTGAATGACTTCTTTTCG	0	0	0	0	2	0	0	2
	†	21UR-10525	TTTGGATATCTCAAAGATAAA	0	0	0	0	1	0	0	1
	†	21UR-10526	TTTGGAATTTCTTTCGGCTTT	0	0	0	0	1	1	0	2
	†	21UR-10527	TTTGAGTTCGTTAAAAAAAAA	0	0	0	0	0	0	0	0
		21UR-10528	TTTGACTTGCAGTTTCCACCA	0	0	0	0	10	7	5	22
		21UR-10529	TTTGACGAGAGTATTATTCAA	0	0	0	0	0	1	0	1
		21UR-10530	TTTGAATTTCCGTTTTGTTCA	0	0	0	0	0	0	0	0
		21UR-10531	TTTGAATATTCATAAGAGAAT	0	0	0	0	0	0	0	0
	†	21UR-10532	TTTGAAATCACCAACCACAAG	1	0	0	0	0	0	0	1
	†	21UR-10533	TTTCTTCGTTTGGGTGAAAAA	0	0	0	0	1	0	0	1
	†	21UR-10534	TTTCTTCATTGGCTGTGCTTG	0	0	0	0	0	0	0	0
	†	21UR-10535	TTTCTCTCCTATCGAGTGGTA	0	0	0	0	0	2	0	2
	†	21UR-10536	TTTCTAAGACCTGTTTATTAT	0	0	0	0	6	2	0	8
		21UR-10537	TTTCGGTAACATCAGACAATT	1	0	0	0	0	1	0	2
	†	21UR-10538	TTTCCTTTCTGTGCTTAACAT	0	0	0	0	0	0	0	0
		21UR-10539	TTTCCCCACAGGTAGTCCACA	0	0	0	0	0	0	0	0
	†	21UR-10540	TTTCCAGTGCGTTTGACATCT	0	0	0	0	1	0	0	1
	†	21UR-10541	TTTCATTGGAAGCATAACTCG	0	0	0	0	7	2	0	9
		21UR-10542	TTTCATTACCTTACATTAGCC	0	0	0	1	2	6	2	11
	†	21UR-10543	TTTCAGTTTGGACTTTGGAGT	0	0	0	0	0	1	0	1
		21UR-10544	TTTCAGTAATCTCTGTTGCAA	0	0	0	0	0	0	0	0
	†	21UR-10545	TTTCAGAAACGCTATGAATAT	7	10	3	3	5	26	2	56
		21UR-10546	TTTCAAACATATACGCAATGT	0	0	0	0	1	0	0	1
		21UR-10547	TTTCAAAAAATTCAATTTCCA	0	0	0	0	0	0	0	0
	†	21UR-10548	TTTATTCCTTCCTTTTGATCC	0	0	0	0	0	0	0	0
		21UR-10549	TTTATTCAAAATTTACTGCAT	0	0	0	0	1	0	0	1
	†	21UR-10550	TTTATGGATTGTTCCGCATGA	0	0	0	0	6	6	6	18
	†	21UR-10551	TTTATGCACGGTCTTGTACAT	2	0	0	0	0	4	0	6
	†	21UR-10552	TTTATGAAGCTTCTAACTGAT	0	0	0	0	0	0	0	0
		21UR-10553	TTTACCTGTATCGAATAACTC	0	0	0	0	3	3	1	7
		21UR-10554	TTTACAAATAAAATGTAGGTT	0	1	0	0	1	1	0	3

†	21UR-10555	TTTAATTCACTTGATTTTCAA	0	0	0	0	0	0	0	0
	21UR-10556	TTTAATTAGTATTCTTCAAGG	0	0	0	1	3	2	0	6
	21UR-10557	TTTAAGGTAGTTATTCTTCTA	0	0	0	0	0	0	0	0
†	21UR-10558	TTTAAGCAATTGCAGGTAATA	1	0	0	0	1	5	0	7
†	21UR-10559	TTTAACATTGTTTTGTCAGAA	0	0	0	0	4	1	0	5
†	21UR-10560	TTTAAAGCACGAGACTTAGAG	0	2	0	0	2	2	0	6
†	21UR-10561	TTGTTTCAAGCAAACGTTGCA	0	0	0	0	0	0	0	0
†	21UR-10562	TTGTTCCCATTGCAGAGTTGC	0	0	0	0	0	1	0	1
†	21UR-10563	TTGTTCCATATATTACCAGAA	0	0	0	0	0	0	0	0
†	21UR-10564	TTGTTATTGTGTTCTTTATCC	0	0	0	0	3	0	0	3
†	21UR-10565	TTGTGTAGTTTGTTCATTAAA	0	0	0	0	0	0	0	0
†	21UR-10566	TTGTCATCTTGATTTCTAAAA	0	0	0	0	0	0	0	0
	21UR-10567	TTGTATGTCTTCAGCCATTTT	0	0	0	0	0	1	0	1
	21UR-10568	TTGTAATTGAAAAATGAATTC	0	1	0	0	0	0	0	1
†	21UR-10569	TTGGTGTAGATTGCCTAGCTT	0	0	0	0	0	0	0	0
	21UR-10570	TTGGACTAAATATAATTAGAC	0	0	0	0	1	0	0	1
†	21UR-10571	TTGCTATAGCGAAATCTTTCA	0	0	0	0	0	0	0	0
	21UR-10572	TTGCAATAATCTTGCCACAAC	0	0	0	0	0	0	0	0
†	21UR-10573	TTGATTTCTATTTCAAATTAA	0	0	0	0	0	0	0	0
	21UR-10574	TTGATTCAAAACTTTTGTTGG	0	0	0	0	0	0	0	0
†	21UR-10575	TTGATGGATTTGAAAAATTGA	0	0	0	0	0	0	0	0
†	21UR-10576	TTGATATTTTTTATTTTAAGC	0	0	0	0	0	0	0	0
	21UR-10577	TTGATAAACTGTTAAAATGCA	0	0	0	0	0	2	0	2
†	21UR-10578	TTGAGGGACTCTCGAAAAATT	0	0	0	0	0	0	0	0
†	21UR-10579	TTGAACAATTGAAAGTAATTG	0	0	0	0	0	0	0	0
	21UR-10580	TTGAAATTTTCAAGCACATTT	0	0	0	0	0	1	0	1
	21UR-10581	TTGAAACGTCATTTAAAAGTG	0	0	0	0	0	0	0	0
†	21UR-10582	TTGAAAAGTAACGGAGTATGC	1	0	0	0	0	0	0	1
	21UR-10583	TTGAAAACGCTAAAATTCATC	0	0	0	0	4	2	0	6
	21UR-10584	TTCTTTTTTTATTTTTCCATG	0	0	0	0	0	0	0	0
†	21UR-10585	TTCTCGTTTTTGTCTTTGAGT	0	0	0	0	0	0	0	0
†	21UR-10586	TTCTCCATCTGACGTTATGAG	0	0	0	0	0	0	0	0
	21UR-10587	TTCTATTTTGTCAAGTTTTGC	0	0	0	0	0	0	0	0
	21UR-10588	TTCTATTCGAGTTGAACTTTT	0	0	0	0	0	1	0	1
†	21UR-10589	TTCTATCGCGGTTATACTATT	1	1	1	0	6	5	2	16
* †	21UR-10590	TTCGTCGAAAACTTTGGCATC	1	0	0	1	1	5	0	8
	21UR-10591	TTCGAATGGGGCTCTAAAACT	0	0	0	0	0	0	0	0
†	21UR-10592	TTCCTTGGGAAATTTATCGTC	0	0	0	0	1	4	3	8
†	21UR-10593	TTCCGTATGGTACAATAGGAA	0	0	0	0	0	0	0	0
	21UR-10594	TTCCGATTGTTGAAACTCGTT	0	0	0	0	0	0	0	0
	21UR-10595	TTCCCTATTCAGGATACTCTG	0	1	0	1	0	1	0	3
	21UR-10596	TTCCAGACATCGAATGAAATT	0	0	0	0	0	0	0	0
	21UR-10597	TTCATTTATTGAATTGACTCA	0	0	0	0	1	0	0	1
†	21UR-10598	TTCATCAGAGGAAAAAACATG	0	0	0	0	0	0	0	0
	21UR-10599	TTCATCACTTCCTTCTCCACC	0	0	0	0	0	0	0	0
†	21UR-10600	TTCATAGGGTAACCGATTTTC	0	0	0	0	2	0	0	2
	21UR-10601	TTCAGACTGTCGAATGTAAAT	0	0	0	0	0	0	0	0
	21UR-10602	TTCAGAATTTGTCAAACGAAG	0	0	0	0	0	0	0	0
†	21UR-10603	TTCACACGGCGACTATTTTGA	0	0	0	0	0	0	1	1
†	21UR-10604	TTCAATAACATTTAAATGAGA	0	0	0	0	0	0	0	0
	21UR-10605	TTCAACATACGAATTCTGTGT	0	0	0	0	0	0	0	0
	21UR-10606	TTCAAAAACTCTCATCAAAAT	0	0	0	0	0	0	0	0
	21UR-10607	TTATTTTTTTTCTGGTCTGAG	0	0	0	0	0	1	0	1
†	21UR-10608	TTATTTGGAGCATGATCAAAT	0	1	0	0	0	2	0	3
	21UR-10609	TTATTTGAGTAGACGTTGATG	0	0	0	1	0	0	3	4
	21UR-10610	TTATTTCAATACGAGTTGATT	0	0	0	0	0	1	0	1
†	21UR-10611	TTATTTACGATAAATTTAAAA	0	0	0	0	0	0	0	0
	21UR-10612	TTATTTAACTGTCCGGTTTTT	0	0	0	0	0	1	4	5
†	21UR-10613	TTATTCTGTCCTTAAGCGTAC	0	0	0	0	0	0	0	0
	21UR-10614	TTATTCATATTCTAGATGATT	0	0	0	0	1	0	0	1
	21UR-10615	TTATTATTTCTAATTTGAATA	0	0	0	0	0	0	0	0
†	21UR-10616	TTATTATGTATCTAGCAGCCG	0	1	0	0	3	0	0	4
†	21UR-10617	TTATTACACGATTGATCTCAC	0	0	0	1	1	2	0	4
†	21UR-10618	TTATTAAGTCCTGTAACTGAA	0	0	0	0	0	0	1	1

†	21UR-10619	TTATTAAGAATGTAAAACCAT	0	0	0	0	0	0	0	0
†	21UR-10620	TTATTAAATGAAGTAGAAAAA	0	0	0	1	3	1	0	5
	21UR-10621	TTATGATAACCTGGGATCATC	0	0	0	0	0	0	0	0
	21UR-10622	TTATCTTTTTCCATTAATCAA	0	0	0	0	0	0	0	0
†	21UR-10623	TTATAGAGTATGTATGCGCTT	0	0	0	0	2	3	0	5
†	21UR-10624	TTAGTTACGAATTTGTCAATT	0	0	0	0	4	0	0	4
†	21UR-10625	TTAGTCTAACTCCAAATATCA	0	0	0	0	0	0	0	0
	21UR-10626	TTAGCAATTTCCGAATGTTTT	0	0	0	0	1	0	0	1
†	21UR-10627	TTACTGTTTTCCTAATCATGG	0	0	0	0	2	7	0	9
	21UR-10628	TTACTCACAGTCAGCTCGGTA	0	0	0	0	0	0	0	0
†	21UR-10629	TTACCAACTTTGCTAATAAGT	0	0	0	0	0	0	0	0
	21UR-10630	TTACATTTTGTCATCAGTTTT	0	0	0	0	0	1	0	1
	21UR-10631	TTACATAATAAAATGTATGAA	0	0	0	0	0	0	0	0
	21UR-10632	TTACAGAGGCAGTTTTATATT	1	0	0	0	6	0	2	9
	21UR-10633	TTAATTGGTTTCAATTGTATG	0	0	0	0	0	0	0	0
†	21UR-10634	TTAATTATCACTCCAATTACT	0	0	0	0	0	0	0	0
†	21UR-10635	TTAATGTCTCTTTGTTTGAGT	0	0	0	0	0	0	0	0
	21UR-10636	TTAAACTTGAATTGCTCAACT	0	0	0	0	0	1	0	1
	21UR-10637	TGTTTTTCTTTCTGATAAATT	0	0	0	0	0	0	0	0
	21UR-10638	TGTTTTTCATTTCAATTCAAA	0	0	0	0	0	0	0	0
†	21UR-10639	TGTTTTCATAGTTATCAGATT	0	0	0	0	0	1	0	1
* †	21UR-10640	TGTTTGTGGAAGCGTGAGCAT	6	2	1	1	8	22	5	45
†	21UR-10641	TGTTTACAATGTTCCAAAAAA	0	0	0	0	0	0	0	0
†	21UR-10642	TGTTGTCTGTTCGATTTAGCA	0	0	0	0	0	0	0	0
†	21UR-10643	TGTTGTCGAAAGTGTACTCGA	0	0	0	0	0	0	0	0
†	21UR-10644	TGTTACTGGAATATGGAAACT	0	0	0	0	0	0	0	0
	21UR-10645	TGTTAACTTACAAGTGTAGCA	0	0	0	0	0	0	0	0
	21UR-10646	TGTGTTGTGTCTCTATTAGCT	0	0	0	0	1	0	0	1
†	21UR-10647	TGTGTGTTAGTCATGTGTTCA	0	0	0	0	0	0	0	0
	21UR-10648	TGTGAATCCAGAGAGTGACAC	0	0	0	0	0	0	1	1
	21UR-10649	TGTCTTTACAAAAAATAATTG	0	0	0	0	0	0	0	0
†	21UR-10650	TGTCGAGTGATGATCAGAACC	0	0	0	0	0	0	0	0
	21UR-10651	TGTCCCATCAATGCTGAAAAA	0	0	0	0	0	0	0	0
	21UR-10652	TGTCATTTCCATATTTCATTC	0	0	0	0	0	1	0	1
	21UR-10653	TGTCAATTTTCTATTTCGTAG	0	0	0	0	0	0	0	0
	21UR-10654	TGTAGTATTGTAAATTCATAA	0	0	0	0	3	5	0	8
	21UR-10655	TGTAATTTTCATAAGAAGTTG	0	0	0	0	0	0	0	0
	21UR-10656	TGGTTTTATTGCAGTTGTTTG	0	0	0	0	1	0	0	1
	21UR-10657	TGGTTTTATCTAACAAACAGT	0	0	0	0	0	0	0	0
	21UR-10658	TGGTTTCCAAGACAATCTTCA	0	0	0	0	0	0	0	0
†	21UR-10659	TGGTTCCTAGGTTTGTACTCG	0	0	0	0	1	0	0	1
	21UR-10660	TGGTTAGGCTTTAGAAGTTTG	0	0	0	0	0	0	0	0
†	21UR-10661	TGGTGCCTATTGCTAGAATCC	0	0	0	0	0	0	0	0
	21UR-10662	TGGCGGAGCATTCATTCTGTC	0	0	0	0	1	1	0	2
	21UR-10663	TGGATTTTTGAATAATCTTGA	1	0	1	1	1	1	0	5
†	21UR-10664	TGGAGAGGAAAGTGAGATATT	0	0	0	0	0	0	0	0
	21UR-10665	TGGAAGGAAAATATTGTTTCA	0	0	0	0	0	0	0	0
	21UR-10666	TGGAAATTTCCAATGTATCTT	0	0	0	0	0	0	0	0
	21UR-10667	TGGAAATTATACATGTCAAAT	0	1	0	0	0	0	1	2
	21UR-10668	TGCTTTTTTGAAAGTTAAGTC	0	0	0	0	0	0	0	0
†	21UR-10669	TGCTTTGTCTTCTAAAGTATC	0	0	0	0	0	0	0	0
	21UR-10670	TGCTTCGAAAAAGCCGATGCT	0	0	0	0	0	0	0	0
†	21UR-10671	TGCTGCTGGTCTATTAAATGC	0	0	0	0	0	0	0	0
†	21UR-10672	TGCTCCTCGGCTATTCCATTG	0	0	0	0	0	0	0	0
†	21UR-10673	TGCGTTTCCAATTCTGAGGAT	1	1	0	0	21	22	4	49
	21UR-10674	TGCGATATTTTTGCGTTTTTT	0	0	0	0	0	0	0	0
†	21UR-10675	TGCCTGCTATTAGAATTCATT	0	0	0	0	0	0	0	0
	21UR-10676	TGCCACAGTCGGTTATTTTAT	0	1	0	0	27	2	0	30
	21UR-10677	TGCATTGTTTCCCACTAATTC	0	0	0	0	0	0	0	0
†	21UR-10678	TGATTTTGGCAAGATAAACTT	6	1	0	0	3	6	0	16
	21UR-10679	TGATTTGAATGTGATCGACCA	0	0	0	0	0	0	0	0
	21UR-10680	TGATTCACATTTTTCCATTTC	0	0	0	0	0	0	0	0
†	21UR-10681	TGATGCTGTCATGTTTCACTG	0	0	0	0	0	0	0	0
	21UR-10682	TGATGAGCGCACAATTTTCAT	0	0	0	0	0	0	0	0

	21UR-10683	TGATCATATTCATTTCATCAA	0	0	0	0	0	0	0	0
†	21UR-10684	TGATAGAATGAATGAGATCAA	0	0	0	0	1	0	0	1
	21UR-10685	TGAGGTGAGCTAAACTAAATC	0	0	0	0	0	0	0	0
	21UR-10686	TGACGGCATAAGTTTGACATC	7	6	0	1	3	46	3	66
	21UR-10687	TGACAGTCTTATCGATTTTGG	0	0	0	0	0	0	0	0
	21UR-10688	TGAATTTTCTGTCAGACAAAA	0	0	0	0	0	2	0	2
	21UR-10689	TGAATTTTCAAATCTGGGAAT	0	0	0	0	0	0	0	0
	21UR-10690	TGAATTATTTCTACTGTTTCT	0	0	0	1	2	0	0	3
	21UR-10691	TGAATCTCAGACTTGAAAAAT	0	0	0	0	0	0	0	0
†	21UR-10692	TGAACTGGTCGCTGTGATGGA	9	5	1	0	5	19	2	41
	21UR-10693	TGAACTCAAAGTTTTTTGAAG	0	0	0	0	0	0	0	0
	21UR-10694	TGAACCGGTAGAATATGAATA	8	7	4	3	5	41	1	69
	21UR-10695	TGAACAGTGATTTTTCAGGAC	0	0	0	0	0	0	0	0
	21UR-10696	TGAAACCGGCAAATTTTTATT	0	0	0	1	0	1	0	2
	21UR-10697	TGAAAATTATCATGAGATTAA	0	0	0	0	0	0	0	0
	21UR-10698	TGAAAAAGCAGCATATTCGGC	0	0	0	0	0	0	0	0
	21UR-10699	TGAAAAAATGATCTCAACAAA	0	0	0	0	0	0	1	1
	21UR-10700	TCTTCGTTGCAAGCTCATTTT	0	0	0	0	2	0	0	2
†	21UR-10701	TCTTCGTTCATGCTTTTCTTT	0	0	0	0	1	1	0	2
	21UR-10702	TCTTCATTCTTGCTGGAGTTT	0	0	0	0	0	0	0	0
†	21UR-10703	TCTGTTGTCAATGGTAACTTG	0	0	0	0	0	0	0	0
	21UR-10704	TCTGTCAAATTCCATAGAAAA	0	0	0	0	0	0	0	0
†	21UR-10705	TCTGCTTATTCGGAATGCAGT	0	0	0	0	0	0	0	0
	21UR-10706	TCTCACTTCTAAACCTTTCAT	0	0	0	0	0	0	0	0
	21UR-10707	TCTACAGGCAAAAATAGATGG	0	0	0	0	0	1	0	1
	21UR-10708	TCTAAATTCTAAAACTCTTTC	0	0	0	0	0	0	0	0
†	21UR-10709	TCGTTCTCATCTAGGAAGAAA	0	0	0	0	0	0	0	0
†	21UR-10710	TCGTGTAGATCGAACTAGTGA	0	0	0	0	1	1	0	2
†	21UR-10711	TCGTACGTTGGAAGCTAAATA	0	0	0	0	8	4	1	13
	21UR-10712	TCGGGTCTCAAAAATTTTATT	0	0	0	0	0	0	0	0
	21UR-10713	TCGCACAAATTCGTGGAAGAA	1	0	0	0	0	3	0	4
†	21UR-10714	TCGATGACAATAGAAATAATT	0	0	0	0	0	0	0	0
†	21UR-10715	TCGATCTATAGATGACATTCA	0	0	0	0	1	0	0	1
†	21UR-10716	TCGACAGGTTTCTGACAAAAT	1	0	0	2	10	8	0	21
†	21UR-10717	TCCTTGCCTGTTTATTCTTTA	0	0	0	0	0	0	0	0
	21UR-10718	TCCTTCAAAGAATTTACCCGG	0	0	0	0	0	0	0	0
	21UR-10719	TCCTGTGCAATTTTGGGATTT	0	0	0	0	0	1	0	1
	21UR-10720	TCCTCGAATATTGGGCAGTCC	0	0	0	0	0	0	0	0
†	21UR-10721	TCCTAGGATCGTTGCACAAAG	2	2	2	1	14	12	5	38
	21UR-10722	TCCGTCACTCTCCACTTCTTC	0	0	0	0	0	0	0	0
†	21UR-10723	TCCGTAATGTTGTATTTTCCA	0	1	0	0	0	0	0	1
	21UR-10724	TCCGTAAAGAAGATTTTTGAC	0	0	0	0	1	0	0	1
	21UR-10725	TCCGGAGAGAACTAAGAGTAT	0	0	0	0	0	0	0	0
	21UR-10726	TCCGATGAGTCATTCTTCAGG	0	0	0	0	0	0	0	0
†	21UR-10727	TCCCATTATTATATGTAGACT	0	0	0	0	0	0	0	0
†	21UR-10728	TCCATGAGGGGGCGAATGAAT	0	0	0	0	0	0	0	0
†	21UR-10729	TCCACTCTACCTAGAGACATC	0	0	0	0	0	0	1	1
†	21UR-10730	TCCAATGTTCTTTGCTTGTTC	0	0	0	0	0	0	0	0
	21UR-10731	TCATTTCCTTATATTCAATTT	0	0	0	0	0	0	0	0
†	21UR-10732	TCATTAAGTTCGATGCTCGTT	0	0	0	0	0	0	0	0
	21UR-10733	TCATGTTTCCATTAAGATTTT	0	0	0	0	0	0	0	0
†	21UR-10734	TCATAGACAATCGTGAAGGCT	2	1	0	0	0	2	1	6
	21UR-10735	TCACTTCTCATTAGGCGGTGA	0	0	0	0	0	0	0	0
	21UR-10736	TCAATATCTCAAATGATAATT	0	0	0	0	0	0	0	0
†	21UR-10737	TCAAGAGTACGATCAGTTTTT	5	2	1	1	1	1	2	13
†	21UR-10738	TCAACATTGGAACAAAATGAA	0	0	0	0	0	1	0	1
	21UR-10739	TCAAATTGCTCCTAGAAATAA	0	0	0	0	0	0	1	1
	21UR-10740	TATTTTTTCTTGGTGTATAGG	1	0	0	0	0	1	0	2
	21UR-10741	TATTTTTCGAAAAAATTGCAT	0	3	0	0	0	0	0	3
†	21UR-10742	TATTTGGCCTAAATCGAATCA	0	0	0	0	0	0	0	0
	21UR-10743	TATTTCCTTTCTGGCCTTTCT	0	0	0	0	0	1	0	1
	21UR-10744	TATTGGCTTTTTTTCTAATAA	0	0	0	1	2	2	0	5
†	21UR-10745	TATTGGAAAAATGGGAATTAC	1	0	0	0	2	2	0	5
†	21UR-10746	TATTCTCGTCCCTCACGGATA	0	0	0	0	0	0	0	0

	21UR-10747	TATTCAAGAGTCGTTTAATAA	0	0	0	0	0	0	0	0
†	21UR-10748	TATTAAGAAAAAGGGGAATTT	0	0	0	0	0	1	0	1
†	21UR-10749	TATTAAAATTCTTCCATTGTT	0	0	0	0	0	0	0	0
†	21UR-10750	TATGTACTATAAGGGTTAGAG	1	0	0	0	0	0	0	1
	21UR-10751	TATGATTTTAGATGTTGAAAT	0	0	0	0	0	0	0	0
†	21UR-10752	TATGAAGCTTCACAAAAGATT	0	0	0	0	0	0	0	0
	21UR-10753	TATCTAGCAATGATTCTTTAC	3	1	0	0	7	0	2	13
	21UR-10754	TATCCATATAAATAAATTTCA	0	0	0	0	1	2	1	4
†	21UR-10755	TATCATTTAATAAGTTACTGA	0	0	0	0	0	0	0	0
	21UR-10756	TATCATGGCATTATTGAATCT	0	0	1	0	21	20	1	43
	21UR-10757	TATATTTTGATCATTTAGTAC	0	0	1	0	5	7	1	14
	21UR-10758	TATATTTTCAATTGTTTTCAA	0	0	0	1	0	0	0	1
	21UR-10759	TATATTTCCAATTCGTTTAAA	0	0	1	0	1	0	2	4
†	21UR-10760	TATATTAAACACATATTGAAC	1	0	0	0	0	0	0	1
	21UR-10761	TATATACAGTTGCACCTTGTT	1	0	0	0	0	4	0	5
	21UR-10762	TATAGAATGTCAAACAAAAAT	0	0	0	0	0	0	0	0
	21UR-10763	TATACTGTTTTTAGTTAGTTG	0	0	0	0	0	0	0	0
	21UR-10764	TATACATTTTTTGCAATTGCT	2	1	2	1	11	8	1	26
	21UR-10765	TATAATTTGAAAATTGAATAC	0	0	0	0	1	0	0	1
	21UR-10766	TAGTTTTTACATTTGACAGTT	0	0	0	0	2	1	0	3
	21UR-10767	TAGTTTATGTAGTCGAATTAT	0	1	2	6	75	60	31	175
†	21UR-10768	TAGTTCCTTTTTAATGGCAAG	0	0	0	0	1	0	0	1
†	21UR-10769	TAGTGCACATGTACTTTCTTG	0	0	0	0	0	0	0	0
†	21UR-10770	TAGTGATAGTGATAGAACATA	0	0	0	0	1	0	0	1
	21UR-10771	TAGTGAAGTTTGAATCGGCCT	0	0	0	0	0	0	0	0
	21UR-10772	TAGCAACTCATTAACAACTTA	0	0	0	0	0	0	0	0
†	21UR-10773	TAGATCGGTATATATATAGGT	3	4	0	6	65	30	7	115
†	21UR-10774	TAGATCATGTAGAGAAAATTT	0	0	0	1	15	12	0	28
†	21UR-10775	TAGACGGTTTTCGTTGTGAAA	0	0	0	0	0	0	0	0
†	21UR-10776	TAGACATATTAGGTCACCAAA	0	0	0	0	0	0	0	0
†	21UR-10777	TAGAACTTTTTGGTTAATTGT	0	0	0	0	0	0	0	0
	21UR-10778	TACTGTTACCCAACGTTTTTA	0	0	0	0	1	0	0	1
†	21UR-10779	TACTGTCTCTCAGTTTTGAAT	0	0	0	0	0	0	0	0
†	21UR-10780	TACTGCAGTCGTTGAATGGTG	0	0	0	0	1	10	1	12
†	21UR-10781	TACTCTATACAAAAAAAAAAC	0	0	0	0	0	0	0	0
	21UR-10782	TACTATACTGGAACTCATAAT	0	0	0	0	0	0	0	0
	21UR-10783	TACTACGTGGGGTCTCGTTTT	0	0	0	0	0	0	2	2
	21UR-10784	TACGTTTGTATTATACAATTT	0	0	0	0	1	1	0	2
	21UR-10785	TACGATAATAAAACTCACCAA	0	0	0	0	0	0	0	0
	21UR-10786	TACGAAATATTGAAACTGATC	0	0	0	0	0	0	0	0
	21UR-10787	TACGAAAGAGGACGCTAGCCA	2	0	0	0	0	5	0	7
	21UR-10788	TACCAATCAAACTTTATTGAA	0	0	0	0	0	0	0	0
	21UR-10789	TACAGTAGTATGATATATGTG	0	0	0	0	0	2	0	2
	21UR-10790	TAATTTATTGAAAAAAACTTT	0	0	1	0	0	1	0	2
†	21UR-10791	TAATTGTCTCTGTGTGGGTAT	16	9	10	9	41	85	11	181
†	21UR-10792	TAATTCTACATTACCGGTAAT	0	0	0	0	1	0	2	3
	21UR-10793	TAATTCATCGAGACAAGAAAA	0	0	0	0	0	0	0	0
	21UR-10794	TAATTAATGACGTGCTCCTTA	1	0	0	0	0	1	1	3
†	21UR-10795	TAATTAACTCAGTCAAAGCGA	0	0	0	0	0	0	1	1
	21UR-10796	TAATGTAAAAGGAAGGTAAAA	1	0	0	0	5	2	0	8
	21UR-10797	TAATATTAAACCATTTTCAAA	0	0	0	0	1	0	0	1
	21UR-10798	TAATATCTGTTTTCTAGTTTT	0	1	0	0	0	1	0	2
†	21UR-10799	TAATATCAAAAATAATGATAT	0	1	0	0	2	1	0	4
†	21UR-10800	TAATAGTGTAGAAAAGTGGTT	2	2	1	0	3	13	0	21
†	21UR-10801	TAATACTCTTGTTTTATTCAG	0	0	0	0	2	0	0	2
†	21UR-10802	TAATACATCACAAACAACTGT	0	0	0	0	0	0	0	0
	21UR-10803	TAATACAGATACACTCATCGC	0	0	0	0	0	0	0	0
	21UR-10804	TAATAATGTGAAACAGGCATT	0	0	0	0	0	0	0	0
	21UR-10805	TAATAATACGATAATCAATGA	0	0	0	0	0	0	0	0
	21UR-10806	TAATAAGTTTTGGCAGCTTTT	0	0	1	1	5	8	1	16
†	21UR-10807	TAATAAACGACTTGTATGAAC	0	0	0	0	11	8	1	20
	21UR-10808	TAAGATTTTTAAGAAAAAAAA	0	0	0	0	0	0	0	0
	21UR-10809	TAACTTGCAGAAGTTTTCACA	0	0	0	0	1	2	0	3
	21UR-10810	TAAATTTATCACTATTTTTAA	0	0	0	0	0	1	0	1

	21UR-10811	TAAATGTTTTTTTTTAGGTTT	0	0	0	0	0	0	0	0
†	21UR-10812	TAAATGATTGATTGCTAGATT	0	0	0	0	0	0	0	0
	21UR-10813	TAAATATAAAAAAACGGAATA	1	2	0	1	13	13	1	31
	21UR-10814	TAAATAATAACAAATATTATT	1	0	0	0	0	0	0	1
	21UR-10815	TAAAGTTTCTTTACAAATATC	0	0	0	0	0	0	0	0
	21UR-10816	TAAAGTATAGATCTATAGACT	0	0	0	0	0	0	0	0
	21UR-10817	TAAACTGTCAATTATATCTTC	0	0	0	0	0	0	0	0
	21UR-10818	TAAACAGCATCTTCTTTTTTA	0	0	0	0	0	0	0	0
* †	21UR-10819	TAAACAAGGGACTGTTAGGGA	8	2	0	1	17	12	2	42
	21UR-10820	TAAAATTTTGTGTGATATGGA	0	0	0	0	0	0	0	0
	21UR-10821	TAAAAGATTTAGGGGCCAAAA	0	0	0	0	0	0	1	1
	21UR-10822	TAAAACGTGCCCTTTCCATTT	0	0	0	0	0	0	0	0
	21UR-10823	TAAAAAAACATTTTTTTAAAA	0	0	0	0	0	0	0	0
	21UR-10824	GTTTTGAATAAAGTTTGCTGA	0	0	0	0	0	0	0	0
	21UR-10825	GGTCGGAGTAAAAATTCAATT	0	0	0	0	0	0	0	0
†	21UR-10826	GGCAGAGCAATCAAATGTTTG	0	0	0	0	0	0	0	0
*	21UR-10827	GAAGTGGAAGAGTAATGAAGA	1	0	0	1	0	0	0	2
†	21UR-10828	CTAGTGATTGAGTAGTAATGC	0	0	0	0	0	0	0	0
	21UR-10829	CGATCTGCTTACAATCAGAAA	0	0	0	0	0	0	0	0
	21UR-10830	CGATAATTGAATACTGAATAT	0	0	0	0	0	0	0	0
†	21UR-10831	CCTTATAACATTGAACAATTT	0	0	0	0	0	0	0	0
†	21UR-10832	CAATAACTTGCCTTGTTCGAA	0	0	0	0	0	0	0	0
†	21UR-10833	CAACAAAAAAAATCAACAAAT	0	0	0	0	0	0	0	0
	21UR-10834	CAAATTTTGAAGAACTTTTGC	0	0	0	0	1	2	0	3
†	21UR-10835	ATTGTTATTTGATTGGCATGT	220	110	69	127	549	1152	60	2287
†	21UR-10836	AGGGCATTTGAACTTAACACT	0	0	0	0	0	0	0	0
†	21UR-10837	AGCGTATTTAAGATTGTTAAA	0	0	0	0	4	1	3	8
†	21UR-10838	AAGAGGCAAATTATATCAAAA	0	0	0	0	0	1	0	1
	21UR-10839	TTTTTTTTATCCGCTTATACG	0	0	0	0	0	0	1	1
	21UR-10840	TTTTTTTCTTGGAGGTTTTTC	0	0	0	0	0	0	0	0
	21UR-10841	TTTTTTCTAATTACCAAGTTT	0	0	0	0	0	1	0	1
	21UR-10842	TTTTTTCATATTCAGGTCGTG	0	0	0	0	0	2	2	4
	21UR-10843	TTTTTTAGCTATAATTGGTTC	0	0	0	0	0	0	0	0
†	21UR-10844	TTTTTCTTCAATATCATCTAC	0	0	0	0	0	0	0	0
	21UR-10845	TTTTTCTCATTATATGACTTT	0	0	0	0	0	0	0	0
†	21UR-10846	TTTTTCAGCCAGTTGTTGTAC	0	0	0	0	0	0	0	0
	21UR-10847	TTTTTCAAAACTTTGTTTATT	0	0	0	0	1	1	0	2
	21UR-10848	TTTTTATTGCTTCATTTTTGA	0	0	0	1	0	0	1	2
	21UR-10849	TTTTTAGTACAGTGGCAATGG	0	0	0	0	0	1	1	2
	21UR-10850	TTTTGTCCTTTTTTTTGGGAAT	0	0	0	1	0	2	0	3
	21UR-10851	TTTTGTATTCGTATTCATTGT	1	0	0	0	1	1	1	4
†	21UR-10852	TTTTGTAGTTGGCTTACAATT	1	1	0	0	0	1	0	3
†	21UR-10853	TTTTGATTGGTTTTTTAAGTA	0	0	0	0	1	0	0	1
	21UR-10854	TTTTCTGGTTATAACCCACCG	0	0	0	0	0	0	0	0
†	21UR-10855	TTTTCTCTCATTGATTCATTT	0	0	0	0	0	0	0	0
†	21UR-10856	TTTTCTCAATCGTCAGTATTT	10	1	3	5	1	9	2	31
	21UR-10857	TTTTCCTTCCAAAATTAGAGA	0	0	0	0	0	0	0	0
	21UR-10858	TTTTCCAGGGTCTCTCGTTCT	0	0	0	0	0	0	0	0
†	21UR-10859	TTTTATTTTCGTTTCTGTCCA	0	0	0	0	0	0	0	0
	21UR-10860	TTTTAGTTTTGTATCGATGTT	2	0	0	3	7	4	9	25
	21UR-10861	TTTTAGCTCTTTAATGTTATT	0	0	0	0	0	0	0	0
	21UR-10862	TTTTACTCAAGGAAAATGTTC	0	0	0	0	0	0	0	0
†	21UR-10863	TTTTACATCAAGAGTTAAGAA	0	0	0	0	0	0	1	1
	21UR-10864	TTTTACAATTAGATTGCTCAA	0	0	0	0	0	0	0	0
†	21UR-10865	TTTTAAGGCACGTAAAGAAAT	0	0	0	0	2	2	2	6
	21UR-10866	TTTTAAAATAAAAAAATTAAA	0	0	0	0	0	0	0	0
	21UR-10867	TTTGTTTGTATAATCAAATCC	0	0	0	0	0	0	0	0
†	21UR-10868	TTTGTTCAGATACTGGTCCTT	0	1	0	0	1	1	0	3
†	21UR-10869	TTTGTTAGGTTGGAATATAAT	0	0	0	0	0	0	0	0
†	21UR-10870	TTTGTGTGTTTTTGAAGAATA	0	0	0	0	0	0	0	0
	21UR-10871	TTTGTGATCAATCATCTCATT	0	0	0	0	0	0	0	0
	21UR-10872	TTTGTGACAAGTGATACATCA	0	0	0	0	0	0	0	0
†	21UR-10873	TTTGTCATTTAATTTGAACTA	0	0	0	0	1	0	0	1
	21UR-10874	TTTGGTAACTGATTATTACCA	0	0	0	0	0	0	1	1

†	21UR-10875	TTTGGGATTTCGCGAAAGCAT	0	0	0	0	0	0	1	1
	21UR-10876	TTTGGGAACTGAATAATGAAT	0	0	0	0	0	0	0	0
	21UR-10877	TTTGGCAGAATGGTCCGAAAA	0	0	1	0	0	0	0	1
†	21UR-10878	TTTGGAGCATGATCAAATAGA	0	0	0	0	0	0	0	0
†	21UR-10879	TTTGCTTTCATTTTTCTGAAA	0	0	0	0	0	0	0	0
	21UR-10880	TTTGCTTTATTTTCCACAATT	0	0	0	0	0	0	0	0
†	21UR-10881	TTTGATTTTGGGCTGATAACA	0	0	0	0	0	1	0	1
	21UR-10882	TTTGATCACTCACCATATGCA	0	0	0	0	0	0	0	0
	21UR-10883	TTTGACCAGCGTTAAAATTTA	0	0	0	0	0	0	0	0
†	21UR-10884	TTTGAAATTTATCGCCTGAAA	0	0	0	0	0	0	0	0
	21UR-10885	TTTGAAAATAATTAGTTTATT	0	0	0	0	0	0	0	0
†	21UR-10886	TTTGAAAAGAAATTCACAAAT	0	0	0	0	0	0	0	0
†	21UR-10887	TTTCTTCTTTGATTCGTTTTT	2	0	0	0	2	1	2	7
†	21UR-10888	TTTCTGGCAACACACAAAAAA	0	0	0	0	0	0	0	0
	21UR-10889	TTTCGTTATCATTATCTTCAT	1	0	0	0	1	1	1	4
	21UR-10890	TTTCGTGAAATTTCAAAGTTT	0	0	0	0	0	0	0	0
	21UR-10891	TTTCGTAGAAAGATTTTAGTT	1	0	0	1	17	11	4	34
†	21UR-10892	TTTCGCCCTGATGAACAAAAA	0	0	0	0	0	0	0	0
†	21UR-10893	TTTCGACTGCAGTTTACTCGC	0	0	0	0	0	0	0	0
†	21UR-10894	TTTCGACAGTACTCAAAATAA	0	0	0	0	2	0	0	2
*	21UR-10895	TTTCATTTTCGGATTTGCGTC	0	0	0	0	1	6	25	32
†	21UR-10896	TTTCATGAGTTTCTGGATTGA	0	0	0	0	0	3	0	3
†	21UR-10897	TTTCATCAGATGGTTCTAGAA	0	0	0	0	0	2	0	2
	21UR-10898	TTTCATATCTGTATGATAATC	0	0	0	0	0	0	0	0
†	21UR-10899	TTTCAGATCCTTGTACTACAT	1	0	0	0	3	3	4	11
†	21UR-10900	TTTCACCGTTTAAGCAGTAAC	0	0	0	0	0	0	0	0
	21UR-10901	TTTCACCAATTCCACAAAAGT	0	0	0	0	0	0	0	0
	21UR-10902	TTTCAATTTCACAAGTTAAGT	0	0	0	0	0	0	0	0
†	21UR-10903	TTTCAATGAAGTTGAATTTAA	0	0	0	0	0	1	0	1
†	21UR-10904	TTTCAATCTAGTTGCTGTTAA	0	0	0	0	0	0	0	0
†	21UR-10905	TTTATTGAAATCTTTAAAAAA	0	0	0	0	0	0	0	0
†	21UR-10906	TTTATCCGAAATTTTCCCAGC	0	0	0	0	1	0	1	2
	21UR-10907	TTTATACCATTCTTTCAGATT	0	0	1	0	0	0	0	1
	21UR-10908	TTTAGAATATTTCGATTTTAA	0	0	0	0	1	0	0	1
	21UR-10909	TTTAGAAAAATGGTATTTTTT	0	0	0	0	0	0	0	0
†	21UR-10910	TTTACTTCGTCTTGGAAAAAA	0	0	0	0	0	0	0	0
†	21UR-10911	TTTACGCGCTAATCTGAGCAG	0	0	0	0	1	2	0	3
†	21UR-10912	TTTAATGACCTTTTCCAAAAA	0	0	0	0	0	0	0	0
†	21UR-10913	TTTAATCGAAGTTCGTGGTAT	2	0	1	0	1	3	0	7
	21UR-10914	TTTAATAAAATTTGGAGCAGC	0	0	0	0	0	2	0	2
	21UR-10915	TTTAAGAATCTTTTTTATATT	0	0	0	0	0	0	0	0
†	21UR-10916	TTGTTTTTGGTGCTTATGGGT	0	0	0	0	0	0	0	0
†	21UR-10917	TTGTTTTTAACAGTTTTTAAC	0	0	0	0	0	0	0	0
	21UR-10918	TTGTTTTTAAATTTCCTATTA	0	0	0	0	0	0	0	0
†	21UR-10919	TTGTTGCACTTGTCATCAACA	0	0	0	0	0	0	0	0
†	21UR-10920	TTGTTCGTGTTTACCAACCAG	0	0	0	0	0	0	0	0
†	21UR-10921	TTGTTATTACGCATTGTGATT	0	0	0	0	0	0	0	0
†	21UR-10922	TTGTGGCACTAGGATCTTGAA	0	0	0	0	0	0	0	0
	21UR-10923	TTGTGAATTGACACTTGTGTG	0	0	0	0	0	0	0	0
†	21UR-10924	TTGTGAAGTTGGAATTTAATT	0	2	1	0	2	4	0	9
†	21UR-10925	TTGTGAACTGCAATTGATGTT	13	36	14	43	805	713	84	1708
†	21UR-10926	TTGTGAACAGATAGTCAACAT	0	0	0	0	0	0	0	0
	21UR-10927	TTGTCGTCTCGATGCTGTAGC	0	0	0	0	0	0	0	0
†	21UR-10928	TTGTCCTAAGAATGCCATTTC	0	0	0	0	0	0	0	0
†	21UR-10929	TTGTCAAATAAACAGTCGAAA	0	0	0	0	0	0	0	0
†	21UR-10930	TTGTAATGTAACTGTATGCCT	0	0	0	0	0	2	0	2
	21UR-10931	TTGTAACAGGACGTTTAGCGA	0	0	1	0	4	1	1	7
†	21UR-10932	TTGGTTTTGCCCTGATCTTGG	0	0	0	0	0	2	0	2
	21UR-10933	TTGGTCTCAATTGGCCATTGA	0	0	0	0	0	0	0	0
†	21UR-10934	TTGGTATCTTACTTATTTCGT	0	0	2	4	3	13	0	22
†	21UR-10935	TTGGGCATATCTCCGGTCACA	0	0	0	0	0	2	0	2
†	21UR-10936	TTGGGAATAATAAATAGTCTA	0	0	0	0	0	0	0	0
†	21UR-10937	TTGGCTGTACTTGTGAATTCA	0	0	0	0	0	0	0	0
†	21UR-10938	TTGGCTCAGGAAATTAGTTAT	0	0	0	0	0	1	0	1

†	21UR-10939	TTGGATTGTAAAAACAATGAT	0	0	0	0	0	0	0	0
†	21UR-10940	TTGCGTCGTTGAATCTCCTTT	0	0	0	0	0	0	0	0
†	21UR-10941	TTGCCTATTCGTCAAATTTGA	0	0	0	0	0	0	0	0
†	21UR-10942	TTGCATTTTTCCATTGCATGA	0	0	0	0	0	0	0	0
†	21UR-10943	TTGCAGGTTTGATTTTCTGTG	0	0	0	0	0	0	0	0
	21UR-10944	TTGATTTGTATTTATATGACC	0	0	0	1	10	1	1	13
†	21UR-10945	TTGATTCTGTGCTATTGATTT	0	0	0	0	0	0	0	0
†	21UR-10946	TTGATCTTTCAAACATCACAT	1	0	0	0	0	1	0	2
†	21UR-10947	TTGATCGGTTTTTCAATTCTG	0	0	0	0	0	0	0	0
†	21UR-10948	TTGATCCATAAAAATAGGATA	0	0	0	0	1	0	0	1
†	21UR-10949	TTGATCACTAGGTTGACAAAA	0	0	0	0	0	0	0	0
	21UR-10950	TTGAGTATTTTCGAAGCTTGT	0	0	0	0	0	1	0	1
†	21UR-10951	TTGAGTAGCATCCGAAGAAAA	0	0	0	0	0	0	0	0
†	21UR-10952	TTGAGACTTTCCTGACTCATT	0	0	0	0	8	2	0	10
†	21UR-10953	TTGACTCAGAAAAAATTTCAT	0	0	0	0	1	0	0	1
†	21UR-10954	TTGACTAATTTGGTACTTCAA	0	0	0	0	1	1	0	2
	21UR-10955	TTGAAATGGAAAAAGTGCAAG	0	0	0	0	0	0	0	0
†	21UR-10956	TTGAAAAATGAGGCCTCAGTG	0	0	0	0	0	0	0	0
	21UR-10957	TTCTTCGAAAACTATTTACAT	0	0	0	0	0	0	0	0
†	21UR-10958	TTCTGGACGTTTTCAGCAATT	0	0	0	0	1	0	2	3
†	21UR-10959	TTCTGAATTGTGAAAGGAAAA	0	0	0	1	0	0	0	1
	21UR-10960	TTCTGAATACCGATTTTCCTA	0	0	0	0	0	0	0	0
†	21UR-10961	TTCTCTTGCGTTTACCTGTAG	0	0	0	0	0	0	0	0
†	21UR-10962	TTCTCCTATCAACAAGCATCT	0	0	0	0	1	3	1	5
†	21UR-10963	TTCTCATTGTATTGTAAAATG	0	0	0	0	0	0	0	0
†	21UR-10964	TTCTATTTGTGATGGACTCTG	0	0	0	0	0	0	0	0
	21UR-10965	TTCTAGTTTCTCAAAATACCG	0	0	0	0	0	0	0	0
†	21UR-10966	TTCTACGGCTTGGAGAAAAAC	0	0	0	0	0	0	0	0
†	21UR-10967	TTCTACATTACCGGTAATAGA	0	0	0	0	1	1	0	2
†	21UR-10968	TTCGTGGTAGAGCTTTGAACA	0	0	0	0	0	0	0	0
†	21UR-10969	TTCGTCCTTCATGGCTGGAGT	0	0	0	0	0	0	0	0
†	21UR-10970	TTCGTATCGTTTTACTTACAA	0	0	0	0	0	1	0	1
†	21UR-10971	TTCGGTAGCTAATTTTCGTAC	0	0	1	0	0	0	0	1
†	21UR-10972	TTCGACTATTGATTTTGTTTG	0	0	0	0	1	6	0	7
†	21UR-10973	TTCGACCTATAAACCTTTGTG	0	0	0	0	0	0	0	0
	21UR-10974	TTCGACCGGAAAGATCCTCTC	0	0	0	0	0	0	0	0
†	21UR-10975	TTCCGAAAAATCGATTATGGA	0	0	0	0	2	1	0	3
†	21UR-10976	TTCCATCGATCTCTTATCTGA	0	1	0	0	0	1	0	2
	21UR-10977	TTCCATCCACTGTTATTCGAC	0	0	0	0	0	0	0	0
	21UR-10978	TTCCACATAGTATATTTAGAA	0	0	0	0	0	0	0	0
	21UR-10979	TTCCAATCGTTTACCCAAAAA	0	0	0	0	0	0	0	0
	21UR-10980	TTCATTGTTTCTTCGTTTTTG	0	0	0	0	0	0	1	1
†	21UR-10981	TTCATGTAAGCAGTGTGAAAA	0	0	0	0	0	0	0	0
†	21UR-10982	TTCATCAAAAAATGTAAAGTT	0	0	0	0	0	0	0	0
	21UR-10983	TTCATATCTCAGTTTATTTTT	0	0	0	0	0	0	1	1
†	21UR-10984	TTCATAAATCAATCCATGTAC	0	0	0	0	0	0	0	0
	21UR-10985	TTCAGTCGTAATGTTTTCTTT	0	0	0	0	1	0	0	1
†	21UR-10986	TTCAGATCAACGAAACAATAA	0	0	0	0	1	0	0	1
	21UR-10987	TTCAGACCATGACTTCAAGGG	0	0	0	0	0	0	0	0
	21UR-10988	TTCAGAACGACACAATTTTGA	0	3	1	0	0	1	0	5
	21UR-10989	TTCAGAAAATTGTGCATGGAA	0	0	0	0	0	0	0	0
	21UR-10990	TTCAATAAAGAGTTAGGAAAA	0	0	0	0	0	0	0	0
†	21UR-10991	TTCAAAAACGCCTCAAATAGT	0	0	0	0	0	0	0	0
	21UR-10992	TTATTTTTTTGGTAACAACTA	0	0	0	0	1	0	0	1
	21UR-10993	TTATTTTTTAGGTCTTCCATT	2	1	0	0	1	2	0	6
	21UR-10994	TTATTTTTTACGACAGTTTTC	0	0	0	0	1	1	0	2
†	21UR-10995	TTATTTCGGAAGTTATTGAAT	0	0	0	0	0	0	0	0
†	21UR-10996	TTATTGGATAGTCTCACGGTA	0	0	0	2	17	19	19	57
†	21UR-10997	TTATTGCTCTGGTTGTTTGGT	1	0	1	1	2	7	0	12
	21UR-10998	TTATTGAAGGGTTTATCCCAA	0	0	0	0	1	0	0	1
	21UR-10999	TTATTGAAAAAATGTTATTGG	0	0	0	0	0	0	0	0
†	21UR-11000	TTATTCTTGAACGGCTAGGAT	3	0	0	1	0	4	0	8
	21UR-11001	TTATTCTGTGTTTCTCATATG	0	0	0	0	5	5	0	10
	21UR-11002	TTATTATTTCGTCACATTACA	2	1	3	10	130	39	39	224

†	21UR-11003	TTATTACTGTTTTATTGAGTA	0	0	0	0	0	0	0	0
†	21UR-11004	TTATTAAGTTTGGTGACCGTT	0	0	0	0	1	0	0	1
†	21UR-11005	TTATTAACTGAAAATTCAAAT	0	0	0	0	2	1	0	3
†	21UR-11006	TTATTAAAACGTACGGAAAAA	2	0	0	0	1	3	1	7
	21UR-11007	TTATGCTTGTTTTGTTCGGTA	0	0	0	0	7	5	6	18
†	21UR-11008	TTATGAATACTTGCTACACTT	0	0	0	0	0	0	0	0
†	21UR-11009	TTATGAAAAAAAGGAACAGTT	0	0	0	0	0	1	0	1
	21UR-11010	TTATCTTTCTTGGAAGTTCAG	0	0	0	0	0	0	0	0
†	21UR-11011	TTATCGATGTTAGAAATTGTG	0	0	0	0	0	0	0	0
†	21UR-11012	TTATCATGTCGAGTTCGTGCA	0	0	1	0	3	0	0	4
	21UR-11013	TTATCAAGGTATTGAAATGGC	0	0	0	0	0	0	0	0
	21UR-11014	TTATAGCTGATAATCACAATG	0	0	0	0	0	1	0	1
	21UR-11015	TTATAATTTCTATACTAAACG	0	0	0	0	0	2	0	2
†	21UR-11016	TTATAAGAAATTTTATTTGCC	0	0	0	0	0	0	0	0
	21UR-11017	TTAGTTATTGATTGAAATTAT	0	0	0	0	0	0	0	0
†	21UR-11018	TTAGCTCTTGGTTTTTAGGAA	0	0	0	0	0	0	0	0
†	21UR-11019	TTAGACTTACAAAAAACTCAT	0	0	0	0	0	0	0	0
†	21UR-11020	TTAGACGTGTTGAAAAAATGC	0	0	0	0	0	1	0	1
	21UR-11021	TTAGAATAGTCTTTAAAAATT	0	0	0	0	0	0	0	0
†	21UR-11022	TTAGAAATGCGGGCGACTACA	1	0	0	0	0	2	0	3
†	21UR-11023	TTAGAAACCAGTAATAGAAAC	0	0	0	0	0	0	0	0
†	21UR-11024	TTACTTCCTTTGTGATCTCCT	1	0	0	0	0	0	0	1
†	21UR-11025	TTACTGATAGTGCGTACGGAA	0	0	0	0	0	1	1	2
	21UR-11026	TTACTCCTACTCACTGAAATT	0	0	0	0	2	1	0	3
†	21UR-11027	TTACTACGGACACCGTTCAAA	0	0	1	0	3	3	0	7
	21UR-11028	TTACTAAACTGAGAAACATTT	0	0	2	1	1	2	0	6
†	21UR-11029	TTACCAGTGGTTTTTGGGATC	0	0	0	0	0	0	0	0
	21UR-11030	TTACAACAACTTCGAAAAGTC	1	2	0	0	2	3	0	8
	21UR-11031	TTACAAAATAGAAAACCGAAT	0	0	0	0	4	1	1	6
	21UR-11032	TTAATTTTTGATGTTTTAACA	0	0	0	0	0	0	0	0
†	21UR-11033	TTAATTTGGTTGGTGTATGAA	0	0	0	0	0	3	0	3
	21UR-11034	TTAATTAGTCAATTACATTAG	0	0	0	0	1	0	1	2
†	21UR-11035	TTAATGGGTAAGGGTTCGATA	0	0	0	0	0	0	0	0
†	21UR-11036	TTAATAGTTTTTATCCGGACA	7	2	1	0	1	10	3	24
	21UR-11037	TTAATAGATTCTGTGCAATTT	0	0	0	0	0	0	0	0
†	21UR-11038	TTAATACATCCTTCTAATCCA	0	0	0	0	0	0	0	0
	21UR-11039	TTAAGATTCACACATAATCTC	7	1	0	1	1	4	0	14
†	21UR-11040	TTAACGACGTTGTATAGGAAT	1	0	2	0	1	1	0	5
†	21UR-11041	TTAACAATTGGTTTCTCCAAT	0	0	0	0	0	0	0	0
†	21UR-11042	TTAACAAGATGCTTTTTTAAA	0	0	0	0	0	0	0	0
†	21UR-11043	TTAACAAATCATCATGCTATA	1	0	0	0	0	0	0	1
†	21UR-11044	TTAAATGGATTCTAAAAAAAT	0	0	0	0	0	0	0	0
†	21UR-11045	TTAAATAAATGCTGAACCCCA	0	0	0	0	0	0	0	0
†	21UR-11046	TTAAAATTCAACAATTGGATC	0	0	0	0	0	0	0	0
†	21UR-11047	TTAAAACGTACGGAAAAAATT	2	0	0	0	1	2	0	5
	21UR-11048	TTAAAAACAACTGAATGCAAA	0	0	0	0	0	0	0	0
	21UR-11049	TTAAAAAACTTTTTTTAAAAC	0	0	0	0	0	0	0	0
	21UR-11050	TGTTTTCTTTACAAAAATATT	0	0	0	0	0	0	0	0
†	21UR-11051	TGTTTTATCATCCCCACAGAT	0	0	0	0	0	0	0	0
	21UR-11052	TGTTTCTCAGAAAAATTGGTT	0	0	0	0	0	0	0	0
†	21UR-11053	TGTTTCGAGTAGCATTTGAGT	0	0	0	0	0	2	0	2
	21UR-11054	TGTTTATCTTCAACTATCTTT	0	0	0	0	0	0	0	0
	21UR-11055	TGTTTAACAATCAGGACTATT	0	0	0	0	0	0	0	0
*	21UR-11056	TGTTGTGACGAATAACGGCAA	1	2	0	0	1	6	3	13
	21UR-11057	TGTTGAAAACATTTTGTGAAT	0	0	0	0	1	0	0	1
†	21UR-11058	TGTTCTTTCTCTTCAAATTAA	0	0	0	0	0	0	0	0
	21UR-11059	TGTTCAATTATGGATTGGAGT	0	0	0	0	0	0	0	0
†	21UR-11060	TGTTAGAACTTTTTGGTTAAT	0	0	0	0	1	1	0	2
†	21UR-11061	TGTTAAAACGTCAAAATCATT	0	0	0	0	0	0	0	0
	21UR-11062	TGTGTTCATAACTATGACTGA	0	0	0	0	0	0	0	0
	21UR-11063	TGTGTGCACTTCTCAAAAGAA	0	0	0	0	0	0	0	0
	21UR-11064	TGTGTATCATATTATATTTAG	0	0	0	0	0	1	0	1
†	21UR-11065	TGTGCATCGATTTGTCATACC	0	0	0	0	0	1	0	1
†	21UR-11066	TGTGCAGGTCGCTTATGAAGA	0	0	0	0	0	1	1	2

	21UR-11067	TGTGAAGACTACTGGAAATTA	0	0	0	0	0	0	0	0
	21UR-11068	TGTGAACTCAATAAATTTTTT	0	0	0	0	0	0	0	0
	21UR-11069	TGTGAAAGAATGTGATAAGGT	0	0	0	0	0	0	0	0
	21UR-11070	TGTCTATCAAATTTCAAAATT	0	0	0	0	0	0	0	0
†	21UR-11071	TGTCTACCTTCTGAACCGGTA	1	0	0	0	0	1	1	3
†	21UR-11072	TGTCGTAATCGCATATTTATT	2	0	0	0	1	2	0	5
	21UR-11073	TGTCGAATATAAAAGTTGGCT	0	0	0	0	1	7	1	9
	21UR-11074	TGTCATAGACAAAAAAGACAA	0	0	0	0	0	0	0	0
†	21UR-11075	TGTATTCATTTGGCGAGGATG	0	0	0	0	0	0	0	0
	21UR-11076	TGTATATAAAGGAATTCTGCC	0	0	0	0	0	0	0	0
	21UR-11077	TGTAGACTGTAAACTTACTTA	0	0	0	0	0	0	0	0
	21UR-11078	TGTACTATGTATTCATTATGT	0	0	0	0	0	0	0	0
	21UR-11079	TGGTTTCTGGATCAATGAGAT	0	0	0	0	0	0	0	0
†	21UR-11080	TGGTTGCCAGTGAAATTTTGA	0	0	0	1	4	0	1	6
†	21UR-11081	TGGTTAGAATTTTTTAAAAAT	0	0	0	0	1	0	0	1
	21UR-11082	TGGGTTTTTACAATTCTTCTT	0	0	0	0	0	0	0	0
	21UR-11083	TGGGCTTCTCGATTTTAGCGT	0	0	0	0	0	1	0	1
	21UR-11084	TGGGCAAATGATTTGAATGTT	0	0	0	0	0	0	0	0
	21UR-11085	TGGGAACCTCGATATTTGAAG	0	0	0	0	0	0	0	0
†	21UR-11086	TGGCTCAAAGAAATTTTAACA	0	0	0	0	0	0	0	0
†	21UR-11087	TGGACTAGAACTTTGAATGCT	0	0	0	0	2	18	0	20
	21UR-11088	TGGAAGGACCAAACTCCAAAC	0	0	0	0	0	0	0	0
	21UR-11089	TGCTTTTGAAAAGTTTTTAAT	0	0	0	0	0	0	0	0
	21UR-11090	TGCTAGGTTCGATTTGTTCTA	0	0	0	0	0	0	0	0
†	21UR-11091	TGCTAGGGTAGGGTTTCGCAT	0	0	0	0	0	0	0	0
	21UR-11092	TGCTAGAGTTTGAATTTCGGG	0	0	0	0	0	0	0	0
†	21UR-11093	TGCTACTTCTTTATGTCCAAA	0	0	0	0	0	0	0	0
†	21UR-11094	TGCTACCCATTTCTCGTAACA	1	0	0	0	0	0	0	1
	21UR-11095	TGCGCCACTGAAAAGTTGTCG	0	0	0	0	0	0	0	0
	21UR-11096	TGCGCCAAAATCCAAAAAAAA	0	0	0	0	0	0	0	0
†	21UR-11097	TGCATCGATAATTGTCAAAAA	0	0	0	0	0	0	0	0
	21UR-11098	TGCAGTTTCAATTGAAAAATT	0	0	0	0	0	0	0	0
†	21UR-11099	TGCAGCCGATTCAAAACAGGA	1	0	0	0	0	0	0	1
	21UR-11100	TGCAGAATATTATTTATATAA	0	0	0	0	0	0	0	0
	21UR-11101	TGCAATGGTTCTTCTTCTCTT	0	0	0	0	0	0	0	0
	21UR-11102	TGCAACCTTCATAGTTGCAAA	0	0	0	0	0	0	0	0
	21UR-11103	TGCAAAAAACTGAATTTCCCC	0	0	0	0	0	0	0	0
	21UR-11104	TGATTTACTGACTTATAAGTT	0	0	0	0	0	0	0	0
	21UR-11105	TGATTGCGAAGATCTTCTCGG	0	0	0	0	0	0	0	0
†	21UR-11106	TGATTCACAATTTTTTAAACG	0	0	0	0	0	0	0	0
†	21UR-11107	TGATGTGACGAAAACTGACGA	0	0	0	0	2	1	0	3
†	21UR-11108	TGATCAATCAAACCCATTAAA	0	0	0	0	0	0	0	0
†	21UR-11109	TGATAGCTCTTCCGAGCGAGT	0	0	0	0	0	0	0	0
†	21UR-11110	TGATAGAATTGATGAATGGGC	0	0	0	0	4	16	4	24
†	21UR-11111	TGATACTGTCTCTCAGTTTTG	0	0	0	0	0	0	0	0
	21UR-11112	TGAGTTCCAAAATCTTCTTTT	0	0	0	0	0	0	0	0
†	21UR-11113	TGAGCGTTAAATATGTATATC	0	0	0	0	0	0	0	0
	21UR-11114	TGAGCGGCAAACGATTGTTTC	0	0	0	0	0	0	0	0
	21UR-11115	TGAGATCCAAAATCCAAAAAT	0	0	0	0	0	0	0	0
†	21UR-11116	TGACTTCATAGTGATTTTTTT	0	0	0	0	0	0	0	0
	21UR-11117	TGACCGATTTTCAGAGCAAAA	0	0	0	0	0	0	0	0
	21UR-11118	TGAATTCGTTAACTTTTTTTC	0	0	0	0	0	0	1	1
†	21UR-11119	TGAAGTGATCCAGCAACTACC	0	0	0	0	0	0	0	0
	21UR-11120	TGAACTTATATAAAATATAAA	0	0	0	0	0	0	0	0
†	21UR-11121	TGAACAATTGAAAGTAATTGA	0	0	0	0	0	0	0	0
†	21UR-11122	TGAAACGGTATGAAATTGTCA	0	0	0	0	0	2	0	2
	21UR-11123	TGAAACATTTTATTTGAGATT	0	0	0	0	0	0	0	0
†	21UR-11124	TGAAAAAATGTACTGTCAGAG	0	0	0	1	3	1	1	6
	21UR-11125	TCTTTTTTGAGACTTAACTGA	0	0	0	0	0	0	0	0
	21UR-11126	TCTTTTGAATTTTCGTAATTT	0	0	0	0	0	0	0	0
	21UR-11127	TCTTTTCACCACATTTAATTG	0	0	0	0	0	0	1	1
	21UR-11128	TCTTGTCAAAATTAGGAATAT	0	0	0	0	0	0	0	0
	21UR-11129	TCTTGGCTCAAAATTGTTATG	0	0	0	0	0	0	0	0
	21UR-11130	TCTTGGAAAGTTGCCCAGATA	0	0	0	0	0	0	0	0

	†	21UR-11131	TCTTGCCATTAACTTTCGAAT	0	0	0	0	0	0	0	0
		21UR-11132	TCTTCTTTTTATTAATTTTGA	0	0	0	0	0	0	0	0
		21UR-11133	TCTTCTTGTTGAAACGTATTC	0	1	1	1	2	5	0	10
		21UR-11134	TCTTCTTCGTGAGAACGACGG	0	0	0	0	0	0	0	0
		21UR-11135	TCTTCCAGCATTATATTTTGT	0	0	0	0	0	0	1	1
	†	21UR-11136	TCTTCAGCATATGGATCAAAA	0	0	0	0	0	0	0	0
		21UR-11137	TCTTATCAGAGCAGTCACGTG	1	0	0	0	0	0	0	1
		21UR-11138	TCTGTCTTTAGTCCAAAATGC	0	0	0	0	0	0	0	0
	†	21UR-11139	TCTGAGTCGAAAAAAAAGCGA	0	0	0	0	0	0	0	0
	†	21UR-11140	TCTCTTCTAGTTAGTGTGTGA	0	0	0	0	0	0	0	0
		21UR-11141	TCTACCAGCAATGTATTTTCA	0	0	0	0	0	0	0	0
		21UR-11142	TCTACAAATTATACCGAAAAA	0	0	0	0	0	0	0	0
	†	21UR-11143	TCGTTTTAGCTACCACCGGAA	0	0	0	0	0	0	0	0
	†	21UR-11144	TCGTTTGTCGTCAGCGAAAGG	0	0	0	0	0	0	0	0
		21UR-11145	TCGGTTTCAATAAATTGTTAA	0	0	0	0	0	0	0	0
		21UR-11146	TCGATTAGTAGACAAAACTAG	0	0	0	0	0	0	0	0
	†	21UR-11147	TCGATATGCTATTTTTCCCGA	0	0	0	0	0	0	0	0
	†	21UR-11148	TCGATAGGTTGTTTGATGAGG	0	0	0	0	0	0	0	0
	†	21UR-11149	TCGATACAGATTCTGACAAAA	0	0	0	0	0	0	0	0
	†	21UR-11150	TCGAGACATCGTGGAAAAATA	0	0	0	0	0	1	0	1
		21UR-11151	TCCTTTTCCTTTTACATAAAT	0	0	0	0	0	0	0	0
	†	21UR-11152	TCCTTCTATTTCACTTGAGAT	0	0	0	0	0	0	0	0
		21UR-11153	TCCTATGATAAATGACTGAAA	0	0	0	0	0	0	0	0
	†	21UR-11154	TCCCTTGCCTGTATTCTTTAA	0	0	0	0	0	0	0	0
	†	21UR-11155	TCCATAACATTCTTCTTTCCA	0	0	0	0	0	0	0	0
		21UR-11156	TCCAGTTTTTTGAAAATGCAT	1	0	0	0	0	0	0	1
	†	21UR-11157	TCCAGAGTTCCAACCTCATCC	0	0	0	0	0	1	0	1
		21UR-11158	TCCAAATGATGGACAGTATAA	0	0	0	0	0	0	0	0
	†	21UR-11159	TCATTCATGTAACGGTTCATG	0	0	0	0	0	0	0	0
	†	21UR-11160	TCATATATGATCAAAAAAAAT	0	0	0	0	0	0	0	0
		21UR-11161	TCATAAAGGAAATTCTGAAAC	0	0	0	0	0	0	0	0
	†	21UR-11162	TCAGTAGGGTACCTCAAATAT	0	0	0	0	0	0	0	0
		21UR-11163	TCAGGGTCGACTACAAACCTT	0	0	0	0	0	0	0	0
	†	21UR-11164	TCAGGATACGAATATACGGAT	0	0	0	0	5	8	0	13
		21UR-11165	TCACTAAGAAATTCTGAGTCA	0	0	0	0	0	0	0	0
	†	21UR-11166	TCACGTAAATTGATAGTCACG	0	0	0	0	0	1	0	1
		21UR-11167	TCACGAAACAAGATTTAGGTA	0	0	0	0	0	1	0	1
	†	21UR-11168	TCAATTAGTAAGCGGTCTAAG	0	0	0	0	0	0	0	0
	†	21UR-11169	TCAATGGATAGATCCCGCTGT	0	0	0	0	0	0	0	0
		21UR-11170	TCAATGCCATGCGTCAGTACA	0	0	0	0	0	0	0	0
	†	21UR-11171	TCAACGAGTACATTTGATATC	0	0	0	0	1	1	0	2
		21UR-11172	TCAACACCGATTTAAAAATCT	0	0	0	0	0	0	0	0
		21UR-11173	TCAAATGATTCCCTTTCAAAT	0	0	0	0	0	0	1	1
		21UR-11174	TCAAATAAGCTGGTGATCAGG	0	0	0	0	0	0	0	0
		21UR-11175	TATTTGATTTGCATTTTTTTT	0	3	2	3	19	11	3	41
	†	21UR-11176	TATTTCTTTTCAACCATTCAA	0	0	0	0	0	0	0	0
		21UR-11177	TATTTCATAGTCAGAAAAATT	0	0	0	0	2	1	0	3
		21UR-11178	TATTTAGATGTTGATAATAAA	0	0	0	0	1	0	0	1
	†	21UR-11179	TATTTAATCGATATCCATTTT	0	0	0	0	0	0	0	0
*		21UR-11180	TATTGTATTGTAAAGGTTTGA	2	1	0	7	46	34	3	93
	†	21UR-11181	TATTGGGTGAAGTTTGGTCTT	2	0	0	0	2	2	1	7
	†	21UR-11182	TATTCTTGTCTTAAACGTTCT	0	0	0	0	0	0	0	0
	†	21UR-11183	TATTCTTCTTCATGTAATTCG	0	0	0	0	0	0	0	0
	†	21UR-11184	TATTCCCCTTATTTTTAATTC	0	0	0	0	0	2	0	2
	†	21UR-11185	TATTCCAATATTGTAGTAAAG	0	0	0	0	0	1	0	1
		21UR-11186	TATTCAAGTTTATTTCATTTT	0	0	0	0	1	0	0	1
	†	21UR-11187	TATTATTCGAATCGAAAGGAG	0	0	0	0	0	0	0	0
*	†	21UR-11188	TATTATTCAAGTAGATAATTT	1	0	1	2	26	11	0	41
	†	21UR-11189	TATTATGAGAAAACAAATAAT	0	0	0	0	0	0	0	0
	†	21UR-11190	TATTATCGTTGCAAATACGAG	0	0	0	0	0	0	0	0
	†	21UR-11191	TATTACGATTTGTCCATTTTT	0	0	0	0	0	0	0	0
	†	21UR-11192	TATTAACCTATCCAAAAAAGT	1	0	0	0	0	0	0	1
	†	21UR-11193	TATGCTGTTTGGCTCAGGAGA	0	0	0	0	0	0	0	0
	†	21UR-11194	TATGCCTATTTCGCCATTGGT	0	0	0	0	0	1	0	1

†	21UR-11195	TATGAAGCTTCTAACTGATAG	0	0	0	0	0	0	0	0
	21UR-11196	TATGAAATGAATATCTAGTAA	0	0	0	0	2	1	0	3
	21UR-11197	TATCTTTTTTTGAATTGTGAG	0	0	0	0	0	0	0	0
	21UR-11198	TATCCGGGAAAATACTGAAAA	0	0	0	0	2	1	0	3
	21UR-11199	TATCACAACTTCAATTGTTAA	0	0	0	0	0	0	0	0
	21UR-11200	TATCAACAGCGTGAGTGCAAG	0	0	0	0	0	0	0	0
	21UR-11201	TATATGTGTGGTTTTCTTATT	0	0	1	1	5	2	0	9
	21UR-11202	TATAGAATTTTGATTGTCATT	0	0	0	2	1	2	0	5
†	21UR-11203	TATACTTCTCCATTCTGATCA	0	0	0	1	1	3	0	5
	21UR-11204	TATACTTCATTTTTTGGATTT	3	0	0	0	0	3	0	6
	21UR-11205	TATAATGATTGTTAGCAATTG	0	0	1	1	3	4	5	14
†	21UR-11206	TATAATAAAATTGTCGTAAAT	18	0	7	1	9	19	7	61
†	21UR-11207	TATAAGAAATTAAAAAAAAAA	0	0	0	0	0	0	0	0
	21UR-11208	TATAACTGATGATGGTAAGTT	0	0	0	0	0	0	0	0
	21UR-11209	TAGTTTTTGAAAGAAATGCTG	0	0	0	0	0	0	0	0
†	21UR-11210	TAGTTCTCTCTCCGTTGTTAC	0	0	0	0	0	0	0	0
†	21UR-11211	TAGTTCTACTCCATTCTTGAT	0	0	0	0	0	0	0	0
†	21UR-11212	TAGTGCAAATCAGAATATTAG	0	0	0	0	0	1	0	1
	21UR-11213	TAGTGAATCACTCAGTTTTTT	0	0	0	0	0	0	0	0
	21UR-11214	TAGGGTTAGTTTTCCACATTT	0	0	0	0	0	0	0	0
	21UR-11215	TAGCAAGTTTACAAATTTTTC	2	0	0	0	1	3	1	7
†	21UR-11216	TAGCAACTTTTCATCATGAAA	0	0	0	0	0	0	0	0
	21UR-11217	TAGATCTACATATCATATACG	0	0	0	0	0	0	0	0
	21UR-11218	TAGATAGAGGTGAGAATTAAA	0	0	0	0	0	0	0	0
	21UR-11219	TAGAGCGTAATTTTCAAACAG	0	0	0	0	2	0	0	2
	21UR-11220	TAGACCAATGACCAAAAATTG	0	0	0	0	0	0	0	0
†	21UR-11221	TAGAAGTAGATTTTACAGCTT	0	0	0	0	2	4	0	6
	21UR-11222	TAGAACATTTTTTTAAAATAT	0	0	0	0	1	0	1	2
†	21UR-11223	TACTTGAAGAAAACAAGATTT	0	0	0	0	0	0	0	0
†	21UR-11224	TACTGGACCAAGGATAATGTC	0	0	0	0	0	1	1	2
†	21UR-11225	TACTGCATTCCGAATAAGCAG	0	0	0	0	0	0	0	0
†	21UR-11226	TACTCACTCCATACTACTAGA	0	0	0	0	0	0	0	0
†	21UR-11227	TACGCCCTGTAGTCTTCAATA	0	0	0	0	0	0	0	0
	21UR-11228	TACGATACCAGCAAATGCGTT	1	0	0	0	0	0	0	1
	21UR-11229	TACCTTGACAAGAGCCATGAC	0	0	0	0	0	0	0	0
	21UR-11230	TACCTTCAAAACTTCCCCTCT	0	0	0	0	0	0	0	0
	21UR-11231	TACCAAAATATCCTGAAAAAA	0	0	0	0	1	0	0	1
†	21UR-11232	TACCAAAAATGGACCAAAAAT	0	0	0	0	0	0	0	0
	21UR-11233	TACATGGTTTCTCCTTTATTT	1	1	0	0	0	2	0	4
†	21UR-11234	TACATCTTCTATTTTAATTGC	1	0	0	1	7	1	5	15
	21UR-11235	TACATCCTTTTTATGTCTATA	0	0	0	0	0	0	0	0
	21UR-11236	TACAGTTTTTCCATATTCTTT	1	0	0	1	0	2	1	5
	21UR-11237	TACACACTTGGCAGCGCAAAA	0	0	0	0	0	2	0	2
	21UR-11238	TACAAAAATATCCTGAAAAAA	0	0	0	0	0	2	0	2
	21UR-11239	TAATTTTTTTCACTGTAACGT	0	0	0	0	0	0	0	0
†	21UR-11240	TAATTTTACGAAACGGTGTGA	1	1	0	0	9	26	2	39
†	21UR-11241	TAATTTGGGTTTCTTTAAATA	0	0	0	0	0	0	0	0
	21UR-11242	TAATTTCTTCAAAAAAAAATA	0	0	0	0	1	1	0	2
	21UR-11243	TAATTTCTAAAAAATAATCTA	0	0	0	0	0	0	0	0
	21UR-11244	TAATTGTGTTTTCTAGCAAAA	0	0	0	0	1	1	0	2
†	21UR-11245	TAATTCTGTCGCGCTTTTGAC	0	0	0	0	0	0	0	0
	21UR-11246	TAATTATTTTTTTAGTAAAAA	0	0	0	0	0	0	0	0
†	21UR-11247	TAATGATCCACCTGTTCTCTC	0	0	0	0	0	0	0	0
†	21UR-11248	TAATGAGCGTTAAATATGTAT	2	1	0	0	7	14	8	32
†	21UR-11249	TAATGAGAGCTTTTGAAATAG	0	0	0	0	0	0	0	0
	21UR-11250	TAATCTTAACATTTGATAAAT	0	0	0	0	3	0	1	4
	21UR-11251	TAATCAAAAGTCATTTTGCAT	0	0	0	0	0	1	0	1
†	21UR-11252	TAATATCATTGGTACACTAAT	0	0	1	0	1	2	1	5
†	21UR-11253	TAATACATCTTTTCTGTTTGG	0	0	0	0	0	0	0	0
	21UR-11254	TAATAATTTGGTCGTTGACTT	0	0	0	0	0	2	1	3
	21UR-11255	TAATAACTTCTACTTTTCATT	1	0	0	0	0	0	0	1
†	21UR-11256	TAAGTGATCGTTACACTCAAA	0	0	0	0	2	2	0	4
	21UR-11257	TAAGTAAATTGTTATAAGTGC	0	0	0	0	0	0	0	0
	21UR-11258	TAAGGCCATAATTCACTCATC	0	0	0	0	1	0	0	1

†	21UR-11259	TAAGCTAGCAAAAATCATTTT	0	0	0	0	0	0	0	0
	21UR-11260	TAAGCAATTGTGGAAGCAGTG	1	2	0	0	2	5	0	10
	21UR-11261	TAAGAGTAATTGGACAAAAAC	0	0	0	0	0	0	1	1
†	21UR-11262	TAACTTTTTTTGATTCACTAA	0	0	0	0	1	0	0	1
	21UR-11263	TAACTGTGGTTCAACTGCCAA	0	0	0	0	1	1	0	2
	21UR-11264	TAACGTTGGTCTGTATTTCAA	0	0	0	0	0	0	0	0
	21UR-11265	TAACAGATGATCTGTTTCACA	0	0	0	0	0	0	0	0
	21UR-11266	TAAATTTTGCACTTTTAGTTT	0	0	0	0	0	0	0	0
	21UR-11267	TAAATTGAAATTTAAAAAAAC	0	0	0	0	0	0	0	0
	21UR-11268	TAAATCATTGTCAAATAATTT	0	0	0	0	0	0	0	0
	21UR-11269	TAAATAGCAACCCATTGTTTT	0	0	0	0	0	1	0	1
	21UR-11270	TAAAGTTTGGAATGTCACAAA	0	0	0	0	0	0	0	0
	21UR-11271	TAAAACTAACCAATTCTCTCT	0	0	0	0	0	0	0	0
†	21UR-11272	TAAAACGTCAAAATCATTTTT	0	0	0	0	0	0	0	0
	21UR-11273	TAAAAATCGGGACCCCAACCA	0	0	0	0	0	0	0	0
	21UR-11274	TAAAAATCAGAAATTGGAGTT	0	0	0	0	0	2	0	2
	21UR-11275	TAAAAAATTTCGGTAACAAGC	0	0	0	0	0	0	0	0
	21UR-11276	TAAAAAAATGCAATGAAAAAA	0	0	0	0	0	0	0	0
	21UR-11277	GTAATAGCAAGTAAGTTGATG	0	0	0	0	0	0	0	0
†	21UR-11278	GATTTTAAATGAAAAATTGGA	0	0	0	0	0	0	0	0
	21UR-11279	CTTTATTGAAATTTGTTAGTA	0	0	0	0	0	0	0	0
	21UR-11280	CTCACTCAATGAAAAGACACG	1	0	0	0	1	0	0	2
	21UR-11281	CGTCAGAACAATTCTAAAAAG	0	0	0	0	0	0	0	0
	21UR-11282	CGGGTTTGCTTTGATGTTAGA	0	0	0	0	0	0	0	0
†	21UR-11283	CGATCAACATGTACACCTTTT	0	0	0	0	1	0	0	1
†	21UR-11284	CGAAATTTACGTTGTCCAGTG	0	0	0	0	0	0	0	0
†	21UR-11285	CATAGCGATACGTTCTTATTT	0	0	0	0	0	0	0	0
	21UR-11286	CAAATAATCTCTCGTTCATTA	0	0	0	0	0	0	0	0
	21UR-11287	TTTTTTTTATGATACGGCAAT	1	1	0	1	0	2	0	5
	21UR-11288	TTTTTTTCAATATTGTTTCTG	0	0	0	0	0	0	0	0
	21UR-11289	TTTTTTTCAAACAAGTTTGAC	0	0	0	0	0	0	1	1
†	21UR-11290	TTTTTTTACCTGACGTTCTCC	0	0	0	0	0	0	0	0
	21UR-11291	TTTTTTGGTAATATAATATCA	0	0	0	0	0	0	0	0
	21UR-11292	TTTTTTGATGTCTAAAATTAG	0	0	0	0	0	0	0	0
	21UR-11293	TTTTTTCCTCAAAGATATTCA	0	0	0	0	0	0	1	1
	21UR-11294	TTTTTTCACTACTGCAGCTTC	0	0	0	0	0	0	0	0
†	21UR-11295	TTTTTTATTAGAAAAGAGTGA	0	0	0	0	0	0	0	0
	21UR-11296	TTTTTTAATCCAATAAATGTG	0	0	0	0	0	0	0	0
†	21UR-11297	TTTTTGATTTTGAGAAACCCT	0	0	0	0	0	0	0	0
†	21UR-11298	TTTTTGAGAGCAAGGTTTTTT	1	0	0	0	0	2	0	3
	21UR-11299	TTTTTGAATGACGAAAAATTT	0	0	0	0	0	0	0	0
†	21UR-11300	TTTTTGAACTGAATGCTAAAC	0	0	0	0	1	0	0	1
†	21UR-11301	TTTTTCTGTTTTCTGGCATTG	0	3	0	2	7	9	2	23
†	21UR-11302	TTTTTCTCATCATTGGAAAGG	0	0	0	0	0	0	0	0
	21UR-11303	TTTTTCCTTTATTTTTTGTGA	0	0	0	0	1	0	0	1
†	21UR-11304	TTTTTCAGACTCCTTGCTATC	0	0	1	0	0	0	0	1
	21UR-11305	TTTTTCACCACAACTATATTT	0	0	0	0	1	0	0	1
†	21UR-11306	TTTTTAGAAGATGACTCACCT	0	2	0	1	2	9	0	14
	21UR-11307	TTTTTAAAAATAAAGTGGCTT	0	0	0	0	0	0	0	0
†	21UR-11308	TTTTGTAACTCCTCCAATTTT	0	1	0	0	1	1	0	3
†	21UR-11309	TTTTGAAGATAATTATTTGAC	0	0	0	0	2	0	0	2
†	21UR-11310	TTTTGAAAAGTCAGATTTGCA	0	0	0	0	0	2	0	2
	21UR-11311	TTTTCTTTCCCTACGCACTTA	0	0	0	0	1	3	8	12
	21UR-11312	TTTTCGCTCTTTTTTAGTCAT	0	0	0	0	0	0	0	0
†	21UR-11313	TTTTCATACTGTTTAAACATG	0	0	0	0	2	0	0	2
†	21UR-11314	TTTTCAGTGTTTTCTCGAGAT	0	0	0	0	0	0	0	0
	21UR-11315	TTTTCACTTTTTTCTAGTAGA	0	0	1	0	2	1	0	4
	21UR-11316	TTTTCACATATTCCGTGGATC	0	0	0	0	0	0	0	0
	21UR-11317	TTTTCAATTTTATTAGCTGTT	0	0	0	0	0	0	0	0
†	21UR-11318	TTTTCAATCTAGTTGCTGTTA	0	0	0	0	0	0	0	0
†	21UR-11319	TTTTCAAAAAGTTGTTTAAAA	0	0	0	0	0	0	0	0
	21UR-11320	TTTTATTTTTGAATGATTGAA	0	0	0	0	0	1	0	1
	21UR-11321	TTTTATTCAAGTCTGCGAGTG	0	0	0	0	0	0	0	0
†	21UR-11322	TTTTATTATCAGTTGTGATAT	0	1	0	0	3	0	0	4

†	21UR-11323	TTTTATGAAATTTTGGAGTTC	0	0	0	0	0	0	0	0
	21UR-11324	TTTTAGTTTTTGGAAATAGCT	0	0	0	0	0	0	0	0
†	21UR-11325	TTTTACCAAATTTCTGAATAA	0	0	0	0	1	1	0	2
	21UR-11326	TTTTAATCATTTCAAGTTTGG	0	0	0	0	0	0	0	0
†	21UR-11327	TTTTAACAGTTTTTAACAGAG	0	0	0	0	0	0	0	0
	21UR-11328	TTTTAACAGTTTTCAGGGCTT	0	0	0	0	0	0	0	0
†	21UR-11329	TTTTAAATCATCCGCAAACTT	0	0	0	0	0	0	0	0
†	21UR-11330	TTTGTTTTGTTTTTGGCATTG	0	0	0	0	1	0	1	2
	21UR-11331	TTTGTTTTGTACAGTTGTACA	0	0	0	0	0	0	0	0
†	21UR-11332	TTTGTTTGGACATTTTTAAAA	0	1	0	5	42	39	7	94
†	21UR-11333	TTTGTTCAGAAATGCGTGTTA	0	1	0	0	0	0	0	1
†	21UR-11334	TTTGTTCACATCAAGAAAGTA	0	0	0	0	0	0	0	0
†	21UR-11335	TTTGTCTGAAAATAAGGAGTT	1	2	1	0	2	12	1	19
	21UR-11336	TTTGTACTCTTTCAGATACAA	0	0	0	0	1	0	0	1
†	21UR-11337	TTTGGTTTTCCATTGCTTTCA	0	0	0	0	0	0	0	0
†	21UR-11338	TTTGGTTTCGATGTTGAAGTA	0	0	0	0	0	1	0	1
	21UR-11339	TTTGGTTGTACCTTTAAAACT	0	0	0	0	1	0	0	1
†	21UR-11340	TTTGGTGACTAAGGAAGAATT	0	0	0	0	0	0	0	0
†	21UR-11341	TTTGGGAAATTTTGTGACTTT	0	0	0	0	0	0	0	0
	21UR-11342	TTTGGCTAATGGGTTTTTCCT	0	0	0	0	0	0	0	0
†	21UR-11343	TTTGGATCAAAAAACAAAAAC	0	0	0	0	0	0	0	0
	21UR-11344	TTTGGAACTTGATTTATTAAT	0	0	0	0	0	0	0	0
†	21UR-11345	TTTGATGTTGAGATAGTAATT	0	0	0	0	0	0	0	0
†	21UR-11346	TTTGATCTCATAGGTCTAAAA	0	0	0	0	0	0	0	0
†	21UR-11347	TTTGAAGATACAAGTTCAGAA	0	0	0	0	0	2	0	2
	21UR-11348	TTTGAACAACATTTGAAATCG	0	1	0	1	1	1	0	4
†	21UR-11349	TTTGAAAAACGAATCAAAGAA	0	0	0	0	0	0	0	0
†	21UR-11350	TTTCTTCCATTATATCAACGG	3	0	0	0	0	0	0	3
†	21UR-11351	TTTCTGCATTGAAAAACTCCC	0	0	0	0	0	0	0	0
	21UR-11352	TTTCTCTCATATTACTAATAA	0	0	0	0	3	0	1	4
†	21UR-11353	TTTCTCGTTTTTGTCTTTGAG	0	0	0	0	0	0	0	0
†	21UR-11354	TTTCGCCCAAACTTCTAAAAA	0	0	0	0	0	0	0	0
†	21UR-11355	TTTCGCACGCGAAAAAATTTC	0	0	0	0	0	0	0	0
†	21UR-11356	TTTCGAGTAGCATTTGAGTGG	1	4	1	1	0	10	0	17
	21UR-11357	TTTCCTTTCAACAAAAATCAA	0	0	0	0	0	0	0	0
	21UR-11358	TTTCCGATATTTTCTGCTATA	0	0	0	0	1	0	0	1
	21UR-11359	TTTCCACTTGAAATATTTTTT	0	0	0	0	0	0	0	0
	21UR-11360	TTTCATTTAAGTTTTAATTAA	0	0	0	0	0	0	0	0
	21UR-11361	TTTCAGAGCATTGGCGATATT	1	1	0	0	0	3	0	5
†	21UR-11362	TTTCAATTCGCTCAGCTCTCG	0	0	0	0	0	0	0	0
	21UR-11363	TTTCAATAGTCTAGTGGTTAT	0	0	0	0	1	0	0	1
†	21UR-11364	TTTCAAGAATAAATTTCAGAG	0	0	0	0	0	0	0	0
	21UR-11365	TTTATTTCAATCACTACTTTA	0	0	0	0	0	1	1	2
†	21UR-11366	TTTATTGCACAATTGATAAGG	0	0	0	0	0	0	0	0
†	21UR-11367	TTTATTCTCCATCTAAGCCAT	0	0	0	0	0	0	0	0
	21UR-11368	TTTATTATAGATTTTTTTACA	0	0	0	0	5	0	0	5
	21UR-11369	TTTATGGTTAATCCAAAACAA	0	0	0	0	1	0	0	1
†	21UR-11370	TTTATAATAGGGTTTAAGATT	0	0	0	0	0	0	0	0
	21UR-11371	TTTATAAATTTCAAATCACTT	0	0	0	0	0	0	0	0
†	21UR-11372	TTTAGAGTAAAATCGCGTTGC	0	0	0	0	0	0	0	0
	21UR-11373	TTTACTTAATTCTTACGTAAA	0	0	0	0	0	0	0	0
†	21UR-11374	TTTACCAAAGCCGATGAATGC	0	0	0	1	1	0	0	2
	21UR-11375	TTTACATCTTTCATCTTTCTC	0	0	0	0	0	0	0	0
	21UR-11376	TTTACAGAATCTGGCTTCAGG	0	0	0	0	0	0	0	0
	21UR-11377	TTTACACAGTTTTGAAGCGCA	1	0	1	0	0	19	1	22
	21UR-11378	TTTAATGTTACCAACCCGTCA	2	0	0	0	0	0	0	2
†	21UR-11379	TTTAATCAATCCGATGATCTT	0	0	0	0	0	0	1	1
	21UR-11380	TTTAATACATGTATGTTTCAG	0	0	0	0	0	0	0	0
	21UR-11381	TTTAATAAAGTCTTGAAAATA	0	0	0	1	0	1	1	3
	21UR-11382	TTTAAATCTTTTAAAAATTAT	0	0	0	0	0	0	0	0
	21UR-11383	TTTAAATCAGTTGAAAAGAAG	0	0	0	0	0	0	0	0
	21UR-11384	TTGTTTTGAACATTTTTTTCA	0	0	0	0	0	0	0	0
	21UR-11385	TTGTTGTAGACGTCAGGGGCA	4	1	2	1	6	21	2	37
†	21UR-11386	TTGTGTTTGCCGCGAGAGAGA	0	0	0	0	0	0	0	0

†	21UR-11387	TTGTGAAGATTTCTCATAGAA	0	0	0	0	0	0	0	0
	21UR-11388	TTGTCTATGACATTCCTCCAT	0	0	0	0	0	0	0	0
†	21UR-11389	TTGTATGCGTAAAAAAAAATG	0	0	0	0	0	0	0	0
†	21UR-11390	TTGGTTTTTTAAGTAAAGATA	0	0	0	0	1	0	0	1
†	21UR-11391	TTGGTTTGTCATTAATGTAGA	0	0	1	0	1	0	1	3
†	21UR-11392	TTGGTTCTTCAGCCTGTGGAG	0	0	0	0	0	0	0	0
†	21UR-11393	TTGGGTGAAGTTTGGTCTTTG	0	0	0	0	1	1	0	2
	21UR-11394	TTGGGTCCCATCCGCTTTTTG	0	0	0	0	0	0	0	0
†	21UR-11395	TTGGCATTAAAGAACGATCAC	0	0	0	0	0	0	0	0
†	21UR-11396	TTGGATTAAGAGCCGGATTTT	0	1	0	0	2	3	2	8
	21UR-11397	TTGGATCTTTCAAATTTTTTT	0	0	0	0	0	0	0	0
†	21UR-11398	TTGGACAATTTAGCTGTTTTA	0	0	0	0	1	0	0	1
	21UR-11399	TTGGAACAGTATTGTTCATCT	0	0	0	0	0	0	0	0
†	21UR-11400	TTGCGTTGTTGTACATATACA	0	0	0	0	0	0	0	0
†	21UR-11401	TTGCCTCGTATGATACCTTTG	0	0	0	0	0	0	0	0
†	21UR-11402	TTGCCACAACAGCTCAGGGAA	0	0	0	0	0	1	0	1
†	21UR-11403	TTGCATATGGATTAGGCGTCA	0	0	0	0	0	1	0	1
†	21UR-11404	TTGATTTGATTACAACAATTT	0	0	0	0	0	0	0	0
†	21UR-11405	TTGATCTGTGAAACTATTCAA	0	0	0	0	0	0	0	0
†	21UR-11406	TTGATCGATTTTTTTGCTTTT	0	0	0	0	0	0	0	0
	21UR-11407	TTGATCGATGTTGTCAAGGTA	0	0	0	0	0	0	0	0
†	21UR-11408	TTGATAGTACAGTGCGTGCAA	0	0	0	0	0	1	0	1
†	21UR-11409	TTGATACCATGGACATTACCA	0	0	0	0	1	0	0	1
†	21UR-11410	TTGATACACGCGATTGATTTT	0	1	0	0	0	0	0	1
†	21UR-11411	TTGAGAAATTTTGAATAGATT	0	0	0	0	0	0	0	0
†	21UR-11412	TTGACGACGTGGCTATCATGA	0	0	0	0	0	0	2	2
†	21UR-11413	TTGACACATTAGGATTCTGCT	0	0	0	1	1	2	1	5
†	21UR-11414	TTGAATCATGTAGAATTGTCA	8	3	1	0	6	12	0	30
†	21UR-11415	TTGAATCAGACGACTGTATTC	0	0	0	0	0	0	0	0
†	21UR-11416	TTGAAACAGCCGTAACGCAGG	0	0	0	0	0	0	0	0
†	21UR-11417	TTGAAAATTTGAGTCTAACAT	0	0	0	0	0	1	0	1
†	21UR-11418	TTGAAAATGGCATCAATTGTA	0	1	0	1	0	1	0	3
	21UR-11419	TTCTTTTATCGCTGATAATGA	0	0	0	0	0	0	0	0
†	21UR-11420	TTCTTTGCGACAAGAATTGTG	0	0	0	0	0	0	0	0
†	21UR-11421	TTCTTTGCATTCTTCGCAAGA	0	0	0	0	0	0	0	0
	21UR-11422	TTCTTGTACATGAAGTGCTTA	0	0	0	0	0	1	0	1
†	21UR-11423	TTCTTGCTTCTTGTGCCTACT	0	0	0	0	1	0	0	1
†	21UR-11424	TTCTTGCAAAATCGGTGTGAA	0	0	0	0	0	3	0	3
†	21UR-11425	TTCTTCTATAGATTGTTGGAA	1	0	1	4	25	15	1	47
†	21UR-11426	TTCTTCCAGTCAAAAGTGAAA	0	0	0	0	0	0	0	0
†	21UR-11427	TTCTTCAATTACCAATTTTCA	0	0	0	0	0	0	0	0
†	21UR-11428	TTCTTATTGCATTCTCTTTCA	0	0	0	0	0	1	0	1
	21UR-11429	TTCTGGGATTATTTTGAATTT	0	0	0	0	0	0	0	0
†	21UR-11430	TTCTGCGATTGTTAGTTTTGC	0	0	0	0	1	0	0	1
†	21UR-11431	TTCTGCCCAGTGGTGTCAAGA	0	0	0	0	0	0	0	0
	21UR-11432	TTCTGCAAAATTCAAGGATCG	0	0	0	0	0	0	0	0
	21UR-11433	TTCTGAGAACATCGTGAAACA	0	0	0	0	0	0	0	0
†	21UR-11434	TTCTGACTATAAGATTTTGAA	0	0	0	0	1	0	0	1
	21UR-11435	TTCTGAATCCAAGATCGGTAC	4	0	5	0	2	8	0	19
	21UR-11436	TTCTCGTGGAATAAATTCTAG	0	0	0	0	0	1	0	1
†	21UR-11437	TTCTCCTCATTCCAATTGAAA	0	0	0	0	0	0	0	0
	21UR-11438	TTCTCAACGTGATAGTTTGAT	0	0	0	0	0	0	0	0
	21UR-11439	TTCTACCACAACAGTACTTAT	0	0	0	0	0	0	0	0
	21UR-11440	TTCGTGTTCTTGATAAGAATG	0	0	0	0	0	0	0	0
†	21UR-11441	TTCGTCAACAAAGTTTTTCTT	0	0	0	0	0	0	0	0
†	21UR-11442	TTCGGACATAATATCGTAGAC	0	0	0	1	2	0	0	3
†	21UR-11443	TTCGATATAGATCTGCCGATT	0	0	0	0	3	1	0	4
†	21UR-11444	TTCGAAGGTTTTTTTTTGGGA	0	0	0	0	0	0	0	0
†	21UR-11445	TTCCTTTGCTGGTTGGCTGGT	0	0	0	0	0	0	0	0
	21UR-11446	TTCCTGTTTGGTATTTTTTTT	0	1	0	2	0	1	0	4
†	21UR-11447	TTCCCGGATCTCCCTTTTTTT	0	0	0	0	0	0	0	0
†	21UR-11448	TTCATTGGTGCATTGTTAAGA	1	0	0	0	0	0	0	1
†	21UR-11449	TTCATTGATTCAATTTCCATT	0	0	0	0	0	0	0	0
	21UR-11450	TTCATGCGCAACTCTTCATGA	1	0	0	0	0	0	0	1

†	21UR-11451	TTCATCATTGAATTGTCTTTC	0	0	0	0	0	0	0	0
	21UR-11452	TTCAGTTTCCACTTGCTTCGT	1	0	0	0	0	0	0	1
†	21UR-11453	TTCAGTTAGCAATGTATAATA	0	0	0	0	2	0	0	2
†	21UR-11454	TTCAGCTTTCTGAAAAATCAA	0	0	0	0	0	0	0	0
†	21UR-11455	TTCAGCAACTGAAATTTTCGA	0	0	0	0	0	0	0	0
	21UR-11456	TTCAGATGGTCTAGGTAAAGC	0	0	0	0	0	0	0	0
	21UR-11457	TTCAGAAACCAATAATTGTTT	0	0	0	0	0	0	0	0
†	21UR-11458	TTCACTTCATTGATGCAAATG	0	0	0	0	0	0	0	0
	21UR-11459	TTCACGTTTCACTTTTTTTTA	0	0	0	0	0	0	0	0
†	21UR-11460	TTCACAATTTTTTAAACGCTT	0	0	0	0	0	0	0	0
†	21UR-11461	TTCACAATTTTCCTAACGTTT	0	0	0	0	0	0	0	0
	21UR-11462	TTCACAAAATTTCACAAAGGA	0	0	0	0	0	0	1	1
†	21UR-11463	TTCAATATCCAAAAAAACTGG	0	0	0	0	0	0	0	0
†	21UR-11464	TTCAAGAATTGATAGCGCTTG	0	0	0	0	0	2	0	2
	21UR-11465	TTCAAGAAAATGACTGGGCTT	0	0	0	1	10	9	1	21
	21UR-11466	TTCAAATCGGGTCATATCTTC	0	0	0	0	0	0	0	0
†	21UR-11467	TTCAAACTTGTGCCTTTGAAA	0	0	0	0	1	1	1	3
	21UR-11468	TTCAAACATCAAATAATGAAC	0	0	0	0	0	0	0	0
	21UR-11469	TTATTTTTTGCTCCTGATGGT	0	1	1	5	36	27	7	77
†	21UR-11470	TTATTTTTGACCCTTTAGAGT	0	0	0	0	0	0	0	0
†	21UR-11471	TTATTTTGATGTCCTTCTTTA	0	0	0	0	0	0	0	0
	21UR-11472	TTATTTTCCAAAACTTTTGTT	0	0	0	0	0	0	0	0
†	21UR-11473	TTATTTGGATTCCATTCTGAA	0	0	0	0	0	0	0	0
	21UR-11474	TTATTTCTATCTAAAAATCTA	0	0	0	0	0	0	0	0
†	21UR-11475	TTATTTCACAGGAGATTTTGC	0	0	0	0	0	0	0	0
†	21UR-11476	TTATTGTTCAAATCATTCAAA	0	0	0	0	0	0	0	0
†	21UR-11477	TTATTGTAGAATAGAATAAAC	0	0	0	0	0	1	0	1
†	21UR-11478	TTATTGCACTGTCTTTGTACG	0	0	0	0	0	0	0	0
†	21UR-11479	TTATTCGAAAAATTGGACTGA	0	0	0	0	2	1	0	3
	21UR-11480	TTATTCCTTTGCATTGTGTTT	0	0	0	0	1	0	0	1
	21UR-11481	TTATTATATTTTTTGCAAAAA	0	0	0	0	1	0	0	1
†	21UR-11482	TTATTACCAGATCTTGAACGT	0	0	0	0	1	1	0	2
†	21UR-11483	TTATTAAGAAAAAGGGGAATT	0	0	0	0	1	1	0	2
	21UR-11484	TTATTAAACGCTTTTAAGTTA	0	0	0	0	1	0	0	1
	21UR-11485	TTATGTGTAAATTTGAAAATC	1	0	0	0	1	2	0	4
	21UR-11486	TTATCTTTAATCAAAAAAAAA	0	0	0	0	0	0	0	0
†	21UR-11487	TTATATGTGTTGCATAGGATG	0	0	0	0	0	0	0	0
	21UR-11488	TTATAACCAGTTTAATGTTGA	0	0	0	0	0	0	0	0
	21UR-11489	TTATAAAACAAAGTAATAGAA	0	0	0	0	0	0	2	2
	21UR-11490	TTAGTTAAAAAATTGTAATTA	0	0	0	0	0	1	0	1
†	21UR-11491	TTAGTAAGTTTCTCTATATTT	0	0	0	0	0	0	0	0
	21UR-11492	TTAGGCATGTAACGTGACTAC	0	0	0	0	0	0	0	0
	21UR-11493	TTAGATGCTTTTCCATTTTTT	0	0	0	0	0	0	0	0
†	21UR-11494	TTAGACAGATTGGCCTGTAAT	0	0	0	0	0	0	0	0
	21UR-11495	TTACTTATCAAGTGTTCCAGT	0	0	0	0	0	0	0	0
†	21UR-11496	TTACTACCAAAAATCGCTTTC	0	0	0	0	0	0	0	0
†	21UR-11497	TTAATTTTGGTGTCGAAAAGT	1	0	0	0	0	0	0	1
	21UR-11498	TTAATTTGGATTTTTTTGATA	0	0	0	0	4	2	0	6
†	21UR-11499	TTAATTGACCGATTTTAAATT	0	0	0	0	0	0	0	0
	21UR-11500	TTAATTCGAAAAACAACAAGA	0	0	0	0	0	0	0	0
†	21UR-11501	TTAATTCAATGCCAAAAACAA	0	0	0	0	0	0	0	0
†	21UR-11502	TTAATTCAAGCAATCCAAACG	0	0	0	0	0	0	0	0
†	21UR-11503	TTAATTCAACATGGTGCTCCT	0	0	0	0	0	0	0	0
†	21UR-11504	TTAATTACACTGCTGTTGTAA	0	0	0	0	0	0	0	0
†	21UR-11505	TTAATGTTGTGAAAGGAAATT	0	0	0	0	0	0	0	0
	21UR-11506	TTAATCGTTCGTAGAATGTAT	4	0	0	2	4	5	0	15
†	21UR-11507	TTAATCGATAGGGTGGCAAAT	0	0	0	0	0	0	0	0
	21UR-11508	TTAATAGATTTAGTTTTGAAA	0	1	0	0	0	0	0	1
†	21UR-11509	TTAATAATGGACGCAGTTTTC	0	0	0	0	1	4	0	5
†	21UR-11510	TTAATAAATGGATAAGATTTT	0	0	0	0	1	0	0	1
†	21UR-11511	TTAAGCTAAAAATAATACCGA	0	0	0	0	0	0	0	0
	21UR-11512	TTAACTTTTATTAATGCACGG	0	0	0	0	3	2	2	7
	21UR-11513	TTAACTTGATAGAACTTGCAT	1	0	0	0	6	11	0	18
†	21UR-11514	TTAAATTCTCCGTTAATATTA	0	0	0	0	0	0	1	1

†	21UR-11515	TTAAATGAAGTAGAAAAAATA	2	4	1	2	12	12	0	33
†	21UR-11516	TTAAAGTTGGTGTGGTCGAAT	0	0	0	0	1	0	0	1
	21UR-11517	TTAAAGCTTTAATTAAACAGT	0	0	0	0	0	1	0	1
†	21UR-11518	TTAAAGACTTTGGAAAAGCGA	0	0	0	0	0	1	0	1
	21UR-11519	TTAAACGGATAAGTATTGTTA	0	0	0	0	0	0	0	0
†	21UR-11520	TTAAACAGTTTCCCGTGATTC	0	0	0	0	0	0	0	0
†	21UR-11521	TTAAACAGTCTTGCCGGTATT	1	0	0	1	1	6	2	11
	21UR-11522	TTAAAAGTGTTCAAATGTAAG	0	0	0	0	0	0	1	1
†	21UR-11523	TGTTTTGGTATTTGAAGATAA	0	0	0	0	0	0	0	0
†	21UR-11524	TGTTTTCGAACGCAATTCTTG	0	0	0	0	0	0	0	0
†	21UR-11525	TGTTTTCCTAATCATGGTTCT	0	0	0	0	0	0	0	0
	21UR-11526	TGTTTTCACTGTTTCAGAAAA	0	0	0	0	0	0	0	0
†	21UR-11527	TGTTTTACTACTAATAAACAT	0	0	0	0	2	0	1	3
†	21UR-11528	TGTTTGGTCATGATAAAAATG	0	0	0	0	1	0	0	1
	21UR-11529	TGTTTATTTTTTTCGTAATGA	0	0	0	0	0	0	0	0
†	21UR-11530	TGTTTATTCTGTCCTTAAGCG	0	0	0	0	0	0	0	0
†	21UR-11531	TGTTTATAAAATAATATGCGA	0	0	0	0	0	0	0	0
	21UR-11532	TGTTGTTTCTGAACTTATTGT	0	0	0	0	0	0	0	0
†	21UR-11533	TGTTGTATCTTGGATTCTATC	0	0	0	0	1	0	0	1
†	21UR-11534	TGTTGGACCTTCCAAATTGAA	0	0	0	0	0	0	1	1
	21UR-11535	TGTTGCAAAATGAGGAAAATG	0	0	0	0	0	0	0	0
	21UR-11536	TGTTCTTTTCATTTTCACAGA	0	0	0	0	0	0	0	0
†	21UR-11537	TGTTCGTGTTTACCAACCAGC	0	0	0	0	0	0	0	0
†	21UR-11538	TGTTAGTTTCGAAATCTTCTT	0	0	0	0	0	0	0	0
	21UR-11539	TGTTAGACACTTAACGACTTA	0	0	0	0	0	0	0	0
†	21UR-11540	TGTTACTCTATACAAAAAAAA	0	0	0	0	0	0	0	0
†	21UR-11541	TGTTAACTTTGATTTTCTAAG	0	0	0	0	0	0	0	0
†	21UR-11542	TGTTAACCGTTTCATCTTATT	0	0	0	0	0	0	0	0
†	21UR-11543	TGTGATTTTGGGTTGAAAAAT	0	0	0	0	0	0	0	0
	21UR-11544	TGTGATCTCAGGATCTCGCTG	0	0	0	0	0	0	0	0
	21UR-11545	TGTGAAAATTGTATTTTCCTG	0	0	0	0	0	0	0	0
	21UR-11546	TGTCTTATGGGAGAACTTACC	0	0	0	0	0	0	0	0
	21UR-11547	TGTCAATTTATCTATTCCTTT	0	0	0	0	0	0	0	0
	21UR-11548	TGTCAATTATTGTCGCTTATG	0	0	0	0	0	0	0	0
	21UR-11549	TGTATTCAAATAAATGAAACC	0	0	0	0	0	0	0	0
†	21UR-11550	TGTATAATGTCCAATAGATGA	0	0	0	0	0	0	0	0
	21UR-11551	TGTATAAAGAAACACTAATTC	0	0	0	0	0	0	0	0
	21UR-11552	TGTAGCGCAAATGACTTTGGG	0	0	0	0	0	0	0	0
†	21UR-11553	TGTACTATGCTACAAAAGTCG	2	0	0	0	0	1	1	4
	21UR-11554	TGTACATCCATTATTCCAAAT	0	0	0	0	0	0	0	0
†	21UR-11555	TGTAACTCTCTGGTTGTTGAT	7	11	4	7	15	62	3	109
†	21UR-11556	TGTAAATTTGCCTTTATGATT	1	1	0	0	2	1	1	6
†	21UR-11557	TGGTCTGTCTAGATTCCAATA	0	0	0	0	1	2	0	3
†	21UR-11558	TGGTAGTTTCGAGTTTGGTTT	0	0	1	0	4	0	0	5
	21UR-11559	TGGGTCAATGTAACGACATTT	0	0	0	0	0	0	0	0
	21UR-11560	TGGGAATTGTTTCATTTTGAC	0	0	0	0	2	0	1	3
	21UR-11561	TGGCACCGTGATGTTTTCTTA	0	0	0	0	0	0	0	0
†	21UR-11562	TGGCAAATAATACTGGTGAGC	0	0	0	0	0	0	0	0
	21UR-11563	TGGAGAGTTTTCAATTAATTT	0	0	0	0	0	0	0	0
	21UR-11564	TGGAATATTAATTTTATAAAT	0	0	0	0	0	0	0	0
	21UR-11565	TGGAAGCATTCGTTTGAGCCA	0	0	0	0	0	0	0	0
	21UR-11566	TGGAAAAGAGGAACAAATTTT	0	0	0	0	0	0	0	0
	21UR-11567	TGCTTGATTTTTGGGCAAGCT	0	0	0	0	0	0	0	0
	21UR-11568	TGCTTCTTTCAACTTTTTTTT	0	0	0	0	0	0	0	0
†	21UR-11569	TGCTTCTCGGTATGACTTTTT	0	0	0	0	0	1	0	1
	21UR-11570	TGCTGTCTCGTTAGATACATC	0	0	0	0	2	0	0	2
†	21UR-11571	TGCTGCTAATAAATGTAAATA	0	0	0	0	0	0	1	1
†	21UR-11572	TGCTGCGATCTAATTTTTTCA	0	0	0	0	0	0	0	0
†	21UR-11573	TGCTGACTTTTTAGTTGCAAC	0	0	0	0	0	0	0	0
	21UR-11574	TGCTACCTTAATTAACTCGGT	0	0	0	0	0	0	0	0
†	21UR-11575	TGCTAACAGGAATATCATTGG	0	0	0	0	0	0	0	0
	21UR-11576	TGCGCAAATGCAACACGCCTT	0	0	0	0	0	0	0	0
	21UR-11577	TGCGAAGACTTCAATTTCACA	0	0	0	0	0	0	0	0
	21UR-11578	TGCGAAAAGTTAGAAAGAAAC	0	0	0	0	0	0	0	0

†	21UR-11579	TGCCTACTACTGGAATCCATT	0	0	0	0	0	0	0	0
	21UR-11580	TGCCATTTCTAAACAAACAAA	0	0	0	0	0	0	0	0
	21UR-11581	TGCCATCGATTTTATAAACTC	0	0	0	0	0	0	0	0
†	21UR-11582	TGCCAAAATTCCTGTATCTGG	0	0	0	0	0	0	0	0
†	21UR-11583	TGCATTACCTCATATTCCTCA	0	0	0	0	0	0	0	0
†	21UR-11584	TGCACGATATTTTTTGCTATA	0	0	0	0	0	0	0	0
	21UR-11585	TGCACCTCGTGAGAAGTTATG	0	0	0	0	0	0	0	0
†	21UR-11586	TGCAAATCCAAAAATCGAAAA	0	0	0	0	0	0	0	0
	21UR-11587	TGATTGATCGACTTTGAGTTC	0	0	0	0	0	0	0	0
†	21UR-11588	TGATTGAATTGTGGAAATATT	0	0	0	0	0	0	0	0
†	21UR-11589	TGATGACTTTCTTATAATTTG	0	0	0	0	0	0	0	0
†	21UR-11590	TGATAGACTAGAACAAATCAA	0	0	0	0	0	0	0	0
†	21UR-11591	TGATACTTCCAGTGTTTGTGA	0	0	0	0	0	0	0	0
	21UR-11592	TGATAAAATGTCGCAGTAGTT	0	0	0	0	0	0	0	0
†	21UR-11593	TGAGTAGGTATTATCGTGATA	0	0	0	0	0	0	0	0
	21UR-11594	TGAGGATGACGTCTACACCTT	0	0	0	0	0	0	0	0
†	21UR-11595	TGAGCAGGTTGGTGTTTTCAG	0	0	0	0	1	0	0	1
†	21UR-11596	TGAGAGCTTTTGAAATAGTAT	0	0	0	0	0	0	0	0
	21UR-11597	TGAGAAATCGATTTTCCTTTG	0	0	0	0	0	0	0	0
†	21UR-11598	TGACTTTCTTATAATTTGCCC	0	0	0	0	0	0	0	0
	21UR-11599	TGAATTTCTCTTTCTACATTG	0	0	0	0	0	0	0	0
†	21UR-11600	TGAAGTTTCAAGTAGGTTTTT	1	0	1	0	5	2	0	9
†	21UR-11601	TGAAGTCACTCACAATGGGTT	0	0	0	0	0	0	0	0
	21UR-11602	TGAAGTATTTTTCCCCCTTTT	0	0	0	0	0	0	0	0
	21UR-11603	TGAAGGAAAAGATGATGAGGG	0	0	0	0	1	0	0	1
	21UR-11604	TGAACTATTTCTAGAACTTCG	0	0	0	0	1	0	1	2
	21UR-11605	TGAACATTTGGTTTTTCTTTC	0	0	0	0	0	0	0	0
	21UR-11606	TGAAATGGAAACATGTTTGGA	0	0	0	0	0	0	0	0
	21UR-11607	TGAAAGATGCTTACAAAATAA	0	0	0	1	3	3	0	7
	21UR-11608	TGAAAGAGCTTCAGATAATGA	0	0	0	0	0	0	0	0
	21UR-11609	TGAAAAGAATAAAAAAATCTC	0	0	0	0	0	0	0	0
†	21UR-11610	TCTTTCAAATTTCTCATGGAT	0	0	0	0	0	0	0	0
	21UR-11611	TCTTTACGAAGTTTCTAAATT	0	0	0	0	0	0	0	0
	21UR-11612	TCTTCTCTATTCTCTTTTTTG	0	0	0	0	0	0	0	0
	21UR-11613	TCTTCGTAGCTCAAATTTGAA	0	0	0	0	5	2	0	7
†	21UR-11614	TCTTATGCGTTACTCGAAAGT	0	0	0	2	8	7	2	19
†	21UR-11615	TCTGGTGTGGGTAAAATGTGT	0	0	0	0	0	0	0	0
	21UR-11616	TCTGGTCTCTGATTATTGGAG	0	0	0	0	0	0	0	0
	21UR-11617	TCTGATAACAGGAGTAGCCAC	0	0	0	0	0	0	0	0
	21UR-11618	TCTGAATTTGATTAAAAATGA	0	0	0	0	0	0	0	0
	21UR-11619	TCTGAAAATTGGACAAATCTT	0	0	0	0	0	0	0	0
	21UR-11620	TCTCTCATAACTTTTTGCTGA	0	0	0	0	0	0	0	0
†	21UR-11621	TCTCCGTATTAGGTACTTGTA	0	0	0	0	0	1	0	1
	21UR-11622	TCTCATATCCTTCTTCTTCTT	0	0	0	0	0	0	0	0
	21UR-11623	TCTATTTCAGAACTAAGACGC	6	4	1	2	6	30	1	50
†	21UR-11624	TCTATTAGGAGGAAGCATTGT	1	0	1	0	0	0	0	2
	21UR-11625	TCTAGCGGAACAGTAAAAAAT	0	0	0	0	0	1	0	1
	21UR-11626	TCTAATTTTTCAAATATTTTT	0	0	0	0	0	0	0	0
†	21UR-11627	TCTAACAGTTTTCTTTGGATT	2	1	0	3	46	28	8	88
†	21UR-11628	TCGTTTGACTTTTCGTTTCTA	0	0	0	0	0	0	0	0
†	21UR-11629	TCGTTCTCAACCAGCTTCGAA	0	0	0	0	0	0	0	0
†	21UR-11630	TCGTCCCGTACCTATTATAAA	0	0	0	0	0	0	0	0
	21UR-11631	TCGGAAGAATTTCGTTGTACC	0	0	0	0	0	0	0	0
	21UR-11632	TCGCCCCGCTGAGAGTATCTA	0	0	0	0	0	0	0	0
†	21UR-11633	TCGCAAAAAATAAAGTATATC	0	0	0	0	0	0	0	0
	21UR-11634	TCGAGTCAATGAGTGTAGTGG	0	0	0	0	0	0	0	0
†	21UR-11635	TCCTTATAACATTGAACAATT	0	0	0	0	0	0	0	0
	21UR-11636	TCCTCTAACAATTGAATGGCT	0	0	0	0	0	0	0	0
	21UR-11637	TCCTCCGTTTTCCATGAACAT	0	0	0	0	0	0	0	0
	21UR-11638	TCCTATTAAAAATTGCCAAGT	0	0	0	0	0	0	0	0
	21UR-11639	TCCTATCAGCAGATAAACTAG	0	0	0	0	0	0	0	0
	21UR-11640	TCCTAGACCAAAACGAAGAAT	0	0	0	0	0	0	0	0
	21UR-11641	TCCGACATCGTTGACCATCTC	0	0	0	0	0	1	1	2
	21UR-11642	TCCCTGATAATTTCCAGGTGC	0	0	0	0	0	0	0	0

	21UR-11643	TCCCCATTCCGTAGAAATCAT	0	0	0	0	0	0	0	0
	21UR-11644	TCCCATCTGAAATTTCAAGAA	0	0	0	0	0	0	0	0
†	21UR-11645	TCCATCGATTTTATATAGGCT	0	0	0	0	0	0	0	0
	21UR-11646	TCCATCAACAGTCTTGCATCT	0	0	0	0	0	0	0	0
†	21UR-11647	TCCAGTATCCAATCATAAAAT	0	0	0	0	0	0	0	0
	21UR-11648	TCCACAAATTTCACTGTTTCA	0	0	0	0	0	0	0	0
†	21UR-11649	TCCAATATTGTAGTAAAGTTG	0	0	0	0	2	2	0	4
†	21UR-11650	TCCAACACCTACGATTGTTTG	0	0	0	0	0	0	0	0
	21UR-11651	TCATTTTGAGGTCTTGGTCTT	0	0	0	0	0	0	0	0
†	21UR-11652	TCATTGGACTTTGATTGGAAA	0	0	0	0	1	0	5	6
	21UR-11653	TCATTACTAGAGACACAAGGA	0	0	0	0	0	0	0	0
†	21UR-11654	TCATCTGTGATCCCCTCAATA	0	0	0	0	0	0	0	0
	21UR-11655	TCATCAAAGTAGAAATCTGGA	0	0	0	0	0	0	0	0
	21UR-11656	TCATATCAATTCTGCTACTAC	0	0	0	0	0	0	0	0
†	21UR-11657	TCAGTTGTCTTTCGGTGTTAT	1	2	0	2	18	18	7	48
†	21UR-11658	TCAGTTGGTAAAATTGAAAAC	0	0	0	0	0	0	0	0
†	21UR-11659	TCAGTTCTCCTTGAATTAAAT	0	0	0	0	0	0	0	0
†	21UR-11660	TCAGTGAAATGAAGGCATACA	0	0	0	0	0	0	0	0
	21UR-11661	TCAGGTTCAATCCTTTAACAT	0	0	0	0	0	0	0	0
	21UR-11662	TCACTTTAAACATGTTTATCC	0	0	0	0	0	0	0	0
†	21UR-11663	TCACGAGCTTTTTATTAAAAA	0	0	0	0	0	0	0	0
†	21UR-11664	TCACGACTTTTATTTGAACTT	0	0	0	0	0	0	0	0
	21UR-11665	TCACATGTAATCCCGGGAGGC	0	0	0	0	0	0	0	0
†	21UR-11666	TCACAGATCATTCAGCGTGAG	0	0	0	0	0	0	0	0
†	21UR-11667	TCAATTGGAGTTTGGTTTAAA	0	0	0	0	0	0	0	0
	21UR-11668	TCAATTATTAATGGAGAATGA	0	0	0	0	0	0	0	0
	21UR-11669	TCAATCAAATTGAAAAGTTTT	0	0	0	0	0	0	0	0
	21UR-11670	TCAATAGAAGGTGTGAAAAAT	0	0	0	0	0	0	0	0
	21UR-11671	TCAACCCTTTTTTTCTATTGG	0	0	0	0	0	0	0	0
†	21UR-11672	TCAAACCAAATTGATACTTCA	0	0	0	0	0	0	0	0
	21UR-11673	TCAAAAGATCAAGTAGTAAAA	0	0	0	0	0	0	1	1
	21UR-11674	TCAAAAATAGCAGTCACGGAA	0	0	0	0	1	2	2	5
	21UR-11675	TATTTTTTTTGAATTGATTTC	0	0	0	0	0	0	0	0
	21UR-11676	TATTTTTTCTAGACCCATATG	0	0	0	0	1	0	0	1
	21UR-11677	TATTTTTTAAAAATTGTAGAA	0	0	0	0	1	1	0	2
	21UR-11678	TATTTTAGAACACCTTCAAAG	0	0	0	0	0	1	2	3
	21UR-11679	TATTTCAGATGCGAGAATAGT	0	0	0	0	1	0	1	2
†	21UR-11680	TATTTAGCAATCGTTCTACAA	0	0	1	0	0	0	0	1
†	21UR-11681	TATTTAAGACACTATGCATAC	0	0	0	0	0	0	0	0
†	21UR-11682	TATTGCGAACAACGGCTCACT	0	0	0	2	23	22	18	65
†	21UR-11683	TATTGATCCCGTTGTTTATAT	0	0	0	0	0	0	0	0
†	21UR-11684	TATTCGTTTCCAATAAATCGT	0	0	0	0	0	0	0	0
†	21UR-11685	TATTCCTTCCTTTTGATCCCC	0	0	0	0	0	0	0	0
†	21UR-11686	TATTCCGTTGCTCAAATCAGG	0	0	0	0	0	0	2	2
	21UR-11687	TATTCATCATGATTTCCATCA	0	0	0	0	0	0	0	0
†	21UR-11688	TATTCACTCTAAAGGAAACTG	0	0	0	0	0	0	0	0
†	21UR-11689	TATTCAAGTGCATGTAAGGAA	0	0	0	0	0	0	0	0
†	21UR-11690	TATTCAAAGCACAGTGCAAAA	0	0	0	0	0	0	1	1
†	21UR-11691	TATTATTTCTCGTACTCAAAA	0	0	0	0	0	0	0	0
†	21UR-11692	TATTATTACTTGAATAATTGC	0	0	0	0	1	0	0	1
†	21UR-11693	TATTATGCCCTTTTCCGTTTT	0	0	0	0	1	0	0	1
†	21UR-11694	TATTATCTGATCGACATTGAT	0	0	0	0	0	0	1	1
†	21UR-11695	TATTAGATCATTAGAGTACGA	0	0	0	1	2	0	1	4
†	21UR-11696	TATTACTGTTTTATTGAGTAC	0	0	0	0	0	1	0	1
	21UR-11697	TATTACGTTGTTTGTACAAAT	0	0	0	0	1	0	0	1
	21UR-11698	TATTACCCACAAAAAAAAATT	0	0	0	0	1	1	0	2
†	21UR-11699	TATGTTCTCGTCACATATTTA	0	0	0	0	0	0	0	0
	21UR-11700	TATGTTCATAAAAATCATCAT	0	0	0	0	0	1	0	1
†	21UR-11701	TATGTATGACATCGAGTGTGG	0	0	0	0	0	0	0	0
	21UR-11702	TATGGAAACAGTACTGAAGTC	0	0	0	0	0	0	0	0
	21UR-11703	TATATTGCTAATTTCTCAAAC	2	0	0	0	0	0	1	3
†	21UR-11704	TATAGATCCATCAATTTCAAA	0	0	0	0	0	0	0	0
†	21UR-11705	TATACCTTTATTCCCAAATTG	0	0	0	0	0	1	0	1
	21UR-11706	TATAATTCAGGAGTGGTACAA	0	0	0	0	0	0	0	0

	†	21UR-11707	TATAATCACTGTATCTTTTTT	0	0	0	0	1	1	0	2
	†	21UR-11708	TAGTTTAACTTTGAGTTTGAA	0	0	0	0	0	0	0	0
		21UR-11709	TAGTGCTATTTTAATATAATC	0	0	0	0	1	0	2	3
	†	21UR-11710	TAGTGCCATTAATTCATCCGA	0	0	0	0	0	0	0	0
	†	21UR-11711	TAGTGCATATCCTTTTAAAAT	0	0	0	0	1	1	0	2
	†	21UR-11712	TAGTCTTCGAATCCATGCAAA	0	0	0	0	0	0	0	0
		21UR-11713	TAGTCTTCAAACGTATTTATA	0	0	0	0	0	0	0	0
		21UR-11714	TAGTCTTATTTAGTTCATTAA	0	0	0	0	0	0	0	0
	†	21UR-11715	TAGTCTCCTTTCTTTCTAATT	0	0	0	0	0	0	0	0
	†	21UR-11716	TAGTCGCTCTTATTGTTTATT	0	0	0	0	0	0	0	0
		21UR-11717	TAGTATTCAAAATGAAAACTG	0	0	0	0	0	0	0	0
	†	21UR-11718	TAGTATGTTCGTGGTGAAAGA	0	0	0	0	0	0	0	0
	†	21UR-11719	TAGTAGTCGCCCTAAATGCTA	0	0	0	0	0	0	0	0
		21UR-11720	TAGTACTGATCCAATTTTCAG	0	0	0	0	0	0	0	0
	†	21UR-11721	TAGTAATCCATGTGTAGTAAT	0	1	0	0	0	0	0	1
		21UR-11722	TAGTAAACATCGTGAGCTACG	0	0	0	0	0	0	0	0
		21UR-11723	TAGGTTACTTTCTTGGTTTTA	0	0	0	0	0	0	0	0
		21UR-11724	TAGCACTCATATAAAAAAGGA	0	0	0	0	0	0	0	0
		21UR-11725	TAGCAATTTTTGAAAAAAAAT	0	0	0	0	0	0	0	0
	†	21UR-11726	TAGAATCTAGAGTTGAGCAAA	0	0	0	0	0	0	0	0
		21UR-11727	TAGAATCGAACTTTTTTTTTC	0	0	0	0	0	0	0	0
		21UR-11728	TAGAATACAGGAAAATAGGAA	0	0	0	0	0	0	1	1
		21UR-11729	TAGAAGGGAGTACCAGAAAGA	0	0	0	0	0	0	0	0
		21UR-11730	TAGAACAGATACTTCCTATAG	0	0	0	0	0	0	0	0
		21UR-11731	TAGAAAATTCAAGAATGTTAT	0	0	0	0	1	0	0	1
	†	21UR-11732	TACTTTTGATCTACGGAGAAA	0	0	0	0	0	0	0	0
	†	21UR-11733	TACTATCTTTCTAGTTCTCTC	0	0	0	0	0	0	0	0
	†	21UR-11734	TACTACTCCTGTGTTTTGTAA	0	0	0	0	0	0	0	0
	†	21UR-11735	TACTACATATAGCATCGAAAT	0	0	0	0	0	0	0	0
		21UR-11736	TACGTGAACTTTTTGAAACTT	0	0	0	0	0	0	0	0
		21UR-11737	TACCTTTCTTCAAGTTTCGTG	0	0	0	0	0	0	0	0
	†	21UR-11738	TACCTTGTTATGATCGAAGAT	4	0	0	2	11	15	17	49
		21UR-11739	TACATTTCCAAAAATGTTTTC	0	0	0	0	0	0	0	0
		21UR-11740	TACATGGCAAAATCAATATTG	0	0	0	0	2	1	0	3
	†	21UR-11741	TACAGCATATGAAAACAAAAA	0	0	0	0	0	2	1	3
		21UR-11742	TACAGATCTTCGATTTATTGA	0	0	0	0	0	0	0	0
		21UR-11743	TACACTCGACTTTATAAAAAG	0	0	0	0	0	0	0	0
		21UR-11744	TACAATTTTTAAAAATTAACA	0	1	1	0	5	0	3	10
		21UR-11745	TACAATACTTTGAAACAGTAA	0	0	0	0	19	10	1	30
		21UR-11746	TACAACACTGTCAACAACTTG	0	0	0	1	0	0	0	1
		21UR-11747	TACAAAACTATCGAAATGATC	0	0	0	0	0	0	0	0
	†	21UR-11748	TAATTGGACTTGGTGATAATT	1	0	0	1	21	9	9	41
	†	21UR-11749	TAATTCTTCTATAGATTGTTG	0	0	0	0	2	4	3	9
	†	21UR-11750	TAATTCTGACATTTCTTCTGA	0	0	0	0	0	1	0	1
		21UR-11751	TAATTCGTCTATACCAAACAG	0	0	0	0	1	0	1	2
		21UR-11752	TAATTCGGTTACATCCAATAG	0	0	0	0	3	2	1	6
	†	21UR-11753	TAATTCCGATCTAGATTTCAT	0	0	0	0	0	0	0	0
	†	21UR-11754	TAATTCAGGAGGTTTACCTTT	0	0	0	0	0	0	0	0
	†	21UR-11755	TAATTCACTGATTAACTGAAT	0	0	0	0	1	1	0	2
		21UR-11756	TAATTACAATTTTCAATACAA	1	0	0	0	3	1	0	5
	†	21UR-11757	TAATGTGCTGTGATTTTGTGT	0	0	0	0	0	0	0	0
	†	21UR-11758	TAATGGCTCAAAGAAATTTTA	0	0	0	0	0	0	0	0
		21UR-11759	TAATGCTAAAATTTTTTACAC	0	0	0	0	3	0	1	4
	†	21UR-11760	TAATCTTTTCGTTCCTCTATC	0	0	0	0	0	0	0	0
	†	21UR-11761	TAATCTGATCAATGAAATATT	0	0	0	0	0	0	0	0
		21UR-11762	TAATCCGAATTTTTTTTCATT	0	1	0	0	1	3	1	6
	†	21UR-11763	TAATATTGTATCGAATGAAAT	0	0	0	0	0	0	0	0
*	†	21UR-11764	TAATATCTTGTGGGCATATAA	1	4	0	0	18	14	2	39
		21UR-11765	TAATAGGAAAAAAAACTTATG	0	0	0	0	1	0	0	1
	†	21UR-11766	TAATACGAGGCACGAATTGTT	0	0	0	0	0	0	0	0
	†	21UR-11767	TAATAATCCGAAGCAATTGAA	0	0	0	0	0	0	0	0
		21UR-11768	TAAGTACTTTTCGAAGCCAAA	0	0	0	0	0	0	0	0
		21UR-11769	TAAGGAATGCTTTCTCAAGAA	0	0	0	0	0	0	0	0
		21UR-11770	TAAGAATTTTGAAAAAAAAAT	0	0	0	0	0	0	0	0

		21UR-11771	TAAGAAACTACTTGATTTATT	0	0	0	0	0	0	0	0
		21UR-11772	TAACTAAAGAAACTCATTTCT	0	0	0	0	0	0	0	0
		21UR-11773	TAACAGCTATAAATATTTTAG	0	0	0	1	0	0	0	1
		21UR-11774	TAACAAATCTTCAACTTACTT	0	0	0	0	0	0	0	0
		21UR-11775	TAAATTTATTCTACGTTTTGA	1	0	0	0	1	0	1	3
	†	21UR-11776	TAAATATTTGAACAATTTTAA	0	1	0	0	3	0	0	4
		21UR-11777	TAAAGTTTTCATTTACGTTTT	0	0	0	0	0	0	0	0
		21UR-11778	TAAACTCAAAAACCGAACCAA	0	0	0	0	0	0	0	0
	†	21UR-11779	TAAAATTTTTTTAAACAATCA	0	0	0	0	0	0	0	0
		21UR-11780	TAAAATGTTGCCAGAGAGCTG	0	0	0	0	0	0	0	0
		21UR-11781	TAAAATAGGATTTATTTTTCA	0	0	0	1	1	0	0	2
*		21UR-11782	TAAAATACTTGAGAAAAATTT	0	0	0	1	9	0	0	10
		21UR-11783	TAAAATAAATATGCAAATATT	0	0	0	0	0	0	0	0
		21UR-11784	TAAAACACGGAATCAGTAGTA	0	0	0	0	1	0	0	1
	†	21UR-11785	TAAAAAGGTAATCTTTAAAAG	0	0	0	0	0	0	0	0
		21UR-11786	TAAAAAACGAGTTACCGATTT	1	0	0	0	0	1	0	2
	†	21UR-11787	GTCATAGCGATACGTTCTTAT	52	7	6	3	16	47	7	138
		21UR-11788	GGTTTCTTAAATAATGTCATA	0	0	0	0	0	0	0	0
	†	21UR-11789	GGCTGATTTTGTAGTAACTGT	0	0	0	0	0	0	0	0
	†	21UR-11790	GCACCTTCCGGAAATCAAGAT	2	0	0	0	0	4	8	14
	†	21UR-11791	GATAGGGAAATAAAGACGATT	0	0	1	0	6	2	1	10
	†	21UR-11792	GACACCGATAAGAGAACAAAA	0	0	0	0	0	0	0	0
	†	21UR-11793	GAAATCTTCATGGTCGACGAT	51	5	5	13	68	137	126	405
	†	21UR-11794	CTCACAGGTCTTAACTATGCA	0	0	0	0	0	0	0	0
		21UR-11795	CTAGTGGAAGGTTTCTGATAT	0	0	0	0	0	1	0	1
	†	21UR-11796	CTAGGAAAACTGAAAACGCAT	0	0	0	1	15	5	2	23
		21UR-11797	CGTTGAACTGTTGCTCCGACA	0	0	0	0	0	0	0	0
	†	21UR-11798	CGGTATTGGTTGCTTTATCTT	0	0	0	0	0	0	0	0
	†	21UR-11799	CGCATAGTTCCGATAGTCTTT	0	0	0	0	3	0	0	3
	†	21UR-11800	CGATTCTCAAGCTCTTGTTCT	1	1	0	0	0	3	0	5
		21UR-11801	CGATGATTTTCAAGACGTGAA	0	0	0	0	0	0	0	0
	†	21UR-11802	CGACAAAATGCAGGCAATGAG	0	0	0	1	0	0	0	1
	†	21UR-11803	CAAGACGTTAAGATTAAAGGT	0	0	0	0	0	0	0	0
		21UR-11804	CAACATCAAAAAGTTCATCAG	0	0	0	0	0	0	0	0
		21UR-11805	CAAAAAAACTACTTTCTCTTT	0	0	0	0	1	0	0	1
	†	21UR-11806	ATTGTATCGAATGAAATTTGT	3	0	0	1	2	4	0	10
		21UR-11807	AGTTTTTAATTACAAAGGCTA	0	0	0	0	0	0	0	0
		21UR-11808	ACTAAAATCATCACTGATAAA	0	0	0	0	0	0	0	0
*	†	21UR-11809	AAGCACGGCCTCTGTGAAATT	98	158	64	68	454	1008	55	1905
		21UR-11810	TTTTTTTTTTATCAGCAAAAC	2	0	0	0	0	3	1	6
		21UR-11811	TTTTTTTGTTTCTTTCTCCTA	0	0	0	0	0	0	0	0
		21UR-11812	TTTTTTTGAATTTAATATTGC	0	0	0	0	0	0	0	0
	†	21UR-11813	TTTTTTTAGTTGGCGAAGGAT	12	3	2	5	50	99	179	350
		21UR-11814	TTTTTTTAGAGATTTTGATGT	0	1	0	3	16	10	4	34
		21UR-11815	TTTTTTCAGGACGTTTGCGGA	0	0	0	0	0	0	0	0
	†	21UR-11816	TTTTTTCAGAGAAAAGTAAAA	0	0	0	0	0	0	1	1
		21UR-11817	TTTTTTACGGTTACTAGAAAC	0	0	0	0	0	1	0	1
		21UR-11818	TTTTTTAATGCAATTAGGTTC	0	0	0	0	0	0	0	0
	†	21UR-11819	TTTTTGTTACAGAGTATAAAC	0	0	0	0	0	0	0	0
	†	21UR-11820	TTTTTGACTATACTCTGTGAA	0	0	0	0	0	1	0	1
		21UR-11821	TTTTTGAAAAAGTACAAAAAA	0	0	0	0	0	0	0	0
	†	21UR-11822	TTTTTCGATCAATCAAATTCG	2	0	0	1	1	4	0	8
	†	21UR-11823	TTTTTCCTTTTGTCACAAATG	0	0	0	0	0	0	0	0
		21UR-11824	TTTTTCCTTTAAAAGGGGAGT	0	0	0	0	0	2	1	3
		21UR-11825	TTTTTCAGTGTTTAACATGAC	0	0	0	0	0	0	0	0
		21UR-11826	TTTTTCAGGCAAGGAATGATG	0	1	0	0	0	0	0	1
		21UR-11827	TTTTTCAGAACACTTTTGGAA	0	0	0	0	0	0	0	0
		21UR-11828	TTTTTCAACTTGTTGCCAAGT	0	0	0	0	0	0	0	0
		21UR-11829	TTTTTCAACGATATTTTTATT	0	0	0	0	0	0	0	0
	†	21UR-11830	TTTTTATTATCAGTTGTGATA	0	0	0	0	1	0	0	1
	†	21UR-11831	TTTTTATATTAACGAAAAAAG	0	0	0	0	0	0	0	0
	†	21UR-11832	TTTTTACTTTATTGTGCAATA	0	0	0	0	0	0	0	0
	†	21UR-11833	TTTTTACGAACAATAACAAAA	0	0	0	0	0	0	0	0
		21UR-11834	TTTTTAAGGTTATAGGTAATC	0	0	0	0	0	0	0	0

†	21UR-11835	TTTTTAAATCCTTCTAGTTGG	0	0	0	0	0	0	2	2
	21UR-11836	TTTTGTCAATTTCTCTCAAAA	0	0	0	0	0	0	0	0
	21UR-11837	TTTTGTAATTAAATAAGAAAG	0	0	0	0	0	0	0	0
	21UR-11838	TTTTGTAATAATTTGAAATTG	0	0	0	0	2	0	0	2
†	21UR-11839	TTTTGGTCGATCGCTAAAATC	0	0	0	0	1	1	1	3
†	21UR-11840	TTTTGGATTGATTTGTGCTAC	0	0	0	1	1	1	1	4
	21UR-11841	TTTTGGACAAAAAGTCTTCTC	0	0	0	0	0	0	0	0
†	21UR-11842	TTTTGCGTACGGAAAACAAAA	0	0	0	0	1	2	0	3
	21UR-11843	TTTTGATTTTCGTTTTACATC	0	0	0	0	1	2	0	3
	21UR-11844	TTTTGATTTATATACGGAGTA	1	1	1	0	0	2	0	5
	21UR-11845	TTTTGATTGCGGATTATTGAG	0	0	0	0	0	0	0	0
†	21UR-11846	TTTTGACTTGTATTTTGACAA	0	0	0	0	0	0	0	0
†	21UR-11847	TTTTGACAGTTGCTAGGTTTT	0	0	0	0	0	1	0	1
†	21UR-11848	TTTTGAACCGTTTCCATAGTT	0	0	0	0	0	0	0	0
	21UR-11849	TTTTGAAATTTCTGTTTTCCA	0	0	0	0	0	0	0	0
†	21UR-11850	TTTTGAAATCGAACTTCAAAA	0	0	0	0	0	0	0	0
	21UR-11851	TTTTGAAATATTTCATCAACT	0	0	0	0	1	0	0	1
†	21UR-11852	TTTTGAAAGTATTTAGAAACT	0	0	1	0	0	0	0	1
†	21UR-11853	TTTTGAAACCCATCTAGATTT	0	0	0	0	0	0	0	0
	21UR-11854	TTTTCTTCAACTGTGGATTAA	0	0	0	0	0	0	0	0
	21UR-11855	TTTTCTGATTTCTCAATAAAA	0	0	0	0	0	0	0	0
	21UR-11856	TTTTCTCTATTTGTATATGTA	0	0	0	0	0	0	0	0
	21UR-11857	TTTTCTCCTTTTACGCAGTGA	0	0	0	0	0	0	0	0
†	21UR-11858	TTTTCTCATCAACTTACAGGC	0	0	0	0	0	0	0	0
	21UR-11859	TTTTCGGGCATAATTTGAACA	0	0	0	0	0	0	0	0
	21UR-11860	TTTTCGGGAAAATTCGAAAGA	0	0	0	0	0	0	0	0
	21UR-11861	TTTTCGATCTTTTTTTCAGCA	0	0	0	0	0	1	1	2
	21UR-11862	TTTTCCTCACAATCAAAATAT	0	0	0	0	0	0	0	0
	21UR-11863	TTTTCCTAGTATCAGGCTAAT	0	0	0	0	0	2	0	2
	21UR-11864	TTTTCCGTTTAAATTGATTTT	0	0	0	0	0	0	0	0
†	21UR-11865	TTTTCACCTTAACTTTAGACC	0	0	0	1	0	0	0	1
	21UR-11866	TTTTATTTAATTTAGATACAT	0	0	0	0	0	0	0	0
†	21UR-11867	TTTTATTGGATAAATGAGAAC	0	0	0	0	2	0	0	2
	21UR-11868	TTTTATTAAATTTGCAATGTT	0	0	0	0	0	0	0	0
†	21UR-11869	TTTTATGGAACAGTTTCCAAT	0	0	0	0	0	0	0	0
†	21UR-11870	TTTTATGCAGTTCTCTAACCG	0	0	0	0	0	0	0	0
	21UR-11871	TTTTATGATTTCTGTTGTCGA	0	0	0	0	0	0	1	1
†	21UR-11872	TTTTAGTTTTTTTTCGAATTA	0	0	0	0	0	0	0	0
	21UR-11873	TTTTAGTTCCGATTTCTTTTT	0	0	0	0	0	1	0	1
	21UR-11874	TTTTAGGGTATCTGGAAGAAG	0	0	0	0	0	2	0	2
†	21UR-11875	TTTTAGAATTTTAGTCGGTAT	2	0	0	0	0	1	0	3
	21UR-11876	TTTTACACACAATTGGTTTTC	0	0	0	0	2	1	4	7
†	21UR-11877	TTTTAAGGTATTTGTTCTATA	0	0	0	0	1	0	0	1
†	21UR-11878	TTTTAACCTTTTTTGTCGTTT	0	0	0	0	0	0	1	1
†	21UR-11879	TTTTAAAGTTTTTTAGTTTCA	0	0	0	0	0	0	0	0
	21UR-11880	TTTTAAAGAATCTTTTGCGGA	6	0	0	1	2	8	1	18
	21UR-11881	TTTGTTTGTTTTGCACCCTTG	0	0	0	0	0	0	0	0
	21UR-11882	TTTGTTTCCCACGTAAACACC	0	0	0	0	0	0	0	0
†	21UR-11883	TTTGTTGATGTTCTGAACACA	0	0	0	0	1	0	0	1
†	21UR-11884	TTTGTTGACGTACGTGGTAAA	0	0	0	0	7	5	1	13
†	21UR-11885	TTTGTTCAGTGTAGGATAATG	0	0	0	0	0	0	0	0
	21UR-11886	TTTGTCCACATTTACTACTCT	0	0	0	0	0	0	0	0
	21UR-11887	TTTGTAAAAAATTATGTTTTT	0	0	0	0	0	0	0	0
†	21UR-11888	TTTGGTCGATCGCTAAAATCG	0	0	0	0	0	1	0	1
†	21UR-11889	TTTGGTACCATATAAGCAGAG	1	0	1	0	0	0	0	2
†	21UR-11890	TTTGCTAATCCAAAAAAACGT	0	0	0	0	0	0	0	0
	21UR-11891	TTTGCATAAGTATTGTTACAT	0	0	0	0	1	0	1	2
	21UR-11892	TTTGCAAAATATTTCCCTTCA	0	0	0	0	0	0	0	0
	21UR-11893	TTTGATTGGACTCGAATGAGG	0	0	0	0	0	0	0	0
†	21UR-11894	TTTGATGTAATTTTGAACAAA	0	0	0	0	1	0	0	1
	21UR-11895	TTTGATACGGCTAGAATTTCA	0	0	0	0	0	0	0	0
†	21UR-11896	TTTGAGATTATTTATCTTTAA	0	0	0	0	0	0	0	0
	21UR-11897	TTTGAATTGAATTTTATGAAT	0	0	0	0	0	0	0	0
	21UR-11898	TTTGAATAAAGTTTGCTGAAA	0	0	0	0	0	0	0	0

†	21UR-11899	TTTGAAGTAGGATTTTGAAAT	0	0	0	0	0	0	0	0
	21UR-11900	TTTGAAATCCACTTTTATAAT	0	0	0	0	0	0	0	0
†	21UR-11901	TTTGAAACGCTAGGGAAAATT	0	0	0	0	0	0	0	0
	21UR-11902	TTTGAAAAGCCATAAAAGATA	0	0	0	0	0	0	0	0
	21UR-11903	TTTGAAAAACTTCCATGGCAC	0	0	0	0	0	0	1	1
†	21UR-11904	TTTCTTTGTTGTTTAAAACTA	0	0	0	0	0	0	0	0
†	21UR-11905	TTTCTTCATTGGGGGGTCGAA	0	0	0	0	0	0	0	0
	21UR-11906	TTTCTTATTTTTGTCATTTAT	0	0	0	0	4	0	0	4
†	21UR-11907	TTTCTGATTCTCCTCCCATAG	0	0	0	0	0	0	0	0
†	21UR-11908	TTTCTCTCATTGATTCATTTT	0	0	0	0	0	0	0	0
†	21UR-11909	TTTCTCGGGAAAAAAATATTG	0	0	0	0	0	0	0	0
	21UR-11910	TTTCTAGAACCCTGAACTATT	0	0	0	0	0	0	0	0
†	21UR-11911	TTTCTAACCAAAACTAATGAT	0	0	0	0	0	0	0	0
†	21UR-11912	TTTCGTTTTCCAAACTTGCAA	0	0	0	0	0	0	0	0
	21UR-11913	TTTCGCGACTTTTATGAACTG	0	0	0	0	0	0	0	0
†	21UR-11914	TTTCGATTCGAGTGGTTGAAG	0	0	0	0	0	0	0	0
†	21UR-11915	TTTCGACGAGATCAAAAGTTT	1	0	0	0	0	0	0	1
	21UR-11916	TTTCCTTTTTCAAAACCATGT	0	0	0	0	1	0	0	1
	21UR-11917	TTTCCTTATGTAAGTTATTTA	0	0	0	0	0	0	0	0
†	21UR-11918	TTTCCTTATGAGTAAAAAAAC	0	0	0	0	0	0	0	0
†	21UR-11919	TTTCCTGATTGTTTGAGTAAG	0	0	0	0	1	0	0	1
†	21UR-11920	TTTCCAGCCATATTTAAATCA	0	0	0	0	2	1	0	3
	21UR-11921	TTTCCACAAATGAAGGAAAAT	0	0	0	0	0	0	0	0
	21UR-11922	TTTCCAATTCAAAAATGTAAA	0	0	0	0	0	0	0	0
	21UR-11923	TTTCATGTGGAGTAGTGGGCT	0	0	0	0	0	0	0	0
†	21UR-11924	TTTCATATCGAAGATTAAATT	0	0	0	0	0	0	0	0
†	21UR-11925	TTTCATAGTTATCAGATTTTC	1	0	0	0	0	0	0	1
†	21UR-11926	TTTCAGTTTGAACCGCCGTGT	0	0	0	0	0	2	1	3
	21UR-11927	TTTCAGTCTCGCTTATAATTC	0	0	0	0	0	0	1	1
	21UR-11928	TTTCAAATTGAAATTGATTAA	0	0	0	0	0	0	0	0
†	21UR-11929	TTTCAAATGGACAACTAGCAA	0	0	0	0	2	0	0	2
	21UR-11930	TTTCAAACTTCTACCGTATCA	0	0	0	0	0	0	0	0
	21UR-11931	TTTCAAAATCCCATGCACTTT	0	0	0	0	0	0	0	0
	21UR-11932	TTTATTTTGTTTTTCTTACGT	0	0	0	0	1	0	0	1
	21UR-11933	TTTATTTTCTAGGACTTATAA	0	0	0	0	0	0	0	0
	21UR-11934	TTTATTTGCCGAACAAATTTT	0	0	0	0	0	0	0	0
†	21UR-11935	TTTATTTGATGAAAACTTTGC	0	0	0	0	1	1	0	2
†	21UR-11936	TTTATTTCACTTCATTGATGC	3	0	0	0	0	1	1	5
†	21UR-11937	TTTATTTAGCAATCGTTCTAC	0	0	0	0	0	0	0	0
†	21UR-11938	TTTATTGATTAGGTTTCAAAT	0	0	0	0	0	0	0	0
	21UR-11939	TTTATTGAAAAATTCGGGATA	0	0	0	0	0	0	1	1
†	21UR-11940	TTTATTATGTATCTAGCAGCC	0	0	0	0	0	2	0	2
	21UR-11941	TTTATTAACAATATCCTTAGC	0	0	0	0	1	0	1	2
	21UR-11942	TTTATGTACTAACAAGTTGGC	0	0	0	0	0	2	0	2
†	21UR-11943	TTTATGGACTTAACTTCAGAA	0	0	0	0	1	0	0	1
†	21UR-11944	TTTATGAGTCCTATTTAATCG	0	0	0	0	1	0	1	2
†	21UR-11945	TTTATGAAAGGAGACAAAAAA	0	0	0	0	0	1	0	1
†	21UR-11946	TTTATCCAGAGTCGGTATAAC	0	0	0	0	2	4	2	8
	21UR-11947	TTTATCATAGTAACGAAGTTA	0	0	0	0	0	1	0	1
	21UR-11948	TTTATAGAGGAACACATATAC	0	0	0	0	0	2	0	2
†	21UR-11949	TTTAGTATTCATCAAAAAAAA	0	0	0	0	0	0	0	0
†	21UR-11950	TTTAGAGCATTTCTTTGAGCT	0	0	0	0	1	0	0	1
	21UR-11951	TTTACTTTTTATGAAAAAAAA	0	0	0	0	0	0	0	0
†	21UR-11952	TTTACTGGTTTTCATTTTAAA	0	0	0	0	0	0	0	0
	21UR-11953	TTTACGGGAAACTTTTTTTGG	0	0	0	0	0	1	0	1
†	21UR-11954	TTTACCGGTGCATACATTAAG	0	0	0	0	0	0	0	0
	21UR-11955	TTTACCAAATCGGTATCGGAG	0	0	0	0	0	0	0	0
	21UR-11956	TTTAATTAAAAAAAAACCAGA	0	0	0	0	0	0	1	1
†	21UR-11957	TTTAATAGGCAATTCTTAATC	0	0	0	0	0	1	0	1
†	21UR-11958	TTTAAGCCATATTTTTTAAAC	0	0	0	0	0	0	0	0
†	21UR-11959	TTTAACTCGTATTGGTTTTTG	0	0	0	0	1	1	0	2
†	21UR-11960	TTTAACAAGATGCTTTTTTAA	0	0	0	0	0	5	2	7
†	21UR-11961	TTTAAAGTTTTTTAGTTTCAA	0	0	0	0	0	0	0	0
†	21UR-11962	TTTAAAAGTATAAACCACTAG	0	0	0	0	0	0	0	0

	21UR-11963	TTGTTTTGAAATTTACAGTTA	0	0	0	2	0	0	0	2
* †	21UR-11964	TTGTTTGTGGAAGCGTGAGCA	0	0	0	0	1	1	0	2
†	21UR-11965	TTGTTTGAAGTAGGATTTTGA	0	0	0	0	0	0	0	0
†	21UR-11966	TTGTTCGCAAATCTGTCCAGC	0	0	0	0	0	0	0	0
	21UR-11967	TTGTTCCACTATTTCTAGTTA	0	0	0	0	0	0	0	0
†	21UR-11968	TTGTTCACTGACAAATTACTC	0	0	0	0	0	0	0	0
	21UR-11969	TTGTTATACAGAAAAAAATTGT	0	0	0	1	0	0	0	1
†	21UR-11970	TTGTTACTGCTATGCTTTTCT	0	0	0	0	0	0	0	0
	21UR-11971	TTGTGTTTATTTAAAACAATT	0	0	0	0	0	0	0	0
†	21UR-11972	TTGTGTGCGCTGTTTTGGTCA	0	0	0	0	0	0	0	0
	21UR-11973	TTGTGGATTTTGACTTTCAAG	0	0	0	0	0	0	0	0
†	21UR-11974	TTGTGCTTCTTTCGAATGATA	0	0	0	0	0	0	0	0
	21UR-11975	TTGTCTAAAATGATTAGGATG	0	0	0	0	0	0	0	0
†	21UR-11976	TTGTCATAATGTTGGGAGAAG	0	0	0	1	1	1	0	3
	21UR-11977	TTGTCACGTCACCACCGTTCA	0	0	0	0	0	0	0	0
	21UR-11978	TTGTACTATGGCATTTTGGCA	0	0	0	0	0	0	0	0
	21UR-11979	TTGTACGACCTGTCTTACAAT	0	0	0	0	0	0	0	0
†	21UR-11980	TTGTAAGGTTGCTCTAGAAAT	2	0	0	0	0	0	0	2
†	21UR-11981	TTGGTTCGCTTGGAAAGTAAC	0	0	0	0	0	0	0	0
†	21UR-11982	TTGGTATTAATTATCAGGGTT	0	0	0	0	0	0	0	0
	21UR-11983	TTGGTATAAACACTTCCAATA	0	0	0	0	0	0	0	0
	21UR-11984	TTGGTAATTTTTTTAATTACG	0	0	1	0	0	0	0	1
†	21UR-11985	TTGGGTTTCGAAGCAAGAGCC	0	0	0	0	0	0	0	0
†	21UR-11986	TTGGGAATGGAAAAATACGAT	0	0	0	0	0	0	0	0
†	21UR-11987	TTGGGAAGTTTGGTCTTACGG	0	0	0	0	0	0	0	0
	21UR-11988	TTGGATCGAGGTTTTATCTTC	0	0	0	0	0	0	0	0
†	21UR-11989	TTGGATAGTACAAAAAAATATT	0	0	0	0	1	0	0	1
†	21UR-11990	TTGGAGTCTATTTCCGTAAAT	0	0	0	0	0	0	0	0
†	21UR-11991	TTGCTTTTGTAGAACTGACTT	0	0	0	0	0	0	0	0
†	21UR-11992	TTGCTTTGGTGGAACTTAAAA	0	0	0	0	0	1	0	1
†	21UR-11993	TTGCTTCTAATGGTTTTCTAT	0	0	0	0	0	0	0	0
†	21UR-11994	TTGCGCATATCAATTGATCTT	0	0	0	0	0	0	0	0
†	21UR-11995	TTGCCACTGTTTAATATTCAT	0	0	0	0	0	0	0	0
†	21UR-11996	TTGCATTGTCTTGCTGAAATA	0	0	0	0	0	0	0	0
	21UR-11997	TTGCATATTATTGTCCATGTA	0	0	0	0	0	0	0	0
†	21UR-11998	TTGCATACACGGTACTCAGAG	0	0	0	0	1	1	0	2
†	21UR-11999	TTGATTTGGAATACGTTGATT	0	0	0	0	0	0	0	0
†	21UR-12000	TTGATTGTACTGCTTTACAGA	0	0	0	0	0	0	0	0
†	21UR-12001	TTGATTCTTCAAACGCCCATC	0	0	0	0	0	0	0	0
†	21UR-12002	TTGATTCGAAGGTTGTCAGAT	0	0	0	0	0	2	0	2
†	21UR-12003	TTGATTCATCGCCATTAAAAA	0	0	0	0	0	0	0	0
†	21UR-12004	TTGATTCACAATTTTTTAAAC	0	0	0	0	0	0	0	0
	21UR-12005	TTGATTACGCCTTGGGTCGAA	0	0	0	0	0	0	0	0
†	21UR-12006	TTGATGTTTCGTAAAACTAAT	0	0	0	0	0	0	0	0
†	21UR-12007	TTGATGTTACTCGTAATTTTA	0	0	0	0	0	0	0	0
†	21UR-12008	TTGATGATGCCTTGGTACTTC	0	0	0	0	0	0	0	0
†	21UR-12009	TTGATCCCGTTGTTTATATTG	0	0	0	0	0	0	0	0
	21UR-12010	TTGATCATACCAGTGTTGATT	0	0	0	0	0	0	0	0
†	21UR-12011	TTGATCACTACGTGGTACAAT	0	0	0	0	0	0	0	0
†	21UR-12012	TTGATATTGATTGTGAGTTCA	0	0	0	0	0	1	0	1
	21UR-12013	TTGATAGCCGGCTAGTTCAAC	0	0	0	0	0	0	0	0
†	21UR-12014	TTGATAAGAACGTCCAAACAA	0	0	0	0	1	2	0	3
†	21UR-12015	TTGAGTGAGGAATATTCGTAG	0	0	0	0	0	0	0	0
†	21UR-12016	TTGAGTCGGTCAATTTAAACA	0	0	0	0	0	0	1	1
†	21UR-12017	TTGAGATTATTTATCTTTAAA	0	0	0	0	0	0	0	0
	21UR-12018	TTGAGAAATTCAACAATGATA	0	0	0	0	0	0	0	0
†	21UR-12019	TTGACTCAACTAACCTGAATT	0	0	0	0	0	0	0	0
	21UR-12020	TTGACGTGATCGATACTAAAA	0	0	0	0	0	0	0	0
†	21UR-12021	TTGAATTCCAAATTCATTACA	0	0	0	0	0	0	0	0
†	21UR-12022	TTGAATAAGACAAATTAGACA	0	0	0	0	11	12	0	23
†	21UR-12023	TTGAAGCTTTTGCAAAAACAC	0	0	0	0	0	0	0	0
†	21UR-12024	TTGAAGCACTGATCCGGTTAA	2	2	1	2	1	1	0	9
†	21UR-12025	TTGAAGAATTTATTTCGGGAA	0	0	0	0	0	0	0	0
†	21UR-12026	TTGAACTTTTTGGAAACATTA	0	0	0	1	0	0	0	1

†	21UR-12027	TTGAAATTCCAAAGTCCATAA	0	0	0	0	0	0	0	0
	21UR-12028	TTGAAACGGTCGATTTTTCAA	0	0	0	0	0	0	0	0
	21UR-12029	TTCTTGTAATCTTTATTGTCT	0	0	0	0	1	1	0	2
†	21UR-12030	TTCTTGATTTCTTCGCGAAGA	0	0	0	0	0	0	0	0
†	21UR-12031	TTCTTGACAAATACAGCAACA	0	0	0	0	0	0	0	0
	21UR-12032	TTCTTCTGATTGTTTTGCTAA	0	0	0	0	0	0	0	0
	21UR-12033	TTCTTACTATGGTTTTTGATG	0	0	0	0	0	0	0	0
	21UR-12034	TTCTGTTTTTAATAATAAAAC	0	0	0	0	0	0	0	0
	21UR-12035	TTCTGTCAAGTGAACCACAAG	0	0	0	0	0	0	0	0
†	21UR-12036	TTCTGACTAAGTTCTGTGAGT	0	0	0	0	0	0	0	0
	21UR-12037	TTCTGAAAATTGAAAAACGTA	0	0	0	0	0	0	0	0
	21UR-12038	TTCTCTCTTTTCCTTTTCAAT	0	0	0	0	0	1	0	1
	21UR-12039	TTCTCGACGAACACCTGGTCG	0	0	0	0	0	0	0	0
	21UR-12040	TTCTCCTCTTAGGTTTGAGGT	0	0	0	0	1	0	0	1
†	21UR-12041	TTCTCCGAATGACAAATTTTT	0	0	0	0	0	0	0	0
†	21UR-12042	TTCTCCAAGCCTTTTCACTTT	0	0	0	0	0	0	0	0
†	21UR-12043	TTCTCAAACTTCTATATGATA	0	0	0	0	0	0	0	0
†	21UR-12044	TTCTAATGGGTTTTTGCAAAC	0	0	0	0	0	0	0	0
†	21UR-12045	TTCTAATCTTTGTGAATCACC	0	0	0	0	0	0	0	0
†	21UR-12046	TTCTAAGGTGATCATTAATTG	0	0	0	0	0	0	0	0
	21UR-12047	TTCGGATCCAATATGTCACCG	0	0	0	0	0	0	0	0
†	21UR-12048	TTCGGATAGGGAAACAGCAAA	0	0	0	0	1	0	0	1
†	21UR-12049	TTCGCGTTTACTTCACGAGAT	0	0	0	0	0	0	0	0
†	21UR-12050	TTCGCGCCATCTAGGAAATAT	1	0	0	0	0	0	0	1
†	21UR-12051	TTCGATTTCGCAAAACATCGG	0	0	0	0	0	1	0	1
†	21UR-12052	TTCGATGGCTATGTGGACTAT	0	0	0	0	0	1	0	1
	21UR-12053	TTCGATGATGAATACAATTCA	0	0	0	0	0	1	0	1
†	21UR-12054	TTCGAAGATGACGATGACCTT	0	0	0	0	3	1	0	4
†	21UR-12055	TTCGAAATGTTCTGTGGTGTT	5	4	4	4	10	20	8	55
	21UR-12056	TTCGAAAATCATCTATGAGAT	0	0	0	0	0	0	0	0
†	21UR-12057	TTCCTTGGCTCTTGATCTGAA	0	0	0	0	0	0	0	0
†	21UR-12058	TTCCTTATGAGTAAAAAAACC	0	0	0	0	0	0	1	1
	21UR-12059	TTCCTGCAAAACCGGACGATC	0	0	0	0	0	0	0	0
†	21UR-12060	TTCCGTAGCGGCTGTAATGCA	0	0	0	0	0	0	0	0
†	21UR-12061	TTCCGGGAATTCAAATTTTAA	0	0	0	0	0	0	0	0
†	21UR-12062	TTCCGCAATCAAATACGGTTT	0	0	0	0	10	1	1	12
†	21UR-12063	TTCCGACCGTTGTTACAGCTA	1	0	0	0	1	2	0	4
	21UR-12064	TTCCGACAAACCTGGTTTTTC	0	0	0	0	0	0	0	0
†	21UR-12065	TTCCGAAGTCGATCAGGTTTA	0	0	0	0	1	0	0	1
†	21UR-12066	TTCCAGCTTTTTTTGTTGTCA	0	0	0	0	0	0	0	0
	21UR-12067	TTCCACTTGTATAAAGCACTT	0	0	0	0	0	0	0	0
†	21UR-12068	TTCATTGGTAAAAGGTACTTC	0	0	0	0	0	0	0	0
	21UR-12069	TTCATCTTCACTATATACAAA	1	0	0	0	0	0	0	1
†	21UR-12070	TTCATCATCAGTCTCTAATTT	0	0	1	0	0	0	0	1
	21UR-12071	TTCATATGCTGTTGTCAAGTA	0	0	0	0	0	0	0	0
	21UR-12072	TTCATAGATACAAGCCTGAAC	0	0	0	0	0	0	0	0
†	21UR-12073	TTCATACGATGGAACTGAGAT	0	0	0	0	0	0	0	0
	21UR-12074	TTCAGGGTTTTCAATTCCTCT	0	0	0	0	0	0	0	0
	21UR-12075	TTCAGCATTAACATTTTGCTG	0	0	0	0	5	2	0	7
†	21UR-12076	TTCAGAGTATTTTATTCAGAA	0	0	0	0	0	0	0	0
†	21UR-12077	TTCAGAAGATAAATTAAAACC	0	0	0	0	0	2	0	2
†	21UR-12078	TTCACTTGCAGAGAAATCAAA	0	0	0	0	0	0	0	0
	21UR-12079	TTCACAGACTTTTGCTTGCTT	0	0	0	0	0	0	0	0
	21UR-12080	TTCAAGCTCAAGAGCCTAGTG	0	0	0	0	0	0	0	0
	21UR-12081	TTCAAGCACCGTATCTAACGA	0	0	0	0	1	0	0	1
†	21UR-12082	TTCAACTCTACGGTGCCTAAA	0	0	0	0	0	1	0	1
†	21UR-12083	TTCAAACGTTTATATCTTAAA	0	0	0	0	0	0	0	0
	21UR-12084	TTATTTTTCACTGAAAAAAGC	0	0	0	0	1	1	0	2
†	21UR-12085	TTATTTAATAGGCAATTCTTA	0	0	0	0	4	1	1	6
†	21UR-12086	TTATTGCTACGCGAATGTTAT	1	0	0	0	0	0	0	1
†	21UR-12087	TTATTGCCACGTAGTTCTCAA	0	0	0	0	0	0	0	0
†	21UR-12088	TTATTGCACTATTCTTTGAAC	0	0	0	2	0	0	0	2
†	21UR-12089	TTATTGAAATTGGGTTGTATC	0	0	0	0	0	0	0	0
†	21UR-12090	TTATTCTGCTCATATCTTGTA	0	0	0	0	0	0	0	0

†	21UR-12091	TTATTCTGATTTGCTCCGGTG	0	0	0	0	0	0	1	1
†	21UR-12092	TTATTCTCGTCCCTCACGGAT	2	0	0	0	0	1	0	3
†	21UR-12093	TTATTCTCCATCTAAGCCATA	0	0	0	0	0	0	0	0
	21UR-12094	TTATTCGTTTTTATTCTTTTT	0	0	0	0	0	0	0	0
	21UR-12095	TTATTCGTTCTTATCGTATTT	0	0	0	0	0	0	0	0
†	21UR-12096	TTATTCGAAAGAGTTGGTTCT	0	0	0	0	0	0	0	0
†	21UR-12097	TTATTACTTCTTCGCAAAAAA	0	0	0	0	0	0	0	0
	21UR-12098	TTATTAATTTTATTCAAAGTT	0	0	0	0	0	0	0	0
†	21UR-12099	TTATGTTATGTATTTGCAGTT	2	0	0	0	1	2	0	5
	21UR-12100	TTATGTTATGCCGAAGGTAGA	0	0	0	0	0	1	0	1
	21UR-12101	TTATGTAATTAATTCCAAATT	0	0	0	0	1	1	0	2
	21UR-12102	TTATGATAAAACGTCGCAAAA	0	0	0	0	0	0	0	0
	21UR-12103	TTATGAATTTTACCGAGTTGG	0	0	0	0	0	0	0	0
†	21UR-12104	TTATGAACATAATGAAACAGT	0	0	0	0	0	0	0	0
	21UR-12105	TTATGAAAAAGGCCTGCGCTT	0	0	0	0	0	0	0	0
†	21UR-12106	TTATCTATTAAATGATTGGCT	0	0	1	0	0	1	0	2
†	21UR-12107	TTATCCGAAATTTTCCCAGCA	0	0	0	0	0	0	0	0
	21UR-12108	TTATCCATTGAAATGAAAAAT	0	0	0	0	0	0	0	0
	21UR-12109	TTATCAAATCGCTGGTCACAA	1	0	1	1	0	0	1	4
	21UR-12110	TTATATCCCGGAATAAGTGGT	0	0	0	0	0	0	0	0
	21UR-12111	TTATAAACCTTTACAACCTTG	0	0	0	0	0	0	0	0
	21UR-12112	TTAGTTTGGGCGTGAATGAAA	0	0	0	0	0	0	0	0
†	21UR-12113	TTAGTTGAGTAGTTAAAATGA	0	0	0	0	0	0	0	0
†	21UR-12114	TTAGTTAGAAAATTTGTGACG	1	0	0	1	0	0	0	2
†	21UR-12115	TTAGTTAAAAATTTGACCGGC	0	0	0	0	0	0	0	0
†	21UR-12116	TTAGTCCTCAATTTTTTCACG	0	0	0	0	0	0	0	0
†	21UR-12117	TTAGTATTAGTATCTAAGGGC	0	0	0	0	0	0	0	0
	21UR-12118	TTAGTAGTGGGACGTGAACTT	0	0	0	0	0	0	0	0
†	21UR-12119	TTAGCATAGATTAATGATTGT	0	0	0	0	0	0	0	0
	21UR-12120	TTAGATGGGTCTCGGCGCGAA	0	0	0	0	0	0	0	0
†	21UR-12121	TTAGATCATATATAGGAATGT	0	0	0	0	0	2	0	2
†	21UR-12122	TTAGAAAATTTGTGACGTTTG	0	0	0	0	1	3	0	4
†	21UR-12123	TTACTTTCCTTGCTCTTTGAA	0	0	0	0	0	0	0	0
†	21UR-12124	TTACGGTCGATCTTGTTTTAG	0	0	0	0	0	0	0	0
	21UR-12125	TTACGCTTGAACCTTAATTTT	0	0	0	0	2	0	0	2
	21UR-12126	TTACGAGAAAATGATTGAGGC	0	0	0	0	0	0	1	1
†	21UR-12127	TTACACGATTGATCTCACTTT	0	0	0	0	0	0	0	0
†	21UR-12128	TTAATTTCAGGATTTGAATGC	0	0	0	0	0	0	0	0
†	21UR-12129	TTAATTTATAACGATTTTGGA	0	0	0	0	0	0	0	0
†	21UR-12130	TTAATTGTTGCACAGAATCAA	0	0	0	0	0	0	0	0
†	21UR-12131	TTAATTGGTTGTTGGCGTGTT	1	1	0	0	0	0	0	2
	21UR-12132	TTAATTCATCATTCATTTCAC	0	0	0	0	0	0	0	0
†	21UR-12133	TTAATGACTTTGGATTTTCTC	0	0	0	0	2	0	0	2
†	21UR-12134	TTAATCTTTCAAATTTCTCAT	0	0	0	0	0	0	0	0
	21UR-12135	TTAATCGAGGAGTATCATTCA	0	0	0	0	0	0	0	0
†	21UR-12136	TTAATCCGTTTACTTTTAGAT	0	0	0	0	0	1	0	1
†	21UR-12137	TTAATATCAAAAAGTTCCAGA	0	0	0	0	0	0	0	0
†	21UR-12138	TTAATAGTTTAACGTCTTCCC	0	0	0	0	0	0	0	0
†	21UR-12139	TTAAGCGTTCGCTTGCACACA	0	0	0	0	0	0	0	0
	21UR-12140	TTAACTTTTGTTACCACAAAA	0	0	0	0	0	0	0	0
	21UR-12141	TTAACTTCAAAATGATTGATG	0	0	0	0	0	0	0	0
	21UR-12142	TTAAATGAAGTTGAACAGTAG	0	0	0	0	0	0	0	0
†	21UR-12143	TTAAATCACACAGAGTAATGA	0	0	0	0	0	0	0	0
	21UR-12144	TTAAATAATCGTAAATCGTAA	4	1	0	0	1	11	0	17
	21UR-12145	TTAAATAAGGTTGATTGGAAA	0	0	1	0	0	0	0	1
	21UR-12146	TTAAAGTCGACCAAAAGAAAA	0	0	0	0	0	0	0	0
†	21UR-12147	TTAAAATCTTTGTATCTGCAA	0	0	0	0	0	0	0	0
†	21UR-12148	TTAAAACTTTGATTGGAAAAA	0	0	0	0	0	0	0	0
	21UR-12149	TTAAAACGAAGTGAAATTCAA	0	0	0	0	0	0	0	0
	21UR-12150	TTAAAAATATAACTGTTTCAA	0	0	0	0	0	0	0	0
	21UR-12151	TTAAAAAAACTTACTTTAGTG	0	0	0	0	0	0	0	0
	21UR-12152	TTAAAAAAAAACCATCACAAG	0	0	0	0	0	0	0	0
†	21UR-12153	TGTTTTTGGCTACAATCACTT	0	0	0	0	1	0	0	1
†	21UR-12154	TGTTTTTCGGGACTTTAAAAG	0	0	0	0	0	0	0	0

†	21UR-12155	TGTTTTGTCATTTAATTTGAA	0	0	0	0	0	0	0	0
	21UR-12156	TGTTTTCTGTAGTGAAGTTTT	1	0	0	0	0	1	0	2
†	21UR-12157	TGTTTGTTCCTACAACAAAAA	0	0	0	0	0	0	0	0
	21UR-12158	TGTTTGTTCACAGTTAGGGTT	0	0	0	0	0	0	0	0
	21UR-12159	TGTTTGATAGTTTTGCAATAA	0	0	0	0	0	0	0	0
	21UR-12160	TGTTTCTTTTGTAGGGGCGAT	0	0	0	0	0	0	0	0
	21UR-12161	TGTTTCTTCAATACAGTTAAT	0	0	0	0	0	1	1	2
	21UR-12162	TGTTTCAGAGAGAAACAGGAA	0	0	0	0	0	0	0	0
†	21UR-12163	TGTTTCAATGTTGGAACTATG	0	0	0	0	0	0	0	0
†	21UR-12164	TGTTTATGAAAGGAGACAAAA	0	0	0	0	0	0	0	0
†	21UR-12165	TGTTTACCCGACATCTTTTGA	0	0	0	0	0	0	1	1
†	21UR-12166	TGTTTAAAGTTGGTGTGGTCG	0	0	1	0	0	1	1	3
†	21UR-12167	TGTTGTTGTTGGTGTAGTGGT	0	0	0	0	1	4	0	5
†	21UR-12168	TGTTGTATCGAATAAGCAATC	0	0	0	0	0	0	0	0
†	21UR-12169	TGTTGGAAAAATCGTCTAATT	75	12	21	7	13	64	15	207
†	21UR-12170	TGTTGCAATTCCGAAATACTT	0	0	0	0	0	0	0	0
†	21UR-12171	TGTTGAAGATGGCAAAAACTT	2	0	0	0	0	0	0	2
	21UR-12172	TGTTGAAAAATAAAGTACTGG	0	1	0	0	0	0	0	1
	21UR-12173	TGTTCTGAAATCATTGGGTTG	0	0	0	0	0	0	0	0
	21UR-12174	TGTTCGGCAATGAACTCGATC	0	0	0	0	0	0	0	0
†	21UR-12175	TGTTCGGAAATTTGAAAAAAT	0	0	0	0	0	0	0	0
†	21UR-12176	TGTTCCAAAGTTGTTTTTGAC	0	0	0	0	0	0	0	0
†	21UR-12177	TGTTCAGATACTGGTCCTTGG	0	0	0	0	0	0	0	0
†	21UR-12178	TGTTCAAATTGGTGCATCGAG	0	0	0	0	0	0	0	0
†	21UR-12179	TGTTATCGTTGGATTAAAAAG	0	0	0	0	1	1	0	2
	21UR-12180	TGTTAGTCAAAATGCATAACT	0	0	0	0	0	0	0	0
†	21UR-12181	TGTTAGTACGATGTCCACTTA	0	0	0	0	0	0	0	0
	21UR-12182	TGTTAGGACAGTCTCCACAAA	0	0	0	0	3	0	0	3
	21UR-12183	TGTTAGGAAAAACAATTTGAA	0	0	0	0	0	0	0	0
†	21UR-12184	TGTTAGAATTGTCACAACCGT	0	0	0	0	1	0	0	1
	21UR-12185	TGTTAGAATTCACTGAAATTC	0	0	0	0	0	0	0	0
	21UR-12186	TGTTAGAAGTTACTTGATGCA	0	0	0	0	0	0	0	0
	21UR-12187	TGTTAATGTAATTCCTGTGAA	0	0	0	0	0	0	0	0
	21UR-12188	TGTGTTCTTTGGAAGCTTTTT	0	0	0	0	0	0	0	0
†	21UR-12189	TGTGTGTTCTTCTGGAATTTT	0	0	0	0	0	0	0	0
†	21UR-12190	TGTGTACTCATGGGAATGGAT	0	0	0	0	0	0	0	0
	21UR-12191	TGTGGCAACTGTCGTAGTTCC	0	0	0	0	0	0	0	0
†	21UR-12192	TGTGGACTTTTCTACATTTTT	0	0	0	0	0	0	0	0
	21UR-12193	TGTGCCTTTTTTTAAGGATAT	0	0	0	0	0	0	0	0
	21UR-12194	TGTGCCCAAAGCAGATGTTGG	0	0	0	0	0	0	0	0
	21UR-12195	TGTGCAATGACCATTTGCAGA	0	0	0	0	0	2	0	2
†	21UR-12196	TGTGATTATTTCCTGGAATGG	0	0	0	0	0	0	0	0
†	21UR-12197	TGTGAGATTCTAAATACACAA	0	0	0	0	0	0	0	0
	21UR-12198	TGTCTTATTTTATTGGACTTG	0	0	0	0	0	0	0	0
	21UR-12199	TGTCGGAAAAGTCGTTATAAT	0	0	0	0	0	2	0	2
†	21UR-12200	TGTCGAACCGATAAAATCGAA	0	0	0	0	0	0	0	0
	21UR-12201	TGTCGAAAAATGTACCTCGTT	0	0	0	0	0	0	0	0
	21UR-12202	TGTCCCTTTTGCCAATTTGCA	0	0	0	0	0	0	0	0
†	21UR-12203	TGTCCAATACTCTCAAAATGA	0	0	0	0	0	0	0	0
†	21UR-12204	TGTCCAAACGTTATTAAAAGT	0	0	0	0	0	0	0	0
	21UR-12205	TGTCAGAACGACTCGATCCAC	0	0	0	0	0	0	0	0
	21UR-12206	TGTCACATATCGGTACGTAAG	0	0	0	0	0	1	0	1
	21UR-12207	TGTATTTTATCCATATTGTAA	0	0	0	0	0	0	0	0
	21UR-12208	TGTATTGTATTCAAGTTCTTG	0	0	0	0	0	0	0	0
	21UR-12209	TGTATTCAATTTTAAAATTGC	0	0	0	0	0	0	0	0
†	21UR-12210	TGTAGTGAGTACTATTGAAGA	0	0	0	0	0	0	0	0
	21UR-12211	TGTAGGCGGCGGTATTACTAT	0	0	0	0	0	0	0	0
†	21UR-12212	TGTAGAAAGACAATTCAATGA	0	0	0	0	0	0	0	0
	21UR-12213	TGTACTTGCTTTTTTCACTTT	0	0	0	0	0	0	0	0
†	21UR-12214	TGTACGTTGCAAAAATTTCTA	0	0	0	0	1	0	0	1
	21UR-12215	TGTACATCTTTTTTTTTAGTT	0	0	0	0	0	0	0	0
	21UR-12216	TGTAAGAAATGTTCCAACATT	0	0	0	0	0	0	0	0
†	21UR-12217	TGTAACATGCGAAAAAAAAAA	0	0	0	0	0	0	0	0
	21UR-12218	TGGTTTTGGATATCTCTGTCC	0	0	0	0	0	0	0	0

	21UR-12219	TGGTTTTGAATAATGTGTCTT	0	0	0	0	0	0	0	0
	21UR-12220	TGGTTTTCTAAAAACTCAAAA	0	0	0	0	0	0	0	0
	21UR-12221	TGGTTGTATTTTGACCTATTT	0	0	0	0	0	0	0	0
†	21UR-12222	TGGTGTTTTTCGGGACTTTAA	0	0	1	1	0	2	0	4
	21UR-12223	TGGTGCCTTCTTAGCACATAA	0	0	0	0	2	0	0	2
	21UR-12224	TGGTAACATGCACTAGGATAT	0	0	0	0	0	0	0	0
	21UR-12225	TGGGTGTTGGTCATTGTTTTC	0	0	0	0	0	0	0	0
*	21UR-12226	TGGGAAGAAGACAAAATCGTC	2	1	0	0	0	0	3	6
	21UR-12227	TGGCCAATTATTTATCTCTGT	0	0	0	0	0	0	0	0
	21UR-12228	TGGCAACCAAATTAAATTTGT	0	0	0	0	0	0	0	0
	21UR-12229	TGGATTTTCGAATTCATAGTG	0	0	0	0	0	0	0	0
†	21UR-12230	TGGATGATTCTTTCTTTTCAA	0	0	0	0	0	0	0	0
	21UR-12231	TGGATAACGTTTTCACAAAAA	0	0	0	0	0	0	0	0
†	21UR-12232	TGGAGTTTTTTTGATTCTGTG	0	0	0	0	0	0	0	0
	21UR-12233	TGGAGTTCAATTTCTAAGAAA	0	0	0	0	0	0	0	0
	21UR-12234	TGGAATGCATGTAGTAGGCCT	3	7	6	2	9	31	0	58
	21UR-12235	TGGAATAAATGTTGAGAAAAA	0	0	0	0	0	0	0	0
	21UR-12236	TGGAAGATCCCATTGATTTGA	0	0	0	0	0	0	0	0
	21UR-12237	TGGAAGAAACCTATTGGAGAT	0	0	0	0	0	0	0	0
	21UR-12238	TGGAACTAAATGTTGTTTTTT	0	0	0	0	1	0	0	1
	21UR-12239	TGGAAATCGATAATAAAAACG	0	0	0	0	0	0	0	0
	21UR-12240	TGGAAATCAAAATTTGTTATT	0	0	0	0	0	0	0	0
†	21UR-12241	TGGAAACCTCAACGCTGAATT	0	0	0	0	0	0	0	0
	21UR-12242	TGGAAAAACATCCGAATTGCA	0	0	0	0	0	0	0	0
	21UR-12243	TGCTTTTTTCAACTCTAGAAT	0	0	0	0	0	0	0	0
	21UR-12244	TGCTTCTTTAACAACAGAAAA	0	0	0	0	0	0	0	0
	21UR-12245	TGCTGGAATAAACAAGACACT	0	0	0	0	0	0	0	0
†	21UR-12246	TGCTGAGAACAGGGGATTCTT	0	0	0	0	0	1	0	1
	21UR-12247	TGCTCAACTCACGTGTTGTCA	0	0	0	0	0	0	0	0
†	21UR-12248	TGCTAGATTTTGGGTCCCATT	0	0	0	0	0	0	0	0
†	21UR-12249	TGCTACTGTGATGGAAAAACG	0	0	0	0	0	1	0	1
†	21UR-12250	TGCTAAAAAGTCCAGGTTTTG	0	0	0	0	0	0	0	0
†	21UR-12251	TGCGGCTAAACCTATTTATCG	0	0	0	0	0	0	0	0
	21UR-12252	TGCGCAGAACCAAAGATCATA	0	0	0	0	0	0	0	0
	21UR-12253	TGCGATATTCAGACAAAATCC	0	0	0	0	0	0	0	0
	21UR-12254	TGCCATATTTCAGGTCACAGT	0	0	0	0	0	0	0	0
	21UR-12255	TGCATACGAAGGTGGCGGCTG	0	0	0	0	0	2	2	4
	21UR-12256	TGCAGGTACAATATATTTGTT	0	0	1	0	0	0	0	1
	21UR-12257	TGCACTGTATTGTCTCCAAAA	0	0	0	0	0	0	0	0
	21UR-12258	TGCACTGACATCTAGACCATT	0	0	0	0	0	0	0	0
	21UR-12259	TGCACAAACAACAAGATGATG	0	0	0	0	0	0	0	0
†	21UR-12260	TGATTTTGAAACCCATCTAGA	0	0	0	0	0	0	0	0
†	21UR-12261	TGATTTTCTTTACGTAGCTAA	0	0	0	0	0	0	0	0
†	21UR-12262	TGATTTGCACGGATTTTCAGC	0	0	0	1	1	1	0	3
†	21UR-12263	TGATTTGAAAAGAAATTCACA	0	0	0	0	0	0	0	0
	21UR-12264	TGATTTCCATTTAGTCTTTTG	0	0	0	0	0	0	0	0
†	21UR-12265	TGATTCTGTAAAATTTATTTA	0	0	0	0	0	0	0	0
	21UR-12266	TGATTCGTCTATAGTAATGGA	0	0	0	0	1	0	0	1
	21UR-12267	TGATTCCTTGTGGTTTGATTG	1	0	0	0	4	8	0	13
†	21UR-12268	TGATTCCATTTGATTGAAAAT	0	0	0	0	0	0	0	0
	21UR-12269	TGATTATATAAGTAGGAACAG	0	0	0	1	0	0	1	2
†	21UR-12270	TGATGAAGGATGCGATACTCT	0	0	0	0	0	0	0	0
	21UR-12271	TGATCTGAATAGCCAACAAGA	0	0	0	0	0	0	0	0
	21UR-12272	TGATCTCGCAATCTCTTGCCT	0	0	0	0	0	0	0	0
†	21UR-12273	TGATATTATTCGGATTTCAGA	0	0	0	0	4	1	0	5
†	21UR-12274	TGATATGGTAAATTGTGAACA	0	0	0	0	2	0	0	2
†	21UR-12275	TGATATCCATGGACACTTTTA	0	0	0	0	0	0	0	0
	21UR-12276	TGATAAGATTTGTACCTCATT	0	0	0	0	1	0	0	1
	21UR-12277	TGATAAGAGTTGGAAAAGCTG	0	0	1	0	5	7	2	15
†	21UR-12278	TGAGTTATACGTCGGCAGAAA	0	0	0	0	0	1	0	1
	21UR-12279	TGAGGATGTTGTCCAAACGCA	0	0	0	0	0	0	0	0
	21UR-12280	TGAGATCGGGATAGAAATACA	0	0	0	0	0	1	0	1
†	21UR-12281	TGAGAACAGGGGATTCTTTGA	0	1	0	0	0	3	0	4
†	21UR-12282	TGACTTATGTATTCTCTACCA	0	0	0	0	0	0	0	0

†	21UR-12283	TGACTCTGTAACGTCGCATTT	0	0	0	0	0	0	0	0
	21UR-12284	TGACACGGCTCTTGATGAGAG	0	0	0	0	0	0	0	0
	21UR-12285	TGACACCAAATCCAGCGGATG	0	0	0	0	0	0	0	0
	21UR-12286	TGACAAGGCAAAGAACATTAA	0	0	0	1	0	2	0	3
†	21UR-12287	TGACAACAAAAAAAGCTGGAA	0	0	0	0	0	0	0	0
†	21UR-12288	TGAATGTTTTTTTCCTGGTAT	0	0	0	0	0	0	0	0
†	21UR-12289	TGAATCCTCGGATTGATTCAT	0	0	0	0	0	5	0	5
	21UR-12290	TGAATAGTTTTCCTAGAAAAG	0	0	0	0	0	0	0	0
†	21UR-12291	TGAAGTCCAATTGGGAATAAT	0	0	0	0	0	0	1	1
	21UR-12292	TGAAGCTTCAGGTAATGCATC	0	0	0	0	0	2	1	3
	21UR-12293	TGAACTTAGCAAAAGTATTTA	0	0	0	0	2	0	0	2
	21UR-12294	TGAACACCCATCTGCCTTTAA	0	0	0	0	0	0	0	0
	21UR-12295	TGAAACTCCAATAAACATTTC	0	0	0	0	0	0	0	0
	21UR-12296	TGAAACTATTTCAGCTGCATG	0	1	0	0	1	2	2	6
	21UR-12297	TGAAAATGCGGCAACAAGAAA	0	0	0	0	0	0	0	0
	21UR-12298	TGAAAAATGAATTCTTAACAT	0	0	0	0	0	0	0	0
†	21UR-12299	TGAAAAAATTTGGGTAACTCG	0	0	0	0	0	0	0	0
	21UR-12300	TCTTTTTTTCAGCCGCTTTTT	0	0	0	0	0	0	0	0
	21UR-12301	TCTTTTTTAAATTTAGTCTTT	0	0	0	0	0	0	0	0
†	21UR-12302	TCTTTTCTCATTTTACGGTTC	0	0	0	0	0	2	1	3
†	21UR-12303	TCTTTCTTTATTTTCAGGACC	0	0	0	0	0	0	0	0
	21UR-12304	TCTTTCGGAATGACTCAGTCT	0	0	0	0	0	1	0	1
	21UR-12305	TCTTTATCGTCTTGCTCAAAT	0	0	0	0	0	0	0	0
	21UR-12306	TCTTTATAGCTTACATGTTGC	0	0	0	0	0	0	0	0
†	21UR-12307	TCTTGGAATATTGTTAACTTT	0	0	0	0	0	0	0	0
†	21UR-12308	TCTTAGGCTGTGAAAAGAAAA	0	1	0	0	1	0	0	2
	21UR-12309	TCTTAATTTAAAATAAGAAAA	0	0	0	0	0	0	0	0
	21UR-12310	TCTGTTCAGTTCTAATTTTTT	0	0	0	0	0	0	0	0
	21UR-12311	TCTGTGACTCGATATCTCCAC	0	0	0	0	0	0	0	0
	21UR-12312	TCTGTGAAAAATGTGGAAAAC	1	0	0	0	0	1	0	2
	21UR-12313	TCTGCGCCCAGCCCACGTTGT	0	0	0	0	0	0	0	0
	21UR-12314	TCTGAGGATTTTTTGTGTCTC	0	0	0	0	0	0	0	0
†	21UR-12315	TCTGACTATAAGATTTTGAAA	0	0	0	1	10	7	2	20
†	21UR-12316	TCTGACAAGCTATGCCCGTGT	0	0	1	0	1	0	0	2
†	21UR-12317	TCTGAATCGTATGTTATTCCC	0	0	0	0	0	0	0	0
	21UR-12318	TCTCGTTTCATTAGCTTCTTT	0	0	0	0	0	0	0	0
†	21UR-12319	TCTCATCCGGTCCAAGAGGTT	3	0	0	0	3	5	6	17
	21UR-12320	TCTATCCAAGATCTGCGTGTC	0	0	0	0	0	0	0	0
	21UR-12321	TCTAGGATGTGTGCAATTTCG	0	0	0	0	1	2	0	3
	21UR-12322	TCTAGAAATTCTCAGAAAGTT	0	0	0	0	0	0	0	0
	21UR-12323	TCTAGAAACTGTTTTTTAGGC	0	0	0	0	0	0	0	0
†	21UR-12324	TCGTTCCGTAGCAGATATTTA	0	3	1	1	1	7	0	13
	21UR-12325	TCGCTGGTTTTCTTCGCGTCT	0	0	0	0	0	0	0	0
†	21UR-12326	TCGCGTTCTAGAATTTCTAAA	0	0	0	0	0	1	0	1
	21UR-12327	TCGATGCAAATAATTGAAAGA	0	0	0	0	0	0	0	0
	21UR-12328	TCGAGTTAGCGACCCTACAAA	0	0	0	0	0	0	1	1
	21UR-12329	TCGAAATGTGGACAGAACATA	0	0	0	0	0	0	0	0
	21UR-12330	TCGAAAGTTGTCTTACCTGAG	0	0	0	0	0	0	0	0
	21UR-12331	TCGAAAAGACGCCTGTGCTTT	0	0	0	0	0	0	0	0
†	21UR-12332	TCCTTTTCAGTGAGAACTAAC	0	0	0	0	1	2	0	3
†	21UR-12333	TCCTGTTCTTAACTTATCAAA	0	0	0	0	0	0	0	0
	21UR-12334	TCCTGCGAAAGACACTGTTTT	0	0	0	0	0	0	0	0
	21UR-12335	TCCTGATTGTATTTTAGATGC	0	0	0	0	0	1	0	1
	21UR-12336	TCCTGAGTTTTTACACCCCTC	0	0	0	0	0	0	0	0
	21UR-12337	TCCGTTCAGAGAAAAAACTTT	0	0	0	0	0	0	0	0
	21UR-12338	TCCGTACAGTTTTCTAACTCA	0	0	0	0	0	0	0	0
	21UR-12339	TCCGATTTTCGCAAAACTCGG	0	0	0	0	0	0	0	0
†	21UR-12340	TCCCATAAAATGAATTGCGGA	0	0	0	0	0	0	0	0
	21UR-12341	TCCATGAGATTCGTTGTGACA	1	0	0	0	3	9	9	22
†	21UR-12342	TCCATAGCTAAAAGCCAATTA	0	0	0	0	0	0	0	0
†	21UR-12343	TCCAGTGAACGATTAAATTTG	0	0	0	0	0	0	0	0
†	21UR-12344	TCCAGTCCACGTATGGTATTT	0	0	0	0	0	0	1	1
†	21UR-12345	TCCACTGATTAGTCATTCCTC	0	0	0	0	0	0	0	0
	21UR-12346	TCCACTCTTTTCCTGCCTTTT	0	0	0	0	0	0	0	0

		21UR-12347	TCCACAGAGAAAGAAAAAACT	0	0	0	0	0	0	0	0
		21UR-12348	TCCAAGATTTTGTTATTGGGA	0	0	0	0	0	0	0	0
	†	21UR-12349	TCCAAAGAACGCGGGATTTCC	0	0	0	0	0	1	0	1
		21UR-12350	TCATTTATGGCTCGAATATAA	0	0	0	0	0	0	0	0
	†	21UR-12351	TCATTCGGACAAAAATTTCCA	0	0	0	0	0	0	0	0
	†	21UR-12352	TCATTCGATCTTCAAAACAGT	0	0	0	0	0	0	0	0
	†	21UR-12353	TCATTAGACACTTTTCTTCTT	0	0	0	0	0	0	0	0
		21UR-12354	TCATGACGTATCGGATGACGT	2	0	0	0	2	0	1	5
	†	21UR-12355	TCAGTTTCGGAGATTCAAACA	0	0	0	0	2	0	0	2
	†	21UR-12356	TCAGTCCCATCTTTTATTAGT	0	0	0	0	0	0	0	0
		21UR-12357	TCAGACGGTGGCTCATAAGGC	0	0	0	0	0	0	0	0
	†	21UR-12358	TCACTTTTTCAAAGTAGTCAT	0	0	0	0	0	0	0	0
		21UR-12359	TCACTCATTTCAATTCATGTT	0	0	0	0	0	0	0	0
	†	21UR-12360	TCACTCAGCCTGTGCATTGAA	0	0	0	0	0	0	0	0
		21UR-12361	TCACACGGTGAACTAATGGGA	0	0	0	0	0	0	0	0
		21UR-12362	TCACAATTGTTAATGGTAGGT	0	0	0	0	0	0	0	0
	†	21UR-12363	TCACAATGCAGCTAGAATTAA	0	0	0	0	1	1	0	2
		21UR-12364	TCACAAGGACAGGCAATATTA	0	0	0	0	2	0	2	4
	†	21UR-12365	TCAATTTTTCCATCGTTTACC	0	0	0	0	0	0	0	0
		21UR-12366	TCAATTGTATGTCATCTTAAT	0	0	0	0	9	1	0	10
		21UR-12367	TCAATTGCTTGACAGCAAAAT	0	0	0	0	0	0	0	0
	†	21UR-12368	TCAATGAATTTCGATTTTGTT	0	0	0	0	0	0	0	0
		21UR-12369	TCAATCAATGTCAAACACTAA	0	0	0	0	0	0	0	0
	†	21UR-12370	TCAATACTAGTGCTTCGGAAA	0	0	0	0	2	1	0	3
	†	21UR-12371	TCAATAATAGTGTCTTTTCGG	0	1	0	0	0	0	0	1
	†	21UR-12372	TCAATAAACGATCCTTCAAAT	0	0	0	0	0	0	0	0
		21UR-12373	TCAAGACGAAGATGAATCAGA	0	0	0	0	0	0	1	1
		21UR-12374	TCAACAATAAAAATTGAGGAG	0	0	0	0	0	0	0	0
		21UR-12375	TCAAATGTTGAATTTCTGATC	0	0	0	0	0	0	0	0
	†	21UR-12376	TATTTTTAACCTTTTTTGTCG	0	0	0	0	0	0	0	0
		21UR-12377	TATTTTGTGATTAAATGTAGC	0	0	0	0	0	0	0	0
	†	21UR-12378	TATTTTGTCTAAAATTTCTCT	0	0	0	0	1	0	0	1
		21UR-12379	TATTTTGTAAATTCGTAGCTT	0	0	0	0	0	1	0	1
		21UR-12380	TATTTTGAATAAAATTTTCGG	0	0	0	0	0	0	0	0
*	†	21UR-12381	TATTTTCACCTTAACTTTAGA	0	0	0	0	3	0	0	3
		21UR-12382	TATTTTAGCGAAAAAATGAGA	0	0	0	0	0	0	0	0
	†	21UR-12383	TATTTGGAATCAATTATTTTT	0	0	0	0	0	0	1	1
	†	21UR-12384	TATTTGCCGTCAAGCTTTCTT	12	0	1	1	3	6	3	26
		21UR-12385	TATTTCTGCACATTCCTGTTG	0	0	0	0	0	0	0	0
		21UR-12386	TATTTCTGAATTTTGTAGGAA	0	0	0	0	0	1	1	2
		21UR-12387	TATTTCTCACTGTTTCAGTAA	0	0	0	0	0	0	0	0
		21UR-12388	TATTTCCCGTGTCTTTTAAAA	0	0	0	0	0	0	0	0
	†	21UR-12389	TATTTATTTCAACACTAAGAG	2	0	0	0	0	0	0	2
		21UR-12390	TATTTATCTATTCAATATTAA	0	0	0	0	0	0	0	0
		21UR-12391	TATTTAAACTGCTATTAATAT	0	0	0	0	1	3	1	5
	†	21UR-12392	TATTGTTTTCGAACGCAATTC	0	0	0	0	2	0	0	2
	†	21UR-12393	TATTGTTTGTTTTGAACCATA	0	0	0	0	1	0	1	2
		21UR-12394	TATTGTTGAAAAACCAATAGG	0	0	0	0	0	0	0	0
*		21UR-12395	TATTGTAATTTTTGTATCATC	0	0	0	0	2	1	0	3
	†	21UR-12396	TATTGGCTCAGGAAATTAGTT	0	0	0	0	0	0	0	0
	†	21UR-12397	TATTGGATAGTCTCACGGTAT	0	0	0	2	26	27	21	76
	†	21UR-12398	TATTGCAATGAATTGAATGTT	0	0	0	0	1	0	1	2
	†	21UR-12399	TATTGCAAAGGCGAAATTTTT	1	0	0	0	1	1	1	4
	†	21UR-12400	TATTGATATCCGTGCTAAAAA	0	0	0	0	0	0	0	0
	†	21UR-12401	TATTGACTCGTATTGGACTAA	12	2	3	0	8	39	15	79
		21UR-12402	TATTGAAACTTGCCGACTTTA	0	0	0	0	0	0	0	0
	†	21UR-12403	TATTCGTCAGCTTTTTGAAAT	0	0	0	0	0	0	0	0
		21UR-12404	TATTCGAAGCCTAAAAGTGAA	0	0	0	0	0	0	0	0
		21UR-12405	TATTCCTCTTTTTCTTATACA	0	0	0	0	0	0	0	0
	†	21UR-12406	TATTCATATGTACTATATAGA	0	0	0	0	0	0	0	0
		21UR-12407	TATTCATAGCAAAATGTTGAA	0	0	0	0	0	0	0	0
	†	21UR-12408	TATTCAGTCCCATCTTTTATT	0	0	0	0	0	1	0	1
	†	21UR-12409	TATTCAATGATAGTTACAATT	0	0	0	0	0	1	0	1
	†	21UR-12410	TATTATTCCCCTTATTTTTAA	0	0	0	0	0	0	0	0

	21UR-12411	TATTATTCCCAAAATTAAAAC	0	0	0	0	0	0	0	0
†	21UR-12412	TATTATGAGGAACATTATCAT	0	0	0	0	0	0	0	0
†	21UR-12413	TATTATAATGTCGAAACAGAC	0	0	0	0	0	0	0	0
†	21UR-12414	TATTAGGTCTATCCTTTTCAA	0	0	0	0	0	0	0	0
†	21UR-12415	TATTAGAAGAATTTGTGCAAA	0	0	0	0	0	0	0	0
†	21UR-12416	TATTACGTCTGAGGTTTTCAT	0	0	0	0	0	0	0	0
†	21UR-12417	TATTACGGGACGAACAACATC	0	0	0	0	0	0	0	0
	21UR-12418	TATTACAATTCTCGACTTAGT	0	0	0	0	0	0	0	0
†	21UR-12419	TATTAACGATGAATAAAACTT	0	0	0	0	0	0	0	0
†	21UR-12420	TATTAAATTGATCCAATCCAA	0	0	0	0	0	0	0	0
†	21UR-12421	TATGTGGTTATTTGAATCAAA	0	0	0	0	0	0	0	0
†	21UR-12422	TATGTCAGATGAAAAAAAAAT	0	1	1	1	0	10	0	13
†	21UR-12423	TATGTATTCCTCGAGTATGTC	0	0	0	0	1	0	0	1
†	21UR-12424	TATGCCTTATCACAATATTTT	0	0	0	0	0	0	0	0
	21UR-12425	TATCTTTAGTGTGAATTCAAC	0	0	0	0	0	0	0	0
†	21UR-12426	TATCCGACAAAAAATTGTGCG	0	0	0	0	0	0	0	0
†	21UR-12427	TATCAGGTAGATACTTTTAGC	0	0	0	0	4	1	0	5
	21UR-12428	TATCAACAGAAGAATATTACT	0	0	0	0	1	0	0	1
†	21UR-12429	TATATTGTATATTGATGAATC	0	0	1	2	2	6	0	11
	21UR-12430	TATATTCATAAAACGACGAAC	0	0	0	0	0	1	0	1
	21UR-12431	TATAGATCGAAGCTCATCGTC	0	0	0	0	0	0	0	0
	21UR-12432	TATACTTTGCTTATGTGCAAA	0	0	0	0	0	0	0	0
	21UR-12433	TATACCGTAATAAGTATTATT	0	0	0	0	0	1	0	1
	21UR-12434	TATACCGATTTTTATTTTTCT	0	0	0	0	7	1	0	8
	21UR-12435	TATACACTTAATGGGAAAGAA	0	0	0	0	0	0	0	0
	21UR-12436	TATAATCATTTTTTCAAACTT	0	0	0	0	0	0	0	0
	21UR-12437	TATAAACTAAAAATAGTGTAT	0	0	0	0	0	0	0	0
	21UR-12438	TAGTTTTTCGAATTTTCTTAG	0	0	0	0	0	0	0	0
	21UR-12439	TAGTTTTGCCCAAACATATTT	0	0	0	0	0	1	0	1
†	21UR-12440	TAGTTTGTTTTTGTTTTAAAT	1	0	0	1	0	0	1	3
	21UR-12441	TAGTTTGAAAAATCAGAAAGT	0	0	0	0	0	0	0	0
	21UR-12442	TAGTTTCGCTTTTAAATTAAG	0	0	0	0	0	0	0	0
	21UR-12443	TAGTTGTAAAAATTTGTAAAA	0	0	0	0	0	0	1	1
†	21UR-12444	TAGTTGATTTTCATTTGTCAG	0	0	0	0	0	1	0	1
†	21UR-12445	TAGTGTAAGTGCTACAACTGA	0	0	0	0	0	0	0	0
†	21UR-12446	TAGTGAGCTTTCTTTATTAAT	0	0	0	0	0	0	0	0
†	21UR-12447	TAGTCTTCAAATTGTTTGATT	0	0	0	0	0	0	0	0
†	21UR-12448	TAGTAGAAGATTTTGAAAGCA	2	0	1	0	6	9	0	18
†	21UR-12449	TAGTAAGGAAGTTATATGTAT	0	0	0	0	1	0	0	1
†	21UR-12450	TAGGTTGTAAGCAAACTATTC	0	0	0	0	0	1	0	1
†	21UR-12451	TAGGTGTTCAAGTTAGGGCTG	1	0	0	0	2	0	0	3
	21UR-12452	TAGGTGAAGTATACCTTCAAT	0	0	0	0	2	0	1	3
†	21UR-12453	TAGGTAGAGAACTTTTTCATT	0	1	0	1	0	0	0	2
	21UR-12454	TAGGTAATCTCCCTCATATAT	0	0	0	0	0	0	0	0
†	21UR-12455	TAGGGATTTCCTCGTTCATTA	0	0	0	0	0	0	0	0
	21UR-12456	TAGGGAGTGCTTTCACTATAC	0	0	0	0	0	0	0	0
	21UR-12457	TAGGATCGGAACGTAAATAGA	0	0	0	0	0	0	0	0
†	21UR-12458	TAGGATATCAGAATTCATTTT	0	0	0	0	0	0	0	0
†	21UR-12459	TAGGACTTTTGTAGACGTTTC	0	0	0	1	7	4	0	12
	21UR-12460	TAGGAATAAAAACTGATATTC	0	0	0	0	0	0	0	0
	21UR-12461	TAGCAAATCCGATCACAAAAG	0	0	0	0	0	0	0	0
†	21UR-12462	TAGATTGCCCGGTACATGTAC	0	0	1	0	0	0	0	1
	21UR-12463	TAGATTATCAGTCATTTTTCA	0	0	0	0	0	0	1	1
†	21UR-12464	TAGATGTGCAAATATCTGGAA	0	0	0	0	0	0	0	0
	21UR-12465	TAGATGATGATTGTTGCATTG	0	0	0	0	0	0	0	0
	21UR-12466	TAGATATATGTGGTGGAAGAT	0	0	0	1	6	4	6	17
†	21UR-12467	TAGACTTGGCGTAATCTCTTG	0	1	0	0	0	0	0	1
	21UR-12468	TAGACTTCAATTATCTCGATG	0	0	0	0	0	0	0	0
	21UR-12469	TAGAATATGCAATTTCGGTGT	0	0	0	0	1	0	0	1
†	21UR-12470	TAGAAGCTCATGCAGTCAGTA	0	0	0	0	0	0	0	0
	21UR-12471	TAGAAGACAACAAAAAAGTAA	0	0	0	0	0	0	0	0
†	21UR-12472	TAGAAGAAGACTCTCAAAAAG	1	2	1	0	3	6	0	13
†	21UR-12473	TAGAAACACATTTAGAATAAA	0	0	0	0	0	0	0	0
	21UR-12474	TAGAAAATCACTGAGAGCCAA	0	0	0	0	0	1	0	1

*		21UR-12475	TACTTGTAGATACAGTAGTGT	2	0	0	0	0	4	0	6
		21UR-12476	TACTTCTATCCAGTTTTTTTC	1	0	0	0	0	0	2	3
		21UR-12477	TACTTCAAATATTTTCCAAGC	0	0	0	0	0	0	0	0
	†	21UR-12478	TACTGTTAGAAGGTTTTTCAA	4	3	2	1	39	20	3	72
	†	21UR-12479	TACTGGGTGAATCCGACAACT	0	0	0	0	0	0	0	0
	†	21UR-12480	TACTGAACTAATTATACACAT	0	0	0	0	0	0	0	0
		21UR-12481	TACTATTTATTCGAAATGGTA	0	0	0	0	0	0	0	0
		21UR-12482	TACTATAATAATCGTGTAGAC	0	0	0	0	0	0	0	0
		21UR-12483	TACGGTCTATTTTCTGTCATT	0	0	0	0	0	0	0	0
	†	21UR-12484	TACGGCCGTACAGCAAATTAA	0	0	2	0	2	3	2	9
		21UR-12485	TACGGAAGTTTTCAGAACATC	0	0	0	1	0	1	0	2
	†	21UR-12486	TACGCATTGTGATTTTCGAAA	0	0	0	0	3	3	0	6
		21UR-12487	TACGATGAGGAAATTTCAAAC	0	0	0	0	0	0	0	0
	†	21UR-12488	TACCCTTCGTTTTCTAATTGT	0	0	0	0	0	0	0	0
		21UR-12489	TACCATTGAAGTAACTGAATA	0	0	0	0	0	1	1	2
		21UR-12490	TACCAAAAAATAATCCGTTAG	0	0	0	0	0	0	0	0
		21UR-12491	TACATACCCTGTTCGCAAAAT	0	0	0	0	0	0	1	1
	†	21UR-12492	TACACAAGTTTTATCCAGTCC	0	0	0	0	0	0	0	0
		21UR-12493	TACAATGTTTTTTTTTGCAGA	0	0	0	0	1	0	0	1
	†	21UR-12494	TACAACGGTATTTCAAGTTTC	0	0	0	0	0	0	0	0
		21UR-12495	TAATTTTTTTGAAGATTTACG	0	0	0	0	0	0	0	0
	†	21UR-12496	TAATTTTTTCACAATCGAGTC	0	0	0	0	0	0	0	0
		21UR-12497	TAATTTGCCCGTTTGAGTATT	0	0	0	0	0	0	0	0
		21UR-12498	TAATTGAATTGGAAATCGGCG	0	0	0	0	0	1	0	1
		21UR-12499	TAATTATGGCGAAAAAAAAAC	0	0	0	0	0	0	0	0
		21UR-12500	TAATTATCAATTTCATAATTT	0	0	0	0	0	0	0	0
		21UR-12501	TAATTAAATTTCTTTCTAGAC	0	0	0	0	0	0	0	0
*	†	21UR-12502	TAATGTTGTAATAGCTACTCG	0	0	0	0	2	0	0	2
	†	21UR-12503	TAATGTTGGTAAAGCTGTGGA	0	0	1	0	0	0	0	1
		21UR-12504	TAATGTCTTTTTCAACTTTTC	0	0	0	0	1	0	0	1
	†	21UR-12505	TAATGTATAATACCCTGAACT	0	0	0	0	0	0	0	0
	†	21UR-12506	TAATGGCACGTTTGATTTCCA	0	0	0	0	0	0	0	0
	†	21UR-12507	TAATGCGAATGGATTTTAGCT	0	0	0	0	0	0	0	0
		21UR-12508	TAATGATTACTGTCAACATTA	0	0	0	0	0	0	0	0
	†	21UR-12509	TAATGATATTTGAACAATGAT	0	0	0	0	0	0	0	0
	†	21UR-12510	TAATGAGTGGTTGTAATTTGG	0	0	1	0	1	4	1	7
		21UR-12511	TAATGAAAATCATACGATGAA	0	0	0	0	0	0	0	0
		21UR-12512	TAATCTTTCATTTTATGTTTT	0	0	0	0	0	0	0	0
	†	21UR-12513	TAATCTTATGTGAATAGCGAA	0	0	0	0	0	0	0	0
		21UR-12514	TAATCATTCTTCTATTGTTTC	0	0	0	0	1	1	0	2
	†	21UR-12515	TAATATTTGTATAACAATCAC	0	0	0	0	0	0	0	0
*		21UR-12516	TAATATGTTTTCAAGAAAATC	1	0	0	0	0	1	0	2
	†	21UR-12517	TAATATCAAATGTTTCAAATC	0	0	0	0	0	1	0	1
	†	21UR-12518	TAATAGTGATAAATACTGGAA	0	0	0	0	0	0	0	0
	†	21UR-12519	TAATAGATAGGCAGGATTGCA	0	0	0	0	0	0	0	0
	†	21UR-12520	TAATAGAACGTTTGGAAAATT	0	0	0	0	0	0	0	0
		21UR-12521	TAATACTTTAACGGTTAATGT	0	0	0	0	0	0	0	0
		21UR-12522	TAATACTAACTGAATATTTAA	0	0	0	0	0	0	0	0
	†	21UR-12523	TAATAAGGGCGTGTTACCATG	0	0	0	0	0	0	0	0
		21UR-12524	TAAGAGAAAATGAGATGAACA	0	0	0	0	0	0	0	0
		21UR-12525	TAACTGGAGACTATTCGGACA	0	0	0	0	0	2	0	2
	†	21UR-12526	TAACTAAAGAGCAGAATATGA	1	0	0	0	0	2	0	3
	†	21UR-12527	TAACGTCTGAAATATGTGGAT	0	0	0	1	0	1	0	2
	†	21UR-12528	TAACGAAATTCTGCTGTTTTT	0	0	0	0	0	1	0	1
		21UR-12529	TAACCTCGTTTGCAGAAATAA	0	1	0	0	0	4	0	5
	†	21UR-12530	TAACCGTTTCATCTTATTTAG	0	0	0	0	0	0	0	0
		21UR-12531	TAACCGATTGAAGTTTCCTGT	0	0	0	0	0	0	1	1
	†	21UR-12532	TAACACATTTAAAAAATAAAC	0	0	0	0	1	0	0	1
		21UR-12533	TAACAACTTCACCGTTTTTTT	0	0	0	0	1	0	0	1
		21UR-12534	TAAATTTTGAATCTAAGAAAA	0	0	0	0	0	0	0	0
		21UR-12535	TAAATTTGGAATATTGCTAAT	0	0	0	0	0	0	0	0
	†	21UR-12536	TAAATTTATATGATTGTGAAG	0	0	0	1	0	0	0	1
	†	21UR-12537	TAAATTTACTTTATGACGATT	0	0	0	0	0	1	0	1
		21UR-12538	TAAATTCTTTTTATCTATGTA	0	0	0	0	0	0	0	0

	21UR-12539	TAAATGTTAGAAATCCATTAA	0	1	0	0	0	1	0	2
	21UR-12540	TAAATGGATTATCAAAAAATT	0	0	0	0	0	1	0	1
	21UR-12541	TAAATATATTATTAGGTAGAA	0	0	0	0	3	4	0	7
	21UR-12542	TAAATACTTAAAAAAAAATTT	0	1	0	0	0	1	0	2
†	21UR-12543	TAAAGTTTAATATACAATTAA	0	0	0	0	0	0	0	0
	21UR-12544	TAAAGTATCAACATTAAATTT	0	0	0	0	0	0	0	0
	21UR-12545	TAAAGAATAAGAACAATTTTA	0	0	0	0	2	2	0	4
†	21UR-12546	TAAAATTTAAAAGTGCAAAAG	0	0	0	0	0	0	0	0
	21UR-12547	TAAAATCGGATTCAGAATATC	0	0	0	0	0	0	0	0
	21UR-12548	TAAAATATATATACTTATGGG	0	0	0	0	0	0	0	0
	21UR-12549	TAAAACGCAAAAATTGTCCGA	0	0	0	0	0	0	1	1
	21UR-12550	TAAAAAATTATTAAGACAAAT	0	0	0	0	0	1	0	1
	21UR-12551	TAAAAAAAATCTCGAACCAAG	0	0	0	0	0	0	0	0
†	21UR-12552	GTGCATCCGTTTGGCAATAGA	1	0	0	0	0	1	1	3
†	21UR-12553	GGTTTGATCAAAAGTGAAAAA	0	0	0	0	0	0	0	0
†	21UR-12554	GGCAAATCGATAAATATTGCA	0	0	0	0	0	0	0	0
	21UR-12555	GGAAAAAATGTAGAAGATACT	0	0	0	0	0	0	0	0
	21UR-12556	GATTTGGTAAAGTTAATTCAA	0	0	0	0	0	0	0	0
	21UR-12557	GATTCTCTCATCTATCTTCTT	0	0	0	0	0	0	0	0
†	21UR-12558	GATAAACTGATAGTTGGTAAA	0	0	0	0	0	0	0	0
	21UR-12559	GAAAATGTCTGATATTTCAAG	0	0	0	0	0	0	0	0
†	21UR-12560	CTTCTCGTTCAATGCATTTCT	0	0	1	0	0	0	0	1
	21UR-12561	CTCTTCGACACTTTGAAAACT	0	0	0	0	0	0	0	0
	21UR-12562	CGTTTCAAACAAACTCACTTC	0	0	0	0	0	0	0	0
	21UR-12563	CGTATTCGTATGGCTTGTGAA	0	0	0	0	0	0	0	0
†	21UR-12564	CGGTTATGGGACTCCTTTTAG	0	0	0	0	0	0	0	0
†	21UR-12565	CGATTCAGCTGATGATAAGAC	0	0	0	0	0	0	0	0
	21UR-12566	CGACAGGCAAGCACATGACTT	0	0	0	0	0	0	0	0
	21UR-12567	CGAATCTGCAATGAATTGTTC	0	0	0	0	0	0	0	0
	21UR-12568	CGAAAAAACTCAATTTAGTGC	0	0	0	0	0	0	0	0
	21UR-12569	CGAAAAAAAAACCGAGTAAGG	0	0	0	0	0	0	0	0
†	21UR-12570	CCTGAATCAGCACTTTTCTTG	0	0	0	0	0	0	0	0
	21UR-12571	CCTAATGTATGTAACGTTGCT	0	0	0	1	0	1	0	2
	21UR-12572	CATTGGATTCTATATAATTCT	0	0	0	0	0	0	0	0
†	21UR-12573	CATGTCGTTAAATGTATCGAT	0	0	0	0	8	2	4	14
†	21UR-12574	CACCAGTAAATCGAATAGCAG	0	0	0	0	0	1	0	1
	21UR-12575	CAACTGCTGCTTTTGTGGAAC	0	0	0	0	0	0	0	0
	21UR-12576	CAAAGCATTATCTGAATTTTC	1	1	1	1	1	5	0	10
	21UR-12577	CAAACTGTATTAAAAATAACC	0	0	0	0	0	0	0	0
	21UR-12578	CAAAATGAATGCCGACGATCT	0	0	0	0	0	0	0	0
†	21UR-12579	ATTTCGCGCCATCTAGGAAAT	10	2	3	1	2	4	7	29
	21UR-12580	ATTGAACAGCATTGCTCCGTC	0	0	0	0	0	0	0	0
†	21UR-12581	ATTATGTGTTCAGCTCTCAGA	0	0	0	0	0	0	0	0
* †	21UR-12582	ATTACGACTCAACGACTAACT	12	14	9	9	56	97	15	212
†	21UR-12583	ATAGCGACGTCTTTTTGTAAT	0	0	0	0	0	0	0	0
†	21UR-12584	AGCGACGTCTTTTTGTAATCC	0	0	0	0	0	0	0	0
	21UR-12585	ACGGCATCATATTGAACAGCT	4	7	6	0	31	40	2	90
†	21UR-12586	ACATATAGCATCGAAATGTGT	0	0	0	0	7	7	1	15
	21UR-12587	AAGAAAGAACAATCTGCTATC	0	0	0	0	0	0	0	0
†	21UR-12588	AACTCTTCTATTCGATTGGTT	1	0	0	1	5	7	7	21
	21UR-12589	TTTTTTTTTCTGTGTTTATTA	0	0	0	0	0	1	0	1
	21UR-12590	TTTTTTTTTCGAATAAATCGA	0	0	0	0	0	0	0	0
	21UR-12591	TTTTTTTTTAATTTTGAAGAA	0	0	0	0	2	0	0	2
	21UR-12592	TTTTTTTTGGGAGATTTGGTC	0	0	0	0	0	0	0	0
	21UR-12593	TTTTTTTTCTGTTCCAGGTGG	0	0	0	0	1	2	0	3
	21UR-12594	TTTTTTTTCTCTTTTTCTCTA	0	0	0	0	0	0	0	0
	21UR-12595	TTTTTTTTCGGTTTCGTCCGT	0	0	0	0	0	0	0	0
†	21UR-12596	TTTTTTTTCAGAGAAAAGTAA	0	0	0	0	0	0	0	0
	21UR-12597	TTTTTTTTACTGAATCTTTTT	0	0	0	0	0	0	0	0
	21UR-12598	TTTTTTTCTTTTACAGTTGTT	0	0	0	0	0	4	0	4
	21UR-12599	TTTTTTTCTTCAACTTGAGAT	0	0	0	0	0	0	0	0
	21UR-12600	TTTTTTTAGGAAAACTCGAAA	0	0	0	0	0	0	0	0
†	21UR-12601	TTTTTTTACGCATTCAACAAA	0	0	0	0	0	0	0	0
	21UR-12602	TTTTTTGTAAGAAGTTTGAAC	0	1	0	0	0	0	0	1

	21UR-12603	TTTTTTGCTCTCAATAAGTCT	0	0	0	0	1	0	0	1
†	21UR-12604	TTTTTTCGATTGATGTGCTAT	3	4	2	12	112	100	9	242
	21UR-12605	TTTTTTCGAACTCTTACAATG	0	0	0	0	0	0	0	0
†	21UR-12606	TTTTTTCCTTTTGTCACAAAT	0	0	0	0	0	0	0	0
	21UR-12607	TTTTTTCCGATCCAAATGCGT	0	0	0	0	0	0	0	0
*	21UR-12608	TTTTTTCATTGGATTGTCGTA	72	25	29	30	78	144	64	442
	21UR-12609	TTTTTTAAGGCTTTAAAACTA	0	0	0	0	0	2	0	2
	21UR-12610	TTTTTGTTCCATTTAACTATC	0	0	0	0	0	0	0	0
†	21UR-12611	TTTTTGTTATAAAAATGAGGC	0	0	0	0	0	0	0	0
	21UR-12612	TTTTTGGTCAACCATCTGATT	0	0	0	0	0	0	0	0
†	21UR-12613	TTTTTGGAGTGTGGTCATCAA	1	0	0	0	0	2	0	3
†	21UR-12614	TTTTTGCGTACGGAAAACAAA	0	0	0	0	0	0	0	0
†	21UR-12615	TTTTTGCAGGCGGAAAATAAA	0	0	0	0	0	1	0	1
	21UR-12616	TTTTTGACGCTACTAATTTGT	2	0	0	0	0	0	0	2
	21UR-12617	TTTTTCTCGACGACTAGCTTT	0	0	0	0	0	0	0	0
	21UR-12618	TTTTTCGTTATTTTTTGTTGA	0	0	0	0	0	0	0	0
†	21UR-12619	TTTTTCGATCTTTGGGTTTCA	0	0	0	0	6	0	1	7
	21UR-12620	TTTTTCCTACATCTTCCATTA	0	0	0	0	0	0	0	0
†	21UR-12621	TTTTTCCAAATTGTGCATTAT	0	1	0	0	0	1	0	2
	21UR-12622	TTTTTCATTTTCAATAATTCG	0	0	0	0	0	0	0	0
†	21UR-12623	TTTTTATTTTGTCTGATTCAG	0	0	0	0	1	0	1	2
†	21UR-12624	TTTTTATGAAATTTTGGAGTT	0	0	0	0	0	0	0	0
†	21UR-12625	TTTTTACAAACTCCGCGCACA	0	0	0	0	0	0	0	0
	21UR-12626	TTTTTAATGTCACGGGCCATT	0	0	0	0	0	0	0	0
	21UR-12627	TTTTTAATAGTAGCCGTCAGA	0	0	0	0	0	0	0	0
	21UR-12628	TTTTTAACTTATGCACTCAAA	0	0	0	0	0	0	0	0
†	21UR-12629	TTTTGTTACAGGATCAGTGAA	0	0	0	0	1	0	0	1
†	21UR-12630	TTTTGTCGAGACTCATTGTTA	0	0	0	0	0	0	0	0
†	21UR-12631	TTTTGTCGAACTTCTTGAATT	0	0	0	0	0	0	0	0
	21UR-12632	TTTTGTCATCAATGCAACAAA	0	0	0	0	0	0	0	0
	21UR-12633	TTTTGTCATATGTAAAAATGA	0	0	0	0	0	0	0	0
	21UR-12634	TTTTGTATGTCCTCATTGTTC	0	0	0	0	0	0	0	0
†	21UR-12635	TTTTGGTTGTCTTTTAGCTTT	0	0	0	0	1	0	0	1
	21UR-12636	TTTTGGTAACTGATTATTACC	0	0	0	0	0	0	1	1
†	21UR-12637	TTTTGGCTACAATCACTTCAT	0	0	0	0	0	0	0	0
	21UR-12638	TTTTGGCACTTTTCCGTTTTC	0	0	0	0	0	0	0	0
	21UR-12639	TTTTGCCAATTGGAAAATATA	0	0	0	0	0	0	0	0
†	21UR-12640	TTTTGCACTTTTTTAAACACA	0	0	0	0	0	0	0	0
	21UR-12641	TTTTGAGAAATTGTAAAAAGT	0	0	0	0	0	0	0	0
	21UR-12642	TTTTGAATAAAATTGTTTGAA	0	0	0	0	0	0	0	0
†	21UR-12643	TTTTCTTTTGATTTTTTTAGG	0	0	0	0	0	0	0	0
	21UR-12644	TTTTCTTTAATTTTTAATTAA	0	0	0	0	0	0	0	0
	21UR-12645	TTTTCTTGCAACGTACTTTTA	0	0	0	1	2	2	0	5
	21UR-12646	TTTTCTTGATTCCAGAAGACA	0	0	0	0	1	0	0	1
	21UR-12647	TTTTCTTACGCGGAAAGCAGA	0	0	0	0	0	0	0	0
	21UR-12648	TTTTCTACGGTTTCTTTGGCC	0	0	0	0	0	1	0	1
†	21UR-12649	TTTTCGTACATTTTCACTGCA	0	0	0	0	0	0	0	0
	21UR-12650	TTTTCGGTTTTTTCGGTTTTT	3	1	1	0	1	3	0	9
	21UR-12651	TTTTCGATATGTACAAATAAG	1	0	1	0	0	3	0	5
	21UR-12652	TTTTCGAGATCGCACATTAAT	0	0	0	0	3	1	4	8
†	21UR-12653	TTTTCGAAATATCCTTGATGT	0	0	0	0	0	0	0	0
†	21UR-12654	TTTTCCTATCAAATTATGTGC	0	0	0	0	0	1	0	1
	21UR-12655	TTTTCCACTTAAAAACGTCTA	0	0	0	0	0	0	0	0
†	21UR-12656	TTTTCATTTCCGTCTTATCAT	0	0	0	0	1	0	1	2
	21UR-12657	TTTTCATGTTCAATATTTTCA	0	0	0	0	0	0	0	0
	21UR-12658	TTTTCATGTCTTTTTTCAACA	0	0	0	0	0	0	0	0
†	21UR-12659	TTTTCAGACGAGCAAAAAACA	3	2	0	0	0	3	0	8
†	21UR-12660	TTTTCAATCCTAACGAAAATG	0	0	0	0	0	0	0	0
	21UR-12661	TTTTCAATCCATATTTGTTTT	0	0	0	0	1	0	0	1
	21UR-12662	TTTTCAAGAACAGGCTATCTG	0	0	0	0	0	0	0	0
	21UR-12663	TTTTCAAATTAATACTAGATT	0	0	0	0	0	1	0	1
	21UR-12664	TTTTCAAAATTTTCTGAAATA	0	0	0	0	0	0	0	0
†	21UR-12665	TTTTATTTTCAAAAATGAATG	0	0	0	0	0	0	0	0
	21UR-12666	TTTTATTTGATTGATAATTTT	0	0	0	0	1	1	0	2

†	21UR-12667	TTTTATTTGAGATTATTTATC	0	0	0	0	0	0	0	0
†	21UR-12668	TTTTATTCATATGCTTAGCTC	0	0	0	1	4	0	0	5
†	21UR-12669	TTTTATCGTACGGTGATTATG	0	0	0	0	0	1	0	1
	21UR-12670	TTTTATCAGTTTCCATAATCT	0	0	0	0	0	0	0	0
†	21UR-12671	TTTTAGTTATCTTCATCAGAT	0	0	0	0	0	0	0	0
	21UR-12672	TTTTAGCCAGGTATTCATATC	0	0	0	0	1	0	3	4
	21UR-12673	TTTTAGACGAGCAAGAGCGAC	0	0	0	0	1	0	0	1
†	21UR-12674	TTTTACTGCATTAAGAAATAA	0	0	0	0	0	0	0	0
†	21UR-12675	TTTTACTGCATGGTTTGCATT	0	0	0	0	1	0	1	2
	21UR-12676	TTTTACTACACTTCTACGACA	1	0	0	0	0	0	0	1
†	21UR-12677	TTTTACAGTCAACCAATAATT	0	0	0	0	0	1	0	1
†	21UR-12678	TTTTACAAGTATTACGCCTAA	2	0	0	0	8	1	0	11
	21UR-12679	TTTTAATGAAAAAAAAAGACT	0	0	0	0	0	0	0	0
	21UR-12680	TTTTAATAAGTTGTGTAGTTG	0	0	0	0	0	0	0	0
	21UR-12681	TTTTAAAATGTCTCATTACGA	0	0	0	0	0	0	0	0
	21UR-12682	TTTTAAAACGATGTTTTCAGA	0	0	0	0	0	0	0	0
	21UR-12683	TTTTAAAAACTAATTGACGTC	0	0	0	0	0	0	0	0
	21UR-12684	TTTGTTTTTCAACATCTAATT	0	0	0	0	0	0	0	0
	21UR-12685	TTTGTTCCCGAGTAATTGACT	0	0	0	0	0	0	0	0
†	21UR-12686	TTTGTTCCATATATTACCAGA	0	0	0	0	0	0	0	0
	21UR-12687	TTTGTTATTTATTGATGAGCA	0	0	0	0	8	7	1	16
	21UR-12688	TTTGTTACTTGCTTTCATTAT	0	0	0	0	0	0	0	0
	21UR-12689	TTTGTGCTTTTTCGAAATTTT	0	0	0	0	0	0	0	0
†	21UR-12690	TTTGTGCCATTAAGAGAAGCT	11	1	1	0	2	12	6	33
†	21UR-12691	TTTGTCGAGACTCATTGTTAT	0	0	0	0	13	5	0	18
†	21UR-12692	TTTGTAGTTGGCTTACAATTA	0	0	0	0	0	0	0	0
†	21UR-12693	TTTGTAGTCTGAAGTTTGCAC	0	0	0	1	2	4	0	7
	21UR-12694	TTTGTAGAAAACGTTAAAAGT	0	6	1	3	15	25	1	51
	21UR-12695	TTTGTACTTGGGTTCCTATAA	0	0	0	0	0	0	0	0
†	21UR-12696	TTTGTACACTGTTAAATACAT	0	0	0	0	2	0	0	2
†	21UR-12697	TTTGTAATTGTTCTTGGTCAC	0	0	0	0	0	0	0	0
	21UR-12698	TTTGGTTATTTCTTTCAATTT	0	0	0	0	0	0	0	0
†	21UR-12699	TTTGGTGTAGTTTGAAAGTAG	0	0	0	0	0	0	0	0
†	21UR-12700	TTTGGCCGAATATTTTATATT	0	0	0	0	0	0	0	0
	21UR-12701	TTTGGCATATTCGAAAAAATC	0	0	0	0	0	0	0	0
	21UR-12702	TTTGGATCCAAAATGATGGAA	0	0	0	0	0	0	0	0
	21UR-12703	TTTGGAGTTTCACCTATGGAT	0	0	0	0	0	0	0	0
†	21UR-12704	TTTGGAGCTTCAACTATGGAT	0	0	0	0	0	0	1	1
†	21UR-12705	TTTGGACAGTCTTGTGTCAAA	0	0	0	0	0	0	0	0
†	21UR-12706	TTTGGAACTAGAGGAATCTTA	0	0	0	0	0	0	0	0
†	21UR-12707	TTTGGAACAAAGCAAATTTAA	0	0	0	0	13	6	2	21
	21UR-12708	TTTGGAAAAAAAAAACTGAAG	0	0	0	0	0	0	0	0
	21UR-12709	TTTGCTGTTGTTCATGTCGTG	0	0	0	0	1	0	0	1
	21UR-12710	TTTGCTCATCTGCACGGTGAC	0	0	0	0	0	0	0	0
†	21UR-12711	TTTGCGGAGGTAATTTCAAAA	0	0	0	0	0	0	0	0
†	21UR-12712	TTTGCGGAGCATTATACAACG	0	0	0	0	1	0	0	1
	21UR-12713	TTTGCCTGGTTGGGACATATG	0	0	0	0	0	0	0	0
	21UR-12714	TTTGCATGTTTCCTAGATTTC	0	0	0	0	0	0	0	0
	21UR-12715	TTTGCACAGTTTTTTTAGTAT	0	0	0	0	0	1	0	1
†	21UR-12716	TTTGATTCCGGCCTGAAAGGT	0	0	0	0	0	0	0	0
	21UR-12717	TTTGATTCAAAAATTATTTCC	0	0	0	0	0	0	0	0
†	21UR-12718	TTTGATCATGAACCGTAAAAT	0	0	0	0	0	0	0	0
	21UR-12719	TTTGATAAATCGAAGTAAATC	0	0	0	0	0	0	0	0
†	21UR-12720	TTTGAGAAACCTTTACCGATT	0	0	0	0	0	0	0	0
	21UR-12721	TTTGACATCAAAGAAAAACAG	0	0	0	0	0	0	0	0
	21UR-12722	TTTGACAATCAACTGGTGTTT	0	0	0	0	0	0	0	0
	21UR-12723	TTTGAATTTTGCAACTACTAA	0	0	0	0	0	0	0	0
†	21UR-12724	TTTGAATCAAATTTATCACAA	0	0	0	0	0	0	0	0
	21UR-12725	TTTGAAGTCCGAAGATACATT	0	0	0	0	0	0	0	0
†	21UR-12726	TTTGAAGATCACGATGTTCAA	0	0	0	0	0	0	0	0
†	21UR-12727	TTTGAAGACTTATTGAAAATG	0	0	0	1	13	2	0	16
†	21UR-12728	TTTGAAGAAACATCTTCTGCC	1	0	0	0	0	0	0	1
	21UR-12729	TTTGAACGGGAAACTGTGAAA	0	0	0	0	0	0	0	0
	21UR-12730	TTTGAAACCGGTTTTTGGTAT	0	0	0	0	0	2	0	2

	21UR-12731	TTTGAAAAATTTATCTGAACA	0	0	0	0	0	0	0	0
	21UR-12732	TTTCTTTTATGAAATCGGGTT	0	0	0	0	0	1	0	1
†	21UR-12733	TTTCTTTTAGATTTTGTGGCA	1	0	0	0	1	1	0	3
†	21UR-12734	TTTCTTTCTAGCTTCAAACAT	1	0	0	0	1	0	0	2
	21UR-12735	TTTCTTTACTAGCTGAGTGGT	0	0	0	0	0	0	0	0
	21UR-12736	TTTCTTCTAATCATGATTTTA	0	0	0	0	0	0	0	0
†	21UR-12737	TTTCTTAGCTTGTGTATGATT	0	0	0	0	0	0	0	0
†	21UR-12738	TTTCTGTTCGGTTCATGTGAA	0	0	0	0	0	0	0	0
†	21UR-12739	TTTCTGTATTTTAGGCTTGTT	0	0	1	2	14	21	2	40
†	21UR-12740	TTTCTGGTTCGTTTAAATTGA	0	0	0	0	0	0	0	0
†	21UR-12741	TTTCTGATTTCTCTTAACTTT	0	0	0	0	1	0	0	1
	21UR-12742	TTTCTCGTTGGAAATCTGTAG	0	0	0	0	0	0	0	0
†	21UR-12743	TTTCTCGACGGTGTGTTGTCC	0	0	0	0	0	0	0	0
†	21UR-12744	TTTCTCAAATTGTTGTAAGGT	0	0	0	0	0	0	0	0
†	21UR-12745	TTTCTATTGGTAGAAACATTT	0	0	0	0	1	0	0	1
†	21UR-12746	TTTCTAATCCAAAATTCTAAT	0	0	0	0	0	0	0	0
	21UR-12747	TTTCGTATCATTACACAGGAG	0	0	0	0	0	0	0	0
†	21UR-12748	TTTCGGTTTGTAACTTTGCCA	0	0	0	0	0	0	0	0
†	21UR-12749	TTTCGACAAGATTTTTCTGCT	0	0	0	0	5	3	0	8
†	21UR-12750	TTTCCTATCAAATTATGTGCC	0	0	0	0	0	0	0	0
	21UR-12751	TTTCCTAAAAATATTTCAATA	0	0	0	0	0	0	0	0
	21UR-12752	TTTCCGCCGTTTGAAATTTCA	0	0	0	0	0	0	0	0
†	21UR-12753	TTTCCGCATTCAAAGTGAACT	0	0	0	0	0	0	0	0
†	21UR-12754	TTTCCCGTTAACTTTCTCTGT	0	0	0	0	0	0	0	0
	21UR-12755	TTTCCAGTTTCAAACAGTTTT	0	0	0	0	0	0	0	0
	21UR-12756	TTTCCAGCACGTTTTCTGAAA	0	0	0	0	0	0	0	0
	21UR-12757	TTTCATTCTCCATCCGAAAAT	0	0	0	0	0	0	0	0
†	21UR-12758	TTTCATTATTATTTCCGGCTG	0	0	0	0	7	5	9	21
	21UR-12759	TTTCATTAGTATTCCGTCGTC	4	1	2	1	1	7	2	18
†	21UR-12760	TTTCATGTCTTCGGTTTCATT	1	1	0	2	3	1	0	8
†	21UR-12761	TTTCATAGCGACGGTTGGAAA	0	0	0	0	0	2	0	2
	21UR-12762	TTTCATACCAAAACGTCCAAC	0	0	0	0	0	0	0	0
	21UR-12763	TTTCAGTTGGTTGGTATAGTG	0	0	0	0	1	1	0	2
†	21UR-12764	TTTCAGATGTGGATCAAACGC	0	0	0	0	0	2	0	2
	21UR-12765	TTTCAGACATAAAGCTGAAAA	0	0	0	0	0	0	0	0
†	21UR-12766	TTTCAATTGTTTAATTCAGGC	0	0	0	0	1	0	0	1
	21UR-12767	TTTCAATAGCGCATTTTGTGC	1	0	0	0	0	0	0	1
	21UR-12768	TTTCAACTTCAGGTACAATCA	0	0	0	0	0	0	0	0
	21UR-12769	TTTCAACTGTAAATTAACAAA	0	0	0	0	0	0	0	0
	21UR-12770	TTTATTTTTAATTTGACAAAA	0	0	0	0	0	1	0	1
†	21UR-12771	TTTATTTCACAGGAGATTTTG	0	0	0	0	0	0	0	0
	21UR-12772	TTTATTTCAATAATGCCTAAA	0	0	0	0	0	0	0	0
	21UR-12773	TTTATTTACTGACTGACTATG	0	0	0	0	0	0	0	0
†	21UR-12774	TTTATTGTCTTTTGTATTTCA	0	0	0	0	0	0	0	0
†	21UR-12775	TTTATTGAGAAATATAGGAAC	0	0	0	0	0	0	0	0
†	21UR-12776	TTTATTCTGTCCTTAAGCGTA	0	0	0	0	0	0	0	0
†	21UR-12777	TTTATTAGTTTGTTTGGACAT	4	0	0	3	7	16	2	32
†	21UR-12778	TTTATGATTTTTGGGTGTCAC	0	0	0	0	0	1	1	2
†	21UR-12779	TTTATCTATATGAGTTCAGTT	0	0	0	0	0	0	0	0
†	21UR-12780	TTTAGTTTATAGATGTCCTCC	0	0	0	0	0	0	0	0
†	21UR-12781	TTTAGTTACGAATTTGTCAAT	0	0	0	0	1	0	0	1
	21UR-12782	TTTAGTGTTTGGAAAAAAAGC	0	0	0	0	0	0	0	0
†	21UR-12783	TTTAGTGCTTTGATCAATTCC	0	0	0	0	0	0	0	0
†	21UR-12784	TTTAGTCTGATAGTAACAAAA	0	2	0	0	4	1	0	7
†	21UR-12785	TTTAGTAAAAAATTTTTTGAG	0	0	0	0	1	0	0	1
	21UR-12786	TTTAGGAAAAAACTACAGAGC	0	0	0	0	0	0	0	0
	21UR-12787	TTTAGCAGAGGATGGTGAAAA	0	0	0	0	0	0	0	0
	21UR-12788	TTTAGAACAATCCATGGAAAA	0	0	0	0	1	1	0	2
	21UR-12789	TTTAGAAATTAGTTAGTGTTG	0	0	0	0	0	0	0	0
†	21UR-12790	TTTACTTTTGATCAATGTCCA	0	0	0	0	2	2	3	7
	21UR-12791	TTTACTGAAATTGAATGTATT	0	0	0	0	0	1	0	1
	21UR-12792	TTTACGATTGGAACCTTGCTT	0	0	0	0	0	0	0	0
†	21UR-12793	TTTACGATAAATTTAAAACTG	0	0	0	0	0	0	0	0
	21UR-12794	TTTACGACAAAACTACGGTAC	0	0	0	0	0	0	0	0

†	21UR-12795	TTTACGAACAATAACAAAAGC	0	0	0	0	0	0	0	0
	21UR-12796	TTTACCTGTATTTCTTAAAGT	0	0	0	0	0	0	0	0
†	21UR-12797	TTTACAGAATAATTGCTTCGA	0	0	0	0	0	0	0	0
	21UR-12798	TTTACACTTGTAGCAACCAAA	0	0	0	0	0	0	0	0
†	21UR-12799	TTTACAACCACTTGTAGTTTC	0	0	0	0	0	0	0	0
†	21UR-12800	TTTAATGTTCATTTTTAAGCG	0	0	0	0	0	0	0	0
†	21UR-12801	TTTAATGGACTGTTGATCTTT	0	0	0	0	0	0	0	0
	21UR-12802	TTTAATCTCTACTATCATATA	0	0	0	0	0	0	0	0
	21UR-12803	TTTAAAGAGTTTTCATGTTTT	0	0	0	0	0	0	0	0
†	21UR-12804	TTTAAACTCTCTTGATGTTTT	0	0	0	0	0	0	0	0
†	21UR-12805	TTTAAAATTCAACAATTGGAT	0	0	0	0	0	0	0	0
†	21UR-12806	TTGTTTTTCAGCGTGTCCTTG	0	0	0	0	0	0	0	0
	21UR-12807	TTGTTTTCTCGCTGTTTTGTC	0	0	0	0	0	0	0	0
†	21UR-12808	TTGTTTTCCTCTGAATTTCCT	0	0	0	0	0	0	0	0
	21UR-12809	TTGTTTTCCATAGCGATGAAA	0	0	0	0	0	1	0	1
†	21UR-12810	TTGTTTTCATTTTCAAAAATT	0	0	0	0	0	0	0	0
	21UR-12811	TTGTTTTCAGAAGCACAATTT	4	0	0	1	10	8	4	27
*	21UR-12812	TTGTTTCTTTAAATGACATAC	0	0	0	0	2	2	0	4
†	21UR-12813	TTGTTTCTGCCGCTCTTTCAC	0	0	0	0	0	1	0	1
†	21UR-12814	TTGTTTCCTTTTTGCGATAAA	0	0	0	0	0	0	0	0
	21UR-12815	TTGTTTCAATCTTATGGATTC	0	0	0	0	0	0	0	0
†	21UR-12816	TTGTTTAATAGGATTCGCAAT	0	0	0	0	0	0	0	0
†	21UR-12817	TTGTTGTGTGTTGTTGTAATA	0	0	0	0	0	0	0	0
†	21UR-12818	TTGTTGGAATGGCGAAAATAC	1	0	0	0	1	0	1	3
†	21UR-12819	TTGTTGATTGTCGTTTTCAAG	0	0	0	2	11	10	11	34
†	21UR-12820	TTGTTGAGACGAATTGAAAAT	0	1	0	0	0	0	0	1
	21UR-12821	TTGTTCTTTTGTAGGGTGTTT	0	0	0	0	1	1	2	4
†	21UR-12822	TTGTTCTCTAGATGGGACCTG	0	0	0	0	0	0	0	0
	21UR-12823	TTGTTCTCCAAGCAAAATTTT	0	0	0	0	0	0	0	0
†	21UR-12824	TTGTTCAGTTTATCGTACAAA	0	0	0	0	0	1	0	1
	21UR-12825	TTGTTATCTCCCAGGAAATTT	0	0	0	0	0	0	0	0
†	21UR-12826	TTGTTACCCTGTTTGTTCAAC	0	0	0	0	0	0	0	0
†	21UR-12827	TTGTTAATAAGTGTGAGGGTA	0	0	0	0	0	0	0	0
	21UR-12828	TTGTGTTGGGTGAGTTTTCAA	0	1	0	0	0	0	1	2
	21UR-12829	TTGTCCTTCTTTTCTTATTAA	0	0	0	0	0	0	0	0
	21UR-12830	TTGTCCCTGTTATTGCCCCTT	0	0	0	0	0	0	0	0
	21UR-12831	TTGTCAGAACATCGTTCATTA	0	0	0	0	0	0	0	0
†	21UR-12832	TTGTATCATCTACTATGTCTA	0	0	0	0	0	0	0	0
†	21UR-12833	TTGTAGAATAGAATAAACAAT	0	0	0	0	1	1	0	2
†	21UR-12834	TTGTACTTCTCCTTATCAGAG	0	0	0	0	0	0	0	0
	21UR-12835	TTGTACGGGTCTAGTATACGT	0	0	0	0	0	0	0	0
†	21UR-12836	TTGTACATTACCCACCAATTT	0	0	0	0	0	0	0	0
†	21UR-12837	TTGTACACTGTTAAATACATT	0	0	0	0	0	0	0	0
†	21UR-12838	TTGTAAGGTTGGACATCTCTT	0	0	0	0	0	0	0	0
†	21UR-12839	TTGGTTTTCGGATTAAAGCAA	0	0	0	0	2	1	0	3
†	21UR-12840	TTGGTTTCCTGATATTCAAAT	0	0	0	0	0	0	0	0
†	21UR-12841	TTGGTTGCAGTCGTAAAATTG	0	0	0	0	0	0	0	0
†	21UR-12842	TTGGTTCGATCGATACGCTAG	0	0	0	0	0	0	0	0
†	21UR-12843	TTGGTTCATCAAAAAATGTAA	0	0	0	0	0	0	0	0
†	21UR-12844	TTGGTTATAGAGTATGTATGC	0	0	0	0	0	0	0	0
†	21UR-12845	TTGGTCCTGCTGTAGAATAAA	0	0	0	0	0	1	0	1
†	21UR-12846	TTGGTCATTAGAATATGAGAA	0	0	0	0	0	0	0	0
†	21UR-12847	TTGGTAAGAATGTATAAGAAT	0	0	0	0	1	1	0	2
†	21UR-12848	TTGGGTGGTGGATGTGTACAA	0	0	0	0	1	3	1	5
†	21UR-12849	TTGGGCATCCAAATTTTACAA	0	0	0	0	0	0	0	0
†	21UR-12850	TTGGGATGCTCTATATCAGTT	0	0	0	0	0	0	0	0
†	21UR-12851	TTGGGAATGTGAAATTTTTCA	0	0	0	0	0	0	0	0
†	21UR-12852	TTGGCAGGTTCTGATTTTAAT	0	0	0	0	0	0	0	0
†	21UR-12853	TTGGACTTTGATTGGAAATTG	0	0	0	0	0	0	0	0
	21UR-12854	TTGGACTGAACAATCAGGTAG	7	5	0	1	10	36	4	63
†	21UR-12855	TTGGAATCTCAAAAATTGTGA	0	0	0	0	0	0	0	0
	21UR-12856	TTGGAACTTTTTTTGTAGAAA	0	1	0	1	7	8	0	17
	21UR-12857	TTGGAAAAGTCATGTTTCATG	0	0	0	0	0	0	0	0
†	21UR-12858	TTGCTTTTGAGCCGTAGCCCA	0	0	0	0	0	0	0	0

	21UR-12859	TTGCTTTGTATGAGACTTTTG	0	0	0	0	0	0	0	0
†	21UR-12860	TTGCTTGTATGTATACTTTGT	0	0	0	0	8	1	0	9
	21UR-12861	TTGCTTCAACACTACCGTAGT	0	0	0	0	0	0	0	0
†	21UR-12862	TTGCTCTATTGGTTTTAGAAA	0	0	0	0	0	0	0	0
†	21UR-12863	TTGCTCTACCGTTAAGCAAAA	0	0	0	0	0	0	0	0
	21UR-12864	TTGCTATAAACAATCAAAAAA	0	0	0	0	0	0	0	0
†	21UR-12865	TTGCTAACGCAATTGATCGAA	0	0	0	0	0	0	0	0
	21UR-12866	TTGCGTCATTTCTAATTGTGA	0	0	0	0	0	0	0	0
†	21UR-12867	TTGCGGTTCTTAGTAATTTCT	0	0	0	1	4	0	0	5
†	21UR-12868	TTGCGGCAAAACTCTATGTCA	0	0	0	0	0	0	0	0
†	21UR-12869	TTGCGAACTATGTTTCCTTCC	0	0	0	0	0	0	0	0
*	21UR-12870	TTGCGAACACAGCTTTTTAGT	0	0	0	0	0	1	0	1
†	21UR-12871	TTGCCCATTGAAAATTCGACA	0	0	0	0	0	0	0	0
	21UR-12872	TTGCCATCTTCCTCTATTAAT	0	0	0	0	0	0	0	0
	21UR-12873	TTGCATTTGTCGTTGGCAACC	1	0	0	0	2	3	2	8
†	21UR-12874	TTGCATTTGCTACCAGATCTC	0	0	0	0	0	0	0	0
†	21UR-12875	TTGCATGTCTTTCTCCGTTTG	0	0	0	0	0	0	0	0
	21UR-12876	TTGCATCCATTCCAAATATGT	0	0	0	0	0	0	0	0
	21UR-12877	TTGCACTTGATAAGTTGGAAC	0	0	0	0	0	0	0	0
†	21UR-12878	TTGCAATGAATTGAATGTTCT	0	0	0	0	0	0	0	0
	21UR-12879	TTGCAAATTTAAATTTAGAGC	0	0	0	0	0	0	0	0
	21UR-12880	TTGCAAAATAACTCTCAGTGC	0	0	0	0	0	0	0	0
†	21UR-12881	TTGCAAAAGTAACAGGCGATG	0	0	0	0	0	0	0	0
†	21UR-12882	TTGATTGACGACGTGGCTATC	0	0	0	0	3	1	0	4
	21UR-12883	TTGATTCGACGGATAGGCGGT	1	0	0	0	0	8	0	9
†	21UR-12884	TTGATTCCGGGAATTCAAATT	0	0	0	0	0	0	0	0
†	21UR-12885	TTGATGTTGTTGTGTTGTTCT	0	0	0	0	0	0	0	0
†	21UR-12886	TTGATGTTACTGGAATATGGA	1	3	0	0	11	10	0	25
	21UR-12887	TTGATGCCAAGCTATTCAAAT	1	0	0	1	0	9	1	12
†	21UR-12888	TTGATGAATAGGTGATTAGGG	0	0	0	0	0	1	1	2
	21UR-12889	TTGATCTCTCCTCTTTTAATA	0	0	0	0	0	0	0	0
†	21UR-12890	TTGATCTCATAGGTCTAAAAA	0	0	0	0	0	0	0	0
†	21UR-12891	TTGATCCAGATTTGATGTTCA	0	0	0	0	0	0	0	0
†	21UR-12892	TTGATCATGTAAGTTTCAAAA	0	0	0	0	0	0	0	0
†	21UR-12893	TTGATCACTATTTTGGCATAT	1	2	0	5	40	35	0	83
†	21UR-12894	TTGATATTCATTCATTGGTTC	0	0	0	0	0	0	0	0
†	21UR-12895	TTGATACTCGTCGAATCGCAT	2	3	0	1	0	1	0	7
†	21UR-12896	TTGATAAGCTCCAATCGTGTC	0	0	0	0	0	0	0	0
†	21UR-12897	TTGAGCTGCAAGTGATAGAAA	0	0	0	0	0	0	0	0
†	21UR-12898	TTGAGAGTGATTTAGTTGGCG	0	0	0	0	1	0	0	1
†	21UR-12899	TTGAGACCGATTAGAATTGAA	0	0	0	0	0	0	0	0
†	21UR-12900	TTGACTTCATAGTGATTTTTT	0	0	0	0	0	0	0	0
†	21UR-12901	TTGACTTATGTATTCTCTACC	0	0	0	0	0	0	0	0
†	21UR-12902	TTGACTCAATCATCGATAATG	0	0	0	0	0	0	0	0
†	21UR-12903	TTGACATTCGACATCTTTAAA	0	0	0	0	0	1	0	1
†	21UR-12904	TTGACATCCAAGTTATCATGG	0	0	0	0	0	0	0	0
†	21UR-12905	TTGACAATGTAGCGTTAGCAA	3	0	2	0	3	1	0	9
	21UR-12906	TTGACAAGTATTCTACAACAG	0	0	0	0	2	1	0	3
†	21UR-12907	TTGAATTTCTAGTGGCGCCAA	0	0	0	0	0	0	0	0
†	21UR-12908	TTGAATTAGTTGTTATTGTTG	0	0	0	0	0	0	0	0
†	21UR-12909	TTGAATTAAATTTGAAAATGT	0	0	0	0	0	0	0	0
†	21UR-12910	TTGAATGTGCAAAGTAAGTAC	0	0	0	0	0	0	0	0
†	21UR-12911	TTGAATACTGCCTTTGTGAAT	0	0	0	0	1	0	0	1
	21UR-12912	TTGAATAATTTTCAGAGGAGA	0	0	0	0	0	0	0	0
	21UR-12913	TTGAATAAATCTAAATCCCTG	0	0	0	0	0	0	0	0
†	21UR-12914	TTGAACTCTTGGTCTACGTTT	0	0	0	0	0	0	0	0
†	21UR-12915	TTGAACTCCACATATCGTTCT	0	0	0	0	0	1	0	1
†	21UR-12916	TTGAACGAAAATGATATTTTT	0	0	0	0	0	0	0	0
	21UR-12917	TTGAACAACGTCTCTGGCAAT	0	0	0	0	0	0	0	0
†	21UR-12918	TTGAAATTTTCACATGCTGTT	0	0	0	0	0	0	0	0
†	21UR-12919	TTGAAATGTGGATTTTTTTAC	0	0	0	0	0	0	0	0
	21UR-12920	TTGAAAGTTGGATTTTTTCAT	0	0	0	0	0	0	0	0
†	21UR-12921	TTGAAAGTGCTGTTGGGCTGA	0	0	0	0	0	3	1	4
	21UR-12922	TTGAAAGCCTGTACATCAAAA	0	0	0	0	0	0	0	0

†	21UR-12923	TTGAAAGCAGAGGTGGGCGGA	1	0	0	0	0	0	0	1
	21UR-12924	TTGAAAAATTAGACTTTACGT	0	0	0	0	0	0	0	0
	21UR-12925	TTGAAAAACTCTCCACATTGC	0	0	0	0	0	0	0	0
	21UR-12926	TTGAAAAAAGTTAAATGCAGA	0	1	0	0	0	0	1	2
	21UR-12927	TTGAAAAAAATCTGTTTACCA	0	0	0	0	0	0	0	0
	21UR-12928	TTCTTTTTCTGAAATTCACAG	0	0	0	0	0	0	0	0
†	21UR-12929	TTCTTTGGATCTTATTCTTGG	0	0	0	0	0	0	0	0
†	21UR-12930	TTCTTTGAACTACTTGCACCA	0	0	0	0	0	1	0	1
†	21UR-12931	TTCTTCTGAGCGGGTTAGATC	0	0	0	0	0	0	0	0
†	21UR-12932	TTCTTCCTTTGTAACCGCCAA	0	0	0	0	0	0	0	0
	21UR-12933	TTCTTCAAGTTTTCCACTGTT	0	0	0	0	0	0	0	0
	21UR-12934	TTCTTAATTTTTATCCGCAGC	0	0	0	0	0	0	0	0
†	21UR-12935	TTCTGTCTGTGTGTTTGAACA	0	0	0	1	5	5	0	11
†	21UR-12936	TTCTGTAAAAACACTAGTTTT	0	0	0	0	0	0	0	0
	21UR-12937	TTCTGGTATAAAATATGACGC	0	0	0	0	1	3	1	5
†	21UR-12938	TTCTGGCGTGGTCCCATAACT	0	0	0	0	0	0	0	0
	21UR-12939	TTCTGGATTTGTTGGTTTTTT	16	5	8	1	6	14	4	54
†	21UR-12940	TTCTGATTATCTTCGTACCTG	0	0	0	0	0	0	0	0
	21UR-12941	TTCTGATCACATACTAATTTC	0	0	0	0	0	0	0	0
†	21UR-12942	TTCTCTGAGCTTTTTTCCAAT	1	0	0	0	0	2	1	4
	21UR-12943	TTCTCTCTCAAAATTTACTTT	0	0	0	0	0	0	0	0
	21UR-12944	TTCTCGGTGAAAAATTTGTGT	0	0	0	0	0	0	0	0
	21UR-12945	TTCTCGAAGGTAGATTATTTT	0	0	0	0	0	0	0	0
	21UR-12946	TTCTCCTCCTTTAAGAATATA	0	0	0	0	0	0	0	0
	21UR-12947	TTCTCCACTTGAGCTTCTGCT	0	0	0	0	0	0	0	0
†	21UR-12948	TTCTCATTAGGATATCATGTT	0	0	0	0	1	0	0	1
†	21UR-12949	TTCTCAAGATAACTTATGAAT	0	0	0	0	1	0	0	1
	21UR-12950	TTCTATTGGTCTATCCAATAT	0	0	0	0	0	1	0	1
	21UR-12951	TTCTATTGCCGAAATGGATTT	0	0	0	0	0	0	0	0
	21UR-12952	TTCTATGCAAAAAACCGTCTC	0	0	0	0	0	0	0	0
	21UR-12953	TTCTATAGAAAAACGGTCAAA	2	1	0	0	2	2	0	7
†	21UR-12954	TTCTAGGCGGATCAAATCATT	0	0	0	0	7	0	2	9
†	21UR-12955	TTCTACGTTTTGAATAGAACT	0	0	0	0	1	0	0	1
	21UR-12956	TTCTAAACCAGATTTTAAAAA	0	0	0	0	0	0	0	0
†	21UR-12957	TTCGTTGTCTCATGTCACAGA	0	0	0	0	0	0	0	0
†	21UR-12958	TTCGGTTATCGATGATTTATA	0	0	0	0	0	0	0	0
	21UR-12959	TTCGCTATCGTAGTTACCGTA	0	0	0	0	0	0	0	0
	21UR-12960	TTCGCGGAAACTGATTGCAGC	0	0	0	0	0	0	0	0
†	21UR-12961	TTCGCATTACGCAGAAAAAAA	0	0	0	0	0	0	0	0
†	21UR-12962	TTCGCATGTTGCTATAGATGA	0	0	0	0	0	0	0	0
	21UR-12963	TTCGCAGCCTATCTTCATTTC	0	0	0	0	0	0	0	0
†	21UR-12964	TTCGATTCTGGATCCACAGTG	0	0	0	0	0	0	0	0
†	21UR-12965	TTCGATTCGAACGATTGAAAA	0	0	0	0	0	0	0	0
†	21UR-12966	TTCGATATGACGATTAAAAAA	0	0	0	0	0	0	0	0
†	21UR-12967	TTCGATAGGTTGTTTGATGAG	0	0	0	0	0	0	0	0
	21UR-12968	TTCGACAATGGCTACGTTCAA	0	0	0	0	0	0	0	0
	21UR-12969	TTCGAATTCTACTTTTTTTTT	0	0	0	0	0	0	0	0
†	21UR-12970	TTCGAATGCATACAGTCCAAA	0	0	0	0	0	1	0	1
	21UR-12971	TTCGAACTGATTATTGAAAAA	0	0	0	0	0	0	0	0
†	21UR-12972	TTCGAAAGAGTTGGTTCTGAA	0	0	0	0	0	1	0	1
†	21UR-12973	TTCCTTTTTAATGGCAAGTTT	0	0	0	0	0	0	0	0
	21UR-12974	TTCCTTTATATTAAAATTTTG	0	0	0	0	2	1	0	3
	21UR-12975	TTCCTTGTCCGCGGGCGAAGA	0	0	0	0	0	0	0	0
†	21UR-12976	TTCCTTCCTTTTGATCCCCAT	0	0	0	0	0	0	0	0
	21UR-12977	TTCCTGGTTTAAGACCTTTTC	0	0	0	0	0	0	0	0
	21UR-12978	TTCCTCTGACGTTGTACCATT	0	0	0	0	0	0	0	0
	21UR-12979	TTCCTCGTTCCTACTGATTGG	0	0	0	0	0	0	0	0
	21UR-12980	TTCCTCGTGTTCTTCCAACTT	0	0	0	0	0	0	0	0
	21UR-12981	TTCCCTGTTCCCAACGATGAA	0	0	0	0	0	0	0	0
	21UR-12982	TTCCATTGTTAATACAACACG	0	0	0	0	0	0	0	0
	21UR-12983	TTCCATTGCTGAAAGTTAAAG	0	0	0	0	0	0	0	0
	21UR-12984	TTCCATTATCAATGAAAACAT	0	0	0	0	0	0	1	1
†	21UR-12985	TTCCATCTTTCTGCGACCAGA	0	0	0	0	0	0	0	0
	21UR-12986	TTCCATATCTTTATGATAATC	0	0	0	0	0	0	0	0

†	21UR-12987	TTCCAATCTATCTTAGATTCC	0	0	0	0	0	0	0	0
†	21UR-12988	TTCCAACAAATGTTGTTTTTT	0	0	1	0	1	0	0	2
	21UR-12989	TTCCAAATAGTAAGATAAGAG	0	0	0	0	0	0	0	0
	21UR-12990	TTCCAAAATGTTCACTGTTTC	0	0	0	0	0	0	0	0
†	21UR-12991	TTCCAAAAATTGAACTTTTCC	0	0	0	0	0	0	0	0
	21UR-12992	TTCATTTTGAAATTCGAGCCC	0	0	0	0	0	0	0	0
†	21UR-12993	TTCATTGTATAACAACTGCTT	0	0	0	1	7	0	0	8
	21UR-12994	TTCATCTAATTGTCTTGCACC	0	0	0	0	0	0	0	0
†	21UR-12995	TTCATCGTACACAATGCCATT	0	0	0	0	6	6	2	14
	21UR-12996	TTCATCAAGTTGGAGAAGGAA	0	0	0	0	0	3	1	4
†	21UR-12997	TTCATATGGTAGAAAAAAGCA	0	0	0	1	1	3	0	5
	21UR-12998	TTCAGTCATCATTAGATCATC	0	0	0	0	1	0	0	1
†	21UR-12999	TTCAGATCAGAAAAAAAATGT	0	0	0	0	0	0	0	0
†	21UR-13000	TTCAGAAAAGGTGTTGTACAA	0	0	0	0	0	1	0	1
†	21UR-13001	TTCACTTTTTTGCTTGCCTAC	0	0	0	0	0	0	0	0
	21UR-13002	TTCACGTCTTTTTTAATTTTT	0	0	0	0	0	0	1	1
†	21UR-13003	TTCACCTCAAAATCCATAACC	0	0	0	0	0	0	2	2
	21UR-13004	TTCACCACTTTGATAAATTTC	0	0	0	0	0	0	0	0
†	21UR-13005	TTCAATTCTAAGGGCTTTGAA	0	0	0	0	0	0	0	0
†	21UR-13006	TTCAATTAGTAAACTGGTAGA	0	0	0	0	0	0	0	0
†	21UR-13007	TTCAATGTTTCAAATCCTTCA	0	0	0	0	0	0	0	0
	21UR-13008	TTCAATGGCCATTGTTTTTTC	0	0	0	0	0	0	0	0
†	21UR-13009	TTCAATGATCAAAGCTTGCAA	0	0	0	0	0	1	0	1
†	21UR-13010	TTCAAGAGGACTTCTCAGGAC	0	0	0	0	0	0	0	0
	21UR-13011	TTCAACTTGTTTGTAAATCCA	0	0	0	0	0	0	0	0
	21UR-13012	TTCAAATTGCAGGGTCACGGA	0	0	0	0	0	0	0	0
	21UR-13013	TTCAAATTGACGCTTTGTGAA	0	0	0	0	0	0	0	0
	21UR-13014	TTCAAATGCTTTTTCTCAAAA	0	0	0	0	0	0	0	0
†	21UR-13015	TTCAAATCCCTTAAAAAGACG	0	0	0	0	0	2	0	2
	21UR-13016	TTCAAAGCGCTGATCTTAGAT	0	0	0	0	0	0	0	0
	21UR-13017	TTCAAACAATTCACAGAATAT	0	0	0	0	0	0	0	0
†	21UR-13018	TTCAAAAACTCCCTAAAACAA	0	0	0	0	0	0	0	0
	21UR-13019	TTATTTTTGTTGAGCGATGTA	0	0	0	0	0	0	0	0
†	21UR-13020	TTATTTTTATGTATATTCGAA	0	0	0	0	1	0	0	1
	21UR-13021	TTATTTTAGCTTTAAGAATAA	0	0	0	0	2	1	0	3
†	21UR-13022	TTATTTCTCCTATCAACAAGC	0	1	0	0	0	1	0	2
†	21UR-13023	TTATTTCCATTATTCCCCTGT	0	0	0	0	0	0	0	0
†	21UR-13024	TTATTTCACCGTTTAAGCAGT	0	0	0	0	0	0	0	0
†	21UR-13025	TTATTTATATGATTTTTGCCA	0	0	0	0	0	0	0	0
†	21UR-13026	TTATTTACCAACTTTGCTAAT	0	0	0	0	0	1	0	1
	21UR-13027	TTATTGTGGTTTAGGAAGTGG	0	0	0	0	0	0	0	0
†	21UR-13028	TTATTGGAATGTAAATCGCGC	0	0	0	0	0	0	0	0
†	21UR-13029	TTATTGCTCGAGAATACAACG	0	0	0	0	0	0	0	0
†	21UR-13030	TTATTGAACTCTTGGTCTACG	0	0	0	0	0	0	0	0
†	21UR-13031	TTATTCTCTTCAGAGATGGCT	0	0	0	1	3	10	2	16
	21UR-13032	TTATTCTCATAATTTGCACAA	0	0	0	0	0	0	0	0
	21UR-13033	TTATTCAAGAAAGAAACAACA	0	0	0	0	0	0	0	0
	21UR-13034	TTATTATGTGAGAGTTTGCGA	0	0	0	0	0	0	0	0
†	21UR-13035	TTATTACGACTCAACGACTAA	1	0	0	1	1	1	1	5
	21UR-13036	TTATGCTTTTTCAAATTTTTA	0	0	0	0	3	0	0	3
†	21UR-13037	TTATCTGATCGACATTGATTG	0	0	0	0	0	0	0	0
†	21UR-13038	TTATCTATACAGGATTAGCTT	0	0	0	0	0	0	0	0
†	21UR-13039	TTATCGCTAAGCTTTTTCTGA	0	0	0	0	0	0	0	0
	21UR-13040	TTATCCTTTCCAATTCCAAAG	0	0	0	0	0	1	0	1
†	21UR-13041	TTATCCCGTCGATAATAAAAA	0	0	0	0	0	0	0	0
†	21UR-13042	TTATATGGTATTTGTTTCATT	0	0	0	0	0	0	0	0
†	21UR-13043	TTATATCGTTAATCTGGCAAT	0	0	0	0	0	1	0	1
	21UR-13044	TTATATACTAGTCAATTATAT	0	0	0	0	0	0	1	1
†	21UR-13045	TTATATAACTCCTCAAAGGAA	0	0	0	0	2	1	0	3
†	21UR-13046	TTATAGTTACCATCAGAGGTG	0	0	0	0	0	0	0	0
	21UR-13047	TTATACAGACTAGCGAGGAAA	0	0	0	0	0	0	0	0
†	21UR-13048	TTATAAGAAATTAAAAAAAAA	0	0	0	0	0	0	0	0
†	21UR-13049	TTAGTTTTCAGTATAGGCCTG	0	0	0	0	0	0	0	0
†	21UR-13050	TTAGTTTGCCGTCTTTCATAA	0	0	0	0	0	0	1	1

	21UR-13051	TTAGTTTCCATTTTACGTGGG	0	0	0	0	0	1	0	1
	21UR-13052	TTAGTTTATTTGTATCAGCCT	0	0	0	0	0	0	0	0
†	21UR-13053	TTAGTTGGACTGTTCCACGTG	0	0	0	0	1	0	0	1
	21UR-13054	TTAGTTGGAATATTTGTGGTT	0	0	0	0	0	0	0	0
†	21UR-13055	TTAGTGGATAGTTTTTTTGGA	0	0	0	0	4	6	0	10
†	21UR-13056	TTAGTGCAAATCAGAATATTA	0	0	0	0	2	1	1	4
†	21UR-13057	TTAGTCAGTGTAGCATTTAAA	0	0	0	0	3	2	0	5
†	21UR-13058	TTAGTAGACTTTTCAAACTTT	0	0	0	0	0	0	0	0
	21UR-13059	TTAGGGTTAACTAGAAACAAT	0	0	0	0	0	0	0	0
†	21UR-13060	TTAGGCAAGAGCGGGTGAAGA	0	0	0	0	1	0	0	1
†	21UR-13061	TTAGGAAACCGATTCCCTAAA	0	0	0	0	0	0	0	0
	21UR-13062	TTAGCAGAGGATGGTGAAAAT	0	0	0	0	0	0	0	0
†	21UR-13063	TTAGATTGCCCGGTACATGTA	0	0	0	0	0	0	0	0
†	21UR-13064	TTAGAGTCCAGTTGATGTCCA	0	0	0	0	0	1	0	1
†	21UR-13065	TTAGAGCTACTCAAAAAAAGC	0	0	0	0	0	0	0	0
†	21UR-13066	TTAGACTGAACCTTTAACACT	0	0	0	0	0	0	0	0
†	21UR-13067	TTAGAAGCTCATGCAGTCAGT	0	0	0	0	0	2	0	2
	21UR-13068	TTAGAAAATCCATAAACACAT	0	0	0	0	0	0	0	0
†	21UR-13069	TTAGAAAAGGAATTGCCTAAC	0	0	0	0	0	0	0	0
†	21UR-13070	TTACTTTAACTGAGATGAAAA	0	0	0	0	0	1	0	1
†	21UR-13071	TTACTTACTTGTTTGTTGAAA	0	0	0	0	0	0	0	0
†	21UR-13072	TTACTGGGACTTCCTAAACGG	0	0	0	0	1	0	0	1
†	21UR-13073	TTACTGCAGTCGTTGAATGGT	0	2	0	0	0	2	0	4
†	21UR-13074	TTACTCTTGCAGTCAAAAAAA	0	0	0	0	0	0	0	0
†	21UR-13075	TTACTCTCACTCAAGTTCAGA	0	0	0	0	1	1	0	2
†	21UR-13076	TTACTCGGATGAAATTGAAAT	0	0	0	0	0	0	0	0
	21UR-13077	TTACTATGTTTACCAAAAAAT	0	0	0	0	0	1	0	1
†	21UR-13078	TTACGTTTTTTTCGGGTTAGT	1	0	0	0	1	0	0	2
†	21UR-13079	TTACCCATGTTTCATCACAAG	0	0	0	0	0	0	0	0
	21UR-13080	TTACATCAATTGTGACGTCAT	0	0	0	0	0	0	0	0
	21UR-13081	TTACAGTTTCACAACTATACA	0	0	0	0	1	0	0	1
†	21UR-13082	TTACACGCGCATAAGTTTCTT	0	0	0	0	0	0	0	0
	21UR-13083	TTAATTTTCTAAACATCAATT	0	0	0	0	1	0	0	1
	21UR-13084	TTAATTTGAAGTTACCTAATA	0	0	0	0	0	0	0	0
	21UR-13085	TTAATTTAAGTTTGAATAGAA	0	0	0	0	0	0	0	0
†	21UR-13086	TTAATTGGTATCTGATCGGTT	0	0	0	0	6	1	9	16
†	21UR-13087	TTAATTGCAACTTTGGTGTCA	0	0	0	0	0	0	0	0
	21UR-13088	TTAATTCTTCATCTGCATTTT	0	0	0	0	1	1	0	2
†	21UR-13089	TTAATTCTACGATCGTATTGA	0	0	0	0	0	0	0	0
†	21UR-13090	TTAATTCAGCTTCAAAGTGCC	0	0	0	0	0	0	0	0
†	21UR-13091	TTAATTCACTGATTAACTGAA	0	0	0	0	0	0	0	0
†	21UR-13092	TTAATTCAAGCAACAATACCT	0	0	0	0	0	1	0	1
†	21UR-13093	TTAATTAGTATTCTGTGCCTT	0	0	0	0	0	0	0	0
†	21UR-13094	TTAATGGACTGTTGATCTTTA	0	0	0	1	0	0	0	1
†	21UR-13095	TTAATGCTATTTCGGTTATAA	3	0	1	0	0	3	2	9
	21UR-13096	TTAATGAATAATTAACTGTTT	0	0	0	0	0	0	0	0
†	21UR-13097	TTAATCTTGTTCTCTCCCAAA	0	0	0	0	0	0	0	0
†	21UR-13098	TTAATCCTCTTGCAATTTTAA	0	0	0	0	0	0	0	0
†	21UR-13099	TTAATCCGTTTCTGTATAACA	0	0	0	0	0	0	0	0
	21UR-13100	TTAATCCACTTTTTTGTATAT	0	0	0	0	0	1	0	1
†	21UR-13101	TTAATCCACACAGAAACAAAC	1	0	0	0	0	0	1	2
†	21UR-13102	TTAATAGTGTAGAAAAGTGGT	1	0	0	0	0	2	0	3
	21UR-13103	TTAATAATAAAAATTAAAATA	0	0	0	0	1	1	0	2
†	21UR-13104	TTAATAAATATTCTTTCGGCT	1	0	0	0	1	1	4	7
	21UR-13105	TTAAGTAAGTAAATCTTCAGC	0	0	0	0	0	0	0	0
	21UR-13106	TTAAGAGTGAACAAAAAAACT	0	0	0	0	0	0	0	0
	21UR-13107	TTAACGATATTCTGTCACTTT	0	0	0	0	0	0	0	0
†	21UR-13108	TTAACATATGCAAATAAGTAC	0	0	0	0	0	0	0	0
	21UR-13109	TTAAATCTTCGTTTATAACAT	0	0	0	0	0	0	0	0
†	21UR-13110	TTAAAGAGGTTTTGAACAATG	0	0	0	0	0	0	0	0
	21UR-13111	TTAAAATGGAGTGAAAAGGTA	0	0	0	0	0	0	0	0
†	21UR-13112	TTAAAAATCGAAATATGGGAA	0	0	0	0	0	0	0	0
†	21UR-13113	TGTTTTTTTCCGAGGAACATT	0	0	0	0	0	0	4	4
†	21UR-13114	TGTTTTTTTAAGGACATTTTA	0	0	0	0	0	2	2	4

	21UR-13115	TGTTTTTTGAAATCATAACTG	0	0	0	0	0	0	0	0
	21UR-13116	TGTTTTTTAATGTAGTACTTG	0	0	0	0	0	0	0	0
	21UR-13117	TGTTTTTACTATTTTTTCAAT	0	0	0	0	0	0	0	0
†	21UR-13118	TGTTTTGAAACACCACATTAA	0	0	0	0	1	0	0	1
†	21UR-13119	TGTTTTATTCCATCTTTTATT	0	0	0	0	0	0	0	0
	21UR-13120	TGTTTGTGTCGGATTTTTTAC	6	0	0	0	2	8	3	19
	21UR-13121	TGTTTGTGCTCACGAATACTT	0	0	0	0	0	0	0	0
	21UR-13122	TGTTTGGTTTTAATTTTGAAC	0	0	0	0	0	0	0	0
†	21UR-13123	TGTTTGGGTTCCTGATCTTCT	0	0	0	0	0	0	0	0
†	21UR-13124	TGTTTGAAGATCACGATGTTC	0	0	0	0	0	0	0	0
	21UR-13125	TGTTTCATGGTTTTATGTTAT	0	0	0	0	0	0	0	0
†	21UR-13126	TGTTTCAGGATTGTAGGTTTT	4	0	0	1	12	9	8	34
†	21UR-13127	TGTTTACACCAGTTCCGCAAA	0	0	0	0	0	0	0	0
	21UR-13128	TGTTTAATGTCTATTGTAATG	0	0	0	1	0	1	0	2
†	21UR-13129	TGTTGCGTCTCTTTGTTGGAG	0	0	0	0	0	0	0	0
†	21UR-13130	TGTTGATCTCCTGGTAATTCT	0	0	0	0	2	2	0	4
†	21UR-13131	TGTTGAACCTTAGGAACATCA	0	0	0	0	0	0	0	0
	21UR-13132	TGTTGAAAAATTATATTACTT	0	0	0	0	0	0	0	0
†	21UR-13133	TGTTCTTGCTAGTCCTCTGTC	0	0	0	0	0	0	0	0
†	21UR-13134	TGTTCTATCGATCAAAACATA	0	0	0	0	0	0	0	0
	21UR-13135	TGTTCGGGCACAAAGTAGGAA	0	0	0	0	0	0	0	0
†	21UR-13136	TGTTCCATTAAAAATTTTCCC	0	0	0	0	0	0	0	0
	21UR-13137	TGTTCATCTCATTCACTCCCA	0	0	0	0	0	0	0	0
†	21UR-13138	TGTTCATATACTGGATAACTT	0	0	0	0	0	0	0	0
†	21UR-13139	TGTTACAGGATCAGTGAAAGG	0	0	0	0	0	0	0	0
†	21UR-13140	TGTTAAGATTTTAGTTCCCTG	0	0	0	0	0	0	0	0
	21UR-13141	TGTTAAATACCGAAACTTGAA	0	0	0	0	0	0	0	0
†	21UR-13142	TGTTAAAGCAGAAATATTTAC	0	0	0	0	0	0	0	0
†	21UR-13143	TGTGTATCGAAACGAATTCCC	0	0	0	0	1	0	0	1
	21UR-13144	TGTGGTGCATCGAATGTATCT	0	0	0	0	0	0	0	0
	21UR-13145	TGTGGGGCACTTTTTTGAAGG	0	0	0	0	0	0	0	0
	21UR-13146	TGTGGGATGTCGAATCAGAGC	0	0	0	0	0	0	0	0
	21UR-13147	TGTGGATATACAGAAAGATAT	0	0	0	0	0	0	0	0
	21UR-13148	TGTGCAAGGACCCGGCAGATG	0	0	0	0	0	0	0	0
	21UR-13149	TGTGAAAAAATATATTCGATG	0	0	0	0	0	0	0	0
	21UR-13150	TGTGAAAAAAAAGTTTGGTTC	0	0	0	0	0	0	0	0
	21UR-13151	TGTCTATTGAAATGTCTTCTA	0	0	0	0	0	0	0	0
	21UR-13152	TGTCAGTTTCAGCCGACTTTT	0	0	0	0	0	0	0	0
†	21UR-13153	TGTCAAGGCACAAATAGAAAA	0	0	0	0	0	0	0	0
†	21UR-13154	TGTATTTGACCCTTTTTCACA	0	0	0	0	0	0	0	0
†	21UR-13155	TGTATTGCACTAAAGGGTATT	0	0	0	0	0	0	0	0
	21UR-13156	TGTATGTAACTTTAGCATAAT	0	0	0	1	1	1	0	3
	21UR-13157	TGTATCTAGAGTCGTATATTA	0	0	0	0	0	0	0	0
	21UR-13158	TGTATCCTTCCGTCATTGTGC	0	0	0	0	0	0	0	0
	21UR-13159	TGTAGTTTCGGTAGGTTTAGA	0	0	0	1	11	13	0	25
	21UR-13160	TGTAGTCGAAGTTTCAATTGT	0	0	0	0	0	0	0	0
†	21UR-13161	TGTAGCCTCTATCATCACAAT	0	0	0	0	0	1	0	1
†	21UR-13162	TGTACTACGATGCAGTAAATA	0	0	0	0	0	0	0	0
	21UR-13163	TGTACATTTCTGTGCAACTTT	0	0	0	0	0	0	0	0
	21UR-13164	TGTAATTAGACGTTAAACGAT	0	0	0	0	0	0	0	0
	21UR-13165	TGTAATAGCGGACTTCATTGC	0	0	0	0	3	3	1	7
	21UR-13166	TGTAAATTCCAACTGTTTCGA	0	0	0	0	0	0	0	0
	21UR-13167	TGGTTTTTTTTTTTCGTTTTC	1	0	0	0	0	1	0	2
	21UR-13168	TGGTTTTCTGGTAATTTGATA	0	0	0	0	0	0	0	0
	21UR-13169	TGGTTTTCGGTAGTTAGTGAA	0	0	0	0	0	0	0	0
†	21UR-13170	TGGTTTATTTAACAGGACACT	0	0	0	0	0	0	0	0
†	21UR-13171	TGGTTGATGTTTCGTCTTTAG	0	0	0	0	0	0	0	0
†	21UR-13172	TGGTGAACAAGAAAAATATGG	0	0	0	0	1	0	0	1
†	21UR-13173	TGGTCGTCGATCAGGCAAAGT	1	0	0	1	6	13	1	22
†	21UR-13174	TGGTCACATCAAAAATTTTGT	0	0	0	0	0	0	0	0
	21UR-13175	TGGGTGTTCTATGGTGCACTT	0	0	0	0	0	0	0	0
†	21UR-13176	TGGGGTAAACTTGAGGGAAAT	6	3	0	2	12	31	6	60
	21UR-13177	TGGGGAAAAACAATGAAAACA	0	0	0	0	0	0	0	0
	21UR-13178	TGGGATTGATGACTTTCATTT	0	0	0	0	0	0	0	0

21UR-13179	TGGGATCTATTATTGTGCATT	0	0	0	0	0	0	0	0
21UR-13180	TGGGATCAAAAGTTATTTTAT	0	0	0	0	0	0	0	0
† 21UR-13181	TGGGAGAAATCTACGGAAATT	0	0	0	0	6	4	5	15
† 21UR-13182	TGGCTGTGAAAAATACTAACA	0	0	0	0	2	0	0	2
† 21UR-13183	TGGCTATGTGGACTATTTAGA	0	0	0	0	1	1	0	2
† 21UR-13184	TGGCTAGTTGGTCCCTCTTTT	0	0	0	0	0	0	0	0
21UR-13185	TGGCCTAAACTTAATTGAACG	0	0	0	0	0	0	0	0
21UR-13186	TGGCATTGCTGTCAAGACAGG	0	0	0	0	0	0	0	0
21UR-13187	TGGCAGTAGTTATTTAGTTTT	0	0	0	0	0	0	0	0
21UR-13188	TGGCAAGTTTTGGATGATTAG	0	0	0	2	0	0	0	2
21UR-13189	TGGATTTTGTGAAACTTTTGC	0	0	0	0	0	0	0	0
21UR-13190	TGGATTGCAAATGTCATGAAC	0	0	0	0	0	0	0	0
† 21UR-13191	TGGATCTAAAGATTTTTCCTT	0	0	0	0	1	0	0	1
† 21UR-13192	TGGAGACATCTGGTAGGTGAT	1	0	2	2	2	5	1	13
21UR-13193	TGGACGATGCCAGGAGGGACC	0	0	0	0	0	1	0	1
21UR-13194	TGGACCATCATGTGATCTTAC	0	0	0	0	0	0	0	0
21UR-13195	TGGAAGCAAATTGTAGGTGTG	0	2	0	0	12	14	28	56
21UR-13196	TGGAACAGTTGATGATCGGGA	0	0	0	0	0	3	4	7
21UR-13197	TGGAAATTATCATCTTGATGT	0	0	0	0	0	0	0	0
21UR-13198	TGGAAATGTGTCAGTGGGGAT	0	0	0	0	0	0	0	0
21UR-13199	TGGAAAGGATCAGTTTTAAAA	0	0	0	0	0	0	0	0
21UR-13200	TGGAAAATTGAAGAGACGCAG	1	0	0	0	0	0	0	1
21UR-13201	TGGAAAACCGATCTGACTACA	0	0	0	0	0	0	0	0
† 21UR-13202	TGCTTTCTTGTGAATGATGGG	0	0	0	0	1	1	0	2
21UR-13203	TGCTTCCACAATTTGCTATAA	0	0	0	0	0	0	0	0
† 21UR-13204	TGCTGCTGTTAGTAGATCCAT	0	0	0	0	1	0	0	1
† 21UR-13205	TGCTGCATCGTTTTTATTGTA	0	0	0	0	0	1	0	1
21UR-13206	TGCTGCAGCCTCTAATAGTCT	0	0	0	0	0	0	0	0
21UR-13207	TGCTGCAATGCACATTTCCTG	0	0	0	0	0	0	0	0
† 21UR-13208	TGCTATTGTTTTGGTATAATG	0	0	0	0	0	0	0	0
† 21UR-13209	TGCTACGATCTTCCTTCAATG	0	0	0	0	0	0	0	0
21UR-13210	TGCGAAAGTTCTGTGAATTTT	0	0	0	0	0	0	0	0
† 21UR-13211	TGCCTTTGGGAAGTTGTAATA	0	0	0	0	0	0	0	0
† 21UR-13212	TGCCTGTTGCTAGAATCCGAT	0	0	0	0	0	0	0	0
21UR-13213	TGCCTATGTTTTTCACGTACC	0	0	0	0	0	0	0	0
21UR-13214	TGCCCTTTTATTTTCCGCAAA	0	0	0	0	0	0	0	0
21UR-13215	TGCCAGACTTCATGGTATTTC	0	0	0	0	0	0	1	1
21UR-13216	TGCCAGAAACTGTTCCTTGTC	0	0	0	0	0	0	0	0
21UR-13217	TGCCAGAAACCAGAAAAAACT	0	0	0	0	0	0	0	0
21UR-13218	TGCCAATTAAAGTTCCAAAAA	0	0	0	0	0	0	0	0
21UR-13219	TGCATTCCACTTTCAAGCACC	0	0	0	0	0	0	0	0
21UR-13220	TGCATTAGTGTTTAAAACAGT	0	0	0	0	0	0	0	0
21UR-13221	TGCATCAAATGGGCTTGTTGC	0	0	0	0	0	0	0	0
21UR-13222	TGCATACTACAAAAATGCATA	0	0	0	0	0	0	0	0
21UR-13223	TGCAGGGTTGGTTTTTTTAAT	0	0	0	0	0	0	0	0
21UR-13224	TGCAGAGGACAATTGGTACAT	0	1	1	1	27	23	4	57
† 21UR-13225	TGCAAGACTTCGAGTGCATAT	0	0	0	0	0	0	0	0
21UR-13226	TGCAAATTCTGCCCAAGCAAA	0	0	0	0	0	0	0	0
21UR-13227	TGATTTTTTAAGGTTTTGACC	0	0	0	0	0	0	0	0
† 21UR-13228	TGATTTTTGGATATAGGAAAT	0	0	0	0	3	1	0	4
† 21UR-13229	TGATTTTGAAACATTTTAAAC	0	0	0	0	0	0	0	0
† 21UR-13230	TGATTTGATTTTGTGTCCTGA	0	0	0	0	1	0	0	1
21UR-13231	TGATTTACCAAAAAAGTGACT	0	0	0	0	0	0	0	0
21UR-13232	TGATTGTTTGTTGTCCAGCAA	0	0	0	0	0	0	0	0
† 21UR-13233	TGATTGGTTTCGGATTCTGCT	0	0	0	0	0	0	0	0
† 21UR-13234	TGATTGAAAGTTGTGCGTTTT	0	0	0	0	0	0	0	0
† 21UR-13235	TGATTCTTTCTTTTCAAAGTG	0	0	0	0	0	0	0	0
† 21UR-13236	TGATTCTTCAAACGCCCATCA	0	0	0	0	0	0	0	0
21UR-13237	TGATTATTCGAAAGAAGTGGT	0	0	0	0	0	0	0	0
† 21UR-13238	TGATTATATAACTCCTCAAAG	0	0	0	0	0	0	0	0
21UR-13239	TGATGTTGTTTTGATAGTAAG	0	0	0	0	0	0	0	0
21UR-13240	TGATGTACTCGGAACTTCAGA	0	0	0	0	0	1	0	1
21UR-13241	TGATGGAATTGACATGTAACT	0	0	0	0	0	0	0	0
† 21UR-13242	TGATGGAAGCTTGATGGAAAA	0	0	0	0	0	0	0	0

		21UR-13243	TGATGCAAATGACGGACAGTA	1	0	0	0	0	2	0	3
	†	21UR-13244	TGATGATCGTAAATTTGTTCG	1	0	0	0	0	0	0	1
		21UR-13245	TGATGACCACGAATATTATCA	1	0	0	0	0	0	0	1
	†	21UR-13246	TGATATTTCGATGGACTTTGG	0	0	0	0	0	0	0	0
		21UR-13247	TGATATCCTGTAATTAGTTTC	0	0	0	0	0	0	0	0
	†	21UR-13248	TGATATAACGGTTAACTTAGA	0	0	0	0	1	0	0	1
	†	21UR-13249	TGATACACACTTGACTTAGGT	0	0	0	0	0	0	0	0
		21UR-13250	TGATAATTTTTAAGGCATTTT	0	0	0	0	0	1	1	2
		21UR-13251	TGATAACTAACCGCTGTCCAC	0	0	0	0	0	0	0	0
	†	21UR-13252	TGAGTTGTTAAAACAGTAAAA	0	0	0	0	0	0	0	0
		21UR-13253	TGAGTATTTTGGTTTTGAGTT	0	0	0	0	0	0	0	0
		21UR-13254	TGAGCTTTCCAAAATTATCAC	0	0	0	0	0	0	0	0
		21UR-13255	TGAGCCCTAATTTTTTGGAAA	0	0	0	0	0	0	0	0
	†	21UR-13256	TGAGCAGTGATTTAAATTGGC	0	0	0	0	0	1	0	1
	†	21UR-13257	TGAGATATGTATTCGGATTCT	0	0	0	0	0	0	0	0
		21UR-13258	TGAGAGCGCAATTGATGTCAG	0	0	0	0	0	0	0	0
	†	21UR-13259	TGAGACCGATTAGAATTGAAT	0	0	0	0	0	0	0	0
		21UR-13260	TGAGAAAAAAAATGTTGCTGG	0	0	0	0	0	0	0	0
	†	21UR-13261	TGACTTTGTTAAAATTTTGAG	0	0	0	0	0	0	0	0
		21UR-13262	TGACTTGAATTATTTTCTTGG	0	0	0	0	0	0	0	0
	†	21UR-13263	TGACTGCCTCTCTTGGAAAAT	0	0	0	0	0	0	0	0
		21UR-13264	TGACTATATCAGAGAAGAGTG	0	0	0	0	0	0	0	0
	†	21UR-13265	TGACGTACGTGGTAAAATACA	0	0	0	0	1	0	0	1
	†	21UR-13266	TGACGATGTGTTTTTTTGCCA	0	0	0	0	0	0	0	0
		21UR-13267	TGACGAATTGAAACTAAAGTT	0	0	0	0	0	0	0	0
		21UR-13268	TGACCTGTAGTAACTTCCAAA	0	0	0	0	0	0	0	0
	†	21UR-13269	TGACAATAAAATTACCGATTA	0	0	0	0	0	0	0	0
		21UR-13270	TGACAAATTGGAATTGGCTCA	0	0	0	0	0	1	0	1
		21UR-13271	TGAATTTATTGGGTACTTGTG	0	0	0	0	0	0	0	0
	†	21UR-13272	TGAATTTAGTCTGTGGAAGGT	15	6	6	13	143	167	18	368
	†	21UR-13273	TGAATTCGGATTCGTTCTCTA	1	1	0	0	0	0	0	2
		21UR-13274	TGAATTCCGAAAAAGCGTCGT	0	0	0	0	0	0	0	0
	†	21UR-13275	TGAATTATCTTCCACCACATT	0	0	0	0	0	0	0	0
	†	21UR-13276	TGAATGTTTCAATACCTCTAA	1	0	0	0	0	0	0	1
		21UR-13277	TGAATGGTACCAAAATGTTCT	0	0	0	0	0	0	0	0
	†	21UR-13278	TGAATACTTTGATTGATTTGA	0	0	0	0	0	0	0	0
	†	21UR-13279	TGAAGCTCTGGATTGAAAAAA	0	0	0	0	0	0	0	0
		21UR-13280	TGAAGCAGCAGATTGAGGGAA	0	1	0	0	0	1	0	2
		21UR-13281	TGAAGATGTGTTGGGAGTGAA	0	0	0	0	0	0	0	0
		21UR-13282	TGAAGAAACTTTATTCGAGAT	0	0	0	0	0	0	0	0
*	†	21UR-13283	TGAACTGTGGCGTGACTTATC	0	0	0	1	1	0	0	2
	†	21UR-13284	TGAACGTTTACTATCGCCATT	0	0	0	0	1	2	0	3
	†	21UR-13285	TGAACATGTTCTTGGGATATT	0	0	0	0	0	0	0	0
		21UR-13286	TGAAATTTGGCTCATAAAAAT	0	0	0	0	0	0	0	0
		21UR-13287	TGAAATTCAAAGATTTGACAA	0	0	0	0	0	0	0	0
		21UR-13288	TGAAAGTTTTTCACAAGTTCA	0	0	0	0	0	0	0	0
	†	21UR-13289	TGAAAGAATTCATGACTGACA	0	0	0	0	0	0	0	0
		21UR-13290	TGAAAATCTAAAAATTTTTGT	0	0	0	0	0	0	0	0
		21UR-13291	TGAAAACGTTTCCTGTTTCAA	0	0	0	0	0	0	0	0
		21UR-13292	TGAAAACAAATTGCAATCAGT	0	0	0	0	0	0	0	0
		21UR-13293	TGAAAAAGATGAGAAAACGGA	0	0	0	0	0	0	1	1
		21UR-13294	TGAAAAACGGGTTTGTTTTTG	0	0	0	0	0	0	0	0
		21UR-13295	TGAAAAAAATACAGAAACATC	0	0	0	0	0	0	0	0
	†	21UR-13296	TCTTTTCTCAAACTAGTGAAA	0	0	0	0	0	0	0	0
		21UR-13297	TCTTTGTTGAGCATTTCGGCC	0	0	0	0	0	0	0	0
		21UR-13298	TCTTTGGACGTTATAGGCCGA	0	0	0	0	0	1	0	1
		21UR-13299	TCTTTGATCTAAATTTTTAAT	0	0	0	0	0	0	0	0
		21UR-13300	TCTTTCTCAAAGTATATTTGA	0	0	0	0	0	0	0	0
		21UR-13301	TCTTTCGCAACTGTTTTCTAT	0	0	0	0	0	1	0	1
		21UR-13302	TCTTTCAGTAGTATATCGCCC	0	0	0	0	0	0	0	0
		21UR-13303	TCTTTCAAATGAGTTTCAGAC	0	0	0	0	0	0	0	0
	†	21UR-13304	TCTTGTTTAATAGGATTCGCA	0	0	0	0	2	3	0	5
		21UR-13305	TCTTGGTATCCCGTATTAAAA	0	0	0	0	0	0	0	0
		21UR-13306	TCTTGGAATTGATATGATTGG	0	0	0	0	0	1	0	1

	21UR-13307	TCTTGCGAATTTTTGGTAAGC	0	0	0	0	0	0	0	0
†	21UR-13308	TCTTGAGTTTAGGCGTTTTTT	0	0	0	0	0	0	0	0
†	21UR-13309	TCTTGAACAAAACGCAGAGAA	0	3	0	1	1	9	0	14
	21UR-13310	TCTTCTCCAGAATTAATCAAT	0	0	0	0	0	0	0	0
†	21UR-13311	TCTTCGAAGTCTTTGTTGTGA	0	0	0	0	0	0	0	0
	21UR-13312	TCTTCAATGTTGGATGAGGAT	0	0	0	0	0	0	0	0
	21UR-13313	TCTTCAAATGAGTCATGAAGA	0	0	0	0	0	0	0	0
	21UR-13314	TCTGTTGAGTGGGGGGACAGT	0	0	0	0	0	0	0	0
	21UR-13315	TCTGTGTAAACGTACATAAGG	0	0	0	0	0	0	0	0
	21UR-13316	TCTGCCATCTCATCCAACACA	0	0	0	0	0	0	0	0
†	21UR-13317	TCTGATAGTGATCCGCTTTCA	0	0	0	0	0	0	0	0
	21UR-13318	TCTGAACTTTTGATCAGAAAT	0	0	0	0	0	0	0	0
	21UR-13319	TCTCTTCTCTTTCTAAGATGT	0	0	0	0	0	0	0	0
	21UR-13320	TCTCTCGAAAAATTTTAGTTA	0	0	0	0	0	0	0	0
	21UR-13321	TCTCGTCTTCTGGTTCTTGAC	0	0	0	0	0	0	0	0
†	21UR-13322	TCTCGGTTTTTGGTAGTGAAT	0	0	0	0	0	0	0	0
†	21UR-13323	TCTCGGATTGATTATCAGATT	0	0	0	0	0	0	0	0
	21UR-13324	TCTCGAAATTCTACGGAAAAA	0	0	0	0	0	0	0	0
	21UR-13325	TCTCCGAATCTATCACTGTCC	0	0	0	0	0	0	0	0
	21UR-13326	TCTCAAATCAATTAGGTTTAG	0	0	0	0	0	0	0	0
	21UR-13327	TCTATGGCTCTCAGAAAATCC	0	0	0	0	0	0	0	0
†	21UR-13328	TCTATGCTTTTTTGTTCAAAA	0	0	0	0	0	0	1	1
†	21UR-13329	TCTATCTTAGATTCCTTCAGA	0	0	0	0	0	0	0	0
	21UR-13330	TCTAGTTTCTCCGTAATTTAG	0	0	0	0	0	0	0	0
†	21UR-13331	TCTAGTATGAAAATAATAGGA	0	0	0	0	0	1	0	1
	21UR-13332	TCTACCAGTGTTAAAAATTCT	0	0	0	0	0	0	0	0
	21UR-13333	TCTACATTGTGATCAATCAGA	0	0	0	0	0	0	0	0
	21UR-13334	TCTAACGTTGTGGTCAGAAGA	0	0	0	0	0	0	0	0
†	21UR-13335	TCGTTTCTTCATTGGCTGTGC	0	0	0	0	0	0	0	0
†	21UR-13336	TCGTTCTTTCGTATATTTGAC	0	0	0	0	0	0	0	0
†	21UR-13337	TCGTTCGCGGTATTAAAAGTG	0	0	0	0	0	0	0	0
†	21UR-13338	TCGTTAGGGTTGATGAGTTAA	0	0	0	0	0	0	0	0
†	21UR-13339	TCGTCTGTGCTAATTATCGTA	0	0	0	1	2	1	0	4
†	21UR-13340	TCGTCGTGTGTAGTTTCGCCA	0	0	0	0	0	0	0	0
†	21UR-13341	TCGTCAAAGAAGTTCGAAAAA	0	0	0	0	0	0	0	0
†	21UR-13342	TCGGTTTCTCCATACAAATTT	8	10	2	7	49	56	13	145
	21UR-13343	TCGGGGGCATATGCGCAATTG	0	0	0	0	0	0	0	0
	21UR-13344	TCGGGCGTTGATATACTTAAA	0	0	0	0	0	0	0	0
†	21UR-13345	TCGGGAGTAAAATGTTGTATT	0	4	0	2	55	34	2	97
	21UR-13346	TCGGAAAAATTGACATTTTGCC	0	0	0	0	0	1	0	1
†	21UR-13347	TCGCTCGTGTTGGCAGATAAT	9	2	0	1	7	20	1	40
	21UR-13348	TCGCATTTCATATCCCGTTTT	0	0	0	0	0	0	0	0
	21UR-13349	TCGCATTGTCAAAATGATAAC	0	0	0	0	0	0	0	0
†	21UR-13350	TCGATGCTGCTGACTTTTTAG	0	0	0	0	0	0	0	0
	21UR-13351	TCGATGATCTCTGAAACTTTA	0	0	0	0	0	0	0	0
†	21UR-13352	TCGAGGGTTATTGATTTAAAG	0	0	0	0	0	0	0	0
	21UR-13353	TCGACTCTATTTAAGGTGGAG	0	0	0	0	0	0	0	0
	21UR-13354	TCGACAATCCATCCTTCCAGA	0	0	0	0	0	0	0	0
	21UR-13355	TCGAATGTTCCCTCTTTGTTT	0	0	0	0	0	0	0	0
	21UR-13356	TCGAATATTTGTGGAAAAAAT	0	0	0	0	0	0	0	0
†	21UR-13357	TCGAAGCAATCGAACTCACAG	0	0	0	0	0	0	0	0
†	21UR-13358	TCGAAATGTCCACTGTCAATA	0	0	0	0	0	0	0	0
	21UR-13359	TCGAAAATGAAAATGATCTCA	0	0	0	0	0	0	0	0
	21UR-13360	TCCTTTTTTGTTGCATTATTT	0	0	0	0	0	0	0	0
†	21UR-13361	TCCTTTTGCAATTGACGAATA	0	0	0	0	0	1	0	1
	21UR-13362	TCCTTTAATGTGACAATTTCA	0	0	0	0	0	0	0	0
†	21UR-13363	TCCTTCCGTTATGGGTCGAAT	3	0	0	0	0	1	0	4
†	21UR-13364	TCCTTCAGAAGAGATTAAAAA	0	0	0	0	0	0	0	0
†	21UR-13365	TCCTGCTGTAGAATAAAATAA	0	0	0	0	0	1	0	1
†	21UR-13366	TCCTGCCTCTCATATCGATTT	0	0	0	0	0	0	0	0
†	21UR-13367	TCCTGATTTTGAACTACAAAA	0	0	0	0	0	0	0	0
	21UR-13368	TCCTGACCATCCAACACACTA	0	0	0	0	0	0	0	0
	21UR-13369	TCCTGAATCATTTCCGCAATA	0	0	0	0	0	0	0	0
†	21UR-13370	TCCTCTTTTCCCTTTGTTCAA	0	0	0	0	0	0	0	0

†	21UR-13371	TCCTCTATATCTCTGACTTTG	0	0	0	0	0	0	0	0
†	21UR-13372	TCCTCAATTTTTTCACGTTAC	0	0	0	0	0	0	0	0
	21UR-13373	TCCTATTCAGGCAAGGTACAG	0	0	0	0	0	0	0	0
†	21UR-13374	TCCTATCAACTGAGATACCCA	0	0	0	0	0	0	0	0
†	21UR-13375	TCCTAGTGTCCCCACAACTAA	0	0	0	0	0	0	0	0
	21UR-13376	TCCTAACTCCCTGAAAGCATG	0	0	0	0	0	0	0	0
	21UR-13377	TCCGTGATTGTTTGCATAAAT	0	0	0	0	1	0	0	1
	21UR-13378	TCCGTCTACTGTCAATCAGTA	0	0	0	0	0	0	0	0
†	21UR-13379	TCCGGAATGCGAAAAAAAGAT	0	0	0	0	0	0	0	0
	21UR-13380	TCCGGAACCAGAAATTTTAAA	0	0	0	0	0	0	1	1
	21UR-13381	TCCGATAGGAAAATGCTTTTT	0	0	0	0	0	0	0	0
	21UR-13382	TCCCTATATTCTTGAAATAAA	0	0	0	0	0	0	0	0
	21UR-13383	TCCCGGAACACTGGATCATCT	0	0	0	0	0	0	0	0
	21UR-13384	TCCCACCGGCGTAGTTGGTTG	0	0	0	0	0	0	0	0
	21UR-13385	TCCCAATTCCTCGAGAATCAG	0	0	0	0	0	0	0	0
†	21UR-13386	TCCATTGTATCAAATTCAAAC	0	0	0	0	0	0	0	0
	21UR-13387	TCCAGAAGGGCCCACAAAAGA	0	0	0	0	0	0	0	0
†	21UR-13388	TCCACGAGATGATTCTTTTGA	0	0	0	0	0	0	0	0
	21UR-13389	TCCAATTCTCGTTCTCGGTTT	0	1	0	0	0	0	0	1
†	21UR-13390	TCCAAGGCGCGATATTGAATT	0	0	0	0	0	0	0	0
	21UR-13391	TCCAAATTTCTCATCTTAACA	0	0	0	0	0	0	0	0
	21UR-13392	TCCAAAGAAAGTAGGACAATG	2	0	0	0	0	4	5	11
	21UR-13393	TCCAAAAGAATTTTCTCAAAA	0	0	0	0	0	0	0	0
	21UR-13394	TCCAAAAATAGTTAAATCTAA	0	0	0	0	0	0	0	0
	21UR-13395	TCATTTTCTTTTGCGAGTTCA	0	0	0	0	0	0	0	0
	21UR-13396	TCATTTTCTGTTTGACCTCCA	0	0	0	0	0	0	0	0
	21UR-13397	TCATTTTACGGAATTTCAATG	0	0	0	0	0	0	0	0
†	21UR-13398	TCATTGTCGATTATCATTGCT	0	0	0	0	3	1	0	4
†	21UR-13399	TCATTGAGCAAATGCGAAAAA	0	0	0	0	0	0	0	0
	21UR-13400	TCATTGACAAATAACGGAAAA	0	0	0	0	0	0	0	0
†	21UR-13401	TCATTCCTCTCCGTTTGTTAT	0	0	0	0	0	0	0	0
†	21UR-13402	TCATTCCATCTTTCTGCGACC	0	0	0	0	0	0	0	0
†	21UR-13403	TCATTCAGTTTTGAACAGCGG	0	1	0	0	1	1	0	3
	21UR-13404	TCATTATAAAATTGTCTGGTC	0	0	0	0	0	0	0	0
	21UR-13405	TCATTACAATGATGATGATGA	0	0	0	0	0	0	0	0
	21UR-13406	TCATGTATTACTTGTGCCTGA	0	0	0	0	1	0	0	1
	21UR-13407	TCAGTTTTGGGAACACAAAGA	0	0	0	0	0	0	0	0
†	21UR-13408	TCAGTGTTTTCGAATTGATCC	0	0	0	0	0	0	0	0
†	21UR-13409	TCAGTCAGCGATCGAGAAATT	0	0	0	0	0	0	0	0
†	21UR-13410	TCAGTAAGATTTGTCACTCGG	0	0	0	1	7	3	3	14
	21UR-13411	TCAGCTTTTTCCCTGCACATG	0	0	0	0	0	0	0	0
	21UR-13412	TCAGCTGAAAAATGTAAACCC	0	0	0	0	0	0	0	0
†	21UR-13413	TCAGATTGATTTGTCCAAAAA	0	0	0	0	0	0	0	0
	21UR-13414	TCAGAGTTTTTCGAAAAAAAA	0	0	0	0	0	0	0	0
†	21UR-13415	TCAGAACTTTTTCATGAGAAA	0	0	0	0	0	0	0	0
	21UR-13416	TCAGAACTGTTCCGATGGTGA	0	0	0	0	0	0	0	0
	21UR-13417	TCAGAAAAAAAAGATTTTCGG	1	0	0	0	0	1	2	4
	21UR-13418	TCACTTCCGAAGTTATCAAAC	0	0	0	0	0	0	0	0
†	21UR-13419	TCACTCGCAGCTGAAAGATGT	1	0	1	2	5	17	1	27
†	21UR-13420	TCACTCCTGAAGAAATTGTGG	0	0	0	0	0	0	0	0
	21UR-13421	TCACTATTTAATTTGAGCTAC	0	0	0	0	0	0	0	0
	21UR-13422	TCACGAAATAGACCTCATCAA	0	0	0	0	0	0	0	0
†	21UR-13423	TCACGAAAAACAGGTGGTTTT	17	3	0	0	1	28	0	49
	21UR-13424	TCACCGAACTAGTGACCCTGC	0	0	0	0	0	0	0	0
	21UR-13425	TCACAAAAAGTGTAAACATGC	0	0	0	0	0	0	0	0
	21UR-13426	TCAATTTTTTGCTGTTTCATC	0	0	0	0	0	0	0	0
	21UR-13427	TCAATTTGTAACTGTTTTCTC	0	0	0	0	0	0	0	0
	21UR-13428	TCAATTTGATTATAGTCGAAT	0	0	0	0	0	0	0	0
†	21UR-13429	TCAATTTCGCACGCGAAAAAA	0	0	0	0	0	0	0	0
	21UR-13430	TCAATTGCGGCAGCAGTCAAG	0	0	0	0	0	0	0	0
	21UR-13431	TCAATTAATTAACCAATTAAT	0	0	0	0	0	0	0	0
	21UR-13432	TCAATGTAACAAAAAACATTG	0	0	0	0	0	0	0	0
	21UR-13433	TCAATCGCCTATGATCAAATT	0	0	0	0	0	0	0	0
	21UR-13434	TCAATCACTCAGCTCAGCTCC	0	0	0	0	0	0	0	0

	21UR-13435	TCAATATATTGAAGATTGGGA	0	0	0	0	2	4	1	7
	21UR-13436	TCAATAACTCAGCTCTATCTA	0	0	0	0	0	0	0	0
†	21UR-13437	TCAAGTAGGTTTAATTTTATT	0	1	0	0	2	3	0	6
	21UR-13438	TCAAGGCGCTTTAAACGTTAT	0	0	0	2	3	3	0	8
	21UR-13439	TCAACTTCTGGAATCGGATCA	0	0	0	0	0	0	0	0
†	21UR-13440	TCAACCATAGGGTTATTGCGG	0	0	0	0	0	0	1	1
	21UR-13441	TCAACCAAGAAGAGATAAACC	0	0	0	0	0	0	1	1
†	21UR-13442	TCAAATTTTGGCGAATCTATT	0	0	0	0	0	0	0	0
†	21UR-13443	TCAAAGCTATGTTCAATAAAC	0	0	0	0	0	0	0	0
	21UR-13444	TCAAACAATATTCGGAGTTTT	0	0	0	0	0	0	0	0
	21UR-13445	TCAAAAATGCGTCCTCCTGTG	0	0	0	0	0	0	0	0
†	21UR-13446	TATTTTTTTATTAGAAAAGAG	0	0	0	0	0	2	0	2
	21UR-13447	TATTTTTGTTTTTTTCGAAAG	0	0	0	0	0	0	0	0
†	21UR-13448	TATTTTTCACGCAGGTTTTTT	2	0	0	0	0	1	2	5
	21UR-13449	TATTTTGATTGAAAAATTTCA	0	0	0	0	0	2	0	2
†	21UR-13450	TATTTTGATGTCCTTCTTTAA	1	0	0	0	0	0	0	1
†	21UR-13451	TATTTTAACGCTACCTCAAAA	0	0	0	0	1	2	0	3
†	21UR-13452	TATTTTAAAAATTGAGAGTTT	0	0	0	0	0	0	0	0
	21UR-13453	TATTTGATCTACTTCCAAAAG	0	0	0	0	0	0	0	0
†	21UR-13454	TATTTGAGATTATTTATCTTT	0	0	0	0	0	0	0	0
†	21UR-13455	TATTTCTGATTCTCCTCCCAT	0	0	0	0	0	0	0	0
	21UR-13456	TATTTCGGGAATTTTCTATTT	0	0	0	1	0	0	0	1
†	21UR-13457	TATTTCCCGTTAACTTTCTCT	0	0	0	0	0	0	0	0
	21UR-13458	TATTTCAATATTGTTCCGTTT	1	0	0	0	0	0	0	1
†	21UR-13459	TATTTAGTTACGAATTTGTCA	0	0	0	0	0	0	0	0
†	21UR-13460	TATTTACTTCTTGGATATGTG	0	0	0	0	0	0	0	0
	21UR-13461	TATTTAATAGCTGTAACTTGT	0	0	0	0	0	2	0	2
†	21UR-13462	TATTTAACTCAAATACTTCAA	0	0	0	0	0	0	0	0
	21UR-13463	TATTGTTTGTGTTTTTTTTTG	0	0	0	0	0	0	0	0
†	21UR-13464	TATTGTTTCGAACTTCAATGC	0	0	0	0	0	0	0	0
†	21UR-13465	TATTGTCTTCATTTTACAAAG	0	0	0	0	0	0	0	0
†	21UR-13466	TATTGTATTATTACTTAACCA	0	0	0	0	0	0	0	0
†	21UR-13467	TATTGGTCTGTCTAGATTCCA	0	0	0	0	0	0	0	0
	21UR-13468	TATTGCGCTTTCCCATCGTGA	0	0	0	0	0	1	0	1
†	21UR-13469	TATTGATTAATGATCATGATA	0	0	0	0	0	0	0	0
†	21UR-13470	TATTGATACGAGTTTTGTGTA	0	0	0	0	0	0	0	0
†	21UR-13471	TATTGAAACCTTGAGAACTAT	0	0	0	0	0	0	0	0
†	21UR-13472	TATTCTTCCTTTGTAACCGCC	1	0	0	0	0	0	0	1
	21UR-13473	TATTCTGTTTATACTTTAACT	0	0	0	1	0	2	0	3
†	21UR-13474	TATTCTCAAGATAACTTATGA	0	0	0	0	0	0	0	0
	21UR-13475	TATTCGTACCCTACTCGGATC	0	0	0	0	0	0	0	0
†	21UR-13476	TATTCGATGGCTATGTGGACT	0	0	0	0	0	0	0	0
†	21UR-13477	TATTCCATTTAAAGTTTATGT	0	0	0	0	2	1	0	3
†	21UR-13478	TATTCCAGTGAACGATTAAAT	0	0	0	0	0	1	0	1
†	21UR-13479	TATTCATTGTCAGTTCTATGC	0	0	0	0	0	0	0	0
	21UR-13480	TATTCAGTTTATATCTGGCGT	0	0	0	0	1	1	0	2
†	21UR-13481	TATTCAAGCAAGGTTACCGAA	0	0	0	0	0	0	0	0
	21UR-13482	TATTCAAATATGTGAGTTTCG	0	0	0	0	0	0	1	1
	21UR-13483	TATTATTGCTATTTTATTTCC	0	0	0	0	0	0	0	0
†	21UR-13484	TATTATTCTCTCGCCGTGTGC	0	0	0	0	0	0	0	0
†	21UR-13485	TATTATGTGTTCAGCTCTCAG	0	0	0	0	0	0	0	0
†	21UR-13486	TATTATCATCTAGTCATTGAT	0	0	0	0	0	0	0	0
†	21UR-13487	TATTATAGTTGGTTTGGGTTT	0	0	0	0	0	0	2	2
†	21UR-13488	TATTATAGTGCTGTTTCAACC	0	0	0	0	0	0	0	0
†	21UR-13489	TATTATAGAAACGTTTCCCTC	0	0	0	0	1	0	0	1
†	21UR-13490	TATTATACCGATTCTTAGTTC	0	0	0	0	0	0	0	0
†	21UR-13491	TATTAGATCGAAGCTCAAACT	0	0	0	0	0	0	0	0
†	21UR-13492	TATTAGATCATATATAGGAAT	0	0	0	0	2	0	0	2
	21UR-13493	TATTAGAATTTTTCGAACAAC	0	0	0	0	2	1	0	3
†	21UR-13494	TATTACTTTGATTCCGGCCTG	0	0	0	0	0	0	0	0
†	21UR-13495	TATTACTTGAATAATTGCATT	0	0	0	0	0	0	0	0
†	21UR-13496	TATTAATTCTTCTTTCTTGGT	0	0	0	0	0	0	0	0
†	21UR-13497	TATTAATTAAATGTCCGGGAA	0	0	0	0	0	0	0	0
	21UR-13498	TATTAATAACATTTAGTTTTT	0	0	0	0	0	0	0	0

†	21UR-13499	TATTAAGTCACTGCTTCTGAA	0	0	0	0	0	0	0	0
†	21UR-13500	TATTAACGCTTGGTTATGGTT	0	0	0	0	0	0	0	0
†	21UR-13501	TATTAACGACGTTGTATAGGA	0	0	0	0	0	0	0	0
†	21UR-13502	TATTAACCAGTGGAATGAAAA	0	1	0	0	0	3	0	4
†	21UR-13503	TATTAAATCCTCTAATCCTAT	0	0	0	0	0	0	0	0
	21UR-13504	TATTAAAATTAGGCGTCTCTA	0	0	0	0	0	0	0	0
	21UR-13505	TATGTTATCTTTGAGGGTTTC	0	0	0	0	2	0	0	2
†	21UR-13506	TATGTCTGATATAAAACAGAT	0	0	0	1	3	1	0	5
	21UR-13507	TATGGTTCCATGGCGGCAATT	0	0	0	0	0	0	0	0
	21UR-13508	TATGGGCCACCTTGTAGTTAA	0	0	0	0	0	0	0	0
	21UR-13509	TATGGATATGGTTTATGTATT	0	0	0	0	0	0	0	0
	21UR-13510	TATGCATTCGGTCCTCTTTCA	0	0	0	0	0	2	0	2
	21UR-13511	TATGCAGAGAAGGCTTTCTTT	0	0	0	0	0	0	0	0
	21UR-13512	TATGATTTTCAGTTTATTCTC	0	0	0	0	2	0	0	2
†	21UR-13513	TATGATGTTGTTCATTTGCAC	0	0	0	0	0	0	0	0
	21UR-13514	TATGAATTTCAGAATGTTCCG	0	0	0	0	0	0	0	0
	21UR-13515	TATGAACTCACTCAGAATGTT	0	0	0	0	0	0	0	0
†	21UR-13516	TATCTCAGTAGGGAATCATAA	0	0	0	0	0	0	0	0
†	21UR-13517	TATCCTAGAATGACAAATCAA	0	0	0	0	0	1	0	1
	21UR-13518	TATCCACTGTTTCGCTTTCTT	0	0	0	0	0	0	0	0
	21UR-13519	TATCATTCTTCTCGTTGGTTT	0	0	0	0	0	0	0	0
	21UR-13520	TATCAGCTGATAGAGAAGTAT	0	1	0	0	0	1	0	2
	21UR-13521	TATCACACAAAGTCATCAAGC	2	0	0	0	0	2	0	4
	21UR-13522	TATATTTTGAGCTCTATTTGA	0	0	0	0	7	4	0	11
	21UR-13523	TATACATTTTGTTTTTTCAAC	0	0	0	0	1	1	0	2
†	21UR-13524	TATACACTTGTTTTCAAACAA	1	0	0	0	0	0	0	1
	21UR-13525	TATAATTTTCAGAAATTTTAG	0	0	0	0	0	0	0	0
	21UR-13526	TATAATTGTTGTCAAGTATCT	0	0	0	0	0	2	0	2
	21UR-13527	TATAACTAAATTCTATTTTAA	3	0	0	0	3	0	0	6
†	21UR-13528	TAGTTTGATATGATTACTTAA	0	0	0	0	0	0	0	0
†	21UR-13529	TAGTTTCGCAAGACAAAAAAC	0	0	0	0	0	0	0	0
	21UR-13530	TAGTTGCTTTATAAAAAAGTA	0	0	0	0	0	0	0	0
	21UR-13531	TAGTTCTATTAGTATTATTAG	0	0	0	0	0	0	0	0
†	21UR-13532	TAGTTCGGATCCAATATTTTT	0	0	0	0	2	0	0	2
†	21UR-13533	TAGTGCGATTGATGACATTGC	0	0	0	0	0	0	0	0
†	21UR-13534	TAGTGATAAATACTGGAAATA	0	0	0	0	0	0	0	0
	21UR-13535	TAGTGAACCGAGTTGGCTGAC	1	0	0	0	0	1	0	2
	21UR-13536	TAGTCTTTACGATACATATGA	0	0	0	0	0	0	0	0
	21UR-13537	TAGTCGAATTAAGAAACTTTT	0	0	0	0	0	0	0	0
	21UR-13538	TAGTCAATTGGCATTACGATT	0	0	0	0	0	0	0	0
	21UR-13539	TAGTCAAAAAGAGAATGCTCA	0	0	0	0	0	0	0	0
†	21UR-13540	TAGTATTCAAAATATCGAAAC	0	0	0	0	0	0	0	0
†	21UR-13541	TAGTAGACTTTTCAAACTTTT	0	0	0	0	0	0	0	0
†	21UR-13542	TAGTAATTGGTCGGCTGCTTC	0	0	0	0	0	0	0	0
†	21UR-13543	TAGTAATGTCAATGTCAGAAA	0	0	1	0	0	0	0	1
	21UR-13544	TAGTAATGGTCGACAATAAAA	0	0	0	0	0	0	0	0
†	21UR-13545	TAGGATTTTTCGGGGGAATGC	0	0	0	0	0	1	1	2
	21UR-13546	TAGCTCGGATTTTAAAATCAT	0	0	0	0	0	0	0	0
	21UR-13547	TAGCATAAATTTCTGAACTAA	11	1	1	0	1	10	1	25
†	21UR-13548	TAGCAACATTGAAAATATTAT	0	0	0	0	2	1	0	3
	21UR-13549	TAGATTTTGGAATGTTTGCGG	0	1	1	1	1	9	0	13
†	21UR-13550	TAGATTTATTCCAGTTTTTTA	0	0	0	0	0	0	0	0
	21UR-13551	TAGATGCCAGATATATCTTAA	0	0	0	0	0	0	0	0
†	21UR-13552	TAGATCTGCGAGGCGTACGGG	0	0	0	0	0	2	0	2
	21UR-13553	TAGAGTCTCAAATTAAAAATT	0	0	0	0	0	0	0	0
	21UR-13554	TAGAGGTTTAATTAATTCAGC	0	0	0	0	1	0	0	1
	21UR-13555	TAGAGGCTATGATCGAGATTA	0	0	0	0	0	0	0	0
	21UR-13556	TAGAGCAATTGAATATTTTCT	0	0	0	0	0	0	0	0
†	21UR-13557	TAGAGAAAATTTAGTTTCCAG	0	0	0	0	0	0	0	0
†	21UR-13558	TAGACTTCCTTAACGCTAGGG	0	0	0	0	0	0	0	0
†	21UR-13559	TAGACTGAACCTTTAACACTG	0	0	0	0	0	0	0	0
	21UR-13560	TAGACACAAGGGACCTGGATC	0	0	0	0	0	0	0	0
	21UR-13561	TAGACACAACAGAAAGGAGAG	0	0	0	0	0	1	0	1
	21UR-13562	TAGAATTACATTGGAAATGCC	0	0	0	0	0	0	0	0

	21UR-13563	TAGAACTGGAAAAAACTACTG	0	0	0	0	0	0	0	0
	21UR-13564	TAGAAAAATAAACTTTTGTGC	0	0	0	0	0	0	0	0
	21UR-13565	TACTTCACGGGAAGGAACCCG	0	0	0	0	0	1	0	1
†	21UR-13566	TACTTACATTAAGTCGGTGTG	0	0	0	0	0	0	0	0
†	21UR-13567	TACTTACAATTAGTCGATCCG	0	0	0	0	0	0	0	0
†	21UR-13568	TACTGCTATGCTTTTCTTGAT	0	0	0	0	0	0	0	0
	21UR-13569	TACTCTACCGTTTTTGTGAAA	0	0	0	0	0	0	0	0
†	21UR-13570	TACTCTACCGTTTTTGTCACA	0	0	0	0	0	0	0	0
†	21UR-13571	TACTCCCTGTTTGGTTAAAAG	0	0	0	0	0	0	0	0
†	21UR-13572	TACTATGAAAGTCGAAGATCG	0	2	0	1	15	15	16	49
†	21UR-13573	TACTACCTGATGTACTGACTC	0	0	0	0	0	0	0	0
†	21UR-13574	TACTAATAACGAAAAATTATT	0	0	0	0	0	0	0	0
†	21UR-13575	TACTAAAAACTAATACAAAAT	0	1	0	0	1	0	0	2
	21UR-13576	TACGTTTTATAAATAATGGAA	0	0	0	0	0	1	0	1
†	21UR-13577	TACGCGAATGTTATTGCTACT	0	0	0	0	0	0	0	0
	21UR-13578	TACGATTTTTTTTTATTGTTT	0	0	0	0	0	0	0	0
	21UR-13579	TACGATCAGTTTCGTGTCAAC	0	0	0	0	0	0	0	0
†	21UR-13580	TACCTCTGGGCTCTTTAATTG	0	0	0	0	0	0	0	0
	21UR-13581	TACCGCAGTTTTTATTCTGAA	0	0	0	0	0	0	0	0
†	21UR-13582	TACCGACATATCAACGGAAAG	0	0	0	0	0	0	0	0
	21UR-13583	TACCCCCAGATTATGTCGAAA	0	0	1	0	0	0	0	1
	21UR-13584	TACCCAAACAGTTCCAGTTTG	0	0	0	0	0	0	0	0
†	21UR-13585	TACATTTGTTTCACACAGATT	0	0	0	0	1	0	0	1
	21UR-13586	TACATTGCGTAGCTTGTAGGG	0	0	0	0	1	3	10	14
	21UR-13587	TACATATTTGAAAATTCTTTG	0	0	0	0	7	3	0	10
	21UR-13588	TACATATCAATTTCCTTATTT	0	0	0	0	6	16	4	26
†	21UR-13589	TACATAGCTTTTCCAGTGATT	0	0	0	0	0	2	0	2
	21UR-13590	TACACTTTTTTCTGAACTTTT	1	0	0	0	0	1	0	2
*	21UR-13591	TACACCAAAATCTGAATTTTT	11	3	5	7	15	37	4	82
	21UR-13592	TACAATTTGTTTATTTCAGTT	0	0	0	0	1	1	0	2
	21UR-13593	TACAAGAAGAATTTTTTTAAA	1	0	0	0	0	2	0	3
	21UR-13594	TACAACTTTCTAGTGTGTCCT	0	0	0	0	0	0	0	0
	21UR-13595	TACAAATTTTAAAGTAAAAAT	0	0	0	0	1	1	0	2
	21UR-13596	TACAAACTACAATCTAAAAAT	0	0	0	0	0	0	0	0
	21UR-13597	TACAAAACTTGTTGAAAATAG	0	0	0	0	0	1	0	1
	21UR-13598	TACAAAAAAAGGACATATTGT	0	0	0	0	0	0	0	0
	21UR-13599	TAATTTTTTTTTATTAATTGG	0	0	0	0	0	0	0	0
	21UR-13600	TAATTTTTGATCGGCAGGCGG	1	0	0	0	0	0	2	3
	21UR-13601	TAATTTTGAACAAAACTTTTG	0	0	0	0	1	0	0	1
	21UR-13602	TAATTTCTTCCTATCTTGTTT	0	0	0	0	0	0	1	1
†	21UR-13603	TAATTGTTGAAACGGTATGAA	1	1	0	0	0	1	0	3
†	21UR-13604	TAATTCTGATCGTAAAAATAT	0	1	0	0	6	3	6	16
†	21UR-13605	TAATTCGCATTCTGAAAGGTT	0	0	0	0	1	0	0	1
	21UR-13606	TAATTCATGGATATACATTAG	0	0	0	0	2	2	0	4
	21UR-13607	TAATGTTGGAACATTTACAGT	0	0	0	0	0	0	0	0
	21UR-13608	TAATGTCAGCGAGCCTTCTCT	0	0	0	0	0	0	0	0
†	21UR-13609	TAATGGTCCTTGGCTATAGCC	0	0	0	0	0	0	0	0
†	21UR-13610	TAATGCTGCCTTGAAACACTT	0	0	0	0	0	0	0	0
†	21UR-13611	TAATCTTTTCGATCACGGCTG	0	0	0	0	0	0	0	0
	21UR-13612	TAATCTCCTATCTTTCCTTTG	0	0	0	0	0	0	0	0
†	21UR-13613	TAATCGTCAGCTTCTATAAAT	0	0	0	0	0	0	0	0
	21UR-13614	TAATCGGTTTTTTGAAAATTT	0	0	0	0	1	0	0	1
	21UR-13615	TAATCCTTTGAAATTATATTC	0	0	0	0	0	0	0	0
	21UR-13616	TAATCCTTCTAAATACTCATG	0	0	0	1	0	0	0	1
†	21UR-13617	TAATCCAGCAGAAAGCAAAAA	1	0	0	0	0	0	0	1
	21UR-13618	TAATCATCGTGTCAATAATGG	0	0	0	0	0	0	0	0
	21UR-13619	TAATATGTTTTCAACAGTAGT	0	0	0	0	0	0	0	0
†	21UR-13620	TAATATGTATTTAAAACAAAA	0	0	0	0	0	0	0	0
†	21UR-13621	TAATAGTTCGTTTGTATGTGT	0	0	0	0	0	0	0	0
†	21UR-13622	TAATAGTGAGTTGTTAAAACA	0	0	0	0	0	0	0	0
†	21UR-13623	TAATAGAATCGGATTATTTAT	0	0	0	0	0	1	0	1
†	21UR-13624	TAATACTTGAAGAAAACAAGA	0	0	0	0	0	0	0	0
†	21UR-13625	TAATACTGAAGAAGACGGACA	0	0	1	1	12	43	5	62
†	21UR-13626	TAATAAGTTCTTCTTCGGGGT	0	0	0	0	0	0	0	0

†	21UR-13627	TAATAAATATTGCAGATACAA	2	0	0	1	1	2	0	6
	21UR-13628	TAATAAATATGAAAGCATTTT	0	0	0	0	0	0	0	0
	21UR-13629	TAATAAAATACTAATATGTAG	0	0	0	0	2	0	0	2
	21UR-13630	TAATAAAAAGAAAATTCGTCA	0	0	0	0	0	0	0	0
	21UR-13631	TAAGTTTTGAATCTATTTTAT	0	0	0	0	1	0	0	1
	21UR-13632	TAAGTTATGTTACCGAATTGA	0	0	0	0	0	0	0	0
†	21UR-13633	TAAGTGTTCATTTGATGCACT	0	0	0	0	0	0	0	0
	21UR-13634	TAAGTGGATTTCCTCAAAAGC	0	0	0	0	2	0	0	2
	21UR-13635	TAAGTAAAAAACTCGAACATT	0	0	0	0	0	0	0	0
†	21UR-13636	TAAGGTGATAAAACATTGTGA	0	0	0	0	0	0	0	0
†	21UR-13637	TAAGGCCGAAATTACAAAAAA	0	0	0	0	0	0	2	2
	21UR-13638	TAAGGATTTTTAAAAAATTTA	0	0	0	0	0	0	0	0
†	21UR-13639	TAAGGATTATCAAATAAGAAT	0	0	0	0	0	0	0	0
	21UR-13640	TAAGATTCTAAAGAAATAGGC	0	0	0	0	0	0	0	0
	21UR-13641	TAAGATGGTATATTTGATACT	0	0	0	0	0	0	0	0
	21UR-13642	TAAGACGTTAATTTTTTGTTA	0	0	0	3	9	12	2	26
	21UR-13643	TAAGAACTGCATGTATGTTAT	0	0	0	0	2	0	0	2
†	21UR-13644	TAAGAAAGGAAACGCAACCTG	0	0	0	1	1	1	0	3
	21UR-13645	TAACTGGTGATTCTCACGAAG	0	0	0	0	0	0	0	0
†	21UR-13646	TAACTATGGGTTGTGCACAAT	0	0	0	0	0	1	0	1
	21UR-13647	TAACTAGTAAATTAAAAAATA	0	0	0	0	0	0	1	1
	21UR-13648	TAACGAAGTTACTTCCCACTT	0	0	0	0	0	0	0	0
†	21UR-13649	TAACACTAATCCAACACCTAC	0	0	0	0	0	0	0	0
	21UR-13650	TAACAAATCATTCAAATTTGG	0	0	0	0	0	0	0	0
	21UR-13651	TAAATTTCAGAAGCTCCAAGA	0	0	0	0	0	0	0	0
	21UR-13652	TAAATTTCACAAGCATGCAAT	0	0	0	0	1	2	0	3
	21UR-13653	TAAATTTAATTTATTCGTTCA	1	0	0	0	0	1	0	2
†	21UR-13654	TAAATCTGCAAAATGCTGCAA	0	0	0	0	0	0	0	0
	21UR-13655	TAAATCACAGAATGAAACAGT	0	0	0	0	0	1	0	1
	21UR-13656	TAAATATTTAACTGCGATCAG	0	0	0	0	0	0	0	0
	21UR-13657	TAAATATTAACTATTGGAAAA	0	0	0	0	0	0	0	0
	21UR-13658	TAAATAAAAATTGGAATGGAA	0	0	0	0	0	0	0	0
	21UR-13659	TAAAGGTCCCCGTTTAATTGC	0	0	0	0	0	0	1	1
*	21UR-13660	TAAAGGGGAAGGCCTTGTTTG	0	0	0	0	1	2	0	3
	21UR-13661	TAAAGACTGCGAAAAAATAAT	1	1	0	0	0	2	0	4
	21UR-13662	TAAACTCACTTAGAACATAAT	0	0	0	0	0	0	0	0
†	21UR-13663	TAAACGAAAAATTTCATTGAA	0	0	0	0	0	0	0	0
	21UR-13664	TAAACAGACACGTGAGAACAA	0	0	0	0	0	0	0	0
	21UR-13665	TAAACAATATATGGTAACTGT	0	0	0	0	0	0	0	0
	21UR-13666	TAAAATTTTCAGATGGAAATG	0	0	0	0	0	0	0	0
	21UR-13667	TAAAATTTGTGTTTTTCTGTA	0	0	0	0	5	0	0	5
†	21UR-13668	TAAAATCTCTGTCTCTCACAA	0	0	0	0	0	1	0	1
†	21UR-13669	TAAAATCGACTCAAACCACTA	0	0	0	0	0	0	0	0
	21UR-13670	TAAAAGTGTCGAAATTCTACT	0	0	0	0	0	0	0	0
†	21UR-13671	TAAAAGGAATAGTCTCCAGTG	0	0	0	0	0	0	0	0
	21UR-13672	TAAAAATGATAAAGAGACATC	0	0	0	0	0	0	0	0
†	21UR-13673	TAAAAATCGACATGTATTTCA	0	0	0	0	1	0	0	1
	21UR-13674	TAAAAATAAAAGATTTTGGGT	0	0	0	0	0	0	0	0
	21UR-13675	TAAAAAAACTGATACTGAGGC	0	0	0	0	0	1	0	1
	21UR-13676	TAAAAAAACCAAGTTGATGAA	0	0	0	0	0	0	0	0
	21UR-13677	TAAAAAAAAATTAACCTAGGA	0	0	0	0	0	0	0	0
†	21UR-13678	GTTTTGTTAGGTTGGAATATA	0	0	0	0	0	0	0	0
†	21UR-13679	GTTCTTTTGCGTTTTGGAGCC	0	0	0	0	0	0	0	0
†	21UR-13680	GTTCTGAATTCTCTCTCCAAC	0	0	0	0	0	0	0	0
†	21UR-13681	GTTATCGAAAGATCTAAAGGT	3	1	0	0	6	7	7	24
†	21UR-13682	GTGATCATCTGTCAACACTCT	0	0	0	0	2	0	0	2
†	21UR-13683	GTCAAAAAAGAGTAGAAGGAA	0	0	0	0	0	0	0	0
	21UR-13684	GTATCACTGTTGCGGATGGTC	0	0	0	0	0	0	0	0
†	21UR-13685	GGCTTCTCCTTTCATGAGCAA	0	0	0	0	0	0	0	0
	21UR-13686	GCTTGAGCTGAAACCCACTGA	0	0	0	0	0	0	0	0
†	21UR-13687	GATTGCTCTGCCGCAATTAAA	0	0	0	0	0	0	0	0
	21UR-13688	GAGTTAAGGCAATTAATATGA	0	0	0	0	0	0	0	0
†	21UR-13689	GAGAGGAAAGTGAGATATTGA	0	0	0	0	0	0	0	0
	21UR-13690	GAGAAATAGAGTGGTCGGATG	0	0	0	0	0	0	0	0

	†	21UR-13691	GACTATTGATTTTGTTTGGAT	0	0	0	0	0	0	0	0
		21UR-13692	CTTTTCCAATAATTCAAAACA	0	0	0	0	0	0	0	0
		21UR-13693	CTCTACAAGGGTTTGGGGTTT	0	0	0	0	0	0	0	0
	†	21UR-13694	CTAGTAATGTCAATGTCAGAA	0	0	0	0	0	0	0	0
	†	21UR-13695	CGTTGAATACTTTGATTGATT	0	0	0	0	1	1	0	2
		21UR-13696	CGTATACTTTAAGAAATTTTT	0	0	0	0	4	1	0	5
		21UR-13697	CGCTTTGAAAAGATCTGTAGT	0	0	0	0	0	0	0	0
	†	21UR-13698	CGCTGATATTTCTACACAGAT	0	0	0	0	0	0	0	0
		21UR-13699	CGCAGTAGGTGGTCTAGGAAT	0	0	0	0	0	0	3	3
	†	21UR-13700	CGATCGGATTAATTTTTGAAA	0	0	0	0	0	0	0	0
	†	21UR-13701	CGATAGATGATAATGATAAGA	0	0	0	0	0	0	0	0
	†	21UR-13702	CGATAACAAGAATACATCGGA	0	0	0	0	1	0	0	1
		21UR-13703	CGAATGCACGCCGAGAAGGAT	0	0	0	0	0	0	0	0
		21UR-13704	CGAAGTCGGAAGAAAACAAGT	0	0	0	0	0	0	0	0
		21UR-13705	CGAAGTACAAATGTCAAAAAC	0	0	0	0	0	0	0	0
		21UR-13706	CCTTTGGCTTAACGTCTGCCT	0	0	0	0	0	0	0	0
	†	21UR-13707	CCGAAATAGATAACCATCAGT	0	0	0	1	0	0	1	2
	†	21UR-13708	CAGTAAAAAATGGTCAGTAAT	0	0	0	0	0	0	0	0
		21UR-13709	CAATTTTCTATATCGGGTCAA	0	0	0	0	0	0	0	0
	†	21UR-13710	CAATTATTGCACTGTCTTTGT	0	0	0	0	0	0	0	0
	†	21UR-13711	CAAGGAAAAATATCCACAGAT	0	0	0	0	0	0	0	0
	†	21UR-13712	CAAGATTTTTCTGCTGATTGT	0	0	0	0	0	0	0	0
	†	21UR-13713	CAAATAAACAGTCGAAAACTA	0	0	0	0	0	0	0	0
		21UR-13714	CAAACGCTCGTTTCCCTTTGA	0	0	0	0	0	0	0	0
	†	21UR-13715	ATTTCACGACTTGAAGGACTC	0	0	0	0	0	0	0	0
*	†	21UR-13716	ATGATTGCACCAGATGTAATG	2	1	0	0	0	0	0	3
	†	21UR-13717	ATGATGATAAAAAACACCAAA	0	0	0	0	0	0	0	0
		21UR-13718	ATAGTGTCATAATCGGTGAAC	0	0	0	0	0	0	0	0
	†	21UR-13719	ATAGGAAAAAAAATTTAAAAAA	0	0	0	0	0	0	0	0
	†	21UR-13720	ATAGCACTTTCGGAAAACAAA	0	0	0	0	0	0	0	0
	†	21UR-13721	AGTGGTCTGCCAAAGAACACG	0	0	0	0	0	0	0	0
	†	21UR-13722	AGTGACCCATAATCGCCAAAA	0	0	0	0	0	0	0	0
		21UR-13723	AGTAGAAGAATTGGTAAGTAT	2	3	1	1	1	5	0	13
	†	21UR-13724	AGTAAATAAACTGTAGACTGT	0	0	0	0	0	0	0	0
	†	21UR-13725	AGGATTCCAGGCGATCTGATT	0	1	0	1	22	17	3	44
		21UR-13726	AGGAAATTAAGAGAAAAAATT	0	0	0	0	0	0	0	0
	†	21UR-13727	AGCTTACTGGAAAAAAATCAA	0	0	0	0	0	0	0	0
		21UR-13728	AGCATATGAACGCGTCTCCAT	0	0	0	0	0	0	1	1
	†	21UR-13729	AGCACTGATCCGGTTAAACCC	1	1	1	0	0	1	0	4
	†	21UR-13730	AGACTGTTGAACATATCAAAA	0	1	0	0	0	1	0	2
		21UR-13731	AATAGTACTTTTTTCAGAAAT	0	0	0	0	0	0	0	0
		21UR-13732	AAGTAACAAACTGTGGAGATA	0	0	0	0	0	0	0	0
		21UR-13733	AACTGTGAATGAAACGTCTGT	0	0	0	0	0	0	0	0
		21UR-13734	AACAATTTGTCTATGATAATA	0	0	0	0	0	0	0	0
		21UR-13735	AAAATAAAAAATTAGCTCGAA	0	0	0	0	0	0	0	0
		21UR-13736	TTTTTTTTTTGGTATCACTTT	0	0	0	0	0	0	0	0
	†	21UR-13737	TTTTTTTTTCTTGCCTTTGAA	0	0	0	0	0	0	0	0
		21UR-13738	TTTTTTTTGTTTTTTTTGGAA	0	0	0	0	0	0	0	0
	†	21UR-13739	TTTTTTTTGAAAGTAAGTTTT	0	0	0	0	0	0	0	0
	†	21UR-13740	TTTTTTTTCGGTGTTTCCATA	0	0	0	0	0	0	0	0
		21UR-13741	TTTTTTTTCGAATTGTTTGGG	0	0	0	0	0	0	0	0
		21UR-13742	TTTTTTTTCCATTAGTTTATT	0	0	0	0	0	0	0	0
	†	21UR-13743	TTTTTTTTCCACGTCAAACTC	0	0	0	0	0	0	0	0
		21UR-13744	TTTTTTTTAAGTTTTAAAACA	0	0	0	0	0	0	0	0
		21UR-13745	TTTTTTTGTCGACCTACTGCC	0	0	0	0	0	0	0	0
	†	21UR-13746	TTTTTTTCTGCAGCAAAGAAT	0	0	0	0	0	0	0	0
		21UR-13747	TTTTTTTCCGATCCAAATGCG	0	0	0	0	0	0	0	0
		21UR-13748	TTTTTTTAAATGGTGGAAAAA	0	0	0	0	0	0	0	0
		21UR-13749	TTTTTTTAAACACTCTTTTAC	0	0	0	0	1	0	1	2
	†	21UR-13750	TTTTTTTAAACAATCATGAAA	0	0	0	0	0	0	0	0
		21UR-13751	TTTTTTGGGTTGCTAGCAGTA	0	0	0	0	0	0	0	0
		21UR-13752	TTTTTTGCGTGAACTTTGGCT	0	0	0	0	0	0	0	0
	†	21UR-13753	TTTTTTGCCACAAAAAGAGAA	0	0	0	0	1	0	0	1
		21UR-13754	TTTTTTGCAAATTTAACCATC	0	0	0	0	2	0	0	2

†	21UR-13755	TTTTTTGAGTCACGGTGATTA	0	0	0	0	0	1	0	1
†	21UR-13756	TTTTTTGAGAGCAAGGTTTTT	1	0	0	0	0	0	0	1
	21UR-13757	TTTTTTCTTGGACCTTCTGCT	0	0	0	0	0	0	0	0
	21UR-13758	TTTTTTCTTCATATTATAGTA	0	0	0	0	2	0	0	2
†	21UR-13759	TTTTTTCTGATATAACGGAGA	0	0	0	0	0	0	0	0
†	21UR-13760	TTTTTTCCGAGGAACATTAAA	0	0	0	0	0	0	0	0
†	21UR-13761	TTTTTTCCAGATCCATTTCAA	0	0	0	0	0	0	0	0
†	21UR-13762	TTTTTTCATGCTCACCATGCT	0	0	0	0	0	0	0	0
	21UR-13763	TTTTTTCAAGATATTACTATG	0	0	0	0	0	0	0	0
	21UR-13764	TTTTTTAGTGAGCTTTGTTCA	0	0	0	0	0	0	0	0
†	21UR-13765	TTTTTTACCTGACGTTCTCCA	0	1	0	0	0	0	0	1
†	21UR-13766	TTTTTTAATGCTCTCGGATTG	0	0	0	0	0	1	0	1
†	21UR-13767	TTTTTGTAGCCAAAACAAAAG	0	0	0	0	0	0	0	0
	21UR-13768	TTTTTGTAAAAGTAGTGGCAT	0	0	0	0	0	0	0	0
	21UR-13769	TTTTTGGTCCAACAGTTTCGG	2	0	0	0	1	3	0	6
†	21UR-13770	TTTTTGGGAAATCAAAATGTG	0	0	0	0	0	0	0	0
	21UR-13771	TTTTTGGAGCTGTCGAATTCC	0	0	0	0	0	0	0	0
	21UR-13772	TTTTTGCATAATTAAAAGCTC	0	0	0	0	0	0	0	0
†	21UR-13773	TTTTTGCACGGTTTCGTATTA	3	2	0	1	4	4	0	14
	21UR-13774	TTTTTGATTTCCCTACTAATT	0	0	0	0	0	0	0	0
†	21UR-13775	TTTTTGATGATGATCCAGCTA	0	0	0	0	0	0	0	0
†	21UR-13776	TTTTTGACACGCTATAAAAAT	0	0	0	0	0	0	1	1
†	21UR-13777	TTTTTGAATTTCTCGGTGCTG	0	0	0	0	0	1	0	1
	21UR-13778	TTTTTCTTTACGTTGTACACT	3	0	0	0	13	6	5	27
	21UR-13779	TTTTTCTTCGTTCAACCATTT	0	0	0	0	0	1	0	1
†	21UR-13780	TTTTTCTTCAGGTTCATAAGA	0	0	0	0	0	0	0	0
	21UR-13781	TTTTTCTTAACCTAAAGCAGA	0	0	0	0	0	0	0	0
	21UR-13782	TTTTTCTGTAACTAGCATGGC	0	0	0	0	0	0	0	0
†	21UR-13783	TTTTTCTCTCAACAGTTGCTG	0	0	0	0	0	0	0	0
	21UR-13784	TTTTTCGTATTCATTTCCGGA	0	0	0	0	0	0	0	0
†	21UR-13785	TTTTTCGGTTCCATTTTCCTC	0	0	1	0	0	0	0	1
	21UR-13786	TTTTTCGATTCCTTGAAATTG	0	0	0	0	0	0	0	0
	21UR-13787	TTTTTCGATGTTATTTGTGAA	0	0	0	0	0	0	0	0
	21UR-13788	TTTTTCGAACTTTCACCAACT	0	0	0	0	0	0	0	0
	21UR-13789	TTTTTCCTTTATTTCTATTTC	0	0	0	0	0	0	0	0
	21UR-13790	TTTTTCCATTAACAACTATTA	0	0	0	0	1	0	0	1
†	21UR-13791	TTTTTCCATCGTTTACCCCAT	0	0	0	0	0	0	0	0
†	21UR-13792	TTTTTCCAGTCCCATTCTTTT	0	0	0	0	0	0	0	0
†	21UR-13793	TTTTTCCAGATCCATTTCAAT	0	0	0	0	0	0	0	0
†	21UR-13794	TTTTTCATTGACGGAGGACTT	0	0	0	0	0	1	0	1
†	21UR-13795	TTTTTCAGGAATACCGTATTT	0	0	0	0	0	0	0	0
†	21UR-13796	TTTTTCAATGTGACTGTTCGA	0	0	0	0	0	0	0	0
	21UR-13797	TTTTTCAAATTTTTATTGAAA	0	0	0	0	1	0	0	1
	21UR-13798	TTTTTATTTGGAAACACATTT	0	0	0	1	1	0	0	2
†	21UR-13799	TTTTTATTGTCGAAATTTAAT	0	0	0	0	1	0	0	1
	21UR-13800	TTTTTATTCTCATAGGCCTGT	0	0	0	0	0	0	0	0
	21UR-13801	TTTTTATTCGAGGTTTCAGAC	0	0	0	0	0	0	0	0
	21UR-13802	TTTTTATCTATCGACTCGGTG	0	0	0	0	0	0	5	5
	21UR-13803	TTTTTAGTAGTTAAAATCTAA	0	0	0	0	0	0	0	0
	21UR-13804	TTTTTACTACTACACTCAAAA	0	0	0	0	0	0	0	0
†	21UR-13805	TTTTTAATTTTGGATACAAGA	0	0	0	0	0	0	0	0
†	21UR-13806	TTTTTAATTGATCAACAAAAT	0	0	0	0	0	0	0	0
	21UR-13807	TTTTTAATTAATTAATTTCTC	0	0	0	0	0	0	0	0
	21UR-13808	TTTTTAACAAAAATTTCACTG	0	0	0	0	0	0	0	0
†	21UR-13809	TTTTTAAATTCTTGAAACAAA	0	0	0	0	0	1	0	1
	21UR-13810	TTTTTAAACTTTATTTTGCAT	0	0	0	0	3	0	1	4
	21UR-13811	TTTTTAAAAAGACTTCCGCAA	0	0	0	0	0	0	0	0
	21UR-13812	TTTTGTTGTTTACAATAAAAT	0	0	0	0	0	0	0	0
†	21UR-13813	TTTTGTTGAGGAGTTGACTCA	0	0	0	0	0	0	0	0
†	21UR-13814	TTTTGTTACCCTGTTTGTTCA	0	0	0	0	0	1	0	1
†	21UR-13815	TTTTGTGTGATTTCAGTTCTT	0	0	0	0	0	0	0	0
	21UR-13816	TTTTGTGATTATTTGAATCCG	0	0	0	0	0	0	0	0
	21UR-13817	TTTTGTATGTGTAATTAACAG	0	0	0	0	0	0	0	0
	21UR-13818	TTTTGTAATTTTTTGGAAAAT	0	0	0	0	1	0	0	1

†	21UR-13819	TTTTGGTTTCAAGTTTTTCTC	0	0	0	0	0	0	0	0
	21UR-13820	TTTTGGTACGAATGGGAAAAA	0	0	0	0	0	0	0	0
†	21UR-13821	TTTTGGGAATGTTCGGTTGGG	0	0	0	0	0	0	0	0
†	21UR-13822	TTTTGGCCCTACGAAATTCAC	0	0	0	0	0	0	0	0
†	21UR-13823	TTTTGGCAAGATAAACTTTAT	0	0	0	0	0	0	0	0
†	21UR-13824	TTTTGGAGCTTCAACTATGGA	0	0	0	0	0	0	0	0
†	21UR-13825	TTTTGGAATATGAAACATTTT	0	0	0	0	0	0	0	0
	21UR-13826	TTTTGGAACAATTTTGGAACA	0	0	0	0	0	0	0	0
	21UR-13827	TTTTGGAAATTATCGGACATT	0	0	0	0	0	0	0	0
	21UR-13828	TTTTGCTTTGTTTCCGACAGT	0	0	0	0	0	0	0	0
†	21UR-13829	TTTTGCGTACATATTGCCAGT	1	0	1	0	0	0	0	2
	21UR-13830	TTTTGCGCCTCTTTTTCTAAA	0	0	0	0	0	0	0	0
	21UR-13831	TTTTGCGAATTTCAAGGAAAA	0	0	0	0	0	0	0	0
	21UR-13832	TTTTGCATATGAACTGTGGTC	0	0	0	0	0	0	0	0
†	21UR-13833	TTTTGCAATTCAAAACTGTTT	1	0	0	0	1	0	0	2
	21UR-13834	TTTTGCAATAAAAATTAAATA	0	0	0	0	0	0	0	0
†	21UR-13835	TTTTGATTTTGAGAAACCCTT	0	0	0	0	0	0	0	0
	21UR-13836	TTTTGATTAAATTTTTTAAGG	0	0	0	0	0	0	0	0
	21UR-13837	TTTTGATGTTTTTCAAGTTTT	0	0	0	0	0	0	0	0
†	21UR-13838	TTTTGATCTGCATTCAATCTT	0	0	0	0	2	0	0	2
†	21UR-13839	TTTTGAGGTGTTATCAGAAAG	0	0	0	0	0	0	0	0
	21UR-13840	TTTTGAGGCAAGTAAATGATT	0	0	0	0	0	0	1	1
†	21UR-13841	TTTTGAGAGCAAGGTTTTTTT	0	0	0	0	0	2	0	2
†	21UR-13842	TTTTGACATTAAATGTTAAAA	0	0	0	0	0	0	0	0
†	21UR-13843	TTTTGACAATCCATTTACAGG	0	0	0	0	0	0	0	0
†	21UR-13844	TTTTGAATCGTTGACAAGAGG	0	0	0	0	0	0	0	0
	21UR-13845	TTTTGAACGAAACATTATAAT	0	0	0	0	0	0	0	0
	21UR-13846	TTTTGAAATTTTCAAGCCAGC	0	0	0	0	0	0	0	0
†	21UR-13847	TTTTGAAATCACATTGATAAC	0	0	0	0	0	0	0	0
	21UR-13848	TTTTGAAAGCAAGGTAAAGAC	0	0	0	0	0	0	0	0
	21UR-13849	TTTTCTTTTTTGTAATCAATT	0	0	0	0	1	0	0	1
	21UR-13850	TTTTCTTTTTCCACTTTAAAA	0	0	0	0	0	0	0	0
	21UR-13851	TTTTCTTTGTGTCAAGCTCTT	0	0	0	0	0	0	0	0
†	21UR-13852	TTTTCTTCCACATTCCACACT	0	0	0	0	0	0	0	0
	21UR-13853	TTTTCTTAATACAAAATTCGA	0	0	0	0	0	0	0	0
	21UR-13854	TTTTCTTAACTCCATGCTTCT	0	0	0	0	0	1	0	1
	21UR-13855	TTTTCTGTCGAGTTACAGAAA	0	0	0	0	0	0	0	0
†	21UR-13856	TTTTCTGTATTTTAGGCTTGT	0	0	0	1	4	4	1	10
	21UR-13857	TTTTCTGTAGTTTCATTATGT	0	0	0	0	0	0	0	0
	21UR-13858	TTTTCTGGAAAATTTTGAATC	0	0	0	0	0	0	0	0
	21UR-13859	TTTTCTGAGATCTAGTTCTGA	0	0	0	0	0	0	0	0
†	21UR-13860	TTTTCTCCGGAACCTAGCTCC	0	0	0	0	0	0	0	0
†	21UR-13861	TTTTCTCATTCCTTTCCCAAA	0	0	0	0	0	0	0	0
†	21UR-13862	TTTTCTATCGTGTTGTAATTT	0	0	0	0	0	0	0	0
†	21UR-13863	TTTTCTATCGGTTTCATTCAA	0	0	0	1	1	0	0	2
	21UR-13864	TTTTCTACCGCGGCAAATTAA	1	0	0	0	4	6	7	18
†	21UR-13865	TTTTCGTTTAATTTCCGTTGG	0	0	0	0	0	0	0	0
	21UR-13866	TTTTCGTTGATGAACTCTTTT	0	0	0	0	0	0	0	0
	21UR-13867	TTTTCGTGGAACAAACACACT	0	0	0	0	1	5	0	6
†	21UR-13868	TTTTCGTATTTATCTTTAATA	0	0	0	0	0	0	0	0
	21UR-13869	TTTTCGGTGAGCTTATATGAA	0	0	0	0	0	0	0	0
	21UR-13870	TTTTCGAAAAAGAAAAAAGCA	0	0	0	0	0	0	0	0
†	21UR-13871	TTTTCCTTTCTGTGCTTAACA	0	0	0	0	0	0	0	0
†	21UR-13872	TTTTCCGTTCAAATTCCAGCT	0	0	0	0	0	0	0	0
	21UR-13873	TTTTCCGTTCAAAAAAAGTCA	0	0	0	0	0	0	0	0
	21UR-13874	TTTTCCGCCACTATTTGAATT	0	0	0	0	0	0	0	0
†	21UR-13875	TTTTCCGATCGAAAACGAGTT	0	0	0	0	0	0	0	0
	21UR-13876	TTTTCCCAATTTCAGCATGCC	0	0	0	0	0	0	0	0
	21UR-13877	TTTTCCATTAAGATCAAAATC	0	0	0	0	0	0	0	0
	21UR-13878	TTTTCAGTAAACAAAGAGAAG	0	0	0	0	0	0	0	0
†	21UR-13879	TTTTCAGGGATTTGTCATTGC	0	1	0	0	0	2	0	3
	21UR-13880	TTTTCACTGTTTCGACGTCTT	0	0	0	1	2	6	0	9
	21UR-13881	TTTTCACACATAGTTAGTTGA	0	0	0	0	0	0	0	0
	21UR-13882	TTTTCAAGAAAAAATCTTTAA	0	0	0	0	0	0	0	0

	21UR-13883	TTTTCAAAACTTTTGAACACG	0	0	0	0	0	0	0	0
	21UR-13884	TTTTATTTGCTGGAAAATATT	0	0	0	0	0	0	0	0
	21UR-13885	TTTTATTTCGACATGATTCTC	0	0	0	0	3	0	0	3
	21UR-13886	TTTTATTTAAGAAGTTCAATT	0	0	0	0	1	0	0	1
†	21UR-13887	TTTTATTCTTCCCAATGGACT	0	0	0	0	0	0	0	0
	21UR-13888	TTTTATTCAAGAACTTATATC	0	0	0	0	9	0	2	11
	21UR-13889	TTTTATTATACTTTTTCAAAA	0	0	0	0	0	0	0	0
	21UR-13890	TTTTATGATAATTTAGATAAT	0	0	0	0	1	0	0	1
†	21UR-13891	TTTTATCCTAAACCCGTTTTT	0	0	0	0	0	0	0	0
	21UR-13892	TTTTATAGTTTTAATTTGATA	0	0	0	0	0	0	0	0
	21UR-13893	TTTTAGTTCATTTTTTTTCTG	0	0	0	0	0	0	0	0
	21UR-13894	TTTTAGCTTATGAATCCGACC	0	0	0	0	0	0	0	0
†	21UR-13895	TTTTAGAATCGTATAGTTTTT	0	0	0	0	0	0	1	1
	21UR-13896	TTTTAGAAGTGTTAATTTTCT	0	0	0	0	0	0	0	0
†	21UR-13897	TTTTACTTGAAGACAACTAAA	0	0	0	0	3	2	0	5
†	21UR-13898	TTTTACGAAAAGTTGTTATTT	0	0	0	0	0	0	0	0
†	21UR-13899	TTTTAATTCTATCAGAAATCT	0	0	0	0	0	0	0	0
	21UR-13900	TTTTAACCAAGAATTCTTGTT	0	0	0	0	0	0	0	0
†	21UR-13901	TTTTAACATTGTTTTGTCAGA	0	0	0	0	0	0	0	0
	21UR-13902	TTTTAACAAATTGCAAGAAAC	0	0	0	0	0	0	0	0
	21UR-13903	TTTTAACAAAAGCGTTGTTTA	0	0	0	0	0	0	0	0
†	21UR-13904	TTTTAAATGAAGATTAAAATG	0	0	0	0	0	0	0	0
†	21UR-13905	TTTTAAATCCTTCTAGTTGGT	1	0	0	0	0	1	3	5
†	21UR-13906	TTTTAAACTCTCTTGATGTTT	0	0	0	0	0	0	0	0
	21UR-13907	TTTTAAAATTTTGATCTCAAG	0	0	0	0	0	0	0	0
	21UR-13908	TTTTAAAAAATAAGGACACAG	0	0	0	0	2	0	0	2
	21UR-13909	TTTGTTTTTTACAGATTGAGT	0	0	0	0	0	0	0	0
	21UR-13910	TTTGTTTCTTGTTTTACAAAG	0	0	0	0	0	0	0	0
†	21UR-13911	TTTGTTGTGTATCGAAACGAA	0	0	0	0	0	0	0	0
†	21UR-13912	TTTGTTCGGAAATTTGAAAAA	1	0	0	0	0	3	0	4
†	21UR-13913	TTTGTTCCATTCCTGGTTGCA	0	0	0	0	0	0	0	0
†	21UR-13914	TTTGTTATTACGCATTGTGAT	0	0	0	0	0	0	0	0
	21UR-13915	TTTGTTAGACTGGTGACTGCT	0	1	0	0	0	1	0	2
	21UR-13916	TTTGTGGGGCTTCAAATGGCA	0	0	0	0	0	1	0	1
†	21UR-13917	TTTGTGATGGACTCTGAAAAT	0	0	0	0	0	0	0	0
†	21UR-13918	TTTGTGAGGACAGTGGCATGG	0	0	0	0	3	1	0	4
	21UR-13919	TTTGTGAGAGAAGAATGAGGA	0	0	0	0	0	0	0	0
	21UR-13920	TTTGTCCTTGTACAAGTGTGA	0	0	0	0	0	0	0	0
	21UR-13921	TTTGTCACAATTTTCTTATTT	0	0	0	0	0	0	0	0
	21UR-13922	TTTGTATCAAATTACCCACAT	0	0	0	0	0	0	0	0
†	21UR-13923	TTTGTACATCTCTATGAAGGG	0	0	0	0	0	0	0	0
	21UR-13924	TTTGTAATACATAGTTGAGAG	0	0	0	0	0	1	0	1
†	21UR-13925	TTTGGTTTTTTCACGACTAGT	0	0	0	0	0	0	0	0
	21UR-13926	TTTGGTTTACCTTTGGTTGTT	0	0	0	0	0	0	0	0
	21UR-13927	TTTGGTTAATGAACTTTAAAA	0	0	0	0	2	0	0	2
†	21UR-13928	TTTGGTCATTAGAATATGAGA	0	0	0	0	0	0	0	0
	21UR-13929	TTTGGTCAAGTTTACCTCGTG	0	0	0	0	0	0	0	0
†	21UR-13930	TTTGGTATGTAATATACACGG	0	0	0	0	0	0	0	0
	21UR-13931	TTTGGTAGAAGGCAATCGTAG	0	0	0	0	0	0	0	0
†	21UR-13932	TTTGGTAATTTGGCCATTGTT	0	0	0	0	0	0	0	0
	21UR-13933	TTTGGGTTGTAACTTGCTGGC	0	0	0	0	0	0	0	0
	21UR-13934	TTTGGGTTAAACATTGCAATT	0	0	0	0	0	0	0	0
†	21UR-13935	TTTGGGTGTTAACATGAAAAT	0	0	0	0	0	0	0	0
†	21UR-13936	TTTGGCTTTTCTTTTAATAGT	0	0	0	0	0	0	0	0
	21UR-13937	TTTGGCTGTCATTCAACATTC	0	0	0	0	0	0	0	0
†	21UR-13938	TTTGGCAAACCTAATACATTC	0	0	0	0	0	0	0	0
	21UR-13939	TTTGGAGCAGATCTAAAATCT	0	0	0	0	0	0	0	0
†	21UR-13940	TTTGGAGAGTGGATCTAATTT	0	0	0	0	14	7	2	23
†	21UR-13941	TTTGGAATATGAAACATTTTG	0	0	0	0	0	0	0	0
†	21UR-13942	TTTGGAATAGCCGCATGAAAA	0	0	0	0	0	0	0	0
†	21UR-13943	TTTGCTTGTTGAGACGAATTG	5	7	2	4	26	53	5	102
	21UR-13944	TTTGCTTCCCATTTTTTTGTT	0	0	0	0	0	0	0	0
	21UR-13945	TTTGCTCAACGGACAGCTCAA	0	0	0	0	0	0	0	0
	21UR-13946	TTTGCTACTCCGTTTATAAAA	0	0	0	0	0	0	0	0

†	21UR-13947	TTTGCGTACATATTGCCAGTG	0	0	0	0	0	0	0	0
†	21UR-13948	TTTGCGGCAAAACTCTATGTC	0	0	0	0	1	1	0	2
†	21UR-13949	TTTGCCTTTAACCTCCTTCAG	0	0	0	0	0	0	0	0
†	21UR-13950	TTTGCCTTGCAACTTTGAAAA	0	0	0	0	0	0	0	0
†	21UR-13951	TTTGCATTTGCTACCAGATCT	0	0	0	0	0	0	0	0
	21UR-13952	TTTGCATCTGAGTGAAGTGAA	0	0	0	0	0	0	0	0
	21UR-13953	TTTGCATATCCTATAAAGCCA	0	0	0	0	0	0	0	0
	21UR-13954	TTTGCAAGTCGAAAATGTGCA	0	0	0	0	0	0	0	0
	21UR-13955	TTTGCAACTGCTTGAAGCCGT	0	0	0	0	0	0	0	0
	21UR-13956	TTTGCAAATTCTACAAAAAAC	0	0	0	0	0	0	0	0
†	21UR-13957	TTTGATTTTTTTAGGACAAAA	0	0	0	0	0	0	0	0
	21UR-13958	TTTGATTGAATTTTGATGGAG	0	0	0	0	1	1	0	2
†	21UR-13959	TTTGATTCTGTGCTATTGATT	0	0	0	0	0	0	0	0
†	21UR-13960	TTTGATTCAAGGTGGTCGAAT	0	0	0	0	0	0	0	0
†	21UR-13961	TTTGATTATTTTATCAAGTTG	0	0	0	0	0	0	0	0
	21UR-13962	TTTGATTAATTCTGAAAATTT	0	0	0	0	0	0	0	0
	21UR-13963	TTTGATGTCTGTAATCGATGG	0	1	0	0	0	3	1	5
†	21UR-13964	TTTGATCAGAGTATGAATATT	0	0	0	0	0	1	0	1
†	21UR-13965	TTTGATCACTACGTGGTACAA	0	0	0	0	0	0	0	0
†	21UR-13966	TTTGATCAAAAGTGAAAAATA	0	0	0	0	0	0	0	0
†	21UR-13967	TTTGATATTTACTCACTGGAA	0	0	0	0	0	0	0	0
†	21UR-13968	TTTGATACATGGTCATTTTAT	0	0	0	0	0	0	0	0
†	21UR-13969	TTTGAGTGAATGTGTTAAAAT	0	0	0	0	0	0	0	0
	21UR-13970	TTTGAGGTAATGAAGGAATTA	0	0	0	0	0	1	0	1
†	21UR-13971	TTTGAGCTCGCCGATTTCTCT	0	0	0	0	0	0	0	0
†	21UR-13972	TTTGACTTTTCGTTTCTAAAT	0	0	0	0	0	0	0	0
	21UR-13973	TTTGACCGCAACCGGTATGAG	0	0	0	0	0	0	0	0
	21UR-13974	TTTGACCAAATTGAAAACAAA	0	0	0	0	0	0	0	0
	21UR-13975	TTTGAATTTCGCGGCGATAAT	0	0	0	0	0	0	0	0
	21UR-13976	TTTGAATGCAGAAAGGAGGCG	0	0	0	0	0	0	0	0
	21UR-13977	TTTGAATATTCTGACTCTGAT	0	0	0	0	0	0	0	0
	21UR-13978	TTTGAACCTCTTGCCACCTTA	0	0	0	0	0	0	0	0
	21UR-13979	TTTGAAATGGTGATTTTATAA	0	0	0	0	0	0	0	0
	21UR-13980	TTTGAAACGGAAACTTATTCA	0	0	0	0	0	1	0	1
	21UR-13981	TTTGAAAATGCAAGAGTGACC	0	0	0	0	0	0	0	0
	21UR-13982	TTTGAAAAAAAAAAGAGTAAA	0	0	0	0	0	0	0	0
†	21UR-13983	TTTCTTTTGATTTTTTTAGGA	0	0	0	0	0	1	0	1
†	21UR-13984	TTTCTTTCAAAATTCCGATCA	0	0	0	0	0	0	0	0
	21UR-13985	TTTCTTGATACGACAAATTGA	0	0	0	0	0	0	0	0
†	21UR-13986	TTTCTTGAGTGTTAATATCAT	0	0	0	0	0	0	0	0
†	21UR-13987	TTTCTTCTATTTCTCCATGGG	0	0	0	0	0	1	0	1
†	21UR-13988	TTTCTTCCACGAACTAAGTAT	0	0	0	0	0	0	2	2
	21UR-13989	TTTCTTATTAGCATTCACTTC	2	0	0	1	1	2	0	6
	21UR-13990	TTTCTGTTTTTTGTCGATCGT	1	0	0	0	0	0	0	1
†	21UR-13991	TTTCTGTTCATGGAGACAATT	13	1	4	6	15	35	19	93
	21UR-13992	TTTCTGCAATGATCTGTTTCC	0	0	0	0	0	0	0	0
	21UR-13993	TTTCTGAGAATGCTCGTGCAA	0	0	0	0	0	0	0	0
	21UR-13994	TTTCTGAAAGTTTAGACTCTA	0	0	0	0	0	0	0	0
†	21UR-13995	TTTCTCTGAAGGCATATTTTT	0	2	0	2	26	11	2	43
†	21UR-13996	TTTCTCGTACTCAAAAGTCAA	0	0	0	0	0	0	0	0
	21UR-13997	TTTCTCCTCCTTAAAATAACA	0	0	0	0	0	0	0	0
†	21UR-13998	TTTCTCCGAATGCATCTAATG	0	0	0	0	0	0	0	0
	21UR-13999	TTTCTCCACGATTTGCAACTA	0	0	0	0	0	0	0	0
	21UR-14000	TTTCTCAAGAAGTCCTCAATT	0	0	1	1	0	0	0	2
	21UR-14001	TTTCTATTTTGTTCATTTTTA	0	0	0	0	0	0	0	0
†	21UR-14002	TTTCTATCGGTTTCATTCAAA	0	0	0	0	0	0	0	0
	21UR-14003	TTTCTATCAAAAGTGCATATA	0	0	0	0	0	0	0	0
†	21UR-14004	TTTCTATAGAAAAACGCTTAA	0	0	0	0	2	1	0	3
	21UR-14005	TTTCTAGTAAATCCTAATACA	0	0	0	0	0	0	0	0
	21UR-14006	TTTCTAAGTTACCTTCACGGA	0	0	0	0	0	0	0	0
†	21UR-14007	TTTCTAAAAATCGCTGACAAT	2	0	0	1	6	8	3	20
†	21UR-14008	TTTCGTTTTGCACCAAGAAAT	0	0	0	0	0	0	0	0
	21UR-14009	TTTCGTTACAGCAATTTTTCA	0	0	0	0	0	0	0	0
	21UR-14010	TTTCGTCTCTTATATCTGTGT	0	0	0	0	0	0	0	0

†	21UR-14011	TTTCGTAGAGCCAACGTACTT	0	0	0	0	0	0	0	0
†	21UR-14012	TTTCGGCAATGTTTCGAAAAG	0	0	0	0	0	0	0	0
	21UR-14013	TTTCGCGCGGAATCATTTGGA	0	0	0	0	0	0	0	0
†	21UR-14014	TTTCGAGTTTGGTTTTTGATT	0	0	0	0	0	0	0	0
†	21UR-14015	TTTCGAACGCAATTCTTGTTT	0	0	0	0	0	0	0	0
	21UR-14016	TTTCGAACCGAAAAAATTATG	0	0	0	0	0	0	0	0
†	21UR-14017	TTTCCTTTTCTGTCCCTCTTT	0	0	0	0	0	0	0	0
†	21UR-14018	TTTCCTGCCATACCTTTACAA	7	0	2	0	1	3	2	15
†	21UR-14019	TTTCCGTTCTTATTTCAACAG	0	0	0	0	0	0	0	0
†	21UR-14020	TTTCCGGTGGCGATTCCTAAA	0	0	0	0	0	0	0	0
	21UR-14021	TTTCCCTTTAAAAATTCATCG	1	0	0	0	0	0	0	1
†	21UR-14022	TTTCCCGGATCTCCCTTTTTT	0	0	0	0	0	0	0	0
	21UR-14023	TTTCCCAACTGTCGCTTCTCT	0	0	0	0	0	0	0	0
	21UR-14024	TTTCCCAAAAATGTATGAATT	0	0	0	0	0	0	0	0
	21UR-14025	TTTCCATTTATTGTAATTAGT	0	0	0	0	0	0	0	0
†	21UR-14026	TTTCCATTGATAATTGATGAT	0	0	0	0	0	0	0	0
†	21UR-14027	TTTCCATGTAGACTGTATTTT	0	0	0	0	2	0	0	2
	21UR-14028	TTTCCAGTTTATCCTGGACAA	0	0	0	0	0	0	0	0
	21UR-14029	TTTCCAGTTCTAATTACCTCC	0	0	0	0	0	0	0	0
†	21UR-14030	TTTCCAATAATTTTTGTTCGA	0	0	0	0	0	0	0	0
	21UR-14031	TTTCATTGAATTCTTGCGATG	0	0	0	0	0	0	0	0
†	21UR-14032	TTTCATTCTTTTTGTTGCCAA	0	0	0	0	0	0	0	0
†	21UR-14033	TTTCATCTTTGATTCTTTGAC	0	0	0	0	0	0	0	0
†	21UR-14034	TTTCATACAATGGGTCTTTTG	0	0	0	0	0	0	0	0
	21UR-14035	TTTCAGTTATGTGATAATGTA	0	0	0	0	0	0	0	0
	21UR-14036	TTTCAGTCTATGGCTAACTTT	0	3	1	1	1	11	0	17
†	21UR-14037	TTTCAGCGAAACATTATCTTT	0	0	0	0	0	0	0	0
	21UR-14038	TTTCACTTTAAAAACTTTTTA	0	0	1	0	4	2	0	7
†	21UR-14039	TTTCACTTCATTGATGCAAAT	0	0	0	0	0	0	0	0
†	21UR-14040	TTTCACTGCACTCGAACTCAC	0	0	0	0	0	0	0	0
†	21UR-14041	TTTCACTATCTTATTGACCAA	0	0	0	0	0	0	0	0
	21UR-14042	TTTCACGTTTCAACGTTTTAG	0	0	0	0	0	0	0	0
†	21UR-14043	TTTCACCTTTCTTCGAAAAAT	0	0	0	0	0	0	0	0
†	21UR-14044	TTTCACAATTTTCCTAACGTT	0	0	0	0	0	0	0	0
†	21UR-14045	TTTCAATTTTTTTGGTTATCC	0	0	0	0	0	0	0	0
†	21UR-14046	TTTCAATTAGTAGGTTGTTCT	0	0	0	0	1	0	0	1
†	21UR-14047	TTTCAATGGAATTCACATGTG	0	0	0	0	0	0	0	0
†	21UR-14048	TTTCAATGCTACCCATTTCTC	0	0	0	0	0	0	0	0
†	21UR-14049	TTTCAATCGCAGTCTGTATTC	0	0	0	0	0	1	0	1
†	21UR-14050	TTTCAATCAGATTGATTTGTC	0	0	0	0	0	0	0	0
†	21UR-14051	TTTCAATACAAGCAAGGGATG	0	0	0	0	1	1	0	2
	21UR-14052	TTTCAAGTATTGTCAATAAGG	0	0	0	0	0	0	0	0
	21UR-14053	TTTCAACTTATTCTTCTGACC	0	0	0	0	0	0	0	0
	21UR-14054	TTTCAAATTTTGAATCGAACT	0	0	0	0	0	0	0	0
	21UR-14055	TTTCAAATTGTGAGTTGAAGA	0	0	0	0	0	0	0	0
†	21UR-14056	TTTCAAATTCTAGACTAGAGA	0	0	0	0	0	0	0	0
†	21UR-14057	TTTCAAATCCCTTAAAAAGAC	0	0	0	0	0	0	0	0
	21UR-14058	TTTCAAATCAACTGTAAGTAA	0	0	0	0	0	0	0	0
	21UR-14059	TTTCAAATAAAGTATAACGGC	0	0	0	0	0	1	0	1
	21UR-14060	TTTCAAACGTTGTTTCTTGCA	0	0	0	0	0	0	0	0
†	21UR-14061	TTTCAAAAACGCCTCAAATAG	0	0	0	0	0	0	0	0
	21UR-14062	TTTATTTTTTGCCTCAGCCGG	0	0	0	0	0	0	0	0
	21UR-14063	TTTATTTTCCTTTTATTTGAT	0	0	0	0	0	0	0	0
	21UR-14064	TTTATTTTCACAAAAATAAAA	0	0	0	0	0	0	0	0
†	21UR-14065	TTTATTTCACCGTTTAAGCAG	0	0	0	0	0	0	0	0
	21UR-14066	TTTATTTATTGAAGCGTTTGA	0	0	0	0	0	0	0	0
†	21UR-14067	TTTATTGTAATATTTGTAGAA	0	0	0	0	0	0	0	0
	21UR-14068	TTTATTGCTCTTCGGCCATAT	0	0	0	0	0	0	1	1
	21UR-14069	TTTATTGAGAGAAATTATATT	0	0	0	0	1	1	0	2
	21UR-14070	TTTATTGAAATAGCGTTAGTA	0	0	0	0	0	0	0	0
	21UR-14071	TTTATTCTGAAGATTCCAATA	0	0	0	0	0	0	0	0
	21UR-14072	TTTATTCTATAAAATTTATGG	0	0	0	0	0	0	0	0
†	21UR-14073	TTTATTCGAAAGAGTTGGTTC	1	0	0	0	0	0	0	1
†	21UR-14074	TTTATTCCATCTTTTATTCAA	0	0	0	0	0	0	0	0

	21UR-14075	TTTATTCATCATACAGTTTAA	0	0	0	1	4	4	2	11
	21UR-14076	TTTATTCAACTTTCCTAAACT	0	0	0	0	0	0	0	0
	21UR-14077	TTTATTATCGTTAATTTTTCA	0	0	0	0	2	1	1	4
	21UR-14078	TTTATGTCAGTTTCTTGAGCT	0	0	0	0	0	0	0	0
	21UR-14079	TTTATGGATTGAATACTCATG	0	0	0	0	0	0	0	0
	21UR-14080	TTTATGGATGTAAATGTCTTT	0	0	0	0	1	0	0	1
	21UR-14081	TTTATGATTTTTTTCTTACAT	0	0	0	0	0	0	0	0
	21UR-14082	TTTATGAACGAAATTACTATT	0	0	0	0	0	0	0	0
†	21UR-14083	TTTATCGATCCAAACAAAAAC	0	0	0	0	0	0	0	0
	21UR-14084	TTTATCAGTGTCGAATCTGGC	0	0	0	0	0	0	0	0
	21UR-14085	TTTATCACGTATCCATGTTCG	0	0	0	0	0	0	0	0
†	21UR-14086	TTTATAGGATAATACCGGATT	0	0	0	0	0	0	0	0
†	21UR-14087	TTTATAAAATTCAGATTTTGC	0	0	0	0	0	0	0	0
†	21UR-14088	TTTAGTTTCCAAACGTTTAAA	0	0	0	0	0	0	0	0
	21UR-14089	TTTAGTGTTGATATTTGATGT	0	0	0	0	0	0	0	0
	21UR-14090	TTTAGTGGCAAAATAATTTTA	2	1	0	0	1	5	0	9
	21UR-14091	TTTAGTGATCGCACGCAAATT	0	0	0	0	0	0	0	0
†	21UR-14092	TTTAGTATCAAGAAAAATACG	0	0	0	0	0	0	0	0
	21UR-14093	TTTAGTAGACCTTAGACCTTC	0	0	0	0	0	0	0	0
	21UR-14094	TTTAGTACTGTAGGGAACTTT	0	0	0	0	0	0	0	0
	21UR-14095	TTTAGCTAGTACTGATAGTGA	0	0	0	0	0	0	0	0
†	21UR-14096	TTTAGAATTTTAGTTGACTAT	0	0	0	0	0	0	0	0
	21UR-14097	TTTAGAAATTCTGATTGAATT	0	0	0	0	0	0	0	0
†	21UR-14098	TTTAGAAACCAGTAATAGAAA	0	0	0	0	0	0	0	0
	21UR-14099	TTTACTTCTGACATTTAACGG	0	0	0	0	17	13	1	31
	21UR-14100	TTTACTTATGCTAATTTAACG	0	0	1	0	0	0	0	1
	21UR-14101	TTTACTGTTCCAGCAAAAAAT	0	0	0	0	0	0	0	0
	21UR-14102	TTTACCTAAGAATGCATATTT	0	0	0	0	9	3	1	13
†	21UR-14103	TTTACCAATTCGGTTCAAAGA	0	0	0	0	0	0	0	0
†	21UR-14104	TTTACACCAGTTCCGCAAACA	0	0	0	0	0	0	0	0
†	21UR-14105	TTTACAATGTTCCAAAAAAAA	0	0	0	0	0	0	0	0
	21UR-14106	TTTAATTTCTATCTTCGAATT	0	0	0	0	0	0	0	0
†	21UR-14107	TTTAATTTATTGGGTATTGGA	0	0	0	0	0	0	0	0
	21UR-14108	TTTAATTGGTTCAGAAGAACG	0	0	0	0	0	0	1	1
	21UR-14109	TTTAATTGCCCTTGTTGTACC	0	0	0	0	0	0	0	0
	21UR-14110	TTTAATTCAAAAACTAACTTG	0	0	0	0	0	0	0	0
†	21UR-14111	TTTAATGTCACGCTGAAGTAA	0	0	0	0	0	0	0	0
	21UR-14112	TTTAATAAAAAATCAAAAATG	0	0	0	0	0	0	0	0
	21UR-14113	TTTAAGAATACCTAATGTGAG	0	0	0	0	0	0	0	0
†	21UR-14114	TTTAACTTTGAGTTTGAAATT	0	0	0	0	0	0	0	0
	21UR-14115	TTTAACTGTAGATTTTTTTGG	0	0	0	0	0	0	0	0
†	21UR-14116	TTTAACGTTAGTAGATGGTTG	1	0	0	1	3	4	0	9
†	21UR-14117	TTTAACGTCTGAATCATGCGA	0	0	0	0	0	0	0	0
†	21UR-14118	TTTAACAGCGGAGAGTTTGTG	0	0	0	0	0	0	0	0
	21UR-14119	TTTAAATGAATTATTGTAATT	0	0	0	0	1	0	0	1
†	21UR-14120	TTTAAAGTTCTCTCGCATTTC	0	0	0	0	0	0	0	0
	21UR-14121	TTTAAACTATGTAGATGAATG	0	0	0	0	0	0	0	0
	21UR-14122	TTTAAACGGGGTAAGTATTGG	0	0	0	0	0	0	0	0
	21UR-14123	TTTAAAATGAAAACTTTGAAT	0	0	0	0	0	0	0	0
	21UR-14124	TTTAAAATCGTTGTTGCTGGG	0	0	0	0	0	0	0	0
	21UR-14125	TTTAAAAAAATTAACCGACAC	0	0	0	0	0	0	0	0
†	21UR-14126	TTGTTTTTCTTGCTGAAAAAG	0	0	0	0	0	0	0	0
	21UR-14127	TTGTTTTGTGGTGCTGAAAAT	0	0	0	0	0	0	0	0
†	21UR-14128	TTGTTTCTCCGTACTTTTGGA	0	0	0	0	0	0	0	0
	21UR-14129	TTGTTTCCTGCTTTATTTTTC	0	0	0	0	0	0	0	0
	21UR-14130	TTGTTTCATTAATTTGTTGCA	0	0	0	0	0	0	0	0
†	21UR-14131	TTGTTTAGCGAAAATAGGAGC	0	0	0	0	0	0	0	0
†	21UR-14132	TTGTTGTTTTTAAGTAACAGA	0	0	0	0	0	0	0	0
†	21UR-14133	TTGTTGTTCTTTTGCCGCTTG	0	0	0	0	0	0	0	0
	21UR-14134	TTGTTGGCGTACTCTCTAAGA	0	0	0	0	0	0	0	0
†	21UR-14135	TTGTTGCAGCGGTTTGATGAT	0	0	0	0	0	0	0	0
	21UR-14136	TTGTTCTTTCGGTATCGATTT	0	0	0	0	0	0	0	0
	21UR-14137	TTGTTCGTGCGGATAAACGAA	0	0	0	0	0	0	1	1
	21UR-14138	TTGTTCAGGGACTATCATAAA	0	0	0	0	0	0	0	0

	21UR-14139	TTGTTCACTGAACACGAAAAA	0	0	0	0	0	0	0	0
	21UR-14140	TTGTTCAAAAATAGGAAATGA	0	0	0	0	0	1	0	1
†	21UR-14141	TTGTTATTTGATTGGCATGTA	0	1	0	0	2	4	0	7
†	21UR-14142	TTGTTATTGTTCTTCAAGGAT	0	0	0	0	0	0	0	0
†	21UR-14143	TTGTTATTGTCGGCAGGAGAT	0	1	0	0	52	91	63	207
	21UR-14144	TTGTTATATCTGAAAGACTTC	0	0	0	0	0	0	0	0
†	21UR-14145	TTGTTACAGGATCAGTGAAAG	0	0	0	0	1	0	0	1
†	21UR-14146	TTGTTAAATCTGCAAAATGCT	0	0	0	0	0	0	0	0
†	21UR-14147	TTGTGTTCGTAACTGAATTGA	0	0	0	0	0	0	0	0
†	21UR-14148	TTGTGTTCATGAAGGAATACA	0	0	0	0	0	0	0	0
†	21UR-14149	TTGTGTAAAGCGTGTACTTCA	0	0	0	0	0	1	0	1
	21UR-14150	TTGTGGTTTGAGCTGAATACT	0	0	0	0	0	0	0	0
	21UR-14151	TTGTGCTTGCTGATTATCAAA	0	0	0	0	0	0	0	0
	21UR-14152	TTGTGCTGACTGAGTTATACG	0	0	0	0	0	0	0	0
†	21UR-14153	TTGTGCTACTTTCCCACAAAT	0	0	0	0	0	0	0	0
	21UR-14154	TTGTGCAGACCGAGTGAGAGT	0	0	0	0	0	0	0	0
†	21UR-14155	TTGTGATTCAAGCTGGTATAA	0	0	0	0	1	0	0	1
†	21UR-14156	TTGTGATCTCTACCGGTATAA	0	0	0	1	0	0	0	1
†	21UR-14157	TTGTCTTTCTTTACGTTGCTT	0	0	0	0	0	0	0	0
†	21UR-14158	TTGTCTTTCGGTGTTATTAAA	0	0	0	0	0	0	0	0
	21UR-14159	TTGTCGACAACACGGAAACGA	0	0	0	0	1	2	0	3
†	21UR-14160	TTGTCATTTTATCGAAGAAAA	0	0	0	0	0	0	0	0
	21UR-14161	TTGTCACTTCCTTTCAAATTT	0	0	0	0	0	0	0	0
†	21UR-14162	TTGTCACTATTCTTTCGTTGA	2	0	0	0	0	0	0	2
†	21UR-14163	TTGTATTTGACCCTTTTTCAC	1	0	0	0	0	0	0	1
†	21UR-14164	TTGTATGTATACTTTGTGTTG	0	0	0	0	0	0	0	0
†	21UR-14165	TTGTATCTGATTTGTGCCTAC	0	0	0	0	0	0	0	0
†	21UR-14166	TTGTATAGACTGTGACTGTAG	1	0	0	0	0	0	0	1
	21UR-14167	TTGTAGCGTTTATTTTTTGCT	0	0	0	0	0	0	0	0
	21UR-14168	TTGTACTTGATTCTGAAAATA	0	0	0	0	0	0	0	0
†	21UR-14169	TTGTACTGATTTAAATGAAGT	0	0	0	0	0	0	0	0
†	21UR-14170	TTGTACTCTTTTTTGTGTTGT	0	0	0	0	0	0	0	0
†	21UR-14171	TTGTACGCACATTGGGTAAAC	0	0	0	0	0	0	0	0
†	21UR-14172	TTGTAATCATGTTCCAATTCA	0	0	0	0	0	0	0	0
	21UR-14173	TTGTAAGTTTATAATTTTACG	0	0	0	0	0	0	0	0
	21UR-14174	TTGTAACCGGTCTTTTTCCAT	0	0	0	0	0	0	0	0
	21UR-14175	TTGTAAAAGTAACATTTAAAT	0	0	0	0	0	0	0	0
†	21UR-14176	TTGGTTTTTTCATCATAGGTA	0	0	0	0	0	0	0	0
†	21UR-14177	TTGGTTTTTATTTAACATAAA	0	0	0	0	0	0	0	0
†	21UR-14178	TTGGTTTCTATCAATCTGACA	0	0	0	0	0	0	0	0
†	21UR-14179	TTGGTTGGTTTCTCTTCTTTT	0	0	0	0	0	0	0	0
†	21UR-14180	TTGGTTGGATTGTCGCAGTCG	0	0	0	0	0	2	2	4
†	21UR-14181	TTGGTTCTTCCAAATGAAAAA	0	0	0	0	0	0	0	0
†	21UR-14182	TTGGTTAATAAAGATAGATCT	0	0	0	0	1	0	0	1
†	21UR-14183	TTGGTCCTGTTCTTAACTTAT	0	0	0	0	0	0	0	0
†	21UR-14184	TTGGTCATGATAAAAATGTTT	0	0	0	0	0	0	0	0
†	21UR-14185	TTGGTCACACACAACAAAGGC	0	0	0	0	0	0	0	0
†	21UR-14186	TTGGTAGTTTCGAGTTTGGTT	0	0	0	0	0	0	0	0
†	21UR-14187	TTGGTAAAATCTCTGTCTCTC	0	0	0	0	0	0	0	0
†	21UR-14188	TTGGGTCGTCGTTTGAGACTG	0	0	0	0	0	2	2	4
†	21UR-14189	TTGGGCAAAAGTTAGGCAGGC	0	0	0	0	1	1	2	4
†	21UR-14190	TTGGGATGTTCAGCTCATATA	0	0	0	0	0	0	0	0
	21UR-14191	TTGGGATAGTTCAACGGAACA	0	0	0	0	0	0	0	0
†	21UR-14192	TTGGGAATCCGATTTGATCGA	0	0	0	0	0	0	0	0
	21UR-14193	TTGGGAATATTCAAAGTTTGA	0	0	0	0	0	0	0	0
	21UR-14194	TTGGCTGTTATTTCATGTGCA	0	0	0	0	0	0	0	0
	21UR-14195	TTGGCGATCAAAATAAAATGA	0	0	0	0	0	0	0	0
†	21UR-14196	TTGGCATTAGATGTTTAGATG	0	0	0	0	0	0	0	0
†	21UR-14197	TTGGATTTGTTGTTAACATTA	0	0	0	0	1	0	0	1
†	21UR-14198	TTGGATTCTACTATATGGTAC	0	0	0	0	0	0	0	0
†	21UR-14199	TTGGATTATAAATAGATATTA	0	0	0	0	0	0	0	0
†	21UR-14200	TTGGATGAATTTTTTGATTTT	0	0	0	0	0	0	0	0
	21UR-14201	TTGGATATTTAAAAATGATAT	0	0	0	0	0	0	0	0
	21UR-14202	TTGGATAGTCTCACGGTCAAT	0	0	1	0	5	6	1	13

†	21UR-14203	TTGGAGTTGTTTCAGTCAGGT	0	0	0	0	0	0	0	0
†	21UR-14204	TTGGACTATTGAGAGTTGTAT	0	0	0	0	0	0	0	0
†	21UR-14205	TTGGACCAAAAAGCTAAAAAA	0	0	0	0	0	0	0	0
†	21UR-14206	TTGGAATTAAAATTGGTGTCG	0	0	0	0	0	0	0	0
	21UR-14207	TTGGAAGATTAAAACTCCGCC	0	0	0	0	0	0	0	0
	21UR-14208	TTGGAAGAAGTCAAAGTCATG	0	0	0	0	0	0	0	0
	21UR-14209	TTGGAAAACGGTGGCGCGATT	0	0	0	0	0	0	0	0
†	21UR-14210	TTGCTTTTTCTGCAAAGTTGT	0	0	0	0	0	0	0	0
	21UR-14211	TTGCTTTTCCGCGTCCTTGTA	0	0	0	0	0	0	0	0
†	21UR-14212	TTGCTTGGATAAAAAGCGTTG	0	0	0	0	0	0	0	0
†	21UR-14213	TTGCTTCTGCAGTAGCGTGTT	0	0	0	0	0	0	0	0
†	21UR-14214	TTGCGTATGCAGTGCACCAAG	0	0	0	0	0	0	0	0
†	21UR-14215	TTGCGGGAGACAGACTTTGCA	0	0	1	1	1	8	1	12
	21UR-14216	TTGCGCGGAGCTGAAAATAGT	0	0	0	0	0	0	1	1
†	21UR-14217	TTGCCGTCAAGCTTTCTTTTG	0	0	0	0	0	0	0	0
	21UR-14218	TTGCCAGAAAAAAATTGGAAA	0	0	0	0	0	0	0	0
†	21UR-14219	TTGCATTGAAGGGTTCCATAG	3	0	0	0	0	3	0	6
	21UR-14220	TTGCATTATTAAATAACCAAA	0	0	0	0	0	1	0	1
	21UR-14221	TTGCATGCATTGAGTATCGCT	0	0	0	0	0	0	0	0
†	21UR-14222	TTGCATCTAAAGTTGATTGAA	0	0	0	0	0	0	0	0
†	21UR-14223	TTGCATACCTCTAATAACCAA	0	0	0	0	1	0	0	1
†	21UR-14224	TTGCACTATTCTTTGAACTGT	0	0	0	0	0	0	0	0
†	21UR-14225	TTGCACGATTTCGGTGATGAT	0	0	0	0	1	3	0	4
†	21UR-14226	TTGCAAGTGTAAAATTCACTG	0	0	0	0	0	0	0	0
†	21UR-14227	TTGCAAGCGATTTTCAAACAA	0	0	0	0	0	1	0	1
†	21UR-14228	TTGATTTGTATCTAAACTTGC	0	0	0	0	0	0	0	0
†	21UR-14229	TTGATTTGATTGCTTCTTGCC	0	0	0	0	0	0	0	0
†	21UR-14230	TTGATTTGAAAAGAAATTCAC	0	0	0	0	0	0	0	0
	21UR-14231	TTGATTTCTAATGTCGAAATT	0	0	0	0	0	0	0	0
†	21UR-14232	TTGATTGGAATCCCTGTCACT	0	0	0	0	3	4	0	7
	21UR-14233	TTGATTGCGGTTAAACTAATG	0	0	0	0	0	0	0	0
†	21UR-14234	TTGATTGAGCTGCAAGTGATA	0	0	0	0	0	0	0	0
†	21UR-14235	TTGATTCCTTCCATTATTTTC	0	0	0	0	0	0	0	0
†	21UR-14236	TTGATTCATCGCTTGACGAGA	0	0	0	0	0	0	0	0
	21UR-14237	TTGATTAGTCTCAAACCCCAA	0	0	0	0	0	0	0	0
†	21UR-14238	TTGATGTGTTGCTAAAAACTT	0	0	0	0	0	0	0	0
†	21UR-14239	TTGATGTGAGTATAGAAAGTT	1	1	0	1	8	9	2	22
†	21UR-14240	TTGATGCTTCGAGCGGATAGT	0	0	0	0	0	0	0	0
†	21UR-14241	TTGATGAGTGACACACACGGC	0	0	0	0	0	0	0	0
	21UR-14242	TTGATGAAAATTGAACTCGGT	0	0	0	0	0	0	0	0
†	21UR-14243	TTGATCTTTCAGTTGACTGAA	0	0	0	0	0	0	0	0
†	21UR-14244	TTGATCGGATTTGCTCACACC	0	0	0	0	0	0	0	0
†	21UR-14245	TTGATCGGATCGGGTCATACC	0	0	0	0	0	0	0	0
†	21UR-14246	TTGATCCAAGAATTCCAGTTT	0	0	0	0	0	0	0	0
†	21UR-14247	TTGATCCAAAATATTCCAAAA	0	0	0	0	0	0	0	0
†	21UR-14248	TTGATATTTTGAACCTGAAAC	0	0	0	0	1	0	0	1
†	21UR-14249	TTGATATGCCTATACTGTTCC	0	0	0	0	0	0	0	0
†	21UR-14250	TTGATATGAGAAAATCAAATT	0	0	0	0	0	0	0	0
†	21UR-14251	TTGATATCCGTGCTAAAAAAG	0	0	0	0	0	0	0	0
†	21UR-14252	TTGATATACAAGCATTTTCCA	0	0	0	0	0	1	0	1
†	21UR-14253	TTGATAGAATTGATGAATGGG	0	0	0	0	0	0	0	0
†	21UR-14254	TTGATACTTTTGCTACACTTT	0	0	0	0	0	0	0	0
	21UR-14255	TTGATACTCATGATCTTTTCT	0	0	0	0	0	0	0	0
	21UR-14256	TTGAGATGAAAATGAGTCGGG	0	1	0	0	1	0	1	3
	21UR-14257	TTGAGATCGGAAATATGTGAT	0	0	0	0	0	1	1	2
	21UR-14258	TTGAGATCCTAGAATTGGTAA	0	0	1	0	0	0	0	1
	21UR-14259	TTGAGATACAATATTTAAAAC	0	0	0	0	0	0	0	0
	21UR-14260	TTGAGATAATTCAAAACTTAT	0	0	0	0	0	0	0	0
	21UR-14261	TTGAGAATCTGCGAAAAGAAT	0	0	0	0	0	0	0	0
	21UR-14262	TTGACTTCGGAAGCAGACGTT	0	0	1	0	2	3	0	6
†	21UR-14263	TTGACTTCCTTCTAATCTACA	0	0	0	0	0	0	0	0
	21UR-14264	TTGACTTATAATTCATTGTTT	0	1	0	1	1	1	1	5
†	21UR-14265	TTGACTGAATTTTTTTGATAG	0	0	0	0	2	0	0	2
†	21UR-14266	TTGACGATAGTTTCGATTGAT	0	0	0	0	0	3	0	3

	21UR-14267	TTGACGAAAAAAAAAATTCACG	0	0	0	0	0	0	0	0
†	21UR-14268	TTGACCCGCAATGATTTCTCC	0	0	0	0	0	0	0	0
	21UR-14269	TTGACCATGCCCACACAGTAG	0	0	0	0	0	0	0	0
	21UR-14270	TTGACATGTTAAAAAAAACGG	0	0	0	0	0	0	0	0
†	21UR-14271	TTGACATCCTAGCAAAATGTT	0	0	0	0	0	0	0	0
†	21UR-14272	TTGACACCGGCAAAAATCAGA	1	0	0	0	0	1	0	2
	21UR-14273	TTGACAAATACATTGGAGTTT	0	0	0	0	0	0	0	0
†	21UR-14274	TTGAATGATATGCCAGATCAC	0	0	0	0	0	0	0	0
†	21UR-14275	TTGAATAGGCTGGCTTGATGG	0	1	0	0	3	9	1	14
†	21UR-14276	TTGAAGTTCTTCGCTGAGTTT	0	0	0	0	0	1	0	1
†	21UR-14277	TTGAAGTCCAATTGGGAATAA	0	0	0	0	0	0	0	0
	21UR-14278	TTGAAGCGAGAAATAAAGAAC	0	0	0	0	0	0	0	0
	21UR-14279	TTGAAGAACAGCGCTCTTTAA	0	0	0	0	0	0	0	0
	21UR-14280	TTGAAGAAAACCCTGTTAAAA	0	0	0	0	0	0	0	0
	21UR-14281	TTGAACCTGAAATAAATTTTA	0	0	0	0	0	0	0	0
	21UR-14282	TTGAAATTAAAGCATTGTCTC	0	0	0	0	0	0	0	0
†	21UR-14283	TTGAAAGAAGGTCAAGTGGGA	0	0	0	0	0	0	0	0
†	21UR-14284	TTGAAAGAACGGAAGGATTTG	0	0	0	0	0	0	0	0
†	21UR-14285	TTGAAACTTCTCATAAAGTCC	0	0	0	0	0	0	0	0
†	21UR-14286	TTGAAACGTAACGTTCAGTTG	0	0	0	0	0	0	0	0
†	21UR-14287	TTGAAAATTTAAGATGAGAAT	0	0	0	0	0	1	0	1
	21UR-14288	TTGAAAATATAGAAAGTGGGT	0	0	0	0	0	0	0	0
	21UR-14289	TTGAAAAGTTGAAAATGTATT	0	0	0	0	0	1	0	1
	21UR-14290	TTGAAAACTGCTCTGAATTTG	0	0	0	0	0	0	0	0
	21UR-14291	TTGAAAAAGTAAGTTGCATTC	0	0	0	0	0	0	0	0
†	21UR-14292	TTGAAAAAATCGCATTGTTTC	0	0	0	0	0	0	0	0
	21UR-14293	TTGAAAAAAACAGTGTAAAGC	0	0	0	0	0	0	0	0
†	21UR-14294	TTCTTTTTTGGTCATTAAAGC	0	0	0	0	0	0	0	0
	21UR-14295	TTCTTTTGGAATTCTTCAGTC	0	0	0	0	0	0	0	0
†	21UR-14296	TTCTTTGTTCCTTATGTTGCA	0	0	0	0	0	0	0	0
†	21UR-14297	TTCTTTCGGTATTATTTTTAG	1	0	0	0	2	0	0	3
†	21UR-14298	TTCTTTCGGTACATTCTTTAT	0	0	0	0	0	1	0	1
†	21UR-14299	TTCTTTCGAGTACCATAGGAC	0	0	1	1	7	4	0	13
†	21UR-14300	TTCTTTCCTGTCCTCTAGTCA	0	0	0	0	0	0	0	0
†	21UR-14301	TTCTTGTACATGAAGTTTAAA	0	0	0	0	0	0	0	0
	21UR-14302	TTCTTGGAATCAAATTGTGAG	0	0	0	0	0	0	0	0
†	21UR-14303	TTCTTGCGAGTTATTTTTCTA	0	0	0	0	0	0	0	0
	21UR-14304	TTCTTGCATCATGCCTCCGAA	0	0	0	0	0	0	0	0
†	21UR-14305	TTCTTGAAGTTTTAGATTAAA	0	0	0	0	0	0	0	0
†	21UR-14306	TTCTTCTTCGTGTAAACATTT	0	0	0	0	0	0	0	0
†	21UR-14307	TTCTTCGAGGCAAAGCAAGAT	0	0	0	0	4	6	0	10
†	21UR-14308	TTCTTCGACTAAACCCAATCA	0	0	0	0	0	0	0	0
†	21UR-14309	TTCTTCATCCTGAACCAAAAA	0	0	0	0	0	0	0	0
	21UR-14310	TTCTTCATAGAGTAGTTAGAT	0	0	0	0	0	0	0	0
†	21UR-14311	TTCTTATTTTGGGGTCAGTGT	0	0	0	0	0	0	0	0
†	21UR-14312	TTCTTAGAGCCTTCACAATAG	0	0	0	0	0	0	0	0
	21UR-14313	TTCTGTTTCGTCGCGCTTTCC	0	0	0	0	0	0	0	0
	21UR-14314	TTCTGTTACAACACGTAAGGT	0	0	0	0	0	0	0	0
†	21UR-14315	TTCTGTCTAGCTCGACATTTA	0	0	0	0	0	0	0	0
	21UR-14316	TTCTGGCAAAAAAAAAACTGT	0	0	0	0	0	0	0	0
†	21UR-14317	TTCTGCTCATATCTTGTAATT	0	0	0	0	0	0	0	0
†	21UR-14318	TTCTGCAATCGATTTCTGCAA	0	1	0	0	2	2	0	5
†	21UR-14319	TTCTGATTTGCTCCGGTGTCA	0	0	0	0	0	0	0	0
	21UR-14320	TTCTGATGCAGTGAATAACGT	0	0	0	0	0	1	0	1
†	21UR-14321	TTCTGACGAGTAGAGTTTAGA	1	1	0	1	54	59	6	122
†	21UR-14322	TTCTCTTTAACTTTTTAGTCA	0	0	0	0	0	0	0	0
	21UR-14323	TTCTCTTCAAATCCTTAATAC	0	0	0	0	0	0	0	0
	21UR-14324	TTCTCTGTGCAAAAATTTTTT	0	0	0	0	0	0	0	0
	21UR-14325	TTCTCTAGTCTGGGGTGTTGA	0	0	0	0	0	0	0	0
†	21UR-14326	TTCTCGTCCCTCACGGATAAG	1	0	0	0	0	0	0	1
†	21UR-14327	TTCTCGGCGACGATTTTTAAC	0	0	0	0	0	0	0	0
†	21UR-14328	TTCTCGAATATACATGTACTG	0	0	0	0	0	0	0	0
†	21UR-14329	TTCTCATGCGATAGAATTAGA	0	0	0	0	0	0	0	0
	21UR-14330	TTCTCATGAGACGCTTCTTGA	0	0	0	0	0	0	0	0

†	21UR-14331	TTCTCAAGCTCTTGTTCTAAA	0	0	0	0	0	0	0	0
	21UR-14332	TTCTATTTTTCTTGCTGACGC	0	0	0	0	0	0	0	0
	21UR-14333	TTCTATTCTAGAAAACTTTCC	0	0	0	0	0	0	0	0
†	21UR-14334	TTCTATGCGAGATCATCTTAA	0	0	1	0	0	1	0	2
	21UR-14335	TTCTACGAGAGAATTTTAAAT	0	0	0	0	1	0	0	1
	21UR-14336	TTCTAACTGTTTGCTTGATAC	0	0	0	0	0	0	0	0
†	21UR-14337	TTCGTTTTTCACATCAGATAA	0	0	0	0	0	0	0	0
†	21UR-14338	TTCGTTTTAGCTACCACCGGA	0	0	0	0	0	0	0	0
†	21UR-14339	TTCGTTGTTTTCTTCAGGTAG	0	0	0	0	0	0	0	0
†	21UR-14340	TTCGTTCTGTTTCTTGTGAAA	2	1	0	1	1	4	0	9
†	21UR-14341	TTCGTGTTTAGAAAAATTTCA	0	0	0	0	0	0	0	0
†	21UR-14342	TTCGTCTAAACCAAACAGTTA	0	0	0	0	0	0	0	0
	21UR-14343	TTCGTACTTTATTCAAAAAGT	0	0	0	0	0	0	0	0
†	21UR-14344	TTCGGTCGTTCATATTAGCTA	0	0	1	0	0	0	0	1
	21UR-14345	TTCGGTCAAAGTGAGTAGAAC	0	0	0	0	0	2	0	2
†	21UR-14346	TTCGGAGAAATAATCATTATT	0	0	0	0	0	2	0	2
†	21UR-14347	TTCGGAATGGGTTCAAGTCAG	0	0	0	0	0	1	0	1
	21UR-14348	TTCGGAAGCGCTGAAATATAG	0	0	0	0	0	0	0	0
	21UR-14349	TTCGGAAACAAGAGAAATAAG	0	0	0	0	0	0	1	1
	21UR-14350	TTCGGAAAAAAAATGCAAAAA	1	1	0	0	0	0	0	2
	21UR-14351	TTCGCACAACGTCACAATAGC	0	0	0	0	0	0	0	0
†	21UR-14352	TTCGATTGTTTGCCTCTAAAC	0	0	0	0	0	0	0	0
	21UR-14353	TTCGATTCTCCTCGTTATCCA	0	0	0	0	0	0	0	0
†	21UR-14354	TTCGAGATAAAAAAATGTTTC	0	0	0	0	1	0	0	1
†	21UR-14355	TTCGAAGACTCAAAAGTGTAG	0	1	0	0	0	1	0	2
†	21UR-14356	TTCGAACTTCAATGCAGGTCC	0	0	0	0	1	8	0	9
†	21UR-14357	TTCCTTTTTTGAATTTTGAAA	0	0	0	0	0	0	0	0
	21UR-14358	TTCCTTTGGTATTCCAAGTAG	0	0	0	0	0	0	0	0
†	21UR-14359	TTCCTTCCATTATTTTCGCTG	0	0	0	0	0	0	0	0
	21UR-14360	TTCCTTCAAAGACCAATGCGC	0	0	0	0	0	0	0	0
	21UR-14361	TTCCTGTTCGAGGCATTAAAA	0	0	0	0	0	0	0	0
	21UR-14362	TTCCTCTTTTTTCCCATACAC	0	0	0	0	1	1	0	2
	21UR-14363	TTCCGTTTTGGTTTGCGTTCA	0	0	0	0	0	0	0	0
†	21UR-14364	TTCCGTTGTTCATTATAGCTA	0	0	0	0	0	0	0	0
†	21UR-14365	TTCCGTTCCATTTTCGTAACA	0	0	0	0	0	0	0	0
†	21UR-14366	TTCCGTCACCATCATTAATCA	0	0	0	0	0	0	0	0
	21UR-14367	TTCCGTACTCGGTTGGACGAT	1	0	0	0	2	2	2	7
	21UR-14368	TTCCGGAGCATTTTACAACGT	0	0	0	0	1	1	0	2
†	21UR-14369	TTCCGGAAAAAAAACGAATTCG	1	2	0	4	15	8	2	32
	21UR-14370	TTCCGCTTGTTCAAAAATTTG	0	0	0	0	0	0	0	0
†	21UR-14371	TTCCGAGTCGAAATGTTTTCC	0	0	0	0	0	0	0	0
†	21UR-14372	TTCCGAAATTTGTGGTTCTTC	0	0	0	0	0	0	0	0
†	21UR-14373	TTCCCATTATTATATGTAGAC	0	0	0	0	0	0	0	0
	21UR-14374	TTCCCAGCCAAGATCTCACAG	0	0	0	0	0	0	0	0
	21UR-14375	TTCCCACCAACCTCTCTCAAA	0	0	0	0	0	0	0	0
	21UR-14376	TTCCCAATGCGACTCACCGAG	0	0	0	0	0	0	0	0
	21UR-14377	TTCCATGATTCTACTAGACAT	0	0	0	0	0	0	0	0
	21UR-14378	TTCCATCATATTTTTAGAGAG	0	0	0	0	0	0	0	0
†	21UR-14379	TTCCAGGGGAAAAGTTGCTGA	0	0	0	0	0	0	0	0
	21UR-14380	TTCCAGCTCTCTAATCTTTGC	0	0	0	0	0	0	0	0
	21UR-14381	TTCCAGAAAATTGTTGGGCGA	0	0	0	0	0	0	0	0
	21UR-14382	TTCCACTATACCACTGTCTGT	0	0	0	0	0	0	0	0
†	21UR-14383	TTCCACGCTGGGTTAAATAAA	0	0	0	0	0	0	0	0
†	21UR-14384	TTCCAATGTACCATAAAACAA	0	0	0	0	0	0	0	0
	21UR-14385	TTCCAAGATTTTCAACTTTGA	0	0	0	0	0	0	0	0
†	21UR-14386	TTCCAAAATGCTGGTGCTTCA	0	0	0	0	0	0	0	0
	21UR-14387	TTCATTTTGTATAAGAGAATA	0	0	0	0	0	0	0	0
†	21UR-14388	TTCATTTTCAACAATGGGTTT	0	0	0	0	0	0	0	0
†	21UR-14389	TTCATTTGCTAATCCAAAAAA	0	0	0	0	0	0	0	0
†	21UR-14390	TTCATTGGTTCTATTTTTCCA	0	0	0	0	0	0	0	0
†	21UR-14391	TTCATTAGACTTGGCGTAATC	0	0	0	0	0	0	0	0
†	21UR-14392	TTCATCTGAAAGAAAACCAGA	0	0	0	0	0	0	0	0
†	21UR-14393	TTCATCGCCATTAAAAATAAA	0	0	0	0	0	0	0	0
	21UR-14394	TTCATATCGCTTGACTCAAAC	0	0	0	0	0	0	0	0

†	21UR-14395	TTCATATACTTGGACAGGGAA	0	0	0	0	0	0	0	0
†	21UR-14396	TTCATAGCACATTTGTGATAT	0	0	0	0	0	0	0	0
†	21UR-14397	TTCATACGCTCAAATTGTATT	0	0	0	0	1	0	0	1
†	21UR-14398	TTCATAATGAGAGCTGATCGA	1	0	0	0	2	3	0	6
†	21UR-14399	TTCAGTTTTGAAATGTCACGA	0	0	0	0	0	0	0	0
	21UR-14400	TTCAGTTGCCTTGTAAAATAA	0	0	0	0	0	0	0	0
†	21UR-14401	TTCAGTTGCAAAATTGTATTG	0	0	0	0	0	0	0	0
	21UR-14402	TTCAGTTAAATGTAGAATTGC	0	0	0	0	0	0	0	0
	21UR-14403	TTCAGTGAGGAAATATAAAAC	0	0	0	0	0	0	0	0
†	21UR-14404	TTCAGTGACAAAAAAACATCT	0	0	0	0	0	0	0	0
†	21UR-14405	TTCAGTCTCATAAAGTCATCA	0	0	0	0	0	0	0	0
†	21UR-14406	TTCAGTCAGGAGAAAAACTTG	0	1	0	0	0	0	0	1
	21UR-14407	TTCAGGAACTTCTGATATCTT	0	0	0	0	0	0	0	0
	21UR-14408	TTCAGCAAGTCTAATTGAATA	0	0	0	0	1	0	0	1
†	21UR-14409	TTCAGATACTGGTCCTTGGAA	0	1	0	0	0	0	0	1
	21UR-14410	TTCAGACTACAATCAGACTAA	0	0	0	0	0	0	0	0
†	21UR-14411	TTCAGACGAGCAAAAAACAAA	3	0	0	0	0	2	0	5
†	21UR-14412	TTCACTTAAAGCTATTTAAAC	0	0	0	0	0	0	0	0
†	21UR-14413	TTCACTGGCGATGAATGTAAT	6	1	2	1	3	13	5	31
†	21UR-14414	TTCACTCTCATTGTTTAGGAT	0	0	0	0	0	0	0	0
	21UR-14415	TTCACTCAAAGCTTCGCAATA	0	0	0	0	0	0	0	0
	21UR-14416	TTCACTATTTCTGGTTCTGTT	0	0	0	0	0	0	0	0
†	21UR-14417	TTCACGAGGTGTTTTTCAGAA	0	0	0	0	0	0	0	0
†	21UR-14418	TTCACCGTTTAAGCAGTAACT	0	0	0	0	1	4	0	5
	21UR-14419	TTCACCCAAAAGAATATGTTC	0	0	0	0	0	0	0	0
†	21UR-14420	TTCACCAGATCCCCAAAATTA	0	0	0	0	0	0	0	0
†	21UR-14421	TTCACATTCTTGTCCTCTGTA	0	0	0	0	0	0	0	0
†	21UR-14422	TTCACATTCCATTGAAATAGT	0	0	0	0	0	0	0	0
†	21UR-14423	TTCACATTAATCAAAGCCAGT	0	0	0	0	0	0	0	0
†	21UR-14424	TTCACATCTTCACTTTTAGAG	0	0	0	0	0	0	0	0
†	21UR-14425	TTCACATCTCTCTCAACCTCA	0	0	0	0	0	0	0	0
	21UR-14426	TTCACAGCAAAAATGTTTTTC	0	0	0	0	0	1	0	1
†	21UR-14427	TTCACAATGGCATGGTGGTAT	0	0	0	0	0	0	0	0
†	21UR-14428	TTCAATTTTACTGCATGGTTT	0	0	0	0	0	0	0	0
†	21UR-14429	TTCAATTTAGTCTAAGCGGCA	1	0	0	0	1	2	1	5
†	21UR-14430	TTCAATTGGTCTGTGGTCTGT	0	0	0	0	0	0	0	0
†	21UR-14431	TTCAATTCCATATTCACTAAA	0	0	0	0	0	0	0	0
†	21UR-14432	TTCAATTAGTCGATTTTGATC	0	0	0	0	0	0	0	0
* †	21UR-14433	TTCAATTAATAAGCAAATTCG	0	1	0	0	1	1	0	3
	21UR-14434	TTCAATGCAATAGTATGTATG	0	0	0	0	1	0	0	1
†	21UR-14435	TTCAATGAAGTTGAATTTAAT	0	0	0	0	0	1	0	1
†	21UR-14436	TTCAATCGTAAATTTGAAGTG	0	0	0	0	0	0	0	0
	21UR-14437	TTCAATCGGTTGGCTGAGACG	0	0	0	0	0	0	0	0
	21UR-14438	TTCAATCATTCCTTCGAACGC	0	0	0	0	0	0	0	0
	21UR-14439	TTCAATAATCAAATAATGAAC	0	0	0	0	0	0	0	0
†	21UR-14440	TTCAAGCAAGGTTACCGAAAT	0	0	0	0	0	1	0	1
	21UR-14441	TTCAACGGTTTAGTGAAACAT	0	1	0	0	0	2	0	3
†	21UR-14442	TTCAACCGCTTGTTAGGGAAT	0	0	0	0	0	0	0	0
†	21UR-14443	TTCAACCAGTAGACATTTATT	0	0	0	0	2	1	0	3
†	21UR-14444	TTCAACATCCTCGATGGAGTA	0	0	0	0	0	0	0	0
†	21UR-14445	TTCAACATCCAGTTTTTGAAC	0	0	0	0	0	0	0	0
	21UR-14446	TTCAACAATTGCAAATTTCCA	0	0	0	0	0	0	0	0
	21UR-14447	TTCAACAAAAAGTTGTGGACC	0	0	0	0	0	0	0	0
	21UR-14448	TTCAAATCTATTGTCTACCTT	0	0	0	0	0	0	0	0
	21UR-14449	TTCAAACATAAAGGAGGAAAT	0	0	0	0	0	0	0	0
†	21UR-14450	TTCAAAATTCCCACCCGATCT	0	0	0	0	0	0	0	0
	21UR-14451	TTCAAAAGTAAGAAGATTGTC	0	0	0	0	0	0	1	1
†	21UR-14452	TTCAAAAATTGAAGGTGTTCA	0	0	0	0	0	0	0	0
	21UR-14453	TTCAAAAAAGTGATCACTTCC	0	0	0	0	0	0	0	0
	21UR-14454	TTCAAAAAACCTGCGATAATT	0	0	0	0	0	0	0	0
	21UR-14455	TTCAAAAAAAATCGTCGTTAC	1	1	0	0	2	15	3	22
†	21UR-14456	TTATTTTTGTCGAACTTCTTG	0	0	0	0	0	0	0	0
	21UR-14457	TTATTTTTCTGGAATTGCTTC	0	0	0	0	0	0	0	0
	21UR-14458	TTATTTTCTGATATCAGGTTT	0	0	1	0	1	2	0	4

	21UR-14459	TTATTTTCTAATCGATCGAGT	0	0	0	0	1	2	0	3
†	21UR-14460	TTATTTTCAATCCTAACGAAA	0	0	0	0	0	0	0	0
†	21UR-14461	TTATTTTAGAAGCCAATTTAG	0	0	0	0	0	0	0	0
†	21UR-14462	TTATTTCTCGTACTCAAAAGT	0	0	0	0	0	0	0	0
	21UR-14463	TTATTTCAAGTGGTGTCTGAT	0	0	0	0	0	0	0	0
†	21UR-14464	TTATTGTGGAAAAGAAAAACA	0	0	0	0	0	0	0	0
†	21UR-14465	TTATTGTGCTTCTTTCGAATG	0	0	0	0	0	0	0	0
†	21UR-14466	TTATTGTCTTTTGTATTTCAT	0	0	0	0	0	0	0	0
†	21UR-14467	TTATTGGCATCTCCGGAAAAA	0	1	0	0	1	1	0	3
†	21UR-14468	TTATTGGCAACTTACGTTAAC	0	0	0	0	0	1	0	1
†	21UR-14469	TTATTGCGTCATTTGATTTTG	0	0	0	0	0	0	0	0
	21UR-14470	TTATTGCAGAAAGAAAAACAA	0	0	0	0	0	0	0	0
†	21UR-14471	TTATTGATACTTTTGCTACAC	0	0	0	0	1	0	0	1
	21UR-14472	TTATTGATAACGATTTGCTCA	0	0	0	0	0	0	0	0
†	21UR-14473	TTATTGAGAGAACGCAATTGA	0	0	0	0	2	3	0	5
†	21UR-14474	TTATTGACAAATTCAGGTAAA	0	0	0	0	0	0	0	0
†	21UR-14475	TTATTCTTTTAGAAGCAGAAT	0	0	0	0	0	0	0	0
†	21UR-14476	TTATTCTGACAAGCTATGCCC	0	0	0	0	0	0	0	0
†	21UR-14477	TTATTCTCTCGCCGTGTGCTT	0	0	0	0	0	0	0	0
†	21UR-14478	TTATTCTCAGATGGTATTTGA	0	0	0	0	0	1	0	1
†	21UR-14479	TTATTCGCGAATATAAAAACT	0	0	0	0	0	0	0	0
	21UR-14480	TTATTCGATTATCAATTTTAT	0	0	0	0	0	0	0	0
†	21UR-14481	TTATTCCGTGTTTGTATGTAA	0	0	0	0	0	0	0	0
†	21UR-14482	TTATTCAATATTTGCATAGGG	0	0	0	0	0	0	0	0
†	21UR-14483	TTATTATCGTCTTCTGAGCAT	0	0	1	0	1	0	0	2
	21UR-14484	TTATTAGAGAGACACAGAGAA	0	0	0	0	0	0	0	0
†	21UR-14485	TTATTAGACGTGTTGAAAAAA	0	0	0	0	0	2	0	2
	21UR-14486	TTATTACTTCCTCAAATAAGA	0	0	0	0	0	0	0	0
†	21UR-14487	TTATTACTGTGTGTCCGTTAG	0	0	0	0	0	0	0	0
†	21UR-14488	TTATTACAAGTTTGATTTTGT	0	0	0	0	0	0	0	0
†	21UR-14489	TTATTAAATCTTCCCCACCAA	0	0	0	0	0	0	0	0
	21UR-14490	TTATGTCTGATATGTCAAAAA	0	0	0	0	0	0	0	0
†	21UR-14491	TTATGTCCTGTAACTGAAACC	0	0	0	0	0	0	0	0
†	21UR-14492	TTATGTCACGAGATTGGTTTA	0	0	0	0	0	0	0	0
†	21UR-14493	TTATGTATTTTTGAACGTCTT	0	0	0	2	2	0	0	4
	21UR-14494	TTATGTAGGGATGAATGTCGA	2	4	1	1	1	13	0	22
†	21UR-14495	TTATGTAGGCTATAGGAATTC	0	0	0	0	0	0	0	0
†	21UR-14496	TTATGGCACGTCATTTTATAA	0	0	0	0	0	0	0	0
†	21UR-14497	TTATGACTACACTTAATGATC	0	0	0	0	0	0	0	0
†	21UR-14498	TTATGAAAAGAAACAAGCAGC	0	0	0	0	0	0	1	1
†	21UR-14499	TTATCTTTCGGAAATACATTT	0	0	0	0	0	0	0	0
†	21UR-14500	TTATCTTTCAACTACTATAGT	0	0	0	0	0	0	0	0
†	21UR-14501	TTATCTGAAGTTGCGATAACT	0	0	0	1	4	5	1	11
	21UR-14502	TTATCTCCTTTAAAGCACAAT	0	0	0	0	0	0	0	0
	21UR-14503	TTATCATTTTTCGGTGCAAAT	0	0	0	0	0	0	0	0
†	21UR-14504	TTATCACTCCAATTACTAAAA	0	0	0	0	0	0	0	0
	21UR-14505	TTATCACAGATCAATTCGTGG	0	0	0	0	0	0	0	0
†	21UR-14506	TTATCACAGACTTAGCAGTAC	0	0	0	0	0	0	0	0
†	21UR-14507	TTATCAATTTTTCGAACACAT	0	0	0	0	0	0	0	0
	21UR-14508	TTATATGGAACATCGAAAAAA	0	0	0	0	0	0	0	0
†	21UR-14509	TTATATCGTTCACAGAACAAA	0	0	0	0	0	0	0	0
	21UR-14510	TTATATCATCATTCAGATGTG	0	0	0	0	2	1	0	3
	21UR-14511	TTATAGTAGTTTATTCTATTT	0	0	0	1	2	2	0	5
	21UR-14512	TTATAGATTAAAATACTTTTT	0	0	0	0	5	0	0	5
†	21UR-14513	TTATACCTTTATTCCCAAATT	0	0	0	0	0	0	0	0
†	21UR-14514	TTATAATTTGAAACGCTAGGG	0	0	0	0	0	1	0	1
	21UR-14515	TTATAATTGTGTCAAAGGGAC	0	0	0	0	0	0	0	0
†	21UR-14516	TTATAAGGTGCAATAGATGAT	0	0	0	0	0	0	0	0
	21UR-14517	TTATAAGAGAACTCTTAAGAT	0	0	0	0	0	1	1	2
	21UR-14518	TTATAACAACCGTGTCAATTT	0	0	0	0	0	0	0	0
	21UR-14519	TTATAAATCCCTGTCATTCTC	0	0	0	0	0	0	0	0
	21UR-14520	TTAGTTGGTTTTTGTTTTGTT	0	0	0	0	0	0	0	0
†	21UR-14521	TTAGTTGGTTGGAATATAGAA	0	0	0	0	0	0	0	0
†	21UR-14522	TTAGTTCGTTAGTGAGAAGGT	0	1	0	0	2	0	3	6

†	21UR-14523	TTAGTTCGGTTGGTTTAGTTC	0	0	0	0	0	1	1	2
	21UR-14524	TTAGTGTGTAGAAGGGCTGTG	0	0	0	0	0	0	0	0
	21UR-14525	TTAGTGGTGTCTGCTTCACAC	0	0	0	0	0	0	0	0
†	21UR-14526	TTAGTGGGCTGATTTTTCGGA	0	0	0	0	0	0	0	0
†	21UR-14527	TTAGTGGGAGAATTATTTTAC	0	0	0	0	0	0	0	0
†	21UR-14528	TTAGTGCTTTGATCAATTCCC	0	0	0	0	0	0	0	0
†	21UR-14529	TTAGTGCGTAGCAATGAAATT	0	0	0	0	0	0	0	0
†	21UR-14530	TTAGTGAATTCGTGCTTTGGA	0	0	0	0	0	0	0	0
	21UR-14531	TTAGTGAATGTGAGTTTTTCT	0	0	0	0	0	0	0	0
†	21UR-14532	TTAGTGAAACATTCACGGTTT	0	0	0	0	0	0	0	0
†	21UR-14533	TTAGTGAAACAAGTATAGTTT	0	0	0	0	0	0	0	0
†	21UR-14534	TTAGTCTCTTGTCCCGCAAAA	0	0	0	0	0	0	0	0
†	21UR-14535	TTAGTATTTCTTCCTCTGATT	0	0	0	0	0	0	0	0
†	21UR-14536	TTAGTAGAGAAATTTGAATAG	0	0	0	0	0	0	0	0
†	21UR-14537	TTAGTACCGCTTCCCACAAAT	1	0	1	0	0	0	1	3
†	21UR-14538	TTAGTAAATTGTTATTAGTGG	0	0	0	0	0	0	0	0
†	21UR-14539	TTAGGTAGTGAATTTCACTGT	0	0	0	0	0	0	0	0
†	21UR-14540	TTAGGATATTTGGGTAGGATA	0	0	0	0	0	0	0	0
†	21UR-14541	TTAGGACCGTTTGTGATGATT	1	3	6	6	144	148	187	495
	21UR-14542	TTAGGAATTAACAGTTTGTAT	0	0	0	0	0	0	0	0
†	21UR-14543	TTAGGAAACTCGGCTCCAGCG	0	0	0	0	0	0	0	0
†	21UR-14544	TTAGCTATTCCGTTATCGATG	0	0	0	0	0	0	0	0
†	21UR-14545	TTAGCAGCCTCTTAACTACAA	0	0	0	0	0	0	0	0
†	21UR-14546	TTAGCACTTCAAAAATTAGGA	0	0	0	0	0	0	0	0
†	21UR-14547	TTAGCAACATTGAAAATATTA	0	0	0	0	0	0	0	0
	21UR-14548	TTAGATAAGAAATGTTGTCAC	0	0	0	0	0	0	0	0
	21UR-14549	TTAGACAGCTGAAAGAATTAG	0	0	0	0	0	0	0	0
	21UR-14550	TTAGAACCAGAAAAAAAAAAC	0	1	0	0	2	0	0	3
†	21UR-14551	TTAGAACACTGCCGTTTTTAA	0	0	0	0	0	0	0	0
	21UR-14552	TTAGAAAGAAATGATGGCACA	0	0	0	0	0	2	0	2
†	21UR-14553	TTACTTTCATTCCTCTTCGTA	0	0	0	0	0	0	0	0
	21UR-14554	TTACTTGTATGCAGTGAGGTA	0	0	0	0	0	0	0	0
†	21UR-14555	TTACTTGCCTTTTTCACCACT	0	0	0	0	0	0	0	0
†	21UR-14556	TTACTTCGTCTTGGAAAAAAC	0	0	0	0	0	0	1	1
	21UR-14557	TTACTTCAACTATATGAACAG	0	0	0	0	0	1	0	1
	21UR-14558	TTACTGTCAAGTCTATTTATC	0	0	0	0	0	0	0	0
†	21UR-14559	TTACTGGATTGATAAGATGCA	0	0	0	0	0	0	0	0
	21UR-14560	TTACTCTTCCAATGGTGTGTG	0	0	0	0	0	0	0	0
†	21UR-14561	TTACTCCTTGTGATTTGGTTT	0	0	0	0	0	0	0	0
†	21UR-14562	TTACTCCTAAGATTTTTGTGT	1	0	0	0	0	0	0	1
†	21UR-14563	TTACTCCACAAATTTCTGTTT	0	0	0	0	5	3	0	8
†	21UR-14564	TTACTCAATTGATGGTTTTTT	0	0	0	0	0	0	0	0
†	21UR-14565	TTACGTTTTCAATGCTGAGCC	0	0	0	0	0	0	0	0
†	21UR-14566	TTACGTTAGCGGTAATAAATT	0	0	0	0	0	0	0	0
†	21UR-14567	TTACGTGTTGAGAACTGTTGT	0	0	0	0	1	1	0	2
†	21UR-14568	TTACGATGGCTACAATAATAG	0	0	0	0	0	0	0	0
†	21UR-14569	TTACGATGAGTGACTTCACAT	0	0	0	0	0	0	0	0
	21UR-14570	TTACGATGACAGATCGGTTTG	0	0	0	0	0	0	1	1
†	21UR-14571	TTACGAGGGACGAAATTTGCT	0	1	0	0	0	0	0	1
†	21UR-14572	TTACGAACACAATTAAATTTT	0	0	0	0	0	0	0	0
	21UR-14573	TTACGAAATTCAGTTAAAATT	0	0	0	0	0	0	0	0
	21UR-14574	TTACCGCAGTGATTGTTGCTC	0	0	0	0	0	0	0	0
	21UR-14575	TTACCCTTGACTGCTCATATC	0	0	0	0	0	0	0	0
	21UR-14576	TTACCAAAGGTGTTGGGAATT	0	0	0	0	0	0	0	0
†	21UR-14577	TTACATTAATATTGTTCGGAA	0	1	0	1	3	5	0	10
†	21UR-14578	TTACATCTGAAACAGGATGAA	0	0	0	0	1	0	0	1
	21UR-14579	TTACAGTAGGCGTTGTAGGCG	0	0	0	0	0	0	0	0
†	21UR-14580	TTACACGTTTTTGTCTTGCCT	0	0	0	0	0	1	0	1
†	21UR-14581	TTACACACTTTAGTCGGGATA	0	0	0	0	1	0	0	1
	21UR-14582	TTACAACGTTGTTGCGGCAAG	0	0	0	0	0	0	0	0
†	21UR-14583	TTACAACAGAGTCACAAAAAA	0	0	0	0	0	0	0	0
†	21UR-14584	TTACAAATGTGTAAACTGAAA	0	0	0	0	0	0	0	0
	21UR-14585	TTACAAATGATAAGGATGATG	0	0	0	0	0	0	0	0
	21UR-14586	TTACAAATCATGCACTCTCTC	0	0	0	0	0	0	0	0

	21UR-14587	TTACAAACCACAGAAAAAGTC	0	0	0	0	0	0	0	0
†	21UR-14588	TTAATTTTGCTGAATTAAAAA	0	0	0	0	0	0	0	0
†	21UR-14589	TTAATTTTGAAGCGAATAATT	0	0	0	0	1	2	0	3
	21UR-14590	TTAATTTTAGCTTTTGTAATT	0	0	0	0	0	0	0	0
†	21UR-14591	TTAATTTGACGATAGTTTCGA	0	0	0	0	0	0	0	0
†	21UR-14592	TTAATTGTTAACTGCTTTTTG	0	0	0	0	0	0	0	0
†	21UR-14593	TTAATTGGTGAATCGGTCCCG	0	0	0	0	0	0	0	0
	21UR-14594	TTAATTCTCCGAGAATTCAAC	0	0	0	0	0	0	0	0
	21UR-14595	TTAATTCTAGCCACTCTTCAA	0	0	0	0	0	0	0	0
†	21UR-14596	TTAATTCACTTGATTTTCAAA	0	0	0	0	0	0	0	0
†	21UR-14597	TTAATTACAAGAACATCGAAC	0	0	0	0	2	0	0	2
†	21UR-14598	TTAATGTTGTAATAGCTACTC	0	0	0	0	0	0	0	0
	21UR-14599	TTAATGGATTCCCAACAGAAA	0	0	0	0	0	0	0	0
†	21UR-14600	TTAATGGACTGCTAAATTGTT	0	0	0	0	0	0	0	0
†	21UR-14601	TTAATCTTTTTCTGATATTTG	0	0	0	0	0	0	0	0
†	21UR-14602	TTAATCTGTCTTTAGGTTGTA	1	0	0	0	0	0	0	1
†	21UR-14603	TTAATCTGCGTGTCATCGTCA	0	0	0	0	2	1	0	3
†	21UR-14604	TTAATCGCGTTCTAGAATTTC	0	0	0	0	0	0	0	0
†	21UR-14605	TTAATCAAACCAATCTTTTTC	0	0	0	0	0	0	0	0
	21UR-14606	TTAATATTTTGATTACTAATT	0	0	0	0	0	0	0	0
	21UR-14607	TTAATATCCTGTTTAAATACG	0	0	0	0	0	0	0	0
†	21UR-14608	TTAATAGTTTTGAAAAAAAAA	0	0	0	0	0	0	0	0
	21UR-14609	TTAATAGTTACAGTTAAAAAA	0	0	0	0	0	2	0	2
†	21UR-14610	TTAATAGGATTCGCAATATTG	0	0	0	0	0	0	0	0
†	21UR-14611	TTAATAAGACTAGCCTATAAT	0	0	0	0	1	0	0	1
†	21UR-14612	TTAATAACTTTGGGTGAAAAT	0	0	0	0	0	0	0	0
†	21UR-14613	TTAATAAAAACTTCGACAGGA	0	0	0	0	0	1	0	1
†	21UR-14614	TTAAGTGTGTTTTCTGTTTTT	0	0	0	0	0	0	0	0
†	21UR-14615	TTAAGTCACTGCTTCTGAATT	0	0	0	0	0	0	0	0
†	21UR-14616	TTAAGGGACCTCTAATATTTC	0	0	0	0	0	0	0	0
†	21UR-14617	TTAAGCTACCGTTTTCGCTCA	0	0	0	0	0	0	0	0
†	21UR-14618	TTAAGATGAAGACATCGTTTG	0	0	0	0	0	0	0	0
†	21UR-14619	TTAAGAATGTAAAACCATGTC	0	1	0	0	0	0	0	1
	21UR-14620	TTAACTGCTTCCCGTCTCTCT	0	0	0	0	0	0	0	0
	21UR-14621	TTAACTATTCAGAAGAAAAAT	0	0	0	1	0	1	0	2
†	21UR-14622	TTAACGCAGGATGCAAATTTA	0	0	0	0	2	2	0	4
	21UR-14623	TTAACGAAAAACATTGAATTT	0	1	0	0	0	0	0	1
	21UR-14624	TTAACCGAACAATCAAGCGAA	0	0	0	0	0	0	0	0
	21UR-14625	TTAACCCGAAGTTGCTATCCT	0	0	0	0	0	0	0	0
†	21UR-14626	TTAACAGTTAGTAAGCGGCAC	0	0	0	0	0	0	0	0
	21UR-14627	TTAAATTTTTAAAAAGTTCTA	0	0	0	0	0	0	0	0
	21UR-14628	TTAAATTCGCAGTGGCTGGAA	0	0	0	0	0	0	0	0
	21UR-14629	TTAAATTCCTCTCTTGCCAAC	0	0	0	0	0	0	0	0
†	21UR-14630	TTAAATCAACTGTTCGAAACA	0	0	0	0	0	0	0	0
†	21UR-14631	TTAAATATCGAGTTCTTGAGA	0	0	0	0	0	0	0	0
	21UR-14632	TTAAAGGAGAAAATGTTTTTA	1	0	1	0	1	1	0	4
	21UR-14633	TTAAAGAGATAATTGGATCAA	0	0	0	0	0	0	0	0
†	21UR-14634	TTAAAGACAATAGTTTGATTC	0	0	0	0	0	0	0	0
†	21UR-14635	TTAAACTGCCTACCTCAGATA	0	0	0	0	0	2	0	2
	21UR-14636	TTAAACTGAAATTTTCAAAAA	0	0	0	0	0	0	0	0
	21UR-14637	TTAAAATGCAAAACACTTGAA	0	0	0	0	0	0	0	0
	21UR-14638	TTAAAATACTATTTGATTCAA	0	0	0	0	0	0	0	0
	21UR-14639	TTAAAATACGAAACAGCAAAA	0	0	0	0	0	0	0	0
	21UR-14640	TTAAAACGCACATATCTGCGA	0	0	0	0	0	0	0	0
†	21UR-14641	TTAAAAATTGTTACTGTTTCT	0	0	0	0	0	0	0	0
	21UR-14642	TTAAAAATGTGTGTGCGTGGT	0	0	0	0	0	0	0	0
	21UR-14643	TTAAAAAAGCACTAAAATAAA	0	0	0	0	0	1	0	1
	21UR-14644	TTAAAAAAAGGTTGAAAACGA	0	0	0	0	0	0	0	0
	21UR-14645	TGTTTTTTATCAGATTAGAGG	0	0	0	0	0	0	0	0
	21UR-14646	TGTTTTTTAACCATATTTTAC	1	0	0	1	0	2	1	5
	21UR-14647	TGTTTTTCTCGCCAAATAATT	0	0	0	0	0	0	0	0
	21UR-14648	TGTTTTTCGAGAACAAAATTC	0	0	0	0	0	0	0	0
	21UR-14649	TGTTTTTCCATTGAAATGACA	0	0	0	0	0	0	0	0
	21UR-14650	TGTTTTGTCCAAATGGGAAAA	0	0	0	0	0	0	0	0

	21UR-14651	TGTTTTCTTAAAGTTCTTAGG	0	0	0	0	0	0	0	0
	21UR-14652	TGTTTTCACTTTATCCTCGCT	0	0	0	0	0	0	0	0
†	21UR-14653	TGTTTTATTCAACATTGGAAC	0	0	0	0	0	0	0	0
†	21UR-14654	TGTTTTAGCGGATTTTTTACA	0	1	0	1	0	3	0	5
†	21UR-14655	TGTTTGTGACAGAACGCATTT	0	0	0	0	0	0	0	0
†	21UR-14656	TGTTTGATTTCAAAATCGGGA	0	0	0	0	0	0	0	0
†	21UR-14657	TGTTTCTTGCTTCTTGTGCCT	0	0	0	0	0	0	0	0
	21UR-14658	TGTTTCTGGTAGTTGTTATTA	0	0	0	0	0	0	0	0
	21UR-14659	TGTTTCGGAGCTCCGTGGATG	0	0	0	0	0	0	0	0
†	21UR-14660	TGTTTCGACTGTCATCGTTAA	0	0	0	0	0	2	0	2
†	21UR-14661	TGTTTCAAGCAAACGTTGCAT	0	0	0	0	0	0	0	0
	21UR-14662	TGTTTCAACTGAAAAACGCAA	0	0	0	0	0	0	0	0
	21UR-14663	TGTTTATAGATATTTCCTCCG	0	0	0	0	0	0	0	0
	21UR-14664	TGTTTAGCGAAATAAATGGGG	0	0	0	0	0	0	0	0
	21UR-14665	TGTTTAGATTGAGTGGATGTT	0	0	0	0	0	0	0	0
	21UR-14666	TGTTTACTATGAGCAGCATCC	0	0	0	0	0	0	0	0
	21UR-14667	TGTTGTTAGAAACGTAAGCCT	0	0	0	0	0	0	0	0
†	21UR-14668	TGTTGGGAGATCGATGCTCAA	0	0	0	0	0	0	0	0
	21UR-14669	TGTTGGCTTACGTGGATAATT	0	0	0	0	0	0	0	0
	21UR-14670	TGTTGGAAAACGTTTTCGAAC	0	0	0	0	0	0	0	0
	21UR-14671	TGTTGCTAAAAATGTCTGGAT	0	0	0	0	0	0	0	0
†	21UR-14672	TGTTGCAGCGGTTTGATGATT	0	0	0	0	4	1	0	5
	21UR-14673	TGTTGCAAAAAAAGGTAAATT	0	0	0	0	0	0	0	0
†	21UR-14674	TGTTGATTGTGTTGTTCATTG	0	0	0	0	0	0	0	0
†	21UR-14675	TGTTGATTCCTTCATATCAGT	0	0	0	0	0	0	0	0
†	21UR-14676	TGTTGATCTCTCTTTTTAAAT	0	0	0	0	0	0	0	0
†	21UR-14677	TGTTGATACAGAAGAAACAGA	0	0	0	0	0	0	0	0
†	21UR-14678	TGTTGAGGTAAAGCCAAGTGA	0	0	0	0	0	0	0	0
†	21UR-14679	TGTTGAGCATACTCGAAGTGC	0	0	0	0	0	0	0	0
†	21UR-14680	TGTTGACGTACGTGGTAAAAT	0	0	0	0	0	0	0	0
†	21UR-14681	TGTTCTTTATTACTGGGCGTC	0	0	0	0	1	0	0	1
	21UR-14682	TGTTCTGTCGATCAAAAGTGC	0	0	0	0	0	0	0	0
†	21UR-14683	TGTTCTGCCTAAAAAAAATCA	0	0	0	0	0	0	0	0
†	21UR-14684	TGTTCGTGTTTAGAAAAAATT	0	0	0	0	0	0	0	0
	21UR-14685	TGTTCCTCATTTTGTGTAAAC	0	0	0	0	0	0	0	0
	21UR-14686	TGTTCCTCATCTTCTGTTGGG	0	0	0	0	0	0	0	0
†	21UR-14687	TGTTCCACTTGTCTTGCCAAT	0	0	0	0	0	0	0	0
†	21UR-14688	TGTTCCACCTGATGCGGGAGT	0	0	0	0	0	0	0	0
	21UR-14689	TGTTCAGACTGATCTGTTGGT	0	1	2	0	2	14	6	25
†	21UR-14690	TGTTATTGTGTTCTTTATCCA	0	0	0	0	0	0	0	0
†	21UR-14691	TGTTATAATACTGCTTCCTTC	0	0	0	0	0	0	0	0
†	21UR-14692	TGTTAGTAGATCCATTGAATT	0	0	0	0	1	0	0	1
	21UR-14693	TGTTACCAATTTGTGATAAAA	0	0	0	0	0	0	0	0
	21UR-14694	TGTTAATTGAGTTTTTACAAC	0	0	0	0	0	0	0	0
†	21UR-14695	TGTTAATGTTACTAAAAATGT	0	0	0	0	0	0	0	0
	21UR-14696	TGTGTGTCTTGTTTCTTCCGA	0	0	0	0	0	0	0	0
	21UR-14697	TGTGTGAAGTGTGCGAAGTCG	1	0	0	0	0	1	0	2
	21UR-14698	TGTGGTTTTTAATCCCGTATA	0	0	0	0	0	0	0	0
	21UR-14699	TGTGGAGTGTGCGTATTTCTA	0	0	0	1	0	3	1	5
	21UR-14700	TGTGCTTGTACCGGGAGAGCC	0	0	0	0	0	0	1	1
	21UR-14701	TGTGCGTAGTATCAATCAAAA	0	0	0	0	0	0	0	0
†	21UR-14702	TGTGCGATTGTTCGTAGAACA	0	0	0	0	0	0	0	0
	21UR-14703	TGTGCATGTCGGGTCTCCTTG	0	0	0	0	0	0	0	0
	21UR-14704	TGTGATGACGACGATGGAGGA	0	0	0	0	0	0	0	0
	21UR-14705	TGTGAAAAGAGTGACATTAGA	0	0	0	0	0	0	0	0
†	21UR-14706	TGTCTTTTGTATTTCATACTT	0	0	0	0	0	0	0	0
	21UR-14707	TGTCTTTCAATTATAGACAAT	0	0	0	0	0	0	0	0
	21UR-14708	TGTCTTCCGGAAAGTTAATTC	0	0	0	0	0	0	0	0
	21UR-14709	TGTCTCAAAAATGATTTCAGG	0	0	0	0	0	0	0	0
	21UR-14710	TGTCTAGAGTGGGGATAAGAG	0	0	0	0	0	0	0	0
†	21UR-14711	TGTCGTACACTATCGCCTCGT	0	0	0	0	10	7	5	22
	21UR-14712	TGTCGGGGAATGAATTGACGG	0	0	0	0	0	0	1	1
	21UR-14713	TGTCGCATTGGTTTATTAAAT	0	0	0	0	0	0	0	0
†	21UR-14714	TGTCCGATTTAGTTGAAGAAA	0	0	0	0	0	0	0	0

	21UR-14715	TGTCCATTTGAACTTTAATCC	0	0	0	0	0	0	0	0
	21UR-14716	TGTCATTGCTTGGCCTCCAGA	0	0	0	0	0	0	0	0
	21UR-14717	TGTCATAAGACCGTCTCCTCC	0	0	0	0	0	0	0	0
†	21UR-14718	TGTCAGGAAATTTAGTGTATG	0	0	0	0	0	0	0	0
	21UR-14719	TGTCAACTTTTCAATAACTTT	0	0	0	0	0	0	0	0
	21UR-14720	TGTATTTTTATTAGAGACCAT	1	0	0	1	3	0	1	6
	21UR-14721	TGTATTTTACGAAAACTCAAA	0	0	0	0	0	0	0	0
	21UR-14722	TGTATTGGAAATCTGAAAATT	0	0	0	0	0	0	0	0
	21UR-14723	TGTATTGCTGATACTGTTAGC	0	0	0	0	0	0	0	0
	21UR-14724	TGTATGCTAGAGGAATCTCAC	0	0	0	0	0	0	0	0
†	21UR-14725	TGTATATAGATCGACTAACAT	0	0	0	0	1	1	0	2
	21UR-14726	TGTATAGTTGTCTGAGAACGC	0	0	0	0	0	0	0	0
†	21UR-14727	TGTATACGGCATTAGTTATAT	0	0	0	0	0	0	0	0
†	21UR-14728	TGTAGTTGATATAATGAAAAT	0	0	1	0	0	0	0	1
	21UR-14729	TGTAGGGGTACAGTGTTATGG	0	0	0	0	0	0	0	0
	21UR-14730	TGTAGCTGTAGATACTTTGAC	0	0	0	0	0	0	0	0
	21UR-14731	TGTAGAACTAACCTGTTGAGC	0	0	0	0	0	0	1	1
	21UR-14732	TGTACTTCTCCGTCAGAAAGT	0	0	0	0	0	0	0	0
	21UR-14733	TGTACTCTATACCTTTACCCT	0	0	0	0	0	0	0	0
†	21UR-14734	TGTACTCGTTGGTTTCTACCC	0	0	0	0	0	0	0	0
†	21UR-14735	TGTAATGCAGCTCGTGCGATT	0	0	0	0	0	0	0	0
	21UR-14736	TGTAATAGGATGCTAAACCCA	0	0	0	0	0	0	0	0
	21UR-14737	TGTAAATCAAATGTGTCGTTT	0	0	0	0	0	0	0	0
	21UR-14738	TGTAAAGTGTTAGGAAATGTG	0	0	0	0	0	0	0	0
	21UR-14739	TGTAAACGTTAATTGAATCTT	0	0	0	0	0	0	0	0
	21UR-14740	TGTAAACAGGAATGAAAAAGG	0	0	0	0	0	0	0	0
†	21UR-14741	TGGTTTGTGAACATCTCATGT	1	1	0	0	0	2	0	4
	21UR-14742	TGGTGCTCAATTGAATGTTGG	0	0	0	0	0	0	0	0
	21UR-14743	TGGTGCATCGAACTTTTGACT	0	0	0	0	0	0	0	0
	21UR-14744	TGGTGAGAATTAAAAATGAGC	0	0	0	0	0	0	0	0
	21UR-14745	TGGTGACTAACAACATCTAGC	0	0	0	0	0	0	0	0
	21UR-14746	TGGTCTTATTCACCGTAATTT	0	0	0	0	0	0	1	1
	21UR-14747	TGGTCTGATTTCTCGAAAAAT	0	0	0	0	0	0	0	0
†	21UR-14748	TGGTCGTATTCGTTAATAGTT	0	0	0	0	0	0	0	0
†	21UR-14749	TGGTCAGGCTCAGTCTTATCA	0	0	0	0	0	0	0	0
	21UR-14750	TGGTATCAGATACGAAAGGGC	0	0	0	0	0	0	0	0
†	21UR-14751	TGGTATAGCACTCGTTTGGGC	0	0	0	0	1	1	0	2
	21UR-14752	TGGTACGTCGTCGTTGTGTTT	0	0	0	0	0	0	0	0
	21UR-14753	TGGTACCGGGAGACTCGGAAG	0	0	0	0	0	0	0	0
	21UR-14754	TGGTAATTCAAGTTGCCGCGC	0	0	0	0	0	0	0	0
	21UR-14755	TGGGTCCAACTGTTCAACAAT	0	0	0	0	0	0	0	0
	21UR-14756	TGGGGTGCTGTAACAATTTTG	0	0	0	0	0	0	0	0
	21UR-14757	TGGGCTTTATTGCAGGTGTGT	0	1	0	0	0	0	0	1
	21UR-14758	TGGGCTCACAGTTTTTTACGA	0	0	0	0	0	0	0	0
	21UR-14759	TGGGCGTTGTATTTGTATTGA	0	0	0	0	0	0	0	0
	21UR-14760	TGGGCATTTTGAGAGTGAATA	0	0	0	0	0	0	0	0
	21UR-14761	TGGGATCACGGTATCAGAGCA	0	0	0	0	0	2	0	2
	21UR-14762	TGGGAGTATCATAAGTTTTCT	0	0	0	0	0	0	1	1
	21UR-14763	TGGGAGTAGTAATTTCATTTT	0	0	0	0	0	0	0	0
	21UR-14764	TGGGAAATTGTTTGAAAACAC	0	0	0	0	0	0	0	0
	21UR-14765	TGGGAAATCCCAAGTTGATGG	0	0	0	0	0	0	0	0
	21UR-14766	TGGCTTGATTCTTTGTAGTAA	0	0	0	0	0	0	0	0
	21UR-14767	TGGCTGATCAAATGAGAATTG	0	0	0	0	0	0	0	0
	21UR-14768	TGGCTCAAGCTATCGATGAAC	0	0	0	0	1	0	0	1
	21UR-14769	TGGCGGTGATTGTATCCGGTG	0	0	0	0	0	0	0	0
	21UR-14770	TGGCAGCCACAAAAATGATGA	0	0	0	0	0	0	0	0
†	21UR-14771	TGGCACGTTTGATTTCCATTC	0	0	0	0	0	0	0	0
	21UR-14772	TGGCACCGTATAAACGGAAAC	0	0	0	0	0	0	0	0
	21UR-14773	TGGCAATCTGAAATGATCGAT	0	0	0	0	0	0	0	0
†	21UR-14774	TGGCAACTTACGTTAACTTAT	0	0	0	0	0	0	0	0
	21UR-14775	TGGATTTCTATTAGCCAGATT	0	0	0	0	0	0	0	0
	21UR-14776	TGGATGAGAGCGAAGGTTGCT	0	0	0	0	0	0	1	1
	21UR-14777	TGGATCCTTTCGAAAATAAAG	0	0	0	0	0	0	0	0
	21UR-14778	TGGATATTTCTTACGTTTCAA	0	0	0	0	1	0	0	1

	21UR-14779	TGGATATGATGTGGATTTGAT	0	0	0	0	1	0	0	1
	21UR-14780	TGGAGTTCTATGAAATTTTGA	0	0	0	0	0	0	0	0
	21UR-14781	TGGAGTACTGGAGTACTGGAA	0	0	0	0	0	0	0	0
†	21UR-14782	TGGAGGTTCTAATTCTATTAA	0	0	0	0	0	0	0	0
†	21UR-14783	TGGAGCAACTTCGGTTTTAAC	0	0	0	0	0	1	0	1
†	21UR-14784	TGGAGATTCCCAATTAAAAAA	0	0	0	0	0	0	0	0
	21UR-14785	TGGAGAAGAGCTTATGCCATT	0	0	0	0	0	0	0	0
	21UR-14786	TGGACTTTGTGATTGAAAAAC	0	0	0	0	0	0	0	0
	21UR-14787	TGGACTTGCCAAGTTAACTAC	0	0	0	0	0	0	0	0
	21UR-14788	TGGACGCTTTCGGATGGATGC	0	0	0	0	0	0	0	0
	21UR-14789	TGGAAGAATTAGTGCGAGAAT	0	0	0	0	0	0	0	0
	21UR-14790	TGGAAGAATACAATGATAGTC	0	0	0	0	0	0	0	0
	21UR-14791	TGGAACGTTTAAGGCTTCTGA	0	0	0	0	0	0	0	0
	21UR-14792	TGGAAACTAACTAGACATTCA	0	0	0	0	0	0	0	0
	21UR-14793	TGGAAAAATTGCAAGAATTAG	0	0	0	0	0	0	0	0
	21UR-14794	TGCTTTCCTTCAACTATGTAT	0	0	0	0	0	0	0	0
†	21UR-14795	TGCTTTACGAAAGTCAAACAT	0	0	0	0	0	0	0	0
	21UR-14796	TGCTTGAGTTGAATTGTATCA	0	0	0	0	0	0	0	0
	21UR-14797	TGCTTCTTATCCAATTCGAAT	0	0	0	0	0	0	0	0
†	21UR-14798	TGCTTCCTCAATTGCAATGTC	0	0	0	0	0	0	0	0
†	21UR-14799	TGCTTCAACATTTTCCCAAAT	0	0	0	0	0	0	0	0
†	21UR-14800	TGCTTAAGATGCCGTTTTTTT	1	1	1	0	15	20	4	42
†	21UR-14801	TGCTGTTAGACATATTAGGTC	0	0	0	0	0	0	0	0
†	21UR-14802	TGCTGGTTTGTTACGTCGATT	0	0	0	0	0	1	0	1
	21UR-14803	TGCTGGACGATGGAAATTTTG	0	0	0	0	0	0	0	0
†	21UR-14804	TGCTGCTTAATGGAAGATAGG	0	0	0	0	0	0	0	0
†	21UR-14805	TGCTGCAATAATCACGCTGCG	0	0	0	0	0	0	0	0
	21UR-14806	TGCTGATGATGAGGAAGACGA	0	0	0	1	0	3	0	4
	21UR-14807	TGCTCCTCTTCAATTCAAAAT	0	0	0	0	0	0	0	0
	21UR-14808	TGCTCAGATGGTCACCATTAT	0	0	0	0	0	0	0	0
†	21UR-14809	TGCTATGGAAAGGACATCATT	0	0	0	0	0	0	0	0
†	21UR-14810	TGCTAACACATTTAAAAAATA	0	0	0	0	0	0	0	0
	21UR-14811	TGCGTTTTCGAAACTAGTGCA	0	0	0	0	0	0	0	0
	21UR-14812	TGCGTGCTTCTACGTCAGGTA	0	0	0	0	0	0	0	0
	21UR-14813	TGCGTCAGCTGAATCTTGATC	0	0	0	0	0	0	0	0
	21UR-14814	TGCGGCACTTCCTTGGATCAA	0	0	0	0	0	0	0	0
†	21UR-14815	TGCGCTTTATCGACACATATT	0	0	0	0	0	0	0	0
	21UR-14816	TGCGCCCAGGGTATCAGCTTC	0	0	0	0	0	0	0	0
	21UR-14817	TGCGACTCTAGATCTTTACAC	0	0	0	0	0	0	0	0
†	21UR-14818	TGCGAACAACGGCTCACTTCA	0	0	0	0	0	0	1	1
	21UR-14819	TGCCTTTTTCCCTTGGAACGA	0	0	0	0	0	0	0	0
	21UR-14820	TGCCTTCTGAATAAATTTAGA	0	0	0	0	0	0	0	0
	21UR-14821	TGCCTGATTTGAAATAGCGTC	0	0	0	0	0	0	0	0
	21UR-14822	TGCCTGATGTCCGTTGTTCGG	0	0	0	0	0	0	0	0
	21UR-14823	TGCCGTGCTAGGATCGGATTT	0	0	0	0	0	0	0	0
	21UR-14824	TGCCATGAGCTCAAGTACCAA	0	0	0	0	0	0	0	0
	21UR-14825	TGCCAATCGAAGGTTCGTATA	0	0	0	0	0	0	0	0
	21UR-14826	TGCCAAGATGCAAGTAGGTTT	0	0	0	0	0	0	1	1
	21UR-14827	TGCATCTTGCAAATCGACACC	0	0	0	0	0	0	0	0
	21UR-14828	TGCATCCTTTACATTGTGCAA	0	0	0	0	0	0	0	0
	21UR-14829	TGCATCCTGAGAACTTCAAAC	0	0	0	0	0	0	0	0
	21UR-14830	TGCATACCTTTGTTATCGTAC	0	1	0	0	0	0	0	1
	21UR-14831	TGCAGTCTGAAATTTATGAAT	0	0	0	0	0	0	0	0
	21UR-14832	TGCAGGCACAAGTATGAAGAT	0	0	0	0	0	0	0	0
	21UR-14833	TGCACATGAAAATGGAGGCAA	0	0	0	0	0	0	0	0
	21UR-14834	TGCAACCCAAATGTTTGAAAC	0	0	0	0	0	0	0	0
	21UR-14835	TGCAAATCCTTGTTAAAGTGA	0	0	0	0	0	0	0	0
	21UR-14836	TGCAAACTTTTTTTGATGTCC	0	0	0	0	0	0	0	0
	21UR-14837	TGCAAAAATAGAAATGACGGC	0	0	0	0	0	1	0	1
	21UR-14838	TGATTTTTTGCGCGCACTAAC	0	0	0	0	0	0	0	0
	21UR-14839	TGATTTTTGGGTAGAAAGGAA	0	0	0	0	0	0	0	0
†	21UR-14840	TGATTTGAAGATTTGTTTTGC	0	0	0	0	1	0	0	1
†	21UR-14841	TGATTTAGTGCATACAATGGA	0	0	0	0	1	0	0	1
	21UR-14842	TGATTGTGAGTGCAAAAAATA	0	0	1	0	0	0	0	1

	21UR-14843	TGATTGGTCTGTCCGGAAGTA	0	0	0	0	0	0	0	0
†	21UR-14844	TGATTGGGCAGTTTTTTGTGA	0	0	0	0	0	0	0	0
	21UR-14845	TGATTGCTAGGAAATTGATAG	0	0	0	0	0	0	0	0
	21UR-14846	TGATTGCAAATTGGGATAACC	0	0	0	0	0	0	0	0
†	21UR-14847	TGATTGAGCTGCAAGTGATAG	0	0	0	0	0	0	0	0
†	21UR-14848	TGATTCTTGTACATGAAGTTT	0	0	0	0	0	1	0	1
†	21UR-14849	TGATTATTAGAGCTTTATCAA	0	0	0	0	0	0	0	0
	21UR-14850	TGATTAGACTCAGAATAAACG	0	0	0	0	0	0	0	0
†	21UR-14851	TGATGTTACTCGTAATTTTAC	0	0	0	0	0	0	0	0
†	21UR-14852	TGATGTGACGTTGAAATGAGT	0	0	0	0	0	0	0	0
†	21UR-14853	TGATGTAATTTTGAACAAAAT	0	0	0	0	9	7	2	18
	21UR-14854	TGATGGTCTAATCAAATCGCA	0	0	0	0	0	0	0	0
†	21UR-14855	TGATGGACGGTTTAAATTAAC	0	0	0	0	0	0	0	0
	21UR-14856	TGATGATGATGGTAGAGGAGC	0	0	0	0	0	0	0	0
	21UR-14857	TGATGACAATCTCGATGATGG	0	0	0	0	0	0	0	0
	21UR-14858	TGATCTTTTTTTTTGGAAAAA	0	0	0	0	0	0	0	0
	21UR-14859	TGATCTATGATATAATCCACT	0	0	0	0	0	0	0	0
	21UR-14860	TGATATTAGATAAAGTTATAG	0	0	0	0	1	0	0	1
†	21UR-14861	TGATATGCCTAAGCATATGGT	2	1	0	0	0	4	0	7
†	21UR-14862	TGATATGAGGAAATCGGGATG	0	0	1	1	1	1	0	4
	21UR-14863	TGATATCATTATTTGGTAAAA	0	0	0	0	0	0	0	0
†	21UR-14864	TGATATACAAGCATTTTCCAT	0	0	0	1	0	2	0	3
†	21UR-14865	TGATAGTGTCACATGGTGTTT	0	0	0	0	0	1	0	1
†	21UR-14866	TGATAGGTAAATAAAAAAAAA	0	0	0	0	0	0	0	0
†	21UR-14867	TGATAGGCCAAGTCGGTTAGC	0	1	0	0	0	0	0	1
†	21UR-14868	TGATAGCTTTCATCTATTCAA	0	0	0	0	0	0	0	0
†	21UR-14869	TGATAGCACGAAAAAAGCGCA	0	0	0	0	0	0	0	0
	21UR-14870	TGATACAGGACACGGACGGAT	0	0	0	0	0	0	0	0
	21UR-14871	TGATAATGAAACATCTGAAAA	0	0	0	0	0	0	0	0
	21UR-14872	TGAGTCCGTGGGTGGAATCTA	0	0	0	0	0	0	0	0
	21UR-14873	TGAGTCAAAAGTGAGTACGGT	0	0	0	0	0	0	0	0
	21UR-14874	TGAGGTACACACAAATTCATA	0	0	0	0	0	0	0	0
	21UR-14875	TGAGCTTGATGAAATTTCAAA	0	0	0	0	0	0	0	0
†	21UR-14876	TGAGAGCTGATCGATTTGAAT	0	0	0	0	0	0	0	0
	21UR-14877	TGAGAGATACCAGTAGCAAAT	0	0	0	0	0	0	0	0
	21UR-14878	TGAGACTGAATTTTTTTAAAA	0	0	0	0	0	0	0	0
	21UR-14879	TGAGAACTCACTCATCATAGC	0	0	0	0	0	0	0	0
†	21UR-14880	TGAGAACATCTGGTTTTAATA	0	0	0	0	0	0	0	0
	21UR-14881	TGAGAAACTGTTTGGGTCATC	0	0	0	0	0	0	0	0
	21UR-14882	TGAGAAAATAAAGTTTGAACG	0	0	0	0	0	0	0	0
†	21UR-14883	TGACTTCCTTTAAACTTCTTA	0	0	0	0	0	0	0	0
†	21UR-14884	TGACTCCTGTGACATTATTTC	0	0	0	0	0	0	0	0
	21UR-14885	TGACGCTGCCGATTCTCCAGT	0	0	0	0	0	0	0	0
†	21UR-14886	TGACCTTCAAGAACAATGCAA	0	1	0	0	3	1	0	5
	21UR-14887	TGACAAGCTGAACACAGGAAT	0	0	0	0	14	18	12	44
	21UR-14888	TGACAACATGGACTTGACACT	0	0	0	0	0	0	0	0
	21UR-14889	TGAATTTGAAAATGACAGAAA	0	0	0	0	1	0	0	1
†	21UR-14890	TGAATTGGCAAAAAAACACAT	0	0	0	0	2	3	1	6
	21UR-14891	TGAATTCATTAAACACCGGCG	0	0	0	0	0	1	0	1
†	21UR-14892	TGAATTCACTCTCAACAATGA	0	0	0	0	0	0	0	0
†	21UR-14893	TGAATTCACACAGTGAAGGGC	0	0	0	0	0	0	0	0
	21UR-14894	TGAATTATTAAAATCTTAAAT	0	0	0	0	0	0	0	0
†	21UR-14895	TGAATGTTGAAATGTATTTTT	2	0	0	1	1	4	1	9
†	21UR-14896	TGAATGTCGCTATGGGTGAAA	0	0	0	0	0	0	0	0
	21UR-14897	TGAATGCTTTCCAGCAAGAAT	0	0	0	0	0	0	0	0
	21UR-14898	TGAATGCAACAACAGGAGGAA	0	0	0	0	0	0	0	0
	21UR-14899	TGAATGAGCTTACAACATTTG	0	0	0	0	0	0	0	0
	21UR-14900	TGAATGACGTGGAGCGGCAGA	0	0	0	0	0	0	5	5
†	21UR-14901	TGAATCCGTTACTAATTTAAT	0	0	0	0	0	1	0	1
	21UR-14902	TGAATAATAAGCAGATAAACA	0	0	0	0	0	0	0	0
	21UR-14903	TGAAGTCTCATCGCACATTTG	0	0	0	0	0	0	0	0
†	21UR-14904	TGAAGGACTCCTAGATTTCAA	0	0	0	0	0	0	0	0
	21UR-14905	TGAAGCAGTGAGATTATGTGC	0	0	0	0	0	0	0	0
	21UR-14906	TGAAGATCCTCCATCAGCAAT	0	0	0	0	0	0	0	0

†	21UR-14907	TGAAGACTGTTGAACATATCA	0	0	0	0	0	0	0	0
†	21UR-14908	TGAACTTTTTGGAAACATTAG	0	0	0	0	0	0	0	0
	21UR-14909	TGAACTGGTGGTCCAGGTATT	0	0	0	0	0	0	0	0
	21UR-14910	TGAACTAGTGATTTTCAATAC	0	0	0	0	0	0	0	0
†	21UR-14911	TGAACCTTAGGAACATCAGTT	0	0	0	0	0	0	0	0
	21UR-14912	TGAACATTGGAGACCGATGCC	0	0	0	0	0	0	0	0
†	21UR-14913	TGAACACTATATGTTTTGAAA	0	0	0	0	0	0	0	0
	21UR-14914	TGAACAATTCATACACGTCAT	0	0	0	0	0	0	0	0
	21UR-14915	TGAACAATGGCCATCGATAAT	0	0	0	0	0	0	0	0
	21UR-14916	TGAACAAGCCGATAACGAAGC	0	0	0	0	0	0	1	1
†	21UR-14917	TGAACAACTTCAACGACGAAT	2	0	0	0	0	1	0	3
	21UR-14918	TGAACAAATGACTTAAAGCTG	0	0	0	0	0	0	0	0
	21UR-14919	TGAAATTGTCAATTTCCGCGG	0	0	0	0	0	0	0	0
	21UR-14920	TGAAATGCATAGTACTGTCCA	0	0	0	0	0	0	0	0
	21UR-14921	TGAAATAGTAGTTTTCTTTAG	0	0	0	0	0	0	0	0
	21UR-14922	TGAAAGGTGTGGAGATAAAGC	0	0	0	0	0	0	1	1
	21UR-14923	TGAAACCGGCTTTTACTCTAA	0	0	0	0	0	0	0	0
†	21UR-14924	TGAAACATTCACGGTTTTAAA	0	0	0	0	0	0	0	0
	21UR-14925	TGAAAATTGAATATTTCGAGC	0	0	0	0	0	0	0	0
	21UR-14926	TGAAAATAGAATGGATTTATG	0	0	0	0	0	0	0	0
	21UR-14927	TGAAAAGAAGCTGAAAATTTT	0	0	0	0	0	0	0	0
	21UR-14928	TGAAAAAAGTTAGAAAACATT	0	0	0	0	0	0	0	0
	21UR-14929	TCTTTTTGCCAGATATGGGTA	0	0	0	0	0	0	0	0
†	21UR-14930	TCTTTTCTGAAAGGTGTCCAA	0	0	0	0	1	0	0	1
†	21UR-14931	TCTTTTAATGTCACGCTGAAG	0	0	0	0	3	5	0	8
	21UR-14932	TCTTTCTCTGAGTCGAGAAAT	0	0	0	0	0	0	0	0
†	21UR-14933	TCTTTACGGGCTTACGAAGTT	0	0	0	0	0	0	0	0
	21UR-14934	TCTTTAAGCCTGGTATCTTTA	0	0	0	0	0	0	0	0
†	21UR-14935	TCTTGTTAGTGAAAAATGTGG	0	0	0	0	0	0	0	0
	21UR-14936	TCTTGTGTTGGATAATCATTA	0	0	0	0	0	0	2	2
†	21UR-14937	TCTTGGAGAAATGAACAAATC	0	0	0	0	0	0	0	0
†	21UR-14938	TCTTGCGTACTTTTTGAATGT	0	0	0	0	0	0	0	0
†	21UR-14939	TCTTGATTGTCAATTGTTTTC	0	0	0	0	0	0	0	0
	21UR-14940	TCTTGATTCGGTCCATGACAA	0	0	0	0	0	0	0	0
	21UR-14941	TCTTGAAACTTGCCTATAAGA	0	0	0	0	0	0	0	0
	21UR-14942	TCTTGAAAACATGTCGATTTA	0	0	0	0	0	0	0	0
†	21UR-14943	TCTTCTTGCGTGTCATGTAAA	0	0	0	0	0	0	0	0
	21UR-14944	TCTTCTATCTTGTAAACATGT	0	0	0	0	0	0	0	0
	21UR-14945	TCTTCCGGTTCCAGCCCACAA	0	0	0	0	0	0	0	0
	21UR-14946	TCTTCCAAGATTGTTGAAATT	0	0	0	0	3	0	2	5
	21UR-14947	TCTTCCAACGCCCCCCTCATT	0	0	0	0	0	0	0	0
	21UR-14948	TCTTCATGTGGGAGTTTTTGG	0	0	0	0	1	0	0	1
	21UR-14949	TCTTCAGGACAATATGGGCGG	0	0	0	0	0	0	0	0
	21UR-14950	TCTTCAAATATTACATGAAAT	0	0	0	0	0	0	0	0
	21UR-14951	TCTTAGTCCGAAATCTATTTT	0	0	0	0	0	0	0	0
	21UR-14952	TCTTAACTTGTTACAAAAACA	0	0	0	0	0	0	0	0
	21UR-14953	TCTGTTTGATTTGATTCTGGC	0	0	0	0	0	0	0	0
†	21UR-14954	TCTGTTGAAAACCGAGTTTCT	0	0	0	0	0	0	0	0
	21UR-14955	TCTGTTCCTCAGTCAGTCGGA	0	0	0	0	1	2	0	3
	21UR-14956	TCTGTTACTATTTACCACTCG	0	0	0	0	0	0	0	0
	21UR-14957	TCTGTGTGACAAAACGTTTAG	0	0	0	0	0	0	0	0
	21UR-14958	TCTGTCAAGAAACAACGCCAA	0	0	0	0	0	0	0	0
†	21UR-14959	TCTGTACTTGAAGCTACGACG	0	0	0	0	0	0	2	2
	21UR-14960	TCTGGTTGCTAGGAAAAATAC	0	0	0	0	0	0	0	0
	21UR-14961	TCTGGATCAGGAACTTCCAAT	0	0	0	0	0	0	1	1
†	21UR-14962	TCTGGATCAATGTTTCCTGTT	0	0	0	0	0	0	0	0
†	21UR-14963	TCTGGACAGAACGTTAGTTAC	0	0	0	0	1	1	0	2
†	21UR-14964	TCTGCTCGATAGAGATACATA	0	0	0	0	0	0	0	0
†	21UR-14965	TCTGCCAAAGCCAATTGGAAA	0	0	0	0	0	0	0	0
†	21UR-14966	TCTGCCAAAGAACACGTTGTT	0	0	0	0	2	4	0	6
†	21UR-14967	TCTGATAGTAACAAAACGGAG	0	1	0	0	3	5	0	9
†	21UR-14968	TCTGAGTTCTATATTGGGAAT	0	0	0	0	0	0	0	0
	21UR-14969	TCTGAGACTGAGTGATAAAAT	0	0	0	0	0	0	0	0
	21UR-14970	TCTGAATTTAGTAGCAAATGC	0	0	0	0	0	0	0	0

†	21UR-14971	TCTCTTTGCTTTTGAGTTGAA	0	0	0	0	0	0	0	0
†	21UR-14972	TCTCTTCAATAAACATTCGAC	0	0	0	0	0	0	0	0
	21UR-14973	TCTCTGGCTGGTGCACTCTTC	0	0	0	0	0	0	0	0
	21UR-14974	TCTCTGCGAATTTGACATTTT	0	0	0	0	0	0	0	0
	21UR-14975	TCTCGAACCCCGAAACAAGGC	0	0	0	0	0	0	0	0
	21UR-14976	TCTCACGGGAACGCAAAAAAA	0	0	0	0	0	0	0	0
	21UR-14977	TCTATTGGATACCTCCATTGT	0	0	0	0	0	0	0	0
	21UR-14978	TCTATTGATTGTTTTTCAAAA	0	0	0	0	0	0	0	0
†	21UR-14979	TCTATTCTATTGAGCGGTCTC	0	0	0	0	0	0	0	0
	21UR-14980	TCTATAATTCCTCAAAAATAC	0	0	0	0	0	0	0	0
	21UR-14981	TCTAGTGAAACGTAATTTTAA	0	0	0	0	0	0	0	0
	21UR-14982	TCTAGCTGTATCTGATACTTT	0	0	0	0	0	0	0	0
†	21UR-14983	TCTACGAGTTATGAAAGTTCT	0	0	0	0	0	0	0	0
	21UR-14984	TCTACCGTTTCAACAAACTGT	0	0	0	0	0	0	0	0
	21UR-14985	TCTAAGAGAAGTGAAAAATGA	0	0	0	0	0	0	0	0
	21UR-14986	TCTAACAAGTGAGTATTCCCC	0	0	0	0	0	0	0	0
	21UR-14987	TCTAAAATGAAGAGAAAACGG	0	0	0	0	0	0	0	0
	21UR-14988	TCTAAAAATTGTCATTTTCTT	0	0	0	0	0	0	0	0
	21UR-14989	TCTAAAAATCTAATGGATGTC	0	0	0	0	0	0	0	0
	21UR-14990	TCTAAAAACTGGGAAAAAAGT	0	0	0	0	0	0	0	0
	21UR-14991	TCTAAAAAAAGTTTGAATGTA	0	0	0	0	0	0	0	0
	21UR-14992	TCGTTTTTTGGAAGACTAGTT	0	1	0	0	1	0	0	2
†	21UR-14993	TCGTTTTCTAGACGACATCAA	0	0	0	0	0	0	0	0
†	21UR-14994	TCGTTGTCTCATGTCACAGAT	0	0	0	0	0	0	0	0
	21UR-14995	TCGTTGGAAAAGTTTATTTTT	0	0	0	0	0	0	0	0
†	21UR-14996	TCGTTGCTTTTGCTTCTTTGC	0	0	0	0	0	0	0	0
†	21UR-14997	TCGTTGCATTTCCTTCTAGGT	0	0	0	0	0	0	0	0
†	21UR-14998	TCGTTGAAGCTCTGACTGTCA	0	0	0	0	0	0	0	0
	21UR-14999	TCGTTCCCGAAAAAAACACAA	0	0	0	0	0	0	0	0
	21UR-15000	TCGTTCACTCTTGTGTTCCTT	0	0	0	0	0	0	0	0
	21UR-15001	TCGTTATGATGGAAAGTGGAA	0	0	0	0	0	0	0	0
†	21UR-15002	TCGTTATAAATTGTGAAAAAT	0	0	0	0	1	0	0	1
†	21UR-15003	TCGTGGCGTGTGAAAGCTATT	0	0	0	0	0	0	0	0
	21UR-15004	TCGTCTCGAATAGGTTTCGCA	0	0	0	0	0	0	0	0
	21UR-15005	TCGGTGTGCCAGTTGAAAGCC	0	0	0	0	0	0	0	0
	21UR-15006	TCGGTACGATATAATGTGAGG	0	0	0	0	0	0	0	0
†	21UR-15007	TCGGTAAACATGCTGTAGAAA	0	0	0	0	0	0	0	0
	21UR-15008	TCGGGCTATGATAGCACCTTA	0	0	0	0	0	0	0	0
*	21UR-15009	TCGGGAAGAAATTATCGGCCA	1	0	0	0	0	1	0	2
	21UR-15010	TCGGGAAAACTTTCTTGGTTT	0	0	0	0	0	0	0	0
	21UR-15011	TCGGATAGCGTATGATTGACG	0	0	0	0	0	0	0	0
†	21UR-15012	TCGGAGAACTTAATTTTGGTC	0	0	0	0	0	0	0	0
	21UR-15013	TCGGAATTTGAGGACGATGTG	0	0	0	0	0	0	0	0
	21UR-15014	TCGCTTCACCACATCGTAACT	0	0	0	0	0	0	0	0
†	21UR-15015	TCGCGTCTGTCGATATTCCAC	0	0	0	0	0	0	0	0
	21UR-15016	TCGCGACCAATTTGATGGATG	0	0	0	0	0	0	0	0
	21UR-15017	TCGATTCTATACCAAATGGGA	0	0	0	0	0	0	0	0
†	21UR-15018	TCGATTCGTCGTGTGTAGTTT	0	0	0	0	0	0	0	0
†	21UR-15019	TCGATTCAGCTGATGATAAGA	0	0	0	0	0	0	0	0
	21UR-15020	TCGATTATTTATTAGAAAATA	0	0	0	0	0	0	0	0
†	21UR-15021	TCGATTATCCTTCTGTATTAC	0	0	0	0	0	0	0	0
	21UR-15022	TCGATGTTTTTTCTGACTTTT	0	0	0	0	0	0	0	0
	21UR-15023	TCGATCAGAAACGGTTGAGCG	0	0	0	0	0	0	0	0
	21UR-15024	TCGAGTGTATAACAAAAGAGA	0	0	0	0	0	0	0	0
†	21UR-15025	TCGAGTAACCATTGGATACGT	0	0	0	0	11	7	3	21
	21UR-15026	TCGACTTTCAAATGAAATCAA	0	0	0	0	0	0	0	0
†	21UR-15027	TCGACTGTTACATCTACGGAT	0	0	0	0	2	2	3	7
†	21UR-15028	TCGACTACTATCCTATTTTCA	0	0	0	0	0	0	0	0
†	21UR-15029	TCGACCTGCAATGACAAATCC	0	0	0	0	0	0	0	0
	21UR-15030	TCGAATTTCCTTTTTTTGTCT	0	0	0	0	0	0	0	0
	21UR-15031	TCGAAGTGACTACAGTTTAAC	0	0	0	0	0	0	0	0
†	21UR-15032	TCCTTTTTTGCTTTCGTGGAA	0	0	0	0	2	1	0	3
	21UR-15033	TCCTTGTGGATTCATGGAAAA	0	0	0	0	0	1	0	1
†	21UR-15034	TCCTTGTGATTTGGTTTTTCA	0	0	0	0	0	0	0	0

	21UR-15035	TCCTTGTAGACAGAAAAATGA	0	0	0	0	0	0	0	0
	21UR-15036	TCCTTGACATTTAGAAATATC	0	0	0	0	0	0	0	0
	21UR-15037	TCCTTCCTGAGATATTGTGAA	0	0	0	0	0	0	0	0
†	21UR-15038	TCCTGTGACATTATTTCATTT	0	0	0	0	0	0	0	0
†	21UR-15039	TCCTCTGTTTTCCATCTGTTT	0	0	0	0	0	0	0	0
†	21UR-15040	TCCTAGTTCCTATTTTTGCGA	0	0	0	0	0	0	0	0
†	21UR-15041	TCCTACCTTTAAAGTGTTTTT	0	0	0	0	0	0	0	0
†	21UR-15042	TCCGTTTTGTATTCTTACCTG	0	0	0	0	0	0	0	0
†	21UR-15043	TCCGTTTGGCAATAGATTGTT	0	0	0	0	0	1	1	2
†	21UR-15044	TCCGTTTCTGTATAACATATT	0	0	0	0	0	0	0	0
†	21UR-15045	TCCGTAATTTCCTTGTTGTTG	0	0	0	0	0	0	0	0
	21UR-15046	TCCGGAAAACTAAGTTTACTG	0	0	0	0	0	0	0	0
	21UR-15047	TCCGCCATAAAATTGATGTAA	0	0	0	0	0	0	0	0
	21UR-15048	TCCGCATCTCCATCAGGAAGT	0	0	0	0	0	0	0	0
	21UR-15049	TCCGATCGCAGAGCACAAGAC	0	0	0	0	0	0	0	0
†	21UR-15050	TCCGAGTCGAAATGTTTTCCA	0	0	0	0	0	0	0	0
	21UR-15051	TCCGAACAAAAACGACAGCGA	0	0	0	0	0	0	0	0
	21UR-15052	TCCCTGTTCAAAATTCTTATC	0	0	0	0	0	0	0	0
†	21UR-15053	TCCCGTTGTTTATATTGTAAT	0	0	0	0	0	0	0	0
	21UR-15054	TCCCGATTGTGATTTGAATGG	0	0	0	0	0	0	0	0
	21UR-15055	TCCCATAGCAACCTGCCAAGC	0	0	0	0	0	0	0	0
	21UR-15056	TCCCAGTGGATCTGTTTATAT	0	0	0	0	0	1	0	1
	21UR-15057	TCCCAGAAAAATGTTGAGTTT	0	0	0	0	0	0	0	0
	21UR-15058	TCCATTTTCTTAAACGAAAAA	0	0	0	0	0	0	0	0
	21UR-15059	TCCATTTAATTTCTCATCGAC	0	0	0	0	0	0	0	0
	21UR-15060	TCCATTGATGAAGGCGACGTC	0	0	0	0	0	0	0	0
	21UR-15061	TCCATACGAACCTACACTCAA	0	0	0	0	0	0	0	0
	21UR-15062	TCCAGTTTTGGCTCTTGTTCC	0	0	0	0	0	0	0	0
	21UR-15063	TCCAGATGATCTATGTCCCGT	0	0	0	0	0	0	0	0
†	21UR-15064	TCCAGAAGAAGAAGTACTAAC	0	0	0	0	1	0	0	1
	21UR-15065	TCCAGAAACAAGAAACCAGAA	0	0	0	0	0	0	0	0
†	21UR-15066	TCCACGTATGGTATTTCAATT	0	0	0	0	0	0	0	0
	21UR-15067	TCCAATGGGTCGTTGTGAACA	0	0	0	0	0	0	1	1
	21UR-15068	TCCAATCGATTTGAGTGGGAG	0	0	0	0	0	0	0	0
	21UR-15069	TCCAATATCATATAAGGAGTG	0	1	0	0	0	0	0	1
	21UR-15070	TCATTGTTTTCCGAGAGGTTA	0	0	0	0	0	0	0	0
	21UR-15071	TCATTGCTTTCAATTATATCA	0	0	0	0	0	0	0	0
†	21UR-15072	TCATTCGCCCCCTCATGGATT	0	0	0	0	0	0	0	0
	21UR-15073	TCATTCATTGATTCGTTGGCG	0	0	0	0	0	1	2	3
	21UR-15074	TCATTAAATTTGACGGTAATA	0	0	0	0	0	0	0	0
†	21UR-15075	TCATTAAAAATCTGAAGAAAC	0	0	0	0	0	0	0	0
†	21UR-15076	TCATGTCTTAATTGCGTGGCA	0	0	0	0	0	0	0	0
†	21UR-15077	TCATGGACATCGAGGAATTTA	0	0	0	0	0	0	0	0
	21UR-15078	TCATGCACAGTAAACTTTACA	0	0	0	0	0	0	0	0
	21UR-15079	TCATGAGAAATCTCACATGGA	0	0	0	0	0	0	0	0
	21UR-15080	TCATGAATTCGAAAATGTTCG	0	0	0	0	0	0	0	0
	21UR-15081	TCATCTTTTGTAGACTGAAAA	0	0	0	0	0	0	0	0
	21UR-15082	TCATCTTCTGACTTAGTTGGA	0	0	0	0	0	0	0	0
†	21UR-15083	TCATCTCTGGGCCAAACTTTC	0	0	0	0	1	0	0	1
	21UR-15084	TCATCCAATTCGTTAGAAAAT	0	0	0	0	0	0	0	0
	21UR-15085	TCATACTCAGTGCTCTTTCGA	0	0	0	0	0	0	0	0
†	21UR-15086	TCATACATTGCAATTACCAGC	0	0	0	0	0	0	0	0
†	21UR-15087	TCATAACGTTTTGATTTCATC	0	0	0	0	0	0	1	1
	21UR-15088	TCATAAACTTCATAAACTTGT	0	0	0	0	1	1	0	2
	21UR-15089	TCAGTTCTATGCAGAGAGGGC	0	0	0	0	0	0	0	0
	21UR-15090	TCAGTTCGACTTCAGGAAATT	0	0	0	0	0	0	0	0
†	21UR-15091	TCAGTTAGCGAGTAAGTGTGC	0	0	0	0	0	0	0	0
†	21UR-15092	TCAGTCGAATGAATTTCAATA	0	0	0	0	0	0	0	0
	21UR-15093	TCAGGACAAAAAAATTTAGAA	0	0	0	0	0	0	0	0
†	21UR-15094	TCAGGAACGTTACTCATTAGA	0	0	0	3	0	1	0	4
	21UR-15095	TCAGCTTTTTCCACTTAGAAA	0	0	0	0	0	0	0	0
†	21UR-15096	TCAGCGATCGAAAGACAACAA	2	1	1	1	9	5	1	20
	21UR-15097	TCAGCCAGAAAGTTCAATGGA	0	0	0	0	0	0	0	0
	21UR-15098	TCAGCATCAACTGATCTGACA	0	0	0	0	0	0	0	0

	21UR-15099	TCAGCATACCAAACAACTCGT	0	0	0	0	0	0	0	0
†	21UR-15100	TCAGATAAGTAGTTCCTTACC	0	0	0	0	0	0	0	0
	21UR-15101	TCAGAGGCTCCAGCAGACTAC	0	0	0	0	0	0	0	0
	21UR-15102	TCAGAGGAAATGGAACACGAC	0	0	0	0	0	0	0	0
	21UR-15103	TCAGACTACCAAGTAGGCAGT	0	0	0	0	0	0	0	0
	21UR-15104	TCAGACGAGCGGAAAACTTAG	0	0	0	0	0	0	0	0
†	21UR-15105	TCAGACGAGCAAAAAACAAAA	0	0	0	0	0	1	0	1
	21UR-15106	TCAGAATTTTTGCCGCTGGAG	0	0	0	0	0	1	0	1
	21UR-15107	TCAGAACTTCAAACTCGTTAG	0	0	0	0	0	0	0	0
	21UR-15108	TCAGAAACTGTTGGATTTTCA	0	0	0	0	0	0	0	0
	21UR-15109	TCACTTTTTCTTGCGTAGCCA	0	0	0	0	0	0	0	0
	21UR-15110	TCACTTGAATACCCCCGAAAG	0	0	0	0	0	0	0	0
†	21UR-15111	TCACTGACTAAAAAATGTAGG	0	0	0	0	0	0	0	0
†	21UR-15112	TCACGGTATTTATTTTGTATG	0	0	0	0	0	0	0	0
	21UR-15113	TCACGGAATTGCACGGAATTT	0	0	0	0	0	0	1	1
†	21UR-15114	TCACGACTTGAAGGACTCGAC	0	0	0	0	0	0	0	0
	21UR-15115	TCACGAAGAAAACCTTAACAA	0	0	0	0	0	0	0	0
	21UR-15116	TCACCGATCATCCAGATTCAG	0	0	0	0	0	0	0	0
†	21UR-15117	TCACCACCCATCATCATAGTT	0	0	0	0	0	0	0	0
	21UR-15118	TCACAATTGCTTTATTTTGAC	0	0	0	0	0	0	0	0
†	21UR-15119	TCACAAAAAAGAGATTTGAAT	0	0	0	2	6	6	1	15
	21UR-15120	TCAATTTTTCAGTTTCGTGCT	0	0	0	0	0	0	0	0
	21UR-15121	TCAATTTGCGTTTTTCTTGAT	0	0	0	0	0	0	0	0
	21UR-15122	TCAATTGGTCCCAAAATGACT	0	0	0	0	0	0	0	0
†	21UR-15123	TCAATTCTCATCGATTTTCAA	0	0	0	0	0	0	0	0
†	21UR-15124	TCAATTCTCAGAATTCCAGAT	0	0	0	0	0	0	0	0
†	21UR-15125	TCAATTAGTAAACTGGTAGAC	0	0	0	0	0	0	0	0
†	21UR-15126	TCAATGTTTCAATGTTGGAAC	0	0	0	0	0	0	0	0
	21UR-15127	TCAATGCTCGAAATTTCTCAA	0	0	0	0	0	0	0	0
†	21UR-15128	TCAATCTTTCTTTGGCCTTGG	0	0	0	0	0	0	0	0
	21UR-15129	TCAATCTATGCTCATCATGCC	0	0	0	0	0	0	0	0
†	21UR-15130	TCAATCGATGTCGTTCTTATG	0	0	0	0	1	0	1	2
†	21UR-15131	TCAATAGCAACACGGGTAATG	0	0	0	0	0	0	0	0
†	21UR-15132	TCAATACATGCTCTTCTCATT	0	0	0	0	0	0	0	0
	21UR-15133	TCAATACACTCAATTCACATT	0	0	0	0	0	0	0	0
†	21UR-15134	TCAATAAACTCTTTAGAACTT	0	0	0	0	0	0	0	0
†	21UR-15135	TCAAGTAGTATGAAATTAAAT	1	0	0	1	0	5	0	7
	21UR-15136	TCAAGCACGGCATGATTTATA	0	0	0	0	0	0	0	0
†	21UR-15137	TCAAGATCTGAGAGTTCTAAT	14	6	10	5	15	26	7	83
	21UR-15138	TCAAGACTAAAACCTTCATGG	0	0	0	0	0	0	0	0
	21UR-15139	TCAAGAACGTTATGCTTTACA	0	0	0	0	0	0	0	0
	21UR-15140	TCAACTGAATCATAACCTGCA	0	0	0	0	1	0	0	1
	21UR-15141	TCAAATAAAGAAATCTAGGCA	1	0	0	2	3	8	0	14
†	21UR-15142	TCAAAGATATTGTAAGGATGA	1	0	0	0	2	0	0	3
	21UR-15143	TCAAACTAAATAAGGCTTGGA	0	0	0	0	0	0	0	0
	21UR-15144	TCAAACCCCCCACAACTGACA	0	0	0	0	0	0	0	0
	21UR-15145	TCAAACACCCAAAGAAGGAAC	0	0	0	0	0	0	0	0
	21UR-15146	TCAAAATGTGTGTGACACTGC	0	0	0	0	0	0	0	0
	21UR-15147	TCAAAATCTGATCAAGTGCTA	0	0	0	0	0	0	0	0
	21UR-15148	TCAAAATCAAAGATATGCGGC	0	0	0	0	0	0	0	0
	21UR-15149	TCAAAAGTTTGGAAAACTGTA	0	0	0	0	0	0	0	0
	21UR-15150	TCAAAAGTCGTGCCTGCAGCA	0	0	0	0	0	0	0	0
	21UR-15151	TCAAAAAGACGTTTACCATCT	0	0	0	0	0	0	0	0
	21UR-15152	TCAAAAACAGACTTGCTGAAT	0	0	0	0	0	3	0	3
	21UR-15153	TCAAAAAAAAAACGTTTTGCC	0	0	0	0	0	0	0	0
	21UR-15154	TATTTTTGATTCATCTAAATC	0	0	0	0	0	0	0	0
†	21UR-15155	TATTTTTACTTTATTGTGCAA	0	0	0	0	0	0	0	0
†	21UR-15156	TATTTTTACATCAAGAGTTAA	0	0	0	0	0	0	0	0
†	21UR-15157	TATTTTGTAGATGGATACACA	0	0	0	0	2	2	0	4
†	21UR-15158	TATTTTGATCTGCATTCAATC	0	0	0	0	0	0	0	0
	21UR-15159	TATTTTCTCTATTTTAAAGTA	0	0	0	0	0	0	0	0
	21UR-15160	TATTTTCTATTCATTATGAAG	0	0	0	0	2	0	0	2
	21UR-15161	TATTTTCGGCAATCGTTGACA	0	0	0	0	0	0	0	0
	21UR-15162	TATTTTAGATCAGTAAAAAAT	0	0	0	0	0	0	0	0

†	21UR-15163	TATTTTAATGCAAATGGTTGT	0	0	0	0	0	0	0	0
	21UR-15164	TATTTTAATAGAAATCTGAAG	0	0	0	0	0	0	0	0
†	21UR-15165	TATTTTAAGGCACGTAAAGAA	0	0	0	0	0	0	0	0
†	21UR-15166	TATTTTAACTCAGTCTAACTC	0	0	0	0	0	0	0	0
	21UR-15167	TATTTGCTCGAATGTTTCCTA	0	0	0	0	0	0	0	0
	21UR-15168	TATTTGCCTAACGCAAGATGG	0	0	0	0	0	0	0	0
	21UR-15169	TATTTGCATTAAAAAAAAACC	1	0	0	0	3	4	0	8
	21UR-15170	TATTTGAAGTTTTTTTGTCGA	0	0	0	0	0	0	0	0
†	21UR-15171	TATTTGAAGACTTATTGAAAA	0	0	0	0	1	1	0	2
†	21UR-15172	TATTTGAACATATACCTCATC	0	0	0	0	1	0	0	1
	21UR-15173	TATTTCTCCCTTTTTATGTAG	0	0	0	1	0	1	0	2
	21UR-15174	TATTTCATAGTCCGTCTATTA	1	0	0	0	0	0	0	1
†	21UR-15175	TATTTATTATGCCGTTGTCCG	0	0	0	0	0	0	0	0
	21UR-15176	TATTTAGTTTTCGTAAAATTA	0	0	0	0	0	0	0	0
†	21UR-15177	TATTTAGAGAGATCTCGTTGT	4	2	3	5	82	60	24	180
†	21UR-15178	TATTTACGATAAATTTAAAAC	0	0	0	0	0	0	0	0
†	21UR-15179	TATTTAATAATGGACGCAGTT	0	0	0	1	6	5	7	19
	21UR-15180	TATTTAAAATGAATACAGTGT	0	0	0	0	0	0	0	0
	21UR-15181	TATTGTTTTTCGTGATCTAGT	0	0	0	0	0	0	0	0
	21UR-15182	TATTGTTGAAAAATGCAATGA	0	0	0	0	0	0	0	0
†	21UR-15183	TATTGTTCTTCAAGGATAAAA	0	0	0	0	0	0	0	0
	21UR-15184	TATTGTCAATTCAGGTAATTC	0	0	0	0	0	0	0	0
†	21UR-15185	TATTGGATTCTACTATATGGT	0	0	0	0	1	0	0	1
†	21UR-15186	TATTGCTTTGGTGGAACTTAA	0	0	0	0	0	0	0	0
	21UR-15187	TATTGCTTCTTTTAGATGCTG	0	0	0	0	0	1	0	1
†	21UR-15188	TATTGCACCGTTTTAGGGTTA	0	0	0	0	0	0	0	0
†	21UR-15189	TATTGATTTCAATCTTATTGG	0	0	0	0	0	0	0	0
†	21UR-15190	TATTGAGTCTTTGGTATTTTT	0	0	0	0	0	0	0	0
†	21UR-15191	TATTGAAGATGCCTTTTATGT	2	0	0	0	1	1	0	4
†	21UR-15192	TATTGAACACAATTTTTGCAA	0	0	0	0	0	0	0	0
†	21UR-15193	TATTCTGTGCGTGGTTTTCAA	0	0	0	0	0	0	0	0
	21UR-15194	TATTCGTATTAGTATTCGTAT	0	0	0	0	0	0	1	1
	21UR-15195	TATTCGCAACCGTTTTTTCTC	0	0	0	0	0	0	0	0
†	21UR-15196	TATTCGATACACTGTTTCAAA	0	0	0	0	0	0	0	0
†	21UR-15197	TATTCCTGCGAATTTCCGGTT	0	0	0	0	0	0	0	0
†	21UR-15198	TATTCCGATTTCTTTTCATCA	1	0	0	0	0	0	0	1
†	21UR-15199	TATTCCATAATTATGTAGTTT	0	0	0	0	0	0	0	0
*	21UR-15200	TATTCCAATGGGTCGTTGTGA	0	0	0	0	1	0	1	2
†	21UR-15201	TATTCACGAGCTTTTTATTAA	0	0	0	0	0	0	0	0
†	21UR-15202	TATTCACATCTCTCTCAACCT	0	0	0	0	0	0	0	0
†	21UR-15203	TATTCAAGGTTTCCAACATAT	0	0	0	0	2	0	0	2
†	21UR-15204	TATTATTTTTGTCGAACTTCT	2	0	2	1	7	8	3	23
†	21UR-15205	TATTATTTCGCGCCATCTAGG	0	0	0	0	0	0	0	0
†	21UR-15206	TATTATTTAATCGATATCCAT	0	0	0	0	0	0	0	0
†	21UR-15207	TATTATTTAACTCAAATACTT	0	0	0	0	5	2	0	7
	21UR-15208	TATTATTCATAATGATAAAAA	0	0	0	0	0	0	0	0
	21UR-15209	TATTATGTGAGAACTCGTATC	0	0	0	0	0	0	0	0
†	21UR-15210	TATTATGTCACGAGATTGGTT	0	0	0	0	0	0	3	3
†	21UR-15211	TATTATGCCATTTTCGATTTC	0	0	0	0	0	0	0	0
†	21UR-15212	TATTATGACAAGGGGAGAAAA	0	1	0	0	0	0	0	1
	21UR-15213	TATTATCTTTAACGTGAAACA	0	0	0	0	0	0	0	0
†	21UR-15214	TATTATAGTATGTTCCAGAGG	0	0	0	0	0	0	0	0
†	21UR-15215	TATTATAGGCGGCCTCTTAAT	0	0	0	0	0	1	0	1
†	21UR-15216	TATTATAATCACTGTATCTTT	0	0	0	0	0	0	0	0
†	21UR-15217	TATTATAAGAACTCATAAAAA	0	0	0	0	0	0	0	0
	21UR-15218	TATTAGTTATCTACACTTTCA	0	0	0	0	1	0	1	2
†	21UR-15219	TATTAGGTAGTGAATTTCACT	0	0	0	0	0	0	0	0
†	21UR-15220	TATTAGAAACTCTATCTCAGA	0	0	0	0	0	0	0	0
†	21UR-15221	TATTACTTGGGGTTCAGCATT	0	0	0	0	1	1	0	2
†	21UR-15222	TATTACTGTGTGTCCGTTAGG	1	0	0	0	0	0	1	2
†	21UR-15223	TATTACTCGGATGAAATTGAA	0	0	0	0	0	0	0	0
†	21UR-15224	TATTACTCCTGTCCTCTCCAA	0	0	0	0	0	0	0	0
	21UR-15225	TATTACGTTTTTGAAATTGGT	0	0	0	0	0	0	1	1
†	21UR-15226	TATTACGTCTTGGTCCTCAAG	0	0	0	0	0	0	0	0

†	21UR-15227	TATTACGTCGGTTGATTGAAT	0	0	0	0	0	0	0	0
†	21UR-15228	TATTACCAGAAATCTACAAAC	0	0	0	0	0	0	0	0
	21UR-15229	TATTACATGCTTTCTGACTGC	0	0	0	0	0	0	0	0
†	21UR-15230	TATTACACATAACAGTAATAA	0	0	0	0	0	0	0	0
	21UR-15231	TATTACAATAAAATTTTAAAA	0	0	0	0	0	0	0	0
†	21UR-15232	TATTACAAGTTTGATTTTGTA	0	0	0	0	0	0	0	0
†	21UR-15233	TATTAATTTTACGAAACGGTG	0	0	0	0	0	0	0	0
†	21UR-15234	TATTAATTTCAGGATTTGAAT	0	0	0	0	0	0	0	0
†	21UR-15235	TATTAATAGCAGAGATAGAGT	0	0	0	0	0	0	1	1
†	21UR-15236	TATTAAGTTTCCAATATGAGA	0	0	0	0	0	0	0	0
†	21UR-15237	TATTAAGAATGTAAAACCATG	0	1	0	0	0	0	0	1
	21UR-15238	TATTAAATGCTCAGAAAACCT	0	0	0	0	0	0	0	0
†	21UR-15239	TATTAAATCACACAGAGTAAT	0	0	0	0	0	0	0	0
†	21UR-15240	TATTAAACTGCCTACCTCAGA	0	0	0	0	0	0	0	0
†	21UR-15241	TATGTATCTAGCAGCCGTGGC	0	0	0	0	0	0	0	0
	21UR-15242	TATGTACTACTACTCACGGTG	0	0	0	0	0	0	0	0
	21UR-15243	TATGTAAGGGATCGCCCAGAC	0	0	0	0	0	0	0	0
	21UR-15244	TATGGTTTTGAAATCTAAAGA	0	0	0	0	0	0	0	0
	21UR-15245	TATGGTGTTAACGAAATGTGG	0	0	0	0	0	0	0	0
	21UR-15246	TATGGGTTTCTGATAGTTGAT	0	0	0	0	0	0	0	0
	21UR-15247	TATGGCCTACATGTATTGGTT	0	0	0	0	0	0	0	0
	21UR-15248	TATGCTATTGAAAAAGTAGGA	0	0	0	0	0	0	0	0
	21UR-15249	TATGCTATGCTAGTAGTATGT	0	0	0	0	0	0	0	0
†	21UR-15250	TATGCGTCCCTAAAAACTGAT	0	0	0	0	1	3	0	4
	21UR-15251	TATGCGCAGAACAAGGAAAAA	0	0	0	0	0	0	0	0
	21UR-15252	TATGATTCGTGTTCAAAAAAT	0	0	0	0	0	0	0	0
†	21UR-15253	TATGATGTTTAAAATTGGTGA	0	0	0	0	0	0	0	0
†	21UR-15254	TATGACGATTAAAAAAGAAGT	0	0	0	0	0	0	0	0
†	21UR-15255	TATGACATTCCTTGTAGAAAA	0	2	0	1	2	4	0	9
	21UR-15256	TATGAACAACTTGAAACAGTT	0	0	0	0	0	0	0	0
	21UR-15257	TATGAAATGCTGAGAGACTGT	0	0	0	0	0	1	0	1
	21UR-15258	TATGAAACAGGAAACATTTCT	0	0	0	0	0	0	0	0
	21UR-15259	TATCTTCCTTCAACCCGTTTT	0	0	0	0	0	0	0	0
	21UR-15260	TATCTGTGAAGACAAAACAAA	0	0	0	0	0	0	0	0
	21UR-15261	TATCTATCTGTCTGTATTTGC	0	0	0	0	0	0	0	0
	21UR-15262	TATCTATAACATTCTCCGTGT	0	0	0	0	0	0	1	1
†	21UR-15263	TATCGGTTCTCGGTGATTGTG	0	0	0	0	0	0	0	0
	21UR-15264	TATCGCGGTGCCGAGATGAAG	0	0	0	0	0	0	0	0
	21UR-15265	TATCGAAATTCCTTGATTCAA	0	0	0	0	0	0	0	0
	21UR-15266	TATCCTTGGCGTCGGGCCTAT	0	0	0	0	0	0	0	0
	21UR-15267	TATCCTCGAGCCATCATTGAC	0	0	0	0	0	0	0	0
	21UR-15268	TATCCGAATCTCGGTGAGGTT	0	0	0	0	0	0	0	0
	21UR-15269	TATCATGTAAATCTAAACTCA	0	0	0	0	0	0	0	0
	21UR-15270	TATCAGAACGAACCTTCATAG	0	0	0	0	0	0	0	0
	21UR-15271	TATCACAAAGTTGGATCAGTG	0	0	0	0	0	0	0	0
	21UR-15272	TATCAACTATTTCAAGTAAAA	0	0	0	0	0	0	0	0
†	21UR-15273	TATCAAATGTTTCAAATCGCT	0	0	0	0	0	0	0	0
	21UR-15274	TATCAAAACACAAAACGTATA	0	0	0	0	0	0	0	0
	21UR-15275	TATATTTTAAACTTCTAGGCT	1	0	0	0	0	1	0	2
†	21UR-15276	TATATTTGAAAAACCGATTCT	0	0	0	0	0	0	0	0
†	21UR-15277	TATATTGACCTAGTAAATAGT	0	0	0	0	0	0	0	0
	21UR-15278	TATATGTAGCTTGAATGCAGT	0	1	1	0	0	2	0	4
	21UR-15279	TATATCTCTAACTGACGATAT	0	0	0	0	0	0	0	0
	21UR-15280	TATACTTAACTTTTATTTAAA	0	0	0	0	4	2	1	7
	21UR-15281	TATACATACTTTGGTTAAATA	0	0	0	0	0	0	1	1
	21UR-15282	TATACACAATTGATTTTCACA	0	0	0	0	0	0	0	0
	21UR-15283	TATAATTTTGAAAACTCGAAA	0	0	0	0	0	0	0	0
	21UR-15284	TATAATTGAAAAGTTCTTAAC	0	0	0	0	0	1	0	1
	21UR-15285	TATAATCTTTATTTTTATCGC	0	0	0	0	0	0	0	0
	21UR-15286	TATAATCATCATTTCAATTTT	1	0	0	0	0	0	0	1
	21UR-15287	TATAATCAATTTTGTCTAATT	0	0	0	0	1	0	1	2
	21UR-15288	TATAATCAAAAAAAAAACAGT	0	0	0	0	0	0	0	0
†	21UR-15289	TATAAGATTTCGGTGCTGTTA	0	0	0	0	0	0	0	0
	21UR-15290	TATAAGATCTTGCGAATCACC	0	0	0	0	0	0	0	0

		21UR-15291	TATAACACCAATTGCTTCAAA	0	0	0	0	0	0	0	0
		21UR-15292	TATAAAACTCATCTTCATGAA	0	0	0	0	0	0	0	0
		21UR-15293	TATAAAACCACTGAATATATT	0	0	0	0	0	0	0	0
		21UR-15294	TATAAAAAATGTCTGGAATTA	0	0	0	0	0	0	0	0
		21UR-15295	TAGTTTTCACTCTCCTTTGAA	0	0	0	0	0	0	0	0
	†	21UR-15296	TAGTTTGTCCTATGGAATCGC	0	0	0	0	0	0	0	0
	†	21UR-15297	TAGTTTCGAAATCTTCTTTTT	0	0	0	0	0	0	0	0
		21UR-15298	TAGTTTCAGCAACTTCACTAT	0	0	0	0	0	0	0	0
	†	21UR-15299	TAGTTTAGTTCTGTACGCACA	0	0	0	0	0	0	0	0
	†	21UR-15300	TAGTTGAAGTTATGGCTAGTT	8	14	6	4	24	286	5	347
		21UR-15301	TAGTTCTTTATTTCGAAACTG	0	0	0	0	0	0	0	0
		21UR-15302	TAGTTCTTCGAAACACGATAA	0	0	0	0	0	0	0	0
	†	21UR-15303	TAGTTCTATATCGAATGACGT	0	0	0	0	0	0	0	0
	†	21UR-15304	TAGTTAGCTTTGCTCTTGCTA	0	0	0	0	0	0	0	0
	†	21UR-15305	TAGTTACAATCGAGTATATAG	0	0	0	0	0	0	0	0
	†	21UR-15306	TAGTTACAAAGCTGATTTCGA	0	0	0	0	0	2	0	2
	†	21UR-15307	TAGTTAATAGAGGATTTTTGA	0	0	0	0	2	1	0	3
	†	21UR-15308	TAGTGTTGTTGTCGAAAGTGT	0	0	0	0	0	0	1	1
	†	21UR-15309	TAGTGCTCCTCGGCTATTCCA	0	0	0	0	0	0	0	0
		21UR-15310	TAGTGCCGCATTTTAATTTTT	0	0	0	0	0	0	0	0
	†	21UR-15311	TAGTGACACATCGGTTTAATG	0	0	0	0	0	0	0	0
		21UR-15312	TAGTGACAATTTCTCCACCGA	0	0	0	0	0	0	0	0
	†	21UR-15313	TAGTGAAAAATGTGGAAAGTT	0	0	0	0	0	0	0	0
		21UR-15314	TAGTCTCGTGTGGCTAATATT	0	0	0	0	0	0	0	0
		21UR-15315	TAGTCGTACAGTCCTACAGTA	0	0	0	0	0	0	0	0
	†	21UR-15316	TAGTCGATTAATTCTACAAGT	0	0	0	0	5	4	0	9
		21UR-15317	TAGTCGACGAATTGAGAAAAG	0	0	0	0	0	0	0	0
	†	21UR-15318	TAGTCCGTTTTTTCGTCAACA	0	1	0	0	0	0	0	1
		21UR-15319	TAGTATTTTTCCTCTTGTTTC	0	0	0	0	0	0	0	0
	†	21UR-15320	TAGTATTCCTCTGTCTCGACT	0	0	0	0	0	0	0	0
		21UR-15321	TAGTATCTTTTTTCAATATTT	0	0	0	0	0	0	0	0
		21UR-15322	TAGTAGATAAGATTCATGAGA	0	0	0	0	0	1	0	1
		21UR-15323	TAGTACATAATTAGATGGGTA	0	0	0	0	0	0	0	0
		21UR-15324	TAGTAAGTTGTGGTAAATATT	0	0	0	0	0	0	0	0
	†	21UR-15325	TAGTAAGCTCTCGGAATTTTC	0	0	0	0	2	0	0	2
	†	21UR-15326	TAGTAACTTCCGCATTTGTGA	0	0	0	0	0	0	0	0
		21UR-15327	TAGTAAACGAGCGAAAGTAAA	0	0	0	0	0	0	0	0
		21UR-15328	TAGTAAACAAGGGGAAAAGTA	0	0	0	0	0	0	0	0
		21UR-15329	TAGGTACTGAACTTGTCCGAA	0	0	0	0	0	1	0	1
		21UR-15330	TAGGATTTTCTACTTGCCAAA	0	0	0	0	0	0	0	0
	†	21UR-15331	TAGGAAGACATAAATAATTGT	5	7	0	5	24	27	2	70
		21UR-15332	TAGGAAAAGAATTTTATTACT	0	0	0	0	0	0	0	0
	†	21UR-15333	TAGCTCTTCCGAGCGAGTTTT	0	0	0	0	0	0	0	0
	†	21UR-15334	TAGCATCCGAAGAAAATTTAA	0	0	0	0	1	0	0	1
	†	21UR-15335	TAGCACTTTCGGAAAACAAAA	0	0	0	0	1	0	0	1
		21UR-15336	TAGATTTCTTGAACTTGAACT	0	0	0	0	0	0	0	0
		21UR-15337	TAGATTTCATGTAAAAATGAA	0	0	0	0	0	0	0	0
	†	21UR-15338	TAGATTGTTGAAATAGTACTT	1	0	1	0	0	1	1	4
		21UR-15339	TAGATTAAAAACCTCGGTCAC	0	0	0	0	0	0	0	0
		21UR-15340	TAGATATCAGGTTACGAGAAA	0	0	0	0	0	0	0	0
	†	21UR-15341	TAGAGTTAGTTAGAAAGCAAC	0	0	0	0	1	1	0	2
		21UR-15342	TAGAGCGTATGATTCAAGGTC	0	0	0	0	0	0	0	0
		21UR-15343	TAGACGGCATCATCAGAAGAA	0	0	0	0	0	0	0	0
		21UR-15344	TAGAACTCCTTGTTCCCGTTT	0	0	0	0	0	1	1	2
	†	21UR-15345	TAGAACTATTGAGAGTTACTG	0	0	0	0	0	0	0	0
	†	21UR-15346	TAGAAACCACTTATTGTGTAT	0	0	0	0	0	0	0	0
		21UR-15347	TAGAAACATGAAAAGGAAGCA	0	0	0	0	0	0	0	0
	†	21UR-15348	TAGAAAACCGGCTCTCAAAGT	0	0	0	0	0	0	0	0
	†	21UR-15349	TACTTTCACAACAAAACCTCT	0	0	0	0	0	0	0	0
		21UR-15350	TACTTTATCCTGCTGAAGTTG	0	0	0	0	0	0	0	0
		21UR-15351	TACTTGTTCAGTTGGTCATTA	0	0	0	0	2	2	0	4
	†	21UR-15352	TACTTGTATTTTATTCTGGTA	0	0	0	0	0	0	0	0
*	†	21UR-15353	TACTTGTAGAACGACAGGAAA	0	0	1	2	34	25	28	90
		21UR-15354	TACTTCGGGAACCTAATAAAT	0	0	0	0	0	0	0	0

	21UR-15355	TACTTCGAGTAAGTTTTTGGG	0	0	0	0	0	0	0	0
†	21UR-15356	TACTTCCTTTGTGATCTCCTA	0	0	0	0	0	0	0	0
†	21UR-15357	TACTGTCATTACGTCTCATAC	0	0	0	0	0	0	0	0
†	21UR-15358	TACTGTAAAACTGCAGTCAGA	0	0	0	0	0	0	0	0
†	21UR-15359	TACTGGGGAAAAATAACTGCA	0	0	0	0	0	0	0	0
†	21UR-15360	TACTCCGTGAAGAGTTTCGGC	0	0	0	0	0	0	0	0
†	21UR-15361	TACTCCACTGTACACCCCCTC	0	0	0	0	0	0	0	0
	21UR-15362	TACTCAAAAATAGCAGTCACG	0	0	0	0	0	0	0	0
	21UR-15363	TACTATTTAGGACGTAGCGTA	0	0	0	0	0	0	0	0
	21UR-15364	TACTATCACGGGAGTTAATAC	0	0	0	0	0	0	0	0
	21UR-15365	TACTATCAAACGGCCGTTACT	0	0	0	0	0	0	0	0
†	21UR-15366	TACTAGAAAGTGGATGAAGTG	0	0	0	0	0	0	0	0
†	21UR-15367	TACTACCTCTCTTTTTTCGAA	0	0	0	0	0	0	0	0
†	21UR-15368	TACTAATTCGTCCTTCATGGC	0	0	0	0	0	0	0	0
	21UR-15369	TACGTTGTCTTGAGTTTCATG	0	0	0	0	0	0	0	0
	21UR-15370	TACGTGATTTCGTCTCGGCCC	0	0	0	0	0	0	0	0
†	21UR-15371	TACGTACGTTAACCGTGCATA	0	0	0	0	0	0	0	0
	21UR-15372	TACGGTAGTTTTTATGGGGAA	0	0	0	0	0	0	1	1
	21UR-15373	TACGGGCCTAACAAACGCCTA	0	0	0	0	0	0	0	0
†	21UR-15374	TACGCTGGATCTATCAAAATG	0	0	0	0	0	0	0	0
	21UR-15375	TACGATTTAAAGAGAAGGAAC	0	0	0	0	0	0	0	0
†	21UR-15376	TACGATGTCCACTTAAAACGG	0	0	0	0	0	1	0	1
	21UR-15377	TACGATCAGTGGACTACGTAG	0	0	0	0	0	0	0	0
* †	21UR-15378	TACGACAGGAGATGAATACAA	0	0	0	0	6	4	0	10
	21UR-15379	TACCTTGAGAATTATGAAGAG	0	0	0	0	0	0	0	0
	21UR-15380	TACCTGAAATACCTGAAAATG	0	0	0	0	0	0	0	0
	21UR-15381	TACCTCTTCAAAGGACCAAAA	0	0	0	0	0	0	0	0
†	21UR-15382	TACCGTGATTTGATTTGGTGC	0	0	0	0	0	0	0	0
	21UR-15383	TACCGCTGGTCAAAAGTCATT	0	0	0	0	0	0	0	0
	21UR-15384	TACCGAAGCATTTAGATCAGG	0	0	0	0	1	0	0	1
	21UR-15385	TACCCCCATATCAGCGTTGTT	0	0	0	0	0	0	0	0
	21UR-15386	TACCCATCTCGCCAAATTTTT	0	0	0	0	0	0	0	0
	21UR-15387	TACCCACGAAAAAATGGTACC	0	0	0	0	0	0	0	0
	21UR-15388	TACCAAAACTTCATCTTTCTC	0	0	0	0	0	0	0	0
	21UR-15389	TACATTCGATTGTGGAGATAC	0	0	0	0	0	0	0	0
†	21UR-15390	TACATATGCATAACCAAAGTT	0	0	0	0	0	0	0	0
	21UR-15391	TACAGTGGAATTCAAAAAAAA	0	5	0	0	3	9	1	18
	21UR-15392	TACAGCCGGAAAACACAAAAA	0	0	0	0	0	0	0	0
†	21UR-15393	TACAGAGTATAAACATTTACA	0	0	0	0	0	2	0	2
†	21UR-15394	TACACTGATGCAAACAAGTTA	0	0	0	0	0	0	0	0
	21UR-15395	TACACGACATACAGTTATGTC	0	0	0	0	0	0	1	1
	21UR-15396	TACACGAAGTTGTCCTTCAAC	0	0	0	0	0	0	0	0
	21UR-15397	TACACATTGGACCCCCTTGCT	0	0	0	0	0	0	0	0
	21UR-15398	TACACAAGTACCAGTTTTTTC	0	0	0	0	0	0	0	0
	21UR-15399	TACAATTCAAGAAAAAAGTAT	0	0	0	1	0	0	0	1
	21UR-15400	TACAATGATGATGATGAGGAT	0	0	0	0	0	0	0	0
*	21UR-15401	TACAATAATAGAAGAACATGG	1	3	0	6	14	18	6	48
	21UR-15402	TACAAGGATTCTATGGTAGAA	0	0	0	0	0	0	0	0
†	21UR-15403	TACAACTTAGGGTTGAAACCA	0	0	0	0	0	0	0	0
	21UR-15404	TACAACTATTGAAAGTACTAG	0	0	0	0	0	0	0	0
	21UR-15405	TACAACGTTCACCTTCAAAAT	0	0	0	0	0	0	1	1
	21UR-15406	TACAACGGTCCTAAAAACTAT	0	0	0	0	0	0	0	0
†	21UR-15407	TACAAAGCTGATTTCGAGTTG	0	0	0	0	0	2	0	2
	21UR-15408	TAATTTTTAGATGTTCGTTTA	0	0	0	0	0	0	0	0
†	21UR-15409	TAATTTTCTTATAACGCTGTT	0	0	0	0	0	0	0	0
	21UR-15410	TAATTTTCAGATGTTGTTGAA	0	0	0	0	0	0	0	0
	21UR-15411	TAATTTTCAATAAATTAAAAA	0	0	0	0	0	0	0	0
	21UR-15412	TAATTTGCCAATATAAAAATC	0	0	0	0	0	0	1	1
	21UR-15413	TAATTTCAGAGATTTCTTTTT	0	0	0	0	0	0	0	0
†	21UR-15414	TAATTTATAGTCTCTTATGAT	0	0	0	0	0	0	0	0
	21UR-15415	TAATTTACACCGCATTATTAG	0	0	0	0	0	0	1	1
†	21UR-15416	TAATTTAAAACGAAGTTTAGG	0	0	0	0	0	0	0	0
	21UR-15417	TAATTGATTTTTCGGTATGCC	0	0	0	0	0	0	0	0
	21UR-15418	TAATTCTGAACTTCTCAGGGT	0	0	0	0	0	0	0	0

†	21UR-15419	TAATTCCATATAATATTTATT	0	0	0	0	0	0	0	0
	21UR-15420	TAATTCCAAGTTATCTTGTTT	0	0	0	0	1	0	0	1
†	21UR-15421	TAATTATGACATTCCTTGTAG	0	0	0	0	0	1	0	1
†	21UR-15422	TAATTAGTATTCTGTGCCTTG	0	0	0	0	0	0	0	0
	21UR-15423	TAATTACTCCATTATTTTTCA	0	0	0	0	0	1	0	1
	21UR-15424	TAATTAAAAAGACAGGAACAC	0	0	0	0	2	1	1	4
	21UR-15425	TAATGTTTCTGGATTGAGGAA	0	0	0	0	0	0	0	0
†	21UR-15426	TAATGTTTCAAATAAACAAAA	0	0	0	0	0	0	0	0
	21UR-15427	TAATGTAGATTTTTGTTTGGA	0	0	0	0	1	1	0	2
†	21UR-15428	TAATGGTTGATTCAAGAATGT	0	0	0	0	0	0	0	0
	21UR-15429	TAATGGTATGATGGTGTGATG	0	0	0	0	0	0	0	0
†	21UR-15430	TAATGGAAGATTAATTTTCTA	0	0	0	0	2	2	0	4
	21UR-15431	TAATGATTATGTCAAAAGGAG	0	0	0	0	0	0	0	0
†	21UR-15432	TAATGAATTTTCTGAATATTC	0	0	0	0	0	0	0	0
†	21UR-15433	TAATGAAACACAAATGTTTGT	0	0	0	0	0	0	0	0
†	21UR-15434	TAATCTTGTCAATTTGTGTTA	0	0	0	0	0	0	0	0
	21UR-15435	TAATCTGACAAAAATTGAAAG	0	0	0	0	0	0	0	0
	21UR-15436	TAATCTCACAAAAAAAATTTA	0	0	0	0	0	0	0	0
†	21UR-15437	TAATCGTCCCGTACCTATTAT	0	0	0	0	0	1	0	1
†	21UR-15438	TAATCGTAGTTTGATAAAATT	0	0	0	0	0	0	0	0
†	21UR-15439	TAATCGACCTGAACTCAGAAA	0	0	0	0	2	1	0	3
†	21UR-15440	TAATCCGTATCTATAATGAAT	0	0	0	0	0	0	0	0
†	21UR-15441	TAATCCACACAGAAACAAACT	1	0	0	0	0	0	1	2
	21UR-15442	TAATCATTGCCCTTTTTTCCA	0	0	0	0	0	0	0	0
†	21UR-15443	TAATCACATCGAAATGAAAAA	1	0	0	0	0	0	0	1
†	21UR-15444	TAATCAATTGAAAGTCTTCAG	0	0	0	0	0	0	0	0
	21UR-15445	TAATCAACCATGGATCTCGTG	0	0	0	0	0	0	0	0
	21UR-15446	TAATCAACCAACTAATGCAAA	0	0	0	0	0	0	0	0
	21UR-15447	TAATATTTTTGAATATTAGAC	0	0	0	0	0	0	0	0
	21UR-15448	TAATATTCGAGCACCTGGGGC	0	0	0	0	0	0	0	0
	21UR-15449	TAATATTACAAAAAATTTAAA	0	0	0	0	0	0	0	0
†	21UR-15450	TAATATGAATTTTAAAAATGA	0	0	0	0	0	0	0	0
	21UR-15451	TAATATGAAATAATATGAATT	0	0	0	0	0	0	0	0
†	21UR-15452	TAATATGAAAGGATACAGCTG	0	0	0	0	0	0	0	0
†	21UR-15453	TAATAGGTACTACTTTGAACT	0	0	0	0	0	0	0	0
†	21UR-15454	TAATAGCTCGCAAAACTTTTT	0	0	0	0	0	0	0	0
	21UR-15455	TAATAGCTATTAAAGCATATA	0	0	0	0	1	0	0	1
†	21UR-15456	TAATAGAGCTAGGTTCCGAAA	0	0	0	0	0	0	0	0
†	21UR-15457	TAATAGAATTAGAACCTCCAA	0	0	0	0	0	0	0	0
†	21UR-15458	TAATAGAAAGTTTGGACAATT	0	0	2	0	11	2	5	20
†	21UR-15459	TAATACGGAGATTGAACAAAA	0	0	0	0	0	1	0	1
†	21UR-15460	TAATACGCCATAAAAAGTAAC	0	0	0	0	0	0	0	0
†	21UR-15461	TAATACATACTCATTGAATGG	0	0	0	0	0	0	0	0
	21UR-15462	TAATAATTTATAGGTATCGCA	0	0	0	0	0	0	0	0
†	21UR-15463	TAATAATTGCACTGTATTAAT	0	0	0	0	0	0	0	0
†	21UR-15464	TAATAATTGATTGTCTCCGGT	0	0	0	0	0	0	0	0
†	21UR-15465	TAATAATGTTTCCTTCCATTT	0	0	0	0	0	0	0	0
†	21UR-15466	TAATAATGGTGTTGCCAGAAG	0	0	0	0	0	0	0	0
	21UR-15467	TAATAATAATACTTTACTTAG	0	0	0	0	0	0	0	0
†	21UR-15468	TAATAAGTTCATCATCGGAAA	0	0	1	0	0	3	0	4
†	21UR-15469	TAATAAGTCTTTGATGGTCAT	1	1	0	0	1	1	0	4
†	21UR-15470	TAATAAGCAAATTCGCAATAT	22	3	3	1	15	36	2	82
†	21UR-15471	TAATAAATTTGAAGTGACAGA	0	0	0	0	0	0	0	0
†	21UR-15472	TAATAAAAAATTTTTCGTTTC	0	0	0	0	0	0	0	0
	21UR-15473	TAAGTTTGTATTTGACAAACT	0	0	0	0	1	0	0	1
†	21UR-15474	TAAGTTTGGTGACCGTTTTTA	0	0	0	0	0	0	0	0
	21UR-15475	TAAGTTTGATAACATCTATTT	0	0	0	0	0	0	0	0
	21UR-15476	TAAGTAACGAACTAACGTATT	0	0	0	0	0	0	0	0
†	21UR-15477	TAAGGCGATTAGGTGAATTAG	0	0	0	0	0	0	0	0
	21UR-15478	TAAGGCCTAGGAATTGTGTGG	0	0	0	0	0	0	0	0
	21UR-15479	TAAGGAGTGTTTGCAACAAAA	0	0	0	0	0	0	0	0
†	21UR-15480	TAAGCAATTGCAGGTAATAGA	1	0	0	0	0	0	0	1
	21UR-15481	TAAGATACTTACAATGAATAA	0	0	0	0	1	7	0	8
	21UR-15482	TAAGATACCTCCCGCTACTCC	0	0	0	0	0	0	0	0

	21UR-15483	TAAGACCAAAAAATCTTCGGT	0	0	0	0	0	0	0	0
	21UR-15484	TAAGAATGAATAGAAGCGACT	0	0	0	0	0	2	2	4
	21UR-15485	TAAGAAACGGAGAACAAGGCA	0	0	0	0	1	3	8	12
	21UR-15486	TAACTTCGGTTCTTTTTTTTC	0	0	0	0	1	0	0	1
	21UR-15487	TAACTGTCGGAATTGTTTTGA	0	0	0	0	0	0	0	0
	21UR-15488	TAACTAACAAAAATTTTTCAC	0	0	0	0	0	0	0	0
†	21UR-15489	TAACGTCTGAATCATGCGAAA	0	0	0	0	0	0	0	0
†	21UR-15490	TAACGGGCTGAAATACTAAAA	0	0	0	0	0	1	0	1
	21UR-15491	TAACGGGAAAACTGATAAAAT	0	0	0	0	0	0	0	0
†	21UR-15492	TAACGACACTTTAGCAAAAAA	0	0	0	0	0	0	0	0
	21UR-15493	TAACGAAAGTAAATTGCTAAA	0	0	0	0	0	0	0	0
	21UR-15494	TAACCCGGCTGGATCCACATG	0	0	0	0	0	0	0	0
†	21UR-15495	TAACCACCATTTTCCAAAATT	0	0	0	0	0	0	2	2
	21UR-15496	TAACCAAAATTGGAGAAGTCA	0	0	0	0	0	0	0	0
	21UR-15497	TAACATTATTTTAAAATAGTT	0	0	0	0	0	0	0	0
	21UR-15498	TAACAGACAGTAGTTTAAATT	0	0	0	0	0	0	0	0
	21UR-15499	TAACACGTCCATCTTCAAGCT	0	0	0	0	0	0	0	0
	21UR-15500	TAACACAAAGTTCGACATTGC	0	0	0	0	0	0	0	0
	21UR-15501	TAACAATTTCTAAGGATACTC	0	0	0	0	0	0	0	0
	21UR-15502	TAACAAGATATTAATTTGAAA	0	0	0	0	0	0	0	0
	21UR-15503	TAAATTTTTATTCCAAATTGC	0	0	0	0	2	2	0	4
†	21UR-15504	TAAATTTTCCACAGCCTAACT	0	0	0	0	1	0	2	3
	21UR-15505	TAAATTTTCAGGCGAATTTTA	0	0	0	0	0	0	0	0
†	21UR-15506	TAAATTTGTTTTTGTCCAGCT	2	0	0	0	1	1	1	5
	21UR-15507	TAAATTGTTCAACTATGGAAA	0	0	0	0	0	0	0	0
	21UR-15508	TAAATTGTCAAGGGCTTTAAA	0	0	0	0	0	0	0	0
†	21UR-15509	TAAATTGAGAAATTTATATTT	0	0	0	0	0	0	0	0
	21UR-15510	TAAATTCAATTGCTTTAGCTT	0	0	0	0	0	0	0	0
	21UR-15511	TAAATGTAACAGATCAGGATT	0	0	0	0	0	0	0	0
†	21UR-15512	TAAATGGATTGAAATTTTCTG	0	0	0	0	0	0	0	0
†	21UR-15513	TAAATGGATAAGATTTTTGAA	0	0	0	0	0	0	0	0
	21UR-15514	TAAATATTTACATTGAAATCA	0	0	0	0	0	0	0	0
	21UR-15515	TAAATACAATATTTTTTGAAA	0	0	0	0	0	0	0	0
	21UR-15516	TAAATACAAAAAAATAGAGGT	0	0	0	0	0	0	0	0
	21UR-15517	TAAATAATTGAATAAGTTTTG	0	0	0	0	0	0	0	0
	21UR-15518	TAAATAACAGTTTAGAATTTT	0	0	0	2	3	4	0	9
†	21UR-15519	TAAATAAACTGTAGACTGTAC	0	0	0	0	0	0	1	1
	21UR-15520	TAAAGTTTTTTGAAGTTTTCT	0	0	0	0	0	0	0	0
	21UR-15521	TAAAGTTTCGACTTGGAATTG	0	0	0	0	0	0	0	0
	21UR-15522	TAAAGTTGCAATTATTAAAGA	0	0	0	0	0	0	0	0
	21UR-15523	TAAAGTGGAGAATGCCTTGAT	0	0	0	0	0	0	0	0
	21UR-15524	TAAAGGCTCTCAATCTCTCAA	0	0	0	0	0	0	0	0
	21UR-15525	TAAAGGAAACGAAGAAAGAAT	0	0	0	0	0	0	0	0
	21UR-15526	TAAAGCATGAAGCTATGAAAA	0	0	0	0	0	0	0	0
	21UR-15527	TAAAGAAAAATTTCCAAATTC	0	0	0	0	0	1	0	1
	21UR-15528	TAAACTGATACAAAAATATAT	0	0	0	0	0	1	0	1
†	21UR-15529	TAAACGACAGTTTGATCAGCA	0	0	0	0	0	0	0	0
	21UR-15530	TAAACCTTTAAAACTATTTTT	0	0	0	0	0	0	0	0
	21UR-15531	TAAACCAAGGAATGTAGAAAT	0	0	0	0	0	0	0	0
	21UR-15532	TAAACATCTGGTAGTTCAGTG	0	0	0	0	0	0	0	0
	21UR-15533	TAAACAATTGTTAATTTCTTG	0	0	0	0	0	1	0	1
	21UR-15534	TAAAATTGAAACAACGTTTTT	0	0	0	0	0	0	0	0
†	21UR-15535	TAAAATCTTTTGATTTAAGAA	0	0	0	0	0	0	0	0
	21UR-15536	TAAAATCGTAGAATGCAAAGT	0	0	0	0	0	0	0	0
	21UR-15537	TAAAATCATCCAACAACCGAT	0	0	0	0	0	0	0	0
	21UR-15538	TAAAATATTTGCTAAGACAAC	0	0	0	0	2	0	0	2
	21UR-15539	TAAAAGTGTTGTTGATCTGTA	0	0	0	0	0	0	0	0
	21UR-15540	TAAAAGTCAAAGTAATGTGAG	0	0	0	0	0	0	0	0
	21UR-15541	TAAAAGCAAACATTTTCAGAA	0	0	0	0	0	0	0	0
	21UR-15542	TAAAACTCAAAAAGCAAGCTC	0	0	0	0	0	0	0	0
	21UR-15543	TAAAACGTATTGAAACTGTAT	0	0	0	0	0	0	0	0
	21UR-15544	TAAAAATGAAGTATGACACAA	0	0	0	0	0	0	0	0
	21UR-15545	TAAAAATCTAGAGCCAGAAGG	0	0	0	0	0	0	0	0
	21UR-15546	TAAAAATCATCATTTGTTTGG	0	0	0	0	0	0	0	0

	21UR-15547	TAAAAATAAACTCGGGCAACA	1	0	0	1	0	0	0	2
	21UR-15548	TAAAAAGTTCAAAAATACTAC	0	0	0	0	1	0	0	1
	21UR-15549	TAAAAAGTGGATCCGTGCATC	0	0	0	0	0	0	0	0
	21UR-15550	TAAAAAGTCATTCCGAGTCGG	0	0	0	0	0	0	0	0
	21UR-15551	TAAAAAGGGAGTAAAAAATGC	0	0	0	0	0	0	0	0
	21UR-15552	TAAAAAGGAAGCGGTTTTGAA	0	0	0	0	0	0	1	1
	21UR-15553	TAAAAACTTCTTCCGTCTTTA	0	0	0	0	0	0	0	0
	21UR-15554	TAAAAACTATAAGCTGGATAA	0	0	0	0	0	0	0	0
	21UR-15555	TAAAAAATTTTCTAAACTGTG	0	0	0	0	0	0	0	0
	21UR-15556	TAAAAAATTCAGAAAAGTGTA	0	0	0	0	0	0	0	0
	21UR-15557	TAAAAAATGTTGCAACGCAGT	0	0	0	0	0	0	0	0
	21UR-15558	TAAAAAATGCGTTTAGTAGTT	0	0	0	0	0	0	0	0
†	21UR-15559	TAAAAAATCGTGTATTCACAG	0	0	0	0	0	0	0	0
	21UR-15560	TAAAAAAGCTTGATCGAAAAA	0	0	0	0	0	0	0	0
	21UR-15561	TAAAAAAAATTGAAACGAAAAT	0	0	0	0	0	0	0	0
	21UR-15562	TAAAAAAAGTGCTGAATAAAT	0	0	0	0	0	1	0	1
	21UR-15563	GTTTTACTTTGTCGTTTGACA	0	0	0	0	0	0	0	0
†	21UR-15564	GTTTGATTTCAAAATCGGGAT	0	0	0	0	0	0	0	0
†	21UR-15565	GTTGTTAATCTAATCATGGAT	0	0	0	0	0	0	0	0
	21UR-15566	GTTACTGAGTGTTCAGTTGAA	0	0	0	0	0	0	0	0
	21UR-15567	GTTAAGTTGTGCAAGTATAAC	0	0	0	0	0	0	0	0
†	21UR-15568	GTTAAAAAATCCTCCAAAGTT	0	0	0	0	0	0	0	0
	21UR-15569	GTCTCTTGACGCTTCGGAAGT	0	0	0	0	0	0	0	0
	21UR-15570	GTCACTTTATTTATGGGGTAC	0	0	0	0	0	0	0	0
†	21UR-15571	GTATTTTTGAACGTCTTTTCC	0	0	0	0	0	0	0	0
†	21UR-15572	GTATATGTCATAAAAACGAAC	0	0	0	0	0	0	0	0
†	21UR-15573	GTACTATAAGGGTTAGAGCGG	0	0	0	0	0	0	0	0
†	21UR-15574	GGTTTGATAAAAAGAAAAAAA	0	0	0	0	0	0	0	0
	21UR-15575	GGTTGTGGAGTAGTCCTCTGA	0	0	0	0	0	0	0	0
	21UR-15576	GGGCTTCGATCAGTTTTCCGC	0	0	0	0	0	0	0	0
†	21UR-15577	GGATCGTCATTTGGTGGTAGT	0	1	0	0	1	3	0	5
*	21UR-15578	GGAATTAAAATTTGATCATGG	6	0	1	0	0	0	4	11
	21UR-15579	GGAAGTTCTCGGAGCATTTGA	0	0	0	0	0	0	0	0
	21UR-15580	GCTCAAATGAAGCTCGTGCGG	0	0	0	0	0	0	0	0
†	21UR-15581	GCATTGATCTGTGGAGGAATT	0	0	0	0	0	0	0	0
†	21UR-15582	GATTTACTGGTGTCGATTTAA	0	0	0	0	0	0	0	0
	21UR-15583	GATTCCACAAGCGTTTTGCAT	0	0	0	0	0	0	0	0
†	21UR-15584	GATTAAGTGTAGGCTATGACC	0	0	0	0	0	0	0	0
†	21UR-15585	GATGCTAAAAAGTCCAGGTTT	0	0	0	0	0	0	0	0
	21UR-15586	GATCCACTCGCAATAAGACAT	0	0	0	0	0	0	0	0
	21UR-15587	GAGTCTCTTGACGCTTCGGAA	0	0	0	0	0	0	0	0
*	21UR-15588	GAGGGATGGTAAACGAACGAA	0	0	0	0	46	55	94	195
	21UR-15589	GAGGAGAGGTGGGGCTCTGCA	0	0	0	0	0	0	0	0
	21UR-15590	GACCAGTGTTTTATTCGAGAA	0	0	0	0	0	0	0	0
	21UR-15591	GAAGAAATCCGAGTCCTCGAC	0	0	0	0	0	0	0	0
	21UR-15592	GAAAAGGTCGACGCAAAAAAT	0	0	0	0	0	0	0	0
	21UR-15593	CTTTTCAGAAGTTTAGACATT	0	0	0	0	0	0	0	0
	21UR-15594	CTTTAAATTCACCACTTGAGA	0	0	0	0	0	0	0	0
	21UR-15595	CTTCGAAAAAAAAACGTGCAAA	0	0	0	0	0	0	0	0
†	21UR-15596	CTTAGTACTGTGCTTTGTGGT	0	0	0	0	1	1	0	2
†	21UR-15597	CTTAACAATTGGTTTCTCCAA	0	0	0	0	0	0	0	0
†	21UR-15598	CTGTCAGTGGCTTTGATTCCG	0	0	0	0	0	0	0	0
†	21UR-15599	CTGAATCCGTATCAAAAACAC	0	0	0	0	0	0	0	0
	21UR-15600	CTCTTTTCCGGATTCGATTTT	0	0	0	0	0	0	0	0
	21UR-15601	CTCTAATGATTGCCAGTATGA	0	0	0	0	0	0	0	0
†	21UR-15602	CTCCAGAAACTTACTTATTGT	0	0	0	5	44	62	8	119
	21UR-15603	CTCCAAAAATTCGAATATGCT	0	0	0	0	0	0	0	0
†	21UR-15604	CTCAATTGAAAAAAACAGGAA	0	0	0	0	0	0	0	0
†	21UR-15605	CTAGGAACATTTTAGTCAAAT	0	0	0	0	0	0	0	0
	21UR-15606	CTACGCTTGGATATTTACACA	0	0	0	0	0	0	0	0
†	21UR-15607	CTAATAATCGGCCCCTTCAAA	0	0	0	0	0	0	0	0
†	21UR-15608	CTAACCACGATTCCTCAAAAA	0	0	0	0	0	0	0	0
†	21UR-15609	CTAAACCGTATGTAGTAAAAG	0	0	0	0	0	0	0	0
	21UR-15610	CGTTTCCCATTATAGATCCTC	0	0	0	0	0	0	0	0

		21UR-15611	CGTGTTGAATGCTTGAGATGA	0	1	0	0	2	8	0	11
	†	21UR-15612	CGTGGTCTTTTTAATTTCAAA	0	0	0	0	0	0	0	0
		21UR-15613	CGTCTTTGAACGCGATGTCCC	0	0	0	0	0	0	0	0
	†	21UR-15614	CGGTTCTCGGTGATTGTGGAA	0	0	0	0	0	0	0	0
		21UR-15615	CGGGATTGGTCGACGACGCAG	0	0	0	0	0	1	0	1
	†	21UR-15616	CGGATTTGCTCACACCGGATT	8	0	2	1	12	18	49	90
		21UR-15617	CGCTGCTTCAGAGCTACTGAA	0	0	0	0	0	0	0	0
		21UR-15618	CGCGGAGATGCAGATTTTTGC	0	0	0	0	0	0	0	0
		21UR-15619	CGCGAATTAGGCACTATGGGG	0	0	0	0	0	0	0	0
	†	21UR-15620	CGATTCAGAAAAGTCGACTAA	0	0	0	0	0	0	0	0
		21UR-15621	CGATCAAGTAGACAGAAACAA	0	4	1	2	56	59	3	125
		21UR-15622	CGATACTAAATCCGATAAAAA	0	0	0	0	0	0	0	0
	†	21UR-15623	CGATAAGCGTTTCAACGAAGA	0	0	0	1	0	0	0	1
		21UR-15624	CGATAACATAAGCTAGAAGAG	0	0	0	0	0	0	0	0
		21UR-15625	CGAGAAGTATCAATTGGTCAG	0	0	0	0	0	0	0	0
	†	21UR-15626	CGACGTTGTATAGGAATAAAA	0	0	0	0	1	0	0	1
		21UR-15627	CGAAAGCCATTGGAGAACCAC	0	0	0	0	0	0	0	0
		21UR-15628	CCTTGTGAAAGAATTGAATTA	0	0	0	0	0	0	0	0
	†	21UR-15629	CCTGCGTTCGCAAGACTTGCT	0	0	0	0	1	1	1	3
		21UR-15630	CCGCCGATTTGTCATCGTTGA	0	0	0	0	0	0	0	0
	†	21UR-15631	CATTTAATAAGTTACTGACAA	0	0	0	0	0	0	0	0
	†	21UR-15632	CATTGGCATATGATTATACAA	0	0	0	0	0	0	0	0
	†	21UR-15633	CATTAAGAATCTTTGCGAATT	0	0	0	0	0	0	0	0
		21UR-15634	CATCAGGAAGAAATAAGATCC	0	1	0	0	0	0	0	1
		21UR-15635	CATATCCCTGAAACATTTTTG	0	0	0	0	0	0	0	0
	†	21UR-15636	CATAAAAATTTTAAGCCCCCA	0	0	0	0	0	0	0	0
	†	21UR-15637	CAGTTGTCCGAGTAGATGTTC	0	0	0	0	0	0	0	0
		21UR-15638	CAGTGTTACAGTCCCTAATGC	0	0	0	0	0	0	0	0
		21UR-15639	CAGGCTTTGCAGAAAGTGGTG	0	0	0	0	0	0	0	0
		21UR-15640	CAGATTTTTAATCATTTAATG	0	0	0	0	0	0	0	0
		21UR-15641	CAGAACATTTAAACACCAAAT	0	0	0	0	0	0	0	0
	†	21UR-15642	CACTACCACTCACCACTTAAA	0	0	0	0	0	0	0	0
	†	21UR-15643	CACTAATCCAACACCTACGAT	0	0	0	0	0	0	0	0
		21UR-15644	CACATGCTTTAACTTTCTTTC	0	0	0	0	0	0	0	0
	†	21UR-15645	CACATGCTTGGACTCGATAGT	1	2	0	1	30	39	16	89
	†	21UR-15646	CACAATGCAGCTAGAATTAAA	0	1	0	0	0	4	0	5
	†	21UR-15647	CACAATAAGTCATGTGATTGA	0	0	0	0	0	0	0	0
		21UR-15648	CACAAAAACCGGAAGAAAAAC	0	0	0	0	0	0	0	0
		21UR-15649	CAAGGTAATTGAAGGAAAAAT	0	0	0	0	0	0	0	0
*	†	21UR-15650	CAAGGCATGTGTAGGAAAGGC	0	0	0	0	1	2	0	3
	†	21UR-15651	CAACTGCATATTGATGATTTG	0	0	0	0	0	0	0	0
	†	21UR-15652	CAACAAGGAATTTACAACAGG	0	0	0	0	0	0	0	0
		21UR-15653	CAAATCTTTCTCTTCTGGACA	0	0	0	0	0	1	0	1
		21UR-15654	CAAAATTCCCAAATCTCACGA	0	0	0	0	0	0	0	0
	†	21UR-15655	ATTTTTGCCAACTTTATACTT	0	0	0	0	0	0	0	0
	†	21UR-15656	ATTTCAGGAACGTTACTCATT	0	0	0	0	0	0	0	0
	†	21UR-15657	ATTGCAAAATCTAGGGTGTTT	0	0	0	0	0	0	0	0
	†	21UR-15658	ATTCCTGTTAGCAATGATTAT	0	0	0	0	0	0	0	0
		21UR-15659	ATTCCCTAAGCGCCGTGGACT	0	0	0	0	0	0	0	0
*	†	21UR-15660	ATTCCAGACACACAGAAGGAA	0	0	0	0	2	0	0	2
	†	21UR-15661	ATTCACTTCAATGTAAGAGGA	0	0	0	0	0	0	0	0
	†	21UR-15662	ATTACTTCTTCGCAAAAAAGT	0	0	0	0	0	5	1	6
	†	21UR-15663	ATGTATATAGATCGACTAACA	0	0	0	0	1	1	0	2
*	†	21UR-15664	ATCTGTTGTCGATGGGTCAAT	0	0	0	0	0	1	0	1
	†	21UR-15665	ATATTTGAACAATTTTAAAAT	0	0	0	0	0	0	0	0
	†	21UR-15666	ATATGATGCCTAATGGAAATC	0	0	0	0	0	0	0	0
	†	21UR-15667	ATACCTACCATGTTCTGAGGT	25	0	1	1	2	14	7	50
		21UR-15668	AGTATCCACATAATTAGTTGA	0	0	0	0	0	0	0	0
	†	21UR-15669	AGTACCGTGATTTGATTTGGT	1	0	0	0	2	3	1	7
		21UR-15670	AGTAAAACTGATATGAAAAAA	0	0	0	0	0	0	0	0
		21UR-15671	AGCTTACCTGTAATGACCCAC	0	0	0	0	0	0	0	0
		21UR-15672	AGCTCTTCCCCGTTAATTACT	0	0	0	0	0	0	0	0
*		21UR-15673	AGAGGAGAGGTAAATCTACGG	0	0	0	0	0	0	1	1
	†	21UR-15674	AGAAGATTTTGGATGGAAACT	10	8	7	11	20	60	8	124

†	21UR-15675	AGAACAGCGTGTTGATGCATT	14	9	0	8	67	140	4	242
†	21UR-15676	ACTAGAGAAGTAGAAGTCATT	22	34	17	103	1588	1253	349	3366
†	21UR-15677	ACATTTTCAAACTGATCAAGT	0	0	0	0	0	0	0	0
†	21UR-15678	ACAGGAAGAAATGGCACTACT	3	3	3	3	85	109	20	226
†	21UR-15679	ACAATTGTATTCAATTTAAAA	0	0	0	0	0	0	0	0
	21UR-15680	ACAATTACTGTAAAATAGTGT	0	0	0	0	0	0	0	0
†	21UR-15681	AATTTCGTCAGCATGGCTAGT	10	6	7	9	211	153	124	520
†	21UR-15682	AATTGATGCCTGCTATTAGAA	0	0	0	0	0	1	0	1
†	21UR-15683	AATTCAGTCAGGAGAAAAACT	0	0	0	0	25	25	5	55
†	21UR-15684	AATTACAATGTAATTGCTTGA	0	0	0	0	0	0	0	0
†	21UR-15685	AATGCATTTTTAATGTCGGAG	0	0	0	0	0	1	0	1
†	21UR-15686	AATCTTTTCGATCACGGCTGA	0	0	0	0	0	0	0	0
†	21UR-15687	AATCGACTGTTACATCTACGG	0	0	0	0	0	0	0	0
†	21UR-15688	AAGGTATCGCAGTACAGGGGT	10	1	3	1	3	13	4	35
	21UR-15689	AAGATACAAATAGCTGAAGAA	0	1	0	0	0	3	0	4
	21UR-15690	AAGAATCTGAACCACTAAAAT	0	0	0	0	0	0	0	0
	21UR-15691	AAGAACACAAGAATATGTATA	0	1	0	0	0	1	0	2
	21UR-15692	AAGAAACCAATAATATGAAGG	0	0	0	0	0	0	0	0
	21UR-15693	AACTTTATCAATCTCGCGAAT	0	0	0	0	1	0	1	2
†	21UR-15694	AACTTTAATAAAAAGTAAAGT	0	0	1	0	0	0	0	1
†	21UR-15695	AAATATCTAGGAATGCGAAAG	0	0	0	0	0	0	0	0
	21UR-15696	AAAGTTGTCCATAAATTGAGA	0	0	0	0	0	0	0	0
†	21UR-15697	AAACACCAGTAACTTGTTGTT	0	0	0	0	4	0	0	4
	21UR-15698	AAAATTACATGGAATCGTTTA	0	0	0	0	0	0	0	0
	21UR-15699	AAAAATTGGCTAAACAACTGA	0	0	0	0	0	0	0	0
	21UR-15700	AAAAATGAATTTATGAATGCA	0	0	0	0	1	0	0	1
	21UR-15701	AAAAAATGAAATAAGTGTGGT	0	0	0	0	0	0	0	0
	21UR-15702	AAAAAAAACTTACCACCGTTT	0	0	0	0	0	0	0	0
	21UR-15703	TGTGTGTGCTAGGAATTTTAG	0	0	0	0	0	0	0	0
	Total number of 21U-RNA reads in each library		74811	51459	27422	34843	262688	443477	67257	961957
