# Supplementary material for: Dynamic expression of small non-coding RNAs, including novel microRNAs and piRNAs/21U-RNAs, during Caenorhabditis elegans development
Source: Genome Biol. 2009 May 21;10(5):R54. doi: 10.1186/gb-2009-10-5-r54 (PMC2718520; doi:10.1186/gb-2009-10-5-r54)
Supplement: Additional data file 10 — The presence of the core consensus motif CTGTTTCA was examined in their possible larger motif regions (-20 to -63 bp upstream of the 5' terminus of each 21nt-U-RNA). This list also contains the novel 21U-RNAs shown in Additional data file 11. [file gb-2009-10-5-r54-S10.pdf]

Sequence Name	21nt-U-RNA Sequence	Chromosome	Starting Position	Ending Position	Strand	21U-RNA motif?
2585699_adh	TTCAACAGAGAAGCCAGGGAA	Chr.1	14615	14595	-	No
2043316_adh	TCTCTTCTGTGGAACAAATCC	Chr.1	15102	15082	-	No
2741477_adh	TTTTGAGTGTATAGGTCTCGA	Chr.1	15876	15896	+	No
2692407_adh	TTTAATTGACGACATTTAGCA	Chr.1	22425	22445	+	No
2729055_adh	TTTGTCGGTTTTTCTATAAAT	Chr.1	26718	26698	-	No
2239115_adh	TGAGGCTTATTATTTTGTCGG	Chr.1	26731	26711	-	No
2137681_adh	TGAATACGAGTAGACGTAATT	Chr.1	37120	37100	-	No
2629632_adh	TTGAATACGAGTAGACGTAAT	Chr.1	37121	37101	-	No
1863981_adh	TAGGAGGAGACAAGACAAATA	Chr.1	86513	86533	+	No
1975641_adh	TCATTTTTGCAGTAGCCTGGT	Chr.1	91746	91726	-	No
2312296_adh	TGCTTTTTTGTGGAAAACACG	Chr.1	92893	92873	-	No
2659436_adh	TTGGAGACTCAGGCGTCGGAA	Chr.1	109947	109927	-	No
2694101_adh	TTTACGTTCGATTTTATGGGT	Chr.1	110657	110637	-	No
1960125_adh	TCAGATCAACAACGGAGTGTT	Chr.1	111778	111798	+	No
2010234_adh	TCGGAAACAGAGAAGATCAAA	Chr.1	112178	112198	+	No
2304817_adh	TGCGTTGAGTCGGCCGTATTG	Chr.1	117742	117762	+	No
2590441_adh	TTCAGAATGAGCGAACAAGCA	Chr.1	117995	117975	-	No
2671494_adh	TTGGTGTTGCCTGTCGTCACC	Chr.1	128452	128472	+	No
2732092_adh	TTTGTTGGAGCGCGTTTGCAT	Chr.1	166886	166866	-	No
2615244_adh	TTCTATAGATTTGTCGTAGAC	Chr.1	182875	182855	-	No
2702686_adh	TTTCACTCTTTTTTCCGTTGA	Chr.1	215573	215553	-	No
2741830_adh	TTTTGATGCGCGGTAATTTCG	Chr.1	215629	215609	-	No
2714572_adh	TTTGAATTTTGATGCGCGGTA	Chr.1	215635	215615	-	No
2740974_adh	TTTTGAATTTTGATGCGCGGT	Chr.1	215636	215616	-	No
2738411_adh	TTTTCGCTCGATTTTCACACA	Chr.1	215971	215991	+	No
2041423_adh	TCTCGTCGAGCAGAATCGCTT	Chr.1	216425	216405	-	No
2556443_adh	TTACCTTACAACCAACACCAA	Chr.1	217302	217322	+	No
2480314_adh	TGTAAAAAAGTGTAGGAAAAT	Chr.1	217865	217885	+	No
1994921_adh	TCGAAAGGACATTGGCGACGG	Chr.1	218904	218924	+	No
2308686_adh	TGCTGCGGACATTTTTTACGA	Chr.1	220757	220737	-	No
2381975_adh	TGGAGGACGCGGCGATCGAAC	Chr.1	231312	231292	-	No
1996069_adh	TCGAAGACTTGATAGACGACA	Chr.1	232704	232684	-	No
2740962_adh	TTTTGAATTTCGATGCGCGGT	Chr.1	251727	251707	-	No
2738411_adh	TTTTCGCTCGATTTTCACACA	Chr.1	252062	252082	+	No
2041423_adh	TCTCGTCGAGCAGAATCGCTT	Chr.1	252518	252498	-	No
1994921_adh	TCGAAAGGACATTGGCGACGG	Chr.1	254078	254098	+	No
2259626_adh	TGATACGGATTTTACTAAATT	Chr.1	272916	272936	+	No
2323372_adh	TGGAAGCCTGAAGCACGTGCG	Chr.1	285502	285522	+	No
1914063_adh	TATGGAGAGAGTAGAATAGAG	Chr.1	289201	289221	+	No
1981061_adh	TCCCCGCGGGCCTGCGAAAAA	Chr.1	323500	323480	-	No
2700917_adh	TTTCAAAAAACAACGTGCGTC	Chr.1	337187	337167	-	No
2689375_adh	TTTAAAGTTAAGTAGGAGTTT	Chr.1	354050	354070	+	No
1900876_adh	TATGAAAAACAGATTTTTCAA	Chr.1	364017	363997	-	No
2546570_adh	TTAACTTGCAGAAAAACTTAA	Chr.1	367608	367628	+	No
2032721_adh	TCTAGCTTGCGGTGATTTTTC	Chr.1	382426	382406	-	No
2033284_adh	TCTAGTCTAGCTTGCGGTGAT	Chr.1	382431	382411	-	No
2721938_adh	TTTGCGAGATTTGAACGCGAT	Chr.1	382452	382432	-	No

2649768_adh	TTGCAGTAGAATTTCTAGAAA	Chr.1	396866	396846	-	No
2025859_adh	TCGTGAATAGAGAAAACTCGG	Chr.1	420186	420166	-	No
2734166_adh	TTTTACGCAAGTCGGAAGTCG	Chr.1	420226	420206	-	No
2640577_adh	TTGAGCACTGAGAAGTTTCAC	Chr.1	421526	421546	+	No
1939630_adh	TCAAGGAGTGAGAAATGGAAT	Chr.1	443436	443416	-	No
2746094_adh	TTTTTCGGCCTATTTTTTCAG	Chr.1	461805	461825	+	No
2070838_adh	TGAAAATTTTCAAAAAATCAA	Chr.1	473168	473148	-	No
2296236_adh	TGCGACAAACAGGAAAACTCA	Chr.1	485794	485774	-	No
2623973_adh	TTCTTTTTCCGTCGTTTTATA	Chr.1	519843	519823	-	No
2736948_adh	TTTTATTTCTTTTTCCGTCGT	Chr.1	519849	519829	-	No
2634610_adh	TTGAATTTTAGTCGTCGAAAA	Chr.1	519991	519971	-	No
2685685_adh	TTGTTGATTTCCGAAGAGCGT	Chr.1	520607	520627	+	No
2032360_adh	TCTAGAATAAGTAGTAATGGG	Chr.1	535600	535620	+	No
1938573_adh	TCAAGACTGTCGAAGAGGACG	Chr.1	536272	536292	+	No
2297094_adh	TGCGAGCTGTTATAGATTTTT	Chr.1	536935	536955	+	No
2524107_adh	TGTTATCAGTTGGAAATCGAT	Chr.1	536971	536991	+	No
1893615_adh	TATCAGTTGGAAATCGATCGA	Chr.1	536974	536994	+	No
2741942_adh	TTTTGATTGGCATATTCTGGT	Chr.1	581254	581234	-	No
2061041_adh	TCTTTCGCGACTGTATATTTA	Chr.1	629673	629653	-	No
1925985_adh	TATTGATCGATGACACATGGT	Chr.1	636864	636884	+	No
2269802_adh	TGATCGATGACACATGGTTTC	Chr.1	636867	636887	+	No
2627312_adh	TTGAAGACCAGAAAAATGATT	Chr.1	653995	653975	-	No
2682834_adh	TTGTTAAGAAAGTCGTCGTTT	Chr.1	656724	656744	+	No
1782177_adh	TAAGCATTTCATGTACGCGCC	Chr.1	772744	772764	+	No
2582299_adh	TTATTGATTGGAGGTTTACGG	Chr.1	781582	781562	-	No
2539370_adh	TTAAACACAGCGGCGCGCGGA	Chr.1	788730	788750	+	No
2517259_adh	TGTGGTATCATAGGCGTGTTT	Chr.1	836555	836535	-	No
2381003_adh	TGGAGCACAATAGGATGCATC	Chr.1	889372	889352	-	No
2748874_adh	TTTTTTCGATTTTATCCCAGA	Chr.1	895190	895210	+	No
2747721_adh	TTTTTGTAGATCACAAACCGC	Chr.1	907061	907041	-	No
1990086_adh	TCCTCCCTGGATCAGTCGGTA	Chr.1	973859	973879	+	No
2271535_adh	TGATGATGATGATGGTGTGGA	Chr.1	986096	986116	+	No
2283545_adh	TGCAATCTGCTTGAAGGAGGT	Chr.1	1016989	1017009	+	No
2749043_adh	TTTTTTCTGAATTTTCGCATA	Chr.1	1022826	1022806	-	No
1967122_adh	TCAGTGTCGGCATCACATTTG	Chr.1	1025158	1025178	+	No
1927055_adh	TATTGGATTCAAACGGCGTGA	Chr.1	1029586	1029566	-	No
2547531_adh	TTAAGCAGGATTTTGACTGAA	Chr.1	1033576	1033556	-	No
2738255_adh	TTTTCGAGATTTAAGCAGGAT	Chr.1	1033586	1033566	-	No
2738382_adh	TTTTCGCCCAAAATCGTCGTT	Chr.1	1038406	1038426	+	No
1758993_adh	TAAAGTGCGAAAAATGTCGGA	Chr.1	1041168	1041188	+	No
1930577_adh	TATTTGTGCATTTTTATCGGA	Chr.1	1044713	1044693	-	No
1867284_adh	TAGGCGAGGAAAATTCGGGAA	Chr.1	1049252	1049232	-	No
1833677_adh	TACTGTAGATCAGAGGTTGGC	Chr.1	1053572	1053552	-	No
2606562_adh	TTCGCGACGAATTTTGGAGGG	Chr.1	1067177	1067197	+	No
2737439_adh	TTTTCAGAAGCGACACAGACA	Chr.1	1145864	1145844	-	No
1930577_adh	TATTTGTGCATTTTTATCGGA	Chr.1	1174715	1174735	+	No
1827206_adh	TACGGTACCAAGGTTGTCGGT	Chr.1	1206478	1206458	-	No
1874318_adh	TAGTAGAATTTCCGGCAAATC	Chr.1	1209884	1209904	+	No
2608133_adh	TTCGGATAGATGCGGCACAGA	Chr.1	1225976	1225996	+	No
2705378_adh	TTTCCCGCTTTTTCGCAGGTA	Chr.1	1226831	1226811	-	No

1972985_adh	TCATGTGAACTAACTCGGAAA	Chr.1	1263874	1263854	-	No
2110417_adh	TGAAATGTAAATGGTTTTTTA	Chr.1	1314388	1314408	+	No
2424674_adh	TGGGAGTGGGAGGGGGAGGGA	Chr.1	1331293	1331313	+	No
2065211_adh	TGAAAAAATAAGAGATTTCGG	Chr.1	1340080	1340060	-	No
1827225_adh	TACGGTAGATTTTGGACAGGT	Chr.1	1340129	1340109	-	No
2607897_adh	TTCGGAGCATCGTTGTAACTA	Chr.1	1394453	1394473	+	No
1929257_adh	TATTTAGCTATGCTTGGTAAG	Chr.1	1432081	1432061	-	No
2183931_adh	TGAGACGAGATTTTCAAGAAC	Chr.1	1435624	1435604	-	No
2113602_adh	TGAAATTAAAAAAGGATTTAA	Chr.1	1469837	1469817	-	No
2450415_adh	TGGGCGACTTGGAGTGCGGCT	Chr.1	1558631	1558611	-	No
2591790_adh	TTCAGCGGCAGCAGCGAATAA	Chr.1	1558914	1558894	-	No
2607300_adh	TTCGGACAGATGCGACACGGA	Chr.1	1572836	1572816	-	No
2733928_adh	TTTTACAACTAGAATCGAGCC	Chr.1	1612394	1612374	-	No
2472963_adh	TGGTGTCAAAATTGGACGACC	Chr.1	1673366	1673346	-	No
2707037_adh	TTTCGAGCGAAATAACTCAGA	Chr.1	1701841	1701821	-	No
2733569_adh	TTTTAAGTCAATTTATCGCGT	Chr.1	1701977	1701997	+	No
2565501_adh	TTAGATGTAAAACTGAATGTA	Chr.1	1725891	1725871	-	No
2635620_adh	TTGACGACAAATTGCACACAT	Chr.1	1751163	1751143	-	No
1981988_adh	TCCCTACTTGTCGTACAATTA	Chr.1	1798694	1798674	-	No
1973761_adh	TCATTCCCTACTTGTCGTACA	Chr.1	1798698	1798678	-	No
2701012_adh	TTTCAAACAAGTTTGGTGGCA	Chr.1	1812900	1812920	+	No
2746811_adh	TTTTTGAGTTTTTTCAGCATT	Chr.1	1832784	1832804	+	No
2533031_adh	TGTTTGCAAGCCGGCGGCGAG	Chr.1	1835511	1835531	+	No
2514794_adh	TGTGGACAAAAAGAAATGGTT	Chr.1	1841909	1841929	+	No
2626557_adh	TTGAACTAGATATGAGCATAT	Chr.1	1858293	1858273	-	No
2376314_adh	TGGACGGCAAGATTTGGTAAA	Chr.1	1898680	1898660	-	No
2710028_adh	TTTCTCGATTTTTCGTCGGAA	Chr.1	1958643	1958663	+	No
2744703_adh	TTTTTAAAGTCGATGGACGGC	Chr.1	1964529	1964509	-	No
2664809_adh	TTGGATTTGTTCGTTGGGGCA	Chr.1	1979249	1979229	-	No
2711763_adh	TTTCTTGATTTTCGGCAATTT	Chr.1	2018031	2018051	+	No
2600777_adh	TTCCTGAGTAGAATTGGCTAT	Chr.1	2039287	2039307	+	No
1808979_adh	TACAAGCTTTGGATTCATTAA	Chr.1	2055012	2054992	-	No
2061324_adh	TCTTTCTGGAAACGACCGCAG	Chr.1	2059253	2059273	+	No
2421323_adh	TGGGACACTGCAGAGCGTCAA	Chr.1	2059533	2059513	-	No
2738274_adh	TTTTCGATAAATATGCTGCTT	Chr.1	2062229	2062249	+	No
2317936_adh	TGGAAATCAAAGAGATCTGGC	Chr.1	2065078	2065098	+	No
1980315_adh	TCCCACGAGGCTGAGCAAAAG	Chr.1	2069782	2069762	-	No
2169729_adh	TGACTGCACCAAGCTTGGATA	Chr.1	2070081	2070061	-	No
2065205_adh	TGAAAAAAGTGTGTGATCTGG	Chr.1	2070787	2070767	-	No
2740261_adh	TTTTCTTGTATTTTCATCGGA	Chr.1	2081549	2081569	+	No
2029212_adh	TCTAAAAAACGGCGTCTGAAT	Chr.1	2082045	2082065	+	No
2537473_adh	TTAAAAAACTTGAATTCGGCG	Chr.1	2084049	2084069	+	No
2738881_adh	TTTTCGTTGCTTTTATCGGTT	Chr.1	2091441	2091461	+	No
2709266_adh	TTTCGTTGCTTTTATCGGTTT	Chr.1	2091442	2091462	+	No
2705775_adh	TTTCCGCTGGAATTGAATCGA	Chr.1	2097043	2097023	-	No
2259331_adh	TGATACACTGGAGAATTGGAT	Chr.1	2102459	2102479	+	No
2732938_adh	TTTTAAAAACTGAATTCGGCG	Chr.1	2104787	2104807	+	No
2732938_adh	TTTTAAAAACTGAATTCGGCG	Chr.1	2104911	2104931	+	No
2585233_adh	TTCAAACTTTAACGATGATCG	Chr.1	2108912	2108892	-	No
2732938_adh	TTTTAAAAACTGAATTCGGCG	Chr.1	2122140	2122160	+	No

1788539_adh	TAAGTGCAAGAAGGAGTACGA	Chr.1	2132835	2132855	+	No
2658146_adh	TTGGACCATATGATAAGAATT	Chr.1	2138934	2138954	+	No
2449910_adh	TGGGCAGAAGAGGTAGATGTC	Chr.1	2147484	2147504	+	No
1770357_adh	TAACTCAAATCGTAGGGGTCA	Chr.1	2156142	2156122	-	No
1851468_adh	TAGATGGCTGAAAAAACAAAT	Chr.1	2210233	2210213	-	No
2728306_adh	TTTGTAGCTAGAGGATTCAAA	Chr.1	2210810	2210830	+	No
1745736_adh	TAAAAAATTTGCAAATCCAAT	Chr.1	2234933	2234913	-	No
2074657_adh	TGAAACTTGTCGATCAATGGA	Chr.1	2268037	2268017	-	No
2747992_adh	TTTTTGTTTAATGATGACGGA	Chr.1	2272212	2272192	-	No
1914950_adh	TATGGATATGAAGAAGAGAAA	Chr.1	2297661	2297681	+	No
2127841_adh	TGAAGACGAAAGAAGATTATC	Chr.1	2304094	2304114	+	No
2744142_adh	TTTTGTTCATCTTTTCGAAAA	Chr.1	2306845	2306825	-	No
2745119_adh	TTTTTAGGCGAATTTCTGGGA	Chr.1	2307204	2307184	-	No
1758046_adh	TAAAGGCATCGGCACGACTTT	Chr.1	2307285	2307305	+	No
2585615_adh	TTCAAATTTCAGGACAAATTT	Chr.1	2309337	2309317	-	No
2566714_adh	TTAGCGCGAACCAGGCAAAAA	Chr.1	2313082	2313062	-	No
2689980_adh	TTTAACGCCTCGATCGGATAA	Chr.1	2316259	2316279	+	No
2740932_adh	TTTTGAATGTAAAATGCTGGA	Chr.1	2318806	2318786	-	No
2647215_adh	TTGCAAAAGCACACTTTTCCG	Chr.1	2318852	2318832	-	No
1829412_adh	TACTAATAAATAGTAGACTCA	Chr.1	2327281	2327261	-	No
2480589_adh	TGTAAACGGTTCTTGTTAAAT	Chr.1	2327466	2327486	+	No
1762885_adh	TAAATTTTCAGGATATTTTGA	Chr.1	2334630	2334650	+	No
2487921_adh	TGTAGATATGGAACAGATTAA	Chr.1	2335635	2335655	+	No
2697875_adh	TTTATAATATTGTTGGGAAGC	Chr.1	2339811	2339791	-	No
1745443_adh	TAAAAAAGACTTTCTGATTGG	Chr.1	2401603	2401583	-	No
1822769_adh	TACGACTTCAAAGGGAGGCGC	Chr.1	2411800	2411820	+	No
2706739_adh	TTTCGAATAGTGACAAAGATA	Chr.1	2417805	2417785	-	No
2706645_adh	TTTCGAAGAAGACATGAGCTG	Chr.1	2420769	2420789	+	No
2602090_adh	TTCGAAGAAGACATGAGCTGA	Chr.1	2420770	2420790	+	No
1810512_adh	TACACACACATGACTCATGAA	Chr.1	2421826	2421806	-	No
2703231_adh	TTTCAGGCTATAAAACACAAA	Chr.1	2421925	2421945	+	No
1979025_adh	TCCAGTCCATTTATTATCTGA	Chr.1	2433821	2433841	+	No
1999780_adh	TCGACATCAGCTGACGAAATT	Chr.1	2485614	2485594	-	No
1921983_adh	TATTATAGAAAAATACGATCG	Chr.1	2493390	2493410	+	No
1921983_adh	TATTATAGAAAAATACGATCG	Chr.1	2493585	2493565	-	No
1833637_adh	TACTGTAGAAAAGGTTGGTAA	Chr.1	2518496	2518476	-	No
2589723_adh	TTCACGGCGTAAATGGTTCAC	Chr.1	2567740	2567760	+	No
1953287_adh	TCACGGCGTAAATGGTTCACG	Chr.1	2567741	2567761	+	No
1878332_adh	TAGTGCAGCTGGATTGAAAAT	Chr.1	2572117	2572097	-	No
2376317_adh	TGGACGGCAAGATTTGGTGAA	Chr.1	2584742	2584722	-	No
2707688_adh	TTTCGGAGATTTCTTTTTCAT	Chr.1	2620556	2620576	+	No
2749048_adh	TTTTTTCTGATTTTCTCGGCC	Chr.1	2620576	2620596	+	No
2003995_adh	TCGATGACAGAAAATGAACGG	Chr.1	2628351	2628371	+	No
2259222_adh	TGATAATTGGACGGTGTACTC	Chr.1	2682038	2682058	+	No
2463037_adh	TGGTAGATCTCGGCCTTCATC	Chr.1	2686988	2687008	+	No
2688970_adh	TTTAAAATTTCAGATTGCGGC	Chr.1	2687987	2687967	-	No
2161038_adh	TGACGATACCTGAAGAATGTG	Chr.1	2688746	2688766	+	No
1814502_adh	TACATTGTGGAATATGTTGGT	Chr.1	2689084	2689104	+	No
2557788_adh	TTACGGTCAGCAGATGCATAG	Chr.1	2689104	2689124	+	No
1819444_adh	TACCGAAGAAGTTCTCGTTTG	Chr.1	2700445	2700465	+	No

2668437_adh	TTGGGTGAACGAGGAAGATCA	Chr.1	2701574	2701594	+	No
2382012_adh	TGGAGGAGAAGCAGAAGTAAA	Chr.1	2702064	2702084	+	No
2739149_adh	TTTTCTAGTCGATTTGAATCG	Chr.1	2723524	2723504	-	No
2534351_adh	TGTTTTCGGTGTATTTTTGGT	Chr.1	2724581	2724601	+	No
2733001_adh	TTTTAAAAGACGGCGTAAATG	Chr.1	2728637	2728617	-	No
1895764_adh	TATCCGGAAAACGGCAAACCG	Chr.1	2737827	2737807	-	No
2240381_adh	TGAGGGTACAAGCAGAGAACA	Chr.1	2761141	2761161	+	No
1999515_adh	TCGACAAAATGCAGGAAATGA	Chr.1	2762809	2762829	+	No
2179790_adh	TGAGAAGACGGTTTGAACACA	Chr.1	2778896	2778916	+	No
1805839_adh	TAATTTTATCGCCGAAGCTGA	Chr.1	2790064	2790044	-	No
2701634_adh	TTTCAAGTTGAACAGTTCATA	Chr.1	2822125	2822105	-	No
2257902_adh	TGAGTTTTGAAGAATTTTAAC	Chr.1	2828823	2828803	-	No
1794972_adh	TAATAGCTGTAGAAGAGGTTA	Chr.1	2831660	2831680	+	No
1858815_adh	TAGCTGTAGAAGAGGTTAAAA	Chr.1	2831663	2831683	+	No
2382012_adh	TGGAGGAGAAGCAGAAGTAAA	Chr.1	2854071	2854051	-	No
2668437_adh	TTGGGTGAACGAGGAAGATCA	Chr.1	2854561	2854541	-	No
2275034_adh	TGATTATTACCGAAGAAGTTA	Chr.1	2855731	2855711	-	No
2694371_adh	TTTACTCTTTTCATCTCTAAA	Chr.1	2861957	2861977	+	No
2720914_adh	TTTGCAAAAGTCACGGATCGA	Chr.1	2870252	2870232	-	No
2534351_adh	TGTTTTCGGTGTATTTTTGGT	Chr.1	2873738	2873718	-	No
2127087_adh	TGAAGAAGAACTGGCAGAACG	Chr.1	2875505	2875485	-	No
2739645_adh	TTTTCTGTAGATTTTTGGATA	Chr.1	2876451	2876431	-	No
2746286_adh	TTTTTCTGTAGATTTTTGGAT	Chr.1	2876452	2876432	-	No
1762683_adh	TAAATTTATACATACACACAC	Chr.1	2878336	2878356	+	No
1759104_adh	TAAAGTGTGGAATTACAAGCA	Chr.1	2900520	2900500	-	No
1988958_adh	TCCGTTTGATGCGCAGGTTTC	Chr.1	2901087	2901067	-	No
2733575_adh	TTTTAAGTGGGAATTGACATA	Chr.1	2901150	2901170	+	No
2070774_adh	TGAAAATTGAGGAAAATGGAG	Chr.1	2901180	2901200	+	No
2624994_adh	TTGAAAGAGACTGAAAAAATC	Chr.1	2925205	2925225	+	No
2690187_adh	TTTAAGAAAGAAGAATACAAC	Chr.1	2929675	2929655	-	No
2688950_adh	TTTAAAATGAAACAGGAAACA	Chr.1	2980476	2980456	-	No
2308088_adh	TGCTGAAAATAGAATTAGAAA	Chr.1	2983076	2983096	+	No
1963434_adh	TCAGGAACAAGTAGTATACAT	Chr.1	2985349	2985369	+	No
2673858_adh	TTGTAACTCTGGCACGGTTTT	Chr.1	3029553	3029533	-	No
2609822_adh	TTCGGTAATTTTCGGGCAGAA	Chr.1	3037958	3037938	-	No
2708306_adh	TTTCGGTAATTTTCGGGCAGA	Chr.1	3037959	3037939	-	No
2692406_adh	TTTAATTGACGACACTTTCTT	Chr.1	3038000	3037980	-	No
1800306_adh	TAATGGAACGTAGAGGAGACA	Chr.1	3096418	3096438	+	No
2320803_adh	TGGAACGTAGAGGAGACAAAA	Chr.1	3096421	3096441	+	No
1996473_adh	TCGAAGCTAAAGATGTCGGTT	Chr.1	3098370	3098350	-	No
2485557_adh	TGTAATTGAAAATTTCCGGCA	Chr.1	3107171	3107191	+	No
2272347_adh	TGATGGCATTGTAGTTTCTAC	Chr.1	3109667	3109687	+	No
2683455_adh	TTGTTCAAGGAGAAAAGCTAA	Chr.1	3131768	3131788	+	Yes
2740670_adh	TTTTGAACAGATTGGTGTTTT	Chr.1	3152098	3152078	-	No
2697674_adh	TTTAGTTTTGAACAGATTGGT	Chr.1	3152103	3152083	-	No
2599045_adh	TTCCGCTTCTTCTCTACGGTT	Chr.1	3162564	3162544	-	No
2304173_adh	TGCGTGCTGAATTCACTTCTC	Chr.1	3162589	3162569	-	No
2709828_adh	TTTCTATTGAAATATATGGGA	Chr.1	3162738	3162758	+	No
2273549_adh	TGATGTGAGTCTGGAACTTGA	Chr.1	3200451	3200431	-	No
1834671_adh	TACTTATTACAGGACCACAAA	Chr.1	3276167	3276147	-	No

2721285_adh	TTTGCAGAATTTTGTGTCGGT	Chr.1	3278955	3278935	-	No
2723068_adh	TTTGGAACTAGAAAATCACGG	Chr.1	3286342	3286362	+	No
2659379_adh	TTGGAGACAGAGGAACGTAAA	Chr.1	3311821	3311801	-	No
1853671_adh	TAGCAATCGAAACAGAGAGAC	Chr.1	3332816	3332796	-	No
2133288_adh	TGAAGGCTCTGAGAGACTGGC	Chr.1	3334517	3334537	+	No
2746015_adh	TTTTTCGCAGAAAATCGTTGA	Chr.1	3338873	3338853	-	No
2300230_adh	TGCGCGTAGAATATGGTGTTT	Chr.1	3338882	3338902	+	No
2159422_adh	TGACCAAGTAGACAGCAATCA	Chr.1	3348130	3348150	+	No
2039593_adh	TCTCGATGGAAAGGAGACGGA	Chr.1	3350077	3350097	+	No
2316670_adh	TGGAAAGGAGACGGAGAAACG	Chr.1	3350083	3350103	+	No
1929858_adh	TATTTCGCCGAAAGATGAGCC	Chr.1	3350558	3350538	-	No
2727104_adh	TTTGTAAAAGAGATTCCTGAA	Chr.1	3363009	3363029	+	No
2566689_adh	TTAGCGATTGAAATAAACAAA	Chr.1	3365937	3365917	-	No
2043625_adh	TCTGAAAACTGGACGGAAAAT	Chr.1	3368826	3368806	-	No
1873517_adh	TAGTAATTCGATCGTACCAGG	Chr.1	3409145	3409165	+	No
2604849_adh	TTCGATCGTACCAGGACCCCA	Chr.1	3409151	3409171	+	No
2725917_adh	TTTGGTAGATTATCTCGCGGA	Chr.1	3411923	3411943	+	No
2725917_adh	TTTGGTAGATTATCTCGCGGA	Chr.1	3412060	3412040	-	No
2555394_adh	TTACATTTTTTCAGTCGACAT	Chr.1	3439090	3439110	+	No
2745501_adh	TTTTTATTGTAAAACAAGCAC	Chr.1	3466221	3466201	-	No
2286001_adh	TGCACAGAGCCATGACGCAAA	Chr.1	3466851	3466831	-	No
2223096_adh	TGAGATGGATGGATTCACAAC	Chr.1	3471168	3471188	+	No
2543434_adh	TTAAATCTGAAGCTAATAACG	Chr.1	3481486	3481466	-	No
1933942_adh	TCAAAGTGAAGATGGAGAAAC	Chr.1	3482533	3482553	+	No
2725917_adh	TTTGGTAGATTATCTCGCGGA	Chr.1	3515006	3515026	+	No
2517872_adh	TGTGGTGTCAAAAAGAATATA	Chr.1	3584795	3584775	-	No
2725917_adh	TTTGGTAGATTATCTCGCGGA	Chr.1	3591640	3591660	+	No
2133703_adh	TGAAGGTTGTGGATGGTTTTG	Chr.1	3594636	3594616	-	No
1767818_adh	TAACGACGGAAAAAGGCTTCT	Chr.1	3594936	3594916	-	No
2396065_adh	TGGATTTGATGAATACGACGA	Chr.1	3642026	3642006	-	No
2250991_adh	TGAGTACTTTTGGAAGATGAA	Chr.1	3677055	3677075	+	No
2617820_adh	TTCTGAAGTTGCACTCGACAG	Chr.1	3680525	3680505	-	No
2004256_adh	TCGATGGGAAGAAAATCAAAC	Chr.1	3683860	3683840	-	No
1967645_adh	TCAGTTGTGAAGAATCCGGTG	Chr.1	3689862	3689882	+	No
1748766_adh	TAAAACTGTAGAGATTGTTCA	Chr.1	3722520	3722540	+	No
2700817_adh	TTTATTTTAGGCCTTTGACTG	Chr.1	3726839	3726859	+	No
2734802_adh	TTTTAGGCCTTTGACTGAAAC	Chr.1	3726843	3726863	+	No
2742696_adh	TTTTGGACAGAGGACAGGGGG	Chr.1	3738246	3738266	+	No
2736870_adh	TTTTATTTAAATACTAATTTA	Chr.1	3742103	3742123	+	No
1931342_adh	TATTTTTCGTCTAATTCTATC	Chr.1	3754785	3754765	-	No
1822135_adh	TACGACATCTCTGAAACAGAC	Chr.1	3757940	3757920	-	No
2736968_adh	TTTTATTTGATTGTATACCGA	Chr.1	3761558	3761578	+	No
2736968_adh	TTTTATTTGATTGTATACCGA	Chr.1	3762320	3762300	-	No
1809244_adh	TACAATAAGAATGATGCGAAA	Chr.1	3774707	3774727	+	No
1983969_adh	TCCGAATAAACTGGCGAGCTG	Chr.1	3781301	3781321	+	No
2534588_adh	TGTTTTGCAGGACGAAATGAG	Chr.1	3781692	3781712	+	No
1886279_adh	TATAATTAAATTTTATTTAAA	Chr.1	3783779	3783799	+	No
1956529_adh	TCACTTTTGACGCTCCTCTTC	Chr.1	3783950	3783970	+	No
2613618_adh	TTCGTTTGTTCGTTGGTAAGT	Chr.1	3788932	3788912	-	No
2624150_adh	TTCTTTTTTGGCTCTGAAGAA	Chr.1	3806213	3806233	+	No

2705948_adh	TTTCCTAATTTGCGTGGATCG	Chr.1	3817493	3817513	+	No
2189530_adh	TGAGAGTTGTTAGATTTTTGA	Chr.1	3817515	3817535	+	No
1866986_adh	TAGGCACTTTTCTAGTCTCCT	Chr.1	3826142	3826162	+	No
2710544_adh	TTTCTGATTCTTGGACAACAA	Chr.1	3829394	3829374	-	No
2042571_adh	TCTCTCGTCAGATGATATCAT	Chr.1	3829438	3829418	-	No
2654230_adh	TTGCTCAAAGACTCATGAAAA	Chr.1	3833434	3833454	+	No
2281379_adh	TGCAACATATCTGACGCGCAA	Chr.1	3835045	3835025	-	No
1919070_adh	TATGTGGATCGTGGAAGACAG	Chr.1	3839163	3839143	-	No
2045803_adh	TCTGACGTCAGCAGTTCTGTA	Chr.1	3863069	3863089	+	No
2047693_adh	TCTGATTTTTAGGCTTTGATT	Chr.1	3866569	3866549	-	No
1995160_adh	TCGAAAGTATAGAAAGCATGC	Chr.1	3866723	3866703	-	No
2272304_adh	TGATGGATGAAGTTGTCAAGG	Chr.1	3868766	3868786	+	No
2051185_adh	TCTGGTGTGGAAATACGCGTT	Chr.1	3876910	3876890	-	No
2550975_adh	TTAATGAAGCTATCTGGACGG	Chr.1	3897610	3897630	+	No
2299986_adh	TGCGCACTATTTGACGACGAA	Chr.1	3908603	3908583	-	No
2149378_adh	TGACAACAAGTAGAAAACATT	Chr.1	3930613	3930633	+	No
2694824_adh	TTTAGAAATTACCAGGTCGGA	Chr.1	3930633	3930653	+	No
2657093_adh	TTGGAAGATCTTTTGGGTATA	Chr.1	3934893	3934913	+	No
2389706_adh	TGGATCGAAACGAAGAATGGT	Chr.1	3941309	3941289	-	No
2653008_adh	TTGCGGTAGAAAATTTGGTCA	Chr.1	3982871	3982851	-	No
1804401_adh	TAATTGTTGAATTTAAGGCTG	Chr.1	3987221	3987201	-	No
2494178_adh	TGTCAAGGTACTGTAGTGTTA	Chr.1	3997301	3997321	+	No
1819454_adh	TACCGACAAGGACGGAGAGCA	Chr.1	4060279	4060299	+	No
2739876_adh	TTTTCTGTTGAGTCGTCTGCT	Chr.1	4066084	4066104	+	No
2727176_adh	TTTGTAAGAATGGATGATGGT	Chr.1	4070557	4070577	+	No
2701743_adh	TTTCAATCGTTCCCAGACAAA	Chr.1	4079766	4079746	-	No
2617753_adh	TTCTGAACTCCATCTCAAAAG	Chr.1	4121905	4121925	+	No
2628457_adh	TTGAAGATGGAACCGAAAAGT	Chr.1	4133567	4133587	+	No
2646251_adh	TTGATTGAAAAGCAGTATGTA	Chr.1	4134427	4134447	+	No
2011274_adh	TCGGACACTATAGAGACGGCA	Chr.1	4138288	4138268	-	No
2738375_adh	TTTTCGCATTTTCCTATCGAC	Chr.1	4139412	4139392	-	No
1775350_adh	TAAGACATGCGGAAGAAGAAT	Chr.1	4172533	4172553	+	No
1748887_adh	TAAAAGAAAAAGAGATTGGTG	Chr.1	4177196	4177176	-	No
2693066_adh	TTTAATTTTTGCAGGAGCTTC	Chr.1	4178547	4178527	-	No
2665833_adh	TTGGCGGCCAATTGACGCGAC	Chr.1	4178671	4178691	+	No
2701542_adh	TTTCAAGATCTGATGGGATTC	Chr.1	4185995	4185975	-	No
2529942_adh	TGTTGGCAAGCTTATGAAGGA	Chr.1	4232212	4232232	+	No
1925815_adh	TATTGAGACTAGTCATCCGGA	Chr.1	4236789	4236769	-	No
1860254_adh	TAGGAAACGCATTCTCAATTC	Chr.1	4254243	4254263	+	No
2577669_adh	TTATCGATTTTTTGGTGAACC	Chr.1	4254768	4254748	-	No
2713205_adh	TTTGAACGAGTGCGTTGCAAT	Chr.1	4254877	4254897	+	No
2712121_adh	TTTCTTTGGCGTTCTCCAATT	Chr.1	4270462	4270442	-	No
2700834_adh	TTTATTTTATTTTCCAGATAT	Chr.1	4270491	4270471	-	No
2694683_adh	TTTACTTGAATTCGTTTGCCG	Chr.1	4274937	4274957	+	No
2481774_adh	TGTAAGAATAGTGGTGACTCT	Chr.1	4274965	4274985	+	No
2124932_adh	TGAACTGAAGAACGATGTCGG	Chr.1	4341504	4341524	+	No
2618465_adh	TTCTGATCTCGACAACAAGAT	Chr.1	4343185	4343205	+	No
2645469_adh	TTGATGTAGACGCCGAGCAAA	Chr.1	4343274	4343294	+	No
2008632_adh	TCGCTGAAGATTTGGATAACA	Chr.1	4343556	4343576	+	No
1940417_adh	TCAAGTCGGACGATTTGAAGA	Chr.1	4343703	4343723	+	No

2618465_adh	TTCTGATCTCGACAACAAGAT	Chr.1	4346225	4346245	+	No
2645469_adh	TTGATGTAGACGCCGAGCAAA	Chr.1	4346314	4346334	+	No
2008632_adh	TCGCTGAAGATTTGGATAACA	Chr.1	4346596	4346616	+	No
1940417_adh	TCAAGTCGGACGATTTGAAGA	Chr.1	4346743	4346763	+	No
2124932_adh	TGAACTGAAGAACGATGTCGG	Chr.1	4347431	4347451	+	No
2618465_adh	TTCTGATCTCGACAACAAGAT	Chr.1	4349112	4349132	+	No
2645469_adh	TTGATGTAGACGCCGAGCAAA	Chr.1	4349201	4349221	+	No
2008632_adh	TCGCTGAAGATTTGGATAACA	Chr.1	4349483	4349503	+	No
1940417_adh	TCAAGTCGGACGATTTGAAGA	Chr.1	4349630	4349650	+	No
2124932_adh	TGAACTGAAGAACGATGTCGG	Chr.1	4350318	4350338	+	No
2618465_adh	TTCTGATCTCGACAACAAGAT	Chr.1	4351999	4352019	+	No
2645469_adh	TTGATGTAGACGCCGAGCAAA	Chr.1	4352088	4352108	+	No
2008632_adh	TCGCTGAAGATTTGGATAACA	Chr.1	4352370	4352390	+	No
1940417_adh	TCAAGTCGGACGATTTGAAGA	Chr.1	4352517	4352537	+	No
2124932_adh	TGAACTGAAGAACGATGTCGG	Chr.1	4353205	4353225	+	No
2618465_adh	TTCTGATCTCGACAACAAGAT	Chr.1	4354886	4354906	+	No
2645469_adh	TTGATGTAGACGCCGAGCAAA	Chr.1	4354975	4354995	+	No
2008632_adh	TCGCTGAAGATTTGGATAACA	Chr.1	4355257	4355277	+	No
1940417_adh	TCAAGTCGGACGATTTGAAGA	Chr.1	4355404	4355424	+	No
2124932_adh	TGAACTGAAGAACGATGTCGG	Chr.1	4356092	4356112	+	No
2652215_adh	TTGCGCACTTTCGGACGGCCA	Chr.1	4367939	4367919	-	No
1968518_adh	TCATAATTTTAGGTCTAAAAC	Chr.1	4373238	4373258	+	No
2733230_adh	TTTTAACAGTACGAAATTCTA	Chr.1	4375058	4375078	+	No
2584956_adh	TTCAAAACGGATGAATAATGC	Chr.1	4381788	4381768	-	No
1883813_adh	TATAAAAACAAAACGTGGACG	Chr.1	4386365	4386385	+	No
2055604_adh	TCTTCAGACGACTATTTGAAA	Chr.1	4389450	4389470	+	No
1958257_adh	TCAGACGACTATTTGAAAAAG	Chr.1	4389453	4389473	+	No
2254507_adh	TGAGTGTGGATCTTGTTGGAA	Chr.1	4393326	4393346	+	No
1770847_adh	TAACTGAAGAAGAACGTGAAT	Chr.1	4396146	4396166	+	No
2037060_adh	TCTCACATTTTAGACCCTGGC	Chr.1	4402095	4402075	-	No
2597135_adh	TTCCATAATTTGTAGAAGTCG	Chr.1	4431156	4431136	-	No
2712127_adh	TTTCTTTGTAGATTTCTTATA	Chr.1	4435903	4435883	-	No
2689532_adh	TTTAAATGTATAACGCTCGGT	Chr.1	4451057	4451037	-	No
2374069_adh	TGGACAGCAGTACAAGATGAC	Chr.1	4457004	4457024	+	No
2374069_adh	TGGACAGCAGTACAAGATGAC	Chr.1	4459828	4459848	+	No
1973831_adh	TCATTCGAGATGTTAGAAGAC	Chr.1	4464672	4464652	-	No
2580691_adh	TTATTACTGTAGATCTAAGGC	Chr.1	4468660	4468640	-	No
2659713_adh	TTGGAGCATATTTTGGACAAT	Chr.1	4478017	4477997	-	No
2584966_adh	TTCAAAACTCGGGGAAGACGA	Chr.1	4480935	4480915	-	No
2047922_adh	TCTGCAGGTTGACGTGATGGT	Chr.1	4511240	4511260	+	No
2710103_adh	TTTCTCTCAGAATTTGCGGAT	Chr.1	4537021	4537001	-	No
2709336_adh	TTTCGTTTATATGGACAACCT	Chr.1	4575105	4575125	+	No
2291044_adh	TGCATTAGTCGGAAGAATTTC	Chr.1	4584797	4584777	-	No
2600554_adh	TTCCTCAGTGGAAAGGCACTC	Chr.1	4585453	4585473	+	No
2736000_adh	TTTTATGCCGGCTTTCTTTCC	Chr.1	4602997	4602977	-	No
2693567_adh	TTTACCATTTTTATGCCGGCT	Chr.1	4603005	4602985	-	No
2665198_adh	TTGGCACTATTCTGAAGAATT	Chr.1	4603105	4603125	+	No
2697723_adh	TTTATAAATTCGGCAATTAAA	Chr.1	4613833	4613853	+	No
2374005_adh	TGGACAGAATCTGAAGGCTTT	Chr.1	4620738	4620718	-	No
2145116_adh	TGAATTAAAGAAAATCGGTCG	Chr.1	4621359	4621339	-	No

2258388_adh	TGATAACAACAAAAAATGGAG	Chr.1	4635479	4635459	-	No
1959113_adh	TCAGAGCTCCAAAGAAGAACC	Chr.1	4645871	4645851	-	No
2616311_adh	TTCTCCCGCATCATCCGTTCC	Chr.1	4645911	4645891	-	No
1883803_adh	TATAAAAAAGATTTTCGGTGA	Chr.1	4647158	4647138	-	No
2285922_adh	TGCACAATCAAAGAATACAAC	Chr.1	4655462	4655482	+	No
2683630_adh	TTGTTCCCAAAACTTTCCAAA	Chr.1	4659570	4659590	+	No
1772143_adh	TAACTTACATGAACAAGACGA	Chr.1	4666394	4666414	+	No
2308233_adh	TGCTGAAGCGGGTTATAGTAA	Chr.1	4694716	4694696	-	No
2537525_adh	TTAAAAAATTTTCGAACACAA	Chr.1	4708759	4708739	-	No
1919070_adh	TATGTGGATCGTGGAAGACAG	Chr.1	4710152	4710132	-	No
1812661_adh	TACAGTAGACAGTGTGCGCTC	Chr.1	4747089	4747069	-	No
2712624_adh	TTTGAAACTTCCACGCAAGCA	Chr.1	4765342	4765362	+	No
2693259_adh	TTTACACGGAAAGGGGACGCG	Chr.1	4766005	4766025	+	No
2693259_adh	TTTACACGGAAAGGGGACGCG	Chr.1	4768135	4768155	+	No
2259797_adh	TGATACGTGAGTGGAGACGGA	Chr.1	4788629	4788609	-	No
2739925_adh	TTTTCTTAATTTTCGTCAGAT	Chr.1	4789797	4789777	-	No
2585756_adh	TTCAACCAACGCTACGCGAAT	Chr.1	4790099	4790119	+	No
2700843_adh	TTTATTTTCTCGTTTGCCGTT	Chr.1	4790128	4790148	+	No
2481283_adh	TGTAACAGATGGAGTAGGTAA	Chr.1	4790592	4790572	-	No
2591924_adh	TTCAGGAAACTCTGACAGCAT	Chr.1	4790838	4790818	-	No
2524438_adh	TGTTATCTTGTGATTCAGGAA	Chr.1	4790851	4790831	-	No
2590248_adh	TTCAGAAACTCGTACAGCTGC	Chr.1	4791067	4791087	+	No
2113954_adh	TGAAATTACGGTATGTCGAAC	Chr.1	4791708	4791688	-	No
2744734_adh	TTTTTAACAGTAGAAGAAAAT	Chr.1	4791894	4791874	-	No
1806111_adh	TAATTTTTAACAGTAGAAGAA	Chr.1	4791897	4791877	-	No
1954596_adh	TCACTGAGTTTTGAAGTTTTG	Chr.1	4818171	4818151	-	No
2625619_adh	TTGAAATTACAGAAGTCTGTA	Chr.1	4845620	4845600	-	No
2653055_adh	TTGCGGTGTGTGGATTTACGG	Chr.1	4895934	4895954	+	No
1973130_adh	TCATTAAAATGAGCTCTTCGG	Chr.1	4895996	4896016	+	No
1983280_adh	TCCCTGATTGTTGTAAAGAAA	Chr.1	4903452	4903472	+	No
1929256_adh	TATTTAGCTACATTTTTAATC	Chr.1	4917952	4917932	-	No
2729246_adh	TTTGTGAAATCGGTTTCTCTC	Chr.1	4959451	4959471	+	No
1921586_adh	TATTAGCATTGAAAGAGCATT	Chr.1	4960326	4960346	+	No
1941106_adh	TCAATAGAATTCAGAGATGAT	Chr.1	4967291	4967271	-	No
2028082_adh	TCGTTCTCGATTCGGCAGTTA	Chr.1	4967402	4967382	-	No
2742836_adh	TTTTGGATGTTTGTAGAGGTT	Chr.1	4995129	4995149	+	No
2000883_adh	TCGAGAAACTCGGCCTGAAAC	Chr.1	4997047	4997067	+	No
2003966_adh	TCGATGAAGAAGGATTCAAAT	Chr.1	5005997	5005977	-	No
1982115_adh	TCCCTCATTTTCGTCGTTAAA	Chr.1	5006044	5006024	-	No
2598382_adh	TTCCCTCATTTTCGTCGTTAA	Chr.1	5006045	5006025	-	No
1932952_adh	TCAAAGAAATCGCCGATGACC	Chr.1	5019130	5019110	-	No
2599053_adh	TTCCGGAAAGCTCGAGTTTGA	Chr.1	5019338	5019318	-	No
1934400_adh	TCAAATCATGCATGGAATGGA	Chr.1	5019823	5019803	-	No
2654515_adh	TTGCTGAAATCGACGGATCCC	Chr.1	5019970	5019950	-	No
1939120_adh	TCAAGCACAAAGGTACAGTTC	Chr.1	5024934	5024914	-	No
2449208_adh	TGGGATTTAATACTATTAGAG	Chr.1	5040100	5040120	+	No
2449208_adh	TGGGATTTAATACTATTAGAG	Chr.1	5040511	5040531	+	No
1821539_adh	TACGAAATCGTAGAAGCTTCT	Chr.1	5040753	5040773	+	No
2486429_adh	TGTACAGAGAAGAACAGGTTA	Chr.1	5048542	5048522	-	No
2740608_adh	TTTTGAACAAGGACGGAATCG	Chr.1	5060056	5060036	-	No

1932383_adh	TCAAAATATGACAGAAGCATC	Chr.1	5103411	5103391	-	No
1933324_adh	TCAAAGATTTGCACGGACAAC	Chr.1	5105063	5105083	+	No
2749012_adh	TTTTTTCTCACTGTACCAAAC	Chr.1	5118889	5118869	-	No
1985244_adh	TCCGCAGTTGAAATTCTAGGC	Chr.1	5118956	5118936	-	No
2601883_adh	TTCGAACAGCTAAAGACAATT	Chr.1	5119048	5119068	+	No
1945463_adh	TCACAGTAGAACAGTTCGGAG	Chr.1	5122794	5122814	+	No
2589429_adh	TTCACCTAAAGAAAGAGCAAA	Chr.1	5124699	5124679	-	No
2605096_adh	TTCGATGACATTTTCTCGGCA	Chr.1	5127533	5127513	-	No
2655221_adh	TTGCTTCAGACTTATTATTCC	Chr.1	5138187	5138207	+	No
2653325_adh	TTGCGTACTCCTCTTGGACAA	Chr.1	5142718	5142738	+	No
2623183_adh	TTCTTGTTGAAATCTTCATAT	Chr.1	5162428	5162408	-	No
1975079_adh	TCATTTCCGTGCACGATTTTT	Chr.1	5162455	5162435	-	No
2703861_adh	TTTCATCAAGCAGTAGATCTA	Chr.1	5162952	5162972	+	No
2568607_adh	TTAGGCTTTTTACGTTTTGAG	Chr.1	5164947	5164927	-	No
2694369_adh	TTTACTCTTGGACCGGCAGGT	Chr.1	5176094	5176074	-	No
1807662_adh	TACAACGATTGTCGCGAATAC	Chr.1	5176271	5176251	-	No
1953226_adh	TCACGGATGGATCGTTGTGCA	Chr.1	5177125	5177145	+	No
1939024_adh	TCAAGATTAGAAGATGTACTT	Chr.1	5194788	5194768	-	No
2704787_adh	TTTCATTTTTCGTTGGTTTTC	Chr.1	5344526	5344546	+	No
1975624_adh	TCATTTTTCGTTGGTTTTCCT	Chr.1	5344528	5344548	+	No
2712659_adh	TTTGAAAGCACTTGGGAAGGT	Chr.1	5353995	5354015	+	No
2114070_adh	TGAAATTATAAGGCATCTGGT	Chr.1	5357350	5357370	+	No
2738020_adh	TTTTCCGGTTTCTTCGTCTGT	Chr.1	5368046	5368066	+	No
2585038_adh	TTCAAAATGAAGGACGGAAAT	Chr.1	5369748	5369768	+	No
1931464_adh	TATTTTTTAAAAGATTGTGGG	Chr.1	5411164	5411184	+	No
1976007_adh	TCCAACATAGTAGAGGAAGAG	Chr.1	5424209	5424189	-	No
2010371_adh	TCGGAAAGTCTGGACAAAAGC	Chr.1	5428931	5428911	-	No
2414757_adh	TGGCTGAGAACGGACGTCTGA	Chr.1	5429263	5429243	-	No
2592732_adh	TTCAGTAGAGCGCATTTGGTG	Chr.1	5429947	5429967	+	No
2625340_adh	TTGAAATAAAATAAAAATTCA	Chr.1	5449090	5449110	+	No
1995400_adh	TCGAACAAATGGACGACGTAC	Chr.1	5452901	5452921	+	No
2745671_adh	TTTTTCACTTTTCGCTAGGCC	Chr.1	5455217	5455237	+	No
2607239_adh	TTCGGAATCGGATAAAGGTAC	Chr.1	5464009	5464029	+	No
1828826_adh	TACGTGTAAGATAATAGCTCA	Chr.1	5470609	5470589	-	No
2707006_adh	TTTCGAGACGAATGGAGAAAT	Chr.1	5472167	5472187	+	No
2150433_adh	TGACAAGAACAAGGAGAGCCG	Chr.1	5484941	5484921	-	No
1938241_adh	TCAAGAAGAAGACTGAGAACA	Chr.1	5485094	5485074	-	No
2586413_adh	TTCAAGAAGAAGACTGAGAAC	Chr.1	5485095	5485075	-	No
2714113_adh	TTTGAATAAAATTTTCGGGGG	Chr.1	5486073	5486093	+	No
2593836_adh	TTCATATTTTTTGGACATTTA	Chr.1	5486112	5486132	+	No
2487594_adh	TGTAGACAGAAGATAGTTTTT	Chr.1	5487575	5487595	+	No
2591771_adh	TTCAGCGCGACAGAAAACACG	Chr.1	5511697	5511677	-	No
1944385_adh	TCACAATTATCCGGCACATAA	Chr.1	5511774	5511794	+	No
2675238_adh	TTGTAGTAAAAGCGGTGACAT	Chr.1	5547467	5547447	-	No
2706480_adh	TTTCCTTTTATTTATCGTTGT	Chr.1	5550557	5550537	-	No
2036667_adh	TCTCAATGATGGTAGTGATAA	Chr.1	5560028	5560008	-	No
1781688_adh	TAAGCAGAAATTTTGGACAAA	Chr.1	5560257	5560277	+	Yes
2309444_adh	TGCTGTCGAAGTAGATGAGCA	Chr.1	5572900	5572880	-	No
2469017_adh	TGGTATTTGCAGAGCGAACGC	Chr.1	5573205	5573225	+	No
1930275_adh	TATTTGCAGAGCGAACGCATA	Chr.1	5573208	5573228	+	No

2609990_adh	TTCGGTCGACTATCGGAGCAA	Chr.1	5579153	5579133	-	No
2144774_adh	TGAATGTCGCTCGAAGAAGAA	Chr.1	5579183	5579163	-	No
1810674_adh	TACACAGAATAAGAGGGATAG	Chr.1	5585539	5585519	-	No
2317863_adh	TGGAAATATAAGTTTGATGAT	Chr.1	5585713	5585733	+	No
2374102_adh	TGGACAGGAGAGGACAAGCCA	Chr.1	5599511	5599491	-	No
2285886_adh	TGCACAAACTGGAACACGGTG	Chr.1	5608744	5608724	-	No
2062602_adh	TCTTTTCGTCAAATTTGTATC	Chr.1	5619249	5619229	-	No
2606057_adh	TTCGATTTTGCAGCTTGAAGT	Chr.1	5619384	5619404	+	No
2706983_adh	TTTCGACTTTAGTTTTGGTTA	Chr.1	5619455	5619475	+	No
1938943_adh	TCAAGATAAGACAACGCCAAG	Chr.1	5619679	5619699	+	No
2512289_adh	TGTGATTGCAACCGTAGGAGA	Chr.1	5644787	5644767	-	No
2500167_adh	TGTCTTCTCGGCTTTCTAAAC	Chr.1	5659093	5659073	-	No
1797782_adh	TAATCAATTATTATGAAATTA	Chr.1	5671427	5671447	+	No
2001567_adh	TCGAGCTGAATACACGTGTTG	Chr.1	5678767	5678747	-	No
2618970_adh	TTCTGGAGCTCTTTTGACGAC	Chr.1	5691676	5691656	-	No
2270478_adh	TGATCTGGATATCAAGTAGAT	Chr.1	5699321	5699301	-	No
1761454_adh	TAAATGATAGGTCTGAGCCAA	Chr.1	5707103	5707123	+	No
2716918_adh	TTTGAGGCGCATAGAAAATCA	Chr.1	5709545	5709565	+	No
1931373_adh	TATTTTTGATCTTGCTGGAAA	Chr.1	5734657	5734677	+	No
1797402_adh	TAATCAAAATTTCACGGCTAT	Chr.1	5735534	5735554	+	No
2334214_adh	TGGAATCGAAAGAAGAATCAA	Chr.1	5738396	5738416	+	No
2060649_adh	TCTTTATCATTTACTGAAGGC	Chr.1	5744027	5744007	-	No
2125499_adh	TGAACTGGGAAGCGAAGAAAG	Chr.1	5762550	5762570	+	No
1893205_adh	TATCACTTTTCAAGTGTTTGT	Chr.1	5774590	5774610	+	No
2584473_adh	TTATTTTAATGTGGAACACGG	Chr.1	5785890	5785870	-	No
2645141_adh	TTGATGGATTAGTAAAAGAAG	Chr.1	5807122	5807102	-	No
1758402_adh	TAAAGTAGAAGAATTCAAGAA	Chr.1	5817527	5817507	-	No
2169412_adh	TGACTGACAAGTTTCAGAATG	Chr.1	5818146	5818126	-	No
2740275_adh	TTTTCTTGTTCGCGGACGTTT	Chr.1	5818194	5818174	-	No
1943310_adh	TCAATTTCCTGAGCAATGCTT	Chr.1	5827990	5828010	+	No
2617653_adh	TTCTGAAAACGAGTCTGAAAG	Chr.1	5847587	5847567	-	No
2557209_adh	TTACGGAAGAAGATCGCGTGT	Chr.1	5851815	5851795	-	No
2161083_adh	TGACGATAGAGTGGGAGACAA	Chr.1	5866099	5866119	+	No
1757644_adh	TAAAGCTGAAGCCATTCAAAC	Chr.1	5876395	5876375	-	No
2541518_adh	TTAAAGCTGAAGCCATTCAAA	Chr.1	5876396	5876376	-	No
2062585_adh	TCTTTTCGAGCAAAGAATTCT	Chr.1	5878373	5878353	-	No
2435439_adh	TGGGATGCAAAGAAAAATCAA	Chr.1	5879262	5879282	+	No
1883975_adh	TATAAAATGATTCTGCAACCT	Chr.1	5883428	5883408	-	No
2254425_adh	TGAGTGTATTTTGTATTTTAG	Chr.1	5900864	5900884	+	No
1830940_adh	TACTCATTGGACTATGTACGG	Chr.1	5936959	5936939	-	No
2689092_adh	TTTAAACGGCTTGCATTTCTC	Chr.1	5937169	5937189	+	No
2539967_adh	TTAAACGGCTTGCATTTCTCC	Chr.1	5937170	5937190	+	No
1837197_adh	TAGAAAGACAAAGGTAACGCT	Chr.1	5964155	5964135	-	No
2480314_adh	TGTAAAAAAGTGTAGGAAAAT	Chr.1	5978955	5978935	-	No
2480314_adh	TGTAAAAAAGTGTAGGAAAAT	Chr.1	5982992	5983012	+	No
2746626_adh	TTTTTGACGCATTTTCATCAA	Chr.1	6003066	6003046	-	No
1943299_adh	TCAATTTCACAACCGTTTTTA	Chr.1	6011987	6012007	+	No
2308845_adh	TGCTGGAGATCAAGGAACAAC	Chr.1	6026080	6026100	+	No
2458272_adh	TGGGTCTTGGTGATCGTCATC	Chr.1	6032403	6032423	+	No
1797906_adh	TAATCATAGAGCTTGTGCAGA	Chr.1	6055301	6055281	-	No

1797906_adh	TAATCATAGAGCTTGTGCAGA	Chr.1	6057705	6057685	-	No
2688729_adh	TTTAAAAATGTTTGTCTGACA	Chr.1	6061306	6061286	-	No
2574441_adh	TTATACGGTGAACCTAGGGGA	Chr.1	6063780	6063760	-	No
2072730_adh	TGAAACCGTAGAAAACTTCAA	Chr.1	6068198	6068178	-	No
2747930_adh	TTTTTGTTGCGTTTGGACATT	Chr.1	6080542	6080522	-	No
2150413_adh	TGACAAGAAAGTATGATGGCT	Chr.1	6082759	6082739	-	No
2691514_adh	TTTAATGAATTCTAAGACCAA	Chr.1	6091080	6091060	-	No
2493618_adh	TGTATTTGAGAAGGCTCGTAT	Chr.1	6093852	6093872	+	No
1996986_adh	TCGAATAATGTGAAGACTCAG	Chr.1	6097572	6097552	-	No
2029610_adh	TCTAACCCTCGTCTTCTCTAC	Chr.1	6098960	6098980	+	No
2706208_adh	TTTCCTCCGAAACATACTTTT	Chr.1	6109712	6109692	-	No
2422785_adh	TGGGAGAATTAGAGAAATCGA	Chr.1	6115119	6115099	-	No
2693097_adh	TTTACAAAGGTAGTTCTAGGT	Chr.1	6115253	6115233	-	No
2309022_adh	TGCTGGCGGCAAACTCTTTTT	Chr.1	6163483	6163463	-	No
1973339_adh	TCATTAGAACTTATAGGCATT	Chr.1	6166666	6166686	+	No
1922470_adh	TATTATGATTGCCTGACGTTT	Chr.1	6176320	6176300	-	No
2658788_adh	TTGGACTCGATGTCGCCGAGC	Chr.1	6207847	6207827	-	No
1873185_adh	TAGTAAGAAGGTACGTGTTTT	Chr.1	6210265	6210245	-	No
2053647_adh	TCTTAAAGGCGCGTAGGAATC	Chr.1	6215612	6215592	-	No
1821995_adh	TACGACAAAGAGACCACTAAT	Chr.1	6219977	6219997	+	No
2487386_adh	TGTAGAAAACAGAAGAGGAGG	Chr.1	6220949	6220969	+	No
2679275_adh	TTGTGAACTGGTTGGTGAATT	Chr.1	6221101	6221121	+	No
1891543_adh	TATCAAGCCGTTCAAGACTTC	Chr.1	6224200	6224220	+	No
1990768_adh	TCCTCTCGGACGATTAATTAA	Chr.1	6224851	6224871	+	No
1934040_adh	TCAAATACAAGTAGACGTAAT	Chr.1	6228705	6228685	-	No
1931519_adh	TATTTTTTGTGAACTGGGGTT	Chr.1	6230189	6230169	-	No
2616069_adh	TTCTCACTTGAACATTAACAT	Chr.1	6232118	6232098	-	No
2616069_adh	TTCTCACTTGAACATTAACAT	Chr.1	6234723	6234743	+	No
2319843_adh	TGGAACCTGAGAAATTAATAG	Chr.1	6238146	6238126	-	No
1896767_adh	TATCGAATATTGAACGGTGTG	Chr.1	6258026	6258006	-	No
2580429_adh	TTATGTTTTTGAATATTGCAA	Chr.1	6261713	6261693	-	No
1974070_adh	TCATTGCAGACATTTGACAAC	Chr.1	6265246	6265266	+	No
2702963_adh	TTTCAGATGATAGTTTTTGTA	Chr.1	6268823	6268803	-	No
2692329_adh	TTTAATTCTCGTCTTGTCTAC	Chr.1	6275291	6275271	-	No
2607136_adh	TTCGGAAATTTGGCGGAATAA	Chr.1	6277250	6277270	+	No
2049141_adh	TCTGGAGGTGTTGCTCGGCGT	Chr.1	6285220	6285240	+	No
2378953_adh	TGGACTTGACAGTTTGGAGCG	Chr.1	6316915	6316935	+	No
1978564_adh	TCCAGCTTCCCGGTATATTCC	Chr.1	6317825	6317805	-	No
2616069_adh	TTCTCACTTGAACATTAACAT	Chr.1	6329540	6329560	+	No
1746068_adh	TAAAAACTCTAGTCGGCATTT	Chr.1	6338183	6338203	+	No
1941082_adh	TCAATAGAAGAACGGTACACA	Chr.1	6340247	6340227	-	No
2737906_adh	TTTTCCATTTTCTCGGCGTTA	Chr.1	6341964	6341984	+	No
2740705_adh	TTTTGAACCGTGATGGTACTC	Chr.1	6342993	6343013	+	No
1848167_adh	TAGAGCTCAACGACAGCAGCC	Chr.1	6343015	6343035	+	No
2590104_adh	TTCACTTGCATGTCGTTGGGT	Chr.1	6348065	6348085	+	No
2579716_adh	TTATGGTAAAGGATCGATTGG	Chr.1	6356018	6356038	+	No
2601895_adh	TTCGAACATTTATTAGTAGAG	Chr.1	6366224	6366204	-	No
2600525_adh	TTCCTCAATTGTAGAGACAAT	Chr.1	6366282	6366262	-	No
2502878_adh	TGTGAATGACGAAGAGACGCA	Chr.1	6412451	6412471	+	No
1973662_adh	TCATTATTGTAGAAGAATAAC	Chr.1	6413898	6413918	+	No

2654236_adh	TTGCTCAAGAATTAGTTAGAA	Chr.1	6460848	6460828	-	No
2712539_adh	TTTGAAACAAATGTTGACTGT	Chr.1	6467187	6467167	-	No
2529784_adh	TGTTGGACTATGGCTATAAAT	Chr.1	6470531	6470511	-	No
2161540_adh	TGACGGAAGCAGCTCTCCATT	Chr.1	6472554	6472534	-	No
2702691_adh	TTTCACTGACGGAAGCAGCTC	Chr.1	6472560	6472540	-	No
2381796_adh	TGGAGCTTGGCATTCTGAAAA	Chr.1	6473235	6473215	-	No
2499371_adh	TGTCTCGCTGGAGCTTGGCAT	Chr.1	6473243	6473223	-	No
2274906_adh	TGATTATAGATTATGGAACAG	Chr.1	6498226	6498246	+	No
2583314_adh	TTATTGTGGAAAGATCAGCGG	Chr.1	6498484	6498504	+	No
1901330_adh	TATGAAAGAATATGGAATACC	Chr.1	6498985	6499005	+	No
2553217_adh	TTACAAATCAGACGACAACTT	Chr.1	6501369	6501349	-	No
1975551_adh	TCATTTTGAAGCAAACTTTGG	Chr.1	6525048	6525068	+	No
2277360_adh	TGATTGTTCTATTCGTCGTCT	Chr.1	6530095	6530075	-	No
1926102_adh	TATTGATTGTTCTATTCGTCG	Chr.1	6530098	6530078	-	No
1923367_adh	TATTCAAGAAGAAAACTGCGT	Chr.1	6553465	6553445	-	No
2646696_adh	TTGATTGTTGTGCCACGTTGC	Chr.1	6554589	6554569	-	No
2745024_adh	TTTTTACTTGATTGTTGTGCC	Chr.1	6554596	6554576	-	No
2240117_adh	TGAGGGCACTCGGCTGACATC	Chr.1	6554758	6554778	+	No
2015777_adh	TCGGCTGACATCAGAGAAGAA	Chr.1	6554767	6554787	+	No
2258741_adh	TGATAAGAATTGATTGATGGA	Chr.1	6556269	6556289	+	No
1852019_adh	TAGATTCCTGTAGAATTTGCA	Chr.1	6558330	6558350	+	No
2527049_adh	TGTTCTGTTGGCATTTTCCAT	Chr.1	6562567	6562587	+	No
2737897_adh	TTTTCCATGCGTCGAATTTCT	Chr.1	6562580	6562600	+	No
1880588_adh	TAGTTCCTTTCTTTCGTCTAA	Chr.1	6565947	6565927	-	No
1745986_adh	TAAAAACGGAAATGGACGTCT	Chr.1	6570515	6570535	+	No
1933909_adh	TCAAAGTCAACGAAAAATAGA	Chr.1	6573123	6573103	-	No
2558143_adh	TTACGTTGAATTTGTGTTTTC	Chr.1	6573936	6573956	+	No
2148991_adh	TGACAAAAGGACGACAGTTGT	Chr.1	6587264	6587284	+	No
1908519_adh	TATGACTCACCGGTCGGAAGA	Chr.1	6593475	6593455	-	Yes
1931922_adh	TCAAAAATGGATCGTCAATTG	Chr.1	6600940	6600920	-	No
2499003_adh	TGTCTAATTAGATCGGGGACA	Chr.1	6609592	6609572	-	No
1763942_adh	TAACAATAAAAGAAAGTGGAA	Chr.1	6682244	6682264	+	No
1989314_adh	TCCTACGATTTCGTTGTTCAA	Chr.1	6682294	6682314	+	No
2696652_adh	TTTAGGATAAGAAAGACGACG	Chr.1	6705673	6705653	-	No
2716327_adh	TTTGAGAACGAGAATGCGGAA	Chr.1	6717653	6717633	-	No
2541811_adh	TTAAAGTTAGGAGACGTAGAG	Chr.1	6721630	6721650	+	No
2578307_adh	TTATGAACTTTTAAAAACGGA	Chr.1	6724638	6724658	+	No
2012775_adh	TCGGAGCTACGTGTCATTTCT	Chr.1	6741292	6741312	+	No
1908660_adh	TATGACTGGATTCGGACAGCC	Chr.1	6763573	6763553	-	No
2499898_adh	TGTCTGTAGTTGACCTTTTTC	Chr.1	6780338	6780358	+	No
1824954_adh	TACGCGATTAAGTATCTTGTA	Chr.1	6787511	6787491	-	No
2587979_adh	TTCAATGAACGATTTAAAAAA	Chr.1	6789135	6789115	-	No
2702975_adh	TTTCAGCAAAGAGCACTTTCA	Chr.1	6790213	6790233	+	No
1886637_adh	TATAATTTTTCCTTAGAACTA	Chr.1	6798462	6798482	+	No
2655935_adh	TTGCTTTCTAGATTTGACATG	Chr.1	6799741	6799761	+	No
2542113_adh	TTAAATACAACGAATAAAGGC	Chr.1	6814262	6814242	-	No
2551668_adh	TTAATTAAATACAACGAATAA	Chr.1	6814266	6814246	-	No
2259028_adh	TGATAAGTTCGGAAATTTGGC	Chr.1	6833489	6833509	+	No
2624576_adh	TTGAAAATTACTAAAGAAAAT	Chr.1	6835882	6835862	-	No
1994646_adh	TCGAAACAGTAGAAATTTCGA	Chr.1	6835968	6835988	+	No

2673666_adh	TTGTAAATTTCTTCGTCATGG	Chr.1	6836059	6836079	+	No
2678224_adh	TTGTCGCTGGATACGGAGATG	Chr.1	6837207	6837227	+	No
2133073_adh	TGAAGGATGGAAAAGACGCTA	Chr.1	6847070	6847050	-	No
1954424_adh	TCACTGAAGGATGGAAAAGAC	Chr.1	6847074	6847054	-	No
2043674_adh	TCTGAAACAAACTCAAGTTCG	Chr.1	6849985	6850005	+	No
2701542_adh	TTTCAAGATCTGATGGGATTC	Chr.1	6854627	6854607	-	No
1919300_adh	TATGTTCAATTGCAGACTTCG	Chr.1	6858371	6858351	-	No
2717444_adh	TTTGATACTTTTGGAACATGC	Chr.1	6882492	6882472	-	No
2734337_adh	TTTTACTGTAGAATGCAAGGT	Chr.1	6884654	6884634	-	No
2279125_adh	TGATTTTACTGTAGAATGCAA	Chr.1	6884657	6884637	-	No
2041953_adh	TCTCTAATTACATTCGGATTG	Chr.1	6893594	6893574	-	No
1924035_adh	TATTCCGAATTTTTAATATTT	Chr.1	6897287	6897307	+	No
2539156_adh	TTAAAATTTGTTTCGTCTTTC	Chr.1	6913866	6913846	-	No
2174527_adh	TGACTTTGAAGGATGTGCTCA	Chr.1	6915156	6915176	+	No
2650907_adh	TTGCATTTGTTCGTCGCCCAT	Chr.1	6923912	6923892	-	No
1773709_adh	TAAGAACATTGATAAGAAGAA	Chr.1	6943077	6943057	-	No
2735532_adh	TTTTATCCGGAATGGTCTCGC	Chr.1	6950793	6950813	+	No
1853753_adh	TAGCAATTTTCCTTCCCAAAG	Chr.1	6955372	6955352	-	No
2741661_adh	TTTTGATCGTTCTTCGAAGTT	Chr.1	6970250	6970230	-	No
2616216_adh	TTCTCCACAATTTTGATCGTT	Chr.1	6970260	6970240	-	No
2513351_adh	TGTGCAGAGAAAACGGTCGGT	Chr.1	6970280	6970260	-	No
2033316_adh	TCTAGTGACTTGATGTGCAGA	Chr.1	6970293	6970273	-	No
2701727_adh	TTTCAATCGGAAAATCGTTGC	Chr.1	6973694	6973674	-	No
2526302_adh	TGTTCCTTAAGAGGGAAGCTT	Chr.1	6992615	6992595	-	No
2636231_adh	TTGACGGTAATTCAAATTGGA	Chr.1	7002435	7002455	+	No
1812687_adh	TACAGTAGGCCGCAAAAATTA	Chr.1	7014412	7014392	-	No
1944131_adh	TCACAAGCAGAATTTTTCGAT	Chr.1	7015706	7015686	-	No
2258922_adh	TGATAAGGATGTGCTCATTGA	Chr.1	7016879	7016859	-	No
1799304_adh	TAATGAAGGAAAGTATCCAAT	Chr.1	7017135	7017115	-	No
2122953_adh	TGAACGACGTGTTTTTGGCTC	Chr.1	7021441	7021421	-	No
2552934_adh	TTAATTTTGTCGGTTTTTTAA	Chr.1	7021943	7021963	+	No
1924104_adh	TATTCCGTACCAGTTCAAGTA	Chr.1	7028988	7029008	+	No
2712824_adh	TTTGAAATTGAACTGCAAAGG	Chr.1	7029690	7029670	-	No
1919752_adh	TATGTTTTAATTCTAGACGGT	Chr.1	7052543	7052563	+	No
2667773_adh	TTGGGGAGTACTGTAAAGAAA	Chr.1	7056630	7056610	-	No
1876825_adh	TAGTCGTGGAACACAGAATGG	Chr.1	7067769	7067749	-	No
2623454_adh	TTCTTTCCTAAATCGAGGCCA	Chr.1	7069369	7069389	+	No
1821574_adh	TACGAACATAAGAAGAACATA	Chr.1	7075863	7075883	+	No
1810667_adh	TACACACTTTATTTCATTGGA	Chr.1	7079601	7079621	+	No
2377771_adh	TGGACTGCGAAGATATTATCA	Chr.1	7080845	7080825	-	No
1957584_adh	TCAGAAGACACTGTTATTCTG	Chr.1	7084672	7084692	+	No
1925023_adh	TATTCTGGTCTGCTTGGAGAG	Chr.1	7084686	7084706	+	No
1921983_adh	TATTATAGAAAAATACGATCG	Chr.1	7086447	7086427	-	No
2728446_adh	TTTGTATATTTTTGAAGCAGA	Chr.1	7086772	7086792	+	No
2624330_adh	TTGAAAACAGATGATTAACAA	Chr.1	7090572	7090592	+	No
1755966_adh	TAAACTTTGTTTTTCTGTCGT	Chr.1	7098387	7098407	+	No
1755966_adh	TAAACTTTGTTTTTCTGTCGT	Chr.1	7099683	7099663	-	No
2544223_adh	TTAAATGTGGATGAAGATGAG	Chr.1	7118380	7118360	-	No
2716934_adh	TTTGAGGGAAAGTGTTAGACG	Chr.1	7128488	7128508	+	No
1806704_adh	TACAAAGAATGCCAAGATTAC	Chr.1	7136669	7136649	-	No

1756594_adh	TAAAGACGTCAGTGGACAAAA	Chr.1	7144589	7144569	-	No
1766211_adh	TAACATGTAAGATTCTTTAAA	Chr.1	7148517	7148537	+	No
2042912_adh	TCTCTGCATACCACTCTAACT	Chr.1	7149605	7149585	-	No
2615958_adh	TTCTCAATAATGTTTGTAGAC	Chr.1	7176441	7176421	-	No
1814404_adh	TACATTCTGGTGTGAGTTGGA	Chr.1	7185698	7185718	+	No
2658422_adh	TTGGACGAGTTGAAGATTTCA	Chr.1	7185714	7185734	+	No
2155026_adh	TGACATACAAGTAGAGAACTG	Chr.1	7188311	7188291	-	No
2386726_adh	TGGATAAATTGCAGAAAAAGG	Chr.1	7193645	7193625	-	No
2674670_adh	TTGTAGAACAAATTCGTCATT	Chr.1	7195672	7195652	-	No
1745979_adh	TAAAAACGCAGAAGTTCAAAT	Chr.1	7218185	7218165	-	No
2567524_adh	TTAGGAACAATTCTACGGAGA	Chr.1	7221867	7221847	-	No
2688381_adh	TTGTTTTTATGCCGCCTCTGA	Chr.1	7235133	7235153	+	No
1925250_adh	TATTCTTTTTCGTCGGCAATA	Chr.1	7235177	7235197	+	No
2639104_adh	TTGACTTGGAAGCAGCTCTAG	Chr.1	7238948	7238928	-	No
2140342_adh	TGAATCACAGAAGCTCATTCA	Chr.1	7245416	7245436	+	No
1882922_adh	TAGTTTCGAAATCGACGGATC	Chr.1	7251490	7251470	-	No
2070791_adh	TGAAAATTGGGCGGTGCTTTA	Chr.1	7251663	7251643	-	No
2702609_adh	TTTCACGTCTCCAATGAACTC	Chr.1	7251842	7251862	+	No
2648972_adh	TTGCACATTCGTTTTGTTTCA	Chr.1	7255406	7255426	+	No
1975964_adh	TCCAAATTCCGACAGATCCAA	Chr.1	7259578	7259558	-	No
1957794_adh	TCAGAAGTCTAAGGCTAAGAT	Chr.1	7262175	7262155	-	No
1824695_adh	TACGCAGAAAGAACAATGACC	Chr.1	7262230	7262210	-	No
2719119_adh	TTTGATGGCTTTGAAGATTTG	Chr.1	7286277	7286297	+	No
2043830_adh	TCTGAAATTTTGCAGGATCGG	Chr.1	7299457	7299437	-	No
2708297_adh	TTTCGGTAACGCTCTTTCAGC	Chr.1	7304763	7304743	-	No
2161216_adh	TGACGCAGTTGATGATGATCG	Chr.1	7318027	7318007	-	No
1958013_adh	TCAGACAAAACAAGCAACTCT	Chr.1	7318874	7318894	+	No
2722884_adh	TTTGGAAACAACATGAGCATA	Chr.1	7330774	7330754	-	No
2605697_adh	TTCGATTCTTTGGTTCCGCGT	Chr.1	7347471	7347491	+	No
1812536_adh	TACAGGCAGATACTCTCTCAT	Chr.1	7352601	7352621	+	No
2007329_adh	TCGCCAAGTTGGAGACCAGAA	Chr.1	7377966	7377946	-	No
1972090_adh	TCATGAGGAACAAGAACACTC	Chr.1	7378094	7378074	-	No
2124652_adh	TGAACTCGAGGAATGCAAGAC	Chr.1	7378352	7378332	-	No
2032386_adh	TCTAGACTGAAGAAGAAGTAC	Chr.1	7378665	7378645	-	No
2723176_adh	TTTGGAATCTGATAGAGGACT	Chr.1	7407271	7407251	-	No
1874738_adh	TAGTAGGATACGGTAAAATTG	Chr.1	7445045	7445065	+	No
1821475_adh	TACGAAACTCGAGCAGACATG	Chr.1	7466741	7466721	-	No
2259546_adh	TGATACGAAACTCGAGCAGAC	Chr.1	7466744	7466724	-	No
1969755_adh	TCATATGGAGAAGAACACGTG	Chr.1	7472453	7472433	-	No
2719722_adh	TTTGATTCAACGATGGAAGGA	Chr.1	7475460	7475480	+	No
1919113_adh	TATGTGGTACTTTCCAGTTGA	Chr.1	7488145	7488125	-	No
2132106_adh	TGAAGCTGTGAAGAAAGTACA	Chr.1	7492613	7492633	+	No
2646009_adh	TTGATTCCAGAAGTAGAGCAA	Chr.1	7493008	7493028	+	No
2596680_adh	TTCCAGAAGTAGAGCAATTCT	Chr.1	7493012	7493032	+	No
2048064_adh	TCTGCATTCCTGTCGAAGTCG	Chr.1	7493777	7493797	+	No
1753056_adh	TAAAATTATCATTTATCGTCA	Chr.1	7509106	7509086	-	No
2160709_adh	TGACGACGGCAACTGTAATGA	Chr.1	7518166	7518146	-	No
2287983_adh	TGCAGATGGTAATATCGTAGA	Chr.1	7542974	7542994	+	No
2003195_adh	TCGATAGTGCGTGACGATATA	Chr.1	7548705	7548725	+	No
2037310_adh	TCTCAGAATTTCGTGGTCTCG	Chr.1	7553370	7553350	-	No

2703548_adh	TTTCAGTCGATTTGACCTTTT	Chr.1	7554852	7554872	+	No
2058078_adh	TCTTGATCTAGAAGAGTGGAA	Chr.1	7567761	7567781	+	No
2380104_adh	TGGAGACCACTTGGAGACTGA	Chr.1	7573835	7573815	-	No
2459351_adh	TGGGTTCAAACTCGACGTGGT	Chr.1	7588125	7588145	+	No
2740564_adh	TTTTGAAACTGTAAATTAATT	Chr.1	7588494	7588474	-	No
2286609_adh	TGCACTCATCTGTCGGTTTTG	Chr.1	7588510	7588490	-	No
2649141_adh	TTGCACTCATCTGTCGGTTTT	Chr.1	7588511	7588491	-	No
2729141_adh	TTTGTCTGCAAGAAACGTATT	Chr.1	7588675	7588695	+	No
1815439_adh	TACCAGCTGAAGATGCTCCAC	Chr.1	7590651	7590671	+	No
2382145_adh	TGGAGGCAGAAGAACCCTCAT	Chr.1	7592233	7592213	-	No
1979779_adh	TCCATGGAAGGGATCTTTGAG	Chr.1	7595459	7595439	-	No
1849557_adh	TAGATAAAAAGATTGGAAATC	Chr.1	7595984	7595964	-	No
2272200_adh	TGATGGACTTGTAAAGGTATA	Chr.1	7599616	7599636	+	No
2265568_adh	TGATATGTAGAGAGGTCAAGT	Chr.1	7614872	7614852	-	No
2172231_adh	TGACTGTGGAGATGGTTCCGA	Chr.1	7621449	7621469	+	No
1911590_adh	TATGATGGAACTGATGAAGAC	Chr.1	7621579	7621599	+	No
1936100_adh	TCAACAGGATTGTGATGATGG	Chr.1	7621818	7621838	+	No
2608305_adh	TTCGGATGTACTAGTGGAAGA	Chr.1	7621855	7621875	+	No
2323172_adh	TGGAAGATGTGCTCGTGACTT	Chr.1	7622173	7622193	+	No
2008629_adh	TCGCTGAAGAGTGTGTCTGTT	Chr.1	7643277	7643257	-	No
2566382_adh	TTAGCAGATATTGGATAACGA	Chr.1	7643368	7643388	+	No
2705274_adh	TTTCCCATGTTGAATTGAGGT	Chr.1	7660262	7660242	-	No
2278604_adh	TGATTTGCATGAGGCTTTTCA	Chr.1	7695278	7695258	-	No
2740132_adh	TTTTCTTCGTTTGTTTCTTTA	Chr.1	7699443	7699463	+	No
2393737_adh	TGGATTGAAGAGTGGAGACAA	Chr.1	7704027	7704047	+	No
2030915_adh	TCTAATTAAGAAACGCGTCTC	Chr.1	7723599	7723619	+	No
2129085_adh	TGAAGAGACGGATTGTTTTAC	Chr.1	7765899	7765919	+	No
2717129_adh	TTTGAGTTGAAATTTTGGTAA	Chr.1	7805041	7805061	+	No
2602662_adh	TTCGAATTTGGAAACGGCAGC	Chr.1	7813489	7813509	+	No
2417923_adh	TGGGAAATTCGGTAATAACTT	Chr.1	7835633	7835613	-	No
2046804_adh	TCTGATCAGTGGAGAGCAACT	Chr.1	7838853	7838873	+	No
2626324_adh	TTGAACGACTGAACGACGACT	Chr.1	7841888	7841908	+	No
2679405_adh	TTGTGAAGTTAGAATAGGTTA	Chr.1	7862652	7862672	+	No
2548496_adh	TTAAGGCTTTAAAATTGTTAT	Chr.1	7914693	7914673	-	No
2279250_adh	TGATTTTCTTCGAGATTAGAT	Chr.1	7914848	7914868	+	No
2702912_adh	TTTCAGAGAGACGATGAAATC	Chr.1	7918874	7918854	-	No
2124023_adh	TGAACTAGAAGATGCTCTCCA	Chr.1	7928499	7928479	-	No
2552900_adh	TTAATTTTCCTGCATTCAACG	Chr.1	7936204	7936224	+	No
1801026_adh	TAATGTAAGAACAACGGTTCC	Chr.1	7985226	7985246	+	No
2375919_adh	TGGACGAAGAAGGACACGTTA	Chr.1	8015189	8015209	+	No
1929212_adh	TATTTAGAACGTCTATTCAAA	Chr.1	8018762	8018782	+	No
2122702_adh	TGAACCTGTAGTGATTGTCAT	Chr.1	8019258	8019278	+	No
2596199_adh	TTCCACGAACGAAGACAGCGC	Chr.1	8019769	8019789	+	No
2635636_adh	TTGACGACTATTGTGGAAGAA	Chr.1	8020649	8020629	-	No
2720420_adh	TTTGATTGTGAAAGGAAGATG	Chr.1	8027083	8027063	-	No
1847982_adh	TAGAGCAGACTGCATTATTGC	Chr.1	8040658	8040678	+	No
2708656_adh	TTTCGTAATGTAGGGTAGGTT	Chr.1	8044436	8044416	-	Yes
2353195_adh	TGGAATGGGACGGAGAAGGCT	Chr.1	8046062	8046042	-	No
2722917_adh	TTTGGAAAGATTCGAAAGCAT	Chr.1	8207674	8207694	+	No
2530143_adh	TGTTGGTGATTGTATACAAAT	Chr.1	8220654	8220634	-	No

2541677_adh	TTAAAGGTGTCATTTTTATTT	Chr.1	8223043	8223023	-	No
2537563_adh	TTAAAAACGGATTCTTATTGA	Chr.1	8223297	8223317	+	No
2018360_adh	TCGGTACATTTTCGTTGGAGA	Chr.1	8224067	8224047	-	No
2605477_adh	TTCGATTATAGAGTTGAGGGA	Chr.1	8226410	8226430	+	No
2513228_adh	TGTGCAAGGATGAAAAAGAAC	Chr.1	8259492	8259472	-	No
2532352_adh	TGTTTCCGCGTGACGAAGACA	Chr.1	8288868	8288888	+	No
2602785_adh	TTCGACAGAAAACTGCTGGAG	Chr.1	8291121	8291101	-	No
2389309_adh	TGGATATTTTCGTTGGAGTTA	Chr.1	8299546	8299526	-	No
2561589_adh	TTAGAAGTTCGTGGCATGTGT	Chr.1	8309421	8309401	-	No
2713741_adh	TTTGAAGACTTTATACAGGAA	Chr.1	8312762	8312782	+	No
1901557_adh	TATGAACAGTACACGACGATA	Chr.1	8317386	8317406	+	No
2657160_adh	TTGGAAGCTGGCAGTGAATTT	Chr.1	8323278	8323298	+	No
2260585_adh	TGATAGATTTTTATCTCGTCG	Chr.1	8324045	8324025	-	No
2704745_adh	TTTCATTTGTCGGGATTTCTT	Chr.1	8324086	8324066	-	No
2737811_adh	TTTTCATTTGTCGGGATTTCT	Chr.1	8324087	8324067	-	No
2529430_adh	TGTTGCCAACAAGATCGGATT	Chr.1	8329133	8329113	-	No
2623832_adh	TTCTTTTCGCGATTTTCGTCG	Chr.1	8330035	8330015	-	No
2301528_adh	TGCGGCATCTCCTGATTGGCT	Chr.1	8370149	8370169	+	No
1861015_adh	TAGGAAGAATCGCGGAAGATG	Chr.1	8381547	8381567	+	No
2556150_adh	TTACCGGGTAAAAGATCTTGA	Chr.1	8386190	8386170	-	No
2584411_adh	TTATTTGTTGGGTGTAAAAAT	Chr.1	8386666	8386646	-	No
2295290_adh	TGCCTTTGGATGCTCAACTCG	Chr.1	8397595	8397615	+	No
2052311_adh	TCTGTCCTGAAAGACGACGAA	Chr.1	8408299	8408279	-	No
2646892_adh	TTGATTTCTCGTATTCACATT	Chr.1	8412721	8412741	+	No
2500012_adh	TGTCTGTTTCTCGGCGTCTCT	Chr.1	8412995	8412975	-	No
2692438_adh	TTTAATTGCGGAACGCTTAGT	Chr.1	8413105	8413125	+	No
2552217_adh	TTAATTGCGGAACGCTTAGTA	Chr.1	8413106	8413126	+	No
1810385_adh	TACAATTTAGAGCCCAACAAA	Chr.1	8416207	8416187	-	No
1834767_adh	TACTTCACAGGATTTACGAAC	Chr.1	8416381	8416401	+	No
1851270_adh	TAGATGAACAGAAGATATTTA	Chr.1	8426323	8426303	-	No
1925246_adh	TATTCTTTTAGCTCGTTTTCA	Chr.1	8434324	8434304	-	No
2740429_adh	TTTTGAAAAACTGCTTCTACA	Chr.1	8438718	8438738	+	No
2622012_adh	TTCTTCTCGTAAAAATGGCTA	Chr.1	8440477	8440457	-	No
2698169_adh	TTTATAGTTATTATTCTGAAT	Chr.1	8463781	8463761	-	No
2321805_adh	TGGAAGAAGCATTATCGACAC	Chr.1	8475706	8475686	-	No
2712429_adh	TTTGAAAAGCTTGAAAACCGA	Chr.1	8487364	8487344	-	No
1931322_adh	TATTTTTCAATGGATTTGGTT	Chr.1	8487530	8487550	+	No
2651361_adh	TTGCCGTGATCTTCGAATATT	Chr.1	8487566	8487586	+	No
2713977_adh	TTTGAAGGATTTATTCGGCGA	Chr.1	8490551	8490571	+	No
2743863_adh	TTTTGTGAAATTTAAACAGTT	Chr.1	8531526	8531506	-	No
2420084_adh	TGGGAATATATACAAAGAATG	Chr.1	8538307	8538327	+	No
1932062_adh	TCAAAACGGGCAAGATTTTAT	Chr.1	8569306	8569326	+	No
2063285_adh	TCTTTTTGTTGAAGAGGCGCA	Chr.1	8590674	8590654	-	No
2008591_adh	TCGCTCTGAAATACGGATTAA	Chr.1	8595951	8595971	+	No
1799852_adh	TAATGCAAAAGATTCGAACAA	Chr.1	8597018	8597038	+	No
2613718_adh	TTCTAAACTGAATACCGCATA	Chr.1	8605381	8605401	+	No
1926701_adh	TATTGCTGGACAAGACAAAGC	Chr.1	8607186	8607166	-	No
1958337_adh	TCAGACGGTAGTTGGGATGAA	Chr.1	8624826	8624806	-	No
1932694_adh	TCAAACCGGCAGTTTTCTCCC	Chr.1	8637393	8637373	-	No
2624759_adh	TTGAAACCCAAAAACAGGACA	Chr.1	8648507	8648487	-	No

2732944_adh	TTTTAAAAAGCAAAATTGACA	Chr.1	8648538	8648518	-	No
2721528_adh	TTTGCCAATAACGAAAACGAC	Chr.1	8648637	8648657	+	No
1829145_adh	TACGTTTGAAGCCGGAAAATG	Chr.1	8648666	8648686	+	No
2747792_adh	TTTTTGTGAAATTTTCGTTTC	Chr.1	8657334	8657314	-	No
2490736_adh	TGTATATTGGCACTTGTTTTC	Chr.1	8657999	8658019	+	No
1973132_adh	TCATTAAACACACAAAATATT	Chr.1	8664280	8664300	+	No
2410520_adh	TGGCCACCTGGTAGACGAGCA	Chr.1	8667137	8667157	+	No
2271495_adh	TGATGATCGGTAGACAAGGCT	Chr.1	8677272	8677292	+	No
2143474_adh	TGAATGAGCACTCATGGGCAA	Chr.1	8692777	8692797	+	No
2308155_adh	TGCTGAACACGATCTCTGGCA	Chr.1	8703045	8703065	+	No
2091036_adh	TGAAATCAGAAGAACAAATGT	Chr.1	8724317	8724297	-	No
2373989_adh	TGGACAGAAGACAAAAGTTCG	Chr.1	8727308	8727328	+	No
2622007_adh	TTCTTCTCGATTCATTTTCAC	Chr.1	8735977	8735997	+	No
1790297_adh	TAATAACGCAATAAAGAGCAT	Chr.1	8758113	8758133	+	No
2739913_adh	TTTTCTTAAATTTTTCGTCCC	Chr.1	8760317	8760337	+	No
2152976_adh	TGACACCGTAGAAGGACTCAA	Chr.1	8763953	8763973	+	No
2526621_adh	TGTTCGTGACTTGGAGACATC	Chr.1	8764307	8764327	+	No
2693525_adh	TTTACATTTTGACGGTGAGCT	Chr.1	8765468	8765448	-	No
1915830_adh	TATGGCTGAAAGCGAAGAGAG	Chr.1	8787139	8787159	+	No
2665346_adh	TTGGCATTCGGAAGATTTGAA	Chr.1	8803952	8803972	+	No
2533384_adh	TGTTTGTAGCGATGAAGGCGA	Chr.1	8813492	8813512	+	No
2675046_adh	TTGTAGCGATGAAGGCGAGCA	Chr.1	8813495	8813515	+	No
2647022_adh	TTGATTTTATTGATTTTTGCC	Chr.1	8813540	8813560	+	No
2309625_adh	TGCTGTGTAGAAGAATGGTAA	Chr.1	8815270	8815290	+	No
2007942_adh	TCGCGGACTCAAGAGAAAGCA	Chr.1	8815948	8815928	-	No
2235408_adh	TGAGCTGAAGAAGAAGAGAAC	Chr.1	8816149	8816129	-	No
2008719_adh	TCGCTGAGCTGAAGAAGAAGA	Chr.1	8816153	8816133	-	No
2746108_adh	TTTTTCGGGCCTGATAAACGG	Chr.1	8816626	8816646	+	No
2681274_adh	TTGTGCATTTCCGTTGTTTAA	Chr.1	8839179	8839159	-	No
2575033_adh	TTATAGGCGGAGTTTTTTGTT	Chr.1	8852304	8852284	-	No
2617951_adh	TTCTGAATTTATAGGCGGAGT	Chr.1	8852312	8852292	-	No
2710449_adh	TTTCTGAATTTATAGGCGGAG	Chr.1	8852313	8852293	-	No
2717186_adh	TTTGATAAAGAACGTTCGGAT	Chr.1	8855290	8855310	+	No
2724888_adh	TTTGGGCTTGTTACGGAAAAA	Chr.1	8907767	8907747	-	No
2001276_adh	TCGAGAGATGGAAGTTGGCTA	Chr.1	8910965	8910945	-	No
2374410_adh	TGGACCAACTCCAGATAAAAA	Chr.1	8915307	8915287	-	No
2048452_adh	TCTGCTCGATTAGTAGACAAA	Chr.1	8923242	8923222	-	No
2513632_adh	TGTGCCAAGAATCGTAAGACG	Chr.1	8948514	8948494	-	No
2742449_adh	TTTTGCGAGTTGGACAGAATT	Chr.1	8950106	8950126	+	No
2742449_adh	TTTTGCGAGTTGGACAGAATT	Chr.1	8952190	8952170	-	No
2042014_adh	TCTCTAGAGATTGAAGTTGGA	Chr.1	8965545	8965525	-	No
1747211_adh	TAAAAATGGAAGATGATGCTC	Chr.1	8970068	8970048	-	No
2603417_adh	TTCGAGAAGAAGGAATGGGAA	Chr.1	8975531	8975551	+	No
1988711_adh	TCCGTTCACGGAATCCTTCTT	Chr.1	8978330	8978310	-	No
2627128_adh	TTGAAGAACGGAGCTCAAATT	Chr.1	8980337	8980317	-	No
2007351_adh	TCGCCAGAAGAATTCAAGTTT	Chr.1	8980525	8980505	-	No
2413624_adh	TGGCGTGTTTGCGTATATTCG	Chr.1	9014093	9014073	-	No
2546519_adh	TTAACTTCAAAATAATATGTA	Chr.1	9034392	9034412	+	No
2704342_adh	TTTCATGTGTCTCGTGTTTAC	Chr.1	9042819	9042799	-	No
2715115_adh	TTTGACGAGAAGAGGTCTGAA	Chr.1	9049324	9049344	+	No

2127047_adh	TGAAGAACTACCAGCAGAGAC	Chr.1	9056547	9056527	-	No
2674823_adh	TTGTAGAGAAGATAAAACGAG	Chr.1	9057841	9057821	-	No
2601842_adh	TTCGAAATTTGGAAAAAGGCT	Chr.1	9058846	9058866	+	No
1995385_adh	TCGAAATTTGGAAAAAGGCTC	Chr.1	9058847	9058867	+	No
2695911_adh	TTTAGCAGGAGGTAGCCAAGA	Chr.1	9059165	9059145	-	No
2147577_adh	TGAATTGGTTTAGCAGGAGGT	Chr.1	9059173	9059153	-	No
1967672_adh	TCAGTTGTTCTGCGAAATTAA	Chr.1	9065037	9065057	+	No
2743960_adh	TTTTGTGGATCAGAGACAACG	Chr.1	9081428	9081408	-	No
2391177_adh	TGGATGCGAATAGAATCAGAC	Chr.1	9086341	9086321	-	No
1999479_adh	TCGAATTTTTAGTTGGCTTAT	Chr.1	9106357	9106337	-	No
2552894_adh	TTAATTTTCAGCATTTTTCCA	Chr.1	9111854	9111874	+	No
2686825_adh	TTGTTTACTTCCTTCGAAGAC	Chr.1	9122370	9122390	+	No
2733799_adh	TTTTAATTAGTTCGTTGGATA	Chr.1	9125998	9125978	-	No
2683552_adh	TTGTTCAGTCAGAATTATGGC	Chr.1	9141852	9141872	+	No
1957975_adh	TCAGAATTATGGCATTTTCCA	Chr.1	9141860	9141880	+	No
2561395_adh	TTAGAACTTTTGAACTAGAAG	Chr.1	9146460	9146480	+	No
2737133_adh	TTTTCAAAGTGATCGTCTCTT	Chr.1	9154730	9154710	-	No
2747985_adh	TTTTTGTTGTTGTATCTGAAG	Chr.1	9163603	9163623	+	No
2041462_adh	TCTCGTCTGAAGTTACTGCTG	Chr.1	9164351	9164371	+	No
1964230_adh	TCAGGCTATTGCTGAGCGTAT	Chr.1	9164451	9164471	+	No
2010286_adh	TCGGAAACGTCGGATTCGGAT	Chr.1	9164690	9164710	+	No
1938506_adh	TCAAGACCAAGAAGTGGAGCA	Chr.1	9164874	9164894	+	No
2341835_adh	TGGAATGAAGGCTCGCATTCA	Chr.1	9165328	9165348	+	No
2527516_adh	TGTTGAACAGAAGAAGAGGAC	Chr.1	9166398	9166418	+	No
2625950_adh	TTGAACAGAAGAAGAGGACAC	Chr.1	9166400	9166420	+	No
2617884_adh	TTCTGAATCAGGAACTGCTGT	Chr.1	9175457	9175477	+	No
2480656_adh	TGTAAAGATGAAGCTGACGGC	Chr.1	9176854	9176834	-	No
2618826_adh	TTCTGGAAAAATGCGAAACGA	Chr.1	9186993	9186973	-	No
1830758_adh	TACTATTGATAGAATATTTTA	Chr.1	9187365	9187345	-	No
2743527_adh	TTTTGTAGAAAATGGGAAGAC	Chr.1	9214052	9214072	+	No
1961775_adh	TCAGATTGGAAAGCATATGCG	Chr.1	9222033	9222013	-	No
2055481_adh	TCTTCAAAACATGTAGAAACG	Chr.1	9236554	9236534	-	No
2613105_adh	TTCGTTCTCCCAGCTGAGCGG	Chr.1	9249378	9249358	-	No
2042307_adh	TCTCTCACATTTGTAGACGTT	Chr.1	9273995	9273975	-	No
2617036_adh	TTCTCTCACATTTGTAGACGT	Chr.1	9273996	9273976	-	No
2548759_adh	TTAAGTAAGAAAGAGACCGGT	Chr.1	9274531	9274511	-	No
2308524_adh	TGCTGATCTGATGTTCGGTGT	Chr.1	9274657	9274637	-	No
2290221_adh	TGCATCATGTAGAATTGTCAC	Chr.1	9284468	9284448	-	No
1931281_adh	TATTTTTAGATATCTGATGAC	Chr.1	9359960	9359980	+	No
2594607_adh	TTCATGAGAGATGGCGTTGTT	Chr.1	9382558	9382538	-	No
2470455_adh	TGGTCTTGTTTAATTTTGGCT	Chr.1	9390714	9390734	+	No
2482174_adh	TGTAAGCTCTACAGAACGGTG	Chr.1	9393457	9393437	-	No
1839650_adh	TAGAAGGGCAATAGAAGAACC	Chr.1	9411972	9411952	-	No
1829382_adh	TACTAAGAACTCACGAGAAGA	Chr.1	9421243	9421223	-	No
2318976_adh	TGGAACAGATGAAGACGTATT	Chr.1	9431794	9431814	+	No
2022032_adh	TCGTAGAAAATGAGACGACGA	Chr.1	9447492	9447512	+	No
2658411_adh	TTGGACGAGAAAAAGAGAAAA	Chr.1	9449921	9449901	-	No
1939031_adh	TCAAGATTGCGGAGTTAAGCC	Chr.1	9458907	9458887	-	No
2704937_adh	TTTCCACGTTTTCGCATTCAA	Chr.1	9488338	9488358	+	No
2628459_adh	TTGAAGATGGACGAACAAAAA	Chr.1	9494802	9494822	+	No

2182461_adh	TGAGACACGGTACCGTATTCT	Chr.1	9501699	9501679	-	No
2573722_adh	TTATAAATAAATTTGTCGGAA	Chr.1	9501885	9501905	+	No
1942049_adh	TCAATGCATTTAATCTGACGC	Chr.1	9506210	9506230	+	No
1898729_adh	TATCGTCCCAATTTTCGCGAT	Chr.1	9512498	9512518	+	No
2144776_adh	TGAATGTCGCTCGTAGAAGAA	Chr.1	9512518	9512538	+	No
2022087_adh	TCGTAGAAGAAGTGGCCTGTT	Chr.1	9512528	9512548	+	No
1844983_adh	TAGACAGAAGGACATTGAAGA	Chr.1	9516176	9516196	+	No
2251129_adh	TGAGTAGAAGAATGTACATGC	Chr.1	9516608	9516628	+	No
2739434_adh	TTTTCTGAACTTGCATCCGGC	Chr.1	9519043	9519063	+	No
1992208_adh	TCCTGTCGGTGTACGGAAAAG	Chr.1	9583164	9583144	-	No
2712826_adh	TTTGAAATTGAATACGAGCGC	Chr.1	9585282	9585302	+	No
2160778_adh	TGACGAGACGCATGAAATGTT	Chr.1	9609901	9609921	+	No
2579056_adh	TTATGCAGAACATGGATCAGA	Chr.1	9610347	9610327	-	No
2025856_adh	TCGTGAAGTGTTGACAAAATA	Chr.1	9611721	9611741	+	No
2046608_adh	TCTGATAAGCGGATTGAACGT	Chr.1	9619194	9619214	+	No
2698684_adh	TTTATCGGTCTTCTACAGTTT	Chr.1	9619923	9619903	-	No
2282882_adh	TGCAATAGAATGATCATTCAA	Chr.1	9622400	9622380	-	No
2695848_adh	TTTAGATGTAGAAAAGCGGAA	Chr.1	9624594	9624574	-	No
2048876_adh	TCTGGACAACAAGCCTAAACA	Chr.1	9625115	9625095	-	No
2746512_adh	TTTTTGAACAGCGGGGGCGGG	Chr.1	9626109	9626129	+	No
2734176_adh	TTTTACGCTTAAAATGGCAAA	Chr.1	9626191	9626211	+	No
2549649_adh	TTAATACATTGTTCGGAAATA	Chr.1	9631225	9631245	+	No
2687300_adh	TTGTTTCTGTGATAAAAACGA	Chr.1	9689008	9689028	+	No
1977907_adh	TCCAGAAGACTCAATGGGCCA	Chr.1	9689794	9689814	+	No
1807117_adh	TACAAATAGCAGTTGAACTCA	Chr.1	9690406	9690426	+	No
2487901_adh	TGTAGATAATCTTACTGTAGA	Chr.1	9691411	9691431	+	No
2639396_adh	TTGAGAAAAACAATCGCGTAC	Chr.1	9693589	9693609	+	No
2687610_adh	TTGTTTGTACTCTCGACGGCC	Chr.1	9698955	9698935	-	No
2381840_adh	TGGAGGAAAAGAGAGATGGCA	Chr.1	9699837	9699857	+	No
1921661_adh	TATTAGGATTTAGAAGAGGAT	Chr.1	9705498	9705478	-	No
2624814_adh	TTGAAACTACAGTACTCGTTA	Chr.1	9709883	9709863	-	No
1883463_adh	TAGTTTTCGTTTTGATTTTTC	Chr.1	9730362	9730342	-	No
2090022_adh	TGAAATATTTCAGAGATCGTC	Chr.1	9741349	9741369	+	No
2470975_adh	TGGTGAATGTGAAAAGGTATG	Chr.1	9744296	9744316	+	No
2653515_adh	TTGCGTCGACGATCAAGTAAT	Chr.1	9744404	9744384	-	No
2414390_adh	TGGCTCAAAACTTGGAAAAAA	Chr.1	9751884	9751864	-	No
2748211_adh	TTTTTTACCTGATTTCGACAT	Chr.1	9772622	9772602	-	No
1855085_adh	TAGCATATGACAGGAGGAAGC	Chr.1	9774094	9774074	-	No
2693142_adh	TTTACAAGTAGACTAGGCCAC	Chr.1	9779601	9779621	+	No
2483533_adh	TGTAATCGGAGGAATCAACAG	Chr.1	9785011	9784991	-	No
2315685_adh	TGGAAAGAAGTCGAAGCTGGA	Chr.1	9786841	9786821	-	No
1866422_adh	TAGGATTGGGAGATGGCTGTC	Chr.1	9788568	9788548	-	No
2701542_adh	TTTCAAGATCTGATGGGATTC	Chr.1	9793547	9793527	-	No
1918992_adh	TATGTGGAGATGCATGGTTAT	Chr.1	9813779	9813759	-	No
2644842_adh	TTGATGAGTGGAAGTTTTAAA	Chr.1	9823123	9823103	-	No
2516249_adh	TGTGGCTTGAGATGAAGAAAA	Chr.1	9830589	9830609	+	No
2601609_adh	TTCGAAAACAGATTGTAAAAT	Chr.1	9853536	9853556	+	No
2513249_adh	TGTGCAATGCGGATTTCAACA	Chr.1	9862154	9862174	+	No
2548100_adh	TTAAGCTGAAAACATTATTTA	Chr.1	9901050	9901030	-	No
2706767_adh	TTTCGAATTAAGCTGAAAACA	Chr.1	9901057	9901037	-	No

2618551_adh	TTCTGCAATTTCGAATTAAGC	Chr.1	9901065	9901045	-	No
1939170_adh	TCAAGCATCGTGGAGCGAAGA	Chr.1	9904173	9904153	-	No
2302298_adh	TGCGGTAGAACAAGTAGCAGA	Chr.1	9920998	9921018	+	No
2470201_adh	TGGTCTGATAATATATGGTGT	Chr.1	9929636	9929656	+	No
2738626_adh	TTTTCGGCTACGGTTTTCAAA	Chr.1	9937509	9937489	-	No
2039305_adh	TCTCCTTTGACCTTTCTCAAC	Chr.1	9955950	9955930	-	No
2593423_adh	TTCAGTTTTTAGAATATATGA	Chr.1	9956051	9956071	+	No
2652711_adh	TTGCGGATTTACGACAGATTT	Chr.1	9962097	9962077	-	No
2053354_adh	TCTGTTGTAGAGGATTTGAGT	Chr.1	9977768	9977788	+	No
2144656_adh	TGAATGTAGAAGAGAGAGAGA	Chr.1	9985098	9985118	+	No
2421919_adh	TGGGACGTATGCAATAAACGA	Chr.1	9987937	9987957	+	No
2124637_adh	TGAACTCGAAGCTTCTGGCAC	Chr.1	9988083	9988063	-	No
2626613_adh	TTGAACTCGAAGCTTCTGGCA	Chr.1	9988084	9988064	-	No
2553217_adh	TTACAAATCAGACGACAACTT	Chr.1	10021031	10021011	-	No
1981180_adh	TCCCCGTAATTTTTCGTTAGA	Chr.1	10051347	10051327	-	No
2602653_adh	TTCGAATTTACGAAGGACGAC	Chr.1	10074383	10074363	-	No
2706785_adh	TTTCGAATTTACGAAGGACGA	Chr.1	10074384	10074364	-	No
2002336_adh	TCGAGTGAATAAATCGATCGG	Chr.1	10078811	10078791	-	No
2710869_adh	TTTCTGTATCGGCGGGAAAAT	Chr.1	10078889	10078869	-	No
2503794_adh	TGTGACCGTAGTAGAAGCGCC	Chr.1	10079128	10079148	+	No
1843963_adh	TAGAATTGAAGAAAACGTTTT	Chr.1	10079591	10079571	-	No
2664138_adh	TTGGATTCAGGAGACGATGAC	Chr.1	10080345	10080365	+	No
2744347_adh	TTTTGTTGGGTAAATCGTTAT	Chr.1	10090515	10090535	+	No
2732179_adh	TTTGTTGGGTAAATCGTTATT	Chr.1	10090516	10090536	+	No
2742785_adh	TTTTGGATAATCGTTTGGAAA	Chr.1	10114749	10114769	+	No
2723872_adh	TTTGGATAATCGTTTGGAAAT	Chr.1	10114750	10114770	+	No
2730064_adh	TTTGTGTCGTGTAAATAAAAT	Chr.1	10118092	10118112	+	No
2396534_adh	TGGATTTTTCAGGTGGAAATC	Chr.1	10122103	10122083	-	No
2704784_adh	TTTCATTTTTCAGGCTTCGGA	Chr.1	10178486	10178506	+	No
2471501_adh	TGGTGATTTGTAGCTGGCAGC	Chr.1	10184737	10184757	+	No
2055594_adh	TCTTCACTGCTCATCATGCGT	Chr.1	10185450	10185430	-	No
1933899_adh	TCAAAGTAGAAAAACTCGTAT	Chr.1	10191123	10191103	-	No
2706785_adh	TTTCGAATTTACGAAGGACGA	Chr.1	10200722	10200742	+	No
2050356_adh	TCTGGGCAAAGTTATGACGGA	Chr.1	10202605	10202585	-	No
2644842_adh	TTGATGAGTGGAAGTTTTAAA	Chr.1	10203955	10203975	+	No
2593292_adh	TTCAGTTGACAATGGTTTCTT	Chr.1	10205273	10205253	-	No
2593292_adh	TTCAGTTGACAATGGTTTCTT	Chr.1	10206206	10206186	-	No
2593298_adh	TTCAGTTGACAATGGTTTTTT	Chr.1	10209430	10209410	-	No
2404164_adh	TGGCAGTAGCAGCAGCACAGA	Chr.1	10235210	10235230	+	No
2139072_adh	TGAATAGTGTATGAACGGAAG	Chr.1	10235295	10235275	-	No
1936062_adh	TCAACAGCAAAAAATGTAGAC	Chr.1	10244047	10244067	+	No
1930190_adh	TATTTGACGTTTTAACTGGCA	Chr.1	10249936	10249956	+	No
2681080_adh	TTGTGATGGAGGAGACAGAGA	Chr.1	10273646	10273626	-	No
2681080_adh	TTGTGATGGAGGAGACAGAGA	Chr.1	10276422	10276402	-	No
2720723_adh	TTTGATTTCGTGAAGTTAGGC	Chr.1	10284856	10284876	+	No
2503853_adh	TGTGACGACAGAACAAGATGA	Chr.1	10303129	10303149	+	No
1838746_adh	TAGAACTCTAAAAGAGAAAAT	Chr.1	10303957	10303977	+	No
2292614_adh	TGCCAGTAGAAAGTATTATGG	Chr.1	10305474	10305454	-	No
2503853_adh	TGTGACGACAGAACAAGATGA	Chr.1	10319024	10319004	-	No
2411141_adh	TGGCCTACGACGATTTTTTCT	Chr.1	10330667	10330687	+	No

2042859_adh	TCTCTGAGATCGAGAAGCAAG	Chr.1	10330925	10330945	+	No
1974007_adh	TCATTGACTTGGAGACCAACG	Chr.1	10330958	10330978	+	No
2026073_adh	TCGTGAGTTGGAGAAGAAGTT	Chr.1	10331190	10331210	+	No
2256831_adh	TGAGTTGGAGAAGAAGTTTGG	Chr.1	10331193	10331213	+	No
2027548_adh	TCGTGTGAAGTTGGACGGAAA	Chr.1	10331433	10331453	+	No
2748038_adh	TTTTTTAAAACATACATTTTT	Chr.1	10333122	10333102	-	No
2177665_adh	TGAGAAATTGTTGAAGGAAGG	Chr.1	10337291	10337311	+	No
1809320_adh	TACAATAGAAAAAATTATGTA	Chr.1	10367602	10367582	-	No
2117949_adh	TGAAATTTAAAAAATATTTTT	Chr.1	10375948	10375968	+	No
2044360_adh	TCTGAAGTTGTGACGTCGCAA	Chr.1	10392244	10392224	-	No
2134068_adh	TGAAGTAGGAGACAGAGTCTT	Chr.1	10398862	10398882	+	No
2715218_adh	TTTGACGCTGAATTGAGATGG	Chr.1	10400847	10400867	+	No
1822924_adh	TACGAGAAGACTGATCCAGCA	Chr.1	10419338	10419318	-	No
2705454_adh	TTTCCCTATAATTTTGTCGTT	Chr.1	10422080	10422060	-	No
2743741_adh	TTTTGTCAGCAGCCAAGAAGC	Chr.1	10422109	10422089	-	No
1799882_adh	TAATGCAGAAAAATTGAATTA	Chr.1	10430247	10430267	+	No
2625899_adh	TTGAACACAGGAATTCGACTA	Chr.1	10432429	10432409	-	No
2039257_adh	TCTCCTGGCTCTGAGCAAAAG	Chr.1	10435785	10435765	-	No
2615904_adh	TTCTCAACGGAAAGGCTAAGC	Chr.1	10436553	10436573	+	No
2288569_adh	TGCAGGGATTGTGAGAAGCAT	Chr.1	10438044	10438064	+	No
2685795_adh	TTGTTGCATTATCGGATTTTC	Chr.1	10446334	10446354	+	No
2731151_adh	TTTGTTGACGGTTTTTCATTA	Chr.1	10446357	10446377	+	No
2405632_adh	TGGCATGAAAAATAGAAAACA	Chr.1	10454191	10454211	+	No
1877082_adh	TAGTGAAACTGTGCTCACGGA	Chr.1	10469346	10469366	+	No
2074194_adh	TGAAACTGTGCTCACGGATTG	Chr.1	10469349	10469369	+	No
1993550_adh	TCCTTGCCGTCTTGTCTTTAT	Chr.1	10474706	10474686	-	No
1924907_adh	TATTCTGAATAACAGAACTGC	Chr.1	10485119	10485139	+	No
2044611_adh	TCTGAATAACAGAACTGCTGA	Chr.1	10485122	10485142	+	No
2676544_adh	TTGTATGAAGAAATTTGTGAA	Chr.1	10485156	10485176	+	No
2048783_adh	TCTGGAAGATGGAAATGTGAA	Chr.1	10485187	10485207	+	No
2273549_adh	TGATGTGAGTCTGGAACTTGA	Chr.1	10500021	10500001	-	No
2677756_adh	TTGTCATTGGATCCGTTGGAC	Chr.1	10516859	10516839	-	No
1804272_adh	TAATTGTCAAGGACTAAAAGA	Chr.1	10518035	10518015	-	No
2550028_adh	TTAATATTAATTGTCAAGGAC	Chr.1	10518042	10518022	-	No
2074041_adh	TGAAACTGGAAGATAAAGCTG	Chr.1	10549766	10549746	-	No
2624865_adh	TTGAAACTGGAAGATAAAGCT	Chr.1	10549767	10549747	-	No
2520033_adh	TGTGTGGAGTTGAAACTGGAA	Chr.1	10549776	10549756	-	No
1847287_adh	TAGAGAATGCGCAGGAATAGA	Chr.1	10553063	10553083	+	No
2117953_adh	TGAAATTTAAAAAATTAGGAT	Chr.1	10553651	10553671	+	No
2062543_adh	TCTTTTCACTTGCGTTCGTCG	Chr.1	10553851	10553831	-	No
1926142_adh	TATTGCAAGACCTGATATTGA	Chr.1	10555943	10555963	+	No
2573757_adh	TTATAAATGTGTCACAAATTA	Chr.1	10565432	10565452	+	No
2054196_adh	TCTTACTCGCGACGACTATCC	Chr.1	10566249	10566269	+	No
2568663_adh	TTAGGGAAGACAGATCTACTT	Chr.1	10567927	10567947	+	No
1946705_adh	TCACCAAGGAAGATCTCGGAG	Chr.1	10568237	10568257	+	No
1977270_adh	TCCACCACAGAACACTGGAAT	Chr.1	10568337	10568357	+	No
1824140_adh	TACGATGATGGAACCCTCTAC	Chr.1	10568590	10568610	+	No
2723104_adh	TTTGGAAGAGACGGAGAAACT	Chr.1	10585356	10585336	-	No
2612086_adh	TTCGTCTGCAGCATCGGTTCC	Chr.1	10589637	10589657	+	No
1930753_adh	TATTTTAGATTCCTAGCGATT	Chr.1	10591682	10591662	-	No

2287389_adh	TGCAGAGGAACGTATAACAAA	Chr.1	10593929	10593909	-	No
2582496_adh	TTATTGCAGAGGAACGTATAA	Chr.1	10593933	10593913	-	No
1794848_adh	TAATAGAGGACAAAAATCAAG	Chr.1	10598494	10598474	-	No
1837672_adh	TAGAAATAGAAATTGCTATAA	Chr.1	10634355	10634335	-	No
2728561_adh	TTTGTATGATGAAGAAGAAAT	Chr.1	10638149	10638129	-	No
1806753_adh	TACAAAGCAGGGTGACTGGGT	Chr.1	10651276	10651296	+	No
2016429_adh	TCGGGACATGGACGCACCTTT	Chr.1	10651330	10651310	-	No
1889899_adh	TATATATTTTCAGTCGGGACA	Chr.1	10651343	10651323	-	No
2287611_adh	TGCAGATAGTGCTATCGCGGC	Chr.1	10653741	10653761	+	No
1990094_adh	TCCTCCGGAATACGAATTTCA	Chr.1	10708142	10708122	-	No
2016372_adh	TCGGGAAGAAAGGAAAATGCA	Chr.1	10720239	10720219	-	No
2561501_adh	TTAGAAGGAGTCGGGAAGAAA	Chr.1	10720249	10720229	-	No
2025849_adh	TCGTGAAGATTGCAAAGAAAA	Chr.1	10731637	10731617	-	No
1802957_adh	TAATTCATGCCTCTGAAATTC	Chr.1	10736449	10736469	+	No
2635654_adh	TTGACGAGAACATGTGATGGA	Chr.1	10750224	10750244	+	No
2702960_adh	TTTCAGATGACGAGGATTTCT	Chr.1	10777754	10777774	+	No
2710372_adh	TTTCTGAACTCTCGCTGGAAT	Chr.1	10788707	10788727	+	No
2681064_adh	TTGTGATGAATTCTTGAAGAA	Chr.1	10788754	10788734	-	No
2639754_adh	TTGAGACTGCCTATGAAGCGA	Chr.1	10790474	10790494	+	No
1956639_adh	TCAGAAAAAGTAGAAACGGAA	Chr.1	10793191	10793171	-	No
2710811_adh	TTTCTGTAAAAAACGCTCAAC	Chr.1	10798318	10798298	-	No
2489689_adh	TGTAGTCTGTCGGCTTTTAAC	Chr.1	10808381	10808361	-	No
2037297_adh	TCTCAGAATTCGGAATTCAGT	Chr.1	10812872	10812852	-	No
1966453_adh	TCAGTCTCAGAATTCGGAATT	Chr.1	10812876	10812856	-	No
1885741_adh	TATAATCGGAGGATTTGCATG	Chr.1	10862956	10862936	-	No
2217566_adh	TGAGATGAAGAGAGTGGAAGA	Chr.1	10875282	10875302	+	No
1824081_adh	TACGATGACTGGGCATACTAT	Chr.1	10906484	10906504	+	Yes
2560189_adh	TTACTTGAATTTCGGAGCGGT	Chr.1	10909707	10909687	-	No
1824081_adh	TACGATGACTGGGCATACTAT	Chr.1	10919406	10919386	-	Yes
1773180_adh	TAAGAAAAGCTTGAAAACAAT	Chr.1	10923721	10923741	+	No
1927362_adh	TATTGGTTGTTTGGAAAATTC	Chr.1	10928269	10928249	-	No
2104133_adh	TGAAATGGAAAAGTGAGAAAA	Chr.1	10930389	10930369	-	No
1980031_adh	TCCATTGGACGCTGAAATGGC	Chr.1	10954087	10954107	+	No
2189153_adh	TGAGAGTAAGCGTTGAATTGA	Chr.1	10959044	10959064	+	No
1819844_adh	TACCGTATACGTGACATTTAG	Chr.1	10990121	10990141	+	No
2651240_adh	TTGCCGACTGTAAGGACGGGA	Chr.1	10995148	10995168	+	No
2016520_adh	TCGGGAGACGTGATAATTGAT	Chr.1	11052932	11052912	-	No
2127868_adh	TGAAGACGACTTTGTGGATCT	Chr.1	11054770	11054790	+	No
2714145_adh	TTTGAATAATGACGCTTCACT	Chr.1	11055207	11055227	+	No
2690674_adh	TTTAAGGGATTTAATTCGGTA	Chr.1	11055318	11055298	-	No
2299652_adh	TGCGATTGATAGATGGTTCCT	Chr.1	11056125	11056145	+	No
1769821_adh	TAACTACTGTAGCGCGGTGTT	Chr.1	11057303	11057283	-	No
2492650_adh	TGTATGTTGGTATCGTGACGG	Chr.1	11062917	11062937	+	No
2737254_adh	TTTTCAATGACGGCGAAGGTG	Chr.1	11072849	11072829	-	No
2634755_adh	TTGACACAACGGCGCCAAAAT	Chr.1	11073374	11073394	+	No
2526009_adh	TGTTCACGGTATTAGTATACA	Chr.1	11083313	11083333	+	No
2623478_adh	TTCTTTCGATTTTTGTAGGTT	Chr.1	11110878	11110858	-	No
2732769_adh	TTTGTTTTAATGGATTTTCGG	Chr.1	11111000	11111020	+	No
2688139_adh	TTGTTTTAATGGATTTTCGGT	Chr.1	11111001	11111021	+	No
2691689_adh	TTTAATGGATTTTCGGTGATT	Chr.1	11111005	11111025	+	No

2748727_adh	TTTTTTCCGGCAATTCGACAA	Chr.1	11159641	11159621	-	No
1891375_adh	TATCAACAACAGCGAAGAACT	Chr.1	11174516	11174496	-	No
2747091_adh	TTTTTGCGGATTTATTTGGTT	Chr.1	11182463	11182443	-	No
1797393_adh	TAATCAAAAAATCGAATCTAT	Chr.1	11245116	11245096	-	No
2601264_adh	TTCCTTCCTTCGATCGAAATT	Chr.1	11252119	11252139	+	No
2706783_adh	TTTCGAATTGTGGACAGCACT	Chr.1	11252147	11252167	+	No
1989367_adh	TCCTACTATTGTTCATCGTCA	Chr.1	11253250	11253270	+	No
2718133_adh	TTTGATCGAAAGAATGTACGC	Chr.1	11271687	11271707	+	No
2333611_adh	TGGAATCCGCAAAGACTGCTA	Chr.1	11292800	11292780	-	No
2589739_adh	TTCACGGTAGCACAGAAACTA	Chr.1	11299918	11299938	+	No
2589739_adh	TTCACGGTAGCACAGAAACTA	Chr.1	11302101	11302081	-	No
2299230_adh	TGCGATGGAGCGATTGGTCGA	Chr.1	11304341	11304361	+	No
2299230_adh	TGCGATGGAGCGATTGGTCGA	Chr.1	11308982	11308962	-	No
2718190_adh	TTTGATCGCAGGGATACGAGA	Chr.1	11319986	11320006	+	No
2721998_adh	TTTGCGGAAAATCTGGCGAAA	Chr.1	11371775	11371755	-	No
2011222_adh	TCGGACAAGCTGGAGAAAATT	Chr.1	11374427	11374407	-	No
1977691_adh	TCCACTCGGACAAGCTGGAGA	Chr.1	11374432	11374412	-	No
2462389_adh	TGGTACGGAAAAAAGGATGGA	Chr.1	11377818	11377838	+	No
2276721_adh	TGATTGGAAAAGAGCAAGAAA	Chr.1	11416850	11416870	+	No
1860665_adh	TAGGAAATACGCAAATGGCGC	Chr.1	11438008	11437988	-	No
2743921_adh	TTTTGTGATGGAATCGTTGGA	Chr.1	11438988	11438968	-	No
1967000_adh	TCAGTGGATTTTGTGATGGAA	Chr.1	11438996	11438976	-	No
2593110_adh	TTCAGTGGATTTTGTGATGGA	Chr.1	11438997	11438977	-	No
2275155_adh	TGATTCAGTGGATTTTGTGAT	Chr.1	11439000	11438980	-	No
2611908_adh	TTCGTCATGATTTTCGTCGAT	Chr.1	11439134	11439154	+	No
1860665_adh	TAGGAAATACGCAAATGGCGC	Chr.1	11446779	11446759	-	No
2589807_adh	TTCACGTTTGGCGGCGATCTT	Chr.1	11487916	11487896	-	No
2733139_adh	TTTTAAATAAAAATTATTTTT	Chr.1	11513972	11513952	-	No
2098815_adh	TGAAATCTGAAAATCCGTCTA	Chr.1	11576331	11576311	-	No
1929890_adh	TATTTCGGAGTGTCGTTGTGC	Chr.1	11578403	11578423	+	No
2707753_adh	TTTCGGAGTGTCGTTGTGCAA	Chr.1	11578405	11578425	+	No
2608092_adh	TTCGGAGTGTCGTTGTGCAAT	Chr.1	11578406	11578426	+	No
2721890_adh	TTTGCGAAAAACGGATCGATA	Chr.1	11586251	11586271	+	No
1930789_adh	TATTTTATCAGTAGAGCGCAT	Chr.1	11609489	11609469	-	No
2747836_adh	TTTTTGTGGTTTTTCGTGGAT	Chr.1	11611403	11611383	-	No
2234879_adh	TGAGCTCGGCACATTTTGAGA	Chr.1	11628081	11628061	-	No
2234879_adh	TGAGCTCGGCACATTTTGAGA	Chr.1	11628728	11628748	+	No
2701005_adh	TTTCAAACAAAACGTTGGGCA	Chr.1	11636151	11636171	+	No
2714561_adh	TTTGAATTTTAGAGAGGATGG	Chr.1	11684798	11684818	+	No
2474770_adh	TGGTTCAGTGGAAACGACGGT	Chr.1	11684816	11684836	+	No
2593087_adh	TTCAGTGGAAACGACGGTTTT	Chr.1	11684819	11684839	+	No
1966944_adh	TCAGTGGAAACGACGGTTTTT	Chr.1	11684820	11684840	+	No
2314372_adh	TGGAAACGACGGTTTTTTCGG	Chr.1	11684824	11684844	+	No
2701617_adh	TTTCAAGTAGATTTTCTCACA	Chr.1	11685548	11685568	+	No
2589723_adh	TTCACGGCGTAAATGGTTCAC	Chr.1	11712555	11712575	+	No
1953287_adh	TCACGGCGTAAATGGTTCACG	Chr.1	11712556	11712576	+	No
2487499_adh	TGTAGAAGGGAAGGCAGATTT	Chr.1	11726877	11726857	-	No
2616137_adh	TTCTCAGTTTTACGATTATCG	Chr.1	11743965	11743945	-	No
2494147_adh	TGTCAAGGAGCTCTTCCGTCA	Chr.1	11775186	11775166	-	No
2730444_adh	TTTGTTAGTTGACAACTTCTT	Chr.1	11885714	11885734	+	No

2490487_adh	TGTATACTGGAAAGACTGGAT	Chr.1	11887010	11887030	+	No
1887649_adh	TATACTGGGAAAAATAATCAT	Chr.1	11896476	11896456	-	No
2749732_adh	TTTTTTGTCGGAAAACCAGTT	Chr.1	11896607	11896627	+	No
2124815_adh	TGAACTCTCGCCTCTGTGACG	Chr.1	11897313	11897293	-	No
1964033_adh	TCAGGATTGGGCATGTTTCAG	Chr.1	11903539	11903519	-	No
1754105_adh	TAAACAATCGGAAACTGGTTA	Chr.1	11909909	11909889	-	No
2057033_adh	TCTTCTCTGTTTACGGTTAGT	Chr.1	11909954	11909934	-	No
1826292_adh	TACGGCCTAAAATCGTCATGT	Chr.1	11909981	11909961	-	No
2745843_adh	TTTTTCCATTTCAAGCTGAAA	Chr.1	11910111	11910131	+	No
2587125_adh	TTCAAGCTGAAAATATGAGGA	Chr.1	11910120	11910140	+	No
1975737_adh	TCCAAAATAAGACCGCGAAGA	Chr.1	11910146	11910166	+	No
2691548_adh	TTTAATGATCTCAATAATAAT	Chr.1	11910377	11910357	-	No
2721922_adh	TTTGCGACCGCGAGACCGGAC	Chr.1	11910562	11910542	-	No
1834776_adh	TACTTCAGCCAGGCTGGAAAC	Chr.1	11910687	11910667	-	No
2587339_adh	TTCAAGTAGAAAACTGGAATA	Chr.1	11910900	11910880	-	No
1923375_adh	TATTCAAGTAGAAAACTGGAA	Chr.1	11910902	11910882	-	No
1852786_adh	TAGCAAACGCTGCGTCTTTTA	Chr.1	11911091	11911111	+	No
2123010_adh	TGAACGAGATAGTAGAACGAG	Chr.1	11912198	11912218	+	No
1848717_adh	TAGAGGTTTAGAGAAGTTGAG	Chr.1	11913012	11913032	+	No
2613239_adh	TTCGTTGGAAAATTAGGTTTT	Chr.1	11929955	11929935	-	No
2629605_adh	TTGAATACAAGTAGACGAAAT	Chr.1	11944871	11944851	-	No
2629605_adh	TTGAATACAAGTAGACGAAAT	Chr.1	11945502	11945522	+	No
2494513_adh	TGTCACATTTTTTCGGGCAAA	Chr.1	11975370	11975350	-	No
2745506_adh	TTTTTATTGTGATTTTGGAAG	Chr.1	12004988	12005008	+	No
1943813_adh	TCACAAACTTAGGATGGAACC	Chr.1	12008156	12008136	-	No
1848736_adh	TAGAGTAAAATATTCTTCACA	Chr.1	12023326	12023346	+	No
2629605_adh	TTGAATACAAGTAGACGAAAT	Chr.1	12023831	12023851	+	No
2046967_adh	TCTGATCGTCCTGGACAGAAA	Chr.1	12033726	12033706	-	No
2581912_adh	TTATTCTGATCGTCCTGGACA	Chr.1	12033730	12033710	-	No
2737419_adh	TTTTCACTTATATGTTTTGTT	Chr.1	12043558	12043578	+	No
1931161_adh	TATTTTGGCTTTTTCCCAAGT	Chr.1	12043971	12043991	+	No
2733928_adh	TTTTACAACTAGAATCGAGCC	Chr.1	12058908	12058888	-	No
2694695_adh	TTTACTTGTGAACAGATGGTG	Chr.1	12065470	12065490	+	No
2189153_adh	TGAGAGTAAGCGTTGAATTGA	Chr.1	12077851	12077831	-	No
1981625_adh	TCCCGGCTTGCTGAAAGAATG	Chr.1	12117691	12117671	-	No
1981625_adh	TCCCGGCTTGCTGAAAGAATG	Chr.1	12125872	12125892	+	No
2711844_adh	TTTCTTGGCGCATGTGACTTT	Chr.1	12147561	12147541	-	No
1930991_adh	TATTTTCTTGGCGCATGTGAC	Chr.1	12147564	12147544	-	No
2180182_adh	TGAGAAGATGACTGCAGTTTT	Chr.1	12163625	12163605	-	No
1919484_adh	TATGTTGCGGAGATTTTGACC	Chr.1	12173673	12173653	-	No
2279222_adh	TGATTTTCGAAAAATGCGGGA	Chr.1	12185929	12185909	-	No
2706178_adh	TTTCCTCACTAAATCGTCGCC	Chr.1	12198848	12198868	+	No
1952620_adh	TCACCTCTTTGTCGGCCATAA	Chr.1	12204871	12204891	+	No
1944430_adh	TCACACACAACAAAACGCGGC	Chr.1	12206771	12206791	+	No
2375930_adh	TGGACGAAGACACCGAAGACG	Chr.1	12209527	12209507	-	No
2624253_adh	TTGAAAAACAATGGAAAAACA	Chr.1	12209670	12209650	-	No
2472960_adh	TGGTGTCAAAATCGGACTACC	Chr.1	12219151	12219131	-	No
2118181_adh	TGAAATTTAAAGAAGTTTTTA	Chr.1	12224636	12224656	+	No
2711071_adh	TTTCTGTTGGAAACGCTAAAA	Chr.1	12259446	12259426	-	No
2711071_adh	TTTCTGTTGGAAACGCTAAAA	Chr.1	12268789	12268769	-	No

2139193_adh	TGAATATAGTTGTGACGTAAT	Chr.1	12286134	12286154	+	No
1967511_adh	TCAGTTGATTGAACATACCAG	Chr.1	12325058	12325078	+	No
2339178_adh	TGGAATGAAAAGAAATTGTAA	Chr.1	12385448	12385428	-	No
2724500_adh	TTTGGCTATGATGCGACGGTT	Chr.1	12454586	12454606	+	No
1924747_adh	TATTCTATCATACTGCACTCT	Chr.1	12473384	12473364	-	No
2287315_adh	TGCAGACTGCAAGAGGGCGTG	Chr.1	12508374	12508394	+	No
2714028_adh	TTTGAAGGTTCGGCAAAGCGG	Chr.1	12513636	12513656	+	No
2084422_adh	TGAAAGTTAGGCCATACTCCT	Chr.1	12522811	12522791	-	No
1762506_adh	TAAATTGCGAAATTTTGCAGG	Chr.1	12532218	12532238	+	No
2566411_adh	TTAGCAGTAAAATTTCGCAAA	Chr.1	12535066	12535046	-	No
2623607_adh	TTCTTTGATCTTTTTGGCAAA	Chr.1	12598961	12598941	-	No
2054992_adh	TCTTATATTAAAATCGTAGAA	Chr.1	12599122	12599142	+	No
1813435_adh	TACATCGTATGCCTTAAGAAT	Chr.1	12606046	12606026	-	No
1766224_adh	TAACATGTATTAGAGCGGAAA	Chr.1	12607514	12607494	-	Yes
2000259_adh	TCGACTCAGGAATCAAGGGAA	Chr.1	12652862	12652882	+	No
1939813_adh	TCAAGGGAATGGCCATGAACT	Chr.1	12652874	12652894	+	No
2513636_adh	TGTGCCAATTGGACGTGGACA	Chr.1	12653166	12653186	+	No
2488730_adh	TGTAGGATCTGCTGCTCAAAC	Chr.1	12653871	12653891	+	No
2429427_adh	TGGGATCGAAAAACGCGTCAA	Chr.1	12728128	12728148	+	No
2624286_adh	TTGAAAAATAGGATTTGACAT	Chr.1	12728471	12728451	-	No
1839805_adh	TAGAAGTCGTAAAAAACAAAC	Chr.1	12754230	12754210	-	No
1879005_adh	TAGTGGTGGAAGAGATGGATA	Chr.1	12792721	12792701	-	No
2742350_adh	TTTTGCATCGATTTTTTCAGT	Chr.1	12806997	12807017	+	No
1968532_adh	TCATACAAGAGAAGAATTTCG	Chr.1	12826750	12826770	+	No
2182023_adh	TGAGACAACGAAGTTTGAATA	Chr.1	12849290	12849310	+	No
2550109_adh	TTAATATTGAGTTGGAGACCA	Chr.1	12859101	12859121	+	No
2318418_adh	TGGAAATTAAAATACTTTTTG	Chr.1	12863602	12863622	+	No
1833685_adh	TACTGTAGATTTAGGTCTTAA	Chr.1	12864127	12864147	+	No
2693033_adh	TTTAATTTTCGTCCGATTTTT	Chr.1	12876318	12876338	+	No
1788110_adh	TAAGTCTTAGAGGAACATCAA	Chr.1	12904227	12904207	-	No
2665571_adh	TTGGCCTAAAATCGATACGGA	Chr.1	12904256	12904236	-	No
2031540_adh	TCTACGACAAGAAGACTTCAT	Chr.1	12905249	12905269	+	No
1954718_adh	TCACTGGAGAAAGCCGAAGGA	Chr.1	12905293	12905313	+	No
1891543_adh	TATCAAGCCGTTCAAGACTTC	Chr.1	12910664	12910644	-	No
2737456_adh	TTTTCAGAGTTTCTTATCGAA	Chr.1	12918342	12918362	+	No
2015229_adh	TCGGCAGAGATGGTGAGCTTC	Chr.1	12919006	12918986	-	No
2709734_adh	TTTCTAGTGGAAAAACGTCGG	Chr.1	12919696	12919676	-	No
2715185_adh	TTTGACGCGCAAAATTTTCTG	Chr.1	12919784	12919804	+	No
2194142_adh	TGAGATAGGTTTGCATACTCT	Chr.1	12923253	12923233	-	No
1830348_adh	TACTAGTTCGCAGTACTTTTC	Chr.1	12929362	12929382	+	No
1805877_adh	TAATTTTCAATGGGAAGGCTG	Chr.1	12933884	12933904	+	No
2745084_adh	TTTTTAGCACATGCATTGACA	Chr.1	12944134	12944154	+	No
2269804_adh	TGATCGATGAGCAGAACAAGC	Chr.1	12964575	12964595	+	No
2604855_adh	TTCGATCGTCATAAACAATGT	Chr.1	12964750	12964730	-	No
1995831_adh	TCGAACTTGTAGTCGAGACGA	Chr.1	12965021	12965041	+	No
2721967_adh	TTTGCGATTTCACAAGACGGT	Chr.1	12966661	12966641	-	No
2675088_adh	TTGTAGGAAATAGGGCGTAAA	Chr.1	12974856	12974836	-	No
2447334_adh	TGGGATGTTTAGTAATTTCTA	Chr.1	13030050	13030030	-	No
2374069_adh	TGGACAGCAGTACAAGATGAC	Chr.1	13085800	13085820	+	No
2292614_adh	TGCCAGTAGAAAGTATTATGG	Chr.1	13098979	13098999	+	No

2374069_adh	TGGACAGCAGTACAAGATGAC	Chr.1	13102046	13102026	-	No
1942763_adh	TCAATGTTGTCACTGGGCATA	Chr.1	13120287	13120267	-	No
2626670_adh	TTGAACTCTGTGGATATGAAA	Chr.1	13147135	13147155	+	No
2626670_adh	TTGAACTCTGTGGATATGAAA	Chr.1	13149243	13149263	+	No
2626670_adh	TTGAACTCTGTGGATATGAAA	Chr.1	13150139	13150159	+	No
2626670_adh	TTGAACTCTGTGGATATGAAA	Chr.1	13150811	13150831	+	No
2626670_adh	TTGAACTCTGTGGATATGAAA	Chr.1	13151707	13151727	+	No
2010194_adh	TCGGAAAATCAGAATGTGCCA	Chr.1	13155183	13155203	+	No
2041708_adh	TCTCGTGGTGTAGTGGTTATT	Chr.1	13156283	13156303	+	No
2702949_adh	TTTCAGATCAATTTCGTCGGT	Chr.1	13160524	13160544	+	No
1931329_adh	TATTTTTCATACTTTTTCAGG	Chr.1	13161896	13161876	-	No
2590455_adh	TTCAGAATTTGGCACACATAC	Chr.1	13231286	13231266	-	No
2590455_adh	TTCAGAATTTGGCACACATAC	Chr.1	13231430	13231410	-	No
2724246_adh	TTTGGCACCTGTAGAGGCTAC	Chr.1	13266066	13266046	-	No
2287282_adh	TGCAGACGTCGCACAAATTGG	Chr.1	13266160	13266180	+	No
1890387_adh	TATATGGGAATGGTGGAAAGA	Chr.1	13294336	13294356	+	No
1812729_adh	TACAGTATTTAGGCGTGAAAA	Chr.1	13331540	13331560	+	No
2737502_adh	TTTTCAGCTTGTTTTGTCGGT	Chr.1	13339097	13339117	+	No
2703129_adh	TTTCAGCTTGTTTTGTCGGTT	Chr.1	13339098	13339118	+	No
1963240_adh	TCAGCTTGTTTTGTCGGTTTT	Chr.1	13339100	13339120	+	No
2703268_adh	TTTCAGGGACTTATCAACAAC	Chr.1	13384601	13384581	-	No
2127078_adh	TGAAGAAGAAAAAAGACGGTT	Chr.1	13384642	13384622	-	No
2446920_adh	TGGGATGTTGAGAAAAATGTT	Chr.1	13413215	13413235	+	No
2016844_adh	TCGGGCGGCATAAACACACAG	Chr.1	13434496	13434476	-	No
2287397_adh	TGCAGAGGAATCGTATTGAGA	Chr.1	13470623	13470643	+	No
2127896_adh	TGAAGACGCAGAGAACGGCTT	Chr.1	13487580	13487560	-	No
2722275_adh	TTTGCTAGATTTCGAATTTTT	Chr.1	13489282	13489262	-	No
2372119_adh	TGGAATTTAATATAGTTTGTA	Chr.1	13500936	13500916	-	No
2623265_adh	TTCTTTAGATCGTTAAACTTC	Chr.1	13530141	13530161	+	No
1971749_adh	TCATGAAGACTGCAGTCATAT	Chr.1	13560010	13560030	+	No
2304006_adh	TGCGTCTGACAAGGAAAGGTT	Chr.1	13562255	13562275	+	No
2738881_adh	TTTTCGTTGCTTTTATCGGTT	Chr.1	13562402	13562422	+	No
2709266_adh	TTTCGTTGCTTTTATCGGTTT	Chr.1	13562403	13562423	+	No
1999418_adh	TCGAATTTCCGCGATTGCACA	Chr.1	13572114	13572094	-	No
2326862_adh	TGGAAGTGCGTAGACGGAGAG	Chr.1	13605846	13605866	+	No
1813071_adh	TACATAATTTTTGAACGAAAA	Chr.1	13615977	13615957	-	No
2633364_adh	TTGAATGTGTGTGTAGACGAA	Chr.1	13626532	13626552	+	No
2217815_adh	TGAGATGAGAAAACTTCTGAA	Chr.1	13628164	13628144	-	No
2721157_adh	TTTGCAATTTTTGATGGTTTT	Chr.1	13628726	13628746	+	No
2403391_adh	TGGCAGACTTCGTTGAGGCTA	Chr.1	13629823	13629843	+	No
2712676_adh	TTTGAAAGCTTAAGATCGCGT	Chr.1	13651323	13651303	-	No
1956864_adh	TCAGAAACGAACAAACAACAG	Chr.1	13659312	13659332	+	No
2279472_adh	TGATTTTTCTGGAGATTTTGC	Chr.1	13659479	13659459	-	No
2742968_adh	TTTTGGGCAATGTGGACGGAA	Chr.1	13660558	13660578	+	No
2395101_adh	TGGATTGTAGTTCAGTGTCAA	Chr.1	13661938	13661958	+	No
1935997_adh	TCAACACGATTTACGGAGGGA	Chr.1	13663390	13663370	-	No
2659713_adh	TTGGAGCATATTTTGGACAAT	Chr.1	13667946	13667926	-	No
1935850_adh	TCAACAACAAGATCATAGGAT	Chr.1	13677939	13677959	+	No
2742945_adh	TTTTGGGAAAAGGCATGAAAT	Chr.1	13762312	13762332	+	No
1844805_adh	TAGAATTTTCGGAAAACGGCT	Chr.1	13783686	13783706	+	No

2742968_adh	TTTTGGGCAATGTGGACGGAA	Chr.1	13800991	13800971	-	No
2015370_adh	TCGGCCGAATGTTGAACGGAT	Chr.1	13817417	13817397	-	No
2291601_adh	TGCATTTTAGTTGGCTTTTCA	Chr.1	13835150	13835130	-	No
2291483_adh	TGCATTGTTTATAAGGATTAT	Chr.1	13835235	13835255	+	No
2531951_adh	TGTTTATAAGGATTATTAGGG	Chr.1	13835240	13835260	+	No
2573986_adh	TTATAAGGATTATTAGGGTTA	Chr.1	13835243	13835263	+	No
1807810_adh	TACAACGGATCATTCGGAACA	Chr.1	13841405	13841425	+	No
1984052_adh	TCCGAATTTGAAGACTGAAAA	Chr.1	13854154	13854134	-	No
2598660_adh	TTCCGAATTTGAAGACTGAAA	Chr.1	13854155	13854135	-	No
1931749_adh	TCAAAAAATGAGGGCATGTAA	Chr.1	13877489	13877469	-	No
1931749_adh	TCAAAAAATGAGGGCATGTAA	Chr.1	13878466	13878446	-	No
1857484_adh	TAGCGGTTCGGATGCATTCGT	Chr.1	13883502	13883522	+	No
2424674_adh	TGGGAGTGGGAGGGGGAGGGA	Chr.1	13894864	13894844	-	No
2044992_adh	TCTGACACGGATAGAATGATT	Chr.1	13933527	13933547	+	No
2148258_adh	TGAATTTCTCATTTTTAGGCG	Chr.1	13951940	13951960	+	No
2745118_adh	TTTTTAGGCGAACTTTTCGGT	Chr.1	13951951	13951971	+	No
2734803_adh	TTTTAGGCGAACTTTTCGGTT	Chr.1	13951952	13951972	+	No
1926142_adh	TATTGCAAGACCTGATATTGA	Chr.1	13959136	13959156	+	No
2710036_adh	TTTCTCGGAAGGTACTGTCGA	Chr.1	13960024	13960004	-	No
2742969_adh	TTTTGGGCAATGTGGACGGTA	Chr.1	13965802	13965822	+	No
2742968_adh	TTTTGGGCAATGTGGACGGAA	Chr.1	13967748	13967768	+	No
2414845_adh	TGGCTGCGTACTCCTCTTGGA	Chr.1	13969686	13969706	+	No
2029595_adh	TCTAACATGCTTTCGAAGGAT	Chr.1	13972408	13972428	+	No
2140342_adh	TGAATCACAGAAGCTCATTCA	Chr.1	13973119	13973099	-	No
2735434_adh	TTTTATCAATGGAGCGCGCTT	Chr.1	13988310	13988330	+	No
2735434_adh	TTTTATCAATGGAGCGCGCTT	Chr.1	13988631	13988611	-	No
2589606_adh	TTCACGCCAAGCTGAGGAACC	Chr.1	14010373	14010393	+	No
2621042_adh	TTCTTCACAGAGAATGCTGCG	Chr.1	14048017	14047997	-	No
2567584_adh	TTAGGAAGGGAAGTAGTTTTT	Chr.1	14058640	14058620	-	No
1747348_adh	TAAAAATTTGCATTAATTTTT	Chr.1	14111925	14111905	-	No
2730444_adh	TTTGTTAGTTGACAACTTCTT	Chr.1	14145462	14145442	-	No
2568854_adh	TTAGGGGTACTGGAGATGGTT	Chr.1	14162798	14162778	-	No
1797735_adh	TAATCAAGTCTAGTGGAATAT	Chr.1	14194185	14194205	+	No
2313962_adh	TGGAAAATGTGAGTTTAGCGG	Chr.1	14200036	14200056	+	No
1985225_adh	TCCGCACTGTGGCATTAATTT	Chr.1	14241305	14241285	-	No
2013754_adh	TCGGATCAGGAGATGGAAAAG	Chr.1	14326504	14326484	-	No
2689879_adh	TTTAACCGAAAATGAGCATTA	Chr.1	14358402	14358382	-	No
2394751_adh	TGGATTGTAAAATGCAAAAAA	Chr.1	14405801	14405821	+	No
1883920_adh	TATAAAAGACCGTCGTCACGC	Chr.1	14428154	14428134	-	No
2513587_adh	TGTGCATCGGGGGCAACTAAT	Chr.1	14464525	14464505	-	No
2479167_adh	TGGTTTTGATTTGTTGTGTTG	Chr.1	14468376	14468356	-	No
2545161_adh	TTAACCATTTACTCGGCTCAT	Chr.1	14508517	14508537	+	No
1999451_adh	TCGAATTTTAAAAGGCGCCAA	Chr.1	14523047	14523027	-	No
2683662_adh	TTGTTCGAATTTTAAAAGGCG	Chr.1	14523051	14523031	-	No
2578358_adh	TTATGAATAGGGGGAAAGGGA	Chr.1	14525621	14525641	+	No
1953599_adh	TCACGTGTTTAATGGATTGAC	Chr.1	14548041	14548021	-	No
2025904_adh	TCGTGACGTCATTTTTCTACA	Chr.1	14595050	14595070	+	No
2270489_adh	TGATCTGGGAAGAAGCGAACG	Chr.1	14612975	14612995	+	No
2733110_adh	TTTTAAAGCGGGGGATTTGAA	Chr.1	14614880	14614900	+	No
2711570_adh	TTTCTTCTCGGATAATAAATT	Chr.1	14621196	14621176	-	No

2497090_adh	TGTCCGAAACTTGACTGAAGA	Chr.1	14622706	14622686	-	No
2186065_adh	TGAGAGAGCTGAGGAGCGTGC	Chr.1	14623851	14623831	-	No
2270660_adh	TGATCTTGAGAGAGCTGAGGA	Chr.1	14623857	14623837	-	No
2025164_adh	TCGTATTGTGCTTGTGGAGGA	Chr.1	14625047	14625027	-	No
2254112_adh	TGAGTGGCGAAATTCGGAAAT	Chr.1	14632312	14632292	-	No
1847393_adh	TAGAGACTGCCGTACTAATTT	Chr.1	14637881	14637901	+	No
2519052_adh	TGTGTATGTAGTGGAACGTGG	Chr.1	14647551	14647531	-	No
2095435_adh	TGAAATCGGAGGAAGGTTATA	Chr.1	14654124	14654144	+	No
2620177_adh	TTCTGTGCGAAACAACAAGGC	Chr.1	14663357	14663337	-	No
2602650_adh	TTCGAATTGTGTATTGACGAC	Chr.1	14664506	14664486	-	No
1880883_adh	TAGTTGAATTTTTTGTCGGAA	Chr.1	14701834	14701854	+	No
1749582_adh	TAAAAGCGAATAAATTTGAAT	Chr.1	14710946	14710926	-	No
2701000_adh	TTTCAAAATTGCACTGCTATT	Chr.1	14716142	14716162	+	No
2580255_adh	TTATGTTACTGTAGTGCAGGC	Chr.1	14716243	14716263	+	No
2614773_adh	TTCTAGAGGGATACTGAGATG	Chr.1	14719040	14719020	-	No
2598705_adh	TTCCGACGACGAACTCGGCAT	Chr.1	14720923	14720943	+	No
2018699_adh	TCGGTCCGTAGATGTGCAAGA	Chr.1	14731153	14731133	-	No
2573430_adh	TTAGTTTGACGCGCGATACCT	Chr.1	14734751	14734731	-	No
1932381_adh	TCAAAATATCGTTAGGTGAAA	Chr.1	14735097	14735117	+	No
2164321_adh	TGACTAGAAGTCGAGTATCTT	Chr.1	14747062	14747042	-	No
2589445_adh	TTCACCTGAGCGGCTCCATTT	Chr.1	14756452	14756432	-	No
2519510_adh	TGTGTGAATCACGGAAGCTGC	Chr.1	14756510	14756530	+	No
2688726_adh	TTTAAAAATGCACGATTATTT	Chr.1	14756761	14756741	-	No
2674078_adh	TTGTAATGGAGAAGGCTCAAC	Chr.1	14757652	14757672	+	No
2705704_adh	TTTCCGAGGCTCTGTGCGGAT	Chr.1	14757963	14757983	+	No
2739560_adh	TTTTCTGCTGGCCTAAACGAT	Chr.1	14759586	14759606	+	No
2723782_adh	TTTGGAGGATGACAGCGATAT	Chr.1	14790402	14790422	+	No
2628343_adh	TTGAAGAGGCAAAGGAGAAAC	Chr.1	14796149	14796169	+	No
1860174_adh	TAGGAAAATAGGAAATGGATA	Chr.1	14801677	14801657	-	No
1822924_adh	TACGAGAAGACTGATCCAGCA	Chr.1	14848976	14848996	+	No
2117529_adh	TGAAATTGTGTAGATTCAAAA	Chr.1	14856040	14856020	-	No
2603407_adh	TTCGAGAACACGAGAAAGAAC	Chr.1	14857441	14857421	-	No
2057946_adh	TCTTGAGATTGAGAAGGAAGA	Chr.1	14857592	14857572	-	No
2046094_adh	TCTGAGAATGGAGACCTTGTC	Chr.1	14858344	14858324	-	No
1785932_adh	TAAGGGACGCCTTCACTCTGA	Chr.1	14858360	14858340	-	No
2127105_adh	TGAAGAAGAAGGAATCTGAGT	Chr.1	14858949	14858929	-	No
2627166_adh	TTGAAGAAGAAGGAATCTGAG	Chr.1	14858950	14858930	-	No
1976127_adh	TCCAAGAAGAACGTCTGGATC	Chr.1	14862791	14862771	-	No
1908531_adh	TATGACTCCAAGAAGAACGTC	Chr.1	14862797	14862777	-	No
1942732_adh	TCAATGTTAGCTATGAATAAT	Chr.1	14953367	14953387	+	No
2111371_adh	TGAAATGTATAAAATTATTTA	Chr.1	14963964	14963984	+	No
2743543_adh	TTTTGTAGACTTATTGATAAT	Chr.1	14990591	14990571	-	No
2700917_adh	TTTCAAAAAACAACGTGCGTC	Chr.1	15014653	15014673	+	No
1999466_adh	TCGAATTTTGACAGCAGTATT	Chr.1	15018268	15018248	-	No
1821207_adh	TACCTTATTTGCAGATCTGCA	Chr.1	15023107	15023127	+	No
2614512_adh	TTCTACGTGACAAGCTTTTGA	Chr.1	15031025	15031045	+	No
2730432_adh	TTTGTTAGGTTTTGCTCATAT	Chr.1	15036009	15036029	+	No
2582305_adh	TTATTGATTTTCTCTCGGCAA	Chr.1	15052575	15052555	-	No
2700098_adh	TTTATTGATTTTCTCTCGGCA	Chr.1	15052576	15052556	-	No
1994133_adh	TCCTTTGATTGAGTTTTTTGT	Chr.1	15061118	15061138	+	No

2154861_adh	TGACAGTTGGTCTGAGACGTG	Chr.1	15061687	15061707	+	No
2670371_adh	TTGGTCTGAGACGTGATGTCT	Chr.1	15061693	15061713	+	No
2530135_adh	TGTTGGTGATGGTAGTGTGTG	Chr.1	15061724	15061744	+	No
2471455_adh	TGGTGATGGTAGTGTGTGTCT	Chr.1	15061727	15061747	+	No
2530725_adh	TGTTGTCGTGGGACTTGTTTA	Chr.1	15061966	15061986	+	No
1970980_adh	TCATCGCCGTGCCTCTGTGCC	Chr.1	15063818	15063838	+	No
1895372_adh	TATCCACAAACCTTAAGATGC	Chr.1	15063844	15063864	+	No
2547294_adh	TTAAGATGCTCGACTGGCTTC	Chr.1	15063856	15063876	+	No
2589742_adh	TTCACGGTCAGTTGAGTGTCG	Chr.1	15063874	15063894	+	No
2398868_adh	TGGCAAGAGTAGTGACTGTCC	Chr.1	15063930	15063950	+	No
1877700_adh	TAGTGACTGTCCGACCCATGG	Chr.1	15063939	15063959	+	No
2171528_adh	TGACTGTCCGACCCATGGAAC	Chr.1	15063942	15063962	+	No
2519187_adh	TGTGTCAGGGCTTAATGATGA	Chr.1	15063988	15064008	+	No
2526938_adh	TGTTCTGCAGTAGACGTTGTT	Chr.1	15064024	15064044	+	No
2618589_adh	TTCTGCAGTAGACGTTGTTCA	Chr.1	15064026	15064046	+	No
2047937_adh	TCTGCAGTAGACGTTGTTCAC	Chr.1	15064027	15064047	+	No
2288722_adh	TGCAGTAGACGTTGTTCACGA	Chr.1	15064029	15064049	+	No
2683500_adh	TTGTTCACGAGTCGTCTCAAC	Chr.1	15064040	15064060	+	No
2589579_adh	TTCACGAGTCGTCTCAACACA	Chr.1	15064043	15064063	+	No
2722039_adh	TTTGCGGAGAGGAAGTTAGTT	Chr.1	15064111	15064131	+	No
1970306_adh	TCATATTTGAATCAGTACACT	Chr.1	15064201	15064221	+	No
1811354_adh	TACACTGATTGCCAAAAGTCT	Chr.1	15064216	15064236	+	No
2589638_adh	TTCACGGACATGCGGTGATTT	Chr.1	15064236	15064256	+	No
1953184_adh	TCACGGACATGCGGTGATTTG	Chr.1	15064237	15064257	+	No
2302864_adh	TGCGGTGATTTGTTGTTGAGA	Chr.1	15064246	15064266	+	No
2278982_adh	TGATTTGTTGTTGAGATTTGC	Chr.1	15064251	15064271	+	No
2732359_adh	TTTGTTGTTGAGATTTGCATC	Chr.1	15064254	15064274	+	No
2650345_adh	TTGCATCTCGATAATACGTAC	Chr.1	15064268	15064288	+	No
2290416_adh	TGCATCTCGATAATACGTACT	Chr.1	15064269	15064289	+	No
2002581_adh	TCGATAATACGTACTAGCTTC	Chr.1	15064275	15064295	+	No
1827717_adh	TACGTACTAGCTTCAGCGATG	Chr.1	15064282	15064302	+	No
1830251_adh	TACTAGCTTCAGCGATGGATC	Chr.1	15064286	15064306	+	No
2051426_adh	TCTGGTTCAGGGTTGTTTAAC	Chr.1	15064424	15064444	+	No
2474744_adh	TGGTTCAGGGTTGTTTAACTC	Chr.1	15064426	15064446	+	No
2546048_adh	TTAACTCAATGCCTTAGGCTT	Chr.1	15064440	15064460	+	No
2056647_adh	TCTTCGGCTTGTCGGGCAACA	Chr.1	15064473	15064493	+	No
2608786_adh	TTCGGCTTGTCGGGCAACATT	Chr.1	15064475	15064495	+	No
2015954_adh	TCGGCTTGTCGGGCAACATTA	Chr.1	15064476	15064496	+	No
2016797_adh	TCGGGCAACATTAGTGAGCTG	Chr.1	15064484	15064504	+	No
2606683_adh	TTCGCGTCTCGGCATACTGAT	Chr.1	15064508	15064528	+	No
2008182_adh	TCGCGTCTCGGCATACTGATC	Chr.1	15064509	15064529	+	No
2040134_adh	TCTCGGCATACTGATCTTGTC	Chr.1	15064514	15064534	+	No
1876258_adh	TAGTCCAGAAGCATCACAAGT	Chr.1	15064536	15064556	+	No
1944269_adh	TCACAAGTCAAGACAGAGCTT	Chr.1	15064549	15064569	+	No
1938495_adh	TCAAGACAGAGCTTCACCGTT	Chr.1	15064556	15064576	+	No
2059294_adh	TCTTGTAATGTGACGCGCTAC	Chr.1	15064580	15064600	+	No
1801790_adh	TAATGTGACGCGCTACTCTTA	Chr.1	15064585	15064605	+	No
2503946_adh	TGTGACGCGCTACTCTTATAT	Chr.1	15064588	15064608	+	No
1831707_adh	TACTCTTATATCGGAGGAGCT	Chr.1	15064598	15064618	+	No
2054978_adh	TCTTATATCGGAGGAGCTGCC	Chr.1	15064601	15064621	+	No

2575599_adh	TTATATCGGAGGAGCTGCCAA	Chr.1	15064603	15064623	+	No
1890071_adh	TATATCGGAGGAGCTGCCAAA	Chr.1	15064604	15064624	+	No
1984345_adh	TCCGACTTGACTCGGCTGATC	Chr.1	15064630	15064650	+	No
2636714_adh	TTGACTCGGCTGATCATCAAG	Chr.1	15064636	15064656	+	No
2168043_adh	TGACTCGGCTGATCATCAAGA	Chr.1	15064637	15064657	+	No
2015791_adh	TCGGCTGATCATCAAGACGTG	Chr.1	15064641	15064661	+	No
2269260_adh	TGATCATCAAGACGTGTTTAA	Chr.1	15064646	15064666	+	No
1970377_adh	TCATCAAGACGTGTTTAACTG	Chr.1	15064649	15064669	+	No
2304328_adh	TGCGTGTCTTATGATGTGAAG	Chr.1	15064668	15064688	+	No
2500067_adh	TGTCTTATGATGTGAAGCTAC	Chr.1	15064672	15064692	+	No
1911757_adh	TATGATGTGAAGCTACTGCAT	Chr.1	15064677	15064697	+	No
2131763_adh	TGAAGCTACTGCATCTATGTC	Chr.1	15064684	15064704	+	No
2494822_adh	TGTCAGAATGTGTCGTCGCTC	Chr.1	15064701	15064721	+	No
1997057_adh	TCGAATACTGGGATTCGTCTA	Chr.1	15064725	15064745	+	No
1805929_adh	TAATTTTCGATTGATGCGGCG	Chr.1	15064772	15064792	+	No
2707209_adh	TTTCGATTGATGCGGCGCTGA	Chr.1	15064776	15064796	+	No
2645007_adh	TTGATGCGGCGCTGAGAAGAG	Chr.1	15064782	15064802	+	No
2271933_adh	TGATGCGGCGCTGAGAAGAGA	Chr.1	15064783	15064803	+	No
2301676_adh	TGCGGCGCTGAGAAGAGAGAC	Chr.1	15064786	15064806	+	No
2179936_adh	TGAGAAGAGAGACGGTGCGTG	Chr.1	15064794	15064814	+	No
1798791_adh	TAATCTCAACCTGAACTCAGT	Chr.1	15064821	15064841	+	No
2035823_adh	TCTCAACCTGAACTCAGTCGT	Chr.1	15064824	15064844	+	No
1994133_adh	TCCTTTGATTGAGTTTTTTGT	Chr.1	15068314	15068334	+	No
2154861_adh	TGACAGTTGGTCTGAGACGTG	Chr.1	15068884	15068904	+	No
2670371_adh	TTGGTCTGAGACGTGATGTCT	Chr.1	15068890	15068910	+	No
2530135_adh	TGTTGGTGATGGTAGTGTGTG	Chr.1	15068921	15068941	+	No
2471455_adh	TGGTGATGGTAGTGTGTGTCT	Chr.1	15068924	15068944	+	No
2530725_adh	TGTTGTCGTGGGACTTGTTTA	Chr.1	15069163	15069183	+	No
1970980_adh	TCATCGCCGTGCCTCTGTGCC	Chr.1	15071015	15071035	+	No
1895372_adh	TATCCACAAACCTTAAGATGC	Chr.1	15071041	15071061	+	No
2547294_adh	TTAAGATGCTCGACTGGCTTC	Chr.1	15071053	15071073	+	No
2589742_adh	TTCACGGTCAGTTGAGTGTCG	Chr.1	15071071	15071091	+	No
2398868_adh	TGGCAAGAGTAGTGACTGTCC	Chr.1	15071127	15071147	+	No
1877700_adh	TAGTGACTGTCCGACCCATGG	Chr.1	15071136	15071156	+	No
2171528_adh	TGACTGTCCGACCCATGGAAC	Chr.1	15071139	15071159	+	No
2519187_adh	TGTGTCAGGGCTTAATGATGA	Chr.1	15071185	15071205	+	No
2526938_adh	TGTTCTGCAGTAGACGTTGTT	Chr.1	15071221	15071241	+	No
2618589_adh	TTCTGCAGTAGACGTTGTTCA	Chr.1	15071223	15071243	+	No
2047937_adh	TCTGCAGTAGACGTTGTTCAC	Chr.1	15071224	15071244	+	No
2288722_adh	TGCAGTAGACGTTGTTCACGA	Chr.1	15071226	15071246	+	No
2683500_adh	TTGTTCACGAGTCGTCTCAAC	Chr.1	15071237	15071257	+	No
2589579_adh	TTCACGAGTCGTCTCAACACA	Chr.1	15071240	15071260	+	No
2722039_adh	TTTGCGGAGAGGAAGTTAGTT	Chr.1	15071308	15071328	+	No
2154861_adh	TGACAGTTGGTCTGAGACGTG	Chr.1	15071976	15071996	+	No
2670371_adh	TTGGTCTGAGACGTGATGTCT	Chr.1	15071982	15072002	+	No
2530135_adh	TGTTGGTGATGGTAGTGTGTG	Chr.1	15072013	15072033	+	No
2471455_adh	TGGTGATGGTAGTGTGTGTCT	Chr.1	15072016	15072036	+	No
2374081_adh	TGGACAGCTGATGGACGAGAC	Chr.2	1515	1495	-	No
2618534_adh	TTCTGATTTTCATTTCTGACG	Chr.2	43779	43759	-	No
2373863_adh	TGGACACGATGACAATTTCAA	Chr.2	43800	43780	-	No

1937466_adh	TCAACTGCGCTCTGTTGGACA	Chr.2	43815	43795	-	No
1929523_adh	TATTTCACGACAGAAGAATAG	Chr.2	43863	43883	+	No
2702556_adh	TTTCACGACAGAAGAATAGTG	Chr.2	43865	43885	+	No
2589541_adh	TTCACGACAGAAGAATAGTGT	Chr.2	43866	43886	+	No
2029220_adh	TCTAAAAACGAACGTGGAGCA	Chr.2	57859	57839	-	No
2688735_adh	TTTAAAAATTGATTTTGACGT	Chr.2	94731	94751	+	No
2608089_adh	TTCGGAGTGTCGTTGGCTAAA	Chr.2	98297	98317	+	No
1858370_adh	TAGCTGAAGAAGAACATAAAG	Chr.2	101599	101579	-	No
2742923_adh	TTTTGGCTGAAAATGGTTGGA	Chr.2	146889	146869	-	No
2520697_adh	TGTGTTAGTAAGAAGGATGAT	Chr.2	167610	167590	-	No
2123264_adh	TGAACGGATCCGGACAAACAT	Chr.2	175105	175125	+	No
1980135_adh	TCCCAAAAGACTGGAATTAAA	Chr.2	192170	192190	+	No
1924095_adh	TATTCCGCGTTTTCGTGCAAA	Chr.2	218877	218857	-	No
1852420_adh	TAGATTTCGGTTTTAGAATGG	Chr.2	218980	219000	+	No
2708570_adh	TTTCGGTTTTAGAATGGCTAA	Chr.2	218984	219004	+	No
2610890_adh	TTCGGTTTTAGAATGGCTAAA	Chr.2	218985	219005	+	No
2526499_adh	TGTTCGGCGTTTATTTCAGAA	Chr.2	233165	233145	-	No
2707345_adh	TTTCGCGCTTTTCGGCCACTG	Chr.2	255302	255282	-	No
1746208_adh	TAAAAAGACGGGAAAAATACA	Chr.2	255449	255469	+	No
1752458_adh	TAAAATGGATTTGTAAGCAAG	Chr.2	273752	273732	-	No
2700882_adh	TTTATTTTTCAATCTTCAAAT	Chr.2	294688	294668	-	No
1992490_adh	TCCTTAACCGTCGGAAAACCA	Chr.2	320905	320925	+	No
1851469_adh	TAGATGGCTGAAAAAATGAGA	Chr.2	339850	339870	+	No
2318774_adh	TGGAACAATGGCAAGAGTGCT	Chr.2	409227	409207	-	No
2314777_adh	TGGAAACTGGTAGAAAAGCGA	Chr.2	409370	409390	+	No
2065922_adh	TGAAAAAGCGCGAGAAGCTAA	Chr.2	411026	411006	-	No
2739588_adh	TTTTCTGGCGAAGATGCTGAA	Chr.2	436521	436541	+	No
2710707_adh	TTTCTGGCGAAGATGCTGAAG	Chr.2	436522	436542	+	No
1979519_adh	TCCATCAAATCAACGTCGTTC	Chr.2	446931	446911	-	No
1833707_adh	TACTGTAGCTCCAGGAGTACG	Chr.2	457455	457435	-	No
1833707_adh	TACTGTAGCTCCAGGAGTACG	Chr.2	461488	461468	-	No
2700173_adh	TTTATTGCTTTATTTCTCTAT	Chr.2	484765	484785	+	No
2625791_adh	TTGAAATTTTTAAAATCTTTT	Chr.2	493388	493408	+	No
1819451_adh	TACCGAATATTCTTATACTTT	Chr.2	500311	500331	+	No
1972321_adh	TCATGCGTGTAGAAAAATGTA	Chr.2	540650	540670	+	No
2292151_adh	TGCCACAGAAGGACCGAGAAG	Chr.2	580315	580295	-	No
2109032_adh	TGAAATGTAAAAATGAATCAA	Chr.2	609918	609938	+	No
2542123_adh	TTAAATACAAGTAGACGCAAT	Chr.2	611576	611556	-	No
2542124_adh	TTAAATACAAGTAGACGTAAT	Chr.2	612207	612227	+	No
1958006_adh	TCAGAATTGTCTCGGCAACAC	Chr.2	634237	634257	+	No
1889703_adh	TATATACTTGGGTGCTCTGAA	Chr.2	650544	650564	+	No
2673761_adh	TTGTAACAGTAGAAGCAGTTG	Chr.2	658859	658879	+	No
1933904_adh	TCAAAGTAGGTGTCGTGGAAT	Chr.2	700987	701007	+	No
2744000_adh	TTTTGTGTAGATTTACGGAGC	Chr.2	714996	714976	-	No
2548098_adh	TTAAGCTCTCCTTCGTTGGAT	Chr.2	719736	719716	-	No
2739419_adh	TTTTCTGAAAACTTCTAGGAT	Chr.2	734028	734048	+	No
2716467_adh	TTTGAGACTTACGAAGAAAAT	Chr.2	736046	736066	+	No
1831630_adh	TACTCTGGATAAAAGACGCGG	Chr.2	741085	741105	+	No
2049221_adh	TCTGGATAAAAGACGCGGTAA	Chr.2	741088	741108	+	No
2084999_adh	TGAAATAAAAATAAAAATCCA	Chr.2	771637	771657	+	No

1824972_adh	TACGCGGAAATGATGGTTCCT	Chr.2	783016	782996	-	No
2307628_adh	TGCTCGTTCTGAGGGACAAGC	Chr.2	783193	783213	+	No
1835655_adh	TACTTGGTTAAAACTTCAACA	Chr.2	783839	783819	-	No
2738340_adh	TTTTCGATTTTCCTGCGGCTC	Chr.2	794668	794648	-	No
1832048_adh	TACTGAAGATGATAAGTGGGT	Chr.2	802243	802223	-	No
1957608_adh	TCAGAAGACTTATTCCTCAGC	Chr.2	806472	806452	-	No
1979016_adh	TCCAGTCAGTCAGAAGACTTA	Chr.2	806481	806461	-	No
2673562_adh	TTGTAAAATAGATCTCAGCAT	Chr.2	812119	812139	+	No
2007851_adh	TCGCGATTTTTAGAGGGCTTT	Chr.2	820985	821005	+	No
2673562_adh	TTGTAAAATAGATCTCAGCAT	Chr.2	823023	823043	+	No
2615880_adh	TTCTCAAAGCATCTGACCGCT	Chr.2	823805	823825	+	No
1830829_adh	TACTCAACGCAATGCGTCGCA	Chr.2	839429	839409	-	No
1815735_adh	TACCAGTTGTATCTATCGGTT	Chr.2	845840	845860	+	No
1830825_adh	TACTCAACATTATGAACGGCT	Chr.2	866918	866898	-	No
2745158_adh	TTTTTAGTGTGAAACGTTGGT	Chr.2	884933	884913	-	No
2745158_adh	TTTTTAGTGTGAAACGTTGGT	Chr.2	885117	885097	-	No
2745158_adh	TTTTTAGTGTGAAACGTTGGT	Chr.2	885301	885281	-	No
2745158_adh	TTTTTAGTGTGAAACGTTGGT	Chr.2	885447	885427	-	No
1795290_adh	TAATAGTCAGTGTGTTCGTCG	Chr.2	896206	896186	-	No
2722052_adh	TTTGCGGATAGGCAGCGTTTT	Chr.2	913361	913341	-	No
2539985_adh	TTAAACGGTAAGGGCCGGCTG	Chr.2	925407	925427	+	No
2691161_adh	TTTAATCAAGACATCTTCGGC	Chr.2	928751	928771	+	No
1814776_adh	TACCAAGGACTGAAGGAAGAG	Chr.2	945540	945520	-	No
2276343_adh	TGATTGACTGTGTGGCGACTA	Chr.2	1005666	1005646	-	No
2276343_adh	TGATTGACTGTGTGGCGACTA	Chr.2	1011494	1011474	-	No
2276343_adh	TGATTGACTGTGTGGCGACTA	Chr.2	1017312	1017292	-	No
2748517_adh	TTTTTTCACTAAATCGTCGGT	Chr.2	1070859	1070879	+	No
2707300_adh	TTTCGCCAGTTTTCCGTCGAT	Chr.2	1123335	1123315	-	No
1936657_adh	TCAACGGACAAAAGGAAAAGA	Chr.2	1135198	1135178	-	No
2639531_adh	TTGAGAAGGATCGGCTGCCAG	Chr.2	1149432	1149452	+	No
2514762_adh	TGTGGAATGGTCGCCTGGATC	Chr.2	1150226	1150246	+	No
2153323_adh	TGACACTGTACACTCCAGAAA	Chr.2	1150248	1150268	+	No
1969042_adh	TCATAGATGGCTGAAAAATAA	Chr.2	1232230	1232250	+	No
1969042_adh	TCATAGATGGCTGAAAAATAA	Chr.2	1232841	1232861	+	No
1925263_adh	TATTGAAAATTCGGTTATCTA	Chr.2	1248931	1248951	+	No
2373136_adh	TGGAATTTTTGTTGGATTTCG	Chr.2	1306702	1306682	-	No
2688478_adh	TTGTTTTTGTTGTCTTAAAGT	Chr.2	1320522	1320502	-	No
1946975_adh	TCACCATCGTCGTCGTCGGAT	Chr.2	1321221	1321201	-	No
2026044_adh	TCGTGAGGAAGACGAGGTCGT	Chr.2	1322315	1322335	+	No
1833707_adh	TACTGTAGCTCCAGGAGTACG	Chr.2	1350285	1350305	+	No
2303332_adh	TGCGTAAGACGCTCGGTGTTT	Chr.2	1350327	1350307	-	No
2723305_adh	TTTGGACGAAAACTGGCAAAT	Chr.2	1369425	1369405	-	No
2742703_adh	TTTTGGACGAAAACTGGCAAA	Chr.2	1369426	1369406	-	No
2126807_adh	TGAACTTTTGTGGATTTCGTA	Chr.2	1377070	1377090	+	No
1746690_adh	TAAAAAGGATTTTCGCTTCAA	Chr.2	1402316	1402296	-	No
2530313_adh	TGTTGTAAACGGGGCAGTGGA	Chr.2	1446854	1446834	-	No
2745747_adh	TTTTTCATATTTTCAAGCTAA	Chr.2	1497241	1497261	+	No
2745747_adh	TTTTTCATATTTTCAAGCTAA	Chr.2	1497695	1497715	+	No
2696635_adh	TTTAGGACTGGGGCACAGGAA	Chr.2	1500482	1500502	+	No
1775379_adh	TAAGACATTTTTACATCTAGA	Chr.2	1501051	1501071	+	No

2693793_adh	TTTACGAGAAGAGAAGTACAA	Chr.2	1548930	1548950	+	No
1883920_adh	TATAAAAGACCGTCGTCACGC	Chr.2	1561511	1561531	+	No
1990086_adh	TCCTCCCTGGATCAGTCGGTA	Chr.2	1591638	1591618	-	No
2742982_adh	TTTTGGGGAGCAGAAATCGGA	Chr.2	1592016	1591996	-	No
2090514_adh	TGAAATCAAACAAAAAATCAA	Chr.2	1754338	1754358	+	No
1762993_adh	TAACAAAAAGCGACGAAAAAG	Chr.2	1814092	1814112	+	No
1850593_adh	TAGATCAAAGTGATATGGGAC	Chr.2	1821882	1821902	+	No
2618481_adh	TTCTGATGGTTGTCGCTTTGG	Chr.2	1855626	1855606	-	No
1999616_adh	TCGACACGGAGTTCTGATGGT	Chr.2	1855637	1855617	-	No
2129901_adh	TGAAGATAACGACTTTTAACA	Chr.2	1855749	1855769	+	No
2618481_adh	TTCTGATGGTTGTCGCTTTGG	Chr.2	1862289	1862269	-	No
1999616_adh	TCGACACGGAGTTCTGATGGT	Chr.2	1862300	1862280	-	No
2129901_adh	TGAAGATAACGACTTTTAACA	Chr.2	1862413	1862433	+	No
2741014_adh	TTTTGACAGGAAAATATCGGA	Chr.2	1918163	1918183	+	No
2689095_adh	TTTAAACGTAGTTCATCGTAT	Chr.2	1922815	1922795	-	No
2701828_adh	TTTCAATGGAAAAATCGTTAA	Chr.2	1928849	1928869	+	No
2600775_adh	TTCCTGAGGATGTGCACAATT	Chr.2	1930546	1930526	-	No
2623607_adh	TTCTTTGATCTTTTTGGCAAA	Chr.2	2017274	2017294	+	No
2270073_adh	TGATCGTTGAAGCCGCTGAAA	Chr.2	2018204	2018184	-	No
2003962_adh	TCGATGAAGAACTGGCGAAAA	Chr.2	2018480	2018460	-	No
2595879_adh	TTCCAAGCGATTTCTGAGTGA	Chr.2	2039722	2039702	-	No
1988983_adh	TCCGTTTTTTAGGACTTTTTC	Chr.2	2042658	2042638	-	No
2740899_adh	TTTTGAATATTCCTGCGGCTT	Chr.2	2047406	2047386	-	No
2748886_adh	TTTTTTCGCGATTTGTCTTGT	Chr.2	2049475	2049455	-	No
2236600_adh	TGAGGAAGGCGAGAACCCCGT	Chr.2	2052964	2052944	-	No
1957820_adh	TCAGAATAAAGCCTTCGAAAC	Chr.2	2059155	2059135	-	No
2618873_adh	TTCTGGAAGAGGGAAAGCTTA	Chr.2	2059260	2059280	+	No
2160577_adh	TGACGAAGAAGGATGTTAACT	Chr.2	2091746	2091726	-	No
2500363_adh	TGTCTTTCTATCGACGATTCA	Chr.2	2091800	2091780	-	No
2651739_adh	TTGCGAAGATGGAAGATCTGA	Chr.2	2105311	2105291	-	No
2000610_adh	TCGACTGGAATTGGACGTGCT	Chr.2	2134256	2134236	-	No
2502984_adh	TGTGAATTAAGTAGTTGATCA	Chr.2	2144982	2145002	+	No
2474515_adh	TGGTTATTGTCGTCGGAATCA	Chr.2	2157748	2157768	+	No
2027013_adh	TCGTGGACTTCTCACCGGTCT	Chr.2	2158272	2158292	+	No
2700706_adh	TTTATTTCGATTTGTCCGTTC	Chr.2	2170202	2170182	-	No
2662502_adh	TTGGATGAGACTCACAAAAAA	Chr.2	2180354	2180374	+	No
2591913_adh	TTCAGCTTGCTGCGTTACTTA	Chr.2	2199248	2199228	-	No
1833743_adh	TACTGTAGTCTTGGCACTTGT	Chr.2	2200798	2200778	-	No
1928872_adh	TATTGTTTTTCTCGCGGATTT	Chr.2	2204226	2204206	-	No
2581183_adh	TTATTATTGTTTTTCTCGCGG	Chr.2	2204230	2204210	-	No
1924720_adh	TATTCTAGAGCAGAATTTGGT	Chr.2	2219971	2219951	-	No
2640994_adh	TTGAGCTGGACGGCGAATATG	Chr.2	2220130	2220150	+	No
2714898_adh	TTTGACATGAATGAAGATTTT	Chr.2	2220418	2220398	-	No
2734498_adh	TTTTAGACTCAGAAAATGGCG	Chr.2	2222822	2222802	-	No
2717776_adh	TTTGATCAGTTTGGAAATCAA	Chr.2	2223598	2223578	-	No
2556964_adh	TTACGCGCTGTCGAAAGGGTA	Chr.2	2232473	2232453	-	No
1761089_adh	TAAATCTGATTTTTTACAAGC	Chr.2	2232566	2232586	+	No
2020739_adh	TCGGTTTTCGAGAAGAATTGA	Chr.2	2232732	2232752	+	No
2717776_adh	TTTGATCAGTTTGGAAATCAA	Chr.2	2255124	2255144	+	No
2717776_adh	TTTGATCAGTTTGGAAATCAA	Chr.2	2265778	2265758	-	No

1807810_adh	TACAACGGATCATTCGGAACA	Chr.2	2272491	2272471	-	No
2721475_adh	TTTGCATTTGTCGTTGGCAAC	Chr.2	2283018	2282998	-	No
2017209_adh	TCGGGGTACTGTAGTAGTGCT	Chr.2	2348753	2348733	-	No
1874650_adh	TAGTAGCTCGTACGGAAGATT	Chr.2	2361781	2361761	-	No
2010464_adh	TCGGAACAAAAAAGAAAAGAT	Chr.2	2434496	2434476	-	No
2590213_adh	TTCAGAAACCGCGTAGACCAC	Chr.2	2437040	2437060	+	No
2707761_adh	TTTCGGATAATTTTTCGTTGA	Chr.2	2474098	2474078	-	No
2142647_adh	TGAATCTAATTGAACTTGAAT	Chr.2	2495356	2495336	-	No
1854552_adh	TAGCAGCATACACCAGAACAC	Chr.2	2549369	2549349	-	No
2292387_adh	TGCCAGAGCTCTGCGAAAGCA	Chr.2	2556797	2556777	-	No
2732937_adh	TTTTAAAAACCGGCAAAAAAT	Chr.2	2570426	2570446	+	No
1804742_adh	TAATTTAGAAAACAGTGACGC	Chr.2	2616449	2616469	+	No
1850533_adh	TAGATATGCAAGTAGGAACGC	Chr.2	2623761	2623741	-	No
2710713_adh	TTTCTGGCGTGAAGAAGGACA	Chr.2	2623811	2623791	-	No
2066166_adh	TGAAAAAGTTCAATATTCCAA	Chr.2	2667622	2667602	-	No
2307306_adh	TGCTCCACTAGAATAATGGCA	Chr.2	2717367	2717347	-	No
2376334_adh	TGGACGGCCAGATTTGGTAAA	Chr.2	2759159	2759139	-	No
2574011_adh	TTATAAGTGACAATGGAGCAT	Chr.2	2787271	2787251	-	No
2747805_adh	TTTTTGTGATTTCGAAGCCAT	Chr.2	2815262	2815282	+	No
1985223_adh	TCCGCACTGTGGCAAATTTTT	Chr.2	2860774	2860794	+	No
2711774_adh	TTTCTTGCAGAAAACATCCAT	Chr.2	2889310	2889290	-	No
2743572_adh	TTTTGTAGATTTTCACGGAAA	Chr.2	2892162	2892142	-	No
2279366_adh	TGATTTTGTAGATTTTCACGG	Chr.2	2892165	2892145	-	No
2743572_adh	TTTTGTAGATTTTCACGGAAA	Chr.2	2909528	2909508	-	No
2597333_adh	TTCCATGGAAACTCTGAGCAA	Chr.2	2915937	2915957	+	No
2496930_adh	TGTCCAAGTGGCGCAGTGGAA	Chr.2	3027106	3027126	+	No
2480354_adh	TGTAAAAATTGTAGGAAGAAC	Chr.2	3056275	3056295	+	No
1999940_adh	TCGACGAACGAGCAATCTGGA	Chr.2	3070591	3070571	-	No
2288460_adh	TGCAGGAATGAGGCGAGATGT	Chr.2	3113565	3113585	+	No
2604197_adh	TTCGAGTTTTTAAATTGAACG	Chr.2	3114245	3114265	+	No
2382742_adh	TGGAGTACTGTAGGAGTACGG	Chr.2	3177026	3177006	-	No
2382742_adh	TGGAGTACTGTAGGAGTACGG	Chr.2	3177149	3177129	-	No
1924747_adh	TATTCTATCATACTGCACTCT	Chr.2	3183415	3183395	-	No
2066166_adh	TGAAAAAGTTCAATATTCCAA	Chr.2	3189874	3189894	+	No
2742617_adh	TTTTGGAAAAGTTGGAGGAGG	Chr.2	3207918	3207898	-	No
2259206_adh	TGATAATTCAAAATCGATCGG	Chr.2	3222892	3222872	-	No
2287161_adh	TGCAGACCAGTGCTGTGCGGC	Chr.2	3235139	3235159	+	No
2357416_adh	TGGAATGTAAAGAAGTGCGGA	Chr.2	3236366	3236386	+	No
1972568_adh	TCATGGAGAACAAATTGATGA	Chr.2	3245748	3245728	-	No
1805384_adh	TAATTTGCTGGAAGCTGGAAA	Chr.2	3246302	3246282	-	No
2492543_adh	TGTATGTATTTGACGGTCGTT	Chr.2	3264230	3264250	+	No
2118344_adh	TGAAATTTAAATTTAAATTTA	Chr.2	3269168	3269148	-	No
2598940_adh	TTCCGATTTGTAAGAATTTCA	Chr.2	3278921	3278941	+	No
1990086_adh	TCCTCCCTGGATCAGTCGGTA	Chr.2	3290091	3290111	+	No
2587826_adh	TTCAATCGGTTGGTGTAGCGG	Chr.2	3291761	3291741	-	No
2035320_adh	TCTCAAAGAATTGTGACGTCA	Chr.2	3310151	3310171	+	No
1860220_adh	TAGGAAAATGTATATGAAAGT	Chr.2	3310552	3310572	+	No
1865140_adh	TAGGATCAATTATATTTGCCA	Chr.2	3314460	3314480	+	No
2649515_adh	TTGCAGATTGGAATATCTATT	Chr.2	3327412	3327432	+	No
1798883_adh	TAATCTGACTACGGTAGACGG	Chr.2	3345143	3345123	-	No

2302652_adh	TGCGGTAGTTGATTTTTAAAA	Chr.2	3346605	3346625	+	No
2070802_adh	TGAAAATTGTGCGAAAACGTT	Chr.2	3348086	3348066	-	No
2480314_adh	TGTAAAAAAGTGTAGGAAAAT	Chr.2	3351357	3351337	-	No
2480314_adh	TGTAAAAAAGTGTAGGAAAAT	Chr.2	3358308	3358328	+	No
2565247_adh	TTAGATGATGAATTCCAAAAG	Chr.2	3370672	3370692	+	No
2618353_adh	TTCTGAGTGGAGTGCGAACAT	Chr.2	3373100	3373080	-	No
2403826_adh	TGGCAGGATCTCAGACAAAAA	Chr.2	3414643	3414663	+	No
2457045_adh	TGGGGTTTTGAAGACGATGTT	Chr.2	3440085	3440105	+	No
2281191_adh	TGCAAATGACGCGCATTGCTC	Chr.2	3445516	3445496	-	No
2042732_adh	TCTCTGAAAAAAGCACAGGTT	Chr.2	3504517	3504537	+	No
2263654_adh	TGATATCTTTTATTCAAAAAA	Chr.2	3523183	3523163	-	No
1795258_adh	TAATAGTAGAAGCCCGGAAAA	Chr.2	3532054	3532034	-	No
2716416_adh	TTTGAGAATTTTTAGATCTGA	Chr.2	3636449	3636429	-	No
2110728_adh	TGAAATGTAACTGAGTATGTA	Chr.2	3707151	3707131	-	No
2737464_adh	TTTTCAGATGGAACGGTGAGT	Chr.2	3713305	3713285	-	No
2710210_adh	TTTCTCTGTGGAGATGGCCTC	Chr.2	3785292	3785312	+	No
2675354_adh	TTGTAGTGTACCCTTAAAAAC	Chr.2	3795868	3795888	+	No
1891039_adh	TATATTTTATCTGGACCTATA	Chr.2	3815578	3815558	-	No
1777610_adh	TAAGAGTGGAAAAACCTTGGT	Chr.2	3831054	3831034	-	No
2707161_adh	TTTCGATGACGACTCAATCTA	Chr.2	3857075	3857095	+	No
1820209_adh	TACCTCTCGTCGAATCGATTC	Chr.2	3906840	3906820	-	No
2011814_adh	TCGGACTATAAGCATCATTGG	Chr.2	3925661	3925641	-	No
2019813_adh	TCGGTTACTAGGCGGAAAAAG	Chr.2	3959328	3959308	-	No
1853760_adh	TAGCACAACAACGACAACAAC	Chr.2	3966392	3966412	+	No
2747142_adh	TTTTTGCTGTAAATTTCGGAT	Chr.2	4044471	4044451	-	No
2711556_adh	TTTCTTCGTTTTTTGCTGTAA	Chr.2	4044480	4044460	-	No
2692406_adh	TTTAATTGACGACACTTTCTT	Chr.2	4048114	4048134	+	No
2608432_adh	TTCGGCAATTTTCGGGCAGAA	Chr.2	4048156	4048176	+	No
2738647_adh	TTTTCGGGCAGAAATTGTGAA	Chr.2	4048164	4048184	+	No
2702431_adh	TTTCACAGAGAATTCAGTGCG	Chr.2	4049971	4049991	+	No
1908722_adh	TATGACTTAGAGAAGAGACAA	Chr.2	4050479	4050459	-	No
1930982_adh	TATTTTCTGTTGACTCGTCTT	Chr.2	4051882	4051902	+	No
1876095_adh	TAGTCACAAGACTGTGTGGCA	Chr.2	4053175	4053155	-	No
1762599_adh	TAAATTGTAGGAGAAAGGTGG	Chr.2	4053200	4053220	+	No
2050761_adh	TCTGGTCATACATGTCGGAGA	Chr.2	4058309	4058289	-	No
2705232_adh	TTTCCATTTACGACTCTTTGA	Chr.2	4075658	4075638	-	No
2728205_adh	TTTGTAGAAGCGGAATGTGAA	Chr.2	4087187	4087207	+	No
2674718_adh	TTGTAGAAGCGGAATGTGAAG	Chr.2	4087188	4087208	+	No
2043781_adh	TCTGAAAGTCGATGGAGGAAT	Chr.2	4093742	4093762	+	No
2723229_adh	TTTGGACAAGCCTTTTTGGCA	Chr.2	4115284	4115304	+	No
2747239_adh	TTTTTGGAGCGAAGGACGATT	Chr.2	4126181	4126161	-	No
2746443_adh	TTTTTCTTGCATATTTTTCCC	Chr.2	4127750	4127770	+	No
1839666_adh	TAGAAGGGTTGAACACAAGTG	Chr.2	4130238	4130218	-	No
2737438_adh	TTTTCAGAAGATGTGCTCCCT	Chr.2	4223298	4223318	+	No
2049099_adh	TCTGGAGGAACACACGGAATA	Chr.2	4235369	4235389	+	No
2602668_adh	TTCGAATTTTAACTGTATTTT	Chr.2	4237820	4237800	-	No
2600842_adh	TTCCTGCAGTAACTTGATGGT	Chr.2	4238055	4238035	-	No
2278820_adh	TGATTTGTCGGAAATTTTCAT	Chr.2	4290029	4290009	-	No
2738327_adh	TTTTCGATTGTTTTCCTTCAA	Chr.2	4323101	4323121	+	No
2492873_adh	TGTATTCAAAAACCAGAAAAA	Chr.2	4339916	4339936	+	No

1897541_adh	TATCGGAACGCTAGAAACAAG	Chr.2	4358834	4358814	-	No
1774568_adh	TAAGAATGAGAGTTCGACAGT	Chr.2	4375506	4375526	+	No
2530034_adh	TGTTGGGATCGGCGGAAAATC	Chr.2	4413372	4413392	+	No
2257300_adh	TGAGTTTAAGACGTATTTCTG	Chr.2	4419184	4419164	-	No
2280720_adh	TGCAAAGGATTATACAGTTTT	Chr.2	4419290	4419310	+	No
2689956_adh	TTTAACGATCAAAAGATGGCC	Chr.2	4419994	4419974	-	No
2044175_adh	TCTGAAGAGCGTGTAGACTTT	Chr.2	4425460	4425480	+	No
2745081_adh	TTTTTAGATTTTTGAACAAAG	Chr.2	4441910	4441930	+	No
1982213_adh	TCCCTGAACTTTTAGAATGAC	Chr.2	4445098	4445078	-	No
2090057_adh	TGAAATATTTGAGAAATTTTT	Chr.2	4448402	4448382	-	No
2289530_adh	TGCAGTTTTTCGATTTCCCAA	Chr.2	4460913	4460933	+	No
2646462_adh	TTGATTGGCAAACTGGTGCGC	Chr.2	4471065	4471045	-	No
2673470_adh	TTGGTTTTTTGTAGATCAGGC	Chr.2	4471122	4471142	+	No
2479378_adh	TGGTTTTTTGTAGATCAGGCG	Chr.2	4471123	4471143	+	No
2749688_adh	TTTTTTGTAGATCAGGCGGGT	Chr.2	4471126	4471146	+	No
2747724_adh	TTTTTGTAGATCAGGCGGGTT	Chr.2	4471127	4471147	+	No
2107304_adh	TGAAATGGCAGAAATGAACGG	Chr.2	4566039	4566059	+	No
1754527_adh	TAAACATTCACCAATCGTCTT	Chr.2	4568547	4568567	+	No
2589123_adh	TTCACACGAGTGCGAGACTTC	Chr.2	4603018	4602998	-	No
1822100_adh	TACGACAGAATGACACTGAAA	Chr.2	4604690	4604710	+	No
2010611_adh	TCGGAACGCACTAGAATGGAC	Chr.2	4622468	4622448	-	No
1878970_adh	TAGTGGTAATGTAGAGGTACT	Chr.2	4626347	4626367	+	No
2496994_adh	TGTCCATAAATCTCGGTTTAA	Chr.2	4641291	4641271	-	No
2496994_adh	TGTCCATAAATCTCGGTTTAA	Chr.2	4641642	4641622	-	No
2496994_adh	TGTCCATAAATCTCGGTTTAA	Chr.2	4642227	4642207	-	No
2496994_adh	TGTCCATAAATCTCGGTTTAA	Chr.2	4642504	4642484	-	No
1759729_adh	TAAATAGGCAGATTGTATCGT	Chr.2	4672604	4672584	-	No
1974353_adh	TCATTGGTAGAAACAAGGCAT	Chr.2	4673120	4673100	-	No
2537487_adh	TTAAAAAAGCAGAACTATATA	Chr.2	4698324	4698304	-	No
2746213_adh	TTTTTCTCATCATTTGCGGAT	Chr.2	4701881	4701901	+	No
2670118_adh	TTGGTCAAAGTCTCGGGATGT	Chr.2	4702273	4702253	-	No
2679044_adh	TTGTCTTGTCTGAGAGTGGCT	Chr.2	4703030	4703010	-	No
2739428_adh	TTTTCTGAACCGCGGCACAGT	Chr.2	4706582	4706602	+	No
2710366_adh	TTTCTGAACCGCGGCACAGTT	Chr.2	4706583	4706603	+	No
2697280_adh	TTTAGTGATGAAATGAAGACA	Chr.2	4706635	4706655	+	No
2326447_adh	TGGAAGTATGATAGACAAATC	Chr.2	4739547	4739567	+	No
2582935_adh	TTATTGGAATTATGAAGAAGA	Chr.2	4744094	4744114	+	No
1833757_adh	TACTGTAGTTTAGACCTTTAG	Chr.2	4790761	4790741	-	No
1941669_adh	TCAATGAAACTAGTAGAAGGC	Chr.2	4823341	4823361	+	No
2125737_adh	TGAACTGTCAAAACGGGACGT	Chr.2	4825256	4825236	-	No
2031121_adh	TCTACAAAGAAGTCGCGATGG	Chr.2	4825385	4825405	+	No
2558046_adh	TTACGTGGACTATCTTTAAAA	Chr.2	4834901	4834881	-	No
1813276_adh	TACATATTTGTATAGACGATC	Chr.2	4842242	4842222	-	No
1813276_adh	TACATATTTGTATAGACGATC	Chr.2	4863657	4863677	+	No
2318528_adh	TGGAAATTGGGAATGGGCACA	Chr.2	4871486	4871466	-	No
1961773_adh	TCAGATTGCTTGACCCGAAAC	Chr.2	4874828	4874808	-	No
1936535_adh	TCAACGAAGTTCGTCCATACT	Chr.2	4882953	4882973	+	No
2538998_adh	TTAAAATCGCTGCGATACTGA	Chr.2	4888721	4888701	-	No
2723262_adh	TTTGGACAGGAGGACACTAAC	Chr.2	4892444	4892424	-	No
2048928_adh	TCTGGACCCAAAGGAAGCTGT	Chr.2	4892496	4892476	-	No

2723262_adh	TTTGGACAGGAGGACACTAAC	Chr.2	4899029	4899049	+	No
2008556_adh	TCGCTCGAACTAGAATTGGTG	Chr.2	4904070	4904050	-	No
1963627_adh	TCAGGAGAACGCTGCTGTGAA	Chr.2	4904671	4904691	+	No
2704027_adh	TTTCATCGTCGGAATCGATAT	Chr.2	4911860	4911880	+	No
2698153_adh	TTTATAGGATTATTTTGTGCA	Chr.2	4914303	4914283	-	No
2615443_adh	TTCTATCGGAATTTCATGCAT	Chr.2	4914324	4914304	-	No
2014186_adh	TCGGATGAAAAAATGTGCATA	Chr.2	4914563	4914583	+	No
1999809_adh	TCGACCACTGAAGTACAATAG	Chr.2	4914696	4914716	+	No
2723262_adh	TTTGGACAGGAGGACACTAAC	Chr.2	4916312	4916292	-	No
2048928_adh	TCTGGACCCAAAGGAAGCTGT	Chr.2	4916364	4916344	-	No
2048928_adh	TCTGGACCCAAAGGAAGCTGT	Chr.2	4933198	4933218	+	No
2723262_adh	TTTGGACAGGAGGACACTAAC	Chr.2	4933250	4933270	+	No
2000854_adh	TCGACTTTGTGGGAAGGGCCA	Chr.2	4935652	4935632	-	No
2161811_adh	TGACGGCTTCTGAAGATGTTC	Chr.2	4939071	4939051	-	No
2104044_adh	TGAAATGGAAAAGAGAGAAAA	Chr.2	4940512	4940492	-	No
1784959_adh	TAAGGCATGTAGTATAGATAA	Chr.2	4946823	4946803	-	No
1909750_adh	TATGAGTCAGTAAGGCATGTA	Chr.2	4946833	4946813	-	No
2126995_adh	TGAAGAACCAACACAAAAGAA	Chr.2	4954517	4954537	+	No
2532675_adh	TGTTTCTCTGTTTTATTAAAT	Chr.2	4955468	4955448	-	No
2488035_adh	TGTAGATCTTCGAGAGATGCA	Chr.2	4959960	4959980	+	No
2712874_adh	TTTGAACAACTTGAGACCGAA	Chr.2	4993451	4993431	-	No
2535106_adh	TGTTTTTCTCTGTTTTGCATG	Chr.2	4999398	4999418	+	No
2549433_adh	TTAATAAAAGTTTCTCGTCGT	Chr.2	4999430	4999450	+	No
2289456_adh	TGCAGTTGGAATTTCTGGAAA	Chr.2	5011813	5011793	-	No
2403271_adh	TGGCACTTACTAGAGACACAA	Chr.2	5040969	5040989	+	No
1989354_adh	TCCTACTAATTGAAAACTGGC	Chr.2	5055200	5055180	-	No
2602102_adh	TTCGAAGACGCCGGCTAGTTT	Chr.2	5055323	5055303	-	No
2281833_adh	TGCAAGAACCCAGTTGAGGAT	Chr.2	5055685	5055705	+	No
2641304_adh	TTGAGGATGGAATTCTCAGAA	Chr.2	5055698	5055718	+	No
1833672_adh	TACTGTAGAGTAAGCGTAGTA	Chr.2	5056733	5056753	+	No
2039579_adh	TCTCGATCAACAACACATAGA	Chr.2	5062793	5062813	+	No
1943786_adh	TCACAAAAGGTAAGTAGGCAA	Chr.2	5073293	5073273	-	No
1829512_adh	TACTACAGAATTTTCGGTGTG	Chr.2	5087497	5087517	+	No
2035348_adh	TCTCAAATCGTAGAAGGTATG	Chr.2	5093111	5093091	-	Yes
1975978_adh	TCCAACAAAGGATGAAGAACG	Chr.2	5094624	5094644	+	No
1754551_adh	TAAACCAAGGAAAAAGTGTGT	Chr.2	5102877	5102857	-	No
2593531_adh	TTCATACATATCTGATATTAA	Chr.2	5148531	5148551	+	No
2018664_adh	TCGGTCAGGAGGATACAAACA	Chr.2	5156827	5156807	-	No
2018664_adh	TCGGTCAGGAGGATACAAACA	Chr.2	5158768	5158748	-	No
2515961_adh	TGTGGCCGACGGACTGAACGG	Chr.2	5184449	5184469	+	No
2636606_adh	TTGACTCATTAGATTGTGAAT	Chr.2	5278413	5278393	-	No
2412176_adh	TGGCGATTTTGGAGATGGACA	Chr.2	5284066	5284046	-	No
1867080_adh	TAGGCATGTAGGTAGGCATTT	Chr.2	5308636	5308616	-	No
1921983_adh	TATTATAGAAAAATACGATCG	Chr.2	5319118	5319138	+	No
2600777_adh	TTCCTGAGTAGAATTGGCTAT	Chr.2	5347186	5347166	-	No
2600777_adh	TTCCTGAGTAGAATTGGCTAT	Chr.2	5349913	5349933	+	No
2448277_adh	TGGGATTGAACTAGTGGACGT	Chr.2	5353796	5353816	+	No
1979999_adh	TCCATTCGATATCATTCGGGA	Chr.2	5358296	5358276	-	No
2742518_adh	TTTTGCTCTGAAATCTGCAGA	Chr.2	5358434	5358454	+	No
2043806_adh	TCTGAAATCTGCAGAAAAACG	Chr.2	5358440	5358460	+	No

2237812_adh	TGAGGAGTTCAAGGCTATCAA	Chr.2	5378994	5379014	+	No
2741511_adh	TTTTGATACTGAAATTCTGAA	Chr.2	5379538	5379558	+	No
2552992_adh	TTACAAAAATGAATGCGTGCG	Chr.2	5414197	5414217	+	No
2598437_adh	TTCCCTGCGCCTATTTCTTCT	Chr.2	5414539	5414559	+	No
2004110_adh	TCGATGCGTAGATGGCGGGAC	Chr.2	5418617	5418597	-	No
1753070_adh	TAAAATTATGTGAAGCCGGCG	Chr.2	5429838	5429818	-	No
1889006_adh	TATAGTCGCTATAAAAACAAT	Chr.2	5433461	5433481	+	No
2584982_adh	TTCAAAAGAAATCGATCAACA	Chr.2	5437855	5437875	+	No
2732081_adh	TTTGTTGGAAGTTAAACGCAT	Chr.2	5453544	5453564	+	No
2727028_adh	TTTGGTTTGTAGAAGAGAGGT	Chr.2	5454369	5454349	-	No
2521414_adh	TGTGTTTCAGAAAGACAATTT	Chr.2	5455974	5455954	-	No
2029237_adh	TCTAAAAATTGCTTTGAACAA	Chr.2	5456142	5456162	+	No
2387387_adh	TGGATAAGAAAAGGGGATTGC	Chr.2	5458787	5458767	-	No
2728977_adh	TTTGTCGATTTTTCAATGGTT	Chr.2	5465963	5465983	+	No
2137811_adh	TGAATACTTCCACTGACGTCG	Chr.2	5493476	5493456	-	No
2672690_adh	TTGGTTGATGAGTATGAGGCG	Chr.2	5544126	5544106	-	No
2744062_adh	TTTTGTTAAAGAACAAGTCGG	Chr.2	5547628	5547608	-	No
1922046_adh	TATTATATAGAGGTCGGAAAG	Chr.2	5599855	5599875	+	No
2741422_adh	TTTTGAGCAGACGCAGCCATT	Chr.2	5661242	5661222	-	No
2148333_adh	TGAATTTGAATAGACTGGCAT	Chr.2	5662020	5662040	+	No
2707855_adh	TTTCGGATTCGTCGGTGTTCC	Chr.2	5666121	5666101	-	No
2714415_adh	TTTGAATGTGAACGCAGCGTT	Chr.2	5714942	5714922	-	No
2740936_adh	TTTTGAATGTGAACGCAGCGT	Chr.2	5714943	5714923	-	No
1806586_adh	TACAAACATTCACAAGAACAA	Chr.2	5716154	5716134	-	No
1957721_adh	TCAGAAGCTGTTGACGTTCAC	Chr.2	5756756	5756736	-	No
1956660_adh	TCAGAAAAAATTTCGGAAGTCG	Chr.2	5757734	5757714	-	No
2590191_adh	TTCAGAAAAAATTTCGGAAGTC	Chr.2	5758010	5758030	+	No
1956660_adh	TCAGAAAAAATTTCGGAAGTCG	Chr.2	5758011	5758031	+	No
1992769_adh	TCCTTCCGGCCGTCGCAATCT	Chr.2	5758492	5758512	+	No
1927558_adh	TATTGTAGAAGTGTGGTTATG	Chr.2	5760682	5760702	+	No
2136265_adh	TGAATAAAAGTAGAAAAAGAC	Chr.2	5764501	5764481	-	No
2742759_adh	TTTTGGAGATGGACGGTGTTT	Chr.2	5778617	5778597	-	No
1991291_adh	TCCTGATTGTGCTGGTCTTCT	Chr.2	5816701	5816721	+	No
1891502_adh	TATCAAGAAACAGGCGAGCAT	Chr.2	5819803	5819783	-	No
1930827_adh	TATTTTATTGTAAATTTTTGA	Chr.2	5835633	5835613	-	No
2007688_adh	TCGCGACGGAGACAGAAATGG	Chr.2	5836381	5836361	-	No
2140207_adh	TGAATCAAAAAAGATGATTCA	Chr.2	5844979	5844999	+	No
2117950_adh	TGAAATTTAAAAAATCATCAA	Chr.2	5862331	5862311	-	No
2031088_adh	TCTAATTTGACGGCTTTTATC	Chr.2	5864978	5864998	+	Yes
2031088_adh	TCTAATTTGACGGCTTTTATC	Chr.2	5866403	5866423	+	Yes
2580514_adh	TTATTAAGAAGAGCTGGCTAA	Chr.2	5893253	5893273	+	No
2694004_adh	TTTACGGTGAACGAATGTGGT	Chr.2	5906380	5906360	-	No
2254452_adh	TGAGTGTCGATTTACGGTGAA	Chr.2	5906390	5906370	-	No
2608087_adh	TTCGGAGTGTCGTTAGATAAC	Chr.2	5918381	5918401	+	No
2710370_adh	TTTCTGAACGTTTGTGCCAAA	Chr.2	5918442	5918462	+	No
2032365_adh	TCTAGAATCCTGAAAGAGAAG	Chr.2	5921690	5921670	-	No
2703442_adh	TTTCAGGTAGAATGCCACCGA	Chr.2	5921927	5921947	+	No
2639235_adh	TTGACTTTAGACACTATTTTC	Chr.2	5931362	5931382	+	No
2318393_adh	TGGAAATGTGGGAGAAAATTC	Chr.2	6000121	6000101	-	No
2402775_adh	TGGCAATGTGGAATACTGGGT	Chr.2	6008428	6008448	+	No

2649221_adh	TTGCACTTTTTACCATGCTGC	Chr.2	6028465	6028445	-	No
1912876_adh	TATGCCTTTCAAGAAGATGTC	Chr.2	6045098	6045078	-	No
2707537_adh	TTTCGGAATTAGCATAATGTC	Chr.2	6045918	6045898	-	No
2738229_adh	TTTTCGACGTCCGATGATTGT	Chr.2	6050443	6050463	+	No
2686686_adh	TTGTTTAAGAAAATTAAAAAA	Chr.2	6060437	6060417	-	No
2665767_adh	TTGGCGCGTAATTCAAAACTA	Chr.2	6061685	6061705	+	No
2744633_adh	TTTTTAAAATCTAACAACACA	Chr.2	6077497	6077477	-	No
1983417_adh	TCCCTGTAGAAGGAGGCTTCT	Chr.2	6091213	6091233	+	No
2742370_adh	TTTTGCATTGATTGAACGACT	Chr.2	6097659	6097679	+	No
2616705_adh	TTCTCGGTTTTGGATTTCTTC	Chr.2	6100163	6100143	-	No
2471480_adh	TGGTGATTGCGTACGTTGACG	Chr.2	6113342	6113322	-	No
2745471_adh	TTTTTATTCGATCGAACGCCG	Chr.2	6122830	6122850	+	No
2747883_adh	TTTTTGTTCGCGATGGCAAAG	Chr.2	6140344	6140364	+	No
2472630_adh	TGGTGGTTTCATTTTGGACGG	Chr.2	6141468	6141488	+	No
2578246_adh	TTATCTTTTCGGTCATATAAC	Chr.2	6197113	6197133	+	No
1980354_adh	TCCCAGAAAGATTGCCTGCCA	Chr.2	6198908	6198928	+	No
2396429_adh	TGGATTTTGCAAACGGACGTT	Chr.2	6245479	6245499	+	No
1999613_adh	TCGACACCATTGTAGAAGAAG	Chr.2	6250443	6250463	+	No
2745376_adh	TTTTTATGCTCTGAAAATGAT	Chr.2	6251021	6251041	+	No
2693259_adh	TTTACACGGAAAGGGGACGCG	Chr.2	6269677	6269657	-	No
2486520_adh	TGTACATGTAGGCGTTCGAAA	Chr.2	6274438	6274458	+	No
2656368_adh	TTGGAAAGAGACGAAGAAAAG	Chr.2	6289280	6289260	-	No
2474676_adh	TGGTTCACACTGGCTGAAGAA	Chr.2	6290645	6290625	-	No
1931214_adh	TATTTTGTCCTGTGATCGTCA	Chr.2	6292553	6292533	-	No
2567409_adh	TTAGCTTAGACGTGTAATTTT	Chr.2	6292620	6292600	-	No
2715602_adh	TTTGACTGAAGATGAGAAACC	Chr.2	6299264	6299244	-	No
2708810_adh	TTTCGTATAGATTTGGAGGAC	Chr.2	6303234	6303214	-	No
2737997_adh	TTTTCCGGAAGTGCTGCCTTT	Chr.2	6321108	6321088	-	No
2647037_adh	TTGATTTTCCGGAAGTGCTGC	Chr.2	6321112	6321092	-	No
2072432_adh	TGAAACATTTCTTAATTTCAA	Chr.2	6348273	6348293	+	No
2315523_adh	TGGAAAGAACAGAAACCGGAT	Chr.2	6352238	6352258	+	No
1894512_adh	TATCATGAAACAGACGAGCAT	Chr.2	6356169	6356189	+	No
2269849_adh	TGATCGCTATGCAGTTTATAT	Chr.2	6365720	6365740	+	No
1932238_adh	TCAAAAGATAAATCAGGAACG	Chr.2	6376441	6376461	+	No
1989406_adh	TCCTACTTTTCTTCTCGGACG	Chr.2	6387949	6387929	-	No
2731036_adh	TTTGTTGAATTGTTGGAAAAT	Chr.2	6391696	6391716	+	No
2261169_adh	TGATAGTTGGCGTTTTCAGTT	Chr.2	6395314	6395334	+	No
2650720_adh	TTGCATTAGTTCAAGCTGTTT	Chr.2	6476869	6476889	+	No
2633791_adh	TTGAATTGAAGAACGGTACTC	Chr.2	6489655	6489635	-	No
2740228_adh	TTTTCTTGACTTAGGACTTTC	Chr.2	6489992	6490012	+	No
2451387_adh	TGGGCTTAATGAACGGCGTTT	Chr.2	6490061	6490081	+	No
2040114_adh	TCTCGGCAACGTCGGACATTT	Chr.2	6492375	6492355	-	No
1819948_adh	TACCTAATTTTAATGCAATCT	Chr.2	6535977	6535957	-	No
1777753_adh	TAAGATAAATAGAAACAACAT	Chr.2	6536001	6535981	-	No
2701173_adh	TTTCAAATATTTCCTTGTCGA	Chr.2	6565354	6565334	-	No
2734979_adh	TTTTAGTTATGAGATTGGCTC	Chr.2	6587336	6587316	-	No
2217499_adh	TGAGATGAACGGTACGGATAG	Chr.2	6587879	6587859	-	No
2129084_adh	TGAAGAGACGCAGACATCAAA	Chr.2	6601113	6601093	-	No
2394032_adh	TGGATTGATGTAGATGTGCAC	Chr.2	6606241	6606221	-	No
2046824_adh	TCTGATCATGATAGAAACTTC	Chr.2	6684079	6684099	+	No

2664125_adh	TTGGATTCAACGTACACAGCA	Chr.2	6686479	6686499	+	No
2515981_adh	TGTGGCCTGAGAAGACAAAAA	Chr.2	6709313	6709293	-	No
2022081_adh	TCGTAGAACGGCAAAAAATAA	Chr.2	6741511	6741491	-	No
2689647_adh	TTTAAATTTTGAACAGAGGCT	Chr.2	6746541	6746521	-	No
1930982_adh	TATTTTCTGTTGACTCGTCTT	Chr.2	6795648	6795668	+	No
1876095_adh	TAGTCACAAGACTGTGTGGCA	Chr.2	6796941	6796921	-	No
1762599_adh	TAAATTGTAGGAGAAAGGTGG	Chr.2	6796966	6796986	+	No
2724089_adh	TTTGGATGTGGGAAAATGGTT	Chr.2	6800452	6800472	+	No
2419168_adh	TGGGAAGCAACGTGGAATTTG	Chr.2	6845929	6845949	+	No
2741650_adh	TTTTGATCGCAAAGCTTCGGA	Chr.2	6852694	6852674	-	No
1852777_adh	TAGCAAACACAACTAGTCGGC	Chr.2	6853045	6853065	+	No
1912450_adh	TATGCAGAACATCTATCGAAA	Chr.2	6857218	6857198	-	No
2132983_adh	TGAAGGAGTACGGTAGTTTTC	Chr.2	6860167	6860147	-	No
2049971_adh	TCTGGATTTGGAGGACAAGTT	Chr.2	6888958	6888938	-	No
2278459_adh	TGATTTGACCGGACAAGTTGT	Chr.2	6889007	6888987	-	No
2020796_adh	TCGTAAAAAACGTGTTCTCAG	Chr.2	6906008	6905988	-	No
2387471_adh	TGGATAAGGAAGAAGACACTT	Chr.2	6914754	6914734	-	No
2500674_adh	TGTGAAACGAGAAGACGCGGA	Chr.2	6926450	6926430	-	No
2598891_adh	TTCCGATGGCAACTGAAACTA	Chr.2	6928229	6928209	-	No
1902160_adh	TATGAATCAAGGACATTGGCA	Chr.2	6940214	6940194	-	No
2596771_adh	TTCCAGCAGAGTTGCAAGCTT	Chr.2	6967665	6967645	-	No
2515841_adh	TGTGGCAGAGAACGAGACGAC	Chr.2	6968591	6968611	+	No
2726293_adh	TTTGGTGCAGTAGCCGTGATA	Chr.2	6975787	6975767	-	No
2651136_adh	TTGCCATGTTTTAAATTAAGA	Chr.2	6983657	6983677	+	No
2689573_adh	TTTAAATTAAGAAATCGACAA	Chr.2	6983666	6983686	+	No
2675089_adh	TTGTAGGAAATCGTCGTTGAT	Chr.2	7011065	7011085	+	No
2616035_adh	TTCTCACATGTTATTATAGGC	Chr.2	7022636	7022616	-	No
1804809_adh	TAATTTATACATATACTCGGC	Chr.2	7023257	7023277	+	No
2623706_adh	TTCTTTGTATATTCTTTTGAG	Chr.2	7024920	7024900	-	No
2693657_adh	TTTACCGTAGACGAGAGGCGT	Chr.2	7025053	7025073	+	No
2712503_adh	TTTGAAAATGAAGGCATGTAT	Chr.2	7036620	7036600	-	No
2271917_adh	TGATGCGCTGGAGAGACGCTA	Chr.2	7043826	7043846	+	No
1791026_adh	TAATACATAAAAAGAACATCG	Chr.2	7045116	7045136	+	No
2271917_adh	TGATGCGCTGGAGAGACGCTA	Chr.2	7046461	7046441	-	No
2260562_adh	TGATAGATCTCGCAGAAAAAT	Chr.2	7061803	7061783	-	No
1953423_adh	TCACGGTTTCTCTTGATTCAA	Chr.2	7061840	7061820	-	No
2667202_adh	TTGGGAGGAATTGAGCATTTT	Chr.2	7072629	7072609	-	No
2463813_adh	TGGTAGTTTTTGGAAGGCTAT	Chr.2	7072717	7072737	+	No
1804160_adh	TAATTGGTGGATGCGTACTTC	Chr.2	7075772	7075792	+	No
2737022_adh	TTTTATTTTCAATGGTTTTAA	Chr.2	7081937	7081957	+	No
2707028_adh	TTTCGAGCAATATAGACAACA	Chr.2	7089509	7089529	+	No
2411140_adh	TGGCCTACGAATTTTTTGAGC	Chr.2	7105198	7105218	+	No
1827979_adh	TACGTATGAATAGTTGACATA	Chr.2	7107056	7107076	+	No
2713470_adh	TTTGAACTGCATTCTGGAGAC	Chr.2	7123095	7123075	-	No
2270910_adh	TGATGAAGAAGATTATGTAGA	Chr.2	7131923	7131903	-	No
2740432_adh	TTTTGAAAAAGTAGAAAGCAA	Chr.2	7132141	7132161	+	No
2321539_adh	TGGAACTTGTTGACGAAGACT	Chr.2	7146992	7146972	-	No
1984389_adh	TCCGAGACAATGTAGTTGAGA	Chr.2	7147338	7147318	-	No
1924038_adh	TATTCCGAGACAATGTAGTTG	Chr.2	7147341	7147321	-	No
1761995_adh	TAAATGTTGTAGGAAATTTAA	Chr.2	7147575	7147555	-	No

2585584_adh	TTCAAATGTGTCGTAAGTTCA	Chr.2	7147664	7147644	-	No
1920665_adh	TATTACAGGACTACAAGGTAA	Chr.2	7155631	7155651	+	No
2160559_adh	TGACGAACAGAAAGAAACTCT	Chr.2	7194466	7194446	-	No
1831584_adh	TACTCTGATCGTCATGCTTTT	Chr.2	7200340	7200360	+	No
2610946_adh	TTCGTAAAAACCTGTTTGAAG	Chr.2	7210681	7210661	-	Yes
2649010_adh	TTGCACCTGTGGTTTGACGGC	Chr.2	7232125	7232145	+	No
2585230_adh	TTCAAACTTACTCTGGCAAAA	Chr.2	7235495	7235515	+	No
2684457_adh	TTGTTGAACAAAAATATCGTC	Chr.2	7235910	7235930	+	No
2607957_adh	TTCGGAGGGTCGTTGGTGGAT	Chr.2	7253231	7253251	+	No
2488086_adh	TGTAGATTGTAGAAACGTGCT	Chr.2	7263978	7263998	+	No
2170324_adh	TGACTGGATAAATCGGATGAC	Chr.2	7283186	7283206	+	No
2160242_adh	TGACCTAGTGAAGACCCTGTG	Chr.2	7298712	7298732	+	No
2127746_adh	TGAAGACCCTGTGTAGATGTA	Chr.2	7298720	7298740	+	No
2036326_adh	TCTCAACTCTTCAGAACTGGC	Chr.2	7298864	7298884	+	No
2290540_adh	TGCATGAGTTCGGCATTTCAA	Chr.2	7301852	7301872	+	No
2618174_adh	TTCTGACGGAAAGTGAAGTAG	Chr.2	7307289	7307309	+	No
2060806_adh	TCTTTCCAACAGTAAGCTCAT	Chr.2	7315877	7315857	-	No
1747352_adh	TAAAAATTTGGCAACAACTCA	Chr.2	7317743	7317723	-	No
1987690_adh	TCCGTAGCAGTTATGAATTAC	Chr.2	7318018	7317998	-	No
2659398_adh	TTGGAGACGATTGGTTGAACA	Chr.2	7350268	7350288	+	No
2593434_adh	TTCATAAAAAGACTATGGCGT	Chr.2	7352718	7352738	+	No
2579875_adh	TTATGTAGAATGTGAAGGAGA	Chr.2	7353323	7353343	+	No
2049976_adh	TCTGGATTTTAGGCAGTATCA	Chr.2	7360265	7360285	+	No
2654543_adh	TTGCTGAAGGAGAACTTGCCA	Chr.2	7363381	7363361	-	No
2414394_adh	TGGCTCAAGCTAAGACTGTTT	Chr.2	7367493	7367473	-	No
2516188_adh	TGTGGCTCAAGCTAAGACTGT	Chr.2	7367495	7367475	-	No
2721686_adh	TTTGCCGATGTTTCTGAGATG	Chr.2	7367529	7367549	+	No
2651255_adh	TTGCCGATGTTTCTGAGATGT	Chr.2	7367530	7367550	+	No
2532685_adh	TGTTTCTGAGATGTCTTCACA	Chr.2	7367537	7367557	+	No
2037256_adh	TCTCAGAAACATCGGCAAATG	Chr.2	7367547	7367527	-	No
2679291_adh	TTGTGAAGACATCTCAGAAAC	Chr.2	7367558	7367538	-	No
2414394_adh	TGGCTCAAGCTAAGACTGTTT	Chr.2	7371715	7371735	+	No
2530481_adh	TGTTGTATCCACTGTTATGGC	Chr.2	7373100	7373080	-	No
2126804_adh	TGAACTTTTGGAAGACTTTGT	Chr.2	7394210	7394190	-	No
1831845_adh	TACTGAACGGATTTGAATGGA	Chr.2	7470991	7470971	-	No
2086561_adh	TGAAATACGATGCTGATGATA	Chr.2	7471156	7471176	+	No
2640010_adh	TTGAGAGGGTTTTTACACCAA	Chr.2	7471371	7471391	+	No
1930166_adh	TATTTGAAGTTAAACATCATG	Chr.2	7489614	7489594	-	No
1770926_adh	TAACTGACAGATGACAATTAA	Chr.2	7537690	7537670	-	No
2546291_adh	TTAACTGACAGATGACAATTA	Chr.2	7537691	7537671	-	No
2693663_adh	TTTACCGTAGTTTGATTCGCA	Chr.2	7537781	7537801	+	No
2272075_adh	TGATGGAAAGGATGGAAGAAA	Chr.2	7544616	7544636	+	No
2031036_adh	TCTAATTTAAATAAACTTTTA	Chr.2	7583764	7583784	+	No
2747239_adh	TTTTTGGAGCGAAGGACGATT	Chr.2	7584741	7584761	+	No
2239290_adh	TGAGGGAAGAAGTAGAAGAAG	Chr.2	7593186	7593206	+	No
2714518_adh	TTTGAATTGTTGAGCGGGATT	Chr.2	7599074	7599094	+	No
1930228_adh	TATTTGATTAGGTTTCGTGGA	Chr.2	7617061	7617041	-	No
2729229_adh	TTTGTCTTTCTATCGGGTTTT	Chr.2	7627497	7627477	-	No
2731840_adh	TTTGTTGAGAACCTGCAGAAA	Chr.2	7664088	7664108	+	No
2624609_adh	TTGAAAATTGTGGGAATTTAA	Chr.2	7667778	7667798	+	No

1981028_adh	TCCCCGATGATTGGCGTTGAC	Chr.2	7668078	7668098	+	No
2252579_adh	TGAGTCAACTACAGAAATTGC	Chr.2	7682837	7682857	+	No
2732936_adh	TTTTAAAAACAGCGACGACTT	Chr.2	7699084	7699064	-	No
1809170_adh	TACAAGTCACGAAAATGCTCT	Chr.2	7708554	7708534	-	No
2695456_adh	TTTAGATCAAACGGTACAAAT	Chr.2	7737163	7737183	+	No
2043945_adh	TCTGAACGAAGCAGTGTAGAA	Chr.2	7737797	7737817	+	No
2396144_adh	TGGATTTGGAAGAAATTGAGA	Chr.2	7744580	7744600	+	No
2559343_adh	TTACTGGATTTGATGAAGAGT	Chr.2	7752947	7752967	+	No
1983677_adh	TCCCTTGTTCTACGAGATCGT	Chr.2	7772509	7772529	+	No
2614431_adh	TTCTACGAGATCGTTGGAAGG	Chr.2	7772516	7772536	+	No
2130331_adh	TGAAGATTTTGCAGAACAGGA	Chr.2	7792114	7792134	+	No
2531588_adh	TGTTTAACGGACATACCTATT	Chr.2	7813285	7813265	-	No
1790776_adh	TAATAATGCATGCTTTGAACT	Chr.2	7815846	7815826	-	No
2616575_adh	TTCTCGCGGCAACAACAAAGA	Chr.2	7816295	7816315	+	No
1929992_adh	TATTTCTCCAGGCGGTACATA	Chr.2	7839740	7839760	+	No
2624241_adh	TTGAAAAAAGTTTCGCCCCGA	Chr.2	7852311	7852291	-	No
2744472_adh	TTTTGTTTCTATAGACTACAT	Chr.2	7857798	7857818	+	No
2615789_adh	TTCTATTGTAAGAATCAGGCC	Chr.2	7863532	7863552	+	No
1824669_adh	TACGCACCGTAGCAGCATAAA	Chr.2	7864315	7864295	-	No
2747776_adh	TTTTTGTCGTTGTGATTCTTT	Chr.2	7864398	7864418	+	No
1839935_adh	TAGAAGTTGAACAGGGAAGGA	Chr.2	7879501	7879521	+	No
1824220_adh	TACGATGGAGTGGAAAAATGA	Chr.2	7879700	7879720	+	No
2218955_adh	TGAGATGCAAAAGAACGGGAA	Chr.2	7880300	7880320	+	No
2669623_adh	TTGGTACTAATGGAAAATAAA	Chr.2	7884349	7884369	+	No
2124930_adh	TGAACTGAAGAAAATTGCTAG	Chr.2	7892610	7892590	-	No
2547382_adh	TTAAGATTTTTGGCCGCGCAA	Chr.2	7892641	7892621	-	No
2127522_adh	TGAAGAATTTTGCCGGCGGAT	Chr.2	7892726	7892706	-	No
2627260_adh	TTGAAGAATTTTGCCGGCGGA	Chr.2	7892727	7892707	-	No
2414680_adh	TGGCTGAAACATGAGTAGACC	Chr.2	7895091	7895111	+	No
2747344_adh	TTTTTGGGACGAAAGAAAAGA	Chr.2	7896470	7896490	+	No
2541156_adh	TTAAAGCACTGTAAAAACCAA	Chr.2	7920910	7920930	+	No
2709868_adh	TTTCTCACAGCTTCCACGGAT	Chr.2	7945237	7945217	-	No
2586005_adh	TTCAACGGAACTTAATGAAGA	Chr.2	7988156	7988176	+	No
2369735_adh	TGGAATTGAAAGAAGAGTAAA	Chr.2	7989157	7989137	-	No
2740978_adh	TTTTGACAAAGTACATGACGA	Chr.2	8011875	8011895	+	No
2714586_adh	TTTGACAAAGTACATGACGAA	Chr.2	8011876	8011896	+	No
2418512_adh	TGGGAACTGTATCAAACAACT	Chr.2	8013441	8013461	+	No
2639467_adh	TTGAGAACTGAATTCGACGAA	Chr.2	8015399	8015419	+	No
1822539_adh	TACGACTGAATGGAATGAAAG	Chr.2	8019968	8019948	-	No
2717585_adh	TTTGATATACCTGTAGATGGG	Chr.2	8020393	8020373	-	No
2381552_adh	TGGAGCTGAAGAGGGAAATCT	Chr.2	8032122	8032102	-	No
2676860_adh	TTGTATGTATGTTGGAACAAT	Chr.2	8041694	8041674	-	No
2728574_adh	TTTGTATGTATGTTGGAACAA	Chr.2	8041695	8041675	-	No
1887674_adh	TATACTGTAGATGTCGTTGGA	Chr.2	8051880	8051860	-	No
2674192_adh	TTGTACACAATCGATTGTTGA	Chr.2	8062005	8062025	+	No
1935511_adh	TCAAATTAAGTGGTGTCTGAA	Chr.2	8062238	8062258	+	No
2010704_adh	TCGGAAGAACGAGTGTTAAAT	Chr.2	8062301	8062281	-	No
1836702_adh	TAGAAAACTGAATTCGGAAGA	Chr.2	8062314	8062294	-	No
2534245_adh	TGTTTTCATACAATCCTCTGC	Chr.2	8062531	8062551	+	No
1956448_adh	TCACTTTGCGGTCGACGTCGA	Chr.2	8070590	8070570	-	No

2173892_adh	TGACTTGATGAAGGAAGTGAT	Chr.2	8085641	8085661	+	No
2162070_adh	TGACGTAGAAAAATGGTTATA	Chr.2	8104240	8104220	-	No
2699517_adh	TTTATTACGTTGGCTTATTTC	Chr.2	8124987	8124967	-	No
2608252_adh	TTCGGATGAAAAGTACGGCAA	Chr.2	8129627	8129607	-	No
2303950_adh	TGCGTCGGAATATTCAGAAAA	Chr.2	8137099	8137119	+	No
2728770_adh	TTTGTCATCATGTAGAGGCAG	Chr.2	8138140	8138160	+	No
2601836_adh	TTCGAAATTTCGGACAAACAA	Chr.2	8143355	8143335	-	No
2706572_adh	TTTCGAAATTTCGGACAAACA	Chr.2	8143356	8143336	-	No
2739403_adh	TTTTCTCTTGAAGCAGTGGAT	Chr.2	8143469	8143489	+	No
2648790_adh	TTGCAATATTTAAGACAATGA	Chr.2	8143590	8143570	-	No
2618570_adh	TTCTGCAGAGGAGTTCCGGAC	Chr.2	8143877	8143897	+	No
2513698_adh	TGTGCCGGAGCTGATCGTCTC	Chr.2	8144174	8144194	+	No
2583412_adh	TTATTTAAAACTTTGCCGGCA	Chr.2	8158154	8158174	+	No
1928977_adh	TATTTAAAACTTTGCCGGCAT	Chr.2	8158155	8158175	+	No
2538646_adh	TTAAAACTTTGCCGGCATTTC	Chr.2	8158158	8158178	+	No
1917342_adh	TATGTAAAAGTTGTCTAGGAT	Chr.2	8173488	8173468	-	No
2023718_adh	TCGTAGTATCCCAGGAGCACA	Chr.2	8182150	8182130	-	No
2531195_adh	TGTTGTTCGTAGAGTTCTGGA	Chr.2	8186708	8186728	+	No
2390457_adh	TGGATCTCGAAGAAGGCACGA	Chr.2	8187139	8187159	+	No
2171128_adh	TGACTGTATGCAGAGTGTTTT	Chr.2	8222754	8222774	+	No
2552670_adh	TTAATTTCTTCCGGATTACTC	Chr.2	8246317	8246297	-	No
2738140_adh	TTTTCCTTTCTGCCTACAAGC	Chr.2	8253369	8253389	+	No
2589789_adh	TTCACGTGAGTTGCGGGTCTG	Chr.2	8276073	8276053	-	No
2145824_adh	TGAATTCACGTGAGTTGCGGG	Chr.2	8276077	8276057	-	No
2057146_adh	TCTTCTGTGCATACTGAAATA	Chr.2	8278729	8278709	-	No
1936528_adh	TCAACGAACTCGCCTTGCATT	Chr.2	8284620	8284640	+	No
2183069_adh	TGAGACATGTTGAACAGACGT	Chr.2	8285675	8285695	+	No
2047162_adh	TCTGATGAGAATGACGCCAAG	Chr.2	8327189	8327169	-	No
2706363_adh	TTTCCTGTTTTGATCTGTTTT	Chr.2	8328967	8328947	-	No
2598042_adh	TTCCCGATACGTCTGACGATT	Chr.2	8399580	8399560	-	No
1751589_adh	TAAAATCGTATAGTAGGAGCA	Chr.2	8413083	8413103	+	No
2581305_adh	TTATTCAGTCTCATCGGAAGA	Chr.2	8442299	8442279	-	No
2738643_adh	TTTTCGGGATATTTCTACATT	Chr.2	8442426	8442446	+	No
1940784_adh	TCAATAAAATATGAAGTGGAA	Chr.2	8454670	8454690	+	No
1751036_adh	TAAAATATGAAGTGGAATGAA	Chr.2	8454674	8454694	+	No
2341541_adh	TGGAATGAAGACAGCTTGAAT	Chr.2	8454686	8454706	+	No
2633605_adh	TTGAATTAGTAGGTCGAAGAA	Chr.2	8454701	8454721	+	No
1754319_adh	TAAACAGAATGGATACTATGA	Chr.2	8463944	8463964	+	No
2552670_adh	TTAATTTCTTCCGGATTACTC	Chr.2	8475736	8475756	+	No
1755792_adh	TAAACTTGAAACGCGGTGATT	Chr.2	8483531	8483551	+	No
2132633_adh	TGAAGGACATAGTACAATTAT	Chr.2	8514150	8514130	-	No
1956916_adh	TCAGAAAGAGCAGTAAAGTGA	Chr.2	8532833	8532813	-	No
2622947_adh	TTCTTGGAAAGTTCCTCGGAA	Chr.2	8545583	8545603	+	No
1932460_adh	TCAAAATGGCTTGCAAGAATC	Chr.2	8545630	8545650	+	No
2596969_adh	TTCCAGGAGGATGCATTCAAT	Chr.2	8568466	8568446	-	No
1867853_adh	TAGGGAAACAGACAGAAAACA	Chr.2	8569341	8569321	-	No
1825806_adh	TACGGACTCAAGGTCGGACTT	Chr.2	8570171	8570191	+	No
2559290_adh	TTACTGGAGAAGACTACAATG	Chr.2	8570334	8570354	+	No
2379565_adh	TGGAGAAGACTACAATGTAGA	Chr.2	8570338	8570358	+	No
2132429_adh	TGAAGGAAGAGGATGAGGATC	Chr.2	8570589	8570609	+	No

1767284_adh	TAACCGTAGAGTACAGTACCT	Chr.2	8573553	8573533	-	No
1787893_adh	TAAGTCGAAGAGCTTCTTTCT	Chr.2	8574915	8574895	-	No
1899581_adh	TATCTCCTTTTTTCCTAAAAT	Chr.2	8575418	8575438	+	No
1939459_adh	TCAAGGAAACATACTTTAATA	Chr.2	8602362	8602342	-	No
2748293_adh	TTTTTTAGGAATTTTGTCGGT	Chr.2	8602532	8602552	+	No
2745107_adh	TTTTTAGGAATTTTGTCGGTA	Chr.2	8602533	8602553	+	No
1861977_adh	TAGGAATTTTGTCGGTATTTT	Chr.2	8602537	8602557	+	No
2271389_adh	TGATGAGGTCGTCGTTATGCG	Chr.2	8603052	8603072	+	No
2683666_adh	TTGTTCGAGAAAATAGTGGCA	Chr.2	8632028	8632048	+	No
1838935_adh	TAGAAGAAAATGAAATGCATA	Chr.2	8649112	8649132	+	No
1916051_adh	TATGGGGCACGGATCGGTTGG	Chr.2	8670202	8670182	-	No
2616437_adh	TTCTCGAAATGAATCGTAGAA	Chr.2	8713731	8713751	+	No
2714164_adh	TTTGAATAGAACGAATATCAC	Chr.2	8716427	8716407	-	No
1764078_adh	TAACAATTGAGAGGATTCCGG	Chr.2	8740511	8740491	-	No
2124482_adh	TGAACTCAAACAGAATGTCAC	Chr.2	8742206	8742186	-	No
2728766_adh	TTTGTCATATTAATTTTCGAG	Chr.2	8759498	8759478	-	No
2058851_adh	TCTTGGAGCTCCAGATGATGT	Chr.2	8763177	8763157	-	No
2617363_adh	TTCTCTGACTGACGGTAAAAC	Chr.2	8787701	8787721	+	No
1924618_adh	TATTCGTATGGTATTCATTTC	Chr.2	8787823	8787843	+	No
2592269_adh	TTCAGGCCATACATTCTGAAA	Chr.2	8788605	8788625	+	No
2627202_adh	TTGAAGAATCGTCATATATGG	Chr.2	8788965	8788985	+	No
2493358_adh	TGTATTGGAGATAGAGACGTT	Chr.2	8790218	8790198	-	No
2048487_adh	TCTGCTCTTTGATACGGCGAC	Chr.2	8808759	8808779	+	No
2747239_adh	TTTTTGGAGCGAAGGACGATT	Chr.2	8824365	8824345	-	No
2654479_adh	TTGCTGAAACAGCAGACGCTA	Chr.2	8859570	8859550	-	No
2736745_adh	TTTTATTGGCGTCGATCCTAA	Chr.2	8884426	8884446	+	No
2165999_adh	TGACTAGAGAGACCACCGGTT	Chr.2	8891094	8891074	-	No
2733741_adh	TTTTAATGGTCTGCACCCGTC	Chr.2	8927315	8927295	-	No
2487310_adh	TGTACTTGTAATATGAAGATT	Chr.2	8933671	8933691	+	No
2502112_adh	TGTGAAGATGGTGCTCTTGAG	Chr.2	8957157	8957177	+	No
2623531_adh	TTCTTTCTGATTTTATCGTTC	Chr.2	8964893	8964873	-	No
2304855_adh	TGCGTTGGAACAAGCCAAAAT	Chr.2	8965020	8965000	-	No
2744572_adh	TTTTGTTTTGGCTGTATTTTC	Chr.2	8965025	8965045	+	No
2301218_adh	TGCGGAGCATCGTTGTACAAT	Chr.2	8973776	8973796	+	No
2126577_adh	TGAACTTGATAAGTGCTGATA	Chr.2	8973937	8973917	-	No
1831889_adh	TACTGAACTTGATAAGTGCTG	Chr.2	8973940	8973920	-	No
1921037_adh	TATTACTGAACTTGATAAGTG	Chr.2	8973943	8973923	-	No
2591010_adh	TTCAGCAGACAGAACTGACAG	Chr.2	8987905	8987925	+	No
2589067_adh	TTCACAAAATTTACAAGACGT	Chr.2	8987979	8987959	-	No
1985200_adh	TCCGCAATCGAGGACTCGTCA	Chr.2	8996335	8996355	+	No
2693139_adh	TTTACAAGATTTTTGCTCGGA	Chr.2	8997625	8997645	+	No
2012487_adh	TCGGAGATTTTTGGCTCAATT	Chr.2	8997641	8997661	+	No
2237496_adh	TGAGGAGAGAAAGAAAAAGAA	Chr.2	9010792	9010812	+	No
2231271_adh	TGAGATTGTTAGAAAAATTAA	Chr.2	9038891	9038911	+	No
1812024_adh	TACAGATCGAACGAAAAGAAA	Chr.2	9044941	9044961	+	No
2472933_adh	TGGTGTATGTTGAAAGAATTC	Chr.2	9051013	9050993	-	No
2703826_adh	TTTCATATCGCTTATTGCTCA	Chr.2	9054637	9054617	-	No
2293896_adh	TGCCGACTTAGAGCAGGAGTT	Chr.2	9056148	9056168	+	No
2063405_adh	TCTTTTTTGGGATCTTTTCGC	Chr.2	9057318	9057298	-	No
2542389_adh	TTAAATAGTTGATTTTCAGAA	Chr.2	9057806	9057826	+	No

2737432_adh	TTTTCAGAAAGTTCAGACGAT	Chr.2	9057818	9057838	+	No
1932659_adh	TCAAACCAAAACGTCATCAAC	Chr.2	9065229	9065209	-	No
2395986_adh	TGGATTTGAACGGAATAAAAT	Chr.2	9067854	9067834	-	No
2713800_adh	TTTGAAGAGCATGAAAAGAGT	Chr.2	9067882	9067862	-	No
1869008_adh	TAGGGGAAGAAGAATAGACGA	Chr.2	9088549	9088569	+	No
1924408_adh	TATTCGGATTATTGACGCGTT	Chr.2	9099136	9099156	+	No
2014764_adh	TCGGATTATTGACGCGTTTCT	Chr.2	9099139	9099159	+	No
1787578_adh	TAAGTAGACGCCAGAAGCCAA	Chr.2	9100893	9100913	+	No
1984100_adh	TCCGACATGAGCAACTTCGAG	Chr.2	9134371	9134351	-	No
2104622_adh	TGAAATGGAAAGAAGGTGCTA	Chr.2	9142679	9142699	+	No
2600820_adh	TTCCTGATTTTATGAATTTCT	Chr.2	9155816	9155836	+	No
2030400_adh	TCTAATAATCATGGAACGACT	Chr.2	9161331	9161351	+	No
2651083_adh	TTGCCAGATTAGAATTGACGA	Chr.2	9172314	9172334	+	No
2608273_adh	TTCGGATGAGAGGATTGCCTT	Chr.2	9181027	9181007	-	No
2180626_adh	TGAGAAGGAGACAATCTGGCT	Chr.2	9204899	9204919	+	No
1986424_adh	TCCGGCTCGACGGAATCTTTT	Chr.2	9211738	9211718	-	No
1896506_adh	TATCCTTAGGTCGCTGGTTCG	Chr.2	9211761	9211741	-	No
2023391_adh	TCGTAGGAGTTGATATAAAAG	Chr.2	9217686	9217706	+	No
1964040_adh	TCAGGATTTTTAGTGGACGGA	Chr.2	9231961	9231981	+	No
2709929_adh	TTTCTCCGACTCGGAAGATGT	Chr.2	9243017	9243037	+	No
2038245_adh	TCTCATCTTTAGTCGTCTGAG	Chr.2	9273041	9273021	-	No
2322414_adh	TGGAAGACGACAGGAACCTAA	Chr.2	9290345	9290325	-	No
2721059_adh	TTTGCAATGTAGAAGTGCACT	Chr.2	9291382	9291362	-	No
2411472_adh	TGGCCTTGTAATGATGACACA	Chr.2	9308615	9308595	-	No
1917554_adh	TATGTAGAACAGAAGAGGTGG	Chr.2	9308917	9308937	+	No
1804608_adh	TAATTGTTGCTCGGATAACTT	Chr.2	9322927	9322907	-	No
2582271_adh	TTATTGATCATCTGATTTCTA	Chr.2	9323001	9322981	-	No
2700083_adh	TTTATTGATCATCTGATTTCT	Chr.2	9323002	9322982	-	No
1762962_adh	TAAATTTTTTCACGGTTTTCG	Chr.2	9323105	9323125	+	No
1811287_adh	TACACTCGGACAAGTGTGCAT	Chr.2	9334130	9334150	+	No
2263604_adh	TGATATCTTTCTGTACTTCAC	Chr.2	9340830	9340810	-	No
2126845_adh	TGAAGAAAAAGCGGAAAAGAA	Chr.2	9346702	9346722	+	No
2603736_adh	TTCGAGATCTATCGTTTCAAC	Chr.2	9351842	9351822	-	No
2515730_adh	TGTGGATTTACGGGCAAACAT	Chr.2	9352807	9352827	+	No
2615930_adh	TTCTCAAGACCGTTCAACAAC	Chr.2	9376377	9376357	-	No
2615930_adh	TTCTCAAGACCGTTCAACAAC	Chr.2	9381421	9381401	-	No
2142249_adh	TGAATCGCGAGAGTGACTGGT	Chr.2	9415907	9415927	+	No
2282540_adh	TGCAAGTAGGGTCGAATTTAC	Chr.2	9464411	9464431	+	Yes
2526351_adh	TGTTCGAGAAGGATATGGTTT	Chr.2	9504642	9504622	-	No
2539010_adh	TTAAAATCTACGAGCAAAAGG	Chr.2	9519007	9519027	+	No
2560189_adh	TTACTTGAATTTCGGAGCGGT	Chr.2	9536779	9536799	+	No
2070007_adh	TGAAAATCTGGAAAAATGGCA	Chr.2	9559876	9559856	-	No
2121530_adh	TGAACATTAAGAACAAAGGTT	Chr.2	9560543	9560563	+	No
2641212_adh	TTGAGGAGAAATGTGTGGTTA	Chr.2	9605359	9605339	-	No
2647138_adh	TTGCAAAAAGGAAAATAAGAA	Chr.2	9611636	9611656	+	No
2488866_adh	TGTAGGGAAGTTGTAGATGAG	Chr.2	9613887	9613867	-	No
2585155_adh	TTCAAACCTAGAATATGGCAA	Chr.2	9617236	9617256	+	No
1789679_adh	TAAGTTTGAAAGATTTTAAGG	Chr.2	9625498	9625518	+	No
1805498_adh	TAATTTGTGAAGTAATGGCAT	Chr.2	9651829	9651849	+	No
2729381_adh	TTTGTGAAGTAATGGCATTGA	Chr.2	9651832	9651852	+	No

2602676_adh	TTCGAATTTTTGGGCACGGCG	Chr.2	9656657	9656637	-	No
2714528_adh	TTTGAATTTCAGATCTCGGTT	Chr.2	9664334	9664314	-	No
2740959_adh	TTTTGAATTTCAGATCTCGGT	Chr.2	9664335	9664315	-	No
2682022_adh	TTGTGTACTCCTCTCGGAAAA	Chr.2	9669077	9669057	-	No
2745502_adh	TTTTTATTGTAGAAGTGGCTT	Chr.2	9689039	9689019	-	No
2489281_adh	TGTAGGTCAGGGCTGTGCGGC	Chr.2	9694109	9694089	-	No
2034080_adh	TCTATCAAGTAGACTGAATGA	Chr.2	9751267	9751287	+	No
2714531_adh	TTTGAATTTCAGGCAGGGACA	Chr.2	9765597	9765617	+	No
2634506_adh	TTGAATTTCAGGCAGGGACAT	Chr.2	9765598	9765618	+	No
1801804_adh	TAATGTGACTGACGTGGCCTC	Chr.2	9770189	9770169	-	No
2732972_adh	TTTTAAAAATCTTCTGACAAC	Chr.2	9829219	9829199	-	No
2595761_adh	TTCCAACAAGAAATGAAGCTC	Chr.2	9831243	9831263	+	No
2063343_adh	TCTTTTTTAGAAGATCTGACA	Chr.2	9834812	9834832	+	No
2071069_adh	TGAAACACAAAGATCAACGGT	Chr.2	9846602	9846582	-	No
1770700_adh	TAACTCTGCCGAAGAAGCCGT	Chr.2	9936691	9936671	-	No
1865602_adh	TAGGATGCAATTTTTTGGAGA	Chr.2	9940503	9940483	-	No
2744629_adh	TTTTTAAAAGTGGAATATTGA	Chr.2	9968369	9968349	-	No
2067684_adh	TGAAAACTGCACACATTTTTC	Chr.2	9968398	9968378	-	No
2618302_adh	TTCTGAGAGAACGCATAGAAA	Chr.2	9970836	9970856	+	No
2145675_adh	TGAATTATGTCAAAATGTCAA	Chr.2	9985534	9985554	+	No
1963300_adh	TCAGGAAACTCAACCGGAAGA	Chr.2	10011259	10011239	-	No
1886711_adh	TATACAAGCTGAAGATCATAA	Chr.2	10013466	10013446	-	No
2305434_adh	TGCTAAGGATGCCAAAGATTC	Chr.2	10015695	10015675	-	No
2624470_adh	TTGAAAAGGAGTACAAGTAGA	Chr.2	10024246	10024226	-	No
2618748_adh	TTCTGCGGAATTTTTGTCGAT	Chr.2	10040791	10040811	+	No
2250803_adh	TGAGTACATTCACATTCACAC	Chr.2	10099331	10099311	-	No
2721280_adh	TTTGCAGAATGAGTTCAATCG	Chr.2	10099783	10099763	-	No
1830805_adh	TACTATTTTGAAGCTGTGCAA	Chr.2	10100128	10100148	+	No
2740848_adh	TTTTGAAGCTGTGCAATTTGT	Chr.2	10100133	10100153	+	No
2713934_adh	TTTGAAGCTGTGCAATTTGTT	Chr.2	10100134	10100154	+	No
1933802_adh	TCAAAGGGAAGAAGAAGGTTA	Chr.2	10100282	10100302	+	No
2305672_adh	TGCTACATACTCGTCCGACAA	Chr.2	10108392	10108372	-	No
2654095_adh	TTGCTACATACTCGTCCGACA	Chr.2	10108393	10108373	-	No
1960185_adh	TCAGATCAGAAGAAGATGGTA	Chr.2	10112614	10112594	-	No
2092862_adh	TGAAATCGAAAAGAATGTGAA	Chr.2	10112874	10112854	-	No
2732958_adh	TTTTAAAAAGTTTTTCTTTTC	Chr.2	10134616	10134596	-	No
2449679_adh	TGGGCAAAAAGAAATCGGAAG	Chr.2	10136534	10136554	+	No
2651856_adh	TTGCGAATGAAAGATGAGCGG	Chr.2	10143698	10143678	-	No
2739533_adh	TTTTCTGCAGTTGTTCACCAA	Chr.2	10171000	10171020	+	No
1956404_adh	TCACTTTAGAAGTCATTTCAA	Chr.2	10172472	10172452	-	No
1845121_adh	TAGACATTCACTTTAGAAGTC	Chr.2	10172479	10172459	-	No
2066913_adh	TGAAAACATTGGAACGGAGCT	Chr.2	10190617	10190597	-	No
2749488_adh	TTTTTTGCTGCGTTGGCTAAC	Chr.2	10197320	10197340	+	No
1771761_adh	TAACTGTACAAAGCATTTTAT	Chr.2	10201079	10201099	+	No
2517221_adh	TGTGGTAGTGTGAGATAGGAG	Chr.2	10240342	10240322	-	No
1754448_adh	TAAACATAATCTGTCGGATAG	Chr.2	10242600	10242620	+	No
2704321_adh	TTTCATGTATTCCTCGTCGGT	Chr.2	10242775	10242795	+	No
2694201_adh	TTTACTCCCGGACCGGCAGGT	Chr.2	10248003	10247983	-	No
2621904_adh	TTCTTCGTTTTACTCCCGGAC	Chr.2	10248011	10247991	-	No
2529122_adh	TGTTGATGACTTCGGTGGATT	Chr.2	10248138	10248158	+	No

2271295_adh	TGATGACTTCGGTGGATTGTC	Chr.2	10248141	10248161	+	No
2173128_adh	TGACTTCGGTGGATTGTCGTG	Chr.2	10248144	10248164	+	No
2395166_adh	TGGATTGTCGTGAGCATTTAG	Chr.2	10248153	10248173	+	No
2694977_adh	TTTAGAATACGGAAAGACTTC	Chr.2	10248169	10248189	+	No
2561670_adh	TTAGAATACGGAAAGACTTCG	Chr.2	10248170	10248190	+	No
1929754_adh	TATTTCCTTGACGCATTTCCT	Chr.2	10248214	10248234	+	No
1807662_adh	TACAACGATTGTCGCGAATAC	Chr.2	10248306	10248286	-	No
2048223_adh	TCTGCCTTAGCGATTTCTGTT	Chr.2	10248532	10248552	+	No
2237441_adh	TGAGGAGAAAACAATAACTGA	Chr.2	10258584	10258604	+	No
2043301_adh	TCTCTTCTACATCGGACAACA	Chr.2	10261203	10261223	+	No
1938552_adh	TCAAGACTATATGAATCGGAA	Chr.2	10263009	10263029	+	No
2544754_adh	TTAAATTTTCAGTGGACAATT	Chr.2	10270116	10270136	+	No
2285829_adh	TGCAATTTGCTAAATTTGGAC	Chr.2	10271114	10271134	+	No
1957839_adh	TCAGAATACTCGGAGAATCAT	Chr.2	10302767	10302747	-	No
2292131_adh	TGCCAATTTGCAGACTCGGAG	Chr.2	10308104	10308084	-	No
2716879_adh	TTTGAGGATCCTGCAGGAGCA	Chr.2	10377794	10377774	-	Yes
2268680_adh	TGATATTTATTTATTTATTTA	Chr.2	10391670	10391690	+	No
2581865_adh	TTATTCTCATTTAAGTTTCGA	Chr.2	10393884	10393904	+	No
1804827_adh	TAATTTATTCTGGATACGGTG	Chr.2	10416568	10416588	+	No
2600742_adh	TTCCTGAGAAATTTAAGACAT	Chr.2	10416693	10416713	+	No
2492601_adh	TGTATGTGAGACTGCGTCAAT	Chr.2	10437822	10437842	+	No
2052601_adh	TCTGTGCGAAGACGTGTAACT	Chr.2	10442325	10442345	+	No
2274249_adh	TGATTACAATGTAGAAATGAC	Chr.2	10493034	10493054	+	No
2553621_adh	TTACAATGTAGAAATGACTAA	Chr.2	10493037	10493057	+	No
2177609_adh	TGAGAAATGAGAAGATGATAT	Chr.2	10557339	10557319	-	No
2530664_adh	TGTTGTCGAAACTCCGAATAC	Chr.2	10561569	10561549	-	No
2500675_adh	TGTGAAACGAGACGAGAGGAG	Chr.2	10563952	10563972	+	No
2678902_adh	TTGTCTGGCGTGTATTTGGTA	Chr.2	10571624	10571644	+	No
2584996_adh	TTCAAAAGAAGACTTAAAATG	Chr.2	10574196	10574176	-	No
2039967_adh	TCTCGGACCGGCAGGTCGCAT	Chr.2	10578428	10578408	-	No
2694242_adh	TTTACTCTCGGACCGGCAGGT	Chr.2	10578433	10578413	-	No
2621909_adh	TTCTTCGTTTTACTCTCGGAC	Chr.2	10578441	10578421	-	No
2173057_adh	TGACTTCCGTGGATTGTCGTG	Chr.2	10578574	10578594	+	No
2395166_adh	TGGATTGTCGTGAGCATTTAG	Chr.2	10578583	10578603	+	No
2694977_adh	TTTAGAATACGGAAAGACTTC	Chr.2	10578599	10578619	+	No
2561670_adh	TTAGAATACGGAAAGACTTCG	Chr.2	10578600	10578620	+	No
1929754_adh	TATTTCCTTGACGCATTTCCT	Chr.2	10578644	10578664	+	No
2308591_adh	TGCTGATTTGAAAATGGATAT	Chr.2	10598007	10597987	-	No
2402911_adh	TGGCACCAGAGATGCTGCAGA	Chr.2	10607680	10607700	+	No
2288041_adh	TGCAGATTTCGAGATGAACAA	Chr.2	10607695	10607715	+	No
2693562_adh	TTTACCAGAGCATTTCCTGAC	Chr.2	10644723	10644703	-	No
2474310_adh	TGGTTAGGAAGAGACACATTC	Chr.2	10657774	10657794	+	No
2602663_adh	TTCGAATTTGGCTGCATTTAT	Chr.2	10658900	10658920	+	No
1999443_adh	TCGAATTTGGCTGCATTTATT	Chr.2	10658901	10658921	+	No
2584524_adh	TTATTTTCGATTTAACCTGAA	Chr.2	10658917	10658937	+	No
2613782_adh	TTCTAAATTGTCGGTTGCAAA	Chr.2	10660923	10660903	-	No
2746012_adh	TTTTTCGATTTTCAGTTTTCC	Chr.2	10712314	10712334	+	No
2618186_adh	TTCTGACGGGTGAACAGGTGA	Chr.2	10730733	10730713	-	No
2738469_adh	TTTTCGGACGACATAATTATT	Chr.2	10753036	10753016	-	No
2712498_adh	TTTGAAAATGAACAACAAGTA	Chr.2	10753369	10753349	-	No

2153394_adh	TGACAGAAACGGACAAAAGAG	Chr.2	10787357	10787337	-	No
2746445_adh	TTTTTCTTGGAACTTTTCGAA	Chr.2	10798536	10798516	-	No
2021926_adh	TCGTACGTAGGTAGACAGGCA	Chr.2	10801007	10800987	-	No
1945018_adh	TCACAGCTGGAGGCCTTTTTA	Chr.2	10820088	10820068	-	No
1964029_adh	TCAGGATTGCGTGGATGAAAA	Chr.2	10820167	10820187	+	No
2651240_adh	TTGCCGACTGTAAGGACGGGA	Chr.2	10848498	10848518	+	No
2570572_adh	TTAGTAGCATATTAAAATAAT	Chr.2	10853297	10853277	-	No
2616133_adh	TTCTCAGTTAGTAGCATATTA	Chr.2	10853304	10853284	-	No
2651240_adh	TTGCCGACTGTAAGGACGGGA	Chr.2	10854447	10854427	-	No
2053507_adh	TCTGTTTTCGGCTTCTTTGTA	Chr.2	10863275	10863255	-	No
2127156_adh	TGAAGAAGAGAGATTACTGTA	Chr.2	10863395	10863415	+	No
2313638_adh	TGGAAAAGATTTGACTTTACT	Chr.2	10863456	10863476	+	No
2606187_adh	TTCGCAACTTTCCTGCTTTCT	Chr.2	10868958	10868978	+	No
1974037_adh	TCATTGATCGTCAGACGCATT	Chr.2	10870132	10870112	-	No
2015339_adh	TCGGCATTTTAATTAAGCTTA	Chr.2	10872598	10872578	-	No
2733212_adh	TTTTAACAATCGGCATTTTAA	Chr.2	10872607	10872587	-	No
2744728_adh	TTTTTAACAATCGGCATTTTA	Chr.2	10872608	10872588	-	No
2167430_adh	TGACTATTGACGTGGATTATC	Chr.2	10872717	10872737	+	No
2691550_adh	TTTAATGATGGAAAAACGCAT	Chr.2	10884462	10884442	-	No
1973033_adh	TCATGTGTCTGGAGGTGTATC	Chr.2	10928547	10928567	+	No
2515714_adh	TGTGGATTGAAGAAGATTGCT	Chr.2	10931548	10931528	-	No
2729962_adh	TTTGTGGGCATAGAGTAGGCG	Chr.2	10932909	10932929	+	No
2727074_adh	TTTGGTTTTTCAGAAGCTACA	Chr.2	10935113	10935093	-	No
2746013_adh	TTTTTCGATTTTTCGGCCTAA	Chr.2	10935373	10935353	-	No
1800787_adh	TAATGGTCACTGTATGCCGGA	Chr.2	10939186	10939166	-	No
2729962_adh	TTTGTGGGCATAGAGTAGGCG	Chr.2	10942110	10942130	+	No
2103700_adh	TGAAATGGAAAAAAGTAGGAA	Chr.2	10965952	10965932	-	No
2295667_adh	TGCGAACTGTCGAGATCGTAA	Chr.2	10972962	10972942	-	No
1797862_adh	TAATCAGGATGTGCAGGACGG	Chr.2	10975652	10975672	+	No
2606309_adh	TTCGCAGTTGGACAAATCATC	Chr.2	10987355	10987375	+	No
2527783_adh	TGTTGAATGACGATGACAAGC	Chr.2	11006259	11006239	-	No
2012891_adh	TCGGAGGCGACAACGGTATTT	Chr.2	11022172	11022192	+	No
2314741_adh	TGGAAACTGAAAGATCGGAAA	Chr.2	11023660	11023680	+	No
2701542_adh	TTTCAAGATCTGATGGGATTC	Chr.2	11050695	11050675	-	No
2535048_adh	TGTTTTTCAAATTGCTTCTAA	Chr.2	11051878	11051858	-	No
1834915_adh	TACTTCGGTATTTGATTAAAC	Chr.2	11092672	11092692	+	No
2749364_adh	TTTTTTGATAAATTGGACTGA	Chr.2	11097355	11097335	-	No
2598685_adh	TTCCGACCGCTGGAACGTCGG	Chr.2	11097526	11097546	+	No
2747064_adh	TTTTTGCCTGATTTTTCAACG	Chr.2	11097565	11097585	+	No
2594529_adh	TTCATGAACAAACCATTCGAT	Chr.2	11098218	11098238	+	No
2739505_adh	TTTTCTGAGTCGTGTCGTCGT	Chr.2	11105775	11105755	-	No
1847085_adh	TAGACTTTTGAATGAAACGGA	Chr.2	11112042	11112062	+	No
1807103_adh	TACAAATAAAATTGTTGGACA	Chr.2	11125372	11125352	-	No
1992729_adh	TCCTTCATCGTTCGTTTGGAC	Chr.2	11125522	11125502	-	No
2128937_adh	TGAAGACTGTGCAATCTAAAG	Chr.2	11126267	11126247	-	No
2044157_adh	TCTGAAGACTGTGCAATCTAA	Chr.2	11126269	11126249	-	No
2747330_adh	TTTTTGGGAAAATCGCTGAAT	Chr.2	11126450	11126430	-	No
2321824_adh	TGGAAGAAGTAGAATATAGGT	Chr.2	11130230	11130210	-	No
2287444_adh	TGCAGAGGTAGGGGTACTGTA	Chr.2	11154573	11154553	-	No
2458787_adh	TGGGTGGATAGAACTAGCAGA	Chr.2	11167371	11167391	+	No

2112720_adh	TGAAATGTTAAAAAATCTCAA	Chr.2	11168433	11168413	-	No
2043675_adh	TCTGAAACAACATCTGCAGAA	Chr.2	11209848	11209868	+	No
2235299_adh	TGAGCTCTCGAAGGATGATCA	Chr.2	11213180	11213200	+	No
2235299_adh	TGAGCTCTCGAAGGATGATCA	Chr.2	11213645	11213665	+	No
2133020_adh	TGAAGGATCAGGAGAAGACAC	Chr.2	11214440	11214460	+	No
2133020_adh	TGAAGGATCAGGAGAAGACAC	Chr.2	11214818	11214838	+	No
2133020_adh	TGAAGGATCAGGAGAAGACAC	Chr.2	11215007	11215027	+	No
2133020_adh	TGAAGGATCAGGAGAAGACAC	Chr.2	11216147	11216167	+	No
2607298_adh	TTCGGACAGAAGCAATGGGAA	Chr.2	11234543	11234523	-	No
2120248_adh	TGAACAGAGAGCTGCTGCATC	Chr.2	11236119	11236099	-	No
1940704_adh	TCAAGTTGAGCAAGTTGTCTA	Chr.2	11237736	11237716	-	No
2294220_adh	TGCCGTCAATTTGTTTTGCTC	Chr.2	11261710	11261730	+	No
1839437_adh	TAGAAGGAAAAACGTAGAAAA	Chr.2	11305086	11305066	-	No
1967451_adh	TCAGTTGAAAGTGAAGTGACA	Chr.2	11307238	11307258	+	No
2372046_adh	TGGAATTTAACTAATTATTTA	Chr.2	11329528	11329548	+	No
1763427_adh	TAACAAGAAAACGCATGACTT	Chr.2	11348056	11348076	+	No
1976624_adh	TCCAATAAATTGTCAGATGTT	Chr.2	11353554	11353534	-	No
1824099_adh	TACGATGAGCAGAGCTAAACA	Chr.2	11376633	11376613	-	No
1812143_adh	TACAGATTTGATGCATGATAT	Chr.2	11376671	11376651	-	No
2518684_adh	TGTGTACTGTGGAAATACAGA	Chr.2	11376686	11376666	-	No
2602960_adh	TTCGACGCCCAGGAGACTGTG	Chr.2	11377351	11377371	+	No
2291672_adh	TGCCAAAACGAGAGGGACCTC	Chr.2	11377405	11377425	+	No
2129273_adh	TGAAGAGATCGAAAATCGGAA	Chr.2	11407561	11407541	-	No
2141983_adh	TGAATCCAAGCACAGAACTGC	Chr.2	11439239	11439219	-	No
1801837_adh	TAATGTGGAAATCGATGGCAC	Chr.2	11443819	11443799	-	No
2042735_adh	TCTCTGAAACAAGCCGACTCC	Chr.2	11443968	11443988	+	No
1913425_adh	TATGGAAAGCTGTCTGATGTC	Chr.2	11452789	11452769	-	No
2729752_adh	TTTGTGCGGCATTTCTTCTTA	Chr.2	11453203	11453223	+	No
1798208_adh	TAATCCTGCATTGAATTTCGC	Chr.2	11453241	11453261	+	No
1838908_adh	TAGAACTTTAAAAAACGCAGA	Chr.2	11453528	11453548	+	No
2488092_adh	TGTAGATTTACGGCACATTTT	Chr.2	11464016	11463996	-	No
2698299_adh	TTTATATGATTTAGTACGGAT	Chr.2	11468576	11468556	-	No
2059770_adh	TCTTGTGATCGTCGAAAACCA	Chr.2	11468602	11468582	-	No
1821945_adh	TACGAATTCGGCGCATTTTTA	Chr.2	11471770	11471750	-	No
2373379_adh	TGGACAAGAAGAAGTAGAGAA	Chr.2	11476254	11476274	+	No
1911583_adh	TATGATGGAAATAATGACGAA	Chr.2	11529094	11529074	-	No
1768275_adh	TAACGATAAGATCTGATTGGA	Chr.2	11532084	11532064	-	No
2723762_adh	TTTGGAGGAAAAAGTGGAAAA	Chr.2	11534530	11534510	-	No
1980198_adh	TCCCAACTATTATTCTCGGAT	Chr.2	11537511	11537531	+	No
2551285_adh	TTAATGGCATTTGAGAATTTT	Chr.2	11540444	11540424	-	No
1839106_adh	TAGAAGACGACTCGATCCTTT	Chr.2	11540992	11541012	+	No
2707038_adh	TTTCGAGCGCAGAGTTCAGGA	Chr.2	11544878	11544898	+	No
2136264_adh	TGAATAAAAGCATGAGCACGA	Chr.2	11545305	11545285	-	No
2057032_adh	TCTTCTCTCTCATGATTCGGC	Chr.2	11545444	11545464	+	No
2734847_adh	TTTTAGGTGATTGTTGCGGTT	Chr.2	11547582	11547562	-	No
2068691_adh	TGAAAAGCAATAGACTATGAC	Chr.2	11549163	11549143	-	No
2549873_adh	TTAATAGATCGTTGATAAACA	Chr.2	11554271	11554251	-	No
2047398_adh	TCTGATTAATAGGATAGGCTC	Chr.2	11563545	11563525	-	No
1784305_adh	TAAGGAGAACACATCGAAAAT	Chr.2	11596267	11596287	+	No
2237880_adh	TGAGGATATTTTCAGGCATGG	Chr.2	11596485	11596505	+	No

2503009_adh	TGTGAATTGAGACTGTGTATA	Chr.2	11600214	11600234	+	No
2468739_adh	TGGTATTGCTCGACGTGCAAA	Chr.2	11609741	11609761	+	No
2183130_adh	TGAGACCAACGATCAATAGAA	Chr.2	11635898	11635918	+	No
2741897_adh	TTTTGATTATTTTTGTAGCAT	Chr.2	11638454	11638434	-	No
1775417_adh	TAAGACCAGTGACGTGCTCTT	Chr.2	11649419	11649399	-	No
2133585_adh	TGAAGGTCGGAGAAGTCGGAT	Chr.2	11652377	11652357	-	No
2519281_adh	TGTGTCGATTTACGGAGAATG	Chr.2	11652912	11652932	+	No
2606808_adh	TTCGCTCTCGTTTGAAATTTA	Chr.2	11676035	11676015	-	No
2573825_adh	TTATAACCAAGGAAACGGTCT	Chr.2	11676236	11676256	+	No
2472743_adh	TGGTGTACTACAACAAAGGAC	Chr.2	11679142	11679122	-	No
1932929_adh	TCAAACTTTGGGACAAGTTGA	Chr.2	11693935	11693915	-	No
2608504_adh	TTCGGCCTAAAATATTTCTTT	Chr.2	11700655	11700635	-	No
1977049_adh	TCCAATTTATTCTGTCGCGTC	Chr.2	11701222	11701242	+	No
1983673_adh	TCCCTTGTTCGTCAGCGGCGG	Chr.2	11709426	11709406	-	No
2281841_adh	TGCAAGAAGAATCTCGAAGAT	Chr.2	11714756	11714736	-	No
1966677_adh	TCAGTGCAAGAAGAATCTCGA	Chr.2	11714760	11714740	-	No
2303297_adh	TGCGTAAAAACTACTATGACA	Chr.2	11723692	11723672	-	No
1839790_adh	TAGAAGTCGCAGAAAACAACA	Chr.2	11724452	11724432	-	No
2618401_adh	TTCTGATAGAAGTCGCAGAAA	Chr.2	11724458	11724438	-	No
2607309_adh	TTCGGACAGCAAAAAACGAGA	Chr.2	11724497	11724477	-	No
2702935_adh	TTTCAGAGTAAAACTTCGGTT	Chr.2	11726491	11726471	-	No
2309878_adh	TGCTGTTGATAAGACGACGGG	Chr.2	11728732	11728712	-	No
2588165_adh	TTCAATGCTGTTGATAAGACG	Chr.2	11728737	11728717	-	No
2642959_adh	TTGAGTGTAGAACTGGATTCC	Chr.2	11730871	11730851	-	No
2726524_adh	TTTGGTGTAGAAAATGAAATT	Chr.2	11744748	11744768	+	No
2623261_adh	TTCTTTAGAGAGGAACTTTGG	Chr.2	11748296	11748316	+	No
2059767_adh	TCTTGTGAGTGTGTGAAGATG	Chr.2	11756753	11756773	+	No
1937408_adh	TCAACTGAAGATGCATCACGT	Chr.2	11757382	11757362	-	No
2132199_adh	TGAAGGAAACAGCTGAGACTT	Chr.2	11758686	11758666	-	No
1958440_adh	TCAGACTGTAAGCATTGGCCA	Chr.2	11758813	11758793	-	No
1958440_adh	TCAGACTGTAAGCATTGGCCA	Chr.2	11759982	11760002	+	No
2132199_adh	TGAAGGAAACAGCTGAGACTT	Chr.2	11760109	11760129	+	No
1937408_adh	TCAACTGAAGATGCATCACGT	Chr.2	11761413	11761433	+	No
1811856_adh	TACAGACGTTACGGAATTGAA	Chr.2	11763205	11763185	-	No
2658420_adh	TTGGACGAGGCACCTTCTTCT	Chr.2	11769377	11769397	+	No
2705130_adh	TTTCCAGCTGAAATCGATCGT	Chr.2	11778066	11778046	-	No
2733031_adh	TTTTAAAATTCGTGCATCTTC	Chr.2	11782669	11782689	+	No
2309166_adh	TGCTGGTTAGCTTGTCGTGGT	Chr.2	11782734	11782754	+	No
1805353_adh	TAATTTGCATGTAAGTGGCCT	Chr.2	11817895	11817915	+	Yes
2063007_adh	TCTTTTTGGAGAACTGACAGA	Chr.2	11860873	11860893	+	No
2296981_adh	TGCGAGATAGAAGTTGTGTAA	Chr.2	11874704	11874684	-	No
2552880_adh	TTAATTTTAGGACGATCGGGA	Chr.2	11875045	11875025	-	No
2151588_adh	TGACAAGTCGTAAACGGTCTG	Chr.2	11880561	11880581	+	No
2391126_adh	TGGATGCAACGAAGAAGGACA	Chr.2	11880602	11880582	-	No
2486805_adh	TGTACGATTTTCCCGCCGGAC	Chr.2	11885352	11885372	+	No
2586286_adh	TTCAACTGGAGACGTTTTAAA	Chr.2	11897781	11897761	-	No
2702872_adh	TTTCAGAATGTCAGACGTTGC	Chr.2	11916423	11916443	+	No
2571304_adh	TTAGTCTGAGCCATCGAAGAA	Chr.2	11917427	11917407	-	No
1931960_adh	TCAAAAATTGTGTGGACATTT	Chr.2	11919317	11919337	+	No
2278054_adh	TGATTTCATTAGCTGAGGCTT	Chr.2	11960856	11960876	+	No

2667897_adh	TTGGGGGGAAACGGTAGAGAT	Chr.2	11972128	11972108	-	No
2674993_adh	TTGTAGATTATGGGTAGAAAA	Chr.2	11980182	11980162	-	No
2737347_adh	TTTTCACCTATTTTCGTCGTT	Chr.2	12006699	12006679	-	No
2457373_adh	TGGGTACTGTACTTGGCGGAT	Chr.2	12023571	12023551	-	No
2714861_adh	TTTGACAGTGGAATATCGAAA	Chr.2	12027487	12027507	+	No
2709569_adh	TTTCTAGAAAAGCGTCATTGT	Chr.2	12027982	12028002	+	No
1855424_adh	TAGCATTAAGAATCTGGAAAA	Chr.2	12059268	12059288	+	No
1884347_adh	TATAACACATTCTCAGTGTAT	Chr.2	12113829	12113809	-	No
1884347_adh	TATAACACATTCTCAGTGTAT	Chr.2	12120255	12120275	+	No
2746153_adh	TTTTTCGTGAAAACTTGTAGA	Chr.2	12132957	12132937	-	No
2732109_adh	TTTGTTGGATTTTTTCGGATT	Chr.2	12196171	12196191	+	No
2379437_adh	TGGAGAACAAGTGGACGGCCG	Chr.2	12213400	12213380	-	No
2553901_adh	TTACACGGCAAAAGGAGAAAT	Chr.2	12213499	12213479	-	No
2689302_adh	TTTAAAGCGAGAACAACGGAA	Chr.2	12223385	12223405	+	No
1938089_adh	TCAAGAAAATCGTTCGAAAAA	Chr.2	12257562	12257542	-	No
2623556_adh	TTCTTTGAAAATGGACAGAAA	Chr.2	12280381	12280361	-	No
2639613_adh	TTGAGAATTCAGCAATAAACT	Chr.2	12280478	12280498	+	No
2132826_adh	TGAAGGAGAAGCTGATGGCCG	Chr.2	12283794	12283814	+	No
2082059_adh	TGAAAGGAAGACTAGTCGAAC	Chr.2	12305516	12305496	-	No
2183142_adh	TGAGACCACGGAAAAGACTGA	Chr.2	12341430	12341410	-	No
1812953_adh	TACAGTTGTGACCTCATAAAT	Chr.2	12413327	12413347	+	No
2304495_adh	TGCGTTCGTAGTTCGTGCTCA	Chr.2	12538864	12538844	-	No
2707437_adh	TTTCGCTCTGAAAATGCTGAA	Chr.2	12599963	12599943	-	No
1929351_adh	TATTTATCAGTTTTCGTTCAA	Chr.2	12604008	12603988	-	No
2606773_adh	TTCGCTCGAATTCGGGGACGG	Chr.2	12671709	12671689	-	No
2736984_adh	TTTTATTTGTCGTCGAATTTA	Chr.2	12678590	12678610	+	No
2150462_adh	TGACAAGAAGAACTACTCGTT	Chr.2	12733089	12733069	-	No
1787848_adh	TAAGTCCACAGAATCGTCCGA	Chr.2	12738647	12738667	+	No
2617115_adh	TTCTCTCGGACAATTGGCGGG	Chr.2	12738878	12738858	-	No
1770350_adh	TAACTCAAAACAAGCTGAAAT	Chr.2	12738970	12738990	+	No
2542021_adh	TTAAATAACATCCCCAAATTA	Chr.2	12750229	12750209	-	No
2601862_adh	TTCGAACAATCTAACCGACGG	Chr.2	12774600	12774620	+	No
2038463_adh	TCTCATTCTCACGCATCCACA	Chr.2	12776127	12776147	+	No
1889112_adh	TATAGTGGGAGATAAGATTTA	Chr.2	12830513	12830533	+	No
2278049_adh	TGATTTCATGGTTAGTTGGAA	Chr.2	12846542	12846522	-	No
1963730_adh	TCAGGAGAGGAATTGGGAAAT	Chr.2	12866294	12866274	-	No
2010276_adh	TCGGAAACGGAACCAGAATCG	Chr.2	12895808	12895828	+	No
2011245_adh	TCGGACAATCATTTAATTAGG	Chr.2	12914826	12914846	+	No
2551829_adh	TTAATTAGGCAATTCTTCGTA	Chr.2	12914838	12914858	+	No
2538910_adh	TTAAAAGTAGAAACAGCGTGA	Chr.2	12914951	12914971	+	No
2526804_adh	TGTTCTCGGTGTAATTTTGCT	Chr.2	12916715	12916695	-	No
2271301_adh	TGATGACTTTAACTTGGGAAG	Chr.2	12945282	12945262	-	No
2680706_adh	TTGTGATCGGCTGAGAGGTTA	Chr.2	12994620	12994600	-	No
2040062_adh	TCTCGGAGCAAAGAAAACGTT	Chr.2	12995777	12995757	-	No
2702686_adh	TTTCACTCTTTTTTCCGTTGA	Chr.2	13043400	13043380	-	No
2714572_adh	TTTGAATTTTGATGCGCGGTA	Chr.2	13043462	13043442	-	No
2740974_adh	TTTTGAATTTTGATGCGCGGT	Chr.2	13043463	13043443	-	No
2738411_adh	TTTTCGCTCGATTTTCACACA	Chr.2	13043798	13043818	+	No
2041423_adh	TCTCGTCGAGCAGAATCGCTT	Chr.2	13044256	13044236	-	No
1994921_adh	TCGAAAGGACATTGGCGACGG	Chr.2	13045828	13045848	+	No

2574074_adh	TTATAATCGGTATATTAGGAC	Chr.2	13058757	13058777	+	No
2693092_adh	TTTACAAACTCGGCAAAACAG	Chr.2	13083697	13083677	-	No
2553576_adh	TTACAATCGTTGTGAAGCTTT	Chr.2	13083921	13083941	+	No
2726975_adh	TTTGGTTTAATAGTGTCGTCG	Chr.2	13083944	13083964	+	No
2673070_adh	TTGGTTTAATAGTGTCGTCGT	Chr.2	13083945	13083965	+	No
2625804_adh	TTGAACAAAAGGTCTTCTTCT	Chr.2	13130486	13130466	-	No
2044808_adh	TCTGAATGTGGAAAATTGCCG	Chr.2	13140769	13140749	-	No
2388873_adh	TGGATATCACTCGAGAAAACT	Chr.2	13145549	13145529	-	No
2746541_adh	TTTTTGAACTGTTTTTCTGGA	Chr.2	13157131	13157151	+	No
2048903_adh	TCTGGACAGCAATTTTTGGCG	Chr.2	13157760	13157780	+	No
1895764_adh	TATCCGGAAAACGGCAAACCG	Chr.2	13177881	13177901	+	No
1809060_adh	TACAAGGCATATTAGAAATCT	Chr.2	13183018	13182998	-	No
2048903_adh	TCTGGACAGCAATTTTTGGCG	Chr.2	13184522	13184542	+	No
2606543_adh	TTCGCGAAGACAATGTTAATG	Chr.2	13184808	13184828	+	No
2007658_adh	TCGCGAAGACAATGTTAATGT	Chr.2	13184809	13184829	+	No
2605180_adh	TTCGATGGACGGCGAGTTTTT	Chr.2	13190110	13190090	-	No
2009814_adh	TCGCTTCCAGATCATCGTCGA	Chr.2	13198226	13198246	+	No
2494513_adh	TGTCACATTTTTTCGGGCAAA	Chr.2	13202828	13202808	-	No
2492035_adh	TGTATGGAAAACTTTTCGGCA	Chr.2	13220617	13220597	-	No
1928450_adh	TATTGTTGGAAAGTTGAAGAA	Chr.2	13237987	13238007	+	No
2529703_adh	TGTTGGAAAGTTGAAGAATTC	Chr.2	13237990	13238010	+	No
2317654_adh	TGGAAAGTTGAAGAATTCTTA	Chr.2	13237993	13238013	+	No
2602619_adh	TTCGAATTCAGAGCTACGAAT	Chr.2	13238022	13238042	+	No
2627281_adh	TTGAAGACAATTATGACGTAG	Chr.2	13244801	13244821	+	No
2580528_adh	TTATTAAGTTCGTTAGATATA	Chr.2	13244877	13244897	+	No
2580528_adh	TTATTAAGTTCGTTAGATATA	Chr.2	13259187	13259207	+	No
2734401_adh	TTTTAGAAATAGGACAATATA	Chr.2	13264402	13264382	-	No
2396234_adh	TGGATTTTAAAAAATATTTAA	Chr.2	13358673	13358653	-	No
2053350_adh	TCTGTTGGTGAAGGTTGGATA	Chr.2	13441651	13441671	+	No
2485374_adh	TGTAATTAAAAGCCATGGCAA	Chr.2	13460793	13460813	+	No
1973991_adh	TCATTGACACATACACAAGAC	Chr.2	13461950	13461930	-	No
2642972_adh	TTGAGTGTATGAAGCCAATGA	Chr.2	13473803	13473783	-	No
2287050_adh	TGCAGAAGAGACGAAGCCGAA	Chr.2	13484063	13484083	+	No
2583653_adh	TTATTTATTTACTTTTTAATC	Chr.2	13494823	13494803	-	No
2735347_adh	TTTTATAGTTGAAAGTCGGGC	Chr.2	13494932	13494952	+	No
2585193_adh	TTCAAACTAAAGTAGATGATC	Chr.2	13513427	13513447	+	No
2628462_adh	TTGAAGATGGCTCTGGATGTT	Chr.2	13513702	13513722	+	No
2337995_adh	TGGAATCTTACGAAAACTAGT	Chr.2	13513864	13513884	+	No
2595647_adh	TTCCAAACGGCGTTCAACTGC	Chr.2	13516191	13516211	+	No
2597538_adh	TTCCATTTCTGGCGGAAGGGG	Chr.2	13521211	13521231	+	No
2619458_adh	TTCTGGCGGAAGGGGCATTGA	Chr.2	13521217	13521237	+	No
2269042_adh	TGATCAAGCAGACTGAGAATT	Chr.2	13637410	13637390	-	No
2722190_adh	TTTGCGTGACATTAGAGGGTA	Chr.2	13638523	13638503	-	No
2414845_adh	TGGCTGCGTACTCCTCTTGGA	Chr.2	13654797	13654817	+	No
2351261_adh	TGGAATGGACACGACGAAGGC	Chr.2	13689817	13689797	-	No
2700399_adh	TTTATTGGATTTTTCGCGTAA	Chr.2	13694764	13694744	-	No
2738368_adh	TTTTCGCATAGTTTTATTGGA	Chr.2	13694776	13694756	-	No
1857893_adh	TAGCTAGAAAATGATGTCATA	Chr.2	13694890	13694910	+	No
2490048_adh	TGTAGTTGACGTATAAAATAC	Chr.2	13695292	13695272	-	No
2102075_adh	TGAAATGATAGTGAGTGTGAG	Chr.2	13705831	13705811	-	No

1926641_adh	TATTGCTGATAAAGGACGACT	Chr.2	13731909	13731889	-	No
1999944_adh	TCGACGAAGCTATGAAACCCT	Chr.2	13737963	13737983	+	No
2644685_adh	TTGATGAAGACTCACAAGTGA	Chr.2	13819856	13819876	+	No
2644685_adh	TTGATGAAGACTCACAAGTGA	Chr.2	13823294	13823314	+	No
2644685_adh	TTGATGAAGACTCACAAGTGA	Chr.2	13825431	13825411	-	No
2625422_adh	TTGAAATCACTTCAAGACTTC	Chr.2	13829147	13829127	-	No
1891025_adh	TATATTTGTAGTTTACTCGTT	Chr.2	13829634	13829614	-	No
1772684_adh	TAACTTGACTACCGGAATTTT	Chr.2	13834376	13834356	-	No
2411545_adh	TGGCGAAAATTGGCTATGTAA	Chr.2	13872451	13872431	-	No
2373239_adh	TGGACAAAGACGACGTGATCA	Chr.2	13873055	13873075	+	No
2010948_adh	TCGGAATATTTGTGTTTGCGG	Chr.2	13888349	13888329	-	No
2728827_adh	TTTGTCCAACTCTACTGCCTC	Chr.2	13888415	13888435	+	No
2513844_adh	TGTGCGGTGAAGCGCGCTTGC	Chr.2	13899231	13899211	-	No
2513844_adh	TGTGCGGTGAAGCGCGCTTGC	Chr.2	13899495	13899475	-	No
2280858_adh	TGCAAAGTTATAGGAAATTTA	Chr.2	13901370	13901350	-	No
2725939_adh	TTTGGTAGGATAGTGTTACGG	Chr.2	13904415	13904395	-	No
1879195_adh	TAGTGTACTGTTTTGTGGTTT	Chr.2	13908763	13908783	+	No
2300799_adh	TGCGGAAGATCATTGAAAAAT	Chr.2	13925998	13925978	-	No
2289010_adh	TGCAGTGCGGAAGATCATTGA	Chr.2	13926003	13925983	-	No
2744347_adh	TTTTGTTGGGTAAATCGTTAT	Chr.2	13947420	13947440	+	No
2732179_adh	TTTGTTGGGTAAATCGTTATT	Chr.2	13947421	13947441	+	No
1866699_adh	TAGGATTTCTGCAGTACACGC	Chr.2	13969693	13969673	-	No
2566090_adh	TTAGATTTTAGAAGACCCGTA	Chr.2	13971966	13971986	+	No
1829145_adh	TACGTTTGAAGCCGGAAAATG	Chr.2	14028713	14028693	-	No
2721528_adh	TTTGCCAATAACGAAAACGAC	Chr.2	14028742	14028722	-	No
2732944_adh	TTTTAAAAAGCAAAATTGACA	Chr.2	14028841	14028861	+	No
2624759_adh	TTGAAACCCAAAAACAGGACA	Chr.2	14028873	14028893	+	No
2601024_adh	TTCCTGTAGAGATAGACTGCT	Chr.2	14038763	14038743	-	No
2459908_adh	TGGGTTGCAAGTAGAGCGATT	Chr.2	14050791	14050811	+	No
2526895_adh	TGTTCTGAAGGCCTGTAAGCA	Chr.2	14080050	14080070	+	No
2041746_adh	TCTCGTTAGGATTTTGGCGGC	Chr.2	14123631	14123611	-	No
1833976_adh	TACTGTGGAATACAATTTAAG	Chr.2	14127116	14127096	-	No
2161716_adh	TGACGGATCACGAGGAGAATA	Chr.2	14139795	14139815	+	No
2728078_adh	TTTGTACTCCTCTCGGACAAA	Chr.2	14140799	14140819	+	No
1749337_adh	TAAAAGATCAGTTTGAAGTTA	Chr.2	14147086	14147066	-	No
1826174_adh	TACGGATTTTGTGAAGGATAA	Chr.2	14151728	14151708	-	No
2410491_adh	TGGCCAAGAGAACTAAGAAGG	Chr.2	14155198	14155218	+	No
2028107_adh	TCGTTCTGCGGAAAGGAGGCC	Chr.2	14155314	14155334	+	No
2260276_adh	TGATAGAAGACACGAATTGCA	Chr.2	14172843	14172863	+	No
1887894_adh	TATAGAAATGTAGAAAACGAG	Chr.2	14192027	14192047	+	No
1790401_adh	TAATAAGAGTAACGGAAGATA	Chr.2	14218399	14218419	+	No
1777548_adh	TAAGAGTAACGGAAGATAAAA	Chr.2	14218402	14218422	+	No
2541722_adh	TTAAAGTATGCTGATTCTGAA	Chr.2	14227129	14227109	-	No
2541722_adh	TTAAAGTATGCTGATTCTGAA	Chr.2	14230666	14230646	-	No
2711745_adh	TTTCTTGAGTTTTTGTCGGTT	Chr.2	14234400	14234380	-	No
2629142_adh	TTGAAGTACGCTGATTCTGAA	Chr.2	14237928	14237908	-	No
2541722_adh	TTAAAGTATGCTGATTCTGAA	Chr.2	14242944	14242964	+	No
2616626_adh	TTCTCGGAGATTCTTTAATAA	Chr.2	14244887	14244867	-	No
1860676_adh	TAGGAAATCGCAGAAATTGAA	Chr.2	14245027	14245047	+	No
2544786_adh	TTAAATTTTTTCTGCGAATTT	Chr.2	14247240	14247220	-	No

1956580_adh	TCACTTTTTCACCCGCCGTGG	Chr.2	14247383	14247403	+	No
2308531_adh	TGCTGATGACGTGGGAAATTA	Chr.2	14251567	14251587	+	No
2488668_adh	TGTAGGAGTACGGTAGGGTAA	Chr.2	14265561	14265581	+	No
2054011_adh	TCTTAATTGAAATTAGCGGAA	Chr.2	14276562	14276582	+	No
2584908_adh	TTCAAAAACCAGCCGGTAAGC	Chr.2	14276629	14276649	+	No
2635753_adh	TTGACGCGCAAAACACTGAAT	Chr.2	14321406	14321426	+	No
2734401_adh	TTTTAGAAATAGGACAATATA	Chr.2	14331996	14331976	-	No
1929576_adh	TATTTCAGTAGATATGGAAAA	Chr.2	14343744	14343724	-	No
2547687_adh	TTAAGCATTTATCGGACTTTC	Chr.2	14349541	14349521	-	No
2533133_adh	TGTTTGCGGACCAAGAAGATT	Chr.2	14358049	14358069	+	No
2652457_adh	TTGCGGACCAAGAAGATTTGA	Chr.2	14358052	14358072	+	No
1866940_adh	TAGGCACCTTTTGATATAAAA	Chr.2	14365149	14365129	-	No
1972111_adh	TCATGATAAATACGTTCCCCA	Chr.2	14365247	14365267	+	No
2707225_adh	TTTCGATTTATTCTCTCGTCT	Chr.2	14383805	14383825	+	No
1962300_adh	TCAGCAGTCACGTGGTTAATT	Chr.2	14399710	14399730	+	No
1814626_adh	TACATTTTTCGTCGATTTTGC	Chr.2	14408036	14408016	-	No
2701660_adh	TTTCAATACATTTTTCGTCGA	Chr.2	14408042	14408022	-	No
2388001_adh	TGGATACTGTACAAGAAGGAA	Chr.2	14408958	14408978	+	No
2546752_adh	TTAAGAAGATACACGACACAG	Chr.2	14440809	14440789	-	No
2531600_adh	TGTTTAAGAAGATACACGACA	Chr.2	14440812	14440792	-	No
1810347_adh	TACAATTTAAAAAATTATTTT	Chr.2	14447511	14447491	-	No
1810441_adh	TACACAAAGAAATTAGGAAGG	Chr.2	14480192	14480212	+	No
2236383_adh	TGAGGAAGAAGGTGTTGGACA	Chr.2	14487098	14487078	-	No
2535317_adh	TGTTTTTTGAAACTAGACGTG	Chr.2	14488737	14488757	+	No
2748222_adh	TTTTTTACGGTTTGTTGAGAT	Chr.2	14496417	14496437	+	No
2272110_adh	TGATGGAACTCCAAGCACAAG	Chr.2	14507090	14507070	-	No
2588931_adh	TTCAATTGTTGCGGTTTTCAT	Chr.2	14512538	14512558	+	No
1984199_adh	TCCGACGTGACGGTGTTTGCA	Chr.2	14561702	14561682	-	No
2707762_adh	TTTCGGATACACGGGGAGGCA	Chr.2	14561719	14561739	+	No
2714576_adh	TTTGAATTTTTTCGTAGAAGT	Chr.2	14608882	14608902	+	No
1968514_adh	TCATAATTTCAAAGACGAGTC	Chr.2	14609294	14609314	+	No
2604202_adh	TTCGAGTTTTTGGCATGCTCT	Chr.2	14632553	14632533	-	No
2523563_adh	TGTTAGGAGTCGTCAAATTTT	Chr.2	14635720	14635700	-	No
1824832_adh	TACGCCTACCTGCCGGAGAAT	Chr.2	14636386	14636406	+	No
1824550_adh	TACGATTGTACTCCATGCCCA	Chr.2	14661966	14661946	-	No
1834427_adh	TACTTAAATCTTTGGATGACA	Chr.2	14698605	14698625	+	No
2543599_adh	TTAAATCTTTGGATGACAATT	Chr.2	14698608	14698628	+	No
2143414_adh	TGAATGAGACGTTGAATGAGT	Chr.2	14708452	14708472	+	No
2151338_adh	TGACAAGGACTGCGACTACGT	Chr.2	14709349	14709369	+	No
1800806_adh	TAATGGTGACAGACCAGTTCA	Chr.2	14710566	14710586	+	No
2291625_adh	TGCATTTTGTAGGAAATCGGA	Chr.2	14741844	14741864	+	No
1936815_adh	TCAACGGGTCGCAAAATGGGC	Chr.2	14753722	14753702	-	No
2161395_adh	TGACGCGGGAAAACTGAAGAA	Chr.2	14753864	14753844	-	No
2003990_adh	TCGATGAATTTGAAGACAATT	Chr.2	14795518	14795538	+	No
2001380_adh	TCGAGATTGACAAGAAGGACG	Chr.2	14796913	14796933	+	No
1891543_adh	TATCAAGCCGTTCAAGACTTC	Chr.2	14802418	14802438	+	No
1830793_adh	TACTATTTCCGTCGAGATTTC	Chr.2	14826195	14826175	-	No
2279355_adh	TGATTTTGGTGCTACGGAATG	Chr.2	14845587	14845607	+	No
2734785_adh	TTTTAGGATGATTGTTTTGAT	Chr.2	14916842	14916822	-	No
2002560_adh	TCGATAAGATTTCGATGATCG	Chr.2	14990208	14990228	+	No

1984555_adh	TCCGAGTAGTTGAGAGAGCGA	Chr.2	14999821	14999841	+	No
2376117_adh	TGGACGATTTTCGCGGCGAGA	Chr.2	15005115	15005095	-	No
1836665_adh	TAGAAAACAGTTGCCGCACTT	Chr.2	15044579	15044559	-	No
2607307_adh	TTCGGACAGATTTGACACGGA	Chr.2	15045624	15045644	+	No
2748705_adh	TTTTTTCCAGATTTTCAGGTT	Chr.2	15089559	15089579	+	No
2596048_adh	TTCCACAGAAAATGCCAATTA	Chr.2	15120119	15120139	+	No
1834770_adh	TACTTCACTGATTTTCTTCAT	Chr.2	15121306	15121286	-	No
2620582_adh	TTCTTACTGTAGTTTTCGGTA	Chr.2	15124696	15124676	-	No
2514967_adh	TGTGGACGAGGTTAATGCTAA	Chr.2	15149627	15149647	+	No
1927998_adh	TATTGTGATTGTGGAATGCAT	Chr.2	15164992	15164972	-	No
2693694_adh	TTTACCTCGTTCGACATTTTT	Chr.2	15165814	15165794	-	No
2116575_adh	TGAAATTGGCGGAAACTCAAA	Chr.2	15232790	15232770	-	No
2711791_adh	TTTCTTGCTGAGAATCTTGCA	Chr.2	15232893	15232913	+	No
2622894_adh	TTCTTGCTGAGAATCTTGCAT	Chr.2	15232894	15232914	+	No
2181459_adh	TGAGAATCTTGCATTTTAAAG	Chr.2	15232901	15232921	+	No
2730444_adh	TTTGTTAGTTGACAACTTCTT	Chr.2	15268670	15268650	-	No
2739231_adh	TTTTCTCAGTTGACAACTTTT	Chr.2	15273575	15273595	+	No
2621022_adh	TTCTTCAACAACTCTCGTCGA	Chr.3	1080	1060	-	No
2378945_adh	TGGACTTGAATGATGATGGAA	Chr.3	18779	18759	-	No
1837651_adh	TAGAAATAAAAGAATAATGAA	Chr.3	48809	48789	-	No
2707946_adh	TTTCGGCTATTTATTGCGGTT	Chr.3	86599	86619	+	No
2745112_adh	TTTTTAGGAGGTTTCCTTGGT	Chr.3	87467	87487	+	No
2117961_adh	TGAAATTTAAAAAATTTTTAA	Chr.3	163642	163662	+	No
2737924_adh	TTTTCCCCGAATTTCATCGAT	Chr.3	188653	188633	-	No
2738305_adh	TTTTCGATGGATTTTCGACGT	Chr.3	235435	235455	+	No
2707167_adh	TTTCGATGGATTTTCGACGTT	Chr.3	235436	235456	+	No
2692406_adh	TTTAATTGACGACACTTTCTT	Chr.3	269840	269860	+	No
2685740_adh	TTGTTGCAGAAAATTAAAAAA	Chr.3	271461	271441	-	No
2510098_adh	TGTGATGCAAAATCACTTCAA	Chr.3	287489	287509	+	No
1798856_adh	TAATCTGAAAATTTTCGTCGA	Chr.3	344367	344347	-	No
2309959_adh	TGCTTAAAATGACTGGAACAA	Chr.3	348715	348735	+	No
2539043_adh	TTAAAATGACTGGAACAAAAG	Chr.3	348718	348738	+	No
2318626_adh	TGGAACAAAAGAAGAATACAT	Chr.3	348728	348748	+	No
1985724_adh	TCCGGAAGACGATCTGTGTTT	Chr.3	399924	399944	+	No
2620582_adh	TTCTTACTGTAGTTTTCGGTA	Chr.3	483434	483414	-	No
1940190_adh	TCAAGTATTGTCGGTAAATTA	Chr.3	484313	484293	-	No
2237052_adh	TGAGGACAACTACGAATGAAA	Chr.3	502880	502900	+	No
1867805_adh	TAGGCTTTAGAAAAAACACAT	Chr.3	502912	502932	+	No
1851347_adh	TAGATGATGAGAGTAGAAGAG	Chr.3	504765	504785	+	No
2741790_adh	TTTTGATGAGTGTTTTCTTCT	Chr.3	556856	556876	+	No
2689489_adh	TTTAAATGAACGAGTGGATAT	Chr.3	599155	599175	+	No
2543654_adh	TTAAATGAACGAGTGGATATA	Chr.3	599156	599176	+	No
2608457_adh	TTCGGCAGTAATGGTAGAGGT	Chr.3	599435	599415	-	No
1827471_adh	TACGGTGTTTGAGAGATGGAG	Chr.3	609156	609136	-	No
2692319_adh	TTTAATTCGCATTGTATTGTA	Chr.3	634810	634830	+	No
1955146_adh	TCACTGTTAATTTTCCGGCAT	Chr.3	640345	640325	-	No
2046223_adh	TCTGAGATTGGAAGAAACAAA	Chr.3	646634	646614	-	No
2520053_adh	TGTGTGGCGGCAATGTAAGCG	Chr.3	654580	654600	+	No
1920442_adh	TATTAATTTTCGGTATTTTTT	Chr.3	665205	665185	-	No
1922417_adh	TATTATGAATTTGCACAGTAA	Chr.3	687566	687546	-	No

2293468_adh	TGCCCCTAGGGATTGTTGGCC	Chr.3	690973	690993	+	No
2590913_adh	TTCAGATTTTCGGAACTTGGC	Chr.3	791466	791446	-	No
2486281_adh	TGTAATTTTGACTGAATCCGG	Chr.3	792473	792453	-	No
2740927_adh	TTTTGAATGGAAAAACGACAG	Chr.3	793716	793696	-	No
1940826_adh	TCAATAAATTGTTGGCATAAT	Chr.3	795006	795026	+	No
1898721_adh	TATCGTCAATGTCTTCCGGTA	Chr.3	795231	795251	+	No
2132491_adh	TGAAGGAAGATGAATCTGAAA	Chr.3	803380	803400	+	No
2546880_adh	TTAAGACGGCTGATGGGATAT	Chr.3	822143	822163	+	No
2701176_adh	TTTCAAATCCTCGTCGTTTTC	Chr.3	828330	828310	-	No
2644495_adh	TTGATCTCTGAAAGAAGATTT	Chr.3	836211	836231	+	No
2629230_adh	TTGAAGTGAAATCGGGGATAA	Chr.3	861961	861941	-	No
2716789_adh	TTTGAGCGTGACGGTGTTTGC	Chr.3	875819	875839	+	No
1789692_adh	TAAGTTTGACGGAGTGATAAT	Chr.3	879668	879648	-	No
2130819_adh	TGAAGCATGCAAATGAGTGAT	Chr.3	879688	879668	-	No
2735321_adh	TTTTATAGGATTCAGGACAAA	Chr.3	881844	881864	+	No
2606561_adh	TTCGCGACATACTTGAATAAC	Chr.3	882275	882255	-	No
2696738_adh	TTTAGGGCATTTGTACGTGTG	Chr.3	889288	889268	-	No
2688130_adh	TTGTTTTAAAGTTGAGAAGCA	Chr.3	889447	889467	+	No
2260884_adh	TGATAGGGTAGATGAATTGAC	Chr.3	890374	890394	+	No
2590456_adh	TTCAGAATTTTGAAGAGCTTG	Chr.3	895921	895941	+	No
2659925_adh	TTGGAGGAGTCTCGCCGAACA	Chr.3	901157	901137	-	No
2709275_adh	TTTCGTTGGGCGGCAATATGA	Chr.3	902082	902102	+	No
2625098_adh	TTGAAAGCGGTACGGTTCATC	Chr.3	910549	910569	+	No
1802031_adh	TAATGTTTTAGACCTAGCAAT	Chr.3	950703	950723	+	No
2643090_adh	TTGAGTTGACGACATCTCAAA	Chr.3	952355	952335	-	No
2709453_adh	TTTCTAAGTATGTTCGGCGGC	Chr.3	964640	964660	+	No
2480514_adh	TGTAAAATGTCCATTTCTCGT	Chr.3	1022110	1022090	-	No
2584978_adh	TTCAAAACTTGAAAATATTGA	Chr.3	1037400	1037420	+	No
2746089_adh	TTTTTCGGCAAATTTTTGGGT	Chr.3	1080649	1080629	-	No
2596723_adh	TTCCAGACCGATGCAGAACGA	Chr.3	1104107	1104127	+	No
1940701_adh	TCAAGTTGAAGGAAGACCTCG	Chr.3	1143263	1143283	+	No
2067479_adh	TGAAAACTAAAGGAACATGTA	Chr.3	1175631	1175611	-	No
2498884_adh	TGTCGTTGAGAAGAAGGAATT	Chr.3	1196210	1196190	-	No
2740234_adh	TTTTCTTGATTTTCTGCGGAA	Chr.3	1202270	1202250	-	No
2737027_adh	TTTTATTTTCGCCGGAATTTT	Chr.3	1202297	1202277	-	No
2688385_adh	TTGTTTTTATTTTCGCCGGAA	Chr.3	1202301	1202281	-	No
2732865_adh	TTTGTTTTTATTTTCGCCGGA	Chr.3	1202302	1202282	-	No
2746149_adh	TTTTTCGTCGATTTTCAGCCA	Chr.3	1208074	1208094	+	No
2146849_adh	TGAATTGAATAGCATTATGTA	Chr.3	1303237	1303217	-	No
2608462_adh	TTCGGCATAAATTTGAAAAAG	Chr.3	1323238	1323218	-	No
2100383_adh	TGAAATGAAAAGAAGAATATA	Chr.3	1333832	1333812	-	No
2595852_adh	TTCCAAGACTGCCTACGGATT	Chr.3	1370474	1370494	+	No
2620325_adh	TTCTGTTCTTGAGCTTTAGAA	Chr.3	1390976	1390996	+	No
2693138_adh	TTTACAAGAGAGATCTGAAAC	Chr.3	1403233	1403253	+	No
2067686_adh	TGAAAACTGCAGAAAAAATTGG	Chr.3	1416823	1416843	+	No
2735613_adh	TTTTATCGATTTTCCAGCTGT	Chr.3	1418794	1418774	-	No
2606844_adh	TTCGCTGCAGATTTGTTTCGC	Chr.3	1418940	1418960	+	No
2585201_adh	TTCAAACTCAGTAATCTATTA	Chr.3	1419740	1419760	+	No
2669696_adh	TTGGTAGAACCTGAGAACGAG	Chr.3	1419875	1419895	+	No
2519198_adh	TGTGTCAGTAGTGTCAGAAGG	Chr.3	1435868	1435888	+	No

2380455_adh	TGGAGAGCAGTGCTGTGCGGC	Chr.3	1437542	1437522	-	No
1824186_adh	TACGATGGAAATAATGAAGAA	Chr.3	1481708	1481688	-	No
2690669_adh	TTTAAGGGAACATGCAGAAAA	Chr.3	1496087	1496107	+	No
2620240_adh	TTCTGTGTAGAAAATGGCTGG	Chr.3	1508023	1508043	+	No
2713986_adh	TTTGAAGGCGCTGAATTCGAA	Chr.3	1520015	1519995	-	No
2711450_adh	TTTCTTCCTGCAAAATAGAGA	Chr.3	1520357	1520377	+	No
2313997_adh	TGGAAAATTGACGGAAAATCG	Chr.3	1521487	1521467	-	No
2713931_adh	TTTGAAGCTGGAGACGTGAAA	Chr.3	1531944	1531924	-	No
2749370_adh	TTTTTTGATATATTTTGCATT	Chr.3	1538359	1538379	+	No
2625127_adh	TTGAAAGCTGGAGATGTGGAG	Chr.3	1564203	1564223	+	No
1806444_adh	TACAAAAATTCCGTGACTAGA	Chr.3	1592260	1592240	-	No
2693390_adh	TTTACATCAAAATTTGCGTCA	Chr.3	1619251	1619271	+	No
2038424_adh	TCTCATTAAGATCGAGGATAT	Chr.3	1623161	1623181	+	No
2629303_adh	TTGAAGTTGGAGAGAAGAAGG	Chr.3	1623406	1623426	+	No
2709715_adh	TTTCTAGGGGTAAATATGAGC	Chr.3	1633103	1633083	-	No
2728380_adh	TTTGTAGTCTGTGACGTCATT	Chr.3	1657920	1657940	+	No
2578521_adh	TTATGACGTCACTGTAATGGC	Chr.3	1732427	1732407	-	No
2072572_adh	TGAAACCGAAAACAAAAACAA	Chr.3	1741741	1741761	+	No
1990259_adh	TCCTCGCATGGTGTAACGGCT	Chr.3	1744686	1744666	-	No
2015016_adh	TCGGATTTCTTAGCAGAAAAA	Chr.3	1750423	1750443	+	No
2029524_adh	TCTAAATGAGCTGAAACTTGA	Chr.3	1781880	1781860	-	No
1927764_adh	TATTGTAGTTTCGAAGAGGTA	Chr.3	1824642	1824622	-	No
2583260_adh	TTATTGTAGTTTCGAAGAGGT	Chr.3	1824643	1824623	-	No
2738379_adh	TTTTCGCCAATTTTCGCCATT	Chr.3	1824770	1824790	+	No
2712672_adh	TTTGAAAGCTGGAGACTTGGA	Chr.3	1837526	1837546	+	No
2745751_adh	TTTTTCATCATTTTCGTTGGG	Chr.3	1859785	1859805	+	No
2557186_adh	TTACGGAAAAATGCAGAATTT	Chr.3	1903159	1903139	-	No
2608091_adh	TTCGGAGTGTCGTTGTGACAA	Chr.3	1905772	1905752	-	No
2608091_adh	TTCGGAGTGTCGTTGTGACAA	Chr.3	1905815	1905835	+	No
2739589_adh	TTTTCTGGCGATTTAGTAGAG	Chr.3	1916504	1916484	-	No
1766715_adh	TAACCAGTGAAAAATGACGCG	Chr.3	1943147	1943127	-	No
2545136_adh	TTAACCAGTGAAAAATGACGC	Chr.3	1943148	1943128	-	No
2737905_adh	TTTTCCATTTTCAGGCGTCGG	Chr.3	1949148	1949128	-	No
2748875_adh	TTTTTTCGATTTTTAGGCATT	Chr.3	1962208	1962188	-	No
2301088_adh	TGCGGACTTAGGCGGTTTTTT	Chr.3	1962296	1962316	+	No
1858198_adh	TAGCTCGTGGAATGCGATGGA	Chr.3	1970238	1970258	+	No
2603668_adh	TTCGAGAATTTTCCGATTGAA	Chr.3	1976738	1976718	-	No
2616704_adh	TTCTCGGTTCTCTCGTTTAAA	Chr.3	1977035	1977055	+	No
2719622_adh	TTTGATTACATTGAGAGCGGA	Chr.3	1978440	1978420	-	No
2502365_adh	TGTGAAGGATGAGATGTCAAA	Chr.3	1979654	1979674	+	No
2100346_adh	TGAAATGAAAAGAAACATATA	Chr.3	1980769	1980789	+	No
1895523_adh	TATCCATCGTGGCCTAGTGGC	Chr.3	2031385	2031365	-	No
2412195_adh	TGGCGCAGTCGGTTACATTTT	Chr.3	2031937	2031917	-	No
2484331_adh	TGTAATGGCGCAGTCGGTTAC	Chr.3	2031942	2031922	-	No
2272936_adh	TGATGTAATGGCGCAGTCGGT	Chr.3	2031945	2031925	-	No
2645453_adh	TTGATGTAATGGCGCAGTCGG	Chr.3	2031946	2031926	-	No
1990086_adh	TCCTCCCTGGATCAGTCGGTA	Chr.3	2077540	2077560	+	No
2532896_adh	TGTTTGACTGTGCGGTGATGT	Chr.3	2090965	2090985	+	No
2128010_adh	TGAAGACGTCCGACATCATCA	Chr.3	2178608	2178588	-	No
2112754_adh	TGAAATGTTAAAAATTATGTA	Chr.3	2227788	2227808	+	No

2717366_adh	TTTGATACAAGTAGACGTAAT	Chr.3	2237222	2237202	-	No
2660798_adh	TTGGATACGAGTAGACGAAAT	Chr.3	2239065	2239085	+	No
1853875_adh	TAGCACATGACATAGAGGTAA	Chr.3	2274967	2274987	+	No
2534352_adh	TGTTTTCGGTGTATTTTTGTC	Chr.3	2278294	2278274	-	No
2285891_adh	TGCACAAATTAGTAGAAACCA	Chr.3	2285883	2285903	+	No
2614899_adh	TTCTAGCGGAAATTAACGAGA	Chr.3	2314220	2314200	-	No
2299894_adh	TGCGATTTTGCGATTGGCTGA	Chr.3	2372019	2371999	-	No
1926475_adh	TATTGCGATTTTGCGATTGGC	Chr.3	2372022	2372002	-	No
1962143_adh	TCAGCAAGTGTAGGATAAGTG	Chr.3	2427369	2427389	+	No
2692223_adh	TTTAATTACCCAAGTTTGAGA	Chr.3	2430998	2431018	+	No
1957676_adh	TCAGAAGATTGCAGGTTCGTG	Chr.3	2431112	2431092	-	No
2587580_adh	TTCAATAGCGGACAAATAAAT	Chr.3	2442909	2442889	-	No
1828779_adh	TACGTGGAAGGTCATCTAAAT	Chr.3	2457346	2457366	+	No
1936585_adh	TCAACGATTATTTCATGAACG	Chr.3	2458763	2458783	+	No
1932375_adh	TCAAAATAGAGGACAATGCAA	Chr.3	2477054	2477034	-	No
2269171_adh	TGATCAGCTGGTAAAATTCTC	Chr.3	2483047	2483067	+	No
2692409_adh	TTTAATTGACGAGCATTAGGT	Chr.3	2487300	2487320	+	No
2635671_adh	TTGACGAGCATTAGGTTTTCA	Chr.3	2487305	2487325	+	No
2737832_adh	TTTTCCAAGCGAACTCGGAAA	Chr.3	2514687	2514667	-	No
1856291_adh	TAGCGAAGCGAGGAACTTTTT	Chr.3	2546504	2546524	+	No
2548805_adh	TTAAGTAGATAGACAGACCCC	Chr.3	2548715	2548735	+	No
2686682_adh	TTGTTTAACTCGCAGCGGGCG	Chr.3	2564620	2564600	-	No
2686682_adh	TTGTTTAACTCGCAGCGGGCG	Chr.3	2565026	2565006	-	No
1990381_adh	TCCTCGGGGAGTACACGATCT	Chr.3	2574368	2574388	+	No
1883920_adh	TATAAAAGACCGTCGTCACGC	Chr.3	2583434	2583414	-	No
2749173_adh	TTTTTTGAAAGAAATCGTTGA	Chr.3	2597359	2597379	+	No
2688961_adh	TTTAAAATGTTGGTGTTGACC	Chr.3	2599095	2599075	-	No
2538933_adh	TTAAAAGTTTAAAATGTTGGT	Chr.3	2599102	2599082	-	No
2123222_adh	TGAACGCTTTGAATTCGGGAG	Chr.3	2605305	2605325	+	No
2048997_adh	TCTGGACTGAAGAAGAAGCAC	Chr.3	2674585	2674605	+	No
2584618_adh	TTATTTTTAAAATTTTCTATT	Chr.3	2695941	2695961	+	No
2288005_adh	TGCAGATTAATCGTCAGAAAC	Chr.3	2711163	2711143	-	No
2620583_adh	TTCTTACTGTAGTTTTCTCTA	Chr.3	2717197	2717217	+	No
2414936_adh	TGGCTGGAATTACGGAGAAAA	Chr.3	2722626	2722606	-	No
2510755_adh	TGTGATGGCTGGAATTACGGA	Chr.3	2722631	2722611	-	No
2649261_adh	TTGCAGAAGTTTCGAGACAAA	Chr.3	2748710	2748690	-	No
2075454_adh	TGAAAGAAGTGAGCAAGACTA	Chr.3	2763075	2763095	+	No
2061703_adh	TCTTTGAAGGACGAACGTATA	Chr.3	2780421	2780401	-	No
2572236_adh	TTAGTTATATTCAAAACGGCT	Chr.3	2788090	2788070	-	No
2577594_adh	TTATCGAAATCTGAAGGAGCA	Chr.3	2788537	2788557	+	No
2679281_adh	TTGTGAACTTTTGTTGCGGCG	Chr.3	2790752	2790772	+	No
2010107_adh	TCGGAAAACGAAGAAAATGGA	Chr.3	2811497	2811477	-	No
2301092_adh	TGCGGACTTTTTGCATCATTT	Chr.3	2815141	2815121	-	No
2348765_adh	TGGAATGGAAAGAGGTATTGA	Chr.3	2891041	2891021	-	No
1990019_adh	TCCTCATATCAATATCGTTTA	Chr.3	2944620	2944600	-	No
1930845_adh	TATTTTCACCGGTTTTTCGCG	Chr.3	2946937	2946957	+	No
2716397_adh	TTTGAGAATATGATTGAACAA	Chr.3	2962656	2962636	-	No
2029595_adh	TCTAACATGCTTTCGAAGGAT	Chr.3	2970031	2970011	-	No
2516392_adh	TGTGGGAGGTGAAGAGACGCA	Chr.3	2973425	2973445	+	No
1901532_adh	TATGAACAACACGTGGAATCA	Chr.3	3052048	3052028	-	No

2737544_adh	TTTTCAGGTTTCAGAAGTGGA	Chr.3	3068007	3067987	-	No
2736583_adh	TTTTATTCTCGCGTTTTGGCC	Chr.3	3068169	3068189	+	No
2699914_adh	TTTATTCTCGCGTTTTGGCCT	Chr.3	3068170	3068190	+	No
2039876_adh	TCTCGCGTTTTGGCCTAAATT	Chr.3	3068175	3068195	+	No
2657545_adh	TTGGAATCGGAATATTCACAA	Chr.3	3070182	3070162	-	No
2618793_adh	TTCTGCTGGAAAATTGGCTAC	Chr.3	3085285	3085265	-	No
2617108_adh	TTCTCTCGATTTTGATTAAAT	Chr.3	3127246	3127226	-	No
2490670_adh	TGTATATGAAGACGTTGAATT	Chr.3	3161890	3161910	+	No
2269450_adh	TGATCCGCGGATTTTGATGGA	Chr.3	3171514	3171494	-	No
2590528_adh	TTCAGACTAAAGGCTAAATTG	Chr.3	3211405	3211425	+	No
1976458_adh	TCCAAGGAGGAATGAGAAGCT	Chr.3	3248883	3248903	+	No
2311978_adh	TGCTTTGTGGCTTTGCTGGTA	Chr.3	3249207	3249227	+	No
2737231_adh	TTTTCAAGTAAATCTACCGCA	Chr.3	3310741	3310761	+	No
1806092_adh	TAATTTTGTGGTGTAGTGGGC	Chr.3	3355914	3355934	+	No
2487548_adh	TGTAGAATGCGGTGCCCGAAG	Chr.3	3387787	3387807	+	No
2594292_adh	TTCATCGATCAAGAGGATCTG	Chr.3	3393514	3393494	-	No
2708877_adh	TTTCGTCGGCTTTTCGCATAT	Chr.3	3396353	3396373	+	No
2706289_adh	TTTCCTGCAGAAACCACAGAA	Chr.3	3398587	3398567	-	No
2005360_adh	TCGATTTTCCTGCAGAAACCA	Chr.3	3398592	3398572	-	No
2736358_adh	TTTTATGTTTGGCTTCGAAAA	Chr.3	3398737	3398757	+	No
2738220_adh	TTTTCGAATTTTGAGGACAAA	Chr.3	3399555	3399535	-	No
1994791_adh	TCGAAAGACGGATCTGTCAAA	Chr.3	3402543	3402523	-	No
2695263_adh	TTTAGACTGTTTCGCCATAGT	Chr.3	3404553	3404533	-	No
2734511_adh	TTTTAGACTGTTTCGCCATAG	Chr.3	3404554	3404534	-	No
2584481_adh	TTATTTTAGACTGTTTCGCCA	Chr.3	3404557	3404537	-	No
2600777_adh	TTCCTGAGTAGAATTGGCTAT	Chr.3	3450741	3450761	+	No
2031209_adh	TCTACACGGCTCGGATGGTGA	Chr.3	3455823	3455803	-	No
2689365_adh	TTTAAAGTGGAAAAGGGCAAA	Chr.3	3460178	3460158	-	No
2721513_adh	TTTGCCAAGAGAACGATGAGA	Chr.3	3471521	3471501	-	No
2583485_adh	TTATTTAATTTTCAGTTGGAC	Chr.3	3503084	3503064	-	No
1984341_adh	TCCGACTTCAACCAAGAAGAA	Chr.3	3503149	3503129	-	No
2498676_adh	TGTCGTCCTCAGAGCTGAAGA	Chr.3	3503479	3503459	-	No
2597271_adh	TTCCATCGTTGCAAAGAAGCT	Chr.3	3503518	3503498	-	No
2734799_adh	TTTTAGGCAGAATTCTTGATT	Chr.3	3508624	3508604	-	No
2338885_adh	TGGAATGAAAAATAGTTTCTT	Chr.3	3519607	3519627	+	No
2580080_adh	TTATGTCCGCATAGCCAGCAA	Chr.3	3525744	3525724	-	No
2723063_adh	TTTGGAACGTAGACAGTTTAT	Chr.3	3528945	3528925	-	No
2688657_adh	TTTAAAAAACTGTTTTTTGTA	Chr.3	3536997	3537017	+	No
1932513_adh	TCAAACAAAAGCTCAAAGACA	Chr.3	3540722	3540702	-	No
1821606_adh	TACGAACGTAGGAATCGTGGT	Chr.3	3540817	3540797	-	No
2607095_adh	TTCGGAAACGTTTGGAAGAGG	Chr.3	3548584	3548604	+	No
2552606_adh	TTAATTTAGTCGGAAACCGCA	Chr.3	3587093	3587113	+	No
2067500_adh	TGAAAACTAGAAAAAATGATG	Chr.3	3650330	3650310	-	No
1878710_adh	TAGTGGAGTGCGCAGAGAAGA	Chr.3	3650486	3650466	-	No
2730057_adh	TTTGTGTCGGAAAAGACGAGA	Chr.3	3652665	3652685	+	No
2065558_adh	TGAAAAACGTCACATTCAGAT	Chr.3	3690850	3690830	-	No
2561501_adh	TTAGAAGGAGTCGGGAAGAAA	Chr.3	3695675	3695695	+	No
2016372_adh	TCGGGAAGAAAGGAAAATGCA	Chr.3	3695685	3695705	+	No
2532809_adh	TGTTTGAAGTCACGGCGTTGA	Chr.3	3699806	3699786	-	No
2290453_adh	TGCATCTTACGACGATTTCTA	Chr.3	3705177	3705197	+	No

2618884_adh	TTCTGGAAGTAGAATGAGGTC	Chr.3	3724933	3724913	-	No
2025980_adh	TCGTGAGAAGATTGACTATTA	Chr.3	3729114	3729094	-	No
2713558_adh	TTTGAAGAAGTTGGAAGTTGA	Chr.3	3729612	3729632	+	No
2041751_adh	TCTCGTTAGTTCTAGTTATCG	Chr.3	3730793	3730813	+	No
1921872_adh	TATTATAAATTTCAGATGATC	Chr.3	3735033	3735013	-	No
2689000_adh	TTTAAACAATTTTTGGTCGGA	Chr.3	3735774	3735794	+	No
2633699_adh	TTGAATTCCTGGCATGTTTCT	Chr.3	3735820	3735840	+	No
2700858_adh	TTTATTTTGGAAGGACGAACG	Chr.3	3735989	3736009	+	No
1802666_adh	TAATTATACGGATATATATTC	Chr.3	3742206	3742186	-	No
2233238_adh	TGAGCACTGTATTTGAGCATA	Chr.3	3767923	3767903	-	No
2720915_adh	TTTGCAAACATGTAGAAAAAG	Chr.3	3782613	3782593	-	No
2047282_adh	TCTGATGGATGGGATAGAACT	Chr.3	3793978	3793958	-	No
2713823_adh	TTTGAAGATAATAAAACAAGT	Chr.3	3799640	3799660	+	No
2462856_adh	TGGTAGAGACGAACTGCGGAT	Chr.3	3801730	3801710	-	No
2166823_adh	TGACTAGGCAGAGGAGGAACG	Chr.3	3831426	3831446	+	No
2711514_adh	TTTCTTCGGCGAAACAAGAGA	Chr.3	3843902	3843882	-	No
2161496_adh	TGACGGAAAAATGCACAGAGT	Chr.3	3844081	3844101	+	No
2743741_adh	TTTTGTCAGCAGCCAAGAAGC	Chr.3	3844609	3844629	+	No
2705454_adh	TTTCCCTATAATTTTGTCGTT	Chr.3	3844638	3844658	+	No
2513334_adh	TGTGCACTTGATCCGACGACA	Chr.3	3863914	3863934	+	No
2337508_adh	TGGAATCTATGGAGATGAGCA	Chr.3	3866284	3866304	+	No
2282178_adh	TGCAAGATCTAAACTTTTAAC	Chr.3	3866915	3866935	+	No
2142754_adh	TGAATCTGAAGGACTATGCTG	Chr.3	3869533	3869553	+	No
2539949_adh	TTAAACGGAGTGTGGACAGCA	Chr.3	3915991	3916011	+	No
2055958_adh	TCTTCGAAATGGTCGAGACGA	Chr.3	3955919	3955939	+	No
2412903_adh	TGGCGGGGAAAAACAAGACGA	Chr.3	3974239	3974219	-	No
2702566_adh	TTTCACGCAACTGAAAATTAA	Chr.3	3985300	3985320	+	No
2662436_adh	TTGGATGAAGATGATGCTATG	Chr.3	4000195	4000175	-	No
2380354_adh	TGGAGAGACTTACGAATCGGA	Chr.3	4016069	4016049	-	No
2674682_adh	TTGTAGAACCATACAATGCCA	Chr.3	4016991	4016971	-	No
2450531_adh	TGGGCGGCAAATTTTGAATTT	Chr.3	4019952	4019932	-	No
2696645_adh	TTTAGGAGCTTTTGGGAAGGA	Chr.3	4058121	4058141	+	No
1774440_adh	TAAGAATAAGAACATTAGCGT	Chr.3	4088457	4088437	-	No
2595487_adh	TTCATTTCTCATTCGCTTCGT	Chr.3	4095544	4095524	-	No
2615020_adh	TTCTAGTAATTTTCGTTCGAA	Chr.3	4098155	4098135	-	No
2603354_adh	TTCGACTTACAAAAATGGCTA	Chr.3	4098309	4098329	+	No
2000762_adh	TCGACTTACAAAAATGGCTAA	Chr.3	4098310	4098330	+	No
2729856_adh	TTTGTGGACTGCGCACAAAAC	Chr.3	4152569	4152589	+	No
1884151_adh	TATAAATACTGCAGAAATCGC	Chr.3	4153026	4153046	+	No
2714177_adh	TTTGAATAGTAGATACTCCTG	Chr.3	4172501	4172481	-	No
2624529_adh	TTGAAAATCTCAATCAGGACG	Chr.3	4176258	4176238	-	No
2678327_adh	TTGTCGGATCTCAAGAAGAAG	Chr.3	4185040	4185020	-	No
2587696_adh	TTCAATCACTCGGAGAAGGCG	Chr.3	4187084	4187104	+	No
1954239_adh	TCACTCGGAGAAGGCGATAAA	Chr.3	4187089	4187109	+	No
2376734_adh	TGGACTACATTAAAAAGACTC	Chr.3	4195373	4195393	+	No
2133378_adh	TGAAGGGAGACGTGTTGAAGT	Chr.3	4200221	4200241	+	No
2742736_adh	TTTTGGAGAATAGATGTGGCA	Chr.3	4206039	4206059	+	No
2749050_adh	TTTTTTCTGCAAAAATCGTTA	Chr.3	4230215	4230195	-	No
2053424_adh	TCTGTTTCAGCTTGAAGAATG	Chr.3	4241938	4241918	-	No
2689573_adh	TTTAAATTAAGAAATCGACAA	Chr.3	4255592	4255572	-	No

1845916_adh	TAGACTGCAGCTGGATGGTAT	Chr.3	4277988	4278008	+	No
2709771_adh	TTTCTATCAAAAATGACATCG	Chr.3	4287456	4287436	-	No
2669789_adh	TTGGTAGCAGTAACTGAAAAT	Chr.3	4288000	4287980	-	No
2737523_adh	TTTTCAGGTAAATTAGGACTA	Chr.3	4340578	4340598	+	No
1839058_adh	TAGAAGACATGAGAGAATAAT	Chr.3	4350798	4350778	-	No
1956940_adh	TCAGAAAGGTTTTTATTTTGA	Chr.3	4352252	4352272	+	No
1994809_adh	TCGAAAGAGCACATTTGCATA	Chr.3	4360515	4360495	-	No
1994809_adh	TCGAAAGAGCACATTTGCATA	Chr.3	4360537	4360557	+	No
1938285_adh	TCAAGAAGATATGGATGTAAG	Chr.3	4373286	4373306	+	No
2044288_adh	TCTGAAGGATTGGCAAAACTT	Chr.3	4401252	4401232	-	No
2307742_adh	TGCTCTGAAGGATTGGCAAAA	Chr.3	4401255	4401235	-	No
2558757_adh	TTACTCGAATTTTGAACGGTA	Chr.3	4404545	4404565	+	No
1831199_adh	TACTCGAATTTTGAACGGTAT	Chr.3	4404546	4404566	+	No
2050088_adh	TCTGGCCGAGAAGATCTGTAT	Chr.3	4407018	4406998	-	No
2590470_adh	TTCAGACATCACTTTGAGAAA	Chr.3	4412196	4412176	-	No
2738699_adh	TTTTCGGTCATTCGTTGTTTT	Chr.3	4419439	4419419	-	No
2025874_adh	TCGTGAATTTGTGGGAGCGGT	Chr.3	4419543	4419563	+	No
2174441_adh	TGACTTTCAGATTCGGCCATT	Chr.3	4433250	4433270	+	No
2385909_adh	TGGAGTGTCAAGAGACGTGCT	Chr.3	4433427	4433447	+	No
2174441_adh	TGACTTTCAGATTCGGCCATT	Chr.3	4438449	4438469	+	No
2385909_adh	TGGAGTGTCAAGAGACGTGCT	Chr.3	4438626	4438646	+	No
2471957_adh	TGGTGGACGTAATACTTGAAA	Chr.3	4441623	4441643	+	No
2123850_adh	TGAACGTGGGACATGTAATGC	Chr.3	4444274	4444254	-	No
2082011_adh	TGAAAGGAAAGAAAATGAGGA	Chr.3	4449143	4449123	-	No
2648281_adh	TTGCAAATTTTAGGACTTTTC	Chr.3	4475670	4475690	+	No
2598383_adh	TTCCCTCCTATTTTTTCAGCA	Chr.3	4475709	4475729	+	No
1954240_adh	TCACTCGGAGACTTGAACAAC	Chr.3	4476161	4476181	+	No
1936233_adh	TCAACCAAGTCAAGAAGACTT	Chr.3	4476393	4476413	+	No
2010129_adh	TCGGAAAAGATATTGAGAAGA	Chr.3	4476580	4476600	+	No
1938958_adh	TCAAGATCATCAAGAGACCAA	Chr.3	4476646	4476666	+	No
2696549_adh	TTTAGGAATTATAGAAAGAAG	Chr.3	4477332	4477312	-	No
2120387_adh	TGAACAGGACGCAAAAACAAG	Chr.3	4478669	4478649	-	No
2060403_adh	TCTTTACATCTGCAGACTTGG	Chr.3	4485341	4485361	+	No
1771373_adh	TAACTGCAACATTTTGTCGAT	Chr.3	4494692	4494672	-	No
2471442_adh	TGGTGATGATAGTGACGACGA	Chr.3	4494852	4494872	+	No
2271473_adh	TGATGATAGTGACGACGACAA	Chr.3	4494855	4494875	+	No
2155376_adh	TGACATCACCTCCACTGAAGA	Chr.3	4500816	4500796	-	No
2379363_adh	TGGAGAAAATGGATGTGCAAC	Chr.3	4507709	4507689	-	No
2681631_adh	TTGTGGAGCCAGTGACTGGAT	Chr.3	4521683	4521703	+	No
1761397_adh	TAAATGAGGACACGAAGAAAG	Chr.3	4530390	4530410	+	No
1839058_adh	TAGAAGACATGAGAGAATAAT	Chr.3	4543592	4543572	-	No
2176888_adh	TGAGAAACGCAGACAGCACAC	Chr.3	4560558	4560578	+	No
2172237_adh	TGACTGTGGCAAGGTATTTTT	Chr.3	4587138	4587118	-	No
1930640_adh	TATTTTAAAGGAGCATAGGCG	Chr.3	4596031	4596011	-	No
1917574_adh	TATGTAGAGAAAGCAAAGAGA	Chr.3	4607864	4607844	-	No
2542226_adh	TTAAATAGAAAAATTAAGAAA	Chr.3	4611961	4611941	-	No
1991109_adh	TCCTGAATTTTGAAACGGTTT	Chr.3	4618640	4618620	-	No
2089616_adh	TGAAATATGAAGAATTTTCAA	Chr.3	4625018	4624998	-	No
2702910_adh	TTTCAGAGACTGACATTCCAA	Chr.3	4626371	4626391	+	No
2013640_adh	TCGGATATTAACGTGCATTTA	Chr.3	4635094	4635074	-	No

1798573_adh	TAATCGGATATTAACGTGCAT	Chr.3	4635097	4635077	-	No
2620521_adh	TTCTTAATTTGAACGAGTTGC	Chr.3	4635205	4635225	+	No
2620521_adh	TTCTTAATTTGAACGAGTTGC	Chr.3	4638284	4638304	+	No
2523708_adh	TGTTAGTGGAACGACAGCAGT	Chr.3	4651123	4651143	+	No
2540109_adh	TTAAACTGTATAGGGAAGACC	Chr.3	4651659	4651679	+	No
1773067_adh	TAAGAAAAACGGCATGTGACT	Chr.3	4652566	4652586	+	No
2172993_adh	TGACTTCATTTAGTTTTTCAA	Chr.3	4654102	4654122	+	No
2565822_adh	TTAGATTGAACTGTGAATGGC	Chr.3	4654324	4654304	-	No
1811111_adh	TACACGGATTTTTGCACCAGA	Chr.3	4655567	4655587	+	No
1971793_adh	TCATGACATTGAGGACAGCTC	Chr.3	4677884	4677864	-	No
2744424_adh	TTTTGTTGTTTCGTCGCATTT	Chr.3	4693488	4693508	+	No
2709954_adh	TTTCTCGAAAATTGTAACGGA	Chr.3	4707054	4707074	+	No
2710651_adh	TTTCTGGAAAATCGGTTTTGC	Chr.3	4707506	4707526	+	No
2569691_adh	TTAGTAACAATTCGAACACGG	Chr.3	4709890	4709910	+	No
1764074_adh	TAACAATTCGAACACGGAATC	Chr.3	4709894	4709914	+	No
1935542_adh	TCAAATTCAGAAAATCGTCTC	Chr.3	4720943	4720923	-	No
2269904_adh	TGATCGGAGATGGAGGTGTCG	Chr.3	4739965	4739945	-	No
1755919_adh	TAAACTTGGTTTTTCTGTCGT	Chr.3	4749501	4749521	+	No
1755919_adh	TAAACTTGGTTTTTCTGTCGT	Chr.3	4752591	4752571	-	No
2608681_adh	TTCGGCTAGACCATACGCCCA	Chr.3	4753708	4753688	-	No
2595572_adh	TTCATTTTGTGTTGCAGCGGG	Chr.3	4757205	4757225	+	No
2589444_adh	TTCACCTGAATAGGCATTTTC	Chr.3	4759586	4759566	-	No
1985203_adh	TCCGCACAATCTGAATATGGT	Chr.3	4769915	4769935	+	No
2688933_adh	TTTAAAAGTTTTTCGTACGGC	Chr.3	4782038	4782018	-	No
2748193_adh	TTTTTTACAATTTTTGTCGTT	Chr.3	4782082	4782062	-	No
2736275_adh	TTTTATGTTATGTGCCGGCAC	Chr.3	4782105	4782085	-	No
2729246_adh	TTTGTGAAATCGGTTTCTCTC	Chr.3	4785185	4785165	-	No
2127343_adh	TGAAGAAGTGAATGAAGAAGA	Chr.3	4790269	4790289	+	No
2608669_adh	TTCGGCTACCCATTCTAAATA	Chr.3	4792775	4792795	+	No
2016662_adh	TCGGGAGGTTAGAGACAGTGT	Chr.3	4796151	4796131	-	No
2148688_adh	TGAATTTTATTTTTTATTGCA	Chr.3	4807517	4807497	-	No
1860663_adh	TAGGAAATAAATGAGCGATGA	Chr.3	4809491	4809511	+	No
2643072_adh	TTGAGTTGAAATATGAATTTG	Chr.3	4840604	4840584	-	No
2185266_adh	TGAGAGAATACGGTATCTATC	Chr.3	4846909	4846889	-	No
2715108_adh	TTTGACGACGATTGATATTCC	Chr.3	4850320	4850340	+	No
2602633_adh	TTCGAATTGCAAAGCGGAGCA	Chr.3	4850347	4850367	+	No
2623967_adh	TTCTTTTTCAGAACGATGGCC	Chr.3	4857339	4857359	+	No
2524033_adh	TGTTATATTATAAGTAGACGA	Chr.3	4862093	4862113	+	No
1866496_adh	TAGGATTTAAATAAAAGAAGA	Chr.3	4876504	4876524	+	No
2734161_adh	TTTTACGATTTTCGCCGGTAT	Chr.3	4878504	4878524	+	No
2512295_adh	TGTGATTGCAGAATTATTCCC	Chr.3	4904345	4904325	-	No
2712317_adh	TTTGAAAAAGAAAGAATGAGG	Chr.3	4905406	4905386	-	No
2724180_adh	TTTGGATTTTAGTTTCAAAGA	Chr.3	4911204	4911224	+	No
1757151_adh	TAAAGCAATATGGGTACTCTT	Chr.3	4912410	4912390	-	No
2716616_adh	TTTGAGATCAAATGGACGCCA	Chr.3	4912656	4912676	+	No
2049706_adh	TCTGGATATGAAGTTTGCTAT	Chr.3	4930539	4930559	+	No
2744192_adh	TTTTGTTGAAGAGCGCTGAAG	Chr.3	4932214	4932234	+	No
2129339_adh	TGAAGAGCGCTGAAGATTTTC	Chr.3	4932220	4932240	+	No
1929435_adh	TATTTATTTTCGGTATTACAT	Chr.3	4932384	4932364	-	No
2581150_adh	TTATTATTATTTATTTTCGGT	Chr.3	4932391	4932371	-	No

2259652_adh	TGATACGGCTTTAATCAATTT	Chr.3	4932422	4932402	-	No
2616046_adh	TTCTCACGATGATACGGCTTT	Chr.3	4932431	4932411	-	No
2258334_adh	TGATAAATCAATCTGAACGGA	Chr.3	4934659	4934639	-	No
2709884_adh	TTTCTCAGGAGCATTGATTAA	Chr.3	4935080	4935100	+	No
2616111_adh	TTCTCAGGAGCATTGATTAAA	Chr.3	4935081	4935101	+	No
2541761_adh	TTAAAGTCGGGCTAAAGTAAA	Chr.3	4935097	4935117	+	No
1930069_adh	TATTTCTGTAGGATTGAACGT	Chr.3	4948951	4948971	+	No
1932150_adh	TCAAAAGAAAAGGAGCATATA	Chr.3	4955021	4955001	-	No
2574110_adh	TTATAATGTAGGAGTTTCGAC	Chr.3	4963147	4963167	+	No
2589453_adh	TTCACCTGGTTCGCCGCCAAA	Chr.3	4984875	4984855	-	No
2487690_adh	TGTAGAGAACATGAAGATGCA	Chr.3	4986515	4986535	+	No
1858942_adh	TAGCTTACCGTAGATTGAACA	Chr.3	4987747	4987727	-	No
2722871_adh	TTTGGAAAAGTGAAGAGAGAA	Chr.3	4998521	4998541	+	No
2740738_adh	TTTTGAACGTTTTCGCGCAGA	Chr.3	4998948	4998928	-	No
2744817_adh	TTTTTAATCGTCAGTTTTGAA	Chr.3	4998962	4998942	-	No
2038617_adh	TCTCCACACGGCTGAAATAAC	Chr.3	4999010	4998990	-	No
1768702_adh	TAACGGAGAACCTATCAACAC	Chr.3	5012530	5012510	-	No
2471757_adh	TGGTGGAAAGATGACTAAAAA	Chr.3	5016498	5016478	-	No
1897255_adh	TATCGATTTTTTGGACAAATA	Chr.3	5018121	5018101	-	No
2713980_adh	TTTGAAGGCCAAGACGGGACA	Chr.3	5018377	5018397	+	No
2646225_adh	TTGATTCTTGCTGTGAAACGG	Chr.3	5024479	5024459	-	No
2709923_adh	TTTCTCCCAGTTTTCTTCAAA	Chr.3	5024615	5024635	+	No
2515589_adh	TGTGGATGGAATGAAGGAAAT	Chr.3	5024789	5024769	-	No
1888035_adh	TATAGAGTGTAGTAAAGCGTG	Chr.3	5030383	5030363	-	No
2608370_adh	TTCGGATTGTCATTGAGATGG	Chr.3	5047348	5047368	+	No
2589095_adh	TTCACAATCAGATTCGGTATA	Chr.3	5056718	5056738	+	No
1961670_adh	TCAGATTCGGTATATTGAGAC	Chr.3	5056725	5056745	+	No
2742889_adh	TTTTGGCATCTTTGGAATCTT	Chr.3	5078817	5078837	+	No
2589071_adh	TTCACAAACAGTGCTTTTATT	Chr.3	5081250	5081270	+	No
2123811_adh	TGAACGTCTTTGGAAGTCCGC	Chr.3	5084895	5084915	+	No
1763008_adh	TAACAAAACATTTTCGTTGGG	Chr.3	5108673	5108653	-	No
2541068_adh	TTAAAGATACAGTGACGTCAA	Chr.3	5158769	5158789	+	No
2710860_adh	TTTCTGTAGTATGGCATATTT	Chr.3	5166287	5166307	+	No
2745990_adh	TTTTTCGAATTCTCATTGCAT	Chr.3	5172628	5172608	-	No
2703987_adh	TTTCATCGATGTTGCGGTGTT	Chr.3	5172775	5172795	+	No
1750624_adh	TAAAATACTTTTCGGATTATC	Chr.3	5173016	5173036	+	No
2259264_adh	TGATACAAACGGTAGACCTGT	Chr.3	5186366	5186346	-	No
2602350_adh	TTCGAATAAAGGAAATGGTGA	Chr.3	5188719	5188739	+	No
2661096_adh	TTGGATATGATTATGGGAGAA	Chr.3	5204564	5204544	-	No
2531105_adh	TGTTGTGTTTTCCTTTCACGA	Chr.3	5207109	5207089	-	No
2724483_adh	TTTGGCGTTAATCAGTTGATT	Chr.3	5207251	5207271	+	No
1774100_adh	TAAGAAGACAAGTAGCAGAAT	Chr.3	5215968	5215988	+	No
2593062_adh	TTCAGTGCAAGTAAGCTAACC	Chr.3	5228171	5228191	+	No
2649148_adh	TTGCACTGAAACAGGCTAGAC	Chr.3	5228180	5228160	-	No
2739225_adh	TTTTCTCAGGCATTTTAGGTG	Chr.3	5228207	5228227	+	Yes
1971732_adh	TCATGAACGTCATTGGAGAGC	Chr.3	5228940	5228960	+	No
2276253_adh	TGATTGAAGGAGGAGTCATTG	Chr.3	5229258	5229278	+	No
2535389_adh	TGTTTTTTGGTAGAGATAAAT	Chr.3	5230244	5230264	+	No
1829907_adh	TACTAGAAACTTGTCAGTTCA	Chr.3	5242913	5242933	+	No
2696448_adh	TTTAGCGTTGAATGCTTTTTT	Chr.3	5256255	5256275	+	No

2586346_adh	TTCAACTTTGAAAGACTAAAT	Chr.3	5263356	5263336	-	No
2488087_adh	TGTAGATTGTCAACAAAAGAA	Chr.3	5268051	5268071	+	No
2423865_adh	TGGGAGGAGCTGGCGGAGCAG	Chr.3	5274169	5274149	-	No
2151570_adh	TGACAAGTACGAAGCAACTCT	Chr.3	5274447	5274427	-	No
2391208_adh	TGGATGCTCGACGAACGGTTT	Chr.3	5282934	5282954	+	No
2272117_adh	TGATGGAAGAAGACTCAAGCG	Chr.3	5286230	5286210	-	No
2706514_adh	TTTCGAAACAAATGAAGACCG	Chr.3	5301845	5301825	-	No
2745917_adh	TTTTTCCTTAGCAACCGGTTG	Chr.3	5308439	5308459	+	No
2560518_adh	TTACTTTGTAGACGCTTCTCC	Chr.3	5310978	5310958	-	No
1809156_adh	TACAAGTAGAAATAAGAAGAT	Chr.3	5318448	5318428	-	No
2534351_adh	TGTTTTCGGTGTATTTTTGGT	Chr.3	5324366	5324386	+	No
2534351_adh	TGTTTTCGGTGTATTTTTGGT	Chr.3	5324605	5324585	-	No
1897609_adh	TATCGGAGCAAAAGTTGCAGA	Chr.3	5326818	5326798	-	No
1890446_adh	TATATGTATGGACCACCACCA	Chr.3	5343886	5343906	+	No
2291625_adh	TGCATTTTGTAGGAAATCGGA	Chr.3	5365846	5365826	-	No
2487020_adh	TGTACTCGAGATTGTTTAATA	Chr.3	5375333	5375353	+	No
2702970_adh	TTTCAGATTTCAGCACTTCCT	Chr.3	5375384	5375404	+	No
2745091_adh	TTTTTAGCGAAGTTTTGTGGT	Chr.3	5380482	5380462	-	No
1995788_adh	TCGAACTGTAAAACACGGAAA	Chr.3	5392813	5392793	-	No
2749062_adh	TTTTTTCTGGTTTCTGGCATT	Chr.3	5394199	5394179	-	No
2295187_adh	TGCCTTCTGCGCATACGTCAA	Chr.3	5397316	5397336	+	No
2702967_adh	TTTCAGATTACGGAATAATGG	Chr.3	5410963	5410983	+	No
1756363_adh	TAAAGAACTCGTAGAAAACGT	Chr.3	5422034	5422014	-	No
2748495_adh	TTTTTTCAATAAAGAACTCGT	Chr.3	5422043	5422023	-	No
2652569_adh	TTGCGGACTTGCATACCGTAT	Chr.3	5442982	5443002	+	No
2391208_adh	TGGATGCTCGACGAACGGTTT	Chr.3	5459942	5459922	-	No
2391208_adh	TGGATGCTCGACGAACGGTTT	Chr.3	5460506	5460526	+	No
2037826_adh	TCTCAGTGGCTGAAGAAACAA	Chr.3	5473228	5473208	-	No
1864191_adh	TAGGAGTATAGACGGCAGAAA	Chr.3	5483915	5483935	+	No
2271889_adh	TGATGCGAAACGGATGAAACT	Chr.3	5486099	5486119	+	No
2689268_adh	TTTAAAGCAAGAACGATAGAG	Chr.3	5488322	5488302	-	No
2641216_adh	TTGAGGAGACTGCAGAGAGAA	Chr.3	5500006	5500026	+	No
2701612_adh	TTTCAAGGTTTTCGCCTACGC	Chr.3	5503900	5503920	+	No
2376091_adh	TGGACGATCAAGACAGCGACA	Chr.3	5505561	5505581	+	No
2550425_adh	TTAATCCACAGTTTGAAGCGC	Chr.3	5506319	5506299	-	No
2737449_adh	TTTTCAGAGAATTCATCGAAA	Chr.3	5516902	5516882	-	No
2235472_adh	TGAGCTGCGTCTGACGGCCAT	Chr.3	5538157	5538177	+	No
2639425_adh	TTGAGAAATATCGGAGGTGTT	Chr.3	5542278	5542298	+	No
2571065_adh	TTAGTCATCATTTTTCCCTCA	Chr.3	5543170	5543150	-	No
2313969_adh	TGGAAAATTAAAGAAGTTGTA	Chr.3	5555006	5554986	-	No
2733496_adh	TTTTAAGATTTTTTGTTGGAA	Chr.3	5555022	5555002	-	No
2573626_adh	TTAGTTTTTTCTTTCGGCGGA	Chr.3	5555133	5555153	+	No
2749139_adh	TTTTTTCTTTCGGCGGAAAAC	Chr.3	5555137	5555157	+	No
2746462_adh	TTTTTCTTTCGGCGGAAAACC	Chr.3	5555138	5555158	+	No
1996060_adh	TCGAAGACTGCATCAGCGAAC	Chr.3	5591060	5591040	-	No
2738122_adh	TTTTCCTTATATTCCCATACT	Chr.3	5591309	5591329	+	No
2123045_adh	TGAACGATGACGTCTTCCTCA	Chr.3	5594091	5594111	+	No
1833638_adh	TACTGTAGAAAATTTAGGTAT	Chr.3	5604270	5604290	+	No
2618498_adh	TTCTGATTCTCATACTCATTA	Chr.3	5614895	5614915	+	No
2667130_adh	TTGGGAGAGAAGATGATTATG	Chr.3	5621201	5621181	-	No

2744468_adh	TTTTGTTTCGTCGGATCAAAT	Chr.3	5635942	5635922	-	No
2748002_adh	TTTTTGTTTCGTCGGATCAAA	Chr.3	5635943	5635923	-	No
2595583_adh	TTCATTTTTGTTTCGTCGGAT	Chr.3	5635947	5635927	-	No
2740775_adh	TTTTGAAGAATGGCTAATTTG	Chr.3	5650158	5650178	+	No
2728387_adh	TTTGTAGTTAGAAGCTGTCTT	Chr.3	5654559	5654539	-	No
1957372_adh	TCAGAACTTCCTGATTCGGCT	Chr.3	5671654	5671674	+	No
1886701_adh	TATACAAGAGTTAAGGTCGGA	Chr.3	5688980	5689000	+	No
2624814_adh	TTGAAACTACAGTACTCGTTA	Chr.3	5689397	5689377	-	No
1762451_adh	TAAATTGACAGCAGAAATTTC	Chr.3	5692733	5692753	+	No
2711365_adh	TTTCTTATTCATTCAGTTCCT	Chr.3	5692789	5692769	-	No
1844717_adh	TAGAATTTCCTCGGATATATT	Chr.3	5692885	5692905	+	No
2706228_adh	TTTCCTCGGATATATTCAAAT	Chr.3	5692890	5692910	+	No
1996104_adh	TCGAAGAGAGGACGTGGAGGA	Chr.3	5706070	5706050	-	No
2546239_adh	TTAACTCTGGTTGGCACTAAA	Chr.3	5706589	5706609	+	No
2589425_adh	TTCACCGTGTGGGATGTCGGA	Chr.3	5716326	5716346	+	No
1953097_adh	TCACGATGCGGTTTTGTTTTT	Chr.3	5728062	5728082	+	No
1805555_adh	TAATTTTAAAAATTAATTTCA	Chr.3	5737135	5737155	+	No
2003962_adh	TCGATGAAGAACTGGCGAAAA	Chr.3	5782532	5782552	+	No
2621121_adh	TTCTTCATCGATTAGCGGCCC	Chr.3	5782542	5782522	-	No
1797890_adh	TAATCAGTTTTTTAACCCATA	Chr.3	5783469	5783449	-	No
2054997_adh	TCTTATATTGAAGTAGTAGAA	Chr.3	5785896	5785916	+	No
2134154_adh	TGAAGTAGTAGAAAAGAAAAA	Chr.3	5785904	5785924	+	No
2521050_adh	TGTGTTGAATGAGGCAAGACA	Chr.3	5802842	5802822	-	No
2744053_adh	TTTTGTGTTTTTAGAACTGAA	Chr.3	5803176	5803196	+	No
2712752_adh	TTTGAAATAAGGTACTGAAGA	Chr.3	5810169	5810149	-	No
2663605_adh	TTGGATGTATAAGGTGCAAGA	Chr.3	5828671	5828691	+	No
2683127_adh	TTGTTACTGAAGGCTTCGAGT	Chr.3	5855135	5855115	-	No
2318537_adh	TGGAAATTGTGGAAAATTGCA	Chr.3	5856482	5856462	-	No
2745647_adh	TTTTTCACGATGGCAACTGTC	Chr.3	5876281	5876261	-	No
2075735_adh	TGAAAGAATTGTGGATTTTGA	Chr.3	5893445	5893465	+	No
2514620_adh	TGTGGAAGACGGAATTTGTGG	Chr.3	5898218	5898238	+	No
2740217_adh	TTTTCTTCTTCGTTTCCGGCC	Chr.3	5906275	5906295	+	No
1927847_adh	TATTGTATTTCGTTCTTTTTA	Chr.3	5925977	5925997	+	No
1912510_adh	TATGCATAGAATATGAAGACA	Chr.3	5934454	5934474	+	No
2705018_adh	TTTCCAGATCTTTCCATCATT	Chr.3	5939266	5939286	+	No
1938586_adh	TCAAGACTTTGACTGGAAAGA	Chr.3	5941181	5941201	+	No
2534143_adh	TGTTTTATCTGTCGTGTCTCG	Chr.3	5953202	5953222	+	No
2602667_adh	TTCGAATTTGTACAAATTTTT	Chr.3	5958564	5958544	-	No
1994589_adh	TCGAAAATAGGCAACGACGTC	Chr.3	5958778	5958798	+	No
1918519_adh	TATGTCATTTCGGAATGGTGT	Chr.3	5987242	5987262	+	No
2061589_adh	TCTTTGAAAATCGCAGAAGCT	Chr.3	5990594	5990574	-	No
2028059_adh	TCGTTCTAAAGTCGGTCTCCA	Chr.3	6020028	6020048	+	No
1994584_adh	TCGAAAATAAACACGAACTTC	Chr.3	6037656	6037636	-	No
2474214_adh	TGGTTAGAACACGGTGCACAA	Chr.3	6039925	6039945	+	No
2678183_adh	TTGTCGATGAAGGGCCCTGCT	Chr.3	6048322	6048342	+	No
2270073_adh	TGATCGTTGAAGCCGCTGAAA	Chr.3	6065961	6065941	-	No
2714190_adh	TTTGAATATTCAGATATGTAG	Chr.3	6067155	6067175	+	No
2729762_adh	TTTGTGCTCTCTGAGACAGCC	Chr.3	6106584	6106604	+	No
2057444_adh	TCTTGAACATCTGGCATTTGG	Chr.3	6111290	6111270	-	No
2332095_adh	TGGAATATATGATATGATGAT	Chr.3	6123954	6123934	-	No

2710559_adh	TTTCTGCAGAAAAGTTGTAAT	Chr.3	6124058	6124078	+	No
2710559_adh	TTTCTGCAGAAAAGTTGTAAT	Chr.3	6124069	6124049	-	No
2056945_adh	TCTTCTCGACATGATTGACGG	Chr.3	6124952	6124932	-	No
2391216_adh	TGGATGCTGAAAGATTTGTTG	Chr.3	6130925	6130905	-	No
2666547_adh	TTGGCTTACAGATGAAATTAC	Chr.3	6140937	6140917	-	No
2701226_adh	TTTCAACAGATTCAACGCCGG	Chr.3	6141047	6141067	+	No
2070833_adh	TGAAAATTTTAATCGGACAGA	Chr.3	6149714	6149734	+	No
2189153_adh	TGAGAGTAAGCGTTGAATTGA	Chr.3	6152189	6152169	-	No
2681786_adh	TTGTGGGAACTGATTATGGAG	Chr.3	6167572	6167552	-	No
1861379_adh	TAGGAAGTGGCAAACTAGCTG	Chr.3	6172883	6172903	+	No
2295414_adh	TGCGAAACTGAATTTTTAGCT	Chr.3	6173112	6173092	-	No
1798636_adh	TAATCGTCCTGGCGCGTTTGC	Chr.3	6173153	6173133	-	No
2554425_adh	TTACAGTATTTTCGGATGAAT	Chr.3	6173213	6173233	+	No
2005806_adh	TCGCAATCATTCTTTTCGTTC	Chr.3	6198904	6198924	+	No
1973935_adh	TCATTCTTTTCGTTCGGTTCA	Chr.3	6198910	6198930	+	No
2062609_adh	TCTTTTCGTTCGGTTCATCCA	Chr.3	6198914	6198934	+	No
2511932_adh	TGTGATTATGACGGACAAAGA	Chr.3	6200248	6200228	-	No
2669692_adh	TTGGTAGAAAATGTGGTGGTG	Chr.3	6208263	6208283	+	No
2691144_adh	TTTAATCAAAAATCGTCATAA	Chr.3	6241706	6241726	+	No
1855193_adh	TAGCATCTAAAAGTGAGGGAA	Chr.3	6248097	6248117	+	No
2583087_adh	TTATTGGATATTCTGGAGATG	Chr.3	6250683	6250703	+	No
2590667_adh	TTCAGAGGAAGTGAAAGAGCA	Chr.3	6278488	6278468	-	No
1799698_adh	TAATGATCACAAAAAGGGCAT	Chr.3	6279947	6279927	-	No
1821669_adh	TACGAAGAACACAAGAAAAAG	Chr.3	6281029	6281049	+	No
2702989_adh	TTTCAGCAGACTATGGACGCA	Chr.3	6301906	6301926	+	No
1993497_adh	TCCTTGATGCTCGGAAGGTGA	Chr.3	6308154	6308134	-	No
2640065_adh	TTGAGAGTGGCCGATTATTTT	Chr.3	6329295	6329275	-	Yes
2005105_adh	TCGATTGGCGACAGAAGAACG	Chr.3	6339654	6339674	+	No
1814245_adh	TACATTATTTTGACAAAATCG	Chr.3	6342358	6342378	+	No
1931913_adh	TCAAAAATCGATCGATAGACA	Chr.3	6344704	6344724	+	No
1805876_adh	TAATTTTCAAGCGTTCGTGTA	Chr.3	6344736	6344756	+	No
2749115_adh	TTTTTTCTTGAATCGGATGGG	Chr.3	6344812	6344792	-	No
2119539_adh	TGAACAAAATTGCGAGACATA	Chr.3	6362365	6362385	+	No
2240140_adh	TGAGGGCATCTGAGCTCAGAA	Chr.3	6367810	6367790	-	No
2593475_adh	TTCATAAGCATTGTTCTGACA	Chr.3	6374080	6374060	-	No
2695946_adh	TTTAGCCAATCATAGGGCACT	Chr.3	6375050	6375030	-	No
2595541_adh	TTCATTTTAGCCAATCATAGG	Chr.3	6375055	6375035	-	No
1972920_adh	TCATGTCATCTGAACTATTGC	Chr.3	6375193	6375213	+	No
2290314_adh	TGCATCGCTTTTCTCAATGTT	Chr.3	6375221	6375241	+	No
2538110_adh	TTAAAACAAGCAAACCGTATT	Chr.3	6379091	6379071	-	No
2137785_adh	TGAATACTGGAGTAGAAGCTT	Chr.3	6395472	6395452	-	No
2306449_adh	TGCTATCGGATATTTGAAAAT	Chr.3	6411333	6411313	-	No
1825270_adh	TACGCTCGTCATCTCGATTAT	Chr.3	6441089	6441069	-	No
1901728_adh	TATGAAGACACACAGACACAC	Chr.3	6444435	6444415	-	No
2635320_adh	TTGACCAACTGCAGACAAAAC	Chr.3	6447377	6447397	+	No
2641159_adh	TTGAGGAATACGTAGAAGAAA	Chr.3	6447544	6447524	-	No
2716850_adh	TTTGAGGAATACGTAGAAGAA	Chr.3	6447545	6447525	-	No
1953401_adh	TCACGGTAGATGTCGGTGGTT	Chr.3	6452904	6452924	+	No
1749552_adh	TAAAAGCCATTAGAATCTCGA	Chr.3	6454247	6454267	+	No
2694120_adh	TTTACTAATGGCTGAAGATTT	Chr.3	6551176	6551156	-	No

2562174_adh	TTAGACGACACTTTGACTATT	Chr.3	6558605	6558585	-	No
2060856_adh	TCTTTCCTGTAGTTAGCCCAA	Chr.3	6558643	6558623	-	No
2589787_adh	TTCACGTGAACTGAATCGTAC	Chr.3	6563700	6563680	-	No
2589787_adh	TTCACGTGAACTGAATCGTAC	Chr.3	6575438	6575418	-	No
1932854_adh	TCAAACTGCCGTCGGTAGCAA	Chr.3	6608213	6608193	-	No
2741367_adh	TTTTGAGAAGGAATGAAAGCA	Chr.3	6608342	6608362	+	No
2716386_adh	TTTGAGAAGGAATGAAAGCAG	Chr.3	6608343	6608363	+	No
2555687_adh	TTACCAGGGTTCATTTACCAG	Chr.3	6615263	6615283	+	No
2526675_adh	TGTTCGTTGGAGATTTTCATC	Chr.3	6625392	6625372	-	No
2683750_adh	TTGTTCGTTGGAGATTTTCAT	Chr.3	6625393	6625373	-	No
2477232_adh	TGGTTGTTCGTTGGAGATTTT	Chr.3	6625396	6625376	-	No
2590041_adh	TTCACTGGTTGTTCGTTGGAG	Chr.3	6625401	6625381	-	No
2526863_adh	TGTTCTCTCCAGATTTGAGGC	Chr.3	6626434	6626414	-	No
1825624_adh	TACGGAAATCTGAAAAAGTGG	Chr.3	6630498	6630518	+	No
2587413_adh	TTCAAGTTCTGAACAAGAAGA	Chr.3	6645539	6645519	-	No
2273278_adh	TGATGTCAGATAGAAGTCAGT	Chr.3	6646781	6646761	-	No
2617731_adh	TTCTGAACCAAACTTTGTATA	Chr.3	6691564	6691584	+	No
2606284_adh	TTCGCAGAAGAACAATATTTA	Chr.3	6710383	6710403	+	No
1765790_adh	TAACATATTCTGCTTCGCATG	Chr.3	6711809	6711829	+	No
2300367_adh	TGCGCTCTGTCGAACTGTCCA	Chr.3	6731426	6731446	+	No
2689803_adh	TTTAACAGGATTCTTGGCATT	Chr.3	6741513	6741493	-	No
1925157_adh	TATTCTTACATATTTCAAAAT	Chr.3	6794506	6794526	+	No
1931992_adh	TCAAAACACTGGCTGAATACT	Chr.3	6796983	6796963	-	No
2624939_adh	TTGAAAGAAAAGAAGGATGTG	Chr.3	6813331	6813351	+	No
1812386_adh	TACAGCTTGTCGGAGCAAGAT	Chr.3	6819983	6820003	+	No
1829905_adh	TACTAGAAACAGGAGGTGACC	Chr.3	6828725	6828705	-	No
2250436_adh	TGAGTAACGACATGACGAACA	Chr.3	6829387	6829367	-	No
1832400_adh	TACTGATGAGAAGCATACTGA	Chr.3	6860436	6860456	+	No
2746017_adh	TTTTTCGCAGGGAATATCGGT	Chr.3	6869357	6869337	-	No
2299277_adh	TGCGATGGATGGGAGAATGGA	Chr.3	6871755	6871735	-	No
1981250_adh	TCCCCTAGGTGGCTTGACTTT	Chr.3	6887679	6887659	-	No
2050059_adh	TCTGGCAGCTCTTGAAGACAA	Chr.3	6900236	6900256	+	No
1967535_adh	TCAGTTGGAAGTTGGTTTGGA	Chr.3	6923392	6923372	-	No
2048754_adh	TCTGGAACTGGAAAGACTGCA	Chr.3	6930344	6930364	+	No
2288393_adh	TGCAGCTGTGAGAAAATGAGC	Chr.3	6946450	6946430	-	No
2486436_adh	TGTACAGCAGAATGGAGGATC	Chr.3	6946477	6946457	-	No
2538534_adh	TTAAAACGGAACGGATCTTGG	Chr.3	6946527	6946547	+	No
1953015_adh	TCACGAATGTCGTCGTCGAAT	Chr.3	6951332	6951352	+	No
2411443_adh	TGGCCTTGCTCAGACATTTAT	Chr.3	6951818	6951798	-	No
2021606_adh	TCGTAAGAGAATTCGGATATT	Chr.3	6953995	6953975	-	No
1999551_adh	TCGACAACTGCTGAAGAATCT	Chr.3	6954034	6954054	+	No
2748912_adh	TTTTTTCGGGTTGATTAAAAA	Chr.3	6961389	6961409	+	No
1747421_adh	TAAAACAAGCAAACGAACCAT	Chr.3	6968454	6968474	+	No
1988792_adh	TCCGTTGAGAAGTCTGATGGA	Chr.3	6970149	6970129	-	No
2308814_adh	TGCTGGAATCGTCGAGCTCAT	Chr.3	6970273	6970253	-	No
1924628_adh	TATTCGTCGTGGATCCGTCTG	Chr.3	6970559	6970539	-	No
2011696_adh	TCGGACGTGTTGAGACCGGAA	Chr.3	6970725	6970705	-	No
2597047_adh	TTCCAGTCGGACGTGTTGAGA	Chr.3	6970731	6970711	-	No
1978684_adh	TCCAGGATGTGTACAAGATCG	Chr.3	6970767	6970747	-	No
2393006_adh	TGGATTCAACGGAGACAACAT	Chr.3	6970934	6970914	-	No

2049936_adh	TCTGGATTCAACGGAGACAAC	Chr.3	6970936	6970916	-	No
2049937_adh	TCTGGATTCATCAAGAAGATC	Chr.3	6970993	6970973	-	No
2294938_adh	TGCCTGCAACAAGATGGACTC	Chr.3	6971066	6971046	-	No
2048813_adh	TCTGGAAGTTCGAGACTGCCA	Chr.3	6971334	6971314	-	No
2668347_adh	TTGGGTCCTCGACAAGCTGAA	Chr.3	6971393	6971373	-	No
2150572_adh	TGACAAGAGAACCATCGAGAA	Chr.3	6971515	6971495	-	No
2025358_adh	TCGTCATCGGACACGTCGACT	Chr.3	6971585	6971565	-	No
1969384_adh	TCATATCAACATCGTCGTCAT	Chr.3	6971599	6971579	-	No
1854559_adh	TAGCAGCCATGGGAAAAGAAA	Chr.3	6971624	6971604	-	No
1973091_adh	TCATGTTGGTTTTTTGTTGGT	Chr.3	6971955	6971935	-	No
2529408_adh	TGTTGCATACTTTTTCTCTCT	Chr.3	6972091	6972111	+	No
2289889_adh	TGCATACTTTTTCTCTCTGGC	Chr.3	6972094	6972114	+	No
2569482_adh	TTAGGTGTACTCTTGCATATT	Chr.3	6977051	6977071	+	No
1996100_adh	TCGAAGAGACCTAGAGACATC	Chr.3	6979026	6979006	-	No
1755919_adh	TAAACTTGGTTTTTCTGTCGT	Chr.3	6982249	6982269	+	No
1755919_adh	TAAACTTGGTTTTTCTGTCGT	Chr.3	6985151	6985131	-	No
2733009_adh	TTTTAAAAGGAGGACCAATTA	Chr.3	7002708	7002728	+	No
2087389_adh	TGAAATAGAAGGTGAACCAAA	Chr.3	7057308	7057328	+	No
2618938_adh	TTCTGGAGACACTGCATTTTC	Chr.3	7064144	7064164	+	No
2488536_adh	TGTAGGAAAACGGATGACACT	Chr.3	7083076	7083096	+	No
2651698_adh	TTGCGAAATGGGAAAGTTGAC	Chr.3	7084929	7084909	-	No
2723473_adh	TTTGGAGAAAGAAGGGTAATT	Chr.3	7088087	7088107	+	No
2646997_adh	TTGATTTTAAAAATGTAGTGC	Chr.3	7088223	7088203	-	No
2319052_adh	TGGAACAGTCGAATGCAGCAA	Chr.3	7102320	7102340	+	No
2049989_adh	TCTGGCAAGAGAGAACACGTG	Chr.3	7102514	7102534	+	No
2292350_adh	TGCCAGAATGGACACGTTGAT	Chr.3	7102520	7102500	-	No
2704750_adh	TTTCATTTGTTTCAGTTGGTT	Chr.3	7104072	7104052	-	No
2595989_adh	TTCCAATTGTAGAGGCGGCAA	Chr.3	7107591	7107571	-	No
1931749_adh	TCAAAAAATGAGGGCATGTAA	Chr.3	7118208	7118228	+	No
2616458_adh	TTCTCGAATACCGAAGAACGT	Chr.3	7138205	7138185	-	No
2084467_adh	TGAAAGTTGAGTAGAAACGTG	Chr.3	7141193	7141173	-	No
2516313_adh	TGTGGGAAGTAGAAGTGTTAC	Chr.3	7145690	7145670	-	No
2374337_adh	TGGACATGATGATGATGATGA	Chr.3	7152722	7152702	-	No
2738348_adh	TTTTCGCAATTTTGACTCGAT	Chr.3	7162344	7162324	-	No
1918901_adh	TATGTGACGTTTCGGGCCTTT	Chr.3	7162460	7162480	+	No
2504450_adh	TGTGACGTTTCGGGCCTTTTC	Chr.3	7162462	7162482	+	No
1979399_adh	TCCATAATCAGTGACTGCAAA	Chr.3	7171512	7171532	+	No
1979399_adh	TCCATAATCAGTGACTGCAAA	Chr.3	7176008	7175988	-	No
2341812_adh	TGGAATGAAGATTGGAAGAAT	Chr.3	7179049	7179029	-	No
1865660_adh	TAGGATGCTGGTTTTTTCGAT	Chr.3	7179838	7179858	+	No
2046038_adh	TCTGACTTTGAAAGAATGCGT	Chr.3	7180597	7180617	+	No
2709898_adh	TTTCTCATCGTCGATATTTTT	Chr.3	7181484	7181464	-	No
2012626_adh	TCGGAGCAGAAGTTGGAACTG	Chr.3	7197833	7197813	-	No
2701650_adh	TTTCAATAAAGATTGTTATAA	Chr.3	7198867	7198847	-	No
2590268_adh	TTCAGAAAGTTATTTAGGCAA	Chr.3	7207928	7207908	-	No
2585244_adh	TTCAAAGAAACGGAACGAAAT	Chr.3	7208759	7208779	+	No
2039966_adh	TCTCGGACATTCCAAGACTGT	Chr.3	7208784	7208804	+	No
2282539_adh	TGCAAGTAGCCTGACAATCTT	Chr.3	7209839	7209819	-	No
2716449_adh	TTTGAGACGATGAGCAGATGC	Chr.3	7217036	7217016	-	No
2664754_adh	TTGGATTTCTCAAGTACAACA	Chr.3	7219761	7219741	-	No

2615825_adh	TTCTATTTCTTTTTCGTTGCT	Chr.3	7238197	7238177	-	No
2698244_adh	TTTATATATGTAGAATCGTCA	Chr.3	7238340	7238360	+	No
1866030_adh	TAGGATGTATGAAGACAAATG	Chr.3	7241147	7241127	-	No
2748354_adh	TTTTTTATATTAATGTCGTCA	Chr.3	7241307	7241327	+	No
2387457_adh	TGGATAAGCATATAGACTCCA	Chr.3	7273712	7273732	+	No
2585091_adh	TTCAAACACAGAAGTAGAACA	Chr.3	7274587	7274607	+	No
1833733_adh	TACTGTAGGTTCCATGGTATT	Chr.3	7281241	7281221	-	No
2689407_adh	TTTAAATATTTTAACGATCTG	Chr.3	7310729	7310709	-	No
2714077_adh	TTTGAAGTGTGAACAAAAAAT	Chr.3	7333821	7333801	-	No
2746614_adh	TTTTTGACATGTTTGCTGCTG	Chr.3	7336364	7336384	+	No
1953132_adh	TCACGCGATTTAAAGACGTAA	Chr.3	7336407	7336427	+	No
2039055_adh	TCTCCTACTTGCATATAAAAT	Chr.3	7337043	7337063	+	No
2148228_adh	TGAATTTCAGCATTCTCTCGG	Chr.3	7359205	7359225	+	No
2029494_adh	TCTAAATAGCTGGAGAACAAT	Chr.3	7362105	7362125	+	No
1821938_adh	TACGAATTATGACGAAGATAT	Chr.3	7377230	7377250	+	No
2589091_adh	TTCACAAGGATTCATACTTTT	Chr.3	7381947	7381967	+	No
1838890_adh	TAGAACTTGAATAGCAAAAGC	Chr.3	7463489	7463469	-	No
2007681_adh	TCGCGAATTGTACATGAGTGT	Chr.3	7470752	7470732	-	No
2379582_adh	TGGAGAAGAGTGTATTGCTGG	Chr.3	7482320	7482300	-	No
2046651_adh	TCTGATAGAAAGGAAGAAGAA	Chr.3	7488292	7488272	-	No
2490443_adh	TGTATAATTTGTAGAAGTTGA	Chr.3	7495355	7495335	-	No
1939463_adh	TCAAGGAAACTGCAGAAACTT	Chr.3	7496646	7496626	-	No
2579361_adh	TTATGGAGCTTGTGATAAAAG	Chr.3	7509626	7509646	+	No
2702868_adh	TTTCAGAATCAGAACACCACG	Chr.3	7514779	7514759	-	No
2615160_adh	TTCTAGTTAGACGATGCTCCT	Chr.3	7515092	7515072	-	No
2712852_adh	TTTGAAATTTGAAGATCTGGC	Chr.3	7571512	7571492	-	No
2706451_adh	TTTCCTTGTACGTTTCAATGA	Chr.3	7571671	7571691	+	No
2667129_adh	TTGGGAGAGAAGATCGTTCGT	Chr.3	7572342	7572322	-	No
1939013_adh	TCAAGATGGAAACAGAACTGT	Chr.3	7572430	7572410	-	No
1874752_adh	TAGTAGGATTTACGCGGGCGT	Chr.3	7584399	7584419	+	No
1971650_adh	TCATGAAAAAGTTTAGACGTG	Chr.3	7593393	7593413	+	No
2713903_adh	TTTGAAGCCGTAGAAAAGAAA	Chr.3	7601177	7601157	-	No
2055462_adh	TCTTATTTTGAAGCCGTAGAA	Chr.3	7601183	7601163	-	No
2584124_adh	TTATTTGGAAGAAGACAAAAA	Chr.3	7605266	7605286	+	No
2161983_adh	TGACGGTGAGGACTCTACTTC	Chr.3	7630503	7630523	+	No
2034703_adh	TCTATGTGATACTGACGACAT	Chr.3	7643141	7643121	-	No
2748789_adh	TTTTTTCCTTCGAAATCATCG	Chr.3	7643942	7643922	-	No
2238880_adh	TGAGGCGAATAATTAAACATA	Chr.3	7650397	7650417	+	No
1985176_adh	TCCGATTTGAGCTGTGTGACG	Chr.3	7662194	7662214	+	No
2677375_adh	TTGTATTTCGTTAGACATTTT	Chr.3	7667541	7667521	-	No
1894842_adh	TATCATTCGTTGGGAAGGCTT	Chr.3	7677143	7677163	+	No
2695873_adh	TTTAGATTTTGCATAATAATT	Chr.3	7682452	7682432	-	No
2737800_adh	TTTTCATTTAGATTTTGCATA	Chr.3	7682458	7682438	-	No
2624050_adh	TTCTTTTTGTCGGTTTTTAAA	Chr.3	7688967	7688947	-	No
2695239_adh	TTTAGACTGAGTTTCACCGAG	Chr.3	7708538	7708518	-	No
2717230_adh	TTTGATAACTTCGGATGGAAA	Chr.3	7712153	7712173	+	No
2146198_adh	TGAATTCGTAGAAAAGAAAAA	Chr.3	7715202	7715182	-	No
2055002_adh	TCTTATATTGAATTCGTAGAA	Chr.3	7715210	7715190	-	No
2737428_adh	TTTTCAGAAACACGGAATCCA	Chr.3	7727556	7727536	-	No
1953050_adh	TCACGACTTAGAGAATCATCA	Chr.3	7727863	7727843	-	No

2007730_adh	TCGCGATCGATTTTTAGAAAA	Chr.3	7766136	7766156	+	No
2699697_adh	TTTATTCAATTGCATTCGTTC	Chr.3	7766166	7766186	+	No
1943089_adh	TCAATTGCATTCGTTCGAATA	Chr.3	7766171	7766191	+	No
2528179_adh	TGTTGACTATGAAGATGCTCC	Chr.3	7768555	7768535	-	No
2010436_adh	TCGGAAATGTGTTCTTTTCGA	Chr.3	7770051	7770031	-	No
1970207_adh	TCATATTCGGAAATGTGTTCT	Chr.3	7770057	7770037	-	No
2259356_adh	TGATACAGCACTGAGGAGACG	Chr.3	7772409	7772429	+	No
1912559_adh	TATGCATTAAATCTGTCGGTA	Chr.3	7777736	7777756	+	No
1854491_adh	TAGCAGAGGGAAGAAGAAACG	Chr.3	7780519	7780499	-	No
2014926_adh	TCGGATTGTGGAGGAGGTCAA	Chr.3	7780736	7780756	+	No
2267971_adh	TGATATTATTAAGTACCAAAT	Chr.3	7811405	7811385	-	No
2723176_adh	TTTGGAATCTGATAGAGGACT	Chr.3	7820614	7820594	-	No
2702601_adh	TTTCACGTCAATATATAAAAC	Chr.3	7820677	7820697	+	No
2657086_adh	TTGGAAGAGTTGAACGAGCGT	Chr.3	7826075	7826055	-	No
1774230_adh	TAAGAAGCTGAAGAAGATTGT	Chr.3	7840956	7840976	+	No
2041982_adh	TCTCTACTGTAAGAAGAAAGG	Chr.3	7847540	7847560	+	No
2020796_adh	TCGTAAAAAACGTGTTCTCAG	Chr.3	7850772	7850752	-	No
1897515_adh	TATCGCTTTGACGGAATCATA	Chr.3	7854521	7854501	-	No
2745378_adh	TTTTTATGGAGGATTTCGCAG	Chr.3	7861722	7861742	+	No
2220806_adh	TGAGATGGAAAGATGTATCTA	Chr.3	7861833	7861813	-	No
2700891_adh	TTTATTTTTGAATTTGGAAAC	Chr.3	7911386	7911366	-	No
1795252_adh	TAATAGTAAGAGGACAATCAA	Chr.3	7911781	7911801	+	No
2607143_adh	TTCGGAACATGTGAACATTTT	Chr.3	7928896	7928916	+	No
2714488_adh	TTTGAATTGAGAACGTTTTCC	Chr.3	7940987	7941007	+	No
2633820_adh	TTGAATTGAGAACGTTTTCCT	Chr.3	7940988	7941008	+	No
2738135_adh	TTTTCCTTGCATTTCTCCAGT	Chr.3	7952143	7952123	-	No
2301472_adh	TGCGGCAAGAGAGATTTCGTG	Chr.3	7954942	7954922	-	No
2738134_adh	TTTTCCTTGCAAATCGTTTGA	Chr.3	7958080	7958060	-	No
1971820_adh	TCATGACTGTCTGCGGTAAAA	Chr.3	7959254	7959234	-	No
1923803_adh	TATTCATGACTGTCTGCGGTA	Chr.3	7959257	7959237	-	No
2537572_adh	TTAAAAACTCGCAAGCATCCT	Chr.3	7959444	7959424	-	No
2286802_adh	TGCACTGTAACGGTAGACTTT	Chr.3	7964221	7964201	-	No
2537572_adh	TTAAAAACTCGCAAGCATCCT	Chr.3	7966257	7966277	+	No
1923803_adh	TATTCATGACTGTCTGCGGTA	Chr.3	7966444	7966464	+	No
1971820_adh	TCATGACTGTCTGCGGTAAAA	Chr.3	7966447	7966467	+	No
1968835_adh	TCATACTGGCTGAGTAGAAGC	Chr.3	7967801	7967821	+	No
2699136_adh	TTTATGCGGAAGAATTTTTAA	Chr.3	7969141	7969121	-	No
1821987_adh	TACGAATTTTATGCGGAAGAA	Chr.3	7969148	7969128	-	No
1990978_adh	TCCTGAAAAGATGGTTGCCGA	Chr.3	7973209	7973189	-	No
2589874_adh	TTCACTCCTGAAAAGATGGTT	Chr.3	7973214	7973194	-	No
1993024_adh	TCCTTCGTGTTTCACTCCTGA	Chr.3	7973224	7973204	-	No
1968634_adh	TCATACCTTCGTCTTCGTCCA	Chr.3	7974602	7974622	+	No
2378915_adh	TGGACTTCTGCCAAGGACGTT	Chr.3	7975133	7975153	+	No
1925278_adh	TATTGAAAGAGACGGACTCTC	Chr.3	7975690	7975710	+	No
2296920_adh	TGCGAGACTCGTCGGCCGAAA	Chr.3	7977945	7977965	+	No
2490187_adh	TGTAGTTGTGAGTAGAATGGC	Chr.3	7985982	7985962	-	No
2674952_adh	TTGTAGATAGAAAGCGAATGG	Chr.3	8024699	8024719	+	No
2389597_adh	TGGATCATTCAGTAGAGTCTT	Chr.3	8028523	8028543	+	No
2308733_adh	TGCTGCTAACACCGTCGTCGT	Chr.3	8029217	8029237	+	No
1967687_adh	TCAGTTTACTTTTGGCAGAAC	Chr.3	8029650	8029670	+	No

2646324_adh	TTGATTGAGAAGTGGCGATGA	Chr.3	8035988	8036008	+	No
2451196_adh	TGGGCTGGCGATGATCGGATC	Chr.3	8048233	8048253	+	No
2576932_adh	TTATCACATTAACACTAAATC	Chr.3	8049055	8049035	-	No
1975559_adh	TCATTTTGATTCTTCCGTCGG	Chr.3	8050200	8050180	-	No
1980608_adh	TCCCAGTTGACGAAAAACAGC	Chr.3	8079027	8079047	+	No
1848124_adh	TAGAGCGCATACTACGGGGCA	Chr.3	8080288	8080268	-	No
2521536_adh	TGTGTTTGTATCAGCTGATGA	Chr.3	8093221	8093241	+	No
2689348_adh	TTTAAAGTAGATCTCGGTGAA	Chr.3	8106297	8106317	+	No
2500192_adh	TGTCTTGAACTAGCATCTCGG	Chr.3	8129020	8129000	-	No
1940096_adh	TCAAGTAGATGAAAACATGTA	Chr.3	8129279	8129299	+	No
1860724_adh	TAGGAAATTGTAAAGAAGCCA	Chr.3	8129368	8129388	+	No
2746440_adh	TTTTTCTTGAGACAAGTTGGA	Chr.3	8162895	8162875	-	No
2373028_adh	TGGAATTTTCATGCACGGAAA	Chr.3	8169612	8169632	+	No
2031128_adh	TCTACAACAAGAAGCTGATGG	Chr.3	8182222	8182242	+	No
2032293_adh	TCTAGAAACTATGAAACGGCC	Chr.3	8194625	8194645	+	No
1895559_adh	TATCCATGTAGAACATCGCAT	Chr.3	8234497	8234477	-	No
1809808_adh	TACAATGAGTTAACGGAACAG	Chr.3	8246405	8246385	-	No
1811096_adh	TACACGGAAGAACTCGGCACT	Chr.3	8247684	8247664	-	No
1888232_adh	TATAGCAGAACACAAAAAGAT	Chr.3	8258064	8258044	-	No
2589761_adh	TTCACGTATCTAGACATTTGA	Chr.3	8264946	8264926	-	No
2742705_adh	TTTTGGACGAGAAGAGACAGA	Chr.3	8268451	8268471	+	No
2140041_adh	TGAATATGGATTATTTGTCAC	Chr.3	8273368	8273388	+	No
2313365_adh	TGGAAAACAGGGAGAATTATG	Chr.3	8310262	8310242	-	No
2656248_adh	TTGGAAAACAGGGAGAATTAT	Chr.3	8310263	8310243	-	No
2701073_adh	TTTCAAAGAAAACGGAGGGAG	Chr.3	8319254	8319274	+	No
2744292_adh	TTTTGTTGAGAAAAAGAGAAT	Chr.3	8319292	8319312	+	No
2050817_adh	TCTGGTCTGGACTTTTCGGTG	Chr.3	8331285	8331265	-	No
1991088_adh	TCCTGAATAAGACTCAATCTA	Chr.3	8341259	8341279	+	No
2161671_adh	TGACGGAGACGTATACACTTG	Chr.3	8343816	8343836	+	No
1991088_adh	TCCTGAATAAGACTCAATCTA	Chr.3	8349454	8349474	+	No
2161671_adh	TGACGGAGACGTATACACTTG	Chr.3	8352011	8352031	+	No
2744159_adh	TTTTGTTCGGAGCACGTGCGA	Chr.3	8353508	8353488	-	No
1977473_adh	TCCACGTATCGTTGCTGACAT	Chr.3	8354229	8354209	-	No
1838965_adh	TAGAAGAAGAACAAGAACGGA	Chr.3	8360777	8360797	+	No
2042028_adh	TCTCTAGATTTCTGACGGCAA	Chr.3	8370167	8370147	-	No
2713225_adh	TTTGAACGCGTCGAAGAAGAA	Chr.3	8370906	8370886	-	No
2054042_adh	TCTTACAATTTGAACGCGTCG	Chr.3	8370914	8370894	-	No
2055634_adh	TCTTCAGGATCGTCTTCATTC	Chr.3	8403783	8403803	+	No
2126942_adh	TGAAGAAATGGATTTTTCTCT	Chr.3	8405625	8405645	+	No
2733895_adh	TTTTAATTTGTCGCCTCGGAA	Chr.3	8411647	8411667	+	No
2576259_adh	TTATATTACAGAATGCCTCGG	Chr.3	8429246	8429266	+	No
1790734_adh	TAATAATCAAGAGAAGTCTAC	Chr.3	8443516	8443496	-	No
1884142_adh	TATAAATAAGTAGGAGATTCA	Chr.3	8443769	8443789	+	No
2124821_adh	TGAACTCTCTTCGCATCGTCA	Chr.3	8446154	8446134	-	No
2036692_adh	TCTCAATGTGCGGTAAGAAGC	Chr.3	8451237	8451217	-	No
2724816_adh	TTTGGGATGGATCAGAAGACT	Chr.3	8459672	8459692	+	No
2256078_adh	TGAGTTCTCAAGAAAACTGCA	Chr.3	8470142	8470122	-	No
2514638_adh	TGTGGAAGATTCGATTCGGCC	Chr.3	8491218	8491238	+	No
1923502_adh	TATTCAGATGGCTCTTGTTTC	Chr.3	8505960	8505980	+	No
2259073_adh	TGATAATAGTAGTTGTAGGTC	Chr.3	8520037	8520057	+	No

2687092_adh	TTGTTTCATATTCTGGTCGGA	Chr.3	8529124	8529144	+	No
2002390_adh	TCGAGTGGTTAGTGCGAATGA	Chr.3	8541132	8541152	+	No
2740094_adh	TTTTCTTCGCGGCATGTCATT	Chr.3	8552191	8552171	-	No
2296254_adh	TGCGACAAGAATTTTCAAAAT	Chr.3	8609339	8609319	-	No
2080222_adh	TGAAAGAGCAATGGTTGAAAA	Chr.3	8610123	8610103	-	No
2130192_adh	TGAAGATGTAGATGATGGATT	Chr.3	8612567	8612547	-	No
1757668_adh	TAAAGCTGGAATGAATGGACT	Chr.3	8628384	8628364	-	No
2046512_adh	TCTGAGTCAAGGTGGAATGAT	Chr.3	8644013	8644033	+	No
2740326_adh	TTTTCTTTGAAGCGCGCTCGT	Chr.3	8717390	8717410	+	No
2046095_adh	TCTGAGAATTAGAATGATGAT	Chr.3	8732664	8732684	+	No
1836729_adh	TAGAAAAGCAAGTAGAATCAT	Chr.3	8743727	8743747	+	No
1975736_adh	TCCAAAATAACTTTGAGAGGG	Chr.3	8754848	8754868	+	No
2657824_adh	TTGGAATTGTATCAAGGCGTA	Chr.3	8767216	8767196	-	No
1926838_adh	TATTGGAATTGTATCAAGGCG	Chr.3	8767218	8767198	-	No
2739406_adh	TTTTCTCTTGTCATCGGTTTA	Chr.3	8767966	8767946	-	No
2738275_adh	TTTTCGATAATCTACTGGACT	Chr.3	8793525	8793545	+	No
1763967_adh	TAACAATATGAGATCAACGAA	Chr.3	8793721	8793701	-	No
1846152_adh	TAGACTTAGAATGTAGAGGAA	Chr.3	8795562	8795542	-	No
2658089_adh	TTGGACATAGTAAGAGTGTTA	Chr.3	8795581	8795561	-	No
2062982_adh	TCTTTTTGCCTCGTCGCGTGT	Chr.3	8795935	8795955	+	No
2131958_adh	TGAAGCTGAATATGAACGAAA	Chr.3	8847129	8847109	-	No
2034207_adh	TCTATCGCAGCATAATGGCTC	Chr.3	8850690	8850670	-	No
2043090_adh	TCTCTGTTCTATCGCAGCATA	Chr.3	8850697	8850677	-	No
2623051_adh	TTCTTGGATTTTGCTCGGACA	Chr.3	8852692	8852712	+	No
2058939_adh	TCTTGGATTTTGCTCGGACAC	Chr.3	8852693	8852713	+	No
2620185_adh	TTCTGTGGACAGGAACTTGAC	Chr.3	8852973	8852993	+	No
1784062_adh	TAAGGAAGCGTTGACAAGAAT	Chr.3	8857317	8857337	+	No
2623051_adh	TTCTTGGATTTTGCTCGGACA	Chr.3	8861065	8861085	+	No
2058939_adh	TCTTGGATTTTGCTCGGACAC	Chr.3	8861066	8861086	+	No
2620185_adh	TTCTGTGGACAGGAACTTGAC	Chr.3	8861346	8861366	+	No
2745519_adh	TTTTTATTTCTATAGACAAAC	Chr.3	8872120	8872100	-	No
2538544_adh	TTAAAACGGTGGCAAATACAA	Chr.3	8887205	8887185	-	No
1933745_adh	TCAAAGGCGAGTATACTGTAG	Chr.3	8887261	8887241	-	No
2235865_adh	TGAGCTTGAAAGTCAAAGGCG	Chr.3	8887273	8887253	-	No
2546983_adh	TTAAGACTGTGTTTCCAATCT	Chr.3	8893241	8893221	-	No
2701009_adh	TTTCAAACAAGGAAAACTCGG	Chr.3	8893979	8893959	-	No
2144898_adh	TGAATGTGGTGGGAAACGGAA	Chr.3	8904261	8904281	+	No
2635160_adh	TTGACAGTCGAACGTCGTGAC	Chr.3	8904300	8904320	+	No
1833124_adh	TACTGGAAGACTGAAAGCTAC	Chr.3	8912595	8912615	+	No
2619493_adh	TTCTGGGAAGAAACGGTGAGA	Chr.3	8918222	8918242	+	No
2722915_adh	TTTGGAAAGAGAAAGAGAGAC	Chr.3	8923355	8923335	-	No
2137748_adh	TGAATACTGAACGGATTTGAC	Chr.3	8935928	8935948	+	No
2057959_adh	TCTTGAGGATCTTAGTACAAG	Chr.3	8974847	8974827	-	No
1845433_adh	TAGACGGTTAGAGAAGATGAA	Chr.3	8985146	8985166	+	No
2300908_adh	TGCGGACCTATTTTGGCGGAA	Chr.3	8985587	8985607	+	No
1961248_adh	TCAGATGACTAATTACTTTCC	Chr.3	8998536	8998516	-	No
2138108_adh	TGAATAGAACGGATAAAATGC	Chr.3	8999133	8999153	+	No
2127955_adh	TGAAGACGGAATCAAATTTTG	Chr.3	9002066	9002086	+	No
2706452_adh	TTTCCTTGTCAGTCGTTGCGA	Chr.3	9002444	9002424	-	No
1763952_adh	TAACAATAAGTCGGGAAGTTC	Chr.3	9035479	9035499	+	No

2169665_adh	TGACTGATGATCTGGTAAGAA	Chr.3	9052763	9052783	+	No
2270497_adh	TGATCTGGTAAGAACGGAAAA	Chr.3	9052770	9052790	+	No
1773789_adh	TAAGAACGGAAAACGAAGAAC	Chr.3	9052778	9052798	+	No
1769572_adh	TAACGTGTAGAAAACAGTTGC	Chr.3	9052923	9052943	+	No
2084203_adh	TGAAAGTGAAGAATATGATAG	Chr.3	9054127	9054147	+	No
2661098_adh	TTGGATATGCAAAATTTTTTC	Chr.3	9054605	9054625	+	No
2318258_adh	TGGAAATGGAAAACAATAAGC	Chr.3	9059978	9059998	+	No
1790468_adh	TAATAAGCTTAATGGTTGGTT	Chr.3	9080349	9080329	-	No
2587066_adh	TTCAAGCCATGGTCGGACAAT	Chr.3	9080415	9080395	-	No
1980220_adh	TCCCAAGATGCCAAGGACTCC	Chr.3	9080540	9080520	-	No
2708831_adh	TTTCGTCAAGAAGGCTATCGC	Chr.3	9080712	9080692	-	No
1977145_adh	TCCACAGAACTTCGACGACAT	Chr.3	9081809	9081829	+	No
2587065_adh	TTCAAGCCATCGTCGGACAAT	Chr.3	9082330	9082350	+	No
2124122_adh	TGAACTATATGCTTTTGGACA	Chr.3	9086633	9086613	-	No
1876086_adh	TAGTCAAGTTTTTGCTCGGTG	Chr.3	9089523	9089503	-	No
2623264_adh	TTCTTTAGATATGAGTAACAG	Chr.3	9101268	9101288	+	No
2597020_adh	TTCCAGGTGTGTAATTCGGTA	Chr.3	9111056	9111076	+	No
1978814_adh	TCCAGGTGTGTAATTCGGTAA	Chr.3	9111057	9111077	+	No
2740248_adh	TTTTCTTGGCACATTTGCATA	Chr.3	9111722	9111702	-	No
2606180_adh	TTCGCAAATTCGAAAATGTAT	Chr.3	9130790	9130810	+	No
2625581_adh	TTGAAATGTGGCCGGCAACAA	Chr.3	9145040	9145060	+	No
2527460_adh	TGTTGAAATAGGAAGTGGAAT	Chr.3	9163957	9163937	-	No
2037351_adh	TCTCAGAGCGGTCTTTTGATG	Chr.3	9173898	9173878	-	No
2033947_adh	TCTATACTGTGGAAGGGTGTC	Chr.3	9176214	9176234	+	No
1962515_adh	TCAGCCGTTAGACGTGTTCAT	Chr.3	9178955	9178975	+	No
1887197_adh	TATACGGGATGAGAAGAAGTG	Chr.3	9186296	9186276	-	No
2674174_adh	TTGTACACAAAATCATCGGCA	Chr.3	9196543	9196563	+	No
2589176_adh	TTCACAGCTGAAGAATTTCAT	Chr.3	9228552	9228572	+	No
1797811_adh	TAATCACTAGGTTTCCCGTCA	Chr.3	9236620	9236600	-	No
2649815_adh	TTGCAGTGAGGTGACCGCTAA	Chr.3	9237419	9237439	+	No
2578832_adh	TTATGATGTCCATGCTCCCTC	Chr.3	9239996	9239976	-	No
2397378_adh	TGGCAACAGACTGTAATATAG	Chr.3	9269182	9269202	+	No
1886957_adh	TATACCACTTTACTCAATAAT	Chr.3	9273644	9273664	+	No
1761111_adh	TAAATCTGTTTGCACACATTT	Chr.3	9322026	9322006	-	No
2411085_adh	TGGCCGTAGTAGATTACTGTC	Chr.3	9331138	9331118	-	No
2132833_adh	TGAAGGAGAATGTGGTGAAGA	Chr.3	9339416	9339396	-	No
1966975_adh	TCAGTGGAGTTTGCAAACAAA	Chr.3	9393437	9393457	+	No
1920705_adh	TATTACATTTTAAGACTGGCG	Chr.3	9396316	9396296	-	No
2747130_adh	TTTTTGCTGATCTCTCTTCTC	Chr.3	9408542	9408562	+	No
2742533_adh	TTTTGCTGATCTCTCTTCTCG	Chr.3	9408543	9408563	+	No
1797890_adh	TAATCAGTTTTTTAACCCATA	Chr.3	9409344	9409324	-	No
2055004_adh	TCTTATATTTAAAGTCGTAGA	Chr.3	9409869	9409889	+	No
2541764_adh	TTAAAGTCGTAGAAAAGAAAA	Chr.3	9409877	9409897	+	No
2401648_adh	TGGCAAGCGGCTGCTGTCTCT	Chr.3	9443514	9443494	-	No
2651870_adh	TTGCGACAAGCATAGTTCTAG	Chr.3	9443643	9443663	+	No
2742554_adh	TTTTGCTGTAAAAACGTCATA	Chr.3	9451383	9451403	+	No
2389708_adh	TGGATCGAAGAAAAGGAATAT	Chr.3	9451439	9451419	-	No
2561689_adh	TTAGAATATGACAATACACGG	Chr.3	9453773	9453793	+	No
2012430_adh	TCGGAGATAGAAGAGAGAAGA	Chr.3	9454841	9454861	+	No
2554454_adh	TTACAGTTGGTTTTTAGTTCG	Chr.3	9456139	9456119	-	No

2577199_adh	TTATCATTCGAAATTGGCATA	Chr.3	9456313	9456333	+	No
1894835_adh	TATCATTCGAAATTGGCATAG	Chr.3	9456314	9456334	+	No
1995377_adh	TCGAAATTGGCATAGGTCTTA	Chr.3	9456320	9456340	+	No
2030545_adh	TCTAATCGCAAGCTTTACATC	Chr.3	9465417	9465397	-	No
1930790_adh	TATTTTATCGAAAACTCCCTC	Chr.3	9465495	9465475	-	No
2017490_adh	TCGGGTCTCGAAAAATCGGCA	Chr.3	9504398	9504418	+	No
2028842_adh	TCGTTTCAGCAATTTTTAGGC	Chr.3	9504526	9504546	+	No
1940760_adh	TCAAGTTTTAGGTCGTCTTGC	Chr.3	9504640	9504660	+	No
1971014_adh	TCATCGGAGAAGTTCAAGGCG	Chr.3	9504680	9504700	+	No
2309544_adh	TGCTGTGGAATTTACATTCGG	Chr.3	9507462	9507442	-	No
2675403_adh	TTGTAGTTTAGTATGAATTTT	Chr.3	9516634	9516614	-	No
2650186_adh	TTGCATCAGATGTTTTTAATT	Chr.3	9521715	9521695	-	No
2706170_adh	TTTCCTATTGCATCAGATGTT	Chr.3	9521722	9521702	-	No
2067082_adh	TGAAAACGAAAGAAACACCAA	Chr.3	9521952	9521932	-	No
2052321_adh	TCTGTCGAATCTGATTGGAGT	Chr.3	9522286	9522306	+	No
2488094_adh	TGTAGATTTACGGCGCTGAAC	Chr.3	9538355	9538375	+	No
2031539_adh	TCTACGACAAACGTTCTCCAT	Chr.3	9542992	9543012	+	No
1839954_adh	TAGAAGTTTTGCATGAATAGA	Chr.3	9545949	9545929	-	No
1751618_adh	TAAAATCGTCGCGTTTTCAGA	Chr.3	9565982	9565962	-	No
2005990_adh	TCGCACTGGCTTGAGGAATTA	Chr.3	9570209	9570189	-	No
2048716_adh	TCTGGAACAAGTATTCATTAC	Chr.3	9573960	9573940	-	No
1984760_adh	TCCGATATCGTTTGTGCAAAG	Chr.3	9580622	9580642	+	No
2054250_adh	TCTTAGAACAACAAATAGAAT	Chr.3	9580652	9580672	+	No
1813263_adh	TACATATGTGCCTATTTCTTG	Chr.3	9614270	9614250	-	No
1996570_adh	TCGAAGGATCGTCTCTTATAT	Chr.3	9615434	9615414	-	No
2608181_adh	TTCGGATCAAGTTACAGCAGC	Chr.3	9618398	9618418	+	No
2526728_adh	TGTTCTACCAGGCTGATGACG	Chr.3	9628614	9628594	-	No
2552572_adh	TTAATTTAACTCGTCGTTCAT	Chr.3	9630345	9630325	-	No
1929356_adh	TATTTATCGAGAATTAGTTTC	Chr.3	9631300	9631280	-	No
1954064_adh	TCACTATGACTGAGATCGCGG	Chr.3	9673228	9673208	-	No
2047699_adh	TCTGCAAAAACATGAATAGGA	Chr.3	9675171	9675151	-	No
2027897_adh	TCGTTATTTTGCACAAATAAA	Chr.3	9723173	9723153	-	No
2748297_adh	TTTTTTAGGATTTCAAGGAAT	Chr.3	9746825	9746845	+	No
2556903_adh	TTACGCCTTTTCTATCGTTGT	Chr.3	9747313	9747333	+	No
2558704_adh	TTACTCATGAATATCGAAGGG	Chr.3	9748708	9748688	-	No
2044135_adh	TCTGAAGAATGATCGCTGCAG	Chr.3	9748832	9748852	+	No
1919947_adh	TATTAAATAACACAGACGCAA	Chr.3	9753100	9753120	+	No
2579270_adh	TTATGGAAATCTGTTTCTCAG	Chr.3	9762386	9762366	-	No
2136613_adh	TGAATAACTTTGAAAATGGCG	Chr.3	9776001	9775981	-	No
1938592_adh	TCAAGAGAAACATTTCTGAAA	Chr.3	9779249	9779269	+	No
2381230_adh	TGGAGCGAACTTGCATTATTA	Chr.3	9780685	9780665	-	No
2738224_adh	TTTTCGACCAGTGACACACAG	Chr.3	9780766	9780786	+	No
2152779_adh	TGACACACAGTCGTATTGCTG	Chr.3	9780777	9780797	+	No
1929504_adh	TATTTCAATTCTCTCTCAATT	Chr.3	9781852	9781872	+	No
2666997_adh	TTGGGACTCTACGGTATGATT	Chr.3	9782599	9782619	+	No
2496561_adh	TGTCATTCAGAAACTTGCTTT	Chr.3	9793979	9793959	-	No
2237873_adh	TGAGGATATGGATGGATCATG	Chr.3	9815714	9815694	-	No
2341509_adh	TGGAATGAAGAAGAAAACGAA	Chr.3	9815776	9815756	-	No
2271452_adh	TGATGATAAGGAAGAATTCGT	Chr.3	9816919	9816899	-	No
2489336_adh	TGTAGGTGTACGGTAGACAAT	Chr.3	9818011	9818031	+	No

2559586_adh	TTACTGTCTGAAATAAACAAT	Chr.3	9827482	9827462	-	No
2656533_adh	TTGGAAATTTCGCAATTAACT	Chr.3	9827725	9827745	+	No
2709399_adh	TTTCTAAAAAATAAATTCGGC	Chr.3	9829883	9829863	-	No
2618978_adh	TTCTGGAGTACTGTAAGCGGG	Chr.3	9832600	9832620	+	No
2641778_adh	TTGAGTAAAATTACAATACTT	Chr.3	9834068	9834048	-	No
1978814_adh	TCCAGGTGTGTAATTCGGTAA	Chr.3	9849099	9849079	-	No
2597020_adh	TTCCAGGTGTGTAATTCGGTA	Chr.3	9849100	9849080	-	No
2623175_adh	TTCTTGTTCGAATGGATTTTT	Chr.3	9868165	9868145	-	No
1803641_adh	TAATTCTTGTTCGAATGGATT	Chr.3	9868168	9868148	-	No
2741897_adh	TTTTGATTATTTTTGTAGCAT	Chr.3	9893859	9893879	+	No
2181112_adh	TGAGAAGTAGTTATGAAAAAT	Chr.3	9916052	9916032	-	No
2181112_adh	TGAGAAGTAGTTATGAAAAAT	Chr.3	9925493	9925473	-	No
2250713_adh	TGAGTACAGTAGTTTCAAACA	Chr.3	9946901	9946921	+	No
2259625_adh	TGATACGGATTATACTGTGGT	Chr.3	9962020	9962000	-	No
2728701_adh	TTTGTCAAGATATAAGATATT	Chr.3	10020377	10020397	+	No
2728953_adh	TTTGTCGAGAATGGCAAACAA	Chr.3	10039993	10039973	-	No
2737298_adh	TTTTCAATTTGTCGAGAATGG	Chr.3	10040000	10039980	-	No
2581950_adh	TTATTCTGTGACACTTTCAGA	Chr.3	10047659	10047639	-	No
2743921_adh	TTTTGTGATGGAATCGTTGGA	Chr.3	10051649	10051629	-	No
1967000_adh	TCAGTGGATTTTGTGATGGAA	Chr.3	10051657	10051637	-	No
2593110_adh	TTCAGTGGATTTTGTGATGGA	Chr.3	10051658	10051638	-	No
2275155_adh	TGATTCAGTGGATTTTGTGAT	Chr.3	10051661	10051641	-	No
2611908_adh	TTCGTCATGATTTTCGTCGAT	Chr.3	10051795	10051815	+	No
2745342_adh	TTTTTATCTTAAATACATAGC	Chr.3	10088548	10088528	-	No
1924387_adh	TATTCGGACACATACAGAAAT	Chr.3	10098239	10098219	-	No
2648949_adh	TTGCACAGGTAGGATTTACGA	Chr.3	10114762	10114782	+	No
2595580_adh	TTCATTTTTCTTGTGTTTAAA	Chr.3	10120578	10120598	+	No
2046018_adh	TCTGACTTGAAGACTTTAACA	Chr.3	10122114	10122134	+	No
1828979_adh	TACGTTCTATGTAGGTTTGGA	Chr.3	10170634	10170654	+	No
2700683_adh	TTTATTTCAATGGACAATTCA	Chr.3	10170992	10171012	+	No
2012015_adh	TCGGAGAATCGAGAAGTTGAA	Chr.3	10182972	10182992	+	No
2747838_adh	TTTTTGTGTAAAACTCGAAAA	Chr.3	10202964	10202984	+	No
1939431_adh	TCAAGCTTGGCAAACAGGAAT	Chr.3	10203320	10203340	+	No
2617105_adh	TTCTCTCGATCAACTGGCTTC	Chr.3	10203344	10203364	+	No
2139247_adh	TGAATATCGGTAGATTGAAGG	Chr.3	10203606	10203586	-	No
2599184_adh	TTCCGGGAAATAGAAGTGAAT	Chr.3	10203622	10203602	-	No
2628396_adh	TTGAAGAGTTTATCGCTGGAC	Chr.3	10207717	10207737	+	No
2032423_adh	TCTAGAGGAAGTGGAATCGGT	Chr.3	10214452	10214472	+	No
1815484_adh	TACCAGGATATGGACATGCCG	Chr.3	10215326	10215306	-	No
2623219_adh	TTCTTTAAACGAGATCCGCTA	Chr.3	10218590	10218570	-	No
2487499_adh	TGTAGAAGGGAAGGCAGATTT	Chr.3	10269181	10269201	+	No
2561196_adh	TTAGAACGAGTCGGGAAGAAA	Chr.3	10270744	10270724	-	No
2707319_adh	TTTCGCGAAAGGAGATGTTTT	Chr.3	10297347	10297367	+	No
1968283_adh	TCATAAGGCAGAGAGAACAAT	Chr.3	10328815	10328835	+	No
1987360_adh	TCCGGTGATTTTTTAGACGGT	Chr.3	10330841	10330861	+	No
2150402_adh	TGACAAGAAACGGAAGTGTTT	Chr.3	10365693	10365713	+	No
2700840_adh	TTTATTTTCGTCGGAATTCCT	Chr.3	10407262	10407242	-	No
2062530_adh	TCTTTTATTTTCGTCGGAATT	Chr.3	10407265	10407245	-	No
2623813_adh	TTCTTTTATTTTCGTCGGAAT	Chr.3	10407266	10407246	-	No
2708946_adh	TTTCGTCTACTGTGGCGGCAT	Chr.3	10436040	10436020	-	No

2692958_adh	TTTAATTTCGTCTACTGTGGC	Chr.3	10436045	10436025	-	No
2729246_adh	TTTGTGAAATCGGTTTCTCTC	Chr.3	10436617	10436637	+	No
2727104_adh	TTTGTAAAAGAGATTCCTGAA	Chr.3	10438614	10438594	-	No
2747081_adh	TTTTTGCGGAAAAACCGAGTT	Chr.3	10460557	10460537	-	No
2704920_adh	TTTCCACCTGTTGTTTTATCG	Chr.3	10469986	10470006	+	No
2055596_adh	TCTTCACTGTATAATGGAAAC	Chr.3	10475767	10475787	+	No
2603667_adh	TTCGAGAATTGTCTGAGGAAT	Chr.3	10483193	10483213	+	No
2126994_adh	TGAAGAACATTTGAAAGCATA	Chr.3	10485002	10484982	-	No
2620488_adh	TTCTTAAGACAAGAGAGATGA	Chr.3	10519333	10519353	+	No
2309360_adh	TGCTGTAGTTGAGACGAAGAA	Chr.3	10520806	10520826	+	No
1873288_adh	TAGTAAGTATGAAGGACAAAA	Chr.3	10542581	10542561	-	No
2607266_adh	TTCGGAATTTGAAGTAAAAAA	Chr.3	10561604	10561584	-	No
2464290_adh	TGGTATCAAAATCGGACGACC	Chr.3	10576443	10576463	+	No
2712054_adh	TTTCTTTCTCGGACGGGATGG	Chr.3	10599831	10599851	+	No
2744782_adh	TTTTTAAGTAGTTGACGACAA	Chr.3	10629293	10629313	+	No
2037310_adh	TCTCAGAATTTCGTGGTCTCG	Chr.3	10639937	10639917	-	No
2589571_adh	TTCACGAGCACAGATGGCGAT	Chr.3	10642379	10642399	+	No
2591047_adh	TTCAGCAGGTAGACCAAAAAC	Chr.3	10680028	10680048	+	No
2712463_adh	TTTGAAAAGTGGAAAATGAAA	Chr.3	10685849	10685829	-	No
2708866_adh	TTTCGTCGATTTCAGCGGCTA	Chr.3	10687792	10687772	-	No
2488945_adh	TGTAGGTAAAGGACATTATAT	Chr.3	10704224	10704244	+	No
2281379_adh	TGCAACATATCTGACGCGCAA	Chr.3	10710754	10710734	-	No
2745820_adh	TTTTTCCACCGAAAATGTTGC	Chr.3	10721044	10721024	-	No
2039967_adh	TCTCGGACCGGCAGGTCGCAT	Chr.3	10730525	10730505	-	No
2694242_adh	TTTACTCTCGGACCGGCAGGT	Chr.3	10730530	10730510	-	No
2529122_adh	TGTTGATGACTTCGGTGGATT	Chr.3	10730657	10730677	+	No
2271295_adh	TGATGACTTCGGTGGATTGTC	Chr.3	10730660	10730680	+	No
2173128_adh	TGACTTCGGTGGATTGTCGTG	Chr.3	10730663	10730683	+	No
2395166_adh	TGGATTGTCGTGAGCATTTAG	Chr.3	10730672	10730692	+	No
2498764_adh	TGTCGTGAGCATTTAGACTTC	Chr.3	10730677	10730697	+	No
2016577_adh	TCGGGAGGAGGCACGATTTTT	Chr.3	10733158	10733138	-	No
1939339_adh	TCAAGCGTATCTCGGAGCAAT	Chr.3	10741080	10741060	-	No
2270249_adh	TGATCTCGAGCCAGGAACGAT	Chr.3	10742065	10742045	-	No
2729051_adh	TTTGTCGGTTTCGCATCGTTA	Chr.3	10742480	10742460	-	No
1975581_adh	TCATTTTGTCGGTTTCGCATC	Chr.3	10742484	10742464	-	No
1994514_adh	TCGAAAACCAATTCCGCGGCA	Chr.3	10742544	10742524	-	No
2644888_adh	TTGATGATTCTGTGGGAGACG	Chr.3	10746386	10746366	-	No
2714501_adh	TTTGAATTGTAGACACCGGTT	Chr.3	10746631	10746651	+	No
2677893_adh	TTGTCCTTGTCGGAGATGGAG	Chr.3	10747574	10747554	-	No
2584901_adh	TTCAAAAACAAGAATGTTCGG	Chr.3	10747760	10747780	+	No
2578652_adh	TTATGAGGCATATTTGGAGTT	Chr.3	10748210	10748230	+	No
1939535_adh	TCAAGGACAGTTGCATCAACG	Chr.3	10749338	10749358	+	No
1921586_adh	TATTAGCATTGAAAGAGCATT	Chr.3	10750602	10750622	+	No
2727104_adh	TTTGTAAAAGAGATTCCTGAA	Chr.3	10752742	10752722	-	No
1925796_adh	TATTGACTTGAATTCCATGTA	Chr.3	10807572	10807552	-	No
2044907_adh	TCTGACAAGACTGGAACTCTC	Chr.3	10815064	10815044	-	No
2048468_adh	TCTGCTCTGACAAGACTGGAA	Chr.3	10815069	10815049	-	No
2376005_adh	TGGACGAATTCGGAGAGCAAC	Chr.3	10815354	10815334	-	No
2658386_adh	TTGGACGAATTCGGAGAGCAA	Chr.3	10815355	10815335	-	No
2623858_adh	TTCTTTTCTTCTTTCCAACTG	Chr.3	10820799	10820779	-	No

2617785_adh	TTCTGAAGAGCAAAAGAACTA	Chr.3	10821277	10821257	-	No
2032974_adh	TCTAGTAGAGCGCGATTGCAT	Chr.3	10839054	10839074	+	No
1990764_adh	TCCTCTCGGACAAGCAACAAT	Chr.3	10844889	10844869	-	No
2376139_adh	TGGACGCAGTTGGAAGTCGAG	Chr.3	10866644	10866624	-	No
2170212_adh	TGACTGGACCAGATGGGGAGA	Chr.3	10867980	10867960	-	No
2693230_adh	TTTACACATGTAAAATGAGCG	Chr.3	10870096	10870076	-	No
2010208_adh	TCGGAAAATTCAGTACGACGA	Chr.3	10875303	10875323	+	No
1822366_adh	TACGACGAATCTCCGGCAAGC	Chr.3	10875316	10875336	+	No
2680397_adh	TTGTGACTGTGGAGATGGCTG	Chr.3	10881820	10881840	+	No
2374041_adh	TGGACAGATGGTTAATGGAAC	Chr.3	10939660	10939680	+	No
2724460_adh	TTTGGCGGTGACGATGGCTCC	Chr.3	11009231	11009211	-	No
2747006_adh	TTTTTGCAGAGTGAGCCATTC	Chr.3	11010055	11010075	+	No
2579743_adh	TTATGGTGGACTTTAGAACGG	Chr.3	11036349	11036369	+	No
2733874_adh	TTTTAATTTAAAATTTGGCGA	Chr.3	11062137	11062157	+	No
2746607_adh	TTTTTGACAATTTCGAGCGAA	Chr.3	11064567	11064547	-	No
1978720_adh	TCCAGGCTGAAAGAAGAAGAA	Chr.3	11078544	11078524	-	No
1992786_adh	TCCTTCGAAGCGAAGAAACAC	Chr.3	11079638	11079658	+	No
2415928_adh	TGGCTTGCTATTTGTAATTTT	Chr.3	11105757	11105777	+	No
2735320_adh	TTTTATAGGAGCGGAGATAGG	Chr.3	11111804	11111784	-	No
2739233_adh	TTTTCTCATAAATACGACTTT	Chr.3	11111932	11111912	-	No
2503676_adh	TGTGACAGTTTTAAAATTTTG	Chr.3	11117894	11117874	-	No
2487457_adh	TGTAGAACTGGCGTCGATGTA	Chr.3	11118434	11118454	+	No
2415928_adh	TGGCTTGCTATTTGTAATTTT	Chr.3	11118456	11118476	+	No
1917338_adh	TATGTAAAACTTGTAGAAGAC	Chr.3	11127381	11127401	+	No
2239040_adh	TGAGGCTCTAAAATTTGAAGT	Chr.3	11129731	11129751	+	No
2160417_adh	TGACCTGTATGACGTCACGGA	Chr.3	11141732	11141712	-	No
2608676_adh	TTCGGCTACGGATGCGGCACG	Chr.3	11143694	11143714	+	No
2682022_adh	TTGTGTACTCCTCTCGGAAAA	Chr.3	11191084	11191064	-	No
1972577_adh	TCATGGAGACTTAGACGAATC	Chr.3	11201320	11201300	-	No
1921714_adh	TATTAGGTTAGTTGGAGGGTC	Chr.3	11205812	11205832	+	No
1973805_adh	TCATTCCTTCTGTGCGAAGAA	Chr.3	11223986	11223966	-	No
2597574_adh	TTCCCAACTTCAGAGGTTTTA	Chr.3	11235342	11235322	-	No
2735439_adh	TTTTATCAATTTTCGTCGGTT	Chr.3	11236928	11236948	+	No
2470481_adh	TGGTCTTTGTAAGGAGGAAAT	Chr.3	11248298	11248318	+	No
2622008_adh	TTCTTCTCGATTTCTTCCGAT	Chr.3	11274593	11274573	-	No
2734551_adh	TTTTAGAGGATTTTTAAGCTA	Chr.3	11274736	11274756	+	No
2720875_adh	TTTGATTTTTGAGCGATTTTT	Chr.3	11280280	11280300	+	No
2744703_adh	TTTTTAAAGTCGATGGACGGC	Chr.3	11307308	11307288	-	No
2375985_adh	TGGACGAAGCATGTATTCCGG	Chr.3	11352661	11352641	-	No
2716938_adh	TTTGAGGGAGCGGTAGAGCAA	Chr.3	11353457	11353437	-	No
2745808_adh	TTTTTCATTGTTTTCATCGGA	Chr.3	11508964	11508984	+	No
2738881_adh	TTTTCGTTGCTTTTATCGGTT	Chr.3	11590543	11590563	+	No
2709266_adh	TTTCGTTGCTTTTATCGGTTT	Chr.3	11590544	11590564	+	No
2618774_adh	TTCTGCTATGATCTTCGACGA	Chr.3	11604197	11604217	+	No
1990767_adh	TCCTCTCGGACAATTGGCAAA	Chr.3	11615899	11615919	+	No
2352015_adh	TGGAATGGATAATAATATATA	Chr.3	11616494	11616474	-	No
2602960_adh	TTCGACGCCCAGGAGACTGTG	Chr.3	11662422	11662402	-	No
2732962_adh	TTTTAAAAATCGATGGACGGC	Chr.3	11682138	11682158	+	No
2701362_adh	TTTCAACGTTTATCGGTTTTT	Chr.3	11719446	11719426	-	No
1990763_adh	TCCTCTCGGAAAACGGTGCGA	Chr.3	11746829	11746849	+	No

2044340_adh	TCTGAAGTGAATATGGCAAAG	Chr.3	11789717	11789697	-	No
1776354_adh	TAAGACTTTTTGAGAATGCAT	Chr.3	11810426	11810446	+	No
1994604_adh	TCGAAAATCGAGGGCAAATTG	Chr.3	11823329	11823309	-	No
2733707_adh	TTTTAATGAGAAATACCCCCC	Chr.3	11847800	11847820	+	No
2481069_adh	TGTAACAAAACATAGGAGGAC	Chr.3	11848500	11848480	-	No
1890982_adh	TATATTTCCATTTTCCTCGGT	Chr.3	11869234	11869214	-	No
2730444_adh	TTTGTTAGTTGACAACTTCTT	Chr.3	11875148	11875128	-	No
2462747_adh	TGGTAGAACCAGCGGCAATGG	Chr.3	11875483	11875463	-	No
2050293_adh	TCTGGGAGACAGAGAATAGGA	Chr.3	11883383	11883403	+	No
2593123_adh	TTCAGTGGCAGCTTACTCGGA	Chr.3	11892651	11892631	-	No
2050293_adh	TCTGGGAGACAGAGAATAGGA	Chr.3	11907447	11907427	-	No
2050293_adh	TCTGGGAGACAGAGAATAGGA	Chr.3	11908886	11908866	-	No
1765440_adh	TAACAGTGTAGACGGTTTTTG	Chr.3	11918985	11919005	+	No
2734219_adh	TTTTACGTGAAACTGTCGAGA	Chr.3	11920406	11920426	+	No
2694075_adh	TTTACGTGAAACTGTCGAGAA	Chr.3	11920407	11920427	+	No
1755919_adh	TAAACTTGGTTTTTCTGTCGT	Chr.3	11939440	11939460	+	No
1755917_adh	TAAACTTGGTATTTCTGTCGT	Chr.3	11940030	11940010	-	No
2487498_adh	TGTAGAAGGGAAGGCAGATAA	Chr.3	12007063	12007043	-	No
2038230_adh	TCTCATCTGGATGAAGTGGGC	Chr.3	12021675	12021655	-	No
2403377_adh	TGGCAGACGGTGTTGAATGAG	Chr.3	12028546	12028526	-	No
2705768_adh	TTTCCGCGGAAAATTGTCGAC	Chr.3	12058491	12058471	-	No
2534349_adh	TGTTTTCGGTGTATTTTTGCA	Chr.3	12068563	12068583	+	No
1927893_adh	TATTGTCGGAAATTTATTATC	Chr.3	12123922	12123902	-	No
2534349_adh	TGTTTTCGGTGTATTTTTGCA	Chr.3	12180581	12180561	-	No
2388151_adh	TGGATAGAAGATCAGAAAAAG	Chr.3	12212954	12212934	-	No
1805516_adh	TAATTTGTGGAACGTGAAGTT	Chr.3	12250465	12250485	+	No
1918978_adh	TATGTGGAAAAGCGAATGGTT	Chr.3	12250805	12250825	+	No
2039500_adh	TCTCGACTCTTCGAATTCGGT	Chr.3	12256562	12256582	+	No
2036736_adh	TCTCAATTGGATGTGACTATG	Chr.3	12264910	12264890	-	No
2040100_adh	TCTCGGATAAGTTGGAATTCA	Chr.3	12273709	12273729	+	No
2487928_adh	TGTAGATCAAAACGTGATGGA	Chr.3	12304661	12304681	+	No
2017209_adh	TCGGGGTACTGTAGTAGTGCT	Chr.3	12306664	12306644	-	No
2677718_adh	TTGTCATCACTTCTCCCGCAA	Chr.3	12316598	12316578	-	No
1811338_adh	TACACTGAATTTATATTTTTG	Chr.3	12351351	12351331	-	No
2584522_adh	TTATTTTCCTTCAGATTTAAC	Chr.3	12363025	12363045	+	No
2584522_adh	TTATTTTCCTTCAGATTTAAC	Chr.3	12363638	12363618	-	No
2595959_adh	TTCCAATGACTCGAAGAAACA	Chr.3	12367232	12367252	+	No
2002517_adh	TCGATAACGTAGGATTTCGGA	Chr.3	12368473	12368493	+	No
2271517_adh	TGATGATGAAATAGGAGGTGT	Chr.3	12368496	12368516	+	No
2737255_adh	TTTTCAATGAGGATTTTTGAA	Chr.3	12369218	12369198	-	No
2682667_adh	TTGTGTTTACGTTAAATTTAA	Chr.3	12370062	12370042	-	No
2503782_adh	TGTGACCGGTGTATCGATGAC	Chr.3	12376899	12376919	+	No
2001503_adh	TCGAGCGACAGGCGGACATCA	Chr.3	12395403	12395423	+	No
1963261_adh	TCAGGAAAAAGAATTTTGGCA	Chr.3	12399019	12399039	+	No
1957607_adh	TCAGAAGACTGTTCGATCGAT	Chr.3	12426866	12426846	-	No
1965937_adh	TCAGTAGATCAACGGAAAATC	Chr.3	12426896	12426916	+	No
2560977_adh	TTAGAACAAAGAAAAGCACGG	Chr.3	12429831	12429851	+	No
1753180_adh	TAAAATTGAAACGGTCTGAAA	Chr.3	12433899	12433919	+	No
2708814_adh	TTTCGTATATTATTAGACGAC	Chr.3	12436395	12436415	+	No
2706426_adh	TTTCCTTCCTATTTTTGACGA	Chr.3	12457398	12457418	+	No

2707483_adh	TTTCGGAAAAAGCATAACAGA	Chr.3	12489466	12489486	+	No
2534353_adh	TGTTTTCGGTGTATTTTTGTT	Chr.3	12493593	12493573	-	No
1884965_adh	TATAAGAAATTTAAATTTCAA	Chr.3	12495690	12495670	-	No
2728078_adh	TTTGTACTCCTCTCGGACAAA	Chr.3	12529588	12529568	-	No
2376312_adh	TGGACGGCAAGATTTGGCTAA	Chr.3	12573120	12573140	+	No
2616105_adh	TTCTCAGATTTATGGATGGAT	Chr.3	12574837	12574817	-	No
2314224_adh	TGGAAACACGTGAAAGTTCGA	Chr.3	12574930	12574910	-	No
2709718_adh	TTTCTAGGTGGAAAACTATGT	Chr.3	12575263	12575243	-	No
1753592_adh	TAAAATTTAAATAATCATTTA	Chr.3	12607120	12607140	+	No
1807295_adh	TACAAATTGGAGATATAACAT	Chr.3	12621335	12621315	-	No
2018691_adh	TCGGTCATTGTGGTATAATAG	Chr.3	12672798	12672818	+	No
1990721_adh	TCCTCTAGGACAAACAGGACA	Chr.3	12673432	12673412	-	No
2709266_adh	TTTCGTTGCTTTTATCGGTTT	Chr.3	12684422	12684402	-	No
2738881_adh	TTTTCGTTGCTTTTATCGGTT	Chr.3	12684423	12684403	-	No
2747833_adh	TTTTTGTGGTTTTCGGTTTTT	Chr.3	12688144	12688164	+	No
2707551_adh	TTTCGGACCAATCAGCGTCAT	Chr.3	12696856	12696876	+	No
2707551_adh	TTTCGGACCAATCAGCGTCAT	Chr.3	12698799	12698779	-	No
2387759_adh	TGGATAATGGCGATGGTGTTT	Chr.3	12711300	12711280	-	No
2609391_adh	TTCGGGTCGAATTTAGAGCGG	Chr.3	12713818	12713798	-	No
1932971_adh	TCAAAGAAGAGAAGACACTCA	Chr.3	12789384	12789364	-	No
2310278_adh	TGCTTCAACACAAGACTCTTT	Chr.3	12805883	12805903	+	No
2042818_adh	TCTCTGACTGGAAAACGGCAA	Chr.3	12816145	12816165	+	No
2689116_adh	TTTAAACTCGACGGAAAATTT	Chr.3	12821481	12821501	+	No
2729246_adh	TTTGTGAAATCGGTTTCTCTC	Chr.3	12878496	12878516	+	No
1921586_adh	TATTAGCATTGAAAGAGCATT	Chr.3	12879359	12879379	+	No
2047784_adh	TCTGCAATTTTGAACGGCAAT	Chr.3	12894076	12894056	-	Yes
2544697_adh	TTAAATTTCGGCACGAGTTTC	Chr.3	12905027	12905007	-	No
1843533_adh	TAGAATGTGGATATGATAATG	Chr.3	12906333	12906353	+	No
2710994_adh	TTTCTGTGTAGAAAAATCGTT	Chr.3	12907219	12907199	-	No
2313640_adh	TGGAAAAGCAAAACAATTTTA	Chr.3	12918038	12918058	+	No
2527581_adh	TGTTGAAGACAGAGAGAGACA	Chr.3	12919927	12919907	-	No
1794678_adh	TAATACTTGAGAACGACCGCA	Chr.3	12926095	12926115	+	No
1776947_adh	TAAGAGCTGAGAGATATTTGG	Chr.3	12971944	12971964	+	No
2711901_adh	TTTCTTGTTCTGTCTTTCGCC	Chr.3	12972333	12972313	-	No
1938245_adh	TCAAGAAGAAGCTGAAGTAAT	Chr.3	12977908	12977888	-	No
2047831_adh	TCTGCAGAAAGTGCTATGCTC	Chr.3	12977992	12977972	-	No
2730056_adh	TTTGTGTCGCTGGGCCTAAAT	Chr.3	12978832	12978812	-	No
2557401_adh	TTACGGGAAGGGGGAGAAGAA	Chr.3	12978907	12978927	+	No
2396143_adh	TGGATTTGGAACTTCGGTAGC	Chr.3	12985098	12985118	+	No
1832596_adh	TACTGCGAGCTCCCGGATGGA	Chr.3	12987161	12987181	+	No
1946298_adh	TCACATTCGAACTTTGCGGAA	Chr.3	12987404	12987384	-	No
2526806_adh	TGTTCTCGGTGTACTTTTGCT	Chr.3	13012566	13012586	+	No
2746931_adh	TTTTTGATGATTTCGGTGTAT	Chr.3	13012804	13012784	-	No
2549248_adh	TTAAGTGTAGTTATAGGACGG	Chr.3	13016545	13016525	-	No
1953149_adh	TCACGCTGGATACGGAGGATA	Chr.3	13019316	13019336	+	No
2697722_adh	TTTATAAATTAAAATCAGCAT	Chr.3	13099703	13099723	+	No
2607300_adh	TTCGGACAGATGCGACACGGA	Chr.3	13103537	13103557	+	No
2065553_adh	TGAAAAACGGCTCTAAAAGAG	Chr.3	13104454	13104434	-	No
2738691_adh	TTTTCGGTAGAAACAGTTATA	Chr.3	13125954	13125974	+	No
2534353_adh	TGTTTTCGGTGTATTTTTGTT	Chr.3	13149161	13149181	+	No

2653005_adh	TTGCGGTAACCGGATAATTTG	Chr.3	13151933	13151953	+	No
2302235_adh	TGCGGTAACCGGATAATTTGT	Chr.3	13151934	13151954	+	No
2608134_adh	TTCGGATAGATGTGACACGGA	Chr.3	13172125	13172105	-	No
2608134_adh	TTCGGATAGATGTGACACGGA	Chr.3	13172338	13172358	+	No
2742880_adh	TTTTGGCAGGAATATGATGGA	Chr.3	13187790	13187770	-	No
2747285_adh	TTTTTGGCAGGAATATGATGG	Chr.3	13187791	13187771	-	No
1811228_adh	TACACGTGGACAAAATCGGTG	Chr.3	13187819	13187799	-	No
1851468_adh	TAGATGGCTGAAAAAACAAAT	Chr.3	13208362	13208342	-	No
1990973_adh	TCCTCTTTTCGGCATCCTCAT	Chr.3	13233480	13233460	-	No
2073650_adh	TGAAACTACTATATTACGGGA	Chr.3	13270085	13270065	-	No
2600777_adh	TTCCTGAGTAGAATTGGCTAT	Chr.3	13295760	13295780	+	No
2411132_adh	TGGCCTAAATTAATTGCGGCA	Chr.3	13296527	13296507	-	No
2550970_adh	TTAATGAAGAAGTTCTTCATC	Chr.3	13307248	13307268	+	No
2230454_adh	TGAGATTGGAATAAGACACAT	Chr.3	13314606	13314586	-	No
2470888_adh	TGGTGAACTATTGAGATTGGA	Chr.3	13314617	13314597	-	No
2716224_adh	TTTGACTTGATATACGGAGAT	Chr.3	13316969	13316949	-	No
2634213_adh	TTGAATTTAAATAATTATTTA	Chr.3	13330760	13330780	+	No
2590762_adh	TTCAGATAGACTCATATTTTA	Chr.3	13331841	13331861	+	No
2585439_adh	TTCAAATAGTACATCAAGCAT	Chr.3	13341502	13341482	-	No
2696735_adh	TTTAGGGCACGATTAGTTGGC	Chr.3	13343384	13343404	+	No
2526801_adh	TGTTCTCGGCTTCTCAGATCA	Chr.3	13343417	13343437	+	No
1983707_adh	TCCCTTTTTCATTATCGTTTG	Chr.3	13343552	13343572	+	No
2132826_adh	TGAAGGAGAAGCTGATGGCCG	Chr.3	13344224	13344244	+	No
2671046_adh	TTGGTGGACGGAAATAAAAAT	Chr.3	13393992	13394012	+	No
2038451_adh	TCTCATTCATCTCTGAACATC	Chr.3	13394612	13394632	+	No
2707485_adh	TTTCGGAAAAAGGCACAAAAC	Chr.3	13426064	13426044	-	No
2738444_adh	TTTTCGGAAAAAGGCACAAAA	Chr.3	13426065	13426045	-	No
2584526_adh	TTATTTTCGGAAAAAGGCACA	Chr.3	13426068	13426048	-	No
2700838_adh	TTTATTTTCGGAAAAAGGCAC	Chr.3	13426069	13426049	-	No
2737219_adh	TTTTCAAGCATTTTCCGTCGT	Chr.3	13436239	13436259	+	No
1956521_adh	TCACTTTTAGTCGTTTCTACA	Chr.3	13455756	13455736	-	No
2587418_adh	TTCAAGTTTTTGCAAAGCTTA	Chr.3	13456189	13456169	-	No
2058031_adh	TCTTGATCACAGAGTAGAAAG	Chr.3	13461792	13461772	-	No
2619431_adh	TTCTGGCAAGCACCGACCACC	Chr.3	13463069	13463089	+	No
1970812_adh	TCATCCGGTACTTCCCAACTC	Chr.3	13463508	13463528	+	No
2011844_adh	TCGGACTCTACAGAGGATTCT	Chr.3	13463837	13463857	+	No
2047267_adh	TCTGATGGACAGAAACTCAAC	Chr.3	13463935	13463955	+	No
2703026_adh	TTTCAGCGAATTCGGAATTCA	Chr.3	13497820	13497800	-	No
2745871_adh	TTTTTCCGCTGATCGATCTGA	Chr.3	13529981	13529961	-	No
1863277_adh	TAGGACTTAAATATGTAAAAT	Chr.3	13534498	13534478	-	No
2728175_adh	TTTGTAGAAAAATAAGTGCTT	Chr.3	13546396	13546376	-	No
2322844_adh	TGGAAGAGGAACGTGAAAAGC	Chr.3	13547686	13547666	-	No
2270929_adh	TGATGAAGATCCTCACCGAGC	Chr.3	13605046	13605026	-	No
2737139_adh	TTTTCAAATGATCGTAACGGC	Chr.3	13625983	13626003	+	No
2741507_adh	TTTTGATACCAAATATAGACG	Chr.3	13640978	13640998	+	No
1848320_adh	TAGAGGAGAATGGGCGGCAGT	Chr.3	13643724	13643744	+	No
2450534_adh	TGGGCGGCAGTTTGAACGATT	Chr.3	13643734	13643754	+	No
2703580_adh	TTTCAGTGGAATAACTCATAA	Chr.3	13643765	13643785	+	No
1912984_adh	TATGCGGATCGATTGGAAAAG	Chr.3	13646540	13646560	+	No
2542059_adh	TTAAATAATATTCGATTTGGA	Chr.3	13683283	13683263	-	No

1889189_adh	TATAGTTCAAGCAGTAAACGA	Chr.3	13698598	13698618	+	No
2039514_adh	TCTCGAGACAGACGAAGAAGC	Chr.3	13700568	13700548	-	No
2457014_adh	TGGGGTTTGGAAATTTCGGTT	Chr.3	13715058	13715038	-	No
2449732_adh	TGGGCAAAGTTGGTTGAGAAG	Chr.3	13728331	13728311	-	No
2035434_adh	TCTCAACCAACTTTGGCCACT	Chr.3	13728374	13728354	-	No
1923444_adh	TATTCACAAGGACAGGCAATA	Chr.3	13755694	13755674	-	No
2640933_adh	TTGAGCGGCTTCAAATTTATT	Chr.3	13762701	13762721	+	No
2739529_adh	TTTTCTGCAGAATGACTAATC	Chr.3	13763512	13763532	+	No
2028612_adh	TCGTTGTCGTCGGCTTCGAGT	Chr.3	13765099	13765119	+	No
2055783_adh	TCTTCCAATCGTCAAATCACT	Chr.3	13765137	13765117	-	No
2062262_adh	TCTTTGTCATTATCTTCTGAA	Chr.3	13768011	13767991	-	No
2281397_adh	TGCAACCACCATTTTGTCGGG	Chr.3	13768104	13768084	-	No
2709264_adh	TTTCGTTGCAACCACCATTTT	Chr.3	13768110	13768090	-	No
2746249_adh	TTTTTCTCTTATTTTCGGGCG	Chr.3	13771159	13771139	-	No
2704921_adh	TTTCCACGAAATTTGAAACGG	Chr.3	13771190	13771170	-	No
2531544_adh	TGTTTAAAACTGCAAGCAGGC	Chr.3	13771332	13771352	+	No
2352759_adh	TGGAATGGATTGGTGTTTCAA	Chr.3	13773058	13773078	+	No
2515739_adh	TGTGGATTTGGTTCGAAATTT	Chr.3	13777555	13777575	+	No
2515739_adh	TGTGGATTTGGTTCGAAATTT	Chr.3	13782756	13782776	+	No
2007681_adh	TCGCGAATTGTACATGAGTGT	Chr.4	66862	66882	+	No
2037135_adh	TCTCACGAGGTTTTGGATAAT	Chr.4	78452	78472	+	No
2018388_adh	TCGGTACTCGGCGATGCACTG	Chr.4	126336	126356	+	No
2711390_adh	TTTCTTCAACAGCAAAAACCA	Chr.4	171217	171197	-	No
2487387_adh	TGTAGAAAACCATGCGTATGA	Chr.4	185748	185728	-	No
1769187_adh	TAACGGTACAGAGAGTGTAGA	Chr.4	206351	206371	+	No
2744000_adh	TTTTGTGTAGATTTACGGAGC	Chr.4	220322	220342	+	No
2001469_adh	TCGAGCATTTAAGACGTTTTT	Chr.4	240430	240410	-	No
2287331_adh	TGCAGAGAACTTTAAGCGCGA	Chr.4	255674	255694	+	No
2526887_adh	TGTTCTGAACGGAAAAATGGC	Chr.4	255985	256005	+	No
2043976_adh	TCTGAACGGAAAAATGGCGGA	Chr.4	255988	256008	+	No
1791581_adh	TAATACGAAATATTGACTTTT	Chr.4	256028	256008	-	No
1764155_adh	TAACACACTAGTTACTTTAAA	Chr.4	275897	275917	+	No
2057677_adh	TCTTGACCACCGGACGGTTTC	Chr.4	278879	278859	-	No
1981607_adh	TCCCGGCCGGGGAGAACTTTT	Chr.4	322645	322625	-	No
2686870_adh	TTGTTTAGTTCCAGCCCCATA	Chr.4	322866	322846	-	No
2676392_adh	TTGTATCGTTTTCTTCAGGAA	Chr.4	327186	327206	+	No
1944405_adh	TCACAATTTTGGAAGTCGACA	Chr.4	329914	329894	-	No
2684504_adh	TTGTTGAAGAAGACTGGCCTG	Chr.4	340371	340391	+	No
2299935_adh	TGCGCAAAGAGAAACGAACGG	Chr.4	341507	341527	+	No
2514606_adh	TGTGGAACTTTAGATGGTATC	Chr.4	357961	357941	-	No
2132919_adh	TGAAGGAGGAGCTCATAAGGT	Chr.4	361925	361905	-	No
1800014_adh	TAATGCCACCAACTCAGAAGA	Chr.4	363824	363804	-	No
2318365_adh	TGGAAATGTCAAGAAAATCGA	Chr.4	367618	367598	-	No
2693896_adh	TTTACGGCACAAAGAAATCTT	Chr.4	379224	379204	-	No
2671237_adh	TTGGTGGCGGCAGTTTGAATA	Chr.4	379771	379751	-	No
2741068_adh	TTTTGACGCAACCTCGATTCC	Chr.4	393533	393513	-	No
2747001_adh	TTTTTGCAGAATGTTCGACGT	Chr.4	403622	403642	+	No
1874570_adh	TAGTAGATTGAGAAACTTCGG	Chr.4	503875	503895	+	No
1852110_adh	TAGATTGAGAAACTTCGGCTT	Chr.4	503878	503898	+	No
1932410_adh	TCAAAATCTGAACGCATTTAA	Chr.4	519559	519539	-	No

1919925_adh	TATTAAAGAACGAACAAACGG	Chr.4	529128	529148	+	No
2042902_adh	TCTCTGCAAAAAAGAACTCGG	Chr.4	580305	580285	-	No
1851712_adh	TAGATGTAGGAACATTCATAA	Chr.4	625696	625716	+	No
1893556_adh	TATCAGTCTGCTGACGTTGCG	Chr.4	648586	648566	-	No
2062546_adh	TCTTTTCATGAAGACTGTGAT	Chr.4	653406	653426	+	No
2010361_adh	TCGGAAAGGGATGGCGTTTCT	Chr.4	653632	653612	-	No
2307964_adh	TGCTCTTCGCTACTTGCTCCG	Chr.4	654521	654501	-	No
1890288_adh	TATATGCAGGAAATCTGGCGC	Chr.4	654556	654536	-	No
1870675_adh	TAGGTAGTGACATAAATGTAC	Chr.4	669928	669948	+	No
2047699_adh	TCTGCAAAAACATGAATAGGA	Chr.4	722112	722092	-	No
2480321_adh	TGTAAAAAATGCACAAAATCC	Chr.4	770102	770082	-	No
2737412_adh	TTTTCACTGTAAAAAATGCAC	Chr.4	770109	770089	-	No
2745665_adh	TTTTTCACTGTAAAAAATGCA	Chr.4	770110	770090	-	No
2714068_adh	TTTGAAGTCTGTCATTTTTCA	Chr.4	770124	770104	-	No
1805156_adh	TAATTTGAAGTCTGTCATTTT	Chr.4	770127	770107	-	No
2743684_adh	TTTTGTATGGAAAATTGATCG	Chr.4	770217	770237	+	No
2700886_adh	TTTATTTTTCTTCGGATTTTC	Chr.4	773162	773182	+	No
1851712_adh	TAGATGTAGGAACATTCATAA	Chr.4	781662	781642	-	No
1833707_adh	TACTGTAGCTCCAGGAGTACG	Chr.4	792910	792930	+	No
2657859_adh	TTGGAATTTTGGTAGAAGATC	Chr.4	804426	804446	+	No
2746582_adh	TTTTTGAATAAAATGTATATA	Chr.4	824064	824084	+	No
2603767_adh	TTCGAGCAGAAATTGGGGTTT	Chr.4	830304	830324	+	No
2738881_adh	TTTTCGTTGCTTTTATCGGTT	Chr.4	867695	867715	+	No
2709266_adh	TTTCGTTGCTTTTATCGGTTT	Chr.4	867696	867716	+	No
2701571_adh	TTTCAAGCGATTTTCTTCATT	Chr.4	874938	874958	+	No
2369919_adh	TGGAATTGAAAGTAGAATAGA	Chr.4	882832	882812	-	No
2143216_adh	TGAATGAATGAAGGAGACACC	Chr.4	958057	958077	+	No
2702854_adh	TTTCAGAACCCCCGAGCGGCC	Chr.4	959446	959466	+	No
2180507_adh	TGAGAAGCTGAGGACGAAAAT	Chr.4	981402	981422	+	No
2704760_adh	TTTCATTTTCGTCGCATTTCC	Chr.4	992215	992235	+	No
2605863_adh	TTCGATTTATGAGCGAAGCCA	Chr.4	1000430	1000450	+	No
1753617_adh	TAAAATTTATAGAAAAATTTA	Chr.4	1007892	1007912	+	No
2609146_adh	TTCGGGATGGATTTTCGTTAT	Chr.4	1012555	1012535	-	No
2606274_adh	TTCGCACTAAAAAGTCGGAAA	Chr.4	1012695	1012715	+	No
1746821_adh	TAAAAAGTCGGAAAAAGTACC	Chr.4	1012702	1012722	+	No
2011236_adh	TCGGACAAGTGAAGACGAGGA	Chr.4	1020666	1020646	-	No
2288050_adh	TGCAGATTTTGGTACACTTCA	Chr.4	1026620	1026640	+	No
1796984_adh	TAATATTAAATTGCGAAGACA	Chr.4	1036625	1036645	+	No
2745603_adh	TTTTTCAAGCATATTTCATGT	Chr.4	1056454	1056434	-	No
1897651_adh	TATCGGAGTGTCGTTGGGAGG	Chr.4	1056724	1056744	+	No
2707142_adh	TTTCGATCGATTTTTAGCAGA	Chr.4	1058651	1058671	+	No
2745085_adh	TTTTTAGCAGAAAAACTCCGA	Chr.4	1058661	1058681	+	No
1936168_adh	TCAACAGTTATGAACGGAATG	Chr.4	1091892	1091872	-	No
2500689_adh	TGTGAAACGGTAACATTTGAG	Chr.4	1097534	1097554	+	No
1919925_adh	TATTAAAGAACGAACAAACGG	Chr.4	1138563	1138583	+	No
1931515_adh	TATTTTTTGGTACAGTGGGAT	Chr.4	1143535	1143515	-	No
2654517_adh	TTGCTGAAATTCGGGAATTTT	Chr.4	1151144	1151164	+	No
2559484_adh	TTACTGTAGTTTTCGAAAAAT	Chr.4	1157415	1157395	-	No
2649247_adh	TTGCAGAACTTAAATATCGAC	Chr.4	1170666	1170686	+	No
2689424_adh	TTTAAATCAGGGCTGTGCGGC	Chr.4	1207169	1207149	-	No

2746991_adh	TTTTTGCAATTTTTCGGGACA	Chr.4	1232858	1232878	+	No
2744637_adh	TTTTTAAAATGTCGGGGGTAT	Chr.4	1264416	1264436	+	No
1766210_adh	TAACATGTAAGAACGATTATG	Chr.4	1264454	1264474	+	No
2707751_adh	TTTCGGAGTGTCGTAGTGCGG	Chr.4	1309993	1309973	-	No
1994097_adh	TCCTTTCTTTTTTAGTCGGCG	Chr.4	1339925	1339905	-	No
2673568_adh	TTGTAAAATTCGAATTTTTAG	Chr.4	1385770	1385790	+	No
1981591_adh	TCCCGGCATGGGAAGTTTATT	Chr.4	1388228	1388208	-	No
1756381_adh	TAAAGAAGAATTTGAAATACA	Chr.4	1395103	1395083	-	No
1764126_adh	TAACAATTTTTCGTACGGAAA	Chr.4	1420720	1420740	+	No
1755436_adh	TAAACTCTCTCTGGACCGGTA	Chr.4	1446943	1446923	-	No
1821762_adh	TACGAAGCGCGGCAAGACTCA	Chr.4	1447382	1447402	+	No
2006565_adh	TCGCAGTAGCGCAGTCGGTTA	Chr.4	1490604	1490584	-	No
2417871_adh	TGGGAAATCAGCTTTTGGGCA	Chr.4	1493213	1493233	+	No
2616501_adh	TTCTCGATCGTTCCAACTTGC	Chr.4	1496107	1496087	-	No
2314326_adh	TGGAAACCTGGAGAATTCGAA	Chr.4	1496203	1496223	+	No
2291625_adh	TGCATTTTGTAGGAAATCGGA	Chr.4	1504240	1504260	+	No
2261258_adh	TGATATAAAATCGTCGAAAAA	Chr.4	1505547	1505567	+	No
2559484_adh	TTACTGTAGTTTTCGAAAAAT	Chr.4	1570071	1570091	+	No
1990765_adh	TCCTCTCGGACAAGCGCAACA	Chr.4	1596025	1596045	+	No
2721317_adh	TTTGCAGAGTGTCGTTGGCAA	Chr.4	1624027	1624047	+	No
2261258_adh	TGATATAAAATCGTCGAAAAA	Chr.4	1647982	1648002	+	No
1977067_adh	TCCACAAAATATATTTTCAAT	Chr.4	1650848	1650828	-	No
2529136_adh	TGTTGATGATTGCGTTGGCCT	Chr.4	1659298	1659318	+	No
2093409_adh	TGAAATCGAAATTTTCAAAAA	Chr.4	1663025	1663005	-	No
2043618_adh	TCTGAAAAAATTTTGACGCATA	Chr.4	1714403	1714423	+	No
1825925_adh	TACGGAGATGAGCAAGTGACC	Chr.4	1722750	1722770	+	No
2159916_adh	TGACCGGAAGACTTGAAAGTT	Chr.4	1739970	1739990	+	No
1911640_adh	TATGATGGGATGGGAGAAGAT	Chr.4	1751797	1751817	+	No
2011151_adh	TCGGAATTGTGAGGGACGTGC	Chr.4	1752544	1752564	+	No
2318544_adh	TGGAAATTTAAAAGAAAGTAG	Chr.4	1763413	1763393	-	No
2072424_adh	TGAAACATTTCAAATTGTCAT	Chr.4	1767774	1767794	+	No
2737125_adh	TTTTCAAAGAATTCGGCTTAA	Chr.4	1803646	1803666	+	No
2703203_adh	TTTCAGGCAAAAACTCCATGA	Chr.4	1846544	1846524	-	No
2291625_adh	TGCATTTTGTAGGAAATCGGA	Chr.4	1855738	1855718	-	No
2650752_adh	TTGCATTCGGCTGGTCATCAC	Chr.4	1923923	1923903	-	No
1826636_adh	TACGGCTCCGCAGTAACGTTT	Chr.4	1929181	1929201	+	No
2303956_adh	TGCGTCGGGAAAAATGCAGAA	Chr.4	1945283	1945263	-	No
2688983_adh	TTTAAACAAATGGTCGAGGAG	Chr.4	1965864	1965884	+	No
2738286_adh	TTTTCGATCGAAAAATACGGG	Chr.4	1970308	1970288	-	No
2518108_adh	TGTGGTTGCTCGGCGCGGTGT	Chr.4	1972342	1972362	+	No
1864035_adh	TAGGAGGCGTGTGAAGTTTGA	Chr.4	1978063	1978043	-	No
2744000_adh	TTTTGTGTAGATTTACGGAGC	Chr.4	1980710	1980690	-	No
2609843_adh	TTCGGTAGAGCGCGATTGCAT	Chr.4	1993620	1993600	-	No
2005002_adh	TCGATTGCAAGTATTGACGAG	Chr.4	2029630	2029650	+	No
2304207_adh	TGCGTGGAGAATTCGGTTTTT	Chr.4	2046184	2046164	-	No
2653587_adh	TTGCGTGGAGAATTCGGTTTT	Chr.4	2046185	2046165	-	No
2276639_adh	TGATTGCGTGGAGAATTCGGT	Chr.4	2046188	2046168	-	No
2411152_adh	TGGCCTAGTGGATAAGAGGGA	Chr.4	2076307	2076327	+	No
2411152_adh	TGGCCTAGTGGATAAGAGGGA	Chr.4	2079061	2079081	+	No
2236276_adh	TGAGGAACAAAAAACGGAGAA	Chr.4	2196400	2196380	-	No

1811193_adh	TACACGTCAAAAGTAGCGAGA	Chr.4	2196430	2196450	+	No
1811193_adh	TACACGTCAAAAGTAGCGAGA	Chr.4	2196530	2196510	-	No
2236276_adh	TGAGGAACAAAAAACGGAGAA	Chr.4	2196560	2196580	+	No
2649595_adh	TTGCAGCGGTGGAAATTTTGA	Chr.4	2203483	2203463	-	No
2649595_adh	TTGCAGCGGTGGAAATTTTGA	Chr.4	2203539	2203559	+	No
2169511_adh	TGACTGAGAAGGTAACTACGA	Chr.4	2206952	2206932	-	No
2618314_adh	TTCTGAGCAATTTTCTGGCGT	Chr.4	2216934	2216914	-	No
2236251_adh	TGAGGAAATGCGACGAAAAAA	Chr.4	2217037	2217057	+	No
1848126_adh	TAGAGCGCGTCTGGCATTCAC	Chr.4	2240974	2240954	-	No
2712089_adh	TTTCTTTGAAAATCGTTTGGA	Chr.4	2277001	2276981	-	No
2700099_adh	TTTATTGATTTTTCGGTTTAA	Chr.4	2277981	2278001	+	No
2648907_adh	TTGCACAAATTGTATATTCAT	Chr.4	2309572	2309552	-	No
2649414_adh	TTGCAGACTGTGGTGAACATT	Chr.4	2311846	2311866	+	No
2004167_adh	TCGATGGATCGAAAGACTGTT	Chr.4	2312741	2312721	-	No
2649535_adh	TTGCAGCATGCGGTCGGTCAG	Chr.4	2313577	2313597	+	No
1825721_adh	TACGGAATGATCGTCGCCCTC	Chr.4	2315497	2315477	-	No
2499479_adh	TGTCTCGTTGGGAAAATGGTG	Chr.4	2317057	2317077	+	No
2523164_adh	TGTTACTAATGGTTTAACTGG	Chr.4	2327519	2327499	-	No
2022038_adh	TCGTAGAAATTGGAAAAAATA	Chr.4	2328440	2328460	+	No
2189153_adh	TGAGAGTAAGCGTTGAATTGA	Chr.4	2331203	2331223	+	No
1930982_adh	TATTTTCTGTTGACTCGTCTT	Chr.4	2368966	2368986	+	No
1876095_adh	TAGTCACAAGACTGTGTGGCA	Chr.4	2370259	2370239	-	No
1762599_adh	TAAATTGTAGGAGAAAGGTGG	Chr.4	2370284	2370304	+	No
2713445_adh	TTTGAACTCTCGGCAAGCTGA	Chr.4	2383159	2383139	-	No
2258151_adh	TGATAAAATGTCGGAAAATGG	Chr.4	2384453	2384473	+	No
2258151_adh	TGATAAAATGTCGGAAAATGG	Chr.4	2384773	2384793	+	No
2258151_adh	TGATAAAATGTCGGAAAATGG	Chr.4	2385116	2385136	+	No
2490300_adh	TGTAGTTTTTGGAGAGTGGCG	Chr.4	2389232	2389252	+	No
2741066_adh	TTTTGACGATTTTTTCGGCGG	Chr.4	2389279	2389299	+	No
1872893_adh	TAGTAACAAGCGGTCGGCGCA	Chr.4	2400174	2400194	+	No
2059589_adh	TCTTGTCGAGAAGGATATCCC	Chr.4	2401057	2401037	-	No
1897697_adh	TATCGGATTCGACATGGGCGG	Chr.4	2409842	2409822	-	No
2701829_adh	TTTCAATGGAACAACTGGAGA	Chr.4	2457010	2456990	-	No
1919595_adh	TATGTTGTTTTAGTTTGTTCA	Chr.4	2488824	2488844	+	No
2376287_adh	TGGACGGAGCATCGAAGAACG	Chr.4	2496724	2496744	+	No
2585694_adh	TTCAACAGAACTCGTCAAGCA	Chr.4	2497344	2497364	+	No
2039776_adh	TCTCGCGACGATAGAAGGATA	Chr.4	2497820	2497840	+	No
1970315_adh	TCATATTTTAGGTTAAATCGT	Chr.4	2502391	2502371	-	No
2176553_adh	TGAGAAAAATGCGGAAACTGT	Chr.4	2551406	2551386	-	No
2643348_adh	TTGATAATGGCATTCACATCA	Chr.4	2552724	2552744	+	No
1875521_adh	TAGTATGGCACTCCCTAATAG	Chr.4	2561066	2561086	+	No
2259371_adh	TGATACAGGTAGAGAAATGAG	Chr.4	2577281	2577261	-	No
1899269_adh	TATCTAGACATCACAGCAGGA	Chr.4	2588102	2588122	+	No
2645917_adh	TTGATTATAGGCGGGAAAATG	Chr.4	2602085	2602065	-	No
2748935_adh	TTTTTTCGTCGGCAGCACGGG	Chr.4	2602227	2602247	+	No
2460933_adh	TGGTAAAAAACATTATTTTCA	Chr.4	2610224	2610204	-	No
2669661_adh	TTGGTACTGTGGATGTTGGTA	Chr.4	2620842	2620862	+	No
2516716_adh	TGTGGGTGGCTCATGAGAAGA	Chr.4	2640803	2640783	-	No
2739281_adh	TTTTCTCGCGACTCGGAAAAG	Chr.4	2641184	2641204	+	No
2568482_adh	TTAGGCAGTTAGACAGTTGTT	Chr.4	2655006	2654986	-	No

2696684_adh	TTTAGGCAGTTAGACAGTTGT	Chr.4	2655007	2654987	-	No
2703099_adh	TTTCAGCGTGGCGCAGTGGCT	Chr.4	2664238	2664258	+	No
2677717_adh	TTGTCATCACTTCGCTCGGAG	Chr.4	2671460	2671440	-	No
2148713_adh	TGAATTTTCTGGCAGTTTTGA	Chr.4	2671747	2671767	+	No
2608090_adh	TTCGGAGTGTCGTTGTGAAAT	Chr.4	2677866	2677846	-	No
2722884_adh	TTTGGAAACAACATGAGCATA	Chr.4	2709876	2709856	-	No
2020055_adh	TCGGTTCGGACAATTTCGGCA	Chr.4	2718625	2718605	-	No
2568197_adh	TTAGGATACACCTGCAAATAT	Chr.4	2729098	2729118	+	No
2568197_adh	TTAGGATACACCTGCAAATAT	Chr.4	2732401	2732381	-	No
2739231_adh	TTTTCTCAGTTGACAACTTTT	Chr.4	2775787	2775767	-	No
2746144_adh	TTTTTCGTATTTTTGAGTGAT	Chr.4	2783113	2783093	-	No
2738619_adh	TTTTCGGCATTTTTAGGCGAT	Chr.4	2783133	2783113	-	No
2711827_adh	TTTCTTGCTTTTCGGCATTTT	Chr.4	2783141	2783121	-	No
2541595_adh	TTAAAGGATTGTAACTTGGCT	Chr.4	2797000	2797020	+	No
2134724_adh	TGAAGTCGTGCATCGGGCCAA	Chr.4	2825731	2825711	-	No
2589767_adh	TTCACGTCCTACTCAGCTGGG	Chr.4	2825853	2825833	-	No
2566585_adh	TTAGCCTCTCTGATTTGTAGT	Chr.4	2846550	2846570	+	No
2677633_adh	TTGTCAGATAATAGACAGCTA	Chr.4	2854105	2854085	-	No
2735892_adh	TTTTATGAACTTTTTGCCTGA	Chr.4	2892905	2892885	-	No
2727078_adh	TTTGGTTTTTTCGGCAAAATT	Chr.4	2893709	2893729	+	No
2748908_adh	TTTTTTCGGCAAAATTTTCTG	Chr.4	2893714	2893734	+	No
1980138_adh	TCCCAAAATTCGTCGTTGCAA	Chr.4	2894881	2894861	-	No
2140342_adh	TGAATCACAGAAGCTCATTCA	Chr.4	2901052	2901072	+	No
2029595_adh	TCTAACATGCTTTCGAAGGAT	Chr.4	2901771	2901751	-	No
1897229_adh	TATCGATTATTCAGAGAGGTG	Chr.4	2914104	2914124	+	No
2043041_adh	TCTCTGTACCGGTAATATTGC	Chr.4	2918214	2918234	+	No
2707876_adh	TTTCGGCAATCGGCGATTCAG	Chr.4	2922439	2922459	+	No
2576931_adh	TTATCACATGTAGAACGGCAT	Chr.4	2927677	2927697	+	No
2615955_adh	TTCTCAATAAAATAATCGATA	Chr.4	2939922	2939902	-	No
1824347_adh	TACGATGTAGACTTTAATATA	Chr.4	2955628	2955608	-	No
2061708_adh	TCTTTGAAGTCTGTCGAGATA	Chr.4	2991332	2991352	+	No
2396515_adh	TGGATTTTTAAAGCGAAGAAT	Chr.4	3009338	3009318	-	No
2749508_adh	TTTTTTGGAACTTTAAAAAAT	Chr.4	3014347	3014367	+	No
2161129_adh	TGACGATGGAGATGAGGATGG	Chr.4	3040683	3040663	-	No
2005888_adh	TCGCACACTGTCGTTGAGAAT	Chr.4	3042107	3042087	-	No
2530982_adh	TGTTGTGGAGACGATGATGAC	Chr.4	3045923	3045903	-	No
2025355_adh	TCGTCATCATGCGTTCGCGGA	Chr.4	3050411	3050391	-	No
2611894_adh	TTCGTCATCATGCGTTCGCGG	Chr.4	3050412	3050392	-	No
1929353_adh	TATTTATCCAGTTCTGCAGAG	Chr.4	3052396	3052376	-	No
1883859_adh	TATAAAAATTTGAGATCTCGT	Chr.4	3054669	3054689	+	No
2612251_adh	TTCGTGATTTCAAGCCTAAAT	Chr.4	3058356	3058376	+	No
2268047_adh	TGATATTCGGAATCAGGAGAA	Chr.4	3058415	3058435	+	No
1991574_adh	TCCTGGAAGACGATGAAGAAG	Chr.4	3063244	3063224	-	No
2005267_adh	TCGATTTGGCGAAGGAAAAAG	Chr.4	3071653	3071673	+	No
1819707_adh	TACCGGCACAGAGAGTGTAGA	Chr.4	3082332	3082312	-	No
2607300_adh	TTCGGACAGATGCGACACGGA	Chr.4	3106733	3106713	-	No
2029160_adh	TCGTTTTTGTTGAGATTTGAA	Chr.4	3142377	3142397	+	No
2612251_adh	TTCGTGATTTCAAGCCTAAAT	Chr.4	3142527	3142547	+	No
2268047_adh	TGATATTCGGAATCAGGAGAA	Chr.4	3142587	3142607	+	No
1918114_adh	TATGTATAGTTTTGTTTTTTG	Chr.4	3151269	3151249	-	No

2649813_adh	TTGCAGTGAGAGCCTAAACAT	Chr.4	3212579	3212559	-	No
2718437_adh	TTTGATCTACGGGAATTGCGG	Chr.4	3231295	3231275	-	No
2578819_adh	TTATGATGATGCATCGTTGGG	Chr.4	3232264	3232244	-	No
1806407_adh	TACAAAAAAGCAGATATTCTA	Chr.4	3250447	3250427	-	No
2623602_adh	TTCTTTGAGCAAACATGCAAT	Chr.4	3271528	3271548	+	No
2417928_adh	TGGGAAATTGATGGATAACTC	Chr.4	3276157	3276177	+	No
2625173_adh	TTGAAAGGGGAAACTGTTGTT	Chr.4	3294687	3294667	-	No
2738647_adh	TTTTCGGGCAGAAATTGTGAA	Chr.4	3313399	3313379	-	No
2608432_adh	TTCGGCAATTTTCGGGCAGAA	Chr.4	3313407	3313387	-	No
2044992_adh	TCTGACACGGATAGAATGATT	Chr.4	3315849	3315869	+	No
1822924_adh	TACGAGAAGACTGATCCAGCA	Chr.4	3322280	3322300	+	No
2048959_adh	TCTGGACGACAGCGAGCTGAG	Chr.4	3336903	3336883	-	No
1976451_adh	TCCAAGGACGAGATGAGCAAG	Chr.4	3337751	3337731	-	No
1773911_adh	TAAGAACTAGATCATGACGGA	Chr.4	3338414	3338394	-	No
2042616_adh	TCTCTCTCGGTTTTCGTCACA	Chr.4	3338827	3338807	-	No
2472039_adh	TGGTGGAGGGTGAGACGCAGT	Chr.4	3350830	3350810	-	No
2689573_adh	TTTAAATTAAGAAATCGACAA	Chr.4	3354407	3354427	+	No
2000633_adh	TCGACTGGTCTCTCGCTCGGC	Chr.4	3368871	3368891	+	No
1908836_adh	TATGAGAAATTGGGCATTTTT	Chr.4	3378635	3378655	+	No
2100848_adh	TGAAATGAAAATTATTGAGAA	Chr.4	3382209	3382229	+	No
2634625_adh	TTGAATTTTGACGGTTTCCGG	Chr.4	3385519	3385539	+	No
2148732_adh	TGAATTTTGACGGTTTCCGGC	Chr.4	3385520	3385540	+	No
1973746_adh	TCATTCCAAGACGATATTTAC	Chr.4	3395799	3395779	-	No
2355692_adh	TGGAATGTAAAATATTATTCA	Chr.4	3421241	3421261	+	No
2702550_adh	TTTCACCTTTTCATGTGATCG	Chr.4	3428845	3428825	-	No
2153271_adh	TGACACTGATCATCAATGAAC	Chr.4	3444522	3444502	-	No
2254774_adh	TGAGTTAGAATTTCGGTGGTT	Chr.4	3560813	3560833	+	No
2529415_adh	TGTTGCATCGTCCCATTACAA	Chr.4	3582956	3582936	-	No
2746291_adh	TTTTTCTGTCAAAACTCGGTA	Chr.4	3599131	3599111	-	No
1861083_adh	TAGGAAGATGTGGTGATGGTT	Chr.4	3648946	3648926	-	No
2737519_adh	TTTTCAGGCGTAATTTCGGAA	Chr.4	3669487	3669507	+	No
2706297_adh	TTTCCTGCCAGAATTTTTTGA	Chr.4	3669818	3669838	+	No
2584580_adh	TTATTTTGGCACGATGATCAT	Chr.4	3678721	3678741	+	No
1849484_adh	TAGAGTTGTCAGAAAATTGTA	Chr.4	3700101	3700121	+	No
2044846_adh	TCTGAATTCCGACGTGACGGT	Chr.4	3726044	3726064	+	No
2657856_adh	TTGGAATTTTCAGATTTTCCC	Chr.4	3726109	3726129	+	No
2641090_adh	TTGAGCTTGTCACGTGGCTCC	Chr.4	3735977	3735997	+	No
2641090_adh	TTGAGCTTGTCACGTGGCTCC	Chr.4	3739687	3739707	+	No
2542289_adh	TTAAATAGATTAAAATTGGCT	Chr.4	3747124	3747104	-	No
2705027_adh	TTTCCAGCAGAATTTCGATGT	Chr.4	3803384	3803364	-	No
2271534_adh	TGATGATGATGATGGTAGAGG	Chr.4	3848990	3848970	-	No
1891511_adh	TATCAAGAACTTAAGACGACA	Chr.4	3854100	3854080	-	No
2601673_adh	TTCGAAACTGCTGAGGAGACG	Chr.4	3875380	3875360	-	No
2577103_adh	TTATCAGTTCCTTAACGGTTT	Chr.4	3881422	3881442	+	No
2668937_adh	TTGGTAACAGTTGGACGGCCA	Chr.4	3891542	3891562	+	No
2530280_adh	TGTTGGTTGTCGTCATTTCGG	Chr.4	3907452	3907432	-	No
2664384_adh	TTGGATTGGTCGGGTTCATTA	Chr.4	3907494	3907474	-	No
2749069_adh	TTTTTTCTGTATTTTAGGCTT	Chr.4	3959984	3960004	+	No
1810809_adh	TACACATGTTTCAAAAACCTT	Chr.4	3979376	3979356	-	No
2039755_adh	TCTCGCCCACGAAGTCTGCAT	Chr.4	4032163	4032183	+	No

2042350_adh	TCTCTCAGTGAACACGGTCAG	Chr.4	4060304	4060284	-	No
1852538_adh	TAGATTTTCTTGGCACCGCAA	Chr.4	4067485	4067505	+	No
2717370_adh	TTTGATACAGAAGTTGGAATT	Chr.4	4068179	4068159	-	No
2285996_adh	TGCACAGAATTCGGATTTTTA	Chr.4	4085999	4086019	+	No
2705139_adh	TTTCCAGGCGTTTTTGCGGAT	Chr.4	4086047	4086067	+	No
1833707_adh	TACTGTAGCTCCAGGAGTACG	Chr.4	4099554	4099534	-	No
2069977_adh	TGAAAATCGGAAATTTTTGAC	Chr.4	4105521	4105541	+	No
2618926_adh	TTCTGGAGAAAGCGGAACAGA	Chr.4	4143473	4143493	+	No
1911848_adh	TATGATTACTGGAGACTGCTC	Chr.4	4156754	4156774	+	No
1989367_adh	TCCTACTATTGTTCATCGTCA	Chr.4	4166442	4166462	+	No
2743684_adh	TTTTGTATGGAAAATTGATCG	Chr.4	4185057	4185077	+	No
2705454_adh	TTTCCCTATAATTTTGTCGTT	Chr.4	4187804	4187784	-	No
2743741_adh	TTTTGTCAGCAGCCAAGAAGC	Chr.4	4187833	4187813	-	No
2711514_adh	TTTCTTCGGCGAAACAAGAGA	Chr.4	4188351	4188371	+	No
2695873_adh	TTTAGATTTTGCATAATAATT	Chr.4	4193155	4193135	-	No
2737800_adh	TTTTCATTTAGATTTTGCATA	Chr.4	4193161	4193141	-	No
2288506_adh	TGCAGGATATGGTTTAACTAG	Chr.4	4222978	4222958	-	No
1931062_adh	TATTTTGAGTTCTGGATGTTT	Chr.4	4224284	4224264	-	No
2000857_adh	TCGACTTTTCGTCTGGAAGTT	Chr.4	4227212	4227192	-	No
1981704_adh	TCCCGGTTTTTATTATCGGAA	Chr.4	4267604	4267624	+	No
1897558_adh	TATCGGAATGCATCTTCCAAC	Chr.4	4267617	4267637	+	No
2676558_adh	TTGTATGAATGATGTTCGGTT	Chr.4	4270199	4270219	+	No
1981694_adh	TCCCGGTTATTGTCGAATGCA	Chr.4	4272450	4272470	+	No
2714049_adh	TTTGAAGTAGACAGTATGAAA	Chr.4	4285319	4285299	-	No
2707262_adh	TTTCGCAATTTTTCCGTCATT	Chr.4	4307478	4307498	+	No
2369081_adh	TGGAATTATCATGTTGGAATT	Chr.4	4307516	4307536	+	No
2370697_adh	TGGAATTGCTTACTGCTGACC	Chr.4	4307530	4307550	+	No
2616640_adh	TTCTCGGATGGCGTATTGCAC	Chr.4	4348172	4348192	+	No
1955371_adh	TCACTTCTTGTTCCCATTTAG	Chr.4	4360756	4360776	+	No
2584895_adh	TTCAAAAAAGCTGTAATCCTC	Chr.4	4366429	4366449	+	No
1757423_adh	TAAAGCGGAGAAGCAAGAATC	Chr.4	4381027	4381047	+	No
2694768_adh	TTTACTTTTGGGCCCGTCGTC	Chr.4	4390586	4390566	-	No
1936555_adh	TCAACGACTGTCCTTGGCCTA	Chr.4	4390622	4390602	-	No
2743806_adh	TTTTGTCGTTGGACCTTGCTT	Chr.4	4390786	4390806	+	No
2729795_adh	TTTGTGCTTTTGAAACGGATA	Chr.4	4393812	4393832	+	No
2046116_adh	TCTGAGACTATAGAGAAGAGA	Chr.4	4404367	4404387	+	No
2230036_adh	TGAGATTGAGCTAGAAACACT	Chr.4	4405541	4405561	+	No
2700712_adh	TTTATTTCGTGATTGCTTGGT	Chr.4	4406224	4406244	+	No
2040115_adh	TCTCGGCAAGTTAAGGTAATT	Chr.4	4435198	4435218	+	No
2040380_adh	TCTCGGTTTGCAATTTTTAAG	Chr.4	4445839	4445819	-	No
2065926_adh	TGAAAAAGCGTGCGTTGGCTG	Chr.4	4447586	4447606	+	No
2687523_adh	TTGTTTGGATGCGAACGGCAG	Chr.4	4448703	4448723	+	No
2643979_adh	TTGATCACAAGGGCCTGTAGT	Chr.4	4511746	4511726	-	No
1939117_adh	TCAAGCAATCTGAAGGGAATA	Chr.4	4532638	4532658	+	No
2381279_adh	TGGAGCGCACTTACTCGGCAA	Chr.4	4548876	4548856	-	No
1990381_adh	TCCTCGGGGAGTACACGATCT	Chr.4	4551329	4551309	-	No
2513770_adh	TGTGCGAGAAAAGTAGAAGAA	Chr.4	4570841	4570821	-	No
2537930_adh	TTAAAAAGTAATTTCTCGATA	Chr.4	4633169	4633189	+	No
2279307_adh	TGATTTTGCTGATTTTGGAAC	Chr.4	4633568	4633548	-	No
2032398_adh	TCTAGACTTTGCGGCATCCAT	Chr.4	4636962	4636942	-	No

1924718_adh	TATTCTAGACTTTGCGGCATC	Chr.4	4636965	4636945	-	No
2706808_adh	TTTCGACAGAATGCAACAGAA	Chr.4	4650742	4650722	-	No
2295903_adh	TGCGAAGCGAGTGAACGGTGA	Chr.4	4660526	4660546	+	No
2590430_adh	TTCAGAATATTGTGAGCATAA	Chr.4	4668537	4668557	+	No
1991153_adh	TCCTGAGACGTCTATCAAACA	Chr.4	4704446	4704426	-	No
2712154_adh	TTTCTTTTACTAAAATATCAT	Chr.4	4712545	4712525	-	No
1770310_adh	TAACTATGCAGAATTGAAACT	Chr.4	4721406	4721386	-	No
2589739_adh	TTCACGGTAGCACAGAAACTA	Chr.4	4725520	4725540	+	No
2692407_adh	TTTAATTGACGACATTTAGCA	Chr.4	4725667	4725687	+	No
2589660_adh	TTCACGGCACGGCGAACGGGA	Chr.4	4728015	4727995	-	No
1888037_adh	TATAGAGTTTCGAGTCGGTAC	Chr.4	4736525	4736545	+	No
1813888_adh	TACATGGAGAAAGATTCTATT	Chr.4	4738196	4738176	-	No
1931291_adh	TATTTTTATACACTTTATGCC	Chr.4	4743888	4743908	+	No
2282910_adh	TGCAATAGCAATTCGTAGTAA	Chr.4	4751915	4751895	-	No
2282910_adh	TGCAATAGCAATTCGTAGTAA	Chr.4	4751941	4751921	-	No
2593338_adh	TTCAGTTGCACAGGTTTTTTG	Chr.4	4784787	4784767	-	No
2712341_adh	TTTGAAAAATTTGACGGTATG	Chr.4	4785009	4785029	+	No
2338597_adh	TGGAATGAAAAAAGAATGTAA	Chr.4	4787721	4787701	-	No
1956384_adh	TCACTTGTTCGTCTTCTTCTT	Chr.4	4799685	4799705	+	No
2668948_adh	TTGGTAACGGTCGTGTTCAAG	Chr.4	4811097	4811117	+	No
2590846_adh	TTCAGATGATTCGGACAATAT	Chr.4	4811553	4811573	+	No
2313907_adh	TGGAAAATCTAACATCGGTGA	Chr.4	4825333	4825313	-	No
2589079_adh	TTCACAACAATCGATAATTCA	Chr.4	4827320	4827300	-	No
1935849_adh	TCAACAACAAGAATATGAACA	Chr.4	4827829	4827849	+	No
2617969_adh	TTCTGACACAGAAGTTAGAAT	Chr.4	4833218	4833198	-	Yes
2615829_adh	TTCTATTTGTATTTTGATTAT	Chr.4	4835467	4835447	-	No
1803564_adh	TAATTCTTCGTTTTGCAATCT	Chr.4	4838711	4838731	+	No
1979309_adh	TCCAGTTGGCTTCAGAAGATG	Chr.4	4838804	4838824	+	No
2588187_adh	TTCAATGGAATTCACATGTGG	Chr.4	4839765	4839785	+	No
2492000_adh	TGTATGGAAAACAAGTAGTAG	Chr.4	4850598	4850618	+	Yes
2735077_adh	TTTTATAAACTAGAAGAACCA	Chr.4	4851653	4851633	-	No
2629291_adh	TTGAAGTTATGGCTAGTTTCT	Chr.4	4852884	4852904	+	Yes
2588915_adh	TTCAATTGTATTCGACAGTGG	Chr.4	4854109	4854089	-	Yes
2740885_adh	TTTTGAATAATCTGTCGCTGC	Chr.4	4854256	4854236	-	Yes
2049220_adh	TCTGGAGTTTTTCTTCGATCG	Chr.4	4856044	4856024	-	Yes
2282320_adh	TGCAAGCTGTTGATATCGAGA	Chr.4	4858879	4858859	-	No
1795806_adh	TAATATCAATATTTTTGTTAT	Chr.4	4862374	4862394	+	No
2269424_adh	TGATCCCTATGAACACGGCTG	Chr.4	4864156	4864176	+	No
2057445_adh	TCTTGAACCAAAAGCTGAACA	Chr.4	4874801	4874821	+	No
1855521_adh	TAGCATTGTTTATACTTTTTT	Chr.4	4887618	4887598	-	No
2034890_adh	TCTATTCATGAAGATGAGTTT	Chr.4	4889299	4889279	-	No
1891446_adh	TATCAACCTTTCTCACATTTA	Chr.4	4893473	4893493	+	No
2745511_adh	TTTTTATTGTTGTAGAGGGCG	Chr.4	4905645	4905625	-	No
2734302_adh	TTTTACTGATTTTCATGCTTT	Chr.4	4913086	4913106	+	No
2537446_adh	TTAAAAAACAAGCGAAAAATT	Chr.4	4915454	4915474	+	No
1745302_adh	TAAAAAACAAGCGAAAAATTA	Chr.4	4915455	4915475	+	No
2576707_adh	TTATATTTGAAGAAACGGATA	Chr.4	4936796	4936776	-	No
2698357_adh	TTTATATTTGAAGAAACGGAT	Chr.4	4936797	4936777	-	No
2721467_adh	TTTGCATTGGAAAGGTAAACT	Chr.4	4946534	4946554	+	Yes
1918012_adh	TATGTAGTAGTTTTGCGGCAA	Chr.4	4947867	4947847	-	No

2699318_adh	TTTATGTAGTAGTTTTGCGGC	Chr.4	4947869	4947849	-	No
1926281_adh	TATTGCAGTAACTATTACTCA	Chr.4	4950753	4950773	+	No
1917684_adh	TATGTAGGGATGAATGTCGAT	Chr.4	4953118	4953098	-	No
2738351_adh	TTTTCGCACAAGTACTTCAAA	Chr.4	4954630	4954610	-	No
2557800_adh	TTACGGTGTCAATTGTGCAAT	Chr.4	4955372	4955392	+	No
1929181_adh	TATTTACGTTGATGCTTTTAG	Chr.4	4958303	4958323	+	Yes
2650807_adh	TTGCATTGCAACAATATATTG	Chr.4	4959851	4959871	+	No
2277678_adh	TGATTTAAGGATTGAAGGTAT	Chr.4	4960523	4960543	+	Yes
2548393_adh	TTAAGGATTGAAGGTATTTTT	Chr.4	4960527	4960547	+	Yes
2721392_adh	TTTGCATATATATCTTACTGT	Chr.4	4961250	4961230	-	No
2001170_adh	TCGAGAATAGGATGGTTGATG	Chr.4	4966383	4966403	+	No
2260352_adh	TGATAGAATGCAGAATGCAGA	Chr.4	4970028	4970048	+	No
1746884_adh	TAAAAATAAAGTAGCTAGACG	Chr.4	4971159	4971179	+	No
1923776_adh	TATTCATATGTAGTAAAGTTA	Chr.4	4975286	4975306	+	No
2643363_adh	TTGATACAAGCACTCGAATAA	Chr.4	4977221	4977241	+	No
2300055_adh	TGCGCATGGTCGGAGACGTAT	Chr.4	4982479	4982459	-	No
2732295_adh	TTTGTTGTCATATTTTTCGGG	Chr.4	4986835	4986815	-	No
2537992_adh	TTAAAAATATGCAGAGTAATA	Chr.4	4989741	4989721	-	No
2716213_adh	TTTGACTTCAGAACATCACGG	Chr.4	4990110	4990130	+	No
1896665_adh	TATCGAAAACGCTGAAAACGC	Chr.4	4990494	4990474	-	No
2381561_adh	TGGAGCTGAGAAGATCATCTT	Chr.4	4994613	4994593	-	No
1942355_adh	TCAATGGTGGAGCTGAGAAGA	Chr.4	4994620	4994600	-	No
2055642_adh	TCTTCAGTACCAAACAACCAA	Chr.4	4994727	4994707	-	No
2516328_adh	TGTGGGACAGGAGAACGCCAT	Chr.4	4995901	4995881	-	No
2381561_adh	TGGAGCTGAGAAGATCATCTT	Chr.4	4999727	4999707	-	No
1942355_adh	TCAATGGTGGAGCTGAGAAGA	Chr.4	4999734	4999714	-	No
2055642_adh	TCTTCAGTACCAAACAACCAA	Chr.4	4999841	4999821	-	No
1863263_adh	TAGGACTGTCGTGGGATAAAG	Chr.4	5001795	5001775	-	No
2488890_adh	TGTAGGGCTGTACAAATCGGC	Chr.4	5011086	5011106	+	No
1760150_adh	TAAATATTGCAGATACAAAGA	Chr.4	5012961	5012941	-	No
2061723_adh	TCTTTGACATGTAAGAAAAAA	Chr.4	5019034	5019054	+	No
2473957_adh	TGGTTAAACTTAGAACAAAAA	Chr.4	5039665	5039645	-	No
2694820_adh	TTTAGAAATGCGGGCGACTAC	Chr.4	5043118	5043138	+	No
2271057_adh	TGATGAATCATATAATATTAA	Chr.4	5044434	5044454	+	No
2580519_adh	TTATTAAGCAGTTCCACCGAC	Chr.4	5047290	5047270	-	No
1943895_adh	TCACAACAATGGAGAGAGAGC	Chr.4	5048369	5048349	-	No
2677753_adh	TTGTCATTGCATACATAATCT	Chr.4	5048647	5048667	+	No
1796904_adh	TAATATGTTGATCGTGTTTAA	Chr.4	5053817	5053837	+	No
2734040_adh	TTTTACATTTTAACGCTTTAT	Chr.4	5053858	5053838	-	No
2126795_adh	TGAACTTTTAGATCTAGGACA	Chr.4	5056107	5056087	-	No
2659091_adh	TTGGACTTGTGAGCAGATTTC	Chr.4	5056279	5056299	+	No
1815748_adh	TACCAGTTTTCAACCGATCAT	Chr.4	5061089	5061069	-	No
2126795_adh	TGAACTTTTAGATCTAGGACA	Chr.4	5062465	5062485	+	No
2707316_adh	TTTCGCCTTTGGACAAGAAGA	Chr.4	5064821	5064801	-	No
2722934_adh	TTTGGAAAGCTTGACAAAAAT	Chr.4	5067170	5067190	+	No
2615969_adh	TTCTCAATCAAGCAGCAATTG	Chr.4	5070875	5070895	+	No
2373369_adh	TGGACAAGAAATAGATACGTT	Chr.4	5072307	5072327	+	No
2052220_adh	TCTGTCAAGGCACAAATAGAA	Chr.4	5075034	5075054	+	Yes
2738765_adh	TTTTCGTAATTTAAATTAATT	Chr.4	5075707	5075727	+	Yes
2160485_adh	TGACCTTTAGAATGACGTCAG	Chr.4	5076661	5076641	-	No

2653000_adh	TTGCGGTAAACTTTCAGCAAC	Chr.4	5078635	5078655	+	No
1755958_adh	TAAACTTTCAGCAACATTTAA	Chr.4	5078641	5078661	+	No
2583429_adh	TTATTTAAAGTTCTCTCGCAT	Chr.4	5079665	5079645	-	Yes
2724462_adh	TTTGGCGTAGACGACAAGAAC	Chr.4	5087705	5087725	+	No
1999471_adh	TCGAATTTTGGACCATTTAGA	Chr.4	5091376	5091396	+	No
1802849_adh	TAATTATTTGTGGAAGAATGA	Chr.4	5096038	5096018	-	No
1885301_adh	TATAATAACTTTAAAAAATTA	Chr.4	5096057	5096037	-	No
2568183_adh	TTAGGAGTTCAGTACTGTTTG	Chr.4	5097975	5097955	-	Yes
2480463_adh	TGTAAAAGGAAGGTAAAACAA	Chr.4	5098451	5098471	+	No
2036394_adh	TCTCAAGAATGTTTGACAATC	Chr.4	5099753	5099733	-	Yes
2618608_adh	TTCTGCATAAAAGAATCTATA	Chr.4	5100104	5100124	+	Yes
2674955_adh	TTGTAGATATAGAAGACTTAT	Chr.4	5101210	5101190	-	Yes
2679699_adh	TTGTGACTAAATTTTTGAAAA	Chr.4	5101925	5101945	+	No
2546884_adh	TTAAGACGTAAACGTATATAT	Chr.4	5102553	5102533	-	No
2308496_adh	TGCTGAGTTTGTGGAAGTATA	Chr.4	5103099	5103119	+	No
2572031_adh	TTAGTGGTTTCCAAGACAATC	Chr.4	5104715	5104695	-	No
2554125_adh	TTACAGAAGTAGAATTCGAAC	Chr.4	5106861	5106841	-	Yes
1923799_adh	TATTCATCTTACGAAATTCTT	Chr.4	5107957	5107937	-	No
2665342_adh	TTGGCATGTTATTTTGTTTAT	Chr.4	5109900	5109920	+	Yes
2593412_adh	TTCAGTTTGGCATAATGGATT	Chr.4	5110330	5110310	-	No
1939190_adh	TCAAGCATTTCCTGACTTGCG	Chr.4	5112028	5112048	+	No
2744854_adh	TTTTTAATTTGTACATATAGC	Chr.4	5118021	5118041	+	Yes
2624348_adh	TTGAAAACGGATAACTGTAGT	Chr.4	5121286	5121306	+	No
1954095_adh	TCACTCAAGAAATACTAGGGG	Chr.4	5121630	5121610	-	No
1890216_adh	TATATGACTAATTTCAGGTAA	Chr.4	5130950	5130970	+	No
2717761_adh	TTTGATCAGCATTGAAAACGT	Chr.4	5132397	5132377	-	No
1762668_adh	TAAATTTAATATGTAAAAATC	Chr.4	5132577	5132557	-	Yes
2740321_adh	TTTTCTTTCTTATGCCTCCAG	Chr.4	5136428	5136408	-	Yes
2733520_adh	TTTTAAGGAAAAAGTTCACGG	Chr.4	5136939	5136919	-	No
2745399_adh	TTTTTATGTAGGCTTATTTGA	Chr.4	5137770	5137750	-	No
2590101_adh	TTCACTTGACTAACTTTGTAA	Chr.4	5138316	5138336	+	Yes
2641935_adh	TTGAGTAGATTAATTGAAACA	Chr.4	5141591	5141611	+	No
2549485_adh	TTAATAACATACGGAAGATAC	Chr.4	5144671	5144691	+	Yes
2590048_adh	TTCACTGTAACGTTGCAAAAA	Chr.4	5149015	5148995	-	No
2664429_adh	TTGGATTGTCGCAGTCGGTTA	Chr.4	5149294	5149274	-	Yes
2730059_adh	TTTGTGTCGGTAAAAATTAAG	Chr.4	5151224	5151204	-	Yes
2573643_adh	TTATAAAAACATATTATCCTC	Chr.4	5152872	5152852	-	No
1868445_adh	TAGGGAGAAGGAGAAGCTGTG	Chr.4	5153257	5153277	+	No
1750242_adh	TAAAAGTCTTATTTTCATTAA	Chr.4	5156076	5156056	-	Yes
1901642_adh	TATGAACTATGATGGGAGAGG	Chr.4	5165183	5165203	+	No
2273044_adh	TGATGTAGGAACATTTATAGG	Chr.4	5168781	5168801	+	No
2660319_adh	TTGGAGTGCTACATTTCTTCA	Chr.4	5172758	5172738	-	No
2566274_adh	TTAGCAATGTATAATATAATC	Chr.4	5172801	5172781	-	Yes
2703612_adh	TTTCAGTTAGCAATGTATAAT	Chr.4	5172807	5172787	-	Yes
2471618_adh	TGGTGCCAAGTAAAAAGTTTC	Chr.4	5179514	5179494	-	Yes
2674591_adh	TTGTAGAAAACGTTAAAAGTA	Chr.4	5179701	5179681	-	No
2702799_adh	TTTCACTTTTCCGTCCGAAAT	Chr.4	5183917	5183937	+	No
2702479_adh	TTTCACATTAAAGGTGGTGTA	Chr.4	5197136	5197156	+	Yes
1819985_adh	TACCTAGAATCTGCATATCAA	Chr.4	5200993	5201013	+	No
1912465_adh	TATGCAGATTCTAGGTACTGA	Chr.4	5201009	5200989	-	No

2643377_adh	TTGATACATAGCTTTTCCAGT	Chr.4	5201594	5201574	-	No
2025481_adh	TCGTCGATCAGGCAAAGTTAG	Chr.4	5202380	5202400	+	No
1936579_adh	TCAACGATCTTCGCGGAAAAT	Chr.4	5206264	5206284	+	No
2395912_adh	TGGATTTCCAGTGGAATGGCA	Chr.4	5209679	5209699	+	No
2559958_adh	TTACTTATTATCAACTTTCTC	Chr.4	5213343	5213323	-	Yes
2704408_adh	TTTCATTAGACTTGGCGTAAT	Chr.4	5216988	5217008	+	No
1973360_adh	TCATTAGACTTGGCGTAATCT	Chr.4	5216990	5217010	+	No
2639508_adh	TTGAGAAGATCGACGACAAAC	Chr.4	5219238	5219258	+	No
2180159_adh	TGAGAAGATCGACGACAAACA	Chr.4	5219239	5219259	+	No
2539217_adh	TTAAACAAGAGAACGAAGTCT	Chr.4	5220192	5220212	+	No
2639508_adh	TTGAGAAGATCGACGACAAAC	Chr.4	5227297	5227317	+	No
2180159_adh	TGAGAAGATCGACGACAAACA	Chr.4	5227298	5227318	+	No
2184674_adh	TGAGACTGCATCTATATAACA	Chr.4	5228616	5228596	-	No
2690914_adh	TTTAATAATGGACGCAGTTTT	Chr.4	5229477	5229497	+	Yes
1930988_adh	TATTTTCTTATGAAAACTTCG	Chr.4	5234438	5234458	+	Yes
2723208_adh	TTTGGAATTTTCAGAAACTGT	Chr.4	5234536	5234556	+	No
2723208_adh	TTTGGAATTTTCAGAAACTGT	Chr.4	5235839	5235819	-	No
1930988_adh	TATTTTCTTATGAAAACTTCG	Chr.4	5235937	5235917	-	Yes
2707316_adh	TTTCGCCTTTGGACAAGAAGA	Chr.4	5241544	5241524	-	No
2499632_adh	TGTCTGACAATCGGAAGATTC	Chr.4	5245325	5245305	-	No
2499632_adh	TGTCTGACAATCGGAAGATTC	Chr.4	5245875	5245895	+	No
2707316_adh	TTTCGCCTTTGGACAAGAAGA	Chr.4	5249656	5249676	+	No
1983869_adh	TCCGAAGAAAAGTTCTTCATT	Chr.4	5252400	5252380	-	No
2373369_adh	TGGACAAGAAATAGATACGTT	Chr.4	5253342	5253322	-	No
2615969_adh	TTCTCAATCAAGCAGCAATTG	Chr.4	5254775	5254755	-	No
2722934_adh	TTTGGAAAGCTTGACAAAAAT	Chr.4	5258481	5258461	-	No
2707316_adh	TTTCGCCTTTGGACAAGAAGA	Chr.4	5260808	5260828	+	No
1765154_adh	TAACAGCTTAGATTCAGTCTC	Chr.4	5266804	5266824	+	No
2036773_adh	TCTCAATTTTGAACGTCGACC	Chr.4	5270064	5270084	+	No
2593298_adh	TTCAGTTGACAATGGTTTTTT	Chr.4	5271206	5271226	+	Yes
2593298_adh	TTCAGTTGACAATGGTTTTTT	Chr.4	5271271	5271291	+	No
1799242_adh	TAATGAACATCTACTTTGGCA	Chr.4	5282100	5282080	-	Yes
2542183_adh	TTAAATACGAATTCATATTAA	Chr.4	5282546	5282526	-	No
2574109_adh	TTATAATGTACATTTACGCGT	Chr.4	5284577	5284597	+	No
2651494_adh	TTGCCTGACAGCTTATTAAAT	Chr.4	5289414	5289394	-	No
1834056_adh	TACTGTTATTACTTAGCAACT	Chr.4	5289664	5289644	-	No
1762170_adh	TAAATTATCATTGCAGAGATC	Chr.4	5291661	5291641	-	No
1929608_adh	TATTTCATCGTTGAATATCCA	Chr.4	5303124	5303104	-	No
2707316_adh	TTTCGCCTTTGGACAAGAAGA	Chr.4	5312870	5312850	-	No
2125737_adh	TGAACTGTCAAAACGGGACGT	Chr.4	5313258	5313278	+	No
2640958_adh	TTGAGCTACTGAGCGATGACA	Chr.4	5316155	5316135	-	No
2640958_adh	TTGAGCTACTGAGCGATGACA	Chr.4	5317610	5317590	-	No
2047438_adh	TCTGATTAGGAGAAAATTGAA	Chr.4	5324952	5324932	-	No
1929362_adh	TATTTATCGTATTTTAAAATC	Chr.4	5326667	5326687	+	Yes
2378769_adh	TGGACTGTTGATCTTTAGAAG	Chr.4	5327693	5327673	-	Yes
2581235_adh	TTATTCAACATATATTTCTAC	Chr.4	5330383	5330403	+	No
2549733_adh	TTAATACTCTTGTTTTATTCA	Chr.4	5336486	5336506	+	Yes
1933941_adh	TCAAAGTGAAGAACTTGTAAC	Chr.4	5340236	5340216	-	No
2710443_adh	TTTCTGAATTGGCAATGTGTT	Chr.4	5340786	5340766	-	Yes
2673527_adh	TTGTAAAACTAGCCAGAAAAA	Chr.4	5342667	5342647	-	No

2282616_adh	TGCAAGTGAGTAGTAGAAGTG	Chr.4	5350765	5350745	-	No
1767569_adh	TAACCTGGAGATTGCAGAAAA	Chr.4	5353720	5353700	-	No
2376713_adh	TGGACTAATAGCAGAAAAAAT	Chr.4	5369632	5369612	-	No
2658538_adh	TTGGACTAATAGCAGAAAAAA	Chr.4	5369633	5369613	-	No
2600825_adh	TTCCTGCAAGCTTCGACATGA	Chr.4	5370042	5370022	-	No
1801984_adh	TAATGTTGTGAAAGGAAATTC	Chr.4	5376574	5376594	+	Yes
2743644_adh	TTTTGTATCTGGTGGCATTAA	Chr.4	5388160	5388180	+	Yes
2744638_adh	TTTTTAAAATTCAAATATCGT	Chr.4	5391719	5391699	-	No
2040346_adh	TCTCGGTGTCGGCATAAAATT	Chr.4	5394915	5394935	+	No
1931349_adh	TATTTTTCTTCCCTCGGCGTT	Chr.4	5394944	5394964	+	No
2710425_adh	TTTCTGAATCGTCGAAAAAAT	Chr.4	5401957	5401937	-	No
2550096_adh	TTAATATTCTTAAATTTTTAT	Chr.4	5402357	5402337	-	No
2276877_adh	TGATTGGGTGATTGGATACAA	Chr.4	5404262	5404242	-	Yes
2575121_adh	TTATAGTGTACAATAATAAAA	Chr.4	5404800	5404820	+	No
1883159_adh	TAGTTTGATGACTTCATAATT	Chr.4	5404877	5404897	+	No
2718853_adh	TTTGATGACTTCATAATTTTT	Chr.4	5404880	5404900	+	No
2644805_adh	TTGATGACTTCATAATTTTTT	Chr.4	5404881	5404901	+	No
2513085_adh	TGTGATTTTTATCTGACGACT	Chr.4	5404914	5404934	+	No
2746814_adh	TTTTTGATAACATAACGGCTG	Chr.4	5405648	5405668	+	No
1765521_adh	TAACATAACGGCTGCATATCA	Chr.4	5405655	5405675	+	No
1832910_adh	TACTGGAAATCACAAGGAAAG	Chr.4	5407588	5407608	+	Yes
1970403_adh	TCATCAAGTATATTGCGGAAT	Chr.4	5410643	5410623	-	No
2004487_adh	TCGATTAAAGAGATGGGATCA	Chr.4	5412125	5412105	-	No
1999559_adh	TCGACAAGACTAAAAGCATTA	Chr.4	5415109	5415129	+	No
2701651_adh	TTTCAATAAATCTCGGAAATT	Chr.4	5416333	5416353	+	No
1833520_adh	TACTGGTATCTGTGATTTGCG	Chr.4	5418452	5418432	-	No
2737250_adh	TTTTCAATCGAGATAAATAGG	Chr.4	5420254	5420274	+	Yes
2555737_adh	TTACCATGTAGTTCTTGGCAT	Chr.4	5427410	5427390	-	Yes
2738781_adh	TTTTCGTAGACGTCATAAACT	Chr.4	5427790	5427770	-	No
1824724_adh	TACGCATACTTGTTGGTTGAT	Chr.4	5428012	5427992	-	Yes
2681187_adh	TTGTGATTTCATATATTCCTT	Chr.4	5431663	5431683	+	No
2069744_adh	TGAAAATAACTGGAACGGACA	Chr.4	5432055	5432035	-	No
2159286_adh	TGACATTTTTCGGAAGATTCA	Chr.4	5432111	5432131	+	No
2393029_adh	TGGATTCAGATAAGAAATGCA	Chr.4	5434815	5434835	+	No
2032762_adh	TCTAGGACATTTTTGCAGAAT	Chr.4	5435576	5435596	+	No
1834772_adh	TACTTCACTTTTTTAAAAACA	Chr.4	5437295	5437315	+	Yes
2728591_adh	TTTGTATTACGAAACTTTATT	Chr.4	5437360	5437380	+	No
2060681_adh	TCTTTATTACTGGGCGTCATA	Chr.4	5438891	5438911	+	Yes
2700714_adh	TTTATTTCGTTTTACAAAACT	Chr.4	5449345	5449325	-	Yes
1934261_adh	TCAAATATTTTTTCGGATGAT	Chr.4	5449438	5449418	-	No
2500038_adh	TGTCTTAAGTTTTAGCTGGTA	Chr.4	5450224	5450244	+	No
2602389_adh	TTCGAATAGTTCTCTTATTGT	Chr.4	5458735	5458755	+	No
2012932_adh	TCGGAGGGTGGTTGTTTCCAT	Chr.4	5462347	5462327	-	No
2005257_adh	TCGATTTGAGAACGACAGCTT	Chr.4	5473048	5473068	+	No
1955183_adh	TCACTGTTTAGAACACGGATA	Chr.4	5473248	5473268	+	No
2531782_adh	TGTTTAGAACACGGATAGACG	Chr.4	5473252	5473272	+	No
1994921_adh	TCGAAAGGACATTGGCGACGG	Chr.4	5484228	5484208	-	No
2041423_adh	TCTCGTCGAGCAGAATCGCTT	Chr.4	5485797	5485817	+	No
1994607_adh	TCGAAAATGAAGAATTTGGCG	Chr.4	5486388	5486368	-	No
2740974_adh	TTTTGAATTTTGATGCGCGGT	Chr.4	5486590	5486610	+	No

2714572_adh	TTTGAATTTTGATGCGCGGTA	Chr.4	5486591	5486611	+	No
2741830_adh	TTTTGATGCGCGGTAATTTCG	Chr.4	5486597	5486617	+	No
2498681_adh	TGTCGTCGAAATCGATTCCTA	Chr.4	5491055	5491075	+	No
2616471_adh	TTCTCGACAATTCGAATACGT	Chr.4	5493980	5494000	+	No
2615660_adh	TTCTATGTACAACGGTTCTCT	Chr.4	5498781	5498801	+	Yes
2639789_adh	TTGAGAGAACCGTTGTACATA	Chr.4	5498804	5498784	-	No
1968003_adh	TCATAAAAAGAACCTAGCGGA	Chr.4	5500560	5500540	-	No
2639781_adh	TTGAGACTTTTATTCAGATAT	Chr.4	5501944	5501924	-	Yes
2716473_adh	TTTGAGACTTTTATTCAGATA	Chr.4	5501945	5501925	-	Yes
2714110_adh	TTTGAAGTTTTTATAAGTTTT	Chr.4	5502158	5502138	-	Yes
1963491_adh	TCAGGAAGCGAAACTCAAGTA	Chr.4	5503606	5503626	+	No
1754064_adh	TAAACAAGTAGTAAGAACTCC	Chr.4	5506621	5506641	+	No
2380139_adh	TGGAGACGCAGAAACGTATTG	Chr.4	5507481	5507501	+	No
2014236_adh	TCGGATGAAATTGAAATCGGG	Chr.4	5507697	5507677	-	Yes
2531569_adh	TGTTTAAAGAGATAATTGGAT	Chr.4	5508124	5508144	+	No
2464720_adh	TGGTATCTGATCGGTTGAAAT	Chr.4	5509212	5509232	+	Yes
2733794_adh	TTTTAATTACAATTTGAATAA	Chr.4	5509355	5509335	-	Yes
1967144_adh	TCAGTGTTTCAGTAGGTGCTT	Chr.4	5523074	5523094	+	No
1934090_adh	TCAAATAGAGAAATGACGCGG	Chr.4	5523231	5523251	+	No
2487525_adh	TGTAGAATAGATTCGGGTGCA	Chr.4	5526188	5526168	-	No
2674740_adh	TTGTAGAATAGATTCGGGTGC	Chr.4	5526189	5526169	-	No
1980050_adh	TCCATTGTAGAATAGATTCGG	Chr.4	5526193	5526173	-	No
2703735_adh	TTTCATACGCCAATGGTTTGT	Chr.4	5526334	5526354	+	No
1876786_adh	TAGTCGTATGTCAACAATTGT	Chr.4	5528813	5528793	-	Yes
2682961_adh	TTGTTACGCAGATTCTAGAAA	Chr.4	5529718	5529738	+	No
2522917_adh	TGTTACGCAGATTCTAGAAAA	Chr.4	5529719	5529739	+	No
2039774_adh	TCTCGCGAAGTGAATTATTTT	Chr.4	5532511	5532491	-	No
1893293_adh	TATCAGAGATTTTCCCGTCGG	Chr.4	5538031	5538051	+	No
2585889_adh	TTCAACGAGAAAATCAACGAT	Chr.4	5541389	5541369	-	No
2268667_adh	TGATATTTATAGCTTATCTTT	Chr.4	5545321	5545341	+	No
1754536_adh	TAAACATTGGACAATGCAAAC	Chr.4	5559939	5559919	-	No
1883520_adh	TAGTTTTGTTATCCGGTGAGT	Chr.4	5560609	5560589	-	Yes
2010494_adh	TCGGAACAATCTTGAGGCGCC	Chr.4	5563491	5563471	-	No
2553597_adh	TTACAATGGTACTTTAAATTC	Chr.4	5573665	5573645	-	No
1855329_adh	TAGCATGGTAGTAGCACTGAC	Chr.4	5574281	5574301	+	No
2737706_adh	TTTTCATCGTTTTTTATATAT	Chr.4	5575744	5575764	+	Yes
2740385_adh	TTTTCTTTTTAACCTGGCAAT	Chr.4	5578553	5578533	-	No
2066789_adh	TGAAAACAGAATATTCGCTAA	Chr.4	5582700	5582680	-	Yes
2598710_adh	TTCCGACGGAAATACATATGA	Chr.4	5587933	5587913	-	No
2039703_adh	TCTCGCAGAATGATCCTTGAA	Chr.4	5590726	5590706	-	Yes
1975539_adh	TCATTTTCGTTTCGGTTTTAA	Chr.4	5591404	5591384	-	No
2594208_adh	TTCATCCAAGGACAATGCGGA	Chr.4	5592169	5592149	-	No
2737960_adh	TTTTCCCTTTAAAAATTCATC	Chr.4	5593029	5593049	+	No
2616772_adh	TTCTCGTAGGAAAAAGCGGTT	Chr.4	5594316	5594336	+	Yes
1790210_adh	TAATAAATGGATAAGATTTTT	Chr.4	5595254	5595234	-	Yes
2584149_adh	TTATTTGGGAAATTTTGTGAC	Chr.4	5596928	5596948	+	Yes
1762096_adh	TAAATTACACAATTCCATTTC	Chr.4	5599553	5599533	-	No
2288712_adh	TGCAGTAGACAGTGGATATAA	Chr.4	5600309	5600289	-	No
2570249_adh	TTAGTACTTCTTCTTCTGGAA	Chr.4	5600786	5600766	-	No
2558721_adh	TTACTCCATTATTTTTCATAA	Chr.4	5602220	5602240	+	No

1980122_adh	TCCATTTTTTATTACAATTTA	Chr.4	5604966	5604946	-	Yes
2746988_adh	TTTTTGCAATCTTTGGACACA	Chr.4	5606955	5606935	-	No
2689286_adh	TTTAAAGCAGCCTAAGTAAAC	Chr.4	5607077	5607057	-	No
1928998_adh	TATTTAAAGCAGCCTAAGTAA	Chr.4	5607079	5607059	-	No
1927996_adh	TATTGTGATTCAAGCTGGTAT	Chr.4	5610797	5610777	-	No
2573659_adh	TTATAAAATGCATCGAATTAT	Chr.4	5619653	5619633	-	No
2526361_adh	TGTTCGAGCAAACACTTTTCA	Chr.4	5625935	5625915	-	No
1762696_adh	TAAATTTATTTCAATTAACTT	Chr.4	5627776	5627796	+	No
2583100_adh	TTATTGGATTCTACTATATGG	Chr.4	5641673	5641653	-	Yes
2043255_adh	TCTCTTCAGAGATGGCTACAT	Chr.4	5642987	5643007	+	Yes
2549503_adh	TTAATAAGAAACAAACATTTG	Chr.4	5644364	5644344	-	No
2732835_adh	TTTGTTTTGATATGTAAGGTT	Chr.4	5645337	5645317	-	No
1890437_adh	TATATGTAGGTAGATCAAATT	Chr.4	5648535	5648515	-	No
2593589_adh	TTCATAGAAGAACAAATAAAT	Chr.4	5649224	5649204	-	No
2657060_adh	TTGGAAGACTTGATTTCATAG	Chr.4	5649238	5649218	-	No
1810326_adh	TACAATTGGAAGACTTGATTT	Chr.4	5649243	5649223	-	No
2486959_adh	TGTACTACAATTGGAAGACTT	Chr.4	5649248	5649228	-	No
1891496_adh	TATCAACTTCCATCGTCTGAA	Chr.4	5649533	5649513	-	No
1813537_adh	TACATCTGTTCGTTGGTGACC	Chr.4	5666564	5666544	-	No
2700716_adh	TTTATTTCTAGTTTGAAAGTA	Chr.4	5667078	5667058	-	No
1890177_adh	TATATGAAAAGGCAGAAAATG	Chr.4	5670913	5670893	-	No
2285019_adh	TGCAATGTATTCAATCGTCCA	Chr.4	5673111	5673091	-	No
2623149_adh	TTCTTGTCTTAAACGTTCTCC	Chr.4	5673824	5673844	+	No
2469208_adh	TGGTCAGAAAATTGCTATGAA	Chr.4	5675067	5675047	-	Yes
2037297_adh	TCTCAGAATTCGGAATTCAGT	Chr.4	5678147	5678127	-	No
1966453_adh	TCAGTCTCAGAATTCGGAATT	Chr.4	5678151	5678131	-	No
1925228_adh	TATTCTTTCGGTACATTCTTT	Chr.4	5680441	5680421	-	Yes
2582038_adh	TTATTCTTTCGGTACATTCTT	Chr.4	5680442	5680422	-	Yes
2699977_adh	TTTATTCTTTCGGTACATTCT	Chr.4	5680443	5680423	-	Yes
2071816_adh	TGAAACAGTAGAAAATTCAGA	Chr.4	5680478	5680498	+	No
1821874_adh	TACGAAGTTTTGGAAACTTCA	Chr.4	5690685	5690665	-	No
2689573_adh	TTTAAATTAAGAAATCGACAA	Chr.4	5695471	5695451	-	No
2739346_adh	TTTTCTCTCCTGAAAACTAGT	Chr.4	5703574	5703554	-	No
2597716_adh	TTCCCATATCAGATATTTTAA	Chr.4	5705879	5705859	-	No
2689643_adh	TTTAAATTTGGCAATTTTTTA	Chr.4	5705947	5705927	-	No
2551858_adh	TTAATTATGCGACTCATTTTG	Chr.4	5706529	5706509	-	Yes
2481289_adh	TGTAACAGGACGTTTAGCGAA	Chr.4	5709793	5709813	+	No
2651143_adh	TTGCCATTTTCCATAGACGCA	Chr.4	5712055	5712075	+	Yes
1919922_adh	TATTAAACTTTTCTTCGAAGC	Chr.4	5714840	5714860	+	No
1750508_adh	TAAAATAATTTGTTAAAAAAT	Chr.4	5716350	5716330	-	No
2644028_adh	TTGATCATATTTTAGGCTCGA	Chr.4	5716799	5716819	+	No
2573364_adh	TTAGTTTATAGATGTCCTCCG	Chr.4	5721849	5721869	+	Yes
2749744_adh	TTTTTTGTCTGAAAATTATCC	Chr.4	5724113	5724133	+	No
2553659_adh	TTACAATTGAATTAAAATTAT	Chr.4	5726042	5726022	-	Yes
2735503_adh	TTTTATCATCTCTTAGTATAC	Chr.4	5728126	5728106	-	No
2040074_adh	TCTCGGAGCGTCGTACCAGAA	Chr.4	5731758	5731738	-	No
1923144_adh	TATTATTTAGGTAGATCTTTC	Chr.4	5733038	5733018	-	No
2557834_adh	TTACGGTTTCAGAGACTTTTC	Chr.4	5733270	5733250	-	No
2744931_adh	TTTTTACGGTTTCAGAGGGTT	Chr.4	5734130	5734110	-	No
1790749_adh	TAATAATCGTTGTGTCTACTT	Chr.4	5736385	5736365	-	Yes

2747284_adh	TTTTTGGCAGAGAGAGTTTGT	Chr.4	5741021	5741001	-	No
2749537_adh	TTTTTTGGCAGAGAGAGTTTG	Chr.4	5741022	5741002	-	No
2014827_adh	TCGGATTGAACTGCAAGTGGA	Chr.4	5742193	5742173	-	No
2584480_adh	TTATTTTAGACTGTGTATTTC	Chr.4	5747124	5747104	-	No
2583659_adh	TTATTTATTTTAGACTGTGTA	Chr.4	5747128	5747108	-	No
2721159_adh	TTTGCACAAGAGTTAGAGGAG	Chr.4	5754299	5754319	+	No
2270002_adh	TGATCGTATTGTTTATGGTAG	Chr.4	5759333	5759353	+	No
2699732_adh	TTTATTCATATGCTTAGCTCT	Chr.4	5762688	5762668	-	Yes
2119778_adh	TGAACAAGGCTTACTAAAAGT	Chr.4	5763651	5763631	-	No
1801872_adh	TAATGTGTTGTAGGGTAAAAT	Chr.4	5769284	5769264	-	Yes
2565372_adh	TTAGATGGCTGAAAATGGACT	Chr.4	5775647	5775627	-	No
2695841_adh	TTTAGATGGCTGAAAATGGAC	Chr.4	5775648	5775628	-	No
2702401_adh	TTTCACAAGTTAATTCTAGCA	Chr.4	5781325	5781345	+	No
2644883_adh	TTGATGATTAATTGCTTTTTT	Chr.4	5789340	5789320	-	No
2650744_adh	TTGCATTCATTGAGTTTTGAG	Chr.4	5789927	5789947	+	No
1805485_adh	TAATTTGTCGCATATTTTTAC	Chr.4	5790888	5790908	+	Yes
2653619_adh	TTGCGTGTTTTCGTTTAAATA	Chr.4	5791428	5791448	+	Yes
2470402_adh	TGGTCTTATGGGTCATTTTTC	Chr.4	5794512	5794532	+	Yes
2581232_adh	TTATTCAAAGCACAGTGCAAA	Chr.4	5797010	5796990	-	No
2744884_adh	TTTTTACAGATATAGAAATTT	Chr.4	5801689	5801669	-	Yes
1797423_adh	TAATCAAATGATTGTCTATGG	Chr.4	5808739	5808719	-	No
2630415_adh	TTGAATCGTACGACAGCAAGC	Chr.4	5814433	5814413	-	No
2607061_adh	TTCGGAAAATTGCATCTTATT	Chr.4	5818618	5818638	+	No
1953999_adh	TCACTAGCAGTTCGAGGAAAT	Chr.4	5819060	5819080	+	Yes
1930444_adh	TATTTGGCAGGGTTTGAAATT	Chr.4	5819519	5819539	+	No
1811697_adh	TACAGAAGAAACAGAAGAATT	Chr.4	5820048	5820028	-	Yes
2685596_adh	TTGTTGATACAGAAGAAACAG	Chr.4	5820055	5820035	-	Yes
2731889_adh	TTTGTTGATACAGAAGAAACA	Chr.4	5820056	5820036	-	Yes
2726041_adh	TTTGGTATTTGAAGATAATAT	Chr.4	5822954	5822934	-	Yes
2463611_adh	TGGTAGTACAGTATACAGATT	Chr.4	5823123	5823103	-	No
2068843_adh	TGAAAAGCTGGCTATGGTCCC	Chr.4	5823239	5823219	-	No
2639655_adh	TTGAGACATTACAATGAATAA	Chr.4	5827929	5827949	+	No
2052278_adh	TCTGTCAGTTGAAATGGACAT	Chr.4	5834436	5834456	+	No
2545067_adh	TTAACATATATTCAAACTGTT	Chr.4	5834994	5834974	-	No
2722751_adh	TTTGCTTGTAGAACAACGGTA	Chr.4	5837288	5837268	-	No
2742591_adh	TTTTGCTTGTAGAACAACGGT	Chr.4	5837289	5837269	-	No
1901097_adh	TATGAAACAGTAGGATTTAGA	Chr.4	5837466	5837446	-	No
1813204_adh	TACATAGTGACGGAACGACAG	Chr.4	5837523	5837503	-	No
2672432_adh	TTGGTTGAATGTCGCTATGGG	Chr.4	5838069	5838089	+	Yes
2130719_adh	TGAAGCAGTGAGAACAAATTA	Chr.4	5839699	5839679	-	No
2579084_adh	TTATGCATTAAGTTACTATAA	Chr.4	5840601	5840581	-	No
2748932_adh	TTTTTTCGTCGGAAAACTTGA	Chr.4	5840763	5840743	-	No
2160220_adh	TGACCGTTTCATCGTCGAAGA	Chr.4	5847043	5847023	-	No
2749218_adh	TTTTTTGAATTTTCGTCGCAT	Chr.4	5848054	5848034	-	No
2396375_adh	TGGATTTTATGAAGAAGAGCT	Chr.4	5855440	5855460	+	No
2610041_adh	TTCGGTCTTGTTGGCAAAATA	Chr.4	5856272	5856252	-	No
2687453_adh	TTGTTTGCCAGATTTATATTT	Chr.4	5856817	5856797	-	No
1930360_adh	TATTTGGAAGCAGAAACTATT	Chr.4	5860166	5860186	+	No
2657129_adh	TTGGAAGCAGAAACTATTCTC	Chr.4	5860169	5860189	+	No
2004521_adh	TCGATTAGGACTCAACAACAA	Chr.4	5863793	5863773	-	No

1920121_adh	TATTAAGACGGCCAAATTATT	Chr.4	5866904	5866924	+	No
2546879_adh	TTAAGACGGCCAAATTATTTT	Chr.4	5866906	5866926	+	No
1930168_adh	TATTTGAATAAAACGTTTGGA	Chr.4	5869851	5869871	+	No
2623347_adh	TTCTTTATACTTACATGTTGA	Chr.4	5873858	5873878	+	No
2694122_adh	TTTACTACAAACTCGTAGAGT	Chr.4	5877398	5877418	+	No
2567294_adh	TTAGCTGAGCGGCAAATGATA	Chr.4	5878765	5878785	+	No
1977908_adh	TCCAGAAGACTGAGTCAAATA	Chr.4	5879597	5879577	-	Yes
1921695_adh	TATTAGGTAGAATAGCGACTA	Chr.4	5879897	5879917	+	No
2423240_adh	TGGGAGATTAGCAGCAATAAG	Chr.4	5882348	5882368	+	No
1894049_adh	TATCATCCTTCTTCATATCAT	Chr.4	5883861	5883881	+	No
1929001_adh	TATTTAAAGTTTGAATTTCAA	Chr.4	5886937	5886917	-	No
2526169_adh	TGTTCATTGTGGGAGTTGGAC	Chr.4	5888334	5888354	+	No
2646759_adh	TTGATTTACGCGCTAATCTGA	Chr.4	5889014	5888994	-	Yes
2739563_adh	TTTTCTGCTGTTTTGAAAAGG	Chr.4	5889332	5889312	-	No
2577174_adh	TTATCATGAAACTCAAGACAA	Chr.4	5890204	5890224	+	No
2479124_adh	TGGTTTTCTATTTATGAATTC	Chr.4	5891385	5891365	-	No
2714163_adh	TTTGAATAGAAAATAAACATG	Chr.4	5892691	5892711	+	Yes
2591888_adh	TTCAGCTGATGATAAGACTTC	Chr.4	5894574	5894594	+	No
1834070_adh	TACTGTTCCTTCAGAAGAGAT	Chr.4	5900623	5900643	+	Yes
2119361_adh	TGAAATTTTCTTTGTAACTTT	Chr.4	5903880	5903860	-	Yes
2593419_adh	TTCAGTTTTGAACAGCGGTAA	Chr.4	5904325	5904345	+	No
2740672_adh	TTTTGAACAGCGGTAACAATA	Chr.4	5904330	5904350	+	No
1762411_adh	TAAATTGAACACAATTCAGAA	Chr.4	5905155	5905175	+	No
2699330_adh	TTTATGTAGTCGAATTATAAT	Chr.4	5905972	5905952	-	No
1921810_adh	TATTAGTTTATGTAGTCGAAT	Chr.4	5905978	5905958	-	No
2526775_adh	TGTTCTCAAACATACTTTATT	Chr.4	5906577	5906557	-	No
2703658_adh	TTTCATAAATACATAACATAA	Chr.4	5908420	5908400	-	No
2172739_adh	TGACTTAGAAATACGCTATAC	Chr.4	5909389	5909369	-	No
2557776_adh	TTACGGTAGATTGCAAAAAAT	Chr.4	5911001	5910981	-	No
2656241_adh	TTGGAAAAAATGTGAATCAAAA	Chr.4	5912676	5912696	+	No
2624502_adh	TTGAAAAGTTTCTGGACGTAT	Chr.4	5913772	5913752	-	No
2527813_adh	TGTTGAATTCGGCTGATTATT	Chr.4	5920598	5920618	+	No
2046684_adh	TCTGATAGGTAACGTGTAGGC	Chr.4	5923200	5923220	+	Yes
1794900_adh	TAATAGCAAGTAAGTTGATGT	Chr.4	5925005	5925025	+	No
1853557_adh	TAGCAAGTAAGTTGATGTTCA	Chr.4	5925008	5925028	+	No
2279263_adh	TGATTTTGAATGGATTTGTGT	Chr.4	5925889	5925909	+	No
2591000_adh	TTCAGCACATTGAATCAATAT	Chr.4	5928717	5928737	+	No
1921918_adh	TATTATAATCGTGGTATTTGC	Chr.4	5932904	5932924	+	No
1994289_adh	TCCTTTTGCTGACAAGTGCTA	Chr.4	5933694	5933674	-	No
2703657_adh	TTTCATAAAGAATTTGGTTGG	Chr.4	5936192	5936172	-	No
2137783_adh	TGAATACTGGAAGGCACTCGA	Chr.4	5943338	5943358	+	No
2699643_adh	TTTATTATCGGATGAATTGGC	Chr.4	5945701	5945721	+	No
2577816_adh	TTATCGGATGAATTGGCGGAA	Chr.4	5945705	5945725	+	No
1897688_adh	TATCGGATGAATTGGCGGAAT	Chr.4	5945706	5945726	+	No
2577907_adh	TTATCGTCGAATAACTTGTTG	Chr.4	5947131	5947111	-	No
2593223_adh	TTCAGTTAGATGACCGTATAA	Chr.4	5947774	5947754	-	No
2057799_adh	TCTTGACGGTTTTTCTTCGAT	Chr.4	5947837	5947817	-	No
2730874_adh	TTTGTTCTTGACGGTTTTTCT	Chr.4	5947842	5947822	-	No
2710648_adh	TTTCTGCTTTGTTCTTGACGG	Chr.4	5947849	5947829	-	No
2739567_adh	TTTTCTGCTTTGTTCTTGACG	Chr.4	5947850	5947830	-	No

2665891_adh	TTGGCGTATTATACTTTAGAT	Chr.4	5949466	5949486	+	No
2496928_adh	TGTCCAAGTCGGACGACCGGA	Chr.4	5950106	5950086	-	No
1975543_adh	TCATTTTCTCTGAAGACGTAA	Chr.4	5950655	5950635	-	No
1968794_adh	TCATACGTTCAAATGATACCA	Chr.4	5951444	5951424	-	No
1891132_adh	TATCAAAATGAACGTTGAAAA	Chr.4	5957756	5957736	-	No
2593596_adh	TTCATAGACAATTCTAATTTT	Chr.4	5961192	5961172	-	No
1829516_adh	TACTACAGCGCTTTTATGACA	Chr.4	5966175	5966195	+	No
1812301_adh	TACAGCGCTTTTATGACAATT	Chr.4	5966178	5966198	+	No
2535029_adh	TGTTTTTATATCGGAATCTGT	Chr.4	5968885	5968865	-	No
1759497_adh	TAAATACAAAAAATACATCAT	Chr.4	5970174	5970154	-	No
2688676_adh	TTTAAAAAATAAATTTCACTC	Chr.4	5971166	5971186	+	Yes
2641001_adh	TTGAGCTGTGATGTGATGAAT	Chr.4	5979478	5979498	+	Yes
1938131_adh	TCAAGAACATCAAGATGACGA	Chr.4	5993409	5993429	+	No
2611825_adh	TTCGTATTTGTTAGAGAAGAG	Chr.4	6001987	6002007	+	No
2714813_adh	TTTGACAGACAAAGAGCAAAT	Chr.4	6011951	6011971	+	No
2707285_adh	TTTCGCATCGAATGGATCTAG	Chr.4	6013578	6013558	-	Yes
1939527_adh	TCAAGGACAAGTGTGAAAGAA	Chr.4	6014746	6014766	+	No
1829931_adh	TACTAGAAGCATATTTAGATG	Chr.4	6017251	6017231	-	No
1969089_adh	TCATAGCTTTACAATAGAAAA	Chr.4	6019878	6019898	+	No
2500730_adh	TGTGAAACTGTAAGACGATAG	Chr.4	6025719	6025699	-	No
2644884_adh	TTGATGATTACTTGAAGTAAG	Chr.4	6028001	6027981	-	No
2639424_adh	TTGAGAAATATAGGAACTTTT	Chr.4	6028549	6028529	-	No
2656470_adh	TTGGAAAGTTCGAGATGAAAT	Chr.4	6034575	6034555	-	No
2553970_adh	TTACACTAACTAAATTCATTT	Chr.4	6035048	6035068	+	Yes
2580981_adh	TTATTAGTACTGGCACTCTAG	Chr.4	6042247	6042267	+	No
1921727_adh	TATTAGTACTGGCACTCTAGA	Chr.4	6042248	6042268	+	No
2570234_adh	TTAGTACTGGCACTCTAGAGA	Chr.4	6042250	6042270	+	No
2373063_adh	TGGAATTTTGCTCGACGTCAT	Chr.4	6047241	6047261	+	No
1899611_adh	TATCTCGGCTTCAATCTATAA	Chr.4	6049686	6049666	-	No
1979942_adh	TCCATTAACAGCAATTTTTTA	Chr.4	6055701	6055721	+	Yes
2743218_adh	TTTTGGTAATTAATCCGAGGC	Chr.4	6057116	6057096	-	No
1878451_adh	TAGTGGAAAATGAGAAAATAT	Chr.4	6059738	6059758	+	No
2480508_adh	TGTAAAATCTTGGGTTAGGAC	Chr.4	6061920	6061900	-	No
1931500_adh	TATTTTTTCTTCAGGTTCATA	Chr.4	6063518	6063498	-	Yes
1814515_adh	TACATTTAGAACGGTAAAATT	Chr.4	6072429	6072409	-	No
1762275_adh	TAAATTCCTCTTTATAGGTAA	Chr.4	6073531	6073551	+	Yes
2038512_adh	TCTCATTGTTTAGGATCGGCA	Chr.4	6076057	6076037	-	Yes
2745825_adh	TTTTTCCAGAAATACATTCCT	Chr.4	6077516	6077496	-	No
2622844_adh	TTCTTGCATGTATGAATAATA	Chr.4	6078402	6078422	+	No
1847688_adh	TAGAGATGGACAGGCTTTTAT	Chr.4	6088417	6088397	-	No
2606606_adh	TTCGCGGAATACAGATGGATG	Chr.4	6089350	6089330	-	No
2561426_adh	TTAGAAGAATTTGTGCAAAAA	Chr.4	6091482	6091502	+	No
2500673_adh	TGTGAAACCTGATGAAAAAAT	Chr.4	6092683	6092663	-	No
2500526_adh	TGTGAAAAATCGGATGTTCAA	Chr.4	6095197	6095177	-	No
2591783_adh	TTCAGCGGAAGTACAGCTAAA	Chr.4	6096435	6096455	+	No
2074052_adh	TGAAACTGGAGAGAATGATGG	Chr.4	6097081	6097061	-	No
2322416_adh	TGGAAGACGACGGAGCCCCAC	Chr.4	6105778	6105758	-	No
1923472_adh	TATTCACTCGCAGCTTTTAGA	Chr.4	6114529	6114549	+	No
2553257_adh	TTACAACAGAATTATGTGAGG	Chr.4	6115465	6115485	+	No
1979913_adh	TCCATGTTACATGTTACATGT	Chr.4	6128686	6128666	-	No

1821416_adh	TACCTTTTTTGCCTGCTTTAT	Chr.4	6129101	6129081	-	Yes
2749128_adh	TTTTTTCTTTACGTTGTACAC	Chr.4	6129917	6129897	-	No
2595946_adh	TTCCAATATATAGAAAAAGGA	Chr.4	6140511	6140491	-	No
1759999_adh	TAAATATAGAGTTGAATTTTG	Chr.4	6151011	6150991	-	No
1953404_adh	TCACGGTATAGGTGCGGTGAA	Chr.4	6151332	6151352	+	No
2704798_adh	TTTCATTTTTTACTACTCGGT	Chr.4	6156933	6156913	-	No
2616469_adh	TTCTCGACAAAAAATGCTGTA	Chr.4	6157074	6157094	+	No
2537516_adh	TTAAAAAATTAAAGTCGTCGT	Chr.4	6157104	6157124	+	No
2743779_adh	TTTTGTCGACGTTATGGAAAG	Chr.4	6162925	6162945	+	No
2586909_adh	TTCAAGAGTGACGTTGTTTAT	Chr.4	6164902	6164922	+	No
2748160_adh	TTTTTTAATATACTATTTACA	Chr.4	6169043	6169023	-	Yes
2162166_adh	TGACGTATCATAGGTAAAATT	Chr.4	6169647	6169667	+	No
2155100_adh	TGACATAGAGCATAAAACAAG	Chr.4	6172353	6172373	+	No
2742523_adh	TTTTGCTGAAAATGTGGCATG	Chr.4	6172434	6172454	+	No
2136065_adh	TGAAGTTTCAGGTTAACGGCA	Chr.4	6173913	6173933	+	No
2004464_adh	TCGATGTTGCATAGATTTATT	Chr.4	6186909	6186929	+	Yes
2678105_adh	TTGTCGAATATAAAAGTTGGC	Chr.4	6189847	6189867	+	No
1880418_adh	TAGTTAGTATGTTCGTGGTGA	Chr.4	6191262	6191282	+	Yes
2728817_adh	TTTGTCATTCGGCGAAAATTT	Chr.4	6193687	6193707	+	Yes
2677748_adh	TTGTCATTCGGCGAAAATTTT	Chr.4	6193688	6193708	+	Yes
2744130_adh	TTTTGTTCAACTGGCTTTTTA	Chr.4	6197502	6197522	+	Yes
2746231_adh	TTTTTCTCGTGTTTGTGGCAT	Chr.4	6206502	6206482	-	No
1869870_adh	TAGGGTTAAAGAAGTTGGTTG	Chr.4	6209585	6209605	+	No
2025371_adh	TCGTCATGTATACGCTCCACA	Chr.4	6217074	6217094	+	Yes
1848225_adh	TAGAGGAAATACGTCAAAGTA	Chr.4	6230958	6230938	-	No
1794847_adh	TAATAGAGGAAATACGTCAAA	Chr.4	6230961	6230941	-	No
2451048_adh	TGGGCTGATGCAGAAGGTGTC	Chr.4	6243523	6243543	+	No
1887182_adh	TATACGGAGCTTCAGTTGTGT	Chr.4	6244502	6244522	+	No
1879145_adh	TAGTGTAAGACGTGGAATGTT	Chr.4	6258765	6258745	-	No
2572034_adh	TTAGTGTAAGACGTGGAATGT	Chr.4	6258766	6258746	-	No
2713212_adh	TTTGAACGCAGAGAAATGTAA	Chr.4	6263080	6263100	+	No
2626356_adh	TTGAACGCAGAGAAATGTAAG	Chr.4	6263081	6263101	+	No
2704747_adh	TTTCATTTGTGGCATACTTGA	Chr.4	6263610	6263630	+	No
1897457_adh	TATCGCTAAGTTTTATAATTT	Chr.4	6267760	6267740	-	Yes
2571887_adh	TTAGTGGAAGGGAGTAGTCGT	Chr.4	6272131	6272151	+	No
1878587_adh	TAGTGGAAGGGAGTAGTCGTA	Chr.4	6272132	6272152	+	No
2126402_adh	TGAACTTACAATTGAATCGGG	Chr.4	6272902	6272922	+	No
1765156_adh	TAACAGCTTGAATTCAGTCTC	Chr.4	6276327	6276347	+	No
2583294_adh	TTATTGTGACGAACACTTTTT	Chr.4	6279647	6279667	+	No
2701152_adh	TTTCAAAGTAGATCTTTTCAC	Chr.4	6280114	6280134	+	No
2737137_adh	TTTTCAAATCGTAAGCTCAAG	Chr.4	6282756	6282736	-	No
2745802_adh	TTTTTCATTGGATTGTCGTAT	Chr.4	6290359	6290379	+	No
2732845_adh	TTTGTTTTGTATTTTCTTTTG	Chr.4	6291602	6291622	+	No
2730108_adh	TTTGTGTGATTTCAGTTCTTT	Chr.4	6301622	6301602	-	Yes
1889896_adh	TATATATTGATTGTCCTGAAA	Chr.4	6305219	6305239	+	No
1853875_adh	TAGCACATGACATAGAGGTAA	Chr.4	6306140	6306160	+	No
1871464_adh	TAGGTGACAGACGAAAAATAG	Chr.4	6307124	6307144	+	No
1812766_adh	TACAGTGAAACTGAAATAGTT	Chr.4	6313948	6313928	-	No
2490487_adh	TGTATACTGGAAAGACTGGAT	Chr.4	6317158	6317138	-	No
2721996_adh	TTTGCGGAAAAATGTGTTCAG	Chr.4	6323748	6323728	-	No

2010654_adh	TCGGAACTCGCGAATGTGACG	Chr.4	6324939	6324959	+	No
2722859_adh	TTTGGAAAACTACGTGATAAC	Chr.4	6337154	6337134	-	No
2739208_adh	TTTTCTCAAGAACTTCGAGAC	Chr.4	6338625	6338605	-	No
2584080_adh	TTATTTGCATACGCTCTCCCC	Chr.4	6343596	6343576	-	No
2724314_adh	TTTGGCAGCAGGTGTGAATTC	Chr.4	6345523	6345543	+	No
2047360_adh	TCTGATGTTGGCAGAAGCACA	Chr.4	6346321	6346341	+	No
2712888_adh	TTTGAACACACGGATATCCAC	Chr.4	6354246	6354226	-	No
1854738_adh	TAGCAGTAAAAGAAGAGGAAA	Chr.4	6376454	6376434	-	No
1799822_adh	TAATGATTGCATTCACTCGGA	Chr.4	6376817	6376837	+	No
1978642_adh	TCCAGGACAAGATGGACAACC	Chr.4	6377711	6377731	+	No
2740596_adh	TTTTGAACAAAACTTTTGAAA	Chr.4	6381361	6381381	+	No
2294333_adh	TGCCTAAGTGTCTCTCATTAA	Chr.4	6383695	6383715	+	No
1828002_adh	TACGTATTATAGTTATTTTGA	Chr.4	6385861	6385841	-	No
2585572_adh	TTCAAATGGTTGTGAAACGGT	Chr.4	6386015	6386035	+	No
2745555_adh	TTTTTCAAAGTAGTCATTTTT	Chr.4	6386954	6386934	-	Yes
2522111_adh	TGTTAAAGTAGATCAGCGGTG	Chr.4	6388190	6388170	-	No
2688678_adh	TTTAAAAAATACAAATAATTA	Chr.4	6388393	6388413	+	No
2733902_adh	TTTTAATTTTGAACGGACTTT	Chr.4	6388471	6388491	+	No
1824455_adh	TACGATTGAAGAGAGAAGACA	Chr.4	6390000	6389980	-	No
2547333_adh	TTAAGATGTTCATGAAACGGT	Chr.4	6393867	6393887	+	No
1760458_adh	TAAATCATGCCAGAAATTGTC	Chr.4	6395077	6395057	-	No
1939123_adh	TCAAGCACAAGATGAATTTTA	Chr.4	6403760	6403740	-	No
1790270_adh	TAATAACATTGCAGATAATCG	Chr.4	6405594	6405574	-	No
2733727_adh	TTTTAATGGAATTTGCACACC	Chr.4	6406377	6406397	+	No
2133369_adh	TGAAGGGACGATTAGAAAATA	Chr.4	6408838	6408818	-	No
2629028_adh	TTGAAGGGACGATTAGAAAAT	Chr.4	6408839	6408819	-	No
2737892_adh	TTTTCCATATTTTTTGTGATT	Chr.4	6410554	6410534	-	Yes
2552877_adh	TTAATTTTACGAAACGGTGTG	Chr.4	6412096	6412116	+	Yes
2734149_adh	TTTTACGAAACGGTGTGACTA	Chr.4	6412100	6412120	+	Yes
2534190_adh	TGTTTTCAAAATATTGTATCT	Chr.4	6414228	6414208	-	No
2311799_adh	TGCTTTGAACGAGATGTCGCA	Chr.4	6416614	6416594	-	No
2487576_adh	TGTAGAATTTTAAGGCGCATT	Chr.4	6421361	6421341	-	No
2749469_adh	TTTTTTGCCTTCGAGGACGTT	Chr.4	6421459	6421479	+	No
2747065_adh	TTTTTGCCTTCGAGGACGTTT	Chr.4	6421460	6421480	+	No
2490755_adh	TGTATCAAGTGGACGATATCC	Chr.4	6424394	6424414	+	No
2681528_adh	TTGTGGAATAGGCATGTAGGT	Chr.4	6426270	6426250	-	No
2703757_adh	TTTCATACTGTTTAAACATGT	Chr.4	6433164	6433184	+	Yes
1900652_adh	TATCTTTGTTTGCATTCATAT	Chr.4	6435132	6435112	-	No
1806293_adh	TAATTTTTGGCAATGGGAGAA	Chr.4	6440351	6440371	+	Yes
1891865_adh	TATCACACGGCCGCTGATAAA	Chr.4	6441950	6441930	-	No
2542498_adh	TTAAATATATTTTTTAAATCG	Chr.4	6443437	6443457	+	Yes
2553551_adh	TTACAATACAGAAAAAGAATT	Chr.4	6446547	6446567	+	No
2140170_adh	TGAATATTGCCGGAAGAAAAA	Chr.4	6450744	6450724	-	No
2544534_adh	TTAAATTGTAGTTAACAAGTC	Chr.4	6456332	6456312	-	No
2487713_adh	TGTAGAGATTCGATTTCGGCA	Chr.4	6457150	6457170	+	No
2747311_adh	TTTTTGGCTACAATCACTTCA	Chr.4	6457776	6457756	-	Yes
2259214_adh	TGATAATTCTGATCGTAAAAA	Chr.4	6468571	6468591	+	Yes
1751114_adh	TAAAATCAAAAGTTTATCAAC	Chr.4	6470980	6470960	-	No
2121471_adh	TGAACATGAAGTCAGAACAGC	Chr.4	6473246	6473266	+	No
1805354_adh	TAATTTGCATGTTTCCTAGAT	Chr.4	6479694	6479714	+	No

1833874_adh	TACTGTCTAGAAAACTTTTTA	Chr.4	6479938	6479958	+	No
2692965_adh	TTTAATTTGAAAGGCTCGTAC	Chr.4	6480163	6480143	-	Yes
1808871_adh	TACAAGCAAGGGATGAACAAA	Chr.4	6482195	6482215	+	No
2738736_adh	TTTTCGGTTCCATTTTCCTCG	Chr.4	6484202	6484182	-	No
2308185_adh	TGCTGAACTTAGCAAAAGTAT	Chr.4	6491595	6491575	-	No
2221592_adh	TGAGATGGAAGTGACCATGAC	Chr.4	6492945	6492925	-	No
2421268_adh	TGGGACACAGCTTTTGTACAC	Chr.4	6503063	6503083	+	No
2595600_adh	TTCCAAAAACATTTTAAATAA	Chr.4	6506475	6506455	-	Yes
2581423_adh	TTATTCATTGGGTATGATATT	Chr.4	6507906	6507926	+	Yes
2697668_adh	TTTAGTTTTAAGGTATTTGTT	Chr.4	6508484	6508504	+	Yes
1968520_adh	TCATACAAAATGAACAGAAGT	Chr.4	6508670	6508650	-	Yes
2618566_adh	TTCTGCAGAATTTTTATTGAT	Chr.4	6525132	6525112	-	No
2039423_adh	TCTCGAATTCGGAAAATAAAT	Chr.4	6530076	6530096	+	No
1923429_adh	TATTCAATTGATCAGTCTACT	Chr.4	6531736	6531756	+	No
1943076_adh	TCAATTGATCAGTCTACTATT	Chr.4	6531739	6531759	+	No
1980659_adh	TCCCATAGTTGATCTCCATCC	Chr.4	6536178	6536198	+	No
2595031_adh	TTCATTAGCAGAAAAGCCTGT	Chr.4	6542638	6542618	-	No
1782982_adh	TAAGCTAGAAGATTGTGACAC	Chr.4	6545503	6545483	-	Yes
2579325_adh	TTATGGAATTTGTTGCGAAAT	Chr.4	6555864	6555844	-	No
2699160_adh	TTTATGGAATTTGTTGCGAAA	Chr.4	6555865	6555845	-	No
2374760_adh	TGGACCGACTTACTTTAAACC	Chr.4	6564367	6564347	-	No
1887703_adh	TATACTTAGAATACTTACTGG	Chr.4	6568854	6568834	-	No
2032682_adh	TCTAGCGTAGATGTATGTAGT	Chr.4	6569916	6569896	-	No
2703930_adh	TTTCATCCGGCATAACTAAAA	Chr.4	6573302	6573282	-	No
2590452_adh	TTCAGAATTCGGGTAATGTTT	Chr.4	6573642	6573662	+	No
2728224_adh	TTTGTAGAATTGCTATTCGGC	Chr.4	6575551	6575531	-	No
1751014_adh	TAAAATATAGAAAACGTGTTA	Chr.4	6575607	6575627	+	No
1886866_adh	TATACATAAACGTACACATAA	Chr.4	6577080	6577060	-	Yes
2034901_adh	TCTATTCCGGATGCGTTCTCT	Chr.4	6585235	6585215	-	No
1778384_adh	TAAGATATCAGTTAGCATTCA	Chr.4	6590878	6590858	-	No
1927891_adh	TATTGTCGGAAAAGTCGTTAT	Chr.4	6592448	6592428	-	No
2583285_adh	TTATTGTCGGAAAAGTCGTTA	Chr.4	6592449	6592429	-	No
2700525_adh	TTTATTGTCGGAAAAGTCGTT	Chr.4	6592450	6592430	-	No
2073903_adh	TGAAACTGCAAGAATCGATGA	Chr.4	6595394	6595414	+	No
1778384_adh	TAAGATATCAGTTAGCATTCA	Chr.4	6598417	6598437	+	No
1778384_adh	TAAGATATCAGTTAGCATTCA	Chr.4	6599378	6599398	+	No
2656930_adh	TTGGAACTGAGCGATTGGCAA	Chr.4	6606808	6606828	+	No
2318533_adh	TGGAAATTGTGAAACGAGCTT	Chr.4	6634086	6634106	+	No
1801563_adh	TAATGTATAGAAGTCCGGATG	Chr.4	6639362	6639342	-	No
2617106_adh	TTCTCTCGATCGTAAACATTA	Chr.4	6642295	6642315	+	Yes
2546586_adh	TTAACTTGTGGAAACGGTATT	Chr.4	6643879	6643899	+	No
2066225_adh	TGAAAAAGTTTCCTCAGTTTG	Chr.4	6657323	6657303	-	No
2561174_adh	TTAGAACGAAGTGATTTGGCC	Chr.4	6659232	6659252	+	No
1984249_adh	TCCGACTGGCGTACCTAAAAT	Chr.4	6664310	6664290	-	No
2607023_adh	TTCGGAAAAAATAAATGTTGC	Chr.4	6668130	6668150	+	No
1854339_adh	TAGCAGAAACGTGTGAAATGA	Chr.4	6677261	6677281	+	No
2716622_adh	TTTGAGATCGGAAATATGTGA	Chr.4	6679661	6679681	+	No
2126803_adh	TGAACTTTTGGAACATGTCCC	Chr.4	6729336	6729356	+	No
2126803_adh	TGAACTTTTGGAACATGTCCC	Chr.4	6729692	6729712	+	No
2126803_adh	TGAACTTTTGGAACATGTCCC	Chr.4	6730563	6730583	+	No

2723765_adh	TTTGGAGGAAACGGTGAATAC	Chr.4	6742926	6742946	+	No
1784189_adh	TAAGGACAGCGTTGCGCTCGG	Chr.4	6747939	6747959	+	No
2270827_adh	TGATGAAATGTTGTGATGAAT	Chr.4	6750783	6750763	-	No
2724457_adh	TTTGGCGGGGAGAAAATAGGC	Chr.4	6756611	6756591	-	No
2319958_adh	TGGAACGAAAGTGAAAGTAGG	Chr.4	6767348	6767328	-	No
1821038_adh	TACCTGTAATGCATTCAAAAC	Chr.4	6774632	6774652	+	No
2488068_adh	TGTAGATGTTTGGCAACTTAT	Chr.4	6790181	6790201	+	No
2734041_adh	TTTTACATTTTACATTTTTCA	Chr.4	6800914	6800894	-	Yes
2737129_adh	TTTTCAAAGCGATTTGTGCAT	Chr.4	6812460	6812480	+	No
2593831_adh	TTCATATTTTAATATCGTCTG	Chr.4	6829940	6829960	+	No
2324126_adh	TGGAAGGAAGAAATCATAGGC	Chr.4	6841640	6841620	-	No
2700650_adh	TTTATTTAGTATTTTTGGCAC	Chr.4	6857328	6857308	-	No
2712463_adh	TTTGAAAAGTGGAAAATGAAA	Chr.4	6901679	6901659	-	No
2062522_adh	TCTTTTATTCGGATTAGATGT	Chr.4	6910132	6910112	-	No
2689252_adh	TTTAAAGATAGATTTTCGCAG	Chr.4	6910338	6910358	+	No
2738362_adh	TTTTCGCAGAATTTATGCATC	Chr.4	6910350	6910370	+	No
2686682_adh	TTGTTTAACTCGCAGCGGGCG	Chr.4	6914512	6914492	-	No
2541813_adh	TTAAAGTTATTTTGGTCGGTC	Chr.4	6919613	6919633	+	No
1940763_adh	TCAAGTTTTTGAAAGTGACTG	Chr.4	6919632	6919652	+	No
1814464_adh	TACATTGATTCTTTAAAAACA	Chr.4	6923126	6923106	-	No
2666050_adh	TTGGCTATATATGAAATCTAA	Chr.4	6940490	6940470	-	Yes
2617753_adh	TTCTGAACTCCATCTCAAAAG	Chr.4	6948839	6948819	-	No
1875521_adh	TAGTATGGCACTCCCTAATAG	Chr.4	6952257	6952237	-	No
2044301_adh	TCTGAAGGGAACATAAGATGG	Chr.4	6959610	6959590	-	No
2587440_adh	TTCAATAAACAAAAACATAAT	Chr.4	6964276	6964296	+	Yes
1940787_adh	TCAATAAACAAAAACATAATT	Chr.4	6964277	6964297	+	Yes
2053443_adh	TCTGTTTGAAAGTTGGACTCA	Chr.4	6964578	6964558	-	No
1802041_adh	TAATGTTTTGTTAGGTTGGAA	Chr.4	6971802	6971822	+	Yes
2534826_adh	TGTTTTGTTAGGTTGGAATAT	Chr.4	6971805	6971825	+	Yes
2550985_adh	TTAATGAATATGAATGACGAA	Chr.4	6994117	6994137	+	Yes
1799338_adh	TAATGAATATGAATGACGAAC	Chr.4	6994118	6994138	+	Yes
2691327_adh	TTTAATCGTTGGAAAAGTTTA	Chr.4	6994893	6994913	+	No
1864926_adh	TAGGATAGTATACGTTCAAAT	Chr.4	6996907	6996927	+	No
2688389_adh	TTGTTTTTCAATTTCGTCGCC	Chr.4	6999483	6999503	+	No
2649268_adh	TTGCAGAATGGAATTAAACAC	Chr.4	7012450	7012430	-	No
1759979_adh	TAAATATAAAAATCGGCAAAT	Chr.4	7012647	7012627	-	No
2746737_adh	TTTTTGACTGTAATTAGCATG	Chr.4	7049250	7049270	+	No
2029541_adh	TCTAAATTTCGAGAATTCAAT	Chr.4	7058074	7058054	-	No
2412176_adh	TGGCGATTTTGGAGATGGACA	Chr.4	7069261	7069241	-	No
2412176_adh	TGGCGATTTTGGAGATGGACA	Chr.4	7069441	7069461	+	No
2534086_adh	TGTTTTAGAAATTTTGCGGAC	Chr.4	7073072	7073052	-	No
2607244_adh	TTCGGAATGAGCAGTCTCGTA	Chr.4	7078097	7078117	+	No
2279147_adh	TGATTTTAGGCCAGACGCAAG	Chr.4	7084893	7084873	-	No
2579231_adh	TTATGCTGCGACACGATTTCG	Chr.4	7085002	7085022	+	No
2612974_adh	TTCGTTATCAGTACATTCAAT	Chr.4	7085034	7085054	+	No
2568224_adh	TTAGGATCAAATATTCGATGG	Chr.4	7103561	7103581	+	No
2679143_adh	TTGTGAAAAGAGTGACATTAG	Chr.4	7124054	7124074	+	No
1761236_adh	TAAATGAAGCAATGAGAACAT	Chr.4	7126666	7126646	-	No
1761236_adh	TAAATGAAGCAATGAGAACAT	Chr.4	7127993	7127973	-	No
2028052_adh	TCGTTCGTGTGTGTTGATGCT	Chr.4	7131482	7131462	-	No

2647101_adh	TTGATTTTTCTCGGTAATAAT	Chr.4	7133354	7133374	+	No
2279206_adh	TGATTTTCAAACGATCCGGAA	Chr.4	7144849	7144829	-	No
2038301_adh	TCTCATGAGCTGATGGTTTCT	Chr.4	7147356	7147336	-	No
1776380_adh	TAAGAGAACGACAGGATTCAC	Chr.4	7151921	7151941	+	No
2545468_adh	TTAACGATGGCAACTCCCGAT	Chr.4	7154701	7154721	+	No
2526097_adh	TGTTCAGGAGTTAGAAGAATA	Chr.4	7165179	7165199	+	No
2313422_adh	TGGAAAACGGAATGTTCGATA	Chr.4	7171057	7171077	+	No
1962152_adh	TCAGCAATGATCGTGACAACA	Chr.4	7180151	7180171	+	No
2609835_adh	TTCGGTAGAAATATTGGTGGA	Chr.4	7190008	7190028	+	No
2585025_adh	TTCAAAATAATCTTACCATTC	Chr.4	7197196	7197176	-	No
2600091_adh	TTCCTAATCTTTTCGGCAACA	Chr.4	7204037	7204057	+	No
2713526_adh	TTTGAAGAAAGAAAGTGTGAA	Chr.4	7208347	7208327	-	No
2681120_adh	TTGTGATTCATTTTTGTTCAC	Chr.4	7230339	7230319	-	No
2723854_adh	TTTGGATAAAAGATATGAAAA	Chr.4	7242778	7242798	+	No
2402775_adh	TGGCAATGTGGAATACTGGGT	Chr.4	7251452	7251472	+	No
2474744_adh	TGGTTCAGGGTTGTTTAACTC	Chr.4	7256475	7256455	-	No
2051426_adh	TCTGGTTCAGGGTTGTTTAAC	Chr.4	7256477	7256457	-	No
2462812_adh	TGGTAGACAAAGATCCGGAGA	Chr.4	7263699	7263679	-	No
1814539_adh	TACATTTATTCATCATCTTTA	Chr.4	7268169	7268189	+	No
2740744_adh	TTTTGAACTCAAGCTACGATA	Chr.4	7292448	7292428	-	No
2745683_adh	TTTTTCAGAGTAGAGGAATTC	Chr.4	7293528	7293548	+	No
2712476_adh	TTTGAAAAGTTCGTGGTGTTT	Chr.4	7312640	7312620	-	No
2011217_adh	TCGGACAAGCCAACGGATACA	Chr.4	7313110	7313090	-	No
1932231_adh	TCAAAAGACTGCTGCTGTTGA	Chr.4	7313245	7313225	-	No
1814028_adh	TACATTAAAGATTTATAGAAA	Chr.4	7314197	7314217	+	No
1757105_adh	TAAAGATTTATAGAAATTCGA	Chr.4	7314202	7314222	+	No
2713600_adh	TTTGAAGACTCCTGCATTTCT	Chr.4	7314748	7314728	-	No
2293117_adh	TGCCATGTGGAAACGATTTCT	Chr.4	7318375	7318395	+	No
1968294_adh	TCATAAGGGATACGTTGGACA	Chr.4	7320445	7320465	+	No
2725847_adh	TTTGGTAATTTCCTTTTATCA	Chr.4	7323091	7323111	+	No
2276438_adh	TGATTGATCGAAGGAACAAAG	Chr.4	7348533	7348513	-	No
1830785_adh	TACTATTTACCACTCGGTCTA	Chr.4	7349574	7349594	+	No
2054651_adh	TCTTAGTTAAACATGAAACGG	Chr.4	7438137	7438157	+	No
1923336_adh	TATTCAAAATGTATGACTGCT	Chr.4	7438422	7438402	-	No
2674069_adh	TTGTAATGAAATGATGGAAAC	Chr.4	7450460	7450440	-	No
2648807_adh	TTGCAATCTGAAGATATCATT	Chr.4	7467950	7467970	+	Yes
2697540_adh	TTTAGTTGGCTAAATTTGTTT	Chr.4	7473359	7473339	-	No
1939042_adh	TCAAGATTTTTAGTTGGCTAA	Chr.4	7473367	7473347	-	No
1883921_adh	TATAAAAGACGTGAATATATA	Chr.4	7487027	7487007	-	No
1798989_adh	TAATCTTCAGAACACGGTGAC	Chr.4	7487368	7487388	+	No
2044224_adh	TCTGAAGCGCCAAGGAAGAAC	Chr.4	7487707	7487687	-	No
2652438_adh	TTGCGGAATCAGAATTCGGTA	Chr.4	7501302	7501322	+	No
1940045_adh	TCAAGTACAAGATCCCACAAT	Chr.4	7507618	7507598	-	No
1930912_adh	TATTTTCGGATGTTTGTTGGT	Chr.4	7508976	7508956	-	No
2737558_adh	TTTTCAGTATTTTCGGATGTT	Chr.4	7508983	7508963	-	No
2535059_adh	TGTTTTTCAGTATTTTCGGAT	Chr.4	7508986	7508966	-	No
2160230_adh	TGACCTACAGGAGAAATTGGA	Chr.4	7512965	7512945	-	No
2745839_adh	TTTTTCCATGTCGGGTCATTT	Chr.4	7513238	7513218	-	No
2722501_adh	TTTGCTGGAGAAAGTTTTGGA	Chr.4	7544373	7544353	-	No
2452776_adh	TGGGGATTGGAGGGACTGGGA	Chr.4	7559176	7559196	+	No

2023222_adh	TCGTAGATTTTAAAACTAGGG	Chr.4	7561978	7561998	+	No
2703274_adh	TTTCAGGGATAGTGGAAGACA	Chr.4	7564097	7564117	+	No
2703274_adh	TTTCAGGGATAGTGGAAGACA	Chr.4	7565724	7565704	-	No
1813275_adh	TACATATTTGATTGTATGGAC	Chr.4	7606373	7606393	+	No
1994546_adh	TCGAAAAGCAGTTGACAAGAT	Chr.4	7632440	7632460	+	No
2525950_adh	TGTTCAATACAAGCGATACTA	Chr.4	7633482	7633502	+	No
2129898_adh	TGAAGATAAAGATGTAGAATA	Chr.4	7654430	7654410	-	No
1854465_adh	TAGCAGACGTAAGCAACTTTG	Chr.4	7662737	7662717	-	No
1770753_adh	TAACTCTTTCGCTCATTTCGG	Chr.4	7673996	7674016	+	Yes
1821873_adh	TACGAAGTTAGCGGGAAATGC	Chr.4	7679799	7679779	-	No
2709963_adh	TTTCTCGAGAAGCAAAAGACC	Chr.4	7684521	7684501	-	No
2059328_adh	TCTTGTAGAAGTGAAAACGGT	Chr.4	7690126	7690146	+	No
1935954_adh	TCAACAAGTGCAACTTCTGGA	Chr.4	7693615	7693595	-	No
1929889_adh	TATTTCGGAGTATCGTTGGGA	Chr.4	7728678	7728658	-	No
2734960_adh	TTTTAGTGGGATTTGACCAAT	Chr.4	7743146	7743126	-	Yes
2568998_adh	TTAGGTAGAGAGATATATTAA	Chr.4	7764367	7764347	-	No
2690671_adh	TTTAAGGGAATTCGGAGAATA	Chr.4	7770909	7770929	+	No
2598870_adh	TTCCGATCGGAAGGAATATAT	Chr.4	7785901	7785921	+	No
1860683_adh	TAGGAAATCTGCACAGCTGAA	Chr.4	7791411	7791391	-	No
2490357_adh	TGTATAACGGATACATGTATT	Chr.4	7791511	7791531	+	No
2702966_adh	TTTCAGATGTTGCTTTCTTTT	Chr.4	7796822	7796802	-	No
2378811_adh	TGGACTTAAATACGGCTCACT	Chr.4	7800482	7800502	+	No
2271722_adh	TGATGATTTGTGAAATAAACT	Chr.4	7803441	7803461	+	No
2718277_adh	TTTGATCGTGCGCACAGCGAG	Chr.4	7816024	7816044	+	No
2589217_adh	TTCACAGTTTGATATCCAAAA	Chr.4	7877842	7877822	-	No
1880679_adh	TAGTTCTATTAGTTCTGCAGC	Chr.4	7889886	7889906	+	Yes
1975992_adh	TCCAACAATTCTTCTCAAAAA	Chr.4	7891953	7891973	+	No
2562173_adh	TTAGACGAATCAATCATTATT	Chr.4	7902561	7902541	-	No
2074643_adh	TGAAACTTGAGAATGAGTATC	Chr.4	7909968	7909948	-	No
2376616_adh	TGGACGTGGAGGACGTGGTGG	Chr.4	7926268	7926248	-	No
2749733_adh	TTTTTTGTCGTTTTGAAGGAA	Chr.4	7926562	7926542	-	No
2712632_adh	TTTGAAAGAAATACAATAACG	Chr.4	7926708	7926728	+	No
2624959_adh	TTGAAAGAAATACAATAACGA	Chr.4	7926709	7926729	+	No
2643993_adh	TTGATCAGACAGCAAAGGACA	Chr.4	7946356	7946336	-	No
2745662_adh	TTTTTCACTGAAGACGTTATT	Chr.4	7948568	7948588	+	No
2625154_adh	TTGAAAGGATGATGGCTGAAG	Chr.4	7953359	7953379	+	No
1957031_adh	TCAGAACAAAATGGAAGAAAC	Chr.4	7953791	7953771	-	No
1830699_adh	TACTATTACGTGGACGTCTAA	Chr.4	7955159	7955179	+	No
2717176_adh	TTTGATAAAATAGAAGACTGA	Chr.4	7958249	7958269	+	No
2697525_adh	TTTAGTTGATCGTTTCTTTCC	Chr.4	7958490	7958510	+	No
2572634_adh	TTAGTTGATCGTTTCTTTCCC	Chr.4	7958491	7958511	+	No
2714571_adh	TTTGAATTTTGATATCATTGC	Chr.4	7958520	7958540	+	No
2018440_adh	TCGGTAGACGAGCTGTTGTAT	Chr.4	7962438	7962418	-	No
2724396_adh	TTTGGCCGCTTATTCGTCGCA	Chr.4	7982133	7982113	-	No
2616505_adh	TTCTCGATGAGATCTCTCGAT	Chr.4	7982153	7982133	-	No
1939536_adh	TCAAGGACATGGACTCTACGA	Chr.4	7983397	7983417	+	No
2737134_adh	TTTTCAAAGTGTCGGAAAAAC	Chr.4	7998891	7998871	-	No
2540770_adh	TTAAAGACGATTTGTGGAAGA	Chr.4	8004272	8004292	+	No
2259602_adh	TGATACGGAAAGAAGAATCAG	Chr.4	8004868	8004888	+	No
1816789_adh	TACCCGCACAACTGGAATACG	Chr.4	8019997	8020017	+	No

2016347_adh	TCGGGAACATATTTTTAAATC	Chr.4	8055786	8055766	-	Yes
2743575_adh	TTTTGTAGCACGAAGAAAGCA	Chr.4	8066221	8066201	-	No
1774419_adh	TAAGAAGTTAGTGCGATTCCC	Chr.4	8085540	8085560	+	No
2549873_adh	TTAATAGATCGTTGATAAACA	Chr.4	8097245	8097225	-	No
2544352_adh	TTAAATTCGGCATTTTATTGA	Chr.4	8099652	8099632	-	Yes
2526584_adh	TGTTCGGTTTTTTCGGTTTTT	Chr.4	8108601	8108621	+	No
1938068_adh	TCAAGAAAAAATTCAAGTGAA	Chr.4	8112889	8112909	+	No
2604401_adh	TTCGATATTTACAAACTCAAC	Chr.4	8119073	8119093	+	No
2709877_adh	TTTCTCACTGGTTTTTTGCTT	Chr.4	8130450	8130430	-	No
2641908_adh	TTGAGTAGAGATGTTAATAAT	Chr.4	8132467	8132447	-	No
2623503_adh	TTCTTTCTACGTCGTATTCTT	Chr.4	8134037	8134017	-	No
1856752_adh	TAGCGGAAAAAAGTGTTTATA	Chr.4	8134335	8134315	-	No
2145906_adh	TGAATTCATTCGTATTCTGTC	Chr.4	8145843	8145823	-	No
2021231_adh	TCGTAACCTTCAAGTGGAACA	Chr.4	8153192	8153172	-	No
1857199_adh	TAGCGGAGTCTAGAGACCATG	Chr.4	8164473	8164493	+	No
2627047_adh	TTGAACTTTCCAAGCTCTGCA	Chr.4	8172424	8172444	+	No
2318257_adh	TGGAAATGGAAAAATAGCAAA	Chr.4	8177369	8177389	+	No
2532541_adh	TGTTTCGCACGTATTAACTAC	Chr.4	8180708	8180728	+	No
2402840_adh	TGGCACAAGTAACTCACTCGA	Chr.4	8212883	8212863	-	No
2699681_adh	TTTATTATTCAGAGAGATCTT	Chr.4	8213706	8213686	-	No
1829481_adh	TACTAATTGTGAAACACTGTT	Chr.4	8216565	8216545	-	No
2722642_adh	TTTGCTTATCAGTTTGTCGTT	Chr.4	8225571	8225591	+	No
2573855_adh	TTATAACGTACGGAATCGAAA	Chr.4	8225834	8225814	-	No
2377114_adh	TGGACTCGAGAAACGATGGTT	Chr.4	8227773	8227753	-	No
2674027_adh	TTGTAATACACGATCGTCGCA	Chr.4	8237289	8237309	+	No
2593479_adh	TTCATAATATTCTGTGCTTCA	Chr.4	8244430	8244450	+	No
1824185_adh	TACGATGGAAATAATAGCGAA	Chr.4	8257028	8257008	-	No
2281248_adh	TGCAAATTCTGCCTCGAAAAG	Chr.4	8257054	8257034	-	No
2576751_adh	TTATATTTTGCAGATCCTGAT	Chr.4	8267443	8267463	+	No
2495770_adh	TGTCATCTAGTAGAAAGCATT	Chr.4	8284619	8284599	-	No
2595419_adh	TTCATTGTTGTTGGTTTTATT	Chr.4	8286751	8286731	-	No
1764169_adh	TAACACAGACTTGAAGAACAT	Chr.4	8287361	8287341	-	No
2127144_adh	TGAAGAAGACGTAGAGGAAGA	Chr.4	8287894	8287874	-	No
2026951_adh	TCGTGGACGCGGTGGAAACGA	Chr.4	8289024	8289004	-	No
2018293_adh	TCGGTAAATCAATGGACGCAG	Chr.4	8291757	8291777	+	No
2387960_adh	TGGATACGGTAGTCAACGATG	Chr.4	8294740	8294760	+	No
2553744_adh	TTACACAGATATACGCGAGAA	Chr.4	8300734	8300714	-	No
2580528_adh	TTATTAAGTTCGTTAGATATA	Chr.4	8325180	8325160	-	No
2651741_adh	TTGCGAAGCAATTCCGGTGAA	Chr.4	8325235	8325215	-	No
2395986_adh	TGGATTTGAACGGAATAAAAT	Chr.4	8325400	8325420	+	No
2740904_adh	TTTTGAATCATCTGGAATGCA	Chr.4	8327120	8327140	+	No
2657915_adh	TTGGACAACTCGGAATTGAAG	Chr.4	8346365	8346385	+	No
2573605_adh	TTAGTTTTTAGGTCGTTTAAG	Chr.4	8350924	8350904	-	No
2546612_adh	TTAACTTTTAGGTCGTTTAAG	Chr.4	8351045	8351065	+	No
2614518_adh	TTCTACGTTGAAACGATGGCA	Chr.4	8360483	8360463	-	No
2645139_adh	TTGATGGATCTTCTGTGACAC	Chr.4	8375178	8375198	+	No
2589218_adh	TTCACATAATAGGCGGAAAAC	Chr.4	8377972	8377952	-	No
2734474_adh	TTTTAGACAGCACTGCTTCGT	Chr.4	8379420	8379400	-	No
2701432_adh	TTTCAACTCTCGTCCTATGAT	Chr.4	8379440	8379420	-	No
1929172_adh	TATTTACGAGATGGTCTTTCA	Chr.4	8379529	8379549	+	No

2470309_adh	TGGTCTGTAGAAGTGGTAGAC	Chr.4	8387406	8387426	+	No
1770310_adh	TAACTATGCAGAATTGAAACT	Chr.4	8395155	8395135	-	No
1834372_adh	TACTGTTTTAGTATCGCGGCA	Chr.4	8397097	8397077	-	No
2747970_adh	TTTTTGTTGTAACATCAATCC	Chr.4	8397155	8397175	+	No
2706258_adh	TTTCCTCTGTTTTTCGAACAA	Chr.4	8398471	8398491	+	No
2038107_adh	TCTCATCAGAAACTCCGACGA	Chr.4	8399188	8399208	+	No
2297446_adh	TGCGATAAGTAGGCGTAGCTT	Chr.4	8400003	8400023	+	No
1762599_adh	TAAATTGTAGGAGAAAGGTGG	Chr.4	8401855	8401835	-	No
1876095_adh	TAGTCACAAGACTGTGTGGCA	Chr.4	8401880	8401900	+	No
1930982_adh	TATTTTCTGTTGACTCGTCTT	Chr.4	8403173	8403153	-	No
2396545_adh	TGGATTTTTGAGCGGTTTTAG	Chr.4	8404207	8404227	+	No
2371144_adh	TGGAATTTAAAAAAGTATAAT	Chr.4	8427234	8427214	-	No
1973146_adh	TCATTAAAGCTTAACGAACAA	Chr.4	8428504	8428484	-	No
2617733_adh	TTCTGAACCAAGTTGCCTAGA	Chr.4	8428603	8428583	-	No
2599179_adh	TTCCGGCTACAATTGATGATT	Chr.4	8428891	8428911	+	No
1824422_adh	TACGATTCGTAGTAAAAGATG	Chr.4	8483737	8483717	-	No
2620304_adh	TTCTGTTCCAAATTGTTAGAA	Chr.4	8484050	8484030	-	No
2620304_adh	TTCTGTTCCAAATTGTTAGAA	Chr.4	8484375	8484355	-	No
2713196_adh	TTTGAACGAGAAATGCGCGCG	Chr.4	8511248	8511268	+	No
2147105_adh	TGAATTGATTGGAAAACGAGA	Chr.4	8517418	8517398	-	No
2721432_adh	TTTGCATGAACTTACGGATGA	Chr.4	8520309	8520289	-	No
2001234_adh	TCGAGACTGTACAAGAAGAGT	Chr.4	8552714	8552734	+	No
2727175_adh	TTTGTAAGAAAAAAGAACGGT	Chr.4	8555500	8555520	+	No
2673909_adh	TTGTAAGAAAAAAGAACGGTA	Chr.4	8555501	8555521	+	No
1765027_adh	TAACAGCAATGATCGTCATTA	Chr.4	8569971	8569951	-	No
2152862_adh	TGACACATGCACTTGGGACAT	Chr.4	8586213	8586193	-	No
1928881_adh	TATTGTTTTTGCATACTGTTT	Chr.4	8589684	8589704	+	No
2535152_adh	TGTTTTTGCATACTGTTTTTT	Chr.4	8589687	8589707	+	No
2742347_adh	TTTTGCATACTGTTTTTTAAA	Chr.4	8589690	8589710	+	No
2013026_adh	TCGGAGTCGTAAGCTGGTTTG	Chr.4	8594769	8594789	+	No
1929754_adh	TATTTCCTTGACGCATTTCCT	Chr.4	8607505	8607485	-	No
2561671_adh	TTAGAATACGGATGGACTTCG	Chr.4	8607549	8607529	-	No
2694978_adh	TTTAGAATACGGATGGACTTC	Chr.4	8607550	8607530	-	No
2395095_adh	TGGATTGTAGTGAGCATTTAG	Chr.4	8607566	8607546	-	No
2610113_adh	TTCGGTGGATTGTAGTGAGCA	Chr.4	8607571	8607551	-	No
2529122_adh	TGTTGATGACTTCGGTGGATT	Chr.4	8607581	8607561	-	No
2621909_adh	TTCTTCGTTTTACTCTCGGAC	Chr.4	8607708	8607728	+	No
2694242_adh	TTTACTCTCGGACCGGCAGGT	Chr.4	8607716	8607736	+	No
1748809_adh	TAAAACTTACCAAGTCGTCGA	Chr.4	8654559	8654579	+	No
2697706_adh	TTTATAAAATCCGGACAGAAT	Chr.4	8665906	8665886	-	No
2735069_adh	TTTTATAAAATCCGGACAGAA	Chr.4	8665907	8665887	-	No
1923845_adh	TATTCATTATGTCGGGCACAC	Chr.4	8728452	8728472	+	No
2601616_adh	TTCGAAAACTGTTGTGGACGG	Chr.4	8734878	8734858	-	No
2562406_adh	TTAGAGAACACAGACGACTAG	Chr.4	8737465	8737445	-	No
2623816_adh	TTCTTTTCCCCATTTTCGGCG	Chr.4	8754263	8754243	-	No
2268841_adh	TGATATTTTAGTAACTTGGAC	Chr.4	8773199	8773179	-	No
1963271_adh	TCAGGAAAACGTAAACATATT	Chr.4	8785204	8785224	+	No
1994668_adh	TCGAAACCAGAATATAGAGCA	Chr.4	8809244	8809224	-	No
1939404_adh	TCAAGCTGGATATGATGCTGG	Chr.4	8823654	8823634	-	No
1939404_adh	TCAAGCTGGATATGATGCTGG	Chr.4	8825288	8825308	+	No

2177125_adh	TGAGAAAGCGGAAGCAGAACT	Chr.4	8837919	8837939	+	No
1873697_adh	TAGTACCGCAGAACCAGTAAA	Chr.4	8849482	8849502	+	No
2742875_adh	TTTTGGCAGAAGAAAGGGAAT	Chr.4	8873505	8873485	-	No
2650417_adh	TTGCATGACTTGATTTTTTGT	Chr.4	8885139	8885159	+	Yes
2737553_adh	TTTTCAGTAGGATCATTTGAT	Chr.4	8888925	8888945	+	No
2058293_adh	TCTTGCATACTTGAATCAGTT	Chr.4	8893976	8893996	+	No
2630211_adh	TTGAATCAGTTTGGAGCATTC	Chr.4	8893986	8894006	+	No
2693316_adh	TTTACAGACTGGCAAAAACAT	Chr.4	8898110	8898090	-	No
2415505_adh	TGGCTTCAGTGGAATTTCTTT	Chr.4	8906861	8906881	+	No
2735610_adh	TTTTATCGATTAATTAAATAA	Chr.4	8926210	8926230	+	No
2581961_adh	TTATTCTGTTAGAAGACAATT	Chr.4	8926239	8926259	+	No
1961409_adh	TCAGATGGATCTGGCTCGTAT	Chr.4	8936962	8936982	+	No
2603887_adh	TTCGAGGATTTTGTAGAGACG	Chr.4	8956655	8956675	+	No
2615217_adh	TTCTATACTCTATAATAAGGC	Chr.4	8961359	8961379	+	No
2396516_adh	TGGATTTTTAAAGCGAATGCA	Chr.4	8973674	8973654	-	No
2215576_adh	TGAGATCTTAGCGAGGACACC	Chr.4	8990795	8990775	-	No
2380904_adh	TGGAGATTTGATGGAGCTTCT	Chr.4	9001188	9001168	-	No
2625020_adh	TTGAAAGATGAAAAGTCGGCA	Chr.4	9013793	9013773	-	No
2641087_adh	TTGAGCTTGGATCGTTTGCTC	Chr.4	9021513	9021533	+	No
2728630_adh	TTTGTATTTGGTTGATCGGCG	Chr.4	9022673	9022653	-	No
1901771_adh	TATGAAGATCTTGTACCACCT	Chr.4	9034171	9034151	-	No
1939411_adh	TCAAGCTGTAAGATAACTAGA	Chr.4	9059255	9059275	+	No
1860675_adh	TAGGAAATCGACAAAAAGGAG	Chr.4	9059807	9059827	+	No
1939411_adh	TCAAGCTGTAAGATAACTAGA	Chr.4	9060252	9060232	-	No
1845389_adh	TAGACGGAAGATAAGGACAGG	Chr.4	9062808	9062788	-	No
2679476_adh	TTGTGAATTTTAAACGCGGCC	Chr.4	9064284	9064264	-	No
2729406_adh	TTTGTGAATTTTAAACGCGGC	Chr.4	9064285	9064265	-	No
2745687_adh	TTTTTCAGATTATGCAGACAT	Chr.4	9074310	9074290	-	No
2748271_adh	TTTTTTAGAAGCATTTTCTGC	Chr.4	9074457	9074477	+	No
2688400_adh	TTGTTTTTCCTAATGGCGCAT	Chr.4	9096752	9096772	+	No
2006116_adh	TCGCAGATCACGAAAACTGAC	Chr.4	9099542	9099522	-	No
1749162_adh	TAAAAGACGCAGCAACGAAGA	Chr.4	9099691	9099671	-	No
2059284_adh	TCTTGTAAGTGATGTTTAGGC	Chr.4	9104475	9104455	-	No
1847357_adh	TAGAGACATGGTTTGTGGTTA	Chr.4	9112812	9112832	+	No
1833713_adh	TACTGTAGGAATGGTCTTGTG	Chr.4	9129987	9129967	-	No
1787684_adh	TAAGTAGTTGTAGTAGGGAAT	Chr.4	9142815	9142795	-	No
2300851_adh	TGCGGAATAACGGAGTGTCGT	Chr.4	9147129	9147149	+	No
1990861_adh	TCCTCTGGACTTGGCAGACTT	Chr.4	9155889	9155869	-	No
2233855_adh	TGAGCGAGAAGTGTGTTCCTG	Chr.4	9166601	9166621	+	No
1838918_adh	TAGAACTTTGGTATCCCAATA	Chr.4	9183286	9183266	-	No
2738647_adh	TTTTCGGGCAGAAATTGTGAA	Chr.4	9196263	9196243	-	No
2608432_adh	TTCGGCAATTTTCGGGCAGAA	Chr.4	9196271	9196251	-	No
2745105_adh	TTTTTAGCTTTTCAAGTTTTA	Chr.4	9196544	9196564	+	No
1832180_adh	TACTGAATGGAGAAGCACGTG	Chr.4	9201651	9201671	+	No
2590769_adh	TTCAGATATGTGAGCAGAAGC	Chr.4	9216528	9216508	-	No
2721346_adh	TTTGCAGGATGTGTGATTCAA	Chr.4	9249094	9249114	+	No
2654102_adh	TTGCTACATTGGAAACAACAC	Chr.4	9250310	9250290	-	No
2746585_adh	TTTTTGAATAAACCAAATCAA	Chr.4	9250674	9250694	+	No
2253409_adh	TGAGTGACAGTAGTCCACGCG	Chr.4	9264648	9264628	-	No
2735082_adh	TTTTATAAATATCAGTATTGA	Chr.4	9271893	9271913	+	No

2741749_adh	TTTTGATCTCGACGTGCATTC	Chr.4	9284785	9284765	-	No
2741749_adh	TTTTGATCTCGACGTGCATTC	Chr.4	9292278	9292258	-	No
2711683_adh	TTTCTTGAATTTTGATACGGT	Chr.4	9316296	9316316	+	No
2622502_adh	TTCTTGAATTTTGATACGGTT	Chr.4	9316297	9316317	+	No
2057656_adh	TCTTGAATTTTGATACGGTTT	Chr.4	9316298	9316318	+	No
2148739_adh	TGAATTTTGATACGGTTTTGT	Chr.4	9316301	9316321	+	No
1975309_adh	TCATTTGGAGAAGAAACGTCA	Chr.4	9325934	9325954	+	No
2584706_adh	TTATTTTTGAAATCTAACTTC	Chr.4	9331000	9330980	-	No
2737448_adh	TTTTCAGAGAAGAGGTTTCAT	Chr.4	9336643	9336623	-	No
1881085_adh	TAGTTGCTCAAGGATTCACTG	Chr.4	9338877	9338857	-	No
1992239_adh	TCCTGTGATACGGTAACTTTC	Chr.4	9342568	9342548	-	No
2162173_adh	TGACGTATTATTCTGTTGAAA	Chr.4	9350601	9350581	-	No
2639668_adh	TTGAGACGAAAGAAGGAAATA	Chr.4	9351674	9351654	-	No
2393726_adh	TGGATTGAAGAAGGAAGTTGA	Chr.4	9353164	9353144	-	No
2032296_adh	TCTAGAAATCGGCGGCGAAAG	Chr.4	9361397	9361417	+	No
2589254_adh	TTCACATTGAATTTCACGACA	Chr.4	9366650	9366670	+	No
2159435_adh	TGACCACAACAAAATACGGCA	Chr.4	9368482	9368502	+	No
2001294_adh	TCGAGAGGATATTAGAAGACG	Chr.4	9398279	9398259	-	No
1753787_adh	TAAACAAATGTGCATTGAATA	Chr.4	9400567	9400587	+	No
2545532_adh	TTAACGGAAATGCGACGAAAA	Chr.4	9403895	9403915	+	No
2704760_adh	TTTCATTTTCGTCGCATTTCC	Chr.4	9403920	9403900	-	No
1899812_adh	TATCTGAAATCAAAGGAAGCA	Chr.4	9437864	9437884	+	No
2589152_adh	TTCACAGAGACGCAGAATGTG	Chr.4	9441184	9441204	+	No
2581972_adh	TTATTCTTCAGGACAATATGG	Chr.4	9447085	9447065	-	No
1900232_adh	TATCTTATGCATCTTCTTTCA	Chr.4	9447751	9447731	-	Yes
2136036_adh	TGAAGTTGTTGAATGTTTATG	Chr.4	9459241	9459221	-	No
1963427_adh	TCAGGAAATGTTGTAAAATGA	Chr.4	9459308	9459288	-	No
2114744_adh	TGAAATTCGTTCGGAAGATTC	Chr.4	9464228	9464248	+	No
2738579_adh	TTTTCGGAGGTTTGTCGTGTT	Chr.4	9464904	9464924	+	No
2488540_adh	TGTAGGAAAAGACGAAGACAG	Chr.4	9465740	9465720	-	No
1927557_adh	TATTGTAGAAGTGTGGCCATG	Chr.4	9481567	9481547	-	No
2538795_adh	TTAAAAGAGTTTTCGACGCAT	Chr.4	9513323	9513303	-	No
1857592_adh	TAGCGTGAAAGGAAACAATGC	Chr.4	9513736	9513716	-	No
2688895_adh	TTTAAAAGAGAGACGCAGAGA	Chr.4	9525327	9525307	-	No
2730667_adh	TTTGTTCAGATCCTGGCGTCT	Chr.4	9528461	9528481	+	No
2668961_adh	TTGGTAAGAAAGGCGTAACTC	Chr.4	9536190	9536170	-	No
1860665_adh	TAGGAAATACGCAAATGGCGC	Chr.4	9548411	9548431	+	No
2106676_adh	TGAAATGGATAGAAATGGCAT	Chr.4	9559424	9559444	+	No
2724115_adh	TTTGGATTAAGAGTAAGCATC	Chr.4	9581210	9581230	+	No
1874582_adh	TAGTAGCAAACAAATTTCGGT	Chr.4	9589297	9589317	+	No
1754211_adh	TAAACACAGAAGCGAGATTTT	Chr.4	9589337	9589357	+	No
2259803_adh	TGATACGTTAGGAGTTGTCGG	Chr.4	9594714	9594694	-	No
1858532_adh	TAGCTGCTCCAAGAGATACGG	Chr.4	9599699	9599679	-	No
2260111_adh	TGATACTTCCTTTATTGATCG	Chr.4	9610321	9610341	+	No
2559644_adh	TTACTGTGGGGTATTTTAGGT	Chr.4	9610482	9610502	+	No
1869133_adh	TAGGGGCAACACTGTATATCA	Chr.4	9610925	9610905	-	No
2721992_adh	TTTGCGCTGTATTACCAAGGG	Chr.4	9618734	9618754	+	Yes
2694182_adh	TTTACTCAAAAGAAAGGATTT	Chr.4	9622774	9622754	-	No
1838851_adh	TAGAACTGTGAAACGGAAGAT	Chr.4	9631429	9631449	+	No
1990987_adh	TCCTGAAAGTCTGAATGCTGA	Chr.4	9633658	9633678	+	No

2730857_adh	TTTGTTCTGTAGATGACAAGG	Chr.4	9647853	9647833	-	No
2028202_adh	TCGTTGAATGGACTGCGCGGT	Chr.4	9648079	9648099	+	No
1875521_adh	TAGTATGGCACTCCCTAATAG	Chr.4	9652941	9652961	+	No
2258813_adh	TGATAAGCAAAATGAGAGAAT	Chr.4	9664398	9664418	+	No
2652198_adh	TTGCGCAAACACATATACGAT	Chr.4	9667209	9667189	-	No
2741369_adh	TTTTGAGAAGGTTAAAGTTGC	Chr.4	9670590	9670610	+	No
1829950_adh	TACTAGACGTTCGCATCATTT	Chr.4	9679579	9679559	-	Yes
1918940_adh	TATGTGATCTGTAATTTCAAT	Chr.4	9684572	9684592	+	No
2125737_adh	TGAACTGTCAAAACGGGACGT	Chr.4	9688827	9688807	-	No
2546035_adh	TTAACTCAAACATGACTTGCT	Chr.4	9718156	9718136	-	No
2494395_adh	TGTCACAAGTGGAGAGGCGAG	Chr.4	9722738	9722758	+	No
2080199_adh	TGAAAGAGATGTGGAAACGTG	Chr.4	9731644	9731624	-	No
1807293_adh	TACAAATTGAAGAGAAAAGAA	Chr.4	9734097	9734117	+	No
2046757_adh	TCTGATCAAGGTGGGAAGCTG	Chr.4	9738539	9738559	+	No
2616006_adh	TTCTCAATGACTGGATGTGTT	Chr.4	9746954	9746974	+	No
1887894_adh	TATAGAAATGTAGAAAACGAG	Chr.4	9754325	9754345	+	No
2480976_adh	TGTAAATGGTTGGAATCTGGT	Chr.4	9757504	9757524	+	No
1761850_adh	TAAATGGTTGGAATCTGGTAT	Chr.4	9757506	9757526	+	No
2699593_adh	TTTATTAGCAAAGGGATAGCA	Chr.4	9758999	9759019	+	No
2747869_adh	TTTTTGTTAATTCGTTTGGTT	Chr.4	9777654	9777634	-	No
2744723_adh	TTTTTAAATTTAATTCTTTGA	Chr.4	9806008	9806028	+	No
2043262_adh	TCTCTTCATGTCGGCTTTTCC	Chr.4	9825281	9825261	-	No
1968104_adh	TCATAAATTAGGATCCAAGGA	Chr.4	9830799	9830819	+	No
2381013_adh	TGGAGCACGGCATATTGGGAA	Chr.4	9850233	9850213	-	No
2381561_adh	TGGAGCTGAGAAGATCATCTT	Chr.4	9850927	9850907	-	No
1942355_adh	TCAATGGTGGAGCTGAGAAGA	Chr.4	9850934	9850914	-	No
2055642_adh	TCTTCAGTACCAAACAACCAA	Chr.4	9851041	9851021	-	No
2236270_adh	TGAGGAAATTGGATTTTCGGG	Chr.4	9852264	9852244	-	No
1920853_adh	TATTACGATCGTTGAGGAAAT	Chr.4	9852276	9852256	-	No
1834747_adh	TACTTATTTGTGGCATGAATT	Chr.4	9856887	9856907	+	No
2590187_adh	TTCAGAAAAAGATATACTCTC	Chr.4	9870403	9870383	-	Yes
2503841_adh	TGTGACGAATAACGGCAATAA	Chr.4	9874681	9874661	-	No
2053350_adh	TCTGTTGGTGAAGGTTGGATA	Chr.4	9877156	9877136	-	No
2250547_adh	TGAGTAATAAAGATTTTAGAG	Chr.4	9890581	9890561	-	No
1791859_adh	TAATACGGATACTAAAGTTTA	Chr.4	9928105	9928085	-	No
1989013_adh	TCCTAAAGCTTATTTGAACGT	Chr.4	9935796	9935776	-	No
2712619_adh	TTTGAAACTGTAAGTATGACG	Chr.4	9952751	9952771	+	No
2743774_adh	TTTTGTCGAAGCAATAGGTAT	Chr.4	9972202	9972222	+	No
1808859_adh	TACAAGCAAACTGAACTGTGT	Chr.4	9990686	9990666	-	No
2667405_adh	TTGGGATTTTGTTGACGGATA	Chr.4	9994321	9994301	-	No
2181590_adh	TGAGAATGCTGACAAGTTGAA	Chr.4	9996158	9996138	-	No
2381427_adh	TGGAGCTAAAGGAGACAATGA	Chr.4	9996314	9996294	-	No
2664096_adh	TTGGATTATAGAGTTTATATC	Chr.4	9996654	9996634	-	No
2699033_adh	TTTATGAGAACTAGAGGACGA	Chr.4	10007808	10007788	-	No
2042812_adh	TCTCTGACGGCCGATCCACCA	Chr.4	10022647	10022627	-	No
2042812_adh	TCTCTGACGGCCGATCCACCA	Chr.4	10029058	10029078	+	No
2042812_adh	TCTCTGACGGCCGATCCACCA	Chr.4	10035613	10035633	+	No
2381997_adh	TGGAGGACTCTTCGATCGTCA	Chr.4	10041144	10041124	-	No
2593850_adh	TTCATCAAGAAGAACAACTGT	Chr.4	10043787	10043807	+	No
2714280_adh	TTTGAATGATAGAAGGAATAA	Chr.4	10045638	10045658	+	No

2739263_adh	TTTTCTCCTTTTTCATACTTT	Chr.4	10083982	10084002	+	No
1973743_adh	TCATTCCAACACAACGGACTG	Chr.4	10112854	10112874	+	No
1821701_adh	TACGAAGAGACAACAAGGATA	Chr.4	10114399	10114379	-	No
1921238_adh	TATTAGAACGGTGAGGAAGGA	Chr.4	10127091	10127111	+	No
2313676_adh	TGGAAAAGGAGGTAGCGAAAA	Chr.4	10128093	10128073	-	No
2311283_adh	TGCTTGGGCAACAAGGGCAGA	Chr.4	10128357	10128337	-	No
1927869_adh	TATTGTCAGTGAAGCGCCTAG	Chr.4	10129343	10129363	+	No
2495087_adh	TGTCAGTGAAGCGCCTAGCAT	Chr.4	10129346	10129366	+	No
2716618_adh	TTTGAGATCAGACAGCTCGAC	Chr.4	10129690	10129670	-	No
1885870_adh	TATAATGAATATTTCTGTCAA	Chr.4	10136052	10136072	+	No
2288211_adh	TGCAGCGATAGAAGAAAAGTC	Chr.4	10151656	10151676	+	No
2734214_adh	TTTTACGTATTTTCCCTAATA	Chr.4	10151687	10151707	+	No
2723957_adh	TTTGGATCCAAGTACTTGACT	Chr.4	10178044	10178024	-	No
2736934_adh	TTTTATTTCCAATTCATATAA	Chr.4	10195074	10195054	-	No
2618414_adh	TTCTGATCAACGAGTCTTAGA	Chr.4	10195296	10195276	-	No
2608409_adh	TTCGGCAAAGAAGAGACGGAG	Chr.4	10197512	10197532	+	No
2130004_adh	TGAAGATCGTTAGTATATATA	Chr.4	10203059	10203039	-	No
2308577_adh	TGCTGATTGTAGATCAACGGA	Chr.4	10203180	10203200	+	No
2674968_adh	TTGTAGATCAACGGATGGCGT	Chr.4	10203186	10203206	+	No
2487937_adh	TGTAGATCAACGGATGGCGTT	Chr.4	10203187	10203207	+	No
2500850_adh	TGTGAAAGGAGTTCCATTAGT	Chr.4	10211403	10211383	-	No
1929258_adh	TATTTAGCTCTGTAGGAGATT	Chr.4	10213316	10213336	+	No
2487427_adh	TGTAGAACAAGGCCAGGAACG	Chr.4	10221050	10221030	-	No
2723881_adh	TTTGGATACTGTAAGTGTGGC	Chr.4	10222135	10222155	+	No
2694961_adh	TTTAGAAGCAAAGAGAACAAG	Chr.4	10222220	10222240	+	No
1831000_adh	TACTCCACGTGGACTCGGACA	Chr.4	10222430	10222410	-	No
1835957_adh	TACTTTATACTTTTTGCAATA	Chr.4	10231032	10231012	-	No
2649239_adh	TTGCAGAAATTTGGAAAGTGG	Chr.4	10232788	10232808	+	No
2286993_adh	TGCAGAAATTTGGAAAGTGGA	Chr.4	10232789	10232809	+	No
2654093_adh	TTGCTACAGGATCTCTCGGAC	Chr.4	10243984	10244004	+	No
2162077_adh	TGACGTAGAGACCTACAAAGC	Chr.4	10244216	10244236	+	No
2038764_adh	TCTCCATGTTGGATCCATCAA	Chr.4	10244565	10244585	+	No
2321088_adh	TGGAACTAGAAGAGACGTGAG	Chr.4	10252693	10252673	-	No
1944763_adh	TCACAGACGCTCAAGACTTCG	Chr.4	10279416	10279436	+	No
1973946_adh	TCATTGAACGGAAAATGGAGA	Chr.4	10285560	10285580	+	No
2626397_adh	TTGAACGGAAAATGGAGAGAA	Chr.4	10285563	10285583	+	No
2123227_adh	TGAACGGAAAATGGAGAGAAT	Chr.4	10285564	10285584	+	No
1836854_adh	TAGAAAATTTTCTCGACTTCA	Chr.4	10294096	10294076	-	No
1829902_adh	TACTAGAAAATTTTCTCGACT	Chr.4	10294099	10294079	-	No
2042190_adh	TCTCTATCAACGGCTTTGAGT	Chr.4	10311328	10311348	+	No
1804327_adh	TAATTGTGAGTTCAAACGGAT	Chr.4	10324095	10324075	-	Yes
2313192_adh	TGGAAAAATAAAAAGTAGAAG	Chr.4	10327073	10327093	+	No
2100848_adh	TGAAATGAAAATTATTGAGAA	Chr.4	10334429	10334449	+	No
2320499_adh	TGGAACGGCCGAACGAGAGAA	Chr.4	10346334	10346354	+	No
2070755_adh	TGAAAATTCATAGAACAGCAA	Chr.4	10348069	10348049	-	No
2673518_adh	TTGTAAAAATCAAAATCGGAG	Chr.4	10357941	10357921	-	No
1901682_adh	TATGAAGAAAAATGAAAGCGG	Chr.4	10373758	10373738	-	No
2616810_adh	TTCTCGTGCTGACGCTTTTAC	Chr.4	10374053	10374073	+	No
1886863_adh	TATACAGTTGAAAAGACCTTC	Chr.4	10377080	10377060	-	No
2557787_adh	TTACGGTCAGATGCCTCAAGC	Chr.4	10386552	10386572	+	No

2579298_adh	TTATGGAAGATCGTCAGAATC	Chr.4	10415938	10415918	-	No
1971767_adh	TCATGAATAGAAAAAACCGGC	Chr.4	10425403	10425423	+	No
1762925_adh	TAAATTTTGACAGCATTATAT	Chr.4	10429454	10429434	-	No
1932228_adh	TCAAAAGACGTCGGTAGTCAC	Chr.4	10435211	10435191	-	No
2176682_adh	TGAGAAAAGTGGAAGTGAACA	Chr.4	10436339	10436319	-	No
2123239_adh	TGAACGGAATAGATTTACGCA	Chr.4	10459552	10459572	+	No
2112218_adh	TGAAATGTCAAGAATTATTTC	Chr.4	10468974	10468994	+	No
2723143_adh	TTTGGAAGTCAATTGGCAGAA	Chr.4	10483836	10483816	-	No
2463797_adh	TGGTAGTTTCAGACGAGGATT	Chr.4	10500958	10500978	+	No
2414238_adh	TGGCTAGGAGAAGGAGGAAAA	Chr.4	10504061	10504081	+	No
2290638_adh	TGCATGCTTAGTTTTCACTGT	Chr.4	10507996	10508016	+	No
2645779_adh	TTGATTACACATTTTTGAATA	Chr.4	10510286	10510266	-	No
1813244_adh	TACATATGAAGGCCTGAAAAA	Chr.4	10519623	10519603	-	No
2608768_adh	TTCGGCTTAGCAAGGTATGTG	Chr.4	10536077	10536097	+	No
1778572_adh	TAAGATCAGCACGGAACGGCA	Chr.4	10549208	10549188	-	No
2449910_adh	TGGGCAGAAGAGGTAGATGTC	Chr.4	10550757	10550737	-	No
2277838_adh	TGATTTAGAAGTACGGTACAG	Chr.4	10564901	10564881	-	No
1808960_adh	TACAAGCTGACTGGTTAAAGA	Chr.4	10571526	10571506	-	No
2740596_adh	TTTTGAACAAAACTTTTGAAA	Chr.4	10582017	10582037	+	No
1802287_adh	TAATTAAGTTTTTGTAGCATA	Chr.4	10589871	10589851	-	No
1764767_adh	TAACACTGCATATGCAACACT	Chr.4	10623246	10623226	-	Yes
1789463_adh	TAAGTTGTGTAGTTGAAGCGA	Chr.4	10655656	10655636	-	No
2606569_adh	TTCGCGAGGATCTTCCTAGAG	Chr.4	10687227	10687247	+	No
2709721_adh	TTTCTAGTAGACGTTAAGGCC	Chr.4	10688233	10688213	-	No
1901690_adh	TATGAAGAACGAGTTCGAATT	Chr.4	10696204	10696184	-	No
2607660_adh	TTCGGACTAGATCGTATTCAA	Chr.4	10699918	10699938	+	No
2634600_adh	TTGAATTTTAAGTAGACCATG	Chr.4	10702937	10702917	-	No
1928972_adh	TATTTAAAAATTGCAAGTTGA	Chr.4	10703031	10703011	-	No
2709772_adh	TTTCTATCAAAATGTCGTCGA	Chr.4	10703098	10703118	+	No
2006167_adh	TCGCAGCATGCATACATTCCT	Chr.4	10709337	10709317	-	No
2580483_adh	TTATTAAAGTTTTGATCGGAT	Chr.4	10734772	10734792	+	No
1759307_adh	TAAAGTTTTGATCGGATCTCC	Chr.4	10734776	10734796	+	No
2589100_adh	TTCACAATTGTCCGACACTGG	Chr.4	10754740	10754720	-	No
2583718_adh	TTATTTCACAATTGTCCGACA	Chr.4	10754744	10754724	-	No
2589624_adh	TTCACGCGTCTTCTCAGCGAT	Chr.4	10758115	10758135	+	No
2483888_adh	TGTAATGAAGTTTTGTAGAGG	Chr.4	10760745	10760765	+	No
1797173_adh	TAATATTGGCAGAACATGGTT	Chr.4	10794897	10794877	-	No
1810774_adh	TACACAGTTCAGCAAAGAAAG	Chr.4	10795082	10795062	-	No
1761137_adh	TAAATCTTTCACAAGGAATTT	Chr.4	10813454	10813474	+	Yes
2595625_adh	TTCCAAAAGAATTTCTTTGTT	Chr.4	10814571	10814551	-	No
1930142_adh	TATTTGAAAAGGAAAACGTAA	Chr.4	10815387	10815367	-	Yes
2515461_adh	TGTGGATAGATTGAGGTGGCA	Chr.4	10817057	10817037	-	No
2308593_adh	TGCTGATTTGGACGGAATGAC	Chr.4	10837078	10837098	+	No
2441374_adh	TGGGATGGTAAAAAATGTCAA	Chr.4	10838182	10838162	-	No
2003356_adh	TCGATCCAGCAAAGGTGACCA	Chr.4	10842484	10842504	+	No
2706635_adh	TTTCGAACTCGTCTCAGCGGA	Chr.4	10864555	10864575	+	No
2262983_adh	TGATATCGTTTTGATTTTCAA	Chr.4	10876432	10876452	+	No
2031425_adh	TCTACAGTTACAGTTTTCACA	Chr.4	10886749	10886769	+	Yes
2487419_adh	TGTAGAAATCGGCAATTGATC	Chr.4	10896683	10896663	-	No
2674649_adh	TTGTAGAAATCGGCAATTGAT	Chr.4	10896684	10896664	-	No

2113629_adh	TGAAATTAAAAAATATTGATT	Chr.4	10912387	10912367	-	No
1758890_adh	TAAAGTCGATTGCTCTACCCA	Chr.4	10914627	10914647	+	No
2176612_adh	TGAGAAAAGAAGAAAGGTCGT	Chr.4	10916231	10916211	-	No
2417254_adh	TGGGAAAAGAACAATCGAAAA	Chr.4	10918958	10918938	-	No
1990941_adh	TCCTCTGTTTGCATGCTATTC	Chr.4	10919393	10919373	-	No
2721152_adh	TTTGCAATTTTCGGCTCCTTT	Chr.4	10933212	10933192	-	No
1967332_adh	TCAGTTCATGATCTTCGAGAC	Chr.4	10938859	10938879	+	No
2069412_adh	TGAAAAGTACGGATATGGTTA	Chr.4	10950929	10950949	+	No
1821467_adh	TACGAAACAAACTAATGGGCA	Chr.4	10951172	10951192	+	No
2583718_adh	TTATTTCACAATTGTCCGACA	Chr.4	10952260	10952280	+	No
2589100_adh	TTCACAATTGTCCGACACTGG	Chr.4	10952264	10952284	+	No
2181480_adh	TGAGAATGAATAGACTTGACC	Chr.4	10966413	10966393	-	No
1988918_adh	TCCGTTGTTGCAAGAGCAGAA	Chr.4	10999217	10999237	+	No
2529348_adh	TGTTGCAAGAGCAGAAATCTG	Chr.4	10999222	10999242	+	No
2282132_adh	TGCAAGAGCAGAAATCTGGTT	Chr.4	10999225	10999245	+	No
2732765_adh	TTTGTTTGTTTTCGGCGCTTT	Chr.4	11020176	11020196	+	No
2403198_adh	TGGCACTCATGGTGTACTCGG	Chr.4	11026187	11026167	-	No
2048360_adh	TCTGCGGTATTTCTTTGGAAA	Chr.4	11038300	11038280	-	No
2748018_adh	TTTTTGTTTTCTCTTTTCGAT	Chr.4	11038424	11038444	+	No
1829129_adh	TACGTTTCCGTTTGATTCACA	Chr.4	11064708	11064728	+	No
1926139_adh	TATTGCAACTGTGATGAATAC	Chr.4	11066680	11066700	+	No
2584549_adh	TTATTTTCTGTCGTCAGCAAA	Chr.4	11081326	11081306	-	No
2452759_adh	TGGGGATTGAAAGCGGAGAGA	Chr.4	11083047	11083067	+	No
2649219_adh	TTGCACTTTTCTGATGGTTAC	Chr.4	11083196	11083216	+	No
2712173_adh	TTTCTTTTCAGTTCGTTTGCG	Chr.4	11083581	11083561	-	No
2038544_adh	TCTCATTTTTCGACGGCTTCT	Chr.4	11103253	11103273	+	No
1986156_adh	TCCGGCAACTGATAAAGTATT	Chr.4	11110158	11110138	-	No
1814285_adh	TACATTCCAGTTAAGGAGAAG	Chr.4	11110790	11110770	-	No
2338053_adh	TGGAATCTTCATTGCTCTCCT	Chr.4	11117364	11117344	-	No
2535252_adh	TGTTTTTGTTTGCCGGATCCA	Chr.4	11119120	11119100	-	No
2040307_adh	TCTCGGTCGTTAGGAAGAGAC	Chr.4	11119398	11119418	+	No
1926851_adh	TATTGGACAATTCGGAGTCGG	Chr.4	11121024	11121044	+	No
2657976_adh	TTGGACAATTCGGAGTCGGAT	Chr.4	11121026	11121046	+	No
2738028_adh	TTTTCCGTTAGACACTTTGAA	Chr.4	11142750	11142730	-	No
1805922_adh	TAATTTTCCGTTAGACACTTT	Chr.4	11142753	11142733	-	No
2056835_adh	TCTTCGTGGATGTGCACCAAT	Chr.4	11148174	11148154	-	No
2586292_adh	TTCAACTGTAGATGAAAGATG	Chr.4	11151971	11151951	-	No
1931486_adh	TATTTTTTCACATCGACTCGG	Chr.4	11159457	11159437	-	No
2707868_adh	TTTCGGCAAGAGAAAAGACGA	Chr.4	11177603	11177583	-	No
1923504_adh	TATTCAGCACGTTGTTGTCCG	Chr.4	11179432	11179452	+	No
2169699_adh	TGACTGATTGCTCCACACAAC	Chr.4	11181803	11181823	+	No
2620283_adh	TTCTGTTAGACTCATCAGCAG	Chr.4	11189630	11189650	+	No
2449910_adh	TGGGCAGAAGAGGTAGATGTC	Chr.4	11190134	11190154	+	No
2291623_adh	TGCATTTTGTAAGAATTCGGA	Chr.4	11191615	11191635	+	No
1966977_adh	TCAGTGGATACGCAGATCGAG	Chr.4	11193953	11193973	+	No
2387949_adh	TGGATACGCAGATCGAGCGCA	Chr.4	11193957	11193977	+	No
2291623_adh	TGCATTTTGTAAGAATTCGGA	Chr.4	11195151	11195131	-	No
1778572_adh	TAAGATCAGCACGGAACGGCA	Chr.4	11196531	11196551	+	No
1990768_adh	TCCTCTCGGACGATTAATTAA	Chr.4	11200468	11200488	+	No
2452080_adh	TGGGGACTCAAAGAAAACGAC	Chr.4	11210612	11210592	-	No

2681461_adh	TTGTGGAAAATATGGCAATTC	Chr.4	11213489	11213509	+	No
2514417_adh	TGTGGAAAATATGGCAATTCA	Chr.4	11213490	11213510	+	No
1860241_adh	TAGGAAACAATTAAAGTAACT	Chr.4	11225605	11225625	+	No
1939404_adh	TCAAGCTGGATATGATGCTGG	Chr.4	11225790	11225810	+	No
1747411_adh	TAAAACAACTGTGGCATTTTC	Chr.4	11230645	11230625	-	No
2708590_adh	TTTCGTAAATTGTTTTTTGGA	Chr.4	11235520	11235500	-	No
2513341_adh	TGTGCAGAAGAACGAAGAATT	Chr.4	11246077	11246057	-	No
2032270_adh	TCTACTTTCGGTATTACAACT	Chr.4	11247976	11247956	-	No
2747737_adh	TTTTTGTATGGTCTGACGTCA	Chr.4	11248363	11248343	-	No
2602674_adh	TTCGAATTTTTACAAGATGGC	Chr.4	11253952	11253972	+	No
2729045_adh	TTTGTCGGTCAGGCGTTGTCT	Chr.4	11260020	11260040	+	No
1810259_adh	TACAATTATATCCTTAGTTGA	Chr.4	11270160	11270140	-	No
2617883_adh	TTCTGAATCAGAGACTTCAGA	Chr.4	11292720	11292740	+	No
2740270_adh	TTTTCTTGTGCTGTTGGATTG	Chr.4	11293474	11293494	+	No
1921528_adh	TATTAGATTAAATTTTCTGGC	Chr.4	11293819	11293799	-	No
2618911_adh	TTCTGGACGAAACTCTCTGCT	Chr.4	11298257	11298237	-	No
1970317_adh	TCATATTTTCTTCGGATTTCT	Chr.4	11320128	11320148	+	No
2710461_adh	TTTCTGACAGAACATGGAAGG	Chr.4	11323841	11323861	+	No
2619983_adh	TTCTGTCAGAAAACGTATGAG	Chr.4	11323852	11323832	-	No
2527014_adh	TGTTCTGTCAGAAAACGTATG	Chr.4	11323854	11323834	-	No
2044224_adh	TCTGAAGCGCCAAGGAAGAAC	Chr.4	11326031	11326011	-	No
1983829_adh	TCCGAACCATCTGAGTACGTC	Chr.4	11328052	11328072	+	No
2023225_adh	TCGTAGATTTTGACACAGAAG	Chr.4	11330118	11330098	-	No
2044224_adh	TCTGAAGCGCCAAGGAAGAAC	Chr.4	11338762	11338782	+	No
1925425_adh	TATTGAATTCAAGCGAACAGA	Chr.4	11340906	11340886	-	No
1995773_adh	TCGAACTGATCGTTGAATTTA	Chr.4	11340946	11340926	-	No
1821443_adh	TACGAAAAAGTAGGACTTCAT	Chr.4	11346144	11346124	-	No
2650211_adh	TTGCATCCAATTTTTTGTTAA	Chr.4	11372463	11372443	-	No
2020611_adh	TCGGTTGTGGAAAGGAGGCTA	Chr.4	11376521	11376541	+	No
2481029_adh	TGTAAATGTGAAAGGGACGCA	Chr.4	11377652	11377672	+	No
2518862_adh	TGTGTAGGGCAAAGTAGGCGC	Chr.4	11382672	11382652	-	No
2233662_adh	TGAGCCACGTAGACGATTCGA	Chr.4	11394328	11394348	+	No
1773076_adh	TAAGAAAAAGCCTCATGACAG	Chr.4	11400224	11400244	+	No
2668808_adh	TTGGGTTGTGGTGGGAGGGCT	Chr.4	11403184	11403204	+	No
2074573_adh	TGAAACTTAGAAGAACGATTT	Chr.4	11406298	11406318	+	No
2044224_adh	TCTGAAGCGCCAAGGAAGAAC	Chr.4	11407356	11407376	+	No
1763162_adh	TAACAAATGTAGAAGTATGAT	Chr.4	11413152	11413172	+	Yes
1932406_adh	TCAAAATCGTCGTAAAATTTT	Chr.4	11424881	11424861	-	No
2474523_adh	TGGTTCAACAGTAGGCTTGGC	Chr.4	11432285	11432305	+	No
2150864_adh	TGACAAGAGTGAAACGCTATG	Chr.4	11482716	11482736	+	No
2577165_adh	TTATCATCTGATTGCAGGCAT	Chr.4	11484698	11484678	-	No
1819707_adh	TACCGGCACAGAGAGTGTAGA	Chr.4	11497159	11497179	+	No
2712630_adh	TTTGAAAGAAAACAACTTTAA	Chr.4	11500332	11500312	-	No
2276571_adh	TGATTGCATGGCCGACTGGTT	Chr.4	11549903	11549883	-	No
2706285_adh	TTTCCTGATTTTCGGCAATTT	Chr.4	11558671	11558691	+	No
2531667_adh	TGTTTAATTTGATGGTGTCTT	Chr.4	11561999	11561979	-	No
2050577_adh	TCTGGGTGTGATGGCGTCAGA	Chr.4	11593770	11593750	-	No
2710733_adh	TTTCTGGGTGTGATGGCGTCA	Chr.4	11593772	11593752	-	No
2690041_adh	TTTAACGTTGAGAAAACTGAA	Chr.4	11602183	11602163	-	No
2043871_adh	TCTGAACAGACAGAAAGGGCC	Chr.4	11602625	11602645	+	No

2654914_adh	TTGCTGGATCTAAGAAGACTC	Chr.4	11602692	11602712	+	No
1938122_adh	TCAAGAACAAGATGACAAGAA	Chr.4	11602897	11602917	+	No
1822928_adh	TACGAGAAGAGACATAAGAAC	Chr.4	11602968	11602988	+	No
2579327_adh	TTATGGACAACAAGTTGGAAT	Chr.4	11624497	11624477	-	No
2579327_adh	TTATGGACAACAAGTTGGAAT	Chr.4	11627163	11627143	-	No
2733216_adh	TTTTAACACAAGTGAATGTAG	Chr.4	11627797	11627817	+	No
2711772_adh	TTTCTTGCAAGGCTCGTTGGA	Chr.4	11637035	11637055	+	No
2695872_adh	TTTAGATTTGCATAGAAACTT	Chr.4	11641270	11641290	+	No
2587702_adh	TTCAATCAGAATCGGATTCTT	Chr.4	11646157	11646137	-	No
1761766_adh	TAAATGGCAGATCTAAATTTA	Chr.4	11652171	11652191	+	No
2742352_adh	TTTTGCATCGTTCTGGATTAA	Chr.4	11656287	11656267	-	No
1975675_adh	TCATTTTTTGCATCGTTCTGG	Chr.4	11656292	11656272	-	No
1917447_adh	TATGTAATGACATTGAGGGAT	Chr.4	11679561	11679541	-	No
1996568_adh	TCGAAGGATACAAGCACTCGG	Chr.4	11695274	11695294	+	No
1750048_adh	TAAAAGTAGAAATTGCAAGAA	Chr.4	11700050	11700070	+	No
1819936_adh	TACCTAATCAATCGCTTGTTT	Chr.4	11703982	11703962	-	No
1823238_adh	TACGAGCTCTCTTAAAGAATC	Chr.4	11704310	11704330	+	No
2624606_adh	TTGAAAATTGAGGGAAAATGG	Chr.4	11729205	11729185	-	No
2736417_adh	TTTTATTACTTATAGTTAGAA	Chr.4	11758472	11758452	-	Yes
1938352_adh	TCAAGAATATTACGTAGATGT	Chr.4	11760884	11760904	+	No
1833978_adh	TACTGTGGACTTTTCATCTGA	Chr.4	11761316	11761296	-	No
1852169_adh	TAGATTGCTGCAGTAAGGGTT	Chr.4	11815893	11815913	+	No
1979954_adh	TCCATTACTCGAAAAACTCCT	Chr.4	11820424	11820444	+	No
2048917_adh	TCTGGACATCTGACGTTGGAG	Chr.4	11821025	11821005	-	No
1747819_adh	TAAAACATGTTATGAAACGAC	Chr.4	11822697	11822717	+	No
2709159_adh	TTTCGTTATTACTGTGTCGGT	Chr.4	11836499	11836519	+	No
2603373_adh	TTCGACTTTATTTTCTGCTCA	Chr.4	11847828	11847808	-	No
1851952_adh	TAGATTATGTATACTGAAACG	Chr.4	11861212	11861192	-	No
1828569_adh	TACGTGAAACTGACATTTGTT	Chr.4	11877848	11877828	-	No
1897538_adh	TATCGGAAATTTATTGGAAAC	Chr.4	11908106	11908086	-	No
2522291_adh	TGTTAACATTCAATGAGGACG	Chr.4	11920362	11920382	+	No
2707879_adh	TTTCGGCACGTTGTTTCTCAA	Chr.4	11961767	11961747	-	No
2015079_adh	TCGGATTTTTTGTTTTCGGCA	Chr.4	11961780	11961760	-	No
2380327_adh	TGGAGAGAAGATTGAGAACAA	Chr.4	11977401	11977381	-	No
2001595_adh	TCGAGGAACAACCAGGACGAC	Chr.4	11981074	11981054	-	No
1825890_adh	TACGGAGAAGCTGAAGCAAAC	Chr.4	11982005	11981985	-	No
2256968_adh	TGAGTTGGGAGAGGATGAAGC	Chr.4	11982888	11982868	-	No
2546360_adh	TTAACTGCTAGCAGAAAAATC	Chr.4	11983109	11983089	-	No
1812929_adh	TACAGTTGATGGCTTGAAACC	Chr.4	11985003	11984983	-	No
2425619_adh	TGGGATAAGGATCATGTTGAC	Chr.4	11986074	11986054	-	No
2515015_adh	TGTGGACGTAGTGGAGATACC	Chr.4	11987346	11987326	-	No
2567523_adh	TTAGGAACAAGGCGCAAGACT	Chr.4	11995541	11995521	-	No
2057043_adh	TCTTCTGAAGAAATCGGCGAG	Chr.4	12003040	12003020	-	No
1775699_adh	TAAGACGGATAAGAGAAGGTA	Chr.4	12006021	12006041	+	No
2259307_adh	TGATACAAGTTGGTTTTCTTA	Chr.4	12029879	12029859	-	No
2320353_adh	TGGAACGGAAACTGCTGAAGC	Chr.4	12035752	12035772	+	No
1919185_adh	TATGTGTTTGACTCATAATCG	Chr.4	12047812	12047832	+	No
1999957_adh	TCGACGACGAAGACGAAGACG	Chr.4	12051911	12051931	+	No
2373786_adh	TGGACACAGACAAACTCTGAA	Chr.4	12078380	12078360	-	No
2413469_adh	TGGCGTGATGGAAAGATAAAT	Chr.4	12078431	12078451	+	No

2293955_adh	TGCCGAGTGGATTTGCAGACA	Chr.4	12089016	12088996	-	No
1896666_adh	TATCGAAAAGCAGAACAAAAT	Chr.4	12089118	12089138	+	No
2616342_adh	TTCTCCGGAGAAACAAGTTGA	Chr.4	12093481	12093461	-	No
1834757_adh	TACTTCAACAATCTAACACCA	Chr.4	12094516	12094536	+	No
1936266_adh	TCAACCAGGACAGAATGGACA	Chr.4	12109306	12109286	-	No
2733020_adh	TTTTAAAATGAGCGAAGAAGA	Chr.4	12121325	12121345	+	No
2749443_adh	TTTTTTGCAGAGAATTAGGGA	Chr.4	12122992	12122972	-	No
2507051_adh	TGTGATAGAACAACAGAAGGA	Chr.4	12124653	12124633	-	No
2389365_adh	TGGATCAAGTATGAAGTCGAT	Chr.4	12124788	12124808	+	No
2635124_adh	TTGACAGTAGCACACAGGTTG	Chr.4	12127272	12127292	+	No
2739336_adh	TTTTCTCTCAACTGATACGAT	Chr.4	12131542	12131562	+	No
2029307_adh	TCTAAACAAGGAACTGATTAA	Chr.4	12135192	12135212	+	No
2609378_adh	TTCGGGTAGAAAATACGACGA	Chr.4	12145920	12145940	+	No
2017394_adh	TCGGGTAGAAAATACGACGAG	Chr.4	12145921	12145941	+	No
2487561_adh	TGTAGAATTAACACGGAGAAT	Chr.4	12145949	12145969	+	No
1829901_adh	TACTAGAAAATGGAATTTAAT	Chr.4	12154907	12154887	-	No
2744831_adh	TTTTTAATGCTGACGTTGATT	Chr.4	12156537	12156517	-	No
2597181_adh	TTCCATCAAGAGAACGGACAC	Chr.4	12178219	12178199	-	No
2705181_adh	TTTCCATCAAGAGAACGGACA	Chr.4	12178220	12178200	-	No
1931912_adh	TCAAAAATCCCATCGGGCACA	Chr.4	12181552	12181532	-	No
2597181_adh	TTCCATCAAGAGAACGGACAC	Chr.4	12186451	12186431	-	No
2705181_adh	TTTCCATCAAGAGAACGGACA	Chr.4	12186452	12186432	-	No
1931912_adh	TCAAAAATCCCATCGGGCACA	Chr.4	12189784	12189764	-	No
2250502_adh	TGAGTAAGGAGAAGAAGGAAC	Chr.4	12192323	12192343	+	No
1848972_adh	TAGAGTCAACAGAAGAATTGA	Chr.4	12213188	12213168	-	No
2747132_adh	TTTTTGCTGATTGTTGAGTTT	Chr.4	12215555	12215535	-	No
2598864_adh	TTCCGATCCATAGTGGACATA	Chr.4	12240248	12240228	-	No
2124637_adh	TGAACTCGAAGCTTCTGGCAC	Chr.4	12250907	12250887	-	No
2626613_adh	TTGAACTCGAAGCTTCTGGCA	Chr.4	12250908	12250888	-	No
2259564_adh	TGATACGAGGCAGATGAAACA	Chr.4	12262156	12262176	+	Yes
2706233_adh	TTTCCTCGTCGAGGAGCAGAA	Chr.4	12269374	12269394	+	No
2603396_adh	TTCGAGAAAATGAACGTTGGT	Chr.4	12270176	12270196	+	No
1944395_adh	TCACAATTGTAGGAGACACCG	Chr.4	12270564	12270584	+	No
2521180_adh	TGTGTTGGAAATGACCGCATC	Chr.4	12270821	12270841	+	No
2043738_adh	TCTGAAAGAAGCTGAATTAAC	Chr.4	12272383	12272363	-	No
2488526_adh	TGTAGCTTTATCGGATTTGGT	Chr.4	12278621	12278601	-	No
2120100_adh	TGAACACTGTAGCTTTATCGG	Chr.4	12278628	12278608	-	No
2042277_adh	TCTCTCAATCGAATATGGCGG	Chr.4	12278731	12278751	+	No
2702976_adh	TTTCAGCAAAGTTTATAGCGT	Chr.4	12361077	12361097	+	No
1778643_adh	TAAGATCATCATCTACAACCA	Chr.4	12365834	12365814	-	No
2695813_adh	TTTAGATGAAGATTTCTCGTT	Chr.4	12372103	12372123	+	No
1961664_adh	TCAGATTCGAATGGAACGGAA	Chr.4	12374530	12374550	+	No
2302908_adh	TGCGGTGGAGAAGGAAGTTTT	Chr.4	12374563	12374583	+	No
2607905_adh	TTCGGAGCCTCGTCATCCACT	Chr.4	12374616	12374596	-	No
1827650_adh	TACGTAATTAAAATTTCTGAA	Chr.4	12385585	12385605	+	No
2679249_adh	TTGTGAACGGAAAACGAATAA	Chr.4	12389399	12389379	-	No
2028839_adh	TCGTTTCAAGATCGTGAAGGT	Chr.4	12390389	12390369	-	No
2649461_adh	TTGCAGAGTAGAGTTTTTGAA	Chr.4	12391382	12391402	+	No
2127972_adh	TGAAGACGGCGTAAATCTGAA	Chr.4	12391888	12391868	-	No
1930322_adh	TATTTGCTATTTTTCCAGGAA	Chr.4	12395667	12395647	-	No

1929075_adh	TATTTAATATTCTGTTCGGAG	Chr.4	12399091	12399111	+	No
1819924_adh	TACCTAAAATAAACTCAAAAA	Chr.4	12402792	12402772	-	No
1883809_adh	TATAAAAAATGTAGACCACGA	Chr.4	12404237	12404217	-	No
2713199_adh	TTTGAACGAGAGAGGGAGACC	Chr.4	12404260	12404240	-	No
2496887_adh	TGTCATTTTCGGCTTCCATTA	Chr.4	12410490	12410470	-	No
1927017_adh	TATTGGAGCTGAAGAGTAGAA	Chr.4	12432562	12432582	+	No
2381553_adh	TGGAGCTGAAGAGTAGAAATA	Chr.4	12432565	12432585	+	No
1823770_adh	TACGATAGTGGTAGCAGAACG	Chr.4	12442904	12442884	-	No
2181387_adh	TGAGAATCCAAAGAGAATGTA	Chr.4	12451460	12451480	+	No
2723171_adh	TTTGGAATCAAGTCGACGCAA	Chr.4	12451873	12451893	+	No
2710865_adh	TTTCTGTATAAATTTTCGGCG	Chr.4	12459611	12459631	+	No
2706580_adh	TTTCGAACCAAGGAATTTGGA	Chr.4	12459649	12459669	+	No
1809207_adh	TACAAGTTTTGGAAACTATAA	Chr.4	12464599	12464579	-	No
1940114_adh	TCAAGTAGGGAAGAATGGAGA	Chr.4	12467841	12467821	-	No
2148654_adh	TGAATTTTAAGATTGCAGTAG	Chr.4	12508572	12508592	+	No
1975366_adh	TCATTTGTCAAAAGATTGCTG	Chr.4	12513366	12513346	-	No
1958431_adh	TCAGACTGATCTGTTGGTTGA	Chr.4	12529571	12529551	-	No
2693272_adh	TTTACACTCGCGGAGAATATG	Chr.4	12531129	12531109	-	No
2620435_adh	TTCTGTTTTACACTCGCGGAG	Chr.4	12531135	12531115	-	No
2711097_adh	TTTCTGTTTTACACTCGCGGA	Chr.4	12531136	12531116	-	No
2554390_adh	TTACAGTAACACCGGTCGAAA	Chr.4	12555065	12555085	+	No
2584782_adh	TTATTTTTTCTTAGGCATTTT	Chr.4	12559239	12559259	+	No
2023745_adh	TCGTAGTCGTAGAAGTGGTAA	Chr.4	12562875	12562855	-	No
2379618_adh	TGGAGAAGGACAGATGGTGTA	Chr.4	12567429	12567449	+	No
1971014_adh	TCATCGGAGAAGTTCAAGGCG	Chr.4	12567841	12567861	+	No
2568198_adh	TTAGGATACATTGAGGACTAT	Chr.4	12570659	12570679	+	No
2463804_adh	TGGTAGTTTGGCACGAAAAAC	Chr.4	12613568	12613548	-	No
1875529_adh	TAGTATGGGACTGAAAAGATA	Chr.4	12638049	12638069	+	No
1875529_adh	TAGTATGGGACTGAAAAGATA	Chr.4	12638145	12638165	+	No
1875529_adh	TAGTATGGGACTGAAAAGATA	Chr.4	12638241	12638261	+	No
1875529_adh	TAGTATGGGACTGAAAAGATA	Chr.4	12638337	12638357	+	No
1875529_adh	TAGTATGGGACTGAAAAGATA	Chr.4	12638433	12638453	+	No
2587851_adh	TTCAATCGTTGGCAGTGGCAT	Chr.4	12639424	12639404	-	No
2701747_adh	TTTCAATCGTTGGCAGTGGCA	Chr.4	12639425	12639405	-	No
2013415_adh	TCGGATAATGTGGCAATTGGA	Chr.4	12676615	12676595	-	No
2587851_adh	TTCAATCGTTGGCAGTGGCAT	Chr.4	12677215	12677195	-	No
2701747_adh	TTTCAATCGTTGGCAGTGGCA	Chr.4	12677216	12677196	-	No
2026450_adh	TCGTGGAATTGAAGAGACGCA	Chr.4	12686044	12686024	-	No
1807123_adh	TACAAATAGTAGAGGAATTTG	Chr.4	12691810	12691790	-	No
2724184_adh	TTTGGATTTTGAGAAGATTTT	Chr.4	12704035	12704055	+	No
2321760_adh	TGGAAGAACTTCGTGAAAAGA	Chr.4	12729819	12729839	+	No
1899750_adh	TATCTCTCTTTTCTCGAGACA	Chr.4	12739492	12739472	-	No
1943105_adh	TCAATTGGAATGTCGGAAACT	Chr.4	12744717	12744697	-	No
1981976_adh	TCCCTACTCGGATTCGTCAAA	Chr.4	12744748	12744728	-	No
2624260_adh	TTGAAAAACTGTGCATTTTTG	Chr.4	12766886	12766906	+	No
2726868_adh	TTTGGTTGCTGAAAACGTAGA	Chr.4	12778081	12778061	-	No
1802895_adh	TAATTCACAAATCCGTTTCAC	Chr.4	12786918	12786898	-	No
2271564_adh	TGATGATGGTTGTGGTGGCAA	Chr.4	12816260	12816280	+	No
1851712_adh	TAGATGTAGGAACATTCATAA	Chr.4	12843773	12843793	+	No
2488575_adh	TGTAGGAACATTCATAAGACA	Chr.4	12843777	12843797	+	No

1978817_adh	TCCAGGTTGAAATTTGAAATG	Chr.4	12865907	12865927	+	No
1828058_adh	TACGTCATGCAGAGCAAACGT	Chr.4	12893452	12893472	+	No
1928553_adh	TATTGTTTGATATGTCGTGAA	Chr.4	12902987	12903007	+	No
2733777_adh	TTTTAATGTGGAAGAAATGAG	Chr.4	12915525	12915505	-	No
1888826_adh	TATAGGTTACTGAAGAAAAAG	Chr.4	12924660	12924640	-	No
2189153_adh	TGAGAGTAAGCGTTGAATTGA	Chr.4	12930673	12930653	-	No
2696475_adh	TTTAGCTGAACGAACGAGCAA	Chr.4	12955747	12955727	-	No
1830816_adh	TACTCAAAAGACGCAGGAAAG	Chr.4	12965079	12965059	-	No
1782003_adh	TAAGCAGTTGGCTTTGAGTCC	Chr.4	12983195	12983175	-	No
1896805_adh	TATCGACGAGAAGAAGACACA	Chr.4	12993438	12993418	-	No
1939522_adh	TCAAGGACAACAGAGGACACA	Chr.4	13028424	13028444	+	No
2742361_adh	TTTTGCATGCAAATTTTTTCT	Chr.4	13098910	13098930	+	No
2713589_adh	TTTGAAGACGACTACTGGATC	Chr.4	13104409	13104389	-	No
2693862_adh	TTTACGCTCGAAGATCGTCAT	Chr.4	13110118	13110098	-	No
2625954_adh	TTGAACAGACAATCATCTGAG	Chr.4	13122244	13122264	+	No
2119656_adh	TGAACAAGATTGAACATTACA	Chr.4	13163966	13163946	-	No
2713174_adh	TTTGAACCTGAAATCAGCATT	Chr.4	13167217	13167197	-	No
1883920_adh	TATAAAAGACCGTCGTCACGC	Chr.4	13189251	13189231	-	No
2387949_adh	TGGATACGCAGATCGAGCGCA	Chr.4	13226950	13226930	-	No
1966977_adh	TCAGTGGATACGCAGATCGAG	Chr.4	13226954	13226934	-	No
2291623_adh	TGCATTTTGTAAGAATTCGGA	Chr.4	13228048	13228028	-	No
2007642_adh	TCGCGAAACAAGAATTTCGAA	Chr.4	13228708	13228728	+	No
1895632_adh	TATCCCTGGTAGACGTCACTC	Chr.4	13240552	13240532	-	No
2743734_adh	TTTTGTCACCGCCGGTATCCC	Chr.4	13240567	13240547	-	No
2624814_adh	TTGAAACTACAGTACTCGTTA	Chr.4	13260582	13260602	+	No
1930982_adh	TATTTTCTGTTGACTCGTCTT	Chr.4	13294365	13294385	+	No
1762599_adh	TAAATTGTAGGAGAAAGGTGG	Chr.4	13295550	13295570	+	No
2039695_adh	TCTCGCAATTCCGTCGGAATT	Chr.4	13297820	13297800	-	No
2583272_adh	TTATTGTCAGAAGTTTAACGT	Chr.4	13298018	13298038	+	No
1958618_adh	TCAGAGAGCGAAGAATCATAG	Chr.4	13298761	13298781	+	No
2004294_adh	TCGATGTAGATGGGAACGGTG	Chr.4	13299224	13299244	+	No
2025531_adh	TCGTCGGTCTTCCAGATGGAT	Chr.4	13324386	13324406	+	No
1832344_adh	TACTGAGGATCGTGTCAAGTG	Chr.4	13324441	13324461	+	No
1826065_adh	TACGGATACAAACGAGAAGTC	Chr.4	13326100	13326120	+	No
2741360_adh	TTTTGACTTTTTACGGCTGAA	Chr.4	13334172	13334192	+	No
2656875_adh	TTGGAACTACGAAAAATCAAA	Chr.4	13349488	13349468	-	No
2595488_adh	TTCATTTCTGCTTCAAAGACG	Chr.4	13353429	13353449	+	No
2618809_adh	TTCTGCTTCAAAGACGTTTTC	Chr.4	13353434	13353454	+	No
2585264_adh	TTCAAAGACGTTTTCTCGAAT	Chr.4	13353440	13353460	+	No
2583875_adh	TTATTTCTAAAGTTTCTGTTC	Chr.4	13364497	13364517	+	No
2649670_adh	TTGCAGGAAAGACAGTCGAAG	Chr.4	13370055	13370035	-	No
2520090_adh	TGTGTGGTGAATGTGAGCAAG	Chr.4	13387083	13387103	+	No
2395674_adh	TGGATTTAGACGACTTATTCT	Chr.4	13390790	13390770	-	No
2028457_adh	TCGTTGGAAAAAGCACGTACA	Chr.4	13395293	13395273	-	No
2618323_adh	TTCTGAGCACTTTGACAGCTT	Chr.4	13400188	13400208	+	No
2668807_adh	TTGGGTTGTGGTGGGAGGGCA	Chr.4	13400349	13400369	+	No
1926476_adh	TATTGCGATTTTTTAATATTT	Chr.4	13400955	13400975	+	No
1995260_adh	TCGAAATCAGTTGACAGCTCT	Chr.4	13412978	13412958	-	No
2665160_adh	TTGGCACATTCTTCTGGAGAT	Chr.4	13416000	13415980	-	No
2372639_adh	TGGAATTTCAGAATTGTTCGT	Chr.4	13416100	13416120	+	No

2626076_adh	TTGAACATTTAAAAATTTTCA	Chr.4	13420164	13420144	-	No
2607166_adh	TTCGGAACTTTTTTGGCGGCG	Chr.4	13453457	13453437	-	No
2707474_adh	TTTCGCTTGCAGACGTTGACG	Chr.4	13453720	13453740	+	No
2488586_adh	TGTAGGAAGAGGGCACATTGA	Chr.4	13454821	13454841	+	No
2414845_adh	TGGCTGCGTACTCCTCTTGGA	Chr.4	13455330	13455350	+	No
2489505_adh	TGTAGTAGTACGGTAGGATTA	Chr.4	13465261	13465241	-	No
1827229_adh	TACGGTAGCTCTTATACGGTA	Chr.4	13469755	13469735	-	No
2707880_adh	TTTCGGCAGAATATCATAATA	Chr.4	13470329	13470349	+	No
2281379_adh	TGCAACATATCTGACGCGCAA	Chr.4	13477672	13477692	+	No
2607300_adh	TTCGGACAGATGCGACACGGA	Chr.4	13493086	13493066	-	No
1978814_adh	TCCAGGTGTGTAATTCGGTAA	Chr.4	13504072	13504052	-	No
2597020_adh	TTCCAGGTGTGTAATTCGGTA	Chr.4	13504073	13504053	-	No
2278422_adh	TGATTTGAACGAGAAGCAGAG	Chr.4	13506866	13506886	+	No
1753852_adh	TAAACAACGGAACGACAATAT	Chr.4	13506909	13506889	-	No
2654136_adh	TTGCTAGAAAATTGTAGAAAG	Chr.4	13510391	13510371	-	No
1975678_adh	TCATTTTTTGTCTTCGAGGAC	Chr.4	13512253	13512233	-	No
2514500_adh	TGTGGAACAGAGAAGAATGCA	Chr.4	13529056	13529076	+	No
2174014_adh	TGACTTGGATCATTACTTGCA	Chr.4	13531744	13531724	-	No
2698250_adh	TTTATATCACGGGTTGTCATC	Chr.4	13558443	13558423	-	No
1769689_adh	TAACTAAATCGTGAATGAATA	Chr.4	13564838	13564818	-	No
2736865_adh	TTTTATTTAAATAACTATTTA	Chr.4	13564926	13564906	-	No
1959815_adh	TCAGATACTCAGTCAAAGACC	Chr.4	13565436	13565456	+	No
2717919_adh	TTTGATCCTACGATGGTGAAT	Chr.4	13568044	13568024	-	Yes
2305333_adh	TGCTAACGTTTGGGCTTCACA	Chr.4	13593514	13593534	+	No
1814621_adh	TACATTTTTACAATTCATATA	Chr.4	13598926	13598946	+	No
2587722_adh	TTCAATCATCAACAAAGACTG	Chr.4	13600049	13600069	+	No
2748349_adh	TTTTTTATACAGTTGACAAGA	Chr.4	13600919	13600899	-	No
1787256_adh	TAAGTAAAACACAAGACATTC	Chr.4	13601249	13601229	-	No
2323319_adh	TGGAAGCATAACTCGATTTGG	Chr.4	13604194	13604174	-	No
1849481_adh	TAGAGTTGTAACAAAACTATC	Chr.4	13605489	13605469	-	No
2745129_adh	TTTTTAGGTGTAGATGAAACA	Chr.4	13605907	13605887	-	No
2134137_adh	TGAAGTAGGGCAATGGGTATA	Chr.4	13608803	13608823	+	No
2747027_adh	TTTTTGCATCGAGGATATTTC	Chr.4	13609044	13609064	+	No
2668867_adh	TTGGGTTTGTCGGCTATTGCT	Chr.4	13610103	13610083	-	No
1833884_adh	TACTGTGAAAGATGATGCAGA	Chr.4	13618360	13618380	+	No
1809195_adh	TACAAGTTCATATTCTGCATC	Chr.4	13618393	13618373	-	No
1926120_adh	TATTGATTTTGAAACATTTTA	Chr.4	13619682	13619662	-	Yes
1806146_adh	TAATTTTTCATGCGTTCAGAA	Chr.4	13624643	13624663	+	No
1957818_adh	TCAGAATAAAAGGTAATATTT	Chr.4	13634354	13634334	-	No
1756597_adh	TAAAGACGTGGAAAAAAGACG	Chr.4	13635910	13635890	-	No
1851531_adh	TAGATGGTAGAAAAATGGGTA	Chr.4	13641226	13641206	-	Yes
2132079_adh	TGAAGCTGGAAGGATTTATAT	Chr.4	13641532	13641512	-	No
2737166_adh	TTTTCAACGATTGAGACTGGC	Chr.4	13641879	13641859	-	No
2674570_adh	TTGTACTTTCAGATTCGGACA	Chr.4	13643818	13643838	+	No
2548322_adh	TTAAGGAGAACATGAAAGATG	Chr.4	13646195	13646175	-	No
2742049_adh	TTTTGATTTAAGTGTTTAGGG	Chr.4	13649411	13649431	+	No
1761862_adh	TAAATGGTTTGCAGACATTTT	Chr.4	13650394	13650414	+	No
2670745_adh	TTGGTGACTGGATAAATGGCA	Chr.4	13654370	13654350	-	No
2643358_adh	TTGATAATTTTCATTTACTTA	Chr.4	13670512	13670532	+	Yes
2593761_adh	TTCATATCATTCTTTCTTACA	Chr.4	13672106	13672086	-	No

2721033_adh	TTTGCAAGGATATATACGGAT	Chr.4	13673722	13673742	+	Yes
2702841_adh	TTTCAGAAAGACGTAAAAATA	Chr.4	13677915	13677935	+	No
2046057_adh	TCTGAGAACATCGTGAAACAG	Chr.4	13681153	13681133	-	No
2576081_adh	TTATATGGGAAATGGGAAATA	Chr.4	13681179	13681199	+	Yes
1957882_adh	TCAGAATCTAGGCTCGGCGAA	Chr.4	13684756	13684776	+	No
2590498_adh	TTCAGACGACGATCCGGTTAT	Chr.4	13689227	13689207	-	Yes
1921491_adh	TATTAGATAGTCGAAAACTCA	Chr.4	13691142	13691122	-	No
1802854_adh	TAATTCAAAAACCGTCTTCAG	Chr.4	13694502	13694522	+	No
2552982_adh	TTACAAAAAAGTGGATGAAGG	Chr.4	13696756	13696736	-	Yes
1867947_adh	TAGGGAACAAATGAAGCGGTA	Chr.4	13697757	13697777	+	No
1804667_adh	TAATTTAAAGCACGAGACTTA	Chr.4	13700851	13700831	-	No
1967089_adh	TCAGTGGTTTTATAGTAGTAG	Chr.4	13704767	13704747	-	No
1935490_adh	TCAAATGTTGATTTCTCGTAG	Chr.4	13705107	13705087	-	No
2605136_adh	TTCGATGCACCACATCGTCGA	Chr.4	13705809	13705789	-	No
2066706_adh	TGAAAACAACTAGATGAATCT	Chr.4	13708384	13708404	+	No
1967414_adh	TCAGTTCTCCAAGACGGACGG	Chr.4	13709237	13709257	+	No
2068186_adh	TGAAAAGAAATCGGAATAAAA	Chr.4	13711225	13711245	+	No
2582274_adh	TTATTGATCGCCTTCTACCAA	Chr.4	13712737	13712717	-	No
2670852_adh	TTGGTGATAGGTTTTTTGAGT	Chr.4	13713147	13713167	+	No
1872737_adh	TAGGTTTTTTGAGTTCTAGGA	Chr.4	13713154	13713174	+	No
2747024_adh	TTTTTGCAGTTGCTGGAAATG	Chr.4	13716365	13716345	-	No
1877037_adh	TAGTCTTTGTCGGAGTTTTAC	Chr.4	13718874	13718854	-	No
1959768_adh	TCAGATAATGCAAATTTTGGC	Chr.4	13719764	13719744	-	No
2549749_adh	TTAATACTTGTTTGTTTCTAA	Chr.4	13720142	13720162	+	No
2046088_adh	TCTGAGAATATTTTTTACATA	Chr.4	13720302	13720322	+	No
1830228_adh	TACTAGCTGTAGTAACCGGAT	Chr.4	13720431	13720451	+	No
1812341_adh	TACAGCTAGTACTAATGTAAG	Chr.4	13720441	13720421	-	No
2703795_adh	TTTCATAGTGTACAAATATAT	Chr.4	13721862	13721882	+	No
2625777_adh	TTGAAATTTGCAGTTGAACTT	Chr.4	13722780	13722800	+	No
2119116_adh	TGAAATTTGCAGTTGAACTTT	Chr.4	13722781	13722801	+	No
1835936_adh	TACTTTACGTAACTGAATAAA	Chr.4	13723092	13723072	-	No
2712547_adh	TTTGAAACACGAGAACAGCAA	Chr.4	13724100	13724080	-	No
2610943_adh	TTCGTAAAAAAGAATTTCAAG	Chr.4	13724644	13724664	+	No
2687603_adh	TTGTTTGTACAATCGTAGGAC	Chr.4	13727050	13727030	-	No
2746508_adh	TTTTTGAAATATGAGAAACGG	Chr.4	13727397	13727417	+	No
2687421_adh	TTGTTTGATCAGAGTATGAAT	Chr.4	13731127	13731147	+	No
2560191_adh	TTACTTGAATTTGAATAAATT	Chr.4	13743954	13743934	-	No
2269046_adh	TGATCAAGGAATCGGCTTTTA	Chr.4	13745385	13745365	-	No
1931710_adh	TCAAAAAACATGTAGTGATTG	Chr.4	13746875	13746855	-	Yes
2649511_adh	TTGCAGATTCATGGGATGTAC	Chr.4	13750135	13750115	-	No
2721332_adh	TTTGCAGCATTCGGACAGAAA	Chr.4	13750848	13750828	-	No
2704380_adh	TTTCATTACATATGTTCCACG	Chr.4	13753338	13753318	-	Yes
1821662_adh	TACGAACTTTTTGTAATTTTT	Chr.4	13754534	13754514	-	No
2032422_adh	TCTAGAGCTTTTGAAACGGTA	Chr.4	13756175	13756195	+	No
2497856_adh	TGTCGATGGGTCAATAAATAT	Chr.4	13757730	13757750	+	Yes
2522444_adh	TGTTAAGACCTAAAAATTCAT	Chr.4	13759721	13759701	-	No
2594205_adh	TTCATCATTGTTTTCGGAATT	Chr.4	13761130	13761150	+	No
2700976_adh	TTTCAAAATCGTTATTTTTCC	Chr.4	13762435	13762455	+	No
1961572_adh	TCAGATGTTAGATAGGTGAAT	Chr.4	13764332	13764312	-	Yes
2550575_adh	TTAATCGGCTTTTAAAAATTT	Chr.4	13768637	13768657	+	No

2190757_adh	TGAGATAAATGCTGGAAGAAT	Chr.4	13770937	13770917	-	No
2602786_adh	TTCGACAGAATTTCGGCAATT	Chr.4	13777605	13777585	-	No
2738221_adh	TTTTCGACAGAATTTCGGCAA	Chr.4	13777607	13777587	-	No
2643661_adh	TTGATATCAACCACAAGCACG	Chr.4	13777922	13777942	+	No
2314376_adh	TGGAAACGAGAACAAGACTGT	Chr.4	13778177	13778197	+	No
2237965_adh	TGAGGATGATTTGACGACGCT	Chr.4	13794664	13794684	+	No
1931058_adh	TATTTTGAGCTCAGCGCGGAA	Chr.4	13798518	13798538	+	No
1886746_adh	TATACACATGATTAATTTTTA	Chr.4	13799709	13799729	+	Yes
1985067_adh	TCCGATTCCGACTAGAAGGCT	Chr.4	13801037	13801017	-	No
1910113_adh	TATGATACGGTTGATAAAAAG	Chr.4	13808565	13808585	+	No
2743556_adh	TTTTGTAGATAATGGAGCAGA	Chr.4	13810882	13810862	-	No
1943558_adh	TCAATTTTGGGATGACGAGCT	Chr.4	13810912	13810892	-	No
1979377_adh	TCCATAAAAATAGGATAAACT	Chr.4	13813059	13813079	+	No
2721842_adh	TTTGCCTACGCTATTATAAAA	Chr.4	13814937	13814957	+	No
2717373_adh	TTTGATACAGTGGAATTCAAA	Chr.4	13818259	13818279	+	No
2259397_adh	TGATACAGTGGAATTCAAAAA	Chr.4	13818261	13818281	+	No
2555755_adh	TTACCCAATTCGAAAGTTTGC	Chr.4	13818340	13818360	+	Yes
2667235_adh	TTGGGAGTACAATGCTGTGGG	Chr.4	13820898	13820878	-	No
1874327_adh	TAGTAGACACAGTTGATACGA	Chr.4	13821623	13821643	+	Yes
2005090_adh	TCGATTGGAACCACTAGACAC	Chr.4	13822207	13822187	-	No
2654666_adh	TTGCTGATTTACTTTCGCGGT	Chr.4	13822338	13822318	-	No
2717373_adh	TTTGATACAGTGGAATTCAAA	Chr.4	13823279	13823299	+	No
2259397_adh	TGATACAGTGGAATTCAAAAA	Chr.4	13823281	13823301	+	No
2655033_adh	TTGCTGTAAAAACGAGTTGAT	Chr.4	13824129	13824149	+	No
2593763_adh	TTCATATCCCAATTCGTTGTA	Chr.4	13825642	13825662	+	No
2558758_adh	TTACTCGACGAAAAAATTGGT	Chr.4	13825864	13825884	+	No
2721247_adh	TTTGCACTGAAGTACAAGTTT	Chr.4	13827540	13827520	-	No
2062674_adh	TCTTTTGAAACTGTAATGCAA	Chr.4	13827574	13827594	+	No
2582223_adh	TTATTGACTTAGTATTTTGTT	Chr.4	13830198	13830178	-	No
2649857_adh	TTGCAGTTTCTAGTGCCATTT	Chr.4	13831209	13831189	-	No
1926515_adh	TATTGCGTATGCAGTGCACCA	Chr.4	13832150	13832130	-	No
2002466_adh	TCGATAAAAATTTTTGCAGAG	Chr.4	13835887	13835867	-	No
2312129_adh	TGCTTTTTAACATACCGTATT	Chr.4	13836660	13836680	+	No
2584121_adh	TTATTTGGAAAACTATATAAA	Chr.4	13837537	13837557	+	No
1923339_adh	TATTCAAACGTTTATATCTTA	Chr.4	13837775	13837795	+	No
2172464_adh	TGACTGTTCGTGTTCAAAAAG	Chr.4	13838626	13838646	+	No
2020162_adh	TCGGTTCTCGGTGATTGTGGA	Chr.4	13841452	13841432	-	No
1789884_adh	TAATAAAAAGTTGGGTTGAAA	Chr.4	13841593	13841613	+	No
2589049_adh	TTCAATTTTGTAACTGACGAC	Chr.4	13843006	13843026	+	No
2701058_adh	TTTCAAACTGAATTCGTTATA	Chr.4	13845128	13845108	-	No
2592743_adh	TTCAGTAGATATGGAAAAAAG	Chr.4	13852085	13852065	-	No
1929576_adh	TATTTCAGTAGATATGGAAAA	Chr.4	13852088	13852068	-	No
2635499_adh	TTGACCGTAGAGGGCGATCGC	Chr.4	13855703	13855723	+	No
2585649_adh	TTCAACAAGTAGAATGACTCA	Chr.4	13863421	13863441	+	No
1935933_adh	TCAACAAGTAGAATGACTCAT	Chr.4	13863422	13863442	+	No
2614595_adh	TTCTACTTGTTGAATAATGGT	Chr.4	13863434	13863414	-	No
2689385_adh	TTTAAATAAATGCTGAACCCC	Chr.4	13863688	13863668	-	No
2650218_adh	TTGCATCCTTTATTTTTTATA	Chr.4	13863904	13863884	-	No
2617897_adh	TTCTGAATCGCATACGCATTT	Chr.4	13870680	13870700	+	Yes
2686490_adh	TTGTTGTTAATTCGGATAAAC	Chr.4	13882554	13882574	+	No

2682931_adh	TTGTTAATTCGGATAAACTTT	Chr.4	13882557	13882577	+	No
2532636_adh	TGTTTCTAGAACAGATCGGAT	Chr.4	13885452	13885472	+	No
2270361_adh	TGATCTCTCTGTCTCGGCGTC	Chr.4	13887669	13887649	-	No
2527565_adh	TGTTGAACTTAGAGCTAGACA	Chr.4	13888392	13888372	-	No
2684499_adh	TTGTTGAACTTAGAGCTAGAC	Chr.4	13888393	13888373	-	No
2645982_adh	TTGATTCATACTCCACCATCA	Chr.4	13893960	13893980	+	No
2538055_adh	TTAAAAATTGTAGAAAACGTT	Chr.4	13894573	13894593	+	No
1806445_adh	TACAAAAATTCTACTGTTTCA	Chr.4	13895820	13895840	+	No
1907722_adh	TATGACATGAGAAACTGTTAG	Chr.4	13896611	13896631	+	No
1925844_adh	TATTGAGGATGGTATTTAATA	Chr.4	13900154	13900174	+	No
2641310_adh	TTGAGGATGGTATTTAATATT	Chr.4	13900156	13900176	+	No
2743799_adh	TTTTGTCGTGAAAATATGGTT	Chr.4	13900945	13900965	+	Yes
1796336_adh	TAATATGGTCCCGGAATCAAT	Chr.4	13903386	13903366	-	No
2622429_adh	TTCTTCTTTTGCGTACCTTTT	Chr.4	13903913	13903893	-	Yes
1920684_adh	TATTACATGAAAAATTTTCAA	Chr.4	13905670	13905690	+	No
2261135_adh	TGATAGTGTGTAATACGAAAA	Chr.4	13905945	13905965	+	No
2170476_adh	TGACTGGCATACACTCCCTTT	Chr.4	13908695	13908715	+	No
1881668_adh	TAGTTGGATGGTTGAAGAAGG	Chr.4	13913682	13913662	-	No
2737930_adh	TTTTCCCGATGGCACATGTGA	Chr.4	13913896	13913876	-	No
2639606_adh	TTGAGAATGGCCAACATTTTA	Chr.4	13914576	13914556	-	No
2712115_adh	TTTCTTTGCTTACAGAATACT	Chr.4	13917192	13917212	+	No
2016434_adh	TCGGGACGAAATTTAAACTGT	Chr.4	13917639	13917619	-	No
2609086_adh	TTCGGGAGGAAATATTCGGGA	Chr.4	13917654	13917634	-	No
2544809_adh	TTAACAAATGACGGTAAATCT	Chr.4	13917847	13917827	-	No
2650358_adh	TTGCATCTGAAAGGAAGATTG	Chr.4	13918081	13918101	+	No
2640334_adh	TTGAGATTATTTTTGTGGTTT	Chr.4	13919198	13919178	-	No
2232264_adh	TGAGATTTCAGATAAGATCAC	Chr.4	13919531	13919551	+	No
2181917_adh	TGAGAATTCTAAAGCTAGGCT	Chr.4	13920829	13920809	-	No
2681242_adh	TTGTGCAGATGTTCATGACCT	Chr.4	13921027	13921047	+	No
1761625_adh	TAAATGCCCAAACTATGCATA	Chr.4	13924974	13924994	+	No
2721273_adh	TTTGCAGAAATCTGGTAAAAG	Chr.4	13926759	13926779	+	No
2027713_adh	TCGTTAACCGGCTTTGAAATG	Chr.4	13928217	13928237	+	No
1790406_adh	TAATAAGATAATCCAGATAGG	Chr.4	13928999	13929019	+	No
1978073_adh	TCCAGATAGGTTTGCTTTACG	Chr.4	13929010	13929030	+	No
1752128_adh	TAAAATGATTTAATGTTTCAA	Chr.4	13934135	13934155	+	No
1925868_adh	TATTGAGTCGGTCAATTTAAA	Chr.4	13937717	13937737	+	Yes
1830666_adh	TACTATGTCGCATGATGGACT	Chr.4	13939081	13939061	-	No
2523355_adh	TGTTAGAACGCGGTATAGCAA	Chr.4	13939636	13939616	-	No
2683194_adh	TTGTTAGAACGCGGTATAGCA	Chr.4	13939637	13939617	-	No
2699380_adh	TTTATGTTCTACAATGACATC	Chr.4	13944779	13944799	+	Yes
1994223_adh	TCCTTTTAATTTTATTTCGGC	Chr.4	13945840	13945860	+	No
2475749_adh	TGGTTGACTCAAATATATTTT	Chr.4	13950174	13950194	+	No
2552677_adh	TTAATTTGAAATCACCAACCA	Chr.4	13954622	13954642	+	No
2533368_adh	TGTTTGTACAAGTTTTTGACC	Chr.4	13955535	13955555	+	No
2727319_adh	TTTGTACAAGTTTTTGACCCA	Chr.4	13955537	13955557	+	No
2532382_adh	TGTTTCCTTTTACTAAGCAGC	Chr.4	13956044	13956024	-	No
2550467_adh	TTAATCCTACTTAATACTGAA	Chr.4	13958089	13958069	-	Yes
2691712_adh	TTTAATGGTAACGATTTTTGG	Chr.4	13960763	13960783	+	No
2386742_adh	TGGATAACAAGTTGATAACCA	Chr.4	13961364	13961344	-	No
2069140_adh	TGAAAAGGGCAGTTTCAAAAA	Chr.4	13972232	13972252	+	No

2126653_adh	TGAACTTGGTGAAACAGTTAA	Chr.4	13981474	13981494	+	No
2742448_adh	TTTTGCGAGTAGAAAATGATT	Chr.4	13984308	13984288	-	Yes
2552923_adh	TTAATTTTGCGAGTAGAAAAT	Chr.4	13984312	13984292	-	Yes
2693036_adh	TTTAATTTTGCGAGTAGAAAA	Chr.4	13984313	13984293	-	Yes
2274214_adh	TGATTAATCGACCCATTCATC	Chr.4	13986094	13986074	-	No
1859918_adh	TAGCTTTTCTCGATTTTATAA	Chr.4	13986125	13986105	-	No
2548913_adh	TTAAGTAGTGTGATGTTATAG	Chr.4	13988697	13988677	-	Yes
1920211_adh	TATTAAGTAGTGTGATGTTAT	Chr.4	13988699	13988679	-	Yes
1989892_adh	TCCTATTGACATGGACTCACT	Chr.4	13989601	13989621	+	No
1884023_adh	TATAAACTAAACACATGAACA	Chr.4	13990392	13990412	+	No
1886686_adh	TATACAACGACTACGACCAAT	Chr.4	13991791	13991811	+	No
2613045_adh	TTCGTTCGATAATGGTTTTAT	Chr.4	13992531	13992551	+	No
1919888_adh	TATTAAAATCGTTTGATATTA	Chr.4	13996118	13996138	+	No
2712346_adh	TTTGAAAACGACTGATCGAAC	Chr.4	13998063	13998083	+	No
2057196_adh	TCTTCTTCCTGATCGTTTGAA	Chr.4	13998322	13998302	-	No
2558218_adh	TTACTAAAATTCATGCCAGTC	Chr.4	13999445	13999465	+	Yes
1829386_adh	TACTAAGACATCGGAACACAA	Chr.4	14000569	14000549	-	Yes
2523414_adh	TGTTAGACTTATTATTAATTT	Chr.4	14000681	14000701	+	No
2149487_adh	TGACAACAGAGTGAATGGTCT	Chr.4	14001027	14001047	+	No
2733943_adh	TTTTACAATTACGAATTTTGC	Chr.4	14004186	14004206	+	No
2581226_adh	TTATTATTTTTGTCGAACTTC	Chr.4	14007257	14007237	-	Yes
1918941_adh	TATGTGATGCTTTTTTCTCTA	Chr.4	14007433	14007453	+	No
2597979_adh	TTCCCCGTTCACACGTTCTAC	Chr.4	14008008	14008028	+	Yes
2733915_adh	TTTTAATTTTTTCGTCGCAAT	Chr.4	14008188	14008208	+	Yes
1922028_adh	TATTATAGTTTCTCGACGGTG	Chr.4	14008849	14008869	+	Yes
2575145_adh	TTATAGTTTCTCGACGGTGTG	Chr.4	14008851	14008871	+	Yes
2047535_adh	TCTGATTGAGAACGTGAAATA	Chr.4	14008899	14008879	-	No
2587902_adh	TTCAATCTGAGGATTTTTTGT	Chr.4	14009098	14009118	+	No
2140192_adh	TGAATATTTATAACTTTGAAA	Chr.4	14009455	14009475	+	Yes
2654538_adh	TTGCTGAAGATACTTGGTTCT	Chr.4	14011057	14011077	+	No
2023692_adh	TCGTAGTAGAAGAATTGGTAA	Chr.4	14014343	14014363	+	No
1839018_adh	TAGAAGAATTGGTAAGTATGA	Chr.4	14014349	14014369	+	No
2055558_adh	TCTTCACAATATAAACTGGCT	Chr.4	14015290	14015310	+	No
2598456_adh	TTCCCTGTTCGATGAAAGCAT	Chr.4	14018568	14018588	+	No
2748135_adh	TTTTTTAAGGACATTTTATTT	Chr.4	14026157	14026177	+	Yes
2702389_adh	TTTCACAAAACTATCACAAAA	Chr.4	14028998	14029018	+	No
2522059_adh	TGTTAAACAAGGCATGTGTAG	Chr.4	14030110	14030090	-	No
2236098_adh	TGAGGAAAAGGAAGAAATATG	Chr.4	14034137	14034157	+	No
2721213_adh	TTTGCACTAGACGACGAACAC	Chr.4	14034540	14034560	+	No
1766840_adh	TAACCCCTCGGCATCGCTCCT	Chr.4	14034980	14034960	-	No
2654153_adh	TTGCTAGAGAAAATTTAGTTT	Chr.4	14036953	14036933	-	No
2025208_adh	TCGTATTTCTTGTTGTGTTTT	Chr.4	14037183	14037163	-	Yes
1806089_adh	TAATTTTGTGAATTTATCGGA	Chr.4	14037754	14037774	+	Yes
1893865_adh	TATCATATTTTATTACGAATG	Chr.4	14038471	14038491	+	Yes
2722404_adh	TTTGCTGACATTCATAATACA	Chr.4	14039273	14039253	-	Yes
2742529_adh	TTTTGCTGACATTCATAATAC	Chr.4	14039274	14039254	-	Yes
1943415_adh	TCAATTTTCAAACTTTGGGAC	Chr.4	14039350	14039370	+	No
2030469_adh	TCTAATCAGGCGAACAATAAT	Chr.4	14042204	14042184	-	No
2582161_adh	TTATTGACATTCGACATCTTT	Chr.4	14042451	14042431	-	No
2653449_adh	TTGCGTATGGCGTATCAGTTC	Chr.4	14043525	14043545	+	Yes

1914268_adh	TATGGAGTCGATATATTTTGA	Chr.4	14045235	14045215	-	No
2583512_adh	TTATTTACTACGAGGATGAAA	Chr.4	14045283	14045263	-	No
1800132_adh	TAATGCGTCATCGTCGCGTCG	Chr.4	14045392	14045412	+	No
2583432_adh	TTATTTAAATCCATGAACACC	Chr.4	14045714	14045694	-	No
2046559_adh	TCTGAGTTGCAACTAGTTGAA	Chr.4	14047246	14047266	+	Yes
2084114_adh	TGAAAGTCGGTTAAGCTACAA	Chr.4	14047949	14047929	-	No
1831793_adh	TACTGAAAGTCGGTTAAGCTA	Chr.4	14047952	14047932	-	No
2559035_adh	TTACTGAAAGTCGGTTAAGCT	Chr.4	14047953	14047933	-	No
2743580_adh	TTTTGTAGCTTTGAAAACAAG	Chr.4	14051033	14051013	-	No
1833309_adh	TACTGGATGGAATGCGTGCTC	Chr.4	14051851	14051871	+	No
2695222_adh	TTTAGACTAGAAAAACAAAGA	Chr.4	14056184	14056164	-	No
2031430_adh	TCTACAGTTTTTCGACGGAGG	Chr.4	14060684	14060664	-	No
2695219_adh	TTTAGACGTTTGCCAAGAATG	Chr.4	14062019	14061999	-	No
2532056_adh	TGTTTATTTTAGACGTTTGCC	Chr.4	14062026	14062006	-	No
2032636_adh	TCTAGCGAGAATGTGGGAAAT	Chr.4	14071013	14070993	-	No
2614892_adh	TTCTAGCGAGAATGTGGGAAA	Chr.4	14071014	14070994	-	No
1761329_adh	TAAATGACGTCCCGTAAATAA	Chr.4	14076659	14076679	+	No
2657807_adh	TTGGAATTGCATTGGAAAACT	Chr.4	14085453	14085433	-	No
2567575_adh	TTAGGAAGGACAAGAAGAAGG	Chr.4	14085845	14085825	-	No
2711912_adh	TTTCTTTAAAAGCTAGTGGAT	Chr.4	14085938	14085918	-	No
1887091_adh	TATACGAAAACAAGCACTTGA	Chr.4	14086339	14086319	-	No
2696411_adh	TTTAGCGGTAGATTCGTTTTC	Chr.4	14088253	14088233	-	No
1746097_adh	TAAAAACTTATTATTTAGTAA	Chr.4	14088618	14088598	-	Yes
1893240_adh	TATCAGAAGCAGAACATGGAA	Chr.4	14089105	14089085	-	No
2237322_adh	TGAGGACGTCATTGTGACATA	Chr.4	14091569	14091549	-	Yes
2690863_adh	TTTAATAAACGGTTTTATTTA	Chr.4	14091679	14091699	+	No
2646464_adh	TTGATTGGCATGTACTCAAGT	Chr.4	14093178	14093158	-	Yes
2720260_adh	TTTGATTGGCATGTACTCAAG	Chr.4	14093179	14093159	-	Yes
1930238_adh	TATTTGATTGGCATGTACTCA	Chr.4	14093181	14093161	-	Yes
2704543_adh	TTTCATTGAACCATATTTTAG	Chr.4	14093662	14093642	-	No
2701209_adh	TTTCAAATTAATACTCCTTTC	Chr.4	14096955	14096975	+	No
2740908_adh	TTTTGAATCCAAAGGAAAGTA	Chr.4	14099485	14099505	+	No
2630253_adh	TTGAATCCAAAGGAAAGTAGA	Chr.4	14099487	14099507	+	No
2733441_adh	TTTTAAGAATGAATGGCAGAA	Chr.4	14099910	14099890	-	No
2692935_adh	TTTAATTTATTGTGAAGACTG	Chr.4	14100337	14100317	-	Yes
2689321_adh	TTTAAAGGCACACACGGTTTT	Chr.4	14100612	14100592	-	No
2699705_adh	TTTATTCACTCTAAAGGAAAC	Chr.4	14100949	14100929	-	Yes
2736909_adh	TTTTATTTATTAGTTGAAGCA	Chr.4	14104279	14104259	-	No
2688721_adh	TTTAAAAATACGGCGTAAAAA	Chr.4	14113694	14113674	-	No
2658074_adh	TTGGACAGTTAGAAGGGAGGA	Chr.4	14114627	14114647	+	No
1795863_adh	TAATATCCATATTTACAAGGG	Chr.4	14118240	14118260	+	No
2574090_adh	TTATAATGAATATTTTGGACA	Chr.4	14118738	14118758	+	Yes
1856440_adh	TAGCGAGTAAGTGTGCGTTAA	Chr.4	14120802	14120822	+	No
2628533_adh	TTGAAGATTTGTTTTGCGCTC	Chr.4	14121628	14121608	-	Yes
2582136_adh	TTATTGAATGAACCTTAGAAA	Chr.4	14122666	14122646	-	Yes
1962526_adh	TCAGCCTGATGAAATTTTATT	Chr.4	14123389	14123369	-	Yes
2582243_adh	TTATTGAGCAATCGATTCGGG	Chr.4	14124691	14124671	-	No
2560332_adh	TTACTTGTGCTAGAAAAATGT	Chr.4	14126397	14126377	-	Yes
1926258_adh	TATTGCAGAATATGGACTACA	Chr.4	14133006	14133026	+	No
1919342_adh	TATGTTCGCTTTACTTGGGAA	Chr.4	14137163	14137143	-	No

2551184_adh	TTAATGGAAAAATCCTGCATT	Chr.4	14143170	14143190	+	No
1769188_adh	TAACGGTAGAAAAATGAAAAT	Chr.4	14143968	14143948	-	No
2258535_adh	TGATAACGGTAGAAAAATGAA	Chr.4	14143971	14143951	-	No
2643252_adh	TTGATAACGGTAGAAAAATGA	Chr.4	14143972	14143952	-	No
1922037_adh	TATTATATAACAAGCAACAAA	Chr.4	14144005	14144025	+	No
2512428_adh	TGTGATTGGTCTTTTCTGAAC	Chr.4	14149825	14149845	+	No
2691087_adh	TTTAATATAAGGTACGGTACA	Chr.4	14150178	14150198	+	No
1782188_adh	TAAGCCAAGTATAGTAAAACA	Chr.4	14152189	14152209	+	No
2153582_adh	TGACAGACATTATGCAATTAT	Chr.4	14160842	14160822	-	No
2669286_adh	TTGGTACAGATTCTTATTATG	Chr.4	14160992	14160972	-	No
2720202_adh	TTTGATTGCATTCCAAAATTA	Chr.4	14164329	14164349	+	Yes
2697138_adh	TTTAGTCGGTTTAGTTTTTGT	Chr.4	14165299	14165279	-	No
2744155_adh	TTTTGTTCGCATAAGACACAA	Chr.4	14167657	14167637	-	No
2745449_adh	TTTTTATTAAAAGCAGAAACT	Chr.4	14174858	14174878	+	Yes
2736390_adh	TTTTATTAAAAGCAGAAACTA	Chr.4	14174859	14174879	+	Yes
2640816_adh	TTGAGCCCGCTTATTGAAAAG	Chr.4	14177678	14177658	-	No
2714217_adh	TTTGAATCGGCTGCAGTGTAT	Chr.4	14181330	14181350	+	No
2088287_adh	TGAAATAGTCCTCGGTAAATT	Chr.4	14181383	14181403	+	No
2580264_adh	TTATGTTAGATTGTTTTTTCG	Chr.4	14181574	14181554	-	No
2699371_adh	TTTATGTTAGATTGTTTTTTC	Chr.4	14181575	14181555	-	No
2300302_adh	TGCGCTCATTAGAAAAAATTT	Chr.4	14182013	14182033	+	No
2420783_adh	TGGGAATGCCAGAATGTGACA	Chr.4	14183855	14183875	+	No
2381411_adh	TGGAGCGTGCTTGAATTATTT	Chr.4	14191157	14191177	+	No
2031218_adh	TCTACACTGACGCGCACACAA	Chr.4	14193236	14193256	+	No
2657609_adh	TTGGAATGAGCCGGAAGACTA	Chr.4	14193765	14193785	+	No
2342675_adh	TGGAATGAGCCGGAAGACTAT	Chr.4	14193766	14193786	+	No
1922109_adh	TATTATCATATCATTCAATTT	Chr.4	14194166	14194186	+	No
1974280_adh	TCATTGGATGATGGAAGAACC	Chr.4	14197654	14197634	-	Yes
2552862_adh	TTAATTTGTTGTTGGCATTTC	Chr.4	14203358	14203378	+	No
1994496_adh	TCGAAAAATTGTAATTTTACA	Chr.4	14203844	14203824	-	No
1969133_adh	TCATAGGGTAACCGATTTTCA	Chr.4	14219655	14219675	+	Yes
2541089_adh	TTAAAGATCAAAAGCACATAT	Chr.4	14228310	14228290	-	Yes
1811804_adh	TACAGAATTTAAAATATTAAA	Chr.4	14230007	14229987	-	No
2656080_adh	TTGCTTTTTAGACTATGCTTC	Chr.4	14232202	14232222	+	Yes
2694953_adh	TTTAGAAGACATTTAAAACAT	Chr.4	14237660	14237640	-	No
2734437_adh	TTTTAGAAGACATTTAAAACA	Chr.4	14237661	14237641	-	No
2072161_adh	TGAAACATCTTAAATCGTTAT	Chr.4	14238711	14238731	+	No
1944386_adh	TCACAATTCAACATTTTGTTT	Chr.4	14243078	14243058	-	No
2052630_adh	TCTGTGCTGACAGTGGAAGTT	Chr.4	14243746	14243766	+	No
2599940_adh	TTCCGTGACCAACTTCCACTG	Chr.4	14243776	14243756	-	No
2688876_adh	TTTAAAAGAAACTCAAACAGC	Chr.4	14244596	14244576	-	Yes
2587340_adh	TTCAAGTAGAAACATGAAAGT	Chr.4	14245318	14245298	-	No
2030587_adh	TCTAATCTGACAGCCGTTCTA	Chr.4	14248796	14248776	-	No
2549873_adh	TTAATAGATCGTTGATAAACA	Chr.4	14252903	14252883	-	No
2572038_adh	TTAGTGTAAGTGCTACAACTG	Chr.4	14254278	14254258	-	No
2748272_adh	TTTTTTAGAAGGATAATATGA	Chr.4	14254961	14254981	+	No
2617411_adh	TTCTCTGCGTCTCTTGGCTCT	Chr.4	14260881	14260861	-	No
2560218_adh	TTACTTGATTACGGCTCCATT	Chr.4	14262202	14262182	-	Yes
1920682_adh	TATTACATCTTCTATTTTAAT	Chr.4	14263266	14263246	-	Yes
1973663_adh	TCATTATTGTAGTTGAGCATG	Chr.4	14267522	14267542	+	No

1927760_adh	TATTGTAGTTGAGCATGTTTT	Chr.4	14267526	14267546	+	No
2648968_adh	TTGCACATTACTTACGAAGAA	Chr.4	14268353	14268333	-	No
1936725_adh	TCAACGGCTGTACAAAAAGAA	Chr.4	14273678	14273658	-	Yes
2625578_adh	TTGAAATGTCTGAAAATACGG	Chr.4	14275284	14275304	+	No
2618819_adh	TTCTGCTTGATTGTCTATCCT	Chr.4	14280519	14280539	+	Yes
1772865_adh	TAACTTTCCAGACACCCTTGC	Chr.4	14283580	14283600	+	No
1864204_adh	TAGGAGTCCGCAAACGTTTTG	Chr.4	14284569	14284589	+	No
2715087_adh	TTTGACCTTATACAACGTGCA	Chr.4	14284609	14284629	+	No
2090936_adh	TGAAATCACAGTTTGATTGGA	Chr.4	14286518	14286498	-	No
2553002_adh	TTACAAAAGTCCATTTATTTT	Chr.4	14286751	14286731	-	Yes
1774750_adh	TAAGAATTGTAAAGCCTTCTA	Chr.4	14287072	14287092	+	Yes
2288543_adh	TGCAGGCGTATTTCCGTAAGC	Chr.4	14288451	14288431	-	No
2552605_adh	TTAATTTAGTCATTGGTGTTA	Chr.4	14291722	14291742	+	No
2586231_adh	TTCAACTATTTATCTGGATAA	Chr.4	14292698	14292718	+	No
2711402_adh	TTTCTTCAATTTTCTTAAAAT	Chr.4	14296168	14296148	-	No
2549805_adh	TTAATAGACTAGGCCCTTTTA	Chr.4	14298403	14298423	+	No
2745191_adh	TTTTTAGTTTTCGTTGTGGAC	Chr.4	14301749	14301769	+	Yes
2735035_adh	TTTTAGTTTTCGTTGTGGACA	Chr.4	14301750	14301770	+	Yes
2697672_adh	TTTAGTTTTCGTTGTGGACAA	Chr.4	14301751	14301771	+	Yes
2573561_adh	TTAGTTTTCGTTGTGGACAAT	Chr.4	14301752	14301772	+	Yes
1883461_adh	TAGTTTTCGTTGTGGACAATT	Chr.4	14301753	14301773	+	Yes
2709284_adh	TTTCGTTGTGGACAATTTGGT	Chr.4	14301757	14301777	+	Yes
2514804_adh	TGTGGACAATTTGGTATTTAT	Chr.4	14301763	14301783	+	Yes
2549424_adh	TTAATAAAAAAGTTATTCATA	Chr.4	14302068	14302088	+	Yes
1873346_adh	TAGTAAGTTAGGCGTAATACT	Chr.4	14302793	14302813	+	No
2650899_adh	TTGCATTTGCTGAATTTGACA	Chr.4	14304349	14304329	-	No
2029318_adh	TCTAAACACGCTACTGCAGAA	Chr.4	14310307	14310327	+	No
1807288_adh	TACAAATTCGAATTTTTGACA	Chr.4	14310719	14310739	+	No
1788645_adh	TAAGTGGAGGTAGCTAAACAT	Chr.4	14318196	14318176	-	No
2582083_adh	TTATTGAAAGCAGAGGTGGGC	Chr.4	14321097	14321117	+	No
2580533_adh	TTATTAATAAATGAAACAAAT	Chr.4	14325234	14325214	-	No
2581851_adh	TTATTCTATACAAAGCTAATG	Chr.4	14326516	14326496	-	No
2148092_adh	TGAATTTAACGAGAATACTGA	Chr.4	14326852	14326832	-	No
1834380_adh	TACTGTTTTCTTTTTATTTAT	Chr.4	14327837	14327857	+	Yes
1983821_adh	TCCGAACACAGCAATGAAAAA	Chr.4	14330081	14330101	+	No
2742228_adh	TTTTGCACTTTCTACTCAAAC	Chr.4	14331427	14331407	-	No
2576902_adh	TTATCACAGGAACAAGTAGGA	Chr.4	14331437	14331457	+	No
1892491_adh	TATCACAGGAACAAGTAGGAA	Chr.4	14331438	14331458	+	No
2046348_adh	TCTGAGGAAGATGTCTGAAGT	Chr.4	14331992	14331972	-	No
2665307_adh	TTGGCATCCACAACTGATGAT	Chr.4	14333153	14333133	-	No
2618572_adh	TTCTGCAGATAGGGAAGCAAA	Chr.4	14334758	14334738	-	No
1756731_adh	TAAAGAGAAGAACGATTAAAA	Chr.4	14338227	14338207	-	No
2560245_adh	TTACTTGGATAGACTAATTAG	Chr.4	14338812	14338832	+	No
2707863_adh	TTTCGGATTTCGAATACGCAT	Chr.4	14340851	14340871	+	No
2608375_adh	TTCGGATTTCGAATACGCATT	Chr.4	14340852	14340872	+	No
1885151_adh	TATAAGGAAATGCGTATTCGA	Chr.4	14340880	14340860	-	No
2154664_adh	TGACAGTATAGGACCTTTTTT	Chr.4	14341541	14341521	-	No
2590921_adh	TTCAGCAAATCGAATTGTGTT	Chr.4	14343971	14343951	-	No
2557363_adh	TTACGGCTAACTTTTGTCATA	Chr.4	14345237	14345257	+	Yes
2585071_adh	TTCAAAATTTTCGGGCATTCA	Chr.4	14350120	14350100	-	No

2011418_adh	TCGGACCAGGGCTGTGCGGCT	Chr.4	14351800	14351780	-	No
2567480_adh	TTAGGAAAATACTTGATGCGC	Chr.4	14352409	14352429	+	No
1983416_adh	TCCCTGTAGAAAAGCACGACA	Chr.4	14354170	14354150	-	No
1989413_adh	TCCTAGAAGAATACGAATTAA	Chr.4	14354627	14354607	-	No
2579544_adh	TTATGGATTTTGGAGACATAA	Chr.4	14356014	14355994	-	No
1922739_adh	TATTATGGATTTTGGAGACAT	Chr.4	14356016	14355996	-	No
1791290_adh	TAATACATTAGAATGAGAAAT	Chr.4	14358560	14358540	-	No
2271079_adh	TGATGACAAAGTGGAGGAAAG	Chr.4	14358720	14358700	-	No
2701002_adh	TTTCAAAATTTGCAGCGAAAT	Chr.4	14359080	14359060	-	No
2611329_adh	TTCGTAGCGTAGCATGATTTT	Chr.4	14359173	14359153	-	Yes
2693407_adh	TTTACATCTGATTTTGAATGA	Chr.4	14361379	14361399	+	No
2526248_adh	TGTTCCGCAGAAATCTCTCTT	Chr.4	14361661	14361681	+	Yes
2666058_adh	TTGGCTATTAAACAACTATTC	Chr.4	14362646	14362626	-	No
2592779_adh	TTCAGTAGTTGTCATGCCAGA	Chr.4	14364010	14364030	+	No
2595094_adh	TTCATTCATATTCCTAGTTGG	Chr.4	14365785	14365805	+	No
2071524_adh	TGAAACAGATAACAGAATTTA	Chr.4	14368291	14368271	-	Yes
2634880_adh	TTGACAGAATTGGGATTTCAG	Chr.4	14369153	14369133	-	No
2549973_adh	TTAATATCATGTAAATCTAAA	Chr.4	14372247	14372227	-	No
2014995_adh	TCGGATTTCAGATAGGAAGAA	Chr.4	14372724	14372704	-	No
2595984_adh	TTCCAATTCCTAATCTAAATA	Chr.4	14374484	14374504	+	No
2741490_adh	TTTTGAGTTTTGTAGGTTTTT	Chr.4	14375244	14375224	-	No
1790384_adh	TAATAAGACCGTGGTAAATAT	Chr.4	14378133	14378113	-	Yes
2736976_adh	TTTTATTTGGAGCATGATCAA	Chr.4	14378871	14378851	-	Yes
2745524_adh	TTTTTATTTGGAGCATGATCA	Chr.4	14378872	14378852	-	Yes
1860009_adh	TAGGAAAAATTAGAAGACATT	Chr.4	14379725	14379705	-	Yes
2488535_adh	TGTAGGAAAAATTAGAAGACA	Chr.4	14379727	14379707	-	Yes
2675080_adh	TTGTAGGAAAAATTAGAAGAC	Chr.4	14379728	14379708	-	Yes
2728309_adh	TTTGTAGGAAAAATTAGAAGA	Chr.4	14379729	14379709	-	Yes
2701210_adh	TTTCAAATTAATTTGACTTAT	Chr.4	14380366	14380346	-	No
2710351_adh	TTTCTGAAATTGGAATTCGAA	Chr.4	14381468	14381488	+	No
2702890_adh	TTTCAGACGAGCAAAAAACAA	Chr.4	14385107	14385087	-	No
2688988_adh	TTTAAACAACTTGAAACACGC	Chr.4	14388775	14388755	-	No
2742197_adh	TTTTGCACGGTTTCTGGTTTT	Chr.4	14393463	14393483	+	Yes
1790814_adh	TAATAATGTCAGTTGAAAATG	Chr.4	14393902	14393922	+	No
1927093_adh	TATTGGCAAAACATCGATAAA	Chr.4	14398596	14398576	-	Yes
2651730_adh	TTGCGAAGACTTTACATATCG	Chr.4	14399862	14399882	+	No
2566239_adh	TTAGCAACGGTATTGTAATCA	Chr.4	14402275	14402295	+	Yes
2592116_adh	TTCAGGAATTCTTTAGCCGGT	Chr.4	14402689	14402669	-	No
1973205_adh	TCATTAAGGTTTAGAAATTTG	Chr.4	14402733	14402713	-	No
2275915_adh	TGATTCGTGGCTGGCATTATT	Chr.4	14404046	14404026	-	No
1926059_adh	TATTGATTCGTGGCTGGCATT	Chr.4	14404049	14404029	-	No
2582240_adh	TTATTGAGATGATTTTTCAAT	Chr.4	14405869	14405849	-	No
2572210_adh	TTAGTTAGGACATAATATGAT	Chr.4	14405952	14405932	-	Yes
2733139_adh	TTTTAAATAAAAATTATTTTT	Chr.4	14406528	14406548	+	No
2721376_adh	TTTGCATACATGCGAACGCAA	Chr.4	14407363	14407343	-	No
2737760_adh	TTTTCATGTGGAGTAGTGGGC	Chr.4	14408275	14408295	+	No
2709936_adh	TTTCTCCTATCAACAAGCATC	Chr.4	14410544	14410524	-	No
1812654_adh	TACAGTAGAAAATGAAGAAAG	Chr.4	14412863	14412843	-	No
1774891_adh	TAAGACAAGTTTAATAAGTTA	Chr.4	14414152	14414172	+	No
2539105_adh	TTAAAATTATAATTAACATTT	Chr.4	14414716	14414696	-	Yes

2703630_adh	TTTCAGTTTAGAACAGGAGAA	Chr.4	14414785	14414805	+	No
2573519_adh	TTAGTTTGTTTGGACATATAA	Chr.4	14434968	14434948	-	No
1921823_adh	TATTAGTTTGTTTGGACATAT	Chr.4	14434970	14434950	-	No
2581021_adh	TTATTAGTTTGTTTGGACATA	Chr.4	14434971	14434951	-	No
2736469_adh	TTTTATTAGTTTGTTTGGACA	Chr.4	14434973	14434953	-	No
2726610_adh	TTTGGTTAGACAGCTGAAAGA	Chr.4	14436070	14436090	+	No
2714410_adh	TTTGAATGTCTAGTGGAATGT	Chr.4	14436185	14436165	-	No
1919638_adh	TATGTTTCCGATGAAAACTCA	Chr.4	14437019	14437039	+	No
1831816_adh	TACTGAACAATTTGGTGATGG	Chr.4	14438721	14438741	+	No
2530295_adh	TGTTGGTTTGTGTTTTGGCTA	Chr.4	14440762	14440782	+	No
1803088_adh	TAATTCCTTCTGAAATAACAA	Chr.4	14444346	14444326	-	Yes
1761887_adh	TAAATGTATTTTTGTTTTTGT	Chr.4	14449127	14449107	-	No
2013748_adh	TCGGATCAGAATCGCTTGTTA	Chr.4	14450719	14450699	-	No
1941271_adh	TCAATCAAAAAATGTCTAGGC	Chr.4	14455510	14455530	+	No
2712756_adh	TTTGAAATAGAGAACTTGGAG	Chr.4	14459117	14459137	+	No
1923398_adh	TATTCAATAAGAATCCTTTTA	Chr.4	14462612	14462592	-	Yes
2554122_adh	TTACAGAAGGACAAACAGAGT	Chr.4	14471798	14471778	-	Yes
2277798_adh	TGATTTACAGAAGGACAAACA	Chr.4	14471802	14471782	-	Yes
1981343_adh	TCCCCTTTCGTAAAACTCTGA	Chr.4	14472972	14472992	+	No
1806557_adh	TACAAAATTAATATCCAAATA	Chr.4	14474276	14474256	-	No
2699657_adh	TTTATTATGTCCTGTAACTGA	Chr.4	14475657	14475677	+	No
1922814_adh	TATTATGTCCTGTAACTGAAA	Chr.4	14475659	14475679	+	No
2710725_adh	TTTCTGGGAGAAAATTGAGTT	Chr.4	14484957	14484937	-	No
2681248_adh	TTGTGCAGTGGATACATTTTT	Chr.4	14487440	14487460	+	Yes
1834049_adh	TACTGTTAGACGAAATTTCAA	Chr.4	14489062	14489082	+	No
2570802_adh	TTAGTAGTTTGTCGATATATA	Chr.4	14489144	14489164	+	No
2687405_adh	TTGTTTGAGCGATCGTTAGGA	Chr.4	14489300	14489320	+	No
2742281_adh	TTTTGCAGTTATATTTTTGTT	Chr.4	14492577	14492597	+	Yes
1830353_adh	TACTAGTTGTAAAAATTTGTA	Chr.4	14493644	14493664	+	No
2492434_adh	TGTATGTAACGTTGCTTAGAA	Chr.4	14493810	14493830	+	No
2259282_adh	TGATACAACGTAGAGTACTGG	Chr.4	14494605	14494625	+	No
2289424_adh	TGCAGTTGAAACACAAGTGAT	Chr.4	14495493	14495513	+	No
1867889_adh	TAGGGAAATAAAGACGATTTA	Chr.4	14495755	14495775	+	No
2533562_adh	TGTTTGTTCAGCTTTTAAATT	Chr.4	14496210	14496230	+	Yes
1771997_adh	TAACTTAAGAAAACGGACTAA	Chr.4	14498200	14498220	+	No
2003270_adh	TCGATATGAGAACAATAATTT	Chr.4	14498347	14498327	-	No
2711788_adh	TTTCTTGCTAGAACACAAATT	Chr.4	14499560	14499580	+	Yes
2242705_adh	TGAGGTAGGCACAGAAATGAC	Chr.4	14500647	14500667	+	No
2706408_adh	TTTCCTTATCTTTTTATGTTA	Chr.4	14501394	14501374	-	Yes
2699890_adh	TTTATTCGTCAATTGCAAAAG	Chr.4	14502017	14502037	+	No
2657883_adh	TTGGACAAATTATAAGGAGAC	Chr.4	14504054	14504034	-	No
2706370_adh	TTTCCTTACATGCACTTGAAT	Chr.4	14505274	14505294	+	No
1867088_adh	TAGGCATGTGTGAGGTAGGCA	Chr.4	14508040	14508060	+	No
2701191_adh	TTTCAAATGAACGGATTTTTT	Chr.4	14508235	14508215	-	No
2602966_adh	TTCGACGCTTCTATTCTCTTC	Chr.4	14508818	14508838	+	Yes
2699696_adh	TTTATTCAATTAGAGCAAAAT	Chr.4	14510772	14510792	+	No
1858904_adh	TAGCTGTTTGATAAGAGCATC	Chr.4	14511658	14511638	-	No
1939699_adh	TCAAGGCATCATTATTCAACT	Chr.4	14512294	14512314	+	No
2580679_adh	TTATTACTCGAGATACAAGCT	Chr.4	14521219	14521239	+	No
1801571_adh	TAATGTATGAAAACGTGCCGT	Chr.4	14526277	14526257	-	Yes

2635656_adh	TTGACGAGAAGAAAAATTAAT	Chr.4	14527016	14527036	+	No
2562511_adh	TTAGAGACTATTGCATTGCTT	Chr.4	14530234	14530254	+	No
2724458_adh	TTTGGCGGGTAGATCAAACGG	Chr.4	14532530	14532550	+	No
2584780_adh	TTATTTTTTCTGATATAACGG	Chr.4	14536504	14536524	+	Yes
2655240_adh	TTGCTTCATTGCCATTCTGCA	Chr.4	14538496	14538516	+	No
1890983_adh	TATATTTCCCATTAATAAATT	Chr.4	14544002	14543982	-	No
2745045_adh	TTTTTAGAAGAACGAATACAT	Chr.4	14544031	14544011	-	No
1749515_adh	TAAAAGCAGTAGTAATTTAAA	Chr.4	14544871	14544891	+	No
1919649_adh	TATGTTTCGTCTTTTTTAAAG	Chr.4	14548368	14548348	-	No
2588855_adh	TTCAATTCTGAAACAGAGTCA	Chr.4	14548767	14548747	-	No
2583716_adh	TTATTTCAATTCTGAAACAGA	Chr.4	14548771	14548751	-	No
1889617_adh	TATATAATACAAAAATATCTT	Chr.4	14551019	14551039	+	No
1943081_adh	TCAATTGATGGTTTTTTGTTT	Chr.4	14552485	14552465	-	Yes
2123754_adh	TGAACGGTTGAAACGGGAGAA	Chr.4	14554052	14554072	+	No
2623512_adh	TTCTTTCTCCTACATTTTAAT	Chr.4	14555003	14554983	-	Yes
2572230_adh	TTAGTTAGTTGATGTGATTGT	Chr.4	14556073	14556093	+	No
2714485_adh	TTTGAATTGAATACGAAGTCA	Chr.4	14560241	14560221	-	No
2623100_adh	TTCTTGTAACTTTAAAAATCG	Chr.4	14561160	14561180	+	No
2561422_adh	TTAGAAGAATATAATTTTTGG	Chr.4	14561395	14561415	+	Yes
2055019_adh	TCTTATCAGGAACGCGAAACA	Chr.4	14564220	14564200	-	No
2737237_adh	TTTTCAATACAATGTACATCT	Chr.4	14564710	14564730	+	No
2701658_adh	TTTCAATACAATGTACATCTT	Chr.4	14564711	14564731	+	No
1763085_adh	TAACAAAGCAGGAGAATAGAA	Chr.4	14565091	14565071	-	No
2029231_adh	TCTAAAAATCGCTGACAATAC	Chr.4	14565282	14565302	+	Yes
2546829_adh	TTAAGACATATATGTGGATTT	Chr.4	14566071	14566051	-	Yes
1885205_adh	TATAAGTAGCTTATTATTTTG	Chr.4	14567311	14567291	-	No
2559071_adh	TTACTGAATATTTACGCTGTA	Chr.4	14568921	14568941	+	No
2656020_adh	TTGCTTTGTCATCGTCGTCAT	Chr.4	14569207	14569187	-	No
1974235_adh	TCATTGCTGTGCAGATTCCTT	Chr.4	14571944	14571964	+	Yes
2739127_adh	TTTTCTAGACGACATCAATTG	Chr.4	14572075	14572055	-	Yes
2554689_adh	TTACATCGGACAGCGAGTGGA	Chr.4	14574062	14574082	+	No
2042746_adh	TCTCTGAAATTTGCATCAGTT	Chr.4	14577619	14577599	-	No
1886609_adh	TATAATTTGGCATCAATCTCT	Chr.4	14580193	14580213	+	No
2701216_adh	TTTCAAATTTCATGTAGTTTG	Chr.4	14585602	14585582	-	No
2712463_adh	TTTGAAAAGTGGAAAATGAAA	Chr.4	14585858	14585878	+	No
1886996_adh	TATACCCTAGTACAAACAATA	Chr.4	14591011	14591031	+	No
2561581_adh	TTAGAAGTCGTATACGCAGCC	Chr.4	14592751	14592771	+	No
2480405_adh	TGTAAAACTTAGGGCTGCGTA	Chr.4	14592783	14592763	-	No
2534777_adh	TGTTTTGTAGTTCAATTTCAT	Chr.4	14597441	14597421	-	No
1925441_adh	TATTGAATTTTGAAATATTTC	Chr.4	14602112	14602132	+	No
1889914_adh	TATATCAATTTTTAAACATTG	Chr.4	14602704	14602724	+	No
2679220_adh	TTGTGAAATTGGTAGGGAGTG	Chr.4	14606267	14606247	-	No
2597548_adh	TTCCATTTGTTTTCTCAACTC	Chr.4	14607457	14607477	+	No
2597486_adh	TTCCATTAGTTTTCTCAATTC	Chr.4	14608025	14608045	+	No
1979979_adh	TCCATTAGTTTTCTCAATTCA	Chr.4	14608026	14608046	+	No
2025374_adh	TCGTCATTAGTATATGCTGCA	Chr.4	14608234	14608254	+	Yes
2279880_adh	TGCAAACAGTGACTGATCAAA	Chr.4	14609057	14609037	-	No
2177595_adh	TGAGAAATCGTCGTCATTTCA	Chr.4	14612876	14612896	+	No
2292533_adh	TGCCAGCTGTAATGTCGACGA	Chr.4	14613144	14613164	+	No
1880692_adh	TAGTTCTGAAAGATCCCAATG	Chr.4	14615317	14615337	+	Yes

2736247_adh	TTTTATGTGCAGTTGCAATAA	Chr.4	14615681	14615701	+	No
1846071_adh	TAGACTGTAGGTACCGGTACT	Chr.4	14620232	14620252	+	No
1909745_adh	TATGAGTATGACGGTAGTTTT	Chr.4	14624603	14624623	+	Yes
1748783_adh	TAAAACTGTTTGATGAAAAAA	Chr.4	14624893	14624873	-	No
2736660_adh	TTTTATTGAGAAAAATGTTCA	Chr.4	14626419	14626439	+	No
1954100_adh	TCACTCAAGTTCAGACGGTAA	Chr.4	14627717	14627737	+	No
2689012_adh	TTTAAACAGCAATATGAACGA	Chr.4	14629507	14629527	+	No
1754338_adh	TAAACAGCAATATGAACGATG	Chr.4	14629509	14629529	+	No
2546321_adh	TTAACTGATGTGTTCAACTTA	Chr.4	14630186	14630166	-	No
2037303_adh	TCTCAGAATTCGGATTTTTTA	Chr.4	14630942	14630962	+	No
1816264_adh	TACCATTGCTAACAGATTTTT	Chr.4	14631339	14631319	-	Yes
2667369_adh	TTGGGATTGATGCAGTTTCAC	Chr.4	14632838	14632818	-	No
2734617_adh	TTTTAGATTTATACAGTTTTA	Chr.4	14632944	14632924	-	No
1886609_adh	TATAATTTGGCATCAATCTCT	Chr.4	14633893	14633873	-	No
2252069_adh	TGAGTAGTTAAAATGAGCATA	Chr.4	14634002	14634022	+	No
2054040_adh	TCTTACAATGTAGTACTAGGC	Chr.4	14634799	14634779	-	No
2623781_adh	TTCTTTGTTTGTATCAATTTT	Chr.4	14635033	14635013	-	Yes
2684508_adh	TTGTTGAAGATGGCAAAAACT	Chr.4	14640251	14640271	+	No
2639622_adh	TTGAGACAAGAAAGTTTTGGT	Chr.4	14640496	14640516	+	No
2740551_adh	TTTTGAAACAAGTGGTTTTGA	Chr.4	14640640	14640660	+	No
2028560_adh	TCGTTGGGTAGACCATGGTTT	Chr.4	14642180	14642160	-	No
2053664_adh	TCTTAAATGAACAATGCGAAG	Chr.4	14644692	14644672	-	No
2635157_adh	TTGACAGTCAGATTGACAACA	Chr.4	14645135	14645115	-	No
2155237_adh	TGACATATTGAAACATCAACA	Chr.4	14645425	14645405	-	No
1956866_adh	TCAGAAACGCTATGAATATCT	Chr.4	14646837	14646857	+	No
2259217_adh	TGATAATTGATGATTAAGGGA	Chr.4	14647441	14647421	-	No
2142617_adh	TGAATCGTGTTTGAGAAAGTT	Chr.4	14648674	14648694	+	No
2142617_adh	TGAATCGTGTTTGAGAAAGTT	Chr.4	14649637	14649657	+	No
2582253_adh	TTATTGAGTAAATTGACACAT	Chr.4	14651723	14651743	+	No
2742246_adh	TTTTGCAGATCTCAAGCCAAG	Chr.4	14652476	14652456	-	No
2276552_adh	TGATTGCAGATATTTCAAAAT	Chr.4	14653837	14653857	+	No
1799197_adh	TAATGAAAGAATTTGCAGAAA	Chr.4	14655433	14655453	+	No
2130416_adh	TGAAGCACAAGCTGAATTCAA	Chr.4	14660603	14660583	-	No
1806545_adh	TACAAAATCGAAAAAGTTTTA	Chr.4	14660675	14660695	+	No
1873153_adh	TAGTAACTAGGGACAGGGCAG	Chr.4	14661092	14661072	-	No
2119647_adh	TGAACAAGAGAGAAATGTCGA	Chr.4	14661725	14661705	-	No
2043850_adh	TCTGAACAAGAGAGAAATGTC	Chr.4	14661727	14661707	-	No
2617707_adh	TTCTGAACAAGAGAGAAATGT	Chr.4	14661728	14661708	-	No
2540560_adh	TTAAAGAAGTGACTAGACATA	Chr.4	14665071	14665051	-	No
2737607_adh	TTTTCATACTCTTTTCCTTTA	Chr.4	14665642	14665622	-	No
2716810_adh	TTTGAGCTCTATTTGACGGAA	Chr.4	14666521	14666501	-	No
2741436_adh	TTTTGAGCTCTATTTGACGGA	Chr.4	14666522	14666502	-	No
2665864_adh	TTGGCGTAGCCAGTAGTTCTC	Chr.4	14666717	14666737	+	No
1764529_adh	TAACACGACGAGGAGGGATAT	Chr.4	14669513	14669493	-	No
1867092_adh	TAGGCATTGCAGGTATCTTAA	Chr.4	14670752	14670732	-	No
2602824_adh	TTCGACATAGATGGCTTGTAT	Chr.4	14674149	14674129	-	No
2692874_adh	TTTAATTTAATAACTCTTCGG	Chr.4	14675148	14675128	-	No
2582184_adh	TTATTGACTATGAGATCTGAA	Chr.4	14675203	14675183	-	Yes
2581165_adh	TTATTATTGACTATGAGATCT	Chr.4	14675206	14675186	-	Yes
2560958_adh	TTAGAAATTAAGGAGTTGGAT	Chr.4	14676569	14676549	-	No

2520855_adh	TGTGTTCGGTGGACTTTCAAA	Chr.4	14678604	14678584	-	Yes
2694341_adh	TTTACTCTGTGTTCGGTGGAC	Chr.4	14678611	14678591	-	Yes
1936221_adh	TCAACATTTGACCATAAATAA	Chr.4	14679710	14679690	-	No
2584081_adh	TTATTTGCATATAATACATAT	Chr.4	14679936	14679956	+	Yes
1970211_adh	TCATATTCGTATTTAACAGTA	Chr.4	14680480	14680460	-	Yes
2737784_adh	TTTTCATTCTGTCGTCCCTTA	Chr.4	14684645	14684665	+	No
2704533_adh	TTTCATTCTGTCGTCCCTTAA	Chr.4	14684646	14684666	+	No
1756068_adh	TAAAGAAATATTTAATTTTGT	Chr.4	14686332	14686352	+	No
2486875_adh	TGTACGGGCTTCTGTTTGGCA	Chr.4	14687970	14687990	+	Yes
2549281_adh	TTAAGTTAGAGTTTGAGACAG	Chr.4	14693298	14693318	+	No
2001326_adh	TCGAGAGTAATTTTTTGCATT	Chr.4	14695087	14695107	+	No
2562181_adh	TTAGACGATACTCGAAAGCTA	Chr.4	14695583	14695603	+	No
2035245_adh	TCTCAAACACTCACAAGCACA	Chr.4	14699681	14699701	+	No
2650922_adh	TTGCATTTTTAACAGCCTGAA	Chr.4	14700896	14700916	+	No
1813785_adh	TACATGCATTTAACAGAACTT	Chr.4	14702227	14702247	+	No
2288095_adh	TGCAGCAGTAGAAACTTTTAT	Chr.4	14704407	14704427	+	No
1937318_adh	TCAACTCCATGAAACTGTAAT	Chr.4	14705383	14705403	+	No
2289567_adh	TGCATAAATGAACGGTTATTT	Chr.4	14708188	14708168	-	Yes
2582656_adh	TTATTGCGAACTATGTTTCCT	Chr.4	14711370	14711350	-	No
1921027_adh	TATTACTCGACGGAAAAAATC	Chr.4	14714852	14714872	+	No
2028613_adh	TCGTTGTCGTTGACGTTTTTT	Chr.4	14715058	14715038	-	No
1966431_adh	TCAGTCGTTGTCGTTGACGTT	Chr.4	14715062	14715042	-	No
2730830_adh	TTTGTTCTATTCCTGACCGGC	Chr.4	14715951	14715931	-	Yes
2740406_adh	TTTTCTTTTTGTTGTTGATTT	Chr.4	14716108	14716088	-	No
2714852_adh	TTTGACAGTATACGATAAATA	Chr.4	14716978	14716998	+	No
2596486_adh	TTCCACTCCAGAAGAAAAGAA	Chr.4	14717254	14717234	-	No
2278092_adh	TGATTTCCACTCCAGAAGAAA	Chr.4	14717258	14717238	-	No
2593445_adh	TTCATAAACTACTTTTGCTGC	Chr.4	14717789	14717769	-	Yes
2676584_adh	TTGTATGATAGCTGTAATAAT	Chr.4	14718160	14718140	-	Yes
2560282_adh	TTACTTGTATGATAGCTGTAA	Chr.4	14718164	14718144	-	Yes
2042041_adh	TCTCTAGCTGGGACTATGTAC	Chr.4	14720117	14720097	-	No
2033376_adh	TCTAGTGGTATGTGCGGGCAA	Chr.4	14723035	14723055	+	No
1799434_adh	TAATGACACATTTTTTGGAAT	Chr.4	14723404	14723384	-	No
2596747_adh	TTCCAGATGATGAACGGTAAT	Chr.4	14723815	14723795	-	Yes
2396522_adh	TGGATTTTTAATACTGTTACA	Chr.4	14724316	14724336	+	No
2730334_adh	TTTGTTAATATCGTAAAAAGT	Chr.4	14725173	14725153	-	No
2480517_adh	TGTAAAATTAGCATTGGACAG	Chr.4	14726905	14726925	+	No
1887449_adh	TATACTGAATTTGCTTAATTT	Chr.4	14727152	14727172	+	No
1884593_adh	TATAACATGTGCATAAATAAT	Chr.4	14727155	14727135	-	No
2695387_adh	TTTAGAGGATTATATTTAAAA	Chr.4	14729050	14729070	+	No
1762961_adh	TAAATTTTTTAGACTTTCAAC	Chr.4	14731139	14731119	-	No
1814585_adh	TACATTTGTTGTGAAAAACCT	Chr.4	14731609	14731589	-	Yes
2233228_adh	TGAGCACTAGAGAAAAGGAAA	Chr.4	14732449	14732429	-	No
1923790_adh	TATTCATCATTCATTGCGTGG	Chr.4	14732543	14732523	-	Yes
2307117_adh	TGCTCAAGAACTACCGACTTT	Chr.4	14733771	14733791	+	No
1867704_adh	TAGGCTTCGAAGAACACAGTC	Chr.4	14733908	14733888	-	No
1794754_adh	TAATAGAACTTCATCTTTAGA	Chr.4	14734172	14734192	+	No
2036331_adh	TCTCAACTGATGGTTCTCGAC	Chr.4	14734890	14734910	+	No
2577898_adh	TTATCGTATGTTGGTTTTTCA	Chr.4	14735340	14735320	-	No
2728968_adh	TTTGTCGATCGGATTAATTTT	Chr.4	14739573	14739593	+	Yes

2710838_adh	TTTCTGTAGAGAAAAACAAAA	Chr.4	14743016	14743036	+	No
2701145_adh	TTTCAAAGGTATAAGGCATTT	Chr.4	14743719	14743699	-	No
2472963_adh	TGGTGTCAAAATTGGACGACC	Chr.4	14747540	14747520	-	No
2631460_adh	TTGAATGAGCAAATTAGAATT	Chr.4	14749879	14749899	+	No
1747066_adh	TAAAAATCAAAGAAAGCGTCA	Chr.4	14751820	14751840	+	No
2597538_adh	TTCCATTTCTGGCGGAAGGGG	Chr.4	14751993	14751973	-	No
2591012_adh	TTCAGCAGAGAACCTAGCAGC	Chr.4	14752063	14752083	+	No
2375908_adh	TGGACGAAATTGCAATATGTT	Chr.4	14752776	14752796	+	Yes
2736622_adh	TTTTATTGAAAACTGCAGAAA	Chr.4	14753092	14753072	-	No
2570583_adh	TTAGTAGGAAAGGAAATGCGG	Chr.4	14753807	14753827	+	No
1777281_adh	TAAGAGGCTGGCCACTTTTTC	Chr.4	14754168	14754188	+	No
2711468_adh	TTTCTTCGATTATCAACTAAA	Chr.4	14755122	14755142	+	No
2616104_adh	TTCTCAGATGGTATTTGAATC	Chr.4	14756812	14756832	+	Yes
1804159_adh	TAATTGGTGCCTGCTATTAGA	Chr.4	14756893	14756913	+	Yes
1940808_adh	TCAATAAACTTGAATAAGCAT	Chr.4	14756949	14756969	+	No
2626498_adh	TTGAACGTAATGAAGTGTGTT	Chr.4	14757055	14757035	-	No
2689131_adh	TTTAAACTGCAAACTTCCAAA	Chr.4	14757202	14757222	+	No
2643605_adh	TTGATAGGATGGTACTATTTC	Chr.4	14763844	14763824	-	No
1836684_adh	TAGAAAACGGATGAAAATGTA	Chr.4	14764734	14764714	-	No
1831749_adh	TACTCTTTGAATTGTTAGACA	Chr.4	14765345	14765365	+	No
2061719_adh	TCTTTGAATTGTTAGACATGA	Chr.4	14765348	14765368	+	No
1845096_adh	TAGACATGACTAGTAGTCGGA	Chr.4	14765360	14765380	+	No
2705710_adh	TTTCCGAGTTTTCTTCGCATA	Chr.4	14767780	14767760	-	No
2129425_adh	TGAAGAGGCAGGGTCCGGTTG	Chr.4	14780819	14780799	-	No
2413512_adh	TGGCGTGGAAGAAGTTGATGA	Chr.4	14780837	14780817	-	No
2272498_adh	TGATGGCGTGGAAGAAGTTGA	Chr.4	14780840	14780820	-	No
2391017_adh	TGGATGATGGCGTGGAAGAAG	Chr.4	14780844	14780824	-	No
2515569_adh	TGTGGATGATGGCGTGGAAGA	Chr.4	14780846	14780826	-	No
2385327_adh	TGGAGTGGATGACGACGACGA	Chr.4	14780885	14780865	-	No
1966973_adh	TCAGTGGAGTGGATGACGACG	Chr.4	14780905	14780925	+	No
2385327_adh	TGGAGTGGATGACGACGACGA	Chr.4	14780909	14780929	+	No
2515569_adh	TGTGGATGATGGCGTGGAAGA	Chr.4	14780948	14780968	+	No
2391017_adh	TGGATGATGGCGTGGAAGAAG	Chr.4	14780950	14780970	+	No
2272498_adh	TGATGGCGTGGAAGAAGTTGA	Chr.4	14780954	14780974	+	No
2413512_adh	TGGCGTGGAAGAAGTTGATGA	Chr.4	14780957	14780977	+	No
2129425_adh	TGAAGAGGCAGGGTCCGGTTG	Chr.4	14780975	14780995	+	No
2489736_adh	TGTAGTGATGATGATGGTTAT	Chr.4	14787247	14787227	-	No
2031172_adh	TCTACAATTCGGACTGAAAAT	Chr.4	14790516	14790536	+	No
2681079_adh	TTGTGATGGAGAATGGAAAGG	Chr.4	14792036	14792016	-	No
2000789_adh	TCGACTTGAATTCCAAGTAGC	Chr.4	14792496	14792516	+	No
2688847_adh	TTTAAAACGCAATTTCAATCA	Chr.4	14795774	14795794	+	No
2716719_adh	TTTGAGCAGGTTAGTAGACGG	Chr.4	14797354	14797334	-	No
2607197_adh	TTCGGAAGCCGTTCAACAACA	Chr.4	14797403	14797383	-	No
2494953_adh	TGTCAGCGGTCGTTCGATTGG	Chr.4	14797527	14797547	+	No
1993874_adh	TCCTTGTCGCACTGATTTGGC	Chr.4	14797549	14797569	+	No
2278695_adh	TGATTTGGCTTTGGATTTCGT	Chr.4	14797561	14797581	+	No
2724647_adh	TTTGGCTTTGGATTTCGTAAT	Chr.4	14797564	14797584	+	No
1754859_adh	TAAACGAGAAAAATCGGACGA	Chr.4	14799560	14799580	+	No
2119546_adh	TGAACAAACGTTGAATTTGAA	Chr.4	14805705	14805685	-	No
2459774_adh	TGGGTTGACGACGATGAAGGC	Chr.4	14806927	14806947	+	No

2008597_adh	TCGCTCTTACGGCACTCTATT	Chr.4	14813694	14813714	+	No
2126223_adh	TGAACTGTTGATTTCGTGCCG	Chr.4	14815871	14815851	-	Yes
2647046_adh	TTGATTTTGAAATTTGAAATT	Chr.4	14816641	14816661	+	No
1885632_adh	TATAATATTTAAATCGTTGTG	Chr.4	14818368	14818388	+	No
2543192_adh	TTAAATCGTTGTGAATGAAAA	Chr.4	14818376	14818396	+	No
2694680_adh	TTTACTTGAAGACAACTAAAT	Chr.4	14819995	14820015	+	Yes
2560176_adh	TTACTTGAAGACAACTAAATT	Chr.4	14819996	14820016	+	Yes
2627270_adh	TTGAAGACAACTAAATTCTCA	Chr.4	14820000	14820020	+	Yes
2735379_adh	TTTTATATGAAGACAACTAAA	Chr.4	14820465	14820485	+	Yes
2748264_adh	TTTTTTAGAAATTCGACGGAA	Chr.4	14822271	14822251	-	No
2576281_adh	TTATATTATCCGAAAGGCAAC	Chr.4	14824892	14824872	-	No
2038618_adh	TCTCCACAGAGGACTCAGACA	Chr.4	14826214	14826234	+	No
1788075_adh	TAAGTCTGATTGTTTACGGGA	Chr.4	14826323	14826343	+	No
1923874_adh	TATTCCAATAATGCACGACAA	Chr.4	14830619	14830639	+	No
1790825_adh	TAATAATTAAAGAGATCTTAT	Chr.4	14831702	14831682	-	No
2686392_adh	TTGTTGTATTTTCGTTCAATT	Chr.4	14832003	14831983	-	No
2644928_adh	TTGATGCAGTTTTTCTTTGGT	Chr.4	14832227	14832247	+	No
2271826_adh	TGATGCAGTTTTTCTTTGGTT	Chr.4	14832228	14832248	+	No
2618313_adh	TTCTGAGATTGTATTAAACCA	Chr.4	14836714	14836694	-	No
1784494_adh	TAAGGATAAGTATTAGTGTTA	Chr.4	14838681	14838701	+	No
2688728_adh	TTTAAAAATGTCTTGTAGAGC	Chr.4	14839852	14839872	+	No
2048819_adh	TCTGGAATAGGATCTAGGATC	Chr.4	14842696	14842716	+	No
2286802_adh	TGCACTGTAACGGTAGACTTT	Chr.4	14842982	14842962	-	No
2721475_adh	TTTGCATTTGTCGTTGGCAAC	Chr.4	14843014	14843034	+	No
2741916_adh	TTTTGATTGAATTCTGCAGGC	Chr.4	14848436	14848456	+	No
2583401_adh	TTATTGTTTTTAGAATTTTAG	Chr.4	14849044	14849064	+	No
2688367_adh	TTGTTTTTAGAATTTTAGTCG	Chr.4	14849047	14849067	+	No
1805957_adh	TAATTTTGAAGCGAATAATTG	Chr.4	14852293	14852313	+	No
2732922_adh	TTTTAAAAAACAATTAACTGT	Chr.4	14853808	14853788	-	Yes
2618192_adh	TTCTGACGTGACTCGTTTCTA	Chr.4	14853938	14853958	+	No
2704764_adh	TTTCATTTTGCACGGGCGATT	Chr.4	14854067	14854047	-	No
2031908_adh	TCTACTGAATTTTGGCAATGA	Chr.4	14855646	14855666	+	No
2698469_adh	TTTATCATTGTTTATAATTAG	Chr.4	14856904	14856924	+	No
2618322_adh	TTCTGAGCACTGGACTTTTCA	Chr.4	14857087	14857107	+	No
2475005_adh	TGGTTCCGCTATAAATTATAT	Chr.4	14860496	14860476	-	No
2734727_adh	TTTTAGCTCGATTTTCACTTA	Chr.4	14861175	14861195	+	No
1920674_adh	TATTACATAAGAAATAAAAAA	Chr.4	14862899	14862879	-	Yes
2735933_adh	TTTTATGATAATTGCTAATAG	Chr.4	14864203	14864223	+	No
2735422_adh	TTTTATCAACCAATTGAACTC	Chr.4	14864591	14864611	+	No
1775353_adh	TAAGACATGTGAAAAAAGACC	Chr.4	14864875	14864895	+	No
2558692_adh	TTACTCACAAGGTAGAAAGCT	Chr.4	14866254	14866234	-	No
2527867_adh	TGTTGACCATATAGACGAAAT	Chr.4	14866734	14866754	+	No
2745602_adh	TTTTTCAAGCACTACGTTTTT	Chr.4	14867769	14867789	+	No
2531636_adh	TGTTTAAGTCGTACTATACAC	Chr.4	14870935	14870915	-	No
2270609_adh	TGATCTGTTTCAAATATTATG	Chr.4	14874408	14874428	+	No
1920000_adh	TATTAAATTTTCCACAGCCTA	Chr.4	14874671	14874691	+	Yes
2743945_adh	TTTTGTGGAAGAACAGGAGAA	Chr.4	14875749	14875769	+	No
2582231_adh	TTATTGAGAACATAGCTTTTT	Chr.4	14876096	14876076	-	Yes
1847229_adh	TAGAGAAGTAGAAGTCATTTG	Chr.4	14876487	14876467	-	Yes
2590847_adh	TTCAGATGCGAGAATAGTGCG	Chr.4	14877457	14877437	-	No

2715699_adh	TTTGACTGGCTGAAATAAAAT	Chr.4	14879459	14879439	-	No
2569930_adh	TTAGTAAGCTCTCGGAATTTT	Chr.4	14880812	14880832	+	No
2742568_adh	TTTTGCTTATTCAGTCAAACT	Chr.4	14881900	14881920	+	No
2742568_adh	TTTTGCTTATTCAGTCAAACT	Chr.4	14882131	14882111	-	No
2700958_adh	TTTCAAAAGAAGAGCGTCGGT	Chr.4	14883574	14883554	-	No
2037855_adh	TCTCATAAATAGGATGCCATT	Chr.4	14891666	14891646	-	No
2695937_adh	TTTAGCATCATAAAAAATATT	Chr.4	14892987	14892967	-	No
2579272_adh	TTATGGAAATGCGGTTATTTG	Chr.4	14893632	14893612	-	Yes
2571285_adh	TTAGTCTATGCTTTGTACCCA	Chr.4	14895131	14895151	+	No
1814170_adh	TACATTACTTTTTGAATTCTA	Chr.4	14897031	14897051	+	No
1929074_adh	TATTTAATAGGCAATTCTTAA	Chr.4	14899085	14899065	-	Yes
2744904_adh	TTTTTACCTGACGTTCTCCAA	Chr.4	14899320	14899340	+	No
2582337_adh	TTATTGCAATGAATTGAATGT	Chr.4	14899514	14899494	-	No
1970343_adh	TCATCAAAATATTCAAATATT	Chr.4	14900641	14900621	-	Yes
2740222_adh	TTTTCTTCTTTGATTCGTTTT	Chr.4	14901566	14901546	-	Yes
2625906_adh	TTGAACACCATACAATCTTTA	Chr.4	14902983	14903003	+	Yes
2376933_adh	TGGACTATCAGTTTCAGGGAT	Chr.4	14904061	14904081	+	No
2706262_adh	TTTCCTCTTTCTTATTACCTG	Chr.4	14908100	14908120	+	No
2029224_adh	TCTAAAAAGTTTCTGAAACGG	Chr.4	14908758	14908778	+	No
2053585_adh	TCTTAAAATTGAACTTTTTAT	Chr.4	14912889	14912869	-	No
2373699_adh	TGGACACAACACGCATTTTTT	Chr.4	14914364	14914344	-	No
2703914_adh	TTTCATCATTTTATTTTTAAT	Chr.4	14918330	14918350	+	Yes
2568602_adh	TTAGGCTTTATTAGGATTATT	Chr.4	14919147	14919167	+	Yes
2693280_adh	TTTACACTGCTTGTAGAAACA	Chr.4	14920481	14920501	+	No
2658032_adh	TTGGACAGATGAAGTTTTTCA	Chr.4	14921240	14921260	+	Yes
2001593_adh	TCGAGGAAATCTGTTTGGCAA	Chr.4	14923206	14923226	+	No
2647095_adh	TTGATTTTTATTTTGTCTGAT	Chr.4	14923691	14923671	-	Yes
2462094_adh	TGGTAATAGATGTTTCAAATA	Chr.4	14924235	14924215	-	No
1806419_adh	TACAAAAACACGGTTCAAGCA	Chr.4	14925039	14925019	-	No
2694991_adh	TTTAGAATTGGATGCTTATAC	Chr.4	14925600	14925580	-	No
1790868_adh	TAATAATTTGATTTCTGATTA	Chr.4	14926845	14926865	+	No
1771691_adh	TAACTGGGCCTATGCTGATTA	Chr.4	14927106	14927126	+	No
2698534_adh	TTTATCCGGCATCGTATCACA	Chr.4	14929467	14929447	-	No
1942816_adh	TCAATTACTGTGGCATTAATT	Chr.4	14930561	14930581	+	No
1804174_adh	TAATTGGTTGTTGGCGTGTTT	Chr.4	14931475	14931455	-	No
1901399_adh	TATGAAAGTCGAAGATCGTGC	Chr.4	14932484	14932504	+	Yes
1811010_adh	TACACGAGTCGCCCGAAAAAA	Chr.4	14937282	14937262	-	No
1771691_adh	TAACTGGGCCTATGCTGATTA	Chr.4	14939122	14939142	+	No
2593731_adh	TTCATAGTTTCTGATATATAC	Chr.4	14940443	14940463	+	No
2620282_adh	TTCTGTTAAAGCAGCTTTCTT	Chr.4	14940484	14940504	+	No
2554521_adh	TTACATAGATATTTTCATATT	Chr.4	14945452	14945432	-	No
2124984_adh	TGAACTGACGGGCTTTTGTTT	Chr.4	14946283	14946263	-	No
2452736_adh	TGGGGATGTTGCAAGCTTGAC	Chr.4	14949152	14949172	+	No
2151575_adh	TGACAAGTAGAATGCGTACAA	Chr.4	14949721	14949741	+	No
2711027_adh	TTTCTGTTCGTGGACACAATT	Chr.4	14950058	14950038	-	Yes
1921990_adh	TATTATAGAACGGACGAAACC	Chr.4	14951897	14951877	-	No
1922928_adh	TATTATTATAGAACGGACGAA	Chr.4	14951900	14951880	-	No
1929403_adh	TATTTATTATTATAGAACGGA	Chr.4	14951904	14951884	-	No
2583625_adh	TTATTTATTATTATAGAACGG	Chr.4	14951905	14951885	-	No
2736910_adh	TTTTATTTATTATTATAGAAC	Chr.4	14951907	14951887	-	No

2590856_adh	TTCAGATGGGTCTCGTCGCGA	Chr.4	14954930	14954950	+	No
2615143_adh	TTCTAGTGAATTTTGTCGTTT	Chr.4	14955113	14955093	-	No
2497891_adh	TGTCGCATGCGATCGCAAAAT	Chr.4	14956895	14956915	+	No
2548710_adh	TTAAGGTTGGCGGGAGACAGT	Chr.4	14961891	14961911	+	No
1809191_adh	TACAAGTTAAAAAACGTCCAA	Chr.4	14963511	14963491	-	No
2590075_adh	TTCACTGTTTCAATGTCATAT	Chr.4	14963636	14963656	+	No
1924388_adh	TATTCGGACAGGCAAGATTTT	Chr.4	14963685	14963705	+	Yes
2607312_adh	TTCGGACAGGCAAGATTTTGT	Chr.4	14963687	14963707	+	Yes
2555651_adh	TTACCAGAAATCTACAAACTT	Chr.4	14963826	14963806	-	No
1833443_adh	TACTGGGACTTCCTAAACGGT	Chr.4	14964363	14964383	+	Yes
1790254_adh	TAATAACAATTGCAGACGGCT	Chr.4	14964485	14964505	+	Yes
2720704_adh	TTTGATTTATGAACTGCAGAA	Chr.4	14965342	14965362	+	No
1901650_adh	TATGAACTGCAGAATGTTGGT	Chr.4	14965349	14965369	+	No
2728339_adh	TTTGTAGGTTCACGACATTAA	Chr.4	14966766	14966786	+	No
1748742_adh	TAAAACTGGAAGCGCTGGGAA	Chr.4	14966980	14967000	+	No
2580888_adh	TTATTAGAAGTTCATCGGGTG	Chr.4	14967082	14967102	+	No
2561588_adh	TTAGAAGTTCATCGGGTGATT	Chr.4	14967085	14967105	+	No
2573719_adh	TTATAAATAAAAACAATTTAA	Chr.4	14968030	14968050	+	No
2501347_adh	TGTGAAATTGCGATTGGAGAA	Chr.4	14968080	14968060	-	Yes
2689755_adh	TTTAACAAGTTGAAACATGCA	Chr.4	14971500	14971480	-	No
2635150_adh	TTGACAGTATGAGGAATTGAG	Chr.4	14972032	14972012	-	No
2738246_adh	TTTTCGACTGTCAATAAAAAT	Chr.4	14972557	14972537	-	No
2127535_adh	TGAAGACAAGAAAAAATGAAT	Chr.4	14974787	14974767	-	No
2656372_adh	TTGGAAAGATCAAATCTGCCA	Chr.4	14975683	14975663	-	No
1782893_adh	TAAGCGTTTCAACGAAGACAC	Chr.4	14975731	14975711	-	No
2724427_adh	TTTGGCGATGCTCTCAGTTGA	Chr.4	14976357	14976377	+	No
2699603_adh	TTTATTAGGTCTATCCTTTTC	Chr.4	14976900	14976880	-	No
2717643_adh	TTTGATATTGTAGATTTCTCC	Chr.4	14979618	14979638	+	Yes
1924188_adh	TATTCGAAATGACAAACTAGA	Chr.4	14980613	14980593	-	No
2014726_adh	TCGGATTAGCGTTAGGTTTTC	Chr.4	14981761	14981741	-	No
1865178_adh	TAGGATCATGAAACAGAAGAA	Chr.4	14982383	14982363	-	No
2624619_adh	TTGAAAATTTTCATTTAACTT	Chr.4	14983291	14983271	-	No
2674732_adh	TTGTAGAAGTTGAAAAACTAA	Chr.4	14984914	14984934	+	No
1978640_adh	TCCAGGACAAGACGGAACTCC	Chr.4	14985760	14985740	-	No
2733151_adh	TTTTAAATATTATTGGGCAAA	Chr.4	14987984	14987964	-	No
2474483_adh	TGGTTATTAAATGGTAGAAAA	Chr.4	14989307	14989327	+	No
1780480_adh	TAAGATTATAGAAGACGACTC	Chr.4	14991702	14991722	+	No
2638007_adh	TTGACTGGGCATATTTAAAAG	Chr.4	14994937	14994957	+	No
2747806_adh	TTTTTGTGCAAAAATACAAAC	Chr.4	14995260	14995280	+	No
2310256_adh	TGCTTATTATGCATGTAGAAT	Chr.4	14995474	14995454	-	No
1945067_adh	TCACAGGAGATTTTGCTCACA	Chr.4	14997252	14997232	-	Yes
1929517_adh	TATTTCACAGGAGATTTTGCT	Chr.4	14997256	14997236	-	Yes
2147970_adh	TGAATTGTTGGTTCCCTGTGT	Chr.4	14997832	14997812	-	No
1766863_adh	TAACCCGTCGGTTTTCCAATT	Chr.4	14999313	14999293	-	No
1891705_adh	TATCAATTAGCTGAAGAATTT	Chr.4	14999340	14999360	+	No
2731036_adh	TTTGTTGAATTGTTGGAAAAT	Chr.4	14999892	14999872	-	No
2278968_adh	TGATTTGTTGAATTGTTGGAA	Chr.4	14999895	14999875	-	No
1925791_adh	TATTGACTGTATTTTGGTTTT	Chr.4	15000593	15000613	+	Yes
2638530_adh	TTGACTGTATTTTGGTTTTTG	Chr.4	15000595	15000615	+	Yes
1789891_adh	TAATAAAAATTCTTAAAAACT	Chr.4	15001526	15001506	-	No

1761449_adh	TAAATGATAATTTGAAAACAG	Chr.4	15002980	15002960	-	No
2580534_adh	TTATTAATAAGAGTTTAAACT	Chr.4	15003004	15003024	+	No
1836677_adh	TAGAAAACCGGGCTAACTAAA	Chr.4	15003956	15003936	-	No
2606569_adh	TTCGCGAGGATCTTCCTAGAG	Chr.4	15007307	15007327	+	No
2708688_adh	TTTCGTACGTAGATCTACAAA	Chr.4	15031195	15031215	+	No
2709694_adh	TTTCTAGCGCTGTTGTTCGGT	Chr.4	15037201	15037221	+	No
2614896_adh	TTCTAGCGCTGTTGTTCGGTT	Chr.4	15037202	15037222	+	No
2531193_adh	TGTTGTTCGGTTTTTTATTTG	Chr.4	15037211	15037231	+	No
2700800_adh	TTTATTTGTTTTGACGCTCGG	Chr.4	15037224	15037244	+	No
2584451_adh	TTATTTGTTTTGACGCTCGGA	Chr.4	15037225	15037245	+	No
2732822_adh	TTTGTTTTGACGCTCGGAAAA	Chr.4	15037228	15037248	+	No
2688275_adh	TTGTTTTGACGCTCGGAAAAT	Chr.4	15037229	15037249	+	No
2715211_adh	TTTGACGCTCGGAAAATTTAT	Chr.4	15037233	15037253	+	No
2513656_adh	TGTGCCAGCATAGTTTATTAT	Chr.4	15037270	15037290	+	No
2686066_adh	TTGTTGGAGCGCGTTTGCATT	Chr.4	15048451	15048431	-	No
2732092_adh	TTTGTTGGAGCGCGTTTGCAT	Chr.4	15048452	15048432	-	No
2688467_adh	TTGTTTTTGGATCTTTTTGTT	Chr.4	15052097	15052077	-	Yes
2572586_adh	TTAGTTGACTTGCTAAAAATG	Chr.4	15053662	15053642	-	No
1919894_adh	TATTAAAATTTCCATTTTGTG	Chr.4	15054702	15054722	+	No
1940041_adh	TCAAGTAATGCTAGGAATTTT	Chr.4	15055643	15055623	-	No
2380770_adh	TGGAGATCGTATCACATTGGT	Chr.4	15057765	15057785	+	No
2712328_adh	TTTGAAAAATTACAGCAGGCG	Chr.4	15058498	15058518	+	No
2700895_adh	TTTATTTTTGCATTCTTCTCC	Chr.4	15061240	15061260	+	Yes
2289640_adh	TGCATAATAATTTGTTTCTTT	Chr.4	15066501	15066481	-	Yes
1774427_adh	TAAGAAGTTGGCATGTTTCAT	Chr.4	15067226	15067246	+	No
2683670_adh	TTGTTCGAGACATCGTGGAAA	Chr.4	15069467	15069487	+	No
1931254_adh	TATTTTTAAATTCTTGAAACA	Chr.4	15071384	15071364	-	No
1922388_adh	TATTATGAAAAGAAACAAGCA	Chr.4	15072402	15072422	+	No
2271619_adh	TGATGATTGATTTTGTTGGTG	Chr.4	15075051	15075031	-	No
2644902_adh	TTGATGATTGATTTTGTTGGT	Chr.4	15075052	15075032	-	No
2745508_adh	TTTTTATTGTTCTGGAATACT	Chr.4	15083912	15083892	-	No
2594989_adh	TTCATTACTGGCATCTTTTTT	Chr.4	15085034	15085014	-	No
1974420_adh	TCATTGTCCAGTTGCGAAAAA	Chr.4	15086618	15086638	+	No
2687400_adh	TTGTTTGACTTAAAATGAAAC	Chr.4	15086798	15086778	-	Yes
2736632_adh	TTTTATTGAAGAATTGGTTTT	Chr.4	15087277	15087257	-	No
1888237_adh	TATAGCAGAACATTACATAAA	Chr.4	15089072	15089052	-	No
1888233_adh	TATAGCAGAACATTAAATTAA	Chr.4	15090069	15090049	-	No
2560156_adh	TTACTTCTTTATTTACTTTAC	Chr.4	15095119	15095139	+	No
1832240_adh	TACTGACCAAAACTCTCGGCA	Chr.4	15095792	15095812	+	No
2047699_adh	TCTGCAAAAACATGAATAGGA	Chr.4	15096139	15096159	+	No
2749455_adh	TTTTTTGCATGATACTGAATA	Chr.4	15097257	15097237	-	No
1875358_adh	TAGTATCTTCGTTTTGGCAAT	Chr.4	15102435	15102415	-	No
2561852_adh	TTAGAATTGGAAGACGAGAAA	Chr.4	15105611	15105591	-	No
2017100_adh	TCGGGGCACGAACGGTTAATT	Chr.4	15108249	15108229	-	Yes
2580495_adh	TTATTAACCAGTGGAATGAAA	Chr.4	15110576	15110596	+	No
2545138_adh	TTAACCAGTGGAATGAAAAAA	Chr.4	15110579	15110599	+	No
1935702_adh	TCAAATTGTTGAGACCGTCAA	Chr.4	15111301	15111321	+	No
1899189_adh	TATCTAAATCATCTCATAAAA	Chr.4	15111736	15111756	+	No
2052475_adh	TCTGTGAACAAAGATTAGGAA	Chr.4	15112865	15112845	-	No
2745370_adh	TTTTTATGCAAGAACTGGTTG	Chr.4	15113457	15113437	-	No

2742687_adh	TTTTGGAATGTAATTCTAACA	Chr.4	15114066	15114046	-	No
2618491_adh	TTCTGATTAGACTAAAAAGAA	Chr.4	15118020	15118000	-	Yes
2278010_adh	TGATTTCACAGCTATCAAAAG	Chr.4	15120597	15120577	-	No
2749722_adh	TTTTTTGTCAGAACACTTTTG	Chr.4	15121360	15121340	-	No
1774217_adh	TAAGAAGCGATTGGCAAAAAG	Chr.4	15122199	15122179	-	No
2682841_adh	TTGTTAAGAAGCGATTGGCAA	Chr.4	15122203	15122183	-	No
1756542_adh	TAAAGACACGAGGAAACTTAC	Chr.4	15124552	15124532	-	No
2272957_adh	TGATGTAGAAAAGAAATGGTG	Chr.4	15125718	15125698	-	No
2738445_adh	TTTTCGGAAACATATTAAAAT	Chr.4	15126513	15126493	-	No
2673557_adh	TTGTAAAAGTTTGAAAATTGC	Chr.4	15126663	15126643	-	No
2572931_adh	TTAGTTGTAAAAGTTTGAAAA	Chr.4	15126667	15126647	-	No
2693352_adh	TTTACAGCCAACATGAAACGG	Chr.4	15126930	15126910	-	No
2655304_adh	TTGCTTCTGAATACACAGTTT	Chr.4	15128079	15128099	+	No
2744113_adh	TTTTGTTAGTTGACATATTTC	Chr.4	15128265	15128245	-	No
1931233_adh	TATTTTGTTAGTTGACATATT	Chr.4	15128267	15128247	-	No
2619977_adh	TTCTGTATTTTAGGCTTGTTA	Chr.4	15129193	15129173	-	No
2633616_adh	TTGAATTATTAATTTTATTTT	Chr.4	15133568	15133588	+	Yes
2710551_adh	TTTCTGCAAAAACTGGAACGA	Chr.4	15136154	15136174	+	No
2593384_adh	TTCAGTTTAAAAAATATGGCT	Chr.4	15136551	15136571	+	Yes
2733076_adh	TTTTAAACTGAATTGTTTGTA	Chr.4	15136562	15136542	-	Yes
2578343_adh	TTATGAAGTTGAATGGGCATT	Chr.4	15138010	15137990	-	No
2736055_adh	TTTTATGGATTGTTCCGCATG	Chr.4	15138555	15138575	+	Yes
1919108_adh	TATGTGGGTTGAAAACGACAT	Chr.4	15142192	15142172	-	No
2236975_adh	TGAGGAATAGAACAATTTCAA	Chr.4	15144931	15144951	+	No
1808666_adh	TACAACTGTGTATGCTATGAA	Chr.4	15147497	15147477	-	No
2693131_adh	TTTACAACTGTGTATGCTATG	Chr.4	15147499	15147479	-	No
1837693_adh	TAGAAATAGGATTAGAAAAAA	Chr.4	15147790	15147810	+	No
2630195_adh	TTGAATCAGAACAATGGTACC	Chr.4	15150502	15150482	-	No
1975225_adh	TCATTTGAAGACCATTTTGAT	Chr.4	15151142	15151122	-	No
2619575_adh	TTCTGGGTTGGAGTCACACGT	Chr.4	15155340	15155360	+	No
1975890_adh	TCCAAAGTCTGGACAAAACGA	Chr.4	15155977	15155957	-	No
2723213_adh	TTTGGACAAAGAAAGTTTCAA	Chr.4	15157412	15157392	-	No
1967418_adh	TCAGTTCTGAAAGCTCAAAAC	Chr.4	15158319	15158299	-	No
2742364_adh	TTTTGCATGGATATATGTCTT	Chr.4	15158535	15158555	+	Yes
2721447_adh	TTTGCATGGATATATGTCTTT	Chr.4	15158536	15158556	+	Yes
2351206_adh	TGGAATGGAATTTGCAGATAA	Chr.4	15159401	15159421	+	No
2351206_adh	TGGAATGGAATTTGCAGATAA	Chr.4	15160662	15160642	-	No
2721447_adh	TTTGCATGGATATATGTCTTT	Chr.4	15161527	15161507	-	Yes
2742364_adh	TTTTGCATGGATATATGTCTT	Chr.4	15161528	15161508	-	Yes
1967418_adh	TCAGTTCTGAAAGCTCAAAAC	Chr.4	15161744	15161764	+	No
2723213_adh	TTTGGACAAAGAAAGTTTCAA	Chr.4	15162651	15162671	+	No
2517603_adh	TGTGGTGAAATGAGCATAGGA	Chr.4	15164552	15164532	-	No
1754392_adh	TAAACAGGCATGGAAAACCAT	Chr.4	15165390	15165410	+	No
2734361_adh	TTTTACTTCAAACTGAGACTT	Chr.4	15165917	15165937	+	No
2585223_adh	TTCAAACTGAGACTTTACTTG	Chr.4	15165923	15165943	+	No
1932370_adh	TCAAAATACTGAGTAGGAAAT	Chr.4	15166073	15166093	+	Yes
2581889_adh	TTATTCTGAACTGAAGGCAAT	Chr.4	15171407	15171427	+	No
1932062_adh	TCAAAACGGGCAAGATTTTAT	Chr.4	15173198	15173218	+	No
2549901_adh	TTAATAGGCGTCTTTCAATTC	Chr.4	15173980	15173960	-	No
1886679_adh	TATACAAATTTGTTAGAATTT	Chr.4	15174718	15174698	-	No

2587849_adh	TTCAATCGTTGCTGAATGGCG	Chr.4	15175852	15175872	+	Yes
2745730_adh	TTTTTCAGTTTCCAACGACAT	Chr.4	15176607	15176587	-	No
2550324_adh	TTAATCATAAAAACTTGGATA	Chr.4	15178593	15178573	-	No
2532677_adh	TGTTTCTCTTCAAGTAGATAT	Chr.4	15180894	15180914	+	Yes
2710224_adh	TTTCTCTTCAAGTAGATATCG	Chr.4	15180896	15180916	+	Yes
1881117_adh	TAGTTGGACTCCGGATGGTAA	Chr.4	15186875	15186895	+	No
1925183_adh	TATTCTTCGTCGCGCTCAAAC	Chr.4	15191632	15191612	-	No
2700604_adh	TTTATTTAAAATCGTCGAAAA	Chr.4	15193886	15193866	-	No
1804836_adh	TAATTTATTTAAAATCGTCGA	Chr.4	15193889	15193869	-	No
2502372_adh	TGTGAAGGCGATGCAGATGAC	Chr.4	15194558	15194578	+	No
2287951_adh	TGCAGATGACGACTTTTAACA	Chr.4	15194569	15194589	+	No
1804339_adh	TAATTGTGGAATTAAACGGGA	Chr.4	15194594	15194614	+	No
2502372_adh	TGTGAAGGCGATGCAGATGAC	Chr.4	15196138	15196158	+	No
2287951_adh	TGCAGATGACGACTTTTAACA	Chr.4	15196149	15196169	+	No
1804339_adh	TAATTGTGGAATTAAACGGGA	Chr.4	15196174	15196194	+	No
2060406_adh	TCTTTACATTCATTGGGCATA	Chr.4	15197113	15197093	-	Yes
2490732_adh	TGTATATTCAAGTGGAACACA	Chr.4	15202811	15202791	-	No
2692730_adh	TTTAATTTAAAACAGTTGAGC	Chr.4	15205908	15205928	+	No
2552532_adh	TTAATTTAAAACAGTTGAGCA	Chr.4	15205909	15205929	+	No
2679383_adh	TTGTGAAGCGAACAACAAATT	Chr.4	15206379	15206359	-	No
1808776_adh	TACAAGACCAATAAAAGTTTC	Chr.4	15206693	15206713	+	No
1807424_adh	TACAACAGCTTGAGGATCATG	Chr.4	15209906	15209886	-	No
2679383_adh	TTGTGAAGCGAACAACAAATT	Chr.4	15210063	15210083	+	No
1974251_adh	TCATTGGACCATTCAGAGCAA	Chr.4	15211020	15211040	+	No
1923441_adh	TATTCACAAAGAAGGTTCTTT	Chr.4	15211786	15211766	-	No
2577122_adh	TTATCATCAGGTTGAAGATTT	Chr.4	15211908	15211928	+	Yes
2620904_adh	TTCTTATAGCAGAATGGGAAA	Chr.4	15214860	15214840	-	No
2158613_adh	TGACATGTTCATTTGAACATC	Chr.4	15218718	15218698	-	No
2700991_adh	TTTCAAAATGAGGGCATGTAA	Chr.4	15220661	15220641	-	No
1749470_adh	TAAAAGCACTGATTAGGACAC	Chr.4	15221086	15221106	+	No
1901098_adh	TATGAAACAGTAGGTTTTTTC	Chr.4	15221713	15221693	-	No
1786209_adh	TAAGGTAAATTTTAAAACATA	Chr.4	15222331	15222351	+	Yes
1786209_adh	TAAGGTAAATTTTAAAACATA	Chr.4	15224651	15224631	-	Yes
1901098_adh	TATGAAACAGTAGGTTTTTTC	Chr.4	15225269	15225289	+	No
2573948_adh	TTATAAGAACTCATAAAAATA	Chr.4	15237882	15237902	+	No
1975364_adh	TCATTTGTAGGCATATCATTA	Chr.4	15242109	15242089	-	No
1892792_adh	TATCACATTTTTGAATTTTTG	Chr.4	15244563	15244583	+	No
2651439_adh	TTGCCTCGGATGTACCATGGT	Chr.4	15245718	15245698	-	Yes
2060020_adh	TCTTGTTATCGAGTAAAAACG	Chr.4	15247302	15247322	+	No
2261261_adh	TGATATAAACTAGGTGAAACG	Chr.4	15252945	15252925	-	No
2719032_adh	TTTGATGGAAGCTGATAATGA	Chr.4	15254115	15254135	+	No
2377033_adh	TGGACTCACGGCATTTAACAC	Chr.4	15258097	15258077	-	No
1922863_adh	TATTATTACCAATTCGTCATG	Chr.4	15258789	15258809	+	No
2555452_adh	TTACCAATTCGTCATGTTTAT	Chr.4	15258794	15258814	+	No
1877069_adh	TAGTGAAACCGGCGATCTTGT	Chr.4	15258876	15258856	-	No
2042492_adh	TCTCTCGCCGTGTGCTTCATG	Chr.4	15259480	15259500	+	Yes
2054568_adh	TCTTAGTACATAGTAGGTGAA	Chr.4	15261336	15261316	-	Yes
2738017_adh	TTTTCCGGTGGCGATTCCTAA	Chr.4	15265680	15265700	+	Yes
1885203_adh	TATAAGTACGGCTCCTTTTTA	Chr.4	15269298	15269318	+	No
1774369_adh	TAAGAAGTCTGTGGAAATGGC	Chr.4	15272340	15272320	-	No

2504076_adh	TGTGACGTCAAGGAGGGAAAC	Chr.4	15272402	15272382	-	No
2528762_adh	TGTTGAGATGGTGATTCTTAA	Chr.4	15273543	15273563	+	No
2489732_adh	TGTAGTGATCAATACGGAAGG	Chr.4	15274108	15274088	-	No
1974284_adh	TCATTGGATTAGACGCTAATT	Chr.4	15275152	15275172	+	No
2664059_adh	TTGGATTAGACGCTAATTTTT	Chr.4	15275155	15275175	+	No
2392776_adh	TGGATTAGACGCTAATTTTTG	Chr.4	15275156	15275176	+	No
2069736_adh	TGAAAATAAAGAAAATCTGGC	Chr.4	15276640	15276620	-	No
1802968_adh	TAATTCATTCTTCAAATGTCT	Chr.4	15277406	15277386	-	No
1940078_adh	TCAAGTAGACAGAAACAAATA	Chr.4	15278937	15278917	-	No
2279685_adh	TGCAAAAAATGAAAAGATTTG	Chr.4	15279254	15279274	+	No
2071828_adh	TGAAACAGTAGAATAATTTGA	Chr.4	15280020	15280040	+	No
2610071_adh	TTCGGTGAATTAATTCGGTGT	Chr.4	15280284	15280304	+	No
2655898_adh	TTGCTTTATAAAAAAGTATAC	Chr.4	15285034	15285014	-	No
2007263_adh	TCGCATTTTTAAAAAATCGCA	Chr.4	15286614	15286634	+	No
2628454_adh	TTGAAGATGCCTTTTATGTTC	Chr.4	15287024	15287004	-	No
2002638_adh	TCGATACTCCGAAACGACATA	Chr.4	15289174	15289154	-	No
2657551_adh	TTGGAATCGTCACTTTCTGCT	Chr.4	15295708	15295728	+	Yes
2558659_adh	TTACTATTAGAATTGTTGAAA	Chr.4	15295771	15295791	+	No
2644027_adh	TTGATCATATTAAGAATATTT	Chr.4	15297226	15297246	+	No
2553878_adh	TTACACGACTCATGTACTGAT	Chr.4	15298053	15298073	+	Yes
1748908_adh	TAAAAGAAATAGATTTGATGT	Chr.4	15300576	15300556	-	No
2133801_adh	TGAAGTAAGCTCACAAAATTA	Chr.4	15300738	15300718	-	No
2274016_adh	TGATGTTTGAAGCTAGAAAGA	Chr.4	15301562	15301582	+	No
2053350_adh	TCTGTTGGTGAAGGTTGGATA	Chr.4	15303683	15303663	-	No
2583235_adh	TTATTGTACCTTTGTAGATTG	Chr.4	15304535	15304555	+	No
1921749_adh	TATTAGTATCTAAGGGCTACC	Chr.4	15307822	15307802	-	Yes
2588858_adh	TTCAATTCTGTTGTGGATGCT	Chr.4	15309381	15309361	-	No
2602033_adh	TTCGAACTATTCCTGGCCAAC	Chr.4	15311658	15311638	-	No
2180230_adh	TGAGAAGATGTAAGAGAGACG	Chr.4	15312171	15312191	+	No
2141992_adh	TGAATCCACGGCATCTCACTT	Chr.4	15313054	15313034	-	No
1889466_adh	TATAGTTTCTCTACTGTTTTT	Chr.4	15318061	15318041	-	No
2550274_adh	TTAATCAGAAAAGACTGAGAC	Chr.4	15318179	15318159	-	No
2602033_adh	TTCGAACTATTCCTGGCCAAC	Chr.4	15319018	15318998	-	No
1791701_adh	TAATACGATCGTTGGGGGTGA	Chr.4	15319367	15319347	-	No
2553689_adh	TTACAATTTTAATTTTAATGT	Chr.4	15322564	15322584	+	No
1934603_adh	TCAAATCGGTTATAAACTTTA	Chr.4	15329903	15329923	+	No
2659282_adh	TTGGAGACAACTACGTCGAAT	Chr.4	15330492	15330512	+	No
2147773_adh	TGAATTGTCACGATACTGAAA	Chr.4	15333983	15333963	-	No
2276277_adh	TGATTGAATTGTCACGATACT	Chr.4	15333987	15333967	-	No
1760527_adh	TAAATCATTTTATTTTGATCG	Chr.4	15334994	15335014	+	No
1893222_adh	TATCAGAAAGTAGCATGTTCC	Chr.4	15335533	15335553	+	No
2595050_adh	TTCATTAGTTTGCCGTCTTTC	Chr.4	15336481	15336461	-	No
2704476_adh	TTTCATTAGTTTGCCGTCTTT	Chr.4	15336482	15336462	-	No
2563465_adh	TTAGATAATTAGGTAGATCAG	Chr.4	15337612	15337632	+	No
2714454_adh	TTTGAATTAATATACTGCAGA	Chr.4	15339048	15339068	+	No
1924985_adh	TATTCTGCAGTATATTAATTC	Chr.4	15339071	15339051	-	Yes
2594992_adh	TTCATTAGAACAACGTTTTTT	Chr.4	15341081	15341101	+	No
1923501_adh	TATTCAGATCTTGGTTTTTTC	Chr.4	15345759	15345739	-	Yes
2581289_adh	TTATTCAGATCTTGGTTTTTT	Chr.4	15345760	15345740	-	Yes
1902084_adh	TATGAATAGAGACAATTTCAA	Chr.4	15346801	15346821	+	No

1925977_adh	TATTGATCCAATTTCAATTTT	Chr.4	15349571	15349551	-	No
1802191_adh	TAATTAAATAGAAAAAAGGCA	Chr.4	15350162	15350182	+	No
2488080_adh	TGTAGATTGGAGAGTAATTTC	Chr.4	15352065	15352085	+	Yes
2420901_adh	TGGGAATGTCATGCTTGAAAT	Chr.4	15356947	15356967	+	No
2643955_adh	TTGATATTTTAATTGTTTTTG	Chr.4	15358283	15358263	-	No
2739493_adh	TTTTCTGAGAATATGACGATG	Chr.4	15361461	15361481	+	No
2040192_adh	TCTCGGCGATGAAAAAATAAC	Chr.4	15362519	15362539	+	No
1790708_adh	TAATAATAAGTTTCTGGACAA	Chr.4	15366394	15366374	-	No
2675092_adh	TTGTAGGAAATTATAACATTA	Chr.4	15367573	15367553	-	No
2124592_adh	TGAACTCCAGAAGAAAACATT	Chr.4	15371909	15371929	+	No
2615497_adh	TTCTATCTTACTATTAAATGT	Chr.4	15371945	15371925	-	No
1946275_adh	TCACATTAAAAAATACAATTG	Chr.4	15377204	15377184	-	No
2171614_adh	TGACTGTGAAGACCAAACCAA	Chr.4	15377607	15377627	+	No
2739209_adh	TTTTCTCAAGAAGTCCTCAAT	Chr.4	15378751	15378771	+	No
2688162_adh	TTGTTTTAGCGGATTTTTTAC	Chr.4	15387734	15387714	-	No
2732775_adh	TTTGTTTTAGCGGATTTTTTA	Chr.4	15387735	15387715	-	No
2744558_adh	TTTTGTTTTAGCGGATTTTTT	Chr.4	15387736	15387716	-	No
2389625_adh	TGGATCCAATTGTTGAATTTT	Chr.4	15388255	15388235	-	No
2650708_adh	TTGCATTACCGACTCAACATA	Chr.4	15389206	15389226	+	No
2044216_adh	TCTGAAGCATACACTGAAAAT	Chr.4	15390084	15390104	+	No
2691291_adh	TTTAATCGGAGTGGCGTTTTG	Chr.4	15390869	15390889	+	Yes
2700867_adh	TTTATTTTGTTTTTAAATACT	Chr.4	15392783	15392803	+	No
2276062_adh	TGATTCTGACACACAAAAGAC	Chr.4	15392927	15392907	-	No
1923028_adh	TATTATTCGTCTAATGAAACT	Chr.4	15393024	15393044	+	No
2640573_adh	TTGAGCACGTCATATTTTTAT	Chr.4	15395074	15395094	+	No
2311593_adh	TGCTTTAGAACGCTCACAGAA	Chr.4	15395713	15395733	+	No
2314244_adh	TGGAAACATAGCATTGATACT	Chr.4	15396477	15396497	+	No
1745581_adh	TAAAAAAGTAGCAGCCGACAT	Chr.4	15398079	15398099	+	No
2553203_adh	TTACAAAGTTGCTTCAATGTG	Chr.4	15399418	15399438	+	No
2071889_adh	TGAAACAGTGGAATTTTTAAA	Chr.4	15400161	15400181	+	No
2029931_adh	TCTAACTTTGCAAAGATTTGA	Chr.4	15400501	15400481	-	Yes
2700877_adh	TTTATTTTTATGTATATTCGA	Chr.4	15401452	15401432	-	Yes
1808737_adh	TACAAGAACATCGAACCAAAT	Chr.4	15403854	15403834	-	Yes
2019164_adh	TCGGTGTCGGACTTAATCGAA	Chr.4	15404251	15404231	-	No
2688470_adh	TTGTTTTTGGATTCATATTTA	Chr.4	15404680	15404700	+	No
1757639_adh	TAAAGCTGAAATTATATAACT	Chr.4	15405896	15405916	+	No
1889918_adh	TATATCACATTTGATGACCAT	Chr.4	15408424	15408404	-	No
1878997_adh	TAGTGGTCTGCCAAAGAACAC	Chr.4	15410455	15410475	+	Yes
1919284_adh	TATGTTATGTTATGTTATGTT	Chr.4	15410987	15411007	+	Yes
1919284_adh	TATGTTATGTTATGTTATGTT	Chr.4	15410992	15411012	+	Yes
1919284_adh	TATGTTATGTTATGTTATGTT	Chr.4	15410997	15411017	+	No
1855565_adh	TAGCCAAAGCATAAATAGCGA	Chr.4	15412180	15412160	-	No
2704485_adh	TTTCATTATTATCTAATCCTT	Chr.4	15412395	15412415	+	Yes
2737799_adh	TTTTCATTTAAGTATCTCGGG	Chr.4	15413053	15413073	+	No
1893296_adh	TATCAGAGCAGAGCAAAATGT	Chr.4	15413858	15413838	-	No
2027708_adh	TCGTTAAAGTTTCCAGACCGT	Chr.4	15414179	15414159	-	No
1957838_adh	TCAGAATACTCCCATTACGAA	Chr.4	15414475	15414455	-	Yes
1814507_adh	TACATTGTTTATAGGCAAACT	Chr.4	15415017	15414997	-	No
1861857_adh	TAGGAATTACTAAAGACTGTT	Chr.4	15416824	15416844	+	No
2704632_adh	TTTCATTGTTTTACATTTTTC	Chr.4	15417211	15417191	-	Yes

2595591_adh	TTCATTTTTTGAGCCCGTCTC	Chr.4	15417755	15417775	+	Yes
2271021_adh	TGATGAATAATAGAAAATGCT	Chr.4	15419703	15419683	-	No
2271021_adh	TGATGAATAATAGAAAATGCT	Chr.4	15420957	15420977	+	No
2595591_adh	TTCATTTTTTGAGCCCGTCTC	Chr.4	15422905	15422885	-	Yes
2704632_adh	TTTCATTGTTTTACATTTTTC	Chr.4	15423449	15423469	+	Yes
1861857_adh	TAGGAATTACTAAAGACTGTT	Chr.4	15423836	15423816	-	No
2136101_adh	TGAAGTTTGGATACAATCAAA	Chr.4	15428412	15428392	-	No
2136101_adh	TGAAGTTTGGATACAATCAAA	Chr.4	15428839	15428859	+	No
2640002_adh	TTGAGAGGGACGAGTTTATTT	Chr.4	15430012	15430032	+	Yes
1891090_adh	TATATTTTTTGTGTTGCATTA	Chr.4	15432450	15432470	+	Yes
1931529_adh	TATTTTTTGTGTTGCATTAAA	Chr.4	15432452	15432472	+	Yes
1789457_adh	TAAGTTGTCAACGGTAATGAA	Chr.4	15433578	15433598	+	No
1965113_adh	TCAGGTTCACTGTGAAACGGT	Chr.4	15435116	15435096	-	No
2468735_adh	TGGTATTGCTAGATTTTGGGT	Chr.4	15435786	15435766	-	No
2625889_adh	TTGAACAATGGACTGAAGACT	Chr.4	15437782	15437802	+	No
1886901_adh	TATACATCTCTATTTTCTATG	Chr.4	15438200	15438220	+	No
1917364_adh	TATGTAAATAAGTTGGATCGC	Chr.4	15439098	15439118	+	No
1931465_adh	TATTTTTTAAAAGGTTTTTTC	Chr.4	15440419	15440399	-	No
2402854_adh	TGGCACAATGGACATTTGATT	Chr.4	15443486	15443466	-	No
2237980_adh	TGAGGATGGACATGAGCTCGT	Chr.4	15445529	15445509	-	No
2044119_adh	TCTGAAGAAAGGGATGATGTG	Chr.4	15471106	15471086	-	No
2690390_adh	TTTAAGATTTAATTGTTTGGT	Chr.4	15474221	15474241	+	No
2590532_adh	TTCAGACTAGAAAATGACGCA	Chr.4	15474460	15474440	-	No
2733976_adh	TTTTACACTCTGATGACGTCA	Chr.4	15476726	15476706	-	No
2028445_adh	TCGTTGCTCGCATGATATAAT	Chr.4	15480387	15480407	+	No
2665319_adh	TTGGCATCTTGAATAAAATTT	Chr.4	15480733	15480713	-	No
1831391_adh	TACTCGTATTTTAGTGAATCA	Chr.4	15480844	15480824	-	No
2546225_adh	TTAACTCTGCATAAAATTAAA	Chr.4	15480952	15480932	-	No
2546225_adh	TTAACTCTGCATAAAATTAAA	Chr.4	15482238	15482258	+	No
1831391_adh	TACTCGTATTTTAGTGAATCA	Chr.4	15482346	15482366	+	No
2665319_adh	TTGGCATCTTGAATAAAATTT	Chr.4	15482457	15482477	+	No
2404161_adh	TGGCAGTAGATATTTAGTTTT	Chr.4	15482691	15482711	+	No
2259598_adh	TGATACGCTGAATTGAATAAT	Chr.4	15483013	15483033	+	No
2700885_adh	TTTATTTTTCTTATTTCCAGG	Chr.4	15487481	15487501	+	No
2584947_adh	TTCAAAACACTACAGTTAGCA	Chr.4	15487784	15487764	-	No
2714201_adh	TTTGAATCAGATCAGGTATTT	Chr.4	15488892	15488912	+	No
1811112_adh	TACACGGCAAATTTTATATAG	Chr.4	15495759	15495779	+	No
2599024_adh	TTCCGCGTGCAGACTTCTTAT	Chr.4	15496102	15496122	+	No
2698316_adh	TTTATATTATATTTCTAGGCA	Chr.4	15497894	15497914	+	Yes
2700971_adh	TTTCAAAATATTTTCATTTTC	Chr.4	15498593	15498573	-	No
2576662_adh	TTATATTTACCCCTAAATTTC	Chr.4	15505065	15505045	-	Yes
1799366_adh	TAATGAATTCGGCCAATTATT	Chr.4	15508271	15508291	+	No
2301260_adh	TGCGGAGGCAAACGATGGAAG	Chr.4	15510917	15510937	+	No
2706258_adh	TTTCCTCTGTTTTTCGAACAA	Chr.4	15513375	15513395	+	No
2706258_adh	TTTCCTCTGTTTTTCGAACAA	Chr.4	15522442	15522422	-	No
1990086_adh	TCCTCCCTGGATCAGTCGGTA	Chr.4	15522628	15522608	-	No
2472274_adh	TGGTGGATTTTAGGCGATTTT	Chr.4	15526577	15526557	-	No
2232558_adh	TGAGATTTTGCGGAAATTGGC	Chr.4	15527048	15527068	+	No
2738647_adh	TTTTCGGGCAGAAATTGTGAA	Chr.4	15530594	15530574	-	No
2608432_adh	TTCGGCAATTTTCGGGCAGAA	Chr.4	15530602	15530582	-	No

2279248_adh	TGATTTTCTGGCGGAAAAAGA	Chr.4	15530794	15530814	+	No
2477774_adh	TGGTTTCGTAGAAAGATTTTA	Chr.4	15531442	15531422	-	No
1800820_adh	TAATGGTGTACGCCTATATCC	Chr.4	15532622	15532602	-	Yes
2626016_adh	TTGAACAGTGAAGCAGAACTT	Chr.4	15535847	15535867	+	No
2733738_adh	TTTTAATGGGTGATCATGGAC	Chr.4	15536497	15536477	-	No
2618508_adh	TTCTGATTGCACAACTTTAAT	Chr.4	15538529	15538509	-	No
1875060_adh	TAGTATAAAAATCACAATTTT	Chr.4	15538620	15538600	-	Yes
2254376_adh	TGAGTGTAGGCGTCTCTTGTT	Chr.4	15540020	15540000	-	No
2305497_adh	TGCTAATGAGTGTAGGCGTCT	Chr.4	15540026	15540006	-	No
2595975_adh	TTCCAATTAAATTGATTTGGA	Chr.4	15540262	15540282	+	Yes
2127346_adh	TGAAGAAGTGCAACTTTTGAT	Chr.4	15541814	15541834	+	No
2732798_adh	TTTGTTTTCGCGTCGTCATCA	Chr.4	15541872	15541852	-	Yes
2138567_adh	TGAATAGATTTTTGGGCCCAA	Chr.4	15544127	15544147	+	No
1866725_adh	TAGGATTTGTGAATGGTTAAT	Chr.4	15544288	15544268	-	No
2745465_adh	TTTTTATTCAAGAACTTATAT	Chr.4	15545640	15545660	+	No
1758223_adh	TAAAGGTGCAGTAGGTCAATG	Chr.4	15546251	15546271	+	No
2683537_adh	TTGTTCAGATACTGGTCCTTG	Chr.4	15547413	15547433	+	No
1824407_adh	TACGATTATTTGAACAAATAA	Chr.4	15548196	15548216	+	No
2540208_adh	TTAAAGAAACCTGAGGATTCA	Chr.4	15561010	15560990	-	Yes
2699250_adh	TTTATGGTGAAATTTAAAGAA	Chr.4	15561023	15561003	-	Yes
1933938_adh	TCAAAGTGAAACAGGACACAG	Chr.4	15561603	15561623	+	No
1851712_adh	TAGATGTAGGAACATTCATAA	Chr.4	15562634	15562614	-	No
1872097_adh	TAGGTGTTTGGAACCTATCTG	Chr.4	15563855	15563835	-	Yes
1938500_adh	TCAAGACAGCAGACAGAATTT	Chr.4	15564717	15564737	+	No
2542660_adh	TTAAATCAGCATTTTGAAAAA	Chr.4	15564834	15564854	+	No
2482836_adh	TGTAATAATGGAAAACGGATT	Chr.4	15567412	15567392	-	No
2549471_adh	TTAATAAATCCCAATTGGCAA	Chr.4	15567812	15567792	-	No
2690875_adh	TTTAATAAATCCCAATTGGCA	Chr.4	15567813	15567793	-	No
2700624_adh	TTTATTTAATACATTTTTTCA	Chr.4	15568622	15568602	-	Yes
1919989_adh	TATTAAATTAATTTTCTACGG	Chr.4	15568940	15568920	-	Yes
2237432_adh	TGAGGACTTTATTTAAAAACA	Chr.4	15569301	15569281	-	No
2617905_adh	TTCTGAATGAGCTTCAACTAA	Chr.4	15569619	15569599	-	No
2699444_adh	TTTATTAAGAAAAAGGGGAAT	Chr.4	15572606	15572626	+	Yes
1752173_adh	TAAAATGCAATAAATGAAATT	Chr.4	15574813	15574793	-	No
2561847_adh	TTAGAATTGACAAGTTGGGAT	Chr.4	15583387	15583367	-	No
2478220_adh	TGGTTTGCAACACTTGTTACA	Chr.4	15583504	15583524	+	No
2579124_adh	TTATGCCTAAGTAGAGTTTAT	Chr.4	15584289	15584269	-	No
2699378_adh	TTTATGTTCGTGGGACTATAA	Chr.4	15587496	15587516	+	No
1954089_adh	TCACTATTTTCTGTGATTGTA	Chr.4	15587747	15587727	-	No
2729397_adh	TTTGTGAATTGAAGTTGCATC	Chr.4	15587887	15587907	+	No
2553297_adh	TTACAACCTGGTTTCTAGTGT	Chr.4	15588464	15588444	-	No
2271542_adh	TGATGATGCTAATGACAAATA	Chr.4	15589996	15589976	-	Yes
1930171_adh	TATTTGAATCAATTTAATTTT	Chr.4	15596897	15596877	-	No
2566435_adh	TTAGCATAAATTTCTGAACTA	Chr.4	15596903	15596923	+	No
2733150_adh	TTTTAAATATTAAAGTAGAAA	Chr.4	15601040	15601060	+	No
2560512_adh	TTACTTTGGCAAACCTAATAC	Chr.4	15602004	15601984	-	No
1796029_adh	TAATATCTGTTTTATCCTGTA	Chr.4	15602876	15602896	+	Yes
2474494_adh	TGGTTATTCCTCTTTTGTCGG	Chr.4	15604652	15604672	+	Yes
1924149_adh	TATTCCTCTTTTGTCGGTTTT	Chr.4	15604656	15604676	+	Yes
2321092_adh	TGGAACTAGAGGAATCTTAAA	Chr.4	15605491	15605511	+	Yes

2612055_adh	TTCGTCTAAAACTGTAAATTC	Chr.4	15605713	15605733	+	Yes
2562164_adh	TTAGACGAAACAGATAGAACG	Chr.4	15605721	15605701	-	No
1814627_adh	TACATTTTTCTCATAGCACGG	Chr.4	15610642	15610622	-	No
1967701_adh	TCAGTTTATCGTACAAATTTT	Chr.4	15611408	15611388	-	No
2550751_adh	TTAATCTTGAATAGTATAATA	Chr.4	15611702	15611682	-	Yes
1831170_adh	TACTCCTTCGGCTATATTTAC	Chr.4	15612107	15612127	+	Yes
2521543_adh	TGTGTTTGTCGAATTAAAAAA	Chr.4	15614825	15614805	-	No
2572084_adh	TTAGTGTGTTTGTCGAATTAA	Chr.4	15614829	15614809	-	No
1921769_adh	TATTAGTGTGTTTGTCGAATT	Chr.4	15614831	15614811	-	No
1977076_adh	TCCACAAATTTCTGTTTTGTT	Chr.4	15620899	15620879	-	No
1901134_adh	TATGAAACCGTAGATAATTGT	Chr.4	15621119	15621099	-	No
2729419_adh	TTTGTGACAGAACGCATTTTT	Chr.4	15621157	15621177	+	No
1922395_adh	TATTATGAACAACAAAAAACA	Chr.4	15621847	15621867	+	No
2747851_adh	TTTTTGTTAAAGTGGACAATA	Chr.4	15624681	15624701	+	No
2611369_adh	TTCGTAGTATAGTAGAAAACC	Chr.4	15625036	15625016	-	No
1829060_adh	TACGTTGGAAACATTATTATT	Chr.4	15628397	15628377	-	No
1859631_adh	TAGCTTCATTAATTTTTTAAT	Chr.4	15629054	15629074	+	No
2606555_adh	TTCGCGAATATAAAAACTTCA	Chr.4	15629309	15629329	+	Yes
2008265_adh	TCGCTAAAACAGACAAAAATG	Chr.4	15631092	15631072	-	No
2530358_adh	TGTTGTACCGTCGGTATAAAT	Chr.4	15657494	15657474	-	No
2747925_adh	TTTTTGTTGATGCATTTTTGA	Chr.4	15657729	15657709	-	No
2675041_adh	TTGTAGCATTTTATAAAAAAC	Chr.4	15658913	15658933	+	No
1807084_adh	TACAAAGTTCAAGATTCGGAT	Chr.4	15661819	15661799	-	No
2592502_adh	TTCAGGTTACTTTTTTACTGA	Chr.4	15661882	15661862	-	No
2634769_adh	TTGACACACGGATGCGAATAG	Chr.4	15662982	15662962	-	No
1912440_adh	TATGCACGGTCTTGTACATTC	Chr.4	15663300	15663280	-	No
2706735_adh	TTTCGAAGTTTGAATCGGCCT	Chr.4	15666203	15666223	+	No
1839506_adh	TAGAAGGAACGGCGGACATAA	Chr.4	15666710	15666690	-	Yes
2561495_adh	TTAGAAGGAACGGCGGACATA	Chr.4	15666711	15666691	-	Yes
2305307_adh	TGCTAACACAGTTGTTCGAAT	Chr.4	15667377	15667357	-	Yes
2742626_adh	TTTTGGAAAGACGACTTTGTT	Chr.4	15667760	15667780	+	No
2558395_adh	TTACTAGACGAATGCTAATAG	Chr.4	15669195	15669175	-	No
2736425_adh	TTTTATTAGACGTGTTGAAAA	Chr.4	15669417	15669437	+	No
2742626_adh	TTTTGGAAAGACGACTTTGTT	Chr.4	15669635	15669655	+	No
2706735_adh	TTTCGAAGTTTGAATCGGCCT	Chr.4	15670283	15670303	+	No
2681216_adh	TTGTGCAATTGCTAAAAACAG	Chr.4	15674806	15674786	-	No
2003875_adh	TCGATGAACTTTCGAACTAAC	Chr.4	15677149	15677129	-	No
2605050_adh	TTCGATGAACTTTCGAACTAA	Chr.4	15677150	15677130	-	No
2127140_adh	TGAAGAAGACGGACAAAGCCA	Chr.4	15677263	15677243	-	Yes
2605050_adh	TTCGATGAACTTTCGAACTAA	Chr.4	15677861	15677881	+	No
2003875_adh	TCGATGAACTTTCGAACTAAC	Chr.4	15677862	15677882	+	No
2332202_adh	TGGAATATGAAAGAGAATGAA	Chr.4	15679109	15679089	-	No
2522440_adh	TGTTAAGAATGCCACTGCAAC	Chr.4	15681611	15681631	+	No
2044797_adh	TCTGAATGGATTTGATGAACC	Chr.4	15684170	15684150	-	No
2723392_adh	TTTGGACTAGAGTTTTACGGA	Chr.4	15684342	15684322	-	No
2723392_adh	TTTGGACTAGAGTTTTACGGA	Chr.4	15686677	15686697	+	No
2044797_adh	TCTGAATGGATTTGATGAACC	Chr.4	15686849	15686869	+	No
2554520_adh	TTACATAGATAACAATATTTC	Chr.4	15690240	15690260	+	Yes
1813158_adh	TACATAGATAACAATATTTCC	Chr.4	15690241	15690261	+	Yes
2733562_adh	TTTTAAGTAGAAATCGTGGCA	Chr.4	15696630	15696650	+	No

2690743_adh	TTTAAGTAGAAATCGTGGCAA	Chr.4	15696631	15696651	+	No
2701163_adh	TTTCAAATAGAACTTTTGGTA	Chr.4	15698281	15698301	+	No
2737451_adh	TTTTCAGAGAGTCAGGATACA	Chr.4	15700046	15700026	-	No
2046572_adh	TCTGATAAAAAAGCGAAATAA	Chr.4	15701830	15701850	+	No
2601724_adh	TTCGAAATCATATAACACTTA	Chr.4	15706299	15706279	-	No
1872769_adh	TAGTAAAAGGGCATTTGAACT	Chr.4	15706992	15706972	-	Yes
2554844_adh	TTACATGAGTCATATCATTAC	Chr.4	15710157	15710137	-	Yes
2042338_adh	TCTCTCAGCCTACGACCAAAA	Chr.4	15711317	15711297	-	Yes
2544369_adh	TTAAATTCGTGTCCTATTCCT	Chr.4	15711596	15711576	-	No
2549243_adh	TTAAGTGTACCTTCAACACTA	Chr.4	15711976	15711956	-	No
2552287_adh	TTAATTGGTTTGGTGTGATTG	Chr.4	15712083	15712063	-	Yes
2699589_adh	TTTATTAGATGAGTGTGTCAG	Chr.4	15712745	15712725	-	Yes
2736428_adh	TTTTATTAGATGAGTGTGTCA	Chr.4	15712746	15712726	-	Yes
2053100_adh	TCTGTTCCGTAGAAGAAAACT	Chr.4	15713985	15713965	-	No
1893624_adh	TATCAGTTGTGATATCAAAAC	Chr.4	15714163	15714143	-	Yes
1923440_adh	TATTCAATTTTTATGTAGAGA	Chr.4	15716601	15716621	+	No
2669374_adh	TTGGTACGTCTGACATCTTAC	Chr.4	15717802	15717782	-	No
1874849_adh	TAGTAGTAAAAAGCCGGATAT	Chr.4	15722728	15722748	+	No
2557869_adh	TTACGTAGGGTTTCGAAACTA	Chr.4	15723242	15723222	-	No
2571401_adh	TTAGTGAAATTTGAGTCGGTT	Chr.4	15724083	15724063	-	Yes
2601078_adh	TTCCTGTTCAATGTCGGCTGC	Chr.4	15724957	15724977	+	No
2664826_adh	TTGGATTTTAAAAAATATTTA	Chr.4	15732058	15732038	-	No
2650144_adh	TTGCATATCCTATAAAGCCAT	Chr.4	15732427	15732447	+	No
2573456_adh	TTAGTTTGATTGTTCTATGGA	Chr.4	15733765	15733785	+	No
2710854_adh	TTTCTGTAGCTAGATTTGCAT	Chr.4	15738296	15738316	+	No
2573456_adh	TTAGTTTGATTGTTCTATGGA	Chr.4	15738830	15738850	+	No
2573456_adh	TTAGTTTGATTGTTCTATGGA	Chr.4	15740913	15740893	-	No
2710854_adh	TTTCTGTAGCTAGATTTGCAT	Chr.4	15741447	15741427	-	No
2134942_adh	TGAAGTGACCGAAGCTTGACG	Chr.4	15745993	15745973	-	No
1968543_adh	TCATACAATGGGTCTTTTGGT	Chr.4	15747097	15747117	+	No
2580477_adh	TTATTAAACTGTGAATTTAGT	Chr.4	15749610	15749630	+	No
2554236_adh	TTACAGCAAATTTTCTGAAGA	Chr.4	15750836	15750856	+	No
2305671_adh	TGCTACAGTTTTTCCATATTC	Chr.4	15755325	15755345	+	No
2700799_adh	TTTATTTGTTTTATTGATTAG	Chr.4	15755664	15755684	+	No
2732780_adh	TTTGTTTTATTGATTAGCATA	Chr.4	15755668	15755688	+	No
2066623_adh	TGAAAACAAAAAATGTATATA	Chr.4	15757734	15757754	+	No
2703802_adh	TTTCATAGTTGATTGCAGAAA	Chr.4	15759919	15759899	-	No
2741897_adh	TTTTGATTATTTTTGTAGCAT	Chr.4	15760654	15760634	-	No
2625967_adh	TTGAACAGCATTGCTCCGTCG	Chr.4	15764124	15764104	-	No
1943358_adh	TCAATTTGCTCAAGAATCATA	Chr.4	15764575	15764555	-	No
2659713_adh	TTGGAGCATATTTTGGACAAT	Chr.4	15769029	15769009	-	No
1771683_adh	TAACTGGGAGAATGACTCGTT	Chr.4	15770760	15770740	-	No
1931221_adh	TATTTTGTGGAAAGTTGTTGC	Chr.4	15771190	15771210	+	No
2654152_adh	TTGCTAGACTTAGATAAAGCT	Chr.4	15772369	15772349	-	No
2479136_adh	TGGTTTTGAAAATGTTTGGCA	Chr.4	15772739	15772719	-	No
1794868_adh	TAATAGATACTTGCATTTTCC	Chr.4	15772947	15772967	+	No
2556081_adh	TTACCGCTGTATTTAAAACTG	Chr.4	15773160	15773180	+	No
1794552_adh	TAATACTGTTTCACTATTTCC	Chr.4	15773389	15773409	+	No
2584773_adh	TTATTTTTTCAGTTTTTGAAT	Chr.4	15777333	15777353	+	Yes
2693312_adh	TTTACAGAATCTACGTTAACT	Chr.4	15779075	15779095	+	Yes

1920793_adh	TATTACGAAGAGATAATACTA	Chr.4	15779896	15779916	+	No
2578090_adh	TTATCTCTTCGTAATAAAACA	Chr.4	15779911	15779891	-	No
2706276_adh	TTTCCTGAGTTCTGCTTGAAA	Chr.4	15786300	15786280	-	No
1992660_adh	TCCTTATGGAAAAGAAGATGA	Chr.4	15792645	15792665	+	No
2527476_adh	TGTTGAAATTTGCGGCCTTGT	Chr.4	15793882	15793902	+	No
2620576_adh	TTCTTACTGAATTAGAAAACG	Chr.4	15794753	15794773	+	No
1832207_adh	TACTGAATTAGAAAACGGATT	Chr.4	15794757	15794777	+	No
2145243_adh	TGAATTAGAAAACGGATTTTC	Chr.4	15794760	15794780	+	No
1746261_adh	TAAAAAGATATATGACAATTC	Chr.4	15804932	15804912	-	No
2318679_adh	TGGAACAAGAAAATTCGAAGA	Chr.4	15805032	15805012	-	No
2631491_adh	TTGAATGATATGACAAATTTT	Chr.4	15807569	15807589	+	No
1926854_adh	TATTGGACACAACACAAGAAC	Chr.4	15810896	15810916	+	Yes
2657985_adh	TTGGACACAACACAAGAACCA	Chr.4	15810898	15810918	+	Yes
2592984_adh	TTCAGTGACTCGTATTAGCGT	Chr.4	15813195	15813175	-	No
2004547_adh	TCGATTATAAGCTCACACGGA	Chr.4	15816625	15816605	-	No
2617667_adh	TTCTGAAACAGTAGAAAAAGT	Chr.4	15817835	15817815	-	No
2722664_adh	TTTGCTTCGTTGGTTTATTAC	Chr.4	15818894	15818874	-	No
2269092_adh	TGATCACCAAGAATTCGGTTA	Chr.4	15821084	15821064	-	Yes
2463291_adh	TGGTAGGCGTCATTAAAACCG	Chr.4	15824887	15824907	+	Yes
2733706_adh	TTTTAATGACGCCTACCAATT	Chr.4	15824904	15824884	-	No
1813240_adh	TACATATCCTTCAAACCAATT	Chr.4	15825197	15825177	-	Yes
2613599_adh	TTCGTTTGTATGTGTTGTTAA	Chr.4	15831766	15831786	+	No
1932390_adh	TCAAAATCAGTCGGGCATTTA	Chr.4	15831867	15831847	-	No
2705964_adh	TTTCCTACGAATTATTTATTA	Chr.4	15834110	15834090	-	No
2691324_adh	TTTAATCGTTCGTAGAATGTA	Chr.4	15837418	15837438	+	No
2028042_adh	TCGTTCGTAGAATGTATTTGT	Chr.4	15837423	15837443	+	No
2500671_adh	TGTGAAACCGTATTTTTTGTT	Chr.4	15837866	15837846	-	No
2620501_adh	TTCTTAAGGTTTTTGTTACGA	Chr.4	15838134	15838154	+	No
1803104_adh	TAATTCGACCTGCAATGACAA	Chr.4	15838636	15838616	-	No
2640371_adh	TTGAGATTTTTAATATCTTCT	Chr.4	15839376	15839356	-	No
2594228_adh	TTCATCCCTCTCGTCAACATA	Chr.4	15841434	15841454	+	Yes
1784621_adh	TAAGGATTTCATCGTTTCAGT	Chr.4	15842130	15842150	+	No
2288055_adh	TGCAGCAAACAAACGTGTTTC	Chr.4	15844233	15844213	-	No
2700420_adh	TTTATTGGGCTAAACGACTGA	Chr.4	15844536	15844556	+	Yes
2743967_adh	TTTTGTGGCTTATCTAAATCC	Chr.4	15844918	15844938	+	Yes
2313380_adh	TGGAAAACATGAGGATGTAGT	Chr.4	15845952	15845972	+	No
1968019_adh	TCATAAAACGTTATACTTACA	Chr.4	15846390	15846410	+	No
1990815_adh	TCCTCTGAAAATTTTTCGCAA	Chr.4	15850038	15850018	-	No
2576589_adh	TTATATTGCATTTTTTATGTG	Chr.4	15850095	15850115	+	No
2579885_adh	TTATGTAGATATCATCCGCAC	Chr.4	15851702	15851682	-	No
2029260_adh	TCTAAAAGTAAACGGATTAAT	Chr.4	15857813	15857833	+	No
1920277_adh	TATTAATCCGTTTACTTTTAG	Chr.4	15857834	15857814	-	No
2643263_adh	TTGATAACTCTTGAAGAGGTT	Chr.4	15860054	15860034	-	No
2413620_adh	TGGCGTGTTCTGCAGAAAACC	Chr.4	15861534	15861554	+	No
1797018_adh	TAATATTAGATTGCCCGGTAC	Chr.4	15864166	15864146	-	No
1809219_adh	TACAATAAAACTTTATTCAAA	Chr.4	15865174	15865194	+	No
2674033_adh	TTGTAATATTTGTAGAAAAAA	Chr.4	15865178	15865158	-	No
1901527_adh	TATGAACAAAGTGAAACAGCA	Chr.4	15866096	15866116	+	No
1747355_adh	TAAAAATTTGTTTTTGAACAG	Chr.4	15866200	15866180	-	No
1953009_adh	TCACGAAGGATATGGCATTAA	Chr.4	15866935	15866915	-	No

2625854_adh	TTGAACAACTTCAACGACGAA	Chr.4	15867051	15867071	+	No
1923377_adh	TATTCAAGTAGAGTCGGAAAT	Chr.4	15869043	15869063	+	No
2577028_adh	TTATCAGCAATCAGAGTGCAA	Chr.4	15876200	15876180	-	No
1755256_adh	TAAACTAATTTTCTTCGCATG	Chr.4	15877916	15877936	+	No
2038406_adh	TCTCATGTAGAAAAGAATTTG	Chr.4	15879330	15879310	-	No
2593255_adh	TTCAGTTCTATGGTTTCATTC	Chr.4	15881116	15881136	+	Yes
1807101_adh	TACAAATAAAAATGGGAATTA	Chr.4	15881549	15881569	+	No
1790929_adh	TAATACACATTTATTTAGAAC	Chr.4	15882394	15882414	+	No
1813926_adh	TACATGGTGCATGCAGCGTAC	Chr.4	15889610	15889590	-	No
2453207_adh	TGGGGCGTGTTTTTGACTGTA	Chr.4	15889759	15889739	-	No
1919985_adh	TATTAAATGTTAGAAATCCAT	Chr.4	15890632	15890652	+	No
1967345_adh	TCAGTTCCCAAAAATAATCGT	Chr.4	15890876	15890856	-	No
2469060_adh	TGGTATTTTTAGTGTGATGGA	Chr.4	15890952	15890972	+	No
2005401_adh	TCGCAAAAATGTTTAATTCTG	Chr.4	15891444	15891464	+	No
2515474_adh	TGTGGATAGGTGTTGGAATTC	Chr.4	15893078	15893058	-	No
2583683_adh	TTATTTCAATATTGTTCCGTT	Chr.4	15895291	15895271	-	No
2691022_adh	TTTAATAGTGTAGAAAAGTGG	Chr.4	15896802	15896822	+	No
2580185_adh	TTATGTGGAGTAATGGTTGAT	Chr.4	15898290	15898270	-	No
2737557_adh	TTTTCAGTATGTTCAAAGGAA	Chr.4	15898518	15898538	+	No
1923368_adh	TATTCAAGAAGAATTTCAAAT	Chr.4	15898893	15898873	-	No
2532141_adh	TGTTTCACATGGTATAATGGT	Chr.4	15899883	15899903	+	No
2551201_adh	TTAATGGAAGATAGGGAAATC	Chr.4	15900516	15900496	-	No
2601090_adh	TTCCTGTTTTGCAAGACAACT	Chr.4	15902383	15902403	+	Yes
2580667_adh	TTATTACGGTCGATCTTGTTT	Chr.4	15903158	15903178	+	No
1883846_adh	TATAAAAATGAGGCGGAACAT	Chr.4	15903634	15903614	-	No
2700851_adh	TTTATTTTGAATCTGTAACAG	Chr.4	15903665	15903685	+	No
2588909_adh	TTCAATTGTAGACTGAGGGCT	Chr.4	15904368	15904388	+	Yes
2325540_adh	TGGAAGGTAAAAAATATTTAA	Chr.4	15905953	15905933	-	No
2703686_adh	TTTCATAATGATAACGGATCT	Chr.4	15906536	15906556	+	Yes
2732166_adh	TTTGTTGGCTGATATTCCTAC	Chr.4	15908526	15908506	-	No
1836686_adh	TAGAAAACGGCTGAAAATGGT	Chr.4	15910197	15910177	-	No
1956979_adh	TCAGAAATACTGTAGTAGGTG	Chr.4	15910426	15910446	+	No
2655039_adh	TTGCTGTAGACTTCACAGATA	Chr.4	15910468	15910488	+	No
2710259_adh	TTTCTCTTGAATCCTGAAGAC	Chr.4	15913073	15913093	+	No
1762881_adh	TAAATTTTATTCATAAGCCAC	Chr.4	15913642	15913622	-	No
2588069_adh	TTCAATGATAGTTACAATTAA	Chr.4	15913876	15913896	+	Yes
2700074_adh	TTTATTGAGACTTGAGACTAC	Chr.4	15914671	15914691	+	No
1762952_adh	TAAATTTTTCAAACAACTTCG	Chr.4	15917210	15917230	+	No
2043631_adh	TCTGAAAAGCCAAAAAGATCG	Chr.4	15919872	15919892	+	No
1867080_adh	TAGGCATGTAGGTAGGCATTT	Chr.4	15926042	15926062	+	No
2595434_adh	TTCATTTAATCTTTAGGAACC	Chr.4	15927665	15927645	-	Yes
2623229_adh	TTCTTTAAGTACATAAGTATC	Chr.4	15927962	15927942	-	Yes
2711920_adh	TTTCTTTAAGTACATAAGTAT	Chr.4	15927963	15927943	-	Yes
1981607_adh	TCCCGGCCGGGGAGAACTTTT	Chr.4	15932029	15932049	+	No
2716897_adh	TTTGAGGCAAGTAAATGATTT	Chr.4	15934383	15934363	-	No
2539370_adh	TTAAACACAGCGGCGCGCGGA	Chr.4	15939918	15939938	+	No
1911845_adh	TATGATTACAGGAGTGGCAGA	Chr.4	15941867	15941887	+	No
2716315_adh	TTTGAGAAAGGAACTCATCGT	Chr.4	15943194	15943174	-	No
2491780_adh	TGTATGATCATGTTGTGCGGT	Chr.4	15943759	15943739	-	No
2740448_adh	TTTTGAAAAGAAATAGACGAC	Chr.4	15944675	15944695	+	No

2048214_adh	TCTGCCTGATTGTGAGGAGTC	Chr.4	15945362	15945342	-	No
2514488_adh	TGTGGAACAAATAGATGACAT	Chr.4	15948534	15948554	+	No
1878664_adh	TAGTGGACGAGAAAAAGGCGG	Chr.4	15951475	15951495	+	No
2486265_adh	TGTAATTTTCAAACGGACATT	Chr.4	15955413	15955393	-	No
2745827_adh	TTTTTCCAGAATGTAGAAGAG	Chr.4	15955841	15955821	-	No
2721202_adh	TTTGCACGTTATCTGACAGAC	Chr.4	15959110	15959090	-	No
2682341_adh	TTGTGTGCCGTCGTTGGTTTT	Chr.4	15959594	15959614	+	Yes
1822903_adh	TACGAGAAATTAGGAATTTAA	Chr.4	15960203	15960183	-	No
2659500_adh	TTGGAGACTTTATGCAGAACA	Chr.4	15960736	15960716	-	Yes
2575243_adh	TTATATAGTAGTTGAACCCCA	Chr.4	15961461	15961481	+	No
1833132_adh	TACTGGAAGATTGATACTTTT	Chr.4	15962012	15962032	+	No
2593405_adh	TTCAGTTTCGGAGATTCAAAC	Chr.4	15963049	15963069	+	No
2734383_adh	TTTTACTTTTGAAGATAATTA	Chr.4	15966053	15966033	-	Yes
1813207_adh	TACATAGTTGAGAGGTTTTTT	Chr.4	15967424	15967404	-	No
1902886_adh	TATGACAATGCAATTCAAATG	Chr.4	15969482	15969462	-	No
2719124_adh	TTTGATGGGACGGAGGCAATC	Chr.4	15970056	15970076	+	No
2708821_adh	TTTCGTATGAGTGCTTAACTT	Chr.4	15970428	15970408	-	No
2698311_adh	TTTATATGTGTTTTCACAGTG	Chr.4	15974404	15974384	-	No
2738606_adh	TTTTCGGATTAAAGCAATACA	Chr.4	15976758	15976738	-	No
1811541_adh	TACACTTATGGAAACAATTTT	Chr.4	15978398	15978378	-	Yes
2029719_adh	TCTAACTCGTAAGATGTCAGT	Chr.4	15982804	15982784	-	No
1877981_adh	TAGTGATTGAGTAGTAATGCA	Chr.4	15986558	15986578	+	No
2276410_adh	TGATTGAGTAGAAATGCAAGG	Chr.4	15988051	15988071	+	Yes
2251064_adh	TGAGTAGAAATGCAAGGTTGA	Chr.4	15988055	15988075	+	Yes
1803911_adh	TAATTGCATAACTTTTATTTT	Chr.4	15988715	15988695	-	Yes
2552213_adh	TTAATTGCATAACTTTTATTT	Chr.4	15988716	15988696	-	Yes
1834640_adh	TACTTATACTTTCCAAGAATT	Chr.4	15989615	15989595	-	No
2700089_adh	TTTATTGATGCCTCTCAAGCC	Chr.4	15989693	15989713	+	No
2677791_adh	TTGTCCATACGTTTCTCACAA	Chr.4	15992093	15992113	+	No
1921662_adh	TATTAGGATTTCACTAGAAGA	Chr.4	15997205	15997185	-	No
1762997_adh	TAACAAAAATATCATACAATT	Chr.4	15998465	15998485	+	No
2749130_adh	TTTTTTCTTTATATATCATTT	Chr.4	15999499	15999479	-	Yes
2062830_adh	TCTTTTGTATTTTGACGAACA	Chr.4	16003941	16003921	-	No
1807120_adh	TACAAATAGCTGAAGAACAAC	Chr.4	16004679	16004699	+	No
1802727_adh	TAATTATTAGAAAATGAATCC	Chr.4	16005853	16005873	+	No
1797324_adh	TAATATTTGTGATAACTACGT	Chr.4	16006043	16006023	-	Yes
1814869_adh	TACCAATCGTCTGACTTCCAG	Chr.4	16006560	16006540	-	No
2031846_adh	TCTACTATCTCACAAGCTGTA	Chr.4	16006834	16006814	-	No
2181581_adh	TGAGAATGCACAGGAAGGAAA	Chr.4	16007291	16007311	+	No
2552022_adh	TTAATTCTAGGCGGATCAAAT	Chr.4	16009687	16009667	-	Yes
2537503_adh	TTAAAAAATATATGAAGAAAG	Chr.4	16010157	16010137	-	No
2500111_adh	TGTCTTCATTTTACAAAGCAT	Chr.4	16011791	16011811	+	Yes
1810468_adh	TACACAAGTTTCAAAAAATAT	Chr.4	16013112	16013092	-	No
2126811_adh	TGAACTTTTTAATCGGCACAA	Chr.4	16013827	16013807	-	No
2749487_adh	TTTTTTGCTGATGGAAACAAA	Chr.4	16017628	16017608	-	No
1834764_adh	TACTTCAATTAAATAAAATTA	Chr.4	16017914	16017894	-	No
2738697_adh	TTTTCGGTCAATGCGGACACA	Chr.4	16021835	16021855	+	No
1944720_adh	TCACAGAAAACATACGCATAA	Chr.4	16022575	16022555	-	No
1890110_adh	TATATCGTTTTTTAATTTAAT	Chr.4	16022907	16022927	+	Yes
1812141_adh	TACAGATTGTTACAGAAAGGT	Chr.4	16023428	16023408	-	No

2692167_adh	TTTAATGTTCATATACTGGAT	Chr.4	16023562	16023542	-	Yes
2691011_adh	TTTAATAGCAGTTTTTATTTA	Chr.4	16024885	16024865	-	No
2151571_adh	TGACAAGTACTGTTTCAGAAT	Chr.4	16027290	16027270	-	No
1833559_adh	TACTGGTGATTGAGAGACGCA	Chr.4	16028773	16028753	-	No
2010955_adh	TCGGAATCAGATATATAAGGC	Chr.4	16029020	16029040	+	No
2743788_adh	TTTTGTCGCTTTAGGTTCAAT	Chr.4	16029885	16029905	+	Yes
2538966_adh	TTAAAATAGATCGCCAATAGA	Chr.4	16035963	16035943	-	No
2672024_adh	TTGGTTCATCGTTTAGTGTAT	Chr.4	16037699	16037679	-	No
1931923_adh	TCAAAAATGTTTCAAAAATTA	Chr.4	16039320	16039340	+	No
2735997_adh	TTTTATGCCATGCAGCAGTTG	Chr.4	16039959	16039939	-	No
2620701_adh	TTCTTAGGGCTTTTGGCAAAT	Chr.4	16040150	16040170	+	No
1929416_adh	TATTTATTCTTGAACGGCTAG	Chr.4	16041040	16041060	+	No
2699973_adh	TTTATTCTTGAACGGCTAGGA	Chr.4	16041042	16041062	+	No
2701509_adh	TTTCAAGACTGTGCCAGTTAA	Chr.4	16041582	16041562	-	No
1745309_adh	TAAAAAACAATATTAATTTTT	Chr.4	16042988	16042968	-	No
2727213_adh	TTTGTAATAACTGAAGCAAAT	Chr.4	16043234	16043254	+	No
1965965_adh	TCAGTAGCACTCGGCATGTTT	Chr.4	16043680	16043660	-	No
2549719_adh	TTAATACTACATTTTCACGAA	Chr.4	16045603	16045623	+	No
1793901_adh	TAATACTACATTTTCACGAAA	Chr.4	16045604	16045624	+	No
2576716_adh	TTATATTTGATTTCTTTTTAG	Chr.4	16050819	16050839	+	No
1830902_adh	TACTCAGTTTGTTACATTTCA	Chr.4	16050906	16050886	-	No
2026241_adh	TCGTGCAGGTAGATGGAAACT	Chr.4	16051881	16051901	+	No
2311514_adh	TGCTTGTTGAGACGAATTGAA	Chr.4	16052145	16052125	-	Yes
2542168_adh	TTAAATACATGTTTTACAGTT	Chr.4	16053068	16053048	-	No
2148194_adh	TGAATTTATGAACTTGTAGAA	Chr.4	16054703	16054683	-	No
2730767_adh	TTTGTTCGGAATACATTGTAA	Chr.4	16055391	16055411	+	No
1993059_adh	TCCTTCTGAACACGGCTGTCT	Chr.4	16056498	16056518	+	No
2558225_adh	TTACTAAACTGATACAAAAAT	Chr.4	16057578	16057558	-	No
2711403_adh	TTTCTTCACAAACAACGGTTT	Chr.4	16062514	16062494	-	No
1863845_adh	TAGGAGGAAGAATTGTGCATA	Chr.4	16062734	16062714	-	No
2607260_adh	TTCGGAATTAAGGTGTTTTAT	Chr.4	16071079	16071099	+	No
2286040_adh	TGCACAGCACGCTATCATTTA	Chr.4	16071791	16071811	+	No
2388879_adh	TGGATATCATAGAAATGTCAT	Chr.4	16072556	16072576	+	No
2620114_adh	TTCTGTCTTGTGGAAAATGGC	Chr.4	16072804	16072784	-	No
2561352_adh	TTAGAACTTCTCAAAAAATAA	Chr.4	16073114	16073094	-	No
1921247_adh	TATTAGAACTTCTCAAAAAAT	Chr.4	16073116	16073096	-	No
2006625_adh	TCGCAGTCTGTATTCCCGTTA	Chr.4	16073193	16073173	-	Yes
2723844_adh	TTTGGAGTGTGGTCATCAAAC	Chr.4	16073769	16073749	-	No
2490729_adh	TGTATATTAGGCTCCGTTTTT	Chr.4	16074313	16074293	-	No
2009407_adh	TCGCTGTATATTAGGCTCCGT	Chr.4	16074317	16074297	-	No
2732861_adh	TTTGTTTTTACTCGCTGGATA	Chr.4	16076043	16076023	-	No
2700844_adh	TTTATTTTCTGATATCAGGTT	Chr.4	16078025	16078045	+	No
2615254_adh	TTCTATAGGTGCATCTAATAA	Chr.4	16078491	16078471	-	No
1941294_adh	TCAATCACATAGACGTATCTT	Chr.4	16078790	16078810	+	Yes
2258840_adh	TGATAAGCGTAATGTGTCGGA	Chr.4	16079217	16079237	+	Yes
2549440_adh	TTAATAAACAACAACAAAAAT	Chr.4	16079645	16079665	+	No
1877912_adh	TAGTGATAGAACATACAATTT	Chr.4	16079804	16079824	+	No
2742517_adh	TTTTGCTCTATACTTTTATAC	Chr.4	16080100	16080120	+	No
1893620_adh	TATCAGTTGGTTATATCGGCA	Chr.4	16080621	16080601	-	No
1917749_adh	TATGTAGTAACGATAGTGCGG	Chr.4	16081103	16081123	+	No

1790228_adh	TAATAAATTTCACCCAAATGT	Chr.4	16082177	16082197	+	No
2639987_adh	TTGAGAGGACCATGTCAAATG	Chr.4	16082279	16082259	-	Yes
1957799_adh	TCAGAAGTCTTTCTTGCCCTT	Chr.4	16088413	16088393	-	No
2745056_adh	TTTTTAGACGAGCAAGAGCGA	Chr.4	16091669	16091649	-	No
2553655_adh	TTACAATTCAAGAAAAAAGTA	Chr.4	16092116	16092096	-	No
1913323_adh	TATGCTTGATTTGAAAACTGT	Chr.4	16096301	16096321	+	No
1804658_adh	TAATTTAAAACAATAAAAAAT	Chr.4	16097388	16097368	-	No
1829122_adh	TACGTTTCAGTTCAGTGCTAA	Chr.4	16098115	16098095	-	No
2715019_adh	TTTGACCACAAGAGGCGATCA	Chr.4	16099768	16099788	+	No
2635327_adh	TTGACCACAAGAGGCGATCAA	Chr.4	16099769	16099789	+	No
2644712_adh	TTGATGACAATGGTTTATGAA	Chr.4	16099957	16099977	+	No
2377631_adh	TGGACTGATACTTCACAGATT	Chr.4	16100280	16100300	+	No
1829372_adh	TACTAACTAGTGCAGTACACG	Chr.4	16100637	16100617	-	Yes
2573367_adh	TTAGTTTATATTTTATTCCCT	Chr.4	16104979	16104999	+	Yes
2655286_adh	TTGCTTCTAAATGAAAACTTG	Chr.4	16105429	16105449	+	No
2748386_adh	TTTTTTATGAAGCTGCAGACA	Chr.4	16105675	16105695	+	No
2745348_adh	TTTTTATGAAGCTGCAGACAA	Chr.4	16105676	16105696	+	No
1829372_adh	TACTAACTAGTGCAGTACACG	Chr.4	16108337	16108357	+	Yes
2377631_adh	TGGACTGATACTTCACAGATT	Chr.4	16108694	16108674	-	No
2644712_adh	TTGATGACAATGGTTTATGAA	Chr.4	16109017	16108997	-	No
2635327_adh	TTGACCACAAGAGGCGATCAA	Chr.4	16109205	16109185	-	No
2715019_adh	TTTGACCACAAGAGGCGATCA	Chr.4	16109206	16109186	-	No
2649223_adh	TTGCAGAAAAAATGGTCGGAA	Chr.4	16112032	16112012	-	No
2007130_adh	TCGCATTCTGAAAGGTTGGTT	Chr.4	16116395	16116415	+	Yes
2744156_adh	TTTTGTTCGGAAATTTGAAAA	Chr.4	16116476	16116456	-	No
2686382_adh	TTGTTGTATCTTGGATTCTAT	Chr.4	16116637	16116657	+	No
1759306_adh	TAAAGTTTTGAATCGTTGACA	Chr.4	16117196	16117176	-	Yes
2029693_adh	TCTAACTAGACACTCGGAAGA	Chr.4	16119073	16119053	-	No
2268086_adh	TGATATTCTAGTTGGAATTCT	Chr.4	16123625	16123645	+	Yes
2747741_adh	TTTTTGTATTAGTTCGCATTG	Chr.4	16125886	16125866	-	No
2686144_adh	TTGTTGGATTTGTTGAGTGCA	Chr.4	16126478	16126458	-	No
2741087_adh	TTTTGACGCTACTAATTTGTT	Chr.4	16126494	16126474	-	No
2071864_adh	TGAAACAGTGACTTGCTTTTC	Chr.4	16134465	16134445	-	No
2069605_adh	TGAAAAGTGACTCGTTTTGAA	Chr.4	16136550	16136570	+	No
2673693_adh	TTGTAACACGATTGAATGGAC	Chr.4	16137088	16137068	-	No
2712558_adh	TTTGAAACAGTAGATTTTTCA	Chr.4	16138715	16138695	-	No
1919557_adh	TATGTTGTAGTCAATTTTTCA	Chr.4	16140258	16140278	+	No
1926136_adh	TATTGCAACGAAGCCTTGACT	Chr.4	16144713	16144733	+	No
1806108_adh	TAATTTTTAAAGACAGTGCGC	Chr.4	16144887	16144867	-	No
2700616_adh	TTTATTTAACCGTCGTTCATT	Chr.4	16146978	16146998	+	No
2583439_adh	TTATTTAACCGTCGTTCATTA	Chr.4	16146979	16146999	+	No
1929030_adh	TATTTAACCGTCGTTCATTAT	Chr.4	16146980	16147000	+	No
2689899_adh	TTTAACCGTCGTTCATTATAT	Chr.4	16146982	16147002	+	No
1829500_adh	TACTACAACTATGGCTAAACT	Chr.4	16147548	16147528	-	No
2626246_adh	TTGAACCTGAGGCATTGGAAT	Chr.4	16147720	16147740	+	No
1825744_adh	TACGGACAACGGATAATGTTG	Chr.4	16148464	16148484	+	No
2551672_adh	TTAATTAAATGTCCGGGAATG	Chr.4	16150384	16150364	-	No
1888449_adh	TATAGGCATATGGATAGGTGT	Chr.4	16155677	16155657	-	No
2482348_adh	TGTAAGTAAAACTAGTATCGA	Chr.4	16156043	16156023	-	No
1887119_adh	TATACGAGATGGGGTTTCCAA	Chr.4	16156241	16156261	+	No

1891055_adh	TATATTTTCAGTCAAGTCGTG	Chr.4	16163543	16163563	+	No
1889898_adh	TATATATTTCTTCGAGAATTT	Chr.4	16163579	16163599	+	No
2688939_adh	TTTAAAATAAGAAAAAATAAT	Chr.4	16163962	16163982	+	No
2699365_adh	TTTATGTGTAGATGCATTGTT	Chr.4	16166601	16166621	+	No
2308455_adh	TGCTGAGGAACCTTGATGGAT	Chr.4	16168404	16168384	-	No
2011026_adh	TCGGAATGGAGCAGGAGTACA	Chr.4	16169026	16169006	-	No
2721367_adh	TTTGCAGTTTCTATCTATAAC	Chr.4	16171174	16171194	+	Yes
1941825_adh	TCAATGAATTTAATAGATTTG	Chr.4	16174725	16174705	-	Yes
1973912_adh	TCATTCTGTGCATTCTTTTTT	Chr.4	16177578	16177598	+	Yes
1989351_adh	TCCTACTAAAATTTATTAATT	Chr.4	16179100	16179080	-	Yes
2699467_adh	TTTATTAATGTGGAGCTCCGA	Chr.4	16179328	16179308	-	No
2561885_adh	TTAGAATTTTCGATGCAGGTG	Chr.4	16179481	16179461	-	No
2696990_adh	TTTAGTAGACACATCTCATCG	Chr.4	16179862	16179842	-	Yes
2734874_adh	TTTTAGTAGACACATCTCATC	Chr.4	16179863	16179843	-	Yes
2702594_adh	TTTCACGGTTCATTTTTCATA	Chr.4	16180670	16180690	+	No
2737469_adh	TTTTCAGCAGATTTCACTCTA	Chr.4	16186112	16186092	-	No
1925925_adh	TATTGATAAGTTTCGACGGAT	Chr.4	16186306	16186286	-	No
1764124_adh	TAACAATTTTATGAAATGTAT	Chr.4	16186369	16186349	-	No
1936702_adh	TCAACGGCAAGAATAGTCTAT	Chr.4	16187423	16187443	+	No
1806564_adh	TACAAAATTTTGAAGGATCGA	Chr.4	16187482	16187462	-	No
2729206_adh	TTTGTCTTATAAAATATTGTC	Chr.4	16188658	16188678	+	No
2286660_adh	TGCACTGAAAAACGGCAATTT	Chr.4	16190248	16190228	-	No
1917305_adh	TATGGTTTTAGGTTTCAATCA	Chr.4	16193618	16193638	+	No
1913183_adh	TATGCTCCGTACAGTTTTCTA	Chr.4	16193908	16193888	-	No
2549385_adh	TTAAGTTTCAGGCATTATTCG	Chr.4	16194768	16194748	-	Yes
2633826_adh	TTGAATTGAGCAAGAAACGTT	Chr.4	16199030	16199050	+	No
2025904_adh	TCGTGACGTCATTTTTCTACA	Chr.4	16203675	16203655	-	No
2311393_adh	TGCTTGTATTTCGTTATGGGG	Chr.4	16203754	16203734	-	No
1941067_adh	TCAATAGAACAAAAATTGAAA	Chr.4	16203909	16203889	-	Yes
2744702_adh	TTTTTAAAGTAGGAGTATATC	Chr.4	16203928	16203908	-	No
2677295_adh	TTGTATTGTTTGAAACGGTAA	Chr.4	16205415	16205395	-	No
2744702_adh	TTTTTAAAGTAGGAGTATATC	Chr.4	16206600	16206580	-	No
1754433_adh	TAAACAGTGGAATTAGGAATT	Chr.4	16208645	16208625	-	Yes
1885790_adh	TATAATCTCAAGTGCATCCTC	Chr.4	16211706	16211686	-	No
1921921_adh	TATTATAATCTCAAGTGCATC	Chr.4	16211709	16211689	-	No
1885790_adh	TATAATCTCAAGTGCATCCTC	Chr.4	16211781	16211761	-	No
1921921_adh	TATTATAATCTCAAGTGCATC	Chr.4	16211784	16211764	-	No
1921780_adh	TATTAGTTGAACAGTAATCTC	Chr.4	16213820	16213840	+	No
2584266_adh	TTATTTGTGGAATAAAATTTT	Chr.4	16217638	16217658	+	No
2722962_adh	TTTGGAAATAGAAGGTGCAAA	Chr.4	16218000	16218020	+	No
1926866_adh	TATTGGACCAAAAAGCTAAAA	Chr.4	16218962	16218942	-	No
2681123_adh	TTGTGATTCGTTAAAACTTTA	Chr.4	16219603	16219583	-	No
2730161_adh	TTTGTGTTTTGGATTTTTGCA	Chr.4	16220101	16220121	+	No
2682719_adh	TTGTGTTTTGGATTTTTGCAT	Chr.4	16220102	16220122	+	No
2718146_adh	TTTGATCGAAGGTTAAATATA	Chr.4	16226444	16226464	+	Yes
1917397_adh	TATGTAAATTTGTAGGAACAA	Chr.4	16227361	16227381	+	No
2688738_adh	TTTAAAAATTGTCGAGGTTTG	Chr.4	16228812	16228832	+	No
2538056_adh	TTAAAAATTGTCGAGGTTTGT	Chr.4	16228813	16228833	+	No
1807235_adh	TACAAATGCTGCGTTCATTGG	Chr.4	16236413	16236433	+	No
2541724_adh	TTAAAGTATGTTTGTCGAACT	Chr.4	16237038	16237018	-	No

1945459_adh	TCACAGTAAAAACTTCTCTGA	Chr.4	16239003	16238983	-	Yes
2127486_adh	TGAAGAATGTGCTTAAATTAT	Chr.4	16239618	16239638	+	No
2047360_adh	TCTGATGTTGGCAGAAGCACA	Chr.4	16241067	16241047	-	No
2724314_adh	TTTGGCAGCAGGTGTGAATTC	Chr.4	16241943	16241923	-	No
2646792_adh	TTGATTTAGTGCATACAATGG	Chr.4	16254966	16254946	-	Yes
1922464_adh	TATTATGATGAAACGTTTCAG	Chr.4	16255472	16255492	+	No
2526346_adh	TGTTCGACTGATATACGACAT	Chr.4	16255844	16255864	+	No
2669693_adh	TTGGTAGAAACACATTTAGAA	Chr.4	16256785	16256805	+	No
1975803_adh	TCCAAAGAAACCCATTCTTTT	Chr.4	16259657	16259677	+	No
1934040_adh	TCAAATACAAGTAGACGTAAT	Chr.4	16266552	16266532	-	No
1931519_adh	TATTTTTTGTGAACTGGGGTT	Chr.4	16268035	16268015	-	No
1745884_adh	TAAAAACCAGTCCGGATGGAA	Chr.4	16269347	16269367	+	No
2594170_adh	TTCATCATCGGAAAACGAGAA	Chr.4	16270364	16270344	-	Yes
1835025_adh	TACTTCTTATCTTTTAAAATC	Chr.4	16270944	16270924	-	No
1942926_adh	TCAATTCAGAATTTTTAGCAA	Chr.4	16273361	16273341	-	No
2269027_adh	TGATCAACTTTGTACGGTATT	Chr.4	16274305	16274285	-	No
1795254_adh	TAATAGTACATTTTTGAAACA	Chr.4	16275773	16275753	-	No
2748007_adh	TTTTTGTTTCTTTAAAACCAC	Chr.4	16275786	16275806	+	No
1830388_adh	TACTATACGATGTTCTCTTAA	Chr.4	16279104	16279124	+	Yes
2056806_adh	TCTTCGTATTGAGAACGTCTT	Chr.4	16279369	16279389	+	No
2601157_adh	TTCCTTAGACTGCGTGTTTCA	Chr.4	16281245	16281265	+	No
2741942_adh	TTTTGATTGGCATATTCTGGT	Chr.4	16285556	16285576	+	No
2741942_adh	TTTTGATTGGCATATTCTGGT	Chr.4	16286043	16286063	+	No
2741942_adh	TTTTGATTGGCATATTCTGGT	Chr.4	16286206	16286226	+	No
1834768_adh	TACTTCACCTTCAATTTACGG	Chr.4	16286594	16286574	-	No
2069041_adh	TGAAAAGGATAAGATTGGAGA	Chr.4	16287398	16287418	+	No
2561143_adh	TTAGAACCGCTGCGATTCAAC	Chr.4	16291948	16291968	+	No
2707873_adh	TTTCGGCAATACTACATATTC	Chr.4	16292356	16292336	-	No
2130605_adh	TGAAGCAGAGTGGCGTAGACC	Chr.4	16294988	16295008	+	No
2061710_adh	TCTTTGAAGTGTTGGGATACT	Chr.4	16295166	16295146	-	No
2053794_adh	TCTTAACGGATATTGTCACAA	Chr.4	16301552	16301572	+	No
1814679_adh	TACCAAATTTCGTAACGAGAC	Chr.4	16304556	16304576	+	No
2738235_adh	TTTTCGACTACAGAAATAAAT	Chr.4	16305581	16305601	+	No
1899592_adh	TATCTCGATCCTCGCGTAAAT	Chr.4	16309392	16309372	-	No
2144161_adh	TGAATGGACGGTATGGAATAA	Chr.4	16316830	16316810	-	No
2669743_adh	TTGGTAGAGCGCATTTTTTGT	Chr.4	16323284	16323304	+	No
2053794_adh	TCTTAACGGATATTGTCACAA	Chr.4	16330865	16330885	+	No
1754120_adh	TAAACAATGCATTTGTATTAA	Chr.4	16338672	16338692	+	No
1866952_adh	TAGGCACGTAAACAGACAAAG	Chr.4	16351785	16351765	-	No
2735565_adh	TTTTATCGAAAAGTTGTCTTA	Chr.4	16356890	16356910	+	No
1900973_adh	TATGAAAAGTAGAAATATGCT	Chr.4	16358869	16358849	-	No
2584088_adh	TTATTTGCATTAATTAAAATA	Chr.4	16359595	16359615	+	No
1979401_adh	TCCATAATTGGTAGAAAACGA	Chr.4	16367923	16367903	-	No
1836401_adh	TACTTTTCTCAGCGGTACAAC	Chr.4	16370413	16370393	-	No
2717780_adh	TTTGATCATAGAGAAGTTCTG	Chr.4	16371054	16371034	-	No
2634736_adh	TTGACAATCGTAAATTATTTT	Chr.4	16372157	16372177	+	Yes
2549882_adh	TTAATAGCAGAGATAGAGTAA	Chr.4	16374108	16374128	+	Yes
2698392_adh	TTTATCAAGGTATTGAAATGG	Chr.4	16374511	16374491	-	No
2744928_adh	TTTTTACGGTTACTAGAAACA	Chr.4	16374535	16374555	+	No
2734209_adh	TTTTACGGTTACTAGAAACAT	Chr.4	16374536	16374556	+	No

2697699_adh	TTTATAAAAATTATCGGAACT	Chr.4	16375543	16375563	+	No
2704649_adh	TTTCATTTAACTCGTATTGGT	Chr.4	16380592	16380612	+	No
2019063_adh	TCGGTGGCAAGGGAAGTTATA	Chr.4	16380780	16380760	-	No
1790844_adh	TAATAATTCGTCGTAAAACCA	Chr.4	16381854	16381874	+	Yes
2121600_adh	TGAACATTTTGAAATAGTGGA	Chr.4	16383013	16382993	-	No
1776279_adh	TAAGACTGTTACGAAATAATT	Chr.4	16384086	16384106	+	No
2667639_adh	TTGGGCTTAAAGGTATGTTAA	Chr.4	16387271	16387291	+	No
2559212_adh	TTACTGCGATGGATTTACAGA	Chr.4	16389819	16389839	+	Yes
2620548_adh	TTCTTACGACGAAACAGTAGA	Chr.4	16391300	16391280	-	Yes
2558897_adh	TTACTCTCTGAAACAGTAATT	Chr.4	16391332	16391352	+	No
2314009_adh	TGGAAAATTTATAGAATTTTA	Chr.4	16392048	16392068	+	No
2671670_adh	TTGGTTAGAATTTTTTAAAAA	Chr.4	16393676	16393656	-	No
2081456_adh	TGAAAGCCTTTTTTATTATAA	Chr.4	16393686	16393706	+	No
1845529_adh	TAGACTAAAGGGAATGGCGGA	Chr.4	16399972	16399992	+	No
2744909_adh	TTTTTACGAATTTCGGGCATT	Chr.4	16415177	16415157	-	No
2714215_adh	TTTGAATCGGAATTTGTCGTC	Chr.4	16415271	16415291	+	No
2037707_adh	TCTCAGTAGAGCGTATTTTCA	Chr.4	16419306	16419326	+	No
2412177_adh	TGGCGATTTTTGGTAGAAAAA	Chr.4	16425830	16425850	+	No
2630071_adh	TTGAATATCATGGTTAGTCTC	Chr.4	16434589	16434569	-	Yes
2748250_adh	TTTTTTACTGTTTCTCGGAAA	Chr.4	16436187	16436167	-	No
2538023_adh	TTAAAAATGCATTCCAATTTC	Chr.4	16436236	16436256	+	No
1919274_adh	TATGTTATCGATAAACAAATT	Chr.4	16438154	16438174	+	No
2629171_adh	TTGAAGTCCTAGCAATTTTAT	Chr.4	16438275	16438255	-	No
1812450_adh	TACAGGAATTGTTGAAATTGT	Chr.4	16439184	16439164	-	No
2488890_adh	TGTAGGGCTGTACAAATCGGC	Chr.4	16440577	16440597	+	No
2145219_adh	TGAATTACTGTAGTTTTCGCC	Chr.4	16442989	16443009	+	No
2586438_adh	TTCAAGAATTACGGATGCCGC	Chr.4	16443686	16443706	+	No
2694827_adh	TTTAGAACAAAAGAAGTGGAT	Chr.4	16444328	16444308	-	No
2538023_adh	TTAAAAATGCATTCCAATTTC	Chr.4	16445880	16445900	+	No
2531705_adh	TGTTTACGCTAGAAAAGTTCA	Chr.4	16446333	16446313	-	No
2703190_adh	TTTCAGGAGGACATTGGCAAT	Chr.4	16446531	16446551	+	No
2699426_adh	TTTATTAAAATATGTATGAAT	Chr.4	16446876	16446896	+	No
1968572_adh	TCATACATATTTTAATAAATT	Chr.4	16446894	16446874	-	No
2033973_adh	TCTATAGACATCATTTCAGAA	Chr.4	16447061	16447041	-	No
2526754_adh	TGTTCTATAGACATCATTTCA	Chr.4	16447064	16447044	-	No
2321171_adh	TGGAACTCTCCGAGATTTGAA	Chr.4	16452261	16452281	+	No
1929766_adh	TATTTCGAACGACATTTATTA	Chr.4	16452491	16452511	+	No
2689858_adh	TTTAACATTCATAATACTATA	Chr.4	16456720	16456740	+	No
2737779_adh	TTTTCATTATTATTTCCGGCT	Chr.4	16456914	16456894	-	Yes
2653500_adh	TTGCGTCATCAACGGCCACTT	Chr.4	16464208	16464228	+	No
1928419_adh	TATTGTTGACTTTCAATTAAA	Chr.4	16464648	16464668	+	No
2578748_adh	TTATGATCAAAATTCGAGTAG	Chr.4	16465212	16465192	-	Yes
2578748_adh	TTATGATCAAAATTCGAGTAG	Chr.4	16465447	16465467	+	Yes
1928419_adh	TATTGTTGACTTTCAATTAAA	Chr.4	16466011	16465991	-	Yes
2618242_adh	TTCTGACTAATAGTGGAAGAA	Chr.4	16467155	16467175	+	No
1977708_adh	TCCACTGAAACGAAGAAGAAG	Chr.4	16470930	16470910	-	No
1924697_adh	TATTCTAAGTGTTTACAGATC	Chr.4	16471499	16471519	+	Yes
2001467_adh	TCGAGCATTCTGTAGGCTATA	Chr.4	16472556	16472536	-	No
2595001_adh	TTCATTAGAGAGTAGGATCAC	Chr.4	16473557	16473577	+	No
2745874_adh	TTTTTCCGTCGTCTTGGACTT	Chr.4	16473690	16473670	-	Yes

1895327_adh	TATCATTTTCTCAAAGAATTT	Chr.4	16476639	16476659	+	Yes
1977708_adh	TCCACTGAAACGAAGAAGAAG	Chr.4	16481167	16481187	+	No
2703174_adh	TTTCAGGAAGGCAACTACGAG	Chr.4	16481653	16481673	+	No
2034525_adh	TCTATGGGATACAGCTGGACA	Chr.4	16486095	16486075	-	No
1746834_adh	TAAAAAGTGGAAATTCGGTTG	Chr.4	16493092	16493112	+	No
2619613_adh	TTCTGGTGGATCGTGTTTTCG	Chr.4	16495101	16495081	-	No
2619613_adh	TTCTGGTGGATCGTGTTTTCG	Chr.4	16495291	16495311	+	No
2352800_adh	TGGAATGGCAAAATGGATGGG	Chr.4	16501983	16501963	-	No
2089932_adh	TGAAATATTGTGGACGACGAG	Chr.4	16502176	16502196	+	No
2664866_adh	TTGGATTTTGATGGTAAATTG	Chr.4	16504348	16504368	+	No
2703174_adh	TTTCAGGAAGGCAACTACGAG	Chr.4	16506620	16506600	-	No
1977708_adh	TCCACTGAAACGAAGAAGAAG	Chr.4	16507147	16507127	-	No
1941011_adh	TCAATACGTTTTCACGCGTTT	Chr.4	16507292	16507312	+	No
2622726_adh	TTCTTGATTCGTTAGTTTTTT	Chr.4	16508476	16508496	+	No
2688730_adh	TTTAAAAATTAATCAAATTTC	Chr.4	16510452	16510472	+	Yes
2551169_adh	TTAATGCTAGTGGATGAGAAG	Chr.4	16522197	16522217	+	Yes
2306098_adh	TGCTAGTGGATGAGAAGCTTC	Chr.4	16522201	16522221	+	Yes
1878728_adh	TAGTGGATGAGAAGCTTCTTC	Chr.4	16522204	16522224	+	Yes
1953421_adh	TCACGGTTTAAATATTTGGGA	Chr.4	16522282	16522262	-	No
2544481_adh	TTAAATTGGATCGAGGTTTTA	Chr.4	16524502	16524522	+	No
2616864_adh	TTCTCTAAGAAAAAATTATTG	Chr.4	16526370	16526350	-	No
2744688_adh	TTTTTAAAGAATCTTTTGCGG	Chr.4	16526667	16526687	+	No
2647100_adh	TTGATTTTTCGGTATGCCAGA	Chr.4	16527534	16527554	+	No
2651863_adh	TTGCGAATTCGACAAGTAATA	Chr.4	16528245	16528225	-	No
2674259_adh	TTGTACATATAAAAGTTTTTT	Chr.4	16528317	16528297	-	No
1973824_adh	TCATTCGACTATTGATTTTGT	Chr.4	16529705	16529685	-	No
1848363_adh	TAGAGGATATACGGTAATTGA	Chr.4	16531281	16531301	+	No
2742527_adh	TTTTGCTGAATTAAAAAATAA	Chr.4	16537796	16537776	-	No
2567593_adh	TTAGGAAGGTTTTCAAGAGAA	Chr.4	16540095	16540115	+	No
2722878_adh	TTTGGAAAATGGGCAAAGCAG	Chr.4	16540639	16540619	-	No
2700382_adh	TTTATTGGATAGTCTCACGGT	Chr.4	16543782	16543802	+	No
2740948_adh	TTTTGAATTCTAGAAGCGTGG	Chr.4	16545061	16545081	+	No
2602379_adh	TTCGAATAGAACAACACGCTG	Chr.4	16546796	16546776	-	No
1974403_adh	TCATTGTACAATATTTTATTT	Chr.4	16549879	16549899	+	No
1918433_adh	TATGTCACTGTCTCTGGCAGC	Chr.4	16550823	16550803	-	No
2707239_adh	TTTCGCAAAACATCGGATTTT	Chr.4	16551223	16551243	+	Yes
2506242_adh	TGTGAGTATAGAAAGTTTGAA	Chr.4	16556673	16556653	-	Yes
2617164_adh	TTCTCTCTGCATATATCTCAT	Chr.4	16556962	16556982	+	Yes
2563894_adh	TTAGATCAGTGCTGTGCGGCT	Chr.4	16557149	16557169	+	Yes
1919992_adh	TATTAAATTGAAAATTGAATA	Chr.4	16557420	16557400	-	No
1890244_adh	TATATGAGGAACGGGGGACAA	Chr.4	16557529	16557509	-	No
2575996_adh	TTATATGAGGAACGGGGGACA	Chr.4	16557530	16557510	-	No
1930162_adh	TATTTGAAGACTGTTGAACAT	Chr.4	16559093	16559073	-	No
1925441_adh	TATTGAATTTTGAAATATTTC	Chr.4	16561738	16561758	+	No
2618331_adh	TTCTGAGGAGAATATTTATAA	Chr.4	16562825	16562805	-	No
2559648_adh	TTACTGTGTGTCCGTTAGGAA	Chr.4	16563236	16563216	-	Yes
2746779_adh	TTTTTGAGGCTTGAAAACTGA	Chr.4	16563971	16563951	-	No
1960096_adh	TCAGATATTAACTGATAATTC	Chr.4	16564351	16564331	-	No
1922931_adh	TATTATTATCGTCGAAATGTT	Chr.4	16566169	16566149	-	Yes
1926273_adh	TATTGCAGATTGAGGTGAATA	Chr.4	16567086	16567066	-	No

1797157_adh	TAATATTGCAGATTGAGGTGA	Chr.4	16567089	16567069	-	No
1923166_adh	TATTATTTCTTTGCTGGCATG	Chr.4	16568413	16568393	-	No
2587342_adh	TTCAAGTAGAAGTTGTGCTAA	Chr.4	16571786	16571806	+	No
2551024_adh	TTAATGACGTGCTCCTTATAT	Chr.4	16573405	16573425	+	No
1925405_adh	TATTGAATAGGCTGGCTTGAT	Chr.4	16577938	16577958	+	No
2160548_adh	TGACGAAATCGGAAGAAAATT	Chr.4	16581979	16581999	+	No
1935967_adh	TCAACAATGAACTAGCTGATT	Chr.4	16587469	16587489	+	No
2124051_adh	TGAACTAGCTGATTGAATGCC	Chr.4	16587476	16587496	+	No
2269065_adh	TGATCAAGTAGTCTTTTAAAT	Chr.4	16588119	16588139	+	No
2563747_adh	TTAGATATTCGGAATTAGAAG	Chr.4	16588908	16588928	+	No
2714574_adh	TTTGAATTTTTCTCTGCAGAC	Chr.4	16592718	16592698	-	No
1872763_adh	TAGTAAAACTTGGTATAAAAT	Chr.4	16593283	16593303	+	No
1923847_adh	TATTCATTCGGACAAAAATTT	Chr.4	16596062	16596042	-	No
2670903_adh	TTGGTGCAGAATAGCTGATCC	Chr.4	16597031	16597051	+	Yes
2594581_adh	TTCATGACTGACATTCATTTT	Chr.4	16598651	16598671	+	Yes
1819346_adh	TACCCTGTTTGTTCAACTTTA	Chr.4	16600370	16600350	-	No
2744726_adh	TTTTTAACAAGATGTTGAAAC	Chr.4	16606708	16606688	-	No
2062248_adh	TCTTTGTAGAGATTTAATCAA	Chr.4	16606971	16606951	-	No
1967963_adh	TCAGTTTTGTTTATTATTTCA	Chr.4	16607451	16607431	-	No
2732976_adh	TTTTAAAAATTTAATAGTTCA	Chr.4	16607975	16607995	+	No
2402127_adh	TGGCAAGTAAAGTAGTATGTA	Chr.4	16608724	16608704	-	No
2025042_adh	TCGTATGTCGTATGGAATTTG	Chr.4	16610275	16610295	+	Yes
2689329_adh	TTTAAAGGTGAAGTAGCGAAA	Chr.4	16610439	16610459	+	No
2541675_adh	TTAAAGGTGAAGTAGCGAAAT	Chr.4	16610440	16610460	+	No
2694795_adh	TTTAGAAAATGAAAAGGTCAC	Chr.4	16613510	16613530	+	No
1931127_adh	TATTTTGCAGGTATAATATAA	Chr.4	16613642	16613662	+	No
1835619_adh	TACTTGGGCATCTTTTCAGAA	Chr.4	16613898	16613918	+	No
2545912_adh	TTAACTAAGTAAAGGGCATTC	Chr.4	16614583	16614563	-	Yes
2580507_adh	TTATTAACTAAGTAAAGGGCA	Chr.4	16614586	16614566	-	Yes
1794798_adh	TAATAGACTGATCTGACTGTA	Chr.4	16616291	16616311	+	Yes
1925229_adh	TATTCTTTCGGTATTATTTTT	Chr.4	16617102	16617122	+	No
2582047_adh	TTATTCTTTTAAAGTTGTTCT	Chr.4	16617739	16617759	+	Yes
2689155_adh	TTTAAAGAAAACATTATGCCA	Chr.4	16619385	16619405	+	No
1935448_adh	TCAAATGTGGCATTTTCTGAG	Chr.4	16620058	16620038	-	No
2739168_adh	TTTTCTATCCCGCATTACTTA	Chr.4	16620205	16620185	-	Yes
1790704_adh	TAATAATAAACAAATGTTCGG	Chr.4	16621905	16621925	+	Yes
2051676_adh	TCTGTAAGCTTGGTTGATTTT	Chr.4	16622345	16622365	+	No
2657703_adh	TTGGAATGTAAATCGCGCCTA	Chr.4	16622993	16623013	+	Yes
2287326_adh	TGCAGACTTGAGGCGCAAATA	Chr.4	16623277	16623297	+	No
2596989_adh	TTCCAGGGAGACATATTTGAT	Chr.4	16629572	16629552	-	No
2580939_adh	TTATTAGATAGAGACTGCATG	Chr.4	16629744	16629764	+	No
1921487_adh	TATTAGATAGAGACTGCATGA	Chr.4	16629745	16629765	+	No
2563549_adh	TTAGATAGAGACTGCATGATT	Chr.4	16629747	16629767	+	No
1847392_adh	TAGAGACTGCATGATTTTTGC	Chr.4	16629752	16629772	+	No
2740833_adh	TTTTGAAGATTTTTGGACGAT	Chr.4	16632288	16632268	-	No
2632851_adh	TTGAATGTAACTTGAAACGGT	Chr.4	16633944	16633924	-	No
2714341_adh	TTTGAATGTAACTTGAAACGG	Chr.4	16633945	16633925	-	No
2709423_adh	TTTCTAAATTTGATAGTCATA	Chr.4	16635375	16635355	-	No
1797176_adh	TAATATTGGTAGAATGAAAAT	Chr.4	16635488	16635508	+	Yes
2554390_adh	TTACAGTAACACCGGTCGAAA	Chr.4	16637596	16637616	+	No

1845094_adh	TAGACATCTCTAACAGCAAAA	Chr.4	16640211	16640191	-	Yes
2369081_adh	TGGAATTATCATGTTGGAATT	Chr.4	16656215	16656195	-	No
1790138_adh	TAATAAATATTCTTTCGGCTA	Chr.4	16660021	16660001	-	No
2699422_adh	TTTATTAAAACGTACGGAAAA	Chr.4	16660961	16660941	-	No
2745447_adh	TTTTTATTAAAACGTACGGAA	Chr.4	16660963	16660943	-	No
2689575_adh	TTTAAATTAATACAGAATCTT	Chr.4	16662272	16662252	-	No
2527415_adh	TGTTGAAAATGAAGGTAGTAT	Chr.4	16662863	16662843	-	Yes
1834077_adh	TACTGTTCTAGCATTTTGAGG	Chr.4	16663159	16663139	-	No
2560195_adh	TTACTTGACTGTCGAAGACCA	Chr.4	16664683	16664703	+	Yes
1927237_adh	TATTGGTAGAAACATTTTCAC	Chr.4	16670340	16670320	-	Yes
2714300_adh	TTTGAATGCGTTCAACTCACA	Chr.4	16672030	16672010	-	No
2311730_adh	TGCTTTCGTGATTTTGTCGTT	Chr.4	16672203	16672183	-	Yes
2584491_adh	TTATTTTATCGAAGAATTTTT	Chr.4	16673020	16673040	+	No
1937328_adh	TCAACTCGAAAAATATTCTTG	Chr.4	16673114	16673134	+	Yes
2572118_adh	TTAGTTAACTTGTAATTCTAG	Chr.4	16674763	16674783	+	No
2649917_adh	TTGCATAACGGCTTGCATTTT	Chr.4	16676325	16676305	-	Yes
2659088_adh	TTGGACTTGGTGATAATTGTA	Chr.4	16677163	16677143	-	No
2550605_adh	TTAATCGTGGCATAACATACT	Chr.4	16678061	16678081	+	No
1834793_adh	TACTTCATCTCGTTCCAAAAT	Chr.4	16678387	16678367	-	No
2305680_adh	TGCTACATTAATAAACTCGGT	Chr.4	16682433	16682453	+	No
2573679_adh	TTATAAACTGTAACAGTGTTT	Chr.4	16683547	16683527	-	No
2060800_adh	TCTTTCATTAGTATTCCGTCG	Chr.4	16685203	16685223	+	No
2001169_adh	TCGAGAATACAACGAAAACAT	Chr.4	16685406	16685426	+	No
2721345_adh	TTTGCAGGAGTGCTTTGATTT	Chr.4	16685728	16685708	-	No
2027377_adh	TCGTGTAATGAAGTTTGCTTA	Chr.4	16687952	16687972	+	Yes
2174259_adh	TGACTTGTTAAAACAGAAGAT	Chr.4	16690845	16690825	-	No
1795778_adh	TAATATATTTTACTTTACAAT	Chr.4	16691937	16691957	+	Yes
1827241_adh	TACGGTATAACAAATTCAACT	Chr.4	16699178	16699198	+	No
2687187_adh	TTGTTTCGACTGTCATCGTTA	Chr.4	16701997	16702017	+	No
2706971_adh	TTTCGACTGTCATCGTTAACC	Chr.4	16702000	16702020	+	No
2034893_adh	TCTATTCATGAGCTCATTAAT	Chr.4	16702236	16702216	-	No
1920097_adh	TATTAACTTAAGTAGATAAAT	Chr.4	16702722	16702742	+	No
2546501_adh	TTAACTTAAGTAGATAAATGA	Chr.4	16702724	16702744	+	No
2538902_adh	TTAAAAGGTATCCAACGAAAT	Chr.4	16703090	16703110	+	No
2052282_adh	TCTGTCATCATGCAACTCAAA	Chr.4	16703437	16703457	+	No
2290556_adh	TGCATGATGACAGAAGAAACA	Chr.4	16703450	16703430	-	No
2060295_adh	TCTTGTTTAGCGAGACGAATA	Chr.4	16704174	16704154	-	No
2645727_adh	TTGATGTTTGAGTAGAATGAA	Chr.4	16705087	16705107	+	No
1831451_adh	TACTCTATAGAATTTACAATA	Chr.4	16705992	16705972	-	Yes
1919925_adh	TATTAAAGAACGAACAAACGG	Chr.4	16706524	16706544	+	No
1923379_adh	TATTCAAGTAGATAATTTCAT	Chr.4	16707657	16707677	+	Yes
2701945_adh	TTTCAATTAGGCGCTGTAACA	Chr.4	16708098	16708118	+	No
2588779_adh	TTCAATTAGGCGCTGTAACAG	Chr.4	16708099	16708119	+	No
2740952_adh	TTTTGAATTGGATACTCATTA	Chr.4	16708605	16708625	+	No
2566220_adh	TTAGCAAAAGTTCACTTATTT	Chr.4	16709810	16709790	-	No
2695877_adh	TTTAGCAAAAGTTCACTTATT	Chr.4	16709811	16709791	-	No
2740952_adh	TTTTGAATTGGATACTCATTA	Chr.4	16716518	16716538	+	No
2717126_adh	TTTGAGTTATTGCAGGTGTGT	Chr.4	16718005	16718025	+	No
1835927_adh	TACTTTAATGAAATCATATTA	Chr.4	16718744	16718724	-	No
2050079_adh	TCTGGCCAACTTTGACTATAA	Chr.4	16719993	16719973	-	Yes

2701160_adh	TTTCAAATAACAGAGACTGTG	Chr.4	16720877	16720857	-	No
1866699_adh	TAGGATTTCTGCAGTACACGC	Chr.4	16722311	16722291	-	No
2254023_adh	TGAGTGGACGAGAAAAAGGAG	Chr.4	16722526	16722546	+	No
1877406_adh	TAGTGAATCCAATATTCGGTA	Chr.4	16724245	16724265	+	No
2697909_adh	TTTATACAGTTTTTGCTGTTT	Chr.4	16724756	16724776	+	No
1923485_adh	TATTCAGAAATTTGGTAAAAT	Chr.4	16726252	16726232	-	No
1974030_adh	TCATTGATATTTTGAACCTGA	Chr.4	16726445	16726465	+	No
1918257_adh	TATGTATTTAAGTATAATTTT	Chr.4	16727106	16727086	-	No
1990838_adh	TCCTCTGACGTTGTACCATTT	Chr.4	16727385	16727405	+	No
2624236_adh	TTGAAAAAAGATGATTTGCAG	Chr.4	16727560	16727540	-	No
2586096_adh	TTCAACGGGATGGTTAAACAG	Chr.4	16728905	16728925	+	No
2554107_adh	TTACAGAAAGCTATATTTTGG	Chr.4	16739890	16739870	-	No
2132079_adh	TGAAGCTGGAAGGATTTATAT	Chr.4	16745150	16745170	+	No
2739905_adh	TTTTCTGTTTTCTGGCATTGT	Chr.4	16748315	16748295	-	Yes
2567276_adh	TTAGCTCTTTTACTACGGCCG	Chr.4	16751732	16751752	+	No
1811747_adh	TACAGAAGTCGACGAAACAAA	Chr.4	16765567	16765587	+	No
1810690_adh	TACACAGCGACGATTTTTGAT	Chr.4	16766486	16766506	+	No
1810690_adh	TACACAGCGACGATTTTTGAT	Chr.4	16769816	16769796	-	No
2119385_adh	TGAAATTTTGGAGCATAGAAA	Chr.4	16771560	16771580	+	No
2474393_adh	TGGTTAGTGTGCATTTTATTT	Chr.4	16771721	16771741	+	No
2601067_adh	TTCCTGTGCTTGCTGAAAATG	Chr.4	16772617	16772637	+	No
1992248_adh	TCCTGTGCTTGCTGAAAATGT	Chr.4	16772618	16772638	+	No
2649765_adh	TTGCAGTACTTTCTTATAGTT	Chr.4	16773832	16773812	-	Yes
1882860_adh	TAGTTTCAAATATTAGGATTG	Chr.4	16773993	16774013	+	No
2088349_adh	TGAAATAGTGGCAATTGATAC	Chr.4	16774312	16774292	-	No
2701667_adh	TTTCAATAGAATACAACATTT	Chr.4	16774938	16774918	-	No
1766759_adh	TAACCATAAGTTCCGTATCAA	Chr.4	16780004	16780024	+	No
1791024_adh	TAATACAGTTTCAATCGCATT	Chr.4	16781041	16781021	-	No
2668348_adh	TTGGGTCCTGACACGAAAAAG	Chr.4	16782164	16782184	+	No
2706011_adh	TTTCCTATAACCTAATTTCCT	Chr.4	16784857	16784877	+	No
2596044_adh	TTCCACACGTAGAAAAAGAAG	Chr.4	16788322	16788342	+	No
1930090_adh	TATTTCTTCCTCTGATTCGTC	Chr.4	16790677	16790697	+	No
2326405_adh	TGGAAGTACTGCAAAGAAGTA	Chr.4	16790797	16790817	+	No
2700172_adh	TTTATTGCTTGTATTATACTT	Chr.4	16791585	16791605	+	No
2649468_adh	TTGCAGAGTTCAAGTGAAAAA	Chr.4	16792401	16792381	-	No
2618324_adh	TTCTGAGCATTGCAGTCGTCA	Chr.4	16793118	16793138	+	No
2628704_adh	TTGAAGCTCGGCATCAAGTAG	Chr.4	16793423	16793403	-	Yes
2538698_adh	TTAAAAGAATTTAAAGCCATA	Chr.4	16795690	16795670	-	No
1995202_adh	TCGAAAGTTAGGCTAATTATA	Chr.4	16796283	16796263	-	No
1781284_adh	TAAGCAAATTCGCAATATTAT	Chr.4	16797228	16797248	+	No
2541623_adh	TTAAAGGCTGGCTTACTGTTA	Chr.4	16799169	16799149	-	No
2518474_adh	TGTGTAAGAAAGACATATCAA	Chr.4	16800648	16800628	-	No
2699154_adh	TTTATGGAAAGTGGACAAAAC	Chr.4	16802402	16802382	-	No
1797194_adh	TAATATTGTAGATCTACGGCA	Chr.4	16802741	16802761	+	No
1927584_adh	TATTGTAGATCTACGGCAGGA	Chr.4	16802744	16802764	+	No
2648399_adh	TTGCAACTGAAGAATCAAAAT	Chr.4	16805986	16805966	-	Yes
2685719_adh	TTGTTGCAACTGAAGAATCAA	Chr.4	16805989	16805969	-	Yes
2120081_adh	TGAACACTGACTGAAAATGGT	Chr.4	16806841	16806861	+	No
2728640_adh	TTTGTCAAAATATGCAGAACA	Chr.4	16807323	16807303	-	No
2549590_adh	TTAATAATTTGAAAAAATGTT	Chr.4	16808344	16808324	-	No

2549590_adh	TTAATAATTTGAAAAAATGTT	Chr.4	16809284	16809304	+	No
2738864_adh	TTTTCGTTCTGTCGAATATTT	Chr.4	16811452	16811432	-	No
2699896_adh	TTTATTCGTTATGTATTTTTT	Chr.4	16811976	16811956	-	Yes
2736574_adh	TTTTATTCGTTATGTATTTTT	Chr.4	16811977	16811957	-	Yes
1919414_adh	TATGTTGACGTAGAGCGAATT	Chr.4	16812360	16812340	-	Yes
1962316_adh	TCAGCATAAAAATTTTAAGCC	Chr.4	16812770	16812750	-	Yes
2560961_adh	TTAGAAATTGAAGAATTGGTA	Chr.4	16814265	16814245	-	Yes
1921983_adh	TATTATAGAAAAATACGATCG	Chr.4	16815642	16815622	-	Yes
2659600_adh	TTGGAGATATATAGTGGTTTG	Chr.4	16820064	16820044	-	No
2583065_adh	TTATTGGAGATATATAGTGGT	Chr.4	16820067	16820047	-	No
1798872_adh	TAATCTGAATGTCGTGATGAT	Chr.4	16820320	16820340	+	Yes
1891245_adh	TATCAAAGGCTGTAAATAAAT	Chr.4	16821272	16821292	+	No
2657627_adh	TTGGAATGCATGTAGTAGGCC	Chr.4	16821392	16821412	+	No
2688922_adh	TTTAAAAGTGTCTGGAAATTA	Chr.4	16822323	16822343	+	No
2538922_adh	TTAAAAGTGTCTGGAAATTAT	Chr.4	16822324	16822344	+	No
2144892_adh	TGAATGTGGCAATTAAAGTTT	Chr.4	16822570	16822550	-	No
1973987_adh	TCATTGAATGTGGCAATTAAA	Chr.4	16822574	16822554	-	No
2699627_adh	TTTATTATAACCTAACTGTCC	Chr.4	16832079	16832059	-	No
1909800_adh	TATGAGTTTTGAGTCTGGGTT	Chr.4	16833878	16833858	-	No
1927976_adh	TATTGTGAGAAGGGTGCAACA	Chr.4	16835691	16835711	+	No
2747756_adh	TTTTTGTCCAGGTCTCGACAC	Chr.4	16844990	16845010	+	No
2458477_adh	TGGGTGACTTGGAGTGCGGCT	Chr.4	16845294	16845274	-	No
2608432_adh	TTCGGCAATTTTCGGGCAGAA	Chr.4	16845681	16845701	+	No
2744839_adh	TTTTTAATGTTTTCGTTGTAT	Chr.4	16849161	16849141	-	No
2747096_adh	TTTTTGCGGGAAATCTTTGGT	Chr.4	16863677	16863697	+	No
1920675_adh	TATTACATACATTAAATTCAG	Chr.4	16870343	16870363	+	Yes
2561153_adh	TTAGAACCTCGGCTAGCTAGT	Chr.4	16872364	16872384	+	No
1890943_adh	TATATTGTTCTGGGATCTTAT	Chr.4	16873086	16873066	-	Yes
2571168_adh	TTAGTCGAGAACGGAACTTCA	Chr.4	16873211	16873191	-	No
2699594_adh	TTTATTAGCTTGTACGATTTT	Chr.4	16874435	16874415	-	No
2674532_adh	TTGTACTTAAAACGTCAGCGA	Chr.4	16875012	16875032	+	No
1937996_adh	TCAACTTGATGGTAAGTAATC	Chr.4	16879317	16879297	-	No
2595511_adh	TTCATTTGCCAAGTATTTGGA	Chr.4	16885229	16885209	-	No
2569954_adh	TTAGTAATATTCGGTGGTTAT	Chr.4	16887528	16887508	-	Yes
2733956_adh	TTTTACACAGTTTTGAAGCGC	Chr.4	16889188	16889168	-	No
1923217_adh	TATTATTTGTGTTCATGAAGG	Chr.4	16889575	16889595	+	Yes
2646803_adh	TTGATTTATGACATGTTGGAG	Chr.4	16890068	16890088	+	No
2578421_adh	TTATGACATGTTGGAGTTTTT	Chr.4	16890073	16890093	+	No
1925273_adh	TATTGAAACTTTTTGATTGTA	Chr.4	16892087	16892067	-	No
2733735_adh	TTTTAATGGGAATTTTGCATG	Chr.4	16894934	16894914	-	No
1941447_adh	TCAATCCTCTGATCGTGAACG	Chr.4	16897126	16897106	-	No
2625889_adh	TTGAACAATGGACTGAAGACT	Chr.4	16898881	16898901	+	No
1917364_adh	TATGTAAATAAGTTGGATCGC	Chr.4	16900155	16900175	+	No
1745768_adh	TAAAAACAATACATTCATTTA	Chr.4	16901531	16901551	+	No
2162608_adh	TGACGTTTTCAGAATTGAGTA	Chr.4	16907941	16907921	-	Yes
2744844_adh	TTTTTAATTCACGATCGTTCA	Chr.4	16911712	16911732	+	No
2733808_adh	TTTTAATTCACGATCGTTCAA	Chr.4	16911713	16911733	+	No
2551909_adh	TTAATTCACGATCGTTCAAAT	Chr.4	16911715	16911735	+	No
2600598_adh	TTCCTCCTGTCAGACTTCGGT	Chr.4	16915265	16915285	+	No
1990134_adh	TCCTCCTGTCAGACTTCGGTT	Chr.4	16915266	16915286	+	No

2113976_adh	TGAAATTACTGTAGTGATGAT	Chr.4	16916543	16916523	-	No
2055669_adh	TCTTCATAAATCAACGACATG	Chr.4	16917982	16917962	-	No
2055669_adh	TCTTCATAAATCAACGACATG	Chr.4	16918246	16918266	+	No
1886710_adh	TATACAAGCTGAACAAAGACG	Chr.4	16918947	16918927	-	No
2566357_adh	TTAGCAGAAACTGTTTTATTT	Chr.4	16919648	16919668	+	No
2559247_adh	TTACTGCTTGTAACATATAAT	Chr.4	16924322	16924342	+	Yes
2697484_adh	TTTAGTTCAAAGATCTGGTTT	Chr.4	16930376	16930356	-	No
2001209_adh	TCGAGACGATGAAGACGACCA	Chr.4	16930813	16930833	+	No
2066326_adh	TGAAAAATATAAAATCCTGAA	Chr.4	16940174	16940154	-	No
2748514_adh	TTTTTTCACGGAGATTGTCGA	Chr.4	16958181	16958161	-	No
1762500_adh	TAAATTGCAGTTCTACGTGTT	Chr.4	16965421	16965401	-	No
1917116_adh	TATGGTTGCATACGCCGTAAA	Chr.4	16968045	16968025	-	No
2712511_adh	TTTGAAAATGTAATTGAATCT	Chr.4	16973240	16973260	+	No
2712511_adh	TTTGAAAATGTAATTGAATCT	Chr.4	16974235	16974255	+	No
1839309_adh	TAGAAGATTTTGAAAGCACAT	Chr.4	16975112	16975132	+	Yes
1929761_adh	TATTTCCTTTTTTGAATTTTG	Chr.4	16978230	16978210	-	Yes
1923482_adh	TATTCACTTGCAGAGAAATCA	Chr.4	16984631	16984611	-	No
2043696_adh	TCTGAAACGGTAGAAAATTTA	Chr.4	16984665	16984685	+	No
2734031_adh	TTTTACATTCACTTAAAATAA	Chr.4	16984943	16984923	-	No
1829105_adh	TACGTTGTTTGTACAAATTAT	Chr.4	16986257	16986237	-	No
2558172_adh	TTACGTTGTTTGTACAAATTA	Chr.4	16986258	16986238	-	No
1921507_adh	TATTAGATCATGTAGAGAAAA	Chr.4	16987486	16987506	+	Yes
2563993_adh	TTAGATCATGTAGAGAAAATT	Chr.4	16987488	16987508	+	Yes
2043696_adh	TCTGAAACGGTAGAAAATTTA	Chr.4	16988247	16988267	+	No
1943408_adh	TCAATTTTAGCATTAATGACA	Chr.4	16988464	16988444	-	No
2500692_adh	TGTGAAACGGTAGAAAATTCA	Chr.4	16990723	16990743	+	No
1805077_adh	TAATTTCTGAAGACCTTTTTT	Chr.4	16991291	16991311	+	No
2128067_adh	TGAAGACTACTCCTGAAAATG	Chr.4	16992265	16992285	+	Yes
2745162_adh	TTTTTAGTTCAATGAAATTTA	Chr.4	16992458	16992478	+	No
1925245_adh	TATTCTTTTAGAAGCAGAATT	Chr.4	16993461	16993441	-	No
2500692_adh	TGTGAAACGGTAGAAAATTCA	Chr.4	16993496	16993516	+	No
1819771_adh	TACCGGTACAGAGAGTGTAGT	Chr.4	17011608	17011588	-	No
2746092_adh	TTTTTCGGCATACAACATCAA	Chr.4	17012182	17012162	-	No
2562222_adh	TTAGACGTACTGAAGAAAAAC	Chr.4	17017888	17017868	-	No
1921414_adh	TATTAGACGTACTGAAGAAAA	Chr.4	17017890	17017870	-	No
1798939_adh	TAATCTGTGTGTCATATTGCC	Chr.4	17018708	17018728	+	Yes
2592910_adh	TTCAGTCGTCTAGGTTTCTAC	Chr.4	17018977	17018997	+	Yes
2721971_adh	TTTGCGCAAGATCGAAAATAA	Chr.4	17025552	17025572	+	No
1883882_adh	TATAAAACTCTAATTGCGGAA	Chr.4	17027588	17027608	+	No
2559722_adh	TTACTTAACAATTGGTTTCTC	Chr.4	17027935	17027915	-	Yes
2641184_adh	TTGAGGACGCGCAGGGAAGCA	Chr.4	17029526	17029506	-	No
1760822_adh	TAAATCGGTATTAACAAACAA	Chr.4	17031584	17031564	-	No
2668929_adh	TTGGTAAATCGGTATTAACAA	Chr.4	17031588	17031568	-	No
2721971_adh	TTTGCGCAAGATCGAAAATAA	Chr.4	17032106	17032126	+	No
2712555_adh	TTTGAAACAGCCGTAACGCAG	Chr.4	17037054	17037034	-	Yes
2631634_adh	TTGAATGCAGATCAAAATAAC	Chr.4	17037853	17037873	+	No
2136996_adh	TGAATAATATAAAATCCTCAA	Chr.4	17041561	17041541	-	No
1940663_adh	TCAAGTTCGATAGCACGTTGT	Chr.4	17041829	17041849	+	Yes
2546310_adh	TTAACTGAGTCTGAATGTTAA	Chr.4	17042732	17042712	-	Yes
1802291_adh	TAATTAATAGCATGAAAGTTT	Chr.4	17042778	17042798	+	No

1996569_adh	TCGAAGGATACATGACCAAAA	Chr.4	17047548	17047528	-	No
1849706_adh	TAGATACATAATAAATACTAA	Chr.4	17052124	17052104	-	No
2305933_adh	TGCTAGATACATAATAAATAC	Chr.4	17052127	17052107	-	No
1797246_adh	TAATATTGTTTGTGGAAGCGT	Chr.4	17052295	17052275	-	No
2549234_adh	TTAAGTGGTTCACGAATTTTT	Chr.4	17058361	17058341	-	No
2644842_adh	TTGATGAGTGGAAGTTTTAAA	Chr.4	17063082	17063062	-	Yes
2644842_adh	TTGATGAGTGGAAGTTTTAAA	Chr.4	17063213	17063193	-	No
2050356_adh	TCTGGGCAAAGTTATGACGGA	Chr.4	17064579	17064599	+	No
2644842_adh	TTGATGAGTGGAAGTTTTAAA	Chr.4	17065996	17066016	+	Yes
2487405_adh	TGTAGAAAGAAGATTCAAAAC	Chr.4	17067311	17067291	-	No
2300031_adh	TGCGCATAGAATTTTTTAATT	Chr.4	17087383	17087363	-	No
2716815_adh	TTTGAGCTCTTCATTTTTCGT	Chr.4	17087687	17087707	+	No
2412748_adh	TGGCGGCAAAAATTAGACATT	Chr.4	17089713	17089693	-	No
2738634_adh	TTTTCGGCTTATTTTCGTTAT	Chr.4	17090226	17090246	+	No
1750044_adh	TAAAAGTACGGGAATGTCTAG	Chr.4	17095287	17095267	-	No
2130280_adh	TGAAGATTGGAATTCGATATA	Chr.4	17115820	17115800	-	No
2578330_adh	TTATGAAGGAAGAGGACGCCG	Chr.4	17118072	17118052	-	No
2749543_adh	TTTTTTGGCTAAAATTCGTGA	Chr.4	17119358	17119338	-	No
2083715_adh	TGAAAGTAGATGAATGAGACA	Chr.4	17126704	17126724	+	No
2748723_adh	TTTTTTCCGATGGGTGGCGTG	Chr.4	17136741	17136761	+	No
1943078_adh	TCAATTGATCTCGGAACGATG	Chr.4	17143221	17143241	+	No
1827620_adh	TACGTAAATTATTATAACCCT	Chr.4	17143391	17143371	-	Yes
2702870_adh	TTTCAGAATCTGAACTTCCAA	Chr.4	17147716	17147736	+	No
2588139_adh	TTCAATGCATTTTGCGACATT	Chr.4	17151913	17151893	-	Yes
2749865_adh	TTTTTTGTTTGATCACGTGAG	Chr.4	17153527	17153547	+	No
2705748_adh	TTTCCGCAATCAAATACGGTT	Chr.4	17153951	17153971	+	Yes
1827600_adh	TACGGTTTTGAAATTGGCTAC	Chr.4	17153965	17153985	+	Yes
2679410_adh	TTGTGAAGTTGGGATAGGTAT	Chr.4	17156582	17156602	+	No
1927964_adh	TATTGTGAAGTTGGAATTTAA	Chr.4	17157207	17157227	+	No
2741373_adh	TTTTGAGACAATACTTTTGCT	Chr.4	17159528	17159548	+	No
2388627_adh	TGGATAGGAGAAAAACAGCAA	Chr.4	17159564	17159544	-	No
2660926_adh	TTGGATAGGAGAAAAACAGCA	Chr.4	17159565	17159545	-	No
2624860_adh	TTGAAACTGCAGGAAATTCGA	Chr.4	17161595	17161575	-	No
2272886_adh	TGATGTAAAATGGTTGGAAGA	Chr.4	17162958	17162978	+	Yes
1824642_adh	TACGCACACCAGATCGTCGTT	Chr.4	17166361	17166381	+	No
2596631_adh	TTCCAGAAACTATTGAAGCAG	Chr.4	17172348	17172328	-	Yes
2159239_adh	TGACATTTGGAGCAGTGGAAC	Chr.4	17174429	17174449	+	No
2269899_adh	TGATCGGAGAGTGCGTTTAGT	Chr.4	17181423	17181403	-	No
2644338_adh	TTGATCGGAGAGTGCGTTTAG	Chr.4	17181424	17181404	-	No
1813556_adh	TACATCTTTCAAATTTAAAAT	Chr.4	17184075	17184095	+	No
1749479_adh	TAAAAGCAGAAAAGGAAGCGT	Chr.4	17184202	17184182	-	No
2488097_adh	TGTAGATTTCGCGGCGAGACC	Chr.4	17188473	17188453	-	No
2712128_adh	TTTCTTTGTAGTTTTCTTAGA	Chr.4	17189824	17189804	-	Yes
2688944_adh	TTTAAAATATTCGGTTTCAAC	Chr.4	17193033	17193013	-	No
1836629_adh	TAGAAAAATAGAATTACATCA	Chr.4	17197399	17197379	-	Yes
1798397_adh	TAATCGACGAATATTTTTACT	Chr.4	17200198	17200178	-	Yes
2564317_adh	TTAGATCGGTATATATATAGG	Chr.4	17202007	17201987	-	Yes
1962150_adh	TCAGCAATGAAGTTATTGCAT	Chr.4	17202562	17202542	-	Yes
2641809_adh	TTGAGTAATTAAAAGACAGAA	Chr.4	17203445	17203465	+	Yes
1979953_adh	TCCATTACCGTTGAATGCATT	Chr.4	17205962	17205942	-	No

2554442_adh	TTACAGTTAGAGAAGCATTAA	Chr.4	17207567	17207547	-	Yes
1766716_adh	TAACCAGTGAAGCAAATTTCC	Chr.4	17208722	17208742	+	No
2676627_adh	TTGTATGCGGTTGATTTTGAG	Chr.4	17213529	17213509	-	No
2548489_adh	TTAAGGCTATTATTTCACTGA	Chr.4	17213684	17213704	+	Yes
2159160_adh	TGACATTGTTGGCATTTTACG	Chr.4	17221729	17221709	-	No
2701353_adh	TTTCAACGTACATAACTTCCT	Chr.4	17223599	17223619	+	Yes
2549553_adh	TTAATAATCCGAAGCAATTGA	Chr.4	17233386	17233366	-	No
2021827_adh	TCGTACACTTTAAGAAGTTTT	Chr.4	17236088	17236108	+	No
1924417_adh	TATTCGGCTCAACTTTTTAAC	Chr.4	17237412	17237432	+	Yes
2583295_adh	TTATTGTGACTATATTTACAA	Chr.4	17238808	17238828	+	Yes
2665147_adh	TTGGCAATTGTGGCAAATGTG	Chr.4	17239816	17239836	+	No
1755465_adh	TAAACTCTTCTAAAAAAGATA	Chr.4	17260771	17260751	-	Yes
1798873_adh	TAATCTGAATTTTCCCAGGAG	Chr.4	17267184	17267204	+	No
2003809_adh	TCGATCTGATCAGAAGTTTGC	Chr.4	17274500	17274480	-	No
2469280_adh	TGGTCATAAACAAAGACATCG	Chr.4	17277904	17277884	-	No
2706537_adh	TTTCGAAATCAAAATGGACTG	Chr.4	17278304	17278284	-	No
2035377_adh	TCTCAACACTTTTCTGTAGGC	Chr.4	17278601	17278621	+	No
2744808_adh	TTTTTAATCATTGTTCTCGAC	Chr.4	17278832	17278852	+	No
2250637_adh	TGAGTACAAAGGAGAGAACAA	Chr.4	17279287	17279307	+	No
2288344_adh	TGCAGCTATCGCTTACGGACT	Chr.4	17279506	17279526	+	No
2620741_adh	TTCTTAGTCGCCTGTACAATT	Chr.4	17280104	17280124	+	No
1999911_adh	TCGACCTCCTCGATCGTTGGT	Chr.4	17281136	17281116	-	No
2749397_adh	TTTTTTGATCTCGGTTTTTAT	Chr.4	17281153	17281173	+	No
2032809_adh	TCTAGGCGAATAACGGTTTGG	Chr.4	17281259	17281239	-	No
2013692_adh	TCGGATATTTGTGTGACGTCT	Chr.4	17281355	17281375	+	No
2634674_adh	TTGACAAAGAAAGAAATGGAT	Chr.4	17281394	17281414	+	No
2609151_adh	TTCGGGATTGTTGTTCAAGGC	Chr.4	17308053	17308073	+	No
2016777_adh	TCGGGATTGTTGTTCAAGGCG	Chr.4	17308054	17308074	+	No
2234739_adh	TGAGCTCCGCATACGCTAAAT	Chr.4	17309165	17309185	+	No
2734615_adh	TTTTAGATTGCGTTTTAAAAA	Chr.4	17311310	17311330	+	No
2046319_adh	TCTGAGCGGTTGACATTCATA	Chr.4	17316114	17316094	-	No
2029161_adh	TCGTTTTTTACTCTAAAAACT	Chr.4	17323870	17323890	+	No
2749032_adh	TTTTTTCTCTTGATTGTCTCA	Chr.4	17331352	17331372	+	No
2463615_adh	TGGTAGTAGACGGAATAGCTG	Chr.4	17342642	17342662	+	No
2148466_adh	TGAATTTGCAGAATGTATCTC	Chr.4	17343577	17343557	-	No
2722851_adh	TTTGGAAAAAGTAGAAAACTC	Chr.4	17348889	17348869	-	No
2707843_adh	TTTCGGATTAGGATGGTTTGA	Chr.4	17364874	17364854	-	No
2005355_adh	TCGATTTTCAGCATGTTTTCC	Chr.4	17380136	17380116	-	No
2553367_adh	TTACAACTAGAGAAACGTTTT	Chr.4	17397974	17397994	+	No
2708950_adh	TTTCGTCTCAAGAAGAAGCTA	Chr.4	17400382	17400402	+	No
1797881_adh	TAATCAGTGACGCAATCTTTA	Chr.4	17415717	17415697	-	No
2560517_adh	TTACTTTGTACAGGTTGCTTG	Chr.4	17422257	17422237	-	No
1922862_adh	TATTATTACATGCTTTCTGAC	Chr.4	17423026	17423006	-	No
1836689_adh	TAGAAAACGTCACGAAGTGGT	Chr.4	17428688	17428708	+	No
2061721_adh	TCTTTGAATTTTGAAGATCAT	Chr.4	17432791	17432771	-	No
2533986_adh	TGTTTGTTTGAAATCTAGGCC	Chr.4	17436860	17436840	-	No
1942148_adh	TCAATGGAGCATCGAACTCTT	Chr.4	17439716	17439696	-	No
2732874_adh	TTTGTTTTTGATCTAGGTAGT	Chr.4	17451324	17451304	-	No
2037275_adh	TCTCAGAAGACGTAGTGGTGC	Chr.4	17463405	17463385	-	No
2233692_adh	TGAGCCAGTTGCCCAAGAAGA	Chr.4	17471176	17471156	-	No

1971778_adh	TCATGAATTTTTGACTTGACG	Chr.4	17474658	17474638	-	No
1973718_adh	TCATTCAGTAGGTGGCATTAA	Chr.4	17482148	17482168	+	No
2508067_adh	TGTGATCATTAGAATCGGCGC	Chr.4	17490993	17490973	-	No
2557208_adh	TTACGGAAGAACGAACTATAA	Chr.5	5099	5079	-	No
2274360_adh	TGATTACGGAAGAACGAACTA	Chr.5	5102	5082	-	No
1852452_adh	TAGATTTGAGTTGGAATTTTT	Chr.5	30758	30778	+	No
2666803_adh	TTGGGAAGTCTGCGGCTAAAC	Chr.5	100493	100513	+	No
2039311_adh	TCTCCTTTTCGTTGTTCTCCT	Chr.5	100648	100628	-	No
2039311_adh	TCTCCTTTTCGTTGTTCTCCT	Chr.5	100934	100954	+	No
1961324_adh	TCAGATGCCACAAGCTTAAGC	Chr.5	103990	103970	-	No
1930522_adh	TATTTGTAGTTTTCGCTCGTA	Chr.5	104522	104502	-	No
2521399_adh	TGTGTTTATCGTAAATCGGCG	Chr.5	104711	104731	+	No
1807595_adh	TACAACGAAGAAAAGAAAACA	Chr.5	131359	131339	-	No
2328196_adh	TGGAATAAAAAGAAGCATGGA	Chr.5	146145	146125	-	No
2119809_adh	TGAACAATACGGATCGTCAAA	Chr.5	157201	157221	+	No
1821444_adh	TACGAAAAATCGACGGGATCA	Chr.5	243087	243107	+	No
1919890_adh	TATTAAAATTAGGGTCGTTAA	Chr.5	290096	290116	+	No
2694253_adh	TTTACTCTGAAAAATTGAAAA	Chr.5	305545	305525	-	No
2640931_adh	TTGAGCGGCAGCAGCGAATAC	Chr.5	311704	311724	+	No
2748722_adh	TTTTTTCCGATGGGTGGCATG	Chr.5	311969	311989	+	No
2458840_adh	TGGGTGGCATGGAGAACGGCT	Chr.5	311979	311999	+	No
2701422_adh	TTTCAACTATTTTCGGCGTTT	Chr.5	347845	347865	+	No
1838843_adh	TAGAACTGGTATTTGAAGGGG	Chr.5	349199	349179	-	No
2733669_adh	TTTTAATCGAATTTCGTGCGG	Chr.5	410499	410519	+	No
2733669_adh	TTTTAATCGAATTTCGTGCGG	Chr.5	411109	411089	-	No
1838899_adh	TAGAACTTGCTCTATTCGGAA	Chr.5	411406	411426	+	No
1866745_adh	TAGGATTTTATAATGGAGCAT	Chr.5	412569	412549	-	No
2046357_adh	TCTGAGGAGAAATTTTAGAAA	Chr.5	517685	517665	-	No
1812953_adh	TACAGTTGTGACCTCATAAAT	Chr.5	553833	553853	+	No
2027977_adh	TCGTTCCCAGAATAAACAGTA	Chr.5	580026	580046	+	No
2718260_adh	TTTGATCGTCAACTGTGAGGT	Chr.5	647070	647090	+	Yes
1849424_adh	TAGAGTTGAGATCACTGGACC	Chr.5	725846	725826	-	No
1971565_adh	TCATCTGGATGGAGACCTCTC	Chr.5	726635	726615	-	No
2574187_adh	TTATACAAAGACACACACAAT	Chr.5	727978	727958	-	No
2560631_adh	TTAGAAAACGGCAATAAATCA	Chr.5	775147	775127	-	No
2588745_adh	TTCAATGTTTGAAGCGAGAAA	Chr.5	775360	775380	+	No
2742371_adh	TTTTGCATTGGAACGTTGACA	Chr.5	776844	776864	+	No
1790133_adh	TAATAAATACATACCTATATT	Chr.5	814682	814702	+	No
2709970_adh	TTTCTCGATTAGAATGGGTTT	Chr.5	856838	856858	+	No
2308276_adh	TGCTGAATCATACTTTGCTCT	Chr.5	862195	862215	+	No
2411275_adh	TGGCCTGAGAAAGTAAAAATC	Chr.5	903962	903942	-	No
2135957_adh	TGAAGTTGGCTCAGAAACAGA	Chr.5	904077	904097	+	No
2666070_adh	TTGGCTCAGAAACAGAAGATG	Chr.5	904082	904102	+	No
2142325_adh	TGAATCGGCAAAAAATTAATC	Chr.5	953783	953803	+	No
1990766_adh	TCCTCTCGGACAAGGCAACAA	Chr.5	965065	965045	-	No
2705373_adh	TTTCCCGCATTTATTGTAGCA	Chr.5	992153	992173	+	No
1806267_adh	TAATTTTTGCAGCTCGACATT	Chr.5	1019990	1019970	-	No
2515733_adh	TGTGGATTTCGAGACTTTGCA	Chr.5	1022729	1022749	+	No
2538093_adh	TTAAAAATTTGAGGAAAGCCA	Chr.5	1066958	1066938	-	No
2447877_adh	TGGGATTATTCAGAAGGTTCT	Chr.5	1084949	1084929	-	No

2656728_adh	TTGGAACGAGTAGAGGATTAT	Chr.5	1094496	1094516	+	No
2656728_adh	TTGGAACGAGTAGAGGATTAT	Chr.5	1097996	1098016	+	No
2733120_adh	TTTTAAAGGTGGTGTATTTAG	Chr.5	1152829	1152809	-	No
2746204_adh	TTTTTCTCAACCAGTGGACGA	Chr.5	1155814	1155834	+	No
2031144_adh	TCTACAAGGAGAAAATGACGA	Chr.5	1178608	1178628	+	No
2537472_adh	TTAAAAAACTTAAGCGGGCAA	Chr.5	1197648	1197668	+	No
2271567_adh	TGATGATGTAGACGCAGACGT	Chr.5	1207945	1207965	+	No
2422369_adh	TGGGACTGTTTTCCAAAGGAG	Chr.5	1270954	1270974	+	No
2717776_adh	TTTGATCAGTTTGGAAATCAA	Chr.5	1276044	1276024	-	No
2051487_adh	TCTGGTTCGGAATCAGGAGAA	Chr.5	1279306	1279286	-	No
2052645_adh	TCTGTGGAAAGTGAAGTACTG	Chr.5	1286189	1286209	+	No
2747718_adh	TTTTTGTAGATCAAACCGGTG	Chr.5	1312607	1312627	+	No
2616777_adh	TTCTCGTAGTAAATCGTTGGC	Chr.5	1319449	1319429	-	No
2472016_adh	TGGTGGAGCTCTTCGACGGCT	Chr.5	1379793	1379773	-	No
1887894_adh	TATAGAAATGTAGAAAACGAG	Chr.5	1383505	1383485	-	No
1999464_adh	TCGAATTTTGAAGCCGTCTCC	Chr.5	1383631	1383611	-	No
1924237_adh	TATTCGAATTTTGAAGCCGTC	Chr.5	1383634	1383614	-	No
1959156_adh	TCAGAGGAATAATTACTTGAA	Chr.5	1383751	1383771	+	No
2469212_adh	TGGTCAGAATTGTACGGTAAA	Chr.5	1426442	1426462	+	No
2734226_adh	TTTTACGTTAAGCAGGGAAAA	Chr.5	1429258	1429238	-	No
2729059_adh	TTTGTCGTATTTTTTCGGTTC	Chr.5	1429786	1429766	-	No
2316293_adh	TGGAAAGATTTTTAGAGCGAA	Chr.5	1471719	1471699	-	No
2553230_adh	TTACAAATGAACTGCAGACAA	Chr.5	1495731	1495751	+	No
2528763_adh	TGTTGAGATGGTGGAGCCGGT	Chr.5	1496083	1496063	-	No
2307252_adh	TGCTCAGGGCTGAAATGGTTG	Chr.5	1504276	1504296	+	No
2742955_adh	TTTTGGGAAGTAACGTGATTA	Chr.5	1505476	1505496	+	No
2546747_adh	TTAAGAAGACTCCCATTGACA	Chr.5	1561622	1561642	+	No
2706258_adh	TTTCCTCTGTTTTTCGAACAA	Chr.5	1587912	1587932	+	No
2746270_adh	TTTTTCTGCGGTAAATCTGAA	Chr.5	1590250	1590230	-	No
2382012_adh	TGGAGGAGAAGCAGAAGTAAA	Chr.5	1658059	1658039	-	No
1819426_adh	TACCGAAAAAGTTCTCGTCTT	Chr.5	1659616	1659596	-	No
2618860_adh	TTCTGGAACTCCACATGGGCA	Chr.5	1685852	1685872	+	No
2296173_adh	TGCGAATTAAGCGGCAGAACT	Chr.5	1773379	1773399	+	No
2585186_adh	TTCAAACGGTTGATCTCGATT	Chr.5	1797126	1797106	-	No
2729426_adh	TTTGTGACAGTGAATAAAAAA	Chr.5	1801851	1801831	-	No
2579737_adh	TTATGGTCTTTGGAACACAAA	Chr.5	1830767	1830787	+	No
2579737_adh	TTATGGTCTTTGGAACACAAA	Chr.5	1841242	1841222	-	No
2043641_adh	TCTGAAAATCGGGGACTAGGT	Chr.5	1843172	1843192	+	No
2733928_adh	TTTTACAACTAGAATCGAGCC	Chr.5	1857771	1857791	+	No
2335573_adh	TGGAATCGGCTTGATTTTGCT	Chr.5	1870346	1870326	-	No
2236763_adh	TGAGGAAGGGGAGAAGTAGAA	Chr.5	1871900	1871880	-	No
2603633_adh	TTCGAGAAGGAATATTTTCGT	Chr.5	1872032	1872052	+	No
1976754_adh	TCCAATCGGAGATGTTGCTAC	Chr.5	1873739	1873759	+	No
2744839_adh	TTTTTAATGTTTTCGTTGTAT	Chr.5	1877525	1877505	-	No
2047711_adh	TCTGCAAAGTGGACTATTTTA	Chr.5	1893164	1893184	+	No
2001415_adh	TCGAGCAATTATGAATATGAG	Chr.5	1919507	1919487	-	No
2744703_adh	TTTTTAAAGTCGATGGACGGC	Chr.5	1931562	1931582	+	No
2749335_adh	TTTTTTGACTGAAAAGGCGTT	Chr.5	1975134	1975114	-	No
2056603_adh	TCTTCGGATTCATTGCAAAGT	Chr.5	2004977	2004997	+	No
2591918_adh	TTCAGGAAAAATTGTCGAAAA	Chr.5	2008673	2008693	+	No

1963262_adh	TCAGGAAAAATTGTCGAAAAC	Chr.5	2008674	2008694	+	No
2604899_adh	TTCGATCGTTGAAGGACGGAT	Chr.5	2065755	2065775	+	No
2673424_adh	TTGGTTTTCGGTTGTTTTCGT	Chr.5	2067676	2067696	+	No
2738748_adh	TTTTCGGTTGTTTTCGTCATT	Chr.5	2067680	2067700	+	No
2031581_adh	TCTACGAGAAGGCTGCCTAAG	Chr.5	2069018	2069038	+	No
1848318_adh	TAGAGGAGAAGCTGACGACTG	Chr.5	2072770	2072750	-	No
2717098_adh	TTTGAGTGTAGAAGAAAAAAG	Chr.5	2090354	2090334	-	No
2171022_adh	TGACTGTACGGAGATTCGGAA	Chr.5	2115218	2115238	+	No
2724350_adh	TTTGGCATCATTCGGAATCAC	Chr.5	2129815	2129835	+	No
1812458_adh	TACAGGAGAAGATGAACAAAA	Chr.5	2161316	2161336	+	No
2710346_adh	TTTCTGAAATACTAAAAATAA	Chr.5	2191713	2191733	+	No
2667318_adh	TTGGGATGAAAGATGAGATGA	Chr.5	2207065	2207045	-	No
2162208_adh	TGACGTCGAGACGGAATTGCT	Chr.5	2255434	2255454	+	No
1851712_adh	TAGATGTAGGAACATTCATAA	Chr.5	2266401	2266381	-	No
2712969_adh	TTTGAACATGAGGGCATGTAA	Chr.5	2282489	2282469	-	No
2692967_adh	TTTAATTTGAACATGAGGGCA	Chr.5	2282494	2282474	-	No
1972742_adh	TCATGGTAGACAGGCGCGGTT	Chr.5	2286225	2286245	+	No
2288744_adh	TGCAGTAGAGCGCGTTTGCAG	Chr.5	2387399	2387419	+	No
2288744_adh	TGCAGTAGAGCGCGTTTGCAG	Chr.5	2495678	2495698	+	No
1935646_adh	TCAAATTGGGAAAATCGAACG	Chr.5	2603237	2603257	+	No
1805139_adh	TAATTTGAAGAAGAGCATTTT	Chr.5	2606368	2606388	+	No
2368351_adh	TGGAATGTTTTGTAGTTTTTA	Chr.5	2606519	2606539	+	No
1836341_adh	TACTTTGGGATTTTCGTCGTT	Chr.5	2654161	2654181	+	No
2584954_adh	TTCAAAACGCGTATTTCTCTA	Chr.5	2682750	2682770	+	No
2699703_adh	TTTATTCACGCGGAATTTTGG	Chr.5	2694578	2694558	-	No
2585574_adh	TTCAAATGTACACTAAATAAC	Chr.5	2697668	2697648	-	No
2734497_adh	TTTTAGACGTGTGGAAGACGA	Chr.5	2698581	2698561	-	No
1847248_adh	TAGAGAAGTTGTTGCTGAAAA	Chr.5	2722017	2722037	+	Yes
1847248_adh	TAGAGAAGTTGTTGCTGAAAA	Chr.5	2724164	2724184	+	No
2048433_adh	TCTGCTCAAATGGACTCGGAC	Chr.5	2810624	2810644	+	No
2745624_adh	TTTTTCAATTGTTGAGCGAAT	Chr.5	2879745	2879725	-	No
2010873_adh	TCGGAAGTAGAATACACGTAA	Chr.5	2954001	2953981	-	No
2299327_adh	TGCGATGGTAAAAAGCAAAAA	Chr.5	3020739	3020719	-	No
2746316_adh	TTTTTCTGTTTTCTGTCATTG	Chr.5	3033452	3033472	+	No
2161827_adh	TGACGGGAGATTTGAGCCATC	Chr.5	3057096	3057116	+	No
2159209_adh	TGACATTTATATAATTTTTTA	Chr.5	3088093	3088073	-	No
2121730_adh	TGAACCAGTCCTTGTTGAACG	Chr.5	3134755	3134735	-	No
2705743_adh	TTTCCGATTGTAGTGATAAAC	Chr.5	3158768	3158748	-	No
1851518_adh	TAGATGGGTGTAGTGTTGGCC	Chr.5	3193307	3193327	+	No
1812606_adh	TACAGGTTGGAAGAACGCATT	Chr.5	3195319	3195339	+	No
2515215_adh	TGTGGAGCTCTAACAGTTGGC	Chr.5	3259359	3259339	-	No
1793922_adh	TAATACTCAAAAAGTCGTCAA	Chr.5	3278399	3278379	-	No
2542024_adh	TTAAATAACGAACTATATTTG	Chr.5	3361988	3362008	+	No
2679187_adh	TTGTGAAAGTGGCAAAAAACC	Chr.5	3480983	3480963	-	No
2043819_adh	TCTGAAATTGGGAAGACTATG	Chr.5	3507819	3507799	-	No
2017209_adh	TCGGGGTACTGTAGTAGTGCT	Chr.5	3520131	3520151	+	No
2048794_adh	TCTGGAAGCTCGGCTATTTTA	Chr.5	3557440	3557460	+	No
1839805_adh	TAGAAGTCGTAAAAAACAAAC	Chr.5	3558826	3558806	-	No
1797890_adh	TAATCAGTTTTTTAACCCATA	Chr.5	3564318	3564298	-	No
1797890_adh	TAATCAGTTTTTTAACCCATA	Chr.5	3571307	3571327	+	No

2042866_adh	TCTCTGAGCACTGCGACACGG	Chr.5	3580859	3580839	-	No
1768071_adh	TAACGAGCTGGTCGTAGAGGT	Chr.5	3585153	3585133	-	No
2552114_adh	TTAATTGAATAGAAATTAATA	Chr.5	3587890	3587910	+	No
2065352_adh	TGAAAAACACGAGGCGTAGAC	Chr.5	3685879	3685859	-	No
2721784_adh	TTTGCCGCAAAATCGATTGAA	Chr.5	3754260	3754240	-	No
2734576_adh	TTTTAGATAAGATGTCCTGAC	Chr.5	3825308	3825288	-	No
2480345_adh	TGTAAAAATACACTGATAGCA	Chr.5	3878837	3878857	+	No
2746012_adh	TTTTTCGATTTTCAGTTTTCC	Chr.5	3885568	3885548	-	No
1919925_adh	TATTAAAGAACGAACAAACGG	Chr.5	3935468	3935488	+	No
2687446_adh	TTGTTTGATTTGTGGCAGGCT	Chr.5	3962536	3962516	-	No
2709428_adh	TTTCTAACGAACGTCATGGAA	Chr.5	4034413	4034433	+	No
2717171_adh	TTTGATAAAAAGCATGCCGTC	Chr.5	4045039	4045059	+	No
2589739_adh	TTCACGGTAGCACAGAAACTA	Chr.5	4063717	4063737	+	No
2263654_adh	TGATATCTTTTATTCAAAAAA	Chr.5	4064326	4064346	+	No
1889977_adh	TATATCATTACGGTAAATTTT	Chr.5	4070794	4070814	+	No
2050362_adh	TCTGGGCAGTGGAGCGCATTT	Chr.5	4147891	4147911	+	No
2737138_adh	TTTTCAAATCTTTTCTCGCAT	Chr.5	4175854	4175874	+	No
2701188_adh	TTTCAAATCTTTTCTCGCATA	Chr.5	4175855	4175875	+	No
2517259_adh	TGTGGTATCATAGGCGTGTTT	Chr.5	4177273	4177253	-	No
1929564_adh	TATTTCAGATCAAAGTTCGGC	Chr.5	4182418	4182398	-	No
2051788_adh	TCTGTACCGGTAATATTTGAA	Chr.5	4185645	4185625	-	No
2559450_adh	TTACTGTAGAGCTGATGGCGA	Chr.5	4192110	4192130	+	No
2737249_adh	TTTTCAATCCGAATATGTGTT	Chr.5	4194626	4194606	-	No
2339184_adh	TGGAATGAAAAGAAATTTTTT	Chr.5	4202001	4201981	-	No
2632749_adh	TTGAATGTAAATCAGTCTGTA	Chr.5	4238525	4238545	+	No
2568697_adh	TTAGGGACGGTTTTGTATAAA	Chr.5	4264995	4264975	-	No
1973739_adh	TCATTCATTCGCATCGACTTC	Chr.5	4284459	4284479	+	No
1942078_adh	TCAATGCGGGAAAATCTGAAA	Chr.5	4332962	4332982	+	No
2738866_adh	TTTTCGTTGAAGAGGACCAAA	Chr.5	4338075	4338055	-	No
2712680_adh	TTTGAAAGGCAATTAACGGGG	Chr.5	4338923	4338943	+	No
2747667_adh	TTTTTGGTTGTTTTTAGATGG	Chr.5	4346872	4346852	-	No
1898611_adh	TATCGGTTTTGTGCTACAAGA	Chr.5	4347069	4347089	+	No
2197550_adh	TGAGATCAGAATACGAATGTG	Chr.5	4349680	4349660	-	No
1963469_adh	TCAGGAAGAAGTCGAAGAAAC	Chr.5	4401330	4401310	-	No
2616476_adh	TTCTCGACTATTTCTGCTTAG	Chr.5	4411842	4411822	-	No
2552615_adh	TTAATTTATGGAGATGTTTAT	Chr.5	4433302	4433282	-	No
2748289_adh	TTTTTTAGCGGATTTTGTCGA	Chr.5	4442763	4442743	-	No
2705783_adh	TTTCCGGAATACGCAAAAATG	Chr.5	4470865	4470885	+	No
1809050_adh	TACAAGGAGGGAAAGCTTAAG	Chr.5	4484419	4484439	+	No
1811203_adh	TACACGTGATTTTCGTATAAT	Chr.5	4570356	4570336	-	No
2410208_adh	TGGCATTGTGAGAGAGAAAGG	Chr.5	4570379	4570359	-	No
2005260_adh	TCGATTTGCAGAGGATTTCTC	Chr.5	4570478	4570498	+	No
2721308_adh	TTTGCAGAGGATTTCTCAGGT	Chr.5	4570482	4570502	+	No
2164535_adh	TGACTAGAGAAACATTCACCA	Chr.5	4589875	4589895	+	No
2015701_adh	TCGGCTCTGAAAGAAGAATGT	Chr.5	4628737	4628757	+	No
2301504_adh	TGCGGCAGGAATATGATATTT	Chr.5	4651114	4651094	-	No
2313874_adh	TGGAAAATATAGAAGTAAAAA	Chr.5	4657537	4657557	+	No
1777367_adh	TAAGAGGGCTAAATCAAACAG	Chr.5	4686808	4686788	-	No
1880641_adh	TAGTTCGGATATTATTTCAAT	Chr.5	4689073	4689093	+	No
2413896_adh	TGGCTACTGGATTTACGGAAC	Chr.5	4735494	4735514	+	No

2723766_adh	TTTGGAGGAAGAACAAGAAGA	Chr.5	4757997	4758017	+	No
2607107_adh	TTCGGAAAGGCGTTTTAATCA	Chr.5	4758807	4758787	-	No
2260225_adh	TGATACTTTCTGCATAGATCA	Chr.5	4788108	4788088	-	No
2281379_adh	TGCAACATATCTGACGCGCAA	Chr.5	4818038	4818018	-	No
1784162_adh	TAAGGAATTCGAGGGATGGAA	Chr.5	4820104	4820084	-	No
2625330_adh	TTGAAAGTTGATGCAAAAGCC	Chr.5	4827087	4827067	-	No
2501307_adh	TGTGAAATGGGCGGAGACGAG	Chr.5	4827122	4827102	-	No
2590979_adh	TTCAGCAATCGAGAGACATTA	Chr.5	4833105	4833085	-	No
2028450_adh	TCGTTGCTGCTTTTCTCGTCG	Chr.5	4890232	4890252	+	No
2308777_adh	TGCTGCTTTTCTCGTCGTGGT	Chr.5	4890236	4890256	+	No
2700622_adh	TTTATTTAATAACCAGGCATG	Chr.5	4891686	4891706	+	No
1991596_adh	TCCTGGACAGACTTAATGACG	Chr.5	4902176	4902196	+	No
2700893_adh	TTTATTTTTGAGCTAATTTCG	Chr.5	4915695	4915675	-	No
2661067_adh	TTGGATATGAAAATGAATGTA	Chr.5	4924330	4924350	+	No
2100793_adh	TGAAATGAAAATAAAAATTTA	Chr.5	4951937	4951957	+	No
1939376_adh	TCAAGCTCGTCACTAATAATT	Chr.5	4994647	4994627	-	No
1756347_adh	TAAAGAACATGGACGGTTGTT	Chr.5	5032428	5032448	+	No
2531543_adh	TGTTTAAAACGGAAAACCTGA	Chr.5	5047098	5047078	-	No
2561501_adh	TTAGAAGGAGTCGGGAAGAAA	Chr.5	5047518	5047538	+	No
2022102_adh	TCGTAGAATTCATCTATAAAA	Chr.5	5052113	5052133	+	No
2274708_adh	TGATTAGAGTCACTGAAAAAT	Chr.5	5054892	5054872	-	No
2311654_adh	TGCTTTCAGAACAGTCCTCTC	Chr.5	5104361	5104341	-	No
1755995_adh	TAAAGAAAAATCGTCGTCATC	Chr.5	5107970	5107950	-	No
2229510_adh	TGAGATTCATAGCGTTCTTCG	Chr.5	5110726	5110706	-	No
1826769_adh	TACGGGACGCAGAAAAACTAA	Chr.5	5124643	5124623	-	No
1883829_adh	TATAAAAACTGACAATGGCAT	Chr.5	5168113	5168133	+	No
1888418_adh	TATAGGAATTCGGTACGACCA	Chr.5	5173117	5173137	+	No
1888418_adh	TATAGGAATTCGGTACGACCA	Chr.5	5173500	5173520	+	No
2140064_adh	TGAATATGTGGAAGAGAAAAC	Chr.5	5173612	5173592	-	No
2723890_adh	TTTGGATAGAATTCAGCATGT	Chr.5	5181647	5181627	-	No
2471149_adh	TGGTGACTGACTGTTTCTTTT	Chr.5	5269269	5269289	+	No
1998319_adh	TCGAATGCCCTTTTCCGCTTG	Chr.5	5315315	5315295	-	No
2062551_adh	TCTTTTCATTTTTGTTTCGGA	Chr.5	5350661	5350681	+	No
2038767_adh	TCTCCATTACTGTAGCAGAGA	Chr.5	5362199	5362179	-	No
2732960_adh	TTTTAAAAATATTCGGCTAAA	Chr.5	5385274	5385294	+	No
2605440_adh	TTCGATTAACAGTATTTGCAT	Chr.5	5434665	5434685	+	No
1890150_adh	TATATCTGTTTCTCGGTTTAT	Chr.5	5437958	5437938	-	No
2697070_adh	TTTAGTCAGGGAAAAGTTGCA	Chr.5	5439379	5439399	+	No
2616498_adh	TTCTCGATCATGTCAGGAGAA	Chr.5	5471762	5471742	-	No
2061324_adh	TCTTTCTGGAAACGACCGCAG	Chr.5	5475218	5475198	-	No
1979270_adh	TCCAGTTGAACAAGTAGTGAA	Chr.5	5478183	5478163	-	No
2287282_adh	TGCAGACGTCGCACAAATTGG	Chr.5	5478274	5478294	+	No
2597686_adh	TTCCCAGTACACAAATAATTA	Chr.5	5481432	5481452	+	No
2616186_adh	TTCTCATTAATTAACGTTCGA	Chr.5	5505082	5505062	-	No
2326436_adh	TGGAAGTAGTGGATGAAACAT	Chr.5	5510095	5510075	-	No
2566621_adh	TTAGCGAATCGCGGACGCAAA	Chr.5	5536389	5536369	-	No
2728631_adh	TTTGTATTTTAATTTGTCGGG	Chr.5	5538154	5538134	-	No
1929559_adh	TATTTCAGAACTTCAAGCCAA	Chr.5	5583888	5583868	-	No
2641785_adh	TTGAGTAAGCGAATTTTGGAA	Chr.5	5583933	5583913	-	No
2561297_adh	TTAGAACTCATTTCAGAGGTT	Chr.5	5584505	5584485	-	No

2015176_adh	TCGGCAAGAATGAAAAGAACA	Chr.5	5600365	5600345	-	No
2708035_adh	TTTCGGGCTTCTAAAGCGCTT	Chr.5	5601725	5601705	-	No
2263779_adh	TGATATGAAGAACATAAAAGT	Chr.5	5602348	5602328	-	No
2462163_adh	TGGTAATGATATGAAGAACAT	Chr.5	5602354	5602334	-	No
2683210_adh	TTGTTAGAGAGTGAGAGACGG	Chr.5	5619507	5619527	+	No
2309841_adh	TGCTGTTCTGACTGATCGTAA	Chr.5	5651535	5651515	-	No
2289340_adh	TGCAGTGTTGATAAGCATGTA	Chr.5	5654186	5654166	-	No
2749535_adh	TTTTTTGGCAAATTTGCATAA	Chr.5	5684669	5684649	-	No
2728078_adh	TTTGTACTCCTCTCGGACAAA	Chr.5	5684763	5684783	+	No
2590109_adh	TTCACTTGGACAGGAAGACAC	Chr.5	5705919	5705899	-	No
2066312_adh	TGAAAAATAGAGACTAGAGAC	Chr.5	5712337	5712317	-	No
1891399_adh	TATCAACATTTGATCGTCCGT	Chr.5	5712804	5712824	+	No
2692408_adh	TTTAATTGACGACATTTTGTG	Chr.5	5717371	5717351	-	No
2658437_adh	TTGGACGCAGAATCGACGCTT	Chr.5	5717562	5717582	+	No
2390782_adh	TGGATGAATTAGGCATTGAAT	Chr.5	5753078	5753058	-	No
2020512_adh	TCGGTTGAACGCGGATTCGCT	Chr.5	5754801	5754821	+	No
1901057_adh	TATGAAAATTGTCGAAAATAA	Chr.5	5756443	5756463	+	No
1935910_adh	TCAACAAGGTAGATCAAAAGT	Chr.5	5761858	5761878	+	No
2390782_adh	TGGATGAATTAGGCATTGAAT	Chr.5	5763257	5763237	-	No
2576718_adh	TTATATTTGCAAACTGAAATT	Chr.5	5763814	5763794	-	No
2390782_adh	TGGATGAATTAGGCATTGAAT	Chr.5	5770931	5770951	+	No
2570016_adh	TTAGTACGAAACATATCGAAT	Chr.5	5776607	5776587	-	No
1753742_adh	TAAAATTTTTCGCAGGTGAGA	Chr.5	5778438	5778458	+	No
1944745_adh	TCACAGAATGCTAACGGAACA	Chr.5	5802485	5802505	+	No
2107235_adh	TGAAATGGCAAAATGTATGTA	Chr.5	5808852	5808832	-	No
2286471_adh	TGCACGGCAGTGAAGAAAAAG	Chr.5	5825601	5825581	-	No
2596969_adh	TTCCAGGAGGATGCATTCAAT	Chr.5	5836334	5836314	-	No
2651390_adh	TTGCCTACATGCGTGCGCGCG	Chr.5	5865766	5865746	-	No
1919615_adh	TATGTTTAGAAAAAGGGATGA	Chr.5	5871914	5871894	-	No
2031990_adh	TCTACTGGTGTGAGAACAGGA	Chr.5	5918268	5918288	+	No
2625230_adh	TTGAAAGTAACTTCTGTCGGT	Chr.5	5939748	5939728	-	No
1835051_adh	TACTTCTTGCACTCTTTGTAA	Chr.5	5950521	5950541	+	No
1812999_adh	TACATAAATCTAGGGTTACGA	Chr.5	5965299	5965279	-	No
2718416_adh	TTTGATCTAAACTGTGATGCA	Chr.5	5991991	5991971	-	No
2600507_adh	TTCCTATTCTGATCTTCCGAC	Chr.5	5999472	5999452	-	No
2549341_adh	TTAAGTTGGAAAATAATAAAT	Chr.5	6003948	6003968	+	No
2144674_adh	TGAATGTAGGAATGATTGGCA	Chr.5	6019149	6019169	+	No
1807361_adh	TACAACAATGAAAGCGGCCTC	Chr.5	6022714	6022694	-	No
2128951_adh	TGAAGACTGTTGAGAAGGAAA	Chr.5	6099309	6099289	-	No
2740979_adh	TTTTGACAAATTGAAGACTGT	Chr.5	6099320	6099300	-	No
1940359_adh	TCAAGTCAGGAACGATCTTTG	Chr.5	6099396	6099376	-	No
2000711_adh	TCGACTGTGGAGGTGGATACG	Chr.5	6100197	6100177	-	No
2742521_adh	TTTTGCTCTTGTTCGGTTTCA	Chr.5	6100664	6100644	-	No
2624386_adh	TTGAAAAGAACAGAAACAGCT	Chr.5	6100998	6101018	+	No
2674725_adh	TTGTAGAAGTCAAAGTAGGAG	Chr.5	6145871	6145851	-	No
1878290_adh	TAGTGCACCAACAGTTGAGAA	Chr.5	6183334	6183314	-	No
1847119_adh	TAGAGAAACTCGAAGAACAAC	Chr.5	6199719	6199699	-	No
2280015_adh	TGCAAACTGTTCGAAGAATTC	Chr.5	6200439	6200419	-	No
1901888_adh	TATGAAGGTGAGAAATGACGT	Chr.5	6208931	6208951	+	No
1811664_adh	TACAGAAAGAAGACAAGAATA	Chr.5	6221163	6221143	-	No

2617382_adh	TTCTCTGATAGTAGCTACCAA	Chr.5	6245626	6245646	+	No
1955306_adh	TCACTTCCGTAGAACAAGCAT	Chr.5	6257836	6257816	-	No
2654556_adh	TTGCTGAATGGGTTCAAACAA	Chr.5	6260791	6260771	-	No
2703121_adh	TTTCAGCTGATTTGCATAAAT	Chr.5	6268230	6268250	+	No
2591891_adh	TTCAGCTGATTTGCATAAATT	Chr.5	6268231	6268251	+	No
2602347_adh	TTCGAAGTTTTCAGATGGGAT	Chr.5	6281391	6281411	+	No
2703120_adh	TTTCAGCTGAGACGACGAGTA	Chr.5	6285546	6285566	+	No
1888893_adh	TATAGTAAGTTAACGAGCTAA	Chr.5	6295536	6295556	+	No
1888893_adh	TATAGTAAGTTAACGAGCTAA	Chr.5	6297087	6297107	+	No
1888893_adh	TATAGTAAGTTAACGAGCTAA	Chr.5	6298463	6298483	+	No
2697473_adh	TTTAGTTAGGCATTATGGAAT	Chr.5	6312392	6312412	+	No
2174623_adh	TGACTTTGGAAAATTGGAAGT	Chr.5	6341658	6341678	+	No
2029396_adh	TCTAAAGAACTTCGGATATTC	Chr.5	6388817	6388837	+	No
2557253_adh	TTACGGAGACTGTGCATCTAT	Chr.5	6393875	6393855	-	No
1977265_adh	TCCACCAAAGAACAAGACTGT	Chr.5	6394841	6394821	-	No
1893118_adh	TATCACTGCATGCCAAACTCG	Chr.5	6395254	6395234	-	No
1985909_adh	TCCGGACTTTTCATCAACGGA	Chr.5	6395653	6395633	-	No
2680679_adh	TTGTGATCCATCGTCTGTCTG	Chr.5	6396116	6396096	-	No
2055096_adh	TCTTATCGTTGACGGATTCAA	Chr.5	6396236	6396216	-	No
2530629_adh	TGTTGTCAACGGAAAGAACGC	Chr.5	6397634	6397614	-	No
2712045_adh	TTTCTTTCATGTACATATGTG	Chr.5	6401489	6401469	-	No
2557253_adh	TTACGGAGACTGTGCATCTAT	Chr.5	6402522	6402502	-	No
1977265_adh	TCCACCAAAGAACAAGACTGT	Chr.5	6403488	6403468	-	No
1893118_adh	TATCACTGCATGCCAAACTCG	Chr.5	6403901	6403881	-	No
1985909_adh	TCCGGACTTTTCATCAACGGA	Chr.5	6404300	6404280	-	No
2680679_adh	TTGTGATCCATCGTCTGTCTG	Chr.5	6404763	6404743	-	No
2055096_adh	TCTTATCGTTGACGGATTCAA	Chr.5	6404883	6404863	-	No
2530629_adh	TGTTGTCAACGGAAAGAACGC	Chr.5	6406281	6406261	-	No
2608209_adh	TTCGGATCAGGAGACACCGTC	Chr.5	6407525	6407505	-	No
2595952_adh	TTCCAATATTTAAGATGGTCG	Chr.5	6425840	6425820	-	No
2610102_adh	TTCGGTGGAACAATTTATCAA	Chr.5	6434882	6434902	+	No
2269038_adh	TGATCAAGACTGTCGATATTG	Chr.5	6435007	6435027	+	No
1927091_adh	TATTGGATTTTATACTTTTAA	Chr.5	6438245	6438225	-	No
2418388_adh	TGGGAACTAGTTCAGAGCAGA	Chr.5	6447833	6447853	+	No
1912441_adh	TATGCACGTAAGAACCGGACA	Chr.5	6473912	6473892	-	No
1989912_adh	TCCTATTTTCGATTCAAAAGT	Chr.5	6484921	6484941	+	No
1891032_adh	TATATTTTAAATTTCTGAAAT	Chr.5	6487441	6487461	+	No
2028554_adh	TCGTTGGGACGGACATGTCGA	Chr.5	6488448	6488428	-	No
1993963_adh	TCCTTTAATAGTTGGATAAGA	Chr.5	6496684	6496704	+	No
2269771_adh	TGATCGAGTTGTGAAGTGGAT	Chr.5	6514176	6514196	+	No
2716806_adh	TTTGAGCTCCAGGATTGTGCT	Chr.5	6516346	6516326	-	No
2741460_adh	TTTTGAGTACGGTAGGCGGCG	Chr.5	6533493	6533473	-	No
2379387_adh	TGGAGAAAGGACAGGCCGGCA	Chr.5	6545084	6545064	-	No
2314256_adh	TGGAAACATGAACAAGAAACA	Chr.5	6589557	6589577	+	No
2695450_adh	TTTAGATATGTTCGGAATTGC	Chr.5	6591094	6591114	+	No
2580528_adh	TTATTAAGTTCGTTAGATATA	Chr.5	6591675	6591655	-	No
2651741_adh	TTGCGAAGCAATTCCGGTGAA	Chr.5	6591731	6591711	-	No
2699872_adh	TTTATTCGGGGCATTCTTCTT	Chr.5	6611613	6611593	-	No
2609123_adh	TTCGGGATAATCGATAATCGG	Chr.5	6656904	6656924	+	No
2053001_adh	TCTGTGTTGTTGGAGAATGCA	Chr.5	6657590	6657610	+	No

2690223_adh	TTTAAGAATTCAAACCACGGC	Chr.5	6671982	6671962	-	No
2124637_adh	TGAACTCGAAGCTTCTGGCAC	Chr.5	6673379	6673359	-	No
2626613_adh	TTGAACTCGAAGCTTCTGGCA	Chr.5	6673380	6673360	-	No
2124637_adh	TGAACTCGAAGCTTCTGGCAC	Chr.5	6673778	6673758	-	No
2626613_adh	TTGAACTCGAAGCTTCTGGCA	Chr.5	6673779	6673759	-	No
2124637_adh	TGAACTCGAAGCTTCTGGCAC	Chr.5	6673978	6673958	-	No
2626613_adh	TTGAACTCGAAGCTTCTGGCA	Chr.5	6673979	6673959	-	No
2707526_adh	TTTCGGAAGGAGATATTAGAT	Chr.5	6708697	6708677	-	No
2616467_adh	TTCTCGAATTGTTGCATTAAA	Chr.5	6709189	6709169	-	No
2054915_adh	TCTTATACTGAAGTCGTAGAA	Chr.5	6714994	6714974	-	No
2568079_adh	TTAGGAGAAGTGGAAAAAGTT	Chr.5	6732383	6732363	-	No
1808830_adh	TACAAGATCACATGTTGTCGG	Chr.5	6746136	6746156	+	No
2554439_adh	TTACAGTGTGATGATGAGCTT	Chr.5	6748726	6748746	+	No
2011298_adh	TCGGACAGCCAAGACAGGTGT	Chr.5	6766214	6766194	-	No
2319227_adh	TGGAACCAACAAGCTCGCATC	Chr.5	6766956	6766936	-	No
2090971_adh	TGAAATCACTGACGAGAAGAA	Chr.5	6767208	6767188	-	No
2716215_adh	TTTGACTTCGAGCAATACAGG	Chr.5	6783186	6783206	+	No
2639037_adh	TTGACTTCGAGCAATACAGGA	Chr.5	6783187	6783207	+	No
2058970_adh	TCTTGGCCTAAAGAAAATCGA	Chr.5	6785210	6785190	-	No
2532159_adh	TGTTTCAGACAAGTAAGACTA	Chr.5	6788138	6788158	+	No
2059754_adh	TCTTGTGACTGTGGCTTCGGC	Chr.5	6831183	6831203	+	No
2680404_adh	TTGTGACTGTGGCTTCGGCCG	Chr.5	6831185	6831205	+	No
2059754_adh	TCTTGTGACTGTGGCTTCGGC	Chr.5	6835651	6835671	+	No
2680404_adh	TTGTGACTGTGGCTTCGGCCG	Chr.5	6835653	6835673	+	No
1813633_adh	TACATGATCGGTCAAAGAAAG	Chr.5	6840090	6840110	+	No
2701583_adh	TTTCAAGCTTGAACGATTCCA	Chr.5	6843854	6843834	-	No
2714048_adh	TTTGAAGTAGAAGTTCCAACG	Chr.5	6846690	6846710	+	No
2701583_adh	TTTCAAGCTTGAACGATTCCA	Chr.5	6848119	6848099	-	No
2043586_adh	TCTCTTTTGAATGCAGGCTTA	Chr.5	6877818	6877838	+	No
2119881_adh	TGAACACAAAAGGAGGATTCA	Chr.5	6912577	6912557	-	No
2488074_adh	TGTAGATTCTCAGAAGCCGAT	Chr.5	6918067	6918047	-	No
2727139_adh	TTTGTAAATGAGGGCATGTAA	Chr.5	6928012	6927992	-	No
1810506_adh	TACACAATTTAAAGCTGATAT	Chr.5	6945925	6945905	-	No
2712321_adh	TTTGAAAAAGGGCATGTAATA	Chr.5	6945966	6945946	-	No
2714431_adh	TTTGAATGTTAGACTGTGAAA	Chr.5	6947893	6947873	-	No
2259596_adh	TGATACGCTAGAATGGCAAAA	Chr.5	7004697	7004717	+	No
2705921_adh	TTTCCGTTCTGCTGTTGGCAT	Chr.5	7060577	7060597	+	No
2405051_adh	TGGCATCGATAAACTGGTCTT	Chr.5	7060592	7060612	+	No
1829296_adh	TACTAAAGAACTTTAGCGGAA	Chr.5	7060700	7060680	-	No
1981591_adh	TCCCGGCATGGGAAGTTTATT	Chr.5	7069743	7069723	-	No
1939616_adh	TCAAGGAGCTGACGAAGAAAC	Chr.5	7076666	7076686	+	No
2621074_adh	TTCTTCACTCCATTGATGACC	Chr.5	7078700	7078720	+	No
1901743_adh	TATGAAGACTGTGGTAGATAG	Chr.5	7078780	7078800	+	No
2237905_adh	TGAGGATGAACAGAAGAAGGA	Chr.5	7080915	7080935	+	No
2711939_adh	TTTCTTTAGAATTCAAGAAAA	Chr.5	7098015	7098035	+	No
2505755_adh	TGTGAGAGAGATAGACATATA	Chr.5	7111606	7111626	+	No
2715259_adh	TTTGACGGGATATGCTCTATG	Chr.5	7132390	7132410	+	No
2679028_adh	TTGTCTTGAAGAATGATGCTC	Chr.5	7180903	7180883	-	No
1936529_adh	TCAACGAAGAAGACCTGGCTT	Chr.5	7190511	7190491	-	No
1802754_adh	TAATTATTCAGAGAGGGCTCA	Chr.5	7193419	7193399	-	No

2569573_adh	TTAGGTTGACGAATGGTCCTA	Chr.5	7196452	7196432	-	No
2714731_adh	TTTGACACTCTTGATTCGACT	Chr.5	7196567	7196547	-	No
1970262_adh	TCATATTGGCAGAGACAGAAT	Chr.5	7199214	7199194	-	No
1827264_adh	TACGGTATTCATATTGGCAGA	Chr.5	7199222	7199202	-	No
2010566_adh	TCGGAACCATTAGAAGGATAC	Chr.5	7243566	7243546	-	No
2374216_adh	TGGACAGTATGAAGTTAAAAG	Chr.5	7243871	7243851	-	No
2658064_adh	TTGGACAGTATGAAGTTAAAA	Chr.5	7243872	7243852	-	No
2747347_adh	TTTTTGGGACTTGGCAATATC	Chr.5	7345439	7345459	+	No
2674742_adh	TTGTAGAATATGGTACAGAAG	Chr.5	7345858	7345838	-	No
2043772_adh	TCTGAAAGTAGAACGACCTTT	Chr.5	7386534	7386554	+	No
2043829_adh	TCTGAAATTTTAGAAGAACAC	Chr.5	7387882	7387862	-	No
2714054_adh	TTTGAAGTCAAAGAAATCAGA	Chr.5	7448003	7448023	+	No
1939169_adh	TCAAGCATCGTCTAAATGAGG	Chr.5	7510237	7510217	-	No
2621812_adh	TTCTTCGGTTTAACTAATAAA	Chr.5	7519173	7519193	+	No
2566695_adh	TTAGCGCAGAATCCAATTTAA	Chr.5	7575735	7575755	+	No
2072971_adh	TGAAACGAAAGACATTTAGAA	Chr.5	7602195	7602215	+	No
1885727_adh	TATAATCGATTGTTTTGGTCA	Chr.5	7622155	7622135	-	No
1888265_adh	TATAGCATTGCAGCGGTCTGT	Chr.5	7643132	7643152	+	No
2686668_adh	TTGTTTAAATCTTGTGCGTCA	Chr.5	7674753	7674733	-	No
1953599_adh	TCACGTGTTTAATGGATTGAC	Chr.5	7695484	7695464	-	No
2601617_adh	TTCGAAAAGAAAAATCATCGG	Chr.5	7732049	7732029	-	No
1745472_adh	TAAAAAAGATTGTATCCGTTC	Chr.5	7774974	7774954	-	No
1774194_adh	TAAGAAGCAAAAGCAAAATCG	Chr.5	7776016	7775996	-	No
2634710_adh	TTGACAAGCAAGACAAGAAAA	Chr.5	7777000	7777020	+	No
2598041_adh	TTCCCGATAAGTTTAGACGCC	Chr.5	7803833	7803853	+	No
2620274_adh	TTCTGTGTTTGGTGAGAACGG	Chr.5	7804135	7804115	-	No
2027525_adh	TCGTGTCTCGGAGCAGGATGA	Chr.5	7812619	7812639	+	No
2062683_adh	TCTTTTGAATGTGGATGAGCA	Chr.5	7824322	7824342	+	No
1953104_adh	TCACGATTGTGATGACGGACT	Chr.5	7837386	7837366	-	No
2058326_adh	TCTTGCCGAAGGAACTACAGA	Chr.5	7837479	7837459	-	No
2740573_adh	TTTTGAAAGGTATAAAACCGC	Chr.5	7890059	7890039	-	No
2741344_adh	TTTTGACTTTAGCTGACTGTT	Chr.5	7893917	7893897	-	No
2527278_adh	TGTTCTTCTCGCGGAAAGGCA	Chr.5	7981002	7980982	-	No
2714573_adh	TTTGAATTTTGGATTTGCATA	Chr.5	7981070	7981090	+	No
2728564_adh	TTTGTATGCATTCAGGTGACT	Chr.5	7984058	7984078	+	No
1995765_adh	TCGAACTCTGCACATGACGAT	Chr.5	8014175	8014155	-	No
2717101_adh	TTTGAGTGTAGCAGTTTTCGA	Chr.5	8014719	8014699	-	No
1931060_adh	TATTTTGAGTGTAGCAGTTTT	Chr.5	8014722	8014702	-	No
2545073_adh	TTAACATCGTATTTGACGACA	Chr.5	8015142	8015122	-	No
2652447_adh	TTGCGGAATGTGGATTTACGG	Chr.5	8024927	8024907	-	No
2595085_adh	TTCATTCAGAAGGATTTTGGC	Chr.5	8029220	8029240	+	No
1957747_adh	TCAGAAGGATTTTGGCCCGTT	Chr.5	8029225	8029245	+	No
1825594_adh	TACGGAAACACAAAATACTGA	Chr.5	8037274	8037294	+	No
2740093_adh	TTTTCTTCGAGTCAAAAAGCA	Chr.5	8045848	8045868	+	No
2677717_adh	TTGTCATCACTTCGCTCGGAG	Chr.5	8057876	8057856	-	No
2016520_adh	TCGGGAGACGTGATAATTGAT	Chr.5	8101829	8101849	+	No
1767226_adh	TAACCGTAAGATAATTTGGCA	Chr.5	8120539	8120559	+	No
2727123_adh	TTTGTAAAGACAATTTGACGG	Chr.5	8147025	8147005	-	No
2610965_adh	TTCGTAAAGTAGAAGAAGCCA	Chr.5	8197971	8197951	-	No
1987627_adh	TCCGTAGAGACTCACTCAAAC	Chr.5	8200888	8200868	-	No

2680581_adh	TTGTGAGCTCACAGGAGAACG	Chr.5	8206061	8206081	+	No
1896506_adh	TATCCTTAGGTCGCTGGTTCG	Chr.5	8247507	8247527	+	No
2481614_adh	TGTAACTCGTAGATTGGGCAA	Chr.5	8247760	8247740	-	No
2595835_adh	TTCCAAGAAAGTATAAGCAGC	Chr.5	8247812	8247832	+	No
2321400_adh	TGGAACTGTAGAAGAGTATAA	Chr.5	8247816	8247796	-	No
2656951_adh	TTGGAACTGTAGAAGAGTATA	Chr.5	8247817	8247797	-	No
2711829_adh	TTTCTTGGAACTGTAGAAGAG	Chr.5	8247821	8247801	-	No
2028223_adh	TCGTTGACAAGCGGAGAGACG	Chr.5	8248074	8248094	+	No
2001246_adh	TCGAGAGAAGAATTTTTGGCG	Chr.5	8265074	8265054	-	No
2739525_adh	TTTTCTGCAGAAATGAATTGC	Chr.5	8267520	8267500	-	No
2314160_adh	TGGAAACAAGAGAATGGGATT	Chr.5	8267635	8267615	-	No
1899604_adh	TATCTCGGAAACATCACCATC	Chr.5	8272582	8272602	+	No
2703849_adh	TTTCATATTTTTCAGAACAAC	Chr.5	8280874	8280854	-	No
2404503_adh	TGGCATAATTGTAGGAACGTA	Chr.5	8299693	8299713	+	No
2038545_adh	TCTCATTTTTGTGACGTGTCT	Chr.5	8302675	8302655	-	No
2310244_adh	TGCTTATGATTGTTTGGCAGA	Chr.5	8313815	8313795	-	No
2310244_adh	TGCTTATGATTGTTTGGCAGA	Chr.5	8317015	8317035	+	No
2702497_adh	TTTCACCACACTACTTGAATA	Chr.5	8322208	8322228	+	No
1953599_adh	TCACGTGTTTAATGGATTGAC	Chr.5	8325167	8325147	-	No
2063331_adh	TCTTTTTTAAAGTGTTCTGTA	Chr.5	8355680	8355700	+	No
2711697_adh	TTTCTTGACGTTGGATAATTT	Chr.5	8359270	8359250	-	No
2029094_adh	TCGTTTTCTTGACGTTGGATA	Chr.5	8359274	8359254	-	No
2527381_adh	TGTTGAAAAACGGAACTGAAA	Chr.5	8359368	8359388	+	No
2577176_adh	TTATCATGAAGGCGGGACATC	Chr.5	8359730	8359710	-	No
2262022_adh	TGATATCATTAGTTGTATCAA	Chr.5	8360045	8360065	+	No
2158686_adh	TGACATTAAAACTTAGATTGC	Chr.5	8401576	8401596	+	No
1972174_adh	TCATGATTTCGAGCGGTATTT	Chr.5	8413846	8413866	+	No
1994270_adh	TCCTTTTCAATTGTCGGGATT	Chr.5	8424939	8424919	-	No
2601553_adh	TTCCTTTTCAATTGTCGGGAT	Chr.5	8424940	8424920	-	No
2617730_adh	TTCTGAACATTTAGAAGCTTC	Chr.5	8460879	8460859	-	No
2745225_adh	TTTTTATAGTAAGCGACACAG	Chr.5	8475414	8475394	-	No
2610906_adh	TTCGGTTTTTATAGTAAGCGA	Chr.5	8475419	8475399	-	No
2737894_adh	TTTTCCATCAAGTCGGCGTGC	Chr.5	8476915	8476895	-	No
2737894_adh	TTTTCCATCAAGTCGGCGTGC	Chr.5	8486207	8486187	-	No
2737894_adh	TTTTCCATCAAGTCGGCGTGC	Chr.5	8487010	8487030	+	No
2610906_adh	TTCGGTTTTTATAGTAAGCGA	Chr.5	8488506	8488526	+	No
2745225_adh	TTTTTATAGTAAGCGACACAG	Chr.5	8488511	8488531	+	No
2326151_adh	TGGAAGGTGAGAATGCTAAAA	Chr.5	8498692	8498712	+	No
2126569_adh	TGAACTTGAGAGCATGGAGCA	Chr.5	8501085	8501105	+	No
2301097_adh	TGCGGAGAACAGTTTACTCGC	Chr.5	8517787	8517767	-	No
2604170_adh	TTCGAGTGTAGGCACAGTTAA	Chr.5	8559141	8559161	+	No
2133172_adh	TGAAGGCCTAGAAGTTGACGA	Chr.5	8605281	8605261	-	No
2738587_adh	TTTTCGGAGTTCTCGTGTGCA	Chr.5	8615157	8615177	+	No
1923404_adh	TATTCAATAGGTTTTCGGCAA	Chr.5	8622731	8622751	+	No
2296915_adh	TGCGAGACCGTGTGATGCATT	Chr.5	8628879	8628899	+	No
1826732_adh	TACGGGAAAACAGTGGGACGA	Chr.5	8629471	8629491	+	No
2043351_adh	TCTCTTGCATAGACTCTTTCA	Chr.5	8641608	8641588	-	No
2004501_adh	TCGATTACATTTGTAGATTTC	Chr.5	8668396	8668416	+	No
1994097_adh	TCCTTTCTTTTTTAGTCGGCG	Chr.5	8726414	8726434	+	No
2035434_adh	TCTCAACCAACTTTGGCCACT	Chr.5	8746616	8746636	+	No

2622016_adh	TTCTTCTCGTTGGGACGGCTG	Chr.5	8785891	8785911	+	No
2424759_adh	TGGGAGTTTAGTTGTCGGGAA	Chr.5	8796396	8796416	+	No
2217299_adh	TGAGATGAAACAATGTATGTA	Chr.5	8808239	8808259	+	No
1853306_adh	TAGCAAGCAGTTTCAGTCTAC	Chr.5	8818401	8818381	-	No
2696463_adh	TTTAGCTCAATTTTTGTGTTT	Chr.5	8828015	8828035	+	No
1933925_adh	TCAAAGTCGCGTCGACGGTGA	Chr.5	8831975	8831955	-	No
2027013_adh	TCGTGGACTTCTCACCGGTCT	Chr.5	8833543	8833523	-	No
1990973_adh	TCCTCTTTTCGGCATCCTCAT	Chr.5	8841439	8841459	+	No
2042523_adh	TCTCTCGTAGAAGGAGCAGAA	Chr.5	8852596	8852616	+	No
1853306_adh	TAGCAAGCAGTTTCAGTCTAC	Chr.5	8860495	8860475	-	No
2696463_adh	TTTAGCTCAATTTTTGTGTTT	Chr.5	8870109	8870129	+	No
1933925_adh	TCAAAGTCGCGTCGACGGTGA	Chr.5	8874069	8874049	-	No
2027013_adh	TCGTGGACTTCTCACCGGTCT	Chr.5	8875637	8875617	-	No
1990973_adh	TCCTCTTTTCGGCATCCTCAT	Chr.5	8883534	8883554	+	No
2042523_adh	TCTCTCGTAGAAGGAGCAGAA	Chr.5	8894691	8894711	+	No
2733200_adh	TTTTAAATTTTTTGACGGACC	Chr.5	8922547	8922567	+	No
2318245_adh	TGGAAATGCAGAAGAAGGAAG	Chr.5	8939768	8939788	+	No
2723278_adh	TTTGGACATTGCAGGAGGTAT	Chr.5	8947898	8947878	-	No
2740567_adh	TTTTGAAAGATGAACCGGCAC	Chr.5	8948811	8948831	+	No
2712653_adh	TTTGAAAGATGAACCGGCACC	Chr.5	8948812	8948832	+	No
2173386_adh	TGACTTGAAAAGAATTATGCA	Chr.5	9024843	9024863	+	No
2504846_adh	TGTGACTGTAAAGACGGATTT	Chr.5	9054495	9054475	-	No
2004067_adh	TCGATGATTATTTAACGGTTT	Chr.5	9065020	9065040	+	No
2374599_adh	TGGACCATAGAGAAAATGATA	Chr.5	9071690	9071710	+	No
2377367_adh	TGGACTGAAAAATAGTAAAAT	Chr.5	9077517	9077497	-	No
1912393_adh	TATGCAAGAAGTACATTGCCG	Chr.5	9087116	9087136	+	No
2414417_adh	TGGCTCAGATGGAACGTCAAT	Chr.5	9087694	9087674	-	No
2414417_adh	TGGCTCAGATGGAACGTCAAT	Chr.5	9090155	9090175	+	No
1912393_adh	TATGCAAGAAGTACATTGCCG	Chr.5	9090733	9090713	-	No
2701613_adh	TTTCAAGTAAAACGTACGTCA	Chr.5	9091063	9091083	+	No
2046621_adh	TCTGATAATGGAGAAAAACTG	Chr.5	9175247	9175227	-	No
2296843_adh	TGCGACTTCTTTTCTCTTCTT	Chr.5	9175436	9175456	+	No
1958603_adh	TCAGAGAGATTATTGGACCAT	Chr.5	9179378	9179358	-	No
1782205_adh	TAAGCCAGAAAATTGCTCAAC	Chr.5	9203373	9203393	+	No
2035065_adh	TCTATTGTTGCATCGCTGGAT	Chr.5	9206752	9206772	+	No
2341732_adh	TGGAATGAAGAGGGGATTGTA	Chr.5	9259782	9259802	+	No
2468565_adh	TGGTATTACGTTGATCTCAAT	Chr.5	9316211	9316231	+	No
1811848_adh	TACAGACGAAACATCATAACT	Chr.5	9323012	9322992	-	No
2748296_adh	TTTTTTAGGATTCATAAAAGT	Chr.5	9323080	9323100	+	No
2733884_adh	TTTTAATTTGAAGTTTAAAAC	Chr.5	9328609	9328589	-	No
2689361_adh	TTTAAAGTGAATTCGTCATGA	Chr.5	9344561	9344581	+	No
2089550_adh	TGAAATATCGGAGTGTCGTCA	Chr.5	9346973	9346953	-	No
2554517_adh	TTACATAGAGCAGAATGAATC	Chr.5	9350002	9349982	-	No
2119924_adh	TGAACACAGAATTTGGGAAGA	Chr.5	9382631	9382611	-	No
1756773_adh	TAAAGAGCTGTAGAAGATGGT	Chr.5	9383541	9383521	-	No
2617955_adh	TTCTGAATTTCTAGAATACTT	Chr.5	9392184	9392204	+	No
2737606_adh	TTTTCATACTATTTTCAAAAT	Chr.5	9396482	9396502	+	No
2539042_adh	TTAAAATGACGGGGGTATAAT	Chr.5	9423319	9423339	+	No
2659273_adh	TTGGAGAATTGGTGTCTTGTT	Chr.5	9425818	9425838	+	No
2500374_adh	TGTCTTTGATTGATTGTGATG	Chr.5	9425846	9425866	+	No

2681063_adh	TTGTGATGAAATCTTTGACAG	Chr.5	9425859	9425879	+	No
2017209_adh	TCGGGGTACTGTAGTAGTGCT	Chr.5	9439805	9439785	-	No
1858889_adh	TAGCTGTTACATCTTGCGGAA	Chr.5	9446454	9446474	+	No
1770084_adh	TAACTAGGCGGCAAGAAGGTG	Chr.5	9483532	9483512	-	No
2669943_adh	TTGGTATAGCAGTGTAAAGTT	Chr.5	9494418	9494438	+	No
2061305_adh	TCTTTCTGAACACTCTGAAGA	Chr.5	9503849	9503869	+	No
2595467_adh	TTCATTTCAAGCGTTCGGACA	Chr.5	9513807	9513827	+	No
2587109_adh	TTCAAGCGTTCGGACACCAGC	Chr.5	9513812	9513832	+	No
2002477_adh	TCGATAAACTGTTTTTGTGCG	Chr.5	9544325	9544305	-	No
2010245_adh	TCGGAAACATGTTCGGACACA	Chr.5	9544463	9544483	+	No
1825754_adh	TACGGACAGCGAGTTGGAGAT	Chr.5	9544854	9544874	+	No
1999550_adh	TCGACAACGGCGGCGAGAAGA	Chr.5	9550460	9550440	-	No
2380618_adh	TGGAGAGTATCAGCTGAAGAA	Chr.5	9551937	9551957	+	No
1985914_adh	TCCGGAGAATAAGAGCATGGT	Chr.5	9568491	9568471	-	No
2054024_adh	TCTTAATTTTCGGTATTTTCT	Chr.5	9583019	9582999	-	No
2620523_adh	TTCTTAATTTTCGGTATTTTC	Chr.5	9583020	9583000	-	No
2513523_adh	TGTGCAGTTTGAATGTAAAAA	Chr.5	9583045	9583025	-	No
2153340_adh	TGACACTGTGCAGTTTGAATG	Chr.5	9583051	9583031	-	No
2309337_adh	TGCTGTAGGTCATGGTGCAAG	Chr.5	9583134	9583154	+	No
2380892_adh	TGGAGATTGTAATGAAGAGCG	Chr.5	9632163	9632143	-	No
2747747_adh	TTTTTGTATTGTTTTGTTGAA	Chr.5	9645705	9645725	+	No
2613634_adh	TTCGTTTTATTTCGTTAAACG	Chr.5	9645759	9645779	+	No
2700713_adh	TTTATTTCGTTAAACGTGGAC	Chr.5	9645764	9645784	+	No
2558680_adh	TTACTCAAAAAATGACGCGAC	Chr.5	9651265	9651285	+	No
1931748_adh	TCAAAAAATGACGCGACTAGC	Chr.5	9651269	9651289	+	No
2172935_adh	TGACTTCATGTAGATGGTTGT	Chr.5	9652585	9652565	-	No
2618863_adh	TTCTGGAACTTTAAGAAGATA	Chr.5	9668634	9668614	-	No
2630147_adh	TTGAATCAAAAAGCATTTTTA	Chr.5	9679782	9679762	-	No
2326403_adh	TGGAAGTACTCTGTTTAAAGC	Chr.5	9692757	9692737	-	No
2234499_adh	TGAGCTATCGAGTACTGGACA	Chr.5	9693779	9693799	+	No
2740564_adh	TTTTGAAACTGTAAATTAATT	Chr.5	9695807	9695827	+	No
2231763_adh	TGAGATTGTTGAGAGTGACGT	Chr.5	9696721	9696741	+	No
2032806_adh	TCTAGGCATGAATGAGAAAGA	Chr.5	9704701	9704721	+	No
2645138_adh	TTGATGGATCTGATTTTGAAG	Chr.5	9727773	9727753	-	No
2119637_adh	TGAACAAGACGTAGCTACTGT	Chr.5	9728036	9728016	-	No
1885910_adh	TATAATGATGAACAAGACGTA	Chr.5	9728044	9728024	-	No
2744021_adh	TTTTGTGTGGACAGACGGACA	Chr.5	9730967	9730947	-	No
1991965_adh	TCCTGGCCAGTAGATGTGTAA	Chr.5	9731379	9731399	+	No
2669943_adh	TTGGTATAGCAGTGTAAAGTT	Chr.5	9760765	9760745	-	No
1944405_adh	TCACAATTTTGGAAGTCGACA	Chr.5	9761871	9761891	+	No
1776541_adh	TAAGAGAATGTGTACTGTTAT	Chr.5	9774775	9774755	-	No
2627313_adh	TTGAAGACCAGTATTGTTGAG	Chr.5	9784591	9784571	-	No
2681808_adh	TTGTGGGAGTTGAGAAATAAC	Chr.5	9786885	9786865	-	No
2711565_adh	TTTCTTCTATTTTTCGTCGAA	Chr.5	9789870	9789850	-	No
2745054_adh	TTTTTAGAATGTCGAAACAAC	Chr.5	9815259	9815239	-	No
1929035_adh	TATTTAACGGTAAGCCTGTCA	Chr.5	9856256	9856236	-	No
1753078_adh	TAAAATTATTCGTAGGTTTCA	Chr.5	9856511	9856491	-	No
2042684_adh	TCTCTCTGCATTGATCAACGG	Chr.5	9856756	9856776	+	No
2558645_adh	TTACTATTAAAAGTTGGCAAG	Chr.5	9946886	9946906	+	No
2311651_adh	TGCTTTCAATGCAGCTTGTCG	Chr.5	9960278	9960258	-	No

2174342_adh	TGACTTTAGCAGTTGTATCCC	Chr.5	9977581	9977601	+	No
2389185_adh	TGGATATGGAAGTCGATGAGG	Chr.5	9980667	9980647	-	No
2035167_adh	TCTATTTTCACGCTGACGCTT	Chr.5	9980699	9980679	-	No
2325187_adh	TGGAAGGGAAAGAGGAAAAAG	Chr.5	9981081	9981101	+	No
2560643_adh	TTAGAAAAGTTACTCTCGGCT	Chr.5	9981158	9981138	-	No
2380917_adh	TGGAGCAAAAGAATACGCTGT	Chr.5	9990087	9990067	-	No
2747183_adh	TTTTTGGAAGATTGAGATGAA	Chr.5	10003400	10003380	-	No
2516266_adh	TGTGGGAAAAGACGCCGATCA	Chr.5	10005555	10005575	+	No
2414225_adh	TGGCTAGCATGACTGTTTTTT	Chr.5	10019074	10019054	-	No
2504495_adh	TGTGACTAGTATATAAAGGCT	Chr.5	10020249	10020269	+	No
1929744_adh	TATTTCCTCAAGACTCACATC	Chr.5	10023448	10023468	+	No
2606179_adh	TTCGCAAATAAGGAAGATTGT	Chr.5	10055965	10055985	+	No
2741038_adh	TTTTGACCCCATTTGTATGAT	Chr.5	10076790	10076810	+	No
2709905_adh	TTTCTCATTGATTTTCGGATA	Chr.5	10078284	10078264	-	No
2526878_adh	TGTTCTCTTTCGTCGTCCGGA	Chr.5	10091260	10091240	-	No
2531659_adh	TGTTTAATTAATTGTAAGAAC	Chr.5	10091798	10091818	+	No
2597746_adh	TTCCCCAAAGATCGAATGGGA	Chr.5	10160892	10160872	-	No
2161703_adh	TGACGGAGTGGATTACATGCA	Chr.5	10171929	10171909	-	No
2692301_adh	TTTAATTCAGTTGAGCTCCTA	Chr.5	10176785	10176765	-	No
2148206_adh	TGAATTTATTCGGGTCTGCAG	Chr.5	10177865	10177845	-	No
2737244_adh	TTTTCAATATTCGGCTTCATG	Chr.5	10200952	10200972	+	No
2287923_adh	TGCAGATCGTGATGGAAAAAT	Chr.5	10202783	10202763	-	No
2699141_adh	TTTATGCGTATAAGTAGAAGA	Chr.5	10204886	10204866	-	No
2502991_adh	TGTGAATTCACTCTTGCAGAT	Chr.5	10212404	10212384	-	No
2554255_adh	TTACAGCAGGATTAGAAAGTT	Chr.5	10212536	10212556	+	No
1866709_adh	TAGGATTTGAGGCATATTTAC	Chr.5	10228822	10228842	+	No
2233756_adh	TGAGCCGTCGTATCCCTGGAC	Chr.5	10238797	10238777	-	No
1776582_adh	TAAGAGACGGAGAATGAGAAA	Chr.5	10247983	10247963	-	No
2046490_adh	TCTGAGTAGGCATTGAGCATT	Chr.5	10254470	10254490	+	No
2587331_adh	TTCAAGTAATAACTGACAGCA	Chr.5	10255335	10255315	-	No
2294699_adh	TGCCTCTGAACATAACAGAAA	Chr.5	10255469	10255449	-	No
2623461_adh	TTCTTTCCTGTCGTTGAAATT	Chr.5	10298145	10298165	+	No
2734588_adh	TTTTAGATCGATTATGGACAT	Chr.5	10310958	10310938	-	No
2034389_adh	TCTATGAAGAACATAAAAATT	Chr.5	10338640	10338620	-	No
2623856_adh	TTCTTTTCTGTATATTTTAAT	Chr.5	10338680	10338660	-	No
2704025_adh	TTTCATCGTCCCATTAACAAT	Chr.5	10342131	10342151	+	No
1830194_adh	TACTAGATTGGCTAGCTTTGA	Chr.5	10342865	10342845	-	No
1938555_adh	TCAAGACTGAAGCATATGAAT	Chr.5	10349818	10349838	+	No
2313652_adh	TGGAAAAGCTGGAACCAAGAA	Chr.5	10351142	10351122	-	No
1783778_adh	TAAGGAAAGATGTATTTATAA	Chr.5	10353063	10353043	-	No
2258892_adh	TGATAAGGAAAGATGTATTTA	Chr.5	10353066	10353046	-	No
2343026_adh	TGGAATGATAAGGAAAGATGT	Chr.5	10353071	10353051	-	No
1833158_adh	TACTGGAATGATAAGGAAAGA	Chr.5	10353074	10353054	-	No
1834661_adh	TACTTATCGATCCAATTTCGG	Chr.5	10353114	10353094	-	No
2011174_adh	TCGGACAAAATAATGGCAGTG	Chr.5	10353229	10353249	+	No
2589623_adh	TTCACGCGGACAAGGTGTTTT	Chr.5	10356071	10356051	-	No
2648778_adh	TTGCAATAGAGGACGAGAAGT	Chr.5	10362173	10362153	-	No
2506640_adh	TGTGATAATGATTGCAGAAAA	Chr.5	10366697	10366677	-	No
2737471_adh	TTTTCAGCAGTATCGTCGTTT	Chr.5	10406390	10406370	-	No
2730039_adh	TTTGTGTATAATTAGTGTGAA	Chr.5	10406492	10406512	+	No

2746486_adh	TTTTTGAAACGAGCACATGAC	Chr.5	10426454	10426474	+	No
1831818_adh	TACTGAACATATTTGACGCGC	Chr.5	10436506	10436486	-	No
2672224_adh	TTGGTTCTAGAACTATAATTA	Chr.5	10441336	10441356	+	No
1774239_adh	TAAGAAGGAAAAAGACAAATA	Chr.5	10459993	10460013	+	No
1773091_adh	TAAGAAAAATAAGTTGTTGAG	Chr.5	10470056	10470036	-	No
1745423_adh	TAAAAAAGAAGAAGAAACAGA	Chr.5	10481309	10481289	-	No
2489567_adh	TGTAGTATGATCTCGTGGCAT	Chr.5	10511874	10511894	+	No
2665322_adh	TTGGCATCTTTTGGCAGTTTC	Chr.5	10526146	10526166	+	No
2602094_adh	TTCGAAGAAGTTGGAAATCAT	Chr.5	10536338	10536358	+	No
2148197_adh	TGAATTTATGCAGAAAATGAG	Chr.5	10542235	10542215	-	No
2616470_adh	TTCTCGACAAATTTATTTACC	Chr.5	10577185	10577205	+	No
2036775_adh	TCTCAATTTTTTGAAAATGAG	Chr.5	10579014	10578994	-	No
1988440_adh	TCCGTGAATTCAGAGCAGAGT	Chr.5	10595256	10595276	+	No
2592752_adh	TTCAGTAGGAATGTCGTCATC	Chr.5	10597510	10597530	+	No
1937341_adh	TCAACTCGGATGGCACCAAAC	Chr.5	10598346	10598366	+	No
2732933_adh	TTTTAAAAAATTGTGGACGGT	Chr.5	10648045	10648025	-	No
2061591_adh	TCTTTGAAAGCACGGATCAAT	Chr.5	10657861	10657841	-	No
2015196_adh	TCGGCAATCTTAGCGGTGTTC	Chr.5	10661294	10661314	+	No
2739652_adh	TTTTCTGTAGGGCGGCAATTC	Chr.5	10661594	10661574	-	No
2053581_adh	TCTTAAAAGCAAAAGTAGCGT	Chr.5	10661880	10661900	+	No
1777967_adh	TAAGATACTGCAGGAATGAAA	Chr.5	10669123	10669103	-	No
2729698_adh	TTTGTGCAACTGTACTCGATC	Chr.5	10686024	10686044	+	No
2394674_adh	TGGATTGGTTACGGTATTCTC	Chr.5	10704159	10704179	+	No
2706976_adh	TTTCGACTGTTTTTGTAGCAT	Chr.5	10706295	10706315	+	No
1969055_adh	TCATAGCACCCGAATGTAGAT	Chr.5	10712075	10712055	-	No
1985362_adh	TCCGCGCAGATAATATCATAA	Chr.5	10720431	10720411	-	No
1875521_adh	TAGTATGGCACTCCCTAATAG	Chr.5	10733439	10733459	+	No
2389348_adh	TGGATCAAGAAAGGACAAAAA	Chr.5	10760509	10760529	+	No
1820249_adh	TACCTGAAGAAGTACAATTAA	Chr.5	10762766	10762786	+	No
2617982_adh	TTCTGACACTGAGAGAATCGT	Chr.5	10771066	10771086	+	No
1821545_adh	TACGAAATTAGAAGAATTGTA	Chr.5	10773709	10773689	-	No
1994548_adh	TCGAAAAGCGGTGGTTTTGAA	Chr.5	10808026	10808006	-	No
2700917_adh	TTTCAAAAAACAACGTGCGTC	Chr.5	10809009	10809029	+	No
1995497_adh	TCGAACCAGAAAACGATACAT	Chr.5	10813935	10813915	-	No
2617385_adh	TTCTCTGATCATGACGGAAGG	Chr.5	10827069	10827089	+	No
2315077_adh	TGGAAAGAAAAGAAGCGAAGA	Chr.5	10855034	10855014	-	No
1901489_adh	TATGAAATTCTGATGATCCAA	Chr.5	10884896	10884876	-	No
2290005_adh	TGCATAGTTTTAGAATTTCGG	Chr.5	10890096	10890116	+	No
2391001_adh	TGGATGATGGACAACAACAAC	Chr.5	10894657	10894637	-	No
2130714_adh	TGAAGCAGTAGAGCGCACTTG	Chr.5	10894743	10894763	+	No
2590069_adh	TTCACTGTTACAACGAAAAAT	Chr.5	10894746	10894726	-	No
1971256_adh	TCATCGTCGGAGCTTAATTGG	Chr.5	10896872	10896852	-	No
2739667_adh	TTTTCTGTCGTCTTTTCGGTT	Chr.5	10897241	10897221	-	No
1994016_adh	TCCTTTCAGTTTTCTGTCGTC	Chr.5	10897250	10897230	-	No
2039134_adh	TCTCCTCGAACATCGTCATTC	Chr.5	10897269	10897249	-	No
1845017_adh	TAGACAGCAAAATACTTCGAA	Chr.5	10897463	10897483	+	No
2127879_adh	TGAAGACGATACATGTAGAAT	Chr.5	10938451	10938431	-	No
2702962_adh	TTTCAGATGAGCTTTACTAAC	Chr.5	10965521	10965501	-	No
2608579_adh	TTCGGCGCCAAGAATCAGGTT	Chr.5	10965607	10965587	-	No
1852046_adh	TAGATTCTCCGACTCGTTGGT	Chr.5	10965774	10965794	+	No

1835668_adh	TACTTGGTTGGAGAACGTCTT	Chr.5	10966393	10966413	+	No
2486540_adh	TGTACCAAAGACTGGAGAAGT	Chr.5	10967301	10967321	+	No
2531761_adh	TGTTTACTCTGAGTCGGAAAG	Chr.5	10967812	10967832	+	No
1884085_adh	TATAAAGGACCCGGTATCACT	Chr.5	10967854	10967874	+	No
2621919_adh	TTCTTCGTTTTCGGTCGTCGT	Chr.5	10967971	10967991	+	No
2693394_adh	TTTACATCAGTGCTGTGCGGC	Chr.5	11003601	11003581	-	No
1971829_adh	TCATGAGAAGACGAAGAATGA	Chr.5	11028649	11028669	+	No
2410975_adh	TGGCCGAACAAACACGTACGG	Chr.5	11029324	11029304	-	No
2604907_adh	TTCGATCGTTTGCGCTGTAGC	Chr.5	11039542	11039522	-	No
2692311_adh	TTTAATTCGATCGTTTGCGCT	Chr.5	11039547	11039527	-	No
2515535_adh	TGTGGATCTCCAAGCAAGAAT	Chr.5	11072053	11072033	-	No
2049822_adh	TCTGGATCGGAGGATCTATCC	Chr.5	11072101	11072081	-	No
1821653_adh	TACGAACTTCCTGACGGACAA	Chr.5	11072452	11072432	-	No
2000776_adh	TCGACTTCGAGCAAGAAATGG	Chr.5	11072508	11072488	-	No
2057873_adh	TCTTGACTTGGCTGGACGTGA	Chr.5	11072639	11072619	-	No
2000395_adh	TCGACTCTGGAGATGGTGTCA	Chr.5	11072712	11072692	-	No
2379589_adh	TGGAGAAGATCTGGCATCACA	Chr.5	11072925	11072905	-	No
1914002_adh	TATGGAGAAGATCTGGCATCA	Chr.5	11072927	11072907	-	No
2434838_adh	TGGGATGATATGGAGAAGATC	Chr.5	11072935	11072915	-	No
1931340_adh	TATTTTTCGCGTCGACTCGGT	Chr.5	11076050	11076030	-	No
2515535_adh	TGTGGATCTCCAAGCAAGAAT	Chr.5	11076941	11076921	-	No
2049822_adh	TCTGGATCGGAGGATCTATCC	Chr.5	11076989	11076969	-	No
1821653_adh	TACGAACTTCCTGACGGACAA	Chr.5	11077339	11077319	-	No
2057873_adh	TCTTGACTTGGCTGGACGTGA	Chr.5	11077526	11077506	-	No
2434838_adh	TGGGATGATATGGAGAAGATC	Chr.5	11081335	11081355	+	No
1914002_adh	TATGGAGAAGATCTGGCATCA	Chr.5	11081343	11081363	+	No
2379589_adh	TGGAGAAGATCTGGCATCACA	Chr.5	11081345	11081365	+	No
2000395_adh	TCGACTCTGGAGATGGTGTCA	Chr.5	11081558	11081578	+	No
2057873_adh	TCTTGACTTGGCTGGACGTGA	Chr.5	11081631	11081651	+	No
2000776_adh	TCGACTTCGAGCAAGAAATGG	Chr.5	11081762	11081782	+	No
1821653_adh	TACGAACTTCCTGACGGACAA	Chr.5	11081818	11081838	+	No
2049822_adh	TCTGGATCGGAGGATCTATCC	Chr.5	11082168	11082188	+	No
2515535_adh	TGTGGATCTCCAAGCAAGAAT	Chr.5	11082216	11082236	+	No
2495878_adh	TGTCATGAGGGCAGAGACGAA	Chr.5	11121398	11121378	-	No
2378871_adh	TGGACTTCAGGAGGCCAAGAA	Chr.5	11123411	11123391	-	No
2391457_adh	TGGATGGAGAAGAAGACACTG	Chr.5	11147516	11147536	+	No
2712436_adh	TTTGAAAAGGGAAGTAGAATG	Chr.5	11171161	11171181	+	No
1991449_adh	TCCTGCGGACGGCATTTCTTT	Chr.5	11174780	11174760	-	No
2648858_adh	TTGCAATTAGGAAATGGAATA	Chr.5	11175794	11175814	+	No
1877212_adh	TAGTGAACTCAAGTGGGTAGA	Chr.5	11177161	11177181	+	No
2066336_adh	TGAAAAATATACATCAAAATT	Chr.5	11180602	11180582	-	No
1811091_adh	TACACGCTTACTCATCGTTCA	Chr.5	11200415	11200395	-	No
1959112_adh	TCAGAGCTCATTAGAACTGCA	Chr.5	11209643	11209663	+	No
2743729_adh	TTTTGTCAACTGTCCTTCGGC	Chr.5	11226219	11226239	+	No
2282500_adh	TGCAAGGTAGATTCATAATAT	Chr.5	11238797	11238777	-	No
2610444_adh	TTCGGTTATCTAAAACGTGGA	Chr.5	11279614	11279594	-	No
2692288_adh	TTTAATTCAAGTATGCGCTAT	Chr.5	11280823	11280843	+	No
1919648_adh	TATGTTTCGGAGTTCTTTTAT	Chr.5	11305914	11305894	-	No
2540751_adh	TTAAAGACATGTTTGCTTTCA	Chr.5	11328830	11328850	+	No
2728634_adh	TTTGTATTTTCTTAGGACACA	Chr.5	11357535	11357515	-	No

1891181_adh	TATCAAACGGCCGTTACTAAA	Chr.5	11369878	11369858	-	Yes
1783818_adh	TAAGGAAATCACGTAGAATGG	Chr.5	11374015	11373995	-	No
1912290_adh	TATGATTTTGTCCATTGAACA	Chr.5	11384125	11384105	-	No
1901583_adh	TATGAACCATTGGAAGAGTAA	Chr.5	11385462	11385442	-	No
2732818_adh	TTTGTTTTGAACATAGGTGGA	Chr.5	11388779	11388799	+	No
1901583_adh	TATGAACCATTGGAAGAGTAA	Chr.5	11389725	11389745	+	No
2618299_adh	TTCTGAGACGCAACGACGAAT	Chr.5	11412664	11412644	-	No
1883999_adh	TATAAACATAGACGGAGGATA	Chr.5	11478318	11478338	+	No
1798528_adh	TAATCGGAACTTCGGATGCAG	Chr.5	11479919	11479899	-	No
1901583_adh	TATGAACCATTGGAAGAGTAA	Chr.5	11484139	11484119	-	No
1891043_adh	TATATTTTCAACATTCAAGAT	Chr.5	11485582	11485602	+	No
2613664_adh	TTCGTTTTTGTGGAGAGAGGG	Chr.5	11504850	11504830	-	No
2709389_adh	TTTCGTTTTTGTGGAGAGAGG	Chr.5	11504851	11504831	-	No
2734977_adh	TTTTAGTTACGCTATGTCCCC	Chr.5	11513412	11513392	-	No
2037480_adh	TCTCAGCGATCGAAGGTCAGA	Chr.5	11517896	11517916	+	No
1976727_adh	TCCAATCAGAACGGTAGTTCG	Chr.5	11523604	11523584	-	No
2441052_adh	TGGGATGGGATGGTAGATGTA	Chr.5	11540640	11540620	-	No
2628672_adh	TTGAAGCGTAGTGTAGTGAAA	Chr.5	11554356	11554336	-	No
2288713_adh	TGCAGTAGACGAATTGAATGT	Chr.5	11573099	11573119	+	No
2648471_adh	TTGCAAGATAGAGGTTCCTAA	Chr.5	11585491	11585511	+	No
1766389_adh	TAACATTGCGAGTGGTAGAAA	Chr.5	11595495	11595475	-	No
2047685_adh	TCTGATTTTATGAAGGTTTGA	Chr.5	11595640	11595660	+	No
2017032_adh	TCGGGGACGAAAGAAGATTGT	Chr.5	11610575	11610555	-	No
2744700_adh	TTTTTAAAGCGATCGTTCGAT	Chr.5	11625356	11625336	-	No
2296554_adh	TGCGACGCGAGCTACATTTAT	Chr.5	11644369	11644389	+	No
2002491_adh	TCGATAAATGTTTTCTGAGAT	Chr.5	11644409	11644429	+	No
2618523_adh	TTCTGATTTCAATGTAGAAGA	Chr.5	11672564	11672544	-	No
1897227_adh	TATCGATTAGGTCGGCAAATA	Chr.5	11673358	11673338	-	No
2003257_adh	TCGATATCGATTAGGTCGGCA	Chr.5	11673362	11673342	-	No
1986390_adh	TCCGGCGGCGTGTTGTTCATT	Chr.5	11673461	11673481	+	No
1992133_adh	TCCTGTACTCACATCGTTGAC	Chr.5	11674208	11674228	+	No
1972152_adh	TCATGATCTTCGAACAATTGC	Chr.5	11732169	11732149	-	No
2623710_adh	TTCTTTGTCAGGTTCGTCGTC	Chr.5	11733756	11733776	+	No
2697340_adh	TTTAGTGGACTGTTGCGACAA	Chr.5	11764751	11764771	+	No
2378772_adh	TGGACTGTTGCGACAATGCAG	Chr.5	11764756	11764776	+	No
2734461_adh	TTTTAGAAGTTTAATGAAAAA	Chr.5	11765371	11765351	-	No
2590193_adh	TTCAGAAAACGAACAAACCAA	Chr.5	11770583	11770563	-	No
2701087_adh	TTTCAAAGCAAACATAATCAC	Chr.5	11770914	11770934	+	No
2088709_adh	TGAAATATAAAAATGTATCTA	Chr.5	11785585	11785565	-	No
2148232_adh	TGAATTTCATATAATAATTCG	Chr.5	11787813	11787793	-	No
2513214_adh	TGTGCAAGAGCTCAACGAAGT	Chr.5	11788517	11788537	+	No
1931749_adh	TCAAAAAATGAGGGCATGTAA	Chr.5	11790233	11790213	-	No
1827799_adh	TACGTAGAAATCTAATGATGG	Chr.5	11813675	11813655	-	No
1827799_adh	TACGTAGAAATCTAATGATGG	Chr.5	11817723	11817703	-	No
1933227_adh	TCAAAGAGGACCAACTGAGTT	Chr.5	11820418	11820398	-	No
2024618_adh	TCGTATCGCCGAAGGAAAATC	Chr.5	11821158	11821138	-	No
1932057_adh	TCAAAACGGATATGTTGAAAA	Chr.5	11826157	11826137	-	No
2547042_adh	TTAAGAGATCACGCGTGAAGG	Chr.5	11836900	11836920	+	No
1766888_adh	TAACCGAACTCGAACTAAAGC	Chr.5	11847179	11847199	+	No
1925832_adh	TATTGAGCAATTGTCGGAAGA	Chr.5	11866808	11866788	-	No

1866704_adh	TAGGATTTGACAGAAGAACAA	Chr.5	11871260	11871280	+	No
1943051_adh	TCAATTGAAGAATACATGAAG	Chr.5	11874496	11874516	+	No
2659713_adh	TTGGAGCATATTTTGGACAAT	Chr.5	11886657	11886637	-	No
2391592_adh	TGGATGGCAACTTGGAGACAA	Chr.5	11888521	11888541	+	No
2057800_adh	TCTTGACGTAGATGAAGCTAT	Chr.5	11889264	11889284	+	No
2131794_adh	TGAAGCTATGGAATTCGTTAC	Chr.5	11889276	11889296	+	No
1867253_adh	TAGGCGAAGATTGAAACAGCT	Chr.5	11916153	11916173	+	No
2487395_adh	TGTAGAAAATTTACGTTCAGA	Chr.5	11917172	11917192	+	No
2487903_adh	TGTAGATAATTTTCATGCAGA	Chr.5	11917337	11917317	-	No
2565625_adh	TTAGATGTTAATTTTGAAATA	Chr.5	11921737	11921757	+	No
2544702_adh	TTAAATTTCTTAACGTAGACG	Chr.5	11924305	11924285	-	No
2449832_adh	TGGGCACAATTGAAACGATGA	Chr.5	11924582	11924562	-	No
1754780_adh	TAAACCTTGCATGAATATTTC	Chr.5	11963565	11963545	-	No
2617474_adh	TTCTCTGTTTACGAAGACTTC	Chr.5	11976432	11976452	+	No
1767506_adh	TAACCTGAACTTTGAAGACGA	Chr.5	11978324	11978344	+	No
2373449_adh	TGGACAAGGAGGTGACAATGA	Chr.5	11981367	11981347	-	No
1991013_adh	TCCTGAAGAAAACAAGTTGAG	Chr.5	11993802	11993782	-	No
1790960_adh	TAATACAGAATAAGCAAACCA	Chr.5	11993902	11993922	+	No
1973928_adh	TCATTCTTTACTCGTCTTTTA	Chr.5	11993956	11993976	+	No
2413159_adh	TGGCGTAAAAAGAAACATGAA	Chr.5	11993993	11994013	+	No
1845609_adh	TAGACTAGAAGAGGTTCTGTA	Chr.5	11995164	11995144	-	No
2657848_adh	TTGGAATTTGAAAGATCATTC	Chr.5	12001628	12001648	+	No
1940721_adh	TCAAGTTGGTATTTAAAAATT	Chr.5	12001647	12001667	+	No
2031536_adh	TCTACGAAGATTTTGAGCATT	Chr.5	12004123	12004143	+	No
2647044_adh	TTGATTTTCTTGTGTAGGATA	Chr.5	12014251	12014231	-	No
2240414_adh	TGAGGGTGGAATAGGAGGACG	Chr.5	12038961	12038981	+	No
1868458_adh	TAGGGAGAGAATTAAGGCGAT	Chr.5	12041447	12041427	-	No
1970973_adh	TCATCGCAAGCACAAAGTGAG	Chr.5	12056067	12056047	-	No
2410466_adh	TGGCCAAATGTAGAAGACAGT	Chr.5	12072113	12072093	-	No
2526888_adh	TGTTCTGAACTACTTTTTAAA	Chr.5	12127902	12127922	+	No
1811747_adh	TACAGAAGTCGACGAAACAAA	Chr.5	12133460	12133480	+	No
1810690_adh	TACACAGCGACGATTTTTGAT	Chr.5	12137130	12137150	+	No
2081337_adh	TGAAAGCCGAACAGCAGATTA	Chr.5	12171757	12171777	+	No
2678337_adh	TTGTCGGCCGATATCGGTATT	Chr.5	12191182	12191202	+	No
1895491_adh	TATCCAGGAAGAGATGGTCAG	Chr.5	12197963	12197943	-	No
2119631_adh	TGAACAAGAATTGGATGGAGA	Chr.5	12227426	12227406	-	No
2590278_adh	TTCAGAACAAGAAGGACGCCA	Chr.5	12255245	12255265	+	No
2276191_adh	TGATTGAAACATTCATAGGCA	Chr.5	12263367	12263387	+	No
1866130_adh	TAGGATTAAAACATTAAATAA	Chr.5	12291444	12291424	-	No
2103625_adh	TGAAATGGAAAAAAGAGAAAA	Chr.5	12333977	12333957	-	No
2008068_adh	TCGCGGTAGACGATGTTCAAC	Chr.5	12354076	12354096	+	No
2523704_adh	TGTTAGTGCTGATTTTCGGAA	Chr.5	12355043	12355063	+	No
2305320_adh	TGCTAACATTGAGACTTCTTT	Chr.5	12386275	12386295	+	No
1999910_adh	TCGACCTATCAGAAGACCTAT	Chr.5	12386656	12386676	+	No
1999910_adh	TCGACCTATCAGAAGACCTAT	Chr.5	12401235	12401215	-	No
1983185_adh	TCCCTGAGTAGAATGGTAGAC	Chr.5	12411460	12411440	-	No
2711748_adh	TTTCTTGATCTCTTCGGTTAT	Chr.5	12412170	12412150	-	Yes
2686846_adh	TTGTTTAGAACAGAATCATCG	Chr.5	12497968	12497988	+	No
2561005_adh	TTAGAACAGAATCATCGCATT	Chr.5	12497972	12497992	+	No
2313974_adh	TGGAAAATTAGATTGTAGTGC	Chr.5	12525176	12525196	+	No

2050077_adh	TCTGGCATTTAGAACGGATTA	Chr.5	12545537	12545557	+	No
2545343_adh	TTAACGAAAAACTTTGATGGC	Chr.5	12560246	12560226	-	No
2688965_adh	TTTAAAATTATTGAACGCGGT	Chr.5	12560602	12560622	+	No
2296307_adh	TGCGACACGGTCTCTCAAATT	Chr.5	12605663	12605643	-	No
2622441_adh	TTCTTGAAACATGGGCAAGAA	Chr.5	12612205	12612185	-	No
2729895_adh	TTTGTGGATATGACGTGACGG	Chr.5	12637007	12636987	-	No
2466884_adh	TGGTATGTAAAGTAGTATGTA	Chr.5	12644885	12644905	+	No
1829145_adh	TACGTTTGAAGCCGGAAAATG	Chr.5	12651829	12651809	-	No
2721528_adh	TTTGCCAATAACGAAAACGAC	Chr.5	12651858	12651838	-	No
2732944_adh	TTTTAAAAAGCAAAATTGACA	Chr.5	12651957	12651977	+	No
2624759_adh	TTGAAACCCAAAAACAGGACA	Chr.5	12651989	12652009	+	No
2094902_adh	TGAAATCGCGCAAGACTTCAA	Chr.5	12660087	12660067	-	No
2624243_adh	TTGAAAAAATACCTTATGTGA	Chr.5	12660671	12660691	+	No
2593730_adh	TTCATAGTTTCGTTTTATTCG	Chr.5	12660912	12660932	+	No
2707009_adh	TTTCGAGACGGTGCTTGACAT	Chr.5	12661203	12661223	+	No
1943338_adh	TCAATTTCTTTTGTCGAGGTA	Chr.5	12661740	12661760	+	No
2590907_adh	TTCAGATTGTTTGGCATTATG	Chr.5	12662089	12662109	+	No
2002373_adh	TCGAGTGGAAGGTGAAAGATC	Chr.5	12692310	12692290	-	No
2603984_adh	TTCGAGTAGATTAACGGGATG	Chr.5	12702461	12702441	-	No
2526087_adh	TGTTCAGCAGATCTGTAAACA	Chr.5	12715690	12715670	-	No
2589870_adh	TTCACTCATCGTTCGGTAACA	Chr.5	12717217	12717237	+	No
2695269_adh	TTTAGACTTCTTGACGGTATT	Chr.5	12725382	12725402	+	No
2729057_adh	TTTGTCGTATATCTTGCCAAC	Chr.5	12743048	12743068	+	No
1992346_adh	TCCTGTTGAAGAATTTAATTA	Chr.5	12749262	12749282	+	No
2604219_adh	TTCGATAAAGCGAGTCTGACA	Chr.5	12763002	12762982	-	No
2737932_adh	TTTTCCCGGCGTTTAATTTAC	Chr.5	12763690	12763710	+	No
2703553_adh	TTTCAGTCGGTTTATTTTGAT	Chr.5	12763740	12763760	+	No
2279693_adh	TGCAAAAACTCTACAGGGACG	Chr.5	12768538	12768518	-	No
2696106_adh	TTTAGCGGAAGGAATCCATGG	Chr.5	12818721	12818701	-	No
1839805_adh	TAGAAGTCGTAAAAAACAAAC	Chr.5	12865643	12865623	-	No
1983849_adh	TCCGAACTAACGTAGCGCGCA	Chr.5	12865698	12865718	+	No
2607253_adh	TTCGGAATGTCGTTGCCATCT	Chr.5	12865703	12865683	-	No
2694796_adh	TTTAGAAAATGGAACAAGACG	Chr.5	12870019	12869999	-	No
2726634_adh	TTTGGTTATTGGTCAACTTTT	Chr.5	12909212	12909192	-	No
2035434_adh	TCTCAACCAACTTTGGCCACT	Chr.5	12919210	12919230	+	No
2620430_adh	TTCTGTTTGGTCAAGATGGAG	Chr.5	12949903	12949923	+	No
2082136_adh	TGAAAGGACAAATGCAGAAAG	Chr.5	12961582	12961602	+	No
2590518_adh	TTCAGACGGTAAGCATTGCAG	Chr.5	12965126	12965146	+	No
2748505_adh	TTTTTTCACAACGAAAACGCG	Chr.5	12970780	12970800	+	No
2591903_adh	TTCAGCTGTATTTTTTATCTA	Chr.5	13000304	13000324	+	No
2744561_adh	TTTTGTTTTCATCAATCGGAT	Chr.5	13000335	13000355	+	No
2623156_adh	TTCTTGTGATCTGTTCAGCGT	Chr.5	13000535	13000555	+	No
1894064_adh	TATCATCGAGACGGCTTGACG	Chr.5	13003010	13002990	-	No
2048087_adh	TCTGCCAGTTGAAGAACTCAA	Chr.5	13006264	13006284	+	No
2722017_adh	TTTGCGGAAGAATCAGAGAAT	Chr.5	13043018	13042998	-	No
1757144_adh	TAAAGCAAGTGGATGGGCTCG	Chr.5	13045366	13045386	+	No
2100792_adh	TGAAATGAAAATAAAAATTAA	Chr.5	13047958	13047938	-	No
1762993_adh	TAACAAAAAGCGACGAAAAAG	Chr.5	13057636	13057656	+	No
1953599_adh	TCACGTGTTTAATGGATTGAC	Chr.5	13061631	13061611	-	No
1994609_adh	TCGAAAATGTTTGTCAGAAGA	Chr.5	13062137	13062157	+	No

2671070_adh	TTGGTGGAGCAAAAGTCATGA	Chr.5	13063476	13063456	-	No
2370208_adh	TGGAATTGAATGAGAAATGTA	Chr.5	13069073	13069053	-	No
2547281_adh	TTAAGATGAAATTATTGAAAG	Chr.5	13085727	13085707	-	No
2692385_adh	TTTAATTGAAGTCTCATCGGA	Chr.5	13085859	13085879	+	No
2629207_adh	TTGAAGTCTCATCGGAATTCT	Chr.5	13085864	13085884	+	No
1942815_adh	TCAATTACTGGTAGAACGGTG	Chr.5	13091253	13091233	-	No
2025310_adh	TCGTCACCGGTCGTCTCCACC	Chr.5	13094995	13095015	+	No
2471153_adh	TGGTGACTGCAGAGCCGTCGG	Chr.5	13115972	13115992	+	No
1847621_adh	TAGAGATCCCATGGCATGAAT	Chr.5	13127156	13127176	+	No
1996574_adh	TCGAAGGATTCCAGAAGCAAG	Chr.5	13127702	13127682	-	No
2683493_adh	TTGTTCACCGTGATGGCCAAA	Chr.5	13129335	13129315	-	No
2010560_adh	TCGGAACCAATCTCGAAACGG	Chr.5	13129870	13129850	-	No
1900731_adh	TATCTTTTTCGTTTCGTACGG	Chr.5	13152622	13152642	+	No
2710233_adh	TTTCTCTTCTTCTGTTGGACA	Chr.5	13159383	13159403	+	No
2706776_adh	TTTCGAATTCTTGTTGGACGG	Chr.5	13162847	13162867	+	No
1762862_adh	TAAATTTTAACAAAAAATAAA	Chr.5	13164478	13164458	-	No
2608180_adh	TTCGGATATTTTTTGTTTTAA	Chr.5	13193719	13193699	-	No
2514215_adh	TGTGCTTCAGGAAAAAGTAGA	Chr.5	13217365	13217385	+	No
2662756_adh	TTGGATGCTGCAGATTTGGAT	Chr.5	13277125	13277105	-	No
1896869_adh	TATCGACTTAGAAACGCGCTT	Chr.5	13315873	13315893	+	No
2075786_adh	TGAAAGACAAAGAAAAATGGA	Chr.5	13316388	13316408	+	No
1978642_adh	TCCAGGACAAGATGGACAACC	Chr.5	13317174	13317154	-	No
2734549_adh	TTTTAGAGGATGGTAGTGTAG	Chr.5	13371448	13371428	-	No
2290018_adh	TGCATATCATTAGCATCGGAT	Chr.5	13373449	13373469	+	No
1747067_adh	TAAAAATCAAAGGACATTTCT	Chr.5	13379684	13379704	+	No
2121587_adh	TGAACATTGGAATCTGGCAAG	Chr.5	13416102	13416122	+	No
2379600_adh	TGGAGAAGATTAAGAAGAACA	Chr.5	13432989	13432969	-	No
2733115_adh	TTTTAAAGGAAAACAGCGGAA	Chr.5	13437699	13437719	+	No
1978642_adh	TCCAGGACAAGATGGACAACC	Chr.5	13443235	13443255	+	No
2075786_adh	TGAAAGACAAAGAAAAATGGA	Chr.5	13444021	13444001	-	No
2272012_adh	TGATGCTCTTCTGAAGACAAT	Chr.5	13466634	13466654	+	No
2011824_adh	TCGGACTCACGTGCATTTTTA	Chr.5	13468379	13468399	+	No
2058259_adh	TCTTGCACAACTGGAGCTGAA	Chr.5	13480043	13480063	+	No
2613702_adh	TTCTAAAATTCAAATTCGAAC	Chr.5	13506072	13506092	+	No
2134154_adh	TGAAGTAGTAGAAAAGAAAAA	Chr.5	13527355	13527335	-	No
2054997_adh	TCTTATATTGAAGTAGTAGAA	Chr.5	13527363	13527343	-	No
2003962_adh	TCGATGAAGAACTGGCGAAAA	Chr.5	13528750	13528730	-	No
2719588_adh	TTTGATGTTTGGATTTCGGAG	Chr.5	13538873	13538853	-	No
2344587_adh	TGGAATGCAAACGATAAGACG	Chr.5	13556989	13557009	+	No
2487570_adh	TGTAGAATTGGCTGACTGAAA	Chr.5	13560543	13560523	-	No
2590716_adh	TTCAGAGTGGGTGTACTTGAG	Chr.5	13565380	13565360	-	No
2713491_adh	TTTGAACTTAAATTGACACAT	Chr.5	13568637	13568617	-	No
1990771_adh	TCCTCTCGGCCAAGTCAGATG	Chr.5	13591695	13591715	+	No
2648770_adh	TTGCAATAAGTAGTCTTCAGC	Chr.5	13591829	13591849	+	No
2588124_adh	TTCAATGCAGAAAACATGACA	Chr.5	13596173	13596193	+	No
2624324_adh	TTGAAAAAATTTCAATCGTCTC	Chr.5	13618253	13618273	+	No
2281379_adh	TGCAACATATCTGACGCGCAA	Chr.5	13620543	13620523	-	No
2546602_adh	TTAACTTTGAAAATGATGGAA	Chr.5	13622238	13622258	+	No
1874886_adh	TAGTAGTAGAGCGCATATTAA	Chr.5	13625975	13625995	+	No
1841099_adh	TAGAATATGAAGCTCAGAGAA	Chr.5	13650849	13650829	-	No

2713197_adh	TTTGAACGAGAACATACAAAC	Chr.5	13656936	13656956	+	No
1891083_adh	TATATTTTTGATGGCACAGCT	Chr.5	13660372	13660392	+	No
1929043_adh	TATTTAAGAATGAGCAAAGGG	Chr.5	13664713	13664693	-	No
1828801_adh	TACGTGGATTGTTTTCGTGTT	Chr.5	13672086	13672066	-	No
2729757_adh	TTTGTGCGTGGAACGGTGTGC	Chr.5	13672111	13672091	-	No
2703637_adh	TTTCAGTTTTATCGGCGCTTC	Chr.5	13715380	13715360	-	No
1747697_adh	TAAAACAGCGACCTGAAAACA	Chr.5	13715694	13715714	+	No
2309944_adh	TGCTGTTTTGTGTTAAAAGAT	Chr.5	13725517	13725537	+	No
2578823_adh	TTATGATGCTACAAATAAATA	Chr.5	13756694	13756714	+	No
2313352_adh	TGGAAAACACAATGTAGGGCA	Chr.5	13782315	13782295	-	No
2273549_adh	TGATGTGAGTCTGGAACTTGA	Chr.5	13923735	13923755	+	No
2005414_adh	TCGCAAAATAGAACAATGGTT	Chr.5	13944541	13944561	+	No
1821564_adh	TACGAACAATTTGATATGGAT	Chr.5	13966272	13966292	+	No
2648777_adh	TTGCAATAGAATAGACTGAAT	Chr.5	14008531	14008551	+	No
1745436_adh	TAAAAAAGACAATGGGAGACA	Chr.5	14011805	14011785	-	No
1978562_adh	TCCAGCTGTACGCAGTGACGT	Chr.5	14012280	14012300	+	No
1944403_adh	TCACAATTTTAACGGAACAAA	Chr.5	14014107	14014087	-	No
1824004_adh	TACGATCTGTGGCTACTATCT	Chr.5	14028380	14028360	-	No
2273549_adh	TGATGTGAGTCTGGAACTTGA	Chr.5	14031476	14031496	+	No
2719708_adh	TTTGATTATTCGGCATTATTT	Chr.5	14038434	14038414	-	No
2743684_adh	TTTTGTATGGAAAATTGATCG	Chr.5	14038968	14038988	+	No
2743684_adh	TTTTGTATGGAAAATTGATCG	Chr.5	14055022	14055002	-	No
1829906_adh	TACTAGAAACGGAAAGTGGGT	Chr.5	14066910	14066890	-	No
1973758_adh	TCATTCCCCGATTGAACGGAC	Chr.5	14067832	14067852	+	No
2177606_adh	TGAGAAATGAAGAATGAAAAT	Chr.5	14118307	14118287	-	No
2343030_adh	TGGAATGATAAGGAACAGGAA	Chr.5	14135226	14135206	-	No
2594853_adh	TTCATGTGCAAAGGACAAAAA	Chr.5	14135265	14135245	-	No
2020772_adh	TCGGTTTTTTGTTCGGCTTCA	Chr.5	14135344	14135324	-	No
2402775_adh	TGGCAATGTGGAATACTGGGT	Chr.5	14137336	14137356	+	No
2732098_adh	TTTGTTGGATGAAAAATGGTT	Chr.5	14168097	14168077	-	No
1998960_adh	TCGAATGTAGGGATGTTTGGT	Chr.5	14170656	14170636	-	No
2692876_adh	TTTAATTTAATCAAGGACAAA	Chr.5	14185593	14185573	-	No
2009971_adh	TCGCTTTAATTTAATCAAGGA	Chr.5	14185597	14185577	-	No
2611812_adh	TTCGTATTCGCTTTAATTTAA	Chr.5	14185604	14185584	-	No
2641403_adh	TTGAGGCGGAATACACAAATT	Chr.5	14185734	14185754	+	No
2044992_adh	TCTGACACGGATAGAATGATT	Chr.5	14202535	14202515	-	No
1935841_adh	TCAACAAATAGAAAGAAACGA	Chr.5	14226761	14226781	+	No
1964001_adh	TCAGGATGCATCAATGACGAG	Chr.5	14275476	14275456	-	No
2628550_adh	TTGAAGCAAGCTGGCGACTGT	Chr.5	14283541	14283521	-	No
1975668_adh	TCATTTTTTATGAAACGTCGG	Chr.5	14293758	14293738	-	No
1755255_adh	TAAACTAATGTGGAAGCGAAG	Chr.5	14350964	14350944	-	No
2271998_adh	TGATGCTATTATTTACGTCGG	Chr.5	14352468	14352488	+	No
2681212_adh	TTGTGCAACTCGTCGGAAAAG	Chr.5	14353517	14353537	+	No
1768631_adh	TAACGCTTTCAGAAACCTCGA	Chr.5	14353869	14353889	+	No
2291625_adh	TGCATTTTGTAGGAAATCGGA	Chr.5	14360732	14360712	-	No
2273183_adh	TGATGTAGTGGAGAGTTCGAA	Chr.5	14393456	14393436	-	Yes
2045921_adh	TCTGACTCTGATACGAAGGTT	Chr.5	14393795	14393815	+	No
2020798_adh	TCGTAAAAAGAAAACTGCATG	Chr.5	14394895	14394875	-	No
2610948_adh	TTCGTAAAAAGAAAACTGCAT	Chr.5	14394896	14394876	-	No
2689321_adh	TTTAAAGGCACACACGGTTTT	Chr.5	14406329	14406309	-	No

2715105_adh	TTTGACGACATAAACAAGAAT	Chr.5	14415385	14415405	+	No
2089044_adh	TGAAATATAAATAATTATTAA	Chr.5	14416682	14416702	+	No
2561404_adh	TTAGAAGAAATGCTGCAGGAA	Chr.5	14429209	14429229	+	No
2725987_adh	TTTGGTAGTTTCATTGATCTG	Chr.5	14439551	14439571	+	No
1754955_adh	TAAACGATTAACTGGGAAACA	Chr.5	14445368	14445388	+	No
2323235_adh	TGGAAGATTTGGAAGAGGACG	Chr.5	14452735	14452755	+	No
1770052_adh	TAACTAGCAGATCTAAAGAGG	Chr.5	14459923	14459903	-	No
2686709_adh	TTGTTTACAAGATGAGGTTGC	Chr.5	14463944	14463964	+	No
2696718_adh	TTTAGGGAATTCAAGTTCGGC	Chr.5	14468859	14468879	+	No
2474691_adh	TGGTTCAGACAAGTGGACAGG	Chr.5	14470351	14470371	+	No
2744632_adh	TTTTTAAAATCAAAGGCGAAT	Chr.5	14471910	14471930	+	No
2129941_adh	TGAAGATATGAAGAACGGTGA	Chr.5	14505982	14506002	+	No
2729485_adh	TTTGTGACTGATTACCAAAAA	Chr.5	14543308	14543288	-	No
2129056_adh	TGAAGAGAAGATTGAGAAGCT	Chr.5	14544099	14544079	-	No
1762701_adh	TAAATTTCAGACACCGAACTC	Chr.5	14545717	14545697	-	No
2043567_adh	TCTCTTTGTCGACGGATAAAC	Chr.5	14546318	14546298	-	No
2191677_adh	TGAGATACTCTACAGTAAGAC	Chr.5	14595748	14595768	+	No
2585368_adh	TTCAAAGGAGAAGGATGAGAG	Chr.5	14597919	14597899	-	No
2525978_adh	TGTTCACACAGCACTTAGCAG	Chr.5	14597987	14598007	+	No
2701690_adh	TTTCAATCAATTCGTTCGTTT	Chr.5	14612434	14612454	+	No
2559092_adh	TTACTGACGACGTGATGACGG	Chr.5	14613829	14613849	+	No
1762355_adh	TAAATTCTGACAGAAACTAGT	Chr.5	14640543	14640523	-	No
2597317_adh	TTCCATGATTGGTCAGTTCGG	Chr.5	14686536	14686556	+	No
2708934_adh	TTTCGTCGTTCGTCCATTCAA	Chr.5	14686659	14686679	+	No
2319987_adh	TGGAACGAAGATTGTCATGCA	Chr.5	14686698	14686718	+	No
1939603_adh	TCAAGGAGATCGTTAAGAAGC	Chr.5	14686760	14686780	+	No
1972570_adh	TCATGGAGAACTGCGAAGAGC	Chr.5	14687258	14687278	+	No
1970395_adh	TCATCAAGGGAGTCGTCGATT	Chr.5	14687300	14687320	+	No
2654824_adh	TTGCTGCTTCAGCTTTCGTCG	Chr.5	14687651	14687671	+	No
1970391_adh	TCATCAAGGACATTTTGGAGA	Chr.5	14687867	14687887	+	No
2723482_adh	TTTGGAGAAGAAGGTTGAGAA	Chr.5	14687880	14687900	+	No
2374062_adh	TGGACAGCAGAAGTATCATAT	Chr.5	14693562	14693542	-	No
2044649_adh	TCTGAATACTTTGCACAACGA	Chr.5	14750097	14750117	+	No
2025887_adh	TCGTGACGAAGAAGGTGCATT	Chr.5	14779718	14779698	-	No
1931153_adh	TATTTTGGATCGTCATTAAAT	Chr.5	14785114	14785134	+	No
2010095_adh	TCGGAAAAAATTGAAGGAAAAT	Chr.5	14821066	14821046	-	No
2714537_adh	TTTGAATTTGAATTTTGGCGC	Chr.5	14832560	14832540	-	No
2701213_adh	TTTCAAATTTAGAGACGCATT	Chr.5	14878158	14878138	-	No
2619734_adh	TTCTGTAAATTTGGCACTTTG	Chr.5	14882124	14882104	-	No
2619734_adh	TTCTGTAAATTTGGCACTTTG	Chr.5	14888492	14888472	-	No
2733939_adh	TTTTACAATAAAGATCGTCGT	Chr.5	14939964	14939984	+	No
2693145_adh	TTTACAATAAAGATCGTCGTA	Chr.5	14939965	14939985	+	No
2585068_adh	TTCAAAATTTGATCGCTGTAC	Chr.5	14944035	14944015	-	No
2228069_adh	TGAGATGTGGAAGAATAACCA	Chr.5	14947886	14947866	-	No
2641006_adh	TTGAGCTTGAACGTAAGGCGC	Chr.5	14992257	14992277	+	No
2618356_adh	TTCTGAGTGTTTTCGGCGCAT	Chr.5	14992833	14992813	-	No
1814871_adh	TACCAATCTTCATCGATATTC	Chr.5	14996514	14996534	+	No
2561858_adh	TTAGAATTGTCGTCCGAATCA	Chr.5	14996538	14996558	+	No
1766126_adh	TAACATGCAGATGAACCTGGC	Chr.5	14996563	14996583	+	No
2379439_adh	TGGAGAACACTCACATCAAGC	Chr.5	15001302	15001282	-	No

2463084_adh	TGGTAGCAGAGGTGGATTTGG	Chr.5	15001825	15001805	-	No
2381239_adh	TGGAGCGAGTCGAGCATTGAA	Chr.5	15002585	15002605	+	No
1986050_adh	TCCGGAGTTGTTCAGATATCG	Chr.5	15002612	15002632	+	No
2724821_adh	TTTGGGATTGATTCGTTCACA	Chr.5	15016877	15016857	-	No
1935979_adh	TCAACAATTTAGTAGATGCGC	Chr.5	15029209	15029229	+	No
2696997_adh	TTTAGTAGATGCGCCTTTGAA	Chr.5	15029216	15029236	+	No
2737100_adh	TTTTCAAAAACTACAACTTCG	Chr.5	15036819	15036799	-	No
2639684_adh	TTGAGACGTAGAGATGGAAAG	Chr.5	15048327	15048347	+	No
2616507_adh	TTCTCGATGGCAATGTTTTAC	Chr.5	15051837	15051817	-	No
1885156_adh	TATAAGGAGCTTCGGAAGTAC	Chr.5	15057189	15057169	-	No
2702813_adh	TTTCAGAAAAAATGGACGGAC	Chr.5	15059581	15059561	-	No
2737925_adh	TTTTCCCCGCTCTTCTCCGTG	Chr.5	15059663	15059643	-	No
1794932_adh	TAATAGCGAAATAATTTTATT	Chr.5	15059804	15059824	+	No
2742061_adh	TTTTGATTTATCGACTTTGGC	Chr.5	15066171	15066191	+	No
2720692_adh	TTTGATTTATCGACTTTGGCA	Chr.5	15066172	15066192	+	No
2698564_adh	TTTATCGACTTTGGCATTCTC	Chr.5	15066177	15066197	+	No
1990119_adh	TCCTCCTAGACAATTGCAGAG	Chr.5	15082460	15082440	-	No
2623210_adh	TTCTTGTTTTGATTTCAACGT	Chr.5	15085495	15085475	-	No
2511744_adh	TGTGATGTTTAATGATTTCAA	Chr.5	15101165	15101145	-	No
2501924_adh	TGTGAAGACAGATGAGAACGG	Chr.5	15103713	15103733	+	No
2737100_adh	TTTTCAAAAACTACAACTTCG	Chr.5	15105908	15105888	-	No
2003962_adh	TCGATGAAGAACTGGCGAAAA	Chr.5	15108825	15108805	-	No
2473245_adh	TGGTGTGAATTTTTAGTAGAA	Chr.5	15136963	15136983	+	No
2503040_adh	TGTGAATTTTTAGTAGAAAGG	Chr.5	15136966	15136986	+	No
2649500_adh	TTGCAGATGGAACGGAATTTT	Chr.5	15145566	15145586	+	No
2687522_adh	TTGTTTGGATGACATACCCAC	Chr.5	15159361	15159381	+	No
2047800_adh	TCTGCACCGTCAAGACTGCCT	Chr.5	15186813	15186793	-	No
2269394_adh	TGATCCAGGGAGGAGACTTCA	Chr.5	15187086	15187066	-	No
2269394_adh	TGATCCAGGGAGGAGACTTCA	Chr.5	15190180	15190200	+	No
2373353_adh	TGGACAACTCAAGGCATAAAT	Chr.5	15190791	15190811	+	No
2245414_adh	TGAGGTAGGTTTAGAAGACGA	Chr.5	15212811	15212831	+	No
1860038_adh	TAGGAAAACGTCTGGAAAAAA	Chr.5	15244326	15244346	+	No
2736498_adh	TTTTATTATGTGTACATTGAT	Chr.5	15297027	15297007	-	No
2522467_adh	TGTTAAGATAGAATTGAATGG	Chr.5	15350986	15350966	-	No
2339050_adh	TGGAATGAAAACTTGAATGCA	Chr.5	15387141	15387161	+	No
2538926_adh	TTAAAAGTTCAGAAAAAAGTG	Chr.5	15395425	15395405	-	No
2712813_adh	TTTGAAATTAATTTGAACAGT	Chr.5	15425437	15425417	-	No
1762993_adh	TAACAAAAAGCGACGAAAAAG	Chr.5	15429811	15429791	-	No
2304367_adh	TGCGTGTTTATGTAGCAAATA	Chr.5	15433169	15433149	-	No
1896506_adh	TATCCTTAGGTCGCTGGTTCG	Chr.5	15480716	15480736	+	No
1757898_adh	TAAAGGAGCAACCAGAGTTGG	Chr.5	15503737	15503757	+	No
1938931_adh	TCAAGATAAAGAAGAACTCCA	Chr.5	15512237	15512257	+	No
2617413_adh	TTCTCTGCTCGTTGTTGGTTT	Chr.5	15512665	15512685	+	No
2418799_adh	TGGGAAGACGAATTCTTGAAG	Chr.5	15516971	15516951	-	No
1774765_adh	TAAGAATTTCGGTATGTTTTG	Chr.5	15577394	15577414	+	No
2734710_adh	TTTTAGCGGTTAATTCGAATA	Chr.5	15578546	15578566	+	No
2595748_adh	TTCCAAATCCCTGTACTTTGA	Chr.5	15578837	15578817	-	No
2412446_adh	TGGCGGAAATTCAAGTGAGAA	Chr.5	15585432	15585452	+	No
2592603_adh	TTCAGTACGAACAAACTTGCC	Chr.5	15611800	15611820	+	No
2737300_adh	TTTTCACAAATTTGGACGAGT	Chr.5	15640868	15640848	-	No

1940162_adh	TCAAGTAGTTTGAGAAATTTC	Chr.5	15661021	15661001	-	No
2497903_adh	TGTCGCGAATTGTGGATGGCA	Chr.5	15668410	15668390	-	No
1993027_adh	TCCTTCGTTCGGTCACGGTCG	Chr.5	15668436	15668416	-	No
2497903_adh	TGTCGCGAATTGTGGATGGCA	Chr.5	15670474	15670494	+	No
1876569_adh	TAGTCGATAGAGGAAGTGTTG	Chr.5	15760754	15760734	-	No
2544884_adh	TTAACAATTATGAAGCTGGCA	Chr.5	15766540	15766560	+	No
2702852_adh	TTTCAGAACATTAAGCTTCAA	Chr.5	15769001	15768981	-	No
2623787_adh	TTCTTTTAATATGCATTTTCA	Chr.5	15769017	15768997	-	No
2643040_adh	TTGAGTTCACGTCGGTAATGT	Chr.5	15844939	15844919	-	No
2411152_adh	TGGCCTAGTGGATAAGAGGGA	Chr.5	15878514	15878494	-	No
2457123_adh	TGGGTAACGAGAGTAGAAGAA	Chr.5	15880982	15881002	+	No
2010998_adh	TCGGAATCTCGACGGTAACTT	Chr.5	15928331	15928311	-	No
2579340_adh	TTATGGACTATGTTAATCGGA	Chr.5	15928347	15928327	-	No
1831247_adh	TACTCGATTGTGATATTTCAA	Chr.5	15970107	15970087	-	No
1828983_adh	TACGTTCTCTTCAAACGATCG	Chr.5	15983281	15983301	+	No
1829214_adh	TACGTTTTCATCAAACAATCA	Chr.5	15988694	15988714	+	No
2733137_adh	TTTTAAAGTTTCTCGTCGTTT	Chr.5	16022851	16022871	+	No
2616800_adh	TTCTCGTCGTTTTCTATCATT	Chr.5	16022860	16022880	+	No
2678739_adh	TTGTCTCCTTAATTCGTTTTC	Chr.5	16024597	16024617	+	No
2032395_adh	TCTAGACTTGCGAGGACTTTT	Chr.5	16031809	16031829	+	No
2032395_adh	TCTAGACTTGCGAGGACTTTT	Chr.5	16036264	16036244	-	No
2590429_adh	TTCAGAATAGCGGTCCAAGAA	Chr.5	16145077	16145057	-	No
2477406_adh	TGGTTTACATATGAAGACTGT	Chr.5	16232452	16232472	+	No
1923400_adh	TATTCAATACTTGTCTCCATA	Chr.5	16237511	16237531	+	No
2145019_adh	TGAATGTTGTGACGTCAGAGA	Chr.5	16245214	16245234	+	No
1972742_adh	TCATGGTAGACAGGCGCGGTT	Chr.5	16270512	16270532	+	No
1833335_adh	TACTGGATTTGTACAGCAAGA	Chr.5	16271548	16271568	+	No
2396187_adh	TGGATTTGTACAGCAAGATTC	Chr.5	16271551	16271571	+	No
2740883_adh	TTTTGAATAAGCAGATCGGCA	Chr.5	16285712	16285692	-	No
2708856_adh	TTTCGTCGAAATTTAGTTGAA	Chr.5	16356902	16356882	-	No
2623195_adh	TTCTTGTTGGAGCATTTGACA	Chr.5	16368791	16368771	-	No
2376820_adh	TGGACTAGAAGTGGCGAATCG	Chr.5	16369840	16369860	+	No
2052286_adh	TCTGTCATTCAGAACTCGCTT	Chr.5	16390447	16390427	-	No
1934888_adh	TCAAATCTTTCAATACATTAA	Chr.5	16424832	16424852	+	No
1883930_adh	TATAAAAGGCGATTGTACGGT	Chr.5	16435789	16435809	+	No
2481541_adh	TGTAACGGTAGACAAATTGTA	Chr.5	16454753	16454773	+	No
2137748_adh	TGAATACTGAACGGATTTGAC	Chr.5	16456373	16456393	+	No
2293986_adh	TGCCGATGTGGACAACTTCCC	Chr.5	16461462	16461482	+	No
2005148_adh	TCGATTGTACAGACGCTAGTA	Chr.5	16473353	16473333	-	No
2293986_adh	TGCCGATGTGGACAACTTCCC	Chr.5	16515163	16515143	-	No
2559484_adh	TTACTGTAGTTTTCGAAAAAT	Chr.5	16546920	16546940	+	No
2748121_adh	TTTTTTAAGAGCTTTGCGAAT	Chr.5	16553200	16553220	+	No
2711763_adh	TTTCTTGATTTTCGGCAATTT	Chr.5	16557804	16557784	-	No
1974009_adh	TCATTGAGAACTAAAAACGTC	Chr.5	16650894	16650914	+	No
1974009_adh	TCATTGAGAACTAAAAACGTC	Chr.5	16664508	16664488	-	No
1965993_adh	TCAGTAGCTGACTCATCTTCT	Chr.5	16666897	16666877	-	No
1912026_adh	TATGATTGATAGAAGAGATAA	Chr.5	16679226	16679246	+	No
1879457_adh	TAGTGTCAAAATCGGACGACC	Chr.5	16684196	16684176	-	No
2045094_adh	TCTGACAGCAAGTTTGATGCT	Chr.5	16693776	16693756	-	No
2013612_adh	TCGGATATAAGTGATCGATAT	Chr.5	16733644	16733624	-	No

1774204_adh	TAAGAAGCAGTACGTGTTTCC	Chr.5	16783054	16783034	-	No
2619467_adh	TTCTGGCGTCGGAAAAGGCAT	Chr.5	16795354	16795374	+	No
1929436_adh	TATTTATTTTCGTCCGGTTTG	Chr.5	16896242	16896262	+	No
2630518_adh	TTGAATCGTAGTTTTATTTTC	Chr.5	16898281	16898261	-	No
2646893_adh	TTGATTTCTGAAGACGTGCAA	Chr.5	16922290	16922310	+	No
1835090_adh	TACTTGAACCGAACAATGATG	Chr.5	16924163	16924183	+	No
2701556_adh	TTTCAAGATTTTTAGGCCAAT	Chr.5	16927860	16927880	+	No
2566461_adh	TTAGCATCGTCTCATCACTAA	Chr.5	16942972	16942992	+	No
1747171_adh	TAAAAATGAAAGAAGTACGTG	Chr.5	16965896	16965916	+	No
2629605_adh	TTGAATACAAGTAGACGAAAT	Chr.5	17035290	17035310	+	No
2461108_adh	TGGTAAATTTCGGACATTCAC	Chr.5	17055112	17055092	-	No
2601617_adh	TTCGAAAAGAAAAATCATCGG	Chr.5	17063116	17063096	-	No
2126664_adh	TGAACTTGTAGAAATGGGGAA	Chr.5	17073611	17073631	+	No
2490060_adh	TGTAGTTGCAGGCCAGTAGAT	Chr.5	17134250	17134270	+	No
1759282_adh	TAAAGTTTGTAGATGACGGGC	Chr.5	17135797	17135817	+	No
1962474_adh	TCAGCCATTCCAGTCGTTGGA	Chr.5	17136458	17136478	+	No
2621033_adh	TTCTTCAATGGGGATTCTGCA	Chr.5	17139533	17139553	+	No
2497427_adh	TGTCCTCGGGCAATCGGTTCT	Chr.5	17142002	17142022	+	No
2729974_adh	TTTGTGGTAACAGGACGTTTT	Chr.5	17180025	17180045	+	No
1833640_adh	TACTGTAGAAATTTCTTCGGC	Chr.5	17210376	17210396	+	No
1836665_adh	TAGAAAACAGTTGCCGCACTT	Chr.5	17224470	17224490	+	No
2055491_adh	TCTTCAAATTGATCGTTTTAA	Chr.5	17228689	17228709	+	No
2681506_adh	TTGTGGAAGAACTAATCAACT	Chr.5	17250635	17250655	+	No
2500731_adh	TGTGAAACTGTTGGAAAAAAT	Chr.5	17334331	17334351	+	No
2748497_adh	TTTTTTCAATCAACAACTAAC	Chr.5	17347868	17347888	+	No
2616014_adh	TTCTCAATTCATCTTCTGGAC	Chr.5	17361420	17361440	+	No
2490060_adh	TGTAGTTGCAGGCCAGTAGAT	Chr.5	17423876	17423896	+	No
1962474_adh	TCAGCCATTCCAGTCGTTGGA	Chr.5	17426084	17426104	+	No
2152884_adh	TGACACCAAGCCCTTGATTTC	Chr.5	17429487	17429507	+	No
2539370_adh	TTAAACACAGCGGCGCGCGGA	Chr.5	17502185	17502205	+	No
1822387_adh	TACGACGCAAACTGAGATTAT	Chr.5	17538116	17538136	+	No
2500731_adh	TGTGAAACTGTTGGAAAAAAT	Chr.5	17560421	17560441	+	No
2733735_adh	TTTTAATGGGAATTTTGCATG	Chr.5	17569156	17569136	-	No
2044992_adh	TCTGACACGGATAGAATGATT	Chr.5	17632666	17632646	-	No
2155378_adh	TGACATCACGGAATCGTCGGG	Chr.5	17649732	17649752	+	No
2693802_adh	TTTACGATGAGCAAGAATTTT	Chr.5	17656700	17656680	-	No
2700734_adh	TTTATTTGACGCGCATCGCAG	Chr.5	17656777	17656797	+	No
1918456_adh	TATGTCAGATGAAATGTGCAA	Chr.5	17701178	17701158	-	No
2722618_adh	TTTGCTTAAAACTGGAGATGA	Chr.5	17701205	17701185	-	No
2510633_adh	TGTGATGGATAGAGGTGGCAA	Chr.5	17709760	17709780	+	No
2001567_adh	TCGAGCTGAATACACGTGTTG	Chr.5	17834115	17834135	+	No
2742771_adh	TTTTGGAGTGAATTTTGACGT	Chr.5	17859469	17859449	-	No
2703112_adh	TTTCAGCTCAAAATTTCCAAT	Chr.5	17970090	17970110	+	No
2737833_adh	TTTTCCAAGCTGAAAACTGTA	Chr.5	17970371	17970351	-	No
2653122_adh	TTGCGTACCCGACCGATGATC	Chr.5	17981850	17981830	-	No
2576606_adh	TTATATTGGAAATTCACGGGA	Chr.5	17985923	17985903	-	No
2589723_adh	TTCACGGCGTAAATGGTTCAC	Chr.5	18018917	18018937	+	No
1953287_adh	TCACGGCGTAAATGGTTCACG	Chr.5	18018918	18018938	+	No
2704197_adh	TTTCATGATTTTCAATATATT	Chr.5	18037259	18037239	-	No
2016520_adh	TCGGGAGACGTGATAATTGAT	Chr.5	18046224	18046204	-	No

2585536_adh	TTCAAATGAGCAAGAACAGCG	Chr.5	18077197	18077177	-	No
2737306_adh	TTTTCACACAGAATTACGCAT	Chr.5	18079779	18079799	+	No
2538913_adh	TTAAAAGTAGAGATTAACGTT	Chr.5	18085499	18085479	-	No
2738218_adh	TTTTCGAATTCGGCGGCGCAT	Chr.5	18086408	18086388	-	No
2377096_adh	TGGACTCGAAACGGTGAAATG	Chr.5	18086944	18086924	-	No
2048530_adh	TCTGCTGTAGGGGAAGAAGCA	Chr.5	18097879	18097859	-	No
2617764_adh	TTCTGAAGAAGCACAGCCTGT	Chr.5	18123705	18123685	-	No
2712850_adh	TTTGAAATTTCTGAAGAAGCA	Chr.5	18123713	18123693	-	No
2595498_adh	TTCATTTGACGAGCACGTAAT	Chr.5	18123779	18123799	+	No
2734948_adh	TTTTAGTGGAAATTTATGGGT	Chr.5	18125427	18125407	-	No
2607167_adh	TTCGGAAGAAAAATGTTTGTC	Chr.5	18134556	18134536	-	No
1999798_adh	TCGACATTCAACTTGACGCGC	Chr.5	18134595	18134615	+	No
2449850_adh	TGGGCACCAAAGCAGCCTGAC	Chr.5	18156621	18156641	+	No
2587262_adh	TTCAAGGATTTTGTGGATTTT	Chr.5	18298304	18298324	+	No
1776767_adh	TAAGAGCAAGTTTTCCGATAA	Chr.5	18310745	18310765	+	No
2739577_adh	TTTTCTGGAGAATTGCAGTGA	Chr.5	18370586	18370606	+	No
2597519_adh	TTCCATTGGGTCGTCTTTATC	Chr.5	18401969	18401989	+	No
2723890_adh	TTTGGATAGAATTCAGCATGT	Chr.5	18438096	18438076	-	No
1834393_adh	TACTTAAAAATATCTTCGGCT	Chr.5	18444250	18444270	+	No
2537989_adh	TTAAAAATATCTTCGGCTCTT	Chr.5	18444253	18444273	+	No
2513825_adh	TGTGCGGCTACCTTTTGGCAT	Chr.5	18451899	18451919	+	No
2098830_adh	TGAAATCTGAAACTTCAAGAA	Chr.5	18664938	18664958	+	No
2254026_adh	TGAGTGGACGAGAACAAGGCG	Chr.5	18755054	18755074	+	No
1762993_adh	TAACAAAAAGCGACGAAAAAG	Chr.5	18951039	18951019	-	No
2734805_adh	TTTTAGGCGATTTACTTAATT	Chr.5	18969949	18969969	+	No
2392500_adh	TGGATGTTTTCGAGCTGACAA	Chr.5	18981103	18981083	-	No
2746026_adh	TTTTTCGCGATTTTCTTTCAG	Chr.5	18981228	18981248	+	No
2705203_adh	TTTCCATGACAGAAAACGACA	Chr.5	18990337	18990317	-	No
2691167_adh	TTTAATCAATTTTCGTCGGTT	Chr.5	19001215	19001235	+	No
1943427_adh	TCAATTTTCGTCGGTTTTCAG	Chr.5	19001220	19001240	+	No
1924184_adh	TATTCGAAAATCACATCGATC	Chr.5	19008496	19008516	+	No
2737514_adh	TTTTCAGGATCTTCAGAGGGT	Chr.5	19014398	19014378	-	No
2005099_adh	TCGATTGGATAGTCTGAAAAA	Chr.5	19016600	19016620	+	No
2429182_adh	TGGGATCCGGAGGATTCGGAC	Chr.5	19049650	19049630	-	No
1946972_adh	TCACCATCGGAGGAGCCCAAT	Chr.5	19049746	19049726	-	No
2011838_adh	TCGGACTCGGATGGCCAGCAC	Chr.5	19050175	19050155	-	No
2005099_adh	TCGATTGGATAGTCTGAAAAA	Chr.5	19071786	19071766	-	No
1883988_adh	TATAAAATTTGGAAAAATCAA	Chr.5	19074430	19074450	+	No
2590706_adh	TTCAGAGGTTTTGGCGTGTCA	Chr.5	19089749	19089769	+	No
2375797_adh	TGGACCTGACAACCGAAGAAA	Chr.5	19091699	19091719	+	No
2054994_adh	TCTTATATTGAAGCCGTAGAA	Chr.5	19094674	19094654	-	No
2003962_adh	TCGATGAAGAACTGGCGAAAA	Chr.5	19097007	19096987	-	No
2011005_adh	TCGGAATCTGAAGACGAGAAG	Chr.5	19202417	19202397	-	No
2742170_adh	TTTTGCACGCGGAATTCTCAT	Chr.5	19205717	19205697	-	No
1835078_adh	TACTTGAAAATTCCAATGGCA	Chr.5	19262411	19262391	-	No
1753799_adh	TAAACAACAGTTGCCGCACTT	Chr.5	19356098	19356078	-	Yes
1985223_adh	TCCGCACTGTGGCAAATTTTT	Chr.5	19360045	19360065	+	No
2119425_adh	TGAAATTTTTATGATTTTTTA	Chr.5	19411364	19411384	+	No
2387835_adh	TGGATAATTGGCTCAGTGGCT	Chr.5	19619815	19619835	+	No
2300902_adh	TGCGGACCAGGCATTTAATTA	Chr.5	19644098	19644118	+	No

2119525_adh	TGAACAAAAATGTAGAAATCG	Chr.5	19645886	19645866	-	No
1941784_adh	TCAATGAACAAAAATGTAGAA	Chr.5	19645890	19645870	-	No
2740548_adh	TTTTGAAAATTTCACGTCGGA	Chr.5	19645955	19645935	-	No
2604195_adh	TTCGAGTTTTCTAAACTTCAA	Chr.5	19646057	19646077	+	No
2029537_adh	TCTAAATTGGGTCTGGAAATA	Chr.5	19673389	19673369	-	No
2606151_adh	TTCGATTTTTGACGGTTGCTT	Chr.5	19739025	19739045	+	No
2303625_adh	TGCGTAGATTTTAGTTTGTTT	Chr.5	19742023	19742043	+	No
2449850_adh	TGGGCACCAAAGCAGCCTGAC	Chr.5	19747995	19747975	-	No
2738647_adh	TTTTCGGGCAGAAATTGTGAA	Chr.5	19766314	19766294	-	No
2605863_adh	TTCGATTTATGAGCGAAGCCA	Chr.5	19768049	19768029	-	No
2654849_adh	TTGCTGGAGAAAAAAGTGTGA	Chr.5	19768261	19768281	+	No
1993935_adh	TCCTTGTTCTGGAGAGCAAAA	Chr.5	19775330	19775310	-	No
2612248_adh	TTCGTGATTCTTGACGGCCGT	Chr.5	19778900	19778880	-	Yes
2708995_adh	TTTCGTGATTCTTGACGGCCG	Chr.5	19778901	19778881	-	Yes
2721537_adh	TTTGCCACGGGGAACTCGAAT	Chr.5	19811180	19811160	-	No
2534353_adh	TGTTTTCGGTGTATTTTTGTT	Chr.5	19812311	19812331	+	No
2534346_adh	TGTTTTCGGTGTATTTTCGCT	Chr.5	19812542	19812522	-	No
2232571_adh	TGAGATTTTGGAATTTTTGAA	Chr.5	19813577	19813557	-	No
1855457_adh	TAGCATTATGACAGATGCATA	Chr.5	19817639	19817659	+	No
2617937_adh	TTCTGAATGTGGGCAAAGGAA	Chr.5	19841765	19841785	+	No
1830529_adh	TACTATCTCGTTTTCGTCTGT	Chr.5	19843587	19843567	-	No
2585061_adh	TTCAAAATTGTAGGGTGCATA	Chr.5	19850278	19850298	+	No
2705240_adh	TTTCCCAAATCCTAATTCGAC	Chr.5	19852774	19852794	+	No
2689573_adh	TTTAAATTAAGAAATCGACAA	Chr.5	19870042	19870022	-	No
2748877_adh	TTTTTTCGCAATTTTCTCCAT	Chr.5	19911398	19911378	-	No
2748877_adh	TTTTTTCGCAATTTTCTCCAT	Chr.5	19911418	19911398	-	No
2568396_adh	TTAGGATTTAGTTGTTTTTAA	Chr.5	19911465	19911445	-	No
2653661_adh	TTGCGTTGAAAATTCTCGGTT	Chr.5	19915553	19915533	-	No
1812624_adh	TACAGTAACCAAAAATCGCCA	Chr.5	19938364	19938344	-	No
2739627_adh	TTTTCTGGTTTGTTGGGTTTT	Chr.5	19944953	19944973	+	No
2404340_adh	TGGCAGTTGATAATAGTTTTT	Chr.5	19982311	19982291	-	No
2516953_adh	TGTGGTACTGTAGGTTTGCAA	Chr.5	20033945	20033965	+	No
2595737_adh	TTCCAAAGTTCTCTCTCGTCA	Chr.5	20042809	20042789	-	No
1985225_adh	TCCGCACTGTGGCATTAATTT	Chr.5	20043578	20043598	+	No
1953192_adh	TCACGGAGAAAAAGCAAAAAT	Chr.5	20083949	20083969	+	No
2711072_adh	TTTCTGTTGGAATTTTGCAGA	Chr.5	20086319	20086339	+	No
2373061_adh	TGGAATTTTGCAGATTTTCAC	Chr.5	20086326	20086346	+	No
1913619_adh	TATGGAACATTTGAAGAAGCT	Chr.5	20100911	20100931	+	No
2050661_adh	TCTGGTAGAAAAAGCTTTCAA	Chr.5	20104260	20104280	+	No
2188834_adh	TGAGAGGGTTTTGTATGGCGA	Chr.5	20147982	20148002	+	No
2617937_adh	TTCTGAATGTGGGCAAAGGAA	Chr.5	20153473	20153453	-	No
2386665_adh	TGGATAAACATGTTGAACGAG	Chr.5	20158792	20158772	-	No
2290110_adh	TGCATATTTGGAACAGAAGCA	Chr.5	20158883	20158863	-	No
2290110_adh	TGCATATTTGGAACAGAAGCA	Chr.5	20158898	20158918	+	No
2055546_adh	TCTTCAATGGTCGTTTTGGAG	Chr.5	20171438	20171418	-	No
2013160_adh	TCGGAGTTCTTCAATGGTCGT	Chr.5	20171445	20171425	-	No
2130058_adh	TGAAGATGACGACTTTTAACA	Chr.5	20171563	20171583	+	No
2055546_adh	TCTTCAATGGTCGTTTTGGAG	Chr.5	20177100	20177080	-	No
2013160_adh	TCGGAGTTCTTCAATGGTCGT	Chr.5	20177107	20177087	-	No
2130058_adh	TGAAGATGACGACTTTTAACA	Chr.5	20177225	20177245	+	No

2682108_adh	TTGTGTATTGCGTACCTTCTA	Chr.5	20192108	20192088	-	No
2682108_adh	TTGTGTATTGCGTACCTTCTA	Chr.5	20194313	20194293	-	No
1929797_adh	TATTTCGATATTGGACCAATT	Chr.5	20245178	20245158	-	No
2519840_adh	TGTGTGATCTCAACGAGCAGA	Chr.5	20263029	20263009	-	No
2414740_adh	TGGCTGACGTGTATTTTTCCA	Chr.5	20265400	20265420	+	No
2128213_adh	TGAAGACTGAAGGCAAGAAAA	Chr.5	20292524	20292544	+	No
2496536_adh	TGTCATTAGATCGCATTTGGT	Chr.5	20312645	20312665	+	No
2615980_adh	TTCTCAATCGAGCGATCGTCA	Chr.5	20313148	20313168	+	No
2036593_adh	TCTCAATCGAGCGATCGTCAA	Chr.5	20313149	20313169	+	No
2001510_adh	TCGAGCGATCGTCAAATCATC	Chr.5	20313155	20313175	+	No
2744596_adh	TTTTGTTTTTTGAGGATCGAC	Chr.5	20364304	20364284	-	No
2579041_adh	TTATGCAACGGAACACGACTA	Chr.5	20376810	20376830	+	No
2047717_adh	TCTGCAACGTCGAGAACAAGT	Chr.5	20464239	20464219	-	No
1774743_adh	TAAGAATTGAGACTCAAGCTC	Chr.5	20468005	20467985	-	No
2556558_adh	TTACGAATTTTGAAGGAAGAA	Chr.5	20474613	20474593	-	No
1919961_adh	TATTAAATCTGAAAAATGAGA	Chr.5	20474639	20474619	-	No
2047717_adh	TCTGCAACGTCGAGAACAAGT	Chr.5	20475646	20475666	+	No
2127831_adh	TGAAGACCTTGAACCAGTAGA	Chr.5	20505357	20505337	-	No
1834881_adh	TACTTCGATTTCCTGTAGCCG	Chr.5	20531689	20531669	-	No
2708553_adh	TTTCGGTTGTAGGCCGCGGTT	Chr.5	20642792	20642812	+	No
1851460_adh	TAGATGGCATAATAAACATCA	Chr.5	20656677	20656697	+	No
1939507_adh	TCAAGGAAGAGGACGATTTGA	Chr.5	20667292	20667312	+	No
1814392_adh	TACATTCTACAATGGCGATTA	Chr.5	20668159	20668179	+	No
1851979_adh	TAGATTCAAGCTCTGAAGAAG	Chr.5	20669258	20669278	+	No
2745530_adh	TTTTTATTTTATTACTCGGCA	Chr.5	20691170	20691190	+	No
2728306_adh	TTTGTAGCTAGAGGATTCAAA	Chr.5	20713025	20713005	-	No
2583818_adh	TTATTTCGAAATGACGGAGCA	Chr.5	20723254	20723234	-	No
2176721_adh	TGAGAAAATCGACCTGGAGAA	Chr.5	20730323	20730303	-	No
2747193_adh	TTTTTGGAATTTTTACTGAAA	Chr.5	20810593	20810613	+	No
2121392_adh	TGAACATAAGATCGATGCATG	Chr.5	20814433	20814453	+	No
1810786_adh	TACACATAGAAACGGAGACAA	Chr.5	20827390	20827370	-	No
1745442_adh	TAAAAAAGACTTGGCAACTGT	Chr.5	20827972	20827952	-	No
2649892_adh	TTGCAGTTTTCGGAGAGCTCA	Chr.5	20828020	20828000	-	No
2279225_adh	TGATTTTCGATGGTAATCTGC	Chr.5	20828331	20828351	+	No
2460674_adh	TGGGTTTGGAGACGAGTTCCA	Chr.5	20832528	20832508	-	No
1824758_adh	TACGCATTAAGAGAACTAATA	Chr.5	20832603	20832623	+	No
2613719_adh	TTCTAAAGAAAAACATTTTAA	Chr.5	20834160	20834140	-	No
2137681_adh	TGAATACGAGTAGACGTAATT	Chr.5	20851694	20851714	+	No
2253236_adh	TGAGTGAAAGATAGTTAGGAC	Chr.5	20856703	20856723	+	No
1762986_adh	TAACAAAAAATATTAAGACTG	Chr.5	20894183	20894163	-	No
2713931_adh	TTTGAAGCTGGAGACGTGAAA	Chr.X	11852	11832	-	No
2068502_adh	TGAAAAGAGTAGAAAATGATG	Chr.X	25218	25238	+	No
2744839_adh	TTTTTAATGTTTTCGTTGTAT	Chr.X	45637	45617	-	No
2020763_adh	TCGGTTTTTGAGAAGAATTGA	Chr.X	47520	47500	-	No
1761089_adh	TAAATCTGATTTTTTACAAGC	Chr.X	47686	47666	-	No
2556964_adh	TTACGCGCTGTCGAAAGGGTA	Chr.X	47779	47799	+	No
2694768_adh	TTTACTTTTGGGCCCGTCGTC	Chr.X	59337	59357	+	No
1757423_adh	TAAAGCGGAGAAGCAAGAATC	Chr.X	68794	68774	-	No
2412446_adh	TGGCGGAAATTCAAGTGAGAA	Chr.X	124424	124404	-	No
2595748_adh	TTCCAAATCCCTGTACTTTGA	Chr.X	132995	133015	+	No

2739627_adh	TTTTCTGGTTTGTTGGGTTTT	Chr.X	163639	163659	+	No
1877373_adh	TAGTGAAGAATTGGGTAAAAG	Chr.X	163670	163690	+	No
2127514_adh	TGAAGAATTGGGTAAAAGCAG	Chr.X	163673	163693	+	No
2748877_adh	TTTTTTCGCAATTTTCTCCAT	Chr.X	166286	166266	-	No
2700817_adh	TTTATTTTAGGCCTTTGACTG	Chr.X	172956	172976	+	No
2734802_adh	TTTTAGGCCTTTGACTGAAAC	Chr.X	172960	172980	+	No
2748877_adh	TTTTTTCGCAATTTTCTCCAT	Chr.X	182654	182674	+	No
2746153_adh	TTTTTCGTGAAAACTTGTAGA	Chr.X	216035	216055	+	No
2703861_adh	TTTCATCAAGCAGTAGATCTA	Chr.X	256150	256130	-	No
1978814_adh	TCCAGGTGTGTAATTCGGTAA	Chr.X	258630	258610	-	No
2597020_adh	TTCCAGGTGTGTAATTCGGTA	Chr.X	258631	258611	-	No
2598382_adh	TTCCCTCATTTTCGTCGTTAA	Chr.X	265371	265391	+	No
1982115_adh	TCCCTCATTTTCGTCGTTAAA	Chr.X	265372	265392	+	No
1751448_adh	TAAAATCGATAAAACGTTTAA	Chr.X	270286	270306	+	No
1953192_adh	TCACGGAGAAAAAGCAAAAAT	Chr.X	303544	303524	-	No
1887942_adh	TATAGAATACAGGGTAAAATT	Chr.X	316711	316731	+	No
1952952_adh	TCACGAAAAGGGAATGTATGT	Chr.X	325791	325771	-	No
1966136_adh	TCAGTCAACATATTCTTGAAC	Chr.X	470936	470916	-	No
1981607_adh	TCCCGGCCGGGGAGAACTTTT	Chr.X	489883	489863	-	No
2694693_adh	TTTACTTGTAGTTTGACGTTT	Chr.X	538576	538596	+	No
2378716_adh	TGGACTGTGCAGAAGTTTGTC	Chr.X	608847	608827	-	No
2573345_adh	TTAGTTTAATTTATTTCTATT	Chr.X	626186	626166	-	No
2718784_adh	TTTGATGACTCTGAATAAAGA	Chr.X	632829	632849	+	No
2007979_adh	TCGCGGATTACATTGAGAAGG	Chr.X	645488	645468	-	No
2089313_adh	TGAAATATATAAAATCATGTA	Chr.X	684581	684601	+	No
2688730_adh	TTTAAAAATTAATCAAATTTC	Chr.X	685813	685833	+	No
1917366_adh	TATGTAAATATGTGTGTCGGC	Chr.X	693645	693625	-	No
2605965_adh	TTCGATTTTGAGAAAACGCAC	Chr.X	745093	745073	-	No
1831303_adh	TACTCGGCAATTCGCAGCATC	Chr.X	754284	754304	+	No
2031124_adh	TCTACAAATATAAACGCTGCA	Chr.X	772013	771993	-	No
2056884_adh	TCTTCTACTGGCCTAAGAGAC	Chr.X	805613	805633	+	No
1938329_adh	TCAAGAAGTCGAACATTGTTT	Chr.X	829952	829972	+	No
2478725_adh	TGGTTTGTTATGTTATAGCCA	Chr.X	849274	849294	+	No
1867080_adh	TAGGCATGTAGGTAGGCATTT	Chr.X	879460	879480	+	No
2589052_adh	TTCAATTTTTAAGCTTAGACA	Chr.X	906200	906220	+	No
2628550_adh	TTGAAGCAAGCTGGCGACTGT	Chr.X	913792	913772	-	No
2262415_adh	TGATATCGATTTTCTCTCCAC	Chr.X	924870	924890	+	No
1810379_adh	TACAATTTAAGAGGATACGGT	Chr.X	932284	932304	+	No
2263759_adh	TGATATGAAACGGCTTCTGCA	Chr.X	937630	937650	+	No
2472158_adh	TGGTGGATAATTTCATGAACA	Chr.X	1143534	1143514	-	No
2139242_adh	TGAATATCGATCGTTCACTAT	Chr.X	1150504	1150484	-	No
1784316_adh	TAAGGAGAAGAGTCAATAGTA	Chr.X	1222683	1222703	+	No
2695020_adh	TTTAGACCAGGGCTGTGCGGC	Chr.X	1235811	1235791	-	No
2703814_adh	TTTCATATCAGAATGCGGAAC	Chr.X	1257911	1257931	+	No
2381287_adh	TGGAGCGCGGAACACGGGAGG	Chr.X	1310789	1310769	-	No
2281104_adh	TGCAAATCTGAAACTTAAAGA	Chr.X	1347920	1347940	+	No
2015176_adh	TCGGCAAGAATGAAAAGAACA	Chr.X	1399891	1399871	-	No
2015176_adh	TCGGCAAGAATGAAAAGAACA	Chr.X	1404258	1404278	+	No
2539370_adh	TTAAACACAGCGGCGCGCGGA	Chr.X	1471472	1471452	-	No
2613199_adh	TTCGTTGACGAATTGAAACTA	Chr.X	1488308	1488328	+	No

2640010_adh	TTGAGAGGGTTTTTACACCAA	Chr.X	1659268	1659248	-	No
2086561_adh	TGAAATACGATGCTGATGATA	Chr.X	1659483	1659463	-	No
1831845_adh	TACTGAACGGATTTGAATGGA	Chr.X	1659647	1659667	+	No
2640010_adh	TTGAGAGGGTTTTTACACCAA	Chr.X	1669860	1669840	-	No
2086561_adh	TGAAATACGATGCTGATGATA	Chr.X	1670075	1670055	-	No
1831845_adh	TACTGAACGGATTTGAATGGA	Chr.X	1670239	1670259	+	No
2561611_adh	TTAGAATAATAGGAAAGGGTA	Chr.X	1675268	1675248	-	No
2737498_adh	TTTTCAGCTGAAAATCGTCGA	Chr.X	1722329	1722349	+	No
2005267_adh	TCGATTTGGCGAAGGAAAAAG	Chr.X	1722435	1722455	+	No
2737300_adh	TTTTCACAAATTTGGACGAGT	Chr.X	1732514	1732494	-	No
2007681_adh	TCGCGAATTGTACATGAGTGT	Chr.X	1755330	1755310	-	No
2056830_adh	TCTTCGTGCATTATCTCCAGT	Chr.X	1773087	1773107	+	No
2654390_adh	TTGCTCTCGAATAGCACGGCT	Chr.X	1781602	1781582	-	No
2354144_adh	TGGAATGGTGTAGGAAGATAG	Chr.X	1796985	1796965	-	No
2531100_adh	TGTTGTGTTTAAGATTGTCGT	Chr.X	1822744	1822724	-	No
2640269_adh	TTGAGATGACGTCACAATATT	Chr.X	1863460	1863440	-	No
1943234_adh	TCAATTGTTTGTGGACTTACA	Chr.X	1873858	1873878	+	No
2710856_adh	TTTCTGTAGTAAAAAATGGTT	Chr.X	1896073	1896093	+	No
2307282_adh	TGCTCATCTTCAGTCGGACAC	Chr.X	1914393	1914373	-	No
2648917_adh	TTGCACAAGGACTCGTCTCCA	Chr.X	1933617	1933597	-	No
2031224_adh	TCTACACTTGCACAAGGACTC	Chr.X	1933624	1933604	-	No
2534420_adh	TGTTTTCTTAAAGTTTATTCT	Chr.X	1942497	1942517	+	No
1874732_adh	TAGTAGGAGGAGTTTGGGTTT	Chr.X	1945196	1945216	+	No
2534420_adh	TGTTTTCTTAAAGTTTATTCT	Chr.X	1951026	1951046	+	No
1874732_adh	TAGTAGGAGGAGTTTGGGTTT	Chr.X	1953725	1953745	+	No
2561212_adh	TTAGAACGGCAATATCAAAAT	Chr.X	1965137	1965157	+	No
2676836_adh	TTGTATGTAGAGAGAGGAGGT	Chr.X	1975551	1975531	-	No
1772688_adh	TAACTTGACTCACCAGAATGC	Chr.X	1975739	1975719	-	Yes
2691472_adh	TTTAATGAAAATGAGTAGGTT	Chr.X	2066074	2066054	-	No
2641316_adh	TTGAGGATTCAGGATAAGGAA	Chr.X	2071702	2071722	+	No
1923484_adh	TATTCACTTTCATGCCCAAAC	Chr.X	2075840	2075860	+	No
2274171_adh	TGATTAACTTCAGATCAGTCA	Chr.X	2076348	2076368	+	No
2139102_adh	TGAATAGTTTGAATTCGACGC	Chr.X	2089714	2089734	+	No
1806157_adh	TAATTTTTGAAATATTTCGAA	Chr.X	2143445	2143425	-	No
1934040_adh	TCAAATACAAGTAGACGTAAT	Chr.X	2170881	2170861	-	No
1931519_adh	TATTTTTTGTGAACTGGGGTT	Chr.X	2172370	2172350	-	No
2560164_adh	TTACTTGAAAATCACGTTAAA	Chr.X	2206212	2206232	+	No
2370709_adh	TGGAATTGGAAATTGGAAAAA	Chr.X	2210783	2210763	-	No
2592118_adh	TTCAGGACAATACGAAAAGAA	Chr.X	2240319	2240339	+	No
1956514_adh	TCACTTTGTGTCGGATGCTTT	Chr.X	2309284	2309304	+	No
2521691_adh	TGTGTTTTTTGTTGAGGTATC	Chr.X	2328983	2329003	+	No
2481452_adh	TGTAACCTTGTAAAAGTCGGC	Chr.X	2354147	2354127	-	No
2178377_adh	TGAGAACCGGTAGAAAAAGAA	Chr.X	2368303	2368283	-	No
2738649_adh	TTTTCGGGCGACGTGAAAAAC	Chr.X	2373821	2373801	-	No
2305815_adh	TGCTACTGTAAGAGATTGGCG	Chr.X	2432663	2432643	-	No
2647307_adh	TTGCAAAGAACGCTCGATCGC	Chr.X	2473690	2473670	-	No
1790874_adh	TAATAATTTTATTCGTGAAGG	Chr.X	2481598	2481618	+	No
2113211_adh	TGAAATGTTTAAATGTATGAA	Chr.X	2485831	2485851	+	No
2559271_adh	TTACTGGAAGCACGGAATTCA	Chr.X	2487898	2487918	+	No
2601715_adh	TTCGAAAGTTGAGAGATGAAT	Chr.X	2489444	2489464	+	No

2058829_adh	TCTTGGACTTTACACGGTAAG	Chr.X	2504149	2504129	-	No
2238140_adh	TGAGGATTGATACGGTGATCG	Chr.X	2547092	2547112	+	No
2739245_adh	TTTTCTCATTCATTTTATGTG	Chr.X	2575932	2575912	-	No
2728768_adh	TTTGTCATCACTTCGCTCGGA	Chr.X	2634162	2634142	-	No
2677717_adh	TTGTCATCACTTCGCTCGGAG	Chr.X	2639300	2639280	-	No
2728768_adh	TTTGTCATCACTTCGCTCGGA	Chr.X	2639301	2639281	-	No
2102330_adh	TGAAATGATTTTAATTTTTTA	Chr.X	2670837	2670857	+	No
2307587_adh	TGCTCGTGACTGGATCGTCGA	Chr.X	2683227	2683207	-	No
2376314_adh	TGGACGGCAAGATTTGGTAAA	Chr.X	2692583	2692563	-	No
2447134_adh	TGGGATGTTGTAGAAGAGCAG	Chr.X	2753517	2753537	+	No
2273044_adh	TGATGTAGGAACATTTATAGG	Chr.X	2883904	2883924	+	No
2014760_adh	TCGGATTATTCTCGGTATCTT	Chr.X	2936995	2937015	+	No
2053290_adh	TCTGTTGATTGTGATACGCAC	Chr.X	2937168	2937188	+	No
1889702_adh	TATATACTGTGGATGTAAAGT	Chr.X	2941234	2941254	+	No
2663579_adh	TTGGATGTAGAATGGGAGCAG	Chr.X	2942162	2942182	+	No
1814679_adh	TACCAAATTTCGTAACGAGAC	Chr.X	2949524	2949504	-	No
2121457_adh	TGAACATCGGACGAAGACAAA	Chr.X	2970277	2970257	-	No
2407493_adh	TGGCATGGAACAAAGTCTGAA	Chr.X	3032530	3032510	-	No
1981038_adh	TCCCCGCAACGGAATAATTTT	Chr.X	3035981	3035961	-	No
2003291_adh	TCGATATTGACGGACCATCGA	Chr.X	3089719	3089699	-	No
2531270_adh	TGTTGTTGGATAGAAAGCATT	Chr.X	3112071	3112051	-	No
2456516_adh	TGGGGTTATTATTCAAAAACA	Chr.X	3157099	3157079	-	No
1772967_adh	TAACTTTTAAAAATCTGGCAT	Chr.X	3189260	3189280	+	No
1794772_adh	TAATAGAAGTGTTTGTAGATT	Chr.X	3189546	3189526	-	No
2664088_adh	TTGGATTAGTCGTCGTCGCAG	Chr.X	3228127	3228107	-	No
2470691_adh	TGGTGAACCAAGTGACGATCC	Chr.X	3239303	3239283	-	No
2276978_adh	TGATTGTAGACACGACGATGG	Chr.X	3239774	3239754	-	No
2161162_adh	TGACGATTAGCAGAGATGGAC	Chr.X	3259003	3258983	-	No
1942082_adh	TCAATGCTCAACTCGAACAAT	Chr.X	3407091	3407111	+	No
2530796_adh	TGTTGTCTCTGAGTACAAGCT	Chr.X	3407212	3407232	+	No
2629160_adh	TTGAAGTAGTAATGAAATATA	Chr.X	3461938	3461958	+	No
2058301_adh	TCTTGCATGCAGATCTGACAC	Chr.X	3496228	3496248	+	No
2119136_adh	TGAAATTTGCGTCTGGACATC	Chr.X	3500200	3500220	+	No
1929560_adh	TATTTCAGAAGAATGACGTAA	Chr.X	3508742	3508762	+	No
2590387_adh	TTCAGAAGAATGACGTAATGA	Chr.X	3508745	3508765	+	No
1842131_adh	TAGAATGCATGAGACGGACTC	Chr.X	3519194	3519174	-	No
2712299_adh	TTTGAAAAAACTCGAAATCGG	Chr.X	3534996	3534976	-	No
2530796_adh	TGTTGTCTCTGAGTACAAGCT	Chr.X	3560216	3560196	-	No
1942082_adh	TCAATGCTCAACTCGAACAAT	Chr.X	3560337	3560317	-	No
1942082_adh	TCAATGCTCAACTCGAACAAT	Chr.X	3568901	3568881	-	No
1979755_adh	TCCATGCCGTTTTTCGTCGAT	Chr.X	3605837	3605817	-	No
1891450_adh	TATCAACGATGAAATGTTGGA	Chr.X	3619964	3619944	-	No
2449494_adh	TGGGATTTTACAATTATTTAA	Chr.X	3658600	3658620	+	No
2391889_adh	TGGATGGTAGCGATGAATGTA	Chr.X	3659700	3659720	+	No
2606322_adh	TTCGCATCCAGAACTTCCGGC	Chr.X	3670160	3670180	+	No
2470387_adh	TGGTCTTAATGCTTTTGTGGG	Chr.X	3676306	3676286	-	No
2702861_adh	TTTCAGAAGAAAAATGTTCAT	Chr.X	3699121	3699141	+	Yes
2691080_adh	TTTAATATAAATTATTATGTA	Chr.X	3731275	3731255	-	No
2368160_adh	TGGAATGTTTCGGTGCATGAA	Chr.X	3754101	3754081	-	No
2616129_adh	TTCTCAGTCAGAAATCGTCGG	Chr.X	3770662	3770642	-	No

2714561_adh	TTTGAATTTTAGAGAGGATGG	Chr.X	3770887	3770907	+	No
2474770_adh	TGGTTCAGTGGAAACGACGGT	Chr.X	3770905	3770925	+	No
2593087_adh	TTCAGTGGAAACGACGGTTTT	Chr.X	3770908	3770928	+	No
1966944_adh	TCAGTGGAAACGACGGTTTTT	Chr.X	3770909	3770929	+	No
2314372_adh	TGGAAACGACGGTTTTTCGG	Chr.X	3770913	3770933	+	No
2044762_adh	TCTGAATGATGACTCTTCGGC	Chr.X	3914454	3914434	-	No
1749569_adh	TAAAAGCCTGAAGGAAAAAAG	Chr.X	3928833	3928853	+	No
2680639_adh	TTGTGATAAGAACGAGCAGTG	Chr.X	3964625	3964605	-	No
2596001_adh	TTCCAATTTTTCGGACATTTC	Chr.X	3971492	3971512	+	No
2705943_adh	TTTCCTAATAATCTTCGGAGC	Chr.X	4137720	4137740	+	No
1919088_adh	TATGTGGGAATGGTCTGGAAC	Chr.X	4176029	4176009	-	No
2710867_adh	TTTCTGTATAATGATTGGCCT	Chr.X	4190853	4190873	+	No
1954728_adh	TCACTGGAGATATTTTGCGTA	Chr.X	4191407	4191427	+	No
1831021_adh	TACTCCCATCCCTTTCCCGAC	Chr.X	4192292	4192312	+	No
2656369_adh	TTGGAAAGAGACTTGCGTCAG	Chr.X	4195077	4195057	-	No
1953599_adh	TCACGTGTTTAATGGATTGAC	Chr.X	4200285	4200305	+	No
2626952_adh	TTGAACTTAAATTTTGGCATG	Chr.X	4210162	4210142	-	No
2559569_adh	TTACTGTCATATTTCACACGA	Chr.X	4211086	4211066	-	No
2534368_adh	TGTTTTCGTGATTGGGGCAGC	Chr.X	4225860	4225880	+	No
1839805_adh	TAGAAGTCGTAAAAAACAAAC	Chr.X	4240458	4240478	+	No
2036693_adh	TCTCAATGTGCTCTGACCAGT	Chr.X	4257006	4257026	+	No
1782192_adh	TAAGCCACACACGTTCGTCGA	Chr.X	4260531	4260551	+	No
1867926_adh	TAGGGAAATTGCTAGGAAGAC	Chr.X	4284948	4284968	+	No
2561226_adh	TTAGAACGTCAACAGTGCATG	Chr.X	4336581	4336601	+	No
2561226_adh	TTAGAACGTCAACAGTGCATG	Chr.X	4339049	4339029	-	No
2157324_adh	TGACATGACCAGTGAGAACGA	Chr.X	4370546	4370526	-	No
1959352_adh	TCAGAGGGTTGGAATTTTTTA	Chr.X	4396947	4396967	+	No
2583249_adh	TTATTGTAGAAATATGGGCAT	Chr.X	4471751	4471771	+	No
1761889_adh	TAAATGTCAAACATCGTTAAC	Chr.X	4526227	4526247	+	No
2566146_adh	TTAGATTTTTAAAAAATTGAA	Chr.X	4564009	4564029	+	No
1943880_adh	TCACAAATGTTGATGTACCGC	Chr.X	4621555	4621535	-	No
2688730_adh	TTTAAAAATTAATCAAATTTC	Chr.X	4650042	4650022	-	No
2441165_adh	TGGGATGGGTAAGGGAGGGGA	Chr.X	4742385	4742365	-	No
2274156_adh	TGATTAACAAGTAGTTGAACA	Chr.X	4802823	4802803	-	No
2667337_adh	TTGGGATGGAAGTCAATTACT	Chr.X	4849771	4849751	-	No
2067907_adh	TGAAAAGAAAAACATAATGTA	Chr.X	4887332	4887312	-	No
1914002_adh	TATGGAGAAGATCTGGCATCA	Chr.X	4962957	4962977	+	No
2379589_adh	TGGAGAAGATCTGGCATCACA	Chr.X	4962959	4962979	+	No
2000395_adh	TCGACTCTGGAGATGGTGTCA	Chr.X	4963172	4963192	+	No
2000776_adh	TCGACTTCGAGCAAGAAATGG	Chr.X	4963376	4963396	+	No
1874744_adh	TAGTAGGATCTGGACATGAAG	Chr.X	4970879	4970859	-	No
1798502_adh	TAATCGCATCAAGCAGCTTGA	Chr.X	4991558	4991578	+	No
2701967_adh	TTTCAATTCAATTATTTGAAT	Chr.X	4992958	4992978	+	No
2734507_adh	TTTTAGACTGTACCAAAGACG	Chr.X	4999340	4999360	+	No
1787502_adh	TAAGTAGAAAAACAAAATAAG	Chr.X	5096067	5096047	-	No
2626618_adh	TTGAACTCGAGGGAACGGAAC	Chr.X	5103688	5103708	+	No
2237486_adh	TGAGGAGACCTACGATTACGA	Chr.X	5105669	5105689	+	No
2636339_adh	TTGACGTGAAGGCTCCAACAA	Chr.X	5113809	5113789	-	No
2068665_adh	TGAAAAGCAAACAAATATAAA	Chr.X	5137292	5137272	-	No
2746757_adh	TTTTTGAGAACACAAGGTTAA	Chr.X	5142080	5142100	+	No

2590306_adh	TTCAGAACGTGCAGATTCATT	Chr.X	5204858	5204838	-	No
1790932_adh	TAATACACCCATACGAATTAC	Chr.X	5216526	5216546	+	No
2717343_adh	TTTGATAATTGAATTTGATAA	Chr.X	5321052	5321032	-	No
2698390_adh	TTTATCAAGCAATCAATCACT	Chr.X	5335435	5335415	-	No
2121650_adh	TGAACCACAGAGAATTCGAGT	Chr.X	5392802	5392782	-	No
2032711_adh	TCTAGCTGTATTACTGGCAAA	Chr.X	5424161	5424181	+	No
1981250_adh	TCCCCTAGGTGGCTTGACTTT	Chr.X	5451184	5451164	-	No
2014783_adh	TCGGATTCCCTGACAGGAGAA	Chr.X	5527738	5527718	-	No
2123931_adh	TGAACTAACGGCTTAGAAACC	Chr.X	5539354	5539374	+	No
2096595_adh	TGAAATCGTTCACTCCTCCAC	Chr.X	5553866	5553846	-	No
2067880_adh	TGAAAAGAAAAACAATAAGCT	Chr.X	5561338	5561358	+	No
2259882_adh	TGATACTCGGAACAGGCAGGA	Chr.X	5591876	5591856	-	No
2267915_adh	TGATATTAAGAAACCATGAGA	Chr.X	5602406	5602426	+	Yes
1886424_adh	TATAATTGATCGGTTACAAAT	Chr.X	5609057	5609077	+	No
2688730_adh	TTTAAAAATTAATCAAATTTC	Chr.X	5642315	5642335	+	No
2745816_adh	TTTTTCCAACGGTTGATCGTC	Chr.X	5680352	5680332	-	No
1981250_adh	TCCCCTAGGTGGCTTGACTTT	Chr.X	5713671	5713691	+	No
2048679_adh	TCTGGAAAGGACGCAACACTA	Chr.X	5772024	5772004	-	No
2439290_adh	TGGGATGGAGACAAGTACAGA	Chr.X	5890507	5890527	+	No
2282533_adh	TGCAAGTACTGAAAGAGCATA	Chr.X	5947675	5947655	-	No
2622206_adh	TTCTTCTTCCTCTTCTTCTTT	Chr.X	5976794	5976814	+	No
2543432_adh	TTAAATCTGAAAATACAGCAA	Chr.X	6003232	6003212	-	No
2437783_adh	TGGGATGGAAAGGAATGTGAT	Chr.X	6010110	6010130	+	No
2079546_adh	TGAAAGACTAAAGAATGGTTT	Chr.X	6025473	6025493	+	No
1842481_adh	TAGAATGGAAAGTGAGAAGAA	Chr.X	6027255	6027235	-	No
2690220_adh	TTTAAGAATGAGGAAGTCGGC	Chr.X	6042997	6042977	-	No
2700991_adh	TTTCAAAATGAGGGCATGTAA	Chr.X	6046798	6046778	-	No
1961410_adh	TCAGATGGATGGTGGTTACAA	Chr.X	6057867	6057847	-	No
2048496_adh	TCTGCTGAGGAGCTTAAGGAG	Chr.X	6089488	6089468	-	No
2184794_adh	TGAGACTGTCGGAGGAGTTAT	Chr.X	6090147	6090127	-	No
2047090_adh	TCTGATCTTAAGAAGGATGAT	Chr.X	6090380	6090360	-	No
2661361_adh	TTGGATCCGACAACAAGCAAT	Chr.X	6091265	6091245	-	No
2748992_adh	TTTTTTCTAGGACTTGCATTT	Chr.X	6127466	6127446	-	No
1938272_adh	TCAAGAAGACTGGAAACCATG	Chr.X	6127785	6127765	-	No
2379892_adh	TGGAGACAAGAGAGAAAAGCA	Chr.X	6220180	6220200	+	No
1977326_adh	TCCACGAACTGGCAAGGAAGA	Chr.X	6240494	6240474	-	No
2388315_adh	TGGATAGATCAGAAGAATATA	Chr.X	6254844	6254864	+	No
1893290_adh	TATCAGAGAACGGTCCTGAAT	Chr.X	6263195	6263175	-	No
2133024_adh	TGAAGGATCTTCAGTTGGACA	Chr.X	6280259	6280279	+	No
2700991_adh	TTTCAAAATGAGGGCATGTAA	Chr.X	6283068	6283088	+	No
2133025_adh	TGAAGGATCTTCCGTTGGACA	Chr.X	6323730	6323750	+	No
2133025_adh	TGAAGGATCTTCCGTTGGACA	Chr.X	6326630	6326650	+	No
1932192_adh	TCAAAAGAACAGAGGGAGGGT	Chr.X	6341503	6341483	-	No
2589069_adh	TTCACAAACAACTAACAAAAC	Chr.X	6361548	6361528	-	No
1967998_adh	TCATAAAAAACGGAATGGAAA	Chr.X	6363200	6363180	-	No
2618789_adh	TTCTGCTGCAACGTCGTCTTC	Chr.X	6378439	6378419	-	No
1929415_adh	TATTTATTCTGTAAGACTGCA	Chr.X	6397600	6397580	-	No
2371062_adh	TGGAATTGTTTGAGCACGGCG	Chr.X	6431840	6431860	+	No
2586242_adh	TTCAACTCTACCACACGGTCA	Chr.X	6603876	6603856	-	No
1836229_adh	TACTTTCTGAGAACGGTCCAC	Chr.X	6604993	6604973	-	No

1924747_adh	TATTCTATCATACTGCACTCT	Chr.X	6629431	6629451	+	No
1922459_adh	TATTATGATATTTGGACATTT	Chr.X	6726907	6726887	-	No
2612220_adh	TTCGTGACTCTTAATGAGAAA	Chr.X	6758266	6758246	-	No
1813212_adh	TACATATACACAAATTTTATC	Chr.X	6772630	6772610	-	No
1926382_adh	TATTGCCTGCAGAATGCACAA	Chr.X	6791537	6791557	+	No
2001347_adh	TCGAGATCCAATACGGAACTG	Chr.X	6791659	6791679	+	No
2295396_adh	TGCGAAACAGAATGAAGATAC	Chr.X	6836317	6836337	+	No
2127959_adh	TGAAGACGGAATTGTTTTACA	Chr.X	6840921	6840901	-	No
2518991_adh	TGTGTATGACGACAACATGTT	Chr.X	6867891	6867911	+	No
2648767_adh	TTGCAAGTTTTTCGTCGACAC	Chr.X	6936627	6936647	+	No
1753487_adh	TAAAATTGTATTGTCGTGCAT	Chr.X	6983971	6983951	-	No
2129004_adh	TGAAGACTTGGCAAACTGAAC	Chr.X	7004965	7004985	+	No
1937375_adh	TCAACTCTGATGATATTTGGA	Chr.X	7068169	7068149	-	No
2229185_adh	TGAGATTAGTGGATCGTCATG	Chr.X	7068408	7068388	-	No
1884021_adh	TATAAACGTTCAATTTTAAAA	Chr.X	7143820	7143800	-	No
2633994_adh	TTGAATTTAAAAAAGTATGTT	Chr.X	7154360	7154340	-	No
1941209_adh	TCAATATCTTGTCGAACAAAT	Chr.X	7179398	7179378	-	No
2492598_adh	TGTATGTGACAGGTGACAGGC	Chr.X	7183175	7183155	-	No
2372855_adh	TGGAATTTGTTGAAATATGTA	Chr.X	7189800	7189780	-	No
2402748_adh	TGGCAATGGGTCGTGGAAAGG	Chr.X	7194838	7194858	+	No
1963796_adh	TCAGGAGTCGGAGTCTACGCC	Chr.X	7232119	7232099	-	No
2616475_adh	TTCTCGACGTCATTCAATCCG	Chr.X	7232157	7232137	-	No
2176975_adh	TGAGAAAGAAAGGCGGAAGAC	Chr.X	7235097	7235117	+	No
1840691_adh	TAGAATACGGCGGAATGATAA	Chr.X	7245034	7245014	-	No
1836959_adh	TAGAAACGGCAATTCTAATGT	Chr.X	7260430	7260410	-	No
2417333_adh	TGGGAAAATACGGGAATTCAA	Chr.X	7295899	7295879	-	No
2737300_adh	TTTTCACAAATTTGGACGAGT	Chr.X	7308862	7308882	+	No
2723087_adh	TTTGGAACTTTTTAGCAGTGA	Chr.X	7311941	7311921	-	No
2501515_adh	TGTGAACATTATGTGAACGAG	Chr.X	7316481	7316461	-	No
2027013_adh	TCGTGGACTTCTCACCGGTCT	Chr.X	7351187	7351167	-	No
2474515_adh	TGGTTATTGTCGTCGGAATCA	Chr.X	7363019	7362999	-	No
2565753_adh	TTAGATTCAACAAAACAGTAG	Chr.X	7418724	7418704	-	No
1825986_adh	TACGGAGATTTCGGTGACAAT	Chr.X	7474385	7474365	-	No
1789920_adh	TAATAAAATAATAAAATTCCA	Chr.X	7491542	7491522	-	No
2041497_adh	TCTCGTGATTCTGTGACAAAA	Chr.X	7508449	7508429	-	No
2067735_adh	TGAAAACTGTATCGCAAAATA	Chr.X	7535134	7535114	-	No
1913938_adh	TATGGACCATGAAGAAGATAT	Chr.X	7568242	7568262	+	No
2290965_adh	TGCATTAAGAGCAAAAGTCAA	Chr.X	7575961	7575941	-	No
2181104_adh	TGAGAAGTACGGAAACTCCAA	Chr.X	7726186	7726206	+	No
2500361_adh	TGTCTTTCTAAGGGTGTCGGC	Chr.X	7736941	7736961	+	No
2615217_adh	TTCTATACTCTATAATAAGGC	Chr.X	7770121	7770141	+	No
2048813_adh	TCTGGAAGTTCGAGACTGCCA	Chr.X	7823848	7823868	+	No
1978684_adh	TCCAGGATGTGTACAAGATCG	Chr.X	7824517	7824537	+	No
2011696_adh	TCGGACGTGTTGAGACCGGAA	Chr.X	7824559	7824579	+	No
2007400_adh	TCGCCCCACAGAACGTGACCA	Chr.X	7824607	7824627	+	No
2308814_adh	TGCTGGAATCGTCGAGCTCAT	Chr.X	7825011	7825031	+	No
2053211_adh	TCTGTTGAGAAGTCCGATGGA	Chr.X	7825135	7825155	+	No
2126890_adh	TGAAGAAACTGCTGCGAACTG	Chr.X	7826759	7826739	-	No
2158848_adh	TGACATTGAAGAAACTGCTGC	Chr.X	7826765	7826745	-	No
1974311_adh	TCATTGGGCAAGCAGCGTATT	Chr.X	7852745	7852725	-	No

1971572_adh	TCATCTGGTAGAAAAAAGCAT	Chr.X	7895856	7895876	+	No
2643103_adh	TTGAGTTGCTTGAAGCTAGTT	Chr.X	7906074	7906054	-	No
1925384_adh	TATTGAAGCGACATAAGACTT	Chr.X	7920054	7920074	+	No
2131321_adh	TGAAGCGACATAAGACTTACC	Chr.X	7920057	7920077	+	No
2417264_adh	TGGGAAAAGATAGAATTGGTC	Chr.X	7929177	7929157	-	No
2380890_adh	TGGAGATTGGGCATTTTTACC	Chr.X	7955712	7955732	+	No
1751868_adh	TAAAATCTTCCATCTCTTAAC	Chr.X	7981433	7981413	-	No
2402508_adh	TGGCAATACTGTACGTTGAAA	Chr.X	8010155	8010135	-	No
2746906_adh	TTTTTGATCGTTTGTAGTCGG	Chr.X	8027759	8027779	+	No
2539042_adh	TTAAAATGACGGGGGTATAAT	Chr.X	8035275	8035295	+	No
2677717_adh	TTGTCATCACTTCGCTCGGAG	Chr.X	8037157	8037177	+	No
1912459_adh	TATGCAGACGACAAATAGTGT	Chr.X	8054450	8054470	+	No
2553538_adh	TTACAAGTGATAGACCTCCTT	Chr.X	8074223	8074243	+	No
1942283_adh	TCAATGGGTAGCACGTGGCAA	Chr.X	8077542	8077522	-	No
2333587_adh	TGGAATCCCACGTGTAGATGA	Chr.X	8082514	8082494	-	No
2742879_adh	TTTTGGCAGCGACTGGCATAA	Chr.X	8110006	8109986	-	No
2404182_adh	TGGCAGTATTAATGACAATGA	Chr.X	8125101	8125121	+	No
1981607_adh	TCCCGGCCGGGGAGAACTTTT	Chr.X	8149157	8149177	+	No
1981607_adh	TCCCGGCCGGGGAGAACTTTT	Chr.X	8152502	8152482	-	No
2598113_adh	TTCCCGGCCGGGAGAACTTTT	Chr.X	8155752	8155772	+	No
1981601_adh	TCCCGGCCGGGAGAACTTTTT	Chr.X	8155753	8155773	+	No
2703281_adh	TTTCAGGGGCTTTACTGAAAC	Chr.X	8158994	8158974	-	No
2229005_adh	TGAGATTAAAAATAAAATGAA	Chr.X	8161221	8161201	-	No
2736503_adh	TTTTATTATTAGTGTCAGACA	Chr.X	8210086	8210106	+	No
1931505_adh	TATTTTTTGAAAAACCTGTTG	Chr.X	8236104	8236084	-	No
2586977_adh	TTCAAGATTGTGGAGATGGTT	Chr.X	8247697	8247717	+	No
1838834_adh	TAGAACTGGCATTGGCTTCAT	Chr.X	8333965	8333985	+	No
2309830_adh	TGCTGTTCAAGAGAATTCGAG	Chr.X	8346806	8346786	-	No
2058830_adh	TCTTGGACTTTTGATAGCATC	Chr.X	8392498	8392478	-	No
1890163_adh	TATATCTTGCAGACACTTTCC	Chr.X	8423683	8423663	-	No
2504698_adh	TGTGACTGCAACTGAAGCGTC	Chr.X	8451730	8451710	-	No
1857587_adh	TAGCGTCTAGTTTCGAGCGAT	Chr.X	8520284	8520264	-	No
2742709_adh	TTTTGGACGTGGAGGACAGGC	Chr.X	8545268	8545248	-	No
2044297_adh	TCTGAAGGCTAGCTGACTGCG	Chr.X	8566113	8566093	-	No
1929723_adh	TATTTCCATGTTCTTTGATTT	Chr.X	8586555	8586535	-	No
2169488_adh	TGACTGACTGAGACTGGTTGC	Chr.X	8601063	8601043	-	No
1968913_adh	TCATAGAATGAGGCATATTAA	Chr.X	8640311	8640331	+	No
2554189_adh	TTACAGAGGATTTGAAATTTT	Chr.X	8680574	8680594	+	No
2382658_adh	TGGAGTAAACGGAGTCAAAGT	Chr.X	8702650	8702670	+	No
2513276_adh	TGTGCACCAGAACGGATATAT	Chr.X	8703754	8703734	-	No
2273573_adh	TGATGTGCACCAGAACGGATA	Chr.X	8703757	8703737	-	No
1936357_adh	TCAACCCAATTCCACTTCAAG	Chr.X	8721881	8721861	-	No
1824596_adh	TACGATTTGGAAAAGAGACGT	Chr.X	8722384	8722364	-	No
2522937_adh	TGTTACGGATGGATAGAGAGA	Chr.X	8738912	8738892	-	No
2723697_adh	TTTGGAGATGAGTATGTTCAC	Chr.X	8745367	8745347	-	No
2534618_adh	TGTTTTGGAAAGTTGTATGTA	Chr.X	8781248	8781228	-	No
1785921_adh	TAAGGGACAACTCGAGAAGAT	Chr.X	8789719	8789739	+	No
2514961_adh	TGTGGACGACAGAGAAATGGA	Chr.X	8791875	8791855	-	No
2273601_adh	TGATGTGGACGACAGAGAAAT	Chr.X	8791878	8791858	-	No
2688730_adh	TTTAAAAATTAATCAAATTTC	Chr.X	8813360	8813340	-	No

2002334_adh	TCGAGTGAACTCGCTAGGCTG	Chr.X	8823085	8823105	+	No
1749706_adh	TAAAAGCTTTTCTGGACAAAA	Chr.X	8835781	8835761	-	No
2538873_adh	TTAAAAGCTTTTCTGGACAAA	Chr.X	8835782	8835762	-	No
1992190_adh	TCCTGTCATTATCGTCTTCAA	Chr.X	8911302	8911322	+	No
1944402_adh	TCACAATTTCGGCTGCTTCCC	Chr.X	8945169	8945189	+	No
2308410_adh	TGCTGACTGCGGTAATTTTTT	Chr.X	8971610	8971630	+	No
1872401_adh	TAGGTTGAAGCTTGGAGTTAG	Chr.X	9043732	9043752	+	No
2711671_adh	TTTCTTGAACTGTATGTGCAT	Chr.X	9075570	9075590	+	No
1994690_adh	TCGAAACGGGAACTAGCGGCT	Chr.X	9108864	9108884	+	No
2520012_adh	TGTGTGGAAAAAGGAAACGCA	Chr.X	9150399	9150379	-	No
2229397_adh	TGAGATTATTGAATAACAAAA	Chr.X	9162596	9162576	-	No
2580300_adh	TTATGTTCTCACTTGATGCCT	Chr.X	9176559	9176539	-	No
2295920_adh	TGCGAAGCTGTTTCTGGTCAA	Chr.X	9191872	9191892	+	No
2737894_adh	TTTTCCATCAAGTCGGCGTGC	Chr.X	9201756	9201776	+	No
2174412_adh	TGACTTTATTTTGTTTTTCTT	Chr.X	9249451	9249471	+	No
2560222_adh	TTACTTGATTTTTCGGCAAAT	Chr.X	9271979	9271959	-	No
2587416_adh	TTCAAGTTTTAGAATGAACGT	Chr.X	9272788	9272808	+	No
1885210_adh	TATAAGTAGTTGTTTCGCATG	Chr.X	9350669	9350649	-	No
1863398_adh	TAGGAGAAAAAGACAAAGAGT	Chr.X	9431089	9431069	-	No
2053350_adh	TCTGTTGGTGAAGGTTGGATA	Chr.X	9444759	9444739	-	No
2029935_adh	TCTAAGAACAAACGAAGAACA	Chr.X	9482407	9482427	+	No
2722395_adh	TTTGCTGAACGAATGCGGTGA	Chr.X	9622923	9622903	-	No
2460008_adh	TGGGTTGGGAGCAGAAACAGA	Chr.X	9627268	9627288	+	No
2367121_adh	TGGAATGTTATTGTTGACGAC	Chr.X	9665888	9665868	-	No
1847181_adh	TAGAGAAGAACTGGATGCAAT	Chr.X	9781440	9781420	-	No
1957841_adh	TCAGAATAGACGGACAACAGA	Chr.X	9837156	9837176	+	No
2636402_adh	TTGACTAAAAGAGCGGCAGAG	Chr.X	9838307	9838327	+	No
1810464_adh	TACACAAGTACCCAAATTATT	Chr.X	9865844	9865824	-	No
2069440_adh	TGAAAAGTAGCTGTTAGAAAA	Chr.X	9883859	9883839	-	No
1834770_adh	TACTTCACTGATTTTCTTCAT	Chr.X	9908470	9908450	-	No
1844143_adh	TAGAATTGATTGATGTAGCTA	Chr.X	9925547	9925527	-	No
2642972_adh	TTGAGTGTATGAAGCCAATGA	Chr.X	9958362	9958382	+	No
2554959_adh	TTACATGTGAAATTGACGACG	Chr.X	9961909	9961929	+	No
1922749_adh	TATTATGGGCACGACCAATAA	Chr.X	10012249	10012229	-	No
2162074_adh	TGACGTAGAATAGAAATGGAG	Chr.X	10025184	10025164	-	No
1831810_adh	TACTGAAATTGTTGTAGACGT	Chr.X	10046292	10046272	-	No
2592672_adh	TTCAGTAGAAGAAAATGTTAT	Chr.X	10081481	10081461	-	No
2706624_adh	TTTCGAACGCTGAATGGTCGG	Chr.X	10083530	10083550	+	No
2290888_adh	TGCATGTAGAAGCAAGGATTT	Chr.X	10118679	10118699	+	No
2585062_adh	TTCAAAATTGTCATTTTATCA	Chr.X	10138067	10138047	-	No
2144891_adh	TGAATGTGGATGAAGAGGGAT	Chr.X	10138792	10138812	+	No
2579097_adh	TTATGCCACAACTTTTCATGC	Chr.X	10158128	10158108	-	No
2646467_adh	TTGATTGGCTTAGAAAAGATG	Chr.X	10197294	10197274	-	No
2737420_adh	TTTTCACTTCATGCAAGCTTC	Chr.X	10220563	10220543	-	No
1766679_adh	TAACCAGGAAACGAGCAAAGT	Chr.X	10276099	10276079	-	No
2619435_adh	TTCTGGCAATTCGGTTTCCTA	Chr.X	10289309	10289289	-	No
1745704_adh	TAAAAAATGGGATGAGCTTAA	Chr.X	10292820	10292800	-	No
2388031_adh	TGGATAGAAAAACAGTAAGTT	Chr.X	10333010	10332990	-	No
2237017_adh	TGAGGAATTCGAGAAGACCCT	Chr.X	10340401	10340381	-	No
1920099_adh	TATTAACTTACCTTATTTTAC	Chr.X	10349615	10349595	-	No

2552101_adh	TTAATTGAAAGCTTGGAATAG	Chr.X	10392152	10392132	-	No
2575177_adh	TTATATAAGTAGGAACAGTTT	Chr.X	10452519	10452499	-	No
1836958_adh	TAGAAACGGATCATTCTGCAT	Chr.X	10487504	10487484	-	No
1835744_adh	TACTTGTATGAAGTACGGAAC	Chr.X	10497774	10497794	+	No
2299109_adh	TGCGATGGAAAAAAGGATGCA	Chr.X	10509029	10509049	+	No
2645883_adh	TTGATTAGACATTTTGAAAAC	Chr.X	10533167	10533147	-	No
2368381_adh	TGGAATGTTTTTGTATTTTTA	Chr.X	10600860	10600840	-	No
1821823_adh	TACGAAGGGAACCGAACACTT	Chr.X	10623296	10623316	+	No
1809568_adh	TACAATCGGCATTATGAAACA	Chr.X	10645023	10645043	+	No
1973008_adh	TCATGTGCATGAGCTCGCTAT	Chr.X	10667917	10667897	-	No
1824571_adh	TACGATTGTTTGAACAAATAA	Chr.X	10674853	10674873	+	No
1956933_adh	TCAGAAAGGATGCAAAATAGA	Chr.X	10679396	10679416	+	No
2025618_adh	TCGTCTATGGAAATTGTGGCA	Chr.X	10708680	10708700	+	No
2720877_adh	TTTGATTTTTGAGTTAGATAT	Chr.X	10737833	10737813	-	No
1845614_adh	TAGACTAGAGAGAGGTGCGGA	Chr.X	10766033	10766013	-	No
2710369_adh	TTTCTGAACGCCAAGAAGGGG	Chr.X	10867580	10867560	-	No
2710369_adh	TTTCTGAACGCCAAGAAGGGG	Chr.X	10873836	10873816	-	No
1829928_adh	TACTAGAAGAAGTGATGGACC	Chr.X	10886221	10886241	+	No
2281858_adh	TGCAAGAATGAGAGGGTGGCA	Chr.X	10900241	10900261	+	No
2532897_adh	TGTTTGACTGTTTTTCAAATT	Chr.X	10938162	10938142	-	No
2481514_adh	TGTAACGGAATTCATTGACGG	Chr.X	10996200	10996220	+	No
2733603_adh	TTTTAATAATAGGTATGTAAT	Chr.X	11013689	11013669	-	Yes
2605752_adh	TTCGATTGCTGAAGATCTCAA	Chr.X	11040408	11040428	+	No
2033741_adh	TCTAGTTGTTTTTCTGGCATA	Chr.X	11044391	11044371	-	No
1880820_adh	TAGTTGAACTCGGAACATAAT	Chr.X	11049521	11049541	+	No
1970551_adh	TCATCAGGGAGAATATGAATA	Chr.X	11063838	11063858	+	No
2576809_adh	TTATATTTTTTCTACGTCTTT	Chr.X	11064391	11064411	+	No
2115706_adh	TGAAATTGAGTTATATGTTTA	Chr.X	11069023	11069003	-	No
2397283_adh	TGGCAAATTGTTAGACGACAT	Chr.X	11072785	11072765	-	No
2689536_adh	TTTAAATGTGATGGTAGAAAA	Chr.X	11095242	11095262	+	No
1978638_adh	TCCAGGACAAGAAGGAGCCCC	Chr.X	11199751	11199771	+	No
2374304_adh	TGGACATCCAGGACGTAACGG	Chr.X	11200138	11200158	+	No
1978653_adh	TCCAGGACGTAACGGACCACC	Chr.X	11200144	11200164	+	No
1978638_adh	TCCAGGACAAGAAGGAGCCCC	Chr.X	11201817	11201837	+	No
2374304_adh	TGGACATCCAGGACGTAACGG	Chr.X	11202204	11202224	+	No
1978653_adh	TCCAGGACGTAACGGACCACC	Chr.X	11202210	11202230	+	No
2129964_adh	TGAAGATCCCATTGAAGAACT	Chr.X	11285920	11285900	-	No
1856064_adh	TAGCCTTCTAGAAACACTGGA	Chr.X	11339497	11339517	+	No
1979856_adh	TCCATGTATTGAAGAAGGACG	Chr.X	11340134	11340154	+	No
1895366_adh	TATCCAATGGTTCAGTTTTCC	Chr.X	11342978	11342998	+	No
1912262_adh	TATGATTTGGAAACGGAACAA	Chr.X	11424037	11424057	+	No
2643486_adh	TTGATAGAAACAGTAATAATT	Chr.X	11451526	11451546	+	No
1958830_adh	TCAGAGATGGCATGAACTTAT	Chr.X	11476082	11476102	+	No
2043930_adh	TCTGAACCTTACAAAAACTCG	Chr.X	11485869	11485889	+	No
1806691_adh	TACAAAGAACTAACAAGTAAC	Chr.X	11497330	11497310	-	No
2727317_adh	TTTGTACAAGAAACTCATCAA	Chr.X	11506730	11506710	-	Yes
1996235_adh	TCGAAGATGAAGACAATCTGC	Chr.X	11521671	11521691	+	No
2541155_adh	TTAAAGCACGGATACTCTAAA	Chr.X	11594890	11594910	+	No
2176596_adh	TGAGAAAAGAAAACTGAAACG	Chr.X	11603527	11603507	-	No
2654344_adh	TTGCTCGACAAGTTCCTCATT	Chr.X	11604952	11604932	-	No

1936212_adh	TCAACATTACTCGCTCGACAA	Chr.X	11605581	11605561	-	No
2414391_adh	TGGCTCAACAGGATGAAGGTT	Chr.X	11667084	11667104	+	No
2530370_adh	TGTTGTAGAAATGGCCAAAAT	Chr.X	11693126	11693106	-	No
2517259_adh	TGTGGTATCATAGGCGTGTTT	Chr.X	11838885	11838865	-	No
2029946_adh	TCTAAGAAGGGGTTTGGAACG	Chr.X	11854648	11854668	+	No
2660478_adh	TTGGAGTGTGACGTTTGCCAG	Chr.X	12018027	12018007	-	No
2529700_adh	TGTTGGAAAGAGGCTGCAAAG	Chr.X	12021503	12021523	+	No
2712803_adh	TTTGAAATGTACAGAACGAGG	Chr.X	12029100	12029120	+	No
1891208_adh	TATCAAAGAAAATTACTATGA	Chr.X	12053164	12053144	-	Yes
2524254_adh	TGTTATCGGTTTTTGGTAGTG	Chr.X	12146030	12146010	-	No
2309151_adh	TGCTGGTCATGAAGGTATGCA	Chr.X	12156085	12156065	-	No
2038508_adh	TCTCATTGTGCAAGAGGGCAA	Chr.X	12158721	12158701	-	No
1922459_adh	TATTATGATATTTGGACATTT	Chr.X	12163108	12163088	-	No
2380116_adh	TGGAGACGAATTAATGAAAAG	Chr.X	12188864	12188884	+	No
1808773_adh	TACAAGACAGCAGACAGTTCG	Chr.X	12191632	12191652	+	No
1808773_adh	TACAAGACAGCAGACAGTTCG	Chr.X	12195411	12195391	-	No
2729906_adh	TTTGTGGATTTTTGGACAAAT	Chr.X	12204312	12204292	-	No
2071337_adh	TGAAACACTTGACTGCATGAG	Chr.X	12221236	12221256	+	No
1749544_adh	TAAAAGCATTTGTACTAACTA	Chr.X	12348309	12348289	-	No
2737740_adh	TTTTCATGATGATATCTGAAA	Chr.X	12352366	12352346	-	No
1783613_adh	TAAGCTGTGAAAAAGGACGTC	Chr.X	12358152	12358172	+	No
2643329_adh	TTGATAATAGAATAATTAATA	Chr.X	12358934	12358914	-	No
1805490_adh	TAATTTGTCTCGAATTCGGCT	Chr.X	12365524	12365544	+	No
2629096_adh	TTGAAGGTCGCTCCGTGAACG	Chr.X	12446158	12446178	+	No
2052291_adh	TCTGTCATTTGGAGAGGGAAA	Chr.X	12452430	12452450	+	No
1908531_adh	TATGACTCCAAGAAGAACGTC	Chr.X	12468260	12468280	+	No
1976127_adh	TCCAAGAAGAACGTCTGGATC	Chr.X	12468266	12468286	+	No
2389634_adh	TGGATCCCAGATTCGGAAGAC	Chr.X	12468281	12468301	+	No
1913915_adh	TATGGACAAGAATAAGAGAAA	Chr.X	12471890	12471910	+	No
2073706_adh	TGAAACTCGAAGACGAGCAAG	Chr.X	12472024	12472044	+	No
2390889_adh	TGGATGAGGAACAAAAGAGAC	Chr.X	12473765	12473785	+	No
2714191_adh	TTTGAATATTCTGTATTGGGC	Chr.X	12568093	12568113	+	No
2670184_adh	TTGGTCAGAGCAATATCAGGC	Chr.X	12600457	12600477	+	No
2389506_adh	TGGATCAGTGACCATAACAAT	Chr.X	12652623	12652603	-	No
2376272_adh	TGGACGGACAATAGACCAGAA	Chr.X	12691758	12691738	-	No
2671228_adh	TTGGTGGATTGGGTACCACAA	Chr.X	12694251	12694271	+	No
2555371_adh	TTACATTTTGAGCTGTTTCAG	Chr.X	12708930	12708950	+	No
2625878_adh	TTGAACAATGCCTGCAAGATG	Chr.X	12765142	12765122	-	No
1788822_adh	TAAGTTAAAATTGAGAGGACT	Chr.X	12781661	12781641	-	No
2284965_adh	TGCAATGTAGAACATATCGGC	Chr.X	12797711	12797691	-	No
1921501_adh	TATTAGATATGATAATCACTA	Chr.X	12859663	12859683	+	No
2688930_adh	TTTAAAAGTTTGAAAATGTGA	Chr.X	12867044	12867064	+	No
2075462_adh	TGAAAGAAGTGCAGTGTAGAA	Chr.X	13060152	13060132	-	No
2657464_adh	TTGGAATAGGAAGAAGGAAGA	Chr.X	13112398	13112378	-	No
2656561_adh	TTGGAACAAGAAACAAGAGAC	Chr.X	13120918	13120938	+	No
1938794_adh	TCAAGAGAAGTCGTACACAAA	Chr.X	13133765	13133785	+	No
2657310_adh	TTGGAAGTAGCATAAATGAAT	Chr.X	13134591	13134611	+	No
2287082_adh	TGCAGAAGCGATTTAGGATAT	Chr.X	13145504	13145524	+	No
2402789_adh	TGGCAATTCAAGATGGACAAG	Chr.X	13219565	13219545	-	No
2665251_adh	TTGGCAGGAAAGGGAAACTTT	Chr.X	13238475	13238455	-	No

1838771_adh	TAGAACTGAGGAGAAGGCACA	Chr.X	13277326	13277346	+	No
2738247_adh	TTTTCGACTTAGCAGAGAATT	Chr.X	13281729	13281709	-	No
1987509_adh	TCCGGTTGGACGGATTTCTTT	Chr.X	13284701	13284681	-	No
2291662_adh	TGCCAAAAAATGTATGAACGG	Chr.X	13284934	13284954	+	No
2740659_adh	TTTTGAACAATGAAGACGAAT	Chr.X	13294054	13294034	-	No
2707020_adh	TTTCGAGATTAGATTTTCGGC	Chr.X	13312656	13312636	-	No
2706360_adh	TTTCCTGTTGAGCGGATAGCT	Chr.X	13342510	13342490	-	No
1833195_adh	TACTGGAGAAGACGTGGAATA	Chr.X	13409357	13409337	-	No
2406532_adh	TGGCATGATAAGTAGTTTGTA	Chr.X	13420286	13420266	-	No
2523286_adh	TGTTACTTGAATTTGAAGAGG	Chr.X	13457231	13457211	-	No
1836600_adh	TAGAAAAACCAGTGGAACACA	Chr.X	13480222	13480242	+	No
1939243_adh	TCAAGCGCACAGACGAATTCA	Chr.X	13506393	13506373	-	No
2263848_adh	TGATATGACTGATATTTTTGG	Chr.X	13622273	13622253	-	No
2330214_adh	TGGAATAGAAAGAGGAGTGGA	Chr.X	13644995	13645015	+	No
2681472_adh	TTGTGGAAACGCTATGAAATT	Chr.X	13692919	13692939	+	No
2273549_adh	TGATGTGAGTCTGGAACTTGA	Chr.X	13699068	13699088	+	No
2258269_adh	TGATAAAGCTCGTGAAGTCGG	Chr.X	13711355	13711335	-	No
2046720_adh	TCTGATATGATCGGAACACGT	Chr.X	13726393	13726413	+	No
2539985_adh	TTAAACGGTAAGGGCCGGCTG	Chr.X	13759186	13759166	-	No
1919906_adh	TATTAAACGGTAAGGGCCGGC	Chr.X	13759188	13759168	-	No
2492700_adh	TGTATTAAACGGTAAGGGCCG	Chr.X	13759190	13759170	-	No
2492700_adh	TGTATTAAACGGTAAGGGCCG	Chr.X	13759211	13759231	+	No
1919906_adh	TATTAAACGGTAAGGGCCGGC	Chr.X	13759213	13759233	+	No
2539985_adh	TTAAACGGTAAGGGCCGGCTG	Chr.X	13759215	13759235	+	No
1872116_adh	TAGGTTAAATATTTTTCGGCG	Chr.X	13826880	13826860	-	No
1826226_adh	TACGGCATATAGAATTTGGAG	Chr.X	13848581	13848561	-	No
1925362_adh	TATTGAAGAACTCGGAAGGTT	Chr.X	13962835	13962855	+	No
1924747_adh	TATTCTATCATACTGCACTCT	Chr.X	13982125	13982105	-	No
2046054_adh	TCTGAGAACAACGAAATTTGG	Chr.X	14046652	14046632	-	No
2728768_adh	TTTGTCATCACTTCGCTCGGA	Chr.X	14110310	14110330	+	No
2677717_adh	TTGTCATCACTTCGCTCGGAG	Chr.X	14110311	14110331	+	No
2561822_adh	TTAGAATTAAATGACAGGCCT	Chr.X	14241284	14241304	+	No
2305514_adh	TGCTAATTAGAATTGAGACAG	Chr.X	14247637	14247657	+	No
2059461_adh	TCTTGTATCAGATTCGTCGGG	Chr.X	14252342	14252322	-	No
2312087_adh	TGCTTTTCTTCCGTCGGTCTC	Chr.X	14312443	14312423	-	No
2560138_adh	TTACTTCTGCAAAAGACCTCA	Chr.X	14318744	14318724	-	No
2507973_adh	TGTGATCATAACTATAAGAAT	Chr.X	14324436	14324416	-	No
1908853_adh	TATGAGAACTCAGGCATGTAA	Chr.X	14331738	14331718	-	No
2600656_adh	TTCCTCGGTTGGCAAGGATCG	Chr.X	14335039	14335059	+	No
2542124_adh	TTAAATACAAGTAGACGTAAT	Chr.X	14446263	14446243	-	No
2049874_adh	TCTGGATGCGTAAGAAGAAGA	Chr.X	14486678	14486698	+	No
2281883_adh	TGCAAGACAGCACTTGAATCT	Chr.X	14523808	14523788	-	No
2281883_adh	TGCAAGACAGCACTTGAATCT	Chr.X	14524786	14524806	+	No
2281883_adh	TGCAAGACAGCACTTGAATCT	Chr.X	14528926	14528906	-	No
2281883_adh	TGCAAGACAGCACTTGAATCT	Chr.X	14529816	14529836	+	No
2069975_adh	TGAAAATCGCTTGACGTGTCA	Chr.X	14567981	14568001	+	No
2372812_adh	TGGAATTTGATTGGGAATGGG	Chr.X	14606356	14606376	+	No
1811889_adh	TACAGAGCAACTTATGGCCAC	Chr.X	14620199	14620219	+	No
2115260_adh	TGAAATTGAAATATACGTCAA	Chr.X	14629928	14629948	+	No
1858370_adh	TAGCTGAAGAAGAACATAAAG	Chr.X	14644376	14644356	-	No

2686852_adh	TTGTTTAGATATCAAGGAATC	Chr.X	14753754	14753774	+	No
1957011_adh	TCAGAAATTCTTTGGCGAGCA	Chr.X	14846147	14846167	+	No
1967933_adh	TCAGTTTTGGATGTTTCGTCG	Chr.X	14873137	14873117	-	No
1836763_adh	TAGAAAAGTAGATGACGATTA	Chr.X	14964385	14964365	-	No
1939524_adh	TCAAGGACAAGCTCCTCCATA	Chr.X	14978270	14978290	+	No
2489596_adh	TGTAGTATTGATCGGCATGAG	Chr.X	15045440	15045420	-	No
2309348_adh	TGCTGTAGTATTGATCGGCAT	Chr.X	15045443	15045423	-	No
2717139_adh	TTTGAGTTTGAATGGCATGGA	Chr.X	15045557	15045577	+	No
2714315_adh	TTTGAATGGCATGGAATGACA	Chr.X	15045563	15045583	+	No
2300416_adh	TGCGCTGGAAGATGTCGTTAT	Chr.X	15084003	15084023	+	No
2733835_adh	TTTTAATTGTAAGAATAGGCG	Chr.X	15100567	15100547	-	No
2733139_adh	TTTTAAATAAAAATTATTTTT	Chr.X	15111944	15111924	-	No
2126869_adh	TGAAGAAAATTTGAATTCGGC	Chr.X	15113689	15113669	-	No
1899936_adh	TATCTGCAGAGTATGAAAAAG	Chr.X	15167192	15167212	+	No
2712597_adh	TTTGAAACGTTCGGGAGAAGC	Chr.X	15178158	15178178	+	No
2046141_adh	TCTGAGAGCGATGGCTATTTA	Chr.X	15215387	15215367	-	No
2308606_adh	TGCTGCAATACTTGGAAGCGA	Chr.X	15345517	15345537	+	No
2282819_adh	TGCAATACTTGGAAGCGATTT	Chr.X	15345520	15345540	+	No
2055485_adh	TCTTCAAAATCTGACGGTATT	Chr.X	15351299	15351279	-	No
2621014_adh	TTCTTCAAAATCTGACGGTAT	Chr.X	15351300	15351280	-	No
2509450_adh	TGTGATCTACAGAAATATTCA	Chr.X	15362072	15362052	-	No
2663473_adh	TTGGATGTAAAATTAGGTGTT	Chr.X	15364032	15364052	+	No
2740661_adh	TTTTGAACACAATCTGAGGAC	Chr.X	15365277	15365257	-	No
2325070_adh	TGGAAGGGAAAGAAGAAGGAA	Chr.X	15380434	15380454	+	No
1879457_adh	TAGTGTCAAAATCGGACGACC	Chr.X	15480275	15480295	+	No
1996365_adh	TCGAAGATGTTGACGTTTTAC	Chr.X	15511956	15511976	+	No
2025717_adh	TCGTCTTATTTTCGTTCGGTA	Chr.X	15590608	15590588	-	No
2017209_adh	TCGGGGTACTGTAGTAGTGCT	Chr.X	15636495	15636475	-	No
2413620_adh	TGGCGTGTTCTGCAGAAAACC	Chr.X	15664049	15664029	-	No
2716804_adh	TTTGAGCTATTTGGAGATGAA	Chr.X	15707100	15707080	-	No
2590775_adh	TTCAGATCAGAGCTGTGCGGC	Chr.X	15765255	15765235	-	No
1831845_adh	TACTGAACGGATTTGAATGGA	Chr.X	15806435	15806415	-	No
2086561_adh	TGAAATACGATGCTGATGATA	Chr.X	15806603	15806623	+	No
2624337_adh	TTGAAAACATGGAGACGTAGA	Chr.X	15838932	15838952	+	No
1934040_adh	TCAAATACAAGTAGACGTAAT	Chr.X	15945162	15945182	+	No
1919925_adh	TATTAAAGAACGAACAAACGG	Chr.X	15973272	15973252	-	No
2372809_adh	TGGAATTTGATTGGAAATTGA	Chr.X	16051760	16051740	-	No
1922186_adh	TATTATCGATCGATGGTTTAT	Chr.X	16059133	16059113	-	No
1922459_adh	TATTATGATATTTGGACATTT	Chr.X	16059447	16059427	-	No
2742449_adh	TTTTGCGAGTTGGACAGAATT	Chr.X	16062273	16062293	+	No
1782928_adh	TAAGCTACTCATTTCACGCTC	Chr.X	16091288	16091268	-	No
2560189_adh	TTACTTGAATTTCGGAGCGGT	Chr.X	16109831	16109811	-	No
2159415_adh	TGACCAAGCTCATAAGACAAG	Chr.X	16150361	16150381	+	No
2697153_adh	TTTAGTCTGAATCATACCTAG	Chr.X	16182100	16182080	-	No
1956762_adh	TCAGAAAATGAAGAGCAGCAG	Chr.X	16188713	16188733	+	No
2472963_adh	TGGTGTCAAAATTGGACGACC	Chr.X	16204383	16204403	+	No
2618988_adh	TTCTGGAGTTGGAGATTAACT	Chr.X	16213476	16213496	+	No
2493745_adh	TGTCAAAATCGTACGACCGGA	Chr.X	16213834	16213854	+	No
2488890_adh	TGTAGGGCTGTACAAATCGGC	Chr.X	16213876	16213856	-	No
1981250_adh	TCCCCTAGGTGGCTTGACTTT	Chr.X	16217826	16217806	-	No

1825624_adh	TACGGAAATCTGAAAAAGTGG	Chr.X	16232689	16232709	+	No
2590779_adh	TTCAGATCAGTGCTGTGCGGC	Chr.X	16243462	16243442	-	No
1981038_adh	TCCCCGCAACGGAATAATTTT	Chr.X	16271226	16271206	-	No
1829331_adh	TACTAAATTTTGGATGGCTCA	Chr.X	16273254	16273274	+	No
1829331_adh	TACTAAATTTTGGATGGCTCA	Chr.X	16274224	16274244	+	No
2602083_adh	TTCGAAGAACAGAAGCAAACA	Chr.X	16274381	16274361	-	No
2085008_adh	TGAAATAAAAATCAATATATA	Chr.X	16275096	16275116	+	No
1829331_adh	TACTAAATTTTGGATGGCTCA	Chr.X	16284741	16284721	-	No
1762555_adh	TAAATTGGATCTGAAGTTGGC	Chr.X	16311297	16311317	+	No
2485170_adh	TGTAATGTCTCTAAAAATTCA	Chr.X	16336311	16336331	+	No
2042576_adh	TCTCTCGTCTAGCAAGTATTA	Chr.X	16359485	16359505	+	No
2376491_adh	TGGACGGTTTGAAGGGAGAGC	Chr.X	16387183	16387163	-	No
2119856_adh	TGAACAATGTAGAGCACAGCT	Chr.X	16396584	16396564	-	No
2047360_adh	TCTGATGTTGGCAGAAGCACA	Chr.X	16453002	16452982	-	No
2724314_adh	TTTGGCAGCAGGTGTGAATTC	Chr.X	16453882	16453862	-	No
2090212_adh	TGAAATCAAAAAATGTATGTA	Chr.X	16542611	16542591	-	No
1781315_adh	TAAGCAACTCGTTTCTCGGCA	Chr.X	16545714	16545694	-	No
2512942_adh	TGTGATTTGACTATATCCATT	Chr.X	16564648	16564668	+	No
2137236_adh	TGAATAATGTCGCCATCCTAT	Chr.X	16564681	16564701	+	No
2293468_adh	TGCCCCTAGGGATTGTTGGCC	Chr.X	16624352	16624372	+	No
1813565_adh	TACATGAAAATTTTGATTAAA	Chr.X	16632968	16632988	+	Yes
2601646_adh	TTCGAAAATGAGAGCATGTGA	Chr.X	16688548	16688568	+	No
1975745_adh	TCCAAAATTCACGTCAGAGCA	Chr.X	16708061	16708041	-	No
2299804_adh	TGCGATTTCAAATTTTGCCCA	Chr.X	16722028	16722008	-	No
1925299_adh	TATTGAAAGTGATGCGATGGT	Chr.X	16727125	16727145	+	No
1833684_adh	TACTGTAGATGTTAGTTAGGC	Chr.X	16770788	16770768	-	No
2459930_adh	TGGGTTGCTCACGTAGACGGT	Chr.X	16792637	16792657	+	No
2701506_adh	TTTCAAGACTGAACTAGATGG	Chr.X	16793365	16793385	+	No
1863262_adh	TAGGACTGTCGATTGGCCGGA	Chr.X	16814729	16814709	-	No
2674734_adh	TTGTAGAAGTTTGATACTAAA	Chr.X	16883442	16883422	-	No
1814948_adh	TACCACACTGAGATTGAACGA	Chr.X	16893080	16893060	-	No
1934952_adh	TCAAATGAAATCAAACGAGGA	Chr.X	16981197	16981177	-	No
1994640_adh	TCGAAACAGAATGAAAACGCT	Chr.X	16993294	16993274	-	No
1867075_adh	TAGGCATGTAGACTGCAGTGT	Chr.X	17097860	17097880	+	No
1867075_adh	TAGGCATGTAGACTGCAGTGT	Chr.X	17105024	17105044	+	No
2474515_adh	TGGTTATTGTCGTCGGAATCA	Chr.X	17127092	17127072	-	No
2579813_adh	TTATGGTTTCTTTTGTGGTTC	Chr.X	17139059	17139079	+	No
2547086_adh	TTAAGAGGAGGACGAATAAGA	Chr.X	17184294	17184274	-	No
2315668_adh	TGGAAAGAAGGAGAAGAACAT	Chr.X	17213981	17213961	-	No
2643721_adh	TTGATATGGTCTCGAATTGTA	Chr.X	17216759	17216739	-	No
2725887_adh	TTTGGTACTTTAATTGAGCAG	Chr.X	17217594	17217614	+	No
2585446_adh	TTCAAATATTGTAGGGGTTCA	Chr.X	17248187	17248207	+	No
2503853_adh	TGTGACGACAGAACAAGATGA	Chr.X	17255998	17256018	+	No
1838746_adh	TAGAACTCTAAAAGAGAAAAT	Chr.X	17257680	17257700	+	No
2292614_adh	TGCCAGTAGAAAGTATTATGG	Chr.X	17259196	17259176	-	No
1829331_adh	TACTAAATTTTGGATGGCTCA	Chr.X	17342765	17342745	-	No
2527828_adh	TGTTGAATTTTGAAATATTTC	Chr.X	17356568	17356548	-	No
1799221_adh	TAATGAAATGATGCGAAGAGA	Chr.X	17392337	17392357	+	No
2618988_adh	TTCTGGAGTTGGAGATTAACT	Chr.X	17406243	17406263	+	No
2310467_adh	TGCTTCGAGTAGGACAGAAAG	Chr.X	17443221	17443241	+	No

2247584_adh	TGAGGTCAAGGCTATGTACAA	Chr.X	17455983	17455963	-	No
2526328_adh	TGTTCGACAAGAAGGACTGCG	Chr.X	17459816	17459836	+	No
2247584_adh	TGAGGTCAAGGCTATGTACAA	Chr.X	17459904	17459924	+	No
2544556_adh	TTAAATTGTTAATTATTTTTT	Chr.X	17461800	17461820	+	No
1821463_adh	TACGAAAATGCGGAAGAATTT	Chr.X	17468994	17468974	-	No
2525920_adh	TGTTCAACGTCGTATAATAAA	Chr.X	17476629	17476609	-	No
2553013_adh	TTACAAAATGAGGGCATGTAA	Chr.X	17490395	17490415	+	No
1924747_adh	TATTCTATCATACTGCACTCT	Chr.X	17579115	17579095	-	No
2690238_adh	TTTAAGACATGTATTTACTAA	Chr.X	17611572	17611592	+	No
1853875_adh	TAGCACATGACATAGAGGTAA	Chr.X	17622334	17622314	-	No
2270469_adh	TGATCTGGAATTTAAGACGAC	Chr.X	17663237	17663217	-	No
1913691_adh	TATGGAAGATCATCGACTGAT	Chr.X	17679853	17679873	+	No
2573719_adh	TTATAAATAAAAACAATTTAA	Chr.X	17699252	17699232	-	No
1860665_adh	TAGGAAATACGCAAATGGCGC	Chr.X	17703670	17703690	+	No
2275155_adh	TGATTCAGTGGATTTTGTGAT	Chr.X	17711885	17711905	+	No
2593110_adh	TTCAGTGGATTTTGTGATGGA	Chr.X	17711888	17711908	+	No
1967000_adh	TCAGTGGATTTTGTGATGGAA	Chr.X	17711889	17711909	+	No
2743921_adh	TTTTGTGATGGAATCGTTGGA	Chr.X	17711897	17711917	+	No
1860665_adh	TAGGAAATACGCAAATGGCGC	Chr.X	17713130	17713150	+	No
2258269_adh	TGATAAAGCTCGTGAAGTCGG	Chr.X	17716314	17716294	-	No
