# Supplementary material for: Dynamic expression of small non-coding RNAs, including novel microRNAs and piRNAs/21U-RNAs, during Caenorhabditis elegans development
Source: Genome Biol. 2009 May 21;10(5):R54. doi: 10.1186/gb-2009-10-5-r54 (PMC2718520; doi:10.1186/gb-2009-10-5-r54)
Supplement: Additional data file 11 — A larger motif of novel 21U-RNAs represents the region -20 to -63 bp upstream of the 5' terminus of each novel 21U-RNA. 21U-RNAs in which we found larger transcripts and overlapping ones within 10 bp of other 21U-RNAs are marked with an asterisk and a dagger, respectively. [file gb-2009-10-5-r54-S11.pdf]

Sequence Name	Novel 21U-RNA Sequence	Accession number	Chromosome	Starting Position	Ending Position	Strand	A larger motif of novel 21U-RNA
† 2617969_adh	TTCTGACACAGAAGTTAGAAT	FJ589865	Chr.4	4833218	4833198	-	gagtcttgaggtcgcatgctgtttcagttgctttaaaaaaatc
2492000_adh	TGTATGGAAAACAAGTAGTAG	FJ589866	Chr.4	4850598	4850618	+	agttactgtttcagaaagttttataaacttggcgaataaaata
† 2629291_adh	TTGAAGTTATGGCTAGTTTCT	FJ589867	Chr.4	4852884	4852904	+	aaaaatatgtcacagctgtttcaagtaactgacaaatgtatag
† 2588915_adh	TTCAATTGTATTCGACAGTGG	FJ589868	Chr.4	4854109	4854089	-	ttattgtgttacaaagttttactgtttcatcgagtttgaaaaa
† 2740885_adh	TTTTGAATAATCTGTCGCTGC	FJ589869	Chr.4	4854256	4854236	-	attcttgttgaatatttactgtttcagagaggttttaaaaata
2049220_adh	TCTGGAGTTTTTCTTCGATCG	FJ589870	Chr.4	4856044	4856024	-	tatttttaaaaattatcctgtttcacaaactactcatatagtt
2721467_adh	TTTGCATTGGAAAGGTAAACT	FJ589871	Chr.4	4946534	4946554	+	aattttgtgtggatgttttgctgtttcaataactaccataagc
† 1929181_adh	TATTTACGTTGATGCTTTTAG	FJ589872	Chr.4	4958303	4958323	+	caaaagtataaactactgtttcaataagatattataataaaaa
† 2277678_adh	TGATTTAAGGATTGAAGGTAT	FJ589873	Chr.4	4960523	4960543	+	ggtgaaatttttagaaatttactgtttcacgtttttcatttat
* † 2548393_adh	TTAAGGATTGAAGGTATTTTT	FJ589874	Chr.4	4960527	4960547	+	aaatttttagaaatttactgtttcacgtttttcatttattttg
† 2052220_adh	TCTGTCAAGGCACAAATAGAA	FJ589875	Chr.4	5075034	5075054	+	ccaaattatttttaaaaatctgtttcagattgaacctaattt
2738765_adh	TTTTCGTAATTTAAATTAATT	FJ589876	Chr.4	5075707	5075727	+	ttatcttacgaatttctgctgtttcaatttgttttcaagtaaa
† 2583429_adh	TTATTTAAAGTTCTCTCGCAT	FJ589877	Chr.4	5079665	5079645	-	aaaaaacggccaaaattttccactgtttcaagtaggtcttata
† 2568183_adh	TTAGGAGTTCAGTACTGTTTG	FJ589878	Chr.4	5097975	5097955	-	aaaaaaaaagaaaaaatatctgtttcaccttgttaccaaatgt
† 2036394_adh	TCTCAAGAATGTTTGACAATC	FJ589879	Chr.4	5099753	5099733	-	aatcttctagatatctgctgtttcaatgagtcgataatagtag
2618608_adh	TTCTGCATAAAAGAATCTATA	FJ589880	Chr.4	5100104	5100124	+	gcaaaaaataaatttactgtttcacagtgtccattaacatttg
* † 2674955_adh	TTGTAGATATAGAAGACTTAT	FJ589881	Chr.4	5101210	5101190	-	aaaaagttttctctgtttcagccagttatgttaaagagttggg
† 2554125_adh	TTACAGAAGTAGAATTCGAAC	FJ589882	Chr.4	5106861	5106841	-	acattttttttagagtttactgtttcagaatttcttcaaagtaa
† 2665342_adh	TTGGCATGTTATTTTGTTTAT	FJ589883	Chr.4	5109900	5109920	+	gaacagtttttcactgtttcacaacttccattaatattttgta
2744854_adh	TTTTTAATTTGTACATATAGC	FJ589884	Chr.4	5118021	5118041	+	aaaactacaaaatttactgtttcatattctcattatataaaaa
1762668_adh	TAAATTTAATATGTAAAAATC	FJ589885	Chr.4	5132577	5132557	-	ttaaaaattttttttactgtttcaagtgtttcataagtttttt
2740321_adh	TTTTCTTTCTTATGCCTCCAG	FJ589886	Chr.4	5136428	5136408	-	aattttaaaaaatctgttctgtttcaccgattttagaatagtt
2590101_adh	TTCACTTGACTAACTTTGTAA	FJ589887	Chr.4	5138316	5138336	+	caaaaaatttagttactgtttcaaaggtttcacatgaaacgtg
† 2549485_adh	TTAATAACATACGGAAGATAC	FJ589888	Chr.4	5144671	5144691	+	acaccgacaccaaatttttcactgtttcaaattgtttattttc
* † 2664429_adh	TTGGATTGTCGCAGTCGGTTA	FJ589889	Chr.4	5149294	5149274	-	ttggaaatttttttctgtttcatcgtgtttttaaaccccgta
† 2730059_adh	TTTGTGTCGGTAAAAATTAAG	FJ589890	Chr.4	5151224	5151204	-	gaaacgcaaacttttatttactgtttcagatacgttatttttc
1750242_adh	TAAAAGTCTTATTTTCATTAA	FJ589891	Chr.4	5156076	5156056	-	tttttttggatttttactgtttcacattttctatttacataca
† 2566274_adh	TTAGCAATGTATAATATAATC	FJ589892	Chr.4	5172801	5172781	-	ttaaaaaaaaataactgtttcaactttgcgatttattacaacg
† 2703612_adh	TTTCAGTTAGCAATGTATAAT	FJ589893	Chr.4	5172807	5172787	-	ttaattttaaaaaaaaataactgtttcaactttgcgatttatt
† 2471618_adh	TGGTGCCAAGTAAAAAGTTTC	FJ589894	Chr.4	5179514	5179494	-	taaatttttgcaaaattttactgtttcaaaaatcttataacac
† 2702479_adh	TTTCACATTAAAGGTGGTGTA	FJ589895	Chr.4	5197136	5197156	+	aggttttaatgtttactgtttcacgatgctcatacatatatgc
† 2559958_adh	TTACTTATTATCAACTTTCTC	FJ589896	Chr.4	5213343	5213323	-	gtttcaatttttctaatttttactgtttcaacacattatttta
† 2690914_adh	TTTAATAATGGACGCAGTTTT	FJ589897	Chr.4	5229477	5229497	+	atgtttatatgaatcactgtttcaacccgaagttttcaagaaa

	1930988_adh	TATTTTCTTATGAAAACTTCG	FJ589898	Chr.4	5234438	5234458	+	atctttttaaaattgtactgtttcattcatttatttaaagcag
	1930988_adh	TATTTTCTTATGAAAACTTCG	FJ589898	Chr.4	5235937	5235917	-	atctttttaaaattgtactgtttcattcatttatttaaagcag
*	2593298_adh	TTCAGTTGACAATGGTTTTTT	FJ589899	Chr.4	5271206	5271226	+	tctgctctccaaaatttcctgtttcagtatactgatttatttg
	1799242_adh	TAATGAACATCTACTTTGGCA	FJ589900	Chr.4	5282100	5282080	-	agtttcaatatggagaactgtttcatttaattttctagtttat
	1929362_adh	TATTTATCGTATTTTAAAATC	FJ589901	Chr.4	5326667	5326687	+	aattgtttgaaaaaaattactgtttcaaaaatgtgacaagaaa
†	2378769_adh	TGGACTGTTGATCTTTAGAAG	FJ589902	Chr.4	5327693	5327673	-	caaataatttaaaactgtttcactgtgccaataagattatttt
†	2549733_adh	TTAATACTCTTGTTTTATTCA	FJ589903	Chr.4	5336486	5336506	+	tcaaaacttgaaaaactttactgtttcataaattctatttaga
†	2710443_adh	TTTCTGAATTGGCAATGTGTT	FJ589904	Chr.4	5340786	5340766	-	aaattaacagagtttactgtttcagatatgaaatatcaaacat
†	1801984_adh	TAATGTTGTGAAAGGAAATTC	FJ589905	Chr.4	5376574	5376594	+	gtattatttttaaaaattactgtttcagagatttattaaaatt
†	2743644_adh	TTTTGTATCTGGTGGCATTAA	FJ589906	Chr.4	5388160	5388180	+	agaaatcaccaaagatgtactgtttcagacaatctatttagaa
	2276877_adh	TGATTGGGTGATTGGATACAA	FJ589907	Chr.4	5404262	5404242	-	agagctgaaaatattactgtttcaaatataaaagaaaaatatg
†	1832910_adh	TACTGGAAATCACAAGGAAAG	FJ589908	Chr.4	5407588	5407608	+	ataatttcgaccgaaaaattactgtttcaaagagcacacttgt
†	2737250_adh	TTTTCAATCGAGATAAATAGG	FJ589909	Chr.4	5420254	5420274	+	tattttcaaaaaaatatcactgtttcaacaaacccttaaaatc
	2555737_adh	TTACCATGTAGTTCTTGGCAT	FJ589910	Chr.4	5427410	5427390	-	attactgtttcagaagtttttcttagaattagtagaaaaataa
†	1824724_adh	TACGCATACTTGTTGGTTGAT	FJ589911	Chr.4	5428012	5427992	-	tagaaacaaaaaatgtactgtttcattgttttttttcatgag
†	1834772_adh	TACTTCACTTTTTTAAAAACA	FJ589912	Chr.4	5437295	5437315	+	tttgagaaaaaataaaataaactgtttcaatatctctataaaa
†	2060681_adh	TCTTTATTACTGGGCGTCATA	FJ589913	Chr.4	5438891	5438911	+	ctaaaaacttttcactgtttcacgttgtaattttaagatgaat
	2700714_adh	TTTATTTCGTTTTACAAAACT	FJ589914	Chr.4	5449345	5449325	-	ctgcaggaaaaaaataactgtttcaaaatgtttttataaattg
†	2615660_adh	TTCTATGTACAACGGTTCTCT	FJ589915	Chr.4	5498781	5498801	+	ttttataataagtattttaactgtttcagatagtttatattat
†	2639781_adh	TTGAGACTTTTATTCAGATAT	FJ589916	Chr.4	5501944	5501924	-	taatttttaaataattactgtttcatattgttttgaaatttt
†	2716473_adh	TTTGAGACTTTTATTCAGATA	FJ589917	Chr.4	5501945	5501925	-	ctaatttttaaataattactgtttcatattgttttgaaatttt
	2714110_adh	TTTGAAGTTTTTATAAGTTTT	FJ589918	Chr.4	5502158	5502138	-	taaaaagttgcaaaaattactgtttcataacattttgaaaaaa
†	2014236_adh	TCGGATGAAATTGAAATCGGG	FJ589919	Chr.4	5507697	5507677	-	ctttcaatatttatactgtttcaaagtgttgttttgtagactt
†	2464720_adh	TGGTATCTGATCGGTTGAAAT	FJ589920	Chr.4	5509212	5509232	+	tttcaaaaatttcttctgtttcagaagtcttataagtatgaac
	2733794_adh	TTTTAATTACAATTTGAATAA	FJ589921	Chr.4	5509355	5509335	-	attttttcataaattttactgtttcatcattgattgaacacaa
†	1876786_adh	TAGTCGTATGTCAACAATTGT	FJ589922	Chr.4	5528813	5528793	-	aaatttataaaatttatttgtctgtttcagtagattcacttta
†	1883520_adh	TAGTTTTGTTATCCGGTGAGT	FJ589923	Chr.4	5560609	5560589	-	gaattgagaaaaaaaactgtttcatgcatattttaaaaaacatt
	2737706_adh	TTTTCATCGTTTTTTATATAT	FJ589924	Chr.4	5575744	5575764	+	tttaaatttaaaaaaacactgtttcattgcatcaacttcacga
	2066789_adh	TGAAAACAGAATATTCGCTAA	FJ589925	Chr.4	5582700	5582680	-	agaaaaaaaattctactgtttcaaacaaatttaaaaattggtt
	2039703_adh	TCTCGCAGAATGATCCTTGAA	FJ589926	Chr.4	5590726	5590706	-	ttgaggaaaaatcccactgtttcaaaagttttcacaaaataga
†	2616772_adh	TTCTCGTAGGAAAAAGCGGTT	FJ589927	Chr.4	5594316	5594336	+	cacattctcctaaatcttactgtttcatttttgtaactaaacc
†	1790210_adh	TAATAAATGGATAAGATTTTT	FJ589928	Chr.4	5595254	5595234	-	aattaaaatcaattcttactgtttcacatttcttacaaacgcg
†	2584149_adh	TTATTTGGGAAATTTTGTGAC	FJ589929	Chr.4	5596928	5596948	+	ttttttattgcaattgatttcactgtttcatgatgttctgaaa
*	1980122_adh	TCCATTTTTTATTACAATTTA	FJ589930	Chr.4	5604966	5604946	-	aaagcattatatcttttgctgtttcatggagaaggtaaattgc
†	2583100_adh	TTATTGGATTCTACTATATGG	FJ589931	Chr.4	5641673	5641653	-	atttgtcaaaaatgagtttactgtttcaagttgtatactaaat
†	2043255_adh	TCTCTTCAGAGATGGCTACAT	FJ589932	Chr.4	5642987	5643007	+	ttcaatttttctcactgtttcaaagaattgctaaatgatggta

†	2469208_adh	TGGTCAGAAAATTGCTATGAA	FJ589933	Chr.4	5675067	5675047	-	tggttttcacaaaataaatctgtttcacacaattgttaagtat
†	1925228_adh	TATTCTTTCGGTACATTCTTT	FJ589934	Chr.4	5680441	5680421	-	ccggttttctgaattttctactgtttcaacaaaccattattat
†	2582038_adh	TTATTCTTTCGGTACATTCTT	FJ589935	Chr.4	5680442	5680422	-	tccggttttctgaattttctactgtttcaacaaaccattatta
†	2699977_adh	TTTATTCTTTCGGTACATTCT	FJ589936	Chr.4	5680443	5680423	-	ttccggttttctgaattttctactgtttcaacaaaccattatt
	2551858_adh	TTAATTATGCGACTCATTTTG	FJ589937	Chr.4	5706529	5706509	-	tgtcttgaatttaatttctgtttcacttctcgccatttcatac
†	2651143_adh	TTGCCATTTTCCATAGACGCA	FJ589938	Chr.4	5712055	5712075	+	ttcaaaattttgattttttactgtttcatctagtaaatttaac
†	2573364_adh	TTAGTTTATAGATGTCCTCCG	FJ589939	Chr.4	5721849	5721869	+	tggttcctaaaaaaaaagttactgtttcaaacgaaacatgaaa
	2553659_adh	TTACAATTGAATTAAAATTAT	FJ589940	Chr.4	5726042	5726022	-	ttttattttaaatttcactgtttcaaaaaatatttatcaaagt
†	1790749_adh	TAATAATCGTTGTGTCTACTT	FJ589941	Chr.4	5736385	5736365	-	tcaatctatgtttttcaatctgtttcaagtccgtttcaaaaat
†	2699732_adh	TTTATTCATATGCTTAGCTCT	FJ589942	Chr.4	5762688	5762668	-	cagttgcaagtttttttctgtttcaattaaagtttttgaagtg
†	1801872_adh	TAATGTGTTGTAGGGTAAAAT	FJ589943	Chr.4	5769284	5769264	-	tactaattttttttcctgtttcacgttatcttcaacaaattcag
†	1805485_adh	TAATTTGTCGCATATTTTTAC	FJ589944	Chr.4	5790888	5790908	+	attccacatttgaactatttactgtttcaaaaaaaatcataatt
†	2653619_adh	TTGCGTGTTTTCGTTTAAATA	FJ589945	Chr.4	5791428	5791448	+	ttggatattcaatttttcactgtttcaagtaactaatgtagt
	2470402_adh	TGGTCTTATGGGTCATTTTTC	FJ589946	Chr.4	5794512	5794532	+	tgaaatttttcaaaaatactgtttcaagttggtaatatatgtt
	2744884_adh	TTTTTACAGATATAGAAATTT	FJ589947	Chr.4	5801689	5801669	-	atgaaaacctctttgatactgtttcaattagctcatagaaagt
†	1953999_adh	TCACTAGCAGTTCGAGGAAAT	FJ589948	Chr.4	5819060	5819080	+	aataatattttaaaaatttactgtttcagaaaattttcatatt
†	1811697_adh	TACAGAAGAAACAGAAGAATT	FJ589949	Chr.4	5820048	5820028	-	ggaaataagattttctgtttcacagggtggtgatatattgaga
†	2685596_adh	TTGTTGATACAGAAGAAACAG	FJ589950	Chr.4	5820055	5820035	-	ttgtgttggaaataagattttctgtttcacagggtggtgatat
†	2731889_adh	TTTGTTGATACAGAAGAAACA	FJ589951	Chr.4	5820056	5820036	-	tttgtgttggaaataagattttctgtttcacagggtggtgata
†	2726041_adh	TTTGGTATTTGAAGATAATAT	FJ589952	Chr.4	5822954	5822934	-	taagtaaaacaattcactgtttcagtatgcaaatacaaaaatg
†	2672432_adh	TTGGTTGAATGTCGCTATGGG	FJ589953	Chr.4	5838069	5838089	+	ggaaatacacgaaaaaaatttactgtttcataattgtttcaaat
†	1977908_adh	TCCAGAAGACTGAGTCAAATA	FJ589954	Chr.4	5879597	5879577	-	atttttacatgatttcactgtttcattaattggttaaggtatt
†	2646759_adh	TTGATTTACGCGCTAATCTGA	FJ589955	Chr.4	5889014	5888994	-	aaactaggtacattttttactgtttcattgggttaaaaaataa
	2714163_adh	TTTGAATAGAAAATAAACATG	FJ589956	Chr.4	5892691	5892711	+	attttcagtaaaaattttgctgtttcaataacttttgaatact
†	1834070_adh	TACTGTTCCTTCAGAAGAGAT	FJ589957	Chr.4	5900623	5900643	+	tttttaaaaatgttttctactgtttcactatagtatatatact
†	2119361_adh	TGAAATTTTCTTTGTAACTTT	FJ589958	Chr.4	5903880	5903860	-	ttaattttttctgtttcacagcttttgtaataaattctttat
†	2046684_adh	TCTGATAGGTAACGTGTAGGC	FJ589959	Chr.4	5923200	5923220	+	aggttacgaaattcctgtttcactatttcaactttttgatata
	2688676_adh	TTTAAAAAATAAATTTCACTC	FJ589960	Chr.4	5971166	5971186	+	ttctattcaaaaaatccctgtttcaggcagacaacaagtagta
†	2641001_adh	TTGAGCTGTGATGTGATGAAT	FJ589961	Chr.4	5979478	5979498	+	acaaaattatcatctgtttcactaagatgttaaattgagctgt
†	2707285_adh	TTTCGCATCGAATGGATCTAG	FJ589962	Chr.4	6013578	6013558	-	aaagtttaaataaaaagctgtttcagaaaatctagataacaat
	2553970_adh	TTACACTAACTAAATTCATTT	FJ589963	Chr.4	6035048	6035068	+	ggaaactaaaagaaaactgtttcagaagttctataaaacattt
	1979942_adh	TCCATTAACAGCAATTTTTTA	FJ589964	Chr.4	6055701	6055721	+	gaaaaaaaaacaaatttgctgtttcacgaaaatttaacaaatt
†	1931500_adh	TATTTTTTCTTCAGGTTCATA	FJ589965	Chr.4	6063518	6063498	-	caaaatgatttgagtattttctgtttcaaattttcaataacta
	1762275_adh	TAAATTCCTCTTTATAGGTAA	FJ589966	Chr.4	6073531	6073551	+	aattgacaatatttgattactgtttcaataatttttaaatagt
†	2038512_adh	TCTCATTGTTTAGGATCGGCA	FJ589967	Chr.4	6076057	6076037	-	ctatattatttttgactgtttcacaatatggtaataaacaccc
	1821416_adh	TACCTTTTTTGCCTGCTTTAT	FJ589968	Chr.4	6129101	6129081	-	atgtaaataccaaaattactgtttcaatttttttcctttaagca

2748160_adh	TTTTTTAATATACTATTTACA	FJ589969	Chr.4	6169043	6169023	-	attgaagaaaaaaaaatgctgtttcaaaaactatgaataaact
† 2004464_adh	TCGATGTTGCATAGATTTATT	FJ589970	Chr.4	6186909	6186929	+	atttaaaaaaaaaaaatattcctgtttcaaaaaaaaaaatgtaa
† 1880418_adh	TAGTTAGTATGTTCGTGGTGA	FJ589971	Chr.4	6191262	6191282	+	atattgcaattttttttccgctgtttcaatccctttgtataaa
† 2728817_adh	TTTGTCATTCGGCGAAAATTT	FJ589972	Chr.4	6193687	6193707	+	taaaaataattttgactgtttcaatttttttgttatagttttag
† 2677748_adh	TTGTCATTCGGCGAAAATTTT	FJ589973	Chr.4	6193688	6193708	+	aaaaataattttgactgtttcaatttttttgttatagttttagt
2744130_adh	TTTTGTTCAACTGGCTTTTTA	FJ589974	Chr.4	6197502	6197522	+	ttgaataatgcaaaaaaaatcctgtttcaagtactgtaaaaaa
2025371_adh	TCGTCATGTATACGCTCCACA	FJ589975	Chr.4	6217074	6217094	+	ttcgattatgaaatgtgctgtttcaacgttttataaataactt
1897457_adh	TATCGCTAAGTTTTATAATTT	FJ589976	Chr.4	6267760	6267740	-	tgtaagttttcttagattactgtttcaaaactataatatacac
† 2730108_adh	TTTGTGTGATTTCAGTTCTTT	FJ589977	Chr.4	6301622	6301602	-	gtaagttaatatgaaactactgtttcaagcaaaacgtttaaag
† 2745555_adh	TTTTTCAAAGTAGTCATTTTT	FJ589978	Chr.4	6386954	6386934	-	aattaagttttattccctgtttcacaaagtctatgaaaactat
2737892_adh	TTTTCCATATTTTTTGTGATT	FJ589979	Chr.4	6410554	6410534	-	tcatgattaatttattactgtttcaggaaaaaagtaaagctag
† 2552877_adh	TTAATTTTACGAAACGGTGTG	FJ589980	Chr.4	6412096	6412116	+	cctgattcatatttttactgtttcaaactttttttgtaagatt
† 2734149_adh	TTTTACGAAACGGTGTGACTA	FJ589981	Chr.4	6412100	6412120	+	attcatatttttactgtttcaaactttttttgtaagattattc
† 2703757_adh	TTTCATACTGTTTAAACATGT	FJ589982	Chr.4	6433164	6433184	+	ttaaaaatattttttttactgtttcaagtttttcataaagaatt
† 1806293_adh	TAATTTTTGGCAATGGGAGAA	FJ589983	Chr.4	6440351	6440371	+	gaactttttgtcactgtttcactgtgttgttaaaagaaaaggg
2542498_adh	TTAAATATATTTTTTAAATCG	FJ589984	Chr.4	6443437	6443457	+	tctccctattttacttttctgtttcaacggttttataagataa
† 2747311_adh	TTTTTGGCTACAATCACTTCA	FJ589985	Chr.4	6457776	6457756	-	ttcattaaaaaaatctactgtttcaaaacactttttttttaata
† 2259214_adh	TGATAATTCTGATCGTAAAAA	FJ589986	Chr.4	6468571	6468591	+	taaattgagaatagttttttctgtttcaacatgcaattttgaa
† 2692965_adh	TTTAATTTGAAAGGCTCGTAC	FJ589987	Chr.4	6480163	6480143	-	atgatcagacgttgctgtttcaaaactgatagaaaacgacgtc
2595600_adh	TTCCAAAAACATTTTAAATAA	FJ589988	Chr.4	6506475	6506455	-	cttttgcctaaaattgttactgtttcaagatagtatcattagg
2581423_adh	TTATTCATTGGGTATGATATT	FJ589989	Chr.4	6507906	6507926	+	ttatagaaaattaaaacactgtttcaatttttattcaaagttt
† 2697668_adh	TTTAGTTTTAAGGTATTTGTT	FJ589990	Chr.4	6508484	6508504	+	agtatccaatatttgaaaaatctgtttcattaaggtgttgaaa
† 1968520_adh	TCATACAAAATGAACAGAAGT	FJ589991	Chr.4	6508670	6508650	-	ctttaaaaaaatctactgtttcaataaatcaataaaagaaaca
1782982_adh	TAAGCTAGAAGATTGTGACAC	FJ589992	Chr.4	6545503	6545483	-	taaattttttgctgtttcaaaaataataatttgataatttgat
1886866_adh	TATACATAAACGTACACATAA	FJ589993	Chr.4	6577080	6577060	-	ttagaattcttcatccctgtttcactgtacccatctattcctc
† 2617106_adh	TTCTCTCGATCGTAAACATTA	FJ589994	Chr.4	6642295	6642315	+	aatgttacaaaaaaaaaattactgtttcacaagttctataattt
2734041_adh	TTTTACATTTTACATTTTTCA	FJ589995	Chr.4	6800914	6800894	-	ctgagtctaatttttcactgtttcaacatttccataaaatctt
† 2666050_adh	TTGGCTATATATGAAATCTAA	FJ589996	Chr.4	6940490	6940470	-	ttcaattttttttactgtttcaaaagattggtaaaacattttgt
† 2587440_adh	TTCAATAAACAAAAACATAAT	FJ589997	Chr.4	6964276	6964296	+	aatttttttcaataaaaaaactgtttcataaaagtcagaaacgt
† 1940787_adh	TCAATAAACAAAAACATAATT	FJ589998	Chr.4	6964277	6964297	+	atttttttcaataaaaaaactgtttcataaaagtcagaaacgtt
† 1802041_adh	TAATGTTTTGTTAGGTTGGAA	FJ589999	Chr.4	6971802	6971822	+	catttcactaaataattttactgtttcaagaaaacatcttata
† 2534826_adh	TGTTTTGTTAGGTTGGAATAT	FJ590000	Chr.4	6971805	6971825	+	ttcactaaataattttactgtttcaagaaaacatcttatagta
† 2550985_adh	TTAATGAATATGAATGACGAA	FJ590001	Chr.4	6994117	6994137	+	ttctatagcaatgaaaatcactgtttcaatagattatctaata
† 1799338_adh	TAATGAATATGAATGACGAAC	FJ590002	Chr.4	6994118	6994138	+	tctatagcaatgaaaatcactgtttcaatagattatctaataa
2648807_adh	TTGCAATCTGAAGATATCATT	FJ590003	Chr.4	7467950	7467970	+	tttcgaaataaaaaattcactgtttcaacttacgcaaaataacc
1770753_adh	TAACTCTTTCGCTCATTTCGG	FJ590004	Chr.4	7673996	7674016	+	cccaaatttaggctgcgccctgtttcaaagctctgctaatagt

	2734960_adh	TTTTAGTGGGATTTGACCAAT	FJ590005	Chr.4	7743146	7743126	-	atgaattcctaaatgtttctgtttcatttattgaataaatagg
	1880679_adh	TAGTTCTATTAGTTCTGCAGC	FJ590006	Chr.4	7889886	7889906	+	gatctgatcaaatttattgctgtttcagttaccgtatttcttc
	2016347_adh	TCGGGAACATATTTTTAAATC	FJ590007	Chr.4	8055786	8055766	-	acagtatttttaaaaatactgtttcaatgaattaacaattgca
†	2544352_adh	TTAAATTCGGCATTTTATTGA	FJ590008	Chr.4	8099652	8099632	-	ttttttaaaaaaagatccctgtttcagttttattttattttta
	2650417_adh	TTGCATGACTTGATTTTTTGT	FJ590009	Chr.4	8885139	8885159	+	gaaatttctattcaatttactgtttcaagtggaaattaaagaa
	1900232_adh	TATCTTATGCATCTTCTTTCA	FJ590010	Chr.4	9447751	9447731	-	atttataaggttttttttctgtttcagaacattttttttta
	2721992_adh	TTTGCGCTGTATTACCAAGGG	FJ590011	Chr.4	9618734	9618754	+	gatttgatttaaaaacgctgtttcaaattttaaagttgaacga
	1829950_adh	TACTAGACGTTCGCATCATTT	FJ590012	Chr.4	9679579	9679559	-	aatttgcaaatttatctgctgtttcaagatatttttaaagata
	2590187_adh	TTCAGAAAAAGATATACTCTC	FJ590013	Chr.4	9870403	9870383	-	aattctaaaaataaatttactgtttcagaaaaggtattttcat
	1804327_adh	TAATTGTGAGTTCAAACGGAT	FJ590014	Chr.4	10324095	10324075	-	cggtgcaaaattttactgtttcatgtatattataagaattttc
	1764767_adh	TAACACTGCATATGCAACACT	FJ590015	Chr.4	10623246	10623226	-	ccttccaaaaaggtatcactgtttcagtgaatagtcccaccaa
	1761137_adh	TAAATCTTTCACAAGGAATTT	FJ590016	Chr.4	10813454	10813474	+	taaaaacaatttttcactgtttcaaaatttcttgatcattcaa
	1930142_adh	TATTTGAAAAGGAAAACGTAA	FJ590017	Chr.4	10815387	10815367	-	aaggaattgaaaattatttctgtttcaacaacaggtgaaatcg
	2031425_adh	TCTACAGTTACAGTTTTCACA	FJ590018	Chr.4	10886749	10886769	+	tttcttgaaaaattttcactgtttcacatattccaaataatga
	1763162_adh	TAACAAATGTAGAAGTATGAT	FJ590019	Chr.4	11413152	11413172	+	aataacatttatttcaactgtttcagtttctggaagaaaactt
	2736417_adh	TTTTATTACTTATAGTTAGAA	FJ590020	Chr.4	11758472	11758452	-	aaaaaaacaaaaaaattcagctgtttcaaagttgagttttcag
	2259564_adh	TGATACGAGGCAGATGAAACA	FJ590021	Chr.4	12262156	12262176	+	ttgaaaaaatacactgtttcattataataattaattgtgacgt
†	2717919_adh	TTTGATCCTACGATGGTGAAT	FJ590022	Chr.4	13568044	13568024	-	taacttttgtatataaattttgctgtttcattaaattaatatg
†	1926120_adh	TATTGATTTTGAAACATTTTA	FJ590023	Chr.4	13619682	13619662	-	aatttccaaaaaaaaaatactgtttcaccatgattttttgaa
	1851531_adh	TAGATGGTAGAAAAATGGGTA	FJ590024	Chr.4	13641226	13641206	-	taattgctgtttcagatttttttgaacaactattgaaaagttt
	2643358_adh	TTGATAATTTTCATTTACTTA	FJ590025	Chr.4	13670512	13670532	+	aaaaacgttggaaatttttctgtttcaatagtcttattaatgc
* †	2721033_adh	TTTGCAAGGATATATACGGAT	FJ590026	Chr.4	13673722	13673742	+	tttcattaattttttctgtttcaaatcgtttaaaaataaact
†	2576081_adh	TTATATGGGAAATGGGAAATA	FJ590027	Chr.4	13681179	13681199	+	tgaaaaatatatttttcactgtttcacgatgttctcagaatct
†	2590498_adh	TTCAGACGACGATCCGGTTAT	FJ590028	Chr.4	13689227	13689207	-	caacttggagaaaaaatttgctgtttcagattgttgttaaagc
†	2552982_adh	TTACAAAAAAGTGGATGAAGG	FJ590029	Chr.4	13696756	13696736	-	agaatgctacatttttactgtttcagggatatcgttaaaatta
†	1931710_adh	TCAAAAAACATGTAGTGATTG	FJ590030	Chr.4	13746875	13746855	-	gtcgcttgcgttttatgtctgtttcacctactttttttcttct
†	2704380_adh	TTTCATTACATATGTTCCACG	FJ590031	Chr.4	13753338	13753318	-	atttcgaaaaatttctcctgtttcaagtattctacatattaat
†	2497856_adh	TGTCGATGGGTCAATAAATAT	FJ590032	Chr.4	13757730	13757750	+	cattctaaaattgactgtttcactaacgtgacaaaagttgcat
†	1961572_adh	TCAGATGTTAGATAGGTGAAT	FJ590033	Chr.4	13764332	13764312	-	gaatttaatacgttttttctgtttcaataatgcgataatataa
	1886746_adh	TATACACATGATTAATTTTTA	FJ590034	Chr.4	13799709	13799729	+	cccctaatattaactttactgtttcactacgttttcaaatatt
†	2555755_adh	TTACCCAATTCGAAAGTTTGC	FJ590035	Chr.4	13818340	13818360	+	caaaaaaaaattttcactgtttcacgttgctgtcaagttgcttg
†	1874327_adh	TAGTAGACACAGTTGATACGA	FJ590036	Chr.4	13821623	13821643	+	attttctttcacaaattttcactgtttcaatgtattgatataa
†	2617897_adh	TTCTGAATCGCATACGCATTT	FJ590037	Chr.4	13870680	13870700	+	ttcgtgtagaaaattttactgtttcaagtttaggataataaca
†	2743799_adh	TTTTGTCGTGAAAATATGGTT	FJ590038	Chr.4	13900945	13900965	+	tgaaaaaatacgaaactgtttcaagttgttaatatatttgagt
†	2622429_adh	TTCTTCTTTTGCGTACCTTTT	FJ590039	Chr.4	13903913	13903893	-	actttttttcaatttttgctgtttcaatgtcgtcaaataaat
†	1925868_adh	TATTGAGTCGGTCAATTTAAA	FJ590040	Chr.4	13937717	13937737	+	tctactttaaaaaaaaagtactgtttcaataaatactgataat

†	2699380_adh	TTTATGTTCTACAATGACATC	FJ590041	Chr.4	13944779	13944799	+	ttgaaagagaaattttacactgtttcaccaacttcatatgaaa
†	2550467_adh	TTAATCCTACTTAATACTGAA	FJ590042	Chr.4	13958089	13958069	-	gagtagtaaaaaaaattactgtttcaagcactgcttataaatt
†	2742448_adh	TTTTGCGAGTAGAAAATGATT	FJ590043	Chr.4	13984308	13984288	-	aaatatttcccctgtttcaaattgtccataactacgtttttat
†	2552923_adh	TTAATTTTGCGAGTAGAAAAT	FJ590044	Chr.4	13984312	13984292	-	ttgtaaatatttcccctgtttcaaattgtccataactacgttt
* †	2693036_adh	TTTAATTTTGCGAGTAGAAAA	FJ590045	Chr.4	13984313	13984293	-	gttgtaaatatttcccctgtttcaaattgtccataactacgtt
†	2548913_adh	TTAAGTAGTGTGATGTTATAG	FJ590046	Chr.4	13988697	13988677	-	agatttttaaaaaattttctgtttcaattttcgaataaaacaa
†	1920211_adh	TATTAAGTAGTGTGATGTTAT	FJ590047	Chr.4	13988699	13988679	-	ttagatttttaaaaaattttctgtttcaattttcgaataaaac
†	2558218_adh	TTACTAAAATTCATGCCAGTC	FJ590048	Chr.4	13999445	13999465	+	tgtgaaaattgaacctgctgtttcatattgtttttaaaaatga
†	1829386_adh	TACTAAGACATCGGAACACAA	FJ590049	Chr.4	14000569	14000549	-	tttttttgaaataaaaaatcactgtttcaaattgaaaatttgt
†	2581226_adh	TTATTATTTTTGTCGAACTTC	FJ590050	Chr.4	14007257	14007237	-	agcaggcaatactatttctcactgtttcagtaagttttttta
†	2597979_adh	TTCCCCGTTCACACGTTCTAC	FJ590051	Chr.4	14008008	14008028	+	ttttgaaaaaaaaaacaactgtttcaacatgtctatatgaaat
†	2733915_adh	TTTTAATTTTTTCGTCGCAAT	FJ590052	Chr.4	14008188	14008208	+	caaagaaaacattgactgtttcaagcttataatttcaatctaa
†	1922028_adh	TATTATAGTTTCTCGACGGTG	FJ590053	Chr.4	14008849	14008869	+	tttcagtatctccaaaattcgactgtttcatattcctaattta
†	2575145_adh	TTATAGTTTCTCGACGGTGTG	FJ590054	Chr.4	14008851	14008871	+	tcagtatctccaaaattcgactgtttcatattcctaatttaaa
	2140192_adh	TGAATATTTATAACTTTGAAA	FJ590055	Chr.4	14009455	14009475	+	cagtctaaaatttcaactgtttcatattcacaatgaaaaaatg
†	2748135_adh	TTTTTTAAGGACATTTTATTT	FJ590056	Chr.4	14026157	14026177	+	tagttattatttttctgtttcaacttcaagatttaaatgttt
†	2025208_adh	TCGTATTTCTTGTTGTGTTTT	FJ590057	Chr.4	14037183	14037163	-	tgaatattcacttttcataaattactgtttcagggcggtttct
†	1806089_adh	TAATTTTGTGAATTTATCGGA	FJ590058	Chr.4	14037754	14037774	+	atgctgttgaactatttttcctgtttcactgtggagttataaa
	1893865_adh	TATCATATTTTATTACGAATG	FJ590059	Chr.4	14038471	14038491	+	tgtgtttgaaagcttttctgtttcactatggaattatatattt
†	2722404_adh	TTTGCTGACATTCATAATACA	FJ590060	Chr.4	14039273	14039253	-	tccgtcaatttttattactgtttcatgttgcattattttggg
†	2742529_adh	TTTTGCTGACATTCATAATAC	FJ590061	Chr.4	14039274	14039254	-	ttccgtcaatttttattactgtttcatgttgcattattttgg
†	2653449_adh	TTGCGTATGGCGTATCAGTTC	FJ590062	Chr.4	14043525	14043545	+	aaattttcggaaaaaatttctgtttcagatcattgacaaaatc
†	2046559_adh	TCTGAGTTGCAACTAGTTGAA	FJ590063	Chr.4	14047246	14047266	+	attaatttacaaattcgactgtttcaacatgtttttttacaa
	1746097_adh	TAAAAACTTATTATTTAGTAA	FJ590064	Chr.4	14088618	14088598	-	aaaaaaggaaaaaattctctgtttcaaaaaaaattacaaaattt
†	2237322_adh	TGAGGACGTCATTGTGACATA	FJ590065	Chr.4	14091569	14091549	-	agaatttttactgtttcacaattttaataaaaatgcaaaaat
†	2646464_adh	TTGATTGGCATGTACTCAAGT	FJ590066	Chr.4	14093178	14093158	-	taaaacctactgtttcatatagttgatataagattgaacgttt
†	2720260_adh	TTTGATTGGCATGTACTCAAG	FJ590067	Chr.4	14093179	14093159	-	ttaaaacctactgtttcatatagttgatataagattgaacgtt
†	1930238_adh	TATTTGATTGGCATGTACTCA	FJ590068	Chr.4	14093181	14093161	-	agttaaaacctactgtttcatatagttgatataagattgaacg
†	2692935_adh	TTTAATTTATTGTGAAGACTG	FJ590069	Chr.4	14100337	14100317	-	catggatagcaagaatttttactgtttcacaattttaataaa
†	2699705_adh	TTTATTCACTCTAAAGGAAAC	FJ590070	Chr.4	14100949	14100929	-	tagggtgttactaagttctactgtttcacatacttgttataat
†	2574090_adh	TTATAATGAATATTTTGGACA	FJ590071	Chr.4	14118738	14118758	+	aattttgaaaaaaattctctgtttcacattttctatgaataaa
†	2628533_adh	TTGAAGATTTGTTTTGCGCTC	FJ590072	Chr.4	14121628	14121608	-	cggagaaaaaaaaattactgtttcaaaatcgtgttaagcatttt
†	2582136_adh	TTATTGAATGAACCTTAGAAA	FJ590073	Chr.4	14122666	14122646	-	ttaaattcaaaaaaaacttgctgtttcaaaaatttaacaaaat
	1962526_adh	TCAGCCTGATGAAATTTTATT	FJ590074	Chr.4	14123389	14123369	-	ttaaaatgaaaattggctgtttcaaaaaaaatgtccatgtattg
†	2560332_adh	TTACTTGTGCTAGAAAAATGT	FJ590075	Chr.4	14126397	14126377	-	aaaaaatttcacaaaatttttctgtttcaaagtggctacatat
	2720202_adh	TTTGATTGCATTCCAAAATTA	FJ590076	Chr.4	14164329	14164349	+	ctgcgttttctttttttactgtttcagtcgtgtaataacaac

† 2745449_adh	TTTTTATTAAAAGCAGAAACT	FJ590077	Chr.4	14174858	14174878	+	caactataataaatatttactgtttcacagtctttgtttaatt
† 2736390_adh	TTTTATTAAAAGCAGAAACTA	FJ590078	Chr.4	14174859	14174879	+	aactataataaatatttactgtttcacagtctttgtttaattt
† 1974280_adh	TCATTGGATGATGGAAGAACC	FJ590079	Chr.4	14197654	14197634	-	ttctaaagaactgatactgtttcaaaatctctatttaatgaac
† 1969133_adh	TCATAGGGTAACCGATTTTCA	FJ590080	Chr.4	14219655	14219675	+	ctaattaaaatcttcactgtttcaccagtgctatataaaattg
† 2541089_adh	TTAAAGATCAAAAGCACATAT	FJ590081	Chr.4	14228310	14228290	-	aacaaaataaaatttttgctgtttcacatattacgtaaaaact
† 2656080_adh	TTGCTTTTTAGACTATGCTTC	FJ590082	Chr.4	14232202	14232222	+	tccaccaaattttcactgtttcaacactgacatatactgcatg
† 2688876_adh	TTTAAAAGAAACTCAAACAGC	FJ590083	Chr.4	14244596	14244576	-	gcaaaataaataattttttctgtttcacaatgttttcatacgg
† 2560218_adh	TTACTTGATTACGGCTCCATT	FJ590084	Chr.4	14262202	14262182	-	aaattgaatactgaagtttccactgtttcatgtagtttataag
† 1920682_adh	TATTACATCTTCTATTTTAAT	FJ590085	Chr.4	14263266	14263246	-	taattgattttaaaattttactgtttcacgaactttataacat
† 1936725_adh	TCAACGGCTGTACAAAAAGAA	FJ590086	Chr.4	14273678	14273658	-	catttcaacgtttcaactgtttcacaatgtctacaagtactgt
† 2618819_adh	TTCTGCTTGATTGTCTATCCT	FJ590087	Chr.4	14280519	14280539	+	agcgtaagtcatattatcactgtttcataatgtttataaaaca
† 2553002_adh	TTACAAAAGTCCATTTATTTT	FJ590088	Chr.4	14286751	14286731	-	tttctttaaatttacctgtttcatgttttcgatatagttttgt
† 1774750_adh	TAAGAATTGTAAAGCCTTCTA	FJ590089	Chr.4	14287072	14287092	+	tttttctacaattttcactgtttcattatctctatttaacagt
† 2745191_adh	TTTTTAGTTTTCGTTGTGGAC	FJ590090	Chr.4	14301749	14301769	+	aattatttttttaaaaagtttccactgtttcaagttgctcaca
† 2735035_adh	TTTTAGTTTTCGTTGTGGACA	FJ590091	Chr.4	14301750	14301770	+	attatttttttaaaaagtttccactgtttcaagttgctcacaa
† 2697672_adh	TTTAGTTTTCGTTGTGGACAA	FJ590092	Chr.4	14301751	14301771	+	ttatttttttaaaaagtttccactgtttcaagttgctcacaaa
† 2573561_adh	TTAGTTTTCGTTGTGGACAAT	FJ590093	Chr.4	14301752	14301772	+	tatttttttaaaaagtttccactgtttcaagttgctcacaaaa
† 1883461_adh	TAGTTTTCGTTGTGGACAATT	FJ590094	Chr.4	14301753	14301773	+	atttttttaaaaagtttccactgtttcaagttgctcacaaaat
† 2709284_adh	TTTCGTTGTGGACAATTTGGT	FJ590095	Chr.4	14301757	14301777	+	ttttaaaaagtttccactgtttcaagttgctcacaaaattcca
† 2514804_adh	TGTGGACAATTTGGTATTTAT	FJ590096	Chr.4	14301763	14301783	+	aaagtttccactgtttcaagttgctcacaaaattccaagaagc
2549424_adh	TTAATAAAAAAGTTATTCATA	FJ590097	Chr.4	14302068	14302088	+	ccaaaaaatcaaaaatcactgtttcaggaaaataatttcagaa
1834380_adh	TACTGTTTTCTTTTTATTTAT	FJ590098	Chr.4	14327837	14327857	+	cacagcttctgtgaaattactgtttcaatacactcataaataa
† 2557363_adh	TTACGGCTAACTTTTGTCATA	FJ590099	Chr.4	14345237	14345257	+	atatttttctatcgaagtctgtttcactgtgttgtcatattgc
† 2611329_adh	TTCGTAGCGTAGCATGATTTT	FJ590100	Chr.4	14359173	14359153	-	ttttttattcaaaaaattactgtttcaaactgttttgaaaag
† 2526248_adh	TGTTCCGCAGAAATCTCTCTT	FJ590101	Chr.4	14361661	14361681	+	aagtgcatacattttttaactgtttcacagttatcattcaaca
2071524_adh	TGAAACAGATAACAGAATTTA	FJ590102	Chr.4	14368291	14368271	-	tttcagaatttttgcctgtttcagcatgttgccttgagagtag
† 1790384_adh	TAATAAGACCGTGGTAAATAT	FJ590103	Chr.4	14378133	14378113	-	ttttcagaatataaacttcaactgtttcactatgctgataagt
† 2736976_adh	TTTTATTTGGAGCATGATCAA	FJ590104	Chr.4	14378871	14378851	-	gagcttttttttcaaaatttcactgtttcagatgtatgattt
† 2745524_adh	TTTTTATTTGGAGCATGATCA	FJ590105	Chr.4	14378872	14378852	-	ggagcttttttttcaaaatttcactgtttcagatgtatgatt
† 1860009_adh	TAGGAAAAATTAGAAGACATT	FJ590106	Chr.4	14379725	14379705	-	taaataaaaagttatactgtttcatgtttctcatataatttgc
† 2488535_adh	TGTAGGAAAAATTAGAAGACA	FJ590107	Chr.4	14379727	14379707	-	tctaaataaaaagttatactgtttcatgtttctcatataattt
† 2675080_adh	TTGTAGGAAAAATTAGAAGAC	FJ590108	Chr.4	14379728	14379708	-	ttctaaataaaaagttatactgtttcatgtttctcatataatt
† 2728309_adh	TTTGTAGGAAAAATTAGAAGA	FJ590109	Chr.4	14379729	14379709	-	tttctaaataaaaagttatactgtttcatgtttctcatataat
† 2742197_adh	TTTTGCACGGTTTCTGGTTTT	FJ590110	Chr.4	14393463	14393483	+	aacagaaaaatttactgtttcaatattattttctttgttgtaa
† 1927093_adh	TATTGGCAAAACATCGATAAA	FJ590111	Chr.4	14398596	14398576	-	ttgacttttttttactgtttcaggttttaaattattaaaggcg
† 2566239_adh	TTAGCAACGGTATTGTAATCA	FJ590112	Chr.4	14402275	14402295	+	gaattctggaattttatactgtttcactttgttttctaattc

†	2572210_adh	TTAGTTAGGACATAATATGAT	FJ590113	Chr.4	14405952	14405932	-	ttagaagttaataatatctgctgtttcaccaacatgttatgaa
	2539105_adh	TTAAAATTATAATTAACATTT	FJ590114	Chr.4	14414716	14414696	-	tttctttatttttttactgtttcaggtttgtgttgaaagtgg
†	1803088_adh	TAATTCCTTCTGAAATAACAA	FJ590115	Chr.4	14444346	14444326	-	tttaaaaatttgaaattactgtttcacaaaagtggtaaatttt
†	1923398_adh	TATTCAATAAGAATCCTTTTA	FJ590116	Chr.4	14462612	14462592	-	atgcgaaaaattatattctgtttcatattattttcaaactgtc
†	2554122_adh	TTACAGAAGGACAAACAGAGT	FJ590117	Chr.4	14471798	14471778	-	ttaaaattaaattcactgtttcaccactataacttgaggtttt
†	2277798_adh	TGATTTACAGAAGGACAAACA	FJ590118	Chr.4	14471802	14471782	-	ttttttaaaattaaattcactgtttcaccactataacttgagg
†	2681248_adh	TTGTGCAGTGGATACATTTTT	FJ590119	Chr.4	14487440	14487460	+	ttttaggaaaaactgttctgtttcaaaattttgttttaagtaa
	2742281_adh	TTTTGCAGTTATATTTTTGTT	FJ590120	Chr.4	14492577	14492597	+	tttgaagcacatttttttgctgtttcaaagtaaacaaaaaagta
†	2533562_adh	TGTTTGTTCAGCTTTTAAATT	FJ590121	Chr.4	14496210	14496230	+	ttgtaaaaaattttgactgtttcatcatgttttgataattacc
†	2711788_adh	TTTCTTGCTAGAACACAAATT	FJ590122	Chr.4	14499560	14499580	+	gaaataattttctcactgtttcaagatcaacatttattgttct
	2706408_adh	TTTCCTTATCTTTTTATGTTA	FJ590123	Chr.4	14501394	14501374	-	ttcacttaatgtttttttactgtttcagaacatctttaatataa
†	2602966_adh	TTCGACGCTTCTATTCTCTTC	FJ590124	Chr.4	14508818	14508838	+	ttttttcaaaaatctctgtttcattaccatgtgataaaacaat
†	1801571_adh	TAATGTATGAAAACGTGCCGT	FJ590125	Chr.4	14526277	14526257	-	tctgtcttttaactgagcactgtttcacattgcttttttgaaa
†	2584780_adh	TTATTTTTTCTGATATAACGG	FJ590126	Chr.4	14536504	14536524	+	ctaacaagtttgaattgcgactgtttcactatcatgagaaagt
†	1943081_adh	TCAATTGATGGTTTTTTGTTT	FJ590127	Chr.4	14552485	14552465	-	ttatgaacaatttttactgtttcacattgtgtttactcaatta
†	2623512_adh	TTCTTTCTCCTACATTTTAAT	FJ590128	Chr.4	14555003	14554983	-	attaattttcagaaaatactgtttcaatcccaccatatttttt
†	2561422_adh	TTAGAAGAATATAATTTTTGG	FJ590129	Chr.4	14561395	14561415	+	atttttcgaaaaaaaaataaactgtttcaatttcatctgatatt
†	2029231_adh	TCTAAAAATCGCTGACAATAC	FJ590130	Chr.4	14565282	14565302	+	gtcattgacaatgttttccgtttctgtttcataacataagtat
†	2546829_adh	TTAAGACATATATGTGGATTT	FJ590131	Chr.4	14566071	14566051	-	atgttttcaaattcactgtttcaaaagtcgaacttgaggttta
	1974235_adh	TCATTGCTGTGCAGATTCCTT	FJ590132	Chr.4	14571944	14571964	+	gaaaaattatgtttgctgtttcaatgtcagtttttgaaaaatg
†	2739127_adh	TTTTCTAGACGACATCAATTG	FJ590133	Chr.4	14572075	14572055	-	aaaaaataattgaaaactctgtttcaataagtctagaaatgtt
†	2025374_adh	TCGTCATTAGTATATGCTGCA	FJ590134	Chr.4	14608234	14608254	+	gtggatatctaatactgtttcaaggttctttcaatagttgatt
†	1880692_adh	TAGTTCTGAAAGATCCCAATG	FJ590135	Chr.4	14615317	14615337	+	tggaaatttttttcactgtttcaaaaattccatgaaaattcct
†	1909745_adh	TATGAGTATGACGGTAGTTTT	FJ590136	Chr.4	14624603	14624623	+	ttttttttcacgactgtttcagaatttccatttaatgcgtaa
†	1816264_adh	TACCATTGCTAACAGATTTTT	FJ590137	Chr.4	14631339	14631319	-	atattctctaaaatatttctgtttcacattgttttgaaatcga
†	2623781_adh	TTCTTTGTTTGTATCAATTTT	FJ590138	Chr.4	14635033	14635013	-	atgatttattttttctgtttcagaacggtcttatatattttt
†	2582184_adh	TTATTGACTATGAGATCTGAA	FJ590139	Chr.4	14675203	14675183	-	ttttgaacattttttttactgtttcaatagttgcattcaatac
†	2581165_adh	TTATTATTGACTATGAGATCT	FJ590140	Chr.4	14675206	14675186	-	tttttttgaacattttttttactgtttcaatagttgcattcaa
* †	2520855_adh	TGTGTTCGGTGGACTTTCAAA	FJ590141	Chr.4	14678604	14678584	-	taaattctgatagcccactgtttcaaggtattaatataattca
†	2694341_adh	TTTACTCTGTGTTCGGTGGAC	FJ590142	Chr.4	14678611	14678591	-	taacgaataaattctgatagcccactgtttcaaggtattaata
	2584081_adh	TTATTTGCATATAATACATAT	FJ590143	Chr.4	14679936	14679956	+	ctaattgttgaattactgtttcagaattctctgttaaatacct
†	1970211_adh	TCATATTCGTATTTAACAGTA	FJ590144	Chr.4	14680480	14680460	-	gatgaaaagaaattgctgtttcaagtacttgatttataatttt
* †	2486875_adh	TGTACGGGCTTCTGTTTGGCA	FJ590145	Chr.4	14687970	14687990	+	gtgttctaaattgatactgtttcatggtattttgatataaatt
†	2289567_adh	TGCATAAATGAACGGTTATTT	FJ590146	Chr.4	14708188	14708168	-	ttaacaaaaattgctgtttcagattttttagataattttcatc
†	2730830_adh	TTTGTTCTATTCCTGACCGGC	FJ590147	Chr.4	14715951	14715931	-	tagaacagtattgcaaatgatactgtttcaatatagtcggaaa
†	2593445_adh	TTCATAAACTACTTTTGCTGC	FJ590148	Chr.4	14717789	14717769	-	ccatgtaaaattgtccactgtttcatcttcactatatacaaat

	† 2676584_adh	TTGTATGATAGCTGTAATAAT	FJ590149	Chr.4	14718160	14718140	-	aaataaataaatactgtttcagcatgctttcataatttgatac
	† 2560282_adh	TTACTTGTATGATAGCTGTAA	FJ590150	Chr.4	14718164	14718144	-	aaataaataaataaatactgtttcagcatgctttcataatttg
	† 2596747_adh	TTCCAGATGATGAACGGTAAT	FJ590151	Chr.4	14723815	14723795	-	atacatttaaaaaaattactgtttcaaatccttatttttgttt
	† 1814585_adh	TACATTTGTTGTGAAAAACCT	FJ590152	Chr.4	14731609	14731589	-	aattaaaaattttactgtttcataaatttgttaaatttttaag
	† 1923790_adh	TATTCATCATTCATTGCGTGG	FJ590153	Chr.4	14732543	14732523	-	ataaaacttcctaaaaaatctactgtttcatattgtcgctata
	† 2728968_adh	TTTGTCGATCGGATTAATTTT	FJ590154	Chr.4	14739573	14739593	+	ttttctattgaaaaattttactgtttcaaaatatttaaaaact
*	† 2375908_adh	TGGACGAAATTGCAATATGTT	FJ590155	Chr.4	14752776	14752796	+	tttaaaaaaatctcaaactgtttcaacatgttcatgtaaacag
	† 2616104_adh	TTCTCAGATGGTATTTGAATC	FJ590156	Chr.4	14756812	14756832	+	tgtaatcaaaagagtttactgtttcaccgtgttaatatcaaaa
	† 1804159_adh	TAATTGGTGCCTGCTATTAGA	FJ590157	Chr.4	14756893	14756913	+	aatccactaataaatttccaactgtttcaatgtgttcatatca
	† 2126223_adh	TGAACTGTTGATTTCGTGCCG	FJ590158	Chr.4	14815871	14815851	-	ttttgaaaaaaaacttactgtttcagctataccatgtaatgac
	† 2694680_adh	TTTACTTGAAGACAACTAAAT	FJ590159	Chr.4	14819995	14820015	+	aaaataaaattaaaatcactgtttcatgaatctcaacggattt
	† 2560176_adh	TTACTTGAAGACAACTAAATT	FJ590160	Chr.4	14819996	14820016	+	aaataaaattaaaatcactgtttcatgaatctcaacggatttg
	† 2627270_adh	TTGAAGACAACTAAATTCTCA	FJ590161	Chr.4	14820000	14820020	+	aaaattaaaatcactgtttcatgaatctcaacggatttggtac
	2735379_adh	TTTTATATGAAGACAACTAAA	FJ590162	Chr.4	14820465	14820485	+	aaaaaaattaaaatcactgtttcataaataaagcttaaaggtt
	† 2732922_adh	TTTTAAAAAACAATTAACTGT	FJ590163	Chr.4	14853808	14853788	-	gttttttcctattaatttatctgtttcaaaatactgttaaatt
	† 1920674_adh	TATTACATAAGAAATAAAAAA	FJ590164	Chr.4	14862899	14862879	-	aaaaaaccacatacagacttactgtttcacagtatcagtaaat
	† 1920000_adh	TATTAAATTTTCCACAGCCTA	FJ590165	Chr.4	14874671	14874691	+	caaattttactcaaaaaattactgtttcaaaaacttaatatta
	† 2582231_adh	TTATTGAGAACATAGCTTTTT	FJ590166	Chr.4	14876096	14876076	-	acttttttaaaaaaattttctgtttcaaaaggtatgaaattat
*	† 1847229_adh	TAGAGAAGTAGAAGTCATTTG	FJ590167	Chr.4	14876487	14876467	-	ttagttacaatttttactgtttcaaaatatttgcaaaatccta
	† 2579272_adh	TTATGGAAATGCGGTTATTTG	FJ590168	Chr.4	14893632	14893612	-	atactgggaaagaaaagcactgtttcaagttgttgatttcaac
	† 1929074_adh	TATTTAATAGGCAATTCTTAA	FJ590169	Chr.4	14899085	14899065	-	tcaagagtttaatttttctgtttcacttctatcatatattaag
	1970343_adh	TCATCAAAATATTCAAATATT	FJ590170	Chr.4	14900641	14900621	-	tatatttaaaataaagtctgtttcaaacctattttatttatct
	† 2740222_adh	TTTTCTTCTTTGATTCGTTTT	FJ590171	Chr.4	14901566	14901546	-	tatagaatttaatgttttaactgtttcaatatgtctagttagt
	† 2625906_adh	TTGAACACCATACAATCTTTA	FJ590172	Chr.4	14902983	14903003	+	aattagaaaaaaaattactgtttcaattattcgataaaagatt
	2703914_adh	TTTCATCATTTTATTTTTAAT	FJ590173	Chr.4	14918330	14918350	+	ttttggttgaaaaattctactgtttcacatggttcttatacgg
	2568602_adh	TTAGGCTTTATTAGGATTATT	FJ590174	Chr.4	14919147	14919167	+	atgtcgtcagcttattttcactgtttcaaattttcaaaatcgt
	† 2658032_adh	TTGGACAGATGAAGTTTTTCA	FJ590175	Chr.4	14921240	14921260	+	gaaaaaatgtatgctctgtttcatattggagtttttttgtgag
	† 2647095_adh	TTGATTTTTATTTTGTCTGAT	FJ590176	Chr.4	14923691	14923671	-	gttcaacttttaaattttgctgtttcacacattcattatcaat
	† 1901399_adh	TATGAAAGTCGAAGATCGTGC	FJ590177	Chr.4	14932484	14932504	+	ttttcaaatttctgctgtttcaagtgtctcacttatgataatt
	† 2711027_adh	TTTCTGTTCGTGGACACAATT	FJ590178	Chr.4	14950058	14950038	-	ttctcaatacattttatctgtttcagtaagtttttaaccacgc
	† 1924388_adh	TATTCGGACAGGCAAGATTTT	FJ590179	Chr.4	14963685	14963705	+	ggcgattcaaaaattttcactgtttcaatgtcatattataaaa
	† 2607312_adh	TTCGGACAGGCAAGATTTTGT	FJ590180	Chr.4	14963687	14963707	+	cgattcaaaaattttcactgtttcaatgtcatattataaaata
	† 1833443_adh	TACTGGGACTTCCTAAACGGT	FJ590181	Chr.4	14964363	14964383	+	gaaattcgaaaagaaattactgtttcaatggcagatttaaata
	† 1790254_adh	TAATAACAATTGCAGACGGCT	FJ590182	Chr.4	14964485	14964505	+	gtattttaaaaattatatcttctgtttcaagttgttaacaaat
	2501347_adh	TGTGAAATTGCGATTGGAGAA	FJ590183	Chr.4	14968080	14968060	-	cattgaaaaaatctactgtttcaactagatgtgataactcaac
	† 2717643_adh	TTTGATATTGTAGATTTCTCC	FJ590184	Chr.4	14979618	14979638	+	gtgggtaggttttagagtttactgtttcatcatatttttagat

†	1945067_adh	TCACAGGAGATTTTGCTCACA	FJ590185	Chr.4	14997252	14997232	-	atgaaaaaaaaaatctgtttcatcgaattgttatagaaagtac
†	1929517_adh	TATTTCACAGGAGATTTTGCT	FJ590186	Chr.4	14997256	14997236	-	aggaatgaaaaaaaaaatctgtttcatcgaattgttatagaaa
†	1925791_adh	TATTGACTGTATTTTGGTTTT	FJ590187	Chr.4	15000593	15000613	+	ttctacttttaaaaaatcaactgtttcattattatgacttatt
†	2638530_adh	TTGACTGTATTTTGGTTTTTG	FJ590188	Chr.4	15000595	15000615	+	ctacttttaaaaaatcaactgtttcattattatgacttattgg
†	2688467_adh	TTGTTTTTGGATCTTTTTGTT	FJ590189	Chr.4	15052097	15052077	-	ttttgcaatacaaatattactgtttcaagtatatcacaaaaca
†	2700895_adh	TTTATTTTTGCATTCTTCTCC	FJ590190	Chr.4	15061240	15061260	+	aatttaattaagatctgtttcacgttgtcgacaagttggtcag
	2289640_adh	TGCATAATAATTTGTTTCTTT	FJ590191	Chr.4	15066501	15066481	-	atattttttttattttcctgtttcactaaaaactaaaaaataa
†	2687400_adh	TTGTTTGACTTAAAATGAAAC	FJ590192	Chr.4	15086798	15086778	-	tgaatatttcagttttttttctgtttcataacctttgtatat
†	2017100_adh	TCGGGGCACGAACGGTTAATT	FJ590193	Chr.4	15108249	15108229	-	atataaatgcaattttactgtttcaaaattgaactaaatttct
†	2618491_adh	TTCTGATTAGACTAAAAAGAA	FJ590194	Chr.4	15118020	15118000	-	ttcggatgtattcaaccactgtttcaagttggtcatatattct
	2633616_adh	TTGAATTATTAATTTTATTTT	FJ590195	Chr.4	15133568	15133588	+	ttttgttttaaaatttttactgtttcacgttgctgtcaaatat
†	2593384_adh	TTCAGTTTAAAAAATATGGCT	FJ590196	Chr.4	15136551	15136571	+	ttaattgtttttcctactgtttcattagttccacaaaaatttt
†	2733076_adh	TTTTAAACTGAATTGTTTGTA	FJ590197	Chr.4	15136562	15136542	-	ataaaactgtttcagtaaaatattttattgaatgctacgcatt
†	2736055_adh	TTTTATGGATTGTTCCGCATG	FJ590198	Chr.4	15138555	15138575	+	taacttctacgaaaatttcctgtttcaaacttttaattttaat
†	2742364_adh	TTTTGCATGGATATATGTCTT	FJ590200	Chr.4	15158535	15158555	+	attttaaagacaaattatatctgtttcaaatgtttcgtaaggt
†	2721447_adh	TTTGCATGGATATATGTCTTT	FJ590199	Chr.4	15158536	15158556	+	ttttaaagacaaattatatctgtttcaaatgtttcgtaaggta
†	2721447_adh	TTTGCATGGATATATGTCTTT	FJ590199	Chr.4	15161527	15161507	-	ttttaaagacaaattatatctgtttcaaatgtttcgtaaggta
†	2742364_adh	TTTTGCATGGATATATGTCTT	FJ590200	Chr.4	15161528	15161508	-	attttaaagacaaattatatctgtttcaaatgtttcgtaaggt
	1932370_adh	TCAAAATACTGAGTAGGAAAT	FJ590201	Chr.4	15166073	15166093	+	cctgtttcaatctgcttgtttcaagctatccattttgcaacaa
* †	2587849_adh	TTCAATCGTTGCTGAATGGCG	FJ590202	Chr.4	15175852	15175872	+	tttcaatttttcaactgtttcaagaaactggtaagtgtttcaa
†	2532677_adh	TGTTTCTCTTCAAGTAGATAT	FJ590203	Chr.4	15180894	15180914	+	ataccataaaagcaaaatttactgtttcagtaacgcatctttt
†	2710224_adh	TTTCTCTTCAAGTAGATATCG	FJ590204	Chr.4	15180896	15180916	+	accataaaagcaaaatttactgtttcagtaacgcatcttttat
	2060406_adh	TCTTTACATTCATTGGGCATA	FJ590205	Chr.4	15197113	15197093	-	atttttcagtcatttttttcctgtttcaataaactttcattaa
†	2577122_adh	TTATCATCAGGTTGAAGATTT	FJ590206	Chr.4	15211908	15211928	+	attgttccaaattttcatctgtttcattatgtttatattaaaa
	1786209_adh	TAAGGTAAATTTTAAAACATA	FJ590207	Chr.4	15222331	15222351	+	ctttgtttaattcttgctgtttcaagcaaccagatagaacgta
	1786209_adh	TAAGGTAAATTTTAAAACATA	FJ590207	Chr.4	15224651	15224631	-	ctttgtttaattcttgctgtttcaagcaaccagatagaacgta
†	2651439_adh	TTGCCTCGGATGTACCATGGT	FJ590208	Chr.4	15245718	15245698	-	tcttttcaaaaaaaaatcactgtttcaaagcaaaaacgaacatg
†	2042492_adh	TCTCTCGCCGTGTGCTTCATG	FJ590209	Chr.4	15259480	15259500	+	tagcgaaaatttttttactgtttcacgtagttcatttattttgt
†	2054568_adh	TCTTAGTACATAGTAGGTGAA	FJ590210	Chr.4	15261336	15261316	-	tttacaaaaaaaatatttctgtttcacaatgtgcatataattaa
†	2738017_adh	TTTTCCGGTGGCGATTCCTAA	FJ590211	Chr.4	15265680	15265700	+	tttttttaaaaatctctgtttcactatcacactatcaataaag
†	2657551_adh	TTGGAATCGTCACTTTCTGCT	FJ590212	Chr.4	15295708	15295728	+	aaaattaaaaatttctgtttcaaaatcttcatgtaaaagtact
†	2553878_adh	TTACACGACTCATGTACTGAT	FJ590213	Chr.4	15298053	15298073	+	tcgtaattcttcaagctggctgtttcagaaagtcgttatcgaa
†	1921749_adh	TATTAGTATCTAAGGGCTACC	FJ590214	Chr.4	15307822	15307802	-	cgaaaaacaaatttgctgtttcatcataccgatgtatttggta
	1924985_adh	TATTCTGCAGTATATTAATTC	FJ590215	Chr.4	15339071	15339051	-	caaaagtttaaaaaaactctgtttcaaaaacatggtttccaat
†	1923501_adh	TATTCAGATCTTGGTTTTTTC	FJ590216	Chr.4	15345759	15345739	-	aatttttcaattaaaaaaaaactgtttcaaaactttaattagtt
†	2581289_adh	TTATTCAGATCTTGGTTTTTT	FJ590217	Chr.4	15345760	15345740	-	gaatttttcaattaaaaaaaaactgtttcaaaactttaattagt

†	2488080_adh	TGTAGATTGGAGAGTAATTTC	FJ590218	Chr.4	15352065	15352085	+	atgatttcgaaaatttactgtttcatgttgcacatttcaaaat
†	2691291_adh	TTTAATCGGAGTGGCGTTTTG	FJ590219	Chr.4	15390869	15390889	+	aacagtttttattttttactgctgtttcatttagatttgata
†	2029931_adh	TCTAACTTTGCAAAGATTTGA	FJ590220	Chr.4	15400501	15400481	-	ataggaaaaaaattactgtttcaaaaagtttagaacaatttta
†	2700877_adh	TTTATTTTTATGTATATTCGA	FJ590221	Chr.4	15401452	15401432	-	aaattgaaaaaaaaaactactgtttcaagaataaaatttactt
†	1808737_adh	TACAAGAACATCGAACCAAAT	FJ590222	Chr.4	15403854	15403834	-	gttgaaaaattgttcactgtttcaaacttatttgaaaagtgca
†	1878997_adh	TAGTGGTCTGCCAAAGAACAC	FJ590223	Chr.4	15410455	15410475	+	ctagatgattttttttgagtctgtttcacgttgttttcatta
†	1919284_adh	TATGTTATGTTATGTTATGTT	FJ590224	Chr.4	15410987	15411007	+	atgaaaacctgtttcaaaattgttatgataagttatgatagtg
†	1919284_adh	TATGTTATGTTATGTTATGTT	FJ590224	Chr.4	15410992	15411012	+	aacctgtttcaaaattgttatgataagttatgatagtgttatg
	2704485_adh	TTTCATTATTATCTAATCCTT	FJ590225	Chr.4	15412395	15412415	+	aaagttttaaaaaaaaacactgtttcaatagcaacatatttag
†	1957838_adh	TCAGAATACTCCCATTACGAA	FJ590226	Chr.4	15414475	15414455	-	tggaaaattgtttactgtttcataatgaacttaaacgtttttc
	2704632_adh	TTTCATTGTTTTACATTTTTC	FJ590228	Chr.4	15417211	15417191	-	tgacaaataaaagtgcatactgtttcaagataattttaatacg
	2595591_adh	TTCATTTTTTGAGCCCGTCTC	FJ590227	Chr.4	15417755	15417775	+	aaattaaactgtttcatttcagtttcactgttccttctaacaa
	2595591_adh	TTCATTTTTTGAGCCCGTCTC	FJ590227	Chr.4	15422905	15422885	-	aaattaaactgtttcatttcagtttcactgttccttctaacaa
	2704632_adh	TTTCATTGTTTTACATTTTTC	FJ590228	Chr.4	15423449	15423469	+	tgacaaataaaagtgcatactgtttcaagataattttaatacg
†	2640002_adh	TTGAGAGGGACGAGTTTATTT	FJ590229	Chr.4	15430012	15430032	+	catttttaaaatggaaactactgtttcaacgaatagataattt
†	1891090_adh	TATATTTTTTGTGTTGCATTA	FJ590230	Chr.4	15432450	15432470	+	aatttttttttgtttttactgtttcagaataaacataaaatt
†	1931529_adh	TATTTTTTGTGTTGCATTAAA	FJ590231	Chr.4	15432452	15432472	+	tttttttttgtttttactgtttcagaataaacataaaattta
†	2698316_adh	TTTATATTATATTTCTAGGCA	FJ590232	Chr.4	15497894	15497914	+	tttagaacaaaaaattccactgtttcaatgtctgcattaataa
	2576662_adh	TTATATTTACCCCTAAATTTC	FJ590233	Chr.4	15505065	15505045	-	aaaaatcgaaaaaatttcctgtttcaaaaacttcattaaaaaa
†	1800820_adh	TAATGGTGTACGCCTATATCC	FJ590234	Chr.4	15532622	15532602	-	atcagattgatttgtagtaactgtttcaactagttaagatatt
	1875060_adh	TAGTATAAAAATCACAATTTT	FJ590235	Chr.4	15538620	15538600	-	tggataaagtttttcactgtttcacctactccttagaagcag
†	2595975_adh	TTCCAATTAAATTGATTTGGA	FJ590236	Chr.4	15540262	15540282	+	ttgatgcatttttctactgtttcaagatgttttcaaaaaatg
†	2732798_adh	TTTGTTTTCGCGTCGTCATCA	FJ590237	Chr.4	15541872	15541852	-	tcatcagcttatgttttcttctactgtttcacgatgttattat
	2540208_adh	TTAAAGAAACCTGAGGATTCA	FJ590238	Chr.4	15561010	15560990	-	tgtttctctgtttcagtactgtttcagtacctgtttcagtaat
	2699250_adh	TTTATGGTGAAATTTAAAGAA	FJ590239	Chr.4	15561023	15561003	-	ttttagtcaaaattgtttctctgtttcagtactgtttcagtac
	1872097_adh	TAGGTGTTTGGAACCTATCTG	FJ590240	Chr.4	15563855	15563835	-	agcttttgaaattttctgctgtttcactaactttttataacac
	2700624_adh	TTTATTTAATACATTTTTTCA	FJ590241	Chr.4	15568622	15568602	-	aaccccaataatctgtcatctgtttcaaaaatttcacaaaaac
†	1919989_adh	TATTAAATTAATTTTCTACGG	FJ590242	Chr.4	15568940	15568920	-	ggatgaaaatttaatttttactgtttcaataagttgatatttg
†	2699444_adh	TTTATTAAGAAAAAGGGGAAT	FJ590243	Chr.4	15572606	15572626	+	gaatgtgtcaaaaatgtttcactgtttcaaggaatttataaaa
†	2271542_adh	TGATGATGCTAATGACAAATA	FJ590244	Chr.4	15589996	15589976	-	ataaaaactgaaagttttagctgtttcaataaatttgaaaaac
†	1796029_adh	TAATATCTGTTTTATCCTGTA	FJ590245	Chr.4	15602876	15602896	+	aatttcaaaaaaaaaagttaactgtttcaccaactgctcataa
†	2474494_adh	TGGTTATTCCTCTTTTGTCGG	FJ590246	Chr.4	15604652	15604672	+	gttcaatgcctaactattactgtttcagaaaatcaattaatac
†	1924149_adh	TATTCCTCTTTTGTCGGTTTT	FJ590247	Chr.4	15604656	15604676	+	aatgcctaactattactgtttcagaaaatcaattaatacgaat
†	2321092_adh	TGGAACTAGAGGAATCTTAAA	FJ590248	Chr.4	15605491	15605511	+	tcaaaaaattaaaagttactgtttcattaaatgaataaagtta
	2612055_adh	TTCGTCTAAAACTGTAAATTC	FJ590249	Chr.4	15605713	15605733	+	ggtttttgaaatttttactgtttcaagtagctttcaagtttc
	2550751_adh	TTAATCTTGAATAGTATAATA	FJ590250	Chr.4	15611702	15611682	-	gggaggtttttgtgaattactgtttcagcgagtttataaaatc

†	1831170_adh	TACTCCTTCGGCTATATTTAC	FJ590251	Chr.4	15612107	15612127	+	aaattctgtataaaaatttgactgtttcaataaggtgctaagt
†	2606555_adh	TTCGCGAATATAAAAACTTCA	FJ590252	Chr.4	15629309	15629329	+	agtcgaaaaactattttctgtttcaaattggatagattgggac
†	1839506_adh	TAGAAGGAACGGCGGACATAA	FJ590253	Chr.4	15666710	15666690	-	agttcacactttcactgtttcaaaatttgctgaaaatgtgtgg
* †	2561495_adh	TTAGAAGGAACGGCGGACATA	FJ590254	Chr.4	15666711	15666691	-	aagttcacactttcactgtttcaaaatttgctgaaaatgtgtg
†	2305307_adh	TGCTAACACAGTTGTTCGAAT	FJ590255	Chr.4	15667377	15667357	-	aatgtgccccctaaaaattgctgtttcattatttttgtaaaac
* †	2127140_adh	TGAAGAAGACGGACAAAGCCA	FJ590256	Chr.4	15677263	15677243	-	tggaatttttattactgtttcattactttcttgaatatgccta
†	2554520_adh	TTACATAGATAACAATATTTC	FJ590257	Chr.4	15690240	15690260	+	aaatcggcaaaatttactgtttcaagtttttgatttaatttct
†	1813158_adh	TACATAGATAACAATATTTCC	FJ590258	Chr.4	15690241	15690261	+	aatcggcaaaatttactgtttcaagtttttgatttaatttcta
†	1872769_adh	TAGTAAAAGGGCATTTGAACT	FJ590259	Chr.4	15706992	15706972	-	aataattttcaattcttccgctgtttcacaaagtttgtaaaac
†	2554844_adh	TTACATGAGTCATATCATTAC	FJ590260	Chr.4	15710157	15710137	-	tgataactaattttttctactgtttcagtattcccattttgat
†	2042338_adh	TCTCTCAGCCTACGACCAAAA	FJ590261	Chr.4	15711317	15711297	-	atatgaaactttaatccactgtttcacaattccaataaaattc
†	2552287_adh	TTAATTGGTTTGGTGTGATTG	FJ590262	Chr.4	15712083	15712063	-	gtcatgaatttcataatttcactgtttcatcaatgaattaaaa
†	2699589_adh	TTTATTAGATGAGTGTGTCAG	FJ590263	Chr.4	15712745	15712725	-	ttttaggaaaaatttttggcaactgtttcattgtgttgatttt
†	2736428_adh	TTTTATTAGATGAGTGTGTCA	FJ590264	Chr.4	15712746	15712726	-	attttaggaaaaatttttggcaactgtttcattgtgttgattt
†	1893624_adh	TATCAGTTGTGATATCAAAAC	FJ590265	Chr.4	15714163	15714143	-	aaatttgtttaactgtttcactacttcaagatattcgctataa
†	2571401_adh	TTAGTGAAATTTGAGTCGGTT	FJ590266	Chr.4	15724083	15724063	-	tttttaaaaaactattgtactgtttcattaacctataaaagag
	2584773_adh	TTATTTTTTCAGTTTTTGAAT	FJ590267	Chr.4	15777333	15777353	+	ggttatcaattaagcattactgtttcactttattttaataact
	2693312_adh	TTTACAGAATCTACGTTAACT	FJ590268	Chr.4	15779075	15779095	+	tgtagcgtgtaaatgttctgtttcaaaatttgcatttttgcaa
†	1926854_adh	TATTGGACACAACACAAGAAC	FJ590269	Chr.4	15810896	15810916	+	aaaaatattttttttaattgctgtttcatagtctttttagata
†	2657985_adh	TTGGACACAACACAAGAACCA	FJ590270	Chr.4	15810898	15810918	+	aaatattttttttaattgctgtttcatagtctttttagataat
†	2269092_adh	TGATCACCAAGAATTCGGTTA	FJ590271	Chr.4	15821084	15821064	-	ttgtgggatttatagaatttactgtttcaacaaattaataaat
†	2463291_adh	TGGTAGGCGTCATTAAAACCG	FJ590272	Chr.4	15824887	15824907	+	aaaagttcaaaaaaagcctgtttcacggtgtcgataataatgt
†	1813240_adh	TACATATCCTTCAAACCAATT	FJ590273	Chr.4	15825197	15825177	-	ctattggaattgatctgctgtttcactatgttgatataagaat
†	2594228_adh	TTCATCCCTCTCGTCAACATA	FJ590274	Chr.4	15841434	15841454	+	attttaaaattttttttcactgtttcaccgccgttttaaggtc
†	2700420_adh	TTTATTGGGCTAAACGACTGA	FJ590275	Chr.4	15844536	15844556	+	ttaataaataagtaaacattgctgtttcattgtgttatcatat
†	2743967_adh	TTTTGTGGCTTATCTAAATCC	FJ590276	Chr.4	15844918	15844938	+	agatttttgttgttcgactgtttcactgtggttttaacatcag
†	2593255_adh	TTCAGTTCTATGGTTTCATTC	FJ590277	Chr.4	15881116	15881136	+	tgacaatttcttgaaaagtttactgtttcataaaattcatatg
†	2601090_adh	TTCCTGTTTTGCAAGACAACT	FJ590278	Chr.4	15902383	15902403	+	ttcaaaaaattataactgtttcagaacgttagtttcagtttta
†	2588909_adh	TTCAATTGTAGACTGAGGGCT	FJ590279	Chr.4	15904368	15904388	+	ctataaaacattttctactgtttcagaatattgattttagtgg
†	2703686_adh	TTTCATAATGATAACGGATCT	FJ590280	Chr.4	15906536	15906556	+	ttttctaacacatactgtttcaccttctctattaattcgttta
†	2588069_adh	TTCAATGATAGTTACAATTAA	FJ590281	Chr.4	15913876	15913896	+	ctatttgaaaaaaaatccactgtttcaataggttatcaattga
†	2595434_adh	TTCATTTAATCTTTAGGAACC	FJ590282	Chr.4	15927665	15927645	-	ctccaaaaaaaaaatttcctgtttcacaacttttgtataagtta
†	2623229_adh	TTCTTTAAGTACATAAGTATC	FJ590283	Chr.4	15927962	15927942	-	aaatgaaaataaaagttactgtttcagataacaattaaaattt
†	2711920_adh	TTTCTTTAAGTACATAAGTAT	FJ590284	Chr.4	15927963	15927943	-	aaaatgaaaataaaagttactgtttcagataacaattaaaatt
* †	2682341_adh	TTGTGTGCCGTCGTTGGTTTT	FJ590285	Chr.4	15959594	15959614	+	ctctcaattttccactgtttcacttaattgagaaaagtagatt
†	2659500_adh	TTGGAGACTTTATGCAGAACA	FJ590286	Chr.4	15960736	15960716	-	aggccgaaatatgtattcctgtttcacaaaactgataaatttt

†	2734383_adh	TTTTACTTTTGAAGATAATTA	FJ590287	Chr.4	15966053	15966033	-	gaatttcgaggaaattttttctgtttcattttctgattatga
†	1811541_adh	TACACTTATGGAAACAATTTT	FJ590288	Chr.4	15978398	15978378	-	ccataatttttactgtttcatgtcatgcttgaaatatatatt
†	2276410_adh	TGATTGAGTAGAAATGCAAGG	FJ590289	Chr.4	15988051	15988071	+	ttattcaataaaaacatattctgtttcacctaactgttatgaa
†	2251064_adh	TGAGTAGAAATGCAAGGTTGA	FJ590290	Chr.4	15988055	15988075	+	tcaataaaaacatattctgtttcacctaactgttatgaaaaca
†	1803911_adh	TAATTGCATAACTTTTATTTT	FJ590291	Chr.4	15988715	15988695	-	catgagaattgaaaatactgtttcaagtgggtactaaaatttc
†	2552213_adh	TTAATTGCATAACTTTTATTT	FJ590292	Chr.4	15988716	15988696	-	acatgagaattgaaaatactgtttcaagtgggtactaaaattt
	2749130_adh	TTTTTTCTTTATATATCATTT	FJ590293	Chr.4	15999499	15999479	-	agaaagtttaaaaaactactgtttcaaattgtttattttcaag
†	1797324_adh	TAATATTTGTGATAACTACGT	FJ590294	Chr.4	16006043	16006023	-	acatttaaaaaaaaaatttactgtttcaagttcaacacttact
†	2552022_adh	TTAATTCTAGGCGGATCAAAT	FJ590295	Chr.4	16009687	16009667	-	tgaaaaataaaataaaatccctgtttcaaagcgtaaacttctt
†	2500111_adh	TGTCTTCATTTTACAAAGCAT	FJ590296	Chr.4	16011791	16011811	+	cactttttacagcttactgtttcaaaaaaatattcttttgtaa
	1890110_adh	TATATCGTTTTTTAATTTAAT	FJ590297	Chr.4	16022907	16022927	+	tttattttcaaattttaaactgtttcaatactttgataaggaa
†	2692167_adh	TTTAATGTTCATATACTGGAT	FJ590298	Chr.4	16023562	16023542	-	ataacaaaaaactgccaagtactgtttcaaacgaaacatataa
†	2743788_adh	TTTTGTCGCTTTAGGTTCAAT	FJ590299	Chr.4	16029885	16029905	+	aaactacaaaaaattactgtttcaaaaagttagtataaaaatt
†	2311514_adh	TGCTTGTTGAGACGAATTGAA	FJ590300	Chr.4	16052145	16052125	-	gttttctcaaattttcactgtttcacgttgttttcaaaaatat
†	2006625_adh	TCGCAGTCTGTATTCCCGTTA	FJ590301	Chr.4	16073193	16073173	-	atttctgtgatgccctgtttcacaatgttctcaaaataaaacg
†	1941294_adh	TCAATCACATAGACGTATCTT	FJ590302	Chr.4	16078790	16078810	+	atttgcggaaattttttcactgtttcacatttccttcaattt
†	2258840_adh	TGATAAGCGTAATGTGTCGGA	FJ590303	Chr.4	16079217	16079237	+	atttccaataaaaatattttctgtttcaacatttacttatagt
†	2639987_adh	TTGAGAGGACCATGTCAAATG	FJ590304	Chr.4	16082279	16082259	-	ttccagtcaaaagtagtttactgtttcagaaaattgatttaag
†	1829372_adh	TACTAACTAGTGCAGTACACG	FJ590306	Chr.4	16100637	16100617	-	aaattagctttaaaaaattatctgtttcacagaaaggtttttt
†	2573367_adh	TTAGTTTATATTTTATTCCCT	FJ590305	Chr.4	16104979	16104999	+	tgcagttttcaaaatttcactgtttcaataatgtggtaagttt
†	1829372_adh	TACTAACTAGTGCAGTACACG	FJ590306	Chr.4	16108337	16108357	+	aaattagctttaaaaaattatctgtttcacagaaaggtttttt
†	2007130_adh	TCGCATTCTGAAAGGTTGGTT	FJ590307	Chr.4	16116395	16116415	+	ccagtactataatgcactgtttcacgcatttttttgaattgaac
†	1759306_adh	TAAAGTTTTGAATCGTTGACA	FJ590308	Chr.4	16117196	16117176	-	tttttttcctgaaaatgttctgtttcatcgtattgatttgag
†	2268086_adh	TGATATTCTAGTTGGAATTCT	FJ590309	Chr.4	16123625	16123645	+	agtataatttgttttgactgtttcaatctgttttcatattttt
†	2721367_adh	TTTGCAGTTTCTATCTATAAC	FJ590310	Chr.4	16171174	16171194	+	ttttttttccaaatttttgctgtttcaaacgttccatttcttc
†	1941825_adh	TCAATGAATTTAATAGATTTG	FJ590311	Chr.4	16174725	16174705	-	atgaaactttttttttactgtttcagaagtgttattttattcc
†	1973912_adh	TCATTCTGTGCATTCTTTTTT	FJ590312	Chr.4	16177578	16177598	+	ttttttgtataactttttctactgtttcagtacatttacttca
	1989351_adh	TCCTACTAAAATTTATTAATT	FJ590313	Chr.4	16179100	16179080	-	tgcatttttcaaacaaaaactgtttcagaatagttatttattt
†	2696990_adh	TTTAGTAGACACATCTCATCG	FJ590314	Chr.4	16179862	16179842	-	ttgaaaaaactttttttttctgtttcaaaatgtttagttatt
†	2734874_adh	TTTTAGTAGACACATCTCATC	FJ590315	Chr.4	16179863	16179843	-	tttgaaaaaactttttttttctgtttcaaaatgtttagttat
†	2549385_adh	TTAAGTTTCAGGCATTATTCG	FJ590316	Chr.4	16194768	16194748	-	ttttcggcaaaatgattgtctgtttcacattacgcatataaaa
†	1941067_adh	TCAATAGAACAAAAATTGAAA	FJ590317	Chr.4	16203909	16203889	-	actgaaacaaaaaatattttactgtttcacagtgtaagtatat
†	1754433_adh	TAAACAGTGGAATTAGGAATT	FJ590318	Chr.4	16208645	16208625	-	gaatttagaaaaaatactgtttcattctaatttgaaattgtta
†	2718146_adh	TTTGATCGAAGGTTAAATATA	FJ590319	Chr.4	16226444	16226464	+	aaatgcactgtttcacattcatataataaacatggcaaaagtc
†	1945459_adh	TCACAGTAAAAACTTCTCTGA	FJ590320	Chr.4	16239003	16238983	-	gtttttaatcgactgtttcaatatgttattttaaggaggagaa
†	2646792_adh	TTGATTTAGTGCATACAATGG	FJ590321	Chr.4	16254966	16254946	-	tagtttgaaatttaatttcactgtttcagatgttttatgaaaa

†	2594170_adh	TTCATCATCGGAAAACGAGAA	FJ590322	Chr.4	16270364	16270344	-	taacaaaaactaatctgtttcaagtttctgataaaacatcatt
†	1830388_adh	TACTATACGATGTTCTCTTAA	FJ590323	Chr.4	16279104	16279124	+	taatttggaaaaaaacggcactgtttcatctagtttttaaatt
†	2634736_adh	TTGACAATCGTAAATTATTTT	FJ590324	Chr.4	16372157	16372177	+	caaaattttaagatttttactgtttcaatattctgacaaatta
†	2549882_adh	TTAATAGCAGAGATAGAGTAA	FJ590325	Chr.4	16374108	16374128	+	aatattcaaaattttaaagctgtttcaatattctgacaaatta
†	1790844_adh	TAATAATTCGTCGTAAAACCA	FJ590326	Chr.4	16381854	16381874	+	tttatattttgaaaaaaaatttctgtttcagaaaaaaaaatgta
†	2559212_adh	TTACTGCGATGGATTTACAGA	FJ590327	Chr.4	16389819	16389839	+	aaatgatttttcgaaaattactgtttcagaaaaccaattaaac
†	2620548_adh	TTCTTACGACGAAACAGTAGA	FJ590328	Chr.4	16391300	16391280	-	aataattttaaaaattactgtttcagagagtaattaaaaattc
	2630071_adh	TTGAATATCATGGTTAGTCTC	FJ590329	Chr.4	16434589	16434569	-	aattgaaacgaaaattctactgtttcaatgttatttttaatgt
†	2737779_adh	TTTTCATTATTATTTCCGGCT	FJ590330	Chr.4	16456914	16456894	-	atttgaaaataaatgtttactgtttcaacggtatttcaagaac
†	2578748_adh	TTATGATCAAAATTCGAGTAG	FJ590331	Chr.4	16465212	16465192	-	aacatttatggaaaattttactgtttcatagaaaacatttcta
†	2578748_adh	TTATGATCAAAATTCGAGTAG	FJ590331	Chr.4	16465447	16465467	+	aacatttatggaaaattttactgtttcatagaaaacatttcta
	1928419_adh	TATTGTTGACTTTCAATTAAA	FJ590332	Chr.4	16466011	16465991	-	gttttctgctatgattactgtttcactaacgttttaaaagata
†	1924697_adh	TATTCTAAGTGTTTACAGATC	FJ590333	Chr.4	16471499	16471519	+	aatatgcaagcaacttatactgtttcagttaaaatttaaatat
†	2745874_adh	TTTTTCCGTCGTCTTGGACTT	FJ590334	Chr.4	16473690	16473670	-	tttaaaaaataaaattactgtttcagaattttttttggttca
	1895327_adh	TATCATTTTCTCAAAGAATTT	FJ590335	Chr.4	16476639	16476659	+	ttttcaaaaaatttttctactgtttcagagaaaacttaagaca
	2688730_adh	TTTAAAAATTAATCAAATTTC	FJ590336	Chr.4	16510452	16510472	+	gaattcggaatttttttactgtttcattacaaaattaggtact
†	2551169_adh	TTAATGCTAGTGGATGAGAAG	FJ590337	Chr.4	16522197	16522217	+	aagtgaattggataaaaatactgtttcaaaaaatctgtttccc
†	2306098_adh	TGCTAGTGGATGAGAAGCTTC	FJ590338	Chr.4	16522201	16522221	+	gaattggataaaaatactgtttcaaaaaatctgtttcccgaac
†	1878728_adh	TAGTGGATGAGAAGCTTCTTC	FJ590339	Chr.4	16522204	16522224	+	ttggataaaaatactgtttcaaaaaatctgtttcccgaactgt
†	2707239_adh	TTTCGCAAAACATCGGATTTT	FJ590340	Chr.4	16551223	16551243	+	gctttttcaatttaactgtttcaactagctgttataaagtgaa
†	2506242_adh	TGTGAGTATAGAAAGTTTGAA	FJ590341	Chr.4	16556673	16556653	-	aattaaaatttcgactgtttcattaacaccatttaacaccatt
†	2617164_adh	TTCTCTCTGCATATATCTCAT	FJ590342	Chr.4	16556962	16556982	+	ttaaaacttaaaactttttctgtttcacaacatactgaactac
* †	2563894_adh	TTAGATCAGTGCTGTGCGGCT	FJ590343	Chr.4	16557149	16557169	+	catgtgaaaatattcgactgtttcaccatcacgatttaataat
†	2559648_adh	TTACTGTGTGTCCGTTAGGAA	FJ590344	Chr.4	16563236	16563216	-	aaaattcaaatttttgcctgtttcaagagctaatgtaattttt
†	1922931_adh	TATTATTATCGTCGAAATGTT	FJ590345	Chr.4	16566169	16566149	-	tgtgaaaattaatattttgactgtttcatcaacgccattttaa
†	2670903_adh	TTGGTGCAGAATAGCTGATCC	FJ590346	Chr.4	16597031	16597051	+	cgattcctggaaaaaatctactgtttcagtaatgagataaagc
†	2594581_adh	TTCATGACTGACATTCATTTT	FJ590347	Chr.4	16598651	16598671	+	ataaaatctgtttcacatatgtttcaatattgtttttagtttt
†	2025042_adh	TCGTATGTCGTATGGAATTTG	FJ590348	Chr.4	16610275	16610295	+	tggttaaaatattaactgtttcacgttttagtcaaattctgaa
†	2545912_adh	TTAACTAAGTAAAGGGCATTC	FJ590349	Chr.4	16614583	16614563	-	aatacctagccagaagttactgtttcaaaaataaaaaaaaaga
†	2580507_adh	TTATTAACTAAGTAAAGGGCA	FJ590350	Chr.4	16614586	16614566	-	aaaaatacctagccagaagttactgtttcaaaaataaaaaaaa
†	1794798_adh	TAATAGACTGATCTGACTGTA	FJ590351	Chr.4	16616291	16616311	+	aatggcgggtactcatttttactgtttcacttttaaattgaat
	2582047_adh	TTATTCTTTTAAAGTTGTTCT	FJ590352	Chr.4	16617739	16617759	+	taaaattgaaataattactgtttcacttgccagataaacacag
†	2739168_adh	TTTTCTATCCCGCATTACTTA	FJ590353	Chr.4	16620205	16620185	-	aatttaaaattttctactgtttcatcgtaattttattagaatg
†	1790704_adh	TAATAATAAACAAATGTTCGG	FJ590354	Chr.4	16621905	16621925	+	attgtagttcaaaatattttctgtttcaccatctactcaatca
†	2657703_adh	TTGGAATGTAAATCGCGCCTA	FJ590355	Chr.4	16622993	16623013	+	gttagttcaaaatatttctgtttcacgttctagtcaagtgcgg
†	1797176_adh	TAATATTGGTAGAATGAAAAT	FJ590356	Chr.4	16635488	16635508	+	aattttaaaaattattctcactgtttcagcttctctttatact

†	1845094_adh	TAGACATCTCTAACAGCAAAA	FJ590357	Chr.4	16640211	16640191	-	agaagaagttttcactgtttcactcacaaattatagatatcaa
†	2527415_adh	TGTTGAAAATGAAGGTAGTAT	FJ590358	Chr.4	16662863	16662843	-	agaagaaaacaatactgtttcagattactgatattgtctgaat
†	2560195_adh	TTACTTGACTGTCGAAGACCA	FJ590359	Chr.4	16664683	16664703	+	aaaaaacataaaaaaaattttactgtttcaaagttttgtgaaa
†	1927237_adh	TATTGGTAGAAACATTTTCAC	FJ590360	Chr.4	16670340	16670320	-	ataaaaaatttttcactgtttcaagaatttgtgagaaaaattg
†	2311730_adh	TGCTTTCGTGATTTTGTCGTT	FJ590361	Chr.4	16672203	16672183	-	aatttttcaaaaaattttccactgtttcaacaggtttttattc
†	1937328_adh	TCAACTCGAAAAATATTCTTG	FJ590362	Chr.4	16673114	16673134	+	ataagacaagatttactgtttcaaaaattccttaaaattttaa
†	2649917_adh	TTGCATAACGGCTTGCATTTT	FJ590363	Chr.4	16676325	16676305	-	tttattgaaaaaattttgctgtttcaaaaacttgatttcatta
†	2027377_adh	TCGTGTAATGAAGTTTGCTTA	FJ590364	Chr.4	16687952	16687972	+	attttaacatatttgctgtttcagaataatataattagttgaa
	1795778_adh	TAATATATTTTACTTTACAAT	FJ590365	Chr.4	16691937	16691957	+	agaaaaatgaaaattttactgtttcagatatgagatttcagaa
†	1831451_adh	TACTCTATAGAATTTACAATA	FJ590366	Chr.4	16705992	16705972	-	tttcttaacaaaaagttactgtttcaccatgctagtaaaaaag
†	1923379_adh	TATTCAAGTAGATAATTTCAT	FJ590367	Chr.4	16707657	16707677	+	atcttaaaaatctgctgtttcaatttcttgataaaattacatg
†	2050079_adh	TCTGGCCAACTTTGACTATAA	FJ590368	Chr.4	16719993	16719973	-	actttttatttactgtttcatcgactagatgaaacatgaaagc
†	2739905_adh	TTTTCTGTTTTCTGGCATTGT	FJ590369	Chr.4	16748315	16748295	-	attccctttaaaaattactgtttcatatactgcttattcgaca
	2649765_adh	TTGCAGTACTTTCTTATAGTT	FJ590370	Chr.4	16773832	16773812	-	aaccgtattttttttgctgtttcaacaatcaataatgtgtgt
†	2628704_adh	TTGAAGCTCGGCATCAAGTAG	FJ590371	Chr.4	16793423	16793403	-	actaattttagtttttactgtttcacatagtttagaaaatctt
* †	2648399_adh	TTGCAACTGAAGAATCAAAAT	FJ590372	Chr.4	16805986	16805966	-	ttaaatattaattttttctgtttcatacatttcagaaaaagaa
†	2685719_adh	TTGTTGCAACTGAAGAATCAA	FJ590373	Chr.4	16805989	16805969	-	agattaaatattaatttttctgtttcatacatttcagaaaaa
†	2699896_adh	TTTATTCGTTATGTATTTTTT	FJ590374	Chr.4	16811976	16811956	-	ttttctcatcaaaaaattgctgtttcaattttattgtttctag
†	2736574_adh	TTTTATTCGTTATGTATTTTT	FJ590375	Chr.4	16811977	16811957	-	attttctcatcaaaaaattgctgtttcaattttattgtttcta
†	1919414_adh	TATGTTGACGTAGAGCGAATT	FJ590376	Chr.4	16812360	16812340	-	ttttctaaaaaatattgctgtttcacaaactattcaatttgaa
†	1962316_adh	TCAGCATAAAAATTTTAAGCC	FJ590377	Chr.4	16812770	16812750	-	aaattccaaatttccaattactgtttcaccttcgcttagtttt
	2560961_adh	TTAGAAATTGAAGAATTGGTA	FJ590378	Chr.4	16814265	16814245	-	ctgtttcaacttgtttatttgaacagactgaattcaatagtcg
	1921983_adh	TATTATAGAAAAATACGATCG	FJ590379	Chr.4	16815642	16815622	-	taaaaaaagaacccactgtttcataggcaaggcacaatggtgt
†	1798872_adh	TAATCTGAATGTCGTGATGAT	FJ590380	Chr.4	16820320	16820340	+	atttatacaaaaacctgctgtttcatcatcattttatagtgaa
†	1920675_adh	TATTACATACATTAAATTCAG	FJ590381	Chr.4	16870343	16870363	+	ataattgttctacaaagaatgctgtttcacaactttgttatta
†	1890943_adh	TATATTGTTCTGGGATCTTAT	FJ590382	Chr.4	16873086	16873066	-	caaaaaaaagttactgtttcagtgactttttgaatcattggac
†	2569954_adh	TTAGTAATATTCGGTGGTTAT	FJ590383	Chr.4	16887528	16887508	-	aaacaataaaataaatacactgtttcaagaaactaacatgata
†	1923217_adh	TATTATTTGTGTTCATGAAGG	FJ590384	Chr.4	16889575	16889595	+	ttttccgaaaaaaaaaatccactgtttcagatattcaatttta
†	2162608_adh	TGACGTTTTCAGAATTGAGTA	FJ590385	Chr.4	16907941	16907921	-	ttacgaaaaaaaaaactgtttcaaaatgtgcataatttcagttc
†	2559247_adh	TTACTGCTTGTAACATATAAT	FJ590386	Chr.4	16924322	16924342	+	attttttgacaaactttttttctgtttcatgtattctatttca
†	1839309_adh	TAGAAGATTTTGAAAGCACAT	FJ590387	Chr.4	16975112	16975132	+	gttggttacaaaactgtttcaacaattgcattaaaggaagaga
†	1929761_adh	TATTTCCTTTTTTGAATTTTG	FJ590388	Chr.4	16978230	16978210	-	tgaaaaatacatttttttactgtttcaaatagctttcatgtaat
†	1921507_adh	TATTAGATCATGTAGAGAAAA	FJ590389	Chr.4	16987486	16987506	+	tcgaaattttgaatattaatgctgtttcattcatttcttttca
†	2563993_adh	TTAGATCATGTAGAGAAAATT	FJ590390	Chr.4	16987488	16987508	+	gaaattttgaatattaatgctgtttcattcatttcttttcaaa
†	2128067_adh	TGAAGACTACTCCTGAAAATG	FJ590391	Chr.4	16992265	16992285	+	tttattatttttgctgtttcactaatttcatttcagaatttca
†	1798939_adh	TAATCTGTGTGTCATATTGCC	FJ590392	Chr.4	17018708	17018728	+	ctttattgtgaaaaaaaatctcctgtttcacgctacgaataagt

† 2592910_adh	TTCAGTCGTCTAGGTTTCTAC	FJ590393	Chr.4	17018977	17018997	+	aaatgaattcaaactttcactgtttcaaataagtaatatattt
† 2559722_adh	TTACTTAACAATTGGTTTCTC	FJ590394	Chr.4	17027935	17027915	-	ataatttgtaaattttttgctgtttcagaatttgaatatttt
† 2712555_adh	TTTGAAACAGCCGTAACGCAG	FJ590395	Chr.4	17037054	17037034	-	gatataaattacaaaaattactgtttcattactacaaagcaat
† 1940663_adh	TCAAGTTCGATAGCACGTTGT	FJ590396	Chr.4	17041829	17041849	+	tgaacataaaatttttctgtttcaggcgtttaatttatttttc
† 2546310_adh	TTAACTGAGTCTGAATGTTAA	FJ590397	Chr.4	17042732	17042712	-	actttcatgctattaattactgtttcaaatgagtcatttgaac
† 2644842_adh	TTGATGAGTGGAAGTTTTAAA	FJ590398	Chr.4	17063082	17063062	-	tttttaattaaaaaaaaaatgcctgtttcaaataccaacggca
† 2644842_adh	TTGATGAGTGGAAGTTTTAAA	FJ590398	Chr.4	17065996	17066016	+	tataaaattcaaaaaaaaattgcctgtttcaaataccaacggc
1827620_adh	TACGTAAATTATTATAACCCT	FJ590399	Chr.4	17143391	17143371	-	actagtaaatattttctcctgtttcatgatgtcctaaaattta
2588139_adh	TTCAATGCATTTTGCGACATT	FJ590400	Chr.4	17151913	17151893	-	caaaaataaaagaaaactgtttcaagctgtacttaaatacatt
† 2705748_adh	TTTCCGCAATCAAATACGGTT	FJ590401	Chr.4	17153951	17153971	+	attaatttaaaaatttactgtttcactaaaattatatcagatt
1827600_adh	TACGGTTTTGAAATTGGCTAC	FJ590402	Chr.4	17153965	17153985	+	ttactgtttcactaaaattatatcagatttctattccgtgtaa
† 2272886_adh	TGATGTAAAATGGTTGGAAGA	FJ590403	Chr.4	17162958	17162978	+	attttaaaaatttactgtttcaacataactttataaaatgtca
† 2596631_adh	TTCCAGAAACTATTGAAGCAG	FJ590404	Chr.4	17172348	17172328	-	actttcttcaaaaattactgtttcaacgtttccatagttaaaa
† 2712128_adh	TTTCTTTGTAGTTTTCTTAGA	FJ590405	Chr.4	17189824	17189804	-	catgcagaagtaaaaacttactgtttcactttgtttttataat
1836629_adh	TAGAAAAATAGAATTACATCA	FJ590406	Chr.4	17197399	17197379	-	aatacataaattattactgtttcatgatgttgttgtacttaaa
† 1798397_adh	TAATCGACGAATATTTTTACT	FJ590407	Chr.4	17200198	17200178	-	aaatttttgaaaatttttcctgtttcaaatagttttaatgaa
† 2564317_adh	TTAGATCGGTATATATATAGG	FJ590408	Chr.4	17202007	17201987	-	aaaatttcaatataaaatttctgtttcactcatttcttaaatt
1962150_adh	TCAGCAATGAAGTTATTGCAT	FJ590409	Chr.4	17202562	17202542	-	ttgtaaaaaatcactgtttcactattgttagcatatttattta
† 2641809_adh	TTGAGTAATTAAAAGACAGAA	FJ590410	Chr.4	17203445	17203465	+	tgaaaattttaaaaaaacctgtttcaaaatgtttttatttatt
† 2554442_adh	TTACAGTTAGAGAAGCATTAA	FJ590411	Chr.4	17207567	17207547	-	aaaattaaaaaatgaaaactctgtttcacatgtagtttctttt
† 2548489_adh	TTAAGGCTATTATTTCACTGA	FJ590412	Chr.4	17213684	17213704	+	gttctaaaaattactgtttcaggaaactagtattttagataat
† 2701353_adh	TTTCAACGTACATAACTTCCT	FJ590413	Chr.4	17223599	17223619	+	tgattaaaaaaaattgtactgtttcaaaacaaatttaaatatc
† 1924417_adh	TATTCGGCTCAACTTTTTAAC	FJ590414	Chr.4	17237412	17237432	+	aataaggatttaaataaattactgtttcaccttttttgatatat
† 2583295_adh	TTATTGTGACTATATTTACAA	FJ590415	Chr.4	17238808	17238828	+	ttctctgaatttccatttttactgtttcaattagtttctaaat
1755465_adh	TAAACTCTTCTAAAAAAGATA	FJ590416	Chr.4	17260771	17260751	-	aattaaacaagaaattactgtttcaatgctttgatataaaaac
