# Supplementary material for: Dynamic expression of small non-coding RNAs, including novel microRNAs and piRNAs/21U-RNAs, during Caenorhabditis elegans development
Source: Genome Biol. 2009 May 21;10(5):R54. doi: 10.1186/gb-2009-10-5-r54 (PMC2718520; doi:10.1186/gb-2009-10-5-r54)
Supplement: Additional data file 13 — The number of reads was obtained from computational output of the SOAP program followed by removal of redundant sequences, and samples of all six developmental stages (embryo to young adult of hermaphrodites) were used as the input. The sequences of longer transcripts, their nucleotide lengths and the number of reads are highlighted in red. [file gb-2009-10-5-r54-S13.pdf]

Sequence name	Sequence of the longer 21U-RNAs	Nt length	Number of reads	Name of 21 nt mature 21U-RNA
1851807_adh	TAGATGTTCTGGAATTTCTGC	21	15	21UR-5
1851808_adh	TAGATGTTCTGGAATTTCTGCA	22	2	21UR-5
2048288_adh	TCTGCGATTGTTAGTTTTGCA	21	41	21UR-15
2048289_adh	TCTGCGATTGTTAGTTTTGCAAA	23	2	21UR-15
1879276_adh	TAGTGTAGAAAAGTGGTTGCT	21	2901	21UR-16
1879285_adh	TAGTGTAGAAAAGTGGTTGCTT	22	4	21UR-16
1879291_adh	TAGTGTAGAAAAGTGGTTGCTTG	23	4	21UR-16
2137276_adh	TGAATAATGTTTTCGTCGTAA	21	437	21UR-30
2137277_adh	TGAATAATGTTTTCGTCGTAAT	22	2	21UR-30
1968336_adh	TCATAATATAGTTGGGACCTA	21	83	21UR-38
1046143_adh	CTCATAATATAGTTGGGACCTA	22	2	21UR-38
2662554_adh	TTGGATGAGTACATATGTGAT	21	411	21UR-59
2662555_adh	TTGGATGAGTACATATGTGATA	22	2	21UR-59
2662556_adh	TTGGATGAGTACATATGTGATAA	23	3	21UR-59
2662557_adh	TTGGATGAGTACATATGTGATAAAT	25	1	21UR-59
2571315_adh	TTAGTCTGATAGTAACAAAAC	21	415	21UR-60
2571319_adh	TTAGTCTGATAGTAACAAAACG	22	1	21UR-60
2571321_adh	TTAGTCTGATAGTAACAAAACGG	23	6	21UR-60
2571322_adh	TTAGTCTGATAGTAACAAAACGGA	24	4	21UR-60
2571323_adh	TTAGTCTGATAGTAACAAAACGGAGC	26	1	21UR-60
1927630_adh	TATTGTAGATTTCTCCGGCGG	21	814	21UR-66
1927647_adh	TATTGTAGATTTCTCCGGCGGT	22	105	21UR-66
1927660_adh	TATTGTAGATTTCTCCGGCGGTT	23	11	21UR-66
1927663_adh	TATTGTAGATTTCTCCGGCGGTTT	24	3	21UR-66
1927664_adh	TATTGTAGATTTCTCCGGCGGTTTTT	26	1	21UR-66
2509509_adh	TGTGATCTCTACCGGTATAAC	21	563	21UR-68
2509510_adh	TGTGATCTCTACCGGTATAACA	22	4	21UR-68
2509512_adh	TGTGATCTCTACCGGTATAACAG	23	1	21UR-68
2616087_adh	TTCTCAGAATTCCAGATGTGT	21	108	21UR-74
248784_adh	AATTCTCAGAATTCCAGATGTGT	23	2	21UR-74
1756964_adh	TAAAGATGGAGTACTGTACTC	21	824	21UR-78
1756967_adh	TAAAGATGGAGTACTGTACTCT	22	1	21UR-78
2291452_adh	TGCATTGTGTGAGCCGGAATC	21	187	21UR-79
229592_adh	AATGCATTGTGTGAGCCGGAATC	23	2	21UR-79
2699855_adh	TTTATTCGGATTAGCGTTAGG	21	158	21UR-93
2699856_adh	TTTATTCGGATTAGCGTTAGGT	22	1	21UR-93
1957892_adh	TCAGAATGACTAGAAAGTTGG	21	23	21UR-97
1957893_adh	TCAGAATGACTAGAAAGTTGGT	22	1	21UR-97
2286363_adh	TGCACCTTGAGGGGAACTAGA	21	16	21UR-103
673008_adh	ATGCACCTTGAGGGGAACTAGA	22	1	21UR-103
1789171_adh	TAAGTTCGAATTCGAGAAAGG	21	319	21UR-108
1789172_adh	TAAGTTCGAATTCGAGAAAGGA	22	1	21UR-108
1789173_adh	TAAGTTCGAATTCGAGAAAGGAGT	24	1	21UR-108
2456765_adh	TGGGGTTGTAGATATAGAAGA	21	6577	21UR-116
2456770_adh	TGGGGTTGTAGATATAGAAGAC	22	1	21UR-116
2456773_adh	TGGGGTTGTAGATATAGAAGACT	23	4	21UR-116
2456774_adh	TGGGGTTGTAGATATAGAAGACTT	24	1	21UR-116

2456775_adh	TGGGGTTGTAGATATAGAAGACTTA	25	3	21UR-116
1834989_adh	TACTTCTGGATAAAAGTTACA	21	55	21UR-124
600533_adh	ATACTTCTGGATAAAAGTTACA	22	1	21UR-124
1746952_adh	TAAAAATAGCGTGTATGTACA	21	52	21UR-130
1746953_adh	TAAAAATAGCGTGTATGTACAAAT	24	1	21UR-130
2473732_adh	TGGTGTTGCCAGAAGATATAT	21	28	21UR-140
239280_adh	AATGGTGTTGCCAGAAGATATAT	23	1	21UR-140
1983723_adh	TCCGAAAAATCGATTATGGAC	21	123	21UR-148
1983724_adh	TCCGAAAAATCGATTATGGACA	22	3	21UR-148
1815942_adh	TACCATGGACATTACCACAAT	21	20	21UR-154
594577_adh	ATACCATGGACATTACCACAAT	22	1	21UR-154
2616162_adh	TTCTCATCCGGTCCAAGAGGT	21	20	21UR-161
2616163_adh	TTCTCATCCGGTCCAAGAGGTTAT	24	1	21UR-161
2707387_adh	TTTCGCGTCGTCATCACAAAC	21	748	21UR-163
2707390_adh	TTTCGCGTCGTCATCACAAACT	22	1	21UR-163
2707392_adh	TTTCGCGTCGTCATCACAAACTT	23	1	21UR-163
1813294_adh	TACATCAATGACAGAAAGATC	21	84	21UR-166
593558_adh	ATACATCAATGACAGAAAGATC	22	1	21UR-166
203236_adh	AATACATCAATGACAGAAAGATC	23	1	21UR-166
2539465_adh	TTAAACAGCAATATGAACGAT	21	1385	21UR-183
2539468_adh	TTAAACAGCAATATGAACGATGGAA	25	1	21UR-183
1971614_adh	TCATCTTATCGTGTACAATCC	21	51	21UR-222
214188_adh	AATCATCTTATCGTGTACAATCC	23	1	21UR-222
2491734_adh	TGTATGATCAGTATGGAAAAT	21	1962	21UR-225
2491737_adh	TGTATGATCAGTATGGAAAATT	22	4	21UR-225
695507_adh	ATGTATGATCAGTATGGAAAAT	22	1	21UR-225
240881_adh	AATGTATGATCAGTATGGAAAAT	23	2	21UR-225
1918535_adh	TATGTCCATTAGAAGAATTCT	21	19	21UR-226
1918539_adh	TATGTCCATTAGAAGAATTCTTGT	24	1	21UR-226
2074125_adh	TGAAACTGTAAAAATTATGGC	21	712	21UR-228
1062000_adh	CTGAAACTGTAAAAATTATGGC	22	1	21UR-228
2074127_adh	TGAAACTGTAAAAATTATGGCT	22	8	21UR-228
1755973_adh	TAAACTTTTGATCCGGTTGCC	21	44	21UR-233
581487_adh	ATAAACTTTTGATCCGGTTGCC	22	1	21UR-233
198971_adh	AATAAACTTTTGATCCGGTTGCC	23	2	21UR-233
1944440_adh	TCACACACAACAAAGGCTAAA	21	54	21UR-266
1944441_adh	TCACACACAACAAAGGCTAAAAA	23	1	21UR-266
2533151_adh	TGTTTGCGTTGTTGTACATAT	21	133	21UR-269
701654_adh	ATGTTTGCGTTGTTGTACATAT	22	1	21UR-269
1822306_adh	TACGACCGAACGAATACTGAA	21	127	21UR-279
1822307_adh	TACGACCGAACGAATACTGAAA	22	4	21UR-279
1822308_adh	TACGACCGAACGAATACTGAAAA	23	34	21UR-279
1822649_adh	TACGACTGGCGTACGTATTCT	21	3816	21UR-294
1822652_adh	TACGACTGGCGTACGTATTCTG	22	2	21UR-294
1822653_adh	TACGACTGGCGTACGTATTCTGT	23	6	21UR-294
1822654_adh	TACGACTGGCGTACGTATTCTGTT	24	2	21UR-294
1822655_adh	TACGACTGGCGTACGTATTCTGTTG	25	1	21UR-294
2032178_adh	TCTACTTGTTGAATAATGGTA	21	2870	21UR-296
2032179_adh	TCTACTTGTTGAATAATGGTAAA	23	1	21UR-296
1835146_adh	TACTTGACGGGACCAATTAAA	21	46	21UR-307

1835147_adh	TACTTGACGGGACCAATTAAAC	22	1	21UR-307
2526186_adh	TGTTCCAAGCATGTGTGATGG	21	30	21UR-312
2526187_adh	TGTTCCAAGCATGTGTGATGGCTC	24	1	21UR-312
2673107_adh	TTGGTTTAGAATTGGATGCTT	21	429	21UR-313
2673108_adh	TTGGTTTAGAATTGGATGCTTA	22	1	21UR-313
1832891_adh	TACTGCTTTTTCACTTCACGA	21	17	21UR-323
1832892_adh	TACTGCTTTTTCACTTCACGAAA	23	1	21UR-323
2047988_adh	TCTGCAGTTTTTGAACGCGTC	21	372	21UR-326
222447_adh	AATCTGCAGTTTTTGAACGCGTC	23	2	21UR-326
1878465_adh	TAGTGGAAACCTCAACGCTGA	21	47	21UR-337
1878466_adh	TAGTGGAAACCTCAACGCTGAA	22	2	21UR-337
2011193_adh	TCGGACAAGAAGAATAGATCG	21	47	21UR-348
2011194_adh	TCGGACAAGAAGAATAGATCGA	22	18	21UR-348
2011195_adh	TCGGACAAGAAGAATAGATCGAG	23	12	21UR-348
2011196_adh	TCGGACAAGAAGAATAGATCGAGA	24	7	21UR-348
2016555_adh	TCGGGAGGAAATATTCGGGAC	21	105	21UR-388
2016557_adh	TCGGGAGGAAATATTCGGGACGA	23	1	21UR-388
2596511_adh	TTCCACTGATATCCATGGACA	21	388	21UR-398
2596512_adh	TTCCACTGATATCCATGGACAC	22	1	21UR-398
713396_adh	ATTCCACTGATATCCATGGACA	22	1	21UR-398
2693295_adh	TTTACAGAAGGACAAACAGAG	21	433	21UR-399
2693297_adh	TTTACAGAAGGACAAACAGAGTGAC	25	1	21UR-399
1821799_adh	TACGAAGGATACATAGATCGT	21	318	21UR-405
596036_adh	ATACGAAGGATACATAGATCGT	22	1	21UR-405
2417091_adh	TGGGAAAAAACGATTCTGCAT	21	1691	21UR-421
2417095_adh	TGGGAAAAAACGATTCTGCATC	22	1	21UR-421
2417096_adh	TGGGAAAAAACGATTCTGCATCTAAT	26	1	21UR-421
1776600_adh	TAAGAGAGAAATAAGAGACTT	21	14	21UR-447
1776602_adh	TAAGAGAGAAATAAGAGACTTTGAT	25	2	21UR-447
1902490_adh	TATGACAACAACGATGACAAC	21	11273	21UR-453
1902491_adh	TATGACAACAACGATGACAACA	22	191	21UR-453
615632_adh	ATATGACAACAACGATGACAAC	22	2	21UR-453
1902492_adh	TATGACAACAACGATGACAACAA	23	70	21UR-453
1902493_adh	TATGACAACAACGATGACAACAAA	24	27	21UR-453
1902494_adh	TATGACAACAACGATGACAACAAAA	25	5	21UR-453
1902495_adh	TATGACAACAACGATGACAACAAAAA	26	1	21UR-453
1809241_adh	TACAATAACGTGTTTTTATCT	21	28	21UR-455
591957_adh	ATACAATAACGTGTTTTTATCT	22	1	21UR-455
2052233_adh	TCTGTCACATGCGAGACCCGG	21	18	21UR-459
1632763_adh	GTCTGTCACATGCGAGACCCGG	22	1	21UR-459
2052237_adh	TCTGTCACATGCGAGACCCGGG	22	1	21UR-459
1007504_adh	CGTCTGTCACATGCGAGACCCGG	23	3	21UR-459
1632764_adh	GTCTGTCACATGCGAGACCCGGG	23	1	21UR-459
1632765_adh	GTCTGTCACATGCGAGACCCGGGT	24	2	21UR-459
2052238_adh	TCTGTCACATGCGAGACCCGGGTT	24	3	21UR-459
1632766_adh	GTCTGTCACATGCGAGACCCGGGTT	25	2	21UR-459
2052239_adh	TCTGTCACATGCGAGACCCGGGTTC	25	1	21UR-459
2052240_adh	TCTGTCACATGCGAGACCCGGGTTCA	26	1	21UR-459
1889887_adh	TATATATTCCCGCGTCAAGAA	21	8	21UR-469
612680_adh	ATATATATTCCCGCGTCAAGAA	22	1	21UR-469

2529325_adh	TGTTGCAACTGAAGAATCAAA	21	466	21UR-480
2529326_adh	TGTTGCAACTGAAGAATCAAAA	22	1	21UR-480
1965831_adh	TCAGTAGACATCTCTAACAGC	21	81	21UR-482
1965832_adh	TCAGTAGACATCTCTAACAGCA	22	1	21UR-482
2296121_adh	TGCGAATGGATTTTAGCTTGA	21	246	21UR-488
2296124_adh	TGCGAATGGATTTTAGCTTGAT	22	2	21UR-488
230345_adh	AATGCGAATGGATTTTAGCTTGA	23	2	21UR-488
1813127_adh	TACATACTCATTGAATGGTAG	21	33	21UR-501
593479_adh	ATACATACTCATTGAATGGTAG	22	1	21UR-501
1754370_adh	TAAACAGGACTTAATCTACAT	21	123	21UR-503
1754371_adh	TAAACAGGACTTAATCTACATA	22	1	21UR-503
1941613_adh	TCAATCTGAGGCTGCAAATGA	21	46	21UR-516
1941615_adh	TCAATCTGAGGCTGCAAATGAG	22	1	21UR-516
1837308_adh	TAGAAAGCATATTCATAGTTA	21	57	21UR-534
1837309_adh	TAGAAAGCATATTCATAGTTAGG	23	1	21UR-534
1861772_adh	TAGGAATGAGTTCAGTAAAGG	21	444	21UR-538
1861774_adh	TAGGAATGAGTTCAGTAAAGGT	22	3	21UR-538
1861775_adh	TAGGAATGAGTTCAGTAAAGGTAG	24	1	21UR-538
1861776_adh	TAGGAATGAGTTCAGTAAAGGTAGGT	26	1	21UR-538
2131852_adh	TGAAGCTCGGCATCAAGTAGA	21	1739	21UR-548
2131854_adh	TGAAGCTCGGCATCAAGTAGAC	22	12	21UR-548
1933527_adh	TCAAAGCTCTGAAGATTTGCC	21	1354	21UR-552
1933528_adh	TCAAAGCTCTGAAGATTTGCCA	22	6	21UR-552
1933531_adh	TCAAAGCTCTGAAGATTTGCCAT	23	2	21UR-552
209681_adh	AATCAAAGCTCTGAAGATTTGCC	23	2	21UR-552
1933532_adh	TCAAAGCTCTGAAGATTTGCCATCT	25	1	21UR-552
2532423_adh	TGTTTCGAACTTCAATGCAGG	21	1106	21UR-603
2532427_adh	TGTTTCGAACTTCAATGCAGGT	22	7	21UR-603
2075225_adh	TGAAAGAACGGAAGGATTTGT	21	624	21UR-607
2075226_adh	TGAAAGAACGGAAGGATTTGTA	22	6	21UR-607
2075228_adh	TGAAAGAACGGAAGGATTTGTAT	23	1	21UR-607
2140633_adh	TGAATCAGAACAATGGTACCC	21	17911	21UR-609
2630196_adh	TTGAATCAGAACAATGGTACCC	22	1	21UR-609
2497807_adh	TGTCGATAACCATACGCATGT	21	48	21UR-611
241276_adh	AATGTCGATAACCATACGCATGT	23	3	21UR-611
1838257_adh	TAGAACATGCAGCTATGATGA	21	564	21UR-632
1032844_adh	CTAGAACATGCAGCTATGATGA	22	1	21UR-632
1838259_adh	TAGAACATGCAGCTATGATGAC	22	4	21UR-632
1838260_adh	TAGAACATGCAGCTATGATGACAC	24	1	21UR-632
1813924_adh	TACATGGTCATTTTATTCTGG	21	12	21UR-662
593978_adh	ATACATGGTCATTTTATTCTGG	22	1	21UR-662
1829630_adh	TACTACGACAGGAGATGAATA	21	978	21UR-681
1031610_adh	CTACTACGACAGGAGATGAATA	22	1	21UR-681
1829632_adh	TACTACGACAGGAGATGAATAC	22	3	21UR-681
2152232_adh	TGACAATGGTTTATGAACGGT	21	24701	21UR-690
2152240_adh	TGACAATGGTTTATGAACGGTT	22	15	21UR-690
2152241_adh	TGACAATGGTTTATGAACGGTTAT	24	2	21UR-690
2152242_adh	TGACAATGGTTTATGAACGGTTATTT	26	1	21UR-690
1761264_adh	TAAATGAAGTAGAAAAAATAC	21	335	21UR-691
1761266_adh	TAAATGAAGTAGAAAAAATACT	22	1	21UR-691

1914227_adh	TATGGAGTAGTATAACCAAAA	21	1083	21UR-692
1914228_adh	TATGGAGTAGTATAACCAAAAA	22	4	21UR-692
1914229_adh	TATGGAGTAGTATAACCAAAAAA	23	2	21UR-692
208666_adh	AATATGGAGTAGTATAACCAAAA	23	3	21UR-692
1914230_adh	TATGGAGTAGTATAACCAAAAAAT	24	1	21UR-692
1833796_adh	TACTGTATTTGTAAGCCATGA	21	50	21UR-700
1833798_adh	TACTGTATTTGTAAGCCATGAAC	23	1	21UR-700
2045513_adh	TCTGACGAGTAGAGTTTAGAA	21	11511	21UR-703
2045514_adh	TCTGACGAGTAGAGTTTAGAAA	22	34	21UR-703
2045519_adh	TCTGACGAGTAGAGTTTAGAAAT	23	24	21UR-703
2618157_adh	TTCTGACGAGTAGAGTTTAGAAA	23	1	21UR-703
2045522_adh	TCTGACGAGTAGAGTTTAGAAATT	24	1	21UR-703
2045523_adh	TCTGACGAGTAGAGTTTAGAAATTTA	26	2	21UR-703
1978871_adh	TCCAGTAGCCTTGATTATCGG	21	731	21UR-711
1978872_adh	TCCAGTAGCCTTGATTATCGGA	22	2	21UR-711
2478704_adh	TGGTTTGTTACGTCGATTCAC	21	155	21UR-712
2478705_adh	TGGTTTGTTACGTCGATTCACC	22	1	21UR-712
1910982_adh	TATGATCAGGCATCCTAAACT	21	3013	21UR-715
1910984_adh	TATGATCAGGCATCCTAAACTC	22	5	21UR-715
616018_adh	ATATGATCAGGCATCCTAAACT	22	1	21UR-715
1910985_adh	TATGATCAGGCATCCTAAACTCG	23	1	21UR-715
208544_adh	AATATGATCAGGCATCCTAAACT	23	4	21UR-715
1910986_adh	TATGATCAGGCATCCTAAACTCGT	24	2	21UR-715
1910988_adh	TATGATCAGGCATCCTAAACTCGTT	25	1	21UR-715
2304263_adh	TGCGTGGTGGAAAGGTTCTGT	21	263	21UR-735
2304264_adh	TGCGTGGTGGAAAGGTTCTGTA	22	5	21UR-735
1874519_adh	TAGTAGATCTTGACAGAAGAA	21	1127	21UR-791
1036214_adh	CTAGTAGATCTTGACAGAAGAA	22	1	21UR-791
1809798_adh	TACAATGAGAAGCAATGTGAA	21	93	21UR-796
203022_adh	AATACAATGAGAAGCAATGTGAA	23	1	21UR-796
2395707_adh	TGGATTTAGCAAGAAACCGAG	21	4977	21UR-798
1082557_adh	CTGGATTTAGCAAGAAACCGAG	22	1	21UR-798
2395716_adh	TGGATTTAGCAAGAAACCGAGT	22	7	21UR-798
2395717_adh	TGGATTTAGCAAGAAACCGAGTA	23	1	21UR-798
2395719_adh	TGGATTTAGCAAGAAACCGAGTAT	24	1	21UR-798
2395720_adh	TGGATTTAGCAAGAAACCGAGTATCT	26	1	21UR-798
2183919_adh	TGAGACGAGAAGAAAAGATTT	21	42	21UR-801
2183920_adh	TGAGACGAGAAGAAAAGATTTC	22	1	21UR-801
2183921_adh	TGAGACGAGAAGAAAAGATTTCATT	25	1	21UR-801
1628374_adh	GTCGGTGGTTCGAGCCCGCCCGA	23	12	21UR-803
1550539_adh	GGTCGGTGGTTCGAGCCCGCCCGA	24	2	21UR-803
1550540_adh	GGTCGGTGGTTCGAGCCCGCCCGAG	25	1	21UR-803
1181997_adh	GAAGGTCGGTGGTTCGAGCCCGCCCG	26	1	21UR-803
2553399_adh	TTACAACTGTGTATGCTATGA	21	265	21UR-812
2553400_adh	TTACAACTGTGTATGCTATGAA	22	7	21UR-812
1963342_adh	TCAGGAAAGCAAGAACTCGAA	21	950	21UR-845
1963343_adh	TCAGGAAAGCAAGAACTCGAAA	22	4	21UR-845
627949_adh	ATCAGGAAAGCAAGAACTCGAA	22	1	21UR-845
213518_adh	AATCAGGAAAGCAAGAACTCGAA	23	1	21UR-845
2278184_adh	TGATTTCGGTTTGTAACTTTG	21	35	21UR-865

2278187_adh	TGATTTCGGTTTGTAACTTTGCC	23	1	21UR-865
2301979_adh	TGCGGGAGACAGACTTTGCAA	21	859	21UR-882
2301981_adh	TGCGGGAGACAGACTTTGCAAT	22	2	21UR-882
2678297_adh	TTGTCGGATAACCACATTGGA	21	507	21UR-888
2678302_adh	TTGTCGGATAACCACATTGGAT	22	1	21UR-888
253577_adh	AATTGTCGGATAACCACATTGGA	23	1	21UR-888
1781170_adh	TAAGATTTTGGTCCCAATGAA	21	48	21UR-911
1781171_adh	TAAGATTTTGGTCCCAATGAAA	22	1	21UR-911
1850557_adh	TAGATATTCTATTACTTGCGG	21	5	21UR-914
1850559_adh	TAGATATTCTATTACTTGCGGC	22	1	21UR-914
1943278_adh	TCAATTTAGTCTAAGCGGCAA	21	120	21UR-916
1943279_adh	TCAATTTAGTCTAAGCGGCAAAA	23	1	21UR-916
1797670_adh	TAATCAAGTAGTATGAAATTA	21	2747	21UR-920
1797671_adh	TAATCAAGTAGTATGAAATTAA	22	1	21UR-920
1797672_adh	TAATCAAGTAGTATGAAATTAAA	23	3	21UR-920
201160_adh	AATAATCAAGTAGTATGAAATTA	23	1	21UR-920
2592017_adh	TTCAGGAACGTTACTCATTAG	21	3118	21UR-928
2592019_adh	TTCAGGAACGTTACTCATTAGATAAA	26	1	21UR-928
2291577_adh	TGCATTTGCTGTCACCTGAAA	21	113	21UR-929
674348_adh	ATGCATTTGCTGTCACCTGAAA	22	1	21UR-929
1918338_adh	TATGTCACGAGATTGGTTTAC	21	409	21UR-944
1918342_adh	TATGTCACGAGATTGGTTTACT	22	1	21UR-944
1804320_adh	TAATTGTGACTAAATTTTTGA	21	19	21UR-950
1804321_adh	TAATTGTGACTAAATTTTTGAAA	23	1	21UR-950
1867720_adh	TAGGCTTCGCAGTAGATTAAG	21	328	21UR-962
1035590_adh	CTAGGCTTCGCAGTAGATTAAG	22	2	21UR-962
1867722_adh	TAGGCTTCGCAGTAGATTAAGT	22	2	21UR-962
1901785_adh	TATGAAGATGATGAGCAATCT	21	124	21UR-970
1901786_adh	TATGAAGATGATGAGCAATCTAT	23	3	21UR-970
1747954_adh	TAAAACCGTAGAATAGAATCA	21	36	21UR-975
1747955_adh	TAAAACCGTAGAATAGAATCAAGA	24	2	21UR-975
1954171_adh	TCACTCCATACTACTAGAACA	21	258	21UR-981
1954173_adh	TCACTCCATACTACTAGAACATGTT	25	1	21UR-981
2693187_adh	TTTACACACTTTAGTCGGGAT	21	467	21UR-998
254752_adh	AATTTACACACTTTAGTCGGGAT	23	2	21UR-998
1880798_adh	TAGTTGAACAGTAATCTCAAA	21	33	21UR-999
1880800_adh	TAGTTGAACAGTAATCTCAAAC	22	1	21UR-999
2143452_adh	TGAATGAGCAAATTAGAATTC	21	176	21UR-1005
2143453_adh	TGAATGAGCAAATTAGAATTCA	22	2	21UR-1005
2130734_adh	TGAAGCAGTTCAAATCTGATG	21	16	21UR-1006
224552_adh	AATGAAGCAGTTCAAATCTGATG	23	2	21UR-1006
2018900_adh	TCGGTGAATTAATTCGGTGTC	21	954	21UR-1011
2018904_adh	TCGGTGAATTAATTCGGTGTCT	22	1	21UR-1011
2168123_adh	TGACTCGTATTGGACTAAAAA	21	1235	21UR-1016
2168125_adh	TGACTCGTATTGGACTAAAAAGA	23	3	21UR-1016
2099269_adh	TGAAATCTTCATGGTCGACGA	21	403	21UR-1017
660311_adh	ATGAAATCTTCATGGTCGACGA	22	4	21UR-1017
2623897_adh	TTCTTTTGGGGTTTGATCGAA	21	94	21UR-1021
2623898_adh	TTCTTTTGGGGTTTGATCGAAGGT	24	1	21UR-1021
1915110_adh	TATGGATTCCAGAAAATTGTC	21	62	21UR-1022

208695_adh	AATATGGATTCCAGAAAATTGTC	23	1	21UR-1022
2501826_adh	TGTGAACTGTTGATTTCGTGC	21	45	21UR-1032
2501829_adh	TGTGAACTGTTGATTTCGTGCCG	23	1	21UR-1032
1971583_adh	TCATCTGTAGGACAACATTTA	21	11	21UR-1036
1971584_adh	TCATCTGTAGGACAACATTTAA	22	1	21UR-1036
1971585_adh	TCATCTGTAGGACAACATTTAATAA	25	1	21UR-1036
2700536_adh	TTTATTGTGAAGACTGTGACG	21	93	21UR-1051
2700537_adh	TTTATTGTGAAGACTGTGACGT	22	3	21UR-1051
2700538_adh	TTTATTGTGAAGACTGTGACGTAAT	25	1	21UR-1051
2233974_adh	TGAGCGCATTTGTATACACTG	21	40	21UR-1063
667880_adh	ATGAGCGCATTTGTATACACTG	22	1	21UR-1063
227181_adh	AATGAGCGCATTTGTATACACTG	23	1	21UR-1063
1886829_adh	TATACAGCTTAACTACCGATC	21	117	21UR-1079
612073_adh	ATATACAGCTTAACTACCGATC	22	1	21UR-1079
207835_adh	AATATACAGCTTAACTACCGATC	23	2	21UR-1079
1808508_adh	TACAACGTCGGTCAAACACAC	21	391	21UR-1087
1808509_adh	TACAACGTCGGTCAAACACACA	22	2	21UR-1087
2550670_adh	TTAATCTGAATGTCGTGATGA	21	262	21UR-1088
2550671_adh	TTAATCTGAATGTCGTGATGAT	22	1	21UR-1088
244101_adh	AATTAATCTGAATGTCGTGATGA	23	1	21UR-1088
2491513_adh	TGTATGAAAACGTGCCGTCTT	21	70	21UR-1091
240875_adh	AATGTATGAAAACGTGCCGTCTT	23	1	21UR-1091
1899008_adh	TATCGTTGGGTAGACCATGGT	21	2129	21UR-1122
1899013_adh	TATCGTTGGGTAGACCATGGTT	22	4	21UR-1122
614928_adh	ATATCGTTGGGTAGACCATGGT	22	1	21UR-1122
208331_adh	AATATCGTTGGGTAGACCATGGT	23	30	21UR-1122
1899017_adh	TATCGTTGGGTAGACCATGGTTTT	24	2	21UR-1122
1772802_adh	TAACTTGTTGAATTTCCGAAT	21	19	21UR-1124
200269_adh	AATAACTTGTTGAATTTCCGAAT	23	1	21UR-1124
2420917_adh	TGGGAATGTGAAATTTTTCAG	21	116	21UR-1125
2420920_adh	TGGGAATGTGAAATTTTTCAGG	22	1	21UR-1125
2485685_adh	TGTAATTGGAAGAATCGTTGC	21	218	21UR-1130
2485687_adh	TGTAATTGGAAGAATCGTTGCGAT	24	1	21UR-1130
1919699_adh	TATGTTTGGAAAAACCGGTTG	21	289	21UR-1134
1919700_adh	TATGTTTGGAAAAACCGGTTGAA	23	1	21UR-1134
1919701_adh	TATGTTTGGAAAAACCGGTTGAAG	24	1	21UR-1134
2476654_adh	TGGTTGGATTGTCGCAGTCGG	21	1468	21UR-1146
2476667_adh	TGGTTGGATTGTCGCAGTCGGT	22	49	21UR-1146
2476675_adh	TGGTTGGATTGTCGCAGTCGGTT	23	6	21UR-1146
1795056_adh	TAATAGGATTTTTCGGGGGAA	21	119	21UR-1147
1795057_adh	TAATAGGATTTTTCGGGGGAAT	22	3	21UR-1147
2482741_adh	TGTAATAAGTGGAGGTAGCTA	21	3818	21UR-1166
2482742_adh	TGTAATAAGTGGAGGTAGCTAA	22	1	21UR-1166
693686_adh	ATGTAATAAGTGGAGGTAGCTA	22	1	21UR-1166
2482743_adh	TGTAATAAGTGGAGGTAGCTAAA	23	2	21UR-1166
2482744_adh	TGTAATAAGTGGAGGTAGCTAAAC	24	1	21UR-1166
2482745_adh	TGTAATAAGTGGAGGTAGCTAAACA	25	1	21UR-1166
2482746_adh	TGTAATAAGTGGAGGTAGCTAAACAT	26	1	21UR-1166
2051841_adh	TCTGTACGAAGAATGGTTGTT	21	2249	21UR-1167
2051845_adh	TCTGTACGAAGAATGGTTGTTT	22	2	21UR-1167

2051849_adh	TCTGTACGAAGAATGGTTGTTTT	23	1	21UR-1167
2567673_adh	TTAGGACATAATATGATCTTA	21	111	21UR-1190
2567674_adh	TTAGGACATAATATGATCTTAA	22	4	21UR-1190
1968147_adh	TCATAACAAACTGGGCATAAA	21	272	21UR-1193
1968150_adh	TCATAACAAACTGGGCATAAAATTAT	26	1	21UR-1193
1997260_adh	TCGAATCAACTTGATCAAATC	21	4	21UR-1194
1997261_adh	TCGAATCAACTTGATCAAATCT	22	1	21UR-1194
670630_adh	ATGATTCCGCAATAAGGCCTTG	22	1	21UR-1198
2123549_adh	TGAACGGCTTTTTTCTGATGG	21	7234	21UR-1200
2123552_adh	TGAACGGCTTTTTTCTGATGGG	22	1	21UR-1200
2123555_adh	TGAACGGCTTTTTTCTGATGGGGACT	26	1	21UR-1200
1775715_adh	TAAGACGGCCAAATTATTTTC	21	135	21UR-1205
1775716_adh	TAAGACGGCCAAATTATTTTCT	22	1	21UR-1205
1761434_adh	TAAATGATAAGTTAGACCACA	21	306	21UR-1213
1761435_adh	TAAATGATAAGTTAGACCACAA	22	1	21UR-1213
1761436_adh	TAAATGATAAGTTAGACCACAACAAG	26	1	21UR-1213
2620064_adh	TTCTGTCTCCCAAGCCAAGTA	21	23	21UR-1215
722082_adh	ATTCTGTCTCCCAAGCCAAGTA	22	1	21UR-1215
1893946_adh	TATCATCAGGTTGAAGATTTG	21	631	21UR-1217
1893948_adh	TATCATCAGGTTGAAGATTTGG	22	1	21UR-1217
1893949_adh	TATCATCAGGTTGAAGATTTGGGT	24	2	21UR-1217
1893950_adh	TATCATCAGGTTGAAGATTTGGGTC	25	2	21UR-1217
1897448_adh	TATCGCTAAGCTTTTTCTGAA	21	53	21UR-1233
1897449_adh	TATCGCTAAGCTTTTTCTGAAAA	23	1	21UR-1233
2137552_adh	TGAATACAATCAATGTTGACG	21	259	21UR-1251
662808_adh	ATGAATACAATCAATGTTGACG	22	2	21UR-1251
224802_adh	AATGAATACAATCAATGTTGACG	23	1	21UR-1251
1932360_adh	TCAAAATAAACACAATTCTGG	21	1	21UR-1255
1932361_adh	TCAAAATAAACACAATTCTGGC	22	2	21UR-1255
1829455_adh	TACTAATGTGTTGTAGGGTAA	21	146	21UR-1257
1829456_adh	TACTAATGTGTTGTAGGGTAAAA	23	1	21UR-1257
1846534_adh	TAGACTTGAGTTAGAACGGTT	21	72024	21UR-1258
1846535_adh	TAGACTTGAGTTAGAACGGTTA	22	6	21UR-1258
602877_adh	ATAGACTTGAGTTAGAACGGTT	22	2	21UR-1258
1846536_adh	TAGACTTGAGTTAGAACGGTTAA	23	11	21UR-1258
1846538_adh	TAGACTTGAGTTAGAACGGTTAAT	24	22	21UR-1258
1846540_adh	TAGACTTGAGTTAGAACGGTTAATT	25	5	21UR-1258
1760845_adh	TAAATCGGTCATATAAGATAA	21	1479	21UR-1285
1760846_adh	TAAATCGGTCATATAAGATAAA	22	1	21UR-1285
1760847_adh	TAAATCGGTCATATAAGATAAAG	23	1	21UR-1285
199436_adh	AATAAATCGGTCATATAAGATAA	23	2	21UR-1285
2572966_adh	TTAGTTGTCCATACGTTTCTC	21	85	21UR-1290
245150_adh	AATTAGTTGTCCATACGTTTCTC	23	1	21UR-1290
2572967_adh	TTAGTTGTCCATACGTTTCTCAC	23	1	21UR-1290
1895455_adh	TATCCAGAAAAAGTTCAGTTT	21	22	21UR-1300
208149_adh	AATATCCAGAAAAAGTTCAGTTT	23	1	21UR-1300
2635427_adh	TTGACCGAAGATTTTGAGAAC	21	281	21UR-1312
1108937_adh	CTTGACCGAAGATTTTGAGAAC	22	1	21UR-1312
2635428_adh	TTGACCGAAGATTTTGAGAACA	22	1	21UR-1312
2635429_adh	TTGACCGAAGATTTTGAGAACAA	23	1	21UR-1312

1987668_adh	TCCGTAGCAGATATTTATCGT	21	312	21UR-1317
1987672_adh	TCCGTAGCAGATATTTATCGTTGA	24	1	21UR-1317
2422406_adh	TGGGACTTCCTAAACGGTTTT	21	362	21UR-1330
2422407_adh	TGGGACTTCCTAAACGGTTTTAT	23	1	21UR-1330
1956987_adh	TCAGAAATACTTCATGAGCAA	21	103	21UR-1336
1956989_adh	TCAGAAATACTTCATGAGCAAG	22	1	21UR-1336
1956990_adh	TCAGAAATACTTCATGAGCAAGACA	25	1	21UR-1336
1890431_adh	TATATGTACTACATCCACCGG	21	47	21UR-1339
1890432_adh	TATATGTACTACATCCACCGGAG	23	1	21UR-1339
2132447_adh	TGAAGGAAGAGTACGAAACTT	21	3028	21UR-1343
2132449_adh	TGAAGGAAGAGTACGAAACTTT	22	5	21UR-1343
224612_adh	AATGAAGGAAGAGTACGAAACTT	23	7	21UR-1343
224613_adh	AATGAAGGAAGAGTACGAAACTTT	24	1	21UR-1343
2132453_adh	TGAAGGAAGAGTACGAAACTTTTGT	25	3	21UR-1343
1923753_adh	TATTCATAGTTTAAGAGCAAT	21	35	21UR-1349
1923754_adh	TATTCATAGTTTAAGAGCAATA	22	1	21UR-1349
1886800_adh	TATACAGAAGACGTCTTGTTA	21	308	21UR-1352
207830_adh	AATATACAGAAGACGTCTTGTTA	23	1	21UR-1352
2073748_adh	TGAAACTCTACTTTCAGGAGG	21	148	21UR-1355
2073749_adh	TGAAACTCTACTTTCAGGAGGA	22	1	21UR-1355
2073751_adh	TGAAACTCTACTTTCAGGAGGAC	23	1	21UR-1355
2617040_adh	TTCTCTCAGCCTACGACCAAA	21	146	21UR-1360
2617041_adh	TTCTCTCAGCCTACGACCAAAA	22	1	21UR-1360
248862_adh	AATTCTCTCAGCCTACGACCAAA	23	1	21UR-1360
2013652_adh	TCGGATATTAGCAAACATTAA	21	353	21UR-1372
2013653_adh	TCGGATATTAGCAAACATTAAA	22	2	21UR-1372
2013654_adh	TCGGATATTAGCAAACATTAAAA	23	1	21UR-1372
2013655_adh	TCGGATATTAGCAAACATTAAAAAT	25	2	21UR-1372
1841352_adh	TAGAATCGGATTATTTATTAT	21	133	21UR-1375
205546_adh	AATAGAATCGGATTATTTATTAT	23	1	21UR-1375
2626853_adh	TTGAACTGGTCGCTGTGATGG	21	1374	21UR-1404
2626854_adh	TTGAACTGGTCGCTGTGATGGA	22	6	21UR-1404
723641_adh	ATTGAACTGGTCGCTGTGATGG	22	1	21UR-1404
2626855_adh	TTGAACTGGTCGCTGTGATGGAAT	24	2	21UR-1404
2626856_adh	TTGAACTGGTCGCTGTGATGGAATT	25	1	21UR-1404
1814098_adh	TACATTAATATTGTTCGGAAA	21	1307	21UR-1415
1814099_adh	TACATTAATATTGTTCGGAAAAA	23	1	21UR-1415
2665203_adh	TTGGCACTCGCGAACACCGCG	21	1	21UR-1430
1114195_adh	CTTGGCACTCGCGAACACCGCG	22	369	21UR-1430
1114196_adh	CTTGGCACTCGCGAACACCGCGA	23	16	21UR-1430
1972216_adh	TCATGCACGGCTAAAAATTTC	21	242	21UR-1442
1972217_adh	TCATGCACGGCTAAAAATTTCT	22	2	21UR-1442
1813182_adh	TACATAGGAATCGAAATATGC	21	16	21UR-1465
203207_adh	AATACATAGGAATCGAAATATGC	23	1	21UR-1465
2472196_adh	TGGTGGATCGTCATTTGGTGG	21	187	21UR-1475
2472199_adh	TGGTGGATCGTCATTTGGTGGT	22	1	21UR-1475
2472200_adh	TGGTGGATCGTCATTTGGTGGTA	23	2	21UR-1475
2472201_adh	TGGTGGATCGTCATTTGGTGGTAGT	25	2	21UR-1475
2671195_adh	TTGGTGGATCGTCATTTGGTGGTAGT	26	1	21UR-1475
1917496_adh	TATGTACGTAACAACACACTG	21	41	21UR-1479

208763_adh	AATATGTACGTAACAACACACTG	23	1	21UR-1479
1941311_adh	TCAATCACGGTATTTATTTTG	21	104	21UR-1483
210484_adh	AATCAATCACGGTATTTATTTTG	23	2	21UR-1483
2289539_adh	TGCAGTTTTTGATGACAGCAA	21	36	21UR-1489
1071421_adh	CTGCAGTTTTTGATGACAGCAA	22	1	21UR-1489
1885898_adh	TATAATGAGATTTGACTTTGT	21	23	21UR-1498
611968_adh	ATATAATGAGATTTGACTTTGT	22	1	21UR-1498
2136911_adh	TGAATAATAGAAAATGCTGGC	21	3111	21UR-1511
2136912_adh	TGAATAATAGAAAATGCTGGCA	22	8	21UR-1511
2136913_adh	TGAATAATAGAAAATGCTGGCAA	23	7	21UR-1511
2136914_adh	TGAATAATAGAAAATGCTGGCAAA	24	2	21UR-1511
2136915_adh	TGAATAATAGAAAATGCTGGCAAAA	25	1	21UR-1511
2004792_adh	TCGATTCGTATAATGCAGAAA	21	1260	21UR-1517
2004795_adh	TCGATTCGTATAATGCAGAAAT	22	3	21UR-1517
2719544_adh	TTTGATGTTGCGTGAAGCATA	21	5	21UR-1531
742961_adh	ATTTGATGTTGCGTGAAGCATA	22	1	21UR-1531
2611766_adh	TTCGTATGTTGAGTTAATGGC	21	1313	21UR-1545
1105099_adh	CTTCGTATGTTGAGTTAATGGC	22	1	21UR-1545
2611768_adh	TTCGTATGTTGAGTTAATGGCGA	23	1	21UR-1545
2611769_adh	TTCGTATGTTGAGTTAATGGCGAAT	25	1	21UR-1545
1827323_adh	TACGGTCGATCTTGTTTTAGG	21	131	21UR-1547
1827324_adh	TACGGTCGATCTTGTTTTAGGAA	23	1	21UR-1547
1811771_adh	TACAGAAGTGGACGTATTTAG	21	546	21UR-1556
1030062_adh	CTACAGAAGTGGACGTATTTAG	22	2	21UR-1556
1811773_adh	TACAGAAGTGGACGTATTTAGGTAGA	26	1	21UR-1556
1806460_adh	TACAAAACCCAAAACTTTTGA	21	68	21UR-1560
1806461_adh	TACAAAACCCAAAACTTTTGAA	22	4	21UR-1560
2272715_adh	TGATGGGTAGTTGATTTTGGT	21	1622	21UR-1585
2272717_adh	TGATGGGTAGTTGATTTTGGTT	22	4	21UR-1585
2272721_adh	TGATGGGTAGTTGATTTTGGTTT	23	2	21UR-1585
2308567_adh	TGCTGATTGAACATTTCAAAA	21	103	21UR-1587
231086_adh	AATGCTGATTGAACATTTCAAAA	23	1	21UR-1587
1768621_adh	TAACGCTTGTGAACGCATGAT	21	73	21UR-1594
1768624_adh	TAACGCTTGTGAACGCATGATT	22	1	21UR-1594
200072_adh	AATAACGCTTGTGAACGCATGAT	23	1	21UR-1594
1838883_adh	TAGAACTTCTCAAAAAATAAC	21	32	21UR-1595
1838884_adh	TAGAACTTCTCAAAAAATAACA	22	1	21UR-1595
1850317_adh	TAGATAGGCAGAAATTTGATG	21	1608	21UR-1598
1850318_adh	TAGATAGGCAGAAATTTGATGA	22	13	21UR-1598
1850319_adh	TAGATAGGCAGAAATTTGATGAA	23	2	21UR-1598
1921734_adh	TATTAGTAGGTCAGCTTTGAA	21	39	21UR-1604
1921735_adh	TATTAGTAGGTCAGCTTTGAAA	22	1	21UR-1604
2081003_adh	TGAAAGCAGAGGTGGGCGGAT	21	135	21UR-1611
2081004_adh	TGAAAGCAGAGGTGGGCGGATA	22	1	21UR-1611
2081006_adh	TGAAAGCAGAGGTGGGCGGATAT	23	1	21UR-1611
2665540_adh	TTGGCCGTACGTAATATGAAT	21	398	21UR-1618
731569_adh	ATTGGCCGTACGTAATATGAAT	22	1	21UR-1618
1913528_adh	TATGGAAATGCGGTTATTTGA	21	1504	21UR-1623
1913529_adh	TATGGAAATGCGGTTATTTGAA	22	7	21UR-1623
1913531_adh	TATGGAAATGCGGTTATTTGAAT	23	6	21UR-1623

1913532_adh	TATGGAAATGCGGTTATTTGAATAG	25	1	21UR-1623
1913533_adh	TATGGAAATGCGGTTATTTGAATAGT	26	4	21UR-1623
1956718_adh	TCAGAAAAGGTGTTGTACAAC	21	341	21UR-1634
1956719_adh	TCAGAAAAGGTGTTGTACAACA	22	1	21UR-1634
2489490_adh	TGTAGTAGCATCTTATTTCAA	21	114	21UR-1642
240769_adh	AATGTAGTAGCATCTTATTTCAAAAT	26	1	21UR-1642
1845344_adh	TAGACGATTGGTTCAATTTGC	21	33	21UR-1649
1845345_adh	TAGACGATTGGTTCAATTTGCAA	23	1	21UR-1649
1758921_adh	TAAAGTCGTGATGACAAATGA	21	69	21UR-1668
1758922_adh	TAAAGTCGTGATGACAAATGAA	22	1	21UR-1668
1976216_adh	TCCAAGAGTCAATGATTCGTT	21	4	21UR-1683
214673_adh	AATCCAAGAGTCAATGATTCGTT	23	1	21UR-1683
2532110_adh	TGTTTCAACTCTACGGTGCCT	21	371	21UR-1684
2532111_adh	TGTTTCAACTCTACGGTGCCTAA	23	1	21UR-1684
1925738_adh	TATTGACTATGAGATCTGAAC	21	33	21UR-1694
1925739_adh	TATTGACTATGAGATCTGAACA	22	2	21UR-1694
1925740_adh	TATTGACTATGAGATCTGAACAA	23	3	21UR-1694
2460129_adh	TGGGTTGTCGATATTCGCAGG	21	36	21UR-1702
2460130_adh	TGGGTTGTCGATATTCGCAGGA	22	1	21UR-1702
2460131_adh	TGGGTTGTCGATATTCGCAGGAA	23	2	21UR-1702
1895737_adh	TATCCGCGTCTGTCACATGCGAGAC	25	1	21UR-1712
1836448_adh	TACTTTTGTCGTGAAAATATG	21	86	21UR-1719
601257_adh	ATACTTTTGTCGTGAAAATATG	22	1	21UR-1719
205328_adh	AATACTTTTGTCGTGAAAATATG	23	1	21UR-1719
2596705_adh	TTCCAGACACACAGAAGGAAG	21	113	21UR-1746
2596706_adh	TTCCAGACACACAGAAGGAAGT	22	1	21UR-1746
1813797_adh	TACATGCCATCGACGGCCATC	21	120	21UR-1749
1813798_adh	TACATGCCATCGACGGCCATCA	22	1	21UR-1749
1825414_adh	TACGCTGGTAGAAAGAAAAAA	21	1550	21UR-1790
1031107_adh	CTACGCTGGTAGAAAGAAAAAA	22	1	21UR-1790
1808128_adh	TACAACGTAGAGTACTGAACT	21	108	21UR-1793
1808129_adh	TACAACGTAGAGTACTGAACTAAT	24	1	21UR-1793
1799258_adh	TAATGAACTGACAAAATTTGT	21	6	21UR-1794
589508_adh	ATAATGAACTGACAAAATTTGT	22	1	21UR-1794
2742302_adh	TTTTGCAGTTGCTGGAAATGG	21	516	21UR-1800
2742303_adh	TTTTGCAGTTGCTGGAAATGGA	22	1	21UR-1800
2742304_adh	TTTTGCAGTTGCTGGAAATGGAT	23	1	21UR-1800
2698490_adh	TTTATCCACACGGCTATCAGT	21	167	21UR-1821
255001_adh	AATTTATCCACACGGCTATCAGT	23	1	21UR-1821
2680597_adh	TTGTGAGGACAGTGGCATGGG	21	136	21UR-1824
2680599_adh	TTGTGAGGACAGTGGCATGGGC	22	2	21UR-1824
1928683_adh	TATTGTTTGTGGAAGCGTGAG	21	6953	21UR-1825
1928685_adh	TATTGTTTGTGGAAGCGTGAGC	22	27	21UR-1825
209387_adh	AATATTGTTTGTGGAAGCGTGAG	23	1	21UR-1825
2689710_adh	TTTAACAAATGACGGTAAATC	21	552	21UR-1832
2689711_adh	TTTAACAAATGACGGTAAATCT	22	1	21UR-1832
2689712_adh	TTTAACAAATGACGGTAAATCTAATC	26	1	21UR-1832
2502236_adh	TGTGAAGCGAGATTGTTCAAC	21	2185	21UR-1833
2502237_adh	TGTGAAGCGAGATTGTTCAACA	22	2	21UR-1833
242153_adh	AATGTGAAGCGAGATTGTTCAAC	23	3	21UR-1833

2502238_adh	TGTGAAGCGAGATTGTTCAACAA	23	2	21UR-1833
2502239_adh	TGTGAAGCGAGATTGTTCAACAAA	24	1	21UR-1833
2683988_adh	TTGTTCTTCGTTCGGTCCAAA	21	4072	21UR-1838
1118902_adh	CTTGTTCTTCGTTCGGTCCAAA	22	1	21UR-1838
1758000_adh	TAAAGGCAGAATTTTATCAAC	21	1646	21UR-1848
1758001_adh	TAAAGGCAGAATTTTATCAACA	22	5	21UR-1848
1758002_adh	TAAAGGCAGAATTTTATCAACAA	23	4	21UR-1848
1758003_adh	TAAAGGCAGAATTTTATCAACAAAAA	26	2	21UR-1848
2302361_adh	TGCGGTAGATAAAGAAGTACT	21	670	21UR-1850
2302369_adh	TGCGGTAGATAAAGAAGTACTT	22	2	21UR-1850
230648_adh	AATGCGGTAGATAAAGAAGTACT	23	1	21UR-1850
2304594_adh	TGCGTTGAAGACTACTCCTGA	21	609	21UR-1861
2304595_adh	TGCGTTGAAGACTACTCCTGAA	22	15	21UR-1861
2304596_adh	TGCGTTGAAGACTACTCCTGAAA	23	18	21UR-1861
2304597_adh	TGCGTTGAAGACTACTCCTGAAAA	24	3	21UR-1861
1942418_adh	TCAATGTACTCGTTGGTTTCT	21	651	21UR-1874
1043680_adh	CTCAATGTACTCGTTGGTTTCT	22	1	21UR-1874
1914299_adh	TATGGAGTCGGTATATTTTGA	21	631	21UR-1876
1914300_adh	TATGGAGTCGGTATATTTTGAA	22	4	21UR-1876
616846_adh	ATATGGAGTCGGTATATTTTGA	22	1	21UR-1876
2554415_adh	TTACAGTATTGGTAAGGTGGA	21	91	21UR-1882
2554416_adh	TTACAGTATTGGTAAGGTGGAAAGT	25	1	21UR-1882
2291205_adh	TGCATTGAAACGAACAACTAC	21	63	21UR-1884
2291207_adh	TGCATTGAAACGAACAACTACT	22	2	21UR-1884
2291208_adh	TGCATTGAAACGAACAACTACTA	23	1	21UR-1884
1889632_adh	TATATAATTAAAGAACATTGA	21	39	21UR-1892
1889633_adh	TATATAATTAAAGAACATTGAA	22	3	21UR-1892
1837639_adh	TAGAAAGTTTGGACAATTTTT	21	14	21UR-1895
1837640_adh	TAGAAAGTTTGGACAATTTTTCGGTA	26	1	21UR-1895
1869158_adh	TAGGGGCATGGTTTATTGAAC	21	531	21UR-1919
1035710_adh	CTAGGGGCATGGTTTATTGAAC	22	2	21UR-1919
1869159_adh	TAGGGGCATGGTTTATTGAACA	22	6	21UR-1919
330801_adh	ACTAGGGGCATGGTTTATTGAACA	24	1	21UR-1919
1901668_adh	TATGAACTTCGTAAGAAAATA	21	9	21UR-1941
615378_adh	ATATGAACTTCGTAAGAAAATA	22	1	21UR-1941
1940291_adh	TCAAGTCAGACAAATGTGCTT	21	3660	21UR-1943
1940293_adh	TCAAGTCAGACAAATGTGCTTC	22	1	21UR-1943
210429_adh	AATCAAGTCAGACAAATGTGCTT	23	7	21UR-1943
1823439_adh	TACGAGGGTGACAAAGAATGG	21	1528	21UR-1949
1823440_adh	TACGAGGGTGACAAAGAATGGA	22	10	21UR-1949
1823441_adh	TACGAGGGTGACAAAGAATGGAA	23	5	21UR-1949
203994_adh	AATACGAGGGTGACAAAGAATGG	23	3	21UR-1949
1823442_adh	TACGAGGGTGACAAAGAATGGAATT	25	1	21UR-1949
2672100_adh	TTGGTTCCTGCTATTAGAACC	21	432	21UR-1956
732928_adh	ATTGGTTCCTGCTATTAGAACC	22	2	21UR-1956
253216_adh	AATTGGTTCCTGCTATTAGAACC	23	3	21UR-1956
2313216_adh	TGGAAAAATCCTGCATTGTAA	21	64	21UR-1960
678600_adh	ATGGAAAAATCCTGCATTGTAA	22	1	21UR-1960
231542_adh	AATGGAAAAATCCTGCATTGTAA	23	2	21UR-1960
1893092_adh	TATCACTCCATTGAAATATTG	21	14	21UR-1995

613448_adh	ATATCACTCCATTGAAATATTG	22	1	21UR-1995
2304533_adh	TGCGTTCTGTATTGTATACCG	21	226	21UR-2000
676836_adh	ATGCGTTCTGTATTGTATACCG	22	3	21UR-2000
2304534_adh	TGCGTTCTGTATTGTATACCGAA	23	2	21UR-2000
1872083_adh	TAGGTGTTGAAGAAAATTAAC	21	17	21UR-2008
1872084_adh	TAGGTGTTGAAGAAAATTAACGAT	24	1	21UR-2008
1946637_adh	TCACCAAGAATTCGGTTACTC	21	2952	21UR-2014
625769_adh	ATCACCAAGAATTCGGTTACTC	22	2	21UR-2014
2031245_adh	TCTACAGAACACTATGGCGAG	21	472	21UR-2055
2031250_adh	TCTACAGAACACTATGGCGAGT	22	2	21UR-2055
651580_adh	ATCTACAGAACACTATGGCGAG	22	1	21UR-2055
2031253_adh	TCTACAGAACACTATGGCGAGTT	23	1	21UR-2055
221316_adh	AATCTACAGAACACTATGGCGAG	23	2	21UR-2055
1945571_adh	TCACATACAAGGCGCGGCTTA	21	278	21UR-2061
625670_adh	ATCACATACAAGGCGCGGCTTA	22	1	21UR-2061
212500_adh	AATCACATACAAGGCGCGGCTTA	23	2	21UR-2061
2269721_adh	TGATCCTTTTGCAATTGACGA	21	112	21UR-2062
228011_adh	AATGATCCTTTTGCAATTGACGA	23	1	21UR-2062
1800825_adh	TAATGGTGTTGCCAGAAGATA	21	19	21UR-2063
589999_adh	ATAATGGTGTTGCCAGAAGATA	22	1	21UR-2063
1761035_adh	TAAATCTATTGAAGAGGACTG	21	691	21UR-2070
199452_adh	AATAAATCTATTGAAGAGGACTG	23	2	21UR-2070
1967490_adh	TCAGTTGAGTTGAAAAAAGGT	21	87	21UR-2091
1967491_adh	TCAGTTGAGTTGAAAAAAGGTT	22	1	21UR-2091
2701676_adh	TTTCAATATCATTTTTGAGTC	21	1	21UR-2094
255136_adh	AATTTCAATATCATTTTTGAGTC	23	1	21UR-2094
2450000_adh	TGGGCAGGTATTAATTCGAGT	21	489	21UR-2102
2450002_adh	TGGGCAGGTATTAATTCGAGTT	22	1	21UR-2102
1852894_adh	TAGCAAATCTGTAAATTTTGC	21	60	21UR-2104
1852895_adh	TAGCAAATCTGTAAATTTTGCA	22	1	21UR-2104
2669825_adh	TTGGTAGGCGTCATTAAAACC	21	332	21UR-2127
732363_adh	ATTGGTAGGCGTCATTAAAACC	22	1	21UR-2127
253029_adh	AATTGGTAGGCGTCATTAAAACC	23	3	21UR-2127
2669827_adh	TTGGTAGGCGTCATTAAAACCGAT	24	1	21UR-2127
1913819_adh	TATGGAATCGCACGGTGTAGA	21	194	21UR-2147
1913821_adh	TATGGAATCGCACGGTGTAGAT	22	1	21UR-2147
1973045_adh	TCATGTTATACTATTAGCATA	21	19	21UR-2148
214448_adh	AATCATGTTATACTATTAGCATA	23	1	21UR-2148
1858958_adh	TAGCTTAGTTCTGTTACTCAT	21	20	21UR-2156
1858959_adh	TAGCTTAGTTCTGTTACTCATTA	23	1	21UR-2156
2519062_adh	TGTGTATGTCGTCCCAAAAAA	21	81	21UR-2157
242664_adh	AATGTGTATGTCGTCCCAAAAAA	23	1	21UR-2157
2725074_adh	TTTGGGTTTGTCGGCTATTGC	21	131	21UR-2161
2725075_adh	TTTGGGTTTGTCGGCTATTGCT	22	2	21UR-2161
2672970_adh	TTGGTTGTGTTTACAAATGTG	21	2	21UR-2163
574752_adh	AGTTGGTTGTGTTTACAAATGTGCTG	26	1	21UR-2163
1861542_adh	TAGGAATATAGCATTAAATAA	21	7176	21UR-2164
1861544_adh	TAGGAATATAGCATTAAATAAG	22	2	21UR-2164
1861545_adh	TAGGAATATAGCATTAAATAAGCAT	25	1	21UR-2164
1766436_adh	TAACATTGTCGTAGAGGAACA	21	226	21UR-2172

1766437_adh	TAACATTGTCGTAGAGGAACAAAA	24	1	21UR-2172
1801630_adh	TAATGTCAGTTGAAAATGTGC	21	64	21UR-2173
1801631_adh	TAATGTCAGTTGAAAATGTGCA	22	1	21UR-2173
1874032_adh	TAGTACTGGCACTCTAGAGAA	21	141	21UR-2179
1874033_adh	TAGTACTGGCACTCTAGAGAAA	22	1	21UR-2179
1874034_adh	TAGTACTGGCACTCTAGAGAAAA	23	1	21UR-2179
1770978_adh	TAACTGACGATACGGCAATAT	21	1635	21UR-2187
584951_adh	ATAACTGACGATACGGCAATAT	22	1	21UR-2187
200208_adh	AATAACTGACGATACGGCAATAT	23	6	21UR-2187
1770980_adh	TAACTGACGATACGGCAATATCTGG	25	1	21UR-2187
2698943_adh	TTTATGACATGTTGGAGTTTT	21	1304	21UR-2200
2698945_adh	TTTATGACATGTTGGAGTTTTT	22	5	21UR-2200
1899393_adh	TATCTCACATCTATTATCATC	21	1	21UR-2206
1899394_adh	TATCTCACATCTATTATCATCA	22	1	21UR-2206
1812489_adh	TACAGGATCAGTGAAAGGTGA	21	296	21UR-2207
1812490_adh	TACAGGATCAGTGAAAGGTGAA	22	1	21UR-2207
1812492_adh	TACAGGATCAGTGAAAGGTGAAG	23	1	21UR-2207
1812493_adh	TACAGGATCAGTGAAAGGTGAAGT	24	1	21UR-2207
1812494_adh	TACAGGATCAGTGAAAGGTGAAGTG	25	1	21UR-2207
1850039_adh	TAGATAGAGACTGCATGATTT	21	15530	21UR-2235
1850044_adh	TAGATAGAGACTGCATGATTTT	22	18	21UR-2235
1850053_adh	TAGATAGAGACTGCATGATTTTT	23	3	21UR-2235
1850054_adh	TAGATAGAGACTGCATGATTTTTG	24	1	21UR-2235
1850055_adh	TAGATAGAGACTGCATGATTTTTGCT	26	1	21UR-2235
1970733_adh	TCATCCAAGGACAATGCGGAT	21	122	21UR-2236
1970735_adh	TCATCCAAGGACAATGCGGATT	22	1	21UR-2236
2309433_adh	TGCTGTCATGTTTCACTGGGT	21	14	21UR-2240
677952_adh	ATGCTGTCATGTTTCACTGGGT	22	1	21UR-2240
1932172_adh	TCAAAAGAAAGCTTGACGGCA	21	210	21UR-2243
209555_adh	AATCAAAAGAAAGCTTGACGGCA	23	1	21UR-2243
2073790_adh	TGAAACTCTTTTCTGATCGTC	21	16	21UR-2246
2073791_adh	TGAAACTCTTTTCTGATCGTCAA	23	1	21UR-2246
2545097_adh	TTAACATTTTTGGCATTTTCG	21	34	21UR-2261
2545098_adh	TTAACATTTTTGGCATTTTCGGACGA	26	1	21UR-2261
1831936_adh	TACTGAAGAAGACGGACAAAG	21	3821	21UR-2268
1831939_adh	TACTGAAGAAGACGGACAAAGC	22	5	21UR-2268
1831940_adh	TACTGAAGAAGACGGACAAAGCC	23	1	21UR-2268
204644_adh	AATACTGAAGAAGACGGACAAAG	23	2	21UR-2268
1812111_adh	TACAGATTCTGACAAAAATAA	21	108	21UR-2275
593108_adh	ATACAGATTCTGACAAAAATAA	22	1	21UR-2275
2018774_adh	TCGGTCGTTGTGCATAAAGTT	21	73	21UR-2286
2018775_adh	TCGGTCGTTGTGCATAAAGTTTAA	24	1	21UR-2286
2018776_adh	TCGGTCGTTGTGCATAAAGTTTAAA	25	1	21UR-2286
2542617_adh	TTAAATCACCATTATCGTCTC	21	362	21UR-2287
2542618_adh	TTAAATCACCATTATCGTCTCAA	23	2	21UR-2287
1986659_adh	TCCGGGAGATTTAATTATCAC	21	3	21UR-2293
215646_adh	AATCCGGGAGATTTAATTATCAC	23	1	21UR-2293
1854239_adh	TAGCACTCGGCATGTTTTTCT	21	2568	21UR-2305
1854240_adh	TAGCACTCGGCATGTTTTTCTAA	23	5	21UR-2305
2006292_adh	TCGCAGCTGAAAGATGTTTTG	21	3337	21UR-2308

2006293_adh	TCGCAGCTGAAAGATGTTTTGA	22	1	21UR-2308
2006294_adh	TCGCAGCTGAAAGATGTTTTGAA	23	15	21UR-2308
2284922_adh	TGCAATGTACAACAGGACAAT	21	421	21UR-2320
229169_adh	AATGCAATGTACAACAGGACAAT	23	1	21UR-2320
1888201_adh	TATAGCACTCGTTTGGGCATT	21	499	21UR-2322
1888203_adh	TATAGCACTCGTTTGGGCATTT	22	1	21UR-2322
2138790_adh	TGAATAGGCTGGCTTGATGGA	21	1303	21UR-2324
2138791_adh	TGAATAGGCTGGCTTGATGGAA	22	3	21UR-2324
2138792_adh	TGAATAGGCTGGCTTGATGGAAA	23	6	21UR-2324
2138793_adh	TGAATAGGCTGGCTTGATGGAAAT	24	1	21UR-2324
1921286_adh	TATTAGAAGGAACGGCGGACA	21	2419	21UR-2325
1921288_adh	TATTAGAAGGAACGGCGGACAT	22	10	21UR-2325
619211_adh	ATATTAGAAGGAACGGCGGACA	22	4	21UR-2325
1773947_adh	TAAGAACTGGCTTCTGTGTGG	21	51	21UR-2327
1773948_adh	TAAGAACTGGCTTCTGTGTGGA	22	1	21UR-2327
1773949_adh	TAAGAACTGGCTTCTGTGTGGAA	23	1	21UR-2327
200311_adh	AATAAGAACTGGCTTCTGTGTGG	23	1	21UR-2327
1966988_adh	TCAGTGGATGTTAATTTCTGG	21	8	21UR-2330
1966989_adh	TCAGTGGATGTTAATTTCTGGA	22	1	21UR-2330
1843859_adh	TAGAATTCGTGATTACAAAAA	21	22	21UR-2343
1843860_adh	TAGAATTCGTGATTACAAAAAA	22	1	21UR-2343
1843861_adh	TAGAATTCGTGATTACAAAAAAGTA	25	1	21UR-2343
2268391_adh	TGATATTGCATATTTTGGTCG	21	139	21UR-2366
227929_adh	AATGATATTGCATATTTTGGTCG	23	4	21UR-2366
2248625_adh	TGAGGTCTGCAACTTGTTCCA	21	424	21UR-2378
2248626_adh	TGAGGTCTGCAACTTGTTCCAA	22	1	21UR-2378
227351_adh	AATGAGGTCTGCAACTTGTTCCA	23	1	21UR-2378
1975142_adh	TCATTTCGTCTTTAGGCTAAA	21	381	21UR-2388
631513_adh	ATCATTTCGTCTTTAGGCTAAA	22	1	21UR-2388
1988286_adh	TCCGTCGATTATTTTTGCATA	21	343	21UR-2392
215748_adh	AATCCGTCGATTATTTTTGCATA	23	16	21UR-2392
2583131_adh	TTATTGGCATATTTTGACAAT	21	657	21UR-2407
2583133_adh	TTATTGGCATATTTTGACAATT	22	1	21UR-2407
245858_adh	AATTATTGGCATATTTTGACAAT	23	3	21UR-2407
1788156_adh	TAAGTCTTTGATGGTCATTTC	21	498	21UR-2422
1788157_adh	TAAGTCTTTGATGGTCATTTCT	22	2	21UR-2422
200795_adh	AATAAGTCTTTGATGGTCATTTC	23	6	21UR-2422
2648419_adh	TTGCAACTTTGGTGTCATAGT	21	25	21UR-2423
251228_adh	AATTGCAACTTTGGTGTCATAGTGT	25	1	21UR-2423
1912733_adh	TATGCCCTTCACTGTGTGAAT	21	79	21UR-2433
1912734_adh	TATGCCCTTCACTGTGTGAATT	22	1	21UR-2433
2289704_adh	TGCATACAACGTTGCCGTATG	21	272	21UR-2436
673799_adh	ATGCATACAACGTTGCCGTATG	22	1	21UR-2436
229498_adh	AATGCATACAACGTTGCCGTATG	23	2	21UR-2436
1918915_adh	TATGTGAGAGAAAGTGACTCT	21	53	21UR-2444
618662_adh	ATATGTGAGAGAAAGTGACTCT	22	1	21UR-2444
2162030_adh	TGACGTAAAAATACTTTCTAA	21	39	21UR-2449
226423_adh	AATGACGTAAAAATACTTTCTAA	23	1	21UR-2449
2117138_adh	TGAAATTGTAGTAGACTGCTG	21	41347	21UR-2502
2117146_adh	TGAAATTGTAGTAGACTGCTGC	22	55	21UR-2502

2117147_adh	TGAAATTGTAGTAGACTGCTGCC	23	2	21UR-2502
224140_adh	AATGAAATTGTAGTAGACTGCTG	23	6	21UR-2502
2117148_adh	TGAAATTGTAGTAGACTGCTGCCA	24	1	21UR-2502
2117149_adh	TGAAATTGTAGTAGACTGCTGCCAA	25	3	21UR-2502
1891155_adh	TATCAAACAGATGGCCCAAAA	21	58	21UR-2504
1891156_adh	TATCAAACAGATGGCCCAAAAT	22	1	21UR-2504
1860435_adh	TAGGAAAGCCCCACTATGCTG	21	2	21UR-2516
1860436_adh	TAGGAAAGCCCCACTATGCTGC	22	1	21UR-2516
2410137_adh	TGGCATTGCAAAATCTAGGGT	21	144	21UR-2523
2410138_adh	TGGCATTGCAAAATCTAGGGTGT	23	1	21UR-2523
1773724_adh	TAAGAACCCGTTAACAAGATC	21	7	21UR-2533
1773725_adh	TAAGAACCCGTTAACAAGATCGA	23	1	21UR-2533
2182153_adh	TGAGACAAGAAAGTTTTGGTT	21	4132	21UR-2543
2182156_adh	TGAGACAAGAAAGTTTTGGTTT	22	4	21UR-2543
2639623_adh	TTGAGACAAGAAAGTTTTGGTTT	23	1	21UR-2543
1760575_adh	TAAATCCATACCAGTAGGACA	21	281	21UR-2549
1760576_adh	TAAATCCATACCAGTAGGACAAAAA	25	1	21UR-2549
1845891_adh	TAGACTGATCTGACTGTAAGA	21	57	21UR-2555
1845892_adh	TAGACTGATCTGACTGTAAGAA	22	2	21UR-2555
2587195_adh	TTCAAGGAACTAACGTTGGGA	21	1070	21UR-2572
2587198_adh	TTCAAGGAACTAACGTTGGGAC	22	2	21UR-2572
711273_adh	ATTCAAGGAACTAACGTTGGGA	22	2	21UR-2572
246019_adh	AATTCAAGGAACTAACGTTGGGA	23	2	21UR-2572
2669863_adh	TTGGTAGGGAGGCAAACTAGT	21	284	21UR-2582
253030_adh	AATTGGTAGGGAGGCAAACTAGT	23	1	21UR-2582
2478982_adh	TGGTTTTATAGTAGTAGGCTC	21	2756	21UR-2589
2478988_adh	TGGTTTTATAGTAGTAGGCTCT	22	2	21UR-2589
2478991_adh	TGGTTTTATAGTAGTAGGCTCTTATG	26	1	21UR-2589
1847324_adh	TAGAGAATTTGTAGATGTCTC	21	585	21UR-2596
1847325_adh	TAGAGAATTTGTAGATGTCTCA	22	9	21UR-2596
1847327_adh	TAGAGAATTTGTAGATGTCTCAT	23	1	21UR-2596
2625919_adh	TTGAACACGGTTTCGGAATGT	21	34	21UR-2604
723275_adh	ATTGAACACGGTTTCGGAATGT	22	1	21UR-2604
2082266_adh	TGAAAGGCAAAATAAGGATAA	21	169	21UR-2621
659943_adh	ATGAAAGGCAAAATAAGGATAA	22	1	21UR-2621
1759752_adh	TAAATAGGTCGAACAAAAAAT	21	64	21UR-2625
1759753_adh	TAAATAGGTCGAACAAAAAATT	22	1	21UR-2625
1788185_adh	TAAGTCTTTTACTCCCGATTT	21	5	21UR-2628
200796_adh	AATAAGTCTTTTACTCCCGATTT	23	1	21UR-2628
2012418_adh	TCGGAGAGTGCGTTTAGTTTT	21	29	21UR-2637
646266_adh	ATCGGAGAGTGCGTTTAGTTTT	22	1	21UR-2637
1848270_adh	TAGAGGAATGATTATGTTAGC	21	174	21UR-2656
1848271_adh	TAGAGGAATGATTATGTTAGCA	22	1	21UR-2656
1939482_adh	TCAAGGAACTAACGTTGGGAC	21	47	21UR-2657
1939483_adh	TCAAGGAACTAACGTTGGGACA	22	1	21UR-2657
2610856_adh	TTCGGTTTCTCCATACAAATT	21	173	21UR-2663
2610858_adh	TTCGGTTTCTCCATACAAATTT	22	1	21UR-2663
718867_adh	ATTCGGTTTCTCCATACAAATT	22	1	21UR-2663
1829027_adh	TACGTTGACTTTGTGCAGTGA	21	277	21UR-2674
1829033_adh	TACGTTGACTTTGTGCAGTGATG	23	1	21UR-2674

1829034_adh	TACGTTGACTTTGTGCAGTGATGT	24	1	21UR-2674
1917818_adh	TATGTAGTAACGGTTGCTGTT	21	4029	21UR-2683
1613110_adh	GTATGTAGTAACGGTTGCTGTT	22	1	21UR-2683
1917822_adh	TATGTAGTAACGGTTGCTGTTG	22	1	21UR-2683
1917823_adh	TATGTAGTAACGGTTGCTGTTGT	23	2	21UR-2683
1917824_adh	TATGTAGTAACGGTTGCTGTTGTG	24	1	21UR-2683
1986252_adh	TCCGGCATTGATCTGTGGAGG	21	1713	21UR-2686
1986253_adh	TCCGGCATTGATCTGTGGAGGA	22	51	21UR-2686
1986254_adh	TCCGGCATTGATCTGTGGAGGAA	23	10	21UR-2686
2595669_adh	TTCCAAAGAACGCGGGATTTC	21	169	21UR-2706
2595670_adh	TTCCAAAGAACGCGGGATTTCC	22	1	21UR-2706
2387649_adh	TGGATAAGTTGTATCTTCGGG	21	4590	21UR-2710
2387653_adh	TGGATAAGTTGTATCTTCGGGT	22	6	21UR-2710
2387656_adh	TGGATAAGTTGTATCTTCGGGTTT	24	1	21UR-2710
2387657_adh	TGGATAAGTTGTATCTTCGGGTTTAT	26	1	21UR-2710
1932555_adh	TCAAACACGCTGATTTCGTGG	21	8	21UR-2711
1932556_adh	TCAAACACGCTGATTTCGTGGA	22	1	21UR-2711
1844920_adh	TAGACACAGTTGATACGATTT	21	146	21UR-2729
1844924_adh	TAGACACAGTTGATACGATTTG	22	1	21UR-2729
1798976_adh	TAATCTTAGGCTGTGAAAAGA	21	48	21UR-2740
1798977_adh	TAATCTTAGGCTGTGAAAAGAA	22	1	21UR-2740
1860537_adh	TAGGAAAGGGGACTGCATTGT	21	437	21UR-2742
206561_adh	AATAGGAAAGGGGACTGCATTGT	23	1	21UR-2742
2658336_adh	TTGGACGAAATTGCAATATGT	21	681	21UR-2799
730524_adh	ATTGGACGAAATTGCAATATGT	22	1	21UR-2799
1787080_adh	TAAGGTTGGCGGGAGACAGTA	21	2209	21UR-2816
1787084_adh	TAAGGTTGGCGGGAGACAGTAT	22	23	21UR-2816
1962018_adh	TCAGATTTTGGACTACGATCG	21	58	21UR-2818
1962019_adh	TCAGATTTTGGACTACGATCGA	22	3	21UR-2818
1962021_adh	TCAGATTTTGGACTACGATCGAG	23	1	21UR-2818
1962022_adh	TCAGATTTTGGACTACGATCGAGTTA	26	1	21UR-2818
1822171_adh	TACGACCAAAAATGAGATTGA	21	1034	21UR-2829
1822173_adh	TACGACCAAAAATGAGATTGAG	22	6	21UR-2829
1822175_adh	TACGACCAAAAATGAGATTGAGT	23	2	21UR-2829
2596280_adh	TTCCACGGCTGAACAGGACAA	21	1696	21UR-2834
2596285_adh	TTCCACGGCTGAACAGGACAAT	22	5	21UR-2834
1568629_adh	GGTTCCACGGCTGAACAGGACAA	23	1	21UR-2834
2596286_adh	TTCCACGGCTGAACAGGACAATA	23	1	21UR-2834
1798705_adh	TAATCTACAAAGCAGGGAAAA	21	138	21UR-2854
589190_adh	ATAATCTACAAAGCAGGGAAAA	22	1	21UR-2854
1964258_adh	TCAGGCTTCGCTTCTAGTTGG	21	9	21UR-2857
1964259_adh	TCAGGCTTCGCTTCTAGTTGGA	22	1	21UR-2857
1964260_adh	TCAGGCTTCGCTTCTAGTTGGAA	23	1	21UR-2857
1814183_adh	TACATTAGAATGAGAAATTAA	21	248	21UR-2861
594054_adh	ATACATTAGAATGAGAAATTAA	22	1	21UR-2861
1812260_adh	TACAGCCCACTGCCATCTAGT	21	4	21UR-2862
1812261_adh	TACAGCCCACTGCCATCTAGTAAA	24	1	21UR-2862
2285404_adh	TGCAATTCAAGAAGATAACGC	21	175	21UR-2863
672823_adh	ATGCAATTCAAGAAGATAACGC	22	1	21UR-2863
2601132_adh	TTCCTTACGAAATCTCCATAA	21	41	21UR-2871

715841_adh	ATTCCTTACGAAATCTCCATAA	22	1	21UR-2871
246804_adh	AATTCCTTACGAAATCTCCATAA	23	1	21UR-2871
2601133_adh	TTCCTTACGAAATCTCCATAATT	23	1	21UR-2871
2309654_adh	TGCTGTGTAGGGCTATAATAA	21	1549	21UR-2872
2309655_adh	TGCTGTGTAGGGCTATAATAAA	22	2	21UR-2872
2309656_adh	TGCTGTGTAGGGCTATAATAAAA	23	4	21UR-2872
2309657_adh	TGCTGTGTAGGGCTATAATAAAAC	24	1	21UR-2872
2123971_adh	TGAACTACTAAAGACAGGAAA	21	434	21UR-2900
2123973_adh	TGAACTACTAAAGACAGGAAAT	22	1	21UR-2900
1834358_adh	TACTGTTTGCTTCTTTTTTGA	21	12	21UR-2904
1834359_adh	TACTGTTTGCTTCTTTTTTGAA	22	3	21UR-2904
2458960_adh	TGGGTGGTGGATGTGTACAAT	21	287	21UR-2906
2458961_adh	TGGGTGGTGGATGTGTACAATATAC	25	1	21UR-2906
2668571_adh	TTGGGTGGTGGATGTGTACAATATA	25	1	21UR-2906
2130932_adh	TGAAGCATGCATATCGGTCTG	21	6942	21UR-2909
2130933_adh	TGAAGCATGCATATCGGTCTGA	22	9	21UR-2909
662004_adh	ATGAAGCATGCATATCGGTCTG	22	2	21UR-2909
2130935_adh	TGAAGCATGCATATCGGTCTGAT	23	1	21UR-2909
224558_adh	AATGAAGCATGCATATCGGTCTG	23	16	21UR-2909
2289365_adh	TGCAGTTAAGGCTATTATTTC	21	96	21UR-2910
229480_adh	AATGCAGTTAAGGCTATTATTTC	23	1	21UR-2910
1823675_adh	TACGAGTTGTGCACTTTGGAA	21	22	21UR-2916
1823678_adh	TACGAGTTGTGCACTTTGGAAAAT	24	1	21UR-2916
2504319_adh	TGTGACGTTGAAATGAGTGGC	21	3007	21UR-2917
2504320_adh	TGTGACGTTGAAATGAGTGGCA	22	102	21UR-2917
2504322_adh	TGTGACGTTGAAATGAGTGGCAC	23	21	21UR-2917
2504323_adh	TGTGACGTTGAAATGAGTGGCACA	24	2	21UR-2917
2671775_adh	TTGGTTCAAAAATGAAGCTGT	21	494	21UR-2929
1115317_adh	CTTGGTTCAAAAATGAAGCTGT	22	1	21UR-2929
2671777_adh	TTGGTTCAAAAATGAAGCTGTC	22	2	21UR-2929
1806070_adh	TAATTTTGTAGGACTTCAATT	21	57	21UR-2934
1806071_adh	TAATTTTGTAGGACTTCAATTT	22	1	21UR-2934
1756867_adh	TAAAGATAGCTGTTTATATTT	21	175	21UR-2940
1024752_adh	CTAAAGATAGCTGTTTATATTT	22	1	21UR-2940
2296435_adh	TGCGACCGCGATACTTCTAAA	21	491	21UR-2943
2296436_adh	TGCGACCGCGATACTTCTAAAA	22	2	21UR-2943
675700_adh	ATGCGACCGCGATACTTCTAAA	22	1	21UR-2943
2296437_adh	TGCGACCGCGATACTTCTAAAAA	23	1	21UR-2943
1889381_adh	TATAGTTTCTCGACGGTGTGT	21	2976	21UR-2949
1889389_adh	TATAGTTTCTCGACGGTGTGTT	22	4	21UR-2949
1911881_adh	TATGATTAGGCGTTAGCAAAA	21	67	21UR-2977
1911882_adh	TATGATTAGGCGTTAGCAAAAAA	23	1	21UR-2977
2525636_adh	TGTTATTTGAATCAGACGACT	21	3586	21UR-3016
700430_adh	ATGTTATTTGAATCAGACGACT	22	1	21UR-3016
2590262_adh	TTCAGAAAGTAAGTGAAGCAA	21	63	21UR-3017
246128_adh	AATTCAGAAAGTAAGTGAAGCAA	23	2	21UR-3017
2296820_adh	TGCGACTTACGTTCTGTCGGA	21	243	21UR-3051
2296821_adh	TGCGACTTACGTTCTGTCGGAA	22	3	21UR-3051
2296822_adh	TGCGACTTACGTTCTGTCGGAAA	23	2	21UR-3051
1937924_adh	TCAACTTCAGATACGTAGTGA	21	696	21UR-3052

1937928_adh	TCAACTTCAGATACGTAGTGAT	22	2	21UR-3052
1829550_adh	TACTACATTTTCACGAAATCA	21	165	21UR-3054
204480_adh	AATACTACATTTTCACGAAATCA	23	4	21UR-3054
2586134_adh	TTCAACGTGTATGTTCGGGTT	21	30	21UR-3059
245971_adh	AATTCAACGTGTATGTTCGGGTT	23	1	21UR-3059
1985580_adh	TCCGCTTCCATTGCTGAATGA	21	9	21UR-3071
1985581_adh	TCCGCTTCCATTGCTGAATGAC	22	2	21UR-3071
2586055_adh	TTCAACGGCTGTACAAAAAGA	21	352	21UR-3074
2586056_adh	TTCAACGGCTGTACAAAAAGAAA	23	4	21UR-3074
2034783_adh	TCTATGTTGGCAATAAGCGGA	21	751	21UR-3078
2034784_adh	TCTATGTTGGCAATAAGCGGAA	22	1	21UR-3078
2034785_adh	TCTATGTTGGCAATAAGCGGAAA	23	5	21UR-3078
1924398_adh	TATTCGGAGGATTCAGCCTGC	21	3	21UR-3079
1924399_adh	TATTCGGAGGATTCAGCCTGCAA	23	1	21UR-3079
2288827_adh	TGCAGTCACAAGAGGATTCAC	21	277	21UR-3084
2288828_adh	TGCAGTCACAAGAGGATTCACGA	23	2	21UR-3084
1827827_adh	TACGTAGACGCCTTTAAAAGC	21	805	21UR-3085
1827828_adh	TACGTAGACGCCTTTAAAAGCAA	23	1	21UR-3085
204228_adh	AATACGTAGACGCCTTTAAAAGC	23	2	21UR-3085
1827829_adh	TACGTAGACGCCTTTAAAAGCAAA	24	1	21UR-3085
1917926_adh	TATGTAGTAATATGGGGATGG	21	3328	21UR-3106
1613111_adh	GTATGTAGTAATATGGGGATGG	22	1	21UR-3106
1917927_adh	TATGTAGTAATATGGGGATGGA	22	3	21UR-3106
1917929_adh	TATGTAGTAATATGGGGATGGAT	23	3	21UR-3106
2273579_adh	TGATGTGCAGGTCGCTTATGA	21	37	21UR-3112
228428_adh	AATGATGTGCAGGTCGCTTATGA	23	2	21UR-3112
2273580_adh	TGATGTGCAGGTCGCTTATGAAGA	24	1	21UR-3112
1875027_adh	TAGTAGTTTGTCGATATATAT	21	675	21UR-3114
1875028_adh	TAGTAGTTTGTCGATATATATA	22	2	21UR-3114
2286064_adh	TGCACAGGAGCAGCTTATTGT	21	34	21UR-3119
672916_adh	ATGCACAGGAGCAGCTTATTGT	22	1	21UR-3119
229204_adh	AATGCACAGGAGCAGCTTATTGT	23	1	21UR-3119
2037772_adh	TCTCAGTCGTTGATACATTGA	21	1406	21UR-3122
653311_adh	ATCTCAGTCGTTGATACATTGA	22	1	21UR-3122
1798941_adh	TAATCTGTTAACATCCGAATA	21	3	21UR-3123
201290_adh	AATAATCTGTTAACATCCGAATA	23	1	21UR-3123
1959850_adh	TCAGATACTGGTCCTTGGAAA	21	385	21UR-3124
1959852_adh	TCAGATACTGGTCCTTGGAAAT	22	1	21UR-3124
1959854_adh	TCAGATACTGGTCCTTGGAAATT	23	1	21UR-3124
2492297_adh	TGTATGTAAAACTTTACGGCA	21	15096	21UR-3129
2492298_adh	TGTATGTAAAACTTTACGGCAA	22	8	21UR-3129
695590_adh	ATGTATGTAAAACTTTACGGCA	22	8	21UR-3129
240900_adh	AATGTATGTAAAACTTTACGGCA	23	15	21UR-3129
2492301_adh	TGTATGTAAAACTTTACGGCAATA	24	2	21UR-3129
2492302_adh	TGTATGTAAAACTTTACGGCAATAT	25	3	21UR-3129
1920898_adh	TATTACGGCCGTACAGCAAAT	21	125	21UR-3168
1920899_adh	TATTACGGCCGTACAGCAAATT	22	1	21UR-3168
1872532_adh	TAGGTTGTTCGTAGCAGTGCT	21	62	21UR-3173
1872533_adh	TAGGTTGTTCGTAGCAGTGCTG	22	1	21UR-3173
206961_adh	AATAGGTTGTTCGTAGCAGTGCT	23	1	21UR-3173

2010522_adh	TCGGAACATAAAGAATGAACA	21	251	21UR-3180
1052088_adh	CTCGGAACATAAAGAATGAACA	22	1	21UR-3180
2010524_adh	TCGGAACATAAAGAATGAACAT	22	1	21UR-3180
334965_adh	ACTCGGAACATAAAGAATGAACAT	24	1	21UR-3180
2010526_adh	TCGGAACATAAAGAATGAACATTAT	25	1	21UR-3180
2295734_adh	TGCGAAGACTTTACATATCGT	21	661	21UR-3183
2295735_adh	TGCGAAGACTTTACATATCGTT	22	1	21UR-3183
2651731_adh	TTGCGAAGACTTTACATATCGT	22	1	21UR-3183
2061430_adh	TCTTTCTGTGGAAGCGTTTGA	21	2168	21UR-3184
2061443_adh	TCTTTCTGTGGAAGCGTTTGAT	22	9	21UR-3184
2061444_adh	TCTTTCTGTGGAAGCGTTTGATA	23	2	21UR-3184
2608649_adh	TTCGGCTACATTTCTCTCTTC	21	196	21UR-3191
2608651_adh	TTCGGCTACATTTCTCTCTTCC	22	1	21UR-3191
247479_adh	AATTCGGCTACATTTCTCTCTTC	23	2	21UR-3191
2558153_adh	TTACGTTGATCGACTAGTTCC	21	109	21UR-3196
2558155_adh	TTACGTTGATCGACTAGTTCCT	22	1	21UR-3196
705725_adh	ATTACGTTGATCGACTAGTTCC	22	1	21UR-3196
1814243_adh	TACATTATTGGCATATATAAC	21	9	21UR-3198
1814244_adh	TACATTATTGGCATATATAACT	22	1	21UR-3198
2475025_adh	TGGTTCCTCATTGAAGATTGA	21	7	21UR-3200
2672078_adh	TTGGTTCCTCATTGAAGATTGAA	23	1	21UR-3200
2421249_adh	TGGGACACACTTTTGGTTTTA	21	59	21UR-3203
237809_adh	AATGGGACACACTTTTGGTTTTA	23	1	21UR-3203
1787340_adh	TAAGTAACGGACTTTTCAATT	21	779	21UR-3213
200737_adh	AATAAGTAACGGACTTTTCAATT	23	4	21UR-3213
1787342_adh	TAAGTAACGGACTTTTCAATTACAT	25	2	21UR-3213
1863917_adh	TAGGAGGAAGCATTGTGCATA	21	1918	21UR-3219
1863918_adh	TAGGAGGAAGCATTGTGCATAA	22	2	21UR-3219
1863920_adh	TAGGAGGAAGCATTGTGCATAAC	23	1	21UR-3219
2119707_adh	TGAACAAGCGACTGCATAAAG	21	2026	21UR-3224
1062890_adh	CTGAACAAGCGACTGCATAAAG	22	3	21UR-3224
2119708_adh	TGAACAAGCGACTGCATAAAGAA	23	3	21UR-3224
2119709_adh	TGAACAAGCGACTGCATAAAGAAT	24	2	21UR-3224
2119710_adh	TGAACAAGCGACTGCATAAAGAATA	25	1	21UR-3224
2119711_adh	TGAACAAGCGACTGCATAAAGAATAA	26	2	21UR-3224
1851864_adh	TAGATTAAGTGTAGGCTATGA	21	141	21UR-3238
604437_adh	ATAGATTAAGTGTAGGCTATGA	22	1	21UR-3238
1789674_adh	TAAGTTTGAAAGATTTGAAGG	21	41	21UR-3242
1789675_adh	TAAGTTTGAAAGATTTGAAGGC	22	1	21UR-3242
2253312_adh	TGAGTGAACTTATCAGCAGGC	21	475	21UR-3246
2642665_adh	TTGAGTGAACTTATCAGCAGGC	22	37	21UR-3246
2253318_adh	TGAGTGAACTTATCAGCAGGCGGG	24	1	21UR-3246
2642668_adh	TTGAGTGAACTTATCAGCAGGCGGG	25	1	21UR-3246
1915304_adh	TATGGCACGTCATTTTATAAA	21	21	21UR-3248
1915305_adh	TATGGCACGTCATTTTATAAAT	22	1	21UR-3248
1929666_adh	TATTTCATGTCTTCGGTTTCA	21	1064	21UR-3260
1929667_adh	TATTTCATGTCTTCGGTTTCAT	22	1	21UR-3260
1897417_adh	TATCGCGCCTTGGAATGGATA	21	77	21UR-3267
1897418_adh	TATCGCGCCTTGGAATGGATAA	22	1	21UR-3267
1882914_adh	TAGTTTCCTCCAAACACGGCA	21	44	21UR-3269

1882915_adh	TAGTTTCCTCCAAACACGGCAA	22	1	21UR-3269
2303995_adh	TGCGTCTGACAAGGAAAGGAA	21	287	21UR-3282
2303998_adh	TGCGTCTGACAAGGAAAGGAAGT	23	2	21UR-3282
2419941_adh	TGGGAAGTTTGGTCTTACGGC	21	458	21UR-3292
2419942_adh	TGGGAAGTTTGGTCTTACGGCA	22	2	21UR-3292
2419943_adh	TGGGAAGTTTGGTCTTACGGCAA	23	9	21UR-3292
2419944_adh	TGGGAAGTTTGGTCTTACGGCAAA	24	1	21UR-3292
2169476_adh	TGACTGACTGACACTACTAGA	21	122	21UR-3324
2169477_adh	TGACTGACTGACACTACTAGAA	22	3	21UR-3324
2277470_adh	TGATTGTTGATTGTCGTTTTC	21	1694	21UR-3330
2277471_adh	TGATTGTTGATTGTCGTTTTCAA	23	3	21UR-3330
1900379_adh	TATCTTCGTTTTGGCAATAAC	21	839	21UR-3336
1900380_adh	TATCTTCGTTTTGGCAATAACT	22	1	21UR-3336
1978231_adh	TCCAGATGATGAACGGTAATT	21	5564	21UR-3339
1978232_adh	TCCAGATGATGAACGGTAATTA	22	1	21UR-3339
1978234_adh	TCCAGATGATGAACGGTAATTAC	23	1	21UR-3339
1978235_adh	TCCAGATGATGAACGGTAATTACAA	25	1	21UR-3339
1908510_adh	TATGACTAGTGCAAATTTCTG	21	20	21UR-3344
1908512_adh	TATGACTAGTGCAAATTTCTGTT	23	1	21UR-3344
1962078_adh	TCAGCAAATGATACAAAATTG	21	2	21UR-3350
1962079_adh	TCAGCAAATGATACAAAATTGA	22	1	21UR-3350
1912002_adh	TATGATTGACCGAATAATTGA	21	29	21UR-3356
1912004_adh	TATGATTGACCGAATAATTGAAAT	24	1	21UR-3356
2004213_adh	TCGATGGCTATGTGGACTATT	21	175	21UR-3363
2004214_adh	TCGATGGCTATGTGGACTATTT	22	3	21UR-3363
2004215_adh	TCGATGGCTATGTGGACTATTTAGA	25	1	21UR-3363
2584324_adh	TTATTTGTGTGCCGTCGTTGG	21	1446	21UR-3375
2584328_adh	TTATTTGTGTGCCGTCGTTGGT	22	3	21UR-3375
2584332_adh	TTATTTGTGTGCCGTCGTTGGTT	23	1	21UR-3375
1830322_adh	TACTAGTCCTATCAACTGAGA	21	26	21UR-3379
1830323_adh	TACTAGTCCTATCAACTGAGATAC	24	1	21UR-3379
2307887_adh	TGCTCTGGACAGAACGTTAGT	21	395	21UR-3381
2307889_adh	TGCTCTGGACAGAACGTTAGTT	22	1	21UR-3381
677408_adh	ATGCTCTGGACAGAACGTTAGT	22	2	21UR-3381
625414_adh	ATCAATTTTGGTAATTAATCCG	22	1	21UR-3386
2126432_adh	TGAACTTAGAGCTAGACAATC	21	526	21UR-3387
2126433_adh	TGAACTTAGAGCTAGACAATCGG	23	2	21UR-3387
2644893_adh	TTGATGATTGAAAGTTGTGCG	21	46	21UR-3395
251029_adh	AATTGATGATTGAAAGTTGTGCG	23	4	21UR-3395
2644895_adh	TTGATGATTGAAAGTTGTGCGTTT	24	1	21UR-3395
2258857_adh	TGATAAGCTATGAAAAGAACG	21	278	21UR-3399
2258858_adh	TGATAAGCTATGAAAAGAACGG	22	2	21UR-3399
227606_adh	AATGATAAGCTATGAAAAGAACG	23	1	21UR-3399
2390247_adh	TGGATCTAAAATTTGTAGAAC	21	201	21UR-3405
233239_adh	AATGGATCTAAAATTTGTAGAAC	23	1	21UR-3405
2593866_adh	TTCATCAAGCTAAAATCAGAA	21	77	21UR-3407
712728_adh	ATTCATCAAGCTAAAATCAGAA	22	2	21UR-3407
246253_adh	AATTCATCAAGCTAAAATCAGAA	23	5	21UR-3407
2593867_adh	TTCATCAAGCTAAAATCAGAAAA	23	1	21UR-3407
1868279_adh	TAGGGAATGACTTTTTTCCCT	21	8	21UR-3445

206771_adh	AATAGGGAATGACTTTTTTCCCT	23	1	21UR-3445
2420769_adh	TGGGAATGATAAATGAATGAA	21	11	21UR-3467
2420770_adh	TGGGAATGATAAATGAATGAAGA	23	1	21UR-3467
1865820_adh	TAGGATGGTACTATTTCATTC	21	660	21UR-3468
607327_adh	ATAGGATGGTACTATTTCATTC	22	2	21UR-3468
1865821_adh	TAGGATGGTACTATTTCATTCAA	23	1	21UR-3468
1777123_adh	TAAGAGGAGATAACGAACTGA	21	3007	21UR-3470
1777131_adh	TAAGAGGAGATAACGAACTGAT	22	7	21UR-3470
586105_adh	ATAAGAGGAGATAACGAACTGA	22	2	21UR-3470
1777138_adh	TAAGAGGAGATAACGAACTGATTG	24	2	21UR-3470
1777139_adh	TAAGAGGAGATAACGAACTGATTGT	25	5	21UR-3470
1814873_adh	TACCAATCTTCTTATTATTGG	21	8	21UR-3486
203523_adh	AATACCAATCTTCTTATTATTGG	23	1	21UR-3486
1830720_adh	TACTATTCTGGCCAACTTTGA	21	26	21UR-3498
1830724_adh	TACTATTCTGGCCAACTTTGAC	22	1	21UR-3498
1928262_adh	TATTGTTATTTGATTGGCATG	21	2851	21UR-3502
621736_adh	ATATTGTTATTTGATTGGCATG	22	2	21UR-3502
209372_adh	AATATTGTTATTTGATTGGCATG	23	45	21UR-3502
2569676_adh	TTAGTAAACTGGTAGACAATA	21	26	21UR-3509
244978_adh	AATTAGTAAACTGGTAGACAATA	23	1	21UR-3509
2486733_adh	TGTACCTTTGTAGATTGCTTT	21	357	21UR-3522
2486734_adh	TGTACCTTTGTAGATTGCTTTA	22	3	21UR-3522
2486735_adh	TGTACCTTTGTAGATTGCTTTAA	23	31	21UR-3522
2486736_adh	TGTACCTTTGTAGATTGCTTTAAA	24	1	21UR-3522
2184689_adh	TGAGACTGCCGAAACAACTCA	21	118	21UR-3527
2184690_adh	TGAGACTGCCGAAACAACTCAG	22	1	21UR-3527
226877_adh	AATGAGACTGCCGAAACAACTCA	23	1	21UR-3527
2182984_adh	TGAGACATAGTTAGGCACAAA	21	654	21UR-3530
2182988_adh	TGAGACATAGTTAGGCACAAAG	22	10	21UR-3530
2182989_adh	TGAGACATAGTTAGGCACAAAGT	23	8	21UR-3530
1833170_adh	TACTGGAATTTTATTGTAACT	21	42	21UR-3538
1833171_adh	TACTGGAATTTTATTGTAACTAT	23	1	21UR-3538
2136401_adh	TGAATAAAGGCCGAAGGCAAA	21	74	21UR-3559
1065031_adh	CTGAATAAAGGCCGAAGGCAAA	22	1	21UR-3559
2136404_adh	TGAATAAAGGCCGAAGGCAAAC	22	1	21UR-3559
1884735_adh	TATAACGGTTAACTTAGAGCC	21	58	21UR-3573
1884736_adh	TATAACGGTTAACTTAGAGCCA	22	2	21UR-3573
1824233_adh	TACGATGGATATTTTGTTGGT	21	288	21UR-3581
596459_adh	ATACGATGGATATTTTGTTGGT	22	1	21UR-3581
204021_adh	AATACGATGGATATTTTGTTGGT	23	1	21UR-3581
2141581_adh	TGAATCATGTAGAATTGTCAA	21	39136	21UR-3588
2141582_adh	TGAATCATGTAGAATTGTCAAA	22	4	21UR-3588
2141583_adh	TGAATCATGTAGAATTGTCAAAA	23	26	21UR-3588
2141585_adh	TGAATCATGTAGAATTGTCAAAAG	24	2	21UR-3588
2141586_adh	TGAATCATGTAGAATTGTCAAAAGT	25	8	21UR-3588
2141587_adh	TGAATCATGTAGAATTGTCAAAAGTA	26	2	21UR-3588
2031713_adh	TCTACGGCTTGGAGAAAAACC	21	687	21UR-3601
2031714_adh	TCTACGGCTTGGAGAAAAACCT	22	1	21UR-3601
2031717_adh	TCTACGGCTTGGAGAAAAACCTTT	24	1	21UR-3601
2031718_adh	TCTACGGCTTGGAGAAAAACCTTTTG	26	1	21UR-3601

2075571_adh	TGAAAGAATGCAGAAATTATG	21	2464	21UR-3604
1062393_adh	CTGAAAGAATGCAGAAATTATG	22	1	21UR-3604
2075574_adh	TGAAAGAATGCAGAAATTATGC	22	2	21UR-3604
2075575_adh	TGAAAGAATGCAGAAATTATGCGAA	25	1	21UR-3604
2731174_adh	TTTGTTGACGTATACGCAATT	21	1789	21UR-3613
1123657_adh	CTTTGTTGACGTATACGCAATT	22	1	21UR-3613
1748207_adh	TAAAACGAGAACTGGCATGAT	21	806	21UR-3636
198766_adh	AATAAAACGAGAACTGGCATGAT	23	2	21UR-3636
1748209_adh	TAAAACGAGAACTGGCATGATACT	24	2	21UR-3636
1863417_adh	TAGGAGAAACAAGTGTGTTGT	21	107	21UR-3639
206612_adh	AATAGGAGAAACAAGTGTGTTGT	23	1	21UR-3639
2320152_adh	TGGAACGATGTGCAACGAAAA	21	2946	21UR-3642
1076015_adh	CTGGAACGATGTGCAACGAAAA	22	7	21UR-3642
2320156_adh	TGGAACGATGTGCAACGAAAAT	22	25	21UR-3642
2320161_adh	TGGAACGATGTGCAACGAAAATT	23	2	21UR-3642
2320162_adh	TGGAACGATGTGCAACGAAAATTAT	25	1	21UR-3642
343495_adh	ACTGGAACGATGTGCAACGAAAATT	25	1	21UR-3642
1813046_adh	TACATAAGCGGTAAGAGTCCA	21	244	21UR-3644
1813047_adh	TACATAAGCGGTAAGAGTCCAGAAT	25	1	21UR-3644
1962084_adh	TCAGCAACCATCGTGTCCCAA	21	2	21UR-3649
213445_adh	AATCAGCAACCATCGTGTCCCAA	23	1	21UR-3649
2041640_adh	TCTCGTGGATAAATAAGCATT	21	1320	21UR-3651
1058260_adh	CTCTCGTGGATAAATAAGCATT	22	1	21UR-3651
2041642_adh	TCTCGTGGATAAATAAGCATTT	22	3	21UR-3651
2041647_adh	TCTCGTGGATAAATAAGCATTTGA	24	2	21UR-3651
2301714_adh	TGCGGCGTAGATATGACATGA	21	43	21UR-3660
2301715_adh	TGCGGCGTAGATATGACATGAT	22	2	21UR-3660
2616367_adh	TTCTCCTACTTTCCAGGCAGA	21	63	21UR-3667
248827_adh	AATTCTCCTACTTTCCAGGCAGA	23	1	21UR-3667
2616368_adh	TTCTCCTACTTTCCAGGCAGAAA	23	2	21UR-3667
2030683_adh	TCTAATGCAGAAATTTTGTTC	21	1000	21UR-3681
2030684_adh	TCTAATGCAGAAATTTTGTTCA	22	1	21UR-3681
2030686_adh	TCTAATGCAGAAATTTTGTTCAC	23	1	21UR-3681
2030687_adh	TCTAATGCAGAAATTTTGTTCACT	24	1	21UR-3681
1812881_adh	TACAGTTAGAGAAGCATTAAC	21	48	21UR-3685
1812882_adh	TACAGTTAGAGAAGCATTAACATTT	25	1	21UR-3685
1853081_adh	TAGCAACGGTATTGTAATCAA	21	1057	21UR-3692
1853082_adh	TAGCAACGGTATTGTAATCAAAAAT	25	2	21UR-3692
2152464_adh	TGACAATGTAGCGTTAGCAAT	21	318	21UR-3708
2152465_adh	TGACAATGTAGCGTTAGCAATA	22	1	21UR-3708
1834944_adh	TACTTCGTGGCTAACTATCGT	21	52	21UR-3716
1834945_adh	TACTTCGTGGCTAACTATCGTA	22	1	21UR-3716
1868955_adh	TAGGGCTTTCTACTTTTTTGA	21	5	21UR-3717
1868956_adh	TAGGGCTTTCTACTTTTTTGAA	22	2	21UR-3717
2738933_adh	TTTTCGTTGTGGACAATTTGG	21	1745	21UR-3730
2738942_adh	TTTTCGTTGTGGACAATTTGGT	22	10	21UR-3730
2738943_adh	TTTTCGTTGTGGACAATTTGGTAT	24	19	21UR-3730
2738945_adh	TTTTCGTTGTGGACAATTTGGTATT	25	12	21UR-3730
1789011_adh	TAAGTTAGGCGTAATACTTGT	21	643	21UR-3742
539978_adh	AGTAAGTTAGGCGTAATACTTGT	23	1	21UR-3742

2323594_adh	TGGAAGCTTGATGGAAAATGC	21	1033	21UR-3766
2323595_adh	TGGAAGCTTGATGGAAAATGCA	22	1	21UR-3766
2323596_adh	TGGAAGCTTGATGGAAAATGCAA	23	2	21UR-3766
2323597_adh	TGGAAGCTTGATGGAAAATGCAAA	24	1	21UR-3766
2149922_adh	TGACAACGGCTTTGTGAGAAA	21	1469	21UR-3775
2149923_adh	TGACAACGGCTTTGTGAGAAAA	22	6	21UR-3775
2149924_adh	TGACAACGGCTTTGTGAGAAAAA	23	5	21UR-3775
1996293_adh	TCGAAGATGACGATGACCTTA	21	2014	21UR-3779
1996294_adh	TCGAAGATGACGATGACCTTAA	22	1	21UR-3779
2296296_adh	TGCGACACCGATAAGAGAACA	21	41	21UR-3788
2296297_adh	TGCGACACCGATAAGAGAACAAA	23	3	21UR-3788
1912228_adh	TATGATTTCATCAGATGGTTC	21	8	21UR-3798
208576_adh	AATATGATTTCATCAGATGGTTC	23	1	21UR-3798
2374477_adh	TGGACCAAGGATAATGTCAGC	21	2502	21UR-3802
2374480_adh	TGGACCAAGGATAATGTCAGCC	22	1	21UR-3802
2568304_adh	TTAGGATTAGTTGTACAGCCC	21	1371	21UR-3805
2568306_adh	TTAGGATTAGTTGTACAGCCCT	22	1	21UR-3805
2670298_adh	TTGGTCGTCGATCAGGCAAAG	21	29	21UR-3808
2670299_adh	TTGGTCGTCGATCAGGCAAAGT	22	1	21UR-3808
2470224_adh	TGGTCTGCCAAAGAACACGTT	21	313	21UR-3819
2470226_adh	TGGTCTGCCAAAGAACACGTTGT	23	1	21UR-3819
2658967_adh	TTGGACTGTTCCACGTGGAAA	21	1604	21UR-3863
2658968_adh	TTGGACTGTTCCACGTGGAAAA	22	10	21UR-3863
2658969_adh	TTGGACTGTTCCACGTGGAAAAA	23	5	21UR-3863
1910009_adh	TATGATAATTTGGTTACTCGA	21	47	21UR-3866
1910010_adh	TATGATAATTTGGTTACTCGAT	22	1	21UR-3866
2529487_adh	TGTTGCGAAATTTTCAAAGTC	21	1	21UR-3891
243142_adh	AATGTTGCGAAATTTTCAAAGTC	23	1	21UR-3891
2251929_adh	TGAGTAGGGCATTGGATTACT	21	398	21UR-3898
2251930_adh	TGAGTAGGGCATTGGATTACTAAT	24	1	21UR-3898
2481985_adh	TGTAAGAGTTGAAAAGGAGAA	21	612	21UR-3901
2481987_adh	TGTAAGAGTTGAAAAGGAGAAT	22	2	21UR-3901
1946314_adh	TCACATTCTTGTCCTCTGTAC	21	21	21UR-3902
1946315_adh	TCACATTCTTGTCCTCTGTACGAC	24	1	21UR-3902
2657503_adh	TTGGAATCAATTGTTGTTGGA	21	186	21UR-3904
2657504_adh	TTGGAATCAATTGTTGTTGGAA	22	1	21UR-3904
1786121_adh	TAAGGGTACCATTTTTTCTAC	21	7	21UR-3916
200641_adh	AATAAGGGTACCATTTTTTCTAC	23	1	21UR-3916
2602304_adh	TTCGAAGGTCAACAAAATATT	21	17	21UR-3920
246902_adh	AATTCGAAGGTCAACAAAATATT	23	1	21UR-3920
1849152_adh	TAGAGTGATATGTTGAGAACA	21	297	21UR-3926
1849153_adh	TAGAGTGATATGTTGAGAACAA	22	1	21UR-3926
1849154_adh	TAGAGTGATATGTTGAGAACAAAT	24	1	21UR-3926
1997060_adh	TCGAATACTTTAAAAATTCAA	21	15	21UR-3927
639556_adh	ATCGAATACTTTAAAAATTCAA	22	1	21UR-3927
217072_adh	AATCGAATACTTTAAAAATTCAA	23	1	21UR-3927
2648650_adh	TTGCAAGGATTTTGGTGATAA	21	656	21UR-3929
2648652_adh	TTGCAAGGATTTTGGTGATAAT	22	1	21UR-3929
1799506_adh	TAATGACGTTTTTTCCTCTGA	21	57	21UR-3937
1799507_adh	TAATGACGTTTTTTCCTCTGAA	22	8	21UR-3937

2611322_adh	TTCGTAGCCTTGTCAGTTAAT	21	18	21UR-3949
247648_adh	AATTCGTAGCCTTGTCAGTTAAT	23	1	21UR-3949
1767872_adh	TAACGACGTTGTATAGGAATA	21	648	21UR-3957
1767873_adh	TAACGACGTTGTATAGGAATAAA	23	1	21UR-3957
1975995_adh	TCCAACACAGAAATTGCCAGG	21	6	21UR-3959
214658_adh	AATCCAACACAGAAATTGCCAGG	23	1	21UR-3959
1877437_adh	TAGTGAATTCGGATTCGTTCT	21	252	21UR-3964
1877439_adh	TAGTGAATTCGGATTCGTTCTC	22	1	21UR-3964
2286867_adh	TGCACTTATTGAATCGACTGA	21	130	21UR-3971
2286868_adh	TGCACTTATTGAATCGACTGAA	22	1	21UR-3971
229306_adh	AATGCACTTATTGAATCGACTGA	23	1	21UR-3971
2502645_adh	TGTGAAGTTGGGATAGGTATC	21	2389	21UR-3972
2502651_adh	TGTGAAGTTGGGATAGGTATCT	22	4	21UR-3972
2493292_adh	TGTATTGCATAAATGAACGGT	21	487	21UR-3979
2493293_adh	TGTATTGCATAAATGAACGGTT	22	1	21UR-3979
2493294_adh	TGTATTGCATAAATGAACGGTTAT	24	1	21UR-3979
2142430_adh	TGAATCGGTAATACTAGAATT	21	5593	21UR-4004
2142431_adh	TGAATCGGTAATACTAGAATTA	22	1	21UR-4004
224847_adh	AATGAATCGGTAATACTAGAATT	23	1	21UR-4004
2142434_adh	TGAATCGGTAATACTAGAATTAGAC	25	1	21UR-4004
2318011_adh	TGGAAATCATGAGAATACTTC	21	1550	21UR-4009
2318012_adh	TGGAAATCATGAGAATACTTCA	22	1	21UR-4009
1878617_adh	TAGTGGAATTTTGCTCGACGT	21	189	21UR-4021
1878619_adh	TAGTGGAATTTTGCTCGACGTC	22	1	21UR-4021
1878620_adh	TAGTGGAATTTTGCTCGACGTCAT	24	7	21UR-4021
1878621_adh	TAGTGGAATTTTGCTCGACGTCATT	25	2	21UR-4021
2184039_adh	TGAGACGCGAAACGACATAAT	21	1137	21UR-4027
2184041_adh	TGAGACGCGAAACGACATAATT	22	3	21UR-4027
226842_adh	AATGAGACGCGAAACGACATAAT	23	2	21UR-4027
2184045_adh	TGAGACGCGAAACGACATAATTTG	24	1	21UR-4027
1889911_adh	TATATCAAGACCAGCCGCTGC	21	7	21UR-4030
1889912_adh	TATATCAAGACCAGCCGCTGCC	22	1	21UR-4030
2373575_adh	TGGACACAACACAAGAACCAA	21	5980	21UR-4031
2373576_adh	TGGACACAACACAAGAACCAAA	22	14	21UR-4031
2657986_adh	TTGGACACAACACAAGAACCAA	22	1	21UR-4031
2373580_adh	TGGACACAACACAAGAACCAAAT	23	4	21UR-4031
2373581_adh	TGGACACAACACAAGAACCAAATT	24	3	21UR-4031
2373582_adh	TGGACACAACACAAGAACCAAATTTT	26	1	21UR-4031
2469498_adh	TGGTCCTTGGCTATAGCCGGC	21	44	21UR-4049
238964_adh	AATGGTCCTTGGCTATAGCCGGC	23	2	21UR-4049
2717116_adh	TTTGAGTGTTGATTTTGTCCA	21	22	21UR-4050
2717117_adh	TTTGAGTGTTGATTTTGTCCAGAT	24	1	21UR-4050
1866962_adh	TAGGCACTACATAACTGAACA	21	193	21UR-4058
1866963_adh	TAGGCACTACATAACTGAACAA	22	1	21UR-4058
2723597_adh	TTTGGAGACTTTATGCAGAAC	21	4331	21UR-4063
2723598_adh	TTTGGAGACTTTATGCAGAACA	22	21	21UR-4063
2723599_adh	TTTGGAGACTTTATGCAGAACAA	23	40	21UR-4063
2723600_adh	TTTGGAGACTTTATGCAGAACAAA	24	5	21UR-4063
2723601_adh	TTTGGAGACTTTATGCAGAACAAAA	25	1	21UR-4063
1847281_adh	TAGAGAATCCATAGCTGTTAC	21	11	21UR-4068

205637_adh	AATAGAGAATCCATAGCTGTTAC	23	1	21UR-4068
2373906_adh	TGGACACTAGTGAATTATTGC	21	553	21UR-4073
1926863_adh	TATTGGACACTAGTGAATTATTGCT	25	1	21UR-4073
2499364_adh	TGTCTCGCCAACTGCAAGCCA	21	2	21UR-4076
241981_adh	AATGTCTCGCCAACTGCAAGCCA	23	1	21UR-4076
2310863_adh	TGCTTGATTGTTAAATTGGAT	21	1140	21UR-4085
2310864_adh	TGCTTGATTGTTAAATTGGATACAC	25	1	21UR-4085
1941467_adh	TCAATCGAAGCAATCGAACTC	21	37	21UR-4091
1941468_adh	TCAATCGAAGCAATCGAACTCA	22	3	21UR-4091
2254298_adh	TGAGTGGTTGTAATTTGGTTG	21	434	21UR-4110
2254300_adh	TGAGTGGTTGTAATTTGGTTGGC	23	1	21UR-4110
227450_adh	AATGAGTGGTTGTAATTTGGTTG	23	1	21UR-4110
1805455_adh	TAATTTGGTTGGTGTATGAAG	21	20	21UR-4126
1805456_adh	TAATTTGGTTGGTGTATGAAGC	22	1	21UR-4126
2306522_adh	TGCTATCTCGGCTTCAATCTA	21	160	21UR-4137
2306524_adh	TGCTATCTCGGCTTCAATCTAT	22	1	21UR-4137
230910_adh	AATGCTATCTCGGCTTCAATCTA	23	1	21UR-4137
1887834_adh	TATACTTTGACATTGACGTTC	21	146	21UR-4141
1887836_adh	TATACTTTGACATTGACGTTCT	22	2	21UR-4141
2617090_adh	TTCTCTCGAAAGTTGGTTTTT	21	104	21UR-4146
2617091_adh	TTCTCTCGAAAGTTGGTTTTTT	22	2	21UR-4146
1781917_adh	TAAGCAGAATGGAAACGATTT	21	45	21UR-4147
1781918_adh	TAAGCAGAATGGAAACGATTTAA	23	1	21UR-4147
2546056_adh	TTAACTCAGTCAAAGCGATCC	21	4	21UR-4149
702949_adh	ATTAACTCAGTCAAAGCGATCC	22	1	21UR-4149
2591418_adh	TTCAGCGATCGAAAGACAACA	21	26402	21UR-4162
2591419_adh	TTCAGCGATCGAAAGACAACAA	22	7	21UR-4162
1725407_adh	GTTTCAGCGATCGAAAGACAACA	23	2	21UR-4162
2591420_adh	TTCAGCGATCGAAAGACAACAAAA	24	1	21UR-4162
2591421_adh	TTCAGCGATCGAAAGACAACAAAAC	25	1	21UR-4162
2667274_adh	TTGGGATCCAATGTGAAATGC	21	4	21UR-4190
2667275_adh	TTGGGATCCAATGTGAAATGCC	22	1	21UR-4190
731975_adh	ATTGGGATCCAATGTGAAATGC	22	1	21UR-4190
2674184_adh	TTGTACACAAACATGTTAGGC	21	106	21UR-4208
2674186_adh	TTGTACACAAACATGTTAGGCC	22	1	21UR-4208
1782404_adh	TAAGCGACAAACCTTTAGTGA	21	24	21UR-4216
1782405_adh	TAAGCGACAAACCTTTAGTGAAA	23	1	21UR-4216
2305365_adh	TGCTAAGACAAGATGGGTAAA	21	186	21UR-4222
230859_adh	AATGCTAAGACAAGATGGGTAAA	23	1	21UR-4222
2305367_adh	TGCTAAGACAAGATGGGTAAACAAT	25	1	21UR-4222
2644965_adh	TTGATGCCTGCTATTAGAATT	21	240	21UR-4230
251036_adh	AATTGATGCCTGCTATTAGAATT	23	1	21UR-4230
2644969_adh	TTGATGCCTGCTATTAGAATTCAT	24	1	21UR-4230
1924914_adh	TATTCTGACAAGCTATGCCCG	21	79	21UR-4231
1924916_adh	TATTCTGACAAGCTATGCCCGT	22	6	21UR-4231
1924917_adh	TATTCTGACAAGCTATGCCCGTGT	24	1	21UR-4231
2660852_adh	TTGGATAGACTAATTAGCAAG	21	449	21UR-4233
1113751_adh	CTTGGATAGACTAATTAGCAAG	22	1	21UR-4233
2660853_adh	TTGGATAGACTAATTAGCAAGA	22	1	21UR-4233
2660854_adh	TTGGATAGACTAATTAGCAAGACAT	25	3	21UR-4233

1845554_adh	TAGACTACAGAAATAGATTGG	21	106	21UR-4238
1845556_adh	TAGACTACAGAAATAGATTGGGAA	24	1	21UR-4238
1772072_adh	TAACTTAAGTAGATAAATGAT	21	1840	21UR-4264
1772075_adh	TAACTTAAGTAGATAAATGATGGA	24	2	21UR-4264
1772076_adh	TAACTTAAGTAGATAAATGATGGAAC	26	1	21UR-4264
1889843_adh	TATATATGATTGTGATCCCTC	21	115	21UR-4274
1889844_adh	TATATATGATTGTGATCCCTCA	22	2	21UR-4274
1938257_adh	TCAAGAAGAATTTCAAATACA	21	140	21UR-4289
1938261_adh	TCAAGAAGAATTTCAAATACATAAC	25	1	21UR-4289
1938262_adh	TCAAGAAGAATTTCAAATACATAACT	26	1	21UR-4289
2651989_adh	TTGCGACTTACGTTCTGTCGG	21	178	21UR-4290
2651990_adh	TTGCGACTTACGTTCTGTCGGA	22	3	21UR-4290
2651991_adh	TTGCGACTTACGTTCTGTCGGAA	23	1	21UR-4290
2513508_adh	TGTGCAGTATGCAACTCTCAA	21	82	21UR-4304
2513509_adh	TGTGCAGTATGCAACTCTCAAT	22	2	21UR-4304
2285546_adh	TGCAATTGATCCCACAATTGA	21	14	21UR-4316
229175_adh	AATGCAATTGATCCCACAATTGA	23	1	21UR-4316
2063200_adh	TCTTTTTGTACTAACGGTTGA	21	137	21UR-4323
2063201_adh	TCTTTTTGTACTAACGGTTGAA	22	1	21UR-4323
2063202_adh	TCTTTTTGTACTAACGGTTGAAA	23	1	21UR-4323
1832420_adh	TACTGATGGCATTTTACATGG	21	516	21UR-4329
598704_adh	ATACTGATGGCATTTTACATGG	22	1	21UR-4329
2257399_adh	TGAGTTTATTTGCACTAAAGT	21	10	21UR-4334
669103_adh	ATGAGTTTATTTGCACTAAAGT	22	1	21UR-4334
1864470_adh	TAGGATAATAATAGGATGATA	21	1227	21UR-4349
1864474_adh	TAGGATAATAATAGGATGATAGGAG	25	2	21UR-4349
1912446_adh	TATGCACTTGAGTAAGCTAAC	21	6	21UR-4351
1912447_adh	TATGCACTTGAGTAAGCTAACTTCT	25	1	21UR-4351
1746907_adh	TAAAAATACATCGGAATCCAA	21	83	21UR-4355
1746908_adh	TAAAAATACATCGGAATCCAAAA	23	1	21UR-4355
1966361_adh	TCAGTCGTATGGGTTTATCCA	21	4	21UR-4364
1966362_adh	TCAGTCGTATGGGTTTATCCAAGT	24	1	21UR-4364
1779814_adh	TAAGATGAAGACATCGTTTGA	21	215	21UR-4371
1779815_adh	TAAGATGAAGACATCGTTTGAAA	23	4	21UR-4371
1779816_adh	TAAGATGAAGACATCGTTTGAAATAA	26	1	21UR-4371
1763180_adh	TAACAACAAGGAATTTACAAC	21	36	21UR-4374
1763181_adh	TAACAACAAGGAATTTACAACA	22	1	21UR-4374
2389780_adh	TGGATCGCGGAAATATGCAAA	21	825	21UR-4382
2389781_adh	TGGATCGCGGAAATATGCAAAA	22	4	21UR-4382
2661497_adh	TTGGATCGCGGAAATATGCAAA	22	1	21UR-4382
2389782_adh	TGGATCGCGGAAATATGCAAAAA	23	5	21UR-4382
2389783_adh	TGGATCGCGGAAATATGCAAAAAT	24	1	21UR-4382
1895015_adh	TATCATTGGTACACTAATTTC	21	15	21UR-4406
1895016_adh	TATCATTGGTACACTAATTTCT	22	1	21UR-4406
208113_adh	AATATCATTGGTACACTAATTTC	23	1	21UR-4406
1895018_adh	TATCATTGGTACACTAATTTCTGC	24	1	21UR-4406
1833926_adh	TACTGTGATGGAAAAACGGTA	21	168	21UR-4407
1833927_adh	TACTGTGATGGAAAAACGGTAAA	23	1	21UR-4407
1938395_adh	TCAAGAATTACGGATGCCGCC	21	411	21UR-4415
1938396_adh	TCAAGAATTACGGATGCCGCCA	22	2	21UR-4415

1938397_adh	TCAAGAATTACGGATGCCGCCAA	23	2	21UR-4415
1827493_adh	TACGGTTAATACGGTTAGGGG	21	99	21UR-4429
1827495_adh	TACGGTTAATACGGTTAGGGGTAA	24	1	21UR-4429
1757693_adh	TAAAGCTGGCTACCGTACTTC	21	241	21UR-4437
1757695_adh	TAAAGCTGGCTACCGTACTTCT	22	1	21UR-4437
1762209_adh	TAAATTCAAATTGCTTGAAGA	21	51	21UR-4447
1025748_adh	CTAAATTCAAATTGCTTGAAGA	22	1	21UR-4447
2469995_adh	TGGTCTAGTGGTATGTGCGGG	21	1201	21UR-4459
2469998_adh	TGGTCTAGTGGTATGTGCGGGC	22	5	21UR-4459
239029_adh	AATGGTCTAGTGGTATGTGCGGG	23	1	21UR-4459
2469999_adh	TGGTCTAGTGGTATGTGCGGGCA	23	1	21UR-4459
2470000_adh	TGGTCTAGTGGTATGTGCGGGCAA	24	1	21UR-4459
2131329_adh	TGAAGCGACTAAAATTACAAC	21	168	21UR-4466
1064441_adh	CTGAAGCGACTAAAATTACAAC	22	1	21UR-4466
2131330_adh	TGAAGCGACTAAAATTACAACAT	23	1	21UR-4466
1835373_adh	TACTTGGATAGACTAATTAGC	21	233	21UR-4467
1835374_adh	TACTTGGATAGACTAATTAGCA	22	2	21UR-4467
1835375_adh	TACTTGGATAGACTAATTAGCAA	23	1	21UR-4467
1952980_adh	TCACGAAATTGTATTAACTGG	21	134	21UR-4471
212796_adh	AATCACGAAATTGTATTAACTGG	23	2	21UR-4471
2614331_adh	TTCTACATCGTAGCGGAAATC	21	559	21UR-4474
2614332_adh	TTCTACATCGTAGCGGAAATCA	22	1	21UR-4474
2614333_adh	TTCTACATCGTAGCGGAAATCAA	23	1	21UR-4474
2038286_adh	TCTCATGACGGTTTCCATTTT	21	236	21UR-4485
221748_adh	AATCTCATGACGGTTTCCATTTT	23	1	21UR-4485
2000276_adh	TCGACTCATTACAGCTGATTA	21	61	21UR-4497
2000278_adh	TCGACTCATTACAGCTGATTAT	22	1	21UR-4497
2000280_adh	TCGACTCATTACAGCTGATTATG	23	1	21UR-4497
2131439_adh	TGAAGCGGAAAATATAGAAAG	21	4872	21UR-4509
2131442_adh	TGAAGCGGAAAATATAGAAAGC	22	32	21UR-4509
2131443_adh	TGAAGCGGAAAATATAGAAAGCAT	24	2	21UR-4509
2131444_adh	TGAAGCGGAAAATATAGAAAGCATA	25	1	21UR-4509
1758574_adh	TAAAGTAGACGTTGCCATGAT	21	6382	21UR-4510
1758579_adh	TAAAGTAGACGTTGCCATGATT	22	7	21UR-4510
2541719_adh	TTAAAGTAGACGTTGCCATGAT	22	1	21UR-4510
1758585_adh	TAAAGTAGACGTTGCCATGATTT	23	1	21UR-4510
1758586_adh	TAAAGTAGACGTTGCCATGATTTT	24	3	21UR-4510
1758587_adh	TAAAGTAGACGTTGCCATGATTTTT	25	1	21UR-4510
1912079_adh	TATGATTGCGTAATTGTTTGA	21	67	21UR-4514
1912080_adh	TATGATTGCGTAATTGTTTGAAA	23	1	21UR-4514
1963184_adh	TCAGCTTAGTAAGTATTTTCA	21	7	21UR-4540
1963185_adh	TCAGCTTAGTAAGTATTTTCAT	22	1	21UR-4540
2659674_adh	TTGGAGCAACTTCGGTTTTAA	21	87	21UR-4543
252523_adh	AATTGGAGCAACTTCGGTTTTAA	23	1	21UR-4543
2659675_adh	TTGGAGCAACTTCGGTTTTAACTGG	25	1	21UR-4543
1916456_adh	TATGGTATTTCTCCCTCTGTG	21	2	21UR-4544
1916457_adh	TATGGTATTTCTCCCTCTGTGCAACT	26	1	21UR-4544
1874450_adh	TAGTAGATACTATAGTAGAAG	21	476	21UR-4545
1874451_adh	TAGTAGATACTATAGTAGAAGAAA	24	1	21UR-4545
1919035_adh	TATGTGGAGTAATGGTTGATG	21	967	21UR-4557

1919036_adh	TATGTGGAGTAATGGTTGATGA	22	9	21UR-4557
1919038_adh	TATGTGGAGTAATGGTTGATGAGA	24	1	21UR-4557
1880382_adh	TAGTTAGGACATAATATGATC	21	77	21UR-4568
1880384_adh	TAGTTAGGACATAATATGATCT	22	1	21UR-4568
1785854_adh	TAAGGGAATCAAATGTCGAAT	21	24	21UR-4570
1785855_adh	TAAGGGAATCAAATGTCGAATTAAA	25	1	21UR-4570
2171344_adh	TGACTGTATTTTGGTTTTTGG	21	12007	21UR-4572
2171351_adh	TGACTGTATTTTGGTTTTTGGT	22	6	21UR-4572
2171355_adh	TGACTGTATTTTGGTTTTTGGTG	23	1	21UR-4572
2171356_adh	TGACTGTATTTTGGTTTTTGGTGT	24	3	21UR-4572
2171357_adh	TGACTGTATTTTGGTTTTTGGTGTC	25	1	21UR-4572
1911935_adh	TATGATTCCATTTCGTCCTCA	21	86	21UR-4573
1911936_adh	TATGATTCCATTTCGTCCTCAT	22	1	21UR-4573
1999743_adh	TCGACATAGATGGCTTGTATG	21	993	21UR-4605
1999745_adh	TCGACATAGATGGCTTGTATGT	22	3	21UR-4605
2034750_adh	TCTATGTTGAGGACGTCATTG	21	191	21UR-4611
652559_adh	ATCTATGTTGAGGACGTCATTG	22	1	21UR-4611
1836648_adh	TAGAAAAATTTGCAATTGAAG	21	16	21UR-4618
205344_adh	AATAGAAAAATTTGCAATTGAAG	23	1	21UR-4618
2638661_adh	TTGACTGTCGAAGACCATTTC	21	5349	21UR-4621
1109438_adh	CTTGACTGTCGAAGACCATTTC	22	4	21UR-4621
2638665_adh	TTGACTGTCGAAGACCATTTCC	22	10	21UR-4621
2638666_adh	TTGACTGTCGAAGACCATTTCCA	23	2	21UR-4621
2638668_adh	TTGACTGTCGAAGACCATTTCCAT	24	7	21UR-4621
2638670_adh	TTGACTGTCGAAGACCATTTCCATT	25	1	21UR-4621
1849056_adh	TAGAGTCCAGTTGATGTCCAT	21	1028	21UR-4625
1849058_adh	TAGAGTCCAGTTGATGTCCATT	22	2	21UR-4625
2054596_adh	TCTTAGTATTGTGAGAAGGGT	21	199	21UR-4632
222906_adh	AATCTTAGTATTGTGAGAAGGGT	23	1	21UR-4632
1884676_adh	TATAACGAGTAGTAGATGGTA	21	348	21UR-4647
1884678_adh	TATAACGAGTAGTAGATGGTAG	22	6	21UR-4647
1861934_adh	TAGGAATTGTGTTCACCAACG	21	123	21UR-4649
1861935_adh	TAGGAATTGTGTTCACCAACGA	22	1	21UR-4649
1861936_adh	TAGGAATTGTGTTCACCAACGAA	23	1	21UR-4649
1770175_adh	TAACTAGTGCAGTACACGGTG	21	433	21UR-4653
1770176_adh	TAACTAGTGCAGTACACGGTGT	22	1	21UR-4653
2310435_adh	TGCTTCGAGCGGATAGTTAAT	21	841	21UR-4655
2310436_adh	TGCTTCGAGCGGATAGTTAATT	22	1	21UR-4655
1309001_adh	GATGCTTCGAGCGGATAGTTAATTT	25	1	21UR-4655
2684337_adh	TTGTTGAAACGGTATGAAATT	21	7575	21UR-4659
2684338_adh	TTGTTGAAACGGTATGAAATTG	22	1	21UR-4659
735686_adh	ATTGTTGAAACGGTATGAAATT	22	6	21UR-4659
253984_adh	AATTGTTGAAACGGTATGAAATT	23	16	21UR-4659
2684339_adh	TTGTTGAAACGGTATGAAATTGT	23	1	21UR-4659
1988427_adh	TCCGTGAAGAGTTTCGGCGTA	21	97	21UR-4667
1049043_adh	CTCCGTGAAGAGTTTCGGCGTA	22	1	21UR-4667
1758322_adh	TAAAGTAATAAGAATGAGTAG	21	284	21UR-4668
199136_adh	AATAAAGTAATAAGAATGAGTAG	23	1	21UR-4668
1823973_adh	TACGATCGTTGGGGGTGAGAA	21	77	21UR-4698
1823974_adh	TACGATCGTTGGGGGTGAGAAAT	23	1	21UR-4698

1776035_adh	TAAGACTAGCCTATAATTTAA	21	195	21UR-4709
200378_adh	AATAAGACTAGCCTATAATTTAA	23	2	21UR-4709
2036916_adh	TCTCACAAATGGACTTTGGTG	21	2941	21UR-4711
2036917_adh	TCTCACAAATGGACTTTGGTGA	22	1	21UR-4711
2036918_adh	TCTCACAAATGGACTTTGGTGAA	23	1	21UR-4711
209791_adh	AATCAACAATCACTATATCCACT	23	1	21UR-4716
1812422_adh	TACAGGAAGAAATGGCACTAC	21	339	21UR-4718
1812424_adh	TACAGGAAGAAATGGCACTACT	22	2	21UR-4718
2449698_adh	TGGGCAAAAGTTAGGCAGGCA	21	124	21UR-4728
2449699_adh	TGGGCAAAAGTTAGGCAGGCAC	22	1	21UR-4728
2449700_adh	TGGGCAAAAGTTAGGCAGGCACGC	24	1	21UR-4728
1919452_adh	TATGTTGATCGTGTTTAAATA	21	74	21UR-4729
618814_adh	ATATGTTGATCGTGTTTAAATA	22	1	21UR-4729
208821_adh	AATATGTTGATCGTGTTTAAATA	23	1	21UR-4729
1992937_adh	TCCTTCGGCTATATTTACAAT	21	2349	21UR-4730
1992938_adh	TCCTTCGGCTATATTTACAATA	22	1	21UR-4730
1918195_adh	TATGTATGTGATTCTAGGTGG	21	99	21UR-4737
1918196_adh	TATGTATGTGATTCTAGGTGGA	22	13	21UR-4737
1918197_adh	TATGTATGTGATTCTAGGTGGAA	23	3	21UR-4737
2151832_adh	TGACAATGACGTAGATTATAA	21	628	21UR-4744
225004_adh	AATGACAATGACGTAGATTATAA	23	2	21UR-4744
1789070_adh	TAAGTTCAAAAAATTGTCAAG	21	9	21UR-4745
1028052_adh	CTAAGTTCAAAAAATTGTCAAG	22	1	21UR-4745
1808170_adh	TACAACGTAGAGTACTGGCTA	21	25460	21UR-4748
1808171_adh	TACAACGTAGAGTACTGGCTAA	22	211	21UR-4748
591641_adh	ATACAACGTAGAGTACTGGCTA	22	3	21UR-4748
1808193_adh	TACAACGTAGAGTACTGGCTAAT	23	97	21UR-4748
1808194_adh	TACAACGTAGAGTACTGGCTAATA	24	2	21UR-4748
1808195_adh	TACAACGTAGAGTACTGGCTAATAA	25	2	21UR-4748
1808196_adh	TACAACGTAGAGTACTGGCTAATAAT	26	8	21UR-4748
1786288_adh	TAAGGTACAGAATAGTTGAAA	21	549	21UR-4755
1786292_adh	TAAGGTACAGAATAGTTGAAATAA	24	1	21UR-4755
1786293_adh	TAAGGTACAGAATAGTTGAAATAAA	25	1	21UR-4755
1842283_adh	TAGAATGCTCGTAGATCTTGG	21	2450	21UR-4773
1033182_adh	CTAGAATGCTCGTAGATCTTGG	22	2	21UR-4773
1842287_adh	TAGAATGCTCGTAGATCTTGGT	22	22	21UR-4773
1842289_adh	TAGAATGCTCGTAGATCTTGGTCAA	25	1	21UR-4773
1842290_adh	TAGAATGCTCGTAGATCTTGGTCAAT	26	1	21UR-4773
1994455_adh	TCGAAAAATTGGAATTGGACA	21	1078	21UR-4782
1994456_adh	TCGAAAAATTGGAATTGGACAA	22	1	21UR-4782
216909_adh	AATCGAAAAATTGGAATTGGACA	23	2	21UR-4782
2495182_adh	TGTCAGTTGAAAATGTGCACT	21	129	21UR-4783
241125_adh	AATGTCAGTTGAAAATGTGCACT	23	1	21UR-4783
2679539_adh	TTGTGACAGAACGCATTTTTT	21	2717	21UR-4797
1731172_adh	GTTTGTGACAGAACGCATTTTTT	23	4	21UR-4797
2679540_adh	TTGTGACAGAACGCATTTTTTAA	23	1	21UR-4797
2422707_adh	TGGGAGAATAGTAAGGACGTG	21	2317	21UR-4807
2422708_adh	TGGGAGAATAGTAAGGACGTGA	22	8	21UR-4807
688652_adh	ATGGGAGAATAGTAAGGACGTG	22	1	21UR-4807
237908_adh	AATGGGAGAATAGTAAGGACGTG	23	6	21UR-4807

2422710_adh	TGGGAGAATAGTAAGGACGTGAT	23	3	21UR-4807
2167641_adh	TGACTCATCGAATTGCATAAT	21	183	21UR-4834
2167642_adh	TGACTCATCGAATTGCATAATA	22	1	21UR-4834
664977_adh	ATGACTCATCGAATTGCATAAT	22	1	21UR-4834
2167643_adh	TGACTCATCGAATTGCATAATAA	23	1	21UR-4834
2167644_adh	TGACTCATCGAATTGCATAATAAAT	25	1	21UR-4834
1901152_adh	TATGAAACTACTTGCATTACC	21	10	21UR-4843
1901153_adh	TATGAAACTACTTGCATTACCGTGAT	26	1	21UR-4843
1846156_adh	TAGACTTAGATAAAGCTAAAC	21	44	21UR-4849
1846157_adh	TAGACTTAGATAAAGCTAAACGACA	25	1	21UR-4849
1813335_adh	TACATCAGCCGCTCGAAGAGG	21	242	21UR-4852
1813336_adh	TACATCAGCCGCTCGAAGAGGT	22	6	21UR-4852
593568_adh	ATACATCAGCCGCTCGAAGAGG	22	1	21UR-4852
203239_adh	AATACATCAGCCGCTCGAAGAGG	23	1	21UR-4852
1944994_adh	TCACAGCTATTTGGCAACACC	21	42	21UR-4855
1944995_adh	TCACAGCTATTTGGCAACACCT	22	1	21UR-4855
2604076_adh	TTCGAGTAGCATTTGAGTGGC	21	813	21UR-4857
2604077_adh	TTCGAGTAGCATTTGAGTGGCA	22	2	21UR-4857
2272183_adh	TGATGGACTTCTACTTGGAAA	21	232	21UR-4881
2272184_adh	TGATGGACTTCTACTTGGAAAA	22	4	21UR-4881
2036719_adh	TCTCAATTGGATAGCATAACA	21	349	21UR-4893
2036720_adh	TCTCAATTGGATAGCATAACAAA	23	2	21UR-4893
2036721_adh	TCTCAATTGGATAGCATAACAAAAG	25	1	21UR-4893
2663227_adh	TTGGATGGTTGAAGAAGGAGT	21	8978	21UR-4895
2663228_adh	TTGGATGGTTGAAGAAGGAGTA	22	4	21UR-4895
2663230_adh	TTGGATGGTTGAAGAAGGAGTAT	23	18	21UR-4895
2663231_adh	TTGGATGGTTGAAGAAGGAGTATC	24	2	21UR-4895
2663232_adh	TTGGATGGTTGAAGAAGGAGTATCG	25	3	21UR-4895
2663233_adh	TTGGATGGTTGAAGAAGGAGTATCGT	26	6	21UR-4895
2708835_adh	TTTCGTCAGCATGGCTAGTAC	21	4	21UR-4902
1805013_adh	TAATTTCGTCAGCATGGCTAGTAC	24	1	21UR-4902
2605141_adh	TTCGATGCACGCGCATGTACC	21	13	21UR-4908
2605142_adh	TTCGATGCACGCGCATGTACCC	22	1	21UR-4908
1999520_adh	TCGACAAAATGCAGGCAATGA	21	306	21UR-4953
217100_adh	AATCGACAAAATGCAGGCAATGA	23	1	21UR-4953
1999521_adh	TCGACAAAATGCAGGCAATGAGGT	24	1	21UR-4953
1814345_adh	TACATTCGGGATTGTTGGTAA	21	634	21UR-4970
1814346_adh	TACATTCGGGATTGTTGGTAAAA	23	1	21UR-4970
1856572_adh	TAGCGATTGCAGAGTAAGATT	21	738	21UR-4976
1856574_adh	TAGCGATTGCAGAGTAAGATTT	22	1	21UR-4976
1856577_adh	TAGCGATTGCAGAGTAAGATTTT	23	1	21UR-4976
206165_adh	AATAGCGATTGCAGAGTAAGATT	23	1	21UR-4976
2376044_adh	TGGACGACCTGGAGTAATAGA	21	168	21UR-4985
2376045_adh	TGGACGACCTGGAGTAATAGAA	22	2	21UR-4985
1882647_adh	TAGTTGTTTAGCGAAAATAGG	21	15	21UR-4989
1882648_adh	TAGTTGTTTAGCGAAAATAGGAG	23	1	21UR-4989
1853166_adh	TAGCAACTAAGAATTCCGTTA	21	130	21UR-5023
604679_adh	ATAGCAACTAAGAATTCCGTTA	22	2	21UR-5023
1862447_adh	TAGGACAGTGGAACGAACGAT	21	13	21UR-5028
1862448_adh	TAGGACAGTGGAACGAACGATA	22	2	21UR-5028

1768292_adh	TAACGATACAGCCTTACATGT	21	45	21UR-5031
1768293_adh	TAACGATACAGCCTTACATGTA	22	2	21UR-5031
1888969_adh	TATAGTATGTTCCAGAGGTGG	21	37	21UR-5041
1888971_adh	TATAGTATGTTCCAGAGGTGGGCGG	25	1	21UR-5041
1937004_adh	TCAACGTATTGTATTCAACTT	21	47	21UR-5042
1937005_adh	TCAACGTATTGTATTCAACTTT	22	1	21UR-5042
2291724_adh	TGCCAAACTCCATTTAACGGC	21	329	21UR-5045
2291725_adh	TGCCAAACTCCATTTAACGGCT	22	1	21UR-5045
229605_adh	AATGCCAAACTCCATTTAACGGC	23	1	21UR-5045
2469173_adh	TGGTCACACACAACAAAGGCT	21	169	21UR-5052
2469174_adh	TGGTCACACACAACAAAGGCTAA	23	1	21UR-5052
2513623_adh	TGTGCATTTCTCTGTTCGTCA	21	10	21UR-5061
2513624_adh	TGTGCATTTCTCTGTTCGTCAAA	23	1	21UR-5061
2275328_adh	TGATTCCATTGTATTGGCATA	21	80	21UR-5069
2275329_adh	TGATTCCATTGTATTGGCATAT	22	1	21UR-5069
228675_adh	AATGATTCCATTGTATTGGCATA	23	1	21UR-5069
2003199_adh	TCGATATAACTGAACTTCAAA	21	14	21UR-5092
2003200_adh	TCGATATAACTGAACTTCAAAA	22	1	21UR-5092
217536_adh	AATCGATATAACTGAACTTCAAA	23	1	21UR-5092
2678818_adh	TTGTCTCTGTGTGGGTATATC	21	164	21UR-5097
253623_adh	AATTGTCTCTGTGTGGGTATATC	23	1	21UR-5097
2678821_adh	TTGTCTCTGTGTGGGTATATCTGT	24	2	21UR-5097
1870506_adh	TAGGTACTACTTTGAACTTGG	21	15	21UR-5101
1870507_adh	TAGGTACTACTTTGAACTTGGA	22	1	21UR-5101
1922269_adh	TATTATCGTCTTCTGAGCATA	21	558	21UR-5105
1922270_adh	TATTATCGTCTTCTGAGCATAAGCG	25	1	21UR-5105
1922271_adh	TATTATCGTCTTCTGAGCATAAGCGC	26	1	21UR-5105
1866600_adh	TAGGATTTCACTAGAAGAAGC	21	3176	21UR-5109
1866601_adh	TAGGATTTCACTAGAAGAAGCA	22	2	21UR-5109
1866603_adh	TAGGATTTCACTAGAAGAAGCAT	23	2	21UR-5109
1866604_adh	TAGGATTTCACTAGAAGAAGCATG	24	2	21UR-5109
1866605_adh	TAGGATTTCACTAGAAGAAGCATGCA	26	1	21UR-5109
2402858_adh	TGGCACAATTGTATTCAATTT	21	17	21UR-5118
686179_adh	ATGGCACAATTGTATTCAATTT	22	1	21UR-5118
1867117_adh	TAGGCCAAGTCGGTTAGCTAA	21	87	21UR-5122
1867118_adh	TAGGCCAAGTCGGTTAGCTAAT	22	1	21UR-5122
1867120_adh	TAGGCCAAGTCGGTTAGCTAATGT	24	1	21UR-5122
1874827_adh	TAGTAGGTGTTCAAGTTAGGG	21	45	21UR-5143
1874829_adh	TAGTAGGTGTTCAAGTTAGGGC	22	1	21UR-5143
1883233_adh	TAGTTTGGTGACTAAGGAAGA	21	139	21UR-5156
1883234_adh	TAGTTTGGTGACTAAGGAAGAA	22	1	21UR-5156
1765746_adh	TAACATATATTTCTGATTAAA	21	140	21UR-5169
1765747_adh	TAACATATATTTCTGATTAAAAA	23	2	21UR-5169
2545795_adh	TTAACTAAAGAGCAGAATATG	21	4660	21UR-5170
243858_adh	AATTAACTAAAGAGCAGAATATG	23	1	21UR-5170
2545796_adh	TTAACTAAAGAGCAGAATATGAA	23	2	21UR-5170
2545797_adh	TTAACTAAAGAGCAGAATATGAAGAT	26	1	21UR-5170
1965615_adh	TCAGTAGAAGAAGACTCTCAA	21	20784	21UR-5174
1965616_adh	TCAGTAGAAGAAGACTCTCAAA	22	3	21UR-5174
628442_adh	ATCAGTAGAAGAAGACTCTCAA	22	2	21UR-5174

1965617_adh	TCAGTAGAAGAAGACTCTCAAAA	23	16	21UR-5174
213619_adh	AATCAGTAGAAGAAGACTCTCAA	23	25	21UR-5174
1965618_adh	TCAGTAGAAGAAGACTCTCAAAAA	24	1	21UR-5174
777568_adh	CAATCAGTAGAAGAAGACTCTCAA	24	1	21UR-5174
1965619_adh	TCAGTAGAAGAAGACTCTCAAAAAG	25	2	21UR-5174
1965620_adh	TCAGTAGAAGAAGACTCTCAAAAAGC	26	1	21UR-5174
2699263_adh	TTTATGGTTAGAAAAACTATA	21	76	21UR-5178
2699264_adh	TTTATGGTTAGAAAAACTATAA	22	2	21UR-5178
2491306_adh	TGTATCGTCGAATGCTCCATC	21	28	21UR-5179
240862_adh	AATGTATCGTCGAATGCTCCATC	23	1	21UR-5179
240863_adh	AATGTATCGTCGAATGCTCCATCAT	25	1	21UR-5179
2271037_adh	TGATGAATAGGTGATTAGGGA	21	152	21UR-5180
2271039_adh	TGATGAATAGGTGATTAGGGAAT	23	1	21UR-5180
1785974_adh	TAAGGGAGTATGCTTAGTAGT	21	37	21UR-5188
587189_adh	ATAAGGGAGTATGCTTAGTAGT	22	1	21UR-5188
1799407_adh	TAATGACAATGACGTAGATTA	21	282	21UR-5195
1799409_adh	TAATGACAATGACGTAGATTAT	22	2	21UR-5195
2646841_adh	TTGATTTCGACAGTACTCAAA	21	567	21UR-5197
2646842_adh	TTGATTTCGACAGTACTCAAAA	22	3	21UR-5197
1988400_adh	TCCGTGAACTTTTTTGCTTGC	21	10	21UR-5213
635385_adh	ATCCGTGAACTTTTTTGCTTGC	22	1	21UR-5213
2270435_adh	TGATCTGATTGAGAACGTGAA	21	101	21UR-5220
2270436_adh	TGATCTGATTGAGAACGTGAAA	22	4	21UR-5220
2052905_adh	TCTGTGTTCGGTGGACTTTCA	21	2066	21UR-5226
2520855_adh	TGTGTTCGGTGGACTTTCAAA	21	1	355_x1/21UR-5226
2052906_adh	TCTGTGTTCGGTGGACTTTCAA	22	1	21UR-5226
2052907_adh	TCTGTGTTCGGTGGACTTTCAAA	23	1	21UR-5226
2060939_adh	TCTTTCGAGTACCATAGGACG	21	2794	21UR-5227
2060943_adh	TCTTTCGAGTACCATAGGACGT	22	9	21UR-5227
2060947_adh	TCTTTCGAGTACCATAGGACGTT	23	2	21UR-5227
2060948_adh	TCTTTCGAGTACCATAGGACGTTT	24	2	21UR-5227
1918767_adh	TATGTCGTTTTGAATCACATA	21	16	21UR-5231
208789_adh	AATATGTCGTTTTGAATCACATA	23	1	21UR-5231
2479108_adh	TGGTTTTCGGATTAAAGCAAT	21	145	21UR-5236
2479109_adh	TGGTTTTCGGATTAAAGCAATACAC	25	2	21UR-5236
2719324_adh	TTTGATGTCCAAACGTTATTA	21	1	21UR-5244
256198_adh	AATTTGATGTCCAAACGTTATTA	23	1	21UR-5244
1870074_adh	TAGGTAACGTGTAGGCGATGC	21	112	21UR-5246
1870075_adh	TAGGTAACGTGTAGGCGATGCA	22	1	21UR-5246
1944223_adh	TCACAAGGTAGAAAGCTATCA	21	354	21UR-5255
1944224_adh	TCACAAGGTAGAAAGCTATCACA	23	1	21UR-5255
2161184_adh	TGACGCAAACTGTTTATTTCC	21	5	21UR-5256
226255_adh	AATGACGCAAACTGTTTATTTCC	23	1	21UR-5256
1772905_adh	TAACTTTGAACATGAAGTAAG	21	492	21UR-5271
1772906_adh	TAACTTTGAACATGAAGTAAGA	22	2	21UR-5271
1772910_adh	TAACTTTGAACATGAAGTAAGAC	23	2	21UR-5271
1772911_adh	TAACTTTGAACATGAAGTAAGACAT	25	1	21UR-5271
2635719_adh	TTGACGCAATCCTTAAAATCA	21	7	21UR-5273
249863_adh	AATTGACGCAATCCTTAAAATCA	23	2	21UR-5273
2641877_adh	TTGAGTAGAAATGCAAGGTTG	21	361	21UR-5305

726289_adh	ATTGAGTAGAAATGCAAGGTTG	22	1	21UR-5305
2641878_adh	TTGAGTAGAAATGCAAGGTTGAA	23	2	21UR-5305
1788061_adh	TAAGTCTCTTTGTCATGCATA	21	113	21UR-5307
200793_adh	AATAAGTCTCTTTGTCATGCATA	23	1	21UR-5307
1967874_adh	TCAGTTTTGAACAGCGGTAAC	21	1687	21UR-5320
1967875_adh	TCAGTTTTGAACAGCGGTAACAA	23	2	21UR-5320
1967876_adh	TCAGTTTTGAACAGCGGTAACAAT	24	1	21UR-5320
2300751_adh	TGCGGAAGAATACCGAGTAGT	21	221	21UR-5322
2300752_adh	TGCGGAAGAATACCGAGTAGTT	22	1	21UR-5322
1825945_adh	TACGGAGATTGAACAAAAAAT	21	694	21UR-5329
1825947_adh	TACGGAGATTGAACAAAAAATC	22	3	21UR-5329
204097_adh	AATACGGAGATTGAACAAAAAAT	23	2	21UR-5329
1924232_adh	TATTCGAATGTACATTTCATC	21	24	21UR-5335
619966_adh	ATATTCGAATGTACATTTCATC	22	1	21UR-5335
2719976_adh	TTTGATTGACAGAATTGGGAT	21	6618	21UR-5338
1122585_adh	CTTTGATTGACAGAATTGGGAT	22	4	21UR-5338
2719981_adh	TTTGATTGACAGAATTGGGATT	22	10	21UR-5338
2719986_adh	TTTGATTGACAGAATTGGGATTTCA	25	1	21UR-5338
2139779_adh	TGAATATGAGTATGACGGTAG	21	16882	21UR-5342
1065124_adh	CTGAATATGAGTATGACGGTAG	22	13	21UR-5342
2139796_adh	TGAATATGAGTATGACGGTAGT	22	116	21UR-5342
2139816_adh	TGAATATGAGTATGACGGTAGTT	23	11	21UR-5342
2139822_adh	TGAATATGAGTATGACGGTAGTTT	24	2	21UR-5342
2139824_adh	TGAATATGAGTATGACGGTAGTTTT	25	1	21UR-5342
2139825_adh	TGAATATGAGTATGACGGTAGTTTTC	26	1	21UR-5342
2721094_adh	TTTGCAATTGGAGCAGTGGAA	21	1177	21UR-5350
2721096_adh	TTTGCAATTGGAGCAGTGGAAC	22	14	21UR-5350
2721098_adh	TTTGCAATTGGAGCAGTGGAACT	23	1	21UR-5350
1844214_adh	TAGAATTGGAAGACGAGAAAA	21	3258	21UR-5357
1844218_adh	TAGAATTGGAAGACGAGAAAACACT	25	1	21UR-5357
2034617_adh	TCTATGTACAACGGTTCTCTC	21	977	21UR-5385
2034618_adh	TCTATGTACAACGGTTCTCTCA	22	3	21UR-5385
2034619_adh	TCTATGTACAACGGTTCTCTCAA	23	2	21UR-5385
1812859_adh	TACAGTGTTGAAAATGAAGGT	21	258	21UR-5389
1812860_adh	TACAGTGTTGAAAATGAAGGTA	22	1	21UR-5389
203178_adh	AATACAGTGTTGAAAATGAAGGT	23	1	21UR-5389
2685724_adh	TTGTTGCACAGAATCAAAAAA	21	12	21UR-5394
254465_adh	AATTGTTGCACAGAATCAAAAAA	23	1	21UR-5394
2571777_adh	TTAGTGCATTACCTCATATTC	21	40	21UR-5409
245040_adh	AATTAGTGCATTACCTCATATTC	23	1	21UR-5409
2063087_adh	TCTTTTTGGAGTGTGGTCATC	21	2622	21UR-5410
2063088_adh	TCTTTTTGGAGTGTGGTCATCA	22	20	21UR-5410
2063089_adh	TCTTTTTGGAGTGTGGTCATCAA	23	2	21UR-5410
2063090_adh	TCTTTTTGGAGTGTGGTCATCAAA	24	1	21UR-5410
1775292_adh	TAAGACATCGGAACACAAATC	21	593	21UR-5413
1775297_adh	TAAGACATCGGAACACAAATCT	22	1	21UR-5413
1775299_adh	TAAGACATCGGAACACAAATCTTTG	25	1	21UR-5413
1926563_adh	TATTGCTAGATTTTGGGTCCC	21	291	21UR-5416
1926564_adh	TATTGCTAGATTTTGGGTCCCA	22	1	21UR-5416
2672506_adh	TTGGTTGAGATCTCACGCGGA	21	804	21UR-5426

2672507_adh	TTGGTTGAGATCTCACGCGGAA	22	14	21UR-5426
2672508_adh	TTGGTTGAGATCTCACGCGGAAA	23	7	21UR-5426
2622255_adh	TTCTTCTTCCTGATCGTTTGA	21	3743	21UR-5432
2622256_adh	TTCTTCTTCCTGATCGTTTGAA	22	6	21UR-5432
2622258_adh	TTCTTCTTCCTGATCGTTTGAATT	24	4	21UR-5432
2697307_adh	TTTAGTGCATACAATGGAGAA	21	73	21UR-5435
2697308_adh	TTTAGTGCATACAATGGAGAAT	22	2	21UR-5435
739271_adh	ATTTAGTGCATACAATGGAGAA	22	1	21UR-5435
2137395_adh	TGAATAATTCGGTGTTGTAGA	21	1220	21UR-5437
2137397_adh	TGAATAATTCGGTGTTGTAGAT	22	1	21UR-5437
2507152_adh	TGTGATAGGGAAATAAAGACG	21	1652	21UR-5450
2507153_adh	TGTGATAGGGAAATAAAGACGA	22	3	21UR-5450
2507155_adh	TGTGATAGGGAAATAAAGACGAT	23	8	21UR-5450
2584653_adh	TTATTTTTCAGAGAGTCAGGA	21	305	21UR-5462
2584654_adh	TTATTTTTCAGAGAGTCAGGAT	22	1	21UR-5462
245910_adh	AATTATTTTTCAGAGAGTCAGGA	23	2	21UR-5462
2584655_adh	TTATTTTTCAGAGAGTCAGGATACA	25	1	21UR-5462
2740633_adh	TTTTGAACAATCGGAAACACA	21	567	21UR-5464
1124443_adh	CTTTTGAACAATCGGAAACACA	22	1	21UR-5464
2740634_adh	TTTTGAACAATCGGAAACACAA	22	1	21UR-5464
1927068_adh	TATTGGATTTAGATCTGGAGA	21	138	21UR-5486
1927069_adh	TATTGGATTTAGATCTGGAGAGA	23	1	21UR-5486
2670942_adh	TTGGTGCCTATTGCTAGAATC	21	48	21UR-5488
2670943_adh	TTGGTGCCTATTGCTAGAATCC	22	1	21UR-5488
253124_adh	AATTGGTGCCTATTGCTAGAATC	23	1	21UR-5488
2702167_adh	TTTCAATTGTAGACTGAGGGC	21	6453	21UR-5503
2702172_adh	TTTCAATTGTAGACTGAGGGCT	22	1	21UR-5503
2670939_adh	TTGGTGCCTACTACTGGAATC	21	33	21UR-5522
253123_adh	AATTGGTGCCTACTACTGGAATC	23	1	21UR-5522
2740484_adh	TTTTGAAAAGCTGACAGGGGG	21	927	21UR-5525
2740488_adh	TTTTGAAAAGCTGACAGGGGGT	22	8	21UR-5525
747154_adh	ATTTTGAAAAGCTGACAGGGGG	22	1	21UR-5525
2740489_adh	TTTTGAAAAGCTGACAGGGGGTA	23	1	21UR-5525
2740490_adh	TTTTGAAAAGCTGACAGGGGGTAAT	25	1	21UR-5525
1996015_adh	TCGAAGACTCAAAAGTGTAGA	21	506	21UR-5527
1996020_adh	TCGAAGACTCAAAAGTGTAGAC	22	2	21UR-5527
1996021_adh	TCGAAGACTCAAAAGTGTAGACA	23	1	21UR-5527
1996022_adh	TCGAAGACTCAAAAGTGTAGACAGT	25	5	21UR-5527
1996023_adh	TCGAAGACTCAAAAGTGTAGACAGTT	26	1	21UR-5527
2307583_adh	TGCTCGTCTAATTTGGTAAGA	21	25	21UR-5541
230974_adh	AATGCTCGTCTAATTTGGTAAGA	23	1	21UR-5541
2146099_adh	TGAATTCGGCTGATTATTATC	21	1236	21UR-5543
2146101_adh	TGAATTCGGCTGATTATTATCT	22	2	21UR-5543
2707792_adh	TTTCGGATCGAAAGTAATACT	21	607	21UR-5548
2707793_adh	TTTCGGATCGAAAGTAATACTA	22	1	21UR-5548
2707794_adh	TTTCGGATCGAAAGTAATACTAA	23	2	21UR-5548
2369299_adh	TGGAATTCACATGTGGAATTT	21	71	21UR-5552
2369300_adh	TGGAATTCACATGTGGAATTTA	22	1	21UR-5552
232244_adh	AATGGAATTCACATGTGGAATTT	23	1	21UR-5552
1958806_adh	TCAGAGATCAAACTACATAGG	21	7	21UR-5564

627406_adh	ATCAGAGATCAAACTACATAGG	22	1	21UR-5564
2719435_adh	TTTGATGTTACTGGAATATGG	21	1904	21UR-5574
2719436_adh	TTTGATGTTACTGGAATATGGA	22	19	21UR-5574
2719437_adh	TTTGATGTTACTGGAATATGGAA	23	3	21UR-5574
2719438_adh	TTTGATGTTACTGGAATATGGAAA	24	1	21UR-5574
2719774_adh	TTTGATTCCATTAGGTCATCA	21	5	21UR-5588
742987_adh	ATTTGATTCCATTAGGTCATCA	22	1	21UR-5588
256202_adh	AATTTGATTCCATTAGGTCATCA	23	1	21UR-5588
2645638_adh	TTGATGTTGCACCAGGTGGAA	21	88	21UR-5599
2719540_adh	TTTGATGTTGCACCAGGTGGAA	22	1	21UR-5599
2037181_adh	TCTCACTCAAGTTCAGACGGT	21	163	21UR-5607
2037182_adh	TCTCACTCAAGTTCAGACGGTAA	23	1	21UR-5607
2037183_adh	TCTCACTCAAGTTCAGACGGTAATAA	26	1	21UR-5607
2540936_adh	TTAAAGAGAAGAACGATTAAA	21	1054	21UR-5610
2540937_adh	TTAAAGAGAAGAACGATTAAAAA	23	4	21UR-5610
2617529_adh	TTCTCTTCAAGTAGATATCGG	21	276	21UR-5638
2617530_adh	TTCTCTTCAAGTAGATATCGGA	22	2	21UR-5638
1826144_adh	TACGGATTCAGTCAACAGTTT	21	276	21UR-5649
1826145_adh	TACGGATTCAGTCAACAGTTTT	22	2	21UR-5649
2727460_adh	TTTGTACGGTGCATTATGTAT	21	185	21UR-5665
256820_adh	AATTTGTACGGTGCATTATGTAT	23	2	21UR-5665
2642051_adh	TTGAGTAGTTAAAATGAGCAT	21	8769	21UR-5675
2642052_adh	TTGAGTAGTTAAAATGAGCATAA	23	9	21UR-5675
2642054_adh	TTGAGTAGTTAAAATGAGCATAAG	24	1	21UR-5675
2642055_adh	TTGAGTAGTTAAAATGAGCATAAGT	25	4	21UR-5675
2722725_adh	TTTGCTTGGAATCGTCACTTT	21	239	21UR-5681
1122839_adh	CTTTGCTTGGAATCGTCACTTT	22	2	21UR-5681
2590368_adh	TTCAGAAGAAGAAATTTGGAA	21	460	21UR-5718
2590370_adh	TTCAGAAGAAGAAATTTGGAAGA	23	1	21UR-5718
2724859_adh	TTTGGGCTGATTTGTCGGAAA	21	191	21UR-5728
2724860_adh	TTTGGGCTGATTTGTCGGAAAGTTCT	26	1	21UR-5728
2614043_adh	TTCTAATCGGTCTCAATCAAT	21	155	21UR-5732
248725_adh	AATTCTAATCGGTCTCAATCAAT	23	1	21UR-5732
2307421_adh	TGCTCGAGAATACAACGAAAA	21	46	21UR-5743
2307423_adh	TGCTCGAGAATACAACGAAAAC	22	1	21UR-5743
2611122_adh	TTCGTACGTTGGAAGCTAAAT	21	1087	21UR-5758
2611123_adh	TTCGTACGTTGGAAGCTAAATA	22	2	21UR-5758
2611125_adh	TTCGTACGTTGGAAGCTAAATAT	23	1	21UR-5758
2159350_adh	TGACCAAAACTCTCGGCATTT	21	348	21UR-5760
1066014_adh	CTGACCAAAACTCTCGGCATTT	22	1	21UR-5760
2159353_adh	TGACCAAAACTCTCGGCATTTCGT	24	2	21UR-5760
2159356_adh	TGACCAAAACTCTCGGCATTTCGTG	25	1	21UR-5760
2159357_adh	TGACCAAAACTCTCGGCATTTCGTGT	26	1	21UR-5760
2035360_adh	TCTCAAATTGATCTGTTGAGG	21	17	21UR-5800
221568_adh	AATCTCAAATTGATCTGTTGAGG	23	6	21UR-5800
2742184_adh	TTTTGCACGGTTTCGTATTAA	21	211	21UR-5810
2742185_adh	TTTTGCACGGTTTCGTATTAAA	22	1	21UR-5810
1830029_adh	TACTAGAGAAGTAGAAGTCAT	21	4118	21UR-5858
1847229_adh	TAGAGAAGTAGAAGTCATTTG	21	134	9_x134/21UR-5858
1830033_adh	TACTAGAGAAGTAGAAGTCATT	22	2	21UR-5858

597710_adh	ATACTAGAGAAGTAGAAGTCAT	22	3	21UR-5858
204508_adh	AATACTAGAGAAGTAGAAGTCAT	23	3	21UR-5858
1830037_adh	TACTAGAGAAGTAGAAGTCATTTGC	25	1	21UR-5858
1865007_adh	TAGGATATAGAATGAATCATT	21	35	21UR-5878
1865008_adh	TAGGATATAGAATGAATCATTAA	23	2	21UR-5878
2167932_adh	TGACTCGATAGATTGCTCACT	21	43	21UR-5883
226560_adh	AATGACTCGATAGATTGCTCACT	23	1	21UR-5883
1868608_adh	TAGGGATATTGCTAGGAAGAC	21	1859	21UR-5886
1868609_adh	TAGGGATATTGCTAGGAAGACA	22	1	21UR-5886
1868610_adh	TAGGGATATTGCTAGGAAGACAC	23	1	21UR-5886
206791_adh	AATAGGGATATTGCTAGGAAGAC	23	1	21UR-5886
1868611_adh	TAGGGATATTGCTAGGAAGACACA	24	2	21UR-5886
1868612_adh	TAGGGATATTGCTAGGAAGACACAA	25	2	21UR-5886
1868613_adh	TAGGGATATTGCTAGGAAGACACAAC	26	2	21UR-5886
1860596_adh	TAGGAAAGTATGTAGGAAAGT	21	841	21UR-5897
1860597_adh	TAGGAAAGTATGTAGGAAAGTAA	23	4	21UR-5897
1860598_adh	TAGGAAAGTATGTAGGAAAGTAAAGT	26	1	21UR-5897
2290769_adh	TGCATGGGCGGATTTCAGTTT	21	89	21UR-5913
229566_adh	AATGCATGGGCGGATTTCAGTTT	23	1	21UR-5913
2285959_adh	TGCACACACGAGGTACGGCAT	21	151	21UR-5921
2285960_adh	TGCACACACGAGGTACGGCATT	22	2	21UR-5921
2562548_adh	TTAGAGAGAGTTTCGGAAATT	21	91	21UR-5929
706624_adh	ATTAGAGAGAGTTTCGGAAATT	22	1	21UR-5929
1835336_adh	TACTTGGACAGATGAAGTTTT	21	108	21UR-5934
600712_adh	ATACTTGGACAGATGAAGTTTT	22	2	21UR-5934
2539584_adh	TTAAACAGTGGAATTAGGAAT	21	916	21UR-5937
2539587_adh	TTAAACAGTGGAATTAGGAATT	22	3	21UR-5937
2609266_adh	TTCGGGGCACGAACGGTTAAT	21	358	21UR-5941
2609267_adh	TTCGGGGCACGAACGGTTAATT	22	4	21UR-5941
2609268_adh	TTCGGGGCACGAACGGTTAATTAT	24	1	21UR-5941
1891799_adh	TATCACAATGCAGCTAGAATT	21	373	21UR-5946
1891800_adh	TATCACAATGCAGCTAGAATTAA	23	2	21UR-5946
208038_adh	AATATCACAATGCAGCTAGAATT	23	1	21UR-5946
2616254_adh	TTCTCCAGTTGACTCTATTTC	21	689	21UR-5950
2616255_adh	TTCTCCAGTTGACTCTATTTCAA	23	1	21UR-5950
2031645_adh	TCTACGGAGCAAGAGGGAAAA	21	200	21UR-5953
2031646_adh	TCTACGGAGCAAGAGGGAAAAT	22	1	21UR-5953
2031647_adh	TCTACGGAGCAAGAGGGAAAATAA	24	1	21UR-5953
1836300_adh	TACTTTGATTATTCCGGCTTC	21	46	21UR-5961
1836302_adh	TACTTTGATTATTCCGGCTTCT	22	1	21UR-5961
2046452_adh	TCTGAGTAGCAATTTTTGAAA	21	951	21UR-5975
2046457_adh	TCTGAGTAGCAATTTTTGAAAG	22	1	21UR-5975
2046458_adh	TCTGAGTAGCAATTTTTGAAAGG	23	2	21UR-5975
2046459_adh	TCTGAGTAGCAATTTTTGAAAGGA	24	4	21UR-5975
2046460_adh	TCTGAGTAGCAATTTTTGAAAGGAA	25	1	21UR-5975
2728674_adh	TTTGTCAAGATAGAAGATAGT	21	656	21UR-6008
2728677_adh	TTTGTCAAGATAGAAGATAGTT	22	3	21UR-6008
256850_adh	AATTTGTCAAGATAGAAGATAGT	23	1	21UR-6008
2182205_adh	TGAGACAAGAATACTTATTCA	21	252	21UR-6020
2182206_adh	TGAGACAAGAATACTTATTCACAA	24	1	21UR-6020

2624026_adh	TTCTTTTTGGAGTGTGGTCAT	21	129	21UR-6041
249354_adh	AATTCTTTTTGGAGTGTGGTCAT	23	1	21UR-6041
1961782_adh	TCAGATTGGAATTAAAATTGG	21	18	21UR-6045
1961783_adh	TCAGATTGGAATTAAAATTGGT	22	1	21UR-6045
2505635_adh	TGTGAGAAGGGAATTTGTCGA	21	64	21UR-6059
2505636_adh	TGTGAGAAGGGAATTTGTCGAA	22	4	21UR-6059
734620_adh	ATTGTGAGAAGGGAATTTGTCGAAAA	26	1	21UR-6059
2055987_adh	TCTTCGAAGGCATTATTTTCT	21	108	21UR-6062
222961_adh	AATCTTCGAAGGCATTATTTTCT	23	1	21UR-6062
1854798_adh	TAGCAGTTCGAGGAAATAAAA	21	15	21UR-6077
1854800_adh	TAGCAGTTCGAGGAAATAAAAGT	23	1	21UR-6077
1780884_adh	TAAGATTGTTAGAGAAATTGG	21	1395	21UR-6085
1026869_adh	CTAAGATTGTTAGAGAAATTGG	22	1	21UR-6085
1780889_adh	TAAGATTGTTAGAGAAATTGGG	22	5	21UR-6085
1780892_adh	TAAGATTGTTAGAGAAATTGGGTTT	25	1	21UR-6085
2605985_adh	TTCGATTTTGAGAAAACTGGC	21	3263	21UR-6098
1103456_adh	CTTCGATTTTGAGAAAACTGGC	22	1	21UR-6098
2605988_adh	TTCGATTTTGAGAAAACTGGCC	22	2	21UR-6098
2689517_adh	TTTAAATGGGCAGAAAAAAGT	21	133	21UR-6106
254646_adh	AATTTAAATGGGCAGAAAAAAGT	23	1	21UR-6106
2711241_adh	TTTCTTAGGGCTTTTGGCAAA	21	856	21UR-6123
2711244_adh	TTTCTTAGGGCTTTTGGCAAAT	22	1	21UR-6123
2739988_adh	TTTTCTTAGGGCTTTTGGCAAA	22	1	21UR-6123
2739990_adh	TTTTCTTAGGGCTTTTGGCAAAT	23	1	21UR-6123
2739991_adh	TTTTCTTAGGGCTTTTGGCAAATT	24	1	21UR-6123
2739992_adh	TTTTCTTAGGGCTTTTGGCAAATTT	25	1	21UR-6123
2742125_adh	TTTTGCAAGGATATATACGGA	21	125	21UR-6156
2721033_adh	TTTGCAAGGATATATACGGAT	21	1	)33_x1/21UR-6156
2742128_adh	TTTTGCAAGGATATATACGGAT	22	5	21UR-6156
747497_adh	ATTTTGCAAGGATATATACGGA	22	1	21UR-6156
2741471_adh	TTTTGAGTCTAATGAAACAAT	21	1	21UR-6157
257450_adh	AATTTTGAGTCTAATGAAACAAT	23	1	21UR-6157
2712406_adh	TTTGAAAAGCTGACAGGGGGT	21	470	21UR-6159
2712407_adh	TTTGAAAAGCTGACAGGGGGTA	22	1	21UR-6159
2712408_adh	TTTGAAAAGCTGACAGGGGGTAA	23	3	21UR-6159
2712409_adh	TTTGAAAAGCTGACAGGGGGTAAT	24	2	21UR-6159
2623045_adh	TTCTTGGATTAAACCGTGAAA	21	3	21UR-6196
249317_adh	AATTCTTGGATTAAACCGTGAAA	23	1	21UR-6196
1883560_adh	TAGTTTTTATCCGGACAACCT	21	859	21UR-6202
207666_adh	AATAGTTTTTATCCGGACAACCT	23	3	21UR-6202
1827279_adh	TACGGTCAATTATTTAAACTC	21	105	21UR-6203
1827280_adh	TACGGTCAATTATTTAAACTCA	22	4	21UR-6203
204169_adh	AATACGGTCAATTATTTAAACTC	23	1	21UR-6203
1869952_adh	TAGGGTTGATGAGTTAAGTTT	21	348	21UR-6207
1869953_adh	TAGGGTTGATGAGTTAAGTTTT	22	2	21UR-6207
2008294_adh	TCGCTACGCTTTTTCCTTTTA	21	5	21UR-6292
218977_adh	AATCGCTACGCTTTTTCCTTTTA	23	1	21UR-6292
2698219_adh	TTTATATATCGTGTTGACTTA	21	241	21UR-6328
739394_adh	ATTTATATATCGTGTTGACTTA	22	1	21UR-6328
2606572_adh	TTCGCGAGGGCGCTTTTTACC	21	49	21UR-6359

247301_adh	AATTCGCGAGGGCGCTTTTTACC	23	1	21UR-6359
2373288_adh	TGGACAAATTATAAGGAGACA	21	726	21UR-6364
2373289_adh	TGGACAAATTATAAGGAGACACTGT	25	1	21UR-6364
1783447_adh	TAAGCTCTCGGAATTTTCTTA	21	39	21UR-6367
1783448_adh	TAAGCTCTCGGAATTTTCTTAAA	23	1	21UR-6367
1854572_adh	TAGCAGCCTCTTAACTACAAA	21	17	21UR-6386
1854573_adh	TAGCAGCCTCTTAACTACAAAATTTG	26	1	21UR-6386
2490518_adh	TGTATAGAAGTCCGGATGATC	21	132	21UR-6394
695363_adh	ATGTATAGAAGTCCGGATGATC	22	1	21UR-6394
2744939_adh	TTTTTACGTATCTGATTTCCA	21	51	21UR-6405
257592_adh	AATTTTTACGTATCTGATTTCCA	23	1	21UR-6405
2582429_adh	TTATTGCAGAATATGGACTAC	21	2628	21UR-6409
1100094_adh	CTTATTGCAGAATATGGACTAC	22	5	21UR-6409
2582430_adh	TTATTGCAGAATATGGACTACA	22	2	21UR-6409
2582431_adh	TTATTGCAGAATATGGACTACAA	23	8	21UR-6409
1923766_adh	TATTCATATGGTAGAAAAAAG	21	162	21UR-6417
1923767_adh	TATTCATATGGTAGAAAAAAGC	22	3	21UR-6417
1923768_adh	TATTCATATGGTAGAAAAAAGCAT	24	1	21UR-6417
2714388_adh	TTTGAATGTCGGCAACGTTTC	21	645	21UR-6424
1422957_adh	GCTTTGAATGTCGGCAACGTTTCCT	25	1	21UR-6424
1850807_adh	TAGATCCAAATTAATCGGCAT	21	373	21UR-6443
205777_adh	AATAGATCCAAATTAATCGGCAT	23	2	21UR-6443
1804130_adh	TAATTGGTCGAGATAAACTTT	21	11	21UR-6445
202725_adh	AATAATTGGTCGAGATAAACTTT	23	1	21UR-6445
1777885_adh	TAAGATAATTCTGCCTACTTT	21	12	21UR-6449
1777886_adh	TAAGATAATTCTGCCTACTTTA	22	1	21UR-6449
2729910_adh	TTTGTGGCACACATGTAAATT	21	38	21UR-6452
256915_adh	AATTTGTGGCACACATGTAAATT	23	1	21UR-6452
1929194_adh	TATTTACTCACTGGAAAATTG	21	6	21UR-6475
1929195_adh	TATTTACTCACTGGAAAATTGA	22	1	21UR-6475
1908070_adh	TATGACGAACAATTCAACTCT	21	29	21UR-6476
1908071_adh	TATGACGAACAATTCAACTCTA	22	1	21UR-6476
1908073_adh	TATGACGAACAATTCAACTCTACTGC	26	1	21UR-6476
2739767_adh	TTTTCTGTTCGTGGACACAAT	21	2988	21UR-6485
2739768_adh	TTTTCTGTTCGTGGACACAATT	22	3	21UR-6485
2739771_adh	TTTTCTGTTCGTGGACACAATTT	23	1	21UR-6485
1916487_adh	TATGGTCCCGGAATCAATCAG	21	9	21UR-6497
1916489_adh	TATGGTCCCGGAATCAATCAGT	22	1	21UR-6497
1787881_adh	TAAGTCCTGGATTGAAATTTT	21	3	21UR-6520
1787882_adh	TAAGTCCTGGATTGAAATTTTA	22	1	21UR-6520
2621301_adh	TTCTTCGAAGATCTTCAAGGG	21	31	21UR-6527
249192_adh	AATTCTTCGAAGATCTTCAAGGG	23	1	21UR-6527
1830344_adh	TACTAGTGCTTCGGAAAACAA	21	55	21UR-6535
597744_adh	ATACTAGTGCTTCGGAAAACAA	22	1	21UR-6535
204526_adh	AATACTAGTGCTTCGGAAAACAA	23	1	21UR-6535
2552551_adh	TTAATTTAAAGCACGAGACTT	21	382	21UR-6576
704487_adh	ATTAATTTAAAGCACGAGACTT	22	1	21UR-6576
2561722_adh	TTAGAATCTAGAGTTGAGCAA	21	68	21UR-6612
2561723_adh	TTAGAATCTAGAGTTGAGCAAAA	23	1	21UR-6612
1964646_adh	TCAGGTAAGTAAGAAAACTAA	21	1262	21UR-6638

1964647_adh	TCAGGTAAGTAAGAAAACTAAA	22	1	21UR-6638
213589_adh	AATCAGGTAAGTAAGAAAACTAA	23	3	21UR-6638
1829132_adh	TACGTTTCCTATATGGAGATC	21	13	21UR-6640
204428_adh	AATACGTTTCCTATATGGAGATC	23	1	21UR-6640
2556850_adh	TTACGCACACGGTGAAGATTT	21	31	21UR-6649
1096268_adh	CTTACGCACACGGTGAAGATTT	22	1	21UR-6649
2066531_adh	TGAAAAATTCCTAAGATGGTG	21	161	21UR-6656
223373_adh	AATGAAAAATTCCTAAGATGGTG	23	1	21UR-6656
2687247_adh	TTGTTTCTACATCGTTGGGGC	21	223	21UR-6689
254585_adh	AATTGTTTCTACATCGTTGGGGC	23	1	21UR-6689
1810898_adh	TACACCCAGAACTAAATTCAG	21	18	21UR-6700
1810899_adh	TACACCCAGAACTAAATTCAGA	22	1	21UR-6700
1810900_adh	TACACCCAGAACTAAATTCAGAA	23	1	21UR-6700
1978601_adh	TCCAGGAACTCAAGGGACGAT	21	565	21UR-6712
1978604_adh	TCCAGGAACTCAAGGGACGATT	22	2	21UR-6712
1773155_adh	TAAGAAAACTGGACGTTCACA	21	15	21UR-6718
1773156_adh	TAAGAAAACTGGACGTTCACAAA	23	1	21UR-6718
200282_adh	AATAAGAAAACTGGACGTTCACA	23	1	21UR-6718
2042660_adh	TCTCTCTGCATATATCTCATT	21	638	21UR-6737
2042661_adh	TCTCTCTGCATATATCTCATTT	22	1	21UR-6737
1931436_adh	TATTTTTGTCGAACTTCTTGA	21	62	21UR-6754
1931437_adh	TATTTTTGTCGAACTTCTTGAA	22	1	21UR-6754
2597326_adh	TTCCATGCATTTACATTTGAA	21	9	21UR-6766
246485_adh	AATTCCATGCATTTACATTTGAA	23	1	21UR-6766
2568507_adh	TTAGGCATTGGTGTACTTGCG	21	28	21UR-6768
2568508_adh	TTAGGCATTGGTGTACTTGCGT	22	7	21UR-6768
2568509_adh	TTAGGCATTGGTGTACTTGCGTG	23	1	21UR-6768
2568510_adh	TTAGGCATTGGTGTACTTGCGTGT	24	2	21UR-6768
1965301_adh	TCAGTAACGTCTCTTTTTCCT	21	13	21UR-6778
628414_adh	ATCAGTAACGTCTCTTTTTCCT	22	1	21UR-6778
2139393_adh	TGAATATGAATGACGAACGGA	21	2177	21UR-6799
2139398_adh	TGAATATGAATGACGAACGGAT	22	6	21UR-6799
662890_adh	ATGAATATGAATGACGAACGGA	22	1	21UR-6799
224823_adh	AATGAATATGAATGACGAACGGA	23	2	21UR-6799
2139400_adh	TGAATATGAATGACGAACGGATGT	24	1	21UR-6799
1941991_adh	TCAATGATTAACGGTACTACT	21	37	21UR-6804
210582_adh	AATCAATGATTAACGGTACTACT	23	1	21UR-6804
1838413_adh	TAGAACGACGCCAACGATATT	21	48	21UR-6818
542353_adh	AGTAGAACGACGCCAACGATATTC	24	1	21UR-6818
1838414_adh	TAGAACGACGCCAACGATATTCTAA	25	2	21UR-6818
1985447_adh	TCCGCGGTCGTACGGCAGATT	21	539	21UR-6828
1985449_adh	TCCGCGGTCGTACGGCAGATTT	22	2	21UR-6828
215550_adh	AATCCGCGGTCGTACGGCAGATT	23	1	21UR-6828
1866854_adh	TAGGCAAGAGCGGGTGAAGAA	21	263	21UR-6834
1866855_adh	TAGGCAAGAGCGGGTGAAGAAAA	23	1	21UR-6834
2648727_adh	TTGCAAGTACGACCACGAAAG	21	33	21UR-6844
1111095_adh	CTTGCAAGTACGACCACGAAAG	22	1	21UR-6844
1805007_adh	TAATTTCGTCAGCATGGCTAG	21	521	21UR-6910
1805012_adh	TAATTTCGTCAGCATGGCTAGT	22	2	21UR-6910
2677238_adh	TTGTATTGATGGTGATGGGCT	21	94	21UR-6913

2677240_adh	TTGTATTGATGGTGATGGGCTT	22	2	21UR-6913
2644073_adh	TTGATCATTCATGTAACGGTT	21	138	21UR-6916
250704_adh	AATTGATCATTCATGTAACGGTT	23	2	21UR-6916
1919972_adh	TATTAAATGAAGTAGAAAAAA	21	100	21UR-6933
1919974_adh	TATTAAATGAAGTAGAAAAAAT	22	4	21UR-6933
1782809_adh	TAAGCGTAATGTGTCGGACGC	21	48	21UR-6937
1782811_adh	TAAGCGTAATGTGTCGGACGCTAAT	25	1	21UR-6937
750583_adh	CAAAAAAGAGTAGAAGGAATC	21	516	21UR-6939
750584_adh	CAAAAAAGAGTAGAAGGAATCA	22	1	21UR-6939
2391989_adh	TGGATGGTTGAAGAAGGAGTA	21	1605	21UR-6953
2391991_adh	TGGATGGTTGAAGAAGGAGTAT	22	4	21UR-6953
2391992_adh	TGGATGGTTGAAGAAGGAGTATCGT	25	1	21UR-6953
2131384_adh	TGAAGCGCTGAAAATCATCAA	21	16	21UR-6955
224570_adh	AATGAAGCGCTGAAAATCATCAA	23	1	21UR-6955
1962326_adh	TCAGCATAACTCATTTTTTAA	21	52	21UR-7003
213475_adh	AATCAGCATAACTCATTTTTTAA	23	1	21UR-7003
2581379_adh	TTATTCATTCGGACAAAAATT	21	632	21UR-7014
1099963_adh	CTTATTCATTCGGACAAAAATT	22	1	21UR-7014
2581380_adh	TTATTCATTCGGACAAAAATTT	22	1	21UR-7014
1821086_adh	TACCTGTGTGTTTGTTACAAA	21	11	21UR-7025
595770_adh	ATACCTGTGTGTTTGTTACAAA	22	1	21UR-7025
1764945_adh	TAACAGACTTATAAAGACATT	21	120	21UR-7029
1025913_adh	CTAACAGACTTATAAAGACATT	22	1	21UR-7029
1929123_adh	TATTTAATTTTGCGAGTAGAA	21	173	21UR-7045
2693036_adh	TTTAATTTTGCGAGTAGAAAA	21	1	)36_x1/21UR-7045
1929124_adh	TATTTAATTTTGCGAGTAGAAAA	23	2	21UR-7045
1824022_adh	TACGATCTTCGGCAAGCTTTC	21	28	21UR-7048
1824024_adh	TACGATCTTCGGCAAGCTTTCTCAGT	26	1	21UR-7048
1901979_adh	TATGAAGTTGAATGGGCATTT	21	26	21UR-7126
1901980_adh	TATGAAGTTGAATGGGCATTTT	22	1	21UR-7126
1803858_adh	TAATTGATTGTCTCCGGTCTT	21	11	21UR-7131
202702_adh	AATAATTGATTGTCTCCGGTCTT	23	1	21UR-7131
2715302_adh	TTTGACGTGACTTCGTTCTGA	21	43	21UR-7141
2715303_adh	TTTGACGTGACTTCGTTCTGAA	22	3	21UR-7141
2715304_adh	TTTGACGTGACTTCGTTCTGAAA	23	4	21UR-7141
2552817_adh	TTAATTTGCGGTACACGAATT	21	5	21UR-7191
244174_adh	AATTAATTTGCGGTACACGAATT	23	1	21UR-7191
2534949_adh	TGTTTTTAGAATTTTAGTCGG	21	866	21UR-7192
2534953_adh	TGTTTTTAGAATTTTAGTCGGT	22	6	21UR-7192
2534954_adh	TGTTTTTAGAATTTTAGTCGGTAT	24	1	21UR-7192
2015448_adh	TCGGCCTGTCATATCGTCATC	21	921	21UR-7199
2015449_adh	TCGGCCTGTCATATCGTCATCA	22	14	21UR-7199
646872_adh	ATCGGCCTGTCATATCGTCATC	22	2	21UR-7199
219943_adh	AATCGGCCTGTCATATCGTCATC	23	7	21UR-7199
1872501_adh	TAGGTTGTAAATTTTTATCGC	21	27	21UR-7205
1872502_adh	TAGGTTGTAAATTTTTATCGCAA	23	1	21UR-7205
2286207_adh	TGCACCACATCGTCGAAATTA	21	977	21UR-7225
2286208_adh	TGCACCACATCGTCGAAATTAC	22	1	21UR-7225
672965_adh	ATGCACCACATCGTCGAAATTA	22	1	21UR-7225
2286209_adh	TGCACCACATCGTCGAAATTACAT	24	1	21UR-7225

1890375_adh	TATATGGGAAATGGGAAATAA	21	59	21UR-7240
1890376_adh	TATATGGGAAATGGGAAATAAT	22	1	21UR-7240
2683601_adh	TTGTTCCAATCTTCATGGAAT	21	32	21UR-7250
253960_adh	AATTGTTCCAATCTTCATGGAAT	23	1	21UR-7250
2561879_adh	TTAGAATTTTAGTTGACTATG	21	11	21UR-7255
2561880_adh	TTAGAATTTTAGTTGACTATGGTA	24	1	21UR-7255
2561881_adh	TTAGAATTTTAGTTGACTATGGTAT	25	2	21UR-7255
2169194_adh	TGACTGAACATCAAAGGAGAA	21	713	21UR-7261
2169195_adh	TGACTGAACATCAAAGGAGAAA	22	1	21UR-7261
1877829_adh	TAGTGAGACTGTTCTGAAAAA	21	47	21UR-7267
1877830_adh	TAGTGAGACTGTTCTGAAAAAT	22	1	21UR-7267
1851135_adh	TAGATCTTGACAGAAGAACAA	21	3210	21UR-7310
1851136_adh	TAGATCTTGACAGAAGAACAAAA	23	2	21UR-7310
1783846_adh	TAAGGAAATTATTAGAATGAA	21	444	21UR-7312
1783847_adh	TAAGGAAATTATTAGAATGAAA	22	1	21UR-7312
1914037_adh	TATGGAGAATGTATGTATTGT	21	243	21UR-7347
1914038_adh	TATGGAGAATGTATGTATTGTA	22	2	21UR-7347
1852716_adh	TAGCAAAAGTGAGCTGAAATA	21	1155	21UR-7348
1852717_adh	TAGCAAAAGTGAGCTGAAATAC	22	1	21UR-7348
205888_adh	AATAGCAAAAGTGAGCTGAAATA	23	3	21UR-7348
1850361_adh	TAGATAGGCAGGATTGCAAAA	21	44	21UR-7349
1850362_adh	TAGATAGGCAGGATTGCAAAAT	22	1	21UR-7349
2644451_adh	TTGATCTCCAATTAAGGATAA	21	4	21UR-7365
726908_adh	ATTGATCTCCAATTAAGGATAA	22	1	21UR-7365
1928108_adh	TATTGTGTTCGAGAGATTTTT	21	233	21UR-7393
1928109_adh	TATTGTGTTCGAGAGATTTTTT	22	1	21UR-7393
1851904_adh	TAGATTACGTGAATAAAGAGA	21	9	21UR-7436
2565705_adh	TTAGATTACGTGAATAAAGAGA	22	1	21UR-7436
1823836_adh	TACGATCACTGGTTTATTTGT	21	39	21UR-7437
1823837_adh	TACGATCACTGGTTTATTTGTAA	23	1	21UR-7437
2590328_adh	TTCAGAACTATTCGACTGCTT	21	568	21UR-7495
711906_adh	ATTCAGAACTATTCGACTGCTT	22	1	21UR-7495
246129_adh	AATTCAGAACTATTCGACTGCTT	23	1	21UR-7495
1946438_adh	TCACATTTTAGATAACTGGTT	21	1112	21UR-7569
212566_adh	AATCACATTTTAGATAACTGGTT	23	2	21UR-7569
1946442_adh	TCACATTTTAGATAACTGGTTTCTTA	26	1	21UR-7569
1860063_adh	TAGGAAAACTGAAAACGCATA	21	899	21UR-7574
1035201_adh	CTAGGAAAACTGAAAACGCATA	22	2	21UR-7574
1860064_adh	TAGGAAAACTGAAAACGCATAT	22	2	21UR-7574
1860065_adh	TAGGAAAACTGAAAACGCATATC	23	1	21UR-7574
2690596_adh	TTTAAGGATTGAAGGTATTTT	21	1031	21UR-7584
2548393_adh	TTAAGGATTGAAGGTATTTTT	21	7	393_x7/21UR-7584
2690598_adh	TTTAAGGATTGAAGGTATTTTT	22	1	21UR-7584
1326459_adh	GATTTAAGGATTGAAGGTATTTTT	24	1	21UR-7584
2059994_adh	TCTTGTGTTTCCGGTTTCAAA	21	1	21UR-7596
223200_adh	AATCTTGTGTTTCCGGTTTCAAA	23	1	21UR-7596
2550935_adh	TTAATGAACTTTTTAATCGGC	21	2938	21UR-7645
2550936_adh	TTAATGAACTTTTTAATCGGCA	22	9	21UR-7645
244109_adh	AATTAATGAACTTTTTAATCGGC	23	2	21UR-7645
2289152_adh	TGCAGTGGACGGATCTGTTTT	21	4922	21UR-7654

1071339_adh	CTGCAGTGGACGGATCTGTTTT	22	2	21UR-7654
2289156_adh	TGCAGTGGACGGATCTGTTTTT	22	15	21UR-7654
2289162_adh	TGCAGTGGACGGATCTGTTTTTG	23	1	21UR-7654
2050301_adh	TCTGGGAGACGGAGAATAAGA	21	180	21UR-7659
2050302_adh	TCTGGGAGACGGAGAATAAGAA	22	1	21UR-7659
2050303_adh	TCTGGGAGACGGAGAATAAGAAA	23	1	21UR-7659
1877079_adh	TAGTGAAACTCTTTTCTGATC	21	4	21UR-7668
1877080_adh	TAGTGAAACTCTTTTCTGATCGTCAA	26	1	21UR-7668
1952995_adh	TCACGAACTGAAACTACATTTT	22	1	21UR-7672
1952996_adh	TCACGAACTGAAACTACATTTTACAT	26	1	21UR-7672
2737659_adh	TTTTCATCCGGCATAACTAAA	21	33	21UR-7676
2737660_adh	TTTTCATCCGGCATAACTAAAAA	23	1	21UR-7676
2737661_adh	TTTTCATCCGGCATAACTAAAAAAT	25	1	21UR-7676
2720350_adh	TTTGATTGTATACCAAATTTG	21	466	21UR-7678
1122632_adh	CTTTGATTGTATACCAAATTTG	22	1	21UR-7678
2720353_adh	TTTGATTGTATACCAAATTTGT	22	1	21UR-7678
1839031_adh	TAGAAGAATTTGTGCAAAAAT	21	210	21UR-7724
1839032_adh	TAGAAGAATTTGTGCAAAAATAA	23	2	21UR-7724
2121240_adh	TGAACAGTGAAGCAGAACTTT	21	834	21UR-7759
2121245_adh	TGAACAGTGAAGCAGAACTTTT	22	2	21UR-7759
2049737_adh	TCTGGATATGTTTAAAGGTGG	21	644	21UR-7761
2049738_adh	TCTGGATATGTTTAAAGGTGGA	22	5	21UR-7761
1836901_adh	TAGAAACAGTCGGAAAAAAGT	21	32	21UR-7772
205378_adh	AATAGAAACAGTCGGAAAAAAGT	23	1	21UR-7772
1753688_adh	TAAAATTTGTCCATACGAACC	21	3	21UR-7775
1753689_adh	TAAAATTTGTCCATACGAACCAA	23	1	21UR-7775
2713962_adh	TTTGAAGGATATAGGGCATTT	21	174	21UR-7783
255975_adh	AATTTGAAGGATATAGGGCATTT	23	1	21UR-7783
2389313_adh	TGGATATTTTGATCAACGGGA	21	122	21UR-7848
684383_adh	ATGGATATTTTGATCAACGGGA	22	1	21UR-7848
2053537_adh	TCTGTTTTGTCGGAAACTGTC	21	15	21UR-7911
657495_adh	ATCTGTTTTGTCGGAAACTGTC	22	1	21UR-7911
1845654_adh	TAGACTCAGAAAAAACATTGA	21	5	21UR-7930
1845655_adh	TAGACTCAGAAAAAACATTGACGA	24	1	21UR-7930
1813023_adh	TACATAAGATGTTTGTTTTCG	21	11	21UR-7933
593443_adh	ATACATAAGATGTTTGTTTTCG	22	1	21UR-7933
1761290_adh	TAAATGAATAAATCATTCATT	21	4	21UR-7981
199459_adh	AATAAATGAATAAATCATTCATT	23	1	21UR-7981
2595499_adh	TTCATTTGACGTTTATGAAAA	21	6	21UR-8005
246349_adh	AATTCATTTGACGTTTATGAAAA	23	1	21UR-8005
1925353_adh	TATTGAAGAAAAATGCACTGG	21	9	21UR-8033
1925354_adh	TATTGAAGAAAAATGCACTGGAA	23	1	21UR-8033
2729926_adh	TTTGTGGCGAAGAAGCATTTT	21	358	21UR-8053
2729928_adh	TTTGTGGCGAAGAAGCATTTTGAAG	25	1	21UR-8053
2729929_adh	TTTGTGGCGAAGAAGCATTTTGAAGA	26	1	21UR-8053
220932_adh	AATCGTGTTAGTAAATAACCACT	23	1	21UR-8084
1900702_adh	TATCTTTTTAAGTCGTACATA	21	618	21UR-8093
1900704_adh	TATCTTTTTAAGTCGTACATAT	22	1	21UR-8093
752781_adh	CAAAATCTTTAGACGACAAAA	21	18	21UR-8107
752782_adh	CAAAATCTTTAGACGACAAAAT	22	1	21UR-8107

2721378_adh	TTTGCATACCTCTAATAACCA	21	12	21UR-8116
256407_adh	AATTTGCATACCTCTAATAACCA	23	1	21UR-8116
1857630_adh	TAGCGTGATGTCATCTATTTG	21	153	21UR-8174
1034743_adh	CTAGCGTGATGTCATCTATTTG	22	1	21UR-8174
2712898_adh	TTTGAACACCATACAATCTTT	21	101	21UR-8191
2712899_adh	TTTGAACACCATACAATCTTTA	22	1	21UR-8191
255938_adh	AATTTGAACACCATACAATCTTT	23	3	21UR-8191
2712900_adh	TTTGAACACCATACAATCTTTAG	23	1	21UR-8191
2132000_adh	TGAAGCTGCAGAGATGAATCT	21	1309	21UR-8227
662191_adh	ATGAAGCTGCAGAGATGAATCT	22	1	21UR-8227
2618340_adh	TTCTGAGGGTTTTGCAATTGCA	22	1	21UR-8229
1782946_adh	TAAGCTAGAAAATGGATAAAT	21	811	21UR-8255
1782947_adh	TAAGCTAGAAAATGGATAAATATT	24	1	21UR-8255
1931031_adh	TATTTTGAATGGGCGCAGGAA	21	41	21UR-8316
1931032_adh	TATTTTGAATGGGCGCAGGAAT	22	1	21UR-8316
2703721_adh	TTTCATACCGTTGGAAAAATT	21	81	21UR-8341
1120726_adh	CTTTCATACCGTTGGAAAAATT	22	2	21UR-8341
1876124_adh	TAGTCAGAAAGCTATCGGTAT	21	99	21UR-8397
609681_adh	ATAGTCAGAAAGCTATCGGTAT	22	1	21UR-8397
1764803_adh	TAACACTTAGAACAGAATTCG	21	244	21UR-8406
1764804_adh	TAACACTTAGAACAGAATTCGAGA	24	1	21UR-8406
1790884_adh	TAATACAAATAGAAATATTGA	21	10	21UR-8490
1790886_adh	TAATACAAATAGAAATATTGAT	22	1	21UR-8490
1923025_adh	TATTATTCGGATTTCAGATAG	21	14	21UR-8580
1923026_adh	TATTATTCGGATTTCAGATAGG	22	1	21UR-8580
2706491_adh	TTTCCTTTTTGAACAACAGCA	21	44	21UR-8613
740651_adh	ATTTCCTTTTTGAACAACAGCA	22	1	21UR-8613
1925173_adh	TATTCTTCAGGCAGTTGCAAA	21	2	21UR-8686
209154_adh	AATATTCTTCAGGCAGTTGCAAA	23	1	21UR-8686
1830215_adh	TACTAGCGATACTTTTTGCAG	21	34	21UR-8695
204521_adh	AATACTAGCGATACTTTTTGCAG	23	1	21UR-8695
2694031_adh	TTTACGTAGAAAACAATTAAA	21	319	21UR-8715
254825_adh	AATTTACGTAGAAAACAATTAAA	23	1	21UR-8715
2041203_adh	TCTCGTAGGAAAAAGCGGTTT	21	314	21UR-8766
2041205_adh	TCTCGTAGGAAAAAGCGGTTTT	22	2	21UR-8766
2041207_adh	TCTCGTAGGAAAAAGCGGTTTTGTGT	26	1	21UR-8766
1837945_adh	TAGAACAAGTAGTCTACGGCT	21	140	21UR-8787
1837946_adh	TAGAACAAGTAGTCTACGGCTT	22	1	21UR-8787
2685958_adh	TTGTTGGAAAAATCGTCTAAT	21	235	21UR-8823
2685959_adh	TTGTTGGAAAAATCGTCTAATT	22	1	21UR-8823
1901659_adh	TATGAACTTAGGTACAACCAA	21	2	21UR-8909
1901660_adh	TATGAACTTAGGTACAACCAAA	22	1	21UR-8909
2692982_adh	TTTAATTTGATCATCCGTTTT	21	26	21UR-8948
575968_adh	AGTTTAATTTGATCATCCGTTTT	23	1	21UR-8948
1929471_adh	TATTTCAAGCTCTTGTTTTTC	21	6	21UR-9038
621924_adh	ATATTTCAAGCTCTTGTTTTTC	22	1	21UR-9038
1909721_adh	TATGAGTAGGTGCTGAAGACG	21	360	21UR-9046
1909722_adh	TATGAGTAGGTGCTGAAGACGT	22	2	21UR-9046
1798950_adh	TAATCTGTTCCGTAGAAGAAA	21	109	21UR-9061
1798951_adh	TAATCTGTTCCGTAGAAGAAAACT	24	1	21UR-9061

383134_adh	AGAAGTCGAAACATACTAGAA	21	96	21UR-9071
383135_adh	AGAAGTCGAAACATACTAGAAT	22	1	21UR-9071
2561511_adh	TTAGAAGGCCCGGTGGAACTG	21	2	21UR-9126
1802524_adh	TAATTAGAAGGCCCGGTGGAACTGC	25	1	21UR-9126
1811383_adh	TACACTGGAGTTTTTAGATTT	21	19	21UR-9190
1811384_adh	TACACTGGAGTTTTTAGATTTA	22	1	21UR-9190
2572527_adh	TTAGTTGAAGTTATGGCTAGT	21	502	21UR-9276
2572530_adh	TTAGTTGAAGTTATGGCTAGTT	22	1	21UR-9276
2572533_adh	TTAGTTGAAGTTATGGCTAGTTT	23	1	21UR-9276
2528767_adh	TGTTGAGCAGGTTGGTGTTTT	21	1	21UR-9284
77732_adh	AAATGTTGAGCAGGTTGGTGTTTT	24	1	21UR-9284
2351418_adh	TGGAATGGACGAAACCCACAA	21	11	21UR-9292
232176_adh	AATGGAATGGACGAAACCCACAA	23	1	21UR-9292
1912915_adh	TATGCGACAGTACGTATGAAA	21	57	21UR-9501
2579154_adh	TTATGCGACAGTACGTATGAAAA	23	1	21UR-9501
1912917_adh	TATGCGACAGTACGTATGAAAATTG	25	1	21UR-9501
1858358_adh	TAGCTGAACGAAGGAGATATC	21	49	21UR-9510
1858359_adh	TAGCTGAACGAAGGAGATATCAATG	25	1	21UR-9510
2617278_adh	TTCTCTGAAGGCATATTTTTG	21	3050	21UR-9596
2617281_adh	TTCTCTGAAGGCATATTTTTGT	22	19	21UR-9596
2617284_adh	TTCTCTGAAGGCATATTTTTGTGT	24	1	21UR-9596
1936301_adh	TCAACCAGTAGACATTTATTA	21	983	21UR-9673
1936302_adh	TCAACCAGTAGACATTTATTAAA	23	1	21UR-9673
1765778_adh	TAACATATGCAAATAAGTACG	21	23	21UR-9720
1765779_adh	TAACATATGCAAATAAGTACGAATG	25	1	21UR-9720
2660719_adh	TTGGATAAGTTGTATCTTCGG	21	16	21UR-9779
2660720_adh	TTGGATAAGTTGTATCTTCGGG	22	1	21UR-9779
2660721_adh	TTGGATAAGTTGTATCTTCGGGT	23	1	21UR-9779
849465_adh	CATTGGATAAGTTGTATCTTCGGG	24	1	21UR-9779
2311836_adh	TGCTTTGACTTAAGAAGAATT	21	2	21UR-9851
678413_adh	ATGCTTTGACTTAAGAAGAATT	22	1	21UR-9851
1866982_adh	TAGGCACTTCAGAGCAAATAG	21	6	21UR-9936
1866983_adh	TAGGCACTTCAGAGCAAATAGAG	23	1	21UR-9936
1826246_adh	TACGGCCAAATCAGATTTCTG	21	14	21UR-9939
1826247_adh	TACGGCCAAATCAGATTTCTGACTGA	26	1	21UR-9939
1884983_adh	TATAAGAAGAAAAACAAAGTTTTA	24	2	21UR-10159
2140638_adh	TGAATCAGAACAATGGTACCCT	22	18	21UR-10193
2140651_adh	TGAATCAGAACAATGGTACCCTT	23	1	21UR-10193
2140652_adh	TGAATCAGAACAATGGTACCCTTTT	25	1	21UR-10193
2140653_adh	TGAATCAGAACAATGGTACCCTTTTT	26	1	21UR-10193
2527431_adh	TGTTGAAACGGTATGAAATTG	21	57	21UR-10324
2527432_adh	TGTTGAAACGGTATGAAATTGT	22	1	21UR-10324
2487392_adh	TGTAGAAAAGTGGTTGCTTGT	21	1	21UR-10334
1879293_adh	TAGTGTAGAAAAGTGGTTGCTTGT	24	10	21UR-10334
2142629_adh	TGAATCGTTGAACCAATTTTA	21	3	21UR-10357
224852_adh	AATGAATCGTTGAACCAATTTTA	23	1	21UR-10357
1788018_adh	TAAGTCGTTGATGATCGTTTT	21	135	21UR-10477
200783_adh	AATAAGTCGTTGATGATCGTTTT	23	2	21UR-10477
902821_adh	CCTTCAAGAACAATGCAAAAG	21	11	21UR-10497
902822_adh	CCTTCAAGAACAATGCAAAAGC	22	1	21UR-10497

2734699_adh	TTTTAGCGGTAGATTCGTTTT	21	177	21UR-10518
746303_adh	ATTTTAGCGGTAGATTCGTTTT	22	1	21UR-10518
2611947_adh	TTCGTCGAAAACTTTGGCATC	21	11	21UR-10590
247673_adh	AATTCGTCGAAAACTTTGGCATC	23	1	21UR-10590
2533467_adh	TGTTTGTGGAAGCGTGAGCAT	21	53	21UR-10640
2533468_adh	TGTTTGTGGAAGCGTGAGCATT	22	1	21UR-10640
1928687_adh	TATTGTTTGTGGAAGCGTGAGCATT	25	1	21UR-10640
1928688_adh	TATTGTTTGTGGAAGCGTGAGCATTT	26	1	21UR-10640
1754038_adh	TAAACAAGGGACTGTTAGGGA	21	21	21UR-10819
2539284_adh	TTAAACAAGGGACTGTTAGGGA	22	21	21UR-10819
2539285_adh	TTAAACAAGGGACTGTTAGGGAC	23	1	21UR-10819
2539286_adh	TTAAACAAGGGACTGTTAGGGACAC	25	1	21UR-10819
1185383_adh	GAAGTGGAAGAGTAATGAAGA	21	1	21UR-10827
1185384_adh	GAAGTGGAAGAGTAATGAAGATGT	24	1	21UR-10827
1686904_adh	GTTAATTTCATTTTCGGATTTGCGTC	26	9	21UR-10895
2530884_adh	TGTTGTGACGAATAACGGCAA	21	13	21UR-11056
701373_adh	ATGTTGTGACGAATAACGGCAA	22	1	21UR-11056
1927828_adh	TATTGTATTGTAAAGGTTTGA	21	102	21UR-11180
1927829_adh	TATTGTATTGTAAAGGTTTGAA	22	6	21UR-11180
1922991_adh	TATTATTCAAGTAGATAATTT	21	51	21UR-11188
1922992_adh	TATTATTCAAGTAGATAATTTCA	23	1	21UR-11188
1796050_adh	TAATATCTTGTGGGCATATAA	21	40	21UR-11764
1796051_adh	TAATATCTTGTGGGCATATAAA	22	1	21UR-11764
1750614_adh	TAAAATACTTGAGAAAAATTT	21	11	21UR-11782
1750615_adh	TAAAATACTTGAGAAAAATTTGAT	24	1	21UR-11782
148853_adh	AAGCACGGCCTCTGTGAAATT	21	1	21UR-11809
1781565_adh	TAAGCACGGCCTCTGTGAAATT	22	1	21UR-11809
2687649_adh	TTGTTTGTGGAAGCGTGAGCA	21	1	21UR-11964
1928686_adh	TATTGTTTGTGGAAGCGTGAGCA	23	1	21UR-11964
1927177_adh	TATTGGGAAGAAGACAAAATCGTCTA	26	1	21UR-12226
1930846_adh	TATTTTCACCTTAACTTTAGA	21	2	21UR-12381
1930847_adh	TATTTTCACCTTAACTTTAGAC	22	1	21UR-12381
1927499_adh	TATTGTAATTTTTGTATCATC	21	4	21UR-12395
1927500_adh	TATTGTAATTTTTGTATCATCA	22	1	21UR-12395
1835704_adh	TACTTGTAGATACAGTAGTGT	21	6	21UR-12475
205281_adh	AATACTTGTAGATACAGTAGTGT	23	1	21UR-12475
1801978_adh	TAATGTTGTAATAGCTACTCG	21	3	21UR-12502
1801979_adh	TAATGTTGTAATAGCTACTCGGAA	24	1	21UR-12502
1796957_adh	TAATATGTTTTCAAGAAAATC	21	1	21UR-12516
201123_adh	AATAATATGTTTTCAAGAAAATC	23	1	21UR-12516
705328_adh	ATTACGACTCAACGACTAACT	21	2	21UR-12582
1920818_adh	TATTACGACTCAACGACTAACT	22	1	21UR-12582
2748637_adh	TTTTTTCATTGGATTGTCGTA	21	453	21UR-12608
257765_adh	AATTTTTTCATTGGATTGTCGTA	23	1	21UR-12608
2687327_adh	TTGTTTCTTTAAATGACATAC	21	2	21UR-12812
2687328_adh	TTGTTTCTTTAAATGACATACA	22	1	21UR-12812
251797_adh	AATTGCGAACACAGCTTTTTAGT	23	1	21UR-12870
2626918_adh	TTGAACTGTGGCGTGACTTATCGA	24	1	21UR-13283
1810844_adh	TACACCAAAATCTGAATTTTT	21	117	21UR-13591
1810845_adh	TACACCAAAATCTGAATTTTTAA	23	1	21UR-13591

203066_adh	AATACACCAAAATCTGAATTTTT	23	1	21UR-13591
1758133_adh	TAAAGGGGAAGGCCTTGTTTG	21	5	21UR-13660
199122_adh	AATAAAGGGGAAGGCCTTGTTTG	23	1	21UR-13660
670824_adh	ATGATTGCACCAGATGTAATGA	22	2	21UR-13716
228837_adh	AATGATTGCACCAGATGTAATGA	23	2	21UR-13716
2588763_adh	TTCAATTAATAAGCAAATTCG	21	3	21UR-14433
246040_adh	AATTCAATTAATAAGCAAATTCG	23	1	21UR-14433
2016373_adh	TCGGGAAGAAATTATCGGCCA	21	1	21UR-15009
718356_adh	ATTCGGGAAGAAATTATCGGCCAA	24	1	21UR-15009
1923877_adh	TATTCCAATGGGTCGTTGTGAA	22	1	21UR-15200
1835688_adh	TACTTGTAGAACGACAGGAAA	21	99	21UR-15353
1835689_adh	TACTTGTAGAACGACAGGAAAA	22	2	21UR-15353
1822112_adh	TACGACAGGAGATGAATACAA	21	17	21UR-15378
1822113_adh	TACGACAGGAGATGAATACAAA	22	1	21UR-15378
1809268_adh	TACAATAATAGAAGAACATGG	21	58	21UR-15401
1809269_adh	TACAATAATAGAAGAACATGGC	22	1	21UR-15401
1443334_adh	GGAATTAAAATTTGATCATGG	21	1	21UR-15578
1443335_adh	GGAATTAAAATTTGATCATGGT	22	1	21UR-15578
515525_adh	AGGGAATTAAAATTTGATCATGGT	24	1	21UR-15578
515526_adh	AGGGAATTAAAATTTGATCATGGTT	25	1	21UR-15578
515528_adh	AGGGAATTAAAATTTGATCATGGTTT	26	1	21UR-15578
1264191_adh	GAGGGATGGTAAACGAACGAA	21	15	21UR-15588
1264196_adh	GAGGGATGGTAAACGAACGAAG	22	54	21UR-15588
1264197_adh	GAGGGATGGTAAACGAACGAAGG	23	28	21UR-15588
1375496_adh	GCGAGGGATGGTAAACGAACGAA	23	1	21UR-15588
1264199_adh	GAGGGATGGTAAACGAACGAAGGG	24	2	21UR-15588
1375498_adh	GCGAGGGATGGTAAACGAACGAAG	24	2	21UR-15588
1487746_adh	GGCGAGGGATGGTAAACGAACGAA	24	1	21UR-15588
1375499_adh	GCGAGGGATGGTAAACGAACGAAGG	25	1	21UR-15588
1375500_adh	GCGAGGGATGGTAAACGAACGAAGGG	26	1	21UR-15588
1487747_adh	GGCGAGGGATGGTAAACGAACGAAGG	26	2	21UR-15588
1753985_adh	TAAACAAGGCATGTGTAGGAAAGGC	25	2	21UR-15650
1753986_adh	TAAACAAGGCATGTGTAGGAAAGGCA	26	1	21UR-15650
713431_adh	ATTCCAGACACACAGAAGGAAG	22	1	21UR-15660
246413_adh	AATTCCAGACACACAGAAGGAAG	23	2	21UR-15660
657440_adh	ATCTGTTGTCGATGGGTCAATA	22	1	21UR-15664
1170544_adh	GAAGAGGAGAGGTAAATCTACGGA	24	1	21UR-15673
1980122_adh	TCCATTTTTTATTACAATTTA	21	2	1980122_adh
214943_adh	AATCCATTTTTTATTACAATTTA	23	1	1980122_adh
2127140_adh	TGAAGAAGACGGACAAAGCCA	21	20	2127140_adh
2127141_adh	TGAAGAAGACGGACAAAGCCAGC	23	1	2127140_adh
2375908_adh	TGGACGAAATTGCAATATGTT	21	1	2375908_adh
2658343_adh	TTGGACGAAATTGCAATATGTT	22	4	2375908_adh
2658344_adh	TTGGACGAAATTGCAATATGTTC	23	1	2375908_adh
2658345_adh	TTGGACGAAATTGCAATATGTTCA	24	1	2375908_adh
2486875_adh	TGTACGGGCTTCTGTTTGGCA	21	1	2486875_adh
1917491_adh	TATGTACGGGCTTCTGTTTGGCA	23	1	2486875_adh
2561495_adh	TTAGAAGGAACGGCGGACATA	21	1	2561495_adh
1921289_adh	TATTAGAAGGAACGGCGGACATAA	24	1	2561495_adh
2563894_adh	TTAGATCAGTGCTGTGCGGCT	21	1	2563894_adh

2695648_adh	TTTAGATCAGTGCTGTGCGGCT	22	105	2563894_adh
2695650_adh	TTTAGATCAGTGCTGTGCGGCTC	23	1	2563894_adh
1422524_adh	GCTTTAGATCAGTGCTGTGCGGCTCT	26	1	2563894_adh
2587849_adh	TTCAATCGTTGCTGAATGGCG	21	2	2587849_adh
2474542_adh	TGGTTCAATCGTTGCTGAATGGCGAA	26	1	2587849_adh
2593298_adh	TTCAGTTGACAATGGTTTTTT	21	526	2593298_adh
712600_adh	ATTCAGTTGACAATGGTTTTTT	22	1	2593298_adh
2648399_adh	TTGCAACTGAAGAATCAAAAT	21	1	2648399_adh
2529328_adh	TGTTGCAACTGAAGAATCAAAATAT	25	1	2648399_adh
2664429_adh	TTGGATTGTCGCAGTCGGTTA	21	7	2664429_adh
2476676_adh	TGGTTGGATTGTCGCAGTCGGTTA	24	1	2664429_adh
2674955_adh	TTGTAGATATAGAAGACTTAT	21	1	2674955_adh
2456776_adh	TGGGGTTGTAGATATAGAAGACTTAT	26	4	2674955_adh
2682341_adh	TTGTGTGCCGTCGTTGGTTTT	21	1	2682341_adh
2584333_adh	TTATTTGTGTGCCGTCGTTGGTTTTT	26	1	2682341_adh
